# The long non-coding RNA MALAT1 encodes a micropeptide that promotes influenza A virus replication by suppressing innate immune responses

**DOI:** 10.1016/j.jbc.2025.111112

**Published:** 2025-12-27

**Authors:** Kul Raj Rai, Faxin Wen, Mohamed Maarouf, Mengjuan Cai, Haowen Sun, Zhihui Yin, Yiming Wang, Xiaojuan Chi, Yongxia Li, Yuhai Chen, Prasha Shrestha, Zhou Yang, Shile Huang, Song Wang, Ji-Long Chen

**Affiliations:** 1 Key Laboratory of Animal Pathogen Infection and Immunology of Fujian Province, College of Animal Sciences, Fujian Agriculture and Forestry University, Fuzhou, China; 2 Key Laboratory of Fujian-Taiwan Animal Pathogen Biology, College of Animal Sciences, Fujian Agriculture and Forestry University, Fuzhou, China; 3 CAS Key Laboratory of Pathogenic Microbiology and Immunology, Institute of Microbiology, Chinese Academy of Sciences, Beijing, China; 4 Department of Biochemistry and Molecular Biology, Louisiana State University Health Sciences Center, Shreveport, Louisiana, USA

**Keywords:** influenza A virus, lncRNA, MALAT1, micropeptide, innate immunity

## Abstract

Long non-coding RNAs (lncRNAs) play critical roles in diverse biological processes and contain structurally distinct domains enabling multifunctional activity. Viral infections dynamically regulate lncRNA expression, leading to modulation of key cellular pathways, including innate immune responses. Metastasis-associated lung adenocarcinoma transcript 1 (MALAT1), an important lncRNA, exerts diverse biological functions through specific RNA motifs; however, its role in influenza A virus (IAV) infection and pathogenesis remains largely unexplored. Here, we investigated the regulation of MALAT1 expression and its role during the IAV infection. We found that IAV infection robustly upregulated the expression of MALAT1 *in vitro* and *in vivo*. The IAV-induced MALAT1 expression was independent of interferon signaling. Furthermore, we demonstrated that MALAT1 expression was regulated *via* the NF-κB/IL-6/STAT3 pathway in host cells infected with IAV. Functional studies revealed that disruption of MALAT1 expression inhibited IAV replication, whereas overexpression of certain MALAT1 fragments enhanced the virus replication. Using ribosome profiling, MS, and antibody validation, we identified a 52-amino acid micropeptide (miPEP-52) encoded by an RNA fragment of MALAT1, which was endogenously expressed and upregulated by IAV infection. Moreover, we observed that miPEP-52 strongly enhanced the replication of IAV, including attenuated strains. Mutating the miPEP-52 start codon or deleting its coding sequence from the MALAT1 RNA fragment abolished these effects. Mechanistically, MALAT1 and the RNA fragment of MALAT1 encoding miPEP-52 significantly suppressed innate immune responses to IAV infection. These findings provide new insights into the role of MALAT1 in viral pathogenesis and suggest a strategy by which virus evades host antiviral innate immunity.

Long non-coding RNAs (lncRNAs), highly abundant transcripts in the human genome, serve vital roles in numerous biological systems (1). Viral infection results in change of lncRNA expression that is involved in modulating various cellular pathways, including innate immunity (2, 3, 4). Many lncRNAs possess structurally distinct domains that enable multifunctional activity, including serving as protein interaction scaffolds, competitive endogenous RNAs that sequester miRNAs (microRNA), or templates for generating regulatory miRNAs and micropeptides (miPEPs) (1, 5). The functional diversity of lncRNAs arises from multivalent RNA sequence motifs that enable distinct molecular interactions (1). A well-characterized example is MIR155HG, which orchestrates immune regulation through its multiple products: the lncRNA-155, miRNA-155, miPEP155 and competitive endogenous RNA (6, 7). Although the immunomodulatory functions of diverse lncRNAs are increasingly reported (1, 4, 5, 8), identification and characterization of lncRNA-encoded miPEPs remain poorly understood. Particularly, extensive studies are required to determine functional involvement of lncRNA-encoded miPEPs in immune regulation during host-virus interactions (9).

Influenza A virus (IAV), an important member of the Orthomyxoviridae family, causes acute respiratory diseases and poses a huge threat to the health of humans and animals worldwide. The innate immune system provides the first line of defense against IAV infection, primarily through recognition of viral infection by host pattern recognition receptors (PRRs) (10, 11, 12, 13). Upon viral detection, PRRs initiate intracellular signaling cascades that activate key transcription factors, including IRF3, IRF7, NF-κB, and AP-1. These factors coordinately upregulate the expression of various immune-modulatory cytokines (14, 15, 16). During IAV infection, cytokine-mediated responses, such as type I and type III interferon (IFN) and interleukin-6 (IL-6) signaling pathways, play critical roles in activating the JAK-STAT pathway by binding to their corresponding receptors (14). IL-6 signaling preferentially induces STAT3 phosphorylation and subsequent nuclear translocation (17, 18). The activated JAK-STAT pathway ultimately regulates the expression of downstream effector genes, which further stimulate the antiviral immune responses (19, 20).

PRR signaling also induces expression of numerous lncRNAs, either through IFN-dependent or independent pathways (2, 8, 21, 22). These lncRNAs subsequently regulate antiviral responses *via* diverse mechanisms. For instance, the expression of metastasis-associated lung adenocarcinoma transcript 1 (MALAT1), an important lncRNA, has been reported to be implicated in viral pathogenesis (23, 24, 25, 26, 27). Studies show that infections with viruses such as HIV-1 (25, 28), herpes simplex virus type 2 (27), human papilloma virus (29), and SARS-CoV-2 virus (30) upregulate the expression of MALAT1 expression (25, 27, 28, 29, 30). However, the regulation of MALAT1 expression by IAV, and the underlying mechanisms remain unexplored experimentally.

MALAT1 is an evolutionarily conserved, highly abundant intergenic lncRNA across species (31). Originally linked to cancer progression (32, 33), it also modulates immune responses through specific RNA sequences functioning as regulatory domains. For instance, MALAT1-associated small cytoplasmic RNA has been documented, which is implicated in immunity (34, 35, 36). Certain murine MALAT1 fragments bind to the transactive response DNA-binding protein (TDP43), and suppress IRF3-initiated IFNs expression (24). MALAT1/miR-145-5p/CHCHD2 axis synergistically inhibits the expression of STAT2 and facilitates HIV-1 replication (28). MALAT1 can also negatively regulate p53, p21, and p27 that have been shown to enhance antiviral immunity (37, 38, 39). Despite these findings, functional involvement of MALAT1 in IAV infection remains elusive. Ribosome profiling studies have predicted putative translationally active sORFs in MALAT1 (40, 41, 42) and a functional miPEP encoded by a sORF of murine MALAT1 has been recently demonstrated (43), but the translational efficiency of these sORFs and biological activity of their encoded miPEPs are largely unknown.

In this study, we demonstrated that IAV infection robustly upregulated MALAT1 through the NF-κB/IL-6/STAT3 axis, independent of IFN signaling. Knockdown of MALAT1 impaired IAV replication, whereas specific MALAT1 fragments enhanced this process. Furthermore, we identified a functional MALAT1-encoded miPEP-52 that significantly promoted the production of IAV and other viruses, including attenuated strains of IAV. Mechanistically, both MALAT1 and the miPEP-52-encoding fragment facilitated viral replication by suppressing innate immune responses. Our results elucidate the previously uncharacterized involvement of MALAT1 in IAV pathogenesis and suggest that induction of MALAT1 and MALAT1-derived miPEPs may be a strategy by which virus evades host innate immunity.

## Results

### IAV infection induces robust expression of MALAT1 *in vivo* and *in vitro*

**Figure 1:**
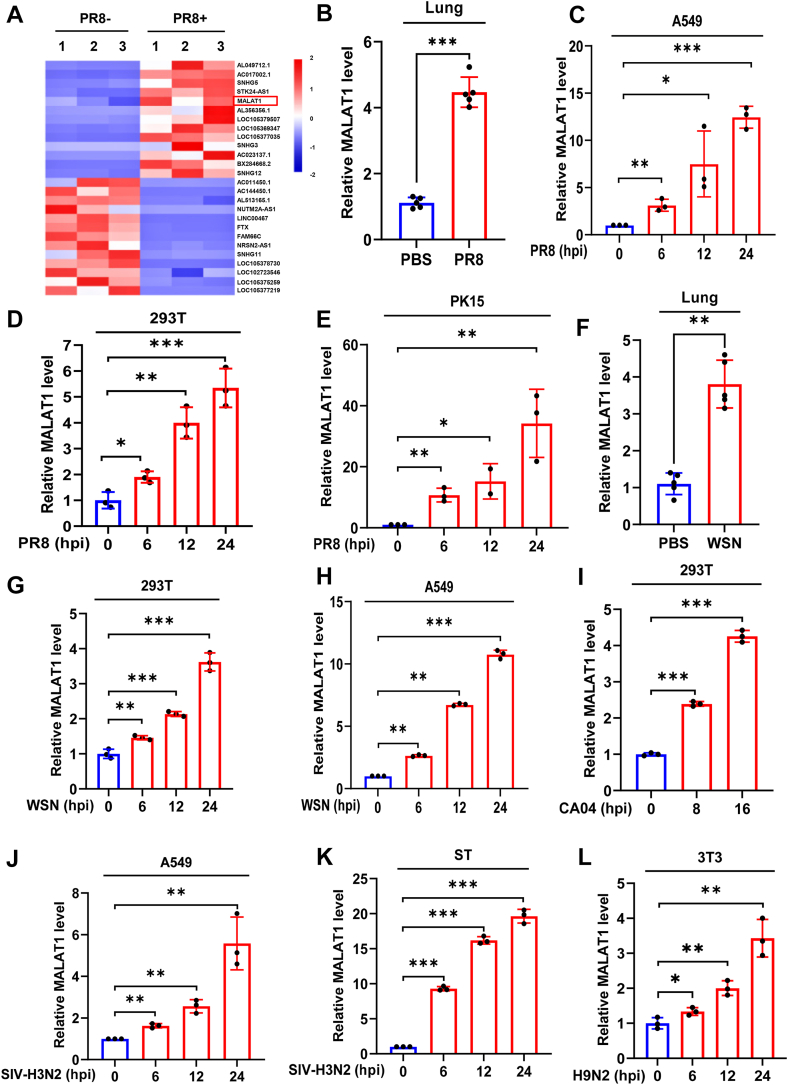
**IAV infection induces robust expression of MALAT1 *in vivo* and *in vitro***. *A*, transcriptome RNA sequencing analysis was conducted on A549 cells infected with or without IAV PR8 influenza virus at a multiplicity of infection (MOI) of 0.5 for 16 h. *B*, wild-type (WT) mice (5–6 weeks old, n = 3–5 each group) were intranasally inoculated with 5 × 10^4^ PFU of PR8 or PBS as a control for 48 h, and the expression level of MALAT1 in the mice lung tissues was examined by RT-qPCR. *C*–*E*, following infection with the PR8 influenza virus (MOI = 1), the expression levels of MALAT1 in different cells were examined by RT-qPCR at the indicated hours post-infection (hpi). *F*, WT mice (5–6 weeks old, n = 3–5 each group) were intranasally inoculated with 5 × 10^4^ PFU of WSN or PBS as a control for 48 h, and the expression level of MALAT1 in the mice lung tissues was examined by RT-qPCR. *G*–*L*, Expression levels of MALAT1 in different cells were examined following infection with various IAV strains by RT-qPCR at the indicated hpi. Shown are representative data from three biologically independent experiments. Statistical analysis was performed using a two-tailed Student's *t* test. Data are presented as means ± SD, ∗*p* < 0.05, ∗∗*p* < 0.01, and ∗∗∗*p* < 0.001. hpi, hours post-infection; IAV, influenza A virus; MOI, multiplicity of infection.

To identify critical lncRNAs implicated in IAV infection and pathogenesis, we foremost employed RNA-Seq to profile differentially expressed lncRNAs in A549 cells mock-infected or infected with the PR8 strain of IAV. The RNA sequencing analysis revealed that 2048 lncRNAs were significantly upregulated, including MALAT1 (NCBI Sequence ID: NR_002819.4), and 2159 lncRNAs were significantly downregulated upon the IAV infection (log2 fold change ≥ 1, and *p* value ≤ 0.05) (Fig. 1*A* and Fig. S1*A*). Since previous studies have shown that MALAT1 is one of the most important lncRNAs involved in various biological processes including innate immunity (23, 24, 34, 35, 44, 45, 46), we specifically focused on MALAT1 for further study. To validate the results from RNA-seq, we performed *in vivo* and *in vitro* experiments using mice and several cell lines. Indeed, the expression of MALAT1 was highly induced in the lung tissues of PR8-infected mice (Fig. 1*B* and S1*B*) and multiple cell lines infected with the PR8 strain of IAV (Fig. 1, *C*–*E* and Fig. S1, *C*–*E*).

To determine whether the expression of MALAT1 can also be regulated by different strains of IAV, we utilized several IAV strains, including A/WSN/1933 (H1N1), A/California/04/2009 (CA04), swine H3N2 (SIV-H3N2), and H9N2. Notably, the WSN infection was found to significantly upregulate MALAT1 in both mouse lung tissues (Fig. 1*F* and S1*F*) and cell lines (Fig. 1, *G* and *E* and Fig. S1*G*). Similarly, other strains of IAV, including CA04, SIV-H3N2, and H9N2, also strongly induced the expression of MALAT1 in different cell lines (Fig. 1, *I*–*L* and Fig. S1, *H*–*L*). These results demonstrate that MALAT1 can be robustly induced by infection with various strains of IAV, suggesting its broad involvement in host cellular responses to IAV infection.

### IFN signaling has no significant effect on IAV-induced MALAT1 expression

**Figure 2:**
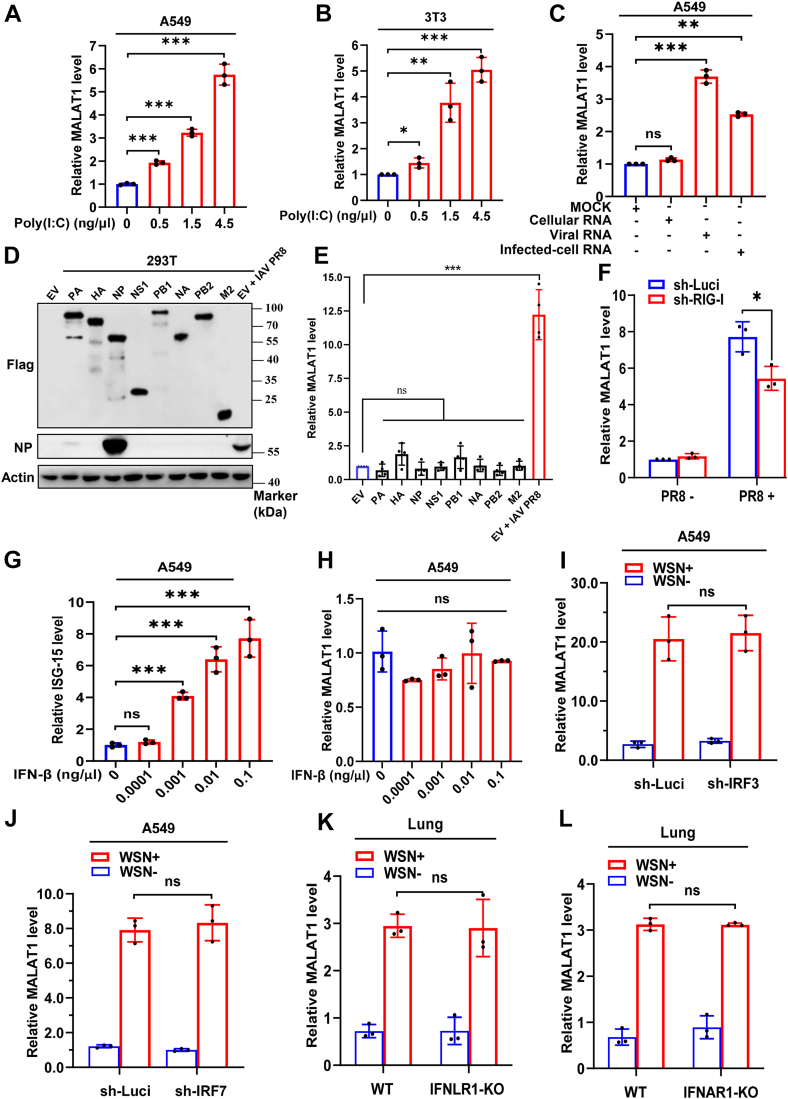
**IFN signaling has no significant effect on IAV-induced MALAT1 expression**. *A* and *B*, cells were treated with Poly (I:C) at indicated concentrations for 4 h, and the expression of MALAT1 was examined by RT-qPCR. *C*, The expression levels of MALAT1 were examined by RT-qPCR after treating A549 cells with the indicated RNAs for 12 h. *D* and *E*, 293T cells were transfected for 48 h with either pCAGGS vectors expressing Flag-tagged IAV PR8 proteins or the pCAGGS empty vector (EV) as a control. During the final 24 h of the 48 h transfection period, the EV-control cells were either mock-infected or infected with IAV PR8 (MOI = 1). Then total protein and RNA were extracted from all samples for analysis. Viral protein expression was assessed by Western blotting (*D*), and MALAT1 expression was quantified by RT-qPCR (*E*). *F*, RIG-I knockdown A549 cells were generated by lentiviral transduction, and the expression of MALAT1 was examined by RT-qPCR with or without IAV PR8 at 16 hpi. *G* and *H*, A549 cells were treated with IFN-β at indicated concentrations for 4 h. ISG-15 and MALAT1 were quantified by RT-qPCR. *I* and *J*, IRF3 and IRF7 knockdown and control A549 cells were infected by influenza virus WSN infection for 16 h, and MALAT1 expression in these cells was examined by RT-qPCR. *K* and *L*, MALAT1 expression was examined by RT-qPCR in wild-type (WT) and IFNLR1-KO or IFNAR1-KO mice (5–6 weeks old, n = 3–5 each group) with/without IAV WSN infection for 48 h. Shown are representative data from three biologically independent experiments. Statistical analysis was performed using a two-tailed Student's *t* test, or a One-way ANOVA with Brown-Forsythe test (*F*). Data are presented as means ± SD, not significant (ns), ∗*p* < 0.05, ∗∗*p* < 0.01, and ∗∗∗*p* < 0.001. EV, empty vector; hpi, hours post-infection; IAV, influenza A virus; MOI, multiplicity of infection.

Previous studies have shown that innate immune signaling can regulate the expression of a variety of lncRNAs (3, 21, 47, 48). To investigate the specific impact of innate immune signaling on MALAT1 expression, we treated the indicated cells with polyinosinic:polycytidylic acid (poly I:C), a structural analog of dsRNA. The experiment demonstrated a dose-dependent induction of MALAT1 upon poly (I:C) treatment (Fig. 2, *A* and *B* and Fig. S2, *A*–*C*). Next, A549 cells were also subjected to transfection with the genomic RNA of PR8 influenza virus (viral RNA), as well as the total RNA isolated from A549 cells infected with PR8 virus (infected-cell RNA). Cellular RNA samples from A549 cells without PR8 virus infection were employed as the negative control. Results showed that both viral RNA and infected-cell RNA induced a significant increase in the expression of MALAT1 (Fig. 2*C*), whereas cellular RNA had no effect on the level of MALAT1. Having known that IAV infection and viral RNA significantly upregulate MALAT1, we sought to determine whether this effect could be attributed to viral proteins. We, therefore, transfected 293T cells with plasmids encoding Flag-tagged IAV proteins. The experiments revealed that ectopic expression of viral proteins had only minimal but not statistically significant effects on MALAT1 levels, whereas a robust upregulation was observed in a positive control with active IAV infection (Fig. 2, *D* and *E*). Moreover, we generated RIG-I-knockdown A549 cells by generating a stable A549 cell line expressing RIG-I-shRNA. Notably, knockdown of RIG-I significantly diminished the expression of MALAT1 induced by IAV infection compared to control cells (Fig. 2*F* and Fig. S2, *D*–*E*).

In order to assess the involvement of type I IFNs, the key cytokine implicated in the innate immune response to IAV, in the regulation of MALAT1 during the viral infection, we treated A549 cells with increasing concentrations of IFN-β. Unexpectedly, while the expression of IFN-stimulated gene (ISG-15), a known target gene of IFN-β, significantly increased with respect to increasing concentrations of IFN-β, the expression of MALAT1 was not significantly changed (Fig. 2, *G* and *H* and Fig. S2*F*). Next, we generated IRF3 or IRF7-knockdown stable A549 cell lines expressing either IRF3-shRNA or IRF7-shRNA (Fig. S2*G*). Knockdown of either IRF3 or IRF7 had no significant effects on IAV-induced expression of MALAT1 compared to control cells (Fig. 2, *I* and *J* and Fig. S2, *H*–*L*). To confirm these findings, we further employed type-I interferon-α/β receptor 1 (IFNAR1) and type-III IFN-λ receptor 1 (IFNLR1) KO mice along with wild-type (WT) control to test the involvement of the IFN signaling in MALAT1 expression. Consistently, we observed no significant difference in the virus-induced expression of MALAT1 between WT mice and those lacking either IFNAR1 or IFNLR1 receptors (Fig. 2, *K* and *L*). These results indicate that type I/III IFNs signaling has no significant effect on IAV-induced MALAT1 expression.

### IAV-induced expression of MALAT1 is regulated by the NF-κB/IL−6/STAT3 axis

**Figure 3:**
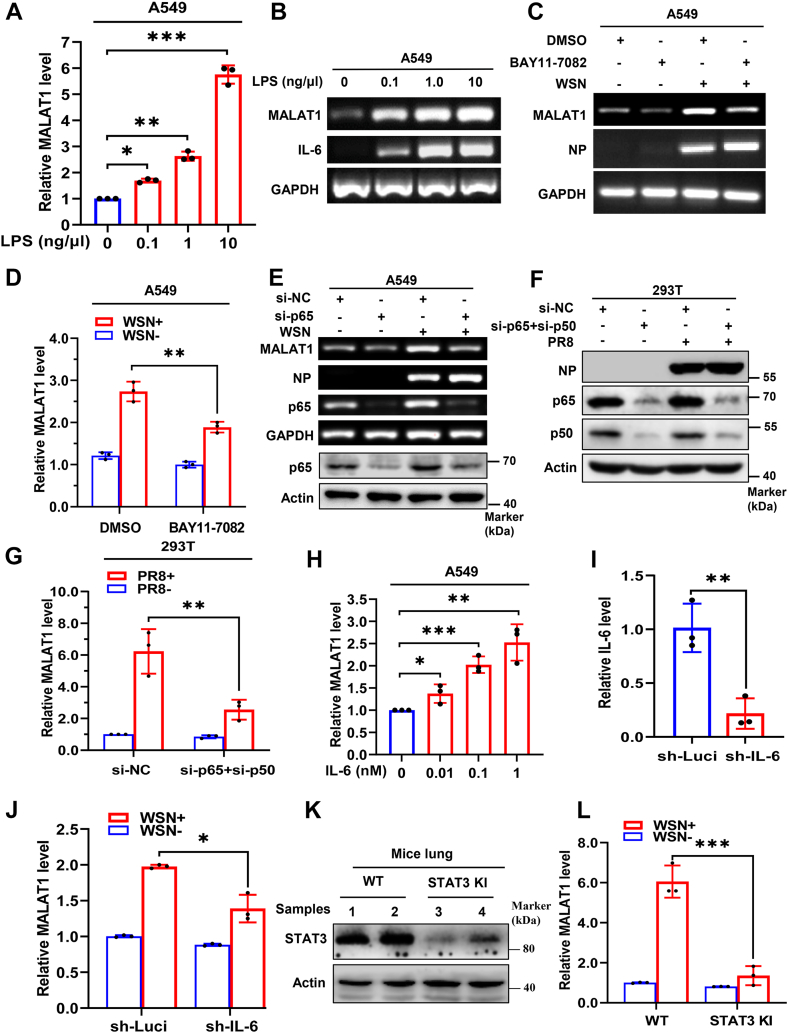
**IAV-induced expression of MALAT1 is regulated by the NF-κB/IL-6/STAT3 axis**. *A* and *B*, cells were treated with LPS at indicated concentrations for 4 h, and the expression of MALAT1 was examined by RT-qPCR (*A*) and RT-PCR (*B*). *C* and *D*, A549 cells were treated with NF-κB inhibitor BAY 11-7082 (50 nM) or dimethyl sulfoxide for 1 h and followed by mock-infection or infection with IAV WSN (MOI = 1) for 16 h. Expression of MALAT1 in the cells was examined by RT-PCR (*C*) and RT-qPCR (*D*). *E*, cells were transfected with 2 μg/ml of p65-targeting siRNA or control siRNA for 24 h, and cells were mock-infected or infected with IAV WSN (MOI = 1) for 16 h. The knockdown effect of p65 was examined by both Western blot & RT-PCR and the expression of MALAT1 was examined by RT-PCR. *F* and *G*, cells were transfected for 24 h with either a control siRNA or siRNAs targeting both p65 and p50 (4 μg/ml total). Following transfection, cells were mock-infected or infected with IAV PR8 (MOI = 1) for 16 h. Knockdown of p65 and p50 was verified at the protein level by Western blotting (*F*), and the relative expression of MALAT1 was examined by RT-qPCR (*G*). *H*, levels of MALAT1 were quantified by RT-qPCR after treating cells with IL-6 at indicated concentrations for 4 h. *I* and *J*, IL-6 knockdown 293T cells were generated by lentivirus transduction and expression levels of IL-6 was examined by RT-qPCR (*I*). IL-6 knockdown and control 293T cells were mock-infected or infected with IAV WSN (MOI = 1) for 16 h, and MALAT1 expression was examined by RT-qPCR (*J*). *K* and *L*, STAT3 protein level was examined by Western blot in the indicated mice (*K*), and MALAT1 expression was examined by RT-qPCR in WT or STAT3^Y705F/+^ knockin (STAT3 KI) mice (5–6 weeks old, n = 3 each group) with/without IAV WSN infection for 48 h (*L*). Shown are representative data from three biologically independent experiments. Statistical analysis was performed using a two-tailed Student's *t* test. Data are presented as means ± SD, ∗*p* < 0.05, ∗∗*p* < 0.01, and ∗∗∗*p* < 0.001. IAV, influenza A virus; MOI, multiplicity of infection.

Since IFN signaling does not significantly affect IAV-induced MALAT1 expression, we investigated whether alternative cytokine signaling pathways regulate MALAT1 upregulation during IAV infection. To assess this, we treated A549 cells with increasing concentrations of lipopolysaccharide (LPS), a known potent agonist of inflammatory response. LPS treatment significantly induced a dose-dependent increase in MALAT1 expression (Fig. 3, *A* and *B*). Given that LPS activates NF-κB signaling pathway (49) and NF-κB serves as a crucial transcriptional regulator of inflammatory cytokines including IL-6 during IAV infection (12, 13, 18), we next investigated the potential role of NF-κB in mediating virus-induced MALAT1 upregulation. Using BAY11-7082, a selective NF-κB inhibitor, we observed a significant reduction in IAV-induced MALAT1 expression compared to controls (Fig. 3, *C* and *D*). Next, we knocked down NF-κB p65 expression in A549 cells by transfecting specific small siRNAs targeting the NF-κB subunit. The knockdown of NF-κB p65 resulted in a clear decrease of MALAT1 expression in cells infected with IAV (Fig. 3*E*). Furthermore, genetic knockdown of both NF-κB subunits p65 and p50 with siRNAs in 293T cells also significantly inhibited IAV PR8-induced MALAT1 expression (Fig. 3, *F* and *G*). Based on the observations presented above, we treated A549 cells with different doses of IL-6, and found that IL-6 treatment significantly elevated the levels of MALAT1 with respect to the increasing concentrations (Figs. 3*H* and S2*M*). To confirm MALAT1 as an IL-6-inducible lncRNA, we generated stable 293T cells with disrupted IL-6 expression (Fig. 3*I*). As expected, MALAT1 expression was significantly lower in IL-6-knockdown cells than that in control cells (Fig. 3*J*). Finally, we utilized STAT3^Y705F/+^ knock-in (KI) mice as previously described (Fig. 3*K*) (17). Interestingly, the IAV-induced expression of MALAT1 was also significantly abrogated in these knock-in mice compared to WT mice upon the viral infection (Fig. 3*L*). Together, these data reveal that IAV-induced innate immune signaling can upregulate the expression of MALAT1 through NF-κB/IL-6/STAT3 pathway but not type I and III IFN signaling.

### Disruption of MALAT1 expression impairs the IAV replication

**Figure 4:**
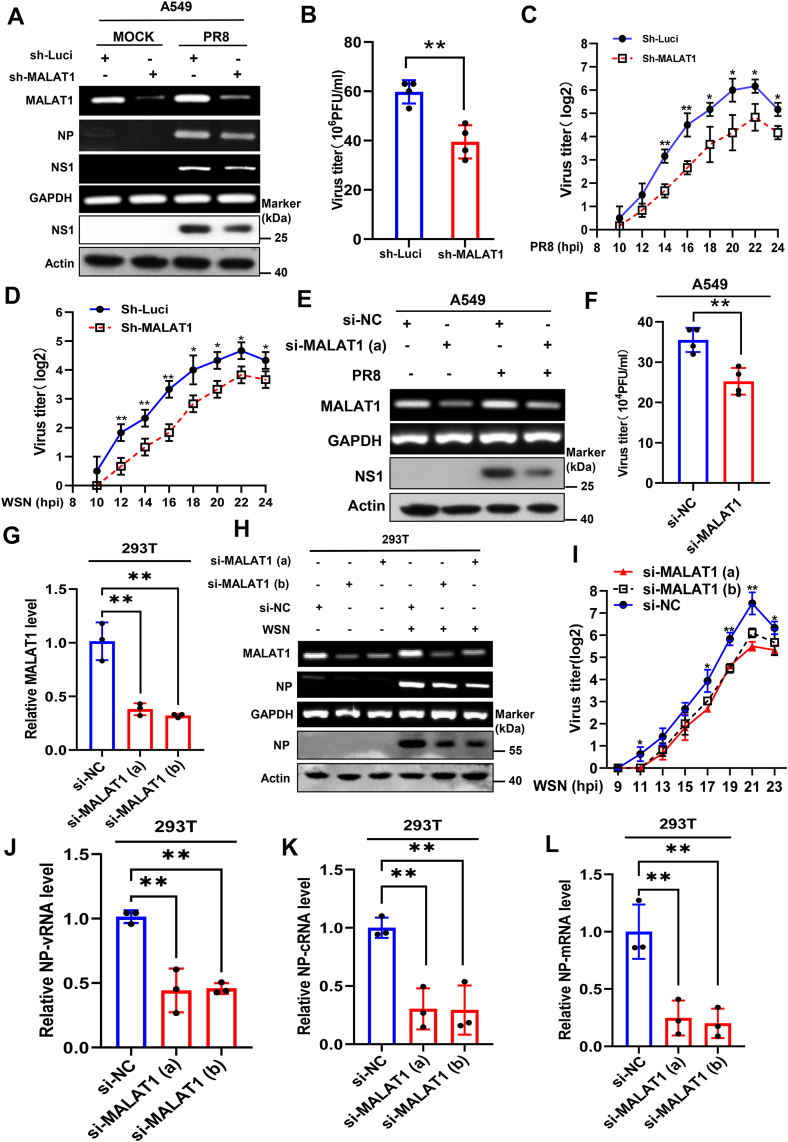
**Disruption of MALAT1 expression impairs the IAV replication**. *A*, A549 cells stably expressing shRNA targeting endogenous MALAT1 or luciferase control were mock-infected or infected with influenza PR8 (MOI = 1) for 16 h. Total protein and RNA were extracted to examine MALAT1 knockdown efficiency and expression of indicated viral genes by RT-PCR and Western blotting. *B* and *C*, the cell culture supernatants were harvested from PR8-infected MALAT1 knockdown and control cells at 17 hpi for plaque assay to measure virus titers (*B*) and at the indicated time points for hemagglutination assay (*C*). *D*, the cell culture supernatants were harvested from WSN-infected MALAT1 knockdown and control cells at the indicated time points for hemagglutination assay (HA). *E*–*I*, the indicated cells were transfected with 2 μg/ml of MALAT1-targeting siRNAs or control siRNA for 24 h, followed by infection with IAV PR8 or WSN (MOI = 1). Then, total protein and RNA extracted at 16 hpi were subjected to Western blot (*E*, *H*), RT-PCR (*E*, *H*) and RT-qPCR (*G*) to assess indicated gene expression. Cell supernatants collected at 16 hpi were used for plaque assay (*F*), and additional supernatants harvested at the indicated time points were analyzed by HA assay (*I*). *J*–*L*, the indicated cells were transfected with 2 μg/ml of MALAT1-targeting or control siRNA for 24 h, followed by infection with IAV PR8 (MOI = 1) for 4 h. Total RNA was then extracted, reverse-transcribed as described in the Materials and Methods, and quantified by real-time PCR to measure the indicated viral RNA species. Shown are representative data from three biologically independent experiments. Statistical analysis was performed using a two-tailed Student's *t* test. Data are presented as means ± SD, ∗*p* < 0.05, and ∗∗*p* < 0.01. HA, hemagglutination assay; hpi, hours post-infection; IAV, influenza A virus; MOI, multiplicity of infection.

To investigate the role of MALAT1 in IAV infection, we generated MALAT1-knockdown A549 cells by introducing lentiviral vectors expressing shRNAs targeting the endogenous MALAT1. Observation of GFP expression by the vector showed that approximately 100% of the cells were positive after infection with the lentiviruses (Fig. S3*A*), and the efficiency of MALAT1 knockdown was examined by RT-PCR and RT-quantitative PCR (RT-qPCR) (Fig. S3, *B*–*C*). MALAT1 knockdown reduced accumulation of IAV PR8 nucleoprotein (NP) and non-structural protein 1 (NS1) levels (Fig. 4*A*). Moreover, we evaluated the PR8 viral loads and viral replication kinetics in the MALAT1 knockdown and control cells using plaque-forming assay and HA assay, respectively. MALAT1 knockdown significantly impaired PR8 viral load and replication kinetics (Fig. 4, *B* and *C*). Similarly, we also investigated the replication kinetics of WSN IAV in these MALAT1 knockdown cells. Consistent with PR8 IAV, MALAT1 knockdown also led to a substantial decrease in viral replication kinetics of WSN (Fig. 4*D*). In addition, we disrupted MALAT1 expression in cells by transfecting specific siRNAs targeting MALAT1 in A549 and 293T cells. The knockdown efficiency of MALAT1 was measured by RT-qPCR and RT-PCR (Fig. 4, *E* and *G*, *H* and Fig. S3, *D*–*E*). Likewise, disruption of MALAT1 caused a decrease in NP expression of IAV (Fig. 4, *E* and *H* and Fig. S3*E*) and also significantly reduced the IAV loads (Fig. 4*F*) and replication kinetics in the infected cells (Fig. 4*I*). We further investigated the effect of MALAT1 knockdown on viral genome replication and mRNA transcription of IAV PR8 by quantifying viral RNA, complementary RNA (cRNA), and mRNA of NP segment. Silencing MALAT1 resulted in a significant reduction in the levels of all three viral RNA species compared to control cells (Fig. 4, *J*–*L*). Together, these data suggest that MALAT1 is required for efficient IAV replication, likely through regulating the viral genome transcription and replication, or upstream stages of IAV life cycle.

### Overexpression of certain MALAT1 fragments enhances the IAV replication

**Figure 5:**
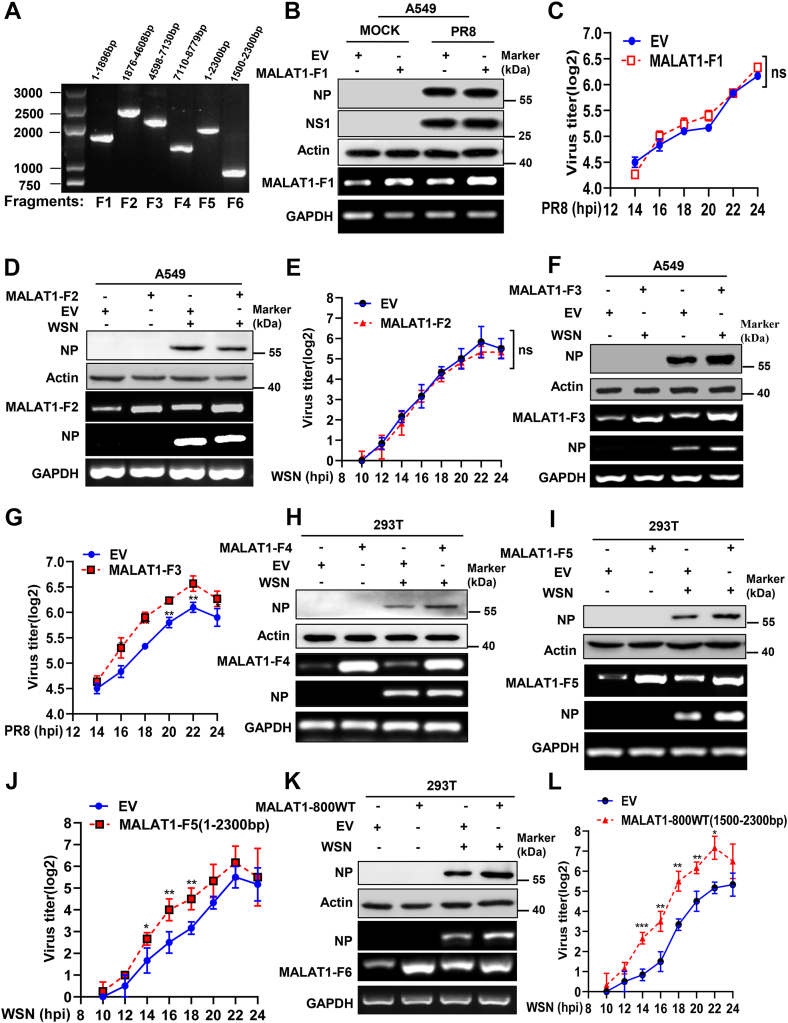
**Overexpression of certain MALAT1 fragments enhances the IAV replication**. *A*, MALAT1 fragments (F1-6) with indicated truncations are depicted *via* agarose gel electrophoresis. *B*–*G*, A549 cells stably expressing MALAT1-F1, MALAT1-F2, MALAT1-F3 or EV were either mock-infected or infected with the indicated IAV strain (MOI = 1) for 16 h. Total protein and RNA were extracted for Western blot (*B*, *D*, and *F*) and RT-PCR (*B*, *D*, and *F*) analysis for the examination of the expression of indicated genes. Cell culture supernatants were collected at the indicated time points for HA assay (*C*, *E* and *G*). *H*, 293T cells expressing MALAT1-F4 or EV were either mock-infected or infected with WSN (MOI = 1). Total RNA and protein were collected for RT-PCR and Western blot analyses to assess MALAT1-F4 overexpression efficiency and viral gene expression. *I* and *J*, 293T cells expressing MALAT1-F5 or EV were mock-infected or infected with IAV WSN (MOI = 1). Total RNA and protein were collected to assess MALAT1-F5 overexpression efficiency and viral gene expression at 16 hpi by RT-PCR and Western blot analyses (*I*), while supernatants from virus-infected cells were harvested for HA assay at the indicated hpi (*J*). *K* and *L*, 293T cells expressing the MALAT1-800WT fragment or EV were infected with IAV WSN (MOI = 1). The overexpression efficiency of MALAT1-800WT and the expression of viral NP at 16 hpi in the indicated cells were determined by Western blot and RT-PCR (*K*). Cell culture supernatants were harvested at the indicated hpi for HA assay (*L*). Shown are representative data from three biologically independent experiments. Statistical analysis was performed using a two-tailed Student's *t* test. Data are presented as means ± SD, not significant (ns), ∗*p* < 0.05, ∗∗*p* < 0.01, and ∗∗∗*p* < 0.001. EV, empty vector; HA, hemagglutination assay; hpi, hours post-infection; IAV, influenza A virus; MOI, multiplicity of infection.

We next investigated effects of MALAT1 overexpression on IAV replication, and determined whether specific domains of MALAT1 regulate IAV replication. Thus, four MALAT1 fragments (F1-4) in a head-to-tail overlap manner were constructed in lentivirus-based expressing vectors as previously described (Fig. 5*A*) (24). We first proceeded to overexpress MALAT1-fragment 1 (MALAT1-F1, 1–1896 bp) in A549 cells, and found that ectopic overexpression of MALAT1-F1 had little impact on viral replication. This was evident from the similar expression levels of viral NP and NS1 genes, as well as comparable viral replication kinetics observed between cells overexpressing MALAT1-F1 and control (Fig. 5, *B* and *C*). Similarly, overexpression of MALAT1-F2 (1876–4608 bp) also did not affect viral replication (Fig. 5, *D* and *E* and Fig. S4*A*). In contrast, overexpression of MALAT1-F3 (4598–7130 bp) in A549 cells positively regulated IAV replication, as indicated by higher expression of viral NP and significantly enhanced viral replication kinetics compared to the empty vector (EV) control (Fig. 5, *F* and *G*). This finding aligns with a prior research demonstrating that the RNA sequences in the MALAT1-F3 interact with TDP43 and inhibit IRF3-mediated antiviral innate immune responses (24). Additionally, MALAT1-F4, which contains the MALAT1-associated small cytoplasmic RNA region known to influence innate immunity (34, 46) also promoted viral replication to some extent (Fig. 5*H* and Figs. S4, *B*–*C*).

Emerging evidence suggests that many small ORFs contained in lncRNAs could be translated into biologically active miPEPs (50). These lncRNAs-encoded miPEPs play critical roles in regulating various biological processes (41, 50, 51). Given this perspective, our focus turned to other unexplored fragments of MALAT1, with a particular emphasis on whether there exist MALAT1-encoded miPEPs in cells. To this end, we conducted Ribo-Seq analysis (Gene Expression Omnibus [GEO] accession number: GSE252920) to identify IAV infection-associated miPEPs encoded by lncRNAs in host cells. Translation efficiency analysis indicated that there existed miPEPs encoded by lncRNAs, including MALAT1, which were induced upon IAV PR8 infection (Fig. S4, *D*–*F*). Simultaneously, using the NCBI ORF viewer, we predicted 64 ORFs in MALAT1 (Fig. S4*G*). Among these, ORF8 (a 159-nucleotide open reading frame spanning positions 1862–2020 within the MALAT1 transcript) was of particular interest, as our Ribo-seq analysis for P-site mapping revealed ribosome occupancy at the ORF8 region of MALAT1 in PR8-infected samples (Fig. S4*F* and Supplementary Datasets 1-2). The MALAT1 ORF8 has been truncated in MALAT1 fragments F1 and F2 (Fig. 5*A*). Supportively, previous ribosome profiling studies also revealed that mammalian MALAT1 ORF8 could be bound to the ribosome (40, 41). Thus, we constructed MALAT1-F5 (1–2300 bp) carrying intact ORF8 (Fig. 5*A*). Surprisingly, overexpression of MALAT1-F5 significantly enhanced IAV replication, indicated by higher expression of viral NP and significantly enhanced viral replication kinetics compared to the EV control (Fig. 5, *I* and *J*). Since MALAT1-F1 & F2 did not exhibit a significant effect on IAV replication, while MALAT1-F5 significantly enhanced it, we further constructed another fragment, MALAT1-F6 (1500–2300 bp) harboring MALAT1 ORF8, here referred to as MALAT1-800WT. Intriguingly, overexpression of MALAT1-800WT also greatly enhanced virus replication, as evidenced by an obvious increase in NP expression and significantly elevated levels of the viral replication compared to the control (Fig. 5, *K* and *L*). These findings demonstrate that certain MALAT1 fragments act as distinct functional domains (Fig. S4*H*), significantly upregulating IAV replication.

### A MALAT1 fragment regulates the IAV replication by encoding a micropeptide

**Figure 6:**
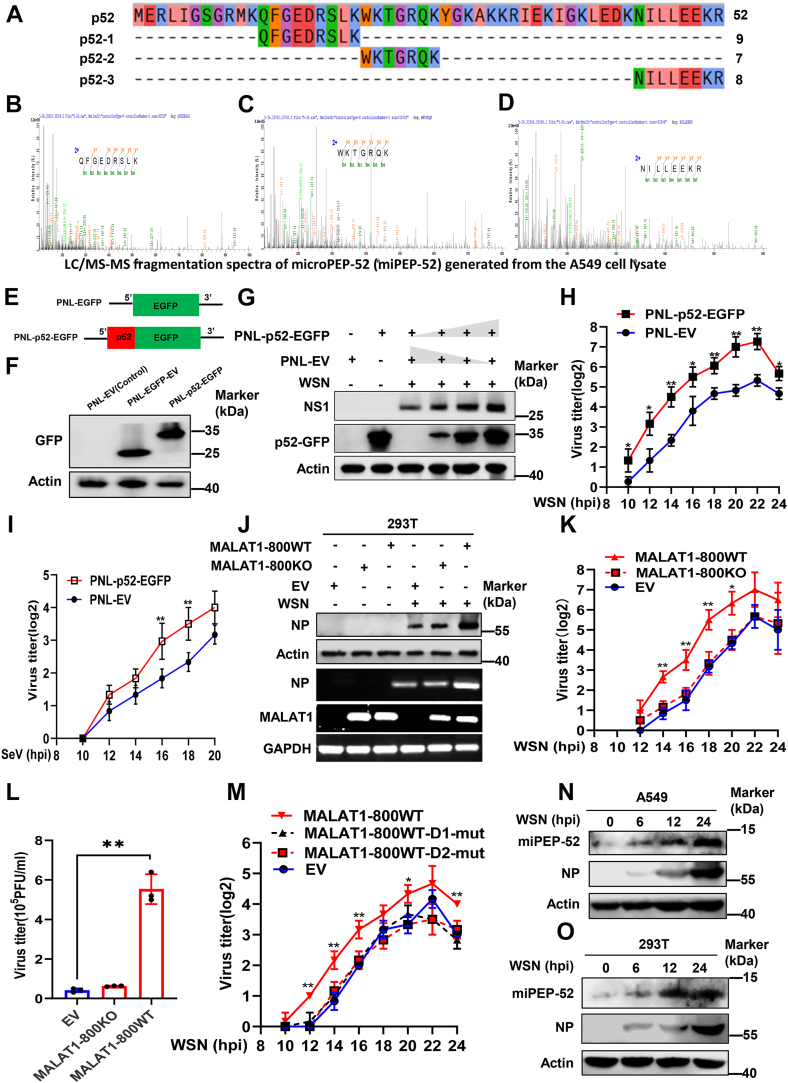
**A MALAT1 fragment regulates the IAV replication by encoding a micropeptide**. *A*–*D*, MS analysis shows three LC/MS-MS fragmentation spectra matching the miPEP-52 sequence generated from the A549 cell lysates. *E*, schematic representation of PNL-p52-EGFP plasmid-vector construct. MALAT1 ORF8 (miPEP-52) sequence was inserted upstream to EGFP of the PNL-EGFP-EV vector. Then, the start codon of EGFP was altered by creating a point mutation. *F*, 293T cells were transfected with PNL-EV, PNL-EGFP-EV, or PNL-p52-EGFP plasmid for 24 h; then total proteins were collected and subjected to Western blotting for the detection of EGFP. *G*, 293T cells were transfected with increasing amounts of PNL-p52-EGFP plasmid, using PNL-EV as both negative control and for maintaining equivalent total DNA amounts across transfection conditions. After 24 h of post-transfection, cells were infected by WSN (MOI = 1) for 16 h. Total proteins were harvested and subjected to Western blotting to detect indicated proteins. *H* and *I*, 293T cells stably expressing miPEP-52 (established *via* PNL-p52-EGFP lentiviral transduction) and EV control cells were infected with WSN or SeV (MOI = 1). Cell supernatants were harvested at indicated hpi and subjected to HA assay. *J*–*L*, 293T cells overexpressing MALAT-800WT, MALAT1-800KO, and EV control using respective vectors were mock-infected or infected with IAV WSN (MOI = 1). Total protein and RNA were extracted at 17 hpi for Western blotting and RT-PCR analysis (*J*); cell supernatants were harvested at indicated hpi for HA assay (*K*) and at 17 hpi for plaque assay (*L*). *M*, 293T cells overexpressing MALAT1-800WT, MALAT1-800WT-D1-mut (single-point mutation altering single ATG of MALAT1-ORF8), MALAT1-800WT-D2-mut (double-point mutation altering double ATGs) and empty vector were infected with WSN, and cell supernatants were collected at indicated hpi for HA assay. *N* and *O*, A549 and 293T cells were infected with IAV WSN (MOI = 1) for the indicated time points, and protein extracts were collected and subjected to Western blotting to detect the expression level of the endogenous miPEP-52. Shown are representative data from three biologically independent experiments. Statistical analysis was performed using a two-tailed Student's *t* test. Data are presented as means ± SD, ∗*p* < 0.05, and ∗∗*p* < 0.01. hpi, hours post-infection; EV, empty vector; HA, hemagglutination assay; IAV, influenza A virus; MOI, multiplicity of infection.

Knowing that MALAT1 fragments harboring MALAT1-ORF8 significantly enhance IAV replication, we employed MS analysis of A549 cell lysates to explore the translation status of MALAT1 ORFs. Interestingly, we found that MS/MS fragmentation spectra belonging to ORF8 were contained in A549 cell lysates (Fig. 6, *A*–*D*), indicating that MALAT1 ORF8 is putatively translated into a micropeptide with 52 amino acids, which we called miPEP-52. Moreover, IAV infection upregulated MALAT1-ORF8 expression (Fig. S5, *A* and *B*). To validate the translation status of ORF8, we built a construct using PNL-EGFP-N1 plasmid encoding GFP, in which MALAT1 ORF8 was inserted upstream to GFP (PNL-EGFP-p52) (Fig. 6*E*). Indeed, the transfection of 293T cells with control vectors (EV or EGFP-EV) or the PNL-p52-EGFP construct resulted in either no detectable expression or produced distinct bands corresponding to GFP and miPEP-52-GFP fusion proteins at their predicted molecular weights (Fig. 6*F*). To examine whether miPEP-52 had any effects on IAV replication, we co-transfected miPEP-52 expressing vector PNL-EGFP-p52 in increasing concentration either with pNL-EV or pNL-EGFP-EV vectors in 293T cells, and the amount of total DNA was kept the same in each sample by equaling with the EV. We observed that ectopic expression of miPEP-52 at increasing concentrations resulted in a concentration-dependent rapid accumulation of IAV NS1 proteins (Figs. 6*G* and S5*C*). Additionally, stable cell lines expressing either miPEP-52 or an EV were generated and the level of miPEP-52 overexpression was examined by Western blotting (Fig. S5*D*). Overexpression of miPEP-52 caused significantly enhanced viral replication kinetics and increased expression of IAV NP as compared to those in control cells (Fig. 6*H* and Figs. S5, *D*–*E*). We also examined role of miPEP-52 in Sendai virus (SeV) infection, another RNA virus. Similarly, overexpression of miPEP-52 enhanced the viral replication kinetics (Fig. 6*I*) and significantly increased SeV-NP expression compared to the control (Fig. S5*F*).

Since MALAT1-800WT and miPEP-52-GFP enhanced IAV replication (Figs. 5, *K*–*L*, Fig. 6, *G* and *H*, and Fig. S5, *C*–*E*), we generated another MALAT1 construct by knocking out MALAT1-ORF8 from the MALAT-800WT, which we called MALAT1-800KO (Fig. S5*G*), to confirm the functional involvement of miPEP-52 in IAV infection. As shown in Figure 6, *J*–*L* and Figure S5*H*, overexpression of MALAT1-800KO exhibited a comparable effect on viral replication and NP expression to the vector control, suggesting that the presence of MALAT1-ORF8 is required for the enhanced viral replication observed in MALAT1-800WT expressing cells. Moreover, knowing that the miPEP-52 sequence contains two closely located translation initiation codons (ATG), we introduced point mutations in the MALAT1-800WT vector and created two different constructs with single- and double-point mutation by altering single ATG and double ATGs, respectively (Fig. S5, *I*–*J*). Of note, viral replication in cells expressing either single- or double-point mutations was also comparable to that in cells expressing EV control (Fig. 6*M* and Fig. S5, *K*–*L*), suggesting miPEP-52 encoded by MALAT1 is essential to promote IAV replication in MALAT1-F6 (MALAT1-800WT) overexpressing cells.

Above LC-MS/MS analysis detected endogenous miPEP-52, which we finally confirmed by immunoblotting. For this, we prepared a polyclonal rabbit antibody against miPEP-52 (Fig. S5*M*). Of note, the antibody was able to detect both ectopically expressed and endogenous miPEP-52 (Fig. S5*N* and Fig. 6, *N* and *O*). As expected, endogenous miPEP-52 level increased markedly following IAV infection (Fig. 6, *N* and *O*). Using this antibody, we confirmed translation of the MALAT1-ORF8 into miPEP-52 in MALAT1-800WT but not in MALAT1-800KO (Fig. S5*O*). Conversely, MALAT1 knockdown in 293T cells by transfecting specific small siRNAs resulted in a clear decrease in the expression of miPEP-52 in cells with or without IAV infection (Fig. S5*P*). Together, these experiments demonstrate the existence of MALAT1-encoded miPEP-52, and MALAT1-800WT fragment regulates the IAV replication by encoding this micropeptide.

### MALAT1 significantly suppresses antiviral immune responses

**Figure 7:**
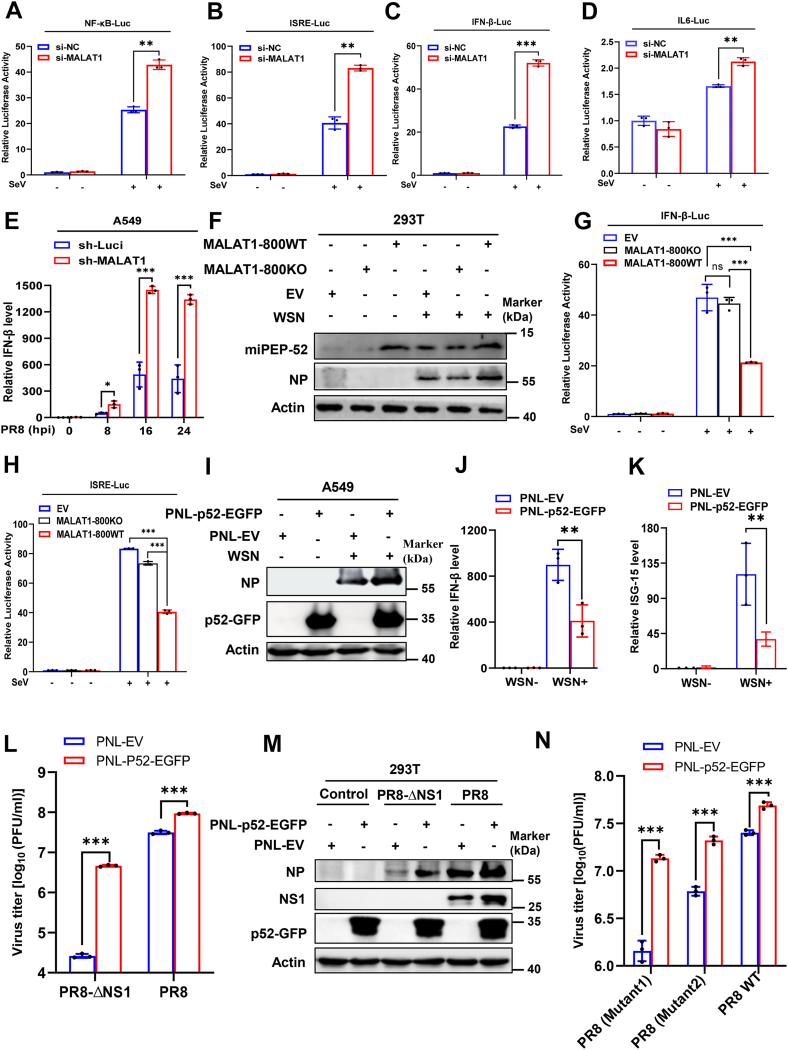
**MALAT1 significantly suppresses antiviral immune responses**. *A–D*, control and MALAT1 knockdown 293T cells were transfected with the indicated luciferase reporter vector for 24 h, followed by SeV (MOI = 0.5) infection for 16 h, and the cells were harvested for luciferase assay. *E*, control and MALAT1 knockdown A549 cells were infected with IAV PR8 (MOI = 1) at indicated hpi, and the expression of IFN-β was quantified by RT-qPCR. *F*, 293T cells stably expressing MALAT1-800WT, MALAT1-800KO, and EV control were generated by lentivirus transduction. Overexpression of MALAT1-800WT with and without WSN infection (MOI = 1) for 16 h was confirmed by Western blotting using polyclonal anti-miPEP-52 serum antibody. *G* and *H*, control, MALAT1-800WT and MALAT1-800KO overexpressing 293T cells were transfected with the indicated luciferase reporter vectors for 24 h, followed by SeV infection (MOI = 0.5) for 16 h, and the cells were harvested for luciferase assay. *I*–*K*, control and miPEP-52 stably expressing A549 cells were mock-infected or infected with IAV WSN (MOI = 1) for 16 h, and total protein was harvested for Western blotting analysis of indicated proteins (*I*), and RNA samples were subjected to RT-qPCR analysis for the relative expression of indicated antiviral genes (*J* and *K*). *L* and *M*, control and miPEP-52 expressing 293T cells were mock-infected or infected with IAV PR8 and PR8-ΔNS1 (MOI = 0.5) for 36 h, and cell supernatants were harvested for plaque assay (*L*), and total cell lysates were collected for Western blotting (*M*). (*N*) 293T cells stably expressing PNL-p52-EGFP vector or EV control were infected with the recombinant IAV viruses Mutant1 (H9N2(HA + NA)/PR8) or Mutant2 (H9N2(HA + NA + M + PB1)/PR8) for 16 h. After infection, cell supernatant samples were collected for plaque assay to determine viral load. Shown are representative data from three biologically independent experiments. Statistical analysis was performed using a two-tailed Student's *t* test. Data are presented as means ± SD, not significant (ns), ∗*p* < 0.05, ∗∗*p* < 0.01, and ∗∗∗*p* < 0.001. EV, empty vector; HA, hemagglutination assay; hpi, hours post-infection; IAV, influenza A virus; MOI, multiplicity of infection.

Next, we explored the mechanism by which MALAT1 and miPEP-52 enhanced the viral replication. For this, we utilized dual-luciferase reporter assays to examine the effects of MALAT1 and miPEP-52 coding sequence on the activity of major elements of antiviral innate immunity, including IFN-β, NF-κB, IL-6, and interferon-stimulated response element (ISRE). We observed that silencing MALAT1 by siRNA significantly enhanced the activation of indicated promoters (IFN-β, NF-κB, IL-6, and ISRE) following SeV infection (Fig. 7, *A*–*D*). Expression of type I IFNs in MALAT1 knockdown and control A549 cells was further measured after IAV infection at the indicated post-hours of infection. It was shown that virus-induced expression of type I IFNs significantly increased in MALAT1 knockdown cells (Figs. 7*E* and S6*A*). Moreover, we generated stable MALAT1 knockdown 293T cells by stably expressing shRNAs that specifically target MALAT1 using lentiviral vectors (Fig. S6*B*). The expression of OAS2, a critical ISG, was quantified by RT-qPCR. Supportively, knockdown of MALAT1 led to a significant increase in virus-induced OAS2 (Fig. S6*C*). Conversely, we generated stable 293T cell lines overexpressing MALAT1-800 WT or MALAT1-800KO by using a lentiviral vector. The overexpression efficiency of these fragments was tested by Western blotting and RT-qPCR (Figs. 7*F* and S6*D*). SeV-induced promoter activity of IFN-β, NF-κB, IL-6, and ISRE was examined by dual-luciferase reporter assays in these cells. Under our experimental conditions, it was observed that SeV-induced promoter activities were significantly diminished in cells overexpressing MALAT1-800WT fragments, but only slightly impaired in cells overexpressing MALAT1-800KO (Fig. 7, *G* and *H* and Fig. S6, *E*–*F*). Additionally, we tested IAV-induced IFN-β and other important ISGs in these cells. As expected, overexpression of MALAT1-800WT caused significantly reduced levels of IFN-β and ISG-15 as compared to control, but MALAT1-800KO had no significant effect on their expression (Fig. S6, *G*–*H*). Furthermore, cell lines overexpressing miPEP-52-GFP were generated (Fig. 7*I*). Ectopic miPEP-52 enhanced IAV NP accumulation, while significantly suppressing the expression of IFN-β (Fig. 7*J*) and multiple ISGs (Fig. 7*K* and Fig. S6, *I*–*J*). Since MALAT1 was regulated by the IAV-induced NF-κB/IL-6/STAT3 axis and miPEP-52 could inhibit activity of the promoters, we examined MALAT1 expression in miPEP-52 overexpressing cells. Forced expression of miPEP-52 appeared to have an inhibitory effect on the IAV-induced expression of MALAT1 (Fig. S6*K*).

It is well established that IAV NS1 is critical for antagonizing innate immunity and its deletion severely compromises IAV replication (52). Given the strong immunosuppressive effect of miPEP-52, we assessed its impact on infection with attenuated IAV strains—referred to here as “attenuated strains”—including NS1-deleted PR8 (PR8-ΔNS1) and low-pathogenicity reassortants containing H9N2 segments, as previously described (53). Interestingly, miPEP-52 overexpression markedly increased PR8-ΔNS1 production (Fig. 7, *L* and *M*) and enhanced replication of H9N2(HA + NA)/PR8 [Mutant1] and H9N2(HA + NA + M + PB1)/PR8 [Mutant2] (Fig. 7*N*) (53). Together, these results reveal that MALAT1 and a MALAT1 fragment encoding miPEP-52 significantly suppress antiviral immune responses.

## Discussion

Although MALAT1 is one of the most important lncRNAs implicated in multiple biological processes, its functional involvement in IAV pathogenesis remains to be determined. Using both *in vitro* and *in vivo* models, we found that MALAT1 expression is robustly upregulated upon IAV infection, consistent with previous observations in infections with a broad ranges of viruses (25, 26, 27, 28, 29). However, nuclear MALAT1 was downregulated after vesicular stomatitis virus infection (24), suggesting that MALAT1 regulation may be virus-specific and potentially influenced by subcellular trafficking, as MALAT1 can be re-localized to the cytoplasm post-transcriptionally (43, 54, 55, 56). Further investigation is needed to clarify the mechanisms underlying virus- and/or tissue-specific regulation of MALAT1.

Upon infection, IAV stimulates innate immune signaling, leading to secretion of various cytokines, including type I and III IFNs and IL-6 (11, 18, 57). These cytokines orchestrate the transcriptional regulation of diverse genes, including lncRNAs (3, 21, 58). Our findings reveal that IAV-induced upregulation of MALAT1 is mediated by the NF-κB/IL-6/STAT3 axis, independent of IFN signaling. This aligns with previous studies demonstrating IFN-independent induction of certain lncRNAs during viral infections (22, 44, 59, 60). Supportively, a previous study demonstrated that LPS upregulates MALAT1 in macrophages in an NF-κB-dependent manner (44), and another study, using chromatin immunoprecipitation and luciferase reporter assays, demonstrated that STAT3 binds the MALAT1 promoter to transcriptionally activate its expression (61). Of note, IAV infection also activates mitogen-activated protein kinase pathways, such as ERK and p38, which are known inducers of IL-6 (62, 63). Since IL-6 can upregulate MALAT1, this MAPK-IL-6 axis might be also implicated in MALAT1 induction during IAV infection. Furthermore, MALAT1 upregulation *via* the PERK axis of the unfolded protein response has been shown in flavivirus infection (26). However, whether these mechanisms are involved in regulation of MALAT1 production during IAV infection requires further experimental validation.

Previous investigations have characterized several specific MALAT1 RNA sequences involved in the regulation of innate immunity (24, 34, 35, 44, 64). For example, a previous study found that certain murine MALAT1 fragments bind to the TDP43, inhibit the cleavage of TDP43 to its active form TDP35, and thereby reduce nuclear IRF3 levels, impairing the antiviral immune response (24). In this study, we observed that MALAT1 knockdown in both A549 and 293T cells significantly impairs IAV replication by enhancing antiviral innate immune responses. However, knockdown approaches do not provide the resolution needed to pinpoint particular functional domains within MALAT1. To address this, we generated and systematically characterized a series of MALAT1 fragments, including MALAT1-800WT, which encodes miPEP-52. Notably, several MALAT1 fragments including MALAT1-800WT significantly enhanced IAV replication. Previous studies have identified some ribosome-associated human MALAT1 ORFs, including MALAT1-ORF8 (40, 41, 42). Although a recent study demonstrated that mouse MALAT1 encodes a biologically active miPEP (43), the translational activity and functional biological significance of human MALAT1 ORFs remain uncharacterized. Given the large size of human MALAT1, it is likely to harbor multiple *bona fide* ORFs. Among them, we detected MALAT1-ORF8 as a ribosome associated human MALAT1 ORF specifically in IAV-infected cells by Ribo-seq analysis. It is worth noting that our use of canonical P-site mapping may limit the detection of weakly translated ORFs compared to alternative approaches. Future work will be required to fully characterize the repertoire of translated ORFs with human MALAT1 and their potential biological functions.

We next focused on characterizing the miPEP encoded by MALAT1-ORF8, which we call miPEP-52, based on Ribo-sequencing and MS analyses. Notably, the previously uncharacterized MALAT1-800 WT fragment (1500–2300 bp) significantly enhanced viral replication, whereas its ORF8-KO counterpart (MALAT1-800KO) failed to do so, highlighting the essential role of MALAT1-ORF8 in promoting IAV infection. To further confirm the existence of miPEP-52, we finally developed a specific polyclonal antibody and successfully detected both ectopically expressed and endogenous miPEP-52. Furthermore, our data reveal that both MALAT1 and its encoded micropeptide, miPEP-52, regulate host innate immune responses during viral infection. While certain MALAT1 RNA domains has been previously reported to play a role in regulation of antiviral innate immunity (24, 44), this study specifically defines previously uncharacterized MALAT1-800WT regulating IAV-induced antiviral responses by encoding miPEP-52. These findings provide the first evidence that miPEP-52, encoded by MALAT1-ORF8, is critical for enhancing IAV replication. Together, our results suggest that induction of MALAT1 and MALAT1-derived miPEPs may be a strategy by which IAV escapes host innate immunity. However, these observations warrant further investigation of the broad immunomodulatory function of MALAT1-800WT fragment and miPEP-52, and underlying mechanism of their action during the viral pathogenesis.

Interestingly, miPEP-52 exhibits modest but statistically significant conservation with a recently identified murine MALAT1-encoded miPEP (43). It would be interesting to study the role of that murine miPEP in the pathogenesis of IAV. In this study, miPEP-52 overexpression also strongly enhanced the replication of “attenuated strains” such as ΔNS1 IAV. This finding may help overcome a major hurdle in vaccine production, as such attenuated viruses typically replicate poorly in standard vaccine manufacturing systems (65). Nonetheless, further investigations are needed to determine whether miPEP-52 could represent a viable strategy to improve the yield of replication-deficient viruses in vaccine development.

In summary, we demonstrate that MALAT1 expression is significantly upregulated during IAV infection *via* the NF-κB/IL-6/STAT3 axis, independently of type I IFNs. MALAT1 knockdown impairs IAV replication, whereas overexpression of specific MALAT1 fragments enhances it. We identify and characterize a novel IAV-inducible functional micropeptide, miPEP-52, encoded by MALAT1, which suppresses antiviral responses. These findings highlight a critical role for MALAT1 in IAV pathogenesis, and suggest that upregulation of MALAT1 and MALAT1-derived miPEPs may be a strategy by which virus escapes host innate immunity.

## Experimental procedures

### Ethics statement

All animal procedures were approved by the Research Ethics Committee of Fujian Agriculture and Forestry University's College of Animal Sciences (Permit No. PZCASFAFU2019003) and complied with China's State Council Regulations for Experimental Animal Administration.

### Cell culture, virus propagation, and infection

Human (293T, A549), murine (3T3), porcine (ST), and canine (MDCK) cell lines were cultured in DMEM supplemented with 10% FBS, 2 mM glutamine, and antibiotics (100 U/ml penicillin, 100 μg/ml streptomycin) at 37 °C with 5% CO_2_. IAV strains (H1N1: PR8, WSN, California/04/2009; H9N2: Fujian/MQ01/2015; SIV-H3N2) were propagated in chicken embryos, and SeV was prepared as previously described (4). For infections, cells were PBS-washed, inoculated at indicated multiplicity of infection (MOI) (45–60 min, 37 °C), then maintained in DMEM containing 2 μg/ml TPCK-trypsin and antibiotics. *In vitro* experiments were done under BSL-2 conditions as described previously (4).

### Mouse experiments

IFNAR1 KO mice, IFNLR1 KO mice, and STAT3^Y705F/+^ KI mice were used on a C57BL/6 background as previously described (17, 20). The mice were anesthetized and inoculated intranasally with 5 × 10^4^ PFU of IAV in 50 μl PBS. Virus infection of mice was carried out under enhanced BSL-2 (BSL-2+) conditions. After infection for the indicated time, mice were euthanized, and their organs were collected for further experiments and analysis as described previously (66).

### RNA extraction, RT-PCR and quantitative real-time PCR (qPCR)

RNA was extracted with Trizol (Invitrogen). For cDNA synthesis, 5 μg of RNA was reverse transcribed with oligo-dT/random primers using Reverse Transcriptase (Promega). PCR was performed with rTaq polymerase; qPCR was carried out using SYBR Premix Ex TaqII (TaKaRa) on a LightCycler (Roche), with ΔΔCT normalization. Using a strand-specific real-time RT-PCR as previously described (67), we quantified viral NP vRNA, cRNA, and mRNA. To ensure specificity, reverse transcription used primers with a unrelated 5′ tag, and the tagged cDNA was amplified with a tag-specific and a segment-specific primer pairs (68). Primers used in this study are listed in Table S1, with GAPDH/actin serving as reference genes.

### Western blotting

Cell lysates were resolved by SDS-PAGE, transferred to nitrocellulose membranes (Whatman), and immunoblotted with: custom anti-miPEP-52 rabbit sera (this study), anti-IAV NP polyclonal antibody (raised against GST-NP, as described previously (66)), or other commercially available antibodies listed in Table S1.

### Plaque forming assay and hemagglutination assay

MDCK cells were infected with virus dilutions (37 °C, 1 h), washed with PBS, and overlaid with α-MEM/3% agarose containing 2 μg/ml TPCK-trypsin. After 72 h incubation, plaques were fixed (4% formaldehyde) and stained with 1% crystal violet for counting. HA assays were performed by incubating 1:1 mixture of virus infected cell-supernatants and 1% chicken erythrocytes in PBS. HA titers were determined based on endpoint hemagglutination assays, as previously described (66).

### Vector construction and transfection of cells

Stable overexpression cell lines were generated *via* lentiviral system as previously described (4, 69). MALAT1 fragments (F1-6; NR_002819.4) were PCR-amplified and cloned into PNL vector. All constructs were verified by sequencing. Vectors were transfected into target cells for functional analysis (primers in Table S1).

### Site-directed mutagenesis

Mutant plasmids were generated using a site-directed mutagenesis kit (Beyotime), with 50 ng plasmid DNA and mutagenic oligonucleotides according to the manufacturer’s protocol. All mutations were verified by Sanger sequencing.

### RNA interference and generation of stable cell lines

shRNA knockdown cell lines were generated as previously described (66). Human MALAT1-targeting shRNA sequences are listed in Table S1. For RIG-I, IRF3, and IRF7 knockdown, previously validated shRNA sequences were used (20). MALAT1 siRNAs and si-NC controls were synthesized by RiboBio and transfected using Lipofectamine 3000 (Invitrogen) according to the manufacturer’s protocols.

### Viral genomic RNA and viral RNA preparation and their transfection

WSN genomic RNA (VG RNA) was extracted from purified virus particles using the Easy Pure Viral RNA Kit (TransGen Biotech), following manufacturer’s instructions. Viral RNA was similarly isolated from WSN-infected A549 cells, with uninfected cell RNA as control (20). For transfection, 2 × 10^6^ A549 cells/well (6-well plates) received 4 μg of VG-RNA, viral RNA, or cellular RNA using Lipofectamine 3000.

### RNA-sequencing and ribo-sequencing

RNA-seq and Ribo-seq were performed by Novogene Co., Ltd as described previously (51, 70). In brief, for RNA-seq, the total RNA from A549 cells was extracted using Trizol reagent. The RNA integrity was then assessed using the RNA Nano 6000 Assay Kit of the Bioanalyzer 2100 system (Agilent Technologies). Following this, sequencing libraries were generated and all samples were sequenced on an Illumina Novaseq platform. Differential expression analysis was performed between the infected and control groups using DESeq2, with differentially expressed genes defined by an adjusted *p*-value < 0.05 and log_2_ fold change ≥ 1. Data are available in GEO under accession GSE211357 (70). For Ribo-seq, A549 cells with or without PR8 infection were lysed using RIPA buffer containing 50 mg/ml cycloheximide and treated with RNase I to generate ribosome-protected fragments. Monosomes were isolated using MicroSpin S-400 HR columns. Ribosome-protected fragments (20–38 nt) underwent PAGE purification, end phosphorylation, adapter ligation, and rRNA depletion (Qiagen). After cDNA synthesis and PCR amplification, libraries were sequenced on an Illumina HiSeq 4000. The Ribo-Seq data are available in GEO under accession number GSE252920 (51).

### Dual-luciferase reporter assay

MALAT1-knockdown (siRNA) or overexpressing (lentiviral) 293T cells were established alongside EV controls. At 70% confluency, cells in 24-well plates were cotransfected in triplicate with NF-κB/ISRE/IFN-β/IL-6 luciferase reporters along with a Renilla plasmid (MiaoLing) using Lipofectamine 3000. After 24 h post-transfection, cells were mock-infected or infected with SeV for 16 h, followed by dual-luciferase assays (Promega).

### LC-MS/MS analysis

Cell lysates from A549 cells were resolved on 12% Bis-Tris/4% stacking gels, stained with Colloidal Blue (Invitrogen), and the protein band corresponding to <15 kDa was excised, destained, and processed for MS analysis at Bioprofile Biotech. The MS proteomics data have been deposited to the ProteomeXchange Consortium *via* the iProX partner repository with the dataset identifier PXD069766.

### Development of anti-miPEP-52 rabbit polyclonal serum antibody

The full-length miPEP-52 cDNA was cloned into pGEX-4T-1, producing a miPEP-52-GST fusion protein in *Escherichia coli* BL21. After purification, the fusion protein was used to immunize New Zealand rabbits, generating anti-miPEP-52 antibodies from the immune sera.

### Quantification and statistical analysis

Comparison between groups was made using Student’s *t* test or One-way ANOVA with Brown-Forsythe test. Data represent the mean ± SD from three independent experiments. Differences were considered statistically significant with *p* < 0.05.

## Data availability

The authors declare that all the data supporting the findings of this study are available within the article and its supporting information. The RNA-sequencing and Ribo-sequencing data are available in the GEO database under the accession numbers provided in the Materials and Methods section. Further inquiries can be directed to the corresponding authors.

## Supporting information

This article contains supporting information.

## Conflict of interest

The authors declare that they have no conflicts of interest with the contents of this article.
