# Supplementary material for: The long non-coding RNA MALAT1 encodes a micropeptide that promotes influenza A virus replication by suppressing innate immune responses
Source: J Biol Chem. 2025 Dec 27;302(2):111112. doi: 10.1016/j.jbc.2025.111112 (PMC12857292; doi:10.1016/j.jbc.2025.111112)
Supplement: Supplementary Material 1 [file mmc1.pdf]

Supporting information

The long non-coding RNA MALAT1 encodes a micropeptide that promotes influenza A virus replication by suppressing innate immune responses

Kul Raj Rai[#], Faxin Wen[#], Mohamed Maarouf[#], Mengjuan Cai, Haowen Sun, Zhihui Yin, Yiming Wang, Xiaojuan Chi, Yongxia Li, Yuhai Chen, Prasha Shrestha, Zhou Yang, Shile Huang, Song Wang[*] and Ji-Long Chen[*]

1. Supplementary Figures and Figure legends

2. Supplementary Tables

3. Supplementary datasets

Figure S1

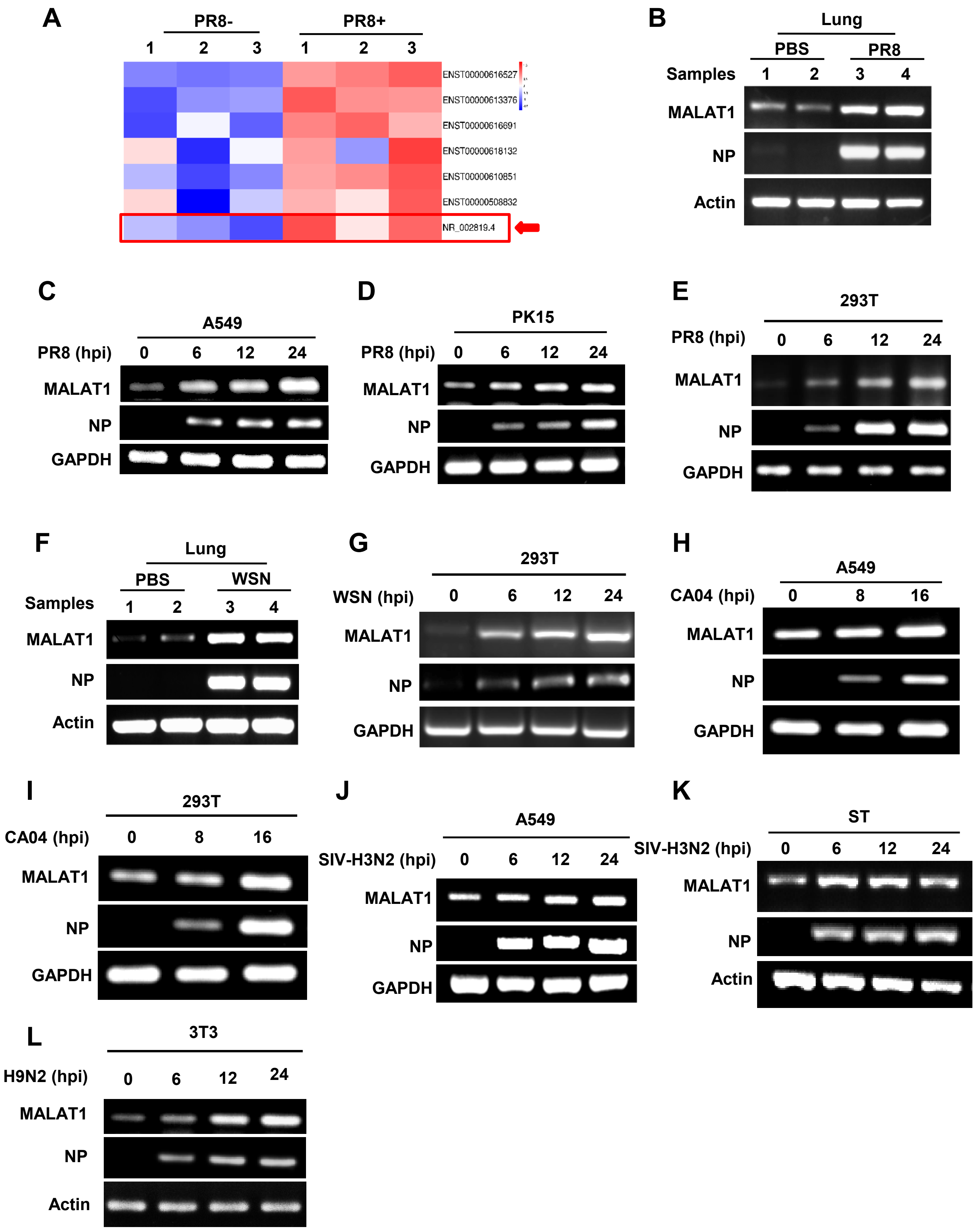
A
PR8-
PR8+
1
2
3
ENST00000616527
ENST00000613376
ENST00000616691
ENST00000618132
ENST00000610851
ENST00000508832
NR_002819.4
B
Lung
PBS
PR8
Samples
MALAT1
NP
Actin
C
A549
PR8 (hpi)
0
6
12
24
GAPDH
D
PK15
E
293T
F
WSN
G
WSN (hpi)
H
CA04 (hpi)
8
16
I
J
SIV-H3N2 (hpi)
K
ST
L
3T3
H9N2 (hpi)

Figure S1. IAV infection induces robust expression of MALAT1 *in vivo* and *in vitro*. (A) Heat map showing expression levels of various MALAT1 transcripts in A549 cells mock-infected or infected with PR8 influenza virus (MOI = 0.5) for16 h. The human transcript (ID NR_002819.4), marked in red, was used for functional and mechanistic analyses in this study. (B) Wild-type mice (5 to 6 weeks old, n=3 to 5 each group) were intranasally inoculated with 5×10^4 PFU PR8 or PBS as a control for 48 h, and the expression level of MALAT1 in the mice lung tissues was examined by RT-PCR. (C-E) MALAT1 expression levels in different cells following PR8 influenza virus infection (MOI = 1) were examined by RT-PCR at the indicated hours post-infection (hpi). (F) Wild-type mice (5 to 6 weeks old, n=3 to 5 each group) were intranasally inoculated with 5×10^4 PFU WSN or PBS as a control for 48 h, and the expression level of MALAT1 in the mice lung tissues was examined by RT-PCR. (G-L) MALAT1 expression levels in different cells following infection with various IAV strains were examined by RT-PCR at the indicated hpi. Shown are representative data from three biologically independent experiments.

Figure S2

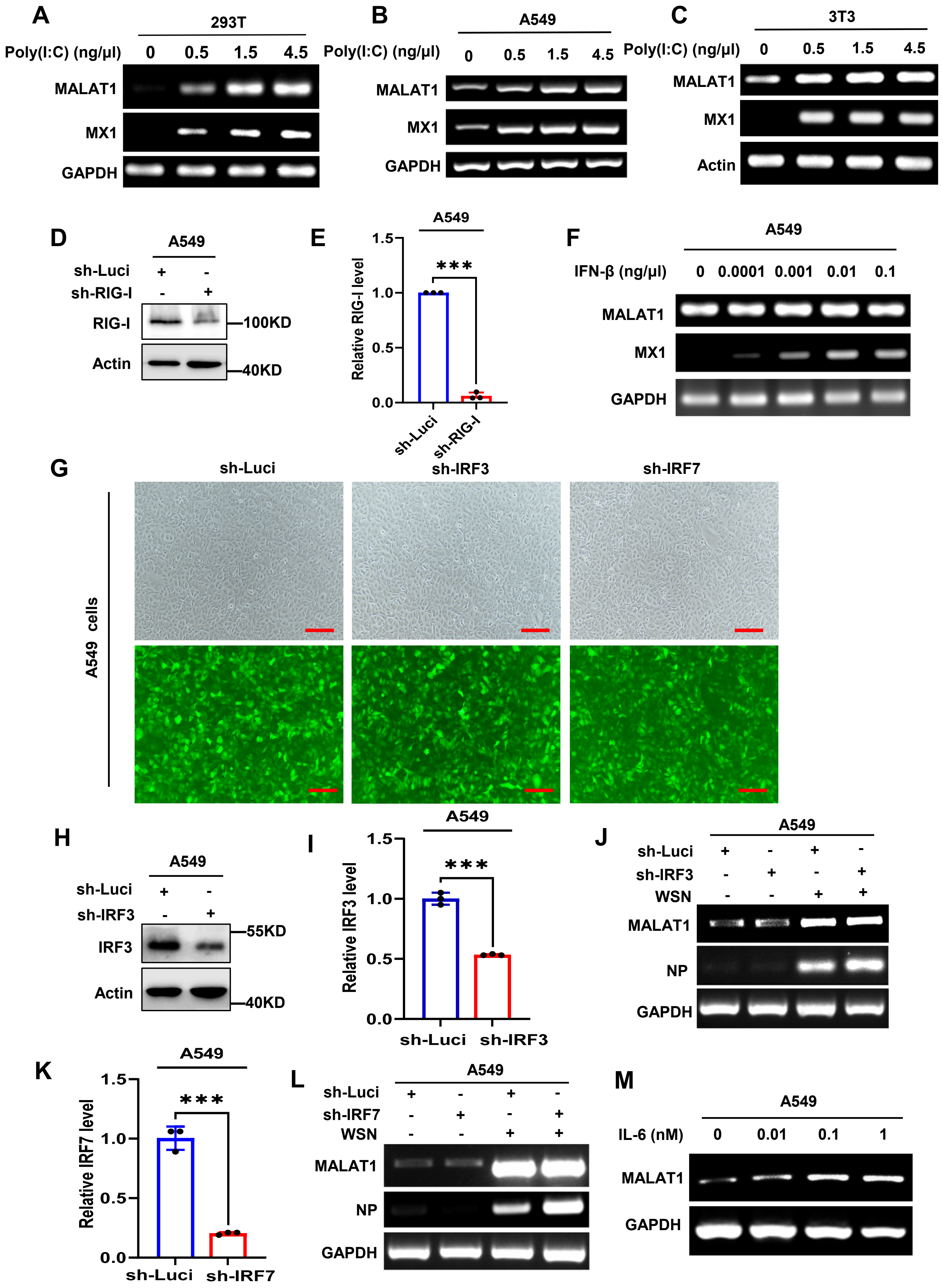

A
293T
Poly(I:C) (ng/μl) 0 0.5 1.5 4.5
MALAT1
MX1
GAPDH
B
A549
Poly(I:C) (ng/μl) 0 0.5 1.5 4.5
MALAT1
MX1
GAPDH
C
3T3
Poly(I:C) (ng/μl) 0 0.5 1.5 4.5
MALAT1
MX1
Actin
D
A549
sh-Luci + -
sh-RIG-I - +
RIG-I
Actin
100KD
40KD
E
A549
Relative RIG-I level
1.5
1.0
0.5
0.0
***
sh-Luci
sh-RIG-I
F
A549
IFN-β (ng/μl) 0 0.0001 0.001 0.01 0.1
MALAT1
MX1
GAPDH
G
sh-Luci
sh-IRF3
sh-IRF7
A549 cells
H
A549
sh-Luci + -
sh-IRF3 - +
IRF3
Actin
55KD
40KD
I
A549
Relative IRF3 level
1.5
1.0
0.5
0.0
***
sh-Luci
sh-IRF3
J
A549
sh-Luci + - + -
sh-IRF3 - + - +
WSN - - + +
MALAT1
NP
GAPDH
K
A549
Relative IRF7 level
1.5
1.0
0.5
0.0
***
sh-Luci
sh-IRF7
L
A549
sh-Luci + - + -
sh-IRF7 - + - +
WSN - - + +
MALAT1
NP
GAPDH
M
A549
IL-6 (nM) 0 0.01 0.1 1
MALAT1
GAPDH

Figure S2. IFN signaling has no significant effect on IAV-induced MALAT1 expression. (A-C) Indicated cells were treated with Poly(I:C) for 4 h and expression of MALAT1 was examined by RT-PCR. (D-E) RIG-I knockdown A549 cells were generated via lentivirus transduction, and knockdown efficiency was verified by Western blotting (D) and RT-qPCR (E). (F) A549 cells were treated with IFN-β at indicated concentrations for 4 h. The expression of indicated genes was examined by RT-PCR. (G-L) IRF3 and IRF7 knockdown A549 cells were generated by lentivirus transduction. Transduction efficiency was examined by microscopic examination for the expression of GFP (G). Knockdown effect of IRF3 was examined by Western blotting (H) & RT-qPCR (I) and IRF7 was examined by RT-qPCR (K), then MALAT1 expression in these cells was examined by RT-PCR following IAV WSN infection for 16 h (J and L). Scale bar, 200 μm. (M) Levels of MALAT1 were examined by RT-PCR after treating cells with IL-6 at indicated concentrations for 4 h. Shown are representative data from three biologically independent experiments. Statistical analysis was performed using a two-tailed Student's *t*-test. Data are presented as means ± SD, $^{***}p < 0.001$.

Figure S3

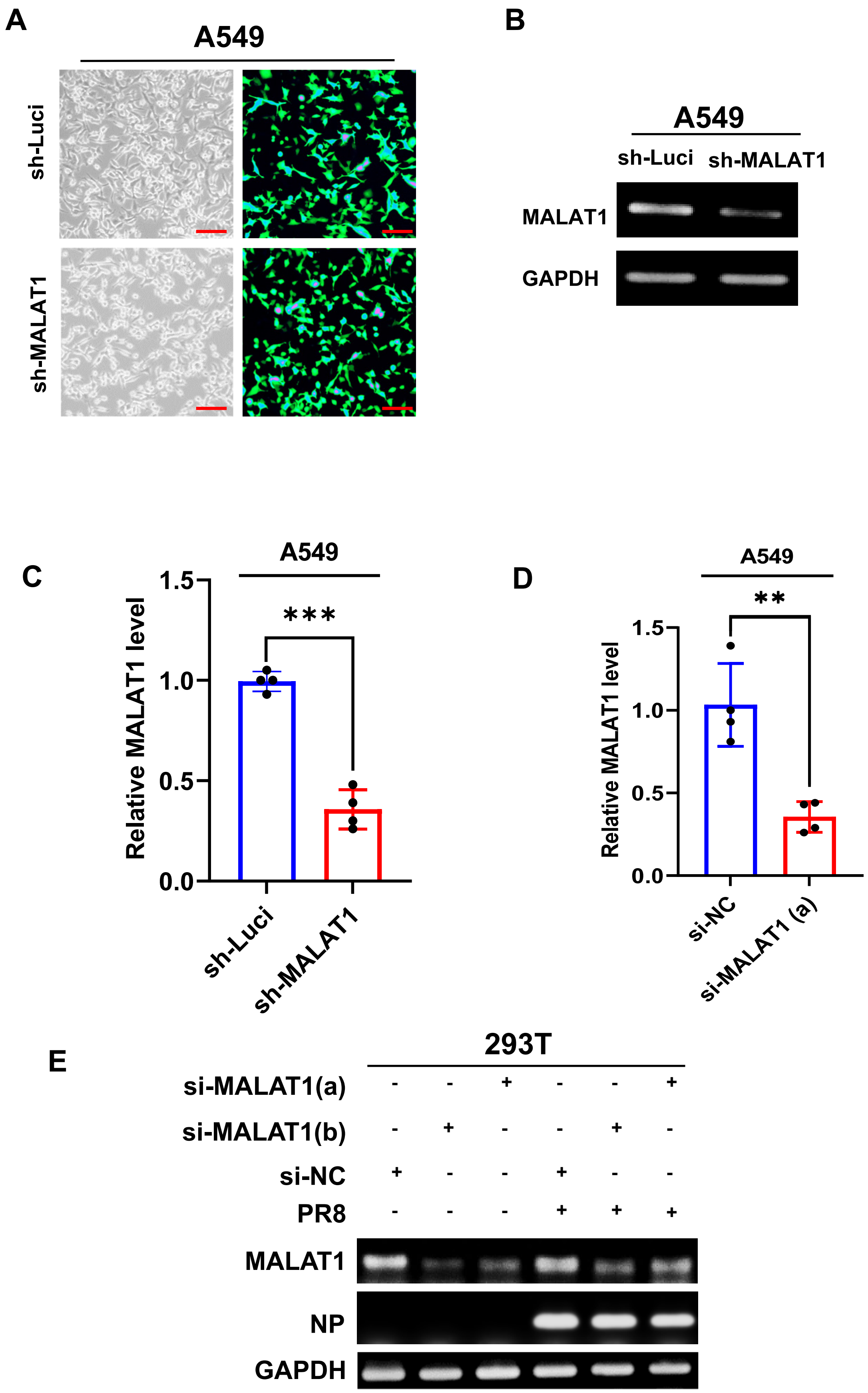
A
A549
sh-Luci
sh-MALAT1
B
A549
sh-Luci
sh-MALAT1
MALAT1
GAPDH
C
A549
***
Relative MALAT1 level
1.5
1.0
0.5
0.0
sh-Luci
sh-MALAT1
D
A549
**
Relative MALAT1 level
1.5
1.0
0.5
0.0
si-NC
si-MALAT1 (a)
E
293T
si-MALAT1(a)
si-MALAT1(b)
si-NC
PR8
MALAT1
NP
GAPDH

Figure S3. Disruption of MALAT1 expression impairs the IAV replication. (A) Fluorescence microscopic images showing the efficiency of MALAT1 knockdown by examining green fluorescent protein expression in lentivirus-transfected A549 cells. Scale bar, 100 μm. (B-C) RT-PCR and RT-qPCR results showing knockdown efficiency of MALAT1 in lentivirus-transfected A549 cells. (D) RT-qPCR results showing knockdown efficiency of MALAT1 in si-MALAT1 transfected A549 cells. (E) Indicated cells were transfected with 2 μg/mL of MALAT1-targeting siRNA or control siRNA for 24 h, followed by either mock infection or infection with IAV WSN (MOI=1). Total RNA was then extracted at 16 hpi to examine the expression of the indicated genes by RT-PCR. Shown are representative data from three biologically independent experiments. Statistical analysis was performed using a two-tailed Student's *t*-test. Data are presented as means ± SD, not significant (ns), $^{**}p < 0.01$, $^{***}p < 0.001$.

Figure S4

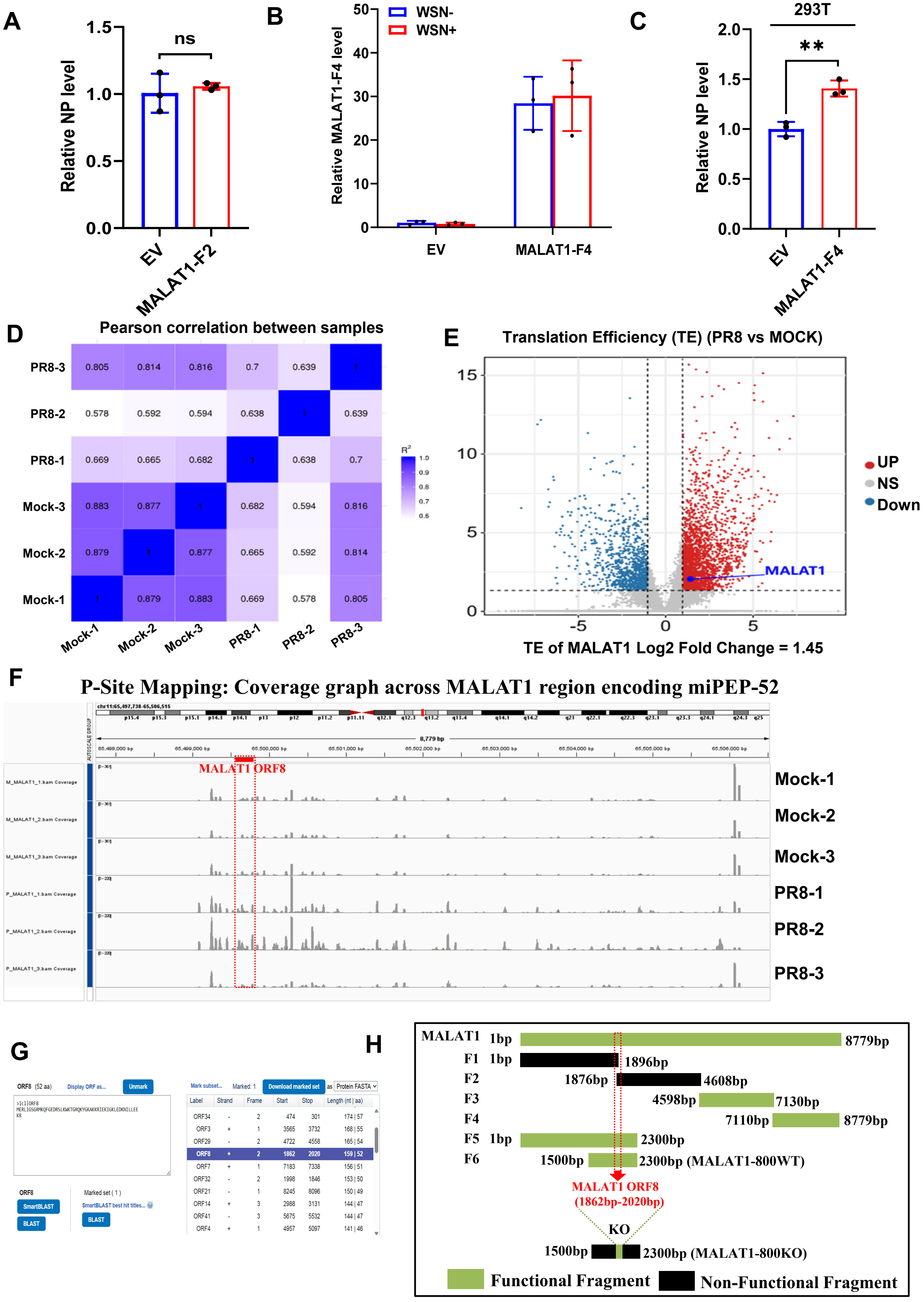

A
Relative NP level
ns
EV
MALAT1-F2
B
Relative MALAT1-F4 level
WSN-
WSN+
EV
MALAT1-F4
C
293T
Relative NP level
**
EV
MALAT1-F4
D
Pearson correlation between samples
PR8-3
PR8-2
PR8-1
Mock-3
Mock-2
Mock-1
E
Translation Efficiency (TE) (PR8 vs MOCK)
UP
NS
Down
MALAT1
TE of MALAT1 Log2 Fold Change = 1.45
F
P-Site Mapping: Coverage graph across MALAT1 region encoding miPEP-52
MALAT1 ORF8
Mock-1
Mock-2
Mock-3
PR8-1
PR8-2
PR8-3
G
H
MALAT1 1bp 8779bp
F1 1bp 1896bp
F2 1876bp 4608bp
F3 4598bp 7130bp
F4 7110bp 8779bp
F5 1bp 2300bp
F6 1500bp 2300bp (MALAT1-800WT)
MALAT1 ORF8
(1862bp-2020bp)
KO
1500bp 2300bp (MALAT1-800KO)
Functional Fragment
Non-Functional Fragment

Figure S4. Overexpression of certain MALAT1 fragments enhances the IAV replication. (A) A549 cells stably expressing the MALAT1-F2 plasmid or empty vector (EV) were either mock-infected or infected with IAV PR8 (MOI=1) for 16 h. Total RNA were collected, and RT-qPCR was performed to examine relative expression of IAV NP. (B and C) 293T cells expressing MALAT1-F4 or EV were either mock-infected or infected with WSN (MOI=1). Total RNA was collected to assess MALAT1-F4 overexpression efficiency and viral NP expression by RT-qPCR. (D and E) Ribo-sequencing analysis of A549 cells infected with PR8 for 12 h (GSE252920). Pearson correlation between samples (n=3 x 2) with (PR8+) or without (PR8-) IAV infection (D); Volcano plot showing differential translation efficiency (TE) of lncRNAs in PR8-infected versus mock-infected A549 cells at 12 hpi (log2 (fold change) > 1; false discovery rate < 0.05) (E). (F) Ribo-seq P-site mapping reveals ribosome occupancy at the 159-nt ORF8 region (nucleotides 1862–2020) of the MALAT1 lncRNA specifically in PR8-infected samples. Track displays ribosome-protected footprints aligned to the MALAT1 genomic locus, with ORF8 position highlighted. Data represent biological replicates (n=3 x 2). In this coverage graph, a higher kurtosis indicates a higher translation activity in this region, and a higher number of ribosomes may correspond to highly expressed genes or active translation processes. Please also refer Supplementary Datasets 1-2 for full Ribo-seq data for all annotated and novel ORFs. (G) The NCBI ORF finder tool was used to identify open reading frames (ORFs) in the MALAT1 sequence. ORFs were predicted using the Standard Genetic Code, with a minimum ORF length of 75 nucleotides. The analysis was performed on both strands and across all six reading frames (+1, +2, +3, -1, -2, -3). ORFs were identified based on the presence of an AUG start codon and terminated at one of the three stop codons (UAA, UAG, or UGA). The results included ORF coordinates, strand orientation, reading frame, and ORF length. (H) A schematic representation of the designed MALAT1 fragments (including the location of MALAT1 ORF8 as well as MALAT1-800WT and MALAT1-800KO) reveals that black-colored fragments exhibit no or a little impact on influenza virus replication, whereas green-colored fragments demonstrate a notable influence on the influenza virus replication. Shown are representative data from three biologically independent experiments. Statistical analysis was performed using a two-tailed Student's *t*-test. Data are presented as means ± SD, not significant (ns), $^{**}p < 0.01$.

Figure S5

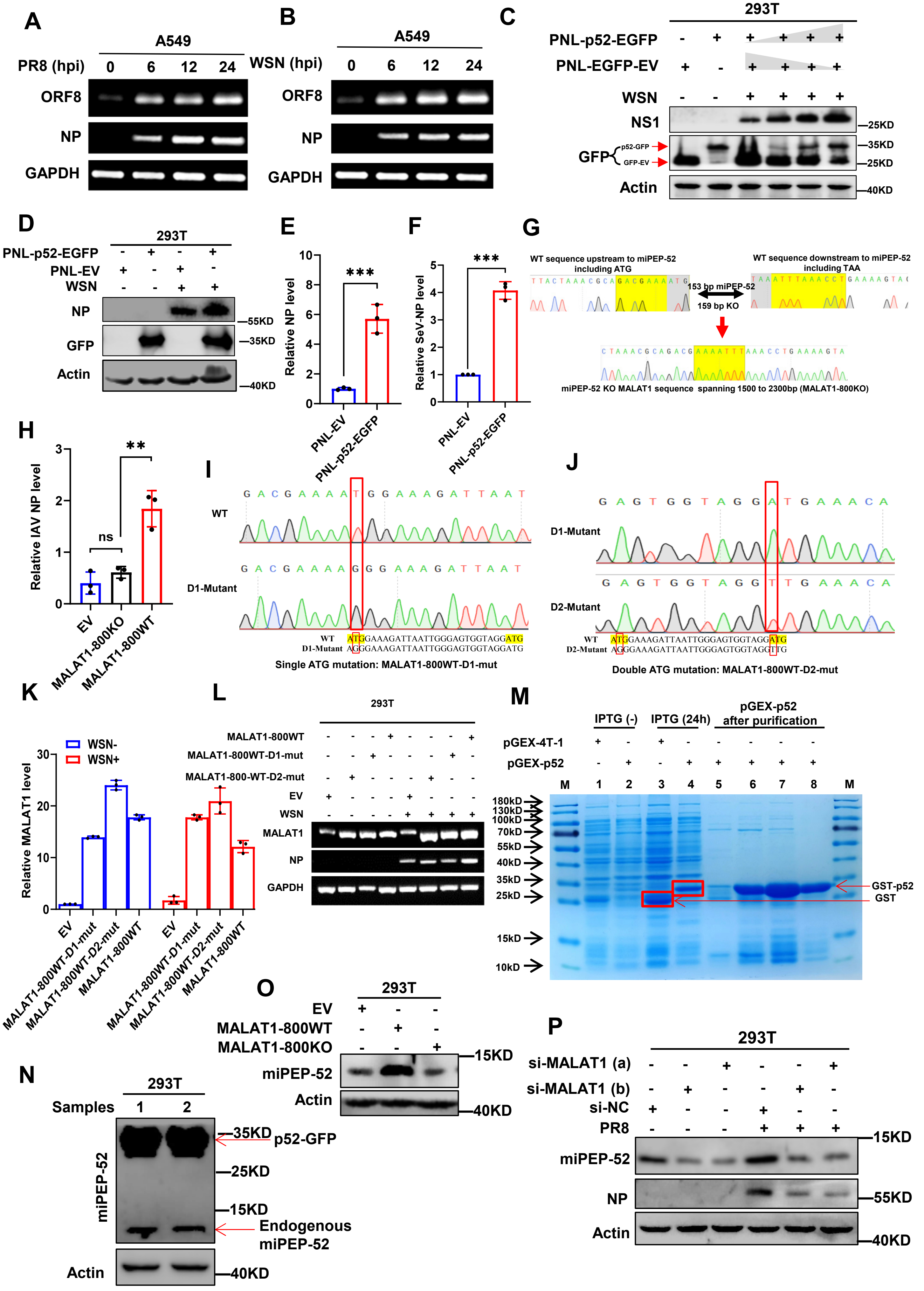

A
A549
PR8 (hpi) 0 6 12 24
ORF8
NP
GAPDH
B
A549
WSN (hpi) 0 6 12 24
ORF8
NP
GAPDH
C
293T
PNL-p52-EGFP
PNL-EGFP-EV
WSN
NS1
GFP
p52-GFP
GFP-EV
Actin
25KD
35KD
25KD
40KD
D
293T
PNL-p52-EGFP
PNL-EV
WSN
NP
GFP
Actin
55KD
35KD
40KD
E
Relative NP level
PNL-EV
PNL-p52-EGFP
***
F
Relative SeV-NP level
PNL-EV
PNL-p52-EGFP
***
G
WT sequence upstream to miPEP-52 including ATG
WT sequence downstream to miPEP-52 including TAA
153 bp miPEP-52
159 bp KO
miPEP-52 KO MALAT1 sequence spanning 1500 to 2300bp (MALAT1-800KO)
H
Relative IAV NP level
EV
MALAT1-800KO
MALAT1-800WT
ns
**
I
WT
D1-Mutant
WT ATGGAAAGATTAATTGGGAGTGGTAGGATG
D1-Mutant AGGGAAAGATTAATTGGGAGTGGTAGGATG
Single ATG mutation: MALAT1-800WT-D1-mut
J
D1-Mutant
D2-Mutant
WT ATGGAAAGATTAATTGGGAGTGGTAGGATG
D2-Mutant AGGGAAAGATTAATTGGGAGTGGTAGGTTG
Double ATG mutation: MALAT1-800WT-D2-mut
K
WSN-
WSN+
Relative MALAT1 level
EV
MALAT1-800WT-D1-mut
MALAT1-800WT-D2-mut
MALAT1-800WT
L
293T
MALAT1-800WT
MALAT1-800WT-D1-mut
MALAT1-800-WT-D2-mut
EV
WSN
MALAT1
NP
GAPDH
M
IPTG (-)
IPTG (24h)
pGEX-p52 after purification
pGEX-4T-1
pGEX-p52
M 1 2 3 4 5 6 7 8 M
180kD
130kD
100kD
70kD
55kD
40kD
35kD
25kD
15kD
10kD
GST-p52
GST
N
293T
Samples 1 2
miPEP-52
35KD
p52-GFP
25KD
15KD
Endogenous miPEP-52
Actin
40KD
O
293T
EV
MALAT1-800WT
MALAT1-800KO
miPEP-52
Actin
15KD
40KD
P
293T
si-MALAT1 (a)
si-MALAT1 (b)
si-NC
PR8
miPEP-52
NP
Actin
15KD
55KD
40KD

Figure S5 A MALAT1 fragment regulates the IAV replication by encoding a micropeptide. (A and B) A549 cells were infected with PR8 and WSN influenza viruses, and RNA was collected at the indicated hpi to examine the expression patterns of MALAT1-ORF8 via RT-PCR. (C) 293T cells were transfected with increasing amounts of the PNL-p52-EGFP plasmid, using PNL-EGFP-EV as both negative control and for maintaining equivalent total DNA amounts across transfection conditions. After 24 hours post-transfection, cells were infected with WSN (MOI=1) for 16 h. Cells were harvested and subjected to Western blotting for the detection of indicated proteins. (D and E) 293T cells stably expressing PNL-p52-EGFP plasmid (overexpressing p52-EGFP) or EV control were either mock-infected or infected with IAV WSN (MOI=1) for 16 h. After infection, total protein and RNA samples were collected for Western blotting (D) and RT-qPCR (E) to assess the expression of p52-EGFP and viral NP. (F) RT-qPCR results showing relative expression of SeV-NP in control and p52-EGFP overexpressing cells infected with SeV for 16h. (G) Schematic diagram showing the sequence of the MALAT1-800KO vector. Briefly, the MALAT1-800KO sequence was synthesized by overlapped PCR, removing MALAT1-ORF8 from the MALAT1-800WT sequence and cloning it into the PNL vector. MALAT1-ORF8 KO was confirmed by Sanger sequencing. (H) 293T cells overexpressing MALAT-800WT, MALAT1-800KO, and EV control were mock-infected or infected with IAV WSN (MOI=1). Total RNA extracted at 17 hpi was subjected to RT-qPCR to assess IAV NP expression. (I and J) Part of the sequencing results shows point mutations in the MALAT1-800WT vector; MALAT1-800WT-D1-mut indicates a single-point mutation altering a single ATG of MALAT1-ORF8 (I); MALAT1-800WT-D2-mut indicates a double-point mutation altering two ATGs of MALAT1-ORF8 (J). (K and L) MALAT1-800WT, MALAT1-800WT-D1-mut, MALAT1-800WT-D2-mut, and PNL-EV were transfected into 293T cells for 24 h, followed by infection with WSN for 16 h. The expression of MALAT1 and viral NP in these cells was examined by RT-qPCR (K) and RT-PCR (L). (M) Coomassie blue staining of the polyacrylamide gel shows the miPEP-52-GST fusion protein expressed by the construct (pGEX-p52-GST vector) in *Escherichia coli* with or without IPTG induction, and after purification. The purified miPEP-52-GST fusion protein was utilized as an antigen to immunize rabbits for the preparation of polyclonal miPEP-52 serum antibody. (N) 293T cells stably overexpressing p52-EGFP were subjected to Western blotting after protein extraction and probed with a polyclonal serum antibody against the miPEP-52. The arrows mark the endogenous and

the ectopically expressed miPEP-52. (O) MALAT1-800WT, MALAT1-800KO and EV control vectors were transfected into 293T cells. Translation of MALAT1-800WT and MALAT1-800KO into miPEP-52 was confirmed by Western blotting using miPEP-52 serum antibody. (P) 293T cells were transfected with 2 μg/ml of MALAT1-targeting siRNAs or control siRNA for 24 h, followed by infection with IAV PR8 (MOI=1). Then, total protein extracted at 16 hpi were subjected to Western blotting. Shown are representative data from three biologically independent experiments. Statistical analysis was performed using a two-tailed Student's *t*-test. Data are presented as means ± SD, not significant (ns), $^{**}p < 0.01$, and $^{***}p < 0.001$.

Figure S6

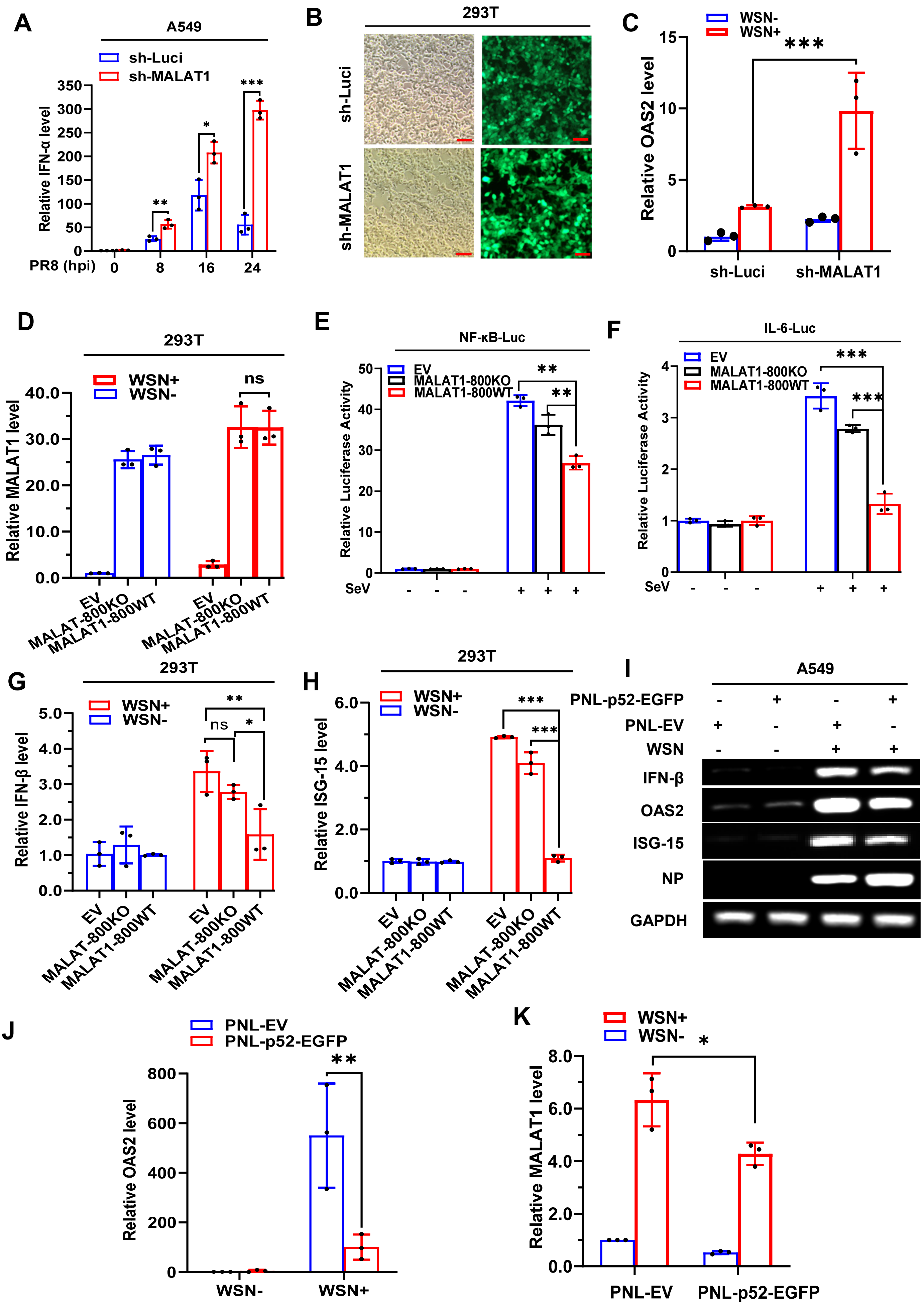

A
A549
sh-Luci
sh-MALAT1
Relative IFN-α level
PR8 (hpi)
B
293T
sh-Luci
sh-MALAT1
C
WSN-
WSN+
Relative OAS2 level
D
293T
Relative MALAT1 level
EV
MALAT-800KO
MALAT1-800WT
ns
E
NF-κB-Luc
Relative Luciferase Activity
MALAT1-800KO
MALAT1-800WT
SeV
F
IL-6-Luc
G
Relative IFN-β level
H
Relative ISG-15 level
I
PNL-p52-EGFP
PNL-EV
WSN
IFN-β
OAS2
ISG-15
NP
GAPDH
J
Relative OAS2 level
K
Relative MALAT1 level

Figure S6 MALAT1 significantly suppresses antiviral immune responses. (A) Control and MALAT1 knockdown A549 cells were infected with IAV PR8 (MOI = 1), and the expression of IFN-α at the indicated hpi was quantified by RT-qPCR. (B) Fluorescence microscopy images showing the efficiency of MALAT1 knockdown by examining green fluorescent protein expression in lentivirus-transfected 293T cells under a microscope. Scale bar, 100 μm. (C) RT-qPCR results showing relative levels of OAS2 in MALAT1 knockdown cells compared with control cells, with or without WSN infection (MOI = 1) for 16 h. (D) 293T cells stably expressing MALAT1-800WT, MALAT1-800KO, and EV control were generated by lentivirus transduction. Overexpression of MALAT1 with and without WSN infection (MOI = 1) for 16 h was confirmed by RT-qPCR. (E and F) MALAT1-800WT and MALAT1-800KO overexpressing 293T cells, along with EV control, were transfected with the indicated luciferase reporter vectors for 24 h, followed by SeV infection (MOI = 0.5) for 16 h. Cells were then harvested for a luciferase assay. (G and H) Control, MALAT1-800WT and MALAT1-800KO overexpressing 293T cells were infected with IAV for 16 h and total RNA was extracted for RT-qPCR to analyze the relative expression of indicated antiviral genes. (I-K) Control EV and miPEP-52 expressing A549 cells were either mock-infected or infected with WSN (MOI = 1) for 16 h, after which total RNA was extracted for RT-PCR and RT-qPCR analysis. Shown are representative data from three biologically independent experiments. Statistical analysis was performed using a two-tailed Student's *t*-test. Data are presented as means ± SD, not significant (ns), *$p < 0.05$, **$p < 0.01$, and ***$p < 0.001$.

2. Supplementary Tables

Table S1: Antibodies, bacterial/viruses' strains, chemicals/reagents, cell lines, animals, oligonucleotides, primers, recombinant DNAs used in this study

Items	Company/Source	ID
**Antibodies**		
anti-Influenza A ns1 (NS1-23-1)	Santa Cruz	Cat#: sc-130568
anti-STAT3 (79D7)	Cell Signaling	Cat#: 4904
anti-NF-κB-p50	Proteintech	Cat#: 14220-1-AP
anti-NF-κB-p65	Cell Signaling	Cat#: 8242S
anti-Actin	Santa Cruz	Cat#: sc-47778
anti-GFP	CST	Cat#: 2955
anti-influenza A virus NP	Our laboratory	N/A
anti-RIG-I	Proteintech	Cat#: 20566-1-AP
anti-IRF3	Selleck	Cat#: F0521
Rabbit polyclonal serum anti-miPEP-52	This manuscript	N/A
**Bacterial and Viruses Strains**		
A/WSN/33 (H1N1)	From Prof. George Fu Gao, Institute of Microbiology, Chinese Academy of Sciences	N/A
A/PR/8/34 (H1N1)	Our laboratory	N/A
A/CA/04/09 (H1N1)	Our laboratory	N/A
H9N2	Our laboratory	N/A
Sendai virus (SeV)	Our laboratory	N/A
Swine influenza virus (SIV-H3N2)	Our laboratory	N/A
Muscovy Duck Reovirus (MDRV)	Our laboratory	N/A
Pseudorabies virus strain Min-A	Our laboratory	N/A
NS1-deleted IAV PR8 strain (PR8-ΔNS1)	Our laboratory	N/A
Recombinant IAV H9N2(HA+NA)/PR8 and H9N2(HA+NA+M+PB1)/PR8 strains	Our laboratory	N/A
**Chemicals, Peptides, and Recombinant Proteins**		
Animal-free Recombinant Human IFN-β	Proteintech	Cat#: HZ-1298
Animal-free Recombinant Human IL-6	Beyotime	Cat#: P5138
Animal-free Recombinant Human LPS	Beyotime	Cat#: ST1470
BAY 11-7082	Beyotime	Cat#: S1523
Poly(I:C) Sodium Salt	Cell Signaling	Cat#: 61401
Lipofectamine 3000	Invitrogen	Cat#: L3000015
Lipo8000™ Transfection Reagent	Beyotime	Cat#: C0533
Synthetic miPEP-52	This manuscript	N/A
Trizol	Invitrogen	Cat#: 15596018CN
**Experimental Models: Cell Lines**		
293T	ATCC	Cat#: CRL-11268
A549	ATCC	Cat#: CCL-185
PK-15	ATCC	Cat#: CCL-33
ST	ATCC	Cat#: CRL-1746
MDCK	ATCC	Cat#: CRL-2935
**Experimental Models: Organisms/Strains**		

Mouse: IFNAR1$^{-/-}$	From Prof. Daniel Portnoy, University of California at Berkeley	N/A
Mouse: IFNLR1$^{-/-}$	Our laboratory	N/A
Mouse: STAT3$^{Y705F/+}$	Our laboratory	N/A
**Oligonucleotides**		
**shRNA and siRNA oligonucleotides**		
Human shRNA: sh-RIG-I<br>5-GCAGAGAAATTGGTGGAATGC-3	This manuscript	N/A
Human shRNA: sh-IRF3<br>5-CATTGTAGATCTGATTACCTTC-3	This manuscript	N/A
Human shRNA: sh-IRF7<br>5-GCCTCTATGACGACATCGAGT-3	This manuscript	N/A
Human shRNA: sh-IL6<br>5-ATATGTGAAGCTGAGTTAATT-3	This manuscript	N/A
Human shRNA: sh-MALAT1<br>5-AAGACCTTGAAATCCATGACGCTC-3	This manuscript	N/A
Human siRNA: si-NF-κB-p65<br>5-GCGACAAGGUGCAGAAAGATT-3	This manuscript	N/A
Human siRNA: si-NF-κB-p50<br>5-GACCAGCAAAGGUUAUUGUTT-3	This manuscript	N/A
Human siRNA: si-MALAT1-1#:<br>5- GAGCAAAGGAAGUGGCUUATT-3	This manuscript	N/A
Human siRNA: si-MALAT1-2#:<br>5-GCGGAAGCUGAUCUCCAAUTT-3	This manuscript	N/A
**Primers for RT-PCR and quantitative real-time PCR**		
Human-Actin<br>Forward: CACCATTGGCAATGAGCGGTTC<br>Reverse: AGGTCTTTGCGGATGTCCACGT	This manuscript	N/A
Human-GAPDH<br>Forward: TGGGTGTGAACCATGAGAAGT<br>Reverse: AAGGCCATGCCAGTGAGCTT	This manuscript	N/A
Human-MALAT1-1<br>Forward: CAGCAGCAGACAGGATTCCA<br>Reverse: ATTGCCGACCTCACGGATTT	This manuscript	N/A
Human-MALAT1-2<br>Forward: CTGAGTGATAAAGGCTGAGTGTTG<br>Reverse: GTGTCTGCTGAGTGTTCCTAA	This manuscript	N/A
Human-IFN-β<br>Forward: GCTCTCCTGTTGTGCTTCTCCAC<br>Reverse: CAATAGTCTCATTCCAGCCAGTGC	This manuscript	N/A
Human-IFN-α<br>Forward: TTTCTCCTGCCTGAAGGACAG<br>Reverse: GCTCATGATTTCTGCTCTGACA	This manuscript	N/A
Human-Mx1<br>Forward: GACATTCGGCTGTTTACC<br>Reverse: GCGGTTCTGTGGAGGTTA	This manuscript	N/A
Human-IL-6<br>Forward: ACATTCCTGGTTGCTGGAGG<br>Reverse: GGTATTGTCAGACCCCAGGC	This manuscript	N/A
Human-P65<br>Forward: GAAGACCCAGGTCCAGATGA<br>Reverse: TGTTTCCTGACTCAGAGGGG	This manuscript	N/A
Human-RIG-I<br>Forward: ATGACCACCGAGCAGCGACG<br>Reverse: TCATTTGGACATTTCTGCTGC	This manuscript	N/A
Human-IRF3<br>Forward: AGAGGCTCGTGATGGTCAAG<br>Reverse: AGGTCCACAGTATTCTCCAGG	This manuscript	N/A
Human-IRF7<br>Forward: CTTCGTGATGCTGCGAGATA<br>Reverse: AAGCCCTTCTTGTCCCTCTC	This manuscript	N/A
Human-MALAT1-F1<br>Forward: CGCAGCCTGCAGCCCGAGAC<br>Reverse: TGTTTCATCCTACCACTCCC	This manuscript	N/A
Human-MALAT1-F2<br>Forward: GGGAGTGGTAGGATGAAACA<br>Reverse: AGCCACTTCCTTTGCTCTGC	This manuscript	N/A

Human-MALAT1-F3<br>Forward: GCAGAGCAAAGGAAGTGGCT<br>Reverse: GGGTCTGGCTTCTCTGGCCCTT	This manuscript	N/A
Human-MALAT1-F4<br>Forward: AAGGGCCAGAGAAGCCAGACCC<br>Reverse: TTTTTTCCCCAATCAAGATT	This manuscript	N/A
Human-MALAT1-F5<br>Forward: CGCAGCCTGCAGCCCGAGAC<br>Reverse: TAAATTGATGGGCTTTTA	This manuscript	N/A
Human-MALAT1-F6<br>Forward: GAAAATATGAAGACTTAGAA<br>Reverse: TAAATTGATGGGCTTTTA	This manuscript	N/A
Human-MALAT1-800KO<br>Forward: CGCAGACGAAAATTTAAACCTG<br>Reverse: AGTTTGTACATTTTGCCCT	This manuscript	N/A
Mouse-Actin<br>Forward: CATTGCTGACAGGATGCAGAAGG<br>Reverse: TGCTGGAAGGTGGACAGTGAGG	This manuscript	N/A
Mouse-MALAT1<br>Forward: GAGTTGTAGGCTTCTGTGTA<br>Reverse: AGGCTTGTGGTAGGTCAT	This manuscript	N/A
Mouse-IFNβ<br>Forward: GCCTTTGCCATCCAAGAGATGC<br>Reverse: ACACTGTCTGCTGGTGGAGTTC	This manuscript	N/A
Mouse-OAS2<br>Forward: CCGGGCCAGTGCACAAGTTAG<br>Reverse: CGATGGCACCGAGGACACC	This manuscript	N/A
Mouse-ISG-15<br>Forward: AGCAAGCAGCCAGAAGCAGACTC<br>Reverse: GGAAAGCCGGCACACCAATC	This manuscript	N/A
Sus-Actin<br>Forward: TCTGGCACCACACCTTCT<br>Reverse: TGATCTGGGTCATCTTCTCAC	This manuscript	N/A
Sus-IFNβ<br>Forward: CATCCTCCAAATCGCTCTCC<br>Reverse: CTGACATGCCAAATTGCTGC	This manuscript	N/A
Sus-MALAT1<br>Forward: TCTAACAGTGCAACCCACCC<br>Reverse: ACCTGAGTGGTGCTTGAAGG	This manuscript	N/A
PRV-gE<br>Forward: CTTCCACTCGCAGCTCTTCT<br>Reverse: TAGATGCAGGGCTCGTACAC	This manuscript	N/A
IAV-NP<br>Forward: TCAAACGTGGGATCAATG<br>Reverse: GTGCAGACCGTGCTAGAA	This manuscript	N/A
CA04-NP<br>Forward: CACTCTCGGTCTGGACATCG<br>Reverse: TTAGAGTCTCCAGCCGTCA	This manuscript	N/A
SIV-H3N2-NP<br>Forward: CCACAAGAGGGGTCCAGATT<br>Reverse: GGAGATTTCGCTGCACTGAG	This manuscript	N/A
MDRV-P10<br>Forward: ATGGCTGACGCTTTGAAGT<br>Reverse: TAGTTAGATCTCGAGAGCCCG	This manuscript	N/A
SEV-NP<br>Forward: ATAAGTCGGGAGGAGGTGCT<br>Reverse: GTTGACCCTGGAAGAGTGGG	This manuscript	N/A
**5' tag RT-PCR and quantitative real time PCR primers used to study viral (IAV PR8) NP-specific RNA species**		
**RT-NP-vRNA**<br>GGCCGTCATGGTGGCGAATGAATGGACGGAG<br>AACAAGGATTGC	This manuscript	N/A
**RT-NP-cRNA**<br>GCTAGCTTCAGCTAGGCATCAGTAGAAACAA<br>GGGTATTTTTCTTT	This manuscript	N/A
**RT-NP-mRNA**<br>CCAGATCGTTCGAGTCGTTTTTTTTTTTTTTTTT<br>CTTTAATTGTC	This manuscript	N/A
**NP-vRNA**<br>Forward: GGCCGTCATGGTGGCGAAT<br>Reverse: CTCAATATGAGTGCAGACCGTGCT	This manuscript	N/A

**NP-cRNA**<br>Forward: CGATCGTGCCCTCCTTTG<br>Reverse: GCTAGCTTCAGCTAGGCATC	This manuscript	N/A
**NP-mRNA**<br>Forward: CGATCGTGCCCTCCTTTG<br>Reverse: CCAGATCGTTCGAGTCGT	This manuscript	N/A
**Recombinant DNA**		
pNL-MALAT1-F1	This manuscript	N/A
pNL-MALAT1-F2	This manuscript	N/A
pNL-MALAT1-F3	This manuscript	N/A
pNL-MALAT1-F4	This manuscript	N/A
pNL-MALAT1-F5	This manuscript	N/A
pNL-MALAT1-F5	This manuscript	N/A
pNL-MALAT1-F6	This manuscript	N/A
pNL-EGFP-p52	This manuscript	N/A
pNL-MALAT1-800KO	This manuscript	N/A

3. Supplementary datasets

Datasets S1 and S2: Summary of annotated and novel ORFs with Ribo-Seq evidence in MOCK-infected (S1) and PR8-infected (S2) cells. The dataset includes ORF identifiers (ORF_ID, gene_name, transcript_id), genomic coordinates (chromosome, strand, ORF start/stop positions), and translation evidence such as P-site counts per reading frame (frame 0, frame 1, frame 2), coverage percentages, and RPKM-normalized values. Statistical significance is indicated by p-values comparing frame 0 (translational signal) to non-coding frames (frame 1, frame 2), with a combined p-value highlighting robust ORF predictions. Annotated protein-coding ORFs (e.g., GSS, POLDIP2) are supported by matching transcript coordinates, while novel ORFs (e.g., within SGCE's retained intron) demonstrate frame 0-biased ribosome occupancy despite lacking canonical annotations. Predicted amino acid sequences (AAseq) are provided for all ORFs. The data were generated from Ribo-seq libraries enriched for ribosome-protected footprints (~30 nt), aligned to the reference genome, and analyzed for reading frame-specific P-site enrichment to distinguish translated ORFs from non-coding RNA or artifacts.
