# Supplementary material for: The long non-coding RNA MALAT1 encodes a micropeptide that promotes influenza A virus replication by suppressing innate immune responses
Source: J Biol Chem. 2025 Dec 27;302(2):111112. doi: 10.1016/j.jbc.2025.111112 (PMC12857292; doi:10.1016/j.jbc.2025.111112)
Supplement: Supplementary Material 2 [file mmc2.pdf]

Ribo-seq datasets S1: Summary of open reading frames (ORFs) with ribosome profiling (Ribo-Seq) evidence in MOCK infected cells. Data highlights translational activity in annotated ORFs and other including ORFs, with statistical support for frame-specific ribosome engagement.

ORF_ID	ORF_type	gene_name	chrom	strand	ORF_length	Psites_sum_frame0	Psites_sum_frame1	Psites_sum_frame2	Psites_coverage_frame0	Psites_frame0_RPKM	pval_frame0_vs_frame1	pval_frame0_vs_frame2	pval_combined
ENSG00000100983_34951852_34929186_450	annotated	GSS	20	-	1350	115	19	0	9.56%	28.1471045	3.46E-06	4.18E-09	2.04E-13
ENSG00000100983_34928980_34928828_50	dORF	GSS	20	-	150	54	13	3	20.00%	118.9521112	0.024605634	0.006200543	0.000791986
ENSG00000147044_41531084_41520420_112	novel	CASK	X	-	336	16	1	0	5.36%	15.73440624	0.042234516	0.011772051	0.002392043
ENSG00000004142_28357448_28348117_350	annotated	POLDIP2	17	-	1050	163	18	5	12.57%	51.29416435	2.13E-06	4.45E-09	1.26E-13
ENSG00000127990_94618900_94585465_278	novel	SGCE	7	-	834	125	8	5	12.95%	49.52376066	1.74E-06	2.28E-07	3.75E-12
ENSG00000100983_34942594_34932034_160	dORF	GSS	20	-	480	64	5	3	13.12%	44.05633748	0.000135637	0.000150486	1.44E-07
ENSG00000106636_44201136_44212282_164	annotated	YKT6	7	+	492	42	6	2	8.54%	28.20680144	0.001501068	0.001196161	1.09E-05
ENSG00000186205_220796752_220813418_151	novel	1-Mar	1	+	453	21	3	0	6.62%	15.31760078	0.011821942	0.002321823	0.000158045
ENSG00000180596_26123904_26123524_126	annotated	HIST1H2BC	6	-	378	1344	135	22	27.78%	1174.835666	2.02E-06	1.37E-07	2.70E-12
ENSG00000123836_207062703_207079019_373	dORF	PFKFB2	1	+	1119	20	1	2	2.68%	5.905675266	0.004911637	0.009469559	0.00024595
ENSG00000092531_42511847_42521641_97	annotated	SNAP23	15	+	291	43	6	1	9.28%	48.82532247	0.004557894	0.006307834	0.000154436
ENSG00000070761_58129115_58116023_97	novel	CFAP20	16	-	291	21	1	1	5.15%	23.84492493	0.028889786	0.028889786	0.003645179
ENSG00000267618_35121293_35011968_272	annotated	AC004223.3	17	-	816	15	2	1	3.31%	6.073943587	0.00640069	0.006024334	0.000203496
ENSG00000134779_36828767_36796805_265	annotated	TPGS2	18	-	795	68	8	7	7.17%	28.26255612	0.000671612	0.000447922	1.96E-06
ENSG00000167468_1104125_1105405_45	annotated	GPX4	19	+	135	58	3	3	24.44%	141.9593097	0.003199141	0.004155764	7.41E-05
ENSG00000060749_32931745_32976474_1702	annotated	QSER1	11	+	5106	24	4	4	0.59%	1.553102379	0.01784439	0.023617361	0.001936773
ENSG00000183808_93736176_93733405_923	annotated	RBM12B	8	-	2769	31	5	6	1.63%	3.699204935	0.003315387	0.003315387	6.17E-05
ENSG00000073712_52950436_52859561_589	annotated	FERMT2	14	-	1767	51	6	4	3.57%	9.536813292	0.000190579	0.000171408	2.28E-07
ENSG00000115129_24084326_24077579_332	annotated	TP53I3	2	-	996	125	15	9	12.95%	41.46869115	9.84E-08	1.85E-07	1.75E-13
ENSG00000127990_94656098_94585499_455	annotated	SGCE	7	-	1365	211	28	12	11.65%	51.07630334	9.25E-08	3.64E-09	3.72E-15
ENSG00000076242_36993650_37050653_722	annotated	MLH1	3	+	2166	50	5	1	3.32%	7.627482251	9.24E-05	1.02E-05	7.76E-09
ENSG00000147454_23529003_23566365_155	annotated	SLC25A37	8	+	465	22	6	3	6.45%	15.63289395	0.0335657	0.018344284	0.002786825
ENSG00000172500_65888418_65887516_164	novel	FIBP	11	-	492	64	9	7	10.37%	42.98179267	0.001064754	0.001182327	7.74E-06
ENSG00000133816_12208064_12260172_784	annotated	MICAL2	11	+	2352	14	6	0	0.77%	1.96680078	0.084246734	0.011772051	0.005012526
ENSG00000133612_151086742_151143943_875	annotated	AGAP3	7	+	2625	12	4	5	1.03%	1.510502999	0.063765378	0.119389439	0.02802983
ENSG00000115425_216058962_216039275_157	novel	PECR	2	-	471	18	3	4	5.73%	12.62761265	0.019264876	0.027941506	0.002438273
ENSG00000107566_100185620_100152131_346	annotated	ERLIN1	10	-	1038	241	76	7	12.14%	76.71659923	3.06E-06	8.88E-08	2.79E-12
ENSG00000142534_49497931_49499635_79	novel	RPS11	19	+	237	1460	86	33	37.97%	2035.514327	2.10E-06	1.01E-06	1.89E-11
ENSG00000142534_49496457_49497690_105	novel	RPS11	19	+	315	711	107	41	13.33%	745.8108559	0.001599741	0.002825752	2.66E-05
ENSG00000182362_46286914_46297634_167	annotated	YBEY	21	+	501	36	2	3	4.19%	23.74293637	0.018151375	0.032206895	0.002651741
ENSG00000184381_38169426_38112161_760	annotated	PLA2G6	22	-	2280	12	0	0	0.66%	1.739065953	0.020613417	0.020613417	0.001946209
ENSG00000159348_202967205_202966478_95	novel	CYB5R1	1	-	285	27	1	1	7.37%	31.30318716	0.008776163	0.01019742	0.000451522
ENSG00000166949_67170580_67190536_214	annotated	SMAD3	15	+	642	55	9	0	7.01%	28.30722619	0.004974812	0.000307311	1.10E-05
ENSG00000105171_29612127_29615380_96	Overlap_dORF	POP4	19	+	288	64	13	0	14.58%	73.42722914	0.0036125	0.000464321	1.11E-05
ENSG00000152952_146070806_146070717_29	novel	PLOD2	3	-	87	14	0	0	17.24%	53.17144179	0.021084099	0.021084099	0.002029891
ENSG00000166471_9314441_9283424_291	annotated	TMEM41B	11	-	873	46	5	0	7.22%	17.4105801	0.000148925	2.28E-05	2.65E-08
ENSG00000135999_148645018_148786377_807	annotated	EPC2	2	+	2421	19	2	7	1.12%	2.593154932	0.014991381	0.075175463	0.005360349
ENSG00000130669_39169554_39178579_501	annotated	PAK4	19	+	1503	141	3	6	6.39%	30.99772248	3.63E-07	1.06E-06	3.54E-12
ENSG00000163322_83485072_83462469_409	annotated	ABRAXAS1	4	-	1227	17	1	2	1.71%	4.577981279	0.008473674	0.032996028	0.00140192
ENSG00000115694_241499298_241495662_245	novel	STK25	2	-	735	10	0	3	2.45%	4.495544641	0.012798403	0.083503598	0.005300298
ENSG00000149636_36771458_36752788_356	annotated	DSN1	20	-	1068	64	2	3	7.02%	19.80060112	6.44E-06	2.89E-05	1.53E-09
ENSG00000154529_42129094_41929428_605	annotated	CNTNAP3B	9	-	1815	47	3	1	1.65%	8.556396123	0.005518828	0.003199141	9.76E-05
ENSG00000161036_102465108_102473049_638	annotated	LRWD1	7	+	1914	50	2	3	2.66%	8.631727563	0.000314831	0.000526879	1.10E-06
ENSG00000148925_13421795_13405627_297	novel	BTBD10	11	-	891	16	0	0	4.04%	5.933513466	0.000745562	0.000745562	3.53E-06
ENSG00000149313_106077750_106091323_125	annotated	AASDHPPT	11	+	375	11	1	0	4.80%	9.692394246	0.026832696	0.011772051	0.001510382
ENSG00000185627_237050_244079_70	annotated	PSMD13	11	+	210	36	1	2	14.29%	56.64386248	0.004467577	0.005109528	0.000123751
ENSG00000185627_244045_252600_345	Overlap_dORF	PSMD13	11	+	1035	144	17	1	12.46%	45.97183042	1.90E-07	5.27E-09	1.11E-14
ENSG00000137776_58933565_58921565_89	annotated	SLTM	15	-	267	18	0	0	5.62%	22.27567626	0.021084099	0.021084099	0.002029891
ENSG00000168807_69270185_69300924_191	dORF	SNTB2	16	+	573	27	5	0	4.71%	15.56964806	0.027805253	0.003554776	0.000563472
ENSG00000168807_69187167_69270152_267	annotated	SNTB2	16	+	801	19	6	0	3.00%	7.837737942	0.088239551	0.005160369	0.002807699
ENSG00000160446_128722761_128721279_256	novel	ZDHHC12	9	-	768	45	4	7	4.30%	19.36069518	0.00372022	0.016132584	0.000328964
ENSG00000160446_128721653_128721181_100	novel	ZDHHC12	9	-	300	23	0	2	8.00%	25.33239405	0.005604867	0.018402302	0.000538358
ENSG00000137210_10749246_10759101_151	annotated	TMEM14B	6	+	453	55	19	0	6.62%	40.11752585	0.036819135	0.002460157	0.000570083
ENSG00000136271_44572972_44566002_447	novel	DDX56	7	-	1341	162	4	10	10.96%	39.91681584	8.71E-10	4.23E-09	4.08E-17
ENSG00000118513_135185940_135217980_706	annotated	MYB	6	+	2118	14	0	0	0.71%	2.184096051	0.019679754	0.019679754	0.001784964
ENSG00000134215_107642744_107573331_251	Overlap_dORF	VAV3	1	-	753	18	7	0	3.19%	7.898546561	0.046544029	0.005554612	0.001427277
ENSG00000198945_130051269_130139753_677	annotated	L3MBTL3	6	+	2031	7	0	1	0.74%	1.13882704	0.019216965	0.041782825	0.003600526
ENSG00000189431_13009577_13011100_507	annotated	RASSF10	11	+	1521	23	3	0	2.56%	4.996527426	0.005114602	0.000539992	1.84E-05
ENSG00000142949_43598896_43622003_960	annotated	PTPRF	1	+	2880	75	10	0	3.23%	8.604753414	2.34E-05	4.73E-07	1.11E-10
ENSG00000142949_43545076_43579285_347	novel	PTPRF	1	+	1041	19	2	1	1.44%	6.030766658	0.020613417	0.035374934	0.003252526
ENSG00000156113_77637360_76877739_1163	annotated	KCNMA1	10	-	3489	71	13	0	3.01%	6.723989599	1.40E-05	8.57E-08	1.38E-11
ENSG00000117620_99993624_100009290_132	annotated	SLC35A3	1	+	396	8	2	0	4.55%	6.675202649	0.080264768	0.009815329	0.004118547
ENSG00000156113_77637360_76877739_1166	annotated	KCNMA1	10	-	3498	71	13	0	3.00%	6.706689454	1.40E-05	8.57E-08	1.38E-11
ENSG00000182307_145052492_145065780_248	annotated	C8orf33	8	+	744	103	15	4	12.10%	45.74397944	0.000114631	3.66E-06	3.83E-09
ENSG00000114353_50252497_50257690_268	Overlap_dORF	GNAI2	3	+	804	61	21	0	8.58%	25.06937114	0.004193927	1.13E-05	5.88E-07
ENSG00000114867_184319757_184334908_1436	annotated	EIF4G1	3	+	4308	151	15	14	4.32%	11.58166253	6.64E-10	1.76E-09	1.29E-17

ENSG00000197771_119853104_119831474_467	novel	MCMBP	10	-	1401	96	6	4	9.21%	22.64137258	5.80E-08	2.23E-08	1.30E-14
ENSG00000163702_9917316_9924107_267	novel	IL17RC	3	+	801	26	13	0	2.25%	10.7253256	0.14059905	0.013428348	0.009976026
ENSG00000117133_84479282_84497470_349	annotated	RPF1	1	+	1047	57	17	0	6.02%	17.98861917	0.003661939	2.54E-05	9.60E-07
ENSG00000185504_81551040_81540819_730	dORF	FAAP100	17	-	2190	13	5	0	0.68%	1.961412285	0.128949518	0.021084099	0.012651688
ENSG00000136811_128460559_128500255_824	annotated	ODF2	9	+	2472	42	2	6	2.55%	5.613975043	0.000117489	0.001697267	1.49E-06
ENSG00000170540_18801466_18793252_203	annotated	ARL6IP1	16	-	609	779	101	17	33.50%	422.6587056	5.39E-12	1.09E-12	7.52E-23
ENSG00000100441_24430731_24437285_678	annotated	KHNYN	14	+	2034	20	1	2	1.62%	3.24899244	0.002559322	0.00718566	0.000103461
ENSG00000205220_67936749_67934560_273	annotated	PSMB10	16	-	819	70	14	2	8.79%	28.24124198	0.00162252	2.08E-05	3.19E-07
ENSG00000125952_65102339_65077803_134	annotated	MAX	14	-	402	21	14	3	5.97%	17.26087849	0.251536638	0.01778219	0.025011259
ENSG00000180228_178444478_178432097_200	dORF	PRKRA	2	-	600	9	6	0	3.50%	4.956337967	0.258817059	0.005706018	0.012340796
ENSG00000180228_178451030_178445535_105	annotated	PRKRA	2	-	315	12	1	0	5.71%	12.58752499	0.023878474	0.01300724	0.001470914
ENSG00000137642_121470034_121546863_579	novel	SORL1	11	+	1737	21	0	0	1.73%	3.994745627	0.002321823	0.002321823	3.12E-05
ENSG00000101966_123885663_123907181_497	annotated	XIAP	X	+	1491	9	3	0	1.41%	1.9945022	0.124106539	0.006937203	0.005289108
ENSG00000159720_67439291_67438839_108	novel	ATP6V0D1	16	-	324	19	9	1	6.48%	19.37662991	0.116114203	0.008776163	0.005800321
ENSG00000092108_30630546_30643372_96	annotated	SCFD1	14	+	288	28	0	0	10.42%	32.12441275	0.002362849	0.002362849	3.23E-05
ENSG00000092108_30643360_30735609_453	Overlap_dORF	SCFD1	14	+	1359	35	6	0	3.97%	8.509778211	0.001466661	8.20E-05	9.28E-07
ENSG00000070770_58167257_58164071_125	annotated	CSNK2A2	16	-	375	30	10	0	12.00%	26.43380249	0.013103679	0.000275394	2.97E-05
ENSG00000106348_128409901_128393007_563	annotated	IMPDH1	7	-	1689	28	5	7	2.49%	5.477697378	0.005045153	0.015383659	0.000409066
ENSG00000145730_102867370_103029065_804	annotated	PAM	5	+	2412	110	22	0	4.98%	15.0690209	9.56E-06	1.36E-08	1.78E-12
ENSG00000100105_31345602_31341405_537	annotated	PATZ1	22	-	1611	12	2	2	1.49%	2.461247904	0.015191411	0.029607079	0.002085318
ENSG00000100104_26483891_26491672_339	annotated	SRRD	22	+	1017	33	6	0	4.72%	10.72167505	0.001871965	0.000186799	2.49E-06
ENSG00000278540_37406281_37289522_108	annotated	ACACA	17	-	324	23	1	10	11.11%	23.45592042	0.001453991	0.028708904	0.000281484
ENSG00000163001_55519802_55545008_342	annotated	CFAP36	2	+	1026	32	5	1	4.97%	10.30557602	0.00120083	0.000180392	1.51E-06
ENSG00000174233_48783305_48768591_1072	annotated	ADCY6	12	-	3216	12	3	0	0.75%	1.232919892	0.047083924	0.004826937	0.001291071
ENSG00000117632_25904676_25901016_149	annotated	STMN1	1	-	447	840	110	15	43.62%	620.9282464	8.38E-12	2.62E-12	2.75E-22
ENSG00000182196_122981140_122982676_181	annotated	ARL6IP4	12	+	543	51	5	2	12.15%	31.03416038	4.15E-05	5.76E-05	1.80E-08
ENSG00000115414_215435697_215361555_2141	annotated	FN1	2	-	6423	328	52	23	5.65%	16.87351552	5.42E-15	9.95E-18	9.17E-31
ENSG00000169118_64300448_64171931_442	annotated	CSNK1G1	15	-	1326	8	2	4	1.13%	1.993499434	0.080264768	0.078649604	0.023170289
ENSG00000138760_76179654_76161713_320	annotated	SCARB2	4	-	960	301	29	9	20.62%	103.6012311	6.10E-11	3.16E-12	2.46E-21
ENSG00000140939_67174170_67175097_181	annotated	AC074143.1	16	+	543	46	8	36	11.05%	27.99159564	0.000639075	0.171625208	0.001601009
ENSG00000120334_173803238_173800448_115	novel	CENPL	1	-	345	16	2	0	4.35%	15.32394347	0.019679754	0.019679754	0.001784964
ENSG00000263001_74689225_74716955_242	annotated	GTF2I	7	+	726	49	8	4	6.20%	22.30124521	0.004006587	0.001207815	2.91E-05
ENSG00000197461_519001_500444_211	annotated	PDGFA	7	-	633	20	5	0	3.32%	10.4398904	0.024135905	0.008776163	0.001049547
ENSG00000103051_70523531_70512268_189	annotated	COG4	16	-	567	19	1	0	4.76%	11.07235995	0.005642788	0.003529166	0.000109245
ENSG00000102978_57462725_57470262_219	novel	POLR2C	16	+	657	138	13	0	12.33%	69.40381932	7.12E-05	2.65E-06	1.72E-09
ENSG00000012822_53725242_53711944_690	annotated	CALCOCO1	12	-	2070	13	3	3	1.01%	2.075117345	0.058290626	0.058290626	0.01323296
ENSG00000110911_51010704_50988325_553	annotated	SLC11A2	12	-	1659	131	12	5	8.68%	26.09123061	1.79E-08	7.17E-09	1.34E-15
ENSG00000123268_50780146_50819779_271	annotated	ATF1	12	+	813	84	5	13	9.59%	34.13959731	2.26E-05	0.000904741	1.79E-07
ENSG00000168385_241346173_241346337_54	novel	2-Sep	2	+	162	58	5	3	16.67%	118.2994247	0.003816221	0.003816221	8.06E-05
ENSG00000091732_130051366_130018664_459	annotated	ZC3HC1	7	-	1377	50	4	6	5.45%	11.99791326	4.85E-05	7.87E-05	2.84E-08
ENSG00000106028_141739167_141750354_148	annotated	SSBP1	7	+	444	456	23	8	32.43%	339.3528698	6.91E-10	1.38E-09	1.06E-17
ENSG00000042429_93788123_93812064_527	dORF	MED17	11	+	1581	29	1	2	2.28%	6.060881342	0.001229744	0.00242368	1.79E-05
ENSG00000136003_108564381_108567315_82	novel	ISCU	12	+	246	63	13	0	15.85%	84.62040431	0.013744474	0.000676852	6.55E-05
ENSG00000111665_6850952_6849043_268	annotated	CDCA3	12	-	804	136	12	2	12.69%	55.89236845	1.85E-06	3.90E-07	6.64E-12
ENSG00000172927_69295625_69296392_255	annotated	MYEOV	11	+	765	21	1	4	3.14%	9.070422423	0.009288069	0.048994651	0.002294763
ENSG00000166741_114296557_114312477_264	annotated	NNMT	11	+	792	693	114	34	33.71%	289.1197147	5.42E-12	9.39E-16	9.47E-26
ENSG00000105323_41268228_41306565_756	annotated	HNRNPUL1	19	+	2268	225	45	20	9.92%	32.78001301	1.26E-09	2.47E-12	4.57E-20
ENSG00000133114_44989655_45028003_340	annotated	GPALPP1	13	+	1020	38	0	0	5.88%	12.309859	2.86E-05	2.86E-05	6.30E-09
ENSG00000105726_19655237_19654067_94	novel	ATP13A1	19	-	282	17	3	2	8.51%	19.91908876	0.036243043	0.02389627	0.003798067
ENSG00000105726_19654061_19645422_572	novel	ATP13A1	19	-	1716	24	1	1	1.75%	4.621294141	0.003133491	0.003133491	5.54E-05
ENSG00000169504_24745566_24797855_57	novel	CLIC4	1	+	171	17	10	0	10.53%	32.84902356	0.295006944	0.013428348	0.02581248
ENSG00000040341_73673120_73561529_278	annotated	STAU2	8	-	834	17	4	3	3.60%	6.73523145	0.006024334	0.013747648	0.00042788
ENSG00000010017_13711505_13622362_729	annotated	RANBP9	6	-	2187	51	6	0	3.02%	7.705326514	0.000374753	1.51E-05	4.82E-08
ENSG00000137274_3127403_3138083_87	novel	BPHL	6	+	261	12	0	0	5.75%	15.19184051	0.021084099	0.021084099	0.002029891
ENSG00000099901_20117650_20122495_127	novel	RANBP1	22	+	381	81	14	6	11.02%	70.24730976	0.009385136	0.000872085	5.32E-05
ENSG00000129810_20184027_20170704_527	annotated	SGO1	3	-	1581	26	4	6	2.47%	5.433893617	0.006379954	0.013853925	0.000453967
ENSG00000123989_219543538_219539383_775	annotated	CHPF	2	-	2325	77	10	3	3.87%	10.94302576	1.05E-05	2.13E-06	1.93E-10
ENSG00000168397_241637715_241651966_88	novel	ATG4B	2	+	264	12	5	0	5.68%	15.01920596	0.121807694	0.019679754	0.01126199
ENSG00000174444_66502750_66499407_333	novel	RPL4	15	-	999	287	35	6	7.21%	94.92619262	3.35E-05	2.13E-05	5.55E-09
ENSG00000185122_144311542_144314330_308	novel	HSF1	8	+	924	61	3	6	7.47%	21.81360866	1.04E-05	0.000119828	1.03E-08
ENSG00000080503_2039681_2192739_1400	annotated	SMARCA2	9	+	4200	48	17	3	1.86%	3.776257498	0.001348316	2.09E-05	2.60E-07
ENSG00000144306_174400003_174427895_344	annotated	SCRN3	2	+	1032	9	0	0	1.74%	2.881591841	0.011772051	0.011772051	0.000681273
ENSG00000152894_128520358_127970227_1462	annotated	PTPRK	6	-	4386	189	27	4	4.51%	14.2384538	2.09E-10	1.13E-12	3.32E-21
ENSG00000185085_62653249_62647020_1019	annotated	INTS5	11	-	3057	48	1	0	2.36%	5.188184983	9.84E-06	7.14E-06	5.76E-10
ENSG00000089682_107116017_107069380_151	annotated	RBM41	X	-	453	8	0	0	3.31%	5.835276488	0.016947427	0.016947427	0.001349898
ENSG00000170027_76358808_76329577_247	annotated	YWHAG	7	-	741	356	48	38	27.53%	158.7455075	1.98E-10	3.53E-10	8.01E-19
ENSG00000184967_132144489_132152401_516	annotated	NOC4L	12	+	1548	56	7	2	4.65%	11.95326986	0.000160058	2.38E-05	2.97E-08
ENSG00000188352_20715354_20995629_1801	annotated	FOCAD	9	+	5403	85	18	5	2.28%	5.198207504	6.64E-05	9.31E-08	8.41E-11

ENSG00000124107_45254543_45252415_132	annotated	SLPI	20	-	396	159	19	1	19.70%	132.6696526	2.98E-05	4.29E-06	1.08E-09
ENSG00000164237_10290762_10280589_169	novel	CMBL	5	-	507	26	16	2	5.92%	16.94474519	0.059781779	0.00771277	0.002450543
ENSG00000072778_7220628_7220863_48	novel	ACADVL	17	+	144	13	2	0	12.50%	29.82981184	0.035772986	0.013428348	0.002259559
ENSG00000125458_75131197_75130488_115	annotated	NT5C	17	-	345	27	3	3	7.83%	25.85915461	0.015487447	0.007790523	0.000606712
ENSG00000161960_7572842_7574527_25	uORF	EIF4A1	17	+	75	14	0	0	24.00%	61.67887247	0.01300724	0.01300724	0.000821865
ENSG00000065485_123067165_123161960_519	annotated	PDIA5	3	+	1557	57	6	2	4.24%	12.09639324	0.00027678	3.27E-05	7.01E-08
ENSG00000115750_9843542_9882737_194	annotated	TAF1B	2	+	582	11	0	1	2.58%	6.245099385	0.020613417	0.02854252	0.002645522
ENSG00000130332_2324131_2321680_49	novel	LSM7	19	-	147	39	8	1	16.33%	87.6631205	0.022007321	0.005088721	0.000595221
ENSG00000119541_63422259_63407409_128	annotated	VPS4B	18	-	384	14	0	0	6.25%	12.04665478	0.004826937	0.004826937	0.000126125
ENSG00000108602_19745129_19738221_453	annotated	ALDH3A1	17	-	1359	4367	672	152	37.75%	1061.777184	7.01E-22	4.19E-27	7.52E-47
ENSG00000185215_103135722_103136003_93	dORF	TNFAIP2	14	+	279	22	0	0	6.45%	26.05482324	0.013428348	0.013428348	0.000872559
ENSG00000185215_103126458_103135360_654	annotated	TNFAIP2	14	+	1962	276	25	10	9.94%	46.48145698	1.37E-09	1.20E-11	2.17E-19
ENSG00000120798_95067339_95022229_588	annotated	NR2C1	12	-	1764	16	6	0	1.53%	2.997029761	0.1205643	0.003229477	0.002937551
ENSG00000172183_88652185_88655531_80	novel	ISG20	15	+	240	20	1	1	12.50%	27.53521093	0.004136993	0.004136993	9.40E-05
ENSG00000173442_65576303_65592302_1523	annotated	EHBP1L1	11	+	4569	69	10	4	1.71%	4.989965998	0.000272687	2.21E-05	4.83E-08
ENSG00000067248_55294112_55283173_436	novel	DHX29	5	-	1308	14	0	0	1.38%	3.536632596	0.013428348	0.013428348	0.000872559
ENSG00000067248_55277195_55256488_637	novel	DHX29	5	-	1911	18	6	1	1.41%	3.112300136	0.057568005	0.005642788	0.001832282
ENSG00000169045_179623133_179621951_40	annotated	HNRNPH1	5	-	120	29	8	5	12.50%	79.85211169	0.021084099	0.085886389	0.008130109
ENSG00000152348_81987571_82242859_171	annotated	ATG10	5	+	513	13	1	2	3.51%	8.373280516	0.023672501	0.061356297	0.006320165
ENSG00000119335_128689990_128694664_265	annotated	SET	9	+	795	36	6	0	4.53%	14.96252971	0.00736895	0.001011481	4.67E-05
ENSG00000114573_113781082_113809427_579	annotated	ATP6V1A	3	+	1737	135	14	1	5.87%	25.6805076	3.47E-05	1.78E-07	7.14E-11
ENSG00000061794_27755220_27755450_76	dORF	MRPS35	12	+	228	16	1	3	7.89%	23.18754604	0.01300724	0.053528973	0.003328217
ENSG00000167985_61437727_61446071_120	dORF	SDHAF2	11	+	360	64	0	2	15.00%	58.74178331	8.70E-05	8.36E-05	5.31E-08
ENSG00000153294_47693673_47718442_701	annotated	ADGRF4	6	+	2103	10	5	0	0.86%	1.571196059	0.150401371	0.012798403	0.010441044
ENSG00000059573_95653377_95606762_793	annotated	ALDH18A1	10	-	2379	147	7	7	6.94%	20.41702903	1.04E-09	8.81E-10	1.02E-17
ENSG00000162600_58539294_58480965_524	annotated	OMA1	1	-	1572	22	14	0	2.86%	4.624233896	0.170834469	0.000225548	0.000807834
ENSG00000059573_95653377_95606762_795	annotated	ALDH18A1	10	-	2385	147	7	7	6.92%	20.36566544	1.04E-09	8.81E-10	1.02E-17
ENSG00000170260_149253570_149254415_281	annotated	ZNF212	7	+	843	6	3	0	1.78%	2.351761787	0.165899918	0.016947427	0.014398895
ENSG00000017260_130934676_131016247_843	annotated	ATP2C1	3	+	2529	93	16	6	5.34%	12.15076923	1.46E-06	7.29E-08	1.08E-12
ENSG00000153113_96662423_96726762_92	novel	CAST	5	+	276	19	6	0	6.52%	22.74647859	0.060667625	0.013428348	0.003899005
ENSG00000081870_53940082_53930019_74	annotated	HSPB11	1	-	222	31	3	0	10.81%	46.14008317	0.041403097	0.005355751	0.001218378
ENSG00000101126_50903996_50891405_1102	annotated	ADNP	20	-	3306	52	4	5	2.36%	5.197208596	2.71E-05	6.11E-05	1.27E-08
ENSG00000099219_5833027_5787144_904	annotated	ERMP1	9	-	2712	324	40	17	9.96%	39.47525814	2.89E-14	1.70E-14	6.75E-27
ENSG00000159720_67481086_67438528_351	annotated	ATP6V0D1	16	-	1053	225	33	2	14.81%	70.60310494	7.37E-09	1.51E-10	1.35E-17
ENSG00000168216_69790322_69676158_467	annotated	LMBRD1	6	-	1401	124	26	0	9.21%	29.24510625	1.30E-05	4.62E-09	9.94E-13
ENSG00000119943_98415135_98383798_581	annotated	PYROXD2	10	-	1743	16	0	5	1.55%	3.033138553	0.003229477	0.039200058	0.000761245
ENSG00000117174_85707846_85654058_324	annotated	ZNHIT6	1	-	972	20	1	0	3.09%	6.798817513	0.005642788	0.002227676	7.16E-05
ENSG00000144579_218403080_218404425_120	novel	CTDSP1	2	+	360	20	5	0	7.50%	18.35680728	0.019194073	0.003529166	0.000376936
ENSG00000144579_218401642_218402355_59	novel	CTDSP1	2	+	177	21	0	3	10.17%	39.20267318	0.013640586	0.032657853	0.002090452
ENSG00000169241_155135953_155138281_175	annotated	SLC50A1	1	+	525	57	6	8	10.86%	35.87444624	0.001097816	0.001226331	8.26E-06
ENSG00000008283_63437460_63434402_222	annotated	CYB561	17	-	666	33	11	0	5.41%	16.37228758	0.011805604	0.001011481	7.74E-05
ENSG00000072210_19671732_19675572_79	Overlap_dORF	ALDH3A2	17	+	237	46	6	0	10.13%	64.13264317	0.047719858	0.005604867	0.001477887
ENSG00000072210_19648972_19671795_393	annotated	ALDH3A2	17	+	1179	212	34	8	12.98%	59.41439915	2.38E-07	3.29E-09	9.10E-15
ENSG00000108312_44216564_44207242_661	annotated	UBTF	17	-	1983	42	19	6	3.63%	6.998359207	0.012167278	0.000147156	1.65E-05
ENSG00000276043_4910907_4960803_786	annotated	UHRF1	19	+	2358	47	1	2	2.29%	6.586030094	8.64E-05	0.000276943	1.72E-07
ENSG00000151348_44107764_44244287_701	annotated	EXT2	11	+	2103	213	24	6	9.13%	33.46647605	2.67E-09	7.07E-12	2.68E-19
ENSG00000135269_116251784_116257482_179	dORF	TES	7	+	537	17	9	0	3.91%	10.46030359	0.236805076	0.008574016	0.014185168
ENSG00000137642_121577325_121629563_1046	annotated	SORL1	11	+	3138	25	5	3	1.05%	2.632429343	0.015652232	0.012970104	0.000976361
ENSG00000111011_122515133_122505527_203	dORF	RSRC2	12	-	609	10	0	5	3.94%	5.425657325	0.004149608	0.049229251	0.001203008
ENSG00000239857_886096_895422_262	annotated	GET4	7	+	786	38	2	3	4.96%	15.97462619	0.001003788	0.002603001	1.59E-05
ENSG00000164587_150447733_150444286_151	annotated	RPS14	5	-	453	903	130	42	31.79%	658.6568335	3.81E-08	5.13E-08	1.94E-14
ENSG00000064199_124675265_124694446_151	annotated	SPA17	11	+	453	32	0	1	8.61%	23.34110595	0.00064467	0.000998504	4.08E-06
ENSG00000255292_112086908_112170527_140	annotated	AP002884.3	11	+	420	77	18	3	6.43%	60.57746404	0.025306216	0.006329843	0.000829929
ENSG00000198816_7519243_7520949_568	annotated	ZNF358	19	+	1704	19	7	2	1.06%	3.684288786	0.043751848	0.030316224	0.005624066
ENSG00000113593_65569946_65587396_490	annotated	PPWD1	5	+	1470	29	1	0	3.47%	6.518539729	0.000253334	0.000110146	1.98E-07
ENSG00000151422_108897846_109187575_411	annotated	FER	5	+	1233	11	0	0	1.22%	2.947808469	0.019679754	0.019679754	0.001784964
ENSG00000136560_161231261_161235518_173	annotated	TANK	2	+	519	31	8	3	6.36%	19.73622055	0.029014944	0.004946235	0.000777204
ENSG00000258315_7014858_7015232_63	novel	C17orf49	17	+	189	48	5	0	14.29%	83.9168333	0.003763158	0.003816221	7.96E-05
ENSG00000258315_7015703_7015879_58	novel	C17orf49	17	+	174	33	5	0	13.79%	62.66634211	0.040465304	0.005604867	0.001233298
ENSG00000129480_31457393_31457187_68	annotated	DTD2	14	-	204	13	5	0	8.82%	21.05633777	0.069457177	0.013428348	0.004505234
ENSG00000117395_43172118_43164443_306	annotated	EBNA1BP2	1	-	918	120	22	13	14.05%	43.19248773	9.81E-07	2.10E-07	1.94E-12
ENSG00000157214_90225083_90232624_490	annotated	STEAP2	7	+	1470	39	8	2	4.49%	8.76631205	0.001091686	6.65E-05	5.63E-07
ENSG00000142192_26140194_25881670_746	annotated	APP	21	-	2238	1826	169	36	27.08%	269.5940759	9.85E-30	1.83E-33	4.29E-61
ENSG00000090863_74607094_74472590_698	annotated	GLG1	16	-	2094	291	24	6	10.89%	45.91831736	2.97E-11	5.57E-14	2.55E-23
ENSG00000054523_10267389_10376587_1670	annotated	KIF1B	1	+	5010	29	9	0	0.96%	1.91262543	0.010286319	0.000174956	1.55E-05
ENSG00000054523_10267389_10378402_1677	annotated	KIF1B	1	+	5031	29	9	0	0.95%	1.904641901	0.010286319	0.000174956	1.55E-05
ENSG00000054523_10267389_10304647_1007	annotated	KIF1B	1	+	3021	21	8	0	1.29%	2.296879561	0.032614614	0.000583356	0.000159364
ENSG00000122203_176361710_176361648_20	uORF	KIAA1191	5	-	60	14	5	5	30.00%	77.09859059	0.101229348	0.135090548	0.046386232

ENSG00000122203_176352640_176347600_200	annotated	KIAA1191	5	-	600	14	0	0	2.50%	7.709859059	0.021084099	0.021084099	0.002029891
ENSG00000048140_176647616_176656960_188	annotated	TSPAN17	5	+	564	27	2	1	5.32%	15.81809989	0.002404282	0.002362849	3.28E-05
ENSG00000077942_45525544_45600446_641	annotated	FBLN1	22	+	1923	140	7	0	7.02%	24.05572249	3.94E-09	2.07E-09	8.81E-17
ENSG00000250317_25862147_25929191_33	novel	SMIM20	4	+	99	28	1	0	18.18%	93.45283708	0.01300724	0.013428348	0.000846864
ENSG00000108528_4939211_4937741_310	novel	SLC25A11	17	-	930	188	15	1	12.26%	66.79509231	1.37E-06	3.71E-08	5.39E-13
ENSG00000133943_91160388_91224833_590	annotated	DGLUCY	14	+	1770	47	12	0	3.56%	8.773931617	0.005195666	2.28E-05	1.33E-06
ENSG00000113068_140303073_140245479_117	annotated	PFDN1	5	-	351	67	3	4	9.40%	63.07210708	0.002961771	0.001818063	3.14E-05
ENSG00000148358_130054008_130135121_575	annotated	GPR107	9	+	1725	109	10	0	7.13%	20.87887298	2.20E-07	9.33E-09	2.20E-14
ENSG00000059758_96334836_96280242_523	annotated	CDK17	12	-	1569	24	0	0	1.91%	5.054264338	0.002335453	0.002335453	3.16E-05
ENSG00000120802_98515868_98537656_248	annotated	TMPO	12	+	744	70	10	5	11.29%	31.08814137	5.11E-05	2.02E-05	8.02E-09
ENSG00000028203_95266541_95299116_411	annotated	VEZT	12	+	1233	16	2	0	1.46%	4.287721409	0.020342454	0.013640586	0.001314121
ENSG00000051825_123202730_123161368_456	annotated	MPHOSPH9	12	-	1368	15	1	0	1.32%	3.623054069	0.015555383	0.011772051	0.000887479
ENSG00000156113_77637360_76877739_1136	annotated	KCNMA1	10	-	3408	71	13	0	3.08%	6.883802732	1.40E-05	8.57E-08	1.38E-11
ENSG00000069966_52133439_52124777_84	novel	GNB5	15	-	252	14	1	5	5.95%	18.35680728	0.020613417	0.145920273	0.014308175
ENSG00000128951_48332252_48341428_143	annotated	DUT	15	+	429	115	27	9	15.38%	88.57480438	0.003136809	1.80E-05	6.03E-07
ENSG00000054116_36149378_36137203_114	annotated	TRAPPC3	1	-	342	86	6	1	23.68%	83.08870665	6.64E-06	2.45E-06	1.39E-10
ENSG00000121766_31319115_31364193_217	annotated	ZCCHC17	1	+	651	20	2	0	4.61%	10.15122983	0.005426623	0.00224099	6.92E-05
ENSG00000167264_68049523_68078986_445	annotated	DUS2	16	+	1335	24	3	0	3.15%	5.940180335	0.001772416	0.000398115	4.63E-06
ENSG00000145349_113761068_113457331_478	annotated	CAMK2D	4	-	1434	40	5	0	3.77%	9.216807005	7.87E-05	8.29E-05	4.77E-08
ENSG00000274210_148522626_148522727_33	novel	RF00003	1	+	99	55	32	6	27.27%	183.5680728	0.091857033	0.02846257	0.01111888
ENSG00000160584_116927357_116870327_444	annotated	SIK3	11	-	1332	13	0	0	1.35%	3.224844523	0.013428348	0.013428348	0.000872559
ENSG00000134138_37098019_36892173_413	annotated	MEIS2	15	-	1239	7	0	2	1.21%	1.866793961	0.019216965	0.119134635	0.010787784
ENSG00000187098_69879285_69965248_435	annotated	MITF	3	+	1305	37	7	1	3.91%	9.368301648	0.003418024	0.000183422	4.67E-06
ENSG00000197461_519001_498564_196	annotated	PDGFA	7	-	588	21	5	0	4.08%	11.80080468	0.018067938	0.005604867	0.000528392
ENSG00000155256_97738478_97759300_318	annotated	ZFYVE27	10	+	954	17	7	4	3.14%	5.888032525	0.106475427	0.017497919	0.008856475
ENSG00000173698_19068834_18990864_987	annotated	ADGRG2	X	-	2961	28	3	0	1.42%	3.124562942	0.003183653	0.000424543	9.00E-06
ENSG00000120727_139363785_139368798_127	annotated	PAIP2	5	+	381	187	24	2	23.62%	162.175888	7.34E-06	1.64E-06	1.05E-10
ENSG00000164180_88206473_88205810_59	annotated	TMEM161B	5	-	177	23	1	0	10.17%	42.93626111	0.013640586	0.013428348	0.00088551
ENSG00000175550_65919502_65921435_205	annotated	DRAP1	11	+	615	188	9	26	21.46%	101.0072128	1.30E-08	3.93E-06	6.44E-13
ENSG00000057663_106316102_106186540_197	Overlap_dORF	ATG5	6	-	591	38	10	1	7.11%	21.24544193	0.013901262	0.000595214	5.97E-05
ENSG00000057663_106316208_106186540_275	annotated	ATG5	6	-	825	45	12	6	6.18%	18.02304715	0.005356506	0.00129387	4.16E-05
ENSG00000197771_119872584_119831474_640	annotated	MCMBP	10	-	1920	120	6	6	8.44%	20.65140819	7.07E-10	9.32E-10	7.33E-18
ENSG00000107651_119892783_119933767_1000	annotated	SEC23IP	10	+	3000	60	5	7	2.80%	6.608450622	2.24E-05	4.93E-05	8.54E-09
ENSG00000005801_3371610_3359118_525	annotated	ZNF195	11	-	1575	6	0	1	0.95%	1.258752499	0.016947427	0.047790352	0.003697512
ENSG00000128585_131387228_131399240_78	annotated	MKLN1	7	+	234	24	2	1	8.97%	33.88949037	0.020342454	0.01208853	0.001177842
ENSG00000129071_129439833_129431501_574	annotated	MBD4	3	-	1722	79	9	7	4.01%	15.15875723	8.50E-05	0.000157066	9.61E-08
ENSG00000198363_61526064_61517482_119	novel	ASPH	8	-	357	133	23	7	23.53%	123.09859	8.78E-05	5.94E-06	4.46E-09
ENSG00000156802_123400784_123349319_539	uORF	ATAD2	8	-	1617	35	9	4	3.34%	7.152002838	0.000400316	0.000950263	2.48E-06
ENSG00000156802_123347248_123321134_705	annotated	ATAD2	8	-	2115	49	1	0	3.26%	7.655179208	1.30E-05	1.02E-05	1.08E-09
ENSG00000114786_51988530_51989075_99	dORF	ABHD14A-ACY1	3	+	297	27	1	4	8.08%	30.03841192	0.005355751	0.025702836	0.000731269
ENSG00000114786_51984156_51986819_194	Overlap_dORF	ABHD14A-ACY1	3	+	582	17	0	0	4.64%	9.651517232	0.003303074	0.003303074	6.12E-05
ENSG00000125818_1118774_1135138_99	novel	PSMF1	20	+	297	48	11	4	13.13%	53.40162119	0.00116429	0.00218081	1.53E-05
ENSG00000135624_73234382_73244053_148	novel	CCT7	2	+	444	156	14	3	13.51%	116.0944028	0.000378757	5.38E-05	1.53E-07
ENSG00000176444_155270977_155263218_497	annotated	CLK2	1	-	1491	13	1	7	1.41%	2.880947622	0.015191411	0.182856148	0.014983237
ENSG00000138663_83035261_83056939_130	novel	COPS4	4	+	390	19	1	5	8.46%	16.09750793	0.002467336	0.023182913	0.000341322
ENSG00000198546_133309432_133310292_120	novel	ZNF511	10	+	360	47	1	0	10.83%	43.13849712	0.000700469	0.000700469	3.13E-06
ENSG00000165915_47410103_47412352_137	internal	SLC39A13	11	+	411	25	4	3	6.57%	20.09869411	0.032506135	0.009341698	0.001500183
ENSG00000146828_100861501_100866605_430	novel	SLC12A9	7	+	1290	31	3	0	3.72%	7.940386407	0.001627676	0.000161701	1.89E-06
ENSG00000108602_19741305_19740328_120	novel	ALDH3A1	17	-	360	541	36	5	25.83%	496.551637	3.11E-06	5.70E-07	1.61E-11
ENSG00000242485_1407217_1406660_148	novel	MRPL20	1	-	444	80	20	5	7.43%	59.53559119	0.01784595	0.003337178	0.000332802
ENSG00000091164_56638440_56603027_289	annotated	TXNL1	18	-	867	73	14	15	10.38%	27.82104357	2.41E-05	0.000250149	4.77E-08
ENSG00000113448_59215808_58974664_604	annotated	PDE4D	5	-	1812	139	25	15	7.95%	25.34698224	3.42E-07	4.86E-07	1.54E-12
ENSG00000100360_36767356_36758311_145	annotated	IFT27	22	-	435	44	8	6	8.97%	33.42204912	0.011217523	0.006208036	0.000359668
ENSG00000104723_15743558_15757809_54	novel	TUSC3	8	+	162	9	2	0	11.11%	18.35680728	0.071345384	0.011772051	0.004172987
ENSG00000149328_134332062_134375058_636	annotated	GLB1L2	11	+	1908	21	4	0	1.57%	3.636725971	0.023704445	0.002201184	0.000318181
ENSG00000135622_74654377_74680209_615	annotated	SEMA4F	2	+	1845	19	6	0	1.46%	3.402725253	0.032474321	0.003606289	0.000675439
ENSG00000154640_17609144_17594093_296	annotated	BTG3	21	-	888	22	7	1	3.38%	8.186143789	0.0690277	0.003737369	0.001640303
ENSG00000257315_203840341_203852399_474	annotated	ZBED6	1	+	1422	15	0	0	1.05%	3.485469738	0.020613417	0.020613417	0.001946209
ENSG00000162521_32651307_32679705_424	annotated	RBBP4	1	+	1272	124	6	2	5.42%	32.21100146	3.48E-05	1.37E-05	3.77E-09
ENSG00000188186_100148973_100153964_99	annotated	LAMTOR4	7	+	297	327	48	11	32.32%	363.7985444	3.90E-06	2.01E-06	6.76E-11
ENSG00000183978_42798681_42798061_106	annotated	COA3	17	-	318	177	17	12	13.21%	183.9144277	0.001309065	0.002236825	1.75E-05
ENSG00000157168_32595851_32728082_153	novel	NRG1	8	+	459	20	1	2	5.23%	14.39749591	0.005554612	0.01299184	0.000376034
ENSG00000125952_65102339_65077412_103	annotated	MAX	14	-	309	21	14	3	7.77%	22.45590017	0.251536638	0.01778219	0.025011259
ENSG00000177302_18307055_18285301_322	novel	TOP3A	17	-	966	21	2	0	2.48%	7.183098503	0.012834473	0.005355751	0.000359536
ENSG00000177302_18285473_18274802_453	novel	TOP3A	17	-	1359	15	1	3	1.99%	3.647047805	0.009234565	0.027614347	0.001257327
ENSG00000112130_37360563_37390852_372	annotated	RNF8	6	+	1116	10	0	0	1.61%	2.960775368	0.012798403	0.012798403	0.000797244
ENSG00000143079_112449005_112457412_585	annotated	CTTNBP2NL	1	+	1755	15	1	2	1.54%	2.824124198	0.006083074	0.016821538	0.000528227
ENSG00000123179_49691459_49669819_77	Overlap_uORF	EBPL	13	-	231	28	8	16	12.99%	40.05121589	0.009621867	0.280254546	0.019377688

ENSG00000123179_49691424_49660968_206	annotated	EBPL	13	-	618	152	26	7	13.59%	81.26897205	0.000451797	1.65E-06	8.72E-09
ENSG00000179262_12946003_12948698_124	annotated	RAD23A	19	+	372	42	1	1	14.52%	37.30576964	0.000113468	8.14E-05	6.69E-08
ENSG00000179262_12948709_12953049_198	dORF	RAD23A	19	+	594	46	6	0	9.09%	25.58827682	0.000207392	8.83E-05	1.31E-07
ENSG00000136937_97633882_97671199_790	annotated	NCBP1	9	+	2370	123	14	2	6.84%	17.14851111	3.57E-08	1.84E-10	8.51E-17
ENSG00000134884_106567919_106543996_273	annotated	ARGLU1	13	-	819	12	3	0	2.93%	4.841355767	0.047083924	0.004826937	0.001291071
ENSG00000100445_24442226_24440083_200	annotated	SDR39U1	14	-	600	19	3	8	4.00%	10.46338015	0.01551626	0.129610511	0.010095816
ENSG00000172530_87984207_88076661_363	annotated	BANP	16	+	1089	15	0	1	1.38%	4.551274533	0.019679754	0.042234516	0.003715148
ENSG00000055732_85034182_85019123_398	annotated	MCOLN3	1	-	1194	17	3	0	1.51%	4.704508399	0.063412829	0.013428348	0.004086769
ENSG00000130202_44846526_44878620_479	annotated	NECTIN2	19	+	1437	100	29	10	7.52%	22.99391309	8.82E-05	1.06E-06	9.54E-10
ENSG00000132879_11655836_11661305_224	annotated	FBXO44	1	+	672	22	2	0	5.80%	10.81740429	0.003468409	0.000539992	1.22E-05
ENSG00000175567_73981622_73981512_36	novel	UCP2	11	-	108	69	2	3	41.67%	211.1032838	0.000297172	0.000282029	5.67E-07
ENSG00000134330_9481342_9488329_135	novel	IAH1	2	+	405	99	7	1	20.00%	80.76995205	7.66E-06	2.44E-06	1.60E-10
ENSG00000177917_152735089_152759840_43	dORF	ARL6IP6	2	+	129	40	13	0	13.95%	102.4565988	0.013640586	0.013640586	0.000898637
ENSG00000136699_130155156_130152555_363	dORF	SMPD4	2	-	1089	9	4	0	1.38%	2.73076472	0.169362074	0.019216965	0.016163992
ENSG00000122218_160343170_160290157_1224	annotated	COPA	1	-	3672	152	25	11	4.74%	13.67762111	8.89E-08	1.26E-09	1.32E-15
ENSG00000100335_39513604_39514323_239	dORF	MIEF1	22	+	717	9	2	0	2.09%	4.147563152	0.083503598	0.020613417	0.007749259
ENSG00000100335_39504231_39504443_70	uORF	MIEF1	22	+	210	21	2	0	12.86%	33.04225311	0.003478238	0.003632191	7.04E-05
ENSG00000100461_22911393_22904161_319	novel	RBM23	14	-	957	86	32	7	10.97%	29.69314282	0.00090819	2.09E-06	2.39E-08
ENSG00000140521_89329071_89316751_941	annotated	POLG	15	-	2823	33	2	5	1.49%	3.862537558	0.001961121	0.009870532	0.000113024
ENSG00000090621_39571256_39561698_471	annotated	PABPC4	1	-	1413	34	2	1	2.76%	7.950719079	0.001588752	0.000640157	6.41E-06
ENSG00000090621_39571256_39561698_484	annotated	PABPC4	1	-	1452	37	2	1	3.10%	8.419857887	0.000676283	0.000279067	1.26E-06
ENSG00000086666_80120345_80120506_53	novel	ZFAND6	15	+	159	22	14	2	9.43%	45.71884078	0.223029775	0.019679754	0.022980859
ENSG00000090621_39571256_39561698_500	annotated	PABPC4	1	-	1500	35	2	1	2.80%	7.709859059	0.001092758	0.000408047	2.90E-06
ENSG00000174130_38829299_38827083_738	annotated	TLR6	4	-	2214	15	0	0	0.95%	2.238635035	0.008473674	0.008473674	0.000366502
ENSG00000187954_144464414_144464127_95	annotated	CYHR1	8	-	285	18	0	0	6.32%	20.86879144	0.01300724	0.01300724	0.000821865
ENSG00000075790_107594044_107615305_155	annotated	BCAP29	7	+	465	80	7	0	18.71%	56.84688707	2.55E-05	1.04E-06	2.49E-10
ENSG00000104177_48178078_48142908_547	annotated	MYEF2	15	-	1641	48	5	0	3.47%	9.665010051	7.24E-05	5.41E-05	2.91E-08
ENSG00000275052_55617285_55549911_849	annotated	PPP4R3B	2	-	2547	78	11	7	3.77%	10.11894677	2.20E-05	1.26E-05	2.20E-09
ENSG00000074582_218660988_218663386_419	annotated	BCS1L	2	+	1257	71	5	2	5.49%	18.66348426	0.000108734	3.17E-05	2.62E-08
ENSG00000047230_16702902_16590793_586	annotated	CTPS2	X	-	1758	17	0	0	1.02%	3.195212189	0.013428348	0.013428348	0.000872559
ENSG00000106028_141739167_141750354_148	annotated	SSBP1	7	+	444	455	23	8	31.76%	338.6086749	9.94E-10	1.86E-09	2.03E-17
ENSG00000106546_17299265_17343064_848	annotated	AHR	7	+	2544	55	3	15	3.07%	7.143568872	1.02E-05	0.000335962	3.02E-08
ENSG00000105655_18436842_18434913_408	dORF	ISYNA1	19	-	1224	27	1	4	3.68%	7.288732304	0.00042308	0.003660151	1.04E-05
ENSG00000136193_29969067_29923957_414	annotated	SCRN1	7	-	1242	74	15	1	6.28%	19.68701071	6.94E-05	4.55E-06	2.74E-09
ENSG00000147804_144413369_144412538_149	novel	SLC39A4	8	-	447	192	25	16	22.15%	141.9264563	3.37E-05	2.38E-05	6.22E-09
ENSG00000254996_140529226_140539098_451	novel	ANKHD1-EIF4EBP3	5	+	1353	16	0	0	2.00%	3.907435697	0.003452921	0.003452921	6.66E-05
ENSG00000187147_44636045_44650029_230	novel	RNF220	1	+	690	29	9	2	6.09%	13.88732377	0.004325203	0.001083027	2.85E-05
ENSG00000148908_119536470_119500113_167	annotated	RGS10	10	-	501	60	14	1	11.38%	39.57156061	0.004623728	6.45E-05	2.71E-06
ENSG00000166401_63978309_63987278_374	annotated	SERPINB8	18	+	1122	13	1	0	1.60%	3.828425049	0.019795882	0.012798403	0.001207991
ENSG00000076984_7910498_7912431_255	annotated	MAP2K7	19	+	765	21	0	0	3.14%	9.070422423	0.005604867	0.005604867	0.000167525
ENSG00000076984_7910498_7912431_262	annotated	MAP2K7	19	+	786	21	0	0	3.05%	8.828082892	0.005604867	0.005604867	0.000167525
ENSG00000019186_54171693_54157179_372	annotated	CYP24A1	20	-	1116	968	135	59	25.81%	286.6030557	3.16E-14	3.83E-14	1.65E-26
ENSG00000204427_31703281_31687212_558	annotated	ABHD16A	6	-	1674	74	11	2	5.02%	14.60649182	0.000141165	4.74E-06	6.03E-09
ENSG00000164924_100948658_100920693_168	annotated	YWHAZ	8	-	504	43	0	3	3.57%	28.19081119	0.013640586	0.020525443	0.001325514
ENSG00000164924_100948658_100920693_168	annotated	YWHAZ	8	-	504	43	0	3	3.57%	28.19081119	0.013640586	0.020525443	0.001325514
ENSG00000099385_30893881_30851969_272	annotated	BCL7C	16	-	816	62	12	3	5.51%	25.10563349	0.003855739	0.000412614	1.07E-05
ENSG00000072134_19309974_19330630_183	novel	EPN2	17	+	549	20	1	0	3.83%	12.03725068	0.011825808	0.008473674	0.000503679
ENSG00000174231_1684571_1650802_2335	annotated	PRPF8	17	-	7005	512	45	17	7.37%	24.15079742	4.00E-25	5.47E-29	4.98E-52
ENSG00000249115_35612795_35622993_633	annotated	HAUS5	19	+	1899	34	9	2	3.16%	5.915937893	0.006392761	9.55E-05	5.45E-06
ENSG00000127990_94656098_94585499_437	annotated	SGCE	7	-	1311	217	28	12	13.04%	54.69236404	2.62E-08	9.06E-10	2.72E-16
ENSG00000147099_72572761_72462089_307	annotated	HDAC8	X	-	921	13	1	0	1.95%	4.663944522	0.023672501	0.012798403	0.001437523
ENSG00000052723_114780607_114779124_141	novel	SIKE1	1	-	423	18	0	2	6.38%	14.06053324	0.003452921	0.014991381	0.000285751
ENSG00000141959_44321777_44326862_367	novel	PFKL	21	+	1101	40	6	1	5.18%	12.00445163	0.000958065	7.30E-05	5.31E-07
ENSG00000206503_29942563_29945455_362	annotated	HLA-A	6	+	1086	182	12	4	9.12%	55.37467833	3.96E-06	1.07E-06	3.74E-11
ENSG00000136261_16665444_16706088_419	annotated	BZW2	7	+	1257	108	12	3	10.50%	28.38952535	1.34E-07	3.26E-09	4.91E-15
ENSG00000111665_6850485_6849385_113	novel	CDCA3	12	-	339	98	5	2	16.81%	95.52037773	0.000151838	0.00015701	1.68E-07
ENSG00000151065_2004431_1993121_153	annotated	DCP1B	12	-	459	11	1	0	4.58%	7.91862275	0.007593099	0.007593099	0.000297833
ENSG00000149357_72098803_72097822_80	Overlap_dORF	LAMTOR1	11	-	240	61	9	2	10.00%	83.98239333	0.0601164	0.005604867	0.001914061
ENSG00000166908_57594058_57601606_352	annotated	PIP4K2C	12	+	1056	99	3	4	7.67%	30.97711229	6.47E-06	1.46E-05	7.74E-10
ENSG00000198431_104311326_104348421_498	annotated	TXNRD1	12	+	1494	893	96	42	22.69%	197.5015531	9.76E-17	9.26E-19	1.49E-33
ENSG00000090581_1351966_1363091_305	annotated	GNPTG	16	+	915	27	6	0	4.92%	9.750173049	0.004754117	0.000257535	8.96E-06
ENSG00000205629_25140216_25174951_144	annotated	LCMT1	16	+	432	17	2	0	5.56%	13.00273849	0.020715809	0.005454749	0.000593634
ENSG00000082212_50895821_50917356_133	annotated	ME2	18	+	399	14	0	1	4.51%	11.59377302	0.011772051	0.026832696	0.001510382
ENSG00000082212_50917407_50947184_408	dORF	ME2	18	+	1224	51	5	1	5.39%	13.76760546	0.000107059	2.04E-05	1.71E-08
ENSG00000219545_7673372_7868410_78	annotated	UMAD1	7	+	234	9	0	0	6.41%	12.70855889	0.019679754	0.019679754	0.001784964
ENSG00000115525_85888905_85840144_365	annotated	ST3GAL5	2	-	1095	143	30	13	12.05%	43.15107027	6.31E-06	4.58E-07	2.71E-11
ENSG00000103202_397223_406365_233	annotated	NME4	16	+	699	37	3	9	6.01%	17.4901769	0.000661391	0.003437197	1.45E-05
ENSG00000176783_179591691_179601990_221	dORF	RUFY1	5	+	663	11	0	0	2.71%	5.482123442	0.012798403	0.012798403	0.000797244

ENSG00000176783_179560072_179589613_293	annotated	RUFY1	5	+	879	43	0	0	4.44%	16.16401461	0.000700469	0.000700469	3.13E-06
ENSG00000109911_31509785_31783524_425	annotated	ELP4	11	+	1275	34	11	0	4.00%	8.811267496	0.007026737	0.000118788	7.27E-06
ENSG00000163468_156325081_156309199_215	novel	CCT3	1	-	645	241	44	5	16.74%	123.4602015	6.18E-06	1.14E-07	7.40E-12
ENSG00000163468_156334775_156309199_497	dORF	CCT3	1	-	1491	658	83	18	20.93%	145.8202719	1.30E-14	1.14E-17	2.60E-30
ENSG00000198715_156295645_156293699_183	annotated	GLMP	1	-	549	61	5	5	9.84%	36.71361457	9.02E-05	8.83E-05	5.79E-08
ENSG00000153914_66144377_66178868_624	annotated	SREK1	5	+	1872	50	4	1	3.85%	8.825388117	3.81E-05	1.02E-05	3.12E-09
ENSG00000090372_46746427_46720602_759	annotated	STRN4	19	-	2277	71	17	9	3.82%	10.30303018	0.000817112	8.50E-05	5.15E-07
ENSG00000196878_209650944_209615271_1172	annotated	LAMB3	1	-	3516	19	7	2	1.02%	1.785559753	0.042090579	0.005489386	0.001265723
ENSG00000165868_116834971_116674781_674	annotated	HSPA12A	10	-	2022	11	0	1	1.04%	1.797550862	0.007882058	0.01551626	0.000614257
ENSG00000085224_77684988_77508351_2288	dORF	ATRX	X	-	6864	30	1	3	0.66%	1.444154419	0.000440588	0.00145172	4.15E-06
ENSG00000164741_13102822_13085811_1017	annotated	DLC1	8	-	3051	13	8	2	0.88%	1.407896724	0.22882902	0.020607861	0.024497847
ENSG00000142192_26170620_25881670_752	annotated	APP	21	-	2256	1837	170	44	27.79%	269.0541621	8.97E-31	1.97E-34	4.25E-63
ENSG00000156976_186787531_186787770_79	novel	EIF4A2	3	+	237	29	18	3	6.33%	40.43144896	0.123780394	0.073562049	0.032681874
ENSG00000138663_83035261_83075324_408	annotated	COPS4	4	+	1224	63	4	5	6.37%	17.00704204	1.39E-05	3.63E-05	3.98E-09
ENSG00000160789_156114919_156137764_487	annotated	LMNA	1	+	1461	490	59	21	18.28%	110.8193294	7.14E-12	2.18E-15	2.77E-25
ENSG00000196776_108090908_108047288_323	annotated	CD47	3	-	969	161	32	8	12.07%	54.89992519	1.57E-05	4.20E-07	6.52E-11
ENSG00000162688_99852670_99921651_1516	Overlap_dORF	AGL	1	+	4548	14	6	0	0.46%	1.017131802	0.12595073	0.007882058	0.005912017
ENSG00000157184_53210029_53213595_540	dORF	CPT2	1	+	1620	56	2	2	3.89%	11.42201342	7.38E-05	4.69E-05	2.58E-08
ENSG00000143164_167936912_168075435_920	annotated	DCAF6	1	+	2760	108	19	3	4.13%	12.9295773	1.98E-06	1.07E-07	2.12E-12
ENSG00000100982_45939000_45947755_704	annotated	PCIF1	20	+	2112	52	10	2	2.98%	8.135403228	0.00163568	8.99E-05	1.13E-06
ENSG00000104375_98825540_98455842_491	annotated	STK3	8	-	1473	45	2	0	4.28%	10.09437468	5.69E-05	2.15E-05	9.48E-09
ENSG00000102225_47223594_47228768_484	annotated	CDK16	X	+	1452	38	2	0	2.89%	8.647421613	0.000705627	0.000428413	1.97E-06
ENSG00000115275_74462963_74461275_562	dORF	MOGS	2	-	1686	74	1	0	4.98%	14.50253102	1.99E-06	1.60E-06	2.79E-11
ENSG00000115275_74465247_74462969_209	annotated	MOGS	2	-	627	31	5	7	4.78%	16.33668017	0.014895507	0.074117384	0.005252102
ENSG00000119965_122954178_122932427_445	annotated	C10orf88	10	-	1335	15	1	0	1.57%	3.712612709	0.013892891	0.008473674	0.00058868
ENSG00000186625_149601743_149598210_96	novel	KATNA1	6	-	288	9	0	0	5.21%	10.3257041	0.020613417	0.020613417	0.001946209
ENSG00000143369_150511469_150513467_300	novel	ECM1	1	+	900	17	2	0	2.67%	6.241314477	0.013892891	0.005306578	0.000386014
ENSG00000100395_41213904_41227355_526	novel	L3MBTL2	22	+	1578	17	6	1	1.90%	3.559685063	0.092011639	0.004053443	0.002465593
ENSG00000163166_127493373_127485905_206	annotated	IWS1	2	-	618	25	7	0	3.88%	13.36660725	0.067931538	0.005454749	0.002154815
ENSG00000188603_28491764_28489349_55	Overlap_uORF	CLN3	16	-	165	18	0	12	14.55%	36.0460943	0.005554612	0.310595281	0.015978689
ENSG00000188603_28489349_28479730_309	annotated	CLN3	16	-	927	134	15	4	11.00%	47.76334322	2.21E-06	1.46E-07	3.16E-12
ENSG00000188603_28477608_28477516_30	dORF	CLN3	16	-	90	23	5	1	23.33%	84.44131351	0.027657845	0.010042643	0.001354611
ENSG00000186184_27648437_27665953_94	annotated	POLR1D	13	+	282	20	1	1	7.45%	23.43422206	0.007905926	0.007905926	0.000321469
ENSG00000055208_149378639_149409719_452	annotated	TAB2	6	+	1356	19	0	0	2.65%	4.629814227	0.000808111	0.000808111	4.12E-06
ENSG00000124299_33391462_33387344_165	novel	PEPD	19	-	495	210	0	3	11.52%	140.1792556	6.14E-05	0.000224904	1.01E-07
ENSG00000164258_53560678_53683130_115	annotated	NDUFS4	5	+	345	159	26	38	29.57%	152.2816883	3.74E-06	6.93E-06	2.18E-10
ENSG00000126088_45012992_45015498_286	annotated	UROD	1	+	858	89	12	9	9.79%	34.27459822	2.01E-06	4.42E-05	8.09E-10
ENSG00000131778_147275365_147295509_470	dORF	CHD1L	1	+	1410	24	0	2	2.34%	5.624213296	0.001092093	0.005502968	3.68E-05
ENSG00000105968_44847971_44834501_90	annotated	H2AFV	7	-	270	136	16	11	16.67%	166.4350527	0.006279813	0.001040517	4.04E-05
ENSG00000075415_98593979_98601528_362	annotated	SLC25A3	12	+	1086	772	111	17	26.24%	234.8859982	2.98E-13	7.17E-16	3.42E-27
ENSG00000182149_71923309_71927813_106	dORF	IST1	16	+	318	35	4	7	9.43%	36.36725971	0.007609621	0.009067228	0.000353271
ENSG00000100201_38507616_38490360_420	novel	DDX17	22	-	1260	120	17	12	9.05%	31.46881249	1.96E-06	5.67E-06	9.57E-11
ENSG00000163466_218217471_218234405_91	novel	ARPC2	2	+	273	75	16	3	18.68%	90.77542064	0.004582173	0.000239323	8.08E-06
ENSG00000144451_213284544_213317372_163	annotated	SPAG16	2	+	489	20	1	0	5.52%	13.51421395	0.00567009	0.003554776	0.000110499
ENSG00000141756_41821026_41822408_137	dORF	FKBP10	17	+	411	114	4	2	23.36%	91.65004513	1.86E-06	8.41E-07	1.39E-11
ENSG00000141756_41813258_41819734_250	Overlap_dORF	FKBP10	17	+	750	205	26	8	13.60%	90.31549184	5.11E-07	9.64E-07	4.48E-12
ENSG00000130402_38729080_38729432_77	novel	ACTN4	19	+	231	90	10	0	24.68%	128.7360511	0.000666858	6.29E-05	3.17E-07
ENSG00000167986_61331584_61323871_286	novel	DDB1	11	-	858	27	5	0	4.90%	10.39791182	0.007336185	0.000375189	1.99E-05
ENSG00000095383_98233542_98199381_710	annotated	TBC1D2	9	-	2130	74	12	1	3.66%	11.47946822	0.000399807	4.44E-06	1.77E-08
ENSG00000095383_98255541_98244088_136	uORF	TBC1D2	9	-	408	25	0	1	7.35%	20.24647862	0.00205871	0.001801897	2.18E-05
ENSG00000015475_17739423_17735580_99	Overlap_dORF	BID	22	-	297	50	11	8	12.12%	55.62668874	0.02055943	0.009965666	0.001000669
ENSG00000116337_109625404_109631152_761	annotated	AMPD2	1	+	2283	8	6	0	0.79%	1.15785381	0.280084718	0.011570466	0.021802746
ENSG00000057663_106316208_106186540_275	annotated	ATG5	6	-	825	45	12	6	6.18%	18.02304715	0.005356506	0.00129387	4.16E-05
ENSG00000119616_74723298_74734930_92	annotated	FCF1	14	+	276	45	8	0	13.04%	53.87323877	0.001015599	0.001015599	6.39E-06
ENSG00000104660_30095513_30104922_155	annotated	LEPROTL1	8	+	465	99	10	0	14.19%	70.34802275	0.000138667	1.88E-05	2.07E-08
ENSG00000164967_34611296_34610805_163	annotated	RPP25L	9	-	489	99	7	2	15.95%	66.89535906	2.85E-05	4.15E-06	9.99E-10
ENSG00000160305_46504471_46549918_634	annotated	DIP2A	21	+	1902	15	0	1	1.10%	2.605855924	0.008473674	0.013892891	0.00058868
ENSG00000108654_64503080_64502922_52	novel	DDX5	17	-	156	98	18	7	11.54%	207.5731285	0.203424872	0.01385614	0.016052972
ENSG00000112812_27251235_27255315_188	novel	PRSS16	6	+	564	9	0	0	2.66%	5.272699965	0.020613417	0.020613417	0.001946209
ENSG00000204351_31959196_31961378_210	novel	SKIV2L	6	+	630	10	1	0	2.86%	5.244802081	0.023672501	0.012798403	0.001437523
ENSG00000158195_27428890_27408325_281	annotated	WASF2	1	-	843	17	0	0	1.78%	6.663325064	0.019679754	0.019679754	0.001784964
ENSG00000063245_55678655_55695356_567	annotated	EPN1	19	+	1701	114	10	3	6.88%	22.1447199	9.42E-07	5.04E-08	4.83E-13
ENSG00000143126_109250239_109274049_2870	annotated	CELSR2	1	+	8610	29	5	0	0.52%	1.112921417	0.007919222	0.000235224	1.47E-05
ENSG00000156976_186783611_186788319_362	annotated	EIF4A2	3	+	1086	100	5	5	7.46%	30.42564743	8.48E-06	2.27E-05	1.56E-09
ENSG00000185201_308193_309165_132	annotated	IFITM2	11	+	396	114	16	0	7.58%	95.12163775	0.002851	0.002460157	4.02E-05
ENSG00000152990_22392552_22387705_448	novel	ADGRA3	4	-	1344	18	5	0	1.79%	4.425301756	0.052370108	0.005454749	0.001603662
ENSG00000157014_10260317_10279025_563	annotated	TATDN2	3	+	1689	13	3	0	1.42%	2.54321664	0.046396937	0.005160369	0.001340235
ENSG00000092199_21234154_21211223_293	annotated	HNRNPC	14	-	879	120	7	7	6.48%	45.10887797	0.000388917	0.000402141	1.04E-06

ENSG00000119723_73950333_73955842_111	novel	COQ6	14	+	333	14	0	0	4.50%	13.89163794	0.021084099	0.021084099	0.002029891
ENSG00000119723_73955835_73958367_104	novel	COQ6	14	+	312	19	2	0	4.81%	20.12188491	0.021557223	0.021557223	0.002115616
ENSG00000165392_31058502_31173102_1414	annotated	WRN	8	+	4242	14	1	3	0.57%	1.090503403	0.00912793	0.040990615	0.001872253
ENSG00000165389_34462198_34435201_68	annotated	SPTSSA	14	-	204	177	8	4	32.35%	286.6901373	0.000111987	6.70E-05	5.49E-08
ENSG00000080503_2161713_2192739_236	annotated	SMARCA2	9	+	708	14	7	2	2.97%	6.533778864	0.106840601	0.037441831	0.016226997
ENSG00000135535_109382378_109368851_178	annotated	CD164	6	-	534	369	85	3	29.78%	228.3256816	7.21E-08	1.12E-10	1.15E-16
ENSG00000054983_87993164_87934732_685	annotated	GALC	14	-	2055	100	5	9	5.55%	16.07895529	8.16E-08	1.17E-06	9.51E-13
ENSG00000087448_27780346_27797290_364	annotated	KLHL42	12	+	1092	14	2	0	1.65%	4.236186296	0.044040756	0.013428348	0.002791708
ENSG00000006530_141601260_141652924_330	annotated	AGK	7	+	990	34	4	0	3.94%	11.3478445	0.000938122	0.000669869	3.98E-06
ENSG00000108389_58508523_58491663_1030	annotated	MTMR4	17	-	3090	44	2	2	1.75%	4.705045751	0.000182163	0.000316339	3.97E-07
ENSG00000122545_35801267_35904293_382	annotated	7-Sep	7	+	1146	108	17	7	5.50%	31.13929613	0.000799946	0.000159373	8.90E-07
ENSG00000166206_26773724_26547793_473	annotated	GABRB3	15	-	1419	167	17	6	8.88%	38.88693636	4.26E-08	2.27E-08	9.74E-15
ENSG00000197912_89550604_89557093_204	novel	SPG7	16	+	612	11	5	0	2.94%	5.938967063	0.168406755	0.012798403	0.011984717
ENSG00000105671_18919742_18928316_483	annotated	DDX49	19	+	1449	66	10	5	4.14%	15.05030162	0.000336035	7.60E-05	1.86E-07
ENSG00000177189_20234787_20155398_708	annotated	RPS6KA3	X	-	2124	48	5	2	3.53%	7.467175844	6.12E-05	1.98E-05	9.41E-09
ENSG00000166352_36610134_36659186_211	annotated	C11orf74	11	+	633	10	0	1	2.37%	5.219945199	0.020613417	0.035374934	0.003252526
ENSG00000175193_183844264_183829598_188	annotated	PARL	3	-	564	7	1	0	2.66%	4.100988861	0.041782825	0.019216965	0.003600526
ENSG00000136940_122826787_122820085_301	annotated	PDCL	9	-	903	74	9	2	8.64%	27.0778154	6.27E-05	6.40E-06	3.36E-09
ENSG00000136933_125203014_125233980_321	annotated	RABEPK	9	+	963	53	3	2	4.98%	18.18524834	0.00027962	0.000633314	1.18E-06
ENSG00000141429_35654663_35709770_559	annotated	GALNT1	18	+	1677	159	38	6	9.12%	31.3280754	1.67E-05	1.05E-09	3.77E-13
ENSG00000101901_111681219_111685119_132	annotated	ALG13	X	+	396	22	1	2	3.79%	18.35680728	0.023199731	0.029237631	0.003013552
ENSG00000162591_3595371_3490528_1427	annotated	MEGF6	1	-	4281	21	0	0	0.91%	1.620853341	0.000577025	0.000577025	2.15E-06
ENSG00000164329_79619649_79685273_480	annotated	TENT2	5	+	1440	16	2	0	1.88%	3.671361457	0.018785664	0.003229477	0.000341578
ENSG00000004779_23596290_23582284_156	annotated	NDUFAB1	16	-	468	468	144	13	33.33%	330.4225311	5.84E-08	3.15E-10	2.36E-16
ENSG00000100823_20455646_20457508_318	annotated	APEX1	14	+	954	460	61	31	20.13%	159.323233	7.70E-10	1.28E-10	1.14E-18
ENSG00000169609_83011526_83004877_126	annotated	C15orf40	15	-	378	27	0	4	5.56%	23.60160937	0.008776163	0.046045458	0.00204958
ENSG00000278053_37642119_37621086_421	novel	DDX52	17	-	1263	54	6	2	4.75%	14.12732912	0.000400927	0.000129283	3.64E-07
ENSG00000176715_89101273_89154207_379	annotated	ACSF3	16	+	1137	19	0	0	2.11%	5.52157264	0.005306578	0.005306578	0.000151001
ENSG00000013375_83188759_83169298_441	annotated	PGM3	6	-	1323	17	1	1	2.49%	4.245792161	0.002415484	0.002415484	3.37E-05
ENSG00000217930_4343264_4340319_115	annotated	PAM16	16	-	345	371	15	7	15.65%	355.3239393	0.000165674	0.000152269	1.77E-07
ENSG00000182979_105420048_105470215_711	annotated	MTA1	14	+	2133	48	15	2	2.81%	7.435668773	0.011757316	4.88E-05	6.60E-06
ENSG00000184009_81512354_81510690_375	annotated	ACTG1	17	-	1125	2149	537	109	29.60%	631.1804617	5.26E-12	3.10E-17	4.09E-27
ENSG00000183828_105181209_105176530_143	novel	NUDT14	14	-	429	36	0	0	7.69%	27.72776485	0.001513502	0.001513502	1.38E-05
ENSG00000154370_228406480_228396648_358	annotated	TRIM11	1	-	1074	12	4	2	1.40%	3.691871856	0.051235217	0.04317391	0.008957297
ENSG00000185129_140114182_140115150_322	annotated	PURA	5	+	966	22	2	1	4.04%	7.525150812	0.000537982	0.00102131	3.51E-06
ENSG00000141576_76240240_76145235_679	annotated	RNF157	17	-	2037	8	0	0	0.74%	1.297682989	0.019679754	0.019679754	0.001784964
ENSG00000247596_52239016_52229034_349	annotated	TWF2	3	-	1047	135	21	13	9.17%	42.60462436	0.000117411	9.76E-05	8.25E-08
ENSG00000134597_130402033_130412848_322	annotated	RBMX2	X	+	966	16	0	4	2.48%	5.472836954	0.005257623	0.058506513	0.001764746
ENSG00000039068_68737416_68833499_821	annotated	CDH1	16	+	2463	191	15	4	8.28%	25.62350932	7.37E-13	1.30E-12	1.23E-23
ENSG00000107554_99957014_99877151_1424	annotated	DNMBP	10	-	4272	66	8	2	1.97%	5.104842475	6.76E-05	2.75E-06	1.69E-09
ENSG00000166200_49155578_49127950_443	annotated	COPS2	15	-	1329	80	15	6	7.22%	19.88999435	0.000215379	8.81E-06	1.65E-08
ENSG00000092098_24147699_24160573_1072	annotated	RNF31	14	+	3216	25	7	3	0.93%	2.568583109	0.047582535	0.017185125	0.003725137
ENSG00000179041_66429132_66430229_365	annotated	RRS1	8	+	1095	39	3	5	4.38%	11.76847371	0.000297378	0.002237678	4.54E-06
ENSG00000263001_74689225_74758838_966	annotated	GTF2I	7	+	2898	58	9	8	1.76%	6.61301132	0.001982699	0.001758679	2.06E-05
ENSG00000266028_206384050_206461420_918	annotated	SRGAP2	1	+	2754	10	1	0	0.76%	1.199791326	0.015191411	0.007593099	0.000581666
ENSG00000173193_122704554_122728597_686	dORF	PARP14	3	+	2058	8	0	0	0.73%	1.284441326	0.019216965	0.019216965	0.001707396
ENSG00000198563_31532908_31531106_103	annotated	DDX39B	6	-	309	56	6	33	14.56%	59.88240046	0.001888962	0.186327884	0.003700202
ENSG00000198563_31531124_31530434_78	Overlap_dORF	DDX39B	6	-	234	53	10	11	19.23%	74.83929124	0.00749654	0.012730008	0.000483434
ENSG00000103335_88717235_88715605_372	novel	PIEZO1	16	-	1116	17	5	2	3.23%	5.033318126	0.03343679	0.004932312	0.000902704
ENSG00000115970_43560266_43230948_1069	annotated	THADA	2	-	3207	34	1	2	1.40%	3.503076413	0.000405022	0.000948585	2.50E-06
ENSG00000070087_149968535_149966489_91	annotated	PFN2	3	-	273	268	20	8	34.07%	324.3708364	1.09E-06	1.54E-06	1.48E-11
ENSG00000070087_149966250_149966167_27	dORF	PFN2	3	-	81	41	1	0	18.52%	167.2509108	0.021084099	0.021084099	0.002029891
ENSG00000274760_49279128_49279205_25	novel	RF02216	20	+	75	139	14	5	44.00%	612.383091	0.002338867	0.001792061	2.46E-05
ENSG00000160256_44940058_44976803_215	annotated	FAM207A	21	+	645	26	0	3	2.79%	13.31935784	0.013640586	0.031254458	0.002000878
ENSG00000147010_19645418_19536417_404	annotated	SH3KBP1	X	-	1212	31	0	2	3.96%	8.451401373	0.000177865	0.000815071	1.00E-06
ENSG00000105726_19655237_19654067_94	novel	ATP13A1	19	-	282	17	3	2	8.51%	19.91908876	0.036243043	0.02389627	0.003798067
ENSG00000281649_37086727_37086870_47	novel	EBLN3P	9	+	141	35	7	2	12.77%	82.01977723	0.013853925	0.024414995	0.001590539
ENSG00000115685_241157830_241167068_212	annotated	PPP1R7	2	+	636	71	2	0	11.79%	36.886792	8.76E-06	5.26E-06	3.82E-10
ENSG00000131100_17594672_17592674_68	novel	ATP6V1E1	22	-	204	11	2	1	8.82%	17.81690119	0.047790352	0.011772051	0.002720069
ENSG00000117500_93160244_93154670_172	novel	TMED5	1	-	516	161	27	1	12.79%	103.0969525	0.000671649	1.89E-05	1.10E-07
ENSG00000048544_42217849_42208289_201	annotated	MRPS10	6	-	603	80	5	5	8.96%	43.83715172	0.000198512	0.000902721	1.23E-06
ENSG00000083444_11934780_11974808_727	annotated	PLOD1	1	+	2181	204	43	2	9.77%	30.90609645	2.14E-08	8.95E-14	5.38E-20
ENSG00000100811_100239911_100277600_139	annotated	YY1	14	+	417	46	5	4	10.07%	36.44948784	0.001127587	0.0011619	8.05E-06
ENSG00000112319_133462632_133528805_442	annotated	EYA4	6	+	1326	18	2	0	1.81%	4.485373726	0.013239535	0.005306578	0.000367823
ENSG00000083937_87245732_87253822_165	annotated	CHMP2B	3	+	495	115	10	2	16.97%	76.76483046	1.28E-05	1.70E-06	1.90E-10
ENSG00000162521_32651307_32657643_126	novel	RBBP4	1	+	378	25	1	0	4.76%	21.85334201	0.013428348	0.013428348	0.000872559
ENSG00000011485_46387047_46390346_213	novel	PPP5C	19	+	639	34	10	6	6.57%	17.58116754	0.004398321	0.004646785	0.000111359
ENSG00000171316_60741679_60824055_1056	annotated	CHD7	8	+	3168	9	0	2	0.47%	0.938700372	0.020613417	0.060322713	0.005529088

ENSG00000134146_35542479_35410884_115	novel	DPH6	15	-	345	19	2	0	5.22%	18.19718287	0.024135905	0.013428348	0.001529427
ENSG00000137074_32989888_32987821_51	annotated	APTX	9	-	153	38	0	1	13.73%	82.06572668	0.008877961	0.008877961	0.000400235
ENSG00000254505_24213439_24211348_141	novel	CHMP4A	14	-	423	45	4	3	12.06%	35.1513331	0.001073184	0.000672326	4.55E-06
ENSG00000040933_98535792_98587608_866	annotated	INPP4A	2	+	2598	11	2	1	0.58%	1.399017645	0.047790352	0.020239728	0.004303932
ENSG00000147408_19506085_19505966_39	novel	CSGALNACT1	8	-	117	24	10	1	20.51%	67.77898074	0.085401204	0.005604867	0.002874515
ENSG00000140650_8813101_8847825_35	dORF	PMM2	16	+	105	12	0	7	17.14%	37.76257498	0.011772051	0.11411552	0.007078638
ENSG00000174227_499336_539369_983	annotated	PIGG	4	+	2949	88	22	2	4.37%	9.860014492	3.39E-05	1.49E-08	8.15E-12
ENSG00000124570_2954670_2948298_259	novel	SERPINB6	6	-	777	175	31	4	18.53%	74.41948899	1.98E-07	1.89E-09	4.48E-15
ENSG00000088836_3234760_3227787_753	annotated	SLC4A11	20	-	2259	7	3	0	0.66%	1.023885665	0.169362074	0.019216965	0.016163992
ENSG00000126602_3658215_3658129_28	annotated	TRAP1	16	-	84	19	1	3	21.43%	74.73842966	0.013428348	0.028889786	0.001825217
ENSG00000142186_65525154_65538540_780	annotated	SCYL1	11	+	2340	65	7	2	3.46%	9.178403642	4.17E-05	4.78E-06	1.69E-09
ENSG00000135525_136525964_136344228_771	annotated	MAP7	6	-	2313	48	7	4	2.85%	6.857017507	0.000416926	0.000160547	4.63E-07
ENSG00000093144_127327136_127289869_225	annotated	ECHDC1	6	-	675	78	7	5	9.78%	38.18215915	0.000198921	0.000111169	1.57E-07
ENSG00000198373_69917851_69939940_488	novel	WWP2	16	+	1464	20	2	4	1.64%	4.513969004	0.015570605	0.036102036	0.002592909
ENSG00000197006_21617995_21625138_95	annotated	METTL9	16	+	285	71	7	4	10.53%	82.31578845	0.00224099	0.017505688	0.000232282
ENSG00000065427_75628685_75627895_71	novel	KARS	16	-	213	47	1	2	18.31%	72.91013597	0.000945593	0.000669869	4.01E-06
ENSG00000166595_66934364_66934164_66	novel	CIAO2B	16	-	198	29	3	0	13.64%	48.3952192	0.005156338	0.003632191	0.000102791
ENSG00000065911_74198642_74214242_222	annotated	MTHFD2	2	+	666	80	2	5	12.16%	39.69039413	4.46E-06	9.83E-06	3.65E-10
ENSG00000183283_51238908_51242458_136	annotated	DAZAP2	12	+	408	120	17	0	10.29%	97.18309739	0.005518847	0.000478119	1.79E-05
ENSG00000139291_71686681_71699159_298	annotated	TMEM19	12	+	894	148	18	0	12.42%	54.70082171	2.54E-06	4.86E-08	1.32E-12
ENSG00000166340_6619400_6618759_81	novel	TPP1	11	-	243	54	9	0	14.81%	73.42722914	0.007666581	0.00104892	5.02E-05
ENSG00000166340_6616817_6614546_320	novel	TPP1	11	-	960	177	39	4	12.50%	60.92165417	1.78E-06	7.56E-08	1.37E-12
ENSG00000101266_489830_483961_167	annotated	CSNK2A1	20	-	501	47	1	0	8.38%	30.99772248	0.000475338	0.000472567	1.47E-06
ENSG00000183765_28719414_28687897_322	dORF	CHEK2	22	-	966	19	0	0	3.11%	6.498993883	0.002254349	0.002254349	2.95E-05
ENSG00000103510_31117682_31130992_467	annotated	KAT8	16	+	1401	62	5	0	5.57%	14.62255313	6.66E-05	2.76E-06	1.66E-09
ENSG00000163875_37514740_37496592_189	annotated	MEAF6	1	-	567	52	11	0	10.05%	30.30330091	0.001670825	5.93E-05	8.07E-07
ENSG00000168672_126557389_126556457_310	annotated	LRATD2	8	-	930	15	0	0	2.58%	5.329395663	0.005160369	0.005160369	0.000143196
ENSG00000244687_50084158_50082768_58	novel	UBE2V1	20	-	174	12	1	2	8.62%	22.78776077	0.051235217	0.051235217	0.010460668
ENSG00000155368_119367052_119368550_123	novel	DBI	2	+	369	57	21	4	10.57%	51.04087879	0.043495005	0.00270297	0.000743435
ENSG00000125166_58734228_58722141_127	novel	GOT2	16	-	381	40	1	0	8.66%	34.69002951	0.001573068	0.001573068	1.48E-05
ENSG00000095787_28590832_28610850_232	novel	WAC	10	+	696	9	0	1	3.45%	4.272705144	0.003327803	0.017404239	0.000323375
ENSG00000118849_158732415_158704776_228	annotated	RARRES1	3	-	684	55	12	1	8.77%	26.56906317	0.00296182	3.86E-05	1.06E-06
ENSG00000144034_73734569_73729943_214	annotated	TPRKB	2	-	642	166	35	0	11.21%	85.4363554	0.002016318	8.71E-06	1.98E-07
ENSG00000091651_46691073_46695709_149	novel	ORC6	16	+	447	44	5	0	6.71%	32.52481291	0.007947031	0.002460157	0.000110549
ENSG00000106144_143292373_143305071_280	Overlap_dORF	CASP2	7	+	840	17	0	0	2.14%	6.687122654	0.013428348	0.013428348	0.000872559
ENSG00000109332_102809680_102797415_110	annotated	UBE2D3	4	-	330	42	2	0	8.18%	42.05377669	0.003710481	0.003710481	7.64E-05
ENSG00000142208_104792643_104770341_480	annotated	AKT1	14	-	1440	43	13	5	3.75%	9.866783915	0.003071899	0.00099268	1.86E-05
ENSG00000133466_37185380_37182188_236	novel	C1QTNF6	22	-	708	82	4	5	8.47%	38.2692762	0.000125937	4.37E-05	4.11E-08
ENSG00000142945_44739939_44756096_227	novel	KIF2C	1	+	681	28	7	0	5.73%	13.58565473	0.013680503	0.000656046	6.35E-05
ENSG00000108671_32477470_32479469_110	novel	PSMD11	17	+	330	91	8	0	9.09%	91.11651616	0.009146401	0.002502537	0.000129518
ENSG00000108671_32480173_32480631_55	novel	PSMD11	17	+	165	22	6	0	16.36%	44.05633748	0.017182073	0.003554776	0.000337701
ENSG00000047410_186375028_186353871_726	annotated	TPR	1	-	2178	22	1	1	1.52%	3.337601324	0.002054387	0.002054387	2.47E-05
ENSG00000134283_42335903_42441509_348	annotated	PPHLN1	12	+	1044	86	19	2	6.32%	27.21871425	0.002878599	3.01E-05	8.35E-07
ENSG00000130733_10923904_10923291_123	internal	YIPF2	19	-	369	18	6	0	6.50%	16.11817225	0.00419316	0.005306578	0.000120897
ENSG00000105341_41439899_41432284_185	annotated	DMAC2	19	-	555	95	9	12	12.43%	56.55881163	1.30E-05	0.000203183	2.20E-08
ENSG00000181038_76729711_76733411_146	annotated	METTL23	17	+	438	32	23	7	8.22%	24.14045889	0.10138588	0.021040203	0.00969386
ENSG00000177426_3450490_3457940_272	annotated	TGIF1	18	+	816	128	28	10	11.40%	51.83098527	0.00032156	1.06E-05	2.98E-08
ENSG00000112419_143753795_143823689_533	annotated	PHACTR2	6	+	1599	14	10	1	1.50%	2.893005276	0.279308269	0.00912793	0.018642435
ENSG00000172732_65860754_65866052_551	annotated	MUS81	11	+	1653	22	5	2	1.63%	4.397638043	0.029401099	0.011768187	0.001654234
ENSG00000119383_129142538_129147464_30	annotated	PTPA	9	+	90	16	1	0	16.67%	58.74178331	0.052029617	0.021084099	0.004853002
ENSG00000081913_62716314_62979431_1507	annotated	PHLPP1	18	+	4521	7	1	0	0.33%	0.511603123	0.041782825	0.019216965	0.003600526
ENSG00000129566_20408370_20368437_2604	annotated	TEP1	14	-	7812	25	0	2	0.54%	1.057419774	0.000390775	0.002088633	5.39E-06
ENSG00000100813_23095112_23059148_1283	annotated	ACIN1	14	-	3849	95	19	10	3.04%	8.155401521	5.56E-06	1.62E-06	7.83E-11
ENSG00000129474_22982266_22973443_538	annotated	AJUBA	14	-	1614	50	5	2	2.79%	10.23613789	0.001103062	0.000523214	3.69E-06
ENSG00000160410_40586917_40590778_108	annotated	SHKBP1	19	+	324	10	4	0	4.63%	10.19822627	0.11290941	0.019679754	0.010350976
ENSG00000160410_40588771_40591207_152	Overlap_dORF	SHKBP1	19	+	456	18	0	1	4.61%	13.04299465	0.008473674	0.011825808	0.000503679
ENSG00000073578_236386_256420_258	novel	SDHA	5	+	774	85	8	6	5.04%	36.28671207	0.001274316	0.00074336	5.92E-06
ENSG00000073578_233560_233655_31	novel	SDHA	5	+	93	49	5	0	16.13%	174.0935917	0.057424805	0.021557223	0.005461773
ENSG00000121073_49707833_49706200_105	novel	SLC35B1	17	-	315	54	7	5	11.43%	56.64386248	0.001993061	0.002793773	3.23E-05
ENSG00000163754_148991641_149026933_333	annotated	GYG1	3	+	999	38	4	1	4.80%	12.56862481	0.001741809	0.000264458	3.14E-06
ENSG00000153113_96663137_96771679_754	annotated	CAST	5	+	2262	487	66	17	14.59%	71.1387147	2.31E-13	1.07E-17	5.20E-29
ENSG00000198563_31540510_31538840_110	annotated	DDX39B	6	-	330	36	6	102	9.09%	36.0460943	0.007635352	0.516161534	0.045829264
ENSG00000165097_18159896_18221992_590	annotated	KDM1B	6	+	1770	14	0	0	1.36%	2.613511546	0.005257623	0.005257623	0.000148366
ENSG00000125746_45630001_45618860_260	annotated	EML2	19	-	780	17	2	1	3.85%	7.201516704	0.012468381	0.004070582	0.000273397
ENSG00000138777_105474050_105369725_232	annotated	PPA2	4	-	696	53	5	0	5.60%	25.16148585	0.00065148	0.00065148	2.72E-06
ENSG00000215114_58411386_58447551_331	annotated	UBXN2B	8	+	993	32	0	0	3.93%	10.6480574	0.000628963	0.000628963	2.54E-06
ENSG00000197157_128089654_128092058_49	novel	SND1	7	+	147	19	1	1	14.29%	42.70767409	0.020342454	0.013892891	0.001336224
ENSG00000163950_1712188_1693597_270	annotated	SLBP	4	-	810	184	6	9	15.56%	75.05894534	8.13E-09	4.81E-08	4.08E-15

ENSG00000196756_38434513_38426436_78	novel	SNHG17	20	-	234	26	5	3	8.97%	36.71361457	0.026714995	0.023878474	0.00284211
ENSG00000172059_10043717_10052507_512	annotated	KLF11	2	+	1536	22	4	3	2.15%	4.732614378	0.018402375	0.009414025	0.000852498
ENSG00000168496_61795362_61796504_380	annotated	FEN1	11	+	1140	83	8	1	7.89%	24.05707902	2.24E-05	9.18E-07	1.94E-10
ENSG00000109736_2933923_2930870_480	annotated	MFSD10	4	-	1440	208	17	22	9.58%	47.72769894	2.80E-07	1.45E-06	3.77E-12
ENSG00000177058_55697949_55669208_178	novel	SLC38A9	5	-	534	14	6	0	4.49%	8.662762988	0.115442043	0.005257623	0.003950847
ENSG00000012660_53295699_53290464_105	novel	ELOVL5	6	-	315	143	12	8	20.95%	150.0013395	0.000255861	5.25E-05	9.99E-08
ENSG00000144848_112561528_112544042_135	novel	ATG3	3	-	405	17	1	0	4.44%	13.86958773	0.013428348	0.01300724	0.000846864
ENSG00000111678_6944122_6945922_126	annotated	C12orf57	12	+	378	209	47	13	22.22%	182.6939392	4.45E-05	1.93E-05	6.67E-09
ENSG00000198925_219226870_219220447_769	annotated	ATG9A	2	-	2307	52	14	1	3.12%	7.447755361	0.002733827	1.03E-05	3.24E-07
ENSG00000146918_158693311_158631666_1055	annotated	NCAPG2	7	-	3165	86	7	2	3.51%	8.978305743	1.69E-06	7.98E-08	1.36E-12
ENSG00000155545_56938897_56923128_450	annotated	MIER3	5	-	1350	23	0	0	2.44%	5.6294209	0.001448134	0.001448134	1.26E-05
ENSG00000143398_151231761_151247865_453	annotated	PIP5K1A	1	+	1359	38	2	0	3.75%	9.239187772	0.000335003	0.000103305	2.46E-07
ENSG00000132507_7309636_7311640_154	annotated	EIF5A	17	+	462	657	241	36	18.18%	469.8865865	0.000502315	1.88E-06	1.10E-08
ENSG00000115419_190930506_190962986_171	annotated	GLS	2	+	513	27	5	3	5.26%	17.39065953	0.030106073	0.008742337	0.001309867
ENSG00000100226_38715969_38730704_547	annotated	GTPBP1	22	+	1641	25	3	2	2.19%	5.033859402	0.007415603	0.003975543	0.000159349
ENSG00000164535_6446076_6409837_502	annotated	DAGLB	7	-	1506	27	2	2	2.99%	5.92390992	0.000805645	0.001407907	7.05E-06
ENSG00000146731_56055714_56055830_38	novel	CCT6A	7	+	114	80	11	2	26.32%	231.8754604	0.006351887	0.002502537	8.96E-05
ENSG00000135473_56333094_56317609_1202	annotated	PAN2	12	-	3606	29	11	1	1.25%	2.657308209	0.03296431	0.00042095	0.000125578
ENSG00000136104_50929501_50950070_207	dORF	RNASEH2B	13	+	621	21	1	0	4.35%	11.17370878	0.00567009	0.003554776	0.000110499
ENSG00000115295_29132200_29189605_571	annotated	CLIP4	2	+	1713	27	0	5	2.45%	5.208060911	0.000398115	0.005109965	1.41E-05
ENSG00000105655_18435607_18434913_155	novel	ISYNA1	19	-	465	11	1	0	3.23%	7.816446973	0.034182656	0.019216965	0.002956053
ENSG00000134333_18402941_18407281_159	novel	LDHA	11	+	477	1564	146	41	28.93%	1083.397985	2.26E-09	2.62E-08	6.46E-16
ENSG00000198755_35468435_35470750_217	annotated	RPL10A	6	+	651	91	22	1	5.07%	46.18809575	0.026832696	0.00252785	0.000408854
ENSG00000132305_86195382_86144268_372	annotated	IMMT	2	-	1116	103	12	4	9.41%	30.49598629	1.23E-05	7.12E-07	8.16E-11
ENSG00000168096_4727029_4697028_550	novel	ANKS3	16	-	1650	25	8	4	1.45%	5.006401987	0.1514727	0.034975232	0.022223091
ENSG00000254788_66552782_66579001_146	annotated	CKLF-CMTM1	16	+	438	35	4	7	5.48%	26.40362692	0.012045688	0.027805253	0.001596836
ENSG00000065000_2111287_2102173_221	novel	AP3D1	19	-	663	49	1	6	4.98%	24.42036806	0.001634015	0.00638574	6.14E-05
ENSG00000180817_70233327_70210394_178	annotated	PPA1	10	-	534	23	0	3	5.06%	14.23168205	0.003554776	0.016170007	0.000317001
ENSG00000169976_144095497_144095237_86	annotated	SF3B5	6	-	258	196	16	2	25.58%	251.018667	6.82E-05	2.39E-05	1.26E-08
ENSG00000076944_7646288_7647810_128	Overlap_dORF	STXBP2	19	+	384	14	0	0	3.91%	12.04665478	0.020613417	0.020613417	0.001946209
ENSG00000134900_102597198_102678316_1196	annotated	TPP2	13	+	3588	47	14	2	2.17%	4.328277303	0.002903514	9.77E-06	3.35E-07
ENSG00000051382_138742725_138655389_919	dORF	PIK3CB	3	-	2757	35	2	3	2.18%	4.19470025	0.000121322	0.00030598	2.61E-07
ENSG00000169045_179623133_179620884_91	novel	HNRNPH1	5	-	273	92	22	11	19.78%	111.3511826	0.000804886	0.000655047	3.35E-06
ENSG00000104412_108475919_108486598_115	novel	EMC2	8	+	345	14	3	0	6.09%	13.40845054	0.032320015	0.008373824	0.001358383
ENSG00000050426_51048357_51059431_360	annotated	LETMD1	12	+	1080	52	0	7	5.00%	15.90923298	8.66E-05	0.000667933	4.24E-07
ENSG00000142687_35470901_35441018_638	novel	KIAA0319L	1	-	1914	28	4	2	2.51%	4.833767435	0.002322932	0.000725652	1.05E-05
ENSG00000132676_155729286_155738242_173	annotated	DAP3	1	+	519	65	7	0	9.25%	41.38239792	0.002181251	0.000206547	3.20E-06
ENSG00000114125_141738342_141742682_68	annotated	RNF7	3	+	204	77	13	9	20.59%	124.7183083	0.00144124	0.000465689	4.33E-06
ENSG00000155189_6708702_6755137_90	internal	AGPAT5	8	+	270	19	4	17	11.11%	23.25195589	0.014728193	0.140113165	0.010635415
ENSG00000155189_6755134_6757388_88	annotated	AGPAT5	8	+	264	21	3	0	10.23%	26.28361043	0.02736363	0.002967235	0.000476755
ENSG00000111641_6566202_6556993_688	annotated	NOP2	12	-	2064	52	3	4	3.20%	8.324598652	5.87E-05	5.77E-05	2.52E-08
ENSG00000141736_39707007_39728044_1225	annotated	ERBB2	17	+	3675	115	21	2	4.33%	10.33975267	1.46E-06	1.29E-10	3.70E-15
ENSG00000109501_6277456_6302468_890	annotated	WFS1	4	+	2670	39	3	1	2.36%	4.826396522	0.00016857	3.42E-05	4.39E-08
ENSG00000137100_34613868_34613782_28	novel	DCTN3	9	-	84	13	0	0	21.43%	51.13682029	0.012798403	0.012798403	0.000797244
ENSG00000116857_201143903_201135663_78	novel	TMEM9	1	-	234	116	8	2	26.92%	163.7992035	0.000253612	4.83E-05	9.16E-08
ENSG00000133067_202300895_202319207_690	annotated	LGR6	1	+	2070	114	12	2	6.09%	18.19718287	3.90E-07	9.05E-09	3.89E-14
ENSG00000113621_134874428_134899685_360	annotated	TXNDC15	5	+	1080	301	49	9	18.61%	92.08998321	4.07E-09	2.49E-12	1.62E-19
ENSG00000137177_17817153_17771896_873	annotated	KIF13A	6	-	2619	19	3	0	0.92%	2.397108855	0.008316137	0.005604867	0.00024458
ENSG00000088038_54142979_54155407_753	annotated	CNOT3	19	+	2259	56	11	4	2.92%	8.191085322	0.000962744	5.35E-05	4.06E-07
ENSG00000037749_154053078_154053713_211	annotated	MFAP3	5	+	633	54	6	7	8.53%	28.18770408	0.000459141	0.001313927	3.90E-06
ENSG00000124664_34544455_34538274_335	annotated	SPDEF	6	-	1005	26	0	0	3.58%	8.548244586	0.000982952	0.000982952	6.00E-06
ENSG00000154719_25607460_25585707_333	annotated	MRPL39	21	-	999	56	1	2	6.61%	18.52218393	2.22E-05	2.99E-05	5.15E-09
ENSG00000092470_43828196_43866392_529	annotated	WDR76	15	+	1587	44	0	0	3.59%	9.161053163	4.97E-05	4.97E-05	1.85E-08
ENSG00000120438_159780023_159779623_98	novel	TCP1	6	-	294	71	6	2	13.27%	79.79591738	0.001384789	0.000905008	7.74E-06
ENSG00000107186_13250315_13106965_2084	annotated	MPDZ	9	-	6252	32	6	4	0.72%	1.691222168	0.007031648	0.002987195	0.000116449
ENSG00000170540_18798783_18793252_174	annotated	ARL6IP1	16	-	522	647	100	8	32.76%	409.5467004	3.73E-10	2.42E-11	1.10E-19
ENSG00000136811_128471335_128494731_508	annotated	ODF2	9	+	1524	22	0	6	2.76%	4.769879058	0.000335351	0.029538586	9.21E-05
ENSG00000165688_136418080_136422260_243	annotated	PMPCA	9	+	729	36	10	1	6.58%	16.31716203	0.003746796	0.000252954	6.81E-06
ENSG00000052126_19260961_19369787_1099	annotated	PLEKHA5	12	+	3297	29	9	1	1.27%	2.906355293	0.024971316	0.000662939	0.000127946
ENSG00000103496_31033806_31034266_64	annotated	STX4	16	+	192	17	2	0	12.50%	29.25616161	0.013091824	0.005554612	0.000378918
ENSG00000245910_66925491_66922703_25	novel	SNHG6	8	-	75	61	0	0	28.00%	268.7436586	0.008877961	0.008877961	0.000400235
ENSG00000112715_43777480_43784562_172	annotated	VEGFA	6	+	516	40	1	3	5.81%	25.6141497	0.002851	0.008025782	0.00012745
ENSG00000243566_76511734_76515204_172	annotated	UPK3B	7	+	516	27	0	16	4.65%	17.28955105	0.005604867	0.286282988	0.014179234
ENSG00000104872_49451550_49446382_282	annotated	PIH1D1	19	-	846	53	8	1	8.87%	20.70022949	0.000183531	6.70E-06	1.09E-08
ENSG00000074054_121503185_121340861_1263	annotated	CLASP1	2	-	3789	31	0	2	0.87%	2.703377795	0.001558047	0.002176803	2.01E-05
ENSG00000164587_150447733_150444286_151	annotated	RPS14	5	-	453	531	130	42	31.13%	387.3164769	6.10E-08	8.34E-08	4.98E-14
ENSG00000066926_57586599_57550712_422	annotated	FECH	18	-	1266	87	11	7	6.87%	22.70676162	1.60E-05	2.84E-05	3.58E-09
ENSG00000197912_89550604_89557093_204	novel	SPG7	16	+	612	11	5	0	2.94%	5.938967063	0.168406755	0.012798403	0.011984717

ENSG00000164074_127982956_128035813_464	annotated	ABHD18	4	+	1392	14	1	0	2.16%	3.323215112	0.004053443	0.001790164	4.21E-05
ENSG00000101266_489830_483961_167	dORF	CSNK2A1	20	-	501	47	1	0	8.38%	30.99772248	0.000475338	0.000472567	1.47E-06
ENSG00000011376_45394454_45547530_903	annotated	LARS2	3	+	2709	27	4	0	1.55%	3.293247818	0.004300893	0.00040306	1.18E-05
ENSG00000188846_40458699_40461518_49	novel	RPL14	3	+	147	68	3	2	10.20%	152.8485178	0.021557223	0.021557223	0.002115616
ENSG00000143624_153751129_153753720_86	novel	INTS3	1	+	258	15	2	7	6.98%	19.21061227	0.044040756	0.117522225	0.020392476
ENSG00000143624_153754707_153773270_734	novel	INTS3	1	+	2202	50	9	1	1.63%	7.502782269	0.020095766	0.001393565	0.000181735
ENSG00000142686_35716321_35715632_229	annotated	C1orf216	1	-	687	14	0	0	2.18%	6.733501362	0.021084099	0.021084099	0.002029891
ENSG00000142684_26170038_26170688_138	annotated	ZNF593	1	+	414	23	3	1	4.35%	18.35680728	0.059897465	0.020342454	0.005428712
ENSG00000160679_153636589_153645269_202	annotated	CHTOP	1	+	606	73	4	1	10.89%	39.80337421	7.66E-05	2.37E-05	1.40E-08
ENSG00000127990_94656098_94588678_435	novel	SGCE	7	-	1305	217	28	12	13.10%	54.94382318	2.62E-08	9.06E-10	2.72E-16
ENSG00000166451_81007268_81011982_20	Overlap_uORF	CENPN	16	+	60	30	9	0	25.00%	165.2112656	0.219194368	0.021084099	0.023589572
ENSG00000168872_70366060_70371986_285	dORF	DDX19A	16	+	855	13	1	4	2.46%	5.023968309	0.016854696	0.049121095	0.003784451
ENSG00000186575_29636751_29683055_523	annotated	NF2	22	+	1569	60	2	2	4.78%	12.63566085	8.64E-06	1.66E-05	1.16E-09
ENSG00000167186_19067665_19074079_136	annotated	COQ7	16	+	408	23	3	0	5.88%	18.62676033	0.021542864	0.005655336	0.000637701
ENSG00000167186_19075756_19078158_83	dORF	COQ7	16	+	249	25	0	0	9.64%	33.17495292	0.005355751	0.005355751	0.00015367
ENSG00000088986_120496458_120498210_77	annotated	DYNLL1	12	+	231	491	158	16	31.17%	702.3266787	6.85E-05	1.81E-05	9.72E-09
ENSG00000155906_151445727_151408766_416	annotated	RMND1	6	-	1248	16	3	0	1.68%	4.236186296	0.041265601	0.008473674	0.001772006
ENSG00000101266_489830_483961_167	annotated	CSNK2A1	20	-	501	47	1	0	8.38%	30.99772248	0.000475338	0.000472567	1.47E-06
ENSG00000144597_15434439_15439155_126	dORF	EAF1	3	+	378	21	0	0	6.35%	18.35680728	0.005355751	0.005355751	0.00015367
ENSG00000163882_184365001_184368294_114	novel	POLR2H	3	+	342	90	9	6	13.16%	86.95329766	0.002041515	0.001799508	2.16E-05
ENSG00000102317_48575181_48575814_118	novel	RBM3	X	+	354	252	146	13	20.34%	235.2160391	0.00041065	1.51E-05	5.30E-08
ENSG00000204386_31862776_31860173_267	annotated	NEU1	6	-	801	169	18	1	11.99%	69.71461643	1.65E-05	5.37E-07	8.59E-11
ENSG00000204386_31860464_31859719_129	Overlap_dORF	NEU1	6	-	387	70	2	0	8.53%	59.7663493	0.001610998	0.001610998	1.55E-05
ENSG00000172428_240133981_240131051_28	novel	COPS9	2	-	84	107	16	3	39.29%	420.895367	0.006678602	0.003368922	0.000123485
ENSG00000164073_127957519_127932981_243	novel	MFSD8	4	-	729	12	0	0	2.88%	5.43905401	0.008175611	0.008175611	0.000342518
ENSG00000168818_4541964_4416149_308	annotated	STX18	4	-	924	23	4	5	3.25%	8.224803264	0.017185125	0.036555921	0.002861401
ENSG00000068745_48715403_48695182_83	internal	IP6K2	3	-	249	19	9	3	7.23%	25.21296422	0.198071955	0.037440157	0.031478411
ENSG00000108669_78698936_78676091_204	annotated	CYTH1	17	-	612	15	0	1	2.45%	8.098591449	0.020613417	0.035374934	0.003252526
ENSG00000134138_37098019_36894754_337	annotated	MEIS2	15	-	1011	7	0	2	1.48%	2.287792006	0.019216965	0.119134635	0.010787784
ENSG00000198911_41833271_41880909_684	annotated	SREBF2	22	+	2052	109	13	4	5.70%	17.55168416	1.81E-06	2.77E-08	5.53E-13
ENSG00000198911_41900370_41905660_215	dORF	SREBF2	22	+	645	41	2	1	3.72%	21.00360275	0.005604867	0.006307834	0.00018747
ENSG00000198911_41884880_41899396_273	dORF	SREBF2	22	+	819	77	12	0	6.96%	31.06536617	0.000797741	6.15E-05	3.76E-07
ENSG00000038210_25252380_25277187_372	annotated	PI4K2B	4	+	1116	19	2	0	1.88%	5.6254732	0.037441831	0.008175611	0.001552076
ENSG00000104064_50309723_50286020_335	annotated	GABPB1	15	-	1005	34	7	0	3.28%	11.17847369	0.026610517	0.001565547	0.000274008
ENSG00000143486_206612342_206605466_139	novel	EIF2D	1	-	417	10	0	1	3.60%	7.923801705	0.016947427	0.047790352	0.003697512
ENSG00000213689_48467073_48467600_175	annotated	TREX1	3	+	525	19	1	0	5.14%	11.95814875	0.003478238	0.003606289	7.00E-05
ENSG00000123472_46643223_46633765_55	annotated	ATPAF1	1	-	165	28	0	0	9.09%	56.07170225	0.021084099	0.021084099	0.002029891
ENSG00000169919_65982183_65960897_651	annotated	GUSB	7	-	1953	202	17	6	10.45%	34.17580711	3.36E-10	2.75E-12	1.26E-20
ENSG00000145431_156970903_156763090_345	annotated	PDGFC	4	-	1035	37	2	0	2.90%	11.81220643	0.002335453	0.002418183	3.27E-05
ENSG00000103121_80997394_80980808_74	annotated	CMC2	16	-	222	33	11	4	10.81%	49.11686273	0.062400078	0.010275151	0.003233385
ENSG00000198055_177426846_177441790_576	annotated	GRK6	5	+	1728	20	5	0	1.74%	3.824334851	0.032024697	0.002201184	0.000445053
ENSG00000150459_21140610_21147342_153	annotated	SAP18	13	+	459	153	13	1	20.26%	110.1408437	7.63E-06	6.27E-07	4.42E-11
ENSG00000115355_55322644_55291711_808	novel	CCDC88A	2	-	2424	36	2	0	2.23%	4.907265314	0.000311843	8.27E-05	1.86E-07
ENSG00000284952_155802558_155763517_245	dORF	AC104472.3	3	-	735	29	2	0	5.71%	13.03707946	0.000673856	0.000405548	1.79E-06
ENSG00000284952_155842476_155828210_243	dORF	AC104472.3	3	-	729	22	3	0	4.94%	9.971599019	0.006392297	0.000943084	3.78E-05
ENSG00000284952_155853997_155842717_351	annotated	AC104472.3	3	-	1053	62	9	1	6.84%	19.45507781	0.000429561	9.85E-06	3.83E-08
ENSG00000026025_17230663_17234854_155	novel	VIM	10	+	465	569	81	41	34.19%	404.3234843	1.05E-09	1.96E-09	2.26E-17
ENSG00000129810_20184027_20161105_561	annotated	SGO1	3	-	1683	24	4	6	2.14%	4.711907752	0.009899987	0.020641078	0.000998683
ENSG00000140577_90530072_90642140_618	annotated	CRTC3	15	+	1854	20	1	2	1.78%	3.564428599	0.002415484	0.005935135	8.11E-05
ENSG00000114982_96619403_96595611_698	dORF	KANSL3	2	-	2094	31	2	0	1.72%	4.891642056	0.003341819	0.001011481	2.06E-05
ENSG00000184432_139371787_139357863_174	annotated	COPB2	3	-	522	20	0	0	4.02%	12.65986709	0.008175611	0.008175611	0.000342518
ENSG00000072042_67695703_67678321_248	annotated	RDH11	14	-	744	485	76	15	27.42%	215.3964081	3.35E-12	4.83E-13	2.11E-23
ENSG00000059691_151760970_151703784_353	annotated	GATB	4	-	1059	15	2	4	2.55%	4.680205823	0.010960145	0.031072489	0.001643991
ENSG00000186001_197791279_197880715_803	annotated	LRCH3	3	+	2409	11	1	3	1.00%	1.508778681	0.010499663	0.043303664	0.002229801
ENSG00000075785_128795368_128813422_98	annotated	RAB7A	3	+	294	133	13	4	24.49%	149.4768593	8.73E-06	1.18E-05	8.38E-10
ENSG00000135930_232556424_232556534_36	novel	EIF4E2	2	+	108	19	1	9	19.44%	58.12988973	0.013892891	0.185427026	0.01431233
ENSG00000114744_149751405_149741521_124	novel	COMMD2	3	-	372	90	9	0	13.71%	79.94093495	0.000574758	0.000128958	5.23E-07
ENSG00000198001_43768112_43786715_460	annotated	IRAK4	12	+	1380	19	1	0	1.74%	4.549295718	0.009341698	0.005355751	0.000262679
ENSG00000138382_169824597_169811820_164	annotated	METTL5	2	-	492	26	2	7	7.32%	17.46135327	0.004389438	0.01237601	0.000290171
ENSG00000125695_63707342_63703599_212	dORF	AC046185.1	17	-	636	10	0	1	2.36%	5.195322816	0.020613417	0.035374934	0.003252526
ENSG00000118777_88139995_88092356_611	annotated	ABCG2	4	-	1833	26	1	4	1.64%	4.686844413	0.003172793	0.012236839	0.000215229
ENSG00000006744_12995061_12992818_223	novel	ELAC2	17	-	669	15	2	0	3.14%	7.408576931	0.031982531	0.008175611	0.001316552
ENSG00000198721_4133671_4115874_364	annotated	ECI2	6	-	1092	82	15	4	5.77%	24.81194831	5.72E-05	3.18E-05	1.39E-08
ENSG00000159176_201496303_201483990_153	annotated	CSRP1	1	-	459	365	39	2	17.65%	262.7543003	6.38E-06	2.67E-06	1.45E-10
ENSG00000106638_73574098_73570507_352	novel	TBL2	7	-	1056	64	2	0	6.82%	20.02560795	2.67E-05	7.40E-06	1.61E-09
ENSG00000196372_5666551_5642549_173	annotated	ASB13	10	-	519	11	1	4	4.62%	7.003175033	0.009847127	0.072063517	0.003660758
ENSG00000175166_184299267_184301786_164	novel	PSMD2	3	+	492	29	2	0	8.54%	19.4761248	0.001083027	0.00040306	2.84E-06
ENSG00000103222_15949752_16141281_1531	annotated	ABCC1	16	+	4593	510	62	9	8.95%	36.68963442	7.17E-18	3.81E-24	8.20E-40

ENSG00000112561_41690695_41684599_331	annotated	TFEB	6	-	993	13	0	0	1.81%	4.325773318	0.012798403	0.012798403	0.000797244
ENSG00000101052_43594780_43646983_410	annotated	IFT52	20	+	1230	44	1	2	3.90%	11.81999298	0.000277134	0.000875286	1.62E-06
ENSG00000131459_180353217_180331477_138	novel	GFPT2	5	-	414	7	0	1	3.62%	5.586854391	0.016947427	0.047790352	0.003697512
ENSG00000169446_135973675_135965024_131	annotated	MMGT1	X	-	393	70	6	3	12.98%	58.85388595	0.000164856	0.000253805	2.90E-07
ENSG00000155957_66138145_66137960_61	dORF	TMBIM4	12	-	183	61	3	0	26.23%	110.1408437	0.000219587	0.00020982	3.18E-07
ENSG00000113734_173144546_173163921_271	annotated	BNIP1	5	+	813	27	5	1	4.43%	10.97344199	0.012305139	0.000982952	7.91E-05
ENSG00000198561_57791641_57816308_879	annotated	CTNND1	11	+	2637	204	34	13	6.60%	25.56169752	2.72E-07	1.92E-09	6.36E-15
ENSG00000113384_32174034_32173753_93	annotated	GOLPH3	5	-	279	23	0	0	6.45%	27.23913339	0.013428348	0.013428348	0.000872559
ENSG00000120889_23068894_23022671_440	annotated	TNFRSF10B	8	-	1320	319	30	6	16.14%	79.85211169	4.07E-10	4.26E-13	2.71E-21
ENSG00000115504_62707273_63045500_1133	annotated	EHBP1	2	+	3399	79	14	0	3.44%	7.679723436	6.05E-06	1.65E-08	1.27E-12
ENSG00000136111_75349290_75286792_861	annotated	TBC1D4	13	-	2583	21	1	1	1.39%	2.686362042	0.00117686	0.00117686	8.49E-06
ENSG00000171453_43517110_43521300_296	annotated	POLR1C	6	+	888	56	4	9	6.08%	20.83745692	0.000339726	0.00231672	5.30E-06
ENSG00000117408_43947601_43967682_963	annotated	IPO13	1	+	2889	30	1	0	1.56%	3.431178932	0.000311218	0.000255448	5.40E-07
ENSG00000097007_130714320_130885683_1149	annotated	ABL1	9	+	3447	35	15	4	1.65%	3.355030052	0.015148384	0.001044465	0.000104576
ENSG00000156976_186787531_186787886_45	novel	EIF4A2	3	+	135	31	0	1	17.78%	75.87480344	0.005454749	0.008219047	0.00023581
ENSG00000156831_125363239_125366885_66	novel	NSMCE2	8	+	198	39	0	0	15.15%	65.08322583	0.002404282	0.002404282	3.34E-05
ENSG00000115239_53750918_53604830_411	annotated	ASB3	2	-	1233	10	0	0	2.19%	2.679825881	0.001946209	0.001946209	2.23E-05
ENSG00000165997_18659638_18675216_179	annotated	ARL5B	10	+	537	46	4	3	9.50%	28.3043509	0.000248273	0.000385725	6.48E-07
ENSG00000083099_89638716_89621838_98	annotated	LYRM2	6	-	294	39	12	0	7.14%	43.83156025	0.044329616	0.008877961	0.001987274
ENSG00000179222_51895191_51901930_717	annotated	MAGED1	X	+	2151	84	11	10	4.32%	12.90352981	4.85E-06	2.82E-05	1.14E-09
ENSG00000104529_143586845_143579792_257	annotated	EEF1D	8	-	771	364	49	6	15.95%	155.9971483	7.97E-08	2.99E-08	2.38E-14
ENSG00000175224_46645957_46672332_401	annotated	ATG13	11	+	1203	60	13	4	6.48%	16.47992674	0.000152942	3.01E-05	3.53E-08
ENSG00000132589_28897574_28880561_428	annotated	FLOT2	17	-	1284	206	13	7	13.55%	53.01171449	2.85E-10	5.03E-11	1.70E-19
ENSG00000170631_144946206_144930738_682	annotated	ZNF16	8	-	2046	13	0	0	0.88%	2.099458898	0.012798403	0.012798403	0.000797244
ENSG00000132589_28897574_28880561_483	annotated	FLOT2	17	-	1449	208	13	7	12.01%	47.4312536	2.68E-10	4.95E-11	1.57E-19
ENSG00000110911_51010704_50988325_553	annotated	SLC11A2	12	-	1659	131	12	5	8.68%	26.09123061	1.79E-08	7.17E-09	1.34E-15
ENSG00000148719_72354897_72335810_375	annotated	DNAJB12	10	-	1125	29	11	1	4.00%	8.51755858	0.045378906	0.000432054	0.000191401
ENSG00000149476_61334729_61346503_575	annotated	TKFC	11	+	1725	40	7	1	2.78%	7.661971736	0.003318537	0.000263972	6.20E-06
ENSG00000167536_28902678_28898441_327	annotated	DHRS13	17	-	981	20	0	0	2.45%	6.73644304	0.005554612	0.005554612	0.000164685
ENSG00000006530_141555555_141652924_394	annotated	AGK	7	+	1182	36	4	0	3.55%	10.06363039	0.000595214	0.000444132	1.72E-06
ENSG00000196628_55350976_55228225_534	annotated	TCF4	18	-	1602	24	0	0	1.87%	4.950150279	0.002404282	0.002404282	3.34E-05
ENSG00000172775_57173854_57154290_254	annotated	FAM192A	16	-	762	69	11	0	9.06%	29.92015046	1.55E-05	1.15E-05	1.44E-09
ENSG00000149925_30068931_30070212_200	novel	ALDOA	16	+	600	1139	150	52	37.50%	627.2521049	1.58E-13	2.65E-14	5.71E-26
ENSG00000149925_30067255_30067724_128	novel	ALDOA	16	+	384	426	59	29	29.69%	366.5624955	1.34E-06	1.33E-07	1.73E-12
ENSG00000198363_61714371_61626273_313	annotated	ASPH	8	-	939	1753	177	105	41.53%	616.8591023	1.21E-20	4.32E-19	8.83E-38
ENSG00000175063_45812696_45816767_179	annotated	UBE2C	20	+	537	409	36	6	22.35%	251.6625982	7.33E-08	4.08E-08	2.97E-14
ENSG00000066629_99865525_99940112_747	annotated	EML1	14	+	2241	36	8	1	2.68%	5.307992468	0.002024956	5.01E-05	8.63E-07
ENSG00000104738_47961145_47976778_742	annotated	MCM4	8	+	2226	199	20	9	8.49%	29.53912116	1.14E-10	1.03E-10	1.36E-19
ENSG00000167767_52191902_52171398_452	annotated	KRT80	12	-	1356	120	20	13	6.64%	29.24093196	0.000203383	2.58E-05	4.10E-08
ENSG00000169764_63856320_63862849_76	annotated	UGP2	2	+	228	14	2	2	7.89%	20.28910279	0.035350572	0.02385744	0.003702948
ENSG00000174775_534322_532636_189	annotated	HRAS	11	-	567	31	3	1	5.82%	18.06542939	0.007459848	0.002467336	0.000103961
ENSG00000143870_10819289_10784258_439	annotated	PDIA6	2	-	1317	1963	268	64	37.13%	492.4976679	4.56E-21	3.71E-27	4.91E-46
ENSG00000138182_89705295_89774048_1780	annotated	KIF20B	10	+	5340	65	9	6	1.85%	4.021997102	2.43E-05	6.05E-06	1.21E-09
ENSG00000074696_65530632_65551852_87	novel	HACD3	15	+	261	79	18	0	12.64%	100.01295	0.004853548	0.001626321	4.62E-05
ENSG00000175482_67352764_67351995_32	annotated	POLD4	11	-	96	25	5	0	21.88%	86.04753414	0.101636223	0.008473674	0.004825296
ENSG00000175482_67351998_67351834_54	Overlap_dORF	POLD4	11	-	162	7	1	1	9.26%	14.27751678	0.041782825	0.041782825	0.007200521
ENSG00000162337_68445591_68449070_151	annotated	LRP5	11	+	453	36	5	1	7.28%	26.25874419	0.009008962	0.001753471	9.32E-05
ENSG00000095066_12773029_12763282_646	annotated	HOOK2	19	-	1938	14	2	2	0.77%	2.386953269	0.045845141	0.044040756	0.008228605
ENSG00000283782_132579369_132642364_1173	annotated	AC116366.3	5	+	3519	98	19	15	3.15%	9.20187782	9.35E-05	3.52E-05	2.48E-08
ENSG00000110321_10803982_10797816_701	annotated	EIF4G2	11	-	2103	317	60	7	10.27%	49.80691506	1.99E-08	3.97E-13	1.82E-19
ENSG00000110321_10804047_10803521_72	internal	EIF4G2	11	-	216	25	2	11	12.50%	38.24334851	0.007312901	0.082500581	0.003382551
ENSG00000110321_10805979_10805911_22	internal	EIF4G2	11	-	66	22	0	12	27.27%	110.1408437	0.013853925	0.14651463	0.010719976
ENSG00000112715_43777480_43784562_166	annotated	VEGFA	6	+	498	40	1	0	6.02%	26.53996234	0.002851	0.002488365	4.06E-05
ENSG00000067596_43484056_43526598_1175	annotated	DHX8	17	+	3525	46	3	10	1.96%	4.31189686	6.94E-05	0.001694812	9.39E-07
ENSG00000115159_156557547_156582918_102	Overlap_dORF	GPD2	2	+	306	7	0	0	5.88%	7.558685352	0.009815329	0.009815329	0.000483714
ENSG00000115159_156496044_156579091_345	annotated	GPD2	2	+	1035	20	3	0	2.90%	6.384976447	0.014216764	0.002227676	0.000185111
ENSG00000165119_83978252_83969350_463	annotated	HNRNPK	9	-	1389	438	46	38	7.34%	104.1937139	6.12E-06	4.02E-06	2.07E-10
ENSG00000130177_114235085_114272443_620	annotated	CDC16	13	+	1860	58	7	1	3.87%	10.30349828	0.000138056	1.01E-05	1.16E-08
ENSG00000196636_97136411_97181215_86	Overlap_dORF	SDHAF3	7	+	258	19	2	0	6.98%	24.33344221	0.035772986	0.01300724	0.002198223
ENSG00000196636_97117724_97136420_68	annotated	SDHAF3	7	+	204	42	3	4	14.71%	68.02816817	0.006953148	0.012185302	0.000432491
ENSG00000123473_47310319_47251136_1287	annotated	STIL	1	-	3861	26	4	0	1.48%	2.22506755	0.000734429	2.98E-05	1.81E-07
ENSG00000129235_6641083_6643019_123	annotated	TXNDC17	17	+	369	335	39	7	27.64%	299.9770946	6.20E-07	6.46E-07	3.65E-12
ENSG00000241973_20838713_20708047_2044	annotated	PI4KA	22	-	6132	26	11	1	0.68%	1.401008775	0.04407608	0.000675451	0.000258286
ENSG00000103042_58680126_58672092_344	novel	SLC38A7	16	-	1032	23	2	4	4.07%	7.364068038	0.002170039	0.004574513	5.67E-05
ENSG00000168872_70365077_70370684_310	novel	DDX19A	16	+	930	13	1	4	2.26%	4.618809575	0.016854696	0.049121095	0.003784451
ENSG00000114491_124737996_124744084_234	Overlap_dORF	UMPS	3	+	702	50	3	5	7.69%	23.53436831	0.000198996	0.000187962	2.60E-07
ENSG00000114491_124730472_124738033_142	annotated	UMPS	3	+	426	45	5	2	5.63%	34.9037885	0.02623975	0.010487024	0.001334343
ENSG00000233608_238848216_238848698_160	annotated	TWIST2	2	+	480	10	0	0	3.75%	6.883802732	0.012798403	0.012798403	0.000797244

ENSG00000143776_227073973_226994268_906	annotated	CDC42BPA	1	-	2718	30	1	0	1.66%	3.647047805	0.000437499	0.000259634	7.65E-07
ENSG00000131873_101178980_101177388_530	dORF	CHSY1	15	-	1590	19	2	0	2.45%	3.94844534	0.000993131	0.000470745	3.02E-06
ENSG00000133422_30946394_30926803_908	annotated	MORC2	22	-	2724	18	6	1	1.10%	2.183408796	0.067734782	0.003646019	0.001573841
ENSG00000171848_10122799_10129386_389	annotated	RRM2	2	+	1167	165	29	11	9.25%	46.71783859	8.86E-06	1.86E-06	1.43E-10
ENSG00000196526_7872078_7763765_730	annotated	AFAP1	4	-	2190	39	16	3	3.01%	5.884236855	0.00845398	0.000112305	8.62E-06
ENSG00000197323_114511076_114397648_1127	annotated	TRIM33	1	-	3381	19	2	1	0.89%	1.856855395	0.01169877	0.003974283	0.000250929
ENSG00000105793_90346742_90385154_308	annotated	GTPBP10	7	+	924	42	3	0	3.90%	15.01920596	0.001245083	0.001053132	8.06E-06
ENSG00000075340_70696385_70672902_532	annotated	ADD2	2	-	1596	16	0	0	2.26%	3.312506578	0.000657764	0.000657764	2.77E-06
ENSG00000198242_28722772_28723881_70	annotated	RPL23A	17	+	210	154	58	13	14.29%	242.3098562	0.005285794	0.002474239	7.38E-05
ENSG00000205250_67192228_67193171_135	novel	E2F4	16	+	405	15	1	2	5.19%	12.23787152	0.013892891	0.023197249	0.001517103
ENSG00000102931_57246138_57252373_141	annotated	ARL2BP	16	+	423	29	7	3	8.51%	22.65308133	0.016891704	0.006005867	0.000524491
ENSG00000103356_23557343_23525127_534	annotated	EARS2	16	-	1602	46	6	1	4.12%	9.487788035	9.19E-05	1.49E-05	1.09E-08
ENSG00000127837_218265460_218264533_106	novel	AAMP	2	-	318	36	12	0	15.09%	37.40632428	0.01136486	0.000163661	1.65E-05
ENSG00000127837_218270086_218266420_233	novel	AAMP	2	-	699	71	7	1	8.15%	33.56223134	0.000352809	6.99E-05	1.81E-07
ENSG00000170035_180982043_181062888_207	annotated	UBE2E3	2	+	621	16	1	1	2.90%	8.513301929	0.01300724	0.023878474	0.001470914
ENSG00000143891_38666162_38733565_194	annotated	GALM	2	+	582	11	5	0	3.09%	6.245099385	0.151766435	0.012798403	0.01055561
ENSG00000187514_231710179_231712851_136	annotated	PTMA	2	+	408	502	62	23	7.35%	406.5492907	0.002531016	0.004962743	7.08E-05
ENSG00000131981_55137374_55145271_250	annotated	LGALS3	14	+	750	76	9	0	5.60%	33.48281649	0.002710732	0.000469808	8.33E-06
ENSG00000143353_219193095_219193274_59	novel	LYPLAL1	1	+	177	10	0	0	8.47%	18.66793961	0.020613417	0.020613417	0.001946209
ENSG00000143353_219173891_219193126_93	novel	LYPLAL1	1	+	279	15	5	2	7.53%	17.76465221	0.069273208	0.023197249	0.007028754
ENSG00000105221_40255258_40234872_450	annotated	AKT2	19	-	1350	67	3	6	7.11%	16.39874784	1.79E-06	3.62E-06	5.61E-11
ENSG00000140521_89327312_89316751_810	Overlap_dORF	POLG	15	-	2430	28	0	2	1.48%	3.807337807	0.000866459	0.005112072	2.78E-05
ENSG00000120690_40982000_40933425_601	annotated	ELF1	13	-	1803	60	2	7	4.49%	10.99575811	2.97E-06	7.24E-05	1.95E-09
ENSG00000167302_81239033_81228928_525	annotated	TEPSIN	17	-	1575	7	0	2	0.95%	1.468544583	0.019216965	0.119134635	0.010787784
ENSG00000104915_13150173_13144583_220	annotated	STX10	19	-	660	28	1	12	5.00%	14.01792556	0.002361478	0.12093302	0.002361276
ENSG00000269190_38950319_38942608_278	annotated	FBXO17	19	-	834	38	3	16	4.32%	15.05522324	0.004244964	0.150736671	0.004775151
ENSG00000109113_28717266_28714643_259	annotated	RAB34	17	-	777	242	36	26	13.13%	102.9115219	2.06E-05	4.79E-05	7.66E-09
ENSG00000167528_48348045_48342973_409	annotated	ZNF641	12	-	1227	11	2	4	1.47%	2.96222318	0.061356297	0.100002646	0.022880377
ENSG00000071859_154444326_154449026_209	novel	FAM50A	X	+	627	19	3	2	4.31%	10.01280397	0.021136549	0.015046252	0.001490789
ENSG00000133138_106802908_106827394_401	annotated	TBC1D8B	X	+	1203	7	0	0	1.25%	1.92265812	0.019216965	0.019216965	0.001707396
ENSG00000124155_45416169_45420616_179	annotated	PIGT	20	+	537	85	48	8	8.94%	52.30151796	0.414287003	0.002735537	0.01711358
ENSG00000130669_39169554_39178579_591	annotated	PAK4	19	+	1773	150	3	6	6.09%	27.95452886	7.81E-08	2.67E-07	2.03E-13
ENSG00000126858_32171069_32224733_597	annotated	RHOT1	17	+	1791	55	7	2	4.36%	10.1469789	0.000124051	1.23E-05	1.24E-08
ENSG00000196497_24182388_24180442_252	Overlap_dORF	IPO4	14	-	756	35	1	0	4.37%	15.2973394	0.001610998	0.001588175	1.53E-05
ENSG00000196497_24188787_24182046_911	annotated	IPO4	14	-	2733	71	21	7	3.51%	8.583973549	0.001558861	1.24E-05	1.98E-07
ENSG00000125779_3908189_3923294_273	annotated	PANK2	20	+	819	25	1	2	4.76%	10.08615785	0.001035025	0.002773194	1.74E-05
ENSG00000108671_32444524_32477616_314	novel	PSMD11	17	+	942	132	28	0	11.46%	46.3012464	3.40E-05	7.39E-08	3.23E-11
ENSG00000114786_51984156_51986623_162	dORF	ABHD14A-ACY1	3	+	486	17	0	0	5.56%	11.55798977	0.003303074	0.003303074	6.12E-05
ENSG00000114786_51987035_51989075_198	dORF	ABHD14A-ACY1	3	+	594	46	3	4	8.08%	25.58827682	0.00030684	0.001054241	2.15E-06
ENSG00000114786_51975136_51984153_167	annotated	ABHD14A-ACY1	3	+	501	9	0	0	3.59%	5.935734092	0.009815329	0.009815329	0.000483714
ENSG00000131473_41913873_41867810_1091	annotated	ACLY	17	-	3273	751	110	42	17.78%	75.81647445	2.55E-24	1.93E-30	1.44E-52
ENSG00000136854_127651608_127695016_560	annotated	STXBP1	9	+	1680	60	6	0	4.29%	11.80080468	0.000136443	7.48E-06	8.76E-09
ENSG00000114770_183989320_183921300_1373	annotated	ABCC5	3	-	4119	18	4	4	0.51%	1.443944054	0.066604992	0.083878283	0.020815868
ENSG00000101160_59006328_58997599_113	novel	CTSZ	20	-	339	192	17	9	27.43%	187.1419645	4.06E-06	5.40E-07	2.01E-11
ENSG00000130717_131530624_131529072_144	novel	UCK1	9	-	432	28	1	1	6.25%	21.41627516	0.003684287	0.005109528	0.000103252
ENSG00000214113_5260733_5138734_70	annotated	LYRM4	6	-	210	30	4	3	12.86%	47.20321873	0.012008015	0.012673659	0.000744064
ENSG00000163479_156020167_156009540_183	annotated	SSR2	1	-	549	1851	78	41	36.07%	1114.04755	8.08E-12	1.19E-12	1.23E-22
ENSG00000010256_48600702_48599447_153	novel	UQCRC1	3	-	459	126	36	0	12.42%	90.70422423	0.000360853	6.29E-05	1.68E-07
ENSG00000167173_75206088_75211116_1034	annotated	C15orf39	15	+	3102	13	1	7	0.68%	1.384749486	0.015625009	0.19866071	0.01693883
ENSG00000083807_58498895_58498515_95	novel	SLC27A5	19	-	285	17	0	0	7.37%	19.70941414	0.007882058	0.007882058	0.000319636
ENSG00000167257_117244571_117285151_435	annotated	RNF214	11	+	1305	11	6	2	1.38%	2.78517076	0.143514872	0.042234516	0.024258209
ENSG00000239672_51155655_51161118_82	annotated	NME1	17	+	246	550	89	13	36.59%	738.7495614	3.36E-05	7.04E-06	1.94E-09
ENSG00000239672_51161199_51161276_25	dORF	NME1	17	+	75	248	19	4	52.00%	1092.59717	0.003397452	0.000717317	1.54E-05
ENSG00000111641_6566202_6556993_688	annotated	NOP2	12	-	2064	52	3	4	3.20%	8.324598652	5.87E-05	5.77E-05	2.52E-08
ENSG00000108587_30477434_30484863_146	novel	GOSR1	17	+	438	13	0	0	5.48%	9.807061426	0.004826937	0.004826937	0.000126125
ENSG00000115866_135911466_135911062_77	novel	DARS	2	-	231	13	1	2	9.09%	18.59520738	0.013794764	0.020159861	0.001316157
ENSG00000160695_119073887_119078424_279	novel	VPS11	11	+	837	6	0	0	1.79%	2.368620295	0.016947427	0.016947427	0.001349898
ENSG00000175727_122078872_122141812_913	annotated	MLXIP	12	+	2739	23	6	1	1.64%	2.774632426	0.007647443	0.000410731	2.25E-05
ENSG00000145242_65669742_65331969_1004	annotated	EPHA5	4	-	3012	40	10	1	1.99%	4.388081423	0.002350966	5.31E-05	1.07E-06
ENSG00000130475_17776253_17788306_493	annotated	FCHO1	19	+	1479	30	4	1	2.84%	6.702282578	0.004367894	0.000335351	1.03E-05
ENSG00000169169_49713020_49713605_76	annotated	CPT1C	19	+	228	9	0	0	6.58%	13.04299465	0.020613417	0.020613417	0.001946209
ENSG00000120727_139363785_139364770_114	novel	PAIP2	5	+	342	151	17	2	21.05%	145.8883105	9.73E-05	1.87E-05	1.43E-08
ENSG00000109381_139084190_139058983_521	annotated	ELF2	4	-	1563	15	2	2	2.30%	3.171041565	0.006096424	0.006096424	0.000196497
ENSG00000220205_8161754_8160855_71	annotated	VAMP2	17	-	213	34	3	0	14.08%	52.74350262	0.002335453	0.002418183	3.27E-05
ENSG00000164144_152829634_152910219_193	annotated	ARFIP1	4	+	579	27	0	0	5.18%	15.40830456	0.002201184	0.002201184	2.82E-05
ENSG00000046647_14020447_14014307_199	novel	GEMIN8	X	-	597	9	0	0	2.51%	4.981244188	0.020613417	0.020613417	0.001946209
ENSG00000167965_2206086_2206749_116	annotated	MLST8	16	+	348	47	3	0	9.48%	44.6260315	0.00368578	0.001580611	3.42E-05
ENSG00000240857_18555727_18555191_178	novel	RDH14	2	-	534	46	8	3	8.99%	28.4633641	0.002272554	0.001187135	1.62E-05

ENSG00000184640_77402556_77482565_189	annotated	9-Sep	17	+	567	16	4	0	4.23%	9.324092589	0.043967804	0.005257623	0.001282071
ENSG00000091129_108160490_108156442_71	novel	NRCAM	7	-	213	7	0	0	8.45%	10.85895642	0.009815329	0.009815329	0.000483714
ENSG00000106028_141739167_141750354_148	annotated	SSBP1	7	+	444	456	23	8	32.43%	339.3528698	6.91E-10	1.38E-09	1.06E-17
ENSG00000176261_32634005_32621862_104	annotated	ZBTB8OS	1	-	312	9	0	0	4.81%	9.531419167	0.019679754	0.019679754	0.001784964
ENSG00000185608_19432555_19435962_206	annotated	MRPL40	22	+	618	88	2	3	13.11%	47.05045751	2.86E-06	2.81E-06	6.90E-11
ENSG00000183963_31083259_31101029_915	annotated	SMTN	22	+	2745	43	10	1	2.62%	5.176017792	0.000493212	9.90E-06	4.47E-08
ENSG00000120705_138542918_138542829_29	uORF	ETF1	5	-	87	19	4	2	20.69%	72.16124243	0.029614852	0.013428348	0.00187062
ENSG00000120705_138518803_138508305_387	annotated	ETF1	5	-	1161	129	14	7	8.53%	36.71361457	9.05E-06	2.01E-06	1.57E-10
ENSG00000138758_77015012_77020686_95	novel	11-Sep	4	+	285	14	4	0	6.32%	16.23128223	0.042234516	0.011772051	0.002392043
ENSG00000276410_26043581_26043519_20	internal	HIST1H2BB	6	-	60	23	12	9	25.00%	126.6619703	0.065398531	0.124431937	0.029796782
ENSG00000276410_26043480_26043277_67	annotated	HIST1H2BB	6	-	201	1107	33	23	34.33%	1819.789761	0.000117476	1.23E-05	1.17E-08
ENSG00000198246_71351617_71362608_329	annotated	SLC29A3	10	+	987	18	0	4	2.74%	6.025942817	0.003529166	0.032126751	0.000655766
ENSG00000130772_28330663_28334970_208	novel	MED18	1	+	624	28	0	1	5.77%	14.82665204	0.000991051	0.001195657	7.31E-06
ENSG00000204217_202513820_202559946_865	Overlap_dORF	BMPR2	2	+	2595	62	1	0	2.77%	7.894488219	6.69E-06	6.69E-06	3.70E-10
ENSG00000135365_46084219_45933968_634	annotated	PHF21A	11	-	1902	18	2	0	1.26%	3.127027108	0.023197249	0.004826937	0.000601035
ENSG00000146007_140700461_140705756_199	annotated	ZMAT2	5	+	597	124	43	9	17.59%	68.63047547	0.000133115	2.79E-06	3.53E-09
ENSG00000135503_51975357_51994110_444	annotated	ACVR1B	12	+	1332	27	5	5	2.93%	6.697754009	0.006483832	0.011542368	0.000384826
ENSG00000163931_53235113_53225756_457	annotated	TKT	3	-	1371	1098	129	30	27.13%	264.627235	1.59E-18	3.30E-22	1.06E-38
ENSG00000163931_53255942_53235065_145	Overlap_uORF	TKT	3	-	435	289	21	88	27.59%	219.5220954	3.17E-07	0.000215632	9.22E-10
ENSG00000232119_120605525_120612264_138	annotated	MCTS1	X	+	414	36	4	3	6.52%	28.73239401	0.007609621	0.01778219	0.000681245
ENSG00000171155_120627166_120626210_318	annotated	C1GALT1C1	X	-	954	158	20	3	12.58%	54.724067	1.66E-06	1.48E-08	2.83E-13
ENSG00000169855_78714439_78598913_1317	annotated	ROBO1	3	-	3951	12	2	1	0.46%	1.003561218	0.050679946	0.019795882	0.004478852
ENSG00000133265_55279608_55262608_359	annotated	HSPBP1	19	-	1077	147	21	3	8.91%	45.09945411	2.24E-06	3.37E-07	7.02E-12
ENSG00000147548_38348138_38275641_1377	annotated	NSD3	8	-	4131	24	3	1	1.02%	1.919666121	0.002438639	0.000671624	1.03E-05
ENSG00000141098_67685709_67675155_383	annotated	GFOD2	16	-	1149	10	0	0	1.83%	2.875740045	0.007593099	0.007593099	0.000297833
ENSG00000068724_46956939_47073923_728	dORF	TTC7A	2	+	2184	34	0	0	1.92%	5.143940503	0.000416874	0.000416874	1.15E-06
ENSG00000111328_123267253_123261736_87	annotated	CDK2AP1	12	-	261	86	18	1	18.39%	108.874857	0.000785347	0.000207945	1.12E-06
ENSG00000011566_39436987_39250618_873	annotated	MAP4K3	2	-	2619	22	10	3	1.37%	2.775599727	0.167014233	0.012970104	0.011976292
ENSG00000138760_76213543_76161713_478	annotated	SCARB2	4	-	1434	354	42	11	17.78%	81.56874199	3.12E-12	1.88E-15	1.00E-25
ENSG00000149761_64226653_64229495_165	annotated	NUDT22	11	+	495	41	3	1	6.67%	27.36833086	0.003783485	0.00140552	3.15E-05
ENSG00000166562_59139949_59155811_156	annotated	SEC11C	18	+	468	123	7	1	18.59%	86.84181908	1.19E-06	1.20E-06	1.26E-11
ENSG00000159921_36246469_36217365_663	annotated	GNE	9	-	1989	89	16	5	5.13%	14.7851208	4.38E-05	1.69E-06	6.84E-10
ENSG00000011105_3201194_3283116_239	annotated	TSPAN9	12	+	717	29	2	0	5.86%	13.36437016	0.000662939	0.000428413	1.85E-06
ENSG00000108582_30445902_30466298_57	annotated	CPD	17	+	171	29	2	0	14.04%	56.0365696	0.005306578	0.005355751	0.00015233
ENSG00000175110_139344162_139349333_124	annotated	MRPS22	3	+	372	26	8	0	12.10%	23.09404787	0.017650719	0.000255448	3.98E-05
ENSG00000083937_87253712_87253822_36	novel	CHMP2B	3	+	108	17	0	0	13.89%	52.01095397	0.021084099	0.021084099	0.002029891
ENSG00000139496_25301774_25338790_540	novel	NUP58	13	+	1620	40	8	4	4.07%	8.158581015	0.000778327	9.81E-05	5.57E-07
ENSG00000165688_136410669_136412261_111	novel	PMPCA	9	+	333	12	4	0	4.50%	11.90711824	0.145920273	0.021084099	0.014551246
ENSG00000239282_30289497_30285620_329	annotated	CASTOR1	22	-	987	12	0	0	2.43%	4.017295211	0.004826937	0.004826937	0.000126125
ENSG00000242372_35284487_35279197_226	annotated	EIF6	20	-	678	243	39	49	16.81%	118.4257744	1.15E-05	7.34E-05	6.81E-09
ENSG00000176697_27658543_27657821_240	annotated	BDNF	11	-	720	54	20	2	10.00%	24.78168983	0.0133993	9.36E-06	2.20E-06
ENSG00000140694_14630125_14551651_524	annotated	PARN	16	-	1572	34	9	6	2.67%	7.146543294	0.004398905	0.004905338	0.000117285
ENSG00000179241_35944099_36229397_296	annotated	LDLRAD3	11	+	888	33	4	1	4.39%	12.27921568	0.002184759	0.00077423	1.05E-05
ENSG00000080822_98521424_98518986_106	annotated	CLDND1	3	-	318	49	4	4	13.21%	50.9141636	0.003265655	0.003265655	5.99E-05
ENSG00000152520_28138658_28272086_687	novel	PAN3	13	+	2061	11	2	3	0.87%	1.763536071	0.060667625	0.071345384	0.016505338
ENSG00000113552_142012035_142002029_289	annotated	GNPDA1	5	-	867	85	4	2	12.11%	32.3943658	1.20E-07	3.33E-07	3.83E-13
ENSG00000259827_56682541_56683277_46	novel	AC026461.1	16	+	138	34	5	0	13.04%	81.4084497	0.013716833	0.013640586	0.000903353
ENSG00000091651_46695593_46697585_92	novel	ORC6	16	+	276	57	2	2	13.04%	68.23943577	0.001087214	0.001082917	7.26E-06
ENSG00000131844_71587426_71656860_563	annotated	MCCC2	5	+	1689	79	15	3	4.97%	15.45493189	0.000646549	2.05E-06	1.58E-08
ENSG00000132463_70836185_70824319_318	annotated	GRSF1	4	-	954	100	11	5	9.12%	34.63548544	3.64E-06	1.43E-05	4.39E-10
ENSG00000145860_159203557_159158670_643	annotated	RNF145	5	-	1929	215	43	6	7.62%	36.82780933	5.09E-07	1.28E-09	8.65E-15
ENSG00000197892_29191027_29070504_1762	annotated	KIF13B	8	-	5286	42	2	5	1.19%	2.625377659	9.33E-05	0.000298466	1.99E-07
ENSG00000147155_48527177_48528457_110	novel	EBP	X	+	330	238	12	6	19.09%	238.3047346	0.000633517	0.000171669	7.52E-07
ENSG00000141002_89873668_89889264_216	annotated	TCF25	16	+	648	52	2	6	7.41%	26.5153883	0.000289115	0.001779035	3.48E-06
ENSG00000133657_194462190_194405919_1226	annotated	ATP13A3	3	-	3678	123	5	7	3.92%	11.05001939	2.46E-09	1.68E-08	4.47E-16
ENSG00000125107_58532305_58520958_381	novel	CNOT1	16	-	1143	34	7	4	3.15%	9.828841695	0.013403288	0.005741161	0.000399569
ENSG00000157077_52238054_52346221_1154	annotated	ZFYVE9	1	+	3462	10	0	0	0.43%	0.954426722	0.019679754	0.019679754	0.001784964
ENSG00000168538_183663916_183712622_1070	annotated	TRAPPC11	4	+	3210	44	1	0	1.96%	4.52915619	3.44E-05	2.27E-05	6.06E-09
ENSG00000198825_119781745_119826713_651	annotated	INPP5F	10	+	1953	10	0	0	0.77%	1.691871639	0.020613417	0.020613417	0.001946209
ENSG00000060069_79695268_79753790_842	annotated	CTDP1	18	+	2526	18	0	0	1.19%	2.354554854	0.002046029	0.002046029	2.45E-05
ENSG00000115204_27322517_27309912_176	annotated	MPV17	2	-	528	124	16	2	18.75%	77.59923079	1.09E-05	3.35E-07	3.59E-11
ENSG00000047634_17750071_17753392_169	annotated	SCML1	X	+	507	12	4	0	5.33%	7.820651624	0.044129916	0.002500085	0.000710788
ENSG00000047634_17750071_17753392_169	annotated	SCML1	X	+	507	12	4	0	5.33%	7.820651624	0.044129916	0.002500085	0.000710788
ENSG00000047634_17750071_17753392_169	annotated	SCML1	X	+	507	12	4	0	5.33%	7.820651624	0.044129916	0.002500085	0.000710788
ENSG00000018699_32630585_32820938_793	annotated	TTC27	2	+	2379	29	10	6	2.27%	4.027849265	0.028044637	0.002691831	0.000452045
ENSG00000134330_9481342_9488329_135	annotated	IAH1	2	+	405	99	7	1	20.00%	80.76995205	7.66E-06	2.44E-06	1.60E-10
ENSG00000145779_119392881_119393381_166	annotated	TNFAIP8	5	+	498	28	2	1	7.83%	18.57797364	0.001585582	0.000993237	9.64E-06
ENSG00000163694_40438758_40425904_479	annotated	RBM47	4	-	1437	82	14	2	5.85%	18.85500873	0.000162123	2.01E-06	3.28E-09

ENSG00000163694_40438758_40425904_548	annotated	RBM47	4	-	1644	91	14	2	5.66%	18.28981164	4.50E-05	6.00E-07	2.78E-10
ENSG00000113368_126777539_126836264_576	annotated	LMNB1	5	+	1728	152	19	6	8.68%	29.06494487	3.17E-07	6.04E-10	2.63E-15
ENSG00000250479_23766242_23766007_44	novel	CHCHD10	22	-	132	115	18	0	38.64%	287.8681142	0.00013364	0.000143673	1.36E-07
ENSG00000164933_103414937_103403258_123	annotated	SLC25A32	8	-	369	47	6	0	9.76%	42.08633865	0.00678006	0.00104892	4.41E-05
ENSG00000164933_103403271_103400411_167	Overlap_dORF	SLC25A32	8	-	501	29	5	0	6.59%	19.1262543	0.018110104	0.001484235	0.000170617
ENSG00000198162_117368184_117522957_641	annotated	MAN1A2	1	+	1923	25	2	1	2.03%	4.295664731	0.003005605	0.000956862	1.76E-05
ENSG00000131238_40092463_40074101_274	annotated	PPT1	1	-	822	385	73	8	13.50%	154.7599446	6.40E-05	5.26E-08	4.95E-11
ENSG00000131238_40074162_40074061_33	Overlap_dORF	PPT1	1	-	99	45	1	0	21.21%	150.1920596	0.008877961	0.008877961	0.000400235
ENSG00000126453_49666693_49673777_185	annotated	BCL2L12	19	+	555	25	15	5	4.86%	14.8838978	0.162090171	0.028645552	0.020605139
ENSG00000079150_178478487_178465770_218	annotated	FKBP7	2	-	654	40	3	0	7.34%	20.20932912	0.000329128	0.000129232	2.98E-07
ENSG00000115380_55922416_55922339_25	novel	EFEMP1	2	-	75	12	0	0	20.00%	52.86760498	0.021084099	0.021084099	0.002029891
ENSG00000105063_55247103_55230528_881	annotated	PPP6R1	19	-	2643	56	12	2	2.61%	7.001007091	0.001000609	1.75E-05	1.60E-07
ENSG00000104723_15540431_15743570_276	annotated	TUSC3	8	+	828	173	25	2	19.93%	69.03755783	1.15E-07	5.42E-11	1.00E-16
ENSG00000101361_2655702_2657390_190	annotated	NOP56	20	+	570	35	4	9	9.47%	20.28910279	0.001292475	0.0037625	2.90E-05
ENSG00000101361_2657932_2658294_120	dORF	NOP56	20	+	360	7	3	0	4.17%	6.424882549	0.170087137	0.019216965	0.016246064
ENSG00000136485_63550678_63589172_142	annotated	DCAF7	17	+	426	51	4	0	5.63%	39.55762696	0.022998895	0.005706018	0.000687556
ENSG00000087460_58891727_58899955_87	annotated	GNAS	20	+	261	129	16	7	17.24%	163.3122855	0.000393727	0.002734313	7.18E-06
ENSG00000013561_141978375_141987790_348	annotated	RNF14	5	+	1044	44	5	0	6.03%	13.9258538	0.000275025	2.18E-05	4.81E-08
ENSG00000198130_190319723_190205117_377	annotated	HIBCH	2	-	1131	22	4	0	2.65%	6.427317139	0.038905521	0.002122793	0.000539938
ENSG00000197183_32474637_32447596_412	annotated	NOL4L	20	-	1236	41	5	4	4.13%	10.96061794	0.001229266	0.000754908	5.80E-06
ENSG00000101079_36721735_36653520_363	annotated	NDRG3	20	-	1089	70	11	0	4.41%	21.23928116	0.004490982	0.000179964	6.20E-06
ENSG00000115414_215435697_215361555_2351	annotated	FN1	2	-	7053	339	59	27	5.27%	15.88164441	1.28E-14	1.73E-17	3.78E-30
ENSG00000170776_85620092_85744677_1434	annotated	AKAP13	15	+	4302	21	3	0	0.98%	1.612941226	0.003103981	0.000241223	5.34E-06
ENSG00000115207_27343554_27333630_584	annotated	GTF3C2	2	-	1752	42	5	4	2.74%	7.921088075	0.001712183	0.001750015	1.78E-05
ENSG00000126012_53224889_53192744_1379	annotated	KDM5C	X	-	4137	41	2	1	1.45%	3.274673381	9.34E-05	5.17E-05	3.58E-08
ENSG00000170889_54201185_54206565_139	annotated	RPS9	19	+	417	549	50	14	12.95%	435.0167136	0.000414357	0.000144574	4.17E-07
ENSG00000047579_15627454_15522975_270	dORF	DTNBP1	6	-	810	6	1	0	1.85%	2.447574305	0.047790352	0.016947427	0.003697512
ENSG00000127080_92669988_92615832_491	annotated	IPPK	9	-	1473	26	6	0	2.24%	5.832305369	0.021076725	0.001558047	0.000210082
ENSG00000170791_56214614_56216505_56	annotated	CHCHD7	8	+	168	23	5	3	8.93%	45.23641795	0.021084099	0.044329616	0.004137993
ENSG00000077463_4179264_4175838_106	novel	AC016586.1	19	-	318	17	5	2	5.66%	17.66409758	0.070946758	0.061356297	0.016586142
ENSG00000145348_106262160_106046570_787	annotated	TBCK	4	-	2361	14	0	1	0.89%	1.959303446	0.008473674	0.013892891	0.00058868
ENSG00000088682_57458261_57460624_111	novel	COQ9	16	+	333	68	4	2	19.82%	67.47367002	6.73E-05	2.50E-05	1.29E-08
ENSG00000166479_68713886_68700481_69	annotated	TMX3	18	-	207	23	1	0	8.70%	36.71361457	0.016983117	0.013640586	0.00110551
ENSG00000145354_102869085_102887430_135	annotated	CISD2	4	+	405	73	8	4	11.85%	59.55764141	0.001823113	0.000267881	3.34E-06
ENSG00000134987_111092289_111126883_951	annotated	WDR36	5	+	2853	43	9	6	2.73%	4.98008021	0.000765753	0.000160797	8.58E-07
ENSG00000164180_88206473_88196378_191	annotated	TMEM161B	5	-	573	24	1	0	3.66%	13.83968717	0.008877961	0.008776163	0.000395901
ENSG00000113648_135389012_135369370_143	novel	H2AFY	5	-	429	106	11	3	13.29%	81.64286317	0.00026227	0.000233082	4.18E-07
ENSG00000090316_1332743_1338875_236	novel	MAEA	4	+	708	30	2	0	5.51%	14.00095471	0.002779203	0.000602625	1.07E-05
ENSG00000115825_37293214_37253177_775	annotated	PRKD3	2	-	2325	13	2	2	0.77%	1.84752383	0.044040756	0.044040756	0.007930666
ENSG00000113163_75511207_75356927_584	annotated	COL4A3BP	5	-	1752	20	11	5	1.71%	3.771946702	0.140831635	0.023449432	0.015138951
ENSG00000285171_71110525_71106579_190	novel	AL590764.2	X	-	570	18	0	0	3.68%	10.43439572	0.008674847	0.008674847	0.000383115
ENSG00000117523_171577574_171591659_493	annotated	PRRC2C	1	+	1479	12	0	0	1.22%	2.680913031	0.012798403	0.012798403	0.000797244
ENSG00000115816_37231567_37201764_1054	annotated	CEBPZ	2	-	3162	75	22	3	3.23%	7.837346563	0.000729293	3.07E-07	3.81E-09
ENSG00000124155_45420349_45425826_316	dORF	PIGT	20	+	948	259	20	2	16.46%	90.2736662	6.03E-10	1.31E-10	9.13E-19
ENSG00000124155_45416169_45419301_119	annotated	PIGT	20	+	357	85	3	4	13.45%	78.67203122	0.000473642	0.00126048	3.85E-06
ENSG00000124155_45424206_45425826_170	novel	PIGT	20	+	510	48	3	1	7.65%	31.09859116	0.000723387	0.000723387	3.33E-06
ENSG00000095203_109320446_109174554_900	annotated	EPB41L4B	9	-	2700	12	1	0	0.78%	1.468544583	0.0135992	0.008175611	0.00055803
ENSG00000255508_62591485_62574805_525	annotated	AP002990.1	11	-	1575	17	0	3	1.90%	3.566465415	0.002008257	0.0280616	0.00035605
ENSG00000255508_62571557_62559679_317	dORF	AP002990.1	11	-	951	168	5	0	2.84%	58.37117269	0.003736771	0.003736771	7.75E-05
ENSG00000168610_42348516_42315745_770	annotated	STAT3	17	-	2310	121	21	10	6.36%	17.30784687	3.22E-06	8.43E-08	2.81E-12
ENSG00000108306_39343241_39261460_422	annotated	FBXL20	17	-	1266	22	3	7	2.13%	5.741939719	0.010815624	0.076961241	0.004240805
ENSG00000198160_66946197_66986473_406	annotated	MIER1	1	+	1218	29	2	0	3.94%	7.867203122	0.000269452	0.000169241	3.16E-07
ENSG00000198160_66946197_66986473_406	annotated	MIER1	1	+	1218	29	2	0	3.94%	7.867203122	0.000269452	0.000169241	3.16E-07
ENSG00000198160_66946197_66986473_426	annotated	MIER1	1	+	1278	29	2	0	3.76%	7.497850863	0.000269452	0.000169241	3.16E-07
ENSG00000086189_62403772_62389010_313	annotated	DIMT1	5	-	939	97	16	5	11.18%	34.13310492	1.87E-05	4.78E-07	8.82E-11
ENSG00000184831_23907702_23840342_198	annotated	APOO	X	-	594	23	1	0	5.56%	12.79413841	0.002296044	0.001448134	1.98E-05
ENSG00000271303_653185_648714_137	annotated	SRXN1	20	-	411	64	2	30	10.22%	51.45265691	0.000562699	0.145389157	0.001143649
ENSG00000169299_37826733_37861612_612	annotated	PGM2	4	+	1836	56	7	2	2.61%	10.07824714	0.001928502	0.000365602	4.68E-06
ENSG00000188986_137255510_137272928_580	annotated	NELFB	9	+	1740	109	16	8	7.59%	20.6988827	1.42E-06	2.45E-07	3.27E-12
ENSG00000138709_128074952_128107402_358	annotated	LARP1B	4	+	1074	15	0	0	1.40%	4.61483982	0.020613417	0.020613417	0.001946209
ENSG00000171928_18781294_18805567_205	annotated	TVP23B	17	+	615	44	3	0	3.41%	23.63998597	0.008980239	0.008980239	0.000408987
ENSG00000157500_57240513_57269687_598	annotated	APPL1	3	+	1794	27	3	5	2.01%	4.972914348	0.003911212	0.010454662	0.000220876
ENSG00000172175_58735260_58747842_313	novel	MALT1	18	+	939	6	1	3	1.60%	2.111326077	0.047790352	0.165899918	0.031107886
ENSG00000075089_100200852_100205756_88	annotated	ACTR6	12	+	264	19	15	0	6.82%	23.78040944	0.220042808	0.013640586	0.017565789
ENSG00000075089_100207732_100223915_288	dORF	ACTR6	12	+	864	31	1	0	4.86%	11.85543804	0.000543162	0.000442807	1.57E-06
ENSG00000258830_57283935_57251174_530	dORF	AC137834.1	12	-	1590	9	2	2	1.13%	1.870316214	0.070229091	0.070229091	0.018549462
ENSG00000204977_50011941_50013164_407	annotated	TRIM13	13	+	1221	24	4	0	2.70%	6.494791766	0.015436708	0.001377539	0.000134539
ENSG00000162613_77979004_77948873_655	annotated	FUBP1	1	-	1965	144	13	7	6.72%	24.21417022	1.97E-07	3.25E-08	6.43E-14

ENSG00000163479_156020896_156012527_136	annotated	SSR2	1	-	408	1151	41	15	38.24%	932.1478758	3.64E-09	2.80E-10	1.17E-17
ENSG00000162613_77979004_77948766_644	annotated	FUBP1	1	-	1932	144	13	7	6.83%	24.62776629	1.97E-07	3.25E-08	6.43E-14
ENSG00000165630_13600328_13630340_266	annotated	PRPF18	10	+	798	12	2	0	2.26%	4.968759866	0.043751848	0.013217227	0.002735927
ENSG00000106484_130495369_130505056_292	annotated	MEST	7	+	876	35	3	2	3.77%	13.20181346	0.001604048	0.003848078	3.62E-05
ENSG00000182325_144358447_144355531_533	annotated	FBXL6	8	-	1599	22	9	2	2.81%	4.546151147	0.023055195	0.00065074	0.000114778
ENSG00000160124_122371785_122359869_122	annotated	CCDC58	3	-	366	59	15	0	9.84%	53.26483425	0.00217482	0.001065834	1.41E-05
ENSG00000179889_14997807_15029032_404	annotated	PDXDC1	16	+	1212	15	1	0	2.23%	4.089387761	0.006432154	0.002967235	0.000105925
ENSG00000134899_102861594_102875903_933	dORF	ERCC5	13	+	2799	19	8	4	1.07%	2.242953945	0.088969337	0.028352861	0.010723907
ENSG00000173221_95822662_95816513_106	annotated	GLRX	5	-	318	104	14	3	23.58%	108.0627146	4.51E-05	5.31E-06	2.02E-09
ENSG00000175756_1374756_1373801_199	annotated	AURKAIP1	1	-	597	204	25	18	19.60%	112.9082016	3.76E-07	8.74E-07	3.02E-12
ENSG00000108679_78977191_78972966_210	annotated	LGALS3BP	17	-	630	296	31	14	20.48%	155.2461416	5.03E-08	1.58E-07	7.82E-14
ENSG00000066117_50094513_50099000_112	annotated	SMARCD1	12	+	336	20	0	0	8.04%	19.6680078	0.003606289	0.003606289	7.24E-05
ENSG00000135451_49323609_49324274_68	annotated	TROAP	12	+	204	20	5	3	8.82%	32.3943658	0.028889786	0.013428348	0.001825217
ENSG00000130332_2324131_2321680_49	dORF	LSM7	19	-	147	39	8	1	16.33%	87.6631205	0.022007321	0.005088721	0.000595221
ENSG00000130332_2328566_2324182_35	annotated	LSM7	19	-	105	26	0	6	14.29%	81.81891247	0.021084099	0.117522225	0.011410931
ENSG00000002330_64284187_64271639_56	internal	BAD	11	-	168	13	7	2	12.50%	25.56841015	0.08847975	0.080264768	0.025767574
ENSG00000002330_64284368_64270209_168	annotated	BAD	11	-	504	44	16	8	8.33%	28.84641145	0.047116331	0.005026755	0.001334859
ENSG00000002330_64284368_64271639_163	annotated	BAD	11	-	489	24	10	2	6.75%	16.21705674	0.046211684	0.009288069	0.002156156
ENSG00000127914_92083305_92108719_1158	annotated	AKAP9	7	+	3474	16	6	1	0.69%	1.521807858	0.066994826	0.009288069	0.003223689
ENSG00000114867_184317438_184334908_1511	annotated	EIF4G1	3	+	4533	153	16	14	4.17%	11.15258047	5.88E-10	1.20E-09	7.87E-18
ENSG00000198563_31540510_31538840_110	internal	DDX39B	6	-	330	36	6	102	9.09%	36.0460943	0.007635352	0.516161534	0.045829264
ENSG00000118849_158732415_158704898_122	novel	RARRES1	3	-	366	29	3	5	7.38%	26.18102023	0.013192068	0.01778219	0.00112011
ENSG00000173436_19597046_19625592_140	novel	MICOS10	1	+	420	161	25	8	15.71%	126.6619703	0.00038241	2.55E-05	7.87E-08
ENSG00000100439_22601716_22603766_138	novel	ABHD4	14	+	414	42	3	0	7.97%	33.52112635	0.003594759	0.001565547	3.31E-05
ENSG00000075415_98593979_98601528_361	annotated	SLC25A3	12	+	1083	940	121	22	29.92%	286.7933326	3.55E-15	4.79E-18	2.94E-31
ENSG00000115211_27369534_27364400_493	annotated	EIF2B4	2	-	1479	88	19	9	6.29%	19.6600289	0.000528117	3.92E-05	1.62E-07
ENSG00000169908_149377547_149371829_101	annotated	TM4SF1	3	-	303	1110	544	78	43.56%	1210.458777	0.002487049	5.23E-08	4.55E-09
ENSG00000169908_149375705_149369866_73	Overlap_dORF	TM4SF1	3	-	219	993	118	60	36.99%	1498.21723	2.71E-05	0.000154537	3.24E-08
ENSG00000144815_101801367_101821954_484	annotated	NXPE3	3	+	1452	19	28	0	1.86%	4.323710807	0.359651006	0.003303074	0.014826993
ENSG00000119650_75985787_76058768_113	annotated	IFT43	14	+	339	52	0	3	11.50%	50.68428206	0.00064467	0.001563962	6.35E-06
ENSG00000213983_23567314_23566417_68	novel	AP1G2	14	-	204	10	1	0	8.82%	16.1971829	0.011772051	0.011772051	0.000681273
ENSG00000075413_103475139_103503227_326	annotated	MARK3	14	+	978	19	6	0	1.84%	6.419251627	0.102109635	0.013428348	0.006888881
ENSG00000105852_95396350_95360089_245	annotated	PON3	7	-	735	168	7	11	13.88%	75.52514997	2.15E-07	4.81E-06	1.02E-11
ENSG00000160584_116862252_116846396_583	novel	SIK3	11	-	1749	8	1	0	1.03%	1.511366637	0.028889786	0.009815329	0.001387795
ENSG00000163161_127289396_127257596_528	dORF	ERCC3	2	-	1584	36	0	0	3.41%	7.50960298	7.14E-05	7.14E-05	3.75E-08
ENSG00000141522_81869930_81868876_204	annotated	ARHGDIA	17	-	612	669	56	11	26.96%	361.1971786	3.87E-09	1.46E-10	6.75E-18
ENSG00000087302_51989640_52004409_193	annotated	RTRAF	14	+	579	379	31	21	22.80%	216.2869418	2.46E-08	1.27E-07	3.16E-14
ENSG00000147804_144414029_144412538_242	annotated	SLC39A4	8	-	726	227	105	16	15.29%	103.3139319	0.000351213	3.60E-06	1.28E-08
ENSG00000104960_49857059_49860279_202	novel	PTOV1	19	+	606	76	1	3	15.84%	41.43912931	3.94E-07	5.87E-07	2.13E-12
ENSG00000117139_202808305_202729036_1539	annotated	KDM5B	1	-	4617	63	8	9	2.34%	4.508689508	1.23E-05	1.66E-05	1.63E-09
ENSG00000104783_43773796_43767543_203	novel	KCNN4	19	-	609	59	6	4	8.87%	32.01137822	0.000688092	0.000315557	1.44E-06
ENSG00000110911_50991621_50981726_102	annotated	SLC11A2	12	-	306	55	6	0	13.73%	59.38967063	0.005056323	0.000449456	1.55E-05
ENSG00000155957_66169951_66138153_162	annotated	TMBIM4	12	-	486	151	34	7	20.37%	102.6621444	6.88E-07	1.30E-06	8.05E-12
ENSG00000155957_66138145_66137960_61	dORF	TMBIM4	12	-	183	61	3	0	26.23%	110.1408437	0.000219587	0.00020982	3.18E-07
ENSG00000256269_119088273_119093283_304	annotated	HMBS	11	+	912	28	1	1	5.26%	10.14455139	0.000278608	0.000278608	5.27E-07
ENSG00000140464_73998130_74045008_797	annotated	PML	15	+	2391	8	3	0	0.75%	1.105554266	0.093731016	0.009815329	0.004911899
ENSG00000140464_73998130_74035951_744	annotated	PML	15	+	2232	8	3	0	0.81%	1.184310147	0.093731016	0.009815329	0.004911899
ENSG00000140463_72686228_72737587_519	annotated	BBS4	15	+	1557	21	3	1	2.12%	4.45656593	0.009129131	0.002532006	0.000130618
ENSG00000136197_42912067_42909635_479	annotated	C7orf25	7	-	1437	12	1	2	1.46%	2.759269571	0.0135992	0.037441831	0.002391926
ENSG00000073584_40642556_40628785_393	annotated	SMARCE1	17	-	1179	57	3	0	3.31%	15.97462619	0.001529613	0.000649205	6.25E-06
ENSG00000105771_43750741_43731596_520	annotated	SMG9	19	-	1560	43	5	4	3.46%	9.107800537	0.000527378	0.000503463	1.73E-06
ENSG00000130511_18419649_18434246_385	annotated	SSBP4	19	+	1155	14	2	1	1.56%	4.005121589	0.044040756	0.020342454	0.003985452
ENSG00000133112_45341069_45337476_197	annotated	TPT1	13	-	591	448	37	13	14.72%	250.4725786	2.05E-05	5.77E-06	9.77E-10
ENSG00000156136_70993836_71029378_260	annotated	DCK	4	+	780	62	9	8	8.85%	26.26435504	0.000140707	0.000341675	3.36E-07
ENSG00000107874_102423710_102423426_66	novel	CUEDC2	10	-	198	18	2	0	9.09%	30.03841192	0.023672501	0.012798403	0.001437523
ENSG00000159363_17011757_17005068_103	Overlap_uORF	ATP13A2	1	-	309	32	1	0	9.71%	34.21851455	0.002474239	0.00244612	3.49E-05
ENSG00000159363_17002303_16986221_971	annotated	ATP13A2	1	-	2913	47	0	0	1.44%	5.331225184	0.000452135	0.000452135	1.34E-06
ENSG00000180423_46616237_46603530_349	annotated	HARBI1	11	-	1047	10	1	0	1.43%	3.1558981	0.042234516	0.019679754	0.003715148
ENSG00000115286_1391131_1395488_73	novel	NDUFS7	19	+	219	58	1	2	17.81%	87.50916349	0.000714894	0.001251551	5.61E-06
ENSG00000178035_49029350_49024383_514	annotated	IMPDH2	3	-	1542	186	25	9	12.45%	39.85641426	7.33E-10	3.47E-11	3.10E-19
ENSG00000197153_27890792_27890382_136	annotated	HIST1H3J	6	-	408	1364	147	42	38.24%	1104.647874	1.37E-10	2.34E-09	3.79E-18
ENSG00000152056_223837450_223770203_137	annotated	AP1S3	2	-	411	32	1	1	8.03%	25.72632846	0.002182477	0.002182477	2.77E-05
ENSG00000196419_41622005_41663815_609	annotated	XRCC6	22	+	1827	167	18	4	9.03%	30.20282578	1.36E-08	9.21E-11	1.62E-17
ENSG00000162073_2971348_2971948_154	annotated	PAQR4	16	+	462	24	0	0	7.79%	17.16480681	0.000943084	0.000943084	5.54E-06
ENSG00000266967_42964440_42956174_230	novel	AARSD1	17	-	690	67	5	1	6.09%	32.08450664	0.003269261	0.000795519	1.62E-05
ENSG00000088727_47277374_47275308_91	annotated	KIF9	3	-	273	9	7	0	5.49%	10.89305048	0.375914817	0.019216965	0.045757765
ENSG00000066136_40738844_40770828_354	annotated	NFYC	1	+	1062	61	3	0	3.95%	18.97907194	0.000738144	0.000460235	2.20E-06
ENSG00000054611_46793523_47173626_470	annotated	TBC1D22A	22	+	1410	26	6	2	2.98%	6.092897737	0.00840293	0.001617456	8.07E-05

ENSG00000137710_110237512_110231869_173	novel	RDX	11	-	519	23	0	3	5.78%	14.64300234	0.002227676	0.013779785	0.000179099
ENSG00000117020_243843170_243505249_479	annotated	AKT3	1	-	1437	30	21	13	2.92%	6.898173927	0.100045546	0.035925772	0.014671657
ENSG00000137497_72035943_72003527_2101	annotated	NUMA1	11	-	6303	108	26	9	2.24%	5.661690205	4.70E-06	5.17E-08	2.69E-12
ENSG00000278259_36499206_36496251_193	novel	MYO19	17	-	579	15	2	0	3.11%	8.5601692	0.04317391	0.012178958	0.002518296
ENSG00000167703_1627760_1585660_451	novel	SLC43A2	17	-	1353	8	1	0	1.11%	1.953717848	0.042234516	0.019679754	0.003715148
ENSG00000126247_36141012_36149839_238	annotated	CAPNS1	19	+	714	2026	235	28	39.92%	937.5855015	1.73E-16	3.84E-17	9.99E-32
ENSG00000122218_160343170_160305747_455	annotated	COPA	1	-	1365	64	8	3	5.27%	15.49233846	5.70E-05	2.91E-05	1.26E-08
ENSG00000122218_160306471_160290157_749	Overlap_dORF	COPA	1	-	2247	85	15	8	4.27%	12.49929468	0.000142213	1.03E-05	1.22E-08
ENSG00000171714_22259519_22279765_444	novel	ANO5	11	+	1332	9	0	0	1.13%	2.23258467	0.019679754	0.019679754	0.001784964
ENSG00000117593_173825230_173857705_594	annotated	DARS2	1	+	1782	80	8	3	5.22%	14.83378366	9.66E-06	9.30E-07	8.15E-11
ENSG00000171793_40983291_40996120_307	novel	CTPS1	1	+	921	11	6	1	1.95%	3.946414595	0.201322915	0.023672501	0.023069683
ENSG00000156261_29063467_29062320_92	novel	CCT8	21	-	276	109	15	8	11.96%	130.4929561	0.013877325	0.003326632	0.000255384
ENSG00000146731_56056386_56058796_158	novel	CCT6A	7	+	474	67	13	0	10.13%	46.70529448	0.000401951	0.000210762	5.77E-07
ENSG00000084676_24665906_24768123_1317	annotated	NCOA1	2	+	3951	9	2	0	0.46%	0.752670914	0.041632258	0.012178958	0.002425608
ENSG00000120885_27610571_27598241_449	annotated	CLU	8	-	1347	1880	175	36	35.86%	461.1687888	1.05E-24	3.77E-27	7.98E-50
ENSG00000168101_4693727_4694568_247	annotated	NUDT16L1	16	+	741	23	3	0	3.24%	10.25602998	0.009939667	0.005554612	0.00028861
ENSG00000086848_111871482_111853705_198	annotated	ALG9	11	-	594	7	0	1	2.53%	3.893868212	0.019216965	0.041782825	0.003600526
ENSG00000075142_88220026_88206478_158	annotated	SRI	7	-	474	112	14	6	17.72%	78.07452212	9.81E-05	5.89E-06	4.98E-09
ENSG00000169019_47463651_47460105_86	novel	COMMD8	4	-	258	11	0	0	5.81%	14.08778233	0.020613417	0.020613417	0.001946209
ENSG00000132394_128153508_128408101_436	annotated	EEFSEC	3	+	1308	15	0	0	1.83%	3.78924921	0.004873904	0.004873904	0.000128468
ENSG00000116133_54887119_54877924_153	annotated	DHCR24	1	-	459	66	13	2	8.50%	47.5117365	0.009191518	0.001023943	5.97E-05
ENSG00000116133_54876020_54852233_378	dORF	DHCR24	1	-	1134	284	31	9	17.46%	82.75132173	6.92E-10	8.45E-12	7.66E-20
ENSG00000115275_74462963_74461275_562	dORF	MOGS	2	-	1686	74	1	0	4.98%	14.50253102	1.99E-06	1.60E-06	2.79E-11
ENSG00000141198_54912675_54942370_326	annotated	TOM1L1	17	+	978	47	6	7	6.75%	15.87920139	0.000278836	0.000133942	2.62E-07
ENSG00000285589_12679664_12675838_186	dORF	AC010422.8	19	-	558	45	18	0	8.06%	26.64697832	0.055107357	0.000288775	0.000183158
ENSG00000285589_12669403_12668359_106	dORF	AC010422.8	19	-	318	532	40	3	36.79%	552.7823477	3.83E-07	3.68E-08	1.42E-13
ENSG00000285589_12681766_12679840_137	annotated	AC010422.8	19	-	411	27	14	1	5.84%	21.70658964	0.37511016	0.008364861	0.027623643
ENSG00000112715_43777480_43784562_172	annotated	VEGFA	6	+	516	40	1	0	5.81%	25.6141497	0.002851	0.002488365	4.06E-05
ENSG00000108433_46935050_46938760_93	annotated	GOSR2	17	+	279	18	8	0	7.53%	21.31758265	0.185865134	0.008473674	0.010168343
ENSG00000132680_155933677_155921385_314	annotated	KHDC4	1	-	942	15	6	4	2.55%	5.261505273	0.139884474	0.048994651	0.026537873
ENSG00000118518_127286653_127287693_346	annotated	RNF146	6	+	1038	40	1	0	5.20%	12.73304552	8.20E-05	8.27E-05	4.95E-08
ENSG00000146374_127119193_127197509_292	annotated	RSPO3	6	+	876	97	13	7	8.22%	36.58788301	0.000178386	0.000154574	1.93E-07
ENSG00000163479_156020944_156014940_146	novel	SSR2	1	-	438	1153	41	15	36.99%	869.8109095	2.17E-09	1.37E-10	3.48E-18
ENSG00000183258_177515793_177511791_468	novel	DDX41	5	-	1404	119	12	4	6.20%	28.00589829	5.66E-06	5.72E-06	2.70E-10
ENSG00000196352_207331218_207370751_155	annotated	CD55	1	+	465	32	3	1	6.45%	22.73875483	0.004400434	0.003133491	7.68E-05
ENSG00000155115_110962457_110967790_109	novel	GTF3C6	6	+	327	19	0	1	5.50%	19.19886266	0.01300724	0.013428348	0.000846864
ENSG00000184924_24792386_24790411_57	novel	PTRHD1	2	-	171	45	10	0	21.05%	86.95329766	0.008030027	0.001019727	5.15E-05
ENSG00000122515_44756268_44767623_914	annotated	ZMIZ2	7	+	2742	32	7	0	1.53%	3.856134572	0.01223992	0.000428413	3.94E-05
ENSG00000015479_139307416_139329358_794	annotated	MATR3	5	+	2382	195	34	12	7.56%	27.04970343	3.03E-08	7.89E-10	2.78E-16
ENSG00000150756_10249873_10227622_158	novel	ATPSCKMT	5	-	474	16	4	0	5.70%	11.15350316	0.029446607	0.002943991	0.000513438
ENSG00000112576_41941649_41937182_136	novel	CCND3	6	-	408	78	6	2	8.82%	63.1690133	0.004762631	0.002231622	6.06E-05
ENSG00000104549_125018088_125018792_91	novel	SQLE	8	+	273	110	24	5	17.58%	133.1372836	0.003241474	0.000416018	9.01E-06
ENSG00000013374_151345350_151360251_267	annotated	NUB1	7	+	801	56	4	5	8.61%	23.1007013	2.87E-05	0.000110184	2.42E-08
ENSG00000130165_11554347_11553746_83	annotated	ELOF1	19	-	249	73	13	0	15.66%	96.87086254	0.00371027	0.000719745	1.69E-05
ENSG00000166946_43190380_43192884_172	novel	CCNDBP1	15	+	516	62	5	0	11.63%	39.70193203	0.00032882	3.67E-05	9.31E-08
ENSG00000186575_29636751_29694802_557	annotated	NF2	22	+	1671	62	2	2	4.67%	12.25984257	5.55E-06	1.10E-05	5.05E-10
ENSG00000113712_149550964_149501235_426	annotated	CSNK1A1	5	-	1278	177	30	3	9.15%	45.76274492	1.86E-05	2.47E-08	6.34E-12
ENSG00000065357_55952342_55953506_188	novel	DGKA	12	+	564	12	0	0	3.19%	7.030266619	0.011772051	0.011772051	0.000681273
ENSG00000163629_86689047_86814551_2332	annotated	PTPN13	4	+	6996	19	1	1	0.47%	0.897373941	0.002559322	0.002559322	3.76E-05
ENSG00000090316_1289947_1339169_344	annotated	MAEA	4	+	1032	94	13	0	10.76%	30.0966259	7.95E-06	4.08E-08	3.84E-12
ENSG00000087274_2875916_2928523_768	annotated	ADD1	4	+	2304	68	6	0	3.91%	9.75205387	1.31E-06	6.18E-07	7.30E-12
ENSG00000175166_184301652_184308890_747	annotated	PSMD2	3	+	2241	218	24	2	8.03%	32.14284328	2.09E-09	1.59E-11	4.40E-19
ENSG00000161057_103347712_103368054_433	annotated	PSMC2	7	+	1299	215	69	2	12.24%	54.68887159	3.09E-05	2.24E-10	2.20E-13
ENSG00000148175_121370187_121341202_123	annotated	STOM	9	-	369	88	3	0	17.89%	78.79995322	2.16E-05	1.80E-05	3.06E-09
ENSG00000124762_36684102_36685800_164	annotated	CDKN1A	6	+	492	125	16	8	17.68%	83.9488138	1.25E-05	1.65E-05	1.65E-09
ENSG00000154511_92876409_92833020_137	annotated	DIPK1A	1	-	411	15	0	0	4.38%	12.05921646	0.013428348	0.013428348	0.000872559
ENSG00000106615_151519511_151467119_184	annotated	RHEB	7	-	552	103	17	7	7.61%	61.65492881	0.000883754	0.001802963	9.81E-06
ENSG00000175893_14674292_14618966_249	annotated	ZDHHC21	9	-	747	11	2	0	2.41%	4.865659762	0.060322713	0.012798403	0.003726043
ENSG00000153914_66162202_66178868_456	annotated	SREK1	5	+	1368	35	3	1	3.95%	8.453792828	0.00047563	0.00012004	4.04E-07
ENSG00000082014_151242768_151239103_347	annotated	SMARCD3	7	-	1041	20	2	1	2.31%	6.34817543	0.018067938	0.005306578	0.000503692
ENSG00000055609_152181940_152136832_2938	annotated	KMT2C	7	-	8814	14	9	2	0.27%	0.52483724	0.310567764	0.019675918	0.035422695
ENSG00000089289_70133948_70165981_339	annotated	IGBP1	X	+	1017	39	4	3	4.72%	12.67107051	0.000281293	0.000880376	1.66E-06
ENSG00000117155_84669800_84647433_512	annotated	SSX2IP	1	-	1536	24	1	1	2.93%	5.162852049	0.000435029	0.000435029	1.25E-06
ENSG00000107242_68780444_68781028_194	annotated	PIP5K1B	9	+	582	19	0	0	3.61%	10.78698985	0.008574016	0.008574016	0.000374746
ENSG00000113595_65624274_65591484_546	annotated	TRIM23	5	-	1638	33	1	0	2.56%	6.65686418	0.000662939	0.0004093	1.77E-06
ENSG00000141424_36127007_36109593_755	annotated	SLC39A6	18	-	2265	162	32	11	7.15%	23.63286978	1.26E-07	6.80E-09	9.19E-15
ENSG00000186468_82278323_82276109_143	annotated	RPS23	5	-	429	3766	262	20	18.18%	2900.632289	0.001322047	2.76E-05	3.23E-07
ENSG00000164251_76819183_76833801_397	annotated	F2RL1	5	+	1191	68	11	6	7.30%	18.86543419	0.000204538	2.55E-05	4.08E-08

ENSG00000164253_77492220_77433135_459	annotated	WDR41	5	-	1377	63	21	12	5.23%	15.1173707	0.015642648	0.002430445	0.000220713
ENSG00000164574_154190867_154416972_603	annotated	GALNT10	5	+	1809	212	25	9	9.45%	38.72281736	5.05E-09	5.58E-10	3.17E-17
ENSG00000100811_100276502_100276693_63	novel	YY1	14	+	189	15	2	0	12.70%	26.22401041	0.020624382	0.005405142	0.000586277
ENSG00000135473_56333094_56319239_1111	novel	PAN2	12	-	3333	28	11	1	1.26%	2.775826844	0.040502927	0.000626688	0.000219663
ENSG00000130731_634621_634526_31	Overlap_dORF	METTL26	16	-	93	12	7	0	19.35%	42.63516531	0.273060506	0.01300724	0.022706215
ENSG00000130731_636290_634597_204	annotated	METTL26	16	-	612	144	36	17	14.22%	77.74647791	0.000634982	0.002029638	8.14E-06
ENSG00000108829_50397317_50382988_307	annotated	LRRC59	17	-	921	383	39	3	18.24%	137.4069809	5.89E-08	3.32E-11	3.08E-17
ENSG00000166986_57512082_57517296_339	Overlap_dORF	MARS	12	+	1017	66	9	0	6.49%	21.4433501	0.000494996	1.62E-05	6.90E-08
ENSG00000166986_57488091_57512308_546	annotated	MARS	12	+	1638	106	6	5	6.78%	21.38265464	4.65E-07	5.77E-07	2.46E-12
ENSG00000130159_11519170_11506184_431	annotated	ECSIT	19	-	1293	63	7	0	4.87%	16.09947367	0.000255159	2.71E-05	5.42E-08
ENSG00000120742_150546135_150544458_66	annotated	SERP1	3	-	198	61	10	0	24.24%	101.7968404	0.004920994	0.000196946	7.41E-06
ENSG00000070087_149970856_149966489_140	annotated	PFN2	3	-	420	378	50	22	32.86%	297.380278	1.92E-07	3.64E-08	6.97E-14
ENSG00000070087_149966250_149966167_27	dORF	PFN2	3	-	81	41	1	0	18.52%	167.2509108	0.021084099	0.021084099	0.002029891
ENSG00000181929_49018740_49002899_331	annotated	PRKAG1	12	-	993	163	14	3	9.37%	54.23854237	6.69E-06	1.56E-06	9.10E-11
ENSG00000101608_3253248_3253451_67	novel	MYL12A	18	+	201	110	8	7	14.93%	180.8282509	0.005134532	0.016039993	0.000433441
ENSG00000132305_86195382_86144268_747	annotated	IMMT	2	-	2241	178	14	8	7.50%	26.24507387	5.52E-09	1.39E-09	8.34E-17
ENSG00000108433_46923193_46964361_180	novel	GOSR2	17	+	540	20	0	0	4.44%	12.23787152	0.005306578	0.005306578	0.000151001
ENSG00000162236_62831081_62807609_267	annotated	STX5	11	-	801	34	11	1	5.99%	14.02542579	0.010666143	0.000263533	2.26E-05
ENSG00000033867_27484099_27376764_1201	annotated	SLC4A7	3	-	3603	78	36	5	2.66%	7.153193846	0.0054847	2.37E-06	2.39E-07
ENSG00000123600_171360506_171325891_241	annotated	METTL8	2	-	723	11	2	0	3.73%	5.027175439	0.006277459	0.002478943	8.78E-05
ENSG00000100461_22911393_22901730_423	annotated	RBM23	14	-	1269	142	43	10	11.35%	36.97399481	0.000113656	5.91E-08	1.06E-10
ENSG00000100461_22919182_22911369_72	Overlap_uORF	RBM23	14	-	216	35	8	7	13.89%	53.54068791	0.036127044	0.037374083	0.005685221
ENSG00000204592_30489532_30490003_113	novel	HLA-E	6	+	339	46	1	0	14.16%	44.83609567	0.00026641	0.000198297	3.63E-07
ENSG00000163754_148991641_149026991_279	annotated	GYG1	3	+	837	22	2	12	4.30%	8.684941081	0.004721554	0.114478235	0.003615235
ENSG00000255723_87340563_87318397_37	novel	AC108516.2	4	-	111	22	2	0	13.51%	65.48915031	0.022899795	0.020613417	0.002147546
ENSG00000145191_184140487_184144943_417	dORF	EIF2B5	3	+	1251	24	8	2	2.64%	6.339041364	0.045595422	0.002484757	0.000734664
ENSG00000131051_35732158_35704481_504	novel	RBM39	20	-	1512	86	13	6	6.94%	18.79387412	2.54E-05	1.36E-06	3.16E-10
ENSG00000100714_64388428_64454926_922	annotated	MTHFD1	14	+	2766	164	14	5	6.40%	19.59121298	2.70E-09	8.99E-11	2.93E-18
ENSG00000136319_20305937_20300526_158	novel	TTC5	14	-	474	61	11	0	5.70%	42.5227308	0.017688833	0.003580484	0.000350285
ENSG00000115275_74464717_74461275_718	novel	MOGS	2	-	2154	112	4	1	5.29%	17.18074442	8.51E-08	3.18E-08	2.70E-14
ENSG00000117139_202808305_202729059_1472	annotated	KDM5B	1	-	4416	59	6	10	2.31%	4.414612621	7.22E-06	5.57E-05	3.33E-09
ENSG00000165458_72224985_72238353_1258	annotated	INPPL1	11	+	3774	36	10	5	1.43%	3.15188424	0.010194533	0.000647459	4.53E-05
ENSG00000102100_48909871_48903438_324	annotated	SLC35A2	X	-	972	53	2	4	5.86%	18.01686641	7.21E-05	0.000112166	5.89E-08
ENSG00000100503_50821942_50729692_1951	annotated	NIN	14	-	5853	34	4	11	0.92%	1.919420136	0.000988313	0.006100767	3.75E-05
ENSG00000103335_88717611_88715615_373	Overlap_dORF	PIEZO1	16	-	1119	12	10	2	2.41%	3.543405159	0.298180466	0.019230617	0.033019667
ENSG00000174130_38829299_38827083_422	annotated	TLR6	4	-	1266	12	0	0	1.42%	3.13196712	0.01300724	0.01300724	0.000821865
ENSG00000274523_75073737_75043032_464	annotated	RCC1L	7	-	1392	84	12	2	6.47%	19.93929067	1.07E-05	1.20E-06	1.15E-10
ENSG00000117593_173825230_173856764_539	annotated	DARS2	1	+	1617	66	3	3	5.01%	13.48663392	7.32E-06	5.05E-06	3.08E-10
ENSG00000103274_10757875_10769105_141	annotated	NUBP1	16	+	423	15	1	0	5.67%	11.71711103	0.004873904	0.005306578	0.000139308
ENSG00000135541_135429895_135285626_342	annotated	AHI1	6	-	1026	14	2	2	1.75%	4.508689508	0.044040756	0.044040756	0.007930666
ENSG00000126456_49662587_49659648_281	annotated	IRF3	19	-	843	54	15	2	6.05%	21.16585609	0.039027222	0.000479151	0.000171011
ENSG00000247077_132710877_132718810_255	annotated	PGAM5	12	+	765	50	10	0	5.88%	21.59624386	0.001698075	0.000302602	3.46E-06
ENSG00000139624_50167297_50130545_392	annotated	CERS5	12	-	1176	120	10	0	9.44%	33.71658481	3.20E-07	4.66E-08	1.48E-13
ENSG00000101871_10567547_10449368_667	annotated	MID1	X	-	2001	66	4	11	5.10%	10.89849428	1.95E-06	8.36E-05	1.55E-09
ENSG00000136521_179618481_179624040_53	novel	NDUFB5	3	+	159	172	23	3	47.17%	357.4382098	2.40E-05	8.02E-06	1.56E-09
ENSG00000106012_2578275_2610162_529	dORF	IQCE	7	+	1587	8	1	0	0.95%	1.66564603	0.042234516	0.019679754	0.003715148
ENSG00000169139_48049949_48060828_58	annotated	UBE2V2	8	+	174	42	13	0	15.52%	79.75716268	0.005134532	0.003710481	0.000104431
ENSG00000104549_124998600_125021945_479	annotated	SQLE	8	+	1437	553	79	18	16.70%	127.1563394	3.13E-11	3.02E-14	1.53E-23
ENSG00000138686_121870313_121828141_672	annotated	BBS7	4	-	2016	38	3	0	1.04%	6.228202471	0.071133254	0.007029303	0.002767761
ENSG00000136485_63550678_63589172_342	annotated	DCAF7	17	+	1026	90	7	8	7.89%	28.98443255	2.45E-05	0.000100567	1.90E-08
ENSG00000213782_12813368_12814253_69	novel	DDX47	12	+	207	9	1	0	7.25%	14.36619701	0.047790352	0.016947427	0.003697512
ENSG00000120265_149749728_149802379_250	annotated	PCMT1	6	+	750	122	25	7	12.00%	53.74873173	6.76E-05	6.08E-06	3.47E-09
ENSG00000140525_89263453_89268543_120	annotated	FANCI	15	+	360	15	0	0	4.17%	13.76760546	0.020613417	0.020613417	0.001946209
ENSG00000214575_82556032_82544592_329	annotated	CPEB1	15	-	987	8	0	0	1.52%	2.678196807	0.019679754	0.019679754	0.001784964
ENSG00000118849_158710780_158698140_84	novel	RARRES1	3	-	252	25	8	1	9.52%	32.78001301	0.06867726	0.005604867	0.002228623
ENSG00000113810_160414137_160433809_991	novel	SMC4	3	+	2973	207	52	18	6.66%	23.00621054	2.68E-07	1.00E-10	4.41E-16
ENSG00000114650_47422501_47413854_883	Overlap_dORF	SCAP	3	-	2649	68	6	0	2.83%	8.481967579	5.18E-05	4.66E-06	2.06E-09
ENSG00000205426_52288073_52286255_235	annotated	KRT81	12	-	705	1537	271	70	31.49%	720.3679863	1.51E-10	1.17E-12	2.45E-21
ENSG00000115884_20224867_20202270_169	annotated	SDC1	2	-	507	192	11	18	16.57%	125.130426	8.26E-06	8.98E-05	6.18E-09
ENSG00000185630_164559823_164560167_70	annotated	PBX1	1	+	210	20	5	0	10.00%	31.46881249	0.045444393	0.008473674	0.001963364
ENSG00000163710_142888896_142818432_303	annotated	PCOLCE2	3	-	909	39	7	0	4.29%	14.17654424	0.003589132	0.00067919	1.55E-05
ENSG00000155957_66169896_66137960_207	Overlap_dORF	TMBIM4	12	-	621	216	40	8	25.60%	114.929576	5.32E-10	6.93E-10	4.12E-18
ENSG00000137497_72013188_72008612_311	annotated	NUMA1	11	-	933	11	2	0	2.25%	3.895656851	0.035227809	0.007029303	0.001282266
ENSG00000111554_68332245_68325206_224	novel	MDM1	12	-	672	10	6	0	2.23%	4.917001951	0.305084881	0.020613417	0.035625003
ENSG00000197102_101964884_102050563_4582	annotated	DYNC1H1	14	+	13746	288	63	35	2.66%	6.92286403	1.91E-12	9.57E-16	3.17E-26
ENSG00000055147_154034953_153992976_435	annotated	FAM114A2	5	-	1305	47	7	6	5.29%	11.90027507	0.000341003	0.000196882	4.60E-07
ENSG00000188603_28489349_28487343_136	novel	CLN3	16	-	408	82	9	2	11.03%	66.40844988	0.000549623	0.000303383	1.11E-06
ENSG00000188603_28491764_28489349_55	novel	CLN3	16	-	165	18	0	12	14.55%	36.0460943	0.005554612	0.310595281	0.015978689

ENSG00000188603_28486378_28479730_140	novel	CLN3	16	-	420	37	2	0	9.29%	29.10865155	0.000998504	0.00064467	4.08E-06
ENSG00000071054_101698081_101891249_1235	annotated	MAP4K4	2	+	3705	40	9	4	1.38%	3.567314776	0.007132807	0.000968136	4.34E-05
ENSG00000152601_152300194_152459327_382	annotated	MBNL1	3	+	1146	67	6	6	5.24%	19.31789667	0.000148948	0.000505146	5.24E-07
ENSG00000163479_156009626_156009540_28	dORF	SSR2	1	-	84	22	0	0	21.43%	86.53923434	0.013640586	0.013640586	0.000898637
ENSG00000163479_156020944_156019233_81	annotated	SSR2	1	-	243	199	12	3	30.86%	270.592937	8.13E-05	5.71E-06	3.98E-09
ENSG00000101452_38992363_39038543_530	dORF	DHX35	20	+	1590	8	2	0	0.94%	1.662503301	0.11777946	0.019679754	0.010847352
ENSG00000115677_241230773_241229601_143	novel	HDLBP	2	-	429	12	0	0	4.90%	9.242588283	0.007029303	0.007029303	0.000257364
ENSG00000165806_113721653_113729540_220	annotated	CASP7	10	+	660	22	0	5	4.55%	11.01408437	0.002281203	0.012808166	0.000169256
ENSG00000115758_10445032_10444518_43	novel	ODC1	2	-	129	8	0	0	11.63%	20.49131976	0.019679754	0.019679754	0.001784964
ENSG00000119321_113221243_113166078_1219	annotated	FKBP15	9	-	3657	18	6	2	1.07%	1.626361925	0.044060476	0.003960945	0.00102279
ENSG00000140264_43792240_43792380_46	Overlap_uORF	SERF2	15	+	138	45	41	5	15.22%	107.7464775	0.157102168	0.01551626	0.012654753
ENSG00000140264_43792377_43799079_108	annotated	SERF2	15	+	324	349	63	2	12.96%	355.9180968	0.00064468	0.000482311	2.02E-06
ENSG00000156469_96251012_96239491_173	annotated	MTERF3	8	-	519	18	3	0	5.20%	11.45974096	0.010499663	0.002967235	0.000173435
ENSG00000110844_49635241_49644192_512	annotated	PRPF40B	12	+	1536	18	1	3	1.37%	3.872139037	0.015625009	0.051745348	0.003744237
ENSG00000028528_64112646_64137618_416	Overlap_dORF	SNX1	15	+	1248	84	7	4	7.45%	22.23997806	1.15E-05	2.64E-06	2.60E-10
ENSG00000158796_161124378_161122147_290	annotated	DEDD	1	-	870	26	8	4	3.79%	9.874696332	0.059631754	0.010831024	0.003212442
ENSG00000136754_26860848_26748570_324	annotated	ABI1	10	-	972	37	3	5	4.94%	12.5778124	0.001252275	0.001880452	1.42E-05
ENSG00000103994_42448692_42417304_1068	annotated	ZNF106	15	-	3204	11	1	1	0.66%	1.134409439	0.013018137	0.013018137	0.00082316
ENSG00000169189_27225816_27225157_56	novel	NSMCE1	16	-	168	28	5	0	16.07%	55.07042185	0.024718994	0.003632191	0.000505352
ENSG00000127804_2502313_2464313_162	annotated	METTL16	17	-	486	23	3	0	5.56%	15.63728028	0.015570605	0.003632191	0.000310569
ENSG00000092208_39114339_39122606_182	novel	GEMIN2	14	+	546	57	5	0	4.40%	34.49465984	0.005756914	0.005756914	0.000176255
ENSG00000134910_125606364_125608333_108	novel	STT3A	11	+	324	63	6	0	15.74%	64.24882549	0.001660335	0.000132663	1.62E-06
ENSG00000112992_43609196_43704404_1086	annotated	NNT	5	+	3258	43	2	0	1.75%	4.361009465	0.000114158	5.53E-05	4.66E-08
ENSG00000128973_68229584_68209686_213	annotated	CLN6	15	-	639	33	2	11	5.63%	17.06407438	0.00077991	0.028046979	0.000166872
ENSG00000091129_108232428_108178385_669	annotated	NRCAM	7	-	2007	53	9	7	4.19%	8.725657274	0.000100857	3.92E-05	2.97E-08
ENSG00000132481_75878548_75874483_638	annotated	AC087289.1	17	-	1914	49	1	7	4.39%	8.459093012	1.77E-06	3.24E-05	5.20E-10
ENSG00000163001_55519802_55545008_367	annotated	CFAP36	2	+	1101	32	5	1	4.63%	9.603561304	0.00120083	0.000180392	1.51E-06
ENSG00000132475_75779174_75778595_136	annotated	H3F3B	17	-	408	248	43	6	14.71%	200.8450679	0.011240137	5.19E-05	6.55E-06
ENSG00000233493_55384259_55383729_176	annotated	TMEM238	19	-	528	33	1	1	5.68%	20.65140819	0.002755835	0.002362849	3.74E-05
ENSG00000164733_11852668_11848147_59	annotated	CTSB	8	-	177	231	10	0	27.12%	431.229405	0.000216957	0.000217439	3.25E-07
ENSG00000164733_11853372_11852665_24	internal	CTSB	8	-	72	32	2	43	37.50%	146.8544583	0.003710481	0.468493666	0.025643776
ENSG00000164733_11850939_11845125_215	Overlap_dORF	CTSB	8	-	645	775	72	148	31.16%	397.0193203	3.71E-11	6.17E-08	3.59E-17
ENSG00000129595_112419039_112164990_686	annotated	EPB41L4A	5	-	2058	27	8	2	1.17%	4.334989475	0.048893856	0.007987976	0.002023419
ENSG00000112541_165987898_165987578_106	novel	PDE10A	6	-	318	45	19	13	14.15%	46.75790535	0.04790541	0.018522412	0.003998427
ENSG00000166352_36610134_36659186_211	annotated	C11orf74	11	+	633	10	0	1	2.37%	5.219945199	0.020613417	0.035374934	0.003252526
ENSG00000148335_129632704_129635464_223	annotated	NTMT1	9	+	669	103	28	11	14.80%	50.87222826	0.000333288	1.94E-06	7.08E-09
ENSG00000113648_135389012_135334976_345	annotated	H2AFY	5	-	1035	197	14	7	14.49%	62.892018	2.87E-09	7.17E-09	2.20E-16
ENSG00000218336_182628718_182800351_2427	annotated	TENM3	4	+	7281	43	2	0	0.91%	1.951403494	3.36E-05	1.32E-05	3.51E-09
ENSG00000129116_168898634_168903963_95	novel	PALLD	4	+	285	20	3	2	6.32%	23.18754604	0.017507491	0.020342454	0.001651864
ENSG00000113328_163441222_163442565_159	novel	CCNG1	5	+	477	39	13	0	9.43%	27.01567864	0.011026486	0.000259634	2.32E-05
ENSG00000015153_42160834_42160589_81	novel	YAF2	12	-	243	21	0	0	9.88%	28.55503355	0.005306578	0.005306578	0.000151001
ENSG00000084072_39748983_39753355_105	dORF	PPIE	1	+	315	20	2	0	8.57%	20.97920832	0.007532653	0.003606289	0.00014802
ENSG00000058673_203840341_203850514_316	annotated	ZC3H11A	1	+	948	15	0	0	1.58%	5.228204606	0.020613417	0.020613417	0.001946209
ENSG00000112992_43624089_43704404_838	dORF	NNT	5	+	2514	34	2	0	1.67%	4.468721582	0.000971426	0.000432307	2.72E-06
ENSG00000140280_51751328_51723607_100	annotated	LYSMD2	15	-	300	26	0	0	7.00%	28.63661936	0.008574016	0.008574016	0.000374746
ENSG00000184277_101650135_101636859_152	annotated	TM2D3	15	-	456	185	8	2	18.42%	134.0530006	1.58E-06	3.11E-06	4.29E-11
ENSG00000243989_51984065_51989075_373	annotated	ACY1	3	+	1119	64	4	4	6.43%	18.89816085	1.41E-05	5.47E-05	6.12E-09
ENSG00000143183_165768742_165754893_98	annotated	TMCO1	1	-	294	173	16	12	20.41%	194.4323057	4.26E-05	6.23E-05	1.99E-08
ENSG00000165322_31908849_31807658_797	annotated	ARHGAP12	10	-	2391	55	7	1	2.51%	7.600685576	0.000841406	5.32E-05	3.50E-07
ENSG00000050130_59484590_59498828_140	annotated	JKAMP	14	+	420	110	44	27	16.43%	86.53923434	0.010647042	0.001944926	0.000121435
ENSG00000104164_45587444_45603122_80	annotated	BLOC1S6	15	+	240	20	1	0	7.50%	27.53521093	0.023672501	0.012798403	0.001437523
ENSG00000100605_92993886_92941561_295	annotated	ITPK1	14	-	885	39	4	6	4.07%	14.5609929	0.008764431	0.011804008	0.000518708
ENSG00000070269_56625412_56647497_231	dORF	TMEM260	14	+	693	9	0	0	2.16%	4.291201703	0.016947427	0.016947427	0.001349898
ENSG00000135446_57749211_57748525_40	annotated	CDK4	12	-	120	104	21	6	32.50%	286.3661936	0.004891619	0.001750551	4.98E-05
ENSG00000135446_57752292_57752176_38	uORF	CDK4	12	-	114	52	13	12	21.05%	150.7190493	0.007356796	0.040643964	0.001550447
ENSG00000165948_94096938_94102568_104	Overlap_dORF	IFI27L1	14	+	312	12	4	3	4.81%	12.70855889	0.169362074	0.050679946	0.033257892
ENSG00000073712_52864511_52859561_129	annotated	FERMT2	14	-	387	23	2	3	6.20%	19.63751477	0.012767383	0.024197104	0.00146555
ENSG00000204316_75904684_75904276_60	novel	MRPL38	17	-	180	40	3	0	11.67%	73.42722914	0.035350572	0.008473674	0.001506136
ENSG00000123297_57782802_57783941_89	annotated	TSFM	12	+	267	19	2	0	10.11%	23.51321382	0.014991381	0.003327803	0.000276729
ENSG00000115540_197540381_197550646_126	dORF	MOB4	2	+	378	41	2	0	8.73%	35.83948089	0.002815105	0.001580611	2.62E-05
ENSG00000115540_197516093_197540125_43	annotated	MOB4	2	+	129	14	3	0	13.95%	35.85980958	0.042234516	0.01300724	0.002602983
ENSG00000140365_75338131_75339382_168	novel	COMMD4	15	+	504	26	2	1	5.36%	17.04560676	0.003684287	0.004512623	9.16E-05
ENSG00000137814_42561296_42566816_141	annotated	HAUS2	15	+	423	30	1	0	6.38%	23.43422206	0.00310098	0.003327803	5.81E-05
ENSG00000174446_66497731_66495258_83	annotated	SNAPC5	15	-	249	18	1	0	8.43%	23.8859661	0.023878474	0.007979402	0.000955388
ENSG00000065054_2027006_2037984_337	annotated	SLC9A3R2	16	+	1011	227	17	4	13.95%	74.18982647	3.68E-09	9.55E-10	3.87E-17
ENSG00000101246_63706690_63705705_69	novel	ARFRP1	20	-	207	15	0	0	7.25%	23.94366168	0.021084099	0.021084099	0.002029891
ENSG00000122390_3443767_3484855_249	annotated	NAA60	16	+	747	21	5	0	3.61%	9.288986819	0.028596741	0.003658191	0.000594887
ENSG00000150403_113495711_113549538_634	annotated	TMCO3	13	+	1902	195	12	5	5.99%	33.87612701	7.77E-07	3.73E-07	2.67E-12

ENSG00000123080_50970369_50974270_168	annotated	CDKN2C	1	+	504	32	4	0	7.14%	20.97920832	0.002280579	0.001003278	1.39E-05
ENSG00000117877_45406697_45409501_512	annotated	CD3EAP	19	+	1536	30	4	3	3.32%	6.453565061	0.000351211	0.000999273	2.31E-06
ENSG00000185624_81860471_81844012_508	annotated	P4HB	17	-	1524	2341	262	59	35.83%	507.5584943	7.24E-28	4.18E-30	6.35E-56
ENSG00000178988_6709527_6709246_93	novel	MRFAP1L1	4	-	279	11	0	0	5.38%	13.02741162	0.020613417	0.020613417	0.001946209
ENSG00000063241_55456486_55453308_221	annotated	ISOC2	19	-	663	106	2	3	11.76%	52.82773499	3.77E-06	9.16E-06	2.90E-10
ENSG00000114867_184320681_184334908_1403	annotated	EIF4G1	3	+	4209	149	16	14	4.35%	11.69706751	1.37E-09	2.56E-09	3.82E-17
ENSG00000198833_89352569_89329679_318	annotated	UBE2J1	6	-	954	56	1	3	5.66%	19.39587185	0.00011814	0.000350362	2.92E-07
ENSG00000167965_2206086_2208735_327	annotated	MLST8	16	+	981	94	11	2	9.17%	31.66128229	1.35E-05	1.91E-06	2.25E-10
ENSG00000167967_2223626_2235572_780	annotated	E4F1	16	+	2340	20	2	1	1.28%	2.824124198	0.007459848	0.003974283	0.000160237
ENSG00000055130_148756998_148800582_666	dORF	CUL1	7	+	1998	68	9	0	2.70%	11.24561167	0.000146042	8.74E-05	9.19E-08
ENSG00000039650_49867204_49861248_521	annotated	PNKP	19	-	1563	28	6	0	2.30%	5.919277589	0.009048123	0.000978918	5.65E-05
ENSG00000105701_18538354_18532169_176	annotated	FKBP8	19	-	528	21	4	4	5.11%	13.14180521	0.02959315	0.02959315	0.003811264
ENSG00000163539_33717987_33498631_1510	annotated	CLASP2	3	-	4530	27	3	0	0.93%	1.969405815	0.002970463	0.000375189	7.50E-06
ENSG00000113621_134875362_134899685_337	annotated	TXNDC15	5	+	1011	276	48	6	18.69%	90.20437051	1.71E-08	1.22E-11	3.23E-18
ENSG00000164070_127782551_127832874_839	annotated	HSPA4L	4	+	2517	19	6	4	1.07%	2.494250334	0.06085885	0.0280616	0.007243609
ENSG00000026297_166956182_166929588_256	annotated	RNASET2	6	-	768	27	6	2	5.08%	11.61641711	0.006735894	0.000917405	3.90E-05
ENSG00000178445_6610256_6533017_830	annotated	GLDC	9	-	2490	8	0	0	0.60%	1.061598494	0.019679754	0.019679754	0.001784964
ENSG00000137411_30915988_30926210_892	annotated	VARS2	6	+	2676	23	12	2	1.79%	2.83995449	0.031365884	0.000557659	0.000146767
ENSG00000120802_98515868_98547858_345	annotated	TMPO	12	+	1035	82	10	5	9.28%	26.17840343	9.23E-06	3.55E-06	2.76E-10
ENSG00000137713_111766361_111741596_474	annotated	PPP2R1B	11	-	1422	33	0	1	3.59%	7.668033423	0.000114888	0.000152976	1.25E-07
ENSG00000178149_49016455_49016035_110	novel	DALRD3	3	-	330	12	0	0	4.55%	12.01536477	0.021084099	0.021084099	0.002029891
ENSG00000167526_89562377_89563042_57	novel	RPL13	16	+	171	199	5	1	19.30%	384.5268052	0.001657226	0.001672809	1.65E-05
ENSG00000128050_56436313_56459538_432	annotated	PAICS	4	+	1296	124	26	4	8.56%	31.61450143	4.60E-06	2.43E-07	1.08E-11
ENSG00000168137_9443332_9478612_876	annotated	SETD5	3	+	2628	19	6	5	1.03%	2.388899578	0.100722125	0.027696082	0.011974134
ENSG00000101220_3758574_3754058_92	annotated	C20orf27	20	-	276	48	3	0	11.96%	57.46478802	0.001618649	0.001626321	1.57E-05
ENSG00000106541_16801796_16799693_126	novel	AGR2	7	-	378	430	32	16	24.60%	375.8774825	2.03E-06	1.80E-06	3.18E-11
ENSG00000148248_133375951_133363729_122	annotated	SURF4	9	-	366	334	44	73	29.51%	301.5331295	1.61E-06	1.92E-05	2.76E-10
ENSG00000148248_133366008_133363493_96	Overlap_dORF	SURF4	9	-	288	226	78	5	32.29%	259.2899029	0.000127413	1.08E-06	1.45E-09
ENSG00000152332_162500086_162522550_286	annotated	UHMK1	1	+	858	31	5	0	4.55%	11.9383432	0.007597585	0.000674519	3.40E-05
ENSG00000158411_99173963_99169375_181	annotated	MITD1	2	-	543	41	5	0	8.84%	24.94903089	0.000191604	0.00019293	2.57E-07
ENSG00000074800_8868018_8861360_341	novel	ENO1	1	-	1023	1716	190	108	41.94%	554.257149	7.08E-23	5.60E-24	7.18E-45
ENSG00000100503_50761879_50727689_1444	annotated	NIN	14	-	4332	23	4	6	0.83%	1.754320918	0.01124389	0.013255529	0.000730165
ENSG00000144747_69052086_69023177_1093	annotated	TMF1	3	-	3279	73	6	2	2.20%	7.35615882	7.37E-05	2.18E-05	1.25E-08
ENSG00000131504_141584219_141516851_1170	annotated	DIAPH1	5	-	3510	91	17	0	4.19%	8.566510066	4.55E-07	2.41E-10	1.72E-15
ENSG00000121753_31742853_31727420_1140	annotated	ADGRB2	1	-	3420	7	3	0	0.53%	0.676303426	0.109745779	0.009815329	0.005898503
ENSG00000128699_189782595_189775561_109	annotated	ORMDL1	2	-	327	36	0	2	8.26%	36.37679242	0.003478238	0.01299184	0.00024813
ENSG00000135930_232556424_232564298_97	internal	EIF4E2	2	+	291	32	5	18	11.34%	36.3351237	0.010798573	0.177532488	0.01135044
ENSG00000135930_232550725_232574308_236	annotated	EIF4E2	2	+	708	28	32	5	6.78%	13.06755773	0.48842348	0.001840117	0.019030224
ENSG00000115919_142885368_143042172_465	annotated	KYNU	2	+	1395	327	44	10	14.84%	77.45388364	7.24E-10	2.15E-12	2.24E-20
ENSG00000102786_51452525_51365752_887	annotated	INTS6	13	-	2661	12	5	0	0.90%	1.490067784	0.127167926	0.004826937	0.004194481
ENSG00000162910_228106918_228106760_52	novel	MRPL55	1	-	156	139	11	4	38.46%	294.4149476	4.79E-05	4.09E-05	1.48E-08
ENSG00000049656_1344763_1318369_476	annotated	CLPTM1L	5	-	1428	123	23	9	7.35%	28.46076423	7.19E-05	5.36E-06	3.30E-09
ENSG00000158828_20645624_20650691_240	novel	PINK1	1	+	720	20	3	0	3.75%	9.178403642	0.022360378	0.003529166	0.000443113
ENSG00000111196_10610636_10609375_44	dORF	MAGOHB	12	-	132	14	7	0	11.36%	35.04481391	0.199537598	0.021084099	0.021025038
ENSG00000111196_10613532_10612827_81	annotated	MAGOHB	12	-	243	22	7	0	6.17%	29.91479706	0.153506949	0.021084099	0.015422968
ENSG00000197321_29550907_29458247_1616	annotated	SVIL	10	-	4848	11	3	1	0.31%	0.74972109	0.083852152	0.027849813	0.009942783
ENSG00000126012_53224889_53192967_1560	annotated	KDM5C	X	-	4680	52	2	1	1.54%	3.671361457	1.59E-05	1.01E-05	1.30E-09
ENSG00000163755_149129724_149172222_839	annotated	HPS3	3	+	2517	63	6	9	4.05%	8.270409003	2.03E-06	1.36E-05	2.41E-10
ENSG00000151632_5003835_5000478_146	novel	AKR1C2	10	-	438	927	242	29	25.34%	699.3189186	4.75E-05	4.60E-07	2.33E-10
ENSG00000151632_4998744_4989996_173	novel	AKR1C2	10	-	519	917	136	12	12.14%	583.8101369	0.000346991	2.81E-05	7.74E-08
ENSG00000005007_18860944_18866163_312	novel	UPF1	19	+	936	10	0	0	1.92%	3.530155247	0.011570466	0.011570466	0.000659484
ENSG00000144677_37971452_37980867_119	novel	CTDSPL	3	+	357	25	5	1	6.72%	23.13883271	0.040990615	0.0135992	0.002622153
ENSG00000133812_9993926_9839494_803	novel	SBF2	11	-	2409	14	4	0	0.87%	1.920263776	0.039615985	0.008373824	0.001680536
ENSG00000103811_78937694_78932021_132	dORF	CTSH	15	-	396	19	0	0	5.30%	15.85360629	0.008473674	0.008473674	0.000366502
ENSG00000103811_78931596_78922130_201	dORF	CTSH	15	-	603	12	1	0	3.98%	6.575572759	0.010020958	0.004826937	0.000255995
ENSG00000100242_38751348_38736267_668	annotated	SUN2	22	-	2004	159	19	6	8.83%	26.21615891	2.55E-09	6.90E-11	2.15E-18
ENSG00000110066_68185791_68166974_294	annotated	KMT5B	11	-	882	16	1	0	3.06%	5.994059521	0.005426623	0.003327803	9.96E-05
ENSG00000182473_76103692_76094370_283	novel	EXOC7	17	-	849	33	3	9	4.59%	12.84327859	0.002492245	0.029663034	0.000451669
ENSG00000100360_36767356_36763803_114	novel	IFT27	22	-	342	28	2	6	7.89%	27.05213705	0.011906648	0.046544029	0.002671302
ENSG00000204852_110614183_110627946_162	annotated	TCTN1	12	+	486	37	0	0	9.26%	25.1556248	0.000284266	0.000284266	5.48E-07
ENSG00000116977_236526074_236539164_138	novel	LGALS8	1	+	414	27	5	1	5.07%	21.54929551	0.020905271	0.013439622	0.00133113
ENSG00000135387_34052421_34099367_709	annotated	CAPRIN1	11	+	2127	112	15	9	7.05%	17.39883568	1.42E-07	1.66E-08	2.43E-14
ENSG00000167996_61965073_61964727_83	dORF	FTH1	11	-	249	35	6	1	6.02%	46.44493409	0.070558069	0.029237631	0.008690646
ENSG00000163374_155680436_155659657_730	annotated	YY1AP1	1	-	2190	38	7	2	2.19%	5.733358987	0.000928267	0.000321274	1.97E-06
ENSG00000171557_154612609_154604340_437	annotated	FGG	4	-	1311	50	8	1	5.72%	12.6019272	0.000208889	6.66E-06	1.24E-08
ENSG00000085511_161048621_161116870_1438	annotated	MAP3K4	6	+	4314	11	5	0	0.49%	0.842523839	0.168406755	0.007882058	0.00850822
ENSG00000111011_122520511_122505527_375	dORF	RSRC2	12	-	1125	44	5	5	5.07%	12.92319233	0.000220923	0.000503463	7.55E-07
ENSG00000100034_21945862_21925468_299	annotated	PPM1F	22	-	897	13	4	0	2.34%	4.788732335	0.016854696	0.007979402	0.000673932

ENSG00000172590_22833438_22834321_64	annotated	MRPL52	14	+	192	56	9	5	20.31%	96.37323824	0.002390059	0.001691112	2.38E-05
ENSG00000128245_31956262_31956792_176	dORF	YWHAH	22	+	528	116	24	0	17.05%	72.59282881	6.64E-05	7.56E-07	5.21E-10
ENSG00000214087_81683753_81681708_173	annotated	ARL16	17	-	519	22	14	5	5.20%	14.00635007	0.199610337	0.02959315	0.026786398
ENSG00000146833_99919401_99902131_294	annotated	TRIM4	7	-	882	27	2	0	2.04%	10.11497544	0.052581193	0.01300724	0.003266469
ENSG00000286140_69120428_69118854_524	annotated	DERPC	16	-	1572	40	4	0	3.63%	8.407697993	0.000360133	4.51E-05	1.23E-07
ENSG00000104231_81721281_81702921_227	annotated	ZFAND1	8	-	681	17	7	4	3.96%	8.248433229	0.035162868	0.028187253	0.004282096
ENSG00000147457_23246786_23258336_135	novel	CHMP7	8	+	405	22	0	12	4.44%	17.94887823	0.013640586	0.19809812	0.015353416
ENSG00000176783_179550570_179609519_708	annotated	RUFY1	5	+	2124	68	0	1	3.53%	10.57849911	5.21E-06	6.26E-06	2.72E-10
ENSG00000215021_6970543_6965685_299	annotated	PHB2	12	-	897	478	59	41	24.08%	176.0780043	1.17E-10	9.77E-12	1.41E-20
ENSG00000143374_150488016_150507064_443	annotated	TARS2	1	+	1329	62	11	2	4.97%	15.41474562	0.000521258	1.93E-05	8.54E-08
ENSG00000143374_150487451_150492478_135	Overlap_uORF	TARS2	1	+	405	19	1	0	7.41%	15.50130393	0.003247793	0.002122793	3.97E-05
ENSG00000130779_122354514_122272875_897	annotated	CLIP1	12	-	2691	32	7	3	1.56%	3.929216275	0.010882675	0.002181097	0.000137292
ENSG00000166986_57516317_57516581_55	dORF	MARS	12	+	165	30	8	0	16.36%	60.07682384	0.053438621	0.003632191	0.00118942
ENSG00000166986_57490577_57516138_635	dORF	MARS	12	+	1905	99	6	5	5.67%	17.17156461	7.69E-07	9.29E-07	6.43E-12
ENSG00000166986_57488091_57490215_140	annotated	MARS	12	+	420	28	2	0	7.86%	22.02816874	0.005625935	0.001506153	5.01E-05
ENSG00000139112_10213130_10221852_117	annotated	GABARAPL1	12	+	351	19	1	2	5.98%	17.88611992	0.020342454	0.032996028	0.003004824
ENSG00000117519_94903458_94897742_288	annotated	CNN3	1	-	864	164	17	3	12.85%	62.71909155	2.71E-07	9.12E-08	2.38E-13
ENSG00000065559_12020887_12141260_410	annotated	MAP2K4	17	+	1230	26	10	0	2.93%	6.984541308	0.060130931	0.001003278	0.00051345
ENSG00000073060_124863720_124778579_552	annotated	SCARB1	12	-	1656	297	52	55	12.14%	59.26056265	1.02E-08	6.35E-09	6.81E-16
ENSG00000101417_33707963_33707706_85	Overlap_dORF	PXMP4	20	-	255	11	3	2	8.24%	14.25352095	0.08791702	0.023672501	0.009148781
ENSG00000008283_63437460_63434402_222	annotated	CYB561	17	-	666	33	11	0	5.41%	16.37228758	0.011805604	0.001011481	7.74E-05
ENSG00000008283_63437460_63434402_222	annotated	CYB561	17	-	666	33	11	0	5.41%	16.37228758	0.011805604	0.001011481	7.74E-05
ENSG00000134759_36129934_36174641_800	annotated	ELP2	18	+	2400	61	9	13	3.50%	8.398239333	8.24E-05	0.000439645	2.63E-07
ENSG00000148841_104315931_104314408_507	annotated	ITPRIP	10	-	1521	44	3	0	3.55%	9.558574207	0.000369137	8.58E-05	2.29E-07
ENSG00000115275_74464717_74463284_89	annotated	MOGS	2	-	267	25	1	1	5.62%	30.93843924	0.021084099	0.021084099	0.002029891
ENSG00000115275_74462963_74461275_562	dORF	MOGS	2	-	1686	74	1	0	4.98%	14.50253102	1.99E-06	1.60E-06	2.79E-11
ENSG00000127054_1324608_1311859_578	annotated	INTS11	1	-	1734	73	7	4	5.19%	13.91052178	6.54E-06	5.04E-06	2.75E-10
ENSG00000133731_81680756_81660528_168	annotated	IMPA1	8	-	504	14	3	0	4.17%	9.178403642	0.031982531	0.007882058	0.001276233
ENSG00000174903_66268680_66276238_201	annotated	RAB1B	11	+	603	189	27	7	19.90%	103.5652709	3.09E-07	1.54E-08	5.02E-14
ENSG00000128513_124842818_124827254_120	dORF	POT1	7	-	360	13	0	0	4.17%	11.93192473	0.019679754	0.019679754	0.001784964
ENSG00000146729_55982257_55991831_66	annotated	NIPSNAP2	7	+	198	63	26	5	21.21%	105.1344417	0.03003023	0.000953102	0.000211902
ENSG00000128708_171965844_171979444_208	dORF	HAT1	2	+	624	61	10	6	8.65%	32.30092051	0.001878153	0.000186489	2.49E-06
ENSG00000128708_171925548_171965796_119	annotated	HAT1	2	+	357	41	1	4	7.56%	37.94768565	0.005159595	0.022138672	0.000606077
ENSG00000171570_40778376_40786963_213	annotated	RAB4B-EGLN2	19	+	639	37	6	0	6.57%	19.13244703	0.006425416	0.000448121	2.00E-05
ENSG00000116337_109625404_109631152_761	annotated	AMPD2	1	+	2283	8	6	0	0.79%	1.15785381	0.280084718	0.011570466	0.021802746
ENSG00000115875_38751256_38745133_226	annotated	SRSF7	2	-	678	416	35	7	24.34%	202.7371282	2.79E-09	6.29E-11	2.16E-18
ENSG00000151131_104986353_104994696_185	annotated	C12orf45	12	+	555	35	2	1	6.49%	20.83745692	0.003350306	0.001494238	2.95E-05
ENSG00000172269_119101655_119101491_54	novel	DPAGT1	11	-	162	16	2	0	9.26%	32.63432406	0.035772986	0.021084099	0.003354605
ENSG00000172269_119100388_119096998_236	novel	DPAGT1	11	-	708	52	21	1	8.47%	24.26832149	0.012498828	5.32E-05	7.62E-06
ENSG00000127603_39231238_39485794_7500	annotated	MACF1	1	+	22500	71	15	0	0.44%	1.042666654	4.63E-05	2.02E-07	1.10E-10
ENSG00000177479_48927571_48961631_102	annotated	ARIH2	3	+	306	15	0	0	4.90%	16.1971829	0.021084099	0.021084099	0.002029891
ENSG00000177479_48968545_48983270_310	dORF	ARIH2	3	+	930	35	10	2	3.87%	12.43525655	0.01243972	0.002296041	0.000165079
ENSG00000114745_39099362_39098236_138	dORF	GORASP1	3	-	414	14	4	0	3.62%	11.17370878	0.065565251	0.021084099	0.006135922
ENSG00000090857_70120496_70156879_878	annotated	PDPR	16	+	2634	15	1	0	1.14%	1.881677284	0.006024334	0.002008257	6.96E-05
ENSG00000005022_119468516_119471058_298	annotated	SLC25A5	X	+	894	729	68	24	18.79%	269.4385069	1.10E-09	1.22E-09	1.47E-17
ENSG00000106692_105601166_105619974_308	dORF	FKTN	9	+	924	14	1	0	2.60%	5.006401987	0.007930666	0.005160369	0.000216377
ENSG00000177731_18256989_18245138_1238	dORF	FLII	17	-	3714	183	27	12	6.38%	16.28091632	1.53E-10	2.71E-13	6.27E-22
ENSG00000178691_31995586_31999003_200	novel	SUZ12	17	+	600	15	0	0	3.00%	8.260563278	0.01300724	0.01300724	0.000821865
ENSG00000182628_59155133_59112277_82	annotated	SKA2	17	-	246	26	3	4	14.63%	34.92270654	0.005435927	0.007322425	0.000210202
ENSG00000197498_110985134_111025582_243	Overlap_dORF	RPF2	6	+	729	30	1	0	4.94%	13.59763503	0.001453991	0.000978918	8.74E-06
ENSG00000132330_238081834_238091287_114	novel	SCLY	2	+	342	8	2	0	4.39%	7.729182014	0.101051022	0.019679754	0.009164498
ENSG00000137804_41332958_41380186_426	annotated	NUSAP1	15	+	1278	124	19	12	10.09%	32.0597761	2.86E-07	2.41E-07	6.47E-13
ENSG00000137801_39581894_39595369_1158	annotated	THBS1	15	+	3474	103	9	2	3.80%	9.796638084	2.06E-07	9.77E-09	2.15E-14
ENSG00000090238_30095368_30092710_119	annotated	YPEL3	16	-	357	17	13	1	6.72%	15.73440624	0.149640738	0.008316137	0.007605951
ENSG00000103042_58675949_58667385_171	dORF	SLC38A7	16	-	513	32	9	0	4.09%	20.61115204	0.028581075	0.008574016	0.001221281
ENSG00000103042_58680126_58676008_260	annotated	SLC38A7	16	-	780	12	0	4	3.08%	5.083423556	0.00419316	0.062411228	0.001592437
ENSG00000104679_23290179_23296097_253	annotated	R3HCC1	8	+	759	21	13	0	3.16%	9.142125367	0.191226008	0.005454749	0.007812091
ENSG00000104611_19319572_19394642_446	annotated	SH2D4A	8	+	1338	35	2	0	4.71%	8.643339753	6.27E-05	1.58E-05	7.81E-09
ENSG00000158856_22067533_22081463_372	annotated	DMTN	8	+	1116	20	6	0	2.69%	5.921550737	0.041018323	0.001801897	0.000504555
ENSG00000054118_36282564_36304017_955	annotated	THRAP3	1	+	2865	65	9	5	2.93%	7.496497216	2.81E-05	1.74E-05	3.84E-09
ENSG00000165609_12186291_12167702_219	annotated	NUDT5	10	-	657	149	23	21	17.35%	74.93600782	8.95E-07	5.84E-06	4.69E-11
ENSG00000178035_49027943_49026949_110	novel	IMPDH2	3	-	330	42	6	0	10.00%	42.05377669	0.002710157	0.001626321	2.59E-05
ENSG00000100722_88563134_88611751_730	annotated	ZC3H14	14	+	2190	69	24	2	5.07%	10.4105729	0.000604545	7.40E-08	9.59E-10
ENSG00000070366_2303720_2061492_1419	annotated	SMG6	17	-	4257	39	4	2	1.34%	3.027126783	0.000429508	0.000124221	3.76E-07
ENSG00000062650_86517949_86437543_1095	annotated	WAPL	10	-	3285	20	10	2	0.91%	2.011704908	0.112672847	0.006101803	0.00427586
ENSG00000114019_134371433_134357705_777	annotated	AMOTL2	3	-	2331	87	7	26	3.86%	12.33237246	1.08E-06	0.001750495	3.07E-08
ENSG00000117305_23820578_23810676_181	novel	HMGCL	1	-	543	9	0	0	2.76%	5.476616538	0.020613417	0.020613417	0.001946209
ENSG00000114098_138239478_138295892_411	novel	ARMC8	3	+	1233	17	7	0	2.19%	4.555703998	0.035256712	0.003303074	0.000688509

ENSG00000223784_6737436_6738367_67	novel	LINP1	10	+	201	39	0	3	10.45%	64.1118344	0.008473674	0.020342454	0.00085717
ENSG00000168807_69187167_69300924_540	annotated	SNTB2	16	+	1620	50	14	0	3.33%	10.19822627	0.007214452	8.27E-05	5.58E-06
ENSG00000147649_97644507_97724670_582	annotated	MTDH	8	+	1746	242	36	9	12.71%	45.79739549	8.41E-12	1.15E-12	1.24E-22
ENSG00000034713_75566496_75577369_114	annotated	GABARAPL2	16	+	342	326	25	19	32.46%	314.9641671	3.85E-07	1.63E-06	5.76E-12
ENSG00000138777_105453627_105396277_129	novel	PPA2	4	-	387	21	2	4	4.65%	17.92990479	0.029237631	0.045644785	0.005671119
ENSG00000204628_181243800_181242319_95	novel	RACK1	5	-	285	281	63	43	21.05%	325.7850219	0.000631852	0.013756376	6.19E-05
ENSG00000005882_50105396_50110097_312	annotated	PDK2	17	+	936	17	2	0	2.88%	6.00126392	0.005561256	0.003452921	0.000105543
ENSG00000169288_77883330_77906983_158	novel	MRPL1	4	+	474	18	9	0	4.43%	12.54769106	0.125735014	0.007979402	0.005952869
ENSG00000123213_65780203_65785915_126	novel	NLN	5	+	378	44	1	3	10.32%	38.46188193	0.001393565	0.002470037	2.05E-05
ENSG00000113648_135389012_135360760_138	annotated	H2AFY	5	-	414	106	11	3	13.77%	84.60093792	0.00026227	0.000233082	4.18E-07
ENSG00000113648_135335071_135334976_31	dORF	H2AFY	5	-	93	16	0	0	22.58%	56.84688707	0.008175611	0.008175611	0.000342518
ENSG00000127191_136898741_136925901_501	annotated	TRAF2	9	+	1503	42	12	0	3.99%	9.233364143	0.001582415	3.51E-05	4.84E-07
ENSG00000163362_200899496_200913629_552	annotated	INAVA	1	+	1656	33	9	0	3.08%	6.584506961	0.006948227	0.000123289	7.40E-06
ENSG00000141030_17276159_17267952_133	novel	COPS3	17	-	399	95	7	6	9.02%	78.67203122	0.001061589	0.002961771	1.91E-05
ENSG00000094841_75274255_75296385_176	annotated	UPRT	X	+	528	8	1	0	3.41%	5.006401987	0.041782825	0.011570466	0.002331326
ENSG00000100941_39175280_39181863_717	annotated	PNN	14	+	2151	66	4	12	4.04%	10.13848771	6.17E-06	0.000285652	1.63E-08
ENSG00000086061_33026485_33038903_397	annotated	DNAJA1	9	+	1191	434	50	20	21.91%	120.4058594	7.10E-13	3.91E-15	4.22E-26
ENSG00000150768_112025473_112062535_647	annotated	DLAT	11	+	1941	135	9	4	7.57%	22.98147434	6.26E-10	5.99E-10	4.20E-18
ENSG00000182944_29268337_29300161_600	annotated	EWSR1	22	+	1800	164	24	5	6.00%	30.10516395	6.27E-06	2.50E-07	1.54E-11
ENSG00000070010_19475590_19450740_261	annotated	UFD1	22	-	783	119	8	3	12.26%	50.2174728	1.64E-06	1.56E-06	2.24E-11
ENSG00000259522_24190408_24189415_140	annotated	AL136295.4	14	-	420	65	2	0	12.86%	51.13682029	0.000148366	8.85E-05	9.44E-08
ENSG00000259522_24188787_24180442_1081	dORF	AL136295.4	14	-	3243	108	22	2	4.16%	11.00389558	1.15E-05	1.94E-09	4.10E-13
ENSG00000147548_38348138_38317956_634	annotated	NSD3	8	-	1902	20	2	1	1.74%	3.474474565	0.004968901	0.002559322	7.16E-05
ENSG00000213928_24162253_24164151_185	novel	IRF9	14	+	555	18	5	0	4.32%	10.71640641	0.044710437	0.005554612	0.001365447
ENSG00000111642_6606316_6570676_1893	annotated	CHD4	12	-	5679	91	9	3	2.17%	5.294673416	1.97E-07	2.05E-08	4.14E-14
ENSG00000085511_161048621_161109756_1326	annotated	MAP3K4	6	+	3978	9	5	0	0.45%	0.747562288	0.23864467	0.011772051	0.01769996
ENSG00000153956_82349627_82050578_270	annotated	CACNA2D1	7	-	810	20	0	0	2.59%	8.158581015	0.006937203	0.006937203	0.000251015
ENSG00000205133_92916989_92886622_86	annotated	TRIQK	8	-	258	21	3	0	10.47%	26.89485718	0.015147584	0.003327803	0.000279725
ENSG00000112514_33417333_33416650_101	novel	CUTA	6	-	303	152	26	2	23.76%	165.7565173	5.66E-05	1.37E-05	6.16E-09
ENSG00000112514_33417668_33417477_63	novel	CUTA	6	-	189	517	38	21	31.75%	903.8542253	0.000141459	7.10E-05	7.31E-08
ENSG00000139218_45928755_45922048_1148	annotated	SCAF11	12	-	3444	59	4	1	2.53%	5.660548588	5.82E-06	1.26E-06	6.45E-11
ENSG00000146263_97267976_97146806_1002	annotated	MMS22L	6	-	3006	11	0	0	0.80%	1.209131019	0.00419316	0.00419316	9.65E-05
ENSG00000147509_53939615_53958458_205	annotated	RGS20	8	+	615	12	0	0	2.93%	6.4472689	0.011772051	0.011772051	0.000681273
ENSG00000256537_11171495_11171563_22	annotated	SMIM10L1	12	+	66	26	10	0	27.27%	130.1664517	0.316140469	0.013428348	0.028480398
ENSG00000111843_10728666_10730666_37	novel	TMEM14C	6	+	111	150	8	0	37.84%	446.5169339	0.001820107	0.000476727	5.61E-06
ENSG00000159377_151401314_151401829_47	novel	PSMB4	1	+	141	69	15	0	23.40%	161.6961322	0.004514955	0.001626321	4.30E-05
ENSG00000109971_123061324_123057734_627	annotated	HSPA8	11	-	1881	923	171	42	15.31%	162.1371591	2.89E-14	6.26E-16	2.70E-28
ENSG00000184677_22490252_22526396_1026	annotated	ZBTB40	1	+	3078	12	2	0	0.78%	1.288197002	0.019435691	0.004826937	0.000499969
ENSG00000138867_24548927_24543006_201	annotated	GUCD1	22	-	603	22	3	8	2.99%	12.05521672	0.060667625	0.136019724	0.030595301
ENSG00000138867_24555016_24548047_59	Overlap_uORF	GUCD1	22	-	177	16	6	0	10.17%	29.86870338	0.128949518	0.013428348	0.009009011
ENSG00000204227_33208823_33212399_406	annotated	RING1	6	+	1218	34	2	0	2.71%	9.223617453	0.002660072	0.001558047	2.44E-05
ENSG00000123106_28257216_28549173_441	annotated	CCDC91	12	+	1323	10	8	0	1.81%	2.497524801	0.323350774	0.004149608	0.014239836
ENSG00000023191_502162_500480_91	novel	RNH1	11	-	273	58	10	4	12.09%	70.19965863	0.01371513	0.004143772	0.000305976
ENSG00000185000_144326636_144316554_488	annotated	DGAT1	8	-	1464	61	15	2	4.30%	13.76760546	0.000257908	4.25E-05	8.29E-08
ENSG00000065615_83864202_83959878_487	annotated	CYB5R4	6	+	1461	29	2	0	2.05%	6.558695005	0.002227676	0.002321823	3.00E-05
ENSG00000100865_102362851_102348709_118	annotated	CINP	14	-	354	58	32	5	15.25%	54.13702487	0.022338596	0.000459896	8.40E-05
ENSG00000100865_102348733_102348557_58	Overlap_dORF	CINP	14	-	174	13	3	0	8.62%	24.68674083	0.068108435	0.020613417	0.00626272
ENSG00000105518_11345476_11342815_176	novel	TMEM205	19	-	528	489	58	15	28.41%	306.0163214	5.82E-09	7.82E-10	5.07E-17
ENSG00000272741_196318152_196270538_106	annotated	AC069257.3	3	-	318	17	8	0	8.49%	17.66409758	0.086894686	0.002967235	0.00182296
ENSG00000137411_30914837_30926210_1063	annotated	VARS2	6	+	3189	25	12	2	1.69%	2.590330285	0.017315147	0.00023325	3.62E-05
ENSG00000054392_210418268_210674379_227	annotated	HHAT	1	+	681	9	0	0	2.20%	4.366817592	0.019679754	0.019679754	0.001784964
ENSG00000174669_66369084_66363436_326	Overlap_dORF	SLC29A2	11	-	978	61	12	11	5.52%	20.60917628	0.003204041	0.004052887	7.24E-05
ENSG00000136193_29969067_29923957_414	annotated	SCRN1	7	-	1242	76	15	1	6.52%	20.21909208	4.77E-05	3.01E-06	1.27E-09
ENSG00000188064_45950016_45922856_282	annotated	WNT7B	22	-	846	37	4	1	3.90%	14.45110361	0.002182477	0.001513502	1.96E-05
ENSG00000135930_232556424_232564298_97	internal	EIF4E2	2	+	291	32	5	18	11.34%	36.3351237	0.010798573	0.177532488	0.01135044
ENSG00000135930_232550725_232567251_233	annotated	EIF4E2	2	+	699	28	32	5	6.87%	13.23580954	0.48842348	0.001840117	0.019030224
ENSG00000115966_175130185_175074609_126	annotated	ATF2	2	-	378	27	0	0	9.52%	23.60160937	0.001003278	0.001003278	6.24E-06
ENSG00000149806_65121813_65120763_87	annotated	FAU	11	-	261	513	130	57	25.29%	649.4511818	0.061918319	0.00418176	0.001574498
ENSG00000149806_65120755_65120681_24	dORF	FAU	11	-	72	158	40	2	20.83%	725.0938877	0.124431937	0.023199731	0.013082704
ENSG00000171792_2885285_2885512_75	Overlap_uORF	RHNO1	12	+	225	11	2	0	9.33%	16.15399041	0.049121095	0.007029303	0.001832503
ENSG00000147799_144548098_144530422_785	annotated	ARHGAP39	8	-	2355	12	0	0	0.89%	1.683681687	0.007029303	0.007029303	0.000257364
ENSG00000179832_144223183_144261740_1202	annotated	MROH1	8	+	3606	16	1	4	0.58%	1.466101081	0.011306644	0.051117422	0.002823276
ENSG00000213614_72375972_72362453_88	annotated	HEXA	15	-	264	40	11	0	21.59%	50.06401987	0.00086852	4.97E-05	3.40E-07
ENSG00000213614_72348106_72346316_22	dORF	HEXA	15	-	66	11	2	0	27.27%	55.07042185	0.035374934	0.012798403	0.002143354
ENSG00000213614_72351228_72348103_86	dORF	HEXA	15	-	258	122	6	3	22.09%	156.2463132	6.32E-05	0.000129595	5.99E-08
ENSG00000058600_22302969_22309044_94	novel	POLR3E	16	+	282	11	1	0	5.32%	12.88882214	0.035772986	0.021084099	0.003354605
ENSG00000092531_42513429_42531478_168	dORF	SNAP23	15	+	504	43	2	1	6.55%	28.19081119	0.001785595	0.00247378	2.59E-05
ENSG00000134287_48941095_48938947_144	annotated	ARF3	12	-	432	76	6	6	14.58%	58.12988973	3.09E-05	0.000171887	4.08E-08

ENSG00000172113_48296784_48294637_141	annotated	NME6	3	-	423	9	0	2	3.55%	7.030266619	0.020613417	0.083503598	0.007749259
ENSG00000149100_32593885_32593953_22	dORF	EIF3M	11	+	66	28	7	0	22.73%	140.1792556	0.298056333	0.021084099	0.035031337
ENSG00000149100_32583888_32589551_59	annotated	EIF3M	11	+	177	33	20	3	15.25%	61.60420072	0.19975566	0.003684287	0.006376824
ENSG00000160877_13135226_13138406_521	annotated	NACC1	19	+	1563	68	4	9	4.80%	14.37538843	1.34E-05	0.00018604	2.06E-08
ENSG00000116977_236526074_236548161_299	annotated	LGALS8	1	+	897	41	5	1	4.35%	15.10292506	0.001679255	0.001000453	1.03E-05
ENSG00000197912_89524038_89526490_123	novel	SPG7	16	+	369	16	2	0	7.32%	14.32726422	0.006083074	0.003229477	0.000108335
ENSG00000100350_36506422_36490008_684	annotated	FOXRED2	22	-	2052	175	15	13	8.04%	28.17930943	7.06E-09	8.38E-09	6.20E-16
ENSG00000145734_71458826_71509634_693	annotated	BDP1	5	+	2079	8	3	0	0.72%	1.271467171	0.11777946	0.016947427	0.009674391
ENSG00000124155_45416169_45425826_437	annotated	PIGT	20	+	1311	323	26	3	16.48%	81.4084497	8.66E-13	6.33E-14	7.44E-25
ENSG00000110801_121888857_121915865_209	annotated	PSMD9	12	+	627	120	5	33	13.40%	63.23876194	2.47E-06	0.000630107	1.80E-08
ENSG00000107872_102421130_102422993_300	annotated	FBXL15	10	+	900	24	16	2	3.67%	8.811267496	0.290650453	0.00345766	0.010730339
ENSG00000157429_71479002_71475170_446	annotated	ZNF19	16	-	1338	16	0	0	1.79%	3.95124103	0.005257623	0.005257623	0.000148366
ENSG00000162063_2443796_2449281_154	annotated	CCNF	16	+	462	9	0	1	3.25%	6.436802554	0.020613417	0.035374934	0.003252526
ENSG00000123992_219386772_219374292_410	annotated	DNPEP	2	-	1230	57	7	2	4.88%	15.31226364	0.000673127	5.33E-05	2.76E-07
ENSG00000147130_71253255_71249592_349	annotated	ZMYM3	X	-	1047	23	0	2	2.01%	7.258565631	0.008473674	0.023197249	0.000977881
ENSG00000114861_71198378_70959247_675	annotated	FOXP1	3	-	2025	18	1	5	1.19%	2.937089165	0.008219047	0.047480794	0.002006772
ENSG00000160710_154589805_154584806_353	dORF	ADAR	1	-	1059	103	10	4	6.80%	32.13741332	2.90E-05	3.71E-05	8.26E-09
ENSG00000160710_154601756_154589900_493	annotated	ADAR	1	-	1479	56	6	2	5.88%	12.51092748	4.18E-05	3.94E-06	1.41E-09
ENSG00000114861_71198378_70959247_675	annotated	FOXP1	3	-	2025	18	1	5	1.19%	2.937089165	0.008219047	0.047480794	0.002006772
ENSG00000116133_54876020_54852233_378	novel	DHCR24	1	-	1134	284	31	9	17.46%	82.75132173	6.92E-10	8.45E-12	7.66E-20
ENSG00000116133_54883737_54879272_55	novel	DHCR24	1	-	165	57	5	0	12.73%	114.1459653	0.008877961	0.008776163	0.000395901
ENSG00000140332_70094543_70050097_692	annotated	TLE3	15	-	2076	21	0	2	1.16%	3.342424448	0.005306578	0.018151375	0.00050607
ENSG00000161513_74864552_74862817_248	novel	FDXR	17	-	744	31	2	0	4.84%	13.76760546	0.003011	0.001007374	1.85E-05
ENSG00000138029_26254255_26289953_474	annotated	HADHB	2	+	1422	239	36	3	15.19%	55.53515115	1.35E-10	1.47E-13	3.12E-22
ENSG00000091157_56695186_57027207_1042	annotated	WDR7	18	+	3126	12	0	0	0.67%	1.268416626	0.007882058	0.007882058	0.000319636
ENSG00000164904_126595198_126577194_136	annotated	ALDH7A1	5	-	408	33	2	3	8.82%	26.72535178	0.003249213	0.003232525	5.91E-05
ENSG00000163719_9671166_9683381_142	dORF	MTMR14	3	+	426	15	0	0	7.75%	11.63459617	0.001203942	0.001203942	8.87E-06
ENSG00000101639_12999425_13124770_2537	annotated	CEP192	18	+	7611	12	1	0	0.24%	0.520965757	0.026832696	0.011772051	0.001510382
ENSG00000269893_118279412_118279714_36	novel	SNHG8	4	+	108	28	18	5	13.89%	85.66510066	0.172615536	0.057424805	0.037344862
ENSG00000106692_105604242_105635264_329	dORF	FKTN	9	+	987	33	2	0	5.17%	11.04756183	0.000185149	0.000112262	1.48E-07
ENSG00000101220_3758574_3754058_133	annotated	C20orf27	20	-	399	60	3	0	10.53%	49.68759866	0.000468432	0.000469808	1.44E-06
ENSG00000115364_75646808_75655285_292	annotated	MRPL19	2	+	876	234	18	0	14.73%	88.26355283	1.56E-07	4.28E-09	7.47E-15
ENSG00000156642_73633215_73561910_282	annotated	NPTN	15	-	846	487	95	25	26.24%	190.2077691	1.49E-11	3.19E-12	5.93E-22
ENSG00000127914_91973855_92110159_3835	annotated	AKAP9	7	+	11505	30	11	7	0.42%	0.861597213	0.025379813	0.007374304	0.000949425
ENSG00000163626_73065298_73058114_150	dORF	COX18	4	-	450	19	1	0	4.67%	13.95117354	0.008473674	0.008373824	0.000362422
ENSG00000163626_73069649_73068083_127	annotated	COX18	4	-	381	10	1	0	4.72%	8.672507378	0.026832696	0.011772051	0.001510382
ENSG00000119777_27033016_27040837_689	annotated	TMEM214	2	+	2067	69	13	0	4.79%	11.03006998	5.78E-05	1.65E-07	1.18E-10
ENSG00000139974_61050574_61052429_127	dORF	SLC38A6	14	+	381	10	6	0	3.94%	8.672507378	0.308985033	0.020613417	0.036244827
ENSG00000139974_61030447_61048279_183	dORF	SLC38A6	14	+	549	26	4	0	4.92%	15.64842588	0.015376781	0.003684287	0.000310392
ENSG00000138303_72213264_72210754_51	annotated	ASCC1	10	-	153	7	0	4	11.76%	15.1173707	0.009815329	0.170566842	0.010088762
ENSG00000262246_4416518_4355158_925	annotated	CORO7	16	-	2775	17	2	5	0.76%	2.024210101	0.032657853	0.059434472	0.008063634
ENSG00000183696_48094803_48108357_173	Overlap_dORF	UPP1	7	+	519	26	0	2	6.94%	16.55295917	0.000978918	0.003975543	2.39E-05
ENSG00000164828_838815_838970_51	internal	SUN1	7	+	153	22	3	16	19.61%	47.5117365	0.010402772	0.229394231	0.015451134
ENSG00000113391_94053185_93737882_305	annotated	FAM172A	5	-	915	29	0	0	4.26%	10.47240809	0.000631191	0.000631191	2.56E-06
ENSG00000186010_19526190_19528126_110	Overlap_dORF	NDUFA13	19	+	330	1129	74	9	50.91%	1130.445569	6.41E-11	4.67E-11	3.55E-20
ENSG00000197747_151986230_151983163_97	annotated	S100A10	1	-	291	1744	152	48	63.92%	1980.264241	1.86E-11	5.81E-12	1.33E-21
ENSG00000009413_111405521_111300016_2959	annotated	REV3L	6	-	8877	8	4	0	0.17%	0.297778557	0.214538039	0.019679754	0.021895271
ENSG00000147202_96758160_97469743_980	annotated	DIAPH2	X	+	2940	22	8	0	1.33%	2.472549553	0.063129387	0.000539992	0.000346183
ENSG00000075711_197297204_197044623_865	annotated	DLG1	3	-	2595	39	4	0	2.08%	4.965887751	0.000762836	7.71E-05	4.39E-07
ENSG00000132305_86195382_86144268_758	annotated	IMMT	2	-	2274	178	14	8	7.39%	25.86420868	5.52E-09	1.39E-09	8.34E-17
ENSG00000115363_75518101_75493236_139	annotated	EVA1A	2	-	417	17	2	0	3.60%	13.4704629	0.070008252	0.021084099	0.006564739
ENSG00000068120_42562623_42565968_564	annotated	COASY	17	+	1692	82	9	5	6.56%	16.01338508	2.07E-06	9.11E-07	1.67E-11
ENSG00000167085_49413264_49404992_272	annotated	PHB	17	-	816	287	31	23	12.13%	116.2147873	6.52E-07	1.58E-06	9.26E-12
ENSG00000088325_31757477_31801080_747	annotated	TPX2	20	+	2241	140	17	5	8.30%	20.64219293	1.66E-09	1.39E-11	3.05E-19
ENSG00000110367_118786251_118754712_483	annotated	DDX6	11	-	1449	96	31	2	6.00%	21.89134782	0.003925684	1.25E-06	9.52E-08
ENSG00000160055_32222322_32216410_164	annotated	TMEM234	1	-	492	7	0	1	3.05%	4.701133573	0.019216965	0.041782825	0.003600526
ENSG00000080493_71397588_71567047_832	annotated	SLC4A4	4	+	2496	10	2	0	0.60%	1.323808218	0.083503598	0.020613417	0.007749259
ENSG00000182504_101724677_101758484_591	novel	CEP97	3	+	1773	23	4	0	1.86%	4.286361092	0.014587973	0.00140552	0.000128651
ENSG00000087460_58895650_58899955_28	dORF	GNAS	20	+	84	9	0	0	17.86%	35.40241405	0.019679754	0.019679754	0.001784964
ENSG00000070087_149968535_149966167_91	annotated	PFN2	3	-	273	285	20	7	36.26%	344.9465984	4.10E-07	6.22E-07	2.34E-12
ENSG00000064545_19132419_19119930_354	novel	TMEM161A	19	-	1062	38	7	3	4.80%	11.82302842	0.00370951	0.000181775	5.07E-06
ENSG00000108846_50663766_50664129_90	novel	ABCC3	17	+	270	73	17	2	21.11%	89.33646212	0.001885431	0.000137497	1.91E-06
ENSG00000160410_40583624_40591207_257	annotated	SHKBP1	19	+	771	35	5	6	6.23%	14.9997258	0.002548497	0.002096929	3.10E-05
ENSG00000104973_49818342_49838824_796	annotated	MED25	19	+	2388	35	10	0	2.14%	4.842876294	0.010488659	0.000118295	1.16E-05
ENSG00000105426_5286095_5159099_1519	novel	PTPRS	19	-	4557	110	10	7	3.49%	7.975966299	7.69E-09	3.48E-09	2.84E-16
ENSG00000141349_44070966_44071628_72	annotated	G6PC3	17	+	216	77	9	6	20.83%	117.7895134	0.000395349	0.000952052	2.46E-06
ENSG00000141349_44074700_44076043_231	dORF	G6PC3	17	+	693	213	22	14	19.48%	101.5584403	1.05E-08	3.29E-08	3.57E-15
ENSG00000137936_93567429_93562241_109	novel	BCAR3	1	-	327	18	6	4	9.17%	18.18839621	0.023814921	0.028206679	0.002983639

ENSG00000166037_95790699_95821984_270	annotated	CEP57	11	+	810	11	9	24	2.22%	4.487219558	0.116253847	0.116253847	0.045661142
ENSG00000117620_99993624_100022476_302	annotated	SLC35A3	1	+	906	16	9	0	3.64%	5.835276488	0.151590816	0.000888449	0.001652192
ENSG00000156875_100038278_100082209_475	annotated	MFSD14A	1	+	1425	151	25	6	9.26%	35.01319453	2.78E-07	1.61E-08	4.68E-14
ENSG00000166171_101588337_101609471_203	annotated	DPCD	10	+	609	48	0	2	8.87%	26.04315516	8.23E-05	0.000273665	1.62E-07
ENSG00000117620_99999317_100022476_244	dORF	SLC35A3	1	+	732	14	7	0	3.69%	6.319556606	0.119389439	0.002500085	0.002417183
ENSG00000168564_183444798_183447427_580	annotated	CDKN2AIP	4	+	1740	21	1	8	1.55%	3.987858134	0.005084584	0.079491076	0.002452229
ENSG00000251537_15651609_15574099_953	annotated	AC005324.3	17	-	2859	83	14	5	1.99%	9.592539378	0.002054494	7.28E-05	1.21E-06
ENSG00000182973_32685461_32773611_717	annotated	CNOT10	3	+	2151	16	4	1	1.53%	2.457815201	0.020272101	0.002532006	0.000301522
ENSG00000065357_55939303_55953768_538	annotated	DGKA	12	+	1614	23	2	0	2.23%	4.70862343	0.001459721	0.000862735	7.79E-06
ENSG00000029363_136278727_136269264_701	annotated	BCLAF1	6	-	2103	10	4	0	1.00%	1.571196059	0.08847975	0.007593099	0.003775554
ENSG00000048828_93451916_93564540_1118	annotated	FAM120A	9	+	3354	221	20	8	6.89%	21.77202724	4.11E-12	4.39E-13	2.36E-23
ENSG00000181744_143972753_143985556_103	novel	DIPK2A	3	+	309	12	0	0	4.85%	12.83194296	0.021084099	0.021084099	0.002029891
ENSG00000162366_47185084_47183971_51	novel	PDZK1IP1	1	-	153	14	5	0	11.76%	30.23474141	0.086207551	0.012798403	0.005490656
ENSG00000165675_130679569_130625314_437	annotated	ENOX2	X	-	1311	7	0	3	1.14%	1.764269808	0.019216965	0.170087137	0.016246064
ENSG00000100650_69768157_69771461_272	annotated	SRSF5	14	+	816	361	60	29	24.26%	146.1795757	2.73E-08	1.19E-10	4.29E-17
ENSG00000124593_41787698_41789328_74	dORF	AL365205.1	6	+	222	408	43	9	44.59%	607.2630302	1.44E-06	4.73E-07	6.20E-12
ENSG00000172819_53220100_53211676_443	annotated	RARG	12	-	1329	24	2	1	2.48%	5.966998305	0.003791764	0.002345022	5.07E-05
ENSG00000134138_37098019_36892173_406	annotated	MEIS2	15	-	1218	7	0	2	1.23%	1.898980064	0.019216965	0.119134635	0.010787784
ENSG00000005889_24179200_24211376_730	annotated	ZFX	X	+	2190	42	2	4	3.01%	6.33687046	4.02E-05	0.000197673	5.99E-08
ENSG00000135390_53672614_53665314_141	annotated	ATP5MC2	12	-	423	370	85	27	30.50%	289.0220721	2.46E-06	4.35E-08	1.15E-12
ENSG00000172534_153954081_153949347_597	annotated	HCFC1	X	-	1791	22	1	1	2.01%	4.05879156	0.002467336	0.001612025	2.34E-05
ENSG00000179918_30445280_30444381_299	annotated	SEPHS2	16	-	897	24	3	6	3.01%	8.840736618	0.003554776	0.063902854	0.001440828
ENSG00000179918_30445727_30445548_59	annotated	SEPHS2	16	-	177	31	3	6	13.56%	57.87061279	0.004873904	0.117178064	0.003809276
ENSG00000032389_3257414_3189334_287	novel	EIPR1	2	-	861	41	5	3	5.92%	15.73440624	0.000876071	0.00046814	2.65E-06
ENSG00000061987_62467256_62592749_1672	annotated	MON2	12	+	5016	35	3	3	0.96%	2.305579862	0.000937835	0.001180291	6.85E-06
ENSG00000005801_3371610_3359118_597	annotated	ZNF195	11	-	1791	6	0	1	0.84%	1.106943153	0.016947427	0.047790352	0.003697512
ENSG00000162910_228108260_228106760_164	annotated	MRPL55	1	-	492	154	11	4	15.24%	103.4249386	6.15E-06	5.55E-06	2.84E-10
ENSG00000162910_228108260_228106760_128	annotated	MRPL55	1	-	384	154	11	4	19.53%	132.5132026	6.15E-06	5.55E-06	2.84E-10
ENSG00000008952_169966823_169982643_85	annotated	SEC62	3	+	255	40	4	0	14.12%	51.83098527	0.005554612	0.001023866	3.51E-05
ENSG00000162910_228106918_228106760_52	annotated	MRPL55	1	-	156	139	11	4	38.46%	294.4149476	4.79E-05	4.09E-05	1.48E-08
ENSG00000162910_228106918_228106760_52	annotated	MRPL55	1	-	156	139	11	4	38.46%	294.4149476	4.79E-05	4.09E-05	1.48E-08
ENSG00000162910_228108260_228106760_128	annotated	MRPL55	1	-	384	154	11	4	19.53%	132.5132026	6.15E-06	5.55E-06	2.84E-10
ENSG00000162910_228108260_228106760_128	annotated	MRPL55	1	-	384	154	11	4	19.53%	132.5132026	6.15E-06	5.55E-06	2.84E-10
ENSG00000162910_228108260_228106760_128	annotated	MRPL55	1	-	384	154	11	4	19.53%	132.5132026	6.15E-06	5.55E-06	2.84E-10
ENSG00000162910_228108260_228106760_128	annotated	MRPL55	1	-	384	154	11	4	19.53%	132.5132026	6.15E-06	5.55E-06	2.84E-10
ENSG00000196352_207321766_207359610_550	annotated	CD55	1	+	1650	259	27	8	11.64%	51.86632458	3.39E-09	2.16E-12	1.17E-19
ENSG00000196352_207321766_207359610_381	annotated	CD55	1	+	1143	262	27	9	17.06%	75.73989777	2.29E-09	1.75E-12	6.31E-20
ENSG00000196352_207321766_207340644_444	annotated	CD55	1	+	1332	247	27	9	13.96%	61.27204594	8.16E-09	5.60E-12	7.19E-19
ENSG00000070669_97869093_97852259_540	annotated	ASNS	7	-	1620	44	1	0	3.15%	8.974439117	0.000166161	0.000127134	1.49E-07
ENSG00000185658_39313491_39187026_2320	annotated	BRWD1	21	-	6960	38	7	0	0.82%	1.804031061	0.002707838	4.92E-05	1.18E-06
ENSG00000105755_43527177_43506850_254	annotated	ETHE1	19	-	762	98	7	2	11.42%	42.49528615	2.50E-05	3.68E-06	7.81E-10
ENSG00000168216_69719072_69676158_325	novel	LMBRD1	6	-	975	85	19	0	8.92%	28.80606682	0.000438301	1.10E-06	5.99E-09
ENSG00000143612_154213923_154207453_150	dORF	C1orf43	1	-	450	142	18	2	20.67%	104.2666654	1.13E-05	1.39E-06	1.40E-10
ENSG00000285827_118472390_118482100_929	annotated	AP001267.5	11	+	2787	6	0	0	0.54%	0.711350982	0.016947427	0.016947427	0.001349898
ENSG00000066379_30061525_30064697_126	annotated	ZNRD1	6	+	378	40	7	5	9.52%	34.96534721	0.004133778	0.003630629	8.30E-05
ENSG00000112983_138178614_138157208_951	annotated	BRD8	5	-	2853	27	9	11	1.58%	3.127027108	0.050716205	0.035013191	0.007357909
ENSG00000177302_18314778_18274802_1001	annotated	TOP3A	17	-	3003	47	3	4	2.20%	5.171448206	0.000100047	0.000138056	9.90E-08
ENSG00000105281_46787965_46775510_541	annotated	SLC1A5	19	-	1623	480	75	7	17.01%	97.72200551	1.73E-14	6.12E-17	1.72E-29
ENSG00000118898_4897692_4883384_1438	annotated	PPL	16	-	4314	7	3	0	0.42%	0.536151534	0.109745779	0.009815329	0.005898503
ENSG00000241370_30345172_30346810_154	annotated	RPP21	6	+	462	88	12	1	9.09%	62.93762497	0.00526969	0.000609242	2.10E-05
ENSG00000119969_94554132_94601622_785	annotated	HELLS	10	+	2355	71	11	0	3.82%	9.961783316	1.70E-05	5.25E-07	8.68E-11
ENSG00000150459_21140940_21147170_60	annotated	SAP18	13	+	180	51	5	0	20.00%	93.61971715	0.005655336	0.001015599	3.55E-05
ENSG00000150459_21146906_21147342_65	Overlap_dORF	SAP18	13	+	195	49	2	0	18.46%	83.02925141	0.001065834	0.001070089	7.04E-06
ENSG00000108587_30477434_30490221_145	novel	GOSR1	17	+	435	27	0	0	7.59%	20.50898469	0.00140552	0.00140552	1.19E-05
ENSG00000074370_3951338_3925422_873	annotated	ATP2A3	17	-	2619	16	2	0	0.69%	2.018617983	0.030316224	0.012798403	0.00183564
ENSG00000074370_3951338_3925422_874	annotated	ATP2A3	17	-	2622	16	2	0	0.69%	2.016308352	0.030316224	0.012798403	0.00183564
ENSG00000106268_2244575_2251001_156	annotated	NUDT1	7	+	468	89	4	2	14.10%	62.8367634	1.82E-05	2.11E-05	3.03E-09
ENSG00000180398_46907920_46905463_80	annotated	MCFD2	2	-	240	51	7	0	13.75%	70.21478786	0.008830848	0.001580611	8.33E-05
ENSG00000115540_197540381_197550646_126	annotated	MOB4	2	+	378	41	2	0	8.73%	35.83948089	0.002815105	0.001580611	2.62E-05
ENSG00000115274_74455117_74457436_184	annotated	INO80B	2	+	552	38	0	0	5.98%	22.74647859	0.001595761	0.001595761	1.52E-05
ENSG00000115524_197434999_197418932_144	annotated	SF3B1	2	-	432	37	13	5	7.64%	28.3000779	0.009797758	0.009821677	0.000483192
ENSG00000187742_89318577_89358824_854	annotated	SECISBP2	9	+	2562	10	4	0	0.82%	1.28970543	0.075513077	0.007029303	0.002963142
ENSG00000099246_27504370_27538051_161	annotated	RAB18	10	+	483	115	14	0	18.63%	78.67203122	2.43E-05	7.69E-07	1.79E-10
ENSG00000163156_151166525_151169085_195	annotated	SCNM1	1	+	585	40	3	6	8.72%	22.59299358	0.000713046	0.001101026	4.93E-06
ENSG00000240682_129159155_129130184_244	annotated	ISY1	3	-	732	72	2	1	6.97%	32.50057683	0.00015434	0.000155626	1.69E-07
ENSG00000240682_129159155_129130081_277	annotated	ISY1	3	-	831	75	1	1	7.22%	29.82152808	4.00E-05	5.10E-05	1.54E-08
ENSG00000114626_127673026_127676003_57	annotated	ABTB1	3	+	171	8	3	0	8.77%	15.45836403	0.102951605	0.019679754	0.009352564
ENSG00000270170_196942717_196943016_99	annotated	NCBP2AS2	3	+	297	46	4	1	11.11%	51.17655364	0.002197839	0.002197839	2.81E-05

ENSG00000089280_31190755_31191438_91	novel	FUS	16	+	273	48	4	0	16.48%	58.09626921	0.001891093	0.000295633	3.79E-06
ENSG00000185624_81860471_81843513_106	annotated	P4HB	17	-	318	153	14	1	17.92%	158.9768782	0.000151707	6.17E-05	6.85E-08
ENSG00000185359_81684067_81690288_173	novel	HGS	17	+	519	27	6	1	3.47%	17.18961145	0.022600676	0.013716833	0.001462305
ENSG00000103544_19555730_19700476_963	annotated	VPS35L	16	+	2889	130	10	13	4.88%	14.86844204	7.09E-08	3.74E-08	2.64E-14
ENSG00000129103_56068502_56078159_121	annotated	SUMF2	7	+	363	274	24	16	19.01%	249.4098444	3.85E-05	0.000266356	7.82E-08
ENSG00000129103_56078432_56079612_53	dORF	SUMF2	7	+	159	82	18	1	16.98%	170.4065884	0.037251511	0.004557894	0.000951259
ENSG00000104763_18071333_18057534_324	Overlap_dORF	ASAH1	8	-	972	285	33	5	16.67%	96.88314956	5.91E-09	3.15E-10	2.16E-17
ENSG00000104763_18084058_18069875_62	annotated	ASAH1	8	-	186	226	3	1	14.52%	401.48114	0.003632191	0.007225456	0.000142984
ENSG00000109046_27294396_27301960_70	novel	WSB1	17	+	210	16	2	0	8.57%	25.17504999	0.035772986	0.013640586	0.002290412
ENSG00000071082_101004219_101023641_64	annotated	RPL31	2	+	192	402	50	7	9.38%	691.8221745	0.021261239	0.013853925	0.001390792
ENSG00000082438_164695723_164685946_572	annotated	COBLL1	2	-	1716	12	1	0	1.05%	2.310647071	0.026602765	0.011570466	0.001474889
ENSG00000111640_6534833_6536794_79	novel	GAPDH	12	+	237	419	12	12	10.13%	584.164728	0.005859343	0.005431112	0.000169616
ENSG00000111640_6536816_6538170_291	novel	GAPDH	12	+	873	1480	213	69	19.59%	560.1664903	3.10E-09	2.60E-09	8.66E-17
ENSG00000163660_157149621_157148241_194	novel	CCNL1	3	-	582	21	6	1	3.09%	11.92246246	0.102671433	0.016983117	0.008311682
ENSG00000144381_197498818_197495282_163	novel	HSPD1	2	-	489	105	12	2	7.36%	70.94962325	0.001691112	0.001423306	1.44E-05
ENSG00000108384_58692671_58693484_83	novel	RAD51C	17	+	249	8	1	0	6.02%	10.61598494	0.016947427	0.019679754	0.001553599
ENSG00000177463_15020926_15043008_413	annotated	NR2C2	3	+	1239	9	5	0	1.45%	2.400163664	0.11411552	0.011772051	0.007078638
ENSG00000257923_101816052_102283090_625	annotated	CUX1	7	+	1875	78	19	0	6.08%	13.74557729	5.39E-05	2.65E-08	2.27E-11
ENSG00000158623_130668668_130506676_871	annotated	COPG2	7	-	2613	65	5	1	4.48%	8.219465948	5.58E-07	2.99E-08	1.72E-13
ENSG00000122966_119876168_119688232_2069	annotated	CIT	12	-	6207	114	17	14	2.32%	6.068659344	1.56E-06	4.41E-07	6.29E-12
ENSG00000179222_51895191_51901930_717	annotated	MAGED1	X	+	2151	84	11	10	4.32%	12.90352981	4.85E-06	2.82E-05	1.14E-09
ENSG00000140650_8801908_8847825_162	Overlap_dORF	PMM2	16	+	486	45	4	10	10.49%	30.59467881	0.000837865	0.005182125	2.73E-05
ENSG00000099364_30927404_30947230_583	annotated	FBXL19	16	+	1749	17	1	0	1.37%	3.211654105	0.010079165	0.004873904	0.000259739
ENSG00000131462_42609738_42615041_451	annotated	TUBG1	17	+	1353	78	3	4	5.32%	19.04874902	2.12E-05	4.84E-05	7.96E-09
ENSG00000100461_22902313_22901730_106	novel	RBM23	14	-	318	52	7	3	10.38%	54.03135729	0.013995673	0.007108701	0.000505528
ENSG00000174744_66341621_66337668_257	annotated	BRMS1	11	-	771	51	7	4	5.06%	21.8567433	0.003226492	0.002805574	5.13E-05
ENSG00000198431_104287428_104348415_547	Overlap_uORF	TXNRD1	12	+	1641	909	103	42	21.94%	183.0311278	6.47E-17	6.99E-20	8.22E-35
ENSG00000149809_65112550_65112873_74	annotated	TM7SF2	11	+	222	13	0	0	8.11%	19.34906714	0.012798403	0.012798403	0.000797244
ENSG00000163755_149129724_149153652_467	novel	HPS3	3	+	1401	25	9	6	3.00%	5.896190777	0.027407609	0.007934483	0.001094312
ENSG00000139116_39369887_39294424_1565	annotated	KIF21A	12	-	4695	25	0	3	0.83%	1.759438398	0.000600464	0.004238779	1.65E-05
ENSG00000114054_136250376_136334307_303	annotated	PCCB	3	+	909	46	11	1	6.93%	16.72105218	0.001238169	2.00E-05	2.27E-07
ENSG00000104763_18063207_18057534_235	novel	ASAH1	8	-	705	259	26	5	19.57%	121.3892703	1.05E-07	7.88E-09	8.78E-15
ENSG00000213190_151067273_151067497_74	annotated	MLLT11	1	+	222	58	2	3	22.97%	86.32660723	0.000184339	0.000515248	6.52E-07
ENSG00000143443_151047977_151050458_298	annotated	C1orf56	1	+	894	46	4	0	3.69%	17.00160675	0.003583465	0.001580611	3.32E-05
ENSG00000171462_43452034_43450539_276	annotated	DLK2	6	-	828	12	2	0	2.54%	4.788732335	0.042435259	0.007029303	0.001563844
ENSG00000026103_88990877_89014450_314	annotated	FAS	10	+	942	31	5	0	4.46%	10.87377756	0.005286514	0.0004093	1.50E-05
ENSG00000169592_29996311_29999283_72	novel	INO80E	16	+	216	56	20	7	18.06%	85.66510066	0.051271272	0.004978722	0.001455956
ENSG00000005810_77326661_77045378_4640	annotated	MYCBP2	13	-	13920	12	5	4	0.15%	0.28484701	0.153960942	0.082535828	0.04432366
ENSG00000198356_12737506_12748104_348	annotated	ASNA1	19	+	1044	90	10	4	6.03%	28.48470096	0.000448577	3.94E-05	1.37E-07
ENSG00000131368_15065188_15053376_108	annotated	MRPS25	3	-	324	61	3	2	13.89%	62.20918024	0.000764563	0.000763157	3.69E-06
ENSG00000015479_139307416_139329395_847	annotated	MATR3	5	+	2541	221	37	12	8.15%	28.73804777	2.25E-09	2.14E-11	6.28E-19
ENSG00000089009_112408455_112405224_221	annotated	AC004086.1	12	-	663	27	0	0	2.26%	13.45612118	0.020613417	0.020613417	0.001946209
ENSG00000132470_75724704_75757555_1752	annotated	ITGB4	17	+	5256	482	67	15	8.28%	30.30130517	1.50E-19	2.43E-24	8.67E-42
ENSG00000105856_107179894_107201431_514	annotated	HBP1	7	+	1542	90	18	7	6.03%	19.28536174	0.000577124	6.76E-06	3.83E-08
ENSG00000188529_23972001_23971142_167	novel	SRSF10	1	-	501	46	6	0	7.19%	30.33819647	0.01345985	0.000958906	8.55E-05
ENSG00000138835_113463803_113541427_602	annotated	RGS3	9	+	1806	13	5	2	1.33%	2.378456758	0.116847432	0.030313544	0.015050175
ENSG00000115310_55050300_54973156_373	annotated	RTN4	2	-	1119	906	165	28	26.81%	267.5270895	1.14E-13	7.35E-17	1.47E-28
ENSG00000128789_12703162_12725531_246	annotated	PSMG2	18	+	738	105	21	0	11.38%	47.01133573	0.00015092	1.57E-06	2.43E-09
ENSG00000176485_63590336_63574945_112	annotated	PLAAT3	11	-	336	55	6	0	12.50%	54.08702146	0.001498272	0.000465689	4.51E-06
ENSG00000187838_7394110_7390385_295	annotated	PLSCR3	17	-	885	18	4	2	2.71%	6.72045826	0.040990615	0.026823708	0.00473838
ENSG00000188677_44093972_44168678_289	annotated	PARVB	22	+	867	26	2	0	4.84%	9.908864832	0.001968141	0.000338646	4.46E-06
ENSG00000178149_49018564_49015588_543	annotated	DALRD3	3	-	1629	43	2	7	3.13%	8.722018931	0.000236112	0.002953465	4.97E-06
ENSG00000100304_43183119_43168008_575	annotated	TTLL12	22	-	1725	84	11	0	4.35%	16.09014065	0.000118892	5.47E-06	5.70E-09
ENSG00000265681_49492316_49491552_24	Overlap_uORF	RPL17	18	-	72	24	51	4	25.00%	110.1408437	0.117522225	0.056923149	0.025132719
ENSG00000100299_50627773_50625145_507	annotated	ARSA	22	-	1521	42	9	2	3.55%	9.124093561	0.003781438	0.000334726	8.77E-06
ENSG00000184117_29581082_29555935_284	annotated	NIPSNAP1	22	-	852	213	11	3	17.96%	82.60563278	8.22E-10	2.02E-10	1.90E-18
ENSG00000100297_35400439_35424255_734	annotated	MCM5	22	+	2202	120	2	1	6.54%	18.00667745	9.19E-10	6.29E-10	6.43E-18
ENSG00000129353_10625634_10643385_706	annotated	SLC44A2	19	+	2118	23	1	0	1.56%	3.588157798	0.004070582	0.001092093	2.70E-05
ENSG00000101608_3253248_3255918_171	annotated	MYL12A	18	+	513	220	24	7	14.04%	141.7016703	0.00035639	8.15E-05	2.10E-07
ENSG00000109929_121306436_121307512_168	novel	SC5D	11	+	504	66	4	0	9.52%	43.26961717	0.000550676	0.000197396	7.43E-07
ENSG00000168564_183444798_183446096_126	annotated	CDKN2AIP	4	+	378	15	0	4	3.97%	13.1120052	0.020613417	0.063071082	0.005786711
ENSG00000171617_74636266_74634716_516	annotated	ENC1	5	-	1548	40	3	6	3.29%	8.5380499	0.000125843	0.001982636	1.87E-06
ENSG00000014216_65182702_65185986_151	novel	CAPN1	11	+	453	30	1	14	5.30%	21.88228683	0.005454749	0.210762106	0.008933617
ENSG00000112695_75243734_75241134_49	annotated	COX7A2	6	-	147	571	94	71	30.61%	1283.477995	0.000247677	0.000559537	9.33E-07
ENSG00000112695_75240319_75237930_25	dORF	COX7A2	6	-	75	196	5	5	32.00%	863.5042146	0.005756914	0.005292868	0.000162773
ENSG00000100219_28800410_28795546_228	annotated	XBP1	22	-	684	44	13	3	7.46%	21.25525054	0.019159789	0.000757695	0.000104674
ENSG00000249884_86620461_86505804_251	annotated	RNF103-CHMP3	2	-	753	97	21	0	9.56%	42.5643898	0.000175904	7.88E-06	1.20E-08
ENSG00000196776_108057558_108047288_47	annotated	CD47	3	-	141	37	8	3	12.77%	86.70662164	0.020342454	0.037178614	0.003374092

ENSG00000169567_131165205_131162625_65	annotated	HINT1	5	-	195	104	9	9	13.85%	176.2253499	0.060942872	0.032746137	0.008282207
ENSG00000280987_139316089_139329395_504	annotated	MATR3	5	+	1512	145	26	9	8.93%	31.68734591	1.79E-06	4.75E-08	8.91E-13
ENSG00000105355_4861394_4839192_434	annotated	PLIN3	19	-	1302	155	13	1	11.75%	39.33601561	7.04E-09	2.33E-10	1.94E-17
ENSG00000121542_123209218_123243834_187	novel	SEC22A	3	+	561	21	3	2	4.81%	12.36875785	0.035106853	0.016228926	0.002614756
ENSG00000197122_37397851_37403379_251	novel	SRC	20	+	753	137	13	3	14.34%	60.11671549	1.99E-06	1.85E-07	3.53E-12
ENSG00000095319_128986603_129003616_824	novel	NUP188	9	+	2472	28	9	0	1.82%	3.742650029	0.004144268	0.000273206	8.12E-06
ENSG00000138028_27104366_27101274_318	annotated	CGREF1	2	-	954	43	2	0	3.46%	14.89325874	0.002667196	0.001565547	2.46E-05
ENSG00000108262_29589378_29574702_761	annotated	GIT1	17	-	2283	58	4	2	2.76%	8.394440125	0.000150234	0.000102592	1.10E-07
ENSG00000183765_28719414_28687897_322	dORF	CHEK2	22	-	966	19	0	0	3.11%	6.498993883	0.002254349	0.002254349	2.95E-05
ENSG00000064490_19193947_19201719_260	annotated	RFXANK	19	+	780	18	16	7	4.23%	7.625135333	0.208395074	0.033788632	0.030976415
ENSG00000196756_38431089_38426436_38	novel	SNHG17	20	-	114	20	1	3	13.16%	57.96886511	0.028889786	0.065398531	0.007975053
ENSG00000064607_19031071_18994366_1082	annotated	SUGP2	19	-	3246	30	0	1	1.39%	3.053812672	0.000281287	0.000366084	6.93E-07
ENSG00000166734_44322995_44413406_317	annotated	CASC4	15	+	951	29	3	3	3.15%	10.07597624	0.004781415	0.008859988	0.000224783
ENSG00000127955_80199276_80217493_236	dORF	GNAI1	7	+	708	96	5	3	11.86%	44.80305507	1.77E-06	2.29E-06	3.53E-11
ENSG00000183283_51238908_51242652_207	annotated	DAZAP2	12	+	621	194	93	9	10.14%	103.2237859	0.0165675	0.000138452	2.28E-05
ENSG00000112851_65992719_66078530_1302	annotated	ERBIN	5	+	3906	78	3	5	2.53%	6.598299392	5.76E-07	2.41E-06	1.26E-11
ENSG00000168884_2747732_2742257_266	annotated	TNIP2	4	-	798	11	9	1	2.26%	4.554696544	0.33568662	0.023672501	0.044346317
ENSG00000151491_15682912_15621317_809	annotated	EPS8	12	-	2427	79	10	0	3.21%	10.75540995	0.000507204	3.18E-06	1.73E-08
ENSG00000135506_57694162_57720909_612	annotated	OS9	12	+	1836	459	61	23	20.59%	82.60563278	1.03E-17	3.08E-21	6.26E-37
ENSG00000182979_105469937_105470215_68	novel	MTA1	14	+	204	22	1	1	8.82%	35.63380238	0.016983117	0.013640586	0.00110551
ENSG00000170917_122922572_122922258_104	novel	NUDT6	4	-	312	21	0	2	6.73%	22.23997806	0.008473674	0.013892891	0.00058868
ENSG00000169756_108659609_108683999_325	annotated	LIMS1	2	+	975	34	2	2	4.00%	11.52242673	0.001811222	0.001811222	1.94E-05
ENSG00000134278_12657866_12449638_742	annotated	SPIRE1	18	-	2226	28	4	1	2.70%	4.156258253	0.001064503	4.64E-05	3.99E-07
ENSG00000138382_169824597_169812313_244	annotated	METTL5	2	-	732	28	2	7	6.56%	12.63911321	0.000736315	0.00290531	1.34E-05
ENSG00000160796_46988048_47009320_2720	annotated	NBEAL2	3	+	8160	35	6	5	0.59%	1.417253504	0.00174801	0.001882578	1.95E-05
ENSG00000185900_43103741_43122877_286	annotated	POMK	8	+	858	22	0	0	2.80%	8.472372593	0.005554612	0.005554612	0.000164685
ENSG00000134900_102616475_102678316_620	Overlap_dORF	TPP2	13	+	1860	31	4	1	2.74%	5.507042185	0.000805645	0.000283906	1.53E-06
ENSG00000114374_12726665_12859416_2379	annotated	USP9Y	Y	+	7137	9	0	0	0.21%	0.416674062	0.019216965	0.019216965	0.001707396
ENSG00000213983_23567314_23566417_68	annotated	AP1G2	14	-	204	10	1	0	8.82%	16.1971829	0.011772051	0.011772051	0.000681273
ENSG00000166888_57108251_57097083_711	annotated	STAT6	12	-	2133	66	7	1	3.52%	10.22404456	8.06E-05	4.63E-06	3.25E-09
ENSG00000040341_73673120_73552006_379	annotated	STAU2	8	-	1137	23	4	3	2.90%	6.684008985	0.003737369	0.007823695	0.000158768
ENSG00000092199_21234154_21211337_275	annotated	HNRNPC	14	-	825	92	6	8	4.00%	36.84711862	0.007480509	0.016114279	0.000608357
ENSG00000075790_107595976_107628866_93	dORF	BCAP29	7	+	279	34	3	0	17.20%	40.26654501	0.001129532	0.000177448	1.40E-06
ENSG00000075790_107580773_107595874_66	annotated	BCAP29	7	+	198	69	20	2	16.67%	115.1472457	0.031528373	0.002237761	0.000443132
ENSG00000142676_23692636_23695932_165	novel	RPL11	1	+	495	388	51	12	29.09%	258.9978628	4.38E-09	2.60E-09	1.22E-16
ENSG00000115355_55296017_55291711_161	dORF	CCDC88A	2	-	483	22	2	0	5.59%	15.05030162	0.009029128	0.003658191	0.000179569
ENSG00000040341_73673120_73552006_379	annotated	STAU2	8	-	1137	23	4	3	2.90%	6.684008985	0.003737369	0.007823695	0.000158768
ENSG00000163637_64198879_64099051_828	annotated	PRICKLE2	3	-	2484	8	1	0	0.72%	1.064162741	0.028889786	0.009815329	0.001387795
ENSG00000129071_129439833_129431501_580	annotated	MBD4	3	-	1740	79	9	7	3.97%	15.0019425	8.50E-05	0.000157066	9.61E-08
ENSG00000084112_108857496_108792100_692	annotated	SSH1	12	-	2076	13	5	4	0.72%	2.069119896	0.083503598	0.15306802	0.044493368
ENSG00000124226_49945442_49952141_111	Overlap_dORF	RNF114	20	+	333	39	0	1	14.41%	38.69813427	0.000176199	0.000423577	5.14E-07
ENSG00000189308_82984727_82928102_710	annotated	LIN54	4	-	2130	13	2	0	1.13%	2.016663335	0.030313544	0.004826937	0.000797936
ENSG00000120253_149746495_149726969_380	annotated	NUP43	6	-	1140	75	8	7	7.37%	21.73832442	1.95E-05	2.19E-05	3.35E-09
ENSG00000039523_67538739_67546463_1162	annotated	RIPOR1	16	+	3486	68	8	0	3.01%	6.445419425	6.21E-06	7.85E-08	5.29E-12
ENSG00000102763_41960952_41568197_1884	annotated	VWA8	13	-	5652	28	2	4	0.42%	1.636912751	0.016228926	0.020990661	0.001587047
ENSG00000144118_120278767_120293260_172	annotated	RALB	2	+	516	46	4	0	7.56%	29.45627215	0.000815216	0.000631191	3.28E-06
ENSG00000065809_14553383_14530515_20	uORF	FAM107B	10	-	60	13	3	0	25.00%	71.59154841	0.138151459	0.019679754	0.01298269
ENSG00000148396_136477582_136441755_2323	annotated	SEC16A	9	-	6969	53	13	3	1.33%	2.512899146	0.000366971	2.67E-06	1.04E-08
ENSG00000080822_98521424_98516659_253	annotated	CLDND1	3	-	759	170	26	5	17.39%	74.00768154	4.66E-08	2.17E-08	1.02E-14
ENSG00000079277_46580540_46558575_325	annotated	MKNK1	1	-	975	15	1	1	1.85%	5.083423556	0.020342454	0.020342454	0.001898761
ENSG00000141522_81869930_81868876_204	annotated	ARHGDIA	17	-	612	669	56	11	26.96%	361.1971786	3.87E-09	1.46E-10	6.75E-18
ENSG00000168256_42021578_42023893_191	annotated	NKIRAS2	17	+	573	29	1	10	6.28%	16.72295533	0.002136761	0.033383459	0.000455494
ENSG00000130023_169756401_169781513_542	annotated	ERMARD	6	+	1626	27	5	0	2.21%	5.486720996	0.008676195	0.001007374	5.53E-05
ENSG00000115221_160200063_160101736_788	annotated	ITGB6	2	-	2364	14	1	0	1.02%	1.95681702	0.010499663	0.00419316	0.000236136
ENSG00000176148_33043774_33072676_509	annotated	TCP11L1	11	+	1527	32	3	5	3.34%	6.924375243	0.000820166	0.002915716	1.48E-05
ENSG00000175482_67352764_67351995_32	annotated	POLD4	11	-	96	25	5	0	21.88%	86.04753414	0.101636223	0.008473674	0.004825296
ENSG00000175482_67351998_67351834_54	Overlap_dORF	POLD4	11	-	162	7	1	1	9.26%	14.27751678	0.041782825	0.041782825	0.007200521
ENSG00000165458_72229532_72238353_1016	annotated	INPPL1	11	+	3048	27	8	5	1.38%	2.92697124	0.02335167	0.003932371	0.000508492
ENSG00000130702_62314425_62310240_670	novel	LAMA5	20	-	2010	186	21	19	7.91%	30.57641333	6.28E-08	1.58E-07	9.68E-14
ENSG00000112208_57182093_57184190_153	annotated	BAG2	6	+	459	67	13	1	13.73%	48.2316113	0.000670871	2.77E-05	1.54E-07
ENSG00000198689_135985575_136044724_677	annotated	SLC9A6	X	+	2031	29	5	0	2.22%	4.717997736	0.003875539	0.00026815	7.43E-06
ENSG00000198689_135985575_136044724_645	annotated	SLC9A6	X	+	1935	29	4	0	2.33%	4.952068942	0.003175962	0.00026815	6.00E-06
ENSG00000139926_51689870_51728029_603	annotated	FRMD6	14	+	1809	14	4	0	1.00%	2.557167184	0.059453662	0.01300724	0.003717979
ENSG00000138757_75662019_75645430_480	annotated	G3BP2	4	-	1440	173	24	8	9.38%	39.69659575	2.94E-06	2.93E-08	9.73E-13
ENSG00000141510_7676594_7673219_346	annotated	TP53	17	-	1038	122	20	8	7.80%	38.83578882	0.000285468	2.01E-05	4.66E-08
ENSG00000141510_7670691_7669609_54	dORF	TP53	17	-	162	181	3	2	24.07%	369.1757909	0.000707658	0.000731334	3.29E-06
ENSG00000145730_102867370_103029065_843	annotated	PAM	5	+	2529	99	18	0	4.39%	12.93468983	3.25E-05	4.32E-08	1.93E-11
ENSG00000112592_170557030_170572265_339	annotated	TBP	6	+	1017	12	0	1	1.47%	3.898790928	0.019216965	0.02170844	0.001914126

ENSG00000071994_170584581_170577559_311	annotated	PDCD2	6	-	933	33	9	4	5.14%	11.68697055	0.00922581	0.001520117	8.43E-05
ENSG00000130023_169756401_169781513_552	annotated	ERMARD	6	+	1656	32	5	0	2.36%	6.384976447	0.005042343	0.00067919	2.21E-05
ENSG00000089057_4912918_4856972_594	annotated	SLC23A2	20	-	1782	97	2	6	6.06%	17.98596269	2.08E-07	5.82E-07	1.14E-12
ENSG00000108774_42128702_42126630_131	annotated	RAB5C	17	-	393	119	11	10	19.85%	100.0516061	1.16E-05	6.79E-05	6.32E-09
ENSG00000102531_49131290_49207395_1063	annotated	FNDC3A	13	+	3189	32	7	6	1.98%	3.315622764	0.003186725	0.001331312	2.53E-05
ENSG00000106603_43729464_43648637_50	uORF	COA1	7	-	150	83	12	2	16.00%	182.8338006	0.028682169	0.005604867	0.000852961
ENSG00000187778_49559963_49556658_169	annotated	MCRS1	12	-	507	35	8	0	9.47%	22.8102339	0.00179234	0.000176199	2.26E-06
ENSG00000115484_61888507_61868692_539	annotated	CCT4	2	-	1617	562	82	17	17.25%	114.8407313	1.53E-12	3.87E-16	1.08E-26
ENSG00000143761_228097115_228098013_181	annotated	ARF1	1	+	543	874	115	29	34.81%	531.8403171	5.82E-12	1.88E-11	1.35E-21
ENSG00000197982_37808685_37809073_47	annotated	C1orf122	1	+	141	34	2	0	12.77%	79.67635502	0.031254458	0.013640586	0.002000878
ENSG00000054611_46894805_47173626_231	dORF	TBC1D22A	22	+	693	15	5	2	3.46%	7.152002838	0.066604992	0.020624382	0.006122828
ENSG00000112739_4040893_4056549_356	novel	PRPF4B	6	+	1068	13	1	1	1.97%	4.021997102	0.015625009	0.015625009	0.001159391
ENSG00000100030_21867404_21769204_348	annotated	MAPK1	22	-	1044	145	27	9	10.92%	45.89201821	4.45E-06	1.85E-07	8.13E-12
ENSG00000100138_41675154_41674933_73	annotated	SNU13	22	-	219	201	12	0	34.25%	303.2645149	1.98E-05	5.88E-06	9.61E-10
ENSG00000011007_23743504_23759573_772	annotated	ELOA	1	+	2316	11	5	1	0.65%	1.569364353	0.145248299	0.035374934	0.021421705
ENSG00000140995_89954260_89955225_57	internal	DEF8	16	+	171	10	2	5	8.77%	19.32295504	0.069457177	0.197776317	0.049762526
ENSG00000134283_42335903_42446618_458	annotated	PPHLN1	12	+	1374	93	21	3	5.68%	22.36484381	0.000954468	8.45E-06	8.15E-08
ENSG00000163918_186804713_186789969_363	annotated	RFC4	3	-	1089	44	5	2	7.71%	13.3504053	5.62E-05	3.10E-06	1.54E-09
ENSG00000173218_115651414_115691379_524	annotated	VANGL1	1	+	1572	34	8	5	3.44%	7.146543294	0.002424173	0.000958065	1.42E-05
ENSG00000103811_78939148_78922130_297	annotated	CTSH	15	-	891	32	1	0	5.39%	11.86702693	0.000282863	0.000177031	3.46E-07
ENSG00000114354_100713686_100747448_284	annotated	TFG	3	+	852	124	7	0	8.45%	48.08966415	4.56E-05	8.46E-06	3.14E-09
ENSG00000067066_230442938_230470112_444	annotated	SP100	2	+	1332	19	3	0	2.93%	4.713234303	0.00596458	0.000528008	2.12E-05
ENSG00000125630_112568018_112575729_536	annotated	POLR1B	2	+	1608	20	5	0	1.87%	4.109732974	0.026101818	0.002321823	0.000369793
ENSG00000138069_65129906_65088493_138	annotated	RAB1A	2	-	414	148	12	3	18.84%	118.1220643	4.11E-05	8.71E-06	2.90E-09
ENSG00000115935_174575240_174567044_386	annotated	WIPF1	2	-	1158	17	6	0	2.07%	4.850762547	0.046396937	0.005405142	0.001391293
ENSG00000144451_213297137_214410315_571	dORF	SPAG16	2	+	1713	18	1	0	1.58%	3.472040607	0.006112544	0.003303074	0.000111133
ENSG00000167130_129081132_129089007_195	annotated	DOLPP1	9	+	585	41	3	0	6.67%	23.15781842	0.001832364	0.00067919	7.81E-06
ENSG00000142546_49560691_49555751_301	annotated	NOSIP	19	-	903	86	16	5	8.97%	31.46881249	0.000806375	6.39E-06	5.32E-08
ENSG00000068400_48990976_48978251_481	novel	GRIPAP1	X	-	1443	7	2	0	1.04%	1.602881301	0.11777946	0.016947427	0.009674391
ENSG00000105048_55141284_55132915_192	annotated	TNNT1	19	-	576	13	2	0	3.65%	7.457452959	0.037995728	0.007979402	0.001544499
ENSG00000116731_13778930_13786527_1304	annotated	PRDM2	1	+	3912	16	4	4	0.54%	1.351421395	0.067757397	0.060590694	0.015722365
ENSG00000085365_78416628_78475668_197	annotated	SCAMP1	5	+	591	86	15	3	13.20%	48.08178964	9.78E-05	2.20E-05	1.67E-08
ENSG00000120708_136049469_136049630_53	novel	TGFBI	5	+	159	201	40	11	13.21%	417.7039544	0.008576801	0.039844013	0.001724932
ENSG00000178057_49022473_49023172_116	annotated	NDUFAF3	3	+	348	42	5	6	14.66%	39.87858134	0.000239652	0.000942043	1.53E-06
ENSG00000101294_31514552_31569219_377	annotated	HM13	20	+	1131	1184	111	11	23.87%	345.9065224	6.27E-15	3.92E-16	3.62E-29
ENSG00000159479_43389764_43384450_301	annotated	MED8	1	-	903	81	14	6	7.31%	29.63923037	0.001014028	0.000223617	1.55E-06
ENSG00000157036_38496368_38524362_318	annotated	EXOG	3	+	954	8	3	0	1.89%	2.770838835	0.113684165	0.011570466	0.006956434
ENSG00000135916_230865026_230878099_267	annotated	ITM2C	2	+	801	128	11	5	12.73%	52.80160297	4.57E-06	5.70E-07	2.39E-11
ENSG00000129003_61931181_61882619_1170	dORF	VPS13C	15	-	3510	13	1	3	0.60%	1.223787152	0.015625009	0.051289318	0.003710285
ENSG00000108518_4946844_4945900_104	annotated	PFN1	17	-	312	1032	78	20	23.08%	1092.936064	0.000152652	1.30E-05	1.63E-08
ENSG00000141456_4704111_4671439_1130	annotated	PELP1	17	-	3390	71	2	2	2.74%	6.920353897	1.36E-06	9.14E-07	1.11E-11
ENSG00000166979_32412854_32515190_393	annotated	EVA1C	21	+	1179	136	16	12	7.38%	38.11489757	2.53E-05	1.82E-05	3.60E-09
ENSG00000184076_29767399_29767587_62	annotated	UQCR10	22	+	186	505	37	4	35.48%	897.1149366	0.000681414	4.39E-05	2.36E-07
ENSG00000104147_41332453_41309754_193	annotated	OIP5	15	-	579	39	2	0	7.25%	22.25643992	0.000595214	0.000444132	1.72E-06
ENSG00000111640_6534833_6536794_79	novel	GAPDH	12	+	237	419	12	12	10.13%	584.164728	0.005859343	0.005431112	0.000169616
ENSG00000111640_6536816_6538170_291	novel	GAPDH	12	+	873	1480	213	69	19.59%	560.1664903	3.10E-09	2.60E-09	8.66E-17
ENSG00000093144_127327136_127316446_63	novel	ECHDC1	6	-	189	47	3	0	19.05%	82.16856594	0.003478991	0.00107863	2.28E-05
ENSG00000112697_75284632_75256102_323	annotated	TMEM30A	6	-	969	484	46	26	24.15%	165.0407689	2.62E-12	1.39E-13	4.93E-24
ENSG00000081320_196143581_196137447_177	annotated	STK17B	2	-	531	11	2	0	3.95%	6.844911191	0.043639483	0.007882058	0.00177109
ENSG00000119041_196799611_196764563_886	annotated	GTF3C3	2	-	2658	51	5	0	2.60%	6.339935699	0.00025487	1.01E-05	2.24E-08
ENSG00000122545_35832891_35904293_384	dORF	7-Sep	7	+	1152	112	17	8	5.99%	32.12441275	0.000395822	8.16E-05	2.34E-07
ENSG00000104763_18084801_18057534_386	annotated	ASAH1	8	-	1158	295	36	5	15.28%	84.17499713	2.55E-09	4.81E-11	1.53E-18
ENSG00000106976_128219081_128234066_274	annotated	DNM1	9	+	822	72	15	12	7.66%	28.94211951	0.000197708	0.001871345	2.62E-06
ENSG00000146648_55152337_55205617_1004	annotated	EGFR	7	+	3012	276	24	10	7.67%	30.27776182	1.54E-12	4.90E-13	9.76E-24
ENSG00000079616_29797027_29805310_597	annotated	KIF22	16	+	1791	177	16	9	7.37%	32.65482301	8.94E-08	1.06E-07	9.20E-14
ENSG00000110713_3705314_3676159_708	annotated	NUP98	11	-	2124	24	1	3	1.98%	3.733587922	0.000674868	0.00358551	1.54E-05
ENSG00000042445_85343687_85343242_62	dORF	RETSAT	2	-	186	20	4	0	12.90%	35.52930442	0.005306578	0.005306578	0.000151001
ENSG00000042445_85350872_85343719_313	annotated	RETSAT	2	-	939	59	14	2	7.67%	20.76137309	0.002290678	3.00E-05	6.42E-07
ENSG00000197713_210002662_210016066_71	Overlap_uORF	RPE	2	+	213	37	8	0	7.04%	57.39734109	0.130723026	0.021557223	0.01306546
ENSG00000197713_210016017_210019791_164	annotated	RPE	2	+	492	49	4	0	7.93%	32.90793501	0.004036058	0.000705256	1.81E-05
ENSG00000156858_30651962_30656311_522	annotated	PRR14	16	+	1566	11	3	0	0.96%	2.320975634	0.12312585	0.020613417	0.011809609
ENSG00000136521_179618481_179624040_53	annotated	NDUFB5	3	+	159	172	23	3	47.17%	357.4382098	2.40E-05	8.02E-06	1.56E-09
ENSG00000137106_37430612_37432558_63	novel	GRHPR	9	+	189	58	0	3	20.63%	101.3995069	0.000681533	0.0014015	6.00E-06
ENSG00000100129_37874420_37888464_297	novel	EIF3L	22	+	891	21	12	2	4.04%	7.787736424	0.070661019	0.004328185	0.00188696
ENSG00000164904_126554349_126549974_117	novel	ALDH7A1	5	-	351	11	0	11	4.27%	10.35512206	0.019679754	0.247542878	0.026229553
ENSG00000165792_20990003_20992204_158	novel	METTL17	14	+	474	36	2	9	5.70%	25.09538211	0.013494345	0.118296658	0.008179919
ENSG00000165516_49768469_49782859_357	annotated	KLHDC2	14	+	1071	42	10	4	7.28%	12.95774632	0.000813023	5.89E-05	3.69E-07
ENSG00000142208_104792643_104770341_480	annotated	AKT1	14	-	1440	43	13	5	3.75%	9.866783915	0.003071899	0.00099268	1.86E-05

ENSG00000138796_107989957_108023492_137	annotated	HADH	4	+	411	26	0	0	7.30%	20.90264187	0.002376615	0.002376615	3.27E-05
ENSG00000138796_108023489_108034357_127	Overlap_dORF	HADH	4	+	381	39	3	4	10.24%	33.82277878	0.003308903	0.004437245	8.14E-05
ENSG00000054654_63954816_64225564_6589	annotated	SYNE2	14	+	19767	33	14	10	0.29%	0.551623591	0.028517757	0.011475639	0.001569511
ENSG00000101558_9950401_9950586_61	novel	VAPA	18	+	183	26	13	1	9.84%	46.94527764	0.130723026	0.016983117	0.010910566
ENSG00000198909_63652645_63693777_541	annotated	MAP3K3	17	+	1623	21	1	0	2.03%	4.275337741	0.002287938	0.001440978	1.96E-05
ENSG00000119383_129147945_129148178_77	dORF	PTPA	9	+	231	49	4	0	6.49%	70.08962781	0.021557223	0.021557223	0.002115616
ENSG00000119383_129142538_129147464_30	annotated	PTPA	9	+	90	16	1	0	16.67%	58.74178331	0.052029617	0.021084099	0.004853002
ENSG00000177042_700163_704646_190	annotated	TMEM80	11	+	570	10	2	0	3.16%	5.796886511	0.030972361	0.011570466	0.001717527
ENSG00000149792_65122347_65125681_140	annotated	MRPL49	11	+	420	64	3	0	16.43%	50.35009998	2.47E-05	1.18E-05	2.32E-09
ENSG00000066697_100441939_100450966_275	annotated	MSANTD3	9	+	825	14	8	2	2.18%	5.607170225	0.274333049	0.012798403	0.022612745
ENSG00000133313_74510849_74513818_169	novel	CNDP2	18	+	507	16	2	0	2.96%	10.4275355	0.039807901	0.021084099	0.003722657
ENSG00000196498_124385846_124325402_1866	annotated	NCOR2	12	-	5598	85	16	13	2.30%	5.017133824	5.14E-06	8.42E-06	3.59E-10
ENSG00000068878_53937467_53866089_1637	annotated	PSME4	2	-	4911	89	4	0	2.44%	5.988109401	5.73E-08	1.19E-08	7.01E-15
ENSG00000142731_127885857_127898541_808	annotated	PLK4	4	+	2424	13	1	0	0.87%	1.77206803	0.015191411	0.007593099	0.000581666
ENSG00000204628_181243800_181243681_39	annotated	RACK1	5	-	117	194	49	8	38.46%	547.8800943	0.000323375	0.000460196	9.91E-07
ENSG00000204628_181242331_181236977_276	dORF	RACK1	5	-	828	2892	266	51	38.77%	1154.084493	4.71E-16	8.95E-18	6.67E-32
ENSG00000169567_131165205_131162382_103	annotated	HINT1	5	-	309	140	10	8	16.50%	149.7060012	0.001088649	0.000644204	4.43E-06
ENSG00000169567_131159596_131159447_49	dORF	HINT1	5	-	147	319	35	12	38.78%	717.0393702	0.000286175	6.46E-05	1.36E-07
ENSG00000145916_178138120_178148032_393	annotated	RMND5B	5	+	1179	45	4	4	5.09%	12.61154699	0.000345962	0.000286109	6.67E-07
ENSG00000157110_30387316_30547423_200	annotated	RBPMS	8	+	600	63	4	1	8.50%	34.69436577	0.000236112	0.000172908	2.83E-07
ENSG00000165792_20990003_20996890_447	annotated	METTL17	14	+	1341	124	15	15	9.40%	30.55361212	3.21E-06	1.86E-06	5.17E-11
ENSG00000168883_85630805_85648808_296	novel	USP39	2	+	888	27	5	0	4.73%	10.04663101	0.004233836	0.000337545	9.97E-06
ENSG00000102805_76992135_77008934_239	novel	CLN5	13	+	717	22	0	1	2.93%	10.13848771	0.008574016	0.010042643	0.000435403
ENSG00000007541_576183_583035_291	dORF	PIGQ	16	+	873	22	1	1	3.44%	8.32679918	0.0034719	0.0034719	6.73E-05
ENSG00000184903_111521447_110663602_175	annotated	IMMP2L	7	-	525	53	13	3	10.86%	33.35694124	0.003582606	0.000150417	4.15E-06
ENSG00000155903_141512204_141612313_795	annotated	RASA2	3	+	2385	16	1	1	1.13%	2.216671068	0.005426623	0.005426623	0.000157554
ENSG00000140365_75336090_75338334_91	novel	COMMD4	15	+	273	101	7	6	8.79%	122.2442331	0.021820178	0.018991317	0.00190254
ENSG00000125841_349647_353635_203	annotated	NRSN2	20	+	609	114	13	7	12.32%	61.85249351	0.000125918	3.64E-05	3.46E-08
ENSG00000151835_23355342_23330136_4156	annotated	SACS	13	-	12468	31	1	1	0.38%	0.821551048	0.000251542	0.000251542	4.32E-07
ENSG00000154957_11992829_11977957_548	annotated	ZNF18	17	-	1644	8	3	0	0.91%	1.607895529	0.136019724	0.019679754	0.012754723
ENSG00000087152_44198055_44194263_349	annotated	ATXN7L3	17	-	1047	10	1	0	1.43%	3.1558981	0.035374934	0.020613417	0.003252526
ENSG00000067829_153786911_153785872_134	annotated	IDH3G	X	-	402	8	0	0	3.73%	6.575572759	0.016947427	0.016947427	0.001349898
ENSG00000166888_57108251_57096572_838	annotated	STAT6	12	-	2514	71	7	1	3.46%	9.331742128	1.92E-05	8.59E-07	1.55E-10
ENSG00000146802_112784567_112767197_611	annotated	TMEM168	7	-	1833	21	6	0	1.96%	3.78552818	0.017317935	0.00077643	9.52E-05
ENSG00000183077_78187422_78197165_40	annotated	AFMID	17	+	120	18	0	0	12.50%	49.56337967	0.019679754	0.019679754	0.001784964
ENSG00000137563_63026179_63023621_112	novel	GGH	8	-	336	33	9	4	10.71%	32.45221288	0.016588179	0.004093256	0.000367894
ENSG00000134014_28106731_28189725_455	dORF	ELP3	8	+	1365	43	1	0	4.62%	10.4089149	3.41E-05	2.41E-05	6.36E-09
ENSG00000074527_95790309_95659086_605	annotated	NTN4	12	-	1815	70	11	0	5.29%	12.74356869	8.08E-05	2.90E-07	2.79E-10
ENSG00000197226_179866024_179863448_324	novel	TBC1D9B	5	-	972	31	1	2	4.01%	10.53816714	0.000982757	0.000585477	3.66E-06
ENSG00000112118_52284649_52264588_762	annotated	MCM3	6	-	2286	113	12	7	4.86%	16.33322223	2.34E-06	9.52E-07	1.97E-11
ENSG00000138592_50494251_50499088_242	novel	USP8	15	+	726	20	2	2	4.13%	9.102549067	0.00574807	0.002767218	8.89E-05
ENSG00000011376_45394454_45500132_575	annotated	LARS2	3	+	1725	22	4	0	1.91%	4.214084455	0.01401587	0.001448134	0.000126331
ENSG00000090989_55870819_55904495_646	annotated	EXOC1	4	+	1938	17	0	0	1.08%	2.898443255	0.008473674	0.008473674	0.000366502
ENSG00000196072_100286127_100275462_95	annotated	BLOC1S2	10	-	285	25	3	0	9.47%	28.98443255	0.004911637	0.003632191	9.81E-05
ENSG00000159346_202948324_202941573_296	annotated	ADIPOR1	1	-	888	49	1	6	7.43%	18.2327748	2.12E-05	0.000178507	2.99E-08
ENSG00000197563_62106857_62082695_271	novel	PIGN	18	-	813	28	2	0	4.06%	11.37986577	0.005435927	0.001506153	4.84E-05
ENSG00000180008_55043423_55044364_313	annotated	SOCS4	14	+	939	8	0	0	1.60%	2.815101437	0.019679754	0.019679754	0.001784964
ENSG00000092148_31114288_31100930_720	novel	HECTD1	14	-	2160	32	4	2	2.22%	4.895148609	0.003631009	0.000919734	2.06E-05
ENSG00000029364_69399370_69458593_307	annotated	SLC39A9	14	+	921	123	6	2	10.10%	44.12809047	4.86E-07	5.35E-07	2.38E-12
ENSG00000203791_124791833_124774763_104	annotated	EEF1AKMT2	10	-	312	21	6	0	8.65%	22.23997806	0.044816497	0.003632191	0.000972814
ENSG00000109046_27294396_27307767_213	annotated	WSB1	17	+	639	23	6	4	4.69%	11.89314275	0.047132153	0.014895507	0.003267468
ENSG00000197756_216499339_216529273_88	annotated	RPL37A	2	+	264	890	113	24	21.59%	1113.924442	0.000836048	0.000125228	7.48E-07
ENSG00000112335_108260921_108212149_130	annotated	SNX3	6	-	390	107	17	1	17.69%	90.65438674	0.001431454	1.05E-05	1.56E-07
ENSG00000136193_29969067_29923957_414	annotated	SCRN1	7	-	1242	74	15	1	6.28%	19.68701071	6.94E-05	4.55E-06	2.74E-09
ENSG00000145781_116092647_116292489_50	annotated	COMMD10	5	+	150	18	3	2	10.00%	39.65070373	0.073562049	0.055917761	0.015799923
ENSG00000013375_83191012_83169234_542	annotated	PGM3	6	-	1626	22	4	1	2.77%	4.470661553	0.00402601	0.000424054	1.15E-05
ENSG00000006534_68015298_68028140_230	annotated	ALDH3B1	11	+	690	152	13	12	14.78%	72.78873149	2.28E-06	2.15E-06	4.24E-11
ENSG00000104691_30754707_30766394_162	dORF	UBXN8	8	+	486	68	2	0	12.35%	46.23195909	3.85E-05	3.37E-05	9.93E-09
ENSG00000123130_23706653_23703894_176	novel	ACOT9	X	-	528	24	10	0	3.98%	15.01920596	0.153960942	0.008175611	0.007787827
ENSG00000123130_23730970_23703894_379	annotated	ACOT9	X	-	1137	79	21	5	6.07%	22.95811782	0.006186301	6.17E-05	3.67E-06
ENSG00000089063_5109430_5100791_120	annotated	TMEM230	20	-	360	109	7	0	10.83%	100.0445997	0.001133048	0.000717317	5.10E-06
ENSG00000172922_65720758_65720068_173	novel	RNASEH2C	11	-	519	35	0	2	5.20%	22.28282965	0.002967235	0.018399684	0.000310873
ENSG00000145391_139556119_139511663_360	annotated	SETD7	4	-	1080	36	7	0	5.56%	11.01408437	0.001300555	2.53E-05	2.94E-07
ENSG00000145390_119256342_119293211_917	annotated	USP53	4	+	2751	21	0	0	1.20%	2.522309398	0.001448134	0.001448134	1.26E-05
ENSG00000163710_142888896_142818335_415	annotated	PCOLCE2	3	-	1245	75	4	2	6.02%	19.90497175	5.96E-06	1.20E-05	5.91E-10
ENSG00000115306_54558779_54660086_2155	annotated	SPTBN1	2	+	6465	157	35	28	3.67%	8.024182117	1.86E-08	6.37E-10	1.37E-16
ENSG00000059588_234400874_234393367_107	novel	TARBP1	1	-	321	19	1	1	6.54%	19.55771991	0.0135992	0.0135992	0.000893524
ENSG00000124783_7313197_7313117_26	novel	SSR1	6	-	78	35	34	4	38.46%	148.2665204	0.280590841	0.01271029	0.023227279

ENSG00000124783_7313120_7303559_88	novel	SSR1	6	-	264	250	18	146	27.27%	312.9001242	0.000190421	0.050920054	0.000121662
ENSG00000144524_231788571_231807645_209	annotated	COPS7B	2	+	627	29	3	0	4.78%	15.2827008	0.002404282	0.002404282	3.34E-05
ENSG00000176845_83085014_83094575_229	annotated	METRNL	17	+	687	20	0	0	3.49%	9.61928766	0.005405142	0.005405142	0.000156372
ENSG00000151532_112447374_112736774_203	annotated	VTI1A	10	+	609	15	5	7	4.43%	8.138485988	0.046396937	0.071812861	0.013120649
ENSG00000131503_140526355_140539418_866	annotated	ANKHD1	5	+	2598	32	0	0	1.73%	4.069869513	0.000276859	0.000276859	5.20E-07
ENSG00000149761_64226425_64227315_227	novel	NUDT22	11	+	681	44	3	1	5.29%	21.348886	0.00236973	0.000958906	1.39E-05
ENSG00000048028_113854326_113813876_561	annotated	USP28	11	-	1683	28	7	1	2.67%	5.497225711	0.010895533	0.000432958	3.48E-05
ENSG00000111639_6492895_6492271_76	annotated	MRPL51	12	-	228	449	24	7	44.74%	650.7005108	1.65E-06	4.28E-07	6.46E-12
ENSG00000111261_12343314_12330027_397	annotated	MANSC1	12	-	1191	27	1	2	2.02%	7.490687103	0.007312901	0.011945875	0.000444205
ENSG00000111276_12717840_12721207_205	annotated	CDKN1B	12	+	615	47	6	6	8.29%	25.25180319	0.000814492	0.000508728	2.67E-06
ENSG00000182196_122982001_122982586_63	novel	ARL6IP4	12	+	189	10	0	0	9.52%	17.4826736	0.012798403	0.012798403	0.000797244
ENSG00000140990_1959625_1961906_172	annotated	NDUFB10	16	+	516	303	66	14	25.00%	194.027184	6.78E-08	7.50E-08	4.98E-14
ENSG00000160867_177089603_177097676_734	annotated	FGFR4	5	+	2202	47	4	9	2.72%	7.052615333	7.38E-05	0.001903138	1.12E-06
ENSG00000172239_43556846_43536878_266	annotated	PAIP1	5	-	798	21	7	0	2.26%	8.695329766	0.012619539	0.01300724	0.00079885
ENSG00000074582_218660988_218663386_419	annotated	BCS1L	2	+	1257	71	5	2	5.49%	18.66348426	0.000108734	3.17E-05	2.62E-08
ENSG00000074582_218660988_218663386_419	annotated	BCS1L	2	+	1257	71	5	2	5.49%	18.66348426	0.000108734	3.17E-05	2.62E-08
ENSG00000107372_72360778_72355953_213	annotated	ZFAND5	9	-	639	174	26	3	12.21%	89.97421035	2.33E-05	5.40E-06	1.04E-09
ENSG00000107372_72360778_72355953_213	annotated	ZFAND5	9	-	639	163	26	3	12.21%	84.28618556	2.33E-05	5.39E-06	1.04E-09
ENSG00000126756_47658927_47657683_92	novel	UXT	X	-	276	40	9	2	13.04%	47.88732335	0.026040673	0.001015599	0.000188703
ENSG00000075303_87858727_87836249_338	annotated	SLC25A40	7	-	1014	73	19	0	7.99%	23.78781536	0.001179718	2.23E-06	3.43E-08
ENSG00000075303_87876195_87858732_62	uORF	SLC25A40	7	-	186	20	0	5	8.06%	35.52930442	0.021557223	0.070008252	0.006686241
ENSG00000120875_29350278_29337026_394	annotated	DUSP4	8	-	1182	202	27	7	14.47%	56.4681483	7.37E-11	6.65E-11	5.77E-20
ENSG00000120875_29345430_29337026_270	annotated	DUSP4	8	-	810	129	13	5	14.81%	52.62284755	6.45E-08	4.54E-08	2.90E-14
ENSG00000118518_127286653_127287693_346	annotated	RNF146	6	+	1038	40	1	0	5.20%	12.73304552	8.20E-05	8.27E-05	4.95E-08
ENSG00000168000_62705595_62702488_118	internal	BSCL2	11	-	354	47	0	15	8.47%	43.86965809	0.002488365	0.018376712	0.00026737
ENSG00000168000_62707195_62690367_464	annotated	BSCL2	11	-	1392	118	51	3	7.54%	28.00995594	0.007372492	3.19E-07	7.80E-08
ENSG00000075711_197297204_197044623_926	annotated	DLG1	3	-	2778	39	4	0	1.94%	4.638761236	0.000762836	7.71E-05	4.39E-07
ENSG00000146676_44885348_44884410_312	annotated	PURB	7	-	936	19	2	6	3.53%	6.707294969	0.003925334	0.040781435	0.000931167
ENSG00000065150_98002888_98021822_648	novel	IPO5	13	+	1944	96	11	9	5.25%	16.31716203	1.14E-05	4.98E-06	4.72E-10
ENSG00000128928_40413036_40435531_200	annotated	IVD	15	+	600	7	0	0	2.50%	3.85492953	0.016947427	0.016947427	0.001349898
ENSG00000164051_48435101_48432408_402	annotated	CCDC51	3	-	1206	40	5	0	4.98%	10.95928793	0.000598085	3.59E-05	1.71E-07
ENSG00000284194_50524411_50523611_266	annotated	SCO2	22	-	798	51	6	20	7.14%	21.11722943	0.00059841	0.017243231	7.66E-05
ENSG00000198363_61714371_61661907_113	annotated	ASPH	8	-	339	405	75	31	33.63%	394.7525814	2.37E-05	3.41E-06	6.88E-10
ENSG00000132906_15507060_15492943_260	dORF	CASP9	1	-	780	22	1	0	4.62%	9.319609852	0.000862735	0.000866459	4.69E-06
ENSG00000173085_83284764_83267173_328	annotated	COQ2	4	-	984	126	22	3	13.41%	42.31020216	7.28E-07	7.62E-09	6.43E-14
ENSG00000187954_144464414_144464127_95	annotated	CYHR1	8	-	285	18	0	0	6.32%	20.86879144	0.01300724	0.01300724	0.000821865
ENSG00000173085_83284764_83264199_371	annotated	COQ2	4	-	1113	146	28	3	14.02%	43.34383607	1.51E-07	3.20E-10	6.62E-16
ENSG00000122783_135168838_135167772_41	Overlap_uORF	CYREN	7	-	123	32	6	1	19.51%	85.96358533	0.024275373	0.006191047	0.000779962
ENSG00000123700_70175094_70176323_409	annotated	KCNJ2	17	+	1227	27	5	0	2.20%	7.270911443	0.019784898	0.003684287	0.000403741
ENSG00000113971_132722355_132681910_1330	annotated	NPHP3	3	-	3990	22	1	6	0.68%	1.821878618	0.005109528	0.049161556	0.001418095
ENSG00000126822_64731134_64743703_948	annotated	PLEKHG3	14	+	2844	14	3	0	0.53%	1.626552544	0.085886389	0.021084099	0.008130109
ENSG00000171311_97445755_97437746_77	novel	EXOSC1	10	-	231	16	1	0	9.09%	22.88640908	0.023878474	0.007979402	0.000955388
ENSG00000188554_43175800_43209914_837	annotated	NBR1	17	+	2511	93	10	3	4.54%	12.23787152	4.43E-06	2.26E-07	9.78E-12
ENSG00000090372_46738813_46720602_634	annotated	STRN4	19	-	1902	70	17	9	4.42%	12.16066098	0.001032735	0.000117222	8.78E-07
ENSG00000139624_50167297_50147340_86	novel	CERS5	12	-	258	22	1	0	5.81%	28.17556467	0.028889786	0.021084099	0.002731533
ENSG00000176697_27658543_27657821_240	annotated	BDNF	11	-	720	54	20	2	10.00%	24.78168983	0.0133993	9.36E-06	2.20E-06
ENSG00000162409_56693779_56707713_389	annotated	PRKAA2	1	+	1167	25	1	2	3.08%	7.078460392	0.00162692	0.004234187	4.03E-05
ENSG00000103512_14864588_14896111_801	annotated	NOMO1	16	+	2403	30	13	0	1.37%	4.125125232	0.046975818	0.001558047	0.000529092
ENSG00000273749_22947285_22870028_1253	annotated	CYFIP1	15	-	3759	158	19	9	4.39%	13.88847032	9.90E-09	1.35E-09	1.46E-16
ENSG00000105655_18437003_18434913_430	annotated	ISYNA1	19	-	1290	28	1	4	3.72%	7.171961916	0.000278081	0.002676324	5.16E-06
ENSG00000013275_39971203_39981305_387	annotated	PSMC4	19	+	1161	289	39	10	16.80%	82.2498807	8.29E-12	1.60E-12	1.68E-22
ENSG00000144579_218398413_218404425_260	annotated	CTDSP1	2	+	780	27	28	0	5.77%	11.437703	0.37130507	0.00026815	0.003679203
ENSG00000144579_218401642_218402355_59	internal	CTDSP1	2	+	177	21	0	3	10.17%	39.20267318	0.013640586	0.032657853	0.002090452
ENSG00000160255_44907035_44886368_700	dORF	ITGB2	21	-	2100	22	2	0	2.00%	3.461569374	0.001303752	0.000335351	2.90E-06
ENSG00000164941_94827285_94859647_587	novel	INTS8	8	+	1761	11	3	0	1.02%	2.06396811	0.051117422	0.012798403	0.003128977
ENSG00000166788_18106041_18080399_474	annotated	SAAL1	11	-	1422	86	3	0	6.54%	19.98335983	1.48E-06	4.85E-07	6.53E-12
ENSG00000143228_163326052_163355469_417	annotated	NUF2	1	+	1251	79	13	1	7.19%	20.86601116	3.39E-05	7.13E-07	2.39E-10
ENSG00000133318_63681637_63758201_1032	annotated	RTN3	11	+	3096	392	50	9	3.88%	41.83644451	1.73E-06	6.30E-08	1.12E-12
ENSG00000204899_72727602_72710322_82	annotated	MZT1	13	-	246	162	18	9	32.93%	217.5953254	6.64E-05	1.48E-05	7.77E-09
ENSG00000136122_72728965_72755216_551	annotated	BORA	13	+	1653	23	1	0	2.00%	4.597530681	0.00140552	0.00140552	1.19E-05
ENSG00000125844_17660456_17614182_1393	annotated	RRBP1	20	-	4179	35	7	5	1.36%	2.767357882	0.002823381	0.000790767	1.39E-05
ENSG00000147123_47144679_47142317_153	annotated	NDUFB11	X	-	459	430	20	10	25.49%	309.546162	2.13E-07	1.28E-07	2.61E-13
ENSG00000072518_63839507_63909237_732	annotated	MARK2	11	+	2196	48	9	3	3.01%	7.222350407	0.001008036	0.00010476	7.74E-07
ENSG00000100364_45203973_45197176_228	annotated	KIAA0930	22	-	684	20	0	1	3.95%	9.661477518	0.003478238	0.004911637	9.42E-05
ENSG00000136758_27154210_27147426_136	annotated	YME1L1	10	-	408	50	9	2	5.15%	40.49295724	0.133816362	0.019903045	0.012627226
ENSG00000156171_111131554_111118160_266	annotated	DRAM2	1	-	798	86	11	0	9.77%	35.60944571	0.000272717	3.62E-06	9.70E-09
ENSG00000169359_155853997_155828210_447	annotated	SLC33A1	3	-	1341	72	15	1	6.71%	17.74080704	0.000336739	8.78E-07	3.65E-09
ENSG00000099956_23787179_23791950_92	novel	SMARCB1	22	+	276	23	3	3	7.61%	27.53521093	0.024275373	0.018738288	0.002078871

ENSG00000100578_58467761_58514227_726	annotated	KIAA0586	14	+	2178	20	0	1	1.38%	3.034183022	0.002227676	0.005642788	7.16E-05
ENSG00000140694_14630125_14540838_529	novel	PARN	16	-	1587	34	9	6	2.65%	7.078995626	0.004398905	0.004905338	0.000117285
ENSG00000101236_3963984_3934063_137	annotated	RNF24	20	-	411	24	0	0	5.84%	19.29474634	0.005454749	0.005454749	0.000159109
ENSG00000125753_45522787_45526177_117	dORF	VASP	19	+	351	20	3	0	9.40%	18.82749465	0.001255833	0.001398493	1.07E-05
ENSG00000013306_44321554_44320001_227	annotated	SLC25A39	17	-	681	116	5	2	15.86%	56.28342674	1.57E-07	7.01E-08	1.07E-13
ENSG00000278615_62663242_62663050_38	novel	C11orf98	11	-	114	24	0	0	18.42%	69.56263813	0.008574016	0.008574016	0.000374746
ENSG00000198561_57795613_57816308_840	annotated	CTNND1	11	+	2520	195	33	13	6.55%	25.56841015	7.00E-07	6.03E-09	4.98E-14
ENSG00000141376_60683995_61392125_881	annotated	BCAS3	17	+	2643	19	2	0	1.14%	2.375341692	0.009621867	0.002227676	0.000123102
ENSG00000147408_19506085_19505966_39	uORF	CSGALNACT1	8	-	117	24	10	1	20.51%	67.77898074	0.085401204	0.005604867	0.002874515
ENSG00000152904_235335265_235342772_300	annotated	GGPS1	1	+	900	28	4	0	4.00%	10.27981208	0.006091863	0.000978918	3.71E-05
ENSG00000155097_103040837_103068747_382	annotated	ATP6V1C1	8	+	1146	116	9	2	9.16%	33.44591065	2.75E-07	1.09E-07	2.87E-13
ENSG00000100483_50116552_50109699_229	annotated	VCPKMT	14	-	687	21	6	5	3.93%	10.10025204	0.028243214	0.036572178	0.004451921
ENSG00000278259_36511380_36498534_231	dORF	MYO19	17	-	693	15	0	0	3.03%	7.152002838	0.008473674	0.008473674	0.000366502
ENSG00000278259_36535375_36535046_109	uORF	MYO19	17	-	327	10	1	3	4.59%	10.10466456	0.042234516	0.150401371	0.025490393
ENSG00000167118_128371381_128389734_101	annotated	URM1	9	+	303	326	40	10	38.61%	355.5041094	2.65E-07	1.15E-07	2.91E-13
ENSG00000167792_67612143_67612458_69	novel	NDUFV1	11	+	207	24	0	0	17.39%	38.30985868	0.00087769	0.00087769	4.83E-06
ENSG00000139174_42469470_42459809_710	annotated	PRICKLE1	12	-	2130	10	0	0	0.70%	1.551279489	0.020613417	0.020613417	0.001946209
ENSG00000164733_11852668_11845125_288	annotated	CTSB	8	-	864	1251	101	26	36.11%	478.4242898	7.01E-17	3.84E-18	4.23E-33
ENSG00000164733_11853372_11852665_24	internal	CTSB	8	-	72	32	2	43	37.50%	146.8544583	0.003710481	0.468493666	0.025643776
ENSG00000100852_32091060_32154948_1372	annotated	ARHGAP5	14	+	4116	36	6	4	1.31%	2.889992984	0.001254322	0.001097093	8.44E-06
ENSG00000168036_41224552_41233459_386	novel	CTNNB1	3	+	1158	137	22	5	11.14%	39.09143935	2.73E-07	1.42E-08	4.09E-14
ENSG00000168036_41233535_41239342_384	novel	CTNNB1	3	+	1152	151	24	8	11.72%	43.31059219	6.45E-07	3.20E-08	2.13E-13
ENSG00000102974_67611139_67637872_623	annotated	CTCF	16	+	1869	54	1	2	4.33%	9.546718395	2.77E-06	4.67E-06	1.10E-10
ENSG00000149231_96384747_96364962_465	novel	CCDC82	11	-	1395	27	0	0	2.80%	6.395274796	0.000635664	0.000635664	2.59E-06
ENSG00000114346_172754531_172820237_914	annotated	ECT2	3	+	2742	339	33	13	9.41%	40.85092562	1.80E-13	3.92E-15	1.03E-26
ENSG00000064309_126023476_125960942_1287	annotated	CDON	11	-	3861	18	4	0	0.85%	1.54043138	0.017669157	0.001343041	0.000152807
ENSG00000153904_85358796_85321452_167	annotated	DDAH1	1	-	501	23	0	0	5.99%	15.16909823	0.002390426	0.002390426	3.30E-05
ENSG00000111731_22544099_22449760_1034	annotated	C2CD5	12	-	3102	13	5	2	1.16%	1.384749486	0.061632282	0.006577294	0.002233751
ENSG00000115919_142885368_143042172_465	annotated	KYNU	2	+	1395	345	44	10	15.05%	81.71740017	4.43E-10	1.41E-12	8.99E-21
ENSG00000141101_69754910_69749626_66	annotated	NOB1	16	-	198	16	0	4	7.58%	26.7008106	0.021084099	0.060667625	0.005667716
ENSG00000006468_13989019_13895866_443	annotated	ETV1	7	-	1329	15	0	0	2.26%	3.72937394	0.002008257	0.002008257	2.37E-05
ENSG00000196924_154359342_154351161_1070	novel	FLNA	X	-	3210	149	9	15	4.67%	15.33736982	5.92E-10	2.22E-08	1.54E-16
ENSG00000196924_154350226_154348849_268	novel	FLNA	X	-	804	21	4	3	3.73%	8.630439246	0.012891076	0.009583527	0.000613108
ENSG00000100280_29363023_29328821_909	annotated	AP1B1	22	-	2727	79	9	6	3.19%	9.572196538	5.30E-05	2.40E-05	9.82E-09
ENSG00000145247_48904549_48885531_99	annotated	OCIAD2	4	-	297	16	1	1	6.06%	17.8005404	0.011772051	0.026832696	0.001510382
ENSG00000141552_81894478_81900065_84	annotated	ANAPC11	17	+	252	694	48	29	25.00%	909.9731611	0.000200778	0.00015937	2.23E-07
ENSG00000163072_168834302_168864970_346	annotated	NOSTRIN	2	+	1038	20	6	0	1.73%	6.366522758	0.196884317	0.01300724	0.01473882
ENSG00000108298_39201214_39202471_86	novel	RPL19	17	+	258	54	11	10	9.30%	69.15820419	0.014786102	0.020905271	0.001451864
ENSG00000108298_39203039_39204648_101	novel	RPL19	17	+	303	412	36	9	18.81%	449.287402	0.000843805	6.87E-05	4.40E-07
ENSG00000145833_134758939_134828706_1032	annotated	DDX46	5	+	3096	139	20	6	5.43%	14.8348617	9.21E-08	3.50E-10	4.23E-16
ENSG00000092621_119726876_119744040_406	dORF	PHGDH	1	+	1218	34	4	1	3.45%	9.223617453	0.001466059	0.000556136	5.19E-06
ENSG00000092621_119712023_119724460_165	annotated	PHGDH	1	+	495	26	4	0	4.24%	17.35552689	0.110834006	0.007029303	0.004652436
ENSG00000116478_32331542_32331823_62	novel	HDAC1	1	+	186	22	1	1	14.52%	39.08223486	0.008219047	0.003529166	0.000158266
ENSG00000100142_37953788_37967174_74	annotated	POLR2F	22	+	222	31	9	15	12.16%	46.14008317	0.049245483	0.08357183	0.015971734
ENSG00000103978_42273398_42258949_181	annotated	TMEM87A	15	-	543	60	9	0	10.50%	36.51077692	0.000349482	5.63E-05	1.47E-07
ENSG00000267855_8316539_8311505_44	dORF	NDUFA7	19	-	132	73	25	0	29.55%	182.7336725	0.017639598	0.000719745	9.13E-05
ENSG00000267855_8321358_8316610_76	annotated	NDUFA7	19	-	228	69	7	0	9.21%	99.99629231	0.053448427	0.008877961	0.00242838
ENSG00000083807_58498895_58498515_95	annotated	SLC27A5	19	-	285	17	0	0	7.37%	19.70941414	0.007882058	0.007882058	0.000319636
ENSG00000147010_19645418_19536417_404	annotated	SH3KBP1	X	-	1212	31	0	2	3.96%	8.451401373	0.000177865	0.000815071	1.00E-06
ENSG00000101247_13785069_13817210_317	annotated	NDUFAF5	20	+	951	56	6	5	6.31%	19.45705756	5.29E-05	0.000837968	3.46E-07
ENSG00000204954_103956678_103951523_51	annotated	C12orf73	12	-	153	16	7	0	17.65%	34.55399018	0.062411228	0.002500085	0.001069711
ENSG00000070610_35744648_35737476_738	annotated	GBA2	9	-	2214	36	9	13	2.44%	5.372724083	0.003263084	0.017523284	0.000320251
ENSG00000072756_3129041_3146449_213	annotated	TRNT1	3	+	639	24	1	0	6.57%	12.41023591	0.000594982	0.000396885	1.55E-06
ENSG00000115525_85863483_85847466_224	annotated	ST3GAL5	2	-	672	100	19	9	11.16%	49.17001951	0.000208105	0.00017171	2.49E-07
ENSG00000115525_85846502_85840144_177	dORF	ST3GAL5	2	-	531	43	4	1	9.60%	26.75738011	0.00076033	0.000186181	9.75E-07
ENSG00000187778_49559533_49559273_46	annotated	MCRS1	12	-	138	24	5	0	17.39%	57.46478802	0.011825808	0.005554612	0.000342496
ENSG00000110955_56644929_56643891_52	novel	ATP5F1B	12	-	156	361	32	6	44.23%	764.6316265	0.000126936	2.18E-05	2.16E-08
ENSG00000181744_143985600_143989841_192	annotated	DIPK2A	3	+	576	16	1	1	4.69%	9.178403642	0.00640069	0.010020958	0.000331324
ENSG00000155229_97461595_97458692_293	novel	MMS19	10	-	879	23	0	1	2.39%	8.645868277	0.008574016	0.013991246	0.000599135
ENSG00000186298_110730669_110720134_169	Overlap_dORF	PPP1CC	12	-	507	236	15	0	13.02%	153.8061486	6.14E-05	1.93E-05	9.22E-09
ENSG00000174437_110332597_110334155_111	novel	ATP2A2	12	+	333	83	13	0	19.82%	82.35756782	0.000478038	1.74E-05	7.11E-08
ENSG00000172819_53215305_53213383_222	novel	RARG	12	-	666	9	0	0	2.25%	4.465169339	0.019679754	0.019679754	0.001784964
ENSG00000140545_88912172_88899395_379	dORF	MFGE8	15	-	1137	134	3	8	11.08%	38.94161756	3.85E-08	7.49E-08	2.86E-14
ENSG00000130164_11089549_11131316_858	annotated	LDLR	19	+	2574	560	59	26	17.37%	71.88679776	6.46E-23	8.92E-24	1.03E-44
ENSG00000157823_89893949_89835515_205	annotated	AP3S2	15	-	615	79	3	8	9.76%	42.44452026	3.96E-05	0.000132446	3.94E-08
ENSG00000160752_155318926_155320609_138	novel	FDPS	1	+	414	122	3	10	17.39%	97.37089081	1.45E-05	4.26E-05	4.89E-09
ENSG00000128654_176270410_176337664_253	dORF	MTX2	2	+	759	124	12	1	13.83%	53.98207359	2.77E-06	1.05E-07	2.93E-12
ENSG00000127952_76021859_75996468_165	Overlap_dORF	STYXL1	7	-	495	16	4	0	3.03%	10.68032424	0.08410207	0.021084099	0.007951576

ENSG00000101773_22936852_23026247_867	annotated	RBBP8	18	+	2601	22	0	8	1.38%	2.794808029	0.000873936	0.043968139	0.000313262
ENSG00000214944_73749846_73941013_1717	annotated	ARHGEF28	5	+	5151	37	6	16	1.22%	2.373448583	0.00066826	0.037249157	0.00020812
ENSG00000088256_3119212_3121179_112	novel	GNA11	19	+	336	22	0	0	7.14%	21.63480858	0.005355751	0.005355751	0.00015367
ENSG00000136603_170360425_170392417_653	annotated	SKIL	3	+	1959	50	12	1	3.83%	8.433448982	0.000499766	6.03E-06	2.95E-08
ENSG00000057294_32878126_32792424_586	annotated	PKP2	12	-	1758	9	2	0	0.85%	1.691582924	0.079862169	0.019216965	0.006984847
ENSG00000085760_55263858_55236648_727	annotated	MTIF2	2	-	2181	62	9	2	4.40%	9.393029312	9.90E-06	5.37E-07	5.02E-11
ENSG00000146223_42879938_42886464_246	annotated	RPL7L1	6	+	738	15	1	0	2.44%	6.715905104	0.015555383	0.011772051	0.000887479
ENSG00000168140_4381073_4382899_608	annotated	VASN	16	+	1824	27	7	0	2.63%	4.891122993	0.010982625	0.00017086	1.64E-05
ENSG00000175193_183844264_183829598_167	annotated	PARL	3	-	501	7	1	0	2.99%	4.616682071	0.041782825	0.019216965	0.003600526
ENSG00000100023_21688812_21695490_153	dORF	PPIL2	22	+	459	8	0	3	3.27%	5.758998364	0.019679754	0.119134635	0.010986432
ENSG00000100023_21670348_21686930_282	dORF	PPIL2	22	+	846	17	0	2	2.13%	6.639696252	0.013428348	0.031019588	0.00195881
ENSG00000119723_73950333_73953577_101	novel	COQ6	14	+	303	14	0	0	4.95%	15.26704764	0.021084099	0.021084099	0.002029891
ENSG00000115234_27374720_27376719_256	dORF	SNX17	2	+	768	139	1	3	11.72%	59.80303623	7.50E-07	1.86E-06	1.25E-11
ENSG00000114770_183989320_183987665_167	annotated	ABCC5	3	-	501	16	2	4	3.59%	10.55241616	0.052581193	0.128949518	0.025842803
ENSG00000099364_30927404_30947230_540	annotated	FBXL19	16	+	1620	17	1	0	1.48%	3.467396931	0.010079165	0.004873904	0.000259739
ENSG00000205707_25203963_25204312_88	annotated	ETFRF1	12	+	264	14	0	0	7.95%	17.52240695	0.007029303	0.007029303	0.000257364
ENSG00000204642_29723464_29726407_254	annotated	HLA-F	6	+	762	13	4	0	2.76%	5.637129796	0.042234516	0.008373824	0.001798314
ENSG00000011243_15403626_15399298_316	novel	AKAP8L	19	-	948	13	1	2	2.85%	4.531110659	0.006467047	0.019435691	0.000645144
ENSG00000085719_86547744_86558410_253	novel	CPNE3	8	+	759	37	5	0	5.93%	16.10755422	0.003320019	0.000283519	6.60E-06
ENSG00000131495_140647583_140645703_76	annotated	NDUFA2	5	-	228	275	18	7	32.89%	398.5359476	1.66E-05	1.22E-05	1.62E-09
ENSG00000151881_43453969_43446222_215	annotated	TMEM267	5	-	645	204	21	2	12.09%	104.5057308	0.003894049	6.82E-06	3.57E-07
ENSG00000142507_4796737_4797732_68	annotated	PSMB6	17	+	204	250	3	0	20.59%	404.9295724	0.000480911	0.000480911	1.51E-06
ENSG00000066136_40738844_40770828_458	annotated	NFYC	1	+	1374	40	2	0	2.84%	9.61928766	0.000867445	0.000683882	3.76E-06
ENSG00000166881_57078745_57059879_444	annotated	NEMP1	12	-	1332	151	8	1	10.81%	37.45780946	1.29E-08	7.34E-10	1.07E-16
ENSG00000150990_124989022_124947802_1157	annotated	DHX37	12	-	3471	24	6	1	1.04%	2.284684744	0.012842162	0.001563962	0.000122737
ENSG00000186832_41612649_41609935_460	annotated	KRT16	17	-	1380	39	11	2	3.04%	9.338028053	0.046661686	0.000675451	0.000277352
ENSG00000163755_149160070_149167148_258	annotated	HPS3	3	+	774	28	1	1	5.81%	11.95326986	0.000287458	0.000287458	5.60E-07
ENSG00000134313_8827093_8730720_1752	annotated	KIDINS220	2	-	5256	49	11	2	1.60%	3.08042314	0.00084268	6.44E-06	5.62E-08
ENSG00000114867_184329016_184334908_252	novel	EIF4G1	3	+	756	45	2	0	7.54%	19.6680078	7.98E-05	4.88E-05	2.89E-08
ENSG00000145781_116087498_116292489_188	annotated	COMMD10	5	+	564	79	20	7	13.83%	46.28258858	0.000838392	3.66E-05	2.50E-07
ENSG00000109819_23889957_23866214_88	novel	PPARGC1A	4	-	264	35	6	0	17.05%	43.80601738	0.003470368	0.000294865	7.16E-06
ENSG00000086289_37920940_37950203_90	annotated	EPDR1	7	+	270	29	3	0	10.00%	35.48982742	0.003554776	0.003710481	7.34E-05
ENSG00000204922_62671764_62672114_87	annotated	UQCC3	11	+	261	58	7	3	21.84%	73.42722914	0.000392695	0.000173036	4.68E-07
ENSG00000099968_17702273_17727534_323	Overlap_dORF	BCL2L13	22	+	969	18	14	8	4.02%	6.137879835	0.254083945	0.034910748	0.04006609
ENSG00000015475_17750116_17735580_195	annotated	BID	22	-	585	113	29	8	13.85%	63.82520687	0.000831817	2.97E-05	2.06E-07
ENSG00000156639_37929972_38152389_177	annotated	ZFAND3	6	+	531	15	6	2	6.21%	9.333969806	0.014229868	0.009234247	0.000651769
ENSG00000135624_73234382_73252861_338	annotated	CCT7	2	+	1014	806	85	20	24.85%	262.6435504	1.12E-11	6.21E-15	1.18E-24
ENSG00000131269_75156257_75053370_747	annotated	ABCB7	X	-	2241	57	2	4	3.08%	8.404321407	1.54E-05	3.94E-05	4.77E-09
ENSG00000214517_74171518_74253510_368	annotated	PPME1	11	+	1104	64	5	4	6.79%	19.15492934	3.81E-05	3.78E-05	1.10E-08
ENSG00000151176_113358613_113388626_553	annotated	PLBD2	12	+	1659	192	18	10	11.21%	38.24058226	3.99E-10	1.17E-10	5.40E-19
ENSG00000114021_100334792_100348841_149	novel	NIT2	3	+	447	114	2	36	12.08%	84.26883344	9.32E-05	0.020787916	2.22E-05
ENSG00000114021_100346216_100348885_40	novel	NIT2	3	+	120	22	1	0	15.00%	60.57746404	0.01300724	0.013428348	0.000846864
ENSG00000066468_121526752_121479857_593	annotated	FGFR2	10	-	1779	10	1	0	0.84%	1.85734981	0.035374934	0.020613417	0.003252526
ENSG00000011478_45692758_45703049_364	annotated	QPCTL	19	+	1092	76	9	4	7.42%	22.99643989	4.35E-05	1.67E-05	5.68E-09
ENSG00000108883_44886750_44851274_937	annotated	EFTUD2	17	-	2811	175	15	11	7.15%	20.57059514	5.34E-12	3.79E-11	2.51E-21
ENSG00000155304_14383107_14373617_467	annotated	HSPA13	21	-	1401	154	22	2	9.42%	36.32053518	6.86E-07	4.33E-09	3.60E-14
ENSG00000117054_75734848_75762763_273	novel	ACADM	1	+	819	31	4	1	5.86%	12.50683573	0.000391303	0.00024364	6.45E-07
ENSG00000144366_188483442_188594011_291	annotated	GULP1	2	+	873	66	5	2	8.59%	24.98039754	1.30E-05	1.60E-05	1.67E-09
ENSG00000082153_200813218_200822178_419	annotated	BZW1	2	+	1257	99	27	14	5.01%	26.02373157	0.004394084	0.000722386	2.02E-05
ENSG00000065911_74198642_74214242_267	annotated	MTHFD2	2	+	801	75	0	2	11.24%	30.93843924	6.32E-07	9.13E-07	5.22E-12
ENSG00000048991_135621596_135724292_965	annotated	R3HDM1	2	+	2895	28	2	3	1.14%	3.195796501	0.004488024	0.008610259	0.000206254
ENSG00000071189_17940295_17882814_189	annotated	SNX13	7	-	567	14	0	0	3.17%	8.158581015	0.012798403	0.012798403	0.000797244
ENSG00000103888_80873919_80948924_1348	annotated	CEMIP	15	+	4044	14	2	0	0.67%	1.143896003	0.012888811	0.002943991	0.000212649
ENSG00000164023_107895554_107910553_365	annotated	SGMS2	4	+	1095	33	4	0	3.29%	9.957939294	0.003283063	0.001057356	2.11E-05
ENSG00000164023_107895554_107910553_365	annotated	SGMS2	4	+	1095	33	4	0	3.29%	9.957939294	0.003283063	0.001057356	2.11E-05
ENSG00000166164_50368774_50319211_652	annotated	BRD7	16	-	1956	26	2	0	1.99%	4.392119534	0.001538479	0.000606963	5.91E-06
ENSG00000166164_50368774_50319211_651	annotated	BRD7	16	-	1953	28	2	0	2.15%	4.737240589	0.000923157	0.000396885	2.39E-06
ENSG00000110075_68564337_68613117_556	annotated	PPP6R3	11	+	1668	86	6	12	7.01%	17.03617367	4.29E-07	1.35E-05	5.64E-11
ENSG00000140859_57771620_57759149_674	annotated	KIFC3	16	-	2022	153	24	8	7.27%	25.00229835	2.20E-06	1.53E-08	3.94E-13
ENSG00000077684_128831759_128872262_842	annotated	JADE1	4	+	2526	41	8	1	2.61%	5.363152722	0.002043451	1.35E-05	2.89E-07
ENSG00000099999_30386759_30379604_310	annotated	RNF215	22	-	930	51	0	0	5.81%	18.11994525	8.35E-05	8.35E-05	5.09E-08
ENSG00000099995_30356792_30334594_793	annotated	SF3A1	22	-	2379	71	12	0	3.40%	9.861286133	9.48E-05	2.31E-06	2.07E-09
ENSG00000167904_55762908_55762552_118	annotated	TMEM68	8	-	354	18	1	0	6.78%	16.80114565	0.005405142	0.005454749	0.000157735
ENSG00000143398_151231761_151247865_425	annotated	PIP5K1A	1	+	1275	38	2	0	4.00%	9.847887202	0.000335003	0.000103305	2.46E-07
ENSG00000162976_11155547_11177828_188	annotated	PQLC3	2	+	564	84	34	3	12.23%	49.21186634	0.003139451	3.08E-05	9.41E-07
ENSG00000100031_24611082_24628839_569	annotated	GGT1	22	+	1707	14	1	1	1.41%	2.709968035	0.010079165	0.010079165	0.000508526
ENSG00000101367_32825985_32848728_249	annotated	MAPRE1	20	+	747	213	28	3	15.66%	94.2168663	5.81E-06	3.26E-08	2.23E-12
ENSG00000114805_155596340_155480968_1646	annotated	PLCH1	3	-	4938	22	3	4	0.49%	1.472113342	0.011428951	0.026799085	0.001469725

ENSG00000177666_824081_824862_170	novel	PNPLA2	11	+	510	19	4	0	5.29%	12.309859	0.026877003	0.003606289	0.000549685
ENSG00000110696_16744670_16748180_32	novel	C11orf58	11	+	96	20	4	0	15.62%	68.83802732	0.170042304	0.021084099	0.017372496
ENSG00000138028_27104366_27101274_318	annotated	CGREF1	2	-	954	43	2	0	3.46%	14.89325874	0.002667196	0.001565547	2.46E-05
ENSG00000137575_58565034_58581740_297	annotated	SDCBP	8	+	891	318	39	6	17.51%	117.9285801	3.43E-09	6.12E-10	2.34E-17
ENSG00000147416_20197407_20212935_318	novel	ATP6V1B2	8	+	954	46	10	0	6.92%	15.9323233	0.000318386	1.43E-05	3.86E-08
ENSG00000147416_20217262_20220402_110	novel	ATP6V1B2	8	+	330	49	7	0	11.82%	49.06273947	0.006640732	0.000719745	3.11E-05
ENSG00000080823_102250855_102229289_237	novel	MOK	14	-	711	19	6	0	3.38%	8.829856668	0.02389627	0.005604867	0.000704504
ENSG00000139624_50137787_50135616_98	novel	CERS5	12	-	294	49	1	0	13.27%	55.07042185	0.00065148	0.000649205	2.71E-06
ENSG00000174243_48845782_48840145_108	annotated	DDX23	12	-	324	17	5	2	10.19%	17.33698466	0.038664476	0.00809034	0.001591478
ENSG00000123349_53297850_53299345_85	dORF	PFDN5	12	+	255	184	11	13	25.88%	238.4225323	0.000132197	0.000120571	1.14E-07
ENSG00000141503_4884369_4897287_1228	annotated	MINK1	17	+	3684	22	6	2	0.90%	1.973207298	0.021097153	0.00595204	0.00065242
ENSG00000196715_65873372_65954300_176	annotated	VKORC1L1	7	+	528	113	14	4	13.07%	70.71542806	0.000390633	2.02E-05	6.52E-08
ENSG00000112319_133462632_133528805_442	annotated	EYA4	6	+	1326	18	2	0	1.81%	4.485373726	0.013239535	0.005306578	0.000367823
ENSG00000151376_86671944_86457650_342	annotated	ME3	11	-	1026	19	4	3	2.34%	6.118935761	0.035955189	0.020990661	0.003357915
ENSG00000175224_46645957_46672332_438	annotated	ATG13	11	+	1314	60	13	4	5.94%	15.08778681	0.000152942	3.01E-05	3.53E-08
ENSG00000169957_30526046_30524517_509	annotated	ZNF768	16	-	1527	23	13	0	1.57%	4.976894706	0.109944916	0.005160369	0.00366851
ENSG00000145191_184142372_184144943_242	dORF	EIF2B5	3	+	726	15	8	2	2.48%	6.8269118	0.253561228	0.026832696	0.033369796
ENSG00000145191_184135386_184141919_402	annotated	EIF2B5	3	+	1206	35	6	3	3.23%	9.58937694	0.020014609	0.001635336	0.000206475
ENSG00000197912_89508547_89530693_85	annotated	SPG7	16	+	255	39	1	1	7.06%	50.53521064	0.012798403	0.020342454	0.001240246
ENSG00000197912_89530761_89557093_482	dORF	SPG7	16	+	1446	31	20	2	3.11%	7.083747209	0.15113843	0.000933393	0.001700359
ENSG00000138685_122827175_122892396_155	annotated	FGF2	4	+	465	65	16	6	11.61%	46.18809575	0.001507119	0.000992855	9.17E-06
ENSG00000168092_117152448_117175962_155	annotated	PAFAH1B2	11	+	465	58	12	2	9.68%	41.21399313	0.002644549	0.00052791	9.02E-06
ENSG00000177239_137087108_137107361_514	annotated	MAN1B1	9	+	1542	157	22	7	7.98%	33.64224214	6.13E-07	1.27E-07	7.43E-13
ENSG00000177239_137107340_137108591_147	Overlap_dORF	MAN1B1	9	+	441	39	8	2	10.20%	29.22104017	0.005031593	0.001101134	3.38E-05
ENSG00000005810_77326661_77045378_4640	annotated	MYCBP2	13	-	13920	12	5	4	0.15%	0.28484701	0.153960942	0.082535828	0.04432366
ENSG00000089053_121352340_121346988_74	Overlap_uORF	ANAPC5	12	-	222	32	3	0	10.81%	47.62847295	0.006346985	0.005756914	0.000193399
ENSG00000118181_119018284_119015845_125	annotated	RPS25	11	-	375	257	123	25	13.60%	226.4495747	0.018857081	0.005734461	0.000563079
ENSG00000164904_126595198_126544965_472	annotated	ALDH7A1	5	-	1416	109	8	3	6.57%	25.43506772	3.53E-06	1.04E-06	3.26E-11
ENSG00000243989_51984156_51989075_356	Overlap_dORF	ACY1	3	+	1068	63	3	4	7.02%	19.49121672	9.13E-06	3.68E-05	2.72E-09
ENSG00000165782_20461325_20458559_277	annotated	PIP4P1	14	-	831	45	3	3	6.86%	17.89291685	0.00022513	0.000339942	5.21E-07
ENSG00000188026_123533482_123472677_386	annotated	RILPL1	12	-	1158	12	2	2	1.55%	3.42406768	0.041632258	0.04317391	0.007396182
ENSG00000128833_52295636_52256701_430	annotated	MYO5C	15	-	1290	19	2	0	1.40%	4.866688443	0.031019588	0.013428348	0.00195881
ENSG00000156113_77108374_76877739_917	Overlap_dORF	KCNMA1	10	-	2751	38	8	0	2.29%	4.56417891	0.000553696	2.22E-05	1.03E-07
ENSG00000156113_77637360_77108332_328	annotated	KCNMA1	10	-	984	33	5	0	4.27%	11.08124342	0.003701066	0.00040306	1.01E-05
ENSG00000184047_122226014_122208557_209	annotated	DIABLO	12	-	627	68	6	13	9.57%	35.83529843	0.000182128	0.000748674	9.40E-07
ENSG00000139687_48303913_48480071_928	annotated	RB1	13	+	2784	65	11	1	3.02%	7.71460651	7.59E-05	1.80E-06	1.30E-09
ENSG00000051180_40698771_40731178_336	annotated	RAD51	15	+	1008	23	4	1	2.38%	7.539402992	0.031969545	0.005998643	0.001012981
ENSG00000171421_1798911_1798624_95	annotated	MRPL36	5	-	285	38	9	1	10.53%	44.05633748	0.051252243	0.00224099	0.000777436
ENSG00000103024_1771534_1770649_169	annotated	NME3	16	-	507	106	13	9	13.02%	69.08242268	1.85E-05	8.15E-05	1.18E-08
ENSG00000204568_30617866_30619642_75	novel	MRPS18B	6	+	225	29	3	0	10.67%	42.5877929	0.012176559	0.005756914	0.000364135
ENSG00000164904_126554349_126544965_160	novel	ALDH7A1	5	-	480	31	4	0	6.88%	21.33978847	0.00850083	0.00140552	7.23E-05
ENSG00000083896_68349753_68314099_727	annotated	YTHDC1	4	-	2181	51	11	1	2.75%	7.726524111	0.002919359	3.85E-05	1.04E-06
ENSG00000142453_10871703_10921757_585	annotated	CARM1	19	+	1755	86	6	1	3.25%	16.1916454	0.00167493	8.09E-05	1.06E-06
ENSG00000196177_123009030_123053765_432	annotated	ACADSB	10	+	1296	64	2	2	5.56%	16.31716203	6.05E-06	3.28E-05	1.64E-09
ENSG00000131748_39653532_39662908_445	annotated	STARD3	17	+	1335	55	15	21	4.49%	13.61291327	0.004836229	0.017094693	0.000438858
ENSG00000135317_85593697_85505967_939	annotated	SNX14	6	-	2817	95	14	3	4.05%	11.14310985	1.44E-05	1.81E-07	2.77E-11
ENSG00000133302_94628859_94695312_1042	annotated	SLF1	5	+	3126	10	10	0	0.67%	1.057013855	0.253561228	0.007029303	0.013708537
ENSG00000138778_103194633_103106222_2525	annotated	CENPE	4	-	7575	45	3	2	0.83%	1.962906125	0.000161753	0.000108195	1.25E-07
ENSG00000072518_63839507_63909237_778	annotated	MARK2	11	+	2334	48	9	3	2.83%	6.795321977	0.001008036	0.00010476	7.74E-07
ENSG00000143621_153662792_153662396_82	novel	ILF2	1	-	246	41	4	0	14.63%	55.07042185	0.003025432	0.001065834	1.96E-05
ENSG00000136155_77568305_77644274_565	annotated	SCEL	13	+	1695	12	0	6	0.88%	2.339274557	0.020613417	0.194271892	0.020032098
ENSG00000085274_169774296_169786678_610	annotated	MYNN	3	+	1830	24	1	1	1.15%	4.333410244	0.01019742	0.01019742	0.000519834
ENSG00000156709_130165656_130129557_613	annotated	AIFM1	X	-	1839	152	13	5	8.16%	27.31061704	2.89E-08	4.50E-10	1.57E-16
ENSG00000132153_47818078_47850120_1166	annotated	DHX30	3	+	3498	88	11	0	2.49%	8.312516506	7.12E-05	1.12E-06	7.95E-10
ENSG00000094631_48806387_48824612_1063	novel	HDAC6	X	+	3189	22	3	5	0.94%	2.279490651	0.016753251	0.01870523	0.001465173
ENSG00000198211_89919475_89935804_725	annotated	AC092143.1	16	+	2175	201	25	7	6.62%	30.53559943	4.05E-09	1.70E-09	7.46E-17
ENSG00000205707_25203963_25204312_88	annotated	ETFRF1	12	+	264	14	0	0	7.95%	17.52240695	0.007029303	0.007029303	0.000257364
ENSG00000088766_6009811_6023387_79	novel	CRLS1	20	+	237	18	1	1	6.33%	25.09538211	0.022899795	0.022899795	0.002367591
ENSG00000120053_99403566_99397547_126	novel	GOT1	10	-	378	89	2	10	11.90%	77.79789754	0.000291809	0.004591011	9.60E-06
ENSG00000167965_2206514_2208877_260	novel	MLST8	16	+	780	87	15	4	12.69%	36.85482078	3.61E-05	1.89E-06	6.18E-10
ENSG00000167965_2206086_2206426_65	novel	MLST8	16	+	195	23	3	0	9.23%	38.97291393	0.060667625	0.011772051	0.003501726
ENSG00000138629_74458867_74446090_380	annotated	UBL7	15	-	1140	74	13	2	7.11%	21.44848009	0.000213959	6.59E-06	1.26E-08
ENSG00000165806_113721653_113729540_220	annotated	CASP7	10	+	660	22	0	5	4.55%	11.01408437	0.002281203	0.012808166	0.000169256
ENSG00000182196_122981140_122982676_170	annotated	ARL6IP4	12	+	510	50	5	6	12.94%	32.3943658	4.61E-05	0.000409905	1.44E-07
ENSG00000196083_190564290_190648705_570	annotated	IL1RAP	3	+	1710	35	4	1	2.63%	6.763034263	0.002150019	0.000344964	4.97E-06
ENSG00000006744_13017902_13013231_143	annotated	ELAC2	17	-	429	13	0	12	4.20%	10.01280397	0.011772051	0.295006944	0.023726452
ENSG00000055070_16367258_16395140_152	annotated	SZRD1	1	+	456	18	8	8	4.61%	13.04299465	0.079942753	0.128936332	0.036418372
ENSG00000127947_77585573_77600846_107	novel	PTPN12	7	+	321	5	1	0	4.67%	5.146768397	0.051235217	0.012673659	0.00311107

ENSG00000135390_53672614_53665314_141	novel	ATP5MC2	12	-	423	370	85	27	30.50%	289.0220721	2.46E-06	4.35E-08	1.15E-12
ENSG00000196497_24188787_24188244_111	novel	IPO4	14	-	333	26	4	0	4.50%	25.79875618	0.087777151	0.021084099	0.008320031
ENSG00000170458_140633071_140631856_375	annotated	CD14	5	-	1125	19	0	0	2.93%	5.580469414	0.001384502	0.001384502	1.16E-05
ENSG00000137216_44136362_44140510_189	novel	TMEM63B	6	+	567	10	2	0	3.17%	5.827557868	0.026832696	0.012798403	0.001626089
ENSG00000138796_108004852_108034357_292	novel	HADH	4	+	876	66	9	4	8.56%	24.89484823	0.000252543	3.59E-05	6.95E-08
ENSG00000104763_18063207_18061715_74	novel	ASAH1	8	-	222	31	14	0	10.81%	46.14008317	0.056358542	0.005706018	0.001804861
ENSG00000121274_50153868_50229328_631	annotated	TENT4B	16	+	1893	17	1	3	1.11%	2.967344442	0.011825808	0.037995728	0.00215378
ENSG00000183283_51240919_51242458_108	Overlap_dORF	DAZAP2	12	+	324	172	22	1	16.67%	175.4094918	0.000636172	9.50E-05	4.39E-07
ENSG00000148450_23095609_23120862_182	annotated	MSRB2	10	+	546	159	15	10	20.33%	96.22194587	8.68E-07	2.08E-06	1.61E-11
ENSG00000062485_56283836_56273084_392	annotated	CS	12	-	1176	190	14	4	12.24%	53.38459261	3.68E-08	1.65E-09	6.81E-16
ENSG00000126581_42818656_42815904_152	novel	BECN1	17	-	456	40	4	1	11.84%	28.98443255	0.000211642	0.000122418	1.83E-07
ENSG00000117450_45519043_45511329_97	annotated	PRDX1	1	-	291	846	82	13	30.93%	960.6098327	2.98E-06	1.04E-06	2.75E-11
ENSG00000183856_156566542_156526486_1588	annotated	IQGAP3	1	-	4764	33	6	2	1.32%	2.288821059	0.001134645	6.25E-05	5.55E-07
ENSG00000186010_19516266_19527356_73	annotated	NDUFA13	19	+	219	465	58	13	50.68%	701.5820866	1.16E-06	1.88E-07	2.06E-12
ENSG00000186010_19527711_19528126_59	dORF	NDUFA13	19	+	177	574	27	1	35.59%	1071.539734	4.48E-05	2.89E-05	9.91E-09
ENSG00000087245_55482984_55505442_584	annotated	MMP2	16	+	1752	134	11	3	8.05%	25.2720429	5.79E-08	3.14E-09	1.99E-15
ENSG00000132522_7313250_7312756_72	novel	GPS2	17	-	216	23	0	2	15.28%	35.18388063	0.001384502	0.005080497	4.19E-05
ENSG00000112592_170557030_170572265_339	annotated	TBP	6	+	1017	12	0	1	1.47%	3.898790928	0.019216965	0.02170844	0.001914126
ENSG00000143537_155051387_155062502_838	annotated	ADAM15	1	+	2514	260	23	12	9.31%	34.17257681	9.67E-12	3.03E-13	3.94E-23
ENSG00000087884_77842497_77900686_147	annotated	AAMDC	11	+	441	24	0	1	4.76%	17.98217856	0.008473674	0.011825808	0.000503679
ENSG00000197217_23448845_23432926_574	annotated	ENTPD4	8	-	1722	66	9	2	4.36%	12.6642782	0.000287186	6.38E-06	1.69E-08
ENSG00000123600_171360506_171324172_357	annotated	METTL8	2	-	1071	11	2	0	2.52%	3.393695464	0.006277459	0.002478943	8.78E-05
ENSG00000182307_145052492_145054157_229	annotated	C8orf33	8	+	687	110	18	4	13.54%	52.90608213	6.76E-05	2.32E-06	1.45E-09
ENSG00000270136_19617297_19626401_70	annotated	MICOS10-NBL1	1	+	210	66	7	5	14.29%	103.8470812	0.005578808	0.00252785	7.94E-05
ENSG00000117984_1763859_1753503_405	annotated	CTSD	11	-	1215	2333	233	30	34.81%	634.4656503	2.48E-19	1.78E-24	1.10E-41
ENSG00000111605_69251225_69249758_208	novel	CPSF6	12	+	624	20	4	0	3.85%	10.59046574	0.043755562	0.005306578	0.001284879
ENSG00000175203_57547063_57535678_90	novel	DCTN2	12	-	270	21	0	0	11.11%	25.6995302	0.00224099	0.00224099	2.92E-05
ENSG00000181284_7435872_7437506_508	annotated	TMEM102	17	+	1524	8	0	0	0.98%	1.734501476	0.019679754	0.019679754	0.001784964
ENSG00000139625_53487316_53481182_867	annotated	MAP3K12	12	-	2601	33	1	6	2.08%	4.192212044	0.000116494	0.00522105	5.09E-06
ENSG00000131469_43000020_43002920_80	annotated	RPL27	17	+	240	95	11	9	18.75%	130.7922519	0.000965649	0.003899626	2.31E-05
ENSG00000138759_78058010_78541124_4012	annotated	FRAS1	4	+	12036	58	8	2	0.70%	1.592265437	0.00027024	6.31E-06	1.56E-08
ENSG00000129116_168681391_168926385_777	annotated	PALLD	4	+	2331	129	17	4	6.18%	18.28593158	1.97E-07	2.83E-09	6.46E-15
ENSG00000012061_45423374_45409675_225	annotated	ERCC1	19	-	675	84	15	7	12.00%	41.11924832	8.97E-05	1.89E-05	1.33E-08
ENSG00000157916_2402238_2403124_64	novel	RER1	1	+	192	54	5	2	20.31%	92.93133688	0.003249213	0.000683882	1.41E-05
ENSG00000150753_10258133_10261871_250	novel	CCT5	5	+	750	507	48	9	18.80%	223.365631	3.30E-08	1.45E-08	4.88E-15
ENSG00000052723_114780194_114779124_81	novel	SIKE1	1	-	243	11	0	0	6.17%	14.95739853	0.019216965	0.019216965	0.001707396
ENSG00000177084_132687315_132624697_2286	annotated	POLE	12	-	6858	42	3	1	0.92%	2.023585055	7.38E-05	3.35E-05	1.87E-08
ENSG00000063761_77819018_77933391_510	annotated	ADCK1	14	+	1530	33	7	0	2.55%	7.126760475	0.009473188	0.000662934	4.26E-05
ENSG00000143870_10819289_10784258_439	annotated	PDIA6	2	-	1317	1963	268	64	37.13%	492.4976679	4.56E-21	3.71E-27	4.91E-46
ENSG00000073350_75558180_75574906_949	novel	LLGL2	17	+	2847	54	9	2	2.63%	6.267234521	0.000734661	2.18E-05	1.38E-07
ENSG00000103496_31033806_31039803_297	annotated	STX4	16	+	891	67	5	2	10.44%	24.84658764	5.40E-06	8.92E-07	4.32E-11
ENSG00000132394_128153508_128408259_596	annotated	EEFSEC	3	+	1788	21	0	0	1.68%	3.88080154	0.00224099	0.00224099	2.92E-05
ENSG00000063241_55456486_55453308_135	annotated	ISOC2	19	-	405	74	2	3	16.30%	60.37349951	1.82E-05	4.80E-05	6.81E-09
ENSG00000163378_69009840_68977618_525	annotated	EOGT	3	-	1575	44	8	3	4.76%	9.230851663	0.000413274	1.13E-05	4.15E-08
ENSG00000100029_30584668_30577046_449	annotated	PES1	22	-	1347	89	4	6	5.57%	21.8319267	6.58E-06	4.29E-05	2.34E-09
ENSG00000100029_30591833_30577046_571	annotated	PES1	22	-	1713	105	17	0	6.13%	20.25357021	4.15E-05	8.89E-08	4.75E-11
ENSG00000144036_72825895_72179335_810	annotated	EXOC6B	2	-	2430	25	7	0	1.60%	3.399408756	0.018877681	0.000544026	7.88E-05
ENSG00000131171_81202201_81297227_114	annotated	SH3BGRL	X	+	342	83	19	3	17.54%	80.1902634	0.000453046	8.28E-05	2.73E-07
ENSG00000168036_41224552_41239342_768	annotated	CTNNB1	3	+	2304	288	46	13	11.46%	41.30281639	2.21E-12	5.94E-15	2.04E-25
ENSG00000168036_41224552_41239342_768	annotated	CTNNB1	3	+	2304	288	46	13	11.46%	41.30281639	2.21E-12	5.94E-15	2.04E-25
ENSG00000177426_3450490_3457940_272	annotated	TGIF1	18	+	816	128	28	10	11.40%	51.83098527	0.00032156	1.06E-05	2.98E-08
ENSG00000149483_61364356_61366169_95	Overlap_uORF	TMEM138	11	+	285	46	4	20	12.63%	53.3313559	0.001946488	0.109098094	0.001796202
ENSG00000185049_2008950_2007101_99	annotated	NELFA	4	-	297	15	0	0	6.06%	16.68800662	0.011772051	0.011772051	0.000681273
ENSG00000113269_180071702_179955654_419	annotated	RNF130	5	-	1257	100	17	4	9.07%	26.28659754	8.49E-07	1.39E-07	1.13E-12
ENSG00000101182_62137387_62136857_38	annotated	PSMA7	20	-	114	7	1	0	13.16%	20.28910279	0.016947427	0.019216965	0.001519081
ENSG00000121022_67059396_67045808_243	novel	COPS5	8	-	729	74	9	0	10.29%	33.54083306	1.25E-05	5.47E-06	5.65E-10
ENSG00000044115_138924641_138931013_232	novel	CTNNA1	5	+	696	95	5	4	8.62%	45.10077652	6.16E-05	0.000110908	5.01E-08
ENSG00000168487_22165406_22199310_717	annotated	BMP1	8	+	2151	86	20	1	4.60%	13.21075671	7.65E-05	2.64E-07	2.42E-10
ENSG00000126107_45008389_45003492_471	annotated	HECTD3	1	-	1413	11	3	0	1.27%	2.572291467	0.067302329	0.012798403	0.004189281
ENSG00000111843_10724614_10730666_112	annotated	TMEM14C	6	+	336	346	39	15	24.11%	340.256535	0.000435838	5.89E-05	1.92E-07
ENSG00000108219_80489234_80517976_253	annotated	TSPAN14	10	+	759	438	57	62	22.13%	190.6786148	2.07E-08	9.19E-06	2.47E-12
ENSG00000167671_4454017_4445498_388	annotated	UBXN6	19	-	1164	29	3	6	3.35%	8.232176463	0.003385412	0.005578142	0.000103936
ENSG00000148660_73874461_73815015_539	annotated	CAMK2G	10	-	1617	27	3	0	2.41%	5.517259332	0.001079221	0.000539992	3.72E-06
ENSG00000054654_64087722_64225526_3040	novel	SYNE2	14	+	9120	19	5	4	0.30%	0.688380273	0.047719858	0.02657605	0.005443062
ENSG00000180228_178443276_178432097_145	novel	PRKRA	2	-	435	8	6	0	4.14%	6.076736204	0.333186701	0.009815329	0.025305041
ENSG00000092036_22951647_22946525_239	annotated	HAUS4	14	-	717	42	9	0	6.28%	19.35529471	0.008713209	0.000307311	2.04E-05
ENSG00000114054_136250376_136330026_482	annotated	PCCB	3	+	1446	76	10	0	6.02%	17.36660606	4.97E-05	1.01E-06	4.91E-10
ENSG00000185722_4242419_4167779_1157	annotated	ANKFY1	17	-	3471	83	10	0	2.85%	7.901201407	1.66E-05	2.11E-07	3.71E-11

ENSG00000214413_110901583_110900360_70	annotated	BBIP1	10	-	210	21	0	9	11.43%	33.04225311	0.005554612	0.108304078	0.003801424
ENSG00000266173_63728369_63704025_299	annotated	STRADA	17	-	897	13	2	1	2.34%	4.788732335	0.007882058	0.013794764	0.000547323
ENSG00000164587_150446935_150445460_119	novel	RPS14	5	-	357	147	46	8	15.97%	136.0563363	0.000546857	5.39E-05	2.24E-07
ENSG00000105341_41438393_41433484_148	novel	DMAC2	19	-	444	81	9	0	12.84%	60.27978608	6.44E-05	6.08E-05	2.90E-08
ENSG00000075234_46273912_46292884_400	annotated	TTC38	22	+	1200	15	4	0	1.50%	4.130281639	0.113790253	0.012798403	0.007514859
ENSG00000137815_41417236_41480687_670	annotated	RTF1	15	+	2010	38	2	0	2.39%	6.246794121	0.000400336	0.000175784	4.85E-07
ENSG00000110651_2386595_2396866_229	annotated	CD81	11	+	687	746	80	9	34.50%	358.7994297	6.26E-13	6.87E-15	6.33E-26
ENSG00000122257_24540627_24572445_952	annotated	RBBP6	16	+	2856	13	4	1	0.63%	1.504024126	0.102109635	0.023672501	0.010721472
ENSG00000080200_97872437_97943314_2556	annotated	CRYBG3	3	+	7668	10	5	0	0.31%	0.430910969	0.167151322	0.003327803	0.004640766
ENSG00000173915_103392376_103392194_31	annotated	ATP5MD	10	-	93	120	3	0	25.81%	426.3516531	0.005808022	0.005859343	0.000180739
ENSG00000168488_28823260_28836777_1068	annotated	ATXN2L	16	+	3204	144	24	9	3.75%	14.85045084	2.13E-05	4.35E-08	1.21E-11
ENSG00000138614_65611148_65599744_203	novel	INTS14	15	-	609	19	1	3	3.45%	10.30874892	0.015625009	0.051745348	0.003744237
ENSG00000108439_47944638_47946782_166	dORF	PNPO	17	+	498	65	14	7	11.45%	43.1274388	0.001370465	0.000481198	4.25E-06
ENSG00000108439_47944638_47946782_148	Overlap_dORF	PNPO	17	+	444	62	12	7	11.49%	46.14008317	0.001313927	0.001007793	8.14E-06
ENSG00000214078_35632923_35626241_481	annotated	CPNE1	20	-	1443	325	32	19	16.01%	74.41948899	3.24E-11	1.77E-12	7.38E-22
ENSG00000100462_22928599_22920904_595	annotated	PRMT5	14	-	1785	125	25	8	9.41%	23.13883271	8.87E-08	8.83E-10	9.52E-16
ENSG00000042429_93797565_93812064_260	dORF	MED17	11	+	780	11	1	0	1.92%	4.659804926	0.035772986	0.021084099	0.003354605
ENSG00000042429_93788123_93796941_163	annotated	MED17	11	+	489	18	0	2	4.29%	12.16279256	0.008473674	0.016947427	0.000715155
ENSG00000159335_6766706_6770442_102	annotated	PTMS	12	+	306	245	53	31	42.16%	264.5539873	1.15E-06	8.19E-07	8.44E-12
ENSG00000111114_93248628_93242273_476	dORF	BTBD7	14	-	1428	16	0	2	1.89%	3.702213234	0.003327803	0.016524459	0.000306274
ENSG00000198399_24310348_24203626_1501	annotated	ITSN2	2	-	4503	26	14	3	1.00%	1.907836067	0.058079157	0.002499833	0.000981325
ENSG00000127955_80134498_80217493_422	annotated	GNAI1	7	+	1266	137	11	5	9.00%	35.75662462	3.33E-08	8.64E-08	2.86E-14
ENSG00000011451_15427294_15423076_538	dORF	WIZ	19	-	1614	16	0	0	0.93%	3.275564125	0.021084099	0.021084099	0.002029891
ENSG00000100664_103335739_103335924_61	Overlap_uORF	EIF5	14	+	183	13	9	0	9.84%	23.47263882	0.182638022	0.012798403	0.013255577
ENSG00000138002_27461469_27444432_1002	annotated	IFT172	2	-	3006	9	0	0	0.50%	0.989289015	0.020613417	0.020613417	0.001946209
ENSG00000109911_31509785_31628298_265	annotated	ELP4	11	+	795	12	2	0	2.26%	4.987509904	0.043751848	0.013217227	0.002735927
ENSG00000109911_31628330_31783524_208	dORF	ELP4	11	+	624	25	9	0	5.77%	13.23808218	0.021085406	0.000943084	0.000139464
ENSG00000144724_61562288_62293307_1445	annotated	PTPRG	3	+	4335	60	6	4	1.52%	4.57332223	0.000108303	4.70E-05	3.78E-08
ENSG00000177225_774104_771187_139	novel	GATD1	11	-	417	17	0	0	5.04%	13.4704629	0.008473674	0.008473674	0.000366502
ENSG00000175376_66000389_65998599_165	annotated	EIF1AD	11	-	495	39	5	5	7.27%	26.03329033	0.004616409	0.006221623	0.000154222
ENSG00000175220_46696107_46688169_106	novel	ARHGAP1	11	-	318	18	2	3	5.66%	18.70316214	0.01300724	0.016503129	0.001029549
ENSG00000131871_101277417_101275249_107	novel	SELENOS	15	-	321	32	2	4	10.28%	32.93931774	0.004514955	0.008711445	0.000209785
ENSG00000196961_49767161_49806758_946	annotated	AP2A1	19	+	2838	111	5	6	4.97%	12.9235028	1.76E-09	1.39E-08	2.67E-16
ENSG00000148985_3811260_3824350_210	novel	PGAP2	11	+	630	33	6	0	4.29%	17.30784687	0.022431136	0.003632191	0.000455556
ENSG00000108187_68296946_68285259_239	annotated	PBLD	10	-	717	24	0	0	3.35%	11.06016841	0.005160369	0.005160369	0.000143196
ENSG00000148090_91361889_91214348_310	annotated	AUH	9	-	930	16	0	2	3.23%	5.684688707	0.002122793	0.0116632	0.000144151
ENSG00000138050_39779149_39736735_473	annotated	THUMPD2	2	-	1419	19	2	0	2.33%	4.424262221	0.004968901	0.001262414	3.77E-05
ENSG00000175505_67367612_67365136_215	annotated	CLCF1	11	-	645	45	11	4	8.84%	23.05273473	0.004588162	7.87E-05	3.17E-06
ENSG00000171004_132958311_132628223_548	annotated	HS6ST2	X	-	1644	14	2	0	1.09%	2.813817175	0.039705313	0.011772051	0.002244513
ENSG00000117054_75724788_75762763_454	annotated	ACADM	1	+	1362	56	7	2	5.51%	13.58565473	1.99E-05	8.83E-06	1.42E-09
ENSG00000171004_132958311_132628223_508	annotated	HS6ST2	X	-	1524	14	2	0	1.18%	3.035377582	0.039705313	0.011772051	0.002244513
ENSG00000196419_41622005_41663815_609	annotated	XRCC6	22	+	1827	167	18	4	9.03%	30.20282578	1.36E-08	9.21E-11	1.62E-17
ENSG00000168014_74139762_74013385_2170	annotated	C2CD3	11	-	6510	18	2	2	0.51%	0.913610685	0.004932312	0.00712681	0.000187059
ENSG00000067064_1049003_1041187_284	annotated	IDI1	10	-	852	282	89	44	21.48%	109.365204	3.14E-06	1.12E-08	4.41E-13
ENSG00000035141_70301966_70297289_123	annotated	FAM136A	2	-	369	23	5	0	7.32%	20.59544232	0.044380821	0.003632191	0.000962121
ENSG00000113161_75342606_75360342_835	annotated	HMGCR	5	+	2505	407	59	25	11.74%	53.68541723	5.44E-12	5.59E-16	5.90E-26
ENSG00000204628_181243800_181236977_269	annotated	RACK1	5	-	807	1737	230	62	35.69%	711.2068607	3.83E-14	8.80E-16	5.00E-28
ENSG00000082068_37392023_37721309_470	novel	WDR70	5	+	1410	35	3	0	2.98%	8.201977723	0.002492245	0.000434916	7.13E-06
ENSG00000113593_65569946_65587396_490	dORF	PPWD1	5	+	1470	29	1	0	3.47%	6.518539729	0.000253334	0.000110146	1.98E-07
ENSG00000112651_43059381_43055932_222	novel	MRPL2	6	-	666	36	5	2	5.86%	17.86067736	0.006053671	0.00183661	6.45E-05
ENSG00000075785_128795368_128813422_160	annotated	RAB7A	3	+	480	193	14	9	23.75%	132.8573927	3.53E-08	4.58E-08	1.61E-14
ENSG00000170248_33798729_33866525_873	annotated	PDCD6IP	3	+	2619	100	9	0	4.58%	12.61636239	3.68E-07	1.19E-08	4.73E-14
ENSG00000123737_121801809_121816856_423	annotated	EXOSC9	4	+	1269	79	7	6	7.09%	20.57003937	1.59E-05	2.29E-05	2.87E-09
ENSG00000156469_96257085_96239491_296	annotated	MTERF3	8	-	888	37	9	0	5.07%	13.76760546	0.009312779	0.000259634	1.91E-05
ENSG00000108654_64504108_64503956_50	novel	DDX5	17	-	150	38	0	4	20.00%	83.70704122	0.002488365	0.004557894	6.41E-05
ENSG00000266412_46016680_46006592_614	annotated	NCOA4	10	-	1842	93	11	4	6.68%	16.68257079	2.20E-07	3.63E-08	7.96E-14
ENSG00000172725_67442628_67440744_282	annotated	CORO1B	11	-	846	76	8	0	7.09%	29.68334795	0.000405405	4.11E-05	1.28E-07
ENSG00000163798_27663915_27694683_742	annotated	SLC4A1AP	2	+	2226	57	11	3	2.96%	8.460954301	0.002319238	9.65E-05	1.76E-06
ENSG00000103064_68291237_68297328_241	Overlap_dORF	SLC7A6	16	+	723	21	1	1	4.15%	9.597334929	0.003068573	0.003068573	5.32E-05
ENSG00000103064_68274898_68291249_172	annotated	SLC7A6	16	+	516	16	6	0	2.91%	10.24565988	0.197313433	0.019679754	0.019753919
ENSG00000108298_39201214_39204648_194	annotated	RPL19	17	+	582	469	48	19	13.92%	266.2683283	7.28E-05	6.69E-06	4.10E-09
ENSG00000162664_90005005_90027701_561	annotated	ZNF326	1	+	1683	28	8	8	2.85%	5.497225711	0.004460232	0.007530381	0.000179816
ENSG00000133193_73212728_73209529_96	annotated	FAM104A	17	-	288	10	0	0	7.29%	11.47300455	0.007593099	0.007593099	0.000297833
ENSG00000132676_155709783_155721690_112	annotated	DAP3	1	+	336	20	1	0	6.25%	19.6680078	0.012000727	0.008674847	0.000522039
ENSG00000172845_173913133_173909941_126	novel	SP3	2	-	378	17	1	0	5.56%	14.86027256	0.007979402	0.007979402	0.000327143
ENSG00000149554_125626769_125635541_241	novel	CHEK1	11	+	723	33	5	1	5.39%	15.08152632	0.006797108	0.000930688	3.99E-05
ENSG00000109971_123061324_123061049_91	novel	HSPA8	11	-	273	153	16	5	12.09%	185.1818581	0.001811542	0.001657226	1.78E-05
ENSG00000166689_17014401_16778998_1271	annotated	PLEKHA7	11	-	3813	15	3	0	0.55%	1.299852601	0.052370108	0.008473674	0.00228699

ENSG00000100504_50944403_50923948_226	novel	PYGL	14	-	678	60	3	6	9.73%	29.24093196	6.41E-05	0.000198658	9.31E-08
ENSG00000204967_140807188_141009937_947	annotated	PCDHA4	5	+	2841	13	2	0	0.95%	1.51196512	0.012888811	0.002943991	0.000212649
ENSG00000163637_64163147_64099051_802	novel	PRICKLE2	3	-	2406	7	1	0	0.62%	0.96132906	0.047790352	0.016947427	0.003697512
ENSG00000172428_240136284_240131051_57	annotated	COPS9	2	-	171	189	27	8	35.09%	365.2038502	0.000521686	0.00011919	4.41E-07
ENSG00000158169_95150086_95101707_384	annotated	FANCC	9	-	1152	16	0	0	2.08%	4.589201821	0.005405142	0.005405142	0.000156372
ENSG00000120686_38349920_38350059_20	novel	UFM1	13	+	60	18	6	9	30.00%	99.12675933	0.11777946	0.123780394	0.048823892
ENSG00000005206_2343260_2351615_176	novel	SPPL2B	19	+	528	26	1	0	3.41%	16.27080646	0.013716833	0.013428348	0.000890162
ENSG00000177646_128879692_128912607_621	annotated	ACAD9	3	+	1863	38	8	2	2.90%	6.73969736	0.004415906	0.000334093	1.03E-05
ENSG00000105254_36116080_36125672_60	annotated	TBCB	19	+	180	132	11	28	25.00%	242.3098562	0.000321743	0.025947115	7.60E-05
ENSG00000073536_35136449_35132437_193	annotated	NLE1	17	-	579	21	4	0	2.59%	11.98423688	0.055414058	0.020613417	0.00507254
ENSG00000105290_35869538_35879441_644	annotated	APLP1	19	+	1932	107	4	7	6.52%	18.29979857	1.33E-08	1.08E-07	1.49E-14
ENSG00000147955_34637697_34635632_223	annotated	SIGMAR1	9	-	669	652	62	16	25.56%	322.0261439	5.81E-09	1.55E-10	1.08E-17
ENSG00000157593_44255605_44254706_299	annotated	SLC35B2	6	-	897	97	12	0	8.36%	35.7313105	0.000163758	5.62E-06	8.22E-09
ENSG00000157895_121016474_121004153_293	annotated	C12orf43	12	-	879	12	6	0	1.71%	4.510887797	0.170042304	0.021084099	0.017372496
ENSG00000273703_27815044_27815424_126	annotated	HIST1H2BM	6	+	378	1012	131	10	23.02%	884.6232844	5.04E-05	1.44E-06	6.87E-10
ENSG00000100823_20455646_20457508_318	novel	APEX1	14	+	954	460	61	31	20.13%	159.323233	7.70E-10	1.28E-10	1.14E-18
ENSG00000040633_7238807_7235626_399	annotated	PHF23	17	-	1197	67	3	4	4.76%	18.49482839	9.75E-05	9.75E-05	6.88E-08
ENSG00000133740_85177421_85213862_346	annotated	E2F5	8	+	1038	19	1	0	3.18%	6.04819662	0.003925334	0.001242734	2.92E-05
ENSG00000213523_140557452_140550700_224	annotated	SRA1	5	-	672	54	4	3	9.38%	26.55181054	8.72E-05	0.000107506	6.79E-08
ENSG00000149547_125572528_125583683_326	annotated	EI24	11	+	978	72	0	0	5.21%	24.32558511	0.000130272	0.000130272	1.21E-07
ENSG00000151503_134223926_134152944_1498	annotated	NCAPD3	11	-	4494	55	5	1	1.60%	4.043889455	0.000110193	1.01E-05	9.23E-09
ENSG00000135108_117189349_117146087_544	annotated	FBXO21	12	-	1632	76	18	2	4.60%	15.38732375	0.000319762	9.49E-06	2.69E-08
ENSG00000148019_78236351_78270851_689	annotated	CEP78	9	+	2067	49	5	0	3.05%	7.832948246	0.000287198	2.41E-05	5.53E-08
ENSG00000153317_130358142_130054731_1109	annotated	ASAP1	8	-	3327	58	15	14	2.80%	5.760296605	0.000197774	0.000672856	9.11E-07
ENSG00000115415_191009922_190970703_723	annotated	STAT1	2	-	2169	104	3	5	5.26%	15.84321956	3.83E-08	1.51E-07	5.73E-14
ENSG00000188677_44069162_44168678_327	annotated	PARVB	22	+	981	29	2	0	4.89%	9.767842408	0.000877879	0.000148081	9.16E-07
ENSG00000106012_2578275_2610162_529	annotated	IQCE	7	+	1587	8	1	0	0.95%	1.66564603	0.042234516	0.019679754	0.003715148
ENSG00000128609_123557645_123542119_116	annotated	NDUFA5	7	-	348	46	6	0	8.62%	43.67654147	0.040756262	0.002254349	0.000597482
ENSG00000123094_26055344_26079073_392	annotated	RASSF8	12	+	1176	20	2	0	2.55%	5.619430801	0.010678933	0.002201184	0.000135702
ENSG00000185800_45792711_45784243_659	annotated	DMWD	19	-	1977	15	0	1	0.91%	2.506999477	0.013428348	0.020342454	0.001295518
ENSG00000108262_29589378_29574950_694	annotated	GIT1	17	-	2082	58	4	2	3.03%	9.204854373	0.000150234	0.000102592	1.10E-07
ENSG00000110080_126406156_126414047_339	annotated	ST3GAL4	11	+	1017	15	2	0	2.06%	4.87348866	0.028426897	0.008473674	0.001202275
ENSG00000110721_68121177_68053988_439	annotated	CHKA	11	-	1317	22	6	0	2.28%	5.5195867	0.026357993	0.002321823	0.000373813
ENSG00000198382_75815408_76141413_699	annotated	UVRAG	11	+	2097	41	3	3	2.58%	6.460335611	0.000268035	0.000376185	6.80E-07
ENSG00000169371_75617494_75601108_190	annotated	SNUPN	15	-	570	37	0	1	6.32%	21.44848009	0.001053132	0.001015599	6.62E-06
ENSG00000132300_86116575_86119052_86	novel	PTCD3	2	+	258	9	0	0	5.81%	11.52636736	0.020613417	0.020613417	0.001946209
ENSG00000184182_237973108_238003414_86	annotated	UBE2F	2	+	258	10	6	0	5.81%	12.80707485	0.171390856	0.020613417	0.017249262
ENSG00000130227_21966293_22005088_1088	annotated	XPO7	8	+	3264	161	17	6	5.42%	16.29841529	4.89E-10	3.71E-11	2.19E-19
ENSG00000136295_2632156_2661739_491	annotated	TTYH3	7	+	1473	120	4	4	8.96%	26.91833247	2.92E-09	7.52E-09	2.34E-16
ENSG00000092531_42511847_42531478_158	annotated	SNAP23	15	+	474	46	2	1	7.59%	32.06632159	0.001268312	0.001805035	1.38E-05
ENSG00000166619_37519174_37518911_87	annotated	BLCAP	20	-	261	35	14	3	11.49%	44.30953482	0.028844093	0.002376615	0.000421153
ENSG00000166619_37519174_37518911_87	annotated	BLCAP	20	-	261	35	14	3	11.49%	44.30953482	0.028844093	0.002376615	0.000421153
ENSG00000166619_37519174_37518911_87	annotated	BLCAP	20	-	261	35	14	3	11.49%	44.30953482	0.028844093	0.002376615	0.000421153
ENSG00000078246_2909554_2940726_479	annotated	TULP3	12	+	1437	41	5	1	3.97%	9.427504367	0.000602308	6.91E-05	3.11E-07
ENSG00000127774_3669147_3669479_110	annotated	EMC6	17	+	330	134	2	5	13.64%	134.1715732	0.000637397	0.001002109	4.05E-06
ENSG00000180979_42548434_42544083_239	annotated	LRRC57	15	-	717	15	3	0	3.77%	6.912605253	0.027614347	0.003278443	0.00052295
ENSG00000166619_37519174_37518911_87	annotated	BLCAP	20	-	261	35	14	3	11.49%	44.30953482	0.028844093	0.002376615	0.000421153
ENSG00000012048_43124045_43045678_1867	annotated	BRCA1	17	-	5601	56	9	3	1.39%	3.303635376	0.000129183	1.56E-05	1.62E-08
ENSG00000125629_118103277_118108362_97	novel	INSIG2	2	+	291	26	11	0	11.34%	29.522288	0.075260163	0.001484235	0.000912245
ENSG00000169599_69415245_69396203_69	novel	NFU1	2	-	207	36	8	0	11.59%	57.46478802	0.024901834	0.005604867	0.000735407
ENSG00000004766_93258384_93340674_454	novel	VPS50	7	+	1362	16	8	0	1.54%	3.881615637	0.141244101	0.008674847	0.007296652
ENSG00000010256_48600702_48599128_112	novel	UQCRC1	3	-	336	158	40	0	21.43%	155.3772617	0.000168168	8.48E-06	1.22E-08
ENSG00000120694_31161582_31137318_814	annotated	HSPH1	13	-	2442	103	17	7	5.53%	13.93674067	1.22E-06	4.94E-08	6.22E-13
ENSG00000142230_47130931_47209251_346	annotated	SAE1	19	+	1038	229	28	12	15.32%	72.89668557	4.04E-07	7.52E-09	3.40E-14
ENSG00000170035_180982043_181062888_207	annotated	UBE2E3	2	+	621	16	1	1	2.90%	8.513301929	0.01300724	0.023878474	0.001470914
ENSG00000163608_113019644_113013949_113	annotated	NEPRO	3	-	339	9	6	0	5.31%	8.772279587	0.261477794	0.011772051	0.020039585
ENSG00000112237_99568527_99549389_202	annotated	CCNC	6	-	606	28	3	4	5.45%	15.26704764	0.001696512	0.004423224	4.38E-05
ENSG00000128989_52568892_52551934_131	annotated	ARPP19	15	-	393	40	5	5	9.16%	33.63079197	0.005757552	0.004681719	0.000145022
ENSG00000051523_88651013_88648029_47	novel	CYBA	16	-	141	165	21	16	25.53%	386.6646641	0.001087214	0.007921151	5.37E-05
ENSG00000137275_3077915_3113339_571	annotated	RIPK1	6	+	1713	14	2	6	1.40%	2.700476028	0.019675918	0.111584927	0.010215361
ENSG00000168005_63813687_63827139_381	annotated	SPINDOC	11	+	1143	11	4	2	1.57%	3.179919372	0.100002646	0.061356297	0.022880377
ENSG00000163463_155172824_155169440_112	novel	KRTCAP2	1	-	336	296	49	14	25.89%	291.0865155	3.90E-05	8.73E-06	2.76E-09
ENSG00000196975_69781572_69810691_140	novel	ANXA4	2	+	420	296	34	17	25.00%	232.8692124	1.64E-06	7.32E-07	1.08E-11
ENSG00000090061_99492678_99510782_580	annotated	CCNK	14	+	1740	69	1	15	3.79%	13.10296244	1.95E-05	0.001416429	2.60E-07
ENSG00000174684_66347476_66346049_392	annotated	B4GAT1	11	-	1176	74	4	1	6.89%	20.79189396	3.68E-06	2.93E-06	9.20E-11
ENSG00000174840_57556380_57560004_609	annotated	PDE12	3	+	1827	76	6	1	4.60%	13.74499856	5.11E-06	2.04E-06	8.98E-11
ENSG00000174775_534322_532636_189	annotated	HRAS	11	-	567	31	3	1	5.82%	18.06542939	0.007459848	0.002467336	0.000103961
ENSG00000105726_19655237_19654067_94	novel	ATP13A1	19	-	282	17	3	2	8.51%	19.91908876	0.036243043	0.02389627	0.003798067

ENSG00000262246_4388068_4358316_601	novel	CORO7	16	-	1803	15	0	5	0.83%	2.748939527	0.021084099	0.123780394	0.012086671
ENSG00000137414_17600410_17608339_413	annotated	FAM8A1	6	+	1239	16	0	0	1.69%	4.266957625	0.008473674	0.008473674	0.000366502
ENSG00000130177_114235085_114272443_569	annotated	CDC16	13	+	1707	48	7	1	3.69%	9.291318977	0.000505336	3.41E-05	1.37E-07
ENSG00000110066_68185791_68165914_303	annotated	KMT5B	11	-	909	16	1	0	2.97%	5.816018149	0.005426623	0.003327803	9.96E-05
ENSG00000197150_151041158_151045349_204	novel	ABCB8	7	+	612	22	2	2	4.90%	11.87793413	0.003646019	0.010187216	0.000202073
ENSG00000168291_58433809_58431404_163	novel	PDHB	3	-	489	27	1	1	6.75%	18.24418883	0.002136761	0.002136761	2.66E-05
ENSG00000137203_10414985_10398417_437	annotated	TFAP2A	6	-	1311	41	6	12	2.29%	10.3335803	0.047296084	0.15245846	0.028228397
ENSG00000003393_201768885_201723031_1237	novel	ALS2	2	-	3711	20	4	0	0.57%	1.780773544	0.060543501	0.008574016	0.002704766
ENSG00000023330_52199350_52214180_604	annotated	ALAS1	3	+	1812	49	9	0	3.81%	8.935267122	0.000396396	1.03E-05	3.67E-08
ENSG00000105669_18919348_18899679_308	annotated	COPE	19	-	924	171	13	4	13.96%	61.14962426	4.79E-08	7.45E-09	3.74E-15
ENSG00000105696_18612977_18620536_336	annotated	AC003112.1	19	+	1008	12	1	0	1.49%	3.933601561	0.035772986	0.021084099	0.003354605
ENSG00000129933_19320860_19355782_613	annotated	MAU2	19	+	1839	15	10	0	1.63%	2.695126681	0.150036677	0.001995757	0.002816621
ENSG00000115275_74465247_74462717_294	annotated	MOGS	2	-	882	40	6	4	4.08%	14.9851488	0.00800348	0.004488024	0.000191963
ENSG00000115275_74462225_74461275_316	dORF	MOGS	2	-	948	61	1	0	7.28%	21.2613654	1.52E-05	1.15E-05	1.40E-09
ENSG00000128944_40383253_40393597_238	annotated	KNSTRN	15	+	714	83	10	1	10.92%	38.4104623	0.000121641	4.56E-06	4.96E-09
ENSG00000100749_96833565_96881208_365	annotated	VRK1	14	+	1095	41	4	1	3.56%	12.37198518	0.002742299	0.000883754	1.49E-05
ENSG00000162191_62678923_62676590_297	annotated	UBXN1	11	-	891	212	23	4	18.52%	78.61905342	1.29E-08	2.35E-10	3.66E-17
ENSG00000115970_43592392_43556334_894	annotated	THADA	2	-	2682	25	8	5	1.79%	3.080001222	0.031129963	0.00257084	0.000488865
ENSG00000119760_27662192_27653485_279	annotated	SUPT7L	2	-	837	32	5	1	5.73%	12.63264157	0.002745971	0.000252954	4.88E-06
ENSG00000172534_153970840_153949347_2080	annotated	HCFC1	X	-	6240	84	13	1	1.92%	4.447995611	1.97E-06	1.59E-08	3.63E-13
ENSG00000169242_155127978_155133693_192	novel	EFNA1	1	+	576	92	11	0	10.94%	52.77582094	0.001235948	2.53E-05	2.77E-07
ENSG00000177854_153982063_153982848_261	annotated	TMEM187	X	+	783	29	10	0	4.60%	12.23787152	0.010725367	0.000966879	6.71E-05
ENSG00000184216_154019713_154011859_679	annotated	IRAK1	X	-	2037	28	7	2	1.62%	4.541890462	0.037701781	0.005625935	0.001145748
ENSG00000196586_75817599_75915012_1278	annotated	MYO6	6	+	3834	18	2	0	0.78%	1.551279489	0.011064574	0.002046029	0.00013212
ENSG00000184209_123465553_123466281_242	annotated	SNRNP35	12	+	726	25	0	1	5.37%	11.37818633	0.000640157	0.000906178	3.68E-06
ENSG00000006282_50548842_50555662_671	annotated	SPATA20	17	+	2013	59	7	8	4.77%	9.684515318	9.43E-06	2.76E-05	2.10E-09
ENSG00000177556_151758551_151746325_68	annotated	ATOX1	5	-	204	196	11	12	48.53%	317.4647848	4.71E-07	1.48E-06	6.35E-12
ENSG00000115875_38751256_38745133_238	annotated	SRSF7	2	-	714	471	40	7	24.37%	217.9678041	3.33E-09	1.97E-11	8.76E-19
ENSG00000037474_6618011_6599926_491	dORF	NSUN2	5	-	1473	92	4	4	6.92%	20.63738823	3.98E-07	4.61E-07	1.70E-12
ENSG00000037474_6632979_6620227_202	annotated	NSUN2	5	-	606	17	6	1	4.46%	9.269278926	0.073313458	0.006542768	0.002709266
ENSG00000135535_109381552_109368851_155	annotated	CD164	6	-	465	218	49	2	21.94%	154.9077673	1.43E-05	1.75E-07	2.67E-11
ENSG00000071655_1585156_1578486_185	novel	MBD3	19	-	555	14	3	6	3.24%	8.334982767	0.062728927	0.023056749	0.006321089
ENSG00000071655_1578477_1578340_45	novel	MBD3	19	-	135	22	0	2	11.11%	53.8466347	0.021084099	0.021084099	0.002029891
ENSG00000161091_3546425_3544723_160	annotated	MFSD12	19	-	480	19	1	0	5.62%	13.07922519	0.005642788	0.003529166	0.000109245
ENSG00000166226_69585522_69589731_230	novel	CCT2	12	+	690	222	29	1	15.22%	106.3098578	2.11E-06	1.14E-07	2.39E-12
ENSG00000171453_43517110_43521300_346	annotated	POLR1C	6	+	1038	56	4	9	5.20%	17.82626372	0.000339726	0.00231672	5.30E-06
ENSG00000116044_177234268_177230785_589	annotated	NFE2L2	2	-	1767	86	6	1	6.28%	16.08168516	1.86E-07	5.34E-08	9.75E-14
ENSG00000102710_37051490_37009709_733	annotated	SUPT20H	13	-	2199	30	2	2	1.64%	4.507810793	0.002631121	0.003604785	5.37E-05
ENSG00000198246_71356089_71362608_269	dORF	SLC29A3	10	+	807	16	0	0	2.97%	6.55112825	0.005306578	0.005306578	0.000151001
ENSG00000182771_86206637_85613573_673	annotated	GRID1	10	-	2019	25	3	0	1.34%	4.091413213	0.014991381	0.003452921	0.000285751
ENSG00000169621_68502887_68578022_403	annotated	APLF	2	+	1209	15	0	3	1.49%	4.099535126	0.013428348	0.062386368	0.004016395
ENSG00000164941_94832070_94880234_762	annotated	INTS8	8	+	2286	13	2	0	1.31%	1.879043265	0.008381168	0.001755235	8.65E-05
ENSG00000044115_138930638_138934089_136	annotated	CTNNA1	5	+	408	9	2	0	3.68%	7.288732304	0.102951605	0.016947427	0.008323174
ENSG00000197081_160089060_160105084_400	novel	IGF2R	6	+	1200	59	3	2	5.75%	16.24577445	6.36E-05	2.38E-05	1.17E-08
ENSG00000197081_160061815_160088238_980	novel	IGF2R	6	+	2940	107	8	7	4.80%	12.02558191	3.33E-08	3.59E-08	1.20E-14
ENSG00000079277_46583327_46558575_412	annotated	MKNK1	1	-	1236	21	1	1	1.94%	5.613975043	0.007181907	0.007181907	0.000268046
ENSG00000169045_179617909_179615546_159	annotated	HNRNPH1	5	-	477	137	8	3	15.09%	94.90123011	1.22E-05	8.28E-06	8.20E-10
ENSG00000060982_24948932_24818008_325	annotated	BCAT1	12	-	975	28	2	0	4.31%	9.489057304	0.002232673	0.000369309	5.49E-06
ENSG00000105928_24749774_24699026_496	annotated	GSDME	7	-	1488	26	3	0	2.42%	5.773511968	0.003872612	0.001007374	2.39E-05
ENSG00000104408_108239971_108201885_342	annotated	EIF3E	8	-	1026	111	14	8	9.94%	35.74746682	1.54E-06	3.19E-06	4.29E-11
ENSG00000146729_55964610_55981505_145	annotated	NIPSNAP2	7	+	435	27	1	3	4.14%	20.50898469	0.020525443	0.037440157	0.00342421
ENSG00000107771_84371440_84513543_705	annotated	CCSER2	10	+	2115	19	3	3	0.71%	2.968334795	0.063412829	0.068289709	0.016495075
ENSG00000072041_84886036_84861632_623	annotated	SLC6A15	12	-	1869	112	18	2	6.58%	19.80060112	3.91E-06	1.48E-08	7.17E-13
ENSG00000111371_46239755_46196127_488	annotated	SLC38A1	12	-	1464	179	38	5	11.27%	40.40002259	2.24E-06	2.83E-10	1.19E-14
ENSG00000204209_33321925_33318747_688	annotated	DAXX	6	-	2064	29	7	3	1.89%	4.642564633	0.01749703	0.003161975	0.00031123
ENSG00000109756_159330355_159358139_1219	annotated	RAPGEF2	4	+	3657	8	2	2	0.49%	0.722827522	0.082663577	0.082663577	0.024878571
ENSG00000075340_70696385_70663425_615	annotated	ADD2	2	-	1845	18	0	3	2.28%	3.22363445	0.000240354	0.007312901	1.36E-05
ENSG00000110931_121274382_121240699_497	annotated	CAMKK2	12	-	1491	21	3	0	1.81%	4.653838466	0.013157114	0.003303074	0.000240229
ENSG00000178694_94063127_94126490_340	annotated	NSUN3	3	+	1020	20	1	5	2.35%	6.478873159	0.009288069	0.041403097	0.001919793
ENSG00000107960_103917486_103882684_332	annotated	STN1	10	-	996	38	4	1	7.23%	12.60648211	6.13E-05	9.14E-06	4.57E-09
ENSG00000055044_202265942_202303436_529	annotated	NOP58	2	+	1587	85	16	0	5.29%	17.69748906	0.000121459	1.65E-06	2.00E-09
ENSG00000089157_120200783_120196773_317	annotated	RPLP0	12	-	951	86	21	7	4.10%	29.88048126	0.004035968	0.002174873	5.02E-05
ENSG00000119632_94128586_94127799_88	novel	IFI27L2	14	-	264	45	1	4	14.77%	56.32202235	0.000804138	0.000804138	4.08E-06
ENSG00000089159_120265629_120212314_591	annotated	PXN	12	-	1773	117	28	8	6.09%	21.80453251	8.33E-06	1.45E-06	1.06E-10
ENSG00000100596_77597311_77535896_403	annotated	SPTLC2	14	-	1209	55	5	0	6.45%	15.03162879	3.77E-05	2.94E-06	9.70E-10
ENSG00000254996_140401968_140549237_2617	annotated	ANKHD1-EIF4EBP3	5	+	7851	104	12	3	2.03%	4.377014805	1.09E-08	3.60E-10	4.59E-17
ENSG00000255292_112086908_112170527_140	annotated	AP002884.3	11	+	420	77	18	3	6.43%	60.57746404	0.025306216	0.006329843	0.000829929
ENSG00000107020_5431914_5358239_126	annotated	PLGRKT	9	-	378	49	22	1	15.87%	42.83255033	0.011974888	4.79E-05	6.65E-06

ENSG00000134283_42335903_42446618_434	annotated	PPHLN1	12	+	1302	89	21	3	5.53%	22.58648638	0.001864975	1.88E-05	3.42E-07
ENSG00000143570_153960721_153960098_207	Overlap_dORF	SLC39A1	1	-	621	215	17	2	22.22%	114.3974947	3.13E-08	2.86E-09	9.73E-16
ENSG00000112304_24687682_24701615_117	annotated	ACOT13	6	+	351	91	15	0	16.24%	85.66510066	0.001796195	6.17E-05	9.04E-07
ENSG00000111581_68687066_68709245_202	novel	NUP107	12	+	606	27	7	5	5.94%	14.72179594	0.009376445	0.015214384	0.000705245
ENSG00000111665_6849424_6849043_85	novel	CDCA3	12	-	255	16	1	0	9.41%	20.73239411	0.005405142	0.005454749	0.000157735
ENSG00000111665_6850485_6849521_118	novel	CDCA3	12	-	354	104	5	2	17.80%	97.07328598	5.88E-05	6.34E-05	2.77E-08
ENSG00000112078_36481642_36487175_173	annotated	KCTD20	6	+	519	15	1	0	2.89%	9.549784136	0.035772986	0.021084099	0.003354605
ENSG00000089693_6752071_6751901_56	novel	MLF2	12	-	168	16	0	0	12.50%	31.46881249	0.008473674	0.008473674	0.000366502
ENSG00000123240_13110300_13136866_513	annotated	OPTN	10	+	1539	117	10	12	7.99%	25.11984155	3.30E-07	1.30E-06	3.96E-12
ENSG00000076864_21619063_21598450_683	annotated	RAP1GAP	1	-	2049	36	2	2	2.78%	5.8053739	0.00025934	0.00025934	4.59E-07
ENSG00000203880_64259966_64273600_361	annotated	PCMTD2	20	+	1083	29	7	1	4.43%	8.847879411	0.002365371	0.000242264	4.01E-06
ENSG00000178999_8206612_8204871_122	annotated	AURKB	17	-	366	19	8	0	7.38%	17.15308222	0.048111409	0.003606289	0.001048508
ENSG00000091527_133575105_133588046_156	annotated	CDV3	3	+	468	26	9	0	5.77%	18.35680728	0.050116035	0.003632191	0.001104829
ENSG00000050426_51056368_51059431_100	Overlap_dORF	LETMD1	12	+	300	11	0	6	5.00%	12.11549281	0.019216965	0.216602931	0.02179448
ENSG00000050426_51048357_51058072_155	annotated	LETMD1	12	+	465	27	4	0	6.45%	19.18582439	0.010908255	0.002335453	0.000146103
ENSG00000131165_89657588_89646066_187	annotated	CHMP1A	16	-	561	67	12	15	9.09%	39.46222742	0.005737435	0.013239506	0.000394476
ENSG00000028203_95256599_95274752_240	novel	VEZT	12	+	720	30	3	1	4.17%	13.76760546	0.006441753	0.003783485	0.000132609
ENSG00000058272_79934925_79775929_972	annotated	PPP1R12A	12	-	2916	43	12	4	2.57%	4.872485884	0.002299191	5.45E-05	1.07E-06
ENSG00000166167_101354181_101550860_605	annotated	BTRC	10	+	1815	25	7	2	1.98%	4.551274533	0.016632829	0.003532672	0.000324694
ENSG00000130713_130698228_130703774_181	novel	EXOSC2	9	+	543	72	3	9	13.26%	43.8129323	7.11E-06	6.20E-05	3.67E-09
ENSG00000130713_130693792_130695597_75	novel	EXOSC2	9	+	225	32	3	0	13.33%	46.99342665	0.007999495	0.002432129	0.000110155
ENSG00000139644_49752494_49762896_237	annotated	TMBIM6	12	+	711	2662	561	87	47.68%	1237.109392	5.70E-17	2.09E-19	2.06E-34
ENSG00000138640_88768056_88728533_508	annotated	FAM13A	4	-	1524	12	1	0	0.98%	2.601752214	0.028889786	0.021084099	0.002731533
ENSG00000114779_51971670_51969426_210	annotated	ABHD14B	3	-	630	38	1	2	6.67%	19.93024791	0.000667595	0.001536049	6.45E-06
ENSG00000237190_134411854_134411064_85	annotated	CDKN2AIPNL	5	-	255	15	0	0	7.06%	19.43661948	0.012798403	0.012798403	0.000797244
ENSG00000160124_122371785_122359869_122	annotated	CCDC58	3	-	366	59	15	0	9.84%	53.26483425	0.00217482	0.001065834	1.41E-05
ENSG00000171853_3443799_3465719_120	novel	TRAPPC12	2	+	360	33	0	0	10.00%	30.28873202	0.001003278	0.001003278	6.24E-06
ENSG00000183291_86914111_86880690_95	annotated	SELENOF	1	-	285	703	115	20	35.79%	815.0422434	4.38E-07	8.77E-07	3.51E-12
ENSG00000075711_197297204_197044623_871	annotated	DLG1	3	-	2613	39	4	0	2.07%	4.931679569	0.000762836	7.71E-05	4.39E-07
ENSG00000108523_4940910_4945016_350	annotated	RNF167	17	+	1050	150	21	5	8.86%	47.20321873	2.29E-06	1.66E-06	3.32E-11
ENSG00000156787_123072770_123151939_970	annotated	TBC1D31	8	+	2910	47	11	1	1.65%	5.336721293	0.01587667	0.000277063	3.74E-05
ENSG00000124067_67957957_67944840_942	annotated	SLC12A4	16	-	2826	70	18	11	3.29%	8.184563757	0.000692127	9.76E-05	4.91E-07
ENSG00000119711_74072316_74060642_457	annotated	ALDH6A1	14	-	1371	18	2	0	1.75%	4.338151393	0.020715809	0.005454749	0.000593634
ENSG00000125630_112559493_112575729_625	annotated	POLR1B	2	+	1875	25	5	0	1.92%	4.405633748	0.011313001	0.001007374	7.37E-05
ENSG00000066279_197143654_197084324_1693	annotated	ASPM	1	-	5079	18	4	6	0.77%	1.171019012	0.01108932	0.036805616	0.001973347
ENSG00000108848_50741124_50750743_356	Overlap_dORF	LUC7L3	17	+	1068	70	9	10	6.74%	21.65690747	0.000142078	6.75E-05	6.99E-08
ENSG00000134531_13213707_13214691_90	annotated	EMP1	12	+	270	20	1	3	10.00%	24.47574305	0.009288069	0.019264876	0.00088118
ENSG00000138614_65605221_65579408_439	annotated	INTS14	15	-	1317	42	0	0	4.10%	10.53739279	7.17E-05	7.17E-05	3.78E-08
ENSG00000234741_173867041_173866752_31	novel	GAS5	1	-	93	25	6	0	19.35%	88.82326105	0.023199731	0.013640586	0.001492437
ENSG00000149809_65113264_65113609_89	novel	TM7SF2	11	+	267	67	14	0	14.61%	82.91501717	0.021252496	0.000698084	0.000110414
ENSG00000124486_41136894_41232524_2395	annotated	USP9X	X	+	7185	52	6	5	1.29%	2.391366961	8.70E-06	7.50E-06	5.35E-10
ENSG00000157800_140345228_140335263_135	annotated	SLC37A3	7	-	405	11	1	0	3.70%	8.974439117	0.035374934	0.020613417	0.003252526
ENSG00000160124_122371785_122368167_88	novel	CCDC58	3	-	264	55	15	0	11.36%	68.83802732	0.005034873	0.00244612	6.96E-05
ENSG00000100316_39318439_39318341_32	novel	RPL3	22	-	96	24	4	0	15.62%	82.60563278	0.021084099	0.021557223	0.002072352
ENSG00000131389_14443635_14485007_620	Overlap_dORF	SLC6A6	3	+	1860	363	41	20	15.48%	64.48568752	2.33E-14	8.79E-16	3.00E-28
ENSG00000197170_67366519_67340843_436	annotated	PSMD12	17	-	1308	99	15	10	8.72%	25.00904479	7.16E-06	7.35E-07	4.81E-11
ENSG00000064042_41360841_41697185_1056	annotated	LIMCH1	4	+	3168	128	38	10	5.30%	13.3504053	1.71E-05	1.57E-09	5.45E-13
ENSG00000160789_156136930_156139106_171	annotated	LMNA	1	+	513	56	2	9	7.02%	36.06951607	0.002008967	0.012441998	0.000147102
ENSG00000080822_98521424_98516856_152	novel	CLDND1	3	-	456	85	22	5	13.82%	61.59191918	0.000203848	0.000208997	2.95E-07
ENSG00000138674_82881936_82819074_1205	annotated	SEC31A	4	-	3615	100	9	6	3.24%	9.14031898	9.09E-08	5.91E-07	5.22E-13
ENSG00000182712_155061980_155061843_45	annotated	CMC4	X	-	135	17	0	0	11.11%	41.60876318	0.021084099	0.021084099	0.002029891
ENSG00000111880_88963409_88612719_574	annotated	RNGTT	6	-	1722	27	0	0	1.57%	5.18084108	0.003529166	0.003529166	6.95E-05
ENSG00000082641_48051119_48059641_761	annotated	NFE2L1	17	+	2283	193	10	5	8.28%	27.93322317	1.27E-11	5.64E-12	8.85E-22
ENSG00000160752_155310161_155318757_160	novel	FDPS	1	+	480	339	45	20	24.38%	233.3609126	7.78E-07	1.54E-07	1.14E-12
ENSG00000132507_7309636_7311640_154	annotated	EIF5A	17	+	462	657	241	36	18.18%	469.8865865	0.000502315	1.88E-06	1.10E-08
ENSG00000013375_83190889_83174477_224	annotated	PGM3	6	-	672	10	0	0	3.12%	4.917001951	0.007593099	0.007593099	0.000297833
ENSG00000091039_76410578_76355889_798	annotated	OSBPL8	12	-	2394	40	7	1	2.38%	5.520844296	0.000820959	7.67E-05	4.72E-07
ENSG00000005436_75702310_75664666_612	annotated	GCFC2	2	-	1836	17	0	2	1.14%	3.059467881	0.008574016	0.028581075	0.001221281
ENSG00000020922_94490910_94420125_682	annotated	MRE11	11	-	2046	15	3	2	0.88%	2.422452574	0.068289709	0.044329616	0.012019874
ENSG00000186283_179082129_179082753_97	annotated	TOR3A	1	+	291	57	1	0	9.28%	64.72193908	0.005159595	0.003736771	0.00010562
ENSG00000111799_75202792_75087557_3062	annotated	COL12A1	6	-	9186	81	20	5	1.21%	2.913588615	6.21E-05	1.35E-07	1.07E-10
ENSG00000184182_237973108_238035887_120	annotated	UBE2F	2	+	360	10	6	0	4.17%	9.178403642	0.171390856	0.020613417	0.017249262
ENSG00000134910_125614099_125620810_183	novel	STT3A	11	+	549	156	12	4	15.85%	93.89055529	5.24E-06	2.42E-06	1.08E-10
ENSG00000163170_74145285_74135593_83	novel	BOLA3	2	-	249	114	19	12	14.46%	151.2777853	0.038740767	0.00470359	0.001018895
ENSG00000108641_19362569_19347265_135	annotated	B9D1	17	-	405	47	6	5	9.63%	38.34533077	0.006371989	0.005347237	0.000181096
ENSG00000155957_66169951_66137960_285	annotated	TMBIM4	12	-	855	236	42	11	20.35%	91.20434777	8.31E-11	2.11E-10	2.04E-19
ENSG00000073464_10197969_10233584_606	annotated	CLCN4	X	+	1818	9	4	1	0.83%	1.635755105	0.171390856	0.035374934	0.025670492
ENSG00000101365_2660289_2659699_76	annotated	IDH3B	20	-	228	36	4	6	9.21%	52.1719786	0.033673326	0.045195857	0.006370694

ENSG00000101365_2659736_2658751_61	Overlap_dORF	IDH3B	20	-	183	57	1	1	21.31%	102.9184933	0.000890803	0.001280026	7.06E-06
ENSG00000104635_22404711_22419698_492	annotated	SLC39A14	8	+	1476	222	27	13	10.37%	49.69769777	3.05E-07	6.78E-09	2.30E-14
ENSG00000175595_13920199_13926760_184	novel	ERCC4	16	+	552	12	0	0	2.72%	7.183098503	0.020613417	0.020613417	0.001946209
ENSG00000106003_2518554_2527212_250	annotated	LFNG	7	+	750	22	5	0	2.40%	9.692394246	0.020342454	0.013428348	0.001295518
ENSG00000196588_40431420_40411390_840	annotated	MRTFA	22	-	2520	33	2	3	2.50%	4.326961717	0.000120269	0.000216633	1.84E-07
ENSG00000174840_57556380_57559420_472	annotated	PDE12	3	+	1416	39	0	1	4.03%	9.10062056	4.86E-05	7.96E-05	2.88E-08
ENSG00000174840_57559585_57560004_139	dORF	PDE12	3	+	417	37	6	0	6.47%	29.31806631	0.009067228	0.003710481	0.000182651
ENSG00000134905_110663486_110644150_165	annotated	CARS2	13	-	495	17	0	5	3.64%	11.3478445	0.013428348	0.086207551	0.005701763
ENSG00000137269_53795257_53897315_197	annotated	LRRC1	6	+	591	5	0	0	2.54%	2.795452886	0.012673659	0.012673659	0.000782701
ENSG00000127914_92093213_92110159_749	novel	AKAP9	7	+	2247	11	5	0	0.80%	1.617555782	0.116253847	0.012798403	0.007703067
ENSG00000176153_64942726_64939488_189	annotated	GPX2	14	-	567	255	112	17	12.70%	148.6027256	0.011220364	0.000799864	6.01E-05
ENSG00000125746_45630001_45609663_464	annotated	EML2	19	-	1392	42	9	2	4.31%	9.969645335	0.003364114	9.33E-05	2.58E-06
ENSG00000167461_16111902_16127661_182	novel	RAB8A	19	+	546	155	18	13	16.48%	93.80126799	2.21E-05	3.01E-05	5.17E-09
ENSG00000116731_13742029_13786527_1597	annotated	PRDM2	1	+	4791	17	12	4	0.50%	1.172444798	0.185187311	0.041403097	0.031444484
ENSG00000160285_46228745_46191104_721	annotated	LSS	21	-	2163	131	15	11	6.80%	20.01172056	7.85E-07	3.07E-08	2.52E-13
ENSG00000133835_119527181_119541994_160	novel	HSD17B4	5	+	480	25	6	0	5.00%	17.20950683	0.067608747	0.005604867	0.002188767
ENSG00000136068_58008565_58170762_2591	annotated	FLNB	3	+	7773	224	35	43	3.74%	9.522018136	6.60E-14	1.25E-10	1.38E-22
ENSG00000160209_43741744_43756063_239	novel	PDXK	21	+	717	332	26	12	21.76%	152.9989963	7.53E-09	5.02E-10	4.31E-17
ENSG00000122779_138460549_138584951_1050	annotated	TRIM24	7	+	3150	30	7	0	1.43%	3.146881249	0.009754973	0.000239176	1.88E-05
ENSG00000166206_26621357_26547793_334	annotated	GABRB3	15	-	1002	153	13	6	10.78%	50.45373978	2.79E-07	2.40E-07	6.29E-13
ENSG00000166881_57063321_57059879_185	dORF	NEMP1	12	-	555	91	1	1	14.59%	54.17738798	2.47E-06	2.71E-06	5.76E-11
ENSG00000166881_57078745_57069300_110	annotated	NEMP1	12	-	330	21	2	0	5.45%	21.02688834	0.044040756	0.012798403	0.002680499
ENSG00000166881_57069265_57063318_50	dORF	NEMP1	12	-	150	13	0	0	12.00%	28.63661936	0.013428348	0.013428348	0.000872559
ENSG00000083896_68349753_68314099_735	annotated	YTHDC1	4	-	2205	46	7	1	2.59%	6.89316845	0.001112459	5.74E-05	5.04E-07
ENSG00000172057_39924203_39922550_153	annotated	ORMDL3	17	-	459	29	16	0	10.46%	20.87636907	0.056044867	0.000177448	0.000131653
ENSG00000204256_32976682_32980718_486	dORF	BRD2	6	+	1458	27	3	9	2.67%	6.118935761	0.004245653	0.023012176	0.000534287
ENSG00000134330_9481342_9488329_135	annotated	IAH1	2	+	405	99	7	1	20.00%	80.76995205	7.66E-06	2.44E-06	1.60E-10
ENSG00000109452_142431259_142028782_924	annotated	INPP4B	4	-	2772	36	10	2	2.27%	4.291201703	0.002758887	0.000120269	2.57E-06
ENSG00000145920_175878740_175879737_70	annotated	CPLX2	5	+	210	131	8	3	15.71%	206.1207218	0.001882885	0.001484235	1.67E-05
ENSG00000135535_109382378_109368279_157	annotated	CD164	6	-	471	239	54	1	21.66%	167.6666347	1.54E-05	1.70E-07	2.82E-11
ENSG00000064042_41360841_41697185_1069	annotated	LIMCH1	4	+	3207	128	33	10	5.24%	13.18805238	4.61E-06	1.57E-09	1.20E-13
ENSG00000138777_105453627_105405758_202	Overlap_dORF	PPA2	4	-	606	49	2	3	7.43%	26.71733337	0.000561382	0.000963047	3.45E-06
ENSG00000075711_197297204_197044623_904	annotated	DLG1	3	-	2712	39	4	0	1.99%	4.751651443	0.000762836	7.71E-05	4.39E-07
ENSG00000115255_1491270_1495715_151	novel	REEP6	19	+	453	20	0	2	5.30%	14.58819122	0.005655336	0.013717442	0.00040343
ENSG00000006534_68015298_68027939_468	annotated	ALDH3B1	11	+	1404	253	21	15	12.82%	59.54195183	4.62E-10	2.60E-10	1.36E-18
ENSG00000125843_3822118_3823878_59	annotated	AP5S1	20	+	177	21	0	0	10.17%	39.20267318	0.013640586	0.013640586	0.000898637
ENSG00000125843_3823884_3824297_137	dORF	AP5S1	20	+	411	28	2	0	5.84%	22.5105374	0.005306578	0.005306578	0.000151001
ENSG00000128585_131387228_131399240_78	annotated	MKLN1	7	+	234	24	2	1	8.97%	33.88949037	0.020342454	0.01208853	0.001177842
ENSG00000128585_131399352_131487728_528	dORF	MKLN1	7	+	1584	63	17	1	4.55%	13.14180521	0.000739367	9.56E-06	6.80E-08
ENSG00000115084_113725436_113717775_126	novel	SLC35F5	2	-	378	13	1	0	4.76%	11.36373784	0.012798403	0.013428348	0.00083412
ENSG00000132640_11919114_11923666_404	annotated	BTBD3	20	+	1212	23	2	2	1.98%	6.270394567	0.010318779	0.015646181	0.000790819
ENSG00000251664_140875473_140877851_792	annotated	PCDHA12	5	+	2376	43	2	3	2.02%	5.97986904	0.000501924	0.00098473	3.18E-06
ENSG00000115234_27375683_27376719_153	dORF	SNX17	2	+	459	49	0	2	9.15%	35.27386498	0.000429708	0.0016988	4.75E-06
ENSG00000127990_94600668_94587820_115	annotated	SGCE	7	-	345	72	4	3	16.52%	68.95774562	0.000219052	9.53E-05	1.49E-07
ENSG00000074201_77625050_77619628_109	dORF	CLNS1A	11	-	327	52	4	9	11.01%	52.54425571	0.005639624	0.00576738	0.000173146
ENSG00000074201_77637714_77625794_49	annotated	CLNS1A	11	-	147	11	1	0	10.20%	24.72549553	0.035772986	0.021084099	0.003354605
ENSG00000087884_77842497_77900601_137	annotated	AAMDC	11	+	411	24	0	1	5.11%	19.29474634	0.008473674	0.011825808	0.000503679
ENSG00000166206_26621357_26547793_334	novel	GABRB3	15	-	1002	153	13	6	10.78%	50.45373978	2.79E-07	2.40E-07	6.29E-13
ENSG00000161981_53952_55489_45	Overlap_uORF	SNRNP25	16	+	135	46	6	16	17.78%	112.588418	0.008127104	0.06867726	0.002981148
ENSG00000113580_143400839_143281889_778	annotated	NR3C1	5	-	2334	125	11	5	6.30%	17.69615098	2.61E-08	3.78E-10	1.20E-16
ENSG00000092036_22955118_22946525_306	annotated	HAUS4	14	-	918	43	19	3	5.88%	15.4773081	0.021948943	0.000479635	8.50E-05
ENSG00000105974_116525063_116526839_114	novel	CAV1	7	+	342	252	37	25	18.42%	243.4692335	0.00012785	0.000113726	1.04E-07
ENSG00000185842_224952748_224967715_213	annotated	DNAH14	1	+	639	32	2	3	3.76%	16.54698121	0.005604867	0.022643694	0.000666224
ENSG00000026297_166952520_166929588_218	annotated	RNASET2	6	-	654	26	6	1	5.50%	13.13606393	0.009834242	0.00097329	6.15E-05
ENSG00000143771_224356925_224375822_92	annotated	CNIH4	1	+	276	230	31	9	30.43%	275.3521093	2.71E-06	1.76E-06	4.14E-11
ENSG00000143771_224356925_224379090_137	annotated	CNIH4	1	+	411	262	29	11	27.74%	210.6343142	4.86E-08	2.70E-08	1.32E-14
ENSG00000143771_224356925_224379090_90	annotated	CNIH4	1	+	270	215	27	8	30.00%	263.1142377	4.10E-06	2.50E-06	8.79E-11
ENSG00000087460_58891727_58910829_379	annotated	GNAS	20	+	1137	1257	208	70	32.72%	365.2956215	1.14E-19	1.45E-19	2.68E-37
ENSG00000124225_57659647_57652053_234	annotated	PMEPA1	20	-	702	215	41	18	16.24%	101.1977837	1.79E-05	2.18E-06	3.41E-10
ENSG00000105852_95396350_95359973_354	annotated	PON3	7	-	1062	206	14	11	12.15%	64.09325933	4.69E-08	1.67E-07	7.74E-14
ENSG00000100612_60153151_60144854_204	annotated	DHRS7	14	-	612	81	7	12	12.25%	43.73239382	7.75E-05	0.000854326	4.97E-07
ENSG00000160679_153638305_153645075_51	novel	CHTOP	1	+	153	20	0	0	9.80%	43.19248773	0.021084099	0.021084099	0.002029891
ENSG00000160679_153645072_153645269_65	novel	CHTOP	1	+	195	23	0	1	12.31%	38.97291393	0.005655336	0.008413705	0.000249423
ENSG00000143815_225424075_225403303_615	annotated	LBR	1	-	1845	214	71	10	13.17%	38.32543179	8.79E-07	8.24E-14	1.19E-18
ENSG00000166747_71789479_71733058_822	annotated	AP1G1	16	-	2466	98	10	1	4.74%	13.13114682	1.37E-06	2.34E-08	3.52E-13
ENSG00000164466_175492104_175509067_56	novel	SFXN1	5	+	168	43	6	0	23.21%	84.57243356	0.002947593	0.000669869	1.25E-05
ENSG00000109180_48832625_48860762_245	annotated	OCIAD1	4	+	735	392	33	9	18.37%	176.2253499	6.07E-08	1.38E-08	8.60E-15
ENSG00000164062_49674402_49683342_732	annotated	APEH	3	+	2196	129	15	8	7.24%	19.41006672	6.29E-09	1.27E-09	8.75E-17

ENSG00000054282_243341039_243499785_227	annotated	SDCCAG8	1	+	681	21	1	1	3.96%	10.18924105	0.007051958	0.004960752	0.000186148
ENSG00000172992_45034786_45033960_99	novel	DCAKD	17	-	297	11	0	0	5.05%	12.23787152	0.020613417	0.020613417	0.001946209
ENSG00000115966_175114798_175074609_329	annotated	ATF2	2	-	987	40	4	0	5.47%	13.39098404	0.000533505	8.50E-05	3.31E-07
ENSG00000171466_9660729_9652949_421	annotated	ZNF562	19	-	1263	12	1	0	1.19%	3.139406471	0.051235217	0.020613417	0.004687384
ENSG00000197555_71624051_71739161_1238	annotated	SIPA1L1	14	+	3714	14	0	1	0.65%	1.245534581	0.005160369	0.00912793	0.000248192
ENSG00000071889_154508625_154506811_189	novel	FAM3A	X	-	567	36	5	0	6.88%	20.97920832	0.006181832	0.000676852	2.73E-05
ENSG00000125450_75231586_75235679_259	annotated	NUP85	17	+	777	66	6	1	9.27%	28.0667787	6.41E-05	1.01E-05	5.25E-09
ENSG00000105974_116526588_116559287_147	dORF	CAV1	7	+	441	1039	121	47	41.50%	778.4784803	3.85E-10	1.36E-11	6.53E-20
ENSG00000018510_177420279_177538195_99	annotated	AGPS	2	+	297	22	4	0	9.09%	24.47574305	0.008316137	0.003554776	0.000161182
ENSG00000126773_60115817_60125844_594	novel	PCNX4	14	+	1782	25	4	0	1.85%	4.635557395	0.010524174	0.00140552	9.06E-05
ENSG00000113391_94053195_93620987_370	annotated	FAM172A	5	-	1110	30	11	0	3.78%	8.930338679	0.035674012	0.000411814	0.000136226
ENSG00000112514_33417668_33416650_156	annotated	CUTA	6	-	468	680	64	23	30.13%	480.1011136	2.16E-08	2.71E-09	6.33E-16
ENSG00000183291_86914111_86863547_123	annotated	SELENOF	1	-	369	686	127	24	26.83%	614.2814535	1.73E-05	2.92E-06	4.33E-10
ENSG00000183291_86880721_86863474_62	Overlap_dORF	SELENOF	1	-	186	21	4	6	11.29%	37.30576964	0.024275373	0.060777753	0.006394763
ENSG00000184349_107427394_107381255_148	annotated	EFNA5	5	-	444	46	4	0	8.78%	34.23296494	0.001791147	0.000698084	7.83E-06
ENSG00000112081_36596763_36601989_164	annotated	SRSF3	6	+	492	267	106	41	24.39%	179.3146663	0.000129568	2.54E-06	3.16E-09
ENSG00000112081_36596860_36596961_33	internal	SRSF3	6	+	99	81	31	39	15.15%	270.3457073	0.023199731	0.256407574	0.030662342
ENSG00000010704_26087477_26094411_325	annotated	HFE	6	+	975	146	12	5	14.46%	49.47865594	5.03E-08	3.46E-09	1.89E-15
ENSG00000085978_233263173_233294350_523	dORF	ATG16L1	2	+	1569	57	6	13	4.21%	12.0038778	0.000355525	0.003277308	7.94E-06
ENSG00000085978_233251828_233294350_588	annotated	ATG16L1	2	+	1764	64	7	13	4.25%	11.98811904	0.000155563	0.00102497	1.12E-06
ENSG00000116260_180154908_180178849_155	annotated	QSOX1	1	+	465	49	0	4	9.03%	34.81871833	0.000469808	0.001700346	5.16E-06
ENSG00000167565_40442080_40441490_196	annotated	SERTAD3	19	-	588	67	8	11	7.65%	37.65018637	0.000860312	0.012137407	6.96E-05
ENSG00000204628_181243800_181236977_273	annotated	RACK1	5	-	819	2744	282	40	38.10%	1107.056685	1.22E-15	8.90E-18	1.76E-31
ENSG00000120942_11273532_11286131_338	annotated	UBIAD1	1	+	1014	89	3	0	9.17%	29.00158311	5.92E-07	4.47E-07	2.43E-12
ENSG00000133028_10695771_10691938_96	dORF	SCO1	17	-	288	25	4	18	10.42%	28.68251138	0.016263223	0.219548803	0.019756175
ENSG00000133028_10697501_10695832_89	annotated	SCO1	17	-	267	26	4	0	11.24%	32.17597681	0.014532329	0.002335453	0.000197301
ENSG00000117500_93180242_93154827_193	annotated	TMED5	1	-	579	70	66	16	7.77%	39.94745627	0.113093502	0.008934022	0.005695998
ENSG00000161714_45113573_45112616_169	novel	PLCD3	17	-	507	10	1	0	2.96%	6.517209687	0.042234516	0.019679754	0.003715148
ENSG00000175745_93585030_93593842_421	annotated	NR2F1	5	+	1263	24	6	1	2.61%	6.278812943	0.015384268	0.002129196	0.000193677
ENSG00000162734_160211545_160213486_130	annotated	PEA15	1	+	390	124	18	6	17.69%	105.0574201	3.31E-05	2.14E-05	5.51E-09
ENSG00000197696_84658152_84655374_121	annotated	NMB	15	-	363	17	0	0	8.26%	15.47433341	0.001790164	0.001790164	1.90E-05
ENSG00000105373_47745560_47752700_285	novel	NOP53	19	+	855	42	12	10	4.91%	16.23128223	0.007562967	0.01959271	0.000746529
ENSG00000160014_46608271_46609153_113	annotated	CALM3	19	+	339	222	26	19	29.20%	216.3828965	3.98E-06	2.08E-06	7.13E-11
ENSG00000118680_3272899_3277937_172	annotated	MYL12B	18	+	516	124	17	1	10.47%	79.40386407	0.00211467	0.000100355	1.65E-06
ENSG00000152767_98385725_98448317_822	annotated	FARP1	13	+	2466	13	1	2	0.73%	1.741886823	0.023672501	0.061356297	0.006320165
ENSG00000145388_118691544_118710302_371	Overlap_dORF	METTL14	4	+	1113	15	2	0	2.16%	4.453133843	0.026714995	0.004873904	0.000703162
ENSG00000142327_240574571_240577892_315	novel	RNPEPL1	2	+	945	29	2	2	4.76%	10.13995069	0.000258233	0.00133156	2.33E-06
ENSG00000107175_35732636_35732824_62	novel	CREB3	9	+	186	23	5	5	12.90%	40.85870008	0.023197249	0.041403097	0.004207174
ENSG00000107175_35733181_35736726_290	novel	CREB3	9	+	870	32	2	1	4.48%	12.15347241	0.002193532	0.000662934	9.16E-06
ENSG00000074800_8874908_8870218_123	novel	ENO1	1	-	369	521	107	26	17.89%	466.5315412	4.46E-05	2.61E-05	8.94E-09
ENSG00000089639_19635441_19629963_459	annotated	GMIP	19	-	1377	11	0	0	1.09%	2.639540917	0.019216965	0.019216965	0.001707396
ENSG00000172992_45034786_45024433_198	annotated	DCAKD	17	-	594	25	0	1	6.06%	13.90667219	0.000966879	0.00117326	7.01E-06
ENSG00000143624_153767762_153773270_382	novel	INTS3	1	+	1146	36	3	0	1.83%	10.37976538	0.024800926	0.008776163	0.00107875
ENSG00000198964_50343661_50307142_262	annotated	SGMS1	10	-	786	8	2	6	2.29%	3.363079197	0.053359408	0.113684165	0.023062811
ENSG00000082996_149912081_149961104_180	annotated	RNF13	3	+	540	86	15	0	15.56%	52.62284755	7.68E-05	1.54E-06	1.14E-09
ENSG00000198894_77105709_77114318_399	annotated	CIPC	14	+	1197	23	0	0	2.76%	6.34897094	0.001484235	0.001484235	1.33E-05
ENSG00000213079_154792961_154833395_1118	annotated	SCAF8	6	+	3354	47	8	1	2.15%	4.630250138	0.000146311	1.01E-05	1.24E-08
ENSG00000170382_204619326_204617851_491	annotated	LRRN2	1	-	1473	5	0	0	1.02%	1.121597186	0.012673659	0.012673659	0.000782701
ENSG00000170382_204619326_204617851_491	annotated	LRRN2	1	-	1473	5	0	0	1.02%	1.121597186	0.012673659	0.012673659	0.000782701
ENSG00000170382_204619326_204617851_491	annotated	LRRN2	1	-	1473	5	0	0	1.02%	1.121597186	0.012673659	0.012673659	0.000782701
ENSG00000178904_32432779_32482104_588	annotated	DPY19L3	19	+	1764	42	13	8	3.40%	7.867203122	0.006739856	0.000573661	2.60E-05
ENSG00000177542_794777_791915_275	annotated	SLC25A22	11	-	825	17	4	1	4.36%	6.808706702	0.022133928	0.001620026	0.00022908
ENSG00000181029_7682254_7682820_188	annotated	TRAPPC5	19	+	564	112	11	2	15.43%	65.61582178	2.01E-06	1.72E-06	3.03E-11
ENSG00000070061_108912405_108869115_983	annotated	ELP1	9	-	2949	41	5	0	2.14%	4.593870389	0.000395354	1.80E-05	5.97E-08
ENSG00000101849_9654265_9740808_526	annotated	TBL1X	X	+	1578	21	2	0	1.90%	4.397258019	0.00376808	0.002201184	4.75E-05
ENSG00000188243_75537805_75526589_85	annotated	COMMD6	13	-	255	162	41	2	18.82%	209.9154904	0.00272923	0.000211707	4.15E-06
ENSG00000162227_62775841_62782337_257	novel	TAF6L	11	+	771	16	2	0	3.11%	6.857017507	0.022789577	0.004149608	0.000518649
ENSG00000105722_42254999_42248465_548	annotated	ERF	19	-	1644	59	19	9	3.10%	11.85822952	0.024591457	0.002615157	0.000382062
ENSG00000213593_57712619_57738725_103	annotated	TMX2	11	+	309	180	75	10	19.42%	192.4791443	0.117537015	0.000234875	0.000462044
ENSG00000075711_197297204_197044623_892	annotated	DLG1	3	-	2676	39	4	0	2.02%	4.815575005	0.000762836	7.71E-05	4.39E-07
ENSG00000137074_33001604_32973498_302	annotated	APTX	9	-	906	72	11	4	6.95%	26.25874419	0.001139536	3.19E-05	3.10E-07
ENSG00000159352_151254783_151266156_268	novel	PSMD4	1	+	804	51	27	2	6.72%	20.95963817	0.003881303	0.00025646	7.16E-06
ENSG00000243989_51984065_51989075_343	annotated	ACY1	3	+	1029	58	2	4	6.12%	18.62439923	2.56E-05	0.00017702	3.53E-08
ENSG00000257341_105487260_105488511_77	annotated	AL928654.3	14	+	231	805	61	46	41.56%	1151.472457	7.11E-07	3.27E-06	2.09E-11
ENSG00000147955_34637697_34635632_192	annotated	SIGMAR1	9	-	576	377	54	13	26.04%	216.2661358	3.00E-08	1.06E-09	3.61E-16
ENSG00000168827_158684611_158690362_85	novel	GFM1	3	+	255	10	1	0	5.88%	12.95774632	0.042234516	0.019679754	0.003715148
ENSG00000144736_72842343_72750284_488	dORF	SHQ1	3	-	1464	19	0	4	2.25%	4.288270554	0.001092093	0.017603896	0.000128243
ENSG00000155755_201643400_201634877_96	annotated	TMEM237	2	-	288	9	3	0	5.21%	10.3257041	0.168144395	0.019679754	0.016305955

ENSG00000119707_73107790_73111259_229	novel	RBM25	14	+	687	9	2	3	2.18%	4.328679447	0.083503598	0.145248299	0.042302052
ENSG00000137504_85664875_85663811_354	annotated	CREBZF	11	-	1062	43	13	9	4.52%	13.37869005	0.005261463	0.004504815	0.000128275
ENSG00000149273_75399548_75404865_243	annotated	RPS3	11	+	729	739	101	57	18.93%	334.9550761	6.56E-09	2.09E-08	1.44E-15
ENSG00000143537_155051387_155054245_145	novel	ADAM15	1	+	435	58	12	4	10.34%	44.05633748	0.014376005	0.000610656	6.33E-05
ENSG00000104691_30766242_30766394_50	novel	UBXN8	8	+	150	34	1	0	12.00%	74.89577372	0.016503129	0.01300724	0.001029549
ENSG00000166900_59773274_59797366_258	annotated	STX3	11	+	774	29	3	0	3.88%	12.38017235	0.009257958	0.002227676	0.000118306
ENSG00000092621_119723428_119744014_243	annotated	PHGDH	1	+	729	25	2	3	3.29%	11.33136252	0.00605881	0.009067228	0.000285832
ENSG00000183258_177513789_177511791_291	novel	DDX41	5	-	873	116	12	4	9.28%	43.90494113	1.22E-05	1.16E-05	1.14E-09
ENSG00000109320_102596143_102616594_534	dORF	NFKB1	4	+	1602	26	2	0	2.25%	5.362662802	0.002254941	0.000982952	1.35E-05
ENSG00000109320_102529899_102582583_246	annotated	NFKB1	4	+	738	14	0	0	2.85%	6.268178097	0.007029303	0.007029303	0.000257364
ENSG00000068724_47046319_47073923_256	novel	TTC7A	2	+	768	15	0	0	1.95%	6.453565061	0.021084099	0.021084099	0.002029891
ENSG00000181938_58403089_58403424_111	novel	GINS3	16	+	333	28	0	0	7.21%	27.78327589	0.005706018	0.005706018	0.00017331
ENSG00000260238_156213016_156242794_142	annotated	PMF1-BGLAP	1	+	426	35	4	0	4.23%	27.14739105	0.062728927	0.01300724	0.003936255
ENSG00000261762_78590401_78590207_64	novel	AC027228.2	15	-	192	6	0	7	7.81%	10.3257041	0.016947427	0.241449849	0.022959401
ENSG00000124570_2968811_2948298_332	annotated	SERPINB6	6	-	996	213	33	4	17.47%	70.66264973	8.20E-09	3.45E-11	3.82E-18
ENSG00000172530_87984207_88076661_405	annotated	BANP	16	+	1215	19	0	0	1.23%	5.16710131	0.021084099	0.021084099	0.002029891
ENSG00000114439_107728954_107805457_693	annotated	BBX	3	+	2079	20	6	0	1.59%	3.178667928	0.040251748	0.001262414	0.000373703
ENSG00000115129_24084326_24080842_159	novel	TP53I3	2	-	477	36	20	6	9.43%	24.93754952	0.152035323	0.004461971	0.00500077
ENSG00000162366_47185084_47183971_51	novel	PDZK1IP1	1	-	153	14	5	0	11.76%	30.23474141	0.086207551	0.012798403	0.005490656
ENSG00000242265_94664609_94665682_357	dORF	PEG10	7	+	1071	99	18	5	8.96%	30.54325918	9.09E-05	1.11E-06	1.03E-09
ENSG00000242265_94663557_94664534_325	annotated	PEG10	7	+	975	161	16	6	15.69%	54.5620795	1.82E-08	1.01E-09	2.08E-16
ENSG00000131844_71587426_71643794_329	annotated	MCCC2	5	+	987	56	9	1	5.78%	18.74737765	0.002093883	5.63E-05	9.89E-07
ENSG00000109066_74788856_74839101_236	annotated	TMEM104	17	+	708	12	0	0	2.12%	5.600381883	0.020613417	0.020613417	0.001946209
ENSG00000124641_41921018_41907072_212	annotated	MED20	6	-	636	43	4	4	4.72%	22.33988811	0.002335453	0.021979756	0.000307648
ENSG00000124571_43575864_43523868_1204	annotated	XPO5	6	-	3612	49	5	4	1.83%	4.482476197	0.000297784	0.000172447	3.55E-07
ENSG00000164258_53646314_53658659_66	novel	NDUFS4	5	+	198	79	16	1	19.70%	131.8352523	0.003066162	0.000707658	1.37E-05
ENSG00000065000_2151298_2102173_1141	annotated	AP3D1	19	-	3423	116	18	11	4.21%	11.19749156	2.74E-07	6.89E-08	1.84E-13
ENSG00000257923_101816052_102249042_1453	annotated	CUX1	7	+	4359	51	11	1	1.79%	3.865920873	0.000542292	3.05E-06	1.81E-08
ENSG00000119977_95693953_95693588_121	novel	TCTN3	10	-	363	26	21	9	10.74%	23.66662757	0.262082048	0.026000317	0.034047039
ENSG00000109180_48842659_48851992_133	novel	OCIAD1	4	+	399	174	12	7	18.05%	144.0940361	0.000173891	3.55E-05	4.69E-08
ENSG00000140043_73860574_73884177_327	annotated	PTGR2	14	+	981	39	3	0	5.20%	13.13606393	0.0007246	0.000121023	6.26E-07
ENSG00000131966_58200218_58215243_179	annotated	ACTR10	14	+	537	67	9	3	5.59%	41.22590239	0.014895507	0.016753251	0.001183975
ENSG00000131966_58215281_58234551_219	dORF	ACTR10	14	+	657	45	10	0	5.48%	22.63168021	0.013132543	0.001044718	8.94E-05
ENSG00000166888_57098871_57096572_185	novel	STAT6	12	-	555	8	0	0	2.70%	4.762847295	0.019679754	0.019679754	0.001784964
ENSG00000122406_92832115_92833798_63	annotated	RPL5	1	+	189	162	14	9	22.22%	283.2193124	0.000672169	0.000475338	2.07E-06
ENSG00000217555_66552782_66566011_130	annotated	CKLF	16	+	390	71	10	12	14.62%	60.15384541	0.00053366	0.002097407	7.18E-06
ENSG00000165030_91410719_91409346_457	annotated	NFIL3	9	-	1371	19	2	1	1.97%	4.579159804	0.013443806	0.008316137	0.000560478
ENSG00000111859_11213721_11200981_168	annotated	NEDD9	6	-	504	23	1	5	3.57%	15.07880598	0.015555383	0.157414378	0.012708044
ENSG00000166526_100077357_100071143_446	annotated	ZNF3	7	-	1338	25	5	4	2.47%	6.173814109	0.018697883	0.010635465	0.000966459
ENSG00000100811_100276502_100277600_109	novel	YY1	14	+	327	23	5	0	11.01%	23.24072849	0.007656911	0.000966879	4.68E-05
ENSG00000144713_12840237_12839345_93	novel	RPL32	3	-	279	134	12	1	11.83%	158.6975597	0.009821677	0.002827043	0.000155209
ENSG00000169032_66387348_66490615_393	annotated	MAP2K1	15	+	1179	50	10	0	4.83%	14.01282999	0.005559485	5.38E-05	2.90E-06
ENSG00000143294_156794791_156800460_56	annotated	PRCC	1	+	168	24	1	0	8.93%	47.20321873	0.035772986	0.021084099	0.003354605
ENSG00000149182_47176853_47175800_104	novel	ARFGAP2	11	-	312	10	0	1	4.81%	10.59046574	0.019679754	0.065398531	0.005779267
ENSG00000145495_10387056_10433684_778	annotated	6-Mar	5	+	2334	173	12	3	9.64%	24.49147296	3.25E-13	7.30E-14	3.18E-25
ENSG00000143537_155051387_155062502_862	annotated	ADAM15	1	+	2586	260	23	12	9.05%	33.22113615	9.67E-12	3.03E-13	3.94E-23
ENSG00000137807_69444775_69447807_172	novel	AC027237.1	15	+	516	25	0	1	6.98%	16.00884356	0.000943084	0.001559221	9.04E-06
ENSG00000124532_24403001_24405171_79	Overlap_uORF	MRS2	6	+	237	48	5	5	15.19%	66.92101896	0.00678006	0.005617464	0.0002013
ENSG00000137135_35665169_35661987_335	annotated	ARHGEF39	9	-	1005	33	1	4	4.48%	10.84969505	0.000377763	0.002237259	5.62E-06
ENSG00000102531_49131290_49145939_191	annotated	FNDC3A	13	+	573	12	2	0	3.14%	6.919843584	0.061356297	0.012798403	0.003794065
ENSG00000132623_10038302_10055660_776	annotated	ANKEF1	20	+	2328	77	0	0	3.09%	10.92892392	8.17E-06	8.17E-06	5.48E-10
ENSG00000150316_94972185_94971328_96	novel	CWC15	11	-	288	15	3	0	6.25%	17.20950683	0.049121095	0.011772051	0.002799454
ENSG00000157916_2402238_2403124_64	Overlap_dORF	RER1	1	+	192	54	5	2	20.31%	92.93133688	0.003249213	0.000683882	1.41E-05
ENSG00000174444_66504790_66501367_227	novel	RPL4	15	-	681	147	24	1	5.73%	71.32468733	0.001388211	0.000727061	6.31E-06
ENSG00000177192_131932280_131943586_291	annotated	PUS1	12	+	873	27	2	0	3.78%	10.21925354	0.005020943	0.001491519	4.43E-05
ENSG00000260269_75601155_75598358_113	novel	AC105036.3	15	-	339	16	4	0	5.31%	15.59516371	0.086597294	0.01300724	0.005588697
ENSG00000172936_38138662_38141286_309	annotated	MYD88	3	+	927	101	9	10	9.06%	36.00072885	7.46E-05	0.000146902	7.95E-08
ENSG00000100578_58428310_58547932_1506	annotated	KIAA0586	14	+	4518	30	6	1	1.13%	2.194040711	0.00486622	0.000284974	1.00E-05
ENSG00000077232_182718162_182736267_276	annotated	DNAJC10	2	+	828	45	15	1	6.52%	17.95774626	0.006569312	0.000100675	5.88E-06
ENSG00000077232_182756320_182777132_240	dORF	DNAJC10	2	+	720	83	2	5	7.92%	38.09037511	6.27E-05	0.000160295	7.35E-08
ENSG00000072518_63895204_63909237_699	annotated	MARK2	11	+	2097	41	9	3	2.72%	6.460335611	0.003317712	0.000358778	8.11E-06
ENSG00000118816_77075543_77066298_59	Overlap_uORF	CCNI	4	-	177	25	0	0	8.47%	46.66984903	0.021084099	0.021084099	0.002029891
ENSG00000118816_77066263_77048219_344	annotated	CCNI	4	-	1032	121	24	3	11.05%	38.74140142	2.94E-05	7.74E-08	2.86E-11
ENSG00000103197_2050439_2088610_1679	annotated	TSC2	16	+	5037	65	5	1	1.55%	4.26393975	6.02E-06	3.46E-06	1.76E-10
ENSG00000157593_44256386_44254706_327	novel	SLC35B2	6	-	981	100	12	0	8.26%	33.6822152	9.49E-05	2.53E-06	2.24E-09
ENSG00000135452_57745215_57746424_108	annotated	TSPAN31	12	+	324	26	1	5	8.33%	26.5153883	0.00567009	0.021936858	0.000651201
ENSG00000184182_237973108_237973674_47	annotated	UBE2F	2	+	141	10	6	0	10.64%	23.43422206	0.171390856	0.020613417	0.017249262
ENSG00000184182_238016628_238041338_93	dORF	UBE2F	2	+	279	15	2	0	5.38%	17.76465221	0.021084099	0.021084099	0.002029891

ENSG00000139620_48681632_48654044_454	annotated	KANSL2	12	-	1362	29	10	1	4.41%	7.035428342	0.014182966	4.79E-05	8.22E-06
ENSG00000121073_49707833_49701458_322	annotated	SLC35B1	17	-	966	111	25	3	11.49%	37.96780637	0.000107472	1.76E-07	2.52E-10
ENSG00000072778_7224858_7225097_55	novel	ACADVL	17	+	165	26	3	0	18.18%	52.06658066	0.00376808	0.002201184	4.75E-05
ENSG00000072778_7223870_7224528_107	novel	ACADVL	17	+	321	191	5	7	23.36%	196.6065528	1.42E-05	1.86E-05	2.10E-09
ENSG00000072778_7224029_7225097_158	Overlap_dORF	ACADVL	17	+	474	74	20	28	15.19%	51.58495212	0.000669494	0.0093796	4.25E-05
ENSG00000072778_7222818_7224645_184	annotated	ACADVL	17	+	552	206	36	5	15.22%	123.3098576	0.000541199	3.61E-06	2.08E-08
ENSG00000144028_96305437_96275012_2136	annotated	SNRNP200	2	-	6408	201	21	10	4.21%	10.36437715	1.93E-12	1.03E-14	2.98E-25
ENSG00000104419_133284299_133238878_390	annotated	NDRG1	8	-	1170	53	1	6	3.85%	14.96785825	0.000431526	0.003997616	1.16E-05
ENSG00000006576_77840256_77940317_546	annotated	PHTF2	7	+	1638	39	8	5	3.48%	7.867203122	0.002777133	0.000682707	1.20E-05
ENSG00000241878_31625812_31619612_374	annotated	PISD	22	-	1122	24	9	2	3.21%	7.067861628	0.015721867	0.003027818	0.000267978
ENSG00000197256_11194511_11192667_470	novel	KANK2	19	-	1410	11	1	0	1.49%	2.577764427	0.013018137	0.007593099	0.000499911
ENSG00000105088_9857777_9854186_355	annotated	OLFM2	19	-	1065	12	0	0	1.97%	3.723070773	0.007029303	0.007029303	0.000257364
ENSG00000239382_36013397_36010849_126	novel	ALKBH6	19	-	378	10	4	0	3.97%	8.741336802	0.121807694	0.019679754	0.01126199
ENSG00000278540_37330399_37289522_77	uORF	ACACA	17	-	231	16	1	0	10.39%	22.88640908	0.009288069	0.005306578	0.000259004
ENSG00000102103_48898510_48903084_265	annotated	PQBP1	X	+	795	45	11	9	6.42%	18.70316214	0.020227839	0.003888751	0.000432783
ENSG00000106263_2366556_2371914_143	novel	EIF3B	7	+	429	54	20	0	11.19%	41.59164727	0.02781034	0.000203771	5.83E-05
ENSG00000129158_18013010_17788085_458	annotated	SERGEF	11	-	1374	15	1	0	1.53%	3.607232872	0.011825808	0.008473674	0.000503679
ENSG00000165688_136410669_136416532_257	novel	PMPCA	9	+	771	41	7	0	5.84%	17.57110736	0.004486581	0.000279805	9.03E-06
ENSG00000108588_63756251_63746881_171	annotated	CCDC47	17	-	513	60	5	1	11.11%	38.64591007	0.000147044	6.08E-05	6.55E-08
ENSG00000108588_63764139_63754490_183	Overlap_uORF	CCDC47	17	-	549	139	19	4	16.39%	83.65889221	0.000147055	3.57E-06	4.89E-09
ENSG00000178467_49001344_49002772_185	novel	P4HTM	3	+	555	30	1	0	4.86%	17.86067736	0.0037806	0.003554776	7.47E-05
ENSG00000102317_48575181_48575814_118	novel	RBM3	X	+	354	252	146	13	20.34%	235.2160391	0.00041065	1.51E-05	5.30E-08
ENSG00000217128_131796921_131644685_1121	annotated	FNIP1	5	-	3363	29	12	4	1.61%	2.849317099	0.040235116	0.000599243	0.000210557
ENSG00000155229_97466851_97458692_574	annotated	MMS19	10	-	1722	42	3	1	2.44%	8.059086125	0.001623839	0.000606419	6.23E-06
ENSG00000170633_121416210_121423576_353	annotated	RNF34	12	+	1059	53	6	0	5.67%	16.53672724	0.000220332	3.93E-05	6.55E-08
ENSG00000152558_102452623_102398867_189	annotated	TMEM123	11	-	567	509	73	51	27.51%	296.6226955	3.03E-09	6.91E-08	2.31E-15
ENSG00000112640_42984678_43011286_594	annotated	PPP2R5D	6	+	1782	56	5	0	4.21%	10.38364856	4.79E-05	3.94E-06	1.63E-09
ENSG00000148399_137578774_137576402_94	novel	DPH7	9	-	282	29	0	0	10.64%	33.97962199	0.002418183	0.002418183	3.38E-05
ENSG00000144711_13073014_12900983_1114	annotated	IQSEC1	3	-	3342	15	2	0	0.81%	1.483045472	0.009018488	0.003327803	0.000164697
ENSG00000137497_72035943_72003527_979	annotated	NUMA1	11	-	2937	70	17	8	2.76%	7.87523908	0.000296034	5.27E-05	1.16E-07
ENSG00000187024_127714279_127714100_59	Overlap_dORF	PTRH1	9	-	177	13	0	0	8.47%	24.26832149	0.019679754	0.019679754	0.001784964
ENSG00000187024_127715591_127714150_166	annotated	PTRH1	9	-	498	34	12	7	6.02%	22.55896799	0.027138166	0.011465974	0.001492509
ENSG00000139263_58914293_58872572_1026	annotated	LRIG3	12	-	3078	30	2	3	1.27%	3.220492506	0.002552269	0.00465809	6.71E-05
ENSG00000118855_158802153_158807060_78	annotated	MFSD1	3	+	234	13	6	1	6.41%	18.35680728	0.101051022	0.021084099	0.009675103
ENSG00000118855_158804334_158828982_368	Overlap_dORF	MFSD1	3	+	1104	105	7	0	10.87%	31.42605595	1.85E-07	1.31E-08	2.54E-14
ENSG00000113761_177022739_177050774_131	annotated	ZNF346	5	+	393	17	0	0	4.58%	14.29308659	0.01300724	0.01300724	0.000821865
ENSG00000116754_70235531_70250805_294	novel	SRSF11	1	+	882	59	8	3	8.16%	22.10309449	5.18E-05	1.00E-05	4.18E-09
ENSG00000016864_52695211_52694845_70	novel	GLT8D1	3	-	210	13	1	0	8.57%	20.45472812	0.012798403	0.012798403	0.000797244
ENSG00000016864_52700460_52695793_259	novel	GLT8D1	3	-	777	13	2	0	2.32%	5.528304896	0.044040756	0.012798403	0.002680499
ENSG00000089127_112907043_112919553_399	annotated	AC004551.1	12	+	1197	129	9	3	7.77%	35.60944571	2.45E-06	1.75E-06	3.73E-11
ENSG00000182199_57229779_57234361_504	annotated	SHMT2	12	+	1512	216	38	11	12.30%	47.20321873	2.37E-08	1.11E-10	3.46E-17
ENSG00000267228_47176277_47156177_82	annotated	AC012254.2	18	-	246	114	33	5	21.95%	153.1226364	0.000633682	0.000178907	7.81E-07
ENSG00000123552_99510220_99435716_814	annotated	USP45	6	-	2442	19	3	3	0.86%	2.570855074	0.013794764	0.032996028	0.002132888
ENSG00000140564_90875741_90881878_794	annotated	FURIN	15	+	2382	86	9	0	4.03%	11.92961279	2.61E-05	3.11E-07	8.57E-11
ENSG00000152683_32165901_32220713_438	annotated	SLC30A6	2	+	1314	115	32	8	9.13%	28.91825805	4.98E-05	2.96E-07	1.66E-10
ENSG00000058272_79796884_79781843_197	annotated	PPP1R12A	12	-	591	10	8	0	2.54%	5.590905772	0.3815123	0.020613417	0.048802575
ENSG00000258315_7014858_7017321_157	annotated	C17orf49	17	+	471	99	14	0	15.92%	69.4518696	9.48E-05	5.56E-06	4.56E-09
ENSG00000075415_98593979_98594347_122	novel	SLC25A3	12	+	366	252	50	0	11.48%	227.5040378	0.005308395	0.000461594	1.67E-05
ENSG00000075415_98595699_98601528_318	novel	SLC25A3	12	+	954	689	71	22	29.87%	238.6384947	5.72E-14	7.39E-16	6.38E-28
ENSG00000077782_38457446_38413628_731	annotated	FGFR1	8	-	2193	99	20	8	5.88%	14.91647541	5.63E-06	2.03E-07	1.14E-11
ENSG00000114209_167720151_167684308_210	annotated	PDCD10	3	-	630	83	7	4	11.90%	43.53185727	2.73E-05	3.49E-05	7.34E-09
ENSG00000197448_143263514_143264696_100	novel	GSTK1	7	+	300	167	70	23	23.00%	183.935209	0.001998878	0.000225235	3.15E-06
ENSG00000197448_143265006_143268837_127	novel	GSTK1	7	+	381	224	26	1	25.98%	194.2641653	1.22E-05	2.41E-07	3.02E-11
ENSG00000163389_119490441_119492438_163	novel	POGLUT1	3	+	489	18	1	0	5.52%	12.16279256	0.006965909	0.003478238	0.00013255
ENSG00000113048_72226107_72220909_152	novel	MRPS27	5	-	456	54	3	1	8.55%	39.12898395	0.001014533	0.000725368	4.63E-06
ENSG00000136444_50478885_50483426_195	annotated	RSAD1	17	+	585	10	2	3	2.56%	5.648248395	0.101051022	0.121807694	0.042132337
ENSG00000186153_78099779_79211796_234	annotated	WWOX	16	+	702	33	0	0	3.42%	15.53268309	0.005604867	0.005604867	0.000167525
ENSG00000166788_18106041_18083713_353	annotated	SAAL1	11	-	1059	63	2	0	7.08%	19.65686446	1.79E-05	4.96E-06	7.39E-10
ENSG00000087365_66053093_66055360_98	novel	SF3B2	11	+	294	14	2	0	6.12%	15.73440624	0.052581193	0.01300724	0.003266469
ENSG00000110330_102350683_102378183_342	annotated	BIRC2	11	+	1026	38	17	1	4.39%	12.23787152	0.023639287	0.000408384	8.18E-05
ENSG00000152422_83104920_83353242_334	annotated	XRCC4	5	+	1002	41	4	0	5.09%	13.52028321	0.001872581	0.000118541	1.67E-06
ENSG00000183891_19901793_19896987_75	Overlap_dORF	TTC32	2	-	225	24	1	0	10.67%	35.24506999	0.006152341	0.005554612	0.000181508
ENSG00000162521_32651307_32680546_410	annotated	RBBP4	1	+	1230	83	5	1	4.39%	22.29680495	0.000306494	0.000102635	2.24E-07
ENSG00000162522_32765770_32772887_1035	annotated	KIAA1522	1	+	3105	42	3	2	2.13%	4.469483513	0.000100256	3.83E-05	2.88E-08
ENSG00000162522_32754090_32772887_1046	annotated	KIAA1522	1	+	3138	42	3	2	2.10%	4.422481296	0.000100256	3.83E-05	2.88E-08
ENSG00000143398_151231761_151247865_404	annotated	PIP5K1A	1	+	1212	36	0	0	3.71%	9.814530627	0.000259634	0.000259634	4.60E-07
ENSG00000131828_19344047_19359653_394	annotated	PDHA1	X	+	1182	132	12	8	9.39%	36.89997809	1.50E-06	3.89E-07	5.36E-12
ENSG00000175550_65919502_65921435_212	annotated	DRAP1	11	+	636	187	9	26	20.28%	97.15253666	1.86E-08	4.72E-06	1.08E-12

ENSG00000156482_98042750_98041801_51	novel	RPL30	8	-	153	1606	28	5	17.65%	3468.356765	0.010303292	0.010183176	0.000524231
ENSG00000100567_58244921_58257751_36	annotated	PSMA3	14	+	108	21	13	0	19.44%	64.24882549	0.247934092	0.008776163	0.015352621
ENSG00000036257_224557850_224474245_744	annotated	CUL3	2	-	2232	63	7	7	3.76%	9.326442411	6.48E-05	2.71E-05	1.34E-08
ENSG00000152133_37089665_37096263_235	annotated	GPATCH11	2	+	705	20	4	0	5.53%	9.373688826	0.017317935	0.000399464	5.56E-05
ENSG00000140854_57741815_57756946_599	annotated	KATNB1	16	+	1797	46	3	5	3.34%	8.458228398	5.06E-05	0.000734458	2.89E-07
ENSG00000156973_231781114_231733017_93	annotated	PDE6D	2	-	279	39	3	0	13.98%	46.18809575	0.000881412	0.000700469	3.91E-06
ENSG00000035403_73998283_74118169_1041	annotated	VCL	10	+	3123	192	17	5	7.40%	20.31416138	3.49E-12	2.68E-14	1.37E-24
ENSG00000243147_27771778_27779556_54	annotated	MRPL33	2	+	162	65	12	3	22.22%	132.5769415	0.008917827	0.003487661	0.000169879
ENSG00000084073_40258308_40292669_463	annotated	ZMPSTE24	1	+	1389	351	30	5	15.33%	83.49770225	8.79E-11	5.18E-13	6.44E-22
ENSG00000107263_131643315_131579497_955	annotated	RAPGEF1	9	-	2865	19	0	2	0.84%	2.191283801	0.005306578	0.012767383	0.000354714
ENSG00000100416_46335765_46337948_83	novel	TRMU	22	+	249	12	2	0	8.43%	15.9239774	0.023672501	0.007882058	0.000936943
ENSG00000133027_17512623_17505791_119	novel	PEMT	17	-	357	65	2	0	11.76%	60.16096505	0.000544626	0.000480911	1.71E-06
ENSG00000159228_36070116_36071197_178	annotated	CBR1	21	+	534	288	16	6	17.98%	178.20541	6.28E-07	3.76E-07	2.17E-12
ENSG00000111707_118376692_118414433_328	annotated	SUDS3	12	+	984	12	3	2	2.13%	4.029543062	0.051745348	0.037441831	0.007959536
ENSG00000166888_57108251_57096572_838	annotated	STAT6	12	-	2514	71	7	1	3.46%	9.331742128	1.92E-05	8.59E-07	1.55E-10
ENSG00000140092_91946712_91870224_443	annotated	FBLN5	14	-	1329	16	2	0	1.81%	3.97799887	0.017404239	0.004873904	0.000450042
ENSG00000147789_144829088_144843168_697	annotated	ZNF7	8	+	2091	25	1	2	1.87%	3.950532414	0.001044243	0.001969684	1.25E-05
ENSG00000132604_69385430_69368530_156	novel	TERF2	16	-	468	39	0	0	7.05%	27.53521093	0.001558047	0.001558047	1.45E-05
ENSG00000198931_88809835_88809698_45	Overlap_dORF	APRT	16	-	135	178	18	1	35.56%	435.6682262	0.000214559	0.000215995	3.20E-07
ENSG00000166340_6619400_6614546_500	annotated	TPP1	11	-	1500	253	46	10	12.20%	55.73126691	2.94E-08	5.47E-10	1.91E-16
ENSG00000147044_41745600_41520420_827	annotated	CASK	X	-	2481	36	8	0	2.06%	4.794522822	0.003393093	0.000110146	3.00E-06
ENSG00000255439_31091310_31091134_58	annotated	AC135050.2	16	-	174	204	55	19	29.31%	387.391933	0.000644384	0.001844079	7.49E-06
ENSG00000284770_235380050_235448762_549	annotated	TBCE	1	+	1647	32	20	5	2.55%	6.419867028	0.050231923	0.004562756	0.001327246
ENSG00000127955_80210970_80217493_157	Overlap_dORF	GNAI1	7	+	471	44	3	3	10.19%	30.8674976	0.000188972	0.000279811	3.64E-07
ENSG00000178537_48879393_48857710_174	dORF	SLC25A20	3	-	522	14	1	0	5.17%	8.861906965	0.006024334	0.003229477	0.000107305
ENSG00000159720_67481086_67445920_115	annotated	ATP6V0D1	16	-	345	90	11	1	14.78%	86.19718203	0.000876727	0.000140303	8.71E-07
ENSG00000130283_18896072_18870577_350	uORF	GDF1	19	-	1050	19	3	0	2.00%	5.979074373	0.016947427	0.008473674	0.000715155
ENSG00000092621_119712023_119744040_533	annotated	PHGDH	1	+	1599	62	8	1	4.13%	12.81188051	0.000414271	2.27E-05	7.69E-08
ENSG00000164073_127965133_127921759_298	annotated	MFSD8	4	-	894	18	0	1	3.36%	6.65280264	0.00224099	0.003372875	4.33E-05
ENSG00000211584_47779069_47780281_87	annotated	SLC48A1	12	+	261	38	11	1	12.64%	48.10749495	0.017505688	0.00236973	0.000243531
ENSG00000100227_42595584_42585791_140	Overlap_dORF	POLDIP3	22	-	420	13	5	0	4.29%	10.22736406	0.060322713	0.013428348	0.003875514
ENSG00000100227_42614857_42595571_281	annotated	POLDIP3	22	-	843	27	6	0	2.85%	10.58292804	0.099838396	0.005405142	0.003373305
ENSG00000165915_47410103_47412352_137	annotated	SLC39A13	11	+	411	25	4	3	6.57%	20.09869411	0.032506135	0.009341698	0.001500183
ENSG00000140538_88256153_87929182_705	annotated	NTRK3	15	-	2115	145	14	7	8.23%	22.65308133	3.99E-09	1.80E-10	8.47E-18
ENSG00000100889_24098504_24100019_274	novel	PCK2	14	+	822	14	4	0	3.28%	5.62763435	0.026731507	0.00308495	0.00047994
ENSG00000196663_102376722_102465304_1267	annotated	TECPR2	14	+	3801	15	0	0	0.63%	1.303956319	0.005160369	0.005160369	0.000143196
ENSG00000118515_134173142_134170975_218	novel	SGK1	6	-	654	17	2	0	3.21%	8.588964876	0.024135905	0.008674847	0.001038937
ENSG00000091140_107904990_107906285_75	dORF	DLD	7	+	225	20	0	2	12.00%	29.37089165	0.003478238	0.003327803	6.48E-05
ENSG00000091140_107891251_107904873_115	annotated	DLD	7	+	345	78	29	20	17.39%	74.70422443	0.015063832	0.009304408	0.000693571
ENSG00000100342_36257084_36266033_383	annotated	APOL1	22	+	1149	26	8	0	2.35%	7.476924116	0.036819135	0.003554776	0.000766908
ENSG00000100342_36257084_36266033_383	annotated	APOL1	22	+	1149	26	8	0	2.35%	7.476924116	0.036819135	0.003554776	0.000766908
ENSG00000099331_17102393_17211785_1797	annotated	MYO9B	19	+	5391	32	5	3	0.83%	1.961328324	0.002266245	0.001871465	2.48E-05
ENSG00000134072_9763146_9757810_192	dORF	CAMK1	3	-	576	5	2	0	2.60%	2.868251138	0.158655254	0.012673659	0.011061611
ENSG00000100836_23321470_23324299_296	annotated	PABPN1	14	+	888	63	10	1	6.42%	23.44213903	0.000962251	6.05E-05	4.52E-07
ENSG00000060339_68722505_68791266_1150	annotated	CCAR1	10	+	3450	82	11	3	2.78%	7.853521029	2.09E-05	7.66E-07	1.52E-10
ENSG00000149925_30069571_30070212_132	novel	ALDOA	16	+	396	579	108	36	33.33%	483.1177917	1.66E-08	4.90E-09	8.57E-16
ENSG00000140464_73998130_74035951_696	annotated	PML	15	+	2088	8	3	0	0.86%	1.265986709	0.093731016	0.009815329	0.004911899
ENSG00000090238_30095368_30092733_75	annotated	YPEL3	16	-	225	10	3	0	6.67%	14.68544583	0.034182656	0.020613417	0.003146197
ENSG00000111642_6587838_6578113_517	annotated	CHD4	12	-	1551	20	2	1	2.13%	4.260767648	0.003734664	0.002296044	4.89E-05
ENSG00000284946_91009831_90984800_475	annotated	AC068831.7	15	-	1425	40	2	4	2.74%	9.275018417	0.002390059	0.002636958	3.63E-05
ENSG00000284946_90984125_90969101_510	dORF	AC068831.7	15	-	1530	199	16	11	9.61%	42.97652529	1.04E-08	6.70E-09	7.31E-16
ENSG00000122481_95244258_95246870_223	novel	RWDD3	1	+	669	14	4	2	3.59%	6.914671802	0.08185299	0.029020025	0.010034774
ENSG00000169855_78714439_78598913_1311	annotated	ROBO1	3	-	3933	12	2	1	0.46%	1.008154176	0.050679946	0.019795882	0.004478852
ENSG00000136908_127936733_127935722_79	novel	DPM2	9	-	237	33	1	0	11.39%	46.00820054	0.003303074	0.003554776	6.57E-05
ENSG00000203666_245082137_245087360_83	novel	EFCAB2	1	+	249	15	2	0	6.02%	19.90497175	0.044329616	0.021557223	0.004218623
ENSG00000104524_143609548_143605700_274	annotated	PYCR3	8	-	822	44	2	0	6.57%	17.68685081	7.13E-05	7.21E-05	3.78E-08
ENSG00000141959_44323829_44325405_189	novel	PFKL	21	+	567	28	5	0	5.29%	16.31716203	0.022431136	0.002201184	0.000299453
ENSG00000125505_54183527_54180703_145	annotated	MBOAT7	19	-	435	27	8	0	8.97%	20.50898469	0.017142865	0.000642411	8.06E-05
ENSG00000117054_75724788_75762763_425	annotated	ACADM	1	+	1275	67	11	2	6.35%	17.36338007	8.10E-06	3.80E-06	2.59E-10
ENSG00000184162_19203060_19201474_167	annotated	NR2C2AP	19	-	501	15	2	0	3.59%	9.892890153	0.032488891	0.013428348	0.002051384
ENSG00000115486_85559067_85558643_56	annotated	GGCX	2	-	168	38	4	0	16.07%	74.73842966	0.003710481	0.003789641	7.80E-05
ENSG00000181744_143972333_143989841_430	annotated	DIPK2A	3	+	1290	33	1	1	3.95%	8.452669401	0.000186516	0.000286582	3.68E-07
ENSG00000131143_85804978_85805115_45	novel	COX4I1	16	+	135	203	18	9	28.89%	496.8575838	0.000825949	0.000823751	4.28E-06
ENSG00000103353_23557952_23559787_181	annotated	UBFD1	16	+	543	43	4	8	7.18%	26.16605679	0.001015857	0.027393145	0.000200166
ENSG00000172270_572635_582577_269	annotated	BSG	19	+	807	4604	409	114	47.58%	1885.087154	5.70E-21	2.70E-21	2.60E-40
ENSG00000148019_78236351_78266762_706	annotated	CEP78	9	+	2118	50	5	0	3.12%	7.800343039	0.00020727	1.57E-05	2.66E-08
ENSG00000117020_243843170_243505249_479	annotated	AKT3	1	-	1437	30	21	13	2.92%	6.898173927	0.100045546	0.035925772	0.014671657
ENSG00000080503_2161713_2192739_254	annotated	SMARCA2	9	+	762	14	7	2	2.76%	6.070755165	0.106840601	0.037441831	0.016226997

ENSG00000035687_244451817_244409586_456	annotated	ADSS	1	-	1368	102	10	13	8.55%	24.63676767	4.40E-07	3.50E-07	1.43E-12
ENSG00000157557_38810059_38822889_461	annotated	ETS2	21	+	1383	172	21	6	11.50%	41.09376381	2.80E-08	1.55E-09	4.85E-16
ENSG00000176946_241637017_241584606_577	annotated	THAP4	2	-	1731	30	4	0	1.91%	5.726560331	0.008742337	0.001506153	7.91E-05
ENSG00000137100_34620464_34613782_186	annotated	DCTN3	9	-	558	177	19	1	20.97%	104.811448	3.67E-06	2.19E-08	9.49E-13
ENSG00000097007_130835447_130885683_1130	annotated	ABL1	9	+	3390	36	15	4	1.77%	3.508911835	0.011325228	0.00073408	5.65E-05
ENSG00000177106_709408_726981_715	annotated	EPS8L2	11	+	2145	102	25	3	5.17%	15.71240008	1.27E-05	5.54E-08	8.44E-12
ENSG00000122406_92832115_92841865_297	annotated	RPL5	1	+	891	184	18	14	7.74%	68.23540485	5.49E-05	1.52E-05	6.57E-09
ENSG00000106346_6111224_6159457_1286	annotated	USP42	7	+	3858	15	2	0	0.78%	1.284691023	0.012926125	0.002008257	0.000153109
ENSG00000140332_70094543_70050097_698	annotated	TLE3	15	-	2094	21	0	2	1.29%	3.313693005	0.003303074	0.014936572	0.000273899
ENSG00000151327_35053278_35054670_61	Overlap_dORF	FAM177A1	14	+	183	6	0	0	8.20%	10.83352561	0.016947427	0.016947427	0.001349898
ENSG00000198015_93469286_93501221_142	annotated	MRPL42	12	+	426	126	32	4	19.72%	97.73060779	0.000197807	3.59E-06	6.77E-09
ENSG00000125954_64914496_65061312_471	annotated	CHURC1-FNTB	14	+	1413	110	116	3	6.16%	25.72291467	0.000110342	1.11E-06	1.28E-09
ENSG00000088986_120496422_120498210_89	annotated	DYNLL1	12	+	267	499	159	25	29.21%	617.5312473	3.54E-05	1.85E-05	5.12E-09
ENSG00000176101_137188727_137189914_119	annotated	SSNA1	9	+	357	120	2	3	21.01%	111.066397	5.85E-06	1.36E-05	6.56E-10
ENSG00000278588_26272976_26273356_126	annotated	HIST1H2BI	6	+	378	873	81	25	23.81%	763.1187028	1.29E-05	1.45E-06	1.67E-10
ENSG00000132436_50447287_50445263_674	annotated	FIGNL1	7	-	2022	21	4	3	1.93%	3.431688009	0.006278716	0.005744972	0.000191041
ENSG00000218739_37199705_37201724_80	annotated	CEBPZOS	2	+	240	86	5	0	18.75%	118.401407	0.001026691	0.000282029	1.94E-06
ENSG00000183495_131960803_131994979_924	annotated	EP400	12	+	2772	7	0	0	0.54%	0.834400331	0.019216965	0.019216965	0.001707396
ENSG00000153048_8859328_8855164_147	annotated	CARHSP1	16	-	441	111	10	3	16.33%	83.16757586	1.54E-05	2.35E-05	2.86E-09
ENSG00000109685_1900838_1939787_568	annotated	NSD2	4	+	1704	24	2	0	1.94%	4.653838466	0.003199288	0.001535678	2.89E-05
ENSG00000175471_94779151_94705135_142	novel	MCTP1	5	-	426	9	0	0	6.34%	6.9807577	0.001349898	0.001349898	1.10E-05
ENSG00000044115_138827710_138887696_98	novel	CTNNA1	5	+	294	20	0	1	9.18%	22.47772321	0.002967235	0.006432154	0.000105925
ENSG00000120896_22566421_22574728_329	novel	SORBS3	8	+	987	45	7	5	5.17%	15.06485704	0.003014023	0.001036014	1.90E-05
ENSG00000120915_27491209_27544522_372	annotated	EPHX2	8	+	1116	6	0	0	1.34%	1.776465221	0.016947427	0.016947427	0.001349898
ENSG00000204463_31651763_31639131_1077	annotated	BAG6	6	-	3231	109	18	3	4.18%	11.14703061	6.48E-07	3.92E-09	3.10E-14
ENSG00000082641_48051119_48059641_772	annotated	NFE2L1	17	+	2316	212	10	5	8.42%	30.24593117	5.48E-12	2.53E-12	1.74E-22
ENSG00000187555_8923294_8903362_466	annotated	USP7	16	-	1398	21	13	5	1.93%	4.963428579	0.072194705	0.058027894	0.01604164
ENSG00000140416_63042857_63071172_275	annotated	TPM1	15	+	825	426	61	50	23.27%	170.6181797	6.61E-09	2.63E-10	2.04E-17
ENSG00000115993_201395418_201380543_649	annotated	TRAK2	2	-	1947	10	4	3	1.23%	1.697085419	0.109006977	0.049229251	0.020703214
ENSG00000105438_48391358_48389535_122	novel	KDELR1	19	-	366	1619	107	70	36.89%	1461.623164	4.02E-08	2.92E-06	1.29E-12
ENSG00000140632_4832860_4805236_467	annotated	GLYR1	16	-	1401	22	10	6	2.36%	5.188647883	0.097568022	0.037352967	0.014765184
ENSG00000182240_41241894_41246102_109	novel	BACE2	21	+	327	16	2	0	7.34%	16.1674633	0.017901377	0.005454749	0.000511125
ENSG00000113851_3174246_3156201_192	novel	CRBN	3	-	576	25	2	0	5.21%	14.34125569	0.002362849	0.002362849	3.23E-05
ENSG00000072364_132934881_132904170_304	novel	AFF4	5	-	912	8	1	0	1.64%	2.898443255	0.042234516	0.019679754	0.003715148
ENSG00000112200_57090254_57154103_1041	annotated	ZNF451	6	+	3123	12	10	0	0.67%	1.269635086	0.376341196	0.007882058	0.026803259
ENSG00000180879_153794688_153795715_39	novel	SSR4	X	+	117	34	1	12	15.38%	96.02022272	0.016822681	0.087374356	0.006913616
ENSG00000153944_57257519_57677028_294	annotated	MSI2	17	+	882	12	5	2	2.04%	4.495544641	0.160188655	0.052581193	0.03227348
ENSG00000171793_40983291_41010245_591	annotated	CTPS1	1	+	1773	40	20	4	3.21%	7.454541029	0.052641935	0.00047468	0.000248456
ENSG00000065427_75644428_75627895_625	annotated	KARS	16	-	1875	215	26	2	12.16%	37.88845023	1.94E-10	9.22E-15	3.67E-23
ENSG00000067182_6341955_6341824_43	uORF	TNFRSF1A	12	-	129	59	7	7	23.26%	151.1234832	0.022350948	0.03211189	0.003185315
ENSG00000067182_6333387_6333097_23	internal	TNFRSF1A	12	-	69	29	1	38	34.78%	138.8732377	0.006307834	0.343734887	0.020265453
ENSG00000067182_6341814_6329312_412	annotated	TNFRSF1A	12	-	1236	694	172	49	24.76%	185.5285086	4.46E-10	2.24E-16	3.45E-24
ENSG00000116062_47783234_47806860_1230	annotated	MSH6	2	+	3690	134	7	7	3.50%	11.99908379	5.82E-08	5.11E-08	2.94E-14
ENSG00000160613_117229739_117206757_556	annotated	PCSK7	11	-	1668	13	2	1	1.26%	2.575235554	0.022898969	0.0135992	0.001469596
ENSG00000149084_43680828_43855248_312	annotated	HSD17B12	11	+	936	366	70	12	20.51%	129.203682	3.08E-09	4.07E-11	1.59E-18
ENSG00000087274_2875916_2928171_632	annotated	ADD1	4	+	1896	57	5	0	4.11%	9.933588752	6.66E-06	2.92E-06	1.65E-10
ENSG00000109674_177335716_177353355_122	Overlap_dORF	NEIL3	4	+	366	15	4	0	5.74%	13.54190701	0.051289318	0.008473674	0.002235953
ENSG00000145782_115841693_115841382_103	annotated	ATG12	5	-	309	15	0	0	4.85%	16.03992869	0.020613417	0.020613417	0.001946209
ENSG00000141425_36067404_35993151_312	annotated	RPRD1A	18	-	936	91	17	4	4.17%	32.12441275	0.050303262	0.002824594	0.000910676
ENSG00000196535_29166541_29074770_1905	annotated	MYO18A	17	-	5715	51	9	2	1.47%	2.948652509	7.34E-05	6.55E-06	4.05E-09
ENSG00000162174_62357038_62392284_180	dORF	ASRGL1	11	+	540	15	0	0	4.44%	9.178403642	0.004826937	0.004826937	0.000126125
ENSG00000188243_75530239_75526589_58	novel	COMMD6	13	-	174	151	36	2	20.69%	286.7459896	0.003496343	0.001082917	2.30E-05
ENSG00000136527_185922105_185917715_107	novel	TRA2B	3	-	321	110	25	0	19.63%	113.2289047	0.004587604	2.74E-05	1.34E-06
ENSG00000138430_174246815_174075426_396	annotated	OLA1	2	-	1188	81	10	0	6.06%	22.52880894	3.23E-05	8.20E-06	2.15E-09
ENSG00000159423_18902523_18872845_563	annotated	ALDH4A1	1	-	1689	31	0	0	3.20%	6.064593526	5.73E-05	5.73E-05	2.44E-08
ENSG00000072501_53415155_53380103_1192	annotated	SMC1A	X	-	3576	41	5	1	2.27%	3.788401503	4.04E-05	2.58E-06	9.25E-10
ENSG00000148110_94440998_94459412_370	annotated	MFSD14B	9	+	1110	143	13	4	12.70%	42.5679477	1.23E-08	2.76E-09	3.64E-16
ENSG00000204348_31971675_31969877_396	annotated	DXO	6	-	1188	10	2	1	1.52%	2.781334437	0.080264768	0.028889786	0.009799991
ENSG00000074054_121605883_121340861_1473	annotated	CLASP1	2	-	4419	41	0	2	1.02%	3.065698976	0.000299491	0.000387696	7.79E-07
ENSG00000065809_14542671_14530438_56	Overlap_uORF	FAM107B	10	-	168	34	0	11	8.93%	66.87122654	0.021084099	0.254711579	0.0285044
ENSG00000065809_14553383_14542668_26	uORF	FAM107B	10	-	78	13	3	0	19.23%	55.07042185	0.138151459	0.019679754	0.01298269
ENSG00000122786_134941200_134947793_107	novel	CALD1	7	+	321	16	0	2	4.67%	16.46965887	0.021557223	0.028889786	0.002787067
ENSG00000174437_110343359_110345091_93	novel	ATP2A2	12	+	279	45	3	3	11.83%	53.29395663	0.001565547	0.008853033	8.29E-05
ENSG00000129422_17715785_17645926_415	annotated	MTUS1	8	-	1245	63	5	0	6.02%	16.72017627	6.92E-05	4.27E-06	2.57E-09
ENSG00000116815_116570972_116514813_250	annotated	CD58	1	-	750	49	4	3	7.20%	21.58760537	0.000262229	0.000475015	8.37E-07
ENSG00000132286_6481517_6482221_71	annotated	TIMM10B	11	+	213	7	1	0	7.04%	10.85895642	0.041782825	0.019216965	0.003600526
ENSG00000078124_76860977_76976299_74	annotated	ACER3	11	+	222	15	0	3	8.11%	22.3258467	0.013428348	0.063412829	0.004086769
ENSG00000078124_76976340_77020327_161	dORF	ACER3	11	+	483	24	0	0	4.97%	16.41851086	0.005355751	0.005355751	0.00015367

ENSG00000146416_143060926_143335091_245	annotated	AIG1	6	+	735	91	6	3	14.29%	40.90945623	2.47E-06	4.76E-07	1.08E-11
ENSG00000257671_52245587_52245342_81	novel	KRT7-AS	12	-	243	250	85	114	30.86%	339.9408756	0.00071961	0.008308056	3.96E-05
ENSG00000165661_136245803_136208728_698	annotated	QSOX2	9	-	2094	192	21	4	8.88%	30.29662176	1.59E-09	9.25E-12	1.99E-19
ENSG00000197837_14771084_14770773_103	annotated	HIST4H4	12	-	309	1063	86	16	33.01%	1136.69628	3.98E-06	2.12E-07	8.22E-12
ENSG00000137868_74195675_74180080_476	novel	STRA6	15	-	1428	160	28	5	10.29%	37.02213234	2.20E-06	2.05E-09	6.57E-14
ENSG00000110090_68815474_68757644_773	annotated	CPT1A	11	-	2319	228	50	6	10.35%	32.48656192	5.58E-09	6.80E-15	1.23E-21
ENSG00000119242_123943476_123937058_314	annotated	CCDC92	12	-	942	13	8	0	1.91%	4.559971236	0.128949518	0.012798403	0.00869264
ENSG00000127311_66310318_66338102_624	annotated	HELB	12	+	1872	17	0	0	1.12%	3.00063196	0.008175611	0.008175611	0.000342518
ENSG00000162378_52726681_52805476_557	annotated	ZYG11B	1	+	1671	21	1	0	1.62%	4.152527321	0.006083074	0.003278443	0.000109846
ENSG00000131725_118346504_118448987_913	annotated	WDR44	X	+	2739	27	2	3	1.42%	3.257177196	0.001318835	0.00465809	3.67E-05
ENSG00000168137_9434119_9464744_912	dORF	SETD5	3	+	2736	21	2	3	0.99%	2.536137848	0.009288069	0.021136549	0.000966013
ENSG00000153310_129879461_129842142_324	annotated	FAM49B	8	-	972	85	7	0	7.72%	28.89497443	1.46E-05	5.30E-06	6.40E-10
ENSG00000204463_31641136_31639131_195	annotated	BAG6	6	-	585	36	5	0	7.69%	20.33369422	0.00084938	0.000299491	1.69E-06
ENSG00000136856_127397231_127407197_411	annotated	SLC2A8	9	+	1233	17	0	0	1.46%	4.555703998	0.012798403	0.012798403	0.000797244
ENSG00000124767_38686953_38677295_149	annotated	GLO1	6	-	447	169	15	1	20.81%	124.9248496	1.32E-06	5.74E-07	6.86E-12
ENSG00000183696_48094803_48108357_173	Overlap_dORF	UPP1	7	+	519	26	0	2	6.94%	16.55295917	0.000978918	0.003975543	2.39E-05
ENSG00000183696_48094784_48108357_310	annotated	UPP1	7	+	930	45	2	0	6.45%	15.98818699	0.00013812	3.72E-05	3.87E-08
ENSG00000134283_42335903_42446618_458	annotated	PPHLN1	12	+	1374	93	21	3	5.68%	22.36484381	0.000954468	8.45E-06	8.15E-08
ENSG00000146109_26596983_26598645_272	annotated	ABT1	6	+	816	43	12	3	6.25%	17.41197162	0.011261142	0.00068298	5.28E-05
ENSG00000161021_179765689_179774877_790	annotated	MAML1	5	+	2370	35	4	0	1.52%	4.879657633	0.002287528	0.001011481	1.41E-05
ENSG00000136238_6391949_6402446_148	novel	RAC1	7	+	444	77	20	1	8.78%	57.30300652	0.003404802	0.000733329	1.57E-05
ENSG00000130558_135090294_135120178_402	annotated	OLFM1	9	+	1206	6	0	0	1.24%	1.64389319	0.016947427	0.016947427	0.001349898
ENSG00000128585_131466324_131471034_94	novel	MKLN1	7	+	282	24	2	0	6.38%	28.12106648	0.01300724	0.013640586	0.000859463
ENSG00000163482_218675415_218702009_1190	annotated	STK36	2	+	3570	9	5	0	0.50%	0.832997978	0.14260114	0.012178958	0.009444992
ENSG00000163481_218671970_218663957_459	annotated	RNF25	2	-	1377	24	5	0	2.61%	5.758998364	0.01202368	0.000950974	7.50E-05
ENSG00000144580_218568955_218593573_258	annotated	CNOT9	2	+	774	36	5	4	5.04%	15.36848982	0.005538404	0.002688791	8.35E-05
ENSG00000163479_156020944_156009540_202	annotated	SSR2	1	-	606	1883	80	41	35.64%	1026.708954	1.65E-12	1.30E-13	2.86E-24
ENSG00000126088_45012992_45013706_100	annotated	UROD	1	+	300	15	4	9	7.00%	16.52112656	0.060419327	0.154611771	0.0346904
ENSG00000134333_18396843_18400848_45	annotated	LDHA	11	+	135	41	10	0	20.00%	100.3505465	0.028682169	0.003710481	0.000603887
ENSG00000117054_75724788_75733166_133	novel	ACADM	1	+	399	14	1	0	3.76%	11.59377302	0.065398531	0.019679754	0.005779267
ENSG00000166452_8911450_8919480_210	annotated	AKIP1	11	+	630	43	5	4	8.10%	22.55264895	0.002240233	0.000581804	8.31E-06
ENSG00000164733_11853372_11852665_24	annotated	CTSB	8	-	72	32	2	43	37.50%	146.8544583	0.003710481	0.468493666	0.025643776
ENSG00000103197_2050439_2088610_1724	annotated	TSC2	16	+	5172	65	6	1	1.51%	4.152642019	7.80E-06	3.46E-06	2.27E-10
ENSG00000124588_3006553_3019655_231	annotated	NQO2	6	+	693	87	8	2	13.42%	41.48161646	9.01E-06	6.11E-07	5.13E-11
ENSG00000124588_3006553_3019655_193	annotated	NQO2	6	+	579	68	8	2	12.95%	38.80610037	9.18E-05	6.67E-06	5.20E-09
ENSG00000102103_48898510_48903084_265	annotated	PQBP1	X	+	795	45	11	9	6.42%	18.70316214	0.020227839	0.003888751	0.000432783
ENSG00000166377_79330063_79359528_363	novel	ATP9B	18	+	1089	49	0	1	4.68%	14.86749681	0.00013293	0.000172339	1.62E-07
ENSG00000066933_72046563_71892923_1779	novel	MYO9A	15	-	5337	15	0	7	0.39%	0.928674905	0.008473674	0.151972721	0.007859489
ENSG00000125386_2662974_2731868_888	annotated	FAM193A	4	+	2664	11	0	0	0.79%	1.364357298	0.007593099	0.007593099	0.000297833
ENSG00000140691_31459302_31466889_967	annotated	ARMC5	16	+	2901	23	5	0	1.34%	2.619689147	0.010842453	0.000606963	4.59E-05
ENSG00000080503_2104121_2192739_509	novel	SMARCA2	9	+	1527	25	8	2	2.55%	5.409668158	0.014976028	0.002809357	0.00023874
ENSG00000141562_82464356_82488157_397	annotated	NARF	17	+	1191	52	1	1	6.05%	14.4265085	1.01E-05	1.01E-05	8.35E-10
ENSG00000144535_232015621_232330699_566	annotated	DIS3L2	2	+	1698	12	1	1	0.88%	2.335141563	0.035374934	0.035374934	0.005300298
ENSG00000130402_38647746_38729432_692	annotated	ACTN4	19	+	2076	648	102	37	19.08%	103.1376687	1.32E-16	7.52E-21	2.12E-35
ENSG00000120948_11013878_11023234_219	annotated	TARDBP	1	+	657	39	1	0	8.22%	19.61412285	0.000123157	7.81E-05	6.98E-08
ENSG00000149925_30067255_30070116_361	annotated	ALDOA	16	+	1083	2009	187	75	36.01%	612.9444737	7.19E-21	4.11E-22	5.14E-41
ENSG00000167967_2232158_2233273_247	novel	E4F1	16	+	741	13	2	1	2.02%	5.796886511	0.060322713	0.035374934	0.008767878
ENSG00000196562_47757363_47658362_870	annotated	SULF2	20	-	2610	106	21	7	5.29%	13.41945912	3.84E-06	4.67E-08	1.97E-12
ENSG00000178035_49025035_49024491_123	novel	IMPDH2	3	-	369	30	1	0	12.20%	26.86362042	0.000273206	0.000281287	5.22E-07
ENSG00000115216_27433274_27441812_543	annotated	NRBP1	2	+	1629	252	18	8	11.23%	51.11508769	7.07E-10	3.78E-11	3.24E-19
ENSG00000113638_40747018_40569404_132	annotated	TTC33	5	-	396	12	0	0	3.79%	10.01280397	0.021084099	0.021084099	0.002029891
ENSG00000261832_28477608_28477516_30	dORF	AC138894.1	16	-	90	23	5	1	23.33%	84.44131351	0.027657845	0.010042643	0.001354611
ENSG00000261832_28491764_28489349_55	Overlap_uORF	AC138894.1	16	-	165	18	0	12	14.55%	36.0460943	0.005554612	0.310595281	0.015978689
ENSG00000261832_28489349_28479730_309	annotated	AC138894.1	16	-	927	134	15	4	11.00%	47.76334322	2.21E-06	1.46E-07	3.16E-12
ENSG00000107201_32489380_32457122_671	annotated	DDX58	9	-	2013	12	0	7	1.19%	1.969731929	0.004873904	0.051745348	0.001446393
ENSG00000134852_55478869_55435415_779	annotated	CLOCK	4	-	2337	12	7	0	0.77%	1.69664971	0.157414378	0.011772051	0.010389467
ENSG00000134013_23368351_23298043_774	annotated	LOXL2	8	-	2322	273	22	16	12.02%	38.84812704	2.84E-14	8.14E-15	3.21E-27
ENSG00000205629_25140216_25178023_155	dORF	LCMT1	16	+	465	19	4	0	5.16%	13.50113568	0.026696365	0.005554612	0.00078491
ENSG00000103042_58678891_58667385_371	annotated	SLC38A7	16	-	1113	41	9	3	4.04%	12.17189917	0.001436042	0.001864451	1.60E-05
ENSG00000161999_684319_682794_264	annotated	JMJD8	16	-	792	55	4	4	9.09%	22.94600911	5.75E-05	6.51E-05	2.78E-08
ENSG00000110931_121274382_121240797_493	annotated	CAMKK2	12	-	1479	26	3	4	2.43%	5.808644901	0.003818382	0.008533454	0.000176458
ENSG00000074582_218660988_218663386_419	annotated	BCS1L	2	+	1257	71	5	2	5.49%	18.66348426	0.000108734	3.17E-05	2.62E-08
ENSG00000095539_100973571_100984131_744	annotated	SEMA4G	10	+	2232	9	2	0	0.81%	1.332348916	0.041632258	0.012178958	0.002425608
ENSG00000124783_7313120_7289864_286	annotated	SSR1	6	-	858	819	105	18	29.72%	315.4033252	2.03E-12	1.75E-15	5.94E-26
ENSG00000124783_7313197_7313117_26	Overlap_uORF	SSR1	6	-	78	34	34	4	34.62%	144.0303341	0.34125222	0.018024648	0.038230595
ENSG00000197822_69504245_69553671_522	annotated	OCLN	5	+	1566	34	4	4	2.49%	7.173924686	0.003908676	0.004756202	0.000101851
ENSG00000197226_179907821_179863448_1233	annotated	TBC1D9B	5	-	3699	89	6	3	3.41%	7.950150113	2.89E-08	6.07E-09	1.84E-15
ENSG00000135316_85641439_85612882_562	annotated	SYNCRIP	6	-	1686	132	8	6	5.87%	25.86937966	2.58E-06	4.02E-06	8.88E-11

ENSG00000082898_61488236_61485744_96	novel	XPO1	2	-	288	19	1	1	9.38%	21.79870865	0.002967235	0.006112544	0.000100709
ENSG00000168397_241653550_241666738_109	novel	ATG4B	2	+	327	18	5	0	6.42%	18.18839621	0.060612546	0.008674847	0.002733668
ENSG00000168397_241637715_241653359_78	novel	ATG4B	2	+	234	12	5	0	6.41%	16.94474519	0.121807694	0.019679754	0.01126199
ENSG00000120756_142664238_142712007_629	annotated	PLS1	3	+	1887	76	16	5	5.56%	13.30795568	2.24E-05	4.37E-07	9.87E-11
ENSG00000137845_58749534_58655345_218	novel	ADAM10	15	-	654	70	13	0	6.88%	35.36632596	0.003731987	0.000293334	7.71E-06
ENSG00000113389_32712236_32786345_387	annotated	NPR3	5	+	1161	44	3	2	4.13%	12.52247319	0.000676283	0.000730067	3.15E-06
ENSG00000137845_58749534_58597547_748	annotated	ADAM10	15	-	2244	328	62	11	11.63%	48.29705446	4.10E-11	1.80E-15	1.53E-24
ENSG00000137842_43133863_43184586_623	annotated	TMEM62	15	+	1869	28	0	1	2.41%	4.950150279	0.00027248	0.000565108	1.03E-06
ENSG00000070950_8963385_8941667_126	annotated	RAD18	3	-	378	21	2	0	4.76%	18.35680728	0.032488891	0.013428348	0.002051384
ENSG00000160345_135499851_135495427_136	annotated	C9orf116	9	-	408	18	3	0	7.35%	14.57746461	0.010645878	0.002201184	0.000135265
ENSG00000153827_229880079_229807609_822	novel	TRIP12	2	-	2466	17	7	0	0.97%	2.277851999	0.0872261	0.005306578	0.002828175
ENSG00000125691_38853710_38850132_140	annotated	RPL23	17	-	420	538	103	31	22.86%	423.255528	0.000137288	2.07E-06	2.80E-09
ENSG00000106992_127871924_127868008_120	novel	AK1	9	-	360	78	11	2	18.33%	71.59154841	0.000230709	4.01E-05	7.01E-08
ENSG00000174891_158122105_158544275_276	annotated	RSRC1	3	+	828	103	23	5	2.90%	41.10328588	0.061782492	0.02600941	0.00689238
ENSG00000153214_112060007_112116143_490	annotated	TMEM87B	2	+	1470	92	11	3	8.57%	20.67950535	6.21E-07	2.73E-08	1.77E-13
ENSG00000183735_64455871_64501381_729	annotated	TBK1	12	+	2187	37	5	1	2.47%	5.590138844	0.001343467	0.000109763	1.09E-06
ENSG00000011376_45394454_45547530_903	annotated	LARS2	3	+	2709	27	4	0	1.55%	3.293247818	0.004300893	0.00040306	1.18E-05
ENSG00000271723_54716624_54741513_307	dORF	MROH7-TTC4	1	+	921	36	0	0	3.91%	12.91553868	0.001032175	0.001032175	6.59E-06
ENSG00000146112_30686018_30677269_613	annotated	PPP1R18	6	-	1839	36	8	7	2.28%	6.468304035	0.00697285	0.005048657	0.000187164
ENSG00000108788_42569599_42571603_88	novel	MLX	17	+	264	29	5	0	10.23%	36.2964144	0.030106073	0.003684287	0.000632811
ENSG00000108788_42567125_42569506_162	novel	MLX	17	+	486	28	2	2	3.09%	19.03668904	0.08410207	0.08410207	0.025660709
ENSG00000132849_61763099_62114250_1516	novel	PATJ	1	+	4548	10	3	0	0.53%	0.726522716	0.041632258	0.004149608	0.000996552
ENSG00000169139_48043161_48060828_97	annotated	UBE2V2	8	+	291	78	36	12	18.56%	88.56686401	0.009975161	0.000895519	5.81E-05
ENSG00000143106_109426330_109413029_109	novel	PSMA5	1	-	327	63	12	11	13.76%	63.65938673	0.005316915	0.004872347	0.000139525
ENSG00000179588_88533594_88534979_461	annotated	ZFPM1	16	+	1383	31	4	2	1.95%	7.406434175	0.005588371	0.003554776	0.000108946
ENSG00000178425_116100931_116244024_455	annotated	NT5DC1	6	+	1365	60	7	5	3.96%	14.5240673	0.000188335	9.55E-05	1.29E-07
ENSG00000185630_164559823_164821583_347	annotated	PBX1	1	+	1041	77	25	1	9.22%	24.4404754	9.71E-05	4.01E-07	4.54E-10
ENSG00000148737_112950757_113165972_579	annotated	TCF7L2	10	+	1737	32	6	0	2.76%	6.087231431	0.004472475	0.000175784	6.05E-06
ENSG00000149547_125572528_125583683_340	annotated	EI24	11	+	1020	72	0	0	5.00%	23.32394337	0.000130272	0.000130272	1.21E-07
ENSG00000103512_14864588_14896111_801	annotated	NOMO1	16	+	2403	30	13	0	1.37%	4.125125232	0.046975818	0.001558047	0.000529092
ENSG00000132463_70836185_70824319_318	annotated	GRSF1	4	-	954	100	11	5	9.12%	34.63548544	3.64E-06	1.43E-05	4.39E-10
ENSG00000164211_111507433_111500919_159	annotated	STARD4	5	-	477	56	7	6	10.06%	38.79174369	0.001563933	0.001879092	1.75E-05
ENSG00000134352_55976278_55941082_918	annotated	IL6ST	5	-	2754	180	23	8	4.90%	21.59624386	2.32E-07	6.69E-08	1.52E-13
ENSG00000187147_44636045_44650775_230	novel	RNF220	1	+	690	30	9	2	6.52%	14.36619701	0.003030417	0.000737506	1.40E-05
ENSG00000008294_51047454_51031639_104	novel	SPAG9	17	-	312	18	0	0	6.73%	19.06283833	0.008473674	0.008473674	0.000366502
ENSG00000132780_45614973_45615711_211	novel	NASP	1	+	633	31	11	2	4.27%	16.18183012	0.068935096	0.006089684	0.002387508
ENSG00000111371_46197943_46197657_61	novel	SLC38A1	12	-	183	22	6	0	9.84%	39.72292724	0.044329616	0.01300724	0.002735782
ENSG00000139546_53501409_53503133_94	annotated	TARBP2	12	+	282	16	3	4	5.32%	18.74737765	0.021084099	0.073562049	0.006910584
ENSG00000139546_53504407_53506148_222	dORF	TARBP2	12	+	666	15	1	3	3.60%	7.441948899	0.010020958	0.030667545	0.001501315
ENSG00000123353_55819048_55820395_137	annotated	ORMDL2	12	+	411	215	13	12	28.47%	172.8487693	2.15E-07	3.36E-08	7.24E-14
ENSG00000179913_17808006_17812122_306	annotated	B3GNT3	19	+	918	31	6	1	3.27%	11.15805933	0.026575195	0.003159663	0.000486445
ENSG00000106853_111586005_111564239_177	dORF	PTGR1	9	-	531	132	13	3	16.38%	82.13893429	6.24E-05	1.02E-06	6.36E-10
ENSG00000106853_111597422_111589370_73	annotated	PTGR1	9	-	219	69	5	8	26.03%	104.105729	6.15E-05	0.000385128	1.76E-07
ENSG00000076043_114446032_114449975_79	novel	REXO2	11	+	237	48	10	0	16.46%	66.92101896	0.010386675	0.000649205	4.63E-05
ENSG00000060982_24948932_24818008_349	annotated	BCAT1	12	-	1047	28	2	0	4.01%	8.836514681	0.002232673	0.000369309	5.49E-06
ENSG00000087088_48956199_48961097_140	annotated	BAX	19	+	420	376	24	5	24.29%	295.8068374	1.21E-06	1.78E-07	2.04E-12
ENSG00000196535_29090056_29074770_229	novel	MYO18A	17	-	687	11	0	0	2.18%	5.290608213	0.020613417	0.020613417	0.001946209
ENSG00000186111_3700390_3633167_640	annotated	PIP5K1C	19	-	1920	25	9	0	2.19%	4.302376707	0.040389295	0.000389561	0.000152689
ENSG00000122786_134891621_134968345_532	annotated	CALD1	7	+	1596	104	14	6	5.64%	21.53129275	8.52E-06	3.75E-06	2.68E-10
ENSG00000162105_70502230_70472869_1088	annotated	SHANK2	11	-	3264	7	3	0	0.46%	0.708626752	0.170087137	0.019216965	0.016246064
ENSG00000134291_47965995_47968349_94	annotated	TMEM106C	12	+	282	22	2	0	8.51%	25.77764427	0.005604867	0.005604867	0.000617525
ENSG00000131174_77902663_77905261_60	Overlap_dORF	COX7B	X	+	180	471	120	13	33.33%	864.6056231	0.005020709	8.70E-05	3.82E-06
ENSG00000023839_99782845_99796587_236	novel	ABCC2	10	+	708	109	16	11	11.86%	50.87013544	7.60E-05	5.86E-05	3.29E-08
ENSG00000147687_124516028_124488594_229	annotated	TATDN1	8	-	687	27	0	0	4.80%	12.98603834	0.001520872	0.001520872	1.39E-05
ENSG00000153339_31931518_31857685_872	annotated	TRAPPC8	18	-	2616	19	1	0	1.03%	2.399857833	0.006432154	0.002967235	0.000105925
ENSG00000204856_110468876_110490137_273	annotated	FAM216A	12	+	819	45	9	0	6.23%	18.15508413	0.002491941	0.00012922	2.45E-06
ENSG00000140632_4832860_4805236_484	dORF	GLYR1	16	-	1452	23	10	6	2.48%	5.233965713	0.077972317	0.02804198	0.009283347
ENSG00000167881_76048018_76045288_122	novel	SRP68	17	-	366	14	0	1	6.56%	12.63911321	0.005257623	0.009234565	0.000255364
ENSG00000118257_205683297_205776526_904	annotated	NRP2	2	+	2712	129	22	2	4.76%	15.71700093	4.36E-06	1.21E-08	6.73E-13
ENSG00000167900_78186994_78174759_267	annotated	TK1	17	-	801	244	30	3	13.48%	100.6530557	1.60E-05	7.62E-08	1.44E-11
ENSG00000105011_14136456_14121671_48	annotated	ASF1B	19	-	144	30	2	1	14.58%	68.83802732	0.020905271	0.012176559	0.001217494
ENSG00000153310_129879461_129842142_324	annotated	FAM49B	8	-	972	85	7	0	7.72%	28.89497443	1.46E-05	5.30E-06	6.40E-10
ENSG00000149196_86306332_86345094_140	novel	HIKESHI	11	+	420	31	5	0	9.29%	24.38832968	0.007740067	0.000602625	3.15E-05
ENSG00000104643_11306196_11322788_350	dORF	MTMR9	8	+	1050	17	3	1	2.57%	5.349698123	0.020607861	0.005849704	0.000627382
ENSG00000167863_75042650_75040015_81	annotated	ATP5PD	17	-	243	88	3	3	17.28%	119.6591882	0.001030441	0.000819367	5.28E-06
ENSG00000148337_128191939_128166197_954	annotated	CIZ1	9	-	2862	102	4	4	3.56%	11.77606505	1.82E-07	1.43E-06	2.49E-12
ENSG00000271723_54716624_54741513_342	novel	MROH7-TTC4	1	+	1026	40	0	0	3.80%	12.88197002	0.00067919	0.00067919	2.95E-06
ENSG00000178982_38624180_38635129_124	annotated	EIF3K	19	+	372	688	62	7	33.06%	611.104036	9.61E-08	2.62E-08	2.53E-14

ENSG00000196072_100286659_100275462_142	annotated	BLOC1S2	10	-	426	84	11	6	14.79%	65.15373853	0.000416936	0.000181591	5.20E-07
ENSG00000102181_150898588_150769034_262	annotated	CD99L2	X	-	786	141	14	0	10.31%	59.27427085	5.94E-05	2.42E-06	1.31E-09
ENSG00000163156_151166153_151167505_162	novel	SCNM1	1	+	486	17	3	0	4.94%	11.55798977	0.032088197	0.005554612	0.000953527
ENSG00000028203_95256599_95299116_569	novel	VEZT	12	+	1707	27	3	1	2.11%	5.226366925	0.002631121	0.001573472	2.44E-05
ENSG00000070087_149970856_149968350_110	annotated	PFN2	3	-	330	410	51	21	36.36%	410.5249629	1.39E-06	2.57E-07	3.34E-12
ENSG00000187736_219158335_219076381_290	annotated	NHEJ1	2	-	870	17	2	0	2.07%	6.456532217	0.042435259	0.01300724	0.002615674
ENSG00000001460_24401388_24358543_334	annotated	STPG1	1	-	1002	8	3	0	1.80%	2.638104041	0.093731016	0.011570466	0.00557496
ENSG00000144357_170001400_170081843_579	annotated	UBR3	2	+	1737	13	0	0	1.04%	2.472937769	0.012798403	0.012798403	0.000797244
ENSG00000076067_56581185_56589012_226	annotated	RBMS2	12	+	678	15	4	1	3.98%	7.310232989	0.065499399	0.010079165	0.003356681
ENSG00000110092_69648023_69651282_94	novel	CCND1	11	+	282	18	1	0	7.45%	21.09079986	0.016854696	0.007029303	0.00060175
ENSG00000139180_4649127_4655741_108	novel	NDUFA9	12	+	324	70	9	2	11.11%	71.38758388	0.001268312	0.00163319	1.25E-05
ENSG00000204463_31651763_31639131_903	annotated	BAG6	6	-	2709	94	13	3	4.21%	11.46538129	1.59E-06	6.22E-08	1.01E-12
ENSG00000164733_11853372_11852665_24	novel	CTSB	8	-	72	32	2	43	37.50%	146.8544583	0.003710481	0.468493666	0.025643776
ENSG00000164733_11852668_11850698_113	novel	CTSB	8	-	339	231	10	0	14.16%	225.1551761	0.000216957	0.000217439	3.25E-07
ENSG00000099917_20582680_20586704_341	novel	MED15	22	+	1023	23	1	2	2.93%	7.428854561	0.00376808	0.008577311	0.000175238
ENSG00000174173_101564782_101565993_403	annotated	TRMT10C	3	+	1209	43	4	4	4.71%	11.75200069	0.000282487	0.00044822	8.49E-07
ENSG00000127947_77537643_77583722_118	novel	PTPN12	7	+	354	12	0	2	5.08%	11.20076377	0.01300724	0.052581193	0.003266469
ENSG00000137161_42929571_42937777_143	annotated	CNPY3	6	+	429	85	5	0	11.89%	65.46833367	0.000988746	0.000114648	8.23E-07
ENSG00000114021_100334792_100355268_276	annotated	NIT2	3	+	828	182	15	22	15.22%	72.62910708	1.55E-07	4.05E-07	5.96E-13
ENSG00000143401_150226625_150220691_65	annotated	ANP32E	1	-	195	26	1	0	16.92%	44.05633748	0.003783485	0.00140552	3.15E-05
ENSG00000154767_14167210_14145941_747	dORF	XPC	3	-	2241	30	5	0	2.28%	4.423327056	0.001616725	9.22E-05	1.14E-06
ENSG00000215271_23277155_23275575_526	annotated	HOMEZ	14	-	1578	17	0	0	1.90%	3.559685063	0.002188006	0.002188006	2.79E-05
ENSG00000128891_40563009_40532299_132	annotated	CCDC32	15	-	396	19	3	0	6.06%	15.85360629	0.031495399	0.005306578	0.000899719
ENSG00000156958_49282047_49318187_159	novel	GALK2	15	+	477	12	0	0	3.14%	8.312516506	0.020613417	0.020613417	0.001946209
ENSG00000068878_53885761_53866089_210	novel	PSME4	2	-	630	30	3	0	3.81%	15.73440624	0.010613888	0.005604867	0.000310358
ENSG00000151338_37267030_37546971_405	annotated	MIPOL1	14	+	1215	12	1	0	1.98%	3.263432406	0.010499663	0.00419316	0.000236136
ENSG00000120333_175014779_175011709_49	novel	MRPS14	1	-	147	30	0	0	16.33%	67.43316962	0.005604867	0.005604867	0.000167525
ENSG00000134248_110407782_110401523_145	annotated	LAMTOR5	1	-	435	103	19	7	17.93%	78.23797863	0.000193738	1.83E-05	2.85E-08
ENSG00000139624_50167297_50153867_93	annotated	CERS5	12	-	279	22	1	0	5.38%	26.05482324	0.028889786	0.021084099	0.002731533
ENSG00000075089_100200852_100208827_144	annotated	ACTR6	12	+	432	20	15	0	4.86%	15.2973394	0.17056305	0.008574016	0.009173812
ENSG00000151239_43800518_43795585_252	dORF	TWF1	12	-	756	25	2	1	1.98%	10.926671	0.021084099	0.023199731	0.002219783
ENSG00000103145_3023941_3022863_119	annotated	HCFC1R1	16	-	357	82	2	2	14.29%	75.89537129	0.000150527	0.000155865	1.65E-07
ENSG00000134375_201955527_201969554_171	annotated	TIMM17A	1	+	513	172	25	9	14.04%	110.7849422	1.86E-05	2.19E-05	3.21E-09
ENSG00000176393_201982667_202005716_611	annotated	RNPEP	1	+	1833	176	16	5	9.66%	31.72633141	1.11E-09	2.40E-11	3.34E-19
ENSG00000163435_202011188_202015323_354	annotated	ELF3	1	+	1062	347	37	47	21.19%	107.9629174	4.33E-12	9.07E-11	4.99E-21
ENSG00000163435_202011188_202015323_354	annotated	ELF3	1	+	1062	347	37	47	21.19%	107.9629174	4.33E-12	9.07E-11	4.99E-21
ENSG00000135373_34642655_34658931_269	annotated	EHF	11	+	807	21	0	3	4.83%	8.598355828	0.000583356	0.005808622	2.25E-05
ENSG00000150712_32312838_32229778_693	annotated	MTMR12	5	-	2079	29	4	3	2.16%	4.609068496	0.004398303	0.002082111	5.25E-05
ENSG00000160818_156599013_156597824_132	annotated	GPATCH4	1	-	396	13	0	0	5.30%	10.8472043	0.007882058	0.007882058	0.000319636
ENSG00000171109_179362388_179392059_483	annotated	MFN1	3	+	1449	16	0	0	1.45%	3.64855797	0.008175611	0.008175611	0.000342518
ENSG00000177239_137099857_137108591_402	novel	MAN1B1	9	+	1206	150	19	3	9.45%	41.09732974	3.10E-06	8.64E-08	2.76E-12
ENSG00000137133_35813753_35813054_157	novel	HINT2	9	-	471	181	28	20	19.11%	126.9776606	5.92E-05	1.07E-05	5.12E-09
ENSG00000134255_111182856_111184310_115	dORF	CEPT1	1	+	345	19	4	0	4.35%	18.19718287	0.100412561	0.021084099	0.009609042
ENSG00000134255_111147787_111182316_256	annotated	CEPT1	1	+	768	49	2	1	7.42%	21.08164587	6.73E-05	6.11E-05	3.04E-08
ENSG00000251493_73448344_73446965_459	annotated	FOXD1	5	-	1377	15	0	0	1.09%	3.599373977	0.021557223	0.021557223	0.002115616
ENSG00000079459_11802833_11838609_417	annotated	FDFT1	8	+	1251	1185	126	52	34.77%	312.9901674	1.76E-22	2.75E-24	8.98E-45
ENSG00000106459_129690424_129755181_342	annotated	NRF1	7	+	1026	12	1	2	2.05%	3.864591007	0.007882058	0.037441831	0.00150521
ENSG00000095261_120842909_120817906_504	annotated	PSMD5	9	-	1512	138	19	11	9.13%	30.15761197	9.16E-07	1.14E-07	1.01E-12
ENSG00000211455_27297727_27322455_462	annotated	STK38L	12	+	1386	35	5	0	3.03%	8.344003311	0.006673533	0.0004093	1.93E-05
ENSG00000009307_114725293_114720488_140	novel	CSDE1	1	-	420	18	2	3	6.43%	14.16096562	0.005727026	0.027614347	0.000834652
ENSG00000115414_215435697_215361555_2261	annotated	FN1	2	-	6783	342	59	23	5.53%	16.65995955	8.17E-15	2.07E-18	3.20E-31
ENSG00000133318_63681637_63758201_255	annotated	RTN3	11	+	765	385	50	9	14.90%	166.2910778	3.67E-06	1.42E-07	5.20E-12
ENSG00000198373_69840134_69939940_754	annotated	WWP2	16	+	2262	37	3	8	1.72%	5.404789413	0.00325503	0.01648003	0.000299646
ENSG00000143353_219179170_219211728_199	novel	LYPLAL1	1	+	597	54	6	0	10.05%	29.88746513	0.000765825	3.81E-05	2.35E-07
ENSG00000137700_119029530_119029333_65	novel	SLC37A4	11	-	195	31	7	0	15.38%	52.52871007	0.019390513	0.002376615	0.000272493
ENSG00000137700_119027649_119024910_228	novel	SLC37A4	11	-	684	39	3	0	6.58%	18.83988116	0.001388942	0.000299491	2.79E-06
ENSG00000101361_2657932_2658294_120	novel	NOP56	20	+	360	8	3	0	5.00%	7.342722914	0.124106539	0.011570466	0.007709042
ENSG00000163636_64013590_64010668_108	novel	PSMD6	3	-	324	49	4	2	10.19%	49.97130872	0.002942571	0.002952162	4.93E-05
ENSG00000118257_205765567_205795058_126	novel	NRP2	2	+	378	18	0	0	5.56%	15.73440624	0.008776163	0.008776163	0.000391611
ENSG00000033867_27484099_27423992_299	novel	SLC4A7	3	-	897	21	13	4	3.01%	7.735644541	0.141565435	0.023983937	0.015488874
ENSG00000184277_101652361_101633677_183	annotated	TM2D3	15	-	549	190	13	2	16.94%	114.3538814	2.80E-06	9.26E-07	2.30E-11
ENSG00000092871_35026553_35011968_363	annotated	RFFL	17	-	1089	24	3	1	4.68%	7.282039253	0.000703076	0.00017104	8.33E-07
ENSG00000177410_49279128_49279205_25	novel	ZFAS1	20	+	75	139	14	5	44.00%	612.383091	0.002338867	0.001792061	2.46E-05
ENSG00000063854_1826787_1809761_236	annotated	HAGH	16	-	708	49	7	15	7.63%	22.86822602	0.000334294	0.026462324	8.02E-05
ENSG00000198563_31540510_31538840_110	internal	DDX39B	6	-	330	36	6	102	9.09%	36.0460943	0.007635352	0.516161534	0.045829264
ENSG00000198563_31539209_31530434_336	annotated	DDX39B	6	-	1008	219	26	15	14.58%	71.78822849	9.72E-07	5.37E-08	5.30E-13
ENSG00000141543_80137411_80135490_92	novel	EIF4A3	17	-	276	82	13	0	16.30%	98.16901287	0.001393661	0.000316881	2.94E-06
ENSG00000196072_100286127_100275462_95	annotated	BLOC1S2	10	-	285	25	3	0	9.47%	28.98443255	0.004911637	0.003632191	9.81E-05

ENSG00000107929_885721_864243_79	annotated	LARP4B	10	-	237	18	4	4	6.33%	25.09538211	0.08410207	0.035772986	0.012269135
ENSG00000181826_37686287_37631388_271	annotated	RELL1	4	-	813	12	3	0	2.21%	4.87708533	0.035220215	0.007152939	0.001300851
ENSG00000159131_33539315_33531411_224	novel	GART	21	-	672	41	0	3	5.80%	20.159708	0.000642411	0.003522801	1.45E-05
ENSG00000131381_15096120_15073782_784	annotated	RBSN	3	-	2352	7	3	0	0.64%	0.98340039	0.170087137	0.019216965	0.016246064
ENSG00000283782_132579932_132642364_1105	novel	AC116366.3	5	+	3315	85	15	15	2.99%	8.472372593	0.000117864	0.000162052	1.36E-07
ENSG00000132507_7309636_7309884_82	novel	EIF5A	17	+	246	578	226	36	26.83%	776.35863	0.004818828	1.99E-05	1.09E-06
ENSG00000187118_28263338_28319629_84	dORF	CMC1	3	+	252	41	2	6	9.52%	53.75922133	0.008413705	0.044620823	0.001914148
ENSG00000143933_47176443_47160776_83	annotated	CALM2	2	-	249	790	49	35	37.35%	1048.328512	4.68E-07	1.14E-06	4.86E-12
ENSG00000120533_109336137_109340498_48	annotated	ENY2	8	+	144	132	36	8	31.25%	302.8873202	0.000447538	0.000655587	1.91E-06
ENSG00000156253_29008544_29006417_271	novel	RWDD2B	21	-	813	42	7	2	7.38%	17.06979866	0.000236553	0.000129425	2.15E-07
ENSG00000168827_158644635_158691467_751	annotated	GFM1	3	+	2253	99	38	4	4.66%	14.51923239	0.001445506	4.12E-07	1.12E-08
ENSG00000232388_18567479_18568739_73	annotated	SMIM26	20	+	219	137	30	9	24.66%	206.7026793	0.002499873	0.000472533	7.70E-06
ENSG00000112319_133462632_133528805_442	annotated	EYA4	6	+	1326	18	2	0	1.81%	4.485373726	0.013239535	0.005306578	0.000367823
ENSG00000131467_42833632_42841578_198	annotated	PSME3	17	+	594	169	31	5	20.71%	94.00910397	8.49E-07	6.62E-08	5.61E-13
ENSG00000104763_18067256_18057534_280	Overlap_dORF	ASAH1	8	-	840	265	30	5	16.79%	104.2404414	8.80E-08	5.15E-09	4.90E-15
ENSG00000104763_18084058_18067253_56	annotated	ASAH1	8	-	168	222	3	1	12.50%	436.6297733	0.008776163	0.016983117	0.000739612
ENSG00000179091_144095100_144097336_325	annotated	CYC1	8	+	975	623	82	16	25.23%	211.131525	3.63E-11	1.22E-14	7.74E-24
ENSG00000107554_99930763_99877151_1209	annotated	DNMBP	10	-	3627	73	7	6	2.65%	6.650356981	2.21E-06	3.25E-06	6.19E-11
ENSG00000102805_76995145_77000969_273	annotated	CLN5	13	+	819	36	2	1	5.86%	14.5240673	0.000412875	0.000270991	7.53E-07
ENSG00000241468_99460131_99460057_24	novel	ATP5MF	7	-	72	80	23	0	20.83%	367.1361457	0.170042304	0.021084099	0.017372496
ENSG00000162032_1778048_1777097_191	novel	SPSB3	16	-	573	14	3	0	4.19%	8.073150848	0.026823708	0.005160369	0.00074112
ENSG00000162032_1781483_1778295_147	novel	SPSB3	16	-	441	19	2	6	6.12%	14.23589136	0.013091824	0.039731909	0.002458944
ENSG00000204344_31972650_31981049_265	novel	STK19	6	+	795	10	1	0	2.64%	4.156258253	0.016854696	0.007029303	0.00060175
ENSG00000167977_2682549_2707327_234	annotated	KCTD5	16	+	702	27	3	0	3.85%	12.70855889	0.003684287	0.003554776	7.29E-05
ENSG00000067369_43446447_43407383_984	dORF	TP53BP1	15	-	2952	34	3	4	1.32%	3.805679559	0.002962915	0.004132712	6.85E-05
ENSG00000105732_42078607_42081297_896	annotated	ZNF574	19	+	2688	39	5	0	1.67%	4.794076902	0.000600369	0.000300266	1.20E-06
ENSG00000128585_131327900_131478787_731	novel	MKLN1	7	+	2193	86	14	2	4.10%	12.95774632	1.96E-05	1.02E-06	1.84E-10
ENSG00000187764_89405393_89378704_841	annotated	SEMA4D	9	-	2523	24	2	1	1.66%	3.143139416	0.00128979	0.000661953	5.38E-06
ENSG00000109814_39521512_39514175_55	annotated	UGDH	4	-	165	141	25	15	36.36%	282.361072	0.000573738	4.30E-05	1.93E-07
ENSG00000132199_712587_674305_443	annotated	ENOSF1	18	-	1329	44	8	3	3.39%	10.93949689	0.002084207	0.001292367	1.61E-05
ENSG00000140474_74842087_74837228_355	dORF	ULK3	15	-	1065	21	4	0	2.54%	6.515373853	0.024209654	0.003529166	0.000482397
ENSG00000006015_18588802_18592180_179	annotated	REX1BD	19	+	537	32	8	9	8.94%	19.68998323	0.005262544	0.022044165	0.000613731
ENSG00000103197_2083709_2088610_508	novel	TSC2	16	+	1524	25	3	0	2.17%	5.420317111	0.00208127	0.001440978	1.79E-05
ENSG00000215301_41343246_41347719_470	annotated	DDX3X	X	+	1410	190	31	7	8.30%	44.52502192	8.60E-07	4.52E-08	3.97E-13
ENSG00000138744_75940949_75917771_323	annotated	NAAA	4	-	969	173	8	8	9.29%	58.99184508	2.54E-06	6.36E-06	1.38E-10
ENSG00000183258_177516945_177514697_312	annotated	DDX41	5	-	936	48	8	4	3.21%	16.94474519	0.005451642	0.003199141	9.64E-05
ENSG00000183258_177513789_177511791_291	dORF	DDX41	5	-	873	116	12	4	9.28%	43.90494113	1.22E-05	1.16E-05	1.14E-09
ENSG00000151693_9207105_9403327_961	annotated	ASAP2	2	+	2883	46	15	1	2.08%	5.272090333	0.009103525	4.75E-05	4.72E-06
ENSG00000185359_81684067_81701618_777	annotated	HGS	17	+	2331	74	6	4	3.60%	10.48960416	3.03E-06	1.10E-05	2.83E-10
ENSG00000181610_57850010_57839783_190	annotated	MRPS23	17	-	570	120	24	2	22.63%	69.56263813	7.04E-06	1.25E-08	1.18E-12
ENSG00000114353_50236387_50257690_338	annotated	GNAI2	3	+	1014	74	24	0	8.28%	24.11367584	0.001622089	1.54E-06	3.71E-08
ENSG00000196611_102798092_102790412_469	annotated	MMP1	11	-	1407	23	0	0	1.28%	5.401363337	0.012798403	0.012798403	0.000797244
ENSG00000162298_65129904_65128679_95	novel	SYVN1	11	-	285	8	4	0	5.26%	9.275018417	0.169362074	0.019216965	0.016163992
ENSG00000128891_40563009_40553971_183	annotated	CCDC32	15	-	549	19	3	0	4.37%	11.43538814	0.031495399	0.005306578	0.000899719
ENSG00000161939_7013669_7014286_71	novel	RNASEK-C17orf49	17	+	213	288	67	5	32.39%	446.7684928	7.97E-05	8.86E-06	5.83E-09
ENSG00000177425_79689956_79604658_152	annotated	PAWR	12	-	456	25	2	0	7.24%	18.11527035	0.001925611	0.001535678	1.76E-05
ENSG00000060138_10704130_10701288_106	novel	YBX3	12	-	318	29	0	3	12.26%	30.13287233	0.000598307	0.000596156	2.30E-06
ENSG00000188641_97920922_97720888_173	annotated	DPYD	1	-	519	14	0	0	3.47%	8.913131861	0.013428348	0.013428348	0.000872559
ENSG00000140691_31459525_31466889_935	annotated	ARMC5	16	+	2805	21	3	0	1.28%	2.47375157	0.00579799	0.000939154	3.40E-05
ENSG00000120159_26892715_26869929_237	annotated	CAAP1	9	-	711	64	12	1	7.59%	29.74267509	0.00112555	0.000101263	8.44E-07
ENSG00000144659_39383725_39396520_304	annotated	SLC25A38	3	+	912	28	3	1	4.28%	10.14455139	0.003727739	0.001573472	3.44E-05
ENSG00000072803_172006502_171868635_542	annotated	FBXW11	5	-	1626	81	8	0	4.43%	16.46016299	0.000329418	7.05E-06	2.14E-08
ENSG00000113732_172983861_173020331_65	annotated	ATP6V0E1	5	+	195	126	38	3	26.15%	213.5037893	0.000574933	0.000143957	5.79E-07
ENSG00000075336_74148809_74155230_128	novel	TIMM21	18	+	384	27	0	0	3.91%	23.23283422	0.021084099	0.021084099	0.002029891
ENSG00000140391_77070954_77046835_228	annotated	TSPAN3	15	-	684	551	98	10	25.88%	266.1737056	1.62E-08	9.04E-12	2.33E-18
ENSG00000196365_5707759_5696592_283	novel	LONP1	19	-	849	29	3	1	3.89%	11.28651755	0.006177293	0.002361478	8.26E-05
ENSG00000196365_5696104_5692032_305	novel	LONP1	19	-	915	31	9	0	5.57%	11.19464313	0.006972929	0.000109913	6.77E-06
ENSG00000165304_36581692_36677337_619	annotated	MELK	9	+	1857	95	26	21	5.98%	16.90368361	7.59E-05	0.000103032	5.70E-08
ENSG00000177192_131941543_131943586_162	novel	PUS1	12	+	486	24	2	0	4.94%	16.31716203	0.011821942	0.005604867	0.000345184
ENSG00000135404_55726940_55725561_145	annotated	CD63	12	-	435	832	87	25	44.83%	631.9805653	1.66E-10	4.12E-12	8.84E-21
ENSG00000141499_7688649_7701666_287	annotated	WRAP53	17	+	861	40	7	24	5.92%	15.35064024	0.004146524	0.163488777	0.005236449
ENSG00000184584_139481212_139476261_260	annotated	TMEM173	5	-	780	16	6	0	3.46%	6.777898074	0.058300982	0.003229477	0.001201035
ENSG00000108312_44216564_44207242_698	annotated	UBTF	17	-	2094	43	19	6	3.44%	6.785180916	0.011404304	0.000148613	1.54E-05
ENSG00000131626_70272299_70343732_541	annotated	PPFIA1	11	+	1623	16	4	5	1.66%	3.257400184	0.035667474	0.06880633	0.010035358
ENSG00000131626_70339211_70382146_675	Overlap_dORF	PPFIA1	11	+	2025	42	2	0	2.52%	6.853208053	0.000261877	0.000127134	2.34E-07
ENSG00000109854_20364238_20367380_133	annotated	HTATIP2	11	+	399	75	13	4	12.78%	62.10949833	0.000141598	0.000294004	2.91E-07
ENSG00000162735_160280168_160279551_75	novel	PEX19	1	-	225	27	0	0	9.33%	39.65070373	0.008674847	0.008674847	0.000383115
ENSG00000148335_129634156_129635464_135	annotated	NTMT1	9	+	405	85	16	5	17.04%	69.34793863	0.000271976	1.12E-05	2.61E-08

ENSG00000140995_89954260_89955225_57	internal	DEF8	16	+	171	10	2	5	8.77%	19.32295504	0.069457177	0.197776317	0.049762526
ENSG00000005889_24179200_24211376_780	annotated	ZFX	X	+	2340	42	2	4	2.82%	5.930660815	4.02E-05	0.000197673	5.99E-08
ENSG00000186638_34311346_34254380_1234	annotated	KIF24	9	-	3702	20	0	0	0.65%	1.785102815	0.005454749	0.005454749	0.000159109
ENSG00000050393_13814429_13790809_359	annotated	MCUR1	6	-	1077	23	2	5	2.51%	7.056377173	0.007609621	0.036603043	0.001426978
ENSG00000082213_31535769_31552902_358	annotated	C5orf22	5	+	1074	28	1	1	3.35%	8.614367664	0.001480749	0.001480749	1.32E-05
ENSG00000092931_76738413_76742222_91	annotated	MFSD11	17	+	273	14	0	2	6.59%	16.94474519	0.011772051	0.071345384	0.004172987
ENSG00000129103_56073037_56079612_213	annotated	SUMF2	7	+	639	423	66	11	23.00%	218.7304079	1.02E-06	1.86E-09	2.58E-14
ENSG00000146733_56021212_56011762_225	annotated	PSPH	7	-	675	26	0	0	5.33%	12.72738638	0.000943084	0.000943084	5.54E-06
ENSG00000146731_56051849_56063085_531	annotated	CCT6A	7	+	1593	443	65	11	14.12%	91.8877472	1.67E-11	1.17E-13	2.81E-23
ENSG00000177410_49279128_49279205_25	novel	ZFAS1	20	+	75	139	14	5	44.00%	612.383091	0.002338867	0.001792061	2.46E-05
ENSG00000173950_195247837_195207458_84	annotated	XXYLT1	3	-	252	13	0	0	5.95%	17.04560676	0.021084099	0.021084099	0.002029891
ENSG00000104524_143609548_143605700_254	annotated	PYCR3	8	-	762	43	2	0	6.69%	18.64589086	0.000112262	0.000113689	9.15E-08
ENSG00000012171_50269241_50276706_748	annotated	SEMA3B	3	+	2244	52	12	0	2.94%	7.656850097	0.002479335	1.68E-05	4.34E-07
ENSG00000159128_33403544_33436962_337	annotated	IFNGR2	21	+	1011	34	18	0	4.15%	11.1121326	0.11381741	0.000428413	0.000662693
ENSG00000131943_29708389_29702712_133	annotated	C19orf12	19	-	399	22	1	0	7.52%	18.21878618	0.004136993	0.002201184	5.20E-05
ENSG00000131023_149702039_149683016_661	annotated	LATS1	6	-	1983	28	1	1	1.21%	4.665572804	0.006191047	0.006191047	0.000202321
ENSG00000164520_149889507_149888498_109	dORF	RAET1E	6	-	327	16	0	0	5.50%	16.1674633	0.013428348	0.013428348	0.000872559
ENSG00000105176_29942548_30015069_535	annotated	URI1	19	+	1605	73	13	12	5.23%	15.02856372	0.000322294	2.80E-05	7.14E-08
ENSG00000009413_111405521_111300016_2959	annotated	REV3L	6	-	8877	8	4	0	0.17%	0.297778557	0.214538039	0.019679754	0.021895271
ENSG00000157593_44255605_44254706_299	annotated	SLC35B2	6	-	897	97	12	0	8.36%	35.7313105	0.000163758	5.62E-06	8.22E-09
ENSG00000157593_44257410_44255565_94	Overlap_uORF	SLC35B2	6	-	282	40	7	4	10.64%	46.86844413	0.009031392	0.006610814	0.00030869
ENSG00000155380_112929308_112913891_500	annotated	SLC16A1	1	-	1500	216	24	1	11.60%	47.58084448	9.51E-09	1.28E-11	1.80E-18
ENSG00000100578_58442716_58537041_1347	novel	KIAA0586	14	+	4041	25	3	1	0.97%	2.044187894	0.004430232	0.001559221	4.06E-05
ENSG00000197565_108438196_108157000_1633	annotated	COL4A6	X	-	4899	19	3	6	0.80%	1.281491752	0.007312901	0.033785934	0.001268482
ENSG00000094914_53309004_53308048_127	novel	AAAS	12	-	381	70	0	0	9.45%	60.70755165	0.001082917	0.001082917	7.23E-06
ENSG00000155729_200507016_200490100_426	annotated	KCTD18	2	-	1278	15	3	0	1.41%	3.878198722	0.062386368	0.012798403	0.003862055
ENSG00000204220_33289857_33290845_129	annotated	PFDN6	6	+	387	174	13	1	25.58%	148.5620683	1.10E-06	2.75E-07	2.82E-12
ENSG00000204220_33289857_33290454_87	novel	PFDN6	6	+	261	160	12	1	31.03%	202.5578735	8.14E-06	2.87E-06	1.98E-10
ENSG00000068001_50320489_50318129_473	annotated	HYAL2	3	-	1419	104	8	2	6.98%	24.21701426	2.59E-06	5.92E-07	1.39E-11
ENSG00000050820_75243015_75229511_693	annotated	BCAR1	16	-	2079	50	8	4	3.03%	7.94666982	0.000310882	0.000199324	4.25E-07
ENSG00000179889_14997807_15138976_473	annotated	PDXDC1	16	+	1419	19	1	0	2.33%	4.424262221	0.002467336	0.001384502	2.03E-05
ENSG00000184831_23858695_23840342_56	annotated	APOO	X	-	168	17	0	0	12.50%	33.43561327	0.008473674	0.008473674	0.000366502
ENSG00000105993_157358573_157410108_334	annotated	DNAJB6	7	+	1002	33	1	2	2.69%	10.88217917	0.003632191	0.007312901	0.000144692
ENSG00000159363_17011757_17005068_103	uORF	ATP13A2	1	-	309	32	1	0	9.71%	34.21851455	0.002474239	0.00244612	3.49E-05
ENSG00000244687_50084158_50082768_58	annotated	UBE2V1	20	-	174	13	4	2	10.34%	24.68674083	0.143893695	0.035374934	0.02120653
ENSG00000022556_54970259_55000898_957	annotated	NLRP2	19	+	2871	52	8	3	3.13%	5.984664444	2.15E-05	3.64E-06	6.63E-10
ENSG00000145817_144165549_144160397_202	annotated	YIPF5	5	-	606	25	1	2	4.95%	13.63129254	0.002201184	0.006423651	8.07E-05
ENSG00000103415_4506896_4509756_287	annotated	HMOX2	16	+	861	46	8	0	5.23%	17.65323627	0.002873497	0.000291809	5.81E-06
ENSG00000006327_3020747_3021826_67	internal	TNFRSF12A	16	+	201	482	28	10	31.34%	792.3565174	4.95E-05	7.47E-05	2.75E-08
ENSG00000070814_150357747_150383755_958	annotated	TCOF1	5	+	2874	25	0	0	1.36%	2.874239136	0.000585477	0.000585477	2.21E-06
ENSG00000103037_58515992_58519029_352	annotated	SETD6	16	+	1056	6	7	0	1.42%	1.877400745	0.165899918	0.016947427	0.014398895
ENSG00000187866_68780444_68781028_194	annotated	FAM122A	9	+	582	19	0	0	3.61%	10.78698985	0.008574016	0.008574016	0.000374746
ENSG00000001631_92235477_92200736_482	novel	KRIT1	7	-	1446	25	8	0	2.49%	5.712699362	0.011220946	0.000947024	6.93E-05
ENSG00000125991_35542098_35543012_145	novel	ERGIC3	20	+	435	172	63	9	21.38%	130.6498284	0.000311537	4.46E-06	1.35E-08
ENSG00000169733_82051766_82048726_331	annotated	RFNG	17	-	993	56	12	0	5.14%	18.63410045	0.003704325	0.000129746	3.80E-06
ENSG00000112282_131628049_131587679_1374	annotated	MED23	6	-	4122	23	4	3	0.87%	1.843696801	0.009497983	0.006392297	0.000314154
ENSG00000029364_69399370_69458593_241	annotated	SLC39A9	14	+	723	80	4	0	9.13%	36.56127592	1.89E-05	1.87E-05	2.79E-09
ENSG00000029364_69398714_69398821_35	uORF	SLC39A9	14	+	105	16	0	9	14.29%	50.35009998	0.020613417	0.361337229	0.04510239
ENSG00000166598_103930516_103942805_550	novel	HSP90B1	12	+	1650	1569	301	44	26.73%	314.2017887	6.94E-22	5.30E-25	7.45E-45
ENSG00000166598_103943293_103947662_182	novel	HSP90B1	12	+	546	942	132	28	35.16%	570.0696416	5.28E-10	2.43E-11	1.57E-19
ENSG00000135905_224853078_224765776_1540	dORF	DOCK10	2	-	4620	13	1	1	0.32%	0.929760369	0.035772986	0.035772986	0.005410795
ENSG00000124570_2956782_2948298_350	Overlap_dORF	SERPINB6	6	-	1050	213	33	4	16.57%	67.0285706	8.20E-09	3.45E-11	3.82E-18
ENSG00000214063_864173_865680_168	novel	TSPAN4	11	+	504	54	12	0	5.36%	35.40241405	0.010303292	0.003789641	0.000211588
ENSG00000144827_111979062_111990741_205	annotated	ABHD10	3	+	615	46	2	3	7.80%	24.71453078	0.000250577	0.000461333	7.79E-07
ENSG00000204371_31884953_31880084_408	novel	EHMT2	6	-	1224	29	1	7	3.19%	7.828638401	0.000995868	0.023425208	0.000164406
ENSG00000089820_153934904_153921704_163	novel	ARHGAP4	X	-	489	34	4	0	7.98%	22.97416372	0.004206461	0.000683882	1.84E-05
ENSG00000137947_88891499_88858771_183	novel	GTF2B	1	-	549	25	2	3	7.65%	15.04656335	0.001081087	0.000368141	2.61E-06
ENSG00000164611_160422313_160422923_101	novel	PTTG1	5	+	303	60	238	65	11.88%	65.43020418	0.067143334	0.016707029	0.005190157
ENSG00000164327_39074377_38971859_313	novel	RICTOR	5	-	939	7	1	0	1.60%	2.463213757	0.047790352	0.016947427	0.003697512
ENSG00000078140_39774844_39778434_97	Overlap_dORF	UBE2K	4	+	291	73	14	2	11.34%	82.88950093	0.022551981	0.004623484	0.000562455
ENSG00000147684_124547045_124549892_66	novel	NDUFB9	8	+	198	119	14	2	28.79%	198.5872788	0.00027502	6.58E-05	1.32E-07
ENSG00000128891_40563009_40553971_183	annotated	CCDC32	15	-	549	19	3	0	4.37%	11.43538814	0.031495399	0.005306578	0.000899719
ENSG00000089094_121549579_121430469_554	annotated	KDM2B	12	-	1662	26	3	0	1.08%	5.169064867	0.013428348	0.013640586	0.00088551
ENSG00000163389_119469022_119478441_187	annotated	POGLUT1	3	+	561	14	1	1	3.74%	8.245838566	0.015625009	0.015625009	0.001159391
ENSG00000163389_119480072_119492438_233	dORF	POGLUT1	3	+	699	50	4	0	8.15%	23.63537419	0.000292227	5.68E-05	1.23E-07
ENSG00000114805_155596340_155480968_1646	annotated	PLCH1	3	-	4938	22	3	4	0.49%	1.472113342	0.011428951	0.026799085	0.001469725
ENSG00000120158_4834156_4860275_215	annotated	RCL1	9	+	645	20	0	5	5.12%	10.24565988	0.001343041	0.034447343	0.00032634
ENSG00000090266_140697330_140704934_93	annotated	NDUFB2	7	+	279	304	26	3	27.96%	360.0302848	3.28E-05	5.44E-06	1.48E-09

ENSG00000169379_93995890_94053263_403	annotated	ARL13B	3	+	1209	15	4	0	1.74%	4.099535126	0.059202727	0.007882058	0.002466793
ENSG00000144560_11601909_11558556_231	annotated	VGLL4	3	-	693	17	0	0	2.60%	8.105603216	0.013428348	0.013428348	0.000872559
ENSG00000204536_31154651_31142592_656	annotated	CCHCR1	6	-	1968	11	7	0	1.07%	1.846873904	0.077087128	0.007593099	0.00322116
ENSG00000110917_120687297_120696545_292	annotated	MLEC	12	+	876	196	39	4	11.64%	73.93015536	7.48E-05	2.37E-07	2.15E-10
ENSG00000103266_681209_682489_231	annotated	STUB1	16	+	693	67	11	11	6.93%	31.94561268	0.003893786	0.003537065	7.65E-05
ENSG00000153317_130358142_130054731_1109	dORF	ASAP1	8	-	3327	58	15	14	2.80%	5.760296605	0.000197774	0.000672856	9.11E-07
ENSG00000133943_91160388_91224833_550	annotated	DGLUCY	14	+	1650	37	10	0	2.91%	7.40947494	0.012777891	0.000176614	2.02E-05
ENSG00000223802_18896072_18870577_350	uORF	CERS1	19	-	1050	19	3	0	2.00%	5.979074373	0.016947427	0.008473674	0.000715155
ENSG00000113300_180550104_180574200_462	annotated	CNOT6	5	+	1386	23	3	3	2.60%	5.483202176	0.002084681	0.002084681	2.54E-05
ENSG00000138760_76213543_76163210_470	annotated	SCARB2	4	-	1410	341	41	7	17.23%	79.91069724	1.36E-11	5.08E-15	1.20E-24
ENSG00000109911_31509785_31714840_450	annotated	ELP4	11	+	1350	26	11	0	2.67%	6.363693192	0.039886809	0.000991051	0.000306424
ENSG00000283977_6481517_6482221_103	annotated	AC084337.2	11	+	309	23	5	2	11.65%	24.59455733	0.00257891	0.000844273	1.35E-05
ENSG00000172845_173955605_173909941_479	annotated	SP3	2	-	1437	59	4	1	4.59%	13.56640872	3.52E-05	2.32E-05	6.32E-09
ENSG00000134910_125595916_125609627_384	novel	STT3A	11	+	1152	225	21	7	15.89%	64.53565061	1.10E-09	4.69E-11	6.30E-19
ENSG00000103275_1314332_1320177_41	novel	UBE2I	16	+	123	112	17	17	24.39%	300.8725487	0.015570605	0.022856675	0.001657969
ENSG00000103495_29808660_29809742_94	annotated	MAZ	16	+	282	16	2	5	8.51%	18.74737765	0.017239393	0.077869819	0.006229023
ENSG00000023171_123577464_123622595_705	annotated	GRAMD1B	11	+	2115	29	0	5	2.13%	4.530616266	0.000256142	0.006313333	1.22E-05
ENSG00000103342_11895000_11891043_47	novel	GSPT1	16	-	141	11	0	0	10.64%	25.77764427	0.021084099	0.021084099	0.002029891
ENSG00000160932_143021655_143021789_44	dORF	LY6E	8	+	132	21	10	0	18.18%	52.56722086	0.141410818	0.005355751	0.005174404
ENSG00000164751_76984178_76983261_305	annotated	PEX2	8	-	915	32	8	1	4.59%	11.55576065	0.007043191	0.000548327	2.62E-05
ENSG00000137547_54147438_54147719_93	annotated	MRPL15	8	+	279	121	15	1	24.73%	143.3015278	0.000139009	1.33E-05	1.51E-08
ENSG00000162734_160211545_160213486_130	novel	PEA15	1	+	390	123	18	6	16.92%	104.2101829	4.77E-05	3.08E-05	1.12E-08
ENSG00000220205_8161754_8160855_71	annotated	VAMP2	17	-	213	34	3	0	14.08%	52.74350262	0.002335453	0.002418183	3.27E-05
ENSG00000100697_95126669_95090498_1651	annotated	DICER1	14	-	4953	28	12	2	0.91%	1.867924666	0.042648284	0.00119113	0.000382887
ENSG00000171148_9791466_9784024_369	annotated	TADA3	3	-	1107	83	22	3	5.96%	24.77422772	0.004357496	1.78E-05	8.86E-07
ENSG00000171148_9791363_9789918_49	internal	TADA3	3	-	147	17	0	3	10.20%	38.21212945	0.021084099	0.08410207	0.007951576
ENSG00000140416_63048576_63069943_245	annotated	TPM1	15	+	735	270	35	41	16.73%	121.3797053	8.05E-07	8.38E-07	6.08E-12
ENSG00000115239_53750918_53670503_445	novel	ASB3	2	-	1335	13	3	0	2.47%	3.217597681	0.013071281	0.000883168	7.72E-05
ENSG00000275052_55573649_55568149_81	novel	PPP4R3B	2	-	243	11	1	1	6.17%	14.95739853	0.020613417	0.051235217	0.004687384
ENSG00000143418_150967454_150967053_70	novel	CERS2	1	-	210	215	14	10	28.57%	338.2897342	8.54E-05	0.000154444	9.50E-08
ENSG00000143418_150966853_150964460_160	novel	CERS2	1	-	480	140	13	0	21.88%	96.37323824	1.31E-06	1.07E-07	1.38E-12
ENSG00000169814_15635440_15642138_159	annotated	BTD	3	+	477	33	8	3	8.18%	22.85942039	0.01179876	0.003168495	0.000207081
ENSG00000144029_95090430_95087357_89	novel	MRPS5	2	-	267	56	10	1	11.24%	69.3021039	0.023966078	0.003500471	0.000473933
ENSG00000159128_33421592_33436962_231	annotated	IFNGR2	21	+	693	30	10	0	5.19%	14.30400568	0.040146923	0.001019727	0.000315721
ENSG00000167460_16086484_16095346_139	annotated	TPM4	19	+	417	71	3	0	10.79%	56.25899211	0.000312071	0.000313669	6.59E-07
ENSG00000198860_184051756_184095070_130	annotated	TSEN15	1	+	390	26	0	1	6.92%	22.02816874	0.003478238	0.005588371	0.000106767
ENSG00000113851_3175246_3167622_202	annotated	CRBN	3	-	606	27	2	0	5.45%	14.72179594	0.001558047	0.001558047	1.45E-05
ENSG00000105699_35250404_35267859_514	annotated	LSR	19	+	1542	37	10	1	2.92%	7.928426492	0.028282463	0.000629624	0.000142422
ENSG00000183258_177516945_177515055_173	novel	DDX41	5	-	519	45	8	4	4.62%	28.64935241	0.013192068	0.007400806	0.000494748
ENSG00000006576_77908873_77937850_317	novel	PHTF2	7	+	951	17	5	1	2.84%	5.90660676	0.027696082	0.006024334	0.000874001
ENSG00000115875_38751256_38749516_132	annotated	SRSF7	2	-	396	307	25	5	26.52%	256.1609016	2.09E-06	2.03E-07	4.05E-12
ENSG00000012061_45423374_45416817_201	novel	ERCC1	19	-	603	46	4	4	6.97%	25.20636224	0.002002002	0.002139792	2.51E-05
ENSG00000150527_39294000_39350264_639	annotated	MIA2	14	+	1917	48	7	0	2.97%	8.273490607	0.000366328	5.54E-05	1.52E-07
ENSG00000125746_45616797_45609663_157	dORF	EML2	19	-	471	15	8	0	3.82%	10.52301055	0.235851115	0.012798403	0.01842735
ENSG00000125746_45630001_45616824_242	annotated	EML2	19	-	726	17	2	1	4.13%	7.737166707	0.012468381	0.004070582	0.000273397
ENSG00000159176_201496303_201485366_40	novel	CSRP1	1	-	120	138	7	2	20.00%	379.9859108	0.017732932	0.005756914	0.000530756
ENSG00000166333_6608119_6610611_65	annotated	ILK	11	+	195	6	4	0	7.69%	10.16684711	0.165899918	0.016947427	0.014398895
ENSG00000149792_65122347_65125872_166	annotated	MRPL49	11	+	498	109	11	0	19.88%	72.32139737	1.62E-06	2.34E-07	3.57E-12
ENSG00000116641_62688264_62455414_2109	annotated	DOCK7	1	-	6327	27	10	3	0.76%	1.410053476	0.032541084	0.001268598	0.000291871
ENSG00000134222_109281627_109280153_163	novel	PSRC1	1	-	489	17	0	6	3.07%	11.48708186	0.021084099	0.153506949	0.015422968
ENSG00000084754_26244596_26191250_792	annotated	HADHA	2	-	2376	196	27	1	7.20%	27.25707748	2.61E-08	1.84E-11	7.48E-18
ENSG00000115216_27434492_27435877_119	novel	NRBP1	2	+	357	37	3	1	7.56%	34.24547241	0.010115653	0.00453523	0.0002442
ENSG00000133835_119479012_119541994_532	dORF	HSD17B4	5	+	1596	90	12	1	4.89%	18.6328495	0.000512167	3.47E-06	1.89E-08
ENSG00000185627_244210_247465_108	novel	PSMD13	11	+	324	67	7	0	14.81%	68.328116	0.001010284	0.000197396	1.38E-06
ENSG00000185627_237050_244165_44	novel	PSMD13	11	+	132	21	0	5	13.64%	52.56722086	0.013428348	0.090097208	0.005987478
ENSG00000079459_11802833_11803360_45	novel	FDFT1	8	+	135	105	4	9	20.00%	256.995302	0.005134532	0.003710481	0.000104431
ENSG00000137812_40621033_40625582_1490	annotated	KNL1	15	+	4470	9	5	2	0.40%	0.665280264	0.157414378	0.071345384	0.040296938
ENSG00000173227_67030145_67048757_421	annotated	SYT12	11	+	1263	18	0	0	1.43%	4.709109707	0.013428348	0.013428348	0.000872559
ENSG00000092020_35108148_35091200_117	dORF	PPP2R3C	14	-	351	12	1	0	5.13%	11.29649679	0.026832696	0.011772051	0.001510382
ENSG00000092020_35096560_35085590_129	dORF	PPP2R3C	14	-	387	12	0	0	4.65%	10.24565988	0.013217227	0.013217227	0.00084697
ENSG00000163378_69009840_68977618_525	annotated	EOGT	3	-	1575	44	8	3	4.76%	9.230851663	0.000413274	1.13E-05	4.15E-08
ENSG00000144749_66500407_66380263_1070	annotated	LRIG1	3	-	3210	10	2	0	0.56%	1.029353679	0.071345384	0.011772051	0.004172987
ENSG00000181090_137757948_137846574_705	annotated	EHMT1	9	+	2115	47	5	6	2.98%	7.342722914	0.000491238	0.000261041	8.62E-07
ENSG00000162385_53238448_53233497_100	novel	MAGOH	1	-	300	83	6	2	14.00%	91.41690028	0.000449456	0.001309571	3.81E-06
ENSG00000179364_105384965_105394672_234	novel	PACS2	14	+	702	27	0	1	2.99%	12.70855889	0.008776163	0.01019742	0.000451522
ENSG00000139624_50167297_50135469_351	annotated	CERS5	12	-	1053	100	6	0	8.55%	31.37915775	4.59E-06	7.52E-07	3.12E-11
ENSG00000134285_48925428_48924219_106	novel	FKBP11	12	-	318	44	7	0	10.38%	45.71884078	0.002205543	0.001595761	2.08E-05
ENSG00000166340_6616465_6616298_55	novel	TPP1	11	-	165	69	15	0	16.36%	138.1766948	0.008127104	0.003710481	0.00016379

ENSG00000166340_6615419_6614546_171	novel	TPP1	11	-	513	66	22	3	11.11%	42.51050108	0.002395438	0.000360511	5.78E-06
ENSG00000101224_3801746_3803151_171	novel	CDC25B	20	+	513	121	32	10	14.62%	77.93591865	0.000942154	3.21E-05	2.54E-07
ENSG00000071462_73683626_73697886_281	annotated	BUD23	7	+	843	254	16	6	16.37%	99.55791566	1.39E-08	5.07E-09	7.41E-16
ENSG00000006704_74508081_74602433_959	annotated	GTF2IRD1	7	+	2877	46	7	6	1.98%	5.283085308	0.000772131	0.000623916	3.07E-06
ENSG00000106682_74174384_74195308_248	annotated	EIF4H	7	+	744	105	29	8	4.84%	46.63221205	0.009897711	0.001091522	6.83E-05
ENSG00000133466_37185380_37184376_74	annotated	C1QTNF6	22	-	222	32	0	0	8.11%	47.62847295	0.013853925	0.013853925	0.000925205
ENSG00000133466_37182631_37182188_147	dORF	C1QTNF6	22	-	441	50	4	5	9.52%	37.46287201	0.001641809	0.000518368	5.45E-06
ENSG00000105974_116526588_116559287_147	annotated	CAV1	7	+	441	1039	121	47	41.50%	778.4784803	3.85E-10	1.36E-11	6.53E-20
ENSG00000204852_110614183_110647892_573	annotated	TCTN1	12	+	1719	138	9	2	5.93%	26.52606707	5.26E-07	4.29E-07	2.07E-12
ENSG00000010278_6200500_6237828_228	annotated	CD9	12	+	684	1188	113	89	42.54%	573.8917646	1.98E-16	9.37E-18	2.89E-32
ENSG00000141428_35972729_35979006_220	annotated	C18orf21	18	+	660	18	2	4	4.09%	9.011523576	0.011064574	0.024710983	0.001317552
ENSG00000070269_56579915_56625385_409	annotated	TMEM260	14	+	1227	16	0	0	1.71%	4.308688262	0.007979402	0.007979402	0.000327143
ENSG00000159423_18902523_18872845_512	annotated	ALDH4A1	1	-	1536	24	0	0	2.73%	5.162852049	0.000292509	0.000292509	5.79E-07
ENSG00000196730_87645916_87707364_1086	dORF	DAPK1	9	+	3258	45	6	0	2.03%	4.563847115	0.000167901	1.49E-05	2.04E-08
ENSG00000104823_38815982_38815613_76	novel	ECH1	19	-	228	110	8	3	34.21%	159.414379	2.69E-05	1.53E-05	3.25E-09
ENSG00000138640_88768056_88728533_536	annotated	FAM13A	4	-	1608	12	1	0	0.93%	2.465839784	0.028889786	0.021084099	0.002731533
ENSG00000109606_24528010_24527924_28	novel	DHX15	4	-	84	14	0	0	17.86%	55.07042185	0.020613417	0.020613417	0.001946209
ENSG00000106609_66945081_66951216_198	novel	TMEM248	7	+	594	92	24	2	11.11%	51.17655364	0.003168741	2.51E-05	8.03E-07
ENSG00000152700_134612730_134606950_130	novel	SAR1B	5	-	390	222	24	5	33.08%	188.0866716	2.47E-08	1.12E-08	2.84E-15
ENSG00000164124_158212668_158253527_345	annotated	TMEM144	4	+	1035	34	8	1	3.48%	10.85445996	0.003849547	0.001083458	2.53E-05
ENSG00000254996_140529226_140549237_532	annotated	ANKHD1-EIF4EBP3	5	+	1596	17	0	0	1.88%	3.519538239	0.002188006	0.002188006	2.79E-05
ENSG00000189067_11556730_11549637_161	annotated	LITAF	16	-	483	339	40	21	26.71%	231.9114659	1.46E-07	1.63E-08	2.45E-14
ENSG00000164120_174522451_174492056_178	annotated	HPGD	4	-	534	114	10	13	18.54%	70.53964147	3.80E-06	3.07E-05	9.95E-10
ENSG00000164120_174522451_174491956_266	annotated	HPGD	4	-	798	206	33	13	18.05%	85.29704437	3.45E-08	5.20E-08	1.79E-14
ENSG00000138061_38074995_38070722_412	annotated	CYP1B1	2	-	1236	40	4	0	3.64%	10.6932858	0.000662939	0.000279805	1.24E-06
ENSG00000197713_210002662_210019684_191	annotated	RPE	2	+	573	54	4	0	5.76%	31.13929613	0.008218057	0.001626321	7.93E-05
ENSG00000197713_210019681_210019791_36	Overlap_dORF	RPE	2	+	108	27	0	0	13.89%	82.60563278	0.021557223	0.021557223	0.002115616
ENSG00000049759_58323278_58396269_803	annotated	NEDD4L	18	+	2409	25	13	6	1.87%	3.429042457	0.014923918	0.010603082	0.00077464
ENSG00000143537_155051387_155062502_839	annotated	ADAM15	1	+	2517	254	23	12	9.18%	33.34418868	1.56E-11	4.68E-13	9.74E-23
ENSG00000124222_58670514_58676291_139	annotated	STX16	20	+	417	13	0	1	5.04%	10.30094222	0.008175611	0.0135992	0.00055803
ENSG00000092931_76738413_76778352_377	annotated	MFSD11	17	+	1131	78	4	4	7.69%	22.78776077	8.74E-06	6.83E-06	4.92E-10
ENSG00000243678_51169976_51171604_63	novel	NME2	17	+	189	173	10	4	19.05%	302.4502533	0.00110451	0.00110017	7.48E-06
ENSG00000114956_73938989_73946846_53	internal	DGUOK	2	+	159	28	1	0	13.21%	58.18761554	0.020342454	0.008473674	0.00085717
ENSG00000114956_73926911_73958736_277	annotated	DGUOK	2	+	831	70	12	32	9.75%	27.83342621	0.000753397	0.011200295	5.70E-05
ENSG00000162144_61356632_61353548_108	novel	CYB561A3	11	-	324	15	0	2	9.26%	15.2973394	0.001790164	0.012430495	0.000133074
ENSG00000158987_131635030_131427266_1601	annotated	RAPGEF6	5	-	4803	25	7	5	0.75%	1.719875761	0.025906943	0.014983743	0.001807818
ENSG00000105974_116526588_116559287_147	annotated	CAV1	7	+	441	1039	121	47	41.50%	778.4784803	3.85E-10	1.36E-11	6.53E-20
ENSG00000105974_116526588_116559287_147	annotated	CAV1	7	+	441	1039	121	47	41.50%	778.4784803	3.85E-10	1.36E-11	6.53E-20
ENSG00000123358_52054329_52055306_325	annotated	NR4A1	12	+	975	25	1	0	2.46%	8.472372593	0.007312901	0.005706018	0.000219657
ENSG00000140836_72959863_72787164_3609	annotated	ZFHX3	16	-	10827	24	7	1	0.39%	0.732441188	0.018568156	0.000649098	8.91E-05
ENSG00000140830_72090749_72086637_149	annotated	TXNL4B	16	-	447	19	1	2	4.03%	14.04480557	0.020342454	0.044040756	0.003985452
ENSG00000140829_72096158_72112497_1227	annotated	DHX38	16	+	3681	38	9	7	1.79%	3.411044874	0.001291623	0.001085462	8.59E-06
ENSG00000157020_10312690_10301261_254	novel	SEC13	3	-	762	136	17	3	17.72%	58.97305017	2.30E-07	6.05E-09	1.54E-14
ENSG00000158417_99337555_99399414_1220	annotated	EIF5B	2	+	3660	76	12	4	2.30%	6.861232887	6.06E-05	1.83E-05	8.66E-09
ENSG00000141526_82236009_82238976_465	annotated	SLC16A3	17	+	1395	443	50	5	16.99%	104.9298791	3.54E-11	1.58E-14	9.53E-24
ENSG00000105676_19042757_19057628_476	annotated	ARMC6	19	+	1428	52	4	3	4.83%	12.03219301	3.17E-05	6.90E-05	1.66E-08
ENSG00000105676_19042757_19057628_476	annotated	ARMC6	19	+	1428	52	4	3	4.83%	12.03219301	3.17E-05	6.90E-05	1.66E-08
ENSG00000141551_82273381_82246007_409	annotated	CSNK1D	17	-	1227	97	12	1	9.54%	26.12142259	1.18E-06	1.88E-08	2.45E-13
ENSG00000198130_190319723_190205227_329	annotated	HIBCH	2	-	987	18	2	0	2.13%	6.025942817	0.037441831	0.008175611	0.001552076
ENSG00000162736_160343397_160358271_709	annotated	NCSTN	1	+	2127	523	71	15	12.83%	81.24634874	8.26E-13	1.45E-15	1.94E-26
ENSG00000074416_127722432_127692198_109	annotated	MGLL	3	-	327	12	1	0	4.59%	12.12559747	0.035374934	0.020613417	0.003252526
ENSG00000196712_31095310_31219133_551	annotated	NF1	17	+	1653	24	3	2	2.00%	4.797423319	0.004665364	0.004042627	0.000103211
ENSG00000175166_184299267_184301662_160	novel	PSMD2	3	+	480	29	2	0	8.75%	19.96302792	0.001083027	0.00040306	2.84E-06
ENSG00000111653_6653232_6651195_158	annotated	ING4	12	-	474	31	5	0	6.96%	21.60991237	0.012181226	0.001580611	0.000117048
ENSG00000256269_119092797_119093283_91	dORF	HMBS	11	+	273	17	0	0	7.69%	20.57576201	0.008473674	0.008473674	0.000366502
ENSG00000256269_119085034_119092515_207	annotated	HMBS	11	+	621	12	1	21	4.83%	6.384976447	0.00398705	0.604245086	0.045595676
ENSG00000012963_93207292_93227035_425	annotated	UBR7	14	+	1275	101	7	0	8.00%	26.17464756	1.00E-06	1.45E-07	1.40E-12
ENSG00000151445_77454102_77427616_444	annotated	VIPAS39	14	-	1332	41	6	2	4.05%	10.1706635	0.001029764	0.000324464	2.21E-06
ENSG00000115421_60781912_60797160_425	dORF	PAPOLG	2	+	1275	24	1	0	2.59%	6.219718233	0.002361478	0.001506153	2.11E-05
ENSG00000241468_99460131_99458327_24	annotated	ATP5MF	7	-	72	96	29	4	29.17%	440.5633748	0.06082143	0.008877961	0.002795448
ENSG00000122390_3476228_3484855_242	annotated	NAA60	16	+	726	20	5	0	3.72%	9.102549067	0.033424799	0.003529166	0.000684983
ENSG00000188690_125816499_125788868_265	annotated	UROS	10	-	795	58	0	3	6.79%	24.10629787	9.07E-05	0.000200711	1.30E-07
ENSG00000091129_108232428_108149910_1103	annotated	NRCAM	7	-	3309	107	15	7	4.90%	10.68456054	1.96E-08	1.15E-09	2.53E-16
ENSG00000101558_9914278_9954211_242	annotated	VAPA	18	+	726	94	19	13	9.09%	42.78198061	0.000402758	0.000183536	5.08E-07
ENSG00000117569_96721865_96813405_532	annotated	PTBP2	1	+	1596	19	5	6	1.50%	3.933601561	0.03662705	0.099097252	0.014761033
ENSG00000114026_9750317_9756843_314	annotated	OGG1	3	+	942	37	7	7	4.14%	12.97837967	0.016638564	0.005685466	0.000492136
ENSG00000128641_191276953_191423960_1059	annotated	MYO1B	2	+	3177	51	9	0	1.79%	5.304233266	0.004106886	5.13E-05	1.96E-06
ENSG00000105701_18541970_18532169_413	annotated	FKBP8	19	-	1239	131	47	13	9.44%	34.93571556	0.000142491	4.66E-07	7.98E-10

ENSG00000149187_47489014_47472230_482	annotated	CELF1	11	-	1446	85	12	3	5.19%	19.42317783	0.000691468	1.62E-05	9.98E-08
ENSG00000146834_100430028_100433554_686	annotated	MEPCE	7	+	2058	43	2	3	2.77%	6.903872127	8.84E-05	0.000391	2.49E-07
ENSG00000115204_27322517_27309912_191	annotated	MPV17	2	-	573	123	16	2	16.75%	70.92839673	1.50E-05	4.79E-07	6.99E-11
ENSG00000106541_16801190_16792951_117	annotated	AGR2	7	-	351	634	96	11	25.64%	596.8315804	2.68E-06	1.13E-06	2.68E-11
ENSG00000147475_37737923_37744893_206	annotated	ERLIN2	8	+	618	69	3	4	9.22%	36.891836	6.12E-05	0.000112727	5.06E-08
ENSG00000117308_23797051_23796505_120	novel	GALE	1	-	360	14	0	1	5.83%	12.8497651	0.008373824	0.011738695	0.000494661
ENSG00000171720_141634860_141621468_351	novel	HDAC3	5	-	1053	45	11	4	5.98%	14.12062099	0.002419462	0.00028196	4.70E-06
ENSG00000060971_38131606_38126490_133	novel	ACAA1	3	-	399	7	0	0	3.76%	5.796886511	0.019216965	0.019216965	0.001707396
ENSG00000051180_40698771_40731178_335	annotated	RAD51	15	+	1005	30	6	1	2.99%	9.863359138	0.017780847	0.002315502	0.000242831
ENSG00000167491_19492608_19505474_436	Overlap_dORF	GATAD2A	19	+	1308	27	0	0	2.06%	6.820648578	0.003684287	0.003684287	7.54E-05
ENSG00000131069_34923350_34927214_117	novel	ACSS2	20	+	351	35	3	2	5.98%	32.94811564	0.020905271	0.01739507	0.00168501
ENSG00000131069_34876646_34927098_212	novel	ACSS2	20	+	636	23	3	24	5.19%	11.94924248	0.005578142	0.332933467	0.017872387
ENSG00000168958_227340328_227357117_89	novel	MFF	2	+	267	32	5	9	7.87%	39.60120223	0.020342454	0.024694694	0.0022779
ENSG00000132376_1509784_1495823_356	annotated	INPP5K	17	-	1068	35	5	5	4.21%	10.82845374	0.006166408	0.002296562	8.04E-05
ENSG00000125885_5952016_5994391_793	annotated	MCM8	20	+	2379	27	2	1	1.77%	3.750066557	0.001083027	0.000655226	4.48E-06
ENSG00000119537_63359759_63331782_255	dORF	KDSR	18	-	765	60	3	0	7.06%	25.91549264	0.00044395	8.70E-05	2.80E-07
ENSG00000160310_46636952_46664334_289	annotated	PRMT2	21	+	867	33	2	10	5.19%	12.57663613	0.000599389	0.013066943	5.60E-05
ENSG00000143933_47162588_47160776_113	annotated	CALM2	2	-	339	735	44	31	24.78%	716.402833	1.84E-06	4.82E-06	7.66E-11
ENSG00000109189_52659150_52597640_339	annotated	USP46	4	-	1017	11	0	0	2.06%	3.573891684	0.007882058	0.007882058	0.000319636
ENSG00000143612_154219886_154213194_110	annotated	C1orf43	1	-	330	43	2	0	9.09%	43.05505708	0.003961388	0.002488365	5.59E-05
ENSG00000143612_154212583_154207453_126	dORF	C1orf43	1	-	378	107	17	2	19.05%	93.53230378	0.000254814	2.48E-05	4.99E-08
ENSG00000105647_18156048_18169294_672	annotated	PIK3R2	19	+	2016	23	3	4	1.34%	3.769701496	0.014094678	0.02192684	0.001455373
ENSG00000105643_18008311_18012966_407	annotated	ARRDC2	19	+	1221	30	6	0	2.21%	8.118489708	0.027540999	0.003763158	0.000584752
ENSG00000105185_32584960_32587300_87	Overlap_dORF	PDCD5	19	+	261	93	26	5	17.24%	117.736764	0.002898308	0.003315387	5.43E-05
ENSG00000062822_50417864_50417947_27	dORF	POLD1	19	+	81	12	0	0	22.22%	48.95148609	0.012798403	0.012798403	0.000797244
ENSG00000062822_50398981_50417184_966	annotated	POLD1	19	+	2898	27	0	8	1.66%	3.078470787	0.000145171	0.013026325	1.77E-05
ENSG00000178401_49348975_49351502_307	annotated	DNAJC22	12	+	921	27	7	0	4.56%	9.686654007	0.012140539	0.000415605	3.81E-05
ENSG00000104824_38846077_38836722_493	annotated	HNRNPL	19	-	1479	303	26	12	8.92%	67.69305404	1.80E-07	4.41E-08	7.85E-14
ENSG00000059804_7936034_7921413_496	annotated	SLC2A3	12	-	1488	21	1	0	2.62%	4.663221205	0.001013137	0.000583356	3.76E-06
ENSG00000164081_51674305_51704016_313	annotated	TEX264	3	+	939	178	24	6	12.46%	62.63600696	2.04E-06	7.66E-08	1.59E-12
ENSG00000070770_58197736_58164071_350	annotated	CSNK2A2	16	-	1050	94	21	0	10.86%	29.58068374	2.45E-05	2.88E-08	9.89E-12
ENSG00000177200_53245444_53324895_1827	novel	CHD9	16	+	5481	28	4	6	0.82%	1.687982279	0.003873118	0.008058973	0.000168899
ENSG00000197713_210016017_210019791_146	annotated	RPE	2	+	438	49	4	0	8.90%	36.96507768	0.004036058	0.000705256	1.81E-05
ENSG00000135525_136550408_136344228_712	annotated	MAP7	6	-	2136	43	5	7	2.67%	6.651764437	0.000468222	0.002122021	6.45E-06
ENSG00000169359_155853997_155845054_265	annotated	SLC33A1	3	-	795	62	9	1	9.06%	25.76880117	0.000429561	9.85E-06	3.83E-08
ENSG00000169359_155829873_155828210_117	dORF	SLC33A1	3	-	351	10	1	0	5.13%	9.413747325	0.023672501	0.012798403	0.001437523
ENSG00000275066_37571958_37541964_713	novel	SYNRG	17	-	2139	26	7	2	1.82%	4.016356152	0.01646004	0.002333533	0.000225053
ENSG00000172922_65720340_65719783_81	novel	RNASEH2C	11	-	243	40	0	2	9.88%	54.3905401	0.005355751	0.020990661	0.000592297
ENSG00000285053_235380050_235448762_527	dORF	TBCE	1	+	1581	34	20	5	2.85%	7.105860884	0.036601443	0.003024558	0.000668515
ENSG00000108439_47941769_47946782_187	annotated	PNPO	17	+	561	66	18	7	9.09%	38.87323895	0.003882003	0.000650933	1.62E-05
ENSG00000138600_50765533_50707800_520	annotated	SPPL2A	15	-	1560	402	56	6	15.96%	85.14734456	4.73E-12	1.46E-14	1.05E-24
ENSG00000131943_29708389_29705348_60	annotated	C19orf12	19	-	180	13	2	0	8.33%	23.86384947	0.08410207	0.021084099	0.007951576
ENSG00000138604_69255807_69269244_617	annotated	GLCE	15	+	1851	73	8	6	4.86%	13.03125055	1.44E-05	1.26E-05	1.45E-09
ENSG00000053747_23873078_23954648_1657	annotated	LAMA3	18	+	4971	15	0	0	0.30%	0.997050486	0.020613417	0.020613417	0.001946209
ENSG00000152942_69372209_69414292_670	annotated	RAD17	5	+	2010	17	1	0	1.19%	2.794618422	0.005454749	0.005554612	0.000161874
ENSG00000102710_37045271_37023031_445	annotated	SUPT20H	13	-	1335	26	1	0	2.02%	6.435195363	0.005642788	0.003529166	0.000109245
ENSG00000211460_121755780_121755890_36	annotated	TSN	2	+	108	11	0	0	16.67%	33.65414669	0.012798403	0.012798403	0.000797244
ENSG00000205758_33631551_33603359_169	novel	CRYZL1	21	-	507	13	3	1	5.33%	8.472372593	0.038777096	0.00650005	0.001330649
ENSG00000007341_112619113_112540834_555	annotated	ST7L	1	-	1665	28	1	3	1.98%	5.556655178	0.002167177	0.008808686	0.000109703
ENSG00000204310_32170017_32169276_74	novel	AGPAT1	6	-	222	12	0	2	8.11%	17.86067736	0.012798403	0.061356297	0.003794065
ENSG00000087302_51999737_52001887_49	novel	RTRAF	14	+	147	205	11	0	12.24%	460.7933257	0.013853925	0.013853925	0.000925205
ENSG00000168487_22165406_22201203_730	annotated	BMP1	8	+	2190	90	20	1	4.79%	13.57900813	3.76E-05	1.20E-07	5.52E-11
ENSG00000129103_56064312_56079612_232	annotated	SUMF2	7	+	696	558	77	11	20.69%	264.9077189	1.94E-06	2.35E-09	6.45E-14
ENSG00000091039_76392690_76390399_122	novel	OSBPL8	12	-	366	10	0	1	4.10%	9.027938009	0.020613417	0.035374934	0.003252526
ENSG00000162623_74733359_74764113_221	annotated	TYW3	1	+	663	21	0	0	4.07%	10.46587203	0.003327803	0.003327803	6.21E-05
ENSG00000105393_17268807_17279048_254	annotated	BABAM1	19	+	762	18	3	0	3.54%	7.805256641	0.003278443	0.003303074	6.08E-05
ENSG00000182985_115231473_115169589_240	annotated	CADM1	11	-	720	50	6	0	8.33%	22.94600911	0.000697572	3.77E-05	2.11E-07
ENSG00000101361_2657932_2658294_120	novel	NOP56	20	+	360	7	3	0	4.17%	6.424882549	0.170087137	0.019216965	0.016246064
ENSG00000134294_46370789_46361111_406	annotated	SLC38A2	12	-	1218	1123	177	55	32.51%	304.6506588	2.35E-18	4.50E-21	1.94E-37
ENSG00000134419_18789104_18783009_127	annotated	RPS15A	16	-	381	162	28	5	8.66%	140.4946195	0.016256388	0.003487661	0.000313547
ENSG00000023228_206153678_206124185_727	annotated	NDUFS1	2	-	2181	172	26	4	8.94%	26.05808132	3.57E-10	4.56E-12	2.15E-20
ENSG00000181929_49004698_49004459_79	novel	PRKAG1	12	-	237	27	5	0	8.86%	37.64307317	0.008574016	0.008574016	0.000374746
ENSG00000181929_49018740_49004960_137	novel	PRKAG1	12	-	411	18	0	0	7.30%	14.47105976	0.002201184	0.002201184	2.82E-05
ENSG00000119801_30156652_30158843_121	novel	YPEL5	2	+	363	82	11	3	19.83%	74.64090235	7.68E-06	2.13E-05	1.33E-09
ENSG00000196465_56153919_56157726_208	annotated	MYL6B	12	+	624	37	10	6	7.21%	19.59236162	0.005320053	0.003041419	8.99E-05
ENSG00000100911_24145273_24143409_162	dORF	PSME2	14	-	486	125	10	4	8.64%	84.98521891	0.000482311	0.000478119	1.51E-06
ENSG00000075711_197297204_197136666_300	annotated	DLG1	3	-	900	8	0	0	1.67%	2.937089165	0.016947427	0.016947427	0.001349898
ENSG00000075711_197130563_197044623_517	dORF	DLG1	3	-	1551	23	4	0	2.13%	4.899882795	0.013619674	0.001440978	0.00012196

ENSG00000077782_38457446_38413628_820	annotated	FGFR1	8	-	2460	101	18	8	5.49%	13.56612831	1.63E-06	9.83E-08	1.59E-12
ENSG00000074842_4670334_4659803_189	annotated	MYDGF	19	-	567	171	23	13	18.52%	99.65123954	6.03E-07	2.32E-06	1.26E-11
ENSG00000135940_97646087_97648108_129	annotated	COX5B	2	+	387	651	52	27	27.13%	555.8270485	1.60E-07	3.48E-06	5.52E-12
ENSG00000244038_20661350_20652379_439	annotated	DDOST	1	-	1317	816	101	13	22.55%	204.7264885	7.47E-14	5.08E-18	7.75E-30
ENSG00000274290_26183796_26184176_126	annotated	HIST1H2BE	6	+	378	176	7	3	14.29%	153.8475277	0.000226501	9.55E-05	1.55E-07
ENSG00000170854_97967593_97945184_464	annotated	RIOX2	3	-	1392	42	16	2	4.53%	9.969645335	0.013138298	3.48E-05	5.82E-06
ENSG00000131943_29708389_29702712_133	annotated	C19orf12	19	-	399	22	1	0	7.52%	18.21878618	0.004136993	0.002201184	5.20E-05
ENSG00000086475_13333956_13319142_252	annotated	SEPHS1	10	-	756	26	2	3	4.37%	11.36373784	0.004803511	0.008742337	0.00022279
ENSG00000196290_200892025_200903678_350	annotated	NIF3L1	2	+	1050	53	6	1	6.57%	16.67847062	0.000150606	9.45E-06	1.20E-08
ENSG00000096384_44248702_44253598_700	annotated	HSP90AB1	6	+	2100	438	84	36	12.43%	68.91669935	1.41E-13	2.89E-13	5.40E-25
ENSG00000054523_10267389_10378402_1663	annotated	KIF1B	1	+	4989	29	9	0	0.96%	1.920676168	0.010286319	0.000174956	1.55E-05
ENSG00000173436_19623479_19626401_39	novel	MICOS10	1	+	117	60	7	4	23.08%	169.4474519	0.009451772	0.003816221	0.000195281
ENSG00000136271_44573612_44566002_483	novel	DDX56	7	-	1449	166	4	10	10.77%	37.85378893	2.76E-10	1.39E-09	4.35E-18
ENSG00000158526_54440422_54444550_191	annotated	TSR2	X	+	573	43	12	0	9.42%	24.79610617	0.003437774	7.61E-05	2.22E-06
ENSG00000040487_19317678_19328643_291	annotated	PQLC2	1	+	873	23	3	2	4.12%	8.705290052	0.006771542	0.004676575	0.000169421
ENSG00000040487_19317678_19328643_291	annotated	PQLC2	1	+	873	23	3	2	4.12%	8.705290052	0.006771542	0.004676575	0.000169421
ENSG00000204315_32129522_32128731_263	annotated	FKBPL	6	-	789	10	2	0	1.90%	4.18786478	0.060322713	0.020613417	0.005529088
ENSG00000127445_9835345_9849199_163	annotated	PIN1	19	+	489	31	3	11	8.59%	20.94703162	0.001096665	0.011457359	8.02E-05
ENSG00000179387_140525429_140527503_59	novel	ELMOD2	4	+	177	18	0	0	13.56%	33.6022913	0.005257623	0.005257623	0.000148366
ENSG00000131446_180792971_180791634_445	annotated	MGAT1	5	-	1335	90	7	9	8.76%	22.27567626	2.32E-07	1.57E-06	3.43E-12
ENSG00000198742_99143780_99030584_757	annotated	SMURF1	7	-	2271	12	5	2	1.19%	1.745957892	0.052098945	0.014696947	0.003581977
ENSG00000198799_113073407_113124101_1065	annotated	LRIG2	1	+	3195	14	2	0	1.03%	1.447860856	0.010137502	0.001068153	6.88E-05
ENSG00000133114_44989655_45028003_338	annotated	GPALPP1	13	+	1014	38	0	0	5.92%	12.3826984	2.86E-05	2.86E-05	6.30E-09
ENSG00000072518_63839507_63909237_719	annotated	MARK2	11	+	2157	48	9	3	3.06%	7.352935324	0.001008036	0.00010476	7.74E-07
ENSG00000156675_37877439_37862895_1122	annotated	RAB11FIP1	8	-	3366	13	1	10	0.45%	1.276141683	0.021084099	0.310960732	0.037095452
ENSG00000259112_78079744_78038664_134	annotated	NDUFC2-KCTD14	11	-	402	601	129	60	29.10%	493.9899035	1.65E-07	1.50E-06	2.38E-12
ENSG00000114346_172754531_172820237_883	annotated	ECT2	3	+	2649	336	31	12	9.63%	41.91089862	1.36E-13	5.51E-15	1.07E-26
ENSG00000114982_96619749_96595611_743	dORF	KANSL3	2	-	2229	38	2	0	1.75%	5.633044496	0.002021422	0.000681533	8.65E-06
ENSG00000203879_154437255_154442672_447	annotated	GDI1	X	+	1341	99	20	7	8.28%	24.39360968	7.56E-05	6.58E-07	5.31E-10
ENSG00000057608_5766074_5766006_22	Overlap_dORF	GDI2	10	-	66	17	0	0	31.82%	85.10883377	0.008473674	0.008473674	0.000366502
ENSG00000120709_138341336_138346959_392	annotated	FAM53C	5	+	1176	15	0	0	1.53%	4.214573101	0.01300724	0.01300724	0.000821865
ENSG00000185624_81860471_81844012_464	annotated	P4HB	17	-	1392	1911	252	48	34.05%	453.6188628	8.94E-24	8.75E-27	1.62E-48
ENSG00000111667_6855428_6866077_812	annotated	USP5	12	+	2436	159	28	10	5.79%	21.56698787	1.32E-06	3.28E-08	4.62E-13
ENSG00000111665_6850952_6849043_243	annotated	CDCA3	12	-	729	111	11	0	12.35%	50.31124959	6.73E-06	7.81E-07	4.78E-11
ENSG00000111666_101697862_101728945_406	annotated	CHPT1	12	+	1218	142	14	6	8.87%	38.52216701	7.17E-06	8.30E-07	5.39E-11
ENSG00000167515_88857151_88859368_75	annotated	TRAPPC2L	16	+	225	113	34	7	20.00%	165.9455378	0.009666645	0.004407029	0.000227452
ENSG00000114062_25375498_25354349_676	annotated	UBE3A	15	-	2028	33	10	0	2.22%	5.376697992	0.002059394	0.000290289	4.07E-06
ENSG00000119801_30156652_30158843_121	annotated	YPEL5	2	+	363	82	11	3	19.83%	74.64090235	7.68E-06	2.13E-05	1.33E-09
ENSG00000103978_42273398_42268003_57	Overlap_uORF	TMEM87A	15	-	171	14	1	0	8.77%	27.05213705	0.035374934	0.020613417	0.003252526
ENSG00000103978_42273276_42211708_494	annotated	TMEM87A	15	-	1482	128	14	2	8.30%	28.53851821	4.33E-07	2.55E-08	1.14E-13
ENSG00000134243_109397892_109314043_831	annotated	SORT1	1	-	2493	177	12	5	6.98%	23.45960209	6.73E-10	3.85E-11	3.14E-19
ENSG00000168394_32852497_32845579_547	novel	TAP1	6	-	1641	38	10	6	3.84%	7.65146629	0.003666291	0.000550757	1.31E-05
ENSG00000110696_16744670_16755104_139	annotated	C11orf58	11	+	417	52	4	8	10.79%	41.20376887	0.001877372	0.008483964	9.30E-05
ENSG00000146063_181224417_181233641_242	annotated	TRIM41	5	+	726	12	4	4	2.07%	5.46152944	0.169362074	0.079862169	0.047393137
ENSG00000075711_197138252_197044623_599	annotated	DLG1	3	-	1797	31	4	0	2.17%	5.700110442	0.004387317	0.000656046	1.85E-05
ENSG00000120915_27491209_27544522_555	annotated	EPHX2	8	+	1665	10	0	0	1.26%	1.984519706	0.007029303	0.007029303	0.000257364
ENSG00000048028_113854326_113830923_237	novel	USP28	11	-	711	11	2	1	2.53%	5.112022282	0.060667625	0.026832696	0.006950196
ENSG00000118518_127286653_127287693_346	annotated	RNF146	6	+	1038	40	1	0	5.20%	12.73304552	8.20E-05	8.27E-05	4.95E-08
ENSG00000108883_44859958_44854093_238	novel	EFTUD2	17	-	714	26	0	4	5.46%	12.03219301	0.000640157	0.007903646	3.39E-05
ENSG00000108883_44853375_44851274_145	novel	EFTUD2	17	-	435	59	9	7	13.79%	44.81592951	0.00036668	0.000452842	1.10E-06
ENSG00000183780_233905533_234323243_471	annotated	SLC35F3	1	+	1413	38	2	2	3.18%	8.886097794	0.000997927	0.000997927	6.18E-06
ENSG00000183780_234214499_234323243_421	annotated	SLC35F3	1	+	1263	32	0	2	2.61%	8.37175059	0.001558047	0.004474589	4.10E-05
ENSG00000118894_5097739_5085632_330	annotated	EEF2KMT	16	-	990	27	2	2	3.03%	9.011523576	0.002390426	0.005340745	7.23E-05
ENSG00000108100_35337054_35569170_316	annotated	CCNY	10	+	948	26	0	0	3.80%	9.062221318	0.000991051	0.000991051	6.10E-06
ENSG00000168275_234374261_234383818_74	annotated	COA6	1	+	222	74	6	3	14.86%	110.1408437	0.005641909	0.006346985	0.000189755
ENSG00000168275_234374261_234383818_74	annotated	COA6	1	+	222	74	6	3	14.86%	110.1408437	0.005641909	0.006346985	0.000189755
ENSG00000168264_234609494_234607137_571	annotated	IRF2BP2	1	-	1713	105	18	7	5.78%	20.25357021	5.19E-05	1.13E-06	5.72E-10
ENSG00000089597_62646599_62625815_852	annotated	GANAB	11	-	2556	1706	241	53	27.93%	220.540234	5.74E-31	1.25E-37	2.31E-66
ENSG00000196628_55350976_55228225_539	annotated	TCF4	18	-	1617	24	0	0	1.86%	4.904230517	0.002404282	0.002404282	3.34E-05
ENSG00000110536_47565942_47571629_131	Overlap_dORF	PTPMT1	11	+	393	40	1	10	12.21%	33.63079197	0.000186366	0.015732779	2.70E-05
ENSG00000111678_6944122_6945922_97	annotated	C12orf57	12	+	291	193	47	13	22.68%	219.1462148	0.000398264	0.000157375	4.34E-07
ENSG00000111678_6943971_6944057_28	uORF	C12orf57	12	+	84	19	0	2	21.43%	74.73842966	0.013640586	0.032657853	0.002090452
ENSG00000138029_26254255_26289953_351	annotated	HADHB	2	+	1053	157	30	2	11.97%	49.26527767	3.25E-06	1.56E-08	6.15E-13
ENSG00000158321_70764912_70790996_801	annotated	AUTS2	7	+	2403	13	4	0	1.00%	1.787554267	0.05158844	0.004826937	0.001430389
ENSG00000168036_41239196_41239342_48	novel	CTNNB1	3	+	144	32	0	0	12.50%	73.42722914	0.013640586	0.013640586	0.000898637
ENSG00000184047_122224668_122208381_186	novel	DIABLO	12	-	558	57	4	0	6.99%	33.7528392	0.00114312	0.000710064	5.10E-06
ENSG00000204356_31954374_31952301_110	novel	NELFE	6	-	330	27	1	0	8.18%	27.03457073	0.003554776	0.003554776	7.04E-05
ENSG00000204356_31958446_31955016_148	novel	NELFE	6	-	444	88	6	4	8.78%	65.48915031	0.002283116	0.001809809	2.42E-05

ENSG00000154813_16264876_16260764_82	annotated	DPH3	3	-	246	18	8	1	6.10%	24.17725837	0.194467655	0.019679754	0.019407797
ENSG00000101444_34290436_34281034_143	novel	AHCY	20	-	429	87	18	1	14.69%	67.00876505	0.001125745	2.76E-05	2.70E-07
ENSG00000101444_34303270_34290547_285	novel	AHCY	20	-	855	317	44	7	17.54%	122.5075349	6.72E-08	2.07E-09	1.57E-15
ENSG00000244005_35674566_35669622_124	novel	NFS1	20	-	372	23	1	2	6.45%	20.42935004	0.009341698	0.009341698	0.000440618
ENSG00000158411_99171402_99169496_96	novel	MITD1	2	-	288	26	5	0	8.33%	29.82981184	0.005604867	0.005604867	0.000167525
ENSG00000163374_155676556_155659657_645	dORF	YY1AP1	1	-	1935	29	7	1	1.86%	4.952068942	0.006392297	0.001476269	5.61E-05
ENSG00000100644_61736965_61740577_59	novel	HIF1A	14	+	177	40	1	7	18.64%	74.67175844	0.001513502	0.019173238	0.000184673
ENSG00000168092_117152448_117163979_165	novel	PAFAH1B2	11	+	495	58	12	2	9.09%	38.71617536	0.002644549	0.00052791	9.02E-06
ENSG00000179913_17808006_17812122_306	annotated	B3GNT3	19	+	918	31	6	1	3.27%	11.15805933	0.026575195	0.003159663	0.000486445
ENSG00000070718_42154688_42167684_273	annotated	AP3M2	8	+	819	11	0	1	1.83%	4.437909453	0.021084099	0.035772986	0.003354605
ENSG00000144713_12840237_12836094_135	annotated	RPL32	3	-	405	76	11	1	8.89%	62.00521572	0.00808812	0.001953476	9.17E-05
ENSG00000091490_25847567_25748425_979	annotated	SEL1L3	4	-	2937	57	4	0	2.66%	6.412694679	1.71E-05	3.38E-06	4.90E-10
ENSG00000159788_3316999_3343062_392	novel	RGS12	4	+	1176	11	0	0	1.28%	3.090686941	0.020613417	0.020613417	0.001946209
ENSG00000141696_41811915_41802957_437	annotated	P3H4	17	-	1311	165	17	0	10.53%	41.58635975	7.71E-08	1.26E-09	1.14E-15
ENSG00000176953_28951012_28952380_211	novel	NFATC2IP	16	+	633	15	2	0	3.32%	7.829917799	0.032488891	0.007882058	0.001297267
ENSG00000152601_152300194_152459327_370	annotated	MBNL1	3	+	1110	67	6	6	5.41%	19.94442305	0.000148948	0.000505146	5.24E-07
ENSG00000243943_27601422_27621461_351	annotated	ZNF512	2	+	1053	13	2	0	2.28%	4.079290508	0.0135992	0.005160369	0.000368527
ENSG00000166888_57106816_57096572_729	dORF	STAT6	12	-	2187	67	3	1	3.84%	10.12268385	4.26E-06	1.23E-06	4.62E-11
ENSG00000009830_77299471_77277376_448	novel	POMT2	14	-	1344	27	3	2	3.35%	6.637952634	0.002026908	0.000437802	5.79E-06
ENSG00000100714_64415361_64458303_854	annotated	MTHFD1	14	+	2562	183	15	5	7.38%	23.60160937	3.29E-10	1.54E-11	6.29E-20
ENSG00000131844_71587426_71643794_329	annotated	MCCC2	5	+	987	56	9	1	5.78%	18.74737765	0.002093883	5.63E-05	9.89E-07
ENSG00000174109_1420443_1420246_65	novel	C16orf91	16	-	195	45	5	4	10.77%	76.25135333	0.032206895	0.01739507	0.002553454
ENSG00000103429_14644347_14667827_325	annotated	BFAR	16	+	975	60	8	2	8.62%	20.33369422	5.81E-05	3.08E-06	1.59E-09
ENSG00000149923_30082782_30085062_228	dORF	PPP4C	16	+	684	222	41	5	20.61%	107.2424004	2.81E-07	2.99E-09	9.91E-15
ENSG00000147364_413080_468804_367	annotated	FBXO25	8	+	1101	21	1	0	2.72%	6.302337106	0.002593574	0.002188006	3.28E-05
ENSG00000048471_11976807_12568629_813	annotated	SNX29	16	+	2439	7	1	0	0.62%	0.948322148	0.041782825	0.019216965	0.003600526
ENSG00000157540_37420375_37512531_754	annotated	DYRK1A	21	+	2262	22	4	0	1.72%	3.21365857	0.007183997	0.000544026	2.66E-05
ENSG00000137404_30690139_30688661_183	annotated	NRM	6	-	549	58	13	3	10.38%	34.90802697	0.00202797	0.000215919	3.08E-06
ENSG00000142599_8557502_8438295_221	annotated	RERE	1	-	663	8	0	1	2.26%	3.986998867	0.019679754	0.042234516	0.003715148
ENSG00000091527_133575105_133587174_110	annotated	CDV3	3	+	330	26	3	0	8.18%	26.03329033	0.008316137	0.003632191	0.000164361
ENSG00000184371_109911024_109925189_256	annotated	CSF1	1	+	768	27	5	1	4.69%	11.61641711	0.010737298	0.001032462	7.11E-05
ENSG00000172845_173955605_173909941_479	annotated	SP3	2	-	1437	59	4	1	4.59%	13.56640872	3.52E-05	2.32E-05	6.32E-09
ENSG00000088256_3118925_3121434_185	annotated	GNA11	19	+	555	25	0	0	5.41%	14.8838978	0.002254349	0.002254349	2.95E-05
ENSG00000099624_1244171_1244437_45	novel	ATP5F1D	19	+	135	380	4	6	48.89%	930.0782357	1.94E-05	2.71E-05	4.11E-09
ENSG00000129351_10670980_10689312_891	annotated	ILF3	19	+	2673	194	24	10	7.18%	23.98128359	1.66E-10	3.31E-11	6.57E-20
ENSG00000158019_27894557_28327433_415	annotated	BABAM2	2	+	1245	66	5	1	7.47%	17.51637514	2.33E-06	5.84E-07	1.23E-11
ENSG00000171858_62387339_62388001_90	annotated	RPS21	20	+	270	916	63	30	23.33%	1120.989031	0.000990638	0.000119985	8.59E-07
ENSG00000184220_100162372_100178308_75	annotated	CMSS1	3	+	225	38	14	0	14.67%	55.80469414	0.036259869	0.001588175	0.000395518
ENSG00000158850_161175991_161171816_370	annotated	B4GALT3	1	-	1110	39	4	5	4.59%	11.60944028	0.000630271	0.001321569	5.26E-06
ENSG00000204130_68404800_68378562_387	annotated	RUFY2	10	-	1161	13	1	0	2.58%	3.699821623	0.004002249	0.001755235	4.09E-05
ENSG00000185917_36057384_36043759_283	annotated	SETD4	21	-	849	8	1	3	1.77%	3.113522084	0.042234516	0.150401371	0.025490393
ENSG00000185917_36058888_36043759_307	annotated	SETD4	21	-	921	8	1	3	1.63%	2.870119706	0.042234516	0.150401371	0.025490393
ENSG00000185917_36057384_36043759_283	annotated	SETD4	21	-	849	8	1	3	1.77%	3.113522084	0.042234516	0.150401371	0.025490393
ENSG00000185917_36058888_36043759_307	annotated	SETD4	21	-	921	8	1	3	1.63%	2.870119706	0.042234516	0.150401371	0.025490393
ENSG00000116698_183515939_183553201_1136	annotated	SMG7	1	+	3408	34	7	3	1.41%	3.296468914	0.005158394	0.000534354	1.84E-05
ENSG00000056097_32390301_32379206_176	annotated	ZFR	5	-	528	26	2	2	3.41%	16.27080646	0.020342454	0.020342454	0.001898761
ENSG00000159461_56369309_56362909_177	annotated	AMFR	16	-	531	80	10	0	16.95%	49.7811723	1.70E-05	7.09E-07	1.14E-10
ENSG00000159461_56403073_56385983_79	internal	AMFR	16	-	237	56	17	4	15.19%	78.07452212	0.075038277	0.004616409	0.002128044
ENSG00000247796_53109990_53112278_65	novel	AC008966.1	5	+	195	17	0	0	9.23%	28.80606682	0.013428348	0.013428348	0.000872559
ENSG00000137513_78528849_78436670_250	annotated	NARS2	11	-	750	38	0	0	4.80%	16.74140824	0.000958906	0.000958906	5.72E-06
ENSG00000164961_125083898_125024617_1159	annotated	WASHC5	8	-	3477	93	9	11	3.71%	8.837876156	3.05E-07	4.85E-07	1.37E-12
ENSG00000147862_14313508_14306015_187	annotated	NFIB	9	-	561	23	3	0	4.81%	13.54673479	0.006191047	0.003303074	0.000112537
ENSG00000170852_32879604_32870210_334	annotated	KBTBD2	7	-	1002	13	1	1	2.40%	4.286919066	0.00912793	0.00912793	0.000421779
ENSG00000155368_119367052_119372318_129	annotated	DBI	2	+	387	667	54	12	23.26%	569.4879283	4.72E-05	5.55E-06	2.20E-09
ENSG00000156860_30666985_30669645_357	dORF	FBRS	16	+	1071	20	0	0	1.68%	6.17035539	0.013428348	0.013428348	0.000872559
ENSG00000144579_218401642_218402355_60	novel	CTDSP1	2	+	180	21	0	3	10.00%	38.5492953	0.013640586	0.032657853	0.002090452
ENSG00000133104_36335830_36304365_666	annotated	SPART	13	-	1998	55	9	2	4.50%	9.095715321	0.000126482	2.55E-06	3.08E-09
ENSG00000111615_75511597_75506728_148	novel	KRR1	12	-	444	15	1	2	5.41%	11.16292335	0.009288069	0.02389627	0.001092027
ENSG00000139624_50167297_50145025_77	annotated	CERS5	12	-	231	22	1	0	6.49%	31.46881249	0.028889786	0.021084099	0.002731533
ENSG00000258388_32155720_32166182_146	Overlap_dORF	PPT2-EGFL8	6	+	438	19	11	0	6.16%	14.33339747	0.192345125	0.003529166	0.005873069
ENSG00000141380_26087577_26068552_55	novel	AC091021.1	18	-	165	11	2	0	9.09%	22.02816874	0.042234516	0.021084099	0.003945154
ENSG00000087191_63829923_63831969_327	dORF	PSMC5	17	+	981	407	52	15	25.99%	137.0866159	1.04E-12	6.14E-15	9.61E-26
ENSG00000136450_58007137_58005747_201	annotated	SRSF1	17	-	603	399	43	66	24.88%	218.6377942	7.06E-08	4.84E-07	3.35E-13
ENSG00000007202_28619865_28618731_122	novel	KIAA0100	17	-	366	7	0	0	4.10%	6.319556606	0.019216965	0.019216965	0.001707396
ENSG00000178999_8207605_8206524_66	novel	AURKB	17	-	198	9	8	0	9.09%	15.01920596	0.356865192	0.011772051	0.031397685
ENSG00000108465_47975293_47981502_350	dORF	CDK5RAP3	17	+	1050	29	2	1	3.71%	9.125955621	0.001480749	0.000995868	9.04E-06
ENSG00000183495_132031959_132050752_576	novel	EP400	12	+	1728	14	3	1	1.39%	2.677034396	0.046396937	0.007930666	0.001900996
ENSG00000141646_51065458_51077320_156	annotated	SMAD4	18	+	468	12	2	0	3.85%	8.472372593	0.052581193	0.01300724	0.003266469

ENSG00000198561_57795613_57816308_861	annotated	CTNND1	11	+	2583	196	34	13	6.50%	25.07271239	6.69E-07	4.54E-09	3.66E-14
ENSG00000152601_152300194_152459327_400	annotated	MBNL1	3	+	1200	73	6	6	5.50%	20.10070398	5.79E-05	0.000178168	7.59E-08
ENSG00000135900_223957473_223967034_332	annotated	MRPL44	2	+	996	111	10	1	11.14%	36.82419774	2.32E-07	7.15E-08	1.61E-13
ENSG00000135899_230216879_230176665_533	annotated	SP110	2	-	1599	9	2	0	1.50%	1.859789106	0.040206608	0.003327803	0.00080239
ENSG00000135899_230216879_230169124_697	annotated	SP110	2	-	2091	8	2	0	1.00%	1.264170373	0.061805201	0.005706018	0.002003224
ENSG00000148841_104315931_104314408_507	annotated	ITPRIP	10	-	1521	44	3	0	3.55%	9.558574207	0.000369137	8.58E-05	2.29E-07
ENSG00000123444_47577999_47575575_237	annotated	KBTBD4	11	-	711	19	4	4	2.95%	8.829856668	0.044241359	0.052998774	0.009449261
ENSG00000139974_61037666_61052429_254	dORF	SLC38A6	14	+	762	22	12	0	3.15%	9.539758116	0.239543855	0.005604867	0.010902226
ENSG00000110719_68044219_68050819_532	novel	TCIRG1	11	+	1596	87	25	6	5.08%	18.01175452	0.000898639	9.02E-06	8.05E-08
ENSG00000145284_82798381_82660876_204	annotated	SCD5	4	-	612	12	2	0	2.45%	6.478873159	0.028915639	0.016947427	0.002246053
ENSG00000160293_133992083_133764062_803	annotated	VAV2	9	-	2409	27	1	5	1.62%	3.703365853	0.000980148	0.011958166	7.64E-05
ENSG00000160293_133992083_133764062_813	annotated	VAV2	9	-	2439	27	1	5	1.60%	3.657813998	0.000980148	0.011958166	7.64E-05
ENSG00000124214_49154033_49114878_496	annotated	STAU1	20	-	1488	70	4	6	7.06%	15.54407068	2.70E-07	3.18E-06	8.17E-12
ENSG00000055130_148730123_148800582_776	annotated	CUL1	7	+	2328	69	10	0	2.45%	9.793451309	0.000153021	5.88E-05	6.60E-08
ENSG00000132591_28855035_28860553_437	annotated	ERAL1	17	+	1311	122	18	4	7.09%	30.74870236	0.000153578	1.46E-06	2.33E-09
ENSG00000221926_15651609_15628615_564	annotated	TRIM16	17	-	1692	98	31	5	3.90%	19.13794802	0.00388623	2.24E-05	9.29E-07
ENSG00000196141_200389245_200478031_558	annotated	SPATS2L	2	+	1674	89	15	3	5.91%	17.56726719	3.82E-05	1.16E-06	4.22E-10
ENSG00000185414_99186204_99194924_101	annotated	MRPL30	2	+	303	20	7	1	4.95%	21.81006806	0.2391991	0.021084099	0.026311969
ENSG00000180398_46941663_46905463_127	annotated	MCFD2	2	-	381	61	7	4	10.24%	52.90229501	0.003903476	0.003000296	6.56E-05
ENSG00000157349_70299298_70333582_479	annotated	DDX19B	16	+	1437	45	1	2	3.55%	10.34726089	0.000125329	0.000478467	4.23E-07
ENSG00000006118_60936206_60936907_233	dORF	TMEM132A	11	+	699	17	2	3	3.00%	8.036027223	0.032826899	0.045243965	0.006233557
ENSG00000182919_93750348_93761688_296	annotated	C11orf54	11	+	888	51	2	1	6.08%	18.97696969	0.000110892	0.000103297	8.24E-08
ENSG00000136861_120550836_120389236_1727	annotated	CDK5RAP2	9	-	5181	52	38	10	1.74%	3.316342717	0.002696287	0.000120187	2.51E-06
ENSG00000197798_126235002_126262133_350	annotated	FAM118B	11	+	1050	44	0	4	4.57%	13.84627749	0.000198297	0.000997799	1.36E-06
ENSG00000178035_49025035_49024655_95	novel	IMPDH2	3	-	285	27	0	0	13.68%	31.30318716	0.000656046	0.000656046	2.76E-06
ENSG00000099331_17102393_17212310_1932	annotated	MYO9B	19	+	5796	32	5	3	0.78%	1.824278985	0.002266245	0.001871465	2.48E-05
ENSG00000124275_7870795_7875267_92	annotated	MTRR	5	+	276	34	5	0	9.78%	40.70422485	0.015932511	0.003763158	0.000327992
ENSG00000092330_24242332_24239797_451	annotated	AL096870.1	14	-	1353	20	16	3	2.22%	4.884294621	0.416713659	0.012468381	0.041431285
ENSG00000158161_28011012_27977282_301	annotated	EYA3	1	-	903	12	0	3	1.99%	4.390997091	0.011772051	0.071345384	0.004172987
ENSG00000166333_6608898_6610611_298	annotated	ILK	11	+	894	94	12	7	10.07%	34.74241379	1.32E-05	2.28E-05	2.38E-09
ENSG00000166326_35725939_35806385_90	novel	TRIM44	11	+	270	9	0	0	6.67%	11.01408437	0.011772051	0.011772051	0.000681273
ENSG00000074201_77637714_77619268_167	annotated	CLNS1A	11	-	501	97	6	0	11.38%	63.97402299	0.000626153	5.71E-05	2.72E-07
ENSG00000091129_108232428_108149910_1081	annotated	NRCAM	7	-	3243	113	16	7	5.09%	11.51333519	1.66E-08	7.48E-10	1.42E-16
ENSG00000122687_2241303_2235122_246	annotated	MRM2	7	-	738	43	13	5	8.13%	19.2522613	0.003004909	0.00044822	8.89E-06
ENSG00000163482_218675415_218702009_1190	annotated	STK36	2	+	3570	9	5	0	0.50%	0.832997978	0.14260114	0.012178958	0.009444992
ENSG00000145920_175878740_175880045_134	annotated	CPLX2	5	+	402	263	11	3	21.64%	216.1719544	1.59E-06	1.16E-06	1.63E-11
ENSG00000115207_27343554_27326675_911	annotated	GTF3C2	2	-	2733	74	8	6	3.18%	8.946676657	3.15E-05	2.76E-05	6.69E-09
ENSG00000164818_729704_750923_147	novel	DNAAF5	7	+	441	30	2	2	6.80%	22.47772321	0.005059698	0.008633544	0.000230818
ENSG00000109381_139084190_139058983_533	annotated	ELF2	4	-	1599	15	2	2	2.25%	3.09964851	0.006096424	0.006096424	0.000196497
ENSG00000089053_121328425_121308480_357	novel	ANAPC5	12	-	1071	66	3	7	6.44%	20.36217279	5.24E-05	0.000205467	7.98E-08
ENSG00000076053_114407466_114407807_113	dORF	RBM7	11	+	339	8	0	0	6.19%	7.797581855	0.005706018	0.005706018	0.00017331
ENSG00000189067_11556730_11549637_161	annotated	LITAF	16	-	483	343	40	21	27.33%	234.6478844	9.36E-08	1.08E-08	1.05E-14
ENSG00000156802_123396357_123321134_1390	annotated	ATAD2	8	-	4170	84	10	6	2.95%	6.655993433	3.18E-08	3.43E-07	1.11E-13
ENSG00000133961_73355751_73276578_603	annotated	NUMB	14	-	1809	26	5	2	2.65%	4.74902477	0.006340142	0.000529469	2.27E-05
ENSG00000146678_45888653_45893091_259	annotated	IGFBP1	7	+	777	158	12	12	12.36%	67.1901672	4.75E-06	4.30E-06	1.72E-10
ENSG00000171130_149873976_149878771_111	annotated	ATP6V0E2	7	+	333	83	4	2	11.71%	82.35756782	0.002787882	0.001699044	2.77E-05
ENSG00000146674_45921140_45914820_291	annotated	IGFBP3	7	-	873	1017	73	61	27.15%	384.9252167	4.04E-12	1.25E-11	6.29E-22
ENSG00000169359_155853388_155828210_234	annotated	SLC33A1	3	-	702	21	3	0	4.70%	9.884434692	0.008780371	0.001484235	7.84E-05
ENSG00000135541_135429895_135302809_318	annotated	AHI1	6	-	954	14	0	0	1.89%	4.848967962	0.013428348	0.013428348	0.000872559
ENSG00000108509_4986222_4968756_1202	annotated	CAMTA2	17	-	3606	42	1	6	1.58%	3.848515337	7.96E-05	0.000709042	4.18E-07
ENSG00000198668_90399082_90404717_150	annotated	CALM1	14	+	450	408	44	14	32.00%	299.5830949	1.16E-08	5.64E-09	6.89E-16
ENSG00000136044_105217719_105177184_517	novel	APPL2	12	-	1551	33	5	4	2.51%	7.030266619	0.005129487	0.003777283	0.000106088
ENSG00000170892_54191365_54191688_107	novel	TSEN34	19	+	321	31	2	1	10.28%	31.90996406	0.001513502	0.001535678	1.40E-05
ENSG00000182899_197950968_197951315_55	novel	RPL35A	3	+	165	240	76	4	25.45%	480.6145907	0.001282041	0.000976573	7.71E-06
ENSG00000068885_160319765_160258525_460	annotated	IFT80	3	-	1380	18	2	1	1.30%	4.309859102	0.028889786	0.016822681	0.002229334
ENSG00000065518_120601195_120602270_41	novel	NDUFB4	3	+	123	31	0	0	14.63%	83.27722329	0.013640586	0.013640586	0.000898637
ENSG00000196305_92289419_92210866_1242	annotated	IARS	9	-	3726	255	35	9	7.81%	22.61345825	1.60E-13	5.25E-17	1.53E-28
ENSG00000198791_17243059_17232421_163	dORF	CNOT7	8	-	489	79	6	4	9.82%	53.38114511	0.002388215	0.00053548	8.22E-06
ENSG00000175662_17972313_17847635_389	annotated	TOM1L2	17	-	1167	10	5	0	1.54%	2.831384157	0.218051256	0.012798403	0.016621114
ENSG00000140538_88256153_87876935_839	annotated	NTRK3	15	-	2517	152	14	7	7.15%	19.95400267	1.64E-09	7.92E-11	1.55E-18
ENSG00000159202_48908504_48927134_354	annotated	UBE2Z	17	+	1062	132	22	5	10.73%	41.06946714	2.87E-07	7.93E-08	2.21E-13
ENSG00000101350_32309934_32331319_695	annotated	KIF3B	20	+	2085	29	8	2	1.73%	4.595804989	0.01117766	0.003018821	0.000187735
ENSG00000104133_44663536_44563121_2293	annotated	SPG11	15	-	6879	51	4	1	0.96%	2.449709127	0.000136339	1.98E-05	2.12E-08
ENSG00000113163_75511207_75379346_596	annotated	COL4A3BP	5	-	1788	22	14	5	1.85%	4.065601613	0.200957424	0.015652232	0.017205167
ENSG00000181481_30971074_30998688_286	annotated	RNF135	17	+	858	25	5	1	1.75%	9.627696128	0.086597294	0.022004978	0.008488695
ENSG00000127990_94618900_94585465_278	novel	SGCE	7	-	834	125	8	5	12.95%	49.52376066	1.74E-06	2.28E-07	3.75E-12
ENSG00000008394_16354253_16376164_87	annotated	MGST1	12	+	261	2294	192	29	33.33%	2904.173511	1.05E-05	3.27E-06	2.91E-10
ENSG00000128739_24942428_24968037_78	uORF	SNRPN	15	+	234	36	4	2	10.26%	50.83423556	0.013786174	0.010327713	0.000700646

ENSG00000128739_24975403_24978444_224	annotated	SNRPN	15	+	672	62	5	0	5.80%	30.4854121	0.006702261	0.000649205	2.87E-05
ENSG00000100124_37832735_37831943_57	annotated	ANKRD54	22	-	171	14	4	0	8.77%	27.05213705	0.044492077	0.016947427	0.003439626
ENSG00000128944_40391543_40393597_71	dORF	KNSTRN	15	+	213	21	5	0	8.45%	32.57686926	0.102951605	0.013640586	0.007037474
ENSG00000067141_73176453_73302696_1099	annotated	NEO1	15	+	3297	69	11	1	3.46%	6.915121215	4.23E-06	3.06E-08	1.50E-12
ENSG00000270757_197500437_197550646_261	annotated	HSPE1-MOB4	2	+	783	335	63	16	13.41%	141.3685159	3.15E-06	1.12E-07	3.57E-12
ENSG00000105852_95372320_95359973_281	dORF	PON3	7	-	843	163	9	5	11.39%	63.88952856	3.10E-06	1.96E-06	5.25E-11
ENSG00000102901_67832088_67828267_458	annotated	CENPT	16	-	1374	12	2	4	1.97%	2.885786298	0.022868511	0.035227809	0.003553359
ENSG00000185627_237050_247278_113	annotated	PSMD13	11	+	339	87	7	2	16.81%	84.79870268	0.000409948	0.000113231	3.29E-07
ENSG00000151233_42144646_42087786_440	annotated	GXYLT1	12	-	1320	14	2	3	1.82%	3.504481391	0.023796156	0.040853127	0.004247274
ENSG00000175066_142225455_142185704_301	annotated	GK5	3	-	903	20	1	0	2.33%	7.318328485	0.0135992	0.008175611	0.00055803
ENSG00000239830_9683718_9683533_61	novel	RPS4XP22	19	-	183	39	8	9	8.20%	70.41791647	0.022899795	0.037440157	0.003772784
ENSG00000159842_1109068_1006080_822	annotated	ABR	17	-	2466	84	16	7	4.99%	11.2552687	1.93E-05	9.64E-07	1.73E-10
ENSG00000125971_34516459_34529916_47	novel	DYNLRB1	20	+	141	40	1	0	21.28%	93.73688826	0.00244612	0.00244612	3.45E-05
ENSG00000125971_34534648_34540624_63	novel	DYNLRB1	20	+	189	160	14	2	25.40%	279.7227777	0.000417968	0.000215516	6.13E-07
ENSG00000163159_151190121_151176644_364	novel	VPS72	1	-	1092	66	14	12	7.69%	19.97059254	0.001397723	0.000100061	1.05E-06
ENSG00000186501_27322330_27334285_136	annotated	TMEM222	1	+	408	52	5	0	14.71%	42.11267553	9.68E-05	3.97E-05	2.88E-08
ENSG00000163608_113011111_113010601_56	novel	NEPRO	3	-	168	13	2	1	8.93%	25.56841015	0.020613417	0.039124093	0.003588156
ENSG00000125148_56608656_56609185_73	annotated	MT2A	16	+	219	42	5	1	6.85%	63.3687046	0.057925749	0.039124093	0.009210429
ENSG00000124570_2959332_2948298_376	annotated	SERPINB6	6	-	1128	227	41	8	16.76%	66.49460511	3.99E-09	2.68E-11	1.42E-18
ENSG00000046647_14020447_14008913_208	annotated	GEMIN8	X	-	624	15	1	0	3.85%	7.942849306	0.005160369	0.004826937	0.000134406
ENSG00000110619_3057367_3001114_831	annotated	CARS	11	-	2493	29	4	2	1.93%	3.843663619	0.001994982	0.000820166	1.01E-05
ENSG00000124570_2959332_2948298_376	annotated	SERPINB6	6	-	1128	227	41	8	16.76%	66.49460511	3.99E-09	2.68E-11	1.42E-18
ENSG00000155876_19049696_19050601_301	annotated	RRAGA	9	+	903	242	18	5	17.94%	88.55177467	1.29E-09	2.24E-10	3.31E-18
ENSG00000148803_133357944_133355499_113	annotated	FUOM	10	-	339	12	2	0	7.08%	11.69637278	0.01551626	0.004826937	0.00039687
ENSG00000283496_133309432_133352440_310	annotated	ZNF511-PRAP1	10	+	930	77	3	1	6.77%	27.3575644	2.81E-05	2.86E-05	6.21E-09
ENSG00000112234_98926778_98874278_551	annotated	FBXL4	6	-	1653	21	2	1	1.63%	4.197745404	0.007532653	0.005034873	0.000201184
ENSG00000111906_125301932_125297555_107	annotated	HDDC2	6	-	321	78	34	6	17.76%	80.289587	0.010669572	0.00036801	2.98E-05
ENSG00000109099_15239585_15230917_92	annotated	PMP22	17	-	276	76	35	0	22.83%	90.98591437	0.001368389	2.68E-05	3.27E-07
ENSG00000134452_5895187_5937280_1043	annotated	FBH1	10	+	3129	54	1	3	2.40%	5.702402263	6.68E-06	2.63E-05	1.44E-09
ENSG00000104365_42272133_42319504_427	novel	IKBKB	8	+	1281	12	5	1	1.87%	3.095293032	0.103962997	0.00419316	0.002939444
ENSG00000068366_109683363_109644029_670	annotated	ACSL4	X	-	2010	94	11	2	7.01%	15.45259598	4.08E-07	2.61E-09	1.30E-14
ENSG00000116793_113726494_113697705_625	annotated	PHTF1	1	-	1875	19	2	0	1.44%	3.348281649	0.011768187	0.003606289	0.000231562
ENSG00000116793_113726494_113697705_625	annotated	PHTF1	1	-	1875	19	2	0	1.44%	3.348281649	0.011768187	0.003606289	0.000231562
ENSG00000177700_842508_840372_67	annotated	POLR2L	11	-	201	1320	117	32	71.64%	2169.93901	1.39E-09	2.51E-09	3.80E-17
ENSG00000119185_9418697_9406834_200	annotated	ITGB1BP1	2	-	600	65	3	4	11.00%	35.7957742	5.52E-05	9.62E-05	3.92E-08
ENSG00000166140_40807815_40814226_396	annotated	ZFYVE19	15	+	1188	21	7	0	2.53%	5.840802318	0.030194312	0.002201184	0.000416483
ENSG00000088035_63370978_63415821_301	annotated	ALG6	1	+	903	50	4	2	6.64%	18.29582121	0.000217231	5.97E-05	9.53E-08
ENSG00000088035_63428929_63437020_131	dORF	ALG6	1	+	393	29	2	2	7.63%	24.38232418	0.004034389	0.004034389	8.97E-05
ENSG00000176953_28951012_28963863_419	annotated	NFATC2IP	16	+	1257	50	3	4	6.21%	13.14329877	1.36E-05	3.20E-05	3.45E-09
ENSG00000132153_47825040_47850120_1222	annotated	DHX30	3	+	3666	89	11	1	2.45%	8.021714476	5.74E-05	8.90E-07	5.14E-10
ENSG00000168291_58433809_58428034_359	annotated	PDHB	3	-	1077	131	14	3	10.58%	40.19066999	3.91E-06	7.08E-08	2.94E-12
ENSG00000168310_184429043_184388758_342	annotated	IRF2	4	-	1026	41	19	0	4.68%	13.20401927	0.026670628	0.000199202	5.44E-05
ENSG00000159202_48916146_48927134_138	novel	UBE2Z	17	+	414	37	3	1	11.59%	29.53051607	0.000262032	0.00024644	4.41E-07
ENSG00000120533_109336137_109343481_96	novel	ENY2	8	+	288	353	56	10	37.50%	404.9970607	2.24E-07	2.31E-07	4.88E-13
ENSG00000085719_86547744_86551193_102	novel	CPNE3	8	+	306	15	1	0	5.88%	16.1971829	0.027849813	0.012798403	0.001687084
ENSG00000145730_103017343_103029065_175	annotated	PAM	5	+	525	34	2	0	7.43%	21.39879249	0.001154796	0.000598307	4.38E-06
ENSG00000161904_33778311_33776950_92	annotated	LEMD2	6	-	276	15	2	5	7.61%	17.95774626	0.026832696	0.059434472	0.006809808
ENSG00000153037_112864630_112867537_78	novel	SRP19	5	+	234	36	3	0	8.97%	50.83423556	0.014786102	0.008877961	0.000652631
ENSG00000113407_33457290_33459889_135	novel	TARS	5	+	405	32	9	0	8.89%	26.10745925	0.037884365	0.001011481	0.000292588
ENSG00000104946_49877724_49888624_648	annotated	TBC1D17	19	+	1944	90	36	12	4.48%	15.2973394	0.000397762	6.17E-05	1.82E-07
ENSG00000119541_63422259_63396986_379	novel	VPS4B	18	-	1137	45	4	0	5.28%	13.07740888	0.000328571	3.42E-05	8.73E-08
ENSG00000128513_124897173_124827465_567	annotated	POT1	7	-	1701	30	3	3	2.12%	5.827557868	0.002631121	0.005604867	8.28E-05
ENSG00000106628_44122053_44114785_469	annotated	POLD2	7	-	1407	157	5	1	9.38%	36.87017582	9.91E-09	3.01E-09	3.19E-16
ENSG00000140575_90388342_90500108_1657	annotated	IQGAP1	15	+	4971	135	26	9	3.74%	8.973454376	3.67E-07	2.18E-10	1.24E-15
ENSG00000144320_175932097_175929967_80	novel	LNPK	2	-	240	14	0	0	6.25%	19.27464765	0.019679754	0.019679754	0.001784964
ENSG00000140577_90530072_90642140_619	annotated	CRTC3	15	+	1857	20	1	2	1.78%	3.558670233	0.002415484	0.005935135	8.11E-05
ENSG00000033011_5075487_5079754_140	novel	ALG1	16	+	420	45	13	0	9.29%	35.40241405	0.053463781	0.000676852	0.000329875
ENSG00000092140_30581080_30616534_706	annotated	G2E3	14	+	2118	24	5	2	1.42%	3.744164659	0.021779045	0.005435927	0.000623251
ENSG00000039319_80434148_80477377_1539	annotated	ZFYVE16	5	+	4617	39	6	3	1.49%	2.791093505	0.000582705	6.85E-05	2.98E-07
ENSG00000257698_57935536_57931610_64	novel	GIHCG	12	-	192	16	0	0	7.81%	27.53521093	0.021084099	0.021084099	0.002029891
ENSG00000187498_110307027_110191626_519	annotated	COL4A1	13	-	1557	46	2	0	4.24%	9.762001562	5.10E-05	1.38E-05	5.59E-09
ENSG00000166226_69586816_69601325_488	annotated	CCT2	12	+	1464	440	42	3	13.93%	99.30731809	4.99E-11	4.27E-13	2.97E-22
ENSG00000104442_65627398_65604394_282	annotated	ARMC1	8	-	846	65	4	1	8.87%	25.3870739	1.09E-05	6.82E-06	6.10E-10
ENSG00000038427_83483549_83512419_344	annotated	VCAN	5	+	1032	202	26	4	14.53%	64.67572799	1.48E-06	7.37E-10	1.73E-14
ENSG00000080822_98521424_98518802_161	novel	CLDND1	3	-	483	57	4	5	11.18%	38.9939633	0.000706925	0.00086212	3.86E-06
ENSG00000129103_56064312_56079612_301	annotated	SUMF2	7	+	903	626	84	12	20.93%	229.0636816	2.56E-08	8.58E-12	3.67E-18
ENSG00000168297_58397099_58424960_284	dORF	PXK	3	+	852	17	0	0	2.11%	6.592937827	0.013640586	0.013640586	0.000898637
ENSG00000113966_97768108_97798049_186	annotated	ARL6	3	+	558	15	6	1	4.30%	8.882326105	0.10760709	0.00912793	0.0054576

ENSG00000092847_35893184_35919607_718	annotated	AGO1	1	+	2154	8	0	0	0.70%	1.22719603	0.019679754	0.019679754	0.001784964
ENSG00000114354_100713686_100748531_400	annotated	TFG	3	+	1200	146	7	0	8.25%	40.20140795	1.49E-06	2.39E-07	3.35E-12
ENSG00000153130_140343639_140381104_122	annotated	SCOC	4	+	366	36	11	0	11.48%	32.50057683	0.04430005	0.000434916	0.000186534
ENSG00000198231_63806601_63818398_674	novel	DDX42	17	+	2022	38	9	1	2.82%	6.209721158	0.005528688	0.00012004	5.57E-06
ENSG00000166226_69585522_69586860_61	novel	CCT2	12	+	183	35	12	1	9.84%	63.19556606	0.145323137	0.013640586	0.010496206
ENSG00000012822_53725242_53714077_541	novel	CALCOCO1	12	-	1623	11	3	0	1.11%	2.239462626	0.098122363	0.011772051	0.005950834
ENSG00000155508_154863279_154875439_292	annotated	CNOT8	5	+	876	86	11	4	10.62%	32.43874164	9.60E-05	7.01E-06	5.71E-09
ENSG00000189171_153626472_153618895_98	annotated	S100A13	1	-	294	240	154	16	30.61%	269.7326785	0.000173759	1.74E-06	3.11E-09
ENSG00000106477_130428012_130398891_272	annotated	CEP41	7	-	816	31	0	0	3.68%	12.55281675	0.002227676	0.002227676	2.89E-05
ENSG00000136450_58005932_58005406_108	dORF	SRSF1	17	-	324	164	44	4	22.22%	167.2509108	0.004072351	1.47E-05	7.00E-07
ENSG00000136450_58007137_58006940_65	annotated	SRSF1	17	-	195	156	16	58	24.62%	264.3380249	0.002063869	0.062021155	0.000917563
ENSG00000119314_112297865_112223851_521	annotated	PTBP3	9	-	1563	83	33	3	7.49%	17.54643	0.000584828	3.22E-08	4.73E-10
ENSG00000119314_112297865_112223851_524	annotated	PTBP3	9	-	1572	83	33	3	7.44%	17.44597333	0.000584828	3.22E-08	4.73E-10
ENSG00000119314_112333477_112297862_20	Overlap_uORF	PTBP3	9	-	60	20	4	3	30.00%	110.1408437	0.013428348	0.012798403	0.00083412
ENSG00000070081_17312112_17331419_119	novel	NUCB2	11	+	357	36	2	0	6.72%	33.3199191	0.006307834	0.005756914	0.000192262
ENSG00000100644_61697851_61747085_850	annotated	HIF1A	14	+	2550	639	88	20	16.82%	82.79999897	1.15E-18	1.61E-23	4.40E-40
ENSG00000176894_132701280_132704687_52	Overlap_dORF	PXMP2	12	+	156	12	3	2	11.54%	25.41711778	0.023672501	0.052581193	0.005415915
ENSG00000113851_3175246_3150865_368	annotated	CRBN	3	-	1104	58	2	1	5.16%	17.35915471	5.54E-05	7.83E-05	3.22E-08
ENSG00000107581_119080676_119036039_1382	annotated	EIF3A	10	-	4146	239	47	25	7.53%	19.04751204	2.30E-12	2.09E-15	8.01E-26
ENSG00000138190_92893440_93058355_740	annotated	EXOC6	10	+	2220	44	4	0	2.43%	6.548915031	0.000715324	8.55E-05	4.50E-07
ENSG00000096384_44248702_44253598_700	annotated	HSP90AB1	6	+	2100	438	84	36	12.43%	68.91669935	1.41E-13	2.89E-13	5.40E-25
ENSG00000077721_119574712_119583255_152	annotated	UBE2A	X	+	456	185	17	0	23.03%	134.0530006	5.69E-06	1.06E-07	6.33E-12
ENSG00000188566_137205778_137216416_590	annotated	NDOR1	9	+	1770	15	4	4	1.02%	2.800190942	0.079167839	0.075513077	0.022064917
ENSG00000094916_54257650_54241755_191	annotated	CBX5	12	-	573	106	33	0	15.18%	61.12528499	2.19E-05	1.14E-06	2.31E-10
ENSG00000247796_53109990_53112278_65	novel	AC008966.1	5	+	195	17	0	0	9.23%	28.80606682	0.013428348	0.013428348	0.000872559
ENSG00000149231_96384747_96353646_495	annotated	CCDC82	11	-	1485	26	0	0	3.03%	5.785175629	0.000253372	0.000253372	4.38E-07
ENSG00000177156_747482_760257_154	novel	TALDO1	11	+	462	480	17	4	28.57%	343.2961362	5.29E-08	6.89E-09	3.84E-15
ENSG00000117519_94926894_94897742_329	annotated	CNN3	1	-	987	173	22	4	12.46%	57.91600596	4.50E-07	2.39E-08	1.12E-13
ENSG00000172339_95072898_94983076_216	annotated	ALG14	1	-	648	18	6	1	2.78%	9.178403642	0.060322713	0.023878474	0.006258656
ENSG00000152078_95143902_95191868_263	annotated	TMEM56	1	+	789	77	12	10	7.98%	32.2465588	0.000131833	0.000546643	5.07E-07
ENSG00000122481_95234231_95246870_267	annotated	RWDD3	1	+	801	15	4	2	3.37%	6.187687849	0.058857695	0.019504171	0.005147486
ENSG00000148719_72340839_72334588_153	annotated	DNAJB12	10	-	459	29	10	1	5.23%	20.87636907	0.17034922	0.007181907	0.008091924
ENSG00000144029_95101694_95087357_166	novel	MRPS5	2	-	498	71	12	1	9.64%	47.10843315	0.003972751	0.000254295	7.29E-06
ENSG00000127837_218270086_218267438_149	novel	AAMP	2	-	447	30	5	1	6.71%	22.1760088	0.016535923	0.003146562	0.00029206
ENSG00000129292_132794453_132845923_975	annotated	PHF20L1	8	+	2925	13	6	0	1.13%	1.468544583	0.111917027	0.000670321	0.000880274
ENSG00000129292_132794453_132824077_512	novel	PHF20L1	8	+	1536	7	6	0	1.37%	1.505831848	0.425777449	0.004075486	0.022580433
ENSG00000204287_32440002_32443910_237	annotated	HLA-DRA	6	+	711	23	9	0	2.11%	10.68877386	0.13552064	0.021084099	0.013379035
ENSG00000136942_124861912_124858469_96	annotated	RPL35	9	-	288	71	9	12	10.42%	81.45833232	0.009211063	0.017902532	0.000813622
ENSG00000136942_124858481_124857918_57	Overlap_dORF	RPL35	9	-	171	1120	75	4	19.30%	2164.170964	0.012553188	0.001665007	0.000126369
ENSG00000147099_72572761_72467987_253	annotated	HDAC8	X	-	759	13	1	0	2.37%	5.659410941	0.023672501	0.012798403	0.001437523
ENSG00000147099_72572761_72330054_377	annotated	HDAC8	X	-	1131	14	2	0	1.86%	4.090110907	0.028902806	0.007882058	0.001149203
ENSG00000112473_33201246_33203813_469	annotated	SLC39A7	6	+	1407	271	14	13	14.29%	63.64215063	2.11E-12	3.34E-11	9.01E-22
ENSG00000004866_116953541_117229857_577	annotated	ST7	7	+	1731	38	9	0	3.29%	7.253643086	0.003400472	5.35E-05	1.64E-06
ENSG00000112473_33201246_33203813_469	annotated	SLC39A7	6	+	1407	272	14	13	14.50%	63.87699251	1.49E-12	2.45E-11	4.72E-22
ENSG00000092098_24157567_24159878_77	annotated	RNF31	14	+	231	18	0	0	6.49%	25.74721022	0.021084099	0.021084099	0.002029891
ENSG00000181827_56142887_56093345_1363	annotated	RFX7	15	-	4089	26	2	2	0.66%	2.10099922	0.011906648	0.011906648	0.000696002
ENSG00000137880_40765875_40767349_56	annotated	GCHFR	15	+	168	22	5	0	14.29%	43.26961717	0.045346668	0.005706018	0.001417412
ENSG00000118513_135185940_135201655_561	annotated	MYB	6	+	1683	14	0	0	0.89%	2.748612855	0.019679754	0.019679754	0.001784964
ENSG00000149503_62128795_62151976_863	annotated	INCENP	11	+	2589	49	13	4	2.55%	6.253651612	0.001669373	0.000170854	2.04E-06
ENSG00000142494_19556006_19577553_282	novel	SLC47A1	17	+	846	50	2	0	4.96%	19.52851839	0.000953102	0.000440166	2.72E-06
ENSG00000119969_94554132_94601622_831	annotated	HELLS	10	+	2493	71	11	0	3.61%	9.410348861	1.70E-05	5.25E-07	8.68E-11
ENSG00000131370_15262205_15256086_262	annotated	SH3BP5	3	-	786	77	6	5	4.96%	32.36963727	0.003324654	0.004059691	7.51E-05
ENSG00000082146_201475660_201480028_222	annotated	STRADB	2	+	666	11	0	0	2.25%	5.457429193	0.019216965	0.019216965	0.001707396
ENSG00000141380_26087577_26018354_395	annotated	AC091021.1	18	-	1185	25	2	6	3.54%	6.970939475	0.000673856	0.01350901	6.40E-05
ENSG00000091164_56638440_56603027_289	annotated	TXNL1	18	-	867	73	14	15	10.38%	27.82104357	2.41E-05	0.000250149	4.77E-08
ENSG00000140691_31459525_31466889_935	annotated	ARMC5	16	+	2805	21	3	0	1.28%	2.47375157	0.00579799	0.000939154	3.40E-05
ENSG00000005100_5462477_5444205_534	dORF	DHX33	17	-	1602	34	3	0	2.62%	7.012712895	0.002063509	0.000424543	5.74E-06
ENSG00000262246_4416518_4355158_840	annotated	CORO7	16	-	2520	17	2	5	0.83%	2.229040885	0.032657853	0.059434472	0.008063634
ENSG00000155363_112674913_112700507_1003	annotated	MOV10	1	+	3009	17	7	1	0.70%	1.866793961	0.0668849	0.007709191	0.002775985
ENSG00000174669_66371591_66363436_456	annotated	SLC29A2	11	-	1368	66	14	15	5.04%	15.9414379	0.001692414	0.005574075	5.51E-05
ENSG00000165060_69035783_69072762_210	annotated	FXN	9	+	630	74	11	0	11.43%	38.8115354	0.000110585	7.95E-06	7.42E-09
ENSG00000132300_86106248_86137559_689	annotated	PTCD3	2	+	2067	97	8	2	5.66%	15.50604041	3.50E-07	2.98E-08	1.06E-13
ENSG00000136827_129824085_129813972_332	annotated	TOR1A	9	-	996	156	14	7	13.25%	51.75292656	4.15E-08	5.90E-08	2.43E-14
ENSG00000104341_97776016_97851474_224	annotated	LAPTM4B	8	+	672	497	61	32	26.79%	244.374997	1.80E-09	1.94E-09	3.79E-17
ENSG00000143776_227034659_227004917_526	annotated	CDC42BPA	1	-	1578	13	1	1	1.14%	2.722112107	0.023672501	0.023672501	0.00251841
ENSG00000184634_71124251_71142218_1565	annotated	MED12	X	+	4695	14	2	1	0.51%	0.985285503	0.023796156	0.009234565	0.001081929
ENSG00000136295_2656485_2660020_126	annotated	TTYH3	7	+	378	15	0	0	5.56%	13.1120052	0.008373824	0.008373824	0.000358384
ENSG00000196628_55275658_55228225_431	annotated	TCF4	18	-	1293	20	0	0	1.62%	5.110944023	0.008776163	0.008776163	0.000391611

ENSG00000181090_137743424_137834953_1006	annotated	EHMT1	9	+	3018	57	6	10	2.78%	6.240584584	5.44E-05	0.000151761	6.08E-08
ENSG00000182220_40605627_40605755_42	dORF	ATP6AP2	X	+	126	26	2	2	16.67%	68.18242706	0.008877961	0.008877961	0.000400235
ENSG00000090020_27154334_27105702_555	annotated	SLC9A1	1	-	1665	10	0	1	1.08%	1.984519706	0.011772051	0.026832696	0.001510382
ENSG00000167632_140451355_139731061_1142	annotated	TRAPPC9	8	-	3426	53	11	5	2.19%	5.111615338	0.000260403	0.00010115	1.87E-07
ENSG00000107625_68906819_68946626_666	annotated	DDX50	10	+	1998	38	5	0	2.40%	6.284312404	0.001338438	0.000178703	1.68E-06
ENSG00000148290_133353936_133351913_191	novel	SURF1	9	-	573	33	15	2	5.76%	19.02956985	0.067244088	0.004569059	0.001856326
ENSG00000165219_125298922_125362746_1478	annotated	GAPVD1	9	+	4434	35	2	4	1.35%	2.608206718	0.000182983	0.00047668	5.99E-07
ENSG00000114744_149752444_149750794_121	annotated	COMMD2	3	-	363	31	13	20	12.40%	28.21790211	0.006479483	0.041566705	0.001430215
ENSG00000163931_53255942_53242053_98	novel	TKT	3	-	294	179	13	8	25.51%	201.1756227	9.94E-05	2.84E-05	2.16E-08
ENSG00000166197_102159553_102162269_418	dORF	NOLC1	10	+	1254	180	12	0	7.89%	47.42907145	1.41E-06	2.25E-07	2.99E-12
ENSG00000166197_102152411_102157494_73	annotated	NOLC1	10	+	219	21	2	3	8.22%	31.6843523	0.032488891	0.044907172	0.006132567
ENSG00000163681_57757652_57925928_746	annotated	SLMAP	3	+	2238	16	3	1	1.21%	2.362270106	0.021084099	0.005561256	0.00061472
ENSG00000177674_11747543_11754428_103	novel	AGTRAP	1	+	309	162	10	9	17.48%	173.2312299	9.38E-05	0.000868713	6.00E-07
ENSG00000129255_7586014_7587444_113	dORF	MPDU1	17	+	339	28	3	11	7.96%	27.29153649	0.015528143	0.109177345	0.00830659
ENSG00000129255_7587444_7587551_35	dORF	MPDU1	17	+	105	13	1	0	17.14%	40.90945623	0.026832696	0.011772051	0.001510382
ENSG00000090006_40593166_40629550_1587	annotated	LTBP4	19	+	4761	59	11	8	1.89%	4.094713156	0.000358599	8.13E-05	2.11E-07
ENSG00000124279_7868032_7861646_591	annotated	FASTKD3	5	-	1773	25	0	1	1.35%	4.659088143	0.005306578	0.009288069	0.000259004
ENSG00000026297_166956182_166929588_256	annotated	RNASET2	6	-	768	27	6	2	5.08%	11.61641711	0.006735894	0.000917405	3.90E-05
ENSG00000232119_120611017_120612264_47	novel	MCTS1	X	+	141	13	1	3	10.64%	30.46448868	0.028889786	0.08410207	0.010278261
ENSG00000156502_69189286_69195279_117	novel	SUPV3L1	10	+	351	8	0	1	4.27%	7.53099786	0.019679754	0.016947427	0.001553599
ENSG00000103496_31039547_31039735_62	novel	STX4	16	+	186	20	0	2	9.68%	35.52930442	0.012798403	0.042234516	0.002567464
ENSG00000266967_42956514_42955328_132	novel	AARSD1	17	-	396	60	3	1	7.58%	50.06401987	0.005939095	0.00390064	0.000126013
ENSG00000131351_17062743_17049873_349	annotated	HAUS8	19	-	1047	17	4	0	2.29%	5.365026771	0.032344272	0.005454749	0.000947183
ENSG00000067900_21045416_20960194_982	annotated	ROCK1	18	-	2946	26	5	7	1.83%	2.916152685	0.002691831	0.00123781	2.00E-05
ENSG00000120708_136049469_136054879_208	annotated	TGFBI	5	+	624	1036	135	33	35.10%	548.5861254	1.11E-10	1.49E-12	2.24E-21
ENSG00000123444_47577999_47572930_518	annotated	KBTBD4	11	-	1554	33	5	4	2.70%	7.016694676	0.003517618	0.003306134	6.51E-05
ENSG00000159322_72755594_72752341_196	novel	ADPGK	15	-	588	85	10	3	13.78%	47.76516181	7.39E-05	1.28E-05	7.57E-09
ENSG00000070761_58129115_58115212_173	novel	CFAP20	16	-	519	45	8	5	8.67%	28.64935241	0.004749354	0.001261294	3.60E-05
ENSG00000106603_43647613_43639582_134	annotated	COA1	7	-	402	36	6	4	9.70%	29.59007741	0.007322027	0.003864916	0.000153348
ENSG00000054116_36140070_36137203_134	annotated	TRAPPC3	1	-	402	105	11	1	22.39%	86.30439246	1.74E-05	7.02E-07	1.16E-10
ENSG00000104835_38930724_38918798_232	annotated	SARS2	19	-	696	33	1	8	6.03%	15.66658553	0.00040306	0.009240278	2.73E-05
ENSG00000100811_100276502_100277600_109	dORF	YY1	14	+	327	23	5	0	11.01%	23.24072849	0.007656911	0.000966879	4.68E-05
ENSG00000100811_100239911_100276035_109	annotated	YY1	14	+	327	38	2	4	9.17%	38.39772533	0.002904374	0.00583526	9.43E-05
ENSG00000267740_5903708_5870571_121	annotated	AC024592.3	19	-	363	59	33	10	11.57%	53.70503949	0.057049371	0.007102572	0.002178391
ENSG00000172053_49103634_49096029_621	dORF	QARS	3	-	1863	67	17	0	5.15%	11.88315061	0.000299426	2.62E-07	1.15E-09
ENSG00000261832_28491764_28489349_55	Overlap_uORF	AC138894.1	16	-	165	18	0	12	14.55%	36.0460943	0.005554612	0.310595281	0.015978689
ENSG00000261832_28489291_28477516_338	Overlap_dORF	AC138894.1	16	-	1014	124	13	32	11.24%	40.40670006	7.63E-07	2.59E-06	1.77E-11
ENSG00000088367_36207493_36229340_456	annotated	EPB41L1	20	+	1368	59	10	0	4.82%	14.25067934	0.000464427	1.74E-05	6.87E-08
ENSG00000163348_154961018_154958779_369	annotated	PYGO2	1	-	1107	22	0	0	2.44%	6.566662768	0.003529166	0.003529166	6.95E-05
ENSG00000163348_154959798_154958779_339	annotated	PYGO2	1	-	1017	17	0	0	2.36%	5.523287147	0.005306578	0.005306578	0.000151001
ENSG00000025156_122412478_122432220_470	annotated	HSF2	6	+	1410	19	2	8	1.49%	4.452502192	0.008674847	0.118250966	0.00587754
ENSG00000160691_154970196_154963803_474	annotated	SHC1	1	-	1422	114	12	8	5.91%	26.48957001	1.29E-05	6.94E-06	7.33E-10
ENSG00000160691_154970196_154963803_473	annotated	SHC1	1	-	1419	114	12	8	5.92%	26.54557332	1.29E-05	6.94E-06	7.33E-10
ENSG00000129657_77190842_77214023_681	annotated	SEC14L1	17	+	2043	57	3	0	2.64%	9.218837138	0.000276151	9.28E-05	1.83E-07
ENSG00000241127_39566419_39572366_87	annotated	YAE1	7	+	261	26	16	2	10.34%	32.91565444	0.16133531	0.011814276	0.010735947
ENSG00000127125_42459615_42459926_103	novel	PPCS	1	+	309	14	1	1	8.74%	14.97060012	0.002500085	0.00640069	9.02E-05
ENSG00000164442_139373944_139373132_213	annotated	CITED2	6	-	639	24	3	0	5.16%	12.41023591	0.007823695	0.001448134	6.80E-05
ENSG00000104973_49818342_49836944_482	annotated	MED25	19	+	1446	18	6	0	2.07%	4.113143541	0.081593387	0.002033393	0.00127441
ENSG00000184640_77320327_77498658_568	annotated	9-Sep	17	+	1704	135	22	6	7.39%	26.17784137	1.48E-05	1.02E-07	1.71E-11
ENSG00000128534_118184224_118192002_96	annotated	LSM8	7	+	288	184	44	4	31.25%	211.1032838	0.000485312	8.80E-07	5.58E-09
ENSG00000133597_140673331_140695804_590	annotated	ADCK2	7	+	1770	41	0	9	3.05%	7.653855241	6.52E-05	0.014098727	1.04E-05
ENSG00000084234_130069978_130143448_751	annotated	APLP2	11	+	2253	677	82	20	20.24%	99.28808414	2.56E-23	1.76E-26	9.38E-48
ENSG00000131051_35740847_35707190_414	annotated	RBM39	20	-	1242	83	12	6	7.97%	22.08137688	4.54E-05	2.60E-06	1.05E-09
ENSG00000113851_3179687_3174055_126	novel	CRBN	3	-	378	34	3	0	8.73%	29.72054513	0.001573068	0.001580611	1.49E-05
ENSG00000196652_99506156_99532249_802	annotated	ZKSCAN5	7	+	2406	16	8	1	0.87%	2.197323565	0.185865134	0.011738695	0.012754119
ENSG00000090316_1289947_1339169_317	annotated	MAEA	4	+	951	68	22	2	8.20%	23.62642704	0.00214985	1.15E-05	2.69E-07
ENSG00000146674_45921140_45914820_263	annotated	IGFBP3	7	-	789	952	55	13	25.86%	398.684727	3.85E-11	8.58E-13	4.40E-22
ENSG00000095794_35167726_35211803_270	annotated	CREM	10	+	810	12	2	0	1.85%	4.895148609	0.070008252	0.021084099	0.006564739
ENSG00000100316_39316750_39312940_251	novel	RPL3	22	-	753	244	28	12	10.76%	107.0691867	9.39E-06	3.53E-06	2.79E-10
ENSG00000100316_39318439_39316844_128	novel	RPL3	22	-	384	501	47	11	17.19%	431.0981461	1.93E-05	2.12E-05	3.22E-09
ENSG00000116171_52927457_53050704_503	annotated	SCP2	1	+	1509	156	13	9	8.75%	34.1589893	6.88E-08	1.26E-08	8.90E-15
ENSG00000124226_49936413_49952242_206	annotated	RNF114	20	+	618	55	2	5	7.77%	29.40653594	0.000437499	0.004727741	1.41E-05
ENSG00000198715_156294893_156294663_76	novel	GLMP	1	-	228	10	0	0	7.89%	14.49221628	0.011570466	0.011570466	0.000659484
ENSG00000113328_163441222_163443703_105	annotated	CCNG1	5	+	315	14	4	0	7.62%	14.68544583	0.050430477	0.004149608	0.001236364
ENSG00000157191_16440762_16458890_202	annotated	NECAP2	1	+	606	18	2	2	2.48%	9.814530627	0.044040756	0.036968718	0.006762352
ENSG00000173221_95822662_95816513_106	annotated	GLRX	5	-	318	109	14	3	24.53%	113.2580374	2.79E-05	3.62E-06	8.62E-10
ENSG00000284194_50524411_50523611_266	annotated	SCO2	22	-	798	51	6	20	7.14%	21.11722943	0.00059841	0.017243231	7.66E-05
ENSG00000134333_18396843_18407281_332	annotated	LDHA	11	+	996	1768	182	49	20.78%	586.5331677	1.74E-12	5.65E-12	1.24E-22

ENSG00000111328_123267253_123261736_87	annotated	CDK2AP1	12	-	261	86	18	1	18.39%	108.874857	0.000785347	0.000207945	1.12E-06
ENSG00000089022_111867587_111893061_406	annotated	MAPKAPK5	12	+	1218	11	1	6	1.48%	2.984111529	0.023672501	0.218051256	0.025408403
ENSG00000131370_15262205_15256086_262	annotated	SH3BP5	3	-	786	77	6	5	4.96%	32.36963727	0.003324654	0.004059691	7.51E-05
ENSG00000182952_26545207_26545479_90	annotated	HMGN4	6	+	270	50	1	0	5.56%	61.18935761	0.021084099	0.021557223	0.002072352
ENSG00000135317_85593697_85505967_658	annotated	SNX14	6	-	1974	75	11	3	4.86%	12.55404753	4.38E-05	2.06E-06	8.19E-10
ENSG00000150347_62059257_62093030_834	annotated	ARID5B	10	+	2502	9	0	1	0.72%	1.188570256	0.011772051	0.026832696	0.001510382
ENSG00000135317_85574362_85565405_91	annotated	SNX14	6	-	273	8	1	0	5.49%	9.682711535	0.065398531	0.019679754	0.005779267
ENSG00000140612_84716075_84670019_179	annotated	SEC11A	15	-	537	628	96	17	27.37%	386.4159209	3.19E-07	8.55E-10	3.63E-15
ENSG00000188917_101042289_101010581_459	annotated	TRMT2B	X	-	1377	21	2	0	1.74%	5.039123568	0.015570605	0.005554612	0.000450864
ENSG00000148337_128185630_128166197_730	annotated	CIZ1	9	-	2190	101	4	4	4.52%	15.23866468	2.71E-07	1.99E-06	5.08E-12
ENSG00000132170_12379712_12434145_475	annotated	PPARG	3	+	1425	42	6	0	4.21%	9.738769338	0.000917199	3.16E-05	2.43E-07
ENSG00000180921_143730321_143725921_1092	annotated	FAM83H	8	-	3276	37	1	7	1.28%	3.731878404	0.000678648	0.01846374	9.18E-05
ENSG00000183735_64464361_64501381_610	annotated	TBK1	12	+	1830	32	5	1	2.62%	5.777880326	0.003657632	0.000263041	6.86E-06
ENSG00000104872_49449547_49447344_97	annotated	PIH1D1	19	-	291	18	11	1	10.31%	20.43850708	0.159347698	0.003737369	0.004708406
ENSG00000125652_6374513_6374973_79	annotated	ALKBH7	19	+	237	31	0	12	11.39%	43.21982475	0.003580484	0.044466451	0.000953251
ENSG00000166949_67138093_67190536_359	annotated	SMAD3	15	+	1077	58	12	0	4.74%	17.79434244	0.006520804	0.000134544	7.40E-06
ENSG00000012171_50269241_50276706_749	annotated	SEMA3B	3	+	2247	52	12	0	2.94%	7.646627333	0.002479335	1.68E-05	4.34E-07
ENSG00000158711_205623882_205616546_431	annotated	ELK4	1	-	1293	10	5	0	1.16%	2.555472012	0.261477794	0.019679754	0.028155188
ENSG00000115204_27313101_27312817_94	novel	MPV17	2	-	282	25	1	1	7.45%	29.29277758	0.008877961	0.012176559	0.000540833
ENSG00000154511_92876409_92843383_344	annotated	DIPK1A	1	-	1032	37	1	1	4.94%	11.84654424	0.000184033	0.000184033	2.36E-07
ENSG00000122140_135500711_135504133_296	annotated	MRPS2	9	+	888	125	17	6	13.51%	46.51218062	8.04E-06	2.72E-07	2.16E-11
ENSG00000285053_235380050_235448762_527	annotated	TBCE	1	+	1581	34	20	5	2.85%	7.105860884	0.036601443	0.003024558	0.000668515
ENSG00000100888_21426172_21395775_1165	novel	CHD8	14	-	3495	27	2	0	1.29%	2.552620412	0.000736315	0.000255448	1.26E-06
ENSG00000100888_21395009_21385613_817	novel	CHD8	14	-	2451	20	0	3	1.10%	2.696226284	0.003478238	0.022431136	0.000439182
ENSG00000152242_46216160_46263118_311	annotated	C18orf25	18	+	933	11	3	0	1.93%	3.895656851	0.042234516	0.012798403	0.002567464
ENSG00000178053_158592471_158605202_240	annotated	MLF1	3	+	720	24	0	0	3.33%	11.01408437	0.005655336	0.005655336	0.0001704
ENSG00000273899_37686393_37691336_213	annotated	NOL12	22	+	639	25	1	1	3.76%	12.92732907	0.005454749	0.006965909	0.000200985
ENSG00000178449_50112278_50120217_133	annotated	COX14	12	+	399	95	24	1	8.27%	78.67203122	0.002863021	0.001634015	2.75E-05
ENSG00000123349_53297850_53297954_34	novel	PFDN5	12	+	102	50	11	6	29.41%	161.971829	0.0199333	0.003920836	0.000429175
ENSG00000117481_46344855_46361846_335	dORF	NSUN4	1	+	1005	18	4	1	2.09%	5.918015483	0.053594825	0.017404239	0.00425017
ENSG00000163320_88055976_88055473_167	annotated	CGGBP1	3	-	501	35	5	2	7.78%	23.08341036	0.002101697	0.001181385	1.50E-05
ENSG00000112339_135039741_134965279_597	annotated	HBS1L	6	-	1791	59	4	1	4.02%	10.884941	7.02E-05	9.74E-06	5.58E-09
ENSG00000213064_168226080_168236766_108	annotated	SFT2D2	1	+	324	39	3	31	9.26%	39.77308245	0.007999495	0.350279879	0.024116756
ENSG00000142252_45090115_45090510_131	annotated	GEMIN7	19	+	393	30	5	0	7.63%	25.22309398	0.002321823	0.002349128	3.16E-05
ENSG00000189067_11556730_11551723_152	annotated	LITAF	16	-	456	183	25	2	16.45%	132.6037789	0.000107855	9.39E-06	8.41E-09
ENSG00000130779_122380446_122272875_1436	annotated	CLIP1	12	-	4308	35	10	7	1.18%	2.684491316	0.015043704	0.007076319	0.000540668
ENSG00000070669_97864301_97852259_413	annotated	ASNS	7	-	1239	38	0	0	3.63%	10.13402436	0.000284266	0.000284266	5.48E-07
ENSG00000184381_38169426_38112161_806	annotated	PLA2G6	22	-	2418	12	0	0	0.62%	1.63981405	0.020613417	0.020613417	0.001946209
ENSG00000136560_161224686_161232848_231	annotated	TANK	2	+	693	41	5	2	6.93%	19.54880776	0.002073811	0.000465311	6.27E-06
ENSG00000196998_49077855_49074803_323	annotated	WDR45	X	-	969	29	4	3	4.33%	9.888806401	0.006519044	0.002747448	0.000100074
ENSG00000149115_57321885_57300540_1729	annotated	TNKS1BP1	11	-	5187	82	14	10	2.20%	5.223568065	6.24E-06	3.37E-06	1.78E-10
ENSG00000149182_47176853_47172328_89	annotated	ARFGAP2	11	-	267	10	0	1	5.62%	12.3753757	0.019679754	0.065398531	0.005779267
ENSG00000136861_120448069_120389236_902	annotated	CDK5RAP2	9	-	2706	22	33	3	1.66%	2.686362042	0.056271034	0.001638072	0.000683855
ENSG00000171130_149874066_149878771_81	annotated	ATP6V0E2	7	+	243	81	4	2	14.81%	110.1408437	0.004274818	0.002516921	6.08E-05
ENSG00000183255_44861832_44851581_159	annotated	PTTG1IP	21	-	477	319	53	3	26.42%	220.9743971	1.84E-05	7.75E-09	2.29E-12
ENSG00000183255_44873616_44851581_107	annotated	PTTG1IP	21	-	321	263	56	3	25.23%	270.7200177	0.000114904	3.16E-06	3.36E-09
ENSG00000080608_2838507_2804331_648	annotated	PUM3	9	-	1944	56	11	0	4.32%	9.518344518	0.000416396	1.29E-06	6.47E-09
ENSG00000158856_22067533_22081463_372	annotated	DMTN	8	+	1116	20	6	0	2.69%	5.921550737	0.041018323	0.001801897	0.000504555
ENSG00000103145_3023324_3022863_75	novel	HCFC1R1	16	-	225	67	0	1	16.00%	98.39248704	0.001044718	0.001118312	7.21E-06
ENSG00000103145_3023941_3023470_51	novel	HCFC1R1	16	-	153	61	6	3	19.61%	131.7370876	0.009098561	0.002488365	0.000128178
ENSG00000143476_212044668_212068647_191	novel	DTL	1	+	573	13	1	1	4.19%	7.496497216	0.00912793	0.00912793	0.000421779
ENSG00000143476_212068679_212102940_431	novel	DTL	1	+	1293	57	8	4	4.18%	14.56619047	0.001699631	0.000504741	5.51E-06
ENSG00000109919_47642402_47617471_124	novel	MTCH2	11	-	372	71	18	5	17.74%	63.06451535	0.000641401	9.02E-05	4.22E-07
ENSG00000076043_114439529_114449975_222	annotated	REXO2	11	+	666	162	34	5	17.57%	80.37304811	8.74E-06	1.04E-07	9.88E-12
ENSG00000132768_43970185_43972539_258	annotated	DPH2	1	+	774	17	1	1	3.49%	7.257342415	0.00640069	0.00640069	0.000215507
ENSG00000160753_155326495_155330571_310	novel	RUSC1	1	+	930	27	8	1	3.87%	9.592912194	0.043841603	0.001453991	0.000461786
ENSG00000166333_6604272_6610611_452	annotated	ILK	11	+	1356	118	21	8	9.29%	28.75358309	4.30E-06	2.90E-07	1.19E-11
ENSG00000257103_34172643_34226431_463	annotated	LSM14A	19	+	1389	122	12	13	7.56%	29.02199337	2.76E-06	1.60E-05	3.79E-10
ENSG00000148842_102918559_103077180_827	annotated	CNNM2	10	+	2481	27	3	1	1.09%	3.595892116	0.015765166	0.004467577	0.000376864
ENSG00000135679_68809227_68839849_486	annotated	MDM2	12	+	1458	55	3	5	5.14%	12.46449877	3.07E-05	5.52E-05	1.29E-08
ENSG00000135679_68809227_68839849_431	annotated	MDM2	12	+	1293	43	3	5	4.41%	10.98852965	0.00035113	0.00059011	1.37E-06
ENSG00000135677_64759276_64716741_552	annotated	GNS	12	-	1656	476	50	10	19.38%	94.97652464	1.57E-14	9.57E-19	3.12E-31
ENSG00000136717_127106934_127048526_494	annotated	BIN1	2	-	1482	35	2	1	2.83%	7.803501072	0.000742993	0.000525339	2.51E-06
ENSG00000136718_130342933_130346468_291	annotated	IMP4	2	+	873	79	3	2	10.31%	29.90077888	6.86E-07	7.81E-07	4.85E-12
ENSG00000136715_128026292_127942006_1056	annotated	SAP130	2	-	3168	40	0	2	1.61%	4.172001656	0.000128696	0.000416383	3.76E-07
ENSG00000136715_128026292_127942006_1022	annotated	SAP130	2	-	3066	31	0	2	1.47%	3.340867079	0.00028802	0.000933393	1.79E-06
ENSG00000138678_83536623_83604767_434	annotated	GPAT3	4	+	1302	29	1	0	3.69%	7.35964163	0.000275071	0.00017086	3.25E-07
ENSG00000166260_54968646_54961265_231	annotated	COX11	17	-	693	87	14	10	10.39%	41.48161646	0.000498484	0.000486898	1.59E-06

ENSG00000172922_65720758_65719783_164	annotated	RNASEH2C	11	-	492	44	0	2	6.71%	29.54998246	0.00140552	0.006771542	5.70E-05
ENSG00000167861_74958464_74951570_370	dORF	HID1	17	-	1110	14	0	0	1.62%	4.167491383	0.013217227	0.013217227	0.00084697
ENSG00000100949_24267764_24265615_151	annotated	RABGGTA	14	-	453	12	2	3	3.31%	8.752914732	0.042234516	0.131599539	0.022155158
ENSG00000231925_33313934_33313182_178	novel	TAPBP	6	-	534	47	14	3	10.67%	29.08213289	0.002806618	0.000171403	3.56E-06
ENSG00000115286_1383927_1393407_236	annotated	NDUFS7	19	+	708	61	8	2	8.90%	28.46860791	0.000176522	6.41E-05	8.27E-08
ENSG00000158793_161119144_161120765_291	annotated	NIT1	1	+	873	66	14	4	7.56%	24.98039754	0.000366912	9.62E-05	2.53E-07
ENSG00000134574_47216251_47238849_224	novel	DDB2	11	+	672	32	5	1	6.25%	15.73440624	0.003539468	0.000629624	1.43E-05
ENSG00000204673_49877311_49877204_35	uORF	AKT1S1	19	-	105	15	6	1	17.14%	47.20321873	0.065834008	0.026832696	0.007541382
ENSG00000204673_49873295_49869917_256	annotated	AKT1S1	19	-	768	66	6	7	10.55%	28.39568627	4.55E-06	7.01E-05	2.76E-09
ENSG00000204673_49873295_49869917_256	annotated	AKT1S1	19	-	768	66	6	7	10.55%	28.39568627	4.55E-06	7.01E-05	2.76E-09
ENSG00000204673_49873295_49869917_256	annotated	AKT1S1	19	-	768	66	6	7	10.55%	28.39568627	4.55E-06	7.01E-05	2.76E-09
ENSG00000204673_49873295_49869917_256	annotated	AKT1S1	19	-	768	66	6	7	10.55%	28.39568627	4.55E-06	7.01E-05	2.76E-09
ENSG00000204673_49873295_49869917_256	annotated	AKT1S1	19	-	768	66	6	7	10.55%	28.39568627	4.55E-06	7.01E-05	2.76E-09
ENSG00000132155_12618721_12584514_648	annotated	RAF1	3	-	1944	61	13	6	2.62%	10.36819671	0.010557975	0.00111378	7.44E-05
ENSG00000122033_27440124_27435675_170	annotated	MTIF3	13	-	510	27	5	0	4.71%	17.49295753	0.0260957	0.005160369	0.000719933
ENSG00000241258_66114829_66152357_141	annotated	CRCP	7	+	423	40	3	0	6.38%	31.24562942	0.016228926	0.003327803	0.000300554
ENSG00000149577_117179264_117196066_829	annotated	SIDT2	11	+	2487	16	2	0	0.84%	2.125758141	0.032488891	0.008473674	0.001379593
ENSG00000162591_3595371_3495941_1209	annotated	MEGF6	1	-	3627	13	0	2	0.74%	1.184310147	0.002943991	0.020677327	0.000349709
ENSG00000135269_116234534_116249350_138	novel	TES	7	+	414	18	2	0	5.80%	14.36619701	0.013991246	0.005355751	0.000391966
ENSG00000151292_123573443_123614396_311	annotated	CSNK1G3	5	+	933	30	1	0	3.22%	10.62451869	0.00345766	0.002321823	4.59E-05
ENSG00000164211_111507433_111501104_55	annotated	STARD4	5	-	165	14	1	5	9.09%	28.03585112	0.028889786	0.145920273	0.018444213
ENSG00000164211_111501104_111499886_107	dORF	STARD4	5	-	321	42	11	1	10.28%	43.23285454	0.0240701	0.002493494	0.000358754
ENSG00000114786_51984156_51986819_194	dORF	ABHD14A-ACY1	3	+	582	17	0	0	4.64%	9.651517232	0.003303074	0.003303074	6.12E-05
ENSG00000114786_51987406_51987645_45	dORF	ABHD14A-ACY1	3	+	135	17	2	0	13.33%	41.60876318	0.022004978	0.011772051	0.001241747
ENSG00000114786_51988530_51989075_99	dORF	ABHD14A-ACY1	3	+	297	27	1	4	8.08%	30.03841192	0.005355751	0.025702836	0.000731269
ENSG00000114786_51975136_51984153_167	annotated	ABHD14A-ACY1	3	+	501	9	0	0	3.59%	5.935734092	0.009815329	0.009815329	0.000483714
ENSG00000099204_114684305_114436260_732	annotated	ABLIM1	10	-	2196	79	6	7	3.96%	11.88678504	1.09E-05	7.77E-06	6.89E-10
ENSG00000099204_114684305_114436260_730	annotated	ABLIM1	10	-	2190	79	6	7	3.97%	11.91935158	1.09E-05	7.77E-06	6.89E-10
ENSG00000099204_114491803_114436260_394	annotated	ABLIM1	10	-	1182	73	5	7	6.09%	20.40680607	4.62E-05	5.09E-05	1.76E-08
ENSG00000151553_114833343_114899514_660	annotated	FAM160B1	10	+	1980	28	0	2	1.97%	4.672641854	0.000598307	0.000544026	2.11E-06
ENSG00000146263_97267976_97146806_962	annotated	MMS22L	6	-	2886	11	0	0	0.83%	1.259406737	0.00419316	0.00419316	9.65E-05
ENSG00000131778_147242704_147295509_897	annotated	CHD1L	1	+	2691	39	3	2	2.01%	4.788732335	0.000311905	0.000210096	4.48E-07
ENSG00000131778_147242704_147295509_693	annotated	CHD1L	1	+	2079	34	0	2	2.16%	5.403735478	0.000259634	0.000743479	1.30E-06
ENSG00000080503_2086868_2192739_717	novel	SMARCA2	9	+	2151	29	11	3	2.23%	4.454790052	0.013693601	0.001273274	0.000110499
ENSG00000109113_28717708_28716053_198	annotated	RAB34	17	-	594	63	83	19	10.61%	35.04481391	0.094246865	0.011096712	0.005431711
ENSG00000104450_100177885_100241022_803	annotated	SPAG1	8	+	2409	24	8	0	1.74%	3.291880758	0.023852945	0.000375189	7.74E-05
ENSG00000187720_71547410_71777374_658	annotated	THSD4	15	+	1974	96	17	2	6.99%	16.06918084	4.00E-07	3.11E-09	1.49E-14
ENSG00000115446_98609760_98618304_259	annotated	UNC50	2	+	777	209	42	9	20.08%	88.87813257	1.18E-07	1.11E-09	1.59E-15
ENSG00000132716_160240344_160217592_572	annotated	DCAF8	1	-	1716	103	2	3	6.12%	19.83305402	1.69E-07	9.25E-08	1.51E-13
ENSG00000115317_74530977_74531566_118	novel	HTRA2	2	+	354	11	1	1	4.24%	10.26736679	0.020613417	0.035374934	0.003252526
ENSG00000115317_74531906_74532985_93	novel	HTRA2	2	+	279	10	0	0	5.38%	11.84310147	0.019679754	0.019679754	0.001784964
ENSG00000174780_56467636_56501861_610	annotated	SRP72	4	+	1830	57	28	2	3.77%	10.29184933	0.019273697	3.32E-05	9.20E-06
ENSG00000113328_163441222_163442565_159	annotated	CCNG1	5	+	477	39	13	0	9.43%	27.01567864	0.011026486	0.000259634	2.32E-05
ENSG00000147526_38745468_38848023_731	annotated	TACC1	8	+	2193	100	8	2	5.47%	15.06714688	1.86E-07	2.16E-08	4.10E-14
ENSG00000136104_50927433_50950098_250	annotated	RNASEH2B	13	+	750	25	2	0	4.00%	11.01408437	0.003500471	0.002362849	4.72E-05
ENSG00000127990_94656098_94585499_429	annotated	SGCE	7	-	1287	217	28	10	13.29%	55.71226826	2.62E-08	3.92E-10	1.24E-16
ENSG00000136717_127106934_127048526_590	annotated	BIN1	2	-	1770	35	2	1	2.37%	6.533778864	0.000742993	0.000525339	2.51E-06
ENSG00000172053_49099226_49096029_228	dORF	QARS	3	-	684	30	14	0	5.26%	14.49221628	0.074841829	0.00092354	0.000639909
ENSG00000172053_49100034_49099338_132	annotated	QARS	3	-	396	14	0	0	6.06%	11.68160464	0.005257623	0.005257623	0.000148366
ENSG00000033011_5073200_5084881_353	dORF	ALG1	16	+	1059	72	16	1	6.52%	22.46498795	0.003715081	1.55E-05	6.52E-07
ENSG00000033011_5071850_5072104_84	annotated	ALG1	16	+	252	9	1	4	5.95%	11.80080468	0.035374934	0.171390856	0.025670492
ENSG00000134779_36818914_36798422_179	dORF	TPGS2	18	-	537	62	7	3	8.38%	38.14934251	0.00182801	0.001194059	1.32E-05
ENSG00000105171_29610472_29615380_179	novel	POP4	19	+	537	108	18	1	13.41%	66.45369341	0.000145583	9.24E-06	1.14E-08
ENSG00000135314_73242511_73241518_126	annotated	KHDC1	6	-	378	9	0	2	3.97%	7.867203122	0.020613417	0.034182656	0.003146197
ENSG00000111361_123633557_123630157_126	novel	EIF2B1	12	-	378	66	12	0	13.49%	57.69282289	0.009148649	0.000129483	1.06E-05
ENSG00000155545_56933291_56923128_316	dORF	MIER3	5	-	948	14	0	0	2.85%	4.879657633	0.00308495	0.00308495	5.38E-05
ENSG00000140682_31473441_31477576_409	Overlap_dORF	TGFB1I1	16	+	1227	37	4	4	4.89%	9.963841607	0.000845406	0.000405757	2.24E-06
ENSG00000106609_66941953_66955522_285	annotated	TMEM248	7	+	855	160	39	2	14.04%	61.83345612	0.000107491	1.80E-08	3.69E-11
ENSG00000171311_97445494_97437746_83	novel	EXOSC1	10	-	249	16	1	0	8.43%	21.23196987	0.023878474	0.007979402	0.000955388
ENSG00000104671_30156384_30183173_190	annotated	DCTN6	8	+	570	123	10	10	12.63%	71.30170408	3.69E-05	0.000299445	8.49E-08
ENSG00000084693_27053896_27069621_687	annotated	AGBL5	2	+	2061	19	0	0	1.75%	3.046107759	0.00077643	0.00077643	3.81E-06
ENSG00000130741_24054969_24062588_101	novel	EIF2S3	X	+	303	44	2	0	9.90%	47.98214973	0.006133031	0.002404282	8.34E-05
ENSG00000103888_80873919_80948924_1348	annotated	CEMIP	15	+	4044	14	2	0	0.67%	1.143896003	0.012888811	0.002943991	0.000212649
ENSG00000114982_96609519_96601771_300	novel	KANSL3	2	-	900	20	2	0	3.33%	7.342722914	0.009621867	0.002227676	0.000123102
ENSG00000108604_63837616_63832938_456	annotated	SMARCD2	17	-	1368	96	8	2	7.46%	23.18754604	1.31E-06	4.25E-07	5.08E-12
ENSG00000108602_19745129_19738221_453	annotated	ALDH3A1	17	-	1359	4367	672	152	37.75%	1061.777184	7.01E-22	4.19E-27	7.52E-47
ENSG00000106608_43906575_43876667_931	annotated	URGCP	7	-	2793	22	7	1	1.72%	2.60268374	0.002909181	0.000256462	5.25E-06
ENSG00000213676_32128195_32116501_623	annotated	ATF6B	6	-	1869	70	8	3	4.33%	12.3753757	9.01E-05	1.24E-05	9.04E-09

ENSG00000114956_73926911_73946846_89	annotated	DGUOK	2	+	267	35	4	4	14.61%	43.31381494	0.010702769	0.003526819	0.000206185
ENSG00000114956_73946758_73958736_179	Overlap_dORF	DGUOK	2	+	537	56	9	18	9.50%	34.45747066	0.005126463	0.01325819	0.000357148
ENSG00000141644_50279992_50270011_550	annotated	MBD1	18	-	1650	17	1	1	1.64%	3.404353351	0.00640069	0.00640069	0.000215507
ENSG00000110321_10805979_10805911_22	internal	EIF4G2	11	-	66	22	0	12	27.27%	110.1408437	0.013853925	0.14651463	0.010719976
ENSG00000110321_10804047_10803521_72	internal	EIF4G2	11	-	216	25	2	11	12.50%	38.24334851	0.007312901	0.082500581	0.003382551
ENSG00000110321_10803982_10797816_701	annotated	EIF4G2	11	-	2103	317	60	7	10.27%	49.80691506	1.99E-08	3.97E-13	1.82E-19
ENSG00000133318_63681637_63758201_1013	annotated	RTN3	11	+	3039	392	50	9	3.95%	42.62113596	1.73E-06	6.30E-08	1.12E-12
ENSG00000072062_14106822_14093112_238	annotated	PRKACA	19	-	714	31	3	0	6.72%	14.34607628	0.001066286	0.000183359	1.36E-06
ENSG00000067836_4802559_4797953_226	annotated	ROGDI	16	-	678	14	3	0	3.10%	6.822884123	0.052370108	0.008473674	0.00228699
ENSG00000171634_67826142_67982288_2965	annotated	BPTF	17	+	8895	40	9	9	0.61%	1.485879848	0.003201028	0.001919608	3.56E-05
ENSG00000048028_113854326_113835277_172	annotated	USP28	11	-	516	10	2	0	2.91%	6.403537425	0.08410207	0.019679754	0.007521961
ENSG00000094631_48802964_48824612_1153	annotated	HDAC6	X	+	3459	25	3	5	1.04%	2.388136247	0.007364879	0.00858612	0.000325247
ENSG00000113328_163439257_163442565_295	annotated	CCNG1	5	+	885	159	26	9	13.22%	59.36404796	4.30E-06	2.46E-07	1.02E-11
ENSG00000144824_111884822_111974563_1005	annotated	PHLDB2	3	+	3015	27	2	4	1.39%	2.959007741	0.001090807	0.003990798	2.64E-05
ENSG00000134333_18396843_18407281_332	annotated	LDHA	11	+	996	1768	182	49	20.78%	586.5331677	1.74E-12	5.65E-12	1.24E-22
ENSG00000172354_100676266_100678801_340	annotated	GNB2	7	+	1020	344	34	6	22.06%	111.4366183	5.84E-12	2.22E-14	1.95E-24
ENSG00000144824_111884822_111974563_962	annotated	PHLDB2	3	+	2886	28	2	4	1.56%	3.205762603	0.000742282	0.002781433	1.29E-05
ENSG00000153786_84990462_84976343_293	annotated	ZDHHC7	16	-	879	76	15	5	10.24%	28.56895605	0.000256308	3.61E-06	9.04E-09
ENSG00000129317_43755245_43730376_701	annotated	PUS7L	12	-	2103	19	3	5	1.71%	2.985272511	0.009022293	0.009834242	0.00044767
ENSG00000166451_81007268_81011982_20	novel	CENPN	16	+	60	54	9	0	35.00%	297.380278	0.037523868	0.008877961	0.001667265
ENSG00000106355_32490365_32487261_91	annotated	LSM5	7	-	273	210	25	7	31.87%	254.1711778	7.99E-06	5.12E-06	3.40E-10
ENSG00000124380_69893985_69904308_155	annotated	SNRNP27	2	+	465	57	6	1	13.55%	40.50340704	0.000199054	3.51E-05	5.33E-08
ENSG00000130255_5690508_5691621_105	annotated	RPL36	19	+	315	417	79	18	33.33%	437.4164936	2.95E-05	2.16E-07	7.09E-11
ENSG00000254505_24213439_24209877_222	annotated	CHMP4A	14	-	666	108	11	5	15.32%	53.58203207	6.24E-06	1.22E-06	6.72E-11
ENSG00000129925_375152_372542_222	annotated	TMEM8A	16	-	666	10	7	1	2.70%	4.961299266	0.261477794	0.011772051	0.020039585
ENSG00000171720_141636790_141636473_70	novel	HDAC3	5	-	210	13	1	2	7.14%	20.45472812	0.020613417	0.052029617	0.004760359
ENSG00000242485_1406917_1402569_39	annotated	MRPL20	1	-	117	63	1	2	23.08%	177.9198244	0.003710481	0.003789641	7.80E-05
ENSG00000100906_35404608_35402653_173	novel	NFKBIA	14	-	519	40	3	7	7.51%	25.46609103	0.00122385	0.006307439	4.69E-05
ENSG00000255508_62574838_62559679_487	novel	AP002990.1	11	-	1461	171	5	0	2.26%	38.67368434	0.001588175	0.001588175	1.51E-05
ENSG00000104518_143559336_143562904_484	annotated	GSDMD	8	+	1452	39	9	1	4.13%	8.87498534	0.001044869	5.15E-05	4.28E-07
ENSG00000166226_69599889_69601325_48	novel	CCT2	12	+	144	66	7	0	14.58%	151.4436601	0.056358542	0.008776163	0.00254826
ENSG00000178449_50120044_50120217_57	annotated	COX14	12	+	171	80	20	0	15.79%	154.5836403	0.007356796	0.003736771	0.000149379
ENSG00000177889_93410908_93410726_60	Overlap_dORF	UBE2N	12	-	180	19	3	1	10.00%	34.87793384	0.053213269	0.012798403	0.003263543
ENSG00000168806_43329226_43328429_265	dORF	LCMT2	15	-	795	6	0	3	1.89%	2.493754952	0.016947427	0.165899918	0.014398895
ENSG00000186468_82278323_82276311_123	annotated	RPS23	5	-	369	3595	271	18	17.89%	3219.15718	0.002205318	7.94E-05	1.41E-06
ENSG00000173540_49723726_49721752_387	annotated	GMPPB	3	-	1161	28	6	1	2.84%	7.968846573	0.018439401	0.001967496	0.000220188
ENSG00000143337_179882866_179918239_462	annotated	TOR1AIP1	1	+	1386	122	6	0	10.82%	29.08481154	2.89E-09	2.52E-10	8.41E-18
ENSG00000197912_89508547_89536981_464	annotated	SPG7	16	+	1392	75	8	2	5.17%	17.8029381	0.000171996	1.31E-05	1.85E-08
ENSG00000117620_99993624_100022476_302	annotated	SLC35A3	1	+	906	16	9	0	3.64%	5.835276488	0.151590816	0.000888449	0.001652192
ENSG00000066427_92096122_92080961_223	novel	ATXN3	14	-	669	31	0	0	5.83%	15.31105899	0.000674519	0.000674519	2.91E-06
ENSG00000258466_67685124_67663181_39	annotated	AL049779.1	14	-	117	19	0	1	12.82%	53.65835975	0.021557223	0.021557223	0.002115616
ENSG00000092199_21234154_21211223_280	annotated	HNRNPC	14	-	840	97	7	8	4.64%	38.15593514	0.004778771	0.007235892	0.000184345
ENSG00000143315_160031739_160030468_423	annotated	PIGM	1	-	1269	55	5	0	4.02%	14.32091348	0.000440234	0.000134274	4.14E-07
ENSG00000072274_196074120_196051942_679	annotated	TFRC	3	-	2037	565	73	22	19.88%	91.64886111	5.11E-21	1.79E-22	1.62E-41
ENSG00000264785_19737876_19738401_36	novel	AC005722.3	17	+	108	104	91	23	16.67%	318.1846596	0.175840341	0.06827946	0.043518162
ENSG00000161960_7572842_7574383_48	novel	EIF4A1	17	+	144	14	0	0	12.50%	32.12441275	0.01300724	0.01300724	0.000821865
ENSG00000101608_3253248_3255918_171	annotated	MYL12A	18	+	513	220	24	7	14.04%	141.7016703	0.00035639	8.15E-05	2.10E-07
ENSG00000007202_28644095_28643178_73	novel	KIAA0100	17	-	219	15	0	0	6.85%	22.63168021	0.021557223	0.021557223	0.002115616
ENSG00000007202_28642954_28639305_269	novel	KIAA0100	17	-	807	13	2	0	2.60%	5.322791703	0.032826899	0.008373824	0.00138048
ENSG00000125733_6751086_6751211_41	internal	TRIP10	19	+	123	11	3	0	12.20%	29.54998246	0.087777151	0.021084099	0.008320031
ENSG00000125733_6739762_6751326_593	annotated	TRIP10	19	+	1779	70	10	13	4.89%	13.00144867	0.000134236	0.000103303	9.93E-08
ENSG00000163806_28783930_28816040_184	novel	SPDYA	2	+	552	146	42	1	15.76%	87.39436511	3.07E-05	1.02E-06	2.98E-10
ENSG00000141699_42609324_42581813_466	annotated	RETREG3	17	-	1398	152	15	0	9.44%	35.92576876	3.93E-08	3.20E-09	1.36E-15
ENSG00000117877_45406697_45409501_510	annotated	CD3EAP	19	+	1530	30	4	3	3.33%	6.478873159	0.000351211	0.000999273	2.31E-06
ENSG00000173511_64234834_64237633_207	annotated	VEGFB	11	+	621	38	2	3	5.31%	20.21909208	0.001398493	0.00718566	6.04E-05
ENSG00000167460_16075707_16101346_247	novel	TPM4	19	+	741	100	5	3	8.91%	44.5914347	1.83E-05	4.10E-05	5.87E-09
ENSG00000139174_42469470_42459809_710	annotated	PRICKLE1	12	-	2130	10	0	0	0.70%	1.551279489	0.020613417	0.020613417	0.001946209
ENSG00000160209_43732438_43737075_128	annotated	PDXK	21	+	384	102	14	5	6.25%	87.76848483	0.01784595	0.005756914	0.000534191
ENSG00000078674_17935704_18027662_1829	annotated	PCM1	8	+	5487	206	29	21	3.88%	12.40514697	1.42E-09	6.83E-11	1.17E-18
ENSG00000055130_148730123_148800582_759	annotated	CUL1	7	+	2277	46	9	0	2.24%	6.675202649	0.000417517	0.000127653	3.75E-07
ENSG00000075711_197138252_197044623_608	dORF	DLG1	3	-	1824	31	4	0	2.14%	5.615733807	0.004387317	0.000656046	1.85E-05
ENSG00000075711_197297204_197270746_118	annotated	DLG1	3	-	354	8	0	0	4.24%	7.467175844	0.016947427	0.016947427	0.001349898
ENSG00000125386_2662974_2720034_875	annotated	FAM193A	4	+	2625	11	0	0	0.80%	1.384627749	0.007593099	0.007593099	0.000297833
ENSG00000168439_64186262_64204126_543	annotated	STIP1	11	+	1629	360	29	21	15.47%	73.02155384	6.30E-15	8.87E-13	8.36E-26
ENSG00000228830_234607288_234607560_90	novel	AL160408.2	1	+	270	32	7	8	10.00%	39.16118887	0.028023387	0.076848202	0.009142281
ENSG00000181991_88467718_88475045_65	novel	MRPS11	15	+	195	42	3	0	16.92%	71.16792978	0.001588175	0.001580611	1.50E-05
ENSG00000161217_196270531_196238688_367	annotated	PCYT1A	3	-	1101	30	0	4	3.27%	9.003338723	0.001007374	0.004044382	2.49E-05
ENSG00000124570_2959332_2948298_376	dORF	SERPINB6	6	-	1128	227	41	8	16.76%	66.49460511	3.99E-09	2.68E-11	1.42E-18

ENSG00000244005_35699288_35669622_457	annotated	NFS1	20	-	1371	81	9	6	7.00%	19.52168127	1.46E-05	3.07E-06	3.82E-10
ENSG00000184007_31919628_31919563_21	uORF	PTP4A2	1	-	63	79	14	9	23.81%	414.3393644	0.097833871	0.130723026	0.043720095
ENSG00000184007_31919026_31908852_154	annotated	PTP4A2	1	-	462	29	2	0	5.19%	20.74080823	0.005756914	0.005706018	0.000174777
ENSG00000186260_14261028_14261444_138	annotated	MRTFB	16	+	414	10	0	0	3.62%	7.981220558	0.020613417	0.020613417	0.001946209
ENSG00000242110_34008019_33989093_367	annotated	AMACR	5	-	1101	37	9	1	3.54%	11.10411776	0.02769848	0.00105275	0.00020861
ENSG00000169925_134036254_134033590_155	novel	BRD3	9	-	465	31	0	3	5.16%	22.02816874	0.005306578	0.018151375	0.00050607
ENSG00000141956_41860336_41801240_1198	annotated	PRDM15	21	-	3594	14	1	0	0.58%	1.287121713	0.011306644	0.007882058	0.000451174
ENSG00000117335_207752091_207752192_33	uORF	CD46	1	+	99	18	6	7	21.21%	60.07682384	0.095084229	0.079167839	0.027185036
ENSG00000117335_207752213_207793591_399	annotated	CD46	1	+	1197	243	27	12	15.04%	67.0782582	1.80E-10	2.92E-10	6.02E-19
ENSG00000100722_88563134_88611751_711	annotated	ZC3H14	14	+	2133	69	24	2	5.20%	10.68877386	0.000604545	7.40E-08	9.59E-10
ENSG00000132388_4366316_4331860_24	uORF	UBE2G1	17	-	72	31	8	12	20.83%	142.2652565	0.021557223	0.112457942	0.011059731
ENSG00000132388_4296750_4282835_99	annotated	UBE2G1	17	-	297	21	1	0	8.08%	23.36320927	0.008316137	0.005554612	0.00024255
ENSG00000166747_71789479_71733058_825	annotated	AP1G1	16	-	2475	98	10	1	4.73%	13.08339719	1.37E-06	2.34E-08	3.52E-13
ENSG00000125877_3223376_3223462_28	novel	ITPA	20	+	84	27	1	0	28.57%	106.2072421	0.005306578	0.005306578	0.000151001
ENSG00000214078_35632923_35632254_146	novel	CPNE1	20	-	438	140	21	10	14.38%	105.6145077	0.003770153	0.000347446	9.03E-06
ENSG00000214078_35632195_35626241_396	novel	CPNE1	20	-	1188	185	16	14	14.14%	51.45468708	2.45E-09	6.79E-10	1.85E-17
ENSG00000185686_22556832_22550763_115	annotated	PRAME	22	-	345	62	0	1	10.43%	59.38028095	0.001091522	0.001157448	7.77E-06
ENSG00000115368_189441493_189451906_127	novel	WDR75	2	+	381	23	0	2	7.87%	19.94676697	0.002227676	0.009621867	0.000123102
ENSG00000114956_73958165_73958736_35	dORF	DGUOK	2	+	105	19	4	0	14.29%	59.79074373	0.112457942	0.021084099	0.010870696
ENSG00000114956_73926911_73946846_89	annotated	DGUOK	2	+	267	35	4	4	14.61%	43.31381494	0.010702769	0.003526819	0.000206185
ENSG00000164068_49713755_49721305_392	annotated	RNF123	3	+	1176	13	3	0	1.79%	3.652630021	0.051117422	0.007593099	0.002037586
ENSG00000104763_18061423_18057534_149	novel	ASAH1	8	-	447	192	22	3	18.79%	141.9264563	6.85E-05	4.60E-06	2.73E-09
ENSG00000104763_18084058_18062204_240	novel	ASAH1	8	-	720	293	16	1	11.25%	134.4636134	2.86E-06	4.02E-06	9.80E-11
ENSG00000196083_190564290_190644386_346	annotated	IL1RAP	3	+	1038	27	2	0	3.47%	8.594805723	0.003532672	0.000978918	2.12E-05
ENSG00000089280_31189689_31191438_206	novel	FUS	16	+	618	156	8	5	19.42%	83.40762921	1.24E-07	3.99E-08	4.91E-14
ENSG00000089280_31180215_31185183_255	novel	FUS	16	+	765	161	23	13	16.86%	69.53990524	1.48E-07	2.89E-08	4.30E-14
ENSG00000078403_21534705_21741983_1048	annotated	MLLT10	10	+	3144	19	2	5	1.34%	1.996828273	0.002665715	0.006524514	9.74E-05
ENSG00000197969_77177705_77416006_3135	annotated	VPS13A	9	+	9405	34	6	4	0.67%	1.194509948	0.001294754	0.000319392	2.75E-06
ENSG00000177674_11747543_11750192_97	Overlap_dORF	AGTRAP	1	+	291	162	10	9	18.56%	183.9465637	9.38E-05	0.000868713	6.00E-07
ENSG00000204592_30489532_30492581_358	annotated	HLA-E	6	+	1074	151	15	1	13.97%	46.45605419	3.10E-09	2.82E-10	1.00E-17
ENSG00000160932_143020940_143021789_131	annotated	LY6E	8	+	393	509	42	18	28.24%	427.9518278	8.03E-06	1.39E-07	1.17E-11
ENSG00000054983_87992321_87933973_586	annotated	GALC	14	-	1758	71	5	7	4.61%	13.34470973	7.60E-06	5.05E-05	3.16E-09
ENSG00000256591_61430147_61481813_133	annotated	AP003108.2	11	+	399	40	2	0	9.02%	33.12506578	0.004244579	0.000881454	2.32E-05
ENSG00000144320_175995584_175929967_459	annotated	LNPK	2	-	1377	49	6	1	5.01%	11.75795499	0.000257178	1.55E-05	3.29E-08
ENSG00000126247_36141012_36149839_94	annotated	CAPNS1	19	+	282	639	71	4	44.68%	748.723395	2.54E-08	1.48E-08	3.84E-15
ENSG00000130024_169721057_169704003_451	annotated	PHF10	6	-	1353	26	0	0	1.77%	6.349583007	0.005306578	0.005306578	0.000151001
ENSG00000123485_233854500_233845789_122	annotated	HJURP	2	-	366	35	10	8	10.66%	31.59778303	0.022522717	0.009433856	0.001043422
ENSG00000120868_98665717_98732566_790	annotated	APAF1	12	+	2370	15	11	0	1.01%	2.091281843	0.127645253	0.005160369	0.004421647
ENSG00000162729_160095244_160091823_591	annotated	IGSF8	1	-	1773	84	3	1	3.21%	15.65453616	0.000212641	5.76E-05	9.03E-08
ENSG00000127554_1984714_1986028_130	annotated	GFER	16	+	390	51	7	0	10.00%	43.20910022	0.000930688	0.000690959	4.06E-06
ENSG00000112983_138177593_138157208_866	annotated	BRD8	5	-	2598	25	9	11	1.50%	3.179585557	0.077723366	0.061281985	0.01802661
ENSG00000113621_134893498_134899685_161	Overlap_dORF	TXNDC15	5	+	483	145	26	1	16.77%	99.1951698	0.000255855	2.51E-06	6.58E-09
ENSG00000182899_197950968_197955773_110	annotated	RPL35A	3	+	330	646	178	21	24.55%	646.8271367	0.000126396	6.54E-06	7.15E-09
ENSG00000141084_67806536_67726428_594	annotated	RANBP10	16	-	1782	41	2	1	2.02%	7.602314128	0.002182856	0.001125366	1.48E-05
ENSG00000145012_188406187_188708440_406	annotated	LPP	3	+	1218	11	3	1	1.23%	2.984111529	0.083852152	0.027849813	0.009942783
ENSG00000143862_202144572_202134467_186	annotated	ARL8A	1	-	558	124	10	5	19.89%	73.42722914	1.07E-07	1.91E-07	1.97E-13
ENSG00000115525_85848138_85840144_290	Overlap_dORF	ST3GAL5	2	-	870	128	20	5	13.45%	48.61388964	1.48E-06	1.67E-07	2.36E-12
ENSG00000073712_52919198_52858377_593	annotated	FERMT2	14	-	1779	39	6	4	2.53%	7.243664257	0.00175022	0.001712183	1.78E-05
ENSG00000136319_20305934_20295308_352	novel	TTC5	14	-	1056	73	11	0	4.83%	22.84170906	0.001696578	0.000121273	1.53E-06
ENSG00000100591_77458190_77462257_122	novel	AHSA1	14	+	366	150	9	9	22.13%	135.4190701	2.94E-06	4.00E-05	1.03E-09
ENSG00000136811_128461020_128500255_762	annotated	ODF2	9	+	2286	40	2	6	2.49%	5.781671586	0.000252295	0.003009807	5.37E-06
ENSG00000127990_94656026_94587779_431	annotated	SGCE	7	-	1293	201	22	8	11.83%	51.36498744	4.60E-08	1.51E-09	7.91E-16
ENSG00000162129_72434474_72337090_327	annotated	CLPB	11	-	981	27	1	0	3.67%	9.094198104	0.002493494	0.00087769	1.35E-05
ENSG00000168036_41224552_41239342_768	annotated	CTNNB1	3	+	2304	288	46	13	11.46%	41.30281639	2.21E-12	5.94E-15	2.04E-25
ENSG00000119760_27661249_27653485_363	annotated	SUPT7L	2	-	1089	43	5	2	6.06%	13.046987	0.000245633	1.94E-05	3.86E-08
ENSG00000119487_125672574_125438887_486	annotated	MAPKAP1	9	-	1458	85	9	4	6.38%	19.26331629	8.29E-06	3.06E-06	2.15E-10
ENSG00000041982_115091009_115021157_2198	annotated	TNC	9	-	6594	33	2	1	0.86%	1.653615943	8.88E-05	6.24E-05	4.08E-08
ENSG00000132376_1509784_1495823_356	Overlap_dORF	INPP5K	17	-	1068	35	5	5	4.21%	10.82845374	0.006166408	0.002296562	8.04E-05
ENSG00000111907_125153952_125262962_209	annotated	TPD52L1	6	+	627	43	12	0	6.22%	22.66055636	0.081224702	0.000548081	0.000490064
ENSG00000126522_66083101_66092912_340	annotated	ASL	7	+	1020	32	5	0	4.12%	10.36619705	0.004127207	0.000434916	1.21E-05
ENSG00000011304_797498_810826_531	annotated	PTBP1	19	+	1593	286	35	22	15.82%	59.32256365	5.18E-13	6.59E-13	4.43E-24
ENSG00000040633_7239267_7236088_154	annotated	PHF23	17	-	462	22	1	1	3.90%	15.73440624	0.013716833	0.013716833	0.000908091
ENSG00000040633_7236505_7235626_142	Overlap_dORF	PHF23	17	-	426	31	2	9	4.23%	24.04483208	0.017507491	0.080200405	0.006508656
ENSG00000073584_40632347_40628785_224	novel	SMARCE1	17	-	672	37	3	0	3.57%	18.19290722	0.009288069	0.005306578	0.000259004
ENSG00000102974_67629456_67637872_180	Overlap_dORF	CTCF	16	+	540	14	1	3	5.00%	8.566510066	0.00640069	0.024022703	0.000794032
ENSG00000102974_67611139_67635594_536	annotated	CTCF	16	+	1608	28	0	1	3.17%	5.753626164	0.000101975	0.000172422	1.25E-07
ENSG00000117632_25904676_25885723_174	annotated	STMN1	1	-	522	554	64	10	25.86%	350.6783185	2.47E-08	6.69E-09	1.73E-15
ENSG00000182134_151781543_151774452_505	annotated	TDRKH	1	-	1515	52	6	0	4.75%	11.34123539	0.000131203	6.54E-06	7.45E-09

ENSG00000165055_128476766_128501916_378	annotated	METTL2B	7	+	1134	20	1	1	1.85%	5.827557868	0.0099656	0.0099656	0.000497776
ENSG00000162775_110339538_110342279_913	annotated	RBM15	1	+	2739	30	4	0	1.42%	3.619085773	0.006750264	0.000662934	2.95E-05
ENSG00000181038_76729711_76733686_190	annotated	METTL23	17	+	570	50	25	15	10.00%	28.98443255	0.017114837	0.007157691	0.00062095
ENSG00000131778_147266075_147295509_603	dORF	CHD1L	1	+	1809	31	0	2	2.32%	5.662298764	0.000378748	0.001209608	3.00E-06
ENSG00000072609_132862423_132841554_488	annotated	CHFR	12	-	1464	10	1	0	1.23%	2.256984502	0.023672501	0.012798403	0.001437523
ENSG00000132640_11919114_11923666_404	annotated	BTBD3	20	+	1212	23	2	2	1.98%	6.270394567	0.010318779	0.015646181	0.000790819
ENSG00000196072_100282936_100275462_95	annotated	BLOC1S2	10	-	285	21	1	0	8.42%	24.34692335	0.008219047	0.005454749	0.00023581
ENSG00000104964_3056347_3053856_145	annotated	TLE5	19	-	435	89	52	1	8.97%	67.60369027	0.125080498	0.000714894	0.001078196
ENSG00000104964_3054064_3053819_81	Overlap_dORF	TLE5	19	-	243	52	2	9	11.11%	70.70770213	0.00310098	0.022714641	0.000404109
ENSG00000048162_176388539_176383981_232	annotated	NOP16	5	-	696	176	26	8	17.24%	83.55512281	4.60E-06	3.12E-08	1.67E-12
ENSG00000167863_75042650_75038932_137	annotated	ATP5PD	17	-	411	295	13	11	18.25%	237.1645905	1.81E-05	2.31E-05	3.29E-09
ENSG00000151923_119596462_119575665_374	annotated	TIAL1	10	-	1122	100	22	4	10.16%	29.44942345	9.48E-06	1.99E-07	1.94E-11
ENSG00000159685_126704313_126935108_192	novel	CHCHD6	3	+	576	18	8	0	3.65%	10.3257041	0.185067256	0.008473674	0.010111413
ENSG00000134851_55417179_55425452_144	novel	TMEM165	4	+	432	149	12	2	20.14%	113.9651786	2.50E-06	1.18E-06	2.60E-11
ENSG00000186184_27665687_27665953_88	dORF	POLR1D	13	+	264	14	1	0	5.68%	17.52240695	0.022600676	0.020613417	0.002121199
ENSG00000185686_22556832_22548067_509	annotated	PRAME	22	-	1527	140	14	2	6.68%	30.29414169	6.97E-06	1.77E-07	1.25E-11
ENSG00000185686_22556832_22548067_509	annotated	PRAME	22	-	1527	140	14	2	6.68%	30.29414169	6.97E-06	1.77E-07	1.25E-11
ENSG00000143369_150509708_150513467_359	annotated	ECM1	1	+	1077	17	2	5	2.23%	5.215583128	0.013892891	0.07477567	0.005013892
ENSG00000163399_116389483_116389722_79	novel	ATP1A1	1	+	237	27	0	0	7.59%	37.64307317	0.012798403	0.012798403	0.000797244
ENSG00000203879_154438574_154440143_164	novel	GDI1	X	+	492	19	12	6	6.10%	12.7602197	0.190891497	0.037150762	0.030022903
ENSG00000203879_154442125_154442259_44	novel	GDI1	X	+	132	10	0	0	11.36%	25.03200993	0.020613417	0.020613417	0.001946209
ENSG00000075790_107594044_107621953_243	annotated	BCAP29	7	+	729	82	7	2	12.35%	37.16686907	1.71E-05	2.25E-06	3.36E-10
ENSG00000104872_49448006_49447344_53	annotated	PIH1D1	19	-	159	11	5	1	13.21%	22.85942039	0.077087128	0.01551626	0.005661469
ENSG00000167774_8321358_8311505_113	annotated	AC010323.1	19	-	339	247	38	1	23.89%	240.7503398	0.000170681	2.73E-06	4.54E-09
ENSG00000111780_120438384_120459959_155	annotated	AL021546.1	12	+	465	299	16	1	18.06%	212.4652404	1.40E-05	1.78E-06	2.18E-10
ENSG00000152556_48130365_48145708_720	annotated	PFKM	12	+	2160	87	14	9	5.83%	13.30868528	2.51E-06	4.48E-07	1.03E-11
ENSG00000160799_46976732_46925151_182	novel	CCDC12	3	-	546	18	3	1	3.85%	10.89305048	0.015625009	0.013991246	0.001045796
ENSG00000106263_2379188_2379497_52	novel	EIF3B	7	+	156	40	7	1	21.15%	84.72372593	0.009282808	0.001603369	8.89E-05
ENSG00000105771_43750741_43738092_312	novel	SMG9	19	-	936	14	4	1	2.24%	4.942217346	0.067796501	0.008838363	0.003140171
ENSG00000134574_47235056_47238849_205	novel	DDB2	11	+	615	21	3	1	3.41%	11.28272057	0.023718446	0.011738695	0.001333341
ENSG00000134574_47216251_47234981_294	novel	DDB2	11	+	882	45	3	0	5.44%	16.8582924	0.000744678	0.000178703	9.19E-07
ENSG00000204387_31834948_31837240_51	novel	SNHG32	6	+	153	12	7	0	11.76%	25.91549264	0.11411552	0.011772051	0.007078638
ENSG00000204386_31862776_31859719_415	annotated	NEU1	6	-	1245	256	22	1	10.84%	67.94230359	8.24E-08	3.31E-09	3.01E-15
ENSG00000068650_112690417_112882064_1191	annotated	ATP11A	13	+	3573	33	2	2	1.26%	3.051761412	0.001028506	0.001028506	6.55E-06
ENSG00000204387_31834948_31835061_37	uORF	SNHG32	6	+	111	12	7	0	16.22%	35.72135472	0.11411552	0.011772051	0.007078638
ENSG00000204387_31834948_31835061_37	novel	SNHG32	6	+	111	12	7	0	16.22%	35.72135472	0.11411552	0.011772051	0.007078638
ENSG00000140750_24954614_24920130_601	novel	ARHGAP17	16	-	1803	26	10	0	1.50%	4.764828513	0.113299699	0.003684287	0.002981121
ENSG00000136104_50929501_50956474_258	novel	RNASEH2B	13	+	774	54	1	0	6.20%	23.05273473	0.000267881	0.000199656	3.68E-07
ENSG00000116337_109625404_109631152_761	annotated	AMPD2	1	+	2283	8	6	0	0.79%	1.15785381	0.280084718	0.011570466	0.021802746
ENSG00000117475_169376731_169387182_327	annotated	BLZF1	1	+	981	13	2	0	1.53%	4.378687976	0.055414058	0.020613417	0.00507254
ENSG00000101311_6087806_6077173_230	novel	FERMT1	20	-	690	11	0	0	2.61%	5.267605569	0.011772051	0.011772051	0.000681273
ENSG00000166295_72233018_72233116_32	novel	ANAPC16	10	+	96	66	3	0	31.25%	227.1654901	0.007635352	0.00244612	0.000105613
ENSG00000112514_33417099_33416650_59	novel	CUTA	6	-	177	116	23	1	27.12%	216.5480995	0.001198213	0.000344013	2.72E-06
ENSG00000168297_58382562_58424935_497	annotated	PXK	3	+	1491	16	4	0	1.41%	3.545781689	0.060590694	0.008473674	0.00268166
ENSG00000089685_78214317_78223657_137	annotated	BIRC5	17	+	411	66	16	6	10.22%	53.06055244	0.007316181	0.003394649	0.000136087
ENSG00000105193_39433667_39433273_66	Overlap_dORF	RPS16	19	-	198	171	6	146	16.67%	285.3649132	0.001657226	0.124359691	0.001911745
ENSG00000105193_39435636_39433409_62	annotated	RPS16	19	-	186	980	120	12	35.48%	1740.935917	3.55E-05	1.99E-05	5.51E-09
ENSG00000083807_58498658_58498515_47	novel	SLC27A5	19	-	141	11	0	0	10.64%	25.77764427	0.019216965	0.019216965	0.001707396
ENSG00000105364_10252254_10258738_263	annotated	MRPL4	19	+	789	101	19	10	9.89%	42.29743427	0.000276117	7.09E-05	1.43E-07
ENSG00000152234_46098231_46084282_531	annotated	ATP5F1A	18	-	1593	923	125	29	25.61%	191.4500918	3.50E-18	1.54E-23	1.38E-39
ENSG00000168397_241637715_241651966_88	novel	ATG4B	2	+	264	12	5	0	5.68%	15.01920596	0.121807694	0.019679754	0.01126199
ENSG00000006530_141555467_141652924_422	annotated	AGK	7	+	1266	42	4	0	4.03%	10.96188492	0.000178977	0.000127394	1.61E-07
ENSG00000115540_197540381_197550646_126	dORF	MOB4	2	+	378	41	2	0	8.73%	35.83948089	0.002815105	0.001580611	2.62E-05
ENSG00000196290_200892025_200903678_350	annotated	NIF3L1	2	+	1050	53	6	1	6.57%	16.67847062	0.000150606	9.45E-06	1.20E-08
ENSG00000007392_229339_189198_371	annotated	LUC7L	16	-	1113	55	6	1	6.47%	16.32815742	0.000191248	1.03E-05	1.66E-08
ENSG00000169169_49713020_49713605_76	novel	CPT1C	19	+	228	9	0	0	6.58%	13.04299465	0.020613417	0.020613417	0.001946209
ENSG00000196476_289586_271207_310	annotated	C20orf96	20	-	930	18	9	1	3.23%	6.395274796	0.152911592	0.003372875	0.004148385
ENSG00000012660_53295699_53269127_326	annotated	ELOVL5	6	-	978	406	37	9	22.39%	137.1692716	9.04E-11	2.24E-13	3.02E-22
ENSG00000162620_74198132_74198019_37	uORF	LRRIQ3	1	-	111	9	0	1	13.51%	26.79101604	0.019216965	0.064489021	0.005584918
ENSG00000171428_18222189_18222920_243	annotated	NAT1	8	+	729	8	0	0	2.88%	3.626036007	0.005706018	0.005706018	0.00017331
ENSG00000102225_47223594_47228768_484	annotated	CDK16	X	+	1452	38	2	0	2.89%	8.647421613	0.000705627	0.000428413	1.97E-06
ENSG00000100997_25390703_25338964_118	annotated	ABHD12	20	-	354	11	3	5	5.93%	10.26736679	0.042234516	0.077869819	0.013082305
ENSG00000115525_85848138_85847466_124	novel	ST3GAL5	2	-	372	77	16	4	14.52%	68.39391101	0.001121188	0.000525374	3.77E-06
ENSG00000115525_85846502_85840144_177	novel	ST3GAL5	2	-	531	43	4	1	9.60%	26.75738011	0.00076033	0.000186181	9.75E-07
ENSG00000231925_33314041_33301760_361	annotated	TAPBP	6	-	1083	334	41	15	18.28%	101.9031629	3.89E-10	8.69E-12	4.33E-20
ENSG00000167702_144467934_144473450_586	annotated	KIFC2	8	+	1758	10	2	1	0.85%	1.879536582	0.078649604	0.047790352	0.014683996
ENSG00000275700_36989298_37021018_116	annotated	AATF	17	+	348	19	2	0	7.76%	18.04031061	0.014772524	0.003229477	0.000265527
ENSG00000137309_34240781_34244884_96	annotated	HMGA1	6	+	288	68	7	3	7.29%	78.01643096	0.047719858	0.018151375	0.003914522

ENSG00000244462_35655274_35652524_916	annotated	RBM12	20	-	2748	43	3	9	2.40%	5.170367117	5.73E-05	0.002569452	1.26E-06
ENSG00000186815_113277300_113280221_82	novel	TPCN1	12	+	246	12	0	0	7.32%	16.11817225	0.01300724	0.01300724	0.000821865
ENSG00000100075_19178673_19176130_311	annotated	SLC25A1	22	-	933	172	45	24	15.76%	60.91390713	1.14E-06	2.90E-07	3.07E-12
ENSG00000152133_37091996_37094222_90	novel	GPATCH11	2	+	270	6	0	0	6.67%	7.342722914	0.007152939	0.007152939	0.000266003
ENSG00000064012_201258358_201286594_496	annotated	CASP8	2	+	1488	45	19	7	4.44%	9.992616868	0.026965083	0.000549121	0.000120839
ENSG00000157191_16440762_16458890_237	annotated	NECAP2	1	+	711	38	2	3	4.64%	17.65971334	0.005064994	0.00736895	0.000198036
ENSG00000011028_62688603_62692451_345	annotated	AC080038.1	17	+	1035	54	0	4	6.09%	17.23943641	2.53E-05	0.000193334	3.82E-08
ENSG00000134313_8827093_8730720_1771	annotated	KIDINS220	2	-	5313	49	11	2	1.58%	3.047375122	0.00084268	6.44E-06	5.62E-08
ENSG00000177054_19117250_19175960_622	annotated	ZDHHC13	11	+	1866	58	11	0	4.02%	10.27036806	0.001747954	4.29E-06	9.36E-08
ENSG00000169919_65980234_65976166_67	Overlap_dORF	GUSB	7	-	201	25	0	0	7.46%	41.09732974	0.021084099	0.021084099	0.002029891
ENSG00000169919_65982183_65979503_144	annotated	GUSB	7	-	432	42	2	2	9.03%	32.12441275	0.001651783	0.000881412	8.98E-06
ENSG00000111711_21501924_21515707_138	annotated	GOLT1B	12	+	414	196	33	13	21.01%	156.4319229	0.000294227	9.99E-05	2.09E-07
ENSG00000265241_145927378_145925882_158	annotated	RBM8A	1	-	474	58	6	0	7.59%	40.43144896	0.001805035	0.001057356	1.16E-05
ENSG00000164609_160408403_160406390_206	novel	SLU7	5	-	618	23	3	1	2.91%	12.29727867	0.028889786	0.011772051	0.001625894
ENSG00000173334_125436046_125436471_141	annotated	TRIB1	8	+	423	20	12	0	5.67%	15.62281471	0.264591651	0.004873904	0.011525983
ENSG00000167186_19067665_19072172_105	novel	COQ7	16	+	315	17	3	0	5.71%	17.83232708	0.053528973	0.013640586	0.003462163
ENSG00000173349_127701529_127702176_215	annotated	SFT2D3	2	+	645	21	1	0	2.79%	10.75794287	0.016983117	0.013640586	0.00110551
ENSG00000152223_45955164_45852467_2500	annotated	EPG5	18	-	7500	10	7	0	0.28%	0.440563375	0.291350991	0.007029303	0.01679328
ENSG00000100567_58252240_58257855_37	novel	PSMA3	14	+	111	55	15	1	27.03%	163.7228758	0.023070074	0.002113288	0.000298587
ENSG00000158987_131635030_131435951_1391	annotated	RAPGEF6	5	-	4173	18	6	5	0.58%	1.425258941	0.069104828	0.060667625	0.016027477
ENSG00000161202_184155780_184170755_668	annotated	DVL3	3	+	2004	10	0	0	1.05%	1.648815026	0.007593099	0.007593099	0.000297833
ENSG00000198252_52730475_52771106_223	annotated	STYX	14	+	669	19	1	0	4.48%	9.384197446	0.004911637	0.002254349	6.30E-05
ENSG00000106346_6146172_6159457_931	dORF	USP42	7	+	2793	12	2	0	0.86%	1.419645676	0.030313544	0.004826937	0.000797936
ENSG00000035681_58589532_58586438_111	novel	NSMAF	8	-	333	15	1	0	5.41%	14.8838978	0.016503129	0.01300724	0.001029549
ENSG00000135919_224001900_224001601_99	novel	SERPINE2	2	-	297	69	4	2	15.15%	76.76483046	0.001271179	0.000706834	5.64E-06
ENSG00000168502_8796277_8831840_976	annotated	MTCL1	18	+	2928	17	3	0	0.72%	1.918436827	0.039615985	0.007882058	0.00159776
ENSG00000129422_17715785_17645926_415	annotated	MTUS1	8	-	1245	63	5	0	6.02%	16.72017627	6.92E-05	4.27E-06	2.57E-09
ENSG00000108055_110567817_110604302_1217	annotated	SMC3	10	+	3651	68	11	2	2.47%	6.154130955	0.000162573	7.83E-07	1.45E-09
ENSG00000129422_17755807_17645926_1216	annotated	MTUS1	8	-	3648	215	21	2	5.92%	19.47391562	2.03E-11	1.63E-13	4.70E-23
ENSG00000083845_58388138_58393646_201	novel	RPS5	19	+	603	773	69	30	16.42%	423.5764785	5.42E-07	8.55E-07	4.21E-12
ENSG00000108883_44894521_44851274_972	annotated	EFTUD2	17	-	2916	185	18	11	7.30%	20.96302066	4.37E-12	7.49E-12	4.07E-22
ENSG00000188603_28477608_28477516_30	dORF	CLN3	16	-	90	19	5	1	16.67%	69.75586768	0.070008252	0.029237631	0.008622834
ENSG00000188603_28489349_28479730_199	annotated	CLN3	16	-	597	115	11	2	13.07%	63.64923129	8.82E-06	3.53E-06	2.62E-10
ENSG00000175203_57547063_57530689_401	annotated	DCTN2	12	-	1203	155	12	6	11.97%	42.57314408	2.09E-08	8.50E-09	1.85E-15
ENSG00000077235_27549890_27461350_2084	annotated	GTF3C1	16	-	6252	67	4	8	1.63%	3.540996415	1.17E-06	5.16E-06	5.34E-11
ENSG00000089006_17954069_17942357_299	novel	SNX5	20	-	897	93	22	11	7.36%	34.2578544	0.000780146	0.000290033	1.51E-06
ENSG00000160584_116859335_116846396_411	novel	SIK3	11	-	1233	7	1	0	1.22%	1.875878117	0.047790352	0.016947427	0.003697512
ENSG00000188186_100153490_100153964_41	novel	LAMTOR4	7	+	123	128	13	4	36.59%	343.8543413	0.000634982	0.000665324	2.71E-06
ENSG00000147684_124539187_124579586_138	annotated	NDUFB9	8	+	414	277	22	9	25.36%	221.0798095	6.03E-07	1.49E-07	8.55E-13
ENSG00000175221_872049_868373_183	dORF	MED16	19	-	549	10	0	0	3.28%	6.018625339	0.012798403	0.012798403	0.000797244
ENSG00000175221_891131_872923_663	annotated	MED16	19	-	1989	31	1	2	2.11%	5.149873537	0.0005964	0.000390775	1.53E-06
ENSG00000164104_173333649_173332080_209	annotated	HMGB2	4	-	627	401	54	21	21.05%	211.3228628	9.69E-08	1.86E-08	1.83E-14
ENSG00000160584_116927357_116846396_1102	annotated	SIK3	11	-	3306	21	1	0	1.09%	2.098872702	0.001621946	0.000873936	8.75E-06
ENSG00000171055_36581407_36553163_181	dORF	FEZ2	2	-	543	20	15	0	4.42%	12.17025897	0.34898553	0.005454749	0.01902107
ENSG00000171055_36590974_36581588_63	annotated	FEZ2	2	-	189	16	1	5	11.11%	27.97227777	0.015625009	0.059434472	0.004322539
ENSG00000174915_488205_490582_278	novel	PTDSS2	11	+	834	67	14	2	10.07%	26.54473571	0.00022294	3.00E-06	6.59E-09
ENSG00000137343_30626886_30643562_310	annotated	ATAT1	6	+	930	12	2	2	1.94%	4.263516531	0.071345384	0.047790352	0.01337936
ENSG00000173465_65571582_65571734_50	dORF	ZNRD2	11	+	150	31	10	0	10.00%	68.2873231	0.112457942	0.021084099	0.010870696
ENSG00000183207_48993912_49015842_463	annotated	RUVBL2	19	+	1389	175	12	11	12.31%	41.62990853	3.66E-10	7.31E-10	3.01E-18
ENSG00000163161_127294081_127257596_807	annotated	ERCC3	2	-	2421	40	0	1	2.48%	5.459273542	3.15E-05	5.30E-05	1.27E-08
ENSG00000134283_42335903_42441509_367	annotated	PPHLN1	12	+	1101	93	20	3	7.08%	27.91035004	0.000841517	8.45E-06	7.07E-08
ENSG00000066427_92096122_92058404_283	annotated	ATXN3	14	-	849	34	1	0	5.30%	13.23246886	0.000407542	0.000290289	7.94E-07
ENSG00000028203_95256599_95296269_565	novel	VEZT	12	+	1695	28	3	1	2.30%	5.458307299	0.00179362	0.001037781	1.13E-05
ENSG00000126432_64318216_64321691_125	annotated	PRDX5	11	+	375	366	36	10	29.60%	322.4923904	1.84E-06	8.01E-08	1.49E-12
ENSG00000086758_53654107_53584278_1672	novel	HUWE1	X	-	5016	46	4	2	1.26%	3.030190676	0.000177463	5.98E-05	7.79E-08
ENSG00000106772_76905963_76823608_259	novel	PRUNE2	9	-	777	7	0	0	1.93%	2.97677956	0.019216965	0.019216965	0.001707396
ENSG00000158864_161213386_161213941_83	novel	NDUFS2	1	+	249	79	6	1	10.84%	104.8328512	0.005697454	0.003800472	0.000118132
ENSG00000006015_18590872_18592180_44	dORF	REX1BD	19	+	132	11	0	2	11.36%	27.53521093	0.019216965	0.083503598	0.007323531
ENSG00000006015_18588802_18590872_66	annotated	REX1BD	19	+	198	16	1	7	10.61%	26.7008106	0.0135992	0.199269954	0.01543597
ENSG00000174775_534322_533296_170	annotated	HRAS	11	-	510	25	3	0	3.53%	16.1971829	0.060667625	0.013428348	0.003899005
ENSG00000174775_533583_532636_110	Overlap_dORF	HRAS	11	-	330	7	0	1	5.45%	7.008962781	0.009815329	0.028889786	0.001387795
ENSG00000174442_66518919_66546679_471	annotated	ZWILCH	15	+	1413	50	1	1	5.10%	11.69223394	1.02E-05	1.02E-05	8.46E-10
ENSG00000169189_27257549_27257370_59	novel	NSMCE1	16	-	177	54	3	6	10.17%	100.8068739	0.013439622	0.060590694	0.003896441
ENSG00000275410_37744884_37731661_293	annotated	HNF1B	17	-	879	32	5	2	3.07%	12.02903412	0.046975818	0.004911637	0.001305882
ENSG00000100445_24442101_24440083_185	annotated	SDR39U1	14	-	555	19	3	3	4.32%	11.31176233	0.01551626	0.038565921	0.002761354
ENSG00000144559_11829777_11809413_159	dORF	TAMM41	3	-	477	8	0	0	3.77%	5.541677671	0.011570466	0.011570466	0.000659484
ENSG00000104960_49851497_49854738_75	novel	PTOV1	19	+	225	22	2	6	13.33%	32.30798082	0.00345766	0.028743211	0.000570893
ENSG00000189057_59109635_59126302_731	annotated	FAM111B	11	+	2193	23	2	0	2.05%	3.465443783	0.001506384	0.000231319	2.40E-06

ENSG00000152082_130182691_130190626_80	annotated	MZT2B	2	+	240	12	4	1	7.50%	16.52112656	0.065565251	0.020239728	0.005930948
ENSG00000022976_102326309_102342179_723	annotated	ZNF839	14	+	2169	11	0	0	0.83%	1.675725146	0.012798403	0.012798403	0.000797244
ENSG00000166181_43312128_43342455_504	annotated	API5	11	+	1512	36	1	2	2.58%	7.867203122	0.000995868	0.002603001	1.58E-05
ENSG00000175756_1374756_1373801_199	annotated	AURKAIP1	1	-	597	204	25	18	19.60%	112.9082016	3.76E-07	8.74E-07	3.02E-12
ENSG00000244687_50084158_50082768_58	annotated	UBE2V1	20	-	174	13	4	2	10.34%	24.68674083	0.143893695	0.035374934	0.02120653
ENSG00000146063_181224015_181234775_625	annotated	TRIM41	5	+	1875	32	4	6	1.76%	5.639211198	0.01008628	0.011228343	0.000563568
ENSG00000106367_101156603_101160566_154	annotated	AP1S1	7	+	462	437	23	5	29.87%	312.542524	4.37E-08	1.57E-09	7.75E-16
ENSG00000285382_32743337_32640582_193	annotated	FO393400.1	20	-	579	36	2	1	6.74%	20.54440608	0.000869763	0.00064467	3.56E-06
ENSG00000163611_113494121_113450259_695	novel	SPICE1	3	-	2085	18	0	3	0.86%	2.852568614	0.013428348	0.063412829	0.004086769
ENSG00000151422_108883436_109187575_501	dORF	FER	5	+	1503	13	0	0	1.20%	2.857946044	0.012798403	0.012798403	0.000797244
ENSG00000077782_38440342_38413628_802	annotated	FGFR1	8	-	2406	96	18	8	5.36%	13.18394139	4.20E-06	2.18E-07	8.93E-12
ENSG00000075415_98593979_98601528_361	annotated	SLC25A3	12	+	1083	873	114	22	29.64%	266.3516802	5.63E-15	7.15E-18	6.96E-31
ENSG00000002919_48112659_48121508_194	Overlap_dORF	SNX11	17	+	582	32	3	0	4.64%	18.16756185	0.012563987	0.003632191	0.000249096
ENSG00000072310_17814850_17814611_79	novel	SREBF1	17	-	237	16	0	3	6.33%	22.30700632	0.021084099	0.085886389	0.008130109
ENSG00000134352_55960410_55941082_612	Overlap_dORF	IL6ST	5	-	1836	153	20	5	6.05%	27.53521093	1.62E-06	3.65E-07	5.46E-12
ENSG00000134352_55976278_55959643_333	annotated	IL6ST	5	-	999	27	3	3	2.40%	8.930338679	0.028352861	0.028352861	0.003520634
ENSG00000138772_78604313_78610115_48	novel	ANXA3	4	+	144	82	8	2	22.92%	188.1572747	0.002009905	0.001603369	1.91E-05
ENSG00000078304_101761894_101925272_555	annotated	PPP2R5C	14	+	1665	57	7	4	4.50%	11.31176233	2.19E-05	6.68E-05	1.13E-08
ENSG00000139637_53299810_53303205_142	novel	C12orf10	12	+	426	35	9	2	7.04%	27.14739105	0.057090446	0.006905573	0.002131637
ENSG00000105221_40255258_40233872_419	Overlap_dORF	AKT2	19	-	1257	74	3	6	8.11%	19.45208218	7.77E-07	1.44E-06	1.00E-11
ENSG00000197170_67366519_67345741_303	novel	PSMD12	17	-	909	84	11	4	11.55%	30.53409528	5.35E-06	1.19E-07	6.59E-12
ENSG00000127952_76030523_75996468_313	annotated	STYXL1	7	-	939	40	13	0	4.15%	14.07550718	0.021077816	0.000674519	0.000106388
ENSG00000117362_150267233_150266522_97	novel	APH1A	1	-	291	117	8	2	23.71%	132.850296	5.31E-05	1.33E-05	5.63E-09
ENSG00000143420_150629470_150625638_117	annotated	ENSA	1	-	351	52	11	9	7.69%	48.95148609	0.029255686	0.014451683	0.001971876
ENSG00000066044_7991791_7963483_318	annotated	ELAVL1	19	-	954	139	21	9	15.09%	48.14332476	2.75E-07	4.41E-08	1.21E-13
ENSG00000100243_42636837_42619773_291	annotated	CYB5R3	22	-	873	324	29	1	16.49%	122.6310425	5.32E-09	7.43E-10	4.40E-17
ENSG00000160445_128755538_128731337_757	annotated	ZER1	9	-	2271	48	4	1	2.38%	6.983831569	0.000263027	0.000111224	2.07E-07
ENSG00000105048_55141284_55132915_192	annotated	TNNT1	19	-	576	13	2	0	3.65%	7.457452959	0.037995728	0.007979402	0.001544499
ENSG00000136854_127663265_127690857_431	novel	STXBP1	9	+	1293	39	5	0	3.71%	9.966340846	0.003116517	0.000176199	4.08E-06
ENSG00000106348_128409901_128393007_598	annotated	IMPDH1	7	-	1794	25	7	7	2.17%	4.604550322	0.021528028	0.024442317	0.002376535
ENSG00000185842_224952748_225399269_4500	annotated	DNAH14	1	+	13500	37	4	3	0.22%	0.905602493	0.004488024	0.008072852	0.000193595
ENSG00000197622_151055730_151054247_79	annotated	CDC42SE1	1	-	237	43	9	1	18.99%	59.95007949	0.002270904	0.000294865	4.57E-06
ENSG00000071553_154428696_154429291_133	annotated	ATP6AP1	X	+	399	72	7	3	8.27%	59.6251184	0.004609847	0.006133031	0.000151899
ENSG00000138074_27206103_27205346_78	novel	SLC5A6	2	-	234	19	4	2	6.41%	26.82917988	0.102951605	0.057424805	0.022249039
ENSG00000100412_41515500_41516045_104	novel	ACO2	22	+	312	10	2	0	4.81%	10.59046574	0.101051022	0.019679754	0.009164498
ENSG00000153317_130180834_130054731_940	annotated	ASAP1	8	-	2820	48	12	14	2.66%	5.624213296	0.000947123	0.004626581	2.71E-05
ENSG00000164808_47260959_47440343_181	annotated	SPIDR	8	+	543	18	3	2	3.31%	10.95323308	0.039104404	0.024275373	0.004143858
ENSG00000167904_55756394_55750916_130	novel	TMEM68	8	-	390	25	0	0	6.15%	21.18093148	0.005706018	0.005706018	0.00017331
ENSG00000125798_22584242_22581850_451	annotated	FOXA2	20	-	1353	28	1	4	2.66%	6.83801247	0.000978918	0.008221728	5.10E-05
ENSG00000100796_91509647_91458759_833	annotated	PPP4R3A	14	-	2499	62	16	8	3.84%	8.197757875	0.000190759	2.83E-05	4.18E-08
ENSG00000134644_31059566_30933211_1091	annotated	PUM1	1	-	3273	60	22	9	2.93%	6.057241634	0.001409591	3.20E-05	3.93E-07
ENSG00000134644_31059566_30933211_1188	annotated	PUM1	1	-	3564	64	23	11	3.11%	5.933513466	0.000491226	2.58E-05	1.03E-07
ENSG00000149636_36771458_36752788_284	annotated	DSN1	20	-	852	54	2	3	7.75%	20.9422731	2.18E-05	9.69E-05	1.64E-08
ENSG00000134644_31059566_30933211_1189	annotated	PUM1	1	-	3567	64	23	11	3.11%	5.928523126	0.000491226	2.58E-05	1.03E-07
ENSG00000005187_20770170_20796972_541	annotated	ACSM3	16	+	1623	31	4	1	3.33%	6.311212856	0.00053339	0.000121504	4.59E-07
ENSG00000092098_24151242_24160573_539	annotated	RNF31	14	+	1617	20	1	0	1.30%	4.086858765	0.015625009	0.007979402	0.000625379
ENSG00000166133_40569440_40574261_450	annotated	RPUSD2	15	+	1350	11	0	3	1.11%	2.692331735	0.020613417	0.114974528	0.0109456
ENSG00000182774_82540428_82539977_52	novel	RPS17	15	-	156	203	30	6	25.00%	429.9729091	0.021700922	0.00273178	0.000345885
ENSG00000137807_69414466_69447807_810	annotated	AC027237.1	15	+	2430	86	7	4	4.44%	11.69396612	1.19E-06	2.25E-07	2.52E-12
ENSG00000143436_151761513_151760050_92	dORF	MRPL9	1	-	276	18	5	3	8.70%	21.54929551	0.037966651	0.032206895	0.005194299
ENSG00000143436_151763479_151762482_122	annotated	MRPL9	1	-	366	66	3	4	13.11%	59.58439086	0.00021836	0.000219177	3.30E-07
ENSG00000145050_51385343_51389089_182	annotated	MANF	3	+	546	559	61	20	36.26%	338.2897342	1.07E-10	3.12E-12	4.33E-21
ENSG00000151348_44206924_44244220_126	novel	EXT2	11	+	378	36	10	4	10.32%	31.46881249	0.012071587	0.00391499	0.000255585
ENSG00000149196_86344615_86345638_53	novel	HIKESHI	11	+	159	22	2	0	15.09%	45.71884078	0.005454749	0.005554612	0.000161874
ENSG00000221968_61879401_61876691_241	novel	FADS3	11	-	723	37	9	15	5.81%	16.90959011	0.004080194	0.017365242	0.000384666
ENSG00000213593_57737945_57740245_202	dORF	TMX2	11	+	606	156	7	0	14.85%	85.05926544	6.71E-07	7.94E-07	4.82E-12
ENSG00000213593_57712619_57737933_115	annotated	TMX2	11	+	345	179	28	6	16.52%	171.4366176	0.001859131	9.17E-05	1.32E-06
ENSG00000117620_99993624_100022476_261	annotated	SLC35A3	1	+	783	10	9	0	3.07%	4.219955698	0.388068955	0.003327803	0.017008244
ENSG00000123600_171360506_171325891_241	annotated	METTL8	2	-	723	9	2	0	3.32%	4.113143541	0.009815329	0.003327803	0.000179363
ENSG00000122218_160311950_160290157_893	dORF	COPA	1	-	2679	109	17	8	4.48%	13.44384318	1.33E-05	4.02E-07	5.27E-11
ENSG00000122218_160332529_160313118_113	annotated	COPA	1	-	339	8	0	1	4.42%	7.797581855	0.019679754	0.042234516	0.003715148
ENSG00000147099_72568850_72462089_241	annotated	HDAC8	X	-	723	10	0	0	2.07%	4.57015949	0.019679754	0.019679754	0.001784964
ENSG00000069702_91797406_91683739_808	annotated	TGFBR3	1	-	2424	33	2	6	2.72%	4.498326537	6.24E-05	0.000695854	3.29E-07
ENSG00000112414_142402769_142405698_97	annotated	ADGRG6	6	+	291	17	4	0	7.22%	19.30303446	0.067302329	0.008175611	0.002928208
ENSG00000106976_128248628_128250944_195	novel	DNM1	9	+	585	18	0	0	5.13%	10.16684711	0.002046029	0.002046029	2.45E-05
ENSG00000106976_128219081_128248034_528	novel	DNM1	9	+	1584	97	19	16	6.25%	20.23420803	9.76E-06	0.000101836	8.21E-09
ENSG00000123358_52054497_52058944_542	annotated	NR4A1	12	+	1626	27	2	2	2.77%	5.486720996	0.001263673	0.001263673	9.73E-06
ENSG00000197969_77177705_77416006_3174	annotated	VPS13A	9	+	9522	34	6	4	0.66%	1.179832604	0.001294754	0.000319392	2.75E-06

ENSG00000100726_1507028_1509936_103	novel	TELO2	16	+	309	16	0	4	6.80%	17.10925727	0.008473674	0.059897465	0.002647935
ENSG00000156515_69300835_69401135_905	annotated	HK1	10	+	2715	183	27	1	6.96%	22.27157392	4.19E-09	1.96E-12	1.34E-19
ENSG00000154723_25729794_25724640_108	annotated	ATP5PF	21	-	324	206	17	2	25.00%	210.0834611	1.07E-05	4.20E-06	3.76E-10
ENSG00000140043_73860574_73884177_327	annotated	PTGR2	14	+	981	39	3	0	5.20%	13.13606393	0.0007246	0.000121023	6.26E-07
ENSG00000077044_233434802_233442217_309	novel	DGKD	2	+	927	22	3	2	2.27%	7.841742918	0.020525443	0.008574016	0.000873936
ENSG00000102081_147928379_147948844_501	annotated	FMR1	X	+	1503	10	1	0	1.80%	2.198420034	0.010460668	0.001946209	0.000119282
ENSG00000184640_77482176_77498658_335	annotated	9-Sep	17	+	1005	113	18	4	9.55%	37.15198609	0.000224578	1.89E-06	4.46E-09
ENSG00000093167_37148983_37053851_424	annotated	LRRFIP2	3	-	1272	45	7	13	4.72%	11.68947634	0.000136071	0.003136164	3.29E-06
ENSG00000062822_50398981_50417947_1064	annotated	POLD1	19	+	3192	48	2	1	2.44%	4.968759866	1.51E-05	4.23E-06	5.35E-10
ENSG00000127955_80135161_80217493_354	annotated	GNAI1	7	+	1062	134	11	5	10.45%	41.6917318	5.07E-08	1.32E-07	6.60E-14
ENSG00000188229_137241361_137243556_445	annotated	TUBB4B	9	+	1335	504	73	21	15.73%	124.743787	1.06E-09	2.47E-12	3.82E-20
ENSG00000009307_114739890_114718169_798	annotated	CSDE1	1	-	2394	202	28	26	9.02%	27.88026369	2.37E-10	1.49E-10	4.08E-19
ENSG00000151353_675775_669580_127	annotated	TMEM18	2	-	381	83	5	1	18.90%	71.98181124	9.53E-06	9.02E-06	7.00E-10
ENSG00000137693_102110849_102229940_504	annotated	YAP1	11	+	1512	90	19	6	5.75%	19.6680078	1.72E-05	8.70E-06	1.21E-09
ENSG00000168488_28823260_28835621_968	novel	ATXN2L	16	+	2904	144	24	7	4.13%	16.38458832	2.13E-05	2.31E-08	6.97E-12
ENSG00000072736_68157905_68214429_589	novel	NFATC3	16	+	1767	13	2	7	0.85%	2.430952408	0.055917761	0.172059248	0.036465499
ENSG00000115109_120042090_120174907_645	annotated	EPB41L5	2	+	1935	19	1	0	1.24%	3.244458962	0.008219047	0.005454749	0.00023581
ENSG00000074054_121605883_121340861_1534	annotated	CLASP1	2	-	4602	41	0	2	0.98%	2.943790477	0.000299491	0.000387696	7.79E-07
ENSG00000118482_63680020_63713708_1951	annotated	PHF3	6	+	5853	42	10	11	1.03%	2.371048404	0.001732619	0.004028513	4.06E-05
ENSG00000163913_129440331_129520265_1131	annotated	IFT122	3	+	3393	20	2	4	0.88%	1.94767186	0.005388912	0.023573843	0.000671609
ENSG00000135622_74654377_74680209_770	annotated	SEMA4F	2	+	2310	27	6	0	1.69%	3.862081533	0.006436307	0.000642411	2.73E-05
ENSG00000149932_29963144_29971545_240	annotated	TMEM219	16	+	720	22	14	10	10.42%	27.99413111	0.000100081	0.000547292	3.94E-07
ENSG00000140365_75336090_75338334_91	novel	COMMD4	15	+	273	101	7	6	8.79%	122.2442331	0.021820178	0.018991317	0.00190254
ENSG00000134247_116967195_116974478_132	novel	PTGFRN	1	+	396	24	1	0	6.06%	20.02560795	0.007312901	0.005706018	0.000219657
ENSG00000168036_41235736_41236507_88	novel	CTNNB1	3	+	264	40	6	0	12.50%	50.06401987	0.005258039	0.001626321	5.01E-05
ENSG00000214160_184245786_184245110_156	novel	ALG3	3	-	468	25	9	0	3.85%	17.65077623	0.397859985	0.012798403	0.039075244
ENSG00000163507_108581486_108551149_746	dORF	CIP2A	3	-	2238	60	5	2	3.49%	8.858512899	1.43E-05	1.13E-05	1.30E-09
ENSG00000163507_108589375_108582130_121	annotated	CIP2A	3	-	363	22	0	4	5.79%	20.02560795	0.008574016	0.053155214	0.002346528
ENSG00000101856_119236377_119236664_95	internal	PGRMC1	X	+	285	74	7	77	17.89%	85.79392036	0.000238305	0.602728603	0.011121732
ENSG00000101856_119236364_119243254_143	annotated	PGRMC1	X	+	429	143	86	10	23.08%	110.1408437	0.088260047	1.97E-06	1.23E-05
ENSG00000162694_100877701_100874420_78	annotated	EXTL2	1	-	234	34	0	1	15.38%	48.01011136	0.00104892	0.001385138	8.90E-06
ENSG00000156958_49201181_49328159_434	annotated	GALK2	15	+	1302	43	6	1	3.00%	10.91257207	0.004866125	0.00084676	2.58E-05
ENSG00000140157_22838748_22838861_37	uORF	NIPA2	15	+	111	43	2	4	18.92%	128.0015211	0.008776163	0.032038753	0.00140113
ENSG00000075711_197297204_197044623_892	annotated	DLG1	3	-	2676	39	4	0	2.02%	4.815575005	0.000762836	7.71E-05	4.39E-07
ENSG00000175455_123949028_123914779_753	annotated	CCDC14	3	-	2259	9	0	0	0.80%	1.316424427	0.011772051	0.011772051	0.000681273
ENSG00000135452_57749009_57749422_137	dORF	TSPAN31	12	+	411	81	56	18	8.03%	65.11976891	0.065714011	0.04343079	0.011382126
ENSG00000135452_57745215_57747290_106	annotated	TSPAN31	12	+	318	54	5	6	14.15%	56.10948642	0.000294865	0.001166459	2.30E-06
ENSG00000272325_34392359_34288753_171	annotated	NUDT3	6	-	513	32	1	5	8.19%	20.61115204	0.000938122	0.004199767	2.43E-05
ENSG00000147099_72572761_72385266_344	annotated	HDAC8	X	-	1032	14	1	0	2.03%	4.482476197	0.01551626	0.007882058	0.000614257
ENSG00000163683_39605145_39556529_38	annotated	SMIM14	4	-	114	7	2	3	15.79%	20.28910279	0.158655254	0.078649604	0.043901103
ENSG00000163683_39556532_39552126_45	Overlap_dORF	SMIM14	4	-	135	28	0	0	24.44%	68.53208053	0.001558047	0.001558047	1.45E-05
ENSG00000166263_54990841_55159911_510	annotated	STXBP4	17	+	1530	18	2	0	1.76%	3.887323895	0.016881136	0.003303074	0.000311189
ENSG00000104980_7941131_7933860_191	novel	TIMM44	19	-	573	37	3	5	6.28%	21.33618438	0.005421488	0.0084311	0.000240355
ENSG00000214046_16659990_16647246_63	annotated	SMIM7	19	-	189	119	14	0	28.57%	208.0438159	0.002793544	9.38E-05	2.11E-06
ENSG00000090372_46728750_46722231_376	novel	STRN4	19	-	1128	49	15	7	4.79%	14.35346101	0.015789902	0.001748065	0.000168491
ENSG00000108465_47971147_47981502_506	annotated	CDK5RAP3	17	+	1518	39	2	1	3.95%	8.489116412	8.04E-05	5.28E-05	3.15E-08
ENSG00000153113_96742661_96771679_422	annotated	CAST	5	+	1266	327	43	9	16.82%	85.34610401	9.67E-10	1.92E-12	2.73E-20
ENSG00000138772_78554474_78582296_105	novel	ANXA3	4	+	315	48	18	12	19.05%	50.35009998	0.009208521	0.000431476	2.88E-05
ENSG00000112576_41940540_41935851_195	annotated	CCND3	6	-	585	48	14	8	8.72%	27.1115923	0.014959642	0.002226557	0.000195302
ENSG00000094841_75274255_75297573_193	novel	UPRT	X	+	579	8	1	0	3.11%	4.565423573	0.041782825	0.011570466	0.002331326
ENSG00000082641_48054726_48059641_584	annotated	NFE2L1	17	+	1752	172	10	5	8.73%	32.43874164	1.06E-09	5.38E-10	6.39E-18
ENSG00000096872_26978128_27036514_332	annotated	IFT74	9	+	996	11	2	0	2.11%	3.649244822	0.022304859	0.007593099	0.000853768
ENSG00000061273_47798925_47783841_952	annotated	HDAC7	12	-	2856	73	7	8	2.84%	8.44567394	2.09E-05	4.28E-05	6.93E-09
ENSG00000139116_39340293_39294424_900	annotated	KIF21A	12	-	2700	15	0	0	0.67%	1.835680728	0.013428348	0.013428348	0.000872559
ENSG00000011465_91178552_91146058_359	annotated	DCN	12	-	1077	11	1	1	1.39%	3.374789083	0.035772986	0.035772986	0.005410795
ENSG00000115561_86510552_86507411_125	novel	CHMP3	2	-	375	15	1	0	4.00%	13.21690124	0.035772986	0.021084099	0.003354605
ENSG00000166510_54942746_54904358_320	annotated	CCDC68	18	-	960	22	1	0	3.44%	7.572183005	0.001856966	0.00146251	1.62E-05
ENSG00000033867_27390077_27376764_188	novel	SLC4A7	3	-	564	17	12	1	3.72%	9.959544378	0.218051256	0.007979402	0.012075627
ENSG00000204435_31666832_31668128_110	novel	CSNK2B	6	+	330	119	43	3	13.64%	119.1523673	0.01214013	0.000571574	4.95E-05
ENSG00000221823_68217104_68180963_160	annotated	PPP3R1	2	-	480	97	35	6	18.12%	66.7728865	0.002154315	2.06E-06	6.64E-08
ENSG00000160199_43028736_43030101_116	novel	PKNOX1	21	+	348	9	5	2	4.31%	8.545410287	0.197554034	0.019216965	0.019453604
ENSG00000245694_54925136_54923546_46	novel	CRNDE	16	-	138	8	14	0	10.87%	19.15492934	0.333186701	0.019216965	0.038471518
ENSG00000129460_23470909_23478026_290	annotated	NGDN	14	+	870	90	13	1	11.03%	34.18164115	1.03E-05	3.23E-07	3.27E-11
ENSG00000164171_52989469_53090599_1181	annotated	ITGA2	5	+	3543	124	16	8	4.83%	11.56432229	3.62E-09	3.90E-11	1.81E-18
ENSG00000196712_31233178_31257719_156	annotated	NF1	17	+	468	22	2	0	3.21%	15.53268309	0.021084099	0.021557223	0.002072352
ENSG00000286053_189661521_189666111_96	annotated	ASDURF	2	+	288	24	0	5	7.29%	27.53521093	0.008776163	0.020525443	0.000892178
ENSG00000164167_146182922_146189656_80	annotated	LSM6	4	+	240	36	1	3	8.75%	49.56337967	0.010042643	0.027811403	0.001362204
ENSG00000163659_156677704_156705131_655	annotated	TIPARP	3	+	1965	28	4	1	2.75%	4.708310876	0.001070883	0.000117931	9.17E-07

ENSG00000116604_156482610_156467645_485	annotated	MEF2D	1	-	1455	35	3	0	2.27%	7.948308309	0.005867783	0.001618649	5.58E-05
ENSG00000167123_128422905_128435905_517	annotated	CERCAM	9	+	1551	97	6	3	6.19%	20.66472309	1.09E-06	1.13E-06	1.09E-11
ENSG00000079277_46580540_46558575_325	novel	MKNK1	1	-	975	15	1	1	1.85%	5.083423556	0.020342454	0.020342454	0.001898761
ENSG00000171634_67826142_67982288_2822	annotated	BPTF	17	+	8466	40	9	9	0.64%	1.561174255	0.003201028	0.001919608	3.56E-05
ENSG00000118482_63680020_63713708_1951	dORF	PHF3	6	+	5853	42	10	11	1.03%	2.371048404	0.001732619	0.004028513	4.06E-05
ENSG00000132356_40798162_40767668_167	novel	PRKAA1	5	-	501	22	3	6	4.79%	14.50957222	0.024700094	0.047572973	0.005090653
ENSG00000143013_87332223_87344796_96	annotated	LMO4	1	+	288	49	2	0	10.42%	56.21772231	0.002484542	0.002460157	3.52E-05
ENSG00000143013_87332223_87344796_96	annotated	LMO4	1	+	288	49	2	0	10.42%	56.21772231	0.002484542	0.002460157	3.52E-05
ENSG00000132676_155729286_155732120_105	novel	DAP3	1	+	315	58	7	0	11.43%	60.83970414	0.009319127	0.001040526	6.14E-05
ENSG00000141295_47840843_47837844_425	annotated	SCRN2	17	-	1275	59	8	7	6.35%	15.29014066	1.45E-05	9.45E-05	1.09E-08
ENSG00000068400_48990976_48974193_644	annotated	GRIPAP1	X	-	1932	10	2	0	0.93%	1.710261548	0.060322713	0.011570466	0.003431813
ENSG00000141522_81869930_81868876_249	novel	ARHGDIA	17	-	747	598	44	11	20.08%	264.514958	9.09E-09	1.02E-09	1.02E-16
ENSG00000177302_18308889_18274802_899	Overlap_dORF	TOP3A	17	-	2697	43	3	3	2.34%	5.268138242	0.000160693	0.000185252	2.08E-07
ENSG00000140950_84495859_84479913_387	annotated	MEAK7	16	-	1161	41	3	1	4.91%	11.6686682	0.000296178	7.68E-05	1.65E-07
ENSG00000047315_57010478_57030988_667	novel	POLR2B	4	+	2001	80	9	7	4.80%	13.2102961	1.14E-05	3.21E-06	3.10E-10
ENSG00000042753_46850664_46838447_144	annotated	AP2S1	19	-	432	314	27	14	29.17%	240.1682286	3.37E-07	3.58E-08	1.22E-13
ENSG00000144524_231794346_231807645_157	annotated	COPS7B	2	+	471	13	0	0	3.82%	9.119942472	0.01300724	0.01300724	0.000821865
ENSG00000269547_38946533_38915606_518	annotated	AC011455.2	19	-	1554	57	7	3	5.21%	12.11974535	3.07E-05	7.66E-06	1.91E-09
ENSG00000177156_747482_756006_74	novel	TALDO1	11	+	222	66	6	0	17.57%	98.23372547	0.001797169	0.00065148	7.37E-06
ENSG00000119707_73106283_73111259_218	novel	RBM25	14	+	654	9	2	3	2.29%	4.547099052	0.083503598	0.145248299	0.042302052
ENSG00000149115_57313040_57299798_778	dORF	TNKS1BP1	11	-	2334	30	4	0	2.06%	4.247076235	0.001053571	0.00017086	1.26E-06
ENSG00000184990_104753202_104766829_169	annotated	SIVA1	14	+	507	202	46	18	15.98%	131.6476357	2.77E-05	7.55E-05	1.60E-08
ENSG00000072042_67695703_67684963_231	novel	RDH11	14	-	693	403	74	13	22.94%	192.1504762	7.90E-10	9.91E-11	9.16E-19
ENSG00000140043_73860574_73877113_106	Overlap_uORF	PTGR2	14	+	318	18	3	0	5.66%	18.70316214	0.038919555	0.013428348	0.002460698
ENSG00000140043_73877052_73884177_217	annotated	PTGR2	14	+	651	13	3	0	3.23%	6.598299392	0.07962816	0.007029303	0.003149529
ENSG00000164104_173333649_173332080_209	annotated	HMGB2	4	-	627	401	54	21	21.05%	211.3228628	9.69E-08	1.86E-08	1.83E-14
ENSG00000197045_54488927_54478088_142	annotated	GMFB	14	-	426	78	41	0	15.49%	60.49990006	0.003518659	1.85E-05	7.08E-07
ENSG00000174437_110327898_110332043_63	novel	ATP2A2	12	+	189	56	3	0	19.05%	97.90297218	0.001397791	0.001082917	9.26E-06
ENSG00000135272_115014497_115015935_94	dORF	MDFIC	7	+	282	14	0	0	6.38%	16.40395545	0.013428348	0.013428348	0.000872559
ENSG00000183527_39180416_39175590_201	Overlap_dORF	PSMG1	21	-	603	64	10	0	9.95%	35.06972138	0.000579485	4.05E-05	1.84E-07
ENSG00000157240_91264881_91266824_647	annotated	FZD1	7	+	1941	24	9	1	1.85%	4.085595439	0.036121129	0.001559221	0.000388074
ENSG00000113161_75342606_75360342_888	annotated	HMGCR	5	+	2664	423	64	25	11.71%	52.46573974	3.25E-12	6.20E-17	4.52E-27
ENSG00000143442_151429672_151404802_1244	annotated	POGZ	1	-	3732	29	7	1	1.37%	2.567592016	0.001937314	0.00017257	2.41E-06
ENSG00000112183_17282811_17292119_178	annotated	RBM24	6	+	534	8	1	0	2.81%	4.950150279	0.042234516	0.019679754	0.003715148
ENSG00000013306_44323562_44320001_359	annotated	SLC25A39	17	-	1077	148	14	2	13.09%	45.40625312	4.89E-08	9.53E-10	5.46E-16
ENSG00000131508_139614609_139626801_110	annotated	UBE2D2	5	+	330	92	8	0	19.09%	92.11779655	0.000218065	2.80E-05	4.75E-08
ENSG00000054523_10267389_10304647_1007	annotated	KIF1B	1	+	3021	21	8	0	1.29%	2.296879561	0.032614614	0.000583356	0.000159364
ENSG00000010361_49812253_49807151_313	dORF	FUZ	19	-	939	6	3	0	1.60%	2.111326077	0.214508587	0.016947427	0.019735972
ENSG00000148950_31433564_31432500_57	dORF	IMMP1L	11	-	171	35	0	1	10.53%	67.63034263	0.013853925	0.017143984	0.001131568
ENSG00000165672_119178790_119168480_256	annotated	PRDX3	10	-	768	1635	187	32	30.47%	703.4385916	5.21E-12	1.81E-14	1.43E-24
ENSG00000141564_80791457_80857936_235	dORF	RPTOR	17	+	705	19	5	0	3.83%	8.905004385	0.036102036	0.003529166	0.000745938
ENSG00000189067_11556730_11549637_161	annotated	LITAF	16	-	483	339	40	21	26.71%	231.9114659	1.46E-07	1.63E-08	2.45E-14
ENSG00000095059_12681766_12675838_370	annotated	DHPS	19	-	1110	86	21	0	8.11%	25.60030421	0.001940972	6.81E-07	2.41E-08
ENSG00000122359_80159175_80156409_115	annotated	ANXA11	10	-	345	45	0	0	9.57%	43.09859102	0.001588175	0.001588175	1.51E-05
ENSG00000165533_88824708_88877410_531	annotated	TTC8	14	+	1593	43	7	2	3.95%	8.919126703	0.000441229	5.63E-05	1.87E-07
ENSG00000123600_171360506_171325891_241	annotated	METTL8	2	-	723	11	2	0	3.73%	5.027175439	0.006277459	0.002478943	8.78E-05
ENSG00000182220_40581066_40600832_262	annotated	ATP6AP2	X	+	786	167	37	3	17.18%	70.20427824	3.30E-06	7.28E-09	3.16E-13
ENSG00000182220_40599680_40605755_118	Overlap_dORF	ATP6AP2	X	+	354	46	2	19	12.71%	42.93626111	0.000309685	0.028510861	8.29E-05
ENSG00000113643_168486499_168492922_147	novel	RARS	5	+	441	11	6	2	4.08%	8.241831842	0.153960942	0.031982531	0.021136544
ENSG00000104738_47961145_47962088_81	annotated	MCM4	8	+	243	14	5	0	6.17%	19.03668904	0.065398531	0.021084099	0.006119907
ENSG00000147419_27765225_27740501_68	annotated	CCDC25	8	-	204	70	12	3	26.47%	113.3802803	0.00070546	0.000143994	7.13E-07
ENSG00000095906_1783021_1788207_251	annotated	NUBP2	16	+	753	63	15	5	7.17%	27.64491296	0.003858763	0.000476024	1.22E-05
ENSG00000082014_151242768_151241387_211	novel	SMARCD3	7	-	633	15	2	1	3.32%	7.829917799	0.032488891	0.007979402	0.001310855
ENSG00000117395_43172118_43171388_137	novel	EBNA1BP2	1	-	411	63	12	10	14.60%	50.64870915	0.000226178	0.000885674	1.36E-06
ENSG00000111142_95474180_95513904_455	annotated	METAP2	12	+	1365	184	32	5	13.41%	44.54047306	1.35E-07	1.06E-11	2.81E-17
ENSG00000108433_46935050_46935280_76	novel	GOSR2	17	+	228	15	4	0	6.58%	21.73832442	0.112457942	0.021084099	0.010870696
ENSG00000120948_11013878_11022654_364	annotated	TARDBP	1	+	1092	73	8	0	6.59%	22.08868569	7.03E-05	7.13E-06	4.19E-09
ENSG00000117620_100007082_100027211_188	Overlap_dORF	SLC35A3	1	+	564	9	7	0	3.19%	5.272699965	0.312211302	0.009815329	0.022960993
ENSG00000138594_51889067_51908810_213	annotated	TMOD3	15	+	639	84	10	2	12.21%	43.43582569	0.000124001	6.80E-06	7.26E-09
ENSG00000084093_56910867_56932152_1021	annotated	REST	4	+	3063	29	3	0	1.67%	3.128388313	0.000306522	0.000114167	2.47E-07
ENSG00000257923_101913334_102283090_692	novel	CUX1	7	+	2076	79	19	1	5.78%	12.57388245	3.07E-05	1.07E-08	5.49E-12
ENSG00000100485_50204316_50118344_1272	annotated	SOS2	14	-	3816	27	11	2	1.26%	2.337895267	0.024841604	0.000450524	9.38E-05
ENSG00000075089_100205736_100223915_314	annotated	ACTR6	12	+	942	32	1	0	4.78%	11.22454458	0.000376977	0.000291809	7.39E-07
ENSG00000156113_77403927_76877869_1024	annotated	KCNMA1	10	-	3072	64	9	0	3.03%	6.883802732	1.02E-05	4.29E-07	2.20E-11
ENSG00000103275_1314031_1324793_158	annotated	UBE2I	16	+	474	313	36	28	22.78%	218.1904056	2.92E-06	1.59E-05	3.96E-10
ENSG00000198121_110942057_110875421_312	annotated	LPAR1	9	-	936	149	18	1	13.14%	52.59931318	1.65E-06	1.18E-08	2.30E-13
ENSG00000135148_112130523_112152791_582	annotated	TRAFD1	12	+	1746	33	3	4	2.92%	6.245099385	0.00086852	0.001223575	6.59E-06
ENSG00000110917_120687297_120696555_217	annotated	MLEC	12	+	651	61	6	20	5.53%	30.961251	0.012413133	0.08452244	0.005244395

ENSG00000179152_44367602_44407682_341	dORF	TCAIM	3	+	1023	11	5	0	2.05%	3.552930442	0.077869819	0.007882058	0.003354422
ENSG00000070367_57246815_57208609_656	annotated	EXOC5	14	-	1968	102	9	0	3.20%	17.12555802	0.000595214	2.76E-05	1.35E-07
ENSG00000104763_18084058_18067074_175	annotated	ASAH1	8	-	525	272	17	1	11.43%	171.1903399	0.000361149	6.31E-05	1.68E-07
ENSG00000101444_34303270_34281034_432	annotated	AHCY	20	-	1296	411	62	8	16.67%	104.7867749	4.37E-10	4.63E-13	3.16E-21
ENSG00000175198_100155060_100530166_374	annotated	PCCA	13	+	1122	44	4	0	4.81%	12.95774632	0.00014238	8.55E-05	8.78E-08
ENSG00000171017_7898910_7900913_667	annotated	LRRC8E	19	+	2001	11	0	0	0.75%	1.816415713	0.021084099	0.021084099	0.002029891
ENSG00000101901_111681219_111713281_296	annotated	ALG13	X	+	888	22	1	4	1.69%	8.186143789	0.023199731	0.044329616	0.004497656
ENSG00000197324_22872304_22877527_713	annotated	LRP10	14	+	2139	433	63	8	13.88%	66.88777745	5.93E-16	4.76E-18	4.59E-32
ENSG00000110931_121274382_121240699_540	annotated	CAMKK2	12	-	1620	26	3	4	2.22%	5.30307766	0.003818382	0.008533454	0.000176458
ENSG00000043143_134559882_134576863_388	annotated	JADE2	5	+	1164	41	4	6	2.84%	11.63859431	0.001558047	0.006296393	5.80E-05
ENSG00000182150_95907078_96004738_1038	annotated	ERCC6L2	9	+	3114	6	3	1	0.48%	0.636652276	0.165899918	0.047790352	0.031107886
ENSG00000122952_56361236_56358418_230	annotated	ZWINT	10	-	690	347	35	3	18.26%	166.169012	1.04E-06	8.10E-09	9.96E-14
ENSG00000133114_45015486_45028003_142	annotated	GPALPP1	13	+	426	20	0	0	7.04%	15.51279489	0.00205871	0.00205871	2.48E-05
ENSG00000072274_196077096_196072145_80	annotated	TFRC	3	-	240	49	9	3	16.25%	67.46126677	0.010358749	0.001494238	9.39E-05
ENSG00000175592_65900339_65892886_271	annotated	FOSL1	11	-	813	27	9	0	3.32%	10.97344199	0.009341698	0.003580484	0.000182232
ENSG00000018408_149657306_149520805_400	annotated	WWTR1	3	-	1200	363	47	14	20.00%	99.95281566	2.02E-12	2.56E-13	6.80E-24
ENSG00000174718_31981886_31992535_1437	annotated	RESF1	12	+	4311	13	5	2	0.35%	0.996402901	0.143211011	0.060322713	0.032066242
ENSG00000163125_150364826_150473334_1424	annotated	RPRD2	1	+	4272	52	0	2	1.54%	4.021997102	1.70E-05	2.95E-05	3.93E-09
ENSG00000143374_150487451_150507064_718	annotated	TARS2	1	+	2154	115	8	2	6.27%	17.64094293	1.61E-08	2.42E-09	4.21E-16
ENSG00000163125_150364826_150441046_115	annotated	RPRD2	1	+	345	12	0	0	4.35%	11.4929576	0.019216965	0.019216965	0.001707396
ENSG00000112297_106512342_106568566_1723	annotated	CRYBG1	6	+	5169	8	3	0	0.35%	0.511391033	0.113684165	0.011570466	0.006956434
ENSG00000130347_106622943_106571996_296	annotated	RTN4IP1	6	-	888	17	6	2	3.72%	6.325656564	0.047400386	0.00809034	0.001977348
ENSG00000112305_70668024_70860334_440	annotated	SMAP1	6	+	1320	23	6	0	3.64%	5.757362285	0.003099838	0.00012627	3.05E-06
ENSG00000092964_26514326_26655706_677	annotated	DPYSL2	8	+	2031	357	54	18	13.44%	58.08017903	1.67E-13	3.72E-16	1.01E-27
ENSG00000104763_18062449_18062204_81	novel	ASAH1	8	-	243	29	2	0	8.64%	39.43314157	0.008776163	0.008776163	0.000391611
ENSG00000104763_18061782_18057534_193	novel	ASAH1	8	-	579	206	24	5	16.58%	117.559657	2.89E-05	1.91E-06	4.93E-10
ENSG00000049618_157148716_157207891_1464	annotated	ARID1B	6	+	4392	16	4	2	0.55%	1.203725068	0.051323546	0.02389627	0.005330496
ENSG00000104763_18071320_18064259_93	dORF	ASAH1	8	-	279	29	7	0	9.68%	34.34499427	0.005059698	0.003554776	9.89E-05
ENSG00000104763_18063207_18057534_235	dORF	ASAH1	8	-	705	259	26	5	19.57%	121.3892703	1.05E-07	7.88E-09	8.78E-15
ENSG00000143801_226883858_226895579_350	annotated	PSEN2	1	+	1050	51	7	0	4.86%	16.04909437	0.000327658	0.000130272	2.99E-07
ENSG00000143801_226883858_226895579_350	annotated	PSEN2	1	+	1050	51	7	0	4.86%	16.04909437	0.000327658	0.000130272	2.99E-07
ENSG00000137274_3118741_3123777_75	novel	BPHL	6	+	225	14	0	1	8.00%	20.55962416	0.013428348	0.013428348	0.000872559
ENSG00000198836_193638000_193644135_184	novel	OPA1	3	+	552	7	1	0	3.26%	4.190140793	0.047790352	0.009815329	0.002338867
ENSG00000080845_36500221_36527031_285	annotated	DLGAP4	20	+	855	72	6	2	6.67%	27.82505525	9.58E-05	9.62E-05	6.67E-08
ENSG00000144029_95121791_95104524_292	novel	MRPS5	2	-	876	83	9	9	7.19%	31.30715763	9.50E-05	0.000662939	4.58E-07
ENSG00000102710_37051490_37009709_811	annotated	SUPT20H	13	-	2433	30	2	2	1.48%	4.074260556	0.002631121	0.003604785	5.37E-05
ENSG00000132463_70827924_70824319_126	dORF	GRSF1	4	-	378	42	2	5	9.52%	36.71361457	0.003613853	0.01127911	0.000222206
ENSG00000132463_70833190_70827936_105	annotated	GRSF1	4	-	315	36	7	11	8.57%	37.76257498	0.003327803	0.036171067	0.000712723
ENSG00000131732_81308630_81312662_120	novel	ZCCHC9	5	+	360	11	1	0	5.00%	10.09624401	0.026832696	0.011772051	0.001510382
ENSG00000144048_73778345_73762661_227	novel	DUSP11	2	-	681	27	1	1	5.73%	13.10045278	0.000923291	0.000923291	5.32E-06
ENSG00000138107_102488209_102484136_141	novel	ACTR1A	10	-	423	45	4	3	8.51%	35.1513331	0.001264423	0.004254522	3.22E-05
ENSG00000197312_15617671_15656633_399	annotated	DDI2	1	+	1197	17	0	3	2.26%	4.692717652	0.003478238	0.02524418	0.000498474
ENSG00000114491_124738140_124742388_170	novel	UMPS	3	+	510	19	1	1	4.12%	12.309859	0.015625009	0.007029303	0.000558033
ENSG00000103707_65029613_65003062_389	annotated	MTFMT	15	-	1167	26	17	0	3.34%	7.361598808	0.132212766	0.000635664	0.001078606
ENSG00000089685_78214317_78222851_120	annotated	BIRC5	17	+	360	91	20	1	15.00%	83.52347314	0.001716442	9.63E-05	1.27E-06
ENSG00000108474_16217236_16299958_98	annotated	PIGL	17	+	294	21	2	14	9.18%	23.60160937	0.010079165	0.116368919	0.006446718
ENSG00000116221_54200244_54225382_483	annotated	MRPL37	1	+	1449	137	20	4	8.90%	31.24077761	2.70E-06	1.42E-08	4.60E-13
ENSG00000148344_129752991_129739611_145	annotated	PTGES	9	-	435	448	36	11	28.28%	340.2972274	1.80E-07	4.33E-08	7.69E-14
ENSG00000139133_34022600_34026915_473	annotated	ALG10	12	+	1419	12	2	0	1.48%	2.794270876	0.033394673	0.006937203	0.001198598
ENSG00000162585_2194749_2187158_195	annotated	FAAP20	1	-	585	31	10	0	5.13%	17.50957002	0.095896152	0.002404282	0.0017686
ENSG00000119321_113221243_113187757_472	annotated	FKBP15	9	-	1416	8	1	0	1.06%	1.866793961	0.047790352	0.016947427	0.003697512
ENSG00000105372_41860775_41871377_145	annotated	RPS19	19	+	435	1476	246	126	36.55%	1121.15783	2.12E-08	8.14E-10	1.97E-16
ENSG00000216490_18175178_18177373_148	novel	IFI30	19	+	444	62	14	0	10.14%	46.14008317	0.006403646	0.000281287	1.34E-05
ENSG00000205189_80486826_80519528_866	annotated	ZBTB10	8	+	2598	17	1	2	0.58%	2.162118179	0.035374934	0.056923149	0.008291339
ENSG00000171530_77776257_77693410_59	annotated	TBCA	5	-	177	279	36	13	20.34%	520.8355151	0.001436707	0.00110451	9.69E-06
ENSG00000105185_32581262_32586889_65	annotated	PDCD5	19	+	195	286	32	19	20.00%	484.6197123	0.020363786	0.002605155	0.000310336
ENSG00000104904_2269599_2271445_68	novel	OAZ1	19	+	204	92	7	3	23.53%	149.0140827	0.001904289	0.000207478	2.78E-06
ENSG00000136448_45061387_45105639_477	annotated	NMT1	17	+	1431	109	15	5	7.13%	25.16845275	6.74E-06	8.69E-07	5.28E-11
ENSG00000165175_38805043_38805498_151	annotated	MID1IP1	X	+	453	38	3	1	5.30%	27.71756332	0.01784272	0.007356796	0.000663314
ENSG00000105971_116499782_116500451_113	annotated	CAV2	7	+	339	313	32	5	30.97%	305.0803901	2.67E-07	2.51E-07	6.29E-13
ENSG00000061273_47791282_47783841_290	annotated	HDAC7	12	-	870	35	2	4	4.14%	13.29286045	0.001015599	0.003249213	2.01E-05
ENSG00000108669_78698936_78676091_204	annotated	CYTH1	17	-	612	15	0	1	2.45%	8.098591449	0.020613417	0.035374934	0.003252526
ENSG00000180694_90645905_90625671_380	annotated	TMEM64	8	-	1140	48	8	1	3.42%	13.91252763	0.000933161	0.000948092	5.52E-06
ENSG00000188554_43190487_43194520_373	novel	NBR1	17	+	1119	41	7	2	4.56%	12.10663429	0.003753256	0.000530244	1.30E-05
ENSG00000165060_69046445_69072762_135	annotated	FXN	9	+	405	62	11	0	15.56%	50.58320229	0.000401923	2.62E-05	8.50E-08
ENSG00000166508_100101294_100092932_719	annotated	MCM7	7	-	2157	423	42	12	13.77%	64.79774254	3.79E-16	6.42E-17	3.64E-31
ENSG00000092201_21357249_21352673_178	novel	SUPT16H	14	-	534	36	2	0	6.18%	22.27567626	0.001696512	0.001580611	1.60E-05
ENSG00000255339_100529851_100524089_172	annotated	AL133352.1	10	-	516	474	56	15	29.07%	303.5276739	7.32E-08	2.40E-09	1.98E-15

ENSG00000100575_58409129_58409034_31	novel	TIMM9	14	-	93	15	0	0	16.13%	53.29395663	0.021084099	0.021084099	0.002029891
ENSG00000126756_47658927_47651842_157	annotated	UXT	X	-	471	145	14	2	22.29%	101.7224353	2.83E-06	1.07E-07	3.05E-12
ENSG00000137936_93584078_93562241_501	annotated	BCAR3	1	-	1503	56	11	7	4.39%	12.31115219	0.001198331	0.000334002	2.65E-06
ENSG00000114796_183650357_183679286_600	annotated	KLHL24	3	+	1800	65	0	5	3.67%	11.93192473	1.82E-05	0.000182998	2.67E-08
ENSG00000172340_67654586_67408957_379	annotated	SUCLG2	3	-	1137	108	9	5	4.49%	31.38578132	0.001775819	0.001009673	1.09E-05
ENSG00000197622_151055730_151054247_79	novel	CDC42SE1	1	-	237	43	9	1	18.99%	59.95007949	0.002270904	0.000294865	4.57E-06
ENSG00000090470_65133781_65118717_485	annotated	PDCD7	15	-	1455	18	0	0	1.86%	4.087701416	0.003303074	0.003303074	6.12E-05
ENSG00000137822_43371355_43405214_667	annotated	TUBGCP4	15	+	2001	10	2	1	0.75%	1.651287012	0.060322713	0.035374934	0.008767878
ENSG00000204642_29724208_29726069_230	novel	HLA-F	6	+	690	10	4	0	2.61%	4.788732335	0.080264768	0.012798403	0.005074154
ENSG00000166130_98644701_98625930_377	annotated	IKBIP	12	-	1131	166	29	5	10.08%	48.49702932	5.97E-06	2.49E-07	1.46E-11
ENSG00000059588_234479103_234391577_1621	annotated	TARBP1	1	-	4863	67	6	0	1.67%	4.552397612	2.83E-05	2.13E-06	5.35E-10
ENSG00000136875_113275744_113291660_522	annotated	PRPF4	9	+	1566	103	9	5	8.05%	21.73277184	1.03E-07	1.43E-07	1.43E-13
ENSG00000078808_1223252_1217670_164	Overlap_dORF	SDF4	1	-	492	90	7	7	12.20%	60.44314594	6.12E-05	0.000428364	1.95E-07
ENSG00000078808_1217673_1217512_53	dORF	SDF4	1	-	159	80	1	0	22.64%	166.2503301	0.001397791	0.001061589	9.08E-06
ENSG00000078808_1228772_1218621_247	annotated	SDF4	1	-	741	199	97	11	14.17%	88.73695505	0.03264552	1.65E-06	2.19E-06
ENSG00000106012_2578275_2610162_529	annotated	IQCE	7	+	1587	8	1	0	0.95%	1.66564603	0.042234516	0.019679754	0.003715148
ENSG00000164120_174522451_174508746_87	annotated	HPGD	4	-	261	73	8	11	17.24%	92.41702978	0.00182801	0.0085369	9.14E-05
ENSG00000164120_174508711_174491956_131	dORF	HPGD	4	-	393	106	25	0	16.79%	89.12159872	0.00020002	1.85E-05	2.97E-08
ENSG00000101639_12999425_13124770_2467	annotated	CEP192	18	+	7401	12	1	0	0.24%	0.535747922	0.026832696	0.011772051	0.001510382
ENSG00000110200_72111276_72109327_110	annotated	ANAPC15	11	-	330	41	1	3	18.18%	41.05249629	5.14E-05	0.00029235	1.12E-07
ENSG00000188554_43175800_43210074_966	annotated	NBR1	17	+	2898	109	10	5	4.87%	12.42790058	1.45E-07	2.54E-08	3.71E-14
ENSG00000119616_74723298_74734930_92	annotated	FCF1	14	+	276	45	8	0	13.04%	53.87323877	0.001015599	0.001015599	6.39E-06
ENSG00000137106_37422769_37434257_352	annotated	GRHPR	9	+	1056	355	49	21	21.88%	111.0795441	8.93E-12	1.13E-13	1.42E-23
ENSG00000168488_28829425_28831031_163	novel	ATXN2L	16	+	489	25	8	0	3.68%	16.89276744	0.129694924	0.013640586	0.00917653
ENSG00000111481_54325164_54350523_154	annotated	COPZ1	12	+	462	457	65	8	31.82%	326.8465297	4.58E-08	5.11E-10	2.86E-16
ENSG00000076043_114439529_114440757_82	novel	REXO2	11	+	246	68	23	5	24.39%	91.33630941	0.002028568	0.00018969	2.75E-06
ENSG00000167123_128422905_128435905_517	annotated	CERCAM	9	+	1551	105	6	3	6.58%	22.36903015	4.72E-07	5.03E-07	2.18E-12
ENSG00000196968_73772334_73775685_492	annotated	FUT11	10	+	1476	37	8	3	2.85%	8.282949628	0.013986977	0.002002002	0.000166026
ENSG00000166788_18106041_18089496_209	novel	SAAL1	11	-	627	31	2	0	6.22%	16.33668017	0.001811222	0.00067919	7.72E-06
ENSG00000131051_35740847_35704481_521	annotated	RBM39	20	-	1563	88	17	6	6.91%	18.60344385	6.33E-05	8.66E-07	5.58E-10
ENSG00000152700_134612730_134606950_130	annotated	SAR1B	5	-	390	222	24	5	33.08%	188.0866716	2.47E-08	1.12E-08	2.84E-15
ENSG00000111271_111692854_111709705_188	novel	ACAD10	12	+	564	7	0	0	2.66%	4.100988861	0.019216965	0.019216965	0.001707396
ENSG00000123213_65788381_65871471_277	annotated	NLN	5	+	831	43	5	1	6.86%	17.0976761	0.000580321	7.47E-05	3.20E-07
ENSG00000067334_93879148_93872194_601	annotated	DNTTIP2	1	-	1803	48	15	2	3.33%	8.796606486	0.027308512	8.90E-05	3.04E-05
ENSG00000183853_157993677_158095120_757	annotated	KIRREL1	1	+	2271	96	11	15	4.62%	13.96766314	4.68E-06	2.10E-05	8.22E-10
ENSG00000148690_93692653_93669826_191	annotated	FRA10AC1	10	-	573	27	1	4	4.71%	15.56964806	0.003554776	0.036243043	0.000753587
ENSG00000163374_155679485_155659657_734	annotated	YY1AP1	1	-	2202	33	7	2	1.91%	4.951836297	0.002477401	0.000833918	1.28E-05
ENSG00000152944_27022580_27030234_159	annotated	MED21	12	+	477	38	5	3	6.29%	26.32296894	0.002815105	0.006133031	9.63E-05
ENSG00000153029_181034053_181055265_234	annotated	MR1	1	+	702	11	0	0	2.56%	5.177561029	0.012798403	0.012798403	0.000797244
ENSG00000153037_112861377_112867403_110	annotated	SRP19	5	+	330	22	5	0	5.45%	22.02816874	0.053624202	0.013640586	0.0034686
ENSG00000179029_8176286_8174203_140	annotated	TMEM107	17	-	420	16	2	1	4.29%	18.09456718	0.01300724	0.023878474	0.001470914
ENSG00000144674_37282231_37366103_2083	annotated	GOLGA4	3	+	6249	69	10	10	1.39%	3.648448495	8.53E-05	3.34E-05	2.16E-08
ENSG00000132330_238081834_238099353_204	annotated	SCLY	2	+	612	13	2	5	3.43%	7.018779256	0.037995728	0.100412561	0.015415463
ENSG00000132470_75731932_75739898_276	novel	ITGB4	17	+	828	118	21	1	9.78%	47.08920129	4.14E-05	4.09E-06	1.45E-09
ENSG00000082996_149812902_149813291_129	uORF	RNF13	3	+	387	24	1	2	5.43%	20.49131976	0.013892891	0.023197249	0.001517103
ENSG00000082996_149846051_149961104_373	annotated	RNF13	3	+	1119	97	19	1	8.85%	28.64252504	3.26E-05	2.70E-07	9.59E-11
ENSG00000136451_57980792_57974473_259	annotated	VEZF1	17	-	777	60	3	2	7.34%	25.51525337	9.35E-05	5.70E-05	3.93E-08
ENSG00000076984_7910498_7912431_255	annotated	MAP2K7	19	+	765	21	0	0	3.14%	9.070422423	0.005604867	0.005604867	0.000167525
ENSG00000104613_19820468_19851805_580	annotated	INTS10	8	+	1740	86	11	7	6.38%	16.33122855	1.87E-06	4.86E-07	8.29E-12
ENSG00000078747_34412633_34507794_720	annotated	ITCH	20	+	2160	51	15	0	2.78%	7.801643096	0.007183613	3.64E-05	2.87E-06
ENSG00000177951_207321_205470_102	annotated	BET1L	11	-	306	19	7	0	7.84%	20.51643167	0.035220215	0.005554612	0.001053497
ENSG00000055130_148756998_148800582_666	dORF	CUL1	7	+	1998	68	9	0	2.70%	11.24561167	0.000146042	8.74E-05	9.19E-08
ENSG00000105221_40239108_40237708_195	novel	AKT2	19	-	585	10	3	0	2.56%	5.648248395	0.11777946	0.016947427	0.009674391
ENSG00000105221_40236373_40233872_200	novel	AKT2	19	-	600	30	0	4	7.00%	16.52112656	0.000431006	0.001692205	4.75E-06
ENSG00000081923_57731807_57648488_1251	annotated	ATP8B1	18	-	3753	43	2	0	1.76%	3.78581637	3.30E-05	1.48E-05	3.85E-09
ENSG00000146909_156949738_156969703_860	annotated	NOM1	7	+	2580	15	0	2	0.93%	1.921061227	0.004826937	0.030313544	0.000797936
ENSG00000231925_33314041_33301760_466	annotated	TAPBP	6	-	1398	350	44	15	15.67%	82.72380965	7.56E-11	7.77E-13	8.07E-22
ENSG00000197375_132390691_132394272_206	novel	SLC22A5	5	+	618	14	5	0	3.88%	7.485300058	0.052998774	0.005257623	0.001578261
ENSG00000157954_5214501_5217271_160	novel	WIPI2	7	+	480	36	3	2	4.38%	24.78168983	0.008776163	0.017282851	0.000752456
ENSG00000109685_1956125_1978909_426	novel	NSD2	4	+	1278	11	3	1	1.64%	2.844012396	0.067796501	0.01551626	0.004936077
ENSG00000169692_136677131_136673752_171	novel	AGPAT2	9	-	513	68	3	1	9.94%	43.79869808	0.000593472	0.000173765	7.11E-07
ENSG00000117139_202733853_202729650_365	novel	KDM5B	1	-	1095	12	2	1	2.19%	3.621068834	0.030313544	0.010020958	0.001483684
ENSG00000168216_69796881_69705581_317	annotated	LMBRD1	6	-	951	85	10	1	9.15%	29.5330338	3.05E-05	1.20E-06	3.41E-10
ENSG00000168216_69705584_69676158_263	Overlap_dORF	LMBRD1	6	-	789	51	18	0	7.22%	21.35811038	0.020519603	5.79E-05	1.52E-05
ENSG00000153914_66144377_66158912_132	annotated	SREK1	5	+	396	15	1	0	4.55%	12.51600497	0.013428348	0.013428348	0.000872559
ENSG00000197217_23437101_23434678_85	novel	ENTPD4	8	-	255	23	0	1	9.41%	29.80281653	0.005554612	0.007181907	0.000210515
ENSG00000147419_27752551_27736216_140	annotated	CCDC25	8	-	420	29	10	0	9.29%	22.81488905	0.032468404	0.000642411	0.000170797
ENSG00000147419_27772540_27752575_26	uORF	CCDC25	8	-	78	23	1	0	19.23%	97.43228482	0.035772986	0.021084099	0.003354605

ENSG00000256861_122263681_122218375_476	annotated	AC048338.1	12	-	1428	38	0	0	3.99%	8.79275643	4.93E-05	4.93E-05	1.82E-08
ENSG00000256861_122218361_122208381_166	dORF	AC048338.1	12	-	498	55	4	0	7.23%	36.49244822	0.001798541	0.001074354	1.17E-05
ENSG00000129562_22589157_22575158_65	annotated	DAD1	14	-	195	249	343	27	33.85%	421.9241551	0.138170586	0.000252106	0.000620216
ENSG00000010270_38207526_38215255_169	novel	STARD3NL	7	+	507	23	2	0	6.51%	14.98958228	0.00345766	0.001513502	3.08E-05
ENSG00000138071_65227910_65246223_65	annotated	ACTR2	2	+	195	25	7	0	12.31%	42.36186296	0.075314168	0.005355751	0.002394811
ENSG00000050165_12008582_11964464_364	annotated	DKK3	11	-	1092	44	2	1	3.57%	13.31372836	0.001023943	0.000812818	5.21E-06
ENSG00000177054_19149203_19166498_398	novel	ZDHHC13	11	+	1194	41	7	0	4.52%	11.34616732	0.004030425	7.78E-05	2.71E-06
ENSG00000114698_146222071_146194411_329	annotated	PLSCR4	3	-	987	11	5	0	1.52%	3.68252061	0.145248299	0.020613417	0.014232745
ENSG00000197548_11299415_11554843_605	annotated	ATG7	3	+	1815	41	3	0	3.47%	7.464090235	9.26E-05	2.11E-05	1.52E-08
ENSG00000178209_143953771_143916177_4525	annotated	PLEC	8	-	13575	247	62	38	2.56%	6.012107933	1.02E-09	1.36E-13	2.60E-21
ENSG00000124067_67968553_67945046_1068	annotated	SLC12A4	16	-	3204	74	19	10	3.09%	7.63148168	0.000416883	2.99E-05	9.93E-08
ENSG00000160783_156213016_156239601_207	annotated	PMF1	1	+	621	54	4	0	7.73%	28.73239401	0.001221317	0.000148081	1.29E-06
ENSG00000160783_156213016_156239601_205	annotated	PMF1	1	+	615	73	10	3	9.27%	39.22088581	0.004006832	0.00026067	7.52E-06
ENSG00000260238_156213016_156243083_220	annotated	PMF1-BGLAP	1	+	660	45	6	5	5.45%	22.52880894	0.007568863	0.005367616	0.00021449
ENSG00000153485_93185406_93186671_163	annotated	TMEM251	14	+	489	21	3	4	5.52%	14.18992465	0.018219708	0.0407781	0.003355576
ENSG00000160783_156213016_156239587_180	annotated	PMF1	1	+	540	45	6	6	6.67%	27.53521093	0.007568863	0.007511169	0.000293902
ENSG00000131378_16493869_16316828_578	annotated	RFTN1	3	-	1734	18	0	6	1.38%	3.429991672	0.004826937	0.138765125	0.004690898
ENSG00000113448_59215808_58974664_604	annotated	PDE4D	5	-	1812	139	25	15	7.95%	25.34698224	3.42E-07	4.86E-07	1.54E-12
ENSG00000283239_1857968_1957263_1352	Overlap_dORF	AC019257.8	8	+	4056	59	8	5	1.92%	4.806442144	7.47E-05	4.39E-05	2.45E-08
ENSG00000182220_40581066_40598678_186	annotated	ATP6AP2	X	+	558	99	28	3	18.28%	58.62335229	0.000240033	7.01E-07	2.05E-09
ENSG00000182220_40600774_40605755_100	dORF	ATP6AP2	X	+	300	41	2	3	12.00%	45.15774592	0.00107863	0.001065834	7.10E-06
ENSG00000079246_216109437_216205202_732	annotated	XRCC5	2	+	2196	487	63	9	11.34%	73.2767635	2.02E-10	1.17E-14	4.77E-23
ENSG00000196655_119019284_119023399_79	Overlap_dORF	TRAPPC4	11	+	237	66	9	5	18.99%	92.01640107	0.005353247	0.002486884	7.51E-05
ENSG00000196655_119018796_119021790_126	annotated	TRAPPC4	11	+	378	54	2	1	14.29%	47.20321873	0.000276943	8.36E-05	1.67E-07
ENSG00000113448_58993423_58974664_488	annotated	PDE4D	5	-	1464	130	23	12	8.40%	29.34079853	1.85E-06	1.32E-06	2.16E-11
ENSG00000138078_44346342_44321356_638	annotated	PREPL	2	-	1914	43	8	1	3.13%	7.423285704	0.001704072	5.31E-05	7.50E-07
ENSG00000171703_64066485_64072180_272	annotated	TCEA2	20	+	816	16	3	3	2.94%	6.478873159	0.025015411	0.035859268	0.003916945
ENSG00000171603_9823733_9730508_971	annotated	CLSTN1	1	-	2913	265	34	4	9.37%	30.05903561	1.73E-13	7.17E-17	2.23E-28
ENSG00000183010_81936814_81933214_319	annotated	PYCR1	17	-	957	123	11	0	11.29%	42.46809961	2.24E-07	6.47E-08	1.41E-13
ENSG00000137449_15007413_15066380_406	annotated	CPEB2	4	+	1218	24	3	0	1.72%	6.51078879	0.018067938	0.008776163	0.000786152
ENSG00000156298_38671382_38687667_157	novel	TSPAN7	X	+	471	14	0	0	3.82%	9.821476509	0.012798403	0.012798403	0.000797244
ENSG00000111319_6355855_6347873_369	Overlap_dORF	SCNN1A	12	-	1107	46	3	1	3.52%	13.73029488	0.002502442	0.000723387	1.13E-05
ENSG00000106799_99105206_99149305_507	annotated	TGFBR1	9	+	1521	58	12	3	4.93%	12.59993873	0.001953051	2.72E-05	4.93E-07
ENSG00000009335_157223163_157267755_446	novel	UBE3C	7	+	1338	53	9	1	4.71%	13.08848591	0.000975633	3.33E-05	2.71E-07
ENSG00000163714_143014353_143021585_204	novel	U2SURP	3	+	612	13	0	3	2.45%	7.018779256	0.021084099	0.063071082	0.005896902
ENSG00000083444_11972929_11974808_74	novel	PLOD1	1	+	222	21	9	0	12.16%	31.25618538	0.08259706	0.003606289	0.001980612
ENSG00000119688_74296397_74286674_433	dORF	ABCD4	14	-	1299	57	1	0	4.85%	14.49891014	2.62E-05	2.61E-05	5.31E-09
ENSG00000091127_105508506_105468442_446	annotated	PUS7	7	-	1338	35	4	2	2.69%	8.643339753	0.006852949	0.000947024	4.08E-05
ENSG00000198689_135985818_136044724_564	annotated	SLC9A6	X	+	1692	27	4	0	2.48%	5.272699965	0.004948692	0.000415605	1.41E-05
ENSG00000183077_78187422_78207011_67	annotated	AFMID	17	+	201	23	0	0	10.45%	37.80954336	0.008574016	0.008574016	0.000374746
ENSG00000187266_11381125_11377984_285	dORF	EPOR	19	-	855	25	10	5	3.86%	9.661477518	0.055247693	0.01585818	0.004056102
ENSG00000140983_668200_670344_135	annotated	RHOT2	16	+	405	14	4	5	5.93%	11.42201342	0.046396937	0.098650979	0.017856065
ENSG00000123213_65777490_65822915_533	novel	NLN	5	+	1599	113	6	4	8.44%	23.35068544	2.78E-08	1.45E-08	4.12E-15
ENSG00000151806_44688041_44698681_345	novel	GUF1	4	+	1035	29	6	0	2.61%	9.258215848	0.024548991	0.003710481	0.000510697
ENSG00000182473_76101830_76083648_609	annotated	EXOC7	17	-	1827	60	7	7	4.11%	10.85131465	0.000176909	0.000167609	2.07E-07
ENSG00000172262_43122054_43175133_589	annotated	ZNF131	5	+	1767	25	2	0	1.87%	4.674908476	0.004803511	0.001484235	4.21E-05
ENSG00000106617_151574893_151557201_235	annotated	PRKAG2	7	-	705	25	0	3	3.40%	11.71711103	0.005604867	0.020990661	0.000616068
ENSG00000143740_227747845_227775881_365	annotated	SNAP47	1	+	1095	15	1	4	1.64%	4.526336043	0.016344022	0.069273208	0.005268498
ENSG00000131378_16482782_16316828_542	annotated	RFTN1	3	-	1626	17	0	6	1.29%	3.454602109	0.007882058	0.184585708	0.009585457
ENSG00000145740_69094256_69129617_765	annotated	SLC30A5	5	+	2295	155	34	17	6.80%	22.31611866	3.08E-06	6.00E-08	1.96E-12
ENSG00000168000_62705595_62702488_118	internal	BSCL2	11	-	354	47	0	15	8.47%	43.86965809	0.002488365	0.018376712	0.00026737
ENSG00000168000_62691399_62690367_167	Overlap_dORF	BSCL2	11	-	501	48	1	2	7.78%	31.65724849	0.000862821	0.001811222	9.64E-06
ENSG00000116977_236543593_236548161_123	novel	LGALS8	1	+	369	12	0	0	4.07%	10.74544817	0.021084099	0.021084099	0.002029891
ENSG00000111144_96009141_96000989_149	novel	LTA4H	12	-	447	24	2	1	6.04%	17.74080704	0.011814276	0.005059698	0.000314551
ENSG00000168002_62761649_62766507_172	annotated	POLR2G	11	+	516	280	17	10	25.00%	179.2990479	7.55E-08	2.06E-08	1.57E-14
ENSG00000180011_75202180_75201416_254	annotated	ZADH2	18	-	762	27	2	0	3.94%	11.70788496	0.00450148	0.002432129	6.20E-05
ENSG00000095794_35175703_35211803_245	annotated	CREM	10	+	735	12	2	0	2.04%	5.394653569	0.070008252	0.021084099	0.006564739
ENSG00000159110_33262853_33263500_215	dORF	IFNAR2	21	+	645	17	3	0	3.72%	8.708810898	0.040990615	0.005160369	0.001169109
ENSG00000073712_52950436_52858377_643	annotated	FERMT2	14	-	1929	51	6	4	3.27%	8.735898956	0.000190579	0.000171408	2.28E-07
ENSG00000183475_100612313_100630050_242	annotated	ASB7	15	+	726	14	2	0	2.89%	6.371784347	0.028902806	0.007882058	0.001149203
ENSG00000110713_3695538_3676159_441	dORF	NUP98	11	-	1323	15	1	3	2.04%	3.746287201	0.006432154	0.030308563	0.001015506
ENSG00000110713_3782117_3696763_1348	annotated	NUP98	11	-	4044	20	2	7	0.96%	1.634137147	0.002141971	0.027267877	0.000363386
ENSG00000176014_12308293_12311100_107	novel	TUBB6	18	+	321	27	11	1	11.21%	27.79254935	0.095721053	0.001631903	0.001333017
ENSG00000167460_16076566_16088101_152	novel	TPM4	19	+	456	72	23	5	13.16%	52.1719786	0.009239293	9.97E-05	8.69E-06
ENSG00000143575_154273565_154275701_185	annotated	HAX1	1	+	555	68	7	5	5.95%	40.48420201	0.017191333	0.009053716	0.000769812
ENSG00000172809_74203956_74209829_67	annotated	RPL38	17	+	201	233	115	44	22.39%	383.0271132	0.030483927	0.00468509	0.000783011
ENSG00000198909_63652645_63693777_537	annotated	MAP3K3	17	+	1611	21	1	0	2.05%	4.307183832	0.002287938	0.001440978	1.96E-05
ENSG00000101473_45857290_45841838_266	Overlap_dORF	ACOT8	20	-	798	32	2	0	3.01%	13.25002631	0.006191047	0.005655336	0.00018572

ENSG00000072062_14106822_14097259_230	novel	PRKACA	19	-	690	27	1	1	6.09%	12.9295773	0.000661391	0.000661391	2.80E-06
ENSG00000170775_124764946_124746525_603	annotated	GPR37	7	-	1809	26	11	0	1.99%	4.74902477	0.030087799	0.001015599	0.000223311
ENSG00000247796_53109990_53112278_65	novel	AC008966.1	5	+	195	17	0	0	9.23%	28.80606682	0.013428348	0.013428348	0.000872559
ENSG00000129810_20184027_20161105_309	annotated	SGO1	3	-	927	19	4	2	2.91%	6.772414338	0.029337119	0.011768187	0.001650627
ENSG00000110768_18338110_18365869_432	annotated	GTF2H1	11	+	1296	26	2	1	2.55%	6.628847075	0.003810897	0.001506153	3.38E-05
ENSG00000134910_125595916_125620810_705	annotated	STT3A	11	+	2115	576	37	13	16.17%	89.98741273	5.42E-18	2.38E-19	2.10E-35
ENSG00000122705_36191057_36211774_248	annotated	CLTA	9	+	744	633	144	14	20.16%	281.1256212	8.80E-07	1.20E-09	1.49E-14
ENSG00000100600_92732786_92716132_135	novel	LGMN	14	-	405	219	24	14	18.52%	178.6729242	9.41E-05	0.000509628	3.46E-07
ENSG00000168056_65548055_65539080_733	annotated	LTBP3	11	-	2199	158	27	8	7.91%	23.74113684	8.85E-09	2.48E-10	2.61E-17
ENSG00000146731_56060845_56061872_73	novel	CCT6A	7	+	219	59	2	0	10.96%	89.01794217	0.008364861	0.005706018	0.000250077
ENSG00000092020_35108148_35085590_289	dORF	PPP2R3C	14	-	867	35	1	0	6.23%	13.3388565	0.00011592	7.87E-05	6.62E-08
ENSG00000140553_90939740_90942748_187	novel	UNC45A	15	+	561	32	0	0	4.81%	18.84763101	0.003684287	0.003684287	7.54E-05
ENSG00000074416_127821818_127692198_303	annotated	MGLL	3	-	909	82	21	2	7.59%	29.80709302	0.00356458	3.15E-05	1.11E-06
ENSG00000176428_73667959_73671376_251	annotated	VPS37D	7	+	753	15	0	0	1.99%	6.582122134	0.021084099	0.021084099	0.002029891
ENSG00000012061_45423374_45409675_273	annotated	ERCC1	19	-	819	69	11	4	7.69%	27.83779566	0.000523044	0.000115218	4.28E-07
ENSG00000169169_49712787_49713605_113	annotated	CPT1C	19	+	339	10	0	0	5.31%	9.746977319	0.012798403	0.012798403	0.000797244
ENSG00000100644_61746963_61747085_40	dORF	HIF1A	14	+	120	12	0	0	12.50%	33.04225311	0.021084099	0.021084099	0.002029891
ENSG00000100644_61695805_61746939_735	annotated	HIF1A	14	+	2205	581	88	21	17.14%	87.06371455	5.20E-16	1.69E-20	1.93E-34
ENSG00000180979_42548434_42544083_239	annotated	LRRC57	15	-	717	15	3	0	3.77%	6.912605253	0.027614347	0.003278443	0.00052295
ENSG00000051382_138712277_138655389_610	annotated	PIK3CB	3	-	1830	29	2	3	2.62%	5.236204045	0.000576177	0.001584658	5.80E-06
ENSG00000143416_151369171_151364543_408	novel	SELENBP1	1	-	1224	11	0	0	1.47%	2.969483531	0.012798403	0.012798403	0.000797244
ENSG00000132716_160240344_160239598_248	annotated	DCAF8	1	-	744	46	1	1	5.24%	20.42935004	0.000725368	0.000700469	3.23E-06
ENSG00000221983_18573301_18575150_128	annotated	UBA52	19	+	384	163	37	5	14.84%	140.2574807	0.002036197	8.13E-05	1.32E-06
ENSG00000166206_26621357_26616021_27	novel	GABRB3	15	-	81	33	0	0	18.52%	134.6165868	0.016947427	0.016947427	0.001349898
ENSG00000129515_34604190_34563122_294	dORF	SNX6	14	-	882	208	30	12	17.35%	77.92277378	3.97E-09	2.89E-09	1.23E-16
ENSG00000184304_29927512_29577238_912	annotated	PRKD1	14	-	2736	15	0	0	0.88%	1.811527035	0.005160369	0.005160369	0.000143196
ENSG00000166228_70888533_70883950_104	annotated	PCBD1	10	-	312	308	42	9	33.65%	326.1863448	1.01E-06	1.20E-06	1.08E-11
ENSG00000175274_44938308_44934884_180	annotated	TP53I11	11	-	540	18	5	1	5.00%	11.01408437	0.035820121	0.005561256	0.001073878
ENSG00000115355_55374859_55291711_1743	annotated	CCDC88A	2	-	5229	69	8	3	1.95%	4.36013667	9.61E-06	6.71E-07	5.99E-11
ENSG00000130703_62260040_62293887_448	annotated	OSBPL2	20	+	1344	36	9	3	3.57%	8.850603512	0.008215072	0.001109096	5.67E-05
ENSG00000285053_235380050_235434253_311	annotated	TBCE	1	+	933	18	9	0	2.57%	6.374711211	0.05393658	0.005554612	0.001681648
ENSG00000105647_18156048_18166140_425	annotated	PIK3R2	19	+	1275	11	3	3	1.18%	2.85070419	0.100412561	0.102951605	0.036012407
ENSG00000278540_37284963_37087316_2268	annotated	ACACA	17	-	6804	101	22	11	1.98%	4.904861206	8.97E-06	1.62E-07	1.51E-11
ENSG00000136930_124415425_124353598_277	annotated	PSMB7	9	-	831	403	31	8	24.55%	160.2410109	3.04E-11	1.90E-12	7.39E-22
ENSG00000141424_36127007_36109593_755	annotated	SLC39A6	18	-	2265	162	32	11	7.15%	23.63286978	1.26E-07	6.80E-09	9.19E-15
ENSG00000127947_77537643_77639280_748	annotated	PTPN12	7	+	2244	73	4	0	4.68%	10.74903956	7.74E-07	7.50E-08	5.73E-13
ENSG00000124226_49945442_49952141_111	dORF	RNF114	20	+	333	39	0	1	14.41%	38.69813427	0.000176199	0.000423577	5.14E-07
ENSG00000106399_7640418_7639028_71	novel	RPA3	7	-	213	94	3	8	16.90%	145.8202719	0.001082917	0.001082917	7.23E-06
ENSG00000005108_11831946_11375794_1657	annotated	THSD7A	7	-	4971	163	16	16	4.65%	10.83461528	4.76E-12	1.91E-11	1.12E-21
ENSG00000130731_636290_634526_205	annotated	METTL26	16	-	615	127	61	5	13.17%	68.23359586	0.05777054	6.41E-05	6.65E-05
ENSG00000064607_19026052_18994993_861	Overlap_dORF	SUGP2	19	-	2583	17	0	3	0.93%	2.174674034	0.005554612	0.02524418	0.000740251
ENSG00000171421_1798911_1798624_95	annotated	MRPL36	5	-	285	38	9	1	10.53%	44.05633748	0.051252243	0.00224099	0.000777436
ENSG00000135317_85543713_85505967_552	novel	SNX14	6	-	1656	43	11	0	2.54%	8.5798121	0.019118278	0.000456173	6.94E-05
ENSG00000135317_85558015_85543620_216	novel	SNX14	6	-	648	20	2	15	4.17%	10.19822627	0.011381273	0.26224294	0.019680263
ENSG00000135317_85574362_85561215_172	novel	SNX14	6	-	516	13	1	0	4.07%	8.324598652	0.023878474	0.007979402	0.000955388
ENSG00000130731_636290_634526_185	annotated	METTL26	16	-	555	127	60	5	14.59%	75.61020081	0.051131245	6.41E-05	5.59E-05
ENSG00000138796_107989957_108034357_309	annotated	HADH	4	+	927	66	9	4	7.77%	23.52522875	0.000341015	5.34E-05	1.36E-07
ENSG00000100348_36480837_36467804_166	annotated	TXN2	22	-	498	59	3	2	12.05%	39.14644445	6.78E-05	4.37E-05	2.22E-08
ENSG00000170310_9505152_9250578_125	Overlap_dORF	STX8	17	-	375	9	4	0	4.80%	7.930140747	0.140443763	0.011772051	0.009046049
ENSG00000172922_65720758_65719783_164	annotated	RNASEH2C	11	-	492	44	0	2	6.71%	29.54998246	0.00140552	0.006771542	5.70E-05
ENSG00000122545_35832669_35904293_417	annotated	7-Sep	7	+	1251	117	17	11	6.00%	30.90282665	0.000195273	0.000121072	1.67E-07
ENSG00000122545_35832891_35904293_384	annotated	7-Sep	7	+	1152	112	17	8	5.99%	32.12441275	0.000395822	8.16E-05	2.34E-07
ENSG00000138029_26261021_26289953_452	annotated	HADHB	2	+	1356	196	29	3	14.60%	47.76018886	1.70E-09	1.52E-12	4.03E-20
ENSG00000103227_879728_854532_321	dORF	LMF1	16	-	963	20	5	2	3.12%	6.862357863	0.037405222	0.006024334	0.001202108
ENSG00000103227_954496_931693_83	annotated	LMF1	16	-	249	13	3	3	9.64%	17.25097552	0.035227809	0.038105097	0.005655927
ENSG00000147533_41490855_41507106_137	annotated	GOLGA7	8	+	411	73	13	0	13.14%	58.68818679	0.000419126	9.35E-05	2.82E-07
ENSG00000083312_72848394_72851243_36	novel	TNPO1	5	+	108	37	1	0	13.89%	113.2003116	0.021084099	0.021084099	0.002029891
ENSG00000084073_40258308_40259412_49	novel	ZMPSTE24	1	+	147	25	0	0	14.29%	56.19430801	0.008776163	0.008776163	0.000391611
ENSG00000108179_79347549_79351609_145	annotated	PPIF	10	+	435	123	8	5	15.86%	93.42981914	1.83E-05	2.21E-05	3.18E-09
ENSG00000159086_32771668_32756342_469	novel	PAXBP1	21	-	1407	16	0	2	1.28%	3.757470148	0.013428348	0.030316224	0.001914614
ENSG00000138413_208239983_208237079_124	novel	IDH1	2	-	372	143	9	1	11.29%	127.0172633	0.000869949	0.000526999	2.94E-06
ENSG00000169710_82096421_82079143_2501	annotated	FASN	17	-	7503	700	87	50	7.68%	30.8271054	8.66E-25	2.54E-26	4.21E-49
ENSG00000178252_49007432_49015288_1121	annotated	WDR6	3	+	3363	130	22	13	3.84%	12.77280079	2.28E-05	1.07E-06	2.26E-10
ENSG00000136717_127106934_127048526_451	annotated	BIN1	2	-	1353	35	2	1	3.10%	8.547515587	0.000742993	0.000525339	2.51E-06
ENSG00000269893_118278820_118278978_52	novel	SNHG8	4	+	156	48	22	4	26.92%	101.6684711	0.02935951	0.001398167	0.000279899
ENSG00000269893_118279412_118279714_36	novel	SNHG8	4	+	108	28	18	5	13.89%	85.66510066	0.172615536	0.057424805	0.037344862
ENSG00000178252_49007432_49009012_61	novel	WDR6	3	+	183	21	0	2	8.20%	37.91733964	0.019679754	0.078649604	0.007006523
ENSG00000266173_63728369_63703640_403	annotated	STRADA	17	-	1209	14	2	5	1.99%	3.826232784	0.004826937	0.073313458	0.002142487

ENSG00000154305_222621164_222645775_1186	novel	MIA3	1	+	3558	44	8	3	1.77%	4.086169581	0.001179889	0.000135154	1.15E-06
ENSG00000134202_109740269_109737071_219	annotated	GSTM3	1	-	657	7	0	3	2.28%	3.520483589	0.019216965	0.169362074	0.016163992
ENSG00000077684_128831759_128872262_830	annotated	JADE1	4	+	2490	41	8	1	2.65%	5.440692279	0.002043451	1.35E-05	2.89E-07
ENSG00000094880_138198277_138192265_118	novel	CDC23	5	-	354	12	0	2	5.08%	11.20076377	0.01300724	0.035772986	0.002198223
ENSG00000198843_150603363_150622394_48	annotated	SELENOT	3	+	144	26	3	0	12.50%	59.65962367	0.062728927	0.01300724	0.003936255
ENSG00000110719_68047689_68048197_143	novel	TCIRG1	11	+	429	23	4	0	3.50%	17.71496088	0.055917761	0.021084099	0.005218013
ENSG00000130255_5690526_5691621_99	annotated	RPL36	19	+	297	412	79	18	34.34%	458.3639152	4.70E-05	3.31E-07	1.72E-10
ENSG00000149932_29963144_29971545_240	annotated	TMEM219	16	+	720	61	14	10	10.42%	27.99413111	0.000100081	0.000547292	3.94E-07
ENSG00000182473_76103692_76083648_735	annotated	EXOC7	17	-	2205	64	8	9	3.95%	9.590495234	8.00E-05	0.000123032	7.13E-08
ENSG00000139163_22625431_22644304_169	annotated	ETNK1	12	+	507	30	3	3	5.92%	19.55162906	0.003514803	0.009929352	0.000190563
ENSG00000133392_15757931_15708811_1448	annotated	MYH11	16	-	4344	6	0	0	0.35%	0.456384711	0.016947427	0.016947427	0.001349898
ENSG00000171298_80104950_80119331_831	annotated	GAA	17	+	2493	252	26	6	10.95%	33.40011145	3.81E-15	1.93E-16	1.10E-29
ENSG00000168872_70366060_70371986_285	novel	DDX19A	16	+	855	13	1	4	2.46%	5.023968309	0.016854696	0.049121095	0.003784451
ENSG00000157870_2586886_2589427_216	annotated	PRXL2B	1	+	648	23	5	4	4.17%	11.72796021	0.030721113	0.029977737	0.003993074
ENSG00000049860_74705225_74721175_331	annotated	HEXB	5	+	993	345	73	3	20.24%	114.7993688	1.59E-08	9.40E-13	3.01E-19
ENSG00000127990_94656026_94585499_441	annotated	SGCE	7	-	1323	195	22	8	10.66%	48.70173361	1.75E-07	6.30E-09	1.20E-14
ENSG00000101347_36919456_36904145_251	novel	SAMHD1	20	-	753	7	0	0	1.99%	3.071656996	0.019216965	0.019216965	0.001707396
ENSG00000085662_134447435_134442728_87	novel	AKR1B1	7	-	261	439	69	4	21.84%	555.7681654	0.000336855	5.70E-05	1.43E-07
ENSG00000085662_134459062_134447929_263	novel	AKR1B1	7	-	789	2429	279	91	30.80%	1017.232355	4.41E-15	1.17E-14	7.25E-28
ENSG00000167965_2206086_2206749_116	annotated	MLST8	16	+	348	47	3	0	9.48%	44.6260315	0.00368578	0.001580611	3.42E-05
ENSG00000038382_14270854_14508422_3035	annotated	TRIO	5	+	9105	38	10	15	0.69%	1.379028686	0.009531323	0.038797375	0.00183283
ENSG00000164078_49903609_49887307_1351	annotated	MST1R	3	-	4053	55	5	0	2.07%	4.483898152	2.72E-05	1.39E-06	3.46E-10
ENSG00000080345_151465269_151475071_556	annotated	RIF1	2	+	1668	34	1	1	1.98%	6.73523145	0.001946488	0.001946488	2.23E-05
ENSG00000167642_38264893_38292006_195	annotated	SPINT2	19	+	585	873	67	18	34.36%	493.0920849	4.26E-11	2.27E-12	1.24E-21
ENSG00000128989_52568892_52551934_112	annotated	ARPP19	15	-	336	41	5	5	11.61%	40.319416	0.004219867	0.00329539	7.73E-05
ENSG00000127990_94656026_94587779_458	annotated	SGCE	7	-	1374	197	23	8	10.48%	47.37499172	1.35E-07	4.13E-09	6.20E-15
ENSG00000133835_119479012_119541994_532	dORF	HSD17B4	5	+	1596	90	12	1	4.89%	18.6328495	0.000512167	3.47E-06	1.89E-08
ENSG00000284946_90984125_90981472_179	dORF	AC068831.7	15	-	537	66	8	5	10.06%	40.61059042	0.000130135	0.000279977	2.56E-07
ENSG00000284946_91009831_90984800_475	annotated	AC068831.7	15	-	1425	40	2	4	2.74%	9.275018417	0.002390059	0.002636958	3.63E-05
ENSG00000054277_241640254_241597961_189	annotated	OPN3	1	-	567	25	3	2	5.82%	14.56889467	0.003107436	0.004461174	7.72E-05
ENSG00000054277_241597913_241594428_143	dORF	OPN3	1	-	429	27	1	0	4.90%	20.79582364	0.008574016	0.008877961	0.000387296
ENSG00000075711_197065774_197044623_182	novel	DLG1	3	-	546	7	0	0	2.75%	4.236186296	0.019216965	0.019216965	0.001707396
ENSG00000124762_36684102_36685800_164	annotated	CDKN1A	6	+	492	125	16	8	17.68%	83.9488138	1.25E-05	1.65E-05	1.65E-09
ENSG00000136279_44044738_44060916_430	annotated	DBNL	7	+	1290	76	5	2	6.51%	19.46675377	7.94E-06	1.53E-06	1.06E-10
ENSG00000075711_197297204_197044623_904	annotated	DLG1	3	-	2712	39	4	0	1.99%	4.751651443	0.000762836	7.71E-05	4.39E-07
ENSG00000131828_19344047_19351439_146	novel	PDHA1	X	+	438	48	9	6	10.27%	36.21068834	0.004124887	0.002453821	5.74E-05
ENSG00000178951_4055232_4047752_584	annotated	ZBTB7A	19	-	1752	38	13	2	3.25%	7.166698734	0.028277153	0.000259645	7.17E-05
ENSG00000135124_121210255_121228546_116	novel	P2RX4	12	+	348	47	1	6	10.34%	44.6260315	0.001573472	0.009994242	9.45E-05
ENSG00000077942_45525544_45563266_621	annotated	FBLN1	22	+	1863	152	12	0	7.73%	26.95878944	9.20E-09	6.53E-10	6.79E-17
ENSG00000124155_45420349_45425826_343	annotated	PIGT	20	+	1029	253	20	2	14.58%	81.24091387	1.46E-09	2.95E-10	4.88E-18
ENSG00000136942_124861912_124858469_96	annotated	RPL35	9	-	288	71	9	17	10.42%	81.45833232	0.009211063	0.034101371	0.001557313
ENSG00000136942_124858019_124857918_33	dORF	RPL35	9	-	99	284	34	4	24.24%	947.8787761	0.033184565	0.005859343	0.00103341
ENSG00000164078_49903609_49895260_1032	annotated	MST1R	3	-	3096	39	3	5	2.03%	4.162299326	0.000134245	0.000296265	2.78E-07
ENSG00000164078_49891530_49887307_266	dORF	MST1R	3	-	798	13	1	0	2.26%	5.382823189	0.020342454	0.013428348	0.001295518
ENSG00000159352_151266331_151267343_115	dORF	PSMD4	1	+	345	37	1	6	7.83%	35.43661928	0.006307834	0.032919989	0.001089933
ENSG00000163755_149129724_149172222_1004	annotated	HPS3	3	+	3012	71	10	9	3.88%	7.788844525	2.47E-06	2.13E-06	4.55E-11
ENSG00000163762_149333382_149321478_201	annotated	TM4SF18	3	-	603	690	43	12	28.36%	378.0954336	7.72E-11	9.87E-11	8.92E-20
ENSG00000175305_94894221_94881632_404	annotated	CCNE2	8	-	1212	17	1	0	1.24%	4.634639463	0.021557223	0.021557223	0.002115616
ENSG00000127419_955417_958496_291	annotated	TMEM175	4	+	873	12	0	0	2.75%	4.541890462	0.004873904	0.004873904	0.000128468
ENSG00000071127_10088363_10077116_377	novel	WDR1	4	-	1131	181	12	14	12.47%	52.87929101	3.19E-08	1.16E-07	3.70E-14
ENSG00000113712_149550964_149549474_89	annotated	CSNK1A1	5	-	267	70	22	0	13.48%	86.62762988	0.119738561	0.001053132	0.001323566
ENSG00000115307_74527304_74527077_33	novel	AUP1	2	-	99	9	2	0	15.15%	30.03841192	0.065398531	0.019216965	0.005666422
ENSG00000163608_113011111_113005536_285	dORF	NEPRO	3	-	855	53	3	7	4.91%	20.48233234	0.000471186	0.002153764	6.59E-06
ENSG00000059691_151760970_151722195_111	annotated	GATB	4	-	333	9	1	0	4.50%	8.930338679	0.042234516	0.019679754	0.003715148
ENSG00000178950_870780_850317_523	novel	GAK	4	-	1569	21	2	2	2.49%	4.422481296	0.003268042	0.003268042	6.00E-05
ENSG00000145348_106262160_106046570_787	annotated	TBCK	4	-	2361	14	0	1	0.89%	1.959303446	0.008473674	0.013892891	0.00058868
ENSG00000088727_47277374_47275308_91	annotated	KIF9	3	-	273	9	7	0	5.49%	10.89305048	0.375914817	0.019216965	0.045757765
ENSG00000149761_64229145_64229990_144	novel	NUDT22	11	+	432	22	1	0	4.86%	16.82707334	0.008175611	0.008473674	0.000354322
ENSG00000134330_9481342_9487473_80	novel	IAH1	2	+	240	70	5	1	21.25%	96.37323824	0.000254751	0.000129483	2.32E-07
ENSG00000163110_94455289_94582562_132	novel	PDLIM5	4	+	396	17	4	5	4.55%	14.18480563	0.044040756	0.073562049	0.012834473
ENSG00000152359_75712937_75674435_547	annotated	POC5	5	-	1641	8	3	0	0.91%	1.610835008	0.169362074	0.019216965	0.016163992
ENSG00000169432_166311549_166198672_1919	annotated	SCN9A	2	-	5757	28	4	0	0.63%	1.607057647	0.007026249	0.000991051	4.36E-05
ENSG00000115966_175130185_175074609_389	annotated	ATF2	2	-	1167	42	3	0	4.37%	11.89181346	0.000506283	0.000132396	4.71E-07
ENSG00000006625_30504709_30497819_166	annotated	GGCT	7	-	498	86	55	7	10.24%	57.06091903	0.186604023	0.001902182	0.003725423
ENSG00000117419_44354921_44221558_302	annotated	ERI3	1	-	906	71	14	1	7.28%	25.89403941	0.002274147	3.27E-05	6.85E-07
ENSG00000138032_44201284_44230001_352	annotated	PPM1B	2	+	1056	53	9	3	6.82%	16.58370658	0.000731329	5.73E-05	3.22E-07
ENSG00000117419_44322727_44221558_175	annotated	ERI3	1	-	525	52	12	1	10.29%	32.72756499	0.005233725	0.000170638	7.03E-06
ENSG00000204843_74374349_74361499_1138	annotated	DCTN1	2	-	3414	94	24	4	3.95%	9.097749832	1.01E-05	1.42E-08	1.97E-12

ENSG00000071994_170584581_170578636_298	annotated	PDCD2	6	-	894	28	9	4	4.70%	10.34880411	0.022265173	0.00386557	0.000476466
ENSG00000158864_161212373_161213837_130	novel	NDUFS2	1	+	390	42	4	0	8.46%	35.58396489	0.002744064	0.001558047	2.52E-05
ENSG00000164181_60787397_60754624_281	annotated	ELOVL7	5	-	843	39	2	11	4.63%	15.28645162	0.001591926	0.022551981	0.000230575
ENSG00000186716_23181027_23315522_1205	annotated	BCR	22	+	3615	11	2	8	0.66%	1.005435088	0.033181033	0.293203098	0.046194389
ENSG00000155438_121736850_121727724_293	annotated	NIFK	2	-	879	65	9	0	6.48%	24.43397557	0.000372365	5.92E-05	1.64E-07
ENSG00000072501_53415155_53380103_1192	annotated	SMC1A	X	-	3576	41	5	1	2.27%	3.788401503	4.04E-05	2.58E-06	9.25E-10
ENSG00000168646_65557993_65529976_569	annotated	AXIN2	17	-	1707	10	4	0	0.88%	1.935691454	0.171888378	0.019679754	0.016736337
ENSG00000100938_24233254_24238778_348	annotated	GMPR2	14	+	1044	78	14	13	7.76%	24.68674083	4.52E-05	0.000297885	1.02E-07
ENSG00000102125_154411916_154413256_71	annotated	TAZ	X	+	213	22	0	1	8.45%	34.12814875	0.01300724	0.023878474	0.001470914
ENSG00000158636_76446939_76550146_1323	annotated	EMSY	11	+	3969	10	4	0	0.45%	0.832508267	0.163744512	0.011772051	0.01090609
ENSG00000134900_102597198_102678316_1209	annotated	TPP2	13	+	3627	47	14	2	2.15%	4.281736687	0.002903514	9.77E-06	3.35E-07
ENSG00000171552_31722218_31665949_170	annotated	BCL2L1	20	-	510	54	18	9	12.35%	34.98591506	0.00512046	0.001187962	3.68E-05
ENSG00000136108_52456597_52475141_634	annotated	CKAP2	13	+	1902	67	17	0	5.84%	11.63948979	1.46E-05	2.98E-08	5.67E-12
ENSG00000105953_44624344_44707937_1001	annotated	OGDH	7	+	3003	204	33	10	7.49%	22.44628583	7.89E-10	1.50E-13	2.13E-21
ENSG00000108448_18727558_18735460_348	novel	TRIM16L	17	+	1044	27	5	1	2.01%	8.545410287	0.016983117	0.010275151	0.000852957
ENSG00000180537_13974243_13976930_47	uORF	RNF182	6	+	141	5	0	0	10.64%	11.71711103	0.012673659	0.012673659	0.000782701
ENSG00000160214_43797399_43803774_328	novel	RRP1	21	+	984	34	0	1	4.27%	11.41703868	0.000416874	0.000617933	1.68E-06
ENSG00000102312_48512346_48520476_324	annotated	PORCN	X	+	972	17	0	0	2.78%	5.778994886	0.003452921	0.003452921	6.66E-05
ENSG00000162783_181088903_181089886_327	annotated	IER5	1	+	981	24	1	6	3.36%	8.083731648	0.00345766	0.018402375	0.000354331
ENSG00000135521_143843478_143863527_475	annotated	LTV1	6	+	1425	37	3	0	4.21%	8.579392036	0.000117504	3.41E-05	3.04E-08
ENSG00000044115_138781925_138810046_37	annotated	CTNNA1	5	+	111	21	0	0	13.51%	62.51237075	0.020613417	0.020613417	0.001946209
ENSG00000044115_138810046_138934089_803	dORF	CTNNA1	5	+	2409	205	20	7	8.34%	28.11814814	2.73E-10	5.19E-12	1.84E-20
ENSG00000023697_15911384_16036746_275	annotated	DERA	12	+	825	148	8	4	15.27%	59.27579952	3.81E-08	2.20E-08	8.46E-15
ENSG00000116473_111697468_111709235_133	annotated	RAP1A	1	+	399	52	9	1	11.28%	43.06258551	0.001210076	0.000503793	3.91E-06
ENSG00000143553_153658744_153659466_53	novel	SNAPIN	1	+	159	8	0	2	9.43%	16.62503301	0.019216965	0.04884498	0.004207729
ENSG00000104231_81721281_81708812_165	annotated	ZFAND1	8	-	495	17	7	4	5.45%	11.3478445	0.035162868	0.028187253	0.004282096
ENSG00000169398_140764351_140659466_680	annotated	PTK2	8	-	2040	69	6	7	3.82%	11.1760562	3.25E-05	8.92E-05	2.20E-08
ENSG00000104660_30101945_30104922_134	annotated	LEPROTL1	8	+	402	91	8	0	13.43%	74.79714013	0.000279974	9.47E-05	1.89E-07
ENSG00000113742_173944987_173956137_295	annotated	CPEB4	5	+	885	22	1	0	3.73%	8.213893429	0.002106591	0.001491519	1.87E-05
ENSG00000153774_75433352_75293952_299	annotated	CFDP1	16	-	897	82	9	5	5.02%	30.20585011	0.005266996	0.003424348	9.93E-05
ENSG00000123066_115997159_115961266_1330	novel	MED13L	12	-	3990	29	6	8	1.43%	2.401567269	0.005403025	0.009298461	0.000263605
ENSG00000145191_184135386_184144943_721	annotated	EIF2B5	3	+	2163	55	14	5	3.05%	8.401867412	0.008817629	6.20E-05	5.61E-06
ENSG00000116489_112619845_112649473_52	novel	CAPZA1	1	+	156	17	2	1	13.46%	36.00758352	0.007029303	0.007979402	0.000290307
ENSG00000049541_74237387_74232106_107	Overlap_dORF	RFC2	7	-	321	22	2	2	8.41%	22.64578095	0.002500085	0.010499663	0.000149096
ENSG00000049541_74239946_74235575_99	annotated	RFC2	7	-	297	19	12	4	10.10%	21.13814172	0.136660839	0.007398015	0.0062439
ENSG00000177058_55697949_55626494_558	annotated	SLC38A9	5	-	1674	34	9	1	3.23%	6.711090835	0.006755084	0.000114873	6.76E-06
ENSG00000137700_119029530_119029333_65	Overlap_uORF	SLC37A4	11	-	195	31	7	0	15.38%	52.52871007	0.019390513	0.002376615	0.000272493
ENSG00000137700_119029267_119024910_395	annotated	SLC37A4	11	-	1185	69	3	0	5.32%	19.23979295	0.000117461	2.60E-05	2.36E-08
ENSG00000164144_152829634_152910219_373	annotated	ARFIP1	4	+	1119	93	1	0	6.43%	27.46138999	1.56E-05	7.19E-06	9.15E-10
ENSG00000120963_101201741_101200002_76	annotated	ZNF706	8	-	228	31	7	1	9.21%	44.92587046	0.061051993	0.016822681	0.004725513
ENSG00000171530_77776257_77691418_131	annotated	TBCA	5	-	393	354	44	13	16.79%	297.6325089	2.44E-05	1.97E-05	3.76E-09
ENSG00000122783_135169443_135168981_33	novel	CYREN	7	-	99	43	3	6	15.15%	143.5168569	0.035772986	0.124431937	0.018327857
ENSG00000122783_135168838_135167772_41	novel	CYREN	7	-	123	32	6	1	19.51%	85.96358533	0.024275373	0.006191047	0.000779962
ENSG00000140262_56919935_57282587_699	annotated	TCF12	15	+	2097	37	6	5	2.58%	5.830058966	0.001593111	0.000999173	9.74E-06
ENSG00000138095_43899326_43896385_143	novel	LRPPRC	2	-	429	12	0	0	3.50%	9.242588283	0.021084099	0.021084099	0.002029891
ENSG00000236015_39569986_39569414_190	novel	AC011290.2	7	-	570	110	9	0	2.63%	63.76575162	0.021557223	0.021557223	0.002115616
ENSG00000168679_110389260_110363766_418	annotated	SLC16A4	1	-	1254	194	36	13	13.88%	51.11799923	3.39E-08	8.48E-10	3.35E-16
ENSG00000101236_3963984_3934063_137	annotated	RNF24	20	-	411	24	0	0	5.84%	19.29474634	0.005454749	0.005454749	0.000159109
ENSG00000286070_24557675_24572026_62	annotated	GGT1	22	+	186	93	42	8	29.03%	165.2112656	0.027971873	0.000611463	0.000137366
ENSG00000137819_69384711_69403822_259	annotated	PAQR5	15	+	777	86	16	2	9.65%	36.57186316	0.002067414	1.00E-05	2.29E-07
ENSG00000196821_34654715_34590565_224	annotated	ILRUN	6	-	672	12	2	4	2.23%	5.900402341	0.083503598	0.15306802	0.044493368
ENSG00000196821_34654715_34590565_224	annotated	ILRUN	6	-	672	12	2	4	2.23%	5.900402341	0.083503598	0.15306802	0.044493368
ENSG00000215021_6970543_6970331_70	novel	PHB2	12	-	210	99	5	33	12.86%	155.7706218	0.003710481	0.091713721	0.002299825
ENSG00000073060_124863720_124782683_481	annotated	SCARB1	12	-	1443	324	54	53	14.55%	74.19050595	2.93E-09	1.11E-09	3.54E-17
ENSG00000145604_36152263_36182031_424	annotated	SKP2	5	+	1272	19	3	0	2.83%	4.935556675	0.00804845	0.000766051	4.04E-05
ENSG00000152795_82429333_82426059_301	annotated	HNRNPDL	4	-	903	206	23	4	14.95%	75.3787834	1.65E-08	2.42E-09	4.31E-16
ENSG00000134030_48619566_48859559_598	annotated	CTIF	18	+	1794	15	3	0	1.17%	2.762730193	0.05158844	0.007882058	0.0021214
ENSG00000163866_34855977_34855699_92	annotated	SMIM12	1	-	276	40	6	3	15.22%	47.88732335	0.003063143	0.001091889	2.02E-05
ENSG00000131446_180792971_180791634_445	annotated	MGAT1	5	-	1335	90	7	9	8.76%	22.27567626	2.32E-07	1.57E-06	3.43E-12
ENSG00000182774_82540428_82538222_136	annotated	RPS17	15	-	408	301	40	9	16.18%	243.7676026	0.000629493	6.02E-05	2.87E-07
ENSG00000132716_160240344_160217592_572	annotated	DCAF8	1	-	1716	103	2	3	6.12%	19.83305402	1.69E-07	9.25E-08	1.51E-13
ENSG00000132676_155731401_155738242_82	annotated	DAP3	1	+	246	38	0	0	13.41%	51.04087879	0.001595761	0.001595761	1.52E-05
ENSG00000144674_37243551_37366103_166	annotated	GOLGA4	3	+	498	19	0	0	4.82%	12.60648211	0.005306578	0.005306578	0.000151001
ENSG00000135926_218282141_218275475_311	annotated	TMBIM1	2	-	933	105	17	0	9.32%	37.1858154	4.63E-05	1.11E-06	4.96E-10
ENSG00000145901_151063724_151030713_583	annotated	TNIP1	5	-	1749	51	5	1	3.77%	9.634962314	0.000174475	2.29E-05	3.14E-08
ENSG00000111206_2874478_2858638_801	annotated	FOXM1	12	-	2403	43	13	12	2.12%	5.9126795	0.015576154	0.008803764	0.000681415
ENSG00000113273_78985248_78885529_398	annotated	ARSB	5	-	1194	29	4	3	3.77%	8.025337858	0.003256993	0.002477401	4.60E-05
ENSG00000115525_85863483_85846391_203	annotated	ST3GAL5	2	-	609	100	19	9	12.32%	54.25657325	0.000208105	0.00017171	2.49E-07

ENSG00000115525_85846397_85840144_142	Overlap_dORF	ST3GAL5	2	-	426	20	0	1	6.34%	15.51279489	0.003303074	0.006112544	0.000111133
ENSG00000185842_224896304_225399269_3493	annotated	DNAH14	1	+	10479	32	6	5	0.20%	1.00902004	0.03713939	0.040465304	0.006276036
ENSG00000104341_97775737_97851474_317	annotated	LAPTM4B	8	+	951	508	66	39	20.19%	176.5033079	1.57E-09	3.42E-09	5.84E-17
ENSG00000115415_190999658_190975808_512	Overlap_dORF	STAT1	2	-	1536	68	2	5	4.49%	14.6280808	1.23E-05	5.24E-05	5.14E-09
ENSG00000115415_191009922_190997967_166	annotated	STAT1	2	-	498	19	1	0	4.22%	12.60648211	0.01208853	0.008776163	0.000531381
ENSG00000102978_57462725_57469775_150	novel	POLR2C	16	+	450	79	5	0	12.00%	58.00751102	0.00048498	9.35E-05	3.27E-07
ENSG00000179958_30429980_30424233_71	annotated	DCTPP1	16	-	213	85	5	2	22.54%	131.8587565	0.00017985	0.000321839	3.99E-07
ENSG00000165272_33447530_33441990_237	annotated	AQP3	9	-	711	177	20	7	15.61%	82.25708581	5.36E-06	3.85E-07	1.95E-11
ENSG00000125247_100668740_100604994_741	annotated	TMTC4	13	-	2223	77	5	0	3.91%	11.44513491	9.24E-06	9.76E-07	8.14E-11
ENSG00000074410_63331802_63326285_87	novel	CA12	15	-	261	9	1	0	6.90%	11.39388038	0.026832696	0.011772051	0.001510382
ENSG00000136381_78484908_78488404_152	novel	IREB2	15	+	456	13	0	0	4.61%	9.41994058	0.008373824	0.008373824	0.000358384
ENSG00000126858_32182830_32224733_484	dORF	RHOT1	17	+	1452	49	7	2	4.75%	11.15062261	0.000399227	4.18E-05	1.28E-07
ENSG00000132780_45614150_45618141_268	novel	NASP	1	+	804	79	12	3	9.33%	32.4668905	0.000232756	1.49E-05	2.86E-08
ENSG00000134905_110663486_110641537_247	novel	CARS2	13	-	741	20	0	5	2.83%	8.91828694	0.008674847	0.060359788	0.00272115
ENSG00000272305_53099403_53077781_106	annotated	AC096887.1	3	-	318	12	0	0	5.66%	12.46877476	0.012798403	0.012798403	0.000797244
ENSG00000049541_74254383_74246755_77	annotated	RFC2	7	-	231	28	4	0	10.39%	40.05121589	0.013786174	0.005706018	0.000408699
ENSG00000142694_36323037_36322295_165	annotated	EVA1B	1	-	495	19	1	1	3.64%	12.68288503	0.023878474	0.023878474	0.002559322
ENSG00000136104_50910077_50959518_261	novel	RNASEH2B	13	+	783	27	3	2	4.60%	11.39388038	0.002631121	0.00398534	5.92E-05
ENSG00000067225_72219097_72199650_531	annotated	PKM	15	-	1593	4146	721	160	35.59%	859.9697514	4.71E-26	6.34E-29	6.35E-53
ENSG00000034713_75568127_75577369_57	annotated	GABARAPL2	16	+	171	133	11	7	24.56%	256.995302	0.003015629	0.002655719	4.56E-05
ENSG00000103197_2050439_2088610_1681	annotated	TSC2	16	+	5043	65	6	1	1.55%	4.258866651	7.80E-06	3.46E-06	2.27E-10
ENSG00000092531_42511847_42521641_97	annotated	SNAP23	15	+	291	43	6	1	9.28%	48.82532247	0.004557894	0.006307834	0.000154436
ENSG00000139620_48681632_48681324_102	novel	KANSL2	12	-	306	11	1	0	7.84%	11.87793413	0.004149608	0.004687384	0.000106268
ENSG00000135720_66729051_66723722_115	annotated	DYNC1LI2	16	-	345	15	3	4	4.35%	14.36619701	0.042234516	0.171513914	0.029347768
ENSG00000065000_2127191_2102173_931	annotated	AP3D1	19	-	2793	103	9	9	4.51%	12.18529205	3.51E-07	2.51E-07	8.24E-13
ENSG00000100938_24233254_24234112_70	annotated	GMPR2	14	+	210	25	8	0	10.00%	39.33601561	0.061282382	0.008776163	0.002792762
ENSG00000140575_90487064_90491611_130	novel	IQGAP1	15	+	390	10	0	1	3.85%	8.472372593	0.020613417	0.035374934	0.003252526
ENSG00000084754_26244596_26199259_499	annotated	HADHA	2	-	1497	117	13	1	6.41%	25.82460664	7.14E-06	3.36E-07	2.32E-11
ENSG00000084754_26195196_26191250_258	dORF	HADHA	2	-	774	77	14	0	9.30%	32.87149211	0.000832075	7.76E-06	6.50E-08
ENSG00000078618_51878468_51789236_1102	annotated	NRDC	1	-	3306	117	14	1	4.72%	11.69371934	4.53E-08	1.04E-10	6.52E-17
ENSG00000126088_45012992_45015498_286	annotated	UROD	1	+	858	89	12	9	9.79%	34.27459822	2.01E-06	4.42E-05	8.09E-10
ENSG00000163507_108581486_108551149_746	annotated	CIP2A	3	-	2238	60	5	2	3.49%	8.858512899	1.43E-05	1.13E-05	1.30E-09
ENSG00000116001_70248418_70216824_210	annotated	TIA1	2	-	630	17	0	2	2.86%	8.916163538	0.012798403	0.030316224	0.00183564
ENSG00000141698_41832402_41825523_199	novel	NT5C3B	17	-	597	22	7	4	4.52%	12.17637468	0.076961241	0.029337119	0.009511591
ENSG00000009844_142147367_142218643_222	annotated	VTA1	6	+	666	49	8	1	6.76%	24.3103664	0.015642785	0.000260336	3.50E-05
ENSG00000131374_17508570_17160963_817	annotated	TBC1D5	3	-	2451	52	25	4	2.82%	7.010188339	0.009115679	8.47E-05	7.50E-06
ENSG00000172354_100676729_100678801_296	annotated	GNB2	7	+	888	319	27	4	22.64%	118.6990849	2.21E-11	4.34E-13	1.30E-22
ENSG00000156983_9739282_9747349_919	annotated	BRPF1	3	+	2757	9	1	0	0.54%	1.078637207	0.035374934	0.020613417	0.003252526
ENSG00000073331_112382445_112441210_1188	annotated	ALPK1	4	+	3564	9	1	0	0.51%	0.834400331	0.026832696	0.011772051	0.001510382
ENSG00000104824_38840296_38836722_245	novel	HNRNPL	19	-	735	220	18	6	11.84%	98.9019821	2.78E-05	4.72E-06	1.10E-09
ENSG00000072682_132218599_132193010_526	annotated	P4HA2	5	-	1578	160	31	4	10.65%	33.50291824	1.93E-08	5.44E-11	1.44E-17
ENSG00000196531_56712866_56712538_31	dORF	NACA	12	-	93	156	10	6	29.03%	554.257149	0.003981745	0.003455215	7.64E-05
ENSG00000105085_16578400_16576027_573	annotated	MED26	19	-	1719	11	0	0	0.87%	2.114396651	0.021084099	0.021084099	0.002029891
ENSG00000115221_160200063_160101736_693	annotated	ITGB6	2	-	2079	8	0	0	1.01%	1.271467171	0.005706018	0.005706018	0.00017331
ENSG00000170892_54191365_54193671_312	annotated	TSEN34	19	+	936	78	6	2	8.97%	27.53521093	9.46E-06	1.49E-06	1.24E-10
ENSG00000026508_35139304_35229333_361	annotated	CD44	11	+	1083	1835	165	66	37.95%	559.8571972	5.54E-18	7.62E-21	7.76E-37
ENSG00000179152_44367602_44407682_341	annotated	TCAIM	3	+	1023	11	5	0	2.05%	3.552930442	0.077869819	0.007882058	0.003354422
ENSG00000163866_34855977_34855699_92	annotated	SMIM12	1	-	276	40	6	3	15.22%	47.88732335	0.003063143	0.001091889	2.02E-05
ENSG00000130734_10551930_10553067_164	annotated	ATG4D	19	+	492	8	2	0	3.05%	5.372724083	0.065398531	0.019679754	0.005779267
ENSG00000100744_96382284_96385684_127	annotated	GSKIP	14	+	381	51	6	2	13.39%	44.22978763	0.000263993	0.000422469	7.51E-07
ENSG00000148019_78236351_78270851_673	annotated	CEP78	9	+	2019	45	2	0	2.82%	7.364543784	0.000218523	5.30E-05	8.58E-08
ENSG00000125257_95247698_95021575_1282	dORF	ABCC4	13	-	3846	147	23	7	4.37%	12.62925431	4.03E-08	6.43E-10	3.09E-16
ENSG00000189308_82984727_82928102_710	annotated	LIN54	4	-	2130	13	2	0	1.13%	2.016663335	0.030313544	0.004826937	0.000797936
ENSG00000177427_18262721_18264764_454	annotated	MIEF2	17	+	1362	21	3	0	2.20%	5.094620524	0.013506184	0.002201184	0.00017355
ENSG00000142002_4705968_4685545_598	novel	DPP9	19	-	1794	35	1	0	2.68%	6.446370451	0.000253427	0.000186366	3.26E-07
ENSG00000142002_4682805_4676564_104	novel	DPP9	19	-	312	7	0	0	4.81%	7.413326019	0.019216965	0.019216965	0.001707396
ENSG00000138696_95114742_95154673_447	annotated	BMPR1B	4	+	1341	65	3	1	6.04%	16.01600636	1.32E-05	3.04E-06	3.42E-10
ENSG00000162413_6599162_6594108_63	annotated	KLHL21	1	-	189	13	4	0	11.11%	22.72747569	0.05226483	0.008373824	0.002260108
ENSG00000156253_29008544_29006417_271	annotated	RWDD2B	21	-	813	42	7	2	7.38%	17.06979866	0.000236553	0.000129425	2.15E-07
ENSG00000124882_74365327_74381141_87	novel	EREG	4	+	261	25	6	4	6.90%	31.64966773	0.079426469	0.053528973	0.016347238
ENSG00000072310_17817860_17817209_137	novel	SREBF1	17	-	411	39	10	0	10.22%	31.35396281	0.028793543	0.000446789	0.000111371
ENSG00000086504_370218_367675_256	annotated	MRPL28	16	-	768	186	16	4	20.31%	80.02420675	2.08E-08	2.04E-10	5.30E-17
ENSG00000149485_61813305_61802411_360	annotated	FADS1	11	-	1080	1133	165	25	28.06%	346.6377109	6.62E-16	4.03E-17	4.05E-31
ENSG00000169714_129171757_129170453_172	annotated	CNBP	3	-	516	533	54	21	30.23%	341.3085447	4.75E-10	5.97E-10	3.19E-18
ENSG00000136731_128187461_128189742_59	novel	UGGT1	2	+	177	23	0	2	16.95%	42.93626111	0.002046029	0.003318031	3.91E-05
ENSG00000186185_44936344_44926080_852	annotated	KIF18B	17	-	2556	29	6	7	1.64%	3.748925431	0.005613246	0.006811055	0.00020204
ENSG00000166562_59139949_59147327_39	novel	SEC11C	18	+	117	23	1	0	12.82%	64.95485654	0.019679754	0.016947427	0.001553599
ENSG00000112319_133462632_133528805_448	annotated	EYA4	6	+	1344	13	2	0	1.56%	3.196051268	0.023025945	0.007882058	0.000911002

ENSG00000160818_156599013_156598221_72	novel	GPATCH4	1	-	216	13	0	0	9.72%	19.88654122	0.007882058	0.007882058	0.000319636
ENSG00000141576_76240240_76145235_657	annotated	RNF157	17	-	1971	8	0	0	0.76%	1.341136605	0.019679754	0.019679754	0.001784964
ENSG00000137955_75786272_75794650_331	annotated	RABGGTB	1	+	993	73	16	2	10.57%	24.29088094	0.000112324	1.75E-07	2.63E-10
ENSG00000122884_73074883_73008222_534	annotated	P4HA1	10	-	1602	155	29	6	9.55%	31.96972055	2.93E-07	3.43E-09	1.17E-14
ENSG00000116954_38859448_38839553_333	annotated	RRAGC	1	-	999	24	4	0	3.90%	7.938078826	0.005969181	0.00060914	2.40E-05
ENSG00000159363_16991743_16986530_422	novel	ATP13A2	1	-	1266	18	0	0	1.18%	4.697950679	0.021084099	0.021084099	0.002029891
ENSG00000185624_81860471_81859146_128	novel	P4HB	17	-	384	294	37	4	24.22%	252.9797504	2.18E-05	9.19E-07	1.88E-10
ENSG00000253729_47778518_47774173_197	novel	PRKDC	8	-	591	21	0	1	5.08%	11.74090212	0.002188006	0.003722062	4.66E-05
ENSG00000189308_82984844_82928102_528	annotated	LIN54	4	-	1584	11	2	0	1.33%	2.294600911	0.049121095	0.007029303	0.001832503
ENSG00000110696_16744670_16754874_113	annotated	C11orf58	11	+	339	38	2	2	9.73%	37.03851381	0.003529166	0.004623484	9.00E-05
ENSG00000134330_9481342_9488329_135	annotated	IAH1	2	+	405	99	7	1	20.00%	80.76995205	7.66E-06	2.44E-06	1.60E-10
ENSG00000134330_9474567_9478230_29	uORF	IAH1	2	+	87	14	1	5	17.24%	53.17144179	0.019679754	0.051235217	0.004505595
ENSG00000148840_102133069_102150029_1664	annotated	PPRC1	10	+	4992	71	5	0	1.98%	4.699519173	2.07E-06	1.80E-07	3.59E-12
ENSG00000244045_28360839_28361252_34	novel	TMEM199	17	+	102	17	1	0	23.53%	55.07042185	0.005306578	0.005306578	0.000151001
ENSG00000115234_27374720_27376719_256	dORF	SNX17	2	+	768	139	1	3	11.72%	59.80303623	7.50E-07	1.86E-06	1.25E-11
ENSG00000242372_35284487_35280046_59	annotated	EIF6	20	-	177	82	3	18	15.25%	153.0771048	0.00567009	0.179855388	0.007382923
ENSG00000242372_35279705_35279197_49	dORF	EIF6	20	-	147	107	21	7	18.37%	240.5116383	0.030620089	0.004355956	0.000740491
ENSG00000130713_130693804_130703774_259	annotated	EXOSC2	9	+	777	90	6	9	11.97%	38.27288005	2.32E-06	3.39E-06	6.74E-11
ENSG00000130713_130693792_130703774_293	annotated	EXOSC2	9	+	879	118	6	9	12.97%	44.35706334	1.41E-07	2.07E-07	2.79E-13
ENSG00000126088_45012992_45013372_62	novel	UROD	1	+	186	15	0	9	11.29%	26.64697832	0.008473674	0.154611771	0.008031369
ENSG00000147905_37126336_37357268_542	novel	ZCCHC7	9	+	1626	9	0	1	0.92%	1.828906999	0.020613417	0.035374934	0.003252526
ENSG00000127527_16471945_16402306_601	annotated	EPS15L1	19	-	1803	15	3	2	1.16%	2.748939527	0.008175611	0.032996028	0.001359906
ENSG00000173614_9972176_9982701_245	annotated	NMNAT1	1	+	735	21	1	1	2.86%	9.440643746	0.01208853	0.01208853	0.000716137
ENSG00000138303_72210826_72102369_361	annotated	ASCC1	10	-	1083	28	9	1	3.32%	8.542780121	0.02984769	0.000978918	0.000214878
ENSG00000138303_72213264_72210754_51	internal	ASCC1	10	-	153	7	0	4	11.76%	15.1173707	0.009815329	0.170566842	0.010088762
ENSG00000138303_72210826_72097334_318	annotated	ASCC1	10	-	954	35	6	1	4.09%	12.1224199	0.003255491	0.000656046	1.36E-05
ENSG00000138303_72213264_72210754_51	internal	ASCC1	10	-	153	7	0	4	11.76%	15.1173707	0.009815329	0.170566842	0.010088762
ENSG00000165506_49633622_49625542_280	annotated	DNAAF2	14	-	840	13	3	0	2.50%	5.113682029	0.067796501	0.007882058	0.002868205
ENSG00000198668_90397231_90404717_83	annotated	CALM1	14	+	249	264	21	9	38.55%	350.3275029	3.73E-06	1.32E-06	4.30E-11
ENSG00000004975_7229381_7228965_74	novel	DVL2	17	-	222	22	0	0	12.16%	32.74457516	0.002967235	0.002967235	4.99E-05
ENSG00000120071_46172143_46031476_1104	annotated	KANSL1	17	-	3312	23	5	3	0.91%	2.294600911	0.032869174	0.010796097	0.001718
ENSG00000167978_2767819_2769886_251	novel	SRRM2	16	+	753	12	0	0	1.99%	5.265697707	0.019679754	0.019679754	0.001784964
ENSG00000125991_35542098_35557504_388	annotated	ERGIC3	20	+	1164	532	108	21	26.29%	151.0178579	3.48E-13	3.90E-18	3.20E-29
ENSG00000054654_63954816_64225564_6595	annotated	SYNE2	14	+	19785	33	14	10	0.29%	0.551121735	0.028517757	0.011475639	0.001569511
ENSG00000171109_179377356_179392059_329	Overlap_dORF	MFN1	3	+	987	15	0	0	1.82%	5.021619014	0.01300724	0.01300724	0.000821865
ENSG00000171109_179348852_179377359_370	annotated	MFN1	3	+	1110	11	1	3	1.62%	3.274457516	0.019795882	0.100002646	0.009103117
ENSG00000106105_30594922_30622466_538	novel	GARS	7	+	1614	228	34	9	8.74%	46.67678878	1.32E-06	2.35E-09	4.21E-14
ENSG00000064933_189791810_189877436_770	annotated	PMS1	2	+	2310	15	10	2	1.04%	2.145600851	0.199697852	0.026606355	0.024831697
ENSG00000198561_57791479_57816308_933	annotated	CTNND1	11	+	2799	208	34	13	6.32%	24.55444318	1.64E-07	1.19E-09	2.41E-15
ENSG00000168958_227340328_227357117_89	annotated	MFF	2	+	267	32	5	9	7.87%	39.60120223	0.020342454	0.024694694	0.0022779
ENSG00000114867_184317438_184334908_1512	annotated	EIF4G1	3	+	4536	153	15	14	4.17%	11.14520442	4.38E-10	1.20E-09	5.91E-18
ENSG00000157191_16440762_16450220_194	annotated	NECAP2	1	+	582	29	3	1	4.12%	16.46435293	0.031423484	0.009341698	0.001448897
ENSG00000187764_89405393_89378704_841	annotated	SEMA4D	9	-	2523	24	2	1	1.66%	3.143139416	0.00128979	0.000661953	5.38E-06
ENSG00000092621_119712023_119744040_445	annotated	PHGDH	1	+	1335	56	6	1	4.04%	13.86042078	0.000782701	0.00010908	6.17E-07
ENSG00000149136_57330426_57330089_61	novel	SSRP1	11	-	183	16	2	0	8.20%	28.88940163	0.070008252	0.021084099	0.006564739
ENSG00000149136_57328335_57327422_100	novel	SSRP1	11	-	300	34	5	0	12.00%	37.44788686	0.01208853	0.001003278	7.89E-05
ENSG00000149136_57335121_57332749_343	novel	SSRP1	11	-	1029	89	14	7	7.00%	28.5788195	0.000572802	0.00013633	5.48E-07
ENSG00000149136_57332927_57330723_320	novel	SSRP1	11	-	960	75	36	3	10.62%	25.81426024	0.001472779	2.65E-07	8.09E-09
ENSG00000149136_57326852_57326407_73	novel	SSRP1	11	-	219	30	12	0	10.96%	45.26336043	0.031850938	0.005355751	0.000917615
ENSG00000111843_10728666_10730666_37	novel	TMEM14C	6	+	111	150	8	0	37.84%	446.5169339	0.001820107	0.000476727	5.61E-06
ENSG00000105443_48473960_48475047_138	novel	CYTH2	19	+	414	11	3	1	4.35%	8.779342614	0.071345384	0.011772051	0.004172987
ENSG00000144354_173364984_173367664_104	novel	CDCA7	2	+	312	12	0	0	7.69%	12.70855889	0.004826937	0.004826937	0.000126125
ENSG00000144354_173354964_173364018_194	novel	CDCA7	2	+	582	19	0	0	3.61%	10.78698985	0.008473674	0.008473674	0.000366502
ENSG00000050344_26152499_26185783_694	annotated	NFE2L3	7	+	2082	25	0	0	1.01%	3.967609644	0.008674847	0.008674847	0.000383115
ENSG00000015171_180013_252470_548	annotated	ZMYND11	10	+	1644	55	12	9	4.20%	11.05428176	0.001287191	0.000405473	3.40E-06
ENSG00000155368_119367052_119372318_87	annotated	DBI	2	+	261	678	54	12	37.93%	858.3389889	1.97E-05	2.06E-06	3.55E-10
ENSG00000015171_180013_252470_522	annotated	ZMYND11	10	+	1566	50	9	5	4.21%	10.54988924	0.000633813	0.000105953	4.83E-07
ENSG00000115310_55050300_54973156_1192	annotated	RTN4	2	-	3576	883	164	28	8.05%	81.58923238	5.85E-13	3.81E-16	3.84E-27
ENSG00000110075_68537665_68614677_791	annotated	PPP6R3	11	+	2373	123	12	10	6.45%	17.12683157	2.04E-08	7.69E-08	1.59E-14
ENSG00000049883_72335817_72358427_198	dORF	PTCD2	5	+	594	15	0	2	3.03%	8.344003311	0.013428348	0.020342454	0.001295518
ENSG00000011485_46387113_46390346_191	novel	PPP5C	19	+	573	27	10	6	6.28%	15.56964806	0.010635465	0.011127573	0.000587249
ENSG00000147457_23246786_23260599_423	annotated	CHMP7	8	+	1269	104	11	0	8.27%	27.0795455	4.22E-06	1.05E-07	4.58E-12
ENSG00000138760_76213543_76161738_449	annotated	SCARB2	4	-	1347	332	41	7	16.93%	81.44044568	7.66E-11	3.56E-14	4.61E-23
ENSG00000132388_4366316_4282835_170	annotated	UBE2G1	17	-	510	82	12	12	9.41%	53.1267599	0.000941925	0.001539238	8.91E-06
ENSG00000180596_26123904_26123524_126	annotated	HIST1H2BC	6	-	378	1380	136	22	28.57%	1206.304479	1.27E-06	9.10E-08	1.14E-12
ENSG00000148985_3811260_3825458_254	annotated	PGAP2	11	+	762	39	7	1	5.12%	16.91138939	0.004964704	0.000705712	2.25E-05
ENSG00000158669_41609798_41621001_330	annotated	GPAT4	8	+	990	30	7	0	5.45%	10.01280397	0.008025811	7.13E-05	5.61E-06
ENSG00000167904_55756394_55740132_143	annotated	TMEM68	8	-	429	25	0	0	5.59%	19.25539226	0.005706018	0.005706018	0.00017331

ENSG00000115525_85863483_85840144_390	annotated	ST3GAL5	2	-	1170	151	23	10	11.79%	42.64427538	2.98E-07	7.02E-08	2.04E-13
ENSG00000072803_172006502_171868635_508	annotated	FBXW11	5	-	1524	81	8	0	4.72%	17.56182744	0.000329418	7.05E-06	2.14E-08
ENSG00000110075_68537665_68613117_873	annotated	PPP6R3	11	+	2619	145	14	12	6.76%	18.29372547	2.65E-09	4.99E-09	1.42E-16
ENSG00000110075_68537665_68613117_879	annotated	PPP6R3	11	+	2637	145	14	12	6.71%	18.16885363	2.65E-09	4.99E-09	1.42E-16
ENSG00000115525_85863483_85840144_390	annotated	ST3GAL5	2	-	1170	151	23	10	11.79%	42.64427538	2.98E-07	7.02E-08	2.04E-13
ENSG00000140157_22851732_22866847_341	annotated	NIPA2	15	+	1023	145	31	5	9.38%	46.8340831	0.000308171	1.89E-06	6.33E-09
ENSG00000140157_22838748_22838861_37	uORF	NIPA2	15	+	111	43	2	4	18.92%	128.0015211	0.008776163	0.032038753	0.00140113
ENSG00000142192_26112077_25881670_653	annotated	APP	21	-	1959	1190	131	36	26.19%	200.7160858	2.75E-26	3.84E-28	2.12E-52
ENSG00000134574_47215137_47238849_427	annotated	DDB2	11	+	1281	66	8	2	5.39%	17.02411167	0.000297009	2.22E-05	5.31E-08
ENSG00000134575_47248789_47240116_423	annotated	ACP2	11	-	1269	87	6	2	8.27%	22.65308133	4.50E-07	1.68E-07	7.14E-13
ENSG00000070010_19475095_19454650_268	novel	UFD1	22	-	804	111	8	3	10.07%	45.61803601	1.02E-05	9.99E-06	8.27E-10
ENSG00000197045_54488927_54482135_55	novel	GMFB	14	-	165	26	33	0	12.73%	52.06658066	0.237626903	0.008877961	0.014585794
ENSG00000107331_137028872_137011121_2024	novel	ABCA2	9	-	6072	79	13	3	1.88%	4.298975619	1.86E-06	1.21E-07	2.22E-12
ENSG00000107331_137011091_137007929_457	novel	ABCA2	9	-	1371	22	2	4	2.41%	5.302185036	0.002484757	0.013501766	0.000192609
ENSG00000063241_55456486_55456343_47	novel	ISOC2	19	-	141	29	2	1	14.89%	67.95924399	0.008776163	0.008776163	0.000391611
ENSG00000145391_139556119_139496353_356	annotated	SETD7	4	-	1068	35	7	0	5.34%	10.82845374	0.001851817	4.05E-05	6.52E-07
ENSG00000127663_5110655_5151511_779	novel	KDM4B	19	+	2337	9	0	0	0.64%	1.272487283	0.019679754	0.019679754	0.001784964
ENSG00000119705_77708112_77717561_107	annotated	SLIRP	14	+	321	559	72	29	37.38%	575.4087068	8.57E-08	1.82E-07	1.51E-13
ENSG00000164172_53102217_53101328_100	novel	MOCS2	5	-	300	12	0	0	5.00%	13.21690124	0.021084099	0.021084099	0.002029891
ENSG00000121073_49708086_49706200_108	novel	SLC35B1	17	-	324	33	4	5	5.56%	33.65414669	0.024275373	0.038919555	0.004124968
ENSG00000121073_49705279_49701458_198	novel	SLC35B1	17	-	594	68	10	2	11.62%	37.82614834	0.000481013	3.95E-05	1.48E-07
ENSG00000109586_173292190_173295541_76	novel	GALNT7	4	+	228	24	2	1	11.84%	34.78131906	0.003303074	0.003478238	6.43E-05
ENSG00000109332_102809680_102797415_110	annotated	UBE2D3	4	-	330	42	2	0	8.18%	42.05377669	0.003710481	0.003710481	7.64E-05
ENSG00000125676_123733022_123610936_1593	annotated	THOC2	X	-	4779	32	4	10	0.75%	2.212496546	0.004206461	0.038094562	0.00091314
ENSG00000145687_81751042_81420504_341	annotated	SSBP2	5	-	1023	17	0	1	1.76%	5.490892501	0.01300724	0.023878474	0.001470914
ENSG00000169299_37839660_37847306_291	Overlap_dORF	PGM2	4	+	873	34	4	0	3.78%	12.86868964	0.011037428	0.001550569	0.000103666
ENSG00000115677_241262839_241229601_1161	annotated	HDLBP	2	-	3483	136	26	28	5.08%	12.90194207	6.57E-08	2.00E-07	1.28E-13
ENSG00000039560_34686920_34830765_951	annotated	RAI14	5	+	2853	65	4	5	3.15%	7.528028224	3.39E-06	2.88E-06	8.37E-11
ENSG00000115363_75518101_75493236_139	annotated	EVA1A	2	-	417	17	2	0	3.60%	13.4704629	0.070008252	0.021084099	0.006564739
ENSG00000000971_196652148_196673186_78	novel	CFH	1	+	234	14	2	1	6.41%	19.76886938	0.027849813	0.027849813	0.003405714
ENSG00000139433_109880374_109852555_209	annotated	GLTP	12	-	627	16	1	0	5.26%	8.431834925	0.002532006	0.001242734	1.89E-05
ENSG00000167552_49186731_49185010_416	annotated	TUBA1A	12	-	1248	35	1	0	2.64%	9.266657523	0.0034719	0.001491519	3.05E-05
ENSG00000167553_49265182_49273227_449	annotated	TUBA1C	12	+	1347	198	10	5	6.46%	48.56990435	1.29E-06	4.09E-06	4.62E-11
ENSG00000170142_23807270_23808733_63	novel	UBE2E1	3	+	189	57	2	1	7.94%	99.65123954	0.029237631	0.021557223	0.002819006
ENSG00000159658_46686870_46678461_166	novel	EFCAB14	1	-	498	28	0	2	5.42%	18.57797364	0.003684287	0.003632191	7.44E-05
ENSG00000116514_32948253_32936806_414	annotated	RNF19B	1	-	1242	19	3	1	1.69%	5.05477302	0.026714995	0.009583875	0.001255763
ENSG00000164167_146182922_146189656_80	annotated	LSM6	4	+	240	36	1	3	8.75%	49.56337967	0.010042643	0.027811403	0.001362204
ENSG00000103018_69424696_69462534_131	annotated	CYB5B	16	+	393	812	251	29	30.53%	682.705077	0.00157765	4.55E-08	2.23E-09
ENSG00000283765_183884869_183884747_40	Overlap_uORF	AC131160.1	3	-	120	11	3	3	12.50%	30.28873202	0.121144048	0.121144048	0.049102245
ENSG00000283765_183884846_183817356_408	annotated	AC131160.1	3	-	1224	43	12	3	4.41%	11.60798108	0.01182161	0.000566655	4.77E-05
ENSG00000156113_77637360_76887266_1114	annotated	KCNMA1	10	-	3342	74	13	0	3.23%	7.316357661	8.97E-06	5.88E-08	6.07E-12
ENSG00000163902_128650800_128641112_154	novel	RPN1	3	-	462	131	7	8	12.34%	93.69123718	0.000239196	6.09E-05	1.07E-07
ENSG00000054148_136849431_136850789_124	novel	PHPT1	9	+	372	718	133	24	36.29%	637.7510144	8.42E-06	4.19E-09	5.56E-13
ENSG00000147164_71068306_71061016_162	annotated	SNX12	X	-	486	72	6	1	16.05%	48.95148609	2.51E-05	3.46E-06	7.41E-10
ENSG00000099817_1091860_1089486_117	Overlap_dORF	POLR2E	19	-	351	67	8	3	11.97%	63.07210708	0.002464758	0.001820107	2.63E-05
ENSG00000099817_1095315_1091809_51	annotated	POLR2E	19	-	153	53	5	25	11.76%	114.4600925	0.016983117	0.237626903	0.022521956
ENSG00000117151_84574415_84554999_385	annotated	CTBS	1	-	1155	60	10	0	4.94%	17.16480681	0.000242888	5.80E-05	1.04E-07
ENSG00000155252_97640743_97673742_479	annotated	PI4K2A	10	+	1437	68	4	0	5.43%	15.6358609	2.03E-05	3.61E-06	6.22E-10
ENSG00000122008_75547023_75597776_461	annotated	POLK	5	+	1383	25	2	2	1.95%	5.972930787	0.007542912	0.007542912	0.000294119
ENSG00000113851_3154822_3150865_189	novel	CRBN	3	-	567	30	0	1	3.70%	17.4826736	0.008473674	0.013892891	0.00058868
ENSG00000265241_145927378_145925882_158	annotated	RBM8A	1	-	474	58	6	0	7.59%	40.43144896	0.001805035	0.001057356	1.16E-05
ENSG00000010244_32362966_32369759_277	annotated	ZNF207	17	+	831	36	6	2	5.42%	14.31433348	0.000673856	0.001158347	4.92E-06
ENSG00000161513_74872944_74862817_497	annotated	FDXR	17	-	1491	81	3	1	5.23%	17.9505198	1.30E-05	4.30E-06	4.66E-10
ENSG00000130159_11507520_11506184_102	dORF	ECSIT	19	-	306	23	0	0	6.86%	24.83568044	0.008776163	0.008776163	0.000391611
ENSG00000164032_99950170_99949447_98	novel	H2AFZ	4	-	294	326	268	34	9.18%	366.3868882	0.106045149	0.006229071	0.004032457
ENSG00000104723_15743558_15748469_49	novel	TUSC3	8	+	147	9	2	0	12.24%	20.22995088	0.071345384	0.011772051	0.004172987
ENSG00000166887_42187330_42160754_717	annotated	VPS39	15	-	2151	13	2	0	1.39%	1.996974851	0.015345264	0.001755235	0.00016394
ENSG00000182544_53252836_53254188_450	annotated	MFSD5	12	+	1350	135	18	0	9.33%	33.04225311	6.86E-07	6.71E-09	5.37E-14
ENSG00000100722_88568068_88611751_569	annotated	ZC3H14	14	+	1707	55	20	2	5.27%	10.64630299	0.001899042	1.29E-06	3.91E-08
ENSG00000196712_31200578_31374155_2488	annotated	NF1	17	+	7464	66	6	7	1.25%	2.921742638	4.32E-06	1.97E-05	7.14E-10
ENSG00000160818_156599013_156596383_131	novel	GPATCH4	1	-	393	13	0	0	5.34%	10.93000739	0.007882058	0.007882058	0.000319636
ENSG00000081760_125065669_125142229_644	annotated	AACS	12	+	1932	29	10	0	2.64%	4.95975849	0.018249365	0.000114167	2.21E-05
ENSG00000198258_9828388_9830008_27	Overlap_dORF	UBL5	19	+	81	848	76	14	74.07%	3459.23835	0.000122208	6.05E-05	5.43E-08
ENSG00000168615_38997064_39090052_628	annotated	ADAM9	8	+	1884	973	105	23	24.20%	170.6481543	5.61E-21	9.31E-26	1.25E-44
ENSG00000168615_39090049_39103700_129	Overlap_dORF	ADAM9	8	+	387	123	10	1	16.28%	105.0180138	0.00010906	2.82E-05	2.35E-08
ENSG00000144746_69085048_69104636_188	annotated	ARL6IP5	3	+	564	420	58	10	22.87%	246.0593317	2.07E-05	9.94E-09	3.26E-12
ENSG00000056097_32444658_32406747_352	novel	ZFR	5	-	1056	29	17	3	2.56%	9.074103601	0.197850305	0.011213018	0.013382089
ENSG00000111358_123659558_123660235_89	novel	GTF2H3	12	+	267	16	0	0	5.62%	19.80060112	0.021084099	0.021084099	0.002029891

ENSG00000109819_23813988_23795822_300	dORF	PPARGC1A	4	-	900	30	6	0	4.00%	11.01408437	0.012038452	0.001003278	7.85E-05
ENSG00000109819_23889957_23824372_271	annotated	PPARGC1A	4	-	813	60	13	3	8.49%	24.38542665	0.001280572	2.93E-05	3.28E-07
ENSG00000123472_46658162_46635776_177	annotated	ATPAF1	1	-	531	63	5	0	11.30%	39.20267318	0.000282485	3.97E-05	8.55E-08
ENSG00000164120_174522451_174492084_143	annotated	HPGD	4	-	429	100	8	13	18.18%	77.02156902	2.29E-05	0.000364218	6.78E-08
ENSG00000119185_9408160_9406834_89	dORF	ITGB1BP1	2	-	267	43	1	0	16.85%	53.2141155	0.000392111	0.000306523	8.05E-07
ENSG00000103274_10743864_10769105_309	annotated	NUBP1	16	+	927	32	3	0	4.21%	11.40617152	0.000635664	0.000676852	2.75E-06
ENSG00000106009_2554431_2543532_286	novel	BRAT1	7	-	858	23	1	3	3.85%	8.857480438	0.002129196	0.009797758	0.00012054
ENSG00000106009_2538698_2538069_209	novel	BRAT1	7	-	627	18	0	0	3.83%	9.48581429	0.005454749	0.005454749	0.000159109
ENSG00000197122_37400168_37401807_110	novel	SRC	20	+	330	20	0	0	4.55%	20.02560795	0.020613417	0.020613417	0.001946209
ENSG00000075336_74148809_74158480_248	annotated	TIMM21	18	+	744	79	17	2	8.47%	35.08518812	0.000513198	2.73E-05	1.14E-07
ENSG00000144366_188483442_188594011_291	annotated	GULP1	2	+	873	66	5	2	8.59%	24.98039754	1.30E-05	1.60E-05	1.67E-09
ENSG00000138382_169824597_169810339_198	annotated	METTL5	2	-	594	28	2	7	8.08%	15.57547285	0.000736315	0.00290531	1.34E-05
ENSG00000086300_26365087_26372572_117	annotated	SNX10	7	+	351	19	7	1	6.84%	17.88611992	0.035859268	0.005454749	0.00105807
ENSG00000149532_61429235_61421330_110	novel	CPSF7	11	-	330	12	8	2	4.55%	12.01536477	0.360078543	0.020613417	0.044877087
ENSG00000213024_49909807_49908239_522	annotated	NUP62	19	-	1566	78	10	7	5.17%	16.45782722	4.18E-06	3.35E-05	1.19E-09
ENSG00000275052_55617285_55549911_817	annotated	PPP4R3B	2	-	2451	70	11	7	3.79%	9.436791994	3.67E-05	2.06E-05	5.89E-09
ENSG00000001629_92391255_92398949_358	dORF	ANKIB1	7	+	1074	8	1	0	1.40%	2.461247904	0.034182656	0.019216965	0.002956053
ENSG00000105976_116759449_116769866_160	annotated	MET	7	+	480	36	2	3	8.75%	24.78168983	0.000725838	0.002537074	1.15E-05
ENSG00000159403_7092388_7089810_144	annotated	C1R	12	-	432	11	1	4	3.47%	8.413536672	0.042234516	0.121807694	0.0204534
ENSG00000149554_125626769_125655320_476	annotated	CHEK1	11	+	1428	47	5	1	3.57%	10.87525137	0.001149898	0.000169511	1.37E-06
ENSG00000136243_23182086_23199465_224	annotated	NUPL2	7	+	672	26	11	6	4.46%	12.78420507	0.038607204	0.006083074	0.001253743
ENSG00000164062_49674402_49683342_737	annotated	APEH	3	+	2211	129	15	8	7.19%	19.27838377	6.29E-09	1.27E-09	8.75E-17
ENSG00000064933_189791810_189877436_932	annotated	PMS1	2	+	2796	16	10	2	0.97%	1.890829935	0.157414378	0.01833556	0.014328391
ENSG00000156928_23299395_23309543_220	annotated	MALSU1	7	+	660	156	12	12	15.45%	78.09987099	1.15E-06	1.12E-06	1.15E-11
ENSG00000175582_73760635_73716282_87	annotated	RAB6A	11	-	261	11	4	2	5.75%	13.9258538	0.171390856	0.070008252	0.043235098
ENSG00000175582_73716288_73677898_87	Overlap_dORF	RAB6A	11	-	261	27	6	0	8.05%	34.18164115	0.042228071	0.008877961	0.001887623
ENSG00000142166_33325059_33355549_556	annotated	IFNAR1	21	+	1668	31	5	1	2.88%	6.140946322	0.001882578	0.000261568	3.38E-06
ENSG00000170456_31500510_31387603_1293	annotated	DENND5B	12	-	3879	49	5	4	2.01%	4.173937619	3.42E-05	2.83E-05	7.46E-09
ENSG00000272398_106974646_106971480_37	annotated	CD24	6	-	111	95	18	6	18.92%	282.7940582	0.008576801	0.008877961	0.000387414
ENSG00000099783_8444999_8488854_730	annotated	HNRNPM	19	+	2190	224	46	11	9.73%	33.79664245	8.39E-09	4.06E-12	5.53E-19
ENSG00000061794_27710844_27716304_41	annotated	MRPS35	12	+	123	49	5	0	14.63%	131.63174	0.013640586	0.013853925	0.000911832
ENSG00000113391_94053195_93620987_370	annotated	FAM172A	5	-	1110	30	11	0	3.78%	8.930338679	0.035674012	0.000411814	0.000136226
ENSG00000006744_13017902_12992818_792	annotated	ELAC2	17	-	2376	89	6	2	4.55%	12.37693824	3.34E-07	2.06E-07	6.48E-13
ENSG00000106853_111597422_111563121_329	annotated	PTGR1	9	-	987	291	34	13	21.58%	97.41940887	2.21E-10	2.24E-12	6.68E-21
ENSG00000006715_38869163_38726246_779	annotated	VPS41	7	-	2337	51	5	4	3.72%	7.21076127	3.63E-06	1.13E-05	3.44E-10
ENSG00000168944_123422998_123390261_380	annotated	CEP120	5	-	1140	14	0	2	1.84%	4.057820558	0.007979402	0.037995728	0.001544499
ENSG00000168944_123386597_123358395_376	dORF	CEP120	5	-	1128	7	1	0	1.33%	2.050494431	0.041782825	0.019216965	0.003600526
ENSG00000084623_32222442_32231196_325	annotated	EIF3I	1	+	975	336	44	13	23.08%	113.8686876	1.43E-12	2.61E-13	4.89E-24
ENSG00000108107_55386358_55386752_87	annotated	RPL28	19	+	261	812	131	38	18.39%	1027.981208	0.003381744	0.00122721	2.49E-05
ENSG00000162144_61356632_61350399_215	annotated	CYB561A3	11	-	645	33	1	2	8.37%	16.9053388	0.000121504	0.000357258	3.06E-07
ENSG00000124787_5002188_4995078_303	annotated	RPP40	6	-	909	37	5	0	3.30%	13.44954197	0.026286608	0.00224099	0.000362009
ENSG00000175198_100155060_100307226_311	annotated	PCCA	13	+	933	34	5	0	4.82%	12.04112118	0.001261454	0.000279067	2.37E-06
ENSG00000088836_3234760_3227787_801	annotated	SLC4A11	20	-	2403	7	3	0	0.62%	0.962529221	0.169362074	0.019216965	0.016163992
ENSG00000105793_90377561_90385154_172	dORF	GTPBP10	7	+	516	26	3	0	4.65%	16.6491973	0.005960501	0.005257623	0.000167207
ENSG00000105793_90346742_90372186_116	annotated	GTPBP10	7	+	348	20	0	0	4.31%	18.98980064	0.021084099	0.021084099	0.002029891
ENSG00000103005_58002534_58012363_112	annotated	USB1	16	+	336	8	1	2	4.46%	7.867203122	0.042234516	0.080264768	0.01347413
ENSG00000116560_35193049_35184456_707	annotated	SFPQ	1	-	2121	289	51	17	10.89%	45.02221192	3.21E-10	7.38E-12	3.04E-20
ENSG00000171453_43517110_43519909_150	novel	POLR1C	6	+	450	24	5	5	4.67%	17.62253499	0.079360157	0.039474169	0.012582898
ENSG00000144867_133815678_133819766_105	novel	SRPRB	3	+	315	137	11	3	15.24%	143.707577	0.002898152	0.000515377	9.70E-06
ENSG00000114786_51984156_51985950_68	dORF	ABHD14A-ACY1	3	+	204	15	0	0	10.29%	24.29577435	0.008473674	0.008473674	0.000366502
ENSG00000114786_51975136_51984153_167	annotated	ABHD14A-ACY1	3	+	501	9	0	0	3.59%	5.935734092	0.009815329	0.009815329	0.000483714
ENSG00000114786_51987406_51987645_45	dORF	ABHD14A-ACY1	3	+	135	17	2	0	13.33%	41.60876318	0.022004978	0.011772051	0.001241747
ENSG00000114786_51988530_51989075_99	dORF	ABHD14A-ACY1	3	+	297	27	1	4	8.08%	30.03841192	0.005355751	0.025702836	0.000731269
ENSG00000153914_66144377_66153657_92	novel	SREK1	5	+	276	13	1	0	5.43%	15.56338009	0.021084099	0.021084099	0.002029891
ENSG00000139531_56002664_56005027_488	annotated	SUOX	12	+	1464	47	5	3	3.89%	10.60782716	5.30E-05	0.000296178	1.17E-07
ENSG00000105443_48469508_48479210_400	annotated	CYTH2	19	+	1200	47	11	2	5.00%	12.94154914	0.001147479	5.34E-05	4.90E-07
ENSG00000116353_29216597_29194022_285	annotated	MECR	1	-	855	31	6	0	4.21%	11.98023212	0.005529566	0.001040526	3.54E-05
ENSG00000164073_127965133_127921759_269	annotated	MFSD8	4	-	807	13	0	1	2.60%	5.322791703	0.008373824	0.013794764	0.000578344
ENSG00000014216_65210078_65211286_73	novel	CAPN1	11	+	219	43	1	0	12.33%	64.87748328	0.003418872	0.003789641	7.22E-05
ENSG00000132199_712587_683012_369	novel	ENOSF1	18	-	1107	28	8	3	2.98%	8.357570796	0.012097975	0.007260877	0.000446786
ENSG00000117335_207752213_207793591_370	annotated	CD46	1	+	1110	240	27	12	15.95%	71.44270943	2.55E-10	4.11E-10	1.19E-18
ENSG00000130024_169721057_169712525_188	novel	PHF10	6	-	564	24	0	0	3.19%	14.06053324	0.013428348	0.013428348	0.000872559
ENSG00000101224_3787132_3804961_489	annotated	CDC25B	20	+	1467	340	50	15	19.02%	76.58054573	2.70E-13	2.16E-16	9.97E-28
ENSG00000100124_37844238_37833728_165	annotated	ANKRD54	22	-	495	14	2	0	3.64%	9.345283708	0.044040756	0.013428348	0.002791708
ENSG00000108651_31887295_31863359_410	novel	UTP6	17	-	1230	41	6	0	4.39%	11.01408437	0.003036458	7.83E-05	1.98E-06
ENSG00000169964_44864233_44865180_83	novel	TMEM42	3	+	249	10	7	0	7.23%	13.26998117	0.196884317	0.012798403	0.01457399
ENSG00000166441_8682814_8685806_113	annotated	RPL27A	11	+	339	128	11	1	6.19%	124.7613097	0.039844013	0.012176559	0.002318225
ENSG00000178252_49007432_49015288_569	annotated	WDR6	3	+	1707	67	12	7	2.99%	12.96913274	0.008325728	0.001758679	8.61E-05

ENSG00000140400_75368583_75355906_1057	annotated	MAN2C1	15	-	3171	36	4	3	1.99%	3.751249171	0.000315783	0.000160798	3.52E-07
ENSG00000188846_40458699_40461961_70	annotated	RPL14	3	+	210	68	3	2	7.14%	106.9939625	0.021557223	0.021557223	0.002115616
ENSG00000134077_9365069_9384688_507	annotated	THUMPD3	3	+	1521	86	11	6	6.11%	18.68266777	2.60E-05	5.52E-06	1.19E-09
ENSG00000113558_134174494_134157733_174	annotated	SKP1	5	-	522	270	26	7	18.39%	170.9082057	7.73E-07	8.78E-07	6.11E-12
ENSG00000125952_65102339_65077803_125	annotated	MAX	14	-	375	18	14	3	6.40%	15.86028149	0.322636366	0.019759574	0.037435152
ENSG00000114446_108222322_108162477_429	annotated	IFT57	3	-	1287	46	9	0	3.73%	11.80997392	0.004792715	0.000202851	7.38E-06
ENSG00000185753_40637117_40630615_149	annotated	CXorf38	X	-	447	11	1	0	4.03%	8.131203227	0.023672501	0.012798403	0.001437523
ENSG00000155975_17247245_17286427_372	annotated	VPS37A	8	+	1116	29	3	0	2.69%	8.586248568	0.014769965	0.002046029	0.000179244
ENSG00000028116_58048901_58154762_373	annotated	VRK2	2	+	1119	62	8	0	5.63%	18.30759332	0.000522303	2.51E-05	1.08E-07
ENSG00000113141_140647909_140662329_557	annotated	IK	5	+	1671	85	10	2	5.39%	16.80784868	2.10E-05	1.77E-06	3.31E-10
ENSG00000131143_85806797_85806874_25	novel	COX4I1	16	+	75	324	43	2	72.00%	1427.425334	0.000337736	9.72E-05	2.35E-07
ENSG00000131143_85804822_85805115_97	novel	COX4I1	16	+	291	204	18	9	14.43%	231.6364136	0.000575979	0.000574642	2.14E-06
ENSG00000131143_85805594_85805872_92	novel	COX4I1	16	+	276	326	30	1	20.65%	390.2816853	9.29E-05	6.54E-05	4.46E-08
ENSG00000163539_33717987_33498631_1508	annotated	CLASP2	3	-	4524	27	3	0	0.93%	1.972017759	0.002970463	0.000375189	7.50E-06
ENSG00000196792_30955638_30895411_566	annotated	STRN3	14	-	1698	41	3	0	3.89%	7.978400339	0.000113475	1.26E-05	1.16E-08
ENSG00000187239_130042975_129895818_592	annotated	FNBP1	9	-	1776	20	2	2	1.35%	3.72097445	0.018151375	0.018151375	0.001534781
ENSG00000139436_109996224_109932978_681	annotated	GIT2	12	-	2043	40	13	0	2.50%	6.469359395	0.036485133	0.000118541	5.51E-05
ENSG00000113649_146482710_146510070_364	novel	TCERG1	5	+	1092	10	0	5	1.92%	3.025847355	0.007029303	0.201322915	0.009948948
ENSG00000067064_1044140_1043970_56	novel	IDI1	10	-	168	50	10	9	25.00%	98.34003902	0.009687038	0.001785915	0.000102131
ENSG00000083312_72848394_72872694_128	novel	TNPO1	5	+	384	46	2	4	7.81%	39.58186571	0.003974283	0.026319981	0.000582913
ENSG00000115806_170929341_170966130_452	annotated	GORASP2	2	+	1356	80	13	1	7.52%	19.49395464	9.15E-06	1.37E-07	1.32E-11
ENSG00000156113_77637360_76877869_1090	annotated	KCNMA1	10	-	3270	71	13	0	3.21%	7.174311838	1.40E-05	8.57E-08	1.38E-11
ENSG00000003989_17543340_17565146_657	annotated	SLC7A2	8	+	1971	164	20	4	8.07%	27.49330041	4.30E-08	2.23E-10	1.24E-16
ENSG00000126226_113197161_113178198_359	annotated	PCID2	13	-	1077	52	4	1	5.57%	15.95354839	0.000105937	4.61E-05	3.64E-08
ENSG00000108349_40140549_40169621_703	annotated	CASC3	17	+	2109	41	11	3	2.42%	6.423576944	0.003393477	0.000438539	9.91E-06
ENSG00000073584_40642556_40630222_345	annotated	SMARCE1	17	-	1035	57	3	0	3.77%	18.19718287	0.001529613	0.000649205	6.25E-06
ENSG00000033627_42460922_42521120_829	annotated	ATP6V0A1	17	+	2487	53	8	7	2.41%	7.041573844	0.001035919	0.001083204	6.93E-06
ENSG00000035403_74070710_74075008_202	novel	VCL	10	+	606	23	0	0	7.92%	12.54078913	0.000141591	0.000141591	1.42E-07
ENSG00000035403_74082466_74118169_801	novel	VCL	10	+	2403	144	14	4	6.37%	19.80060112	1.34E-08	6.06E-10	9.35E-17
ENSG00000137509_82824995_82824906_29	novel	PRCP	11	-	87	10	1	0	17.24%	37.97960128	0.051235217	0.020613417	0.004687384
ENSG00000137073_33926640_33922504_290	novel	UBAP2	9	-	870	9	0	0	1.72%	3.418164115	0.016947427	0.016947427	0.001349898
ENSG00000148429_11573678_11462441_851	annotated	USP6NL	10	-	2553	12	0	1	0.71%	1.553102379	0.012178958	0.023056749	0.001340064
ENSG00000145014_194633215_194588621_396	annotated	TMEM44	3	-	1188	13	0	7	1.26%	3.615734768	0.021084099	0.083503598	0.007891846
ENSG00000107249_4117806_3828272_373	annotated	GLIS3	9	-	1119	11	2	1	1.61%	3.248121396	0.043020198	0.014014728	0.002826223
ENSG00000137073_34017145_33922504_1118	annotated	UBAP2	9	-	3354	38	7	10	2.06%	3.743606494	0.001150476	0.003592539	2.49E-05
ENSG00000184047_122224668_122208381_186	Overlap_dORF	DIABLO	12	-	558	57	4	0	6.99%	33.7528392	0.00114312	0.000710064	5.10E-06
ENSG00000132676_155709783_155738242_397	annotated	DAP3	1	+	1191	147	14	1	11.34%	40.78262979	1.48E-07	2.35E-09	4.04E-15
ENSG00000204713_28923299_28904070_402	annotated	TRIM27	6	-	1206	55	1	0	6.47%	15.0690209	4.43E-06	2.73E-06	1.03E-10
ENSG00000171603_9744584_9730508_633	Overlap_dORF	CLSTN1	1	-	1899	219	30	4	10.74%	38.10559995	3.78E-10	5.70E-13	3.28E-21
ENSG00000082701_120093359_119826788_445	annotated	GSK3B	3	-	1335	87	10	1	6.97%	21.53315371	1.45E-05	6.05E-07	8.37E-11
ENSG00000167600_41193265_41203462_165	annotated	CYP2S1	19	+	495	52	16	0	9.09%	34.71105377	0.043651554	0.000262451	0.000125589
ENSG00000167600_41201276_41206488_211	Overlap_dORF	CYP2S1	19	+	633	29	1	2	7.58%	15.13784108	0.000263041	0.000813801	1.44E-06
ENSG00000104067_29800693_29701595_1676	annotated	TJP1	15	-	5028	61	7	6	1.91%	4.008706125	4.41E-06	1.11E-05	4.08E-10
ENSG00000135250_105132793_105117838_116	novel	SRPK2	7	-	348	13	0	1	6.90%	12.34337042	0.004826937	0.010020958	0.000255995
ENSG00000101928_134899433_134889161_159	annotated	MOSPD1	X	-	477	14	0	0	3.14%	9.697935924	0.021084099	0.021084099	0.002029891
ENSG00000162585_2194749_2189709_180	annotated	FAAP20	1	-	540	34	10	0	6.11%	20.80438159	0.056742382	0.001580611	0.000672369
ENSG00000136156_48233361_48261224_160	annotated	ITM2B	13	+	480	634	49	17	28.12%	436.4330932	1.43E-08	3.95E-09	5.99E-16
ENSG00000160113_17245214_17232352_402	annotated	NR2F6	19	-	1206	44	0	1	4.23%	12.05521672	0.000114167	0.000286023	2.31E-07
ENSG00000074054_121605883_121340861_1474	annotated	CLASP1	2	-	4422	41	0	2	1.02%	3.063619126	0.000299491	0.000387696	7.79E-07
ENSG00000099364_30927404_30947230_583	annotated	FBXL19	16	+	1749	17	1	0	1.37%	3.211654105	0.010079165	0.004873904	0.000259739
ENSG00000171310_104457412_104757803_347	annotated	CHST11	12	+	1041	21	1	2	2.88%	6.665584201	0.002281203	0.00666989	8.65E-05
ENSG00000181929_49013076_49002899_280	annotated	PRKAG1	12	-	840	155	14	3	9.29%	60.97082419	4.03E-05	1.08E-05	3.49E-09
ENSG00000144401_207624375_207613046_218	annotated	METTL21A	2	-	654	31	4	2	3.67%	15.66223007	0.039465372	0.016228926	0.002937831
ENSG00000176261_32650529_32621862_167	annotated	ZBTB8OS	1	-	501	10	0	0	3.59%	6.595260102	0.011772051	0.011772051	0.000681273
ENSG00000222041_87521255_87521413_52	novel	CYTOR	2	+	156	31	3	20	11.54%	65.66088759	0.025538366	0.220433518	0.027149935
ENSG00000204427_31691887_31687212_339	dORF	ABHD16A	6	-	1017	43	11	0	5.31%	13.97066749	0.011487324	7.75E-05	9.23E-06
ENSG00000123130_23730970_23707915_136	annotated	ACOT9	X	-	408	45	8	5	8.82%	36.44366152	0.009965666	0.004036058	0.000216554
ENSG00000171557_154606966_154604340_148	novel	FGG	4	-	444	24	3	0	7.43%	17.86067736	0.007790523	0.001506153	7.01E-05
ENSG00000149743_64225860_64223876_255	annotated	TRPT1	11	-	765	35	4	5	3.92%	15.1173707	0.01421337	0.011657423	0.000806901
ENSG00000149743_64225508_64223876_204	annotated	TRPT1	11	-	612	16	4	2	3.43%	8.638497546	0.066604992	0.032488891	0.008974512
ENSG00000173486_64242388_64244029_142	annotated	FKBP2	11	+	426	240	31	9	27.46%	186.1535387	1.24E-07	7.53E-08	9.04E-14
ENSG00000117410_43976115_43977411_214	novel	ATP6V0B	1	+	642	170	18	3	14.95%	87.49506276	4.36E-06	8.03E-07	3.14E-11
ENSG00000165568_4826325_4847530_320	annotated	AKR1E2	10	+	960	25	1	2	3.44%	8.604753414	0.001671554	0.00379998	3.71E-05
ENSG00000076242_36993650_37050653_653	annotated	MLH1	3	+	1959	38	5	1	2.91%	6.409421226	0.000980065	7.99E-05	5.90E-07
ENSG00000165060_69046445_69072762_135	annotated	FXN	9	+	405	62	11	0	15.56%	50.58320229	0.000401923	2.62E-05	8.50E-08
ENSG00000076043_114439529_114446035_95	novel	REXO2	11	+	285	65	23	5	20.00%	75.35952464	0.003185595	0.000290209	6.45E-06
ENSG00000175567_73981622_73981512_36	novel	UCP2	11	-	108	69	2	3	41.67%	211.1032838	0.000297172	0.000282029	5.67E-07
ENSG00000275066_37586480_37518940_1005	annotated	SYNRG	17	-	3015	34	7	2	1.79%	3.726157896	0.002651267	0.00030161	5.49E-06

ENSG00000101246_63707058_63700683_110	annotated	ARFRP1	20	-	330	30	5	3	7.27%	30.03841192	0.013192068	0.018738288	0.001177804
ENSG00000160695_119069198_119081623_878	annotated	VPS11	11	+	2634	19	0	2	1.14%	2.383457893	0.002227676	0.009621867	0.000123102
ENSG00000197747_151986230_151984118_65	novel	S100A10	1	-	195	324	20	6	33.85%	549.009744	1.97E-05	3.24E-05	4.97E-09
ENSG00000168827_158644635_158682169_591	annotated	GFM1	3	+	1773	81	30	4	4.74%	15.09544558	0.002297235	6.73E-06	1.87E-07
ENSG00000157181_186398999_186411815_78	novel	ODR4	1	+	234	18	0	5	6.41%	25.41711778	0.021084099	0.133791954	0.013186697
ENSG00000163430_120450746_120396952_308	annotated	FSTL1	3	-	924	284	29	6	19.48%	101.5584403	5.07E-10	2.52E-11	1.57E-19
ENSG00000133858_71663610_71611782_1910	annotated	ZFC3H1	12	-	5730	21	0	3	0.68%	1.210972627	0.000470745	0.006674189	2.17E-05
ENSG00000215193_18078377_18088075_256	annotated	PEX26	22	+	768	16	0	0	3.52%	6.883802732	0.002943991	0.002943991	4.92E-05
ENSG00000101132_54215423_54219150_49	novel	PFDN4	20	+	147	44	19	7	14.29%	98.9019821	0.039659082	0.02520501	0.004343134
ENSG00000144827_111979062_111991721_306	annotated	ABHD10	3	+	918	72	2	5	8.82%	25.91549264	3.01E-06	7.63E-06	1.95E-10
ENSG00000198668_90397231_90401545_106	novel	CALM1	14	+	318	174	19	3	22.64%	180.797234	7.72E-05	9.81E-06	6.19E-09
ENSG00000198805_20475084_20476601_128	novel	PNP	14	+	384	132	16	6	14.06%	113.5827451	0.004105948	0.00031277	9.03E-06
ENSG00000179832_144223183_144261740_1211	annotated	MROH1	8	+	3633	16	1	4	0.58%	1.455205202	0.011306644	0.051117422	0.002823276
ENSG00000151718_183245540_183315729_950	dORF	WWC2	4	+	2850	18	0	3	1.37%	2.086879144	0.000470745	0.005746658	1.85E-05
ENSG00000151718_183208006_183245421_77	annotated	WWC2	4	+	231	12	0	0	7.79%	17.16480681	0.013217227	0.013217227	0.00084697
ENSG00000205189_80486826_80519528_842	annotated	ZBTB10	8	+	2526	17	1	2	0.59%	2.223746251	0.035374934	0.056923149	0.008291339
ENSG00000114439_107728954_107805457_743	annotated	BBX	3	+	2229	20	6	0	1.48%	2.964760261	0.040251748	0.001262414	0.000373703
ENSG00000214046_16660110_16647246_75	annotated	SMIM7	19	-	225	194	19	3	33.33%	284.8976491	0.00010461	9.05E-06	7.88E-09
ENSG00000073849_187042869_187075803_351	annotated	ST6GAL1	3	+	1053	17	0	0	1.99%	5.334456818	0.008473674	0.008473674	0.000366502
ENSG00000185722_4242419_4167779_1156	annotated	ANKFY1	17	-	3468	83	10	0	2.85%	7.908036356	1.66E-05	2.11E-07	3.71E-11
ENSG00000177885_75393628_75320368_217	annotated	GRB2	17	-	651	98	16	4	11.52%	49.74102619	0.000212837	1.69E-05	2.93E-08
ENSG00000214575_82556032_82544592_329	annotated	CPEB1	15	-	987	8	0	0	1.52%	2.678196807	0.019679754	0.019679754	0.001784964
ENSG00000022556_54994156_55000898_197	novel	NLRP2	19	+	591	18	1	2	6.09%	10.06363039	0.001620026	0.005878501	5.60E-05
ENSG00000163930_52409878_52402288_711	annotated	BAP1	3	-	2133	13	1	4	1.41%	2.013826959	0.004004658	0.044982488	0.00105613
ENSG00000232119_120605525_120612264_138	annotated	MCTS1	X	+	414	36	4	3	6.52%	28.73239401	0.007609621	0.01778219	0.000681245
ENSG00000031691_49470177_49492275_268	annotated	CENPQ	6	+	804	49	0	0	5.60%	20.13769157	0.000311274	0.000311274	6.53E-07
ENSG00000140455_63529142_63590826_498	annotated	USP3	15	+	1494	48	1	10	3.82%	10.61598494	7.28E-05	0.002100078	1.24E-06
ENSG00000187109_76067450_76048429_322	annotated	NAP1L1	12	-	966	168	19	13	9.94%	57.46478802	5.80E-06	2.10E-06	1.05E-10
ENSG00000158528_94910546_95290303_1152	annotated	PPP1R9A	7	+	3456	14	2	1	0.61%	1.338517198	0.028902806	0.01551626	0.002075277
ENSG00000138434_181892067_181928265_1259	annotated	ITPRID2	2	+	3777	72	6	10	1.99%	6.298761515	4.59E-05	0.0002111	7.25E-08
ENSG00000166226_69585522_69587648_95	novel	CCT2	12	+	285	83	17	1	12.63%	96.22831608	0.004462641	0.001082917	2.94E-05
ENSG00000134575_47248502_47240116_391	annotated	ACP2	11	-	1173	80	6	2	8.18%	22.53521099	1.60E-06	5.92E-07	8.54E-12
ENSG00000171163_248855682_248850210_241	novel	ZNF692	1	-	723	9	1	0	2.07%	4.113143541	0.041782825	0.019216965	0.003600526
ENSG00000114745_39107541_39098236_285	annotated	GORASP1	3	-	855	19	9	1	3.51%	7.342722914	0.064560935	0.003974283	0.001589267
ENSG00000013375_83188759_83169234_420	annotated	PGM3	6	-	1260	13	4	1	2.14%	3.409121353	0.046217795	0.005849704	0.001476854
ENSG00000163082_222521773_222558898_271	annotated	SGPP2	2	+	813	17	0	0	2.95%	6.909204218	0.004826937	0.004826937	0.000126125
ENSG00000101266_489830_483961_167	annotated	CSNK2A1	20	-	501	47	1	0	8.38%	30.99772248	0.000475338	0.000472567	1.47E-06
ENSG00000135932_230790905_230818704_292	annotated	CAB39	2	+	876	21	3	1	4.11%	7.921088075	0.003725191	0.001410049	3.11E-05
ENSG00000141127_18882588_18930698_225	annotated	PRPSAP2	17	+	675	32	3	2	7.11%	15.66447555	0.001349473	0.000280249	2.55E-06
ENSG00000170291_7252503_7258788_279	annotated	ELP5	17	+	837	76	21	7	9.32%	30.00252373	0.000906985	4.66E-05	3.37E-07
ENSG00000080503_2039681_2182174_1238	annotated	SMARCA2	9	+	3714	41	17	1	1.94%	3.647636989	0.003281882	9.72E-06	3.86E-07
ENSG00000137185_28227157_28233178_421	annotated	ZSCAN9	6	+	1263	12	0	0	1.19%	3.139406471	0.020613417	0.020613417	0.001946209
ENSG00000105357_50260713_50309790_1229	annotated	MYH14	19	+	3687	8	1	0	0.49%	0.716946094	0.028889786	0.009815329	0.001387795
ENSG00000121644_244653353_244705789_148	annotated	DESI2	1	+	444	10	5	0	3.38%	7.441948899	0.124106539	0.012673659	0.00825064
ENSG00000196230_30720507_30724397_444	annotated	TUBB	6	+	1332	740	93	40	13.29%	183.5680728	2.11E-10	9.64E-10	2.32E-18
ENSG00000275835_23031961_22999820_866	annotated	TUBGCP5	15	-	2598	24	8	4	1.04%	3.052402135	0.077766915	0.022856675	0.007822532
ENSG00000183077_78187422_78206937_291	annotated	AFMID	17	+	873	25	11	2	2.75%	9.462271796	0.095896152	0.005642788	0.003317787
ENSG00000171530_77776257_77693122_129	annotated	TBCA	5	-	387	299	38	13	11.63%	255.287692	0.000399699	0.000323375	8.63E-07
ENSG00000065665_12129788_12164454_476	annotated	SEC61A2	10	+	1428	57	15	1	3.99%	13.18913465	0.044883311	7.98E-05	5.45E-05
ENSG00000106348_128405864_128393007_513	annotated	IMPDH1	7	-	1539	24	5	7	2.53%	5.15278801	0.007819181	0.025321803	0.000996466
ENSG00000071282_8532737_8537512_138	novel	LMCD1	3	+	414	16	3	0	5.07%	12.76995289	0.027197958	0.008175611	0.001114136
ENSG00000184009_81512354_81511434_198	annotated	ACTG1	17	-	594	766	363	372	22.22%	426.1004357	0.006868219	0.045437505	0.001651974
ENSG00000127955_80134498_80217493_422	annotated	GNAI1	7	+	1266	137	11	5	9.00%	35.75662462	3.33E-08	8.64E-08	2.86E-14
ENSG00000147099_72572761_72462089_307	annotated	HDAC8	X	-	921	13	1	0	1.95%	4.663944522	0.023672501	0.012798403	0.001437523
ENSG00000255639_4649127_4772597_567	annotated	AC005833.1	12	+	1701	208	21	3	6.17%	40.40440122	7.17E-07	2.26E-07	1.52E-12
ENSG00000075239_108121607_108147390_427	annotated	ACAT1	11	+	1281	182	20	6	11.24%	46.94527764	1.23E-07	6.88E-09	9.11E-15
ENSG00000138134_88905593_88923224_376	annotated	STAMBPL1	10	+	1128	27	2	0	2.39%	7.909049947	0.00567009	0.003554776	0.000110499
ENSG00000153922_98901322_98856380_1560	annotated	CHD1	5	-	4680	29	4	1	0.90%	2.047490043	0.004727741	0.000678648	2.06E-05
ENSG00000162407_56512077_56496551_75	novel	PLPP3	1	-	225	24	0	0	8.00%	35.24506999	0.013853925	0.013853925	0.000925205
ENSG00000169604_69013500_69145376_333	annotated	ANTXR1	2	+	999	524	73	14	19.82%	173.314721	6.24E-08	6.37E-12	7.57E-18
ENSG00000135469_56267396_56270317_215	annotated	COQ10A	12	+	645	21	4	0	5.58%	10.75794287	0.01616783	0.00092354	0.000101542
ENSG00000173918_79043969_79048088_281	annotated	C1QTNF1	17	+	843	33	5	6	4.27%	12.93468983	0.003908676	0.010294619	0.000217355
ENSG00000005469_87349069_87398644_640	annotated	CROT	7	+	1920	18	3	1	1.25%	3.097711229	0.010678933	0.005622352	0.000313115
ENSG00000087087_100875591_100888549_872	annotated	SRRT	7	+	2616	128	14	13	4.36%	16.1674633	8.39E-06	3.02E-06	2.15E-10
ENSG00000143850_204248949_204223470_483	annotated	PLEKHA6	1	-	1449	13	2	0	1.86%	2.96445335	0.020607861	0.00308495	0.0003625
ENSG00000100031_24627444_24628839_252	annotated	GGT1	22	+	756	12	1	0	1.98%	5.244802081	0.035374934	0.020613417	0.003252526
ENSG00000145191_184135386_184144943_729	annotated	EIF2B5	3	+	2187	51	14	5	2.74%	7.705326514	0.018006709	0.000147131	2.64E-05
ENSG00000196139_5077963_5107503_300	annotated	AKR1C3	10	+	900	2873	343	84	32.00%	1054.782147	2.63E-15	1.39E-17	5.94E-31

ENSG00000112715_43777480_43784546_131	annotated	VEGFA	6	+	393	39	1	0	6.87%	32.79002217	0.003961388	0.003789641	8.30E-05
ENSG00000100911_24146581_24145641_70	novel	PSME2	14	-	210	13	3	0	7.14%	20.45472812	0.035374934	0.021084099	0.003318418
ENSG00000125733_6739762_6751211_545	annotated	TRIP10	19	+	1635	78	13	2	6.06%	15.76327671	0.000100144	2.69E-07	3.34E-10
ENSG00000160803_156050449_156036978_407	annotated	UBQLN4	1	-	1221	15	0	0	1.97%	4.059244854	0.005160369	0.005160369	0.000143196
ENSG00000013810_1723578_1745013_786	annotated	TACC3	4	+	2358	110	22	2	6.23%	15.41411299	4.33E-06	9.56E-10	7.26E-14
ENSG00000116586_156054890_156058371_125	annotated	LAMTOR2	1	+	375	168	13	13	20.80%	148.0292939	3.90E-06	2.20E-05	7.24E-10
ENSG00000116586_156054890_156058371_95	annotated	LAMTOR2	1	+	285	115	9	13	20.00%	133.3283897	6.34E-05	0.000406626	1.91E-07
ENSG00000116586_156054890_156058409_150	annotated	LAMTOR2	1	+	450	166	15	13	16.67%	121.8892004	1.11E-05	3.10E-05	2.76E-09
ENSG00000160789_156114919_156137764_572	annotated	LMNA	1	+	1716	540	61	24	18.01%	103.9791182	4.96E-14	1.52E-17	1.39E-29
ENSG00000160789_156114919_156139106_664	annotated	LMNA	1	+	1992	544	61	30	15.81%	90.23587195	2.81E-14	4.10E-17	1.95E-29
ENSG00000164032_99950170_99949356_34	novel	H2AFZ	4	-	102	326	268	34	26.47%	1056.056325	0.106045149	0.006229071	0.004032457
ENSG00000259316_64381371_64366005_65	annotated	AC087632.1	15	-	195	212	82	168	15.38%	359.2285979	0.015298565	0.181583589	0.014929221
ENSG00000198498_163494802_163517480_144	annotated	TMA16	4	+	432	17	1	1	4.17%	13.00273849	0.020342454	0.020342454	0.001898761
ENSG00000103550_19714855_19706910_398	annotated	KNOP1	16	-	1194	20	3	0	2.26%	5.534715764	0.022360378	0.003478238	0.000437704
ENSG00000106397_101210612_101206281_216	annotated	PLOD3	7	-	648	43	3	1	7.87%	21.92618648	0.000591655	0.000121775	5.11E-07
ENSG00000114686_131469731_131462723_88	novel	MRPL3	3	-	264	12	1	1	9.09%	15.01920596	0.010499663	0.010499663	0.000549256
ENSG00000145439_169006800_168894674_90	dORF	CBR4	4	-	270	22	0	0	11.11%	26.92331735	0.00224099	0.00224099	2.92E-05
ENSG00000150756_10249873_10227441_216	annotated	ATPSCKMT	5	-	648	23	2	2	6.48%	11.72796021	0.001444156	0.000995993	8.83E-06
ENSG00000155506_154803320_154804837_156	annotated	LARP1	5	+	468	8	2	5	3.85%	5.648248395	0.053359408	0.218864129	0.045572689
ENSG00000147536_41530203_41542087_223	annotated	GINS4	8	+	669	47	0	0	5.83%	23.21354105	0.000712477	0.000712477	3.23E-06
ENSG00000104365_42316253_42330979_475	dORF	IKBKB	8	+	1425	21	3	6	3.37%	4.869384669	0.00129704	0.003895439	3.02E-05
ENSG00000054118_36282564_36304017_955	annotated	THRAP3	1	+	2865	65	9	5	2.93%	7.496497216	2.81E-05	1.74E-05	3.84E-09
ENSG00000171853_3443799_3467471_126	novel	TRAPPC12	2	+	378	31	0	0	8.73%	27.09814409	0.001491519	0.001491519	1.34E-05
ENSG00000143321_156751429_156744511_120	novel	HDGF	1	-	360	136	7	7	20.83%	124.8262895	3.51E-05	6.12E-06	1.78E-09
ENSG00000108219_80489234_80509501_159	novel	TSPAN14	10	+	477	268	46	59	23.27%	185.646202	7.04E-05	0.005355874	3.46E-06
ENSG00000183077_78187422_78206937_286	annotated	AFMID	17	+	858	25	11	2	2.80%	9.627696128	0.095896152	0.005642788	0.003317787
ENSG00000167272_120581362_120579894_103	novel	POP5	12	-	309	65	3	1	11.65%	69.50635768	0.001010108	0.001057356	6.61E-06
ENSG00000111077_53050186_53063882_1344	annotated	TNS2	12	+	4032	30	5	4	1.04%	2.458500976	0.003987731	0.003311621	7.35E-05
ENSG00000105926_24623668_24687614_540	annotated	MPP6	7	+	1620	15	0	2	1.67%	3.059467881	0.002874854	0.014775609	0.000240126
ENSG00000147854_6413491_6492317_503	annotated	UHRF2	9	+	1509	22	8	7	2.19%	4.817293363	0.063850092	0.020090055	0.00573578
ENSG00000147854_6493925_6506179_270	dORF	UHRF2	9	+	810	7	0	1	2.22%	2.855503355	0.009815329	0.028889786	0.001387795
ENSG00000157870_2586886_2589427_198	annotated	PRXL2B	1	+	594	22	5	4	4.04%	12.23787152	0.039807901	0.03638408	0.006066533
ENSG00000116288_7962786_7985054_189	annotated	PARK7	1	+	567	598	52	12	33.86%	348.4879605	4.67E-11	1.50E-12	9.16E-22
ENSG00000152422_83104920_83353242_336	annotated	XRCC4	5	+	1008	42	4	0	5.36%	13.76760546	0.001368483	7.61E-05	8.07E-07
ENSG00000100813_23069595_23059148_568	annotated	ACIN1	14	-	1704	57	10	7	4.23%	11.05286636	0.000135942	0.000101314	9.87E-08
ENSG00000162624_75137178_75160895_295	annotated	LHX8	1	+	885	10	6	1	2.03%	3.733587922	0.23864467	0.023672501	0.028399548
ENSG00000160325_133465323_133468653_107	annotated	CACFD1	9	+	321	14	0	0	4.67%	14.41095151	0.020613417	0.020613417	0.001946209
ENSG00000156976_186783611_186785965_96	annotated	EIF4A2	3	+	288	33	1	0	5.21%	37.86091502	0.047790352	0.016947427	0.003697512
ENSG00000156976_186785916_186787487_178	Overlap_dORF	EIF4A2	3	+	534	24	3	0	6.74%	14.85045084	0.000939154	0.000978918	5.72E-06
ENSG00000130921_123253675_123257031_166	annotated	C12orf65	12	+	498	25	2	0	5.42%	16.58747646	0.010529248	0.003684287	0.000210871
ENSG00000161204_184186208_184193698_709	annotated	ABCF3	3	+	2127	66	6	4	3.24%	10.25288531	0.000103632	5.03E-05	3.86E-08
ENSG00000071553_154428759_154434218_122	annotated	ATP6AP1	X	+	366	82	12	3	10.66%	74.02909167	0.007860495	0.003257575	0.000140784
ENSG00000089157_120199407_120196773_273	novel	RPLP0	12	-	819	75	19	4	4.03%	30.25847355	0.009165995	0.002322952	0.000121513
ENSG00000135476_53268767_53293474_2120	annotated	ESPL1	12	+	6360	33	11	2	1.04%	1.714456529	0.007550115	8.36E-05	5.94E-06
ENSG00000135454_57629168_57628701_155	novel	B4GALNT1	12	-	465	15	6	2	3.87%	10.65879133	0.027849813	0.035350572	0.004256925
ENSG00000090989_55870819_55904495_631	annotated	EXOC1	4	+	1893	17	0	0	1.11%	2.967344442	0.008473674	0.008473674	0.000366502
ENSG00000157911_2412502_2405766_326	annotated	PEX10	1	-	978	8	0	0	1.53%	2.70284279	0.019679754	0.019679754	0.001784964
ENSG00000115677_241262839_241229601_1092	annotated	HDLBP	2	-	3276	129	23	28	5.13%	13.01114362	5.76E-08	5.99E-07	3.44E-13
ENSG00000100852_32091060_32154948_1371	annotated	ARHGAP5	14	+	4113	36	6	4	1.31%	2.892100929	0.001254322	0.001097093	8.44E-06
ENSG00000162972_199956126_200008480_123	annotated	MAIP1	2	+	369	21	3	0	4.88%	18.80453429	0.044329616	0.01300724	0.002735782
ENSG00000166965_90956735_90961769_376	annotated	RCCD1	15	+	1128	53	7	1	6.91%	15.52517212	4.98E-05	4.46E-06	1.90E-09
ENSG00000172057_39924155_39922550_121	annotated	ORMDL3	17	-	363	21	10	0	9.09%	19.11535304	0.063898042	0.001484235	0.000742443
ENSG00000146112_30686018_30677269_613	annotated	PPP1R18	6	-	1839	36	8	7	2.28%	6.468304035	0.00697285	0.005048657	0.000187164
ENSG00000101546_80034496_80045937_242	annotated	RBFA	18	+	726	19	4	4	3.72%	8.647421613	0.01505303	0.023182913	0.00163004
ENSG00000140284_50182428_50236096_567	annotated	SLC27A2	15	+	1701	58	14	2	4.76%	11.26661188	0.000528945	7.20E-06	3.67E-08
ENSG00000150527_39276958_39350264_775	annotated	MIA2	14	+	2325	51	7	0	2.71%	7.247978102	0.000171144	2.42E-05	3.23E-08
ENSG00000170638_50193061_50198519_376	annotated	TRABD	22	+	1128	45	9	5	5.05%	13.18174991	0.000728767	0.000886929	4.08E-06
ENSG00000124225_57659647_57652053_234	annotated	PMEPA1	20	-	702	215	41	18	16.24%	101.1977837	1.79E-05	2.18E-06	3.41E-10
ENSG00000123143_14440291_14471784_948	annotated	PKN1	19	+	2844	118	13	3	5.38%	13.7095143	1.97E-08	1.16E-09	2.56E-16
ENSG00000163870_127580146_127573597_293	annotated	TPRA1	3	-	879	42	39	10	5.80%	15.78810729	0.326767785	0.013381335	0.029809296
ENSG00000163870_127575986_127573521_131	Overlap_dORF	TPRA1	3	-	393	27	7	0	5.34%	22.70078458	0.019795882	0.008473674	0.000834197
ENSG00000281649_37086727_37086870_47	novel	EBLN3P	9	+	141	35	7	2	12.77%	82.01977723	0.013853925	0.024414995	0.001590539
ENSG00000149782_64251650_64267556_1234	annotated	PLCB3	11	+	3702	44	16	2	2.11%	3.927226194	0.010293993	6.61E-06	1.19E-06
ENSG00000137100_34613868_34613782_28	annotated	DCTN3	9	-	84	13	0	0	21.43%	51.13682029	0.012798403	0.012798403	0.000797244
ENSG00000115234_27370744_27376719_445	annotated	SNX17	2	+	1335	213	1	6	12.13%	52.71910047	6.51E-11	6.25E-10	4.82E-19
ENSG00000122966_119784039_119688232_1583	novel	CIT	12	-	4749	70	13	8	2.15%	4.870410019	5.91E-05	7.65E-06	3.74E-09
ENSG00000108826_50373170_50368092_148	annotated	MRPL27	17	-	444	154	11	8	22.97%	114.606013	4.42E-06	1.95E-06	7.44E-11
ENSG00000142444_10928823_10929702_260	annotated	TIMM29	19	+	780	16	6	6	3.46%	6.777898074	0.042056994	0.035350572	0.006220118

ENSG00000149273_75404048_75404865_117	annotated	RPS3	11	+	351	365	54	19	19.66%	343.6017774	1.68E-05	6.19E-05	8.15E-09
ENSG00000150779_112085450_112085295_51	dORF	TIMM8B	11	-	153	104	6	0	25.49%	224.6009362	0.000731334	0.000710064	3.30E-06
ENSG00000150779_112086768_112086296_47	annotated	TIMM8B	11	-	141	49	9	1	14.89%	114.8276881	0.008776163	0.008877961	0.000395901
ENSG00000131844_71587426_71641095_363	annotated	MCCC2	5	+	1089	61	9	1	5.51%	18.50851644	0.001212082	3.84E-05	3.90E-07
ENSG00000161513_74872838_74862817_489	Overlap_dORF	FDXR	17	-	1467	81	3	1	5.32%	18.24418883	1.30E-05	4.30E-06	4.66E-10
ENSG00000073350_75568691_75574193_578	novel	LLGL2	17	+	1734	19	9	0	2.08%	3.620546765	0.113155271	0.000844273	0.001049093
ENSG00000114861_71041530_70959247_455	annotated	FOXP1	3	-	1365	15	0	5	1.54%	3.631016825	0.008473674	0.081055481	0.00371419
ENSG00000162236_62824503_62807469_108	dORF	STX5	11	-	324	15	4	0	6.48%	15.2973394	0.059287765	0.008373824	0.002593538
ENSG00000162236_62831081_62824503_196	annotated	STX5	11	-	588	18	8	9	5.61%	10.11497544	0.022132147	0.050423034	0.004902976
ENSG00000102317_48575181_48575814_118	novel	RBM3	X	+	354	252	146	13	20.34%	235.2160391	0.00041065	1.51E-05	5.30E-08
ENSG00000130529_49183078_49211498_678	dORF	TRPM4	19	+	2034	14	2	0	1.18%	2.274294708	0.019504171	0.004873904	0.000506014
ENSG00000156697_129906211_129929608_719	annotated	UTP14A	X	+	2157	18	1	2	1.81%	2.757350746	0.000918126	0.003906744	2.21E-05
ENSG00000143614_153828230_153810177_538	annotated	GATAD2B	1	-	1614	52	4	2	3.53%	10.64558341	0.000246643	0.000239183	4.04E-07
ENSG00000155313_15818787_15878475_747	annotated	USP25	21	+	2241	36	5	0	2.14%	5.307992468	0.002415531	0.000183786	3.22E-06
ENSG00000037637_16315382_16250670_705	annotated	FBXO42	1	-	2115	19	9	0	1.28%	2.968334795	0.109311583	0.003478238	0.002732156
ENSG00000151876_41925310_41941211_305	annotated	FBXO4	5	+	915	23	1	0	2.62%	8.305702968	0.008219047	0.005454749	0.00023581
ENSG00000100888_21431628_21385613_2576	annotated	CHD8	14	-	7728	54	2	3	1.09%	2.30885309	3.00E-06	1.00E-05	2.56E-10
ENSG00000172845_173964524_173909941_769	annotated	SP3	2	-	2307	68	5	2	3.25%	9.739372395	1.46E-05	9.46E-06	1.12E-09
ENSG00000023839_99782845_99796384_217	annotated	ABCC2	10	+	651	109	15	11	12.90%	55.3242026	6.19E-05	5.86E-05	2.69E-08
ENSG00000012171_50274055_50276706_371	annotated	SEMA3B	3	+	1113	28	8	0	3.23%	8.312516506	0.026534973	0.000991051	0.00018913
ENSG00000128016_39406905_39408699_326	annotated	ZFP36	19	+	978	22	1	1	1.84%	7.432817673	0.016983117	0.016983117	0.001355223
ENSG00000175029_125003077_124989518_365	annotated	CTBP2	10	-	1095	28	2	4	4.11%	8.449160613	0.000924855	0.004886527	2.81E-05
ENSG00000100522_52784650_52778311_184	annotated	GNPNAT1	14	-	552	89	10	2	8.15%	53.27464723	0.001816677	0.00031447	3.84E-06
ENSG00000168802_69121458_69120122_75	annotated	CHTF8	16	-	225	11	0	3	9.33%	16.15399041	0.007593099	0.065814109	0.00269374
ENSG00000147533_41497641_41507106_56	novel	GOLGA7	8	+	168	18	6	0	10.71%	35.40241405	0.067757397	0.013640586	0.004442878
ENSG00000117682_26442863_26469131_160	annotated	DHDDS	1	+	480	14	0	0	4.38%	9.637323824	0.008373824	0.008373824	0.000358384
ENSG00000174485_65756450_65661851_1863	annotated	DENND4A	15	-	5589	22	3	0	0.43%	1.30064335	0.028352861	0.005604867	0.000842634
ENSG00000196531_56724521_56712538_215	annotated	NACA	12	-	645	466	71	22	22.33%	238.7238752	2.73E-06	1.29E-08	4.29E-13
ENSG00000105357_50260713_50309790_1229	annotated	MYH14	19	+	3687	8	1	0	0.49%	0.716946094	0.028889786	0.009815329	0.001387795
ENSG00000138778_103194633_103106222_2488	annotated	CENPE	4	-	7464	45	3	2	0.84%	1.992097254	0.000161753	0.000108195	1.25E-07
ENSG00000165688_136417068_136419202_202	novel	PMPCA	9	+	606	65	1	1	10.40%	35.4413606	4.63E-05	3.19E-05	1.13E-08
ENSG00000165688_136416314_136416532_72	novel	PMPCA	9	+	216	10	0	0	6.94%	15.2973394	0.019679754	0.019679754	0.001784964
ENSG00000139436_109996224_109948823_421	annotated	GIT2	12	-	1263	33	9	0	3.80%	8.633367796	0.019456837	0.000172082	3.28E-05
ENSG00000131652_3025741_3027657_317	annotated	THOC6	16	+	951	71	3	0	8.52%	24.66876941	6.84E-06	2.41E-06	1.41E-10
ENSG00000118961_20774836_20684896_223	annotated	LDAH	2	-	669	14	2	0	4.04%	6.914671802	0.010960145	0.003229477	0.000195317
ENSG00000163001_55519802_55535898_223	annotated	CFAP36	2	+	669	21	5	1	4.93%	10.3720077	0.015553826	0.002296044	0.000208974
ENSG00000153294_47707270_47718442_687	annotated	ADGRF4	6	+	2061	10	5	0	0.87%	1.60321461	0.150401371	0.012798403	0.010441044
ENSG00000184500_93973749_93874245_676	annotated	PROS1	3	-	2028	35	5	0	2.37%	5.702558476	0.001581821	0.000188972	2.11E-06
ENSG00000138802_109462918_109539675_1183	annotated	SEC24B	4	+	3549	23	4	6	1.35%	2.141368897	0.002559514	0.009321808	0.000134722
ENSG00000185808_37072515_37065582_134	annotated	PIGP	21	-	402	15	0	0	5.97%	12.32919892	0.004873904	0.004873904	0.000128468
ENSG00000185808_37072515_37065582_134	annotated	PIGP	21	-	402	15	0	0	5.97%	12.32919892	0.004873904	0.004873904	0.000128468
ENSG00000182158_138001615_137912827_218	annotated	CREB3L2	7	-	654	34	0	5	5.50%	17.17792975	0.000982952	0.009821677	6.19E-05
ENSG00000153786_84990462_84976343_256	annotated	ZDHHC7	16	-	768	73	13	5	10.55%	31.40734996	0.000249696	1.07E-05	2.30E-08
ENSG00000130703_62260040_62293887_448	annotated	OSBPL2	20	+	1344	36	9	3	3.57%	8.850603512	0.008215072	0.001109096	5.67E-05
ENSG00000157837_120791547_120764999_347	annotated	SPPL3	12	-	1041	142	15	0	8.65%	45.07204555	8.63E-07	8.19E-07	6.36E-12
ENSG00000174177_88706531_88715220_485	annotated	CTU2	16	+	1455	32	1	0	2.68%	7.267024739	0.000669869	0.00067919	2.91E-06
ENSG00000104763_18084801_18067074_191	novel	ASAH1	8	-	573	52	16	0	7.85%	29.98598886	0.006971677	0.000294865	1.53E-05
ENSG00000114786_51984156_51987112_183	dORF	ABHD14A-ACY1	3	+	549	17	0	0	4.92%	10.23166308	0.003303074	0.003303074	6.12E-05
ENSG00000114786_51975136_51984153_167	annotated	ABHD14A-ACY1	3	+	501	9	0	0	3.59%	5.935734092	0.009815329	0.009815329	0.000483714
ENSG00000114786_51988530_51989075_99	dORF	ABHD14A-ACY1	3	+	297	27	1	4	8.08%	30.03841192	0.005355751	0.025702836	0.000731269
ENSG00000196628_55350976_55228225_539	annotated	TCF4	18	-	1617	24	0	0	1.86%	4.904230517	0.002404282	0.002404282	3.34E-05
ENSG00000149577_117179264_117196066_832	annotated	SIDT2	11	+	2496	17	2	0	0.96%	2.25047397	0.023197249	0.005306578	0.000651782
ENSG00000162076_2896645_2899149_75	annotated	FLYWCH2	16	+	225	41	3	2	17.33%	60.21032789	0.000862821	0.00067919	3.71E-06
ENSG00000156261_29073590_29066883_189	novel	CCT8	21	-	567	72	5	0	8.47%	41.95841665	0.000331296	0.000207012	4.69E-07
ENSG00000124155_45416169_45416698_118	novel	PIGT	20	+	354	85	3	4	13.56%	79.33874335	0.000473642	0.00126048	3.85E-06
ENSG00000124155_45420633_45425826_254	novel	PIGT	20	+	762	214	16	2	15.35%	92.79582895	5.59E-08	2.12E-08	1.20E-14
ENSG00000144815_101801367_101821954_484	annotated	NXPE3	3	+	1452	19	28	0	1.86%	4.323710807	0.359651006	0.003303074	0.014826993
ENSG00000138760_76179654_76161713_320	novel	SCARB2	4	-	960	301	29	9	20.62%	103.6012311	6.10E-11	3.16E-12	2.46E-21
ENSG00000138760_76213543_76180849_175	novel	SCARB2	4	-	525	50	13	2	10.29%	31.46881249	0.008848649	8.33E-05	7.15E-06
ENSG00000169045_179623133_179620012_140	novel	HNRNPH1	5	-	420	195	39	13	24.29%	153.4104609	1.27E-05	2.86E-06	3.11E-10
ENSG00000213923_38302953_38298090_251	annotated	CSNK1E	22	-	753	74	5	0	7.17%	32.47180253	0.000341912	9.27E-05	2.27E-07
ENSG00000150433_125102332_125101229_91	Overlap_uORF	TMEM218	11	-	273	21	10	12	12.09%	25.41711778	0.067026977	0.146153925	0.035608098
ENSG00000148824_133395724_133420165_293	annotated	MTG1	10	+	879	86	14	7	10.24%	32.32802921	0.000150324	2.83E-05	3.27E-08
ENSG00000109255_55636192_55599146_174	annotated	NMU	4	-	522	26	5	0	7.47%	16.45782722	0.012036615	0.000596156	5.08E-05
ENSG00000166797_64093784_64093701_27	Overlap_uORF	CIAO2A	15	-	81	74	36	3	33.33%	301.8674976	0.296306413	0.007356796	0.017723142
ENSG00000166797_64088758_64072931_71	Overlap_dORF	CIAO2A	15	-	213	52	13	12	16.90%	80.66653342	0.01436208	0.066614395	0.004549201
ENSG00000166797_64093768_64075518_102	annotated	CIAO2A	15	-	306	64	13	27	16.67%	69.10798036	0.001267207	0.069032629	0.000727375
ENSG00000168958_227347269_227357117_130	novel	MFF	2	+	390	31	9	9	6.15%	26.26435504	0.041403097	0.021542864	0.003942439

ENSG00000127054_1324608_1319816_75	novel	INTS11	1	-	225	38	6	0	17.33%	55.80469414	0.005147642	0.000690959	2.29E-05
ENSG00000167962_2002678_2000387_258	novel	ZNF598	16	-	774	13	3	5	2.33%	5.549732435	0.086207551	0.100002646	0.030671193
ENSG00000179818_70088801_70088577_74	novel	PCBP1-AS1	2	-	222	27	13	16	10.81%	40.18652405	0.032088197	0.099690431	0.013336729
ENSG00000104388_60584796_60618687_79	annotated	RAB2A	8	+	237	38	3	1	11.39%	52.97914001	0.007558271	0.005059698	0.000202771
ENSG00000104870_49513401_49524527_235	novel	FCGRT	19	+	705	25	2	5	3.40%	11.71711103	0.010402772	0.033503887	0.001696852
ENSG00000018408_149527942_149520805_134	Overlap_dORF	WWTR1	3	-	402	234	28	4	35.82%	192.3355032	7.82E-08	2.18E-09	1.93E-15
ENSG00000182185_67823544_68477664_350	annotated	RAD51B	14	+	1050	15	0	0	1.71%	4.720321873	0.012798403	0.012798403	0.000797244
ENSG00000074201_77637714_77619628_237	annotated	CLNS1A	11	-	711	152	8	9	15.19%	70.63885335	7.62E-07	3.49E-07	2.45E-12
ENSG00000108578_30291370_30249017_368	Overlap_dORF	BLMH	17	-	1104	53	0	0	5.71%	15.86267586	2.56E-05	2.56E-05	5.10E-09
ENSG00000125458_75131707_75131177_92	novel	NT5C	17	-	276	72	5	2	18.48%	86.19718203	0.000887211	0.000123036	7.83E-07
ENSG00000116273_6614067_6621637_300	annotated	PHF13	1	+	900	24	7	7	4.00%	8.811267496	0.0365699	0.010737298	0.001907167
ENSG00000111700_20858564_20916247_585	annotated	SLCO1B3	12	+	1755	59	15	5	4.44%	11.10822184	0.001681264	3.42E-05	5.07E-07
ENSG00000060982_24948932_24818008_386	annotated	BCAT1	12	-	1158	30	2	0	3.89%	8.5601692	0.001434496	0.000249257	2.44E-06
ENSG00000132964_26254642_26404081_464	annotated	CDK8	13	+	1392	9	2	1	1.08%	2.136352572	0.083503598	0.035374934	0.012068608
ENSG00000169764_63856320_63891227_497	annotated	UGP2	2	+	1491	119	13	5	9.26%	26.37175131	1.66E-07	5.28E-09	9.66E-15
ENSG00000188725_61160171_61159947_74	annotated	SMIM15	5	-	222	25	1	0	13.51%	37.2097445	0.002390426	0.002390426	3.30E-05
ENSG00000188725_61161196_61161110_28	uORF	SMIM15	5	-	84	37	2	5	28.57%	145.5432578	0.005655336	0.011883851	0.000349796
ENSG00000115355_55296017_55291711_161	annotated	CCDC88A	2	-	483	22	2	0	5.59%	15.05030162	0.009029128	0.003658191	0.000179569
ENSG00000108602_19745129_19738221_453	annotated	ALDH3A1	17	-	1359	4367	672	152	37.75%	1061.777184	7.01E-22	4.19E-27	7.52E-47
ENSG00000105819_103299518_103329052_403	dORF	PMPCB	7	+	1209	129	8	1	7.69%	35.25600208	3.22E-06	4.95E-07	1.46E-11
ENSG00000138073_27133640_27131414_345	Overlap_dORF	PREB	2	-	1035	39	7	5	5.51%	12.45070407	0.001242154	0.000642168	5.03E-06
ENSG00000131508_139614609_139623465_96	novel	UBE2D2	5	+	288	84	8	0	18.75%	96.37323824	0.000663691	9.27E-05	4.48E-07
ENSG00000119977_95693953_95664067_625	annotated	TCTN3	10	-	1875	259	51	13	9.44%	45.64236563	6.73E-07	8.05E-10	7.76E-15
ENSG00000095564_91924077_92028933_1849	annotated	BTAF1	10	+	5547	24	0	0	0.92%	1.42962696	7.88E-05	7.88E-05	4.55E-08
ENSG00000196591_113970908_113941058_488	annotated	HDAC2	6	-	1464	145	34	1	8.81%	32.72627528	2.61E-05	4.29E-09	2.08E-12
ENSG00000136636_215620109_215620618_169	novel	KCTD3	1	+	507	31	5	0	8.28%	20.20335003	0.001666677	0.000428413	4.65E-06
ENSG00000103507_31111173_31112265_146	dORF	BCKDK	16	+	438	39	0	0	8.22%	29.42118428	0.001053132	0.001053132	6.85E-06
ENSG00000103507_31109224_31111102_217	annotated	BCKDK	16	+	651	39	6	0	6.45%	19.79489818	0.000976691	0.000440166	2.79E-06
ENSG00000005339_3879916_3727718_2404	annotated	CREBBP	16	-	7212	44	2	2	0.96%	2.015888986	1.94E-05	1.94E-05	2.97E-09
ENSG00000242110_33998746_33989093_171	Overlap_dORF	AMACR	5	-	513	10	1	0	4.68%	6.440985012	0.010437297	0.004149608	0.000232539
ENSG00000273294_33998746_33989093_171	dORF	C1QTNF3-AMACR	5	-	513	10	1	0	4.68%	6.440985012	0.010437297	0.004149608	0.000232539
ENSG00000257698_57935536_57931610_64	novel	GIHCG	12	-	192	16	0	0	7.81%	27.53521093	0.021084099	0.021084099	0.002029891
ENSG00000157193_53271061_53247018_498	annotated	LRP8	1	-	1494	50	18	6	5.02%	11.05831764	0.014939106	9.46E-05	1.49E-05
ENSG00000135480_52248596_52248760_54	novel	KRT7	12	+	162	471	35	9	44.44%	960.6729145	2.83E-05	4.31E-05	9.33E-09
ENSG00000126456_49664838_49659589_452	annotated	IRF3	19	-	1356	32	50	7	3.54%	7.797581855	0.488106427	0.008138029	0.042713933
ENSG00000115966_175130185_175092614_377	annotated	ATF2	2	-	1131	19	6	0	2.12%	5.550864802	0.076557256	0.005604867	0.00252778
ENSG00000115966_175074863_175074609_84	dORF	ATF2	2	-	252	21	0	0	8.33%	27.53521093	0.008776163	0.008776163	0.000391611
ENSG00000092036_22955118_22947993_188	novel	HAUS4	14	-	564	39	14	3	7.45%	22.84836651	0.016769851	0.001851891	0.000188796
ENSG00000054983_87993164_87987046_112	annotated	GALC	14	-	336	9	0	0	4.46%	8.850603512	0.020613417	0.020613417	0.001946209
ENSG00000138443_203328530_203427352_502	annotated	ABI2	2	+	1506	19	4	0	2.59%	4.168677351	0.002264699	0.000474427	6.98E-06
ENSG00000089916_76154364_76178213_365	annotated	GPATCH2L	14	+	1095	41	3	0	4.38%	12.37198518	0.000648066	0.000188101	8.37E-07
ENSG00000164933_103414937_103401988_123	annotated	SLC25A32	8	-	369	49	6	4	10.57%	43.87724668	0.004253235	0.003989005	9.33E-05
ENSG00000198399_24310348_24203626_1474	annotated	ITSN2	2	-	4422	26	14	3	1.02%	1.94278286	0.058079157	0.002499833	0.000981325
ENSG00000065675_6515135_6428207_706	annotated	PRKCQ	10	-	2118	18	1	5	1.27%	2.808123494	0.003229477	0.027941506	0.000523148
ENSG00000099956_23787179_23834180_336	annotated	SMARCB1	22	+	1008	116	20	5	11.31%	38.02481509	1.91E-06	1.67E-07	3.08E-12
ENSG00000106785_98119188_98087470_442	annotated	TRIM14	9	-	1326	22	5	2	1.81%	5.482123442	0.031969545	0.018402302	0.002665059
ENSG00000139579_56224497_56229213_227	annotated	NABP2	12	+	681	130	15	10	14.10%	63.0762541	2.67E-05	1.04E-05	2.22E-09
ENSG00000077782_38421911_38413628_500	dORF	FGFR1	8	-	1500	86	13	8	7.20%	18.94422512	5.60E-06	2.83E-06	1.35E-10
ENSG00000077782_38457446_38421928_367	annotated	FGFR1	8	-	1101	13	3	0	1.91%	3.90144678	0.058290626	0.007979402	0.002448908
ENSG00000097096_85200384_85158750_990	annotated	SYDE2	1	-	2970	11	0	0	0.51%	1.223787152	0.019679754	0.019679754	0.001784964
ENSG00000104691_30766242_30766394_50	novel	UBXN8	8	+	150	34	1	0	12.00%	74.89577372	0.016503129	0.01300724	0.001029549
ENSG00000184922_45234079_45246596_936	annotated	FMNL1	17	+	2808	22	8	10	1.07%	2.588780514	0.055474537	0.096388797	0.020278665
ENSG00000182768_90265713_90265907_64	novel	NGRN	15	+	192	68	2	6	25.00%	117.0246464	0.000255231	0.000207012	3.63E-07
ENSG00000183598_149813681_149813271_136	annotated	HIST2H3D	1	-	408	33	5	0	6.62%	26.72535178	0.018151375	0.003303074	0.000335828
ENSG00000154277_41256977_41268073_207	annotated	UCHL1	4	+	621	1095	142	35	37.68%	582.6291008	3.06E-11	1.28E-13	5.73E-23
ENSG00000126226_113197161_113178198_359	annotated	PCID2	13	-	1077	52	4	1	5.57%	15.95354839	0.000105937	4.61E-05	3.64E-08
ENSG00000063245_55678655_55695356_541	annotated	EPN1	19	+	1623	111	10	3	7.02%	22.59821377	1.53E-06	7.72E-08	1.19E-12
ENSG00000141503_4891086_4897287_736	dORF	MINK1	17	+	2208	14	4	0	1.09%	2.095070397	0.042379653	0.005160369	0.001212668
ENSG00000110514_47274705_47329150_1519	annotated	MADD	11	+	4557	32	4	0	1.12%	2.320281105	0.001207856	0.000112736	1.00E-06
ENSG00000167972_2326466_2276674_1704	annotated	ABCA3	16	-	5112	72	5	4	1.94%	4.653838466	1.45E-06	1.65E-06	2.11E-11
ENSG00000205937_2264643_2253964_305	annotated	RNPS1	16	-	915	21	9	3	3.28%	7.583467927	0.034881441	0.009583527	0.001648848
ENSG00000137878_57618171_57684498_338	annotated	GCOM1	15	+	1014	12	0	0	1.78%	3.910325812	0.011772051	0.011772051	0.000681273
ENSG00000123992_219387157_219385364_263	novel	DNPEP	2	-	789	29	6	3	6.08%	12.14480786	0.001446343	0.000918318	8.19E-06
ENSG00000003402_201133036_201136136_87	novel	CFLAR	2	+	261	8	0	1	5.75%	10.12789367	0.019679754	0.065398531	0.005779267
ENSG00000167775_8302942_8302463_102	dORF	CD320	19	-	306	55	5	3	8.82%	59.38967063	0.005518828	0.006870388	0.000200515
ENSG00000167775_8308290_8305702_81	annotated	CD320	19	-	243	81	8	20	17.28%	110.1408437	0.000893161	0.021613042	0.00013729
ENSG00000196235_39464951_39476399_828	annotated	SUPT5H	19	+	2484	62	3	1	2.78%	8.247261244	4.60E-05	1.50E-05	5.43E-09
ENSG00000115275_74462963_74461275_562	novel	MOGS	2	-	1686	74	1	0	4.98%	14.50253102	1.99E-06	1.60E-06	2.79E-11

ENSG00000111540_55986961_55992212_215	annotated	AC034102.1	12	+	645	100	28	1	12.56%	51.2282994	0.000182409	2.55E-06	4.59E-09
ENSG00000108395_59104361_58982918_946	annotated	TRIM37	17	-	2838	23	6	0	1.27%	2.677842923	0.028585895	0.000873936	0.000186716
ENSG00000100697_95126669_95090498_1651	annotated	DICER1	14	-	4953	28	12	2	0.91%	1.867924666	0.042648284	0.00119113	0.000382887
ENSG00000171564_154567760_154570650_272	annotated	FGB	4	+	816	22	0	1	3.31%	8.908450594	0.003554776	0.00567009	0.000110499
ENSG00000186184_27621984_27665953_122	annotated	POLR1D	13	+	366	21	1	1	6.56%	18.95866982	0.005960501	0.005960501	0.000188269
ENSG00000197756_216499339_216520753_60	annotated	RPL37A	2	+	180	890	113	24	31.67%	1633.755848	0.000836048	0.000125228	7.48E-07
ENSG00000104728_1859962_1903505_500	annotated	ARHGEF10	8	+	1500	23	6	3	2.00%	5.06647881	0.028118902	0.010805327	0.00146817
ENSG00000104728_1857968_1957263_1330	annotated	ARHGEF10	8	+	3990	59	8	7	1.95%	4.885947202	7.47E-05	0.000116744	6.34E-08
ENSG00000083799_50749699_50796508_953	annotated	CYLD	16	+	2859	26	7	4	1.36%	3.004891853	0.012643165	0.005714521	0.000375455
ENSG00000175054_142562807_142449429_2382	annotated	ATR	3	-	7146	33	9	5	0.63%	1.525880706	0.011394337	0.003156623	0.000199157
ENSG00000126858_32171069_32224733_638	annotated	RHOT1	17	+	1914	55	7	2	4.08%	9.494900319	0.000124051	1.23E-05	1.24E-08
ENSG00000155760_202034960_202036372_470	annotated	FZD7	2	+	1410	35	1	0	2.98%	8.201977723	0.000680681	0.000434916	1.93E-06
ENSG00000167257_117244571_117285151_400	annotated	RNF214	11	+	1200	11	6	2	1.50%	3.028873202	0.143514872	0.042234516	0.024258209
ENSG00000104529_143586845_143581107_136	novel	EEF1D	8	-	408	145	4	4	15.44%	117.429576	2.90E-05	2.89E-05	6.47E-09
ENSG00000114544_126068219_126006407_471	annotated	SLC41A3	3	-	1413	43	4	1	4.25%	10.05532119	0.000530755	5.29E-05	2.14E-07
ENSG00000100266_42912080_42871357_486	annotated	PACSIN2	22	-	1458	59	3	14	4.94%	13.37100777	1.61E-05	0.002699151	4.63E-07
ENSG00000154743_12489930_12532721_422	annotated	TSEN2	3	+	1266	24	2	0	2.37%	6.263934239	0.008661718	0.002335453	0.000115215
ENSG00000134318_11227392_11183437_1145	annotated	ROCK2	2	-	3435	104	15	5	3.58%	10.00405917	7.20E-06	8.76E-08	6.85E-12
ENSG00000115652_106194241_106094026_420	annotated	UXS1	2	-	1260	118	13	0	9.52%	30.94433228	1.52E-06	1.33E-08	2.35E-13
ENSG00000197448_143263514_143268837_214	annotated	GSTK1	7	+	642	334	85	24	23.36%	171.9020645	6.07E-07	9.11E-09	6.25E-14
ENSG00000143442_151429672_151404802_1235	annotated	POGZ	1	-	3705	29	7	1	1.38%	2.586303213	0.001937314	0.00017257	2.41E-06
ENSG00000182307_145052492_145061264_236	annotated	C8orf33	8	+	708	103	15	4	12.71%	48.0699445	0.000114631	3.66E-06	3.83E-09
ENSG00000221914_26359008_26370413_340	annotated	PPP2R2A	8	+	1020	89	1	0	8.82%	28.83098556	6.96E-07	7.01E-07	4.42E-12
ENSG00000169679_110650741_110641755_181	annotated	BUB1	2	-	543	14	2	0	3.31%	8.519181281	0.011570466	0.011772051	0.000670297
ENSG00000169679_110641380_110637964_182	dORF	BUB1	2	-	546	19	4	0	4.95%	11.49821995	0.02089895	0.003529166	0.000412398
ENSG00000108469_75666443_75627422_953	annotated	RECQL5	17	-	2859	22	2	0	1.36%	2.542600799	0.001253735	0.000564499	4.50E-06
ENSG00000188938_93452346_93447611_135	annotated	FAM120AOS	9	-	405	9	4	1	5.19%	7.342722914	0.105872578	0.017404239	0.008764339
ENSG00000106397_101210612_101210003_117	novel	PLOD3	7	-	351	13	3	0	4.27%	12.23787152	0.086207551	0.020613417	0.008015435
ENSG00000101901_111681219_111759999_1137	annotated	ALG13	X	+	3411	24	3	2	0.62%	2.32487269	0.02846257	0.020905271	0.002672295
ENSG00000016864_52700460_52694845_371	annotated	GLT8D1	3	-	1113	42	6	0	5.12%	12.46877476	0.000869598	5.53E-05	3.75E-07
ENSG00000164038_103066375_103026370_463	annotated	SLC9B2	4	-	1389	12	6	0	1.08%	2.854622299	0.222621372	0.021084099	0.024047755
ENSG00000149809_65113264_65114865_135	novel	TM7SF2	11	+	405	79	22	0	11.11%	64.45279002	0.039432762	0.000312071	0.000125349
ENSG00000149809_65112016_65112873_103	novel	TM7SF2	11	+	309	17	0	0	6.80%	18.17858585	0.008175611	0.008175611	0.000342518
ENSG00000196419_41622042_41663815_559	annotated	XRCC6	22	+	1677	145	16	7	8.77%	28.56962851	1.18E-07	5.90E-09	7.53E-15
ENSG00000083857_186709827_186588592_4588	annotated	FAT1	4	-	13764	433	56	24	3.75%	10.39472217	5.48E-23	1.50E-26	1.76E-47
ENSG00000187735_53979168_53968104_74	annotated	TCEA1	8	-	222	39	22	2	12.16%	58.04720141	0.040165756	0.013443806	0.002543326
ENSG00000160753_155327044_155330571_127	annotated	RUSC1	1	+	381	23	0	0	7.09%	19.94676697	0.003606289	0.003606289	7.24E-05
ENSG00000171130_149874066_149878771_81	annotated	ATP6V0E2	7	+	243	81	4	2	14.81%	110.1408437	0.004274818	0.002516921	6.08E-05
ENSG00000078237_4337146_4352691_211	annotated	TIGAR	12	+	633	31	6	4	7.11%	16.18183012	0.005969645	0.002374643	8.03E-05
ENSG00000138629_74458867_74446090_380	annotated	UBL7	15	-	1140	74	13	2	7.11%	21.44848009	0.000213959	6.59E-06	1.26E-08
ENSG00000107951_30349275_30313609_582	annotated	MTPAP	10	-	1746	80	17	6	4.12%	15.13963487	0.001933908	8.54E-05	1.30E-06
ENSG00000108433_46929545_46963884_192	annotated	GOSR2	17	+	576	33	7	0	6.77%	18.93045751	0.019941106	0.000662934	9.84E-05
ENSG00000118961_20801463_20684896_370	annotated	LDAH	2	-	1110	22	4	0	3.78%	6.548915031	0.002435384	0.000368141	5.99E-06
ENSG00000141198_54900866_54960626_476	annotated	TOM1L1	17	+	1428	52	7	8	5.46%	12.03219301	0.000101698	4.62E-05	3.49E-08
ENSG00000257594_89524549_89522813_578	annotated	GALNT4	12	-	1734	47	9	1	3.63%	8.956089367	0.000378209	2.94E-05	8.83E-08
ENSG00000198561_57795613_57816308_832	annotated	CTNND1	11	+	2496	194	34	13	6.61%	25.68187942	9.34E-07	6.67E-09	7.43E-14
ENSG00000110172_90205564_90202405_144	annotated	CHORDC1	11	-	432	13	0	0	4.17%	9.943270612	0.012798403	0.012798403	0.000797244
ENSG00000115685_241157830_241167068_212	annotated	PPP1R7	2	+	636	71	2	0	11.79%	36.886792	8.76E-06	5.26E-06	3.82E-10
ENSG00000023839_99782845_99794011_195	annotated	ABCC2	10	+	585	109	15	11	14.36%	61.56590751	6.19E-05	5.86E-05	2.69E-08
ENSG00000183765_28734586_28687897_469	annotated	CHEK2	22	-	1407	21	0	0	2.56%	4.931679569	0.000885228	0.000885228	4.91E-06
ENSG00000145191_184140650_184143146_227	Overlap_dORF	EIF2B5	3	+	681	12	2	5	3.08%	5.822423456	0.01551626	0.091912668	0.006854887
ENSG00000145191_184135386_184141928_389	annotated	EIF2B5	3	+	1167	32	6	3	3.08%	9.060429302	0.036157847	0.002558966	0.000576384
ENSG00000285839_52028617_52020933_115	dORF	AL445685.3	1	-	345	230	105	0	16.52%	220.2816874	7.67E-05	6.36E-05	3.59E-08
ENSG00000147439_22630576_22621422_199	annotated	BIN3	8	-	597	20	0	1	5.03%	11.06943153	0.002201184	0.003737369	4.71E-05
ENSG00000180694_90631677_90625671_105	annotated	TMEM64	8	-	315	22	5	0	7.62%	23.07712916	0.00912793	0.005160369	0.000248192
ENSG00000134333_18396843_18407132_241	annotated	LDHA	11	+	723	852	95	15	18.26%	389.3775885	1.49E-08	5.74E-09	8.97E-16
ENSG00000107186_13136160_13106965_604	annotated	MPDZ	9	-	1812	12	0	1	0.83%	2.188228683	0.019679754	0.042234516	0.003715148
ENSG00000115414_215435697_215361555_2205	annotated	FN1	2	-	6615	338	55	24	5.62%	16.88326765	3.28E-15	3.87E-18	2.22E-31
ENSG00000133243_2015703_1986488_525	annotated	BTBD2	19	-	1575	67	5	1	5.33%	14.05606958	3.87E-06	1.96E-06	6.55E-11
ENSG00000103121_81007019_80976093_186	annotated	CMC2	16	-	558	71	26	9	10.22%	42.04301023	0.00693992	0.000168234	9.55E-06
ENSG00000115241_27409422_27381599_546	annotated	PPM1G	2	-	1638	232	22	10	13.55%	46.79977242	1.41E-11	7.29E-13	1.35E-22
ENSG00000136045_107688670_107712220_439	annotated	PWP1	12	+	1317	110	21	12	9.11%	27.5979335	5.04E-06	1.66E-06	7.27E-11
ENSG00000100722_88563134_88611751_736	annotated	ZC3H14	14	+	2208	69	24	2	5.03%	10.3257041	0.000604545	7.40E-08	9.59E-10
ENSG00000144524_231788571_231807645_264	annotated	COPS7B	2	+	792	36	3	2	5.30%	15.01920596	0.000434916	0.00157599	4.45E-06
ENSG00000132780_45584153_45618141_786	annotated	NASP	1	+	2358	109	12	7	4.83%	15.27398469	1.75E-06	6.70E-07	1.05E-11
ENSG00000247077_132717516_132720828_140	annotated	PGAM5	12	+	420	36	11	0	8.57%	28.32193124	0.018431869	0.000943084	0.000119708
ENSG00000169814_15635440_15645488_523	annotated	BTD	3	+	1569	56	8	3	4.40%	11.79328346	0.000235687	5.63E-05	9.80E-08
ENSG00000182872_47147482_47186599_853	annotated	RBM10	X	+	2559	24	6	3	1.17%	3.098921746	0.030614564	0.00972614	0.001460559

ENSG00000079459_11802833_11826102_33	novel	FDFT1	8	+	99	97	3	9	15.15%	323.7473285	0.021557223	0.021557223	0.002115616
ENSG00000079459_11802847_11838609_250	novel	FDFT1	8	+	750	549	69	19	26.40%	241.8692928	8.58E-11	2.87E-12	3.18E-21
ENSG00000106603_43647613_43639582_134	annotated	COA1	7	-	402	36	6	4	9.70%	29.59007741	0.007322027	0.003864916	0.000153348
ENSG00000106603_43729464_43648611_25	Overlap_uORF	COA1	7	-	75	100	8	2	32.00%	440.5633748	0.010275151	0.005756914	0.000308065
ENSG00000164168_147617957_147634434_438	annotated	TMEM184C	4	+	1314	80	15	1	5.71%	20.11704908	0.000430481	5.28E-06	2.23E-08
ENSG00000115648_237493427_237511148_163	novel	MLPH	2	+	489	39	7	23	7.98%	26.35271721	0.010383622	0.06058233	0.003157965
ENSG00000138443_203328530_203427352_470	annotated	ABI2	2	+	1410	21	4	0	3.19%	4.921186634	0.000912974	0.000177563	1.13E-06
ENSG00000138443_203391121_203427352_267	annotated	ABI2	2	+	801	17	3	0	4.12%	7.012712895	0.002549574	0.001255833	1.92E-05
ENSG00000138442_202911476_202880860_423	annotated	WDR12	2	-	1269	31	7	3	4.49%	8.0717876	0.004642795	0.000494574	1.53E-05
ENSG00000138443_203328530_203427352_508	annotated	ABI2	2	+	1524	19	4	0	2.56%	4.119441005	0.002264699	0.000474427	6.98E-06
ENSG00000136717_127106934_127048526_547	annotated	BIN1	2	-	1641	35	2	1	2.56%	7.047403162	0.000742993	0.000525339	2.51E-06
ENSG00000260269_75601155_75598358_113	novel	AC105036.3	15	-	339	16	4	0	5.31%	15.59516371	0.086597294	0.01300724	0.005588697
ENSG00000100888_21428134_21385613_2136	annotated	CHD8	14	-	6408	49	2	3	1.22%	2.526639205	6.79E-06	2.38E-05	1.32E-09
ENSG00000127990_94656098_94644645_43	annotated	SGCE	7	-	129	21	6	4	18.60%	53.78971437	0.044816497	0.037011271	0.00688081
ENSG00000043514_39857348_39848092_153	annotated	TRIT1	1	-	459	8	0	0	3.27%	5.758998364	0.019216965	0.019216965	0.001707396
ENSG00000073584_40642556_40628785_393	Overlap_dORF	SMARCE1	17	-	1179	57	3	0	3.31%	15.97462619	0.001529613	0.000649205	6.25E-06
ENSG00000158796_161123174_161122147_158	novel	DEDD	1	-	474	15	5	0	3.80%	10.45640921	0.091912668	0.012798403	0.005896977
ENSG00000171159_128160408_128162026_33	novel	C9orf16	9	+	99	33	0	0	15.15%	110.1408437	0.021084099	0.021084099	0.002029891
ENSG00000167642_38264893_38292006_252	annotated	SPINT2	19	+	756	1048	82	23	34.92%	458.0460484	6.11E-14	9.97E-16	9.09E-28
ENSG00000213722_31729161_31728566_158	novel	DDAH2	6	-	474	31	1	4	6.96%	21.60991237	0.001988635	0.00626331	7.18E-05
ENSG00000241468_99460131_99458327_63	annotated	ATP5MF	7	-	189	184	36	5	28.57%	321.6811943	0.000504986	9.60E-05	3.49E-07
ENSG00000144524_231794346_231807645_157	novel	COPS7B	2	+	471	13	0	0	3.82%	9.119942472	0.01300724	0.01300724	0.000821865
ENSG00000163539_33658983_33498631_1292	annotated	CLASP2	3	-	3876	24	3	0	0.85%	2.045959945	0.009583527	0.001484235	8.60E-05
ENSG00000165280_35061609_35060777_114	novel	VCP	9	-	342	83	15	13	17.54%	80.1902634	0.005141917	0.000602137	2.03E-05
ENSG00000134255_111147787_111159562_149	novel	CEPT1	1	+	447	26	0	0	6.04%	19.21920763	0.003710481	0.003710481	7.64E-05
ENSG00000080189_46355193_46350702_189	novel	SLC35C2	20	-	567	24	1	5	5.29%	13.98613888	0.002953574	0.019491326	0.000329227
ENSG00000176454_34367100_34364146_212	novel	LPCAT4	15	-	636	24	1	0	3.77%	12.46877476	0.008364861	0.005604867	0.00024597
ENSG00000103275_1314031_1324793_158	annotated	UBE2I	16	+	474	313	36	28	22.78%	218.1904056	2.92E-06	1.59E-05	3.96E-10
ENSG00000174197_41669483_41767280_2660	annotated	MGA	15	+	7980	20	0	6	0.49%	0.828126644	0.000564499	0.017213168	7.29E-05
ENSG00000145375_122923143_123102216_790	novel	SPATA5	4	+	2370	14	10	0	1.14%	1.951863053	0.270647753	0.002500085	0.007828708
ENSG00000129103_56079529_56079612_27	internal	SUMF2	7	+	81	23	8	1	18.52%	93.82368167	0.230298309	0.028889786	0.031203709
ENSG00000129103_56064312_56079647_352	annotated	SUMF2	7	+	1056	608	99	19	17.33%	190.2432755	3.88E-07	1.83E-10	1.13E-15
ENSG00000196756_38431089_38426436_38	novel	SNHG17	20	-	114	20	1	3	13.16%	57.96886511	0.028889786	0.065398531	0.007975053
ENSG00000082153_200813218_200815552_91	novel	BZW1	2	+	273	68	24	12	14.29%	82.30304804	0.048189693	0.01381117	0.003136476
ENSG00000198836_193637210_193692127_642	dORF	OPA1	3	+	1926	52	7	3	4.05%	8.921065222	0.000244656	1.91E-05	3.78E-08
ENSG00000016864_52700460_52694845_371	annotated	GLT8D1	3	-	1113	42	6	0	5.12%	12.46877476	0.000869598	5.53E-05	3.75E-07
ENSG00000258881_70994748_70942315_194	annotated	AC007040.2	2	-	582	84	10	1	8.76%	47.68984985	0.000689281	0.000181655	8.63E-07
ENSG00000171130_149874066_149879677_164	annotated	ATP6V0E2	7	+	492	11	3	2	3.05%	7.387495614	0.087777151	0.070008252	0.022678706
ENSG00000125457_75269377_75266740_205	annotated	MIF4GD	17	-	615	77	18	3	12.68%	41.36997544	0.00065207	6.51E-06	4.25E-08
ENSG00000125458_75131707_75130488_201	annotated	NT5C	17	-	603	98	8	5	12.44%	53.70051086	9.02E-05	1.49E-05	1.07E-08
ENSG00000126773_60118638_60133664_462	annotated	PCNX4	14	+	1386	22	0	0	1.73%	5.244802081	0.005604867	0.005604867	0.000167525
ENSG00000100567_58244921_58267524_197	novel	PSMA3	14	+	591	394	70	13	27.41%	220.2816874	3.66E-06	1.39E-10	1.15E-14
ENSG00000110200_72112742_72111464_41	uORF	ANAPC15	11	-	123	18	4	1	17.07%	48.35451675	0.053017408	0.01208853	0.003100937
ENSG00000110200_72111276_72109881_125	annotated	ANAPC15	11	-	375	48	2	5	19.20%	42.29408398	9.60E-06	0.000119092	9.52E-09
ENSG00000162298_65132346_65131444_83	novel	SYVN1	11	-	249	43	4	3	15.66%	57.06091903	0.001937037	0.003735293	4.18E-05
ENSG00000206527_123585027_123494888_254	annotated	HACD2	3	-	762	168	36	3	11.81%	72.84906198	0.00060395	7.87E-07	6.57E-09
ENSG00000197885_23910904_23893095_192	annotated	NKIRAS1	3	-	576	26	1	0	7.29%	14.91490592	0.000872559	0.000398115	2.27E-06
ENSG00000243678_51166831_51171604_152	annotated	NME2	17	+	456	925	119	26	35.53%	670.2650028	1.26E-09	1.37E-10	2.00E-18
ENSG00000132432_54757588_54755775_66	novel	SEC61G	7	-	198	188	28	3	37.88%	313.7345245	5.88E-06	8.54E-06	4.15E-10
ENSG00000119953_110303587_110297700_102	novel	SMNDC1	10	-	306	25	1	11	7.84%	26.99530483	0.01019742	0.132371794	0.007583147
ENSG00000124357_71068684_71078508_344	annotated	NAGK	2	+	1032	91	17	2	6.69%	29.13609528	0.000965067	2.30E-05	1.95E-07
ENSG00000007376_787737_786839_131	annotated	RPUSD1	16	-	393	11	0	6	4.58%	9.248467792	0.012798403	0.196285373	0.014517613
ENSG00000177200_53222720_53324895_2278	annotated	CHD9	16	+	6834	39	4	7	0.83%	1.885642188	0.000648887	0.001717592	7.02E-06
ENSG00000137106_37422769_37436782_322	annotated	GRHPR	9	+	966	421	59	22	27.02%	144.0040224	8.92E-13	2.40E-15	3.36E-26
ENSG00000184056_91009831_90998975_493	annotated	VPS33B	15	-	1479	40	2	4	2.64%	8.936376771	0.002390059	0.002636958	3.63E-05
ENSG00000106459_129690424_129755181_361	annotated	NRF1	7	+	1083	12	1	2	1.94%	3.661191481	0.007882058	0.037441831	0.00150521
ENSG00000011304_797498_810826_197	annotated	PTBP1	19	+	591	141	14	14	18.27%	78.83177138	3.21E-07	2.39E-06	7.15E-12
ENSG00000163913_129466909_129520265_1047	dORF	IFT122	3	+	3141	17	2	4	0.76%	1.788342257	0.013091824	0.044710437	0.002775155
ENSG00000143418_150967837_150966148_230	novel	CERS2	1	-	690	397	29	10	28.26%	190.1126737	2.61E-11	6.59E-12	2.12E-21
ENSG00000204967_140807188_141010236_901	annotated	PCDHA4	5	+	2703	12	2	0	0.89%	1.466914678	0.019435691	0.004826937	0.000499969
ENSG00000064042_41360841_41599030_115	novel	LIMCH1	4	+	345	16	2	0	6.96%	15.32394347	0.013892891	0.005454749	0.000395614
ENSG00000118193_200618492_200553388_1571	annotated	KIF14	1	-	4713	48	12	3	1.27%	3.365219922	0.005407187	0.00012101	5.47E-06
ENSG00000162702_200409705_200407018_895	annotated	ZNF281	1	-	2685	41	1	5	1.79%	5.045558203	0.000284799	0.001434505	2.75E-06
ENSG00000162702_200409705_200407018_859	annotated	ZNF281	1	-	2577	41	1	5	1.86%	5.257013495	0.000284799	0.001434505	2.75E-06
ENSG00000100949_24271115_24265615_567	annotated	RABGGTA	14	-	1701	18	10	4	1.76%	3.496534721	0.121961391	0.03472485	0.017528997
ENSG00000150782_112155053_112143596_193	annotated	IL18	11	-	579	148	23	2	17.10%	84.46033611	8.75E-06	2.20E-07	1.95E-11
ENSG00000081177_69228858_69241100_496	annotated	EXD2	14	+	1488	43	7	3	3.83%	9.548500563	0.000966593	0.000363136	2.30E-06
ENSG00000081177_69209471_69210147_122	uORF	EXD2	14	+	366	7	0	0	4.10%	6.319556606	0.019216965	0.019216965	0.001707396

ENSG00000049618_157148716_157207891_1421	annotated	ARID1B	6	+	4263	16	4	2	0.56%	1.240150246	0.051323546	0.02389627	0.005330496
ENSG00000173726_235113800_235112064_25	novel	TOMM20	1	-	75	66	13	0	24.00%	290.7718274	0.037701781	0.013853925	0.002447522
ENSG00000170142_23810476_23889378_167	annotated	UBE2E1	3	+	501	95	7	4	10.78%	62.65497097	0.000232202	0.000286961	4.55E-07
ENSG00000113448_59768509_58974664_745	annotated	PDE4D	5	-	2235	142	25	15	6.58%	20.99328833	2.17E-07	3.27E-07	6.67E-13
ENSG00000112996_44808963_44815202_439	annotated	MRPS30	5	+	1317	105	6	2	9.11%	26.34348198	1.71E-08	2.37E-08	4.13E-15
ENSG00000130479_17720400_17734428_1030	annotated	MAP1S	19	+	3090	22	4	1	1.17%	2.352522875	0.00077296	0.001621946	7.80E-06
ENSG00000134531_13213707_13213991_94	annotated	EMP1	12	+	282	8	0	2	5.32%	9.373688826	0.019679754	0.080264768	0.007158564
ENSG00000092108_30630546_30735609_575	annotated	SCFD1	14	+	1725	65	6	0	5.04%	12.45070407	1.18E-05	1.03E-06	1.10E-10
ENSG00000139428_109559136_109557028_49	dORF	MMAB	12	-	147	8	0	0	10.20%	17.98217856	0.019679754	0.019679754	0.001784964
ENSG00000010219_4612562_4613753_132	novel	DYRK4	12	+	396	20	3	0	5.30%	16.68800662	0.071345384	0.007029303	0.002777152
ENSG00000177706_248364_259980_249	novel	FAM20C	7	+	747	34	2	0	4.42%	15.03931199	0.003224199	0.001558047	2.95E-05
ENSG00000118816_77075543_77066298_59	novel	CCNI	4	-	177	25	0	0	8.47%	46.66984903	0.021084099	0.021084099	0.002029891
ENSG00000113460_34915739_34922291_129	novel	BRIX1	5	+	387	53	8	1	9.30%	45.25166447	0.010255697	0.001097317	7.12E-05
ENSG00000039560_34688204_34830765_983	annotated	RAI14	5	+	2949	65	4	5	3.05%	7.28296525	3.39E-06	2.88E-06	8.37E-11
ENSG00000172053_49099226_49098206_162	novel	QARS	3	-	486	15	12	0	3.09%	10.19822627	0.336071985	0.019679754	0.039528703
ENSG00000136152_45492180_45511860_232	novel	COG3	13	+	696	13	2	0	3.02%	6.171685208	0.030804354	0.007882058	0.001227455
ENSG00000147536_41530203_41542087_223	annotated	GINS4	8	+	669	47	0	0	5.83%	23.21354105	0.000712477	0.000712477	3.23E-06
ENSG00000169727_82053265_82057127_483	Overlap_dORF	GPS1	17	+	1449	116	9	10	8.49%	26.45204528	4.10E-08	2.57E-07	1.05E-13
ENSG00000141522_81869930_81868379_235	annotated	ARHGDIA	17	-	705	475	38	13	17.02%	222.6251096	1.06E-06	1.08E-07	1.11E-12
ENSG00000003147_8221318_8113923_371	annotated	ICA1	7	-	1113	14	1	1	2.16%	4.156258253	0.005160369	0.007930666	0.000216377
ENSG00000102393_101398612_101397809_177	novel	GLA	X	-	531	83	7	0	11.86%	51.64796626	0.000100394	2.78E-05	2.13E-08
ENSG00000161204_184186208_184187548_144	novel	ABCF3	3	+	432	24	0	1	4.86%	18.35680728	0.008776163	0.008776163	0.000391611
ENSG00000070061_108922844_108869115_1149	annotated	ELP1	9	-	3447	51	7	0	2.09%	4.888758076	0.000348693	5.66E-06	1.88E-08
ENSG00000187109_76074219_76049463_383	annotated	NAP1L1	12	-	1149	196	35	16	11.23%	56.36450487	3.03E-06	1.31E-07	3.96E-12
ENSG00000111684_6981056_6977146_203	novel	LPCAT3	12	-	609	111	14	0	15.27%	60.22479631	2.83E-05	5.13E-07	1.46E-10
ENSG00000177239_137106191_137107361_110	novel	MAN1B1	9	+	330	23	2	5	5.45%	23.02944914	0.037440157	0.086597294	0.013121962
ENSG00000177239_137107340_137108591_147	novel	MAN1B1	9	+	441	39	8	2	10.20%	29.22104017	0.005031593	0.001101134	3.38E-05
ENSG00000003402_201129923_201145385_224	annotated	CFLAR	2	+	672	38	11	3	7.14%	18.68460741	0.002223054	0.001125346	1.51E-05
ENSG00000165671_177135479_177295459_2571	annotated	NSD1	5	+	7713	39	8	3	0.74%	1.670747921	0.003381975	0.000396446	9.03E-06
ENSG00000100650_69770482_69771461_145	novel	SRSF5	14	+	435	78	24	3	13.10%	59.24817799	0.018632935	0.000108871	2.18E-05
ENSG00000108559_5419650_5386206_741	annotated	NUP88	17	-	2223	65	3	5	3.51%	9.661477518	1.55E-05	3.37E-05	4.11E-09
ENSG00000122390_3479556_3484855_177	annotated	NAA60	16	+	531	18	4	0	3.95%	11.20076377	0.041678091	0.008674847	0.001826095
ENSG00000149792_65122347_65125872_166	annotated	MRPL49	11	+	498	109	11	0	19.88%	72.32139737	1.62E-06	2.34E-07	3.57E-12
ENSG00000014216_65182702_65211286_714	annotated	CAPN1	11	+	2142	251	18	11	10.50%	38.71898007	3.65E-12	3.08E-13	1.49E-23
ENSG00000149798_65320899_65321531_210	annotated	CDC42EP2	11	+	630	28	3	1	3.81%	14.68544583	0.009288069	0.005021538	0.000246239
ENSG00000139370_128808935_128800840_105	novel	SLC15A4	12	-	315	13	4	0	4.76%	13.63648541	0.065398531	0.021084099	0.006119907
ENSG00000103187_84590215_84566845_73	novel	COTL1	16	-	219	300	28	7	36.99%	452.6336043	2.24E-06	2.70E-06	5.21E-11
ENSG00000105135_15123390_15115252_525	annotated	ILVBL	19	-	1575	110	34	8	6.48%	23.07712916	0.000981139	5.74E-06	6.07E-08
ENSG00000179950_143829303_143821438_151	novel	PUF60	8	-	453	21	4	3	5.30%	15.31760078	0.035092496	0.008219047	0.001456623
ENSG00000130299_17337612_17341703_492	annotated	GTPBP3	19	+	1476	20	6	0	2.24%	4.477270069	0.032335844	0.00146251	0.000324341
ENSG00000131143_85801206_85805115_83	novel	COX4I1	16	+	249	433	69	21	32.53%	574.5901846	1.10E-05	2.79E-06	2.61E-10
ENSG00000103121_80997460_80976093_101	novel	CMC2	16	-	303	58	9	6	14.85%	63.24919738	0.002849205	0.00091795	1.61E-05
ENSG00000175656_73976282_73978869_107	novel	TMEM70	8	+	321	76	5	7	14.02%	78.23087964	0.000568618	0.003543277	1.30E-05
ENSG00000196655_119018796_119019321_117	annotated	TRAPPC4	11	+	351	54	2	1	15.38%	50.83423556	0.000276943	8.36E-05	1.67E-07
ENSG00000164733_11853372_11852665_24	novel	CTSB	8	-	72	32	2	43	37.50%	146.8544583	0.003710481	0.468493666	0.025643776
ENSG00000110660_107815934_107792659_310	annotated	SLC35F2	11	-	930	76	10	8	10.00%	27.00227136	1.44E-05	4.86E-05	5.54E-09
ENSG00000115648_237525778_237537540_97	novel	MLPH	2	+	291	24	0	1	9.28%	27.25134277	0.002943991	0.002967235	4.95E-05
ENSG00000084754_26244596_26191250_763	annotated	HADHA	2	-	2289	196	28	1	7.60%	28.29306077	3.36E-08	1.21E-11	6.76E-18
ENSG00000172409_57659597_57661436_385	annotated	CLP1	11	+	1155	22	4	0	2.34%	6.293762497	0.023871233	0.003303074	0.000449475
ENSG00000141858_14090420_14088417_432	annotated	SAMD1	19	-	1296	24	6	8	2.55%	6.118935761	0.023421242	0.032869174	0.00339725
ENSG00000172269_119100388_119098399_71	novel	DPAGT1	11	-	213	22	11	1	12.68%	34.12814875	0.085005563	0.006112544	0.003053487
ENSG00000172269_119100804_119100622_60	novel	DPAGT1	11	-	180	59	3	0	21.67%	108.305163	0.002333599	0.000700469	1.03E-05
ENSG00000044459_17135165_17298382_358	annotated	CNTLN	9	+	1074	13	5	0	2.23%	3.999527844	0.049386833	0.00419316	0.001217431
ENSG00000044459_17135165_17502652_1373	annotated	CNTLN	9	+	4119	17	11	1	0.73%	1.36372494	0.101528246	0.003974283	0.00274395
ENSG00000122033_27440124_27435675_170	annotated	MTIF3	13	-	510	27	5	0	4.71%	17.49295753	0.0260957	0.005160369	0.000719933
ENSG00000136280_45027786_45076057_438	annotated	CCM2	7	+	1314	14	0	7	1.60%	3.520483589	0.008175611	0.164061516	0.00844186
ENSG00000049167_60922154_60874615_338	dORF	ERCC8	5	-	1014	13	2	2	1.48%	4.236186296	0.060322713	0.083503598	0.01901092
ENSG00000121940_108950437_108934670_366	annotated	CLCC1	1	-	1098	53	9	1	5.74%	15.94935715	0.001332918	3.16E-05	3.66E-07
ENSG00000121940_108950437_108934670_430	annotated	CLCC1	1	-	1290	70	9	1	5.81%	17.92990479	0.000227048	6.35E-06	1.30E-08
ENSG00000085433_109023368_108971430_871	annotated	WDR47	1	-	2613	11	1	0	0.69%	1.390986545	0.023672501	0.012798403	0.001437523
ENSG00000085433_109023368_108971430_872	annotated	WDR47	1	-	2616	11	1	0	0.69%	1.389391377	0.023672501	0.012798403	0.001437523
ENSG00000077150_102395960_102402376_900	annotated	NFKB2	10	+	2700	35	4	0	2.00%	4.283255033	0.000120212	7.13E-05	6.24E-08
ENSG00000197114_63708581_63735919_511	annotated	ZGPAT	20	+	1533	8	3	0	1.17%	1.724318492	0.124106539	0.011570466	0.007709042
ENSG00000058600_22302969_22333700_687	annotated	POLR3E	16	+	2061	59	6	0	3.35%	9.458966199	7.06E-05	1.14E-05	6.50E-09
ENSG00000122378_80420501_80432099_218	annotated	PRXL2A	10	+	654	360	67	6	22.94%	181.8839621	5.98E-09	3.17E-10	2.20E-17
ENSG00000123992_219387157_219374292_471	dORF	DNPEP	2	-	1413	65	8	4	5.10%	15.19990412	0.000133128	4.04E-05	4.03E-08
ENSG00000163507_108589375_108551149_906	annotated	CIP2A	3	-	2718	82	5	6	3.64%	9.968597333	8.61E-07	4.48E-06	3.45E-11
ENSG00000132507_7309636_7309884_82	novel	EIF5A	17	+	246	578	226	36	26.83%	776.35863	0.004818828	1.99E-05	1.09E-06

ENSG00000116133_54887119_54879791_133	annotated	DHCR24	1	-	399	66	13	2	9.77%	54.65635853	0.009191518	0.001023943	5.97E-05
ENSG00000116133_54879763_54852233_392	dORF	DHCR24	1	-	1176	290	31	9	17.09%	81.48174662	4.28E-10	5.52E-12	3.11E-20
ENSG00000186575_29604095_29683055_475	annotated	NF2	22	+	1425	55	1	2	4.63%	12.75315032	2.11E-05	5.21E-05	8.53E-09
ENSG00000134480_87409351_87394987_245	annotated	CCNH	5	-	735	23	0	0	4.90%	10.33975267	0.000943084	0.000943084	5.54E-06
ENSG00000168137_9436883_9476091_1255	annotated	SETD5	3	+	3765	27	8	5	1.12%	2.369563968	0.034331991	0.00386557	0.000760048
ENSG00000173698_19068834_18990864_898	annotated	ADGRG2	X	-	2694	28	3	0	1.56%	3.434235661	0.003183653	0.000424543	9.00E-06
ENSG00000144591_219500186_219506798_385	annotated	GMPPA	2	+	1155	32	8	0	2.34%	9.154563633	0.056711303	0.003554776	0.001252885
ENSG00000102471_79481486_79551120_242	annotated	NDFIP2	13	+	726	54	9	0	8.26%	24.57688248	0.001382279	3.41E-05	4.06E-07
ENSG00000092531_42511847_42521641_97	annotated	SNAP23	15	+	291	43	6	1	9.28%	48.82532247	0.004557894	0.006307834	0.000154436
ENSG00000135083_160311920_160253378_434	annotated	CCNJL	5	-	1302	19	1	0	1.61%	4.821834171	0.01208853	0.008776163	0.000531381
ENSG00000239779_74458603_74460681_303	annotated	WBP1	2	+	909	19	3	0	3.63%	6.906521552	0.003722062	0.001384502	3.06E-05
ENSG00000239306_66616721_66626483_156	annotated	RBM14	11	+	468	18	0	0	5.13%	12.70855889	0.005257623	0.005257623	0.000148366
ENSG00000053371_19311974_19304225_309	annotated	AKR7A2	1	-	927	50	0	3	6.47%	17.82214299	3.16E-05	8.02E-05	1.92E-08
ENSG00000132780_45584153_45618141_447	annotated	NASP	1	+	1341	115	12	6	8.28%	28.33601124	2.23E-06	5.83E-07	1.18E-11
ENSG00000198642_21334832_21333006_608	annotated	KLHL9	9	-	1824	91	8	5	4.11%	16.48489602	7.10E-06	2.95E-05	1.71E-09
ENSG00000104064_50309723_50286020_323	annotated	GABPB1	15	-	969	36	7	0	3.72%	12.27575967	0.018994618	0.001023866	0.000132458
ENSG00000196961_49767161_49806758_968	annotated	AP2A1	19	+	2904	111	5	6	4.86%	12.62978683	1.76E-09	1.39E-08	2.67E-16
ENSG00000198546_133309432_133311950_197	annotated	ZNF511	10	+	591	77	3	1	10.66%	43.04997444	2.81E-05	2.86E-05	6.21E-09
ENSG00000119048_134371623_134390353_134	annotated	UBE2B	5	+	402	123	9	6	13.43%	101.0994312	0.000340265	0.000420806	9.53E-07
ENSG00000163734_74038611_74038089_103	novel	CXCL3	4	-	309	27	6	0	5.83%	28.87187165	0.01300724	0.011772051	0.000748533
ENSG00000173928_11374744_11376016_229	annotated	SWSAP1	19	+	687	13	0	0	2.18%	6.252536979	0.020613417	0.020613417	0.001946209
ENSG00000132470_75724704_75757555_1752	annotated	ITGB4	17	+	5256	482	67	15	8.28%	30.30130517	1.50E-19	2.43E-24	8.67E-42
ENSG00000205268_65841508_65719290_482	annotated	PDE7A	8	-	1446	25	3	4	1.66%	5.712699362	0.01778219	0.017600937	0.001462862
ENSG00000143742_225778007_225786969_60	novel	SRP9	1	+	180	25	1	0	8.33%	45.89201821	0.029237631	0.021557223	0.002819006
ENSG00000175390_7987353_7996022_357	annotated	EIF3F	11	+	1071	56	8	1	4.20%	17.27699509	0.00507103	0.000412614	1.44E-05
ENSG00000113522_132579932_132594589_399	novel	RAD50	5	+	1197	21	4	1	2.76%	5.796886511	0.014139202	0.002106591	0.00017543
ENSG00000163683_39605145_39552118_54	annotated	SMIM14	4	-	162	7	2	2	11.11%	14.27751678	0.158655254	0.029390861	0.020505786
ENSG00000198189_87372791_87337276_142	novel	HSD17B11	4	-	426	82	20	2	14.79%	63.60245904	0.00172783	2.88E-05	4.51E-07
ENSG00000155229_97498384_97458692_987	annotated	MMS19	10	-	2961	110	5	4	3.75%	12.2750687	2.86E-07	2.31E-07	6.21E-13
ENSG00000197451_178204838_178210623_285	annotated	HNRNPAB	5	+	855	234	111	9	17.89%	90.43142957	0.000263173	6.51E-09	4.84E-11
ENSG00000197747_151986230_151983163_97	annotated	S100A10	1	-	291	1744	152	48	63.92%	1980.264241	1.86E-11	5.81E-12	1.33E-21
ENSG00000167088_21612430_21629138_119	annotated	SNRPD1	18	+	357	229	22	4	15.97%	211.9517076	0.000562364	6.67E-05	2.80E-07
ENSG00000148356_127454591_127502899_702	annotated	LRSAM1	9	+	2106	21	3	0	1.28%	3.294811564	0.016912105	0.003452921	0.000323936
ENSG00000088448_110914934_110893150_220	annotated	ANKRD10	13	-	660	22	1	3	4.09%	11.01408437	0.005588371	0.011768187	0.000342715
ENSG00000204427_31703281_31701020_65	annotated	ABHD16A	6	-	195	21	0	0	9.23%	35.58396489	0.01300724	0.01300724	0.000821865
ENSG00000144036_72825895_72465094_676	annotated	EXOC6B	2	-	2028	21	7	0	1.63%	3.421535086	0.041410799	0.001262414	0.000386596
ENSG00000177951_207321_168958_152	annotated	BET1L	11	-	456	18	7	0	4.61%	13.04299465	0.050639737	0.008674847	0.00224805
ENSG00000164808_47260959_47440343_181	annotated	SPIDR	8	+	543	18	3	2	3.31%	10.95323308	0.039104404	0.024275373	0.004143858
ENSG00000088682_57447506_57460624_207	annotated	COQ9	16	+	621	105	8	2	14.01%	55.86854391	5.74E-06	1.39E-06	7.00E-11
ENSG00000013810_1739489_1741121_209	novel	TACC3	4	+	627	17	8	0	3.83%	8.958824608	0.12775441	0.005160369	0.004426437
ENSG00000163754_148991641_149024165_193	annotated	GYG1	3	+	579	23	0	6	6.22%	13.12559277	0.000939154	0.012936105	8.04E-05
ENSG00000173698_19068834_18990864_898	annotated	ADGRG2	X	-	2694	28	3	0	1.56%	3.434235661	0.003183653	0.000424543	9.00E-06
ENSG00000142327_240576895_240577892_129	novel	RNPEPL1	2	+	387	9	0	2	4.65%	7.68424491	0.011772051	0.071345384	0.004172987
ENSG00000158092_136928134_136948453_333	annotated	NCK1	3	+	999	35	1	3	3.90%	11.57636495	0.000862821	0.002681474	1.43E-05
ENSG00000172936_38138662_38140608_240	novel	MYD88	3	+	720	87	7	7	9.17%	39.92605584	0.000399021	0.000374905	9.94E-07
ENSG00000172936_38140767_38141286_78	novel	MYD88	3	+	234	10	2	3	6.41%	14.12062099	0.102951605	0.11777946	0.041532788
ENSG00000120725_138947473_138947117_118	novel	SIL1	5	-	354	55	4	0	12.71%	51.33683393	0.000540414	0.000316076	1.14E-06
ENSG00000145685_78510183_78488897_218	annotated	LHFPL2	5	-	654	113	11	2	12.84%	57.09135476	7.69E-06	2.59E-06	1.70E-10
ENSG00000153044_69189653_69209799_222	annotated	CENPH	5	+	666	24	3	2	6.76%	11.90711824	0.003217849	0.001398313	2.67E-05
ENSG00000153130_140379186_140381104_77	annotated	SCOC	4	+	231	34	10	0	15.58%	48.6336193	0.040244294	0.001040526	0.000321625
ENSG00000255073_58610981_58622749_311	annotated	ZFP91-CNTF	11	+	933	26	6	5	2.89%	9.207916194	0.00640069	0.018399684	0.000604834
ENSG00000121741_19993073_20019684_462	novel	ZMYM2	13	+	1386	15	1	0	1.52%	3.576001419	0.011738695	0.008373824	0.000494661
ENSG00000185049_2008950_1983319_525	annotated	NELFA	4	-	1575	45	1	0	3.62%	9.440643746	7.24E-05	5.47E-05	2.94E-08
ENSG00000121741_20051562_20086014_570	novel	ZMYM2	13	+	1710	14	2	0	1.40%	2.705213705	0.022898969	0.005160369	0.000627835
ENSG00000123136_14413220_14410592_208	novel	DDX39A	19	-	624	77	18	19	9.62%	40.7732931	0.013296842	0.005839452	0.000402497
ENSG00000164291_95555279_95600879_395	annotated	ARSK	5	+	1185	22	7	0	2.28%	6.134426738	0.057935965	0.003606289	0.00129889
ENSG00000104973_49818342_49836944_534	annotated	MED25	19	+	1602	19	9	0	2.06%	3.918868971	0.162415925	0.001255833	0.002305453
ENSG00000137996_100266412_100291504_354	annotated	RTCA	1	+	1062	25	4	6	3.11%	7.778308171	0.011044978	0.018846099	0.001007752
ENSG00000244754_32527488_32436382_730	annotated	N4BP2L2	13	-	2190	16	0	6	0.96%	2.414045889	0.008473674	0.118250966	0.005776373
ENSG00000134480_87401753_87394446_87	novel	CCNH	5	-	261	46	1	0	10.34%	58.23538863	0.005059698	0.003632191	0.000100924
ENSG00000164096_119300535_119298779_66	annotated	C4orf3	4	-	198	116	14	10	19.70%	193.5808768	0.075921787	0.012785158	0.004770161
ENSG00000136143_47996933_47943371_403	annotated	SUCLA2	13	-	1209	71	9	1	5.21%	19.40446626	0.000138971	3.46E-05	3.65E-08
ENSG00000090266_140702960_140704934_41	annotated	NDUFB2	7	+	123	264	18	1	43.90%	709.199579	0.000312009	0.000119373	2.63E-07
ENSG00000090266_140696745_140700880_34	novel	NDUFB2	7	+	102	349	15	8	41.18%	1130.563366	0.000972459	0.000489353	3.06E-06
ENSG00000090266_140702960_140704934_41	novel	NDUFB2	7	+	123	264	18	1	43.90%	709.199579	0.000312009	0.000119373	2.63E-07
ENSG00000181722_114351606_114339005_584	annotated	ZBTB20	3	-	1752	10	4	0	1.20%	1.885973351	0.095107389	0.005706018	0.003312541
ENSG00000267735_12881036_12881143_35	novel	AC020934.2	19	+	105	32	9	2	17.14%	100.7002	0.013428348	0.017620112	0.001128599
ENSG00000123159_14479522_14478416_114	annotated	GIPC1	19	-	342	67	13	4	21.05%	64.73189937	0.000383829	1.12E-05	3.79E-08

ENSG00000123200_46045507_45963822_1564	annotated	ZC3H13	13	-	4692	35	6	1	1.21%	2.464788702	0.003090362	7.41E-05	1.93E-06
ENSG00000198382_75879912_76141413_598	annotated	UVRAG	11	+	1794	36	3	2	2.51%	6.630552464	0.001057803	0.000948822	6.23E-06
ENSG00000166136_100529851_100529376_71	annotated	NDUFB8	10	-	213	156	27	4	30.99%	241.9996003	8.74E-05	4.41E-05	2.88E-08
ENSG00000166136_100526554_100524089_68	dORF	NDUFB8	10	-	204	47	9	4	16.18%	76.12675962	0.004810144	0.002815105	7.58E-05
ENSG00000198625_204537468_204549682_363	Overlap_dORF	MDM4	1	+	1089	13	3	0	1.38%	3.944437929	0.063071082	0.021084099	0.005896902
ENSG00000172845_173964524_173909941_728	annotated	SP3	2	-	2184	68	5	1	3.43%	10.28788101	1.46E-05	6.84E-06	8.20E-10
ENSG00000133895_64810109_64804334_615	annotated	MEN1	11	-	1845	47	1	7	3.09%	8.41726773	7.80E-05	0.003400472	2.24E-06
ENSG00000133895_64810109_64804334_615	annotated	MEN1	11	-	1845	47	1	7	3.09%	8.41726773	7.80E-05	0.003400472	2.24E-06
ENSG00000145868_148402396_148442147_955	annotated	FBXO38	5	+	2865	14	0	0	0.84%	1.61463017	0.005160369	0.005160369	0.000143196
ENSG00000167005_56451202_56447785_106	novel	NUDT21	16	-	318	62	3	0	16.04%	64.42200292	0.000493731	0.00011345	3.98E-07
ENSG00000089685_78214680_78216062_68	annotated	BIRC5	17	+	204	52	7	0	13.24%	84.22535107	0.006307834	0.003789641	0.000130107
ENSG00000169045_179623133_179614841_472	annotated	HNRNPH1	5	-	1416	531	74	23	18.86%	123.9084492	2.29E-13	5.89E-15	1.95E-26
ENSG00000007376_787098_785950_183	annotated	RPUSD1	16	-	549	12	5	0	3.28%	7.222350407	0.138644629	0.01300724	0.009584248
ENSG00000168894_85595830_85597565_165	annotated	RNF181	2	+	495	61	7	78	6.67%	40.71873616	0.002268044	0.405205082	0.014755871
ENSG00000147099_72572761_72422398_359	novel	HDAC8	X	-	1077	14	1	0	1.95%	4.295186105	0.01551626	0.007882058	0.000614257
ENSG00000063177_48619143_48616582_146	novel	RPL18	19	-	438	133	25	4	13.70%	100.3337823	0.001057838	7.76E-05	6.23E-07
ENSG00000143774_228145513_228148697_214	annotated	GUK1	1	+	642	11	4	1	2.80%	5.661445237	0.279004417	0.028889786	0.039555724
ENSG00000146433_158573430_158631888_469	annotated	TMEM181	6	+	1407	41	10	8	4.69%	9.628517254	0.004221183	0.001022429	2.64E-05
ENSG00000130338_158449095_158506694_1329	annotated	TULP4	6	+	3987	12	2	0	0.38%	0.994499717	0.101051022	0.019679754	0.009164498
ENSG00000215301_41334253_41364342_733	annotated	DDX3X	X	+	2199	224	48	10	7.64%	33.65832059	1.60E-07	2.03E-10	4.68E-16
ENSG00000141428_35975060_35979006_132	annotated	C18orf21	18	+	396	11	2	4	4.55%	9.178403642	0.042234516	0.100002646	0.016744483
ENSG00000033867_27403173_27376764_461	dORF	SLC4A7	3	-	1383	45	13	1	3.90%	10.75127542	0.004302989	7.80E-05	2.93E-06
ENSG00000033867_27484099_27404954_454	annotated	SLC4A7	3	-	1362	26	23	4	2.64%	6.30762541	0.266259065	0.007910857	0.015872659
ENSG00000173039_65654839_65654378_153	dORF	RELA	11	-	459	31	0	0	4.58%	22.31611866	0.008877961	0.008877961	0.000400235
ENSG00000173039_65662832_65654952_246	annotated	RELA	11	-	738	44	6	3	6.50%	19.69998831	0.001973325	0.000735397	9.05E-06
ENSG00000141526_82237209_82261095_293	annotated	SLC16A3	17	+	879	237	18	0	14.33%	89.09003399	1.31E-07	7.67E-09	1.08E-14
ENSG00000075188_102077500_102074354_145	dORF	NUP37	12	-	435	31	4	0	8.28%	23.54735279	0.003791764	0.000982952	2.28E-05
ENSG00000110851_107761008_107760611_70	novel	PRDM4	12	-	210	7	0	0	7.14%	11.01408437	0.019216965	0.019216965	0.001707396
ENSG00000139116_39369887_39294424_1540	annotated	KIF21A	12	-	4620	25	0	3	0.84%	1.78800071	0.000600464	0.004238779	1.65E-05
ENSG00000285815_39380385_39405984_173	annotated	WRB-SH3BGR	21	+	519	143	24	9	19.08%	91.04127543	2.42E-06	4.11E-07	9.18E-12
ENSG00000145191_184140487_184144943_417	dORF	EIF2B5	3	+	1251	24	8	2	2.64%	6.339041364	0.045595422	0.002484757	0.000734664
ENSG00000156671_75150529_75176692_415	annotated	SAMD8	10	+	1245	50	11	5	3.61%	13.26998117	0.013376962	0.002457464	0.00018893
ENSG00000144228_138550507_138569080_358	annotated	SPOPL	2	+	1074	8	1	0	1.40%	2.461247904	0.034182656	0.019216965	0.002956053
ENSG00000100526_54397069_54420078_172	annotated	CDKN3	14	+	516	87	9	10	15.70%	55.7107756	5.28E-06	2.61E-05	1.15E-09
ENSG00000164830_106679254_106750941_785	annotated	OXR1	8	+	2355	9	0	3	0.89%	1.262761265	0.006937203	0.075249618	0.002921248
ENSG00000136942_124861912_124857918_123	annotated	RPL35	9	-	369	1191	84	21	17.07%	1066.485731	0.00064184	0.000462249	1.93E-06
ENSG00000239900_40346559_40366522_484	annotated	ADSL	22	+	1452	155	41	13	9.71%	35.27237763	1.98E-06	1.38E-08	3.23E-13
ENSG00000117385_42766878_42746833_666	annotated	P3H1	1	-	1998	145	9	2	5.71%	23.97961312	1.49E-06	8.53E-08	1.27E-12
ENSG00000106355_32489303_32487261_62	annotated	LSM5	7	-	186	163	19	3	37.10%	289.563831	2.09E-05	1.95E-05	3.21E-09
ENSG00000014641_63589044_63606987_169	annotated	MDH1	2	+	507	346	40	11	30.18%	225.4954552	3.00E-09	2.62E-09	8.47E-17
ENSG00000103363_2777239_2771990_118	annotated	ELOB	16	-	354	1688	162	26	35.59%	1575.574103	2.70E-08	2.69E-08	7.33E-15
ENSG00000142534_49496457_49499635_158	annotated	RPS11	19	+	474	2099	192	75	27.85%	1463.200196	3.10E-08	2.47E-08	7.73E-15
ENSG00000087191_63828138_63831969_398	annotated	PSMC5	17	+	1194	413	53	16	22.11%	114.2918805	4.23E-13	2.30E-15	1.49E-26
ENSG00000111665_6850952_6846788_219	annotated	CDCA3	12	-	657	119	11	3	11.42%	59.84822101	5.10E-05	1.34E-05	5.44E-09
ENSG00000067560_49375589_49360366_187	annotated	RHOA	3	-	561	334	30	8	20.86%	196.7221487	2.13E-07	4.42E-08	9.34E-14
ENSG00000067560_49360352_49360209_47	dORF	RHOA	3	-	141	340	43	6	51.06%	796.7635502	0.000131449	8.97E-06	9.93E-09
ENSG00000168288_149587097_149569974_330	annotated	MMADHC	2	-	990	222	34	2	13.94%	74.0947494	5.78E-06	2.43E-09	2.26E-13
ENSG00000163870_127580146_127575258_225	annotated	TPRA1	3	-	675	46	12	3	8.00%	22.5176836	0.004434571	0.000438498	1.32E-05
ENSG00000102882_30123047_30118023_184	novel	MAPK3	16	-	552	17	6	0	4.89%	10.17605621	0.035820121	0.003452921	0.000726466
ENSG00000118785_87977833_87982896_289	novel	SPP1	4	+	867	1612	169	70	45.67%	614.3496196	7.02E-21	5.44E-22	6.61E-41
ENSG00000198498_163515422_163519514_87	annotated	TMA16	4	+	261	41	0	0	8.05%	51.90545508	0.008877961	0.008877961	0.000400235
ENSG00000142949_43545076_43622003_1898	annotated	PTPRF	1	+	5694	154	22	1	3.32%	8.936612187	5.88E-09	1.97E-12	1.95E-19
ENSG00000169689_82022861_82019186_131	annotated	CENPX	17	-	393	164	11	152	17.56%	137.8862471	1.30E-05	0.139186173	9.18E-05
ENSG00000160888_13153253_13153858_201	annotated	IER2	19	+	603	34	7	0	4.98%	18.63078948	0.020131593	0.002474239	0.000293518
ENSG00000179409_752142_747226_184	annotated	GEMIN4	17	-	552	48	1	2	4.89%	28.73239401	0.003736771	0.006901532	0.000140271
ENSG00000161203_184176994_184179157_124	novel	AP2M1	3	+	372	88	13	3	10.48%	78.16446973	0.001505615	0.001538257	1.39E-05
ENSG00000213983_23567314_23566417_68	novel	AP1G2	14	-	204	10	1	0	8.82%	16.1971829	0.011772051	0.011772051	0.000681273
ENSG00000149136_57335121_57326407_709	annotated	SSRP1	11	-	2127	267	64	8	12.41%	41.47758148	5.31E-10	1.39E-15	2.08E-23
ENSG00000104388_60584796_60620769_98	Overlap_dORF	RAB2A	8	+	294	40	9	1	11.22%	44.95544641	0.019176024	0.002345022	0.000266228
ENSG00000112715_43777480_43778540_88	novel	VEGFA	6	+	264	22	0	0	5.68%	27.53521093	0.021557223	0.021557223	0.002115616
ENSG00000106462_148846595_148807646_655	annotated	EZH2	7	-	1965	23	8	7	1.83%	3.867541077	0.056326529	0.051344725	0.011431832
ENSG00000154001_63453814_63376009_386	annotated	PPP2R5E	14	-	1158	38	4	3	5.44%	10.84288099	0.000138283	0.00018599	1.81E-07
ENSG00000114867_184317438_184334908_1511	annotated	EIF4G1	3	+	4533	153	16	14	4.17%	11.15258047	5.88E-10	1.20E-09	7.87E-18
ENSG00000213145_105487260_105488511_77	annotated	CRIP1	14	+	231	805	61	46	41.56%	1151.472457	7.11E-07	3.27E-06	2.09E-11
ENSG00000135090_118239266_118150997_798	annotated	TAOK3	12	-	2394	22	0	0	1.00%	3.036464363	0.005355751	0.005355751	0.00015367
ENSG00000103932_41531080_41520238_1020	annotated	RPAP1	15	-	3060	13	3	1	0.69%	1.403755851	0.067796501	0.011306644	0.003821731
ENSG00000164828_832525_873331_785	annotated	SUN1	7	+	2355	162	37	3	7.26%	22.72970278	1.02E-06	9.80E-11	1.95E-15
ENSG00000164828_838815_838970_51	internal	SUN1	7	+	153	22	3	16	19.61%	47.5117365	0.010402772	0.229394231	0.015451134

ENSG00000112029_152972406_152971163_128	novel	FBXO5	6	-	384	24	5	0	5.47%	20.65140819	0.052585976	0.008776163	0.002363736
ENSG00000160014_46608271_46608618_68	novel	CALM3	19	+	204	164	5	4	33.82%	265.6337995	2.83E-05	1.19E-05	2.68E-09
ENSG00000100473_30874939_30894920_494	annotated	COCH	14	+	1482	75	17	2	5.47%	16.72178801	0.000157445	6.57E-06	9.07E-09
ENSG00000168710_109985053_110012430_139	novel	AHCYL1	1	+	417	17	2	0	4.32%	13.4704629	0.024135905	0.013428348	0.001529427
ENSG00000175183_76866260_76858952_193	annotated	CSRP2	12	-	579	8	3	2	2.59%	4.565423573	0.143893695	0.101051022	0.049102245
ENSG00000145919_173616436_173609239_185	annotated	BOD1	5	-	555	20	0	2	4.32%	11.90711824	0.005554612	0.010276881	0.000298213
ENSG00000166997_100119811_100124888_226	annotated	CNPY4	7	+	678	24	6	0	4.42%	11.69637278	0.0220327	0.002349128	0.00030996
ENSG00000143418_150969090_150966148_380	annotated	CERS2	1	-	1140	651	53	43	30.26%	188.6886559	8.67E-18	1.15E-17	1.54E-33
ENSG00000132382_4542930_4539415_370	novel	MYBBP1A	17	-	1110	12	3	0	1.62%	3.572135472	0.051431483	0.013217227	0.003234637
ENSG00000143321_156751429_156743449_240	annotated	HDGF	1	-	720	325	34	13	23.75%	149.1490592	1.65E-10	9.13E-11	1.75E-19
ENSG00000105726_19663666_19645422_1204	annotated	ATP13A1	19	-	3612	64	13	4	2.41%	5.854662788	0.000510747	6.68E-06	3.31E-08
ENSG00000131652_3025741_3027657_317	annotated	THOC6	16	+	951	71	3	0	8.52%	24.66876941	6.84E-06	2.41E-06	1.41E-10
ENSG00000105197_39486289_39489820_155	novel	TIMM50	19	+	465	73	16	6	12.90%	51.87278445	0.002643479	0.000222804	4.19E-06
ENSG00000165271_33464102_33462664_200	annotated	NOL6	9	-	600	8	4	1	3.00%	4.405633748	0.113684165	0.041782825	0.018894059
ENSG00000135164_87182316_87195140_494	dORF	DMTF1	7	+	1482	13	0	0	1.21%	2.898443255	0.01300724	0.01300724	0.000821865
ENSG00000167543_29571893_29572102_69	novel	TP53I13	17	+	207	12	4	0	11.59%	19.15492934	0.065131088	0.004826937	0.001866503
ENSG00000167543_29572334_29572789_151	novel	TP53I13	17	+	453	29	1	0	7.28%	21.15287727	0.002493494	0.00140552	2.08E-05
ENSG00000164904_126550022_126544965_74	novel	ALDH7A1	5	-	222	21	7	0	9.46%	31.25618538	0.128936332	0.008473674	0.00641402
ENSG00000100292_35381174_35393598_288	annotated	HMOX1	22	+	864	25	1	0	4.17%	9.560837127	0.00092354	0.000978918	5.63E-06
ENSG00000134480_87411304_87394446_270	annotated	CCNH	5	-	810	63	1	0	6.67%	25.6995302	0.000119103	8.14E-05	7.02E-08
ENSG00000197147_89582753_89593043_769	annotated	LRRC8B	1	+	2307	12	1	0	0.91%	1.718712776	0.010460668	0.006937203	0.000372001
ENSG00000157426_56384257_56338402_1084	annotated	AASDH	4	-	3252	7	5	0	0.46%	0.711241611	0.282609087	0.019216965	0.030706309
ENSG00000134779_36828767_36796805_300	annotated	TPGS2	18	-	900	75	10	10	7.67%	27.53521093	0.000391748	0.000392833	1.02E-06
ENSG00000179950_143829303_143816520_513	annotated	PUF60	8	-	1539	243	29	3	11.89%	52.1719786	3.20E-09	5.75E-12	2.68E-19
ENSG00000171940_53582625_53571744_981	annotated	ZNF217	20	-	2943	82	3	10	3.87%	9.206472155	7.59E-08	3.38E-06	2.69E-12
ENSG00000061273_47789524_47785283_249	novel	HDAC7	12	-	747	40	2	4	4.02%	17.69330823	0.002376615	0.007635352	0.00010292
ENSG00000284770_235380050_235435792_235	annotated	TBCE	1	+	705	18	9	0	3.40%	8.436319943	0.05393658	0.005554612	0.001681648
ENSG00000271601_145957927_145936310_337	annotated	LIX1L	1	-	1011	124	32	0	9.50%	40.52660124	0.001248459	3.25E-07	7.72E-09
ENSG00000127415_1000893_1004393_521	annotated	IDUA	4	+	1563	20	1	1	2.30%	4.228055421	0.001607079	0.001607079	1.54E-05
ENSG00000221914_26363816_26370413_148	novel	PPP2R2A	8	+	444	63	0	0	12.16%	46.88427806	9.02E-05	9.02E-05	5.91E-08
ENSG00000109332_102809680_102797415_110	annotated	UBE2D3	4	-	330	42	2	0	8.18%	42.05377669	0.003710481	0.003710481	7.64E-05
ENSG00000164889_151064864_151066671_85	internal	SLC4A2	7	+	255	13	0	7	5.88%	16.84507021	0.021084099	0.229044117	0.024915513
ENSG00000164889_151061988_151076367_1241	annotated	SLC4A2	7	+	3723	297	57	2	7.49%	26.35925107	5.87E-10	2.34E-17	6.73E-25
ENSG00000041357_78544194_78548944_190	annotated	PSMA4	15	+	570	612	51	8	22.11%	354.7694545	2.77E-08	6.69E-08	1.86E-14
ENSG00000041357_78540431_78541915_39	uORF	PSMA4	15	+	117	19	13	1	15.38%	53.65835975	0.276345604	0.020342454	0.030944751
ENSG00000105341_41439899_41432231_257	annotated	DMAC2	19	-	771	113	16	0	11.67%	48.42768614	7.48E-06	7.71E-07	5.25E-11
ENSG00000204427_31691626_31688245_171	novel	ABHD16A	6	-	513	26	7	0	5.26%	16.74656103	0.06712464	0.003303074	0.001443674
ENSG00000114125_141738342_141742795_108	annotated	RNF7	3	+	324	74	16	9	12.04%	75.46687439	0.004253235	0.000700469	1.90E-05
ENSG00000100029_30591833_30577046_583	annotated	PES1	22	-	1749	105	17	6	6.00%	19.83668712	4.15E-05	8.72E-07	3.56E-10
ENSG00000088888_3861463_3866147_399	annotated	MAVS	20	+	1197	28	7	4	3.01%	7.729182014	0.017338935	0.00842386	0.000727592
ENSG00000169241_155135953_155138281_166	Overlap_dORF	SLC50A1	1	+	498	48	19	8	8.43%	31.84795481	0.10076033	0.007445065	0.004332801
ENSG00000169241_155135912_155137594_63	annotated	SLC50A1	1	+	189	26	3	14	15.87%	45.45495137	0.00444514	0.140484886	0.00449641
ENSG00000119689_74889332_74902330_349	dORF	DLST	14	+	1047	85	6	2	5.16%	26.82513385	0.000364535	0.000167777	4.22E-07
ENSG00000101266_489830_483961_167	novel	CSNK2A1	20	-	501	47	1	0	8.38%	30.99772248	0.000475338	0.000472567	1.47E-06
ENSG00000168036_41235736_41235858_40	novel	CTNNB1	3	+	120	16	3	0	12.50%	44.05633748	0.045845141	0.021557223	0.00436099
ENSG00000168036_41233346_41233960_176	novel	CTNNB1	3	+	528	88	24	7	14.20%	55.07042185	0.000272256	2.58E-05	5.55E-08
ENSG00000121073_49705279_49704035_115	novel	SLC35B1	17	-	345	45	7	2	11.30%	43.09859102	0.011198964	0.002395345	0.000153508
ENSG00000148498_34372518_34346442_179	novel	PARD3	10	-	537	20	0	0	2.79%	12.30623952	0.021084099	0.021084099	0.002029891
ENSG00000100578_58457777_58499312_915	annotated	KIAA0586	14	+	2745	21	3	0	1.09%	2.527822642	0.013506184	0.002201184	0.00017355
ENSG00000110719_68041281_68050819_827	annotated	TCIRG1	11	+	2481	121	36	6	4.96%	16.11492393	7.40E-05	4.60E-08	5.23E-11
ENSG00000110721_68121177_68053988_457	annotated	CHKA	11	-	1371	31	10	0	2.84%	7.471260733	0.022861026	0.000667552	0.000115982
ENSG00000003402_201129923_201163080_443	annotated	CFLAR	2	+	1329	57	11	3	5.42%	14.17162097	9.26E-05	4.58E-05	3.16E-08
ENSG00000196628_55350976_55228225_538	annotated	TCF4	18	-	1614	24	0	0	1.86%	4.913346188	0.002404282	0.002404282	3.34E-05
ENSG00000117868_158798064_158734207_738	annotated	ESYT2	7	-	2214	193	26	11	7.59%	28.80377078	5.73E-08	1.80E-09	1.17E-15
ENSG00000129197_5426074_5432571_59	Overlap_dORF	RPAIN	17	+	177	20	1	1	8.47%	37.33587922	0.035772986	0.035772986	0.005410795
ENSG00000129197_5421311_5428083_113	annotated	RPAIN	17	+	339	61	17	0	9.73%	59.45656165	0.017540787	0.001618649	0.000177083
ENSG00000089157_120200783_120196773_317	annotated	RPLP0	12	-	951	86	21	7	4.10%	29.88048126	0.004035968	0.002174873	5.02E-05
ENSG00000065357_55939303_55953768_538	annotated	DGKA	12	+	1614	23	2	0	2.23%	4.70862343	0.001459721	0.000862735	7.79E-06
ENSG00000101972_124031120_124100597_1137	annotated	STAG2	X	+	3411	71	7	1	2.64%	6.877748376	1.32E-05	8.77E-07	1.07E-10
ENSG00000132849_61763099_62160935_1765	annotated	PATJ	1	+	5295	21	5	1	0.74%	1.31045763	0.013145698	0.000988864	8.54E-05
ENSG00000145979_13326898_13305101_293	annotated	TBC1D7	6	-	879	112	4	2	8.87%	42.10161944	3.54E-05	3.32E-06	1.02E-09
ENSG00000123130_23743144_23703894_448	annotated	ACOT9	X	-	1344	87	21	5	5.58%	21.38895849	0.002905975	2.73E-05	7.79E-07
ENSG00000145979_13326898_13305101_266	annotated	TBC1D7	6	-	798	105	1	2	8.27%	43.47664883	3.31E-05	1.69E-05	4.39E-09
ENSG00000149380_74302515_74273580_260	dORF	P4HA3	11	-	780	20	2	0	2.69%	8.472372593	0.013786174	0.008776163	0.000603312
ENSG00000008283_63437460_63434402_222	annotated	CYB561	17	-	666	33	11	0	5.41%	16.37228758	0.011805604	0.001011481	7.74E-05
ENSG00000125166_58734228_58708171_387	annotated	GOT2	16	-	1161	258	23	2	11.63%	73.42722914	1.03E-08	2.42E-09	2.68E-16
ENSG00000004799_95596293_95585641_411	annotated	PDK4	7	-	1233	427	49	21	20.19%	114.4285651	3.51E-11	6.96E-15	4.45E-24
ENSG00000013441_200861862_200853306_484	annotated	CLK1	2	-	1452	12	3	4	1.45%	2.73076472	0.043639483	0.067796501	0.011775

ENSG00000110321_10805979_10805911_22	internal	EIF4G2	11	-	66	22	0	12	27.27%	110.1408437	0.013853925	0.14651463	0.010719976
ENSG00000104635_22404711_22434004_481	annotated	SLC39A14	8	+	1443	191	25	10	8.94%	43.73576122	4.71E-06	5.41E-08	2.81E-12
ENSG00000110080_126406156_126414047_329	annotated	ST3GAL4	11	+	987	13	2	0	1.82%	4.352069812	0.044040756	0.012798403	0.002680499
ENSG00000104290_28503149_28563011_621	annotated	FZD3	8	+	1863	51	5	7	2.90%	9.045383299	0.000386364	0.000909079	2.30E-06
ENSG00000213246_58351436_58346246_70	annotated	SUPT4H1	17	-	210	74	3	3	18.57%	116.4346062	0.002524177	0.001397791	2.10E-05
ENSG00000198909_63652645_63693777_537	annotated	MAP3K3	17	+	1611	21	1	0	2.05%	4.307183832	0.002287938	0.001440978	1.96E-05
ENSG00000177731_18245960_18245138_173	annotated	FLII	17	-	519	20	0	4	6.94%	12.73304552	0.000844273	0.009745921	5.39E-05
ENSG00000108306_39343241_39261460_422	annotated	FBXL20	17	-	1266	22	3	7	2.13%	5.741939719	0.010815624	0.076961241	0.004240805
ENSG00000240682_129159155_129130081_299	annotated	ISY1	3	-	897	75	1	1	6.69%	27.62730193	4.00E-05	5.10E-05	1.54E-08
ENSG00000141198_54912675_54915925_183	annotated	TOM1L1	17	+	549	25	3	2	7.10%	15.04656335	0.004584507	0.001786536	4.76E-05
ENSG00000142875_84164413_84235305_355	annotated	PRKACB	1	+	1065	19	1	0	2.82%	5.894862057	0.003737369	0.002201184	4.71E-05
ENSG00000146143_57318446_57326063_158	annotated	PRIM2	6	+	474	11	0	0	3.80%	7.668033423	0.012798403	0.012798403	0.000797244
ENSG00000142875_84144548_84235305_336	annotated	PRKACB	1	+	1008	19	1	0	2.98%	6.228202471	0.003737369	0.002201184	4.71E-05
ENSG00000142875_84078326_84235305_351	annotated	PRKACB	1	+	1053	19	4	0	2.85%	5.962039973	0.023182913	0.002201184	0.000310488
ENSG00000090621_39571256_39561698_488	annotated	PABPC4	1	-	1464	35	2	1	2.87%	7.899445758	0.001092758	0.000408047	2.90E-06
ENSG00000025770_50516489_50523259_539	annotated	NCAPH2	22	+	1617	34	2	1	2.60%	6.9476599	0.000844722	0.000593823	3.20E-06
ENSG00000149084_43680828_43754886_98	annotated	HSD17B12	11	+	294	57	9	5	13.27%	64.06151113	0.000881412	0.003066162	1.67E-05
ENSG00000177427_18262721_18264764_454	annotated	MIEF2	17	+	1362	21	3	0	2.20%	5.094620524	0.013506184	0.002201184	0.00017355
ENSG00000177427_18264246_18264764_172	dORF	MIEF2	17	+	516	9	0	0	2.91%	5.763183682	0.019216965	0.019216965	0.001707396
ENSG00000167004_43746681_43765529_100	annotated	PDIA3	15	+	300	39	15	36	13.00%	42.95492905	0.008071484	0.433797573	0.034459678
ENSG00000068903_38893429_38879155_319	annotated	SIRT2	19	-	957	34	3	0	5.64%	11.73914949	0.000218269	6.41E-05	1.02E-07
ENSG00000127824_219252188_219250352_433	annotated	TUBA4A	2	-	1299	87	0	3	7.16%	22.12991548	4.09E-07	6.03E-07	2.27E-12
ENSG00000115641_105386516_105361283_279	annotated	FHL2	2	-	837	117	12	0	10.04%	46.18809575	2.51E-05	1.64E-06	3.70E-10
ENSG00000168214_26415539_26430799_310	annotated	RBPJ	4	+	930	13	0	0	1.61%	4.618809575	0.021084099	0.021084099	0.002029891
ENSG00000180198_28531958_28535629_150	novel	RCC1	1	+	450	25	2	1	6.00%	18.35680728	0.003303074	0.006112544	0.000111133
ENSG00000164120_174522451_174508746_87	Overlap_uORF	HPGD	4	-	261	73	8	11	17.24%	92.41702978	0.00182801	0.0085369	9.14E-05
ENSG00000164120_174508711_174491956_131	annotated	HPGD	4	-	393	106	25	0	16.79%	89.12159872	0.00020002	1.85E-05	2.97E-08
ENSG00000234741_173867041_173866181_50	novel	GAS5	1	-	150	20	0	0	10.00%	44.05633748	0.021557223	0.021557223	0.002115616
ENSG00000163347_190322206_190308277_211	annotated	CLDN1	3	-	633	108	18	5	13.27%	56.37540815	0.000347759	2.12E-05	6.01E-08
ENSG00000143793_228103227_228101131_263	annotated	C1orf35	1	-	789	18	5	1	3.04%	7.538156603	0.087887166	0.009288069	0.004373107
ENSG00000168000_62705512_62690367_398	annotated	BSCL2	11	-	1194	119	51	2	8.79%	32.9315588	0.006736553	2.56E-07	5.85E-08
ENSG00000168000_62705595_62702488_118	Overlap_uORF	BSCL2	11	-	354	47	0	15	8.47%	43.86965809	0.002488365	0.018376712	0.00026737
ENSG00000177628_155240684_155234995_467	annotated	GBA	1	-	1401	248	23	6	7.92%	58.4902125	3.92E-07	1.23E-07	4.59E-13
ENSG00000007541_578418_582256_233	annotated	PIGQ	16	+	699	17	1	2	3.43%	8.036027223	0.009288069	0.02389627	0.001092027
ENSG00000106070_50626921_50592952_407	annotated	GRB10	7	-	1221	23	2	1	2.95%	6.224175443	0.003782216	0.001559221	3.46E-05
ENSG00000106070_50732322_50592952_594	annotated	GRB10	7	-	1782	25	2	1	2.36%	4.635557395	0.001783	0.000673856	7.55E-06
ENSG00000092036_22955118_22946525_351	annotated	HAUS4	14	-	1053	65	21	3	7.12%	20.39645254	0.002329998	2.61E-05	5.82E-07
ENSG00000160710_154588654_154586820_119	annotated	ADAR	1	-	357	38	7	0	4.20%	35.17102572	0.035772986	0.020613417	0.003288067
ENSG00000160710_154586740_154584806_255	dORF	ADAR	1	-	765	62	1	4	6.67%	26.77934239	0.000148772	0.000690444	7.19E-07
ENSG00000151914_56640580_56615517_2502	annotated	DST	6	-	7506	27	1	5	0.48%	1.188570256	0.001476269	0.010215516	9.15E-05
ENSG00000144524_231788571_231791888_105	novel	COPS7B	2	+	315	18	3	0	5.71%	18.88128749	0.013428348	0.013428348	0.000872559
ENSG00000144357_169827508_170081843_1888	annotated	UBR3	2	+	5664	46	1	0	1.06%	2.683516319	5.30E-05	3.64E-05	1.46E-08
ENSG00000174886_5903708_5892917_228	annotated	NDUFA11	19	-	684	59	33	10	6.14%	28.50135868	0.057049371	0.007102572	0.002178391
ENSG00000068028_50340805_50330581_344	annotated	RASSF1	3	-	1032	11	0	0	1.74%	3.521945584	0.012798403	0.012798403	0.000797244
ENSG00000141447_24280946_24163179_558	annotated	OSBPL1A	18	-	1674	17	3	2	1.61%	3.355545418	0.028023387	0.015046252	0.001959107
ENSG00000162616_78005111_78016247_337	annotated	DNAJB4	1	+	1011	29	4	0	4.15%	9.477995452	0.003008559	0.000378748	7.67E-06
ENSG00000134851_55396190_55403259_76	annotated	TMEM165	4	+	228	13	0	1	6.58%	18.83988116	0.021084099	0.028889786	0.002731533
ENSG00000134851_55403333_55425452_257	dORF	TMEM165	4	+	771	173	14	3	14.40%	74.14150179	1.18E-07	5.73E-08	6.62E-14
ENSG00000115109_120042090_120174900_599	annotated	EPB41L5	2	+	1797	16	4	0	1.17%	2.941992486	0.059453662	0.008473674	0.002626388
ENSG00000173218_115651414_115691379_522	annotated	VANGL1	1	+	1566	33	8	5	3.26%	6.962926901	0.003274181	0.001388273	2.70E-05
ENSG00000166888_57108251_57096572_838	annotated	STAT6	12	-	2514	71	7	1	3.46%	9.331742128	1.92E-05	8.59E-07	1.55E-10
ENSG00000166801_59148873_59153504_611	annotated	FAM111A	11	+	1833	38	4	0	2.78%	6.850003373	0.000938981	0.000125329	8.45E-07
ENSG00000007968_23530730_23509880_416	annotated	E2F2	1	-	1248	9	1	0	1.44%	2.382854792	0.026832696	0.011772051	0.001510382
ENSG00000214063_862679_866630_174	annotated	TSPAN4	11	+	522	318	49	1	28.16%	201.2918868	7.64E-09	5.20E-10	4.53E-17
ENSG00000214063_850341_866630_226	annotated	TSPAN4	11	+	678	377	52	6	28.32%	183.7305225	7.22E-11	2.87E-12	2.67E-21
ENSG00000133424_33604482_33274427_567	annotated	LARGE1	22	-	1701	8	0	0	0.88%	1.554015431	0.019679754	0.019679754	0.001784964
ENSG00000071189_17940295_17796819_888	annotated	SNX13	7	-	2664	47	0	1	2.14%	5.829526638	5.26E-05	7.48E-05	2.92E-08
ENSG00000118518_127286653_127287693_346	dORF	RNF146	6	+	1038	40	1	0	5.20%	12.73304552	8.20E-05	8.27E-05	4.95E-08
ENSG00000126773_60115290_60168748_712	annotated	PCNX4	14	+	2136	29	5	0	1.83%	4.48607369	0.006988802	0.000598307	2.80E-05
ENSG00000107938_125723859_125763472_1094	annotated	EDRF1	10	+	3282	24	0	2	0.73%	2.416252513	0.005706018	0.013786174	0.000408699
ENSG00000125450_75225822_75232038_156	Overlap_dORF	NUP85	17	+	468	20	5	1	4.49%	14.12062099	0.060359788	0.012000727	0.003537692
ENSG00000149485_61803755_61802411_146	annotated	FADS1	11	-	438	708	131	15	23.97%	534.107653	1.98E-06	4.18E-07	7.61E-12
ENSG00000163281_44722207_44707741_259	annotated	GNPDA2	4	-	777	15	2	0	2.32%	6.378813342	0.023718446	0.012798403	0.001440254
ENSG00000121211_153350106_153361607_111	annotated	MND1	4	+	333	31	0	0	9.01%	30.76005545	0.00244612	0.00244612	3.45E-05
ENSG00000121211_153363020_153414857_115	dORF	MND1	4	+	345	34	5	4	7.83%	32.56337988	0.01778219	0.02437921	0.001989998
ENSG00000140395_78288374_78278251_102	annotated	WDR61	15	-	306	79	12	0	11.76%	85.30516326	0.003257575	0.001061589	2.10E-05
ENSG00000101294_31564202_31569219_87	dORF	HM13	20	+	261	217	2	0	17.24%	274.7191159	0.000364255	0.000307311	7.51E-07
ENSG00000101294_31514552_31564139_365	annotated	HM13	20	+	1095	926	97	10	19.18%	279.4258117	3.68E-12	8.18E-13	3.86E-23

ENSG00000067141_73289208_73302696_224	novel	NEO1	15	+	672	18	2	1	4.46%	8.850603512	0.007439212	0.003957932	0.000159198
ENSG00000128833_52256641_52239855_340	dORF	MYO5C	15	-	1020	10	0	0	2.06%	3.23943658	0.007593099	0.007593099	0.000297833
ENSG00000010626_6873689_6883901_96	uORF	LRRC23	12	+	288	16	0	3	5.21%	18.35680728	0.021084099	0.068043226	0.0063746
ENSG00000185721_31400620_31423313_221	novel	DRG1	22	+	663	57	9	0	7.24%	28.40736693	0.003132423	0.000207012	4.72E-06
ENSG00000124596_41071634_41071049_88	annotated	OARD1	6	-	264	20	0	1	7.95%	25.03200993	0.008473674	0.013892891	0.00058868
ENSG00000182093_39390904_39396939_90	Overlap_dORF	WRB	21	+	270	60	13	1	20.00%	73.42722914	0.000371152	9.04E-05	2.41E-07
ENSG00000182093_39390698_39390907_69	annotated	WRB	21	+	207	85	9	8	23.19%	135.6807495	0.000414314	0.000179964	5.13E-07
ENSG00000174891_158132173_158298067_97	dORF	RSRC1	3	+	291	28	0	12	7.22%	31.79323323	0.008776163	0.123271564	0.006232237
ENSG00000101882_119936810_119925220_302	novel	NKAP	X	-	906	27	3	1	3.31%	9.847029073	0.012563987	0.00224099	0.000163338
ENSG00000020256_52164722_52088967_197	novel	ZFP64	20	-	591	6	0	0	2.54%	3.354543463	0.016947427	0.016947427	0.001349898
ENSG00000170445_140691304_140674257_489	annotated	HARS	5	-	1467	122	19	7	6.54%	27.4789017	1.28E-05	4.23E-06	4.53E-10
ENSG00000143575_154272724_154273657_124	novel	HAX1	1	+	372	38	5	1	5.65%	33.7528392	0.03713939	0.010275151	0.001866575
ENSG00000115944_42361187_42353337_34	Overlap_uORF	COX7A2L	2	-	102	81	7	14	23.53%	262.3943629	0.005655336	0.1806996	0.007416216
ENSG00000115944_42361161_42351219_114	annotated	COX7A2L	2	-	342	116	29	4	19.30%	112.0731392	0.000105878	2.22E-05	1.83E-08
ENSG00000003147_8221318_8113923_400	annotated	ICA1	7	-	1200	14	1	1	2.00%	3.85492953	0.005160369	0.007930666	0.000216377
ENSG00000013563_154405062_154402707_250	annotated	DNASE1L1	X	-	750	31	6	0	6.00%	13.65746462	0.004766409	0.000266003	9.24E-06
ENSG00000089685_78214317_78214793_74	annotated	BIRC5	17	+	222	65	16	0	16.22%	96.74533569	0.007661138	0.00107863	5.14E-05
ENSG00000152234_46091840_46089784_123	novel	ATP5F1A	18	-	369	294	21	3	26.83%	263.2634801	3.32E-06	2.54E-07	8.05E-12
ENSG00000163703_9934475_9944391_410	annotated	CRELD1	3	+	1230	30	5	0	2.93%	8.059086125	0.008855938	0.000954935	5.40E-05
ENSG00000119599_72945995_72958805_281	annotated	DCAF4	14	+	843	6	0	0	1.78%	2.351761787	0.016947427	0.016947427	0.001349898
ENSG00000108559_5419650_5388771_557	novel	NUP88	17	-	1671	56	2	4	3.59%	11.07340619	0.000108871	0.000223784	1.73E-07
ENSG00000106541_16801796_16794847_188	annotated	AGR2	7	-	564	1048	128	26	30.85%	613.9766181	2.31E-10	5.25E-11	1.43E-19
ENSG00000128309_37024216_37029514_297	annotated	MPST	22	+	891	100	14	8	7.41%	37.08445916	0.001171666	0.000379722	2.91E-06
ENSG00000127483_20780440_20744797_553	annotated	HP1BP3	1	-	1659	117	13	19	6.33%	23.30285482	1.49E-05	8.15E-05	9.67E-09
ENSG00000175376_66000389_65998599_165	annotated	EIF1AD	11	-	495	39	5	5	7.27%	26.03329033	0.004616409	0.006221623	0.000154222
ENSG00000205643_46247199_46245092_78	novel	CDPF1	22	-	234	8	0	0	6.41%	11.29649679	0.019679754	0.019679754	0.001784964
ENSG00000168591_44190529_44190864_111	annotated	TMUB2	17	+	333	22	1	0	9.91%	21.82971677	0.003990669	0.001269017	3.03E-05
ENSG00000101811_100820417_100837409_438	annotated	CSTF2	X	+	1314	12	1	0	1.60%	3.017557362	0.01551626	0.007882058	0.000614257
ENSG00000169118_64300448_64171931_413	annotated	CSNK1G1	15	-	1239	8	2	4	1.21%	2.133478813	0.080264768	0.078649604	0.023170289
ENSG00000167131_44900999_44902817_242	annotated	CCDC103	17	+	726	9	1	0	2.07%	4.09614708	0.041782825	0.019216965	0.003600526
ENSG00000144724_61562288_62293307_1416	annotated	PTPRG	3	+	4248	62	6	4	1.62%	4.822551066	6.99E-05	2.96E-05	1.58E-08
ENSG00000153187_244863998_244854450_697	annotated	HNRNPU	1	-	2091	469	55	26	13.77%	74.11198809	2.53E-13	2.20E-14	7.66E-26
ENSG00000163611_113506605_113445307_855	annotated	SPICE1	3	-	2565	20	0	3	0.94%	2.576394005	0.005306578	0.031850938	0.000910573
ENSG00000119004_203239063_203296908_470	annotated	CYP20A1	2	+	1410	53	2	3	4.26%	12.42013769	0.000124227	5.18E-05	4.76E-08
ENSG00000164828_838815_838970_51	novel	SUN1	7	+	153	22	3	16	19.61%	47.5117365	0.010402772	0.229394231	0.015451134
ENSG00000164828_832525_849689_341	novel	SUN1	7	+	1023	59	24	3	6.74%	19.05662692	0.007451799	5.70E-05	4.29E-06
ENSG00000177830_904816_869858_418	annotated	CHID1	11	-	1254	142	14	0	11.48%	37.41626748	8.34E-09	5.45E-10	5.18E-17
ENSG00000204843_74378227_74361499_1256	annotated	DCTN1	2	-	3768	103	27	4	3.98%	9.032250718	5.69E-06	2.04E-09	1.91E-13
ENSG00000169371_75621051_75598358_360	annotated	SNUPN	15	-	1080	66	6	1	6.39%	20.19248801	0.000177483	1.20E-05	1.78E-08
ENSG00000140718_53711428_54008434_450	annotated	FTO	16	+	1350	41	5	3	4.44%	10.03505465	0.000603442	0.000250038	1.02E-06
ENSG00000114786_51987035_51987645_103	dORF	ABHD14A-ACY1	3	+	309	18	2	0	6.80%	19.24791443	0.016503129	0.007979402	0.00066003
ENSG00000114786_51984065_51986623_214	Overlap_dORF	ABHD14A-ACY1	3	+	642	28	1	0	5.14%	14.41095151	0.001506153	0.001506153	1.36E-05
ENSG00000114786_51988530_51989075_99	dORF	ABHD14A-ACY1	3	+	297	27	1	4	8.08%	30.03841192	0.005355751	0.025702836	0.000731269
ENSG00000114786_51975136_51984153_167	annotated	ABHD14A-ACY1	3	+	501	9	0	0	3.59%	5.935734092	0.009815329	0.009815329	0.000483714
ENSG00000104763_18084058_18069875_42	annotated	ASAH1	8	-	126	222	3	1	16.67%	582.173031	0.008776163	0.016983117	0.000739612
ENSG00000104763_18069848_18057534_313	dORF	ASAH1	8	-	939	275	33	5	15.97%	96.76911188	2.73E-08	1.44E-09	4.40E-16
ENSG00000130635_134642188_134842303_1838	annotated	COL5A1	9	+	5514	59	6	8	1.25%	3.535533068	0.000209508	0.00030639	4.39E-07
ENSG00000063177_48619143_48615873_164	annotated	RPL18	19	-	492	757	33	14	29.88%	508.3940164	7.93E-09	8.93E-10	7.89E-17
ENSG00000108788_42570079_42571603_53	novel	MLX	17	+	159	25	2	0	13.21%	51.95322816	0.019778611	0.008473674	0.000833471
ENSG00000108788_42567125_42569593_90	novel	MLX	17	+	270	28	2	2	5.56%	34.26604026	0.08410207	0.08410207	0.025660709
ENSG00000167792_67608700_67611931_230	novel	NDUFV1	11	+	690	143	65	3	12.61%	68.47887239	0.003025788	2.92E-06	1.33E-07
ENSG00000143416_151369171_151364543_408	novel	SELENBP1	1	-	1224	11	0	0	1.47%	2.969483531	0.012798403	0.012798403	0.000797244
ENSG00000130429_99385715_99394489_372	annotated	ARPC1B	7	+	1116	335	32	23	16.67%	99.18597484	2.13E-10	1.57E-09	3.84E-18
ENSG00000248487_51975136_51981018_273	annotated	ABHD14A	3	+	819	12	0	1	2.93%	4.841355767	0.00419316	0.010499663	0.000236136
ENSG00000184677_22490252_22526396_1138	annotated	ZBTB40	1	+	3414	13	7	0	0.79%	1.258199445	0.072063517	0.002943991	0.001440602
ENSG00000168214_26415539_26430110_293	novel	RBPJ	4	+	879	13	0	0	1.71%	4.886795113	0.021084099	0.021084099	0.002029891
ENSG00000062194_57246335_57262752_302	annotated	GPBP1	5	+	906	13	2	0	2.98%	4.741162146	0.020607861	0.00308495	0.0003625
ENSG00000129055_134482904_134482785_39	novel	ANAPC13	3	-	117	15	4	2	15.38%	42.36186296	0.080264768	0.035772986	0.011710952
ENSG00000132294_131944766_132010895_785	annotated	EFR3A	8	+	2355	114	17	4	5.99%	15.99497603	1.93E-07	7.49E-09	1.57E-14
ENSG00000169398_140890737_140659466_1006	annotated	PTK2	8	-	3018	80	14	9	3.28%	8.758715205	5.46E-05	8.62E-06	3.85E-09
ENSG00000187079_12764287_12937222_354	annotated	TEAD1	11	+	1062	34	1	0	5.08%	10.57849911	0.000180392	7.57E-05	9.88E-08
ENSG00000102805_76995908_77000969_243	Overlap_dORF	CLN5	13	+	729	29	2	1	6.17%	13.14438052	0.000660022	0.000415728	1.79E-06
ENSG00000114062_25375498_25340081_724	annotated	UBE3A	15	-	2172	33	10	1	2.07%	5.020231826	0.002059394	0.000407542	5.52E-06
ENSG00000162910_228106918_228106760_52	annotated	MRPL55	1	-	156	139	11	4	38.46%	294.4149476	4.79E-05	4.09E-05	1.48E-08
ENSG00000164327_39074377_38978628_257	annotated	RICTOR	5	-	771	23	1	0	2.33%	9.856962666	0.023878474	0.01300724	0.001470914
ENSG00000164327_38975570_38942304_1385	dORF	RICTOR	5	-	4155	27	2	7	0.94%	2.147150022	0.001588752	0.016567905	0.000163807
ENSG00000117523_171513048_171591847_2764	annotated	PRRC2C	1	+	8292	62	3	12	1.12%	2.470597797	1.15E-06	0.000107548	1.27E-09
ENSG00000139637_53305908_53307149_213	annotated	C12orf10	12	+	639	58	2	2	9.39%	29.99140345	7.94E-05	0.00013812	7.93E-08

ENSG00000197912_89508547_89529209_187	annotated	SPG7	16	+	561	50	2	2	6.95%	29.44942345	0.001027359	0.001765381	1.11E-05
ENSG00000143376_151612202_151693601_407	annotated	SNX27	1	+	1221	35	2	1	3.69%	9.471571326	0.000743479	0.000437499	2.11E-06
ENSG00000186298_110722672_110721076_141	novel	PPP1CC	12	-	423	234	15	1	16.31%	182.7869321	4.75E-05	1.29E-05	4.86E-09
ENSG00000139644_49753061_49758539_115	novel	TMBIM6	12	+	345	961	367	46	38.26%	920.3943548	1.42E-06	5.99E-09	1.06E-13
ENSG00000104763_18084058_18057534_370	annotated	ASAH1	8	-	1110	515	37	6	17.30%	153.3041473	1.13E-10	1.03E-11	1.43E-20
ENSG00000143179_165896284_165907823_111	novel	UCK2	1	+	333	49	2	0	10.81%	48.62073281	0.001003278	0.001040526	6.47E-06
ENSG00000078618_51878468_51862367_81	novel	NRDC	1	-	243	10	1	0	6.17%	13.59763503	0.020613417	0.019679754	0.001864002
ENSG00000257923_101816052_102283090_671	annotated	CUX1	7	+	2013	82	19	1	6.11%	13.45983485	2.03E-05	7.46E-09	2.49E-12
ENSG00000213983_23567314_23566417_68	novel	AP1G2	14	-	204	10	1	0	8.82%	16.1971829	0.011772051	0.011772051	0.000681273
ENSG00000135090_118239266_118150997_798	annotated	TAOK3	12	-	2394	22	0	0	1.00%	3.036464363	0.005355751	0.005355751	0.00015367
ENSG00000125520_63739913_63742990_387	annotated	SLC2A4RG	20	+	1161	47	4	3	4.13%	13.37627818	0.00093424	0.000381693	2.33E-06
ENSG00000157168_32722003_32763338_308	annotated	NRG1	8	+	924	21	2	1	2.92%	7.50960298	0.007181907	0.004400434	0.000169559
ENSG00000156603_57712179_57704622_194	annotated	MED19	11	-	582	19	3	2	6.19%	10.78698985	0.006220149	0.005380042	0.000177983
ENSG00000101849_9654265_9716246_526	annotated	TBL1X	X	+	1578	28	2	0	2.28%	5.863010692	0.001573472	0.000947024	9.15E-06
ENSG00000103351_3504767_3536271_409	annotated	CLUAP1	16	+	1227	29	8	0	3.18%	7.809497475	0.023199731	0.000642411	0.000114443
ENSG00000288053_49077855_49071869_255	annotated	AC231657.3	X	-	765	40	8	1	7.06%	17.27699509	0.001198386	0.00010536	9.34E-07
ENSG00000134590_135032384_135033184_83	novel	RTL8C	X	+	249	89	5	1	14.46%	118.1028324	0.002797218	0.001029593	1.75E-05
ENSG00000126214_103675572_103685018_149	annotated	KLC1	14	+	447	47	2	1	9.40%	34.74241379	0.001003788	0.000667595	4.24E-06
ENSG00000100938_24233254_24238778_320	annotated	GMPR2	14	+	960	81	18	0	8.12%	27.87940106	0.000225465	3.61E-06	7.85E-09
ENSG00000048649_77820684_77666917_1431	annotated	RSF1	11	-	4293	28	4	4	1.19%	2.155096872	0.00259673	0.001546981	2.37E-05
ENSG00000100902_35292477_35317272_203	annotated	PSMA6	14	+	609	154	19	2	12.32%	83.55512281	4.97E-05	8.84E-06	3.58E-09
ENSG00000100612_60165309_60152864_235	novel	DHRS7	14	-	705	196	18	9	17.02%	91.86215049	4.43E-08	4.04E-08	1.78E-14
ENSG00000178252_49013983_49015288_305	annotated	WDR6	3	+	915	41	4	5	3.28%	14.80581833	0.009076127	0.009008962	0.000414356
ENSG00000241978_110048700_110168497_961	annotated	AKAP2	9	+	2883	24	4	0	1.35%	2.750655826	0.004644735	0.000633425	1.91E-05
ENSG00000167703_1627760_1585660_451	novel	SLC43A2	17	-	1353	8	1	0	1.11%	1.953717848	0.042234516	0.019679754	0.003715148
ENSG00000143321_156751429_156743449_240	annotated	HDGF	1	-	720	325	34	13	23.75%	149.1490592	1.65E-10	9.13E-11	1.75E-19
ENSG00000070159_109457369_109379556_857	annotated	PTPN3	9	-	2571	38	6	0	1.75%	4.883724692	0.001971466	0.000299491	4.00E-06
ENSG00000168944_123422998_123346519_986	annotated	CEP120	5	-	2958	25	1	2	1.52%	2.792617741	0.000433186	0.001508934	4.25E-06
ENSG00000151665_46615164_46588105_206	annotated	PIGF	2	-	618	38	6	1	8.25%	20.31724301	0.00080433	0.000172552	9.63E-07
ENSG00000204209_33321925_33318743_740	annotated	DAXX	6	-	2220	32	7	2	2.03%	4.762847295	0.009011833	0.001095361	6.19E-05
ENSG00000182944_29268337_29288790_325	novel	EWSR1	22	+	975	52	11	3	3.69%	17.62253499	0.016435635	0.003322445	0.000304131
ENSG00000132906_15524086_15492943_378	annotated	CASP9	1	-	1134	27	3	0	4.23%	7.867203122	0.001009322	0.000145171	1.04E-06
ENSG00000136861_120536400_120408300_1347	novel	CDK5RAP2	9	-	4041	43	3	6	1.78%	3.516003177	5.13E-05	0.000148092	5.62E-08
ENSG00000121060_56904440_56899107_139	novel	TRIM25	17	-	417	22	0	3	5.76%	17.43236375	0.005604867	0.024498662	0.000722998
ENSG00000128609_123557795_123542119_101	annotated	NDUFA5	7	-	303	43	5	0	8.91%	46.89164633	0.060543501	0.003327803	0.000128497
ENSG00000127922_96709763_96645933_57	annotated	SEM1	7	-	171	133	15	43	26.32%	256.995302	0.000315272	0.026244752	7.59E-05
ENSG00000149428_119056160_119045593_999	annotated	HYOU1	11	-	2997	321	51	9	10.31%	35.39060143	9.22E-14	1.38E-17	2.45E-29
ENSG00000166452_8911450_8919480_183	annotated	AKIP1	11	+	549	43	5	4	9.29%	25.88008896	0.002240233	0.000581804	8.31E-06
ENSG00000204316_75904684_75898850_346	annotated	MRPL38	17	-	1038	149	13	11	14.16%	47.43059454	7.03E-08	6.52E-08	4.50E-14
ENSG00000111206_2864370_2858638_358	novel	FOXM1	12	-	1074	24	0	0	2.23%	7.383743712	0.005604867	0.005604867	0.000167525
ENSG00000124356_73830857_73849431_209	annotated	STAMBP	2	+	627	16	32	4	4.78%	8.431834925	0.04578229	0.023141261	0.004631938
ENSG00000072864_15664779_15724251_335	annotated	NDE1	16	+	1005	66	13	1	9.25%	21.6993901	3.00E-05	5.86E-07	1.76E-10
ENSG00000072864_15664779_15724251_335	annotated	NDE1	16	+	1005	66	13	1	9.25%	21.6993901	3.00E-05	5.86E-07	1.76E-10
ENSG00000055044_202282365_202290335_85	novel	NOP58	2	+	255	14	0	0	9.41%	18.14084485	0.004149608	0.004149608	9.46E-05
ENSG00000175662_17869449_17847635_240	annotated	TOM1L2	17	-	720	10	0	1	2.50%	4.589201821	0.012798403	0.023672501	0.001437523
ENSG00000141012_88856877_88814439_522	annotated	GALNS	16	-	1566	130	19	3	7.66%	27.42971203	1.08E-05	8.79E-08	1.06E-11
ENSG00000135250_105388806_105117838_699	annotated	SRPK2	7	-	2097	59	3	5	3.72%	9.296580513	2.18E-05	4.05E-05	6.84E-09
ENSG00000157349_70317575_70333582_354	annotated	DDX19B	16	+	1062	41	1	0	4.24%	12.7564254	0.000279067	0.000279067	5.29E-07
ENSG00000099956_23787179_23834180_391	annotated	SMARCB1	22	+	1173	136	23	5	10.49%	38.30985868	1.20E-06	4.55E-08	5.64E-13
ENSG00000105357_50260713_50309790_1229	annotated	MYH14	19	+	3687	8	1	0	0.49%	0.716946094	0.028889786	0.009815329	0.001387795
ENSG00000172270_571548_582577_205	annotated	BSG	19	+	615	3397	370	85	46.34%	1825.114371	1.02E-15	2.31E-16	3.46E-30
ENSG00000141556_82763973_82942463_1149	annotated	TBCD	17	+	3447	113	16	0	3.83%	10.83195417	1.54E-06	2.83E-09	5.90E-14
ENSG00000143514_223802248_223780853_770	novel	TP53BP2	1	-	2310	10	0	0	0.91%	1.430400568	0.007029303	0.007029303	0.000257364
ENSG00000141959_44326018_44326862_98	novel	PFKL	21	+	294	6	0	1	5.10%	6.743316962	0.016947427	0.047790352	0.003697512
ENSG00000141959_44321777_44324921_213	novel	PFKL	21	+	639	31	2	0	5.63%	16.02988805	0.001414407	0.00092354	8.05E-06
ENSG00000163002_183128430_183161131_265	annotated	NUP35	2	+	795	28	5	0	3.77%	11.63752311	0.012925525	0.002432129	0.000180628
ENSG00000162591_3531245_3490528_1229	annotated	MEGF6	1	-	3687	20	0	0	0.98%	1.792365235	0.000919662	0.000919662	5.28E-06
ENSG00000038532_10962474_11178690_977	annotated	CLEC16A	16	+	2931	9	2	3	0.72%	1.014603473	0.056175599	0.065144778	0.01417143
ENSG00000005448_74421751_74425657_244	annotated	WDR54	2	+	732	67	5	2	11.07%	30.24359233	3.89E-05	7.14E-06	2.28E-09
ENSG00000181555_47123969_47017093_2342	annotated	SETD2	3	-	7026	29	1	6	0.68%	1.363827697	0.000169241	0.0033	4.20E-06
ENSG00000147642_109586100_109574906_500	annotated	SYBU	8	-	1500	22	9	1	2.00%	4.846197123	0.02437921	0.00345766	0.000477745
ENSG00000166557_79311250_79411504_173	annotated	TMED3	15	+	519	86	14	0	14.45%	54.75209571	7.69E-05	5.44E-06	3.59E-09
ENSG00000116685_11926237_11920315_634	annotated	KIAA2013	1	-	1902	18	3	0	1.74%	3.127027108	0.003957932	0.001377539	3.24E-05
ENSG00000249158_140869104_140871536_810	annotated	PCDHA11	5	+	2430	14	0	2	0.62%	1.903668904	0.021084099	0.055917761	0.005218013
ENSG00000170889_54201185_54206526_156	novel	RPS9	19	+	468	549	50	16	11.54%	387.6110461	0.000414357	0.000194404	5.51E-07
ENSG00000006744_12995061_12992818_223	novel	ELAC2	17	-	669	15	2	0	3.14%	7.408576931	0.031982531	0.008175611	0.001316552
ENSG00000006744_13017084_12996418_501	novel	ELAC2	17	-	1503	65	4	2	4.99%	14.28973022	1.33E-05	1.71E-05	1.81E-09
ENSG00000109452_142431259_142028782_924	annotated	INPP4B	4	-	2772	36	10	2	2.27%	4.291201703	0.002758887	0.000120269	2.57E-06

ENSG00000101266_489830_483961_167	annotated	CSNK2A1	20	-	501	47	1	0	8.38%	30.99772248	0.000475338	0.000472567	1.47E-06
ENSG00000180901_75059579_75063047_60	dORF	KCTD2	17	+	180	11	0	0	8.33%	20.19248801	0.019216965	0.019216965	0.001707396
ENSG00000170088_165112755_165079658_265	annotated	TMEM192	4	-	795	56	8	1	6.42%	23.27504622	0.001375745	0.000187107	1.80E-06
ENSG00000168944_123418486_123346519_960	annotated	CEP120	5	-	2880	20	1	0	1.46%	2.294600911	0.000661953	0.000328827	1.44E-06
ENSG00000189067_11556730_11549738_75	annotated	LITAF	16	-	225	112	12	0	14.67%	164.4769933	0.002017024	0.001626321	1.94E-05
ENSG00000173801_41771836_41755744_739	annotated	JUP	17	-	2217	209	12	8	7.17%	31.14944024	9.72E-10	6.43E-10	6.96E-18
ENSG00000100162_41939175_41939056_39	annotated	CENPM	22	-	117	48	4	2	20.51%	135.5579615	0.011575476	0.011575476	0.000660022
ENSG00000100300_43159263_43159424_53	novel	TSPO	22	+	159	89	9	2	16.98%	184.9534923	0.010698784	0.003792958	0.000219936
ENSG00000145901_151065095_151030713_572	annotated	TNIP1	5	-	1716	59	6	2	4.55%	11.36068143	4.86E-05	9.77E-06	3.84E-09
ENSG00000134014_28099925_28112534_102	novel	ELP3	8	+	306	18	1	0	6.86%	19.43661948	0.0099656	0.008473674	0.000427489
ENSG00000109390_139295798_139292550_76	annotated	NDUFC1	4	-	228	129	21	8	26.32%	186.94959	0.000474984	7.52E-05	2.62E-07
ENSG00000139192_6457486_6462149_253	novel	TAPBPL	12	+	759	7	1	1	1.98%	3.047375122	0.041782825	0.064489021	0.010808225
ENSG00000120533_109334469_109340498_53	annotated	ENY2	8	+	159	140	39	8	30.19%	290.9380777	0.000403636	0.000420215	1.12E-06
ENSG00000196591_113946098_113941058_191	novel	HDAC2	6	-	573	76	21	1	10.47%	43.82567603	0.004655379	4.03E-05	1.86E-06
ENSG00000196591_113953333_113949072_81	novel	HDAC2	6	-	243	15	1	0	6.17%	20.39645254	0.021084099	0.021084099	0.002029891
ENSG00000196591_113949066_113948911_51	novel	HDAC2	6	-	153	20	0	0	11.76%	43.19248773	0.01300724	0.01300724	0.000821865
ENSG00000156170_95025009_95034072_121	annotated	NDUFAF6	8	+	363	12	2	1	4.13%	10.92305888	0.060667625	0.035772986	0.00890249
ENSG00000145901_151065095_151030642_635	annotated	TNIP1	5	-	1905	59	6	2	4.09%	10.23355871	4.86E-05	9.77E-06	3.84E-09
ENSG00000126768_48895519_48894089_122	dORF	TIMM17B	X	-	366	36	3	0	11.48%	32.50057683	0.000436224	0.000448121	1.29E-06
ENSG00000108375_58415526_58354943_766	annotated	RNF43	17	-	2298	6	2	0	0.65%	0.862722013	0.158655254	0.016947427	0.0136533
ENSG00000168385_241326104_241350174_321	annotated	2-Sep	2	+	963	333	31	9	19.31%	114.2582584	4.85E-09	5.13E-12	3.79E-19
ENSG00000010278_6235323_6237828_81	Overlap_dORF	CD9	12	+	243	379	34	7	39.51%	515.3503675	1.03E-05	3.73E-07	3.73E-11
ENSG00000010278_6200500_6236202_182	annotated	CD9	12	+	546	800	351	93	35.16%	484.1355767	2.33E-05	2.45E-11	2.59E-14
ENSG00000097033_86704900_86743235_365	annotated	SH3GLB1	1	+	1095	101	11	0	8.49%	30.47732935	3.65E-05	4.71E-07	1.79E-10
ENSG00000105221_40242066_40241809_85	novel	AKT2	19	-	255	12	0	0	5.88%	15.54929558	0.021084099	0.021084099	0.002029891
ENSG00000182180_73251778_73250852_51	novel	MRPS16	10	-	153	73	12	0	27.45%	157.6525802	0.001564278	0.000476727	4.81E-06
ENSG00000124702_43017193_43020733_382	annotated	KLHDC3	6	+	1146	30	0	1	4.71%	8.649804479	6.79E-05	0.000117477	5.83E-08
ENSG00000144579_218401642_218402355_59	novel	CTDSP1	2	+	177	21	0	3	10.17%	39.20267318	0.013640586	0.032657853	0.002090452
ENSG00000015532_50346141_50360291_865	annotated	XYLT2	17	+	2595	58	7	2	2.77%	7.385166399	5.25E-05	1.23E-05	5.16E-09
ENSG00000168002_62765209_62766507_69	dORF	POLR2G	11	+	207	203	11	7	37.68%	324.0375547	2.11E-05	7.41E-06	1.27E-09
ENSG00000133789_9664180_9740465_490	novel	SWAP70	11	+	1470	37	3	4	2.65%	8.316757586	0.00211153	0.004567961	5.52E-05
ENSG00000175575_73887063_73927362_375	annotated	PAAF1	11	+	1125	12	7	0	2.13%	3.524506999	0.233427135	0.004873904	0.009587304
ENSG00000137710_110272594_110231869_447	annotated	RDX	11	-	1341	68	1	3	5.82%	16.75520665	4.11E-06	1.65E-05	5.70E-10
ENSG00000174669_66371591_66363436_456	annotated	SLC29A2	11	-	1368	66	14	15	5.04%	15.9414379	0.001692414	0.005574075	5.51E-05
ENSG00000132155_12618588_12584514_566	Overlap_dORF	RAF1	3	-	1698	56	13	6	2.47%	10.89732729	0.023438118	0.002856574	0.000390246
ENSG00000214413_110918157_110900360_67	annotated	BBIP1	10	-	201	21	0	8	10.45%	34.52175698	0.008776163	0.127803999	0.006510564
ENSG00000103005_58002534_58011129_135	annotated	USB1	16	+	405	9	1	2	4.44%	7.342722914	0.026832696	0.053594825	0.006148603
ENSG00000074582_218662227_218663386_189	novel	BCS1L	2	+	567	24	5	0	5.29%	13.98613888	0.02591725	0.002201184	0.000351167
ENSG00000074582_218661800_218662578_93	novel	BCS1L	2	+	279	26	1	0	5.38%	30.79206383	0.022899795	0.021084099	0.002192856
ENSG00000175866_81035255_81108998_521	annotated	BAIAP2	17	+	1563	11	0	0	1.34%	2.325430481	0.007882058	0.007882058	0.000319636
ENSG00000133816_12208064_12262520_763	annotated	MICAL2	11	+	2289	14	6	0	0.79%	2.020932912	0.084246734	0.011772051	0.005012526
ENSG00000004897_47189172_47120935_823	annotated	CDC27	17	-	2469	11	7	1	0.85%	1.472113342	0.213136039	0.016735684	0.019407396
ENSG00000149806_65121813_65120681_133	annotated	FAU	11	-	399	809	84	58	26.32%	669.9544553	2.87E-06	2.37E-06	5.86E-11
ENSG00000048707_12363243_12509024_907	novel	VPS13D	1	+	2721	20	3	1	0.99%	2.428684536	0.015528143	0.005034873	0.00041222
ENSG00000095002_47403192_47607456_938	annotated	MSH2	2	+	2814	76	11	9	4.05%	8.923991601	2.85E-06	2.21E-06	5.42E-11
ENSG00000103021_58254034_58279777_289	annotated	CCDC113	16	+	867	8	1	1	2.08%	3.048881487	0.026602765	0.026602765	0.003128246
ENSG00000112531_163478879_163570710_205	dORF	QKI	6	+	615	23	6	0	3.90%	12.35726539	0.042503047	0.005706018	0.001320328
ENSG00000111530_67281932_67312830_1200	annotated	CAND1	12	+	3600	166	27	11	4.92%	15.23615005	1.69E-07	9.59E-10	2.03E-15
ENSG00000123159_14482976_14478416_333	annotated	GIPC1	19	-	999	114	25	17	12.31%	37.70587442	1.06E-05	1.34E-06	1.26E-10
ENSG00000113643_168495354_168519190_454	dORF	RARS	5	+	1362	114	6	1	9.25%	27.65651141	8.64E-09	7.31E-09	6.61E-16
ENSG00000084693_27053896_27070263_757	annotated	AGBL5	2	+	2271	19	0	0	1.59%	2.764433329	0.00077643	0.00077643	3.81E-06
ENSG00000141179_55767410_55773711_64	annotated	PCTP	17	+	192	22	0	0	9.38%	37.86091502	0.01300724	0.01300724	0.000821865
ENSG00000130159_11519170_11506184_217	annotated	ECSIT	19	-	651	36	0	0	4.15%	18.2722137	0.003789641	0.003789641	7.96E-05
ENSG00000106603_43647613_43639582_134	annotated	COA1	7	-	402	36	6	4	9.70%	29.59007741	0.007322027	0.003864916	0.000153348
ENSG00000103353_23557952_23570590_300	annotated	UBFD1	16	+	900	84	11	9	9.33%	30.83943624	1.55E-05	6.93E-05	8.51E-09
ENSG00000215301_41334253_41345417_420	novel	DDX3X	X	+	1260	106	35	9	7.14%	27.79745103	0.000950113	1.52E-05	1.34E-07
ENSG00000215301_41345404_41347719_272	novel	DDX3X	X	+	816	123	13	4	9.56%	49.80633741	7.97E-06	4.42E-06	2.94E-10
ENSG00000095002_47403192_47632806_918	annotated	MSH2	2	+	2754	76	11	9	4.14%	9.118414076	2.85E-06	2.21E-06	5.42E-11
ENSG00000177426_3456398_3457940_252	annotated	TGIF1	18	+	756	105	16	5	10.32%	45.89201821	7.42E-05	1.54E-05	9.06E-09
ENSG00000135272_114942346_115015935_191	annotated	MDFIC	7	+	573	28	0	2	4.71%	16.1463017	0.003632191	0.011814276	0.000233964
ENSG00000178209_143939461_143916177_4547	annotated	PLEC	8	-	13641	244	62	38	2.55%	5.910350971	1.36E-09	1.66E-13	4.21E-21
ENSG00000166340_6619400_6617622_127	novel	TPP1	11	-	381	62	13	0	14.17%	53.76954575	0.006041044	8.33E-05	4.57E-06
ENSG00000166340_6616817_6616581_78	novel	TPP1	11	-	234	24	1	1	6.41%	33.88949037	0.029237631	0.029237631	0.003726901
ENSG00000166340_6616465_6616298_55	novel	TPP1	11	-	165	69	15	0	16.36%	138.1766948	0.008127104	0.003710481	0.00016379
ENSG00000166340_6615582_6614546_188	novel	TPP1	11	-	564	67	22	3	10.64%	39.25232196	0.001714251	0.000250708	2.95E-06
ENSG00000136104_50927433_50948072_203	novel	RNASEH2B	13	+	609	18	2	0	3.94%	9.766183186	0.008316137	0.005554612	0.00024255
ENSG00000101266_489830_483961_167	novel	CSNK2A1	20	-	501	47	1	0	8.38%	30.99772248	0.000475338	0.000472567	1.47E-06
ENSG00000170142_23807270_23807680_136	novel	UBE2E1	3	+	408	57	2	1	3.68%	46.16197126	0.029237631	0.021557223	0.002819006

ENSG00000120948_11022340_11022654_104	annotated	TARDBP	1	+	312	34	7	0	5.77%	36.00758352	0.063412829	0.013428348	0.004086769
ENSG00000143799_226364061_226361460_125	novel	PARP1	1	-	375	44	1	0	8.00%	38.76957698	0.002432129	0.002362849	3.32E-05
ENSG00000071073_98726332_98625566_535	annotated	MGAT4A	2	-	1605	10	2	0	1.12%	2.058707359	0.056853419	0.011570466	0.003221159
ENSG00000106603_43729464_43648637_50	uORF	COA1	7	-	150	83	12	2	16.00%	182.8338006	0.028682169	0.005604867	0.000852961
ENSG00000106603_43647613_43639582_134	annotated	COA1	7	-	402	36	6	4	9.70%	29.59007741	0.007322027	0.003864916	0.000153348
ENSG00000182240_41168264_41275624_468	annotated	BACE2	21	+	1404	63	13	5	5.77%	14.82665204	0.001357039	5.57E-05	6.09E-07
ENSG00000128708_171925548_171983352_413	annotated	HAT1	2	+	1239	137	17	8	8.96%	36.53582467	4.07E-06	1.58E-07	6.41E-12
ENSG00000091409_172427789_172501879_1073	annotated	ITGA6	2	+	3219	351	39	11	8.95%	36.02929743	9.40E-14	6.86E-16	9.90E-28
ENSG00000196756_38434513_38426436_49	novel	SNHG17	20	-	147	26	3	3	14.29%	58.44208033	0.009583875	0.023878474	0.001121714
ENSG00000103534_19440039_19497966_948	annotated	TMC5	16	+	2844	22	2	1	0.84%	2.556011141	0.012834473	0.005998643	0.000398124
ENSG00000178996_54517953_54543432_624	annotated	SNX18	5	+	1872	13	0	1	0.80%	2.294600911	0.021084099	0.035772986	0.003354605
ENSG00000168453_22128957_22115700_1118	annotated	HR	8	-	3354	33	10	0	1.52%	3.251026693	0.015338427	0.000115129	1.79E-05
ENSG00000149311_108227625_108365508_3056	annotated	ATM	11	+	9168	31	5	3	0.59%	1.117266412	0.002379531	0.0006246	9.42E-06
ENSG00000168792_29566966_29562561_468	annotated	ABHD15	17	-	1404	17	4	0	2.14%	4.000842613	0.038905521	0.002122793	0.000539938
ENSG00000176225_70030080_70004151_334	annotated	RTTN	18	-	1002	19	0	0	1.50%	6.265497097	0.020613417	0.020613417	0.001946209
ENSG00000171130_149875611_149878771_42	novel	ATP6V0E2	7	+	126	76	4	2	23.81%	199.3024791	0.009088823	0.005538787	0.000263745
ENSG00000170315_16381908_16382597_229	annotated	UBB	17	+	687	683	95	21	12.66%	328.4986736	4.19E-05	1.51E-05	4.99E-09
ENSG00000100938_24238371_24238778_74	novel	GMPR2	14	+	222	17	2	0	6.76%	25.30262626	0.021084099	0.021084099	0.002029891
ENSG00000166803_64381371_64373707_115	annotated	PCLAF	15	-	345	334	93	11	20.87%	319.88732	0.000576203	0.000129182	5.25E-07
ENSG00000101247_13785069_13814443_267	annotated	NDUFAF5	20	+	801	55	6	5	7.12%	22.68818878	7.97E-05	0.001172763	7.13E-07
ENSG00000103429_14644574_14667827_306	Overlap_dORF	BFAR	16	+	918	70	8	1	10.46%	25.19561784	8.47E-06	3.71E-07	3.03E-11
ENSG00000168036_41233535_41233960_141	novel	CTNNB1	3	+	423	30	8	5	8.51%	23.43422206	0.006549171	0.006753026	0.00023172
ENSG00000168036_41235736_41235858_40	novel	CTNNB1	3	+	120	16	3	0	12.50%	44.05633748	0.045845141	0.021557223	0.00436099
ENSG00000263001_74689225_74758838_966	annotated	GTF2I	7	+	2898	58	9	8	1.76%	6.61301132	0.001982699	0.001758679	2.06E-05
ENSG00000173638_45537959_45514976_489	annotated	SLC19A1	21	-	1467	64	4	2	4.70%	14.41516155	4.37E-05	3.54E-05	1.18E-08
ENSG00000182718_60397913_60347630_357	annotated	ANXA2	15	-	1071	1399	140	79	11.48%	431.6163595	3.77E-07	3.69E-07	1.29E-12
ENSG00000131263_74595977_74591440_624	annotated	RLIM	X	-	1872	33	3	5	2.56%	5.824756157	0.000590613	0.002056461	7.72E-06
ENSG00000135480_52245529_52251778_46	novel	KRT7	12	+	138	252	29	9	50.00%	603.3802742	1.74E-05	2.59E-05	3.53E-09
ENSG00000131165_89649410_89647038_117	novel	CHMP1A	16	-	351	40	2	3	6.84%	37.6549893	0.018067938	0.027859662	0.002294714
ENSG00000114391_101686676_101681179_121	annotated	RPL24	3	-	363	261	11	21	9.92%	237.5765306	0.001475176	0.001859036	1.64E-05
ENSG00000197771_119853104_119843174_186	novel	MCMBP	10	-	558	29	1	0	8.06%	17.17249714	0.000436588	0.000258933	7.61E-07
ENSG00000106799_99128965_99149305_434	annotated	TGFBR1	9	+	1302	53	10	3	5.53%	13.45037953	0.001488312	4.32E-05	5.41E-07
ENSG00000171130_149875611_149878771_42	novel	ATP6V0E2	7	+	126	76	4	2	23.81%	199.3024791	0.009088823	0.005538787	0.000263745
ENSG00000139624_50167297_50165940_66	annotated	CERS5	12	-	198	22	1	0	7.58%	36.71361457	0.028889786	0.021084099	0.002731533
ENSG00000162105_70502230_70472869_1098	annotated	SHANK2	11	-	3294	7	3	0	0.46%	0.702172956	0.170087137	0.019216965	0.016246064
ENSG00000258643_23307903_23325286_288	annotated	BCL2L2-PABPN1	14	+	864	48	8	1	4.51%	18.35680728	0.005208023	0.000710064	2.38E-05
ENSG00000113580_143400839_143281889_777	annotated	NR3C1	5	-	2331	125	11	5	6.31%	17.71892595	2.61E-08	3.78E-10	1.20E-16
ENSG00000171224_69631744_69633059_121	novel	FAM241B	10	+	363	17	4	0	4.96%	15.47433341	0.074296157	0.013428348	0.004845297
ENSG00000169228_177303047_177301743_154	annotated	RAB24	5	-	462	77	14	1	15.58%	55.07042185	0.000446769	9.95E-06	4.04E-08
ENSG00000168936_1720837_1717410_232	annotated	TMEM129	4	-	696	24	0	1	5.17%	11.39388038	0.000947024	0.001563962	9.10E-06
ENSG00000104419_133262129_133238878_313	Overlap_dORF	NDRG1	8	-	939	48	1	6	4.15%	16.89060862	0.001040543	0.007860495	5.12E-05
ENSG00000104412_108443659_108455963_131	novel	EMC2	8	+	393	27	2	1	8.40%	22.70078458	0.006771542	0.002493494	9.52E-05
ENSG00000120438_159779908_159779045_76	Overlap_dORF	TCP1	6	-	228	98	21	1	6.58%	142.0237195	0.021084099	0.022899795	0.002192856
ENSG00000120438_159789468_159779250_433	annotated	TCP1	6	-	1299	122	25	8	8.55%	31.03275504	4.74E-05	1.52E-06	6.78E-10
ENSG00000119723_73950333_73956448_166	novel	COQ6	14	+	498	34	0	0	6.63%	22.55896799	0.001610998	0.001610998	1.55E-05
ENSG00000100883_34996780_35029152_450	annotated	SRP54	14	+	1350	45	7	3	5.33%	11.01408437	0.000431313	2.26E-05	8.00E-08
ENSG00000119632_94128586_94127799_88	novel	IFI27L2	14	-	264	45	1	4	14.77%	56.32202235	0.000804138	0.000804138	4.08E-06
ENSG00000168303_40134567_40133059_179	annotated	MPLKIP	7	-	537	39	19	3	7.82%	23.99716706	0.031289992	0.001757437	0.000361658
ENSG00000132003_13809184_13831050_781	annotated	ZSWIM4	19	+	2343	18	2	0	0.90%	2.538457345	0.023296852	0.008574016	0.000992302
ENSG00000105426_5286095_5273319_113	annotated	PTPRS	19	-	339	15	0	0	7.96%	14.62046598	0.003327803	0.003327803	6.21E-05
ENSG00000111145_96255521_96267180_83	novel	ELK3	12	+	249	9	0	0	6.02%	11.94298305	0.016947427	0.016947427	0.001349898
ENSG00000105388_41708732_41727316_702	annotated	CEACAM5	19	+	2106	10	0	0	0.71%	1.568957888	0.019679754	0.019679754	0.001784964
ENSG00000114127_142447944_142311511_1706	annotated	XRN1	3	-	5118	33	0	4	0.76%	2.1305087	0.000546051	0.004669158	1.68E-05
ENSG00000121211_153350106_153409047_165	annotated	MND1	4	+	495	42	1	2	9.70%	28.03585112	0.000387287	0.000546144	1.39E-06
ENSG00000113328_163439257_163442565_295	annotated	CCNG1	5	+	885	159	26	9	13.22%	59.36404796	4.30E-06	2.46E-07	1.02E-11
ENSG00000065883_39950642_40094980_1452	annotated	CDK13	7	+	4356	20	1	1	0.76%	1.517091511	0.002559322	0.002559322	3.76E-05
ENSG00000115355_55374859_55291711_1744	annotated	CCDC88A	2	-	5232	69	8	3	1.95%	4.357636592	9.61E-06	6.71E-07	5.99E-11
ENSG00000115183_159097759_159231012_1800	annotated	TANC1	2	+	5400	15	5	0	0.56%	0.917840364	0.022027531	0.001790164	0.000247384
ENSG00000115239_53750918_53670503_445	annotated	ASB3	2	-	1335	13	3	0	2.47%	3.217597681	0.013071281	0.000883168	7.72E-05
ENSG00000163681_57757652_57927388_811	annotated	SLMAP	3	+	2433	14	3	1	0.99%	1.901321593	0.034881441	0.009234565	0.001597643
ENSG00000163681_57757652_57927388_811	annotated	SLMAP	3	+	2433	14	3	1	0.99%	1.901321593	0.034881441	0.009234565	0.001597643
ENSG00000136068_58008565_58170762_2602	annotated	FLNB	3	+	7806	224	35	43	3.73%	9.48176364	6.60E-14	1.25E-10	1.38E-22
ENSG00000163683_39605145_39552126_99	annotated	SMIM14	4	-	297	39	4	2	19.19%	43.38881722	0.000813783	7.97E-05	4.84E-07
ENSG00000105223_40366484_40378173_490	annotated	PLD3	19	+	1470	323	42	10	17.76%	72.60304595	6.46E-13	6.64E-15	6.33E-26
ENSG00000144136_112646829_112663025_679	annotated	SLC20A1	2	+	2037	247	43	15	11.34%	40.06596229	9.70E-10	5.38E-13	8.51E-21
ENSG00000165916_47426231_47418835_423	annotated	PSMC3	11	-	1269	175	7	3	12.06%	45.5665429	3.46E-10	4.84E-10	1.89E-18
ENSG00000125375_50321491_50325631_111	dORF	DMAC2L	14	+	333	29	0	5	8.11%	28.77553574	0.003710481	0.011906648	0.000240324
ENSG00000179933_23097683_23098081_132	annotated	C14orf119	14	+	396	157	5	0	15.15%	131.000852	5.63E-05	4.27E-05	1.81E-08

ENSG00000196155_67280165_67288808_1151	annotated	PLEKHG4	16	+	3453	28	5	1	1.56%	2.679360229	0.001093313	9.54E-05	7.75E-07
ENSG00000108592_63826902_63819802_847	annotated	FTSJ3	17	-	2541	81	6	0	3.54%	10.53294963	4.33E-06	7.17E-07	2.81E-11
ENSG00000126214_103654586_103685643_544	annotated	KLC1	14	+	1632	150	28	13	8.64%	30.36971793	1.43E-05	6.48E-08	1.11E-11
ENSG00000104381_74351361_74364367_290	annotated	GDAP1	8	+	870	19	0	0	3.10%	7.216124243	0.003606289	0.003606289	7.24E-05
ENSG00000114735_50571123_50580417_332	annotated	HEMK1	3	+	996	12	2	0	1.81%	3.980994351	0.052581193	0.01300724	0.003266469
ENSG00000064545_19134839_19121093_308	annotated	TMEM161A	19	-	924	48	1	3	6.17%	17.16480681	4.55E-05	8.05E-05	2.74E-08
ENSG00000134333_18396843_18400670_94	annotated	LDHA	11	+	282	92	13	0	12.77%	107.7974215	0.00864514	0.001087214	5.89E-05
ENSG00000125629_118096641_118108362_210	annotated	INSIG2	2	+	630	45	14	0	9.05%	23.60160937	0.009976494	5.58E-05	5.99E-06
ENSG00000168591_44189047_44190864_301	annotated	TMUB2	17	+	903	49	8	6	6.64%	17.92990479	0.000773154	0.000872496	4.25E-06
ENSG00000166454_81043186_81044970_594	novel	ATMIN	16	+	1782	22	0	0	1.18%	4.079290508	0.008473674	0.008473674	0.000366502
ENSG00000136819_129833487_129828987_256	novel	C9orf78	9	-	768	27	5	1	4.69%	11.61641711	0.004918174	0.001946488	5.52E-05
ENSG00000148337_128167622_128166197_161	novel	CIZ1	9	-	483	57	3	0	6.83%	38.9939633	0.001626321	0.001626321	1.58E-05
ENSG00000129636_47461045_47155719_612	annotated	ITFG1	16	-	1836	349	34	5	13.73%	62.8090759	8.80E-14	6.95E-16	9.37E-28
ENSG00000197894_99076901_99072417_252	dORF	ADH5	4	-	756	88	21	6	9.92%	38.46188193	0.00129755	3.86E-05	4.22E-07
ENSG00000102901_67832088_67828267_458	dORF	CENPT	16	-	1374	12	2	4	1.97%	2.885786298	0.022868511	0.035227809	0.003553359
ENSG00000213983_23567314_23559749_785	annotated	AP1G2	14	-	2355	56	13	1	3.18%	7.857181207	0.000482493	6.49E-06	3.03E-08
ENSG00000062485_56283836_56274820_223	novel	CS	12	-	669	104	15	2	11.21%	51.36613339	0.000312438	1.27E-05	3.41E-08
ENSG00000062485_56275043_56273503_145	novel	CS	12	-	435	54	4	0	8.97%	41.01796938	0.002518734	0.000712477	1.12E-05
ENSG00000166046_106311076_106323693_272	annotated	TCP11L2	12	+	816	7	1	2	1.84%	2.834507007	0.047790352	0.11777946	0.021834536
ENSG00000155463_22766702_22770407_217	novel	OXA1L	14	+	651	234	20	7	19.82%	118.7693891	7.95E-08	1.68E-08	1.36E-14
ENSG00000128604_128947526_128948818_255	annotated	IRF5	7	+	765	7	0	0	1.96%	3.023474141	0.016947427	0.016947427	0.001349898
ENSG00000049860_74685261_74689544_171	novel	HEXB	5	+	513	89	3	0	12.87%	57.32476661	5.93E-05	1.52E-05	7.12E-09
ENSG00000159352_151254783_151262428_97	novel	PSMD4	1	+	291	18	10	2	7.22%	20.43850708	0.077869819	0.032488891	0.010480844
ENSG00000111653_6656754_6651346_152	annotated	ING4	12	-	456	17	1	1	5.26%	12.31838384	0.008316137	0.008316137	0.000353732
ENSG00000093183_42561243_42548616_95	annotated	SEC22C	3	-	285	20	0	1	7.37%	23.18754604	0.007029303	0.007882058	0.000286931
ENSG00000093183_42569065_42568979_28	Overlap_uORF	SEC22C	3	-	84	12	0	0	17.86%	47.20321873	0.019679754	0.019679754	0.001784964
ENSG00000000460_169804192_169852987_710	annotated	C1orf112	1	+	2130	25	0	3	1.69%	3.878198722	0.000943084	0.007322425	4.36E-05
ENSG00000243678_51166831_51171604_152	annotated	NME2	17	+	456	935	132	26	36.84%	677.511111	3.42E-09	6.32E-11	2.68E-18
ENSG00000011052_51155655_51171604_267	annotated	NME1-NME2	17	+	801	1750	233	45	38.95%	721.8969157	9.20E-16	4.02E-18	6.14E-32
ENSG00000117305_23820578_23804346_177	novel	HMGCL	1	-	531	12	0	0	3.95%	7.467175844	0.007882058	0.007882058	0.000319636
ENSG00000176018_90519160_90518819_113	dORF	LYSMD3	5	-	339	16	0	0	7.08%	15.59516371	0.005306578	0.005306578	0.000151001
ENSG00000164180_88206473_88202958_97	annotated	TMEM161B	5	-	291	23	1	0	6.19%	26.11587016	0.013640586	0.013428348	0.00088551
ENSG00000104723_15540431_15733399_289	annotated	TUSC3	8	+	867	176	25	2	20.07%	67.07539271	4.68E-08	1.74E-11	1.35E-17
ENSG00000125870_16731703_16741005_225	annotated	SNRPB2	20	+	675	151	19	5	12.89%	73.916744	6.50E-05	1.23E-06	7.84E-10
ENSG00000147535_38267333_38267199_44	novel	PLPP5	8	-	132	19	1	0	20.45%	47.56081887	0.003529166	0.003606289	7.09E-05
ENSG00000173039_65661862_65654378_498	novel	RELA	11	-	1494	103	10	3	6.02%	22.78013434	3.02E-05	2.28E-06	6.09E-10
ENSG00000149196_86337408_86345638_98	novel	HIKESHI	11	+	294	38	6	0	14.29%	42.70767409	0.003908676	0.000456173	1.19E-05
ENSG00000141279_47603876_47619182_291	novel	NPEPPS	17	+	873	57	7	14	6.87%	21.57397969	0.000766381	0.003595597	1.73E-05
ENSG00000015475_17739423_17735580_99	annotated	BID	22	-	297	50	11	8	12.12%	55.62668874	0.02055943	0.009965666	0.001000669
ENSG00000015475_17739423_17735580_99	annotated	BID	22	-	297	50	11	8	12.12%	55.62668874	0.02055943	0.009965666	0.001000669
ENSG00000204178_25431099_25498466_437	annotated	MACO1	1	+	1311	44	1	0	3.20%	11.08969593	0.000662939	0.0004093	1.77E-06
ENSG00000156639_37929972_38142293_159	annotated	ZFAND3	6	+	477	12	11	2	5.66%	8.312516506	0.175468548	0.022079251	0.018640136
ENSG00000094841_75274255_75303511_309	annotated	UPRT	X	+	927	14	1	0	2.59%	4.990200038	0.01551626	0.004826937	0.00039687
ENSG00000141646_51047047_51078467_552	annotated	SMAD4	18	+	1656	40	6	0	3.26%	7.981220558	0.001354029	7.71E-05	8.07E-07
ENSG00000132463_70839827_70824319_424	annotated	GRSF1	4	-	1272	141	17	5	9.91%	36.62702585	3.40E-08	7.92E-08	2.68E-14
ENSG00000171428_18222189_18222920_243	annotated	NAT1	8	+	729	8	0	0	2.88%	3.626036007	0.005706018	0.005706018	0.00017331
ENSG00000204922_62671764_62672114_87	annotated	UQCC3	11	+	261	58	7	3	21.84%	73.42722914	0.000392695	0.000173036	4.68E-07
ENSG00000173599_66872039_66848899_1138	annotated	PC	11	-	3414	30	4	0	1.14%	2.90353718	0.004866374	0.000628963	1.99E-05
ENSG00000198925_219226870_219220447_769	annotated	ATG9A	2	-	2307	52	14	1	3.12%	7.447755361	0.002733827	1.03E-05	3.24E-07
ENSG00000140264_43793010_43793773_45	annotated	SERF2	15	+	135	376	54	13	35.56%	920.2879385	0.000782012	0.000496034	2.50E-06
ENSG00000140264_43792377_43800501_40	annotated	SERF2	15	+	120	349	63	2	35.00%	960.9788613	0.00064468	0.000482311	2.02E-06
ENSG00000140264_43793050_43801806_175	Overlap_dORF	SERF2	15	+	525	184	21	218	17.71%	115.80523	0.00011892	0.000325884	2.73E-07
ENSG00000115762_131120942_131146773_222	annotated	PLEKHB2	2	+	666	64	29	2	11.71%	31.7523153	0.005449528	1.35E-05	9.31E-07
ENSG00000105472_50725153_50725467_104	Overlap_dORF	CLEC11A	19	+	312	13	1	0	5.77%	13.76760546	0.023672501	0.012798403	0.001437523
ENSG00000136448_45096248_45098556_109	dORF	NMT1	17	+	327	15	0	0	4.59%	15.15699684	0.020613417	0.020613417	0.001946209
ENSG00000136448_45061387_45096222_118	annotated	NMT1	17	+	354	15	13	0	5.08%	14.00095471	0.386986	0.012798403	0.037413289
ENSG00000170525_6203261_6231305_526	annotated	PFKFB3	10	+	1578	114	22	6	6.65%	23.87082924	3.00E-05	2.67E-07	8.68E-11
ENSG00000142188_33468885_33456015_151	annotated	TMEM50B	21	-	453	16	4	0	4.64%	11.67055298	0.060590694	0.008473674	0.00268166
ENSG00000040341_73673120_73421372_476	annotated	STAU2	8	-	1428	36	13	3	3.36%	8.329979776	0.003677301	0.000813866	1.87E-05
ENSG00000113558_134174022_134157733_163	annotated	SKP1	5	-	489	242	28	6	16.56%	163.5219888	9.45E-06	3.80E-06	3.02E-10
ENSG00000198625_204525519_204549682_392	annotated	MDM4	1	+	1176	18	3	5	2.04%	5.057487721	0.022360378	0.053624202	0.005259544
ENSG00000149591_117203041_117204359_192	annotated	TAGLN	11	+	576	16	0	0	2.60%	9.178403642	0.020613417	0.020613417	0.001946209
ENSG00000150782_112155053_112143596_189	annotated	IL18	11	-	567	126	19	2	16.40%	73.42722914	1.88E-05	4.87E-07	9.01E-11
ENSG00000148943_27506752_27498649_173	annotated	LIN7C	11	-	519	42	9	0	9.25%	26.73939558	0.003657276	0.000198297	5.38E-06
ENSG00000101290_5127093_5190234_445	annotated	CDS2	20	+	1335	361	30	6	12.58%	89.35021253	2.29E-09	9.77E-11	2.67E-18
ENSG00000074266_86245272_86277168_370	novel	EED	11	+	1110	19	0	2	2.70%	5.655881163	0.002254349	0.009469559	0.00012237
ENSG00000112130_37368448_37390758_417	dORF	RNF8	6	+	1251	14	0	0	1.92%	3.697774129	0.005160369	0.005160369	0.000143196
ENSG00000006530_141601260_141624272_134	annotated	AGK	7	+	402	11	1	0	5.22%	9.041412543	0.01551626	0.007882058	0.000614257

ENSG00000006530_141633928_141652924_217	dORF	AGK	7	+	651	23	3	0	2.76%	11.67391431	0.013640586	0.013640586	0.000898637
ENSG00000110321_10804047_10803521_72	annotated	EIF4G2	11	-	216	25	2	11	12.50%	38.24334851	0.007312901	0.082500581	0.003382551
ENSG00000110321_10805979_10805911_22	annotated	EIF4G2	11	-	66	22	0	12	27.27%	110.1408437	0.013853925	0.14651463	0.010719976
ENSG00000198945_130051269_130139753_677	annotated	L3MBTL3	6	+	2031	7	0	1	0.74%	1.13882704	0.019216965	0.041782825	0.003600526
ENSG00000145819_143056047_143222446_258	annotated	ARHGAP26	5	+	774	17	2	0	2.71%	7.257342415	0.024135905	0.008674847	0.001038937
ENSG00000185432_50925039_50930150_244	annotated	METTL7A	12	+	732	15	1	0	3.28%	6.770953506	0.010499663	0.00419316	0.000236136
ENSG00000139116_39337185_39322664_441	annotated	KIF21A	12	-	1323	12	0	0	1.13%	2.997029761	0.020613417	0.020613417	0.001946209
ENSG00000163710_142888896_142818432_401	annotated	PCOLCE2	3	-	1203	64	7	0	5.49%	17.57858852	9.34E-05	1.82E-05	1.34E-08
ENSG00000136111_75349290_75286792_806	annotated	TBC1D4	13	-	2418	21	1	1	1.49%	2.869674588	0.00117686	0.00117686	8.49E-06
ENSG00000100938_24233254_24238778_348	annotated	GMPR2	14	+	1044	91	18	2	9.20%	28.80119764	2.89E-05	6.16E-07	1.77E-10
ENSG00000154518_175181393_175178288_142	annotated	ATP5MC3	2	-	426	756	120	43	34.51%	586.3836468	2.47E-06	1.59E-08	4.63E-13
ENSG00000151136_107319115_107657718_1046	annotated	BTBD11	12	+	3138	89	19	1	3.25%	9.371448461	0.000257057	1.78E-07	6.98E-10
ENSG00000170540_18801466_18793252_88	annotated	ARL6IP1	16	-	264	236	11	10	27.27%	295.3777172	3.85E-05	5.89E-05	1.71E-08
ENSG00000117593_173825230_173857705_624	annotated	DARS2	1	+	1872	90	8	5	5.61%	15.88569861	1.98E-06	4.96E-07	8.97E-12
ENSG00000151135_106971140_106971391_83	annotated	TMEM263	12	+	249	17	10	0	10.84%	22.55896799	0.17974379	0.003303074	0.005106915
ENSG00000077152_202335754_202335584_56	novel	UBE2T	1	-	168	36	5	4	14.29%	70.8048281	0.013991246	0.02876187	0.001884322
ENSG00000077152_202333278_202331835_83	novel	UBE2T	1	-	249	120	20	7	26.51%	159.239774	0.000262792	3.00E-05	6.13E-08
ENSG00000172922_65720340_65719783_81	novel	RNASEH2C	11	-	243	40	0	2	9.88%	54.3905401	0.005355751	0.020990661	0.000592297
ENSG00000117479_169485766_169465849_464	annotated	SLC19A2	1	-	1392	21	4	0	2.59%	4.984822668	0.010725367	0.000931326	6.50E-05
ENSG00000136104_50927433_50950109_229	annotated	RNASEH2B	13	+	687	25	2	0	4.37%	12.02410957	0.003500471	0.002362849	4.72E-05
ENSG00000136104_50950271_50956474_81	dORF	RNASEH2B	13	+	243	33	0	0	8.64%	44.87219558	0.008674847	0.008674847	0.000383115
ENSG00000131941_33002370_32979996_359	novel	RHPN2	19	-	1077	24	5	0	2.23%	7.363176181	0.041815588	0.005554612	0.001268857
ENSG00000109911_31509785_31719517_396	annotated	ELP4	11	+	1188	27	11	0	3.28%	7.50960298	0.032335844	0.000635664	0.000168567
ENSG00000139618_32316461_32398770_3418	annotated	BRCA2	13	+	10254	13	5	0	0.20%	0.418909002	0.092312258	0.008175611	0.004196557
ENSG00000111321_6385229_6387898_148	annotated	AC005840.1	12	+	444	43	7	5	11.49%	32.00038027	0.001807146	0.001079561	1.19E-05
ENSG00000140391_77052426_77046835_44	annotated	TSPAN3	15	-	132	110	11	1	25.00%	275.3521093	0.001817257	0.001409965	1.53E-05
ENSG00000159176_201490405_201484713_176	novel	CSRP1	1	-	528	313	34	5	17.61%	195.8754777	5.28E-07	1.06E-06	5.09E-12
ENSG00000176148_33043774_33085120_595	annotated	TCP11L1	11	+	1785	32	3	5	2.86%	5.923541174	0.000820166	0.002915716	1.48E-05
ENSG00000129559_24232267_24217127_81	annotated	NEDD8	14	-	243	118	28	10	25.93%	160.4520933	0.004006097	0.000238242	6.95E-06
ENSG00000157184_53196944_53213365_598	annotated	CPT2	1	+	1794	50	2	6	3.68%	9.209100644	5.96E-05	0.000269212	1.18E-07
ENSG00000129562_22589157_22575103_113	annotated	DAD1	14	-	339	812	70	24	47.79%	791.4545583	4.14E-09	1.65E-10	8.10E-18
ENSG00000146410_136249069_136231275_375	annotated	MTFR2	6	-	1125	16	1	0	1.87%	4.699342665	0.011825808	0.008473674	0.000503679
ENSG00000100225_32483984_32498530_354	dORF	FBXO7	22	+	1062	76	4	5	6.78%	23.64605684	1.87E-05	5.51E-05	8.03E-09
ENSG00000013588_12909072_12912181_65	annotated	GPRC5A	12	+	195	127	4	7	18.46%	215.1982639	0.001070089	0.001378275	9.03E-06
ENSG00000103037_58515992_58519029_397	annotated	SETD6	16	+	1191	6	7	0	1.26%	1.664597134	0.165899918	0.016947427	0.014398895
ENSG00000103035_74296915_74305733_324	annotated	PSMD7	16	+	972	397	53	36	14.51%	134.9565276	5.63E-09	9.43E-09	5.57E-16
ENSG00000121766_31310099_31364193_241	annotated	ZCCHC17	1	+	723	21	2	0	4.56%	9.597334929	0.003646019	0.001440978	3.11E-05
ENSG00000143322_179229397_179107718_1161	annotated	ABL2	1	-	3483	19	4	1	0.95%	1.802477201	0.015721867	0.002458651	0.000224099
ENSG00000176871_118061048_118035210_315	novel	WSB2	12	-	945	18	6	0	2.54%	6.293762497	0.082182474	0.005554612	0.002728198
ENSG00000167642_38283671_38292006_202	annotated	SPINT2	19	+	606	989	78	22	36.63%	539.2539328	3.95E-12	1.89E-13	1.00E-23
ENSG00000182326_7062524_7067014_312	annotated	C1S	12	+	936	22	2	1	3.21%	7.766341543	0.005034873	0.003068573	8.58E-05
ENSG00000182326_7067021_7070651_365	dORF	C1S	12	+	1095	72	5	2	6.85%	21.726413	3.08E-05	1.38E-05	3.35E-09
ENSG00000085982_233565554_233477392_1235	annotated	USP40	2	-	3705	28	4	0	1.54%	2.497120343	0.000348371	3.95E-05	1.06E-07
ENSG00000178921_8253938_8269264_1338	annotated	PFAS	17	+	4014	74	13	10	2.32%	6.091496588	7.40E-05	3.67E-05	2.05E-08
ENSG00000138801_107701282_107614249_603	annotated	PAPSS1	4	-	1809	44	5	0	3.65%	8.036811149	9.32E-05	1.62E-05	1.20E-08
ENSG00000105393_17268807_17279048_329	annotated	BABAM1	19	+	987	30	5	0	4.26%	10.04323803	0.001809669	0.0004093	4.85E-06
ENSG00000188352_20715354_20995629_1801	annotated	FOCAD	9	+	5403	85	18	5	2.28%	5.198207504	6.64E-05	9.31E-08	8.41E-11
ENSG00000089280_31180215_31191438_525	annotated	FUS	16	+	1575	374	49	28	17.90%	78.46223913	1.07E-14	1.90E-15	2.89E-28
ENSG00000047230_16702902_16590793_586	annotated	CTPS2	X	-	1758	17	0	0	1.02%	3.195212189	0.013428348	0.013428348	0.000872559
ENSG00000073756_186674751_186674353_132	novel	PTGS2	1	-	396	39	0	1	7.58%	32.54161291	0.002474239	0.002839008	4.02E-05
ENSG00000073756_186680290_186676417_339	novel	PTGS2	1	-	1017	30	11	0	3.54%	9.746977319	0.058332232	0.001003278	0.000494148
ENSG00000136068_58008565_58170762_2633	annotated	FLNB	3	+	7899	224	36	43	3.68%	9.370128747	9.28E-14	1.25E-10	1.89E-22
ENSG00000143549_154183119_154157639_248	annotated	TPM3	1	-	744	283	35	9	12.50%	125.6849144	3.97E-06	1.85E-06	6.36E-11
ENSG00000277791_38762447_38764167_35	novel	PSMB3	17	+	105	252	8	4	25.71%	793.0140747	0.012070269	0.008127104	0.000494332
ENSG00000169738_82036410_82035960_82	novel	DCXR	17	-	246	30	6	0	10.98%	40.29543062	0.022643694	0.003710481	0.000468526
ENSG00000134824_61840382_61840564_60	uORF	FADS2	11	+	180	125	11	1	31.67%	229.4600911	0.000104909	7.63E-05	5.83E-08
ENSG00000147130_71253255_71247488_797	novel	ZMYM3	X	-	2391	28	0	1	1.13%	3.869439929	0.003554776	0.00567009	0.000110499
ENSG00000147130_71245830_71240916_464	novel	ZMYM3	X	-	1392	13	0	0	1.72%	3.085842604	0.004687384	0.004687384	0.000119282
ENSG00000105281_46782522_46775510_313	annotated	SLC1A5	19	-	939	341	29	7	18.85%	119.9936987	4.77E-11	2.03E-11	1.17E-20
ENSG00000020426_60734863_60808440_143	novel	MNAT1	14	+	429	13	3	2	4.90%	10.01280397	0.059202727	0.043639483	0.010354425
ENSG00000183010_81936814_81933214_319	annotated	PYCR1	17	-	957	123	11	0	11.29%	42.46809961	2.24E-07	6.47E-08	1.41E-13
ENSG00000164548_23516395_23507416_113	novel	TRA2A	7	-	339	31	9	2	9.73%	30.21562969	0.042627965	0.00507972	0.001204969
ENSG00000156515_69384682_69386493_134	novel	HK1	10	+	402	34	2	0	5.22%	27.94618422	0.008776163	0.008776163	0.000391611
ENSG00000156515_69343848_69384547_566	novel	HK1	10	+	1698	89	12	1	5.48%	17.31896659	2.98E-05	5.94E-07	1.76E-10
ENSG00000136490_63699298_63698552_127	annotated	LIMD2	17	-	381	8	4	0	3.94%	6.938005903	0.170042304	0.019679754	0.016523685
ENSG00000115128_24076223_24067762_123	annotated	SF3B6	2	-	369	186	22	7	22.76%	166.5544466	3.17E-05	4.04E-06	1.09E-09
ENSG00000154229_66302852_66696435_308	annotated	PRKCA	17	+	924	33	3	0	4.22%	11.80080468	0.00278522	0.000602625	1.07E-05
ENSG00000003402_201133036_201140475_117	annotated	CFLAR	2	+	351	8	0	1	4.27%	7.53099786	0.019679754	0.065398531	0.005779267

ENSG00000170464_139439445_139414148_358	annotated	DNAJC18	5	-	1074	9	1	1	1.68%	2.768903892	0.023056749	0.023056749	0.002397885
ENSG00000129422_17676329_17654535_151	novel	MTUS1	8	-	453	22	1	0	4.64%	16.04701034	0.013892891	0.008473674	0.00058868
ENSG00000129422_17653535_17645926_211	novel	MTUS1	8	-	633	40	4	0	8.06%	20.8797808	0.001292475	0.000112262	1.07E-06
ENSG00000138119_93482194_93306963_2061	annotated	MYOF	10	-	6183	295	54	27	6.31%	15.76494366	1.20E-13	2.56E-19	8.37E-31
ENSG00000118777_88139995_88092234_655	annotated	ABCG2	4	-	1965	33	7	0	2.14%	5.549080675	0.009363059	0.000450794	3.04E-05
ENSG00000160305_46504471_46567622_1312	annotated	DIP2A	21	+	3936	17	0	3	0.69%	1.427129835	0.003303074	0.019264876	0.000357606
ENSG00000141580_82648340_82619086_174	annotated	WDR45B	17	-	522	23	0	3	5.75%	14.55884716	0.002321823	0.003068573	4.08E-05
ENSG00000163629_86689047_86814551_2356	annotated	PTPN13	4	+	7068	19	1	1	0.47%	0.888232611	0.002559322	0.002559322	3.76E-05
ENSG00000102100_48911636_48904727_393	annotated	SLC35A2	X	-	1179	54	2	3	5.09%	15.13385639	5.24E-05	6.63E-05	2.59E-08
ENSG00000071626_1429969_1434912_172	novel	DAZAP1	19	+	516	28	8	0	5.23%	17.92990479	0.035987166	0.003478238	0.000734577
ENSG00000071626_1417366_1428852_192	novel	DAZAP1	19	+	576	36	6	2	8.33%	20.65140819	0.001761276	0.000415934	4.79E-06
ENSG00000198755_35468435_35469043_58	novel	RPL10A	6	+	174	38	9	0	10.34%	72.16124243	0.052585976	0.013640586	0.003398505
ENSG00000160294_46256943_46235268_721	novel	MCM3AP	21	-	2163	16	1	1	1.11%	2.444179611	0.006542768	0.006542768	0.000224664
ENSG00000169814_15635440_15642138_159	novel	BTD	3	+	477	33	8	3	8.18%	22.85942039	0.01179876	0.003168495	0.000207081
ENSG00000101052_43613893_43624067_138	novel	IFT52	20	+	414	13	0	0	4.35%	10.37558673	0.012798403	0.012798403	0.000797244
ENSG00000133318_63681637_63758201_236	annotated	RTN3	11	+	708	462	68	14	18.22%	215.6147025	2.23E-06	2.00E-08	5.10E-13
ENSG00000173436_19597046_19617268_24	novel	MICOS10	1	+	72	105	25	2	41.67%	481.8661912	0.020380127	0.002516754	0.00030172
ENSG00000173436_19617297_19626401_70	novel	MICOS10	1	+	210	66	7	5	14.29%	103.8470812	0.005578808	0.00252785	7.94E-05
ENSG00000127481_19081673_19081389_94	internal	UBR4	1	-	282	20	10	11	12.77%	23.43422206	0.09799506	0.04583453	0.01755968
ENSG00000127481_19077984_19074832_78	annotated	UBR4	1	-	234	145	7	0	24.36%	204.7490043	9.43E-05	6.44E-05	4.46E-08
ENSG00000197070_137613972_137615092_317	novel	ARRDC1	9	+	951	16	3	2	2.52%	5.559159304	0.031495399	0.016170007	0.002341768
ENSG00000157224_90384918_90385154_78	uORF	CLDN12	7	+	234	22	3	0	7.69%	31.06536617	0.016822681	0.013217227	0.001064153
ENSG00000147119_46573932_46575392_486	annotated	CHST7	X	+	1458	7	2	1	1.23%	1.586390753	0.093731016	0.028889786	0.011492152
ENSG00000112578_41921563_41932706_437	annotated	BYSL	6	+	1311	38	0	10	3.89%	9.57746467	0.000120773	0.007538926	8.00E-06
ENSG00000178982_38619269_38626156_135	novel	EIF3K	19	+	405	573	51	19	20.74%	467.4866922	6.13E-06	1.17E-05	5.92E-10
ENSG00000068438_48481180_48482851_205	novel	FTSJ1	X	+	615	62	6	0	9.76%	33.31088932	0.00019612	4.06E-05	6.00E-08
ENSG00000070010_19465339_19451990_149	novel	UFD1	22	-	447	57	7	3	10.74%	42.13441672	0.001057438	0.000786552	5.21E-06
ENSG00000130939_10132477_10144941_68	novel	UBE4B	1	+	204	13	2	0	7.35%	21.05633777	0.065398531	0.019679754	0.005779267
ENSG00000082781_124766289_124763623_108	novel	ITGB5	3	-	324	40	5	5	15.74%	40.79290508	0.000258724	0.001679424	2.97E-06
ENSG00000091844_153024441_153011574_122	annotated	RGS17	6	-	366	15	1	0	4.10%	13.54190701	0.042234516	0.019679754	0.003715148
ENSG00000184357_27867529_27866849_226	annotated	HIST1H1B	6	-	678	26	2	2	4.87%	12.67107051	0.003224199	0.00507972	9.07E-05
ENSG00000104853_44955056_44992897_655	annotated	CLPTM1	19	+	1965	701	74	21	18.93%	117.8759259	2.37E-19	4.78E-21	1.95E-38
ENSG00000134574_47215137_47217121_175	novel	DDB2	11	+	525	16	4	1	4.57%	10.07002	0.058290626	0.009288069	0.002767457
ENSG00000065518_120596360_120596569_69	annotated	NDUFB4	3	+	207	137	11	8	31.88%	218.6854433	1.89E-05	1.93E-05	2.87E-09
ENSG00000101040_47356670_47210761_1188	annotated	ZMYND8	20	-	3564	24	10	0	0.84%	2.22506755	0.060116676	0.002321823	0.000966448
ENSG00000143578_153968526_153969737_194	novel	CREB3L4	1	+	582	12	0	0	2.58%	6.812835693	0.021084099	0.021084099	0.002029891
ENSG00000157734_64156036_64156287_83	dORF	SNX22	15	+	249	218	66	104	16.87%	289.2855895	0.005220547	0.019232304	0.000529385
ENSG00000157734_64156726_64156857_43	dORF	SNX22	15	+	129	59	14	15	23.26%	151.1234832	0.007635352	0.105704367	0.004674262
ENSG00000163931_53255942_53225756_623	annotated	TKT	3	-	1869	1941	229	77	29.70%	343.151489	7.30E-26	2.12E-30	3.80E-54
ENSG00000114383_50328099_50320308_164	novel	TUSC2	3	-	492	10	7	0	4.27%	6.715905104	0.277188338	0.007593099	0.01638696
ENSG00000106089_73719631_73704314_130	novel	STX1A	7	-	390	20	2	1	6.92%	16.94474519	0.009288069	0.003478238	0.000176522
ENSG00000146731_56060845_56063085_114	novel	CCT6A	7	+	342	60	6	1	7.02%	57.96886511	0.040965401	0.005706018	0.001268341
ENSG00000106484_130495369_130505056_326	annotated	MEST	7	+	978	43	4	2	4.91%	14.52778	0.000362935	0.000678439	1.62E-06
ENSG00000156599_57673091_57700031_715	annotated	ZDHHC5	11	+	2145	32	11	4	2.52%	4.929380418	0.034695306	0.00123135	0.000307341
ENSG00000165304_36607590_36677337_457	Overlap_dORF	MELK	9	+	1371	64	12	2	5.91%	15.42453829	0.000135361	8.79E-06	1.00E-08
ENSG00000177119_45216322_45429311_910	annotated	ANO6	12	+	2730	109	14	7	4.62%	13.19269447	3.38E-06	6.15E-08	2.21E-12
ENSG00000124783_7313197_7313117_26	Overlap_uORF	SSR1	6	-	78	34	34	4	34.62%	144.0303341	0.34125222	0.018024648	0.038230595
ENSG00000124783_7313120_7289864_291	annotated	SSR1	6	-	873	819	105	18	29.21%	309.984024	2.03E-12	1.75E-15	5.94E-26
ENSG00000049323_33020941_33398545_1522	annotated	LTBP1	2	+	4566	16	0	0	0.39%	1.15785381	0.01300724	0.01300724	0.000821865
ENSG00000162910_228108260_228106760_128	annotated	MRPL55	1	-	384	154	11	4	19.53%	132.5132026	6.15E-06	5.55E-06	2.84E-10
ENSG00000101150_63865366_63889945_186	annotated	TPD52L2	20	+	558	211	28	6	23.12%	124.9447205	3.90E-07	1.81E-08	7.44E-14
ENSG00000148737_112950757_113160661_476	annotated	TCF7L2	10	+	1428	32	6	1	3.36%	7.404426468	0.004472475	0.0004227	1.29E-05
ENSG00000166741_114312268_114312477_69	novel	NNMT	11	+	207	163	21	7	36.23%	260.1877902	3.64E-05	9.21E-06	2.71E-09
ENSG00000106012_2559182_2610162_679	annotated	IQCE	7	+	2037	10	1	0	0.88%	1.622103736	0.023672501	0.012798403	0.001437523
ENSG00000166908_57594058_57601606_352	annotated	PIP4K2C	12	+	1056	99	3	4	7.67%	30.97711229	6.47E-06	1.46E-05	7.74E-10
ENSG00000162144_61356538_61350399_114	Overlap_dORF	CYB561A3	11	-	342	16	1	0	6.14%	15.45836403	0.013892891	0.008473674	0.00058868
ENSG00000010219_4604991_4613753_234	annotated	DYRK4	12	+	702	56	8	0	6.84%	26.35849251	0.001108861	0.000178703	1.38E-06
ENSG00000092853_35769870_35736496_1275	annotated	CLSPN	1	-	3825	18	4	5	0.94%	1.554929558	0.015765093	0.013786308	0.001040291
ENSG00000172731_70376533_70301354_184	annotated	LRRC20	10	-	552	15	1	0	2.72%	8.978873128	0.02854252	0.020613417	0.002645522
ENSG00000136877_127804381_127813604_509	annotated	FPGS	9	+	1527	41	10	0	3.73%	8.87185578	0.001014686	4.44E-05	3.64E-07
ENSG00000136877_127804381_127810095_297	annotated	FPGS	9	+	891	26	7	6	3.37%	9.641959382	0.019243136	0.048170141	0.004154176
ENSG00000124207_49058464_49096438_915	annotated	CSE1L	20	+	2745	218	57	5	8.63%	26.24120648	3.60E-08	3.35E-14	4.22E-20
ENSG00000151116_18588654_18532320_471	annotated	UEVLD	11	-	1413	36	5	3	3.82%	8.418408436	0.001361005	0.000558901	4.84E-06
ENSG00000151116_18566419_18532320_331	dORF	UEVLD	11	-	993	29	5	0	3.63%	9.649802016	0.009712977	0.001003278	6.22E-05
ENSG00000164949_94260461_94250310_282	annotated	GEM	8	-	846	14	0	2	2.84%	5.467985148	0.005160369	0.022898969	0.000627835
ENSG00000106153_56104351_56101851_93	annotated	CHCHD2	7	-	279	188	7	0	11.83%	222.6503077	0.001665007	0.001657226	1.64E-05
ENSG00000071564_1625648_1611707_567	annotated	TCF3	19	-	1701	21	0	2	1.23%	4.079290508	0.008776163	0.020779354	0.000903181
ENSG00000126787_55189179_55148454_842	annotated	DLGAP5	14	-	2526	76	13	2	4.63%	9.941453826	2.07E-05	3.48E-08	9.64E-12

ENSG00000245910_66925491_66922376_23	novel	SNHG6	8	-	69	37	0	0	26.09%	177.1830964	0.013640586	0.013640586	0.000898637
ENSG00000141012_88837632_88814439_337	novel	GALNS	16	-	1011	101	16	3	8.31%	33.00957037	0.000610546	1.02E-05	5.81E-08
ENSG00000141012_88842829_88842629_66	novel	GALNS	16	-	198	19	1	0	9.09%	31.70721258	0.016983117	0.013640586	0.00110551
ENSG00000177548_28924508_28910867_313	novel	RABEP2	16	-	939	12	1	0	2.24%	4.222652155	0.01551626	0.007882058	0.000614257
ENSG00000102897_20915556_20924116_122	annotated	LYRM1	16	+	366	17	1	0	8.20%	15.34749461	0.002008257	0.002122793	2.50E-05
ENSG00000148429_11532232_11462441_845	annotated	USP6NL	10	-	2535	12	0	1	0.71%	1.564130325	0.012178958	0.023056749	0.001340064
ENSG00000185088_63157405_63155553_97	annotated	RPS27L	15	-	291	145	59	0	14.43%	164.6435293	0.015293003	0.000461594	5.40E-05
ENSG00000148426_11852114_11870406_423	annotated	PROSER2	10	+	1269	22	0	2	1.89%	5.728365394	0.005604867	0.018402302	0.000538358
ENSG00000149357_72103224_72098777_89	novel	LAMTOR1	11	-	267	139	20	13	19.10%	172.0177222	0.000475147	0.000989796	3.03E-06
ENSG00000062598_46389175_46367360_624	annotated	ELMO2	20	-	1872	12	3	1	1.60%	2.118093148	0.019400218	0.00398705	0.000423191
ENSG00000110906_109456297_109451595_132	dORF	KCTD10	12	-	396	17	0	0	4.55%	14.18480563	0.013428348	0.013428348	0.000872559
ENSG00000124688_43635876_43640533_274	annotated	MAD2L1BP	6	+	822	42	7	0	4.01%	16.88290305	0.012521771	0.00140552	0.000109062
ENSG00000179241_35944099_36229397_335	annotated	LDLRAD3	11	+	1005	33	4	1	3.88%	10.84969505	0.002184759	0.00077423	1.05E-05
ENSG00000162909_223712641_223774857_700	annotated	CAPN2	1	+	2100	437	41	26	13.86%	68.75935528	9.19E-15	9.35E-16	1.24E-28
ENSG00000185875_25023512_25025455_647	annotated	THNSL1	10	+	1941	34	1	0	2.01%	5.787926872	0.000874411	0.000690959	3.82E-06
ENSG00000149499_62612457_62602475_896	annotated	EML3	11	-	2688	26	14	3	1.45%	3.196051268	0.131068782	0.004649221	0.004243971
ENSG00000149499_62611531_62602302_889	annotated	EML3	11	-	2667	22	13	3	1.12%	2.725645176	0.205190919	0.01405519	0.016391528
ENSG00000181381_168472492_168358147_1694	annotated	DDX60L	4	-	5082	25	0	0	0.71%	1.62545519	0.000991051	0.000991051	6.10E-06
ENSG00000166171_101588337_101608902_170	annotated	DPCD	10	+	510	39	0	2	8.24%	25.26760532	0.000450794	0.001336373	3.90E-06
ENSG00000095739_28681949_28682401_150	novel	BAMBI	10	+	450	31	0	0	8.67%	22.76244103	0.000683882	0.000683882	2.99E-06
ENSG00000114354_100713686_100748531_400	annotated	TFG	3	+	1200	146	7	0	8.25%	40.20140795	1.49E-06	2.39E-07	3.35E-12
ENSG00000076053_114407466_114407807_113	annotated	RBM7	11	+	339	8	0	0	6.19%	7.797581855	0.005706018	0.005706018	0.00017331
ENSG00000164051_48433856_48432408_302	annotated	CCDC51	3	-	906	31	3	0	4.97%	11.30584819	0.00157596	0.000276859	2.95E-06
ENSG00000141956_41860336_41801240_1132	annotated	PRDM15	21	-	3396	14	1	0	0.62%	1.362165912	0.011306644	0.007882058	0.000451174
ENSG00000107186_13250315_13106965_2037	annotated	MPDZ	9	-	6111	32	6	4	0.74%	1.730243986	0.007031648	0.002987195	0.000116449
ENSG00000100416_46337935_46357006_306	Overlap_dORF	TRMU	22	+	918	9	0	3	1.96%	3.23943658	0.009815329	0.080264768	0.004118547
ENSG00000100416_46335765_46346473_99	annotated	TRMU	22	+	297	12	2	0	7.07%	13.3504053	0.023672501	0.007882058	0.000936943
ENSG00000180389_27945260_27945415_51	novel	ATP5F1EP2	13	+	153	8	24	0	9.80%	17.27699509	0.36944134	0.019679754	0.045262858
ENSG00000173915_103392376_103392194_31	annotated	ATP5MD	10	-	93	120	3	0	25.81%	426.3516531	0.005808022	0.005859343	0.000180739
ENSG00000118402_79947279_79916608_314	annotated	ELOVL4	6	-	942	23	2	0	2.87%	8.067641418	0.009181916	0.003632191	0.000181437
ENSG00000176153_64942726_64942607_39	annotated	GPX2	14	-	117	325	65	9	53.85%	917.8403642	0.02530186	0.000122292	3.51E-05
ENSG00000173915_103392376_103392194_31	annotated	ATP5MD	10	-	93	120	3	0	25.81%	426.3516531	0.005808022	0.005859343	0.000180739
ENSG00000108433_46929545_46938760_147	annotated	GOSR2	17	+	441	14	1	0	5.44%	10.48960416	0.007930666	0.005160369	0.000216377
ENSG00000188428_8064376_8015649_187	annotated	BLOC1S5	6	-	561	24	8	0	4.81%	14.13572326	0.067508691	0.003303074	0.001453527
ENSG00000138796_108004852_108034357_292	dORF	HADH	4	+	876	66	9	4	8.56%	24.89484823	0.000252543	3.59E-05	6.95E-08
ENSG00000123505_110892816_110893616_102	novel	AMD1	6	+	306	8	1	1	4.90%	8.638497546	0.019216965	0.019216965	0.001707396
ENSG00000123505_110892187_110892494_70	novel	AMD1	6	+	210	9	3	1	10.00%	14.16096562	0.053359408	0.016735684	0.004093888
ENSG00000197879_1484273_1465726_1028	annotated	MYO1C	17	-	3084	245	36	10	7.39%	26.24952014	2.41E-09	3.02E-13	1.36E-20
ENSG00000181991_88467718_88468028_60	novel	MRPS11	15	+	180	57	5	0	16.67%	104.6338015	0.004326528	0.002502537	6.12E-05
ENSG00000127980_92517434_92487457_923	novel	PEX1	7	-	2769	20	5	0	1.19%	2.386583829	0.021896615	0.001440978	0.000205599
ENSG00000140395_78295686_78283432_212	annotated	WDR61	15	-	636	105	15	0	10.38%	54.55088957	0.000183739	1.79E-05	2.64E-08
ENSG00000139641_56128359_56143862_1091	annotated	ESYT1	12	+	3273	300	31	8	8.80%	30.28620817	1.47E-14	9.75E-17	2.26E-29
ENSG00000137942_93448282_93551125_551	annotated	FNBP1L	1	+	1653	44	1	7	3.63%	8.795276085	5.20E-05	0.001228821	5.15E-07
ENSG00000137936_93674723_93562241_756	annotated	BCAR3	1	-	2268	63	11	8	3.44%	9.178403642	0.00029407	0.000102133	2.14E-07
ENSG00000137944_88988350_88936183_454	annotated	KYAT3	1	-	1362	33	11	0	3.96%	8.005832252	0.015600477	7.87E-05	1.36E-05
ENSG00000196914_120421814_120477540_1078	novel	ARHGEF12	11	+	3234	18	4	3	1.02%	1.839086444	0.014484381	0.014185331	0.000986312
ENSG00000109861_88337672_88326377_141	annotated	CTSC	11	-	423	182	19	1	16.31%	142.1676139	0.001739089	1.55E-05	2.70E-07
ENSG00000215440_58694422_58712760_281	novel	NPEPL1	20	+	843	10	0	6	1.78%	3.919602979	0.019679754	0.261804282	0.02820101
ENSG00000074211_6472229_6323302_447	annotated	PPP2R2C	4	-	1341	14	0	2	1.79%	3.449601369	0.005160369	0.026823708	0.00074112
ENSG00000150457_20991401_20974870_973	annotated	LATS2	13	-	2919	21	2	1	0.92%	2.377140512	0.008364861	0.00567009	0.000248618
ENSG00000164828_852875_873331_460	novel	SUN1	7	+	1380	103	11	0	7.17%	24.66197153	8.34E-06	2.16E-07	1.82E-11
ENSG00000164828_832525_852882_505	novel	SUN1	7	+	1515	88	31	3	6.34%	19.19285989	0.001227904	1.53E-06	2.64E-08
ENSG00000164828_838815_838970_51	novel	SUN1	7	+	153	22	3	16	19.61%	47.5117365	0.010402772	0.229394231	0.015451134
ENSG00000105835_106284884_106261570_368	annotated	NAMPT	7	-	1104	73	8	1	2.99%	21.84859128	0.002642383	0.001811542	2.80E-05
ENSG00000145833_134758939_134828706_1031	annotated	DDX46	5	+	3093	139	20	6	5.43%	14.84925051	9.21E-08	3.50E-10	4.23E-16
ENSG00000170456_31590832_31433110_738	annotated	DENND5B	12	-	2214	37	0	4	2.57%	5.521966419	5.02E-05	0.000440768	1.68E-07
ENSG00000177225_774104_770897_134	annotated	GATD1	11	-	402	13	0	0	4.48%	10.68530573	0.012798403	0.012798403	0.000797244
ENSG00000134330_9481342_9484586_86	novel	IAH1	2	+	258	70	5	1	19.77%	89.64952395	0.000254751	0.000129483	2.32E-07
ENSG00000134330_9474567_9478230_29	novel	IAH1	2	+	87	14	1	5	17.24%	53.17144179	0.019679754	0.051235217	0.004505595
ENSG00000171792_2885285_2885512_75	Overlap_uORF	RHNO1	12	+	225	11	2	0	9.33%	16.15399041	0.049121095	0.007029303	0.001832503
ENSG00000048405_127391557_127371814_664	annotated	ZNF800	7	-	1992	12	3	0	1.20%	1.990497175	0.047083924	0.004826937	0.001291071
ENSG00000163660_157150374_157148241_299	dORF	CCNL1	3	-	897	26	8	1	3.68%	9.57746467	0.067865885	0.002493494	0.001181394
ENSG00000143437_150876567_150812021_774	annotated	ARNT	1	-	2322	37	2	0	2.45%	5.265130771	0.000140958	4.53E-05	4.76E-08
ENSG00000183655_85769591_85768041_516	annotated	KLHL25	15	-	1548	18	0	0	1.36%	3.842122455	0.008473674	0.008473674	0.000366502
ENSG00000174891_158122105_158544275_276	annotated	RSRC1	3	+	828	103	23	5	2.90%	41.10328588	0.061782492	0.02600941	0.00689238
ENSG00000175334_66003293_66003772_75	annotated	BANF1	11	+	225	667	71	11	29.33%	979.5192367	5.17E-05	3.80E-05	1.48E-08
ENSG00000107874_102425188_102424165_169	novel	CUEDC2	10	-	507	77	14	12	10.06%	50.18251459	0.001606234	0.002085392	1.98E-05
ENSG00000137955_75786272_75790101_152	annotated	RABGGTB	1	+	456	42	13	2	13.16%	30.43365418	0.007696563	7.79E-05	5.74E-06

ENSG00000182446_81636930_81559259_608	annotated	NPLOC4	17	-	1824	46	13	8	2.80%	8.333024359	0.027594279	0.002895495	0.000471538
ENSG00000166548_66550061_66511968_247	annotated	TK2	16	-	741	25	3	2	4.45%	11.14785867	0.001398493	0.006753026	5.66E-05
ENSG00000138640_88768056_88728533_536	annotated	FAM13A	4	-	1608	12	1	0	0.93%	2.465839784	0.028889786	0.021084099	0.002731533
ENSG00000138674_82881906_82875818_131	novel	SEC31A	4	-	393	11	0	0	3.82%	9.248467792	0.019679754	0.019679754	0.001784964
ENSG00000197265_30653598_30578921_291	annotated	GTF2E2	8	-	873	83	8	2	8.59%	31.41474236	2.20E-05	1.34E-05	2.34E-09
ENSG00000143933_47162588_47160776_113	novel	CALM2	2	-	339	735	44	31	24.78%	716.402833	1.84E-06	4.82E-06	7.66E-11
ENSG00000116815_116570972_116519227_248	annotated	CD58	1	-	744	42	4	0	6.85%	18.65288482	0.000416547	0.000126358	3.70E-07
ENSG00000143401_150235780_150220691_134	annotated	ANP32E	1	-	402	81	2	0	19.40%	66.57767418	9.45E-06	3.35E-06	2.67E-10
ENSG00000003137_72147675_72132227_459	annotated	CYP26B1	2	-	1377	21	5	0	2.18%	5.039123568	0.026101818	0.002321823	0.000369793
ENSG00000198363_61689688_61626273_256	annotated	ASPH	8	-	768	1212	123	79	38.67%	521.4480569	4.69E-15	6.64E-15	4.37E-28
ENSG00000113643_168486499_168493922_113	annotated	RARS	5	+	339	11	6	2	5.31%	10.72167505	0.153960942	0.031982531	0.021136544
ENSG00000198542_101452834_101715654_445	annotated	ITGBL1	13	+	1335	286	25	6	14.38%	70.78714899	6.75E-10	7.51E-12	6.68E-20
ENSG00000100504_50944403_50905392_847	annotated	PYGL	14	-	2541	187	17	16	7.56%	24.31680965	3.74E-11	9.39E-11	4.17E-20
ENSG00000277775_26250605_26250195_136	annotated	HIST1H3F	6	-	408	665	45	7	24.26%	538.5563314	2.17E-06	5.56E-07	1.09E-11
ENSG00000176022_1232402_1233268_288	annotated	B3GALT6	1	+	864	65	1	3	7.64%	24.85817653	1.79E-05	8.88E-05	1.25E-08
ENSG00000116731_13742029_13816547_1633	annotated	PRDM2	1	+	4899	17	12	4	0.49%	1.146597883	0.185187311	0.041403097	0.031444484
ENSG00000134109_5187896_5211252_541	novel	EDEM1	3	+	1623	30	8	7	3.70%	6.107625344	0.003443648	0.002546018	4.98E-05
ENSG00000114021_100334792_100345707_152	novel	NIT2	3	+	456	123	2	17	15.13%	89.1271301	1.27E-05	9.89E-05	1.01E-08
ENSG00000114021_100346216_100355268_121	novel	NIT2	3	+	363	58	13	4	14.88%	52.79478459	0.003669394	0.000620107	1.46E-05
ENSG00000168061_65041332_65044727_345	annotated	SAC3D1	11	+	1035	29	7	0	2.03%	9.258215848	0.044620823	0.008877961	0.002001142
ENSG00000137710_110279692_110199612_200	annotated	RDX	11	-	600	16	1	0	2.50%	8.811267496	0.028889786	0.021084099	0.002731533
ENSG00000130402_38647746_38730020_102	annotated	ACTN4	19	+	306	157	30	11	19.61%	169.5305143	0.000945089	0.000743668	4.42E-06
ENSG00000182093_39380385_39390907_103	annotated	WRB	21	+	309	92	9	8	17.48%	98.37822933	0.000173194	8.38E-05	1.05E-07
ENSG00000182093_39393175_39405984_58	dORF	WRB	21	+	174	40	10	1	17.24%	75.95920256	0.008605408	0.002390426	0.000116841
ENSG00000185432_50925189_50930150_194	annotated	METTL7A	12	+	582	14	0	0	3.61%	7.948308309	0.007029303	0.007029303	0.000257364
ENSG00000166908_57594058_57597960_102	annotated	PIP4K2C	12	+	306	45	0	0	8.82%	48.59154869	0.003327803	0.003327803	6.21E-05
ENSG00000087470_32731470_32737960_125	novel	DNM1L	12	+	375	13	0	0	6.40%	11.45464775	0.004826937	0.004826937	0.000126125
ENSG00000148840_102141608_102150029_631	annotated	PPRC1	10	+	1893	38	5	0	2.85%	6.632887577	0.00086394	8.12E-05	5.24E-07
ENSG00000112701_75602525_75715594_1105	annotated	SENP6	6	+	3315	44	5	1	1.63%	4.385698754	0.000396068	0.000111557	3.13E-07
ENSG00000102030_153934904_153930269_169	annotated	NAA10	X	-	507	36	4	0	8.88%	23.46195487	0.002554839	0.000290289	5.10E-06
ENSG00000122034_27434867_27435597_130	annotated	GTF3A	13	+	390	12	2	1	4.62%	10.16684711	0.071345384	0.026832696	0.008177618
ENSG00000148634_68073108_67922931_1057	annotated	HERC4	10	-	3171	31	6	7	1.80%	3.230242341	0.002675044	0.005331125	8.00E-05
ENSG00000102882_30123047_30116837_303	annotated	MAPK3	16	-	909	41	6	1	5.28%	14.90354651	0.001964449	0.000263972	3.56E-06
ENSG00000132507_7309636_7311640_154	annotated	EIF5A	17	+	462	657	241	36	18.18%	469.8865865	0.000502315	1.88E-06	1.10E-08
ENSG00000111678_6943662_6945922_109	novel	C12orf57	12	+	327	197	47	9	23.85%	199.0618918	0.000107587	1.63E-05	1.39E-08
ENSG00000184304_29927512_29577238_912	annotated	PRKD1	14	-	2736	17	0	0	0.99%	2.053063973	0.003327803	0.003327803	6.21E-05
ENSG00000119812_33599206_33584776_513	annotated	FAM98A	2	-	1539	42	6	4	3.12%	9.017379017	0.001624713	0.001052931	1.04E-05
ENSG00000065809_14553383_14530515_20	uORF	FAM107B	10	-	60	13	3	0	25.00%	71.59154841	0.138151459	0.019679754	0.01298269
ENSG00000120708_136029056_136063226_683	annotated	TGFBI	5	+	2049	2370	307	73	33.53%	382.1871151	7.82E-32	8.92E-38	2.08E-67
ENSG00000141699_42585248_42581813_265	dORF	RETREG3	17	-	795	89	9	0	8.68%	36.99069845	3.41E-05	1.22E-05	3.30E-09
ENSG00000141699_42609324_42586836_133	annotated	RETREG3	17	-	399	37	3	4	9.02%	30.64068584	0.001003278	0.005630074	3.50E-05
ENSG00000100650_69768157_69769126_99	annotated	SRSF5	14	+	297	222	29	21	38.38%	246.982498	1.97E-06	7.87E-07	1.39E-11
ENSG00000100445_24440454_24440083_123	novel	SDR39U1	14	-	369	13	0	0	4.07%	11.64090218	0.016947427	0.016947427	0.001349898
ENSG00000067248_55294112_55256482_1142	annotated	DHX29	5	-	3426	39	9	1	1.49%	3.761377324	0.002049849	0.000170356	2.53E-06
ENSG00000204427_31687887_31687212_97	novel	ABHD16A	6	-	291	15	4	0	7.22%	17.03208923	0.051745348	0.007882058	0.00212842
ENSG00000140262_57231230_57282587_487	annotated	TCF12	15	+	1461	30	3	5	3.29%	6.784856902	0.000857837	0.002150019	1.14E-05
ENSG00000131023_149702039_149661729_1101	annotated	LATS1	6	-	3303	42	4	1	1.18%	4.201558071	0.001926349	0.000808471	9.65E-06
ENSG00000040933_98535792_98587608_861	annotated	INPP4A	2	+	2583	11	2	1	0.58%	1.407142022	0.047790352	0.020239728	0.004303932
ENSG00000105819_103297460_103309125_340	novel	PMPCB	7	+	1020	133	13	1	9.71%	43.08450651	5.18E-06	3.60E-07	1.77E-11
ENSG00000075711_197297204_197044623_893	annotated	DLG1	3	-	2679	39	4	0	2.02%	4.810182424	0.000762836	7.71E-05	4.39E-07
ENSG00000120265_149749728_149810628_286	annotated	PCMT1	6	+	858	182	31	7	15.03%	70.08962781	1.00E-06	3.76E-08	3.92E-13
ENSG00000151414_198232581_198319522_302	annotated	NEK7	1	+	906	47	1	0	5.63%	17.14112468	0.000283388	0.000112974	2.26E-07
ENSG00000120265_149749728_149802379_250	annotated	PCMT1	6	+	750	181	31	7	16.80%	79.74197084	1.28E-06	5.07E-08	6.67E-13
ENSG00000085433_109011706_108971430_808	annotated	WDR47	1	-	2424	10	1	0	0.62%	1.363129254	0.035374934	0.020613417	0.003252526
ENSG00000113249_157057943_157029588_401	annotated	HAVCR1	5	-	1203	844	114	26	22.69%	231.8176361	1.30E-13	3.07E-15	5.83E-27
ENSG00000117620_99993624_100022476_302	annotated	SLC35A3	1	+	906	16	9	0	3.64%	5.835276488	0.151590816	0.000888449	0.001652192
ENSG00000082898_61526515_61478820_1027	annotated	XPO1	2	-	3081	251	36	10	9.15%	26.9185509	2.16E-13	2.64E-16	9.52E-28
ENSG00000175602_65890919_65891392_157	annotated	CCDC85B	11	+	471	24	0	0	5.73%	16.83681687	0.003554776	0.003554776	7.04E-05
ENSG00000081692_227735273_227732392_417	annotated	JMJD4	1	-	1251	19	0	6	1.92%	5.018407747	0.005554612	0.075179667	0.002457728
ENSG00000213983_23567314_23559749_785	annotated	AP1G2	14	-	2355	56	13	1	3.18%	7.857181207	0.000482493	6.49E-06	3.03E-08
ENSG00000197905_3017440_3040478_302	annotated	TEAD4	12	+	906	24	5	2	3.64%	8.752914732	0.014881989	0.005035606	0.000394897
ENSG00000132356_40798162_40762778_550	annotated	PRKAA1	5	-	1650	95	14	7	5.27%	19.02432755	0.000176173	9.83E-06	1.47E-08
ENSG00000134897_102807268_102839865_503	annotated	BIVM	13	+	1509	17	1	0	1.59%	3.722453962	0.00912793	0.005160369	0.000248192
ENSG00000104067_29800693_29701595_1756	annotated	TJP1	15	-	5268	62	8	6	1.88%	3.888799721	4.23E-06	7.89E-06	2.79E-10
ENSG00000256646_42924697_42912534_65	dORF	AC010132.3	7	-	195	87	14	1	20.00%	147.4192831	0.000741347	0.000722178	3.40E-06
ENSG00000256646_42932158_42924769_48	annotated	AC010132.3	7	-	144	100	4	19	27.08%	229.4600911	0.000707658	0.008542715	4.02E-05
ENSG00000130202_44846526_44888379_538	annotated	NECTIN2	19	+	1614	95	28	10	5.95%	19.44866199	0.000163964	5.06E-06	7.50E-09
ENSG00000135966_105308298_105267383_859	annotated	TGFBRAP1	2	-	2577	24	4	0	1.51%	3.077276192	0.007497815	0.000585477	2.97E-05

ENSG00000115641_105386516_105361283_279	annotated	FHL2	2	-	837	117	12	0	10.04%	46.18809575	2.51E-05	1.64E-06	3.70E-10
ENSG00000113300_180550104_180574200_462	annotated	CNOT6	5	+	1386	23	3	3	2.60%	5.483202176	0.002084681	0.002084681	2.54E-05
ENSG00000116793_113726494_113697705_625	annotated	PHTF1	1	-	1875	19	2	0	1.44%	3.348281649	0.011768187	0.003606289	0.000231562
ENSG00000106089_73705198_73700827_182	novel	STX1A	7	-	546	14	6	2	3.30%	8.472372593	0.159270956	0.023672501	0.017530873
ENSG00000116539_155521498_155337660_2957	annotated	ASH1L	1	-	8871	37	5	1	0.51%	1.378157327	0.002868264	0.000311218	6.13E-06
ENSG00000100321_39350011_39374651_144	annotated	SYNGR1	22	+	432	14	10	0	5.56%	10.70813758	0.308446548	0.005257623	0.01527812
ENSG00000068400_48990976_48974193_613	annotated	GRIPAP1	X	-	1839	10	2	0	0.98%	1.796751121	0.060322713	0.011570466	0.003431813
ENSG00000162298_65133580_65128382_609	annotated	SYVN1	11	-	1827	134	11	10	6.24%	24.23460272	1.03E-06	3.30E-06	3.00E-11
ENSG00000111142_95512882_95513904_95	dORF	METAP2	12	+	285	77	15	2	17.89%	89.27205227	0.00192925	0.000141837	2.02E-06
ENSG00000111142_95474180_95512823_328	annotated	METAP2	12	+	984	103	13	3	12.80%	34.58691129	3.74E-06	1.52E-08	7.00E-13
ENSG00000158042_6683296_6682118_175	annotated	MRPL17	11	-	525	146	7	12	21.71%	91.88893246	8.70E-08	4.75E-08	4.08E-14
ENSG00000174446_66497731_66494436_98	annotated	SNAPC5	15	-	294	28	2	2	11.22%	31.46881249	0.011099465	0.003990669	0.00023884
ENSG00000079691_25495169_25612396_916	annotated	CARMIL1	6	+	2748	13	0	2	0.87%	1.563134245	0.004826937	0.026606355	0.000694433
ENSG00000137210_10755171_10756518_37	novel	TMEM14B	6	+	111	41	9	0	16.22%	122.0479619	0.057424805	0.013640586	0.003726901
ENSG00000178974_55350469_55352526_685	annotated	FBXO34	14	+	2055	16	1	1	1.17%	2.572632846	0.011306644	0.007930666	0.000453741
ENSG00000173786_41971921_41973924_186	novel	CNP	17	+	558	46	4	2	6.99%	27.23913339	0.000709615	0.001451832	6.45E-06
ENSG00000005007_18832210_18866163_1118	annotated	UPF1	19	+	3354	42	1	0	1.79%	4.137670336	5.26E-05	3.61E-05	1.44E-08
ENSG00000105879_107744164_107758148_123	annotated	CBLL1	7	+	369	18	4	0	4.07%	16.11817225	0.101636223	0.021084099	0.009735732
ENSG00000071051_105855064_105893007_83	annotated	NCK2	2	+	249	8	3	0	6.02%	10.61598494	0.128949518	0.019216965	0.011791668
ENSG00000184428_143329415_143309441_503	annotated	TOP1MT	8	-	1509	24	10	0	2.78%	5.255229123	0.071922295	0.000375189	0.000316796
ENSG00000109320_102578871_102616594_782	annotated	NFKB1	4	+	2346	38	2	0	2.43%	5.35211261	0.000110102	5.25E-05	4.27E-08
ENSG00000163072_168834302_168864970_346	annotated	NOSTRIN	2	+	1038	20	6	0	1.73%	6.366522758	0.196884317	0.01300724	0.01473882
ENSG00000166352_36610134_36659186_211	annotated	C11orf74	11	+	633	10	0	1	2.37%	5.219945199	0.020613417	0.035374934	0.003252526
ENSG00000100711_103715842_103733018_252	annotated	ZFYVE21	14	+	756	42	0	0	6.35%	18.35680728	0.000199202	0.000199202	2.75E-07
ENSG00000168077_27634201_27671351_606	annotated	SCARA3	8	+	1818	100	1	3	6.93%	18.17505672	6.48E-09	4.10E-08	2.79E-15
ENSG00000168078_27833113_27810305_322	annotated	PBK	8	-	966	253	38	10	16.15%	86.53923434	8.06E-08	1.68E-10	1.88E-16
ENSG00000124702_43017681_43020733_238	annotated	KLHDC3	6	+	714	22	0	0	5.88%	10.18108639	0.00035774	0.00035774	8.55E-07
ENSG00000188603_28489349_28477516_360	annotated	CLN3	16	-	1080	125	15	3	10.56%	38.24334851	7.97E-07	3.81E-08	3.13E-13
ENSG00000111642_6595423_6570676_1251	annotated	CHD4	12	-	3753	68	6	2	2.32%	5.9868724	9.14E-06	1.85E-06	1.47E-10
ENSG00000112855_140696548_140698552_253	dORF	HARS2	5	+	759	32	0	0	5.14%	13.9308577	0.000662934	0.000662934	2.81E-06
ENSG00000163608_113008280_113005536_192	dORF	NEPRO	3	-	576	31	0	3	3.65%	17.78315706	0.008574016	0.016101939	0.000687297
ENSG00000115355_55322644_55291711_828	annotated	CCDC88A	2	-	2484	37	2	0	2.29%	4.921752678	0.000218523	5.40E-05	8.73E-08
ENSG00000070159_109448830_109379556_782	annotated	PTPN3	9	-	2346	32	5	0	1.53%	4.507042198	0.004957106	0.001040526	3.16E-05
ENSG00000187720_71215047_71777374_981	annotated	THSD4	15	+	2943	154	21	2	6.42%	17.2902038	7.89E-10	3.72E-12	4.08E-20
ENSG00000118961_20774836_20686903_178	annotated	LDAH	2	-	534	14	2	0	5.06%	8.662762988	0.010960145	0.003229477	0.000195317
ENSG00000089157_120200783_120196773_255	annotated	RPLP0	12	-	765	78	19	5	4.31%	33.69014043	0.00782527	0.003487661	0.000149111
ENSG00000137074_32989888_32987654_84	annotated	APTX	9	-	252	46	0	3	11.90%	60.31522393	0.002376615	0.004130613	5.58E-05
ENSG00000137074_32984722_32973498_116	dORF	APTX	9	-	348	16	1	1	5.17%	15.19184051	0.012798403	0.01300724	0.000809468
ENSG00000131437_132726451_132696634_617	annotated	KIF3A	5	-	1851	24	2	0	2.27%	4.284246757	0.002072853	0.000375189	5.16E-06
ENSG00000100201_38506000_38485935_650	annotated	DDX17	22	-	1950	182	29	13	8.62%	30.83943624	2.15E-08	8.17E-09	1.83E-15
ENSG00000148660_73847290_73815015_267	annotated	CAMK2G	10	-	801	12	0	0	1.87%	4.950150279	0.019679754	0.019679754	0.001784964
ENSG00000170445_140679874_140674257_406	annotated	HARS	5	-	1218	89	17	3	6.40%	24.1441751	9.41E-05	9.66E-06	7.49E-09
ENSG00000144848_112534324_112532699_45	novel	ATG3	3	-	135	10	3	0	11.11%	24.47574305	0.065398531	0.020613417	0.006006025
ENSG00000129351_10670980_10677169_76	annotated	ILF3	19	+	228	35	3	1	10.53%	50.72275697	0.012834473	0.005604867	0.000374516
ENSG00000163608_113019644_113011362_142	novel	NEPRO	3	-	426	9	6	0	4.23%	6.9807577	0.261477794	0.011772051	0.020039585
ENSG00000198522_27629059_27631208_128	novel	GPN1	2	+	384	18	1	0	4.69%	15.48855615	0.016822681	0.013428348	0.001079822
ENSG00000099250_33263694_33249421_166	annotated	NRP1	10	-	498	65	3	3	12.65%	43.1274388	0.000114435	0.000105979	8.71E-08
ENSG00000204316_75904684_75904379_101	novel	MRPL38	17	-	303	40	3	0	6.93%	43.62013612	0.035350572	0.008473674	0.001506136
ENSG00000273345_134321863_134205868_467	novel	AC104109.4	5	-	1401	45	2	6	2.57%	10.6131434	0.001023866	0.004659898	2.93E-05
ENSG00000164919_99892021_99887505_75	annotated	COX6C	8	-	225	1085	161	46	42.67%	1593.370872	8.42E-06	2.45E-06	1.77E-10
ENSG00000137700_119029530_119029333_65	novel	SLC37A4	11	-	195	31	7	0	15.38%	52.52871007	0.019390513	0.002376615	0.000272493
ENSG00000137700_119027649_119024910_250	novel	SLC37A4	11	-	750	33	3	0	5.20%	14.53859137	0.003275177	0.000669869	1.39E-05
ENSG00000148396_136461211_136441755_685	annotated	SEC16A	9	-	2055	23	6	0	2.19%	3.698159716	0.006096968	0.000207642	9.84E-06
ENSG00000187601_55452375_55453034_219	annotated	MAGEH1	X	+	657	31	0	0	4.11%	15.59071304	0.003684287	0.003684287	7.54E-05
ENSG00000117620_100007082_100015441_127	Overlap_dORF	SLC35A3	1	+	381	9	7	0	4.72%	7.805256641	0.312211302	0.009815329	0.022960993
ENSG00000015475_17750116_17740031_122	novel	BID	22	-	366	35	18	0	4.92%	31.59778303	0.22110303	0.013428348	0.017487823
ENSG00000015475_17739423_17735580_99	novel	BID	22	-	297	50	11	8	12.12%	55.62668874	0.02055943	0.009965666	0.001000669
ENSG00000012171_50274447_50275777_132	novel	SEMA3B	3	+	396	13	8	0	3.79%	10.8472043	0.311391534	0.021084099	0.037165365
ENSG00000145555_16818050_16666692_1979	annotated	MYO10	5	-	5937	105	19	22	2.58%	5.843753708	4.04E-07	2.54E-06	9.46E-12
ENSG00000182923_134495321_134561535_703	annotated	CEP63	3	+	2109	10	3	0	0.85%	1.566726084	0.100002646	0.012798403	0.006484129
ENSG00000151552_17512054_17490723_152	annotated	QDPR	4	-	456	34	3	2	7.89%	24.63676767	0.004779044	0.003734664	9.80E-05
ENSG00000151552_17490711_17460794_49	dORF	QDPR	4	-	147	19	2	0	12.24%	42.70767409	0.013428348	0.013428348	0.000872559
ENSG00000175595_13935283_13948347_466	novel	ERCC4	16	+	1398	17	1	1	1.72%	4.018013612	0.008219047	0.008219047	0.000345967
ENSG00000026297_166956182_166929588_306	annotated	RNASET2	6	-	918	27	6	1	4.25%	9.718309739	0.006735894	0.000706601	3.11E-05
ENSG00000109738_157122395_157170728_399	annotated	GLRB	4	+	1197	15	4	0	3.01%	4.140633222	0.019400218	0.000648448	9.36E-05
ENSG00000154889_11893566_11886653_170	novel	MPPE1	18	-	510	23	1	0	5.29%	14.90140827	0.00650005	0.002500085	9.16E-05
ENSG00000133243_1987330_1986488_157	novel	BTBD2	19	-	471	22	2	0	5.73%	15.4337488	0.00567009	0.003684287	0.000114233
ENSG00000092094_20448156_20448094_20	novel	OSGEP	14	-	60	14	2	0	25.00%	77.09859059	0.051235217	0.021084099	0.004778761

ENSG00000258724_10839756_10725738_440	annotated	PINX1	8	-	1320	12	2	2	1.82%	3.003841192	0.030313544	0.030313544	0.0039848
ENSG00000126215_103703271_103698798_192	annotated	XRCC3	14	-	576	17	0	0	4.69%	9.75205387	0.002943991	0.002943991	4.92E-05
ENSG00000197119_100292858_100292283_191	annotated	SLC25A29	14	-	573	21	2	0	4.19%	12.10972627	0.005355751	0.005604867	0.000160457
ENSG00000162194_62667669_62663050_159	Overlap_dORF	LBHD1	11	-	477	82	8	6	11.95%	56.80219612	0.000203551	0.000130081	1.86E-07
ENSG00000129810_20184027_20161105_292	annotated	SGO1	3	-	876	17	4	2	2.74%	6.412309394	0.044710437	0.018318558	0.003695494
ENSG00000115163_26786197_26793279_140	annotated	CENPA	2	+	420	46	10	13	7.86%	36.18913436	0.026567016	0.069437446	0.007890574
ENSG00000198258_9828473_9828679_68	novel	UBL5	19	+	204	75	19	5	11.76%	121.4788717	0.04845384	0.012132638	0.002829919
ENSG00000127995_94528221_94555758_654	annotated	CASD1	7	+	1962	22	6	1	2.14%	3.705043672	0.006657659	0.000671624	2.94E-05
ENSG00000078618_51840459_51789236_1087	annotated	NRDC	1	-	3261	107	13	1	4.32%	10.84183098	2.94E-07	7.39E-10	2.92E-15
ENSG00000065613_103967746_104025720_1204	annotated	SLK	10	+	3612	106	6	6	3.41%	9.696785243	2.72E-07	2.50E-07	6.40E-13
ENSG00000166510_54942746_54904358_320	annotated	CCDC68	18	-	960	22	1	0	3.44%	7.572183005	0.001856966	0.00146251	1.62E-05
ENSG00000188010_38881442_38882515_79	annotated	MORN2	2	+	237	28	3	0	8.86%	39.03726106	0.028352861	0.008776163	0.001235904
ENSG00000170802_48346302_48375443_402	annotated	FOXN2	2	+	1206	31	1	1	3.98%	8.493448146	0.00024364	0.00024364	4.06E-07
ENSG00000125772_5593410_5547661_623	annotated	GPCPD1	20	-	1869	21	3	1	1.93%	3.712612709	0.008291198	0.002559322	0.000119561
ENSG00000065665_12129788_12153782_168	annotated	SEC61A2	10	+	504	42	4	1	7.14%	27.53521093	0.008338546	0.001635336	8.09E-05
ENSG00000110422_33287399_33353568_866	annotated	HIPK3	11	+	2598	16	7	1	0.92%	2.034934757	0.132308109	0.009234565	0.007047991
ENSG00000123240_13110300_13136866_507	annotated	OPTN	10	+	1521	117	10	9	8.09%	25.41711778	3.30E-07	3.76E-07	1.15E-12
ENSG00000159055_32279014_32269037_233	annotated	MIS18A	21	-	699	30	1	1	6.01%	14.18122451	0.000593823	0.000593823	2.27E-06
ENSG00000163964_196716869_196733902_217	annotated	PIGX	3	+	651	34	4	0	4.15%	17.25709072	0.004533158	0.003327803	8.36E-05
ENSG00000072274_196077096_196051942_759	annotated	TFRC	3	-	2277	619	82	25	19.63%	89.82500956	2.31E-22	1.50E-24	6.56E-45
ENSG00000173156_67065926_67071602_156	annotated	RHOD	11	+	468	19	0	0	3.85%	13.41458994	0.01300724	0.01300724	0.000821865
ENSG00000129255_7587444_7587551_35	novel	MPDU1	17	+	105	13	1	0	17.14%	40.90945623	0.026832696	0.011772051	0.001510382
ENSG00000129255_7583863_7586857_155	novel	MPDU1	17	+	465	50	8	10	11.61%	35.52930442	0.002307488	0.002389806	3.19E-05
ENSG00000197045_54488927_54478088_142	annotated	GMFB	14	-	426	71	41	0	14.79%	55.07042185	0.005683792	2.73E-05	1.72E-06
ENSG00000126453_49666693_49673748_250	annotated	BCL2L12	19	+	750	81	1	5	8.40%	35.68563336	2.77E-05	9.55E-05	2.03E-08
ENSG00000128973_68256868_68208140_343	annotated	CLN6	15	-	1029	117	21	4	7.29%	37.56990879	1.38E-05	5.60E-06	6.38E-10
ENSG00000128739_24955049_24967032_37	uORF	SNRPN	15	+	111	16	2	2	13.51%	47.62847295	0.052029617	0.070008252	0.014157655
ENSG00000175727_122133555_122141812_486	annotated	MLXIP	12	+	1458	6	0	1	1.03%	1.359763503	0.016947427	0.047790352	0.003697512
ENSG00000205730_19114462_19116069_535	annotated	ITPRIPL2	16	+	1605	13	0	3	1.87%	2.676319567	0.001755235	0.021861499	0.000241364
ENSG00000177239_137108457_137108591_44	annotated	MAN1B1	9	+	132	18	0	0	11.36%	45.05761788	0.020613417	0.020613417	0.001946209
ENSG00000117569_96721865_96813405_536	annotated	PTBP2	1	+	1608	20	5	6	1.68%	4.109732974	0.02524418	0.075587118	0.008243784
ENSG00000117569_96721865_96813405_537	annotated	PTBP2	1	+	1611	20	5	6	1.68%	4.10207984	0.02524418	0.075587118	0.008243784
ENSG00000188641_97920922_97078976_1025	annotated	DPYD	1	-	3075	39	3	2	1.76%	4.190724785	0.000320827	0.000328713	7.09E-07
ENSG00000162688_99851085_99921651_1518	annotated	AGL	1	+	4554	23	6	0	0.53%	1.668800662	0.06712464	0.005306578	0.00208032
ENSG00000123106_28304699_28549173_409	dORF	CCDC91	12	+	1227	10	8	0	1.96%	2.692930164	0.323350774	0.004149608	0.014239836
ENSG00000164053_48466710_48467600_296	dORF	ATRIP	3	+	888	25	1	1	3.38%	9.302436124	0.002335453	0.003146562	4.21E-05
ENSG00000164053_48454342_48465554_593	annotated	ATRIP	3	+	1779	14	0	0	1.35%	2.600289733	0.004873904	0.004873904	0.000128468
ENSG00000162032_1781483_1777097_355	annotated	SPSB3	16	-	1065	39	5	1	5.35%	12.09998001	0.000656618	7.95E-05	3.86E-07
ENSG00000009307_114739890_114718169_798	annotated	CSDE1	1	-	2394	202	28	26	9.02%	27.88026369	2.37E-10	1.49E-10	4.08E-19
ENSG00000106009_2554431_2538069_821	annotated	BRAT1	7	-	2463	67	4	10	3.65%	8.988351435	6.75E-06	0.000111399	6.44E-09
ENSG00000127022_179705682_179728644_592	annotated	CANX	5	+	1776	3664	499	96	44.26%	681.6825191	3.00E-36	3.56E-43	3.60E-77
ENSG00000164713_98281796_98291243_125	annotated	BRI3	7	+	375	113	14	16	15.20%	99.56732271	0.001301672	0.000746792	6.08E-06
ENSG00000143546_153390535_153390103_93	annotated	S100A8	1	-	279	39	10	13	10.75%	46.18809575	0.02524418	0.079620228	0.008696174
ENSG00000056586_122858785_122849627_469	dORF	RC3H2	9	-	1407	6	3	0	1.07%	1.409051305	0.214508587	0.016947427	0.019735972
ENSG00000100714_64388428_64440136_535	novel	MTHFD1	14	+	1605	123	8	0	8.22%	25.32210052	1.26E-07	2.75E-09	3.95E-15
ENSG00000100714_64441439_64458303_312	novel	MTHFD1	14	+	936	42	6	5	5.13%	14.82665204	0.00107137	0.002145389	1.39E-05
ENSG00000133884_65333887_65341586_162	novel	DPF2	11	+	486	18	1	0	4.94%	12.23787152	0.005622352	0.004873904	0.000147222
ENSG00000160688_154983695_154993037_587	annotated	FLAD1	1	+	1761	65	23	2	5.45%	12.1961752	0.000661966	5.96E-07	5.83E-09
ENSG00000017260_130934676_131001350_823	annotated	ATP2C1	3	+	2469	90	13	6	5.22%	12.04456371	6.15E-07	1.57E-07	9.16E-13
ENSG00000141971_17420136_17424993_273	annotated	MVB12A	19	+	819	103	16	8	9.89%	41.55497034	5.72E-05	3.53E-05	1.53E-08
ENSG00000185515_155071528_155120150_316	annotated	BRCC3	X	+	948	34	2	0	3.80%	11.85059711	0.002225493	0.001053132	1.42E-05
ENSG00000116830_117060347_117101524_1162	annotated	TTF2	1	+	3486	21	10	0	0.77%	1.990497175	0.170643609	0.003303074	0.004750867
ENSG00000100804_23034881_23026089_263	annotated	PSMB5	14	-	789	465	53	15	26.62%	194.7357123	1.07E-11	1.17E-12	1.61E-22
ENSG00000166171_101587367_101596639_72	novel	DPCD	10	+	216	17	0	2	8.33%	26.00547699	0.013428348	0.037178614	0.002349204
ENSG00000205937_2264643_2253964_305	annotated	RNPS1	16	-	915	21	9	3	3.28%	7.583467927	0.034881441	0.009583527	0.001648848
ENSG00000178381_1160132_1158247_59	uORF	ZFAND2A	7	-	177	24	0	0	8.47%	44.80305507	0.021084099	0.021084099	0.002029891
ENSG00000151881_43453969_43446222_215	annotated	TMEM267	5	-	645	204	21	2	12.09%	104.5057308	0.003894049	6.82E-06	3.57E-07
ENSG00000075420_172112480_172186740_71	annotated	FNDC3B	3	+	213	10	1	1	7.04%	15.51279489	0.047790352	0.047790352	0.009211063
ENSG00000091039_76410578_76355889_798	annotated	OSBPL8	12	-	2394	40	7	1	2.38%	5.520844296	0.000820959	7.67E-05	4.72E-07
ENSG00000138078_44346342_44321356_638	annotated	PREPL	2	-	1914	43	8	1	3.13%	7.423285704	0.001704072	5.31E-05	7.50E-07
ENSG00000176697_27658543_27657821_240	annotated	BDNF	11	-	720	54	20	2	10.00%	24.78168983	0.0133993	9.36E-06	2.20E-06
ENSG00000079459_11808548_11838609_466	annotated	FDFT1	8	+	1398	977	119	40	27.90%	230.9176058	7.02E-20	1.67E-21	2.03E-39
ENSG00000117151_84574415_84566002_108	novel	CTBS	1	-	324	10	0	0	4.63%	10.19822627	0.020613417	0.020613417	0.001946209
ENSG00000117151_84565919_84554999_179	novel	CTBS	1	-	537	41	10	0	5.59%	25.22779102	0.015186081	0.002376615	0.000209829
ENSG00000100347_43976046_43996383_256	novel	SAMM50	22	+	768	51	7	4	8.20%	21.94212121	0.000312056	2.35E-05	5.88E-08
ENSG00000159210_48944734_48937068_53	annotated	SNF8	17	-	159	64	5	1	11.32%	133.0002641	0.016983117	0.016983117	0.001355223
ENSG00000175203_57547063_57532083_315	annotated	DCTN2	12	-	945	146	12	6	13.97%	51.04940692	1.03E-07	4.03E-08	4.11E-14
ENSG00000285025_39504231_39504443_70	annotated	AL022312.1	22	+	210	21	2	0	12.86%	33.04225311	0.003478238	0.003632191	7.04E-05

ENSG00000104763_18061423_18057534_149	novel	ASAH1	8	-	447	192	22	3	18.79%	141.9264563	6.85E-05	4.60E-06	2.73E-09
ENSG00000157954_5190420_5230947_436	annotated	WIPI2	7	+	1308	81	11	2	5.96%	20.46194573	5.08E-05	9.42E-06	3.88E-09
ENSG00000115355_55374859_55291711_1771	annotated	CCDC88A	2	-	5313	69	8	3	1.92%	4.291201703	9.61E-06	6.71E-07	5.99E-11
ENSG00000136522_179604624_179588872_250	annotated	MRPL47	3	-	750	112	16	3	12.40%	49.34309798	5.79E-06	1.26E-06	6.45E-11
ENSG00000182670_37124713_37201574_1624	novel	TTC3	21	+	4872	16	0	0	0.49%	1.085131465	0.005454749	0.005454749	0.000159109
ENSG00000162976_11155547_11160707_102	novel	PQLC3	2	+	306	34	29	2	9.80%	36.71361457	0.128427924	0.006133031	0.005046434
ENSG00000134779_36828767_36783042_248	annotated	TPGS2	18	-	744	67	10	10	7.66%	29.75579245	0.001860701	0.001567163	1.74E-05
ENSG00000175931_76398516_76390943_676	annotated	UBE2O	17	-	2028	22	3	1	1.48%	3.584465328	0.012527503	0.00348617	0.00023953
ENSG00000055163_157311716_157393000_905	annotated	CYFIP2	5	+	2715	35	5	0	2.21%	4.259590641	0.00044257	3.28E-05	1.15E-07
ENSG00000156113_77586415_77039271_541	dORF	KCNMA1	10	-	1623	38	2	0	2.96%	7.736325436	0.000260584	0.00019293	3.46E-07
ENSG00000283782_132587682_132595107_306	novel	AC116366.3	5	+	918	17	4	1	2.94%	6.118935761	0.032542254	0.00488717	0.000870041
ENSG00000283782_132595575_132642364_655	novel	AC116366.3	5	+	1965	53	11	14	3.21%	8.912159872	0.002861991	0.009184027	0.000146559
ENSG00000086589_150700985_150695487_254	novel	RBM22	5	-	762	30	14	1	5.91%	13.00876107	0.019358733	0.000440588	6.85E-05
ENSG00000165240_77971690_78046570_1406	annotated	ATP7A	X	+	4218	21	0	0	0.78%	1.645062388	0.001491519	0.001491519	1.34E-05
ENSG00000143514_223821367_223780853_1125	annotated	TP53BP2	1	-	3375	12	1	0	0.80%	1.174835666	0.00650005	0.002500085	9.16E-05
ENSG00000155366_112703799_112703587_70	novel	RHOC	1	-	210	54	16	11	20.00%	84.96579372	0.045462691	0.000525339	0.000222131
ENSG00000095203_109320446_109241662_518	annotated	EPB41L4B	9	-	1554	16	1	1	1.74%	3.402033782	0.004669668	0.004669668	0.000118426
ENSG00000101391_33370531_33359043_134	novel	CDK5RAP1	20	-	402	18	0	0	5.22%	14.79503871	0.008373824	0.008373824	0.000358384
ENSG00000114520_125457340_125449202_129	novel	SNX4	3	-	387	17	2	0	5.43%	14.51468483	0.023097734	0.008373824	0.000963586
ENSG00000092621_119740467_119744040_191	novel	PHGDH	1	+	573	11	1	0	3.66%	6.343189952	0.01551626	0.007882058	0.000614257
ENSG00000104805_48900797_48922424_461	annotated	NUCB1	19	+	1383	480	35	13	24.73%	114.6802711	6.45E-18	8.25E-20	8.96E-36
ENSG00000032444_7535945_7561562_1314	annotated	PNPLA6	19	+	3942	103	14	10	3.35%	8.633566896	3.10E-07	2.16E-07	6.31E-13
ENSG00000064545_19134839_19119930_359	annotated	TMEM161A	19	-	1077	41	7	3	5.01%	12.57875931	0.002083948	0.000121504	1.91E-06
ENSG00000005884_50076638_50090318_216	annotated	ITGA3	17	+	648	136	10	3	15.28%	69.34793863	1.34E-06	6.88E-07	8.29E-12
ENSG00000172992_45034786_45024433_198	annotated	DCAKD	17	-	594	25	0	1	6.06%	13.90667219	0.000966879	0.00117326	7.01E-06
ENSG00000082153_200813218_200822178_419	annotated	BZW1	2	+	1257	99	27	14	5.01%	26.02373157	0.004394084	0.000722386	2.02E-05
ENSG00000157181_186379786_186419076_422	annotated	ODR4	1	+	1266	39	1	12	3.32%	10.17889314	0.0005994	0.041601378	0.000219524
ENSG00000176273_93894034_93901490_365	annotated	SLC35G1	10	+	1095	13	1	0	2.47%	3.92282457	0.005849704	0.00308495	9.99E-05
ENSG00000144647_43081431_43079689_580	annotated	POMGNT2	3	-	1740	21	5	0	1.38%	3.987858134	0.039365406	0.005306578	0.001144392
ENSG00000184381_38169426_38112161_752	annotated	PLA2G6	22	-	2256	12	0	0	0.66%	1.757566655	0.020613417	0.020613417	0.001946209
ENSG00000177189_20266596_20155398_728	annotated	RPS6KA3	X	-	2184	52	5	2	3.57%	7.867203122	3.73E-05	1.28E-05	3.79E-09
ENSG00000181220_149477720_149474384_460	annotated	ZNF746	7	-	1380	28	3	1	1.96%	6.704225269	0.009105393	0.004490072	0.000218038
ENSG00000111845_10694986_10709452_392	annotated	PAK1IP1	6	+	1176	67	4	4	5.36%	18.82509318	9.28E-05	0.000159319	1.06E-07
ENSG00000163517_13480348_13504683_247	annotated	HDAC11	3	+	741	7	0	0	2.43%	3.121400429	0.009815329	0.009815329	0.000483714
ENSG00000137261_24596538_24547165_1027	annotated	KIAA0319	6	-	3081	32	6	4	1.46%	3.431847126	0.003741986	0.002484866	5.28E-05
ENSG00000105323_41264504_41306565_752	annotated	HNRNPUL1	19	+	2256	204	41	51	9.57%	29.87863313	9.90E-09	6.26E-09	6.50E-16
ENSG00000027697_137207006_137198031_437	annotated	IFNGR1	6	-	1311	130	16	3	8.24%	32.76501071	3.22E-06	2.88E-07	8.77E-12
ENSG00000157764_140850194_140734597_714	annotated	BRAF	7	-	2142	16	0	0	0.70%	2.468142156	0.019679754	0.019679754	0.001784964
ENSG00000180488_77783253_77875064_601	annotated	MIGA1	1	+	1803	27	12	0	1.83%	4.948091148	0.082854473	0.001506153	0.001042829
ENSG00000134278_12635073_12449638_622	annotated	SPIRE1	18	-	1866	27	4	1	3.05%	4.781033408	0.001546987	7.31E-05	8.90E-07
ENSG00000138162_122088731_122254056_971	annotated	TACC2	10	+	2913	41	9	0	2.16%	4.650643246	0.001990914	2.15E-05	4.14E-07
ENSG00000027697_137207006_137198031_437	dORF	IFNGR1	6	-	1311	130	16	3	8.24%	32.76501071	3.22E-06	2.88E-07	8.77E-12
ENSG00000065000_2151298_2102173_1203	annotated	AP3D1	19	-	3609	126	18	12	4.32%	11.53594872	5.88E-08	1.77E-08	1.06E-14
ENSG00000015171_180013_252470_517	annotated	ZMYND11	10	+	1551	47	12	9	3.87%	10.01280397	0.004593347	0.001479289	4.01E-05
ENSG00000065559_12095620_12141260_213	dORF	MAP2K4	17	+	639	15	3	0	3.76%	7.756397444	0.029020025	0.005306578	0.000824676
ENSG00000197858_144083224_144084570_214	novel	GPAA1	8	+	642	188	49	15	13.08%	96.75924587	0.002966	9.08E-06	3.24E-07
ENSG00000162298_65133580_65132286_142	annotated	SYVN1	11	-	426	34	4	6	7.04%	26.37175131	0.013506184	0.052316126	0.00335256
ENSG00000182220_40591300_40599762_96	novel	ATP6AP2	X	+	288	57	9	0	12.50%	65.39612595	0.005395979	0.001091522	3.60E-05
ENSG00000182220_40600774_40605755_100	novel	ATP6AP2	X	+	300	41	2	3	12.00%	45.15774592	0.00107863	0.001065834	7.10E-06
ENSG00000196628_55350976_55228225_499	annotated	TCF4	18	-	1497	13	0	0	1.40%	2.869400738	0.008175611	0.008175611	0.000342518
ENSG00000147804_144413369_144409915_143	novel	SLC39A4	8	-	429	133	22	16	18.88%	102.4386868	0.000291868	0.000330204	6.50E-07
ENSG00000103326_546839_553516_1086	annotated	CAPN15	16	+	3258	12	3	2	0.64%	1.217025897	0.065131088	0.028902806	0.007945652
ENSG00000198589_150928602_150265722_2698	annotated	LRBA	4	-	8094	33	6	4	0.67%	1.347163767	0.003497224	0.000863283	1.87E-05
ENSG00000183735_64455871_64490226_515	novel	TBK1	12	+	1545	19	2	0	1.75%	4.063448603	0.013443806	0.003529166	0.000260295
ENSG00000104343_73878813_73794102_159	annotated	UBE2W	8	-	477	29	1	2	7.55%	20.08858156	0.00092354	0.002061799	1.17E-05
ENSG00000100991_35092781_35003146_797	annotated	TRPC4AP	20	-	2391	109	10	1	5.90%	15.06317687	4.80E-08	1.06E-09	5.93E-16
ENSG00000101452_38962368_39038543_703	annotated	DHX35	20	+	2109	10	2	0	1.00%	1.566726084	0.053594825	0.007029303	0.002016282
ENSG00000149809_65112550_65116053_422	novel	TM7SF2	11	+	1266	99	28	0	6.64%	25.83872874	0.00353785	1.54E-06	9.80E-08
ENSG00000131408_50377606_50382602_430	annotated	NR1H2	19	+	1290	57	7	4	6.05%	14.60006533	6.37E-05	7.19E-06	3.81E-09
ENSG00000278637_26021679_26021990_103	annotated	HIST1H4A	6	+	309	2076	153	33	39.81%	2219.926131	1.51E-07	3.82E-08	5.72E-14
ENSG00000135250_105268843_105117838_688	annotated	SRPK2	7	-	2064	54	3	5	3.63%	8.644775523	3.44E-05	6.63E-05	1.73E-08
ENSG00000139436_109996224_109932978_631	annotated	GIT2	12	-	1893	40	11	2	2.69%	6.981986923	0.023262949	0.000538554	9.98E-05
ENSG00000111540_55986961_55992212_215	annotated	AC034102.1	12	+	645	100	28	1	12.56%	51.2282994	0.000182409	2.55E-06	4.59E-09
ENSG00000164904_126595198_126544965_475	annotated	ALDH7A1	5	-	1425	101	8	3	6.53%	23.4194215	6.02E-06	1.35E-06	7.13E-11
ENSG00000213625_65420725_65427603_37	novel	LEPROT	1	+	111	43	2	0	24.32%	128.0015211	0.005134532	0.003710481	0.000104431
ENSG00000159086_32751207_32734950_411	novel	PAXBP1	21	-	1233	9	0	0	1.22%	2.411843293	0.019216965	0.019216965	0.001707396
ENSG00000139174_42469470_42459809_710	annotated	PRICKLE1	12	-	2130	10	0	0	0.70%	1.551279489	0.020613417	0.020613417	0.001946209
ENSG00000135390_53672614_53665314_141	annotated	ATP5MC2	12	-	423	370	85	27	30.50%	289.0220721	2.46E-06	4.35E-08	1.15E-12

ENSG00000181852_56214047_56207642_201	annotated	RNF41	12	-	603	20	0	2	4.98%	10.95928793	0.002227676	0.006423651	8.15E-05
ENSG00000076067_56562441_56589012_314	annotated	RBMS2	12	+	942	15	4	1	2.87%	5.261505273	0.065499399	0.010079165	0.003356681
ENSG00000143771_224365930_224375822_76	novel	CNIH4	1	+	228	129	8	4	26.32%	186.94959	4.20E-05	3.38E-05	1.08E-08
ENSG00000143756_224114364_224157746_290	annotated	FBXO28	1	+	870	73	10	0	8.97%	27.72510893	0.000162234	3.60E-06	5.48E-09
ENSG00000136044_105208015_105197853_153	novel	APPL2	12	-	459	13	0	0	3.27%	9.358372341	0.016947427	0.016947427	0.001349898
ENSG00000139546_53502025_53504743_187	annotated	TARBP2	12	+	561	18	4	4	3.74%	10.60179244	0.013892891	0.046544029	0.003041163
ENSG00000139546_53504782_53506148_173	dORF	TARBP2	12	+	519	14	1	3	4.05%	8.913131861	0.01551626	0.044241359	0.003171415
ENSG00000067829_153790253_153786149_322	annotated	IDH3G	X	-	966	20	5	4	4.04%	6.841046193	0.019827586	0.005367352	0.000559559
ENSG00000142396_58312274_58314783_74	annotated	ERVK3-1	19	+	222	51	3	4	21.62%	75.90787877	0.000494352	0.001141744	3.63E-06
ENSG00000004864_96277305_96121191_641	annotated	SLC25A13	7	-	1923	90	7	4	6.86%	15.46439303	1.14E-07	1.42E-08	1.67E-14
ENSG00000110075_68537665_68613117_827	annotated	PPP6R3	11	+	2481	138	14	12	7.13%	18.37900415	3.06E-09	5.86E-09	1.91E-16
ENSG00000110075_68537665_68613117_793	annotated	PPP6R3	11	+	2379	125	12	11	6.56%	17.36141925	1.37E-08	6.57E-08	9.25E-15
ENSG00000134996_75127617_75146741_171	annotated	OSTF1	9	+	513	38	0	3	8.19%	24.47574305	0.000428413	0.002947593	8.37E-06
ENSG00000139620_48679778_48662584_245	annotated	KANSL2	12	-	735	7	6	1	2.45%	3.146881249	0.333113823	0.028889786	0.049816245
ENSG00000131389_14472288_14478627_109	dORF	SLC6A6	3	+	327	84	10	3	13.76%	84.8791823	0.001213019	0.000309685	2.50E-06
ENSG00000131389_14443635_14472313_359	Overlap_dORF	SLC6A6	3	+	1077	190	72	15	15.04%	58.29181143	5.14E-06	6.33E-09	4.56E-13
ENSG00000166260_54968646_54963261_230	annotated	COX11	17	-	690	87	16	10	10.43%	41.66197131	0.001114231	0.000486898	3.49E-06
ENSG00000164073_127965133_127920630_518	annotated	MFSD8	4	-	1554	28	2	1	2.70%	5.953559119	0.000573217	0.000573217	2.12E-06
ENSG00000115904_39067669_38985824_1276	annotated	SOS1	2	-	3828	16	1	7	0.94%	1.38107641	0.001589662	0.067483894	0.000836597
ENSG00000186260_14140607_14261444_1099	annotated	MRTFB	16	+	3297	33	1	2	1.73%	3.307231886	7.93E-05	0.000241806	1.38E-07
ENSG00000185787_78873018_78897067_323	annotated	MORF4L1	15	+	969	105	8	5	4.33%	35.80429904	0.002121389	0.004630651	5.62E-05
ENSG00000168758_96867886_96860626_833	annotated	SEMA4C	2	-	2499	42	4	0	1.92%	5.553319851	0.001319996	0.000199656	1.83E-06
ENSG00000148175_121370187_121339621_168	annotated	STOM	9	-	504	236	12	3	20.83%	154.7216614	2.12E-06	1.76E-07	3.61E-12
ENSG00000029364_69442218_69458593_189	novel	SLC39A9	14	+	567	45	1	2	7.41%	26.22401041	0.000449456	0.000449456	1.33E-06
ENSG00000137700_119029530_119028360_101	uORF	SLC37A4	11	-	303	12	7	0	4.95%	13.08604084	0.170042304	0.021084099	0.017372496
ENSG00000137700_119028355_119024910_356	annotated	SLC37A4	11	-	1068	66	3	0	5.62%	20.4193699	0.000176337	3.82E-05	5.09E-08
ENSG00000117481_46344855_46361846_335	annotated	NSUN4	1	+	1005	18	4	1	2.09%	5.918015483	0.053594825	0.017404239	0.00425017
ENSG00000006757_7921841_7900686_159	annotated	PNPLA4	X	-	477	12	2	0	4.40%	8.312516506	0.031982531	0.008175611	0.001316552
ENSG00000167460_16086484_16101346_139	dORF	TPM4	19	+	417	70	3	0	10.07%	55.46661194	0.000468432	0.000471186	1.45E-06
ENSG00000104723_15623143_15650850_86	novel	TUSC3	8	+	258	29	6	2	12.79%	37.14051706	0.021123297	0.004389438	0.000502364
ENSG00000145833_134785473_134790515_95	novel	DDX46	5	+	285	10	0	0	5.26%	11.59377302	0.020613417	0.020613417	0.001946209
ENSG00000131467_42833632_42841578_170	annotated	PSME3	17	+	510	125	20	5	17.06%	80.98591449	3.09E-05	7.63E-06	1.92E-09
ENSG00000171130_149873976_149879677_194	annotated	ATP6V0E2	7	+	582	13	3	2	3.09%	7.380572001	0.055917761	0.044040756	0.009894017
ENSG00000186501_27322258_27335466_188	annotated	TMEM222	1	+	564	62	9	0	12.77%	36.3230442	5.77E-05	8.00E-06	3.80E-09
ENSG00000101247_13801528_13817210_130	novel	NDUFAF5	20	+	390	12	0	3	6.15%	10.16684711	0.004826937	0.04536668	0.001238719
ENSG00000146701_76048161_76066410_296	annotated	MDH2	7	+	888	684	64	14	27.70%	254.5146523	1.16E-12	3.11E-14	5.06E-25
ENSG00000047579_15662869_15522975_316	annotated	DTNBP1	6	-	948	11	2	0	2.53%	3.834016711	0.019230617	0.004687384	0.000482115
ENSG00000114745_39107541_39098236_345	annotated	GORASP1	3	-	1035	22	9	1	3.48%	7.023474091	0.031722546	0.001612025	0.00034285
ENSG00000057608_5766624_5766490_44	novel	GDI2	10	-	132	54	3	1	11.36%	135.1728536	0.070008252	0.035772986	0.010232754
ENSG00000057608_5766074_5766006_22	novel	GDI2	10	-	66	17	0	0	31.82%	85.10883377	0.008473674	0.008473674	0.000366502
ENSG00000215440_58694422_58715326_411	annotated	NPEPL1	20	+	1233	24	0	6	2.92%	6.431582114	0.000978918	0.015620679	0.000102533
ENSG00000110660_107815934_107792615_327	annotated	SLC35F2	11	-	981	88	11	4	11.01%	29.64034938	2.35E-06	7.05E-07	1.49E-11
ENSG00000168958_227330816_227357117_221	annotated	MFF	2	+	663	80	12	10	7.69%	39.86998867	0.000792786	0.000448054	2.30E-06
ENSG00000206503_29942563_29945455_368	annotated	HLA-A	6	+	1104	180	12	4	8.42%	53.87323877	6.87E-06	2.03E-06	1.20E-10
ENSG00000120942_11273532_11294883_179	annotated	UBIAD1	1	+	537	52	2	0	8.38%	31.99622275	0.000275394	0.000279805	5.23E-07
ENSG00000206503_29942563_29945455_296	annotated	HLA-A	6	+	888	157	12	0	9.46%	58.41929886	2.78E-05	1.61E-06	4.05E-10
ENSG00000206503_29942563_29945455_362	annotated	HLA-A	6	+	1086	182	12	4	9.12%	55.37467833	3.96E-06	1.07E-06	3.74E-11
ENSG00000010244_32350286_32360971_184	novel	ZNF207	17	+	552	170	10	11	15.76%	101.7605621	1.89E-06	6.53E-06	1.07E-10
ENSG00000142192_25897626_25881670_100	novel	APP	21	-	300	152	17	7	24.00%	167.4140824	1.73E-05	2.32E-05	3.16E-09
ENSG00000111237_110502051_110492992_144	novel	VPS29	12	-	432	153	22	7	18.75%	117.0246464	4.09E-05	1.16E-05	3.79E-09
ENSG00000167118_128371381_128387901_63	annotated	URM1	9	+	189	152	19	9	30.16%	265.7366388	0.000435347	0.000294433	8.58E-07
ENSG00000196950_195680055_195735041_827	annotated	SLC39A10	2	+	2481	465	55	16	11.61%	61.92925311	3.02E-12	3.98E-16	2.29E-26
ENSG00000091409_172427789_172504252_1091	annotated	ITGA6	2	+	3273	357	44	11	9.17%	36.04058772	1.48E-13	1.64E-16	4.10E-28
ENSG00000158796_161124416_161122147_235	novel	DEDD	1	-	705	24	7	0	3.83%	11.24842659	0.054516989	0.003529166	0.001190074
ENSG00000122687_2242169_2238768_155	annotated	MRM2	7	-	465	29	8	6	7.10%	20.60699656	0.011978812	0.018230749	0.001050407
ENSG00000122687_2238060_2235122_162	dORF	MRM2	7	-	486	14	5	1	5.56%	9.518344518	0.05444419	0.005162103	0.001603268
ENSG00000156973_231781114_231737942_111	novel	PDE6D	2	-	333	39	3	0	11.71%	38.69813427	0.000881412	0.000700469	3.91E-06
ENSG00000130669_39169554_39178579_591	annotated	PAK4	19	+	1773	150	3	6	6.09%	27.95452886	7.81E-08	2.67E-07	2.03E-13
ENSG00000139117_38907353_38653882_543	annotated	CPNE8	12	-	1629	18	0	0	1.66%	3.651077692	0.003529166	0.003529166	6.95E-05
ENSG00000128739_24975403_24978444_224	annotated	SNRPN	15	+	672	62	5	0	5.80%	30.4854121	0.006702261	0.000649205	2.87E-05
ENSG00000128739_24920123_24968037_67	uORF	SNRPN	15	+	201	36	4	2	11.94%	59.18015483	0.013786174	0.010327713	0.000700646
ENSG00000007168_2666995_2670342_127	novel	PAFAH1B1	17	+	381	40	2	3	9.45%	34.69002951	0.001480749	0.001735366	1.54E-05
ENSG00000069275_205749973_205718280_243	annotated	NUCKS1	1	-	729	293	45	22	17.70%	132.8035687	6.82E-06	8.96E-07	5.50E-11
ENSG00000215712_157323499_157318722_128	annotated	TMEM242	6	-	384	8	3	0	5.47%	6.883802732	0.105872578	0.005706018	0.003771426
ENSG00000152683_32165901_32187236_122	annotated	SLC30A6	2	+	366	26	7	1	5.74%	23.47263882	0.053017408	0.008776163	0.002384715
ENSG00000115694_241500775_241495662_332	annotated	STK25	2	-	996	24	2	3	3.01%	7.961988702	0.002321823	0.009985438	0.000132651
ENSG00000125347_132487065_132483951_241	annotated	IRF1	5	-	723	15	2	2	3.73%	6.855239235	0.018785664	0.018785664	0.00163652
ENSG00000148848_126118181_126042132_582	annotated	ADAM12	10	-	1746	5	0	1	0.86%	0.94622718	0.012673659	0.051235217	0.00311107

ENSG00000143624_153767762_153773270_316	novel	INTS3	1	+	948	36	3	0	2.22%	12.54769106	0.024800926	0.008776163	0.00107875
ENSG00000148848_126118181_126017279_753	annotated	ADAM12	10	-	2259	9	0	1	0.80%	1.316424427	0.009815329	0.028889786	0.001387795
ENSG00000102786_51395378_51365752_709	dORF	INTS6	13	-	2127	10	2	0	0.85%	1.553467471	0.061356297	0.012798403	0.003794065
ENSG00000145901_151065095_151030713_572	annotated	TNIP1	5	-	1716	59	6	2	4.55%	11.36068143	4.86E-05	9.77E-06	3.84E-09
ENSG00000113712_149550964_149529713_78	annotated	CSNK1A1	5	-	234	70	22	0	15.38%	98.84434692	0.119738561	0.001053132	0.001323566
ENSG00000105699_35249023_35267859_601	annotated	LSR	19	+	1803	53	10	1	3.49%	9.712919661	0.002378218	5.15E-05	1.06E-06
ENSG00000198561_57791479_57816308_939	annotated	CTNND1	11	+	2817	208	34	13	6.28%	24.39754578	1.64E-07	1.19E-09	2.41E-15
ENSG00000198945_130051269_130139753_677	annotated	L3MBTL3	6	+	2031	7	0	1	0.74%	1.13882704	0.019216965	0.041782825	0.003600526
ENSG00000187147_44636045_44650775_230	annotated	RNF220	1	+	690	30	9	2	6.52%	14.36619701	0.003030417	0.000737506	1.40E-05
ENSG00000133119_33818179_33966125_305	annotated	RFC3	13	+	915	57	9	7	7.21%	20.58369866	0.000792293	0.000160416	8.87E-07
ENSG00000111596_70278227_70353915_540	annotated	AC092881.1	12	+	1620	35	8	1	3.70%	7.138758388	0.00041117	5.05E-05	1.57E-07
ENSG00000144554_10028658_10098950_1471	annotated	FANCD2	3	+	4413	54	3	6	2.04%	4.043239674	1.94E-06	4.59E-05	8.17E-10
ENSG00000119408_124313935_124350947_232	annotated	NEK6	9	+	696	22	3	0	6.03%	10.44439035	0.002827043	0.000241223	4.83E-06
ENSG00000169738_82036266_82035960_59	novel	DCXR	17	-	177	29	6	0	13.56%	54.13702487	0.02846257	0.005756914	0.000865442
ENSG00000176595_2001967_2003064_365	annotated	KBTBD11	8	+	1095	12	0	0	1.37%	3.621068834	0.021084099	0.021084099	0.002029891
ENSG00000100280_29363023_29328821_879	annotated	AP1B1	22	-	2637	79	9	6	3.30%	9.898892665	5.30E-05	2.40E-05	9.82E-09
ENSG00000015475_17773665_17735580_233	annotated	BID	22	-	699	116	29	10	12.45%	54.83406811	0.000470013	2.91E-05	1.10E-07
ENSG00000197912_89524038_89557093_659	annotated	SPG7	16	+	1977	54	22	2	4.10%	9.025198118	0.00613783	6.87E-06	6.33E-07
ENSG00000144747_69052086_69023177_985	annotated	TMF1	3	-	2955	62	1	2	1.83%	6.932723157	9.99E-05	0.000200711	1.43E-07
ENSG00000101266_489830_483961_167	annotated	CSNK2A1	20	-	501	47	1	0	8.38%	30.99772248	0.000475338	0.000472567	1.47E-06
ENSG00000178057_49022473_49023172_116	annotated	NDUFAF3	3	+	348	42	5	6	14.66%	39.87858134	0.000239652	0.000942043	1.53E-06
ENSG00000127990_94628318_94585499_337	dORF	SGCE	7	-	1011	145	9	7	12.17%	47.38997726	2.60E-07	7.38E-08	1.87E-13
ENSG00000197912_89507690_89557093_751	annotated	SPG7	16	+	2253	94	23	4	4.53%	13.78593783	0.000598809	7.92E-07	6.54E-09
ENSG00000136717_127106934_127048526_406	annotated	BIN1	2	-	1218	35	2	1	3.45%	9.494900319	0.000742993	0.000525339	2.51E-06
ENSG00000141258_2361705_2379520_939	annotated	SGSM2	17	+	2817	23	4	5	1.28%	2.697805543	0.007352087	0.009754926	0.000366944
ENSG00000163762_149333382_149321478_201	annotated	TM4SF18	3	-	603	690	43	12	28.36%	378.0954336	7.72E-11	9.87E-11	8.92E-20
ENSG00000204392_31797992_31797757_42	novel	LSM2	6	-	126	16	0	0	11.90%	41.95841665	0.021557223	0.021557223	0.002115616
ENSG00000204392_31797992_31797757_42	novel	LSM2	6	-	126	16	0	0	11.90%	41.95841665	0.021557223	0.021557223	0.002115616
ENSG00000105723_42242465_42230794_483	annotated	GSK3A	19	-	1449	154	14	4	8.90%	35.11737046	3.35E-07	1.83E-08	6.40E-14
ENSG00000185000_144318342_144317183_189	novel	DGAT1	8	-	567	43	13	2	6.35%	25.05849883	0.003299553	0.001632324	3.16E-05
ENSG00000137100_34620464_34613782_176	annotated	DCTN3	9	-	528	145	18	0	17.61%	90.74103601	6.99E-05	5.11E-07	3.90E-10
ENSG00000204316_75904684_75900978_203	novel	MRPL38	17	-	609	98	3	9	15.76%	53.17144179	1.43E-06	2.01E-05	2.59E-10
ENSG00000068400_48990976_48974193_644	annotated	GRIPAP1	X	-	1932	10	2	0	0.93%	1.710261548	0.060322713	0.011570466	0.003431813
ENSG00000143228_163338074_163345857_165	novel	NUF2	1	+	495	24	7	0	4.24%	16.02048636	0.04555867	0.008877961	0.002045901
ENSG00000137185_28227157_28228055_237	annotated	ZSCAN9	6	+	711	12	0	0	2.11%	5.57675158	0.020613417	0.020613417	0.001946209
ENSG00000168002_62765209_62766507_69	novel	POLR2G	11	+	207	203	11	7	37.68%	324.0375547	2.11E-05	7.41E-06	1.27E-09
ENSG00000119707_73077390_73119805_784	annotated	RBM25	14	+	2352	60	5	12	3.19%	8.429146202	5.25E-05	0.001685494	7.33E-07
ENSG00000139637_53299810_53307149_301	annotated	C12orf10	12	+	903	75	9	2	8.31%	27.44373182	0.000279604	1.67E-05	3.86E-08
ENSG00000175387_47896756_47845234_431	annotated	SMAD2	18	-	1293	117	15	2	9.28%	29.89902254	2.48E-06	2.58E-08	7.20E-13
ENSG00000123607_165949601_165871001_1226	annotated	TTC21B	2	-	3678	16	3	0	0.65%	1.437400897	0.025015411	0.005405142	0.000716373
ENSG00000053371_19311974_19304225_274	annotated	AKR7A2	1	-	822	41	0	1	6.57%	16.48092917	6.51E-05	0.000187149	8.90E-08
ENSG00000141458_23535606_23529338_143	annotated	NPC1	18	-	429	31	2	3	10.49%	23.8766864	0.000440588	0.001690947	4.84E-06
ENSG00000109099_15260727_15230917_160	annotated	PMP22	17	-	480	250	52	0	30.00%	172.0950683	9.94E-08	7.34E-10	9.05E-16
ENSG00000124733_43013378_43012470_172	annotated	MEA1	6	-	516	158	12	13	19.19%	101.1758913	5.55E-07	3.27E-05	1.84E-10
ENSG00000113163_75511207_75374043_605	annotated	COL4A3BP	5	-	1815	20	11	5	1.65%	3.641019627	0.140831635	0.023449432	0.015138951
ENSG00000227057_33287139_33280642_164	novel	WDR46	6	-	492	14	3	0	3.66%	9.402267146	0.062728927	0.01300724	0.003936255
ENSG00000044090_43050353_43037688_1272	annotated	CUL7	6	-	3816	12	3	0	0.71%	1.039064563	0.028282463	0.002500085	0.000429458
ENSG00000072364_132934881_132881059_1102	annotated	AFF4	5	-	3306	35	6	0	1.54%	3.49812117	0.003107721	0.000120275	2.93E-06
ENSG00000053108_133603983_133199095_842	annotated	FSTL4	5	-	2526	71	9	0	4.28%	9.287410811	1.47E-06	5.15E-08	7.86E-13
ENSG00000069011_135033803_135028779_288	annotated	PITX1	5	-	864	24	2	4	3.12%	9.178403642	0.007405129	0.035914078	0.001367073
ENSG00000122515_44756268_44767623_888	annotated	ZMIZ2	7	+	2664	35	7	0	1.69%	4.341136858	0.007310804	0.000290289	1.60E-05
ENSG00000112078_36481642_36487175_173	dORF	KCTD20	6	+	519	15	1	0	2.89%	9.549784136	0.035772986	0.021084099	0.003354605
ENSG00000130811_10119859_10118417_146	novel	EIF3G	19	-	438	66	19	4	11.64%	49.78969647	0.006736569	0.000949214	4.02E-05
ENSG00000179335_74619197_74629883_490	annotated	CLK3	15	+	1470	30	7	1	2.86%	6.743316962	0.014135576	0.000565108	5.83E-05
ENSG00000163754_148991641_149026933_350	annotated	GYG1	3	+	1050	38	4	1	4.57%	11.95814875	0.001741809	0.000264458	3.14E-06
ENSG00000075624_5529657_5529045_125	novel	ACTB	7	-	375	2723	155	43	22.40%	2399.308139	4.64E-05	3.05E-05	1.08E-08
ENSG00000100416_46335765_46353981_328	novel	TRMU	22	+	984	21	9	0	3.66%	7.051700359	0.032486551	0.000862735	0.000215067
ENSG00000166340_6616817_6616581_78	novel	TPP1	11	-	234	24	1	1	6.41%	33.88949037	0.029237631	0.029237631	0.003726901
ENSG00000166340_6619400_6618759_81	novel	TPP1	11	-	243	54	9	0	14.81%	73.42722914	0.007666581	0.00104892	5.02E-05
ENSG00000111642_6606316_6570676_1886	annotated	CHD4	12	-	5658	91	9	3	2.17%	5.314324908	1.97E-07	2.05E-08	4.14E-14
ENSG00000135905_224853078_224765721_1542	annotated	DOCK10	2	-	4626	13	1	1	0.32%	0.928554454	0.035772986	0.035772986	0.005410795
ENSG00000047457_149221792_149177801_1018	novel	CP	3	-	3054	12	2	0	0.79%	1.298320358	0.019435691	0.004826937	0.000499969
ENSG00000112992_43615995_43704404_910	annotated	NNT	5	+	2730	39	2	0	1.87%	4.720321873	0.000289254	0.000121524	2.48E-07
ENSG00000133961_73355751_73276578_651	annotated	NUMB	14	-	1953	26	5	2	2.46%	4.398866262	0.006340142	0.000529469	2.27E-05
ENSG00000078304_101909635_101921230_127	annotated	PPP2R5C	14	+	381	9	0	0	4.72%	7.805256641	0.012178958	0.012178958	0.000726245
ENSG00000058272_79934925_79775929_993	annotated	PPP1R12A	12	-	2979	43	12	4	2.52%	4.769442376	0.002299191	5.45E-05	1.07E-06
ENSG00000176209_42546494_42552608_100	annotated	SMIM19	8	+	300	121	16	2	16.00%	133.2704209	0.000215919	0.000199231	2.98E-07
ENSG00000149380_74311611_74267248_544	annotated	P4HA3	11	-	1632	32	4	0	2.39%	6.478873159	0.001735343	0.000642411	7.03E-06

ENSG00000095059_12681766_12679840_139	annotated	DHPS	19	-	417	27	14	1	5.76%	21.3942646	0.37511016	0.008364861	0.027623643
ENSG00000178802_74890571_74893445_244	annotated	MPI	15	+	732	21	0	0	2.87%	9.479334909	0.008776163	0.008776163	0.000391611
ENSG00000167526_89560981_89561796_147	novel	RPL13	16	+	441	190	28	1	7.48%	142.3589136	0.010020066	0.001665007	9.95E-05
ENSG00000123374_55971152_55971625_66	novel	CDK2	12	+	198	13	0	0	9.09%	21.69440861	0.013428348	0.013428348	0.000872559
ENSG00000133961_73355751_73276578_592	annotated	NUMB	14	-	1776	26	5	2	2.70%	4.837266784	0.006340142	0.000529469	2.27E-05
ENSG00000100902_35308980_35317306_167	dORF	PSMA6	14	+	501	92	14	2	9.58%	60.67639294	0.002237421	0.000300676	4.58E-06
ENSG00000180901_75059579_75063047_60	novel	KCTD2	17	+	180	11	0	0	8.33%	20.19248801	0.019216965	0.019216965	0.001707396
ENSG00000276043_4910907_4960803_786	annotated	UHRF1	19	+	2358	47	1	2	2.29%	6.586030094	8.64E-05	0.000276943	1.72E-07
ENSG00000145495_10381814_10433684_842	annotated	6-Mar	5	+	2526	179	16	3	9.26%	23.41473993	6.63E-13	2.26E-14	2.10E-25
ENSG00000172432_43625809_43621614_451	annotated	GTPBP2	6	-	1353	7	0	0	1.33%	1.709503117	0.009815329	0.009815329	0.000483714
ENSG00000122884_73074883_73008222_534	annotated	P4HA1	10	-	1602	137	29	8	8.43%	28.25710784	3.23E-06	1.03E-07	3.40E-12
ENSG00000104093_51563425_51448984_2226	annotated	DMXL2	15	-	6678	13	4	3	0.27%	0.643230444	0.100696627	0.059897465	0.022567502
ENSG00000104133_44663536_44573512_2042	annotated	SPG11	15	-	6126	40	3	1	0.93%	2.157509181	0.000438498	6.99E-05	2.26E-07
ENSG00000067225_72219097_72210423_69	internal	PKM	15	-	207	121	72	4	23.19%	193.1455375	0.064399577	0.000389877	0.000280513
ENSG00000067225_72221201_72199650_516	annotated	PKM	15	-	1548	3763	618	268	31.01%	803.2170443	8.41E-23	2.40E-19	4.04E-40
ENSG00000140395_78295686_78283432_305	annotated	WDR61	15	-	915	273	26	5	13.77%	98.58508305	2.14E-07	1.54E-08	3.44E-14
ENSG00000160404_127735270_127733493_72	annotated	TOR2A	9	-	216	10	2	0	6.94%	15.2973394	0.08410207	0.019679754	0.007521961
ENSG00000132623_10038302_10055660_776	annotated	ANKEF1	20	+	2328	77	0	0	3.09%	10.92892392	8.17E-06	8.17E-06	5.48E-10
ENSG00000183763_49856453_49829425_284	annotated	TRAIP	3	-	852	21	1	0	2.82%	8.144217316	0.006152341	0.005554612	0.000181508
ENSG00000133226_24643327_24672286_904	annotated	SRRM1	1	+	2712	44	13	0	2.32%	5.360837525	0.003672231	2.01E-05	7.95E-07
ENSG00000196235_39464951_39476399_828	annotated	SUPT5H	19	+	2484	62	3	1	2.78%	8.247261244	4.60E-05	1.50E-05	5.43E-09
ENSG00000259075_89525895_89522813_575	annotated	POC1B-GALNT4	12	-	1725	47	9	0	3.65%	9.00281679	0.000378209	2.51E-05	7.66E-08
ENSG00000092841_56158402_56160174_124	novel	MYL6	12	+	372	3523	139	35	37.10%	3129.243487	7.84E-08	5.02E-09	4.25E-15
ENSG00000092841_56160251_56160331_26	novel	MYL6	12	+	78	523	41	11	53.85%	2215.525433	0.000980698	0.000542016	3.40E-06
ENSG00000119681_74611944_74500884_1777	annotated	LTBP2	14	-	5331	15	2	2	0.62%	0.929720121	0.009234247	0.009234247	0.000431101
ENSG00000136536_159729023_159767342_704	annotated	7-Mar	2	+	2112	48	7	11	3.55%	7.50960298	0.000180082	0.00088607	1.11E-06
ENSG00000005486_75879083_75888349_364	annotated	RHBDD2	7	+	1092	63	0	0	6.87%	19.06283833	3.17E-06	3.17E-06	8.58E-11
ENSG00000127554_1984219_1986028_205	annotated	GFER	16	+	615	70	9	3	10.24%	37.60906858	7.32E-05	7.00E-05	3.77E-08
ENSG00000072778_7224858_7225097_55	dORF	ACADVL	17	+	165	26	3	0	18.18%	52.06658066	0.00376808	0.002201184	4.75E-05
ENSG00000100225_32475003_32498530_522	annotated	FBXO7	22	+	1566	89	5	9	5.75%	18.77880285	1.94E-06	2.19E-05	3.76E-10
ENSG00000169169_49712787_49713605_131	novel	CPT1C	19	+	393	8	0	0	3.82%	6.726158394	0.019679754	0.019679754	0.001784964
ENSG00000145012_188406187_188874479_590	annotated	LPP	3	+	1770	45	3	3	3.05%	8.400572825	0.000297078	0.000168883	3.47E-07
ENSG00000167900_78186994_78175564_87	annotated	TK1	17	-	261	24	6	66	10.34%	30.38368102	0.042090579	0.036196037	0.006359536
ENSG00000095066_12773029_12763282_644	annotated	HOOK2	19	-	1932	14	2	2	0.78%	2.394366168	0.045845141	0.044040756	0.008228605
ENSG00000111321_6375556_6390937_416	annotated	AC005840.1	12	+	1248	286	28	12	17.07%	75.72183005	4.36E-11	2.76E-12	1.53E-21
ENSG00000188559_20619345_20393099_1433	annotated	RALGAPA2	20	-	4299	17	6	0	0.77%	1.306625501	0.054793613	0.001343041	0.000569314
ENSG00000167658_3985380_3984132_73	novel	EEF2	19	-	219	50	8	3	17.81%	75.43893404	0.007274525	0.001272209	5.63E-05
ENSG00000155463_22766702_22771558_435	annotated	OXA1L	14	+	1305	394	36	15	16.78%	99.75975269	5.74E-11	1.24E-13	1.08E-22
ENSG00000122966_119876168_119688057_482	annotated	CIT	12	-	1446	44	4	6	2.90%	10.05435088	0.003269261	0.009574786	0.00017217
ENSG00000117632_25901583_25901449_44	novel	STMN1	1	-	132	170	13	6	31.82%	425.5441689	0.000486528	0.000753479	2.37E-06
ENSG00000107829_101676435_101611291_325	annotated	FBXW4	10	-	975	15	2	2	2.77%	5.083423556	0.010960145	0.018785664	0.000997625
ENSG00000171320_27775515_27803438_601	annotated	ESCO2	8	+	1803	48	9	0	3.16%	8.796606486	0.003730909	5.35E-05	1.82E-06
ENSG00000067365_8639114_8644595_117	annotated	METTL22	16	+	351	11	2	2	4.27%	10.35512206	0.101051022	0.078649604	0.028587196
ENSG00000103363_2777239_2772040_66	annotated	ELOB	16	-	198	252	29	8	43.94%	420.5377669	1.99E-06	2.00E-06	3.47E-11
ENSG00000159479_43386646_43385922_120	novel	MED8	1	-	360	17	6	0	5.00%	15.60328619	0.145323137	0.01300724	0.010137493
ENSG00000129103_56064312_56079612_301	annotated	SUMF2	7	+	903	626	84	12	20.93%	229.0636816	2.56E-08	8.58E-12	3.67E-18
ENSG00000114988_96740040_96737200_107	annotated	LMAN2L	2	-	321	27	3	0	7.48%	27.79254935	0.012185302	0.005756914	0.000364393
ENSG00000114988_96712001_96707256_171	dORF	LMAN2L	2	-	513	19	0	0	6.43%	12.23787152	0.001377539	0.001377539	1.15E-05
ENSG00000217128_131796921_131644685_1138	annotated	FNIP1	5	-	3414	36	12	4	1.85%	3.484244616	0.014577573	0.000175021	2.35E-05
ENSG00000249859_127794565_127890868_149	novel	PVT1	8	+	447	10	1	0	3.36%	7.392002933	0.035374934	0.020613417	0.003252526
ENSG00000198774_85836201_85804702_435	annotated	RASSF9	12	-	1305	15	8	4	2.07%	3.797960128	0.09530924	0.031072489	0.012404459
ENSG00000167220_47136439_47108682_259	annotated	HDHD2	18	-	777	11	2	2	2.70%	4.677796451	0.043639483	0.043639483	0.007798642
ENSG00000198853_35546522_35561382_1516	annotated	RUSC2	9	+	4548	31	8	6	0.79%	2.252220419	0.029084344	0.010877193	0.001527702
ENSG00000082014_151242768_151242400_83	novel	SMARCD3	7	-	249	10	2	1	6.02%	13.26998117	0.069457177	0.020613417	0.006391023
ENSG00000168090_100089304_100089787_94	novel	COPS6	7	+	282	33	3	0	14.89%	38.66646641	0.000872559	0.000415605	2.36E-06
ENSG00000239672_51154374_51160183_134	novel	NME1	17	+	402	497	69	13	23.13%	408.5074576	2.95E-05	5.30E-06	1.30E-09
ENSG00000145715_87269250_87390883_880	annotated	RASA1	5	+	2640	48	0	2	2.16%	6.007682384	5.93E-05	0.000164899	7.18E-08
ENSG00000132254_6480053_6477113_303	annotated	ARFIP2	11	-	909	46	14	3	5.28%	16.72105218	0.0112192	0.001135171	8.08E-05
ENSG00000155256_97743101_97759300_348	annotated	ZFYVE27	10	+	1044	25	5	4	3.16%	7.912416933	0.0189538	0.009254025	0.000864282
ENSG00000089327_35155551_35169615_178	annotated	FXYD5	19	+	534	553	55	17	28.09%	342.179138	4.81E-09	3.58E-10	1.97E-17
ENSG00000173264_64286525_64289365_417	annotated	GPR137	11	+	1251	38	1	1	4.08%	10.03681549	0.000181297	0.000181297	2.29E-07
ENSG00000089006_17968425_17942357_404	annotated	SNX5	20	-	1212	107	24	13	7.18%	29.17096603	8.87E-05	5.53E-05	3.63E-08
ENSG00000138674_82881906_82819074_1057	annotated	SEC31A	4	-	3171	95	8	3	3.41%	9.899129756	1.96E-07	3.71E-07	6.83E-13
ENSG00000080815_73170959_73219289_384	annotated	PSEN1	14	+	1152	130	19	8	10.16%	37.2872648	9.28E-07	2.87E-07	2.47E-12
ENSG00000149016_62591485_62575094_874	annotated	TUT1	11	-	2622	21	0	3	1.60%	2.646404711	0.000288544	0.006674189	1.43E-05
ENSG00000180263_95208985_95085762_608	annotated	FGD6	12	-	1824	9	2	0	0.99%	1.630374331	0.071345384	0.011772051	0.004172987
ENSG00000106617_151574893_151557201_235	dORF	PRKAG2	7	-	705	25	0	3	3.40%	11.71711103	0.005604867	0.020990661	0.000616068
ENSG00000011021_11824512_11838993_751	annotated	CLCN6	1	+	2253	24	0	4	2.00%	3.519813913	0.000248576	0.003660151	6.54E-06

ENSG00000158985_131385570_131391091_57	annotated	CDC42SE2	5	+	171	102	2	0	14.04%	197.0941414	0.005604867	0.005655336	0.000168956
ENSG00000133835_119493868_119625993_453	novel	HSD17B4	5	+	1359	74	10	1	4.42%	17.9921025	0.002315052	3.88E-05	8.07E-07
ENSG00000133835_119452576_119492174_262	novel	HSD17B4	5	+	786	28	0	6	4.96%	11.77077719	0.000585477	0.016404287	7.11E-05
ENSG00000215301_41334253_41347719_662	annotated	DDX3X	X	+	1986	238	48	10	8.91%	39.59746345	4.34E-08	5.58E-11	3.54E-17
ENSG00000116337_109625635_109631152_760	annotated	AMPD2	1	+	2280	8	6	0	0.79%	1.159377302	0.280084718	0.011570466	0.021802746
ENSG00000258947_89923402_89934499_148	annotated	TUBB3	16	+	444	109	14	5	14.86%	81.117243	2.33E-05	3.46E-05	6.22E-09
ENSG00000184216_154019713_154011859_649	annotated	IRAK1	X	-	1947	25	7	0	1.54%	4.242713548	0.059781779	0.00224099	0.000934347
ENSG00000084090_96195509_96186730_260	annotated	STARD7	2	-	780	32	1	0	6.15%	13.55579615	0.000273058	0.000128239	2.46E-07
ENSG00000103534_19460312_19497966_652	annotated	TMC5	16	+	1956	19	2	1	1.23%	3.209625813	0.017321761	0.006965909	0.000613451
ENSG00000177156_764251_764845_71	novel	TALDO1	11	+	213	166	32	4	21.13%	257.5123951	0.028741062	0.000595517	0.000138998
ENSG00000079459_11808887_11838609_353	annotated	FDFT1	8	+	1059	956	118	38	35.41%	298.2851178	4.09E-19	6.01E-21	4.24E-38
ENSG00000162521_32651307_32679705_425	annotated	RBBP4	1	+	1275	125	6	2	5.65%	32.3943658	2.57E-05	9.90E-06	2.05E-09
ENSG00000119487_125672574_125438887_522	annotated	MAPKAP1	9	-	1566	85	9	4	5.94%	17.93481171	8.29E-06	3.06E-06	2.15E-10
ENSG00000198815_42311093_42179710_622	annotated	FOXJ3	1	-	1866	25	9	0	2.25%	4.426882786	0.024416325	0.000390775	8.22E-05
ENSG00000141756_41813035_41822408_582	annotated	FKBP10	17	+	1746	438	34	10	16.32%	82.88950093	2.84E-16	6.64E-17	2.82E-31
ENSG00000060138_10723111_10701288_372	annotated	YBX3	12	-	1116	120	19	3	9.14%	35.52930442	2.52E-06	1.35E-07	3.36E-12
ENSG00000164252_77030767_77063252_714	annotated	AGGF1	5	+	2142	23	6	0	1.96%	3.547954349	0.028132332	0.000334258	8.65E-05
ENSG00000115548_86442048_86492119_1321	annotated	KDM3A	2	+	3963	30	0	0	1.21%	2.501306064	0.000174956	0.000174956	2.14E-07
ENSG00000102312_48512346_48520476_330	annotated	PORCN	X	+	990	17	0	0	2.73%	5.673922251	0.003452921	0.003452921	6.66E-05
ENSG00000204574_30571488_30590701_845	annotated	ABCF1	6	+	2535	28	1	0	1.89%	3.649637425	0.000276617	0.000172082	3.29E-07
ENSG00000004534_50048254_50077133_601	annotated	RBM6	3	+	1803	11	5	1	1.00%	2.015888986	0.216331417	0.026832696	0.027478972
ENSG00000147140_71290638_71300076_471	annotated	NONO	X	+	1413	133	12	6	8.07%	31.10134228	1.43E-06	1.23E-07	1.72E-12
ENSG00000253710_52012419_52028590_492	annotated	ALG11	13	+	1476	28	0	0	2.24%	6.268178097	0.001513502	0.001513502	1.38E-05
ENSG00000121022_67059396_67043223_270	novel	COPS5	8	-	810	82	14	0	10.00%	33.45018216	1.48E-05	2.47E-06	3.15E-10
ENSG00000244274_45408824_45409000_58	annotated	DBNDD2	20	+	174	11	2	0	8.62%	20.8887807	0.083503598	0.019216965	0.007323531
ENSG00000244274_45409934_45410140_68	dORF	DBNDD2	20	+	204	14	0	2	7.35%	22.67605606	0.020613417	0.056923149	0.005212417
ENSG00000244274_45408824_45410140_107	annotated	DBNDD2	20	+	321	27	2	2	10.28%	27.79254935	0.00505029	0.00505029	0.000137448
ENSG00000244274_45408824_45410140_107	annotated	DBNDD2	20	+	321	27	2	2	10.28%	27.79254935	0.00505029	0.00505029	0.000137448
ENSG00000105926_24649678_24687614_428	annotated	MPP6	7	+	1284	13	0	2	1.87%	3.345399458	0.004687384	0.024771279	0.000628108
ENSG00000172115_25124083_25123701_93	annotated	CYCS	7	-	279	106	11	3	15.05%	125.5368756	0.000473951	0.000785846	2.41E-06
ENSG00000163939_52679711_52548063_1597	annotated	PBRM1	3	-	4791	24	7	3	1.00%	1.655216186	0.013944777	0.000838746	7.95E-05
ENSG00000168883_85616196_85647969_488	annotated	USP39	2	+	1464	46	21	4	3.89%	10.38212871	0.126621815	0.000458434	0.000811229
ENSG00000148337_128185630_128166197_730	annotated	CIZ1	9	-	2190	101	4	4	4.52%	15.23866468	2.71E-07	1.99E-06	5.08E-12
ENSG00000134285_48924233_48921984_99	dORF	FKBP11	12	-	297	30	7	3	8.08%	33.37601324	0.06845324	0.021542864	0.006529639
ENSG00000160584_116927357_116846396_1162	annotated	SIK3	11	-	3486	21	1	0	1.03%	1.990497175	0.001621946	0.000873936	8.75E-06
ENSG00000126088_45013615_45014115_127	novel	UROD	1	+	381	39	6	0	11.81%	33.82277878	0.001188948	0.000294865	2.34E-06
ENSG00000126088_45012992_45013706_119	novel	UROD	1	+	357	13	4	2	5.04%	12.03219301	0.102488202	0.044040756	0.017763347
ENSG00000108187_68296946_68284177_247	annotated	PBLD	10	-	741	24	0	0	3.24%	10.70194433	0.005160369	0.005160369	0.000143196
ENSG00000130177_114235085_114272443_619	annotated	CDC16	13	+	1857	56	7	1	3.72%	9.964276652	0.000204766	1.54E-05	2.58E-08
ENSG00000143252_161314406_161362433_135	annotated	SDHC	1	+	405	94	11	4	5.93%	76.69066154	0.005756914	0.005808022	0.00017774
ENSG00000125149_67110176_67147131_422	annotated	C16orf70	16	+	1266	25	8	0	2.61%	6.524931499	0.040804041	0.001535678	0.000442457
ENSG00000151491_15682912_15621317_809	annotated	EPS8	12	-	2427	79	10	0	3.21%	10.75540995	0.000507204	3.18E-06	1.73E-08
ENSG00000140416_63059567_63061730_92	novel	TPM1	15	+	276	190	24	10	19.57%	227.4647859	0.001202536	0.00038556	3.03E-06
ENSG00000124782_7211681_7248968_1461	annotated	RREB1	6	+	4383	11	1	3	0.34%	0.829260288	0.035374934	0.114974528	0.016723634
ENSG00000156642_73633215_73561910_278	annotated	NPTN	15	-	834	445	92	15	25.18%	176.3045879	2.38E-11	1.74E-12	5.30E-22
ENSG00000103479_53451714_53454331_149	novel	RBL2	16	+	447	11	0	0	3.36%	8.131203227	0.021084099	0.021084099	0.002029891
ENSG00000103479_53454256_53490300_839	novel	RBL2	16	+	2517	63	2	0	2.62%	8.270409003	3.04E-05	1.77E-05	4.22E-09
ENSG00000124782_7211681_7248968_1516	annotated	RREB1	6	+	4548	11	1	3	0.33%	0.799174987	0.035374934	0.114974528	0.016723634
ENSG00000172915_35159256_35670991_1915	annotated	NBEA	13	+	5745	12	0	0	0.26%	0.690177611	0.020613417	0.020613417	0.001946209
ENSG00000213694_89001420_89002337_305	annotated	S1PR3	9	+	915	25	0	0	3.61%	9.027938009	0.001520872	0.001520872	1.39E-05
ENSG00000131143_85805768_85806874_77	novel	COX4I1	16	+	231	673	80	6	49.35%	962.659582	2.95E-07	3.63E-08	1.07E-13
ENSG00000137497_72035943_72003527_979	annotated	NUMA1	11	-	2937	70	17	8	2.76%	7.87523908	0.000296034	5.27E-05	1.16E-07
ENSG00000139324_88153390_88172987_310	annotated	TMTC3	12	+	930	78	6	3	8.06%	27.71285745	1.48E-05	1.16E-05	1.38E-09
ENSG00000139625_53487316_53481182_867	annotated	MAP3K12	12	-	2601	33	1	6	2.08%	4.192212044	0.000116494	0.00522105	5.09E-06
ENSG00000109911_31509785_31783538_475	annotated	ELP4	11	+	1425	30	15	0	2.95%	6.956263813	0.066423533	0.000424543	0.000310968
ENSG00000283761_99999317_100049921_218	dORF	AC118553.2	1	+	654	14	7	0	4.13%	7.073265192	0.119389439	0.002500085	0.002417183
ENSG00000283761_100058702_100082209_418	dORF	AC118553.2	1	+	1254	148	21	6	10.05%	38.99723653	1.41E-07	3.29E-08	4.65E-14
ENSG00000152700_134624019_134606950_198	annotated	SAR1B	5	-	594	273	32	11	28.28%	151.8608603	5.59E-10	4.38E-10	2.75E-18
ENSG00000151967_159886220_159896774_190	annotated	SCHIP1	3	+	570	13	0	0	3.68%	7.535952464	0.008373824	0.008373824	0.000358384
ENSG00000137700_119029530_119029333_65	novel	SLC37A4	11	-	195	31	7	0	15.38%	52.52871007	0.019390513	0.002376615	0.000272493
ENSG00000140199_34252377_34233881_775	annotated	SLC12A6	15	-	2325	7	4	0	0.77%	0.994820524	0.170566842	0.009815329	0.010088762
ENSG00000259494_88459792_88459613_59	novel	MRPL46	15	-	177	29	0	0	11.86%	54.13702487	0.007979402	0.007979402	0.000327143
ENSG00000259494_88465635_88464680_81	novel	MRPL46	15	-	243	40	1	0	12.35%	54.3905401	0.002851	0.002488365	4.06E-05
ENSG00000100227_42614857_42585791_392	annotated	POLDIP3	22	-	1176	48	11	0	3.83%	13.48663392	0.014749822	0.000294865	3.61E-05
ENSG00000115919_142918683_142967328_166	annotated	KYNU	2	+	498	97	11	2	15.66%	64.35940867	5.07E-05	3.55E-06	1.57E-09
ENSG00000140931_66608420_66612637_96	annotated	CMTM3	16	+	288	86	10	3	18.75%	98.66783915	0.003347287	0.000392271	8.85E-06
ENSG00000011198_43699352_43707759_162	novel	ABHD5	3	+	486	8	0	0	3.70%	5.43905401	0.009815329	0.009815329	0.000483714
ENSG00000011198_43715000_43718532_111	novel	ABHD5	3	+	333	26	0	0	5.41%	25.79875618	0.011772051	0.011772051	0.000681273

ENSG00000104687_30709899_30707012_82	novel	GSR	8	-	246	65	12	1	9.76%	87.30676635	0.049832653	0.005808022	0.001595621
ENSG00000104687_30708100_30687595_245	novel	GSR	8	-	735	129	17	7	12.65%	57.99252587	7.33E-06	3.17E-06	1.97E-10
ENSG00000104687_30680936_30679520_60	novel	GSR	8	-	180	48	14	4	20.00%	88.11267496	0.010009985	0.002775311	0.000155676
ENSG00000284770_235414605_235448762_345	dORF	TBCE	1	+	1035	20	11	5	2.32%	6.384976447	0.116207132	0.045028592	0.020525495
ENSG00000111846_10528915_10626607_401	annotated	GCNT2	6	+	1203	34	5	1	2.99%	9.338625152	0.005946344	0.001252797	4.50E-05
ENSG00000154814_16271663_16303562_271	annotated	OXNAD1	3	+	813	25	3	1	3.69%	10.16059444	0.003737369	0.003783485	7.84E-05
ENSG00000096433_33621612_33695780_2668	annotated	ITPR3	6	+	8004	67	17	6	1.20%	2.765905745	0.000564669	1.19E-05	6.10E-08
ENSG00000171467_43369365_43331775_1117	annotated	ZNF318	6	-	3351	9	0	0	0.45%	0.887437416	0.020613417	0.020613417	0.001946209
ENSG00000169814_15635440_15645488_523	annotated	BTD	3	+	1569	56	8	3	4.40%	11.79328346	0.000235687	5.63E-05	9.80E-08
ENSG00000116191_178893893_178916359_150	novel	RALGPS2	1	+	450	28	4	0	8.00%	20.55962416	0.011884585	0.00087769	6.92E-05
ENSG00000117859_51617156_51787789_721	annotated	OSBPL9	1	+	2163	44	17	2	2.77%	6.721493929	0.012269464	0.000132928	1.53E-05
ENSG00000084774_27241977_27243518_274	annotated	CAD	2	+	822	21	3	1	4.01%	8.441451525	0.007260592	0.002106591	8.78E-05
ENSG00000139324_88153390_88195649_818	annotated	TMTC3	12	+	2454	159	19	4	6.48%	21.4087948	4.24E-09	2.36E-10	1.17E-17
ENSG00000104731_87765830_87708351_500	annotated	KLHDC4	16	-	1500	26	2	3	2.20%	5.727323873	0.001398493	0.00856855	7.26E-05
ENSG00000184047_122226014_122208381_195	annotated	DIABLO	12	-	585	83	7	1	10.26%	46.88046168	0.000198774	6.62E-05	9.60E-08
ENSG00000101199_63275581_63287873_414	annotated	ARFGAP1	20	+	1242	57	18	5	5.07%	15.16431906	0.005022345	0.000366365	1.29E-05
ENSG00000132604_69385845_69370441_251	annotated	TERF2	16	-	753	55	0	0	5.58%	24.13444782	0.000456173	0.000456173	1.37E-06
ENSG00000168497_191846841_191835923_397	annotated	CAVIN2	2	-	1191	11	1	0	2.02%	3.051761412	0.010499663	0.00419316	0.000236136
ENSG00000249915_271721_314515_189	annotated	PDCD6	5	+	567	318	50	14	22.22%	185.3163402	8.17E-06	1.82E-08	1.93E-12
ENSG00000136450_58007137_58005747_201	annotated	SRSF1	17	-	603	399	43	66	24.88%	218.6377942	7.06E-08	4.84E-07	3.35E-13
ENSG00000214575_82556032_82544592_329	annotated	CPEB1	15	-	987	8	0	0	1.52%	2.678196807	0.019679754	0.019679754	0.001784964
ENSG00000143622_155910498_155900388_181	annotated	RIT1	1	-	543	38	0	1	8.84%	23.12349205	0.000197846	0.000245984	3.36E-07
ENSG00000172594_122803750_122809408_235	annotated	SMPDL3A	6	+	705	13	3	1	3.40%	6.092897737	0.0400283	0.010020958	0.001975105
ENSG00000139990_69122328_69053857_860	annotated	DCAF5	14	-	2580	17	1	0	1.05%	2.177202724	0.005588371	0.003478238	0.000106767
ENSG00000100441_24430731_24437285_678	annotated	KHNYN	14	+	2034	20	1	2	1.62%	3.24899244	0.002559322	0.00718566	0.000103461
ENSG00000172590_22833438_22834321_64	annotated	MRPL52	14	+	192	56	9	5	20.31%	96.37323824	0.002390059	0.001691112	2.38E-05
ENSG00000175166_184303358_184303800_88	novel	PSMD2	3	+	264	7	2	0	5.68%	8.761203477	0.064489021	0.019216965	0.005584918
ENSG00000181019_69718478_69710976_253	annotated	NQO1	16	-	759	3800	320	247	56.52%	1654.289352	3.87E-21	3.28E-21	2.15E-40
ENSG00000076928_41901919_41903673_185	novel	ARHGEF1	19	+	555	12	3	1	3.24%	7.144270943	0.051117422	0.019795882	0.004517762
ENSG00000076928_41903686_41906468_284	novel	ARHGEF1	19	+	852	15	5	4	2.82%	5.817298083	0.081055481	0.059861443	0.018364919
ENSG00000163072_168802647_168864970_563	annotated	NOSTRIN	2	+	1689	26	8	0	1.60%	5.086333279	0.13068706	0.003327803	0.003333927
ENSG00000164323_185191196_185162798_532	annotated	CFAP97	4	-	1596	48	6	3	3.76%	9.937519733	0.001016074	0.000264373	1.81E-06
ENSG00000122741_37800867_37861508_559	annotated	DCAF10	9	+	1677	86	13	1	5.37%	16.94474519	0.000110192	1.38E-06	1.54E-09
ENSG00000158406_26285499_26285188_103	annotated	HIST1H4H	6	-	309	3522	418	57	52.43%	3766.175258	3.90E-08	5.88E-10	2.75E-16
ENSG00000101040_47356670_47210761_1107	annotated	ZMYND8	20	-	3321	24	10	0	0.90%	2.38787737	0.060116676	0.002321823	0.000966448
ENSG00000135801_229602890_229594297_497	annotated	TAF5L	1	-	1491	18	0	2	2.01%	3.9890044	0.002201184	0.010678933	0.000135702
ENSG00000155254_97713877_97714398_173	annotated	MARVELD1	10	+	519	14	0	0	3.47%	8.913131861	0.012798403	0.012798403	0.000797244
ENSG00000156467_96231935_96231553_76	annotated	UQCRB	8	-	228	809	35	17	35.53%	1172.420297	5.12E-06	5.39E-06	2.31E-10
ENSG00000118680_3272899_3277937_172	annotated	MYL12B	18	+	516	124	17	1	10.47%	79.40386407	0.00211467	0.000100355	1.65E-06
ENSG00000143420_150629470_150627332_105	annotated	ENSA	1	-	315	42	3	9	9.52%	44.05633748	0.010831024	0.026610517	0.001392092
ENSG00000120549_24473393_24546324_1606	annotated	KIAA1217	10	+	4818	23	1	1	0.75%	1.57735953	0.001559221	0.001559221	1.46E-05
ENSG00000120549_24473393_24545118_1410	annotated	KIAA1217	10	+	4230	23	1	1	0.85%	1.796623692	0.001559221	0.001559221	1.46E-05
ENSG00000152683_32165901_32220713_461	annotated	SLC30A6	2	+	1383	117	32	8	9.11%	27.95331608	2.57E-05	1.50E-07	4.41E-11
ENSG00000077150_102395960_102398959_403	novel	NFKB2	10	+	1209	11	3	0	1.49%	3.006325759	0.011570466	0.011772051	0.000670297
ENSG00000128609_123550481_123542119_59	novel	NDUFA5	7	-	177	41	4	0	11.86%	76.53855241	0.052585976	0.008574016	0.002319313
ENSG00000196504_152693053_152679290_108	novel	PRPF40A	2	-	324	19	4	0	9.26%	19.37662991	0.024209654	0.002281203	0.000335406
ENSG00000160214_43793351_43803774_359	novel	RRP1	21	+	1077	37	0	1	3.90%	11.35156328	0.000434916	0.000585523	1.66E-06
ENSG00000155957_66169951_66137960_238	annotated	TMBIM4	12	-	714	236	42	11	24.37%	109.2152904	8.31E-11	2.11E-10	2.04E-19
ENSG00000136802_128907165_128916371_810	annotated	LRRC8A	9	+	2430	93	8	10	5.43%	12.64580057	3.27E-07	2.03E-07	6.25E-13
ENSG00000133393_15888585_15867440_100	annotated	FOPNL	16	-	300	71	15	3	20.00%	78.19999903	0.002717299	7.70E-05	1.73E-06
ENSG00000077150_102395960_102402376_899	annotated	NFKB2	10	+	2697	35	4	0	2.00%	4.288019499	0.000120212	7.13E-05	6.24E-08
ENSG00000170270_93207037_93203688_100	annotated	GON7	14	-	300	69	9	1	17.00%	75.99718216	0.010701222	0.000103528	1.07E-05
ENSG00000111319_6355855_6347873_369	annotated	SCNN1A	12	-	1107	46	3	1	3.52%	13.73029488	0.002502442	0.000723387	1.13E-05
ENSG00000067208_92736546_92513656_777	annotated	EVI5	1	-	2331	11	2	1	0.77%	1.559265484	0.061356297	0.023672501	0.006320165
ENSG00000105048_55141284_55132915_192	annotated	TNNT1	19	-	576	13	2	0	3.65%	7.457452959	0.037995728	0.007979402	0.001544499
ENSG00000162521_32669128_32679705_173	annotated	RBBP4	1	+	519	72	2	2	6.94%	45.83896385	0.001164653	0.001394911	9.90E-06
ENSG00000151726_184803424_184757125_668	annotated	ACSL1	4	-	2004	51	1	0	3.29%	8.40895663	2.17E-05	1.69E-05	2.89E-09
ENSG00000143418_150967837_150967658_24	novel	CERS2	1	-	72	20	0	0	25.00%	91.78403642	0.013640586	0.013640586	0.000898637
ENSG00000154518_175181393_175178288_142	annotated	ATP5MC3	2	-	426	756	120	43	34.51%	586.3836468	2.47E-06	1.59E-08	4.63E-13
ENSG00000099999_30380359_30379600_115	novel	RNF215	22	-	345	26	0	0	9.57%	24.90140814	0.001491519	0.001491519	1.34E-05
ENSG00000152795_82429333_82426059_301	annotated	HNRNPDL	4	-	903	206	23	4	14.95%	75.3787834	1.65E-08	2.42E-09	4.31E-16
ENSG00000078140_39698328_39778434_149	annotated	UBE2K	4	+	447	94	24	2	12.08%	69.48482757	0.020287549	0.000248144	4.60E-05
ENSG00000129128_176320077_176328330_180	annotated	SPCS3	4	+	540	241	46	5	23.89%	147.4663518	4.07E-06	1.30E-08	6.67E-13
ENSG00000089693_6752334_6750515_155	novel	MLF2	12	-	465	74	7	5	14.19%	52.58337054	0.000342856	0.000100737	2.46E-07
ENSG00000164808_47279972_47335450_845	annotated	SPIDR	8	+	2535	23	5	6	1.30%	2.997916456	0.026171386	0.026818362	0.003104381
ENSG00000164741_13276376_13085811_1117	annotated	DLC1	8	-	3351	16	8	2	0.90%	1.577666517	0.134720114	0.0116632	0.008547783
ENSG00000104852_49104447_49108443_341	novel	SNRNP70	19	+	1023	147	17	6	13.49%	47.48007045	3.68E-07	1.42E-08	5.56E-14
ENSG00000179950_143829303_143820624_129	novel	PUF60	8	-	387	38	4	4	8.53%	32.44458962	0.010805327	0.004474589	0.000257631

ENSG00000146409_132787493_132770270_309	annotated	SLC18B1	6	-	927	22	3	2	4.21%	7.841742918	0.005926165	0.002999187	9.86E-05
ENSG00000137210_10749246_10755347_135	annotated	TMEM14B	6	+	405	55	19	0	7.41%	44.87219558	0.036819135	0.002460157	0.000570083
ENSG00000175061_16439055_16440252_53	novel	SNHG29	17	+	159	158	20	7	24.53%	328.344402	0.003166886	0.001327289	2.50E-05
ENSG00000167491_19465346_19505474_633	annotated	GATAD2A	19	+	1899	40	4	0	2.37%	6.959926932	0.002094425	0.000291049	4.15E-06
ENSG00000198276_63946178_63940685_288	annotated	UCKL1	20	-	864	10	6	0	2.43%	3.824334851	0.232300293	0.007029303	0.012111047
ENSG00000198276_63940994_63939976_191	Overlap_dORF	UCKL1	20	-	573	16	4	1	5.24%	9.226458111	0.023533756	0.003646019	0.000481004
ENSG00000198604_34874604_34753508_1524	annotated	BAZ1A	14	-	4572	17	4	2	0.72%	1.228605212	0.026447338	0.005489386	0.000769467
ENSG00000072571_163460693_163491164_724	annotated	HMMR	5	+	2172	87	13	8	4.42%	13.23515663	5.06E-05	5.57E-06	2.37E-09
ENSG00000131732_81304827_81312662_248	annotated	ZCCHC9	5	+	744	18	7	0	3.23%	7.994093495	0.104244116	0.004873904	0.003294081
ENSG00000166548_66517813_66511968_94	annotated	TK2	16	-	282	16	3	0	7.45%	18.74737765	0.008373824	0.008473674	0.000362422
ENSG00000151366_78079744_78069873_88	annotated	NDUFC2	11	-	264	288	49	52	22.73%	360.460943	0.000218832	0.001639617	2.49E-06
ENSG00000116750_193029302_193020343_180	annotated	UCHL5	1	-	540	49	3	1	7.22%	29.98278523	0.000604792	0.001037781	3.99E-06
ENSG00000243335_66629065_66640394_288	annotated	KCTD7	7	+	864	15	3	0	2.43%	5.736502276	0.043639483	0.007882058	0.00177109
ENSG00000145907_151786627_151791089_123	novel	G3BP1	5	+	369	46	12	0	12.20%	41.19088464	0.018737302	0.000301822	4.88E-05
ENSG00000104671_30156384_30165741_37	annotated	DCTN6	8	+	111	13	3	0	16.22%	38.69813427	0.083503598	0.012798403	0.005300298
ENSG00000161013_179801456_179798007_336	annotated	MGAT4B	5	-	1008	81	43	14	5.65%	26.55181054	0.223811263	0.003401347	0.007130012
ENSG00000113742_173889734_173953305_639	annotated	CPEB4	5	+	1917	19	2	0	1.41%	3.274923365	0.007379738	0.003452921	0.000139406
ENSG00000108515_4951836_4953950_180	novel	ENO3	17	+	540	31	4	0	6.11%	18.96870086	0.001506153	0.001513502	1.37E-05
ENSG00000102390_76173139_76177908_233	annotated	PBDC1	X	+	699	46	8	1	6.44%	21.74454425	0.002910421	0.000400846	7.79E-06
ENSG00000102390_76173139_76178017_232	annotated	PBDC1	X	+	696	36	7	13	5.17%	17.09082057	0.009991572	0.072336194	0.003718743
ENSG00000151239_43800518_43795585_252	annotated	TWF1	12	-	756	25	2	1	1.98%	10.926671	0.021084099	0.023199731	0.002219783
ENSG00000109016_21172043_21191153_310	annotated	DHRS7B	17	+	930	39	2	4	5.16%	13.85642872	0.000380129	0.000815366	2.03E-06
ENSG00000086062_33167169_33104752_225	annotated	B4GALT1	9	-	675	311	28	8	22.22%	152.2391217	5.08E-08	4.76E-10	2.98E-16
ENSG00000174780_56467636_56501861_671	annotated	SRP72	4	+	2013	66	28	2	3.87%	10.83352561	0.00674652	9.20E-06	8.95E-07
ENSG00000127990_94656098_94597920_436	novel	SGCE	7	-	1308	197	28	11	10.78%	49.76547296	1.02E-06	3.22E-08	3.47E-13
ENSG00000173409_230979106_230979360_84	novel	ARV1	1	+	252	11	5	2	5.95%	14.42320572	0.166999128	0.019216965	0.015897431
ENSG00000206053_1678313_1698998_218	annotated	JPT2	16	+	654	123	13	0	15.14%	62.14368704	1.69E-05	2.33E-07	4.15E-11
ENSG00000196628_55350976_55232505_418	novel	TCF4	18	-	1254	17	0	0	1.67%	4.479412304	0.008674847	0.008674847	0.000383115
ENSG00000149571_126562844_126424580_725	annotated	KIRREL3	11	-	2175	33	0	0	1.93%	5.013307369	0.000444132	0.000444132	1.30E-06
ENSG00000133997_70592891_70584813_56	Overlap_dORF	MED6	14	-	168	15	0	0	10.71%	29.50201171	0.013428348	0.013428348	0.000872559
ENSG00000133997_70600637_70584892_181	annotated	MED6	14	-	543	27	20	0	6.08%	16.42984961	0.32233595	0.001506153	0.007681248
ENSG00000138303_72203481_72097443_275	annotated	ASCC1	10	-	825	19	5	1	2.91%	7.60973102	0.05393658	0.005454749	0.001657528
ENSG00000197312_15617671_15633569_211	novel	DDI2	1	+	633	14	0	3	2.84%	7.307923279	0.01300724	0.073562049	0.004672677
ENSG00000153066_11742730_11679195_958	annotated	TXNDC11	16	-	2874	44	11	4	1.98%	5.05866088	0.014821548	0.000892578	8.96E-05
ENSG00000140612_84670748_84670019_24	dORF	SEC11A	15	-	72	99	7	3	25.00%	454.3309803	0.037701781	0.029237631	0.004727254
ENSG00000140612_84716075_84670757_164	annotated	SEC11A	15	-	492	412	70	21	23.78%	276.6952903	8.75E-07	1.93E-07	1.59E-12
ENSG00000067141_73052676_73302696_1408	annotated	NEO1	15	+	4224	93	13	4	3.41%	7.274927887	1.70E-07	5.02E-09	9.44E-15
ENSG00000028528_64129967_64134858_185	dORF	SNX1	15	+	555	38	2	4	8.65%	22.62352465	0.000498598	0.001124817	3.60E-06
ENSG00000028528_64112646_64130019_160	Overlap_dORF	SNX1	15	+	480	26	7	2	5.00%	17.8978871	0.082712367	0.018318558	0.006975982
ENSG00000167965_2206086_2206521_72	annotated	MLST8	16	+	216	22	3	0	6.94%	33.65414669	0.08410207	0.019679754	0.007521961
ENSG00000099341_38376420_38382333_172	annotated	PSMD8	19	+	516	62	3	7	11.05%	39.70193203	0.000106337	0.000690748	5.29E-07
ENSG00000213064_168226080_168236766_108	annotated	SFT2D2	1	+	324	39	3	31	9.26%	39.77308245	0.007999495	0.350279879	0.024116756
ENSG00000141504_7627635_7627408_29	novel	SAT2	17	-	87	12	1	0	20.69%	45.57552153	0.023878474	0.01300724	0.001470914
ENSG00000141504_7626958_7626447_74	novel	SAT2	17	-	222	10	10	0	8.11%	14.8838978	0.330203535	0.011772051	0.027944305
ENSG00000215305_2840775_2866574_695	annotated	VPS16	20	+	2085	66	5	0	3.88%	10.45941825	1.27E-05	2.28E-06	2.49E-10
ENSG00000166851_23678933_23679454_173	annotated	PLK1	16	+	519	83	15	1	9.83%	52.84213889	0.000289115	0.00014114	2.85E-07
ENSG00000085721_15094233_15060168_638	annotated	RRN3	16	-	1914	52	3	0	2.98%	8.976996666	0.000213727	5.82E-05	9.16E-08
ENSG00000140682_31474420_31477576_300	dORF	TGFB1I1	16	+	900	28	2	0	5.00%	10.27981208	0.001508934	0.000231967	2.41E-06
ENSG00000108439_47942095_47946782_228	novel	PNPO	17	+	684	71	20	7	9.21%	34.29824519	0.001760832	0.000186632	2.33E-06
ENSG00000013364_29830550_29847989_893	annotated	MVP	16	+	2679	164	14	6	6.61%	20.22743378	3.77E-10	7.93E-11	3.49E-19
ENSG00000114861_71198378_70959247_676	annotated	FOXP1	3	-	2028	18	1	5	1.18%	2.932744359	0.008219047	0.047480794	0.002006772
ENSG00000113368_126777539_126819146_377	annotated	LMNB1	5	+	1131	85	13	1	7.43%	24.83281622	0.00018553	2.00E-06	3.79E-09
ENSG00000105866_21428721_21511269_767	annotated	SP4	7	+	2301	5	0	0	0.65%	0.717997677	0.012673659	0.012673659	0.000782701
ENSG00000127955_80135161_80217446_148	annotated	GNAI1	7	+	444	23	2	0	4.05%	17.11648247	0.011772051	0.011772051	0.000681273
ENSG00000204351_31962047_31969715_894	dORF	SKIV2L	6	+	2682	21	1	0	1.34%	2.587201027	0.001410049	0.000939154	8.16E-06
ENSG00000039650_49867204_49861248_490	annotated	PNKP	19	-	1470	28	6	0	2.45%	6.293762497	0.009048123	0.000978918	5.65E-05
ENSG00000188186_100148973_100153422_30	novel	LAMTOR4	7	+	90	124	49	8	16.67%	455.2488206	0.086477459	0.075027848	0.023773918
ENSG00000188186_100153490_100153964_41	novel	LAMTOR4	7	+	123	128	13	4	36.59%	343.8543413	0.000634982	0.000665324	2.71E-06
ENSG00000114054_136283882_136327653_209	novel	PCCB	3	+	627	26	3	1	5.74%	13.70173175	0.00724517	0.001535641	6.61E-05
ENSG00000156256_29046934_29054187_282	novel	USP16	21	+	846	11	0	0	2.48%	4.296274045	0.007593099	0.007593099	0.000297833
ENSG00000089775_64490383_64486923_385	annotated	ZBTB25	14	-	1155	10	0	0	2.34%	2.860801135	0.001946209	0.001946209	2.23E-05
ENSG00000160216_43971480_43981207_101	novel	AGPAT3	21	+	303	26	4	0	4.95%	28.35308848	0.060322713	0.020613417	0.005529088
ENSG00000121864_179328294_179334422_485	annotated	ZNF639	3	+	1455	14	3	0	1.24%	3.179323323	0.012798403	0.01300724	0.000809468
ENSG00000173698_19068834_18990864_993	annotated	ADGRG2	X	-	2979	28	3	0	1.41%	3.105683408	0.003183653	0.000424543	9.00E-06
ENSG00000173698_19068834_18990864_966	annotated	ADGRG2	X	-	2898	27	6	0	1.35%	3.078470787	0.019433552	0.000656046	9.47E-05
ENSG00000066654_20741739_20736880_353	annotated	THUMPD1	16	-	1059	23	0	0	3.12%	7.176315596	0.00140552	0.00140552	1.19E-05
ENSG00000169093_1452840_1403269_605	annotated	ASMTL	X	-	1815	23	1	1	1.65%	4.187172571	0.003783485	0.003783485	7.93E-05
ENSG00000173698_19068834_18990864_1001	annotated	ADGRG2	X	-	3003	28	3	0	1.40%	3.080862761	0.003183653	0.000424543	9.00E-06

ENSG00000122965_113962414_113823224_948	annotated	RBM19	12	-	2844	20	0	3	0.95%	2.323646492	0.003452921	0.022360378	0.000435012
ENSG00000104763_18063207_18057534_235	novel	ASAH1	8	-	705	259	26	5	19.57%	121.3892703	1.05E-07	7.88E-09	8.78E-15
ENSG00000111300_112093110_112029531_944	annotated	NAA25	12	-	2832	54	12	2	2.75%	6.300429619	0.000146977	1.40E-05	1.67E-08
ENSG00000086666_80120345_80137628_208	annotated	ZFAND6	15	+	624	70	25	2	10.58%	37.06663009	0.005217009	1.52E-05	9.71E-07
ENSG00000152558_102402099_102398867_120	annotated	TMEM123	11	-	360	163	13	4	25.00%	149.6079794	4.56E-06	1.64E-06	6.52E-11
ENSG00000009335_157139273_157188961_404	annotated	UBE3C	7	+	1212	12	2	0	1.73%	3.271510209	0.037811058	0.007882058	0.001520873
ENSG00000064703_111755976_111766899_807	annotated	DDX20	1	+	2421	33	3	0	1.61%	4.503900672	0.002487786	0.000676852	1.06E-05
ENSG00000065833_83430954_83211924_572	annotated	ME1	6	-	1716	250	50	17	14.16%	48.13848064	4.31E-10	2.06E-13	1.47E-21
ENSG00000116473_111697468_111709235_133	annotated	RAP1A	1	+	399	52	9	1	11.28%	43.06258551	0.001210076	0.000503793	3.91E-06
ENSG00000149929_29995587_29993500_169	annotated	HIRIP3	16	-	507	20	7	0	3.55%	13.03441937	0.074100693	0.01300724	0.004709878
ENSG00000205771_43746682_43745119_66	novel	CATSPER2P1	15	-	198	49	33	1	10.61%	81.77123245	0.044040756	0.007979402	0.001806733
ENSG00000188428_8064376_8027380_114	annotated	BLOC1S5	6	-	342	15	5	0	4.39%	14.49221628	0.229044117	0.019679754	0.023761959
ENSG00000124795_18263915_18225719_317	annotated	DEK	6	-	951	67	14	2	6.62%	23.27897958	0.000352638	4.34E-05	1.17E-07
ENSG00000156970_40165129_40220759_1027	annotated	BUB1B	15	+	3081	56	3	0	2.34%	6.005732471	2.22E-05	7.27E-06	1.32E-09
ENSG00000234741_173867041_173866752_31	novel	GAS5	1	-	93	25	6	0	19.35%	88.82326105	0.023199731	0.013640586	0.001492437
ENSG00000168268_52525097_52524470_113	novel	NT5DC2	3	-	339	20	11	3	5.31%	19.49395464	0.101051022	0.026832696	0.011714757
ENSG00000168488_28823260_28836800_1044	annotated	ATXN2L	16	+	3132	145	24	8	3.93%	15.2973394	1.70E-05	2.17E-08	5.12E-12
ENSG00000175832_43545617_43528519_484	annotated	ETV4	17	-	1452	38	4	13	4.13%	8.647421613	0.000402776	0.018863433	6.19E-05
ENSG00000141759_79988383_79977543_100	annotated	TXNL4A	18	-	300	35	12	1	8.00%	38.5492953	0.152224769	0.007312901	0.007092059
ENSG00000152240_46118204_46128125_202	dORF	HAUS1	18	+	606	86	6	17	7.92%	46.89164633	0.000316705	0.004092318	9.10E-06
ENSG00000063241_55456486_55455624_119	novel	ISOC2	19	-	357	62	2	1	10.08%	57.38430512	0.001070089	0.001070089	7.07E-06
ENSG00000185896_113297435_113322421_417	annotated	LAMP1	13	+	1251	916	183	24	23.74%	241.9400787	1.12E-15	7.46E-18	1.36E-31
ENSG00000100938_24238371_24238778_74	annotated	GMPR2	14	+	222	17	2	0	6.76%	25.30262626	0.021084099	0.021084099	0.002029891
ENSG00000092439_50648830_50561678_1806	annotated	TRPM7	15	-	5418	33	5	0	1.00%	2.012540333	0.000862922	6.99E-05	4.58E-07
ENSG00000183735_64464361_64501381_644	dORF	TBK1	12	+	1932	32	5	1	2.48%	5.472836954	0.003657632	0.000263041	6.86E-06
ENSG00000237188_147254969_147295509_727	novel	AC242426.2	1	+	2181	31	1	2	1.93%	4.696514656	0.000671624	0.001303752	5.51E-06
ENSG00000213246_58352135_58346246_117	annotated	SUPT4H1	17	-	351	97	12	6	19.66%	91.31334905	0.000747038	8.50E-05	4.69E-07
ENSG00000112837_84762566_84742545_177	novel	TBX18	6	-	531	20	3	0	5.08%	12.44529307	0.009288069	0.003303074	0.000168518
ENSG00000108946_68515400_68530449_381	annotated	PRKAR1A	17	+	1143	541	40	14	22.05%	156.3942164	2.74E-14	1.46E-14	5.51E-27
ENSG00000105220_34394016_34400036_221	novel	GPI	19	+	663	433	29	26	23.08%	215.7963137	4.49E-09	1.04E-08	4.92E-16
ENSG00000173153_64314055_64315966_280	annotated	ESRRA	11	+	840	41	8	4	5.36%	16.1277664	0.006556828	0.001679061	6.46E-05
ENSG00000037474_6604760_6599926_213	novel	NSUN2	5	-	639	41	2	2	7.51%	21.20081968	0.000273511	0.000273511	5.08E-07
ENSG00000145284_82798381_82631327_278	annotated	SCD5	4	-	834	34	2	0	4.68%	13.4704629	0.00099184	0.000676852	4.24E-06
ENSG00000037757_13765024_13772281_227	annotated	MRI1	19	+	681	8	0	0	2.20%	3.881615637	0.019679754	0.019679754	0.001784964
ENSG00000143198_165632240_165655504_166	annotated	MGST3	1	+	498	1214	291	47	39.76%	805.487857	2.92E-09	6.55E-12	2.75E-19
ENSG00000143198_165649848_165655504_152	annotated	MGST3	1	+	456	1205	291	47	42.11%	873.1560307	6.08E-09	1.48E-11	1.28E-18
ENSG00000143183_165768742_165728023_185	annotated	TMCO1	1	-	555	348	49	19	30.27%	207.1838573	4.60E-10	6.04E-11	3.29E-19
ENSG00000143198_165632240_165655504_166	annotated	MGST3	1	+	498	1214	291	47	39.76%	805.487857	2.92E-09	6.55E-12	2.75E-19
ENSG00000143198_165649848_165655504_152	annotated	MGST3	1	+	456	1475	291	47	42.76%	1068.80095	3.71E-09	9.76E-12	5.12E-19
ENSG00000143198_165649848_165661496_129	annotated	MGST3	1	+	387	949	251	36	36.43%	810.2609355	6.25E-06	1.55E-08	1.25E-12
ENSG00000049883_72320383_72338626_148	Overlap_uORF	PTCD2	5	+	444	15	3	4	4.73%	11.16292335	0.07962816	0.049121095	0.015214415
ENSG00000049883_72326711_72358427_216	annotated	PTCD2	5	+	648	16	0	2	3.24%	8.158581015	0.008473674	0.013892891	0.00058868
ENSG00000171793_40983291_41008797_451	annotated	CTPS1	1	+	1353	32	14	4	3.33%	7.814871394	0.051026698	0.002377639	0.000809725
ENSG00000122085_241099852_241095998_360	annotated	MTERF4	2	-	1080	9	0	3	1.67%	2.753521093	0.011772051	0.097937785	0.005938109
ENSG00000157978_25543699_25566992_308	annotated	LDLRAP1	1	+	924	8	2	0	1.62%	2.860801135	0.065398531	0.019679754	0.005779267
ENSG00000179241_36001138_36229215_121	novel	LDLRAD3	11	+	363	19	2	3	4.96%	17.29484323	0.016503129	0.039104404	0.002953538
ENSG00000077514_74612937_74640766_360	annotated	POLD3	11	+	1080	15	0	0	2.50%	4.589201821	0.003278443	0.003278443	6.04E-05
ENSG00000137942_93448282_93551125_609	annotated	FNBP1L	1	+	1827	44	1	7	3.28%	7.957630744	5.20E-05	0.001228821	5.15E-07
ENSG00000166471_9314441_9283424_291	annotated	TMEM41B	11	-	873	46	5	0	7.22%	17.4105801	0.000148925	2.28E-05	2.65E-08
ENSG00000136156_48233361_48261224_266	annotated	ITM2B	13	+	798	1161	79	40	31.58%	480.7275171	5.76E-14	1.29E-14	1.02E-26
ENSG00000076928_41888083_41907413_933	annotated	ARHGEF1	19	+	2799	78	13	1	3.75%	9.207916194	3.93E-05	1.17E-07	5.68E-11
ENSG00000152465_15168530_15109195_485	annotated	NMT2	10	-	1455	22	6	4	2.27%	4.996079508	0.022851748	0.012837471	0.001392576
ENSG00000147044_41745600_41520420_816	annotated	CASK	X	-	2448	35	8	0	1.96%	4.724178345	0.004784059	0.00017249	6.42E-06
ENSG00000147044_41745600_41520420_815	annotated	CASK	X	-	2445	36	8	0	2.09%	4.865117023	0.003393093	0.000110146	3.00E-06
ENSG00000148331_129641999_129637790_421	annotated	ASB6	9	-	1263	24	13	3	2.85%	6.278812943	0.157518271	0.00092354	0.001794399
ENSG00000148331_129635373_129635221_50	dORF	ASB6	9	-	150	17	12	0	14.00%	37.44788686	0.129839126	0.008674847	0.006580666
ENSG00000102554_73059349_73075886_450	annotated	KLF5	13	+	1350	74	13	0	6.67%	18.11204985	8.66E-05	6.55E-07	6.15E-10
ENSG00000148331_129638356_129637790_188	Overlap_dORF	ASB6	9	-	564	15	4	3	3.72%	8.787833274	0.060281869	0.008175611	0.002591214
ENSG00000154814_16271663_16303562_271	annotated	OXNAD1	3	+	813	25	3	1	3.69%	10.16059444	0.003737369	0.003783485	7.84E-05
ENSG00000174744_66341621_66338493_259	novel	BRMS1	11	-	777	48	7	4	4.63%	20.41220269	0.005187512	0.003882003	0.00011003
ENSG00000055208_149378639_149409719_452	annotated	TAB2	6	+	1356	19	0	0	2.65%	4.629814227	0.000808111	0.000808111	4.12E-06
ENSG00000167770_63986457_63997546_271	annotated	OTUB1	11	+	813	278	45	11	22.88%	112.9858101	1.04E-08	1.46E-10	1.88E-17
ENSG00000248098_41397828_41410972_147	novel	BCKDHA	19	+	441	31	9	0	6.12%	23.22698065	0.044716345	0.003684287	0.000981445
ENSG00000135297_73466259_73500735_628	annotated	MTO1	6	+	1884	22	6	37	2.23%	3.8584372	0.026337154	0.022773487	0.002684778
ENSG00000143033_93079527_93137027_536	annotated	MTF2	1	+	1608	10	0	3	0.93%	2.054866487	0.019679754	0.131599539	0.01228543
ENSG00000135297_73461855_73500735_717	annotated	MTO1	6	+	2151	29	6	37	2.23%	4.454790052	0.008344617	0.009321449	0.000395415
ENSG00000135297_73461855_73479848_313	novel	MTO1	6	+	939	17	3	37	2.56%	5.982090553	0.037966651	0.154611771	0.024191651
ENSG00000135297_73482172_73500735_228	novel	MTO1	6	+	684	12	2	0	3.51%	5.796886511	0.033181033	0.00419316	0.000782812

ENSG00000123444_47577999_47572930_518	annotated	KBTBD4	11	-	1554	33	5	4	2.70%	7.016694676	0.003517618	0.003306134	6.51E-05
ENSG00000204256_32974574_32980718_789	annotated	BRD2	6	+	2367	36	5	12	2.15%	5.025437736	0.001554277	0.014660983	0.000140809
ENSG00000178537_48898794_48857710_228	annotated	SLC25A20	3	-	684	17	1	2	4.82%	8.21225589	0.002415484	0.00809034	0.000110745
ENSG00000099246_27504370_27538051_142	annotated	RAB18	10	+	426	106	16	0	19.01%	82.21781291	0.000113464	2.58E-06	2.77E-09
ENSG00000112715_43777480_43784562_189	annotated	VEGFA	6	+	567	40	1	3	5.29%	23.31023147	0.002851	0.008025782	0.00012745
ENSG00000166484_19378901_19379603_80	annotated	MAPK7	17	+	240	8	0	0	6.25%	11.01408437	0.016947427	0.016947427	0.001349898
ENSG00000101935_110317684_110198520_204	annotated	AMMECR1	X	-	612	22	0	0	5.39%	11.87793413	0.001448134	0.001448134	1.26E-05
ENSG00000144306_174400003_174427895_344	annotated	SCRN3	2	+	1032	9	0	0	1.74%	2.881591841	0.011772051	0.011772051	0.000681273
ENSG00000214253_101240151_101239806_35	dORF	FIS1	7	-	105	31	2	3	17.14%	97.55331871	0.044329616	0.045195857	0.008170094
ENSG00000132780_45584153_45618141_420	annotated	NASP	1	+	1260	115	12	6	8.81%	30.15761197	2.23E-06	5.83E-07	1.18E-11
ENSG00000121542_123209218_123271722_307	annotated	SEC22A	3	+	921	47	3	3	6.84%	16.86195327	0.000152817	0.000164151	1.76E-07
ENSG00000124222_58670514_58676291_139	Overlap_dORF	STX16	20	+	417	13	0	1	5.04%	10.30094222	0.008175611	0.0135992	0.00055803
ENSG00000139726_122753702_122753821_39	novel	DENR	12	+	117	34	11	0	15.38%	96.02022272	0.117522225	0.013428348	0.008088185
ENSG00000080815_73170959_73173782_101	annotated	PSEN1	14	+	303	37	0	0	6.93%	40.34862591	0.008776163	0.008776163	0.000391611
ENSG00000140937_64947887_64947603_94	dORF	CDH11	16	-	282	29	0	0	12.77%	33.97962199	0.001003278	0.001003278	6.24E-06
ENSG00000140937_65004824_64948091_678	annotated	CDH11	16	-	2034	134	7	1	5.46%	21.76824935	3.00E-07	4.90E-08	1.46E-13
ENSG00000254858_18193282_18194072_131	novel	MPV17L2	19	+	393	39	1	0	8.40%	32.79002217	0.00199571	0.001610998	1.91E-05
ENSG00000167700_144510868_144511164_69	novel	MFSD3	8	+	207	63	4	3	8.70%	100.563379	0.026832696	0.023878474	0.002853849
ENSG00000083099_89638716_89637338_72	annotated	LYRM2	6	-	216	40	12	0	11.11%	61.18935761	0.032657853	0.005655336	0.000986292
ENSG00000186501_27322330_27335466_189	annotated	TMEM222	1	+	567	56	6	0	11.64%	32.63432406	5.55E-05	1.77E-05	7.67E-09
ENSG00000110871_120529141_120527353_75	novel	COQ5	12	-	225	18	0	0	12.00%	26.43380249	0.003278443	0.003278443	6.04E-05
ENSG00000134283_42387440_42399006_122	novel	PPHLN1	12	+	366	7	0	0	4.10%	6.319556606	0.019216965	0.019216965	0.001707396
ENSG00000105993_157358573_157416098_211	annotated	DNAJB6	7	+	633	15	1	0	2.84%	7.829917799	0.012798403	0.013428348	0.00083412
ENSG00000176101_137188727_137188978_83	novel	SSNA1	9	+	249	19	1	2	7.23%	25.21296422	0.016503129	0.028889786	0.002191483
ENSG00000162959_32010247_31868361_297	annotated	MEMO1	2	-	891	16	4	5	2.02%	5.933513466	0.044040756	0.090097208	0.015632667
ENSG00000103121_80997394_80980808_74	novel	CMC2	16	-	222	33	11	4	10.81%	49.11686273	0.062400078	0.010275151	0.003233385
ENSG00000124942_62536098_62433884_149	annotated	AHNAK	11	-	447	41	2	0	6.71%	30.30721203	0.005451642	0.002376615	7.34E-05
ENSG00000134809_57530138_57528717_73	annotated	TIMM10	11	-	219	667	50	16	54.79%	1006.35538	5.46E-07	1.20E-07	6.30E-13
ENSG00000102974_67616777_67637872_397	novel	CTCF	16	+	1191	40	1	2	4.53%	11.09731423	0.000120705	0.000222345	1.89E-07
ENSG00000140284_50182428_50236096_620	annotated	SLC27A2	15	+	1860	69	14	2	5.00%	12.25761003	0.000113644	1.49E-06	1.71E-09
ENSG00000148908_119500247_119500113_44	novel	RGS10	10	-	132	25	3	0	18.18%	62.58002483	0.018570095	0.005706018	0.000551937
ENSG00000198363_61646807_61626273_112	annotated	ASPH	8	-	336	871	72	58	41.96%	856.5417399	2.20E-09	1.90E-07	4.96E-15
ENSG00000120694_31140297_31137318_236	novel	HSPH1	13	-	708	45	11	2	8.90%	21.00143206	0.00453164	8.41E-05	3.31E-06
ENSG00000120694_31150954_31141065_336	novel	HSPH1	13	-	1008	38	5	0	4.17%	12.45640494	0.000997927	0.000410556	2.67E-06
ENSG00000123080_50970369_50974270_168	annotated	CDKN2C	1	+	504	32	4	0	7.14%	20.97920832	0.002280579	0.001003278	1.39E-05
ENSG00000165322_31908849_31807658_839	annotated	ARHGAP12	10	-	2517	59	9	1	2.62%	7.745303669	0.000962744	2.27E-05	1.92E-07
ENSG00000130520_18323020_18307464_125	annotated	LSM4	19	-	375	246	45	13	24.00%	216.7571804	0.000293956	7.46E-07	2.72E-09
ENSG00000158773_161043275_161040555_244	novel	USF1	1	-	732	25	7	0	4.92%	11.28492251	0.02611945	0.00092354	0.000175529
ENSG00000068438_48481180_48483018_197	novel	FTSJ1	X	+	591	62	6	0	10.15%	34.66361579	0.00019612	4.06E-05	6.00E-08
ENSG00000198860_184051756_184072863_129	annotated	TSEN15	1	+	387	26	0	1	6.98%	22.19892974	0.003478238	0.005588371	0.000106767
ENSG00000102908_69566302_69653304_135	annotated	NFAT5	16	+	405	11	5	0	3.70%	8.974439117	0.145248299	0.020613417	0.014232745
ENSG00000149428_119056160_119055746_62	novel	HYOU1	11	-	186	20	6	0	11.29%	35.52930442	0.011738695	0.008473674	0.000500106
ENSG00000184009_81512354_81510690_375	annotated	ACTG1	17	-	1125	2286	538	109	29.87%	671.4185832	2.67E-12	1.76E-17	1.16E-27
ENSG00000141543_80146961_80135490_390	annotated	EIF4A3	17	-	1170	228	35	1	13.59%	64.3900317	3.51E-08	1.07E-10	5.08E-17
ENSG00000147874_19093198_19057950_802	annotated	HAUS6	9	-	2406	24	0	0	1.37%	3.295985348	0.001506153	0.001506153	1.36E-05
ENSG00000158321_70764912_70790996_786	annotated	AUTS2	7	+	2358	10	4	0	0.89%	1.401282999	0.075513077	0.007029303	0.002963142
ENSG00000169714_129171757_129170453_179	annotated	CNBP	3	-	537	532	54	21	29.05%	327.3459712	4.87E-10	5.97E-10	3.26E-18
ENSG00000122515_44756268_44767623_914	annotated	ZMIZ2	7	+	2742	32	7	0	1.53%	3.856134572	0.01223992	0.000428413	3.94E-05
ENSG00000121057_57105510_57108013_578	annotated	AKAP1	17	+	1734	58	5	0	3.98%	11.05219539	9.42E-05	1.15E-05	8.83E-09
ENSG00000170581_56356556_56343389_846	annotated	STAT2	12	-	2538	19	4	0	0.83%	2.473612329	0.058290626	0.007979402	0.002448908
ENSG00000172432_43625809_43621614_451	novel	GTPBP2	6	-	1353	7	0	0	1.33%	1.709503117	0.009815329	0.009815329	0.000483714
ENSG00000177674_11747543_11750192_104	dORF	AGTRAP	1	+	312	162	10	9	17.31%	171.565545	9.38E-05	0.000868713	6.00E-07
ENSG00000140553_90934999_90953716_961	annotated	UNC45A	15	+	2883	85	9	12	3.64%	9.741906051	4.58E-06	1.85E-05	7.06E-10
ENSG00000153187_244860523_244859328_70	annotated	HNRNPU	1	-	210	35	5	0	12.86%	55.07042185	0.020349766	0.003763158	0.000423483
ENSG00000153187_244860372_244854450_491	Overlap_dORF	HNRNPU	1	-	1473	368	45	22	14.05%	82.54955292	2.62E-10	1.38E-10	4.18E-19
ENSG00000110906_109469722_109451595_310	annotated	KCTD10	12	-	930	33	0	6	4.52%	11.72467046	0.000413076	0.004245653	1.19E-05
ENSG00000175581_73787972_73843351_101	uORF	MRPL48	11	+	303	29	9	5	5.94%	31.62459869	0.152428873	0.089010362	0.046680747
ENSG00000132361_2703447_2690594_1233	annotated	CLUH	17	-	3699	105	12	4	3.41%	9.379390583	7.47E-07	3.69E-08	2.84E-13
ENSG00000122882_73160534_73134583_527	annotated	ECD	10	-	1581	31	5	0	3.04%	6.478873159	0.003845964	0.000184214	5.33E-06
ENSG00000146648_55019278_55205617_1165	annotated	EGFR	7	+	3495	316	29	10	7.81%	29.87511297	1.25E-14	2.53E-15	4.49E-28
ENSG00000153391_35480530_35454822_134	annotated	INO80C	18	-	402	39	4	3	7.46%	32.0559172	0.002418183	0.011381896	0.000157411
ENSG00000140521_89326694_89317504_640	dORF	POLG	15	-	1920	23	0	2	1.25%	3.958186571	0.005306578	0.02389627	0.000672269
ENSG00000138674_82881906_82819074_1210	annotated	SEC31A	4	-	3630	99	9	6	3.14%	9.011523576	1.27E-07	8.49E-07	1.04E-12
ENSG00000071994_170584581_170582258_188	annotated	PDCD2	6	-	564	17	2	4	4.26%	9.959544378	0.010020958	0.0400283	0.001975105
ENSG00000119787_38377260_38296483_579	annotated	ATL2	2	-	1737	67	2	1	4.32%	12.74514081	6.80E-06	6.82E-06	3.83E-10
ENSG00000168040_70203460_70206473_208	annotated	FADD	11	+	624	79	10	3	8.65%	41.83233968	0.00164523	0.000173219	2.03E-06
ENSG00000174099_65278769_65453697_160	annotated	MSRB3	12	+	480	8	3	0	3.12%	5.507042185	0.150401371	0.019679754	0.014313628
ENSG00000137710_110279692_110200362_590	annotated	RDX	11	-	1770	83	2	3	5.08%	15.49438988	9.15E-07	3.22E-06	2.62E-11

ENSG00000075624_5529657_5527748_375	annotated	ACTB	7	-	1125	5496	336	114	26.93%	1614.224205	1.38E-15	1.48E-16	3.03E-30
ENSG00000169902_66240630_66356842_302	annotated	TPST1	7	+	906	16	0	0	3.31%	5.835276488	0.002008257	0.002008257	2.37E-05
ENSG00000145216_53377839_53428191_378	annotated	FIP1L1	4	+	1134	29	3	0	3.17%	8.449958909	0.003995149	0.000954935	2.35E-05
ENSG00000182809_105474863_105479653_208	annotated	CRIP2	14	+	624	38	5	2	6.73%	20.12188491	0.00098021	0.000742275	4.58E-06
ENSG00000256053_103563002_103587334_177	annotated	APOPT1	14	+	531	42	2	9	6.21%	26.13511546	0.005625935	0.001506153	5.01E-05
ENSG00000167323_3856271_4091326_540	annotated	STIM1	11	+	1620	33	6	7	2.96%	6.730829338	0.004440344	0.003309619	8.15E-05
ENSG00000069849_141902161_141921998_193	dORF	ATP1B3	3	+	579	443	90	2	16.58%	252.8103304	1.01E-05	6.64E-07	6.27E-11
ENSG00000069849_141922019_141925701_71	dORF	ATP1B3	3	+	213	35	4	0	11.27%	54.29478211	0.01208853	0.005756914	0.000361529
ENSG00000069849_141876802_141902161_44	annotated	ATP1B3	3	+	132	63	13	3	20.45%	157.7016626	0.020547428	0.007096745	0.000740516
ENSG00000134248_110406378_110401523_79	annotated	LAMTOR5	1	-	237	73	4	4	20.25%	101.7757163	0.000398525	0.000440825	1.16E-06
ENSG00000172379_80470258_80593698_639	annotated	ARNT2	15	+	1917	11	1	0	0.94%	1.896008264	0.019795882	0.012798403	0.001207991
ENSG00000205213_27385491_27384332_104	novel	LGR4	11	-	312	39	4	1	7.69%	41.30281639	0.01019742	0.005604867	0.000298393
ENSG00000112851_65992719_66078530_610	annotated	ERBIN	5	+	1830	37	1	0	2.46%	6.680674126	0.000427981	0.000279067	8.02E-07
ENSG00000090266_140702960_140704934_41	novel	NDUFB2	7	+	123	264	18	1	43.90%	709.199579	0.000312009	0.000119373	2.63E-07
ENSG00000258289_64914415_64932230_140	annotated	CHURC1	14	+	420	203	123	43	20.71%	159.7042234	0.000232659	5.13E-05	8.87E-08
ENSG00000107372_72360694_72355953_185	annotated	ZFAND5	9	-	555	157	26	3	13.51%	93.47087817	3.75E-05	8.08E-06	2.47E-09
ENSG00000126226_113185526_113178198_232	novel	PCID2	13	-	696	40	4	0	6.90%	18.98980064	0.000754144	0.0001956	1.01E-06
ENSG00000169972_1310918_1311379_67	novel	PUSL1	1	+	201	15	2	0	7.46%	24.65839784	0.030784922	0.020613417	0.002844155
ENSG00000104365_42290241_42319504_437	novel	IKBKB	8	+	1311	11	4	1	1.83%	2.772423983	0.077023474	0.004149608	0.002023916
ENSG00000104365_42320810_42330979_205	novel	IKBKB	8	+	615	12	1	4	3.90%	6.4472689	0.010499663	0.043303664	0.002229801
ENSG00000147419_27765225_27752575_24	annotated	CCDC25	8	-	72	27	1	0	29.17%	123.9084492	0.013991246	0.008574016	0.000599135
ENSG00000083454_3691823_3673868_362	annotated	P2RX5	17	-	1086	14	2	0	1.38%	4.259590641	0.035374934	0.020613417	0.003252526
ENSG00000187109_76067450_76048429_349	annotated	NAP1L1	12	-	1047	191	27	16	11.17%	60.27765372	9.80E-07	4.03E-07	3.62E-12
ENSG00000131143_85801206_85806874_169	annotated	COX4I1	16	+	507	1162	151	28	42.01%	757.2997656	3.41E-12	1.14E-13	5.32E-24
ENSG00000164465_117503811_117532299_131	novel	DCBLD1	6	+	393	35	4	1	9.92%	29.42694297	0.003996827	0.000950595	2.34E-05
ENSG00000198825_119781745_119827780_1036	annotated	INPP5F	10	+	3108	11	0	0	0.58%	1.169449113	0.012798403	0.012798403	0.000797244
ENSG00000150316_94972185_94971328_96	novel	CWC15	11	-	288	15	3	0	6.25%	17.20950683	0.049121095	0.011772051	0.002799454
ENSG00000150316_94966377_94963385_70	novel	CWC15	11	-	210	47	5	1	17.14%	73.95170934	0.002776122	0.00114312	1.91E-05
ENSG00000198382_76016871_76141413_327	annotated	UVRAG	11	+	981	20	2	1	2.45%	6.73644304	0.01299184	0.011825808	0.000750884
ENSG00000138758_76949904_77036794_432	annotated	11-Sep	4	+	1296	48	6	0	5.09%	12.23787152	3.36E-05	1.27E-05	3.40E-09
ENSG00000109743_15707574_15723456_164	annotated	BST1	4	+	492	51	0	4	10.37%	34.25111603	0.000134544	0.000596718	5.65E-07
ENSG00000056097_32444658_32355760_1074	annotated	ZFR	5	-	3222	118	35	8	4.10%	12.10113553	0.000233621	9.01E-08	3.54E-10
ENSG00000075785_128795368_128813422_207	annotated	RAB7A	3	+	621	257	26	10	25.12%	136.7449122	2.46E-09	2.09E-10	5.96E-18
ENSG00000172819_53215305_53211676_300	novel	RARG	12	-	900	19	0	1	2.67%	6.975586768	0.005355751	0.009341698	0.000262679
ENSG00000111319_6374540_6347873_588	annotated	SCNN1A	12	-	1764	70	3	3	3.40%	13.1120052	0.000150216	7.65E-05	8.32E-08
ENSG00000149231_96384747_96353646_544	annotated	CCDC82	11	-	1632	37	0	0	3.49%	7.49119709	4.84E-05	4.84E-05	1.76E-08
ENSG00000095002_47403192_47562111_924	annotated	MSH2	2	+	2772	76	11	9	4.11%	9.059203595	2.85E-06	2.21E-06	5.42E-11
ENSG00000163161_127286733_127271450_140	annotated	ERCC3	2	-	420	5	0	0	3.57%	3.933601561	0.012673659	0.012673659	0.000782701
ENSG00000094914_53321465_53315629_134	novel	AAAS	12	-	402	18	7	1	5.22%	14.79503871	0.102694323	0.013892891	0.007117292
ENSG00000186468_82278323_82275254_134	annotated	RPS23	5	-	402	3766	262	20	19.40%	3095.450876	0.001322047	2.76E-05	3.23E-07
ENSG00000070087_149968535_149966489_91	annotated	PFN2	3	-	273	268	20	8	34.07%	324.3708364	1.09E-06	1.54E-06	1.48E-11
ENSG00000143321_156745364_156743449_208	annotated	HDGF	1	-	624	250	34	12	23.56%	132.3808218	3.96E-09	1.94E-09	8.30E-17
ENSG00000164405_132866882_132867109_75	novel	UQCRQ	5	+	225	139	15	38	18.67%	204.127697	0.002295866	0.018068137	0.000245278
ENSG00000174720_112647303_112657327_332	annotated	LARP7	4	+	996	16	4	2	1.81%	5.307992468	0.125333036	0.071345384	0.032244416
ENSG00000140416_63059567_63069943_155	annotated	TPM1	15	+	465	172	12	18	12.26%	122.2208072	0.000105163	0.000251724	1.88E-07
ENSG00000134480_87412794_87392860_325	annotated	CCNH	5	-	975	28	0	0	4.92%	9.489057304	0.000161701	0.000161701	1.84E-07
ENSG00000184304_29666116_29577238_747	dORF	PRKD1	14	-	2241	13	0	0	0.94%	1.916775058	0.007882058	0.007882058	0.000319636
ENSG00000110395_119206418_119299781_862	annotated	CBL	11	+	2586	34	1	0	1.86%	4.34430242	0.000283388	0.000177448	3.47E-07
ENSG00000259316_64300448_64181892_357	novel	AC087632.1	15	-	1071	8	2	4	1.40%	2.468142156	0.080264768	0.078649604	0.023170289
ENSG00000149428_119056160_119045114_678	annotated	HYOU1	11	-	2034	188	30	7	9.00%	30.54052893	1.68E-08	9.61E-11	2.11E-17
ENSG00000077097_25636130_25598307_1407	annotated	TOP2B	3	-	4221	128	15	16	3.34%	10.01992039	9.70E-08	1.03E-06	9.84E-13
ENSG00000108953_1400110_1345447_255	annotated	YWHAE	17	-	765	720	99	37	20.39%	310.9859116	1.35E-07	7.95E-09	1.15E-14
ENSG00000259316_64381371_64300704_48	annotated	AC087632.1	15	-	144	198	78	3	14.58%	454.3309803	0.024834768	0.014886438	0.001724932
ENSG00000120889_23068894_23022671_411	annotated	TNFRSF10B	8	-	1233	317	27	6	17.03%	84.95048042	2.35E-10	6.40E-13	2.20E-21
ENSG00000082805_1028168_1410415_860	annotated	ERC1	12	+	2580	9	3	2	0.70%	1.152636736	0.110834006	0.053594825	0.022573869
ENSG00000132646_5119681_5115283_222	annotated	PCNA	20	-	666	879	67	31	41.44%	436.0982055	8.09E-15	1.57E-16	1.94E-29
ENSG00000109911_31509785_31783538_476	annotated	ELP4	11	+	1428	27	15	0	2.73%	6.247484832	0.109935855	0.000635664	0.000827344
ENSG00000067992_24465456_24534072_406	annotated	PDK3	X	+	1218	26	1	0	1.48%	7.053354523	0.013428348	0.013640586	0.00088551
ENSG00000072736_68085682_68191807_1045	novel	NFATC3	16	+	3135	26	2	8	0.96%	2.740346351	0.008605408	0.030772499	0.001321679
ENSG00000148153_112694168_112687538_74	novel	INIP	9	-	222	19	4	4	10.81%	28.27940582	0.03195078	0.008219047	0.001321144
ENSG00000066651_125996018_126038836_400	annotated	TRMT11	6	+	1200	12	2	2	2.25%	3.304225311	0.012769128	0.022974703	0.001393205
ENSG00000143797_9003614_8858679_520	annotated	MBOAT2	2	-	1560	26	1	0	2.69%	5.507042185	0.000639977	0.000390775	1.64E-06
ENSG00000185104_50959787_50441440_490	annotated	FAF1	1	-	1470	52	1	0	3.27%	11.68841607	0.000164055	0.000164055	1.89E-07
ENSG00000173486_64242388_64243556_129	novel	FKBP2	11	+	387	224	32	10	24.81%	191.2523178	1.60E-06	8.30E-07	1.19E-11
ENSG00000241837_33907388_33903612_59	annotated	ATP5PO	21	-	177	116	12	15	27.12%	216.5480995	0.000642246	0.003833557	1.58E-05
ENSG00000241837_33903615_33903526_29	Overlap_dORF	ATP5PO	21	-	87	53	9	0	31.03%	201.2918868	0.018991317	0.003789641	0.000396202
ENSG00000133740_85177421_85213862_345	annotated	E2F5	8	+	1035	19	1	0	3.19%	6.065727624	0.003925334	0.001242734	2.92E-05
ENSG00000038358_67873262_67884148_1401	annotated	EDC4	16	+	4203	44	15	7	2.00%	3.459098589	0.00423645	3.38E-05	1.44E-06

ENSG00000155366_112703799_112701540_193	annotated	RHOC	1	-	579	299	26	25	29.53%	170.6327061	3.86E-10	9.22E-11	4.15E-19
ENSG00000064012_201266487_201277805_278	annotated	CASP8	2	+	834	25	11	2	4.68%	9.904752132	0.085783639	0.002773194	0.001706423
ENSG00000064012_201266487_201286594_464	annotated	CASP8	2	+	1392	43	19	7	4.31%	10.20701784	0.036241528	0.00109903	0.000296024
ENSG00000172927_69295355_69296392_313	annotated	MYEOV	11	+	939	26	1	4	3.19%	9.149079669	0.00345766	0.019607004	0.000378813
ENSG00000116670_11680601_11674775_211	annotated	MAD2L2	1	-	633	76	1	0	8.06%	39.67158351	0.000145688	0.000142825	1.47E-07
ENSG00000111615_75511597_75506469_177	novel	KRR1	12	-	531	20	1	2	6.21%	12.44529307	0.002484757	0.006753026	9.46E-05
ENSG00000241978_110136238_110168497_859	annotated	AKAP2	9	+	2577	21	1	0	1.28%	2.692616668	0.002106591	0.001491519	1.87E-05
ENSG00000115525_85863483_85840144_390	annotated	ST3GAL5	2	-	1170	151	23	10	11.79%	42.64427538	2.98E-07	7.02E-08	2.04E-13
ENSG00000111206_2874478_2858638_763	annotated	FOXM1	12	-	2289	43	13	12	2.23%	6.207151087	0.015576154	0.008803764	0.000681415
ENSG00000138760_76179654_76161713_320	Overlap_dORF	SCARB2	4	-	960	301	29	9	20.62%	103.6012311	6.10E-11	3.16E-12	2.46E-21
ENSG00000156113_77027863_76887266_562	annotated	KCNMA1	10	-	1686	31	8	0	3.02%	6.075384617	0.002705267	0.000116339	2.45E-06
ENSG00000100592_59263478_59368859_1078	annotated	DAAM1	14	+	3234	14	0	0	0.65%	1.430400568	0.008473674	0.008473674	0.000366502
ENSG00000013583_12987318_12983732_56	annotated	HEBP1	12	-	168	13	1	0	10.71%	25.56841015	0.012798403	0.012798403	0.000797244
ENSG00000179632_144105684_144107109_286	annotated	MAF1	8	+	858	108	9	6	9.79%	41.59164727	3.50E-06	1.97E-05	5.84E-10
ENSG00000084234_130069978_130120810_116	annotated	APLP2	11	+	348	172	18	14	24.14%	163.3122855	4.60E-06	2.99E-05	1.16E-09
ENSG00000138376_214809569_214728676_758	annotated	BARD1	2	-	2274	17	1	2	0.92%	2.470177233	0.012000727	0.024135905	0.001383386
ENSG00000070423_663121_651889_74	annotated	AC004156.1	19	-	222	21	3	0	9.46%	31.25618538	0.016983117	0.008574016	0.000724265
ENSG00000070423_649726_648128_135	dORF	AC004156.1	19	-	405	27	3	0	9.63%	22.02816874	0.003810897	0.000544026	1.35E-05
ENSG00000197879_1484273_1465726_1028	annotated	MYO1C	17	-	3084	245	36	10	7.39%	26.24952014	2.41E-09	3.02E-13	1.36E-20
ENSG00000175595_13920199_13932302_361	annotated	ERCC4	16	+	1083	13	4	0	1.66%	3.966290771	0.086207551	0.012798403	0.005490656
ENSG00000005100_5461088_5444205_474	novel	DHX33	17	-	1422	22	3	0	2.11%	5.112022282	0.012454647	0.002281203	0.00016436
ENSG00000066777_67258125_67175555_1000	annotated	ARFGEF1	8	-	3000	35	6	2	1.70%	3.85492953	0.004004658	0.000536279	1.41E-05
ENSG00000136699_130155156_130152555_363	annotated	SMPD4	2	-	1089	9	4	0	1.38%	2.73076472	0.169362074	0.019216965	0.016163992
ENSG00000181555_47123126_47103420_1069	annotated	SETD2	3	-	3207	14	1	3	0.84%	1.442443229	0.00308495	0.0280616	0.000506087
ENSG00000140553_90939740_90940620_132	novel	UNC45A	15	+	396	23	0	0	4.55%	19.19120762	0.013640586	0.013640586	0.000898637
ENSG00000143570_153962715_153960098_324	annotated	SLC39A1	1	-	972	265	40	2	18.21%	90.08433204	9.96E-09	2.02E-11	2.87E-18
ENSG00000158769_161021073_160998871_299	annotated	F11R	1	-	897	101	9	3	10.70%	37.2047666	1.80E-05	1.17E-06	1.93E-10
ENSG00000158773_161043275_161039920_310	annotated	USF1	1	-	930	52	12	1	7.10%	18.4752383	0.002095583	1.65E-05	3.51E-07
ENSG00000158773_161043275_161039920_310	annotated	USF1	1	-	930	52	12	1	7.10%	18.4752383	0.002095583	1.65E-05	3.51E-07
ENSG00000113282_157817534_157787646_625	annotated	CLINT1	5	-	1875	98	21	7	5.76%	17.27008429	1.70E-05	6.84E-07	1.11E-10
ENSG00000104231_81721281_81702695_261	annotated	ZFAND1	8	-	783	18	8	4	3.83%	7.595920256	0.032452171	0.019047079	0.002788336
ENSG00000104760_17882152_17868532_234	novel	FGL1	8	-	702	234	52	4	14.53%	110.1408437	6.94E-05	4.81E-07	3.67E-10
ENSG00000188313_146533542_146528539_121	novel	PLSCR1	3	-	363	17	2	2	4.96%	15.47433341	0.037178614	0.037178614	0.005809068
ENSG00000147457_23255229_23260588_278	novel	CHMP7	8	+	834	50	5	20	6.12%	19.80950426	0.000683223	0.038454062	0.000220111
ENSG00000198959_38132918_38130219_288	novel	TGM2	20	-	864	213	20	10	12.50%	81.45833232	1.61E-06	3.49E-07	5.19E-12
ENSG00000198805_20469525_20476601_289	annotated	PNP	14	+	867	282	43	13	15.22%	107.4730724	1.24E-05	8.75E-08	1.23E-11
ENSG00000178149_49018564_49017371_286	novel	DALRD3	3	-	858	21	1	5	2.80%	8.087264748	0.007181907	0.043580564	0.00163802
ENSG00000178149_49016455_49015588_170	novel	DALRD3	3	-	510	14	1	0	4.12%	9.070422423	0.015625009	0.007979402	0.000625379
ENSG00000122068_197750065_197773408_98	annotated	FYTTD1	3	+	294	17	4	0	7.14%	19.10606472	0.023878474	0.008473674	0.001006868
ENSG00000250151_9793122_9803864_47	annotated	ARPC4-TTLL3	3	+	141	89	6	5	23.40%	208.5645764	0.008072852	0.014761051	0.000597653
ENSG00000099899_20114991_20112907_223	novel	TRMT2A	22	-	669	17	2	0	3.59%	8.396387188	0.020715809	0.005454749	0.000593634
ENSG00000180900_143815372_143791163_1630	annotated	SCRIB	8	-	4890	93	26	15	2.27%	6.284109487	0.000314366	2.12E-05	5.41E-08
ENSG00000073282_189737795_189894231_471	annotated	TP63	3	+	1413	11	0	0	1.27%	2.572291467	0.012798403	0.012798403	0.000797244
ENSG00000138796_108033232_108034357_59	novel	HADH	4	+	177	24	3	0	11.86%	44.80305507	0.033503887	0.008776163	0.001467425
ENSG00000102606_111115527_111292332_782	annotated	ARHGEF7	13	+	2346	11	1	11	0.64%	1.549295755	0.020239728	0.361337229	0.04459564
ENSG00000164171_52989469_53080514_942	annotated	ITGA2	5	+	2826	80	12	3	4.35%	9.353787151	2.84E-07	6.14E-09	1.95E-14
ENSG00000164171_53080538_53090599_196	dORF	ITGA2	5	+	588	41	2	5	7.65%	23.03966629	0.000600369	0.00096618	3.69E-06
ENSG00000078674_17935704_17955657_499	annotated	PCM1	8	+	1497	78	3	7	4.21%	17.21640443	0.000111202	0.000484078	3.83E-07
ENSG00000112715_43777480_43778922_98	novel	VEGFA	6	+	294	30	1	0	6.12%	33.71658481	0.017143984	0.013853925	0.001131568
ENSG00000109814_39521512_39500143_494	annotated	UGDH	4	-	1482	1533	130	43	30.16%	341.793347	3.84E-22	2.25E-25	1.78E-45
ENSG00000179029_8176286_8174203_146	annotated	TMEM107	17	-	438	23	2	1	4.11%	17.35095483	0.01300724	0.023878474	0.001470914
ENSG00000105849_19709016_19698316_338	annotated	TWISTNB	7	-	1014	34	4	0	4.14%	11.07925647	0.001957387	0.000428413	5.48E-06
ENSG00000156521_70146535_70139924_549	annotated	TYSND1	10	-	1647	41	0	12	4.01%	8.22545463	1.38E-05	0.008898291	1.74E-06
ENSG00000100401_41281044_41246603_587	annotated	RANGAP1	22	-	1761	103	17	3	5.79%	19.32624685	4.87E-05	8.09E-07	3.95E-10
ENSG00000124228_49236348_49243707_252	novel	DDX27	20	+	756	42	3	0	5.16%	18.35680728	0.002280579	0.000642411	9.26E-06
ENSG00000136270_45109237_45107767_140	novel	TBRG4	7	-	420	28	0	2	7.14%	22.02816874	0.002404282	0.00688184	9.36E-05
ENSG00000148180_121326778_121332603_207	Overlap_dORF	GSN	9	+	621	49	1	2	8.21%	26.07198716	0.000166161	0.000330633	3.80E-07
ENSG00000110921_109574823_109596577_396	annotated	MVK	12	+	1188	231	16	17	12.37%	64.24882549	1.83E-08	5.99E-08	1.11E-14
ENSG00000137106_37422769_37424897_29	novel	GRHPR	9	+	87	39	7	7	24.14%	148.120445	0.020905271	0.028902005	0.002711627
ENSG00000137145_19346079_19372046_791	annotated	DENND4C	9	+	2373	33	1	1	1.90%	4.595003593	0.000388494	0.000388494	1.00E-06
ENSG00000113194_176448408_176506950_445	annotated	FAF2	5	+	1335	155	14	7	8.31%	38.36366466	9.02E-07	5.84E-07	4.78E-12
ENSG00000110435_34916656_34995172_501	annotated	PDHX	11	+	1503	51	5	1	4.59%	11.21194217	9.59E-05	1.10E-05	8.58E-09
ENSG00000189091_70555198_70556379_69	novel	SF3B3	16	+	207	14	3	3	10.14%	22.34741756	0.095107389	0.006937203	0.003836779
ENSG00000163382_156593411_156594084_115	novel	NAXE	1	+	345	88	7	0	12.17%	84.2816891	0.008928169	0.000436224	2.81E-05
ENSG00000143294_156791109_156800460_148	annotated	PRCC	1	+	444	25	1	0	4.05%	18.60487225	0.023878474	0.01300724	0.001470914
ENSG00000139146_31298180_31282712_213	annotated	SINHCAF	12	-	639	18	0	0	2.35%	9.307676933	0.021084099	0.021084099	0.002029891
ENSG00000114796_183650357_183679286_600	annotated	KLHL24	3	+	1800	65	0	5	3.67%	11.93192473	1.82E-05	0.000182998	2.67E-08
ENSG00000103254_721275_722557_235	annotated	FAM173A	16	+	705	119	18	8	9.79%	55.77344851	0.000112855	5.12E-05	4.28E-08

ENSG00000127990_94656098_94587779_482	annotated	SGCE	7	-	1446	217	28	12	11.83%	49.58623046	2.62E-08	9.06E-10	2.72E-16
ENSG00000131149_85648638_85672539_1113	annotated	GSE1	16	+	3339	51	5	2	2.25%	5.046885022	6.07E-05	1.02E-05	5.01E-09
ENSG00000093000_45171615_45184655_440	annotated	NUP50	22	+	1320	12	3	0	1.14%	3.003841192	0.150401371	0.019679754	0.014313628
ENSG00000114062_25375498_25339137_763	annotated	UBE3A	15	-	2289	36	11	1	2.10%	5.196684631	0.001850845	0.000263486	3.34E-06
ENSG00000258643_23307903_23325286_288	annotated	BCL2L2-PABPN1	14	+	864	48	8	1	4.51%	18.35680728	0.005208023	0.000710064	2.38E-05
ENSG00000116337_109625404_109631152_761	novel	AMPD2	1	+	2283	8	6	0	0.79%	1.15785381	0.280084718	0.011570466	0.021802746
ENSG00000110075_68587954_68613117_326	novel	PPP6R3	11	+	978	63	4	8	7.36%	21.28488697	3.74E-05	0.000439267	1.28E-07
ENSG00000137807_69426113_69447807_597	novel	AC027237.1	15	+	1791	91	4	4	6.70%	16.78863782	3.20E-08	4.17E-08	1.34E-14
ENSG00000140199_34252377_34233881_775	annotated	SLC12A6	15	-	2325	7	4	0	0.77%	0.994820524	0.170566842	0.009815329	0.010088762
ENSG00000113716_150000046_150052192_1538	annotated	HMGXB3	5	+	4614	30	14	2	0.98%	2.14839097	0.063385505	0.000591584	0.000372033
ENSG00000213626_30234430_30257621_88	novel	LBH	2	+	264	125	5	0	18.18%	156.4500621	0.000210762	0.000210762	3.07E-07
ENSG00000186480_155298286_155308270_277	annotated	INSIG1	7	+	831	416	46	16	18.77%	165.4100757	1.51E-07	4.68E-09	7.82E-15
ENSG00000138459_112563452_112582736_408	annotated	SLC35A5	3	+	1224	137	6	2	8.82%	36.98356762	1.69E-07	1.18E-07	1.91E-13
ENSG00000138459_112562063_112562236_57	uORF	SLC35A5	3	+	171	21	6	0	8.77%	40.57820558	0.078130071	0.021084099	0.007358915
ENSG00000175416_176416363_176406538_134	annotated	CLTB	5	-	402	36	1	9	6.72%	29.59007741	0.002967235	0.010499663	0.000173435
ENSG00000150093_32935558_32933573_25	uORF	ITGB1	10	-	75	556	122	16	40.00%	2449.532364	0.012930838	0.003703923	0.000260989
ENSG00000205707_25203963_25204312_88	annotated	ETFRF1	12	+	264	14	0	0	7.95%	17.52240695	0.007029303	0.007029303	0.000257364
ENSG00000127990_94656098_94623369_45	annotated	SGCE	7	-	135	21	6	4	17.78%	51.3990604	0.044816497	0.037011271	0.00688081
ENSG00000127990_94618900_94585499_264	dORF	SGCE	7	-	792	131	8	5	15.15%	54.65322169	4.36E-07	4.89E-08	2.13E-13
ENSG00000147155_48523772_48528457_230	annotated	EBP	X	+	690	501	37	33	25.65%	239.91549	5.37E-10	2.06E-09	1.24E-17
ENSG00000144824_111911674_111974563_730	annotated	PHLDB2	3	+	2190	21	1	4	1.51%	3.16843523	0.002336818	0.014065161	0.000190703
ENSG00000206503_29943288_29945513_262	novel	HLA-A	6	+	786	155	11	10	7.25%	65.15965944	0.000314949	0.000597751	1.25E-06
ENSG00000206503_29942563_29943034_113	novel	HLA-A	6	+	339	16	3	0	8.85%	15.59516371	0.020027431	0.002008257	0.000245088
ENSG00000186591_129952555_129834937_152	annotated	UBE2H	7	-	456	74	0	3	11.18%	53.62120022	0.000138364	0.000273634	2.65E-07
ENSG00000135506_57694162_57715841_298	novel	OS9	12	+	894	222	33	44	14.77%	82.05123256	2.20E-07	3.95E-05	9.90E-11
ENSG00000100316_39318439_39316844_128	annotated	RPL3	22	-	384	501	47	11	17.19%	431.0981461	1.93E-05	2.12E-05	3.22E-09
ENSG00000169241_155135672_155138281_156	annotated	SLC50A1	1	+	468	48	11	8	8.97%	33.88949037	0.015721867	0.007445065	0.00059116
ENSG00000163681_57757652_57897015_506	annotated	SLMAP	3	+	1518	9	2	3	0.99%	1.959026864	0.083503598	0.097833871	0.029236747
ENSG00000168297_58332998_58420555_512	annotated	PXK	3	+	1536	17	0	0	1.56%	3.657020201	0.005454749	0.005454749	0.000159109
ENSG00000168297_58382562_58420555_432	annotated	PXK	3	+	1296	16	0	0	1.62%	4.079290508	0.008473674	0.008473674	0.000366502
ENSG00000168291_58433809_58428034_341	annotated	PDHB	3	-	1023	123	14	2	9.97%	39.72822222	2.27E-05	2.59E-07	6.25E-11
ENSG00000197956_153535339_153535142_65	novel	S100A6	1	-	195	1446	198	58	21.54%	2450.210154	0.009391052	0.00113916	6.69E-05
ENSG00000136717_127050810_127048526_72	novel	BIN1	2	-	216	12	2	0	6.94%	18.35680728	0.051235217	0.020613417	0.004687384
ENSG00000105819_103297460_103308968_202	annotated	PMPCB	7	+	606	98	15	1	10.89%	53.43466675	0.00117585	2.78E-05	2.85E-07
ENSG00000089327_35155551_35169615_178	annotated	FXYD5	19	+	534	549	55	17	27.53%	339.7040629	7.54E-09	5.26E-10	4.51E-17
ENSG00000099949_20989635_20997348_639	dORF	LZTR1	22	+	1917	17	8	3	1.56%	2.93019459	0.138719553	0.018949244	0.012679138
ENSG00000143476_212044668_212102940_668	annotated	DTL	1	+	2004	73	9	5	4.34%	12.03634969	6.18E-05	1.15E-05	5.71E-09
ENSG00000143493_212035437_211941824_942	annotated	INTS7	1	-	2826	35	17	0	1.91%	4.092281879	0.039131696	7.13E-05	4.16E-05
ENSG00000143493_212035437_211941824_948	annotated	INTS7	1	-	2844	27	17	0	1.48%	3.136922764	0.128619445	0.00040306	0.00076114
ENSG00000143493_212035437_211941824_962	annotated	INTS7	1	-	2886	35	17	0	1.87%	4.007203253	0.039131696	7.13E-05	4.16E-05
ENSG00000123684_211829296_211749899_370	annotated	LPGAT1	1	-	1110	189	34	6	12.70%	56.26113368	2.27E-07	4.88E-09	1.24E-14
ENSG00000123684_211829296_211749899_370	annotated	LPGAT1	1	-	1110	189	34	6	12.70%	56.26113368	2.27E-07	4.88E-09	1.24E-14
ENSG00000117650_211675479_211667062_384	annotated	NEK2	1	-	1152	107	19	5	10.42%	30.69028718	2.94E-06	9.12E-08	2.74E-12
ENSG00000167985_61430147_61438169_141	annotated	SDHAF2	11	+	423	40	2	0	8.51%	31.24562942	0.004244579	0.000881454	2.32E-05
ENSG00000111652_6724657_6731039_275	annotated	COPS7A	12	+	825	89	17	7	11.64%	35.64558214	4.59E-05	6.26E-06	2.39E-09
ENSG00000178999_8207605_8204871_287	annotated	AURKB	17	-	861	59	17	5	9.76%	22.64219435	0.000991292	1.51E-05	1.40E-07
ENSG00000006715_38909174_38796467_209	annotated	VPS41	7	-	627	18	2	0	2.87%	9.48581429	0.044040756	0.013428348	0.002791708
ENSG00000131374_17508570_17160963_795	annotated	TBC1D5	3	-	2385	52	25	4	2.89%	7.204180972	0.009115679	8.47E-05	7.50E-06
ENSG00000131381_15096120_15073782_784	annotated	RBSN	3	-	2352	7	3	0	0.64%	0.98340039	0.170087137	0.019216965	0.016246064
ENSG00000141480_4710722_4721039_406	annotated	ARRB2	17	+	1218	35	10	10	3.94%	9.494900319	0.013497182	0.01002772	0.000668125
ENSG00000113558_134174494_134173744_50	annotated	SKP1	5	-	150	95	12	3	30.00%	209.267603	0.000366528	0.000526999	1.27E-06
ENSG00000083857_186709827_186588592_4590	annotated	FAT1	4	-	13770	431	54	24	3.70%	10.34220123	5.67E-23	2.97E-26	3.52E-47
ENSG00000122786_134891621_134968345_557	annotated	CALD1	7	+	1671	101	13	6	5.21%	19.97167902	1.07E-05	5.96E-06	5.24E-10
ENSG00000168411_74661434_74623928_769	annotated	RFWD3	16	-	2307	71	11	3	4.29%	10.16905059	5.50E-05	1.18E-06	6.31E-10
ENSG00000197766_859690_863238_260	annotated	CFD	19	+	780	252	19	3	18.85%	106.7518947	3.42E-09	5.60E-10	2.15E-17
ENSG00000178467_49002536_49004264_75	annotated	P4HTM	3	+	225	16	0	0	9.33%	23.49671332	0.008373824	0.008373824	0.000358384
ENSG00000129055_134482904_134478590_74	annotated	ANAPC13	3	-	222	93	7	4	29.73%	138.4202495	0.000124939	4.91E-05	4.55E-08
ENSG00000115525_85888905_85861154_114	novel	ST3GAL5	2	-	342	27	10	8	7.89%	26.0859893	0.096103384	0.055297605	0.020166199
ENSG00000153046_4892085_4954056_412	annotated	CDYL	6	+	1236	12	7	0	1.46%	3.207985739	0.194271892	0.012798403	0.014328705
ENSG00000148180_121301972_121332603_731	annotated	GSN	9	+	2193	69	7	5	3.69%	10.39633135	7.24E-05	1.83E-05	1.03E-08
ENSG00000109685_1900838_1918668_423	novel	NSD2	4	+	1269	11	1	0	1.18%	2.864182697	0.035374934	0.020613417	0.003252526
ENSG00000117602_24514388_24535277_236	annotated	RCAN3	1	+	708	15	7	4	3.81%	7.000477354	0.086894686	0.031072489	0.011283286
ENSG00000164111_121696589_121668468_320	annotated	ANXA5	4	-	960	909	81	21	31.87%	312.8688342	1.61E-16	1.30E-17	3.21E-32
ENSG00000132522_7314562_7314069_92	novel	GPS2	17	-	276	12	9	0	6.52%	14.36619701	0.258817059	0.009815329	0.017540961
ENSG00000132522_7314562_7313882_124	novel	GPS2	17	-	372	5	5	0	4.03%	4.441163053	0.430378112	0.012673659	0.044081029
ENSG00000132522_7313109_7312756_54	novel	GPS2	17	-	162	11	0	0	11.11%	22.43609779	0.009815329	0.009815329	0.000483714
ENSG00000118961_20801463_20686903_283	annotated	LDAH	2	-	849	22	3	0	4.95%	8.56218573	0.001301419	0.000368141	3.15E-06
ENSG00000141580_82644006_82619086_180	annotated	WDR45B	17	-	540	21	0	2	5.56%	12.8497651	0.002321823	0.002254349	3.04E-05

ENSG00000122390_3476228_3484855_242	annotated	NAA60	16	+	726	20	5	0	3.72%	9.102549067	0.033424799	0.003529166	0.000684983
ENSG00000164338_73570609_73580094_328	annotated	UTP15	5	+	984	53	4	0	5.18%	17.79714853	0.000563971	0.000131066	5.21E-07
ENSG00000214087_81683060_81681708_111	annotated	ARL16	17	-	333	13	10	1	5.41%	12.89937809	0.33664499	0.020342454	0.040457907
ENSG00000174547_66438754_66436837_181	annotated	MRPL11	11	-	543	75	15	4	12.15%	45.63847115	0.001621467	5.84E-05	7.70E-07
ENSG00000115289_74506834_74505143_176	annotated	PCGF1	2	-	528	18	0	5	3.41%	11.26440447	0.012798403	0.086207551	0.005490656
ENSG00000185686_22556832_22548067_509	annotated	PRAME	22	-	1527	140	14	2	6.68%	30.29414169	6.97E-06	1.77E-07	1.25E-11
ENSG00000175387_47896756_47841827_467	annotated	SMAD2	18	-	1401	151	18	5	11.13%	35.61299229	1.08E-07	1.22E-09	1.57E-15
ENSG00000167114_128343133_128360491_643	annotated	SLC27A4	9	+	1929	66	5	13	4.20%	11.305281	3.68E-05	0.000221183	6.20E-08
ENSG00000167114_128340763_128345319_135	Overlap_uORF	SLC27A4	9	+	405	17	19	2	6.67%	13.86958773	0.429928525	0.012941946	0.044542694
ENSG00000142856_63454913_63446807_74	novel	ITGB3BP	1	-	222	23	3	0	6.76%	34.23296494	0.064489021	0.020613417	0.005920197
ENSG00000206560_15859403_15670270_1053	annotated	ANKRD28	3	-	3159	53	10	7	2.18%	5.543651203	0.000206076	0.000318626	4.49E-07
ENSG00000163050_226984602_226986737_163	novel	COQ8A	1	+	489	17	1	4	4.29%	11.48708186	0.011825808	0.032488891	0.001836837
ENSG00000106772_76905963_76788394_298	annotated	PRUNE2	9	-	894	7	0	0	1.68%	2.587201027	0.019216965	0.019216965	0.001707396
ENSG00000155636_178112547_178126176_476	annotated	RBM45	2	+	1428	11	2	0	1.05%	2.545271598	0.08410207	0.019679754	0.007521961
ENSG00000277258_38740402_38735223_344	annotated	PCGF2	17	-	1032	42	13	3	5.23%	13.44742859	0.010096393	0.000145442	1.31E-05
ENSG00000141012_88832048_88814439_205	dORF	GALNS	16	-	615	49	12	0	8.29%	26.32634801	0.005978704	0.000128696	6.46E-06
ENSG00000135404_55728341_55725561_238	annotated	CD63	12	-	714	1521	140	43	45.38%	703.8832911	5.12E-17	4.74E-19	4.04E-34
ENSG00000107164_130623646_130631184_130	novel	FUBP3	9	+	390	13	0	1	4.62%	11.01408437	0.013428348	0.020342454	0.001295518
ENSG00000134684_32781140_32775981_179	novel	YARS	1	-	537	33	4	0	8.38%	20.30529521	0.00096618	0.000279067	1.80E-06
ENSG00000164091_52261463_52257490_199	novel	WDR82	3	-	597	67	2	2	7.04%	37.08259562	0.000948092	0.000872085	5.17E-06
ENSG00000072310_17817860_17817645_71	novel	SREBF1	17	-	213	24	5	0	12.68%	37.23070773	0.044380821	0.003632191	0.000962121
ENSG00000261582_35279705_35272097_62	annotated	AL121753.1	20	-	186	103	21	7	12.90%	182.9759178	0.045876598	0.007225456	0.001739968
ENSG00000042429_93797565_93812064_260	novel	MED17	11	+	780	11	1	0	1.92%	4.659804926	0.035772986	0.021084099	0.003354605
ENSG00000042429_93784619_93793891_231	novel	MED17	11	+	693	23	2	2	3.90%	10.96640435	0.004638749	0.006592935	0.000163848
ENSG00000156113_77637360_76877869_1034	annotated	KCNMA1	10	-	3102	66	11	0	3.09%	7.030266619	3.24E-05	2.89E-07	1.01E-10
ENSG00000188690_125816499_125788868_265	annotated	UROS	10	-	795	58	0	3	6.79%	24.10629787	9.07E-05	0.000200711	1.30E-07
ENSG00000128513_124897173_124825314_580	annotated	POT1	7	-	1740	30	3	3	2.07%	5.696940192	0.002631121	0.005604867	8.28E-05
ENSG00000148429_11597634_11462441_828	annotated	USP6NL	10	-	2484	12	0	1	0.72%	1.596244112	0.012178958	0.023056749	0.001340064
ENSG00000132600_68321442_68357224_655	annotated	PRMT7	16	+	1965	26	1	7	1.83%	4.372002956	0.000982952	0.010921516	6.94E-05
ENSG00000105053_50016162_49979094_424	annotated	VRK3	19	-	1272	45	2	2	3.77%	11.68947634	0.000638407	0.000638407	2.61E-06
ENSG00000154059_24426757_24450847_320	annotated	IMPACT	18	+	960	35	4	3	4.69%	12.04665478	0.002477401	0.001998878	2.88E-05
ENSG00000138685_122827175_122892396_155	annotated	FGF2	4	+	465	65	16	6	11.61%	46.18809575	0.001507119	0.000992855	9.17E-06
ENSG00000162063_2429482_2435935_135	novel	CCNF	16	+	405	16	5	0	5.19%	13.05372962	0.067234256	0.008473674	0.00300901
ENSG00000110911_51010704_50981726_560	annotated	SLC11A2	12	-	1680	153	13	5	9.11%	30.09205194	8.36E-09	2.24E-09	2.02E-16
ENSG00000197912_89544650_89557093_353	dORF	SPG7	16	+	1059	19	15	2	2.83%	5.928260709	0.299302749	0.009621867	0.021310549
ENSG00000197912_89508547_89541011_413	annotated	SPG7	16	+	1239	66	5	2	5.08%	17.6012002	0.000142442	5.22E-05	5.49E-08
ENSG00000063244_55655105_55674068_471	annotated	U2AF2	19	+	1413	455	51	28	16.77%	106.3993288	7.52E-12	6.06E-14	6.56E-24
ENSG00000169139_48008455_48060828_145	annotated	UBE2V2	8	+	435	119	58	14	21.38%	90.39145104	0.002770985	9.48E-06	3.09E-07
ENSG00000157193_53271061_53247018_557	annotated	LRP8	1	-	1671	53	18	6	4.85%	10.480188	0.009009024	4.38E-05	4.37E-06
ENSG00000086061_33036599_33037136_70	novel	DNAJA1	9	+	210	95	9	5	15.71%	149.4768593	0.003816364	0.002025913	4.45E-05
ENSG00000106263_2378671_2378802_43	novel	EIF3B	7	+	129	44	8	1	16.28%	112.7022587	0.060322713	0.008776163	0.002744782
ENSG00000106263_2379188_2379497_52	novel	EIF3B	7	+	156	40	7	1	21.15%	84.72372593	0.009282808	0.001603369	8.89E-05
ENSG00000078747_34412633_34507794_793	annotated	ITCH	20	+	2379	55	14	0	2.90%	7.639024469	0.001650653	1.04E-05	1.81E-07
ENSG00000116918_231529344_231542637_95	novel	TSNAX	1	+	285	56	9	0	6.32%	64.92512892	0.061512597	0.013640586	0.004007738
ENSG00000100711_103715842_103729208_183	novel	ZFYVE21	14	+	549	31	0	0	6.01%	18.65773855	0.001565547	0.001565547	1.47E-05
ENSG00000213930_34647878_34650449_238	novel	GALT	9	+	714	10	0	0	2.10%	4.627766542	0.020613417	0.020613417	0.001946209
ENSG00000110619_3042204_3028992_251	novel	CARS	11	-	753	12	2	0	3.19%	5.265697707	0.033181033	0.00419316	0.000782812
ENSG00000140497_74851374_74845083_179	novel	SCAMP2	15	-	537	189	9	3	16.20%	116.2939635	1.41E-05	2.31E-06	2.81E-10
ENSG00000134897_102805131_102839865_534	annotated	BIVM	13	+	1602	17	1	0	1.50%	3.506356448	0.00912793	0.005160369	0.000248192
ENSG00000178802_74890571_74897531_267	annotated	MPI	15	+	801	22	0	0	3.00%	9.075275511	0.005604867	0.005604867	0.000167525
ENSG00000124733_43013378_43012470_172	annotated	MEA1	6	-	516	158	12	13	19.19%	101.1758913	5.55E-07	3.27E-05	1.84E-10
ENSG00000149925_30067255_30070212_364	annotated	ALDOA	16	+	1092	2409	280	109	40.66%	728.9266277	1.39E-23	3.79E-25	9.85E-47
ENSG00000136104_50927433_50956474_282	annotated	RNASEH2B	13	+	846	58	2	0	6.03%	22.65308133	0.000174338	0.000133467	1.64E-07
ENSG00000100528_54441327_54427814_121	annotated	CNIH1	14	-	363	235	26	8	26.45%	213.9099031	1.58E-06	6.73E-07	9.54E-12
ENSG00000164180_88206473_88205810_59	dORF	TMEM161B	5	-	177	23	1	0	10.17%	42.93626111	0.013640586	0.013428348	0.00088551
ENSG00000099381_30958732_30984023_1707	annotated	SETD1A	16	+	5121	33	8	7	1.00%	2.129260599	0.006378369	0.007856095	0.00026112
ENSG00000080603_30704064_30739733_3212	annotated	SRCAP	16	+	9636	44	19	0	0.75%	1.508778681	0.015157785	5.55E-06	1.75E-06
ENSG00000171307_97452908_97456891_163	annotated	ZDHHC16	10	+	489	37	11	3	8.59%	25.00129581	0.009291403	0.001376174	7.79E-05
ENSG00000087245_55479492_55505442_656	annotated	MMP2	16	+	1968	163	11	3	8.23%	27.3673133	2.64E-09	1.85E-10	5.69E-18
ENSG00000139546_53502025_53506148_345	annotated	TARBP2	12	+	1035	32	5	7	4.06%	10.21596231	0.001090807	0.009334498	6.41E-05
ENSG00000131408_50377606_50382602_363	annotated	NR1H2	19	+	1089	47	1	2	4.68%	14.2606602	0.00017957	0.000262933	3.26E-07
ENSG00000278311_36554826_36585458_424	novel	GGNBP2	17	+	1272	32	6	0	4.01%	8.312516506	0.002925324	0.000117804	2.69E-06
ENSG00000109332_102809680_102797415_110	annotated	UBE2D3	4	-	330	42	2	0	8.18%	42.05377669	0.003710481	0.003710481	7.64E-05
ENSG00000169855_78714439_78598913_1311	annotated	ROBO1	3	-	3933	12	2	1	0.46%	1.008154176	0.050679946	0.019795882	0.004478852
ENSG00000174720_112647303_112657327_332	annotated	LARP7	4	+	996	16	4	2	1.81%	5.307992468	0.125333036	0.071345384	0.032244416
ENSG00000101246_63707091_63700443_201	annotated	ARFRP1	20	-	603	50	8	3	6.47%	27.39821983	0.004020061	0.002081625	4.80E-05
ENSG00000102974_67628469_67637872_188	novel	CTCF	16	+	564	33	1	2	6.91%	19.3332332	0.00105275	0.001981405	1.27E-05
ENSG00000215301_41343246_41347719_471	annotated	DDX3X	X	+	1413	189	30	7	8.70%	44.19664429	3.70E-07	2.15E-08	8.26E-14

ENSG00000197912_89526341_89548160_359	novel	SPG7	16	+	1077	33	11	0	4.18%	10.12436725	0.012248613	0.000281287	2.80E-05
ENSG00000168036_41224552_41239342_768	annotated	CTNNB1	3	+	2304	288	46	13	11.46%	41.30281639	2.21E-12	5.94E-15	2.04E-25
ENSG00000115525_85848138_85840144_290	novel	ST3GAL5	2	-	870	128	20	5	13.45%	48.61388964	1.48E-06	1.67E-07	2.36E-12
ENSG00000128989_52568892_52551934_112	annotated	ARPP19	15	-	336	43	5	5	12.50%	42.28621678	0.002873497	0.002329389	3.84E-05
ENSG00000054116_36140070_36139675_48	novel	TRAPPC3	1	-	144	26	5	0	12.50%	59.65962367	0.157414378	0.011772051	0.010389467
ENSG00000156502_69207892_69209035_161	novel	SUPV3L1	10	+	483	11	0	0	4.35%	7.525150812	0.007593099	0.007593099	0.000297833
ENSG00000108433_46923193_46940613_213	annotated	GOSR2	17	+	639	40	7	0	6.57%	20.68372652	0.011149146	0.000448121	3.68E-05
ENSG00000197122_37384154_37403379_536	annotated	SRC	20	+	1608	273	35	9	11.01%	56.0978551	4.54E-09	1.22E-10	6.71E-18
ENSG00000164938_94940920_94930479_233	annotated	TP53INP1	8	-	699	12	0	0	2.15%	5.672489805	0.021084099	0.021084099	0.002029891
ENSG00000241370_30345172_30346773_144	annotated	RPP21	6	+	432	72	12	1	7.64%	55.07042185	0.024040328	0.00236973	0.000343473
ENSG00000107290_132300630_132264239_857	annotated	SETX	9	-	2571	10	0	3	0.82%	1.285190708	0.007029303	0.065565251	0.002523831
ENSG00000170889_54201185_54206565_139	annotated	RPS9	19	+	417	549	50	14	12.95%	435.0167136	0.000414357	0.000144574	4.17E-07
ENSG00000150093_32935558_32933573_25	uORF	ITGB1	10	-	75	556	122	16	40.00%	2449.532364	0.012930838	0.003703923	0.000260989
ENSG00000105404_41958772_41956846_75	Overlap_dORF	RABAC1	19	-	225	169	19	14	38.67%	248.1840345	3.05E-05	1.06E-05	2.58E-09
ENSG00000133114_44989655_45001746_43	novel	GPALPP1	13	+	129	12	0	0	11.63%	30.73697964	0.020613417	0.020613417	0.001946209
ENSG00000104442_65605472_65604394_105	annotated	ARMC1	8	-	315	36	3	1	12.38%	37.76257498	0.001029525	0.001029525	6.56E-06
ENSG00000104442_65627398_65605505_102	Overlap_uORF	ARMC1	8	-	306	10	0	0	4.90%	10.79812193	0.019679754	0.019679754	0.001784964
ENSG00000144455_4467245_4362144_242	annotated	SUMF1	3	-	726	48	6	0	7.44%	21.84611776	0.000276738	8.81E-05	1.75E-07
ENSG00000135404_55728341_55725561_238	annotated	CD63	12	-	714	1521	140	43	45.38%	703.8832911	5.12E-17	4.74E-19	4.04E-34
ENSG00000005020_26864429_26670100_359	annotated	SKAP2	7	-	1077	41	7	1	3.90%	12.57875931	0.005505676	0.000560067	2.05E-05
ENSG00000104825_38900033_38907707_338	annotated	NFKBIB	19	+	1014	43	5	1	5.03%	14.01200083	0.000611847	0.000142864	6.12E-07
ENSG00000124222_58670514_58676291_139	novel	STX16	20	+	417	13	0	1	5.04%	10.30094222	0.008175611	0.0135992	0.00055803
ENSG00000092199_21234154_21211223_292	annotated	HNRNPC	14	-	876	120	7	7	6.51%	45.26336043	0.000388917	0.000402141	1.04E-06
ENSG00000085978_233251828_233294350_444	annotated	ATG16L1	2	+	1332	54	6	9	4.28%	13.39550802	0.001050447	0.002327441	1.48E-05
ENSG00000119650_76082636_76083577_79	novel	IFT43	14	+	237	22	7	0	11.39%	30.67213369	0.019504171	0.002943991	0.000328552
ENSG00000119650_75985787_76082371_123	novel	IFT43	14	+	369	63	6	3	13.82%	56.41360287	0.001317961	0.000276044	2.45E-06
ENSG00000100823_20455646_20455777_43	novel	APEX1	14	+	129	13	2	13	11.63%	33.29839461	0.020613417	0.263544628	0.029339597
ENSG00000120705_138542918_138542829_29	uORF	ETF1	5	-	87	19	4	2	20.69%	72.16124243	0.029614852	0.013428348	0.00187062
ENSG00000168887_85611924_85608945_146	annotated	C2orf68	2	-	438	13	5	1	5.48%	9.807061426	0.08489385	0.010020958	0.004458787
ENSG00000149474_18150836_18187459_651	annotated	KAT14	20	+	1953	17	0	0	1.23%	2.876181786	0.005306578	0.005306578	0.000151001
ENSG00000128944_40383253_40393597_238	annotated	KNSTRN	15	+	714	83	10	1	10.92%	38.4104623	0.000121641	4.56E-06	4.96E-09
ENSG00000073417_85064400_85137903_757	annotated	PDE8A	15	+	2271	35	10	2	2.38%	5.092377186	0.005989556	0.000309814	1.35E-05
ENSG00000064042_41360841_41598941_62	novel	LIMCH1	4	+	186	13	2	0	8.06%	23.09404787	0.039124093	0.019679754	0.003445121
ENSG00000124067_67957957_67944840_942	annotated	SLC12A4	16	-	2826	70	18	11	3.29%	8.184563757	0.000692127	9.76E-05	4.91E-07
ENSG00000242110_33989443_33989093_116	novel	AMACR	5	-	348	5	0	0	4.31%	4.74745016	0.012673659	0.012673659	0.000782701
ENSG00000113851_3179687_3150865_442	annotated	CRBN	3	-	1326	74	3	1	5.20%	18.43986976	1.13E-05	1.51E-05	1.38E-09
ENSG00000170860_14178861_14198116_102	annotated	LSM3	3	+	306	151	13	2	15.69%	163.0516412	0.000215037	0.000215037	3.19E-07
ENSG00000166347_74291875_74256845_98	annotated	CYB5A	18	-	294	168	24	18	14.29%	188.8128749	0.032982218	0.012138338	0.001907616
ENSG00000166598_103938307_103941559_139	novel	HSP90B1	12	+	417	314	173	20	25.18%	248.8073736	2.89E-05	1.43E-07	4.82E-11
ENSG00000135956_96265381_96253808_238	annotated	TMEM127	2	-	714	79	5	4	11.76%	36.55935568	1.81E-05	1.11E-05	1.61E-09
ENSG00000245937_128083022_128082768_84	novel	LINC01184	5	-	252	19	4	1	7.14%	24.91280989	0.060590694	0.020342454	0.005492863
ENSG00000163626_73069649_73065357_129	annotated	COX18	4	-	387	10	0	0	4.65%	8.5380499	0.011772051	0.011772051	0.000681273
ENSG00000163626_73065298_73058114_150	dORF	COX18	4	-	450	19	1	0	4.67%	13.95117354	0.008473674	0.008373824	0.000362422
ENSG00000157326_23953789_23968807_188	annotated	DHRS4	14	+	564	31	3	0	3.72%	18.1615221	0.010275151	0.008776163	0.0004548
ENSG00000160710_154601756_154601037_239	novel	ADAR	1	-	717	38	6	0	7.11%	17.51193331	0.00285968	0.000127134	2.80E-06
ENSG00000124787_5002188_4995078_280	annotated	RPP40	6	-	840	37	5	0	3.57%	14.55432578	0.026286608	0.00224099	0.000362009
ENSG00000131467_42834817_42841578_193	annotated	PSME3	17	+	579	171	24	0	21.24%	97.58592888	1.55E-07	9.90E-09	1.62E-14
ENSG00000141458_23533427_23522890_29	annotated	NPC1	18	-	87	8	1	0	17.24%	30.38368102	0.016947427	0.016947427	0.001349898
ENSG00000196365_5720143_5719805_112	novel	LONP1	19	-	336	8	1	3	4.46%	7.867203122	0.019216965	0.079862169	0.006984847
ENSG00000196365_5719790_5692032_829	annotated	LONP1	19	-	2487	70	17	1	4.10%	9.300191869	9.72E-05	1.56E-07	2.02E-10
ENSG00000151491_15682912_15621317_826	annotated	EPS8	12	-	2478	78	10	0	3.03%	10.40070921	0.000661686	4.90E-06	3.39E-08
ENSG00000257923_101816052_102249042_1509	annotated	CUX1	7	+	4527	56	11	1	1.79%	4.087400429	0.000342185	2.11E-06	7.84E-09
ENSG00000196526_7872078_7763765_730	annotated	AFAP1	4	-	2190	39	11	3	3.01%	5.884236855	0.002099703	0.000112305	1.80E-06
ENSG00000159884_35658380_35660823_155	annotated	CCDC107	9	+	465	12	2	0	3.23%	8.527033061	0.080264768	0.019679754	0.007158564
ENSG00000185024_105256547_105210551_530	annotated	BRF1	14	-	1590	26	0	1	2.64%	5.403135729	0.000415605	0.000616482	1.68E-06
ENSG00000104529_143589937_143580019_583	annotated	EEF1D	8	-	1749	392	49	7	7.72%	74.05696524	1.67E-08	6.40E-09	1.12E-15
ENSG00000169740_43646185_43646060_41	novel	ZNF32	10	-	123	10	4	2	12.20%	26.86362042	0.152627529	0.065565251	0.036531536
ENSG00000221988_32154595_32162950_302	annotated	PPT2	6	+	906	32	14	0	4.64%	11.67055298	0.042702904	0.000428413	0.000176022
ENSG00000077549_19485438_19342762_277	annotated	CAPZB	1	-	831	744	34	23	21.30%	295.8295585	1.16E-10	2.18E-10	2.94E-19
ENSG00000184470_19880687_19877111_150	annotated	TXNRD2	22	-	450	20	1	0	6.00%	14.68544583	0.005034873	0.003606289	9.98E-05
ENSG00000077549_19419717_19339530_260	annotated	CAPZB	1	-	780	767	40	24	25.38%	324.9154889	8.17E-11	3.78E-11	3.66E-20
ENSG00000124275_7870795_7872241_46	annotated	MTRR	5	+	138	16	5	0	10.87%	38.30985868	0.102951605	0.021084099	0.009872288
ENSG00000138640_88768056_88728533_536	annotated	FAM13A	4	-	1608	12	1	0	0.93%	2.465839784	0.028889786	0.021084099	0.002731533
ENSG00000125877_3209552_3223462_194	annotated	ITPA	20	+	582	144	15	5	20.62%	81.75402832	5.11E-07	8.55E-08	4.24E-13
ENSG00000140479_101312183_101305258_71	novel	PCSK6	15	-	213	10	0	2	7.04%	15.51279489	0.019679754	0.065565251	0.005794489
ENSG00000134138_37098019_36894754_330	annotated	MEIS2	15	-	990	7	0	2	1.52%	2.336320927	0.019216965	0.119134635	0.010787784
ENSG00000177082_84654274_84643470_378	annotated	WDR73	15	-	1134	15	13	1	2.38%	4.370668401	0.159811165	0.006083074	0.006630957
ENSG00000198729_150214753_150248820_60	annotated	PPP1R14C	6	+	180	15	0	0	8.33%	27.53521093	0.021084099	0.021084099	0.002029891

ENSG00000198887_70259079_70352331_1101	annotated	SMC5	9	+	3303	13	2	1	1.00%	1.30048226	0.006209665	0.002254349	7.97E-05
ENSG00000160752_155310161_155318336_144	novel	FDPS	1	+	432	274	26	20	20.83%	209.5735498	5.39E-06	8.80E-06	3.93E-10
ENSG00000154743_12489930_12532721_422	annotated	TSEN2	3	+	1266	24	2	0	2.37%	6.263934239	0.008661718	0.002335453	0.000115215
ENSG00000204130_68341286_68341203_27	novel	RUFY2	10	-	81	19	5	6	22.22%	77.50651964	0.028915639	0.157414378	0.020079681
ENSG00000036257_224506992_224505923_114	novel	CUL3	2	-	342	12	0	1	7.02%	11.59377302	0.00419316	0.010499663	0.000236136
ENSG00000150093_32935558_32926080_128	novel	ITGB1	10	-	384	1104	248	120	35.16%	949.964777	2.31E-07	4.30E-06	9.65E-12
ENSG00000129197_5421311_5426246_75	annotated	RPAIN	17	+	225	67	17	0	16.00%	98.39248704	0.009810084	0.001065834	6.63E-05
ENSG00000254505_24213439_24210760_123	annotated	CHMP4A	14	-	369	45	4	3	13.82%	40.29543062	0.001073184	0.000672326	4.55E-06
ENSG00000254505_24210743_24209877_94	dORF	CHMP4A	14	-	282	49	4	2	15.96%	57.41384406	0.000850936	0.000503793	2.76E-06
ENSG00000115525_85848138_85847466_124	dORF	ST3GAL5	2	-	372	77	16	4	14.52%	68.39391101	0.001121188	0.000525374	3.77E-06
ENSG00000115525_85846502_85840144_177	dORF	ST3GAL5	2	-	531	43	4	1	9.60%	26.75738011	0.00076033	0.000186181	9.75E-07
ENSG00000115525_85888905_85848361_112	annotated	ST3GAL5	2	-	336	27	10	8	8.04%	26.55181054	0.096103384	0.055297605	0.020166199
ENSG00000144677_37971452_37984266_150	annotated	CTDSPL	3	+	450	28	5	1	5.33%	20.55962416	0.041815588	0.011825808	0.002376669
ENSG00000100162_41947076_41939056_180	annotated	CENPM	22	-	540	89	7	12	12.22%	54.45852828	9.91E-05	0.000974653	7.11E-07
ENSG00000049245_7771384_7779645_100	annotated	VAMP3	1	+	300	189	10	3	23.00%	208.1661946	1.26E-05	1.47E-05	1.49E-09
ENSG00000111669_6867567_6870383_249	annotated	TPI1	12	+	747	1319	85	15	16.06%	583.4368387	2.48E-08	2.43E-08	6.09E-15
ENSG00000111674_6915833_6922800_434	annotated	ENO2	12	+	1302	77	8	1	6.91%	19.54111743	3.10E-05	6.52E-07	2.00E-10
ENSG00000107959_3172779_3138031_939	annotated	PITRM1	10	-	2817	111	14	4	5.22%	13.01984414	6.14E-08	2.58E-09	1.76E-15
ENSG00000170619_144851338_144850664_224	annotated	COMMD5	8	-	672	14	6	0	2.68%	6.883802732	0.145248299	0.012798403	0.010012163
ENSG00000143924_42280894_42330207_744	annotated	EML4	2	+	2232	64	14	2	3.36%	9.474481179	0.000905305	8.00E-06	7.33E-08
ENSG00000178999_8210224_8207082_163	novel	AURKB	17	-	489	23	4	0	8.59%	15.54134604	0.005573589	0.000369309	1.45E-05
ENSG00000265241_145926129_145925882_44	annotated	RBM8A	1	-	132	38	1	0	11.36%	95.12163775	0.021084099	0.021084099	0.002029891
ENSG00000147586_80003120_79918980_96	annotated	MRPS28	8	-	288	175	29	9	26.04%	200.7775797	6.91E-05	3.57E-05	1.87E-08
ENSG00000120526_109329811_109313835_71	annotated	NUDCD1	8	-	213	9	0	0	7.04%	13.9615154	0.020613417	0.020613417	0.001946209
ENSG00000104549_125009289_125020875_160	annotated	SQLE	8	+	480	230	32	5	17.50%	158.3274628	3.56E-05	2.95E-06	9.18E-10
ENSG00000151914_56640580_56624416_996	annotated	DST	6	-	2988	25	1	4	1.10%	2.76457941	0.002336818	0.011821942	0.000158933
ENSG00000076554_80171443_80038116_207	annotated	TPD52	8	-	621	230	21	4	13.04%	122.3787152	1.80E-05	2.75E-06	4.26E-10
ENSG00000198792_38247859_38221469_373	annotated	TMEM184B	22	-	1119	75	7	5	6.97%	22.14628225	5.09E-05	1.82E-05	7.23E-09
ENSG00000149187_47489014_47472230_483	annotated	CELF1	11	-	1449	85	12	3	5.18%	19.38296421	0.000691468	1.62E-05	9.98E-08
ENSG00000119685_75734035_75776746_384	novel	TTLL5	14	+	1152	6	3	1	1.56%	1.720950683	0.182856148	0.029390861	0.024082579
ENSG00000122786_134867785_134968345_521	annotated	CALD1	7	+	1563	94	9	4	5.37%	19.87186048	2.70E-06	4.91E-06	1.13E-10
ENSG00000087299_50312150_50282497_236	annotated	L2HGDH	14	-	708	7	1	2	2.12%	3.266889432	0.041782825	0.119134635	0.019821075
ENSG00000198836_193593378_193692127_997	annotated	OPA1	3	+	2991	78	19	3	3.61%	8.616836318	0.000333469	3.75E-07	1.76E-09
ENSG00000140931_66604806_66612637_182	annotated	CMTM3	16	+	546	91	12	3	11.54%	55.07042185	0.001697097	0.000117229	1.49E-06
ENSG00000139112_10213130_10220711_146	annotated	GABARAPL1	12	+	438	16	1	2	4.11%	12.07022945	0.035374934	0.061356297	0.008913099
ENSG00000069329_46677332_46672458_79	novel	VPS35	16	-	237	37	1	1	15.19%	51.58495212	0.001360042	0.001023866	8.54E-06
ENSG00000242498_89903837_89903138_100	annotated	ARPIN	15	-	300	15	0	0	5.00%	16.52112656	0.021084099	0.021084099	0.002029891
ENSG00000110697_67504111_67492033_1221	annotated	PITPNM1	11	-	3663	57	6	0	2.29%	5.141710148	4.79E-05	1.42E-06	6.42E-10
ENSG00000041357_78544194_78548944_190	novel	PSMA4	15	+	570	612	51	8	22.11%	354.7694545	2.77E-08	6.69E-08	1.86E-14
ENSG00000270106_231528811_231616693_138	annotated	TSNAX-DISC1	1	+	414	65	9	0	5.80%	51.87793363	0.020415603	0.005655336	0.000603396
ENSG00000215301_41334253_41347719_662	annotated	DDX3X	X	+	1986	235	49	10	8.46%	39.09833576	1.38E-07	1.77E-10	3.52E-16
ENSG00000115355_55322644_55291711_755	annotated	CCDC88A	2	-	2265	25	0	0	1.85%	3.647047805	0.00040182	0.00040182	1.07E-06
ENSG00000136631_150068645_150144796_534	annotated	VPS45	1	+	1602	53	8	6	3.93%	10.93158187	0.00100786	0.000237917	1.63E-06
ENSG00000139437_109903027_109916623_498	annotated	TCHP	12	+	1494	10	1	0	1.00%	2.211663528	0.042234516	0.019679754	0.003715148
ENSG00000151136_107319115_107649307_949	annotated	BTBD11	12	+	2847	76	12	0	3.06%	8.820552288	0.000220274	9.15E-07	2.33E-09
ENSG00000171130_149875611_149878771_42	novel	ATP6V0E2	7	+	126	76	4	2	23.81%	199.3024791	0.009088823	0.005538787	0.000263745
ENSG00000170791_56214614_56216505_56	novel	CHCHD7	8	+	168	23	5	3	8.93%	45.23641795	0.021084099	0.044329616	0.004137993
ENSG00000104325_90006269_90051897_315	annotated	DECR1	8	+	945	163	18	3	12.38%	56.99351595	1.03E-06	5.16E-08	5.40E-13
ENSG00000107959_3172772_3138031_1037	annotated	PITRM1	10	-	3111	133	15	5	5.59%	14.12606771	2.27E-09	9.83E-11	2.67E-18
ENSG00000204852_110614183_110634681_263	annotated	TCTN1	12	+	789	62	1	0	7.22%	25.96476163	6.91E-05	5.61E-05	2.88E-08
ENSG00000204852_110634747_110647892_324	dORF	TCTN1	12	+	972	76	11	2	4.63%	25.83550655	0.004341878	0.00103761	2.75E-05
ENSG00000205659_74084975_74198977_112	annotated	LIN52	14	+	336	11	4	2	4.46%	10.81740429	0.15306802	0.070008252	0.038603597
ENSG00000138363_215334930_215347638_195	novel	ATIC	2	+	585	51	6	1	11.28%	28.80606682	0.00016234	2.04E-05	2.62E-08
ENSG00000103197_2086755_2088610_183	dORF	TSC2	16	+	549	12	2	0	2.73%	7.222350407	0.02854252	0.019679754	0.002536889
ENSG00000177105_3828138_3827563_191	annotated	RHOG	11	-	573	64	10	1	11.52%	36.90583245	0.000496609	2.32E-05	9.52E-08
ENSG00000120451_130915852_130905829_774	annotated	SNX19	11	-	2322	22	0	0	1.81%	3.130618297	0.00037283	0.00037283	9.26E-07
ENSG00000099204_114684305_114436260_702	annotated	ABLIM1	10	-	2106	79	7	7	4.13%	12.39476731	1.58E-05	7.77E-06	9.97E-10
ENSG00000181019_69718478_69718003_119	novel	NQO1	16	-	357	1148	88	77	42.86%	1062.535198	4.10E-08	5.37E-07	2.23E-13
ENSG00000166173_70854088_70832052_491	annotated	LARP6	15	-	1473	7	1	0	1.22%	1.570236061	0.028889786	0.009815329	0.001387795
ENSG00000164211_111501104_111499886_107	novel	STARD4	5	-	321	42	11	1	10.28%	43.23285454	0.0240701	0.002493494	0.000358754
ENSG00000188603_28489349_28477516_336	annotated	CLN3	16	-	1008	165	16	3	12.80%	54.08702146	3.39E-08	5.34E-09	1.91E-15
ENSG00000168214_26415539_26431007_414	annotated	RBPJ	4	+	1242	16	0	0	1.69%	4.256650964	0.008473674	0.008473674	0.000366502
ENSG00000100603_77738824_77718088_374	annotated	SNW1	14	-	1122	45	11	0	4.55%	13.25224055	0.005286563	0.000130272	5.67E-06
ENSG00000106462_148846595_148807646_706	annotated	EZH2	7	-	2118	26	8	7	2.12%	4.05617838	0.026792051	0.022294426	0.002675736
ENSG00000111319_6374540_6347873_588	annotated	SCNN1A	12	-	1764	70	3	3	3.40%	13.1120052	0.000150216	7.65E-05	8.32E-08
ENSG00000197798_126235002_126262133_275	annotated	FAM118B	11	+	825	30	0	1	4.36%	12.01536477	0.001003278	0.001206953	7.46E-06
ENSG00000125149_67110176_67147131_422	annotated	C16orf70	16	+	1266	25	8	0	2.61%	6.524931499	0.040804041	0.001535678	0.000442457
ENSG00000175938_30949290_30954123_280	annotated	ORAI3	16	+	840	13	2	0	2.50%	5.113682029	0.026832696	0.007882058	0.001064673

ENSG00000103121_80991768_80976093_57	novel	CMC2	16	-	171	41	8	6	19.30%	79.22411565	0.017142865	0.005198269	0.00046884
ENSG00000140365_75336090_75338334_91	novel	COMMD4	15	+	273	101	7	6	8.79%	122.2442331	0.021820178	0.018991317	0.00190254
ENSG00000111145_96223567_96267180_407	annotated	ELK3	12	+	1221	40	11	0	4.18%	10.82465294	0.014216871	0.000118541	1.67E-05
ENSG00000131584_1302924_1293564_742	annotated	ACAP3	1	-	2226	17	1	1	0.94%	2.523442511	0.012000727	0.012000727	0.000706384
ENSG00000148803_133357944_133355370_133	annotated	FUOM	10	-	399	13	2	0	6.77%	10.76564638	0.010020958	0.002943991	0.000164225
ENSG00000131269_75156257_75097524_150	annotated	ABCB7	X	-	450	7	1	0	3.33%	5.13990604	0.041782825	0.019216965	0.003600526
ENSG00000215301_41345217_41347719_307	novel	DDX3X	X	+	921	143	16	5	9.77%	51.30338974	4.91E-06	1.04E-06	4.54E-11
ENSG00000106615_151471431_151467119_70	annotated	RHEB	7	-	210	79	8	0	14.29%	124.3018093	0.005134532	0.002376615	6.91E-05
ENSG00000221978_1399306_1392782_226	novel	CCNL2	1	-	678	23	5	1	4.87%	11.20902392	0.029590254	0.002467336	0.00044676
ENSG00000169756_108659609_108683999_325	annotated	LIMS1	2	+	975	34	2	2	4.00%	11.52242673	0.001811222	0.001811222	1.94E-05
ENSG00000003056_8946404_8941818_191	annotated	M6PR	12	-	573	242	19	29	23.04%	139.5501789	8.47E-08	6.31E-06	5.77E-12
ENSG00000003056_8949729_8949517_70	uORF	M6PR	12	-	210	38	3	8	10.00%	59.79074373	0.024275373	0.086158131	0.009141461
ENSG00000113712_149525134_149496853_276	novel	CSNK1A1	5	-	828	104	12	2	10.14%	41.5023469	6.05E-05	1.72E-06	9.82E-10
ENSG00000163608_113019644_113005536_566	annotated	NEPRO	3	-	1698	62	10	7	3.53%	12.06489807	0.000625639	0.000501237	2.03E-06
ENSG00000088992_117099282_117048752_135	annotated	TESC	12	-	405	29	13	4	10.37%	23.65988494	0.072118755	0.004464153	0.001979282
ENSG00000084090_96195509_96193161_104	novel	STARD7	2	-	312	19	1	0	6.73%	20.12188491	0.016854696	0.007029303	0.00060175
ENSG00000139926_51689870_51728029_611	annotated	FRMD6	14	+	1833	14	4	0	0.98%	2.523685453	0.059453662	0.01300724	0.003717979
ENSG00000141349_44075330_44076043_161	Overlap_dORF	G6PC3	17	+	483	136	14	10	19.25%	93.03822822	3.08E-06	5.88E-06	1.54E-10
ENSG00000125107_58526078_58520958_205	dORF	CNOT1	16	-	615	22	7	0	3.41%	11.81999298	0.105141107	0.007979402	0.004800443
ENSG00000125107_58599337_58528091_2150	annotated	CNOT1	16	-	6450	144	32	7	2.56%	7.376875113	1.56E-06	5.79E-10	1.47E-14
ENSG00000114861_71041530_70959247_455	annotated	FOXP1	3	-	1365	15	0	5	1.54%	3.631016825	0.008473674	0.081055481	0.00371419
ENSG00000185917_36058888_36036117_440	annotated	SETD4	21	-	1320	8	1	3	1.14%	2.002560795	0.042234516	0.150401371	0.025490393
ENSG00000179195_124012202_124012930_242	annotated	ZNF664	12	+	726	12	1	0	2.07%	5.46152944	0.035772986	0.021084099	0.003354605
ENSG00000185917_36057384_36036117_416	annotated	SETD4	21	-	1248	8	1	3	1.20%	2.118093148	0.042234516	0.150401371	0.025490393
ENSG00000165688_136416314_136423264_340	annotated	PMPCA	9	+	1020	116	13	1	10.29%	37.57746432	7.38E-06	1.18E-07	9.19E-12
ENSG00000165688_136410669_136416294_146	Overlap_uORF	PMPCA	9	+	438	18	4	0	4.79%	13.57900813	0.053017408	0.008776163	0.002384715
ENSG00000152700_134612730_134606950_130	annotated	SAR1B	5	-	390	222	24	5	33.08%	188.0866716	2.47E-08	1.12E-08	2.84E-15
ENSG00000188725_61161196_61161110_28	uORF	SMIM15	5	-	84	37	2	5	28.57%	145.5432578	0.005655336	0.011883851	0.000349796
ENSG00000188725_61160171_61159947_74	annotated	SMIM15	5	-	222	25	1	0	13.51%	37.2097445	0.002390426	0.002390426	3.30E-05
ENSG00000120727_139363785_139363991_68	novel	PAIP2	5	+	204	10	4	0	7.35%	16.1971829	0.097833871	0.020613417	0.009177766
ENSG00000080822_98521424_98516834_252	novel	CLDND1	3	-	756	109	33	5	10.32%	47.64028557	0.000317982	2.55E-05	6.47E-08
ENSG00000257698_57935536_57931610_64	novel	GIHCG	12	-	192	16	0	0	7.81%	27.53521093	0.021084099	0.021084099	0.002029891
ENSG00000048162_176388363_176386906_49	novel	NOP16	5	-	147	17	0	0	16.33%	38.21212945	0.005454749	0.005454749	0.000159109
ENSG00000048162_176388539_176388429_36	novel	NOP16	5	-	108	60	23	5	27.78%	183.5680728	0.132490593	0.004490072	0.00419766
ENSG00000196756_38434513_38426436_49	novel	SNHG17	20	-	147	26	3	3	14.29%	58.44208033	0.009583875	0.023878474	0.001121714
ENSG00000161960_7577836_7578142_40	novel	EIF4A1	17	+	120	338	13	11	17.50%	930.6901293	0.008980239	0.010353108	0.000468033
ENSG00000105852_95363975_95359973_160	novel	PON3	7	-	480	78	9	5	10.00%	53.69366131	0.001957414	0.000908545	1.09E-05
ENSG00000106066_29121049_28995772_472	annotated	CPVL	7	-	1416	40	6	1	4.45%	9.333969806	0.000427585	3.35E-05	1.13E-07
ENSG00000106049_27662788_27526214_336	annotated	HIBADH	7	-	1008	186	27	1	11.90%	60.97082419	2.46E-06	1.71E-08	4.94E-13
ENSG00000106052_27748525_27828829_747	annotated	TAX1BP1	7	+	2241	109	15	2	5.76%	16.07142164	8.97E-08	5.03E-09	4.88E-15
ENSG00000186298_110742707_110722406_270	annotated	PPP1CC	12	-	810	273	38	4	16.67%	111.3646309	8.17E-07	9.76E-09	9.11E-14
ENSG00000175061_16439055_16439685_39	novel	SNHG29	17	+	117	132	18	6	20.51%	372.7843941	0.066537981	0.015820889	0.00491662
ENSG00000112984_138179681_138181770_138	novel	KIF20A	5	+	414	22	0	0	5.80%	17.55868523	0.005604867	0.005604867	0.000167525
ENSG00000106638_73574098_73570507_352	dORF	TBL2	7	-	1056	64	2	0	6.82%	20.02560795	2.67E-05	7.40E-06	1.61E-09
ENSG00000100814_20316505_20311550_271	annotated	CCNB1IP1	14	-	813	7	4	1	1.85%	2.844966443	0.218864129	0.041782825	0.038173193
ENSG00000124228_49219449_49243834_765	annotated	DDX27	20	+	2295	77	15	3	3.66%	11.08607185	0.000270435	9.73E-06	2.30E-08
ENSG00000131778_147254969_147295509_784	dORF	CHD1L	1	+	2352	35	1	2	2.04%	4.917001951	0.000286582	0.000501115	9.60E-07
ENSG00000171560_154590678_154585484_286	annotated	FGA	4	-	858	19	0	1	4.20%	7.317049057	0.000862735	0.001607079	8.56E-06
ENSG00000100814_20316505_20311550_271	annotated	CCNB1IP1	14	-	813	7	4	1	1.85%	2.844966443	0.218864129	0.041782825	0.038173193
ENSG00000048392_102232190_102208133_297	annotated	RRM2B	8	-	891	65	11	3	6.40%	24.10489845	0.000979232	0.0001756	1.20E-06
ENSG00000117448_45561834_45569956_312	annotated	AKR1A1	1	+	936	143	11	14	12.82%	50.48122003	3.22E-07	2.39E-07	7.20E-13
ENSG00000176953_28958747_28963863_127	annotated	NFATC2IP	16	+	381	20	1	0	9.45%	17.34501476	0.000885228	0.000919662	5.09E-06
ENSG00000117318_23559426_23558960_119	annotated	ID3	1	-	357	760	83	25	33.61%	703.4205144	1.25E-07	2.25E-07	2.68E-13
ENSG00000130414_240025301_240018494_201	annotated	NDUFA10	2	-	603	62	3	3	7.46%	33.97379259	0.000916317	0.00031447	1.91E-06
ENSG00000104823_38831772_38817349_191	novel	ECH1	19	-	573	365	50	30	27.23%	210.4785757	4.62E-08	1.75E-09	9.13E-16
ENSG00000136270_45111669_45100325_642	annotated	TBRG4	7	-	1926	175	16	6	8.88%	30.02281565	3.45E-10	1.19E-10	4.74E-19
ENSG00000126602_3717508_3658129_704	annotated	TRAP1	16	-	2112	355	20	18	13.35%	55.53977204	9.64E-15	7.85E-15	1.05E-27
ENSG00000150753_10254154_10264783_503	annotated	CCT5	5	+	1509	808	113	17	21.87%	176.9260471	3.93E-15	1.79E-18	1.29E-31
ENSG00000249859_127794565_127890868_149	novel	PVT1	8	+	447	10	1	0	3.36%	7.392002933	0.035374934	0.020613417	0.003252526
ENSG00000265681_49492316_49491779_37	uORF	RPL17	18	-	111	35	2	6	24.32%	104.1872846	0.008413705	0.051745348	0.002244442
ENSG00000155621_71911735_71982873_179	annotated	C9orf85	9	+	537	21	5	0	2.79%	12.9215515	0.021084099	0.021084099	0.002029891
ENSG00000160049_10472458_10463034_268	annotated	DFFA	1	-	804	48	2	7	7.09%	19.72671828	0.000110561	0.001021437	8.24E-07
ENSG00000078061_47563244_47565354_148	annotated	ARAF	X	+	444	11	0	0	3.38%	8.186143789	0.019679754	0.019679754	0.001784964
ENSG00000160049_10472458_10461490_331	annotated	DFFA	1	-	993	60	4	7	7.25%	19.96510762	2.72E-05	0.000141665	2.97E-08
ENSG00000161888_11149227_11146239_118	annotated	SPC24	19	-	354	17	3	0	7.63%	15.86774867	0.030308563	0.002967235	0.000533479
ENSG00000170653_53552589_53543240_85	annotated	ATF7	12	-	255	29	4	0	7.06%	37.57746432	0.024913475	0.013640586	0.001599859
ENSG00000070423_663121_651607_148	novel	AC004156.1	19	-	444	27	4	4	7.43%	20.09326203	0.0034719	0.009821677	0.000186419
ENSG00000132471_75855297_75846734_261	annotated	WBP2	17	-	783	112	13	1	11.11%	47.26350381	1.36E-05	1.18E-06	1.44E-10

ENSG00000183161_22625810_22624686_374	annotated	FANCF	11	-	1122	25	2	0	4.28%	7.362355863	0.000950038	0.000128901	8.77E-07
ENSG00000111832_116588866_116593101_145	annotated	RWDD1	6	+	435	22	5	0	5.52%	16.71102456	0.044816497	0.005554612	0.00136901
ENSG00000111832_116571561_116571644_27	uORF	RWDD1	6	+	81	24	0	1	22.22%	97.90297218	0.013640586	0.020525443	0.001325514
ENSG00000163507_108589375_108563122_545	novel	CIP2A	3	-	1635	51	2	6	3.49%	10.30675785	0.000103144	0.001149682	8.77E-07
ENSG00000011454_122984539_123103213_1001	annotated	RABGAP1	9	+	3003	32	8	5	1.80%	3.520986013	0.007789526	0.000920614	4.56E-05
ENSG00000161960_7572842_7574383_48	novel	EIF4A1	17	+	144	14	0	0	12.50%	32.12441275	0.01300724	0.01300724	0.000821865
ENSG00000161960_7576539_7576700_53	novel	EIF4A1	17	+	159	59	4	0	22.64%	122.6096185	0.008603705	0.001019727	5.54E-05
ENSG00000196715_65954076_65954300_74	internal	VKORC1L1	7	+	222	98	13	4	24.32%	145.8621984	0.002372747	0.000143994	2.55E-06
ENSG00000149084_43854862_43855248_35	novel	HSD17B12	11	+	105	71	11	0	22.86%	223.4285687	0.018151375	0.005756914	0.000543483
ENSG00000126858_32171069_32221064_604	annotated	RHOT1	17	+	1812	55	3	2	4.30%	10.02938146	1.88E-05	1.23E-05	1.84E-09
ENSG00000215301_41343817_41347719_409	novel	DDX3X	X	+	1227	169	20	7	9.29%	45.51051977	1.82E-07	7.77E-08	1.37E-13
ENSG00000215301_41334253_41341620_95	novel	DDX3X	X	+	285	23	9	2	9.47%	26.66567795	0.064380244	0.010487024	0.003400624
ENSG00000101333_9384236_9478989_879	novel	PLCB4	20	+	2637	14	0	3	0.80%	1.754234143	0.008473674	0.052370108	0.00228699
ENSG00000198692_20589462_20592346_39	novel	EIF1AY	Y	+	117	25	2	1	17.95%	70.60310494	0.007882058	0.01551626	0.000614257
ENSG00000066136_40738844_40758342_202	novel	NFYC	1	+	606	55	2	0	5.45%	29.98884358	0.001988635	0.001603369	1.89E-05
ENSG00000101040_47276605_47210761_792	annotated	ZMYND8	20	-	2376	20	2	0	1.01%	2.781334437	0.013091824	0.005554612	0.000378918
ENSG00000077454_100576826_100575107_210	novel	LRCH4	7	-	630	6	0	2	2.38%	3.146881249	0.016947427	0.139742684	0.011764961
ENSG00000154845_9584738_9562972_381	dORF	PPP4R1	18	-	1143	32	1	0	3.15%	9.250674537	0.001480749	0.001003278	9.11E-06
ENSG00000154845_9559565_9547789_323	dORF	PPP4R1	18	-	969	30	1	2	3.41%	10.22979972	0.002190151	0.003240864	4.08E-05
ENSG00000100461_22919182_22911369_72	uORF	RBM23	14	-	216	35	8	7	13.89%	53.54068791	0.036127044	0.037374083	0.005685221
ENSG00000101384_10672964_10639498_1177	annotated	JAG1	20	-	3531	264	27	10	7.73%	24.70448831	1.27E-13	1.27E-15	2.45E-27
ENSG00000137478_73001073_72843140_470	annotated	FCHSD2	11	-	1410	13	2	0	1.28%	3.046448868	0.036394529	0.012798403	0.002205885
ENSG00000132612_69311512_69324309_437	annotated	VPS4A	16	+	1311	66	13	2	7.09%	16.6345439	0.000140486	1.86E-06	2.61E-09
ENSG00000143373_151286292_151291209_1237	annotated	ZNF687	1	+	3711	20	1	2	1.21%	1.780773544	0.000409346	0.00089198	2.38E-06
ENSG00000101310_18510836_18560740_767	annotated	SEC23B	20	+	2301	150	18	1	7.04%	21.53993032	2.15E-08	5.19E-11	1.56E-17
ENSG00000136536_159729023_159767342_704	annotated	7-Mar	2	+	2112	48	7	11	3.55%	7.50960298	0.000180082	0.00088607	1.11E-06
ENSG00000091436_173091032_173266750_800	annotated	MAP3K20	2	+	2400	43	11	6	2.62%	5.920070349	0.001923517	0.000399956	5.06E-06
ENSG00000115641_105386516_105361283_279	annotated	FHL2	2	-	837	117	12	0	10.04%	46.18809575	2.51E-05	1.64E-06	3.70E-10
ENSG00000173744_227472467_227554495_545	annotated	AGFG1	2	+	1635	56	3	2	4.40%	11.31722431	1.74E-05	1.74E-05	2.40E-09
ENSG00000131149_85633949_85672539_1203	annotated	GSE1	16	+	3609	52	5	2	2.16%	4.760867725	4.11E-05	6.91E-06	2.34E-09
ENSG00000131143_85801206_85806874_169	annotated	COX4I1	16	+	507	1164	151	28	42.01%	758.6032075	3.18E-12	1.04E-13	4.54E-24
ENSG00000131148_85799295_85779708_210	annotated	EMC8	16	-	630	52	5	3	9.52%	27.27297082	0.000182394	7.68E-05	1.01E-07
ENSG00000108651_31901627_31880547_330	novel	UTP6	17	-	990	18	6	1	2.73%	6.007682384	0.09587281	0.002943991	0.002049631
ENSG00000134900_102654999_102678316_268	novel	TPP2	13	+	804	23	4	1	4.10%	9.45238584	0.009197853	0.003386928	0.000170669
ENSG00000000419_50936189_50935132_48	novel	DPM1	20	-	144	19	4	0	12.50%	43.5974173	0.029237631	0.013640586	0.001872687
ENSG00000000419_50958523_50940883_188	novel	DPM1	20	-	564	104	34	6	17.55%	60.92897737	0.001022382	7.83E-07	1.23E-08
ENSG00000037897_57769410_57768996_87	Overlap_dORF	METTL1	12	-	261	14	1	2	6.90%	17.72381393	0.016344022	0.023718446	0.001795277
ENSG00000196943_24299955_24305095_636	annotated	NOP9	14	+	1908	27	6	0	1.73%	4.675790535	0.020843735	0.001448134	0.000195372
ENSG00000136261_16689838_16706088_225	Overlap_dORF	BZW2	7	+	675	62	7	2	10.67%	30.34992138	5.92E-05	6.57E-06	3.25E-09
ENSG00000136261_16665444_16689877_161	annotated	BZW2	7	+	483	47	5	1	13.04%	32.15291711	0.000252823	3.42E-05	6.65E-08
ENSG00000148985_3811260_3822914_56	annotated	PGAP2	11	+	168	27	1	0	12.50%	53.10362107	0.008473674	0.008473674	0.000366502
ENSG00000134852_55478869_55435415_779	annotated	CLOCK	4	-	2337	12	7	0	0.77%	1.69664971	0.157414378	0.011772051	0.010389467
ENSG00000123374_55967009_55971625_238	annotated	CDK2	12	+	714	57	15	2	9.24%	26.37826929	0.000301277	3.06E-05	7.21E-08
ENSG00000147642_109586100_109574906_500	annotated	SYBU	8	-	1500	22	9	1	2.00%	4.846197123	0.02437921	0.00345766	0.000477745
ENSG00000164758_117521048_117539978_121	annotated	MED30	8	+	363	23	2	0	5.79%	20.93586285	0.013991246	0.008574016	0.000599135
ENSG00000198074_134527912_134541089_316	annotated	AKR1B10	7	+	948	1440	175	80	25.95%	501.9076422	8.48E-13	1.34E-13	1.51E-24
ENSG00000180822_3259113_3267712_93	annotated	PSMG4	6	+	279	48	13	3	10.75%	56.84688707	0.025878367	0.007609621	0.000995195
ENSG00000169504_24797796_24840937_211	annotated	CLIC4	1	+	633	82	12	5	9.95%	42.80355063	5.08E-05	5.23E-05	1.99E-08
ENSG00000157036_38503621_38524362_215	dORF	EXOG	3	+	645	7	3	0	2.33%	3.585980958	0.169362074	0.019216965	0.016163992
ENSG00000106003_2519862_2528886_361	annotated	LFNG	7	+	1083	27	5	0	2.49%	8.237680831	0.00567009	0.003554776	0.000110499
ENSG00000196363_134139878_134157993_334	annotated	WDR5	9	+	1002	71	8	3	7.78%	23.41317336	9.06E-05	2.27E-05	1.59E-08
ENSG00000172932_67289461_67290606_121	novel	ANKRD13D	11	+	363	8	0	0	4.13%	7.282039253	0.019679754	0.019679754	0.001784964
ENSG00000165533_88841528_88877410_317	annotated	TTC8	14	+	951	12	2	2	2.84%	4.169369478	0.014696947	0.014696947	0.001033656
ENSG00000165533_88824708_88841526_186	uORF	TTC8	14	+	558	29	5	0	5.91%	17.17249714	0.010934044	0.001520872	0.00010094
ENSG00000172572_20572127_20680271_819	novel	PDE3A	12	+	2457	159	27	1	6.35%	21.38265464	1.89E-07	2.25E-10	6.17E-16
ENSG00000133216_22781429_22891149_459	annotated	EPHB2	1	+	1377	81	6	2	4.79%	19.43661948	0.000124394	5.53E-05	5.07E-08
ENSG00000111737_120097373_120097245_42	dORF	RAB35	12	-	126	16	0	0	16.67%	41.95841665	0.008473674	0.008473674	0.000366502
ENSG00000026025_17229423_17237271_466	annotated	VIM	10	+	1398	1392	190	98	34.55%	329.0044087	5.61E-25	1.12E-23	1.16E-46
ENSG00000168610_42345636_42315745_672	annotated	STAT3	17	-	2016	108	21	10	6.85%	17.70120702	1.49E-05	3.43E-07	5.13E-11
ENSG00000156795_123416850_123441415_205	annotated	WDYHV1	8	+	615	55	6	0	7.32%	29.54998246	0.001213543	0.000307311	2.49E-06
ENSG00000158941_22605774_22619682_923	annotated	CCAR2	8	+	2769	64	11	11	3.58%	7.637068253	3.29E-05	1.63E-05	4.20E-09
ENSG00000156787_123072770_123151939_1066	annotated	TBC1D31	8	+	3198	47	11	1	1.50%	4.856115998	0.01587667	0.000277063	3.74E-05
ENSG00000125826_408758_430430_510	annotated	RBCK1	20	+	1530	55	26	24	3.14%	11.87793413	0.042306784	0.037822699	0.006650275
ENSG00000125826_408241_408705_154	uORF	RBCK1	20	+	462	42	10	15	8.44%	30.03841192	0.009263777	0.064710135	0.003096171
ENSG00000129250_5000247_5024151_1103	annotated	KIF1C	17	+	3309	28	17	6	1.36%	2.795959768	0.135539483	0.008234549	0.006678272
ENSG00000168653_39028725_39034496_106	annotated	NDUFS5	1	+	318	580	89	47	33.02%	602.6574467	4.52E-07	4.27E-06	1.79E-11
ENSG00000196655_119018796_119023399_176	annotated	TRAPPC4	11	+	528	112	13	3	17.61%	70.08962781	7.35E-05	1.50E-06	1.07E-09
ENSG00000167333_4602265_4600276_262	dORF	TRIM68	11	-	786	10	3	0	1.91%	4.203848996	0.042234516	0.020613417	0.003868181

ENSG00000163584_170870167_170868109_64	annotated	RPL22L1	3	-	192	23	5	3	10.94%	39.58186571	0.05158844	0.032657853	0.007032595
ENSG00000114416_180947925_180976292_535	annotated	FXR1	3	+	1605	57	8	0	5.23%	11.73463195	0.000109344	1.30E-06	1.45E-09
ENSG00000089280_31180215_31191438_526	annotated	FUS	16	+	1578	377	43	28	18.25%	78.9412511	2.72E-15	9.14E-16	3.57E-29
ENSG00000174485_65756450_65661851_1906	annotated	DENND4A	15	-	5718	22	3	0	0.42%	1.2713004	0.028352861	0.005604867	0.000842634
ENSG00000166340_6619400_6618759_81	annotated	TPP1	11	-	243	54	9	0	14.81%	73.42722914	0.007666581	0.00104892	5.02E-05
ENSG00000156510_69220454_69266757_911	annotated	HKDC1	10	+	2733	126	25	3	5.71%	15.23353052	9.39E-07	2.00E-10	3.29E-15
ENSG00000169045_179621263_179620884_57	novel	HNRNPH1	5	-	171	57	12	8	22.81%	110.1408437	0.003642531	0.002944096	6.03E-05
ENSG00000253719_74538113_74538406_97	annotated	ATXN7L3B	12	+	291	34	4	5	10.31%	38.60606893	0.010100577	0.016039993	0.000794438
ENSG00000060138_10702428_10701288_85	novel	YBX3	12	-	255	19	0	3	9.41%	24.619718	0.004873904	0.004873904	0.000128468
ENSG00000101974_139796386_139732436_758	novel	ATP11C	X	-	2274	20	0	0	1.32%	2.906090863	0.002201184	0.002201184	2.82E-05
ENSG00000100804_23034716_23016631_114	annotated	PSMB5	14	-	342	238	21	4	30.70%	229.9431649	2.12E-06	1.65E-07	3.40E-12
ENSG00000162852_246621458_246648052_480	annotated	CNST	1	+	1440	22	3	0	2.92%	5.048122003	0.002843489	0.000291514	5.74E-06
ENSG00000162852_246621458_246665905_589	annotated	CNST	1	+	1767	26	3	0	2.72%	4.861904816	0.001205335	0.000128239	1.12E-06
ENSG00000115947_147975958_147935510_436	annotated	ORC4	2	-	1308	41	1	2	3.90%	10.35728117	0.00016923	0.000419418	4.90E-07
ENSG00000111642_6606316_6570676_1901	annotated	CHD4	12	-	5703	90	9	3	2.10%	5.214453411	2.68E-07	2.95E-08	8.01E-14
ENSG00000142192_26170620_25881670_770	annotated	APP	21	-	2310	1894	173	48	27.79%	270.9178675	1.29E-31	7.82E-35	2.37E-64
ENSG00000108641_19347312_19343319_84	annotated	B9D1	17	-	252	31	0	0	11.90%	40.64721613	0.002335453	0.002335453	3.16E-05
ENSG00000080822_98521424_98516659_253	annotated	CLDND1	3	-	759	170	26	5	17.39%	74.00768154	4.66E-08	2.17E-08	1.02E-14
ENSG00000117862_52055096_52034042_55	annotated	TXNDC12	1	-	165	39	7	14	18.18%	78.09987099	0.016263223	0.072833036	0.005533782
ENSG00000122952_56359749_56358418_110	dORF	ZWINT	10	-	330	256	21	2	19.09%	256.3277817	0.000144209	2.65E-05	2.95E-08
ENSG00000122952_56361236_56360278_48	annotated	ZWINT	10	-	144	11	13	0	10.42%	25.24061002	0.361504355	0.019679754	0.043859366
ENSG00000106443_11051667_11103374_97	novel	PHF14	7	+	291	15	0	1	6.19%	17.03208923	0.012798403	0.019795882	0.001207991
ENSG00000168906_85541275_85542409_204	novel	MAT2A	2	+	612	83	30	6	11.76%	44.81220602	0.005547873	0.000143404	6.49E-06
ENSG00000121057_57110042_57120324_326	dORF	AKAP1	17	+	978	66	0	0	7.06%	22.29845302	1.16E-05	1.16E-05	1.09E-09
ENSG00000121057_57105510_57108013_578	annotated	AKAP1	17	+	1734	58	5	0	3.98%	11.05219539	9.42E-05	1.15E-05	8.83E-09
ENSG00000048707_12277942_12509024_2936	annotated	VPS13D	1	+	8808	24	5	1	0.41%	0.900333872	0.011014422	0.001453991	9.79E-05
ENSG00000103351_3504767_3532913_364	annotated	CLUAP1	16	+	1092	24	7	0	3.02%	7.262033651	0.049241666	0.001484235	0.00053941
ENSG00000129292_132794453_132807748_222	annotated	PHF20L1	8	+	666	6	3	0	2.70%	2.97677956	0.263544628	0.007152939	0.014656016
ENSG00000111358_123645485_123659859_200	annotated	GTF2H3	12	+	600	40	25	3	8.50%	22.02816874	0.028889786	0.000604349	0.000141481
ENSG00000173692_231057026_231170712_953	annotated	PSMD1	2	+	2859	173	14	5	5.56%	19.9940881	1.34E-09	2.75E-10	4.18E-18
ENSG00000090863_74462613_74461983_112	novel	GLG1	16	-	336	22	6	2	8.04%	21.63480858	0.005588371	0.011814276	0.000344044
ENSG00000090863_74467854_74463246_156	novel	GLG1	16	-	468	22	7	1	5.77%	15.53268309	0.069104828	0.006142079	0.002410281
ENSG00000137776_58933565_58879999_1034	annotated	SLTM	15	-	3102	104	10	6	4.06%	11.07799589	3.22E-07	1.05E-07	3.26E-13
ENSG00000079616_29797027_29805310_597	annotated	KIF22	16	+	1791	177	16	9	7.37%	32.65482301	8.94E-08	1.06E-07	9.20E-14
ENSG00000143549_154172514_154157639_233	novel	TPM3	1	-	699	164	20	2	9.01%	77.52402733	0.000142782	2.90E-05	3.18E-08
ENSG00000127884_133373333_133362868_290	annotated	ECHS1	10	-	870	301	13	11	22.41%	114.3185998	9.51E-12	5.19E-12	6.11E-22
ENSG00000152904_235335265_235342772_300	annotated	GGPS1	1	+	900	28	4	0	4.00%	10.27981208	0.006091863	0.000978918	3.71E-05
ENSG00000178802_74890571_74896501_319	annotated	MPI	15	+	957	24	0	0	3.13%	8.286458461	0.00224099	0.00224099	2.92E-05
ENSG00000168701_67228543_67229113_88	novel	TMEM208	16	+	264	42	6	0	14.77%	52.56722086	0.003934467	0.000665241	1.67E-05
ENSG00000143793_228103227_228102426_141	novel	C1orf35	1	-	423	12	1	1	3.55%	9.373688826	0.035374934	0.035374934	0.005300298
ENSG00000125124_56519862_56484761_721	annotated	BBS2	16	-	2163	39	4	2	2.50%	5.957687801	0.000762611	0.000313065	1.58E-06
ENSG00000163832_47504433_47496069_193	annotated	ELP6	3	-	579	48	7	4	8.81%	27.39254144	0.003881303	0.00054256	1.38E-05
ENSG00000113838_186570154_186551150_461	annotated	TBCCD1	3	-	1383	10	0	4	1.95%	2.389172315	0.001946209	0.029390861	0.000365676
ENSG00000114988_96740040_96711973_169	annotated	LMAN2L	2	-	507	33	4	0	7.10%	21.50679197	0.004213161	0.001044718	2.69E-05
ENSG00000114988_96734450_96707256_204	Overlap_dORF	LMAN2L	2	-	612	20	0	0	5.88%	10.79812193	0.000862735	0.000862735	4.67E-06
ENSG00000115761_10689861_10572071_638	annotated	NOL10	2	-	1914	20	4	0	2.04%	3.452691025	0.013711343	0.000537982	5.41E-05
ENSG00000137831_70699624_70657056_1378	annotated	UACA	15	-	4134	7	0	0	0.44%	0.559496303	0.009815329	0.009815329	0.000483714
ENSG00000164292_95731683_95793174_611	annotated	RHOBTB3	5	+	1833	32	1	0	2.29%	5.768423893	0.000626688	0.000424543	1.74E-06
ENSG00000133026_8623246_8475804_1985	annotated	MYH10	17	-	5955	27	3	4	0.60%	1.498137421	0.005441582	0.006751002	0.000194582
ENSG00000155366_112703799_112701540_193	annotated	RHOC	1	-	579	299	26	25	29.53%	170.6327061	3.86E-10	9.22E-11	4.15E-19
ENSG00000135317_85593697_85549877_176	annotated	SNX14	6	-	528	28	1	0	7.95%	17.52240695	0.000812992	0.000415605	2.20E-06
ENSG00000155366_112701683_112701540_47	internal	RHOC	1	-	141	59	1	0	27.66%	138.2619102	0.000705256	0.000719745	3.23E-06
ENSG00000155366_112703799_112701461_199	annotated	RHOC	1	-	597	167	18	84	17.09%	92.42975326	3.73E-06	0.011333581	8.78E-07
ENSG00000155366_112703799_112701540_193	annotated	RHOC	1	-	579	299	26	25	29.53%	170.6327061	3.86E-10	9.22E-11	4.15E-19
ENSG00000155366_112703799_112701540_193	annotated	RHOC	1	-	579	299	26	25	29.53%	170.6327061	3.86E-10	9.22E-11	4.15E-19
ENSG00000155366_112703799_112701540_193	annotated	RHOC	1	-	579	299	26	25	29.53%	170.6327061	3.86E-10	9.22E-11	4.15E-19
ENSG00000142687_35554491_35466676_344	annotated	KIAA0319L	1	-	1032	7	1	0	1.45%	2.241238099	0.047790352	0.016947427	0.003697512
ENSG00000175110_139346949_139357014_279	annotated	MRPS22	3	+	837	68	16	0	10.39%	26.84436334	0.000369233	9.44E-07	4.31E-09
ENSG00000118263_207167142_207081213_269	annotated	KLF7	2	-	807	10	0	0	2.23%	4.094455156	0.009815329	0.009815329	0.000483714
ENSG00000119335_128689583_128694664_277	annotated	SET	9	+	831	49	8	1	6.14%	19.48339834	0.002368388	0.000181356	3.11E-06
ENSG00000127415_1000893_1004053_527	novel	IDUA	4	+	1581	11	8	1	1.14%	2.298954992	0.325055061	0.023672501	0.042436587
ENSG00000196998_49077855_49074803_334	annotated	WDR45	X	-	1002	28	4	3	3.89%	9.233364143	0.008855938	0.004081271	0.000194486
ENSG00000085491_108200138_108136653_477	annotated	SLC25A24	1	-	1431	57	14	1	4.40%	13.16148447	0.002697602	3.11E-05	7.97E-07
ENSG00000103496_31033806_31034266_64	novel	STX4	16	+	192	17	2	0	12.50%	29.25616161	0.013091824	0.005554612	0.000378918
ENSG00000167378_43595338_43592026_623	annotated	IRGQ	19	-	1869	25	2	0	1.93%	4.419777035	0.001886381	0.000978918	1.13E-05
ENSG00000095383_98255541_98199381_928	annotated	TBC1D2	9	-	2784	99	12	2	3.88%	11.74993915	4.66E-06	7.14E-08	3.57E-12
ENSG00000197965_167722152_167773372_202	novel	MPZL1	1	+	606	164	23	6	14.85%	89.42127905	4.40E-05	3.14E-06	1.21E-09
ENSG00000162521_32651307_32652065_55	novel	RBBP4	1	+	165	21	1	0	9.09%	42.05377669	0.021084099	0.021084099	0.002029891

ENSG00000183011_7857423_7856731_173	annotated	NAA38	17	-	519	353	144	15	21.97%	224.7382533	0.000824732	6.40E-08	1.26E-09
ENSG00000143179_165827834_165907823_261	annotated	UCK2	1	+	783	135	9	1	13.79%	56.96940192	1.46E-06	8.27E-08	1.21E-12
ENSG00000172409_57659597_57661436_321	annotated	CLP1	11	+	963	22	4	0	2.80%	7.54859365	0.023871233	0.003303074	0.000449475
ENSG00000173482_7567819_8406162_1465	annotated	PTPRM	18	+	4395	108	17	27	3.69%	8.119598034	9.08E-08	6.10E-07	5.39E-13
ENSG00000071127_10087838_10084432_76	novel	WDR1	4	-	228	26	1	2	17.11%	37.67976232	0.000980148	0.001453991	8.75E-06
ENSG00000134352_55960471_55956011_125	novel	IL6ST	5	-	375	22	4	4	8.80%	19.38478849	0.009376445	0.012837471	0.000598466
ENSG00000065809_14530459_14521190_131	annotated	FAM107B	10	-	393	106	26	3	14.50%	89.12159872	0.004452946	8.01E-05	3.11E-06
ENSG00000103051_70523531_70481010_785	annotated	COG4	16	-	2355	86	6	5	4.84%	12.06638543	3.74E-07	4.17E-07	1.44E-12
ENSG00000104976_7920367_7922764_334	annotated	SNAPC2	19	+	1002	39	5	2	5.39%	12.8607572	0.00072211	0.000118568	6.12E-07
ENSG00000204852_110614183_110647892_587	annotated	TCTN1	12	+	1761	138	14	2	5.79%	25.89341811	6.32E-06	4.29E-07	2.56E-11
ENSG00000138796_107989957_108034357_263	annotated	HADH	4	+	789	52	9	4	7.60%	21.77689685	0.001841996	0.000297833	3.71E-06
ENSG00000143373_151287705_151290746_632	annotated	ZNF687	1	+	1896	9	1	1	1.27%	1.568461382	0.010460668	0.010460668	0.000545418
ENSG00000181029_7682254_7682820_188	annotated	TRAPPC5	19	+	564	112	11	2	15.43%	65.61582178	2.01E-06	1.72E-06	3.03E-11
ENSG00000071282_8532737_8549894_233	annotated	LMCD1	3	+	699	21	6	0	4.29%	9.926857158	0.042966333	0.002188006	0.000620411
ENSG00000146457_159753494_159755611_234	dORF	WTAP	6	+	702	34	5	1	5.98%	16.00337045	0.002181097	0.000638487	8.80E-06
ENSG00000124795_18258349_18226147_313	annotated	DEK	6	-	939	67	14	1	6.71%	23.57647453	0.000352638	3.34E-05	9.19E-08
ENSG00000060237_753746_908792_2773	annotated	WNK1	12	+	8319	101	32	9	1.77%	4.011621065	0.000691109	2.40E-08	4.64E-10
ENSG00000120211_5231524_5233877_139	annotated	INSL4	9	+	417	206	21	3	17.99%	163.2303151	2.02E-05	5.85E-06	9.72E-10
ENSG00000111445_118026898_118031278_116	novel	RFC5	12	+	348	39	0	0	8.62%	37.03011125	0.002418183	0.002418183	3.38E-05
ENSG00000103544_19579078_19608228_96	Overlap_uORF	VPS35L	16	+	288	13	0	0	5.21%	14.91490592	0.020613417	0.020613417	0.001946209
ENSG00000103544_19601693_19700476_712	annotated	VPS35L	16	+	2136	76	6	1	4.21%	11.75660691	1.31E-05	8.97E-07	1.08E-10
ENSG00000111652_6724657_6731039_275	annotated	COPS7A	12	+	825	89	17	7	11.64%	35.64558214	4.59E-05	6.26E-06	2.39E-09
ENSG00000023839_99782845_99794011_195	annotated	ABCC2	10	+	585	109	15	11	14.36%	61.56590751	6.19E-05	5.86E-05	2.69E-08
ENSG00000274290_26183796_26184176_126	annotated	HIST1H2BE	6	+	378	176	7	3	14.29%	153.8475277	0.000226501	9.55E-05	1.55E-07
ENSG00000168216_69796881_69676158_540	annotated	LMBRD1	6	-	1620	136	26	1	8.89%	27.73917545	2.30E-06	7.20E-10	2.76E-14
ENSG00000104904_2269599_2273108_226	annotated	OAZ1	19	+	678	111	10	9	7.52%	54.09572412	0.00144604	0.000136991	1.44E-06
ENSG00000151233_42144646_42087786_409	annotated	GXYLT1	12	-	1227	15	2	3	2.20%	4.039395246	0.016821538	0.027614347	0.002133626
ENSG00000178971_8248036_8228180_1217	annotated	CTC1	17	-	3651	18	1	2	0.82%	1.629034664	0.003737369	0.010678933	0.000216647
ENSG00000171903_15934408_15913877_454	annotated	CYP4F11	19	-	1362	50	7	0	5.07%	12.13004887	0.000325378	1.10E-05	3.12E-08
ENSG00000151445_77454102_77427616_493	annotated	VIPAS39	14	-	1479	42	6	3	3.85%	9.38319561	0.000713309	0.000306583	1.45E-06
ENSG00000153046_4892085_4954056_412	annotated	CDYL	6	+	1236	12	7	0	1.46%	3.207985739	0.194271892	0.012798403	0.014328705
ENSG00000177119_45216322_45439938_929	annotated	ANO6	12	+	2787	108	14	7	4.41%	12.80431768	4.24E-06	8.91E-08	3.95E-12
ENSG00000104522_143616691_143612910_314	annotated	TSTA3	8	-	942	97	14	0	7.32%	34.02440076	0.000535387	1.24E-05	5.97E-08
ENSG00000163808_44802973_44852735_832	annotated	KIF15	3	+	2496	8	0	1	0.60%	1.059046574	0.019679754	0.042234516	0.003715148
ENSG00000133030_17136336_17184894_793	annotated	MPRIP	17	+	2379	33	5	4	1.89%	4.583414681	0.00237684	0.00342233	4.64E-05
ENSG00000108465_47975891_47981502_281	novel	CDK5RAP3	17	+	843	22	2	1	3.91%	8.623126553	0.00376808	0.002484757	5.31E-05
ENSG00000108465_47975086_47975657_131	novel	CDK5RAP3	17	+	393	12	0	0	3.82%	10.08923759	0.020613417	0.020613417	0.001946209
ENSG00000108439_47941769_47946782_217	annotated	PNPO	17	+	651	88	22	7	11.52%	44.66541127	0.000523536	2.85E-05	1.21E-07
ENSG00000111670_101830675_101767972_490	annotated	GNPTAB	12	-	1470	12	8	2	1.63%	2.697326785	0.248121237	0.030313544	0.035318603
ENSG00000152556_48130365_48145708_665	annotated	PFKM	12	+	1995	78	14	7	5.71%	12.91877565	1.17E-05	6.48E-07	7.09E-11
ENSG00000161202_184155780_184170755_651	annotated	DVL3	3	+	1953	10	0	0	1.08%	1.691871639	0.007593099	0.007593099	0.000297833
ENSG00000157150_12158840_12153515_224	annotated	TIMP4	3	-	672	31	8	0	6.70%	15.24270605	0.010449648	0.000259634	2.18E-05
ENSG00000078668_42394212_42405462_284	annotated	VDAC3	8	+	852	529	159	11	22.89%	205.1567124	3.53E-10	2.83E-12	1.36E-20
ENSG00000148153_112716485_112687538_104	annotated	INIP	9	-	312	39	4	4	10.58%	41.30281639	0.011978812	0.002345022	0.000161626
ENSG00000109971_123061324_123060047_210	novel	HSPA8	11	-	630	237	16	6	7.14%	124.3018093	0.000315505	0.000325833	6.91E-07
ENSG00000117713_26779173_26780756_527	dORF	ARID1A	1	+	1581	28	0	6	2.47%	5.851885434	0.000642411	0.011011012	4.90E-05
ENSG00000092931_76738413_76742206_94	annotated	MFSD11	17	+	282	14	0	2	6.38%	16.40395545	0.011772051	0.071345384	0.004172987
ENSG00000099622_1270934_1272443_172	annotated	CIRBP	19	+	516	139	14	12	20.35%	89.0091702	1.38E-06	2.48E-06	3.00E-11
ENSG00000196535_29166541_29074770_1906	annotated	MYO18A	17	-	5718	51	9	2	1.47%	2.947105472	7.34E-05	6.55E-06	4.05E-09
ENSG00000096060_35642824_35575835_457	annotated	FKBP5	6	-	1371	34	3	5	3.06%	8.194285965	0.0016955	0.003530286	3.49E-05
ENSG00000151338_37267030_37546971_405	annotated	MIPOL1	14	+	1215	12	1	0	1.98%	3.263432406	0.010499663	0.00419316	0.000236136
ENSG00000168014_74139762_74129539_323	annotated	C2CD3	11	-	969	8	0	0	1.55%	2.727946593	0.019679754	0.019679754	0.001784964
ENSG00000013503_106393067_106509549_880	annotated	POLR3B	12	+	2640	10	1	1	0.57%	1.251600497	0.035374934	0.035374934	0.005300298
ENSG00000136536_159729023_159767352_635	annotated	7-Mar	2	+	1905	39	4	7	3.31%	6.764555755	0.000171526	0.000797853	9.50E-07
ENSG00000115286_1390949_1395488_111	dORF	NDUFS7	19	+	333	70	5	2	15.32%	69.45818972	0.000219998	0.000231683	3.51E-07
ENSG00000112531_163415257_163564710_296	annotated	QKI	6	+	888	54	15	4	6.76%	20.09326203	0.003791753	0.000196649	5.55E-06
ENSG00000105053_50016162_49992901_284	novel	VRK3	19	-	852	19	4	2	2.82%	7.368577572	0.066994826	0.023296852	0.006817054
ENSG00000066855_65670010_65708125_329	novel	MTFR1	8	+	987	55	11	1	6.69%	18.41260305	0.00147859	2.30E-05	3.13E-07
ENSG00000184709_137169943_137168854_334	annotated	LRRC26	9	-	1002	15	0	2	1.50%	4.946445077	0.020613417	0.083503598	0.007749259
ENSG00000134744_52525770_52423935_1474	annotated	TUT4	1	-	4422	21	6	6	1.02%	1.569170772	0.015666274	0.006605717	0.000529246
ENSG00000123472_46668322_46635776_328	annotated	ATPAF1	1	-	984	82	8	0	7.93%	27.53521093	0.000116214	3.47E-06	3.70E-09
ENSG00000130449_61332273_61544317_1215	annotated	ZSWIM6	5	+	3645	27	2	8	0.91%	2.447574305	0.003810897	0.037922586	0.000838503
ENSG00000185963_92764744_92715154_855	annotated	BICD2	9	-	2565	37	4	12	1.99%	4.766328909	0.000893538	0.008340344	4.79E-05
ENSG00000149925_30067255_30070212_368	annotated	ALDOA	16	+	1104	2409	280	109	40.22%	721.0035122	1.39E-23	3.79E-25	9.85E-47
ENSG00000134265_10539849_10550220_197	dORF	NAPG	18	+	591	19	0	1	3.55%	10.62272097	0.008574016	0.010042643	0.000435403
ENSG00000124942_62536098_62433838_131	annotated	AHNAK	11	-	393	27	2	0	3.82%	22.70078458	0.060667625	0.021084099	0.005667716
ENSG00000254995_58652007_58691486_382	annotated	STX16-NPEPL1	20	+	1146	16	0	1	2.09%	4.613229056	0.005454749	0.008219047	0.00023581
ENSG00000254995_58707138_58715326_244	dORF	STX16-NPEPL1	20	+	732	18	0	0	3.28%	8.125144208	0.005257623	0.005257623	0.000148366

ENSG00000106683_74093250_74121301_613	annotated	LIMK1	7	+	1839	28	7	1	2.45%	5.030903138	0.011272488	0.000387679	3.31E-05
ENSG00000122642_32957574_33005351_623	annotated	FKBP9	7	+	1869	171	13	10	5.14%	30.23127492	7.15E-06	2.82E-06	1.71E-10
ENSG00000206503_29942563_29943034_113	novel	HLA-A	6	+	339	16	3	0	8.85%	15.59516371	0.020027431	0.002008257	0.000245088
ENSG00000129292_132794453_132811134_269	annotated	PHF20L1	8	+	807	6	6	0	2.23%	2.456673094	0.529186544	0.007152939	0.046452128
ENSG00000108064_58388757_58394534_111	annotated	TFAM	10	+	333	39	0	2	12.61%	38.69813427	0.000432307	0.001566718	4.40E-06
ENSG00000197122_37384154_37403379_536	annotated	SRC	20	+	1608	274	35	9	11.19%	56.30334174	3.55E-09	8.81E-11	3.82E-18
ENSG00000204120_232747715_232856860_1252	annotated	GIGYF2	2	+	3756	33	7	2	1.52%	2.903073356	0.002130337	0.00013672	2.17E-06
ENSG00000147099_72572761_72551590_417	annotated	HDAC8	X	-	1251	14	1	0	1.68%	3.697774129	0.01551626	0.007882058	0.000614257
ENSG00000108515_4951836_4957047_432	annotated	ENO3	17	+	1296	55	10	0	4.40%	14.02256112	0.000616925	5.91E-05	2.76E-07
ENSG00000136381_78463005_78473390_280	annotated	IREB2	15	+	840	42	0	0	5.71%	16.52112656	0.000196946	0.000196946	2.69E-07
ENSG00000137831_70699624_70657056_1376	annotated	UACA	15	-	4128	7	0	0	0.44%	0.560309525	0.009815329	0.009815329	0.000483714
ENSG00000160199_43013068_43030101_319	Overlap_dORF	PKNOX1	21	+	957	13	7	2	1.88%	4.488498333	0.198071955	0.012798403	0.014686028
ENSG00000090661_8251230_8262109_343	annotated	CERS4	19	+	1029	6	0	0	1.46%	1.926661989	0.016947427	0.016947427	0.001349898
ENSG00000156958_49201181_49328159_434	annotated	GALK2	15	+	1302	43	6	1	3.00%	10.91257207	0.004866125	0.00084676	2.58E-05
ENSG00000167792_67608469_67609836_212	novel	NDUFV1	11	+	636	85	6	1	8.49%	44.16024394	0.000174625	0.000118844	1.47E-07
ENSG00000075239_108134253_108139065_110	annotated	ACAT1	11	+	330	87	3	0	15.45%	87.11139457	0.000140025	0.000140025	1.39E-07
ENSG00000147789_144829088_144843168_686	annotated	ZNF7	8	+	2058	25	1	2	1.90%	4.013879144	0.001044243	0.001969684	1.25E-05
ENSG00000139613_56189461_56163689_1152	annotated	SMARCC2	12	-	3456	77	10	7	2.78%	7.361844588	8.34E-06	1.50E-05	1.01E-09
ENSG00000119723_73950093_73962999_443	annotated	COQ6	14	+	1329	75	8	0	5.87%	18.6468697	2.68E-05	3.58E-06	8.17E-10
ENSG00000186666_49843087_49838371_292	annotated	BCDIN3D	12	-	876	45	3	2	4.11%	16.97376016	0.002962805	0.002213263	3.77E-05
ENSG00000069966_52179879_52141261_100	annotated	GNB5	15	-	300	13	0	0	5.00%	14.31830968	0.021084099	0.021084099	0.002029891
ENSG00000117899_80989791_80979219_234	annotated	MESD	15	-	702	248	64	0	20.94%	116.7304668	7.47E-06	5.01E-10	7.85E-14
ENSG00000032444_7535945_7561562_1287	annotated	PNPLA6	19	+	3861	102	14	10	3.34%	8.729111156	4.15E-07	3.00E-07	1.16E-12
ENSG00000173918_79043969_79048088_281	annotated	C1QTNF1	17	+	843	33	5	6	4.27%	12.93468983	0.003908676	0.010294619	0.000217355
ENSG00000070081_17295324_17317598_338	annotated	NUCB2	11	+	1014	84	9	8	10.36%	27.37228068	3.34E-06	4.12E-06	1.17E-10
ENSG00000141696_41803431_41802957_55	novel	P3H4	17	-	165	70	1	0	21.82%	140.1792556	0.000934493	0.000958906	5.58E-06
ENSG00000213625_65420725_65430201_143	annotated	LEPROT	1	+	429	174	18	6	18.88%	134.0175301	8.10E-05	1.49E-05	9.57E-09
ENSG00000108671_32444524_32447126_90	novel	PSMD11	17	+	270	30	12	0	10.00%	36.71361457	0.102774365	0.003684287	0.002635658
ENSG00000119899_73641807_73635265_175	novel	SLC17A5	6	-	525	16	0	0	4.00%	10.07002	0.008473674	0.008473674	0.000366502
ENSG00000130429_99390975_99394489_178	novel	ARPC1B	7	+	534	174	16	14	23.03%	107.6657686	1.16E-07	5.31E-07	5.91E-13
ENSG00000136854_127651608_127690857_580	annotated	STXBP1	9	+	1740	60	6	0	4.14%	11.39388038	0.000136443	7.48E-06	8.76E-09
ENSG00000147140_71291892_71300076_382	annotated	NONO	X	+	1146	132	12	6	9.69%	38.05913971	1.93E-06	1.78E-07	3.30E-12
ENSG00000181031_327543_213852_286	annotated	RPH3AL	17	-	858	22	7	4	3.15%	8.472372593	0.058211678	0.024209654	0.006109982
ENSG00000075651_171737991_171603078_1016	annotated	PLD1	3	-	3048	34	1	0	1.28%	3.685815636	0.000993237	0.000640157	4.03E-06
ENSG00000198146_34983410_34981359_683	annotated	ZNF770	15	-	2049	17	2	0	1.46%	2.741426564	0.007398015	0.002008257	8.57E-05
ENSG00000162384_53218923_53214659_130	novel	CZIB	1	-	390	76	12	0	14.62%	64.3900317	0.000192256	6.09E-05	8.58E-08
ENSG00000144840_120742507_120689823_212	annotated	RABL3	3	-	636	50	2	0	7.08%	25.97661408	0.000399893	0.000313669	8.39E-07
ENSG00000133393_15888585_15873605_82	annotated	FOPNL	16	-	246	67	15	3	21.95%	89.99312839	0.005357763	0.000181655	7.61E-06
ENSG00000180340_44557689_44559386_565	annotated	FZD2	17	+	1695	165	19	2	7.79%	32.16502515	1.57E-07	4.56E-09	7.97E-15
ENSG00000128581_101321709_101315134_185	annotated	IFT22	7	-	555	50	2	3	6.49%	29.7677956	0.001485237	0.001003278	9.13E-06
ENSG00000148337_128166857_128166197_102	novel	CIZ1	9	-	306	27	3	0	6.86%	29.15492922	0.008776163	0.008776163	0.000391611
ENSG00000130429_99394087_99394489_23	Overlap_dORF	ARPC1B	7	+	69	18	0	0	34.78%	86.19718203	0.005160369	0.005160369	0.000143196
ENSG00000130429_99385715_99394102_352	annotated	ARPC1B	7	+	1056	301	24	25	13.92%	94.18293737	6.26E-09	1.88E-07	1.29E-14
ENSG00000083312_72896466_72905410_181	annotated	TNPO1	5	+	543	18	5	0	2.76%	10.95323308	0.246358339	0.020613417	0.02689978
ENSG00000214655_73789438_73801519_1661	annotated	ZSWIM8	10	+	4983	22	2	2	0.78%	1.458819122	0.001489127	0.001730453	1.54E-05
ENSG00000151292_123545664_123614396_423	annotated	CSNK1G3	5	+	1269	34	6	0	3.31%	8.852928336	0.011011012	0.000375189	3.13E-05
ENSG00000164253_77492220_77433135_404	annotated	WDR41	5	-	1212	60	13	10	5.20%	16.35755105	0.003596403	0.002140485	4.42E-05
ENSG00000134056_69228289_69229225_38	novel	MRPS36	5	+	114	84	7	2	21.05%	243.4692335	0.005355751	0.005998643	0.000171188
ENSG00000113621_134887796_134899685_292	dORF	TXNDC15	5	+	876	233	48	6	18.49%	87.88635816	7.28E-07	4.20E-10	4.74E-15
ENSG00000101132_54215423_54219150_49	novel	PFDN4	20	+	147	44	19	7	14.29%	98.9019821	0.039659082	0.02520501	0.004343134
ENSG00000180398_46909165_46908893_90	novel	MCFD2	2	-	270	136	7	0	14.44%	166.4350527	0.00273178	0.000688595	1.18E-05
ENSG00000120509_70289341_70287082_140	annotated	PDZD11	X	-	420	71	0	0	15.71%	55.85714216	1.63E-05	1.63E-05	2.11E-09
ENSG00000162923_224402006_224389835_229	dORF	WDR26	1	-	687	21	3	2	5.24%	10.10025204	0.005259714	0.004665364	0.000132541
ENSG00000099795_14566910_14566133_92	Overlap_dORF	NDUFB7	19	-	276	201	17	1	25.00%	240.6337998	0.000166988	1.23E-05	1.70E-08
ENSG00000176986_73759740_73771095_975	Overlap_dORF	SEC24C	10	+	2925	132	14	3	4.51%	14.91137576	1.10E-07	1.45E-08	1.65E-14
ENSG00000140299_59677989_59664069_195	annotated	BNIP2	15	-	585	28	3	2	7.69%	15.81509551	0.00418119	0.000657225	1.76E-05
ENSG00000110075_68544884_68551204_120	novel	PPP6R3	11	+	360	44	3	0	11.67%	40.38497603	0.000446789	0.000444132	1.31E-06
ENSG00000205336_57650288_57663582_687	annotated	ADGRG1	16	+	2061	289	28	21	11.21%	46.33290223	1.16E-11	1.43E-12	2.12E-22
ENSG00000148399_137578774_137555239_451	annotated	DPH7	9	-	1353	37	1	1	3.10%	9.035945049	0.000639972	0.000639972	2.63E-06
ENSG00000153487_110719759_110719932_57	annotated	ING1	13	+	171	11	0	1	12.28%	21.25525054	0.007593099	0.013018137	0.000499911
ENSG00000140264_43792377_43793972_133	annotated	SERF2	15	+	399	361	67	210	13.53%	298.9537186	0.000340829	0.001527053	3.45E-06
ENSG00000198954_68988833_69016416_646	annotated	KIF1BP	10	+	1938	69	7	1	4.95%	11.76426968	1.20E-05	3.96E-07	4.65E-11
ENSG00000165322_31908849_31807658_844	annotated	ARHGAP12	10	-	2532	59	9	1	2.61%	7.699419169	0.000962744	2.27E-05	1.92E-07
ENSG00000131778_147266075_147295509_603	dORF	CHD1L	1	+	1809	31	0	2	2.32%	5.662298764	0.000378748	0.001209608	3.00E-06
ENSG00000131778_147254969_147272183_252	annotated	CHD1L	1	+	756	11	1	0	1.98%	4.807735241	0.02854252	0.020613417	0.002645522
ENSG00000131778_147275365_147295509_470	dORF	CHD1L	1	+	1410	24	0	2	2.34%	5.624213296	0.001092093	0.005502968	3.68E-05
ENSG00000136827_129824085_129818194_283	annotated	TOR1A	9	-	849	101	4	7	11.66%	39.3082163	2.73E-07	4.44E-06	1.16E-11
ENSG00000136827_129814118_129813972_48	dORF	TOR1A	9	-	144	41	10	0	16.67%	94.07863733	0.090563067	0.005604867	0.003082269

ENSG00000162402_55159682_55069045_2288	annotated	USP24	1	-	6864	31	4	2	0.96%	1.4922929	0.000220462	4.01E-05	6.69E-08
ENSG00000123159_14479522_14478416_114	novel	GIPC1	19	-	342	67	13	4	21.05%	64.73189937	0.000383829	1.12E-05	3.79E-08
ENSG00000105171_29610472_29615380_179	annotated	POP4	19	+	537	108	18	1	13.41%	66.45369341	0.000145583	9.24E-06	1.14E-08
ENSG00000108946_68515400_68523761_131	annotated	PRKAR1A	17	+	393	86	10	13	12.98%	72.30620274	0.000319304	0.003229477	7.11E-06
ENSG00000172037_49123094_49122621_157	novel	LAMB2	3	-	471	10	4	1	4.46%	7.015340363	0.099364848	0.016854696	0.007969224
ENSG00000136247_6580607_6588910_329	annotated	ZDHHC4	7	+	987	36	2	0	6.38%	12.05188563	0.000116791	1.35E-05	1.28E-08
ENSG00000182944_29292142_29300161_317	novel	EWSR1	22	+	951	116	13	4	7.89%	40.30390495	2.80E-05	1.42E-05	3.15E-09
ENSG00000196352_207331218_207372871_175	annotated	CD55	1	+	525	32	3	1	5.71%	20.14003999	0.004400434	0.003133491	7.68E-05
ENSG00000161526_75699809_75706521_197	novel	SAP30BP	17	+	591	52	4	3	8.12%	29.07271001	0.00020011	0.000786552	1.08E-06
ENSG00000188153_108440126_108696378_1691	annotated	COL4A5	X	+	5073	36	1	2	1.18%	2.344808027	5.22E-05	8.36E-05	3.24E-08
ENSG00000065923_46759029_46606952_725	annotated	SLC9A7	X	-	2175	25	1	0	1.93%	3.797960128	0.000673856	0.000375189	1.66E-06
ENSG00000138785_105700005_105682733_462	annotated	INTS12	4	-	1386	16	2	0	1.52%	3.814401514	0.017404239	0.007882058	0.000688104
ENSG00000114439_107728954_107805457_713	annotated	BBX	3	+	2139	22	6	0	1.68%	3.398455205	0.029833576	0.000873936	0.000196364
ENSG00000144381_197498818_197487046_563	annotated	HSPD1	2	-	1689	327	32	6	7.46%	63.97168009	9.20E-08	2.33E-08	2.16E-14
ENSG00000070018_12203348_12121126_1446	annotated	LRP6	12	-	4338	102	14	6	3.18%	7.769271133	7.52E-07	7.64E-09	6.67E-14
ENSG00000279073_137040373_137032039_93	internal	AL807752.6	9	-	279	22	3	0	7.53%	26.05482324	0.023672501	0.008175611	0.000967328
ENSG00000151726_184776947_184757125_527	annotated	ACSL1	4	-	1581	43	1	0	3.61%	8.986824059	7.83E-05	5.56E-05	3.23E-08
ENSG00000169045_179618370_179617960_108	novel	HNRNPH1	5	-	324	153	22	7	20.37%	156.0328619	0.000239675	6.19E-05	1.09E-07
ENSG00000179886_143598051_143599832_593	annotated	TIGD5	8	+	1779	27	4	7	1.85%	5.014844486	0.00809034	0.016752628	0.000678245
ENSG00000105856_107195883_107200319_142	annotated	HBP1	7	+	426	50	3	0	7.75%	38.78198722	0.004678229	0.001618649	4.44E-05
ENSG00000185813_81908981_81904833_311	annotated	PCYT2	17	-	933	38	4	2	4.82%	13.45772367	0.001015395	0.000339173	2.27E-06
ENSG00000265817_94407640_94372170_717	annotated	FSBP	8	-	2151	18	3	0	1.12%	2.765042101	0.027477461	0.005454749	0.000796727
ENSG00000103811_78931892_78922130_179	novel	CTSH	15	-	537	12	1	0	4.47%	7.383743712	0.010020958	0.004826937	0.000255995
ENSG00000111481_54325164_54347848_132	novel	COPZ1	12	+	396	500	70	12	40.91%	417.2001656	7.30E-09	1.03E-10	9.35E-18
ENSG00000110583_63945897_63954479_216	annotated	NAA40	11	+	648	26	10	1	5.56%	13.25769415	0.055693975	0.001563962	0.000651954
ENSG00000133657_194462190_194405919_1229	annotated	ATP13A3	3	-	3687	122	5	7	3.82%	10.93342793	3.55E-09	2.44E-08	9.25E-16
ENSG00000010278_6232667_6237828_158	annotated	CD9	12	+	474	763	56	16	42.41%	531.8826819	7.88E-12	1.22E-12	1.23E-22
ENSG00000124733_43013378_43012470_172	annotated	MEA1	6	-	516	158	12	13	19.19%	101.1758913	5.55E-07	3.27E-05	1.84E-10
ENSG00000080822_98517142_98515803_66	annotated	CLDND1	3	-	198	47	2	0	16.67%	78.43363112	0.00326373	0.001618649	3.10E-05
ENSG00000148337_128185630_128166197_674	annotated	CIZ1	9	-	2022	101	4	4	4.90%	16.50478518	2.71E-07	1.99E-06	5.08E-12
ENSG00000166557_79311250_79314564_146	annotated	TMED3	15	+	438	82	13	0	15.75%	61.85992592	0.00013633	1.19E-05	1.33E-08
ENSG00000072756_3140639_3148154_277	dORF	TRNT1	3	+	831	20	2	0	2.17%	7.952407488	0.025538366	0.013640586	0.001639124
ENSG00000072756_3129041_3140517_51	annotated	TRNT1	3	+	153	5	0	0	9.80%	10.79812193	0.012673659	0.012673659	0.000782701
ENSG00000182180_73252482_73250852_137	annotated	MRPS16	10	-	411	182	26	17	24.09%	146.3184931	2.11E-06	4.16E-07	8.06E-12
ENSG00000182180_73252482_73249293_99	annotated	MRPS16	10	-	297	109	14	17	19.19%	121.2661815	0.000162446	0.000125119	1.44E-07
ENSG00000178053_158571301_158605185_218	annotated	MLF1	3	+	654	29	0	0	4.59%	14.65176361	0.002376615	0.002376615	3.27E-05
ENSG00000144278_154242763_154409181_316	dORF	GALNT13	2	+	948	18	0	0	2.22%	6.273845528	0.008373824	0.008373824	0.000358384
ENSG00000116678_65570544_65633169_859	annotated	LEPR	1	+	2577	15	2	0	0.93%	1.92329762	0.009656373	0.004826937	0.000246791
ENSG00000007541_574075_576503_396	annotated	PIGQ	16	+	1188	22	1	0	2.78%	6.118935761	0.002106591	0.001491519	1.87E-05
ENSG00000080503_2161713_2192739_236	Overlap_dORF	SMARCA2	9	+	708	14	7	2	2.97%	6.533778864	0.106840601	0.037441831	0.016226997
ENSG00000134574_47234613_47235496_154	novel	DDB2	11	+	462	29	0	0	3.90%	20.74080823	0.013640586	0.013640586	0.000898637
ENSG00000147862_14146773_14088197_281	annotated	NFIB	9	-	843	18	1	0	2.49%	7.055285362	0.0135992	0.008175611	0.00055803
ENSG00000134333_18396843_18400935_46	annotated	LDHA	11	+	138	39	12	14	19.57%	93.38028053	0.032563252	0.036760328	0.00509069
ENSG00000110801_121888857_121903048_179	annotated	PSMD9	12	+	537	101	12	40	11.17%	62.14650958	0.000406552	0.036685252	0.000139698
ENSG00000119537_63367118_63331782_332	annotated	KDSR	18	-	996	66	11	1	6.63%	21.89546893	0.001349558	2.35E-05	2.87E-07
ENSG00000168036_41224552_41239342_768	annotated	CTNNB1	3	+	2304	288	46	13	11.46%	41.30281639	2.21E-12	5.94E-15	2.04E-25
ENSG00000153310_129871436_129849289_217	annotated	FAM49B	8	-	651	27	5	27	3.69%	13.70416028	0.041403097	0.251296779	0.044506808
ENSG00000104783_43780861_43774274_129	Overlap_uORF	KCNN4	19	-	387	51	3	11	9.30%	43.54405449	0.003019138	0.02726739	0.000481776
ENSG00000104783_43776610_43767543_295	annotated	KCNN4	19	-	885	66	35	4	7.46%	24.64168029	0.009802304	7.74E-05	7.62E-06
ENSG00000152601_152300194_152459327_340	annotated	MBNL1	3	+	1020	64	5	8	5.29%	20.73239411	0.000248806	0.002168115	3.74E-06
ENSG00000114120_140942055_140976453_311	annotated	SLC25A36	3	+	933	50	3	9	5.79%	17.70753114	0.000372365	0.002139951	5.29E-06
ENSG00000153015_64769147_65018321_472	annotated	CWC27	5	+	1416	21	0	0	2.12%	4.900334148	0.002254349	0.002254349	2.95E-05
ENSG00000118515_134173142_134170268_288	annotated	SGK1	6	-	864	17	2	0	2.43%	6.501369246	0.024135905	0.008674847	0.001038937
ENSG00000197965_167772389_167787844_85	annotated	MPZL1	1	+	255	120	16	5	22.35%	155.4929558	0.000267156	0.000178746	3.30E-07
ENSG00000163435_202011188_202011352_54	novel	ELF3	1	+	162	86	5	12	27.78%	175.4094918	0.00031447	0.002397262	5.12E-06
ENSG00000118515_134173142_134170268_288	annotated	SGK1	6	-	864	17	2	0	2.43%	6.501369246	0.024135905	0.008674847	0.001038937
ENSG00000170876_14125194_14141795_400	annotated	TMEM43	3	+	1200	209	18	12	14.25%	57.54859084	5.15E-10	5.14E-10	2.98E-18
ENSG00000111907_125229199_125262843_59	novel	TPD52L1	6	+	177	30	7	0	10.17%	56.00381883	0.130134391	0.013640586	0.009212927
ENSG00000111907_125220146_125262962_175	annotated	TPD52L1	6	+	525	38	7	0	6.29%	23.91629749	0.027389432	0.001412569	0.000260603
ENSG00000149970_21374955_21652521_985	annotated	CNKSR2	X	+	2955	13	1	2	0.51%	1.453635501	0.025787932	0.033389602	0.003761814
ENSG00000091129_108232428_108149910_1196	annotated	NRCAM	7	-	3588	107	15	7	4.52%	9.853737689	1.96E-08	1.15E-09	2.53E-16
ENSG00000127564_2977008_2972953_488	annotated	PKMYT1	16	-	1464	31	2	4	3.48%	6.996651957	0.000742282	0.002054176	9.51E-06
ENSG00000077684_128831759_128872262_842	annotated	JADE1	4	+	2526	41	8	1	2.61%	5.363152722	0.002043451	1.35E-05	2.89E-07
ENSG00000183048_81714998_81720077_241	dORF	SLC25A10	17	+	723	80	8	0	8.30%	36.56127592	0.000275782	4.05E-05	8.49E-08
ENSG00000184900_44817968_44808347_147	annotated	SUMO3	21	-	441	54	5	0	8.16%	40.45990177	0.010817194	0.00087769	6.24E-05
ENSG00000196712_31095310_31374155_2839	annotated	NF1	17	+	8517	86	9	9	1.41%	3.336425699	5.03E-07	1.46E-06	6.68E-12
ENSG00000184900_44817968_44806905_135	annotated	SUMO3	21	-	405	58	5	59	10.37%	47.31976989	0.004367894	0.108884544	0.003208371
ENSG00000184900_44807034_44806951_27	internal	SUMO3	21	-	81	59	1	0	18.52%	240.6781399	0.039124093	0.021084099	0.003660118

ENSG00000006576_77840256_77954878_747	annotated	PHTF2	7	+	2241	42	15	5	2.81%	6.192657879	0.015414795	0.000311891	3.98E-05
ENSG00000130414_240025301_239983517_429	annotated	NDUFA10	2	-	1287	92	13	6	5.36%	23.61994783	0.00030413	2.18E-05	5.35E-08
ENSG00000154719_25607460_25585751_348	annotated	MRPL39	21	-	1044	52	1	2	5.75%	16.45782722	5.00E-05	6.70E-05	2.50E-08
ENSG00000077147_96576799_96576626_57	novel	TM9SF3	10	-	171	100	19	0	24.56%	193.2295504	0.000853138	0.000461594	2.55E-06
ENSG00000136935_124899331_124880530_331	dORF	GOLGA1	9	-	993	28	1	2	3.32%	9.317050223	0.002493494	0.004400313	6.21E-05
ENSG00000184254_100894017_100914773_312	annotated	ALDH1A3	15	+	936	16	2	0	2.24%	5.648248395	0.030804354	0.007882058	0.001227455
ENSG00000205084_75556209_75539994_316	annotated	TMEM231	16	-	948	14	0	0	1.58%	4.879657633	0.016947427	0.016947427	0.001349898
ENSG00000106245_99411093_99419441_144	annotated	BUD31	7	+	432	208	30	3	25.00%	159.0923298	1.37E-06	1.58E-07	2.09E-12
ENSG00000107036_5629310_5774246_1386	annotated	RIC1	9	+	4158	31	4	0	1.30%	2.463467644	0.001511532	7.40E-05	8.77E-07
ENSG00000168090_100089304_100091789_297	annotated	COPS6	7	+	891	475	50	14	19.87%	176.151181	2.05E-10	9.15E-11	2.19E-19
ENSG00000143569_154259007_154270919_244	annotated	UBAP2L	1	+	732	22	5	4	3.28%	9.930731809	0.047132153	0.005454749	0.001426183
ENSG00000243989_51987406_51989075_140	novel	ACY1	3	+	420	45	3	4	10.71%	35.40241405	0.0004258	0.001413543	3.91E-06
ENSG00000157184_53210029_53213595_540	dORF	CPT2	1	+	1620	56	2	2	3.89%	11.42201342	7.38E-05	4.69E-05	2.58E-08
ENSG00000198026_45967758_45948953_1079	annotated	ZNF335	20	-	3237	18	0	4	0.74%	1.837382008	0.005554612	0.040065279	0.001211065
ENSG00000113448_59215808_58974664_604	annotated	PDE4D	5	-	1812	139	25	15	7.95%	25.34698224	3.42E-07	4.86E-07	1.54E-12
ENSG00000114520_125504744_125480308_155	annotated	SNX4	3	-	465	25	2	0	5.81%	17.76465221	0.007558271	0.003632191	0.000149492
ENSG00000196756_38434513_38426436_49	novel	SNHG17	20	-	147	26	3	3	14.29%	58.44208033	0.009583875	0.023878474	0.001121714
ENSG00000136710_130342125_130339120_180	annotated	CCDC115	2	-	540	23	0	1	5.00%	14.07355225	0.002967235	0.006432154	0.000105925
ENSG00000179409_748009_744866_1047	annotated	GEMIN4	17	-	3141	132	12	1	4.68%	13.88595164	1.13E-07	4.74E-10	6.97E-16
ENSG00000176095_49728216_49727122_215	annotated	IP6K1	3	-	645	20	0	0	4.19%	10.24565988	0.003478238	0.003478238	6.76E-05
ENSG00000106305_6009433_6023691_228	annotated	AIMP2	7	+	684	129	19	2	15.35%	62.31652999	3.39E-06	9.76E-08	3.40E-12
ENSG00000082212_50917407_50932449_325	novel	ME2	18	+	975	25	4	0	3.38%	8.472372593	0.007779339	0.001484235	6.91E-05
ENSG00000082212_50908093_50940313_434	annotated	ME2	18	+	1302	43	4	0	4.38%	10.91257207	0.000348514	5.47E-05	1.42E-07
ENSG00000082212_50932343_50947184_94	Overlap_dORF	ME2	18	+	282	23	0	0	9.57%	26.94935537	0.003684287	0.003684287	7.54E-05
ENSG00000124155_45420349_45425826_316	novel	PIGT	20	+	948	259	20	2	16.46%	90.2736662	6.03E-10	1.31E-10	9.13E-19
ENSG00000153187_244859284_244857574_176	novel	HNRNPU	1	-	528	141	18	18	17.05%	88.23783501	5.96E-06	0.000138944	7.27E-09
ENSG00000153187_244860523_244860317_68	novel	HNRNPU	1	-	204	36	5	0	14.71%	58.30985843	0.016039993	0.00244612	0.000227917
ENSG00000100664_103334576_103335924_97	Overlap_uORF	EIF5	14	+	291	23	9	0	9.28%	26.11587016	0.055721081	0.003529166	0.001220604
ENSG00000140157_22838748_22838861_37	novel	NIPA2	15	+	111	43	2	4	18.92%	128.0015211	0.008776163	0.032038753	0.00140113
ENSG00000160193_42863454_42850049_266	novel	WDR4	21	-	798	18	3	0	3.76%	7.4531398	0.00841109	0.00224099	0.00010778
ENSG00000221968_61891381_61877820_380	novel	FADS3	11	-	1140	30	8	8	2.89%	8.695329766	0.016975308	0.013368856	0.001084704
ENSG00000137700_119029530_119029333_65	novel	SLC37A4	11	-	195	31	7	0	15.38%	52.52871007	0.019390513	0.002376615	0.000272493
ENSG00000137700_119027649_119024910_228	novel	SLC37A4	11	-	684	39	3	0	6.58%	18.83988116	0.001388942	0.000299491	2.79E-06
ENSG00000151304_121962033_122027126_429	annotated	SRFBP1	5	+	1287	35	9	3	4.66%	8.98584972	0.003669781	0.000244777	6.47E-06
ENSG00000136938_97983562_98015431_249	annotated	ANP32B	9	+	747	282	17	20	21.69%	124.737823	9.75E-10	1.16E-09	1.25E-17
ENSG00000188739_235161211_235160215_127	novel	RBM34	1	-	381	21	6	2	5.51%	18.21226549	0.059950845	0.027657845	0.00704866
ENSG00000138760_76166264_76161713_70	novel	SCARB2	4	-	210	83	2	6	24.29%	130.5955718	0.000173194	0.000531956	6.35E-07
ENSG00000129696_33503815_33474246_185	novel	TTI2	8	-	555	26	0	0	5.41%	15.47925371	0.00224099	0.00224099	2.92E-05
ENSG00000111671_6872901_6872098_267	annotated	SPSB2	12	-	801	22	6	3	3.00%	9.075275511	0.099542334	0.027300312	0.011691174
ENSG00000151553_114833343_114861540_687	annotated	FAM160B1	10	+	2061	32	0	2	2.04%	5.130286752	0.0004093	0.000378748	1.03E-06
ENSG00000156467_96235530_96231105_140	annotated	UQCRB	8	-	420	866	41	17	24.29%	681.2997903	4.75E-07	3.32E-07	1.46E-12
ENSG00000112234_98926778_98874278_551	annotated	FBXL4	6	-	1653	21	2	1	1.63%	4.197745404	0.007532653	0.005034873	0.000201184
ENSG00000198959_38165198_38130219_687	annotated	TGM2	20	-	2061	817	82	37	24.02%	130.9826336	4.59E-24	1.98E-26	1.80E-48
ENSG00000181191_69163238_69161307_643	annotated	PJA1	X	-	1929	16	4	0	1.09%	2.740674182	0.069763484	0.008674847	0.003194238
ENSG00000196199_19633947_19671875_794	annotated	MPHOSPH8	13	+	2382	11	4	0	0.76%	1.525880706	0.095107389	0.012798403	0.006127298
ENSG00000188603_28489349_28477516_384	annotated	CLN3	16	-	1152	182	20	5	13.02%	52.20217071	1.25E-08	3.38E-10	5.00E-17
ENSG00000188603_28491764_28489349_55	Overlap_uORF	CLN3	16	-	165	18	0	12	14.55%	36.0460943	0.005554612	0.310595281	0.015978689
ENSG00000225663_81824506_81823247_97	annotated	MCRIP1	17	-	291	18	8	2	8.25%	20.43850708	0.118715845	0.013892891	0.008395717
ENSG00000156113_77217544_76877869_1026	novel	KCNMA1	10	-	3078	54	8	0	2.63%	5.796886511	4.98E-05	2.14E-06	9.72E-10
ENSG00000204356_31958446_31957400_36	annotated	NELFE	6	-	108	62	6	2	13.89%	189.6870086	0.070558069	0.028889786	0.008603545
ENSG00000152952_146124173_146070717_703	annotated	PLOD2	3	-	2109	657	75	16	19.35%	102.9339037	2.74E-18	2.74E-24	2.14E-40
ENSG00000152952_146160989_146124218_70	uORF	PLOD2	3	-	210	40	3	3	7.14%	62.93762497	0.039807901	0.039124093	0.006483478
ENSG00000127054_1324608_1311859_499	annotated	INTS11	1	-	1497	64	7	4	5.61%	14.12628056	1.52E-05	1.25E-05	1.52E-09
ENSG00000160310_46648473_46658924_163	novel	PRMT2	21	+	489	24	1	4	6.13%	16.21705674	0.003500471	0.010944759	0.000209505
ENSG00000147044_41745600_41520420_804	annotated	CASK	X	-	2412	36	7	0	2.11%	4.931679569	0.002457774	0.000110146	2.10E-06
ENSG00000198836_193637210_193692127_694	dORF	OPA1	3	+	2082	52	7	3	3.75%	8.252628059	0.000244656	1.91E-05	3.78E-08
ENSG00000198836_193593378_193628514_209	annotated	OPA1	3	+	627	13	8	0	2.39%	6.850865876	0.305084881	0.021084099	0.036148407
ENSG00000140025_89954660_89797243_163	annotated	EFCAB11	14	-	489	57	6	4	6.75%	38.51550976	0.014765682	0.009156138	0.000670339
ENSG00000096746_68337222_68337371_49	novel	HNRNPH3	10	+	147	18	6	1	10.20%	40.45990177	0.124431937	0.029237631	0.015647409
ENSG00000096746_68338645_68342054_215	novel	HNRNPH3	10	+	645	140	17	1	15.35%	71.71961916	4.72E-05	2.19E-07	1.21E-10
ENSG00000211460_121755780_121755890_36	novel	TSN	2	+	108	11	0	0	16.67%	33.65414669	0.012798403	0.012798403	0.000797244
ENSG00000090905_24789453_24823807_1692	dORF	TNRC6A	16	+	5076	27	6	10	0.89%	1.757566655	0.004952923	0.015836789	0.000414507
ENSG00000198945_130051269_130139753_677	annotated	L3MBTL3	6	+	2031	7	0	1	0.74%	1.13882704	0.019216965	0.041782825	0.003600526
ENSG00000144559_11844205_11790505_269	annotated	TAMM41	3	-	807	14	0	0	3.35%	5.732237219	0.00308495	0.00308495	5.38E-05
ENSG00000132471_75855297_75846747_231	annotated	WBP2	17	-	693	102	13	1	11.69%	48.6336193	3.21E-05	2.57E-06	7.24E-10
ENSG00000267281_53552589_53506858_431	annotated	ATF7-NPFF	12	-	1293	24	4	0	2.55%	6.133132828	0.009583527	0.001513502	8.74E-05
ENSG00000104763_18071320_18057534_305	dORF	ASAH1	8	-	915	282	30	5	17.70%	101.8351407	5.91E-09	3.14E-10	2.15E-17
ENSG00000162910_228108260_228106760_128	annotated	MRPL55	1	-	384	154	11	4	19.53%	132.5132026	6.15E-06	5.55E-06	2.84E-10

ENSG00000196756_38431089_38426436_38	novel	SNHG17	20	-	114	20	1	3	13.16%	57.96886511	0.028889786	0.065398531	0.007975053
ENSG00000116337_109625635_109631152_760	novel	AMPD2	1	+	2280	8	6	0	0.79%	1.159377302	0.280084718	0.011570466	0.021802746
ENSG00000102119_154379485_154381197_254	annotated	EMD	X	+	762	163	15	1	16.14%	70.68093513	3.16E-07	1.15E-08	3.92E-14
ENSG00000180329_44689753_44678856_224	annotated	CCDC43	17	-	672	36	7	1	7.14%	17.70120702	0.005335206	0.000284799	1.11E-05
ENSG00000021574_32064090_32154496_530	annotated	SPAST	2	+	1590	55	9	0	3.77%	11.4297102	0.002232171	3.76E-05	7.54E-07
ENSG00000124782_7211681_7248968_1461	annotated	RREB1	6	+	4383	11	1	3	0.34%	0.829260288	0.035374934	0.114974528	0.016723634
ENSG00000110881_50059781_50081649_446	annotated	ASIC1	12	+	1338	11	0	0	1.79%	2.716478208	0.004687384	0.004687384	0.000119282
ENSG00000133706_146182493_146142974_142	uORF	LARS	5	-	426	26	4	0	5.63%	20.16663335	0.013648825	0.005454749	0.000388655
ENSG00000133706_146133019_146114106_418	annotated	LARS	5	-	1254	56	4	5	5.26%	14.75571112	7.93E-05	0.00012722	7.31E-08
ENSG00000145882_149358069_149368654_494	annotated	PCYOX1L	5	+	1482	86	4	1	5.47%	19.17431692	1.76E-05	3.09E-06	4.64E-10
ENSG00000108599_19977613_19906227_640	annotated	AKAP10	17	-	1920	28	8	3	2.34%	4.818661912	0.016606353	0.002254447	0.00022063
ENSG00000106682_74174384_74189935_141	novel	EIF4H	7	+	423	78	20	8	4.26%	60.92897737	0.025747276	0.013853925	0.001675292
ENSG00000115364_75655076_75655285_69	annotated	MRPL19	2	+	207	50	10	0	17.39%	79.81220558	0.015193372	0.000962887	9.81E-05
ENSG00000243156_17906812_17864654_1073	annotated	MICAL3	22	-	3219	17	4	0	0.65%	1.745008707	0.082535828	0.007882058	0.003585402
ENSG00000105426_5286095_5206774_1933	annotated	PTPRS	19	-	5799	169	14	11	3.41%	9.629489181	8.84E-11	2.14E-11	2.28E-20
ENSG00000186185_44936344_44926400_833	annotated	KIF18B	17	-	2499	30	6	7	1.80%	3.966657036	0.004228386	0.005279354	0.000121249
ENSG00000177697_836070_838192_253	annotated	CD151	11	+	759	1246	99	44	39.53%	542.4327718	2.53E-15	1.13E-16	4.29E-30
ENSG00000088836_3234760_3227787_778	annotated	SLC4A11	20	-	2334	7	3	0	0.64%	0.990984455	0.169362074	0.019216965	0.016163992
ENSG00000131238_40097238_40074061_203	annotated	PPT1	1	-	609	253	20	0	20.69%	137.2691303	8.52E-08	7.02E-09	6.35E-15
ENSG00000197563_62161260_62045856_900	annotated	PIGN	18	-	2700	90	12	0	3.89%	11.01408437	1.48E-05	8.70E-08	1.49E-11
ENSG00000182199_57230833_57234361_483	annotated	SHMT2	12	+	1449	197	37	5	11.59%	44.92287	2.66E-07	1.80E-10	7.35E-16
ENSG00000141552_81894478_81900065_84	annotated	ANAPC11	17	+	252	694	48	29	25.00%	909.9731611	0.000200778	0.00015937	2.23E-07
ENSG00000167965_2206086_2208877_326	annotated	MLST8	16	+	978	109	18	4	11.66%	36.82623302	1.36E-05	2.83E-07	3.92E-11
ENSG00000213614_72375972_72346662_373	annotated	HEXA	15	-	1119	341	48	20	21.98%	100.6917633	3.11E-11	4.48E-13	1.91E-22
ENSG00000136152_45465037_45493494_444	annotated	COG3	13	+	1332	21	1	1	2.70%	5.209364229	0.001635336	0.001635336	1.60E-05
ENSG00000083799_50749699_50796508_953	annotated	CYLD	16	+	2859	26	7	4	1.36%	3.004891853	0.012643165	0.005714521	0.000375455
ENSG00000007923_6701800_6653833_194	novel	DNAJC11	1	-	582	38	9	3	5.67%	21.57397969	0.001747087	0.003807034	3.87E-05
ENSG00000101019_35381953_35347071_122	dORF	UQCC1	20	-	366	15	0	1	6.56%	13.54190701	0.005405142	0.008170681	0.00023249
ENSG00000128944_40383253_40391989_163	annotated	KNSTRN	15	+	489	58	11	4	11.04%	39.19122046	0.00241785	0.000526389	8.20E-06
ENSG00000106459_129690424_129755181_342	annotated	NRF1	7	+	1026	12	1	2	2.05%	3.864591007	0.007882058	0.037441831	0.00150521
ENSG00000100225_32493041_32498530_221	novel	FBXO7	22	+	663	45	4	0	8.14%	22.42686863	0.000261983	8.81E-05	1.66E-07
ENSG00000100211_38668055_38671257_123	novel	CBY1	22	+	369	16	8	4	6.50%	14.32726422	0.099279224	0.034975232	0.014242308
ENSG00000204356_31958446_31956976_57	annotated	NELFE	6	-	171	63	6	2	10.53%	121.7346167	0.053528973	0.020342454	0.004843608
ENSG00000020426_60818845_60968349_81	annotated	MNAT1	14	+	243	12	1	0	6.17%	16.31716203	0.035374934	0.020613417	0.003252526
ENSG00000140105_100361879_100334875_424	annotated	WARS	14	-	1272	141	24	4	7.55%	36.62702585	3.08E-05	1.38E-06	3.93E-10
ENSG00000184752_94996976_94971440_113	Overlap_dORF	NDUFA12	12	-	339	201	32	1	18.58%	195.9142441	0.000433578	3.12E-05	1.07E-07
ENSG00000087884_77842497_77872315_122	annotated	AAMDC	11	+	366	32	0	1	8.20%	28.88940163	0.002335453	0.003081498	4.12E-05
ENSG00000182134_151778838_151774452_318	dORF	TDRKH	1	-	954	34	4	0	4.72%	11.77606505	0.001790076	0.000237194	2.94E-06
ENSG00000111596_70310907_70353915_520	annotated	AC092881.1	12	+	1560	28	6	0	3.46%	5.930660815	0.001089199	6.79E-05	5.72E-07
ENSG00000063177_48619143_48615372_133	annotated	RPL18	19	-	399	164	33	4	19.55%	135.8127697	0.000246522	7.75E-06	1.70E-08
ENSG00000204520_31412115_31415139_124	Overlap_dORF	MICA	6	+	372	39	1	11	8.87%	34.64107181	0.0020028	0.015073811	0.000179882
ENSG00000112378_138107340_138092042_193	annotated	PERP	6	-	579	997	180	23	34.20%	568.9659128	2.64E-11	6.00E-13	2.13E-22
ENSG00000248643_66616721_66639806_147	annotated	RBM14-RBM4	11	+	441	21	1	0	6.12%	15.73440624	0.004333803	0.003529166	8.46E-05
ENSG00000248643_66639712_66644132_364	Overlap_dORF	RBM14-RBM4	11	+	1092	40	4	0	4.95%	12.10338942	0.000663461	5.74E-05	2.91E-07
ENSG00000120733_138415143_138435700_691	dORF	KDM3B	5	+	2073	19	7	1	1.88%	3.028474718	0.051125687	0.001035355	0.000429181
ENSG00000165152_101476912_101475881_343	annotated	TMEM246	9	-	1029	26	2	0	2.92%	8.348868619	0.003737369	0.002227676	4.76E-05
ENSG00000240065_32856177_32859532_186	annotated	PSMB9	6	+	558	28	2	0	6.45%	16.58034206	0.00236973	0.000958906	1.39E-05
ENSG00000174953_154324416_154276171_979	annotated	DHX36	3	-	2937	42	7	2	1.84%	4.725143448	0.003449312	0.000370858	8.70E-06
ENSG00000094631_48802964_48824612_1153	annotated	HDAC6	X	+	3459	25	3	5	1.04%	2.388136247	0.007364879	0.00858612	0.000325247
ENSG00000115355_55322644_55291711_754	annotated	CCDC88A	2	-	2262	25	0	0	1.86%	3.651884738	0.00040182	0.00040182	1.07E-06
ENSG00000135679_68809227_68835836_180	annotated	MDM2	12	+	540	13	0	0	3.89%	7.95461649	0.007593099	0.007593099	0.000297833
ENSG00000135679_68839304_68839849_181	dORF	MDM2	12	+	543	15	0	4	3.87%	9.12769423	0.008473674	0.069273208	0.003110985
ENSG00000073584_40631717_40628785_181	novel	SMARCE1	17	-	543	33	3	0	3.87%	20.08092731	0.01551626	0.007882058	0.000614257
ENSG00000139154_19439943_19518117_422	annotated	AEBP2	12	+	1266	53	5	2	4.74%	13.83285478	0.000409332	0.000140482	4.01E-07
ENSG00000104835_38930724_38921582_131	annotated	SARS2	19	-	393	29	1	7	7.63%	24.38232418	0.002335453	0.022401729	0.000314101
ENSG00000013306_44323562_44320001_351	annotated	SLC25A39	17	-	1053	148	14	2	13.39%	46.44115347	4.89E-08	9.53E-10	5.46E-16
ENSG00000168096_4714099_4697028_436	dORF	ANKS3	16	-	1308	23	4	4	1.38%	5.810182122	0.061051993	0.059897465	0.01414689
ENSG00000157193_53266654_53247018_414	dORF	LRP8	1	-	1242	48	16	2	5.56%	12.76995289	0.016154166	2.21E-05	5.37E-06
ENSG00000075711_197297204_197044623_871	annotated	DLG1	3	-	2613	39	4	0	2.07%	4.931679569	0.000762836	7.71E-05	4.39E-07
ENSG00000137074_32989888_32973498_341	annotated	APTX	9	-	1023	71	11	4	6.16%	22.93255104	0.000977537	2.97E-05	2.46E-07
ENSG00000177337_3596842_3597228_128	novel	DLGAP1-AS1	18	+	384	8	0	0	3.91%	6.883802732	0.019679754	0.019679754	0.001784964
ENSG00000145414_163166727_163128897_494	annotated	NAF1	4	-	1482	28	3	2	3.64%	6.242800858	0.000656474	4.85E-05	2.47E-07
ENSG00000167965_2206613_2207522_150	novel	MLST8	16	+	450	33	2	0	9.33%	24.23098562	0.001186298	0.000446789	3.43E-06
ENSG00000133142_103586676_103587323_215	annotated	TCEAL4	X	+	645	59	11	1	7.44%	30.22469664	0.000389694	0.000251967	6.63E-07
ENSG00000151967_159886220_159887923_106	novel	SCHIP1	3	+	318	11	0	0	5.66%	11.4297102	0.012798403	0.012798403	0.000797244
ENSG00000142541_49487630_49490915_130	novel	RPL13A	19	+	390	37	4	0	4.62%	31.34777859	0.031019588	0.013428348	0.00195881
ENSG00000205336_57650288_57663582_687	annotated	ADGRG1	16	+	2061	289	28	21	11.21%	46.33290223	1.16E-11	1.43E-12	2.12E-22
ENSG00000122691_19117318_19116713_201	annotated	TWIST1	7	-	603	10	0	2	2.49%	5.479643965	0.019679754	0.101051022	0.009164498

ENSG00000167526_89562377_89563042_57	novel	RPL13	16	+	171	199	5	1	19.30%	384.5268052	0.001657226	0.001672809	1.65E-05
ENSG00000167526_89560981_89561506_120	novel	RPL13	16	+	360	255	162	55	12.50%	234.0492929	0.123191463	0.009779265	0.006745202
ENSG00000112306_132814769_132815191_77	novel	RPS12	6	+	231	60	16	6	10.39%	85.82403406	0.039465372	0.041065427	0.006725392
ENSG00000112306_132816507_132817542_73	novel	RPS12	6	+	219	27	3	0	9.59%	40.73702438	0.008574016	0.008776163	0.000383092
ENSG00000100316_39318439_39317457_70	novel	RPL3	22	-	210	501	47	11	31.43%	788.2937528	1.93E-05	2.12E-05	3.22E-09
ENSG00000244005_35698507_35669622_397	annotated	NFS1	20	-	1191	76	9	6	7.05%	21.08489703	5.58E-05	1.31E-05	5.81E-09
ENSG00000115275_74464929_74461275_637	annotated	MOGS	2	-	1911	77	3	0	4.71%	13.31372836	2.24E-06	7.18E-07	1.44E-11
ENSG00000090020_27103309_27100307_319	novel	SLC9A1	1	-	957	12	0	0	2.19%	4.14322923	0.008175611	0.008175611	0.000342518
ENSG00000137074_32984722_32973498_116	dORF	APTX	9	-	348	16	1	1	5.17%	15.19184051	0.012798403	0.01300724	0.000809468
ENSG00000137074_32989888_32984844_67	annotated	APTX	9	-	201	38	0	1	10.45%	62.46794121	0.008877961	0.008877961	0.000400235
ENSG00000242612_410305_411578_159	annotated	DECR2	16	+	477	24	0	0	5.66%	16.62503301	0.003554776	0.003554776	7.04E-05
ENSG00000183283_51238908_51242458_86	annotated	DAZAP2	12	+	258	31	7	0	6.98%	39.70193203	0.130723026	0.013640586	0.009261742
ENSG00000163950_1699682_1693597_150	annotated	SLBP	4	-	450	136	2	7	18.67%	99.86103163	1.74E-06	1.05E-05	1.59E-10
ENSG00000007923_6701800_6635675_559	annotated	DNAJC11	1	-	1677	86	22	3	5.55%	16.94474519	0.00015955	1.53E-06	2.54E-09
ENSG00000129925_377092_371987_578	annotated	TMEM8A	16	-	1734	27	9	2	2.60%	5.144987509	0.017772798	0.000437802	6.17E-05
ENSG00000129084_14610986_14505192_269	annotated	PSMA1	11	-	807	268	36	7	21.56%	109.7313982	1.08E-08	7.25E-11	1.02E-17
ENSG00000243989_51988530_51989075_99	novel	ACY1	3	+	297	27	1	4	8.08%	30.03841192	0.005355751	0.025702836	0.000731269
ENSG00000128973_68209731_68208140_121	dORF	CLN6	15	-	363	85	19	4	11.57%	77.37166707	0.000858719	0.000480911	2.66E-06
ENSG00000128973_68229584_68211645_171	annotated	CLN6	15	-	513	32	2	0	6.43%	20.61115204	0.001269017	0.001412569	1.09E-05
ENSG00000180900_143815372_143791163_1655	annotated	SCRIB	8	-	4965	93	26	15	2.24%	6.189183362	0.000314366	2.12E-05	5.41E-08
ENSG00000116514_32948253_32937288_270	annotated	RNF19B	1	-	810	10	2	1	1.85%	4.079290508	0.065565251	0.027849813	0.007750595
ENSG00000135597_138930024_138905064_366	dORF	REPS1	6	-	1098	19	1	3	2.46%	5.717694072	0.006112544	0.021725767	0.000688083
ENSG00000177674_11736209_11750192_152	annotated	AGTRAP	1	+	456	203	23	12	17.11%	147.0959952	8.53E-06	4.51E-05	3.14E-09
ENSG00000177674_11747543_11750192_104	Overlap_dORF	AGTRAP	1	+	312	162	10	9	17.31%	171.565545	9.38E-05	0.000868713	6.00E-07
ENSG00000204590_30556203_30546072_607	annotated	GNL1	6	-	1821	21	4	1	1.98%	3.810474	0.010716902	0.001410049	9.26E-05
ENSG00000182944_29268337_29300161_661	annotated	EWSR1	22	+	1983	171	24	7	5.90%	28.49331963	2.00E-06	1.62E-07	3.13E-12
ENSG00000175063_45812696_45816767_161	annotated	UBE2C	20	+	483	177	27	3	13.66%	121.0865176	0.000114103	3.87E-05	3.32E-08
ENSG00000120533_109334469_109343481_101	annotated	ENY2	8	+	303	361	59	10	36.63%	393.6717285	2.14E-07	1.53E-07	3.11E-13
ENSG00000175581_73844903_73864370_113	dORF	MRPL48	11	+	339	71	8	2	7.08%	69.20353897	0.021450603	0.012008015	0.001232895
ENSG00000187109_76056041_76048429_208	annotated	NAP1L1	12	-	624	117	17	12	9.62%	61.95422458	0.000544045	0.00027863	1.01E-06
ENSG00000161011_179820937_179821567_73	annotated	SQSTM1	5	+	219	60	3	3	21.92%	90.52672085	0.000940126	0.000984975	5.76E-06
ENSG00000258881_70994748_70948370_48	annotated	AC007040.2	2	-	144	34	7	1	14.58%	78.01643096	0.037440157	0.013991246	0.002451139
ENSG00000068912_53794387_53817969_415	annotated	ERLEC1	2	+	1245	92	22	0	10.36%	24.41676535	1.01E-05	3.59E-09	6.01E-13
ENSG00000104343_73810626_73795889_84	Overlap_dORF	UBE2W	8	-	252	14	1	0	8.33%	18.35680728	0.008175611	0.007882058	0.000330894
ENSG00000104343_73878813_73810554_57	annotated	UBE2W	8	-	171	16	1	9	8.77%	30.91672806	0.035772986	0.286758969	0.047240597
ENSG00000182372_1771055_1780567_286	annotated	CLN8	8	+	858	44	5	1	4.90%	16.94474519	0.004245653	0.000410556	1.19E-05
ENSG00000078674_17935704_18027662_1985	annotated	PCM1	8	+	5955	214	31	21	3.88%	11.87412622	3.28E-10	1.03E-11	4.27E-20
ENSG00000158710_159920509_159918800_199	annotated	TAGLN2	1	-	597	803	97	45	25.63%	444.4376759	1.03E-08	9.22E-09	9.89E-16
ENSG00000158710_159920509_159918800_199	annotated	TAGLN2	1	-	597	803	97	45	25.63%	444.4376759	1.03E-08	9.22E-09	9.89E-16
ENSG00000113742_173910577_173956137_328	annotated	CPEB4	5	+	984	25	1	0	3.66%	8.39488138	0.001302568	0.000966879	7.76E-06
ENSG00000154582_73959768_73946630_112	annotated	ELOC	8	-	336	83	32	2	17.86%	81.62223239	0.020681041	9.81E-05	2.29E-05
ENSG00000057663_106248206_106186540_103	Overlap_dORF	ATG5	6	-	309	22	7	1	7.77%	23.52522875	0.058487168	0.007225456	0.002270273
ENSG00000053770_57269315_57275024_284	novel	AP5M1	14	+	852	28	6	1	4.58%	10.85895642	0.0123411	0.000993237	8.00E-05
ENSG00000053770_57269196_57269291_31	novel	AP5M1	14	+	93	24	1	0	16.13%	85.27033061	0.021084099	0.021084099	0.002029891
ENSG00000159593_66830899_66823561_85	annotated	NAE1	16	-	255	11	1	4	5.88%	14.25352095	0.035374934	0.12312585	0.017966277
ENSG00000162032_1777975_1777097_191	novel	SPSB3	16	-	573	11	4	0	3.14%	6.343189952	0.08791702	0.012798403	0.005611737
ENSG00000162032_1781483_1778295_147	novel	SPSB3	16	-	441	19	2	6	6.12%	14.23589136	0.013091824	0.039731909	0.002458944
ENSG00000080822_98522855_98516659_158	annotated	CLDND1	3	-	474	118	22	0	18.35%	82.25708581	3.16E-06	1.13E-06	3.13E-11
ENSG00000142599_8361310_8355087_834	annotated	RERE	1	-	2502	10	0	0	0.60%	1.320633618	0.020613417	0.020613417	0.001946209
ENSG00000197912_89508547_89557093_737	annotated	SPG7	16	+	2211	92	21	4	4.48%	13.74892486	0.000412676	1.20E-06	5.99E-09
ENSG00000108641_19362569_19343710_183	annotated	B9D1	17	-	549	48	6	5	7.65%	28.88940163	0.004428796	0.003666397	8.95E-05
ENSG00000108641_19343402_19343319_27	dORF	B9D1	17	-	81	10	0	0	18.52%	40.79290508	0.020613417	0.020613417	0.001946209
ENSG00000243147_27774440_27779482_46	novel	MRPL33	2	+	138	323	25	15	39.13%	773.3802721	0.003479145	0.000915487	1.96E-05
ENSG00000137947_88887291_88853319_194	annotated	GTF2B	1	-	582	26	1	3	7.73%	14.761144	0.000432054	0.000231319	6.77E-07
ENSG00000101150_63865366_63889945_209	annotated	TPD52L2	20	+	627	191	28	6	18.66%	100.6550294	2.10E-06	8.83E-08	1.88E-12
ENSG00000078808_1218796_1217512_126	novel	SDF4	1	-	378	156	8	2	23.81%	136.3648541	1.34E-06	8.98E-07	1.07E-11
ENSG00000079335_100351757_100499379_624	annotated	CDC14A	1	+	1872	12	0	0	0.80%	2.118093148	0.021084099	0.021084099	0.002029891
ENSG00000077463_4182539_4174898_187	annotated	AC016586.1	19	-	561	21	3	2	3.21%	12.36875785	0.051745348	0.037440157	0.00795922
ENSG00000143870_10812696_10784258_440	annotated	PDIA6	2	-	1320	1990	297	74	37.95%	498.1369977	1.16E-20	8.72E-28	3.48E-46
ENSG00000170653_53552589_53517137_462	annotated	ATF7	12	-	1386	27	4	0	2.81%	6.436802554	0.004587372	0.000656046	1.94E-05
ENSG00000089916_76154364_76173697_351	novel	GPATCH2L	14	+	1053	40	3	0	4.27%	12.5516631	0.000906967	0.000291049	1.76E-06
ENSG00000143401_150235780_150220691_266	annotated	ANP32E	1	-	798	248	33	3	21.80%	102.6877039	1.12E-08	4.17E-11	6.31E-18
ENSG00000099949_20989635_20997348_639	annotated	LZTR1	22	+	1917	17	8	3	1.56%	2.93019459	0.138719553	0.018949244	0.012679138
ENSG00000133835_119471700_119541994_699	annotated	HSD17B4	5	+	2097	109	10	7	5.01%	17.17503857	1.44E-05	2.07E-06	2.59E-10
ENSG00000147044_41745600_41520420_805	annotated	CASK	X	-	2415	35	7	0	1.99%	4.788732335	0.003568196	0.00017249	4.64E-06
ENSG00000215301_41334253_41347468_641	annotated	DDX3X	X	+	1923	224	48	10	8.74%	38.48915599	1.60E-07	2.03E-10	4.68E-16
ENSG00000151491_15682912_15621317_817	annotated	EPS8	12	-	2451	79	10	0	3.18%	10.65009382	0.000507204	3.18E-06	1.73E-08
ENSG00000104998_14031873_14052290_636	annotated	IL27RA	19	+	1908	52	3	0	2.67%	9.005226215	0.000538851	0.000130272	4.94E-07

ENSG00000086544_40717205_40739560_660	annotated	ITPKC	19	+	1980	14	2	1	1.67%	2.336320927	0.010137502	0.002521511	0.000144889
ENSG00000141867_15273099_15238377_1362	annotated	BRD4	19	-	4086	43	5	4	1.69%	3.477280675	0.000125939	0.000232778	2.06E-07
ENSG00000168092_117167580_117167699_39	annotated	PAFAH1B2	11	+	117	28	2	0	15.38%	79.07547753	0.016983117	0.013640586	0.00110551
ENSG00000105968_44847971_44834501_102	annotated	H2AFV	7	-	306	193	14	11	17.65%	208.4037533	0.00043473	0.00024186	7.11E-07
ENSG00000103197_2050439_2088610_1725	annotated	TSC2	16	+	5175	65	6	1	1.51%	4.15023469	7.80E-06	3.46E-06	2.27E-10
ENSG00000168701_67227149_67227879_39	Overlap_uORF	TMEM208	16	+	117	41	3	0	30.77%	115.7890921	0.00507972	0.000962887	3.03E-05
ENSG00000168701_67227219_67229113_173	annotated	TMEM208	16	+	519	75	13	11	12.72%	47.74892068	0.000354446	0.000481433	1.13E-06
ENSG00000165171_73842140_73840100_90	novel	METTL27	7	-	270	22	11	0	6.67%	26.92331735	0.127877374	0.013640586	0.009026432
ENSG00000185344_123712593_123737352_363	annotated	ATP6V0A2	12	+	1089	26	1	2	2.75%	7.888875858	0.003247793	0.005426623	9.74E-05
ENSG00000117868_158798064_158734207_717	annotated	ESYT2	7	-	2151	192	26	11	7.67%	29.49378241	7.32E-08	2.53E-09	2.08E-15
ENSG00000144677_37862200_37980867_276	annotated	CTDSPL	3	+	828	30	8	1	3.99%	11.97183084	0.055693975	0.003247793	0.001143427
ENSG00000168385_241324233_241350174_361	annotated	2-Sep	2	+	1083	341	37	9	17.73%	104.0388579	9.15E-09	2.44E-12	3.85E-19
ENSG00000213123_196316149_196291327_77	dORF	TCTEX1D2	3	-	231	51	1	0	9.09%	72.95042895	0.008877961	0.008877961	0.000400235
ENSG00000144560_11601909_11558556_231	annotated	VGLL4	3	-	693	17	0	0	2.60%	8.105603216	0.013428348	0.013428348	0.000872559
ENSG00000072756_3129041_3129944_57	annotated	TRNT1	3	+	171	5	0	0	8.77%	9.661477518	0.012673659	0.012673659	0.000782701
ENSG00000136536_159752538_159767352_108	annotated	7-Mar	2	+	324	12	4	2	6.48%	12.23787152	0.071345384	0.053594825	0.014791652
ENSG00000103187_84617914_84566845_142	annotated	COTL1	16	-	426	356	45	27	30.28%	276.127749	1.23E-07	1.74E-07	2.06E-13
ENSG00000213024_49909807_49908239_522	annotated	NUP62	19	-	1566	78	10	7	5.17%	16.45782722	4.18E-06	3.35E-05	1.19E-09
ENSG00000096063_35886775_35835304_513	novel	SRPK1	6	-	1539	37	8	2	3.51%	7.943881515	0.001428885	0.000176878	1.78E-06
ENSG00000182220_40581066_40605755_350	annotated	ATP6AP2	X	+	1050	215	42	6	16.86%	67.65794685	1.24E-08	2.95E-11	5.13E-18
ENSG00000099622_1270934_1274311_168	annotated	CIRBP	19	+	504	139	14	12	20.83%	91.12843616	1.38E-06	2.48E-06	3.00E-11
ENSG00000047579_15662869_15522975_351	annotated	DTNBP1	6	-	1053	12	2	0	2.56%	3.76549893	0.012769128	0.002874854	0.000206279
ENSG00000002549_17577559_17607589_488	annotated	LAP3	4	+	1464	74	6	0	5.12%	16.70168532	4.37E-05	5.46E-06	2.00E-09
ENSG00000211455_27315300_27322455_202	dORF	STK38L	12	+	606	14	4	0	2.48%	7.633523821	0.121807694	0.021084099	0.011872686
ENSG00000099282_69495265_69506978_228	novel	TSPAN15	10	+	684	140	34	7	13.16%	67.63034263	0.000120586	4.61E-06	4.96E-09
ENSG00000213923_38303150_38293287_358	annotated	CSNK1E	22	-	1074	83	7	5	6.15%	25.535447	0.000124209	0.000118485	1.05E-07
ENSG00000108654_64504108_64499923_509	dORF	DDX5	17	-	1527	321	51	28	14.15%	69.46013915	1.46E-09	2.38E-12	5.18E-20
ENSG00000086189_62403772_62398492_154	annotated	DIMT1	5	-	462	38	3	0	11.04%	27.17761078	0.00069231	0.000120773	5.96E-07
ENSG00000124596_41071634_41070022_98	novel	OARD1	6	-	294	34	0	1	15.31%	38.21212945	0.000259634	0.000437499	7.65E-07
ENSG00000055332_37147776_37107273_500	annotated	EIF2AK2	2	-	1500	65	15	5	6.60%	14.31830968	1.91E-05	2.56E-06	4.22E-10
ENSG00000267261_42130502_42120326_161	annotated	AC099811.2	17	-	483	174	14	24	21.74%	119.0342038	2.32E-06	3.41E-05	7.03E-10
ENSG00000167645_38308847_38305352_153	annotated	YIF1B	19	-	459	254	3	10	17.65%	182.848198	9.27E-06	2.97E-05	2.22E-09
ENSG00000182093_39390698_39396939_140	annotated	WRB	21	+	420	150	26	9	25.71%	118.0080468	6.64E-07	1.23E-07	7.81E-13
ENSG00000152234_46098231_46084282_553	annotated	ATP5F1A	18	-	1659	976	133	29	26.22%	194.3896265	2.26E-19	4.69E-25	2.87E-42
ENSG00000041880_51943356_51948480_533	annotated	PARP3	3	+	1599	10	3	0	0.94%	2.06643234	0.114974528	0.020613417	0.0109456
ENSG00000164211_111507433_111499886_205	annotated	STARD4	5	-	615	94	18	6	11.71%	50.50360638	0.000544766	6.60E-05	2.69E-07
ENSG00000138772_78604313_78610115_48	novel	ANXA3	4	+	144	82	8	2	22.92%	188.1572747	0.002009905	0.001603369	1.91E-05
ENSG00000075624_5529657_5527748_375	annotated	ACTB	7	-	1125	5496	336	114	26.93%	1614.224205	1.38E-15	1.48E-16	3.03E-30
ENSG00000175029_125003077_124989518_365	annotated	CTBP2	10	-	1095	28	2	4	4.11%	8.449160613	0.000924855	0.004886527	2.81E-05
ENSG00000125166_58716156_58709243_155	novel	GOT2	16	-	465	114	5	2	9.68%	81.00681408	0.000314841	0.000325012	6.88E-07
ENSG00000085449_223945284_223878671_410	annotated	WDFY1	2	-	1230	38	9	3	4.39%	10.20817576	0.009070584	0.000524078	3.33E-05
ENSG00000151135_106971140_106971391_83	annotated	TMEM263	12	+	249	17	10	0	10.84%	22.55896799	0.17974379	0.003303074	0.005106915
ENSG00000076108_56617530_56598618_1903	annotated	BAZ2A	12	-	5709	78	8	6	2.15%	4.51444341	9.66E-07	2.52E-07	2.27E-12
ENSG00000221914_26361001_26370413_285	novel	PPP2R2A	8	+	855	86	1	0	9.82%	33.23548266	1.56E-06	1.57E-06	2.15E-11
ENSG00000106028_141739167_141750354_148	annotated	SSBP1	7	+	444	456	23	8	32.43%	339.3528698	6.91E-10	1.38E-09	1.06E-17
ENSG00000137210_10749202_10756518_103	annotated	TMEM14B	6	+	309	79	21	0	12.62%	84.47695779	0.008966975	0.000710064	4.25E-05
ENSG00000142534_49497931_49499635_79	Overlap_dORF	RPS11	19	+	237	1460	86	33	37.97%	2035.514327	2.10E-06	1.01E-06	1.89E-11
ENSG00000140564_90875741_90881878_794	annotated	FURIN	15	+	2382	86	9	0	4.03%	11.92961279	2.61E-05	3.11E-07	8.57E-11
ENSG00000132031_20012631_19993111_444	annotated	MATN3	2	-	1332	29	3	0	2.25%	7.193883936	0.012954922	0.002254349	0.00016955
ENSG00000108219_80489234_80517976_253	annotated	TSPAN14	10	+	759	438	57	62	22.13%	190.6786148	2.07E-08	9.19E-06	2.47E-12
ENSG00000188603_28489349_28477516_287	annotated	CLN3	16	-	861	138	18	5	11.50%	52.95970882	6.17E-06	2.62E-07	1.58E-11
ENSG00000188603_28491764_28489349_55	Overlap_uORF	CLN3	16	-	165	18	0	12	14.55%	36.0460943	0.005554612	0.310595281	0.015978689
ENSG00000039650_49867204_49861248_485	annotated	PNKP	19	-	1455	26	6	0	2.27%	5.904457601	0.013398591	0.001513502	0.000124923
ENSG00000101940_48598952_48605032_393	novel	WDR13	X	+	1179	23	3	0	2.04%	6.445901794	0.005554612	0.005604867	0.000166099
ENSG00000127054_1313734_1312783_142	novel	INTS11	1	-	426	13	7	0	4.23%	10.08331668	0.19866071	0.012798403	0.014741692
ENSG00000104960_49851329_49860279_416	annotated	PTOV1	19	+	1248	142	10	10	12.02%	37.59615338	5.90E-09	8.23E-09	5.10E-16
ENSG00000076944_7642293_7647810_310	dORF	STXBP2	19	+	930	23	4	1	3.87%	8.171740017	0.009176002	0.001635336	8.94E-05
ENSG00000141646_51054795_51078467_396	novel	SMAD4	18	+	1188	34	4	0	3.54%	9.456537086	0.001743983	0.00040306	4.61E-06
ENSG00000141429_35663759_35709770_469	dORF	GALNT1	18	+	1407	131	27	6	8.74%	30.76428683	5.88E-05	4.95E-08	4.26E-11
ENSG00000141429_35654663_35659871_50	annotated	GALNT1	18	+	150	18	5	0	14.00%	39.65070373	0.044329616	0.008473674	0.001912029
ENSG00000129083_14498928_14457824_953	annotated	COPB1	11	-	2859	130	24	2	4.93%	15.02445927	6.06E-07	1.42E-09	1.15E-14
ENSG00000141526_82236009_82238976_465	annotated	SLC16A3	17	+	1395	443	50	5	16.99%	104.9298791	3.54E-11	1.58E-14	9.53E-24
ENSG00000169604_69013500_69172384_368	annotated	ANTXR1	2	+	1104	597	73	14	21.47%	178.6795753	7.07E-10	4.63E-14	6.52E-22
ENSG00000160294_46267052_46246620_612	novel	MCM3AP	21	-	1836	20	0	0	1.47%	3.599373977	0.003529166	0.003529166	6.95E-05
ENSG00000115084_113755876_113742677_248	annotated	SLC35F5	2	-	744	123	12	2	10.08%	54.62630555	0.000172687	6.11E-06	9.38E-09
ENSG00000067066_230416297_230470112_480	annotated	SP100	2	+	1440	21	3	0	3.12%	4.818661912	0.002808772	0.000204068	4.14E-06
ENSG00000138382_169824597_169811820_110	annotated	METTL5	2	-	330	21	0	6	7.27%	21.02688834	0.005604867	0.042228071	0.001292002
ENSG00000115446_98609760_98618304_259	annotated	UNC50	2	+	777	209	42	9	20.08%	88.87813257	1.18E-07	1.11E-09	1.59E-15

ENSG00000157483_59272383_59137380_1085	annotated	MYO1E	15	-	3255	98	21	2	3.87%	9.948205238	2.37E-05	1.08E-08	4.07E-12
ENSG00000161981_53952_55489_45	Overlap_uORF	SNRNP25	16	+	135	46	6	16	17.78%	112.588418	0.008127104	0.06867726	0.002981148
ENSG00000161980_53586_47430_108	annotated	POLR3K	16	-	324	49	1	4	13.89%	49.97130872	0.000291049	0.002453821	4.90E-06
ENSG00000171853_3443799_3460703_98	novel	TRAPPC12	2	+	294	29	0	0	9.18%	32.59269865	0.003327803	0.003327803	6.21E-05
ENSG00000168291_58433809_58429886_251	annotated	PDHB	3	-	753	36	2	2	5.58%	15.79709312	0.001188759	0.000850936	6.29E-06
ENSG00000168291_58428581_58428034_84	dORF	PDHB	3	-	252	22	9	1	14.29%	28.84641145	0.054241581	0.001640459	0.000654692
ENSG00000177239_137106191_137108591_259	novel	MAN1B1	9	+	777	88	11	2	9.27%	37.42237161	0.000172084	2.23E-05	3.02E-08
ENSG00000159403_7090146_7089272_151	novel	C1R	12	-	453	9	2	0	3.31%	6.564686049	0.101051022	0.019679754	0.009164498
ENSG00000159403_7086394_7080532_338	novel	C1R	12	-	1014	60	3	1	7.69%	19.55162906	1.89E-05	4.52E-06	7.13E-10
ENSG00000167491_19465346_19505474_633	annotated	GATAD2A	19	+	1899	40	4	0	2.37%	6.959926932	0.002094425	0.000291049	4.15E-06
ENSG00000198604_34874604_34753508_1556	annotated	BAZ1A	14	-	4668	17	4	2	0.71%	1.203338267	0.026447338	0.005489386	0.000769467
ENSG00000065150_97985430_98021822_1037	annotated	IPO5	13	+	3111	172	16	12	5.79%	18.26829809	1.42E-09	2.72E-10	4.39E-18
ENSG00000196924_154371164_154348849_2612	annotated	FLNA	X	-	7836	496	54	33	6.36%	20.91495348	2.65E-24	4.84E-25	2.37E-47
ENSG00000144120_119680854_119681789_311	annotated	TMEM177	2	+	933	12	1	0	2.89%	4.249807474	0.00650005	0.002500085	9.16E-05
ENSG00000107537_13302007_13278301_321	annotated	PHYH	10	-	963	84	10	2	10.28%	28.82190303	2.60E-05	7.24E-07	1.82E-10
ENSG00000070718_42154688_42169061_418	annotated	AP3M2	8	+	1254	14	0	0	1.91%	3.68892778	0.004873904	0.004873904	0.000128468
ENSG00000166197_102159553_102162269_418	dORF	NOLC1	10	+	1254	180	12	0	7.89%	47.42907145	1.41E-06	2.25E-07	2.99E-12
ENSG00000011295_16026612_16026710_32	annotated	TTC19	17	+	96	28	4	0	15.62%	96.37323824	0.021084099	0.021084099	0.002029891
ENSG00000145860_159203557_159158670_643	annotated	RNF145	5	-	1929	215	43	6	7.62%	36.82780933	5.09E-07	1.28E-09	8.65E-15
ENSG00000164754_116852052_116847500_176	annotated	RAD21	8	-	528	95	11	2	13.07%	59.45102359	0.000123261	1.32E-05	1.32E-08
ENSG00000120885_27610571_27608842_113	novel	CLU	8	-	339	235	7	4	26.55%	229.053967	7.82E-07	1.30E-06	9.13E-12
ENSG00000120756_142664238_142712007_629	annotated	PLS1	3	+	1887	76	16	5	5.56%	13.30795568	2.24E-05	4.37E-07	9.87E-11
ENSG00000169251_161222023_161250910_500	annotated	NMD3	3	+	1500	57	12	3	4.60%	12.55605618	0.002145907	6.48E-05	1.15E-06
ENSG00000143493_211967969_211952449_137	novel	INTS7	1	-	411	13	0	0	3.65%	10.45132094	0.019216965	0.019216965	0.001707396
ENSG00000088833_1452575_1443749_136	novel	NSFL1C	20	-	408	33	9	1	8.82%	26.72535178	0.026494379	0.001380936	0.000246363
ENSG00000160404_127735270_127734953_105	novel	TOR2A	9	-	315	10	0	0	4.76%	10.48960416	0.019679754	0.019679754	0.001784964
ENSG00000256500_103563002_103685018_719	annotated	AL139300.1	14	+	2157	196	30	22	8.34%	30.02448591	3.60E-07	4.82E-10	2.47E-15
ENSG00000168066_64776579_64776415_54	novel	SF1	11	-	162	31	0	3	9.26%	63.22900287	0.021084099	0.028889786	0.002731533
ENSG00000173163_62000719_62135941_124	novel	COMMD1	2	+	372	25	1	0	9.68%	22.20581526	0.001314757	0.000978918	7.92E-06
ENSG00000127990_94618900_94585465_278	dORF	SGCE	7	-	834	125	8	5	12.95%	49.52376066	1.74E-06	2.28E-07	3.75E-12
ENSG00000127990_94656098_94644645_43	annotated	SGCE	7	-	129	21	6	4	18.60%	53.78971437	0.044816497	0.037011271	0.00688081
ENSG00000127990_94628318_94621973_70	dORF	SGCE	7	-	210	20	0	0	7.14%	31.46881249	0.021557223	0.021557223	0.002115616
ENSG00000182134_151781543_151774452_505	annotated	TDRKH	1	-	1515	52	6	0	4.75%	11.34123539	0.000131203	6.54E-06	7.45E-09
ENSG00000179010_6640863_6641246_127	annotated	MRFAP1	4	+	381	161	38	4	24.41%	139.6273688	0.000425717	8.49E-07	4.65E-09
ENSG00000111653_6653232_6652658_76	novel	ING4	12	-	228	15	1	0	7.89%	21.73832442	0.016822681	0.013428348	0.001079822
ENSG00000111653_6652402_6651195_77	novel	ING4	12	-	231	16	4	0	6.49%	22.88640908	0.139258512	0.021084099	0.013797358
ENSG00000117676_26529957_26574201_712	annotated	RPS6KA1	1	+	2136	17	3	0	1.40%	2.629767336	0.010627882	0.002122793	0.000130816
ENSG00000117676_26554667_26574201_507	dORF	RPS6KA1	1	+	1521	14	3	0	1.58%	3.04136452	0.027110498	0.005160369	0.000749488
ENSG00000117676_26529957_26574201_723	annotated	RPS6KA1	1	+	2169	19	3	0	1.52%	2.894434344	0.005976737	0.001377539	4.92E-05
ENSG00000148229_113410293_113408811_147	annotated	POLE3	9	-	441	99	9	0	19.73%	74.17648658	1.28E-06	1.16E-06	1.32E-11
ENSG00000259956_51391721_51394072_783	annotated	RBM15B	3	+	2349	14	1	0	1.02%	1.969312659	0.00912793	0.005160369	0.000248192
ENSG00000072736_68157905_68214429_589	novel	NFATC3	16	+	1767	13	2	7	0.85%	2.430952408	0.055917761	0.172059248	0.036465499
ENSG00000099804_535900_541552_83	annotated	CDC34	19	+	249	18	5	0	9.64%	23.8859661	0.041265601	0.005454749	0.001232244
ENSG00000108406_59565672_59607172_779	annotated	DHX40	17	+	2337	27	2	6	1.80%	3.817461849	0.002141971	0.009519009	0.000177611
ENSG00000173875_12627884_12629260_458	dORF	ZNF791	19	+	1374	5	0	0	1.09%	1.202410957	0.012673659	0.012673659	0.000782701
ENSG00000105173_29812993_29823777_365	annotated	CCNE1	19	+	1095	14	0	1	2.19%	4.224580307	0.00419316	0.010499663	0.000236136
ENSG00000184990_104753202_104756764_92	novel	SIVA1	14	+	276	49	7	0	17.39%	58.6619711	0.000599389	0.000202851	8.30E-07
ENSG00000167632_140439100_139731061_912	annotated	TRAPPC9	8	-	2736	42	11	5	2.19%	5.072275697	0.001811619	0.000764132	8.61E-06
ENSG00000083312_72848394_72872694_128	annotated	TNPO1	5	+	384	46	2	4	7.81%	39.58186571	0.003974283	0.026319981	0.000582913
ENSG00000163159_151190121_151176644_375	annotated	VPS72	1	-	1125	59	13	12	6.67%	17.32882608	0.003099134	0.000318563	6.79E-06
ENSG00000087088_48954929_48961636_143	annotated	BAX	19	+	429	438	37	5	31.47%	337.3544723	3.74E-08	2.42E-09	9.95E-16
ENSG00000067221_73992223_73983937_347	annotated	STOML1	15	-	1041	12	0	2	1.44%	3.808905258	0.021084099	0.055917761	0.005218013
ENSG00000174547_66438754_66436007_192	annotated	MRPL11	11	-	576	130	17	4	15.10%	74.57452959	0.000165562	2.86E-06	4.57E-09
ENSG00000182196_122981140_122982676_226	annotated	ARL6IP4	12	+	678	102	15	7	12.39%	49.70958433	4.94E-05	4.00E-05	1.49E-08
ENSG00000281649_37086727_37086870_47	novel	EBLN3P	9	+	141	35	7	2	12.77%	82.01977723	0.013853925	0.024414995	0.001590539
ENSG00000170430_129536253_129536381_42	novel	MGMT	10	+	126	12	0	0	11.90%	31.46881249	0.021084099	0.021084099	0.002029891
ENSG00000164244_127523483_127548050_417	annotated	PRRC1	5	+	1251	41	6	0	3.36%	10.82919566	0.002717205	0.000440166	7.88E-06
ENSG00000155508_154870668_154875439_186	Overlap_dORF	CNOT8	5	+	558	70	4	2	11.83%	41.45085516	8.80E-05	6.07E-05	3.93E-08
ENSG00000177830_883197_869858_90	annotated	CHID1	11	-	270	38	1	0	16.67%	46.50391179	0.000275394	0.000275394	5.15E-07
ENSG00000147526_38820520_38848023_391	annotated	TACC1	8	+	1173	60	2	2	5.37%	16.90140824	4.67E-05	4.67E-05	1.64E-08
ENSG00000156787_123105357_123151939_620	annotated	TBC1D31	8	+	1860	16	3	1	0.97%	2.842344354	0.044040756	0.019795882	0.003892451
ENSG00000075142_88226914_88210855_76	novel	SRI	7	-	228	59	8	2	15.79%	85.50407603	0.008252457	0.001065834	5.51E-05
ENSG00000066855_65670010_65709044_281	annotated	MTFR1	8	+	843	57	9	0	7.83%	22.34173698	0.000639325	1.68E-05	9.43E-08
ENSG00000145860_159203557_159158670_643	annotated	RNF145	5	-	1929	215	43	6	7.62%	36.82780933	5.09E-07	1.28E-09	8.65E-15
ENSG00000125818_1164334_1186481_94	annotated	PSMF1	20	+	282	43	7	1	11.70%	50.38357744	0.014295976	0.00140552	0.000125855
ENSG00000110651_2393896_2396866_242	annotated	CD81	11	+	726	636	70	9	28.51%	289.4610603	1.84E-11	3.22E-13	8.09E-23
ENSG00000183814_226309139_226233092_473	annotated	LIN9	1	-	1419	13	3	2	1.48%	3.027126783	0.065131088	0.043639483	0.011333421
ENSG00000035928_39366241_39288761_1148	annotated	RFC1	4	-	3444	34	1	3	1.31%	3.262011051	0.000427981	0.001582481	4.41E-06

ENSG00000108010_130136421_130179392_335	annotated	GLRX3	10	+	1005	219	30	9	12.54%	72.00252171	5.03E-08	3.00E-08	1.51E-14
ENSG00000032389_3377689_3196978_132	annotated	EIPR1	2	-	396	24	3	2	3.79%	20.02560795	0.131599539	0.060667625	0.029607079
ENSG00000102897_20915556_20924116_122	annotated	LYRM1	16	+	366	16	1	0	7.38%	14.44470081	0.003229477	0.003327803	6.04E-05
ENSG00000167196_75913236_75933102_299	dORF	FBXO22	15	+	897	51	14	1	7.69%	18.78656531	0.002045111	1.52E-05	3.20E-07
ENSG00000149923_30076378_30085062_307	annotated	PPP4C	16	+	921	300	56	17	19.87%	107.629489	2.33E-08	2.36E-10	6.84E-17
ENSG00000144224_135770661_135782887_259	novel	UBXN4	2	+	777	49	10	2	8.11%	20.83745692	0.001981969	5.07E-05	8.52E-07
ENSG00000136877_127804381_127807766_195	novel	FPGS	9	+	585	24	7	0	4.62%	13.55579615	0.027941506	0.003303074	0.000532997
ENSG00000180537_13974243_13976930_47	uORF	RNF182	6	+	141	5	0	0	10.64%	11.71711103	0.012673659	0.012673659	0.000782701
ENSG00000122705_36191057_36265473_260	annotated	CLTA	9	+	780	547	130	11	15.00%	231.7193904	1.54E-05	9.14E-08	1.63E-11
ENSG00000078618_51878468_51840199_169	novel	NRDC	1	-	507	19	6	0	6.51%	12.3826984	0.040733279	0.001343041	0.000397735
ENSG00000117597_209828064_209851447_756	annotated	UTP25	1	+	2268	15	3	3	1.06%	2.185334201	0.030313544	0.030313544	0.0039848
ENSG00000065809_14667643_14521190_153	annotated	FAM107B	10	-	459	112	56	3	14.38%	80.62597709	0.042844126	2.33E-05	2.11E-05
ENSG00000166965_90956735_90961769_374	annotated	RCCD1	15	+	1122	54	7	1	6.68%	15.90268866	7.23E-05	6.62E-06	4.03E-09
ENSG00000140750_25015240_24920130_796	annotated	ARHGAP17	16	-	2388	32	11	4	1.76%	4.427772611	0.03007929	0.003430523	0.000596032
ENSG00000159063_78106811_78100964_135	annotated	ALG8	11	-	405	84	13	3	14.07%	68.53208053	0.003295579	0.000277817	6.44E-06
ENSG00000119523_99221047_99217934_323	annotated	ALG2	9	-	969	48	5	5	5.57%	16.36767956	0.000537782	0.000791258	2.73E-06
ENSG00000149480_62601450_62598309_129	novel	MTA2	11	-	387	30	4	1	9.30%	25.6141497	0.009354457	0.001453991	8.23E-05
ENSG00000198780_74834531_74781460_482	annotated	FAM169A	5	-	1446	18	3	0	1.87%	4.113143541	0.02524418	0.003478238	0.000498474
ENSG00000143811_225924110_225920455_246	annotated	PYCR2	1	-	738	40	2	1	4.47%	17.90908028	0.002717354	0.0020028	3.16E-05
ENSG00000011422_43670120_43648890_286	annotated	PLAUR	19	-	858	119	17	4	12.94%	45.82783357	2.35E-06	1.85E-07	4.19E-12
ENSG00000156976_186786208_186787886_173	novel	EIF4A2	3	+	519	54	3	1	10.98%	34.37922289	5.22E-05	7.24E-05	2.81E-08
ENSG00000140299_59682457_59678806_107	novel	BNIP2	15	-	321	23	3	1	9.35%	23.67513463	0.00205871	0.00224099	2.69E-05
ENSG00000103994_42448692_42417304_1068	annotated	ZNF106	15	-	3204	11	1	1	0.66%	1.134409439	0.013018137	0.013018137	0.00082316
ENSG00000103121_80997394_80980808_74	annotated	CMC2	16	-	222	33	11	4	10.81%	49.11686273	0.062400078	0.010275151	0.003233385
ENSG00000100528_54441327_54436765_33	annotated	CNIH1	14	-	99	23	0	4	15.15%	76.76483046	0.021084099	0.111400496	0.01075863
ENSG00000100528_54432178_54427814_80	dORF	CNIH1	14	-	240	204	21	4	31.25%	280.8591514	2.37E-05	5.87E-06	1.15E-09
ENSG00000165533_88824795_88877410_418	annotated	TTC8	14	+	1254	41	8	2	4.07%	10.8032885	0.002594683	0.000288868	5.16E-06
ENSG00000100796_91509647_91458759_820	annotated	PPP4R3A	14	-	2460	62	16	8	3.90%	8.327722329	0.000190759	2.83E-05	4.18E-08
ENSG00000104835_38919974_38918090_139	novel	SARS2	19	-	417	17	4	0	5.04%	13.4704629	0.045444393	0.008574016	0.001982537
ENSG00000196628_55321693_55228225_496	annotated	TCF4	18	-	1488	21	0	0	1.61%	4.663221205	0.005706018	0.005706018	0.00017331
ENSG00000184432_139389550_139357863_906	annotated	COPB2	3	-	2718	83	5	3	3.64%	10.09016559	8.98E-07	4.06E-07	3.34E-12
ENSG00000137177_17987421_17764110_1743	annotated	KIF13A	6	-	5229	32	8	0	0.80%	2.022092369	0.007799506	0.000434916	2.41E-05
ENSG00000213930_34647151_34650449_331	annotated	GALT	9	+	993	11	0	0	1.81%	3.66026973	0.012798403	0.012798403	0.000797244
ENSG00000183049_12349819_12825725_357	annotated	CAMK1D	10	+	1071	41	1	0	5.60%	12.64922855	5.05E-05	3.70E-05	1.41E-08
ENSG00000167397_31091310_31091134_58	dORF	VKORC1	16	-	174	204	55	19	29.31%	387.391933	0.000644384	0.001844079	7.49E-06
ENSG00000051382_138755916_138655389_992	annotated	PIK3CB	3	-	2976	43	2	5	2.52%	4.774250282	1.56E-05	0.000156031	1.96E-08
ENSG00000115221_160200017_160101736_746	annotated	ITGB6	2	-	2238	14	1	0	1.07%	2.066986343	0.010499663	0.00419316	0.000236136
ENSG00000130175_11436148_11449412_515	annotated	PRKCSH	19	+	1545	759	99	23	24.27%	162.3240784	8.40E-18	8.91E-22	1.56E-37
ENSG00000134463_11755450_11763544_159	novel	ECHDC3	10	+	477	15	0	0	3.77%	10.39064563	0.01300724	0.01300724	0.000821865
ENSG00000117410_43975041_43976383_93	novel	ATP6V0B	1	+	279	236	10	8	24.73%	279.4971948	4.41E-05	1.25E-05	4.39E-09
ENSG00000215301_41343246_41347719_471	annotated	DDX3X	X	+	1413	189	30	7	8.70%	44.19664429	3.70E-07	2.15E-08	8.26E-14
ENSG00000118564_15655236_15612252_653	novel	FBXL5	4	-	1959	59	11	0	3.52%	9.951469799	0.001629088	1.15E-05	1.95E-07
ENSG00000123374_55967009_55971625_298	annotated	CDK2	12	+	894	73	16	2	10.07%	26.98081071	1.90E-05	1.28E-06	2.21E-10
ENSG00000102081_147928379_147948844_507	annotated	FMR1	X	+	1521	13	1	0	2.17%	2.824124198	0.00398705	0.000883168	2.18E-05
ENSG00000104904_2269599_2271445_68	novel	OAZ1	19	+	204	92	7	3	23.53%	149.0140827	0.001904289	0.000207478	2.78E-06
ENSG00000104889_12807013_12807333_64	novel	RNASEH2A	19	+	192	30	2	1	9.38%	51.62852049	0.016983117	0.017143984	0.001367233
ENSG00000104889_12810092_12813466_155	novel	RNASEH2A	19	+	465	88	1	0	8.39%	62.53157578	0.000722178	0.000722178	3.32E-06
ENSG00000112305_70668024_70860234_436	annotated	SMAP1	6	+	1308	23	6	0	3.67%	5.810182122	0.003099838	0.00012627	3.05E-06
ENSG00000101546_80042145_80046155_176	novel	RBFA	18	+	528	14	2	0	5.11%	8.761203477	0.00912793	0.003229477	0.000162278
ENSG00000171488_89712712_89714982_756	annotated	LRRC8C	1	+	2268	39	4	0	2.65%	5.681868921	0.00039975	3.30E-05	1.04E-07
ENSG00000112305_70668024_70860334_467	annotated	SMAP1	6	+	1401	23	6	0	3.43%	5.424495514	0.003099838	0.00012627	3.05E-06
ENSG00000070814_150375390_150391699_599	novel	TCOF1	5	+	1797	20	0	0	1.50%	3.677490608	0.003278443	0.003278443	6.04E-05
ENSG00000205726_33765954_33888300_1427	annotated	ITSN1	21	+	4281	19	0	2	0.70%	1.466486356	0.002046029	0.010448364	0.000124448
ENSG00000272822_48941095_48903660_210	annotated	AC073610.2	12	-	630	102	8	6	10.95%	53.49698123	1.40E-05	6.90E-05	7.66E-09
ENSG00000100902_35308980_35317306_167	annotated	PSMA6	14	+	501	92	14	2	9.58%	60.67639294	0.002237421	0.000300676	4.58E-06
ENSG00000272921_4604991_4631333_35	annotated	AC005832.4	12	+	105	32	5	0	17.14%	100.7002	0.013640586	0.013640586	0.000898637
ENSG00000182149_71916578_71921537_143	novel	IST1	16	+	429	43	2	0	7.69%	33.11927468	0.001580611	0.001588175	1.50E-05
ENSG00000196839_44651607_44622860_274	novel	ADA	20	-	822	29	5	0	2.92%	11.65724258	0.068160782	0.005355751	0.002132648
ENSG00000163636_64023419_64010668_442	annotated	PSMD6	3	-	1326	258	18	7	14.25%	64.29035673	2.68E-11	2.25E-11	7.25E-21
ENSG00000131584_1302924_1293564_709	novel	ACAP3	1	-	2127	17	1	1	0.99%	2.640894701	0.012000727	0.012000727	0.000706384
ENSG00000184903_111521447_111483647_106	novel	IMMP2L	7	-	318	37	2	1	10.38%	38.44538884	0.002923439	0.001785595	3.05E-05
ENSG00000099624_1244171_1244437_45	novel	ATP5F1D	19	+	135	381	4	6	51.11%	932.52581	1.30E-05	1.91E-05	1.98E-09
ENSG00000127463_19219616_19219303_75	novel	EMC1	1	-	225	11	0	0	8.00%	16.15399041	0.012798403	0.012798403	0.000797244
ENSG00000144524_231794346_231807619_171	novel	COPS7B	2	+	513	13	0	0	3.51%	8.373280516	0.01300724	0.01300724	0.000821865
ENSG00000143418_150969090_150966148_380	annotated	CERS2	1	-	1140	649	53	20	30.00%	188.1089673	1.25E-17	4.47E-19	9.08E-35
ENSG00000099783_8444999_8463601_99	novel	HNRNPM	19	+	297	23	13	2	9.09%	25.58827682	0.150419977	0.008171138	0.007560092
ENSG00000168487_22165406_22211728_986	annotated	BMP1	8	+	2958	120	26	2	4.67%	13.40456516	3.63E-06	1.90E-09	1.08E-13
ENSG00000134779_36828767_36807827_90	novel	TPGS2	18	-	270	28	8	7	11.11%	34.26604026	0.046020931	0.017322097	0.003626081

ENSG00000117114_81907220_81991145_1339	annotated	ADGRL2	1	+	4017	25	0	0	0.75%	2.056401115	0.002362849	0.002362849	3.23E-05
ENSG00000136261_16689838_16706088_225	annotated	BZW2	7	+	675	62	7	2	10.67%	30.34992138	5.92E-05	6.57E-06	3.25E-09
ENSG00000144959_172645660_172633475_275	annotated	NCEH1	3	-	825	150	12	4	15.27%	60.07682384	4.74E-07	1.58E-08	8.01E-14
ENSG00000185236_8390417_8403558_218	annotated	RAB11B	19	+	654	141	18	6	18.81%	71.23788515	1.67E-06	4.55E-08	7.97E-13
ENSG00000137269_53795257_53922793_524	annotated	LRRC1	6	+	1572	21	1	2	2.29%	4.414041446	0.001635336	0.003276402	3.14E-05
ENSG00000259040_8026413_7883144_360	Overlap_dORF	BLOC1S5-TXNDC5	6	-	1080	965	54	34	24.44%	295.2386505	8.76E-14	2.89E-14	3.44E-26
ENSG00000164305_184635393_184629272_251	annotated	CASP3	4	-	753	36	1	3	5.58%	15.79709312	0.000605011	0.00194169	7.44E-06
ENSG00000163754_148991641_148996748_91	novel	GYG1	3	+	273	5	0	1	5.49%	6.051694709	0.012673659	0.051235217	0.00311107
ENSG00000136986_123015524_123015447_25	novel	DERL1	8	-	75	30	2	0	40.00%	132.1690124	0.002335453	0.002418183	3.27E-05
ENSG00000136986_123025015_123019191_106	novel	DERL1	8	-	318	108	15	6	24.53%	112.2189728	3.81E-05	1.89E-05	5.62E-09
ENSG00000163166_127526208_127481044_819	annotated	IWS1	2	-	2457	48	10	4	2.56%	6.455141023	0.000704641	0.000232274	1.11E-06
ENSG00000163171_37646587_37645823_254	annotated	CDC42EP3	2	-	762	25	6	0	4.33%	10.84063422	0.019903045	0.001550569	0.00019636
ENSG00000153914_66144377_66156072_100	novel	SREK1	5	+	300	15	1	0	6.00%	16.52112656	0.013428348	0.013428348	0.000872559
ENSG00000136158_80337705_80336758_315	annotated	SPRY2	13	-	945	17	4	2	2.22%	5.944109025	0.060543501	0.028581075	0.007320449
ENSG00000102226_47233044_47247930_920	annotated	USP11	X	+	2760	68	3	3	3.26%	8.14084497	8.30E-07	3.31E-06	2.45E-11
ENSG00000078403_21534705_21556942_159	annotated	MLLT10	10	+	477	7	0	0	3.14%	4.848967962	0.019216965	0.019216965	0.001707396
ENSG00000178726_23049672_23047777_631	annotated	THBD	20	-	1893	54	3	2	3.33%	9.425682346	5.10E-05	6.80E-05	2.58E-08
ENSG00000136158_80337705_80336758_315	annotated	SPRY2	13	-	945	17	4	2	2.22%	5.944109025	0.060543501	0.028581075	0.007320449
ENSG00000175198_100089121_100530166_681	annotated	PCCA	13	+	2043	61	6	0	4.26%	9.865773078	4.77E-06	9.35E-07	3.98E-11
ENSG00000145247_48904549_48885531_99	annotated	OCIAD2	4	-	297	16	1	1	6.06%	17.8005404	0.011772051	0.026832696	0.001510382
ENSG00000244687_50084158_50082768_58	annotated	UBE2V1	20	-	174	13	4	2	10.34%	24.68674083	0.143893695	0.035374934	0.02120653
ENSG00000106052_27769694_27828829_590	annotated	TAX1BP1	7	+	1770	90	14	2	6.95%	16.80114565	1.60E-07	1.10E-08	1.85E-14
ENSG00000114209_167720151_167687230_184	annotated	PDCD10	3	-	552	70	7	4	11.96%	41.90140793	0.000100029	0.000133993	9.61E-08
ENSG00000068885_160319765_160258525_460	novel	IFT80	3	-	1380	18	2	1	1.30%	4.309859102	0.028889786	0.016822681	0.002229334
ENSG00000149636_36771458_36752788_249	annotated	DSN1	20	-	747	39	2	3	7.23%	17.25097552	0.000113298	0.000535009	4.31E-07
ENSG00000112078_36470098_36487175_419	annotated	KCTD20	6	+	1257	26	3	2	1.67%	6.834515361	0.036499023	0.023296852	0.003738066
ENSG00000150787_112226444_112228711_66	novel	PTS	11	+	198	16	4	0	7.58%	26.7008106	0.019679754	0.019216965	0.001745788
ENSG00000136874_99915240_99968673_302	annotated	STX17	9	+	906	14	12	0	2.32%	5.105866927	0.446609873	0.008473674	0.037262288
ENSG00000100664_103334576_103335924_97	novel	EIF5	14	+	291	23	9	0	9.28%	26.11587016	0.055721081	0.003529166	0.001220604
ENSG00000185630_164559823_164846676_339	annotated	PBX1	1	+	1017	58	21	0	7.37%	18.84415615	0.001208174	4.97E-06	6.84E-08
ENSG00000185033_90225706_90227919_80	annotated	SEMA4B	15	+	240	17	0	0	8.75%	23.40492929	0.008574016	0.008574016	0.000374746
ENSG00000163382_156592168_156592688_94	novel	NAXE	1	+	282	86	8	0	12.77%	100.7671549	0.003000296	0.001082917	1.97E-05
ENSG00000132356_40798162_40768883_207	annotated	PRKAA1	5	-	621	23	3	6	4.35%	12.23787152	0.01778219	0.032390891	0.002618309
ENSG00000164347_74763715_74721655_770	annotated	GFM2	5	-	2310	106	8	7	5.45%	15.16224602	1.86E-07	1.43E-07	2.54E-13
ENSG00000160948_144423903_144423805_32	novel	VPS28	8	-	96	34	3	2	18.75%	117.0246464	0.068043226	0.032488891	0.009165526
ENSG00000160948_144425732_144423887_185	novel	VPS28	8	-	555	183	29	8	16.76%	108.9501319	2.69E-05	3.42E-06	7.88E-10
ENSG00000175224_46645957_46656362_116	novel	ATG13	11	+	348	11	0	0	4.31%	10.44439035	0.019216965	0.019216965	0.001707396
ENSG00000172409_57659597_57661436_385	annotated	CLP1	11	+	1155	22	4	0	2.34%	6.293762497	0.023871233	0.003303074	0.000449475
ENSG00000023171_123577464_123622595_698	annotated	GRAMD1B	11	+	2094	29	0	5	2.15%	4.576052246	0.000256142	0.006313333	1.22E-05
ENSG00000166136_100529851_100526951_111	novel	NDUFB8	10	-	333	400	40	9	33.33%	396.9039413	1.46E-06	2.70E-07	3.68E-12
ENSG00000125991_35548618_35557485_145	annotated	ERGIC3	20	+	435	101	20	2	13.79%	76.71879458	0.002384345	4.78E-05	9.95E-07
ENSG00000172269_119100804_119096998_301	Overlap_dORF	DPAGT1	11	-	903	112	25	1	11.30%	40.98263952	0.000270534	1.87E-07	7.71E-10
ENSG00000172269_119101655_119100801_102	annotated	DPAGT1	11	-	306	23	6	5	6.86%	24.83568044	0.0601164	0.076476367	0.017460088
ENSG00000104738_47961596_47976778_813	Overlap_dORF	MCM4	8	+	2439	218	22	13	8.73%	29.53346117	5.85E-11	2.78E-11	1.94E-20
ENSG00000163956_3532412_3516630_279	annotated	LRPAP1	4	-	837	695	17	1	21.86%	274.3651841	1.92E-11	6.73E-12	1.59E-21
ENSG00000110063_126330391_126345613_227	annotated	DCPS	11	+	681	46	0	0	5.29%	22.31928991	0.001070089	0.001070089	7.07E-06
ENSG00000198825_119781745_119827780_1036	dORF	INPP5F	10	+	3108	11	0	0	0.58%	1.169449113	0.012798403	0.012798403	0.000797244
ENSG00000060339_68757372_68791266_512	Overlap_dORF	CCAR1	10	+	1536	32	5	1	3.12%	6.883802732	0.003932371	0.000277134	7.76E-06
ENSG00000060339_68722505_68757375_568	annotated	CCAR1	10	+	1704	42	7	1	2.29%	8.144217316	0.00551913	0.000862821	3.00E-05
ENSG00000188186_100153791_100153964_57	dORF	LAMTOR4	7	+	171	53	8	4	17.54%	102.4116617	0.004799398	0.004691965	0.000122105
ENSG00000188186_100148973_100153729_137	annotated	LAMTOR4	7	+	411	199	35	7	12.41%	159.9856051	0.000894725	0.000418914	2.44E-06
ENSG00000154144_124623084_124632238_411	annotated	TBRG1	11	+	1233	9	2	0	1.22%	2.411843293	0.060322713	0.020613417	0.005529088
ENSG00000064607_19031071_18994993_1040	annotated	SUGP2	19	-	3120	29	0	1	1.35%	3.071235065	0.000424543	0.000525339	1.46E-06
ENSG00000065057_2044770_2039924_177	annotated	NTHL1	16	-	531	46	5	1	10.17%	28.62417407	0.000936709	7.61E-05	5.39E-07
ENSG00000107960_103897652_103882684_152	novel	STN1	10	-	456	26	1	1	9.87%	18.83988116	0.000231967	0.000437802	6.88E-07
ENSG00000107960_103917486_103898323_175	novel	STN1	10	-	525	14	3	0	5.71%	8.811267496	0.023268551	0.001790164	0.000262902
ENSG00000127463_19251509_19219303_992	annotated	EMC1	1	-	2976	168	34	5	6.75%	18.65288482	8.03E-09	6.56E-12	8.15E-19
ENSG00000085662_134459062_134442728_316	annotated	AKR1B1	7	-	948	2865	348	93	31.33%	998.5870798	3.13E-17	8.67E-18	4.17E-33
ENSG00000114988_96711764_96707679_87	novel	LMAN2L	2	-	261	15	0	0	10.34%	18.98980064	0.003278443	0.003278443	6.04E-05
ENSG00000066136_40738844_40770828_335	annotated	NFYC	1	+	1005	42	3	0	4.48%	13.80870279	0.000599389	0.000290289	1.16E-06
ENSG00000184182_237973108_237986190_48	annotated	UBE2F	2	+	144	10	6	0	10.42%	22.94600911	0.171390856	0.020613417	0.017249262
ENSG00000130396_167864572_167969935_1789	annotated	AFDN	6	+	5367	12	6	0	0.39%	0.738787101	0.176150259	0.008175611	0.009250017
ENSG00000177105_3828138_3827563_191	annotated	RHOG	11	-	573	64	10	1	11.52%	36.90583245	0.000496609	2.32E-05	9.52E-08
ENSG00000178904_32463870_32469017_92	novel	DPY19L3	19	+	276	12	4	5	6.52%	14.36619701	0.065131088	0.11411552	0.027308284
ENSG00000170917_122922572_122892828_316	annotated	NUDT6	4	-	948	38	3	5	4.75%	13.244785	0.001474044	0.002042192	1.79E-05
ENSG00000012660_53295699_53269127_299	annotated	ELOVL5	6	-	897	406	37	9	24.41%	149.5557945	9.04E-11	2.24E-13	3.02E-22
ENSG00000213390_97292627_97226122_494	annotated	ARHGAP19	10	-	1482	16	3	3	1.62%	3.567314776	0.03662705	0.03662705	0.00565129
ENSG00000136068_58008565_58170762_2578	annotated	FLNB	3	+	7734	224	35	43	3.76%	9.570034519	6.60E-14	1.25E-10	1.38E-22

ENSG00000164050_48424611_48404486_2135	annotated	PLXNB1	3	-	6405	35	4	2	0.70%	1.805587602	0.002075178	0.000877671	1.12E-05
ENSG00000005483_105073671_105113333_1675	annotated	KMT2E	7	+	5025	26	10	2	0.96%	1.709648917	0.047921686	0.000975372	0.000378668
ENSG00000124370_71130219_71109970_176	annotated	MCEE	2	-	528	38	2	4	6.82%	23.78040944	0.001458431	0.006140302	5.33E-05
ENSG00000166689_17014401_16789087_1121	annotated	PLEKHA7	11	-	3363	15	3	0	0.62%	1.473784706	0.052370108	0.008473674	0.00228699
ENSG00000083896_68349753_68314099_709	annotated	YTHDC1	4	-	2127	51	11	1	2.82%	7.922684103	0.002919359	3.85E-05	1.04E-06
ENSG00000182670_37087258_37201574_2025	annotated	TTC3	21	+	6075	17	0	0	0.44%	0.924639182	0.003478238	0.003478238	6.76E-05
ENSG00000079805_10718258_10831047_865	annotated	DNM2	19	+	2595	96	6	9	5.20%	12.22372369	3.18E-08	2.06E-07	6.57E-14
ENSG00000103353_23562655_23570590_89	novel	UBFD1	16	+	267	35	7	1	13.48%	43.31381494	0.00604633	0.001114796	4.13E-05
ENSG00000103024_1771534_1771265_63	novel	NME3	16	-	189	27	2	3	11.11%	47.20321873	0.008877961	0.024694694	0.00108496
ENSG00000132603_69340204_69341652_129	novel	NIP7	16	+	387	54	9	3	13.18%	46.10546946	0.001679424	0.000413969	4.54E-06
ENSG00000011295_16001924_16027522_273	dORF	TTC19	17	+	819	71	9	3	5.49%	28.64468829	0.000337613	0.000668477	1.49E-06
ENSG00000114867_184328662_184334908_271	novel	EIF4G1	3	+	813	54	2	0	8.49%	21.94688399	1.50E-05	8.79E-06	1.07E-09
ENSG00000166548_66517813_66511968_94	annotated	TK2	16	-	282	16	3	0	7.45%	18.74737765	0.008373824	0.008473674	0.000362422
ENSG00000116353_29230906_29194022_373	annotated	MECR	1	-	1119	41	12	0	4.02%	12.10663429	0.009780668	0.000306523	2.32E-05
ENSG00000119912_92537525_92480826_568	dORF	IDE	10	-	1704	50	0	5	4.23%	9.695496805	6.42E-06	0.000155968	8.80E-09
ENSG00000088179_119920102_119977070_639	annotated	PTPN4	2	+	1917	10	0	0	0.94%	1.723643876	0.011570466	0.011570466	0.000659484
ENSG00000124789_17706387_17616097_1475	annotated	NUP153	6	-	4425	43	2	3	1.42%	3.210885613	3.76E-05	0.000107619	3.05E-08
ENSG00000049860_74720698_74721175_35	annotated	HEXB	5	+	105	67	15	0	34.29%	210.8410437	0.004791578	0.00107863	3.15E-05
ENSG00000127184_86618056_86619469_63	annotated	COX7C	5	+	189	283	22	4	19.05%	494.759663	0.003770571	0.002557486	5.46E-05
ENSG00000007541_574075_582256_560	annotated	PIGQ	16	+	1680	40	2	2	3.39%	7.867203122	0.000102211	0.000194697	1.42E-07
ENSG00000076928_41888083_41906786_912	annotated	ARHGEF1	19	+	2736	83	12	1	4.28%	10.02378292	7.71E-06	2.24E-08	2.18E-12
ENSG00000158092_136945762_136946973_192	novel	NCK1	3	+	576	9	0	0	2.60%	5.162852049	0.019216965	0.019216965	0.001707396
ENSG00000109736_2932467_2930838_263	dORF	MFSD10	4	-	789	111	7	5	9.13%	46.48529905	0.000171446	2.90E-05	3.83E-08
ENSG00000109736_2933923_2933143_100	annotated	MFSD10	4	-	300	53	5	10	13.00%	58.37464716	0.003050214	0.029407445	0.000527835
ENSG00000075711_197138252_197044623_599	novel	DLG1	3	-	1797	31	4	0	2.17%	5.700110442	0.004387317	0.000656046	1.85E-05
ENSG00000100897_24117382_24119217_150	novel	DCAF11	14	+	450	16	0	2	4.00%	11.74835666	0.013640586	0.037440157	0.002398086
ENSG00000137807_69414466_69421763_108	novel	AC027237.1	15	+	324	15	4	0	4.63%	15.2973394	0.133791954	0.021084099	0.013186697
ENSG00000172183_88639406_88655531_168	annotated	ISG20	15	+	504	28	3	1	7.74%	18.35680728	0.003472886	0.001203181	2.51E-05
ENSG00000074603_65455668_65450972_149	novel	DPP8	15	-	447	13	0	1	3.36%	9.609603813	0.021084099	0.028889786	0.002731533
ENSG00000067082_3784999_3779539_278	annotated	KLF6	10	-	834	54	4	1	6.47%	21.3942646	0.000459894	8.75E-05	2.91E-07
ENSG00000088833_1466824_1443749_339	annotated	NSFL1C	20	-	1017	79	13	5	8.55%	25.66704027	0.000524512	2.10E-05	9.24E-08
ENSG00000138381_189661521_189666111_96	uORF	ASNSD1	2	+	288	24	0	5	7.29%	27.53521093	0.008776163	0.020525443	0.000892178
ENSG00000196139_5094445_5097598_138	novel	AKR1C3	10	+	414	901	125	15	25.36%	719.1079723	5.08E-07	9.76E-08	4.79E-13
ENSG00000166997_100122201_100124888_228	novel	CNPY4	7	+	684	22	6	0	3.95%	10.62762527	0.032214773	0.003529166	0.000657741
ENSG00000008282_106112208_106091805_241	annotated	SYPL1	7	-	723	1063	95	15	26.14%	485.8079538	8.72E-10	8.92E-12	1.03E-19
ENSG00000182087_1012108_1011637_101	novel	TMEM259	19	-	303	28	1	7	6.93%	30.53409528	0.013991246	0.081617439	0.005545949
ENSG00000182087_1010889_1010350_179	novel	TMEM259	19	-	537	63	1	0	7.82%	38.76465449	0.001003788	0.000413076	2.70E-06
ENSG00000062716_59735462_59839911_172	Overlap_dORF	VMP1	17	+	516	77	13	10	10.47%	49.30723817	0.003800892	0.001936658	4.25E-05
ENSG00000070950_8963385_8912329_310	annotated	RAD18	3	-	930	37	6	0	4.52%	13.14584264	0.004228448	0.000432307	1.23E-05
ENSG00000070950_8912369_8881357_172	Overlap_dORF	RAD18	3	-	516	11	0	0	5.23%	7.043891167	0.002478943	0.002478943	3.54E-05
ENSG00000100650_69768157_69769561_138	annotated	SRSF5	14	+	414	278	33	26	32.61%	221.8779315	1.67E-07	1.91E-07	3.04E-13
ENSG00000134215_107688431_107573331_287	annotated	VAV3	1	-	861	20	7	0	3.48%	7.675320119	0.025616856	0.002227676	0.000350081
ENSG00000142230_47130931_47150205_33	uORF	SAE1	19	+	99	13	3	6	15.15%	43.38881722	0.08410207	0.143893695	0.042170358
ENSG00000003056_8943899_8943401_77	novel	M6PR	12	-	231	98	7	1	28.57%	140.1792556	0.000106348	2.20E-05	1.82E-08
ENSG00000136718_130345474_130346468_193	annotated	IMP4	2	+	579	64	3	1	11.92%	36.52338859	1.10E-05	1.15E-05	1.02E-09
ENSG00000128283_37566386_37568820_379	annotated	CDC42EP1	22	+	1137	12	8	0	2.11%	3.487309036	0.236105842	0.004873904	0.00974688
ENSG00000138758_76995936_77034512_421	annotated	11-Sep	4	+	1263	42	6	0	4.99%	10.98792265	4.98E-05	1.80E-05	7.02E-09
ENSG00000078747_34477872_34507794_321	dORF	ITCH	20	+	963	25	11	0	2.80%	8.577947329	0.175398352	0.003327803	0.004960525
ENSG00000078747_34507813_34540624_127	dORF	ITCH	20	+	381	208	15	3	21.26%	180.3881535	6.32E-06	2.55E-06	1.37E-10
ENSG00000078747_34412633_34477864_443	annotated	ITCH	20	+	1329	30	5	0	3.16%	7.458747881	0.003978619	0.000424543	1.14E-05
ENSG00000168056_65557917_65539080_1242	annotated	LTBP3	11	-	3726	211	32	9	6.52%	18.7115282	6.26E-12	6.12E-14	5.48E-24
ENSG00000105552_48811007_48795426_392	annotated	BCAT2	19	-	1176	238	7	2	7.65%	66.87122654	7.77E-07	1.27E-06	8.88E-12
ENSG00000080822_98521424_98521071_117	novel	CLDND1	3	-	351	49	4	4	11.97%	46.12736189	0.003265655	0.003265655	5.99E-05
ENSG00000108469_75666443_75651182_372	annotated	RECQL5	17	-	1116	19	2	0	2.96%	5.6254732	0.002979763	0.001343041	2.38E-05
ENSG00000163110_94575691_94664067_474	dORF	PDLIM5	4	+	1422	450	54	30	13.92%	104.5640921	5.46E-10	1.38E-10	8.71E-19
ENSG00000177463_15003915_15043008_615	annotated	NR2C2	3	+	1845	10	5	0	1.14%	1.790908028	0.075513077	0.007029303	0.002963142
ENSG00000064419_129054770_128957255_957	annotated	TNPO3	7	-	2871	102	6	6	4.60%	11.73914949	4.38E-08	6.81E-08	2.95E-14
ENSG00000189079_45848888_45905078_1291	novel	ARID2	12	+	3873	17	1	3	0.85%	1.450344185	0.002098277	0.010690846	0.000130295
ENSG00000107745_72477224_72368195_248	annotated	MICU1	10	-	744	40	11	4	7.26%	17.76465221	0.007031052	0.000313993	1.63E-05
ENSG00000285314_20989635_20997348_639	novel	AC002470.2	22	+	1917	17	8	3	1.56%	2.93019459	0.138719553	0.018949244	0.012679138
ENSG00000182473_76103692_76083648_697	annotated	EXOC7	17	-	2091	63	8	7	4.02%	9.955341684	0.000105489	6.13E-05	4.75E-08
ENSG00000010361_49813106_49811150_234	novel	FUZ	19	-	702	9	1	3	2.14%	4.236186296	0.042234516	0.150401371	0.025490393
ENSG00000136273_47979519_47978363_136	novel	HUS1	7	-	408	10	0	0	3.68%	8.098591449	0.020613417	0.020613417	0.001946209
ENSG00000124193_43458034_43463350_276	annotated	SRSF6	20	+	828	150	15	9	10.51%	59.85915419	1.50E-05	3.53E-06	4.47E-10
ENSG00000135506_57694162_57694771_75	annotated	OS9	12	+	225	72	24	12	17.33%	105.73521	0.014931386	0.013648863	0.000979036
ENSG00000145912_178153817_178149713_153	annotated	NHP2	5	-	459	176	27	19	13.73%	126.697964	7.11E-05	0.000163631	8.46E-08
ENSG00000134709_59836876_59872965_569	annotated	HOOK1	1	+	1707	16	0	0	0.88%	3.097106326	0.021084099	0.021084099	0.002029891
ENSG00000119977_95693899_95683393_443	annotated	TCTN3	10	-	1329	215	45	13	10.16%	53.45435981	1.58E-05	1.40E-07	2.43E-11

ENSG00000177830_884096_883144_62	novel	CHID1	11	-	186	19	2	0	9.68%	33.7528392	0.012619539	0.011772051	0.000727418
ENSG00000072274_196065548_196062562_131	novel	TFRC	3	-	393	114	7	1	20.61%	95.84775712	2.52E-06	2.41E-06	5.25E-11
ENSG00000279605_143580525_143580761_78	novel	AC067930.5	8	+	234	42	11	8	11.54%	59.30660815	0.0220327	0.037251511	0.003627898
ENSG00000002586_2691361_2733364_160	annotated	CD99	X	+	480	271	113	13	23.12%	186.551054	6.10E-05	1.50E-07	1.15E-10
ENSG00000132466_73177491_73076231_2458	annotated	ANKRD17	4	-	7374	45	3	1	1.10%	2.01641089	1.56E-05	2.77E-06	3.71E-10
ENSG00000101974_139796386_139728900_749	annotated	ATP11C	X	-	2247	20	0	0	1.34%	2.941010513	0.002201184	0.002201184	2.82E-05
ENSG00000183291_86914111_86880690_95	annotated	SELENOF	1	-	285	703	115	20	35.79%	815.0422434	4.38E-07	8.77E-07	3.51E-12
ENSG00000153936_86915106_87097939_206	annotated	HS2ST1	1	+	618	69	16	1	8.74%	36.891836	0.000235155	0.000116567	1.94E-07
ENSG00000153936_86915106_87104696_333	annotated	HS2ST1	1	+	999	74	21	4	6.31%	24.47574305	0.000788831	0.000166258	9.12E-07
ENSG00000035141_70301966_70297289_230	annotated	FAM136A	2	-	690	23	5	0	3.91%	11.01408437	0.044380821	0.003632191	0.000962121
ENSG00000011422_43670120_43648890_335	annotated	PLAUR	19	-	1005	135	20	4	12.24%	44.38511612	4.79E-07	3.87E-08	1.88E-13
ENSG00000173821_80273288_80393498_5159	annotated	AC124319.1	17	+	15477	163	26	27	1.61%	3.479929739	1.73E-10	1.30E-10	2.61E-19
ENSG00000104870_49513401_49526119_99	annotated	FCGRT	19	+	297	9	1	0	6.06%	10.01280397	0.026832696	0.011772051	0.001510382
ENSG00000185515_155071528_155120150_293	annotated	BRCC3	X	+	879	20	2	0	2.73%	7.518146328	0.018402302	0.005604867	0.000538358
ENSG00000105671_18919742_18921942_172	novel	DDX49	19	+	516	32	7	1	3.49%	20.49131976	0.016724853	0.012619539	0.001014093
ENSG00000172500_65888418_65885797_178	novel	FIBP	11	-	534	70	9	7	10.11%	43.31381494	0.00073803	0.000698972	3.28E-06
ENSG00000157450_59031054_59094900_917	annotated	RNF111	15	+	2751	34	7	0	1.64%	4.083739025	0.005958567	0.000277593	1.22E-05
ENSG00000110330_102349969_102378183_580	annotated	BIRC2	11	+	1740	39	17	1	2.76%	7.406022249	0.017220256	0.000275536	4.10E-05
ENSG00000149792_65125529_65125872_76	novel	MRPL49	11	+	228	63	10	0	23.68%	91.30096255	0.000805931	8.75E-05	5.21E-07
ENSG00000158042_6682327_6681198_60	novel	MRPL17	11	-	180	49	5	4	13.33%	89.94835569	0.005808022	0.006346985	0.000195019
ENSG00000254986_66482201_66509251_737	annotated	DPP3	11	+	2211	78	10	1	4.75%	11.65669716	5.37E-06	1.21E-07	6.71E-12
ENSG00000007202_28644095_28643178_73	internal	KIAA0100	17	-	219	15	0	0	6.85%	22.63168021	0.021557223	0.021557223	0.002115616
ENSG00000007202_28642954_28615064_2092	annotated	KIAA0100	17	-	6276	89	28	6	2.10%	4.68572423	5.01E-05	8.39E-08	5.59E-11
ENSG00000090520_186570898_186585408_358	annotated	DNAJB11	3	+	1074	171	17	11	12.85%	52.60917395	3.75E-08	4.99E-08	1.87E-14
ENSG00000010244_32350286_32373409_493	annotated	ZNF207	17	+	1479	205	16	13	8.72%	45.79893095	1.47E-08	8.06E-08	1.22E-14
ENSG00000167632_140451355_139731061_1142	annotated	TRAPPC9	8	-	3426	53	11	5	2.19%	5.111615338	0.000260403	0.00010115	1.87E-07
ENSG00000121064_56981156_57006235_402	Overlap_dORF	SCPEP1	17	+	1206	195	6	10	11.44%	53.42652866	1.24E-08	2.13E-08	2.71E-15
ENSG00000189067_11553630_11549637_68	dORF	LITAF	16	-	204	227	28	21	44.12%	367.6760518	1.11E-05	2.04E-06	1.97E-10
ENSG00000155463_22767037_22771558_419	annotated	OXA1L	14	+	1257	346	35	14	15.75%	90.9516275	1.32E-09	2.24E-12	4.41E-20
ENSG00000104687_30727706_30679520_426	annotated	GSR	8	-	1278	242	46	17	14.79%	62.56827272	3.14E-08	4.12E-11	1.89E-17
ENSG00000175390_7992114_7996022_202	dORF	EIF3F	11	+	606	53	8	1	6.44%	28.89834018	0.0100415	0.000950595	6.16E-05
ENSG00000145191_184135386_184142809_530	annotated	EIF2B5	3	+	1590	38	9	4	3.02%	7.896890681	0.029349866	0.000860419	0.000190257
ENSG00000028116_58048901_58154770_374	annotated	VRK2	2	+	1122	62	8	0	5.61%	18.25864254	0.000522303	2.51E-05	1.08E-07
ENSG00000112245_63576890_63580174_170	annotated	PTP4A1	6	+	510	134	7	2	11.76%	86.81690033	5.96E-05	4.42E-05	1.98E-08
ENSG00000112245_63532525_63576531_33	uORF	PTP4A1	6	+	99	577	49	8	36.36%	1925.795964	0.001049313	0.00258473	1.65E-05
ENSG00000136156_48253859_48261224_210	novel	ITM2B	13	+	630	969	74	31	31.90%	508.2213217	4.74E-11	7.31E-12	4.27E-21
ENSG00000115902_65010624_65021146_234	annotated	SLC1A4	2	+	702	25	1	2	5.56%	11.76718416	0.001013137	0.003263237	2.01E-05
ENSG00000163660_157150374_157148241_299	dORF	CCNL1	3	-	897	26	8	1	3.68%	9.57746467	0.067865885	0.002493494	0.001181394
ENSG00000185753_40647520_40630615_319	annotated	CXorf38	X	-	957	17	1	0	2.82%	5.869574743	0.005561256	0.003452921	0.000105543
ENSG00000121152_96364490_96373351_178	annotated	NCAPH	2	+	534	17	4	0	4.49%	10.51906934	0.015191411	0.005160369	0.000412029
ENSG00000114805_155596340_155483366_975	annotated	PLCH1	3	-	2925	16	3	4	0.51%	1.807439486	0.050997316	0.086597294	0.017028761
ENSG00000002834_38870190_38918778_261	annotated	LASP1	17	+	783	426	27	14	21.07%	179.7701127	6.14E-11	4.69E-10	3.40E-19
ENSG00000100714_64454747_64458303_72	novel	MTHFD1	14	+	216	22	1	0	9.72%	33.65414669	0.008473674	0.008674847	0.000374723
ENSG00000118600_63779961_63809092_443	annotated	RXYLT1	12	+	1329	59	14	2	4.29%	14.66887083	0.003064381	0.000206647	4.60E-06
ENSG00000067182_6341814_6333886_65	novel	TNFRSF1A	12	-	195	59	41	5	16.92%	99.97399659	0.188614681	0.002275573	0.004261891
ENSG00000067182_6341955_6341824_43	novel	TNFRSF1A	12	-	129	59	7	7	23.26%	151.1234832	0.022350948	0.03211189	0.003185315
ENSG00000163607_112991000_113000919_307	annotated	GTPBP8	3	+	921	50	1	0	6.19%	17.93824816	0.000111058	5.79E-05	4.73E-08
ENSG00000012171_50274055_50276706_371	novel	SEMA3B	3	+	1113	28	8	0	3.23%	8.312516506	0.026534973	0.000991051	0.00018913
ENSG00000012171_50269241_50271429_202	novel	SEMA3B	3	+	606	12	3	0	2.48%	6.543020418	0.121807694	0.019679754	0.01126199
ENSG00000100462_22922554_22920904_109	novel	PRMT5	14	-	327	24	6	3	11.01%	24.25119494	0.012837471	0.007560076	0.000491183
ENSG00000091140_107891251_107919259_509	annotated	DLD	7	+	1527	221	51	33	12.77%	47.82146652	1.02E-07	2.63E-09	3.04E-15
ENSG00000100473_30874939_30889791_615	annotated	COCH	14	+	1845	87	17	2	5.04%	15.58089984	3.64E-05	1.38E-06	4.68E-10
ENSG00000100479_49688203_49643652_527	annotated	POLE2	14	-	1581	27	3	1	2.47%	5.642889526	0.003379684	0.000906178	1.89E-05
ENSG00000149925_30067255_30067440_61	novel	ALDOA	16	+	183	131	19	15	18.03%	236.5319758	0.00664636	0.005395979	0.00019018
ENSG00000184588_66355536_66372678_484	annotated	PDE4B	1	+	1452	71	12	5	5.79%	16.15702459	8.96E-05	4.68E-06	3.67E-09
ENSG00000177239_137107340_137108591_147	novel	MAN1B1	9	+	441	39	8	2	10.20%	29.22104017	0.005031593	0.001101134	3.38E-05
ENSG00000112561_41690695_41684599_331	annotated	TFEB	6	-	993	13	0	0	1.81%	4.325773318	0.012798403	0.012798403	0.000797244
ENSG00000144824_111884822_111974563_962	annotated	PHLDB2	3	+	2886	28	2	4	1.56%	3.205762603	0.000742282	0.002781433	1.29E-05
ENSG00000213923_38303150_38296561_278	annotated	CSNK1E	22	-	834	77	5	1	6.83%	30.50663657	0.000204612	6.99E-05	1.04E-07
ENSG00000204536_31154651_31144664_546	novel	CCHCR1	6	-	1638	11	1	0	1.28%	2.218954727	0.007593099	0.007593099	0.000297833
ENSG00000143374_150488016_150507064_443	Overlap_dORF	TARS2	1	+	1329	62	11	2	4.97%	15.41474562	0.000521258	1.93E-05	8.54E-08
ENSG00000143374_150487451_150492478_135	annotated	TARS2	1	+	405	19	1	0	7.41%	15.50130393	0.003247793	0.002122793	3.97E-05
ENSG00000143374_150487451_150489237_178	novel	TARS2	1	+	534	30	1	0	6.74%	18.56306355	0.001318835	0.000982952	7.98E-06
ENSG00000143374_150487451_150507064_588	annotated	TARS2	1	+	1764	106	7	2	6.63%	19.85532216	1.08E-07	2.22E-08	2.42E-14
ENSG00000243989_51984065_51989075_408	annotated	ACY1	3	+	1224	74	4	4	6.62%	19.97652557	4.09E-06	1.50E-05	5.15E-10
ENSG00000188603_28482191_28477516_115	novel	CLN3	16	-	345	54	5	1	16.52%	51.71830922	0.000208869	7.59E-05	1.15E-07
ENSG00000153048_8859328_8855164_147	annotated	CARHSP1	16	-	441	111	10	3	16.33%	83.16757586	1.54E-05	2.35E-05	2.86E-09
ENSG00000284917_184249695_184292238_883	annotated	ECE2	3	+	2649	28	0	0	1.25%	3.492574885	0.001558047	0.001558047	1.45E-05

ENSG00000105829_94004216_93975952_127	annotated	BET1	7	-	381	20	2	0	3.94%	17.34501476	0.044040756	0.021084099	0.004111364
ENSG00000172172_120445094_120425341_58	annotated	MRPL13	8	-	174	43	5	43	18.97%	81.65614275	0.005546388	0.026610517	0.000781277
ENSG00000172172_120419881_120396104_57	dORF	MRPL13	8	-	171	50	5	0	19.30%	96.61477518	0.00850083	0.00140552	7.23E-05
ENSG00000169410_75578746_75468769_583	annotated	PTPN9	15	-	1749	39	6	2	2.57%	7.367912358	0.003957724	0.000802897	1.99E-05
ENSG00000144354_173354964_173367664_450	annotated	CDCA7	2	+	1350	48	3	1	4.89%	11.74835666	9.91E-05	2.32E-05	1.78E-08
ENSG00000139350_96917657_96952053_571	annotated	NEDD1	12	+	1713	31	8	3	2.28%	5.97962549	0.013839786	0.00210825	0.000171608
ENSG00000140718_53704185_53979452_481	annotated	FTO	16	+	1443	44	5	3	4.57%	10.07525389	0.000267507	0.000111699	2.11E-07
ENSG00000135018_83707571_83661787_525	annotated	UBQLN1	9	-	1575	161	12	13	8.57%	33.7765254	2.76E-08	2.63E-07	7.38E-14
ENSG00000059804_7936034_7930451_233	novel	SLC2A3	12	-	699	15	1	0	3.43%	7.090612256	0.008170681	0.005405142	0.00023249
ENSG00000122566_26196593_26195800_75	novel	HNRNPA2B1	7	-	225	364	61	18	48.00%	534.5502281	1.97E-06	2.59E-07	4.81E-12
ENSG00000078269_157981962_158096364_1496	annotated	SYNJ2	6	+	4488	63	16	3	1.74%	4.638284193	0.002704619	7.83E-06	2.57E-07
ENSG00000148985_3811260_3824350_81	annotated	PGAP2	11	+	243	27	1	0	8.64%	36.71361457	0.008473674	0.008473674	0.000366502
ENSG00000137207_43516071_43512167_315	annotated	YIPF3	6	-	945	327	22	8	19.37%	114.3366854	2.54E-11	7.99E-12	2.49E-21
ENSG00000170522_110198329_110059708_96	novel	ELOVL6	4	-	288	41	10	31	16.67%	47.03931867	0.01796334	0.17325314	0.015817231
ENSG00000106355_32490365_32488621_57	novel	LSM5	7	-	171	152	20	7	28.07%	293.7089165	0.000579912	0.000932425	3.45E-06
ENSG00000112514_33417668_33417247_83	novel	CUTA	6	-	249	533	38	21	30.12%	707.2899963	2.32E-05	9.78E-06	1.83E-09
ENSG00000108861_43769746_43769609_45	dORF	DUSP3	17	-	135	16	1	0	11.11%	39.16118887	0.019679754	0.019679754	0.001784964
ENSG00000101343_20048422_20036195_562	dORF	CRNKL1	20	-	1686	38	2	4	4.09%	7.44724566	5.56E-05	9.13E-05	3.75E-08
ENSG00000196139_5094445_5099647_190	novel	AKR1C3	10	+	570	570	77	10	12.63%	330.4225311	7.98E-05	7.75E-06	5.15E-09
ENSG00000109255_55636192_55599146_149	annotated	NMU	4	-	447	25	0	0	8.05%	18.48000733	0.000943084	0.000943084	5.54E-06
ENSG00000115963_150487372_150469987_229	annotated	RND3	2	-	687	37	15	0	6.99%	17.79568217	0.0177461	0.000184214	3.10E-05
ENSG00000148090_91361889_91214348_339	annotated	AUH	9	-	1017	16	1	2	2.95%	5.198387903	0.003646019	0.0116632	0.000231703
ENSG00000118965_19990015_19913558_1170	annotated	WDR35	2	-	3510	30	10	6	1.45%	2.824124198	0.033238485	0.005319561	0.000955053
ENSG00000116809_15948495_15941969_810	annotated	ZBTB17	1	-	2430	11	1	4	0.74%	1.495739853	0.019795882	0.116253847	0.010739827
ENSG00000165915_47410380_47415122_230	annotated	SLC39A13	11	+	690	15	0	0	2.61%	7.183098503	0.013428348	0.013428348	0.000872559
ENSG00000165915_47410103_47412352_137	internal	SLC39A13	11	+	411	25	4	3	6.57%	20.09869411	0.032506135	0.009341698	0.001500183
ENSG00000162300_65087047_65088114_110	novel	ZFPL1	11	+	330	23	8	3	8.18%	23.02944914	0.031335694	0.013091824	0.00193526
ENSG00000151694_9527915_9521286_129	novel	ADAM17	2	-	387	45	3	1	10.08%	38.42122455	0.003044359	0.000883754	1.66E-05
ENSG00000151694_9521214_9501859_264	novel	ADAM17	2	-	792	45	3	4	6.44%	18.77400745	0.000132663	0.000419659	3.90E-07
ENSG00000167779_53100855_53102167_81	annotated	IGFBP6	12	+	243	212	10	3	25.93%	288.2698625	2.94E-05	2.58E-05	5.87E-09
ENSG00000112541_165987898_165987578_106	novel	PDE10A	6	-	318	45	19	13	14.15%	46.75790535	0.04790541	0.018522412	0.003998427
ENSG00000125755_45817019_45815560_262	novel	SYMPK	19	-	786	41	3	0	4.96%	17.23578089	0.003358805	0.00065148	1.39E-05
ENSG00000071889_154512893_154506811_244	annotated	FAM3A	X	-	732	38	5	2	6.15%	17.15308222	0.003481598	0.001021259	2.17E-05
ENSG00000120948_11016999_11023183_153	annotated	TARDBP	1	+	459	35	1	0	10.46%	25.19561784	0.00028444	0.000178284	3.50E-07
ENSG00000102804_44436046_44434626_86	annotated	TSC22D1	13	-	258	155	24	5	31.40%	198.5096602	1.25E-05	2.78E-06	2.97E-10
ENSG00000198130_190249720_190205117_163	annotated	HIBCH	2	-	489	7	2	0	3.07%	4.729974883	0.119134635	0.019216965	0.010787784
ENSG00000110108_60919751_60922165_224	annotated	TMEM109	11	+	672	289	22	9	20.09%	142.1013564	1.04E-07	1.41E-08	1.50E-14
ENSG00000101049_43569397_43585016_287	annotated	SGK2	20	+	861	13	1	0	2.09%	4.988958077	0.016344022	0.012798403	0.001004962
ENSG00000160999_102306790_102321630_366	annotated	SH2B2	7	+	1098	9	0	1	1.37%	2.708381403	0.020613417	0.035374934	0.003252526
ENSG00000145979_13326898_13305101_247	annotated	TBC1D7	6	-	741	99	4	2	8.50%	44.14552035	0.00037684	2.19E-05	6.75E-08
ENSG00000160199_43007491_43030101_419	annotated	PKNOX1	21	+	1257	19	8	2	2.15%	4.994453533	0.076476367	0.003478238	0.001755739
ENSG00000125844_17643062_17614182_751	annotated	RRBP1	20	-	2253	30	7	5	2.26%	4.399767392	0.006545952	0.001903084	7.21E-05
ENSG00000241489_149496423_149482746_283	annotated	AC244197.3	X	-	849	15	3	4	3.18%	5.837853907	0.025557109	0.038440602	0.004267345
ENSG00000196756_38434513_38426436_78	novel	SNHG17	20	-	234	26	5	3	8.97%	36.71361457	0.026714995	0.023878474	0.00284211
ENSG00000086189_62403772_62373015_317	annotated	DIMT1	5	-	951	97	16	5	11.04%	33.70240328	1.87E-05	4.78E-07	8.82E-11
ENSG00000033011_5071850_5073286_139	novel	ALG1	16	+	417	22	6	4	6.47%	17.43236375	0.042385694	0.028926347	0.005232822
ENSG00000126261_34460491_34469221_166	annotated	UBA2	19	+	498	43	12	0	9.64%	28.53045951	0.0094274	0.000193373	1.52E-05
ENSG00000062716_59839789_59839911_40	novel	VMP1	17	+	120	31	3	5	15.00%	85.35915387	0.023199731	0.046045458	0.004668342
ENSG00000136560_161179663_161219783_128	annotated	TANK	2	+	384	10	3	0	4.69%	8.604753414	0.100002646	0.012798403	0.006484129
ENSG00000162623_74733359_74764113_188	annotated	TYW3	1	+	564	14	0	0	3.19%	8.201977723	0.01300724	0.01300724	0.000821865
ENSG00000170477_52811794_52807069_305	dORF	KRT4	12	-	915	29	2	1	7.54%	10.47240809	3.66E-05	1.46E-05	4.22E-09
ENSG00000136603_170360425_170392417_653	annotated	SKIL	3	+	1959	50	12	1	3.83%	8.433448982	0.000499766	6.03E-06	2.95E-08
ENSG00000131165_89657588_89647038_181	annotated	CHMP1A	16	-	543	56	11	9	7.18%	34.07672512	0.016657955	0.012607423	0.00100932
ENSG00000170293_32238973_32369967_115	annotated	CMTM8	3	+	345	25	1	7	10.43%	23.94366168	0.000991051	0.013219333	8.61E-05
ENSG00000168610_42348516_42316827_722	annotated	STAT3	17	-	2166	106	17	7	6.37%	16.17026237	3.14E-06	6.83E-08	2.26E-12
ENSG00000152240_46123320_46128125_71	Overlap_dORF	HAUS1	18	+	213	53	2	15	9.86%	82.21781291	0.008473674	0.060590694	0.00268166
ENSG00000050130_59495064_59504072_212	novel	JKAMP	14	+	636	255	41	3	21.70%	132.4807318	2.96E-08	1.31E-09	4.36E-16
ENSG00000075413_103386030_103503227_744	annotated	MARK3	14	+	2232	39	10	5	2.02%	5.773511968	0.0148119	0.002392647	0.00020554
ENSG00000273259_94614451_94623814_420	dORF	AL049839.2	14	+	1260	8	0	0	1.43%	2.097920832	0.011570466	0.011570466	0.000659484
ENSG00000182199_57230833_57231742_65	novel	SHMT2	12	+	195	55	15	1	10.77%	93.19609852	0.0839881	0.01208853	0.005100402
ENSG00000197114_63708581_63735919_511	annotated	ZGPAT	20	+	1533	8	3	0	1.17%	1.724318492	0.124106539	0.011570466	0.007709042
ENSG00000136436_48848100_48863005_374	annotated	CALCOCO2	17	+	1122	75	11	0	7.49%	22.08706759	5.43E-05	1.62E-06	8.26E-10
ENSG00000100906_35403755_35402013_227	annotated	NFKBIA	14	-	681	44	3	8	8.81%	21.348886	0.00013589	0.000816124	7.86E-07
ENSG00000161904_33784490_33780096_99	novel	LEMD2	6	-	297	20	1	0	7.07%	22.2506755	0.008574016	0.008776163	0.000383092
ENSG00000139990_69152948_69053857_931	annotated	DCAF5	14	-	2793	16	1	0	0.97%	1.892860901	0.005426623	0.003327803	9.96E-05
ENSG00000177485_120253416_120255434_672	annotated	ZBTB33	X	+	2016	14	4	0	1.34%	2.294600911	0.061503838	0.003229477	0.001279093
ENSG00000100416_46346542_46356954_198	dORF	TRMU	22	+	594	8	1	3	2.53%	4.450135099	0.047790352	0.11777946	0.021834536
ENSG00000100416_46335765_46346473_99	annotated	TRMU	22	+	297	12	2	0	7.07%	13.3504053	0.023672501	0.007882058	0.000936943

ENSG00000231925_33314041_33304300_156	annotated	TAPBP	6	-	468	50	14	3	12.82%	35.30155247	0.001465813	8.67E-05	9.75E-07
ENSG00000286075_6348758_6331215_68	uORF	AC009412.1	7	-	204	13	1	2	7.35%	21.05633777	0.042234516	0.065398531	0.011057031
ENSG00000286075_6330962_6330375_195	annotated	AC009412.1	7	-	585	42	1	0	4.62%	23.72264326	0.003554776	0.003554776	7.04E-05
ENSG00000204842_111552304_111452812_953	annotated	ATXN2	12	-	2859	22	3	0	0.94%	2.542600799	0.008219047	0.003632191	0.000162452
ENSG00000127946_75611695_75538172_1007	annotated	HIP1	7	-	3021	30	5	1	1.49%	3.281256516	0.003178116	0.000414862	8.81E-06
ENSG00000135624_73234382_73244004_82	annotated	CCT7	2	+	246	59	18	5	8.54%	79.24768023	0.360377459	0.024275373	0.049724095
ENSG00000197930_52695481_52657949_257	annotated	ERO1A	14	-	771	232	30	7	12.45%	99.42675385	0.000156552	3.54E-07	7.02E-10
ENSG00000166135_100535959_100548137_349	annotated	HIF1AN	10	+	1047	30	5	0	4.30%	9.467694301	0.003834926	0.000281287	7.66E-06
ENSG00000166136_100529851_100523837_186	annotated	NDUFB8	10	-	558	521	59	15	32.80%	308.5127934	3.48E-09	3.90E-11	1.74E-18
ENSG00000102893_47461351_47699366_1093	annotated	PHKB	16	+	3279	88	14	9	3.93%	8.867698304	2.91E-06	9.35E-08	2.78E-12
ENSG00000113971_132692704_132681910_522	dORF	NPHP3	3	-	1566	14	0	0	0.96%	2.953968988	0.021084099	0.021084099	0.002029891
ENSG00000240972_23894475_23895005_113	novel	MIF	22	+	339	1847	158	33	37.17%	1800.266711	5.98E-08	5.41E-08	3.19E-14
ENSG00000088448_110914934_110900631_161	annotated	ANKRD10	13	-	483	22	1	3	5.59%	15.05030162	0.005588371	0.011768187	0.000342715
ENSG00000154781_14651837_14666988_254	novel	CCDC174	3	+	762	33	3	0	4.72%	14.30963717	0.000978918	0.000982952	5.98E-06
ENSG00000110906_109469722_109451595_287	annotated	KCTD10	12	-	861	24	0	0	3.83%	9.210384143	0.001269017	0.001269017	9.81E-06
ENSG00000154582_73959768_73946630_112	annotated	ELOC	8	-	336	83	32	2	17.86%	81.62223239	0.020681041	9.81E-05	2.29E-05
ENSG00000276043_4910886_4960803_793	annotated	UHRF1	19	+	2379	48	2	2	2.40%	6.666784991	7.47E-05	0.00019657	1.06E-07
ENSG00000106976_128203486_128253122_846	annotated	DNM1	9	+	2538	133	27	17	5.91%	17.3152863	2.09E-07	2.83E-07	5.56E-13
ENSG00000125726_6591002_6583300_208	annotated	CD70	19	-	624	64	10	0	6.25%	33.88949037	0.005063995	0.000688595	2.25E-05
ENSG00000179115_12933696_12922748_477	annotated	FARSA	19	-	1431	98	6	5	8.18%	22.62851716	4.13E-07	1.28E-07	5.04E-13
ENSG00000135678_68885806_68856437_362	annotated	CPM	12	-	1086	14	0	0	1.93%	4.259590641	0.008175611	0.008175611	0.000342518
ENSG00000123562_103677027_103676161_288	annotated	MORF4L2	X	-	864	192	23	11	16.67%	73.42722914	1.11E-07	1.33E-08	1.53E-14
ENSG00000134686_33354458_33324865_359	annotated	PHC2	1	-	1077	56	7	9	4.74%	17.18074442	0.002794823	0.005189626	8.11E-05
ENSG00000198792_38231272_38221469_267	dORF	TMEM184B	22	-	801	63	4	4	7.87%	25.98828896	0.000138666	0.0001178	1.16E-07
ENSG00000198792_38247859_38246859_38	annotated	TMEM184B	22	-	114	12	0	0	13.16%	34.78131906	0.019679754	0.019679754	0.001784964
ENSG00000084234_130140481_130143448_111	novel	APLP2	11	+	333	64	3	0	13.51%	63.5046306	0.001034407	0.000306523	2.11E-06
ENSG00000185475_62787432_62789901_144	annotated	TMEM179B	11	+	432	265	24	4	20.14%	202.6897471	6.84E-06	3.22E-06	1.86E-10
ENSG00000142186_65525154_65538540_787	annotated	SCYL1	11	+	2361	55	7	12	3.05%	7.697263537	0.000182411	0.001845592	2.40E-06
ENSG00000166471_9287827_9283424_144	novel	TMEM41B	11	-	432	17	1	0	5.56%	13.00273849	0.006965909	0.005306578	0.000195878
ENSG00000125445_75262823_75262918_31	novel	MRPS7	17	+	93	19	2	3	16.13%	67.5056784	0.039124093	0.052029617	0.008320031
ENSG00000171681_14424099_14481226_1044	annotated	ATF7IP	12	+	3132	24	6	0	1.15%	2.531973419	0.022156796	0.000978918	0.000152118
ENSG00000185686_22556832_22548067_509	annotated	PRAME	22	-	1527	140	14	2	6.68%	30.29414169	6.97E-06	1.77E-07	1.25E-11
ENSG00000068024_239190027_239053097_1041	annotated	HDAC4	2	-	3123	20	0	1	1.15%	2.116058477	0.000866459	0.001612025	8.62E-06
ENSG00000058600_22302969_22333700_666	annotated	POLR3E	16	+	1998	58	6	0	3.30%	9.591845248	9.75E-05	1.74E-05	1.34E-08
ENSG00000121578_119230099_119212549_344	annotated	B4GALT4	3	-	1032	142	24	2	13.95%	45.46511572	1.55E-06	9.52E-10	2.27E-14
ENSG00000184939_68558091_68564698_594	annotated	ZFP90	16	+	1782	16	7	0	1.35%	2.966756733	0.131833324	0.005454749	0.004793496
ENSG00000173950_195175673_195069715_190	annotated	XXYLT1	3	-	570	22	2	0	2.63%	12.75315032	0.021557223	0.021557223	0.002115616
ENSG00000134748_52412577_52416690_125	novel	PRPF38A	1	+	375	31	0	2	4.80%	27.31492924	0.013640586	0.037440157	0.002398086
ENSG00000144524_231794346_231807645_157	novel	COPS7B	2	+	471	13	0	0	3.82%	9.119942472	0.01300724	0.01300724	0.000821865
ENSG00000140105_100361879_100334875_424	annotated	WARS	14	-	1272	141	24	4	7.55%	36.62702585	3.08E-05	1.38E-06	3.93E-10
ENSG00000109390_139295798_139292550_76	annotated	NDUFC1	4	-	228	129	21	8	26.32%	186.94959	0.000474984	7.52E-05	2.62E-07
ENSG00000141959_44306683_44326862_751	dORF	PFKL	21	+	2253	73	6	2	4.79%	10.70610065	1.02E-06	1.08E-07	1.07E-12
ENSG00000015171_180013_252470_602	annotated	ZMYND11	10	+	1806	68	17	9	4.65%	12.44115843	0.001177563	5.29E-05	5.00E-07
ENSG00000141699_42609324_42586125_83	annotated	RETREG3	17	-	249	13	3	0	6.02%	17.25097552	0.020613417	0.020613417	0.001946209
ENSG00000142949_43545076_43579285_353	novel	PTPRF	1	+	1059	19	2	1	1.42%	5.928260709	0.020613417	0.035374934	0.003252526
ENSG00000103351_3515565_3536271_229	annotated	CLUAP1	16	+	687	26	5	0	4.80%	12.50507396	0.01277527	0.001506153	0.000118195
ENSG00000135002_76392471_76387399_95	novel	RFK	9	-	285	36	7	0	10.53%	41.73758288	0.007000869	0.002404282	9.53E-05
ENSG00000147475_37737923_37745603_152	annotated	ERLIN2	8	+	456	72	3	4	13.16%	52.1719786	4.14E-05	7.41E-05	2.30E-08
ENSG00000169919_65976050_65974522_72	novel	GUSB	7	-	216	8	2	0	6.94%	12.23787152	0.101051022	0.019679754	0.009164498
ENSG00000114416_180947925_180976292_564	novel	FXR1	3	+	1692	57	8	0	4.96%	11.13125548	0.000109344	1.30E-06	1.45E-09
ENSG00000093144_127290273_127289869_134	novel	ECHDC1	6	-	402	31	4	5	7.46%	25.48034444	0.009941611	0.015997149	0.000781011
ENSG00000002586_2691361_2733364_160	annotated	CD99	X	+	480	299	113	13	24.38%	205.8257017	3.34E-05	7.16E-08	3.08E-11
ENSG00000113758_177473521_177467554_172	novel	DBN1	5	-	516	18	8	2	4.65%	11.52636736	0.085005563	0.02389627	0.00889931
ENSG00000113758_177467675_177457433_555	novel	DBN1	5	-	1665	52	6	2	4.68%	10.31950247	9.28E-05	1.40E-05	1.04E-08
ENSG00000132842_78034410_78003044_106	annotated	AP3B1	5	-	318	7	0	1	4.72%	7.273451943	0.019216965	0.041782825	0.003600526
ENSG00000164808_47260959_47388355_179	novel	SPIDR	8	+	537	18	3	2	3.35%	11.07561557	0.039104404	0.024275373	0.004143858
ENSG00000078269_158063813_158096364_1113	annotated	SYNJ2	6	+	3339	38	8	2	1.62%	3.760424134	0.005677483	0.00016368	7.44E-06
ENSG00000196588_40417042_40411390_194	Overlap_dORF	MRTFA	22	-	582	12	0	0	3.09%	6.812835693	0.013217227	0.013217227	0.00084697
ENSG00000196588_40431420_40411799_757	annotated	MRTFA	22	-	2271	22	10	3	2.11%	3.200922803	0.033169574	0.001657596	0.000369034
ENSG00000187118_28263338_28319629_84	novel	CMC1	3	+	252	41	2	6	9.52%	53.75922133	0.008413705	0.044620823	0.001914148
ENSG00000138796_107989957_108034357_168	annotated	HADH	4	+	504	42	3	4	8.33%	27.53521093	0.002121694	0.003076592	3.76E-05
ENSG00000164904_126582881_126559328_111	dORF	ALDH7A1	5	-	333	28	1	0	6.31%	27.78327589	0.008877961	0.008877961	0.000400235
ENSG00000164904_126595198_126593406_77	annotated	ALDH7A1	5	-	231	26	2	3	9.09%	37.19041476	0.017620112	0.017620112	0.001451864
ENSG00000164904_126554349_126544965_160	dORF	ALDH7A1	5	-	480	31	4	0	6.88%	21.33978847	0.00850083	0.00140552	7.23E-05
ENSG00000100591_77458190_77468612_288	annotated	AHSA1	14	+	864	239	17	11	18.75%	91.40160294	2.07E-10	1.18E-09	2.80E-18
ENSG00000213983_23566346_23559749_708	novel	AP1G2	14	-	2124	45	13	1	2.54%	7.000477354	0.007601334	0.000120586	8.07E-06
ENSG00000010626_6905634_6913003_343	annotated	LRRC23	12	+	1029	10	0	0	1.46%	3.211103315	0.020613417	0.020613417	0.001946209
ENSG00000080824_102086345_102081712_721	annotated	HSP90AA1	14	-	2163	850	119	26	16.23%	129.8470418	1.26E-16	3.84E-20	9.32E-35

ENSG00000075413_103386030_103503227_713	annotated	MARK3	14	+	2139	39	10	4	2.10%	6.024534228	0.0148119	0.00190111	0.000168882
ENSG00000243989_51984065_51989075_408	annotated	ACY1	3	+	1224	74	4	4	6.62%	19.97652557	4.09E-06	1.50E-05	5.15E-10
ENSG00000102763_41960952_41719587_1018	annotated	VWA8	13	-	3054	19	2	4	0.59%	2.0556739	0.053594825	0.060667625	0.012723061
ENSG00000164631_6697718_6690848_623	annotated	ZNF12	7	-	1869	9	0	0	1.12%	1.591119733	0.005706018	0.005706018	0.00017331
ENSG00000141556_82938059_82942463_95	novel	TBCD	17	+	285	9	5	0	5.26%	10.43439572	0.246358339	0.019216965	0.025652408
ENSG00000178209_143950385_143916177_4577	annotated	PLEC	8	-	13731	245	62	38	2.53%	5.895675488	1.16E-09	1.50E-13	3.23E-21
ENSG00000182196_122981140_122982676_229	annotated	ARL6IP4	12	+	687	104	15	7	12.66%	50.02029583	3.23E-05	2.71E-05	6.77E-09
ENSG00000172725_67442628_67441011_289	novel	CORO1B	11	-	867	76	8	0	6.92%	28.96437412	0.000405405	4.11E-05	1.28E-07
ENSG00000124155_45420349_45425826_316	dORF	PIGT	20	+	948	259	20	2	16.46%	90.2736662	6.03E-10	1.31E-10	9.13E-19
ENSG00000124155_45416169_45418829_136	annotated	PIGT	20	+	408	88	3	4	12.50%	71.26760475	0.000289196	0.000866705	1.67E-06
ENSG00000138796_108004852_108023617_207	dORF	HADH	4	+	621	36	6	0	6.76%	19.15492934	0.00479213	0.000436224	1.42E-05
ENSG00000132716_160218371_160217592_54	novel	DCAF8	1	-	162	16	1	0	12.96%	32.63432406	0.016854696	0.007029303	0.00060175
ENSG00000214078_35655274_35632880_38	Overlap_uORF	CPNE1	20	-	114	11	0	2	13.16%	31.88287581	0.019679754	0.101051022	0.009164498
ENSG00000099814_104878451_104894958_1460	annotated	CEP170B	14	+	4380	9	2	1	0.41%	0.678950406	0.053594825	0.026832696	0.006148603
ENSG00000143321_156745364_156743449_208	annotated	HDGF	1	-	624	254	34	12	24.04%	134.4989149	2.64E-09	1.28E-09	3.68E-17
ENSG00000152894_128520358_127970227_1472	annotated	PTPRK	6	-	4416	189	27	4	4.48%	14.14172518	2.09E-10	1.13E-12	3.32E-21
ENSG00000109113_28717266_28714643_237	annotated	RAB34	17	-	711	231	35	21	12.24%	107.3524679	0.000102906	0.000116395	8.61E-08
ENSG00000177410_49279128_49279205_25	novel	ZFAS1	20	+	75	139	14	5	44.00%	612.383091	0.002338867	0.001792061	2.46E-05
ENSG00000125743_45688538_45687553_108	annotated	SNRPD2	19	-	324	1043	156	17	40.74%	1063.675	5.36E-08	1.32E-08	7.24E-15
ENSG00000074603_65512541_65446884_778	annotated	DPP8	15	-	2334	21	2	0	1.29%	2.972953365	0.010448364	0.002046029	0.000124448
ENSG00000198060_92291217_92311190_122	Overlap_uORF	5-Mar	10	+	366	37	2	13	7.38%	33.40337063	0.009335736	0.070191664	0.003408368
ENSG00000198060_92291519_92351207_270	annotated	5-Mar	10	+	810	92	38	0	11.11%	37.52947267	0.00536185	7.27E-07	9.41E-08
ENSG00000011422_43670120_43646457_281	annotated	PLAUR	19	-	843	104	21	0	11.39%	40.76387098	1.90E-05	3.55E-07	6.84E-11
ENSG00000100814_20316505_20311550_271	annotated	CCNB1IP1	14	-	813	7	4	1	1.85%	2.844966443	0.218864129	0.041782825	0.038173193
ENSG00000068903_38893429_38879480_201	annotated	SIRT2	19	-	603	24	3	0	5.47%	13.15114552	0.004968901	0.001262414	3.77E-05
ENSG00000103227_954496_934105_63	annotated	LMF1	16	-	189	13	3	3	12.70%	22.72747569	0.035227809	0.038105097	0.005655927
ENSG00000239382_36013397_36010580_133	annotated	ALKBH6	19	-	399	10	4	0	3.76%	8.281266444	0.121807694	0.019679754	0.01126199
ENSG00000171311_97445985_97436477_139	annotated	EXOSC1	10	-	417	17	2	1	5.76%	13.4704629	0.029020025	0.010079165	0.001426583
ENSG00000123136_14413220_14409910_267	annotated	DDX39A	19	-	801	94	19	19	9.36%	38.77617718	0.002464745	0.000912614	1.38E-05
ENSG00000112237_99568527_99544222_282	annotated	CCNC	6	-	846	28	3	4	3.90%	10.9359703	0.001696512	0.004423224	4.38E-05
ENSG00000114098_138187585_138295892_663	annotated	ARMC8	3	+	1989	28	10	0	2.11%	4.651498678	0.018017259	0.000377559	5.57E-05
ENSG00000101346_32228372_32231091_118	novel	POFUT1	20	+	354	83	4	0	12.71%	77.47194939	0.000312071	0.000312071	6.56E-07
ENSG00000204525_31272062_31269169_369	annotated	HLA-C	6	-	1107	77	12	4	5.42%	22.98331969	0.000120424	0.000121007	1.04E-07
ENSG00000106484_130500844_130505056_101	annotated	MEST	7	+	303	20	0	0	6.93%	21.81006806	0.008776163	0.008776163	0.000391611
ENSG00000148660_73874461_73815015_529	annotated	CAMK2G	10	-	1587	27	3	0	2.46%	5.62155535	0.001079221	0.000539992	3.72E-06
ENSG00000175470_131943995_131955963_285	dORF	PPP2R2D	10	+	855	26	0	0	3.51%	10.04793662	0.002227676	0.002227676	2.89E-05
ENSG00000007541_574075_576049_299	annotated	PIGQ	16	+	897	21	1	0	3.34%	7.735644541	0.003029975	0.002281203	3.97E-05
ENSG00000111300_112039231_112029531_90	novel	NAA25	12	-	270	14	2	0	5.56%	17.13302013	0.052029617	0.021084099	0.004853002
ENSG00000111300_112093110_112040439_831	novel	NAA25	12	-	2493	33	8	2	2.29%	4.373824118	0.000769494	0.000275798	1.42E-06
ENSG00000183527_39178620_39175590_127	novel	PSMG1	21	-	381	48	9	0	11.81%	41.62803542	0.003418076	0.000300266	7.16E-06
ENSG00000163374_155679485_155659657_714	annotated	YY1AP1	1	-	2142	33	7	2	1.96%	5.090543196	0.002477401	0.000833918	1.28E-05
ENSG00000197256_11194511_11169853_841	annotated	KANK2	19	-	2523	37	2	1	2.26%	4.845673266	6.84E-05	6.84E-05	3.45E-08
ENSG00000111424_47865170_47844746_376	annotated	VDR	12	-	1128	12	0	0	2.13%	3.51513331	0.004826937	0.004826937	0.000126125
ENSG00000205155_35745931_35746651_97	annotated	PSENEN	19	+	291	72	64	21	20.62%	81.75402832	0.00290636	0.000991013	1.76E-05
ENSG00000205155_35746701_35746847_48	dORF	PSENEN	19	+	144	135	5	0	18.75%	309.7711229	0.004626223	0.003789641	9.63E-05
ENSG00000119048_134371623_134390238_130	annotated	UBE2B	5	+	390	101	6	6	9.23%	85.57096319	0.001381425	0.005303083	4.37E-05
ENSG00000185658_39313491_39194723_2199	annotated	BRWD1	21	-	6597	38	7	0	0.86%	1.90329789	0.002707838	4.92E-05	1.18E-06
ENSG00000117868_158798064_158734207_717	annotated	ESYT2	7	-	2151	193	26	11	7.81%	29.64739586	5.73E-08	1.80E-09	1.17E-15
ENSG00000159110_33241974_33263500_498	annotated	IFNAR2	21	+	1494	40	3	1	2.61%	8.846654113	0.004253235	0.00090861	2.39E-05
ENSG00000108946_68530294_68530449_51	dORF	PRKAR1A	17	+	153	127	9	0	29.41%	274.2722971	0.000485796	0.000317688	1.03E-06
ENSG00000139372_103980914_103987090_267	annotated	TDG	12	+	801	17	0	1	3.37%	7.012712895	0.003452921	0.005561256	0.000105543
ENSG00000071994_170583108_170578636_96	dORF	PDCD2	6	-	288	9	7	0	6.25%	10.3257041	0.365323432	0.011772051	0.03254298
ENSG00000099622_1270934_1272443_297	annotated	CIRBP	19	+	891	142	16	12	12.12%	52.65993201	1.69E-06	1.57E-06	2.32E-11
ENSG00000102900_56839482_56844609_120	novel	NUP93	16	+	360	7	0	0	5.00%	6.424882549	0.009815329	0.009815329	0.000483714
ENSG00000148824_133394221_133420165_283	annotated	MTG1	10	+	849	86	11	7	9.89%	33.4703624	6.89E-05	4.39E-05	2.27E-08
ENSG00000100852_32091060_32154948_1372	annotated	ARHGAP5	14	+	4116	36	6	4	1.31%	2.889992984	0.001254322	0.001097093	8.44E-06
ENSG00000140988_1964625_1962098_293	annotated	RPS2	16	-	879	23	3	1	2.05%	8.645868277	0.020342454	0.013428348	0.001295518
ENSG00000060749_32893126_32976474_1864	annotated	QSER1	11	+	5592	26	4	4	0.59%	1.536299322	0.009915672	0.015331882	0.000747715
ENSG00000164331_73562803_73562588_71	novel	ANKRA2	5	-	213	15	0	0	7.04%	23.26919233	0.021557223	0.021557223	0.002115616
ENSG00000164347_74763715_74721655_770	annotated	GFM2	5	-	2310	106	8	7	5.45%	15.16224602	1.86E-07	1.43E-07	2.54E-13
ENSG00000131507_142108975_142131903_52	novel	NDFIP1	5	+	156	37	5	12	13.46%	78.36944648	0.01982494	0.162952049	0.015799539
ENSG00000099377_30985659_30988183_369	annotated	HSD3B7	16	+	1107	25	1	3	2.71%	7.462116782	0.003133491	0.009991303	0.000173073
ENSG00000177565_177064977_177025498_514	annotated	TBL1XR1	3	-	1542	42	6	5	3.70%	8.999835478	0.000835535	0.000112625	6.79E-07
ENSG00000141295_47838673_47837844_138	annotated	SCRN2	17	-	414	17	3	0	6.52%	13.56807495	0.00912793	0.003278443	0.000164484
ENSG00000125691_38853710_38850333_122	annotated	RPL23	17	-	366	691	108	30	22.95%	623.8305164	0.000244353	9.13E-06	1.95E-08
ENSG00000133316_62839570_62835241_210	novel	WDR74	11	-	630	71	6	3	10.00%	37.23809478	0.000167427	8.16E-05	9.85E-08
ENSG00000133997_70600637_70584813_253	annotated	MED6	14	-	759	71	18	0	9.49%	30.90909053	0.000646101	8.26E-06	5.16E-08
ENSG00000160888_13153253_13153858_201	annotated	IER2	19	+	603	34	7	0	4.98%	18.63078948	0.020131593	0.002474239	0.000293518

ENSG00000214078_35626794_35626516_92	novel	CPNE1	20	-	276	50	10	5	15.22%	59.85915419	0.00452525	0.000731725	2.11E-05
ENSG00000005448_74421751_74423226_54	novel	WDR54	2	+	162	15	2	0	9.26%	30.59467881	0.08410207	0.019679754	0.007521961
ENSG00000100664_103336078_103341052_393	annotated	EIF5	14	+	1179	79	5	7	5.34%	22.14027138	0.000112264	0.000328201	2.61E-07
ENSG00000100664_103335739_103335924_61	Overlap_uORF	EIF5	14	+	183	13	9	0	9.84%	23.47263882	0.182638022	0.012798403	0.013255577
ENSG00000082438_164695723_164685946_572	annotated	COBLL1	2	-	1716	12	1	0	1.05%	2.310647071	0.026602765	0.011570466	0.001474889
ENSG00000108946_68515400_68530449_381	annotated	PRKAR1A	17	+	1143	541	35	14	22.05%	156.3942164	6.83E-15	1.46E-14	1.39E-27
ENSG00000108946_68523749_68530449_257	Overlap_dORF	PRKAR1A	17	+	771	442	26	14	25.29%	189.4251086	6.19E-12	1.97E-11	1.50E-21
ENSG00000143367_151540367_151581707_335	annotated	TUFT1	1	+	1005	22	2	0	3.58%	7.233130034	0.002296044	0.000943084	1.33E-05
ENSG00000162959_32010247_31868361_297	annotated	MEMO1	2	-	891	16	4	5	2.02%	5.933513466	0.044040756	0.090097208	0.015632667
ENSG00000091490_25862749_25748425_1103	annotated	SEL1L3	4	-	3309	65	4	0	2.72%	6.490620889	4.11E-06	6.67E-07	2.48E-11
ENSG00000144867_133805849_133819766_271	annotated	SRPRB	3	+	813	273	42	17	16.24%	110.9536913	8.19E-06	4.61E-07	3.58E-11
ENSG00000101246_63706690_63701731_124	novel	ARFRP1	20	-	372	16	1	0	4.84%	14.21172177	0.023878474	0.01300724	0.001470914
ENSG00000102531_49131290_49207395_1063	annotated	FNDC3A	13	+	3189	32	7	6	1.98%	3.315622764	0.003186725	0.001331312	2.53E-05
ENSG00000110651_2394127_2396866_165	annotated	CD81	11	+	495	518	70	9	38.79%	345.7754972	1.83E-10	2.32E-12	5.67E-21
ENSG00000172590_22834199_22834321_40	annotated	MRPL52	14	+	120	40	5	3	17.50%	110.1408437	0.008473674	0.008776163	0.000378864
ENSG00000100749_96860574_96931390_73	annotated	VRK1	14	+	219	15	0	0	6.85%	22.63168021	0.021557223	0.021557223	0.002115616
ENSG00000165410_34714540_34712865_166	annotated	CFL2	14	-	498	292	47	9	18.67%	193.7417251	3.24E-06	5.39E-07	1.60E-11
ENSG00000198805_20469525_20474632_113	novel	PNP	14	+	339	121	25	3	16.81%	117.9384256	0.007599539	0.000156755	1.00E-05
ENSG00000112419_143753795_143823689_453	annotated	PHACTR2	6	+	1359	14	10	1	1.77%	3.403911284	0.279308269	0.00912793	0.018642435
ENSG00000136295_2632156_2661739_523	annotated	TTYH3	7	+	1569	139	7	4	9.37%	29.27261429	2.44E-09	1.01E-09	2.70E-17
ENSG00000139323_89497316_89461295_305	novel	POC1B	12	-	915	18	0	2	2.30%	6.500115366	0.008674847	0.024135905	0.001038937
ENSG00000126749_6974567_6975809_99	annotated	EMG1	12	+	297	24	5	0	10.10%	26.7008106	0.020822301	0.00224099	0.000280219
ENSG00000075151_20941797_20807319_1226	annotated	EIF4G3	1	-	3678	19	5	0	0.98%	1.706913565	0.021519587	0.000885228	0.000135628
ENSG00000142227_48326845_48327634_63	novel	EMP3	19	+	189	748	117	63	44.44%	1307.703986	0.0002222	2.55E-05	4.45E-08
ENSG00000076928_41895390_41928954_698	annotated	ARHGEF1	19	+	2094	40	13	1	3.15%	6.311796201	0.00907039	2.24E-05	2.60E-06
ENSG00000119638_75106009_75086997_420	novel	NEK9	14	-	1260	46	1	4	4.52%	12.06304479	7.80E-05	0.000446986	2.55E-07
ENSG00000119638_75126921_75106431_532	novel	NEK9	14	-	1596	47	11	3	3.20%	9.730488072	0.00825943	0.000336729	2.07E-05
ENSG00000077232_182718162_182777132_722	annotated	DNAJC10	2	+	2166	196	21	9	7.62%	29.89973042	3.84E-09	5.11E-10	2.21E-17
ENSG00000278053_37643420_37614296_599	annotated	DDX52	17	-	1797	67	6	8	4.51%	12.31959354	2.68E-05	7.88E-05	1.61E-08
ENSG00000176715_89101273_89154207_379	annotated	ACSF3	16	+	1137	19	0	0	2.11%	5.52157264	0.005306578	0.005306578	0.000151001
ENSG00000127922_96709763_96688924_70	annotated	SEM1	7	-	210	192	44	44	27.14%	302.1005999	0.000600114	0.006579979	2.63E-05
ENSG00000127920_93922186_93926216_57	annotated	GNG11	7	+	171	875	82	28	38.60%	1690.758566	3.32E-05	4.29E-05	1.09E-08
ENSG00000174775_533583_532636_110	novel	HRAS	11	-	330	7	0	1	5.45%	7.008962781	0.009815329	0.028889786	0.001387795
ENSG00000141504_7627635_7627368_41	annotated	SAT2	17	-	123	11	3	0	17.07%	29.54998246	0.053594825	0.007882058	0.002211477
ENSG00000219200_7013669_7013746_25	novel	RNASEK	17	+	75	173	50	3	28.00%	762.1746384	0.014157027	0.010439632	0.000725899
ENSG00000219200_7012684_7013321_26	novel	RNASEK	17	+	78	68	27	8	34.62%	288.0606682	0.012008015	0.003478238	0.000228926
ENSG00000157538_37267294_37232571_168	novel	VPS26C	21	-	504	25	1	0	4.17%	16.3900065	0.01208853	0.008776163	0.000531381
ENSG00000157538_37231519_37225544_163	novel	VPS26C	21	-	489	11	0	3	3.68%	7.432817673	0.012798403	0.100002646	0.006484129
ENSG00000157538_37267294_37225544_172	annotated	VPS26C	21	-	516	32	0	3	6.40%	20.49131976	0.001506153	0.008742337	7.91E-05
ENSG00000145723_103108707_103087898_522	annotated	GIN1	5	-	1566	14	1	0	1.15%	2.953968988	0.012798403	0.012798403	0.000797244
ENSG00000132640_11919114_11923666_404	annotated	BTBD3	20	+	1212	23	2	2	1.98%	6.270394567	0.010318779	0.015646181	0.000790819
ENSG00000168763_96816278_96832616_707	annotated	CNNM3	2	+	2121	39	5	0	2.26%	6.075661817	0.002182134	0.000183359	2.88E-06
ENSG00000055950_100987443_100986972_44	annotated	MRPL43	10	-	132	71	34	2	25.00%	177.7272705	0.043966541	0.001565547	0.000490705
ENSG00000184216_154018637_154010802_204	novel	IRAK1	X	-	612	16	3	0	3.43%	8.638497546	0.052370108	0.008473674	0.00228699
ENSG00000181038_76733095_76733526_71	annotated	METTL23	17	+	213	17	1	0	7.04%	26.37175131	0.019679754	0.020613417	0.001864002
ENSG00000046647_14020447_14008913_208	annotated	GEMIN8	X	-	624	15	1	0	3.85%	7.942849306	0.005160369	0.004826937	0.000134406
ENSG00000132522_7314983_7313882_144	novel	GPS2	17	-	432	7	5	0	4.17%	5.354068791	0.30740494	0.009815329	0.022441687
ENSG00000186260_14200488_14240825_378	annotated	MRTFB	16	+	1134	12	0	0	1.85%	3.496534721	0.007029303	0.007029303	0.000257364
ENSG00000078747_34412633_34463148_386	novel	ITCH	20	+	1158	28	4	0	3.11%	7.989491253	0.005030886	0.001007374	3.12E-05
ENSG00000109911_31603822_31783524_235	annotated	ELP4	11	+	705	27	9	0	5.53%	12.65447992	0.012673659	0.000635664	5.68E-05
ENSG00000108433_46923193_46955562_214	annotated	GOSR2	17	+	642	40	7	0	6.54%	20.58707359	0.011149146	0.000448121	3.68E-05
ENSG00000052723_114780607_114774271_207	annotated	SIKE1	1	-	621	46	1	2	7.73%	24.47574305	0.000176199	0.000824	1.01E-06
ENSG00000143761_228097115_228098013_181	novel	ARF1	1	+	543	874	115	29	34.81%	531.8403171	5.82E-12	1.88E-11	1.35E-21
ENSG00000141378_59697957_59697439_172	annotated	PTRH2	17	-	516	108	22	4	20.93%	69.15820419	2.11E-06	3.67E-07	7.17E-12
ENSG00000023287_52685469_52623782_1591	annotated	RB1CC1	8	-	4773	79	24	6	2.26%	5.468967098	0.000928458	9.95E-07	1.33E-08
ENSG00000143842_204112916_204126134_622	annotated	SOX13	1	+	1866	13	2	0	1.13%	2.301979049	0.030804354	0.007882058	0.001227455
ENSG00000132376_1509784_1495823_356	annotated	INPP5K	17	-	1068	35	5	5	4.21%	10.82845374	0.006166408	0.002296562	8.04E-05
ENSG00000100124_37840198_37831943_177	annotated	ANKRD54	22	-	531	19	6	1	4.52%	11.82302842	0.035538344	0.006542768	0.001218901
ENSG00000136699_130176592_130152555_798	annotated	SMPD4	2	-	2394	11	4	0	0.88%	1.518232181	0.075249618	0.006937203	0.002921248
ENSG00000074047_120968812_120990675_1339	annotated	GLI2	2	+	4017	27	3	0	0.82%	2.220913204	0.007322425	0.001520872	6.63E-05
ENSG00000120656_28613361_28589750_112	novel	TAF12	1	-	336	27	0	0	8.04%	26.55181054	0.003710481	0.003710481	7.64E-05
ENSG00000198431_104215803_104348415_647	annotated	TXNRD1	12	+	1941	909	103	42	18.55%	154.7419272	6.47E-17	6.99E-20	8.22E-35
ENSG00000203705_212796574_212815156_221	novel	TATDN3	1	+	663	26	6	3	4.52%	12.95774632	0.051818085	0.013819019	0.003383266
ENSG00000174165_66546003_66539529_284	annotated	ZDHHC24	11	-	852	94	4	0	7.04%	36.45506799	8.19E-05	4.17E-05	2.56E-08
ENSG00000197261_49550850_49551527_225	annotated	C6orf141	6	+	675	15	2	1	2.22%	7.342722914	0.044040756	0.028889786	0.005421999
ENSG00000143537_155056417_155062502_548	novel	ADAM15	1	+	1644	133	8	6	7.85%	26.73126316	7.40E-08	7.65E-08	5.54E-14
ENSG00000143537_155051387_155055789_204	novel	ADAM15	1	+	612	84	12	4	9.80%	45.35211211	0.00128145	7.46E-05	7.39E-07
ENSG00000103507_31109224_31109792_127	novel	BCKDK	16	+	381	12	1	0	3.94%	10.40700885	0.042234516	0.019679754	0.003715148

ENSG00000103507_31111173_31112265_146	novel	BCKDK	16	+	438	39	0	0	8.22%	29.42118428	0.001053132	0.001053132	6.85E-06
ENSG00000103507_31110002_31110538_126	novel	BCKDK	16	+	378	31	5	0	7.94%	27.09814409	0.003146562	0.002418183	4.35E-05
ENSG00000065427_75648640_75634110_362	novel	KARS	16	-	1086	88	16	0	8.29%	26.77456974	0.000290129	6.57E-07	2.40E-09
ENSG00000225697_48632322_48630695_212	annotated	SLC26A6	3	-	636	18	0	3	3.77%	9.351581069	0.005257623	0.031732377	0.000899959
ENSG00000204856_110486421_110486730_76	dORF	FAM216A	12	+	228	12	0	0	6.58%	17.39065953	0.016947427	0.016947427	0.001349898
ENSG00000204856_110468876_110486374_77	annotated	FAM216A	12	+	231	19	4	0	6.49%	27.17761078	0.022600676	0.020613417	0.002121199
ENSG00000168036_41224552_41239342_768	annotated	CTNNB1	3	+	2304	288	46	13	11.46%	41.30281639	2.21E-12	5.94E-15	2.04E-25
ENSG00000082515_154941180_154966897_209	annotated	MRPL22	5	+	627	120	9	5	13.40%	63.23876194	5.46E-06	9.69E-06	4.38E-10
ENSG00000197912_89524038_89557093_657	annotated	SPG7	16	+	1971	54	22	2	4.11%	9.052672085	0.00613783	6.87E-06	6.33E-07
ENSG00000215301_41340781_41347719_622	annotated	DDX3X	X	+	1866	229	40	8	8.68%	40.55024632	3.29E-08	1.74E-10	7.45E-17
ENSG00000127990_94628318_94585465_369	novel	SGCE	7	-	1107	151	9	7	12.20%	45.07118536	6.55E-08	1.72E-08	1.15E-14
ENSG00000140455_63588795_63590826_84	annotated	USP3	15	+	252	20	1	2	9.52%	26.22401041	0.010079165	0.019504171	0.000959722
ENSG00000137868_74202418_74195602_175	novel	STRA6	15	-	525	20	1	0	5.14%	12.58752499	0.007181907	0.003606289	0.000141205
ENSG00000100605_93115163_92941561_414	annotated	ITPK1	14	-	1242	55	4	6	4.35%	14.63223769	0.000821977	0.001050559	5.39E-06
ENSG00000115641_105386516_105361283_279	annotated	FHL2	2	-	837	117	12	0	10.04%	46.18809575	2.51E-05	1.64E-06	3.70E-10
ENSG00000151640_132192719_132204930_509	annotated	DPYSL4	10	+	1527	15	1	0	1.18%	3.245800895	0.020342454	0.013428348	0.001295518
ENSG00000143376_151638856_151693601_435	novel	SNX27	1	+	1305	44	2	1	3.91%	11.14068304	0.000262229	0.000178977	3.24E-07
ENSG00000136052_104834108_104805152_67	novel	SLC41A2	12	-	201	22	0	0	13.43%	36.16565017	0.003478238	0.003478238	6.76E-05
ENSG00000111300_112081065_112049567_433	dORF	NAA25	12	-	1299	22	5	2	2.31%	5.596070581	0.002767218	0.008132971	0.000125749
ENSG00000143569_154225127_154270295_1086	annotated	UBAP2L	1	+	3258	114	16	33	4.42%	11.56174602	8.21E-07	2.08E-05	1.61E-10
ENSG00000171858_62387339_62388368_81	novel	RPS21	20	+	243	1019	67	29	39.51%	1385.599009	1.91E-05	1.73E-06	2.94E-10
ENSG00000176087_140564844_140566762_103	uORF	SLC35A4	5	+	309	69	14	3	13.59%	73.783672	0.007208094	0.000804138	3.74E-05
ENSG00000277443_113857746_113860579_332	annotated	MARCKS	6	+	996	18	5	0	2.71%	5.971491526	0.055437684	0.003452921	0.001192892
ENSG00000197930_52653100_52643570_127	dORF	ERO1A	14	-	381	93	10	0	11.81%	80.65431862	0.004434961	0.000282773	9.00E-06
ENSG00000197930_52695481_52657949_257	annotated	ERO1A	14	-	771	232	30	7	12.45%	99.42675385	0.000156552	3.54E-07	7.02E-10
ENSG00000070367_57231567_57208609_346	novel	EXOC5	14	-	1038	25	2	0	2.60%	7.958153447	0.013494345	0.003554776	0.000262963
ENSG00000126804_64521805_64523646_613	annotated	ZBTB1	14	+	1839	46	5	3	2.94%	8.265055156	0.000559723	0.000435314	1.59E-06
ENSG00000132912_150756451_150711149_403	annotated	DCTN4	5	-	1209	61	8	2	7.44%	16.67144284	9.47E-05	1.40E-06	1.32E-09
ENSG00000130402_38647746_38729432_521	annotated	ACTN4	19	+	1563	479	69	25	16.70%	101.2619273	7.60E-13	2.18E-14	2.33E-25
ENSG00000048828_93543374_93564540_385	annotated	FAM120A	9	+	1155	69	1	0	7.01%	19.73952783	2.48E-06	2.24E-06	4.81E-11
ENSG00000125944_23341008_23310454_595	annotated	HNRNPR	1	-	1785	133	21	3	7.56%	24.619718	5.94E-07	7.93E-09	5.38E-14
ENSG00000156113_77637360_76887266_1113	annotated	KCNMA1	10	-	3339	74	13	0	3.23%	7.322931208	8.97E-06	5.88E-08	6.07E-12
ENSG00000137497_72007197_72003527_297	novel	NUMA1	11	-	891	13	3	1	2.02%	4.820979691	0.062043408	0.019795882	0.005501028
ENSG00000075275_46536321_46363178_2731	annotated	CELSR1	22	-	8193	111	15	5	1.79%	4.476614299	5.43E-09	4.35E-09	2.50E-16
ENSG00000105223_40366484_40366919_82	novel	PLD3	19	+	246	28	5	0	10.98%	37.60906858	0.019243136	0.003554776	0.000380295
ENSG00000100364_45199933_45197176_86	novel	KIAA0930	22	-	258	14	0	1	5.81%	17.92990479	0.021084099	0.028889786	0.002731533
ENSG00000146674_45921140_45914820_270	annotated	IGFBP3	7	-	810	965	55	32	25.93%	393.651534	1.96E-11	2.96E-12	7.27E-22
ENSG00000131469_42998763_43002920_132	annotated	RPL27	17	+	396	1316	158	18	21.97%	1098.070836	1.52E-05	9.08E-06	1.12E-09
ENSG00000197912_89508547_89520042_103	annotated	SPG7	16	+	309	39	1	1	5.83%	41.70381461	0.012798403	0.020342454	0.001240246
ENSG00000197912_89544650_89557093_353	dORF	SPG7	16	+	1059	19	15	2	2.83%	5.928260709	0.299302749	0.009621867	0.021310549
ENSG00000197912_89524038_89541207_334	annotated	SPG7	16	+	1002	35	7	0	5.09%	11.54170518	0.002655719	0.000109913	2.28E-06
ENSG00000117523_171513048_171591847_2764	annotated	PRRC2C	1	+	8292	62	3	12	1.12%	2.470597797	1.15E-06	0.000107548	1.27E-09
ENSG00000240184_141476534_141511173_795	annotated	PCDHGC3	5	+	2385	19	6	0	1.51%	2.632296894	0.037729629	0.00077643	0.000237279
ENSG00000131368_15065188_15053376_108	annotated	MRPS25	3	-	324	61	3	2	13.89%	62.20918024	0.000764563	0.000763157	3.69E-06
ENSG00000124795_18258349_18244508_218	annotated	DEK	6	-	654	62	14	1	8.26%	31.32446014	0.001089259	0.000113737	9.04E-07
ENSG00000112659_43199300_43224445_1489	novel	CUL9	6	+	4467	11	5	0	0.40%	0.813666407	0.116253847	0.012798403	0.007703067
ENSG00000116044_177234268_177233178_141	annotated	NFE2L2	2	-	423	12	0	0	4.96%	9.373688826	0.007882058	0.007882058	0.000319636
ENSG00000163931_53255942_53225756_631	annotated	TKT	3	-	1893	1941	229	77	29.32%	338.8009154	7.30E-26	2.12E-30	3.80E-54
ENSG00000117650_211671278_211671198_26	novel	NEK2	1	-	78	7	1	0	19.23%	29.65330407	0.016947427	0.016947427	0.001349898
ENSG00000117650_211670295_211663426_187	novel	NEK2	1	-	561	33	7	0	6.95%	19.43661948	0.005112425	0.000690959	2.28E-05
ENSG00000173821_80273288_80393498_5208	annotated	AC124319.1	17	+	15624	171	26	27	1.63%	3.616375629	6.97E-11	5.13E-11	4.22E-20
ENSG00000151552_17512054_17487184_162	annotated	QDPR	4	-	486	36	3	16	8.02%	24.47574305	0.002923597	0.100893847	0.002175314
ENSG00000183258_177516945_177515305_201	annotated	DDX41	5	-	603	46	8	4	4.48%	25.20636224	0.00931822	0.005159595	0.000253228
ENSG00000184584_139481212_139476362_164	novel	TMEM173	5	-	492	14	3	0	4.88%	9.402267146	0.016735684	0.004687384	0.000417841
ENSG00000196141_200309073_200478031_588	annotated	SPATS2L	2	+	1764	93	18	3	5.95%	17.42023548	6.09E-05	5.27E-07	3.44E-10
ENSG00000183309_143649993_143651483_496	annotated	ZNF623	8	+	1488	22	4	4	2.22%	4.885279358	0.025075573	0.009920635	0.001214374
ENSG00000105928_24749774_24744558_135	novel	GSDME	7	-	405	11	0	0	3.70%	8.974439117	0.020613417	0.020613417	0.001946209
ENSG00000123545_96897801_96896145_82	novel	NDUFAF4	6	-	246	21	2	0	10.98%	28.20680144	0.010360743	0.003658191	0.000206155
ENSG00000144034_73737304_73729171_145	novel	TPRKB	2	-	435	80	15	0	9.66%	60.76736204	0.012638456	0.000462957	4.36E-05
ENSG00000170606_133101849_133104436_131	novel	HSPA4	5	+	393	62	13	12	10.69%	52.12772756	0.007908138	0.005271173	0.000220064
ENSG00000164073_127965133_127920630_518	annotated	MFSD8	4	-	1554	28	2	1	2.70%	5.953559119	0.000573217	0.000573217	2.12E-06
ENSG00000154582_73959768_73946630_112	annotated	ELOC	8	-	336	83	32	2	17.86%	81.62223239	0.020681041	9.81E-05	2.29E-05
ENSG00000172757_65858099_65855931_104	novel	CFL1	11	-	312	2141	340	40	44.23%	2267.418715	4.17E-08	3.28E-09	1.48E-15
ENSG00000015475_17750116_17735580_195	annotated	BID	22	-	585	113	29	8	13.85%	63.82520687	0.000831817	2.97E-05	2.06E-07
ENSG00000166206_26621357_26547793_334	annotated	GABRB3	15	-	1002	153	13	6	10.78%	50.45373978	2.79E-07	2.40E-07	6.29E-13
ENSG00000166206_26772962_26642505_87	uORF	GABRB3	15	-	261	13	2	0	6.90%	16.45782722	0.012798403	0.01300724	0.000809468
ENSG00000172115_25124083_25123701_93	annotated	CYCS	7	-	279	106	11	3	15.05%	125.5368756	0.000473951	0.000785846	2.41E-06
ENSG00000187147_44636045_44650775_230	annotated	RNF220	1	+	690	30	9	2	6.52%	14.36619701	0.003030417	0.000737506	1.40E-05

ENSG00000101844_108131319_108153712_314	dORF	ATG4A	X	+	942	9	0	0	1.59%	3.156903164	0.020613417	0.020613417	0.001946209
ENSG00000126106_44674391_44636410_111	annotated	TMEM53	1	-	333	11	1	0	5.41%	10.91485839	0.012798403	0.012798403	0.000797244
ENSG00000126106_44674391_44636410_201	annotated	TMEM53	1	-	603	18	1	0	3.98%	9.863359138	0.005454749	0.005554612	0.000161874
ENSG00000136717_127106934_127048526_436	annotated	BIN1	2	-	1308	35	2	1	3.21%	8.84158149	0.000742993	0.000525339	2.51E-06
ENSG00000134278_12657866_12449638_756	annotated	SPIRE1	18	-	2268	27	2	1	2.51%	3.933601561	0.000290884	7.31E-05	1.55E-07
ENSG00000013441_200861862_200854685_355	annotated	CLK1	2	-	1065	13	3	1	2.25%	4.033326671	0.030313544	0.004826937	0.000797936
ENSG00000173933_66639712_66644132_364	annotated	RBM4	11	+	1092	40	4	0	4.95%	12.10338942	0.000663461	5.74E-05	2.91E-07
ENSG00000141456_4674889_4671439_683	novel	PELP1	17	-	2049	40	1	0	2.20%	6.450415444	0.000400846	0.000284266	7.65E-07
ENSG00000141456_4704111_4675560_434	novel	PELP1	17	-	1302	27	1	1	3.00%	6.852080138	0.00162692	0.00105275	1.04E-05
ENSG00000163659_156677704_156705131_655	annotated	TIPARP	3	+	1965	28	4	1	2.75%	4.708310876	0.001070883	0.000117931	9.17E-07
ENSG00000131669_93126563_93124908_102	novel	NINJ1	9	-	306	54	5	0	12.75%	58.30985843	0.001248772	0.000686235	5.39E-06
ENSG00000137133_35814979_35813258_135	novel	HINT2	9	-	405	147	36	11	22.96%	119.9311409	0.000242009	1.71E-06	4.43E-09
ENSG00000112651_43059381_43054440_225	annotated	MRPL2	6	-	675	41	8	2	7.56%	20.0701093	0.004373898	0.000450598	1.33E-05
ENSG00000143409_151002332_150997287_374	annotated	MINDY1	1	-	1122	12	1	0	1.87%	3.533930814	0.01551626	0.007882058	0.000614257
ENSG00000130396_167864572_167952262_1570	novel	AFDN	6	+	4710	12	6	0	0.45%	0.841840844	0.176150259	0.008175611	0.009250017
ENSG00000151657_7787933_7756080_393	annotated	KIN	10	-	1179	28	19	0	3.31%	7.847184793	0.15748365	0.000642411	0.001408823
ENSG00000197448_143263514_143268837_183	annotated	GSTK1	7	+	549	333	54	24	26.23%	200.4202238	4.65E-07	1.73E-08	8.56E-14
ENSG00000131269_75156257_75053370_708	annotated	ABCB7	X	-	2124	55	2	4	3.11%	8.556138988	2.39E-05	6.10E-05	1.12E-08
ENSG00000263001_74689225_74758838_925	annotated	GTF2I	7	+	2775	58	9	8	1.84%	6.906128578	0.001982699	0.001758679	2.06E-05
ENSG00000184007_31919628_31919563_21	uORF	PTP4A2	1	-	63	79	14	9	23.81%	414.3393644	0.097833871	0.130723026	0.043720095
ENSG00000130475_17776253_17788306_493	annotated	FCHO1	19	+	1479	30	4	1	2.84%	6.702282578	0.004367894	0.000335351	1.03E-05
ENSG00000099797_14529697_14565043_248	novel	TECR	19	+	744	306	48	57	14.92%	135.8995894	3.05E-07	0.000363043	1.64E-09
ENSG00000168066_64778392_64765389_571	annotated	SF1	11	-	1713	214	36	13	9.98%	41.278705	1.76E-07	7.35E-10	1.66E-15
ENSG00000168066_64778392_64765818_639	annotated	SF1	11	-	1917	207	37	24	8.76%	35.67942824	2.09E-06	9.17E-09	2.36E-13
ENSG00000171621_9356018_9367575_231	annotated	SPSB1	1	+	693	35	0	0	6.49%	16.68800662	0.000299491	0.000299491	6.06E-07
ENSG00000135040_85958533_85986480_221	annotated	NAA35	9	+	663	35	0	0	3.62%	17.44312004	0.005604867	0.005604867	0.000167525
ENSG00000012061_45423374_45409686_213	annotated	ERCC1	19	-	639	44	4	7	6.10%	22.75209917	0.003183653	0.010796097	0.000189918
ENSG00000175390_7987353_7996022_357	annotated	EIF3F	11	+	1071	56	8	1	4.20%	17.27699509	0.00507103	0.000412614	1.44E-05
ENSG00000118513_135185940_135217980_585	annotated	MYB	6	+	1755	14	0	0	0.85%	2.635849251	0.019679754	0.019679754	0.001784964
ENSG00000103111_77191486_77198308_547	annotated	MON1B	16	+	1641	29	11	6	2.74%	5.839276906	0.022643536	0.010953216	0.001197539
ENSG00000136193_29969067_29947221_140	annotated	SCRN1	7	-	420	22	8	0	5.00%	17.30784687	0.028426897	0.008473674	0.001202275
ENSG00000174891_158122105_158469369_169	novel	RSRC1	3	+	507	98	23	5	3.55%	63.86865493	0.129839126	0.05323297	0.026281881
ENSG00000152818_144421902_144850997_3378	annotated	UTRN	6	+	10134	32	8	4	0.56%	1.043370929	0.004109798	0.001088536	2.72E-05
ENSG00000111907_125220146_125252040_105	novel	TPD52L1	6	+	315	31	7	0	6.67%	32.5177729	0.104741188	0.008574016	0.005043772
ENSG00000156976_186783611_186784719_77	novel	EIF4A2	3	+	231	34	0	0	6.49%	48.6336193	0.019679754	0.019679754	0.001784964
ENSG00000124155_45416169_45420152_92	annotated	PIGT	20	+	276	25	6	0	9.78%	29.92957709	0.035861481	0.003478238	0.000731728
ENSG00000124155_45420349_45425826_316	dORF	PIGT	20	+	948	259	20	2	16.46%	90.2736662	6.03E-10	1.31E-10	9.13E-19
ENSG00000197563_62146983_62045856_667	dORF	PIGN	18	-	2001	42	6	0	2.55%	6.935405451	0.002742059	0.000122026	2.59E-06
ENSG00000197563_62161260_62148248_153	annotated	PIGN	18	-	459	27	6	0	8.50%	19.43661948	0.009573277	0.000596156	3.94E-05
ENSG00000117620_100007082_100022476_195	Overlap_dORF	SLC35A3	1	+	585	9	7	0	3.08%	5.083423556	0.312211302	0.009815329	0.022960993
ENSG00000182307_145052492_145061264_236	annotated	C8orf33	8	+	708	103	15	4	12.71%	48.0699445	0.000114631	3.66E-06	3.83E-09
ENSG00000170043_7931829_7930606_145	annotated	TRAPPC1	17	-	435	279	49	15	31.03%	211.9261751	5.60E-07	7.92E-09	5.04E-14
ENSG00000128915_60477965_60423634_978	annotated	ICE2	15	-	2934	33	3	4	1.74%	3.716408837	0.000381209	0.00146261	3.66E-06
ENSG00000146828_100854348_100865324_581	annotated	SLC12A9	7	+	1743	44	5	2	3.44%	8.341131021	0.000645978	3.40E-05	1.78E-07
ENSG00000140538_88256153_87979345_612	annotated	NTRK3	15	-	1836	153	16	7	9.80%	27.53521093	2.22E-09	7.76E-11	2.08E-18
ENSG00000134444_62187506_62305534_1216	annotated	RELCH	18	+	3648	34	2	0	1.15%	3.079595959	0.001588752	0.0004093	4.25E-06
ENSG00000182220_40589096_40605755_301	annotated	ATP6AP2	X	+	903	174	29	5	15.95%	63.66945782	4.45E-08	1.09E-09	5.59E-16
ENSG00000090006_40601388_40629550_1557	annotated	LTBP4	19	+	4671	56	11	8	1.86%	3.961391938	0.000598396	0.000132838	5.60E-07
ENSG00000157379_24299106_24290859_313	annotated	DHRS1	14	-	939	12	3	2	2.56%	4.222652155	0.035031293	0.030313544	0.004558897
ENSG00000100211_38668055_38673236_126	annotated	CBY1	22	+	378	21	9	4	9.52%	18.35680728	0.029921291	0.007963342	0.001201538
ENSG00000110911_51009138_50988325_557	annotated	SLC11A2	12	-	1671	126	12	5	8.44%	24.91516393	2.77E-08	1.11E-08	3.17E-15
ENSG00000131584_1302924_1293564_709	annotated	ACAP3	1	-	2127	17	1	1	0.99%	2.640894701	0.012000727	0.012000727	0.000706384
ENSG00000125826_417617_430430_424	annotated	RBCK1	20	+	1272	44	17	12	2.83%	11.4297102	0.031146433	0.012057173	0.001790263
ENSG00000125826_408241_408705_154	uORF	RBCK1	20	+	462	42	10	15	8.44%	30.03841192	0.009263777	0.064710135	0.003096171
ENSG00000165458_72228490_72238130_1082	annotated	INPPL1	11	+	3246	26	9	5	1.29%	2.646637649	0.031109587	0.004091257	0.000715144
ENSG00000166741_114312268_114312477_69	novel	NNMT	11	+	207	163	21	7	36.23%	260.1877902	3.64E-05	9.21E-06	2.71E-09
ENSG00000187098_69879285_69965248_435	annotated	MITF	3	+	1305	37	7	1	3.91%	9.368301648	0.003418024	0.000183422	4.67E-06
ENSG00000107672_121970962_121967651_59	novel	NSMCE4A	10	-	177	41	2	1	13.56%	76.53855241	0.005604867	0.005355751	0.000160457
ENSG00000076924_7629518_7619586_852	annotated	XAB2	19	-	2556	43	2	6	2.35%	5.558751502	0.000140943	0.000999125	1.00E-06
ENSG00000148343_129059256_129070453_334	annotated	MIGA2	9	+	1002	13	0	2	1.80%	4.286919066	0.012798403	0.03508989	0.002125904
ENSG00000133895_64810109_64804334_575	annotated	MEN1	11	-	1725	40	1	7	2.61%	7.661971736	0.000415728	0.015001358	4.86E-05
ENSG00000065665_12129788_12169327_437	annotated	SEC61A2	10	+	1311	57	13	1	4.35%	14.36619701	0.0239488	7.98E-05	2.36E-05
ENSG00000014914_149936211_149929129_681	annotated	MTMR11	1	-	2043	27	2	7	1.62%	4.366817592	0.005530543	0.03630415	0.001084539
ENSG00000213347_177311830_177307588_206	annotated	MXD3	5	-	618	87	4	0	10.19%	46.51579322	0.000179195	2.37E-05	3.33E-08
ENSG00000126858_32171069_32224733_670	annotated	RHOT1	17	+	2010	61	7	2	4.33%	10.02774846	3.84E-05	3.61E-06	1.19E-09
ENSG00000060642_26790816_26797844_493	annotated	PIGV	1	+	1479	77	5	11	6.69%	17.20252528	2.68E-07	3.23E-05	9.48E-11
ENSG00000104524_143609548_143605700_274	annotated	PYCR3	8	-	822	44	2	0	6.57%	17.68685081	7.13E-05	7.21E-05	3.78E-08
ENSG00000157193_53250842_53248559_120	annotated	LRP8	1	-	360	16	3	1	8.33%	14.68544583	0.025557109	0.003957932	0.000562893

ENSG00000187720_71547410_71738171_329	novel	THSD4	15	+	987	41	6	1	5.78%	13.72575864	7.77E-05	8.02E-05	4.56E-08
ENSG00000159593_66823582_66817356_161	dORF	NAE1	16	-	483	55	8	1	9.32%	37.62575406	0.001151302	0.000309685	2.37E-06
ENSG00000134046_54224112_54159777_262	annotated	MBD2	18	-	786	9	0	3	1.91%	3.783464097	0.019216965	0.131156788	0.012020478
ENSG00000102048_15269853_15248715_245	annotated	ASB9	X	-	735	14	0	0	3.67%	6.293762497	0.003229477	0.003229477	5.87E-05
ENSG00000131051_35725100_35704481_367	dORF	RBM39	20	-	1101	50	11	6	5.45%	15.00556454	0.005933528	0.000710061	2.73E-05
ENSG00000087191_63827462_63828213_42	novel	PSMC5	17	+	126	32	5	22	11.90%	83.9168333	0.075027848	0.172059248	0.045827822
ENSG00000087191_63829467_63831969_383	novel	PSMC5	17	+	1149	411	53	15	22.72%	118.1929158	6.13E-13	2.92E-15	2.74E-26
ENSG00000129255_7583863_7587444_259	novel	MPDU1	17	+	777	140	15	21	11.20%	59.53559119	2.84E-05	0.000274835	6.12E-08
ENSG00000129255_7587444_7587551_35	novel	MPDU1	17	+	105	13	1	0	17.14%	40.90945623	0.026832696	0.011772051	0.001510382
ENSG00000082269_70475684_70559921_1239	annotated	FAM135A	6	+	3717	16	6	0	0.40%	1.422319208	0.129839126	0.021084099	0.01274956
ENSG00000089902_102592896_102726506_482	annotated	RCOR1	14	+	1446	87	9	3	4.98%	19.88019378	8.03E-05	6.72E-06	4.55E-09
ENSG00000135093_109057967_109085931_439	annotated	USP30	12	+	1317	12	0	0	1.82%	3.010683655	0.004873904	0.004873904	0.000128468
ENSG00000116001_70248418_70212719_281	annotated	TIA1	2	-	843	25	1	3	3.56%	9.799007447	0.003247793	0.008413705	0.000150284
ENSG00000102908_69566302_69646563_119	annotated	NFAT5	16	+	357	19	5	3	6.72%	17.58551286	0.035092496	0.042481711	0.006237397
ENSG00000140688_31508373_31490835_423	annotated	C16orf58	16	-	1269	113	2	9	8.27%	29.4229677	1.19E-07	1.23E-06	1.42E-12
ENSG00000140525_89247648_89264611_252	annotated	FANCI	15	+	756	8	1	3	1.98%	3.496534721	0.047790352	0.190227563	0.036034263
ENSG00000140995_89954260_89955225_57	internal	DEF8	16	+	171	10	2	5	8.77%	19.32295504	0.069457177	0.197776317	0.049762526
ENSG00000185215_103130020_103135360_323	annotated	TNFAIP2	14	+	969	213	12	3	13.93%	72.63157805	4.91E-08	2.50E-09	1.36E-15
ENSG00000115355_55322644_55291711_856	annotated	CCDC88A	2	-	2568	37	2	0	2.22%	4.760760768	0.000218523	5.40E-05	8.73E-08
ENSG00000148019_78243471_78270851_502	dORF	CEP78	9	+	1506	30	5	0	2.79%	6.582122134	0.006495295	0.000405548	1.86E-05
ENSG00000113163_75511207_75379346_567	annotated	COL4A3BP	5	-	1701	20	14	5	1.59%	3.885038579	0.285863894	0.031728005	0.04342502
ENSG00000221926_15651609_15628615_564	annotated	TRIM16	17	-	1692	98	31	5	3.90%	19.13794802	0.00388623	2.24E-05	9.29E-07
ENSG00000143512_222548044_222522601_724	annotated	HHIPL2	1	-	2172	13	1	0	1.24%	1.977667083	0.00650005	0.002500085	9.16E-05
ENSG00000183087_113826996_113820864_186	novel	GAS6	13	-	558	13	0	0	3.76%	7.698015958	0.007882058	0.007882058	0.000319636
ENSG00000171793_40983291_41008718_451	novel	CTPS1	1	+	1353	32	14	4	3.33%	7.814871394	0.051026698	0.002377639	0.000809725
ENSG00000140497_74851374_74845371_168	annotated	SCAMP2	15	-	504	161	9	3	16.07%	105.5516419	3.76E-05	5.39E-06	1.70E-09
ENSG00000168701_67227149_67227633_31	novel	TMEM208	16	+	93	31	3	0	22.58%	110.1408437	0.037440157	0.008574016	0.001615304
ENSG00000166454_81042293_81044970_665	annotated	ATMIN	16	+	1995	28	0	0	1.50%	4.637509209	0.002321823	0.002321823	3.12E-05
ENSG00000198721_4117442_4115874_96	novel	ECI2	6	-	288	53	14	3	8.33%	60.80692413	0.010042643	0.005454749	0.00028675
ENSG00000198721_4133671_4126131_195	novel	ECI2	6	-	585	26	0	1	5.13%	14.68544583	0.00224099	0.002639378	3.41E-05
ENSG00000003402_201129923_201141433_202	annotated	CFLAR	2	+	606	42	11	3	8.91%	22.90057146	0.001006713	0.000463595	3.01E-06
ENSG00000234741_173867041_173866752_31	novel	GAS5	1	-	93	25	6	0	19.35%	88.82326105	0.023199731	0.013640586	0.001492437
ENSG00000114098_138221930_138295892_631	annotated	ARMC8	3	+	1893	20	10	0	1.90%	3.490993461	0.04552565	0.00077643	0.000298859
ENSG00000241837_33907388_33907305_27	novel	ATP5PO	21	-	81	59	3	0	18.52%	240.6781399	0.021084099	0.021084099	0.002029891
ENSG00000241837_33906487_33903526_79	novel	ATP5PO	21	-	237	139	11	0	27.85%	193.7921174	0.000162615	1.92E-05	2.49E-08
ENSG00000174442_66518919_66546679_471	annotated	ZWILCH	15	+	1413	50	1	1	5.10%	11.69223394	1.02E-05	1.02E-05	8.46E-10
ENSG00000120925_42887864_42850791_200	annotated	RNF170	8	-	600	15	1	0	3.00%	8.260563278	0.023672501	0.012798403	0.001437523
ENSG00000121749_71861494_71923204_665	annotated	TBC1D15	12	+	1995	37	4	3	3.01%	6.128137169	0.000182952	0.00040921	5.14E-07
ENSG00000221983_18573301_18575150_128	annotated	UBA52	19	+	384	124	35	5	13.28%	106.6989423	0.005536205	0.000198245	8.49E-06
ENSG00000175061_16439055_16439470_49	novel	SNHG29	17	+	147	142	15	5	14.29%	319.1836695	0.036854422	0.012353295	0.002167239
ENSG00000074181_15200863_15160662_2307	annotated	NOTCH3	19	-	6921	44	10	1	0.87%	2.100648948	0.000978189	5.29E-05	4.09E-07
ENSG00000112118_52273354_52264664_316	annotated	MCM3	6	-	948	34	7	2	4.11%	11.85059711	0.008417183	0.002779203	0.000130695
ENSG00000011451_15427294_15423076_538	annotated	WIZ	19	-	1614	16	0	0	0.93%	3.275564125	0.021084099	0.021084099	0.002029891
ENSG00000105135_15125059_15115252_632	annotated	ILVBL	19	-	1896	125	44	8	6.17%	21.78418586	0.000903723	6.35E-07	8.92E-09
ENSG00000105011_14136456_14120459_202	annotated	ASF1B	19	-	606	136	18	7	17.82%	74.15423141	2.60E-05	3.24E-07	8.84E-11
ENSG00000105058_16185443_16190955_112	annotated	FAM32A	19	+	336	47	4	1	16.96%	46.21981834	0.000505336	5.60E-05	2.14E-07
ENSG00000140740_21962771_21976247_242	novel	UQCRC2	16	+	726	106	21	1	11.98%	48.24351005	0.000282053	1.33E-06	4.24E-09
ENSG00000067064_1044140_1041187_227	annotated	IDI1	10	-	681	270	72	35	24.23%	131.0045278	7.22E-07	9.85E-09	8.07E-14
ENSG00000114480_81761517_81490407_702	annotated	GBE1	3	-	2106	79	15	2	4.13%	12.39476731	0.000112635	3.37E-06	3.49E-09
ENSG00000183624_129279733_129304825_312	annotated	HMCES	3	+	936	47	3	0	7.05%	16.59172966	9.49E-05	1.51E-05	1.14E-08
ENSG00000162929_61076927_61122162_572	annotated	KIAA1841	2	+	1716	11	2	0	0.87%	2.118093148	0.043751848	0.020613417	0.004005334
ENSG00000198826_32625165_32637845_811	annotated	ARHGAP11A	15	+	2433	40	4	2	1.73%	5.432347408	0.00054445	0.001306909	4.54E-06
ENSG00000119723_73950333_73955969_173	novel	COQ6	14	+	519	34	0	0	6.36%	21.64617738	0.001610998	0.001610998	1.55E-05
ENSG00000065883_40045877_40094980_670	annotated	CDK13	7	+	2010	18	1	1	1.49%	2.959007741	0.004053443	0.004053443	9.05E-05
ENSG00000147044_41739449_41586892_315	novel	CASK	X	-	945	11	6	0	1.90%	3.846188193	0.116253847	0.012798403	0.007703067
ENSG00000106771_109038092_109036202_84	novel	TMEM245	9	-	252	30	3	0	10.71%	39.33601561	0.009181916	0.003632191	0.000181437
ENSG00000167004_43746540_43771218_505	annotated	PDIA3	15	+	1515	431	32	19	8.91%	94.00139334	2.53E-07	2.83E-08	7.26E-14
ENSG00000005981_95485972_95528375_349	annotated	ASB4	7	+	1047	31	17	5	4.30%	9.783284111	0.139807983	0.007124906	0.00625253
ENSG00000079277_46583327_46558762_335	annotated	MKNK1	1	-	1005	24	1	0	3.28%	7.89068731	0.002336818	0.001484235	2.06E-05
ENSG00000214046_16660110_16655346_47	annotated	SMIM7	19	-	141	116	10	2	31.91%	271.836976	0.001842419	0.000395349	4.79E-06
ENSG00000089006_17950309_17947481_128	annotated	SNX5	20	-	384	73	17	10	10.16%	62.81469993	0.005102439	0.005133296	0.000140962
ENSG00000101166_59037211_59035007_104	dORF	PRELID3B	20	-	312	76	10	11	16.35%	80.48753963	0.001929683	0.001189598	1.39E-05
ENSG00000101166_59042730_59037294_68	annotated	PRELID3B	20	-	204	33	0	0	13.24%	53.45070356	0.003710481	0.003710481	7.64E-05
ENSG00000122515_44760512_44767623_534	novel	ZMIZ2	7	+	1602	23	6	0	1.69%	4.743894017	0.052476578	0.003632191	0.001164783
ENSG00000115944_42361161_42353176_79	annotated	COX7A2L	2	-	237	31	7	0	10.13%	43.21982475	0.110454359	0.004826937	0.003513488
ENSG00000185432_50925039_50930150_244	annotated	METTL7A	12	+	732	15	1	0	3.28%	6.770953506	0.010499663	0.00419316	0.000236136
ENSG00000117479_169485766_169465849_497	annotated	SLC19A2	1	-	1491	23	4	0	2.62%	5.097061178	0.007109228	0.000622309	2.95E-05
ENSG00000022840_120546530_120576666_722	annotated	RNF10	12	+	2166	42	4	1	2.35%	6.407085091	0.000355906	0.000127164	3.18E-07

ENSG00000129128_176322126_176328330_147	novel	SPCS3	4	+	441	194	25	1	22.45%	145.3559434	9.24E-06	2.49E-07	2.30E-11
ENSG00000161217_196248252_196242554_94	novel	PCYT1A	3	-	282	12	0	4	5.32%	14.06053324	0.020613417	0.122468289	0.011739344
ENSG00000108424_47677101_47678418_93	novel	KPNB1	17	+	279	92	5	0	18.28%	108.9565336	0.000137814	0.000137814	1.35E-07
ENSG00000178074_199911439_199925886_385	annotated	C2orf69	2	+	1155	20	8	0	1.56%	5.72160227	0.155688889	0.013640586	0.011400674
ENSG00000125319_44142143_44161932_647	annotated	C17orf53	17	+	1941	12	3	3	0.93%	2.042797719	0.086207551	0.086207551	0.026823708
ENSG00000183431_37989965_37958186_501	annotated	SF3A3	1	-	1503	88	19	5	6.79%	19.3460963	0.000120762	1.41E-06	1.73E-09
ENSG00000183386_37999412_37997405_280	annotated	FHL3	1	-	840	16	2	0	2.86%	6.293762497	0.020715809	0.005454749	0.000593634
ENSG00000183520_38012803_38023628_253	annotated	UTP11	1	+	759	107	20	0	11.46%	46.58130544	0.000156549	1.04E-06	1.77E-09
ENSG00000172167_120445471_120523336_904	annotated	MTBP	8	+	2712	13	1	4	0.88%	1.583883814	0.010020958	0.04536668	0.002251992
ENSG00000264772_7580301_7581511_174	novel	AC016876.2	17	+	522	18	1	0	4.60%	11.39388038	0.008219047	0.005454749	0.00023581
ENSG00000264772_7572842_7577934_377	novel	AC016876.2	17	+	1131	586	32	17	12.20%	171.2003565	1.37E-08	2.87E-09	4.23E-16
ENSG00000168159_228487816_228493885_126	annotated	RNF187	1	+	378	128	8	4	24.60%	111.8891111	2.44E-06	2.51E-06	5.28E-11
ENSG00000133315_64165994_64000108_250	novel	MACROD1	11	-	750	22	3	2	4.00%	9.692394246	0.008764431	0.00681086	0.000308338
ENSG00000134333_18396843_18398800_42	annotated	LDHA	11	+	126	41	10	0	21.43%	107.5184427	0.028682169	0.003710481	0.000603887
ENSG00000159403_7092388_7090048_143	novel	C1R	12	-	429	11	1	4	3.50%	8.472372593	0.042234516	0.121807694	0.0204534
ENSG00000129480_31457393_31448129_168	annotated	DTD2	14	-	504	22	5	2	5.95%	14.42320572	0.015528143	0.007532653	0.000590128
ENSG00000184922_45239003_45245642_569	annotated	FMNL1	17	+	1707	13	3	10	1.23%	2.51639889	0.035227809	0.29517333	0.04847789
ENSG00000110080_126406156_126408592_240	novel	ST3GAL4	11	+	720	10	2	0	2.08%	4.589201821	0.08410207	0.019679754	0.007521961
ENSG00000110442_36289412_36277075_121	novel	COMMD9	11	-	363	34	10	4	7.44%	30.94866683	0.009029128	0.012138701	0.000547697
ENSG00000105576_12720977_12701346_887	annotated	TNPO2	19	-	2661	31	4	8	1.92%	3.849341775	0.00052153	0.005788985	2.03E-05
ENSG00000131149_85663431_85672411_397	annotated	GSE1	16	+	1191	22	4	0	3.02%	6.103522825	0.009754926	0.000943084	5.93E-05
ENSG00000147439_22669051_22621422_253	annotated	BIN3	8	-	759	23	0	1	4.74%	10.01280397	0.000943084	0.001559221	9.04E-06
ENSG00000080189_46358506_46350394_365	annotated	SLC35C2	20	-	1095	48	8	7	6.30%	14.48427534	0.000355596	0.000254008	6.11E-07
ENSG00000244405_186105912_186105676_41	Overlap_uORF	ETV5	3	-	123	15	2	4	19.51%	40.29543062	0.026823708	0.042234516	0.004876395
ENSG00000011638_21161183_21178812_163	annotated	TMEM159	16	+	489	8	0	0	3.68%	5.405685581	0.009815329	0.009815329	0.000483714
ENSG00000108094_35071317_35010311_745	annotated	CUL2	10	-	2235	37	2	1	2.95%	5.470082171	2.20E-05	2.25E-05	3.87E-09
ENSG00000204237_81666577_81665201_147	annotated	OXLD1	17	-	441	29	5	2	8.84%	21.72846576	0.002948808	0.001765381	3.04E-05
ENSG00000108094_35071317_35010311_706	annotated	CUL2	10	-	2118	37	2	1	3.12%	5.772253849	2.20E-05	2.25E-05	3.87E-09
ENSG00000182446_81636930_81565320_617	annotated	NPLOC4	17	-	1851	46	11	8	2.76%	8.21147295	0.014811166	0.002895495	0.000242223
ENSG00000161016_144792129_144789804_257	annotated	RPL8	8	-	771	2151	291	109	31.52%	921.8402911	3.47E-12	1.37E-13	6.47E-24
ENSG00000162441_9942735_9942631_34	uORF	LZIC	1	-	102	32	4	0	14.71%	103.6619705	0.039807901	0.021084099	0.003722657
ENSG00000162441_9936550_9930399_167	annotated	LZIC	1	-	501	61	4	3	8.98%	40.23108662	0.000561382	0.0010268	3.68E-06
ENSG00000133773_82358376_82353151_241	annotated	CCDC59	12	-	723	51	15	14	5.81%	23.3078134	0.026847077	0.041183811	0.004763492
ENSG00000141552_81894478_81900100_196	annotated	ANAPC11	17	+	588	486	58	25	7.14%	273.1043369	0.007700797	0.007104815	0.000283483
ENSG00000141425_36067404_35993151_312	annotated	RPRD1A	18	-	936	91	17	4	4.17%	32.12441275	0.050303262	0.002824594	0.000910676
ENSG00000113810_160400827_160433809_1288	annotated	SMC4	3	+	3864	265	58	21	6.91%	22.66096551	1.83E-10	3.86E-14	1.26E-22
ENSG00000104687_30727706_30679520_450	annotated	GSR	8	-	1350	262	52	14	13.33%	64.12644678	6.97E-08	1.05E-10	1.05E-16
ENSG00000131002_19587225_19587647_140	novel	TXLNGY	Y	+	420	12	2	2	3.57%	9.440643746	0.020613417	0.052029617	0.004760359
ENSG00000156298_38646304_38687667_225	annotated	TSPAN7	X	+	675	18	4	0	4.00%	8.811267496	0.061503838	0.003229477	0.001279093
ENSG00000186153_78115085_79211796_301	dORF	WWOX	16	+	903	8	1	0	1.66%	2.927331394	0.034182656	0.019216965	0.002956053
ENSG00000134058_69262263_69277135_151	dORF	CDK7	5	+	453	8	3	0	3.97%	5.835276488	0.113684165	0.011570466	0.006956434
ENSG00000171634_67826142_67982288_2907	annotated	BPTF	17	+	8721	40	9	9	0.62%	1.515525885	0.003201028	0.001919608	3.56E-05
ENSG00000177352_49164100_49162805_431	annotated	CCDC71	3	-	1293	10	0	0	1.16%	2.555472012	0.020613417	0.020613417	0.001946209
ENSG00000137411_30914837_30926210_1063	annotated	VARS2	6	+	3189	25	12	2	1.69%	2.590330285	0.017315147	0.00023325	3.62E-05
ENSG00000123352_49461013_49526255_545	annotated	SPATS2	12	+	1635	30	3	5	2.94%	6.062798736	0.001336806	0.004595209	3.66E-05
ENSG00000066027_212286111_212360770_486	annotated	PPP2R5A	1	+	1458	33	8	3	3.50%	7.478699264	0.010865102	0.001067625	7.41E-05
ENSG00000103356_23557343_23525138_506	annotated	EARS2	16	-	1518	47	6	1	4.55%	10.23047362	6.46E-05	9.56E-06	5.03E-09
ENSG00000100726_1502072_1506514_219	novel	TELO2	16	+	657	14	1	2	2.28%	7.040967178	0.042234516	0.101051022	0.016920389
ENSG00000163468_156338181_156335761_51	novel	CCT3	1	-	153	35	3	1	15.69%	75.58685352	0.005756914	0.005604867	0.000171838
ENSG00000284686_56512077_56483358_106	annotated	AC119674.2	1	-	318	24	0	0	5.66%	24.93754952	0.013853925	0.013853925	0.000925205
ENSG00000120656_28622081_28603539_161	annotated	TAF12	1	-	483	43	5	0	10.56%	29.41649863	0.000508728	0.000121023	4.35E-07
ENSG00000168216_69719072_69709121_126	novel	LMBRD1	6	-	378	34	3	0	7.94%	29.72054513	0.00645193	0.002432129	8.87E-05
ENSG00000168216_69700809_69676158_159	novel	LMBRD1	6	-	477	47	16	0	10.06%	32.55735632	0.023460746	0.000202392	4.69E-05
ENSG00000101294_31514675_31567643_304	annotated	HM13	20	+	912	963	109	11	24.34%	348.9001069	2.37E-12	1.64E-13	5.21E-24
ENSG00000188690_125816499_125802999_139	annotated	UROS	10	-	417	43	0	1	10.07%	34.07234733	0.000457524	0.000457524	1.37E-06
ENSG00000114861_71198378_70959247_692	annotated	FOXP1	3	-	2076	15	1	5	0.87%	2.387446034	0.019795882	0.092312258	0.008348861
ENSG00000213694_88991174_88991920_101	internal	S1PR3	9	+	303	8	1	1	6.93%	8.724027224	0.028889786	0.017404239	0.002298137
ENSG00000140367_75869002_75897010_74	annotated	UBE2Q2	15	+	222	12	2	0	6.76%	17.86067736	0.051235217	0.020613417	0.004687384
ENSG00000178691_31937472_31999003_664	annotated	SUZ12	17	+	1992	31	5	0	1.96%	5.142117703	0.004949514	0.000676852	2.16E-05
ENSG00000159140_33543093_33557551_2108	annotated	SON	21	+	6324	118	10	1	2.47%	6.165379297	1.31E-08	1.36E-10	2.25E-17
ENSG00000177051_45713495_45711684_603	annotated	FBXO46	19	-	1809	10	2	0	0.83%	1.826547988	0.069457177	0.020613417	0.006391023
ENSG00000153187_244864307_244848809_804	annotated	HNRNPU	1	-	2412	516	60	30	13.93%	70.68740715	2.79E-15	1.68E-16	7.00E-30
ENSG00000138160_92606395_92653796_987	annotated	KIF11	10	+	2961	126	12	9	4.66%	14.06053324	5.08E-08	5.93E-08	2.97E-14
ENSG00000138162_122163991_122254056_1004	annotated	TACC2	10	+	3012	41	9	0	2.09%	4.497783458	0.001990914	2.15E-05	4.14E-07
ENSG00000172531_67401782_67401033_73	novel	PPP1CA	11	-	219	242	43	26	28.77%	365.1244408	4.81E-05	0.000347646	1.26E-07
ENSG00000110172_90205564_90202405_144	annotated	CHORDC1	11	-	432	13	0	0	4.17%	9.943270612	0.012798403	0.012798403	0.000797244
ENSG00000132768_43970185_43971570_229	novel	DPH2	1	+	687	7	1	1	2.18%	3.366750681	0.047790352	0.047790352	0.009211063
ENSG00000115520_197453561_197473924_188	annotated	COQ10B	2	+	564	57	5	2	9.04%	33.39376644	0.000181655	0.000181655	2.30E-07

ENSG00000108825_42970303_42950593_404	annotated	PTGES3L-AARSD1	17	-	1212	77	12	1	4.95%	20.99219051	0.006259751	7.60E-05	4.41E-06
ENSG00000135930_232550725_232580943_189	annotated	EIF4E2	2	+	567	25	20	1	6.88%	14.56889467	0.265788852	0.000539992	0.002945676
ENSG00000135930_232556424_232564298_52	internal	EIF4E2	2	+	156	20	1	15	15.38%	42.36186296	0.009288069	0.351779283	0.026584329
ENSG00000006625_30504709_30497178_166	annotated	GGCT	7	-	498	76	55	7	7.83%	50.42592844	0.409582882	0.008853033	0.032979045
ENSG00000071073_98726332_98625566_535	annotated	MGAT4A	2	-	1605	10	2	0	1.12%	2.058707359	0.056853419	0.011570466	0.003221159
ENSG00000213145_105487260_105488511_77	annotated	CRIP1	14	+	231	805	61	46	41.56%	1151.472457	7.11E-07	3.27E-06	2.09E-11
ENSG00000148985_3811260_3822914_56	Overlap_uORF	PGAP2	11	+	168	27	1	0	12.50%	53.10362107	0.008473674	0.008473674	0.000366502
ENSG00000148985_3822943_3825458_216	annotated	PGAP2	11	+	648	12	6	1	2.78%	6.118935761	0.128949518	0.019795882	0.012059958
ENSG00000132170_12379712_12399373_184	annotated	PPARG	3	+	552	14	2	0	2.72%	8.380281586	0.035374934	0.021084099	0.003318418
ENSG00000157954_5190420_5230947_443	annotated	WIPI2	7	+	1329	90	11	2	6.09%	22.37624364	3.10E-05	6.13E-06	1.57E-09
ENSG00000185344_123712593_123737352_363	novel	ATP6V0A2	12	+	1089	26	1	2	2.75%	7.888875858	0.003247793	0.005426623	9.74E-05
ENSG00000068120_42562623_42565968_564	annotated	COASY	17	+	1692	82	9	5	6.56%	16.01338508	2.07E-06	9.11E-07	1.67E-11
ENSG00000179912_57283935_57254773_637	novel	R3HDM2	12	-	1911	9	2	2	0.94%	1.556150068	0.070229091	0.070229091	0.018549462
ENSG00000157593_44255605_44254706_299	Overlap_dORF	SLC35B2	6	-	897	97	12	0	8.36%	35.7313105	0.000163758	5.62E-06	8.22E-09
ENSG00000157593_44257410_44255565_94	annotated	SLC35B2	6	-	282	40	7	4	10.64%	46.86844413	0.009031392	0.006610814	0.00030869
ENSG00000110090_68815474_68754816_756	annotated	CPT1A	11	-	2268	224	46	6	10.45%	32.63432406	2.63E-09	1.01E-14	7.33E-22
ENSG00000174243_48837948_48830146_211	annotated	DDX23	12	-	633	14	7	0	2.84%	7.307923279	0.236391543	0.011772051	0.017476259
ENSG00000170889_54201185_54231754_77	annotated	RPS9	19	+	231	356	50	14	10.39%	509.2226021	0.023426642	0.017704709	0.001908124
ENSG00000119185_9418697_9407425_184	annotated	ITGB1BP1	2	-	552	37	2	4	8.15%	22.14788705	0.001019452	0.001735173	1.08E-05
ENSG00000140682_31472189_31474069_138	novel	TGFB1I1	16	+	414	18	4	2	4.35%	14.36619701	0.059453662	0.01300724	0.003717979
ENSG00000005889_24179200_24211376_780	annotated	ZFX	X	+	2340	42	2	4	2.82%	5.930660815	4.02E-05	0.000197673	5.99E-08
ENSG00000114098_138188528_138245249_385	annotated	ARMC8	3	+	1155	18	10	0	2.34%	5.149442043	0.101438872	0.003303074	0.002393861
ENSG00000243566_76511734_76515251_161	annotated	UPK3B	7	+	483	21	14	0	4.97%	14.36619701	0.19727072	0.005706018	0.008403738
ENSG00000109917_116787640_116778925_372	annotated	ZPR1	11	-	1116	36	1	2	4.03%	10.65879133	0.000235224	0.001347597	2.17E-06
ENSG00000148737_112950757_113165634_489	annotated	TCF7L2	10	+	1467	34	6	0	3.27%	7.658054573	0.003731783	0.000174956	4.94E-06
ENSG00000167131_44900999_44902817_242	annotated	CCDC103	17	+	726	9	1	0	2.07%	4.09614708	0.041782825	0.019216965	0.003600526
ENSG00000163939_52679711_52548063_1609	annotated	PBRM1	3	-	4827	24	7	3	0.99%	1.642871503	0.013944777	0.000838746	7.95E-05
ENSG00000109911_31509785_31719517_395	annotated	ELP4	11	+	1185	30	11	0	3.54%	8.36512737	0.017920224	0.000424543	6.07E-05
ENSG00000136243_23182086_23188055_155	annotated	NUPL2	7	+	465	21	11	6	5.81%	14.92230786	0.071226447	0.010020958	0.003664133
ENSG00000115282_74483096_74492233_340	annotated	TTC31	2	+	1020	11	4	0	1.76%	3.563380238	0.111584927	0.012798403	0.007347425
ENSG00000092621_119712023_119723445_119	novel	PHGDH	1	+	357	26	4	0	5.88%	24.06438602	0.110834006	0.007029303	0.004652436
ENSG00000167325_4095013_4138383_792	annotated	RRM1	11	+	2376	138	11	9	6.82%	19.19120762	3.23E-09	3.50E-10	1.29E-17
ENSG00000148985_3811260_3825458_250	annotated	PGAP2	11	+	750	39	7	1	5.20%	17.18197162	0.004964704	0.000705712	2.25E-05
ENSG00000124486_41136894_41232524_2379	annotated	USP9X	X	+	7137	52	6	5	1.30%	2.407450136	8.70E-06	7.50E-06	5.35E-10
ENSG00000167323_3856271_4091798_685	annotated	STIM1	11	+	2055	37	3	7	2.77%	5.949213456	0.000344257	0.001064008	2.41E-06
ENSG00000167548_49051537_49021780_4822	annotated	KMT2D	12	-	14466	45	11	0	0.46%	1.027859388	0.001153288	1.60E-05	1.74E-07
ENSG00000163349_113952812_113973512_836	annotated	HIPK1	1	+	2508	29	11	2	1.20%	3.8206752	0.10289491	0.006133031	0.003843395
ENSG00000196812_28125444_28129950_348	annotated	ZSCAN16	6	+	1044	6	2	0	1.44%	1.898980064	0.102951605	0.016947427	0.008323174
ENSG00000181722_114351606_114339005_584	annotated	ZBTB20	3	-	1752	10	4	0	1.20%	1.885973351	0.095107389	0.005706018	0.003312541
ENSG00000164465_117503811_117508076_76	novel	DCBLD1	6	+	228	27	0	0	10.53%	39.12898395	0.005756914	0.005756914	0.000176255
ENSG00000182979_105469937_105470215_68	novel	MTA1	14	+	204	22	1	1	8.82%	35.63380238	0.016983117	0.013640586	0.00110551
ENSG00000231500_33272120_33276271_128	novel	RPS18	6	+	384	197	6	8	7.03%	169.5136423	0.005396391	0.010869341	0.000306953
ENSG00000270181_102807268_102875903_1640	annotated	BIVM-ERCC5	13	+	4920	36	9	5	1.10%	2.417725837	0.006120049	0.001353812	4.97E-05
ENSG00000115525_85863483_85840144_390	annotated	ST3GAL5	2	-	1170	151	23	10	11.79%	42.64427538	2.98E-07	7.02E-08	2.04E-13
ENSG00000100526_54411657_54418273_81	novel	CDKN3	14	+	243	21	3	0	9.88%	28.55503355	0.006965909	0.005306578	0.000195878
ENSG00000054983_87993116_87963321_391	annotated	GALC	14	-	1173	53	5	5	5.12%	14.92957728	0.00012712	0.000320429	2.86E-07
ENSG00000129292_132794453_132845923_975	annotated	PHF20L1	8	+	2925	13	6	0	1.13%	1.468544583	0.111917027	0.000670321	0.000880274
ENSG00000186871_72238911_72205014_1250	annotated	ERCC6L	X	-	3750	21	3	0	0.48%	1.850366174	0.031019588	0.013428348	0.00195881
ENSG00000168890_85601948_85599076_271	annotated	TMEM150A	2	-	813	16	0	1	2.21%	6.50278044	0.013428348	0.013428348	0.000872559
ENSG00000119986_97679905_97677869_147	annotated	AVPI1	10	-	441	77	44	1	13.61%	57.69282289	0.059619188	6.58E-05	7.09E-05
ENSG00000165650_117375212_117283268_1149	annotated	PDZD8	10	-	3447	201	32	11	5.66%	19.2674583	9.79E-09	2.10E-11	2.93E-18
ENSG00000165650_117375319_117375209_36	Overlap_uORF	PDZD8	10	-	108	15	0	1	13.89%	45.89201821	0.021084099	0.035772986	0.003354605
ENSG00000065809_14530459_14521190_131	annotated	FAM107B	10	-	393	106	26	3	14.50%	89.12159872	0.004452946	8.01E-05	3.11E-06
ENSG00000065809_14553383_14530515_20	uORF	FAM107B	10	-	60	13	3	0	25.00%	71.59154841	0.138151459	0.019679754	0.01298269
ENSG00000130749_47085148_47066356_881	annotated	AC008755.1	19	-	2643	22	7	9	1.70%	2.750395643	0.004398026	0.004429039	0.000106375
ENSG00000130299_17337612_17341703_471	annotated	GTPBP3	19	+	1413	20	5	0	2.34%	4.676893576	0.024135035	0.00146251	0.00023217
ENSG00000161960_7576539_7577934_257	Overlap_dORF	EIF4A1	17	+	771	440	26	13	11.67%	188.5567814	2.28E-06	1.46E-06	2.92E-11
ENSG00000213246_58351550_58346246_108	annotated	SUPT4H1	17	-	324	86	4	5	15.74%	87.70474591	0.000938371	0.000681399	4.04E-06
ENSG00000099622_1270934_1272443_297	annotated	CIRBP	19	+	891	142	16	12	12.12%	52.65993201	1.69E-06	1.57E-06	2.32E-11
ENSG00000164902_126608482_126624844_118	novel	PHAX	5	+	354	13	2	0	5.08%	12.13416075	0.071345384	0.011772051	0.004172987
ENSG00000094804_40289421_40302001_560	annotated	CDC6	17	+	1680	51	5	3	4.11%	10.03068398	0.000127493	6.65E-05	6.19E-08
ENSG00000183648_92121777_92116193_105	annotated	NDUFB1	14	-	315	450	45	21	20.00%	472.0321873	0.000645766	0.000599348	2.49E-06
ENSG00000151465_12237258_12250337_134	novel	CDC123	10	+	402	53	1	0	11.94%	43.56316953	0.000286552	0.000179964	3.56E-07
ENSG00000116096_72887433_72888427_103	novel	SPR	2	+	309	17	5	0	6.80%	18.17858585	0.069518469	0.008574016	0.003152467
ENSG00000116096_72888544_72891537_83	novel	SPR	2	+	249	36	8	1	14.46%	47.77193221	0.006086362	0.001368377	4.99E-05
ENSG00000127054_1313914_1313494_93	novel	INTS11	1	-	279	15	0	3	8.60%	17.76465221	0.005306578	0.036243043	0.001046215
ENSG00000113368_126819039_126836264_234	dORF	LMNB1	5	+	702	78	8	5	11.11%	36.71361457	9.27E-05	5.73E-06	4.57E-09
ENSG00000113368_126777539_126815220_319	annotated	LMNB1	5	+	957	59	11	1	5.96%	20.37087705	0.001886428	7.78E-05	1.17E-06

ENSG00000148303_133350630_133351366_90	novel	RPL7A	9	+	270	86	12	0	5.56%	105.2456951	0.021084099	0.021557223	0.002072352
ENSG00000239282_30289497_30286698_252	novel	CASTOR1	22	-	756	8	0	0	1.98%	3.496534721	0.019679754	0.019679754	0.001784964
ENSG00000148248_133366008_133363493_96	novel	SURF4	9	-	288	226	78	5	32.29%	259.2899029	0.000127413	1.08E-06	1.45E-09
ENSG00000198860_184054366_184054867_69	novel	TSEN15	1	+	207	12	0	0	7.25%	19.15492934	0.020613417	0.020613417	0.001946209
ENSG00000132300_86106248_86119587_193	novel	PTCD3	2	+	579	19	1	0	5.18%	10.84288099	0.002940943	0.002188006	3.71E-05
ENSG00000160633_5623377_5668291_859	annotated	SAFB	19	+	2577	58	14	1	3.61%	7.436750797	0.000296445	5.69E-07	2.18E-09
ENSG00000142910_31577149_31577469_106	novel	TINAGL1	1	+	318	49	0	3	14.15%	50.9141636	0.000306523	0.000532835	1.09E-06
ENSG00000276368_27814740_27814354_128	annotated	HIST1H2AJ	6	-	384	134	8	17	7.81%	115.3036958	0.005518828	0.002376615	7.43E-05
ENSG00000115306_54612257_54668569_2234	annotated	SPTBN1	2	+	6702	148	34	27	3.36%	7.29670764	7.93E-08	2.47E-09	2.20E-15
ENSG00000132182_13353963_13351810_143	novel	NUP210	3	-	429	14	3	0	3.50%	10.78301966	0.068043226	0.021084099	0.0063746
ENSG00000114388_50349440_50347606_249	novel	NPRL2	3	-	747	5	1	0	2.01%	2.211663528	0.051235217	0.012673659	0.00311107
ENSG00000137764_67543369_67806750_437	annotated	MAP2K5	15	+	1311	16	9	2	2.29%	4.032616703	0.191016305	0.012468381	0.013771834
ENSG00000124608_44312221_44300547_890	annotated	AARS2	6	-	2670	28	3	0	1.46%	3.465105195	0.002942496	0.000667552	1.24E-05
ENSG00000113716_150004892_150052192_1279	annotated	HMGXB3	5	+	3837	24	11	0	0.94%	2.066755472	0.095796813	0.000991051	0.000934147
ENSG00000005893_120469169_120439154_410	annotated	LAMP2	X	-	1230	177	16	17	12.44%	47.54860814	9.68E-08	8.42E-08	7.93E-14
ENSG00000119969_94562707_94601622_700	annotated	HELLS	10	+	2100	62	11	0	3.71%	9.755331871	8.90E-05	2.46E-06	2.04E-09
ENSG00000102908_69566302_69646563_99	annotated	NFAT5	16	+	297	24	5	1	10.10%	26.7008106	0.016685545	0.003990669	0.00036205
ENSG00000130733_10927830_10924136_87	novel	YIPF2	19	-	261	12	1	1	5.75%	15.19184051	0.035772986	0.035772986	0.005410795
ENSG00000017260_130934676_131016247_853	annotated	ATP2C1	3	+	2559	92	16	6	5.16%	11.87920002	2.05E-06	1.06E-07	2.17E-12
ENSG00000144366_188483442_188594011_188	annotated	GULP1	2	+	564	52	5	2	9.04%	30.46448868	0.000327658	0.000329639	7.25E-07
ENSG00000115641_105386516_105361283_279	annotated	FHL2	2	-	837	117	12	0	10.04%	46.18809575	2.51E-05	1.64E-06	3.70E-10
ENSG00000180398_46907920_46905463_80	annotated	MCFD2	2	-	240	51	7	0	13.75%	70.21478786	0.008830848	0.001580611	8.33E-05
ENSG00000111859_11213721_11185162_828	annotated	NEDD9	6	-	2484	119	11	5	4.59%	15.82942077	1.58E-06	4.42E-07	6.39E-12
ENSG00000227500_1915020_1924284_229	annotated	SCAMP4	19	+	687	137	32	2	16.16%	65.89212047	5.68E-05	1.12E-07	8.24E-11
ENSG00000163359_237388017_237324774_2885	annotated	COL6A3	2	-	8655	19	4	0	0.38%	0.725364309	0.017088526	0.001262414	0.000139885
ENSG00000139180_4649169_4654946_99	novel	NDUFA9	12	+	297	78	9	2	11.11%	86.77763443	0.001811542	0.002060045	2.20E-05
ENSG00000241489_149496423_149482746_283	annotated	AC244197.3	X	-	849	15	3	4	3.18%	5.837853907	0.025557109	0.038440602	0.004267345
ENSG00000135241_108515491_108472401_782	annotated	PNPLA8	7	-	2346	53	12	0	2.69%	7.46478864	0.000761151	2.38E-05	1.55E-07
ENSG00000148737_112950757_113165972_607	annotated	TCF7L2	10	+	1821	32	6	0	2.64%	5.806436571	0.004472475	0.000175784	6.05E-06
ENSG00000183735_64464361_64488489_351	novel	TBK1	12	+	1053	14	2	0	1.99%	4.393082085	0.037995728	0.007979402	0.001544499
ENSG00000164898_139341385_139345704_113	annotated	FMC1	7	+	339	62	4	0	12.39%	60.43125938	0.001905473	0.000452135	5.60E-06
ENSG00000164896_151080766_151076725_549	annotated	FASTK	7	-	1647	32	1	10	2.91%	6.419867028	0.00027351	0.01162175	2.57E-05
ENSG00000114745_39107541_39100563_249	novel	GORASP1	3	-	747	16	0	1	2.01%	7.07732329	0.019679754	0.042234516	0.003715148
ENSG00000114745_39100523_39098236_258	novel	GORASP1	3	-	774	19	9	0	3.49%	8.111147405	0.080798099	0.003478238	0.001876682
ENSG00000167699_783265_760173_502	annotated	GLOD4	17	-	1506	102	15	11	6.77%	22.37921525	9.46E-06	1.29E-06	1.08E-10
ENSG00000115947_147958839_147935510_352	annotated	ORC4	2	-	1056	37	1	2	3.98%	11.57730459	0.000556136	0.001216039	4.31E-06
ENSG00000165533_88824708_88877410_475	annotated	TTC8	14	+	1425	45	8	2	4.21%	10.43439572	0.000863386	8.45E-05	5.43E-07
ENSG00000165124_110579474_110366539_3548	annotated	SVEP1	9	-	10644	51	8	5	0.82%	1.583197021	0.000138319	9.68E-06	1.12E-08
ENSG00000169826_43155426_43183542_450	annotated	CSGALNACT2	10	+	1350	24	8	1	2.22%	5.874178331	0.015528143	0.002708919	0.000240346
ENSG00000165124_110579474_110476196_845	annotated	SVEP1	9	-	2535	12	1	4	1.07%	1.564130325	0.00650005	0.031984458	0.001084244
ENSG00000099250_33334382_33180076_906	annotated	NRP1	10	-	2718	456	43	24	12.69%	55.43512663	1.56E-17	3.42E-17	8.10E-33
ENSG00000104870_49513401_49515253_225	annotated	FCGRT	19	+	675	25	2	5	3.56%	12.23787152	0.010402772	0.033503887	0.001696852
ENSG00000104870_49524518_49526082_157	dORF	FCGRT	19	+	471	16	0	0	5.10%	11.22454458	0.005306578	0.005306578	0.000151001
ENSG00000197756_216499339_216520753_60	annotated	RPL37A	2	+	180	890	113	24	31.67%	1633.755848	0.000836048	0.000125228	7.48E-07
ENSG00000077312_40751409_40763086_203	novel	SNRPA	19	+	609	44	1	4	8.37%	23.87289223	0.000149522	0.001081978	1.15E-06
ENSG00000140479_101431427_101347764_440	annotated	PCSK6	15	-	1320	11	0	0	1.36%	2.753521093	0.011772051	0.011772051	0.000681273
ENSG00000127603_39332133_39485794_5650	annotated	MACF1	1	+	16950	60	13	0	0.48%	1.169637278	0.000262497	2.26E-06	6.17E-09
ENSG00000117602_24514388_24535277_226	annotated	RCAN3	1	+	678	15	7	4	3.98%	7.310232989	0.086894686	0.031072489	0.011283286
ENSG00000163412_71725367_71684682_224	annotated	EIF4E3	3	-	672	27	0	1	3.12%	13.27590527	0.008877961	0.008776163	0.000395901
ENSG00000084112_108857496_108787988_1049	annotated	SSH1	12	-	3147	23	5	4	0.86%	2.414908871	0.013494345	0.029977737	0.001901549
ENSG00000103051_70523531_70509893_284	novel	COG4	16	-	852	29	3	0	4.23%	11.24677629	0.003911212	0.001028015	2.45E-05
ENSG00000051523_88651013_88647097_68	novel	CYBA	16	-	204	204	21	17	32.35%	330.4225311	1.94E-05	0.000164686	2.54E-08
ENSG00000126777_55664009_55684103_281	annotated	KTN1	14	+	843	24	5	4	4.27%	9.407047149	0.016833283	0.007169783	0.00061168
ENSG00000149657_62122667_62133461_385	annotated	LSM14B	20	+	1155	47	4	6	5.19%	13.44576534	0.000112879	0.000158386	1.27E-07
ENSG00000112992_43609196_43704404_1086	annotated	NNT	5	+	3258	43	2	0	1.75%	4.361009465	0.000114158	5.53E-05	4.66E-08
ENSG00000116649_11055108_11054865_55	novel	SRM	1	-	165	33	8	5	14.55%	66.08450622	0.027811403	0.032563252	0.003935341
ENSG00000100296_29544504_29536585_185	novel	THOC5	22	-	555	14	0	0	3.78%	8.334982767	0.007882058	0.007882058	0.000319636
ENSG00000108641_19347847_19343319_123	Overlap_dORF	B9D1	17	-	369	20	0	33	6.50%	17.90908028	0.005604867	0.562114616	0.046212349
ENSG00000156411_103915153_103912666_46	annotated	ATP5MPL	14	-	138	240	24	3	41.30%	574.6478802	6.43E-05	8.30E-05	3.93E-08
ENSG00000108559_5416586_5386206_626	annotated	NUP88	17	-	1878	52	3	4	3.35%	9.149079669	0.000119005	0.000188904	1.59E-07
ENSG00000104763_18084058_18067074_175	novel	ASAH1	8	-	525	272	17	1	11.43%	171.1903399	0.000361149	6.31E-05	1.68E-07
ENSG00000057663_106316102_106046821_153	annotated	ATG5	6	-	459	38	4	0	9.15%	27.35524223	0.002211307	0.000444132	6.42E-06
ENSG00000125434_8294807_8289616_295	annotated	SLC25A35	17	-	885	9	1	0	1.69%	3.36022913	0.035374934	0.020613417	0.003252526
ENSG00000096872_26978128_27062736_560	annotated	IFT74	9	+	1680	29	10	5	2.50%	5.703722263	0.02396179	0.004113232	0.000542871
ENSG00000147862_14321943_14088309_419	annotated	NFIB	9	-	1257	35	3	0	3.58%	9.20030914	0.000601831	0.00028802	1.16E-06
ENSG00000096060_35642824_35575835_457	annotated	FKBP5	6	-	1371	34	3	5	3.06%	8.194285965	0.0016955	0.003530286	3.49E-05
ENSG00000103253_728560_733471_49	novel	HAGHL	16	+	147	12	0	4	10.20%	26.97326785	0.019679754	0.113684165	0.010429585
ENSG00000104964_3056347_3055403_119	novel	TLE5	19	-	357	60	2	0	5.88%	55.53319851	0.008877961	0.008877961	0.000400235

ENSG00000104964_3054064_3053819_81	novel	TLE5	19	-	243	52	2	9	11.11%	70.70770213	0.00310098	0.022714641	0.000404109
ENSG00000169684_78565720_78593253_468	annotated	CHRNA5	15	+	1404	190	16	4	9.19%	44.71529979	1.08E-07	1.29E-08	1.44E-14
ENSG00000166439_74748881_74836730_708	annotated	RNF169	11	+	2124	22	9	0	1.69%	3.422455595	0.045348857	0.000919662	0.000338306
ENSG00000261832_28477608_28477516_30	dORF	AC138894.1	16	-	90	23	5	1	23.33%	84.44131351	0.027657845	0.010042643	0.001354611
ENSG00000243989_51988530_51989075_99	novel	ACY1	3	+	297	27	1	4	8.08%	30.03841192	0.005355751	0.025702836	0.000731269
ENSG00000147862_14313508_14093461_444	annotated	NFIB	9	-	1332	48	4	0	4.05%	11.90711824	0.000188654	8.74E-05	1.18E-07
ENSG00000179222_51900270_51901930_134	novel	MAGED1	X	+	402	26	0	0	7.46%	21.37061147	0.002227676	0.002227676	2.89E-05
ENSG00000168487_22170986_22176942_147	novel	BMP1	8	+	441	14	6	1	4.08%	10.48960416	0.162003278	0.020342454	0.015990656
ENSG00000164924_100924973_100920693_125	annotated	YWHAZ	8	-	375	23	0	1	4.00%	20.26591524	0.021084099	0.035772986	0.003354605
ENSG00000072803_171914393_171868635_510	dORF	FBXW11	5	-	1530	79	8	0	4.51%	17.06103265	0.000497011	1.05E-05	4.77E-08
ENSG00000147419_27765225_27748245_74	annotated	CCDC25	8	-	222	70	16	5	24.32%	104.1872846	0.001976402	0.00025821	3.51E-06
ENSG00000160932_143020940_143023697_66	annotated	LY6E	8	+	198	422	28	18	31.82%	704.2338794	0.000434557	9.54E-05	2.98E-07
ENSG00000182010_62268610_62197908_609	annotated	RTKN2	10	-	1827	26	8	0	2.30%	4.702236349	0.004635804	0.000377559	1.21E-05
ENSG00000181915_62805075_62805872_265	annotated	ADO	10	+	795	61	1	4	5.66%	25.35317534	0.000309685	0.000312071	6.51E-07
ENSG00000136848_121757101_121783545_1010	annotated	DAB2IP	9	+	3030	10	0	2	0.50%	1.090503403	0.020613417	0.069457177	0.006391023
ENSG00000119541_63422259_63403787_111	novel	VPS4B	18	-	333	6	5	0	4.50%	5.953559119	0.241449849	0.016947427	0.022959401
ENSG00000119541_63410299_63396986_261	novel	VPS4B	18	-	783	30	4	0	4.21%	12.65986709	0.008711445	0.001580611	8.22E-05
ENSG00000242114_30426813_30428534_128	Overlap_dORF	MTFP1	22	+	384	46	7	4	10.16%	39.58186571	0.002619468	0.001052136	1.67E-05
ENSG00000132024_13906442_13930395_950	annotated	CC2D1A	19	+	2850	25	7	2	0.95%	2.898443255	0.044742753	0.009067228	0.002042347
ENSG00000139289_76031033_76030536_165	annotated	PHLDA1	12	-	495	68	7	5	12.12%	45.39137801	0.000368286	0.000187148	4.73E-07
ENSG00000101752_21791549_21864666_645	novel	MIB1	18	+	1935	29	7	0	1.86%	4.952068942	0.021040203	0.001032175	0.0001497
ENSG00000170315_16382364_16382597_77	novel	UBB	17	+	231	368	57	1	10.39%	526.3874089	0.007152939	0.005808022	0.000218635
ENSG00000006576_77840256_77922832_352	annotated	PHTF2	7	+	1056	24	0	4	3.41%	7.50960298	0.000943084	0.008887078	5.37E-05
ENSG00000158856_22067533_22081463_350	annotated	DMTN	8	+	1050	21	6	0	3.14%	6.608450622	0.02826768	0.001092093	0.000219937
ENSG00000066926_57586599_57573242_104	novel	FECH	18	-	312	21	1	4	5.77%	22.23997806	0.020525443	0.079426469	0.007325331
ENSG00000170142_23807270_23887768_134	novel	UBE2E1	3	+	402	90	6	4	10.45%	73.97519353	0.001298872	0.001032612	8.24E-06
ENSG00000164081_51674305_51704016_313	annotated	TEX264	3	+	939	178	24	6	12.46%	62.63600696	2.04E-06	7.66E-08	1.59E-12
ENSG00000168872_70346992_70371986_478	annotated	DDX19A	16	+	1434	27	4	4	2.30%	6.221344728	0.002559322	0.007205324	0.000103746
ENSG00000185630_164559823_164849402_420	annotated	PBX1	1	+	1260	72	21	0	7.38%	18.88128749	0.000107625	4.68E-07	5.83E-10
ENSG00000111142_95474180_95513904_442	annotated	METAP2	12	+	1326	192	32	5	14.03%	47.84398641	6.45E-08	6.90E-12	8.42E-18
ENSG00000186283_179085822_179088141_100	novel	TOR3A	1	+	300	32	0	1	11.00%	35.24506999	0.001398493	0.00376808	3.12E-05
ENSG00000127990_94656098_94585465_485	annotated	SGCE	7	-	1455	210	28	11	11.13%	47.68984985	8.39E-08	2.42E-09	2.29E-15
ENSG00000215301_41343246_41347719_471	novel	DDX3X	X	+	1413	189	30	7	8.70%	44.19664429	3.70E-07	2.15E-08	8.26E-14
ENSG00000215301_41334253_41342657_148	novel	DDX3X	X	+	444	49	18	3	12.16%	36.46554961	0.013604033	0.000328142	3.58E-05
ENSG00000198042_33485207_33489143_131	novel	MAK16	8	+	393	78	4	1	14.50%	65.58004434	0.000264075	7.19E-05	1.38E-07
ENSG00000120314_140668985_140669654_165	novel	WDR55	5	+	495	20	12	2	4.85%	13.3504053	0.250378122	0.02389627	0.030354635
ENSG00000104365_42288721_42330979_692	annotated	IKBKB	8	+	2076	26	6	6	2.75%	4.138239792	0.003052945	0.001155174	2.12E-05
ENSG00000125246_99859011_99865072_131	annotated	CLYBL	13	+	393	11	0	0	3.82%	9.248467792	0.020613417	0.020613417	0.001946209
ENSG00000169139_48043161_48060828_97	annotated	UBE2V2	8	+	291	78	36	12	18.56%	88.56686401	0.009975161	0.000895519	5.81E-05
ENSG00000176095_49747923_49727122_402	annotated	IP6K1	3	-	1206	38	4	0	2.99%	10.41132353	0.002710157	0.001019727	1.68E-05
ENSG00000104343_73878813_73794102_159	annotated	UBE2W	8	-	477	29	1	2	7.55%	20.08858156	0.00092354	0.002061799	1.17E-05
ENSG00000134897_102807268_102835772_391	novel	BIVM	13	+	1173	13	1	0	1.53%	3.661971786	0.023672501	0.012798403	0.001437523
ENSG00000101266_489830_483961_167	annotated	CSNK2A1	20	-	501	47	1	0	8.38%	30.99772248	0.000475338	0.000472567	1.47E-06
ENSG00000166340_6616817_6614546_320	annotated	TPP1	11	-	960	177	39	4	12.50%	60.92165417	1.78E-06	7.56E-08	1.37E-12
ENSG00000133657_194450232_194419892_845	novel	ATP13A3	3	-	2535	102	4	7	4.50%	13.29510776	4.76E-08	7.79E-07	3.76E-13
ENSG00000127990_94618900_94524260_254	novel	SGCE	7	-	762	125	8	5	14.17%	54.20317111	1.74E-06	2.28E-07	3.75E-12
ENSG00000179262_12946003_12953049_344	annotated	RAD23A	19	+	1032	108	7	1	13.37%	34.57910209	4.05E-09	1.16E-09	5.13E-17
ENSG00000105011_14136456_14120459_143	annotated	ASF1B	19	-	429	110	15	4	19.58%	84.72372593	0.0001456	2.77E-06	3.87E-09
ENSG00000187741_89816014_89799079_280	novel	FANCA	16	-	840	28	0	1	3.57%	11.01408437	0.002362849	0.003500471	4.72E-05
ENSG00000066933_72010381_71897457_1299	annotated	MYO9A	15	-	3897	9	0	3	0.38%	0.763100534	0.019216965	0.131156788	0.012020478
ENSG00000166946_43190120_43192884_201	novel	CCNDBP1	15	+	603	73	5	0	11.44%	40.00140095	9.01E-05	1.05E-05	7.72E-09
ENSG00000261067_28982291_28984299_228	novel	AC109460.3	16	+	684	115	18	1	15.35%	55.55349573	3.67E-06	1.22E-07	4.54E-12
ENSG00000261067_28975371_28981619_197	novel	AC109460.3	16	+	591	79	5	0	10.15%	44.1681556	0.0001178	3.87E-05	3.42E-08
ENSG00000140464_73998130_74034495_475	annotated	PML	15	+	1425	8	3	0	1.26%	1.855003683	0.093731016	0.009815329	0.004911899
ENSG00000141562_82458804_82483783_278	novel	NARF	17	+	834	39	1	0	6.47%	15.45141333	8.42E-05	8.42E-05	5.18E-08
ENSG00000141562_82484781_82488157_189	novel	NARF	17	+	567	22	0	1	4.23%	12.82062731	0.005454749	0.008219047	0.00023581
ENSG00000245937_128083022_128082768_84	novel	LINC01184	5	-	252	19	4	1	7.14%	24.91280989	0.060590694	0.020342454	0.005492863
ENSG00000181924_73873378_73873115_87	annotated	COA4	11	-	261	148	58	8	25.29%	187.366033	0.01103701	0.000392935	3.26E-05
ENSG00000183605_119165647_119141242_337	annotated	SFXN4	10	-	1011	84	16	15	7.42%	27.45350407	0.000303607	0.000148449	3.14E-07
ENSG00000114054_136250376_136330026_423	annotated	PCCB	3	+	1269	68	11	1	6.38%	17.70585667	6.09E-05	2.03E-06	1.15E-09
ENSG00000143376_151658250_151693601_342	novel	SNX27	1	+	1026	34	2	1	4.09%	10.94967452	0.001092758	0.000662939	4.57E-06
ENSG00000141068_27597283_27623392_490	annotated	KSR1	17	+	1470	11	1	4	1.63%	2.472549553	0.010499663	0.069043051	0.003674424
ENSG00000285130_69035922_69099934_92	Overlap_dORF	AL358113.1	9	+	276	35	6	5	11.96%	41.90140793	0.007990369	0.01106333	0.000447413
ENSG00000131508_139561792_139626801_147	annotated	UBE2D2	5	+	441	166	10	11	19.73%	124.3767351	9.20E-06	2.94E-05	2.18E-09
ENSG00000174891_158122105_158544275_334	annotated	RSRC1	3	+	1002	116	51	5	4.49%	38.25250859	0.37156367	0.002288824	0.012655602
ENSG00000174891_158123898_158298067_98	internal	RSRC1	3	+	294	30	0	14	8.16%	33.71658481	0.005756914	0.142861984	0.005518563
ENSG00000198663_36899445_36923441_347	annotated	C6orf89	6	+	1041	51	1	0	6.05%	16.18784735	2.20E-05	2.21E-05	3.80E-09
ENSG00000182208_1486478_1470172_242	annotated	MOB2	11	-	726	30	4	0	4.13%	13.6538236	0.007259965	0.002376615	9.78E-05

ENSG00000079805_10812306_10831047_337	novel	DNM2	19	+	1011	41	1	4	5.34%	13.39992461	0.000120178	0.000849056	7.32E-07
ENSG00000037474_6632979_6599926_767	annotated	NSUN2	5	-	2301	122	16	4	6.39%	17.51914333	2.40E-07	1.19E-09	3.61E-15
ENSG00000188026_123499510_123472538_273	novel	RILPL1	12	-	819	10	1	2	1.83%	4.034463139	0.035374934	0.069457177	0.010057191
ENSG00000132780_45584153_45618141_722	annotated	NASP	1	+	2166	91	12	7	4.57%	13.8820177	1.28E-05	5.06E-06	5.38E-10
ENSG00000205208_158671801_158669613_113	annotated	C4orf46	4	-	339	71	2	0	4.42%	69.20353897	0.021084099	0.021084099	0.002029891
ENSG00000120539_27155427_27186536_840	annotated	MASTL	10	+	2520	72	2	2	3.21%	9.440643746	4.46E-06	4.46E-06	1.67E-10
ENSG00000184640_77281536_77498658_567	annotated	9-Sep	17	+	1701	136	22	6	7.58%	26.41826233	1.17E-05	7.30E-08	9.81E-12
ENSG00000223784_6737436_6738367_67	novel	LINP1	10	+	201	47	0	3	13.43%	77.26297991	0.003710481	0.007356796	0.000148417
ENSG00000143363_151008633_151034234_454	annotated	PRUNE1	1	+	1362	20	7	0	1.98%	4.852019546	0.082426618	0.003478238	0.001922849
ENSG00000149474_18142661_18187459_782	annotated	KAT14	20	+	2346	29	1	2	1.53%	4.084506992	0.001480749	0.003224199	2.82E-05
ENSG00000100372_40799068_40776282_216	annotated	SLC25A17	22	-	648	66	5	6	3.24%	33.65414669	0.053017408	0.062029681	0.012861168
ENSG00000151914_56843145_56459005_5375	annotated	DST	6	-	16125	69	7	11	0.56%	1.413901063	1.78E-05	0.000168399	2.40E-08
ENSG00000101928_134899433_134889161_213	annotated	MOSPD1	X	-	639	25	0	0	5.16%	12.92732907	0.001558047	0.001558047	1.45E-05
ENSG00000114030_122452025_122426985_337	novel	KPNA1	3	-	1011	38	2	0	4.15%	12.41944232	0.000548327	0.000444132	1.59E-06
ENSG00000158467_129397228_129427045_369	annotated	AHCYL2	7	+	1107	18	0	0	2.44%	5.372724083	0.003529166	0.003529166	6.95E-05
ENSG00000128567_131556359_131504311_558	annotated	PODXL	7	-	1674	64	6	2	5.56%	12.63264157	4.81E-06	8.76E-07	3.77E-11
ENSG00000138032_44201284_44232345_359	annotated	PPM1B	2	+	1077	53	9	3	6.69%	16.2603474	0.000731329	5.73E-05	3.22E-07
ENSG00000166484_19378901_19383231_816	annotated	MAPK7	17	+	2448	21	0	6	1.35%	2.834507007	0.001269017	0.034800501	0.000315837
ENSG00000085224_77684988_77508351_2288	annotated	ATRX	X	-	6864	30	1	3	0.66%	1.444154419	0.000440588	0.00145172	4.15E-06
ENSG00000100523_53103734_53046768_559	annotated	DDHD1	14	-	1677	16	5	0	1.25%	3.152510732	0.07477567	0.008473674	0.003389727
ENSG00000166484_19378901_19383231_816	annotated	MAPK7	17	+	2448	21	0	6	1.35%	2.834507007	0.001269017	0.034800501	0.000315837
ENSG00000130299_17337612_17339215_151	annotated	GTPBP3	19	+	453	11	1	0	3.31%	8.023505171	0.02854252	0.020613417	0.002645522
ENSG00000187098_69879285_69965248_435	annotated	MITF	3	+	1305	37	7	1	3.91%	9.368301648	0.003418024	0.000183422	4.67E-06
ENSG00000135678_68885806_68856437_362	annotated	CPM	12	-	1086	14	0	0	1.93%	4.259590641	0.008175611	0.008175611	0.000342518
ENSG00000088992_117046827_117046591_43	internal	TESC	12	-	129	29	4	1	13.95%	74.28103413	0.013640586	0.016983117	0.00110551
ENSG00000175066_142225455_142185704_301	annotated	GK5	3	-	903	20	1	0	2.33%	7.318328485	0.0135992	0.008175611	0.00055803
ENSG00000157191_16440762_16458890_263	annotated	NECAP2	1	+	789	43	3	3	4.56%	18.00781855	0.004269801	0.004269801	9.99E-05
ENSG00000073169_50210259_50217360_327	novel	SELENOO	22	+	981	56	1	1	7.03%	18.86204051	1.53E-05	1.53E-05	1.87E-09
ENSG00000125457_75270195_75266740_170	annotated	MIF4GD	17	-	510	48	17	2	10.59%	31.09859116	0.010949191	0.00016215	1.57E-05
ENSG00000258947_89923402_89933236_87	annotated	TUBB3	16	+	261	42	9	2	10.34%	53.17144179	0.003684287	0.007121296	0.000142801
ENSG00000130734_10551930_10553067_141	Overlap_dORF	ATG4D	19	+	423	8	2	0	3.55%	6.249125884	0.065398531	0.019679754	0.005779267
ENSG00000214575_82556032_82544592_329	Overlap_dORF	CPEB1	15	-	987	8	0	0	1.52%	2.678196807	0.019679754	0.019679754	0.001784964
ENSG00000198862_28992805_28930448_1766	annotated	LTN1	21	-	5298	19	3	1	0.51%	1.184980765	0.020770034	0.006083074	0.00065433
ENSG00000110841_27682695_27692882_218	annotated	PPFIBP1	12	+	654	22	4	0	3.21%	11.11513102	0.031850938	0.008574016	0.001365212
ENSG00000123091_51236757_51271322_154	annotated	RNF11	1	+	462	6	0	0	3.25%	4.291201703	0.016947427	0.016947427	0.001349898
ENSG00000163428_120349243_120331200_371	annotated	LRRC58	3	-	1113	44	3	3	5.39%	13.06252594	7.77E-05	0.000210555	1.18E-07
ENSG00000182809_105474863_105478736_96	novel	CRIP2	14	+	288	18	1	9	7.29%	20.65140819	0.008574016	0.170989522	0.009203223
ENSG00000151092_25732330_25719460_183	novel	NGLY1	3	-	549	15	0	2	4.37%	9.027938009	0.005405142	0.012941946	0.000365537
ENSG00000151092_25783390_25737305_343	novel	NGLY1	3	-	1029	22	4	0	2.92%	7.064427293	0.011768187	0.002321823	0.000157296
ENSG00000050426_51048357_51059431_243	annotated	LETMD1	12	+	729	46	0	7	6.17%	20.84970704	0.000279805	0.001936335	3.69E-06
ENSG00000163867_35011946_34990259_397	novel	ZMYM6	1	-	1191	5	3	0	1.26%	1.387164278	0.158655254	0.012673659	0.011061611
ENSG00000119632_94128586_94127799_88	annotated	IFI27L2	14	-	264	45	1	4	14.77%	56.32202235	0.000804138	0.000804138	4.08E-06
ENSG00000134001_67364828_67374556_89	novel	EIF2S1	14	+	267	31	13	4	14.61%	38.36366466	0.006771889	0.005077325	0.000182917
ENSG00000198668_90404421_90404717_40	novel	CALM1	14	+	120	122	3	6	35.00%	335.9295733	0.001141632	0.001538329	1.07E-05
ENSG00000097033_86704900_86743235_394	annotated	SH3GLB1	1	+	1182	98	10	0	7.36%	27.39543828	4.14E-05	1.06E-06	4.22E-10
ENSG00000110768_18347687_18365869_236	annotated	GTF2H1	11	+	708	19	1	1	2.97%	8.867271315	0.013892891	0.008473674	0.00058868
ENSG00000125375_50321491_50322769_120	annotated	DMAC2L	14	+	360	29	0	5	7.50%	26.61737056	0.003710481	0.011906648	0.000240324
ENSG00000117410_43976115_43978007_158	novel	ATP6V0B	1	+	474	173	20	3	21.52%	120.5972529	4.29E-06	3.63E-07	1.46E-11
ENSG00000055163_157302858_157393000_1067	annotated	CYFIP2	5	+	3201	36	5	2	1.97%	3.716092196	0.000301599	0.000112078	2.39E-07
ENSG00000141510_7676594_7669609_393	annotated	TP53	17	-	1179	335	24	10	11.20%	93.88596092	2.17E-07	1.62E-08	3.65E-14
ENSG00000149084_43830976_43855248_145	novel	HSD17B12	11	+	435	171	39	4	22.76%	129.8902364	0.000192067	1.78E-06	3.55E-09
ENSG00000104960_49857059_49858794_179	novel	PTOV1	19	+	537	52	1	3	12.85%	31.99622275	1.51E-05	2.29E-05	2.74E-09
ENSG00000134371_193141905_193250712_342	dORF	CDC73	1	+	1026	58	5	4	5.56%	18.67885653	0.000202062	0.000175048	2.46E-07
ENSG00000131023_149701958_149676206_922	novel	LATS1	6	-	2766	29	2	1	0.87%	3.464299856	0.008092227	0.006307834	0.000265871
ENSG00000099622_1270934_1271465_142	novel	CIRBP	19	+	426	73	68	5	10.56%	56.62170134	0.514981381	0.001265823	0.017490757
ENSG00000122008_75547023_75598018_870	annotated	POLK	5	+	2610	44	2	4	1.95%	5.570341521	0.000309112	0.000912852	1.87E-06
ENSG00000109854_20383104_20383205_33	novel	HTATIP2	11	+	99	60	3	0	36.36%	200.2560795	0.001621946	0.000954935	9.50E-06
ENSG00000112118_52284674_52264588_808	annotated	MCM3	6	-	2424	123	13	5	5.20%	16.76648982	5.09E-07	9.44E-08	4.65E-13
ENSG00000101255_388011_396690_358	annotated	TRIB3	20	+	1074	239	41	19	15.08%	73.52978113	6.62E-07	2.18E-09	1.86E-14
ENSG00000090273_26921849_26946181_331	annotated	NUDC	1	+	993	113	14	11	10.88%	37.60095268	1.10E-06	2.46E-06	2.39E-11
ENSG00000180902_241735225_241767969_521	annotated	D2HGDH	2	+	1563	17	3	10	1.15%	3.593847107	0.027657845	0.235737162	0.031142163
ENSG00000084072_39738901_39753355_301	annotated	PPIE	1	+	903	42	4	0	5.98%	15.36848982	0.000360295	8.58E-05	2.23E-07
ENSG00000181852_56214047_56206447_317	annotated	RNF41	12	-	951	30	6	4	4.10%	10.42342369	0.01553064	0.006519044	0.000518524
ENSG00000188986_137255510_137272928_580	annotated	NELFB	9	+	1740	109	16	8	7.59%	20.6988827	1.42E-06	2.45E-07	3.27E-12
ENSG00000197885_23910904_23893095_192	annotated	NKIRAS1	3	-	576	26	1	0	7.29%	14.91490592	0.000872559	0.000398115	2.27E-06
ENSG00000104427_78666149_78717493_325	annotated	ZC2HC1A	8	+	975	16	9	0	3.08%	5.422318459	0.179648419	0.002008257	0.003654173
ENSG00000110048_59615664_59576577_807	annotated	OSBP	11	-	2421	93	23	0	5.08%	12.69281098	5.73E-05	7.37E-09	8.30E-12
ENSG00000169118_64300448_64189406_376	annotated	CSNK1G1	15	-	1128	8	2	4	1.33%	2.343422206	0.080264768	0.078649604	0.023170289

ENSG00000136143_47996933_47988499_131	annotated	SUCLA2	13	-	393	15	0	0	4.58%	12.61154699	0.01300724	0.01300724	0.000821865
ENSG00000135052_86079317_86046564_104	annotated	GOLM1	9	-	312	74	7	3	20.19%	78.36944648	0.000126298	7.91E-05	7.24E-08
ENSG00000124422_21043760_21003031_513	annotated	USP22	17	-	1539	200	26	8	10.53%	42.93990008	1.80E-07	3.20E-10	8.01E-16
ENSG00000153406_4463818_4461780_106	annotated	NMRAL1	16	-	318	35	2	2	11.32%	36.36725971	0.003166711	0.000943084	1.83E-05
ENSG00000123066_115991347_115982351_533	novel	MED13L	12	-	1599	22	3	3	2.44%	4.546151147	0.008291198	0.005721603	0.000248571
ENSG00000077549_19484279_19339530_301	annotated	CAPZB	1	-	903	774	41	25	22.59%	283.2193124	3.63E-11	1.90E-11	8.32E-21
ENSG00000251357_23894805_23895106_68	Overlap_dORF	AP000350.4	22	+	204	1921	141	35	51.47%	3111.478835	1.48E-06	2.42E-06	3.12E-11
ENSG00000124155_45419234_45425826_434	dORF	PIGT	20	+	1302	310	28	3	15.44%	78.67203122	1.36E-11	4.20E-13	7.65E-23
ENSG00000099917_20554941_20586704_667	dORF	MED15	22	+	2001	28	1	2	1.95%	4.623603634	0.001029525	0.002513196	1.57E-05
ENSG00000124155_45420349_45425826_316	dORF	PIGT	20	+	948	259	20	2	16.46%	90.2736662	6.03E-10	1.31E-10	9.13E-19
ENSG00000124155_45416169_45419525_124	annotated	PIGT	20	+	372	85	3	4	12.90%	75.49977189	0.000473642	0.00126048	3.85E-06
ENSG00000283761_100058702_100082209_418	dORF	AC118553.2	1	+	1254	148	21	6	10.05%	38.99723653	1.41E-07	3.29E-08	4.65E-14
ENSG00000283761_99993624_100049931_195	annotated	AC118553.2	1	+	585	14	5	0	4.62%	7.907547753	0.078000663	0.002500085	0.001404042
ENSG00000168159_228487816_228493885_126	annotated	RNF187	1	+	378	128	8	4	24.60%	111.8891111	2.44E-06	2.51E-06	5.28E-11
ENSG00000119403_120866883_120857944_348	annotated	PHF19	9	-	1044	11	1	0	2.01%	3.48146345	0.007593099	0.007593099	0.000297833
ENSG00000131389_14472288_14485007_227	Overlap_dORF	SLC6A6	3	+	681	143	17	5	15.86%	69.38387951	3.73E-06	1.78E-07	6.55E-12
ENSG00000131389_14443635_14472313_359	annotated	SLC6A6	3	+	1077	190	72	15	15.04%	58.29181143	5.14E-06	6.33E-09	4.56E-13
ENSG00000156298_38563015_38687667_275	Overlap_dORF	TSPAN7	X	+	825	18	4	0	3.27%	7.209218861	0.061503838	0.003229477	0.001279093
ENSG00000181722_114351606_114339005_584	annotated	ZBTB20	3	-	1752	10	4	0	1.20%	1.885973351	0.095107389	0.005706018	0.003312541
ENSG00000147140_71294272_71297465_212	novel	NONO	X	+	636	95	7	6	11.79%	49.35556675	2.81E-05	1.82E-05	4.01E-09
ENSG00000106392_7182814_7243727_371	annotated	C1GALT1	7	+	1113	41	10	1	6.47%	12.17189917	0.000477403	1.44E-05	6.01E-08
ENSG00000135480_52233297_52248760_469	annotated	KRT7	12	+	1407	2890	261	101	40.72%	678.6930454	1.00E-28	3.67E-31	7.94E-58
ENSG00000138074_27212205_27211486_77	uORF	SLC5A6	2	-	231	8	4	0	6.49%	11.44320454	0.169362074	0.019679754	0.016445545
ENSG00000026508_35139304_35176744_78	annotated	CD44	11	+	234	861	70	20	43.59%	1215.785467	3.21E-05	4.45E-06	1.21E-09
ENSG00000198331_125899408_125900268_286	annotated	HYLS1	11	+	858	70	11	0	7.34%	26.95754916	0.000500733	2.72E-05	1.11E-07
ENSG00000049618_156935469_157207891_1606	annotated	ARID1B	6	+	4818	18	6	2	0.62%	1.234455284	0.074038859	0.01169877	0.004324138
ENSG00000141576_76240240_76145235_680	annotated	RNF157	17	-	2040	8	0	0	0.74%	1.295774632	0.019679754	0.019679754	0.001784964
ENSG00000146409_132787493_132770270_309	annotated	SLC18B1	6	-	927	22	3	2	4.21%	7.841742918	0.005926165	0.002999187	9.86E-05
ENSG00000168216_69790322_69676158_467	novel	LMBRD1	6	-	1401	124	26	0	9.21%	29.24510625	1.30E-05	4.62E-09	9.94E-13
ENSG00000184500_93973749_93874379_556	annotated	PROS1	3	-	1668	29	5	0	2.34%	5.744756236	0.004949029	0.000669869	2.14E-05
ENSG00000103423_4425882_4442283_98	annotated	DNAJA3	16	+	294	30	1	17	6.12%	33.71658481	0.016983117	0.237137175	0.022462032
ENSG00000103423_4425992_4455568_300	Overlap_dORF	DNAJA3	16	+	900	69	32	1	7.67%	25.33239405	0.005436316	1.17E-05	8.25E-07
ENSG00000108433_46923193_46938760_210	annotated	GOSR2	17	+	630	39	8	0	6.67%	20.45472812	0.012656337	0.000448121	4.25E-05
ENSG00000184009_81511106_81510690_107	novel	ACTG1	17	-	321	719	52	13	28.04%	740.1052956	5.64E-06	8.06E-07	4.10E-11
ENSG00000184009_81512354_81511129_286	novel	ACTG1	17	-	858	1567	486	96	28.67%	603.4639933	3.68E-08	2.25E-12	1.67E-18
ENSG00000108433_46923193_46966724_257	annotated	GOSR2	17	+	771	40	7	0	5.45%	17.14254377	0.011149146	0.000448121	3.68E-05
ENSG00000075303_87876195_87858740_92	uORF	SLC25A40	7	-	276	23	1	5	6.52%	27.53521093	0.017143984	0.037440157	0.002923797
ENSG00000140332_70094543_70050097_698	annotated	TLE3	15	-	2094	24	0	2	1.43%	3.787077721	0.00224099	0.008605408	0.000110331
ENSG00000140297_59618239_59619555_438	annotated	GCNT3	15	+	1314	247	28	8	12.33%	62.11138903	2.13E-08	5.12E-10	1.29E-16
ENSG00000108107_55386358_55388216_163	annotated	RPL28	19	+	489	901	134	39	15.95%	608.8153385	0.000133428	2.85E-05	2.91E-08
ENSG00000142166_33325059_33348587_235	annotated	IFNAR1	21	+	705	13	3	0	2.55%	6.092897737	0.071345384	0.012798403	0.004461831
ENSG00000196535_29166541_29074770_1921	annotated	MYO18A	17	-	5763	51	9	2	1.46%	2.924093196	7.34E-05	6.55E-06	4.05E-09
ENSG00000221968_61877522_61873814_163	annotated	FADS3	11	-	489	9	1	5	3.68%	6.081396278	0.026832696	0.109745779	0.012786835
ENSG00000010278_6200500_6237828_228	annotated	CD9	12	+	684	1188	113	89	42.54%	573.8917646	1.98E-16	9.37E-18	2.89E-32
ENSG00000103534_19460312_19497966_631	annotated	TMC5	16	+	1893	19	2	1	1.27%	3.316443788	0.017321761	0.006965909	0.000613451
ENSG00000153551_32452422_32454254_21	dORF	CMTM7	3	+	63	31	0	0	28.57%	162.5888645	0.013853925	0.013853925	0.000925205
ENSG00000153551_32391907_32452089_151	annotated	CMTM7	3	+	453	28	3	0	6.62%	20.42346771	0.008661718	0.002362849	0.000116415
ENSG00000120549_24473393_24545118_877	annotated	KIAA1217	10	+	2631	17	1	1	1.14%	2.134999251	0.003722062	0.003722062	7.69E-05
ENSG00000120727_139363785_139368798_67	annotated	PAIP2	5	+	201	42	9	0	14.93%	69.04351396	0.006649741	0.00244612	9.19E-05
ENSG00000102743_40793227_40809667_301	annotated	SLC25A15	13	+	903	19	0	0	3.32%	6.952412061	0.002254349	0.002254349	2.95E-05
ENSG00000125505_54183527_54174044_310	annotated	MBOAT7	19	-	930	135	28	1	10.65%	47.96456097	6.52E-05	2.48E-07	1.92E-10
ENSG00000158987_131635030_131464037_827	annotated	RAPGEF6	5	-	2481	13	6	0	0.73%	1.731355463	0.168624199	0.012798403	0.012003794
ENSG00000120709_138341336_138346959_392	annotated	FAM53C	5	+	1176	15	0	0	1.53%	4.214573101	0.01300724	0.01300724	0.000821865
ENSG00000158019_27894557_27929916_70	novel	BABAM2	2	+	210	24	1	0	11.43%	37.76257498	0.008316137	0.005554612	0.00024255
ENSG00000090263_141010603_141006430_96	annotated	MRPS33	7	-	288	153	35	5	21.88%	175.5369697	0.000327195	4.15E-05	1.04E-07
ENSG00000135679_68839304_68839849_181	dORF	MDM2	12	+	543	15	0	4	3.87%	9.12769423	0.008473674	0.069273208	0.003110985
ENSG00000150527_39234115_39350264_1339	annotated	MIA2	14	+	4017	57	7	0	1.72%	4.688594542	7.21E-05	1.08E-05	6.30E-09
ENSG00000104626_9003064_9009044_99	annotated	ERI1	8	+	297	37	7	0	5.05%	41.16374967	0.022600676	0.020613417	0.002121199
ENSG00000205133_92916989_92886622_86	annotated	TRIQK	8	-	258	21	3	0	10.47%	26.89485718	0.015147584	0.003327803	0.000279725
ENSG00000125844_17616818_17614182_150	novel	RRBP1	20	-	450	12	2	0	3.33%	8.811267496	0.069457177	0.020613417	0.006391023
ENSG00000170191_25623948_25615925_248	annotated	NANP	20	-	744	32	0	2	4.84%	14.21172177	0.000982952	0.003541644	2.13E-05
ENSG00000130770_28236184_28246637_75	annotated	ATP5IF1	1	+	225	86	23	6	26.67%	126.2948341	0.002486673	0.000142862	2.66E-06
ENSG00000105409_41986127_41966937_860	annotated	ATP1A3	19	-	2580	15	2	0	0.93%	1.921061227	0.013892891	0.005306578	0.000386014
ENSG00000105618_54118279_54131322_491	annotated	PRPF31	19	+	1473	117	10	7	7.94%	26.24537416	9.13E-07	1.56E-07	1.36E-12
ENSG00000147274_136879427_136872338_290	annotated	RBMX	X	-	870	20	1	0	3.79%	7.595920256	0.004004658	0.000888449	2.21E-05
ENSG00000183283_51240919_51242458_108	novel	DAZAP2	12	+	324	172	22	1	16.67%	175.4094918	0.000636172	9.50E-05	4.39E-07
ENSG00000014138_65262293_65297269_598	annotated	POLA2	11	+	1794	41	9	2	3.85%	7.551462528	0.0020173	5.12E-05	8.75E-07
ENSG00000126432_64318216_64321691_214	annotated	PRDX5	11	+	642	703	86	21	31.31%	361.8178183	2.86E-11	7.48E-13	2.85E-22

ENSG00000124920_61766142_61786143_1014	annotated	MYRF	11	+	3042	59	15	6	2.96%	6.408589525	0.00054572	2.57E-05	1.15E-07
ENSG00000140521_89329071_89323529_428	dORF	POLG	15	-	1284	11	2	3	1.17%	2.830722619	0.070008252	0.102951605	0.026316151
ENSG00000140521_89322848_89316751_466	dORF	POLG	15	-	1398	19	0	0	1.50%	4.490721095	0.007882058	0.007882058	0.000319636
ENSG00000029364_69399370_69461702_232	annotated	SLC39A9	14	+	696	117	6	2	12.50%	55.54516687	1.09E-06	1.18E-06	1.14E-11
ENSG00000029364_69398714_69398821_35	uORF	SLC39A9	14	+	105	16	0	9	14.29%	50.35009998	0.020613417	0.361337229	0.04510239
ENSG00000243943_27601422_27621461_351	annotated	ZNF512	2	+	1053	13	2	0	2.28%	4.079290508	0.0135992	0.005160369	0.000368527
ENSG00000100605_93115163_92941561_414	annotated	ITPK1	14	-	1242	55	4	6	4.35%	14.63223769	0.000821977	0.001050559	5.39E-06
ENSG00000136504_49798447_49827502_425	annotated	KAT7	17	+	1275	36	3	2	3.29%	9.329577349	0.000436224	0.000846273	2.40E-06
ENSG00000140025_89950124_89797243_100	annotated	EFCAB11	14	-	300	38	5	4	7.00%	41.85352061	0.056005225	0.042090579	0.009516873
ENSG00000159202_48910845_48916153_81	annotated	UBE2Z	17	+	243	26	6	7	11.11%	35.35385107	0.050452032	0.037011271	0.007691656
ENSG00000138674_82881936_82877998_144	novel	SEC31A	4	-	432	12	0	0	4.17%	9.178403642	0.011772051	0.011772051	0.000681273
ENSG00000113456_34914892_34908765_282	annotated	RAD1	5	-	846	29	2	3	3.55%	11.32654066	0.003500471	0.011179354	0.000214062
ENSG00000175387_47896756_47841827_467	annotated	SMAD2	18	-	1401	151	18	5	11.13%	35.61299229	1.08E-07	1.22E-09	1.57E-15
ENSG00000130726_58544758_58550553_753	annotated	TRIM28	19	+	2259	431	76	18	11.02%	63.0421031	1.11E-09	1.12E-13	2.37E-21
ENSG00000168575_42472366_42417803_644	annotated	SLC20A2	8	-	1932	56	3	4	2.80%	9.57746467	0.000293483	0.000265125	5.28E-07
ENSG00000119655_74493274_74480618_174	annotated	NPC2	14	-	522	299	71	1	20.69%	189.265013	1.03E-05	6.85E-08	8.05E-12
ENSG00000182871_45496503_45512398_503	annotated	COL18A1	21	+	1509	132	17	0	7.55%	28.90376018	3.35E-06	2.97E-08	1.13E-12
ENSG00000104365_42316253_42330979_475	dORF	IKBKB	8	+	1425	21	3	6	3.37%	4.869384669	0.00129704	0.003895439	3.02E-05
ENSG00000173230_121727014_121664480_3080	annotated	GOLGB1	3	-	9240	38	6	2	0.75%	1.358880539	0.000647885	5.00E-05	2.51E-07
ENSG00000161618_49453332_49470467_802	annotated	ALDH16A1	19	+	2406	17	5	0	1.00%	2.334656288	0.051323546	0.005306578	0.001533731
ENSG00000164237_10290762_10280453_245	annotated	CMBL	5	-	735	77	9	3	8.16%	34.61569374	0.000536706	0.000201887	7.39E-07
ENSG00000134294_46365266_46361111_344	annotated	SLC38A2	12	-	1032	960	152	37	30.81%	307.3697964	1.02E-14	1.31E-17	2.30E-30
ENSG00000168000_62705512_62690367_398	annotated	BSCL2	11	-	1194	119	51	2	8.79%	32.9315588	0.006736553	2.56E-07	5.85E-08
ENSG00000168000_62705595_62702488_118	Overlap_uORF	BSCL2	11	-	354	47	0	15	8.47%	43.86965809	0.002488365	0.018376712	0.00026737
ENSG00000108773_42121304_42113649_837	annotated	KAT2A	17	-	2511	48	9	8	2.99%	6.316320786	0.000610572	0.000521099	2.06E-06
ENSG00000134900_102657200_102678316_217	annotated	TPP2	13	+	651	22	4	1	4.61%	11.16635282	0.01299184	0.004911637	0.000336826
ENSG00000169714_129171757_129170453_177	annotated	CNBP	3	-	531	589	57	21	30.51%	366.513881	2.32E-10	3.01E-10	7.98E-19
ENSG00000172331_134661610_134679031_225	annotated	BPGM	7	+	675	29	10	0	4.00%	14.19593097	0.151657651	0.003327803	0.004064471
ENSG00000130021_7148071_7050296_171	annotated	PUDP	X	-	513	24	0	0	4.68%	15.45836403	0.005604867	0.005604867	0.000167525
ENSG00000119599_72945995_72958805_281	dORF	DCAF4	14	+	843	6	0	0	1.78%	2.351761787	0.016947427	0.016947427	0.001349898
ENSG00000110080_126406156_126414047_333	annotated	ST3GAL4	11	+	999	15	2	0	2.10%	4.961299266	0.028426897	0.008473674	0.001202275
ENSG00000134954_128490526_128462361_397	annotated	ETS1	11	-	1191	20	4	0	2.02%	5.548657114	0.038066506	0.005355751	0.001111773
ENSG00000141380_26087577_26079065_45	annotated	AC091021.1	18	-	135	11	2	0	11.11%	26.92331735	0.042234516	0.021084099	0.003945154
ENSG00000135473_56333094_56317609_1202	annotated	PAN2	12	-	3606	29	11	1	1.25%	2.657308209	0.03296431	0.00042095	0.000125578
ENSG00000108788_42567125_42571603_214	annotated	MLX	17	+	642	57	7	2	6.54%	29.33657987	0.009806659	0.001344478	8.09E-05
ENSG00000136754_26860848_26748570_491	annotated	ABI1	10	-	1473	64	8	6	5.70%	14.35644399	6.55E-05	2.29E-05	1.16E-08
ENSG00000108094_35071317_35010311_745	annotated	CUL2	10	-	2235	37	2	1	2.95%	5.470082171	2.20E-05	2.25E-05	3.87E-09
ENSG00000072041_84873319_84861859_316	annotated	SLC6A15	12	-	948	43	2	0	5.38%	14.98751987	0.000226663	0.000124817	1.99E-07
ENSG00000138085_27213252_27215396_40	novel	ATRAID	2	+	120	293	23	15	25.00%	806.7816801	0.005303083	0.008052106	0.000225206
ENSG00000170471_38488433_38574967_1491	annotated	RALGAPB	20	+	4473	28	2	5	1.07%	2.068372652	0.000534963	0.002571927	8.87E-06
ENSG00000155330_46831149_46802952_154	annotated	C16orf87	16	-	462	11	5	0	3.25%	7.867203122	0.117097152	0.019216965	0.010582135
ENSG00000170471_38488433_38574967_1494	annotated	RALGAPB	20	+	4482	28	2	5	1.07%	2.064219293	0.000534963	0.002571927	8.87E-06
ENSG00000161904_33789116_33772628_503	annotated	LEMD2	6	-	1509	101	16	8	6.36%	22.11575589	4.93E-06	5.21E-06	2.15E-10
ENSG00000197563_62161260_62045856_900	annotated	PIGN	18	-	2700	90	12	0	3.89%	11.01408437	1.48E-05	8.70E-08	1.49E-11
ENSG00000188312_92325999_92613149_288	annotated	CENPP	9	+	864	11	1	0	3.12%	4.206768336	0.006467047	0.002478943	9.04E-05
ENSG00000074219_49360075_49341324_450	annotated	TEAD2	19	-	1350	22	6	0	2.89%	5.38466347	0.01219545	0.000577025	5.02E-05
ENSG00000128524_128862905_128865578_147	annotated	ATP6V1F	7	+	441	116	5	0	12.93%	86.91386306	0.000171259	6.26E-05	7.84E-08
ENSG00000026103_88990877_89012001_132	annotated	FAS	10	+	396	16	5	0	6.82%	13.3504053	0.0682837	0.002943991	0.001349864
ENSG00000106268_2244575_2251001_156	annotated	NUDT1	7	+	468	89	4	2	14.10%	62.8367634	1.82E-05	2.11E-05	3.03E-09
ENSG00000100749_96860574_96860852_92	annotated	VRK1	14	+	276	15	0	0	5.43%	17.95774626	0.021557223	0.021557223	0.002115616
ENSG00000165795_21021845_21017596_245	Overlap_dORF	NDRG2	14	-	735	16	0	2	3.27%	7.192871426	0.005454749	0.020715809	0.000593634
ENSG00000141552_81894478_81900065_84	annotated	ANAPC11	17	+	252	694	48	29	25.00%	909.9731611	0.000200778	0.00015937	2.23E-07
ENSG00000122085_241099852_241095998_360	annotated	MTERF4	2	-	1080	9	0	3	1.67%	2.753521093	0.011772051	0.097937785	0.005938109
ENSG00000164713_98281796_98292690_98	annotated	BRI3	7	+	294	36	4	2	14.29%	40.45990177	0.00182368	0.000664488	7.62E-06
ENSG00000076248_109098327_109105098_258	annotated	UNG	12	+	774	122	13	0	10.47%	52.08210439	4.85E-06	2.52E-06	1.04E-10
ENSG00000060982_24902988_24818008_398	annotated	BCAT1	12	-	1194	30	2	0	3.77%	8.302073646	0.001434496	0.000249257	2.44E-06
ENSG00000148834_104255213_104267405_213	annotated	GSTO1	10	+	639	331	34	7	23.00%	171.1578369	5.99E-09	3.13E-09	2.00E-16
ENSG00000149600_32743337_32703382_182	annotated	COMMD7	20	-	546	38	2	1	8.24%	22.99643989	0.000408384	0.000284266	7.79E-07
ENSG00000083845_58388138_58394750_204	annotated	RPS5	19	+	612	1132	88	51	26.47%	611.1737013	3.15E-10	1.01E-09	3.60E-18
ENSG00000068796_62306473_62385569_744	annotated	KIF2A	5	+	2232	54	10	10	3.49%	7.994093495	0.000314361	0.00063535	1.33E-06
ENSG00000185339_30610930_30626521_359	annotated	TCN2	22	+	1077	31	7	0	3.62%	9.510769234	0.015036239	0.000662934	7.12E-05
ENSG00000163001_55519802_55545008_313	annotated	CFAP36	2	+	939	30	5	1	5.11%	10.55663039	0.001894943	0.000280249	3.62E-06
ENSG00000005022_119470375_119471058_98	novel	SLC25A5	X	+	294	467	34	14	29.59%	524.8548368	1.35E-05	3.85E-06	4.36E-10
ENSG00000170791_56214614_56217435_85	annotated	CHCHD7	8	+	255	17	5	4	9.41%	22.02816874	0.004149608	0.055297605	0.001373491
ENSG00000100532_54509960_54538383_332	annotated	CGRRF1	14	+	996	15	1	3	1.81%	4.976242938	0.019795882	0.044843011	0.003962908
ENSG00000013441_200861862_200861232_131	novel	CLK1	2	-	393	10	1	1	4.58%	8.407697993	0.011772051	0.011772051	0.000681273
ENSG00000159479_43387505_43386545_89	novel	MED8	1	-	267	29	6	2	8.99%	35.88858952	0.028426897	0.016025708	0.002101493
ENSG00000105854_95434951_95416188_84	annotated	PON2	7	-	252	969	182	26	45.24%	1270.553304	6.72E-06	5.16E-08	3.95E-12

ENSG00000124191_44051432_44068686_327	annotated	TOX2	20	+	981	10	0	0	1.53%	3.36822152	0.020613417	0.020613417	0.001946209
ENSG00000128563_102376289_102425111_127	annotated	PRKRIP1	7	+	381	44	5	0	7.87%	38.15903246	0.006487086	0.002488365	9.10E-05
ENSG00000156261_29073590_29056475_548	annotated	CCT8	21	-	1644	406	50	22	13.50%	81.60069807	3.04E-11	3.66E-12	1.40E-21
ENSG00000068028_50337289_50330581_260	annotated	RASSF1	3	-	780	16	0	3	2.69%	6.777898074	0.008473674	0.044843011	0.001935645
ENSG00000091542_18184292_18208396_378	annotated	ALKBH5	17	+	1134	83	1	1	7.94%	24.18436515	8.95E-07	8.95E-07	7.20E-12
ENSG00000121988_135504489_135271315_428	annotated	ZRANB3	2	-	1284	20	3	0	1.87%	5.146768397	0.015358648	0.005554612	0.000444679
ENSG00000184436_21002096_21000771_87	Overlap_uORF	THAP7	22	-	261	15	1	2	6.90%	18.98980064	0.013428348	0.037178614	0.002349204
ENSG00000137821_70873206_71049612_676	annotated	LRRC49	15	+	2028	12	0	0	0.74%	1.955162906	0.019679754	0.019679754	0.001784964
ENSG00000130164_11089549_11131316_682	annotated	LDLR	19	+	2046	486	49	21	16.57%	78.4874634	1.30E-17	5.25E-18	1.05E-33
ENSG00000164327_39074377_38942304_1708	annotated	RICTOR	5	-	5124	57	6	7	1.35%	3.675660475	0.000175106	0.000290887	3.52E-07
ENSG00000175221_891131_868373_860	annotated	MED16	19	-	2580	36	1	2	2.09%	4.610546946	0.000117875	6.99E-05	6.01E-08
ENSG00000104695_30794072_30791193_92	annotated	PPP2CB	8	-	276	76	4	0	8.70%	90.98591437	0.007400806	0.005808022	0.000225904
ENSG00000089177_16528406_16273253_1239	annotated	KIF16B	20	-	3717	32	4	2	1.13%	2.844638417	0.002051131	0.000721766	9.25E-06
ENSG00000176390_30824651_30784187_442	annotated	CRLF3	17	-	1326	30	9	0	3.39%	7.475622876	0.009284659	0.000284266	2.05E-05
ENSG00000188603_28491764_28489349_55	novel	CLN3	16	-	165	18	0	12	14.55%	36.0460943	0.005554612	0.310595281	0.015978689
ENSG00000188603_28489349_28482459_253	novel	CLN3	16	-	759	115	13	4	10.28%	50.06401987	4.09E-05	3.60E-06	1.27E-09
ENSG00000188603_28477608_28477516_30	novel	CLN3	16	-	90	23	5	1	23.33%	84.44131351	0.027657845	0.010042643	0.001354611
ENSG00000141956_41860336_41801240_1132	annotated	PRDM15	21	-	3396	14	1	0	0.62%	1.362165912	0.011306644	0.007882058	0.000451174
ENSG00000105676_19051622_19057628_408	annotated	ARMC6	19	+	1224	42	2	3	4.66%	11.33802803	7.47E-05	0.000366328	2.00E-07
ENSG00000168101_4693778_4695179_192	annotated	NUDT16L1	16	+	576	25	6	0	3.65%	14.34125569	0.060777753	0.008776163	0.002767513
ENSG00000163071_52051705_52094887_538	annotated	SPATA18	4	+	1614	14	0	0	1.30%	2.866118609	0.008175611	0.008175611	0.000342518
ENSG00000255872_38068321_38015425_189	annotated	AL138752.2	9	-	567	16	4	2	3.70%	9.324092589	0.043639483	0.043639483	0.007798642
ENSG00000255872_38015383_37899929_179	dORF	AL138752.2	9	-	537	23	3	0	2.79%	14.15217545	0.068289709	0.021557223	0.006517186
ENSG00000068024_239190027_239053097_1036	annotated	HDAC4	2	-	3108	20	0	1	1.16%	2.126271114	0.000866459	0.001612025	8.62E-06
ENSG00000074319_18483977_18481501_158	novel	TSG101	11	-	474	17	8	1	6.33%	11.85059711	0.066656377	0.004004658	0.001659678
ENSG00000196704_68251115_68256969_186	dORF	AMZ2	17	+	558	79	7	8	10.22%	46.78025082	7.23E-05	0.000468646	2.49E-07
ENSG00000163960_196407325_196356685_442	annotated	UBXN7	3	-	1326	24	5	2	3.39%	5.980498301	0.004389517	0.000800452	2.21E-05
ENSG00000115091_113916937_113957455_367	annotated	ACTR3	2	+	1101	141	9	14	11.44%	42.315692	4.89E-08	2.32E-07	1.12E-13
ENSG00000134330_9474567_9478295_40	annotated	IAH1	2	+	120	14	1	5	12.50%	38.5492953	0.019679754	0.051235217	0.004505595
ENSG00000134330_9481342_9488329_135	dORF	IAH1	2	+	405	99	7	1	20.00%	80.76995205	7.66E-06	2.44E-06	1.60E-10
ENSG00000266173_63728369_63703599_336	annotated	STRADA	17	-	1008	13	2	2	1.79%	4.261401691	0.012798403	0.031982531	0.001936542
ENSG00000108433_46923193_46940613_197	annotated	GOSR2	17	+	591	20	0	0	4.06%	11.18181154	0.005306578	0.005306578	0.000151001
ENSG00000124155_45424269_45425826_121	annotated	PIGT	20	+	363	30	3	1	5.79%	27.3076472	0.010275151	0.010275151	0.000527329
ENSG00000117298_21290156_21219955_753	annotated	ECE1	1	-	2259	364	28	8	12.35%	53.24205459	1.38E-14	1.39E-16	2.98E-29
ENSG00000077549_19448914_19339567_260	annotated	CAPZB	1	-	780	686	58	32	19.62%	290.6023799	9.66E-07	5.07E-08	4.98E-13
ENSG00000077549_19485438_19339530_272	annotated	CAPZB	1	-	816	797	49	25	25.74%	322.7288692	1.73E-11	8.17E-12	1.73E-21
ENSG00000175193_183884846_183829598_379	annotated	PARL	3	-	1137	43	13	3	4.75%	12.49619071	0.014424689	0.000566655	5.97E-05
ENSG00000175193_183884869_183884747_40	Overlap_uORF	PARL	3	-	120	11	3	3	12.50%	30.28873202	0.121144048	0.121144048	0.049102245
ENSG00000130985_47203586_47206337_78	novel	UBA1	X	+	234	9	0	0	7.69%	12.70855889	0.009815329	0.009815329	0.000483714
ENSG00000182093_39393175_39396939_59	novel	WRB	21	+	177	54	12	1	25.42%	100.8068739	0.000815603	0.000295633	1.60E-06
ENSG00000132768_43970185_43971224_169	novel	DPH2	1	+	507	7	1	1	2.96%	4.562046781	0.047790352	0.047790352	0.009211063
ENSG00000091039_76410578_76355889_798	annotated	OSBPL8	12	-	2394	40	7	1	2.38%	5.520844296	0.000820959	7.67E-05	4.72E-07
ENSG00000187109_76074219_76048429_391	annotated	NAP1L1	12	-	1173	198	34	18	11.00%	55.7746472	1.87E-06	2.10E-07	3.74E-12
ENSG00000118596_59771299_59779679_379	annotated	SLC16A7	12	+	1137	21	0	0	3.43%	6.102790812	0.000583356	0.000583356	2.20E-06
ENSG00000164073_127965133_127921759_269	annotated	MFSD8	4	-	807	13	0	1	2.60%	5.322791703	0.008373824	0.013794764	0.000578344
ENSG00000140396_70162813_70113632_1173	annotated	NCOA2	8	-	3519	23	4	0	1.02%	2.159624386	0.011525313	0.00087769	6.69E-05
ENSG00000131002_19587225_19591938_177	novel	TXLNGY	Y	+	531	12	2	2	2.82%	7.467175844	0.020613417	0.052029617	0.004760359
ENSG00000025800_32108131_32160639_186	annotated	KPNA6	1	+	558	32	5	0	5.91%	18.94896236	0.00972614	0.001558047	9.11E-05
ENSG00000025800_32161975_32170894_311	dORF	KPNA6	1	+	933	26	9	3	3.54%	9.207916194	0.035596039	0.008742337	0.00155761
ENSG00000114054_136250376_136333476_550	annotated	PCCB	3	+	1650	94	16	1	6.91%	18.82407147	6.69E-06	2.37E-08	1.96E-12
ENSG00000159176_201496303_201490172_94	novel	CSRP1	1	-	282	341	35	2	22.34%	399.5534862	6.76E-05	2.87E-05	1.48E-08
ENSG00000136068_58168574_58170762_158	novel	FLNB	3	+	474	5	1	1	3.16%	3.485469738	0.051235217	0.051235217	0.010460668
ENSG00000141562_82458804_82478927_167	novel	NARF	17	+	501	22	1	0	5.99%	14.50957222	0.002321823	0.002321823	3.12E-05
ENSG00000100321_39364195_39377690_192	annotated	SYNGR1	22	+	576	38	10	3	9.90%	21.79870865	0.001627605	0.000357202	3.85E-06
ENSG00000197535_52425891_52313696_1697	annotated	MYO5A	15	-	5091	21	8	1	0.77%	1.362968602	0.054187382	0.001044243	0.000464309
ENSG00000119705_77708112_77717494_92	annotated	SLIRP	14	+	276	503	63	28	38.04%	602.1830911	6.69E-07	1.18E-06	7.10E-12
ENSG00000275714_26020490_26020900_136	annotated	HIST1H3A	6	+	408	299	40	14	27.94%	242.1478843	5.86E-07	4.73E-07	2.54E-12
ENSG00000148516_31319235_31527264_1125	annotated	ZEB1	10	+	3375	10	0	0	0.62%	0.979029722	0.007593099	0.007593099	0.000297833
ENSG00000124357_71068684_71078508_344	annotated	NAGK	2	+	1032	91	17	2	6.69%	29.13609528	0.000965067	2.30E-05	1.95E-07
ENSG00000137714_110430121_110462468_184	annotated	FDX1	11	+	552	46	5	0	5.98%	27.53521093	0.002875906	0.001580611	2.67E-05
ENSG00000072958_16198027_16234435_435	annotated	AP1M1	19	+	1305	49	5	2	5.06%	12.40666975	3.39E-05	6.31E-05	1.62E-08
ENSG00000163072_168802647_168864970_563	annotated	NOSTRIN	2	+	1689	26	8	0	1.60%	5.086433279	0.13068706	0.003327803	0.003333927
ENSG00000156113_77184864_76877869_993	novel	KCNMA1	10	-	2979	52	8	0	2.62%	5.767697757	7.51E-05	3.18E-06	2.15E-09
ENSG00000115525_85848191_85847466_98	Overlap_dORF	ST3GAL5	2	-	294	24	12	5	7.14%	26.97326785	0.06845324	0.042090579	0.011516761
ENSG00000115525_85846502_85840144_177	dORF	ST3GAL5	2	-	531	43	4	1	9.60%	26.75738011	0.00076033	0.000186181	9.75E-07
ENSG00000115525_85863483_85847969_112	annotated	ST3GAL5	2	-	336	24	3	5	7.14%	23.60160937	0.021450603	0.032206895	0.003078374
ENSG00000183475_100612313_100648462_286	annotated	ASB7	15	+	858	14	2	0	2.45%	5.391509832	0.028902806	0.007882058	0.001149203
ENSG00000119912_92537525_92454444_978	annotated	IDE	10	-	2934	62	8	5	3.27%	6.982343875	6.78E-05	6.89E-06	3.91E-09

ENSG00000110315_10534158_10514916_230	annotated	RNF141	11	-	690	26	3	1	4.35%	12.45070407	0.00611134	0.002404282	8.31E-05
ENSG00000168476_22140684_22139155_210	novel	REEP4	8	-	630	107	13	6	10.00%	56.11938227	0.000166432	0.000292455	3.37E-07
ENSG00000078668_42394212_42396752_63	novel	VDAC3	8	+	189	45	96	1	14.29%	78.67203122	0.054560643	0.003684287	0.001231964
ENSG00000099341_38374881_38383390_257	annotated	PSMD8	19	+	771	153	13	21	15.56%	65.57022991	1.36E-07	1.18E-06	1.55E-12
ENSG00000112511_33412264_33415266_419	annotated	PHF1	6	+	1257	11	0	7	1.43%	2.89152573	0.012798403	0.217184882	0.016535204
ENSG00000112759_44229418_44233528_437	annotated	SLC29A1	6	+	1311	146	48	11	11.67%	36.79762742	9.25E-06	5.48E-09	7.79E-13
ENSG00000069956_52019251_52042759_43	novel	MAPK6	15	+	129	38	2	0	11.63%	97.33376886	0.021084099	0.021557223	0.002072352
ENSG00000170365_145514755_145557934_418	annotated	SMAD1	4	+	1254	14	4	0	2.15%	3.68892778	0.041018323	0.002943991	0.000744057
ENSG00000164347_74763715_74736764_504	annotated	GFM2	5	-	1512	61	5	7	5.56%	13.33053862	2.89E-05	4.29E-05	9.49E-09
ENSG00000132600_68339385_68357224_429	novel	PRMT7	16	+	1287	25	1	7	2.56%	6.418464085	0.001520872	0.016229229	0.000154399
ENSG00000140464_73998130_74034495_338	annotated	PML	15	+	1014	7	3	0	1.48%	2.28102339	0.139742684	0.016947427	0.011764961
ENSG00000067369_43492337_43407383_1931	annotated	TP53BP1	15	-	5793	65	3	5	1.35%	3.707485676	1.32E-05	2.22E-05	2.32E-09
ENSG00000100503_50821942_50725872_1339	annotated	NIN	14	-	4017	22	4	6	0.90%	1.809632981	0.011226498	0.012351267	0.000681837
ENSG00000135535_109382378_109368279_189	annotated	CD164	6	-	567	273	73	2	22.22%	159.0923298	5.95E-06	7.57E-09	6.29E-13
ENSG00000006634_87900321_87908163_414	dORF	DBF4	7	+	1242	18	0	1	1.93%	4.788732335	0.004826937	0.00419316	0.00011038
ENSG00000112715_43777480_43784562_166	annotated	VEGFA	6	+	498	40	1	3	6.02%	26.53996234	0.002851	0.008025782	0.00012745
ENSG00000142784_27261055_27306383_677	annotated	WDTC1	1	+	2031	11	4	1	1.03%	1.789585348	0.091912668	0.01551626	0.006854887
ENSG00000134954_128490526_128462361_397	annotated	ETS1	11	-	1191	20	4	0	2.02%	5.548657114	0.038066506	0.005355751	0.001111773
ENSG00000066084_50505141_50678911_382	novel	DIP2B	12	+	1146	9	3	0	1.57%	2.594941344	0.114974528	0.012178958	0.007325749
ENSG00000167779_53100855_53102167_81	annotated	IGFBP6	12	+	243	212	10	3	25.93%	288.2698625	2.94E-05	2.58E-05	5.87E-09
ENSG00000121864_179328294_179334422_485	annotated	ZNF639	3	+	1455	14	3	0	1.24%	3.179323323	0.012798403	0.01300724	0.000809468
ENSG00000071189_17821571_17794045_363	novel	SNX13	7	-	1089	10	1	1	2.20%	3.034183022	0.010460668	0.010460668	0.000545418
ENSG00000171953_18021823_18018549_110	novel	ATPAF2	17	-	330	12	0	0	4.55%	12.01536477	0.020613417	0.020613417	0.001946209
ENSG00000026297_166952520_166933896_131	novel	RNASET2	6	-	393	21	5	1	6.87%	17.65616578	0.031984458	0.002515381	0.000495063
ENSG00000134602_132023654_132074159_342	annotated	STK26	X	+	1026	19	0	0	2.63%	6.118935761	0.003606289	0.003606289	7.24E-05
ENSG00000066427_92096122_92080961_172	novel	ATXN3	14	-	516	30	0	0	6.98%	19.21061227	0.001040526	0.001040526	6.70E-06
ENSG00000099998_24244725_24219970_554	annotated	GGT5	22	-	1662	61	11	4	5.42%	12.12742142	5.43E-05	5.92E-06	2.70E-09
ENSG00000100228_23146617_23161564_267	annotated	RAB36	22	+	801	6	0	0	1.87%	2.475075139	0.016947427	0.016947427	0.001349898
ENSG00000099991_24042910_24178196_2103	annotated	CABIN1	22	+	6309	33	1	3	0.90%	1.728315664	7.82E-05	0.000430115	2.45E-07
ENSG00000185347_105491376_105498946_426	annotated	TEDC1	14	+	1278	76	10	0	6.57%	19.64954019	1.19E-05	1.62E-06	1.70E-10
ENSG00000185347_105491376_105498946_495	annotated	TEDC1	14	+	1485	76	10	0	5.66%	16.91051338	1.19E-05	1.62E-06	1.70E-10
ENSG00000185347_105491103_105498946_352	annotated	TEDC1	14	+	1056	74	10	0	7.39%	23.15460919	2.30E-05	3.66E-06	7.15E-10
ENSG00000249115_35612795_35614110_89	annotated	HAUS5	19	+	267	12	0	0	5.62%	14.85045084	0.019679754	0.019679754	0.001784964
ENSG00000249115_35615123_35622993_533	dORF	HAUS5	19	+	1599	22	9	2	2.81%	4.546151147	0.04130915	0.00085483	0.000285346
ENSG00000100796_91509647_91466415_540	annotated	PPP4R3A	14	-	1620	48	13	5	4.44%	9.790297218	0.000964638	0.000100389	7.12E-07
ENSG00000064042_41605996_41697185_1467	annotated	LIMCH1	4	+	4401	109	34	10	3.14%	8.183607337	0.000129032	7.27E-08	1.47E-10
ENSG00000149806_65121813_65121492_75	annotated	FAU	11	-	225	506	27	40	24.00%	743.0835589	0.000194777	0.000678234	9.05E-07
ENSG00000197070_137605718_137613149_123	annotated	ARRDC1	9	+	369	27	0	0	8.13%	24.17725837	0.002404282	0.002404282	3.34E-05
ENSG00000104388_60517208_60620769_212	annotated	RAB2A	8	+	636	102	26	4	11.32%	52.99229273	0.000989217	2.98E-05	2.50E-07
ENSG00000124333_155889467_155942139_260	annotated	VAMP7	X	+	780	14	8	0	2.69%	5.930660815	0.190378872	0.007882058	0.099983433
ENSG00000076650_33094203_33130160_769	annotated	GPATCH1	19	+	2307	18	1	0	1.04%	2.578069163	0.009341698	0.005355751	0.000262679
ENSG00000120705_138542918_138513681_95	annotated	ETF1	5	-	285	40	4	2	15.79%	46.37509209	0.000759415	0.000299491	1.51E-06
ENSG00000102125_154411916_154421004_224	annotated	TAZ	X	+	672	24	1	1	3.57%	11.80080468	0.010079165	0.010079165	0.000508526
ENSG00000148180_121301972_121332603_731	annotated	GSN	9	+	2193	69	7	5	3.69%	10.39633135	7.24E-05	1.83E-05	1.03E-08
ENSG00000145817_144169955_144164075_154	novel	YIPF5	5	-	462	32	2	9	7.79%	22.88640908	0.003182738	0.037701781	0.000720122
ENSG00000129055_134482904_134478590_74	novel	ANAPC13	3	-	222	93	7	4	29.73%	138.4202495	0.000124939	4.91E-05	4.55E-08
ENSG00000127948_75982288_75986481_415	annotated	POR	7	+	1245	262	29	5	13.25%	69.53470133	5.68E-09	5.52E-11	4.01E-18
ENSG00000031823_5978082_5917610_562	annotated	RANBP3	19	-	1686	51	9	0	3.38%	9.994987596	0.000374172	5.82E-05	1.62E-07
ENSG00000109099_15260727_15239412_125	annotated	PMP22	17	-	375	205	32	0	28.80%	180.6309837	3.21E-06	7.85E-08	2.62E-12
ENSG00000096063_35921056_35835304_655	annotated	SRPK1	6	-	1965	55	11	6	3.66%	9.248467792	0.000286838	0.000103515	2.11E-07
ENSG00000187109_76074219_76048429_391	annotated	NAP1L1	12	-	1173	198	34	18	11.00%	55.7746472	1.87E-06	2.10E-07	3.74E-12
ENSG00000167978_2761969_2770867_2272	annotated	SRRM2	16	+	6816	147	26	16	3.04%	7.126190152	7.10E-09	2.02E-10	1.71E-17
ENSG00000163738_74158139_74174511_60	uORF	MTHFD2L	4	+	180	14	3	1	8.33%	25.6995302	0.086207551	0.035374934	0.012460195
ENSG00000151532_112447374_112815383_217	annotated	VTI1A	10	+	651	17	5	7	4.61%	8.628545359	0.031423484	0.044978913	0.005961824
ENSG00000168917_136854605_136855699_364	annotated	SLC35G2	3	+	1092	26	5	1	3.85%	7.867203122	0.005965858	0.000617933	2.43E-05
ENSG00000196136_94614451_94623814_420	annotated	SERPINA3	14	+	1260	8	0	0	1.43%	2.097920832	0.011570466	0.011570466	0.000659484
ENSG00000120708_136055749_136056455_24	dORF	TGFBI	5	+	72	161	11	4	41.67%	738.8614932	0.003792958	0.003792958	7.97E-05
ENSG00000163512_28340617_28331899_232	annotated	AZI2	3	-	696	14	1	7	3.45%	6.646430224	0.005160369	0.114955097	0.003876984
ENSG00000140688_31508373_31490835_466	annotated	C16orf58	16	-	1398	129	6	9	7.94%	30.4896327	5.36E-08	5.04E-07	2.69E-13
ENSG00000067225_72206790_72203018_77	novel	PKM	15	-	231	372	21	5	14.29%	532.1090111	0.009228435	0.008114228	0.000381513
ENSG00000174197_41669483_41767280_2869	annotated	MGA	15	+	8607	21	0	6	0.49%	0.806189515	0.00035774	0.012463746	3.47E-05
ENSG00000131389_14443635_14485007_620	annotated	SLC6A6	3	+	1860	363	41	20	15.48%	64.48568752	2.33E-14	8.79E-16	3.00E-28
ENSG00000160404_127735270_127733216_253	annotated	TOR2A	9	-	759	15	0	2	3.56%	6.530089548	0.002967235	0.010079165	0.000166355
ENSG00000160404_127735270_127732034_321	annotated	TOR2A	9	-	963	25	0	2	4.36%	8.577947329	0.000338646	0.00105275	2.35E-06
ENSG00000155792_119873934_120049704_380	annotated	DEPTOR	8	+	1140	19	0	6	2.11%	5.507042185	0.005554612	0.05393658	0.001681648
ENSG00000121864_179328294_179334422_485	annotated	ZNF639	3	+	1455	14	3	0	1.24%	3.179323323	0.012798403	0.01300724	0.000809468
ENSG00000148985_3811260_3825458_315	annotated	PGAP2	11	+	945	39	7	1	4.13%	13.63648541	0.004964704	0.000705712	2.25E-05
ENSG00000180104_453441_464448_458	annotated	EXOC3	5	+	1374	58	3	4	5.46%	13.94796711	8.93E-06	4.95E-05	3.60E-09

ENSG00000180104_465222_466898_116	dORF	EXOC3	5	+	348	9	2	1	7.76%	8.545410287	0.028889786	0.005706018	0.000872559
ENSG00000147677_116755797_116645006_352	annotated	EIF3H	8	-	1056	225	31	7	14.49%	70.40252794	4.40E-08	1.20E-09	6.09E-16
ENSG00000115904_39067669_38985824_1276	annotated	SOS1	2	-	3828	16	1	7	0.94%	1.38107641	0.001589662	0.067483894	0.000836597
ENSG00000147684_124539187_124549892_179	annotated	NDUFB9	8	+	537	341	32	9	26.82%	209.8213838	1.38E-08	8.46E-10	1.31E-16
ENSG00000170458_140633071_140631856_375	annotated	CD14	5	-	1125	19	0	0	2.93%	5.580469414	0.001384502	0.001384502	1.16E-05
ENSG00000214063_850341_866630_226	annotated	TSPAN4	11	+	678	377	52	6	28.32%	183.7305225	7.22E-11	2.87E-12	2.67E-21
ENSG00000091409_172467516_172501879_954	annotated	ITGA6	2	+	2862	283	24	9	8.28%	32.67280793	1.56E-12	2.06E-13	4.25E-24
ENSG00000157985_235744785_236124122_643	annotated	AGAP1	2	+	1929	10	0	2	0.93%	1.712921364	0.011570466	0.056853419	0.003221159
ENSG00000101849_9654265_9716246_526	annotated	TBL1X	X	+	1578	28	2	0	2.28%	5.863010692	0.001573472	0.000947024	9.15E-06
ENSG00000116337_109625404_109631152_761	dORF	AMPD2	1	+	2283	8	6	0	0.79%	1.15785381	0.280084718	0.011570466	0.021802746
ENSG00000173039_65662832_65654378_551	annotated	RELA	11	-	1653	106	12	3	5.81%	21.18861966	2.37E-05	1.05E-06	2.33E-10
ENSG00000243667_68144696_68134701_187	annotated	WDR92	2	-	561	18	1	1	3.74%	10.60179244	0.013892891	0.013892891	0.000930096
ENSG00000143801_226883858_226895579_350	annotated	PSEN2	1	+	1050	51	7	0	4.86%	16.04909437	0.000327658	0.000130272	2.99E-07
ENSG00000124155_45420633_45425826_187	dORF	PIGT	20	+	561	59	5	1	8.56%	34.75031967	0.000215514	0.000196974	2.94E-07
ENSG00000124155_45416169_45420543_296	annotated	PIGT	20	+	888	171	13	8	13.85%	63.62866309	3.87E-07	8.74E-08	3.28E-13
ENSG00000116750_193059230_193020343_316	annotated	UCHL5	1	-	948	75	3	1	7.28%	26.14102303	1.12E-05	1.41E-05	1.28E-09
ENSG00000149480_62601450_62593875_668	annotated	MTA2	11	-	2004	103	6	1	6.14%	16.98279476	7.64E-08	1.14E-08	9.03E-15
ENSG00000149483_61366091_61368709_104	annotated	TMEM138	11	+	312	61	34	4	16.35%	64.60184102	0.078364698	0.000207962	0.000234999
ENSG00000149483_61364356_61366169_95	Overlap_uORF	TMEM138	11	+	285	46	4	20	12.63%	53.3313559	0.001946488	0.109098094	0.001796202
ENSG00000221968_61891381_61873814_445	annotated	FADS3	11	-	1335	53	11	15	5.17%	13.11789824	0.000234439	0.000614699	9.74E-07
ENSG00000204356_31958446_31952301_380	annotated	NELFE	6	-	1140	223	8	9	15.79%	64.6352846	2.87E-11	1.34E-10	4.59E-20
ENSG00000204356_31958446_31952301_380	annotated	NELFE	6	-	1140	223	8	9	15.79%	64.6352846	2.87E-11	1.34E-10	4.59E-20
ENSG00000064651_128158069_128171766_147	novel	SLC12A2	5	+	441	29	8	1	8.84%	21.72846576	0.005234647	0.000980148	3.17E-05
ENSG00000103253_727510_729168_290	annotated	HAGHL	16	+	870	22	4	18	3.79%	8.355512281	0.01282155	0.164974643	0.011700116
ENSG00000136718_130346361_130346468_35	novel	IMP4	2	+	105	15	0	1	20.00%	47.20321873	0.008473674	0.008473674	0.000366502
ENSG00000136718_130345844_130346179_83	novel	IMP4	2	+	249	18	0	0	7.23%	23.8859661	0.013428348	0.013428348	0.000872559
ENSG00000163938_52686792_52694275_537	annotated	GNL3	3	+	1611	79	24	0	5.03%	16.20321537	0.000946368	2.37E-06	2.78E-08
ENSG00000196968_73772334_73775515_476	annotated	FUT11	10	+	1428	27	8	1	2.73%	6.247484832	0.024969202	0.000980148	0.000174707
ENSG00000132470_75724704_75757555_1822	annotated	ITGB4	17	+	5466	474	67	15	7.85%	28.65354551	3.55E-19	5.21E-24	4.40E-41
ENSG00000120727_139363785_139368798_127	annotated	PAIP2	5	+	381	187	24	2	23.62%	162.175888	7.34E-06	1.64E-06	1.05E-10
ENSG00000143061_116616278_116577312_1140	annotated	IGSF3	1	-	3420	36	11	4	1.58%	3.478131906	0.007102339	0.00097077	4.33E-05
ENSG00000069702_91797406_91683739_809	annotated	TGFBR3	1	-	2427	39	2	6	2.97%	5.309632762	2.61E-05	0.000294428	6.09E-08
ENSG00000100416_46346542_46357006_243	dORF	TRMU	22	+	729	9	0	3	2.47%	4.079290508	0.009815329	0.080264768	0.004118547
ENSG00000100416_46335765_46346473_99	annotated	TRMU	22	+	297	12	2	0	7.07%	13.3504053	0.023672501	0.007882058	0.000936943
ENSG00000155508_154863279_154867734_117	annotated	CNOT8	5	+	351	15	7	2	6.84%	14.12062099	0.189368274	0.030313544	0.025642234
ENSG00000104365_42322414_42329427_170	novel	IKBKB	8	+	510	8	1	4	2.94%	5.183098527	0.047790352	0.139742684	0.025994162
ENSG00000008952_169966823_169982714_101	annotated	SEC62	3	+	303	40	4	0	11.88%	43.62013612	0.005554612	0.001023866	3.51E-05
ENSG00000103174_5028175_5027174_149	novel	NAGPA	16	-	447	15	0	1	5.37%	11.0880044	0.005405142	0.008170681	0.00023249
ENSG00000132612_69321103_69322037_75	novel	VPS4A	16	+	225	25	2	0	13.33%	36.71361457	0.009621867	0.002227676	0.000123102
ENSG00000139684_46781549_46777435_113	annotated	ESD	13	-	339	84	17	5	14.16%	81.87460948	0.001570009	0.000207012	2.27E-06
ENSG00000110711_67489366_67490585_178	annotated	AIP	11	+	534	18	10	4	6.74%	11.13783813	0.123365516	0.01294611	0.008324473
ENSG00000148985_3811260_3825458_254	annotated	PGAP2	11	+	762	39	7	1	5.12%	16.91138939	0.004964704	0.000705712	2.25E-05
ENSG00000138867_24555825_24543006_295	annotated	GUCD1	22	-	885	28	3	19	3.73%	10.45404618	0.010677183	0.414088267	0.037457019
ENSG00000146674_45921140_45914820_297	annotated	IGFBP3	7	-	891	1017	73	61	26.60%	377.1489497	4.04E-12	1.25E-11	6.29E-22
ENSG00000060642_26790816_26797844_493	annotated	PIGV	1	+	1479	77	5	11	6.69%	17.20252528	2.68E-07	3.23E-05	9.48E-11
ENSG00000162337_68312715_68403486_471	annotated	LRP5	11	+	1413	119	23	0	9.55%	27.82751677	9.33E-06	1.98E-09	3.28E-13
ENSG00000162337_68406631_68449070_979	dORF	LRP5	11	+	2937	204	36	13	7.76%	22.95069675	1.09E-09	2.24E-13	4.36E-21
ENSG00000162627_98661732_98760131_396	annotated	SNX7	1	+	1188	41	3	3	3.28%	11.40347119	0.001390209	0.003042349	2.51E-05
ENSG00000172893_71444952_71444253_32	uORF	DHCR7	11	-	96	106	1	3	15.62%	364.8415448	0.021557223	0.037701781	0.003600199
ENSG00000106348_128409901_128393007_589	annotated	IMPDH1	7	-	1767	28	7	7	2.38%	5.235897494	0.013641843	0.015383659	0.001006554
ENSG00000163104_94208395_94289534_1026	annotated	SMARCAD1	4	+	3078	63	0	1	2.92%	6.763034263	5.76E-07	8.76E-07	4.57E-12
ENSG00000116885_36450321_36428320_207	annotated	OSCP1	1	-	621	5	2	0	2.42%	2.660406853	0.128419629	0.012673659	0.008587904
ENSG00000126858_32171069_32224733_597	annotated	RHOT1	17	+	1791	55	7	2	4.36%	10.1469789	0.000124051	1.23E-05	1.24E-08
ENSG00000147457_23246786_23256466_133	annotated	CHMP7	8	+	399	22	0	12	4.51%	18.21878618	0.013640586	0.19809812	0.015353416
ENSG00000147419_27765225_27748245_74	annotated	CCDC25	8	-	222	70	16	5	24.32%	104.1872846	0.001976402	0.00025821	3.51E-06
ENSG00000104490_101719629_101689309_193	annotated	NCALD	8	-	579	16	5	0	4.66%	9.130847147	0.038516527	0.003229477	0.000746335
ENSG00000133422_30946394_30926803_908	annotated	MORC2	22	-	2724	18	6	1	1.10%	2.183408796	0.067734782	0.003646019	0.001573841
ENSG00000103199_4765978_4752376_480	annotated	ZNF500	16	-	1440	8	0	0	1.04%	1.835680728	0.019679754	0.019679754	0.001784964
ENSG00000103202_397223_400342_187	annotated	NME4	16	+	561	40	3	9	7.49%	23.55953876	0.000638487	0.003260153	1.33E-05
ENSG00000103197_2050439_2088610_1748	annotated	TSC2	16	+	5244	65	6	1	1.49%	4.095626339	7.80E-06	3.46E-06	2.27E-10
ENSG00000133265_55279608_55262608_359	annotated	HSPBP1	19	-	1077	147	21	3	8.91%	45.09945411	2.24E-06	3.37E-07	7.02E-12
ENSG00000099204_114491803_114436260_448	annotated	ABLIM1	10	-	1344	73	6	7	5.36%	17.94705712	6.24E-05	5.09E-05	2.37E-08
ENSG00000149591_117203041_117204359_192	annotated	TAGLN	11	+	576	16	0	0	2.60%	9.178403642	0.020613417	0.020613417	0.001946209
ENSG00000266173_63728369_63704283_348	annotated	STRADA	17	-	1044	15	2	3	2.30%	4.74745016	0.005160369	0.020613417	0.000562924
ENSG00000099204_114684305_114436260_702	annotated	ABLIM1	10	-	2106	79	7	7	4.13%	12.39476731	1.58E-05	7.77E-06	9.97E-10
ENSG00000100347_43955578_43996383_469	annotated	SAMM50	22	+	1407	95	11	11	7.68%	22.309979	2.84E-06	8.76E-07	2.22E-11
ENSG00000176454_34367100_34359127_524	annotated	LPCAT4	15	-	1572	107	10	1	5.73%	22.49059213	1.79E-06	8.42E-07	1.35E-11
ENSG00000165264_32573060_32553876_113	annotated	NDUFB6	9	-	339	652	99	35	33.63%	635.5029212	1.36E-07	2.29E-07	2.96E-13

ENSG00000143498_222588482_222558660_423	annotated	TAF1A	1	-	1269	8	0	0	1.18%	2.083041961	0.019679754	0.019679754	0.001784964
ENSG00000049618_156935469_157207891_1606	annotated	ARID1B	6	+	4818	18	6	2	0.62%	1.234455284	0.074038859	0.01169877	0.004324138
ENSG00000197345_68896655_68891331_120	annotated	MRPL21	11	-	360	150	33	3	22.50%	137.6760546	2.58E-05	2.84E-06	6.35E-10
ENSG00000072134_19283147_19334254_632	annotated	EPN2	17	+	1896	38	2	0	2.69%	6.622392501	0.000289254	0.000121524	2.48E-07
ENSG00000150753_10263194_10264783_82	novel	CCT5	5	+	246	142	39	1	31.71%	190.731705	4.15E-05	3.97E-06	1.41E-09
ENSG00000125826_408241_408705_154	uORF	RBCK1	20	+	462	42	10	15	8.44%	30.03841192	0.009263777	0.064710135	0.003096171
ENSG00000125826_417835_430430_335	Overlap_dORF	RBCK1	20	+	1005	29	15	7	2.99%	9.5345805	0.108672843	0.030759455	0.014102976
ENSG00000166734_44289030_44413406_380	annotated	CASC4	15	+	1140	41	9	5	3.16%	11.88361735	0.008136829	0.003601996	0.000159622
ENSG00000132432_54757576_54752411_64	annotated	SEC61G	7	-	192	195	29	3	42.19%	335.5853832	2.67E-06	3.67E-06	8.40E-11
ENSG00000176974_18347600_18328750_345	annotated	SHMT1	17	-	1035	80	7	3	8.99%	25.53990579	1.78E-05	2.25E-06	3.48E-10
ENSG00000122861_73911892_73916565_414	annotated	PLAU	10	+	1242	182	43	2	13.04%	48.41940472	4.59E-07	6.14E-11	5.21E-16
ENSG00000070159_109448830_109379556_737	annotated	PTPN3	9	-	2211	32	5	0	1.63%	4.782234733	0.004957106	0.001040526	3.16E-05
ENSG00000157593_44257410_44254706_432	annotated	SLC35B2	6	-	1296	151	17	4	9.49%	38.49830417	4.69E-07	3.68E-08	1.75E-13
ENSG00000112695_75243734_75241134_49	novel	COX7A2	6	-	147	571	94	71	30.61%	1283.477995	0.000247677	0.000559537	9.33E-07
ENSG00000134825_61792558_61789795_79	annotated	TMEM258	11	-	237	350	51	6	37.97%	487.9657633	6.21E-05	8.15E-07	5.17E-10
ENSG00000115694_241500775_241495662_332	annotated	STK25	2	-	996	24	2	3	3.01%	7.961988702	0.002321823	0.009985438	0.000132651
ENSG00000142192_26170620_25881670_751	annotated	APP	21	-	2253	1851	173	45	27.83%	271.4656481	9.36E-31	5.49E-34	1.20E-62
ENSG00000108107_55386358_55388332_137	annotated	RPL28	19	+	411	985	146	39	22.63%	791.8885478	1.60E-05	2.79E-06	3.81E-10
ENSG00000108107_55391557_55391739_60	dORF	RPL28	19	+	180	35	3	0	16.67%	64.24882549	0.002432129	0.002404282	3.38E-05
ENSG00000162032_1778048_1777097_191	novel	SPSB3	16	-	573	14	3	0	4.19%	8.073150848	0.026823708	0.005160369	0.00074112
ENSG00000162032_1781483_1778295_147	novel	SPSB3	16	-	441	19	2	6	6.12%	14.23589136	0.013091824	0.039731909	0.002458944
ENSG00000118263_207134084_207081213_274	annotated	KLF7	2	-	822	10	0	0	2.19%	4.019738821	0.009815329	0.009815329	0.000483714
ENSG00000178741_74926861_74923657_69	annotated	COX5A	15	-	207	81	19	3	23.19%	129.295773	0.014848125	0.000229184	2.97E-05
ENSG00000065057_2046303_2039924_194	Overlap_dORF	NTHL1	16	-	582	56	5	12	9.79%	31.79323323	0.000559163	0.000537782	1.95E-06
ENSG00000121390_19782754_19703175_522	annotated	PSPC1	13	-	1566	39	0	0	3.45%	8.22891361	7.41E-05	7.41E-05	4.04E-08
ENSG00000152242_46216160_46263118_250	annotated	C18orf25	18	+	750	13	3	0	2.40%	5.727323873	0.035772986	0.013428348	0.002259559
ENSG00000101447_38926443_38952520_585	annotated	FAM83D	20	+	1755	132	13	3	7.35%	24.85229294	1.16E-07	5.17E-09	6.51E-15
ENSG00000168275_234374261_234383818_74	annotated	COA6	1	+	222	74	6	3	14.86%	110.1408437	0.005641909	0.006346985	0.000189755
ENSG00000101266_489830_483961_167	dORF	CSNK2A1	20	-	501	47	1	0	8.38%	30.99772248	0.000475338	0.000472567	1.47E-06
ENSG00000111786_120469609_120464812_119	annotated	SRSF9	12	-	357	296	75	22	22.69%	273.9637793	0.00453619	2.48E-05	1.21E-06
ENSG00000111786_120464742_120462019_108	dORF	SRSF9	12	-	324	82	10	1	19.44%	83.62545541	0.000186124	2.87E-05	4.13E-08
ENSG00000109083_28331985_28329038_115	annotated	IFT20	17	-	345	8	4	0	4.35%	7.661971736	0.139742684	0.016947427	0.011764961
ENSG00000023228_206153678_206124185_727	annotated	NDUFS1	2	-	2181	172	26	4	8.94%	26.05808132	3.57E-10	4.56E-12	2.15E-20
ENSG00000143537_155050916_155055767_201	novel	ADAM15	1	+	603	63	10	0	7.96%	34.52175698	0.004142377	0.000205155	6.34E-06
ENSG00000254553_32593082_32600419_391	dORF	AL033529.1	1	+	1173	9	0	0	1.28%	2.535211236	0.019679754	0.019679754	0.001784964
ENSG00000149179_47137656_47161638_234	annotated	C11orf49	11	+	702	60	14	0	7.69%	28.24124198	0.006539492	8.88E-05	5.27E-06
ENSG00000115457_216661906_216664104_85	Overlap_dORF	IGFBP2	2	+	255	122	5	1	28.24%	158.0845051	1.33E-05	8.22E-06	8.89E-10
ENSG00000129667_76479090_76474530_305	novel	RHBDF2	17	-	915	11	0	0	2.30%	3.972292724	0.007029303	0.007029303	0.000257364
ENSG00000181038_76729711_76733253_119	novel	METTL23	17	+	357	32	23	7	10.08%	29.61770587	0.10138588	0.021040203	0.00969386
ENSG00000104419_133262129_133238878_313	annotated	NDRG1	8	-	939	48	1	6	4.15%	16.89060862	0.001040543	0.007860495	5.12E-05
ENSG00000112531_163562018_163570710_147	annotated	QKI	6	+	441	16	6	0	3.40%	11.98811904	0.123780394	0.021084099	0.012086671
ENSG00000137261_24596538_24547165_966	annotated	KIAA0319	6	-	2898	32	6	4	1.55%	3.64855797	0.003741986	0.002484866	5.28E-05
ENSG00000131779_145918445_145912161_245	annotated	PEX11B	1	-	735	58	11	1	9.39%	26.07415892	0.000955423	1.46E-05	1.30E-07
ENSG00000125450_75209834_75235679_610	annotated	NUP85	17	+	1830	87	11	3	5.25%	15.70861214	1.50E-05	1.20E-06	1.63E-10
ENSG00000182199_57231811_57234361_377	dORF	SHMT2	12	+	1131	124	19	5	10.88%	36.2266966	7.19E-07	7.10E-08	5.05E-13
ENSG00000182199_57230833_57231742_65	annotated	SHMT2	12	+	195	55	15	1	10.77%	93.19609852	0.0839881	0.01208853	0.005100402
ENSG00000149483_61364356_61364595_79	novel	TMEM138	11	+	237	22	0	5	7.59%	30.67213369	0.013428348	0.145023522	0.010350889
ENSG00000113838_186564080_186551150_474	annotated	TBCCD1	3	-	1422	12	2	4	2.11%	2.78837579	0.01617279	0.020489117	0.00154601
ENSG00000181104_76716308_76733503_425	annotated	F2R	5	+	1275	64	2	0	5.41%	16.58591529	2.10E-05	1.14E-05	1.92E-09
ENSG00000166938_66308770_66333312_893	annotated	DIS3L	15	+	2679	43	1	7	2.69%	5.303534467	9.71E-06	0.000293999	2.51E-08
ENSG00000108828_43022322_43016061_325	annotated	VAT1	17	-	975	191	20	5	14.77%	64.72892661	1.33E-07	8.35E-09	1.18E-14
ENSG00000154945_50699750_50695997_226	annotated	ANKRD40	17	-	678	18	0	6	3.54%	8.772279587	0.005355751	0.053311599	0.001612295
ENSG00000159873_28772850_28786326_204	annotated	CCDC117	22	+	612	9	1	2	2.45%	4.859154869	0.035374934	0.060322713	0.008767878
ENSG00000104783_43774409_43769438_195	novel	KCNN4	19	-	585	29	6	0	5.13%	16.37992035	0.024832451	0.002376615	0.000356787
ENSG00000104783_43767701_43767543_52	novel	KCNN4	19	-	156	28	0	1	15.38%	59.30660815	0.005306578	0.005960501	0.000168675
ENSG00000159720_67443134_67438528_176	annotated	ATP6V0D1	16	-	528	103	17	1	15.91%	64.45742558	2.02E-05	1.74E-06	3.12E-10
ENSG00000013364_29830550_29831685_120	novel	MVP	16	+	360	12	3	1	5.00%	11.01408437	0.077869819	0.023878474	0.008119336
ENSG00000196628_55350976_55228225_539	annotated	TCF4	18	-	1617	24	0	0	1.86%	4.904230517	0.002404282	0.002404282	3.34E-05
ENSG00000120256_149864020_149842612_325	annotated	LRP11	6	-	975	141	15	8	11.08%	47.78418142	2.23E-06	1.28E-06	2.52E-11
ENSG00000203722_149923010_149916912_334	annotated	RAET1G	6	-	1002	22	3	0	3.29%	7.254786112	0.007306945	0.001513502	6.59E-05
ENSG00000203722_149923010_149918274_213	annotated	RAET1G	6	-	639	20	3	0	4.69%	10.34186326	0.009512533	0.002281203	0.000124232
ENSG00000164520_149889924_149888498_161	annotated	RAET1E	6	-	483	17	0	0	4.35%	11.62977853	0.008473674	0.008473674	0.000366502
ENSG00000198563_31540510_31538840_110	novel	DDX39B	6	-	330	36	6	102	9.09%	36.0460943	0.007635352	0.516161534	0.045829264
ENSG00000198563_31539209_31535229_198	novel	DDX39B	6	-	594	87	12	0	6.06%	48.3952192	0.038274767	0.001019727	0.000298084
ENSG00000198563_31532908_31530434_182	novel	DDX39B	6	-	546	131	14	15	19.78%	79.27720069	4.55E-06	8.01E-06	3.04E-10
ENSG00000126602_3677574_3658129_495	annotated	TRAP1	16	-	1485	214	18	17	13.13%	47.61644556	5.13E-10	4.77E-10	2.75E-18
ENSG00000118096_118557842_118545004_266	annotated	IFT46	11	-	798	8	0	0	1.88%	3.312506578	0.016947427	0.016947427	0.001349898
ENSG00000150787_112226444_112233555_145	annotated	PTS	11	+	435	71	21	2	11.03%	53.93103381	0.020051186	0.000197396	3.79E-05

ENSG00000103227_954496_934236_52	annotated	LMF1	16	-	156	14	3	3	17.31%	29.65330407	0.022868511	0.02510048	0.00257613
ENSG00000163607_112995192_113000919_120	annotated	GTPBP8	3	+	360	12	1	0	5.00%	11.01408437	0.035374934	0.012798403	0.002143354
ENSG00000078618_51792092_51789236_208	Overlap_dORF	NRDC	1	-	624	25	3	0	4.81%	13.23808218	0.004638749	0.002321823	6.12E-05
ENSG00000078618_51878468_51792073_900	annotated	NRDC	1	-	2700	92	11	1	4.67%	11.2588418	1.25E-06	4.95E-09	7.76E-14
ENSG00000256591_61437727_61481813_87	novel	AP003108.2	11	+	261	34	0	0	10.34%	43.04354811	0.003327803	0.003327803	6.21E-05
ENSG00000134987_111092457_111094985_75	novel	WDR36	5	+	225	7	4	0	6.67%	10.27981208	0.301439101	0.019216965	0.033495552
ENSG00000100294_43143348_43133043_390	annotated	MCAT	22	-	1170	32	3	1	3.08%	9.037197432	0.002336818	0.001480749	2.05E-05
ENSG00000146281_89146145_89162263_436	annotated	PM20D2	6	+	1308	26	4	4	2.98%	6.568031964	0.003750423	0.001825217	3.97E-05
ENSG00000129255_7583863_7586683_109	annotated	MPDU1	17	+	327	31	8	8	10.09%	31.32446014	0.03120188	0.015831738	0.002277356
ENSG00000198832_31107505_31105951_47	annotated	SELENOM	22	-	141	56	14	1	14.89%	131.2316436	0.116830194	0.008574016	0.00574301
ENSG00000079134_225097_214626_281	novel	THOC1	18	-	843	12	4	1	1.78%	4.703523575	0.100412561	0.035374934	0.014542509
ENSG00000141551_82273381_82255296_122	novel	CSNK1D	17	-	366	28	3	0	7.38%	25.27822642	0.010318779	0.003632191	0.000204008
ENSG00000164054_48504094_48469107_240	annotated	SHISA5	3	-	720	75	16	0	10.00%	34.41901366	0.007659089	6.23E-06	7.77E-07
ENSG00000105618_54128315_54131322_130	novel	PRPF31	19	+	390	46	3	2	13.08%	38.97291393	0.000384591	0.000207677	5.45E-07
ENSG00000105618_54121862_54128204_278	novel	PRPF31	19	+	834	60	7	4	6.83%	23.77140512	0.001185535	0.000270608	2.16E-06
ENSG00000118412_89846590_89873817_1967	novel	CASP8AP2	6	+	5901	17	3	0	0.51%	0.951903581	0.019264876	0.001790164	0.000213432
ENSG00000159788_3316999_3430972_1100	annotated	RGS12	4	+	3300	15	0	0	0.73%	1.501920596	0.005160369	0.005160369	0.000143196
ENSG00000165233_93112223_93113107_60	dORF	CARD19	9	+	180	32	2	5	11.67%	58.74178331	0.007979402	0.013439622	0.000539562
ENSG00000165233_93096346_93111104_228	annotated	CARD19	9	+	684	20	1	10	4.82%	9.661477518	0.002532006	0.10681658	0.002109156
ENSG00000072518_63895204_63909237_691	annotated	MARK2	11	+	2073	41	9	3	2.75%	6.535129655	0.003317712	0.000358778	8.11E-06
ENSG00000142634_15409972_15428724_240	annotated	EFHD2	1	+	720	197	7	4	12.50%	90.40727587	1.36E-06	1.35E-06	1.62E-11
ENSG00000116171_53047884_53050704_49	Overlap_dORF	SCP2	1	+	147	52	0	6	24.49%	116.8841607	0.001082917	0.001590443	1.05E-05
ENSG00000170473_55927761_55901872_204	annotated	PYM1	12	-	612	30	3	8	6.37%	16.1971829	0.003178226	0.020990601	0.000379123
ENSG00000183741_38872190_38866209_412	annotated	CBX6	22	-	1236	53	6	6	4.13%	14.16860368	0.003203492	0.000187021	4.43E-06
ENSG00000133216_22781429_22913570_964	annotated	EPHB2	1	+	2892	128	7	3	4.67%	14.62451037	2.01E-08	8.23E-09	1.71E-15
ENSG00000133216_22781429_22913570_963	annotated	EPHB2	1	+	2889	128	7	3	4.67%	14.63969677	2.01E-08	8.23E-09	1.71E-15
ENSG00000162599_61088263_61455320_462	annotated	NFIA	1	+	1386	12	0	0	1.73%	2.860801135	0.00419316	0.00419316	9.65E-05
ENSG00000116133_54887119_54852233_528	annotated	DHCR24	1	-	1584	360	43	11	15.34%	75.0960298	1.70E-11	2.77E-14	7.38E-24
ENSG00000141219_73235867_73247341_481	annotated	C17orf80	17	+	1443	23	2	1	2.49%	5.26660999	0.002493494	0.000866459	1.33E-05
ENSG00000138071_65246584_65268734_321	annotated	ACTR2	2	+	963	103	18	3	11.21%	35.341143	3.08E-06	1.19E-07	3.68E-12
ENSG00000166801_59148873_59153504_611	annotated	FAM111A	11	+	1833	38	4	0	2.78%	6.850003373	0.000938981	0.000125329	8.45E-07
ENSG00000141971_17420136_17420720_165	novel	MVB12A	19	+	495	41	7	0	4.24%	27.36833086	0.024800926	0.008776163	0.00107875
ENSG00000112183_17282811_17292119_178	annotated	RBM24	6	+	534	8	1	0	2.81%	4.950150279	0.042234516	0.019679754	0.003715148
ENSG00000070814_150357747_150399048_1451	annotated	TCOF1	5	+	4353	48	7	0	1.52%	3.643528944	0.000209577	1.56E-05	2.67E-08
ENSG00000204580_30888931_30891233_189	novel	DDR1	6	+	567	32	9	0	5.29%	18.64818518	0.042274398	0.002376615	0.000649843
ENSG00000101843_108091520_108085120_110	annotated	PSMD10	X	-	330	57	2	3	12.73%	57.07298265	0.000641032	0.001098965	4.45E-06
ENSG00000101843_108091520_108084974_185	annotated	PSMD10	X	-	555	61	2	3	8.65%	36.31671063	0.000294451	0.000519196	1.02E-06
ENSG00000149782_64251650_64267556_1234	annotated	PLCB3	11	+	3702	44	16	2	2.11%	3.927226194	0.010293993	6.61E-06	1.19E-06
ENSG00000146670_65079424_65079107_50	Overlap_dORF	CDCA5	11	-	150	24	0	0	10.00%	52.86760498	0.021557223	0.021557223	0.002115616
ENSG00000124357_71070780_71075570_144	annotated	NAGK	2	+	432	44	10	2	6.25%	33.65414669	0.061949988	0.007987976	0.002621525
ENSG00000149554_125626769_125655320_476	annotated	CHEK1	11	+	1428	47	5	1	3.57%	10.87525137	0.001149898	0.000169511	1.37E-06
ENSG00000171204_85628608_85635782_170	annotated	TMEM126B	11	+	510	38	16	6	7.65%	24.619718	0.02526281	0.005837456	0.000772927
ENSG00000136854_127653778_127684412_549	annotated	STXBP1	9	+	1647	58	5	0	4.19%	11.63600899	0.000154694	1.10E-05	1.42E-08
ENSG00000164904_126595198_126544965_554	annotated	ALDH7A1	5	-	1662	111	8	3	6.32%	22.06793078	1.15E-06	2.64E-07	2.82E-12
ENSG00000183605_119156735_119141242_151	novel	SFXN4	10	-	453	59	10	4	10.60%	43.0351641	0.002715282	0.000216324	4.20E-06
ENSG00000117360_150324943_150352979_683	annotated	PRPF3	1	+	2049	27	11	5	2.34%	4.354030425	0.033230463	0.003252115	0.000636619
ENSG00000159792_67909263_67927642_253	annotated	PSKH1	16	+	759	10	2	0	2.77%	4.353393032	0.049338282	0.007593099	0.001960563
ENSG00000176454_34362580_34360009_155	novel	LPCAT4	15	-	465	67	9	1	10.32%	47.60926792	0.000338775	0.000217137	5.02E-07
ENSG00000162032_1781483_1778295_147	novel	SPSB3	16	-	441	19	2	6	6.12%	14.23589136	0.013091824	0.039731909	0.002458944
ENSG00000162032_1778048_1777097_191	novel	SPSB3	16	-	573	12	3	0	3.66%	6.919843584	0.043639483	0.007882058	0.00177109
ENSG00000034713_75577286_75577369_27	Overlap_dORF	GABARAPL2	16	+	81	30	7	0	25.93%	122.3787152	0.046045458	0.008776163	0.00204958
ENSG00000103494_53700723_53602076_1281	annotated	RPGRIP1L	16	-	3843	31	7	0	0.94%	2.665391222	0.03744259	0.000954935	0.000275897
ENSG00000143537_155051387_155058474_633	annotated	ADAM15	1	+	1899	197	16	13	8.85%	34.27764014	1.35E-09	1.18E-09	1.74E-17
ENSG00000105518_11345619_11342815_189	annotated	TMEM205	19	-	567	510	75	20	31.22%	297.2054513	9.39E-09	7.07E-11	8.58E-18
ENSG00000175348_8956273_8948320_124	annotated	TMEM9B	11	-	372	40	6	0	11.29%	35.52930442	0.002578662	0.000379939	6.54E-06
ENSG00000074201_77637714_77619628_179	annotated	CLNS1A	11	-	537	114	4	3	16.20%	70.14556526	2.07E-06	5.04E-06	8.99E-11
ENSG00000148926_10305725_10306641_177	annotated	ADM	11	+	531	17	3	3	5.65%	10.57849911	0.018949244	0.016824507	0.001489035
ENSG00000052749_97400439_97357094_1119	annotated	RRP12	10	-	3357	17	2	3	1.07%	1.673274659	0.002845702	0.005110181	8.12E-05
ENSG00000026025_17229423_17230788_233	novel	VIM	10	+	699	410	43	41	25.75%	193.8100683	9.52E-09	1.35E-08	1.33E-15
ENSG00000136518_179570124_179588010_376	annotated	ACTL6A	3	+	1128	80	0	0	9.57%	23.43422206	5.24E-08	5.24E-08	2.72E-14
ENSG00000075239_108121607_108136123_162	annotated	ACAT1	11	+	486	44	5	1	6.17%	29.91479706	0.012983979	0.003514803	0.000250267
ENSG00000100934_39096118_39094978_86	annotated	SEC23A	14	-	258	27	0	0	15.12%	34.57910209	0.000585477	0.000585477	2.21E-06
ENSG00000172922_65720340_65719783_81	novel	RNASEH2C	11	-	243	40	0	2	9.88%	54.3905401	0.005355751	0.020990661	0.000592297
ENSG00000165533_88824708_88877410_515	annotated	TTC8	14	+	1545	48	8	2	4.27%	10.26555436	0.000395186	3.63E-05	1.12E-07
ENSG00000184216_154019713_154016616_306	annotated	IRAK1	X	-	918	9	1	0	1.63%	3.23943658	0.035374934	0.020613417	0.003252526
ENSG00000121940_108950437_108934670_501	annotated	CLCC1	1	-	1503	115	10	2	6.59%	25.28183039	7.39E-06	3.17E-07	2.28E-11
ENSG00000196586_75817599_75915012_1268	annotated	MYO6	6	+	3804	18	2	0	0.79%	1.563513554	0.011064574	0.002046029	0.00013212
ENSG00000121940_108950437_108931356_262	annotated	CLCC1	1	-	786	62	10	1	5.73%	26.06386378	0.005400616	0.000388891	1.47E-05

ENSG00000196586_75817599_75915012_1236	annotated	MYO6	6	+	3708	18	2	0	0.81%	1.603992869	0.011064574	0.002046029	0.00013212
ENSG00000184216_154019713_154011859_600	annotated	IRAK1	X	-	1800	28	7	2	1.83%	5.13990604	0.037701781	0.005625935	0.001145748
ENSG00000103653_74800698_74802010_209	novel	CSK	15	+	627	25	5	0	5.26%	13.17474207	0.002467336	0.001506153	2.20E-05
ENSG00000134686_33354458_33324865_359	annotated	PHC2	1	-	1077	56	7	9	4.74%	17.18074442	0.002794823	0.005189626	8.11E-05
ENSG00000144591_219500186_219506798_438	annotated	GMPPA	2	+	1314	29	4	0	1.83%	7.292430291	0.022572793	0.005604867	0.000664064
ENSG00000122406_92832115_92834928_112	novel	RPL5	1	+	336	164	14	9	14.29%	161.277664	0.000342584	0.000211707	4.95E-07
ENSG00000123066_116019401_115961266_1933	novel	MED13L	12	-	5799	40	12	8	1.40%	2.279169037	0.004841578	0.000433127	1.43E-05
ENSG00000073584_40642556_40630222_345	Overlap_dORF	SMARCE1	17	-	1035	57	3	0	3.77%	18.19718287	0.001529613	0.000649205	6.25E-06
ENSG00000168301_58498756_58501632_237	annotated	KCTD6	3	+	711	16	0	2	2.53%	7.435668773	0.012798403	0.012619539	0.000786774
ENSG00000092445_41561207_41578276_822	annotated	TYRO3	15	+	2466	21	5	0	1.34%	2.813817175	0.027642496	0.001448134	0.00026868
ENSG00000068308_48957495_48923174_546	annotated	OTUD5	X	-	1638	41	0	0	3.48%	8.270649436	5.43E-05	5.43E-05	2.20E-08
ENSG00000266412_46016680_46009147_634	annotated	NCOA4	10	-	1902	91	11	4	6.15%	15.80885927	4.42E-07	7.81E-08	3.36E-13
ENSG00000164181_60787397_60754624_281	annotated	ELOVL7	5	-	843	39	2	11	4.63%	15.28645162	0.001591926	0.022551981	0.000230575
ENSG00000138036_43774139_43809767_351	annotated	DYNC2LI1	2	+	1053	30	3	0	3.13%	9.413747325	0.004433848	0.001513502	3.95E-05
ENSG00000138035_55693823_55680832_179	annotated	PNPT1	2	-	537	28	6	0	6.70%	17.22873533	0.013238288	0.001032175	8.93E-05
ENSG00000138035_55640671_55636237_82	dORF	PNPT1	2	-	246	30	1	0	9.76%	40.29543062	0.007181907	0.005554612	0.000210515
ENSG00000138035_55672039_55643198_380	dORF	PNPT1	2	-	1140	33	3	0	3.16%	9.564862743	0.002296041	0.001044718	1.46E-05
ENSG00000058673_203840341_203852399_474	dORF	ZC3H11A	1	+	1422	15	0	0	1.05%	3.485469738	0.020613417	0.020613417	0.001946209
ENSG00000138031_24918987_24819932_1144	annotated	ADCY3	2	-	3432	90	16	2	3.58%	8.664926515	6.70E-06	3.08E-08	2.48E-12
ENSG00000137575_58565034_58581740_298	annotated	SDCBP	8	+	894	320	39	6	17.79%	118.2720469	2.29E-09	4.26E-10	1.10E-17
ENSG00000100578_58428310_58547976_1617	annotated	KIAA0586	14	+	4851	27	6	4	0.93%	1.839086444	0.009732377	0.004428467	0.000229997
ENSG00000139289_76031033_76030536_165	annotated	PHLDA1	12	-	495	68	7	5	12.12%	45.39137801	0.000368286	0.000187148	4.73E-07
ENSG00000173599_66872039_66848899_1138	annotated	PC	11	-	3414	30	4	0	1.14%	2.90353718	0.004866374	0.000628963	1.99E-05
ENSG00000185019_3123365_3110106_541	annotated	UBOX5	20	-	1623	22	0	0	1.85%	4.478925252	0.002335453	0.002335453	3.16E-05
ENSG00000163719_9662322_9701973_417	annotated	MTMR14	3	+	1251	68	7	4	7.43%	17.9606172	1.91E-05	4.61E-06	7.35E-10
ENSG00000007384_59340_58340_193	annotated	RHBDF1	16	-	579	7	0	0	2.59%	3.994745627	0.019216965	0.019216965	0.001707396
ENSG00000196655_119018796_119023399_181	annotated	TRAPPC4	11	+	543	95	13	3	16.57%	57.80873012	0.000101754	2.17E-06	2.11E-09
ENSG00000198646_34757391_34715322_1611	annotated	NCOA6	20	-	4833	20	5	2	0.81%	1.367359947	0.009325538	0.003587255	0.000182225
ENSG00000109390_139295798_139292550_76	annotated	NDUFC1	4	-	228	129	21	8	26.32%	186.94959	0.000474984	7.52E-05	2.62E-07
ENSG00000131373_15601516_15601391_41	Overlap_uORF	HACL1	3	-	123	16	1	0	12.20%	42.98179267	0.019679754	0.020613417	0.001864002
ENSG00000163466_218217471_218235362_144	novel	ARPC2	2	+	432	75	13	3	11.81%	57.36502276	0.001361001	0.000239323	2.23E-06
ENSG00000258466_67685124_67662505_46	novel	AL049779.1	14	-	138	19	0	3	10.87%	45.49295718	0.021557223	0.046295798	0.004403397
ENSG00000006695_14074454_14209528_257	annotated	COX10	17	+	771	17	1	0	3.50%	7.285581101	0.005561256	0.003452921	0.000105543
ENSG00000183597_20053502_20064662_166	annotated	TANGO2	22	+	498	10	1	0	3.01%	6.634990585	0.042234516	0.019679754	0.003715148
ENSG00000143549_154191290_154171486_168	annotated	TPM3	1	-	504	125	13	8	7.74%	81.95003252	0.000719745	0.002573466	1.16E-05
ENSG00000143549_154191290_154167937_239	annotated	TPM3	1	-	717	156	18	8	7.11%	71.89109464	0.000141701	0.000490578	4.86E-07
ENSG00000177150_13682077_13666480_272	annotated	FAM210A	18	-	816	36	6	1	5.15%	14.57746461	0.00526969	0.00054445	1.91E-05
ENSG00000125952_65102339_65070367_48	annotated	MAX	14	-	144	17	14	3	14.58%	39.00821548	0.359958927	0.024096744	0.049415486
ENSG00000152422_83104920_83353242_336	annotated	XRCC4	5	+	1008	42	4	0	5.36%	13.76760546	0.001368483	7.61E-05	8.07E-07
ENSG00000073578_236386_256420_258	novel	SDHA	5	+	774	85	8	6	5.04%	36.28671207	0.001274316	0.00074336	5.92E-06
ENSG00000165092_72930953_72923989_179	novel	ALDH1A1	9	-	537	2901	300	73	49.72%	1785.020042	2.78E-13	4.09E-15	1.67E-26
ENSG00000154723_25729794_25724640_108	annotated	ATP5PF	21	-	324	206	17	2	25.00%	210.0834611	1.07E-05	4.20E-06	3.76E-10
ENSG00000177189_20218838_20155398_711	annotated	RPS6KA3	X	-	2133	48	5	2	3.52%	7.435668773	6.12E-05	1.98E-05	9.41E-09
ENSG00000062716_59731447_59839911_406	annotated	VMP1	17	+	1218	263	43	22	12.81%	71.34739383	2.51E-06	4.14E-08	1.12E-12
ENSG00000068489_59169753_59201631_360	annotated	PRR11	17	+	1080	145	15	0	11.11%	44.36228427	2.69E-07	1.60E-08	4.50E-14
ENSG00000104356_98123374_98158271_1012	annotated	POP1	8	+	3036	9	4	1	0.49%	0.979513432	0.159270956	0.035374934	0.02367768
ENSG00000163349_113940819_113972038_930	annotated	HIPK1	1	+	2790	23	11	2	1.08%	2.723913339	0.140907465	0.00681086	0.006113599
ENSG00000125434_8294807_8289616_295	annotated	SLC25A35	17	-	885	9	1	0	1.69%	3.36022913	0.035374934	0.020613417	0.003252526
ENSG00000136631_150068645_150098868_398	annotated	VPS45	1	+	1194	45	6	6	4.27%	12.45311047	0.001496428	0.001195441	1.09E-05
ENSG00000196642_136829405_136840522_604	annotated	RABL6	9	+	1812	68	15	9	4.64%	12.39996254	0.000130403	3.38E-05	3.34E-08
ENSG00000054148_136849431_136850789_124	annotated	PHPT1	9	+	372	733	133	24	37.90%	651.0745035	3.00E-06	1.80E-09	8.30E-14
ENSG00000131724_118727639_118791854_427	annotated	IL13RA1	X	+	1281	95	7	3	7.03%	24.50440317	1.19E-05	1.73E-06	1.80E-10
ENSG00000051382_138707263_138655389_578	novel	PIK3CB	3	-	1734	25	1	3	2.25%	4.763877323	0.000906178	0.005078217	2.87E-05
ENSG00000100335_39504231_39504443_70	novel	MIEF1	22	+	210	21	2	0	12.86%	33.04225311	0.003478238	0.003632191	7.04E-05
ENSG00000105254_36115561_36116316_129	novel	TBCB	19	+	387	137	11	0	11.63%	116.9712836	0.000321743	0.000320929	6.94E-07
ENSG00000113448_59039047_58974664_565	novel	PDE4D	5	-	1695	137	25	15	8.14%	26.70671785	5.87E-07	8.74E-07	4.66E-12
ENSG00000113141_140659318_140660827_81	novel	IK	5	+	243	16	0	0	6.17%	21.75621604	0.019679754	0.019679754	0.001784964
ENSG00000164111_121696589_121668468_220	annotated	ANXA5	4	-	660	318	32	9	28.18%	159.2035832	2.70E-11	3.26E-11	1.06E-20
ENSG00000064393_139716692_139583725_804	annotated	HIPK2	7	-	2412	15	0	6	0.87%	2.054866487	0.008473674	0.155043753	0.008059628
ENSG00000122359_80172861_80155853_505	annotated	ANXA11	10	-	1515	229	12	3	11.49%	49.94505586	3.93E-10	4.58E-11	2.15E-19
ENSG00000074054_121605883_121340861_1534	annotated	CLASP1	2	-	4602	41	0	2	0.98%	2.943790477	0.000299491	0.000387696	7.79E-07
ENSG00000129255_7583863_7586082_101	annotated	MPDU1	17	+	303	33	8	10	11.88%	35.9866123	0.022794215	0.021145531	0.002189268
ENSG00000219545_7673372_7868410_78	annotated	UMAD1	7	+	234	9	0	0	6.41%	12.70855889	0.019679754	0.019679754	0.001784964
ENSG00000197563_62161260_62113095_459	annotated	PIGN	18	-	1377	55	9	0	4.36%	13.19770458	0.001284535	3.81E-05	4.12E-07
ENSG00000163626_73069649_73068552_146	novel	COX18	4	-	438	10	0	0	4.11%	7.543893404	0.011772051	0.011772051	0.000681273
ENSG00000128585_131387228_131487728_643	annotated	MKLN1	7	+	1929	89	19	2	4.98%	15.24500014	4.68E-05	4.67E-07	2.32E-10
ENSG00000109790_39062653_39121066_709	annotated	KLHL5	4	+	2127	91	17	5	4.51%	14.13655399	0.000161368	5.29E-06	7.66E-09
ENSG00000121067_49622004_49600378_327	annotated	SPOP	17	-	981	31	1	0	4.59%	10.44148671	0.000441519	0.000237853	7.10E-07

ENSG00000070010_19475590_19450670_302	annotated	UFD1	22	-	906	127	8	3	11.92%	46.31750712	3.80E-07	3.26E-07	1.15E-12
ENSG00000134815_47353031_47382113_1143	annotated	DHX34	19	+	3429	19	3	3	1.05%	1.830862669	0.009022293	0.007322157	0.000339172
ENSG00000175470_131901149_131955963_453	annotated	PPP2R2D	10	+	1359	49	1	0	3.53%	11.9136895	0.000428866	0.000178703	5.27E-07
ENSG00000127452_9811651_9810896_251	dORF	FBXL12	19	-	753	6	0	0	1.99%	2.632848854	0.016947427	0.016947427	0.001349898
ENSG00000105401_10395101_10391551_156	annotated	CDC37	19	-	468	171	6	6	14.74%	120.7313094	1.92E-05	3.85E-05	5.75E-09
ENSG00000105254_36116080_36118560_45	annotated	TBCB	19	+	135	125	11	0	26.67%	305.9467881	0.001095841	0.001095841	7.40E-06
ENSG00000188211_17356761_17372512_394	annotated	NCR3LG1	11	+	1182	46	2	0	2.79%	12.85908328	0.002213263	0.001603369	2.10E-05
ENSG00000152253_168889519_168871431_224	annotated	SPC25	2	-	672	86	7	1	9.38%	42.28621678	8.23E-05	2.84E-05	1.78E-08
ENSG00000137700_119029530_119028360_101	novel	SLC37A4	11	-	303	12	7	0	4.95%	13.08604084	0.170042304	0.021084099	0.017372496
ENSG00000137700_119027649_119024910_228	novel	SLC37A4	11	-	684	39	3	0	6.58%	18.83988116	0.001388942	0.000299491	2.79E-06
ENSG00000101843_108091520_108088725_79	novel	PSMD10	X	-	237	48	2	3	15.19%	66.92101896	0.001975805	0.0036383	4.15E-05
ENSG00000149273_75399548_75404780_91	annotated	RPS3	11	+	273	299	36	32	20.88%	361.8913436	0.000461104	0.000269987	8.36E-07
ENSG00000129595_112419039_112164990_686	annotated	EPB41L4A	5	-	2058	27	8	2	1.17%	4.334989475	0.048893856	0.007987976	0.002023419
ENSG00000070047_581513_611777_1648	annotated	PHRF1	11	+	4944	15	0	3	0.67%	1.002495543	0.001203942	0.015137476	0.000117639
ENSG00000135390_53672614_53665314_141	annotated	ATP5MC2	12	-	423	370	85	27	30.50%	289.0220721	2.46E-06	4.35E-08	1.15E-12
ENSG00000071967_171522546_171551133_185	novel	CYBRD1	2	+	555	65	3	0	11.35%	38.69813427	5.73E-05	2.69E-05	1.18E-08
ENSG00000078403_21669029_21670769_114	novel	MLLT10	10	+	342	5	0	0	4.39%	4.830738759	0.012673659	0.012673659	0.000782701
ENSG00000012048_43124045_43045784_682	annotated	BRCA1	17	-	2046	24	10	2	1.61%	3.875924119	0.059183319	0.004803511	0.001665416
ENSG00000186480_155301646_155302421_71	novel	INSIG1	7	+	213	200	10	5	25.35%	310.2558978	9.40E-05	9.40E-05	6.40E-08
ENSG00000175130_32336033_32334597_195	annotated	MARCKSL1	1	-	585	26	3	0	6.15%	14.68544583	0.001563962	0.000982952	9.42E-06
ENSG00000167645_38309458_38305352_233	annotated	YIF1B	19	-	699	270	5	10	14.16%	127.6310206	1.54E-06	2.56E-06	3.44E-11
ENSG00000136811_128460559_128494731_576	annotated	ODF2	9	+	1728	24	0	6	2.78%	4.589201821	0.000128239	0.016663301	2.16E-05
ENSG00000117155_84669800_84647433_512	annotated	SSX2IP	1	-	1536	24	1	1	2.93%	5.162852049	0.000435029	0.000435029	1.25E-06
ENSG00000137700_119029267_119024910_395	annotated	SLC37A4	11	-	1185	69	3	0	5.32%	19.23979295	0.000117461	2.60E-05	2.36E-08
ENSG00000137700_119029530_119029333_65	Overlap_uORF	SLC37A4	11	-	195	31	7	0	15.38%	52.52871007	0.019390513	0.002376615	0.000272493
ENSG00000116521_155256736_155256273_69	novel	SCAMP3	1	-	207	27	6	0	13.04%	43.09859102	0.032563252	0.003554776	0.000669524
ENSG00000116521_155258883_155256694_51	novel	SCAMP3	1	-	153	21	2	10	11.76%	45.35211211	0.031982531	0.268546989	0.040379691
ENSG00000159840_143381572_143390682_572	annotated	ZYX	7	+	1716	141	12	13	8.04%	27.15010308	1.03E-08	2.56E-08	2.70E-15
ENSG00000121067_49619237_49600378_258	dORF	SPOP	17	-	774	30	1	0	5.43%	12.80707485	0.00068025	0.000379939	1.70E-06
ENSG00000152795_82429333_82426059_301	annotated	HNRNPDL	4	-	903	206	23	4	14.95%	75.3787834	1.65E-08	2.42E-09	4.31E-16
ENSG00000081692_227735273_227732392_417	annotated	JMJD4	1	-	1251	19	0	6	1.92%	5.018407747	0.005554612	0.075179667	0.002457728
ENSG00000143740_227747845_227780673_383	annotated	SNAP47	1	+	1149	15	1	4	1.57%	4.313610067	0.016344022	0.069273208	0.005268498
ENSG00000160633_5623377_5668291_858	annotated	SAFB	19	+	2574	57	14	1	3.50%	7.317049057	0.000386308	8.63E-07	4.21E-09
ENSG00000154040_24143371_24156983_441	annotated	CABYR	18	+	1323	7	0	0	1.13%	1.74826736	0.016947427	0.016947427	0.001349898
ENSG00000123836_207061994_207072371_463	annotated	PFKFB2	1	+	1389	21	4	2	2.38%	4.995589023	0.021450603	0.006753026	0.00074068
ENSG00000184014_9265069_9139671_1287	annotated	DENND5A	11	-	3861	25	8	12	0.85%	2.139488028	0.027891304	0.130258759	0.015852154
ENSG00000141401_11981670_12008360_85	annotated	IMPA2	18	+	255	35	2	11	10.59%	45.35211211	0.00390064	0.033767299	0.000751883
ENSG00000119596_74763868_74835411_2020	annotated	YLPM1	14	+	6060	41	2	4	1.04%	2.235531976	9.35E-05	0.000133047	8.95E-08
ENSG00000110955_56643918_56640165_145	novel	ATP5F1B	12	-	435	961	114	457	35.86%	729.9679366	1.66E-06	0.014702012	6.90E-07
ENSG00000061273_47785874_47785399_78	annotated	HDAC7	12	-	234	23	2	4	7.69%	32.47742827	0.013428348	0.044040756	0.002791708
ENSG00000100030_21867404_21769204_304	annotated	MAPK1	22	-	912	120	22	9	11.51%	43.47664883	1.50E-05	6.13E-07	8.77E-11
ENSG00000002330_64284187_64270209_44	internal	BAD	11	-	132	16	0	1	11.36%	40.05121589	0.021084099	0.028889786	0.002731533
ENSG00000169221_30370183_30357944_808	annotated	TBC1D10B	16	-	2424	90	6	5	4.46%	12.26816328	9.49E-07	1.79E-07	1.61E-12
ENSG00000142655_10474967_10629987_377	annotated	PEX14	1	+	1131	21	5	3	3.71%	6.13516636	0.014335716	0.00365071	0.00028644
ENSG00000173698_19068834_18990864_1003	annotated	ADGRG2	X	-	3009	28	3	0	1.40%	3.074719465	0.003183653	0.000424543	9.00E-06
ENSG00000150627_176131703_176179579_929	annotated	WDR17	4	+	2787	9	1	0	0.54%	1.067026473	0.035374934	0.020613417	0.003252526
ENSG00000163975_197029702_197003372_738	annotated	MELTF	3	-	2214	88	17	2	5.15%	13.13332554	1.14E-05	4.12E-08	5.74E-12
ENSG00000163975_197029702_197019605_302	annotated	MELTF	3	-	906	41	10	2	5.96%	14.952896	0.004032546	0.000180389	5.52E-06
ENSG00000150527_39276958_39350264_775	annotated	MIA2	14	+	2325	51	7	0	2.71%	7.247978102	0.000171144	2.42E-05	3.23E-08
ENSG00000032219_58299855_58371989_1188	annotated	ARID4A	14	+	3564	14	0	3	0.59%	1.297956071	0.008373824	0.059666052	0.002611735
ENSG00000213965_32692126_32711957_320	annotated	NUDT19	19	+	960	76	1	1	7.81%	26.15845038	9.46E-06	6.49E-06	5.05E-10
ENSG00000116044_177264573_177230785_604	annotated	NFE2L2	2	-	1812	87	7	1	6.29%	15.86465795	2.00E-07	3.52E-08	7.02E-14
ENSG00000116044_177234268_177230785_589	annotated	NFE2L2	2	-	1767	86	6	1	6.28%	16.08168516	1.86E-07	5.34E-08	9.75E-14
ENSG00000218739_37199705_37201724_80	annotated	CEBPZOS	2	+	240	121	5	0	20.00%	166.5880261	0.000555138	0.000201477	7.63E-07
ENSG00000162063_2429482_2457020_786	annotated	CCNF	16	+	2358	64	9	4	3.82%	8.968211192	8.34E-05	6.30E-06	4.46E-09
ENSG00000187498_110307027_110150363_1669	annotated	COL4A1	13	-	5007	86	11	0	2.46%	5.675322084	3.94E-06	6.21E-09	3.34E-13
ENSG00000164111_121671628_121668468_107	novel	ANXA5	4	-	321	194	19	4	29.91%	199.6946138	1.83E-06	1.76E-06	2.82E-11
ENSG00000109819_23877827_23820610_261	novel	PPARGC1A	4	-	783	32	8	3	4.21%	13.50385823	0.02455477	0.00357219	0.000494785
ENSG00000164548_23516395_23505559_180	annotated	TRA2A	7	-	540	62	9	4	12.22%	37.93740172	0.000498765	0.0001433	4.99E-07
ENSG00000088986_120496422_120498210_89	annotated	DYNLL1	12	+	267	499	159	25	29.21%	617.5312473	3.54E-05	1.85E-05	5.12E-09
ENSG00000088986_120496458_120498210_77	annotated	DYNLL1	12	+	231	491	158	16	31.17%	702.3266787	6.85E-05	1.81E-05	9.72E-09
ENSG00000138448_186668894_186675957_130	annotated	ITGAV	2	+	390	73	10	0	13.85%	61.84831993	0.000186904	9.35E-05	1.25E-07
ENSG00000149150_57501192_57485096_462	annotated	SLC43A1	11	-	1386	9	1	1	1.08%	2.145600851	0.035374934	0.035374934	0.005300298
ENSG00000213593_57712619_57740245_296	annotated	TMX2	11	+	888	336	35	6	16.89%	125.0247415	8.74E-09	5.53E-10	5.50E-17
ENSG00000206562_15426511_15411256_284	annotated	METTL6	3	-	852	23	1	2	3.87%	8.919857061	0.002129196	0.005530543	6.73E-05
ENSG00000172053_49104733_49103886_100	annotated	QARS	3	-	300	31	4	5	7.00%	34.14366155	0.052370108	0.090418715	0.018157952
ENSG00000154380_225652690_225497775_802	annotated	ENAH	1	-	2406	69	20	3	3.74%	9.475957875	0.001610555	3.87E-06	7.80E-08
ENSG00000270647_35809570_35846945_589	annotated	TAF15	17	+	1767	110	9	6	5.60%	20.5695973	4.67E-06	3.71E-06	1.46E-10

ENSG00000122008_75573783_75597776_310	dORF	POLK	5	+	930	17	2	2	2.26%	6.039981752	0.022200631	0.022200631	0.002234769
ENSG00000075188_102118518_102074354_326	annotated	NUP37	12	-	978	94	17	0	9.51%	31.75840279	9.25E-06	4.75E-07	4.18E-11
ENSG00000077254_77741697_77697317_911	annotated	USP33	1	-	2733	61	3	1	2.96%	7.37496319	1.03E-05	3.03E-06	2.64E-10
ENSG00000114302_48847596_48751585_404	annotated	PRKAR2A	3	-	1212	40	3	4	3.71%	10.90503403	0.000759157	0.001563879	7.40E-06
ENSG00000127481_19076779_19074832_34	novel	UBR4	1	-	102	140	4	0	44.12%	453.5211211	0.000323375	0.000325833	7.08E-07
ENSG00000136718_130346361_130346468_35	novel	IMP4	2	+	105	17	0	1	22.86%	53.49698123	0.005306578	0.005454749	0.000155005
ENSG00000196517_44008606_43997545_521	annotated	SLC6A9	1	-	1563	14	4	1	1.15%	2.959638794	0.086207551	0.019795882	0.007759185
ENSG00000130305_73308790_73303398_470	annotated	NSUN5	7	-	1410	45	14	0	3.62%	10.54539993	0.006528965	0.000130801	7.24E-06
ENSG00000261832_28489349_28479730_309	annotated	AC138894.1	16	-	927	134	15	4	11.00%	47.76334322	2.21E-06	1.46E-07	3.16E-12
ENSG00000261832_28477608_28477516_30	dORF	AC138894.1	16	-	90	23	5	1	23.33%	84.44131351	0.027657845	0.010042643	0.001354611
ENSG00000261832_28491764_28489349_55	Overlap_uORF	AC138894.1	16	-	165	18	0	12	14.55%	36.0460943	0.005554612	0.310595281	0.015978689
ENSG00000139579_56224857_56229213_211	annotated	NABP2	12	+	633	129	15	10	14.69%	67.33729307	3.44E-05	1.39E-05	3.79E-09
ENSG00000131732_81304827_81312662_248	annotated	ZCCHC9	5	+	744	18	7	0	3.23%	7.994093495	0.104244116	0.004873904	0.003294081
ENSG00000180488_77779656_77875064_632	annotated	MIGA1	1	+	1896	36	12	0	2.53%	6.273845528	0.017934771	0.000178284	3.06E-05
ENSG00000162521_32651307_32668249_62	annotated	RBBP4	1	+	186	21	1	0	8.06%	37.30576964	0.021084099	0.021084099	0.002029891
ENSG00000129255_7583925_7587551_160	Overlap_dORF	MPDU1	17	+	480	132	9	6	16.88%	90.86619606	9.51E-06	1.16E-05	8.92E-10
ENSG00000164930_103329785_103331509_149	dORF	FZD6	8	+	447	23	4	0	8.72%	17.00160675	0.009947071	0.000596156	4.11E-05
ENSG00000164930_103300114_103329666_465	annotated	FZD6	8	+	1395	92	8	3	7.53%	21.79130671	3.66E-06	3.96E-07	1.35E-11
ENSG00000114867_184317438_184334908_1512	annotated	EIF4G1	3	+	4536	153	15	14	4.17%	11.14520442	4.38E-10	1.20E-09	5.91E-18
ENSG00000185033_90201594_90228643_832	annotated	SEMA4B	15	+	2496	185	3	5	8.17%	24.49045203	1.48E-12	2.01E-12	3.77E-23
ENSG00000186635_72712275_72685664_1136	annotated	ARAP1	11	-	3408	38	5	8	1.94%	3.684288786	0.000250684	0.000258518	4.42E-07
ENSG00000140022_81398382_81261782_977	annotated	STON2	14	-	2931	13	0	0	0.61%	1.46553835	0.01300724	0.01300724	0.000821865
ENSG00000125691_38852646_38850132_79	annotated	RPL23	17	-	237	218	36	13	22.78%	303.9329611	0.001874099	0.000245063	3.17E-06
ENSG00000214078_35632923_35626241_537	annotated	CPNE1	20	-	1611	341	37	24	15.46%	69.94046127	1.75E-11	4.17E-13	9.84E-23
ENSG00000079246_216109437_216117760_112	novel	XRCC5	2	+	336	187	11	0	13.39%	183.895873	0.005704554	0.000300266	1.25E-05
ENSG00000157184_53197476_53213595_618	dORF	CPT2	1	+	1854	64	2	2	4.21%	11.40617152	1.08E-05	6.44E-06	5.73E-10
ENSG00000130703_62284062_62293887_184	dORF	OSBPL2	20	+	552	18	1	0	5.43%	10.77464775	0.003737369	0.002201184	4.71E-05
ENSG00000133794_13358525_13386756_521	annotated	ARNTL	11	+	1563	14	0	0	1.15%	2.959638794	0.013428348	0.013428348	0.000872559
ENSG00000138768_75752570_75813295_901	annotated	USO1	4	+	2703	55	7	5	3.11%	6.723358939	2.86E-05	3.36E-05	7.41E-09
ENSG00000198780_74804576_74781460_394	dORF	FAM169A	5	-	1182	17	3	0	2.03%	4.752269906	0.036243043	0.005306578	0.001046215
ENSG00000203667_244835715_244843176_118	annotated	COX20	1	+	354	14	0	0	5.08%	13.06755773	0.012798403	0.012798403	0.000797244
ENSG00000088766_6006247_6032055_205	annotated	CRLS1	20	+	615	30	8	1	5.85%	16.11817225	0.025321606	0.001480749	0.000247593
ENSG00000088766_6006126_6006206_26	uORF	CRLS1	20	+	78	19	1	1	19.23%	80.48753963	0.020613417	0.021084099	0.00198765
ENSG00000164754_116848963_116847500_69	annotated	RAD21	8	-	207	18	2	0	8.70%	28.73239401	0.030550465	0.013640586	0.001956061
ENSG00000166974_35070202_35140369_284	annotated	MAPRE2	18	+	852	23	3	1	3.52%	8.919857061	0.017276406	0.00376808	0.000357157
ENSG00000135637_74472660_74472119_112	dORF	CCDC142	2	-	336	216	42	11	31.25%	212.4144843	1.13E-05	1.55E-07	1.85E-11
ENSG00000163291_78935198_78920539_221	annotated	PAQR3	4	-	663	13	1	0	2.26%	6.478873159	0.035772986	0.021084099	0.003354605
ENSG00000186318_117315609_117289566_439	annotated	BACE1	11	-	1317	12	0	1	1.37%	3.010683655	0.01300724	0.023878474	0.001470914
ENSG00000168827_158652205_158655954_144	novel	GFM1	3	+	432	7	7	1	3.47%	5.354068791	0.241449849	0.041782825	0.042744447
ENSG00000134058_69258103_69277135_227	annotated	CDK7	5	+	681	10	3	0	3.52%	4.852019546	0.049229251	0.004149608	0.001203008
ENSG00000117151_84570624_84554999_294	dORF	CTBS	1	-	882	51	10	0	5.10%	19.10606472	0.001520117	0.000293334	3.00E-06
ENSG00000087269_2963319_2938831_857	annotated	NOP14	4	-	2571	53	1	2	2.92%	6.811510754	4.70E-06	1.78E-05	6.98E-10
ENSG00000167972_2326466_2276674_1646	annotated	ABCA3	16	-	4938	71	4	2	1.94%	4.750911241	1.34E-06	1.18E-06	1.40E-11
ENSG00000204264_32843203_32840959_265	novel	PSMB8	6	-	795	57	9	2	6.79%	23.69067204	0.000626122	0.000298936	1.25E-06
ENSG00000117139_202773219_202729036_1386	Overlap_dORF	KDM5B	1	-	4158	59	5	7	2.45%	4.688535194	4.59E-06	9.86E-06	3.77E-10
ENSG00000010704_26087441_26094226_242	annotated	HFE	6	+	726	99	6	5	12.40%	45.05761788	2.92E-06	2.93E-06	7.34E-11
ENSG00000119669_77027717_77025402_771	annotated	IRF2BPL	14	-	2313	14	1	2	1.17%	1.99996344	0.00640069	0.01833556	0.000602704
ENSG00000090316_1289947_1339169_423	annotated	MAEA	4	+	1269	96	13	0	8.98%	24.99650354	5.23E-06	2.74E-08	1.71E-12
ENSG00000113621_134893498_134899685_161	novel	TXNDC15	5	+	483	145	28	1	16.77%	99.1951698	0.000467847	2.51E-06	1.30E-08
ENSG00000008294_51047454_50989652_775	novel	SPAG9	17	-	2325	49	15	1	2.71%	6.963743667	0.003544134	3.20E-05	1.12E-06
ENSG00000087053_95865604_95835290_557	annotated	MTMR2	11	-	1671	43	9	0	3.23%	8.502794038	0.006698967	7.25E-05	4.60E-06
ENSG00000136104_50927433_50956474_279	novel	RNASEH2B	13	+	837	58	2	0	6.09%	22.89666285	0.000174338	0.000133467	1.64E-07
ENSG00000070814_150357747_150383755_994	annotated	TCOF1	5	+	2982	25	0	0	1.31%	2.770141944	0.000585477	0.000585477	2.21E-06
ENSG00000154380_225554942_225519124_187	annotated	ENAH	1	-	561	23	4	3	5.35%	13.54673479	0.025616856	0.014895507	0.001778675
ENSG00000113569_37370950_37291900_1318	annotated	NUP155	5	-	3954	64	10	5	2.35%	5.348265552	8.11E-05	5.81E-06	4.03E-09
ENSG00000145431_156778227_156763090_189	dORF	PDGFC	4	-	567	25	0	0	2.65%	14.56889467	0.019679754	0.019679754	0.001784964
ENSG00000167460_16086484_16101346_139	dORF	TPM4	19	+	417	70	3	0	10.07%	55.46661194	0.000468432	0.000471186	1.45E-06
ENSG00000167460_16076566_16086461_56	annotated	TPM4	19	+	168	18	15	2	12.50%	35.40241405	0.360290169	0.008473674	0.02610587
ENSG00000115109_120042090_120104240_417	annotated	EPB41L5	2	+	1251	12	0	0	1.20%	3.169520682	0.021084099	0.021084099	0.002029891
ENSG00000080822_98521163_98515765_115	annotated	CLDND1	3	-	345	60	20	1	10.43%	57.46478802	0.001992103	0.001245083	1.49E-05
ENSG00000006534_68018428_68027939_447	novel	ALDH3B1	11	+	1341	231	21	15	11.63%	56.91842259	9.92E-09	5.01E-09	5.23E-16
ENSG00000006534_68015298_68015708_136	novel	ALDH3B1	11	+	408	23	0	0	5.88%	18.62676033	0.005604867	0.005604867	0.000167525
ENSG00000105655_18437979_18434913_504	annotated	ISYNA1	19	-	1512	35	2	4	3.77%	7.648669702	0.000193958	0.000713918	9.50E-07
ENSG00000174227_505724_521765_341	Overlap_dORF	PIGG	4	+	1023	32	3	3	4.11%	10.33579765	0.000369309	0.001741424	4.25E-06
ENSG00000108591_18103808_18107240_93	Overlap_dORF	DRG2	17	+	279	11	0	2	6.45%	13.02741162	0.011772051	0.042234516	0.002392043
ENSG00000167792_67608700_67612458_363	annotated	NDUFV1	11	+	1089	220	65	3	15.15%	66.75202649	1.32E-07	1.25E-10	2.44E-16
ENSG00000104522_143616691_143614602_154	novel	TSTA3	8	-	462	79	10	0	10.39%	56.50082242	0.002726437	0.000207012	4.06E-06
ENSG00000165916_47426231_47424414_139	novel	PSMC3	11	-	417	79	6	1	16.55%	62.59803347	2.79E-05	1.23E-05	2.73E-09

ENSG00000162607_62439868_62450981_785	annotated	USP1	1	+	2355	37	4	3	2.68%	5.191351869	0.000248701	0.000171648	2.96E-07
ENSG00000101972_124031120_124100597_1174	annotated	STAG2	X	+	3522	71	7	1	2.56%	6.660987992	1.32E-05	8.77E-07	1.07E-10
ENSG00000101972_124031120_124100597_1137	annotated	STAG2	X	+	3411	71	7	1	2.64%	6.877748376	1.32E-05	8.77E-07	1.07E-10
ENSG00000149532_61429235_61410943_471	annotated	CPSF7	11	-	1413	63	17	6	5.94%	14.73221476	0.002147321	1.42E-05	3.20E-07
ENSG00000077147_96586835_96522263_589	annotated	TM9SF3	10	-	1767	626	107	8	21.22%	117.0597083	7.18E-19	2.00E-22	2.89E-39
ENSG00000124222_58652007_58676291_325	annotated	STX16	20	+	975	16	0	1	2.46%	5.422318459	0.005454749	0.008219047	0.00023581
ENSG00000188647_69759938_69718342_402	annotated	PTAR1	9	-	1206	26	1	6	2.74%	7.123537155	0.002361478	0.030759455	0.000450196
ENSG00000174938_29899019_29871699_879	annotated	SEZ6L2	16	-	2637	34	3	2	2.28%	4.260282919	0.000346931	0.000162351	3.90E-07
ENSG00000123131_23667571_23675191_186	annotated	PRDX4	X	+	558	228	40	5	28.49%	135.0113568	4.23E-09	1.48E-10	7.48E-18
ENSG00000089057_4912918_4856972_594	annotated	SLC23A2	20	-	1782	97	2	6	6.06%	17.98596269	2.08E-07	5.82E-07	1.14E-12
ENSG00000164104_173332898_173332800_32	novel	HMGB2	4	-	96	56	0	0	18.75%	192.7464765	0.013853925	0.013853925	0.000925205
ENSG00000164104_173333649_173333065_99	novel	HMGB2	4	-	297	233	48	13	16.16%	259.2203695	0.001976395	0.000879156	1.07E-05
ENSG00000114988_96712001_96707256_171	dORF	LMAN2L	2	-	513	19	0	0	6.43%	12.23787152	0.001377539	0.001377539	1.15E-05
ENSG00000177479_48927571_48927855_94	novel	ARIH2	3	+	282	15	0	0	5.32%	17.57566655	0.021084099	0.021084099	0.002029891
ENSG00000124920_61779275_61779427_50	novel	MYRF	11	+	150	11	0	0	14.00%	24.23098562	0.007593099	0.007593099	0.000297833
ENSG00000169435_73611786_73576235_300	annotated	RASSF6	4	-	900	10	0	3	2.33%	3.671361457	0.007593099	0.077087128	0.00322116
ENSG00000113441_96979176_97028533_1006	annotated	LNPEP	5	+	3018	137	15	0	5.96%	14.99929979	1.72E-09	5.04E-12	1.23E-19
ENSG00000119383_129136484_129137827_115	annotated	PTPA	9	+	345	46	10	1	13.04%	44.05633748	0.003343197	0.0003635	8.27E-06
ENSG00000205758_33631551_33599266_198	annotated	CRYZL1	21	-	594	14	3	1	5.05%	7.787736424	0.026447338	0.004004658	0.000589688
ENSG00000103145_3023941_3022863_138	annotated	HCFC1R1	16	-	414	129	7	5	16.67%	102.9577452	6.99E-05	1.31E-05	7.34E-09
ENSG00000058063_182793760_182918104_1177	annotated	ATP11B	3	+	3531	40	4	0	1.61%	3.743104289	0.00047468	5.53E-05	1.98E-07
ENSG00000123810_41363519_41354700_175	annotated	B9D2	19	-	525	12	1	0	2.86%	7.552514997	0.042234516	0.019679754	0.003715148
ENSG00000133316_62839570_62834423_254	novel	WDR74	11	-	762	64	6	4	7.87%	27.75202361	0.000356911	0.000201552	4.92E-07
ENSG00000160688_154988534_154992662_170	annotated	FLAD1	1	+	510	16	0	3	3.53%	10.36619705	0.013428348	0.062386368	0.004016395
ENSG00000092208_39114339_39135988_275	annotated	GEMIN2	14	+	825	68	5	0	4.73%	27.23482681	0.000719745	0.000722178	3.31E-06
ENSG00000104133_44663536_44563258_2228	annotated	SPG11	15	-	6684	49	3	1	0.94%	2.422307604	0.000175616	3.08E-05	4.15E-08
ENSG00000112992_43609196_43704404_1086	annotated	NNT	5	+	3258	43	2	0	1.75%	4.361009465	0.000114158	5.53E-05	4.66E-08
ENSG00000178149_49017829_49017371_119	novel	DALRD3	3	-	357	11	1	3	4.20%	10.18108639	0.02854252	0.073194094	0.008836695
ENSG00000148824_133394323_133399639_142	novel	MTG1	10	+	426	28	3	0	8.45%	21.71791284	0.00522105	0.000881454	2.88E-05
ENSG00000076944_7637150_7645202_408	annotated	STXBP2	19	+	1224	10	9	0	1.72%	2.699530483	0.280222137	0.007029303	0.015850883
ENSG00000285982_103489697_103400411_264	annotated	AC012213.5	8	-	792	43	11	0	6.06%	17.93960712	0.016049839	0.000190285	2.81E-05
ENSG00000068308_48957570_48923174_542	annotated	OTUD5	X	-	1626	41	0	0	3.51%	8.331687439	5.43E-05	5.43E-05	2.20E-08
ENSG00000167578_40778376_40786963_213	annotated	RAB4B	19	+	639	37	6	0	6.57%	19.13244703	0.006425416	0.000448121	2.00E-05
ENSG00000152683_32165901_32220713_432	annotated	SLC30A6	2	+	1296	92	32	7	8.33%	23.45592042	0.000269736	1.88E-06	5.43E-09
ENSG00000163904_185586414_185629844_589	annotated	SENP2	3	+	1767	12	2	3	1.02%	2.243956069	0.043751848	0.059666052	0.010454552
ENSG00000163902_128650800_128620411_607	annotated	RPN1	3	-	1821	701	69	23	23.72%	127.1972511	2.94E-19	1.15E-24	8.73E-42
ENSG00000163900_185498961_185491752_92	annotated	TMEM41A	3	-	276	42	0	0	11.96%	50.28168952	0.001412569	0.001412569	1.21E-05
ENSG00000163898_185552471_185508790_451	annotated	LIPH	3	-	1353	54	3	3	4.43%	13.18759548	0.00017499	0.000197105	2.40E-07
ENSG00000125818_1118774_1166254_263	annotated	PSMF1	20	+	789	221	45	8	20.15%	92.55181163	1.98E-07	1.81E-09	4.31E-15
ENSG00000052723_114780607_114774271_211	annotated	SIKE1	1	-	633	43	1	2	6.64%	22.44576436	0.0004093	0.001820171	4.88E-06
ENSG00000052723_114780185_114774271_106	novel	SIKE1	1	-	318	34	1	0	6.60%	35.32819515	0.008776163	0.008776163	0.000391611
ENSG00000010165_171781968_171796756_699	annotated	EEF1AKNMT	1	+	2097	41	6	3	2.15%	6.460335611	0.000854054	0.000740896	4.00E-06
ENSG00000176396_39540079_39539369_236	annotated	EID2	19	-	708	24	21	3	4.24%	11.20076377	0.317957529	0.015528143	0.031476219
ENSG00000152942_69372209_69414292_670	annotated	RAD17	5	+	2010	17	1	0	1.19%	2.794618422	0.005454749	0.005554612	0.000161874
ENSG00000141376_60683995_61392125_896	annotated	BCAS3	17	+	2688	19	2	0	1.12%	2.335575927	0.009621867	0.002227676	0.000123102
ENSG00000143409_151002332_150997287_374	annotated	MINDY1	1	-	1122	12	1	0	1.87%	3.533930814	0.01551626	0.007882058	0.000614257
ENSG00000071282_8548652_8567598_208	annotated	LMCD1	3	+	624	28	0	0	6.73%	14.82665204	0.000415605	0.000415605	1.14E-06
ENSG00000130529_49196579_49211498_431	novel	TRPM4	19	+	1293	11	2	0	1.39%	2.811019213	0.053594825	0.011772051	0.003068673
ENSG00000186187_74999672_75106539_227	annotated	ZNRF1	16	+	681	12	7	1	2.64%	5.822423456	0.255658113	0.019795882	0.027452066
ENSG00000128242_30557359_30554953_412	annotated	GAL3ST1	22	-	1236	31	4	0	3.64%	8.287296492	0.002082111	0.000281287	4.00E-06
ENSG00000096063_35891039_35835304_639	annotated	SRPK1	6	-	1917	49	8	6	3.44%	8.445854995	0.000262933	6.49E-05	1.25E-07
ENSG00000161888_11155776_11149107_134	annotated	SPC24	19	-	402	19	0	9	3.73%	15.6169853	0.021084099	0.219478022	0.023627369
ENSG00000161888_11149227_11147183_140	Overlap_dORF	SPC24	19	-	420	19	3	0	7.14%	14.94768593	0.018382224	0.002046029	0.000226775
ENSG00000112715_43777480_43784562_189	annotated	VEGFA	6	+	567	40	1	0	5.29%	23.31023147	0.002851	0.002488365	4.06E-05
ENSG00000141562_82484920_82488157_136	novel	NARF	17	+	408	14	0	1	3.68%	11.33802803	0.020613417	0.035374934	0.003252526
ENSG00000109065_74773672_74771724_176	annotated	NAT9	17	-	528	13	0	0	2.84%	8.135403228	0.021084099	0.021084099	0.002029891
ENSG00000101972_124031120_124100597_1137	annotated	STAG2	X	+	3411	71	7	1	2.64%	6.877748376	1.32E-05	8.77E-07	1.07E-10
ENSG00000103202_399454_400190_73	annotated	NME4	16	+	219	27	2	5	10.96%	40.73702438	0.005306578	0.005355751	0.00015233
ENSG00000116005_70258165_70277392_505	annotated	PCYOX1	2	+	1515	260	9	19	13.86%	56.70617696	6.41E-13	7.85E-11	7.00E-22
ENSG00000164073_127930792_127920630_222	dORF	MFSD8	4	-	666	12	2	0	2.25%	5.953559119	0.02854252	0.019679754	0.002536889
ENSG00000128245_31956262_31956792_176	novel	YWHAH	22	+	528	116	24	0	17.05%	72.59282881	6.64E-05	7.56E-07	5.21E-10
ENSG00000143321_156745364_156744511_88	novel	HDGF	1	-	264	65	7	6	20.45%	81.35403228	0.000710968	8.56E-05	4.48E-07
ENSG00000022267_136206433_136210025_280	annotated	FHL1	X	+	840	11	1	0	2.50%	4.326961717	0.01551626	0.007882058	0.000614257
ENSG00000180398_46909165_46907669_147	novel	MCFD2	2	-	441	177	10	0	13.61%	132.6185669	0.000273681	4.08E-05	8.49E-08
ENSG00000170248_33798729_33825194_113	novel	PDCD6IP	3	+	339	28	3	0	6.19%	27.29153649	0.016854696	0.007029303	0.00060175
ENSG00000101255_388011_396690_358	annotated	TRIB3	20	+	1074	239	41	19	15.08%	73.52978113	6.62E-07	2.18E-09	1.86E-14
ENSG00000177917_152718625_152759840_226	annotated	ARL6IP6	2	+	678	90	28	2	11.06%	43.86139794	8.55E-05	1.51E-05	1.03E-08
ENSG00000068885_160384600_160258525_777	annotated	IFT80	3	-	2331	25	9	2	1.42%	3.54378519	0.084773141	0.003801324	0.002126544

ENSG00000143622_155910761_155900388_220	annotated	RIT1	1	-	660	40	0	4	7.73%	20.02560795	0.000130009	0.000834171	7.72E-07
ENSG00000144120_119680854_119681789_311	annotated	TMEM177	2	+	933	12	1	0	2.89%	4.249807474	0.00650005	0.002500085	9.16E-05
ENSG00000141252_714706_532827_421	annotated	VPS53	17	-	1263	28	0	0	2.14%	7.325281767	0.003710481	0.003710481	7.64E-05
ENSG00000114982_96637135_96598143_903	annotated	KANSL3	2	-	2709	52	2	0	1.99%	6.342551354	0.000277675	8.39E-05	1.68E-07
ENSG00000112992_43609196_43704404_1086	annotated	NNT	5	+	3258	43	2	0	1.75%	4.361009465	0.000114158	5.53E-05	4.66E-08
ENSG00000104164_45587444_45606514_172	annotated	BLOC1S6	15	+	516	27	1	0	5.23%	17.28955105	0.005642788	0.003529166	0.000109245
ENSG00000198246_71351617_71362608_329	Overlap_dORF	SLC29A3	10	+	987	18	0	4	2.74%	6.025942817	0.003529166	0.032126751	0.000655766
ENSG00000111110_62934688_62648489_498	annotated	PPM1H	12	-	1494	36	1	0	2.41%	7.961988702	0.001485237	0.001007374	9.17E-06
ENSG00000127445_9835345_9848196_145	annotated	PIN1	19	+	435	24	2	7	7.59%	18.23020861	0.003810897	0.006387516	0.000132396
ENSG00000036054_100295186_100323668_466	annotated	TBC1D23	3	+	1398	20	3	2	2.36%	4.727074837	0.008727216	0.006139464	0.000278842
ENSG00000116138_15529106_15566190_595	annotated	DNAJC16	1	+	1785	20	2	1	1.68%	3.702213234	0.006423651	0.002201184	8.07E-05
ENSG00000141429_35654663_35663806_105	annotated	GALNT1	18	+	315	35	13	0	10.48%	36.71361457	0.033673326	0.001603369	0.000365703
ENSG00000185024_105219187_105210551_203	novel	BRF1	14	-	609	10	0	1	2.96%	5.425657325	0.012798403	0.023672501	0.001437523
ENSG00000138668_82358757_82355334_181	dORF	HNRNPD	4	-	543	104	8	3	9.94%	63.28534666	0.000118791	0.000175951	1.48E-07
ENSG00000138668_82373678_82358793_86	annotated	HNRNPD	4	-	258	12	3	2	8.14%	15.36848982	0.028902806	0.01551626	0.002075277
ENSG00000040487_19325534_19328643_180	dORF	PQLC2	1	+	540	8	3	0	2.78%	4.895148609	0.138151459	0.019679754	0.01298269
ENSG00000040487_19317678_19325534_100	annotated	PQLC2	1	+	300	15	0	2	7.00%	16.52112656	0.007979402	0.037995728	0.001544499
ENSG00000249915_306604_314515_119	novel	PDCD6	5	+	357	263	40	11	26.89%	243.4205201	8.31E-05	7.63E-07	6.71E-10
ENSG00000249915_271625_304493_191	novel	PDCD6	5	+	573	40	4	4	5.24%	23.06614528	0.008052198	0.008052198	0.000332809
ENSG00000078114_21172426_20785747_230	annotated	NEBL	10	-	690	23	0	0	2.61%	11.01408437	0.013640586	0.013640586	0.000898637
ENSG00000115084_113755876_113717775_517	annotated	SLC35F5	2	-	1551	167	14	3	7.74%	35.57740986	5.12E-07	1.42E-08	7.88E-14
ENSG00000205758_33631551_33599266_183	annotated	CRYZL1	21	-	549	12	3	1	4.37%	7.222350407	0.056043583	0.010499663	0.002930046
ENSG00000167397_31091310_31091134_58	Overlap_dORF	VKORC1	16	-	174	204	55	19	29.31%	387.391933	0.000644384	0.001844079	7.49E-06
ENSG00000012061_45423374_45409675_297	annotated	ERCC1	19	-	891	90	15	7	9.76%	33.37601324	3.57E-05	7.87E-06	2.29E-09
ENSG00000104529_143586845_143579792_281	annotated	EEF1D	8	-	843	390	49	7	15.66%	152.8645162	2.49E-08	9.14E-09	2.35E-15
ENSG00000076513_109999689_110021159_261	novel	ANKRD13A	12	+	783	6	3	0	1.92%	2.531973419	0.214508587	0.016947427	0.019735972
ENSG00000141295_47840843_47840160_128	novel	SCRN2	17	-	384	18	1	7	5.47%	15.48855615	0.013892891	0.17034922	0.012888103
ENSG00000104626_9003064_9030034_349	annotated	ERI1	8	+	1047	82	9	2	7.16%	25.87836442	1.04E-05	1.57E-05	1.32E-09
ENSG00000101654_13734581_13759979_298	annotated	RNMT	18	+	894	15	5	0	2.68%	5.5440022	0.090418715	0.005306578	0.002952369
ENSG00000170340_62222293_62223414_373	annotated	B3GNT2	2	+	1119	52	8	0	5.90%	15.35475569	4.01E-05	1.69E-05	5.30E-09
ENSG00000104886_2234301_2233857_118	novel	PLEKHJ1	19	-	354	18	0	5	5.08%	16.80114565	0.013217227	0.063071082	0.004011161
ENSG00000104886_2236248_2235758_108	novel	PLEKHJ1	19	-	324	26	5	5	6.48%	26.5153883	0.047719858	0.076476367	0.014276811
ENSG00000186854_84881039_84821917_456	annotated	TRABD2A	2	-	1368	9	1	0	1.10%	2.173832442	0.041782825	0.019216965	0.003600526
ENSG00000153246_160028931_159941778_1172	annotated	PLA2R1	2	-	3516	19	2	0	0.60%	1.785559753	0.013892891	0.008473674	0.00058868
ENSG00000146670_65083978_65079107_307	annotated	CDCA5	11	-	921	52	0	1	4.89%	18.65577809	0.000312071	0.000365769	7.65E-07
ENSG00000157212_155002896_154944149_1058	annotated	PAXIP1	7	-	3174	11	4	0	0.66%	1.145131645	0.091912668	0.007882058	0.004060665
ENSG00000102805_76992099_77000969_358	annotated	CLN5	13	+	1074	42	2	1	5.31%	12.9215515	9.78E-05	7.00E-05	5.01E-08
ENSG00000140694_14630125_14436717_664	annotated	PARN	16	-	1992	38	9	6	2.71%	6.303241055	0.001367275	0.001695253	1.39E-05
ENSG00000167461_16111902_16112493_82	novel	RAB8A	19	+	246	14	5	0	7.32%	18.80453429	0.097833871	0.013428348	0.006564717
ENSG00000143322_179229397_179107718_1079	annotated	ABL2	1	-	3237	19	4	1	1.02%	1.939458786	0.015721867	0.002458651	0.000224099
ENSG00000070756_100713158_100709644_111	annotated	PABPC1	8	-	333	43	4	5	9.91%	42.66717369	0.002176803	0.002225493	2.82E-05
ENSG00000073584_40630897_40628785_130	novel	SMARCE1	17	-	390	31	3	0	4.62%	26.26435504	0.023672501	0.012798403	0.001437523
ENSG00000120694_31152870_31137318_688	dORF	HSPH1	13	-	2064	87	17	3	5.38%	13.9276939	2.36E-05	1.24E-07	3.39E-11
ENSG00000120694_31161582_31152909_115	annotated	HSPH1	13	-	345	11	0	7	5.22%	10.53521114	0.012798403	0.274691701	0.022653639
ENSG00000172071_88595591_88557736_946	novel	EIF2AK3	2	-	2838	25	0	0	1.27%	2.910698829	0.000943084	0.000943084	5.54E-06
ENSG00000100578_58442716_58512659_1319	Overlap_dORF	KIAA0586	14	+	3957	25	3	1	0.99%	2.08758233	0.004430232	0.001559221	4.06E-05
ENSG00000163156_151168181_151169085_85	novel	SCNM1	1	+	255	31	0	6	14.12%	40.16901359	0.000991051	0.006976718	4.33E-05
ENSG00000187522_14838403_14871606_509	annotated	HSPA14	10	+	1527	66	1	0	5.11%	14.28152394	4.38E-06	3.55E-06	1.32E-10
ENSG00000067560_49362504_49360209_60	dORF	RHOA	3	-	180	356	43	6	48.33%	653.5023393	2.48E-05	1.24E-06	2.84E-10
ENSG00000067560_49375589_49362510_90	annotated	RHOA	3	-	270	67	10	168	16.67%	81.9937392	0.001598122	0.65831425	0.036224138
ENSG00000204954_103956678_103951523_51	annotated	C12orf73	12	-	153	16	7	0	17.65%	34.55399018	0.062411228	0.002500085	0.001069711
ENSG00000139644_49759248_49762896_57	novel	TMBIM6	12	+	171	656	99	12	49.12%	1267.58585	1.72E-05	3.95E-06	5.72E-10
ENSG00000143376_151658250_151693504_346	dORF	SNX27	1	+	1038	34	2	1	4.05%	10.82308869	0.001092758	0.000662939	4.57E-06
ENSG00000102030_153930774_153929987_82	novel	NAA10	X	-	246	59	5	2	12.20%	79.24768023	0.003798923	0.00205871	4.50E-05
ENSG00000102030_153934904_153932300_118	novel	NAA10	X	-	354	34	4	0	11.02%	31.73549734	0.004206461	0.00683882	1.84E-05
ENSG00000075089_100212341_100241784_151	dORF	ACTR6	12	+	453	14	1	0	4.64%	10.21173385	0.013892891	0.008473674	0.00058868
ENSG00000075089_100200852_100210299_127	annotated	ACTR6	12	+	381	20	15	0	5.51%	17.34501476	0.17056305	0.008574016	0.009173812
ENSG00000178971_8248036_8228848_1031	annotated	CTC1	17	-	3093	15	1	2	0.68%	1.602437105	0.011825808	0.027657845	0.001562883
ENSG00000168256_42021578_42023893_135	annotated	NKIRAS2	17	+	405	18	1	10	6.67%	14.68544583	0.008219047	0.114155756	0.005413346
ENSG00000149483_61364449_61366169_64	novel	TMEM138	11	+	192	29	4	18	12.50%	49.9075698	0.0099656	0.236467188	0.01564402
ENSG00000101193_62941587_62944912_200	annotated	GID8	20	+	600	38	3	5	7.50%	20.9267603	0.000275394	0.005113145	1.03E-05
ENSG00000101216_63595760_63590089_374	annotated	GMEB2	20	-	1122	20	2	0	1.34%	5.88988469	0.020613417	0.021084099	0.00198765
ENSG00000116171_53015021_53047967_121	annotated	SCP2	1	+	363	94	8	6	17.36%	85.56396123	0.000102372	4.39E-05	3.36E-08
ENSG00000072756_3129041_3148154_434	annotated	TRNT1	3	+	1302	44	3	0	4.61%	11.16635282	0.000160693	3.71E-05	4.51E-08
ENSG00000074219_49360075_49341324_451	annotated	TEAD2	19	-	1353	22	6	0	2.88%	5.372724083	0.01219545	0.000577025	5.02E-05
ENSG00000039650_49866479_49861248_482	dORF	PNKP	19	-	1446	27	6	0	2.49%	6.169715311	0.008798119	0.00092354	5.21E-05
ENSG00000104883_7485876_7477202_171	annotated	PEX11G	19	-	513	13	0	0	2.92%	8.373280516	0.021084099	0.021084099	0.002029891
ENSG00000182208_1480409_1470172_152	annotated	MOB2	11	-	456	19	2	0	4.61%	13.76760546	0.018151375	0.008776163	0.000789738

ENSG00000149428_119056160_119045114_677	annotated	HYOU1	11	-	2031	188	30	7	9.01%	30.5856405	1.68E-08	9.61E-11	2.11E-17
ENSG00000157851_26898500_26946995_564	annotated	DPYSL5	2	+	1692	11	2	0	1.24%	2.148137023	0.026832696	0.007029303	0.00096477
ENSG00000143545_153983523_153982099_122	annotated	RAB13	1	-	366	61	1	1	13.11%	55.07042185	0.000251967	0.000251967	4.34E-07
ENSG00000197102_102028049_102043027_1138	novel	DYNC1H1	14	+	3414	74	13	9	2.72%	7.162058378	9.91E-05	5.85E-05	4.27E-08
ENSG00000126778_60649183_60646283_282	annotated	SIX1	14	-	846	11	4	0	2.13%	4.296274045	0.11290941	0.012798403	0.007447865
ENSG00000106608_43879063_43876667_798	novel	URGCP	7	-	2394	18	7	1	1.50%	2.484379933	0.01499044	0.001620026	0.000149374
ENSG00000138326_78033902_78037480_143	novel	RPS24	10	+	429	363	73	13	16.78%	279.5882956	0.000529471	8.82E-06	4.38E-08
ENSG00000006327_3020398_3021826_94	annotated	TNFRSF12A	16	+	282	751	35	18	34.04%	879.9550385	5.51E-07	9.71E-07	4.86E-12
ENSG00000080815_73206409_73219289_170	novel	PSEN1	14	+	510	33	10	0	6.47%	21.38028143	0.00956428	0.001595761	9.14E-05
ENSG00000116127_73432246_73609612_3706	annotated	ALMS1	2	+	11118	11	6	1	0.16%	0.326915618	0.143514872	0.023672501	0.015574534
ENSG00000113712_149550964_149520385_77	annotated	CSNK1A1	5	-	231	70	22	0	15.58%	100.1280397	0.119738561	0.001053132	0.001323566
ENSG00000113712_149511835_149496853_114	dORF	CSNK1A1	5	-	342	36	3	0	12.28%	34.78131906	0.001356947	0.000456173	4.00E-06
ENSG00000169047_226798666_226795010_1218	annotated	IRS1	2	-	3654	44	0	2	1.81%	3.978815372	1.37E-05	6.49E-05	7.09E-09
ENSG00000164118_174302124_174331595_341	annotated	CEP44	4	+	1023	27	2	0	2.64%	8.720829267	0.007225456	0.003632191	0.000142984
ENSG00000168778_123671530_123707713_661	annotated	TCTN2	12	+	1983	57	5	0	3.33%	9.497773209	0.000133349	1.71E-05	1.81E-08
ENSG00000102225_47223594_47228768_484	annotated	CDK16	X	+	1452	38	2	0	2.89%	8.647421613	0.000705627	0.000428413	1.97E-06
ENSG00000140153_102139951_102209880_499	annotated	WDR20	14	+	1497	33	3	0	2.00%	7.283863411	0.004490072	0.00244612	6.22E-05
ENSG00000092199_21234154_21211287_218	annotated	HNRNPC	14	-	654	101	3	7	5.50%	51.02855603	0.004676575	0.00706123	0.000176449
ENSG00000149177_47980748_48167362_1397	annotated	PTPRJ	11	+	4191	181	13	3	4.65%	14.27021669	1.18E-11	2.67E-12	3.94E-22
ENSG00000011295_16025052_16026710_96	novel	TTC19	17	+	288	64	8	0	11.46%	73.42722914	0.001626321	0.001626321	1.58E-05
ENSG00000125734_6731475_6730312_94	novel	GPR108	19	-	282	31	8	0	13.83%	36.3230442	0.011823317	0.000656046	5.39E-05
ENSG00000111319_6374540_6347873_588	annotated	SCNN1A	12	-	1764	70	3	3	3.40%	13.1120052	0.000150216	7.65E-05	8.32E-08
ENSG00000033627_42460922_42521120_779	annotated	ATP6V0A1	17	+	2337	48	7	7	2.44%	6.786598842	0.001210582	0.00186649	1.36E-05
ENSG00000013375_83191012_83169680_566	annotated	PGM3	6	-	1698	22	1	1	2.65%	4.281092865	0.000424054	0.000424054	1.19E-06
ENSG00000115652_106194241_106145470_93	novel	UXS1	2	-	279	22	6	0	6.45%	26.05482324	0.15306802	0.012798403	0.010665221
ENSG00000107789_87504916_87552185_312	annotated	MINPP1	10	+	936	104	6	3	9.94%	36.71361457	2.69E-06	1.65E-06	3.85E-11
ENSG00000107789_87552209_87552478_89	dORF	MINPP1	10	+	267	43	2	1	14.61%	53.2141155	0.001380936	0.000876741	7.49E-06
ENSG00000115457_216633524_216664104_325	annotated	IGFBP2	2	+	975	188	11	2	14.46%	63.7122419	9.63E-09	9.30E-10	9.98E-17
ENSG00000110717_68032152_68033427_171	novel	NDUFS8	11	+	513	235	24	8	21.05%	151.3631478	6.39E-07	1.23E-07	7.54E-13
ENSG00000167601_41219414_41259904_887	annotated	AXL	19	+	2661	311	42	13	9.81%	38.61759007	3.09E-12	2.08E-14	9.45E-25
ENSG00000136273_47978569_47965356_212	annotated	HUS1	7	-	636	24	3	0	5.19%	12.46877476	0.010677183	0.001448134	9.44E-05
ENSG00000179715_47235064_47236362_432	annotated	PCED1B	12	+	1296	87	7	2	6.94%	22.18114214	1.26E-05	2.40E-06	2.60E-10
ENSG00000137074_33001604_32987654_99	annotated	APTX	9	-	297	55	0	3	13.13%	61.18935761	0.000700469	0.001348057	5.92E-06
ENSG00000165060_69035783_69099934_171	annotated	FXN	9	+	513	59	11	0	9.94%	38.00181157	0.001444597	0.000138364	1.45E-06
ENSG00000165060_69035783_69072712_196	annotated	FXN	9	+	588	59	11	9	8.67%	33.15464173	0.001444597	0.002326287	2.00E-05
ENSG00000166783_15636147_15596693_1629	annotated	MARF1	16	-	4887	41	6	0	1.17%	2.772114544	0.002061438	3.95E-05	7.20E-07
ENSG00000105671_18919742_18921947_199	novel	DDX49	19	+	597	30	7	3	3.02%	16.60414729	0.035350572	0.028581075	0.004358154
ENSG00000124783_7313120_7287913_288	annotated	SSR1	6	-	864	814	105	18	28.82%	311.3008569	4.36E-12	3.85E-15	2.78E-25
ENSG00000124783_7313197_7313117_26	Overlap_uORF	SSR1	6	-	78	35	34	4	38.46%	148.2665204	0.280590841	0.01271029	0.023227279
ENSG00000114861_71041530_70959247_455	annotated	FOXP1	3	-	1365	15	0	5	1.54%	3.631016825	0.008473674	0.081055481	0.00371419
ENSG00000079999_10499706_10486652_515	annotated	KEAP1	19	-	1545	111	7	2	7.57%	23.73909447	1.42E-07	6.24E-08	8.68E-14
ENSG00000179832_144223183_144261740_1211	annotated	MROH1	8	+	3633	16	1	4	0.58%	1.455205202	0.011306644	0.051117422	0.002823276
ENSG00000174851_66288985_66284637_293	annotated	YIF1A	11	-	879	214	9	9	12.97%	80.44416571	3.78E-08	2.41E-07	9.11E-14
ENSG00000275379_27872315_27871905_136	annotated	HIST1H3I	6	-	408	562	46	8	18.38%	455.1408394	1.11E-05	9.43E-06	8.51E-10
ENSG00000141526_82236009_82237182_127	novel	SLC16A3	17	+	381	82	21	7	13.39%	71.1145605	0.012288339	0.001635047	0.000121623
ENSG00000085721_15083566_15061744_447	annotated	RRN3	16	-	1341	52	3	0	4.25%	12.81280508	0.000213727	5.82E-05	9.16E-08
ENSG00000115649_219176828_219172586_410	annotated	CNPPD1	2	-	1230	31	5	3	3.17%	8.327722329	0.005068734	0.003709388	0.000103107
ENSG00000135932_230790905_230818704_292	annotated	CAB39	2	+	876	21	3	1	4.11%	7.921088075	0.003725191	0.001410049	3.11E-05
ENSG00000106355_32489303_32487261_62	annotated	LSM5	7	-	186	163	19	3	37.10%	289.563831	2.09E-05	1.95E-05	3.21E-09
ENSG00000176407_84971452_85053409_381	annotated	KCMF1	2	+	1143	71	33	0	6.30%	20.52493413	0.023699309	7.71E-06	4.13E-06
ENSG00000138069_65129906_65088493_202	annotated	RAB1A	2	-	606	215	19	5	20.79%	117.2291158	2.70E-07	1.90E-08	5.33E-14
ENSG00000101150_63865366_63890042_222	annotated	TPD52L2	20	+	666	173	30	19	15.77%	85.8304773	2.23E-05	1.94E-05	3.39E-09
ENSG00000171262_38454195_38481555_319	annotated	FAM98B	15	+	957	68	8	1	5.96%	23.47829897	0.000671142	6.14E-05	3.13E-07
ENSG00000083444_11965489_11975339_110	novel	PLOD1	1	+	330	25	0	0	4.55%	25.03200993	0.021084099	0.021084099	0.002029891
ENSG00000099860_2476359_2477601_160	annotated	GADD45B	19	+	480	22	8	1	3.75%	15.14436601	0.155259175	0.020159861	0.015117627
ENSG00000283900_38303150_38293287_358	annotated	TPTEP2-CSNK1E	22	-	1074	83	7	5	6.15%	25.535447	0.000124209	0.000118485	1.05E-07
ENSG00000100335_39504231_39504443_70	novel	MIEF1	22	+	210	21	2	0	12.86%	33.04225311	0.003478238	0.003632191	7.04E-05
ENSG00000185236_8390417_8402594_179	annotated	RAB11B	19	+	537	85	17	2	17.88%	52.30151796	6.46E-05	3.12E-07	2.32E-10
ENSG00000072506_53434345_53431571_169	annotated	HSD17B10	X	-	507	120	5	1	20.71%	78.20651624	2.34E-07	1.27E-07	2.83E-13
ENSG00000079819_130956485_130858136_935	annotated	EPB41L2	6	-	2805	65	4	0	2.89%	7.656850097	8.39E-06	2.29E-06	1.65E-10
ENSG00000077721_119574956_119583255_119	annotated	UBE2A	X	+	357	148	7	0	23.53%	136.9818897	4.24E-06	1.60E-06	5.90E-11
ENSG00000138674_82881906_82819074_1096	annotated	SEC31A	4	-	3288	96	8	3	3.38%	9.647373171	1.39E-07	2.52E-07	3.33E-13
ENSG00000113312_160036732_160065065_148	novel	TTC1	5	+	444	124	6	0	14.19%	92.28016635	5.11E-05	2.72E-05	1.06E-08
ENSG00000156471_96299703_96333850_301	annotated	PTDSS1	8	+	903	102	5	0	11.30%	37.32347528	5.39E-07	1.56E-07	7.99E-13
ENSG00000104299_28859500_28771151_501	annotated	INTS9	8	-	1503	29	3	2	2.79%	6.375418099	0.000337545	0.001209608	2.70E-06
ENSG00000105339_141188998_141194233_246	annotated	DENND3	8	+	738	7	2	0	2.03%	3.134089049	0.102951605	0.016947427	0.008323174
ENSG00000061987_62501630_62592749_1477	dORF	MON2	12	+	4431	30	3	0	1.02%	2.237119371	0.001565127	0.000273206	2.90E-06
ENSG00000160014_46601435_46609153_83	annotated	CALM3	19	+	249	92	26	13	25.30%	122.0838268	0.005732753	0.000525248	2.02E-05

ENSG00000168137_9428939_9476091_1461	annotated	SETD5	3	+	4383	31	9	8	1.16%	2.337006266	0.017298378	0.006183677	0.000551262
ENSG00000135119_116750028_116852712_330	annotated	RNFT2	12	+	990	31	4	4	4.24%	10.34656411	0.002040495	0.004518151	5.29E-05
ENSG00000125450_75234711_75235133_31	annotated	NUP85	17	+	93	14	1	0	16.13%	49.74102619	0.019679754	0.021084099	0.001903851
ENSG00000125450_75235133_75235679_56	dORF	NUP85	17	+	168	24	1	0	12.50%	47.20321873	0.01208853	0.008776163	0.000531381
ENSG00000109083_28331985_28329169_106	annotated	IFT20	17	-	318	8	4	0	4.72%	8.312516506	0.139742684	0.016947427	0.011764961
ENSG00000234741_173867041_173866181_50	novel	GAS5	1	-	150	20	0	0	10.00%	44.05633748	0.021557223	0.021557223	0.002115616
ENSG00000157349_70294846_70333582_453	annotated	DDX19B	16	+	1359	43	1	0	3.53%	10.45487037	0.000187666	0.000187666	2.45E-07
ENSG00000162616_78013374_78016247_159	novel	DNAJB4	1	+	477	24	4	0	6.92%	16.62503301	0.010312081	0.001398493	8.82E-05
ENSG00000213563_144528916_144527342_216	annotated	C8orf82	8	-	648	11	0	2	2.31%	5.609024448	0.019679754	0.08410207	0.007521961
ENSG00000176102_33161325_33085087_749	annotated	CSTF3	11	-	2247	123	10	0	2.14%	18.08721465	0.001009009	0.000192487	1.34E-06
ENSG00000138303_72213264_72210754_51	annotated	ASCC1	10	-	153	7	0	4	11.76%	15.1173707	0.009815329	0.170566842	0.010088762
ENSG00000169756_108534563_108683999_337	annotated	LIMS1	2	+	1011	36	3	2	4.15%	11.76578746	0.001477695	0.001203591	1.08E-05
ENSG00000065911_74198642_74206004_102	novel	MTHFD2	2	+	306	36	0	2	9.80%	38.87323895	0.002460157	0.003185952	4.47E-05
ENSG00000174437_110346029_110346470_119	novel	ATP2A2	12	+	357	101	23	6	17.65%	93.48088415	0.001926703	3.24E-05	5.63E-07
ENSG00000101311_6119428_6077173_635	annotated	FERMT1	20	-	1905	21	1	0	2.20%	3.642453099	0.00065074	0.000288544	1.25E-06
ENSG00000198815_42311093_42179710_622	annotated	FOXJ3	1	-	1866	25	9	0	2.25%	4.426882786	0.024416325	0.000390775	8.22E-05
ENSG00000073584_40642556_40632183_223	novel	SMARCE1	17	-	669	24	0	0	2.69%	11.85372309	0.013428348	0.013428348	0.000872559
ENSG00000073584_40630897_40630222_82	novel	SMARCE1	17	-	246	31	3	0	7.32%	41.63861164	0.023672501	0.012798403	0.001437523
ENSG00000010278_6232667_6237828_158	annotated	CD9	12	+	474	763	56	16	42.41%	531.8826819	7.88E-12	1.22E-12	1.23E-22
ENSG00000100983_34932022_34928828_159	dORF	GSS	20	-	477	73	14	3	11.95%	50.56780875	0.001023072	0.000183564	1.30E-06
ENSG00000100983_34951852_34941806_124	annotated	GSS	20	-	372	19	9	0	6.45%	16.8764196	0.169705346	0.005554612	0.006736088
ENSG00000116209_54053188_54045644_145	annotated	TMEM59	1	-	435	101	20	6	16.55%	76.71879458	0.000521217	5.69E-05	2.24E-07
ENSG00000132170_12381414_12434145_371	annotated	PPARG	3	+	1113	29	5	0	4.31%	8.609392096	0.005289692	0.000146985	6.28E-06
ENSG00000103005_58002534_58020245_214	annotated	USB1	16	+	642	8	1	2	2.34%	4.117414718	0.042234516	0.080264768	0.01347413
ENSG00000101146_57354087_57378099_352	annotated	RAE1	20	+	1056	86	5	5	8.52%	26.90941068	8.12E-06	8.12E-06	5.41E-10
ENSG00000101146_57354087_57378099_352	annotated	RAE1	20	+	1056	86	5	5	8.52%	26.90941068	8.12E-06	8.12E-06	5.41E-10
ENSG00000108474_16217236_16325906_241	annotated	PIGL	17	+	723	32	4	13	5.39%	14.62451037	0.006188524	0.02308716	0.000740329
ENSG00000141027_16064948_16032296_766	annotated	NCOR1	17	-	2298	16	2	1	1.17%	2.300592036	0.012888811	0.00640069	0.000423911
ENSG00000105185_32584960_32587300_87	annotated	PDCD5	19	+	261	93	26	5	17.24%	117.736764	0.002898308	0.003315387	5.43E-05
ENSG00000143353_219179170_219211728_208	novel	LYPLAL1	1	+	624	54	6	0	9.62%	28.5942575	0.000765825	3.81E-05	2.35E-07
ENSG00000085063_33722445_33710328_91	annotated	CD59	11	-	273	173	42	0	18.68%	209.3886369	0.019140625	0.000141701	2.77E-05
ENSG00000085063_33713388_33710126_75	Overlap_dORF	CD59	11	-	225	160	34	1	30.67%	234.9671332	0.000178424	1.32E-05	1.94E-08
ENSG00000188603_28482191_28477516_115	novel	CLN3	16	-	345	54	5	1	16.52%	51.71830922	0.000208869	7.59E-05	1.15E-07
ENSG00000135503_51951744_51992032_476	annotated	ACVR1B	12	+	1428	34	9	5	3.36%	7.867203122	0.009192346	0.003360686	0.000169379
ENSG00000131051_35740847_35704481_515	annotated	RBM39	20	-	1545	87	17	6	6.99%	18.60631729	6.68E-05	8.71E-07	5.94E-10
ENSG00000041802_194672114_194642068_642	annotated	LSG1	3	-	1926	69	10	4	3.89%	11.83756731	0.00030618	2.64E-05	6.41E-08
ENSG00000179195_124012202_124012930_242	annotated	ZNF664	12	+	726	12	1	0	2.07%	5.46152944	0.035772986	0.021084099	0.003354605
ENSG00000100983_34928932_34928828_34	dORF	GSS	20	-	102	50	13	3	26.47%	161.971829	0.036196037	0.010327713	0.001825598
ENSG00000100983_34935599_34928932_106	annotated	GSS	20	-	318	16	0	0	4.72%	16.62503301	0.016947427	0.016947427	0.001349898
ENSG00000196352_207321766_207359669_439	annotated	CD55	1	+	1317	244	35	9	13.90%	61.21723431	1.18E-07	8.47E-12	1.99E-17
ENSG00000168036_41224552_41239342_768	annotated	CTNNB1	3	+	2304	288	46	13	11.46%	41.30281639	2.21E-12	5.94E-15	2.04E-25
ENSG00000101343_20048422_20036195_562	dORF	CRNKL1	20	-	1686	38	2	4	4.09%	7.44724566	5.56E-05	9.13E-05	3.75E-08
ENSG00000158467_129397228_129427045_369	annotated	AHCYL2	7	+	1107	18	0	0	2.44%	5.372724083	0.003529166	0.003529166	6.95E-05
ENSG00000122566_26197458_26192516_301	novel	HNRNPA2B1	7	-	903	1270	161	63	44.85%	464.7138588	3.98E-20	1.39E-23	1.13E-41
ENSG00000137274_3118741_3138201_274	novel	BPHL	6	+	822	32	0	1	5.11%	12.86316423	0.000434916	0.000434916	1.25E-06
ENSG00000114698_146222071_146194411_329	annotated	PLSCR4	3	-	987	11	5	0	1.52%	3.68252061	0.145248299	0.020613417	0.014232745
ENSG00000168591_44189047_44190864_264	annotated	TMUB2	17	+	792	44	3	2	6.82%	18.35680728	0.00022947	0.000313473	4.90E-07
ENSG00000143740_227747845_227780673_383	annotated	SNAP47	1	+	1149	15	1	4	1.57%	4.313610067	0.016344022	0.069273208	0.005268498
ENSG00000115966_175130185_175074609_487	annotated	ATF2	2	-	1461	46	6	0	4.11%	10.40344725	0.000666429	3.78E-05	2.01E-07
ENSG00000162775_110339538_110346354_925	annotated	RBM15	1	+	2775	30	4	0	1.41%	3.572135472	0.006750264	0.000662934	2.95E-05
ENSG00000103769_65869586_65887752_155	annotated	RAB11A	15	+	465	190	28	2	22.58%	135.0113568	3.85E-06	1.10E-07	4.34E-12
ENSG00000074696_65556886_65576379_245	annotated	HACD3	15	+	735	307	23	17	21.63%	138.0132205	1.33E-08	1.38E-09	2.02E-16
ENSG00000169855_78714439_78598913_1256	annotated	ROBO1	3	-	3768	12	2	1	0.48%	1.052301055	0.050679946	0.019795882	0.004478852
ENSG00000170004_7884849_7911585_2045	annotated	CHD3	17	+	6135	69	15	2	1.71%	3.716243626	4.55E-05	3.55E-07	1.77E-10
ENSG00000173848_5446639_5446719_26	uORF	NET1	10	+	78	13	3	0	19.23%	55.07042185	0.083503598	0.019679754	0.007465082
ENSG00000173848_5446766_5456994_542	annotated	NET1	10	+	1626	132	14	12	7.20%	26.82396932	5.02E-07	6.85E-07	3.14E-12
ENSG00000134014_28106731_28189725_455	annotated	ELP3	8	+	1365	43	1	0	4.62%	10.4089149	3.41E-05	2.41E-05	6.36E-09
ENSG00000196323_130261807_130236164_445	annotated	ZBTB44	11	-	1335	9	1	1	1.12%	2.227567626	0.035374934	0.035374934	0.005300298
ENSG00000139180_4668459_4682304_80	novel	NDUFA9	12	+	240	33	0	0	8.75%	45.43309803	0.008574016	0.008574016	0.000374746
ENSG00000131238_40097238_40075138_301	annotated	PPT1	1	-	903	454	38	7	15.95%	166.1260566	7.36E-09	7.69E-10	6.33E-17
ENSG00000131238_40074162_40074061_33	dORF	PPT1	1	-	99	45	1	0	21.21%	150.1920596	0.008877961	0.008877961	0.000400235
ENSG00000130270_1810673_1799651_529	annotated	ATP8B3	19	-	1587	30	3	0	3.59%	6.246172611	0.000461789	4.02E-05	1.44E-07
ENSG00000011243_15401191_15397752_186	novel	AKAP8L	19	-	558	8	1	2	3.76%	4.737240589	0.017404239	0.061805201	0.004922815
ENSG00000108312_44216564_44207242_661	annotated	UBTF	17	-	1983	42	18	6	3.63%	6.998359207	0.009542311	0.000147156	1.24E-05
ENSG00000125691_38853710_38847939_114	annotated	RPL23	17	-	342	562	67	18	13.16%	542.9750365	0.001499876	0.000794465	7.40E-06
ENSG00000104980_7941131_7935146_102	annotated	TIMM44	19	-	306	21	2	0	6.86%	22.67605606	0.042435259	0.007029303	0.001563844
ENSG00000144426_203049824_203217354_2643	annotated	NBEAL1	2	+	7929	14	6	3	0.30%	0.583417258	0.139258512	0.040853127	0.022899425
ENSG00000184640_77402556_77498658_395	annotated	9-Sep	17	+	1185	115	18	4	8.61%	32.06632159	0.000137499	9.24E-07	1.38E-09

ENSG00000151093_25791021_25791783_102	novel	OXSM	3	+	306	23	1	0	5.88%	24.83568044	0.020525443	0.013640586	0.001325514
ENSG00000103549_30765232_30766582_164	novel	RNF40	16	+	492	10	3	0	3.05%	6.715905104	0.100002646	0.020613417	0.009397837
ENSG00000063854_1822969_1809353_258	annotated	HAGH	16	-	774	37	0	2	7.36%	15.79539231	5.40E-05	0.000218523	8.73E-08
ENSG00000172270_572635_582540_55	annotated	BSG	19	+	165	1058	40	22	34.55%	2118.709321	0.000380784	0.000158194	4.17E-07
ENSG00000172270_579563_582577_24	Overlap_dORF	BSG	19	+	72	300	70	7	41.67%	1376.760546	0.002531016	0.004889814	6.98E-05
ENSG00000104343_73878813_73794102_159	annotated	UBE2W	8	-	477	29	1	2	7.55%	20.08858156	0.00092354	0.002061799	1.17E-05
ENSG00000072756_3137409_3144631_53	annotated	TRNT1	3	+	159	12	0	0	9.43%	24.93754952	0.019216965	0.019216965	0.001707396
ENSG00000131778_147275365_147295509_470	novel	CHD1L	1	+	1410	24	0	2	2.34%	5.624213296	0.001092093	0.005502968	3.68E-05
ENSG00000227227_186641550_186641386_54	novel	AC017101.1	2	-	162	41	3	6	11.11%	83.62545541	0.017143984	0.061512597	0.004837825
ENSG00000172046_49119145_49108412_1372	annotated	USP19	3	-	4116	40	7	1	1.38%	3.211103315	0.001639071	7.26E-05	9.41E-07
ENSG00000106244_99408548_99403511_39	annotated	PDAP1	7	-	117	24	3	3	15.38%	67.77898074	0.013428348	0.063412829	0.004086769
ENSG00000204520_31412115_31415139_124	Overlap_dORF	MICA	6	+	372	39	1	11	8.87%	34.64107181	0.0020028	0.015073811	0.000179882
ENSG00000204520_31410764_31412431_235	annotated	MICA	6	+	705	20	9	1	3.40%	9.373688826	0.184585708	0.009288069	0.010738847
ENSG00000162813_220074026_220067290_106	novel	BPNT1	1	-	318	14	2	1	5.66%	14.54690389	0.042234516	0.023672501	0.004366627
ENSG00000127419_950475_958496_422	annotated	TMEM175	4	+	1266	14	9	0	2.13%	3.65396164	0.093607421	0.00308495	0.002058411
ENSG00000198793_11144737_11132972_229	novel	MTOR	1	-	687	12	0	0	2.18%	5.771572596	0.020613417	0.020613417	0.001946209
ENSG00000115648_237510536_237553592_496	novel	MLPH	2	+	1488	146	16	20	9.68%	32.42049028	7.32E-08	2.64E-08	1.94E-14
ENSG00000009844_142170350_142198629_123	novel	VTA1	6	+	369	12	1	1	6.50%	10.74544817	0.010020958	0.00419316	0.00022538
ENSG00000131748_39660491_39662908_139	novel	STARD3	17	+	417	15	0	6	5.04%	11.88570256	0.008473674	0.079426469	0.003629372
ENSG00000184277_101645142_101642479_73	novel	TM2D3	15	-	219	64	8	0	20.55%	96.56183558	0.000295633	0.000295633	5.91E-07
ENSG00000139697_123350441_123295908_1393	annotated	SBNO1	12	-	4179	146	16	15	4.09%	11.54383574	1.74E-08	1.39E-08	2.49E-15
ENSG00000129515_34586235_34575735_37	novel	SNX6	14	-	111	30	7	4	13.51%	89.30338679	0.022899795	0.019679754	0.002057638
ENSG00000100650_69770482_69771461_145	dORF	SRSF5	14	+	435	78	24	3	13.10%	59.24817799	0.018632935	0.000108871	2.18E-05
ENSG00000100650_69768157_69769561_138	annotated	SRSF5	14	+	414	278	33	26	32.61%	221.8779315	1.67E-07	1.91E-07	3.04E-13
ENSG00000176463_92126159_92163135_286	novel	SLCO3A1	15	+	858	25	0	0	3.50%	9.627696128	0.002321823	0.002321823	3.12E-05
ENSG00000141447_24377452_24163179_923	annotated	OSBPL1A	18	-	2769	24	3	2	1.19%	2.863900595	0.008808686	0.005064994	0.000235624
ENSG00000105372_41860775_41871377_145	annotated	RPS19	19	+	435	1883	259	138	36.55%	1430.311784	1.01E-08	5.46E-10	6.32E-17
ENSG00000196365_5720143_5719805_112	novel	LONP1	19	-	336	8	1	3	4.46%	7.867203122	0.019216965	0.079862169	0.006984847
ENSG00000092931_76738413_76778352_429	annotated	MFSD11	17	+	1287	79	4	4	6.99%	20.28234651	5.91E-06	4.79E-06	2.37E-10
ENSG00000114988_96740040_96707256_348	annotated	LMAN2L	2	-	1044	53	6	0	6.90%	16.7743239	9.31E-05	7.30E-06	5.73E-09
ENSG00000071073_98726332_98625566_535	annotated	MGAT4A	2	-	1605	10	2	0	1.12%	2.058707359	0.056853419	0.011570466	0.003221159
ENSG00000079462_42302240_42297078_184	annotated	PAFAH1B3	19	-	552	135	19	6	15.22%	80.80985815	0.000209232	3.06E-05	4.93E-08
ENSG00000151914_56535208_56459005_2163	annotated	DST	6	-	6489	19	5	4	0.46%	0.967487763	0.045791795	0.046151733	0.00857947
ENSG00000104946_49883667_49888195_216	annotated	TBC1D17	19	+	648	17	1	0	2.78%	8.668492329	0.042234516	0.011772051	0.002392043
ENSG00000108469_75666443_75650640_397	annotated	RECQL5	17	-	1191	19	2	0	2.77%	5.271224258	0.002979763	0.001343041	2.38E-05
ENSG00000163686_58256608_58293765_330	annotated	ABHD6	3	+	990	39	1	0	6.06%	13.01664517	4.92E-05	3.59E-05	1.34E-08
ENSG00000138078_44346342_44321356_638	annotated	PREPL	2	-	1914	43	8	1	3.13%	7.423285704	0.001704072	5.31E-05	7.50E-07
ENSG00000196756_38434513_38426436_49	novel	SNHG17	20	-	147	26	3	3	14.29%	58.44208033	0.009583875	0.023878474	0.001121714
ENSG00000144161_112254981_112230918_291	annotated	ZC3H8	2	-	873	8	5	0	1.72%	3.027926975	0.301439101	0.019216965	0.033495552
ENSG00000133997_70600637_70592872_157	novel	MED6	14	-	471	27	10	0	7.01%	18.94141898	0.056644149	0.001506153	0.000646833
ENSG00000100596_77597311_77578951_94	novel	SPTLC2	14	-	282	14	2	0	5.32%	16.40395545	0.055414058	0.019216965	0.004780439
ENSG00000258790_35278700_35317306_227	dORF	AL121594.1	14	+	681	181	23	2	11.89%	87.82155379	3.60E-05	3.81E-06	1.18E-09
ENSG00000258790_35123246_35273566_583	annotated	AL121594.1	14	+	1749	83	3	1	5.15%	15.68042886	3.79E-06	8.96E-07	3.03E-11
ENSG00000169432_166288475_166272439_541	annotated	SCN9A	2	-	1623	17	0	0	1.11%	3.460987695	0.013428348	0.013428348	0.000872559
ENSG00000167315_49813820_49783847_393	annotated	ACAA2	18	-	1179	90	8	4	6.87%	25.22309398	1.23E-05	1.19E-05	1.18E-09
ENSG00000170312_60780166_60792147_224	annotated	CDK1	10	+	672	105	19	1	12.95%	51.62852049	0.000111058	1.28E-06	1.46E-09
ENSG00000170312_60792014_60793975_100	Overlap_dORF	CDK1	10	+	300	76	6	12	18.00%	83.70704122	8.75E-05	0.001323541	8.81E-07
ENSG00000166855_65185153_65150823_633	annotated	CLPX	15	-	1899	64	13	0	4.27%	11.13588309	0.000789293	1.88E-06	1.86E-08
ENSG00000111653_6652402_6651195_77	novel	ING4	12	-	231	16	4	0	6.49%	22.88640908	0.139258512	0.021084099	0.013797358
ENSG00000186283_179087920_179091716_73	novel	TOR3A	1	+	219	28	0	1	12.33%	42.24580307	0.003303074	0.009288069	0.000168518
ENSG00000122515_44761744_44767623_442	novel	ZMIZ2	7	+	1326	16	5	0	1.36%	3.986998867	0.145023522	0.013428348	0.010350889
ENSG00000106211_76302713_76303273_186	annotated	HSPB1	7	+	558	380	68	6	17.74%	225.018928	1.47E-05	2.60E-07	3.94E-11
ENSG00000106211_76304060_76304173_37	dORF	HSPB1	7	+	111	43	15	2	16.22%	128.0015211	0.053844448	0.021261239	0.005058937
ENSG00000266967_42956514_42956317_65	novel	AARSD1	17	-	195	15	2	1	9.23%	25.41711778	0.044040756	0.028889786	0.005421999
ENSG00000143569_154225127_154263152_975	annotated	UBAP2L	1	+	2925	109	18	33	4.82%	12.3131815	2.32E-06	3.59E-05	7.41E-10
ENSG00000136270_45109237_45100325_521	annotated	TBRG4	7	-	1563	121	6	3	8.45%	25.57973529	5.41E-08	1.12E-08	6.26E-15
ENSG00000164258_53560678_53683221_170	annotated	NDUFS4	5	+	510	226	30	39	26.47%	146.4225334	4.15E-08	9.09E-08	3.73E-14
ENSG00000040633_7239267_7235626_332	annotated	PHF23	17	-	996	59	3	4	4.82%	19.57322222	0.000403006	0.000403006	1.08E-06
ENSG00000099910_20489211_20442073_634	annotated	KLHL22	22	-	1902	29	4	0	1.89%	5.037988119	0.006154857	0.000982952	3.77E-05
ENSG00000132382_4542930_4539415_370	novel	MYBBP1A	17	-	1110	12	3	0	1.62%	3.572135472	0.051431483	0.013217227	0.003234637
ENSG00000110911_51010704_50981726_560	annotated	SLC11A2	12	-	1680	153	13	5	9.11%	30.09205194	8.36E-09	2.24E-09	2.02E-16
ENSG00000092841_56158402_56159789_170	novel	MYL6	12	+	510	949	45	11	8.24%	614.8450628	0.001118811	0.00049077	3.53E-06
ENSG00000154743_12489930_12529912_359	novel	TSEN2	3	+	1077	18	2	0	1.95%	5.522382136	0.032996028	0.008473674	0.00140192
ENSG00000188529_23972001_23971569_69	novel	SRSF10	1	-	207	34	6	0	7.25%	54.2722998	0.091177563	0.021084099	0.008663519
ENSG00000100813_23061439_23059148_189	dORF	ACIN1	14	-	567	15	1	0	4.23%	8.741336802	0.010020958	0.004826937	0.000255995
ENSG00000178252_49007432_49009012_67	novel	WDR6	3	+	201	21	0	2	7.46%	34.52175698	0.019679754	0.078649604	0.007006523
ENSG00000116898_36456327_36455788_92	novel	MRPS15	1	-	276	61	2	0	18.48%	73.02816811	0.000297145	0.000136717	2.84E-07
ENSG00000172530_87984207_88076661_424	annotated	BANP	16	+	1272	19	0	1	1.18%	4.935556675	0.021084099	0.035772986	0.003354605

ENSG00000243678_51166831_51171507_82	annotated	NME2	17	+	246	659	101	19	40.24%	885.1562927	5.89E-06	5.44E-07	2.97E-11
ENSG00000106484_130495369_130505056_326	annotated	MEST	7	+	978	43	4	2	4.91%	14.52778	0.000362935	0.000678439	1.62E-06
ENSG00000136997_127738263_127740958_439	annotated	MYC	8	+	1317	212	38	14	9.79%	53.18874457	1.01E-05	3.51E-07	3.49E-11
ENSG00000175592_65900339_65892886_235	annotated	FOSL1	11	-	705	26	9	0	3.40%	12.18579547	0.013991246	0.005655336	0.000411477
ENSG00000134910_125602808_125620810_613	annotated	STT3A	11	+	1839	526	24	9	15.82%	94.50910895	2.22E-16	5.75E-17	1.91E-31
ENSG00000183020_1009173_1009922_117	novel	AP2A2	11	+	351	12	0	0	4.27%	11.29649679	0.019679754	0.019679754	0.001784964
ENSG00000140983_668200_668468_50	novel	RHOT2	16	+	150	10	4	0	10.00%	22.02816874	0.145248299	0.020613417	0.014232745
ENSG00000106003_2513269_2527212_255	annotated	LFNG	7	+	765	22	5	0	2.35%	9.5023473	0.020342454	0.013428348	0.001295518
ENSG00000100280_29363023_29328821_899	annotated	AP1B1	22	-	2697	79	9	6	3.23%	9.678672584	5.30E-05	2.40E-05	9.82E-09
ENSG00000138081_47839647_47808118_809	annotated	FBXO11	2	-	2427	18	13	3	1.61%	2.450599736	0.232686791	0.006674189	0.011730973
ENSG00000122390_3479556_3484855_177	annotated	NAA60	16	+	531	18	4	0	3.95%	11.20076377	0.041678091	0.008674847	0.001826095
ENSG00000204954_103956678_103951523_51	annotated	C12orf73	12	-	153	16	7	0	17.65%	34.55399018	0.062411228	0.002500085	0.001069711
ENSG00000198000_92319259_92297836_1041	dORF	NOL8	9	-	3123	40	1	2	1.92%	4.232116953	3.14E-05	0.000158386	3.80E-08
ENSG00000149273_75399548_75401660_44	annotated	RPS3	11	+	132	225	20	9	36.36%	563.2202235	0.000749784	0.00039459	1.93E-06
ENSG00000149273_75400758_75404865_196	Overlap_dORF	RPS3	11	+	588	441	73	28	14.29%	247.8168983	3.36E-06	1.08E-05	3.07E-10
ENSG00000177697_836070_838192_253	annotated	CD151	11	+	759	1246	99	44	39.53%	542.4327718	2.53E-15	1.13E-16	4.29E-30
ENSG00000137500_83285972_83265913_208	annotated	CCDC90B	11	-	624	26	7	13	5.77%	13.76760546	0.030396003	0.108436089	0.013938015
ENSG00000159596_45691069_45693905_247	annotated	TMEM69	1	+	741	41	11	0	4.86%	18.28248823	0.019937236	0.001065834	0.000144572
ENSG00000101040_47356670_47210761_1125	annotated	ZMYND8	20	-	3375	22	8	0	0.80%	2.153865388	0.048111409	0.003606289	0.001048508
ENSG00000148296_133336132_133331869_361	annotated	SURF6	9	-	1083	47	7	1	4.71%	14.33966663	0.002791083	0.000166438	3.45E-06
ENSG00000164953_93763916_93816452_835	annotated	TMEM67	8	+	2505	23	4	0	1.44%	3.033819647	0.007963342	0.00092354	4.69E-05
ENSG00000115419_190927345_190932862_169	annotated	GLS	2	+	507	60	5	3	12.43%	39.10325812	7.48E-05	5.07E-05	2.82E-08
ENSG00000172366_641992_646487_65	annotated	MCRIP2	16	+	195	9	0	0	7.69%	15.25027067	0.016947427	0.016947427	0.001349898
ENSG00000168036_41224552_41239342_768	annotated	CTNNB1	3	+	2304	288	46	13	11.46%	41.30281639	2.21E-12	5.94E-15	2.04E-25
ENSG00000010278_6232667_6232765_32	novel	CD9	12	+	96	58	1	0	18.75%	199.6302792	0.013640586	0.013640586	0.000898637
ENSG00000099795_14572000_14566133_137	annotated	NDUFB7	19	-	411	220	30	1	21.17%	176.8685081	0.000115074	1.09E-06	1.32E-09
ENSG00000181744_143985600_143989841_192	annotated	DIPK2A	3	+	576	16	1	1	4.69%	9.178403642	0.00640069	0.010020958	0.000331324
ENSG00000149577_117179264_117195984_855	annotated	SIDT2	11	+	2565	17	2	0	0.94%	2.189934904	0.023197249	0.005306578	0.000651782
ENSG00000149182_47176853_47175393_94	annotated	ARFGAP2	11	-	282	10	0	1	5.32%	11.71711103	0.019679754	0.065398531	0.005779267
ENSG00000079819_130880483_130858136_383	annotated	EPB41L2	6	-	1149	20	0	0	2.61%	5.751480089	0.002321823	0.002321823	3.12E-05
ENSG00000114491_124730472_124731550_66	annotated	UMPS	3	+	198	48	6	1	13.64%	80.10243179	0.020460432	0.004580614	0.000504034
ENSG00000114491_124737792_124744084_302	dORF	UMPS	3	+	906	52	3	5	6.29%	18.96464859	0.0001259	0.000123342	1.11E-07
ENSG00000082898_61495460_61493843_84	novel	XPO1	2	-	252	21	1	1	9.52%	27.53521093	0.010079165	0.010079165	0.000508526
ENSG00000115163_26786197_26793279_140	novel	CENPA	2	+	420	46	10	13	7.86%	36.18913436	0.026567016	0.069437446	0.007890574
ENSG00000117385_42754941_42746697_312	novel	P3H1	1	-	936	30	4	3	5.13%	10.59046574	0.00162318	0.001331916	1.30E-05
ENSG00000117385_42766878_42755161_377	novel	P3H1	1	-	1131	115	5	0	5.84%	33.59733959	6.96E-05	1.87E-05	1.02E-08
ENSG00000132254_6480388_6478581_191	annotated	ARFIP2	11	-	573	33	14	1	5.24%	19.02956985	0.089622512	0.003783485	0.002268102
ENSG00000161016_144791457_144791248_69	novel	RPL8	8	-	207	882	102	17	31.88%	1407.887306	4.25E-05	2.63E-05	8.59E-09
ENSG00000161016_144792129_144791768_94	novel	RPL8	8	-	282	770	139	66	30.85%	902.2175495	3.45E-05	6.91E-05	1.80E-08
ENSG00000160746_43605852_43366906_660	annotated	ANO10	3	-	1980	28	4	4	2.27%	4.672641854	0.001646163	0.002356052	2.29E-05
ENSG00000134215_107642744_107609482_94	Overlap_dORF	VAV3	1	-	282	10	7	0	5.32%	11.71711103	0.226483201	0.020613417	0.024188353
ENSG00000075945_170055336_169921676_748	annotated	KIFAP3	1	-	2244	23	1	2	1.60%	3.386683697	0.001559221	0.004359749	3.99E-05
ENSG00000075945_170074467_169921676_748	annotated	KIFAP3	1	-	2244	23	1	2	1.60%	3.386683697	0.001559221	0.004359749	3.99E-05
ENSG00000120370_170532149_170552462_394	annotated	GORAB	1	+	1182	15	1	3	1.52%	4.193179329	0.022004978	0.073562049	0.007157727
ENSG00000063854_1826787_1819074_193	novel	HAGH	16	-	579	55	7	4	10.88%	31.38728707	9.59E-05	0.000173529	1.19E-07
ENSG00000159176_201489070_201488783_95	novel	CSRP1	1	-	285	24	4	0	6.32%	27.82505525	0.013428348	0.013640586	0.00088551
ENSG00000159176_201490405_201490172_77	novel	CSRP1	1	-	231	196	27	0	14.29%	280.3585112	0.001626321	0.001634015	1.59E-05
ENSG00000085063_33722445_33710275_119	annotated	CD59	11	-	357	176	43	56	15.97%	162.8973823	0.01680466	0.072704487	0.005672071
ENSG00000215301_41334253_41345417_420	novel	DDX3X	X	+	1260	106	35	9	7.14%	27.79745103	0.000950113	1.52E-05	1.34E-07
ENSG00000215301_41345404_41347719_271	novel	DDX3X	X	+	813	124	14	4	8.86%	50.39654841	2.15E-05	9.54E-06	1.65E-09
ENSG00000168216_69752278_69676158_389	Overlap_dORF	LMBRD1	6	-	1167	95	20	0	8.23%	26.89814949	0.000122546	3.42E-07	5.16E-10
ENSG00000168216_69796881_69741848_144	annotated	LMBRD1	6	-	432	32	6	1	9.03%	24.47574305	0.01035193	0.000874411	5.93E-05
ENSG00000168216_69790322_69676158_467	novel	LMBRD1	6	-	1401	124	26	0	9.21%	29.24510625	1.30E-05	4.62E-09	9.94E-13
ENSG00000117139_202733853_202729036_392	novel	KDM5B	1	-	1176	15	2	1	2.55%	4.214573101	0.012926125	0.003925334	0.000274612
ENSG00000103540_19536363_19551248_781	annotated	CCP110	16	+	2343	10	3	0	0.77%	1.410254081	0.08791702	0.012798403	0.005611737
ENSG00000179151_74656003_74632612_325	annotated	EDC3	15	-	975	11	4	0	2.15%	3.727843941	0.121794855	0.007593099	0.005521741
ENSG00000167965_2206086_2208877_326	annotated	MLST8	16	+	978	109	18	4	11.66%	36.82623302	1.36E-05	2.83E-07	3.92E-11
ENSG00000087253_55528552_55541978_168	annotated	LPCAT2	16	+	504	18	0	2	4.76%	11.80080468	0.005257623	0.017239393	0.00047625
ENSG00000164073_127965133_127921759_336	annotated	MFSD8	4	-	1008	18	0	1	2.98%	5.900402341	0.00224099	0.003372875	4.33E-05
ENSG00000168216_69790322_69676158_467	annotated	LMBRD1	6	-	1401	124	26	0	9.21%	29.24510625	1.30E-05	4.62E-09	9.94E-13
ENSG00000213380_69339552_69330839_612	annotated	COG8	16	-	1836	35	8	0	3.10%	6.29890446	0.003301094	3.53E-05	1.12E-06
ENSG00000100138_41675154_41674933_73	novel	SNU13	22	-	219	201	12	0	34.25%	303.2645149	1.98E-05	5.88E-06	9.61E-10
ENSG00000137700_119028246_119024910_286	novel	SLC37A4	11	-	858	41	3	0	5.94%	15.78942165	0.000671423	0.000127134	6.05E-07
ENSG00000137700_119029530_119029333_65	novel	SLC37A4	11	-	195	31	7	0	15.38%	52.52871007	0.019390513	0.002376615	0.000272493
ENSG00000114107_138571122_138495015_532	annotated	CEP70	3	-	1596	15	0	0	1.13%	3.105474917	0.013428348	0.013428348	0.000872559
ENSG00000115317_74530977_74531664_89	novel	HTRA2	2	+	267	11	1	1	5.62%	13.61291327	0.020613417	0.035374934	0.003252526
ENSG00000140382_77458420_77479315_343	annotated	HMG20A	15	+	1029	29	5	0	3.21%	9.312199614	0.009797758	0.001513502	8.95E-05
ENSG00000167508_88663080_88652525_400	annotated	MVD	16	-	1200	142	33	8	13.75%	39.09999952	1.20E-06	2.62E-09	4.20E-14

ENSG00000101940_48598952_48605032_393	annotated	WDR13	X	+	1179	23	3	0	2.04%	6.445901794	0.005554612	0.005604867	0.000166099
ENSG00000143420_150629470_150625638_140	annotated	ENSA	1	-	420	52	11	9	6.43%	40.90945623	0.029255686	0.014451683	0.001971876
ENSG00000108433_46923193_46963884_210	annotated	GOSR2	17	+	630	40	7	0	6.67%	20.97920832	0.011149146	0.000448121	3.68E-05
ENSG00000283761_100058702_100082209_418	dORF	AC118553.2	1	+	1254	148	21	6	10.05%	38.99723653	1.41E-07	3.29E-08	4.65E-14
ENSG00000283761_99993624_100049921_276	annotated	AC118553.2	1	+	828	16	9	0	3.99%	6.384976447	0.151590816	0.000888449	0.001652192
ENSG00000121766_31310099_31364193_241	annotated	ZCCHC17	1	+	723	21	2	0	4.56%	9.597334929	0.003646019	0.001440978	3.11E-05
ENSG00000073008_44647366_44661811_343	dORF	PVR	19	+	1029	149	14	1	9.91%	47.84543939	1.25E-05	1.81E-07	2.39E-11
ENSG00000065559_12095620_12141260_253	Overlap_dORF	MAP2K4	17	+	759	15	3	0	3.16%	6.530089548	0.029020025	0.005306578	0.000824676
ENSG00000177963_209296_214350_524	annotated	RIC8A	11	+	1572	78	6	6	4.96%	16.39501109	3.79E-05	4.56E-05	1.31E-08
ENSG00000221968_61879401_61873814_310	annotated	FADS3	11	-	930	43	10	15	5.81%	15.2776009	0.00115278	0.004107349	2.86E-05
ENSG00000147162_71536192_71573794_1029	annotated	OGT	X	+	3087	19	0	3	1.07%	2.033698766	0.001440978	0.009470507	8.28E-05
ENSG00000197756_216498875_216499958_58	annotated	RPL37A	2	+	174	52	5	27	18.97%	98.74696332	0.001565547	0.024946005	0.000254712
ENSG00000197756_216499955_216501404_46	Overlap_dORF	RPL37A	2	+	138	1252	149	27	63.04%	2997.746442	1.22E-05	1.82E-06	1.94E-10
ENSG00000197563_62161260_62045856_839	annotated	PIGN	18	-	2517	90	9	0	4.17%	11.81487	4.20E-06	8.70E-08	3.83E-12
ENSG00000180185_1827239_1839306_223	annotated	FAHD1	16	+	669	104	19	0	12.11%	51.36613339	0.000147752	2.56E-06	3.66E-09
ENSG00000180185_1827239_1838126_245	annotated	FAHD1	16	+	735	104	19	0	11.02%	46.75366427	0.000147752	2.56E-06	3.66E-09
ENSG00000172500_65888418_65887822_131	novel	FIBP	11	-	393	62	9	6	12.21%	52.12772756	0.001576668	0.001460542	1.38E-05
ENSG00000105983_156892993_156684078_490	annotated	LMBR1	7	-	1470	88	22	5	6.94%	19.78039642	0.000314892	1.30E-06	4.71E-09
ENSG00000169567_131165205_131159447_126	annotated	HINT1	5	-	378	464	45	20	30.95%	405.5980276	4.98E-07	5.80E-08	2.86E-13
ENSG00000172057_39924203_39922550_153	annotated	ORMDL3	17	-	459	29	16	0	10.46%	20.87636907	0.056044867	0.000177448	0.000131653
ENSG00000109111_28673402_28701625_1726	annotated	SUPT6H	17	+	5178	59	13	5	1.80%	3.764953522	0.000394318	6.44E-06	2.41E-08
ENSG00000165097_18159896_18221992_822	annotated	KDM1B	6	+	2466	16	1	0	1.09%	2.143860705	0.005561256	0.003452921	0.000105543
ENSG00000138794_109698328_109689330_275	annotated	CASP6	4	-	825	15	7	2	2.91%	6.007682384	0.094176549	0.020624382	0.0088127
ENSG00000166803_64381371_64366005_65	annotated	PCLAF	15	-	195	212	82	168	15.38%	359.2285979	0.015298565	0.181583589	0.014929221
ENSG00000185621_197960246_198038667_647	annotated	LMLN	3	+	1941	10	0	0	0.77%	1.702331433	0.019679754	0.019679754	0.001784964
ENSG00000038002_177442375_177438821_105	novel	AGA	4	-	315	29	1	2	10.48%	30.41985207	0.002361478	0.005625935	7.53E-05
ENSG00000100722_88563134_88611751_605	annotated	ZC3H14	14	+	1815	61	24	2	5.12%	11.10510986	0.003602255	8.76E-07	6.45E-08
ENSG00000213983_23567314_23566417_68	annotated	AP1G2	14	-	204	10	1	0	8.82%	16.1971829	0.011772051	0.011772051	0.000681273
ENSG00000133997_70600637_70584813_246	annotated	MED6	14	-	738	74	18	0	10.16%	33.13179851	0.00044774	5.54E-06	2.43E-08
ENSG00000095794_35148378_35179329_135	annotated	CREM	10	+	405	14	2	0	3.70%	11.42201342	0.045845141	0.021084099	0.004277929
ENSG00000160752_155319665_155320609_154	novel	FDPS	1	+	462	106	1	7	12.99%	75.81123008	4.34E-05	6.54E-05	2.13E-08
ENSG00000173272_131491960_131484061_80	novel	MZT2A	2	-	240	20	3	0	7.50%	27.53521093	0.062728927	0.01300724	0.003936255
ENSG00000175198_100368473_100530166_133	annotated	PCCA	13	+	399	19	0	0	6.02%	15.73440624	0.005306578	0.005306578	0.000151001
ENSG00000149925_30069625_30070212_114	annotated	ALDOA	16	+	342	572	104	36	35.96%	552.636514	1.96E-08	1.39E-08	2.80E-15
ENSG00000188603_28482191_28477516_115	novel	CLN3	16	-	345	54	5	1	16.52%	51.71830922	0.000208869	7.59E-05	1.15E-07
ENSG00000132600_68321442_68352496_442	novel	PRMT7	16	+	1326	24	1	7	2.49%	5.980498301	0.001491519	0.01636533	0.00015329
ENSG00000214447_44900999_44902817_242	annotated	FAM187A	17	+	726	9	1	0	2.07%	4.09614708	0.041782825	0.019216965	0.003600526
ENSG00000107362_71875080_71866787_288	annotated	ABHD17B	9	-	864	40	8	2	5.21%	15.2973394	0.005456048	0.000262451	1.06E-05
ENSG00000152242_46216160_46216618_152	novel	C18orf25	18	+	456	10	3	0	3.29%	7.246108139	0.065398531	0.020613417	0.006006025
ENSG00000174437_110281790_110282736_50	annotated	ATP2A2	12	+	150	20	6	2	12.00%	44.05633748	0.020342454	0.02385744	0.002204905
ENSG00000174437_110296650_110346470_917	dORF	ATP2A2	12	+	2751	875	107	29	20.94%	105.0962249	6.72E-27	5.45E-32	9.60E-57
ENSG00000171566_154548875_154536685_482	annotated	PLRG1	4	-	1446	81	8	4	5.81%	18.50914593	7.38E-05	1.18E-05	7.02E-09
ENSG00000168710_110018438_110018695_57	novel	AHCYL1	1	+	171	13	0	0	10.53%	25.11984155	0.01300724	0.01300724	0.000821865
ENSG00000175221_885781_868450_462	annotated	MED16	19	-	1386	16	0	4	1.52%	3.814401514	0.008473674	0.060419327	0.002673316
ENSG00000076554_80080438_80052624_146	annotated	TPD52	8	-	438	87	9	0	6.16%	65.63187262	0.022356462	0.003816221	0.00047335
ENSG00000076554_80044171_80038116_57	dORF	TPD52	8	-	171	29	2	0	14.04%	56.0365696	0.012000727	0.005706018	0.000356061
ENSG00000106829_79627422_79725144_684	annotated	TLE4	9	+	2052	21	0	1	1.75%	3.381517131	0.00077643	0.001635336	7.89E-06
ENSG00000140521_89329071_89318013_870	annotated	POLG	15	-	2610	29	2	5	1.26%	3.671361457	0.006208285	0.026171386	0.000845245
ENSG00000164733_11853372_11852665_24	internal	CTSB	8	-	72	32	2	43	37.50%	146.8544583	0.003710481	0.468493666	0.025643776
ENSG00000259030_74198279_74464742_843	annotated	FPGT-TNNI3K	1	+	2529	17	2	0	0.71%	2.221108355	0.052581193	0.01300724	0.003266469
ENSG00000099956_23787179_23801397_259	novel	SMARCB1	22	+	777	60	7	3	5.41%	25.51525337	0.002239807	0.000773031	1.08E-05
ENSG00000127955_80188989_80217493_302	novel	GNAI1	7	+	906	119	9	5	11.26%	43.39986888	1.78E-07	4.72E-07	7.92E-13
ENSG00000106638_73574098_73570507_352	dORF	TBL2	7	-	1056	64	2	0	6.82%	20.02560795	2.67E-05	7.40E-06	1.61E-09
ENSG00000121064_56978160_56987294_122	novel	SCPEP1	17	+	366	16	0	3	5.74%	14.44470081	0.007882058	0.065131088	0.002742388
ENSG00000177663_17102285_17109820_618	annotated	IL17RA	22	+	1854	23	1	0	1.62%	4.099092889	0.002767218	0.002008257	3.22E-05
ENSG00000120868_98665717_98732566_875	annotated	APAF1	12	+	2625	15	11	0	0.91%	1.888128749	0.127645253	0.005160369	0.004421647
ENSG00000161011_179823917_179833291_217	novel	SQSTM1	5	+	651	708	98	40	37.33%	359.3535361	2.41E-12	7.21E-13	2.23E-23
ENSG00000178105_108691963_108940423_521	annotated	DDX10	11	+	1563	16	2	6	1.34%	3.382444336	0.028902806	0.097833871	0.012019898
ENSG00000087365_66052385_66068745_895	annotated	SF3B2	11	+	2685	311	35	10	11.06%	38.27240491	7.03E-14	1.57E-16	1.80E-28
ENSG00000091483_241519722_241497828_510	annotated	FH	1	-	1530	288	37	9	14.71%	62.19718233	2.07E-11	3.42E-13	9.66E-23
ENSG00000171307_97452908_97456891_179	annotated	ZDHHC16	10	+	537	43	11	3	8.94%	26.45841497	0.004093768	0.000600369	1.59E-05
ENSG00000058673_203840341_203852399_474	annotated	ZC3H11A	1	+	1422	15	0	0	1.05%	3.485469738	0.020613417	0.020613417	0.001946209
ENSG00000100324_39417798_39436571_296	annotated	TAB1	22	+	888	9	5	0	2.03%	3.348877005	0.113684165	0.012178958	0.007230548
ENSG00000185721_31400620_31433971_353	annotated	DRG1	22	+	1059	75	9	0	5.95%	23.40102912	0.000482564	2.76E-05	1.08E-07
ENSG00000183763_49856453_49829103_469	annotated	TRAIP	3	-	1407	31	4	0	2.56%	7.280098411	0.005757552	0.001040526	3.69E-05
ENSG00000167283_118401666_118409012_34	annotated	ATP5MG	11	+	102	329	19	2	35.29%	1065.774635	0.001818063	0.001087214	1.20E-05
ENSG00000187954_144453442_144452180_203	novel	CYHR1	8	-	609	21	0	0	3.45%	11.39388038	0.008473674	0.008473674	0.000366502
ENSG00000147642_109586100_109574906_500	annotated	SYBU	8	-	1500	22	9	1	2.00%	4.846197123	0.02437921	0.00345766	0.000477745

ENSG00000069345_46971413_46957029_313	dORF	DNAJA2	16	-	939	149	27	7	15.97%	52.43126426	5.32E-07	3.57E-09	2.30E-14
ENSG00000168291_58431954_58428034_317	novel	PDHB	3	-	951	115	14	2	10.41%	39.9564575	3.80E-05	3.90E-07	1.58E-10
ENSG00000160410_40586917_40591207_270	annotated	SHKBP1	19	+	810	29	0	0	4.81%	11.82994247	0.000628963	0.000628963	2.54E-06
ENSG00000124795_18255838_18237103_236	novel	DEK	6	-	708	47	13	1	4.66%	21.93482904	0.010009985	0.001811542	0.000107014
ENSG00000112983_138177612_138157208_830	Overlap_dORF	BRD8	5	-	2490	24	9	11	1.45%	3.184795481	0.094084112	0.079598314	0.027051275
ENSG00000020129_35558191_35565663_729	annotated	NCDN	1	+	2187	13	1	0	0.69%	1.964102837	0.028889786	0.021084099	0.002731533
ENSG00000215440_58692901_58715326_523	annotated	NPEPL1	20	+	1569	30	2	6	2.87%	6.317830423	0.001099207	0.004859907	3.26E-05
ENSG00000100299_50627773_50625145_507	annotated	ARSA	22	-	1521	42	9	2	3.55%	9.124093561	0.003781438	0.000334726	8.77E-06
ENSG00000106723_88448967_88475277_236	novel	SPIN1	9	+	708	28	9	0	4.24%	13.06755773	0.060590694	0.002254349	0.000953926
ENSG00000005448_74422154_74425701_334	annotated	WDR54	2	+	1002	83	9	5	9.28%	27.37032942	2.00E-05	4.90E-06	8.16E-10
ENSG00000005448_74421751_74422302_71	Overlap_uORF	WDR54	2	+	213	23	17	0	11.27%	35.67942824	0.251860071	0.005355751	0.011383491
ENSG00000114956_73938989_73946846_53	internal	DGUOK	2	+	159	28	1	0	13.21%	58.18761554	0.020342454	0.008473674	0.00085717
ENSG00000114956_73926911_73958736_189	annotated	DGUOK	2	+	567	41	11	32	8.99%	23.89298726	0.023744495	0.172898806	0.019316983
ENSG00000118197_200666740_200624901_580	annotated	DDX59	1	-	1740	22	0	0	1.72%	4.177756141	0.00205871	0.00205871	2.48E-05
ENSG00000153048_8859328_8855164_147	annotated	CARHSP1	16	-	441	111	10	3	16.33%	83.16757586	1.54E-05	2.35E-05	2.86E-09
ENSG00000139370_128808935_128794196_207	novel	SLC15A4	12	-	621	31	7	0	7.25%	16.49452249	0.000671624	0.000236536	1.07E-06
ENSG00000167528_48348045_48342973_409	annotated	ZNF641	12	-	1227	11	2	4	1.47%	2.96222318	0.061356297	0.100002646	0.022880377
ENSG00000149357_72103224_72086409_142	annotated	LAMTOR1	11	-	426	139	20	13	11.97%	107.8139245	0.000475147	0.000989796	3.03E-06
ENSG00000107036_5769001_5774246_367	dORF	RIC1	9	+	1101	15	2	0	2.45%	4.501669361	0.016524459	0.003327803	0.000306274
ENSG00000074319_18514691_18483846_110	novel	TSG101	11	-	330	19	0	1	5.45%	19.02432755	0.013640586	0.020525443	0.001325514
ENSG00000074319_18526816_18484063_152	novel	TSG101	11	-	456	47	24	4	9.21%	34.05670825	0.071310785	0.002591458	0.001291851
ENSG00000132382_4555324_4539415_1328	annotated	MYBBP1A	17	-	3984	53	3	4	2.03%	4.395681262	5.76E-06	2.68E-05	1.28E-09
ENSG00000132383_1830094_1897175_616	annotated	RPA1	17	+	1848	141	15	1	8.77%	25.21081	3.75E-08	7.04E-11	3.74E-17
ENSG00000064042_41360841_41697185_1083	annotated	LIMCH1	4	+	3249	127	38	9	5.08%	12.91586995	2.19E-05	1.85E-09	8.36E-13
ENSG00000110811_6828441_6839461_736	annotated	P3H3	12	+	2208	67	3	3	3.80%	10.02640833	8.88E-06	1.07E-05	7.70E-10
ENSG00000011143_58219230_58206283_456	annotated	MKS1	17	-	1368	22	5	1	2.63%	5.313812635	0.012970104	0.002467336	0.000183552
ENSG00000003756_50090435_50093041_81	annotated	RBM5	3	+	243	10	0	6	6.17%	13.59763503	0.019679754	0.175320778	0.017134007
ENSG00000009307_114739890_114718169_798	annotated	CSDE1	1	-	2394	202	28	26	9.02%	27.88026369	2.37E-10	1.49E-10	4.08E-19
ENSG00000123485_233854500_233837577_663	annotated	HJURP	2	-	1989	105	10	8	6.64%	17.44312004	1.10E-07	4.60E-08	5.00E-14
ENSG00000257103_34172643_34226431_422	annotated	LSM14A	19	+	1266	109	10	13	7.11%	28.44870134	6.22E-06	9.30E-05	4.94E-09
ENSG00000124177_41533378_41404593_2640	annotated	CHD6	20	-	7920	18	7	3	0.30%	0.750960298	0.104333894	0.020665591	0.009859959
ENSG00000180817_70233327_70203155_289	annotated	PPA1	10	-	867	67	0	3	7.27%	25.53438245	2.69E-05	6.77E-05	1.39E-08
ENSG00000180817_70233327_70210394_178	annotated	PPA1	10	-	534	23	0	3	5.06%	14.23168205	0.003554776	0.016170007	0.000317001
ENSG00000126067_35641432_35603267_201	annotated	PSMB2	1	-	603	313	31	13	24.38%	171.5128561	5.29E-08	1.13E-09	6.96E-16
ENSG00000079332_70161915_70157746_121	annotated	SAR1A	10	-	363	119	11	1	22.31%	108.3203339	1.28E-05	2.68E-06	2.94E-10
ENSG00000079332_70161915_70152476_198	annotated	SAR1A	10	-	594	221	19	4	23.74%	122.9349821	6.78E-09	2.31E-09	1.68E-16
ENSG00000129103_56064312_56079612_217	annotated	SUMF2	7	+	651	547	71	11	20.74%	277.636136	1.41E-06	6.46E-09	1.12E-13
ENSG00000129103_56064312_56079612_286	annotated	SUMF2	7	+	858	603	78	12	20.28%	232.2200306	3.63E-08	4.94E-11	2.61E-17
ENSG00000104946_49877724_49888624_615	annotated	TBC1D17	19	+	1845	73	4	6	3.74%	13.0736286	4.17E-05	6.53E-05	2.04E-08
ENSG00000149357_72103224_72094744_99	annotated	LAMTOR1	11	-	297	139	20	13	17.17%	154.6421947	0.000475147	0.000989796	3.03E-06
ENSG00000136631_150068645_150144921_538	annotated	VPS45	1	+	1614	46	10	6	3.35%	9.41724686	0.006441634	0.00079247	3.27E-05
ENSG00000119185_9423391_9406834_106	novel	ITGB1BP1	2	-	318	44	3	0	12.26%	45.71884078	0.001796133	0.000698084	7.85E-06
ENSG00000188130_50257099_50253401_231	novel	MAPK12	22	-	693	18	8	0	2.60%	8.582403406	0.169274096	0.013640586	0.012622088
ENSG00000206560_15735413_15670270_871	novel	ANKRD28	3	-	2613	48	7	5	2.30%	6.069759469	0.000326565	0.000443866	9.65E-07
ENSG00000153048_8859328_8859167_53	novel	CARHSP1	16	-	159	62	9	0	15.09%	128.8440058	0.013892891	0.005604867	0.000405324
ENSG00000137700_119026008_119024910_115	novel	SLC37A4	11	-	345	13	0	0	4.35%	12.45070407	0.021084099	0.021084099	0.002029891
ENSG00000167283_118401666_118431041_76	annotated	ATP5MG	11	+	228	341	19	3	19.74%	494.184575	0.000528239	0.000526999	1.81E-06
ENSG00000162972_199955799_199963811_291	annotated	MAIP1	2	+	873	34	6	4	4.12%	12.86868964	0.021545124	0.00522105	0.000595256
ENSG00000120915_27503616_27544522_489	annotated	EPHX2	8	+	1467	9	0	0	1.23%	2.027132093	0.011772051	0.011772051	0.000681273
ENSG00000156261_29071954_29056475_529	annotated	CCT8	21	-	1587	383	50	22	13.42%	79.74280367	9.13E-11	1.11E-11	1.24E-20
ENSG00000198689_136010500_136044724_382	novel	SLC9A6	X	+	1146	19	2	0	2.36%	5.478209504	0.011768187	0.003606289	0.000231562
ENSG00000147164_71068306_71060357_126	novel	SNX12	X	-	378	31	3	1	10.32%	27.09814409	0.000930688	0.000646935	3.82E-06
ENSG00000073584_40642556_40630222_345	annotated	SMARCE1	17	-	1035	57	3	0	3.77%	18.19718287	0.001529613	0.000649205	6.25E-06
ENSG00000064999_34981783_35087071_997	annotated	ANKS1A	6	+	2991	8	0	1	0.50%	0.883778084	0.019679754	0.042234516	0.003715148
ENSG00000105700_18557907_18562105_25	Overlap_uORF	KXD1	19	+	75	52	4	3	40.00%	229.0929549	0.002376615	0.002362849	3.25E-05
ENSG00000105700_18562108_18568631_159	annotated	KXD1	19	+	477	33	7	3	6.29%	22.85942039	0.003146562	0.002404282	4.33E-05
ENSG00000144746_69085048_69101846_93	annotated	ARL6IP5	3	+	279	72	40	0	11.83%	85.27033061	0.307410296	0.001618649	0.007390023
ENSG00000115241_27409422_27387063_71	novel	PPM1G	2	-	213	18	1	2	8.45%	27.9230308	0.023878474	0.035772986	0.003748301
ENSG00000105341_41439899_41433268_172	annotated	DMAC2	19	-	516	46	1	0	11.05%	29.45627215	5.79E-05	5.79E-05	2.50E-08
ENSG00000184584_139481704_139476362_283	annotated	TMEM173	5	-	849	22	3	0	3.89%	8.56218573	0.003925334	0.001343041	3.14E-05
ENSG00000122008_75573783_75593998_311	annotated	POLK	5	+	933	17	2	0	2.25%	6.020560588	0.022200631	0.007979402	0.000887484
ENSG00000122008_75594046_75598018_362	dORF	POLK	5	+	1086	19	0	4	2.21%	5.780873012	0.004826937	0.0400283	0.001078664
ENSG00000006695_14074454_14207213_385	annotated	COX10	17	+	1155	34	1	0	3.38%	9.72672386	0.000938122	0.000669869	3.98E-06
ENSG00000164172_53102217_53100466_97	dORF	MOCS2	5	-	291	12	0	0	5.15%	13.62567139	0.021084099	0.021084099	0.002029891
ENSG00000164172_53109729_53107095_88	annotated	MOCS2	5	-	264	56	3	6	14.77%	70.08962781	0.002090977	0.000812818	1.05E-05
ENSG00000136504_49788835_49827502_442	annotated	KAT7	17	+	1326	28	4	2	2.49%	6.977248018	0.013487927	0.002638766	0.000202686
ENSG00000072310_17836817_17812622_1147	annotated	SREBF1	17	-	3441	455	59	25	11.33%	43.69144192	4.79E-18	4.59E-20	3.75E-36
ENSG00000011295_15999849_16027522_380	annotated	TTC19	17	+	1140	76	9	4	4.47%	22.02816874	0.000142075	0.000320828	3.18E-07

ENSG00000013563_154405062_154402707_250	annotated	DNASE1L1	X	-	750	31	6	0	6.00%	13.65746462	0.004766409	0.000266003	9.24E-06
ENSG00000124920_61777770_61786143_537	annotated	MYRF	11	+	1611	44	10	4	3.91%	9.024575648	0.002849334	0.000219066	4.48E-06
ENSG00000184371_109911024_109925189_554	annotated	CSF1	1	+	1662	32	7	1	2.89%	6.36192599	0.005250307	0.000230371	9.09E-06
ENSG00000184371_109911024_109925189_438	annotated	CSF1	1	+	1314	28	7	1	2.97%	7.040967178	0.014383886	0.000742993	7.44E-05
ENSG00000013563_154405062_154402707_250	annotated	DNASE1L1	X	-	750	31	6	0	6.00%	13.65746462	0.004766409	0.000266003	9.24E-06
ENSG00000013563_154405062_154402707_250	annotated	DNASE1L1	X	-	750	31	6	0	6.00%	13.65746462	0.004766409	0.000266003	9.24E-06
ENSG00000186187_75093634_75106539_65	novel	ZNRF1	16	+	195	11	4	0	7.69%	18.6392197	0.133791954	0.019679754	0.012517628
ENSG00000070018_12266735_12121126_1613	annotated	LRP6	12	-	4839	106	14	11	3.04%	7.238021967	2.96E-07	2.40E-08	7.29E-14
ENSG00000103978_42228796_42226766_95	novel	TMEM87A	15	-	285	42	1	0	10.53%	48.69384669	0.002404282	0.002362849	3.28E-05
ENSG00000270617_43881673_43866699_159	Overlap_dORF	URGCP-MRPS24	7	-	477	148	6	0	22.01%	102.5210369	7.65E-07	1.13E-07	8.34E-13
ENSG00000179222_51895191_51897179_446	novel	MAGED1	X	+	1338	28	5	10	3.14%	6.914671802	0.004934346	0.021067771	0.000553959
ENSG00000179222_51900270_51901930_134	novel	MAGED1	X	+	402	26	0	0	7.46%	21.37061147	0.002227676	0.002227676	2.89E-05
ENSG00000111247_4538940_4558993_335	annotated	RAD51AP1	12	+	1005	29	3	2	2.99%	9.5345805	0.00453523	0.005399795	0.000132344
ENSG00000156976_186783611_186785965_96	novel	EIF4A2	3	+	288	33	1	0	5.21%	37.86091502	0.047790352	0.016947427	0.003697512
ENSG00000197563_62161260_62045856_880	annotated	PIGN	18	-	2640	90	12	0	3.98%	11.26440447	1.48E-05	8.70E-08	1.49E-11
ENSG00000124155_45416169_45424321_425	annotated	PIGT	20	+	1275	338	36	8	16.71%	87.59436511	1.57E-10	5.83E-13	1.32E-21
ENSG00000124155_45424269_45425826_149	Overlap_dORF	PIGT	20	+	447	36	3	1	6.71%	26.61121056	0.002815105	0.002815105	4.51E-05
ENSG00000164520_149890823_149889340_183	annotated	RAET1E	6	-	549	19	5	0	4.37%	11.43538814	0.041815588	0.005454749	0.001250208
ENSG00000166452_8911594_8919480_108	annotated	AKIP1	11	+	324	10	1	0	7.41%	10.19822627	0.003327803	0.004149608	7.68E-05
ENSG00000197302_31713440_31754286_250	Overlap_dORF	ZNF720	16	+	750	19	2	2	2.80%	8.370704122	0.007979402	0.023197249	0.000927766
ENSG00000163728_180604901_180617567_403	annotated	TTC14	3	+	1209	9	0	0	1.24%	2.459721075	0.020613417	0.020613417	0.001946209
ENSG00000179010_6640863_6641246_127	annotated	MRFAP1	4	+	381	161	38	4	24.41%	139.6273688	0.000425717	8.49E-07	4.65E-09
ENSG00000183765_28734586_28687897_541	annotated	CHEK2	22	-	1623	25	0	0	2.59%	5.089687787	0.000398115	0.000398115	1.05E-06
ENSG00000131373_15589566_15560865_460	annotated	HACL1	3	-	1380	27	0	2	2.83%	6.464788652	0.000642411	0.002603001	1.06E-05
ENSG00000131373_15601516_15601391_41	Overlap_uORF	HACL1	3	-	123	16	1	0	12.20%	42.98179267	0.019679754	0.020613417	0.001864002
ENSG00000157764_140850194_140726494_755	annotated	BRAF	7	-	2265	17	0	0	0.79%	2.479992507	0.011772051	0.011772051	0.000681273
ENSG00000105472_50725153_50725467_104	annotated	CLEC11A	19	+	312	13	1	0	5.77%	13.76760546	0.023672501	0.012798403	0.001437523
ENSG00000090487_64983569_64963620_281	annotated	SPG21	15	-	843	223	32	13	11.03%	87.40714643	0.000408133	4.15E-05	1.30E-07
ENSG00000171735_6785531_7766491_1673	annotated	CAMTA1	1	+	5019	66	11	1	0.60%	4.345066159	0.007635352	0.003514803	0.000146547
ENSG00000171843_20620750_20346443_536	annotated	MLLT3	9	-	1608	28	10	1	2.61%	5.753626164	0.043199594	0.000678648	0.000252817
ENSG00000204351_31959196_31969715_1245	annotated	SKIV2L	6	+	3735	31	2	0	1.45%	2.742462775	0.000171705	7.57E-05	9.41E-08
ENSG00000130119_54532567_54560602_582	annotated	GNL3L	X	+	1746	54	6	0	4.81%	10.21925354	2.96E-05	1.29E-06	3.55E-10
ENSG00000181789_129249710_129277424_874	annotated	COPG1	3	+	2622	155	14	6	5.72%	19.53298716	1.30E-08	1.41E-09	2.02E-16
ENSG00000164070_127798638_127832874_720	annotated	HSPA4L	4	+	2160	15	4	3	0.97%	2.294600911	0.065131088	0.043639483	0.011333421
ENSG00000111684_7018424_6981166_158	annotated	LPCAT3	12	-	474	23	5	4	5.06%	16.03316079	0.059950845	0.031850938	0.007957846
ENSG00000101182_62143303_62139679_149	annotated	PSMA7	20	-	447	376	66	12	28.86%	277.9393103	4.30E-07	3.34E-08	1.46E-13
ENSG00000171307_97451676_97456891_377	annotated	ZDHHC16	10	+	1131	62	12	6	6.10%	18.1133483	0.000305754	0.000143626	3.06E-07
ENSG00000130731_634621_634526_31	Overlap_dORF	METTL26	16	-	93	12	7	0	19.35%	42.63516531	0.273060506	0.01300724	0.022706215
ENSG00000130731_636290_634597_127	annotated	METTL26	16	-	381	118	27	16	14.96%	102.3355871	0.004743311	0.035652892	0.000936956
ENSG00000188191_584544_550430_137	annotated	PRKAR1B	7	-	411	13	1	2	3.65%	10.45132094	0.078649604	0.11777946	0.032976561
ENSG00000110066_68185791_68157688_786	annotated	KMT5B	11	-	2358	18	3	1	1.40%	2.522309398	0.007480509	0.002415484	0.000102283
ENSG00000137207_43516071_43513468_151	novel	YIPF3	6	-	453	144	4	2	15.23%	105.0349768	1.25E-05	1.87E-05	1.87E-09
ENSG00000196756_38434513_38426436_49	novel	SNHG17	20	-	147	26	3	3	14.29%	58.44208033	0.009583875	0.023878474	0.001121714
ENSG00000115738_8682166_8682899_134	annotated	ID2	2	+	402	121	5	9	17.16%	99.45553797	1.29E-05	8.13E-05	8.38E-09
ENSG00000184270_149887916_149887524_130	annotated	HIST2H2AB	1	-	390	1382	139	20	30.77%	1170.881892	2.18E-06	1.32E-07	2.83E-12
ENSG00000164237_10290762_10286268_183	novel	CMBL	5	-	549	26	4	0	5.46%	15.64842588	0.011575476	0.002321823	0.000154612
ENSG00000159199_48893418_48895769_136	annotated	ATP5MC1	17	+	408	260	17	5	16.91%	210.5633777	1.87E-05	1.30E-05	1.94E-09
ENSG00000101166_59042730_59035007_194	annotated	PRELID3B	20	-	582	145	10	13	15.98%	82.32176463	5.14E-06	5.16E-06	2.22E-10
ENSG00000167920_40834517_40835293_258	novel	TMEM99	17	+	774	12	1	0	2.71%	5.12282994	0.016854696	0.007029303	0.00060175
ENSG00000160310_46648473_46660977_210	novel	PRMT2	21	+	630	29	1	5	5.71%	15.20992604	0.001376742	0.004903596	4.02E-05
ENSG00000097033_86704909_86743235_383	annotated	SH3GLB1	1	+	1149	77	9	0	7.31%	22.14319834	6.91E-05	1.54E-06	1.02E-09
ENSG00000065665_12143135_12149737_67	annotated	SEC61A2	10	+	201	9	4	0	8.96%	14.79503871	0.11777946	0.011772051	0.007344096
ENSG00000168397_241653550_241671545_341	novel	ATG4B	2	+	1023	31	3	4	3.81%	10.01280397	0.002078934	0.006186427	7.38E-05
ENSG00000168397_241637715_241653359_78	novel	ATG4B	2	+	234	12	5	0	6.41%	16.94474519	0.121807694	0.019679754	0.01126199
ENSG00000175215_57846435_57823602_98	annotated	CTDSP2	12	-	294	16	6	1	7.14%	17.98217856	0.084260146	0.013892891	0.005711046
ENSG00000122406_92841782_92841865_27	novel	RPL5	1	+	81	18	4	4	18.52%	73.42722914	0.08410207	0.019679754	0.007521961
ENSG00000149182_47176853_47171779_70	annotated	ARFGAP2	11	-	210	10	0	1	7.14%	15.73440624	0.019679754	0.065398531	0.005779267
ENSG00000149182_47171445_47165482_214	dORF	ARFGAP2	11	-	642	16	0	0	4.67%	8.234829436	0.002008257	0.002008257	2.37E-05
ENSG00000176623_86508620_86494887_108	annotated	RMDN1	8	-	324	25	2	0	7.41%	25.49556567	0.008364861	0.005355751	0.000235847
ENSG00000104613_19823896_19826432_51	novel	INTS10	8	+	153	14	0	0	11.76%	30.23474141	0.012798403	0.012798403	0.000797244
ENSG00000176697_27658543_27657821_240	annotated	BDNF	11	-	720	54	20	2	10.00%	24.78168983	0.0133993	9.36E-06	2.20E-06
ENSG00000158636_76446939_76550146_1231	annotated	EMSY	11	+	3693	6	4	0	0.41%	0.536835956	0.303883016	0.016947427	0.03123584
ENSG00000188895_40122619_40134369_596	annotated	MSL1	17	+	1788	21	1	2	1.85%	3.88080154	0.002296044	0.003734664	4.89E-05
ENSG00000162129_72434474_72380352_149	annotated	CLPB	11	-	447	19	4	0	4.70%	14.04480557	0.068043226	0.007882058	0.002879912
ENSG00000162129_72402994_72293367_506	Overlap_dORF	CLPB	11	-	1518	43	4	0	4.15%	9.359795018	0.000245079	1.80E-05	3.59E-08
ENSG00000162819_222715700_222732714_379	annotated	BROX	1	+	1137	167	33	7	10.03%	48.53171741	4.78E-06	2.95E-07	1.35E-11
ENSG00000110921_109591316_109596577_115	annotated	MVK	12	+	345	41	9	0	10.43%	39.26760515	0.011245276	0.001070089	7.70E-05
ENSG00000122971_120725886_120737222_148	novel	ACADS	12	+	444	12	1	0	3.38%	8.930338679	0.020613417	0.019679754	0.001864002

ENSG00000167671_4457697_4445498_441	annotated	UBXN6	19	-	1323	37	6	6	3.63%	9.240841762	0.001258243	0.001095142	8.45E-06
ENSG00000005889_24179200_24211376_780	annotated	ZFX	X	+	2340	42	2	4	2.82%	5.930660815	4.02E-05	0.000197673	5.99E-08
ENSG00000165006_34179061_34179147_28	uORF	UBAP1	9	+	84	51	1	1	17.86%	200.6136796	0.021084099	0.039124093	0.003660118
ENSG00000165006_34220915_34251532_441	annotated	UBAP1	9	+	1323	51	13	0	4.54%	12.73737648	0.004992761	3.53E-05	1.81E-06
ENSG00000265354_45972625_46003318_209	annotated	TIMM23	10	+	627	92	12	10	15.79%	48.48305082	3.14E-06	6.41E-06	1.71E-10
ENSG00000071894_144409107_144394964_131	annotated	CPSF1	8	-	393	12	0	0	3.82%	10.08923759	0.020613417	0.020613417	0.001946209
ENSG00000130332_2324131_2321680_49	novel	LSM7	19	-	147	39	8	1	16.33%	87.6631205	0.022007321	0.005088721	0.000595221
ENSG00000103051_70508440_70481010_447	novel	COG4	16	-	1341	54	3	2	5.15%	13.30560528	3.32E-05	3.24E-05	8.25E-09
ENSG00000103051_70517694_70509207_241	novel	COG4	16	-	723	22	3	3	4.56%	10.05435088	0.009376445	0.009254025	0.00043825
ENSG00000158856_22067533_22081463_372	annotated	DMTN	8	+	1116	20	6	0	2.69%	5.921550737	0.041018323	0.001801897	0.000504555
ENSG00000123358_52054497_52058944_542	annotated	NR4A1	12	+	1626	27	2	2	2.77%	5.486720996	0.001263673	0.001263673	9.73E-06
ENSG00000102030_153934904_153929987_220	annotated	NAA10	X	-	660	95	9	2	11.36%	47.56081887	6.44E-05	4.66E-06	2.59E-09
ENSG00000116266_108753114_108808877_542	annotated	STXBP3	1	+	1626	31	10	3	3.51%	6.299568551	0.015162296	0.000308054	3.86E-05
ENSG00000159140_33543093_33576424_454	annotated	SON	21	+	1362	19	1	3	2.42%	4.609418569	0.002458651	0.011412214	0.000160153
ENSG00000075420_172112480_172337439_629	novel	FNDC3B	3	+	1887	65	11	1	3.02%	11.3818042	0.000291725	7.60E-05	1.61E-07
ENSG00000166197_102159553_102162269_418	annotated	NOLC1	10	+	1254	180	12	0	7.89%	47.42907145	1.41E-06	2.25E-07	2.99E-12
ENSG00000172301_31856811_31852139_133	annotated	COPRS	17	-	399	130	21	1	22.56%	107.6564638	0.000174072	7.37E-07	1.48E-09
ENSG00000173457_64245239_64244754_45	annotated	PPP1R14B	11	-	135	79	11	4	11.11%	193.3583701	0.335103451	0.019679754	0.039368637
ENSG00000181004_122741932_122744025_697	annotated	BBS12	4	+	2091	7	0	0	0.72%	1.106149076	0.019216965	0.019216965	0.001707396
ENSG00000142188_33468885_33450818_158	annotated	TMEM50B	21	-	474	34	8	2	8.23%	23.70119422	0.011080924	0.00199398	0.000129393
ENSG00000137449_15007413_15066380_417	annotated	CPEB2	4	+	1251	24	3	0	1.68%	6.339041364	0.018067938	0.008776163	0.000786152
ENSG00000137054_37486048_37492364_141	annotated	POLR1E	9	+	423	8	0	0	4.26%	6.249125884	0.009815329	0.009815329	0.000483714
ENSG00000137054_37493559_37503202_285	dORF	POLR1E	9	+	855	78	4	10	10.18%	30.14380986	7.20E-06	4.83E-05	2.87E-09
ENSG00000141510_7676594_7673307_341	annotated	TP53	17	-	1023	122	20	8	7.92%	39.40522854	0.000285468	2.01E-05	4.66E-08
ENSG00000141510_7670691_7669609_54	dORF	TP53	17	-	162	181	3	2	24.07%	369.1757909	0.000707658	0.000731334	3.29E-06
ENSG00000142186_65536618_65538566_246	annotated	SCYL1	11	+	738	28	3	1	4.07%	12.53635619	0.004512623	0.003146562	7.90E-05
ENSG00000111237_110501503_110492005_214	annotated	VPS29	12	-	642	162	25	7	13.55%	83.37764804	3.91E-05	5.43E-06	1.78E-09
ENSG00000132305_86195382_86144268_757	annotated	IMMT	2	-	2271	178	14	8	7.40%	25.8983754	5.52E-09	1.39E-09	8.34E-17
ENSG00000072042_67685124_67678321_70	novel	RDH11	14	-	210	101	2	3	28.57%	158.9175031	6.73E-05	6.74E-05	3.35E-08
ENSG00000072042_67691200_67690988_70	novel	RDH11	14	-	210	52	14	3	7.14%	81.81891247	0.021557223	0.021084099	0.002072352
ENSG00000171135_9892988_9893377_129	dORF	JAGN1	3	+	387	79	2	3	11.63%	67.45059421	0.000612073	0.001040517	4.04E-06
ENSG00000137074_32989891_32973498_342	annotated	APTX	9	-	1026	74	11	4	6.73%	23.83164454	0.000540996	1.32E-05	6.38E-08
ENSG00000133789_9728062_9750418_277	dORF	SWAP70	11	+	831	20	3	0	2.53%	7.952407488	0.038066506	0.008574016	0.001643654
ENSG00000111321_6388409_6388942_79	novel	AC005840.1	12	+	237	63	2	0	10.13%	87.83383739	0.012070269	0.005808022	0.000363885
ENSG00000256269_119088273_119092193_209	novel	HMBS	11	+	627	10	1	1	3.83%	5.269896828	0.010437297	0.010437297	0.000543123
ENSG00000256269_119092389_119093283_149	novel	HMBS	11	+	447	37	0	0	8.05%	27.35041085	0.001015599	0.001015599	6.39E-06
ENSG00000077721_119574712_119583255_122	annotated	UBE2A	X	+	366	175	17	0	26.23%	157.9889152	2.82E-05	3.43E-07	1.02E-10
ENSG00000149532_61429235_61421330_110	novel	CPSF7	11	-	330	12	8	2	4.55%	12.01536477	0.360078543	0.020613417	0.044877087
ENSG00000101391_33387486_33359043_186	novel	CDK5RAP1	20	-	558	18	0	0	5.38%	10.65879133	0.002201184	0.002201184	2.82E-05
ENSG00000206560_15796521_15686218_687	novel	ANKRD28	3	-	2061	27	4	7	1.75%	4.328679447	0.003224199	0.021440337	0.000392538
ENSG00000023228_206132961_206127791_117	novel	NDUFS1	2	-	351	30	10	0	12.82%	28.24124198	0.005958567	0.000282773	1.24E-05
ENSG00000186081_52520296_52516633_480	novel	KRT5	12	-	1440	37	31	2	4.17%	8.490023369	0.011876315	8.13E-05	9.98E-06
ENSG00000178209_143942515_143916177_4551	annotated	PLEC	8	-	13653	248	62	38	2.55%	6.001962039	1.02E-09	1.35E-13	2.56E-21
ENSG00000203724_197902650_197907220_145	annotated	C1orf53	1	+	435	56	4	1	6.90%	42.53715343	0.00224099	0.00232587	3.02E-05
ENSG00000172500_65888418_65885634_146	annotated	FIBP	11	-	438	64	10	7	11.64%	48.28091779	0.001035032	0.001182327	7.54E-06
ENSG00000172732_65861063_65866052_476	annotated	MUS81	11	+	1428	17	4	0	1.68%	3.933601561	0.054233792	0.005454749	0.001667789
ENSG00000113318_80670232_80875862_899	annotated	MSH3	5	+	2697	18	0	1	1.11%	2.205267171	0.002188006	0.003722062	4.66E-05
ENSG00000161091_3546284_3542781_150	annotated	MFSD12	19	-	450	14	1	0	4.00%	10.27981208	0.023672501	0.012798403	0.001437523
ENSG00000128309_37024216_37029514_297	annotated	MPST	22	+	891	100	14	8	7.41%	37.08445916	0.001171666	0.000379722	2.91E-06
ENSG00000157020_10318055_10301106_284	annotated	SEC13	3	-	852	132	17	3	15.49%	51.19222313	4.30E-07	9.77E-09	4.63E-14
ENSG00000133466_37185380_37182188_236	annotated	C1QTNF6	22	-	708	4	5	8.47%	38.2692762	0.000125937	4.37E-05	4.11E-08
ENSG00000077782_38457446_38413628_820	annotated	FGFR1	8	-	2460	101	18	8	5.49%	13.56612831	1.63E-06	9.83E-08	1.59E-12
ENSG00000134905_110706093_110641537_564	annotated	CARS2	13	-	1692	43	1	8	3.19%	8.397262907	0.000116567	0.003185106	2.93E-06
ENSG00000113318_80670232_80848475_708	annotated	MSH3	5	+	2124	16	0	1	1.13%	2.489058615	0.005405142	0.008170681	0.00023249
ENSG00000075785_128795368_128813422_134	annotated	RAB7A	3	+	402	148	13	5	20.90%	121.648096	1.80E-06	2.48E-06	3.87E-11
ENSG00000135535_109382378_109368851_184	annotated	CD164	6	-	552	345	78	3	29.89%	206.5140819	1.20E-08	5.10E-11	8.19E-18
ENSG00000213689_48467073_48467600_175	annotated	TREX1	3	+	525	19	1	0	5.14%	11.95814875	0.003478238	0.003606289	7.00E-05
ENSG00000175455_123961173_123914779_864	annotated	CCDC14	3	-	2592	15	1	0	1.39%	1.912167425	0.001504058	0.000529717	5.09E-06
ENSG00000055044_202287706_202297425_157	annotated	NOP58	2	+	471	20	0	0	4.46%	14.03068073	0.008473674	0.008473674	0.000366502
ENSG00000066926_57586599_57550712_416	annotated	FECH	18	-	1248	87	11	7	6.97%	23.03426299	1.60E-05	2.84E-05	3.58E-09
ENSG00000132357_40843190_40854446_930	annotated	CARD6	5	+	2790	9	1	4	0.65%	1.065879133	0.026832696	0.140443763	0.016715238
ENSG00000071051_105855064_105893176_380	annotated	NCK2	2	+	1140	8	3	0	1.32%	2.318754604	0.128949518	0.019216965	0.011791668
ENSG00000167085_49413264_49408934_205	novel	PHB	17	-	615	125	15	14	8.78%	67.15905104	0.000129918	9.32E-05	8.71E-08
ENSG00000073921_86031486_85959046_567	annotated	PICALM	11	-	1701	66	1	1	3.88%	12.82062731	2.22E-05	2.22E-05	3.85E-09
ENSG00000131446_180792971_180791634_445	annotated	MGAT1	5	-	1335	90	7	9	8.76%	22.27567626	2.32E-07	1.57E-06	3.43E-12
ENSG00000182196_122981140_122982586_215	annotated	ARL6IP4	12	+	645	95	15	5	12.09%	48.66688443	0.00012784	3.30E-05	3.20E-08
ENSG00000198836_193593378_193692127_978	annotated	OPA1	3	+	2934	75	19	3	3.48%	8.44638372	0.000597489	8.04E-07	6.61E-09
ENSG00000145014_194633215_194588529_475	annotated	TMEM44	3	-	1425	14	0	7	1.26%	3.246256446	0.013428348	0.060322713	0.003875514

ENSG00000182196_122982001_122982586_63	novel	ARL6IP4	12	+	189	10	0	0	9.52%	17.4826736	0.012798403	0.012798403	0.000797244
ENSG00000182196_122981140_122981412_90	novel	ARL6IP4	12	+	270	25	3	4	8.89%	30.59467881	0.010275151	0.02857234	0.001428016
ENSG00000140995_89954260_89955225_57	internal	DEF8	16	+	171	10	2	5	8.77%	19.32295504	0.069457177	0.197776317	0.049762526
ENSG00000125124_56506021_56501733_125	novel	BBS2	16	-	375	14	4	0	4.80%	12.33577449	0.101229348	0.013428348	0.006821841
ENSG00000105204_39831867_39825715_589	annotated	DYRK1B	19	-	1767	21	3	0	1.53%	3.92692312	0.017322097	0.003554776	0.000340576
ENSG00000181649_2929364_2928906_152	annotated	PHLDA2	11	-	456	30	7	2	6.58%	21.73832442	0.017961475	0.003372875	0.000338139
ENSG00000173273_9580197_9776736_1090	annotated	TNKS	8	+	3270	48	8	4	2.02%	4.850238989	0.000301525	0.000155253	3.25E-07
ENSG00000137312_30730565_30728116_110	novel	FLOT1	6	-	330	19	4	0	8.18%	19.02432755	0.034975232	0.003303074	0.000682399
ENSG00000137312_30740729_30730915_161	novel	FLOT1	6	-	483	20	8	0	6.21%	13.68209239	0.066928101	0.002321823	0.001100583
ENSG00000116691_12029752_12031808_154	novel	MIIP	1	+	462	11	4	0	3.90%	7.867203122	0.116253847	0.012798403	0.007703067
ENSG00000102226_47245051_47247285_120	novel	USP11	X	+	360	12	0	0	4.17%	11.01408437	0.021084099	0.021084099	0.002029891
ENSG00000183011_7857463_7856731_125	annotated	NAA38	17	-	375	376	141	11	34.40%	331.3036579	0.000135745	7.09E-09	2.28E-11
ENSG00000137210_10755171_10756518_37	novel	TMEM14B	6	+	111	41	9	0	16.22%	122.0479619	0.057424805	0.013640586	0.003726901
ENSG00000250644_1763859_1735280_557	annotated	AC068580.4	11	-	1671	1990	220	28	22.26%	393.5013985	2.19E-17	7.04E-22	3.44E-37
ENSG00000108100_35529989_35569170_202	annotated	CCNY	10	+	606	25	0	0	5.45%	13.63129254	0.001535678	0.001535678	1.41E-05
ENSG00000170234_160119388_160078155_520	annotated	PWWP2A	5	-	1560	11	1	4	1.35%	2.329902463	0.01551626	0.077869819	0.005723364
ENSG00000170791_56214614_56216505_56	annotated	CHCHD7	8	+	168	23	5	3	8.93%	45.23641795	0.021084099	0.044329616	0.004137993
ENSG00000104671_30156384_30179435_73	annotated	DCTN6	8	+	219	37	4	0	12.33%	55.82481119	0.02532052	0.003554776	0.00050929
ENSG00000104671_30179425_30183173_90	Overlap_dORF	DCTN6	8	+	270	62	0	2	12.22%	75.87480344	0.001626321	0.003274169	3.12E-05
ENSG00000185340_29308190_29312497_653	annotated	GAS2L1	22	+	1959	16	6	2	0.92%	2.698703674	0.093901624	0.024913475	0.010216879
ENSG00000141002_89873668_89911238_676	annotated	TCF25	16	+	2028	81	4	16	5.03%	13.19734962	5.42E-07	0.0001081	6.66E-10
ENSG00000133943_91160388_91224833_585	annotated	DGLUCY	14	+	1755	47	12	0	3.59%	8.848922486	0.005195666	2.28E-05	1.33E-06
ENSG00000129810_20184027_20161105_309	annotated	SGO1	3	-	927	17	4	2	2.59%	6.059528618	0.044710437	0.018318558	0.003695494
ENSG00000089234_111685792_111644199_592	annotated	BRAP	12	-	1776	30	3	1	2.87%	5.581461674	0.000896331	0.000182983	1.14E-06
ENSG00000112679_292552_348951_201	annotated	DUSP22	6	+	603	10	0	0	2.49%	5.479643965	0.019679754	0.019679754	0.001784964
ENSG00000130669_39177711_39178579_84	novel	PAK4	19	+	252	68	3	2	17.86%	89.16163538	0.00033832	0.000638607	1.43E-06
ENSG00000090372_46733196_46725641_273	annotated	STRN4	19	-	819	48	7	0	7.33%	19.36542307	0.001328724	3.10E-05	3.59E-07
ENSG00000170903_106010665_106009535_261	annotated	MSANTD4	11	-	783	16	0	0	3.07%	6.751929116	0.005257623	0.005257623	0.000148366
ENSG00000049618_157167059_157207891_1336	dORF	ARID1B	6	+	4008	9	4	2	0.37%	0.741966761	0.159270956	0.083503598	0.046240841
ENSG00000069020_67133584_67167051_1902	annotated	MAST4	5	+	5706	13	0	0	0.32%	0.752802822	0.013428348	0.013428348	0.000872559
ENSG00000285171_71110525_71106663_199	annotated	AL590764.2	X	-	597	14	0	0	3.02%	7.74860207	0.013428348	0.013428348	0.000872559
ENSG00000165171_73842140_73840100_90	annotated	METTL27	7	-	270	22	11	0	6.67%	26.92331735	0.127877374	0.013640586	0.009026432
ENSG00000140718_53704185_54061665_455	annotated	FTO	16	+	1365	44	5	3	4.84%	10.65098269	0.000267507	0.000111699	2.11E-07
ENSG00000114786_51984156_51985950_68	dORF	ABHD14A-ACY1	3	+	204	15	0	0	10.29%	24.29577435	0.008473674	0.008473674	0.000366502
ENSG00000114786_51975136_51984153_167	annotated	ABHD14A-ACY1	3	+	501	9	0	0	3.59%	5.935734092	0.009815329	0.009815329	0.000483714
ENSG00000152620_36241798_36195144_410	annotated	NADK2	5	-	1230	51	0	6	4.63%	13.70044641	5.67E-05	0.000166548	6.95E-08
ENSG00000145741_73502922_73505238_99	novel	BTF3	5	+	297	772	182	20	18.18%	858.8760741	0.001446775	0.000197453	1.99E-06
ENSG00000068366_109659399_109644029_67	dORF	ACSL4	X	-	201	15	0	0	10.45%	24.65839784	0.008473674	0.008473674	0.000366502
ENSG00000068366_109668224_109659419_114	annotated	ACSL4	X	-	342	16	0	2	5.26%	15.45836403	0.01300724	0.052581193	0.003266469
ENSG00000170525_6223963_6232942_56	annotated	PFKFB3	10	+	168	31	9	1	10.71%	60.97082419	0.070252771	0.01300724	0.004445345
ENSG00000111653_6652402_6651195_77	dORF	ING4	12	-	231	16	4	0	6.49%	22.88640908	0.139258512	0.021084099	0.013797358
ENSG00000183963_31082921_31104466_1002	annotated	SMTN	22	+	3006	42	10	1	2.30%	4.616682071	0.000668217	1.47E-05	8.81E-08
ENSG00000176597_183269925_183270935_336	annotated	B3GNT5	3	+	1008	21	0	1	2.38%	6.883802732	0.005454749	0.008219047	0.00023581
ENSG00000124570_2955568_2948298_287	novel	SERPINB6	6	-	861	191	31	4	17.42%	73.29930713	8.88E-08	8.67E-10	9.36E-16
ENSG00000092096_23352214_23346648_520	annotated	SLC22A17	14	-	1560	19	8	0	1.54%	4.024376982	0.113508073	0.005604867	0.004054109
ENSG00000109323_102669034_102632057_464	dORF	MANBA	4	-	1392	48	0	0	3.23%	11.39388038	0.000301822	0.000301822	6.15E-07
ENSG00000109323_102723876_102671000_292	annotated	MANBA	4	-	876	18	4	0	3.77%	6.789504064	0.016291574	0.001262414	0.000132698
ENSG00000168036_41224552_41239342_768	annotated	CTNNB1	3	+	2304	288	46	13	11.46%	41.30281639	2.21E-12	5.94E-15	2.04E-25
ENSG00000132570_134905221_134960681_103	annotated	PCBD2	5	+	309	9	0	4	6.80%	9.623957217	0.005706018	0.179313335	0.007385043
ENSG00000134779_36828767_36798422_227	annotated	TPGS2	18	-	681	67	10	10	8.37%	32.50853096	0.001860701	0.001567163	1.74E-05
ENSG00000134333_18396843_18407281_274	annotated	LDHA	11	+	822	1703	174	42	22.26%	684.5615213	3.72E-11	8.06E-11	3.56E-20
ENSG00000130349_107040103_107040384_43	novel	C6orf203	6	+	129	37	1	1	13.95%	94.77235389	0.013428348	0.01300724	0.000846864
ENSG00000138443_203411341_203436774_133	novel	ABI2	2	+	399	10	3	0	5.26%	8.281266444	0.017404239	0.007029303	0.000621356
ENSG00000136279_44044738_44056806_128	annotated	DBNL	7	+	384	24	2	0	7.81%	20.65140819	0.002281203	0.002362849	3.12E-05
ENSG00000132541_98108702_98102842_99	annotated	RIDA	8	-	297	30	2	0	12.12%	33.37601324	0.002190151	0.001011481	1.35E-05
ENSG00000175305_94894221_94881632_404	annotated	CCNE2	8	-	1212	17	1	0	1.24%	4.634639463	0.021557223	0.021557223	0.002115616
ENSG00000104450_100177885_100194423_293	annotated	SPAG1	8	+	879	16	5	0	2.73%	6.014517062	0.060419327	0.005306578	0.001843925
ENSG00000067533_218285317_218331091_282	annotated	RRP15	1	+	846	68	5	4	8.51%	26.55878501	4.83E-05	4.36E-05	1.59E-08
ENSG00000092969_218346702_218441362_414	annotated	TGFB2	1	+	1242	99	16	1	7.25%	26.33802784	1.04E-05	7.08E-07	6.82E-11
ENSG00000092978_217620483_217610160_352	annotated	GPATCH2	1	-	1056	16	2	0	2.27%	5.006401987	0.005160369	0.005160369	0.000143196
ENSG00000092978_217620483_217431145_504	annotated	GPATCH2	1	-	1512	17	2	0	1.79%	3.715068141	0.003229477	0.003229477	5.87E-05
ENSG00000161981_53952_55489_45	Overlap_uORF	SNRNP25	16	+	135	46	6	16	17.78%	112.588418	0.008127104	0.06867726	0.002981148
ENSG00000042493_85398131_85394893_88	annotated	CAPG	2	-	264	34	5	3	14.77%	42.55441689	0.006485538	0.003934467	0.000138405
ENSG00000140105_100361879_100354443_134	novel	WARS	14	-	402	30	0	1	5.97%	24.65839784	0.005655336	0.006229864	0.00018683
ENSG00000183258_177516945_177516009_160	novel	DDX41	5	-	480	45	8	4	5.00%	30.97711229	0.013192068	0.007400806	0.000494748
ENSG00000183258_177513451_177511791_245	novel	DDX41	5	-	735	112	12	4	9.80%	50.35009998	3.48E-05	3.20E-05	8.53E-09
ENSG00000169045_179623133_179621168_108	novel	HNRNPH1	5	-	324	145	29	6	22.22%	147.8742809	0.000216263	5.01E-05	8.06E-08
ENSG00000112514_33417099_33416650_59	dORF	CUTA	6	-	177	116	23	1	27.12%	216.5480995	0.001198213	0.000344013	2.72E-06

ENSG00000112514_33417668_33417247_83	annotated	CUTA	6	-	249	533	38	21	30.12%	707.2899963	2.32E-05	9.78E-06	1.83E-09
ENSG00000180891_57879663_57866477_249	annotated	CUEDC1	17	-	747	26	3	4	4.82%	11.50065035	0.006765524	0.008221728	0.000288206
ENSG00000196531_56724521_56712538_925	annotated	NACA	12	-	2775	466	70	22	5.19%	55.48717099	2.15E-06	1.29E-08	3.31E-13
ENSG00000102974_67616777_67637872_429	novel	CTCF	16	+	1287	40	1	2	4.20%	10.26954254	0.000120705	0.000222345	1.89E-07
ENSG00000113194_176494230_176496721_93	novel	FAF2	5	+	279	48	3	2	11.83%	56.84688707	0.005625935	0.005625935	0.000168722
ENSG00000133835_119479012_119541994_532	annotated	HSD17B4	5	+	1596	90	12	1	4.89%	18.6328495	0.000512167	3.47E-06	1.89E-08
ENSG00000157168_32728038_32763338_270	dORF	NRG1	8	+	810	20	3	1	2.96%	8.158581015	0.013580321	0.006075267	0.000425911
ENSG00000125863_10420692_10420546_48	uORF	MKKS	20	-	144	78	28	27	22.92%	178.978871	0.027766886	0.084277666	0.009970178
ENSG00000125863_10420750_10420619_43	uORF	MKKS	20	-	129	75	62	16	32.56%	192.1061227	0.070254996	0.018930945	0.006029729
ENSG00000125863_10413022_10405247_406	annotated	MKKS	20	-	1218	11	4	2	1.72%	2.984111529	0.070229091	0.041632258	0.011692616
ENSG00000214575_82556032_82544592_329	annotated	CPEB1	15	-	987	8	0	0	1.52%	2.678196807	0.019679754	0.019679754	0.001784964
ENSG00000145247_48904549_48885531_99	annotated	OCIAD2	4	-	297	16	1	1	6.06%	17.8005404	0.011772051	0.026832696	0.001510382
ENSG00000184584_139481704_139476261_379	annotated	TMEM173	5	-	1137	24	6	0	3.17%	6.974618071	0.013501766	0.00092354	8.31E-05
ENSG00000152133_37091996_37096263_127	annotated	GPATCH11	2	+	381	12	1	0	7.87%	10.40700885	0.003763158	0.001140969	2.59E-05
ENSG00000036257_224557850_224474245_744	annotated	CUL3	2	-	2232	63	7	7	3.76%	9.326442411	6.48E-05	2.71E-05	1.34E-08
ENSG00000130208_44914892_44919223_129	annotated	APOC1	19	+	387	43	14	1	11.63%	36.71361457	0.081834039	0.000667595	0.000569959
ENSG00000065665_12129788_12164454_410	annotated	SEC61A2	10	+	1230	52	15	1	4.15%	13.96908262	0.089758266	0.000185568	0.000263867
ENSG00000091129_108232428_108149910_1196	annotated	NRCAM	7	-	3588	102	15	3	4.43%	9.393282657	3.19E-08	1.81E-10	7.45E-17
ENSG00000091129_108232428_108149910_1084	annotated	NRCAM	7	-	3252	107	15	7	4.98%	10.87183605	1.96E-08	1.15E-09	2.53E-16
ENSG00000091129_108232428_108149910_1196	annotated	NRCAM	7	-	3588	107	15	7	4.52%	9.853737689	1.96E-08	1.15E-09	2.53E-16
ENSG00000182704_76795617_76796678_353	annotated	TSKU	11	+	1059	242	45	8	15.01%	75.50732061	1.61E-06	3.94E-10	1.09E-14
ENSG00000164692_94395032_94430393_1366	annotated	COL1A2	7	+	4098	77	10	4	2.20%	6.208524865	8.73E-05	6.80E-06	5.02E-09
ENSG00000164695_81732627_81758544_233	annotated	CHMP4C	8	+	699	22	3	0	3.86%	10.39956464	0.019194073	0.003529166	0.000376936
ENSG00000257103_34215168_34227388_269	novel	LSM14A	19	+	807	61	5	13	7.43%	24.97617645	5.15E-05	0.003895732	1.85E-06
ENSG00000108679_78976139_78973324_108	novel	LGALS3BP	17	-	324	206	19	3	22.22%	210.0834611	2.74E-05	1.47E-05	3.18E-09
ENSG00000156261_29060636_29056475_57	annotated	CCT8	21	-	171	67	14	0	17.54%	129.4637987	0.015820889	0.002254349	0.000209496
ENSG00000134138_37098019_36894754_330	annotated	MEIS2	15	-	990	7	0	2	1.52%	2.336320927	0.019216965	0.119134635	0.010787784
ENSG00000122591_22991111_22945589_521	annotated	FAM126A	7	-	1563	16	5	0	1.54%	3.382444336	0.066604992	0.005306578	0.002061766
ENSG00000101558_9914278_9954211_287	annotated	VAPA	18	+	861	78	17	9	6.97%	29.93374846	0.000585236	0.000462659	1.76E-06
ENSG00000196628_55350976_55228225_539	annotated	TCF4	18	-	1617	24	0	0	1.86%	4.904230517	0.002404282	0.002404282	3.34E-05
ENSG00000121281_50290532_50305800_351	annotated	ADCY7	16	+	1053	8	0	2	1.71%	2.51033262	0.011570466	0.082663577	0.00484172
ENSG00000121281_50307140_50310498_120	dORF	ADCY7	16	+	360	7	0	0	4.17%	6.424882549	0.019216965	0.019216965	0.001707396
ENSG00000182149_71921346_71923392_139	novel	IST1	16	+	417	53	7	2	10.79%	41.99614904	0.000619427	0.000679571	2.70E-06
ENSG00000082641_48055019_48059641_616	annotated	NFE2L1	17	+	1848	172	10	5	8.28%	30.7536122	1.06E-09	5.38E-10	6.39E-18
ENSG00000152990_22515784_22387705_1321	annotated	ADGRA3	4	-	3963	49	6	0	1.74%	4.085466572	0.000147433	1.06E-05	1.30E-08
ENSG00000120805_101405934_101395640_164	annotated	ARL1	12	-	492	164	14	2	18.90%	110.1408437	1.07E-06	1.08E-06	1.03E-11
ENSG00000175115_66193529_66216829_210	novel	PACS1	11	+	630	29	4	2	5.24%	15.20992604	0.004400313	0.00505029	0.000120637
ENSG00000118096_118557842_118544916_324	annotated	IFT46	11	-	972	12	0	0	2.47%	4.079290508	0.00419316	0.00419316	9.65E-05
ENSG00000110400_119678730_119664747_479	annotated	NECTIN1	11	-	1437	16	0	0	2.09%	3.679026095	0.002008257	0.002008257	2.37E-05
ENSG00000141552_81894478_81900065_84	annotated	ANAPC11	17	+	252	694	48	29	25.00%	909.9731611	0.000200778	0.00015937	2.23E-07
ENSG00000129255_7586750_7586857_35	novel	MPDU1	17	+	105	16	0	0	14.29%	50.35009998	0.020613417	0.020613417	0.001946209
ENSG00000129255_7587444_7587551_35	novel	MPDU1	17	+	105	13	1	0	17.14%	40.90945623	0.026832696	0.011772051	0.001510382
ENSG00000079459_11808887_11838609_353	annotated	FDFT1	8	+	1059	956	118	38	35.41%	298.2851178	4.09E-19	6.01E-21	4.24E-38
ENSG00000171307_97452908_97456891_179	novel	ZDHHC16	10	+	537	43	11	3	8.94%	26.45841497	0.004093768	0.000600369	1.59E-05
ENSG00000125691_38853710_38852596_77	novel	RPL23	17	-	231	562	67	18	19.48%	803.885119	0.001499876	0.000794465	7.40E-06
ENSG00000112541_165987898_165987578_106	novel	PDE10A	6	-	318	45	19	13	14.15%	46.75790535	0.04790541	0.018522412	0.003998427
ENSG00000168003_62885528_62886520_54	novel	SLC3A2	11	+	162	74	7	0	22.22%	150.9337488	0.00513589	0.001023866	3.23E-05
ENSG00000140455_63558163_63590826_351	dORF	USP3	15	+	1053	40	1	4	3.99%	12.5516631	0.000600799	0.00256119	9.80E-06
ENSG00000139613_56189461_56163689_1245	annotated	SMARCC2	12	-	3735	77	10	7	2.57%	6.811923667	8.34E-06	1.50E-05	1.01E-09
ENSG00000134291_47964237_47966733_181	annotated	TMEM106C	12	+	543	30	6	10	6.63%	18.25538846	0.01031092	0.022772171	0.001141307
ENSG00000000460_169804192_169852987_710	dORF	C1orf112	1	+	2130	25	0	3	1.69%	3.878198722	0.000943084	0.007322425	4.36E-05
ENSG00000221983_18573301_18575150_128	annotated	UBA52	19	+	384	749	48	5	14.06%	644.4960307	0.003166282	0.000126706	3.13E-06
ENSG00000111247_4548127_4558993_233	dORF	RAD51AP1	12	+	699	21	3	2	2.58%	9.926857158	0.022899795	0.020342454	0.002121458
ENSG00000184990_104753202_104756764_157	annotated	SIVA1	14	+	471	203	46	18	17.83%	142.4114094	2.19E-05	5.42E-05	9.20E-09
ENSG00000137868_74202267_74180080_667	annotated	STRA6	15	-	2001	188	29	5	9.15%	31.04419583	4.53E-08	1.84E-11	1.37E-17
ENSG00000154723_25729794_25724640_108	annotated	ATP5PF	21	-	324	206	17	2	25.00%	210.0834611	1.07E-05	4.20E-06	3.76E-10
ENSG00000128739_24975403_24978444_224	annotated	SNRPN	15	+	672	62	5	0	5.80%	30.4854121	0.006702261	0.000649205	2.87E-05
ENSG00000128739_24920123_24968037_67	uORF	SNRPN	15	+	201	36	4	2	11.94%	59.18015483	0.013786174	0.010327713	0.000700646
ENSG00000154723_25729794_25724640_108	annotated	ATP5PF	21	-	324	206	17	2	25.00%	210.0834611	1.07E-05	4.20E-06	3.76E-10
ENSG00000154723_25729794_25724640_108	annotated	ATP5PF	21	-	324	206	17	2	25.00%	210.0834611	1.07E-05	4.20E-06	3.76E-10
ENSG00000154723_25729794_25717689_119	annotated	ATP5PF	21	-	357	191	16	2	20.17%	176.7806819	4.33E-05	1.43E-05	4.89E-09
ENSG00000119397_121123979_121177186_1759	annotated	CNTRL	9	+	5277	21	3	0	0.51%	1.314927639	0.007532653	0.003606289	0.00014802
ENSG00000181513_45137058_45143641_230	annotated	ACBD4	17	+	690	18	1	1	2.61%	8.619718203	0.020525443	0.020525443	0.001930745
ENSG00000213563_144528916_144527342_216	annotated	C8orf82	8	-	648	11	0	2	2.31%	5.609024448	0.019679754	0.08410207	0.007521961
ENSG00000198931_88811899_88810144_63	annotated	APRT	16	-	189	58	5	1	23.81%	101.3995069	0.001796133	0.000567986	6.49E-06
ENSG00000198931_88809835_88809698_45	dORF	APRT	16	-	135	198	20	1	35.56%	484.6197123	0.000215516	0.000217439	3.23E-07
ENSG00000106692_105601166_105623068_334	dORF	FKTN	9	+	1002	20	2	0	3.29%	6.595260102	0.002287938	0.001448134	1.97E-05
ENSG00000166548_66550061_66511968_265	annotated	TK2	16	-	795	27	3	2	4.53%	11.22189728	0.000943084	0.004359749	2.54E-05

ENSG00000089876_125852347_125836687_311	annotated	DHX32	10	-	933	31	6	2	4.50%	10.97866931	0.00386557	0.001765381	3.97E-05
ENSG00000197956_153535339_153534696_90	annotated	S100A6	1	-	270	12809	980	230	56.67%	15675.48963	3.23E-09	2.91E-10	1.08E-17
ENSG00000159788_3316999_3439684_1171	annotated	RGS12	4	+	3513	15	0	0	0.68%	1.410856239	0.005160369	0.005160369	0.000143196
ENSG00000188153_108440126_108696378_1685	annotated	COL4A5	X	+	5055	36	1	2	1.19%	2.353157492	5.22E-05	8.36E-05	3.24E-08
ENSG00000198912_3900186_3889960_243	annotated	C1orf174	1	-	729	19	4	3	3.29%	8.611835516	0.041265601	0.02736363	0.004854213
ENSG00000140983_668200_668468_50	novel	RHOT2	16	+	150	10	4	0	10.00%	22.02816874	0.145248299	0.020613417	0.014232745
ENSG00000122008_75547023_75598018_672	annotated	POLK	5	+	2016	33	0	4	1.93%	5.408702146	0.000642411	0.005116237	2.14E-05
ENSG00000112855_140691649_140698552_481	annotated	HARS2	5	+	1443	41	1	0	4.16%	9.388304765	5.03E-05	3.42E-05	1.31E-08
ENSG00000176476_28581070_28591706_293	annotated	SGF29	16	+	879	18	1	0	2.39%	6.766331695	0.013892891	0.008473674	0.00058868
ENSG00000108061_110964359_111011818_582	annotated	SHOC2	10	+	1746	70	0	1	4.64%	13.24718051	2.17E-06	3.06E-06	5.73E-11
ENSG00000185515_155071528_155120150_247	annotated	BRCC3	X	+	741	34	2	0	4.86%	15.1610878	0.002225493	0.001053132	1.42E-05
ENSG00000108474_16217236_16279759_142	annotated	PIGL	17	+	426	23	2	13	7.75%	17.83971412	0.004070582	0.058059782	0.001431049
ENSG00000172053_49099226_49098349_148	novel	QARS	3	-	444	15	12	0	3.38%	11.16292335	0.336071985	0.019679754	0.039528703
ENSG00000157020_10319141_10315317_55	novel	SEC13	3	-	165	16	0	0	10.91%	32.04097271	0.013640586	0.013640586	0.000898637
ENSG00000146540_1127385_997625_194	annotated	C7orf50	7	-	582	56	11	5	6.19%	31.79323323	0.01578201	0.005972369	0.00048739
ENSG00000077782_38457446_38413628_820	annotated	FGFR1	8	-	2460	101	18	8	5.49%	13.56612831	1.63E-06	9.83E-08	1.59E-12
ENSG00000077782_38457446_38429674_121	novel	FGFR1	8	-	363	11	0	0	4.13%	10.01280397	0.021084099	0.021084099	0.002029891
ENSG00000147099_72572761_72461956_350	annotated	HDAC8	X	-	1050	14	1	0	2.00%	4.405633748	0.01551626	0.007882058	0.000614257
ENSG00000183765_28712463_28687897_383	novel	CHEK2	22	-	1149	19	0	0	2.61%	5.463906085	0.002254349	0.002254349	2.95E-05
ENSG00000114902_52706674_52707812_80	annotated	SPCS1	3	+	240	284	46	15	43.75%	390.9999952	5.27E-07	1.72E-07	8.54E-13
ENSG00000187446_41262784_41272182_54	annotated	CHP1	15	+	162	65	4	1	20.37%	132.5769415	0.003731737	0.001634015	3.57E-05
ENSG00000157617_41918203_41889124_489	novel	C2CD2	21	-	1467	13	3	0	1.64%	2.92807969	0.0400283	0.004826937	0.001078664
ENSG00000198860_184054366_184072120_91	annotated	TSEN15	1	+	273	13	0	0	6.59%	15.73440624	0.012798403	0.012798403	0.000797244
ENSG00000145741_73499134_73505238_162	annotated	BTF3	5	+	486	1402	219	30	16.67%	953.1942153	0.000278343	1.23E-05	2.91E-08
ENSG00000136122_72729044_72755216_489	annotated	BORA	13	+	1467	22	1	0	2.04%	4.955211782	0.00224099	0.00224099	2.92E-05
ENSG00000124635_27132750_27132370_126	annotated	HIST1H2BJ	6	-	378	404	168	21	15.87%	353.1500068	0.022276963	0.000256733	5.29E-05
ENSG00000014138_65287730_65305501_251	annotated	POLA2	11	+	753	7	0	0	1.99%	3.071656996	0.016947427	0.016947427	0.001349898
ENSG00000073111_127619225_127621773_167	dORF	MCM2	3	+	501	25	5	1	5.39%	16.48815026	0.026877003	0.005034873	0.000727371
ENSG00000073111_127605147_127619121_443	dORF	MCM2	3	+	1329	19	2	1	2.26%	4.723873658	0.003737369	0.003386928	7.06E-05
ENSG00000187134_4963445_4967115_146	novel	AKR1C1	10	+	438	331	51	7	13.01%	249.7028717	0.000615378	0.000206985	8.68E-07
ENSG00000187134_4968897_4977742_149	novel	AKR1C1	10	+	447	172	51	0	6.71%	127.1424505	0.022646393	0.002531016	0.000339852
ENSG00000101365_2664188_2658509_387	annotated	IDH3B	20	-	1161	209	9	8	14.47%	59.48174763	8.13E-11	3.43E-10	3.25E-19
ENSG00000163608_113008280_113005536_192	novel	NEPRO	3	-	576	31	0	3	3.65%	17.78315706	0.008574016	0.016101939	0.000687297
ENSG00000204120_232811347_232856860_632	novel	GIGYF2	2	+	1896	18	5	1	1.27%	3.136922764	0.040990615	0.007930666	0.001665162
ENSG00000103197_2062584_2088610_1358	annotated	TSC2	16	+	4074	45	5	0	1.47%	3.649733407	0.00010655	3.81E-05	3.05E-08
ENSG00000002549_17577559_17607589_488	annotated	LAP3	4	+	1464	74	6	0	5.12%	16.70168532	4.37E-05	5.46E-06	2.00E-09
ENSG00000187838_7394110_7390385_271	annotated	PLSCR3	17	-	813	16	4	2	2.58%	6.50278044	0.059202727	0.043639483	0.010354425
ENSG00000116786_15684559_15733934_978	annotated	PLEKHM2	1	+	2934	20	10	5	1.23%	2.252368992	0.080933993	0.025109881	0.008805675
ENSG00000140718_53704185_54118490_533	annotated	FTO	16	+	1599	50	5	3	4.69%	10.3321617	7.15E-05	3.11E-05	1.69E-08
ENSG00000132294_131944766_132010895_785	annotated	EFR3A	8	+	2355	108	15	4	5.86%	15.15313518	3.06E-07	1.15E-08	3.78E-14
ENSG00000118894_5097739_5085632_269	annotated	EEF2KMT	16	-	807	17	2	2	1.86%	6.960573766	0.020613417	0.036968718	0.003394961
ENSG00000117308_23798659_23798145_41	novel	GALE	1	-	123	16	7	3	12.20%	42.98179267	0.154380157	0.060322713	0.034593895
ENSG00000079150_178478487_178465928_70	novel	FKBP7	2	-	210	19	1	0	10.00%	29.89537186	0.007322157	0.007029303	0.000267517
ENSG00000106261_100023615_100024330_164	novel	ZKSCAN1	7	+	492	8	2	0	3.05%	5.372724083	0.080264768	0.019679754	0.007158564
ENSG00000169762_16226457_16163308_567	annotated	TAPT1	4	-	1701	22	4	0	2.29%	4.273542437	0.005573589	0.000544026	2.03E-05
ENSG00000100105_31345602_31327276_537	annotated	PATZ1	22	-	1611	12	2	2	1.49%	2.461247904	0.015191411	0.029607079	0.002085318
ENSG00000204628_181243800_181236977_317	annotated	RACK1	5	-	951	3163	318	65	39.43%	1098.976305	1.02E-18	1.34E-20	2.35E-37
ENSG00000160818_156599013_156597810_174	novel	GPATCH4	1	-	522	13	0	0	4.02%	8.22891361	0.007882058	0.007882058	0.000319636
ENSG00000164828_838815_838970_51	internal	SUN1	7	+	153	22	3	16	19.61%	47.5117365	0.010402772	0.229394231	0.015451134
ENSG00000164828_832525_873331_682	annotated	SUN1	7	+	2046	145	34	3	7.18%	23.41704155	6.49E-06	2.16E-09	2.32E-13
ENSG00000106628_44122053_44114785_455	annotated	POLD2	7	-	1365	154	5	1	9.23%	37.27843941	2.17E-08	6.69E-09	1.52E-15
ENSG00000115109_120042090_120174907_644	annotated	EPB41L5	2	+	1932	19	1	0	1.24%	3.249496942	0.008219047	0.005454749	0.00023581
ENSG00000106635_73557578_73537298_202	annotated	BCL7B	7	-	606	15	3	4	3.96%	8.178775523	0.020613417	0.049443454	0.004523201
ENSG00000160410_40576900_40580335_141	annotated	SHKBP1	19	+	423	16	0	8	4.26%	12.49825177	0.013640586	0.191268497	0.014688674
ENSG00000180329_44689753_44678856_227	annotated	CCDC43	17	-	681	31	7	1	5.73%	15.04126059	0.016248112	0.00105275	0.000113778
ENSG00000105576_12720977_12701346_887	annotated	TNPO2	19	-	2661	31	4	8	1.92%	3.849341775	0.00052153	0.005788985	2.03E-05
ENSG00000163466_218217471_218253915_300	annotated	ARPC2	2	+	900	251	30	10	20.33%	92.15117257	2.37E-10	1.99E-11	5.74E-20
ENSG00000121741_19993073_20086014_1377	annotated	ZMYM2	13	+	4131	30	5	1	1.16%	2.399582652	0.002726455	0.000249199	4.78E-06
ENSG00000121741_20051562_20086014_570	novel	ZMYM2	13	+	1710	14	2	0	1.40%	2.705213705	0.022898969	0.005160369	0.000627835
ENSG00000154710_66795574_66809284_299	dORF	RABGEF1	7	+	897	10	0	2	2.01%	3.683640258	0.011772051	0.071345384	0.004172987
ENSG00000136153_75760709_75857943_1285	annotated	LMO7	13	+	3855	25	7	2	1.17%	2.142817971	0.011291018	0.000796571	6.03E-05
ENSG00000160948_144424801_144424625_58	dORF	VPS28	8	-	174	44	1	5	10.34%	83.55512281	0.016822681	0.08410207	0.006633963
ENSG00000160948_144424253_144423805_82	dORF	VPS28	8	-	246	86	5	2	23.17%	115.5135678	0.00012122	7.45E-05	6.56E-08
ENSG00000109381_139125215_139058983_531	annotated	ELF2	4	-	1593	13	2	2	2.07%	2.696480166	0.009367993	0.009367993	0.000442962
ENSG00000182197_118111046_117799712_746	annotated	EXT1	8	-	2238	26	1	0	1.47%	3.838688923	0.001535678	0.001535678	1.41E-05
ENSG00000152464_15103435_15104166_243	annotated	RPP38	10	+	729	21	1	3	4.12%	9.518344518	0.002953574	0.008132971	0.000133338
ENSG00000152464_15103435_15104166_243	annotated	RPP38	10	+	729	21	1	3	4.12%	9.518344518	0.002953574	0.008132971	0.000133338
ENSG00000152464_15103435_15106059_105	annotated	RPP38	10	+	315	13	0	1	4.76%	13.63648541	0.021084099	0.021084099	0.002029891

ENSG00000133104_36335830_36304365_666	annotated	SPART	13	-	1998	55	9	2	4.50%	9.095715321	0.000126482	2.55E-06	3.08E-09
ENSG00000184281_2402787_2403623_278	annotated	TSSC4	11	+	834	18	0	0	1.80%	7.131421535	0.019679754	0.019679754	0.001784964
ENSG00000197912_89508547_89545958_468	annotated	SPG7	16	+	1404	82	13	2	5.77%	19.29818202	0.000169073	3.89E-06	6.13E-09
ENSG00000197912_89550604_89557093_204	dORF	SPG7	16	+	612	11	5	0	2.94%	5.938967063	0.168406755	0.012798403	0.011984717
ENSG00000106367_101156696_101159122_46	novel	AP1S1	7	+	138	50	4	25	17.39%	119.7183084	0.008576801	0.215762384	0.012496299
ENSG00000168522_43056497_43077264_134	novel	FNTA	8	+	402	39	7	3	8.96%	32.0559172	0.009174191	0.003668512	0.000182918
ENSG00000074410_63340783_63340207_100	novel	CA12	15	-	300	266	28	3	26.00%	292.9746443	1.74E-05	4.05E-06	5.92E-10
ENSG00000128513_124897173_124823962_634	annotated	POT1	7	-	1902	30	9	3	1.89%	5.211711847	0.016168792	0.005604867	0.0004721
ENSG00000123415_54183889_54182096_253	annotated	SMUG1	12	-	759	7	3	0	2.37%	3.047375122	0.152320961	0.009815329	0.008755903
ENSG00000258311_55719107_55721706_44	annotated	AC009779.3	12	+	132	61	8	1	20.45%	152.6952606	0.013855018	0.004557894	0.000336301
ENSG00000148337_128185630_128162939_652	annotated	CIZ1	9	-	1956	89	4	4	4.60%	15.03456302	8.86E-07	6.97E-06	5.57E-11
ENSG00000106617_151565858_151557201_149	novel	PRKAG2	7	-	447	22	0	0	4.70%	16.26240645	0.008776163	0.008776163	0.000391611
ENSG00000214413_110901583_110900360_70	annotated	BBIP1	10	-	210	21	0	9	11.43%	33.04225311	0.005554612	0.108304078	0.003801424
ENSG00000025708_50529586_50525770_441	annotated	TYMP	22	-	1323	45	4	1	4.31%	11.2388616	0.000725098	8.13E-05	4.37E-07
ENSG00000215021_6970543_6965692_267	annotated	PHB2	12	-	801	440	56	40	23.60%	181.5055102	3.01E-09	2.09E-10	7.32E-18
ENSG00000104872_49448006_49447344_53	annotated	PIH1D1	19	-	159	9	5	1	11.32%	18.70316214	0.113684165	0.026832696	0.013278195
ENSG00000101166_59042730_59035007_164	annotated	PRELID3B	20	-	492	131	10	13	16.46%	87.97835686	3.11E-05	2.33E-05	5.63E-09
ENSG00000118898_4897692_4883384_1438	annotated	PPL	16	-	4314	7	3	0	0.42%	0.536151534	0.109745779	0.009815329	0.005898503
ENSG00000180957_27919191_27854892_271	annotated	PITPNB	22	-	813	66	10	1	7.38%	26.82396932	0.000420919	3.87E-05	1.27E-07
ENSG00000203485_104707957_104711055_195	uORF	INF2	14	+	585	26	7	3	5.64%	14.68544583	0.019437421	0.00345766	0.000375355
ENSG00000203485_104712954_104715339_337	uORF	INF2	14	+	1011	36	6	1	4.15%	11.76578746	0.004595668	0.000446789	1.39E-05
ENSG00000162129_72434474_72293367_648	annotated	CLPB	11	-	1944	62	5	0	4.32%	10.53816714	2.85E-05	1.01E-06	2.72E-10
ENSG00000135441_55719107_55719609_75	Overlap_dORF	BLOC1S1	12	+	225	92	10	2	20.00%	135.1061016	0.001026106	0.00036881	2.48E-06
ENSG00000164054_48469394_48469107_37	novel	SHISA5	3	-	111	37	0	0	16.22%	110.1408437	0.01300724	0.01300724	0.000821865
ENSG00000115935_174575240_174562101_403	annotated	WIPF1	2	-	1209	18	6	0	2.23%	4.919442151	0.031423484	0.003452921	0.000628643
ENSG00000141349_44070966_44071187_73	novel	G6PC3	17	+	219	77	9	6	20.55%	116.1759584	0.000395349	0.000952052	2.46E-06
ENSG00000144320_175995584_175929967_428	annotated	LNPK	2	-	1284	49	6	1	5.37%	12.60958257	0.000257178	1.55E-05	3.29E-08
ENSG00000249115_35612795_35619762_469	novel	HAUS5	19	+	1407	29	0	0	3.20%	6.810414643	0.000255448	0.000255448	4.45E-07
ENSG00000152082_130182283_130190626_158	annotated	MZT2B	2	+	474	71	16	3	9.49%	49.49367027	0.017157529	0.000820094	9.85E-05
ENSG00000102786_51395378_51365752_709	annotated	INTS6	13	-	2127	10	2	0	0.85%	1.553467471	0.061356297	0.012798403	0.003794065
ENSG00000110090_68815474_68757644_773	annotated	CPT1A	11	-	2319	228	50	6	10.35%	32.48656192	5.58E-09	6.80E-15	1.23E-21
ENSG00000180185_1827239_1827904_221	annotated	FAHD1	16	+	663	104	21	0	12.22%	51.83098527	0.000272725	2.56E-06	7.18E-09
ENSG00000136167_46151034_46127591_366	annotated	LCP1	13	-	1098	15	0	0	1.37%	4.513969004	0.020613417	0.020613417	0.001946209
ENSG00000131459_180353217_180301564_682	annotated	GFPT2	5	-	2046	63	8	6	5.43%	10.17430081	8.27E-06	1.01E-06	7.53E-11
ENSG00000182022_124046059_124010149_510	annotated	CHST15	10	-	1530	23	7	6	2.94%	4.967136089	0.017483412	0.006166959	0.000555882
ENSG00000101150_63865366_63889945_206	annotated	TPD52L2	20	+	618	215	30	7	23.30%	114.9528223	1.40E-07	2.95E-09	4.70E-15
ENSG00000275066_37586480_37568787_492	novel	SYNRG	17	-	1476	10	0	2	1.42%	2.238635035	0.007593099	0.049338282	0.001960563
ENSG00000106853_111597422_111549773_301	annotated	PTGR1	9	-	903	279	28	13	21.93%	102.0906824	1.50E-10	1.44E-11	2.64E-20
ENSG00000172197_20212234_20102286_495	annotated	MBOAT1	6	-	1485	12	2	0	1.41%	2.67008106	0.022898969	0.008175611	0.000935377
ENSG00000169100_1392009_1390231_57	novel	SLC25A6	X	-	171	38	7	1	14.04%	73.42722914	0.010327713	0.007356796	0.000387901
ENSG00000100151_38057810_38074084_364	novel	PICK1	22	+	1092	13	3	0	2.20%	3.933601561	0.047083924	0.004826937	0.001291071
ENSG00000133657_194431884_194430939_124	novel	ATP13A3	3	-	372	12	0	0	5.65%	10.65879133	0.007882058	0.007882058	0.000319636
ENSG00000135318_85487487_85491126_116	annotated	NT5E	6	+	348	26	2	5	12.93%	24.68674083	0.001203162	0.004687549	3.40E-05
ENSG00000204070_45363532_45374294_76	annotated	SYS1	20	+	228	21	1	0	10.53%	30.43365418	0.008316137	0.005554612	0.00024255
ENSG00000164054_48473014_48469107_137	annotated	SHISA5	3	-	411	42	7	0	7.30%	33.7658061	0.081676868	0.002046029	0.001281895
ENSG00000129347_10565890_10553951_705	novel	KRI1	19	-	2115	25	4	2	1.99%	3.905703677	0.006280399	0.002328995	8.30E-05
ENSG00000119326_109013442_108942769_734	annotated	CTNNAL1	9	-	2202	196	24	2	6.40%	29.41090649	7.34E-07	2.62E-09	2.46E-14
ENSG00000204410_31753401_31761237_333	annotated	MSH5	6	+	999	9	0	3	1.80%	2.97677956	0.011772051	0.110834006	0.006843142
ENSG00000137522_71982779_71995526_150	annotated	RNF121	11	+	450	21	5	15	7.33%	15.41971812	0.019903045	0.108246426	0.009967809
ENSG00000137168_36856016_36855813_67	novel	PPIL1	6	-	201	21	3	0	13.43%	34.52175698	0.017321761	0.003478238	0.000334242
ENSG00000159069_136943501_136942436_195	novel	FBXW5	9	-	585	11	0	9	4.10%	6.213073235	0.00419316	0.271401227	0.010885465
ENSG00000159069_136942385_136940928_314	novel	FBXW5	9	-	942	35	0	0	3.50%	12.27684564	0.001580611	0.001580611	1.50E-05
ENSG00000159069_136944091_136944026_21	novel	FBXW5	9	-	63	55	14	4	23.81%	288.4641145	0.198166381	0.052029617	0.040132401
ENSG00000115363_75518101_75493236_139	annotated	EVA1A	2	-	417	17	2	0	3.60%	13.4704629	0.070008252	0.021084099	0.006564739
ENSG00000110881_50077282_50080466_227	novel	ASIC1	12	+	681	7	0	0	2.20%	3.396413683	0.019216965	0.019216965	0.001707396
ENSG00000115365_210476396_210434487_399	annotated	LANCL1	2	-	1197	106	8	2	7.27%	29.26047477	2.60E-05	1.37E-06	3.26E-10
ENSG00000135451_49323609_49325826_158	annotated	TROAP	12	+	474	32	22	3	6.96%	22.30700632	0.220133141	0.001506153	0.004104836
ENSG00000247077_132710877_132720828_289	annotated	PGAM5	12	+	867	62	15	0	7.27%	23.62883152	0.001564522	2.54E-05	3.63E-07
ENSG00000115738_8682166_8682899_134	annotated	ID2	2	+	402	121	5	9	17.16%	99.45553797	1.29E-05	8.13E-05	8.38E-09
ENSG00000151338_37267030_37546971_405	annotated	MIPOL1	14	+	1215	12	1	0	1.98%	3.263432406	0.010499663	0.00419316	0.000236136
ENSG00000174953_154284906_154280655_92	annotated	DHX36	3	-	276	10	0	0	5.43%	11.97183084	0.019679754	0.019679754	0.001784964
ENSG00000106080_30026508_30022633_126	novel	FKBP14	7	-	378	27	2	0	6.35%	23.60160937	0.019435691	0.004826937	0.000499969
ENSG00000150787_112226444_112233185_61	annotated	PTS	11	+	183	16	4	0	8.20%	28.88940163	0.019679754	0.019216965	0.001745788
ENSG00000150787_112233172_112233555_61	Overlap_dORF	PTS	11	+	183	21	0	1	9.84%	37.91733964	0.013428348	0.01300724	0.000846864
ENSG00000143322_179229397_179110805_542	annotated	ABL2	1	-	1626	15	4	1	1.29%	3.048178331	0.079167839	0.011738695	0.004667806
ENSG00000117054_75740079_75762763_232	dORF	ACADM	1	+	696	22	4	0	4.74%	10.44439035	0.003640501	0.001448134	3.12E-05
ENSG00000133313_74501401_74509174_119	annotated	CNDP2	18	+	357	15	1	0	8.40%	13.88329963	0.00640069	0.001790164	6.67E-05
ENSG00000036257_224557850_224474245_744	annotated	CUL3	2	-	2232	63	7	7	3.76%	9.326442411	6.48E-05	2.71E-05	1.34E-08

ENSG00000155368_119367585_119372318_104	annotated	DBI	2	+	312	668	54	12	29.81%	707.4431115	4.17E-05	4.55E-06	1.61E-09
ENSG00000144524_231794346_231807645_157	annotated	COPS7B	2	+	471	13	0	0	3.82%	9.119942472	0.01300724	0.01300724	0.000821865
ENSG00000135930_232550725_232580943_234	annotated	EIF4E2	2	+	702	28	32	5	6.84%	13.17924626	0.48842348	0.001840117	0.019030224
ENSG00000135930_232556424_232564298_97	internal	EIF4E2	2	+	291	32	5	18	11.34%	36.3351237	0.010798573	0.177532488	0.01135044
ENSG00000138674_82881906_82819074_1241	annotated	SEC31A	4	-	3723	99	9	6	3.06%	8.786417024	1.27E-07	8.49E-07	1.04E-12
ENSG00000137947_88863992_88860089_69	novel	GTF2B	1	-	207	6	0	0	7.25%	9.57746467	0.016947427	0.016947427	0.001349898
ENSG00000012983_50532049_50420019_846	annotated	MAP4K5	14	-	2538	23	7	0	1.30%	2.994372819	0.036215591	0.001498825	0.000377312
ENSG00000135972_105038093_105099761_396	annotated	MRPS9	2	+	1188	48	5	0	5.05%	13.3504053	0.000333761	3.78E-05	9.73E-08
ENSG00000156958_49201181_49328159_434	annotated	GALK2	15	+	1302	43	6	1	3.00%	10.91257207	0.004866125	0.00084676	2.58E-05
ENSG00000156802_123396357_123344782_939	novel	ATAD2	8	-	2817	53	9	6	2.66%	6.216682339	1.87E-05	0.000189409	2.84E-08
ENSG00000123562_103677027_103676161_288	annotated	MORF4L2	X	-	864	192	23	11	16.67%	73.42722914	1.11E-07	1.33E-08	1.53E-14
ENSG00000134901_102798670_102791254_282	novel	POGLUT2	13	-	846	19	5	0	3.90%	7.420836987	0.032024697	0.001384502	0.000307051
ENSG00000134901_102791122_102784495_215	novel	POGLUT2	13	-	645	19	4	1	4.65%	9.733376886	0.016957522	0.002201184	0.000221103
ENSG00000268790_16659990_16647246_63	annotated	AC008764.4	19	-	189	119	14	0	28.57%	208.0438159	0.002793544	9.38E-05	2.11E-06
ENSG00000214046_16660110_16654122_23	novel	SMIM7	19	-	69	65	5	2	43.48%	311.2676018	0.010444866	0.005697454	0.0003101
ENSG00000125734_6731475_6730074_126	annotated	GPR108	19	-	378	27	3	0	7.94%	23.60160937	0.005034873	0.002362849	6.74E-05
ENSG00000130311_17315804_17319656_100	novel	DDA1	19	+	300	42	3	5	14.00%	46.25915436	0.000780578	0.003660423	1.79E-05
ENSG00000064012_201258358_201276997_293	novel	CASP8	2	+	879	27	11	2	5.12%	10.14949754	0.057624207	0.001299835	0.00059105
ENSG00000064012_201284932_201286594_173	novel	CASP8	2	+	519	15	7	0	2.89%	9.549784136	0.116253847	0.020613417	0.011080196
ENSG00000154429_229342432_229325235_259	annotated	CCSAP	1	-	777	9	2	0	1.93%	3.827288005	0.080264768	0.019679754	0.007158564
ENSG00000145996_20546351_21231039_579	annotated	CDKAL1	6	+	1737	8	3	0	0.86%	1.521807858	0.119134635	0.019216965	0.010787784
ENSG00000163545_205321760_205303450_672	annotated	NUAK2	1	-	2016	28	2	3	2.08%	4.589201821	0.000960952	0.002154443	1.27E-05
ENSG00000166263_54990841_55043629_299	annotated	STXBP4	17	+	897	12	2	0	1.67%	4.420368309	0.08410207	0.019679754	0.007521961
ENSG00000170264_61842198_61826455_545	annotated	FAM161A	2	-	1635	11	2	0	0.92%	2.223026203	0.070008252	0.021084099	0.006564739
ENSG00000143621_153670922_153667496_150	annotated	ILF2	1	-	450	103	14	3	17.33%	75.63004601	4.36E-05	8.09E-06	2.88E-09
ENSG00000160679_153638350_153642449_101	annotated	CHTOP	1	+	303	32	3	0	10.89%	34.8961089	0.010817194	0.00140552	9.32E-05
ENSG00000160679_153638305_153645269_223	annotated	CHTOP	1	+	669	65	4	1	10.76%	32.10383337	5.08E-05	1.04E-05	4.26E-09
ENSG00000143553_153658744_153661301_136	annotated	SNAPIN	1	+	408	33	0	2	10.29%	26.72535178	0.000446789	0.000867445	2.51E-06
ENSG00000155189_6732609_6757388_213	dORF	AGPAT5	8	+	639	72	10	7	8.92%	37.23070773	0.000806097	0.000340102	1.81E-06
ENSG00000155189_6708702_6730758_77	annotated	AGPAT5	8	+	231	15	2	7	10.39%	21.45600851	0.017404239	0.182268951	0.016438769
ENSG00000134824_61857477_61865689_168	novel	FADS2	11	+	504	395	78	17	33.93%	258.9621028	1.72E-08	1.49E-10	3.24E-17
ENSG00000182372_1780295_1780567_90	novel	CLN8	8	+	270	35	4	1	10.00%	42.83255033	0.02089895	0.003554776	0.000414939
ENSG00000182504_101724677_101765551_865	annotated	CEP97	3	+	2595	32	7	0	1.85%	4.074574565	0.013783116	0.000148081	1.92E-05
ENSG00000155229_97498348_97458692_1018	annotated	MMS19	10	-	3054	107	5	4	3.63%	11.57668986	2.93E-07	2.37E-07	6.53E-13
ENSG00000087077_100872664_100873303_70	novel	TRIP6	7	+	210	30	1	1	12.86%	47.20321873	0.003941085	0.003941085	8.58E-05
ENSG00000213920_24215955_24214024_176	annotated	MDP1	14	-	528	80	10	11	10.80%	50.06401987	0.000445436	0.000494631	1.44E-06
ENSG00000168214_26415539_26431007_414	annotated	RBPJ	4	+	1242	16	0	0	1.69%	4.256650964	0.008473674	0.008473674	0.000366502
ENSG00000146576_6600083_6607624_133	annotated	C7orf26	7	+	399	13	0	0	6.02%	10.76564638	0.00419316	0.00419316	9.65E-05
ENSG00000198561_57801746_57816308_610	annotated	CTNND1	11	+	1830	127	19	9	6.39%	22.93096254	3.30E-05	9.92E-07	3.13E-10
ENSG00000167792_67611168_67612458_173	novel	NDUFV1	11	+	519	81	14	2	16.18%	51.56883434	6.80E-05	4.18E-06	2.48E-09
ENSG00000167792_67608700_67610611_145	novel	NDUFV1	11	+	435	122	44	1	12.41%	92.67022712	0.000422097	0.000121804	3.63E-07
ENSG00000148950_31433564_31432500_57	annotated	IMMP1L	11	-	171	35	0	1	10.53%	67.63034263	0.013853925	0.017143984	0.001131568
ENSG00000052841_43396793_43445965_707	novel	TTC17	11	+	2121	23	3	0	2.12%	3.58308261	0.001835088	0.000178099	2.34E-06
ENSG00000185803_144359294_144360180_87	annotated	SLC52A2	8	+	261	18	0	2	12.64%	22.78776077	0.001242734	0.004932312	3.69E-05
ENSG00000127990_94656026_94587779_381	annotated	SGCE	7	-	1143	150	9	7	11.29%	43.36253689	1.08E-07	3.10E-08	3.34E-14
ENSG00000114861_71041530_70972536_334	novel	FOXP1	3	-	1002	12	0	5	1.80%	3.957156061	0.01300724	0.138644629	0.009584248
ENSG00000150593_110876028_110898088_469	annotated	PDCD4	10	+	1407	99	15	2	6.82%	23.24934654	8.03E-05	3.91E-07	3.60E-10
ENSG00000103197_2079153_2088610_755	novel	TSC2	16	+	2265	32	4	0	1.99%	4.66822119	0.000589487	0.000276859	1.09E-06
ENSG00000109323_102723876_102682745_244	annotated	MANBA	4	-	732	10	3	0	2.46%	4.513969004	0.102951605	0.011772051	0.006286113
ENSG00000109323_102671361_102632057_496	dORF	MANBA	4	-	1488	49	1	0	3.23%	10.88084948	0.000213096	0.000202392	2.98E-07
ENSG00000115525_85863483_85847466_224	dORF	ST3GAL5	2	-	672	100	19	9	11.16%	49.17001951	0.000208105	0.00017171	2.49E-07
ENSG00000115525_85846502_85840144_177	dORF	ST3GAL5	2	-	531	43	4	1	9.60%	26.75738011	0.00076033	0.000186181	9.75E-07
ENSG00000278615_62665169_62663050_123	annotated	C11orf98	11	-	369	92	8	4	17.89%	82.38176928	4.74E-05	1.89E-05	6.98E-09
ENSG00000183020_986876_988710_78	novel	AP2A2	11	+	234	17	0	0	6.41%	24.00505568	0.021084099	0.021084099	0.002029891
ENSG00000111906_125292903_125234355_86	Overlap_dORF	HDDC2	6	-	258	16	0	0	9.30%	20.49131976	0.005454749	0.005454749	0.000159109
ENSG00000111906_125301932_125292900_71	annotated	HDDC2	6	-	213	77	32	3	26.76%	119.4485206	0.010669572	0.000114197	1.15E-05
ENSG00000162191_62678697_62677769_146	novel	UBXN1	11	-	438	82	10	3	16.44%	61.85992592	6.48E-05	2.55E-05	1.27E-08
ENSG00000120925_42887864_42856159_258	annotated	RNF170	8	-	774	30	4	4	4.65%	12.80707485	0.006403707	0.014055347	0.000462055
ENSG00000117859_51772638_51784063_163	novel	OSBPL9	1	+	489	15	2	0	4.29%	10.13566046	0.032996028	0.008473674	0.00140192
ENSG00000198382_76016871_76141413_327	annotated	UVRAG	11	+	981	20	2	1	2.45%	6.73644304	0.01299184	0.011825808	0.000750884
ENSG00000075239_108138904_108142582_176	Overlap_dORF	ACAT1	11	+	528	70	8	3	11.36%	43.80601738	0.0002746	0.000148113	2.83E-07
ENSG00000075239_108121607_108138989_141	annotated	ACAT1	11	+	423	43	11	1	6.38%	33.58905163	0.080285538	0.005134532	0.002499763
ENSG00000083845_58388478_58394750_169	annotated	RPS5	19	+	507	1074	85	34	30.77%	699.9483204	6.34E-10	5.99E-10	4.24E-18
ENSG00000184584_139481212_139476362_164	annotated	TMEM173	5	-	492	14	3	0	4.88%	9.402267146	0.016735684	0.004687384	0.000417841
ENSG00000140694_14628215_14555696_390	Overlap_dORF	PARN	16	-	1170	17	9	4	2.05%	4.801011136	0.094580636	0.038565921	0.014690309
ENSG00000140694_14555690_14436717_212	dORF	PARN	16	-	636	15	0	0	3.77%	7.792984224	0.004826937	0.004826937	0.000126125
ENSG00000203791_124791833_124760409_236	annotated	EEF1AKMT2	10	-	708	27	8	3	4.66%	12.60085924	0.032911453	0.008031566	0.001335972
ENSG00000169499_38917930_38969783_425	annotated	PLEKHA2	8	+	1275	13	9	0	1.88%	3.369014043	0.10003353	0.004826937	0.003109159

ENSG00000109685_1956125_1978909_426	dORF	NSD2	4	+	1278	11	3	1	1.64%	2.844012396	0.067796501	0.01551626	0.004936077
ENSG00000109685_1900838_1942401_586	annotated	NSD2	4	+	1758	24	2	0	1.88%	4.510887797	0.003199288	0.001535678	2.89E-05
ENSG00000102897_20915556_20920286_107	annotated	LYRM1	16	+	321	12	1	0	5.61%	12.35224415	0.012798403	0.012798403	0.000797244
ENSG00000103197_2050439_2088610_1701	annotated	TSC2	16	+	5103	65	6	1	1.53%	4.208791794	7.80E-06	3.46E-06	2.27E-10
ENSG00000115355_55334663_55291711_1104	annotated	CCDC88A	2	-	3312	39	5	0	1.81%	3.890845022	0.000630522	3.59E-05	1.81E-07
ENSG00000127990_94656026_94597920_457	novel	SGCE	7	-	1371	189	23	7	10.07%	45.55058963	4.05E-07	9.86E-09	4.39E-14
ENSG00000077684_128831759_128872262_842	annotated	JADE1	4	+	2526	41	8	1	2.61%	5.363152722	0.002043451	1.35E-05	2.89E-07
ENSG00000100242_38751348_38736267_668	annotated	SUN2	22	-	2004	159	19	6	8.83%	26.21615891	2.55E-09	6.90E-11	2.15E-18
ENSG00000119048_134371596_134390353_152	annotated	UBE2B	5	+	456	128	9	6	13.82%	92.75018417	9.80E-05	0.000153436	1.08E-07
ENSG00000213585_133993012_133972771_283	annotated	VDAC1	5	-	849	175	7	6	7.77%	68.10829558	7.50E-05	0.000116041	6.33E-08
ENSG00000115355_55344472_55336814_84	dORF	CCDC88A	2	-	252	10	0	2	5.95%	13.1120052	0.019679754	0.065565251	0.005794489
ENSG00000197102_102017383_102042059_1280	annotated	DYNC1H1	14	+	3840	96	27	14	2.81%	8.260563278	0.000671664	3.48E-05	1.89E-07
ENSG00000197102_102042037_102050563_604	Overlap_dORF	DYNC1H1	14	+	1812	38	3	1	2.98%	6.929390829	0.000426906	0.00010993	3.33E-07
ENSG00000055732_85034182_85025891_225	dORF	MCOLN3	1	-	675	12	3	0	2.22%	5.874178331	0.12312585	0.020613417	0.011809609
ENSG00000100461_22904988_22901730_189	annotated	RBM23	14	-	567	63	12	4	8.47%	36.71361457	0.00966045	0.00131215	7.79E-05
ENSG00000285920_41358244_41380186_225	novel	AC087721.2	15	+	675	61	9	4	9.33%	29.86040652	0.000285673	3.18E-05	7.06E-08
ENSG00000153113_96742661_96771679_399	annotated	CAST	5	+	1197	328	41	9	18.05%	90.54184645	5.14E-10	1.36E-12	1.01E-20
ENSG00000196961_49767161_49796199_287	novel	AP2A1	19	+	861	52	2	5	6.27%	19.95583231	8.97E-05	0.000708619	4.66E-07
ENSG00000105287_46710946_46674523_721	annotated	PRKD2	19	-	2163	19	5	4	1.39%	2.902463288	0.025495061	0.02160994	0.002481423
ENSG00000135862_183023717_183142790_1609	annotated	LAMC1	1	+	4827	524	81	29	10.94%	35.86936116	1.21E-22	3.88E-27	1.11E-47
ENSG00000062822_50398981_50417947_1090	annotated	POLD1	19	+	3270	49	2	1	2.48%	4.951285634	1.01E-05	2.77E-06	2.39E-10
ENSG00000169169_49712787_49713605_113	novel	CPT1C	19	+	339	10	0	0	5.31%	9.746977319	0.012798403	0.012798403	0.000797244
ENSG00000125734_6732495_6730312_198	dORF	GPR108	19	-	594	42	9	0	9.60%	23.36320927	0.001268577	5.09E-05	5.24E-07
ENSG00000213983_23561616_23559749_201	novel	AP1G2	14	-	603	11	0	0	2.99%	6.027608362	0.012798403	0.012798403	0.000797244
ENSG00000213930_34647878_34650449_238	dORF	GALT	9	+	714	10	0	0	2.10%	4.627766542	0.020613417	0.020613417	0.001946209
ENSG00000133392_15757931_15708811_1448	annotated	MYH11	16	-	4344	6	0	0	0.35%	0.456384711	0.016947427	0.016947427	0.001349898
ENSG00000117625_211259573_211312994_445	annotated	RCOR3	1	+	1335	8	1	2	1.12%	1.980060112	0.042234516	0.101051022	0.016920389
ENSG00000136854_127651608_127684477_589	annotated	STXBP1	9	+	1767	60	6	0	4.07%	11.21978034	0.000136443	7.48E-06	8.76E-09
ENSG00000172432_43625809_43621614_451	annotated	GTPBP2	6	-	1353	7	0	0	1.33%	1.709503117	0.009815329	0.009815329	0.000483714
ENSG00000143545_153983523_153982099_122	novel	RAB13	1	-	366	61	1	1	13.11%	55.07042185	0.000251967	0.000251967	4.34E-07
ENSG00000204520_31412115_31415139_124	Overlap_dORF	MICA	6	+	372	39	1	11	8.87%	34.64107181	0.0020028	0.015073811	0.000179882
ENSG00000204520_31403633_31412431_332	annotated	MICA	6	+	996	50	18	1	4.82%	16.58747646	0.079114997	0.000260584	0.000279086
ENSG00000064601_45891845_45892329_79	novel	CTSA	20	+	237	91	11	1	12.66%	126.8710984	0.003778043	0.002516754	5.39E-05
ENSG00000064601_45891569_45891805_78	novel	CTSA	20	+	234	36	5	0	17.95%	50.83423556	0.000867445	0.000450794	2.53E-06
ENSG00000137871_56704241_56700837_140	annotated	ZNF280D	15	-	420	5	2	0	3.57%	3.933601561	0.089856247	0.012673659	0.005706018
ENSG00000171241_46621359_46603930_378	novel	SHCBP1	16	-	1134	95	1	0	6.88%	27.68089987	3.85E-06	3.84E-06	1.25E-10
ENSG00000103035_74296915_74300244_67	novel	PSMD7	16	+	201	152	36	26	8.96%	249.8717648	0.013640586	0.013853925	0.000911832
ENSG00000149925_30067255_30068229_123	novel	ALDOA	16	+	369	426	59	29	30.89%	381.4634099	1.34E-06	1.33E-07	1.73E-12
ENSG00000161204_184186208_184186679_81	novel	ABCF3	3	+	243	12	0	0	6.17%	16.31716203	0.019679754	0.019679754	0.001784964
ENSG00000089159_120216278_120212314_316	dORF	PXN	12	-	948	50	13	2	6.01%	17.42734869	0.002247034	0.000208869	3.33E-06
ENSG00000089159_120265629_120216712_706	annotated	PXN	12	-	2118	67	15	6	2.41%	10.45245967	0.00053548	0.000893859	3.07E-06
ENSG00000159110_33241974_33262716_314	annotated	IFNAR2	21	+	942	37	0	1	1.91%	12.97837967	0.013640586	0.020525443	0.001325514
ENSG00000136240_6484057_6482564_38	annotated	KDELR2	7	-	114	146	10	3	31.58%	423.1727153	0.00115027	0.001042714	7.39E-06
ENSG00000131408_50377606_50382602_416	annotated	NR1H2	19	+	1248	57	7	4	6.25%	15.09141368	6.37E-05	7.19E-06	3.81E-09
ENSG00000087274_2875916_2928171_663	annotated	ADD1	4	+	1989	55	5	0	3.77%	9.136872404	1.00E-05	4.27E-06	3.57E-10
ENSG00000166986_57488091_57489402_111	novel	MARS	12	+	333	23	2	0	6.31%	22.82197662	0.020779354	0.008776163	0.000903181
ENSG00000196998_49077855_49074803_324	annotated	WDR45	X	-	972	29	4	3	4.32%	9.858285393	0.006519044	0.002747448	0.000100074
ENSG00000213523_140552041_140550700_126	novel	SRA1	5	-	378	40	2	1	11.11%	34.96534721	0.000448121	0.000603605	1.76E-06
ENSG00000136527_185922105_185917715_87	annotated	TRA2B	3	-	261	102	23	0	21.84%	129.1306443	0.005783554	6.17E-05	3.39E-06
ENSG00000041357_78542509_78548944_237	annotated	PSMA4	15	+	711	684	60	15	20.68%	317.8748401	2.71E-09	3.13E-09	9.10E-17
ENSG00000176087_140564844_140566762_103	annotated	SLC35A4	5	+	309	69	14	3	13.59%	73.783672	0.007208094	0.000804138	3.74E-05
ENSG00000099875_2050851_2037739_414	annotated	MKNK2	19	-	1242	65	3	3	5.80%	17.29264454	1.72E-05	2.66E-05	3.59E-09
ENSG00000165959_95319792_95191564_1002	annotated	CLMN	14	-	3006	34	0	0	1.50%	3.737314058	0.000276859	0.000276859	5.20E-07
ENSG00000188906_40243570_40367765_2285	annotated	LRRK2	12	+	6855	15	3	1	0.39%	0.723025232	0.022973464	0.005426623	0.000657751
ENSG00000162836_147670027_147647423_421	annotated	ACP6	1	-	1263	27	2	0	3.09%	7.063664561	0.002546667	0.000640157	1.03E-05
ENSG00000128534_118184224_118188409_67	annotated	LSM8	7	+	201	160	37	1	31.34%	263.0229103	0.004287614	3.24E-05	1.41E-06
ENSG00000163430_120450746_120396952_273	annotated	FSTL1	3	-	819	272	20	6	20.51%	109.7373974	3.61E-10	1.20E-10	4.99E-19
ENSG00000169432_166311549_166198672_1919	annotated	SCN9A	2	-	5757	28	5	0	0.63%	1.607057647	0.008400647	0.000991051	5.27E-05
ENSG00000111361_123632420_123622665_202	annotated	EIF2B1	12	-	606	71	10	6	8.91%	38.71287081	0.002747656	0.001147965	1.90E-05
ENSG00000111361_123622746_123621756_91	Overlap_dORF	EIF2B1	12	-	273	39	2	0	12.09%	47.20321873	0.001565547	0.001588175	1.49E-05
ENSG00000163626_73069649_73058114_333	annotated	COX18	4	-	999	32	2	0	4.50%	10.5841051	0.000432958	0.000255448	7.46E-07
ENSG00000163634_63863790_63834132_204	annotated	THOC7	3	-	612	143	8	11	15.20%	77.20657181	2.73E-06	1.03E-05	2.40E-10
ENSG00000108010_130136421_130179392_335	annotated	GLRX3	10	+	1005	219	30	9	12.54%	72.00252171	5.03E-08	3.00E-08	1.51E-14
ENSG00000100577_77327502_77331195_161	annotated	GSTZ1	14	+	483	25	0	0	8.07%	17.10261548	0.00060914	0.00060914	2.39E-06
ENSG00000087008_8416521_8366961_700	annotated	ACOX3	4	-	2100	38	0	0	1.57%	5.979074373	0.001384502	0.001384502	1.16E-05
ENSG00000114554_127027991_127034017_425	novel	PLXNA1	3	+	1275	17	2	0	1.65%	4.405633748	0.017901377	0.008473674	0.000754898
ENSG00000168884_2756289_2742257_346	annotated	TNIP2	4	-	1038	14	4	0	2.60%	4.45656593	0.047083924	0.002943991	0.000871348
ENSG00000137166_41584820_41598936_563	annotated	FOXP4	6	+	1689	19	1	0	1.78%	3.717008935	0.003646019	0.002122793	4.44E-05

ENSG00000108439_47941769_47946782_212	annotated	PNPO	17	+	636	86	20	7	11.32%	44.67977622	0.00039525	4.38E-05	1.32E-07
ENSG00000198917_128828822_128822765_336	annotated	SPOUT1	9	-	1008	26	8	0	3.27%	8.522803382	0.037150013	0.001412569	0.000371036
ENSG00000038358_67876067_67880240_638	novel	EDC4	16	+	1914	26	7	6	2.04%	4.488498333	0.020157702	0.008844056	0.000882278
ENSG00000186184_27648437_27665953_94	novel	POLR1D	13	+	282	20	1	1	7.45%	23.43422206	0.007905926	0.007905926	0.000321469
ENSG00000160055_32222322_32216410_164	annotated	TMEM234	1	-	492	7	0	1	3.05%	4.701133573	0.019216965	0.041782825	0.003600526
ENSG00000082898_61488236_61485744_96	novel	XPO1	2	-	288	19	1	1	9.38%	21.79870865	0.002967235	0.006112544	0.000100709
ENSG00000133027_17591626_17522362_97	annotated	PEMT	17	-	291	40	0	0	11.34%	45.41890462	0.001535678	0.001535678	1.41E-05
ENSG00000083857_186589194_186588592_200	annotated	FAT1	4	-	600	29	1	5	6.00%	15.97042234	0.001032175	0.006167354	3.94E-05
ENSG00000156735_38176870_38210493_457	annotated	BAG4	8	+	1371	47	10	5	3.50%	11.3273953	0.007459848	0.001664233	7.32E-05
ENSG00000114054_136326890_136330026_113	Overlap_dORF	PCCB	3	+	339	29	5	0	7.96%	28.26623423	0.013577462	0.003763158	0.000278201
ENSG00000114054_136250376_136327653_405	annotated	PCCB	3	+	1215	66	14	2	7.16%	17.94887823	0.000208288	1.27E-06	2.90E-09
ENSG00000189308_82939667_82928102_312	dORF	LIN54	4	-	936	9	0	0	1.60%	3.177139722	0.019679754	0.019679754	0.001784964
ENSG00000043355_99982065_99985682_532	annotated	ZIC2	13	+	1596	18	1	0	1.88%	3.7265699	0.003974283	0.002046029	4.67E-05
ENSG00000139620_48681632_48671925_155	annotated	KANSL2	12	-	465	18	5	2	7.10%	12.79054959	0.030636454	0.00809034	0.001248001
ENSG00000110880_108652315_108647403_155	annotated	CORO1C	12	-	465	56	5	0	10.32%	39.79282095	0.000804205	0.000199656	1.10E-06
ENSG00000166896_57951797_57956890_128	novel	ATP23	12	+	384	8	0	0	3.91%	6.883802732	0.019679754	0.019679754	0.001784964
ENSG00000169223_177351647_177332202_325	annotated	LMAN2	5	-	975	748	72	17	30.77%	253.493388	5.46E-16	1.14E-17	9.80E-32
ENSG00000100889_24098330_24103964_506	annotated	PCK2	14	+	1518	98	8	0	6.52%	21.33162586	4.26E-06	2.23E-07	9.27E-12
ENSG00000137497_72035943_72003527_2115	annotated	NUMA1	11	-	6345	108	26	9	2.22%	5.624213296	4.70E-06	5.17E-08	2.69E-12
ENSG00000128891_40563009_40539235_171	annotated	CCDC32	15	-	513	19	3	0	4.68%	12.23787152	0.031495399	0.005306578	0.000899719
ENSG00000132522_7314983_7311579_280	annotated	GPS2	17	-	840	43	5	3	7.50%	16.91448671	0.000610706	0.000134926	5.80E-07
ENSG00000104529_143586845_143580630_130	annotated	EEF1D	8	-	390	236	10	4	17.69%	199.9479932	1.35E-05	1.30E-05	1.41E-09
ENSG00000149269_77392454_77323137_500	annotated	PAK1	11	-	1500	46	5	3	2.60%	10.13295762	0.002894165	0.002293196	3.81E-05
ENSG00000254692_24213439_24189415_815	annotated	AL136295.1	14	-	2445	374	42	17	12.88%	50.54316018	4.84E-15	2.43E-16	1.75E-29
ENSG00000132792_37777351_37872013_311	annotated	CTNNBL1	20	+	933	29	1	0	3.54%	10.27036806	0.002568463	0.001269017	1.95E-05
ENSG00000115310_55050300_54973156_345	annotated	RTN4	2	-	1035	874	137	24	26.96%	279.0234707	8.24E-14	9.01E-16	1.12E-27
ENSG00000100243_42636837_42619773_291	annotated	CYB5R3	22	-	873	324	29	1	16.49%	122.6310425	5.32E-09	7.43E-10	4.40E-17
ENSG00000121900_32898284_32894805_205	annotated	TMEM54	1	-	615	14	0	2	3.90%	7.521813716	0.00419316	0.033181033	0.000782812
ENSG00000132170_12379712_12399373_184	annotated	PPARG	3	+	552	14	2	0	2.72%	8.380281586	0.035374934	0.021084099	0.003318418
ENSG00000100983_34951852_34942859_140	novel	GSS	20	-	420	19	9	0	5.71%	14.94768593	0.169705346	0.005554612	0.006736088
ENSG00000075151_20941797_20807319_1189	annotated	EIF4G3	1	-	3567	19	5	0	1.01%	1.760030303	0.021519587	0.000885228	0.000135628
ENSG00000163001_55519802_55538781_218	annotated	CFAP36	2	+	654	22	5	1	5.50%	11.11513102	0.011220946	0.001535641	0.000104623
ENSG00000134905_110701485_110646046_339	novel	CARS2	13	-	1017	18	0	2	2.06%	5.848186391	0.008473674	0.028426897	0.001202275
ENSG00000061936_131711230_131714299_148	novel	SFSWAP	12	+	444	13	0	0	3.38%	9.674533569	0.021084099	0.021084099	0.002029891
ENSG00000104131_44537195_44561149_204	annotated	EIF3J	15	+	612	61	2	3	9.80%	32.93427189	4.82E-05	8.06E-05	2.89E-08
ENSG00000130396_167864572_167969935_1701	annotated	AFDN	6	+	5103	12	6	0	0.41%	0.777007716	0.176150259	0.008175611	0.009250017
ENSG00000171004_132958311_132628223_508	annotated	HS6ST2	X	-	1524	14	2	0	1.18%	3.035377582	0.039705313	0.011772051	0.002244513
ENSG00000157045_15047485_15038031_205	annotated	NTAN1	16	-	615	11	0	0	2.44%	5.909996492	0.020613417	0.020613417	0.001946209
ENSG00000177106_722539_726981_316	novel	EPS8L2	11	+	948	37	1	0	4.11%	12.89623803	0.001037781	0.000602625	3.97E-06
ENSG00000131653_2165885_2176574_641	annotated	TRAF7	16	+	1923	110	6	5	6.55%	18.90092482	1.24E-08	7.97E-08	1.02E-14
ENSG00000013563_154405062_154402707_250	annotated	DNASE1L1	X	-	750	41	6	0	6.00%	13.65746462	0.004766409	0.000266003	9.24E-06
ENSG00000013583_13000105_12975308_186	annotated	HEBP1	12	-	558	62	3	12	13.44%	36.71361457	9.97E-06	0.000487092	4.44E-08
ENSG00000166479_68714981_68691257_197	annotated	TMX3	18	-	591	41	9	9	6.60%	22.92271366	0.016135515	0.006396123	0.000529648
ENSG00000197892_29191027_29146333_679	annotated	KIF13B	8	-	2037	17	0	4	1.47%	2.757576352	0.002122793	0.023533756	0.000306342
ENSG00000178605_318787_305074_516	annotated	GTPBP6	X	-	1548	44	10	4	4.26%	9.39185489	0.000670049	1.43E-05	8.64E-08
ENSG00000197043_151129469_151101448_382	annotated	ANXA6	5	-	1146	47	3	0	3.14%	13.55136035	0.003261738	0.001044718	2.07E-05
ENSG00000103544_19555730_19579118_184	annotated	VPS35L	16	+	552	41	4	12	6.52%	24.54225322	0.004465797	0.021336303	0.000515222
ENSG00000106399_7639126_7637000_82	annotated	RPA3	7	-	246	213	11	2	19.51%	286.0975574	0.00073378	0.000223299	1.11E-06
ENSG00000197694_128566741_128633334_2477	annotated	SPTAN1	9	+	7431	119	31	31	2.06%	5.291384901	3.73E-05	3.09E-05	8.82E-09
ENSG00000124145_45348384_45327264_198	annotated	SDC4	20	-	594	92	7	9	16.16%	51.17655364	8.05E-07	5.24E-06	3.80E-11
ENSG00000138069_65129906_65088493_170	annotated	RAB1A	2	-	510	165	12	3	19.41%	106.9014071	4.09E-06	6.71E-07	2.49E-11
ENSG00000221823_68217104_68180963_160	annotated	PPP3R1	2	-	480	97	35	6	18.12%	66.7728865	0.002154315	2.06E-06	6.64E-08
ENSG00000108298_39201214_39204648_194	annotated	RPL19	17	+	582	469	48	19	13.92%	266.2683283	7.28E-05	6.69E-06	4.10E-09
ENSG00000130640_133302941_133279766_902	annotated	TUBGCP2	10	-	2706	92	9	2	3.88%	11.23387763	3.37E-06	3.12E-07	9.92E-12
ENSG00000130638_45672106_45843671_461	annotated	ATXN10	22	+	1383	387	73	9	16.27%	92.46096858	1.86E-13	3.06E-13	7.50E-25
ENSG00000073350_75543427_75574878_1019	annotated	LLGL2	17	+	3057	63	9	0	2.75%	6.80949279	0.000223824	1.10E-06	2.78E-09
ENSG00000076248_109098327_109102856_141	annotated	UNG	12	+	423	28	3	0	8.51%	21.87194059	0.000943084	0.001003278	5.88E-06
ENSG00000140691_31461993_31466889_561	annotated	ARMC5	16	+	1683	9	1	0	1.07%	1.766965407	0.026832696	0.011772051	0.001510382
ENSG00000100711_103727815_103733018_166	novel	ZFYVE21	14	+	498	29	0	0	6.63%	19.2414727	0.001558047	0.001558047	1.45E-05
ENSG00000140983_668200_668655_87	novel	RHOT2	16	+	261	12	4	0	8.05%	15.19184051	0.067796501	0.007882058	0.002868205
ENSG00000140983_670322_673606_484	novel	RHOT2	16	+	1452	39	12	10	2.89%	8.87498534	0.072280784	0.025093412	0.007836268
ENSG00000100865_102362851_102350033_105	novel	CINP	14	-	315	58	32	1	17.14%	60.83970414	0.022338596	9.28E-05	2.43E-05
ENSG00000164733_11853372_11852665_24	internal	CTSB	8	-	72	32	2	43	37.50%	146.8544583	0.003710481	0.468493666	0.025643776
ENSG00000196497_24184380_24180442_525	annotated	IPO4	14	-	1575	52	14	1	4.19%	10.90918833	0.003292166	2.12E-05	7.31E-07
ENSG00000122729_32405507_32450111_889	annotated	ACO1	9	+	2667	82	10	4	4.05%	10.15922293	4.95E-06	4.12E-07	1.91E-11
ENSG00000184164_49918770_49927307_402	annotated	CRELD2	22	+	1206	80	14	1	6.72%	21.91857586	7.03E-05	3.02E-06	1.91E-09
ENSG00000140416_63048576_63071172_248	annotated	TPM1	15	+	744	413	49	46	23.79%	183.4200341	6.03E-09	1.02E-09	6.77E-17
ENSG00000197976_1593463_1599621_446	annotated	AKAP17A	X	+	1338	10	1	0	1.79%	2.469525644	0.010437297	0.004149608	0.000232539

ENSG00000006007_19521964_19517120_108	annotated	GDE1	16	-	324	45	0	1	10.19%	45.89201821	0.001588175	0.002205543	2.07E-05
ENSG00000006007_19517120_19503470_221	dORF	GDE1	16	-	663	98	14	0	13.12%	48.84073612	1.96E-05	1.13E-06	2.05E-10
ENSG00000140682_31474160_31474775_132	novel	TGFB1I1	16	+	396	15	0	0	5.30%	12.51600497	0.008373824	0.008373824	0.000358384
ENSG00000103121_80997394_80976093_55	annotated	CMC2	16	-	165	45	3	1	16.36%	90.11523576	0.005285794	0.003710481	0.00010741
ENSG00000111358_123645485_123648140_84	novel	GTF2H3	12	+	252	17	2	3	9.52%	22.29040885	0.013892891	0.031335694	0.002038539
ENSG00000166263_54999396_55043629_245	annotated	STXBP4	17	+	735	12	2	0	2.04%	5.394653569	0.08410207	0.019679754	0.007521961
ENSG00000167770_63986457_63997546_280	annotated	OTUB1	11	+	840	272	48	12	20.71%	106.9939625	8.50E-08	6.44E-10	6.79E-16
ENSG00000092621_119712023_119727234_213	novel	PHGDH	1	+	639	37	5	0	5.16%	19.13244703	0.016752628	0.001491519	0.000157264
ENSG00000092621_119712023_119724800_123	annotated	PHGDH	1	+	369	26	4	0	5.69%	23.28180436	0.110834006	0.007029303	0.004652436
ENSG00000108825_42970303_42950593_404	annotated	PTGES3L-AARSD1	17	-	1212	77	12	1	4.95%	20.99219051	0.006259751	7.60E-05	4.41E-06
ENSG00000198585_131381805_131382591_227	annotated	NUDT16	3	+	681	13	6	2	3.08%	6.30762541	0.113790253	0.022898969	0.011741768
ENSG00000003989_17543340_17565146_658	annotated	SLC7A2	8	+	1974	165	20	4	8.21%	27.61890458	3.28E-08	1.54E-10	6.61E-17
ENSG00000115525_85863483_85848122_182	annotated	ST3GAL5	2	-	546	21	10	5	3.85%	12.70855889	0.197389325	0.044843011	0.035793473
ENSG00000115525_85848138_85840144_290	Overlap_dORF	ST3GAL5	2	-	870	128	20	5	13.45%	48.61388964	1.48E-06	1.67E-07	2.36E-12
ENSG00000171960_42658856_42659348_57	annotated	PPIH	1	+	171	30	3	0	10.53%	57.96886511	0.01300724	0.01300724	0.000821865
ENSG00000116977_236526074_236548161_316	annotated	LGALS8	1	+	948	57	5	1	6.33%	19.8671775	0.000119632	5.91E-05	5.19E-08
ENSG00000137500_83285972_83274609_151	novel	CCDC90B	11	-	453	25	7	10	7.28%	18.23523902	0.04039794	0.055693975	0.00912827
ENSG00000184209_123465541_123466281_246	annotated	SNRNP35	12	+	738	27	0	1	5.69%	12.08862919	0.000415605	0.000565108	1.54E-06
ENSG00000070081_17310969_17331419_181	annotated	NUCB2	11	+	543	45	8	0	4.97%	27.38308269	0.01289399	0.003710481	0.000260636
ENSG00000004700_21499570_21470194_649	annotated	RECQL	12	-	1947	50	4	2	4.01%	8.485427096	4.23E-06	6.52E-06	2.31E-10
ENSG00000119723_73961218_73962999_156	novel	COQ6	14	+	468	20	6	0	3.85%	14.12062099	0.087374356	0.013428348	0.005787157
ENSG00000133597_140673331_140694803_626	annotated	ADCK2	7	+	1878	41	0	9	2.88%	7.213697431	6.52E-05	0.014098727	1.04E-05
ENSG00000025770_50516489_50519359_249	annotated	NCAPH2	22	+	747	11	0	0	2.41%	4.865659762	0.012798403	0.012798403	0.000797244
ENSG00000206053_1685443_1698998_174	annotated	JPT2	16	+	522	111	13	0	17.24%	70.26226236	5.14E-05	7.42E-07	3.87E-10
ENSG00000206053_1685443_1698998_174	annotated	JPT2	16	+	522	111	13	0	17.24%	70.26226236	5.14E-05	7.42E-07	3.87E-10
ENSG00000102977_67660220_67657606_455	annotated	ACD	16	-	1365	42	10	5	4.62%	10.16684711	0.003061625	0.000246473	5.36E-06
ENSG00000087263_56453280_56476205_485	annotated	OGFOD1	16	+	1455	75	14	1	3.92%	17.03208923	0.005584637	7.78E-05	3.94E-06
ENSG00000127837_218269517_218264533_388	annotated	AAMP	2	-	1164	213	15	10	10.57%	60.46391678	4.27E-07	3.68E-07	1.45E-12
ENSG00000171729_15215091_15219743_252	annotated	TMEM51	1	+	756	35	4	2	3.97%	15.2973394	0.002388631	0.004585802	6.21E-05
ENSG00000114650_47418222_47413854_493	Overlap_dORF	SCAP	3	-	1479	41	3	0	3.04%	9.159786191	0.000887233	0.000295633	1.75E-06
ENSG00000114650_47442993_47417788_659	annotated	SCAP	3	-	1977	28	7	2	1.67%	4.679732358	0.017712405	0.003182738	0.00031704
ENSG00000155100_91070385_91076603_108	novel	OTUD6B	8	+	324	13	1	0	5.56%	13.25769415	0.023672501	0.012798403	0.001437523
ENSG00000155100_91078437_91078727_96	novel	OTUD6B	8	+	288	11	3	0	6.25%	12.62030501	0.053594825	0.011772051	0.003068673
ENSG00000167965_2206086_2206521_71	annotated	MLST8	16	+	213	19	3	0	7.04%	29.47431029	0.101051022	0.019679754	0.009164498
ENSG00000101266_489830_483961_167	annotated	CSNK2A1	20	-	501	47	1	0	8.38%	30.99772248	0.000475338	0.000472567	1.47E-06
ENSG00000215301_41343246_41347719_471	novel	DDX3X	X	+	1413	189	30	7	8.70%	44.19664429	3.70E-07	2.15E-08	8.26E-14
ENSG00000164024_98995754_99005801_48	annotated	METAP1	4	+	144	14	0	0	14.58%	32.12441275	0.007979402	0.007979402	0.000327143
ENSG00000165416_52652921_52687835_365	annotated	SUGT1	13	+	1095	60	2	7	6.30%	18.10534417	3.01E-05	0.000120593	2.77E-08
ENSG00000283103_58326846_58314875_61	novel	AC010642.2	19	-	183	8	2	1	8.20%	14.44470081	0.158655254	0.047790352	0.029673219
ENSG00000170631_144946206_144930738_682	annotated	ZNF16	8	-	2046	13	0	0	0.88%	2.099458898	0.012798403	0.012798403	0.000797244
ENSG00000139644_49752494_49762896_237	annotated	TMBIM6	12	+	711	2662	561	87	47.68%	1237.109392	5.70E-17	2.09E-19	2.06E-34
ENSG00000145439_169010089_168990150_237	annotated	CBR4	4	-	711	46	8	0	7.17%	21.37754772	0.005027802	0.000121023	5.04E-06
ENSG00000150093_32912060_32908533_106	annotated	ITGB1	10	-	318	752	67	19	41.51%	781.3765516	5.22E-09	7.31E-09	4.02E-16
ENSG00000126088_45012992_45013706_119	novel	UROD	1	+	357	13	4	2	5.04%	12.03219301	0.102488202	0.044040756	0.017763347
ENSG00000126088_45013615_45014115_127	novel	UROD	1	+	381	39	6	0	11.81%	33.82277878	0.001188948	0.000294865	2.34E-06
ENSG00000163453_57110351_57032415_279	annotated	IGFBP7	4	-	837	50	5	0	5.38%	19.73850246	0.001252275	0.000300266	2.51E-06
ENSG00000108465_47975891_47981734_316	novel	CDK5RAP3	17	+	948	22	2	1	3.48%	7.668033423	0.00376808	0.002484757	5.31E-05
ENSG00000108465_47975086_47975657_131	novel	CDK5RAP3	17	+	393	12	0	0	3.82%	10.08923759	0.020613417	0.020613417	0.001946209
ENSG00000172270_572635_582577_385	annotated	BSG	19	+	1155	4604	409	114	33.25%	1317.112843	5.70E-21	2.70E-21	2.60E-40
ENSG00000125871_17969866_17990109_342	annotated	MGME1	20	+	1026	63	14	1	6.73%	20.28910279	0.00303678	1.46E-05	4.90E-07
ENSG00000240184_141476534_141478708_724	annotated	PCDHGC3	5	+	2172	16	4	0	1.38%	2.434051795	0.025739926	0.001801897	0.000295857
ENSG00000167110_128275940_128257067_990	annotated	GOLGA2	9	-	2970	21	1	8	1.41%	2.336320927	0.000483714	0.057770204	0.000284309
ENSG00000117500_93156458_93154670_125	dORF	TMED5	1	-	375	120	23	1	13.60%	105.73521	0.002571752	0.000139232	2.70E-06
ENSG00000117500_93180242_93156479_120	annotated	TMED5	1	-	360	16	1	3	6.67%	14.68544583	0.010020958	0.030313544	0.001483684
ENSG00000143033_93079527_93137027_593	annotated	MTF2	1	+	1779	10	0	3	0.84%	1.85734981	0.019679754	0.131599539	0.01228543
ENSG00000133895_64810109_64804334_610	annotated	MEN1	11	-	1830	47	1	7	3.11%	8.486261728	7.80E-05	0.003400472	2.24E-06
ENSG00000002822_2230109_1816070_710	annotated	MAD1L1	7	-	2130	11	2	5	1.13%	1.706407438	0.019230617	0.186781093	0.018181624
ENSG00000154059_24426757_24451429_332	annotated	IMPACT	18	+	996	30	4	3	4.22%	9.952485877	0.00423191	0.003385412	7.95E-05
ENSG00000104904_2269599_2271445_68	novel	OAZ1	19	+	204	92	7	3	23.53%	149.0140827	0.001904289	0.000207478	2.78E-06
ENSG00000165105_83062867_82982677_740	annotated	RASEF	9	-	2220	50	6	5	3.38%	7.441948899	3.85E-05	6.64E-05	1.93E-08
ENSG00000204560_30672841_30653242_1041	annotated	DHX16	6	-	3123	25	3	0	1.25%	2.645073096	0.003052949	0.000596156	1.17E-05
ENSG00000175573_65918214_65917459_251	annotated	C11orf68	11	-	753	12	1	0	1.99%	5.265697707	0.035374934	0.020613417	0.003252526
ENSG00000142687_35454452_35453553_75	novel	KIAA0319L	1	-	225	11	0	0	9.33%	16.15399041	0.007029303	0.007029303	0.000257364
ENSG00000122952_56359520_56358429_116	novel	ZWINT	10	-	348	77	7	230	14.66%	73.11073246	0.000393725	0.20760072	0.001588821
ENSG00000179862_40862112_40861573_179	annotated	CITED4	1	-	537	51	8	4	6.15%	31.38091078	0.013118615	0.004769506	0.000331341
ENSG00000166681_103377534_103377827_97	annotated	BEX3	X	+	291	56	8	0	14.43%	63.58646647	0.00098021	0.000456173	2.89E-06
ENSG00000067048_12913748_12918122_471	annotated	DDX3Y	Y	+	1413	26	9	3	3.18%	6.079961648	0.026994742	0.001992104	0.000338672
ENSG00000119408_124313935_124350947_232	annotated	NEK6	9	+	696	22	3	0	6.03%	10.44439035	0.002827043	0.000241223	4.83E-06

ENSG00000108523_4940910_4944570_133	annotated	RNF167	17	+	399	58	0	3	9.77%	48.03134538	0.000676852	0.00211153	8.98E-06
ENSG00000022556_54983562_54986328_171	annotated	NLRP2	19	+	513	10	1	0	3.51%	6.440985012	0.018714028	0.011570466	0.001043918
ENSG00000110583_63945897_63952569_116	novel	NAA40	11	+	348	23	10	1	7.76%	21.83827073	0.109280257	0.005059698	0.003588888
ENSG00000127527_16471945_16361967_756	annotated	EPS15L1	19	-	2268	21	3	4	1.32%	3.059467881	0.00224099	0.023573843	0.000320974
ENSG00000090316_1289947_1312233_96	annotated	MAEA	4	+	288	21	2	0	9.38%	24.09330956	0.008219047	0.003478238	0.000156195
ENSG00000054654_63954816_64225526_6678	annotated	SYNE2	14	+	20034	33	14	10	0.28%	0.544271914	0.028517757	0.011475639	0.001569511
ENSG00000071462_73683626_73692799_220	novel	BUD23	7	+	660	212	11	2	15.91%	106.1357221	4.00E-07	1.18E-07	4.51E-13
ENSG00000071462_73693377_73697886_95	novel	BUD23	7	+	285	37	5	2	9.47%	42.89696018	0.011011597	0.007815503	0.000436379
ENSG00000198919_108632995_108690897_962	annotated	DZIP3	3	+	2886	21	4	0	0.94%	2.404321952	0.026696365	0.003452921	0.000526287
ENSG00000140740_21953424_21959405_132	annotated	UQCRC2	16	+	396	73	7	2	15.91%	60.91122417	6.09E-05	6.25E-05	2.82E-08
ENSG00000170917_122922536_122922258_92	annotated	NUDT6	4	-	276	19	0	2	6.52%	22.74647859	0.01300724	0.023878474	0.001470914
ENSG00000119725_73893923_73904969_162	novel	ZNF410	14	+	486	21	8	1	4.94%	14.27751678	0.12595073	0.009288069	0.006668486
ENSG00000100697_95126669_95090614_1558	annotated	DICER1	14	-	4674	27	12	2	0.90%	1.908730924	0.055652875	0.001740566	0.00070664
ENSG00000060138_10704130_10702087_74	novel	YBX3	12	-	222	10	0	0	6.76%	14.8838978	0.020613417	0.020613417	0.001946209
ENSG00000135119_116750028_116849448_354	annotated	RNFT2	12	+	1062	38	6	4	5.37%	11.82302842	0.000742139	0.000621739	2.95E-06
ENSG00000198369_65431987_65313501_418	annotated	SPRED2	2	-	1254	21	9	0	2.39%	5.533391669	0.113812147	0.002227676	0.002090819
ENSG00000186350_134401685_134436614_435	novel	RXRA	9	+	1305	15	7	4	2.07%	3.797960128	0.05158844	0.061503838	0.012452199
ENSG00000198382_76016871_76141413_327	annotated	UVRAG	11	+	981	20	2	1	2.45%	6.73644304	0.01299184	0.011825808	0.000750884
ENSG00000171843_20414248_20363472_245	novel	MLLT3	9	-	735	12	3	0	2.86%	5.394653569	0.065131088	0.007882058	0.002742388
ENSG00000168385_241324233_241337520_159	novel	2-Sep	2	+	477	153	23	1	13.84%	105.9845855	0.007921937	2.09E-05	2.08E-06
ENSG00000146828_100858870_100866605_650	novel	SLC12A9	7	+	1950	39	7	0	2.92%	6.608450622	0.002970271	4.51E-05	1.21E-06
ENSG00000126821_63727944_63686105_441	annotated	SGPP1	14	-	1323	73	9	2	6.80%	18.23193104	2.86E-06	9.06E-07	2.30E-11
ENSG00000244687_50084158_50082768_58	novel	UBE2V1	20	-	174	13	4	2	10.34%	24.68674083	0.143893695	0.035374934	0.02120653
ENSG00000102897_20915556_20924116_122	annotated	LYRM1	16	+	366	17	1	0	8.20%	15.34749461	0.002008257	0.002122793	2.50E-05
ENSG00000131080_66616020_66599484_318	annotated	EDA2R	X	-	954	11	1	0	1.89%	3.809903399	0.023672501	0.012798403	0.001437523
ENSG00000173542_70958986_70982057_174	annotated	MOB1B	4	+	522	26	1	0	6.90%	16.45782722	0.001007374	0.000991051	6.20E-06
ENSG00000090266_140696745_140721744_96	annotated	NDUFB2	7	+	288	351	21	8	15.62%	402.7024598	0.001872676	0.000325833	4.09E-06
ENSG00000108176_67838011_67797116_228	annotated	DNAJC12	10	-	684	35	9	2	6.58%	16.90758566	0.018238157	0.001103062	0.000134525
ENSG00000255150_104303935_104304936_333	annotated	EID3	12	+	999	21	1	1	3.00%	6.945818972	0.005034873	0.005034873	0.000136652
ENSG00000119185_9408160_9406834_39	dORF	ITGB1BP1	2	-	117	28	1	0	17.95%	79.07547753	0.01019742	0.008776163	0.000451522
ENSG00000119185_9418697_9412233_107	annotated	ITGB1BP1	2	-	321	17	2	4	5.61%	17.49901255	0.037178614	0.063412829	0.009607434
ENSG00000213722_31728669_31727237_136	novel	DDAH2	6	-	408	8	0	5	5.88%	6.478873159	0.002338867	0.124106539	0.002427528
ENSG00000123600_171360506_171324172_357	Overlap_dORF	METTL8	2	-	1071	11	2	0	2.52%	3.393695464	0.006277459	0.002478943	8.78E-05
ENSG00000164953_93795931_93816452_394	novel	TMEM67	8	+	1182	19	1	0	2.03%	5.311360483	0.005454749	0.005306578	0.000155005
ENSG00000076944_7637150_7647810_604	annotated	STXBP2	19	+	1812	33	9	1	2.48%	6.017628878	0.009747438	0.000436588	3.10E-05
ENSG00000161011_179823011_179836593_354	annotated	SQSTM1	5	+	1062	1898	202	84	41.53%	590.5291564	1.29E-21	3.76E-22	8.37E-42
ENSG00000115355_55374859_55291711_1697	annotated	CCDC88A	2	-	5091	57	6	3	1.71%	3.699486206	2.72E-05	5.46E-06	1.23E-09
ENSG00000127081_92877840_92846228_854	annotated	ZNF484	9	-	2562	12	0	0	0.59%	1.547646516	0.019679754	0.019679754	0.001784964
ENSG00000136631_150068645_150144796_534	dORF	VPS45	1	+	1602	53	8	6	3.93%	10.93158187	0.00100786	0.000237917	1.63E-06
ENSG00000111276_12717885_12718946_96	annotated	CDKN1B	12	+	288	30	3	1	8.33%	34.41901366	0.032206895	0.005454749	0.000942887
ENSG00000172269_119097523_119096998_93	Overlap_dORF	DPAGT1	11	-	279	18	11	0	5.38%	21.31758265	0.241112522	0.021557223	0.026983717
ENSG00000172269_119100804_119097520_169	annotated	DPAGT1	11	-	507	82	14	4	13.61%	53.44111943	0.00117618	7.41E-05	6.70E-07
ENSG00000170946_31370749_31414966_88	novel	DNAJC24	11	+	264	20	2	1	9.09%	25.03200993	0.020715809	0.008219047	0.000849657
ENSG00000143614_153828347_153810177_593	annotated	GATAD2B	1	-	1779	58	10	2	3.37%	10.7726289	0.00095811	0.000150397	1.02E-06
ENSG00000114786_51984156_51987112_183	dORF	ABHD14A-ACY1	3	+	549	17	0	0	4.92%	10.23166308	0.003303074	0.003303074	6.12E-05
ENSG00000114786_51984065_51986623_214	Overlap_dORF	ABHD14A-ACY1	3	+	642	28	1	0	5.14%	14.41095151	0.001506153	0.001506153	1.36E-05
ENSG00000114786_51987035_51989075_198	dORF	ABHD14A-ACY1	3	+	594	46	3	4	8.08%	25.58827682	0.00030684	0.001054241	2.15E-06
ENSG00000114786_51975136_51984153_167	annotated	ABHD14A-ACY1	3	+	501	9	0	0	3.59%	5.935734092	0.009815329	0.009815329	0.000483714
ENSG00000153037_112861377_112891607_101	annotated	SRP19	5	+	303	22	5	0	5.94%	23.99107487	0.053624202	0.013640586	0.0034686
ENSG00000128989_52557219_52551934_96	annotated	ARPP19	15	-	288	39	5	5	11.46%	44.74471776	0.007714041	0.006557658	0.000263348
ENSG00000164073_127965133_127920630_518	annotated	MFSD8	4	-	1554	28	2	1	2.70%	5.953559119	0.000573217	0.000573217	2.12E-06
ENSG00000134371_193122201_193156975_370	novel	CDC73	1	+	1110	86	6	3	6.49%	25.60030421	3.84E-05	1.61E-05	4.86E-09
ENSG00000123066_116008435_115961266_1550	annotated	MED13L	12	-	4650	32	11	8	1.35%	2.273875483	0.023364097	0.004201685	0.000538147
ENSG00000115275_74462963_74461275_562	dORF	MOGS	2	-	1686	74	1	0	4.98%	14.50253102	1.99E-06	1.60E-06	2.79E-11
ENSG00000115275_74464717_74462969_100	annotated	MOGS	2	-	300	29	1	4	8.00%	31.94084467	0.005306578	0.034975232	0.001006765
ENSG00000170322_129892774_129864725_1274	annotated	NFRKB	11	-	3822	19	0	1	0.71%	1.64260285	0.003606289	0.005034873	9.98E-05
ENSG00000138073_27134421_27133443_137	novel	PREB	2	-	411	13	1	4	3.65%	10.45132094	0.028889786	0.087777151	0.010739216
ENSG00000176225_70030080_70004151_334	dORF	RTTN	18	-	1002	19	0	0	1.50%	6.265497097	0.020613417	0.020613417	0.001946209
ENSG00000120948_11020489_11023327_100	annotated	TARDBP	1	+	300	12	0	0	6.00%	13.21690124	0.012798403	0.012798403	0.000797244
ENSG00000064601_45897005_45897196_63	novel	CTSA	20	+	189	30	0	0	7.94%	52.44802081	0.020613417	0.020613417	0.001946209
ENSG00000064601_45898005_45898450_62	novel	CTSA	20	+	186	77	10	1	17.74%	136.787822	0.004488024	0.001953476	5.05E-05
ENSG00000128463_34225115_34229847_149	annotated	EMC4	15	+	447	64	48	6	13.42%	47.30881877	0.060170361	0.000298992	0.000211307
ENSG00000134453_6097066_6115556_401	annotated	RBM17	10	+	1203	76	8	1	6.23%	20.87457387	0.000214401	6.66E-06	1.28E-08
ENSG00000107201_32500895_32457122_875	annotated	DDX58	9	-	2625	15	0	7	1.03%	1.888128749	0.003327803	0.035350572	0.000694744
ENSG00000180694_90645905_90625671_328	annotated	TMEM64	8	-	984	30	4	1	2.44%	10.07385766	0.008364861	0.008413705	0.000359633
ENSG00000076513_109999689_110037554_590	annotated	ANKRD13A	12	+	1770	16	11	0	1.53%	2.986870338	0.251330862	0.003229477	0.008201988
ENSG00000148834_104255213_104267405_213	annotated	GSTO1	10	+	639	331	34	7	23.00%	171.1578369	5.99E-09	3.13E-09	2.00E-16
ENSG00000127838_218271527_218272762_71	novel	PNKD	2	+	213	53	3	1	22.54%	82.21781291	0.000250577	0.000202392	3.49E-07

ENSG00000148834_104255213_104267405_180	annotated	GSTO1	10	+	540	294	33	5	22.78%	179.8967114	1.64E-07	7.19E-08	1.15E-13
ENSG00000167004_43746540_43756712_76	novel	PDIA3	15	+	228	76	4	6	9.21%	110.1408437	0.008574016	0.015721867	0.000671382
ENSG00000140650_8797883_8806423_120	novel	PMM2	16	+	360	22	2	3	5.83%	20.19248801	0.013580321	0.044843011	0.002871182
ENSG00000058729_97179181_97163061_526	annotated	RIOK2	5	-	1578	19	1	0	1.90%	3.978471541	0.00376808	0.002227676	4.80E-05
ENSG00000065243_88741057_88805810_565	annotated	PKN2	1	+	1695	9	3	2	0.88%	1.754455917	0.114974528	0.060322713	0.025806112
ENSG00000101972_124031120_124100597_1174	annotated	STAG2	X	+	3522	71	7	1	2.56%	6.660987992	1.32E-05	8.77E-07	1.07E-10
ENSG00000168488_28823260_28836777_1062	annotated	ATXN2L	16	+	3186	144	24	9	3.77%	14.93435169	2.13E-05	4.35E-08	1.21E-11
ENSG00000140538_88256153_87876935_817	annotated	NTRK3	15	-	2451	147	14	7	7.22%	19.81726319	2.66E-09	1.20E-10	3.80E-18
ENSG00000185104_50705860_50452086_464	annotated	FAF1	1	-	1392	41	1	1	1.51%	9.732272827	0.008473674	0.0083057	0.000359638
ENSG00000078070_183092489_183034036_408	annotated	MCCC1	3	-	1224	10	3	0	1.47%	2.699530483	0.113684165	0.011570466	0.006956434
ENSG00000183891_19897953_19896987_74	annotated	TTC32	2	-	222	25	0	0	10.81%	37.2097445	0.005405142	0.005405142	0.000156372
ENSG00000128928_40405828_40418263_393	annotated	IVD	15	+	1179	32	0	0	3.56%	8.968211192	0.000408047	0.000408047	1.10E-06
ENSG00000166979_32412854_32515190_438	annotated	EVA1C	21	+	1314	137	16	12	6.85%	34.45044655	1.96E-05	1.31E-05	2.05E-09
ENSG00000143569_154259007_154270295_280	annotated	UBAP2L	1	+	840	25	5	4	3.21%	9.834003902	0.025622446	0.003478238	0.000506528
ENSG00000177885_75393628_75320382_156	annotated	GRB2	17	-	468	40	7	34	8.33%	28.24124198	0.007174979	0.210913373	0.01074523
ENSG00000079805_10783085_10795530_157	novel	DNM2	19	+	471	7	1	1	3.18%	4.910738254	0.041782825	0.041782825	0.007200521
ENSG00000174917_5680486_5679484_102	novel	MICOS13	19	-	306	55	6	0	8.82%	59.38967063	0.003763158	0.003763158	7.85E-05
ENSG00000114738_50617566_50648046_382	annotated	MAPKAPK3	3	+	1146	32	11	0	2.62%	9.226458111	0.060535796	0.00224099	0.000948575
ENSG00000104325_90001493_90051897_335	annotated	DECR1	8	+	1005	169	21	3	12.54%	55.56358981	8.31E-07	1.63E-08	1.49E-13
ENSG00000104324_96784898_97143183_472	annotated	CPQ	8	+	1416	8	1	0	1.06%	1.866793961	0.042234516	0.019679754	0.003715148
ENSG00000062716_59839789_59839911_40	novel	VMP1	17	+	120	31	3	5	15.00%	85.35915387	0.023199731	0.046045458	0.004668342
ENSG00000131469_42998763_42998939_58	novel	RPL27	17	+	174	1138	125	2	8.62%	2161.039313	0.021557223	0.023199731	0.002265826
ENSG00000154380_225514670_225497775_210	novel	ENAH	1	-	630	19	10	0	6.19%	9.965123954	0.062971854	0.000528008	0.000339438
ENSG00000102882_30116983_30116668_70	novel	MAPK3	16	-	210	13	0	0	8.57%	20.45472812	0.01300724	0.01300724	0.000821865
ENSG00000013375_83188759_83169234_461	annotated	PGM3	6	-	1383	17	4	1	2.39%	4.061592935	0.019251948	0.002415484	0.000274112
ENSG00000131778_147252707_147295509_791	Overlap_dORF	CHD1L	1	+	2373	35	1	2	2.02%	4.87348866	0.000286582	0.000501115	9.60E-07
ENSG00000141510_7676594_7669609_393	annotated	TP53	17	-	1179	335	24	10	11.20%	93.88596092	2.17E-07	1.62E-08	3.65E-14
ENSG00000134574_47233353_47237621_181	dORF	DDB2	11	+	543	34	1	0	4.97%	20.68944025	0.00567009	0.003554776	0.000110499
ENSG00000104973_49818342_49836944_482	annotated	MED25	19	+	1446	18	6	0	2.07%	4.113143541	0.081593387	0.002033393	0.00127441
ENSG00000263766_47604057_47603938_39	novel	AC025682.1	17	-	117	6	3	0	12.82%	16.94474519	0.294344067	0.016947427	0.029893607
ENSG00000204178_25431099_25498466_664	annotated	MACO1	1	+	1992	47	1	0	2.26%	7.796113937	0.000427981	0.000279067	8.02E-07
ENSG00000078124_76860977_77020327_267	annotated	ACER3	11	+	801	41	0	3	5.99%	16.91301345	0.000179122	0.001077035	1.34E-06
ENSG00000087884_77842497_77918099_88	annotated	AAMDC	11	+	264	24	0	1	7.95%	30.03841192	0.008473674	0.011825808	0.000503679
ENSG00000119707_73106283_73111563_220	novel	RBM25	14	+	660	9	2	3	2.27%	4.505761788	0.083503598	0.145248299	0.042302052
ENSG00000126457_49680535_49688245_325	annotated	PRMT1	19	+	975	486	31	7	16.62%	164.7029232	2.81E-09	2.11E-10	6.88E-18
ENSG00000132906_15506076_15492943_145	annotated	CASP9	1	-	435	14	1	0	4.14%	10.63428836	0.012798403	0.01300724	0.000809468
ENSG00000105221_40265267_40233872_438	annotated	AKT2	19	-	1314	76	3	6	7.53%	19.11119662	1.12E-06	2.05E-06	2.03E-11
ENSG00000144659_39384688_39396520_287	annotated	SLC25A38	3	+	861	16	3	1	3.48%	6.140256095	0.015743816	0.006432154	0.000519376
ENSG00000125352_119871613_119870582_343	annotated	RNF113A	X	-	1029	11	6	0	1.75%	3.532213647	0.128949518	0.012798403	0.00869264
ENSG00000182154_137552133_137552495_120	annotated	MRPL41	9	+	360	315	22	11	38.33%	289.1197147	3.21E-08	2.69E-08	8.69E-15
ENSG00000147010_19645418_19588411_248	annotated	SH3KBP1	X	-	744	9	0	2	2.42%	3.997046747	0.012178958	0.070229091	0.004215017
ENSG00000018699_32702906_32820938_437	dORF	TTC27	2	+	1311	13	4	0	2.06%	3.276501071	0.042927328	0.002500085	0.000688452
ENSG00000088035_63370978_63411939_194	annotated	ALG6	1	+	582	27	1	2	5.67%	15.32888031	0.002190151	0.003240864	4.08E-05
ENSG00000088035_63411255_63437020_306	Overlap_dORF	ALG6	1	+	918	35	3	2	4.58%	12.59780892	0.001566718	0.001073644	1.03E-05
ENSG00000183309_143649993_143651483_496	annotated	ZNF623	8	+	1488	22	4	4	2.22%	4.885279358	0.025075573	0.009920635	0.001214374
ENSG00000147010_19645418_19536417_404	annotated	SH3KBP1	X	-	1212	31	0	2	3.96%	8.451401373	0.000177865	0.000815071	1.00E-06
ENSG00000170190_75093637_75106033_505	annotated	SLC16A5	17	+	1515	47	14	6	4.75%	10.25073199	0.001780586	0.000415213	4.84E-06
ENSG00000011143_58219230_58214875_146	annotated	MKS1	17	-	438	16	3	1	6.16%	12.07022945	0.010499663	0.010020958	0.000525659
ENSG00000137124_38395749_38397302_517	annotated	ALDH1B1	9	+	1551	161	15	7	7.93%	34.29917957	1.59E-06	8.22E-08	1.31E-12
ENSG00000137124_38392735_38395780_37	Overlap_uORF	ALDH1B1	9	+	111	24	0	4	13.51%	71.44270943	0.020613417	0.036968718	0.003394961
ENSG00000099875_2041201_2037743_172	Overlap_dORF	MKNK2	19	-	516	24	0	0	4.07%	15.36848982	0.008776163	0.008776163	0.000391611
ENSG00000111652_6724657_6731152_262	annotated	COPS7A	12	+	786	71	9	8	10.31%	29.84732787	5.49E-05	0.000112801	4.57E-08
ENSG00000135124_121210255_121228546_116	annotated	P2RX4	12	+	348	47	1	6	10.34%	44.6260315	0.001573472	0.009994242	9.45E-05
ENSG00000010278_6200500_6225725_121	novel	CD9	12	+	363	374	44	73	18.18%	340.4353351	3.54E-05	1.48E-05	4.13E-09
ENSG00000164430_73452088_73414010_416	annotated	CGAS	6	-	1248	9	3	0	1.20%	2.382854792	0.121807694	0.019679754	0.01126199
ENSG00000154511_92876409_92843383_403	annotated	DIPK1A	1	-	1209	44	2	1	4.96%	12.02530303	7.95E-05	5.29E-05	3.12E-08
ENSG00000164430_73452088_73425227_491	annotated	CGAS	6	-	1473	20	3	0	1.43%	4.486388746	0.039104404	0.008574016	0.001690775
ENSG00000163660_157160094_157146553_428	annotated	CCNL1	3	-	1284	11	8	2	2.10%	2.830722619	0.154920533	0.024779192	0.017572165
ENSG00000144560_11601909_11558556_231	annotated	VGLL4	3	-	693	17	0	0	2.60%	8.105603216	0.013428348	0.013428348	0.000872559
ENSG00000149187_47489014_47472230_485	annotated	CELF1	11	-	1455	84	12	3	4.95%	19.07593994	0.000883111	2.40E-05	1.83E-07
ENSG00000119977_95693899_95664067_459	annotated	TCTN3	10	-	1377	221	45	13	10.24%	53.0307766	1.50E-05	9.44E-08	1.62E-11
ENSG00000175581_73787972_73864370_121	annotated	MRPL48	11	+	363	93	17	7	9.09%	84.65370632	0.040368571	0.01371513	0.002600452
ENSG00000082805_1028168_1410415_888	annotated	ERC1	12	+	2664	9	3	2	0.68%	1.116292335	0.110834006	0.053594825	0.022573869
ENSG00000091317_32502745_32483960_183	annotated	CMTM6	3	-	549	129	9	2	17.49%	77.64026687	4.36E-06	3.79E-07	1.55E-11
ENSG00000174989_116928029_117028172_490	annotated	FBXW8	12	+	1470	20	4	5	2.04%	4.495544641	0.027671636	0.043904042	0.005203232
ENSG00000154240_66183230_65635971_890	annotated	CEP112	17	-	2670	10	0	0	0.56%	1.23753757	0.020613417	0.020613417	0.001946209
ENSG00000166313_6411188_6395534_657	annotated	APBB1	11	-	1971	21	3	0	1.37%	3.520483589	0.014936572	0.003303074	0.000273899
ENSG00000161217_196270531_196238688_367	annotated	PCYT1A	3	-	1101	30	0	4	3.27%	9.003338723	0.001007374	0.004044382	2.49E-05

ENSG00000196639_11259353_11260501_382	annotated	HRH1	3	+	1146	19	0	0	1.83%	5.478209504	0.008674847	0.008674847	0.000383115
ENSG00000172057_39924203_39922550_153	annotated	ORMDL3	17	-	459	29	16	0	10.46%	20.87636907	0.056044867	0.000177448	0.000131653
ENSG00000100938_24233254_24234112_70	annotated	GMPR2	14	+	210	25	8	0	10.00%	39.33601561	0.061282382	0.008776163	0.002792762
ENSG00000100938_24234191_24238778_357	dORF	GMPR2	14	+	1071	60	10	2	6.16%	18.51106617	0.000213619	3.41E-05	5.57E-08
ENSG00000134255_111147787_111184310_392	novel	CEPT1	1	+	1176	71	6	1	6.63%	19.94897934	1.82E-05	3.80E-06	5.82E-10
ENSG00000179242_61252514_61936943_916	annotated	CDH4	20	+	2748	10	0	0	0.55%	1.202410957	0.016947427	0.016947427	0.001349898
ENSG00000259075_89523625_89522813_270	dORF	POC1B-GALNT4	12	-	810	28	5	0	4.81%	11.42201342	0.005386392	0.000635664	2.24E-05
ENSG00000089157_120200783_120198735_142	annotated	RPLP0	12	-	426	78	19	5	7.75%	60.49990006	0.00782527	0.003487661	0.000149111
ENSG00000135452_57746619_57747290_96	annotated	TSPAN31	12	+	288	69	5	4	18.75%	79.16373141	8.97E-05	0.000473864	3.08E-07
ENSG00000103248_86554707_86532011_363	annotated	MTHFSD	16	-	1089	18	2	3	1.65%	5.46152944	0.030316224	0.044843011	0.005757811
ENSG00000196367_98970232_99012355_1329	annotated	TRRAP	7	+	3987	25	5	6	1.05%	2.071874411	0.010001685	0.012635673	0.000625714
ENSG00000197548_11299415_11554843_632	annotated	ATG7	3	+	1896	42	3	0	3.48%	7.319486449	6.36E-05	1.35E-05	6.82E-09
ENSG00000116793_113713332_113697705_519	novel	PHTF1	1	-	1557	11	2	0	1.16%	2.334391678	0.050679946	0.012798403	0.00310099
ENSG00000140391_77070954_77056028_96	novel	TSPAN3	15	-	288	281	60	8	23.96%	322.3914279	0.001890839	1.22E-05	2.44E-07
ENSG00000082269_70475684_70559921_1213	annotated	FAM135A	6	+	3639	16	6	0	0.41%	1.452805853	0.129839126	0.021084099	0.01274956
ENSG00000107521_98443240_98417564_700	annotated	HPS1	10	-	2100	11	1	1	1.00%	1.730784687	0.013018137	0.013018137	0.00082316
ENSG00000074047_120968812_120990675_1339	annotated	GLI2	2	+	4017	27	3	0	0.82%	2.220913204	0.007322425	0.001520872	6.63E-05
ENSG00000131504_141584219_141516851_1170	annotated	DIAPH1	5	-	3510	91	17	0	4.19%	8.566510066	4.55E-07	2.41E-10	1.72E-15
ENSG00000171560_154590678_154585494_641	annotated	FGA	4	-	1923	56	6	1	4.99%	9.622288998	1.29E-05	3.46E-07	4.42E-11
ENSG00000198053_1895448_1937568_504	annotated	SIRPA	20	+	1512	46	6	0	3.97%	10.05253732	0.00038443	3.51E-05	1.05E-07
ENSG00000183765_28734586_28687897_469	annotated	CHEK2	22	-	1407	21	0	0	2.56%	4.931679569	0.000885228	0.000885228	4.91E-06
ENSG00000060656_29259520_29325661_1223	annotated	PTPRU	1	+	3669	35	1	0	1.06%	3.152027416	0.000795519	0.000693329	3.50E-06
ENSG00000171723_66508543_67180937_731	annotated	GPHN	14	+	2193	19	1	0	1.09%	2.862757908	0.009288069	0.005306578	0.000259004
ENSG00000177628_155239931_155234995_449	annotated	GBA	1	-	1347	240	22	0	8.02%	58.87261133	4.97E-07	7.37E-08	3.59E-13
ENSG00000150527_39276958_39350264_780	annotated	MIA2	14	+	2340	51	7	0	2.69%	7.201516704	0.000171144	2.42E-05	3.23E-08
ENSG00000185104_50959787_50441440_642	annotated	FAF1	1	-	1926	60	1	1	2.96%	10.29353679	5.33E-05	8.06E-05	3.18E-08
ENSG00000113273_78985248_78816086_413	annotated	ARSB	5	-	1239	32	4	3	4.12%	8.533915251	0.001482835	0.001119705	1.01E-05
ENSG00000127054_1324608_1320798_107	novel	INTS11	1	-	321	25	0	0	7.48%	25.73384199	0.005604867	0.005604867	0.000167525
ENSG00000168090_100089304_100090756_165	novel	COPS6	7	+	495	45	4	0	10.91%	30.03841192	0.000247732	8.02E-05	1.43E-07
ENSG00000072849_5480531_5477957_92	dORF	DERL2	17	-	276	56	0	1	19.57%	67.04225269	8.66E-05	0.00011709	7.33E-08
ENSG00000127804_2502313_2464313_115	annotated	METTL16	17	-	345	21	3	0	6.96%	20.11267581	0.02532052	0.005604867	0.00074832
ENSG00000127804_2441546_2419970_315	dORF	METTL16	17	-	945	18	2	0	2.54%	6.293762497	0.018318558	0.005554612	0.000531652
ENSG00000133318_63681637_63758201_920	annotated	RTN3	11	+	2760	392	50	9	4.35%	46.92957688	1.73E-06	6.30E-08	1.12E-12
ENSG00000088367_36175560_36229340_779	annotated	EPB41L1	20	+	2337	151	21	5	7.70%	21.34950886	8.96E-09	4.87E-11	5.77E-18
ENSG00000168894_85595764_85597196_139	annotated	RNF181	2	+	417	126	5	6	17.99%	99.83990149	1.34E-05	1.71E-05	1.82E-09
ENSG00000168894_85597427_85597504_25	dORF	RNF181	2	+	75	71	25	5	28.00%	312.7999961	0.044329616	0.021261239	0.004168196
ENSG00000117859_51617156_51787789_704	annotated	OSBPL9	1	+	2112	33	15	2	2.41%	5.162852049	0.0206376	0.000472825	7.81E-05
ENSG00000181830_45805964_45811335_310	annotated	SLC35C1	11	+	930	17	3	2	2.58%	6.039981752	0.028023387	0.020715809	0.002611326
ENSG00000176340_63974681_63976320_69	annotated	COX8A	11	+	207	1102	110	52	62.32%	1759.061011	4.24E-08	3.94E-08	1.67E-14
ENSG00000166441_8682814_8685806_148	annotated	RPL27A	11	+	444	126	11	1	4.05%	93.76855613	0.053844448	0.020525443	0.004909623
ENSG00000142856_63490146_63449778_133	novel	ITGB3BP	1	-	399	15	4	0	3.76%	12.42189967	0.12312585	0.020613417	0.011809609
ENSG00000114744_149752444_149750377_135	annotated	COMMD2	3	-	405	93	18	2	15.56%	75.87480344	0.00051337	0.000102899	3.79E-07
ENSG00000198034_72276183_72275014_114	novel	RPS4X	X	-	342	77	16	6	6.14%	74.39337689	0.01208853	0.024135905	0.001392393
ENSG00000174886_5901347_5894742_113	novel	NDUFA11	19	-	339	85	14	3	18.58%	82.84930721	0.000852362	3.19E-05	2.26E-07
ENSG00000107745_72566793_72368195_480	annotated	MICU1	10	-	1440	127	17	4	9.17%	29.14143156	2.66E-06	7.09E-09	2.43E-13
ENSG00000160360_136335982_136358220_573	annotated	GPSM1	9	+	1719	19	0	0	1.22%	3.652139669	0.008473674	0.008473674	0.000366502
ENSG00000050820_75243015_75229511_841	dORF	BCAR1	16	-	2523	53	8	4	2.62%	6.941099544	0.000186965	0.000127269	1.68E-07
ENSG00000166548_66517813_66511968_94	novel	TK2	16	-	282	16	3	0	7.45%	18.74737765	0.008373824	0.008473674	0.000362422
ENSG00000205336_57650288_57663582_687	annotated	ADGRG1	16	+	2061	289	28	21	11.21%	46.33290223	1.16E-11	1.43E-12	2.12E-22
ENSG00000171208_47128397_47083221_344	annotated	NETO2	16	-	1032	65	15	2	8.14%	20.81149663	0.000639998	5.85E-06	3.80E-08
ENSG00000151491_15682912_15621317_809	annotated	EPS8	12	-	2427	79	10	0	3.21%	10.75540995	0.000507204	3.18E-06	1.73E-08
ENSG00000100600_92732786_92704319_433	annotated	LGMN	14	-	1299	352	40	14	12.24%	89.53712929	6.56E-09	7.95E-09	5.47E-16
ENSG00000167397_31091310_31091134_58	Overlap_dORF	VKORC1	16	-	174	204	55	19	29.31%	387.391933	0.000644384	0.001844079	7.49E-06
ENSG00000267673_10315811_10310572_111	novel	FDX2	19	-	333	13	10	0	7.21%	12.89937809	0.294631094	0.004826937	0.013491844
ENSG00000123178_49936163_49915063_133	annotated	SPRYD7	13	-	399	20	13	0	4.51%	16.56253289	0.129147156	0.013428348	0.009025177
ENSG00000161526_75667373_75702950_163	novel	SAP30BP	17	+	489	34	5	10	9.20%	22.97416372	0.004431353	0.021538037	0.000516885
ENSG00000145907_151786627_151804091_464	annotated	G3BP1	5	+	1392	183	36	13	11.64%	43.43916896	3.21E-07	5.26E-09	1.92E-14
ENSG00000141646_51047116_51076645_297	annotated	SMAD4	18	+	891	21	2	0	3.03%	7.787736424	0.016881136	0.003303074	0.000311189
ENSG00000102805_76992135_77016746_236	annotated	CLN5	13	+	708	22	0	1	2.97%	10.26736679	0.008574016	0.010042643	0.000435403
ENSG00000173153_64314055_64315966_280	annotated	ESRRA	11	+	840	41	8	4	5.36%	16.1277664	0.006556828	0.001679061	6.46E-05
ENSG00000133065_205799788_205791533_339	novel	SLC41A1	1	-	1017	18	1	0	3.24%	5.848186391	0.002559322	0.001262414	1.93E-05
ENSG00000136636_215573811_215581068_204	annotated	KCTD3	1	+	612	14	0	0	2.94%	7.558685352	0.01300724	0.01300724	0.000821865
ENSG00000214253_101245004_101239806_152	annotated	FIS1	7	-	456	182	34	6	19.08%	131.8791681	0.000224883	4.87E-06	1.02E-08
ENSG00000120948_11016999_11018930_68	novel	TARDBP	1	+	204	27	1	0	14.71%	43.73239382	0.003500471	0.002362849	4.72E-05
ENSG00000144711_12936529_12901509_760	annotated	IQSEC1	3	-	2280	14	1	0	1.05%	2.028910279	0.007930666	0.005160369	0.000216377
ENSG00000115163_26792651_26793279_105	dORF	CENPA	2	+	315	34	6	11	7.62%	35.66465415	0.023197249	0.165177397	0.018009935
ENSG00000183814_226309139_226232501_542	annotated	LIN9	1	-	1626	15	3	2	1.48%	3.048178331	0.038565921	0.026823708	0.004469878
ENSG00000171557_154612609_154604340_445	annotated	FGG	4	-	1335	49	8	1	5.39%	12.12786818	0.000272568	9.92E-06	2.36E-08

ENSG00000121900_32898284_32894805_152	annotated	TMEM54	1	-	456	13	0	2	4.61%	9.41994058	0.007029303	0.049121095	0.001832503
ENSG00000168028_39407654_39412368_295	annotated	RPSA	3	+	885	211	31	8	3.73%	78.77870516	0.004760946	0.001672809	4.65E-05
ENSG00000177189_20234787_20155398_708	annotated	RPS6KA3	X	-	2124	48	5	2	3.53%	7.467175844	6.12E-05	1.98E-05	9.41E-09
ENSG00000161202_184165153_184170755_503	novel	DVL3	3	+	1509	7	0	0	0.99%	1.532775161	0.019216965	0.019216965	0.001707396
ENSG00000150401_113483894_113458029_204	annotated	DCUN1D2	13	-	612	13	0	0	2.45%	7.018779256	0.021084099	0.021084099	0.002029891
ENSG00000119977_95693899_95692912_168	novel	TCTN3	10	-	504	119	33	13	16.07%	78.01643096	0.001406515	0.000179923	1.78E-06
ENSG00000171055_36598142_36555734_305	annotated	FEZ2	2	-	915	38	15	5	5.25%	13.72246577	0.037412304	0.001565127	0.000405534
ENSG00000109323_102664780_102632057_416	novel	MANBA	4	-	1248	43	0	0	3.12%	11.38475067	0.000698084	0.000698084	3.11E-06
ENSG00000109323_102689627_102668933_146	novel	MANBA	4	-	438	11	1	0	4.11%	8.298282745	0.023672501	0.012798403	0.001437523
ENSG00000109323_102723876_102690953_104	novel	MANBA	4	-	312	10	2	0	5.77%	10.59046574	0.047790352	0.011772051	0.002720069
ENSG00000114805_155596340_155480968_1666	annotated	PLCH1	3	-	4998	22	3	4	0.48%	1.454440913	0.011428951	0.026799085	0.001469725
ENSG00000113575_134199117_134197772_34	novel	PPP2CA	5	-	102	50	6	2	23.53%	161.971829	0.005998643	0.007971974	0.000250074
ENSG00000120705_138542918_138529672_30	uORF	ETF1	5	-	90	19	4	2	20.00%	69.75586768	0.029614852	0.013428348	0.00187062
ENSG00000108654_64500303_64499923_126	novel	DDX5	17	-	378	66	16	2	9.52%	57.69282289	0.010908255	0.002775311	0.000170005
ENSG00000108654_64506119_64502005_406	novel	DDX5	17	-	1218	251	49	29	14.29%	68.09199943	1.30E-06	3.39E-10	7.50E-15
ENSG00000131966_58200218_58207171_90	annotated	ACTR10	14	+	270	14	0	1	5.56%	17.13302013	0.019679754	0.042234516	0.003715148
ENSG00000120694_31138535_31137089_104	annotated	HSPH1	13	-	312	25	0	1	12.50%	26.47616435	0.000539992	0.001612025	5.56E-06
ENSG00000156515_69288744_69401135_921	annotated	HK1	10	+	2763	183	27	1	6.84%	21.88466276	4.19E-09	1.96E-12	1.34E-19
ENSG00000180488_77779656_77878800_535	annotated	MIGA1	1	+	1605	27	12	0	2.24%	5.558509869	0.074721747	0.000947024	0.000650353
ENSG00000104529_143580715_143579792_147	novel	EEF1D	8	-	441	152	39	3	12.93%	113.8871309	0.00038264	0.000165413	4.37E-07
ENSG00000121057_57105510_57120324_888	annotated	AKAP1	17	+	2664	149	6	0	5.29%	18.48083977	7.78E-09	8.89E-10	7.70E-17
ENSG00000149503_62128795_62151976_859	annotated	INCENP	11	+	2577	49	13	4	2.56%	6.282772225	0.001669373	0.000170854	2.04E-06
ENSG00000115271_162344249_162356928_158	novel	GCA	2	+	474	26	2	0	5.70%	18.12444264	0.006839307	0.003554776	0.000132796
ENSG00000135002_76388644_76388537_35	novel	RFK	9	-	105	14	2	0	14.29%	44.05633748	0.039124093	0.020613417	0.003588156
ENSG00000130638_45738548_45843671_180	novel	ATXN10	22	+	540	124	17	3	15.56%	75.87480344	1.67E-06	2.44E-06	3.56E-11
ENSG00000106144_143291559_143303787_312	novel	CASP2	7	+	936	18	0	0	2.24%	6.354279445	0.007979402	0.007979402	0.000327143
ENSG00000198001_43768112_43786715_460	annotated	IRAK4	12	+	1380	19	1	0	1.74%	4.549295718	0.009341698	0.005355751	0.000262679
ENSG00000104529_143586845_143579792_281	annotated	EEF1D	8	-	843	390	49	7	15.66%	152.8645162	2.49E-08	9.14E-09	2.35E-15
ENSG00000163956_3532412_3523464_128	novel	LRPAP1	4	-	384	499	10	1	15.62%	429.3771954	3.99E-05	6.86E-05	2.06E-08
ENSG00000171793_40983291_40996120_307	novel	CTPS1	1	+	921	11	6	1	1.95%	3.946414595	0.201322915	0.023672501	0.023069683
ENSG00000174132_100562055_100581365_50	novel	FAM174A	5	+	150	16	15	0	14.00%	35.24506999	0.116253847	0.008175611	0.005511306
ENSG00000113389_32712236_32786345_388	annotated	NPR3	5	+	1164	44	3	2	4.12%	12.49019877	0.000676283	0.000730067	3.15E-06
ENSG00000135525_136383755_136344228_565	annotated	MAP7	6	-	1695	41	4	4	3.19%	7.992521402	0.000517318	0.000762611	2.54E-06
ENSG00000103510_31117682_31131259_458	annotated	KAT8	16	+	1374	60	5	0	5.46%	14.42893149	0.000105666	3.96E-06	3.77E-09
ENSG00000107077_6792989_7076451_813	annotated	KDM4C	9	+	2439	19	0	2	1.23%	2.574017258	0.002227676	0.005642788	7.16E-05
ENSG00000105409_41986127_41966937_860	annotated	ATP1A3	19	-	2580	15	2	0	0.93%	1.921061227	0.013892891	0.005306578	0.000386014
ENSG00000104093_51563425_51448984_2863	annotated	DMXL2	15	-	8589	20	4	3	0.31%	0.769408618	0.023141261	0.012200825	0.001347041
ENSG00000215301_41345217_41347016_236	novel	DDX3X	X	+	708	121	14	5	10.59%	56.47051732	2.71E-05	8.12E-06	1.79E-09
ENSG00000115355_55374859_55301816_1476	novel	CCDC88A	2	-	4428	47	6	3	1.69%	3.507194888	0.000157004	3.02E-05	3.63E-08
ENSG00000285053_235414605_235448762_408	dORF	TBCE	1	+	1224	23	11	5	2.45%	6.208920111	0.063328909	0.021696829	0.006065144
ENSG00000138767_77744762_77720431_331	annotated	CNOT6L	4	-	993	10	0	0	1.81%	3.327517937	0.012798403	0.012798403	0.000797244
ENSG00000164040_128272398_128271316_44	annotated	PGRMC2	4	-	132	99	16	2	34.09%	247.8168983	0.001384343	0.000282519	2.63E-06
ENSG00000161277_36054217_36035440_274	annotated	THAP8	19	-	822	18	1	3	2.55%	7.235529878	0.012000727	0.033334524	0.001909261
ENSG00000109919_47627109_47618833_86	novel	MTCH2	11	-	258	71	11	0	20.93%	90.93023143	0.002793789	8.94E-05	2.02E-06
ENSG00000235173_144137836_144139552_390	annotated	HGH1	8	+	1170	56	1	2	5.64%	15.81509551	2.29E-05	6.64E-05	1.18E-08
ENSG00000144354_173354964_173367664_371	annotated	CDCA7	2	+	1113	47	3	1	5.66%	13.95315271	0.000147046	3.49E-05	3.89E-08
ENSG00000141452_23506971_23531684_501	annotated	RMC1	18	+	1503	13	3	1	1.20%	2.857946044	0.059897465	0.01300724	0.003747438
ENSG00000149792_65122382_65125872_104	Overlap_dORF	MRPL49	11	+	312	72	25	0	20.19%	76.25135333	0.01337826	2.64E-05	4.81E-06
ENSG00000164978_34339040_34343440_147	annotated	NUDT2	9	+	441	31	4	0	8.16%	23.22698065	0.002558966	0.000660888	1.07E-05
ENSG00000164978_34339040_34343440_147	annotated	NUDT2	9	+	441	31	4	0	8.16%	23.22698065	0.002558966	0.000660888	1.07E-05
ENSG00000133114_44989655_45028003_340	annotated	GPALPP1	13	+	1020	38	0	0	5.88%	12.309859	2.86E-05	2.86E-05	6.30E-09
ENSG00000103064_68274898_68291249_172	annotated	SLC7A6	16	+	516	16	6	0	2.91%	10.24565988	0.197313433	0.019679754	0.019753919
ENSG00000103064_68291237_68297328_241	Overlap_dORF	SLC7A6	16	+	723	21	1	1	4.15%	9.597334929	0.003068573	0.003068573	5.32E-05
ENSG00000161904_33789116_33772628_503	annotated	LEMD2	6	-	1509	101	16	8	6.36%	22.11575589	4.93E-06	5.21E-06	2.15E-10
ENSG00000125746_45630001_45609663_464	annotated	EML2	19	-	1392	42	9	2	4.31%	9.969645335	0.003364114	9.33E-05	2.58E-06
ENSG00000164654_7572476_7607092_875	annotated	MIOS	7	+	2625	40	2	4	2.29%	5.035009998	8.97E-05	8.02E-05	5.24E-08
ENSG00000169429_73740659_73742464_99	annotated	CXCL8	4	+	297	308	40	4	16.16%	342.6604026	0.001965993	0.000675871	8.35E-06
ENSG00000179195_124012202_124012930_242	annotated	ZNF664	12	+	726	12	1	0	2.07%	5.46152944	0.035772986	0.021084099	0.003354605
ENSG00000069943_55320400_55355432_459	annotated	PIGB	15	+	1377	17	1	4	1.53%	4.079290508	0.012000727	0.061051993	0.003581135
ENSG00000170312_60780166_60785799_109	novel	CDK1	10	+	327	29	3	0	12.84%	29.30352722	0.000626688	0.000424543	1.74E-06
ENSG00000124228_49219449_49243834_765	annotated	DDX27	20	+	2295	77	15	3	3.66%	11.08607185	0.000270435	9.73E-06	2.30E-08
ENSG00000123080_50970369_50974270_168	annotated	CDKN2C	1	+	504	32	4	0	7.14%	20.97920832	0.002280579	0.001003278	1.39E-05
ENSG00000100599_92652385_92688252_540	novel	RIN3	14	+	1620	9	1	0	0.93%	1.835680728	0.041782825	0.019216965	0.003600526
ENSG00000126464_49591655_49625607_2036	annotated	PRR12	19	+	6108	40	7	4	1.13%	2.163867263	0.000667639	0.000116734	5.56E-07
ENSG00000124160_46079424_46062300_579	annotated	NCOA5	20	-	1737	32	4	1	2.59%	6.087231431	0.002455139	0.000423577	6.85E-06
ENSG00000133393_15888585_15879840_124	annotated	FOPNL	16	-	372	68	76	5	15.32%	60.39981752	0.091876091	0.00038931	0.000456529
ENSG00000137055_26947045_26905511_795	annotated	PLAA	9	-	2385	102	8	4	5.79%	14.13127806	2.79E-08	1.25E-08	3.58E-15
ENSG00000178904_32432779_32482240_616	annotated	DPY19L3	19	+	1848	46	13	8	3.41%	8.224803264	0.00389058	0.000374543	9.97E-06

ENSG00000064012_201266487_201276939_235	annotated	CASP8	2	+	705	21	12	0	4.68%	9.842373267	0.187228333	0.001398493	0.003055986
ENSG00000111859_11213721_11185162_828	annotated	NEDD9	6	-	2484	119	11	5	4.59%	15.82942077	1.58E-06	4.42E-07	6.39E-12
ENSG00000101945_48696785_48707570_412	annotated	SUV39H1	X	+	1236	23	1	1	3.40%	6.148639333	0.000669086	0.000669086	2.86E-06
ENSG00000147416_20197407_20220402_511	annotated	ATP6V1B2	8	+	1533	116	20	0	7.83%	25.00261814	1.02E-05	1.27E-08	1.81E-12
ENSG00000197056_35093988_35115259_1142	annotated	ZMYM1	1	+	3426	20	2	1	0.79%	1.928911448	0.010402772	0.005034873	0.000276005
ENSG00000162441_9935527_9922255_138	annotated	LZIC	1	-	414	48	0	3	7.25%	38.30985868	0.002376615	0.006381879	8.59E-05
ENSG00000162704_183635659_183627532_151	annotated	ARPC5	1	-	453	742	150	64	44.37%	541.2218942	4.16E-09	4.98E-10	2.34E-17
ENSG00000106089_73719631_73700472_251	annotated	STX1A	7	-	753	32	8	3	5.18%	14.04186055	0.011243323	0.00090861	6.70E-05
ENSG00000106089_73719631_73700882_280	annotated	STX1A	7	-	840	32	8	3	4.64%	12.58752499	0.011243323	0.00090861	6.70E-05
ENSG00000106089_73719631_73700827_260	annotated	STX1A	7	-	780	23	8	3	4.23%	9.743228482	0.036024968	0.002215716	0.000511439
ENSG00000284431_40346559_40349857_142	annotated	AL022238.4	22	+	426	18	6	7	3.52%	13.9615154	0.134659074	0.065398531	0.032192193
ENSG00000284431_40400807_40408917_658	dORF	AL022238.4	22	+	1974	17	0	0	1.67%	2.845584108	0.001343041	0.001343041	1.09E-05
ENSG00000284431_40350019_40400704_324	dORF	AL022238.4	22	+	972	106	25	6	10.49%	36.03373282	2.61E-05	1.55E-06	3.66E-10
ENSG00000042429_93784619_93812227_617	annotated	MED17	11	+	1851	27	3	5	1.94%	4.819777601	0.002233752	0.01212589	0.000156932
ENSG00000270800_34425077_34288753_243	annotated	RPS10-NUDT3	6	-	729	32	1	5	5.76%	14.50414403	0.000938122	0.004199767	2.43E-05
ENSG00000100575_58411945_58409034_89	annotated	TIMM9	14	-	267	32	4	1	13.48%	39.60120223	0.008373135	0.001351739	6.88E-05
ENSG00000166441_8684746_8685806_91	dORF	RPL27A	11	+	273	110	11	1	5.49%	133.1372836	0.103580224	0.035772986	0.015149602
ENSG00000149823_65110701_65111587_113	novel	VPS51	11	+	339	26	0	1	7.08%	25.34214103	0.005706018	0.007312901	0.000219657
ENSG00000149823_65109337_65110352_268	novel	VPS51	11	+	804	24	0	0	2.61%	9.863359138	0.008776163	0.008776163	0.000391611
ENSG00000185627_237050_244079_70	novel	PSMD13	11	+	210	36	1	2	14.29%	56.64386248	0.004467577	0.005109528	0.000123751
ENSG00000100227_42614857_42584984_229	annotated	POLDIP3	22	-	687	25	6	0	3.06%	12.02410957	0.128725186	0.008373824	0.006346004
ENSG00000120948_11016999_11023248_167	annotated	TARDBP	1	+	501	35	1	0	9.58%	23.08341036	0.00028444	0.000178284	3.50E-07
ENSG00000131143_85801206_85804595_42	annotated	COX4I1	16	+	126	23	8	5	14.29%	60.31522393	0.103580224	0.01300724	0.006832148
ENSG00000242265_94663557_94664534_325	annotated	PEG10	7	+	975	161	16	6	15.69%	54.5620795	1.82E-08	1.01E-09	2.08E-16
ENSG00000242265_94664609_94665682_357	dORF	PEG10	7	+	1071	99	18	5	8.96%	30.54325918	9.09E-05	1.11E-06	1.03E-09
ENSG00000091640_4967771_4959716_194	annotated	SPAG7	17	-	582	78	5	1	11.86%	44.28343201	3.22E-05	1.44E-05	3.67E-09
ENSG00000108953_1400110_1361898_124	novel	YWHAE	17	-	372	306	36	20	11.29%	271.7991788	0.006230522	0.001868374	6.75E-05
ENSG00000175567_73978036_73975007_247	annotated	UCP2	11	-	741	62	14	60	4.86%	27.64668951	0.01732615	0.011251891	0.000944548
ENSG00000175567_73981622_73981512_36	uORF	UCP2	11	-	108	69	2	3	41.67%	211.1032838	0.000297172	0.000282029	5.67E-07
ENSG00000100360_36767356_36760917_113	annotated	IFT27	22	-	339	43	8	6	10.62%	41.91200247	0.01373432	0.008052198	0.000555677
ENSG00000089597_62639071_62625815_847	annotated	GANAB	11	-	2541	1841	228	58	28.81%	239.3970405	3.10E-33	1.12E-38	9.96E-70
ENSG00000089597_62646599_62639095_52	uORF	GANAB	11	-	156	24	16	0	17.31%	50.83423556	0.251130497	0.003303074	0.008310705
ENSG00000168938_123036625_123029137_132	novel	PPIC	5	-	396	33	0	1	9.85%	27.53521093	0.000642411	0.000642411	2.65E-06
ENSG00000121022_67047741_67043233_96	novel	COPS5	8	-	288	22	5	0	5.21%	25.24061002	0.045845141	0.021557223	0.00436099
ENSG00000198546_133309432_133312866_187	annotated	ZNF511	10	+	561	81	3	2	12.83%	47.70806599	8.41E-06	1.06E-05	7.30E-10
ENSG00000135365_46084219_45933968_680	annotated	PHF21A	11	-	2040	18	2	0	1.18%	2.915492922	0.023197249	0.004826937	0.000601035
ENSG00000105696_18612977_18620536_336	annotated	AC003112.1	19	+	1008	12	1	0	1.49%	3.933601561	0.035772986	0.021084099	0.003354605
ENSG00000130414_240011621_239895219_116	annotated	NDUFA10	2	-	348	22	6	0	5.17%	20.8887807	0.090908082	0.013428348	0.006047419
ENSG00000170266_33072698_33058265_84	annotated	GLB1	3	-	252	29	23	7	11.90%	38.02481509	0.16996692	0.039800835	0.027787806
ENSG00000170917_122897669_122892828_147	Overlap_dORF	NUDT6	4	-	441	14	3	0	4.08%	10.48960416	0.059897465	0.013428348	0.003846583
ENSG00000175416_176416363_176406538_134	annotated	CLTB	5	-	402	39	12	10	7.46%	32.0559172	0.052378745	0.012888811	0.003228679
ENSG00000198171_3195245_3190814_38	novel	DDRGK1	20	-	114	55	5	0	26.32%	159.414379	0.009117593	0.002488365	0.000128452
ENSG00000180228_178450347_178445535_62	novel	PRKRA	2	-	186	8	1	0	8.06%	14.21172177	0.042234516	0.019679754	0.003715148
ENSG00000008256_6187064_6164944_321	annotated	CYTH3	7	-	963	20	5	0	3.43%	6.862357863	0.020350677	0.001262414	0.000169936
ENSG00000105856_107179894_107201431_514	annotated	HBP1	7	+	1542	90	18	7	6.03%	19.28536174	0.000577124	6.76E-06	3.83E-08
ENSG00000105854_95435014_95405330_375	annotated	PON2	7	-	1125	1918	281	66	35.73%	563.3337019	8.20E-19	5.35E-22	8.47E-39
ENSG00000155868_157139431_157138730_233	annotated	MED7	5	-	699	19	7	2	4.72%	8.981442191	0.069752393	0.007962493	0.002985036
ENSG00000171453_43519384_43521300_282	annotated	POLR1C	6	+	846	47	1	9	4.96%	18.35680728	0.000567986	0.009799807	3.88E-05
ENSG00000284770_235380050_235419429_127	annotated	TBCE	1	+	381	11	9	0	3.94%	9.539758116	0.171390856	0.020613417	0.017249262
ENSG00000284770_235419441_235448762_414	dORF	TBCE	1	+	1242	23	11	5	2.42%	6.118935761	0.063328909	0.021696829	0.006065144
ENSG00000123933_2261980_2250544_209	annotated	MXD4	4	-	627	22	4	4	3.83%	11.59377302	0.03195078	0.041403097	0.005594446
ENSG00000173914_66677079_66668624_359	annotated	RBM4B	11	-	1077	11	4	0	1.67%	3.374789083	0.159270956	0.012798403	0.01119263
ENSG00000090905_24789453_24823807_1643	annotated	TNRC6A	16	+	4929	27	6	10	0.91%	1.809983433	0.004952923	0.015836789	0.000414507
ENSG00000163933_53106855_53103903_120	novel	RFT1	3	-	360	26	9	0	8.33%	23.86384947	0.017276406	0.002362849	0.000239527
ENSG00000154359_12737053_12723096_373	dORF	LONRF1	8	-	1119	15	2	0	1.61%	4.429256449	0.052581193	0.01300724	0.003266469
ENSG00000109099_15260727_15260488_79	novel	PMP22	17	-	237	30	6	0	6.33%	41.82563685	0.021557223	0.021557223	0.002115616
ENSG00000188313_146533542_146516045_311	annotated	PLSCR1	3	-	933	67	14	4	7.72%	23.72809173	0.000433491	3.54E-05	1.20E-07
ENSG00000140983_668200_670344_135	annotated	RHOT2	16	+	405	14	4	5	5.93%	11.42201342	0.046396937	0.098650979	0.017856065
ENSG00000134014_28099925_28189725_475	annotated	ELP3	8	+	1425	46	2	0	4.63%	10.66627118	3.18E-05	1.63E-05	4.06E-09
ENSG00000113273_78781960_78780397_124	novel	ARSB	5	-	372	8	1	0	4.84%	7.105860884	0.028889786	0.009815329	0.001387795
ENSG00000108518_4948394_4945900_140	annotated	PFN1	17	-	420	5244	935	69	40.71%	4125.561317	4.96E-10	1.15E-10	6.63E-19
ENSG00000103248_86554707_86532011_363	annotated	MTHFSD	16	-	1089	17	2	3	1.38%	5.158111138	0.035772986	0.052581193	0.007761385
ENSG00000196083_190564290_190648705_570	annotated	IL1RAP	3	+	1710	35	4	1	2.63%	6.763034263	0.002150019	0.000344964	4.97E-06
ENSG00000119681_74611944_74500884_1821	annotated	LTBP2	14	-	5463	18	4	2	0.71%	1.088706857	0.017648298	0.003902032	0.000376352
ENSG00000071462_73697633_73697886_38	annotated	BUD23	7	+	114	12	1	0	13.16%	34.78131906	0.035374934	0.020613417	0.003252526
ENSG00000176225_70135188_70061366_633	annotated	RTTN	18	-	1899	13	5	0	0.95%	2.261976253	0.178080356	0.012798403	0.012843613
ENSG00000156113_77637360_76877869_1146	annotated	KCNMA1	10	-	3438	71	13	0	3.05%	6.823734645	1.40E-05	8.57E-08	1.38E-11
ENSG00000119203_9423774_9473017_684	annotated	CPSF3	2	+	2052	138	3	11	5.12%	22.22139829	1.53E-07	4.35E-06	6.67E-12

ENSG00000077721_119574712_119576988_64	annotated	UBE2A	X	+	192	90	11	0	23.44%	154.8855615	0.005941113	0.000315272	1.36E-05
ENSG00000137288_33697841_33697653_62	novel	UQCC2	6	-	186	39	0	0	11.29%	69.28214362	0.008473674	0.008473674	0.000366502
ENSG00000204120_232747715_232856860_1274	annotated	GIGYF2	2	+	3822	33	7	2	1.49%	2.852941791	0.002130337	0.00013672	2.17E-06
ENSG00000131724_118740153_118791854_425	annotated	IL13RA1	X	+	1275	77	7	3	5.88%	19.95492933	8.69E-05	1.35E-05	9.37E-09
ENSG00000110697_67496340_67492033_526	novel	PITPNM1	11	-	1578	27	2	0	2.28%	5.653617453	0.003668512	0.001003278	2.25E-05
ENSG00000110697_67497919_67497220_125	novel	PITPNM1	11	-	375	10	0	0	4.00%	8.811267496	0.019679754	0.019679754	0.001784964
ENSG00000120318_141680024_141653950_1390	annotated	ARAP3	5	-	4170	9	0	1	0.36%	0.713142153	0.019679754	0.042234516	0.003715148
ENSG00000065268_984366_994344_405	annotated	WDR18	19	+	1215	146	5	3	11.36%	39.70509427	1.19E-08	2.77E-09	3.55E-16
ENSG00000068097_60078914_60066396_413	annotated	HEATR6	17	-	1239	16	4	0	2.42%	4.266957625	0.030636454	0.002008257	0.000393092
ENSG00000198682_87727298_87745970_321	annotated	PAPSS2	10	+	963	20	2	2	2.49%	6.862357863	0.012941946	0.013580321	0.000851862
ENSG00000132406_4248202_4237836_165	annotated	TMEM128	4	-	495	80	6	0	8.48%	53.40162119	0.004273704	0.000464321	1.33E-05
ENSG00000163866_34855977_34855699_92	annotated	SMIM12	1	-	276	40	6	3	15.22%	47.88732335	0.003063143	0.001091889	2.02E-05
ENSG00000169490_38995428_38991332_146	annotated	TM2D2	8	-	438	149	37	19	23.29%	112.4040117	2.24E-07	4.99E-06	1.10E-11
ENSG00000137413_42050542_42086734_311	annotated	TAF8	6	+	933	18	2	0	2.57%	6.374711211	0.011825808	0.005454749	0.000336929
ENSG00000155363_112691744_112700507_698	annotated	MOV10	1	+	2094	14	7	1	0.86%	2.20912867	0.100696627	0.012484591	0.006411599
ENSG00000117118_17044789_17027665_150	novel	SDHB	1	-	450	86	21	3	10.67%	63.14741706	0.006723651	0.000655924	2.91E-05
ENSG00000135318_85450140_85471469_264	annotated	NT5E	6	+	792	108	14	1	14.77%	45.05761788	1.15E-06	2.45E-08	3.05E-13
ENSG00000073578_225446_235435_338	novel	SDHA	5	+	1014	102	12	2	6.51%	33.2377694	0.000104567	2.47E-05	1.99E-08
ENSG00000155366_112703799_112701540_193	annotated	RHOC	1	-	579	299	26	25	29.53%	170.6327061	3.86E-10	9.22E-11	4.15E-19
ENSG00000074219_49360075_49341324_451	annotated	TEAD2	19	-	1353	22	6	0	2.88%	5.372724083	0.01219545	0.000577025	5.02E-05
ENSG00000159322_72756295_72752341_232	dORF	ADPGK	15	-	696	86	10	3	12.07%	40.82807137	5.51E-05	9.16E-06	4.10E-09
ENSG00000159322_72783691_72760419_113	annotated	ADPGK	15	-	339	21	3	0	6.19%	20.46865237	0.024197104	0.008574016	0.001031003
ENSG00000168036_41224552_41236742_689	annotated	CTNNB1	3	+	2067	227	39	10	10.60%	36.28733167	6.89E-11	6.51E-13	6.20E-22
ENSG00000103197_2050439_2068560_513	annotated	TSC2	16	+	1539	22	1	1	1.36%	4.723389009	0.007322157	0.007979402	0.000301664
ENSG00000111642_6606316_6580630_1626	annotated	CHD4	12	-	4878	77	9	2	2.28%	5.215771811	1.12E-06	7.24E-08	8.09E-13
ENSG00000147894_27567120_27548236_492	annotated	C9orf72	9	-	1476	30	0	2	2.64%	6.715905104	0.000564499	0.000646991	2.35E-06
ENSG00000100519_52707220_52727617_389	annotated	PSMC6	14	+	1167	76	21	2	5.14%	21.51851959	0.000638157	0.000112692	5.14E-07
ENSG00000107929_885721_812926_738	annotated	LARP4B	10	-	2214	62	10	8	3.93%	9.25302481	8.02E-05	3.52E-05	2.13E-08
ENSG00000175745_93585030_93593842_370	annotated	NR2F1	5	+	1110	24	6	1	2.97%	7.144270943	0.015384268	0.002129196	0.000193677
ENSG00000125821_18588073_18620080_127	annotated	DTD1	20	+	381	48	1	2	11.81%	41.62803542	0.000309685	0.000463371	9.57E-07
ENSG00000125821_18628147_18744252_79	dORF	DTD1	20	+	237	88	3	0	24.05%	122.6885348	7.41E-05	6.12E-05	3.35E-08
ENSG00000103202_399454_400342_87	annotated	NME4	16	+	261	33	2	5	11.49%	41.77756141	0.002362849	0.002376615	3.25E-05
ENSG00000091140_107891251_107904670_114	annotated	DLD	7	+	342	78	29	20	17.54%	75.35952464	0.015063832	0.009304408	0.000693571
ENSG00000114686_131500480_131488781_89	novel	MRPL3	3	-	267	37	6	0	12.36%	45.78889008	0.01385829	0.001506153	0.000129016
ENSG00000152620_36225612_36195144_301	annotated	NADK2	5	-	903	37	0	4	4.32%	13.5389077	0.00067919	0.000943098	4.06E-06
ENSG00000135506_57694162_57720909_667	annotated	OS9	12	+	2001	478	63	23	19.79%	78.93151918	1.37E-18	3.10E-22	8.60E-39
ENSG00000131143_85804978_85805115_45	novel	COX4I1	16	+	135	203	18	9	28.89%	496.8575838	0.000825949	0.000823751	4.28E-06
ENSG00000131143_85801206_85804948_26	novel	COX4I1	16	+	78	24	8	5	26.92%	101.6684711	0.075027848	0.008574016	0.003434129
ENSG00000119689_74881954_74889360_94	novel	DLST	14	+	282	17	3	0	5.32%	19.91908876	0.055414058	0.020613417	0.00507254
ENSG00000100632_69398233_69380538_104	annotated	ERH	14	-	312	1563	111	32	27.88%	1655.289795	0.000137654	3.54E-05	3.69E-08
ENSG00000006712_39390864_39389608_107	novel	PAF1	19	-	321	11	0	2	5.61%	11.32289047	0.011772051	0.080264768	0.004750049
ENSG00000167747_50802319_50798925_66	novel	C19orf48	19	-	198	28	0	2	13.64%	46.72641854	0.003554776	0.011906648	0.000231368
ENSG00000117906_76943866_76947540_41	novel	RCN2	15	+	123	22	4	0	21.95%	59.09996492	0.023197249	0.003554776	0.00046365
ENSG00000168883_85612326_85648808_487	annotated	USP39	2	+	1461	42	17	3	3.90%	9.498799662	0.068655119	0.00036536	0.000291791
ENSG00000168488_28823260_28836777_1044	annotated	ATXN2L	16	+	3132	144	24	9	3.83%	15.19184051	2.13E-05	4.35E-08	1.21E-11
ENSG00000168679_110389270_110363766_425	Overlap_dORF	SLC16A4	1	-	1275	231	71	18	15.06%	59.86478799	1.07E-06	5.34E-11	1.21E-15
ENSG00000168679_110389260_110381709_36	annotated	SLC16A4	1	-	108	33	0	7	13.89%	100.9624401	0.021084099	0.087374356	0.008279507
ENSG00000136527_185925496_185917715_188	novel	TRA2B	3	-	564	183	34	3	21.28%	107.2115659	6.28E-05	3.96E-08	3.80E-11
ENSG00000071127_10116666_10075378_606	annotated	WDR1	4	-	1818	281	15	11	12.71%	51.07190937	4.70E-13	1.80E-13	1.12E-24
ENSG00000071127_10116666_10075378_466	annotated	WDR1	4	-	1398	193	13	10	12.23%	45.61627218	7.13E-10	4.36E-10	3.49E-18
ENSG00000167965_2206086_2208877_325	annotated	MLST8	16	+	975	106	18	4	11.69%	35.92285979	1.50E-05	2.99E-07	4.58E-11
ENSG00000161203_184176994_184183616_433	annotated	AP2M1	3	+	1299	377	39	15	18.48%	95.89630041	2.87E-13	1.42E-13	5.42E-25
ENSG00000131778_147284359_147295509_326	dORF	CHD1L	1	+	978	18	0	2	2.15%	6.081396278	0.007029303	0.035227809	0.001282266
ENSG00000132170_12379712_12434145_475	annotated	PPARG	3	+	1425	42	6	0	4.21%	9.738769338	0.000917199	3.16E-05	2.43E-07
ENSG00000105197_39480854_39489820_157	annotated	TIMM50	19	+	471	71	20	9	12.74%	49.80891658	0.007205211	0.000539984	2.65E-05
ENSG00000136104_50929501_50969932_227	annotated	RNASEH2B	13	+	681	40	1	0	5.73%	19.40807819	0.000953102	0.000683882	4.12E-06
ENSG00000172922_65720340_65720068_90	novel	RNASEH2C	11	-	270	31	0	2	6.67%	37.93740172	0.011772051	0.060667625	0.003501726
ENSG00000121749_71839782_71923204_691	annotated	TBC1D15	12	+	2073	37	4	3	2.89%	5.897556031	0.000182952	0.00040921	5.14E-07
ENSG00000134291_47966707_47968229_58	dORF	TMEM106C	12	+	174	29	8	0	17.24%	55.07042185	0.017397629	0.00224099	0.000230743
ENSG00000161813_50401011_50475864_653	annotated	LARP4	12	+	1959	38	2	4	2.45%	6.409421226	0.000327164	0.000635348	1.38E-06
ENSG00000162704_183633147_183627532_101	novel	ARPC5	1	-	303	534	43	21	39.60%	582.3288172	4.19E-07	2.32E-07	9.07E-13
ENSG00000101365_2664188_2660613_204	novel	IDH3B	20	-	612	78	7	1	13.73%	42.11267553	4.63E-06	2.05E-06	8.17E-11
ENSG00000173915_103392376_103392194_31	annotated	ATP5MD	10	-	93	120	3	0	25.81%	426.3516531	0.005808022	0.005859343	0.000180739
ENSG00000236287_10854840_10852864_658	annotated	ZBED5	11	-	1974	40	0	0	2.89%	6.695492019	5.30E-05	5.30E-05	2.10E-08
ENSG00000132170_12379712_12434145_475	annotated	PPARG	3	+	1425	42	6	0	4.21%	9.738769338	0.000917199	3.16E-05	2.43E-07
ENSG00000117632_25904676_25901016_149	annotated	STMN1	1	-	447	840	110	15	43.62%	620.9282464	8.38E-12	2.62E-12	2.75E-22
ENSG00000127990_94656098_94623369_45	annotated	SGCE	7	-	135	21	6	4	17.78%	51.3990604	0.044816497	0.037011271	0.00688081
ENSG00000050748_180280561_180236384_424	annotated	MAPK9	5	-	1272	59	4	4	3.77%	15.32620231	0.00053733	0.002284129	7.88E-06

ENSG00000215301_41343246_41347719_471	novel	DDX3X	X	+	1413	189	30	7	8.70%	44.19664429	3.70E-07	2.15E-08	8.26E-14
ENSG00000165097_18212563_18222267_265	novel	KDM1B	6	+	795	10	0	0	2.26%	4.156258253	0.012798403	0.012798403	0.000797244
ENSG00000116717_67685495_67687774_131	annotated	GADD45A	1	+	393	25	2	1	9.16%	21.01924498	0.002336818	0.000943084	1.35E-05
ENSG00000277791_38760525_38764167_75	novel	PSMB3	17	+	225	348	26	7	29.33%	511.0535148	0.000128473	4.91E-05	4.68E-08
ENSG00000112651_43059381_43058039_96	annotated	MRPL2	6	-	288	28	0	0	6.25%	32.12441275	0.013640586	0.013640586	0.000898637
ENSG00000146678_45888653_45893091_216	annotated	IGFBP1	7	+	648	93	9	10	10.19%	47.42175215	0.000356053	0.000353845	8.42E-07
ENSG00000117691_212432944_212446006_172	annotated	NENF	1	+	516	78	10	3	13.37%	49.94759191	0.000158987	4.32E-05	5.14E-08
ENSG00000170892_54191365_54193362_310	annotated	TSEN34	19	+	930	80	6	2	9.35%	28.42344354	6.54E-06	1.01E-06	5.92E-11
ENSG00000079805_10718258_10831047_864	annotated	DNM2	19	+	2592	96	6	9	5.21%	12.23787152	3.18E-08	2.06E-07	6.57E-14
ENSG00000141699_42592110_42581813_369	annotated	RETREG3	17	-	1107	132	12	0	9.76%	39.39997661	6.88E-07	7.41E-08	5.02E-13
ENSG00000130159_11507520_11506184_102	novel	ECSIT	19	-	306	23	0	0	6.86%	24.83568044	0.008776163	0.008776163	0.000391611
ENSG00000163050_226982134_226986737_368	novel	COQ8A	1	+	1104	29	3	4	3.53%	8.679577357	0.003975389	0.002613653	5.87E-05
ENSG00000168291_58433809_58428034_341	annotated	PDHB	3	-	1023	131	14	3	11.14%	42.31217163	3.91E-06	7.08E-08	2.94E-12
ENSG00000058600_22302969_22333700_708	annotated	POLR3E	16	+	2124	59	6	0	3.25%	9.178403642	7.06E-05	1.14E-05	6.50E-09
ENSG00000131368_15065188_15052441_171	annotated	MRPS25	3	-	513	67	5	2	11.11%	43.15459958	0.000469128	0.000177203	5.72E-07
ENSG00000105339_141136647_141194233_1146	annotated	DENND3	8	+	3438	26	2	6	1.48%	2.498832405	0.000365346	0.0056817	1.47E-05
ENSG00000103994_42457205_42417304_1883	annotated	ZNF106	15	-	5649	16	1	3	0.48%	0.935875464	0.004669668	0.018219708	0.000454465
ENSG00000134202_109740269_109737071_219	annotated	GSTM3	1	-	657	7	0	3	2.28%	3.520483589	0.019216965	0.169362074	0.016163992
ENSG00000159063_78139588_78102894_467	annotated	ALG8	11	-	1401	293	32	11	15.63%	69.1033559	2.71E-10	5.34E-13	2.16E-21
ENSG00000100911_24146581_24143409_254	annotated	PSME2	14	-	762	231	20	4	11.81%	100.1674602	2.75E-06	8.24E-07	2.02E-11
ENSG00000154518_175181393_175180068_49	novel	ATP5MC3	2	-	147	525	34	8	20.41%	1180.080468	0.006351887	0.004326528	0.000148117
ENSG00000110931_121274382_121240699_540	annotated	CAMKK2	12	-	1620	26	3	4	2.22%	5.30307766	0.003818382	0.008533454	0.000176458
ENSG00000071894_144409107_144393318_1426	annotated	CPSF1	8	-	4278	39	6	14	1.47%	3.012267114	0.000500906	0.005421904	1.83E-05
ENSG00000204580_30888931_30899296_877	annotated	DDR1	6	+	2631	109	19	2	5.02%	13.68911284	2.20E-06	9.53E-09	2.58E-13
ENSG00000183048_81716010_81720077_161	novel	SLC25A10	17	+	483	34	6	0	4.97%	23.25955706	0.036196037	0.005756914	0.001117562
ENSG00000092108_30643360_30735609_453	dORF	SCFD1	14	+	1359	35	6	0	3.97%	8.509778211	0.001466661	8.20E-05	9.28E-07
ENSG00000119431_113374354_113373599_251	annotated	HDHD3	9	-	753	27	4	3	5.18%	11.84781984	0.006948862	0.004890191	0.000181085
ENSG00000107643_48401661_48434895_384	annotated	MAPK8	10	+	1152	28	4	2	3.39%	8.031103187	0.000585477	0.002815409	1.06E-05
ENSG00000164104_173333649_173332080_209	annotated	HMGB2	4	-	627	401	54	21	21.05%	211.3228628	9.69E-08	1.86E-08	1.83E-14
ENSG00000117682_26438201_26447748_177	annotated	DHDDS	1	+	531	23	1	3	5.65%	14.31208704	0.00345766	0.010728371	0.000203071
ENSG00000134779_36828767_36787069_130	annotated	TPGS2	18	-	390	35	10	10	10.77%	29.65330407	0.02538804	0.015018191	0.001776247
ENSG00000125885_5952016_5994391_840	annotated	MCM8	20	+	2520	27	3	1	1.67%	3.540241405	0.00179234	0.000655226	7.39E-06
ENSG00000065675_6511105_6428207_637	annotated	PRKCQ	10	-	1911	12	1	5	1.26%	2.074866757	0.004687384	0.05158844	0.001397404
ENSG00000009307_114739890_114718169_798	annotated	CSDE1	1	-	2394	202	28	26	9.02%	27.88026369	2.37E-10	1.49E-10	4.08E-19
ENSG00000179104_82895899_83132389_591	dORF	TMTC2	12	+	1773	19	2	1	2.03%	3.540906989	0.005100032	0.001607079	4.81E-05
ENSG00000111231_110469054_110468305_132	novel	GPN3	12	-	396	20	0	9	3.79%	16.68800662	0.021084099	0.133791954	0.013186697
ENSG00000163870_127580146_127575258_239	annotated	TPRA1	3	-	717	42	12	10	7.11%	19.35529471	0.007026767	0.006975324	0.000255405
ENSG00000011638_21161183_21179556_161	annotated	TMEM159	16	+	483	12	0	0	5.59%	8.209255431	0.002500085	0.002500085	3.60E-05
ENSG00000108468_48077004_48071435_185	annotated	CBX1	17	-	555	92	2	1	12.43%	54.7727439	1.25E-05	1.25E-05	1.25E-09
ENSG00000115286_1383927_1395488_213	annotated	NDUFS7	19	+	639	112	9	2	15.02%	57.91443425	1.84E-06	7.55E-07	1.24E-11
ENSG00000115282_74483096_74493218_519	annotated	TTC31	2	+	1557	18	4	0	1.93%	3.819913655	0.017621446	0.002201184	0.000230417
ENSG00000130714_131504219_131523106_725	annotated	POMT1	9	+	2175	71	4	2	4.28%	10.78620676	2.12E-06	1.58E-06	2.93E-11
ENSG00000182326_7064377_7070651_521	annotated	C1S	12	+	1563	87	7	3	5.95%	18.39204108	5.50E-06	1.55E-06	7.45E-11
ENSG00000183137_109145237_109162970_455	annotated	CEP57L1	6	+	1365	8	0	0	1.10%	1.936542307	0.019679754	0.019679754	0.001784964
ENSG00000198546_133310227_133312866_88	novel	ZNF511	10	+	264	44	2	2	15.91%	55.07042185	0.000454824	0.000858744	2.53E-06
ENSG00000108474_16217308_16217609_71	annotated	PIGL	17	+	213	10	1	1	8.45%	15.51279489	0.009815329	0.026832696	0.001287874
ENSG00000249859_127794565_127890868_149	novel	PVT1	8	+	447	10	1	0	3.36%	7.392002933	0.035374934	0.020613417	0.003252526
ENSG00000103496_31033806_31034333_79	novel	STX4	16	+	237	23	2	0	15.19%	32.06632159	0.002613653	0.000943084	1.51E-05
ENSG00000103496_31035026_31039803_176	novel	STX4	16	+	528	43	3	2	10.23%	26.90941068	0.000490038	0.000181496	6.11E-07
ENSG00000128928_40405828_40418263_423	annotated	IVD	15	+	1269	34	3	0	3.55%	8.852928336	0.001699351	0.000276859	3.19E-06
ENSG00000158373_26158170_26158550_126	annotated	HIST1H2BD	6	+	378	718	54	32	23.02%	627.6279824	1.22E-06	1.04E-05	1.13E-10
ENSG00000204619_30068677_30069306_74	annotated	PPP1R11	6	+	222	35	0	1	9.46%	52.09364229	0.008574016	0.020525443	0.000873936
ENSG00000132879_11655836_11661305_224	annotated	FBXO44	1	+	672	22	2	0	5.80%	10.81740429	0.003468409	0.000539992	1.22E-05
ENSG00000204619_30068677_30069306_74	annotated	PPP1R11	6	+	222	35	0	1	9.46%	52.09364229	0.008574016	0.020525443	0.000873936
ENSG00000132879_11655836_11661305_224	annotated	FBXO44	1	+	672	22	2	0	5.80%	10.81740429	0.003468409	0.000539992	1.22E-05
ENSG00000274641_27893463_27893843_126	annotated	HIST1H2BO	6	+	378	536	220	40	16.67%	468.5356526	0.00014212	3.26E-05	3.53E-08
ENSG00000132879_11655836_11661305_256	annotated	FBXO44	1	+	768	22	2	0	5.08%	9.465228756	0.003468409	0.000539992	1.22E-05
ENSG00000043143_134559882_134579317_670	annotated	JADE2	5	+	2010	48	8	8	2.24%	7.89068731	0.000685965	0.003513668	1.53E-05
ENSG00000143621_153670922_153662396_390	annotated	ILF2	1	-	1170	330	31	9	16.67%	93.19609852	1.31E-10	3.14E-12	5.35E-21
ENSG00000037897_57769669_57768996_120	Overlap_dORF	METTL1	12	-	360	14	1	2	5.00%	12.8497651	0.016344022	0.023718446	0.001795277
ENSG00000108506_61926593_61867557_1179	annotated	INTS2	17	-	3537	35	11	0	1.44%	3.269660331	0.010239425	0.000125586	1.18E-05
ENSG00000180398_46941663_46905463_127	annotated	MCFD2	2	-	381	61	7	4	10.24%	52.90229501	0.003903476	0.003000296	6.56E-05
ENSG00000156113_77637360_76887266_1084	annotated	KCNMA1	10	-	3252	74	13	0	3.32%	7.518839884	8.97E-06	5.88E-08	6.07E-12
ENSG00000115525_85848138_85840356_104	dORF	ST3GAL5	2	-	312	77	16	6	17.31%	81.5465862	0.001121188	0.00071069	5.01E-06
ENSG00000173918_79046646_79048088_199	annotated	C1QTNF1	17	+	597	22	2	6	4.02%	12.17637468	0.013786174	0.048013541	0.003119878
ENSG00000159322_72783691_72752341_496	annotated	ADPGK	15	-	1488	119	23	3	8.47%	26.42492016	1.92E-05	3.61E-08	9.15E-12
ENSG00000182473_76103692_76083648_707	annotated	EXOC7	17	-	2121	64	8	7	4.10%	9.970316828	8.00E-05	4.33E-05	2.59E-08
ENSG00000116350_29154904_29148410_371	novel	SRSF4	1	-	1113	17	2	1	2.96%	5.046885022	0.005502968	0.002558966	7.92E-05

ENSG00000141698_41832402_41825523_132	annotated	NT5C3B	17	-	396	16	0	4	4.55%	13.3504053	0.01300724	0.100696627	0.006616977
ENSG00000103248_86554707_86532011_363	annotated	MTHFSD	16	-	1089	18	2	3	1.65%	5.46152944	0.030316224	0.044843011	0.005757811
ENSG00000184220_100147011_100178388_245	annotated	CMSS1	3	+	735	76	20	2	10.61%	34.16613927	0.000778141	6.74E-06	5.34E-08
ENSG00000153310_129854286_129842142_159	annotated	FAM49B	8	-	477	26	2	0	8.18%	18.01045243	0.000980148	0.000631191	3.92E-06
ENSG00000073060_124863720_124778467_506	annotated	SCARB1	12	-	1518	295	52	56	13.04%	64.21254722	1.44E-08	9.51E-09	1.42E-15
ENSG00000073060_124863901_124863596_101	Overlap_uORF	SCARB1	12	-	303	45	14	3	9.90%	49.07265314	0.130421861	0.008853033	0.006757697
ENSG00000154124_14664826_14693048_352	annotated	OTULIN	5	+	1056	14	5	0	1.42%	4.380601738	0.129819886	0.019216965	0.011881757
ENSG00000154127_122656188_122809886_603	annotated	UBASH3B	11	+	1809	83	6	0	6.47%	15.1603483	7.29E-07	1.66E-08	1.32E-13
ENSG00000186566_44436489_44397568_1419	novel	GPATCH8	17	-	4257	19	1	3	0.78%	1.474754074	0.002559322	0.007459848	0.000107439
ENSG00000111906_125292903_125277040_87	novel	HDDC2	6	-	261	16	2	0	9.20%	20.25578735	0.020715809	0.005454749	0.000593634
ENSG00000111906_125301932_125292900_71	novel	HDDC2	6	-	213	77	32	3	26.76%	119.4485206	0.010669572	0.000114197	1.15E-05
ENSG00000134419_18789104_18784734_97	annotated	RPS15A	16	-	291	162	28	5	11.34%	183.9465637	0.016256388	0.003487661	0.000313547
ENSG00000066427_92096122_92064320_293	annotated	ATXN3	14	-	879	36	1	0	5.46%	13.53266339	0.000260102	0.000192487	3.45E-07
ENSG00000285238_6606316_6578908_1652	annotated	AC006064.6	12	-	4956	77	9	2	2.24%	5.133683393	1.12E-06	7.24E-08	8.09E-13
ENSG00000285238_6578439_6566816_248	dORF	AC006064.6	12	-	744	15	0	1	2.02%	6.661744579	0.021084099	0.035772986	0.003354605
ENSG00000285238_6566202_6556993_688	dORF	AC006064.6	12	-	2064	52	3	4	3.20%	8.324598652	5.87E-05	5.77E-05	2.52E-08
ENSG00000116906_231260573_231277542_571	dORF	GNPAT	1	+	1713	132	14	3	7.88%	25.46163112	1.81E-07	1.22E-08	2.31E-14
ENSG00000112715_43777480_43784546_131	annotated	VEGFA	6	+	393	39	1	0	6.87%	32.79002217	0.003961388	0.003789641	8.30E-05
ENSG00000133872_30069825_30063888_143	annotated	SARAF	8	-	429	312	27	3	30.77%	240.3072954	2.72E-07	9.05E-09	2.67E-14
ENSG00000137871_56704241_56631498_961	annotated	ZNF280D	15	-	2883	11	4	0	0.94%	1.260717254	0.065285009	0.001946209	0.00093453
ENSG00000166562_59139949_59158650_161	annotated	SEC11C	18	+	483	123	7	1	18.01%	84.14486817	1.19E-06	1.20E-06	1.26E-11
ENSG00000141376_60947221_61392125_550	annotated	BCAS3	17	+	1650	11	2	0	1.27%	2.202816874	0.043639483	0.007882058	0.00177109
ENSG00000149016_62591485_62575094_874	annotated	TUT1	11	-	2622	21	0	3	1.60%	2.646404711	0.000288544	0.006674189	1.43E-05
ENSG00000117155_84651943_84647433_133	dORF	SSX2IP	1	-	399	7	0	0	3.76%	5.796886511	0.019216965	0.019216965	0.001707396
ENSG00000117155_84669800_84651984_406	annotated	SSX2IP	1	-	1218	14	1	1	2.22%	3.797960128	0.00640069	0.00640069	0.000215507
ENSG00000256053_103563002_103574175_129	novel	APOPT1	14	+	387	42	2	9	8.53%	35.85980958	0.005625935	0.001506153	5.01E-05
ENSG00000075413_103466349_103503227_436	annotated	MARK3	14	+	1308	27	9	0	2.29%	6.820648578	0.060706083	0.002362849	0.000991025
ENSG00000100902_35292477_35310287_107	annotated	PSMA6	14	+	321	92	9	0	11.21%	94.70053851	0.001798541	0.001091522	1.19E-05
ENSG00000215301_41340781_41347719_622	dORF	DDX3X	X	+	1866	229	40	8	8.68%	40.55024632	3.29E-08	1.74E-10	7.45E-17
ENSG00000153048_8859328_8859167_53	novel	CARHSP1	16	-	159	62	9	0	15.09%	128.8440058	0.013892891	0.005604867	0.000405324
ENSG00000157540_37490315_37512531_495	novel	DYRK1A	21	+	1485	20	1	0	2.22%	4.450135099	0.002484757	0.001398493	2.07E-05
ENSG00000170445_140679874_140674257_406	dORF	HARS	5	-	1218	89	17	3	6.40%	24.1441751	9.41E-05	9.66E-06	7.49E-09
ENSG00000116815_116570972_116519295_237	annotated	CD58	1	-	711	42	4	0	7.17%	19.51863053	0.000416547	0.000126358	3.70E-07
ENSG00000166925_100478038_100474270_310	novel	TSC22D4	7	-	930	16	2	0	2.26%	5.684688707	0.01965437	0.007882058	0.000776762
ENSG00000104824_38846077_38836722_456	annotated	HNRNPL	19	-	1368	303	26	12	9.65%	73.1856922	1.80E-07	4.41E-08	7.85E-14
ENSG00000104872_49449547_49446382_169	annotated	PIH1D1	19	-	507	28	8	1	8.28%	18.24818712	0.015584354	0.000629624	7.11E-05
ENSG00000138629_74452355_74446090_174	annotated	UBL7	15	-	522	30	1	0	8.05%	18.98980064	0.000662939	0.0004093	1.77E-06
ENSG00000111875_118894414_118907614_204	annotated	ASF1A	6	+	612	31	13	12	7.84%	16.73708899	0.040804684	0.02681062	0.004715717
ENSG00000285130_69046445_69099934_96	annotated	AL358113.1	9	+	288	47	11	0	14.58%	53.9231214	0.005313426	0.000468432	1.69E-05
ENSG00000197102_102008259_102016143_543	novel	DYNC1H1	14	+	1629	23	7	2	2.03%	4.66526594	0.023900277	0.004354582	0.000568182
ENSG00000092621_119712023_119744040_526	annotated	PHGDH	1	+	1578	62	8	1	4.18%	12.98238082	0.000414271	2.27E-05	7.69E-08
ENSG00000171132_45652101_46184881_737	annotated	PRKCE	2	+	2211	15	3	1	1.22%	2.241672531	0.031423484	0.005426623	0.000914478
ENSG00000172725_67442628_67438376_489	annotated	CORO1B	11	-	1467	116	21	3	7.98%	26.12748031	2.67E-05	1.27E-07	3.96E-11
ENSG00000065057_2047823_2039924_304	annotated	NTHL1	16	-	912	159	18	7	13.82%	57.6065597	5.94E-07	3.12E-08	1.91E-13
ENSG00000115464_61229633_61188102_1092	annotated	USP34	2	-	3276	23	2	1	0.82%	2.319816305	0.007558271	0.005059698	0.000202771
ENSG00000119383_129136511_129142900_213	annotated	PTPA	9	+	639	50	12	8	7.51%	25.85465815	0.003244288	0.003675608	6.67E-05
ENSG00000100348_36480837_36468664_146	annotated	TXN2	22	-	438	57	3	3	11.64%	43.00019241	0.000235673	0.000152044	2.50E-07
ENSG00000040199_71676630_71648890_894	annotated	PHLPP2	16	-	2682	15	1	4	0.67%	1.848000733	0.020342454	0.069273208	0.006304262
ENSG00000140983_668200_671768_322	novel	RHOT2	16	+	966	26	17	10	4.35%	8.893360051	0.260410251	0.041285488	0.046330129
ENSG00000112679_292552_350868_180	annotated	DUSP22	6	+	540	11	1	0	3.33%	6.730829338	0.042234516	0.011772051	0.002392043
ENSG00000127054_1324608_1311859_389	annotated	INTS11	1	-	1167	65	7	3	6.94%	18.40399702	2.29E-05	1.45E-05	2.63E-09
ENSG00000154889_11893566_11885029_243	annotated	MPPE1	18	-	729	29	1	0	4.53%	13.14438052	0.002558966	0.001092093	1.69E-05
ENSG00000077454_100577093_100575107_231	annotated	LRCH4	7	-	693	6	0	2	2.16%	2.860801135	0.016947427	0.139742684	0.011764961
ENSG00000109180_48832625_48848496_96	novel	OCIAD1	4	+	288	171	18	2	13.54%	196.1883779	0.00203158	0.001391532	1.69E-05
ENSG00000214274_20693571_20694008_145	annotated	ANG	14	+	435	16	4	0	3.45%	12.15347241	0.070558069	0.021557223	0.006740452
ENSG00000109606_24584393_24527924_795	annotated	DHX15	4	-	2385	164	24	10	7.30%	22.72087845	3.32E-08	8.95E-10	3.44E-16
ENSG00000012171_50269241_50276706_754	annotated	SEMA3B	3	+	2262	52	12	0	2.92%	7.595920256	0.002479335	1.68E-05	4.34E-07
ENSG00000183208_90241017_90242066_349	annotated	GDPGP1	15	+	1047	22	1	3	2.01%	6.942975821	0.01208853	0.033503887	0.001931204
ENSG00000054267_235326919_235168525_1312	annotated	ARID4B	1	-	3936	16	1	0	0.61%	1.343181021	0.010499663	0.00419316	0.000236136
ENSG00000101407_38013816_37983456_1089	annotated	TTI1	20	-	3267	79	4	2	2.94%	7.990015292	2.22E-06	5.99E-07	1.20E-11
ENSG00000169439_96494272_96609548_201	annotated	SDC2	8	+	603	8	0	0	2.49%	4.383715172	0.019679754	0.019679754	0.001784964
ENSG00000177830_904816_869858_393	annotated	CHID1	11	-	1179	143	15	0	12.47%	40.07669377	8.11E-09	3.68E-10	3.46E-17
ENSG00000123219_65554907_65518475_269	annotated	CENPK	5	-	807	65	19	18	8.18%	26.61395852	0.00608235	0.003697976	0.000122748
ENSG00000136279_44050254_44060916_331	annotated	DBNL	7	+	993	52	3	2	5.44%	17.30309327	0.000412241	8.24E-05	2.47E-07
ENSG00000155957_66169951_66138145_161	annotated	TMBIM4	12	-	483	151	34	10	20.50%	103.2997975	6.88E-07	3.81E-06	2.37E-11
ENSG00000155957_66138145_66137960_61	dORF	TMBIM4	12	-	183	61	3	0	26.23%	110.1408437	0.000219587	0.00020982	3.18E-07
ENSG00000100823_20455646_20457508_318	annotated	APEX1	14	+	954	460	61	31	20.13%	159.323233	7.70E-10	1.28E-10	1.14E-18
ENSG00000100554_67359698_67349017_108	novel	ATP6V1D	14	-	324	20	8	1	4.63%	20.39645254	0.17410084	0.035374934	0.026121667

ENSG00000131966_58200218_58215243_126	annotated	ACTR10	14	+	378	16	3	3	5.56%	13.98613888	0.057109797	0.07962816	0.017335158
ENSG00000119682_74676750_74670110_343	annotated	AREL1	14	-	1029	11	1	0	1.75%	3.532213647	0.019795882	0.012798403	0.001207991
ENSG00000108666_32342115_32338186_162	novel	C17orf75	17	-	486	11	4	0	3.09%	7.478699264	0.10818374	0.019679754	0.009874411
ENSG00000143761_228097115_228098013_181	novel	ARF1	1	+	543	874	115	29	34.81%	531.8403171	5.82E-12	1.88E-11	1.35E-21
ENSG00000013288_6575319_6617543_918	annotated	MAN2B2	4	+	2754	35	2	1	1.63%	4.19926964	0.000437499	0.000423577	1.22E-06
ENSG00000168502_8706678_8828946_1566	annotated	MTCL1	18	+	4698	29	5	8	0.89%	2.039645254	0.006888593	0.01959031	0.000687437
ENSG00000118705_37236582_37241315_46	annotated	RPN2	20	+	138	87	8	2	26.09%	208.3098566	0.00592392	0.001822449	6.27E-05
ENSG00000171017_7898910_7900913_667	annotated	LRRC8E	19	+	2001	11	0	0	0.75%	1.816415713	0.021084099	0.021084099	0.002029891
ENSG00000102225_47223594_47228768_484	annotated	CDK16	X	+	1452	38	2	0	2.89%	8.647421613	0.000705627	0.000428413	1.97E-06
ENSG00000099331_17102393_17211785_1797	annotated	MYO9B	19	+	5391	32	5	3	0.83%	1.961328324	0.002266245	0.001871465	2.48E-05
ENSG00000185728_63168878_63209706_583	annotated	YTHDF3	8	+	1749	53	5	5	3.09%	10.01280397	0.000823917	0.000259772	1.44E-06
ENSG00000149428_119056160_119045593_937	annotated	HYOU1	11	-	2811	308	51	9	10.35%	36.20424745	7.40E-13	1.30E-16	1.81E-27
ENSG00000121060_56908536_56891700_422	dORF	TRIM25	17	-	1266	65	6	3	5.45%	16.9648219	0.000122536	5.21E-05	4.72E-08
ENSG00000107438_95290915_95237925_329	annotated	PDLIM1	10	-	987	119	16	9	12.16%	39.83817751	4.85E-06	3.11E-07	1.44E-11
ENSG00000137145_19341102_19372173_961	dORF	DENND4C	9	+	2883	42	5	1	1.77%	4.813647696	0.001156971	0.000166161	1.35E-06
ENSG00000135919_224001900_223975867_398	annotated	SERPINE2	2	-	1194	312	38	6	14.82%	86.34156592	3.86E-08	4.20E-11	2.41E-17
ENSG00000204852_110614183_110619857_100	annotated	TCTN1	12	+	300	16	4	0	6.00%	17.62253499	0.088737635	0.011772051	0.005312172
ENSG00000204852_110614412_110647892_531	Overlap_dORF	TCTN1	12	+	1593	109	14	2	4.33%	22.60894908	0.000573442	3.85E-05	1.74E-07
ENSG00000139684_46791413_46785706_56	novel	ESD	13	-	168	22	4	4	12.50%	43.26961717	0.028352861	0.033503887	0.004113665
ENSG00000184470_19880687_19877111_150	novel	TXNRD2	22	-	450	20	1	0	6.00%	14.68544583	0.005034873	0.003606289	9.98E-05
ENSG00000166908_57594058_57601606_334	annotated	PIP4K2C	12	+	1002	99	3	4	8.08%	32.64653751	6.47E-06	1.46E-05	7.74E-10
ENSG00000106397_101217969_101212894_333	novel	PLOD3	7	-	999	153	20	6	11.11%	50.60525251	1.46E-06	3.92E-07	5.25E-12
ENSG00000100359_40404291_40406851_390	novel	SGSM3	22	+	1170	8	0	0	1.28%	2.259299358	0.019679754	0.019679754	0.001784964
ENSG00000115875_38751256_38749319_137	annotated	SRSF7	2	-	411	307	25	5	25.55%	246.8119636	2.09E-06	2.03E-07	4.05E-12
ENSG00000115875_38748932_38745133_118	dORF	SRSF7	2	-	354	127	14	1	13.56%	118.5414165	0.003612198	0.000211707	5.61E-06
ENSG00000105323_41276293_41306565_532	annotated	HNRNPUL1	19	+	1596	187	24	15	11.28%	38.71492063	4.11E-10	1.24E-10	5.90E-19
ENSG00000254858_18193282_18196055_206	annotated	MPV17L2	19	+	618	70	3	2	8.74%	37.42650029	0.000117789	0.000148919	1.25E-07
ENSG00000173120_67207521_67255055_1056	annotated	KDM2A	11	+	3168	33	2	1	1.61%	3.441901366	0.000500835	0.000171689	5.93E-07
ENSG00000123472_46668322_46635776_328	annotated	ATPAF1	1	-	984	82	8	0	7.93%	27.53521093	0.000116214	3.47E-06	3.70E-09
ENSG00000064933_189844003_189877436_725	annotated	PMS1	2	+	2175	14	7	0	0.97%	2.126857672	0.102488202	0.007882058	0.004614559
ENSG00000115464_61470692_61188102_3546	annotated	USP34	2	-	10638	95	17	13	1.16%	2.950755824	1.40E-05	2.19E-06	2.66E-10
ENSG00000081177_69209471_69241100_621	annotated	EXD2	14	+	1863	50	7	3	3.86%	8.868022843	0.000150921	5.40E-05	6.01E-08
ENSG00000204843_74378227_74361499_1236	annotated	DCTN1	2	-	3708	104	27	4	4.13%	9.267514357	4.26E-06	1.37E-09	9.71E-14
ENSG00000068724_46956860_47073923_735	annotated	TTC7A	2	+	2205	35	0	0	2.04%	5.244802081	0.000273206	0.000273206	5.07E-07
ENSG00000243449_2042382_2043926_85	annotated	C4orf48	4	+	255	138	6	2	14.12%	178.8168992	0.002878557	0.001095841	1.91E-05
ENSG00000173698_19068834_18990864_995	annotated	ADGRG2	X	-	2985	28	3	0	1.41%	3.099440828	0.003183653	0.000424543	9.00E-06
ENSG00000147065_65716839_65739893_566	annotated	MSN	X	+	1698	154	8	8	7.77%	29.96765005	1.91E-08	6.14E-08	1.19E-14
ENSG00000257218_120446481_120459959_136	annotated	GATC	12	+	408	94	4	0	17.65%	76.12675962	2.08E-05	8.27E-06	1.39E-09
ENSG00000140612_84716075_84670019_129	annotated	SEC11A	15	-	387	461	73	13	28.68%	393.6041004	2.93E-05	8.94E-08	3.24E-11
ENSG00000099998_24244725_24219970_586	annotated	GGT5	22	-	1758	61	11	4	5.12%	11.46517315	5.43E-05	5.92E-06	2.70E-09
ENSG00000106524_16645573_16600761_441	annotated	ANKMY2	7	-	1323	29	7	3	3.40%	7.242821922	0.011451789	0.001933934	0.000130436
ENSG00000133606_140479344_140454517_482	annotated	MKRN1	7	-	1446	29	7	5	3.11%	6.62673126	0.004779643	0.002747171	7.36E-05
ENSG00000130414_240011687_239961118_129	novel	NDUFA10	2	-	387	39	7	2	8.53%	33.29839461	0.013523718	0.002252091	0.000177243
ENSG00000130414_240025301_240014697_236	novel	NDUFA10	2	-	708	65	6	4	7.20%	30.33540187	0.001164547	0.000152552	1.26E-06
ENSG00000158747_19655034_19657129_180	annotated	NBL1	1	+	540	22	6	4	5.56%	13.46165868	0.023197249	0.019260489	0.002042005
ENSG00000221988_32154595_32162950_302	annotated	PPT2	6	+	906	32	14	0	4.64%	11.67055298	0.042702904	0.000428413	0.000176022
ENSG00000136937_97666803_97669814_141	annotated	NCBP1	9	+	423	37	3	0	11.35%	28.90220721	0.001513751	0.000162875	1.76E-06
ENSG00000132507_7309636_7311640_186	annotated	EIF5A	17	+	558	667	243	36	16.13%	394.9674341	0.00035902	8.55E-07	3.84E-09
ENSG00000157734_64156726_64156857_43	novel	SNX22	15	+	129	59	14	15	23.26%	151.1234832	0.007635352	0.105704367	0.004674262
ENSG00000157734_64156036_64156287_83	novel	SNX22	15	+	249	218	66	104	16.87%	289.2855895	0.005220547	0.019232304	0.000529385
ENSG00000172493_87046734_87135701_1151	annotated	AFF1	4	+	3453	28	3	2	1.30%	2.679360229	0.001448134	0.000805645	7.25E-06
ENSG00000181026_88626372_88626815_147	novel	AEN	15	+	441	22	0	0	3.40%	16.48366368	0.021084099	0.021084099	0.002029891
ENSG00000068305_99645690_99712771_436	annotated	MEF2A	15	+	1308	15	0	0	1.61%	3.78924921	0.007979402	0.007979402	0.000327143
ENSG00000169228_177303047_177302275_135	novel	RAB24	5	-	405	47	8	0	9.63%	38.34533077	0.004496616	0.000710064	2.04E-05
ENSG00000166888_57105209_57104374_119	novel	STAT6	12	-	357	12	0	1	4.20%	11.1066397	0.020613417	0.019679754	0.001864002
ENSG00000005187_20770170_20786251_393	annotated	ACSM3	16	+	1179	21	2	1	3.56%	5.885388595	0.001035355	0.000670006	4.38E-06
ENSG00000149177_47980748_48131515_594	annotated	PTPRJ	11	+	1782	70	2	3	4.71%	12.97956071	2.88E-06	6.83E-06	1.67E-10
ENSG00000120314_140668751_140669654_210	annotated	WDR55	5	+	630	28	12	2	5.71%	14.68544583	0.075155916	0.004370296	0.002044684
ENSG00000185722_4182261_4181217_78	novel	ANKFY1	17	-	234	13	0	0	7.69%	18.35680728	0.012798403	0.012798403	0.000797244
ENSG00000150471_61732873_62070169_1112	annotated	ADGRL3	4	+	3336	49	7	0	2.25%	4.853328545	0.000214099	4.12E-06	8.34E-09
ENSG00000257923_101817640_102249042_1505	annotated	CUX1	7	+	4515	53	11	1	1.73%	3.878714097	0.000523536	3.07E-06	1.75E-08
ENSG00000196531_56712866_56712759_35	novel	NACA	12	-	105	98	7	5	20.00%	308.3943624	0.008980239	0.00864514	0.000394577
ENSG00000086589_150700985_150691751_371	annotated	RBM22	5	-	1113	39	12	4	5.12%	11.57814799	0.003561876	0.000466311	1.10E-05
ENSG00000143437_150876567_150812021_789	annotated	ARNT	1	-	2367	37	2	1	2.41%	5.165033228	0.000140958	7.99E-05	8.15E-08
ENSG00000141030_17276159_17247098_345	annotated	COPS3	17	-	1035	214	18	9	10.72%	68.31924798	1.71E-07	3.62E-07	5.84E-13
ENSG00000149187_47484455_47472230_360	annotated	CELF1	11	-	1080	57	4	3	5.83%	17.43896692	0.000128902	8.57E-05	7.98E-08
ENSG00000197111_53454801_53479442_365	annotated	PCBP2	12	+	1095	382	78	41	16.71%	115.2706912	9.47E-08	4.91E-10	5.94E-16
ENSG00000108946_68515400_68530449_381	annotated	PRKAR1A	17	+	1143	541	35	14	22.05%	156.3942164	6.83E-15	1.46E-14	1.39E-27

ENSG00000185324_89693299_89695692_190	annotated	CDK10	16	+	570	20	2	0	3.16%	11.59377302	0.044040756	0.012798403	0.002680499
ENSG00000111602_56434155_56417716_1202	annotated	TIMELESS	12	-	3606	77	8	7	3.58%	7.055611452	2.31E-08	7.86E-08	1.83E-14
ENSG00000148358_130054008_130135121_575	annotated	GPR107	9	+	1725	109	10	0	7.13%	20.87887298	2.20E-07	9.33E-09	2.20E-14
ENSG00000075711_197138252_197044623_587	novel	DLG1	3	-	1761	31	4	0	2.21%	5.816637402	0.004387317	0.000656046	1.85E-05
ENSG00000060069_79679948_79753671_867	annotated	CTDP1	18	+	2601	24	0	0	1.50%	3.048881487	0.000596156	0.000596156	2.29E-06
ENSG00000115204_27312534_27309912_36	dORF	MPV17	2	-	108	46	15	2	19.44%	140.7355225	0.116114203	0.01208853	0.007368666
ENSG00000151116_18575356_18532472_318	annotated	UEVLD	11	-	954	34	3	1	5.03%	11.77606505	0.001129532	0.000283388	2.14E-06
ENSG00000006118_60935348_60936779_93	annotated	TMEM132A	11	+	279	40	5	0	7.53%	47.37240589	0.047132153	0.008574016	0.002061348
ENSG00000089053_121330702_121308480_421	annotated	ANAPC5	12	-	1263	67	3	7	5.70%	17.5283528	3.73E-05	0.000141959	4.00E-08
ENSG00000134759_36129934_36138479_165	novel	ELP2	18	+	495	28	4	13	4.85%	18.69056742	0.032478985	0.19809812	0.028394422
ENSG00000089916_76154364_76201851_477	annotated	GPATCH2L	14	+	1431	48	3	0	4.19%	11.08335534	0.000121218	3.60E-05	3.30E-08
ENSG00000100938_24233254_24238778_348	annotated	GMPR2	14	+	1044	91	18	2	9.20%	28.80119764	2.89E-05	6.16E-07	1.77E-10
ENSG00000086666_80120345_80137628_208	annotated	ZFAND6	15	+	624	70	25	2	10.58%	37.06663009	0.005217009	1.52E-05	9.71E-07
ENSG00000140416_63059567_63061730_92	annotated	TPM1	15	+	276	190	24	10	19.57%	227.4647859	0.001202536	0.00038556	3.03E-06
ENSG00000055163_157287106_157393000_1159	annotated	CYFIP2	5	+	3477	39	5	2	1.90%	3.70620613	0.000192577	7.09E-05	9.92E-08
ENSG00000123562_103686696_103685228_32	uORF	MORF4L2	X	-	96	10	2	0	15.62%	34.41901366	0.016947427	0.019679754	0.001553599
ENSG00000162688_99851085_99921651_1518	annotated	AGL	1	+	4554	23	6	0	0.53%	1.668800662	0.06712464	0.005306578	0.00208032
ENSG00000066427_92096118_92080961_193	novel	ATXN3	14	-	579	30	0	0	6.22%	17.1203384	0.001040526	0.001040526	6.70E-06
ENSG00000067955_67029408_67098778_187	annotated	CBFB	16	+	561	158	2	6	13.90%	93.0601781	3.58E-06	3.31E-05	1.02E-09
ENSG00000103494_53700723_53695311_125	annotated	RPGRIP1L	16	-	375	26	2	0	6.40%	22.90929549	0.016025708	0.005604867	0.00046788
ENSG00000051108_56940238_56943290_92	annotated	HERPUD1	16	+	276	35	4	2	11.96%	41.90140793	0.004514955	0.001573068	4.17E-05
ENSG00000137500_83285972_83274734_117	annotated	CCDC90B	11	-	351	25	7	8	9.40%	23.53436831	0.04039794	0.023617361	0.004173475
ENSG00000102858_4624961_4686299_530	annotated	MGRN1	16	+	1590	58	9	3	5.09%	12.05314893	0.000306935	6.70E-06	1.89E-08
ENSG00000112576_41936681_41935940_96	annotated	CCND3	6	-	288	32	7	0	11.46%	36.71361457	0.014511286	0.001565547	0.000140108
ENSG00000055044_202265942_202284485_145	novel	NOP58	2	+	435	22	8	0	6.90%	16.71102456	0.021861499	0.002008257	0.00026979
ENSG00000107960_103917486_103882684_332	annotated	STN1	10	-	996	38	4	1	7.23%	12.60648211	6.13E-05	9.14E-06	4.57E-09
ENSG00000141258_2361705_2379520_984	annotated	SGSM2	17	+	2952	23	4	5	1.22%	2.57443029	0.007352087	0.009754926	0.000366944
ENSG00000162923_224401048_224389835_221	annotated	WDR26	1	-	663	20	3	2	4.98%	9.967497168	0.00758668	0.006753026	0.000266441
ENSG00000156411_103915153_103913964_87	annotated	ATP5MPL	14	-	261	155	13	0	12.64%	196.2279399	0.001657226	0.001665007	1.64E-05
ENSG00000170348_75134965_75134885_26	novel	TMED10	14	-	78	253	21	1	23.08%	1071.755133	0.017143984	0.017143984	0.001379341
ENSG00000170348_75147699_75135719_67	novel	TMED10	14	-	201	118	2	0	14.93%	193.9793964	0.002863021	0.002502537	4.10E-05
ENSG00000117308_23799007_23796166_360	novel	GALE	1	-	1080	90	3	10	7.22%	27.53521093	5.18E-06	0.000197271	9.34E-09
ENSG00000117308_23796532_23795949_65	novel	GALE	1	-	195	17	1	0	7.69%	28.80606682	0.020613417	0.021084099	0.00198765
ENSG00000022267_136206433_136210025_280	annotated	FHL1	X	+	840	11	1	0	2.50%	4.326961717	0.01551626	0.007882058	0.000614257
ENSG00000060138_10704130_10701288_106	novel	YBX3	12	-	318	29	0	3	12.26%	30.13287233	0.000598307	0.000596156	2.30E-06
ENSG00000164305_184635393_184629436_156	annotated	CASP3	4	-	468	21	0	3	5.13%	14.82665204	0.005604867	0.021450603	0.000629982
ENSG00000076864_21619063_21597720_672	annotated	RAP1GAP	1	-	2016	40	2	7	2.98%	6.556002602	0.000158386	0.001441211	1.63E-06
ENSG00000166741_114312268_114312477_69	novel	NNMT	11	+	207	163	21	7	36.23%	260.1877902	3.64E-05	9.21E-06	2.71E-09
ENSG00000104824_38840176_38838890_68	novel	HNRNPL	19	-	204	102	16	3	19.12%	165.2112656	0.006612777	0.001141632	4.63E-05
ENSG00000188603_28491764_28489349_55	Overlap_uORF	CLN3	16	-	165	18	0	12	14.55%	36.0460943	0.005554612	0.310595281	0.015978689
ENSG00000124299_33521760_33387344_493	annotated	PEPD	19	-	1479	285	10	6	8.11%	63.67168449	1.94E-07	1.46E-07	2.71E-13
ENSG00000103495_29808660_29810231_78	novel	MAZ	16	+	234	14	3	5	8.97%	19.76886938	0.059666052	0.116253847	0.025850842
ENSG00000126698_28205322_28200198_96	annotated	DNAJC8	1	-	288	24	1	0	6.25%	27.53521093	0.01300724	0.01300724	0.000821865
ENSG00000111331_112938531_112969973_1087	annotated	OAS3	12	+	3261	71	14	0	2.94%	7.194112146	9.99E-05	2.84E-07	3.49E-10
ENSG00000111325_122975252_122979346_350	annotated	OGFOD2	12	+	1050	22	4	0	2.86%	6.923138747	0.009583527	0.002227676	0.000122596
ENSG00000125170_57479462_57473377_350	annotated	DOK4	16	-	1050	12	4	0	1.71%	3.776257498	0.117100642	0.013217227	0.007958958
ENSG00000141499_7688649_7703486_548	annotated	WRAP53	17	+	1644	69	11	13	5.11%	13.86809893	0.000151733	0.000502919	5.31E-07
ENSG00000090686_21782957_21728562_485	annotated	USP48	1	-	1455	48	14	0	4.12%	10.90053711	0.002419812	3.87E-05	8.46E-07
ENSG00000106636_44211032_44212282_42	novel	YKT6	7	+	126	10	0	0	11.90%	26.22401041	0.019679754	0.019679754	0.001784964
ENSG00000181929_49018740_49002899_340	annotated	PRKAG1	12	-	1020	162	14	3	8.82%	52.47887259	9.29E-06	2.27E-06	1.81E-10
ENSG00000175866_81035255_81115839_534	annotated	BAIAP2	17	+	1602	11	0	0	1.31%	2.268818878	0.007882058	0.007882058	0.000319636
ENSG00000151552_17512054_17487131_213	annotated	QDPR	4	-	639	79	11	0	11.74%	40.85035987	1.50E-05	5.14E-06	6.40E-10
ENSG00000093010_19963730_19967427_141	annotated	COMT	22	+	423	81	5	2	8.51%	63.27239958	0.004758488	0.001272209	3.63E-05
ENSG00000012817_19744534_19705995_1482	annotated	KDM5D	Y	-	4446	22	4	0	0.88%	1.635019272	0.006943373	0.000589733	2.75E-05
ENSG00000096092_52677103_52686049_99	annotated	TMEM14A	6	+	297	130	58	29	21.21%	144.6293907	0.048521932	0.000561589	0.000253636
ENSG00000134602_132023654_132074159_380	annotated	STK26	X	+	1140	18	0	0	2.37%	5.21719786	0.003529166	0.003529166	6.95E-05
ENSG00000198836_193593378_193648799_569	annotated	OPA1	3	+	1707	51	16	0	3.87%	9.872026413	0.010479115	1.52E-05	2.30E-06
ENSG00000198836_193654944_193692127_317	dORF	OPA1	3	+	951	20	3	3	3.15%	6.94894913	0.015358648	0.015358648	0.001122628
ENSG00000111642_6606316_6570676_1887	annotated	CHD4	12	-	5661	90	9	3	2.12%	5.253140399	2.68E-07	2.95E-08	8.01E-14
ENSG00000215301_41343246_41347719_470	novel	DDX3X	X	+	1410	190	31	7	8.30%	44.52502192	8.60E-07	4.52E-08	3.97E-13
ENSG00000215301_41334253_41342657_148	novel	DDX3X	X	+	444	49	18	3	12.16%	36.46554961	0.013604033	0.000328142	3.58E-05
ENSG00000137337_30715175_30700465_2089	annotated	MDC1	6	-	6267	23	4	0	0.62%	1.212656489	0.010500707	0.000596156	4.36E-05
ENSG00000119688_74292906_74289225_215	novel	ABCD4	14	-	645	38	1	0	5.58%	19.46675377	0.001053132	0.001053132	6.85E-06
ENSG00000113621_134888174_134896036_62	novel	TXNDC15	5	+	186	72	13	0	19.35%	127.9054959	0.008373135	0.001061589	5.57E-05
ENSG00000083312_72848394_72905410_890	annotated	TNPO1	5	+	2670	195	19	7	6.29%	24.13198261	3.67E-08	4.53E-10	2.02E-16
ENSG00000163069_52033610_52031890_83	annotated	SGCB	4	-	249	23	3	1	6.02%	30.52095669	0.044329616	0.021557223	0.004218623
ENSG00000198925_219226870_219220447_769	annotated	ATG9A	2	-	2307	52	14	1	3.12%	7.447755361	0.002733827	1.03E-05	3.24E-07
ENSG00000131732_81308630_81312662_120	novel	ZCCHC9	5	+	360	11	1	0	5.00%	10.09624401	0.026832696	0.011772051	0.001510382

ENSG00000181264_120327442_120330515_245	annotated	TMEM136	11	+	735	24	3	0	3.27%	10.78930714	0.010068376	0.005306578	0.000280339
ENSG00000179134_39356897_39383527_693	annotated	SAMD4B	19	+	2079	38	4	3	2.31%	6.039469063	0.002374643	0.001068966	1.54E-05
ENSG00000168701_67227219_67228635_100	novel	TMEM208	16	+	300	54	11	10	14.00%	59.4760556	0.003418076	0.008473358	0.000158497
ENSG00000168701_67227149_67227879_39	novel	TMEM208	16	+	117	41	3	0	30.77%	115.7890921	0.00507972	0.000962887	3.03E-05
ENSG00000140365_75336090_75340005_113	annotated	COMMD4	15	+	339	140	10	7	17.70%	136.4576825	0.000184405	0.000182185	2.34E-07
ENSG00000136868_113258732_113260473_110	annotated	SLC31A1	9	+	330	44	0	0	7.27%	44.05633748	0.005454749	0.005454749	0.000159109
ENSG00000062725_60525931_60466398_224	novel	APPBP2	17	-	672	8	5	0	2.23%	3.933601561	0.170042304	0.019679754	0.016523685
ENSG00000063177_48617357_48615372_136	annotated	RPL18	19	-	408	769	36	14	34.56%	622.7816824	8.12E-09	1.82E-09	1.60E-16
ENSG00000156931_184824633_185052025_1426	annotated	VPS8	3	+	4278	12	7	0	0.63%	0.92685142	0.235318089	0.002874854	0.006889537
ENSG00000138674_82881936_82863297_509	novel	SEC31A	4	-	1527	46	2	0	3.73%	9.953789411	0.000119012	4.95E-05	4.37E-08
ENSG00000168958_227330726_227346276_167	novel	MFF	2	+	501	75	15	1	8.38%	49.46445077	0.009740623	0.000471186	3.30E-05
ENSG00000115414_215379333_215361555_640	novel	FN1	2	-	1920	101	14	4	5.62%	17.3816019	7.12E-06	3.69E-07	2.52E-11
ENSG00000115414_215391764_215384828_213	novel	FN1	2	-	639	19	0	5	4.23%	9.824770096	0.003327803	0.031423484	0.000609882
ENSG00000081177_69209471_69234800_247	novel	EXD2	14	+	741	25	3	3	5.26%	11.14785867	0.001969684	0.005331506	6.05E-05
ENSG00000081177_69234871_69241100_325	novel	EXD2	14	+	975	25	4	0	3.38%	8.472372593	0.010716902	0.001513502	9.84E-05
ENSG00000064419_129054770_128957255_923	annotated	TNPO3	7	-	2769	103	6	6	4.88%	12.29090672	3.04E-08	4.62E-08	1.41E-14
ENSG00000127054_1324608_1313787_278	annotated	INTS11	1	-	834	45	7	0	6.12%	17.82855384	0.001400237	0.000131066	1.34E-06
ENSG00000151726_184765959_184757125_258	annotated	ACSL1	4	-	774	27	0	0	4.65%	11.52636736	0.000947024	0.000947024	5.59E-06
ENSG00000136518_179570124_179588010_376	annotated	ACTL6A	3	+	1128	80	0	0	9.57%	23.43422206	5.24E-08	5.24E-08	2.72E-14
ENSG00000005700_82224092_82171425_1047	dORF	IBTK	6	-	3141	89	18	0	3.34%	9.362497698	7.23E-05	9.38E-08	9.33E-11
ENSG00000005700_82240474_82225613_234	annotated	IBTK	6	-	702	44	7	0	3.85%	20.71024412	0.044860784	0.003632191	0.000973902
ENSG00000072110_68877216_68874859_97	novel	ACTN1	14	-	291	48	7	0	16.49%	54.50268554	0.002318734	0.000190724	3.18E-06
ENSG00000163808_44780919_44852735_1269	annotated	KIF15	3	+	3807	14	0	7	0.79%	1.215107811	0.001790164	0.033944577	0.00040295
ENSG00000101294_31514552_31569219_426	annotated	HM13	20	+	1278	1186	111	11	21.36%	306.6362456	4.33E-15	2.73E-16	1.75E-29
ENSG00000187634_939310_944153_496	annotated	SAMD11	1	+	1488	31	10	4	2.62%	6.883802732	0.038557528	0.006702261	0.00135658
ENSG00000021574_32089578_32154496_430	annotated	SPAST	2	+	1290	53	7	0	4.42%	13.57549934	0.001434474	5.69E-05	6.59E-07
ENSG00000204217_202377475_202559702_530	annotated	BMPR2	2	+	1590	23	0	0	2.26%	4.779696991	0.00092354	0.00092354	5.32E-06
ENSG00000181191_69163073_69161307_588	annotated	PJA1	X	-	1764	11	0	0	0.85%	2.06045796	0.021084099	0.021084099	0.002029891
ENSG00000116729_68153545_68142800_216	novel	WLS	1	-	648	23	2	0	6.02%	11.72796021	0.001621946	0.000596156	6.13E-06
ENSG00000187109_76067450_76049738_326	annotated	NAP1L1	12	-	978	188	27	14	11.66%	63.51680557	1.47E-06	3.39E-07	4.61E-12
ENSG00000131748_39660491_39662908_139	novel	STARD3	17	+	417	15	0	6	5.04%	11.88570256	0.008473674	0.079426469	0.003629372
ENSG00000051128_18938982_18929443_361	annotated	HOMER3	19	-	1083	26	5	0	2.77%	7.932581541	0.007867321	0.002390426	0.000106645
ENSG00000106070_50626921_50592952_407	annotated	GRB10	7	-	1221	23	2	1	2.95%	6.224175443	0.003782216	0.001559221	3.46E-05
ENSG00000145293_82430830_82460120_261	annotated	ENOPH1	4	+	783	64	3	0	9.58%	27.00771646	1.62E-05	4.32E-06	5.86E-10
ENSG00000104388_60584796_60620769_98	novel	RAB2A	8	+	294	40	9	1	11.22%	44.95544641	0.019176024	0.002345022	0.000266228
ENSG00000082146_201475660_201480175_263	annotated	STRADB	2	+	789	11	0	0	1.90%	4.606651258	0.019216965	0.019216965	0.001707396
ENSG00000047634_17750071_17753392_169	novel	SCML1	X	+	507	12	4	0	5.33%	7.820651624	0.044129916	0.002500085	0.000710788
ENSG00000198818_166328344_166324533_55	novel	SFT2D1	6	-	165	87	7	2	20.00%	174.2227891	0.00164173	0.001626321	1.59E-05
ENSG00000140157_22838748_22838861_37	uORF	NIPA2	15	+	111	43	2	4	18.92%	128.0015211	0.008776163	0.032038753	0.00140113
ENSG00000140157_22851732_22866847_360	annotated	NIPA2	15	+	1080	159	33	6	10.28%	48.6455393	9.37E-05	3.00E-07	3.40E-10
ENSG00000153832_229932884_230010884_157	annotated	FBXO36	2	+	471	12	1	0	3.82%	8.418408436	0.026832696	0.011772051	0.001510382
ENSG00000161513_74863163_74862817_72	dORF	FDXR	17	-	216	15	0	0	8.33%	22.94600911	0.01300724	0.01300724	0.000821865
ENSG00000003096_117920277_117898908_544	annotated	KLHL13	X	-	1632	9	0	0	1.10%	1.822183076	0.009815329	0.009815329	0.000483714
ENSG00000123473_47310319_47251136_1288	annotated	STIL	1	-	3864	26	4	0	1.48%	2.223340013	0.000734429	2.98E-05	1.81E-07
ENSG00000003096_117920277_117898908_544	annotated	KLHL13	X	-	1632	9	0	0	1.10%	1.822183076	0.009815329	0.009815329	0.000483714
ENSG00000154839_50375831_50392247_255	annotated	SKA1	18	+	765	14	2	7	3.14%	6.046948282	0.019504171	0.197103475	0.019603754
ENSG00000162368_47333946_47376745_228	annotated	CMPK1	1	+	684	67	23	1	10.96%	32.36594969	0.007415706	6.80E-06	7.98E-07
ENSG00000131778_147242704_147275402_377	annotated	CHD1L	1	+	1131	15	3	0	1.86%	4.382261686	0.023718446	0.008373824	0.000989736
ENSG00000131778_147275365_147295509_470	Overlap_dORF	CHD1L	1	+	1410	24	0	2	2.34%	5.624213296	0.001092093	0.005502968	3.68E-05
ENSG00000151348_44130073_44198032_133	novel	EXT2	11	+	399	17	0	0	3.76%	14.07815295	0.021557223	0.021557223	0.002115616
ENSG00000204370_112086908_112094893_85	annotated	SDHD	11	+	255	74	14	16	9.41%	95.88732276	0.024974988	0.041252132	0.004474685
ENSG00000167700_144510868_144511164_69	novel	MFSD3	8	+	207	63	4	3	8.70%	100.563379	0.026832696	0.023878474	0.002853849
ENSG00000167700_144509334_144510617_233	novel	MFSD3	8	+	699	28	3	2	5.15%	13.23580954	0.006549171	0.003955987	0.000140438
ENSG00000132603_69339830_69341652_133	annotated	NIP7	16	+	399	61	9	1	12.78%	50.51572531	0.000929831	0.000120469	8.07E-07
ENSG00000132603_69339830_69341652_180	annotated	NIP7	16	+	540	72	10	3	12.78%	44.05633748	0.000163602	3.69E-05	4.57E-08
ENSG00000132604_69385845_69356898_500	annotated	TERF2	16	-	1500	83	5	0	4.80%	18.28338006	7.99E-06	8.11E-06	5.32E-10
ENSG00000135930_232550725_232567251_188	annotated	EIF4E2	2	+	564	25	20	1	6.91%	14.64638879	0.265788852	0.000539992	0.002945676
ENSG00000135930_232556424_232564298_52	internal	EIF4E2	2	+	156	20	1	15	15.38%	42.36186296	0.009288069	0.351779283	0.026584329
ENSG00000164008_42767312_42775394_199	annotated	C1orf50	1	+	597	13	3	0	3.02%	7.195130493	0.035772986	0.013428348	0.002259559
ENSG00000243667_68157338_68134701_253	annotated	WDR92	2	-	759	22	1	1	3.56%	9.57746467	0.005588371	0.005588371	0.00016659
ENSG00000168612_45883067_45884050_327	annotated	ZSWIM1	20	+	981	14	0	2	2.45%	4.715510128	0.004826937	0.030313544	0.000797936
ENSG00000134759_36129934_36139675_152	novel	ELP2	18	+	456	28	4	13	5.26%	20.28910279	0.032478985	0.19809812	0.028394422
ENSG00000105245_40684542_40667468_568	annotated	NUMBL	19	-	1704	31	0	0	2.46%	6.011208019	0.000444132	0.000444132	1.30E-06
ENSG00000180228_178451030_178447501_106	novel	PRKRA	2	-	318	12	1	0	5.66%	12.46877476	0.023878474	0.01300724	0.001470914
ENSG00000198799_113110301_113124101_553	novel	LRIG2	1	+	1659	10	2	0	1.45%	1.991696993	0.037556183	0.004149608	0.000889008
ENSG00000050426_51048357_51052113_99	novel	LETMD1	12	+	297	21	0	0	7.07%	23.36320927	0.008473674	0.008473674	0.000366502
ENSG00000181789_129263967_129267103_118	novel	COPG1	3	+	354	25	2	0	9.32%	23.33492451	0.0034719	0.001491519	3.05E-05
ENSG00000182809_105474863_105479653_280	novel	CRIP2	14	+	840	41	5	2	5.36%	16.1277664	0.000605286	0.000461039	1.82E-06

ENSG00000092841_56158402_56161415_151	annotated	MYL6	12	+	453	4533	234	49	47.68%	3306.41354	5.38E-12	1.90E-13	1.38E-23
ENSG00000087299_50312150_50247058_463	annotated	L2HGDH	14	-	1389	22	1	3	2.81%	5.233474215	0.000993131	0.007312901	4.56E-05
ENSG00000100162_41947076_41946413_46	novel	CENPM	22	-	138	29	3	7	17.39%	69.43661886	0.020021357	0.051011568	0.004552118
ENSG00000185813_81907595_81904833_198	annotated	PCYT2	17	-	594	16	2	0	3.54%	8.900270198	0.017404239	0.007882058	0.000688104
ENSG00000128581_101316517_101315134_108	novel	IFT22	7	-	324	9	1	0	4.63%	9.178403642	0.042234516	0.019679754	0.003715148
ENSG00000160299_46334757_46445327_3127	annotated	PCNT	21	+	9381	37	4	3	0.51%	1.30323352	0.000502938	0.000641195	2.09E-06
ENSG00000120265_149749728_149772042_120	annotated	PCMT1	6	+	360	72	15	5	11.67%	66.08450622	0.006208942	0.001978304	7.08E-05
ENSG00000173264_64286525_64289196_396	annotated	GPR137	11	+	1188	41	1	1	4.55%	11.40347119	0.00010993	0.00010993	8.68E-08
ENSG00000106105_30594922_30633860_739	annotated	GARS	7	+	2217	288	39	9	9.47%	42.92363056	2.29E-09	4.12E-13	1.70E-20
ENSG00000168003_62882768_62884024_55	uORF	SLC3A2	11	+	165	372	13	5	20.00%	744.9526156	0.001619961	0.00164173	1.59E-05
ENSG00000070413_19122206_19060762_269	uORF	DGCR2	22	-	807	21	7	0	2.97%	8.598355828	0.103874448	0.005355751	0.003516099
ENSG00000070413_19057115_19038865_326	annotated	DGCR2	22	-	978	62	8	0	4.91%	20.94703162	0.001824635	0.00020935	2.68E-06
ENSG00000008838_40053598_40019529_976	annotated	MED24	17	-	2928	100	22	1	3.79%	11.28492251	0.000122222	5.48E-08	1.09E-10
ENSG00000108641_19362569_19337720_187	annotated	B9D1	17	-	561	48	6	5	7.49%	28.27144651	0.004428796	0.003666397	8.95E-05
ENSG00000169241_155136321_155138281_187	Overlap_dORF	SLC50A1	1	+	561	63	6	14	11.76%	37.10627355	0.000358776	0.001757693	4.18E-06
ENSG00000163875_37514740_37496592_179	novel	MEAF6	1	-	537	52	11	0	10.61%	31.99622275	0.001670825	5.93E-05	8.07E-07
ENSG00000160058_32386874_32366622_343	annotated	BSDC1	1	-	1029	23	4	1	3.50%	7.385537625	0.008887078	0.001559221	8.29E-05
ENSG00000069849_141903682_141925580_38	internal	ATP1B3	3	+	114	38	0	0	15.79%	110.1408437	0.013640586	0.013640586	0.000898637
ENSG00000069849_141903710_141925701_69	annotated	ATP1B3	3	+	207	30	3	5	7.25%	47.88732335	0.021084099	0.074662305	0.007018177
ENSG00000129691_38107048_38139071_534	annotated	ASH2L	8	+	1602	49	9	1	4.31%	10.10655682	0.000906107	2.29E-05	1.81E-07
ENSG00000131844_71604445_71641095_125	novel	MCCC2	5	+	375	23	0	0	8.80%	20.26591524	0.001262414	0.001262414	9.71E-06
ENSG00000091947_44014952_44011928_257	annotated	TMEM101	17	-	771	121	36	6	12.45%	51.8561949	0.000374045	2.61E-06	1.04E-08
ENSG00000173327_65608323_65598291_559	annotated	MAP3K11	11	-	1677	19	13	0	1.25%	3.743606494	0.304091105	0.008473674	0.020134645
ENSG00000103174_5033914_5031873_161	novel	NAGPA	16	-	483	5	0	0	3.11%	3.420523096	0.012673659	0.012673659	0.000782701
ENSG00000111642_6578439_6570848_217	novel	CHD4	12	-	651	16	0	1	2.30%	8.120983868	0.021557223	0.029237631	0.002819006
ENSG00000158006_25989553_25961989_346	annotated	PAFAH2	1	-	1038	45	3	0	5.20%	14.3246762	0.000513719	7.75E-05	2.92E-07
ENSG00000213614_72356657_72355449_102	novel	HEXA	15	-	306	46	2	2	9.80%	49.67136089	0.00244612	0.004853548	6.71E-05
ENSG00000213614_72351228_72346299_198	novel	HEXA	15	-	594	190	22	0	18.18%	105.6907086	2.19E-06	7.50E-08	1.68E-12
ENSG00000213614_72346281_72345442_51	novel	HEXA	15	-	153	43	2	0	29.41%	92.86384861	0.000294865	0.000294865	5.88E-07
ENSG00000072609_132887328_132841554_572	annotated	CHFR	12	-	1716	10	1	0	1.05%	1.925539226	0.023672501	0.012798403	0.001437523
ENSG00000196628_55350976_55228225_535	annotated	TCF4	18	-	1605	24	0	0	1.87%	4.940897662	0.002404282	0.002404282	3.34E-05
ENSG00000135686_84657042_84662133_538	annotated	KLHL36	16	+	1614	15	2	0	1.12%	3.070841367	0.042234516	0.012798403	0.002567464
ENSG00000067225_72206880_72206398_160	novel	PKM	15	-	480	579	27	8	15.00%	398.5721782	1.38E-05	2.67E-05	2.93E-09
ENSG00000110801_121888857_121903022_85	annotated	PSMD9	12	+	255	91	4	26	18.82%	117.9154915	0.00024007	0.016432371	3.48E-05
ENSG00000173715_66744743_66843234_677	annotated	C11orf80	11	+	2031	17	5	3	1.33%	2.765722811	0.063328909	0.02524418	0.006888858
ENSG00000111674_6915833_6922800_434	annotated	ENO2	12	+	1302	77	8	1	6.91%	19.54111743	3.10E-05	6.52E-07	2.00E-10
ENSG00000167965_2206086_2208877_326	novel	MLST8	16	+	978	109	18	4	11.66%	36.82623302	1.36E-05	2.83E-07	3.92E-11
ENSG00000128989_52557219_52551934_96	annotated	ARPP19	15	-	288	39	5	5	11.46%	44.74471776	0.007714041	0.006557658	0.000263348
ENSG00000160949_144440789_144433553_833	novel	TONSL	8	-	2499	25	4	0	1.32%	3.30554753	0.010805327	0.001506153	9.88E-05
ENSG00000179818_70087970_70051267_99	novel	PCBP1-AS1	2	-	297	41	13	1	14.14%	45.61388477	0.012576416	0.000566661	5.12E-05
ENSG00000099917_20582680_20586704_341	novel	MED15	22	+	1023	23	1	2	2.93%	7.428854561	0.00376808	0.008577311	0.000175238
ENSG00000132763_45500333_45509215_282	annotated	MMACHC	1	+	846	10	0	1	1.77%	3.905703677	0.019679754	0.042234516	0.003715148
ENSG00000197448_143263514_143264196_60	novel	GSTK1	7	+	180	114	31	23	25.00%	209.267603	0.00453292	0.00417846	0.000103593
ENSG00000122958_69124278_69157045_63	annotated	VPS26A	10	+	189	8	0	0	7.94%	13.98613888	0.019679754	0.019679754	0.001784964
ENSG00000173436_19597046_19600884_53	novel	MICOS10	1	+	159	79	17	2	16.98%	164.172201	0.045876598	0.003816221	0.001039053
ENSG00000173436_19600956_19626401_62	novel	MICOS10	1	+	186	66	7	5	16.13%	117.2467046	0.005578808	0.00252785	7.94E-05
ENSG00000117385_42752629_42750064_153	novel	P3H1	1	-	459	7	4	0	3.27%	5.039123568	0.169362074	0.019216965	0.016163992
ENSG00000111785_106774746_106886015_520	annotated	RIC8B	12	+	1560	13	5	2	0.96%	2.753521093	0.17410084	0.055917761	0.036918467
ENSG00000051180_40698771_40731123_276	annotated	RAD51	15	+	828	28	6	1	3.26%	11.17370878	0.025932707	0.0037806	0.000550193
ENSG00000164961_125081734_125024617_1011	annotated	WASHC5	8	-	3033	80	6	5	3.66%	8.715398117	6.01E-07	2.44E-07	1.37E-12
ENSG00000106976_128203486_128254714_859	annotated	DNM1	9	+	2577	128	27	17	5.47%	16.41213969	6.88E-07	8.36E-07	5.20E-12
ENSG00000102054_16858763_16853678_122	novel	RBBP7	X	-	366	55	2	0	12.30%	49.65365905	0.000571973	0.000308892	1.17E-06
ENSG00000162645_89120192_89108175_453	annotated	GBP2	1	-	1359	12	0	5	1.32%	2.917638244	0.013217227	0.117100642	0.007958958
ENSG00000089685_78214317_78222843_78	annotated	BIRC5	17	+	234	64	16	0	15.38%	90.37197432	0.009124611	0.001091522	6.26E-05
ENSG00000125912_3204609_3207688_207	annotated	NCLN	19	+	621	38	4	0	8.70%	20.21909208	0.00022296	8.02E-05	1.29E-07
ENSG00000169592_29996311_29996621_51	novel	INO80E	16	+	153	24	13	6	13.73%	51.83098527	0.220042808	0.058290626	0.048904081
ENSG00000147874_19096765_19056343_911	annotated	HAUS6	9	-	2733	32	1	0	1.65%	3.868833149	0.000427981	0.000279067	8.02E-07
ENSG00000187555_8904446_8902041_131	novel	USP7	16	-	393	11	0	1	3.82%	9.248467792	0.019679754	0.042234516	0.003715148
ENSG00000143545_153986236_153982099_203	annotated	RAB13	1	-	609	65	3	1	9.36%	35.26677261	0.000214822	7.69E-05	1.20E-07
ENSG00000153975_116667144_116635832_562	annotated	ZUP1	6	-	1686	6	0	0	0.89%	1.175880894	0.016947427	0.016947427	0.001349898
ENSG00000115204_27312694_27309912_88	dORF	MPV17	2	-	264	79	16	1	19.32%	98.87643924	0.006914262	0.000172339	9.71E-06
ENSG00000103502_29862857_29859275_157	annotated	CDIPT	16	-	471	106	13	38	12.10%	74.36260785	0.001122155	0.039026669	0.000328653
ENSG00000141994_5791141_5785203_650	annotated	DUS3L	19	-	1950	19	0	4	2.00%	3.219501585	0.000528008	0.009412849	3.49E-05
ENSG00000151292_123545664_123614396_423	annotated	CSNK1G3	5	+	1269	34	6	0	3.31%	8.852928336	0.011011012	0.000375189	3.13E-05
ENSG00000151292_123512228_123512335_35	uORF	CSNK1G3	5	+	105	11	3	2	14.29%	34.61569374	0.100412561	0.070008252	0.025705542
ENSG00000163466_218249839_218253977_85	annotated	ARPC2	2	+	255	33	6	6	9.41%	42.76056285	0.005454749	0.011825808	0.000336929
ENSG00000213145_105487260_105488511_77	annotated	CRIP1	14	+	231	805	61	46	41.56%	1151.472457	7.11E-07	3.27E-06	2.09E-11
ENSG00000181852_56210361_56207307_102	annotated	RNF41	12	-	306	16	0	0	7.84%	17.27699509	0.005257623	0.005257623	0.000148366

ENSG00000135317_85593697_85505967_930	annotated	SNX14	6	-	2790	94	14	3	3.98%	11.13251539	1.90E-05	2.63E-07	5.21E-11
ENSG00000155380_112929308_112913891_500	annotated	SLC16A1	1	-	1500	216	24	1	11.60%	47.58084448	9.51E-09	1.28E-11	1.80E-18
ENSG00000135316_85641439_85614756_623	annotated	SYNCRIP	6	-	1869	130	8	6	5.30%	22.98284058	2.98E-06	4.78E-06	1.21E-10
ENSG00000160211_154546155_154532000_561	annotated	G6PD	X	-	1683	1087	118	53	25.49%	213.4101553	8.04E-21	7.40E-23	1.08E-41
ENSG00000177628_155236349_155235677_90	novel	GBA	1	-	270	147	13	0	16.67%	179.8967114	0.000297943	0.000297943	6.00E-07
ENSG00000114735_50571123_50580417_332	annotated	HEMK1	3	+	996	12	2	0	1.81%	3.980994351	0.052581193	0.01300724	0.003266469
ENSG00000141644_50279992_50271501_605	annotated	MBD1	18	-	1815	22	1	3	1.82%	4.005121589	0.002467336	0.011220946	0.000157857
ENSG00000184897_129315902_129315261_213	annotated	H1FX	3	-	639	170	14	5	16.43%	87.9058377	5.46E-07	2.84E-07	1.44E-12
ENSG00000163545_205321760_205303450_672	annotated	NUAK2	1	-	2016	28	2	3	2.08%	4.589201821	0.000960952	0.002154443	1.27E-05
ENSG00000197451_178204838_178210623_285	annotated	HNRNPAB	5	+	855	234	111	9	17.89%	90.43142957	0.000263173	6.51E-09	4.84E-11
ENSG00000170085_176296309_176345445_317	annotated	SIMC1	5	+	951	11	1	0	1.89%	3.821922021	0.014014728	0.009815329	0.000679217
ENSG00000122550_23123801_23125819_118	novel	KLHL7	7	+	354	30	0	0	8.47%	28.00190942	0.002335453	0.002335453	3.16E-05
ENSG00000197933_11738819_11721701_610	annotated	ZNF823	19	-	1830	8	0	2	0.82%	1.444470081	0.019679754	0.101051022	0.009164498
ENSG00000124191_43914892_44068686_506	annotated	TOX2	20	+	1518	12	5	0	1.38%	2.612035819	0.11290941	0.007882058	0.005179079
ENSG00000120899_27397660_27458509_942	annotated	PTK2B	8	+	2826	31	5	5	1.38%	3.624592521	0.008628846	0.010171135	0.000443344
ENSG00000105197_39480854_39489820_353	annotated	TIMM50	19	+	1059	179	39	16	14.45%	55.85045616	5.85E-07	2.25E-08	1.39E-13
ENSG00000104964_3062927_3053819_264	annotated	TLE5	19	-	792	260	33	18	14.02%	108.472043	1.02E-06	1.29E-06	1.17E-11
ENSG00000104969_2769068_2757343_313	annotated	SGTA	19	-	939	100	7	2	11.18%	35.18876796	8.95E-07	1.25E-07	1.07E-12
ENSG00000142227_48329378_48330470_94	dORF	EMP3	19	+	282	214	18	17	36.17%	250.7461761	1.75E-06	8.78E-07	1.37E-11
ENSG00000142227_48326845_48327634_63	annotated	EMP3	19	+	189	748	117	63	44.44%	1307.703986	0.0002222	2.55E-05	4.45E-08
ENSG00000092841_56158402_56160331_145	annotated	MYL6	12	+	435	4058	180	46	42.76%	3082.42444	1.77E-10	1.01E-11	2.23E-20
ENSG00000185033_90201594_90228643_832	annotated	SEMA4B	15	+	2496	185	3	5	8.17%	24.49045203	1.48E-12	2.01E-12	3.77E-23
ENSG00000186834_45149191_45150270_359	annotated	HEXIM1	17	+	1077	167	24	8	16.71%	51.23543426	3.83E-09	1.67E-11	8.72E-19
ENSG00000113318_80670232_80875862_899	annotated	MSH3	5	+	2697	18	0	1	1.11%	2.205267171	0.002188006	0.003722062	4.66E-05
ENSG00000119912_92537525_92454444_978	annotated	IDE	10	-	2934	62	3	5	3.27%	6.982343875	2.94E-06	6.89E-06	1.72E-10
ENSG00000138750_76132583_76115366_225	dORF	NUP54	4	-	675	12	5	0	2.67%	5.874178331	0.175009948	0.012798403	0.012568728
ENSG00000179941_76348346_76345813_719	annotated	BBS10	12	-	2157	18	0	1	0.97%	2.757350746	0.008776163	0.01208853	0.000531381
ENSG00000101084_36605952_36612223_143	annotated	RAB5IF	20	+	429	107	7	7	9.79%	82.41307886	0.000482311	0.001700838	5.29E-06
ENSG00000131503_140401968_140472312_581	annotated	ANKHD1	5	+	1743	40	9	1	3.10%	7.582846382	0.003119927	0.000108589	2.70E-06
ENSG00000133318_63681637_63750202_117	novel	RTN3	11	+	351	138	34	7	12.82%	129.9097131	0.018001669	0.000316881	4.84E-05
ENSG00000132950_19852096_19824477_641	annotated	ZMYM5	13	-	1923	8	0	0	0.78%	1.374612714	0.019216965	0.019216965	0.001707396
ENSG00000189159_75148589_75147538_58	novel	JPT1	17	-	174	193	30	4	31.03%	366.5031523	0.000135682	9.65E-05	9.40E-08
ENSG00000107949_125823558_125836274_314	annotated	BCCIP	10	+	942	109	15	10	13.38%	38.23360498	3.09E-06	8.93E-08	2.84E-12
ENSG00000172936_38138662_38141286_309	annotated	MYD88	3	+	927	101	9	10	9.06%	36.00072885	7.46E-05	0.000146902	7.95E-08
ENSG00000157168_32647829_32763338_485	annotated	NRG1	8	+	1455	21	3	2	1.86%	4.768984985	0.010276881	0.006003419	0.000320165
ENSG00000157168_32595851_32854547_355	annotated	NRG1	8	+	1065	26	1	3	3.10%	8.066653342	0.001535678	0.004862286	4.40E-05
ENSG00000077782_38457446_38413628_820	annotated	FGFR1	8	-	2460	101	18	8	5.49%	13.56612831	1.63E-06	9.83E-08	1.59E-12
ENSG00000075239_108139039_108144062_147	novel	ACAT1	11	+	441	31	7	3	8.16%	23.22698065	0.011841039	0.005421488	0.000335502
ENSG00000042445_85343687_85343242_62	dORF	RETSAT	2	-	186	20	4	0	12.90%	35.52930442	0.005306578	0.005306578	0.000151001
ENSG00000042445_85344691_85343719_95	dORF	RETSAT	2	-	285	16	4	0	6.32%	18.55003683	0.042234516	0.012798403	0.002567464
ENSG00000182979_105420048_105464563_426	annotated	MTA1	14	+	1278	17	10	0	2.58%	4.395291885	0.206946044	0.001343041	0.003465222
ENSG00000182979_105464722_105470215_251	dORF	MTA1	14	+	753	27	5	2	3.19%	11.84781984	0.021984885	0.006191047	0.000704259
ENSG00000196151_159283069_159236033_384	annotated	WDSUB1	2	-	1152	13	0	3	1.82%	3.72872648	0.007029303	0.057109797	0.002162931
ENSG00000113761_177022739_177064599_294	annotated	ZNF346	5	+	882	22	1	1	3.40%	8.241831842	0.003990669	0.003990669	8.78E-05
ENSG00000234741_173867041_173866181_50	novel	GAS5	1	-	150	20	0	0	10.00%	44.05633748	0.021557223	0.021557223	0.002115616
ENSG00000125746_45630001_45620943_276	novel	EML2	19	-	828	13	2	1	2.90%	5.187793363	0.033181033	0.010499663	0.001693572
ENSG00000175482_67353399_67351967_79	annotated	POLD4	11	-	237	28	1	4	12.66%	39.03726106	0.002321823	0.014292659	0.000192891
ENSG00000175482_67351998_67351834_54	Overlap_dORF	POLD4	11	-	162	7	1	1	9.26%	14.27751678	0.041782825	0.041782825	0.007200521
ENSG00000130021_7105752_7050296_179	annotated	PUDP	X	-	537	29	0	0	5.03%	17.8440473	0.003554776	0.003554776	7.04E-05
ENSG00000005007_18832210_18866163_1129	annotated	UPF1	19	+	3387	41	1	0	1.68%	3.999800347	7.84E-05	5.57E-05	3.23E-08
ENSG00000162377_52698317_52687720_228	annotated	COA7	1	-	684	14	4	0	3.51%	6.763034263	0.050311155	0.004149608	0.001233042
ENSG00000115204_27313101_27312392_132	novel	MPV17	2	-	396	71	2	2	13.64%	59.24242351	0.000232202	0.000150215	2.44E-07
ENSG00000111684_7018424_6977146_487	annotated	LPCAT3	12	-	1461	241	25	8	11.91%	54.50501711	4.81E-09	9.40E-11	5.56E-18
ENSG00000138744_75940949_75914904_359	annotated	NAAA	4	-	1077	173	8	8	8.36%	53.0762283	2.54E-06	6.36E-06	1.38E-10
ENSG00000161956_7565540_7571483_159	annotated	SENP3	17	+	477	27	2	1	6.29%	18.70316214	0.006112544	0.003974283	0.000131863
ENSG00000128791_9337230_9388044_75	annotated	TWSG1	18	+	225	51	3	2	22.67%	74.89577372	0.000285157	0.00029133	5.63E-07
ENSG00000128791_9396282_9399527_148	dORF	TWSG1	18	+	444	91	6	0	10.81%	67.72173498	0.000804886	0.000210762	1.16E-06
ENSG00000110057_68004043_67999515_185	novel	UNC93B1	11	-	555	45	1	4	5.95%	26.79101604	0.002197839	0.008093529	0.000101846
ENSG00000137522_71982779_71996315_240	annotated	RNF121	11	+	720	86	7	1	11.25%	39.46713566	2.53E-05	2.90E-06	6.35E-10
ENSG00000105204_39831867_39825715_601	annotated	DYRK1B	19	-	1803	16	3	0	1.33%	2.932202162	0.031335694	0.005306578	0.00089485
ENSG00000137074_32989888_32988108_48	annotated	APTX	9	-	144	38	1	1	14.58%	87.1948346	0.010275151	0.008877961	0.000459728
ENSG00000107789_87552209_87552478_89	novel	MINPP1	10	+	267	43	2	1	14.61%	53.2141155	0.001380936	0.000876741	7.49E-06
ENSG00000163479_156020896_156011768_163	novel	SSR2	1	-	489	1740	69	41	36.20%	1175.736614	1.97E-10	1.81E-11	4.35E-20
ENSG00000213639_28752125_28799303_327	annotated	PPP1CB	2	+	981	474	71	17	21.71%	159.6537	4.22E-11	8.57E-13	4.85E-22
ENSG00000160055_32222322_32217044_180	annotated	TMEM234	1	-	540	7	0	1	2.78%	4.283255033	0.019216965	0.041782825	0.003600526
ENSG00000111676_6934182_6941780_1179	annotated	ATN1	12	+	3537	31	7	4	1.10%	2.895984864	0.019625165	0.004918174	0.000513261
ENSG00000156976_186783611_186789269_407	annotated	EIF4A2	3	+	1221	115	12	18	7.86%	31.12087721	6.87E-06	2.30E-05	1.29E-09
ENSG00000106399_7640418_7637000_121	annotated	RPA3	7	-	363	293	13	10	20.66%	266.7046877	1.94E-05	5.96E-06	9.52E-10

ENSG00000196547_90904208_90913744_938	annotated	MAN2A2	15	+	2814	28	4	1	1.60%	3.287786379	0.002868236	0.00042095	8.01E-06
ENSG00000140299_59682457_59677897_161	novel	BNIP2	15	-	483	41	6	4	10.56%	28.04828939	0.000539314	0.000374489	1.33E-06
ENSG00000124067_67968553_67945365_1011	novel	SLC12A4	16	-	3033	74	19	6	3.26%	8.061743258	0.000416883	3.39E-06	1.47E-08
ENSG00000243678_51168317_51171604_85	novel	NME2	17	+	255	478	33	10	30.59%	619.380274	7.26E-06	4.86E-06	2.94E-10
ENSG00000108107_55386379_55388332_130	annotated	RPL28	19	+	390	981	146	35	23.08%	831.1397513	2.47E-05	2.09E-06	4.56E-10
ENSG00000107077_6792989_7170040_1047	annotated	KDM4C	9	+	3141	20	0	2	1.05%	2.103932067	0.001398493	0.00376808	3.12E-05
ENSG00000100387_40951399_40972488_108	annotated	RBX1	22	+	324	268	31	14	36.11%	273.312464	1.92E-06	1.87E-07	3.44E-12
ENSG00000168569_62791811_62772106_76	annotated	TMEM223	11	-	228	30	1	0	7.89%	43.47664883	0.013640586	0.013640586	0.000898637
ENSG00000074201_77637714_77619628_237	annotated	CLNS1A	11	-	711	152	8	9	15.19%	70.63885335	7.62E-07	3.49E-07	2.45E-12
ENSG00000205531_2964682_2954521_145	novel	NAP1L4	11	-	435	72	9	11	12.41%	54.69062584	0.001571697	0.00104976	1.01E-05
ENSG00000165916_47426231_47424586_120	novel	PSMC3	11	-	360	79	6	1	19.17%	72.50938877	2.79E-05	1.23E-05	2.73E-09
ENSG00000171204_85628608_85631186_40	annotated	TMEM126B	11	+	120	15	8	2	15.00%	41.30281639	0.160796588	0.037178614	0.024843756
ENSG00000171204_85631759_85634305_89	dORF	TMEM126B	11	+	267	26	8	4	8.99%	32.17597681	0.035987166	0.01778219	0.002903079
ENSG00000163636_64018895_64010909_77	annotated	PSMD6	3	-	231	64	0	0	18.18%	91.54563633	0.000465689	0.000465689	1.42E-06
ENSG00000163636_64018630_64010668_68	Overlap_dORF	PSMD6	3	-	204	26	15	2	7.35%	42.11267553	0.238211026	0.022899795	0.027697336
ENSG00000100097_37675703_37677525_30	novel	LGALS1	22	+	90	226	8	9	56.67%	829.7276892	0.000486477	0.00015692	5.30E-07
ENSG00000111540_55986961_55990155_123	novel	AC034102.1	12	+	369	19	7	1	5.69%	17.01362626	0.105552673	0.008776163	0.005179823
ENSG00000257390_55820382_55820278_34	Overlap_dORF	AC023055.1	12	-	102	18	3	0	17.65%	58.30985843	0.022004978	0.011772051	0.001241747
ENSG00000015676_44485263_44484600_106	novel	NUDCD3	7	-	318	25	0	4	6.60%	25.97661408	0.008776163	0.024197104	0.001052232
ENSG00000050426_51056368_51059431_100	novel	LETMD1	12	+	300	11	0	6	5.00%	12.11549281	0.019216965	0.216602931	0.02179448
ENSG00000079432_42284765_42295191_1588	annotated	CIC	19	+	4764	60	2	2	1.70%	4.161492835	3.46E-06	2.52E-06	7.47E-11
ENSG00000181852_56210361_56206447_218	annotated	RNF41	12	-	654	26	6	2	5.05%	13.13606393	0.034237244	0.006753026	0.001203038
ENSG00000100664_103335739_103335924_61	Overlap_uORF	EIF5	14	+	183	13	9	0	9.84%	23.47263882	0.182638022	0.012798403	0.013255577
ENSG00000128915_60453677_60442490_374	novel	ICE2	15	-	1122	14	1	0	1.87%	4.122919283	0.011738695	0.008373824	0.000494661
ENSG00000196465_56155129_56157726_116	novel	MYL6B	12	+	348	28	2	6	10.34%	26.58572089	0.002336818	0.012521771	0.000168776
ENSG00000204104_238325860_238398919_544	annotated	TRAF3IP1	2	+	1632	7	0	0	1.10%	1.417253504	0.009815329	0.009815329	0.000483714
ENSG00000198924_113852804_113835152_914	annotated	DCLRE1A	10	-	2742	7	3	0	0.55%	0.843529438	0.170087137	0.019216965	0.016246064
ENSG00000020256_52191636_52084557_645	annotated	ZFP64	20	-	1935	10	0	0	1.09%	1.70760998	0.007593099	0.007593099	0.000297833
ENSG00000198886_11164_11412_82	internal	MT-ND4	MT	+	246	147	73	147	43.90%	197.4476101	0.003395035	0.368107553	0.015686132
ENSG00000198886_11557_11856_99	internal	MT-ND4	MT	+	297	338	138	103	32.32%	376.0364159	0.200495704	0.005256724	0.008131147
ENSG00000132792_37732930_37872013_536	annotated	CTNNBL1	20	+	1608	53	8	1	3.54%	10.89079238	0.001391185	7.69E-05	8.30E-07
ENSG00000100577_77327502_77331195_161	annotated	GSTZ1	14	+	483	25	0	0	8.07%	17.10261548	0.00060914	0.00060914	2.39E-06
ENSG00000100207_42215032_42168653_1869	annotated	TCF20	22	-	5607	33	1	0	0.59%	1.944701895	0.001753471	0.001580611	1.65E-05
ENSG00000081692_227735273_227732392_401	annotated	JMJD4	1	-	1203	19	0	6	2.00%	5.218643467	0.005554612	0.075179667	0.002457728
ENSG00000185803_144359294_144361015_445	annotated	SLC52A2	8	+	1335	171	17	4	12.13%	42.32378488	2.17E-08	1.89E-10	5.16E-17
ENSG00000129197_5421311_5428078_74	annotated	RPAIN	17	+	222	62	17	0	16.22%	92.28016635	0.016702523	0.00107863	0.000119708
ENSG00000215271_23285952_23275575_550	annotated	HOMEZ	14	-	1650	19	0	0	2.00%	3.80486551	0.001440978	0.001440978	1.25E-05
ENSG00000196642_136829405_136840968_394	annotated	RABL6	9	+	1182	25	13	1	3.05%	6.988632215	0.131547658	0.001573472	0.001992047
ENSG00000121774_32014284_32042624_347	novel	KHDRBS1	1	+	1041	217	13	9	17.29%	68.87770341	4.08E-11	1.24E-10	5.98E-20
ENSG00000122203_176352640_176347600_200	annotated	KIAA1191	5	-	600	14	0	0	2.50%	7.709859059	0.021084099	0.021084099	0.002029891
ENSG00000122203_176361710_176361648_20	uORF	KIAA1191	5	-	60	14	5	5	30.00%	77.09859059	0.101229348	0.135090548	0.046386232
ENSG00000017260_130934676_131001350_823	annotated	ATP2C1	3	+	2469	90	13	6	5.22%	12.04456371	6.15E-07	1.57E-07	9.16E-13
ENSG00000141682_59900178_59902753_54	annotated	PMAIP1	18	+	162	37	18	0	16.67%	75.46687439	0.077021773	0.003632191	0.001829731
ENSG00000100316_39315411_39314080_110	novel	RPL3	22	-	330	164	22	12	17.27%	164.2099852	0.000240426	9.23E-05	1.59E-07
ENSG00000168118_229271355_229288847_71	novel	RAB4A	1	+	213	13	0	0	8.45%	20.16663335	0.013428348	0.013428348	0.000872559
ENSG00000188603_28489291_28477516_338	Overlap_dORF	CLN3	16	-	1014	124	13	32	11.24%	40.40670006	7.63E-07	2.59E-06	1.77E-11
ENSG00000198954_68989719_69016416_487	Overlap_dORF	KIF1BP	10	+	1461	55	4	1	4.93%	12.43890432	5.44E-05	1.03E-05	4.55E-09
ENSG00000243989_51987406_51987645_45	novel	ACY1	3	+	135	17	2	0	13.33%	41.60876318	0.022004978	0.011772051	0.001241747
ENSG00000188603_28489349_28474155_316	annotated	CLN3	16	-	948	110	13	4	9.18%	38.34016711	1.52E-05	1.01E-06	1.40E-10
ENSG00000188603_28491764_28489349_55	Overlap_uORF	CLN3	16	-	165	18	0	12	14.55%	36.0460943	0.005554612	0.310595281	0.015978689
ENSG00000198954_68989845_69016416_484	Overlap_dORF	KIF1BP	10	+	1452	55	4	1	4.96%	12.51600497	5.44E-05	1.03E-05	4.55E-09
ENSG00000198954_68988833_69000424_189	annotated	KIF1BP	10	+	567	14	3	0	4.23%	8.158581015	0.038565921	0.005160369	0.001093753
ENSG00000116337_109621108_109631152_804	annotated	AMPD2	1	+	2412	8	6	0	0.75%	1.095928793	0.280084718	0.011570466	0.021802746
ENSG00000197535_52425891_52313696_1697	annotated	MYO5A	15	-	5091	21	8	1	0.77%	1.362968602	0.054187382	0.001044243	0.000464309
ENSG00000151665_46615164_46614916_82	novel	PIGF	2	-	246	22	4	0	8.54%	29.54998246	0.013239535	0.007979402	0.000531733
ENSG00000179335_74625815_74629883_269	dORF	CLK3	15	+	807	20	0	1	2.97%	8.188910313	0.004873904	0.005622352	0.000147222
ENSG00000111907_125220146_125262843_102	annotated	TPD52L1	6	+	306	31	7	0	6.86%	33.47417799	0.104741188	0.008574016	0.005043772
ENSG00000105223_40371694_40374642_113	novel	PLD3	19	+	339	95	21	3	20.35%	92.59628453	0.000300308	2.77E-05	6.56E-08
ENSG00000133316_62839142_62835608_112	novel	WDR74	11	-	336	42	3	1	12.50%	41.30281639	0.000834706	0.000507411	2.73E-06
ENSG00000157985_235744785_236124122_696	annotated	AGAP1	2	+	2088	10	0	2	0.86%	1.582483387	0.011570466	0.056853419	0.003221159
ENSG00000017260_130934676_131001350_823	annotated	ATP2C1	3	+	2469	90	13	6	5.22%	12.04456371	6.15E-07	1.57E-07	9.16E-13
ENSG00000120053_99430565_99420609_104	novel	GOT1	10	-	312	29	0	0	11.54%	30.71235065	0.000978918	0.000978918	5.96E-06
ENSG00000084754_26244596_26234287_82	novel	HADHA	2	-	246	24	3	1	7.32%	32.2363445	0.030784922	0.013428348	0.001944056
ENSG00000084754_26234354_26212501_242	novel	HADHA	2	-	726	73	11	0	7.44%	33.22430409	0.001513887	9.02E-05	1.04E-06
ENSG00000178053_158592471_158605202_255	annotated	MLF1	3	+	765	24	0	0	3.14%	10.36619705	0.005655336	0.005655336	0.0001704
ENSG00000116871_36179389_36180258_154	novel	MAP7D1	1	+	462	11	1	0	3.25%	7.867203122	0.035772986	0.021084099	0.003354605
ENSG00000106290_100111297_100110146_95	novel	TAF6	7	-	285	33	0	4	10.53%	38.25945097	0.002390426	0.005380077	7.28E-05
ENSG00000127483_20780440_20776593_117	novel	HP1BP3	1	-	351	74	11	6	13.68%	69.66173021	0.005154867	0.001291985	4.00E-05

ENSG00000173418_20026786_20033187_121	novel	NAA20	20	+	363	202	28	0	28.10%	183.8714911	1.53E-05	1.71E-07	2.82E-11
ENSG00000145349_113513352_113457331_171	annotated	CAMK2D	4	-	513	10	0	0	3.51%	6.440985012	0.012798403	0.012798403	0.000797244
ENSG00000214413_110900497_110900360_45	novel	BBIP1	10	-	135	15	0	4	11.11%	36.71361457	0.021084099	0.11411552	0.011046874
ENSG00000157193_53250842_53247037_77	annotated	LRP8	1	-	231	13	6	1	10.39%	18.59520738	0.175505527	0.010020958	0.010614415
ENSG00000185651_21592888_21621669_136	annotated	UBE2L3	22	+	408	38	2	5	7.35%	30.77464751	0.005059698	0.016039993	0.000427873
ENSG00000082438_164695723_164685946_572	annotated	COBLL1	2	-	1716	12	1	0	1.05%	2.310647071	0.026602765	0.011570466	0.001474889
ENSG00000129103_56079529_56079612_27	internal	SUMF2	7	+	81	23	8	1	18.52%	93.82368167	0.230298309	0.028889786	0.031203709
ENSG00000129103_56064312_56079871_339	annotated	SUMF2	7	+	1017	504	56	33	13.86%	163.749219	2.63E-07	3.28E-07	8.06E-13
ENSG00000114850_156555089_156543250_174	annotated	SSR3	3	-	522	256	32	10	19.54%	162.0462988	1.77E-06	1.05E-06	1.64E-11
ENSG00000130414_240011687_239961118_129	novel	NDUFA10	2	-	387	39	7	2	8.53%	33.29839461	0.013523718	0.002252091	0.000177243
ENSG00000106443_10974324_11169490_948	annotated	PHF14	7	+	2844	68	1	5	3.16%	7.900398072	8.84E-07	7.76E-06	6.20E-11
ENSG00000132004_12694598_12689216_196	dORF	FBXW9	19	-	588	5	0	0	2.55%	2.809715401	0.012673659	0.012673659	0.000782701
ENSG00000130175_11448275_11449412_142	novel	PRKCSH	19	+	426	182	14	0	19.01%	141.1664335	1.35E-05	2.68E-06	3.10E-10
ENSG00000147526_38757333_38840338_209	novel	TACC1	8	+	627	22	2	0	4.78%	11.59377302	0.006423651	0.002227676	8.15E-05
ENSG00000164949_94260461_94250310_282	annotated	GEM	8	-	846	14	0	2	2.84%	5.467985148	0.005160369	0.022898969	0.000627835
ENSG00000134108_5122466_5178713_177	Overlap_dORF	ARL8B	3	+	531	56	10	4	9.04%	34.84682061	0.001058985	0.000673753	4.50E-06
ENSG00000172331_134661610_134679031_225	annotated	BPGM	7	+	675	29	10	0	4.00%	14.19593097	0.151657651	0.003327803	0.004064471
ENSG00000154305_222621164_222665619_1861	annotated	MIA3	1	+	5583	96	10	4	2.58%	5.681634065	7.21E-08	4.09E-09	3.21E-15
ENSG00000131778_147254969_147294454_775	annotated	CHD1L	1	+	2325	34	1	2	1.94%	4.831985401	0.000437499	0.000743479	2.11E-06
ENSG00000245060_180831248_180831430_60	novel	LINC00847	5	+	180	13	0	5	8.33%	23.86384947	0.020613417	0.102951605	0.009698738
ENSG00000188157_1040676_1054981_1894	annotated	AGRN	1	+	5682	514	64	14	9.24%	29.89038736	4.49E-23	1.71E-28	2.03E-49
ENSG00000286192_2238060_2235122_162	dORF	AC069288.1	7	-	486	14	5	1	5.56%	9.518344518	0.05444419	0.005162103	0.001603268
ENSG00000286192_2242169_2238768_155	annotated	AC069288.1	7	-	465	29	8	6	7.10%	20.60699656	0.011978812	0.018230749	0.001050407
ENSG00000001036_143511634_143495707_467	annotated	FUCA2	6	-	1401	272	32	10	16.06%	64.15055565	2.38E-11	3.17E-13	1.04E-22
ENSG00000100031_24611082_24628839_569	annotated	GGT1	22	+	1707	14	1	1	1.41%	2.709968035	0.010079165	0.010079165	0.000508526
ENSG00000156869_99748666_99708898_592	annotated	FRRS1	1	-	1776	30	3	5	1.69%	5.581461674	0.012673659	0.018267211	0.001107983
ENSG00000110514_47274705_47328681_1513	annotated	MADD	11	+	4539	29	4	0	0.99%	2.111093501	0.002572424	0.000258933	4.65E-06
ENSG00000104723_15540431_15743570_276	annotated	TUSC3	8	+	828	173	25	2	19.93%	69.03755783	1.15E-07	5.42E-11	1.00E-16
ENSG00000104723_15743558_15764218_53	Overlap_dORF	TUSC3	8	+	159	9	2	0	11.32%	18.70316214	0.071345384	0.011772051	0.004172987
ENSG00000163931_53231526_53225899_289	Overlap_dORF	TKT	3	-	867	333	50	157	17.30%	126.9096919	5.61E-08	0.039208904	2.92E-07
ENSG00000163931_53255942_53230566_334	annotated	TKT	3	-	1002	1185	126	49	26.65%	390.7691611	2.09E-13	6.02E-15	1.80E-26
ENSG00000163931_53225818_53225756_20	dORF	TKT	3	-	60	44	0	0	30.00%	242.3098562	0.013428348	0.013428348	0.000872559
ENSG00000050748_180280561_180236384_339	annotated	MAPK9	5	-	1017	47	4	4	3.54%	15.27026447	0.003545596	0.013408897	0.000260661
ENSG00000139613_56189461_56163689_1130	annotated	SMARCC2	12	-	3390	77	10	7	2.83%	7.505172536	8.34E-06	1.50E-05	1.01E-09
ENSG00000169258_176599225_176596808_805	annotated	GPRIN1	5	-	2415	15	0	2	0.87%	2.052313858	0.008473674	0.027657845	0.001169005
ENSG00000184924_24793335_24790411_126	annotated	PTRHD1	2	-	378	53	13	1	11.90%	46.32908505	0.006053996	0.000414314	1.75E-05
ENSG00000008988_56074383_56069738_142	annotated	RPS20	8	-	426	141	24	4	9.86%	109.365204	0.003376726	0.001388211	2.78E-05
ENSG00000100934_39096118_39094978_85	annotated	SEC23A	14	-	255	27	0	0	15.29%	34.98591506	0.000585477	0.000585477	2.21E-06
ENSG00000259030_74198279_74543982_936	annotated	FPGT-TNNI3K	1	+	2808	17	2	0	0.64%	2.000421307	0.052581193	0.01300724	0.003266469
ENSG00000138796_108033232_108034357_59	dORF	HADH	4	+	177	24	3	0	11.86%	44.80305507	0.033503887	0.008776163	0.001467425
ENSG00000138796_107989957_108027967_237	annotated	HADH	4	+	711	39	6	0	6.33%	18.12444264	0.003337626	0.000294865	6.87E-06
ENSG00000160131_151397309_151405058_101	annotated	VMA21	X	+	303	34	12	0	11.88%	37.0771157	0.034399923	0.000958906	0.000250084
ENSG00000165675_130679569_130625314_437	annotated	ENOX2	X	-	1311	7	0	3	1.14%	1.764269808	0.019216965	0.170087137	0.016246064
ENSG00000109332_102809680_102797415_110	annotated	UBE2D3	4	-	330	42	2	0	8.18%	42.05377669	0.003710481	0.003710481	7.64E-05
ENSG00000176422_56468592_56469577_207	annotated	SPRYD4	12	+	621	22	6	2	4.83%	11.70579015	0.035475746	0.006441753	0.001200923
ENSG00000142676_23692636_23696373_167	annotated	RPL11	1	+	501	389	51	12	29.34%	256.555618	3.61E-09	1.93E-09	7.52E-17
ENSG00000185414_99186204_99195705_161	annotated	MRPL30	2	+	483	24	7	1	4.35%	16.41851086	0.136660839	0.008473674	0.006887839
ENSG00000170677_70324888_70326276_462	annotated	SOCS6	18	+	1386	78	13	4	6.28%	18.59520738	6.52E-05	8.28E-06	4.45E-09
ENSG00000248919_99460131_99441444_29	annotated	ATP5MF-PTCD1	7	-	87	80	21	0	17.24%	303.8368102	0.065398531	0.021084099	0.006119907
ENSG00000115211_27369534_27364400_492	annotated	EIF2B4	2	-	1476	86	19	9	6.10%	19.2522613	0.000752018	5.57E-05	3.23E-07
ENSG00000067066_230416297_230542946_885	annotated	SP100	2	+	2655	23	6	0	1.81%	2.862417407	0.010324917	0.000141591	1.31E-05
ENSG00000198677_95520795_95516901_138	novel	TTC37	5	-	414	10	1	0	4.35%	7.981220558	0.026832696	0.011772051	0.001510382
ENSG00000049656_1338924_1318369_324	annotated	CLPTM1L	5	-	972	108	13	2	9.26%	36.71361457	1.62E-05	2.06E-06	2.90E-10
ENSG00000170004_7889001_7911585_1966	annotated	CHD3	17	+	5898	68	15	2	1.73%	3.809551054	5.97E-05	5.10E-07	3.27E-10
ENSG00000205531_2969823_2954399_177	annotated	NAP1L4	11	-	531	75	7	11	11.86%	46.66984903	0.00039037	0.000415588	1.07E-06
ENSG00000117523_171513048_171591847_2764	annotated	PRRC2C	1	+	8292	62	3	12	1.12%	2.470597797	1.15E-06	0.000107548	1.27E-09
ENSG00000198467_35685547_35683159_158	novel	TPM2	9	-	474	131	16	16	20.89%	91.31930712	8.03E-06	5.60E-06	3.72E-10
ENSG00000198824_114323843_114326281_812	annotated	CHAMP1	13	+	2436	42	15	7	2.09%	5.696940192	0.012310942	0.003034945	0.000208285
ENSG00000134324_11765542_11824791_926	annotated	LPIN1	2	+	2778	84	14	4	3.89%	9.991178047	2.54E-05	4.77E-07	1.22E-10
ENSG00000122705_36191057_36211774_230	annotated	CLTA	9	+	690	633	144	14	21.74%	303.1267568	8.80E-07	1.20E-09	1.49E-14
ENSG00000157193_53271061_53247018_557	annotated	LRP8	1	-	1671	53	18	6	4.85%	10.480188	0.009009024	4.38E-05	4.37E-06
ENSG00000127022_179705682_179708253_61	annotated	CANX	5	+	183	450	52	20	50.82%	812.5144208	2.14E-06	1.39E-06	2.62E-11
ENSG00000017260_130964044_130992947_204	annotated	ATP2C1	3	+	612	27	4	0	7.35%	14.57746461	0.000665918	0.000231967	1.04E-06
ENSG00000113580_143400839_143281889_778	annotated	NR3C1	5	-	2334	125	11	5	6.30%	17.69615098	2.61E-08	3.78E-10	1.20E-16
ENSG00000120254_150865823_151092556_979	annotated	MTHFD1L	6	+	2937	50	7	0	2.25%	5.625170771	0.000310012	1.40E-05	3.68E-08
ENSG00000185813_81909550_81904833_342	annotated	PCYT2	17	-	1026	41	4	2	4.97%	13.20401927	0.000508205	0.000153188	5.41E-07
ENSG00000145734_71458826_71545240_2101	novel	BDP1	5	+	6303	24	4	2	0.67%	1.258153379	0.004783653	0.002228483	6.07E-05
ENSG00000023228_206153678_206124185_727	annotated	NDUFS1	2	-	2181	172	26	4	8.94%	26.05808132	3.57E-10	4.56E-12	2.15E-20

ENSG00000112855_140691649_140698552_473	annotated	HARS2	5	+	1419	39	1	1	4.02%	9.081380348	7.80E-05	7.80E-05	4.45E-08
ENSG00000021574_32064090_32147257_433	annotated	SPAST	2	+	1299	43	6	0	3.46%	10.93777432	0.006425416	0.000284266	1.36E-05
ENSG00000127990_94656098_94628187_134	novel	SGCE	7	-	402	76	19	5	10.45%	62.46794121	0.01361479	0.001958185	0.000158305
ENSG00000127990_94618900_94585512_245	novel	SGCE	7	-	735	117	8	4	13.88%	52.5978723	5.55E-06	4.67E-07	2.42E-11
ENSG00000127990_94656098_94597920_436	novel	SGCE	7	-	1308	197	28	11	10.78%	49.76547296	1.02E-06	3.22E-08	3.47E-13
ENSG00000141756_41821026_41822408_137	novel	FKBP10	17	+	411	114	4	2	23.36%	91.65004513	1.86E-06	8.41E-07	1.39E-11
ENSG00000196526_7793808_7763765_386	novel	AFAP1	4	-	1158	8	0	2	1.55%	2.282711787	0.009815329	0.065834008	0.0033046
ENSG00000172845_173955605_173907955_408	annotated	SP3	2	-	1224	43	3	1	3.92%	11.60798108	0.000412614	0.00026641	7.40E-07
ENSG00000213551_73247189_73243400_260	annotated	DNAJC9	10	-	780	28	10	5	2.69%	11.86132163	0.171390856	0.039474169	0.027868881
ENSG00000148337_128185630_128166197_674	annotated	CIZ1	9	-	2022	101	4	4	4.90%	16.50478518	2.71E-07	1.99E-06	5.08E-12
ENSG00000109390_139295798_139292550_76	novel	NDUFC1	4	-	228	129	21	8	26.32%	186.94959	0.000474984	7.52E-05	2.62E-07
ENSG00000173702_124922235_124908147_277	annotated	MUC13	3	-	831	10	10	0	2.17%	3.976203744	0.320606914	0.012798403	0.028197542
ENSG00000168268_52533524_52524470_486	annotated	NT5DC2	3	-	1458	63	16	7	5.56%	14.27751678	0.000121629	3.60E-05	3.31E-08
ENSG00000124570_2959332_2948298_376	annotated	SERPINB6	6	-	1128	227	41	8	16.76%	66.49460511	3.99E-09	2.68E-11	1.42E-18
ENSG00000275395_39934618_39863487_4204	annotated	FCGBP	19	-	12612	25	2	0	0.21%	0.654976473	0.009257958	0.003529166	0.000178263
ENSG00000140990_1959625_1961734_168	annotated	NDUFB10	16	+	504	285	64	14	23.81%	186.8460741	2.45E-07	3.08E-07	7.05E-13
ENSG00000100485_50204316_50118344_1239	annotated	SOS2	14	-	3717	27	11	2	1.29%	2.400163664	0.024841604	0.000450524	9.38E-05
ENSG00000106003_2524655_2527212_248	annotated	LFNG	7	+	744	22	5	0	2.42%	9.770558716	0.020342454	0.013428348	0.001295518
ENSG00000161847_10333507_10317474_606	annotated	RAVER1	19	-	1818	44	1	1	2.48%	7.997024956	0.000299491	0.00036124	7.27E-07
ENSG00000142892_77219402_77154436_332	annotated	PIGK	1	-	996	111	13	2	13.55%	36.82419774	3.27E-07	5.70E-09	2.11E-14
ENSG00000144366_188483442_188594011_291	annotated	GULP1	2	+	873	66	5	2	8.59%	24.98039754	1.30E-05	1.60E-05	1.67E-09
ENSG00000144366_188292859_188292996_45	uORF	GULP1	2	+	135	10	0	0	11.11%	24.47574305	0.020613417	0.020613417	0.001946209
ENSG00000118257_205683297_205743579_553	annotated	NRP2	2	+	1659	86	16	2	4.88%	17.12859414	0.000276422	7.15E-06	1.79E-08
ENSG00000162909_223771817_223772269_65	novel	CAPN2	1	+	195	60	1	4	18.46%	101.6684711	0.001355887	0.008897471	7.35E-05
ENSG00000143164_167936912_168075435_951	annotated	DCAF6	1	+	2853	108	19	3	4.00%	12.50810843	1.98E-06	1.07E-07	2.12E-12
ENSG00000143164_167936912_168075435_880	annotated	DCAF6	1	+	2640	113	19	3	4.55%	14.14308561	8.48E-07	4.64E-08	4.00E-13
ENSG00000143158_167935775_167918323_105	annotated	MPC2	1	-	315	86	26	1	16.19%	90.2105958	0.006376796	0.000181954	9.26E-06
ENSG00000077150_102395960_102402376_900	annotated	NFKB2	10	+	2700	35	4	0	2.00%	4.283255033	0.000120212	7.13E-05	6.24E-08
ENSG00000249859_127794565_127890868_149	novel	PVT1	8	+	447	10	1	0	3.36%	7.392002933	0.035374934	0.020613417	0.003252526
ENSG00000165912_47182727_47178969_320	annotated	PACSIN3	11	-	960	19	7	2	1.88%	6.539612595	0.130134391	0.030550465	0.016991467
ENSG00000257923_101816052_102283090_669	annotated	CUX1	7	+	2007	76	19	1	5.83%	12.51226326	4.58E-05	1.57E-08	1.21E-11
ENSG00000172171_30906198_30900501_171	annotated	TEFM	17	-	513	19	0	1	4.09%	12.23787152	0.008473674	0.013892891	0.00058868
ENSG00000091640_4967804_4959534_227	annotated	SPAG7	17	-	681	95	6	1	11.89%	46.09418569	7.44E-06	2.88E-06	1.82E-10
ENSG00000161813_50401011_50475864_653	annotated	LARP4	12	+	1959	31	1	2	2.14%	5.228738369	0.000631096	0.0004093	1.69E-06
ENSG00000178381_1160132_1158247_59	uORF	ZFAND2A	7	-	177	24	0	0	8.47%	44.80305507	0.021084099	0.021084099	0.002029891
ENSG00000136560_161179663_161204826_119	annotated	TANK	2	+	357	10	3	0	5.04%	9.255533084	0.100002646	0.012798403	0.006484129
ENSG00000072778_7220628_7225097_579	dORF	ACADVL	17	+	1737	430	53	11	14.34%	81.79717235	3.07E-11	5.88E-15	3.29E-24
ENSG00000170291_7254674_7258930_170	annotated	ELP5	17	+	510	36	5	0	6.47%	23.32394337	0.002944156	0.001558047	2.70E-05
ENSG00000141524_78126295_78113148_721	annotated	TMC6	17	-	2163	30	0	0	2.08%	4.58283677	0.000259634	0.000259634	4.60E-07
ENSG00000117335_207752091_207752192_33	uORF	CD46	1	+	99	18	6	7	21.21%	60.07682384	0.095084229	0.079167839	0.027185036
ENSG00000117335_207752213_207793595_349	annotated	CD46	1	+	1047	224	29	14	14.90%	70.69211745	1.24E-08	1.22E-08	1.56E-15
ENSG00000089195_5950405_5938535_497	annotated	TRMT6	20	-	1491	64	7	1	5.43%	14.18312675	5.68E-05	2.95E-06	1.49E-09
ENSG00000163702_9917316_9933593_720	annotated	IL17RC	3	+	2160	42	22	0	1.67%	6.424882549	0.106041272	0.001040526	0.001109876
ENSG00000110514_47274705_47328681_1411	annotated	MADD	11	+	4233	30	4	0	1.06%	2.341761383	0.002488688	0.000276859	4.76E-06
ENSG00000168385_241324233_241336177_139	novel	2-Sep	2	+	417	70	15	0	12.95%	55.46661194	0.004214721	9.14E-05	3.26E-06
ENSG00000003096_117920277_117898908_544	annotated	KLHL13	X	-	1632	9	0	0	1.10%	1.822183076	0.009815329	0.009815329	0.000483714
ENSG00000197713_210002662_210019791_228	annotated	RPE	2	+	684	86	4	0	7.89%	41.54435333	0.000466044	9.40E-05	3.15E-07
ENSG00000116127_73432246_73609612_3706	annotated	ALMS1	2	+	11118	11	6	1	0.16%	0.326915618	0.143514872	0.023672501	0.015574534
ENSG00000135698_82170175_82148731_160	annotated	MPHOSPH6	16	-	480	44	0	0	8.75%	30.28873202	0.000448121	0.000448121	1.32E-06
ENSG00000203668_241640254_241636033_137	annotated	CHML	1	-	411	14	1	2	3.65%	11.2552687	0.021084099	0.045845141	0.004277929
ENSG00000126768_48897749_48893729_222	annotated	TIMM17B	X	-	666	47	4	1	8.56%	23.31810655	7.81E-05	5.66E-05	3.27E-08
ENSG00000221968_61891381_61873814_417	annotated	FADS3	11	-	1251	53	11	15	5.52%	13.99871635	0.000234439	0.000614699	9.74E-07
ENSG00000076706_119313157_119310998_461	novel	AP002956.1	11	-	1383	15	0	0	1.08%	3.583758472	0.021557223	0.021557223	0.002115616
ENSG00000134809_57530138_57528717_73	annotated	TIMM10	11	-	219	667	50	16	54.79%	1006.35538	5.46E-07	1.20E-07	6.30E-13
ENSG00000188342_45120656_45283561_249	annotated	GTF2F2	13	-	747	65	7	2	6.43%	28.75162587	0.001513887	0.000517075	5.01E-06
ENSG00000156113_77019031_76877739_567	novel	KCNMA1	10	-	1701	28	8	0	2.82%	5.43905401	0.00411642	0.00017249	5.43E-06
ENSG00000047315_56994753_57030988_1020	annotated	POLR2B	4	+	3060	150	16	7	5.29%	16.1971829	4.48E-08	5.52E-10	2.99E-16
ENSG00000196497_24187579_24186203_199	novel	IPO4	14	-	597	10	1	0	4.02%	5.534715764	0.010437297	0.004149608	0.000232539
ENSG00000196497_24186173_24180442_743	novel	IPO4	14	-	2229	70	17	2	4.17%	10.37666091	0.000375143	5.17E-07	2.65E-09
ENSG00000243989_51984065_51989075_373	annotated	ACY1	3	+	1119	58	3	4	6.17%	17.12645827	2.12E-05	8.41E-05	1.38E-08
ENSG00000138028_27104366_27099784_132	annotated	CGREF1	2	-	396	17	1	0	6.06%	14.18480563	0.008219047	0.005454749	0.00023581
ENSG00000137693_102110849_102229940_454	annotated	YAP1	11	+	1362	85	19	6	5.73%	20.62108307	5.17E-05	2.60E-05	1.03E-08
ENSG00000065518_120596360_120601293_120	annotated	NDUFB4	3	+	360	362	25	8	25.00%	332.2582118	3.34E-06	8.32E-07	2.48E-11
ENSG00000165832_114942653_114959824_81	novel	TRUB1	10	+	243	9	0	0	6.17%	12.23787152	0.020613417	0.020613417	0.001946209
ENSG00000160633_5623377_5668291_791	annotated	SAFB	19	+	2373	53	10	1	3.54%	7.379854256	0.00016243	1.93E-06	3.17E-09
ENSG00000248643_66616721_66638578_118	novel	RBM14-RBM4	11	+	354	18	0	0	6.78%	16.80114565	0.005257623	0.005257623	0.000148366
ENSG00000158716_159781101_159782338_150	annotated	DUSP23	1	+	450	104	20	10	17.33%	76.3643183	5.85E-05	3.25E-05	1.44E-08
ENSG00000158716_159781101_159782338_150	annotated	DUSP23	1	+	450	104	20	10	17.33%	76.3643183	5.85E-05	3.25E-05	1.44E-08

ENSG00000014123_96521874_96553503_794	annotated	UFL1	6	+	2382	76	4	0	2.52%	10.54244852	6.70E-05	3.94E-05	1.99E-08
ENSG00000126088_45012266_45015358_298	annotated	UROD	1	+	894	63	6	10	9.06%	23.28480924	8.48E-06	8.42E-05	5.92E-09
ENSG00000198718_44962638_45073561_1701	annotated	TOGARAM1	14	+	5103	12	0	0	0.41%	0.777007716	0.008175611	0.008175611	0.000342518
ENSG00000198408_101813051_101786451_807	annotated	OGA	10	-	2421	59	9	7	2.97%	8.052428474	0.000295173	0.000114555	2.39E-07
ENSG00000104763_18084058_18057534_376	annotated	ASAH1	8	-	1128	515	37	6	17.02%	150.8578045	1.13E-10	1.03E-11	1.43E-20
ENSG00000188603_28491764_28489349_55	Overlap_uORF	CLN3	16	-	165	18	0	12	14.55%	36.0460943	0.005554612	0.310595281	0.015978689
ENSG00000188603_28489291_28477516_338	Overlap_dORF	CLN3	16	-	1014	120	13	32	10.65%	39.10325812	1.64E-06	5.92E-06	8.45E-11
ENSG00000103363_2777115_2771990_136	annotated	ELOB	16	-	408	253	31	4	24.26%	204.8943637	1.52E-06	3.72E-07	5.19E-12
ENSG00000132507_7309636_7311640_154	annotated	EIF5A	17	+	462	657	241	36	18.18%	469.8865865	0.000502315	1.88E-06	1.10E-08
ENSG00000109606_24532869_24517586_80	novel	DHX15	4	-	240	21	3	0	8.75%	28.91197147	0.045243965	0.008373824	0.001935094
ENSG00000127418_1022214_1025347_474	annotated	FGFRL1	4	+	1422	34	0	5	1.90%	7.900398072	0.003684287	0.006030645	0.000121311
ENSG00000111642_6591574_6587488_182	novel	CHD4	12	-	546	12	0	1	4.40%	7.262033651	0.004873904	0.008811045	0.00022752
ENSG00000102974_67611139_67637872_625	annotated	CTCF	16	+	1875	54	1	2	4.32%	9.516168896	2.77E-06	4.67E-06	1.10E-10
ENSG00000168036_41239196_41239342_48	dORF	CTNNB1	3	+	144	32	0	0	12.50%	73.42722914	0.013640586	0.013640586	0.000898637
ENSG00000168036_41224552_41238381_702	annotated	CTNNB1	3	+	2106	245	41	13	11.11%	38.43946824	1.99E-11	3.03E-13	8.30E-23
ENSG00000177830_904816_869858_362	annotated	CHID1	11	-	1086	134	13	0	12.71%	40.77036756	1.25E-08	1.19E-09	1.64E-16
ENSG00000187498_110210176_110205278_175	novel	COL4A1	13	-	525	18	1	0	5.71%	11.3287725	0.006432154	0.001801897	6.75E-05
ENSG00000088833_1466824_1463429_71	annotated	NSFL1C	20	-	213	37	1	0	15.49%	57.39734109	0.003990669	0.001269017	3.03E-05
ENSG00000104687_30727706_30679520_479	annotated	GSR	8	-	1437	314	52	17	15.45%	72.20088711	4.37E-10	5.50E-13	3.70E-21
ENSG00000104915_13150173_13144857_113	annotated	STX10	19	-	339	25	4	4	7.96%	24.3674433	0.029977737	0.024498662	0.003242696
ENSG00000171903_15922373_15913732_199	annotated	CYP4F11	19	-	597	33	0	0	6.53%	18.26456202	0.000669869	0.000669869	2.87E-06
ENSG00000129347_10565999_10553951_703	annotated	KRI1	19	-	2109	25	4	3	1.99%	3.91681521	0.006280399	0.003854732	0.000131611
ENSG00000214413_110918157_110900360_92	annotated	BBIP1	10	-	276	28	0	9	10.87%	33.52112635	0.002362849	0.039337095	0.000595371
ENSG00000026103_88990877_89014450_335	annotated	FAS	10	+	1005	31	5	0	4.18%	10.19213778	0.005286514	0.0004093	1.50E-05
ENSG00000106617_151574893_151557201_235	annotated	PRKAG2	7	-	705	25	0	3	3.40%	11.71711103	0.005604867	0.020990661	0.000616068
ENSG00000182379_57216970_57225747_308	annotated	NXPH4	12	+	924	41	5	1	3.25%	14.66160582	0.017322097	0.003783485	0.000359405
ENSG00000168438_110180445_110210919_280	novel	CDC40	6	+	840	9	3	0	1.79%	3.540241405	0.145248299	0.020613417	0.014232745
ENSG00000168438_110212210_110230131_311	novel	CDC40	6	+	933	18	0	5	2.89%	6.374711211	0.003529166	0.053174765	0.001156256
ENSG00000103253_727510_729092_227	annotated	HAGHL	16	+	681	42	4	7	5.73%	20.3784821	0.003908676	0.00391499	8.45E-05
ENSG00000030582_44350711_44352652_368	annotated	GRN	17	+	1104	993	97	9	24.73%	297.2007005	6.89E-13	4.95E-17	6.88E-28
ENSG00000011198_43690993_43718532_349	annotated	ABHD5	3	+	1047	36	0	0	4.01%	11.36123316	0.00040306	0.00040306	1.08E-06
ENSG00000185591_53381682_53411240_775	annotated	SP1	12	+	2325	38	19	8	2.71%	5.400454272	0.047957351	0.000278151	0.000148267
ENSG00000116237_6235911_6234445_116	annotated	ICMT	1	-	348	18	6	0	8.62%	17.09082057	0.053111416	0.002046029	0.000756049
ENSG00000116237_6233639_6225080_188	dORF	ICMT	1	-	564	74	2	2	12.77%	43.35331082	7.89E-06	1.26E-05	8.10E-10
ENSG00000113851_3179687_3150865_441	annotated	CRBN	3	-	1323	74	3	1	5.22%	18.48168352	1.13E-05	1.51E-05	1.38E-09
ENSG00000160211_154533662_154533510_50	novel	G6PD	X	-	150	32	0	0	18.00%	70.49013997	0.003736771	0.003736771	7.75E-05
ENSG00000078061_47563244_47565143_115	novel	ARAF	X	+	345	11	0	0	4.35%	10.53521114	0.019679754	0.019679754	0.001784964
ENSG00000171617_74636266_74634716_516	annotated	ENC1	5	-	1548	40	3	6	3.29%	8.5380499	0.000125843	0.001982636	1.87E-06
ENSG00000108587_30477434_30522378_250	annotated	GOSR1	17	+	750	67	13	0	9.60%	29.51774611	0.000498291	7.42E-06	3.52E-08
ENSG00000108588_63766175_63746881_483	annotated	CCDC47	17	-	1449	329	37	14	15.73%	75.02347325	2.60E-11	4.74E-14	1.91E-23
ENSG00000108591_18088024_18107240_364	annotated	DRG2	17	+	1092	40	4	2	4.95%	12.10338942	0.000703701	0.000178682	8.68E-07
ENSG00000108590_6651510_6644467_125	annotated	MED31	17	-	375	10	2	1	4.80%	8.811267496	0.060322713	0.023672501	0.006212924
ENSG00000138433_174381734_174348497_380	annotated	CIR1	2	-	1140	6	0	0	1.32%	1.739065953	0.016947427	0.016947427	0.001349898
ENSG00000106617_151574893_151557201_235	annotated	PRKAG2	7	-	705	25	0	3	3.40%	11.71711103	0.005604867	0.020990661	0.000616068
ENSG00000162836_147670027_147652612_264	annotated	ACP6	1	-	792	12	0	0	2.65%	5.006401987	0.008175611	0.008175611	0.000342518
ENSG00000111271_111692854_111756473_1042	annotated	ACAD10	12	+	3126	21	9	4	1.44%	2.219729096	0.004417975	0.007937724	0.000187685
ENSG00000108679_78976126_78971576_459	Overlap_dORF	LGALS3BP	17	-	1377	415	46	13	18.30%	99.58268004	3.74E-12	1.00E-14	5.78E-25
ENSG00000197256_11170230_11166558_108	novel	KANK2	19	-	324	15	4	1	5.56%	15.2973394	0.08410207	0.026832696	0.009673585
ENSG00000101266_489830_483961_167	annotated	CSNK2A1	20	-	501	47	1	0	8.38%	30.99772248	0.000475338	0.000472567	1.47E-06
ENSG00000134313_8818775_8730720_1672	dORF	KIDINS220	2	-	5016	48	10	2	1.61%	3.161938097	0.000833919	9.74E-06	7.90E-08
ENSG00000127483_20749723_20744797_173	novel	HP1BP3	1	-	519	12	2	5	4.05%	7.639827309	0.043639483	0.114955097	0.019792762
ENSG00000100284_35299929_35347209_492	annotated	TOM1	22	+	1476	50	0	0	4.88%	11.19317517	6.14E-06	6.14E-06	3.13E-10
ENSG00000116213_3649999_3630975_460	annotated	WRAP73	1	-	1380	47	2	2	4.78%	11.25352099	6.08E-05	2.24E-05	1.05E-08
ENSG00000179335_74622469_74629883_343	novel	CLK3	15	+	1029	23	3	1	3.21%	7.385537625	0.010677183	0.002061799	0.000128146
ENSG00000166747_71789479_71772991_176	annotated	AP1G1	16	-	528	17	2	0	3.98%	10.63860422	0.037995728	0.007979402	0.001544499
ENSG00000058272_79934925_79775929_1028	annotated	PPP1R12A	12	-	3084	43	12	4	2.43%	4.607058637	0.002299191	5.45E-05	1.07E-06
ENSG00000077232_182718162_182739556_307	novel	DNAJC10	2	+	921	46	17	1	6.19%	16.50318831	0.00752581	6.99E-05	5.12E-06
ENSG00000077232_182756320_182777132_240	novel	DNAJC10	2	+	720	83	2	5	7.92%	38.09037511	6.27E-05	0.000160295	7.35E-08
ENSG00000137776_58933565_58897141_177	annotated	SLTM	15	-	531	21	0	4	3.95%	13.06755773	0.008473674	0.052370108	0.00228699
ENSG00000137776_58894166_58879999_566	dORF	SLTM	15	-	1698	42	3	1	3.53%	8.172995469	7.84E-05	5.20E-05	3.03E-08
ENSG00000171735_6785531_6887725_101	annotated	CAMTA1	1	+	303	60	11	1	6.93%	65.43020418	0.024414995	0.01208853	0.001408254
ENSG00000005893_120469169_120431323_410	annotated	LAMP2	X	-	1230	181	14	18	12.93%	48.62315295	2.09E-08	6.41E-08	1.35E-14
ENSG00000166598_103942563_103943415_191	novel	HSP90B1	12	+	573	408	56	7	27.75%	235.2746818	1.47E-08	1.05E-10	1.98E-17
ENSG00000131165_89657588_89647038_181	novel	CHMP1A	16	-	543	56	11	9	7.18%	34.07672512	0.016657955	0.012607423	0.00100932
ENSG00000135655_62294252_62355375_167	novel	USP15	12	+	501	18	4	1	4.19%	11.87146818	0.053624202	0.011825808	0.00308201
ENSG00000166226_69585522_69588272_151	novel	CCT2	12	+	453	126	27	1	15.89%	91.90560468	0.000172052	8.57E-06	1.27E-08
ENSG00000161204_184186208_184187374_103	annotated	ABCF3	3	+	309	18	0	0	5.83%	19.24791443	0.01300724	0.01300724	0.000821865
ENSG00000164654_7572476_7607092_875	annotated	MIOS	7	+	2625	40	2	4	2.29%	5.035009998	8.97E-05	8.02E-05	5.24E-08

ENSG00000088970_21161973_21246576_504	annotated	KIZ	20	+	1512	5	0	0	0.99%	1.0926671	0.012673659	0.012673659	0.000782701
ENSG00000023171_123577464_123622595_698	annotated	GRAMD1B	11	+	2094	29	0	5	2.15%	4.576052246	0.000256142	0.006313333	1.22E-05
ENSG00000122783_135169443_135168981_33	uORF	CYREN	7	-	99	43	3	6	15.15%	143.5168569	0.035772986	0.124431937	0.018327857
ENSG00000129484_20343642_20357797_570	annotated	PARP2	14	+	1710	75	16	0	4.91%	14.49221628	0.000641603	1.47E-06	1.19E-08
ENSG00000165152_101476912_101475881_343	annotated	TMEM246	9	-	1029	26	2	0	2.92%	8.348868619	0.003737369	0.002227676	4.76E-05
ENSG00000125971_34516775_34540624_129	annotated	DYNLRB1	20	+	387	208	15	2	20.93%	177.5914379	6.32E-06	2.73E-06	1.47E-10
ENSG00000165152_101476912_101475881_343	annotated	TMEM246	9	-	1029	26	2	0	2.92%	8.348868619	0.003737369	0.002227676	4.76E-05
ENSG00000113845_119498734_119523756_151	annotated	TIMMDC1	3	+	453	129	19	4	16.56%	94.09383336	0.000205903	1.22E-05	2.10E-08
ENSG00000100823_20455646_20455854_36	novel	APEX1	14	+	108	12	2	13	13.89%	36.71361457	0.019679754	0.298056333	0.033498464
ENSG00000198805_20469525_20472569_90	novel	PNP	14	+	270	111	18	3	16.67%	135.8403739	0.007366305	0.000853138	4.03E-05
ENSG00000112592_170557030_170572265_288	annotated	TBP	6	+	864	12	0	1	1.74%	4.589201821	0.019216965	0.02170844	0.001914126
ENSG00000127990_94628318_94618957_77	novel	SGCE	7	-	231	20	0	0	6.49%	28.60801135	0.021557223	0.021557223	0.002115616
ENSG00000141349_44070966_44076043_346	annotated	G6PC3	17	+	1038	553	40	20	19.36%	176.0343542	2.22E-12	1.90E-11	5.34E-22
ENSG00000087191_63828138_63831969_398	annotated	PSMC5	17	+	1194	413	53	16	22.11%	114.2918805	4.23E-13	2.30E-15	1.49E-26
ENSG00000141551_82273381_82244781_415	annotated	CSNK1D	17	-	1245	97	12	1	9.40%	25.74376347	1.18E-06	1.88E-08	2.45E-13
ENSG00000139163_22625431_22684954_363	annotated	ETNK1	12	+	1089	63	4	8	6.89%	19.11535304	8.63E-06	5.69E-05	4.02E-09
ENSG00000178397_6348758_6341911_81	novel	FAM220A	7	-	243	13	1	2	6.17%	17.67692553	0.042234516	0.065398531	0.011057031
ENSG00000130726_58550255_58550553_68	novel	TRIM28	19	+	204	64	7	4	16.18%	103.6619705	0.001588175	0.005538787	5.17E-05
ENSG00000129667_76472919_76471633_217	novel	RHBDF2	17	-	651	12	0	0	2.76%	6.090737901	0.011772051	0.011772051	0.000681273
ENSG00000129667_76477283_76473001_365	novel	RHBDF2	17	-	1095	22	4	0	3.01%	6.638626196	0.012837471	0.001398493	0.00011155
ENSG00000099864_736069_746814_179	annotated	PALM	19	+	537	6	0	0	2.79%	3.691871856	0.016947427	0.016947427	0.001349898
ENSG00000089234_111683185_111644199_524	annotated	BRAP	12	-	1572	27	3	0	2.86%	5.675196145	0.002026908	0.000255448	3.57E-06
ENSG00000100294_43133396_43133043_117	dORF	MCAT	22	-	351	18	0	1	5.13%	16.94474519	0.01300724	0.023878474	0.001470914
ENSG00000100294_43143348_43133455_180	annotated	MCAT	22	-	540	12	3	0	2.78%	7.342722914	0.065398531	0.021084099	0.006119907
ENSG00000196704_68250188_68256969_360	annotated	AMZ2	17	+	1080	87	7	11	6.94%	26.61737056	8.60E-06	0.00022034	1.65E-08
ENSG00000105220_34399246_34399512_88	novel	GPI	19	+	264	97	1	10	11.36%	121.4052482	0.002460157	0.011089255	0.000155541
ENSG00000105220_34399721_34399789_22	novel	GPI	19	+	66	116	17	2	31.82%	580.7426304	0.053528973	0.01385614	0.003507582
ENSG00000160439_55063032_55045074_331	annotated	RDH13	19	-	993	32	5	1	3.32%	10.6480574	0.005065652	0.001779143	5.24E-05
ENSG00000214941_15999594_15978047_140	annotated	ZSWIM7	17	-	420	11	0	0	5.00%	8.653923434	0.007882058	0.007882058	0.000319636
ENSG00000145388_118691544_118710302_371	annotated	METTL14	4	+	1113	15	2	0	2.16%	4.453133843	0.026714995	0.004873904	0.000703162
ENSG00000133112_45339619_45337386_80	novel	TPT1	13	-	240	55	5	5	11.25%	75.72183005	0.008092227	0.004490072	0.000194132
ENSG00000120265_149749728_149772088_124	annotated	PCMT1	6	+	372	72	15	5	11.29%	63.95274796	0.006208942	0.001978304	7.08E-05
ENSG00000130429_99385715_99390016_167	novel	ARPC1B	7	+	501	150	12	9	11.38%	98.92890153	0.000178936	0.000839461	1.04E-06
ENSG00000171793_40983291_41009593_564	annotated	CTPS1	1	+	1692	39	20	4	3.19%	7.616122171	0.063011061	0.000674021	0.000406215
ENSG00000140443_98649582_98957442_1366	annotated	IGF1R	15	+	4098	129	21	11	3.88%	10.4012949	5.31E-08	1.46E-08	7.91E-15
ENSG00000171130_149873976_149878771_111	annotated	ATP6V0E2	7	+	333	83	4	2	11.71%	82.35756782	0.002787882	0.001699044	2.77E-05
ENSG00000168411_74661449_74623928_774	annotated	RFWD3	16	-	2322	75	11	3	4.39%	10.67256237	3.25E-05	7.64E-07	2.43E-10
ENSG00000107077_6792989_7174729_1056	annotated	KDM4C	9	+	3168	20	0	2	1.04%	2.086000828	0.001398493	0.00376808	3.12E-05
ENSG00000184924_24790518_24790411_35	novel	PTRHD1	2	-	105	30	9	0	20.00%	94.40643746	0.045644785	0.008574016	0.001991869
ENSG00000173193_122700559_122720490_1012	novel	PARP14	3	+	3036	17	2	0	0.89%	1.850192039	0.012941946	0.003452921	0.000245571
ENSG00000115286_1383735_1393407_270	annotated	NDUFS7	19	+	810	62	8	2	8.15%	25.29160115	0.000131318	4.63E-05	4.52E-08
ENSG00000273088_155172824_155172642_60	internal	AL713999.1	1	-	180	25	8	10	10.00%	45.89201821	0.060777753	0.117522225	0.026528193
ENSG00000273088_155169547_155169440_35	internal	AL713999.1	1	-	105	109	10	3	37.14%	343.0100561	0.003522457	0.000722178	1.60E-05
ENSG00000215301_41334253_41347719_662	annotated	DDX3X	X	+	1986	238	48	10	8.91%	39.59746345	4.34E-08	5.58E-11	3.54E-17
ENSG00000147687_124516028_124488594_229	annotated	TATDN1	8	-	687	27	0	0	4.80%	12.98603834	0.001520872	0.001520872	1.39E-05
ENSG00000138073_27134421_27131414_359	annotated	PREB	2	-	1077	48	5	9	6.13%	14.72635236	0.0001103	0.000744251	5.91E-07
ENSG00000154529_42129094_41893989_1288	annotated	CNTNAP3B	9	-	3864	54	6	1	1.09%	4.61770618	0.002519088	0.000610656	9.78E-06
ENSG00000173064_112273769_112162387_3807	annotated	HECTD4	12	-	11421	20	7	3	0.32%	0.578622767	0.022086057	0.005086831	0.000597245
ENSG00000173852_35037394_34931573_748	annotated	DPY19L1	7	-	2244	179	42	11	5.35%	26.35723399	7.75E-05	8.93E-08	9.69E-11
ENSG00000173852_35037402_35037313_29	Overlap_uORF	DPY19L1	7	-	87	13	0	1	17.24%	49.37348166	0.021084099	0.035772986	0.003354605
ENSG00000023171_123577464_123622595_698	annotated	GRAMD1B	11	+	2094	29	0	5	2.15%	4.576052246	0.000256142	0.006313333	1.22E-05
ENSG00000175334_66003293_66003772_75	annotated	BANF1	11	+	225	667	71	11	29.33%	979.5192367	5.17E-05	3.80E-05	1.48E-08
ENSG00000142634_15410269_15428720_177	annotated	EFHD2	1	+	531	105	5	3	8.47%	65.33778864	0.000533716	0.0005994	2.07E-06
ENSG00000157168_32595851_32763338_421	annotated	NRG1	8	+	1263	37	3	3	3.56%	9.67983662	0.000549623	0.000610154	2.17E-06
ENSG00000155097_103055914_103063249_76	novel	ATP6V1C1	8	+	228	30	2	0	10.53%	43.47664883	0.010275151	0.005756914	0.000308065
ENSG00000156787_123084185_123151939_927	annotated	TBC1D31	8	+	2781	29	3	0	1.29%	3.445614312	0.004573123	0.001003278	2.82E-05
ENSG00000124762_36684102_36685800_164	annotated	CDKN1A	6	+	492	199	22	8	18.29%	133.6465116	7.83E-06	1.02E-05	6.54E-10
ENSG00000198522_27629059_27650200_374	annotated	GPN1	2	+	1122	110	11	7	10.70%	32.3943658	2.12E-07	1.89E-07	3.78E-13
ENSG00000071889_154515772_154506811_230	annotated	FAM3A	X	-	690	42	5	2	7.39%	20.11267581	0.001484342	0.000439046	4.23E-06
ENSG00000102312_48512346_48520476_324	annotated	PORCN	X	+	972	17	0	0	2.78%	5.778994886	0.003452921	0.003452921	6.66E-05
ENSG00000147853_4722566_4712976_157	annotated	AK3	9	-	471	118	14	2	12.10%	82.78101629	0.000329232	0.000157394	3.59E-07
ENSG00000128989_52568892_52551934_112	annotated	ARPP19	15	-	336	41	5	5	11.61%	40.319416	0.004219867	0.00329539	7.73E-05
ENSG00000188690_125816499_125796854_160	annotated	UROS	10	-	480	44	0	1	9.38%	30.28873202	0.000300266	0.000300266	6.09E-07
ENSG00000133316_62839570_62832952_366	annotated	WDR74	11	-	1098	96	8	4	9.02%	28.88940163	1.74E-06	8.00E-07	1.24E-11
ENSG00000023734_15882714_15902978_361	annotated	STRAP	12	+	1083	198	41	3	13.57%	60.40965943	1.11E-06	1.00E-09	1.63E-14
ENSG00000100983_34951852_34928828_474	annotated	GSS	20	-	1422	165	32	3	10.97%	38.34016711	8.48E-07	4.73E-10	6.24E-15
ENSG00000197102_102039237_102048175_640	novel	DYNC1H1	14	+	1920	34	9	0	2.66%	5.851232322	0.004962094	0.000115129	4.76E-06
ENSG00000157193_53327912_53247018_790	annotated	LRP8	1	-	2370	75	21	13	4.81%	10.45640921	0.000598235	1.64E-05	8.57E-08

ENSG00000198363_61684117_61680939_58	novel	ASPH	8	-	174	341	57	19	41.38%	647.5522018	0.000854159	0.000331459	1.87E-06
ENSG00000070610_35739053_35738626_109	novel	GBA2	9	-	327	11	0	0	5.50%	11.11513102	0.009815329	0.009815329	0.000483714
ENSG00000107951_30341602_30313609_517	novel	MTPAP	10	-	1551	38	17	6	2.71%	8.095458532	0.06216577	0.005056323	0.001834163
ENSG00000101365_2664188_2660613_204	novel	IDH3B	20	-	612	76	6	1	13.24%	41.03286334	6.83E-06	3.08E-06	1.78E-10
ENSG00000101365_2660659_2658751_231	novel	IDH3B	20	-	693	137	3	7	13.85%	65.32162592	4.13E-07	2.92E-06	1.12E-11
ENSG00000140043_73860574_73884177_327	annotated	PTGR2	14	+	981	39	3	0	5.20%	13.13606393	0.0007246	0.000121023	6.26E-07
ENSG00000125952_65102339_65077412_94	annotated	MAX	14	-	282	18	14	3	8.51%	21.09079986	0.322636366	0.019759574	0.037435152
ENSG00000187720_71660710_71728763_79	novel	THSD4	15	+	237	18	1	0	10.13%	25.09538211	0.005306578	0.005160369	0.00014705
ENSG00000149554_125629285_125655320_362	annotated	CHEK1	11	+	1086	35	2	0	3.04%	10.6489766	0.003185952	0.001580611	2.96E-05
ENSG00000127990_94656098_94624214_141	novel	SGCE	7	-	423	76	19	5	9.93%	59.3666959	0.01361479	0.001958185	0.000158305
ENSG00000127990_94618900_94585465_278	novel	SGCE	7	-	834	125	8	5	12.95%	49.52376066	1.74E-06	2.28E-07	3.75E-12
ENSG00000147408_19506085_19505966_39	uORF	CSGALNACT1	8	-	117	24	10	1	20.51%	67.77898074	0.085401204	0.005604867	0.002874515
ENSG00000147408_19505744_19405780_502	annotated	CSGALNACT1	8	-	1506	122	4	13	7.37%	26.76729668	7.08E-08	1.45E-06	1.04E-12
ENSG00000001617_50173885_50188115_686	annotated	SEMA3F	3	+	2058	138	16	4	6.56%	22.15661287	3.22E-06	1.30E-08	5.16E-13
ENSG00000161999_684319_684050_63	novel	JMJD8	16	-	189	22	2	0	14.29%	38.46188193	0.013443806	0.003529166	0.000260295
ENSG00000114503_196941958_196937011_138	annotated	NCBP2	3	-	414	46	7	2	11.59%	36.71361457	0.00189368	0.000581555	7.00E-06
ENSG00000111057_52949174_52950414_167	novel	KRT18	12	+	501	352	15	16	8.38%	232.1531556	0.000482311	0.000483714	1.53E-06
ENSG00000111057_52950769_52952346_218	novel	KRT18	12	+	654	2132	133	172	16.97%	1077.157242	1.37E-06	4.25E-07	5.30E-12
ENSG00000065357_55939303_55953768_538	novel	DGKA	12	+	1614	23	2	0	2.23%	4.70862343	0.001459721	0.000862735	7.79E-06
ENSG00000168036_41224552_41239342_746	annotated	CTNNB1	3	+	2238	278	46	13	11.53%	41.0444431	4.82E-12	1.28E-14	9.50E-25
ENSG00000177189_20234787_20155398_708	annotated	RPS6KA3	X	-	2124	48	5	2	3.53%	7.467175844	6.12E-05	1.98E-05	9.41E-09
ENSG00000143878_20447466_20448056_196	annotated	RHOB	2	+	588	64	10	3	8.67%	35.96435713	0.000714149	0.000254593	1.22E-06
ENSG00000042753_46850184_46838447_158	annotated	AP2S1	19	-	474	314	27	14	26.58%	218.8874995	3.37E-07	3.58E-08	1.22E-13
ENSG00000090060_96547823_96552626_80	novel	PAPOLA	14	+	240	9	0	2	6.25%	12.39084492	0.020613417	0.051235217	0.004687384
ENSG00000198431_104309938_104348415_563	annotated	TXNRD1	12	+	1689	896	96	42	20.43%	175.2863161	5.01E-17	4.57E-19	3.82E-34
ENSG00000014216_65182702_65187230_201	annotated	CAPN1	11	+	603	74	3	0	9.95%	40.54936534	3.98E-05	4.01E-05	1.21E-08
ENSG00000040933_98535792_98587608_866	annotated	INPP4A	2	+	2598	11	2	1	0.58%	1.399017645	0.047790352	0.020239728	0.004303932
ENSG00000127990_94656098_94587779_446	annotated	SGCE	7	-	1338	211	28	12	11.88%	52.10699108	9.25E-08	3.64E-09	3.72E-15
ENSG00000090013_40465688_40447889_206	annotated	BLVRB	19	-	618	673	87	40	31.07%	359.8290671	4.27E-09	1.44E-10	7.35E-18
ENSG00000074266_86245230_86278525_441	annotated	EED	11	+	1323	22	0	2	2.49%	5.494554561	0.001513502	0.005530543	4.95E-05
ENSG00000105323_41268228_41306565_756	annotated	HNRNPUL1	19	+	2268	225	45	20	9.92%	32.78001301	1.26E-09	2.47E-12	4.57E-20
ENSG00000093183_42569065_42568979_28	novel	SEC22C	3	-	84	12	0	0	17.86%	47.20321873	0.019679754	0.019679754	0.001784964
ENSG00000204316_75904684_75901577_207	novel	MRPL38	17	-	621	73	3	9	11.11%	38.84194005	5.35E-05	0.000692788	2.86E-07
ENSG00000115355_55322644_55295064_1012	annotated	CCDC88A	2	-	3036	38	2	0	1.98%	4.13572338	0.000151706	3.51E-05	4.03E-08
ENSG00000106261_100023615_100034197_527	annotated	ZKSCAN1	7	+	1581	13	4	0	1.71%	2.716946809	0.04159736	0.002943991	0.00075602
ENSG00000104859_45040213_45052896_100	novel	CLASRP	19	+	300	17	1	0	7.00%	18.72394343	0.007979402	0.007979402	0.000327143
ENSG00000188157_1040676_1054981_1871	annotated	AGRN	1	+	5613	519	64	14	9.51%	30.55216349	1.55E-23	5.56E-29	2.31E-50
ENSG00000067955_67066700_67098778_87	novel	CBFB	16	+	261	136	2	6	14.94%	172.1741925	0.000712477	0.00450513	2.05E-05
ENSG00000067955_67029025_67066743_128	novel	CBFB	16	+	384	18	25	0	7.81%	15.48855615	0.207913715	0.002008257	0.004532299
ENSG00000070404_680318_681708_152	novel	FSTL3	19	+	456	52	9	3	13.16%	37.67976232	0.000121361	0.00010699	9.31E-08
ENSG00000183527_39180416_39175590_201	dORF	PSMG1	21	-	603	64	10	0	9.95%	35.06972138	0.000579485	4.05E-05	1.84E-07
ENSG00000145592_40835185_40832504_97	annotated	RPL37	5	-	291	1360	247	61	32.99%	1544.242757	8.98E-05	3.67E-06	2.95E-09
ENSG00000092931_76738413_76778352_377	annotated	MFSD11	17	+	1131	78	4	4	7.69%	22.78776077	8.74E-06	6.83E-06	4.92E-10
ENSG00000165861_73024460_72970882_747	annotated	ZFYVE1	14	-	2241	22	0	0	1.34%	3.243773175	0.002321823	0.002321823	3.12E-05
ENSG00000163946_56633964_56623037_728	novel	TASOR	3	-	2184	32	0	1	2.06%	4.841355767	0.000283519	0.0003684	7.03E-07
ENSG00000164466_175492104_175511550_177	annotated	SFXN1	5	+	531	95	9	5	16.38%	59.1151421	3.22E-06	2.25E-06	6.23E-11
ENSG00000161956_7563077_7571483_574	annotated	SENP3	17	+	1722	50	5	1	3.83%	9.594150149	0.000155523	2.31E-05	2.81E-08
ENSG00000129116_168898634_168926385_379	novel	PALLD	4	+	1137	81	11	3	8.97%	23.53933599	4.60E-06	5.82E-07	2.45E-11
ENSG00000123144_12734657_12730961_150	annotated	TRIR	19	-	450	47	10	3	10.00%	34.51079769	0.006514663	0.001618607	6.21E-05
ENSG00000081870_53940082_53939981_33	novel	HSPB11	1	-	99	10	3	0	18.18%	33.37601324	0.150401371	0.011772051	0.009827015
ENSG00000166484_19378901_19379951_133	novel	MAPK7	17	+	399	8	0	0	3.76%	6.625013155	0.016947427	0.016947427	0.001349898
ENSG00000055130_148730123_148800582_678	annotated	CUL1	7	+	2034	69	10	0	2.80%	11.20902392	0.000153021	5.88E-05	6.60E-08
ENSG00000138796_107989756_108034357_390	annotated	HADH	4	+	1170	67	9	4	6.41%	18.92163212	0.000241403	3.62E-05	6.67E-08
ENSG00000087460_58903762_58909635_123	novel	GNAS	20	+	369	594	123	48	41.46%	531.8996842	1.75E-09	2.10E-09	3.99E-17
ENSG00000136824_104129654_104135898_166	annotated	SMC2	9	+	498	7	1	1	4.22%	4.644493409	0.029390861	0.016947427	0.002281933
ENSG00000011638_21161183_21179556_161	annotated	TMEM159	16	+	483	12	0	0	5.59%	8.209255431	0.002500085	0.002500085	3.60E-05
ENSG00000125875_441682_438586_226	novel	TBC1D20	20	-	678	30	9	2	3.98%	14.62046598	0.061949988	0.008953125	0.002871793
ENSG00000125875_462405_441847_177	novel	TBC1D20	20	-	531	9	0	0	3.95%	5.600381883	0.006937203	0.006937203	0.000251015
ENSG00000125954_64924066_64932230_75	annotated	CHURC1-FNTB	14	+	225	67	9	0	17.33%	98.39248704	0.011149146	0.000676852	5.19E-05
ENSG00000160410_40588771_40591207_153	annotated	SHKBP1	19	+	459	18	0	1	4.58%	12.95774632	0.008473674	0.011825808	0.000503679
ENSG00000015479_139307416_139329395_283	annotated	MATR3	5	+	849	93	16	4	9.89%	36.19469422	9.98E-05	7.47E-06	6.29E-09
ENSG00000141503_4891086_4897287_716	novel	MINK1	17	+	2148	14	4	0	1.12%	2.153591916	0.042379653	0.005160369	0.001212668
ENSG00000175354_12884141_12785823_410	annotated	PTPN2	18	-	1230	15	0	3	1.71%	4.029543062	0.007882058	0.015555383	0.000615783
ENSG00000136048_101877790_101921260_238	annotated	DRAM1	12	+	714	37	9	1	5.88%	17.12273621	0.01550722	0.000585523	6.66E-05
ENSG00000136052_104928395_104805152_529	annotated	SLC41A2	12	-	1587	44	1	2	3.21%	9.161053163	0.000126875	0.000442235	3.95E-07
ENSG00000178982_38619269_38636920_218	annotated	EIF3K	19	+	654	900	90	22	27.52%	454.7099052	1.72E-10	8.08E-11	1.63E-19
ENSG00000067606_2146036_2185009_405	novel	PRKCZ	1	+	1215	17	3	0	2.47%	4.623195909	0.012468381	0.002046029	0.000149774
ENSG00000203722_149923010_149919008_221	annotated	RAET1G	6	-	663	17	3	0	4.07%	8.472372593	0.016912105	0.003452921	0.000323936

ENSG00000158604_44582206_44579479_178	annotated	TMED4	7	-	534	139	21	4	21.35%	86.0088611	5.66E-07	9.80E-08	5.36E-13
ENSG00000104783_43772038_43767543_167	dORF	KCNN4	19	-	501	54	6	4	10.18%	35.61440455	0.00116681	0.000523506	3.91E-06
ENSG00000104783_43780861_43772089_57	annotated	KCNN4	19	-	171	16	3	5	8.77%	30.91672806	0.073562049	0.123780394	0.032681874
ENSG00000136754_26860848_26748570_503	annotated	ABI1	10	-	1509	63	8	6	5.37%	13.79497645	9.43E-05	3.34E-05	2.39E-08
ENSG00000105341_41438393_41433268_159	annotated	DMAC2	19	-	477	43	1	0	11.32%	29.78651748	8.59E-05	8.59E-05	5.38E-08
ENSG00000164828_838815_838970_51	novel	SUN1	7	+	153	22	3	16	19.61%	47.5117365	0.010402772	0.229394231	0.015451134
ENSG00000164828_832525_843636_257	novel	SUN1	7	+	771	46	23	3	7.39%	19.71392533	0.033694833	0.000318089	0.000104199
ENSG00000137133_35814979_35813054_163	annotated	HINT2	9	-	489	232	45	12	24.54%	156.7648818	2.04E-05	5.14E-08	1.33E-11
ENSG00000204469_31636303_31637357_215	novel	PRRC2A	6	+	645	9	0	1	2.33%	4.610546946	0.020613417	0.035374934	0.003252526
ENSG00000166484_19378901_19379951_133	novel	MAPK7	17	+	399	8	0	0	3.76%	6.625013155	0.016947427	0.016947427	0.001349898
ENSG00000146918_158693311_158641464_1068	annotated	NCAPG2	7	-	3204	72	7	2	3.28%	7.425225418	4.48E-06	1.79E-07	7.95E-12
ENSG00000198612_237087196_237097742_124	annotated	COPS8	2	+	372	42	4	0	8.87%	37.30576964	0.004514955	0.001603369	4.24E-05
ENSG00000184182_237973108_238041338_163	annotated	UBE2F	2	+	489	25	8	0	6.13%	16.89276744	0.024548991	0.002362849	0.000350622
ENSG00000104320_89981448_89935582_672	annotated	NBN	8	-	2016	68	4	1	4.32%	11.14520442	5.91E-06	1.30E-06	6.75E-11
ENSG00000157470_59460105_59521378_304	annotated	FAM81A	15	+	912	15	0	0	2.30%	5.434581104	0.008373824	0.008373824	0.000358384
ENSG00000104859_45040213_45070845_655	annotated	CLASRP	19	+	1965	19	7	0	1.37%	3.194925237	0.078444815	0.002967235	0.001606924
ENSG00000105568_52212720_52225981_410	annotated	PPP2R1A	19	+	1230	295	32	6	16.83%	79.24768023	2.67E-10	1.95E-12	7.14E-21
ENSG00000168003_62856270_62888693_568	annotated	SLC3A2	11	+	1704	2363	195	69	35.74%	458.209179	4.83E-32	4.74E-32	4.81E-62
ENSG00000132664_18469059_18483558_257	novel	POLR3F	20	+	771	12	1	1	3.50%	5.14276313	0.009847127	0.006306719	0.000321333
ENSG00000156831_125102442_125156047_66	annotated	NSMCE2	8	+	198	27	4	0	13.64%	45.05761788	0.017642875	0.003554776	0.000347173
ENSG00000182196_122981140_122981412_90	novel	ARL6IP4	12	+	270	25	3	4	8.89%	30.59467881	0.010275151	0.02857234	0.001428016
ENSG00000038274_163503404_163512202_73	novel	MAT2B	5	+	219	28	12	6	13.70%	42.24580307	0.141410818	0.040339912	0.023044532
ENSG00000044115_138886260_138934089_536	annotated	CTNNA1	5	+	1608	161	9	7	8.77%	33.08335044	8.11E-09	1.22E-08	1.03E-15
ENSG00000174669_66371591_66366153_301	annotated	SLC29A2	11	-	903	35	12	7	4.65%	12.80707485	0.025421411	0.013844643	0.00165355
ENSG00000174669_66365977_66363436_117	dORF	SLC29A2	11	-	351	21	2	5	5.13%	19.76886938	0.042435259	0.089010362	0.014973172
ENSG00000186501_27322258_27332477_93	annotated	TMEM222	1	+	279	19	3	0	7.53%	22.5018928	0.024275373	0.008574016	0.001034373
ENSG00000058063_182884758_182913868_314	Overlap_dORF	ATP11B	3	+	942	10	0	0	1.59%	3.507670182	0.020613417	0.020613417	0.001946209
ENSG00000196531_56724521_56712538_215	annotated	NACA	12	-	645	468	69	22	22.33%	239.7484412	1.15E-06	1.21E-08	1.59E-13
ENSG00000100897_24117302_24123309_418	annotated	DCAF11	14	+	1254	30	0	3	3.35%	7.904845242	0.000434916	0.00090861	2.56E-06
ENSG00000083168_42048977_41932205_2004	annotated	KAT6A	8	-	6012	18	5	3	0.40%	0.989289015	0.074117384	0.034975232	0.010617713
ENSG00000168802_69121458_69118490_58	annotated	CHTF8	16	-	174	10	0	2	10.34%	18.98980064	0.011570466	0.041439533	0.002311527
ENSG00000104611_19313778_19394642_409	annotated	SH2D4A	8	+	1227	31	2	0	4.40%	8.348083508	0.000230673	5.73E-05	9.74E-08
ENSG00000131504_141584219_141516851_1170	annotated	DIAPH1	5	-	3510	90	17	0	4.10%	8.472372593	6.52E-07	3.72E-10	3.78E-15
ENSG00000106268_2244575_2251001_156	annotated	NUDT1	7	+	468	89	4	2	14.10%	62.8367634	1.82E-05	2.11E-05	3.03E-09
ENSG00000030582_44349165_44352798_593	annotated	GRN	17	+	1779	1464	156	21	23.78%	271.9160121	5.80E-19	2.75E-24	4.08E-41
ENSG00000163466_218238738_218238854_38	novel	ARPC2	2	+	114	15	0	0	13.16%	43.47664883	0.021084099	0.021084099	0.002029891
ENSG00000162735_160283019_160279551_209	dORF	PEX19	1	-	627	49	0	0	7.66%	25.82249446	0.000188101	0.000188101	2.46E-07
ENSG00000136854_127651608_127690857_580	annotated	STXBP1	9	+	1740	60	6	0	4.14%	11.39388038	0.000136443	7.48E-06	8.76E-09
ENSG00000065911_74198642_74205939_111	novel	MTHFD2	2	+	333	36	0	2	9.01%	35.72135472	0.002460157	0.003185952	4.47E-05
ENSG00000176171_131973863_131973789_24	novel	BNIP3	10	-	72	16	0	0	25.00%	73.42722914	0.01300724	0.01300724	0.000821865
ENSG00000107643_48401661_48434895_384	annotated	MAPK8	10	+	1152	28	4	2	3.39%	8.031103187	0.000585477	0.002815409	1.06E-05
ENSG00000084090_96195509_96195306_67	novel	STARD7	2	-	201	17	1	0	7.46%	27.94618422	0.042234516	0.019679754	0.003715148
ENSG00000148229_113410293_113408811_147	annotated	POLE3	9	-	441	99	9	0	19.73%	74.17648658	1.28E-06	1.16E-06	1.32E-11
ENSG00000107643_48401661_48425219_218	novel	MAPK8	10	+	654	7	0	2	2.75%	3.536632596	0.009815329	0.093731016	0.004911899
ENSG00000107643_48401661_48435029_427	annotated	MAPK8	10	+	1281	31	4	2	3.28%	7.996173665	0.00040182	0.0016955	4.46E-06
ENSG00000198715_156294893_156293044_325	annotated	GLMP	1	-	975	84	5	6	9.23%	28.46717191	1.28E-06	8.35E-07	9.57E-12
ENSG00000198715_156295645_156295499_48	uORF	GLMP	1	-	144	20	3	0	12.50%	45.89201821	0.013640586	0.013640586	0.000898637
ENSG00000127324_71157645_71125334_226	annotated	TSPAN8	12	-	678	47	8	2	8.41%	22.9053967	0.00065891	0.00019657	8.86E-07
ENSG00000089157_120200783_120198800_172	novel	RPLP0	12	-	516	79	19	5	6.98%	50.58794566	0.005871691	0.002781999	9.12E-05
ENSG00000134324_11765542_11824791_890	annotated	LPIN1	2	+	2670	84	14	4	4.04%	10.39531559	2.54E-05	4.77E-07	1.22E-10
ENSG00000187239_130042975_129890539_617	annotated	FNBP1	9	-	1851	19	2	2	1.13%	3.391695349	0.024275373	0.024275373	0.002638996
ENSG00000138413_208251500_208237079_397	annotated	IDH1	2	-	1191	272	39	1	10.83%	75.46173675	6.90E-07	5.19E-09	4.27E-14
ENSG00000109819_23831604_23795822_671	annotated	PPARGC1A	4	-	2013	47	11	0	2.98%	7.714783389	0.002452398	3.75E-05	8.36E-07
ENSG00000126254_35629154_35637554_458	annotated	RBM42	19	+	1374	122	19	7	7.42%	29.33882736	2.30E-05	2.03E-06	4.12E-10
ENSG00000144366_188584274_188588075_116	annotated	GULP1	2	+	348	26	3	0	6.03%	24.68674083	0.013892891	0.008674847	0.000601427
ENSG00000087470_32679364_32743410_699	annotated	DNM1L	12	+	2097	115	16	6	6.58%	18.12045354	3.30E-07	2.12E-08	7.25E-14
ENSG00000163110_94455289_94664067_625	annotated	PDLIM5	4	+	1875	459	54	35	10.72%	80.88743562	1.91E-10	3.02E-10	6.62E-19
ENSG00000145819_143056047_143222446_305	annotated	ARHGAP26	5	+	915	17	2	0	2.30%	6.138997846	0.024135905	0.008674847	0.001038937
ENSG00000158941_22614456_22619400_592	annotated	CCAR2	8	+	1776	45	11	11	3.89%	8.372192511	0.001476796	0.000803506	7.37E-06
ENSG00000156831_125102331_125182312_157	annotated	NSMCE2	8	+	471	54	7	4	11.46%	37.88283796	0.000975024	0.00053821	3.36E-06
ENSG00000113742_173889734_173956137_704	annotated	CPEB4	5	+	2112	27	2	0	1.99%	4.224151676	0.000943654	0.000411814	2.53E-06
ENSG00000116729_68232299_68126226_541	annotated	WLS	1	-	1623	87	5	5	6.47%	17.7121135	1.18E-06	8.37E-07	8.83E-12
ENSG00000136942_124861912_124858469_96	annotated	RPL35	9	-	288	71	9	17	10.42%	81.45833232	0.009211063	0.034101371	0.001557313
ENSG00000156113_77637360_76877739_1167	annotated	KCNMA1	10	-	3501	71	13	0	3.00%	6.700942505	1.40E-05	8.57E-08	1.38E-11
ENSG00000124155_45416169_45424583_491	annotated	PIGT	20	+	1473	385	28	8	16.90%	86.36298335	7.57E-14	5.46E-15	5.84E-27
ENSG00000128564_101164843_101162996_615	annotated	VGF	7	-	1845	63	7	4	3.74%	11.28272057	0.000264877	9.93E-05	1.87E-07
ENSG00000072778_7224441_7225097_133	novel	ACADVL	17	+	399	58	16	1	12.78%	48.03134538	0.001775498	0.000165128	2.11E-06
ENSG00000161513_74872944_74862817_534	annotated	FDXR	17	-	1602	81	3	1	4.87%	16.70675719	1.30E-05	4.30E-06	4.66E-10

ENSG00000150593_110887800_110890694_107	novel	PDCD4	10	+	321	49	3	1	11.21%	50.43833029	0.004278096	0.001498751	3.78E-05
ENSG00000084207_67584484_67584783_61	novel	GSTP1	11	+	183	420	53	53	34.43%	758.3467927	3.23E-05	0.000203179	5.04E-08
ENSG00000065911_74208652_74214242_186	dORF	MTHFD2	2	+	558	55	2	3	12.37%	32.56852905	2.32E-05	4.17E-05	7.50E-09
ENSG00000147654_109550825_109557763_153	novel	EBAG9	8	+	459	47	2	5	8.50%	33.83411539	0.000795519	0.002325971	1.15E-05
ENSG00000187555_8923294_8893998_1001	annotated	USP7	16	-	3003	80	8	7	3.20%	8.802465031	5.65E-06	9.87E-06	4.60E-10
ENSG00000205808_4662649_4663263_204	annotated	PLPP6	9	+	612	47	0	2	8.33%	25.37558654	0.000121023	0.000284967	2.43E-07
ENSG00000197622_151055730_151054247_79	annotated	CDC42SE1	1	-	237	43	9	1	18.99%	59.95007949	0.002270904	0.000294865	4.57E-06
ENSG00000127824_219253858_219251472_155	novel	TUBA4A	2	-	465	15	0	1	3.87%	10.65879133	0.01300724	0.012798403	0.000809468
ENSG00000100503_50821942_50725872_2052	annotated	NIN	14	-	6156	34	6	11	0.88%	1.824945753	0.002721991	0.006100767	9.29E-05
ENSG00000166479_68714981_68700481_89	annotated	TMX3	18	-	267	29	7	9	8.99%	35.88858952	0.053965854	0.042503047	0.009271285
ENSG00000159322_72783691_72760419_113	novel	ADPGK	15	-	339	21	3	0	6.19%	20.46865237	0.024197104	0.008574016	0.001031003
ENSG00000072041_84867122_84861632_208	novel	SLC6A15	12	-	624	32	4	2	6.73%	16.94474519	0.002703862	0.00084676	1.41E-05
ENSG00000012822_53725242_53713096_633	annotated	CALCOCO1	12	-	1899	13	3	3	1.11%	2.261976253	0.058290626	0.058290626	0.01323296
ENSG00000167460_16081341_16095346_205	novel	TPM4	19	+	615	100	5	3	10.73%	53.72724083	1.83E-05	4.10E-05	5.87E-09
ENSG00000160813_100436368_100435762_176	novel	PPP1R35	7	-	528	59	6	7	6.82%	36.92221465	0.006487086	0.00757204	0.000256101
ENSG00000100483_50116552_50115843_144	annotated	VCPKMT	14	-	432	21	6	5	6.25%	16.06220637	0.028243214	0.036572178	0.004451921
ENSG00000044115_138886260_138904456_97	novel	CTNNA1	5	+	291	11	0	1	6.19%	12.49019877	0.011772051	0.026832696	0.001510382
ENSG00000198363_61689688_61626273_298	annotated	ASPH	8	-	894	1723	172	105	41.95%	636.8210527	8.83E-20	3.63E-18	5.32E-36
ENSG00000070367_57246815_57208609_653	dORF	EXOC5	14	-	1959	102	9	0	3.22%	17.20423592	0.000595214	2.76E-05	1.35E-07
ENSG00000137944_88962097_88936183_287	novel	KYAT3	1	-	861	17	9	0	3.48%	6.524022101	0.161946107	0.002188006	0.003337896
ENSG00000137944_88988350_88964662_167	novel	KYAT3	1	-	501	11	2	0	3.59%	7.254786112	0.027849813	0.012798403	0.001687084
ENSG00000127080_92635240_92615832_163	annotated	IPPK	9	-	489	12	3	0	3.07%	8.108528371	0.100412561	0.021084099	0.009609042
ENSG00000149823_65109813_65110799_112	annotated	VPS51	11	+	336	24	0	1	6.25%	23.60160937	0.008776163	0.01208853	0.000531381
ENSG00000179409_752142_744866_1058	annotated	GEMIN4	17	-	3174	134	12	1	4.73%	13.9497855	7.72E-08	3.19E-10	3.21E-16
ENSG00000177156_747482_764845_337	annotated	TALDO1	11	+	1011	978	78	18	27.89%	319.6372259	5.09E-13	9.28E-17	8.90E-28
ENSG00000108654_64506119_64499923_535	annotated	DDX5	17	-	1605	310	61	31	13.83%	63.81992813	2.16E-08	4.09E-12	1.57E-18
ENSG00000137804_41332958_41380186_378	annotated	NUSAP1	15	+	1134	117	19	12	10.85%	34.09121353	4.55E-07	3.10E-07	1.31E-12
ENSG00000141644_50279992_50271501_549	annotated	MBD1	18	-	1647	14	1	3	1.64%	2.808691825	0.006024334	0.0280616	0.000886089
ENSG00000090372_46746427_46720602_752	annotated	STRN4	19	-	2256	71	17	9	3.86%	10.39893604	0.000817112	8.50E-05	5.15E-07
ENSG00000115694_241500775_241495662_332	annotated	STK25	2	-	996	24	2	3	3.01%	7.961988702	0.002321823	0.009985438	0.000132651
ENSG00000158636_76446939_76550146_1322	annotated	EMSY	11	+	3966	10	4	0	0.45%	0.833138001	0.163744512	0.011772051	0.01090609
ENSG00000129810_20184027_20170704_275	annotated	SGO1	3	-	825	21	4	2	3.64%	8.410755337	0.019145333	0.007532653	0.000726845
ENSG00000103544_19555730_19700476_963	annotated	VPS35L	16	+	2889	130	10	13	4.88%	14.86844204	7.09E-08	3.74E-08	2.64E-14
ENSG00000169045_179618370_179617500_193	novel	HNRNPH1	5	-	579	282	28	10	22.28%	160.931181	1.47E-07	2.29E-08	3.41E-14
ENSG00000095906_1786802_1788714_211	novel	NUBP2	16	+	633	56	13	5	9.48%	29.23169312	0.001539739	0.000350298	3.57E-06
ENSG00000179151_74656003_74632612_325	dORF	EDC3	15	-	975	11	4	0	2.15%	3.727843941	0.121794855	0.007593099	0.005521741
ENSG00000127527_16471945_16361967_754	annotated	EPS15L1	19	-	2262	21	3	4	1.33%	3.06758318	0.00224099	0.023573843	0.000320974
ENSG00000154710_66771900_66809284_491	annotated	RABGEF1	7	+	1473	25	2	6	2.44%	5.607985932	0.004359749	0.029833576	0.000720531
ENSG00000128891_40563009_40557212_132	annotated	CCDC32	15	-	396	24	3	0	6.82%	20.02560795	0.017480387	0.003554776	0.000343829
ENSG00000109118_28950948_28911356_701	annotated	PHF12	17	-	2103	20	0	0	1.00%	3.142392117	0.008574016	0.008574016	0.000374746
ENSG00000168385_241337698_241350174_166	annotated	2-Sep	2	+	498	100	6	5	15.66%	66.34990585	2.41E-05	6.76E-06	1.33E-09
ENSG00000145725_103129590_103201634_1278	annotated	PPIP5K2	5	+	3834	74	3	11	2.43%	6.377482343	6.58E-07	0.000100017	7.24E-10
ENSG00000145901_151065095_151030713_636	annotated	TNIP1	5	-	1908	59	6	2	4.09%	10.21746821	4.86E-05	9.77E-06	3.84E-09
ENSG00000070961_89655877_89590984_1217	annotated	ATP2B1	12	-	3651	127	38	12	5.09%	11.49374458	9.19E-06	1.27E-09	2.21E-13
ENSG00000168936_1720837_1717180_362	annotated	TMEM129	4	-	1086	28	3	1	3.87%	8.519181281	0.003178226	0.000617933	1.26E-05
ENSG00000004059_127590135_127591299_71	novel	ARF5	7	+	213	57	8	7	15.49%	88.42293086	0.001786287	0.004045672	4.20E-05
ENSG00000004059_127588499_127589621_94	novel	ARF5	7	+	282	216	20	0	37.23%	253.0895983	6.63E-07	1.15E-07	7.35E-13
ENSG00000107537_13300042_13295444_98	novel	PHYH	10	-	294	30	0	0	8.16%	33.71658481	0.005756914	0.005756914	0.000176255
ENSG00000153558_33277513_33385608_348	novel	FBXL2	3	+	1044	10	0	3	1.44%	3.164966773	0.020613417	0.100002646	0.009397837
ENSG00000197586_25225252_25225597_54	novel	ENTPD6	20	+	162	49	3	8	22.22%	99.94261744	0.001385138	0.004361926	3.59E-05
ENSG00000109572_169687691_169719997_701	annotated	CLCN3	4	+	2103	68	11	1	4.56%	10.6841332	2.62E-05	5.15E-07	1.35E-10
ENSG00000197756_216498875_216529273_72	annotated	RPL37A	2	+	216	916	113	51	36.11%	1401.236289	6.40E-05	7.22E-05	3.42E-08
ENSG00000138448_186668798_186669853_91	novel	ITGAV	2	+	273	25	0	0	9.89%	30.25847355	0.003684287	0.003684287	7.54E-05
ENSG00000075711_197138252_197050598_584	internal	DLG1	3	-	1752	29	4	0	2.05%	5.469322718	0.007169783	0.000991051	4.46E-05
ENSG00000058799_53866835_53860064_116	annotated	YIPF1	1	-	348	11	0	0	4.31%	10.44439035	0.019679754	0.019679754	0.001784964
ENSG00000181090_137743424_137792120_564	dORF	EHMT1	9	+	1692	27	3	9	2.30%	5.272699965	0.004360764	0.030856737	0.000747356
ENSG00000196683_22822779_22817987_54	novel	TOMM7	7	-	162	92	9	12	24.07%	187.6473633	0.000661127	0.001013339	4.24E-06
ENSG00000156261_29062187_29056475_164	novel	CCT8	21	-	492	171	23	7	20.73%	114.8419773	4.78E-06	1.47E-06	6.15E-11
ENSG00000127980_92528435_92487457_1283	annotated	PEX1	7	-	3849	24	5	3	1.09%	2.060311963	0.00840293	0.003994934	0.00018107
ENSG00000113407_33454984_33467708_559	Overlap_dORF	TARS	5	+	1677	201	30	12	11.63%	39.60341607	2.85E-08	9.77E-12	4.64E-18
ENSG00000075539_48620766_48550563_1491	annotated	FRYL	4	-	4473	9	0	0	0.34%	0.664834067	0.020613417	0.020613417	0.001946209
ENSG00000139508_28717950_28701497_445	annotated	SLC46A3	13	-	1335	11	0	0	1.80%	2.722582653	0.003327803	0.003327803	6.21E-05
ENSG00000116584_155969210_155947942_935	annotated	ARHGEF2	1	-	2805	16	1	0	1.18%	1.884763101	0.002532006	0.001242734	1.89E-05
ENSG00000092203_21477279_21487810_144	novel	TOX4	14	+	432	10	0	1	3.47%	7.648669702	0.020613417	0.035374934	0.003252526
ENSG00000178982_38619269_38630851_167	novel	EIF3K	19	+	501	573	51	19	16.77%	377.9084039	6.13E-06	1.17E-05	5.92E-10
ENSG00000129351_10683780_10685028_56	annotated	ILF3	19	+	168	9	3	1	10.71%	17.70120702	0.059285716	0.023056749	0.0059707
ENSG00000162664_90005005_90027701_472	annotated	ZNF326	1	+	1416	14	4	7	1.69%	3.266889432	0.022789577	0.132518593	0.01383925
ENSG00000010404_149505137_149487288_343	annotated	IDS	X	-	1029	13	4	0	2.62%	4.17443431	0.07279348	0.002943991	0.001458313

ENSG00000122482_90941278_90916798_1302	annotated	ZNF644	1	-	3906	22	1	1	1.00%	1.861058803	0.000918835	0.000918835	5.27E-06
ENSG00000182087_1010889_1010350_179	dORF	TMEM259	19	-	537	63	1	0	7.82%	38.76465449	0.001003788	0.000413076	2.70E-06
ENSG00000182087_1020996_1011092_406	annotated	TMEM259	19	-	1218	84	5	1	7.39%	22.78776077	9.41E-06	9.22E-07	7.86E-11
ENSG00000205726_33765954_33836988_860	annotated	ITSN1	21	+	2580	18	0	2	1.05%	2.305273473	0.003303074	0.014936572	0.000273899
ENSG00000177054_19149203_19175960_492	annotated	ZDHHC13	11	+	1476	46	10	0	4.47%	10.29772116	0.004045928	1.39E-05	6.63E-07
ENSG00000205726_33765954_33836988_926	annotated	ITSN1	21	+	2778	19	0	2	1.08%	2.25990932	0.002046029	0.010448364	0.000124448
ENSG00000205726_33765954_33836988_926	annotated	ITSN1	21	+	2778	19	0	2	1.08%	2.25990932	0.002046029	0.010448364	0.000124448
ENSG00000160209_43719295_43737075_87	annotated	PDXK	21	+	261	102	14	5	10.34%	129.1306443	0.014200919	0.003789641	0.000293103
ENSG00000047634_17750071_17753392_169	annotated	SCML1	X	+	507	12	4	0	5.33%	7.820651624	0.044129916	0.002500085	0.000710788
ENSG00000160209_43719295_43743697_91	novel	PDXK	21	+	273	157	21	8	21.98%	190.0232139	0.000847762	7.95E-05	5.04E-07
ENSG00000171155_120627166_120626210_318	annotated	C1GALT1C1	X	-	954	165	20	3	12.89%	57.14855098	1.01E-06	1.01E-08	1.18E-13
ENSG00000117118_17054019_17032947_132	novel	SDHB	1	-	396	140	6	7	7.58%	116.8160464	0.002863021	0.006229071	9.93E-05
ENSG00000198000_92319259_92297836_1041	dORF	NOL8	9	-	3123	40	1	2	1.92%	4.232116953	3.14E-05	0.000158386	3.80E-08
ENSG00000087191_63830397_63830949_81	novel	PSMC5	17	+	243	120	19	0	37.04%	163.1716203	5.59E-05	8.04E-07	4.56E-10
ENSG00000123131_23667571_23671431_92	novel	PRDX4	X	+	276	97	20	5	22.83%	116.1267591	7.55E-05	5.89E-05	3.29E-08
ENSG00000162191_62678923_62678218_136	novel	UBXN1	11	-	408	66	16	1	14.71%	53.45070356	0.001803023	5.81E-05	8.62E-07
ENSG00000110048_59600540_59593971_109	novel	OSBP	11	-	327	14	8	0	7.34%	14.14653038	0.250200285	0.005160369	0.011006471
ENSG00000137710_110247751_110199612_257	annotated	RDX	11	-	771	55	0	3	8.17%	23.57099768	2.53E-05	0.000122961	2.40E-08
ENSG00000132286_6481787_6482221_80	novel	TIMM10B	11	+	240	22	4	2	13.75%	30.28873202	0.002287938	0.001343041	1.83E-05
ENSG00000164331_73562803_73557549_153	novel	ANKRA2	5	-	459	17	1	0	4.58%	12.23787152	0.013991246	0.008574016	0.000599135
ENSG00000145715_87372124_87390883_509	dORF	RASA1	5	+	1527	41	0	2	2.95%	8.87185578	0.000307311	0.00075476	1.54E-06
ENSG00000145715_87268455_87366360_538	annotated	RASA1	5	+	1614	12	0	3	1.12%	2.456673094	0.012178958	0.070229091	0.004215017
ENSG00000278311_36554826_36589411_664	annotated	GGNBP2	17	+	1992	53	11	3	4.07%	8.791362525	0.00074084	1.47E-05	9.85E-08
ENSG00000121064_56987806_56995731_151	novel	SCPEP1	17	+	453	38	3	0	9.93%	27.71756332	0.001216039	0.000299491	2.43E-06
ENSG00000204237_81665371_81665201_56	novel	OXLD1	17	-	168	19	1	0	8.93%	37.36921483	0.021084099	0.021084099	0.002029891
ENSG00000115053_231458474_231455191_350	novel	NCL	2	-	1050	184	28	19	16.00%	57.90261498	1.99E-09	2.09E-09	4.52E-17
ENSG00000115053_231463286_231458993_374	novel	NCL	2	-	1122	183	20	23	13.37%	53.89244491	2.08E-08	1.13E-09	2.63E-16
ENSG00000136819_129835221_129830902_65	novel	C9orf78	9	-	195	26	4	4	15.38%	44.05633748	0.009257958	0.015358648	0.000703553
ENSG00000214046_16660110_16652862_103	annotated	SMIM7	19	-	309	173	19	3	22.33%	184.9938443	0.000306593	2.05E-05	5.11E-08
ENSG00000126456_49664838_49659648_427	annotated	IRF3	19	-	1281	64	23	6	5.39%	16.5082295	0.03718269	0.000326414	0.000120321
ENSG00000130731_634621_634526_31	Overlap_dORF	METTL26	16	-	93	12	7	0	19.35%	42.63516531	0.273060506	0.01300724	0.022706215
ENSG00000130731_636290_634597_184	annotated	METTL26	16	-	552	144	35	17	15.76%	86.19718203	0.000514244	0.002029638	6.71E-06
ENSG00000090520_186570898_186585408_358	annotated	DNAJB11	3	+	1074	171	17	11	12.85%	52.60917395	3.75E-08	4.99E-08	1.87E-14
ENSG00000113013_138556845_138556037_96	novel	HSPA9	5	-	288	47	10	2	17.71%	53.9231214	0.005969999	0.000125586	6.32E-06
ENSG00000134014_28099925_28189725_475	annotated	ELP3	8	+	1425	46	2	0	4.63%	10.66627118	3.18E-05	1.63E-05	4.06E-09
ENSG00000155508_154871749_154875439_128	annotated	CNOT8	5	+	384	59	2	0	10.94%	50.76804515	0.001117289	0.000457524	3.30E-06
ENSG00000160752_155310065_155318336_176	novel	FDPS	1	+	528	376	37	28	20.45%	235.3008934	3.74E-06	9.19E-06	2.89E-10
ENSG00000132823_44206957_44197052_292	annotated	OSER1	20	-	876	112	7	1	10.62%	42.24580307	4.05E-06	6.20E-07	2.29E-11
ENSG00000204536_31154651_31142592_656	annotated	CCHCR1	6	-	1968	11	7	0	1.07%	1.846873904	0.077087128	0.007593099	0.00322116
ENSG00000165119_83978252_83969407_464	annotated	HNRNPK	9	-	1392	441	48	38	7.33%	104.681276	7.62E-06	3.35E-06	2.16E-10
ENSG00000179262_12949325_12953049_120	Overlap_dORF	RAD23A	19	+	360	31	6	0	10.00%	28.45305129	0.00265296	0.001003278	1.62E-05
ENSG00000170921_63009581_63421955_1993	annotated	TANC2	17	+	5979	10	1	2	0.40%	0.552638453	0.010437297	0.037556183	0.001913486
ENSG00000196628_55350976_55228225_545	annotated	TCF4	18	-	1635	24	0	0	1.83%	4.850238989	0.002404282	0.002404282	3.34E-05
ENSG00000125166_58734228_58716168_237	novel	GOT2	16	-	711	126	19	0	10.97%	58.55589159	2.54E-05	3.85E-06	8.29E-10
ENSG00000167515_88857151_88859368_75	annotated	TRAPPC2L	16	+	225	113	34	7	20.00%	165.9455378	0.009666645	0.004407029	0.000227452
ENSG00000167515_88859700_88860959_65	dORF	TRAPPC2L	16	+	195	23	2	0	10.77%	38.97291393	0.013991246	0.008574016	0.000599135
ENSG00000197912_89524038_89529153_122	annotated	SPG7	16	+	366	16	2	0	7.38%	14.44470081	0.006083074	0.003229477	0.000108335
ENSG00000060688_31269325_31260072_127	annotated	SNRNP40	1	-	381	23	3	1	5.51%	19.94676697	0.059897465	0.013892891	0.003955494
ENSG00000148187_122280532_122322617_171	annotated	MRRF	9	+	513	58	12	4	7.60%	37.35771307	0.005068734	0.002208321	6.38E-05
ENSG00000158480_49906770_49905619_383	annotated	SPATA2	20	-	1149	9	0	0	1.57%	2.58816604	0.009815329	0.009815329	0.000483714
ENSG00000161057_103347712_103368054_433	annotated	PSMC2	7	+	1299	215	69	2	12.24%	54.68887159	3.09E-05	2.24E-10	2.20E-13
ENSG00000100889_24094069_24103867_509	annotated	PCK2	14	+	1527	77	12	1	5.50%	16.66177793	7.95E-05	1.77E-06	1.35E-09
ENSG00000140455_63537128_63590826_435	annotated	USP3	15	+	1305	47	1	10	4.14%	11.90027507	0.000106	0.002765105	2.31E-06
ENSG00000185215_103133368_103136003_199	annotated	TNFAIP2	14	+	597	76	1	0	7.04%	42.06383981	0.000603605	0.000452135	1.78E-06
ENSG00000134255_111182208_111184310_171	novel	CEPT1	1	+	513	24	4	0	4.68%	15.45836403	0.028023387	0.005454749	0.000813426
ENSG00000151876_41925310_41934334_307	annotated	FBXO4	5	+	921	26	1	0	2.93%	9.327889043	0.005059698	0.003632191	0.000100924
ENSG00000139496_25301774_25340134_587	annotated	NUP58	13	+	1761	52	8	4	3.92%	9.756940158	0.000349424	5.69E-05	1.48E-07
ENSG00000133112_45340184_45337386_138	novel	TPT1	13	-	414	113	15	5	9.42%	90.18779231	0.005944871	0.00074336	2.84E-05
ENSG00000120833_93574739_93575179_146	annotated	SOCS2	12	+	438	8	0	5	4.11%	6.035114724	0.011570466	0.214508587	0.015188371
ENSG00000138796_108027733_108034357_87	novel	HADH	4	+	261	30	3	4	12.64%	37.97960128	0.008897471	0.01106333	0.000494329
ENSG00000138796_107989957_108023617_221	novel	HADH	4	+	663	39	6	0	6.79%	19.43661948	0.003337626	0.000294865	6.87E-06
ENSG00000142867_85276352_85267627_233	annotated	BCL10	1	-	699	64	15	0	9.44%	30.25327896	0.00047076	1.52E-05	6.20E-08
ENSG00000106399_7640418_7637000_121	annotated	RPA3	7	-	363	293	13	10	20.66%	266.7046877	1.94E-05	5.96E-06	9.52E-10
ENSG00000204569_30609141_30601549_897	annotated	PPP1R10	6	-	2691	39	1	3	1.67%	4.788732335	0.000415728	0.000655226	1.78E-06
ENSG00000257046_20858564_21090106_631	annotated	AC011604.2	12	+	1893	46	15	4	3.49%	8.029284961	0.009436119	0.000234244	1.78E-05
ENSG00000175063_45812696_45816767_150	annotated	UBE2C	20	+	450	363	29	5	22.67%	266.5408418	2.61E-07	4.75E-07	1.16E-12
ENSG00000196531_56713680_56712538_136	annotated	NACA	12	-	408	411	56	16	25.00%	332.8521086	8.79E-06	6.22E-07	5.09E-11
ENSG00000154710_66771900_66809284_491	annotated	RABGEF1	7	+	1473	25	2	6	2.44%	5.607985932	0.004359749	0.029833576	0.000720531

ENSG00000123159_14479522_14478416_114	Overlap_dORF	GIPC1	19	-	342	67	13	4	21.05%	64.73189937	0.000383829	1.12E-05	3.79E-08
ENSG00000123159_14480775_14479519_62	annotated	GIPC1	19	-	186	22	9	2	14.52%	39.08223486	0.07341731	0.011814276	0.004317997
ENSG00000136603_170360425_170392417_607	annotated	SKIL	3	+	1821	47	12	1	3.79%	8.528203714	0.001034354	1.38E-05	1.36E-07
ENSG00000173575_92901241_92949080_500	annotated	CHD2	15	+	1500	14	4	4	1.40%	3.083943624	0.065834008	0.065834008	0.016503129
ENSG00000047315_56978986_57030988_1174	annotated	POLR2B	4	+	3522	167	26	7	4.86%	15.66739429	1.16E-07	1.58E-10	2.65E-16
ENSG00000181019_69718478_69710976_219	annotated	NQO1	16	-	657	2776	280	191	56.16%	1396.123206	6.62E-18	4.93E-18	5.05E-34
ENSG00000104331_56993576_56963793_356	annotated	IMPAD1	8	-	1068	176	21	1	13.48%	54.45165307	8.39E-08	6.61E-10	6.86E-16
ENSG00000117640_25826667_25832026_195	annotated	MTFR1L	1	+	585	19	5	0	6.67%	10.73167195	0.013266614	0.000528008	5.13E-05
ENSG00000105173_29812993_29823777_365	annotated	CCNE1	19	+	1095	14	0	1	2.19%	4.224580307	0.00419316	0.010499663	0.000236136
ENSG00000102931_57245368_57252267_123	annotated	ARL2BP	16	+	369	53	9	16	14.63%	47.45906273	0.003159519	0.005050419	8.85E-05
ENSG00000140650_8842296_8847825_61	novel	PMM2	16	+	183	10	0	7	8.20%	18.05587602	0.019679754	0.170042304	0.016523685
ENSG00000272897_35674384_35655234_134	annotated	AL109827.1	20	-	402	25	9	2	5.97%	20.54866487	0.076203142	0.007181907	0.003044496
ENSG00000272897_35655274_35632880_38	Overlap_dORF	AL109827.1	20	-	114	11	0	2	13.16%	31.88287581	0.019679754	0.101051022	0.009164498
ENSG00000110911_51010704_50988325_553	annotated	SLC11A2	12	-	1659	131	12	5	8.68%	26.09123061	1.79E-08	7.17E-09	1.34E-15
ENSG00000174106_65169597_65210928_274	novel	LEMD3	12	+	822	18	1	0	2.92%	7.235529878	0.009288069	0.005306578	0.000259004
ENSG00000146963_139398602_139422340_339	annotated	LUC7L2	7	+	1017	85	24	2	9.44%	27.61643574	0.00187783	2.64E-07	1.10E-08
ENSG00000186416_119590857_119589352_501	annotated	NKRF	X	-	1503	9	0	0	1.00%	1.978578031	0.019679754	0.019679754	0.001784964
ENSG00000120526_109334010_109243009_583	annotated	NUDCD1	8	-	1749	67	3	0	3.95%	12.65769559	3.93E-05	1.17E-05	3.66E-09
ENSG00000080371_71773989_71785673_106	novel	RAB21	12	+	318	54	4	4	17.92%	56.10948642	0.000228786	0.000365929	5.69E-07
ENSG00000132549_99013819_99875666_4012	annotated	VPS13B	8	+	12036	31	5	0	0.52%	0.851038423	0.001522844	1.54E-05	2.31E-07
ENSG00000101150_63865366_63889945_200	annotated	TPD52L2	20	+	600	191	28	6	19.50%	105.1845057	2.10E-06	8.83E-08	1.88E-12
ENSG00000166987_57524304_57529234_1003	annotated	MBD6	12	+	3009	11	1	0	0.70%	1.207925504	0.01551626	0.007882058	0.000614257
ENSG00000107815_100988550_100993510_571	annotated	TWNK	10	+	1713	14	0	0	1.40%	2.700476028	0.005257623	0.005257623	0.000148366
ENSG00000174206_64222237_64187579_468	annotated	C12orf66	12	-	1404	17	0	0	2.14%	4.000842613	0.002008257	0.002008257	2.37E-05
ENSG00000132716_160240344_160239598_248	annotated	DCAF8	1	-	744	46	1	1	5.24%	20.42935004	0.000725368	0.000700469	3.23E-06
ENSG00000092203_21488677_21495396_467	novel	TOX4	14	+	1401	28	2	1	2.57%	6.60373367	0.004265403	0.001414407	3.58E-05
ENSG00000092203_21477279_21487810_144	novel	TOX4	14	+	432	10	0	1	3.47%	7.648669702	0.020613417	0.035374934	0.003252526
ENSG00000116199_179044118_179072144_319	annotated	FAM20B	1	+	957	49	3	0	5.02%	16.91818602	0.000427981	0.000187666	5.51E-07
ENSG00000116016_46297912_46384660_870	annotated	EPAS1	2	+	2610	107	24	8	5.40%	13.54605779	2.47E-05	3.60E-08	1.21E-11
ENSG00000068784_45605441_45389310_995	annotated	SRBD1	2	-	2985	31	1	5	1.31%	3.431523774	0.000676852	0.007403996	3.32E-05
ENSG00000106537_16753968_16783491_204	annotated	TSPAN13	7	+	612	63	10	0	12.75%	34.01408409	0.000347557	3.32E-06	1.18E-08
ENSG00000167657_3960097_3959101_191	novel	DAPK3	19	-	573	11	0	0	2.62%	6.343189952	0.019679754	0.019679754	0.001784964
ENSG00000111481_54325164_54350523_177	annotated	COPZ1	12	+	531	535	72	12	35.03%	332.9115897	5.57E-10	5.17E-12	3.86E-20
ENSG00000140937_64998706_64947603_670	annotated	CDH11	16	-	2010	128	5	0	6.42%	21.04183283	3.98E-08	4.20E-09	1.79E-15
ENSG00000188603_28489349_28482512_262	novel	CLN3	16	-	786	102	13	4	9.16%	42.87925976	9.76E-05	7.76E-06	6.37E-09
ENSG00000188603_28482605_28479730_104	novel	CLN3	16	-	312	19	2	0	7.69%	20.12188491	0.009341698	0.005355751	0.000262679
ENSG00000140995_89954260_89955225_57	annotated	DEF8	16	+	171	10	2	5	8.77%	19.32295504	0.069457177	0.197776317	0.049762526
ENSG00000107077_6792989_6893418_368	novel	KDM4C	9	+	1104	16	0	2	2.17%	4.788732335	0.005306578	0.013892891	0.000386014
ENSG00000138326_78035385_78040626_121	novel	RPS24	10	+	363	221	52	0	13.22%	201.1663344	0.00100915	0.000211234	1.46E-06
ENSG00000136830_127531704_127506845_703	annotated	FAM129B	9	-	2109	240	20	15	9.96%	37.60142602	4.27E-11	5.97E-11	3.02E-20
ENSG00000174928_155802558_155763517_245	Overlap_dORF	C3orf33	3	-	735	29	2	0	5.71%	13.03707946	0.000673856	0.000405548	1.79E-06
ENSG00000219545_7673372_7967773_76	annotated	UMAD1	7	+	228	10	0	0	7.89%	14.49221628	0.011772051	0.011772051	0.000681273
ENSG00000010219_4604991_4613753_234	annotated	DYRK4	12	+	702	56	8	0	6.84%	26.35849251	0.001108861	0.000178703	1.38E-06
ENSG00000204628_181243800_181241574_116	novel	RACK1	5	-	348	639	180	47	32.76%	606.7241304	0.000272006	3.41E-06	9.18E-09
ENSG00000204628_181242252_181241483_79	novel	RACK1	5	-	237	1367	90	128	30.38%	1905.854852	3.99E-05	0.001080869	3.56E-07
ENSG00000138796_107989957_108023617_221	novel	HADH	4	+	663	39	6	0	6.79%	19.43661948	0.003337626	0.000294865	6.87E-06
ENSG00000141552_81894478_81894612_44	annotated	ANAPC11	17	+	132	495	32	20	40.91%	1239.084492	0.000164926	0.000597751	6.83E-07
ENSG00000125691_38850368_38850132_29	novel	RPL23	17	-	87	89	0	1	17.24%	338.0184514	0.021557223	0.021557223	0.002115616
ENSG00000177302_18299627_18274802_694	dORF	TOP3A	17	-	2082	33	3	3	2.31%	5.23724473	0.001384196	0.001286757	1.08E-05
ENSG00000161999_684319_683168_221	novel	JMJD8	16	-	663	39	13	0	7.24%	19.43661948	0.012458414	0.000188972	2.07E-05
ENSG00000198373_69930131_69939940_431	annotated	WWP2	16	+	1293	17	2	1	1.39%	4.34430242	0.030316224	0.016822681	0.002336478
ENSG00000089280_31180215_31191438_527	annotated	FUS	16	+	1581	349	35	19	17.27%	72.93957202	1.52E-14	4.95E-16	1.12E-28
ENSG00000090238_30095368_30095099_54	novel	YPEL3	16	-	162	10	3	0	9.26%	20.39645254	0.034182656	0.020613417	0.003146197
ENSG00000108107_55386358_55403131_170	annotated	RPL28	19	+	510	901	134	39	15.29%	583.7464716	0.000133428	2.85E-05	2.91E-08
ENSG00000136243_23182086_23187154_150	novel	NUPL2	7	+	450	21	11	6	6.00%	15.41971812	0.071226447	0.010020958	0.003664133
ENSG00000105339_141128708_141194233_1278	annotated	DENND3	8	+	3834	27	2	6	1.41%	2.326919233	0.000232235	0.003975933	6.75E-06
ENSG00000151353_669813_669580_50	novel	TMEM18	2	-	150	40	1	1	22.00%	88.11267496	0.001626321	0.001792061	1.73E-05
ENSG00000257698_57935536_57931610_64	novel	GIHCG	12	-	192	16	0	0	7.81%	27.53521093	0.021084099	0.021084099	0.002029891
ENSG00000118518_127286653_127287693_346	annotated	RNF146	6	+	1038	40	1	0	5.20%	12.73304552	8.20E-05	8.27E-05	4.95E-08
ENSG00000116977_236526074_236548161_316	annotated	LGALS8	1	+	948	57	5	1	6.33%	19.8671775	0.000119632	5.91E-05	5.19E-08
ENSG00000119725_73893923_73931541_425	annotated	ZNF410	14	+	1275	29	10	1	3.06%	7.515492865	0.041860335	0.001032273	0.00033523
ENSG00000166579_8444272_8467023_345	annotated	NDEL1	17	+	1035	41	12	1	5.51%	13.08920172	0.00427928	7.77E-05	2.90E-06
ENSG00000156113_77637360_77636387_74	annotated	KCNMA1	10	-	222	8	4	0	6.76%	11.90711824	0.217578036	0.019216965	0.021916959
ENSG00000173540_49723726_49721752_360	annotated	GMPPB	3	-	1080	31	6	1	3.33%	9.48435043	0.010999263	0.001233568	8.49E-05
ENSG00000187147_44644712_44650029_186	novel	RNF220	1	+	558	21	8	2	5.91%	12.43525655	0.010960145	0.004367728	0.000255702
ENSG00000169241_155135953_155138281_214	Overlap_dORF	SLC50A1	1	+	642	64	23	8	10.75%	32.93931774	0.02694425	0.000285334	7.26E-05
ENSG00000169241_155135912_155136380_53	annotated	SLC50A1	1	+	159	24	1	12	16.98%	49.87509904	0.003658191	0.150132612	0.004281933
ENSG00000105404_41959292_41956846_151	annotated	RABAC1	19	-	453	127	25	6	23.18%	92.63501424	4.33E-05	2.85E-07	1.38E-10

ENSG00000185236_8402207_8402594_60	annotated	RAB11B	19	+	180	19	8	0	13.33%	34.87793384	0.113688485	0.005306578	0.003902773
ENSG00000119013_201078883_201085615_98	annotated	NDUFB3	2	+	294	146	28	39	20.41%	164.0873794	0.00136591	0.001371602	1.13E-05
ENSG00000084731_25981293_25934144_464	annotated	KIF3C	2	-	1392	19	2	0	1.94%	4.510077652	0.01292879	0.002967235	0.000214802
ENSG00000143416_151369719_151368312_105	annotated	SELENBP1	1	-	315	13	0	0	4.76%	13.63648541	0.019679754	0.019679754	0.001784964
ENSG00000095321_129098370_129095397_200	annotated	CRAT	9	-	600	39	0	0	6.00%	21.47746452	0.000943084	0.000943084	5.54E-06
ENSG00000092203_21488677_21496606_486	dORF	TOX4	14	+	1458	28	2	1	2.47%	6.345563012	0.004265403	0.001414407	3.58E-05
ENSG00000092203_21477279_21488608_81	annotated	TOX4	14	+	243	10	0	1	6.17%	13.59763503	0.020613417	0.035374934	0.003252526
ENSG00000105983_156796442_156684078_408	annotated	LMBR1	7	-	1224	72	20	5	6.86%	19.43661948	0.001464228	1.38E-05	2.00E-07
ENSG00000178234_152094228_152103301_202	annotated	GALNT11	7	+	606	13	3	0	2.48%	7.08827212	0.063071082	0.021084099	0.005896902
ENSG00000091640_4967771_4960455_70	novel	SPAG7	17	-	210	27	3	0	12.86%	42.48289686	0.003684287	0.003684287	7.54E-05
ENSG00000100526_54418283_54420078_36	dORF	CDKN3	14	+	108	30	4	3	22.22%	91.78403642	0.011825808	0.018654142	0.001061508
ENSG00000100526_54397069_54418273_203	annotated	CDKN3	14	+	609	77	18	14	12.32%	41.77756141	8.15E-05	0.000204361	1.20E-07
ENSG00000052841_43358955_43493904_1141	annotated	TTC17	11	+	3423	53	4	0	2.37%	5.116095282	1.09E-05	1.97E-06	1.86E-10
ENSG00000256269_119085034_119089764_115	novel	HMBS	11	+	345	9	1	0	6.09%	8.619718203	0.016735684	0.006937203	0.00059053
ENSG00000105552_48800228_48799671_109	novel	BCAT2	19	-	327	58	0	0	11.93%	58.60705445	0.000712477	0.000712477	3.23E-06
ENSG00000154277_41261759_41264227_93	novel	UCHL1	4	+	279	414	72	9	32.26%	490.304401	8.04E-06	8.49E-07	6.19E-11
ENSG00000167110_128275940_128257067_990	annotated	GOLGA2	9	-	2970	21	1	8	1.41%	2.336320927	0.000483714	0.057770204	0.000284309
ENSG00000266173_63728369_63704025_314	annotated	STRADA	17	-	942	14	2	2	2.55%	4.910738254	0.004826937	0.016111152	0.000412375
ENSG00000087191_63827462_63829497_42	Overlap_uORF	PSMC5	17	+	126	34	5	24	14.29%	89.16163538	0.05323297	0.180773536	0.036997037
ENSG00000241837_33903615_33903526_29	dORF	ATP5PO	21	-	87	53	9	0	31.03%	201.2918868	0.018991317	0.003789641	0.000396202
ENSG00000167112_128322408_128315859_117	novel	TRUB2	9	-	351	42	5	0	6.84%	39.53773877	0.032801035	0.005706018	0.000998319
ENSG00000121578_119226883_119216339_109	novel	B4GALT4	3	-	327	29	11	0	13.76%	29.30352722	0.031438028	0.000258933	8.17E-05
ENSG00000139496_25301774_25309296_99	novel	NUP58	13	+	297	10	2	0	5.05%	11.12533775	0.069457177	0.020613417	0.006391023
ENSG00000121775_32073048_32076608_131	novel	TMEM39B	1	+	393	12	0	1	3.82%	10.08923759	0.021084099	0.028889786	0.002731533
ENSG00000121775_32076641_32102673_377	novel	TMEM39B	1	+	1131	51	3	3	5.04%	14.89968973	0.000133667	0.000344766	3.23E-07
ENSG00000112062_36052814_36108447_283	annotated	MAPK14	6	+	849	40	0	0	6.01%	15.56761042	0.000130009	0.000130009	1.20E-07
ENSG00000198792_38247859_38231364_41	annotated	TMEM184B	22	-	123	12	0	0	12.20%	32.2363445	0.019679754	0.019679754	0.001784964
ENSG00000247596_52239016_52229197_254	annotated	TWF2	3	-	762	127	18	12	9.84%	55.07042185	0.000256119	0.000398586	6.89E-07
ENSG00000151148_109521187_109534064_338	novel	UBE3B	12	+	1014	12	0	1	1.78%	3.910325812	0.013217227	0.020159861	0.001265933
ENSG00000135341_90523750_90516501_143	novel	MAP3K7	6	-	429	6	0	2	3.50%	4.621294141	0.016947427	0.139742684	0.011764961
ENSG00000269891_97292627_97163444_517	annotated	ARHGAP19-SLIT1	10	-	1551	16	3	0	1.55%	3.408614119	0.03662705	0.005454749	0.001082538
ENSG00000112110_159791009_159791071_20	novel	MRPL18	6	+	60	38	1	14	30.00%	209.267603	0.01300724	0.295504895	0.025350795
ENSG00000100902_35292477_35310287_107	annotated	PSMA6	14	+	321	92	9	0	11.21%	94.70053851	0.001798541	0.001091522	1.19E-05
ENSG00000100138_41675154_41674933_73	annotated	SNU13	22	-	219	201	12	0	34.25%	303.2645149	1.98E-05	5.88E-06	9.61E-10
ENSG00000182240_41241894_41275624_287	novel	BACE2	21	+	861	49	9	7	9.06%	18.80453429	0.000419099	9.87E-05	2.96E-07
ENSG00000065357_55940942_55953768_381	novel	DGKA	12	+	1143	19	2	0	2.36%	5.492588006	0.004257536	0.002943991	7.01E-05
ENSG00000018236_40908439_41070035_1016	annotated	CNTN1	12	+	3048	433	65	8	12.01%	46.93994619	3.29E-17	6.96E-22	5.25E-37
ENSG00000184640_77320327_77498658_568	annotated	9-Sep	17	+	1704	135	22	6	7.39%	26.17784137	1.48E-05	1.02E-07	1.71E-11
ENSG00000119977_95693899_95664067_607	annotated	TCTN3	10	-	1821	256	51	13	9.56%	46.45149257	9.76E-07	1.22E-09	1.69E-14
ENSG00000154040_24143371_24156983_441	annotated	CABYR	18	+	1323	7	0	0	1.13%	1.74826736	0.016947427	0.016947427	0.001349898
ENSG00000102125_154411916_154413256_71	novel	TAZ	X	+	213	22	0	1	8.45%	34.12814875	0.01300724	0.023878474	0.001470914
ENSG00000152782_89612031_89584406_358	annotated	PANK1	10	-	1074	21	1	0	1.96%	6.460775748	0.0135992	0.008175611	0.00055803
ENSG00000113532_100902955_100811847_359	annotated	ST8SIA4	5	-	1077	82	16	6	10.58%	25.15751862	1.84E-05	2.24E-07	4.40E-11
ENSG00000234741_173867041_173866181_50	novel	GAS5	1	-	150	20	0	0	10.00%	44.05633748	0.021557223	0.021557223	0.002115616
ENSG00000115204_27313101_27309912_118	annotated	MPV17	2	-	354	86	14	1	20.34%	80.27214033	0.000159216	9.81E-06	1.32E-08
ENSG00000177707_111072018_111133692_366	annotated	NECTIN3	3	+	1098	83	12	0	7.10%	24.97729516	0.000176894	3.44E-06	5.77E-09
ENSG00000070404_676424_681708_263	annotated	FSTL3	19	+	789	78	20	3	11.03%	32.66534528	8.07E-05	2.96E-06	2.17E-09
ENSG00000149311_108227625_108365508_3056	annotated	ATM	11	+	9168	31	5	3	0.59%	1.117266412	0.002379531	0.0006246	9.42E-06
ENSG00000149308_108222536_108158942_1427	annotated	NPAT	11	-	4281	16	3	4	0.56%	1.234935879	0.012941946	0.053467929	0.003310398
ENSG00000242265_94663557_94665682_707	annotated	PEG10	7	+	2121	252	37	10	11.60%	39.25812251	9.38E-11	2.20E-14	3.69E-23
ENSG00000081692_227735273_227732392_417	annotated	JMJD4	1	-	1251	19	0	6	1.92%	5.018407747	0.005554612	0.075179667	0.002457728
ENSG00000106261_100023615_100034197_527	annotated	ZKSCAN1	7	+	1581	13	4	0	1.71%	2.716946809	0.04159736	0.002943991	0.00075602
ENSG00000118418_79234560_79201688_130	annotated	HMGN3	6	-	390	48	8	9	11.54%	40.66738845	0.001592597	0.005217234	4.88E-05
ENSG00000143457_150694838_150648321_285	annotated	GOLPH3L	1	-	855	35	2	3	6.67%	13.52606853	7.18E-05	0.000316732	1.66E-07
ENSG00000099194_100347505_100360933_359	annotated	SCD	10	+	1077	2373	376	90	33.43%	728.0340449	1.20E-16	5.11E-21	1.33E-35
ENSG00000072756_3129041_3148154_434	annotated	TRNT1	3	+	1302	44	3	0	4.61%	11.16635282	0.000160693	3.71E-05	4.51E-08
ENSG00000007341_112619113_112526013_575	annotated	ST7L	1	-	1725	28	1	3	1.91%	5.363380215	0.002167177	0.008808686	0.000109703
ENSG00000152942_69391863_69414292_324	annotated	RAD17	5	+	972	13	1	0	2.16%	4.419231383	0.008175611	0.008373824	0.000350368
ENSG00000157259_92447730_92456562_269	annotated	GATAD1	7	+	807	31	4	0	4.46%	12.69281098	0.002336818	0.000978918	1.40E-05
ENSG00000033867_27484099_27376764_1237	annotated	SLC4A7	3	-	3711	79	36	5	2.67%	7.034055499	0.004239202	1.62E-06	1.28E-07
ENSG00000108306_39343241_39261460_422	annotated	FBXL20	17	-	1266	22	3	7	2.13%	5.741939719	0.010815624	0.076961241	0.004240805
ENSG00000145191_184135734_184144943_661	dORF	EIF2B5	3	+	1983	51	14	5	3.03%	8.498007608	0.018006709	0.000147131	2.64E-05
ENSG00000197121_196913008_196841234_748	dORF	PGAP1	2	-	2244	11	5	1	1.20%	1.61971829	0.082893342	0.006277459	0.003025516
ENSG00000165689_136410135_136402788_354	annotated	ENTR1	9	-	1062	21	4	1	3.67%	6.533778864	0.010017752	0.000399464	2.97E-05
ENSG00000103152_78310_79709_102	novel	MPG	16	+	306	38	12	0	8.82%	41.03286334	0.090576482	0.003580484	0.00220563
ENSG00000147044_41745600_41520420_799	annotated	CASK	X	-	2397	35	7	0	2.00%	4.824692778	0.003568196	0.00017249	4.64E-06
ENSG00000100526_54397069_54420078_207	annotated	CDKN3	14	+	621	104	21	17	14.98%	55.33646254	1.19E-05	5.22E-05	4.97E-09
ENSG00000127081_92875029_92846228_852	annotated	ZNF484	9	-	2556	12	0	0	0.59%	1.551279489	0.019679754	0.019679754	0.001784964

ENSG00000011243_15403509_15397752_356	annotated	AKAP8L	19	-	1068	12	1	2	2.53%	3.712612709	0.00650005	0.022079251	0.000737903
ENSG00000065325_9857454_9889705_339	annotated	GLP2R	17	+	1017	10	4	1	2.06%	3.24899244	0.099364848	0.016854696	0.007969224
ENSG00000119013_201078883_201085615_98	annotated	NDUFB3	2	+	294	116	26	38	18.37%	130.3707946	0.003628498	0.003298058	6.69E-05
ENSG00000134108_5122466_5178713_186	annotated	ARL8B	3	+	558	100	12	5	11.29%	59.21550737	0.000235522	0.000116357	1.94E-07
ENSG00000026508_35139304_35229333_493	annotated	CD44	11	+	1479	1839	171	66	28.60%	410.8499221	3.61E-18	2.36E-21	1.63E-37
ENSG00000117118_17054019_17018881_280	annotated	SDHB	1	-	840	374	41	11	17.14%	147.1166984	1.66E-07	1.13E-08	1.97E-14
ENSG00000078618_51878468_51789236_1170	annotated	NRDC	1	-	3510	117	14	1	4.44%	11.01408437	4.53E-08	1.04E-10	6.52E-17
ENSG00000145242_65669742_65324114_1015	annotated	EPHA5	4	-	3045	42	9	1	2.07%	4.557552153	0.001148303	3.51E-05	3.40E-07
ENSG00000147526_38787583_38848023_805	annotated	TACC1	8	+	2415	106	8	3	5.22%	14.50301793	8.51E-08	9.95E-09	8.84E-15
ENSG00000126814_60980973_60975109_509	annotated	TRMT5	14	-	1527	50	5	4	3.73%	10.81933632	0.000227153	0.000365873	5.65E-07
ENSG00000020426_60734863_60968349_309	annotated	MNAT1	14	+	927	54	4	2	5.83%	19.24791443	0.000628016	0.000313473	1.31E-06
ENSG00000166025_94799704_94870795_785	annotated	AMOTL1	11	+	2355	6	3	0	0.64%	0.841840844	0.214508587	0.016947427	0.019735972
ENSG00000121073_49705279_49705118_53	novel	SLC35B1	17	-	159	25	4	2	13.21%	51.95322816	0.013892891	0.024275373	0.00158566
ENSG00000065485_123150349_123209216_79	annotated	PDIA5	3	+	237	15	1	2	7.59%	20.91281843	0.020342454	0.032488891	0.002960206
ENSG00000136167_46151034_46127591_366	annotated	LCP1	13	-	1098	15	0	0	1.37%	4.513969004	0.020613417	0.020613417	0.001946209
ENSG00000104765_26391353_26505319_137	annotated	BNIP3L	8	+	411	125	3	0	16.79%	100.4934705	2.20E-05	1.21E-05	2.12E-09
ENSG00000198663_36899445_36923441_347	annotated	C6orf89	6	+	1041	51	1	0	6.05%	16.18784735	2.20E-05	2.21E-05	3.80E-09
ENSG00000204842_111552304_111452812_953	annotated	ATXN2	12	-	2859	22	3	0	0.94%	2.542600799	0.008219047	0.003632191	0.000162452
ENSG00000173692_231060356_231170712_953	annotated	PSMD1	2	+	2859	172	14	5	5.46%	19.87851534	1.86E-09	3.93E-10	8.21E-18
ENSG00000086848_111860565_111788446_429	dORF	ALG9	11	-	1287	35	7	1	3.96%	8.98584972	0.001612731	0.000270991	2.97E-06
ENSG00000100949_24267764_24265691_123	annotated	RABGGTA	14	-	369	12	2	3	4.07%	10.74544817	0.042234516	0.131599539	0.022155158
ENSG00000140332_70094543_70054645_463	annotated	TLE3	15	-	1389	14	4	0	1.51%	3.330392682	0.059434472	0.007979402	0.002501923
ENSG00000157778_1569339_1567698_122	annotated	PSMG3	7	-	366	26	5	0	9.02%	23.47263882	0.013458127	0.001550569	0.000128127
ENSG00000134146_35542479_35538253_93	novel	DPH6	15	-	279	19	2	0	6.45%	22.5018928	0.024135905	0.013428348	0.001529427
ENSG00000137804_41332958_41380186_441	annotated	NUSAP1	15	+	1323	130	20	12	9.98%	32.46782241	2.18E-07	1.36E-07	2.82E-13
ENSG00000087053_95865604_95835290_557	annotated	MTMR2	11	-	1671	43	9	0	3.23%	8.502794038	0.006698967	7.25E-05	4.60E-06
ENSG00000136861_120550836_120460601_663	annotated	CDK5RAP2	9	-	1989	18	5	7	1.66%	2.99024915	0.043438817	0.054573114	0.009553036
ENSG00000136861_120458619_120389236_1158	dORF	CDK5RAP2	9	-	3474	37	33	3	1.90%	3.519180671	0.004981868	7.46E-05	3.34E-06
ENSG00000174891_158123898_158225721_110	internal	RSRC1	3	+	330	22	0	13	5.45%	22.02816874	0.013853925	0.286699644	0.025305605
ENSG00000137642_121470034_121629563_2110	annotated	SORL1	11	+	6330	60	7	3	1.23%	3.13196712	0.000110358	1.59E-05	1.40E-08
ENSG00000134882_99200909_99385335_344	annotated	UBAC2	13	+	1032	179	8	2	10.76%	57.31165995	7.86E-08	5.34E-08	4.13E-14
ENSG00000197712_38878136_38943557_544	annotated	FAM114A1	4	+	1632	54	4	1	3.31%	10.93309846	0.00068505	0.000116047	5.68E-07
ENSG00000171204_85628608_85636229_230	annotated	TMEM126B	11	+	690	38	16	6	5.65%	18.19718287	0.02526281	0.005837456	0.000772927
ENSG00000092871_35026553_35011968_355	annotated	RFFL	17	-	1065	24	3	1	4.79%	7.446141546	0.000703076	0.00017104	8.33E-07
ENSG00000167767_52191902_52171523_422	annotated	KRT80	12	-	1266	116	20	13	6.64%	30.27568216	0.000408466	6.10E-05	1.85E-07
ENSG00000171634_67948103_67981442_394	annotated	BPTF	17	+	1182	11	0	1	1.27%	3.074998175	0.020613417	0.02854252	0.002645522
ENSG00000179085_155140240_155139962_92	annotated	DPM3	1	-	276	311	25	9	31.52%	372.323939	1.91E-06	1.29E-06	2.17E-11
ENSG00000107669_121927949_121743680_518	annotated	ATE1	10	-	1554	51	11	2	3.86%	10.84398268	0.004661249	0.000158386	5.80E-06
ENSG00000107669_121913838_121743680_422	annotated	ATE1	10	-	1266	34	10	0	2.84%	8.873906839	0.04432297	0.000982952	0.000346111
ENSG00000143369_150511469_150513467_300	annotated	ECM1	1	+	900	17	2	0	2.67%	6.241314477	0.013892891	0.005306578	0.000386014
ENSG00000130348_106629682_106666002_528	annotated	QRSL1	6	+	1584	47	1	0	3.41%	9.80420389	9.60E-05	8.08E-05	5.65E-08
ENSG00000130348_106629682_106652693_319	annotated	QRSL1	6	+	957	16	0	0	1.57%	5.52430564	0.021084099	0.021084099	0.002029891
ENSG00000143369_150511469_150513467_300	annotated	ECM1	1	+	900	17	2	0	2.67%	6.241314477	0.013892891	0.005306578	0.000386014
ENSG00000120915_27491209_27538805_462	novel	EPHX2	8	+	1386	9	0	0	1.30%	2.145600851	0.011772051	0.011772051	0.000681273
ENSG00000168487_22177929_22201203_461	dORF	BMP1	8	+	1383	58	14	0	4.56%	13.85719942	0.000683749	2.66E-05	1.52E-07
ENSG00000168487_22165406_22176942_189	annotated	BMP1	8	+	567	20	6	1	4.76%	11.65511574	0.058487168	0.005034873	0.001704942
ENSG00000168487_22207315_22211728_195	dORF	BMP1	8	+	585	28	2	1	5.64%	15.81509551	0.006753026	0.002296044	8.81E-05
ENSG00000198689_135985818_136044724_596	annotated	SLC9A6	X	+	1788	27	5	0	2.35%	4.98960198	0.006139455	0.000415605	1.78E-05
ENSG00000166979_32412854_32515190_441	annotated	EVA1C	21	+	1323	139	16	12	7.03%	34.71559473	1.36E-05	9.17E-06	1.01E-09
ENSG00000172500_65888418_65883974_357	annotated	FIBP	11	-	1071	195	17	17	12.32%	60.16096505	1.17E-07	9.90E-07	1.13E-12
ENSG00000001461_24419548_24469185_406	annotated	NIPAL3	1	+	1218	20	2	0	2.22%	5.425657325	0.016170007	0.003303074	0.000297492
ENSG00000141510_7676594_7669609_393	annotated	TP53	17	-	1179	335	24	10	11.20%	93.88596092	2.17E-07	1.62E-08	3.65E-14
ENSG00000163161_127289396_127257596_528	dORF	ERCC3	2	-	1584	36	0	0	3.41%	7.50960298	7.14E-05	7.14E-05	3.75E-08
ENSG00000119820_32278156_32305626_244	annotated	YIPF4	2	+	732	223	16	7	13.11%	100.6615088	5.25E-05	4.54E-06	2.04E-09
ENSG00000101019_35347256_35303935_139	dORF	UQCC1	20	-	417	32	4	3	10.07%	25.35616546	0.005958567	0.002675253	8.93E-05
ENSG00000112715_43777480_43784562_166	annotated	VEGFA	6	+	498	40	1	0	6.02%	26.53996234	0.002851	0.002488365	4.06E-05
ENSG00000130724_58554212_58551649_222	annotated	CHMP2A	19	-	666	212	24	23	20.72%	105.1795444	1.49E-07	4.01E-07	5.67E-13
ENSG00000112763_26458637_26468549_527	annotated	BTN2A1	6	+	1581	31	0	0	1.90%	6.478873159	0.002376615	0.002376615	3.27E-05
ENSG00000080823_102304968_102233684_231	novel	MOK	14	-	693	12	4	0	2.16%	5.72160227	0.019679754	0.021084099	0.001903851
ENSG00000080823_102232608_102229289_155	novel	MOK	14	-	465	13	0	0	3.87%	9.237619149	0.013428348	0.013428348	0.000872559
ENSG00000136003_108562623_108568916_167	annotated	ISCU	12	+	501	134	29	0	16.77%	88.37648537	0.00025606	1.62E-06	4.50E-09
ENSG00000172590_22834199_22834321_40	Overlap_dORF	MRPL52	14	+	120	40	5	3	17.50%	110.1408437	0.008473674	0.008776163	0.000378864
ENSG00000175595_13920199_13948347_905	annotated	ERCC4	16	+	2715	32	5	8	1.77%	3.894482871	0.002139621	0.002818126	3.48E-05
ENSG00000109736_2933923_2931907_242	annotated	MFSD10	4	-	726	106	10	40	10.33%	48.24351005	5.93E-05	0.039656989	3.71E-05
ENSG00000109736_2931623_2930838_137	dORF	MFSD10	4	-	411	76	5	3	9.49%	61.10003008	0.006656811	0.002742299	0.00010201
ENSG00000147687_124516028_124503921_139	novel	TATDN1	8	-	417	12	0	0	4.32%	9.508562047	0.012178958	0.012178958	0.000726245
ENSG00000132294_131985022_132000798_118	novel	EFR3A	8	+	354	10	0	0	4.24%	9.333969806	0.019679754	0.019679754	0.001784964
ENSG00000170266_33072698_32997045_516	annotated	GLB1	3	-	1548	228	58	7	13.37%	48.66688443	2.99E-07	1.47E-12	1.27E-17

ENSG00000159377_151399588_151400899_209	novel	PSMB4	1	+	627	243	40	9	23.92%	128.0584929	6.60E-08	3.44E-10	2.92E-16
ENSG00000204386_31862776_31861979_123	novel	NEU1	6	-	369	80	8	1	12.20%	71.63632111	0.004797061	0.000600799	1.88E-05
ENSG00000204386_31861295_31861059_78	novel	NEU1	6	-	234	42	5	0	8.97%	59.30660815	0.008473674	0.008877961	0.000383024
ENSG00000160404_127735270_127733493_72	annotated	TOR2A	9	-	216	10	2	0	6.94%	15.2973394	0.08410207	0.019679754	0.007521961
ENSG00000109323_102723876_102722811_81	novel	MANBA	4	-	243	9	2	0	6.17%	12.23787152	0.078649604	0.019679754	0.007006523
ENSG00000113013_138575318_138570956_137	novel	HSPA9	5	-	411	89	4	2	13.87%	71.55135102	0.000291516	0.000187875	3.77E-07
ENSG00000107959_3157085_3138031_595	annotated	PITRM1	10	-	1785	79	5	4	5.38%	14.62374227	1.38E-06	1.73E-06	2.10E-11
ENSG00000054611_46793523_47173626_470	annotated	TBC1D22A	22	+	1410	26	6	2	2.98%	6.092897737	0.00840293	0.001617456	8.07E-05
ENSG00000070087_149970856_149965191_188	annotated	PFN2	3	-	564	354	49	21	22.87%	207.3928653	4.70E-07	8.26E-08	3.78E-13
ENSG00000168000_62705595_62702488_118	Overlap_uORF	BSCL2	11	-	354	47	0	15	8.47%	43.86965809	0.002488365	0.018376712	0.00026737
ENSG00000168000_62705512_62690648_287	annotated	BSCL2	11	-	861	72	52	6	8.01%	27.63115243	0.166281593	0.000154735	0.000606372
ENSG00000160948_144424801_144423805_115	dORF	VPS28	8	-	345	147	6	7	24.35%	140.7887306	2.47E-06	8.35E-06	1.76E-10
ENSG00000163785_134250654_134158153_607	annotated	RYK	3	-	1821	20	4	0	2.31%	3.629022857	0.003562607	0.000328827	8.10E-06
ENSG00000225733_14947392_14947210_60	novel	FGD5-AS1	3	-	180	22	7	10	11.67%	40.38497603	0.060359788	0.162611181	0.036494482
ENSG00000084764_27022219_27026348_266	annotated	MAPRE3	2	+	798	23	0	0	4.51%	9.523456411	0.000966879	0.000966879	5.82E-06
ENSG00000075420_172112480_172397475_1204	annotated	FNDC3B	3	+	3612	129	12	4	3.74%	11.80080468	8.93E-09	1.06E-08	9.81E-16
ENSG00000139624_50167297_50144945_73	uORF	CERS5	12	-	219	23	1	0	8.22%	34.70190966	0.020342454	0.013428348	0.001295518
ENSG00000139624_50144788_50130545_333	annotated	CERS5	12	-	999	93	8	0	9.31%	30.76005545	2.63E-06	4.73E-07	1.14E-11
ENSG00000089053_121309878_121308480_129	novel	ANAPC5	12	-	387	23	0	6	8.53%	19.63751477	0.001377539	0.023141261	0.000210881
ENSG00000092871_35026553_35011968_327	annotated	RFFL	17	-	981	21	1	1	4.59%	7.073265192	0.000145094	0.000409346	4.14E-07
ENSG00000087191_63831745_63831969_39	novel	PSMC5	17	+	117	17	0	5	17.95%	48.01011136	0.006937203	0.125735014	0.005374792
ENSG00000006744_13013234_12992818_624	annotated	ELAC2	17	-	1872	56	3	2	3.69%	9.884434692	4.11E-05	4.04E-05	1.26E-08
ENSG00000275066_37586480_37518940_1156	annotated	SYNRG	17	-	3468	34	7	3	1.56%	3.23943658	0.002651267	0.000379218	6.72E-06
ENSG00000110811_6837749_6839461_160	novel	P3H3	12	+	480	25	0	0	4.38%	17.20950683	0.008473674	0.008473674	0.000366502
ENSG00000211460_121755780_121765207_148	annotated	TSN	2	+	444	93	30	4	14.86%	69.21012476	0.004918794	0.000119954	4.88E-06
ENSG00000149532_61429235_61421098_101	annotated	CPSF7	11	-	303	12	8	2	4.95%	13.08604084	0.360078543	0.020613417	0.044877087
ENSG00000151376_86671944_86464082_321	annotated	ME3	11	-	963	19	4	3	2.49%	6.51923997	0.035955189	0.020990661	0.003357915
ENSG00000135047_87727604_87731107_333	annotated	CTSL	9	+	999	1892	185	72	38.44%	625.7852141	1.07E-18	1.13E-18	1.91E-35
ENSG00000116747_193069055_193084731_538	annotated	RO60	1	+	1614	58	15	3	4.65%	11.87391995	0.001197538	1.45E-05	1.67E-07
ENSG00000171617_74636266_74634716_516	annotated	ENC1	5	-	1548	40	3	6	3.29%	8.5380499	0.000125843	0.001982636	1.87E-06
ENSG00000132780_45615198_45618141_128	annotated	NASP	1	+	384	22	6	2	8.59%	18.93045751	0.019530325	0.004400313	0.000463889
ENSG00000075415_98593979_98601528_361	annotated	SLC25A3	12	+	1083	869	114	22	29.36%	265.131283	8.76E-15	1.04E-17	1.58E-30
ENSG00000111424_47865170_47844746_376	annotated	VDR	12	-	1128	12	0	0	2.13%	3.51513331	0.004826937	0.004826937	0.000126125
ENSG00000113649_146457252_146510070_914	annotated	TCERG1	5	+	2742	35	0	6	2.08%	4.217647188	4.88E-05	0.001776495	7.29E-07
ENSG00000033627_42460922_42521120_785	annotated	ATP6V0A1	17	+	2355	48	7	7	2.42%	6.734726749	0.001210582	0.00186649	1.36E-05
ENSG00000011114_93294026_93242273_706	annotated	BTBD7	14	-	2118	20	0	2	1.56%	3.120137215	0.001448134	0.006753026	5.84E-05
ENSG00000168175_55063095_55063905_94	novel	MAPK1IP1L	14	+	282	20	0	2	6.38%	23.43422206	0.01300724	0.035772986	0.002198223
ENSG00000165914_90617972_90541368_252	annotated	TTC7B	14	-	756	10	3	0	1.98%	4.370668401	0.131599539	0.019679754	0.01228543
ENSG00000160691_154970526_154963803_584	annotated	SHC1	1	-	1752	125	27	8	5.48%	23.57466689	8.14E-05	1.47E-06	1.17E-09
ENSG00000163818_45913122_45826327_248	Overlap_dORF	LZTFL1	3	-	744	13	1	0	2.82%	5.773511968	0.019795882	0.008175611	0.000808111
ENSG00000149636_36771179_36752788_340	annotated	DSN1	20	-	1020	61	1	3	7.06%	19.76056314	9.87E-06	4.59E-05	3.66E-09
ENSG00000011454_122984539_123065557_599	annotated	RABGAP1	9	+	1797	25	2	5	2.17%	4.59686326	0.002773194	0.007087426	0.000109717
[truncated: 5,060,134 more chars]
