# Supplementary material for: The long non-coding RNA MALAT1 encodes a micropeptide that promotes influenza A virus replication by suppressing innate immune responses
Source: J Biol Chem. 2025 Dec 27;302(2):111112. doi: 10.1016/j.jbc.2025.111112 (PMC12857292; doi:10.1016/j.jbc.2025.111112)
Supplement: Supplementary Material 3 [file mmc3.pdf]

Ribo-seq datasets S2: Summary of open reading frames (ORFs) with ribosome profiling (Ribo-Seq) evidence in PR8 infected cells. Data set highlights translational activity in annotated ORFs and other including ORFs, with statistical support for frame-specific ribosome engagement.

ORF_ID	gene_name	chrom	strand	ORF_length	Psites_sum_frame0	Psites_sum_frame1	Psites_sum_frame2	Psites_coverage_frame0	Psites_frame0_RPKM	pval_frame0_vs_frame1	pval_frame0_vs_frame2	pval_combined
ENSG00000100983_34951852_34929186_450	GSS	20	-	1350	32	0	0	3.11%	13.908869305635559	0.0004030602366301852	0.0004030602366301852	1.0760858435364246e-06
ENSG00000004142_28357448_28348117_350	POLDIP2	17	-	1050	42	1	0	4.29%	23.471216953260004	0.00042975130913337566	0.0002805452199820376	8.091127106498412e-07
ENSG00000127990_94618900_94585465_278	SGCE	7	-	834	11	0	2	2.52%	7.739310146458635	0.007029302661868638	0.03522780890193623	0.0012822660609675817
ENSG00000106636_44201136_44212282_164	YKT6	7	+	492	14	0	0	4.88%	16.697003930164637	0.005160369442893307	0.005160369442893307	0.00014319601891785326
ENSG00000180596_26123904_26123524_126	HIST1H2BC	6	-	378	231	9	2	17.46%	358.58803678591676	1.9446687077430367e-05	1.633254835252562e-05	2.5122404435040085e-09
ENSG00000123836_207063386_207079019_333	PFKFB2	1	+	999	12	2	0	1.50%	7.048413499477479	0.06945717695039447	0.020613416668581838	0.006391023138248565
ENSG00000134779_36818914_36796805_217	TPGS2	18	-	651	8	0	0	2.30%	7.210819340479264	0.019216965118390855	0.019216965118390855	0.0017073955890589052
ENSG00000167468_1104125_1105405_45	GPX4	19	+	135	18	0	1	20.00%	78.23738984420002	0.0034782380676658805	0.004911637253759624	9.416876808028951e-05
ENSG00000183808_93736410_93733405_1001	RBM12B	8	-	3003	14	1	3	0.70%	2.735573071475525	0.011306643944114857	0.0272062339958007	0.001477966747020601
ENSG00000073712_52950547_52859561_626	FERMT2	14	-	1878	35	0	1	2.72%	10.935737398350641	0.00011297380978831068	0.00018702083254192264	1.498932361695684e-07
ENSG00000115129_24084326_24077579_332	TP53I3	2	-	996	27	5	1	3.31%	15.906698236396087	0.017902906441311938	0.002484757332982506	0.0002596761963296964
ENSG00000127990_94656098_94585499_455	SGCE	7	-	1365	25	3	2	2.42%	10.746894209368135	0.007887772776385514	0.0038108971419065062	0.00016292425881891603
ENSG00000076242_36993650_37050653_722	MLH1	3	+	2166	22	0	3	1.66%	5.9599119687409985	0.0009430837296224684	0.0072760284411492815	4.334637034690379e-05
ENSG00000172500_65888418_65887516_164	FIBP	11	-	492	10	1	0	3.66%	11.926431378689028	0.026832695913080365	0.011772051206276392	0.0015103817605807263
ENSG00000107566_100185620_100152131_346	ERLIN1	10	-	1038	75	0	1	5.20%	42.39742946759394	8.99016647834184e-05	0.0001127271246253546	7.322784784629293e-08
ENSG00000142534_49497931_49499635_79	RPS11	19	+	237	493	8	1	39.24%	1220.6023162402091	5.703516416371565e-07	5.112362712138789e-07	2.667508397423284e-12
ENSG00000142534_49496457_49497690_105	RPS11	19	+	315	403	37	9	16.19%	750.706383505062	0.00014339038551129576	0.0004081272808206081	4.0802313357111404e-07
ENSG00000182362_46286914_46297634_167	YBEY	21	+	501	10	0	0	2.99%	11.712184108413176	0.019679754444124863	0.019679754444124863	0.0017849636115922674
ENSG00000105171_29612127_29615380_96	POP4	19	+	288	8	0	0	5.21%	16.29945621754167	0.019679754444124863	0.019679754444124863	0.0017849636115922674
ENSG00000166471_9314441_9283424_291	TMEM41B	11	-	873	10	1	1	2.41%	6.72142524434708	0.016854695534768073	0.016854695534768073	0.0013361081351157898
ENSG00000130669_39172987_39178579_410	PAK4	19	+	1230	27	0	0	3.17%	12.88054588898415	0.0006424109906750971	0.0006424109906750971	2.6462455329680906e-06
ENSG00000149636_36771458_36752788_356	DSN1	20	-	1068	23	1	0	2.81%	12.636657067532306	0.0030814981005195803	0.0023354528714849923	4.1239111951852285e-05
ENSG00000185627_244045_252600_345	PSMD13	11	+	1035	57	17	2	5.80%	32.315443631300006	0.0072931885163897	7.41110692143134e-05	5.1712914819673005e-06
ENSG00000137210_10749246_10759101_151	TMEM14B	6	+	453	21	0	0	4.64%	27.201741502122523	0.00877616296920843	0.00877616296920843	0.00039161135667564577
ENSG00000136271_44572734_44566002_416	DDX56	7	-	1248	66	5	0	4.81%	31.031657029550487	0.0005511066486141615	3.193916253061629e-05	1.4156817129151907e-07
ENSG00000142949_43605575_43622003_773	PTPRF	1	+	2319	11	1	0	0.65%	2.7833482803564045	0.04223451612662591	0.019679754444124863	0.003715148010809822
ENSG00000156113_77637360_76877739_1163	KCNMA1	10	-	3489	15	4	2	0.69%	2.5227017361629414	0.048994651353958696	0.02389626957043539	0.005091569865673637
ENSG00000156113_77637360_76877739_1166	KCNMA1	10	-	3498	15	4	2	0.69%	2.5162110798949406	0.048994651353958696	0.02389626957043539	0.005091569865673637
ENSG00000114353_50252497_50257690_268	GNAI2	3	+	804	20	0	4	2.99%	14.596527956007465	0.005306578019052857	0.019778610614370123	0.0005526454465689531
ENSG00000114867_184320681_184334908_1404	EIF4G1	3	+	4212	86	7	5	2.71%	11.980796877851143	7.385814168744835e-07	2.993515018880905e-07	2.0477869100525147e-12
ENSG00000197771_119853104_119831474_467	MCMBP	10	-	1401	43	1	2	4.71%	18.009677533729125	2.2506448441443177e-05	6.535472090912878e-05	1.136356564208478e-08
ENSG00000117133_84479477_84497470_284	RPF1	1	+	852	15	0	0	2.82%	10.330641264639087	0.004193160480899131	0.004193160480899131	9.649553577281792e-05
ENSG00000136811_128460559_128500255_824	ODF2	9	+	2472	18	0	0	1.21%	4.272672989064321	0.002046028879997488	0.002046028879997488	2.4518070358754455e-05
ENSG00000170540_18801466_18793252_203	ARL6IP1	16	-	609	186	13	4	17.73%	179.21372550518723	1.2031547920890415e-07	1.7082149509358175e-07	1.9706210064305358e-13
ENSG00000205220_67936749_67934560_273	PSMB10	16	-	819	17	2	0	3.66%	12.179813437283885	0.010645877633714064	0.0021880057261679567	0.0001345568947401665
ENSG00000070770_58167287_58164071_135	CSNK2A2	16	-	405	9	1	1	3.70%	13.039564974033336	0.04223451612662591	0.04223451612662591	0.007344095972923886
ENSG00000145730_102866196_103029065_866	PAM	5	+	2598	10	0	2	0.92%	2.2585851571651276	0.00414960799764038	0.022789577393962826	0.0005186485208373894
ENSG00000117632_25904676_25901016_149	STMN1	1	-	447	233	7	18	23.49%	305.86093680702356	2.043479731736036e-07	1.4662072863005735e-06	2.8395761137540576e-12
ENSG00000182196_122981140_122982676_181	ARL6IP4	12	+	543	22	3	1	6.63%	23.77379249409393	0.006723915400800463	0.0016120252826907794	6.392944801064204e-05
ENSG00000115414_215435802_215361555_2176	FN1	2	-	6528	103	6	10	2.53%	9.258330829449221	4.1283047366718684e-10	4.182912146227761e-09	1.961823424431738e-17
ENSG00000138760_76179654_76161713_320	SCARB2	4	-	960	72	5	0	9.38%	44.00853178736251	4.09601459398266e-06	6.893507041361946e-07	2.554533397957012e-11
ENSG00000263001_74689207_74716955_248	GTF2I	7	+	744	16	0	0	4.03%	12.618933845838713	0.0017901637611834264	0.0017901637611834264	1.8982104156792192e-05
ENSG00000102978_57462725_57470262_219	POLR2C	16	+	657	39	0	0	6.39%	34.83171465666439	0.0004323067881491664	0.0004323067881491664	1.2320451301178172e-06
ENSG00000063046_53027859_53040223_363	EIF4B	12	+	1089	10	5	0	1.38%	5.388249989269974	0.24515715983453445	0.019679754444124863	0.02590570419655819
ENSG00000110911_51010704_50988325_553	SLC11A2	12	-	1659	22	0	0	1.63%	7.7812955541247755	0.0033030736763512156	0.0033030736763512156	6.124050385493983e-05
ENSG00000123268_50780146_50819779_271	ATF1	12	+	813	37	10	2	4.43%	26.70464413501292	0.030799869687811805	0.0039853401752523695	0.0006922265581398149
ENSG00000168385_241346173_241346337_54	SEPT2	2	+	162	18	3	1	14.81%	65.19782487016668	0.005160369442893307	0.005306578019052857	0.00014705008385520126
ENSG00000091732_130051366_130018664_459	ZC3HC1	7	-	1377	19	0	0	2.40%	8.096461911981484	0.001092092816906976	0.001092092816906976	7.350803965414657e-06
ENSG00000106028_141739167_141750354_148	SSBP1	7	+	444	109	3	4	17.57%	144.05195089557097	1.3567009445878335e-05	3.4303114258667915e-06	3.929288911145101e-10
ENSG00000111665_6850952_6849043_268	CDCA3	12	-	804	22	2	0	4.85%	16.056180751608213	0.003181324321652496	0.0005440261073422468	1.1213842753597438e-05
ENSG00000166741_114296557_114312477_264	NNMT	11	+	792	139	7	5	15.91%	102.98292791992237	9.371246260523021e-08	7.579959529567668e-09	7.527926071253008e-15
ENSG00000105323_41276293_41306565_596	HNRNPUL1	19	+	1788	83	5	3	5.03%	27.238688578307894	2.5349936190846526e-06	1.9066683571442567e-06	4.193633978817017e-11
ENSG00000133114_44989655_45028003_340	GPALPP1	13	+	1020	10	0	0	2.06%	5.752749253250001	0.007593099278532274	0.007593099278532274	0.00029783286705462064
ENSG00000040341_73688813_73561529_372	STAU2	8	-	1116	16	0	0	1.88%	8.412622563892475	0.00797940233269714	0.00797940233269714	0.000327142761693177
ENSG00000010017_13711505_13622362_729	RANBP9	6	-	2187	11	0	0	0.96%	2.951341866550755	0.007029302661868638	0.007029302661868638	0.0002573639808771639
ENSG00000123989_219543538_219539383_775	CHPF	2	-	2325	13	1	0	1.03%	3.2809227999180655	0.00912793029372963	0.005160369442893307	0.00024819240235076056
ENSG00000174444_66502750_66499407_333	RPL4	15	-	999	162	14	1	6.31%	95.15358224294597	2.7304291930629852e-05	3.23863734002329e-05	6.820103537240181e-09
ENSG00000080503_2029239_2192739_1518	SMARCA2	9	+	4554	22	4	6	0.72%	2.834688037833334	0.014015870411971724	0.03286917352493041	0.0021544021968327126
ENSG00000152894_128520358_127970227_1462	PTPRK	6	-	4386	26	1	1	0.96%	3.478406525220931	0.0006179331360801681	0.0006179331360801681	2.4552533592415123e-06
ENSG00000185085_62649512_62647020_830	INTS5	11	-	2490	13	3	2	0.72%	3.063512252935543	0.07134538375819317	0.012798402692974279	0.004461830587596429
ENSG00000170027_76358730_76329577_221	YWHAG	7	-	663	138	13	13	16.74%	122.1352918382308	2.431640334080204e-06	3.7238681197911607e-06	7.764955718593046e-11
ENSG00000184967_132145594_132152401_425	NOC4L	12	+	1275	18	0	0	2.35%	8.283958924680002	0.0021227925778456486	0.0021227925778456486	2.6309315010939275e-05
ENSG00000188352_20740279_20995629_1691	FOCAD	9	+	5073	21	2	0	0.47%	2.429014173164105	0.018151375140008384	0.005306578019052857	0.0005060696292973746
ENSG00000124107_45254543_45252415_132	SLPI	20	-	396	27	0	3	6.06%	40.00775617032956	0.004873903550586949	0.016170007243128042	0.0004174451818308815
ENSG00000108602_19745129_19738221_453	ALDH3A1	17	-	1359	1460	90	42	29.14%	630.3895649698236	3.0830000355996146e-20	4.26820705775165e-22	2.336312221601433e-40

ENSG00000185215_103126458_103135360_654	TNFAIP2	14	+	1962	79	14	7	3.36%	23.626734700656733	0.0016703017665603984	0.00018379948185116388	2.174579713992858e-06
ENSG00000120798_95051844_95022229_309	NR2C1	12	-	927	11	0	0	2.27%	6.962874500697413	0.0078820576498062	0.0078820576498062	0.00031963642907451164
ENSG00000173442_65579395_65592302_1451	EHBP1L1	11	+	4353	17	7	4	0.69%	2.29158447165989	0.07752278569762952	0.02285348025923311	0.007796148856273221
ENSG00000067248_55277195_55256488_637	DHX29	5	-	1911	13	0	0	1.26%	3.9917035634795925	0.005160369442893307	0.005160369442893307	0.00014319601891785326
ENSG00000114573_113781082_113809427_579	ATP6V1A	3	+	1737	55	1	0	2.94%	18.579691025177034	0.00018075915371093578	0.000128958049060277	1.645343788752155e-07
ENSG00000061794_27755220_27755450_76	MRPS35	12	+	228	12	0	0	7.89%	30.8831802016579	0.013217226988443315	0.013217226988443315	0.0008469697265466234
ENSG00000167985_61437727_61446071_120	SDHAF2	11	+	360	15	0	0	6.67%	24.449184326312505	0.005306578019052857	0.005306578019052857	0.00015100114740534233
ENSG00000177606_58783070_58782075_331	JUN	1	-	993	23	9	1	3.02%	13.591087359641998	0.0525859756543302	0.003457660202585786	0.0011233057048498421
ENSG00000059573_95653377_95606762_793	ALDH18A1	10	-	2379	42	0	0	2.27%	10.359301303456496	7.234804392511998e-05	7.234804392511998e-05	3.850196372848201e-08
ENSG00000162600_58539294_58480965_524	OMA1	1	-	1572	12	0	0	1.34%	4.479239876576337	0.0078820576498062	0.0078820576498062	0.00031963642907451164
ENSG00000059573_95653377_95606762_795	ALDH18A1	10	-	2385	42	0	0	2.26%	10.33324016810189	7.234804392511998e-05	7.234804392511998e-05	3.850196372848201e-08
ENSG00000017260_130934676_131016247_843	ATP2C1	3	+	2529	17	3	1	1.19%	3.9443523942805467	0.011168917508652896	0.00324779312858951	0.00020011016427610653
ENSG00000101126_50903996_50891405_1102	ADNP	20	-	3306	11	1	0	0.54%	1.9523849552772237	0.026832695913080365	0.011772051206276392	0.0015103817605807263
ENSG00000099219_5833027_5787144_904	ERMP1	9	-	2712	48	4	4	2.88%	10.385494227106197	3.4979833320767105e-05	3.4979833320767105e-05	9.355731560550685e-09
ENSG00000159720_67481086_67438528_351	ATP6V0D1	16	-	1053	44	4	0	4.27%	24.518840122113964	0.0009915726665809088	0.0003010435303081121	1.986855280261905e-06
ENSG00000168216_69790322_69676158_467	LMBRD1	6	-	1401	21	3	0	2.14%	8.795423911821201	0.01424822060979566	0.0022409900782254734	0.00018649561876446586
ENSG00000169241_155135953_155138281_175	SLC50A1	1	+	525	10	0	1	4.57%	11.17676997774286	0.00414960799764038	0.010437296944230427	0.00023253924594002017
ENSG00000008283_63437460_63434402_222	CYB561	17	-	666	14	0	3	2.70%	12.334723624085589	0.013217226988443315	0.06307108214216126	0.0040111611771127525
ENSG00000072210_19648972_19671795_393	ALDH3A2	17	+	1179	64	11	1	5.34%	31.852372455653953	0.0017398152242529606	2.5423809763485554e-05	4.08966102026247e-07
ENSG00000108312_44213282_44207242_569	UBTF	17	-	1707	33	4	3	2.46%	11.34373402837698	0.0038766098248878157	0.002532570608603844	5.56157809650073e-05
ENSG00000276043_4910907_4960803_786	UHRF1	19	+	2358	26	0	2	1.27%	6.470013155054708	0.0020082573300163734	0.009197853169380737	0.00010725224857862305
ENSG00000151348_44107836_44244287_677	EXT2	11	+	2031	49	1	1	2.66%	14.156691663093799	0.0001191030451631177	0.0001191030451631177	1.014320640544434e-07
ENSG00000239857_886593_895422_241	GET4	7	+	723	13	0	0	2.07%	10.550685352433613	0.02108409854857798	0.02108409854857798	0.002029891263157455
ENSG00000164587_150447733_150444286_151	RPS14	5	-	453	413	31	9	17.22%	534.9675828750762	9.032029441910137e-05	9.261347683862905e-06	6.906200470174873e-09
ENSG00000064199_124675265_124694446_151	SPA17	11	+	453	16	0	3	4.64%	20.72513638256954	0.00847367416878415	0.052370108323589605	0.002286989911380386
ENSG00000255292_112086908_112170527_140	AP002884.3	11	+	420	16	0	1	4.29%	22.35353995548572	0.013007239811447113	0.016503128830616266	0.001029548967667127
ENSG00000113593_65569985_65587396_477	PPWD1	5	+	1431	13	2	0	1.26%	5.330639769258911	0.044040755831095145	0.012798402692974279	0.0026804992160797854
ENSG00000136560_161231261_161235518_173	TANK	2	+	519	13	0	0	2.89%	14.697775548765899	0.02108409854857798	0.02108409854857798	0.002029891263157455
ENSG00000258315_7015802_7015879_25	C17orf49	17	+	75	18	0	0	20.00%	140.82730171956004	0.02108409854857798	0.02108409854857798	0.002029891263157455
ENSG00000117395_43172421_43164443_361	EBNA1BP2	1	-	1083	72	9	2	7.76%	39.01033288630472	2.143295495861036e-05	4.457902109835598e-06	7.996695600833288e-10
ENSG00000157214_90225101_90232624_484	STEAP2	7	+	1452	14	0	0	1.24%	5.6576624887334725	0.013428347753762199	0.013428347753762199	0.0008725593497644525
ENSG00000142192_26140194_25881670_746	APP	21	-	2238	399	7	8	13.40%	104.61366805575003	5.890478718947262e-18	5.099748989628326e-18	4.650522206698195e-34
ENSG00000090863_74607067_74472590_689	GLG1	16	-	2067	54	13	2	3.48%	15.329532117513791	0.0006414216153611048	2.960700812686211e-05	1.5569392721909822e-07
ENSG00000054523_10261953_10376587_1679	KIF1B	1	+	5037	8	1	1	0.30%	0.9319522316164386	0.0477903522728147	0.0477903522728147	0.009211062727049486
ENSG00000054523_10261953_10378402_1686	KIF1B	1	+	5058	8	1	1	0.30%	0.9280829163013051	0.0477903522728147	0.0477903522728147	0.009211062727049486
ENSG00000077942_45518729_45600446_661	FBLN1	22	+	1983	28	4	2	1.36%	8.285351420717097	0.015783443557198514	0.00798797567349413	0.0006322349400539041
ENSG00000108528_4939211_4937741_310	SLC25A11	17	-	930	28	0	0	3.23%	17.6665073841742	0.002446120450917513	0.002446120450917513	3.450690187599895e-05
ENSG00000148358_130086423_130135121_411	GPR107	9	+	1233	17	1	0	2.19%	8.090241042283457	0.004669667620227399	0.0032294771334464936	8.365569901249867e-05
ENSG00000120802_98515868_98537656_248	TMPO	12	+	744	32	0	2	5.65%	25.237867691677426	0.0003799387260530251	0.0019165825227435488	4.811222291338649e-06
ENSG00000156113_77637360_76877739_1136	KCNMA1	10	-	3408	15	4	2	0.70%	2.582660316159772	0.048994651353958696	0.02389626957043539	0.005091569865673637
ENSG00000128951_48332252_48341428_143	DUT	15	+	429	29	0	1	6.29%	39.665809536395116	0.0036321914711260127	0.005059698366439319	0.00010092379077801036
ENSG00000054116_36149378_36137203_114	TRAPPC3	1	-	342	23	0	5	7.89%	39.461841368785095	0.0035291660242640502	0.028679681212872853	0.0005792560557308559
ENSG00000145349_113759386_113457331_447	CAMK2D	4	-	1341	8	0	0	1.34%	3.500554355445191	0.011570465654371866	0.011570465654371866	0.0006594843062896773
ENSG00000155256_97738529_97759300_301	ZFYVE27	10	+	903	5	0	2	1.66%	3.249061040041529	0.012673659338734126	0.15865525393145707	0.011061610944025602
ENSG00000120727_139363893_139368798_91	PAIP2	5	+	273	72	1	0	17.58%	154.75527661490113	0.00021123378834695988	0.00021123378834695988	3.082076744927865e-07
ENSG00000175550_65919502_65921435_205	DRAP1	11	+	615	24	3	0	6.83%	22.89874824708293	0.002604389851012134	0.00039688527256343887	6.87393210415695e-06
ENSG00000057663_106316102_106186540_197	ATG5	6	-	591	11	0	0	2.54%	10.92146304931726	0.02108409854857798	0.02108409854857798	0.002029891263157455
ENSG00000057663_106316208_106186540_275	ATG5	6	-	825	13	0	0	2.18%	9.246236981587275	0.013428347753762199	0.013428347753762199	0.0008725593497644525
ENSG00000197771_119872584_119831474_640	MCMBP	10	-	1920	50	1	2	4.06%	15.280740203945316	4.092664829715607e-06	1.244462736205032e-05	4.2602396203460535e-10
ENSG00000107651_119892783_119933767_1000	SEC23IP	10	+	3000	29	0	0	1.20%	5.672210763704501	0.0008776897154483972	0.0008776897154483972	4.827634122642857e-06
ENSG00000198363_61526064_61517482_119	ASPH	8	-	357	22	2	1	8.40%	36.16013816328572	0.0023218226288164415	0.0034718995429267267	4.605086959766457e-05
ENSG00000156802_123400784_123349319_539	ATAD2	8	-	1617	15	4	3	1.30%	5.443232132017626	0.17931333536745808	0.053594824633710306	0.03686942949289057
ENSG00000156802_123347248_123321134_705	ATAD2	8	-	2115	25	0	3	1.56%	6.935938815975179	0.0012690167775026367	0.009191647611799117	7.182050815832313e-05
ENSG00000135624_73234382_73244053_148	CCT7	2	+	444	54	0	0	11.49%	71.36518668220947	0.00011392800213026252	0.00011392800213026252	9.304949505818572e-08
ENSG00000198546_133309432_133310292_120	ZNF511	10	+	360	11	1	1	5.00%	17.92940183929584	0.023672501042627626	0.023672501042627626	0.0025184098398201636
ENSG00000108602_19741305_19740328_120	ALDH3A1	17	-	360	229	6	4	16.67%	373.2575473817042	4.4655447013031034e-05	6.802234984171572e-05	2.2732989917144153e-08
ENSG00000091164_56626417_56603027_243	TXNL1	18	-	729	30	0	0	4.94%	24.147342544506177	0.000978917897162665	0.000978917897162665	5.956062365741233e-06
ENSG00000113448_58993456_58974664_499	PDE4D	5	-	1497	37	1	0	3.61%	14.502922967111227	0.0001185681716428482	8.091994981866961e-05	6.953223258948248e-08
ENSG00000100360_36767356_36758311_145	IFT27	22	-	435	22	0	0	6.90%	29.6762513202138	0.0022543491824521134	0.0022543491824521134	2.9517890247939808e-05
ENSG00000154640_17609144_17594093_296	BTG3	21	-	888	14	0	2	2.70%	9.251042718064191	0.004826937407846361	0.030313543781140352	0.0007979355218377862
ENSG00000162521_32652003_32679705_390	RBBP4	1	+	1170	33	5	0	3.33%	16.550217082426926	0.0032701130750291308	0.0006311913977015671	1.3176275336358013e-05
ENSG00000188186_100148973_100153964_99	LAMTOR4	7	+	297	50	1	1	13.13%	98.78458313661618	0.0007004692736203066	0.000883754130767968	3.915224658583823e-06
ENSG00000183978_42798681_42798061_106	COA3	17	-	318	24	0	0	5.66%	44.28531500615095	0.013428347753762199	0.013428347753762199	0.0008725593497644525
ENSG00000157168_32595851_32728082_153	NRG1	8	+	459	19	0	1	3.27%	24.28938573594445	0.02108409854857798	0.028889785561798623	0.002731532589007073
ENSG00000123179_49691424_49660968_206	EBPL	13	-	618	35	1	5	3.88%	33.23190102605583	0.008413704741378425	0.037139389724084426	0.0015764790226658531
ENSG00000179262_12946003_12948698_124	RAD23A	19	+	372	24	0	0	7.26%	37.85680153751614	0.0036321914711260127	0.0036321914711260127	7.340009712338726e-05
ENSG00000136937_97633882_97671199_790	NCBP1	9	+	2370	42	0	1	2.28%	10.398640422330383	8.641221921650504e-05	0.00011691540433181747	7.30503501382025e-08

ENSG00000134884_106567919_106543996_273	ARGLU1	13	-	819	8	2	2	1.83%	5.731676911663005	0.10105102209468636	0.10105102209468636	0.035619536184904606
ENSG00000130202_44846526_44878620_479	NECTIN2	19	+	1437	24	0	7	2.71%	9.80009058591232	0.000596156229600823	0.01907291812977645	8.578797820436054e-05
ENSG00000175567_73981586_73981512_24	UCP2	11	-	72	19	0	0	20.83%	154.84483406664586	0.02108409854857798	0.02108409854857798	0.002029891263157455
ENSG00000134330_9481342_9488329_135	IAH1	2	+	405	18	0	0	5.19%	26.079129948066672	0.008674846900444944	0.008674846900444944	0.00038311532916634116
ENSG00000122218_160340217_160290157_1185	COPA	1	-	3555	48	6	0	2.36%	7.922773655108863	1.934280799951444e-05	1.2321133142975722e-06	2.1724054605677885e-10
ENSG00000100461_22911357_22904161_307	RBM23	14	-	921	22	2	2	3.58%	14.01647049326059	0.004275489618562656	0.0031006807887314905	7.392649036285615e-05
ENSG00000090621_39572527_39561698_547	PABPC4	1	-	1641	11	0	1	1.10%	3.9333239866828165	0.012798402692974279	0.023672501042627626	0.0014375227604752335
ENSG00000090621_39572527_39561698_560	PABPC4	1	-	1680	11	0	1	1.07%	3.842014679849108	0.012798402692974279	0.023672501042627626	0.0014375227604752335
ENSG00000090621_39572527_39561698_576	PABPC4	1	-	1728	11	0	1	1.04%	3.7352920498532995	0.012798402692974279	0.023672501042627626	0.0014375227604752335
ENSG00000075790_107594044_107615305_155	BCAP29	7	+	465	22	0	0	5.16%	27.76165446084517	0.005306578019052857	0.005306578019052857	0.00015100114740534233
ENSG00000104177_48178078_48142908_547	MYEF2	15	-	1641	9	1	0	1.28%	3.218174170922304	0.017404239405933563	0.005706018193000826	0.0005167232679833428
ENSG00000275052_55598964_55549911_725	PPP4R3B	2	-	2175	14	2	3	0.97%	3.7769774407544836	0.02750441681463286	0.03282689904363471	0.003925293300512299
ENSG00000074582_218660988_218663386_419	BCS1L	2	+	1257	20	0	0	2.86%	9.336204038687352	0.00091966234969362	0.00091966234969362	5.2817946186748165e-06
ENSG00000106028_141739167_141750354_148	SSBP1	7	+	444	106	1	4	16.22%	140.0872183021149	1.4804923484658253e-05	7.785150634569525e-06	9.37847911391775e-10
ENSG00000106546_17299265_17343064_848	AHR	7	+	2544	19	0	0	1.18%	4.382400964150355	0.00228120298834518	0.00228120298834518	3.0194288573932866e-05
ENSG00000105655_18436788_18434913_390	ISYNA1	19	-	1170	11	2	0	1.79%	5.5167390274756425	0.028902805874428627	0.0078820576498062	0.0011492025149225664
ENSG00000136193_29969067_29923957_414	SCRN1	7	-	1242	20	0	1	2.90%	9.448960126111114	0.0007764301431553706	0.0016353363669355965	7.894496093549459e-06
ENSG00000147804_144413369_144412538_149	SLC39A4	8	-	447	42	2	0	6.04%	55.133731098261755	0.013494345032394814	0.0035547763465261306	0.00026296295714225076
ENSG00000148908_119515532_119500113_56	RGS10	10	-	168	17	0	0	14.29%	59.376590506758944	0.005405141725979036	0.005405141725979036	0.00015637213408961292
ENSG00000019186_54171693_54157179_372	CYP24A1	20	-	1116	284	31	12	13.44%	149.32405050909142	1.3279869519869776e-07	6.936855247376608e-09	9.908431707275139e-15
ENSG00000204427_31703281_31687212_558	ABHD16A	6	-	1674	9	4	0	1.43%	3.1547334614596783	0.04607018573370898	0.0033278027414746884	0.0009363067526309808
ENSG00000164924_100948658_100920693_168	YWHAZ	8	-	504	23	0	0	3.57%	26.777678071675602	0.013007239811447113	0.013007239811447113	0.0008218654148058079
ENSG00000164924_100948658_100920693_168	YWHAZ	8	-	504	23	0	0	3.57%	26.777678071675602	0.013007239811447113	0.013007239811447113	0.0008218654148058079
ENSG00000174231_1682280_1650802_2241	PRPF8	17	-	6723	162	13	9	3.44%	14.139287321240968	4.230064835534824e-12	1.2475742370049311e-12	6.713749630955908e-23
ENSG00000028310_878410_864468_192	BRD9	5	-	576	12	0	0	2.60%	12.224592163156252	0.019679754444124863	0.019679754444124863	0.0017849636115922674
ENSG00000127990_94656098_94585499_437	SGCE	7	-	1311	26	3	2	2.75%	11.637140365842107	0.005454749182134642	0.0026311212314026142	8.05834708867572e-05
ENSG00000206503_29942563_29945455_362	HLA-A	6	+	1086	69	1	3	4.97%	37.281629138465476	0.00011621995324350891	8.56333565309349e-05	7.199417312878499e-08
ENSG00000136261_16665444_16706088_419	BZW2	7	+	1257	14	0	0	2.63%	6.535342827081147	0.001068153208390582	0.001068153208390582	7.044127641813182e-06
ENSG00000111665_6850485_6849385_113	CDCA3	12	-	339	17	2	0	7.96%	29.42556697680089	0.014936572009614516	0.0033030736763512156	0.0002738986320205498
ENSG00000166908_57594058_57601606_352	PIP4K2C	12	+	1056	14	0	5	2.27%	7.779285922008524	0.005160369442893307	0.05268573235456097	0.0015446541073930182
ENSG00000198431_104311326_104348421_498	TXNRD1	12	+	1494	246	16	13	11.45%	96.61846336181327	4.301115028239964e-09	3.5953447909585565e-09	1.649442573520257e-16
ENSG00000090581_1351966_1363091_305	GNPTG	16	+	915	10	0	0	1.64%	6.412900806901641	0.019679754444124863	0.019679754444124863	0.0017849636115922674
ENSG00000082212_50908093_50917356_87	ME2	18	+	261	6	0	0	5.75%	13.489205145551727	0.016947426762344633	0.016947426762344633	0.0013498980316300959
ENSG00000082212_50921158_50947184_242	ME2	18	+	726	15	0	0	2.48%	12.12356247585744	0.013428347753762199	0.013428347753762199	0.0008725593497644525
ENSG00000115525_85863399_85840144_309	ST3GAL5	2	-	927	14	1	3	2.27%	8.861840273614888	0.015516259750072458	0.0581241478854986	0.004198806874037122
ENSG00000103202_397223_406365_233	NME4	16	+	699	10	2	0	2.15%	8.394569725772534	0.019679754444124863	0.019679754444124863	0.0017849636115922674
ENSG00000163468_156325081_156309199_215	CCT3	1	-	645	119	5	5	12.09%	108.25871385418374	1.6127806568958507e-05	2.170917256314447e-05	2.7648446744602324e-09
ENSG00000163468_156334775_156309199_497	CCT3	1	-	1491	327	10	8	12.88%	128.69027403950406	7.260120596143719e-12	3.278451378216868e-11	2.9281949516707194e-21
ENSG00000153914_66144377_66178868_624	SREK1	5	+	1872	11	0	2	1.12%	3.4479618921722763	0.0078820576498062	0.04363948305378087	0.0017710903275458955
ENSG00000142192_26170620_25881670_752	APP	21	-	2256	413	7	8	13.70%	107.42035241241558	1.870476249983353e-18	1.623920392565233e-18	4.771632591367041e-35
ENSG00000138663_83035261_83075324_408	COPS4	4	+	1224	22	3	0	3.19%	10.546706964291669	0.005301672691061329	0.0005897333285925991	2.059257203002657e-05
ENSG00000160789_156114919_156137764_487	LMNA	1	+	1461	200	19	11	12.53%	80.32586226303903	7.7715807493968e-10	2.148925859376437e-10	1.9056545015844746e-18
ENSG00000196776_108090908_108047288_323	CD47	3	-	969	56	0	1	6.19%	33.91094296652632	3.297819501010363e-05	5.185709832851827e-05	1.2998795509045118e-08
ENSG00000143164_167993255_168075435_712	DCAF6	1	+	2136	20	0	2	1.54%	5.494198725014046	0.0014409783181973444	0.003092538603222271	2.637025864246733e-05
ENSG00000104375_98825540_98455842_491	STK3	8	-	1473	8	0	0	1.02%	3.1868590567902246	0.016947426762344633	0.016947426762344633	0.0013498980316300959
ENSG00000115275_74462963_74461275_562	MOGS	2	-	1686	22	0	3	1.96%	7.656684059485767	0.0013845019718837994	0.01157547599262317	9.8960288269354e-05
ENSG00000115275_74465247_74462969_209	MOGS	2	-	627	9	0	3	2.87%	8.422685509543063	0.011772051206276392	0.11083400608850136	0.0068431417097640475
ENSG00000188603_28491759_28479730_363	CLN3	16	-	1089	21	2	0	2.20%	11.315324977466945	0.018151375140008384	0.005604866691947602	0.0005308776856007569
ENSG00000164258_53560678_53683130_115	NDUFS4	5	+	345	41	0	20	11.30%	69.73332573070002	0.0006980837449708158	0.024636660839484018	0.000131198986132053
ENSG00000126088_45012992_45015498_286	UROD	1	+	858	34	0	1	5.24%	23.252371107541965	0.00027320647912852615	0.0004209495667581447	7.730741880533374e-07
ENSG00000131778_147275365_147295509_470	CHD1L	1	+	1410	7	0	1	1.28%	2.9130943027095753	0.009815328628645334	0.0477903522728147	0.0023388674905236288
ENSG00000105968_44847971_44834501_90	H2AFV	7	-	270	71	0	1	10.00%	154.3015188593945	0.0037896409716948643	0.0037896409716948643	7.95824840892005e-05
ENSG00000075415_98593979_98601528_362	SLC25A3	12	+	1086	331	24	10	15.19%	178.84375717147933	2.6450888385778688e-08	1.265862928146527e-09	3.768545692194208e-16
ENSG00000100201_38507616_38490360_420	DDX17	22	-	1260	67	2	5	5.48%	31.201816187865482	1.4960527508149594e-05	6.395460654134617e-05	7.5665475963956e-09
ENSG00000071462_73693377_73694054_48	BUD23	7	+	144	14	0	0	10.42%	57.04809676139585	0.019679754444124863	0.019679754444124863	0.0017849636115922674
ENSG00000071462_73683626_73693338_140	BUD23	7	+	420	20	1	33	7.86%	27.94192494435715	0.0033869280463461717	0.5972002566814318	0.0408722032296197
ENSG00000141756_41821026_41822408_137	FKBP10	17	+	411	12	1	0	5.84%	17.13227514836497	0.016854695534768073	0.004193160480899131	0.0003819250574820159
ENSG00000141756_41813258_41819734_250	FKBP10	17	+	750	40	0	2	6.40%	31.294955937680008	0.00018853614083995916	0.0007312163512578176	9.47782637843207e-07
ENSG00000130402_38729080_38729432_77	ACTN4	19	+	231	26	1	4	11.69%	66.04454986848053	0.006142079104304129	0.028023386503852714	0.0008994912242666182
ENSG00000167986_61332968_61323871_342	DDB1	11	-	1026	26	1	0	2.92%	14.869679356353805	0.0033869280463461717	0.0022543491824521134	4.372784971036962e-05
ENSG00000095383_98213212_98199381_468	TBC1D2	9	-	1404	14	2	0	1.71%	5.851086847322651	0.019504170898671437	0.004873903550586949	0.0005060140340053911
ENSG00000015475_17739423_17735580_99	BID	22	-	297	12	0	0	5.05%	23.708299952787883	0.020613416668581838	0.020613416668581838	0.001946208561389314
ENSG00000116337_109625404_109631152_761	AMPD2	1	+	2283	7	0	1	0.66%	1.7991515404382397	0.019216965118390855	0.04178282491678763	0.003600525600033436
ENSG00000057663_106316208_106186540_275	ATG5	6	-	825	13	0	0	2.18%	9.246236981587275	0.013428347753762199	0.013428347753762199	0.0008725593497644525
ENSG00000119616_74723298_74734930_92	FCF1	14	+	276	16	0	3	7.61%	34.01625645400001	0.0078820576498062	0.059202726829632964	0.002466792732126766
ENSG00000104660_30095513_30104922_155	LEPROTL1	8	+	465	20	0	1	6.45%	25.237867691677426	0.001801896632038876	0.0040705821972625445	4.2571970118849174e-05
ENSG00000164967_34611215_34610805_136	RPP25L	9	-	408	24	0	0	7.35%	34.516495519500005	0.0022543491824521134	0.0022543491824521134	2.9517890247939808e-05

ENSG00000108654_64503080_64502922_52	DDX5	17	-	156	95	7	1	17.31%	357.33423246149044	0.028462570115697007	0.003710480643933249	0.0005988771752778722
ENSG00000063245_55678802_55695356_518	EPN1	19	+	1554	23	4	2	2.32%	8.684652347570465	0.008780371388832966	0.003975543265698643	0.00018829814118630052
ENSG00000156976_186783611_186788319_362	EIF4A2	3	+	1086	34	2	3	3.59%	18.37065783634531	0.0014584306145854712	0.0033854124258957835	2.917440139394502e-05
ENSG00000092199_21233974_21211223_233	HNRNPC	14	-	699	33	2	9	4.29%	27.70208009504936	0.0020841258324441106	0.015856692345517474	0.00019643434173390785
ENSG00000165389_34462198_34435201_68	SPTSSA	14	-	204	15	0	0	7.35%	43.14561939937501	0.019679754444124863	0.019679754444124863	0.0017849636115922674
ENSG00000135535_109382378_109368851_178	CD164	6	-	534	83	9	1	16.85%	91.20369883523317	3.789060808062779e-05	6.645159185897709e-07	2.5273488114931544e-10
ENSG00000054983_87993116_87934732_669	GALC	14	-	2007	19	0	1	1.94%	5.5549716257092685	0.0005280076130035933	0.0010273594880659044	3.4729681009304174e-06
ENSG00000006530_141601260_141652924_330	AGK	7	+	990	12	0	0	1.52%	7.1124899858363655	0.019679754444124863	0.019679754444124863	0.0017849636115922674
ENSG00000108389_58508523_58491663_1030	MTMR4	17	-	3090	11	0	0	0.49%	2.0888623502092236	0.019679754444124863	0.019679754444124863	0.0017849636115922674
ENSG00000122545_35873720_35904293_285	SEPT7	7	+	855	20	7	0	1.75%	13.725857867403512	0.13072302616481507	0.02108409854857798	0.01284698802284646
ENSG00000166206_26621357_26547793_334	GABRB3	15	-	1002	27	0	1	2.99%	15.811448546357788	0.0024321290225875155	0.0031727927861210656	4.407497946231007e-05
ENSG00000105671_18919742_18928316_483	DDX49	19	+	1449	33	3	0	2.28%	13.363529321214289	0.004659898275907902	0.0016033688297201391	4.381300645331322e-05
ENSG00000177189_20187899_20155398_506	RPS6KA3	X	-	1518	20	2	0	2.37%	7.730967375909093	0.004498799248433914	0.0008852284070317903	2.4782952419168947e-05
ENSG00000136940_122826787_122820085_301	PDCL	9	-	903	16	1	0	1.99%	10.396995328132892	0.02034245439037237	0.013428347753762199	0.0012955184805712684
ENSG00000136933_125232700_125233980_112	RABEPK	9	+	336	13	0	0	4.46%	22.702814017290184	0.02108409854857798	0.02108409854857798	0.002029891263157455
ENSG00000141429_35683456_35709770_377	GALNT1	18	+	1131	24	0	0	2.39%	12.451573980509286	0.0035547763465261306	0.0035547763465261306	7.044676146010672e-05
ENSG00000101901_111681219_111685119_132	ALG13	X	+	396	10	3	0	3.79%	14.817687470492428	0.11497452839710681	0.020613416668581838	0.010945600217888082
ENSG00000004779_23596218_23582284_132	NDUFAB1	16	-	396	225	5	11	31.82%	333.39796808607963	2.0469302697238314e-08	7.356461747733742e-08	1.5229218894484512e-14
ENSG00000100823_20455646_20457508_318	APEX1	14	+	954	109	11	4	9.43%	67.0430463287563	1.257751652788525e-05	2.805509405993715e-06	3.0087131975870506e-10
ENSG00000278053_37642119_37621086_421	DDX52	17	-	1263	28	0	1	2.14%	13.008592135615205	0.0035547763465261306	0.005670089768331009	0.00011049933550063637
ENSG00000217930_4343264_4340319_115	PAM16	16	-	345	79	0	0	4.35%	134.36421299330004	0.019679754444124863	0.019679754444124863	0.0017849636115922674
ENSG00000182979_105460864_105470215_431	MTA1	14	+	1293	17	2	0	1.86%	7.714823824544085	0.02671499548383061	0.004873903550586949	0.0007031623440447889
ENSG00000184009_81512354_81510690_375	ACTG1	17	-	1125	677	106	22	21.33%	353.11141949682275	1.1716238881727553e-10	1.211718474150954e-13	2.2486586420399057e-22
ENSG00000154370_228406333_228396648_309	TRIM11	1	-	927	6	0	0	1.62%	3.7979315458349525	0.016947426762344633	0.016947426762344633	0.0013498980316300959
ENSG00000247596_52239016_52229034_349	TWF2	3	-	1047	38	3	1	5.16%	21.296710702575936	0.00048530225960600776	0.00011662196331848013	4.0110702304161126e-07
ENSG00000039068_68737416_68833499_821	CDH1	16	+	2463	18	0	0	1.46%	4.288285679645555	0.0008296862941421134	0.0008296862941421134	4.332176089688835e-06
ENSG00000107554_99972124_99877151_1577	DNMBP	10	-	4731	32	5	3	0.76%	3.9689227568395697	0.007980324840312225	0.004244963795959635	0.00018188834843764156
ENSG00000166200_49137437_49127950_319	COPS2	15	-	957	19	0	0	3.13%	11.649768080249219	0.001801896632038876	0.001801896632038876	1.9221247299282243e-05
ENSG00000179041_66429132_66430229_365	RRS1	8	+	1095	10	1	0	1.64%	5.358725331794521	0.026832695913080365	0.011772051206276392	0.0015103817605807263
ENSG00000263001_74689207_74758838_972	GTF2I	7	+	2916	19	0	0	1.13%	3.8233292362134783	0.0012624143894574274	0.0012624143894574274	9.711623744831373e-06
ENSG00000198563_31535426_31531106_124	DDX39B	6	-	372	27	2	9	8.06%	42.588901729705654	0.006133030693106486	0.03803677030715626	0.001242185656257546
ENSG00000115970_43541206_43230948_807	THADA	2	-	2421	16	0	1	0.87%	3.877937538745974	0.008674846900444944	0.012000726946169103	0.0005220387231428462
ENSG00000070087_149968535_149966489_91	PFN2	3	-	273	64	3	8	17.58%	137.56024587991212	0.0006674249601086224	0.001736918071338517	7.284967500522852e-06
ENSG00000274760_49279128_49279205_25	RF02216	20	+	75	60	3	1	28.00%	469.4243390652001	0.027657845067597595	0.016503128830616266	0.0021005858213874354
ENSG00000147010_19645418_19536417_404	SH3KBP1	X	-	1212	26	0	0	2.23%	12.587698861071786	0.003684287313187465	0.003684287313187465	7.541963121699725e-05
ENSG00000115685_241157830_241167068_212	PPP1R7	2	+	636	20	0	0	3.77%	18.45221458589623	0.005306578019052857	0.005306578019052857	0.00015100114740534233
ENSG00000117500_93160244_93154670_172	TMED5	1	-	516	44	0	0	5.23%	50.035540016639544	0.0036321914711260127	0.0036321914711260127	7.340009712338726e-05
ENSG00000083444_11934780_11974808_727	PLOD1	1	+	2181	55	0	0	3.30%	14.797305506984184	7.344941444126607e-06	7.344941444126607e-06	4.44409566375512e-10
ENSG00000100811_100239911_100277600_139	YY1	14	+	417	27	0	5	5.76%	37.99297708261512	0.005604866691947602	0.032563251835678174	0.0009759088692525751
ENSG00000083937_87245732_87253822_165	CHMP2B	3	+	495	28	0	0	7.88%	33.19161993390304	0.0006606329705481882	0.0006606329705481882	2.7928664278936094e-06
ENSG00000011485_46387113_46390346_191	PPP5C	19	+	573	16	0	0	3.14%	16.384793684649217	0.013428347753762199	0.013428347753762199	0.0008725593497644525
ENSG00000174227_505724_539369_861	PIGG	4	+	2583	18	2	0	0.93%	4.089062186979095	0.02389626957043539	0.005306578019052857	0.0006722688034155889
ENSG00000124570_2954670_2948298_259	SERPINB6	6	-	777	52	1	2	6.95%	39.26973235423167	9.707904216161918e-05	0.0001955312910985679	1.3557960306751437e-07
ENSG00000142186_65525154_65538540_780	SCYL1	11	+	2340	8	1	0	0.90%	2.0060869190820516	0.017404239405933563	0.005706018193000826	0.0005167232679833428
ENSG00000135525_136525964_136344228_771	MAP7	6	-	2313	8	0	0	0.78%	2.029504276114138	0.011570465654371866	0.011570465654371866	0.0006594843062896773
ENSG00000093144_127327136_127289869_225	ECHDC1	6	-	675	12	0	1	3.11%	10.431651979226668	0.008175610787902942	0.013599199788808391	0.0005580304998096723
ENSG00000065427_75628685_75627895_71	KARS	16	-	213	22	0	1	14.08%	60.60642875254931	0.00228120298834518	0.003415127931522041	4.457419682640551e-05
ENSG00000065911_74198642_74214242_222	MTHFD2	2	+	666	42	0	0	6.31%	37.004170872256765	0.00046705916373637345	0.00046705916373637345	1.430492622033892e-06
ENSG00000183283_51238908_51242458_136	DAZAP2	12	+	408	20	0	0	5.15%	28.763746266250006	0.008574016250729465	0.008574016250729465	0.0003747459614100891
ENSG00000139291_71686789_71699159_262	TMEM19	12	+	786	11	0	0	3.05%	8.211939773723284	0.004687384229717438	0.004687384229717438	0.00011928172701435494
ENSG00000166340_6616817_6614546_320	TPP1	11	-	960	19	0	0	3.12%	11.61336255499844	0.0017901637611834264	0.0017901637611834264	1.8982104156792192e-05
ENSG00000101266_489860_483961_177	CSNK2A1	20	-	531	9	0	0	2.82%	9.945430912398308	0.019216965118390855	0.019216965118390855	0.0017073955890589052
ENSG00000103510_31117682_31130992_467	KAT8	16	+	1401	14	2	2	1.50%	5.863615941214134	0.03299602752967379	0.03299602752967379	0.004661188023718761
ENSG00000163875_37514740_37496592_189	MEAF6	1	-	567	13	0	0	4.23%	13.453519417653443	0.004826937407846361	0.004826937407846361	0.00012612451166366307
ENSG00000155368_119367052_119368550_123	DBI	2	+	369	14	4	3	4.07%	22.262671906886183	0.12378039365238508	0.1029516053660342	0.04344365798954223
ENSG00000125166_58734228_58722141_127	GOT2	16	-	381	11	0	0	3.94%	16.941167092248037	0.020613416668581838	0.020613416668581838	0.001946208561389314
ENSG00000095787_28590832_28610850_232	WAC	10	+	696	9	0	0	2.16%	7.587677894372846	0.020613416668581838	0.020613416668581838	0.001946208561389314
ENSG00000144034_73734569_73729943_214	TPRKB	2	-	642	27	4	0	4.67%	24.677681376091126	0.01980017951014062	0.0022276761777358703	0.00026398470517929683
ENSG00000091651_46691100_46695709_140	ORC6	16	+	420	11	0	4	3.57%	15.368058719396432	0.02108409854857798	0.06539853090342929	0.006119906528703008
ENSG00000109332_102809680_102797415_110	UBE2D3	4	-	330	19	0	1	5.45%	33.78432743272273	0.013428347753762199	0.013428347753762199	0.0008725593497644525
ENSG00000142208_104776759_104770341_418	AKT1	14	-	1254	19	0	0	1.91%	8.890612482295456	0.0053557511377634245	0.0053557511377634245	0.00015366972994913032
ENSG00000142945_44739933_44756096_229	KIF2C	1	+	687	17	1	0	3.93%	14.520039599906116	0.003819354202256993	0.002943990679779778	6.312952134771863e-05
ENSG00000108671_32477470_32479469_110	PSMD11	17	+	330	87	0	0	8.18%	154.69665719194094	0.0035547763465261306	0.0035547763465261306	7.044676146010672e-05
ENSG00000108671_32480173_32480631_55	PSMD11	17	+	165	19	1	4	10.91%	67.56865486544547	0.019795881618345192	0.06688490016463032	0.005944028397503504
ENSG00000134283_42335903_42441509_348	PPHLN1	12	+	1044	15	1	1	2.01%	8.43075321596983	0.013892891352654344	0.013892891352654344	0.0009300961024180418
ENSG00000177426_3456398_3457940_252	TGIF1	18	+	756	34	0	2	2.78%	26.38959578078175	0.008574016250729465	0.013648824643852793	0.0005848802985757839
ENSG00000129566_20405585_20368437_2382	TEP1	14	-	7146	13	0	2	0.21%	1.0674706842722506	0.02108409854857798	0.03577298629450316	0.003354604907114977

ENSG00000100813_23090621_23059148_1211	ACIN1	14	-	3633	45	11	5	1.40%	7.268130765873247	0.006607599257270876	0.0011210739524526398	4.554783231696604e-05
ENSG00000129474_22982266_22973443_538	AJUBA	14	-	1614	19	2	3	1.49%	6.907576240891266	0.01006837577517317	0.03624304254342325	0.0017889048977041129
ENSG00000163754_148991641_149026933_333	GYG1	3	+	999	12	0	0	1.80%	7.048413499477479	0.013217226988443315	0.013217226988443315	0.0008469697265466234
ENSG00000153113_96663137_96771679_754	CAST	5	+	2262	121	0	4	5.70%	31.388342742533826	4.0077545563218955e-09	4.42202571771872e-08	1.9053817941938333e-15
ENSG00000125746_45638587_45618860_413	EML2	19	-	1239	9	0	0	1.21%	4.262327533884989	0.020613416668581838	0.020613416668581838	0.001946208561389314
ENSG00000138777_105473957_105369725_201	PPA2	4	-	603	7	0	2	2.49%	6.811713046136817	0.019216965118390855	0.1191346346731873	0.010787784428637617
ENSG00000197157_128089654_128092058_49	SND1	7	+	147	13	0	0	10.20%	51.892146325234705	0.02108409854857798	0.02108409854857798	0.002029891263157455
ENSG00000163950_1712188_1693597_270	SLBP	4	-	810	52	1	0	8.89%	37.669854369429636	6.4153405496546115e-06	6.676689036413407e-06	3.5466033930174656e-10
ENSG00000168496_61795362_61796504_380	FEN1	11	+	1140	36	0	1	3.42%	18.52990812099474	0.0006606329705481882	0.000844481639333518	3.542401178992254e-06
ENSG00000109736_2933923_2930870_480	MFSD10	4	-	1440	35	7	2	2.71%	14.262024190348962	0.008429359628173896	0.0011887586598383483	6.192528206631547e-05
ENSG00000012660_53295699_53290464_105	ELOVL5	6	-	315	29	0	1	10.48%	54.02105489242382	0.0014481341328822738	0.0014532536214945922	1.2682655484270462e-05
ENSG00000111678_6944529_6945922_91	C12orf57	12	+	273	51	3	0	12.09%	109.61832093555498	0.0014055202957965589	0.0014915192737735051	1.2636475669781728e-05
ENSG00000198925_219225073_219220447_668	ATG9A	2	-	2004	8	0	0	1.05%	2.3424368216826355	0.005706018193000826	0.005706018193000826	0.0001733096755673334
ENSG00000146918_158701899_158631666_1143	NCAPG2	7	-	3429	50	0	3	1.92%	8.556144996084866	1.4965834940431001e-05	0.0001066924919191799	1.2766188626507607e-08
ENSG00000155545_56938897_56923128_450	MIER3	5	-	1350	16	1	4	2.00%	6.954434652817779	0.006112543793728684	0.031335693934481226	0.001007533225434303
ENSG00000143398_151231761_151247865_453	PIP5K1A	1	+	1359	12	0	1	1.10%	5.181284095642385	0.019679754444124863	0.02784981298332479	0.002477909089056847
ENSG00000132507_7309636_7311640_154	EIF5A	17	+	462	179	71	8	11.69%	227.34566204726954	0.007938263751100936	9.685105774909149e-05	7.0979032624909975e-06
ENSG00000164535_6446076_6409837_502	DAGLB	7	-	1506	7	0	0	1.00%	2.727399048353586	0.016947426762344633	0.016947426762344633	0.0013498980316300959
ENSG00000146731_56055714_56055830_38	CCT6A	7	+	114	30	0	3	23.68%	154.4159010082895	0.0036321914711260127	0.003684287313187465	7.440341320821008e-05
ENSG00000135473_56333046_56317609_1186	PAN2	12	-	3558	8	0	4	0.42%	1.3193489012512651	0.019679754444124863	0.2463583386135439	0.026068547507937054
ENSG00000134333_18402941_18407281_159	LDHA	11	+	477	494	20	8	21.38%	607.6929336955159	1.6871854087431992e-07	5.793694942921583e-07	9.241568657874308e-13
ENSG00000198755_35468435_35470750_217	RPL10A	6	+	651	62	9	2	5.99%	55.8838498887143	0.009775943147511958	0.0007004692736203066	4.6185824074800754e-05
ENSG00000132305_86195382_86144268_372	IMMT	2	-	1116	40	0	0	4.84%	21.031556409731188	8.25698237868128e-05	8.25698237868128e-05	4.9782384041413114e-08
ENSG00000065000_2111353_2102173_243	AP3D1	19	-	729	16	0	0	2.88%	12.878582690403295	0.00797940233269714	0.00797940233269714	0.000327142761693177
ENSG00000169976_144095497_144095237_86	SF3B5	6	-	258	54	0	0	10.47%	122.81450731356979	0.003684287313187465	0.003684287313187465	7.541963121699725e-05
ENSG00000134900_102634053_102678316_800	TPP2	13	+	2400	18	3	0	0.75%	4.400853178736251	0.06272892732212154	0.013007239811447113	0.003936255353260731
ENSG00000169045_179623133_179620884_91	HNRNPH1	5	-	273	54	4	6	13.19%	116.06645746117584	0.0009829516685359422	0.008711444927384192	5.441942251909839e-05
ENSG00000142687_35470901_35441018_638	KIAA0319L	1	-	1914	15	0	1	0.78%	4.59859266325627	0.02108409854857798	0.03577298629450316	0.003354604907114977
ENSG00000132676_155729286_155738242_173	DAP3	1	+	519	23	0	0	4.05%	26.003756740124285	0.00847367416878415	0.00847367416878415	0.0003665024264111182
ENSG00000114125_141738342_141742682_68	RNF7	3	+	204	22	0	0	13.24%	63.280241785750015	0.0036062891729786097	0.0036062891729786097	7.240558534568731e-05
ENSG00000111641_6567918_6556993_808	NOP2	12	-	2424	26	4	2	2.10%	6.293849430535893	0.0010891986948716168	0.0002707893389517208	1.9824595550127703e-06
ENSG00000141736_39707049_39728044_1211	ERBB2	17	+	3633	9	0	1	0.41%	1.4536261531746493	0.020613416668581838	0.03537493353818881	0.003252526348827943
ENSG00000121897_39465049_39477115_240	LIAS	4	+	720	14	0	0	2.08%	11.409619352279169	0.02108409854857798	0.02108409854857798	0.002029891263157455
ENSG00000109501_6277456_6302468_890	WFS1	4	+	2670	10	0	0	0.79%	2.1976794900056187	0.007593099278532274	0.007593099278532274	0.0002978328670546206 4
ENSG00000116857_201143891_201135663_74	TMEM9	1	-	222	11	1	1	8.11%	29.0747056853446	0.019795881618345192	0.019795881618345192	0.001804673467972721
ENSG00000133067_202225433_202319207_797	LGR6	1	+	2391	14	0	0	0.63%	3.435769942969888	0.02108409854857798	0.02108409854857798	0.002029891263157455
ENSG00000113621_134874428_134899685_360	TXNDC15	5	+	1080	38	3	0	4.72%	20.64597787555278	0.0007671900197604208	0.00011297380978831068	6.233850222657666e-07
ENSG00000088038_54142979_54155407_753	CNOT3	19	+	2259	8	3	0	0.80%	2.0780183225551134	0.11368416502207135	0.011570465654371866	0.006956434060967934
ENSG00000154719_25607460_25585707_333	MRPL39	21	-	999	24	0	0	3.90%	14.096826998954958	0.000609140140830996	0.000609140140830996	2.3883185421048832e-06
ENSG00000092470_43828097_43866392_562	WDR76	15	+	1686	5	0	0	0.89%	1.740155468064947	0.012673659338734126	0.012673659338734126	0.000782701129001274
ENSG00000120438_159780023_159779623_98	TCP1	6	-	294	25	0	0	6.12%	49.89629454349491	0.013640585738808999	0.013640585738808999	0.00089863692579514
ENSG00000170540_18798783_18793252_174	ARL6IP1	16	-	522	182	13	4	19.54%	204.58627804086785	2.6069271415592943e-07	3.546892249393905e-07	8.640010002378163e-13
ENSG00000136811_128471335_128494731_508	ODF2	9	+	1524	12	0	0	1.38%	4.620318297885828	0.007029302661868638	0.007029302661868638	0.0002573639808771639
ENSG00000104872_49451550_49446382_282	PIH1D1	19	-	846	11	0	0	2.84%	7.629532697572697	0.004193160480899131	0.004193160480899131	9.649553577281792e-05
ENSG00000074054_121469859_121340861_1223	CLASP1	2	-	3669	9	3	0	0.57%	1.4393632636913332	0.06180520118411761	0.005706018193000826	0.0020032236666657145
ENSG00000164587_150447733_150444286_151	RPS14	5	-	453	289	31	9	16.56%	374.34777591016234	0.00015161219108303402	1.4727994620521913e-05	1.8097672615244504e-08
ENSG00000066926_57586599_57550712_422	FECH	18	-	1266	15	0	0	2.13%	6.952374689946684	0.0033278027414746945	0.0033278027414746945	6.211806171561056e-05
ENSG00000101266_489860_483961_177	CSNK2A1	20	-	531	9	0	0	2.82%	9.945430912398308	0.019216965118390855	0.019216965118390855	0.0017073955890589052
ENSG00000011376_45419682_45547530_747	LARS2	3	+	2241	13	0	0	0.80%	3.4039025032617145	0.012798402692974279	0.012798402692974279	0.0007972436888943567
ENSG00000143624_153759604_153773270_633	INTS3	1	+	1899	15	0	2	1.11%	4.634916459964456	0.007029302661868638	0.015555382717448078	0.000555563984002246
ENSG00000160679_153636589_153645269_202	CHTOP	1	+	606	19	2	3	2.97%	18.3974060277203	0.013428347753762199	0.02034245439037237	0.0012955184805712684
ENSG00000127990_94656098_94588678_435	SGCE	7	-	1305	26	3	2	2.76%	11.690644459478163	0.005454749182134642	0.0026311212314026142	8.05834708867572e-05
ENSG00000186575_29636751_29683055_523	NF2	22	+	1569	14	0	0	1.34%	5.235771786896751	0.007029302661868638	0.007029302661868638	0.0002573639808771639
ENSG00000088986_120496422_120498210_89	DYNLL1	12	+	267	251	5	9	15.73%	551.6175519914102	0.00048083181382083286	0.000745376529342948	2.3179087696108e-06
ENSG00000101266_489860_483961_177	CSNK2A1	20	-	531	9	0	0	2.82%	9.945430912398308	0.019216965118390855	0.019216965118390855	0.0017073955890589052
ENSG00000163882_184365001_184368294_114	POLR2H	3	+	342	14	1	7	6.14%	24.020251267956144	0.023878473930440184	0.2074676260485629	0.024066247317836537
ENSG00000102317_48575181_48575814_118	RBM3	X	+	354	53	2	2	8.47%	87.85130639285171	0.0061547572324231374	0.0062201494882096935	0.00020209328853378674
ENSG00000204386_31862776_31860173_267	NEU1	6	-	801	13	1	1	2.62%	9.523277790024347	0.013599199788808391	0.013599199788808391	0.0008935241583747976
ENSG00000172428_240133981_240131051_28	COPS9	2	-	84	41	0	3	35.71%	286.40473067966076	0.002418182715931848	0.011381896039302706	0.00015741134072802708
ENSG00000198911_41884880_41899396_273	SREBF2	22	+	819	10	7	0	1.83%	7.164596139578756	0.27598757962657733	0.020613416668581838	0.031163316063832016
ENSG00000104064_50309723_50286020_335	GABPB1	15	-	1005	14	0	7	1.79%	8.174055655364182	0.012798402692974279	0.2405700776118731	0.018919609472776648
ENSG00000169919_65976050_65960897_359	GUSB	7	-	1077	14	0	3	2.79%	7.627600681189417	0.0017901637611834264	0.019679754444124863	0.00021847779822210372
ENSG00000198055_177430916_177441790_544	GRK6	5	+	1632	6	4	0	0.92%	2.1572809699687503	0.165899918248885	0.016947426762344633	0.014398895220848395
ENSG00000150459_21140610_21147342_153	SAP18	13	+	459	39	6	1	9.15%	49.85716019483335	0.004314308702745973	0.0005561355897365262	1.572761018517368e-05
ENSG00000284952_155842476_155828210_243	AC104472.3	3	-	729	11	1	0	2.06%	8.854025599652266	0.04223451612662591	0.019679754444124863	0.003715148010809822
ENSG00000284952_155853997_155842717_351	AC104472.3	3	-	1053	18	0	0	1.99%	10.03043459541026	0.008674846900444944	0.008674846900444944	0.0003831153291663411 6
ENSG00000026025_17230663_17234854_155	VIM	10	+	465	332	16	17	25.81%	418.94860368184527	9.090437681470475e-08	5.830304873158548e-07	5.148657087106556e-13

ENSG00000184432_139371787_139357863_174	COPB2	3	-	522	10	0	2	3.45%	11.241004287959772	0.012798402692974279	0.061356297364084836	0.0037940651817215715
ENSG00000072042_67693048_67678321_222	RDH11	14	-	666	160	0	7	13.51%	140.96826998954958	6.434050522204316e-07	2.8321435931818703e-06	1.6458430809204054e-11
ENSG00000075785_128795368_128813422_98	RAB7A	3	+	294	34	0	3	11.22%	67.85896057915308	0.0015806110104534784	0.005089012094854324	4.727447707517822e-05
ENSG00000114744_149751405_149741521_124	COMMD2	3	-	372	14	0	0	4.84%	22.08313423021775	0.013007239811447113	0.013007239811447113	0.0008218654148058079
ENSG00000138382_169822052_169811820_126	METTL5	2	-	378	13	0	0	5.56%	20.180279126480162	0.00797940233269714	0.00797940233269714	0.000327142761693177
ENSG00000198721_4133671_4115874_364	ECI2	6	-	1092	37	0	0	2.75%	19.88175428733105	0.0023766146074493525	0.0023766146074493525	3.265611492547055e-05
ENSG00000159176_201496303_201483990_153	CSRP1	1	-	459	124	4	5	7.84%	158.52020164511114	0.001806384452599662	0.001811541904428426	1.936593110542893e-05
ENSG00000175166_184299267_184301786_164	PSMD2	3	+	492	9	1	0	3.05%	10.733788240820124	0.03537493353818881	0.020613416668581838	0.003252526348827943
ENSG00000103222_15949752_16141281_1531	ABCC1	16	+	4593	85	5	8	2.48%	10.859206624358265	1.7781853253748688e-07	1.5928185811945543e-06	2.709907289268901e-12
ENSG00000101052_43603877_43646983_329	IFT52	20	+	987	12	0	0	2.13%	7.134108496431613	0.006937202941512724	0.006937202941512724	0.00025101522741487095
ENSG00000131459_180338586_180331477_131	GFPT2	5	-	393	9	1	0	3.82%	13.437719629729012	0.03537493353818881	0.020613416668581838	0.003252526348827943
ENSG00000169446_135973675_135965024_131	MMGT1	X	-	393	12	0	0	4.58%	17.916959506305346	0.013217226988443315	0.013217226988443315	0.0008469697265466234
ENSG00000198561_57791641_57816308_879	CTNND1	11	+	2637	64	7	2	2.73%	14.241163111572245	0.0002556913665467353	1.5363660848022635e-05	3.2544366602591786e-08
ENSG00000120889_23068894_23022671_440	TNFRSF10B	8	-	1320	83	1	8	4.32%	36.896041801526145	7.457932414905961e-05	0.0009689468400989585	5.484167037042924e-07
ENSG00000115504_62707273_63045500_1133	EHBP1	2	+	3399	27	5	0	1.50%	4.661097806251987	0.0009758619863385294	0.00010000342828152886	7.17841410011521e-07
ENSG00000103126_346269_293424_497	AXIN1	16	-	1491	12	1	0	1.01%	4.722578863835011	0.03537493353818881	0.020613416668581838	0.003252526348827943
ENSG00000156831_125363239_125366885_66	NSMCE2	8	+	198	12	0	0	7.58%	35.562449929181824	0.019679754444124863	0.019679754444124863	0.0017849636115922674
ENSG00000165997_18659638_18675216_179	ARL5B	10	+	537	26	0	1	5.03%	28.410225362419002	0.003684287313187465	0.00510952817114065	0.00010325221210932265
ENSG00000179222_51894305_51901930_834	MAGED1	X	+	2502	18	0	2	0.96%	4.221441898068346	0.005554612275049112	0.018318557839637383	0.0005316522383050482
ENSG00000104529_143586845_143579792_257	EEF1D	8	-	771	158	0	4	9.34%	120.24812835976267	8.279378352254905e-06	3.52747712715908e-05	2.3742621826643e-09
ENSG00000175224_46657603_46672332_255	ATG13	11	+	765	11	6	1	1.96%	8.437365571433336	0.172059248067656	0.03577298629450316	0.02599789514137937
ENSG00000132589_28884299_28880561_379	FLOT2	17	-	1137	32	0	6	3.96%	16.514488621467024	0.00028351878813536103	0.0036118516004294207	7.226275455663078e-06
ENSG00000132589_28884299_28880561_379	FLOT2	17	-	1137	32	0	6	3.96%	16.514488621467024	0.00028351878813536103	0.0036118516004294207	7.226275455663078e-06
ENSG00000110911_51010704_50988325_553	SLC11A2	12	-	1659	22	0	0	1.63%	7.7812955541247755	0.0033030736763512156	0.0033030736763512156	6.124050385493983e-05
ENSG00000149476_61344153_61346503_202	TKFC	11	+	606	8	0	0	2.48%	7.746276222198022	0.019216965118390855	0.019216965118390855	0.0017073955890589052
ENSG00000006530_141555555_141652924_394	AGK	7	+	1182	16	0	0	1.52%	7.942882217685281	0.013007239811447113	0.013007239811447113	0.0008218654148058079
ENSG00000172775_57173854_57154290_254	FAM192A	16	-	762	24	0	0	4.72%	18.481273191543313	0.0009313263635146231	0.0009313263635146231	5.411461678703094e-06
ENSG00000149925_30068931_30070212_200	ALDOA	16	+	600	269	48	15	27.00%	263.0732233511226	4.8302786019336356e-08	4.76568555482099e-10	2.83008912382655e-16
ENSG00000149925_30067255_30067724_128	ALDOA	16	+	384	134	16	11	23.44%	204.76191873286723	1.1970365748366949e-05	3.367522327008567e-06	3.402952953596963e-10
ENSG00000198363_61714371_61626273_313	ASPH	8	-	939	376	19	31	18.85%	234.96212924456236	2.4787263172953206e-10	5.626848283165933e-09	1.633962855191027e-17
ENSG00000175063_45812696_45816767_179	UBE2C	20	+	537	134	3	0	15.08%	146.4219307140056	2.328039183165384e-06	2.3336565222259586e-06	4.698709817896599e-11
ENSG00000104738_47961145_47976778_742	MCM4	8	+	2226	49	4	3	4.04%	12.916550210127362	7.406184732479553e-06	2.0116003228725672e-06	1.28520500620118e-10
ENSG00000167767_52191902_52171398_452	KRT80	12	-	1356	63	2	7	4.65%	27.261922346153767	0.00010819541046960865	0.0017091408758760419	1.3956394432117088e-06
ENSG00000143870_10819289_10784258_439	PDIA6	2	-	1317	602	19	19	27.56%	268.2170198531231	3.82016279060415e-21	4.275049111014097e-20	2.764494454277351e-39
ENSG00000138182_89705295_89774048_1780	KIF20B	10	+	5340	26	0	4	0.79%	2.856983337007304	0.00040182005258836655	0.004021069509754137	1.0981592821747484e-05
ENSG00000283782_132575861_132642364_1213	AC116366.3	5	+	3639	38	3	1	1.48%	6.1274130545746095	0.00040966467025599373	0.00011295777531333699	3.2792132837715125e-07
ENSG00000110321_10803521_10797816_650	EIF4G2	11	-	1950	98	2	1	6.00%	29.48947771050616	6.683999657642746e-08	2.5043965068970413e-08	1.681434228220179e-14
ENSG00000110321_10804047_10803521_72	EIF4G2	11	-	216	20	0	3	9.72%	54.3315207251389	0.008674846900444944	0.020779353648479937	0.000893938215340485
ENSG00000067596_43490430_43526598_1090	DHX8	17	+	3270	27	0	4	1.47%	4.844975976590368	0.00016643457103121306	0.0019307453316667101	2.321420159408011e-06
ENSG00000165119_83978252_83969350_463	HNRNPK	9	-	1389	209	5	5	5.83%	88.29165484577648	9.329072057653913e-06	8.072931950304863e-06	6.159359715239583e-10
ENSG00000130177_114239476_114272443_498	CDC16	13	+	1494	36	0	5	3.01%	14.139287321240968	0.00028877520139654507	0.0025792363906108322	5.131036494031878e-06
ENSG00000129235_6641083_6643019_123	TXNDC17	17	+	369	82	6	2	15.45%	130.39564974033337	0.00015198091919181207	6.167689547256311e-05	6.864474087791452e-08
ENSG00000241973_20813381_20708047_1775	PI4KA	22	-	5325	15	0	0	0.28%	1.652902602342254	0.02108409854857798	0.02108409854857798	0.002029891263157455
ENSG00000114491_124730472_124738033_142	UMPS	3	+	426	10	0	0	3.52%	13.774188352852116	0.019679754444124863	0.019679754444124863	0.0017849636115922674
ENSG00000171848_10122799_10129386_389	RRM2	2	+	1167	52	1	1	4.37%	26.1461714132288	0.00012152394927894852	0.00018495144114449941	1.589708426019472e-07
ENSG00000105793_90346742_90385154_308	GTPBP10	7	+	924	8	0	2	1.62%	5.080349989883118	0.019216965118390855	0.09718295540155453	0.008620840042435133
ENSG00000198242_28722772_28723881_70	RPL23A	17	+	210	113	19	2	8.57%	315.74375187123576	0.013439622348536238	0.013856139512579892	0.0008993698691259064
ENSG00000103356_23557343_23525127_534	EARS2	16	-	1602	10	0	0	0.94%	3.662799150009364	0.020613416668581838	0.020613416668581838	0.001946208561389314
ENSG00000127837_218265460_218264533_106	AAMP	2	-	318	14	0	0	6.60%	25.833100420254723	0.00797940233269714	0.00797940233269714	0.000327142761693177
ENSG00000127837_218270086_218266420_233	AAMP	2	-	699	27	1	0	4.29%	22.66533825958584	0.0035004709947243163	0.0023628488670607213	4.7187726630981656e-05
ENSG00000187514_231710179_231712851_136	PTMA	2	+	408	178	8	9	7.35%	255.99734176962505	0.0025025372014526115	0.004926212446124825	6.957895768210636e-05
ENSG00000131981_55137374_55145271_250	LGALS3	14	+	750	38	0	0	4.00%	29.730208140796005	0.002446120450917513	0.002446120450917513	3.450690187599895e-05
ENSG00000120690_40958911_40933425_560	ELF1	13	-	1680	14	0	0	1.79%	4.8898368652625015	0.0014903523101034196	0.0014903523101034196	1.3356391343355004e-05
ENSG00000269190_38950319_38942608_278	FBXO17	19	-	834	9	0	3	2.16%	6.332162847102519	0.012178957859018494	0.07986216920813294	0.004852065185980775
ENSG00000109113_28718165_28717320_49	RAB34	17	-	147	17	3	0	10.20%	67.85896057915308	0.0682897090290637	0.021557223391537678	0.006517186294745621
ENSG00000109113_28717266_28714643_259	RAB34	17	-	777	76	7	3	8.11%	57.3942242100309	0.0006038791504987458	0.00011732921528396223	5.042084532308569e-07
ENSG00000071859_154444326_154449026_209	FAM50A	X	+	627	19	2	0	3.35%	17.781224964590912	0.03299602752967379	0.00847367416878415	0.0014019198044660757
ENSG00000130669_39172987_39178579_500	PAK4	19	+	1500	29	0	0	3.00%	11.344421527409002	0.00026886811829191485	0.00026886811829191485	4.91773397748167e-07
ENSG00000126858_32192201_32224733_438	RHOT1	17	+	1314	7	0	1	1.14%	3.125923110213471	0.019216965118390855	0.04178282491678763	0.003600525600033436
ENSG00000196497_24187138_24182046_711	IPO4	14	-	2133	28	1	8	2.25%	7.702696609133616	0.00043205369195467813	0.009509567664187285	2.9858015919478986e-05
ENSG00000125779_3908189_3923294_273	PANK2	20	+	819	14	2	0	2.56%	10.03043459541026	0.02750441681463286	0.00837382380159347	0.0011505359199841657
ENSG00000108671_32444524_32477616_314	PSMD11	17	+	942	34	1	4	5.41%	21.17891126355733	0.00017168856529097966	0.00111867268915165	1.3459853116853916e-06
ENSG00000131473_41913873_41867810_1091	ACLY	17	-	3273	333	28	19	9.72%	59.6999331304276	6.536951109028016e-17	1.5133401133889646e-17	1.507319833723374e-32
ENSG00000101160_59006328_58997599_113	CTSZ	20	-	339	19	0	3	5.31%	32.88739838583629	0.013428347753762199	0.013007239811447113	0.0008468635297504387
ENSG00000163479_156020167_156009540_183	SSR2	1	-	549	482	4	5	19.13%	515.1696981544318	3.916593558687141e-07	2.9831695092088113e-07	1.0870913852094975e-12
ENSG00000010256_48600720_48599447_159	UQCRC1	3	-	477	30	0	1	5.03%	36.90442917179246	0.005604866691947602	0.008364861494636245	0.00024596970711672695
ENSG00000239672_51155655_51161118_82	NME1	17	+	246	181	14	0	21.95%	431.7368159085428	0.0012475315725757993	9.070903305645646e-05	8.53415363079334e-07

ENSG00000239672_51161199_51161276_25	NME1	17	+	75	81	3	2	36.00%	633.7228577380201	0.0053878105512546945	0.0038428970276066315	0.00011306125532763197
ENSG00000111641_6567918_6556993_812	NOP2	12	−	2436	26	4	2	2.09%	6.262845246149016	0.0010891986948716168	0.0002707893389517208	1.9824595550127703e-06
ENSG00000120727_139363893_139364770_78	PAIP2	5	+	234	60	1	0	16.67%	150.45651893115388	0.0007148938932365558	0.0007076576786684489	3.22010567298996e-06
ENSG00000240857_18555727_18555191_178	RDH14	2	−	534	11	0	0	3.93%	12.087237195030902	0.0078820576498062	0.0078820576498062	0.00031963642907451164
ENSG00000106028_141739167_141750354_148	SSBP1	7	+	444	109	3	4	17.57%	144.05195089557097	1.3567009445878335e-05	3.4303114258667915e-06	3.929288911145101e-10
ENSG00000185608_19432555_19435962_206	MRPL40	22	+	618	28	0	2	5.83%	26.585520820844668	0.0009549346450480869	0.0039853401752523695	2.3409618695501826e-05
ENSG00000120705_138518803_138508305_387	ETF1	5	−	1161	33	0	0	3.62%	16.67851333887985	0.00044280702707823286	0.00044280702707823286	1.290505655922651e-06
ENSG00000276410_26043480_26043277_67	HIST1H2BB	6	−	201	140	2	0	16.42%	408.70278276820903	0.0016417304930345215	0.0016340149330375125	1.6007320882608168e-05
ENSG00000204217_202518954_202559946_787	BMPR2	2	+	2361	10	0	2	1.02%	2.485304632916138	0.00414960799764038	0.03755618289012509	0.0008890075989244122
ENSG00000146007_140700461_140705756_199	ZMAT2	5	+	597	22	5	1	7.04%	21.62339920317086	0.004606456612177916	0.0006175442218579485	1.847587981627041e-05
ENSG00000163931_53235113_53225756_457	TKT	3	−	1371	332	28	4	16.19%	142.09416536255145	1.46068562328868e-11	3.8297745198965425e-14	8.518083228020116e-24
ENSG00000163931_53255942_53235065_145	TKT	3	−	435	49	8	15	11.72%	66.09710521320346	0.0009301095727979364	0.010235139143676047	6.174416878967672e-05
ENSG00000232119_120604854_120612264_182	MCTS1	X	+	546	17	2	1	3.85%	18.269720155925828	0.03299602752967379	0.013892891352654344	0.0021461203908066497
ENSG00000171155_120627097_120626210_295	C1GALT1C1	X	−	885	36	1	3	4.75%	23.869034189755936	0.0006325693003332578	0.002713573254137601	1.0896677684161084e-05
ENSG00000133265_55279608_55262608_359	HSPBP1	19	−	1077	46	2	1	6.13%	25.062116523908085	3.3624968141452445e-05	2.158727805327666e-05	5.638215995335599e-09
ENSG00000147548_38348138_38275641_1377	NSD3	8	−	4131	7	2	0	0.36%	0.9943023400679014	0.1191346346731873	0.019216965118390855	0.010787784428637617
ENSG00000068724_46956939_47073923_728	TTC7A	2	+	2184	16	3	0	0.82%	4.298757683747254	0.06341282918854552	0.013428347753762199	0.004086769345423957
ENSG00000111328_123267253_123261736_87	CDK2AP1	12	−	261	16	0	0	8.05%	35.97121372147127	0.00847367416878415	0.00847367416878415	0.0003665024264111182
ENSG00000011566_39356301_39250618_809	MAP4K3	2	−	2427	12	0	0	0.62%	2.9012629114042032	0.019679754444124863	0.019679754444124863	0.0017849636115922674
ENSG00000138760_76213543_76161713_478	SCARB2	4	−	1434	87	5	1	8.16%	35.59964914458892	1.2993260804208283e-07	2.437884849652543e-08	3.1972940475432517e-14
ENSG00000166562_59139949_59155811_156	SEC11C	18	+	468	30	3	0	5.77%	37.61412973278847	0.00699534207690609	0.003684287313187465	0.00014031246620721993
ENSG00000159921_36246469_36217365_663	GNE	9	−	1989	15	0	3	1.36%	4.425191733269232	0.00327844299461777	0.027614347364305233	0.0005229495287998096
ENSG00000139496_25301774_25338790_540	NUP58	13	+	1620	30	2	0	2.04%	10.86630414502778	0.004118201609641029	0.001573068226067653	3.8031009531462414e-05
ENSG00000242372_35284487_35279197_226	EIF6	20	−	678	112	8	15	11.95%	96.93127945299118	1.8755282191312573e-05	8.860707314829683e-05	1.3019391600462996e-08
ENSG00000176697_27658504_27657821_227	BDNF	11	−	681	10	1	0	2.64%	8.616452626013217	0.023672501042627626	0.012798402692974279	0.0014375227604752335
ENSG00000140694_14630125_14551651_524	PARN	16	−	1572	18	0	2	1.53%	6.718859814864506	0.0053557511377634245	0.016228925652561413	0.00045523357042063324
ENSG00000113552_142012035_142002029_289	GNPDA1	5	−	867	13	0	2	2.77%	8.798322387323532	0.004687384229717438	0.013018137044106636	0.0003237395824878957
ENSG00000091651_46695593_46697585_92	ORC6	16	+	276	12	0	4	6.52%	25.512192340500008	0.013007239811447113	0.04223451612662591	0.0026029828050034526
ENSG00000131844_71592941_71656860_515	MCCC2	5	+	1545	20	0	2	2.14%	7.595863091669905	0.0013845019718837994	0.007443125733849675	6.20801023785377e-05
ENSG00000132463_70836185_70824319_318	GRSF1	4	−	954	41	4	3	3.46%	25.218026600724848	0.0027029703238042042	0.004224170501915509	6.426303143589004e-05
ENSG00000145860_159203617_159158670_663	RNF145	5	−	1989	34	2	1	2.71%	10.03043459541026	0.00041617781144845243	0.00011977551446593921	3.5184401231069343e-07
ENSG00000147155_48527177_48528457_110	EBP	X	+	330	34	0	0	5.45%	60.456164879609105	0.013640585738808999	0.013640585738808999	0.00089863692579514
ENSG00000141002_89873668_89889264_216	TCF25	16	+	648	17	1	0	4.63%	15.393930872122688	0.00392533363377418	0.0020082573300163734	4.538242985436214e-05
ENSG00000133657_194460735_194405919_1177	ATP13A3	3	−	3531	37	0	1	1.02%	6.148647884951998	0.0010447178140462346	0.0017534708537449623	1.1163276398224378e-05
ENSG00000168538_183666308_183712622_1001	TRAPPC11	4	+	3003	9	0	0	0.60%	1.7585826888056948	0.012178957859018494	0.012178957859018494	0.0007262454987071801
ENSG00000115204_27322517_27309912_176	MPV17	2	−	528	25	1	3	7.95%	27.7831640071733	0.0006738559393031653	0.003231801322599742	1.3802715270828426e-05
ENSG00000018699_32664305_32820938_629	TTC27	2	+	1887	11	4	0	1.27%	3.420553610040541	0.040206607807213995	0.0033278027414746884	0.0008023897325825505
ENSG00000134330_9481342_9488329_135	IAH1	2	+	405	18	0	0	5.19%	26.079129948066672	0.008674846900444944	0.008674846900444944	0.00038311532916634116
ENSG00000163694_40438758_40425904_479	RBM47	4	−	1437	14	0	0	1.46%	5.716719508448853	0.00797940233269714	0.00797940233269714	0.000327142761693177
ENSG00000163694_40438758_40425904_548	RBM47	4	−	1644	14	0	0	1.28%	4.996913584939782	0.00797940233269714	0.00797940233269714	0.000327142761693177
ENSG00000113368_126777539_126836264_576	LMNB1	5	+	1728	43	2	0	3.47%	14.601596194881079	0.00013249008517324145	3.592951532853599e-05	3.5962012264093225e-08
ENSG00000250479_23766242_23766007_44	CHCHD10	22	−	132	11	5	0	15.91%	48.89836865262501	0.16840675538759814	0.0078820576498062	0.008508220424389708
ENSG00000131238_40092463_40074101_274	PPT1	1	−	822	50	27	0	6.93%	35.69223989242702	0.029409789393099052	5.3019663895075235e-05	2.2801154976199626e-05
ENSG00000131238_40074162_40074061_33	PPT1	1	−	99	26	0	0	15.15%	154.10394969312125	0.019679754444124863	0.019679754444124863	0.0017849636115922674
ENSG00000105063_55247103_55230528_881	PPP6R1	19	−	2643	20	5	6	1.25%	4.440260490590239	0.025291128564067122	0.02118350383597727	0.002418053026064387
ENSG00000104723_15540431_15743570_276	TUSC3	8	+	828	30	0	0	4.35%	21.260160283750004	0.0010405263639310788	0.0010405263639310788	6.697970672456085e-06
ENSG00000101361_2655702_2657390_190	NOP56	20	+	570	9	2	3	2.63%	9.26495406049737	0.08350359792828288	0.13115678761539135	0.0383832462008165
ENSG00000136485_63550678_63589172_142	DCAF7	17	+	426	27	0	0	4.23%	37.190308552700714	0.013640585738808999	0.013640585738808999	0.00089863692579514
ENSG00000013561_141978375_141987790_348	RNF14	5	+	1044	28	0	0	3.16%	15.73740600314368	0.00151350169064872	0.00151350169064872	1.3757299738441384e-05
ENSG00000101079_36721735_36653520_363	NDRG3	20	−	1089	18	1	0	1.65%	9.698849980685953	0.023672501042627626	0.012798402692974279	0.0014375227604752335
ENSG00000115414_215435802_215361555_2386	FN1	2	−	7158	111	6	10	2.51%	9.099277318426449	5.430240679044199e-11	5.795991638210809e-10	3.7472105056715723e-19
ENSG00000115207_27343497_27333630_565	GTF3C2	2	−	1695	16	0	0	1.06%	5.538930254456639	0.013007239811447113	0.013007239811447113	0.0008218654148058079
ENSG00000126012_53214694_53192744_1074	KDM5C	X	−	3222	13	7	0	0.47%	2.3675187802015834	0.2226213721087219	0.02108409854857798	0.02404775518195576
ENSG00000170889_54201185_54206565_139	RPS9	19	+	417	135	11	0	8.63%	189.9648854130756	0.004183873134198697	0.001100170263453246	2.7943281629777465e-05
ENSG00000088682_57458318_57460624_92	COQ9	16	+	276	16	0	0	8.70%	34.01625645400001	0.004873903550586949	0.004873903550586949	0.00012846764509679302
ENSG00000134987_111092457_111126883_895	WDR36	5	+	2685	37	0	1	2.35%	8.085987218534639	2.160433169782662e-05	2.133701000158462e-05	3.611990854309992e-09
ENSG00000113648_135389093_135369370_170	H2AFY	5	−	510	35	1	0	6.47%	40.26924477275001	0.001558047347358915	0.001558047347358915	1.4544595481456893e-05
ENSG00000113163_75511207_75356927_584	COL4A3BP	5	−	1752	14	4	0	1.20%	4.688884665320207	0.0750151653743942	0.008175610787902942	0.0033080285523474006
ENSG00000115816_37231567_37201764_1054	CEBPZ	2	−	3162	34	1	2	1.42%	6.3094669229193565	0.0006384869324604317	0.0006629387121614735	2.7117238785804994e-06
ENSG00000124155_45420349_45425826_316	PIGT	20	+	948	52	2	0	4.11%	32.18626797387975	0.0022083206986541785	0.0006768518708446028	9.39609878359303e-06
ENSG00000255508_62571557_62559679_317	AP002990.1	11	−	951	174	0	2	1.89%	107.36045609535334	0.013640585738808999	0.013640585738808999	0.00089863692579514
ENSG00000168610_42345636_42315745_672	STAT3	17	−	2016	31	1	0	2.08%	9.022913263281996	0.0005938231711225875	0.00044280702707823286	1.7158342196872716e-06
ENSG00000198160_66925029_66986473_486	MIER1	1	+	1458	12	1	2	1.65%	4.829468508901235	0.008811045481162216	0.029607078753113344	0.0012962940759966696
ENSG00000198160_66958068_66986473_370	MIER1	1	+	1110	11	0	2	1.89%	5.81494113706892	0.007593099278532274	0.0416322583317752	0.0016329495508977223
ENSG00000198160_66958068_66986473_390	MIER1	1	+	1170	11	0	2	1.79%	5.5167390274756425	0.007593099278532274	0.0416322583317752	0.0016329495508977223
ENSG00000086189_62403341_62389010_285	DIMT1	5	−	855	40	2	1	5.26%	27.451715734807024	0.0006660403445293448	0.00042798108473948845	1.8585197475478207e-06
ENSG00000184831_23907702_23840342_198	APOO	X	−	594	12	0	0	3.03%	11.854149976393941	0.013007239811447113	0.013007239811447113	0.0008218654148058079

ENSG00000169299_37826733_37861612_612	PGM2	4	+	1836	10	0	0	0.82%	3.195971807361112	0.020613416668581838	0.020613416668581838	0.001946208561389314
ENSG00000188986_137255510_137272928_580	NELFB	9	+	1740	21	0	4	1.55%	7.0818327014146565	0.0033030736763512156	0.0360990988508288	0.0007068562907806174
ENSG00000162613_77966722_77948873_507	FUBP1	1	−	1521	44	0	6	3.94%	16.974581623001978	3.77282131074845e−05	0.000691895620082398	2.0925469637748268e−07
ENSG00000163479_156020896_156012527_136	SSR2	1	−	408	305	4	3	20.59%	438.6471305603126	6.88154288683408e−06	5.175318599856146e−06	2.9634539867614995e−10
ENSG00000162613_77966722_77948766_496	FUBP1	1	−	1488	44	0	6	4.03%	17.35103403802823	3.77282131074845e−05	0.000691895620082398	2.0925469637748268e−07
ENSG00000182325_144358447_144355531_533	FBXL6	8	−	1599	12	0	0	1.69%	4.403605432131333	0.0025000845279814113	0.0025000845279814113	3.597708735589818e−05
ENSG00000160124_122383224_122359869_144	CCDC58	3	−	432	12	0	0	4.86%	16.29945621754167	0.0078820576498062	0.0078820576498062	0.00031963642907451164
ENSG00000175756_1374756_1373801_199	AURKAIP1	1	−	597	71	5	5	9.55%	69.78460651932414	0.00036318786265457023	0.0004249631561444351	1.0245332642003257e−06
ENSG00000108679_78977191_78972966_210	LGALS3BP	17	−	630	72	3	9	12.38%	67.06061986645716	1.8182011455729523e−05	0.00011731883236475379	1.685098450841827e−08
ENSG00000002330_64284368_64270209_168	BAD	11	−	504	7	0	0	2.98%	8.149728108770836	0.019216965118390855	0.019216965118390855	0.0017073955890589052
ENSG00000114867_184320681_184334908_1403	EIF4G1	3	+	4209	90	7	5	2.78%	12.546979839590167	4.60942195086692e−07	1.9011841143914044e−07	8.246422000810537e−13
ENSG00000173436_19597046_19625592_140	MICOS10	1	+	420	17	0	0	3.57%	23.75063620270358	0.02108409854857798	0.02108409854857798	0.002029891263157455
ENSG00000100439_22598307_22603766_162	ABHD4	14	+	486	18	0	1	3.70%	21.73260829005556	0.013428347753762199	0.02034245439037237	0.0012955184805712684
ENSG00000075415_98593979_98601528_361	SLC25A3	12	+	1083	372	26	11	18.56%	201.55338657924105	6.063592031365678e−10	1.71753698610492e−11	1.3066797127050397e−19
ENSG00000115211_27369534_27364400_493	EIF2B4	2	−	1479	51	0	0	3.45%	20.23380771832759	0.0001294826452477558	0.0001294826452477558	1.1929552110054982e−07
ENSG00000169908_149377547_149371829_101	TM4SF1	3	−	303	225	18	11	30.69%	435.7280374986387	5.381626697199e−06	2.7317486244990195e−06	1.2517684303451258e−10
ENSG00000169908_149375705_149369866_73	TM4SF1	3	−	219	119	3	16	20.55%	318.84415724177404	0.0017782831266420149	0.027696813277064525	0.00031786470673432726
ENSG00000105852_95396293_95360089_226	PON3	7	−	678	32	0	2	4.42%	27.694651272283192	0.0023766146074493525	0.005451642374001699	7.337249061775614e−05
ENSG00000141522_81869930_81868876_204	ARHGDIA	17	−	612	196	7	0	17.16%	187.9231422728334	3.0930198244530585e−07	1.0062128811291726e−07	2.99248989215105e−13
ENSG00000087302_51989640_52004409_193	RTRAF	14	+	579	127	4	6	11.92%	128.70658691986273	2.2656324292883543e−05	0.0001570867839260124	2.787505104680662e−08
ENSG00000147804_144413369_144412538_149	SLC39A4	8	−	447	42	2	0	6.04%	55.133731098261755	0.013494345032394814	0.0035547763465261306	0.00026296295714225076
ENSG00000104960_49857059_49860279_202	PTOV1	19	+	606	23	5	0	4.46%	22.27054413881931	0.028679681212872853	0.003327802741474694 5	0.0005517678081496619
ENSG00000117139_202808305_202729036_1539	KDM5B	1	−	4617	24	5	0	0.78%	3.0501906372007803	0.0111945450140469 5	0.000978917897162665	7.106965662114738e−05
ENSG00000155957_66169858_66138153_131	TMBIM4	12	−	393	39	0	1	8.40%	58.23011839549238	0.001588175367824666	0.001759868229841518	1.6653375375657385e−05
ENSG00000256269_119088273_119093283_304	HMBS	11	+	912	15	0	0	1.64%	9.650993813018093	0.019679754444124863	0.019679754444124863	0.0017849636115922674
ENSG00000133112_45341069_45337476_197	TPT1	13	−	591	219	12	5	10.66%	217.43640070913457	4.394701515060627e−05	4.7119642833443635e−05	1.5619215085792663e−08
ENSG00000156136_70993836_71029378_260	DCK	4	+	780	21	1	1	3.46%	15.797934487771158	0.0050845836060656145	0.0050845836060656145	0.0001392267422259072
ENSG00000115286_1391131_1395488_73	NDUFS7	19	+	219	18	2	0	12.33%	48.228527986150695	0.011630309792725807	0.0035291660242640502	0.00022446083915843008
ENSG00000178035_49029350_49024383_514	IMPDH2	3	−	1542	96	1	5	7.59%	36.531076970054485	2.4292373466780832e−08	3.1905032254399335e−07	7.963744970094789e−14
ENSG00000197153_27890792_27890382_136	HIST1H3J	6	−	408	266	28	0	18.38%	382.55782534112507	0.000363826001714688	5.898571273469576e−06	2.0403572714680864e−08
ENSG00000152056_223837450_223770203_137	AP1S3	2	−	411	10	2	0	3.65%	14.276895956970806	0.06556525143482543	0.019679754444124863	0.00579448895258773
ENSG00000196419_41622005_41663815_609	XRCC6	22	+	1827	46	10	6	3.45%	14.773891349889986	0.0017348806905588586	0.00027598514109279714	3.254294054705697e−06
ENSG00000266967_42961207_42956174_125	AARSD1	17	−	375	15	0	0	4.80%	23.471216953260004	0.012798402692974279	0.012798402692974279	0.0007972436888943567
ENSG00000117020_243843170_243505249_479	AKT3	1	−	1437	14	3	0	1.46%	5.716719508448853	0.05989746521295916	0.008175610787902942	0.002573001276540562
ENSG00000137497_72022389_72003527_1994	NUMA1	11	−	5982	37	5	3	0.90%	3.6293673824415755	0.0010297636725339206	0.0004366288061172337	2.913957752380149e−06
ENSG00000126247_36141012_36149839_238	CAPNS1	19	+	714	497	21	14	23.53%	408.4451969807501	3.6771565918670896e−11	5.697776399594709e−11	2.491134717731067e−20
ENSG00000122218_160340217_160305747_416	COPA	1	−	1248	14	1	0	2.16%	6.582472703237983	0.00912793029372963	0.0032294771334464936	0.0001622779595684381
ENSG00000122218_160305718_160290157_725	COPA	1	−	2175	32	5	0	2.48%	8.633091293153106	0.0005837204194495279	7.484487117395401e−05	3.231691175812666e−07
ENSG00000117593_173825230_173857705_594	DARS2	1	+	1782	22	0	1	2.69%	7.244202763351853	0.00010834798793830713	0.00025750057329094263	1.978171745478083e−07
ENSG00000171793_40983291_40996120_307	CTPS1	1	+	921	10	3	0	1.63%	6.371122951482086	0.12114404826813535	0.020613416668581838	0.011598140826794287
ENSG00000156261_29063467_29062320_92	CCT8	21	−	276	28	2	2	11.96%	59.528448794500015	0.0013984928336429232	0.0013984928336429232	1.1821238358622835e−05
ENSG00000146731_56056380_56058796_160	CCT6A	7	+	480	36	1	0	10.00%	44.00853178736251	0.0002316811405554319	0.0001837862173985288	2.947987176325142e−07
ENSG00000120885_27610571_27598241_449	CLU	8	−	1347	460	12	4	17.15%	200.38529692835195	1.5830288136621456e−14	2.9827041344232324e−14	6.49000258331343e−27
ENSG00000075142_88220026_88206478_158	SRI	7	−	474	26	0	4	6.33%	32.18626797387975	0.0024042815392806777	0.008339388582650115	0.00011374251558657479
ENSG00000116133_54887119_54877924_153	DHCR24	1	−	459	10	0	0	3.92%	12.783887229444447	0.011570465654371866	0.011570465654371866	0.0006594843062896773
ENSG00000116133_54876020_54852233_378	DHCR24	1	−	1134	49	0	4	5.82%	25.354709671731488	1.492853633392576e−05	0.00014421184258919996	1.7393667732461633e−08
ENSG00000115275_74462963_74461275_562	MOGS	2	−	1686	22	0	3	1.96%	7.656684059485767	0.0013845019718837994	0.01157547599262317	9.8960288269354e−05
ENSG00000141198_54912675_54942370_326	TOM1L1	17	+	978	17	3	1	3.07%	10.199659718952457	0.01664033820237559	0.00397428314897603	0.0003597475883221794
ENSG00000285589_12669403_12668359_106	AC010422.8	19	−	318	66	5	0	20.75%	121.78461626691512	0.00013744675240103787	1.6926289629330058e−05	1.8569686374469487e−08
ENSG00000163479_156020896_156014940_130	SSR2	1	−	390	305	4	3	21.54%	458.89238274001934	6.88154288683408e−06	5.175318599856146e−06	2.9634539867614995e−10
ENSG00000183258_177514993_177511791_382	DDX41	5	−	1146	20	1	5	2.36%	10.240496052905762	0.006142079104304129	0.04227848696273928	0.0013942017966237796
ENSG00000015479_139307593_139329358_735	MATR3	5	+	2205	95	12	5	4.90%	25.280789235370754	6.160484141620512e−06	4.192770642272378e−07	2.4378752091924582e−11
ENSG00000112576_41941649_41937182_136	CCND3	6	−	408	16	0	0	4.41%	23.010997013000004	0.011570465654371866	0.011570465654371866	0.0006594843062896773
ENSG00000104549_125018088_125018792_91	SQLE	8	+	273	20	3	0	7.69%	42.98757683747254	0.05989746521295916	0.00847367416878415	0.0026479354932912483
ENSG00000013374_151345350_151360251_267	NUB1	7	+	801	18	0	3	3.00%	13.186076940033711	0.005306578019052857	0.03149539866061481	0.0008997191470395496
ENSG00000130165_11554302_11553746_68	ELOF1	19	−	204	22	0	0	7.35%	63.280241785750015	0.020613416668581838	0.020613416668581838	0.001946208561389314
ENSG00000166946_43190380_43192884_172	CCNDBP1	15	+	516	21	0	0	5.23%	23.880598644305238	0.0033030736763512156	0.0033030736763512156	6.124050385493983e−05
ENSG00000186575_29636751_29694802_557	NF2	22	+	1671	14	0	0	1.26%	4.916173509061043	0.007029302661868638	0.007029302661868638	0.0002573639808771639
ENSG00000113712_149525134_149501235_259	CSNK1A1	5	−	777	29	0	0	4.63%	21.900427659090738	0.000978917897162665	0.000978917897162665	5.956062365741233e−06
ENSG00000090316_1289914_1339169_355	MAEA	4	+	1065	34	0	0	3.66%	18.732896159878877	0.0006909589238230294	0.0006909589238230294	3.0452559464443365e−06
ENSG00000087274_2881994_2928523_671	ADD1	4	+	2013	13	0	1	1.04%	3.789441385896424	0.00797940233269714	0.015625008558487224	0.0006253789790324684
ENSG00000175166_184301652_184308890_747	PSMD2	3	+	2241	87	1	1	4.95%	22.77996290644378	5.174064629976779e−08	5.174064629976779e−08	2.6496459359340756e−14
ENSG00000161057_103347712_103368054_433	PSMC2	7	+	1299	83	14	1	5.77%	37.49251360894112	0.00042670678534371807	6.332363126702123e−06	2.5872920010490292e−08
ENSG00000148175_121370187_121341202_123	STOM	9	−	369	19	0	0	6.50%	30.213626159345534	0.005554612275049112	0.005554612275049112	0.0001646845769100730 6
ENSG00000124762_36684102_36685800_164	CDKN1A	6	+	492	29	2	8	6.71%	34.58665099819818	0.007205323565060537	0.012371379225907928	0.0004534421781350234 7
ENSG00000106615_151519511_151467119_184	RHEB	7	−	552	27	0	0	3.80%	28.701216383062505	0.008574016250729465	0.008574016250729465	0.0003747459614100891
ENSG00000089289_70133948_70165981_339	IGBP1	X	+	1017	15	4	0	2.06%	8.654578522588498	0.058290625655499875	0.007029302661868638	0.00221264198215177

ENSG00000141424_36127007_36109593_755	SLC39A6	18	-	2265	35	1	1	2.25%	9.067247167374175	0.00018129710408843058	0.00018129710408843058	2.2919265383054332e-07
ENSG00000186468_82278323_82276109_143	RPS23	5	-	429	828	27	20	9.79%	1132.5272515908673	0.012180114527427986	0.014519426903687729	0.0008576173033568209
ENSG00000164251_76833322_76833801_159	F2RL1	5	+	477	8	1	0	3.14%	9.84118111247799	0.04223451612662591	0.019679754444124863	0.003715148010809822
ENSG00000164253_77492220_77433135_459	WDR41	5	-	1377	27	1	0	2.61%	11.505498506500002	0.001600023614510982	0.0006608884069239612	6.6501872000915935e-06
ENSG00000164574_154297970_154416972_506	GALNT10	5	+	1518	33	9	2	2.77%	12.756096170250004	0.026473509998160383	0.0009306875619951529	0.00017940172874903487
ENSG00000130731_636290_634597_204	METTL26	16	-	612	16	1	1	4.41%	15.340664675333336	0.006432154271512831	0.006432154271512831	0.00021751960674370818
ENSG00000108829_50397317_50382988_307	LRRC59	17	-	921	96	1	0	8.79%	61.162780334228025	3.0626218387986296e-06	2.4134746032240404e-06	6.356555054681895e-11
ENSG00000166986_57512813_57517296_294	MARS	12	+	882	30	5	4	3.06%	19.958517817397965	0.004274506774981852	0.02193685758795414	0.0005109559657840735
ENSG00000166986_57488091_57512308_546	MARS	12	+	1638	39	5	2	3.85%	13.970962472178575	0.0004847635194590202	4.771265688604687e-05	1.7716182690730894e-07
ENSG00000120742_150546135_150544458_66	SERP1	3	-	198	29	3	0	13.64%	85.94258732885608	0.01748038679108207	0.0035547763465261306	0.00034382947735276184
ENSG00000070087_149970856_149966489_140	PFN2	3	-	420	117	7	8	17.86%	163.46026092448932	5.398461708679084e-05	4.6277835475447794e-05	1.8755319313621784e-08
ENSG00000181929_49005362_49002899_247	PRKAG1	12	-	741	29	0	0	4.05%	22.964416047386646	0.0023628488670607213	0.0023628488670607213	3.2295294885504634e-05
ENSG00000132305_86195382_86144268_747	IMMT	2	-	2241	55	1	2	3.35%	14.401125975338022	6.688252526459028e-06	1.717267842574393e-05	9.398810624366696e-10
ENSG00000235027_1761117_1761687_148	AC068580.3	11	+	444	9	0	8	4.05%	11.894197780368247	0.011772051206276392	0.2802221369599416	0.022062594115594084
ENSG00000033867_27434087_27376764_1012	SLC4A7	3	-	3036	24	0	2	1.38%	4.638580425545456	0.0003577397812417709	0.0017304525386319881	4.101878901667356e-06
ENSG00000100461_22911357_22901730_411	RBM23	14	-	1233	32	2	2	3.41%	15.22868902076886	0.0009603792052290822	0.0007056271418515881	4.275063095079128e-06
ENSG00000204592_30489532_30490003_113	HLA-E	6	+	339	8	2	0	4.42%	13.847325636141596	0.10105102209468636	0.019679754444124863	0.009164497669452725
ENSG00000163754_148991641_149026991_279	GYG1	3	+	837	9	0	0	1.79%	6.3094669229193565	0.020613416668581838	0.020613416668581838	0.001946208561389314
ENSG00000145191_184140487_184144943_417	EIF2B5	3	+	1251	7	0	0	1.20%	3.2833436984976028	0.016947426762344633	0.016947426762344633	0.0013498980316300959
ENSG00000131051_35732158_35704481_504	RBM39	20	-	1512	31	1	0	2.78%	12.030551017709328	0.0006179331360801681	0.0004168743852437731	1.684212386012298e-06
ENSG00000100714_64388428_64454926_922	MTHFD1	14	+	2766	51	5	1	3.15%	10.819161827695773	1.6361887884711613e-05	1.249978300523906e-06	1.849646658037528e-10
ENSG00000136319_20305934_20300526_157	TTC5	14	-	471	10	0	0	3.18%	12.458183096210194	0.019679754444124863	0.019679754444124863	0.0017849636115922674
ENSG00000115275_74464717_74461275_718	MOGS	2	-	2154	26	0	3	1.95%	7.082772061104459	0.00036930943616753716	0.003539467718397726	8.906402084963395e-06
ENSG00000117139_202755365_202729059_1032	KDM5B	1	-	3096	21	5	0	0.97%	3.980099774050873	0.023966077693362193	0.0023218226288164415	0.0003365714315363229
ENSG00000100503_50821942_50729692_1951	NIN	14	-	5853	14	0	3	0.41%	1.4035410787016918	0.005257622967929454	0.04085312694387945	0.0011826962990484254
ENSG00000274523_75073737_75043032_464	RCC1L	7	-	1392	8	0	1	1.08%	3.372301286387932	0.019216965118390855	0.03418265644467716	0.0029560527921161815
ENSG00000117593_173825230_173856764_539	DARS2	1	+	1617	19	0	1	2.41%	6.89476070055566	0.00047442668928020845	0.0010353552647448026	3.165909631142535e-06
ENSG00000103274_10757875_10769105_141	NUBP1	16	+	423	6	0	4	3.55%	8.323126579170214	0.016947426762344633	0.165899918248885	0.014398895220848395
ENSG00000126456_49662587_49659648_281	IRF3	19	-	843	13	0	3	2.14%	9.048808433937724	0.013428347753762199	0.05989746521295916	0.003846583043074148
ENSG00000139624_50167297_50130545_392	CERS5	12	-	1176	15	1	0	2.81%	7.484444181524236	0.0022543491824521134	0.0006703205586147392	9.509667761901958e-06
ENSG00000101871_10567547_10449368_667	MID1	X	-	2001	34	2	0	3.15%	9.97028206410345	0.00012098605422020245	1.97867302464402e-05	1.8808280444408308e-08
ENSG00000136521_179618481_179624040_53	NDUFB5	3	+	159	37	2	0	22.64%	136.5463879356321	0.0017658144955514316	0.0010405263639310788	1.1198112383099405e-05
ENSG00000104549_124998600_125021945_479	SQLE	8	+	1437	113	3	3	7.93%	46.14209317533717	1.6427632435488223e-07	9.512485346719043e-08	1.5073320395018563e-13
ENSG00000136485_63550678_63589172_342	DCAF7	17	+	1026	53	2	0	5.85%	30.311269457182757	7.960803452267643e-05	3.64134137370543e-05	2.1851719407149344e-08
ENSG00000120265_149749728_149802379_250	PCMT1	6	+	750	25	1	4	4.40%	19.559347461050006	0.0022960440039733582	0.014015870411971724	0.000187143072015702
ENSG00000113810_160414137_160433809_991	SMC4	3	+	2973	76	6	3	3.23%	15.000105015537844	7.896245186011905e-06	2.505859899057593e-06	1.6900215309256496e-10
ENSG00000205426_52288073_52286255_235	KRT81	12	-	705	621	64	16	21.28%	516.8661605664703	2.0699607060581504e-08	1.525909622977858e-09	3.497454644147486e-16
ENSG00000115884_20224867_20202270_169	SDC1	2	-	507	23	1	0	5.33%	26.61923027243492	0.00510952817114065	0.003684287313187465	0.00010325221210932265
ENSG00000155957_66169896_66137960_207	TMBIM4	12	-	621	48	0	4	6.76%	45.355008605333346	0.0004561727980981236	0.0021227925778456486	6.304516214572376e-06
ENSG00000197102_101964884_102050563_4582	DYNC1H1	14	+	13746	143	19	12	1.66%	6.104292201942712	4.3952280105592036e-10	6.687290193278446e-12	3.834136364227799e-20
ENSG00000055147_154034953_153992976_435	FAM114A2	5	-	1305	9	0	0	1.38%	4.046761543665518	0.011772051206276392	0.011772051206276392	0.000681272511389508
ENSG00000188603_28491759_28487343_190	CLN3	16	-	570	17	2	0	3.16%	17.500468780939478	0.044040755831095145	0.013428347753762199	0.0027917083341200354
ENSG00000071054_101823961_101891249_1164	MAP4K4	2	+	3492	23	5	3	1.12%	3.8648195154995713	0.007949119477744631	0.004175186551296234	0.00017845908989950217
ENSG00000163479_156020896_156019233_65	SSR2	1	-	195	44	1	0	18.46%	132.4017366594154	0.0013893479493583228	0.001053132412899783	8.960332326514618e-06
ENSG00000165806_113721653_113729540_220	CASP7	10	+	660	14	0	0	3.64%	12.446857475213639	0.005160369442893307	0.005160369442893307	0.00014319601891785326
ENSG00000140264_43792377_43799079_108	SERF2	15	+	324	50	0	0	6.48%	90.55253454189817	0.00847367416878415	0.00847367416878415	0.0003665024264111182
ENSG00000028528_64112646_64137618_416	SNX1	15	+	1248	20	0	3	2.88%	9.403532433197118	0.0008852284070317903	0.00947050714314387	5.4426809642256264e-05
ENSG00000158796_161124462_161122147_318	DEDD	1	-	954	12	0	0	1.89%	7.380885834358493	0.013007239811447113	0.013007239811447113	0.0008218654148058079
ENSG00000134910_125606364_125608333_108	STT3A	11	+	324	20	2	1	7.41%	36.22101381675927	0.01006837577517317	0.0069659089849165145	0.00035985969576590374
ENSG00000112992_43644650_43704404_707	NNT	5	+	2121	7	0	0	0.71%	1.9365690555495054	0.019216965118390855	0.019216965118390855	0.0017073955890589052
ENSG00000091129_108232428_108178385_669	NRCAM	7	-	2007	13	0	1	1.05%	3.8007700596958154	0.007593099278532274	0.015191410988288745	0.0005816659162195647
ENSG00000132481_75878548_75874483_638	AC087289.1	17	-	1914	17	3	2	1.25%	5.21173835169044	0.01956379767784387	0.0153445488034536	0.0014097326775257904
ENSG00000132475_75779174_75778595_136	H3F3B	17	-	408	56	2	1	5.88%	80.53848954550001	0.013141913862703638	0.008364861494636245	0.0005511868195615003
ENSG00000164733_11853454_11848147_110	CTSB	8	-	330	68	9	0	15.45%	120.91232975921821	0.0014545096389273744	0.0001329304023902354	1.4091186835065798e-06
ENSG00000164733_11850939_11845125_215	CTSB	8	-	645	177	5	20	19.53%	161.02346514445819	1.0882868655944395e-08	1.0633847244440042e-06	1.3326584154636204e-13
ENSG00000148335_129632791_129635464_194	NTMT1	9	+	582	32	4	0	4.64%	32.26284117286599	0.03624304254342322	0.0035547763465261306	0.0007535874658647828
ENSG00000113648_135389093_135334976_372	H2AFY	5	-	1116	65	1	0	6.72%	34.17627916581318	5.066429166445862e-06	5.066429166445862e-06	2.1491773541407047e-10
ENSG00000218336_182681918_182800351_2053	TENM3	4	+	6159	18	0	0	0.29%	1.714896513876766	0.013428347753762199	0.013428347753762199	0.0008725593497644525
ENSG00000113328_163441222_163442565_159	CCNG1	5	+	477	36	1	1	6.29%	44.28531500615095	0.0031859520777271916	0.0031859520777271916	5.71642936696178e-05
ENSG00000084072_39748983_39753355_105	PPIE	1	+	315	10	0	0	4.76%	18.627949962904765	0.019679754444124863	0.019679754444124863	0.0017849636115922674
ENSG00000112992_43644650_43704404_707	NNT	5	+	2121	7	0	0	0.71%	1.9365690555495054	0.019216965118390855	0.019216965118390855	0.0017073955890589052
ENSG00000184277_101650135_101636859_152	TM2D3	15	-	456	10	5	0	3.95%	12.867991750690793	0.11411551976453232	0.011772051206276392	0.007078637642502531
ENSG00000243989_51986441_51989075_254	ACY1	3	+	762	7	1	0	1.97%	5.390371347533466	0.0477903522728147	0.016947426762344633	0.0036975118224100542
ENSG00000143183_165768742_165754893_98	TMCO1	1	-	294	66	1	10	13.27%	131.72621759482655	0.0007173166069655031	0.004240015136745302	1.9333067330003084e-05
ENSG00000165322_31908573_31807658_705	ARHGAP12	10	-	2115	23	1	1	1.28%	6.381063710697164	0.005059698366439319	0.005059698366439319	0.000137934786740623
ENSG00000050130_59484590_59498828_140	JKAMP	14	+	420	16	12	5	6.43%	22.35353995548572	0.20203352444242845	0.05408878139336595	0.042180973455118266
ENSG00000100605_92993886_92941561_295	ITPK1	14	-	885	7	0	0	1.69%	4.641201092452543	0.019216965118390855	0.019216965118390855	0.0017073955890589052

ENSG00000135446_57752292_57752176_38	CDK4	12	-	114	17	0	2	15.79%	87.50234390469738	0.013428347753762199	0.037178613843887286	0.0023492041693324566
ENSG00000115540_197540381_197550646_126	MOB4	2	+	378	13	1	0	4.76%	20.180279126480162	0.023672501042627626	0.012798402692974279	0.0014375227604752335
ENSG00000065054_2027006_2037984_337	SLC9A3R2	16	+	1011	50	0	1	4.75%	29.0198033546736	0.00019829743686022551	0.0002464402706279408	3.3685352217424857e-07
ENSG00000150403_113498432_113549538_499	TMCO3	13	+	1497	11	0	0	1.20%	4.311679801033067	0.012798402692974279	0.012798402692974279	0.0007972436888943567
ENSG00000117877_45406697_45409501_512	CD3EAP	19	+	1536	11	6	0	1.37%	4.202203556084962	0.25452096168535077	0.007593099278532274	0.01449162980351053
ENSG00000185624_81860471_81844012_508	P4HB	17	-	1524	478	17	20	19.88%	184.04267886578546	1.0439556986060714e-17	5.802259410771935e-17	9.284747261988608e-33
ENSG00000063241_55456486_55453308_221	ISOC2	19	-	663	21	0	0	4.52%	18.585805279730774	0.0022409900782254734	0.0022409900782254734	2.9184102569313086e-05
ENSG00000114867_184320681_184334908_1403	EIF4G1	3	+	4209	90	7	5	2.78%	12.546979839590167	4.60942195086692e-07	1.9011841143914044e-07	8.246422000810537e-13
ENSG00000167965_2206086_2208735_327	MLST8	16	+	981	14	0	4	2.45%	8.374032552131501	0.005257622967929454	0.052998774212472885	0.001578260860379592
ENSG00000055130_148756998_148800582_666	CUL1	7	+	1998	26	0	0	1.20%	7.635781291100602	0.005604866691947602	0.005604866691947602	0.00016752456572620382
ENSG00000113621_134875362_134899685_337	TXNDC15	5	+	1011	28	3	0	4.15%	16.251089878617215	0.003188107704916696	0.0003386460863230731	7.387651400636725e-06
ENSG00000164070_127782551_127832874_839	HSPA4L	4	+	2517	19	3	2	1.19%	4.429411224790823	0.015358647673375043	0.009621867080838033	0.0007286998036562927
ENSG00000120802_98515868_98547858_345	TMPO	12	+	1035	38	0	2	5.22%	21.54362908753334	7.139403444741481e-05	0.0003848889106694383	2.0138745832249654e-07
ENSG00000137713_111766361_111741596_474	PPP2R1B	11	-	1422	13	0	0	1.05%	5.364377995646626	0.020613416668581838	0.020613416668581838	0.001946208561389314
ENSG00000167526_89562377_89563042_57	RPL13	16	+	171	102	1	6	12.28%	350.00937561878953	0.008877961307018026	0.010353108236244411	0.0004630494296106523
ENSG00000128050_56436313_56459538_432	PAICS	4	+	1296	49	1	0	5.32%	22.185370962765052	1.202122360114406e-05	9.932719860816285e-06	9.665014861240016e-10
ENSG00000106541_16801796_16799693_126	AGR2	7	-	378	52	0	0	11.11%	80.72111650592065	0.00045213489585846177	0.00045213489585846177	1.3435289073568847e-06
ENSG00000148248_133375969_133363729_128	SURF4	9	-	384	66	2	19	13.28%	100.85288534603909	0.0002111189685664936	0.00658316006445003	1.0871851942004051e-05
ENSG00000148248_133366008_133363493_96	SURF4	9	-	288	46	18	1	12.50%	93.7218732508646	0.033590309857166684	0.00220554346506264	0.0004705272927290935
ENSG00000158411_99173963_99169375_181	MITD1	2	-	543	14	0	2	2.76%	15.128777041696136	0.020613416668581838	0.08350359792828288	0.007749259071793911
ENSG00000074800_8868018_8861360_341	ENO1	1	-	1023	686	29	36	28.45%	393.4813008293344	1.0352831986293368e-16	1.664726312205538e-17	2.627059814704888e-32
ENSG00000144747_69048086_69023177_887	TMF1	3	-	2661	8	0	2	0.68%	1.7640899626651638	0.011570465654371866	0.08266357711566169	0.004841719827405853
ENSG00000131504_141618914_141516851_1272	DIAPH1	5	-	3816	28	2	5	1.26%	4.30551673670912	0.00043205369195467813	0.0025251310032153905	7.181156752337885e-06
ENSG00000115919_142885368_143042172_465	KYNU	2	+	1395	86	4	0	6.24%	36.17427702473764	7.501376655071619e-06	9.369490065467012e-07	6.326299570499205e-11
ENSG00000162910_228106918_228106760_52	MRPL55	1	-	156	34	1	1	17.31%	127.8880410914808	0.004355956481189797	0.004355956481189797	0.0001037507970309169
ENSG00000049656_1344763_1318369_476	CLPTM1L	5	-	1428	46	4	0	3.99%	18.90189040353572	0.00020444120666883157	5.5341223974656866e-05	8.351841923724843e-08
ENSG00000197321_29550907_29458247_1616	SVIL	10	-	4848	11	0	0	0.31%	1.331391225690285	0.02108409854857798	0.02108409854857798	0.002029891263157455
ENSG00000126012_53224889_53192967_1560	KDM5C	X	-	4680	14	7	0	0.38%	1.7553260541967952	0.17988303231784347	0.013007239811447113	0.013155422755515516
ENSG00000163755_149129724_149172222_839	HPS3	3	+	2517	10	3	0	0.60%	2.331269065679381	0.12114404826813535	0.020613416668581838	0.011598140826794287
ENSG00000151632_5003835_5000478_146	AKR1C2	10	-	438	283	20	9	18.49%	379.12981722446244	2.1397363459111005e-05	8.740067697097048e-06	1.5119007451169545e-09
ENSG00000151632_4998744_4989996_173	AKR1C2	10	-	519	217	6	9	9.25%	245.33979185247694	0.00021408175522948358	0.0004871590686550402	7.091477209263242e-07
ENSG00000005007_18860374_18866163_373	UPF1	19	+	1119	11	0	0	1.88%	5.768172173500001	0.007029302661868638	0.007029302661868638	0.0002573639808771639
ENSG00000100242_38752628_38736267_717	SUN2	22	-	2151	24	1	3	1.81%	6.547061911648537	0.0010295252912250277	0.004890190720947476	3.090191282706422e-05
ENSG00000110066_68190049_68166974_384	KMT5B	11	-	1152	5	0	1	1.30%	2.546790033990886	0.012673659338734126	0.051235217429874705	0.0031110695322510533
ENSG00000100360_36767356_36763803_114	IFT27	22	-	342	11	0	0	4.39%	18.87305456767983	0.02108409854857798	0.02108409854857798	0.002029891263157455
ENSG00000135387_34071753_34099367_628	CAPRIN1	11	+	1884	50	2	0	4.30%	15.572728870262742	6.524192347581214e-06	1.940453335491528e-06	1.0930444540327216e-10
ENSG00000111011_122520511_122505527_375	RSRC2	12	-	1125	8	0	0	1.60%	4.172660791690667	0.011570465654371866	0.011570465654371866	0.0006594843062896773
ENSG00000172590_22829913_22834321_122	MRPL52	14	+	366	38	3	7	13.11%	60.922557665565584	0.0009450514886518204	0.0043669525455147925	2.5476722512411666e-05
ENSG00000128245_31956262_31956792_176	YWHAH	22	+	528	10	0	11	3.98%	11.11326560286932	0.007593099278532274	0.47947610046037925	0.039785224825561294
ENSG00000286140_69120272_69118854_472	DERPC	16	-	1416	12	0	0	1.06%	4.972715456199154	0.02108409854857798	0.02108409854857798	0.002029891263157455
ENSG00000176783_179560072_179609519_589	RUFY1	5	+	1767	17	0	0	1.87%	5.645312509980477	0.0013430411379358334	0.0013430411379358334	1.0937773364376948e-05
ENSG00000215021_6970543_6965685_299	PHB2	12	-	897	137	8	4	11.37%	89.61975258073079	1.963459915289962e-06	4.0521171592324965e-07	7.316547206432241e-12
ENSG00000166986_57516317_57516581_55	MARS	12	+	165	15	0	0	9.09%	53.343674893772736	0.02108409854857798	0.02108409854857798	0.002029891263157455
ENSG00000166986_57490577_57516138_635	MARS	12	+	1905	43	5	5	2.83%	13.244912453939373	0.00012753051567450572	0.0008437572633493226	7.678492906071766e-07
ENSG00000166986_57488091_57490215_140	MARS	12	+	420	8	5	1	3.57%	11.17676997774286	0.28185143082538655	0.03418265644467716	0.04484691569225287
ENSG00000117519_94903458_94897742_288	CNN3	1	-	864	34	1	0	4.86%	23.090896308184032	0.0006786479905456451	0.00037874750290730355	1.686250983246043e-06
ENSG00000073060_124863630_124778579_522	SCARB1	12	-	1566	46	1	2	3.45%	17.23620657487165	0.00012374249556490374	0.00031567046080049535	2.747431735905352e-07
ENSG00000008283_63437460_63434402_222	CYB561	17	-	666	14	0	3	2.70%	12.334723624085589	0.013217226988443315	0.06307108214216126	0.0040111611771127525
ENSG00000008283_63437460_63434402_222	CYB561	17	-	666	14	0	3	2.70%	12.334723624085589	0.013217226988443315	0.06307108214216126	0.0040111611771127525
ENSG00000134759_36129934_36174641_800	ELP2	18	+	2400	24	0	0	1.62%	5.867804238315001	0.000609140140830996	0.000609140140830996	2.3883185421048832e-06
ENSG00000115275_74465079_74463284_152	MOGS	2	-	456	6	0	0	3.29%	7.720795050414475	0.016947426762344633	0.016947426762344633	0.0013498980316300959
ENSG00000115275_74462963_74461275_562	MOGS	2	-	1686	22	0	3	1.96%	7.656684059485767	0.0013845019718837994	0.01157547599262317	9.8960288269354e-05
ENSG00000127054_1324608_1311859_578	INTS11	1	-	1734	12	0	2	1.56%	4.060764178764707	0.002874854311667729	0.022974702734688237	0.0003839431943786049
ENSG00000174903_66268680_66276238_201	RAB1B	11	+	603	49	0	0	6.97%	47.68199132295772	0.00041307551203580774	0.00041307551203580774	1.1283521396441709e-06
ENSG00000146729_55983761_55991831_47	NIPSNAP2	7	+	141	13	1	0	10.64%	54.100322764606396	0.019679754444124863	0.020613416668581838	0.001864001753513706
ENSG00000128708_171965844_171979444_208	HAT1	2	+	624	40	0	0	6.25%	37.61412973278847	0.000649205117157464	0.000649205117157464	2.700471097049339e-06
ENSG00000116337_109625404_109631152_761	AMPD2	1	+	2283	7	0	1	0.66%	1.7991515404382397	0.019216965118390855	0.04178282491678763	0.003600525600033436
ENSG00000115875_38751256_38745133_226	SRSF7	2	-	678	165	6	3	15.93%	142.80054562271022	1.549171611938806e-07	1.388052534701795e-07	2.0583178254734377e-13
ENSG00000151131_104986353_104994696_185	C12orf45	12	+	555	9	0	0	3.24%	9.515358224294596	0.012178957859018494	0.012178957859018494	0.0007262454987071801
ENSG00000172269_119098449_119096998_181	DPAGT1	11	-	543	33	0	0	6.63%	35.660688741140895	0.001032175130546352	0.001032175130546352	6.594970400795017e-06
ENSG00000127603_39284144_39485794_7224	MACF1	1	+	21672	26	1	9	0.19%	0.7039632253423312	0.0005610828647514753	0.013230226475135486	5.373688940890212e-05
ENSG00000005022_119468516_119471058_298	SLC25A5	X	+	894	292	7	4	11.41%	191.65535096062422	3.859956327932165e-07	3.877286428296764e-07	1.3854875314754485e-12
ENSG00000177731_18256989_18245138_1238	FLII	17	-	3714	52	4	3	2.10%	8.215557899633282	3.547753859250034e-05	2.4420059611515583e-05	6.694857887323595e-09
ENSG00000197498_110999716_111025582_199	RPF2	6	+	597	17	0	0	4.02%	16.7089902933593	0.005405141725979036	0.005405141725979036	0.00015637213408961292
ENSG00000137804_41332958_41380186_426	NUSAP1	15	+	1278	42	3	2	3.99%	19.283863693992963	0.00024409963436993035	0.0002147825895300285	3.604154209561318e-07
ENSG00000137801_39581894_39595369_1158	THBS1	15	+	3474	72	0	0	2.68%	12.161252307388605	3.1634127684446915e-07	3.1634127684446915e-07	9.330048080339862e-13
ENSG00000069424_6082210_6098574_295	KCNAB2	1	+	885	8	0	0	1.69%	5.304229819945764	0.019679754444124863	0.019679754444124863	0.0017849636115922674

ENSG00000104611_19333014_19394642_374	SH2D4A	8	+	1122	11	0	3	1.60%	5.752749253250001	0.012798402692974279	0.05111742236774389	0.003128977431720179
ENSG00000054118_36282564_36304017_955	THRAP3	1	+	2865	26	0	2	1.15%	5.325057947510996	0.001484234770590415	0.004803510583362958	4.2126168520311525e-05
ENSG00000165609_12186291_12167702_219	NUDT5	10	-	657	56	0	1	11.87%	50.014769763415536	2.490694691282911e-06	4.2831309097391446e-06	9.127971527841244e-11
ENSG00000178035_49027943_49026949_110	IMPDH2	3	-	330	29	0	0	10.00%	51.565552397313645	0.001565547007472977	0.001565547007472977	1.467919054732344e-05
ENSG00000100722_88563134_88611751_730	ZC3H14	14	+	2190	27	3	3	2.33%	7.234279197922604	0.0010108714401354142	0.0011294498960164967	7.050473514997274e-06
ENSG00000114019_134371433_134357705_777	AMOTL2	3	-	2331	42	4	13	2.57%	10.572620249216218	0.00036942355516503625	0.014762025815636752	4.331749224354655e-05
ENSG00000117305_23820578_23810676_181	HMGCL	1	-	543	15	0	0	3.31%	16.20940397324586	0.012798402692974279	0.012798402692974279	0.0007972436888943567
ENSG00000147649_97644576_97724670_559	MTDH	8	+	1677	48	1	0	3.94%	16.795146299291595	2.3217555979206773e-05	1.59616126746164e-05	2.924352848578089e-09
ENSG00000034713_75566496_75577369_114	GABARAPL2	16	+	342	97	5	2	18.42%	166.42602664226757	2.7197082363645283e-05	3.347165177043602e-05	7.0170920672660714e-09
ENSG00000204628_181243800_181242319_95	RACK1	5	-	285	95	6	24	10.53%	195.59347461050004	0.00319914062860709	0.06582688924469236	0.0013764206932317701
ENSG00000113648_135389093_135360760_165	H2AFY	5	-	495	35	1	0	6.67%	41.489524917378795	0.001558047347358915	0.001558047347358915	1.4544595481456893e-05
ENSG00000100941_39175280_39181863_717	PNN	14	+	2151	30	2	4	2.09%	8.18382738956067	0.0006719212067857174	0.002306200888048942	9.75250200186307e-06
ENSG00000086061_33026485_33038903_397	DNAJA1	9	+	1191	347	14	0	18.14%	170.95953574267887	1.2992344057708813e-12	5.4000378012101005e-14	9.654307308269323e-25
ENSG00000150768_112025473_112062535_647	DLAT	11	+	1941	21	0	3	1.85%	6.348474446399538	0.0008664586529590942	0.007725504039740604	4.293397515197606e-05
ENSG00000182944_29268337_29300161_600	EWSR1	22	+	1800	126	6	8	4.00%	41.074629668205006	0.00012487302501014574	0.0001440551817339119	1.2777049173942254e-07
ENSG00000070010_19475590_19450740_261	UFD1	22	-	783	47	0	1	7.28%	35.22181343560729	5.896622986852109e-05	6.736774575813048e-05	2.9449687597533134e-08
ENSG00000259522_24190408_24189415_140	AL136295.4	14	-	420	14	0	0	4.29%	19.559347461050006	0.013428347753762199	0.013428347753762199	0.0008725593497644525
ENSG00000259522_24187138_24180442_881	AL136295.4	14	-	2643	38	5	5	2.16%	8.436494932121455	0.00019610576897910605	0.0006587863671996574	8.843603158782651e-07
ENSG00000213928_24162145_24164151_221	IRF9	14	+	663	23	0	2	4.52%	20.355881973038468	0.0023218226288164415	0.007712769865834601	0.00010183073869623003
ENSG00000111642_6601370_6570676_1673	CHD4	12	-	5019	21	5	1	0.66%	2.4551482168682015	0.02946433998583899	0.001323351237915142	0.00026896173468470523
ENSG00000205133_92916989_92886622_86	TRIQK	8	-	258	7	0	0	5.81%	15.920399096203491	0.019216965118390855	0.019216965118390855	0.0017073955890589052
ENSG00000112514_33417333_33416650_101	CUTA	6	-	303	47	1	0	11.88%	91.01874561082676	0.001053132412899783	0.0010447178140462346	6.802228069098471e-06
ENSG00000112514_33417668_33417477_63	CUTA	6	-	189	113	1	6	17.46%	350.8263909680398	0.0023697301888609516	0.011684784968151578	0.00015893655968699872
ENSG00000139218_45928755_45922048_1148	SCAF11	12	-	3444	16	0	5	0.61%	2.7260414579860632	0.007029302661868638	0.17645041170550768	0.008347141929788618
ENSG00000146263_97282477_97146806_1243	MMS22L	6	-	3729	17	0	2	0.56%	2.6750515433455355	0.008175610787902942	0.027965089620958453	0.0011463419454493871
ENSG00000111843_10728672_10730666_35	TMEM14C	6	+	105	24	0	0	14.29%	134.12123973291432	0.021557223391537678	0.021557223391537678	0.002115616449879074
ENSG00000159377_151401314_151401829_47	PSMB4	1	+	141	26	0	1	12.77%	108.20064552921279	0.013640585738808999	0.013640585738808999	0.00089863692579514
ENSG00000109971_123061324_123057734_627	HSPA8	11	-	1881	440	9	18	12.44%	137.2585786740351	1.4901234441039318e-14	6.144632957048658e-13	1.3015464808773914e-25
ENSG00000023191_502162_500480_91	RNH1	11	-	273	12	1	0	7.69%	25.792546102483524	0.0078820576498062	0.0078820576498062	0.00031963642907451164
ENSG00000185000_144326636_144316554_488	DGAT1	8	-	1464	11	2	0	1.43%	4.408869304744878	0.04223451612662591	0.006937202941512724	0.0015390746531944901
ENSG00000065615_83864202_83959878_487	CYB5R4	6	+	1461	6	0	1	1.03%	2.409775867891171	0.016947426762344633	0.0477903522728147	0.0036975118224100542
ENSG00000105518_11345476_11342815_176	TMEM205	19	-	528	72	6	5	10.80%	80.01551234065911	0.0006159525080452096	0.00013311120503951327	5.776971514218554e-07
ENSG00000136193_29969067_29923957_414	SCRN1	7	-	1242	20	0	1	2.90%	9.448960126111114	0.0007764301431553706	0.0016353363669355965	7.894496093549459e-06
ENSG00000149806_65121813_65120763_87	FAU	11	-	261	85	46	6	10.34%	191.09707289531613	0.19669302953072448	0.012930838315446122	0.014660475072810054
ENSG00000149806_65120755_65120681_24	FAU	11	-	72	59	7	0	20.83%	480.83395841747927	0.08588638879785576	0.02108409854857798	0.008130108632843268
ENSG00000181163_171387973_171410565_257	NPM1	5	+	771	25	1	1	1.95%	19.026602588570043	0.05202961726446396	0.03577298629450316	0.007683857857448879
ENSG00000213614_72351228_72348103_86	HEXA	15	-	258	31	0	3	13.95%	70.50462456890118	0.001032175130546352	0.0026814738672924004	1.6819346216870764e-05
ENSG00000134287_48941032_48938947_123	ARF3	12	-	369	32	0	3	7.32%	50.88610721573985	0.003684287313187465	0.013900915060036254	0.0002797463677970316
ENSG00000149100_32587136_32593953_63	EIF3M	11	+	189	12	0	0	9.52%	37.25589992580953	0.011772051206276392	0.011772051206276392	0.000681272511389508
ENSG00000149100_32583888_32589551_59	EIF3M	11	+	177	28	0	0	11.86%	92.82402184905087	0.008574016250729465	0.008574016250729465	0.0003747459614100891
ENSG00000160877_13135226_13138406_521	NACC1	19	+	1563	9	2	0	1.15%	3.3787740335786958	0.07134538375819317	0.011772051206276392	0.0041729868335021346
ENSG00000116977_236539066_236548161_193	LGALS8	1	+	579	10	1	0	2.59%	10.134376922823837	0.03537493353818881	0.020613416668581838	0.003252526348827943
ENSG00000100350_36506290_36490008_640	FOXRED2	22	-	1920	32	4	1	1.88%	9.779673730525003	0.005391414071672784	0.000871358954992987	2.9501686946772053e-05
ENSG00000124155_45416169_45425826_437	PIGT	20	+	1311	65	4	0	4.35%	29.09285091460527	0.00034806401812914183	5.830279403891538e-05	1.5078639154760736e-07
ENSG00000110801_121888857_121915865_209	PSMD9	12	+	627	29	2	3	4.31%	27.139764419638762	0.006112543793728684	0.014936572009614516	0.0004708578184995048
ENSG00000123992_219386772_219374292_410	DNPEP	2	-	1230	17	2	2	1.95%	8.109973337508539	0.02671499548383061	0.02671499548383061	0.003152782508864385
ENSG00000160710_154589889_154584806_381	ADAR	1	-	1143	74	4	3	5.77%	37.98928378261681	0.00015853131810226044	7.408924655856144e-05	8.517404373768503e-08
ENSG00000160710_154601756_154589900_493	ADAR	1	-	1479	29	2	0	3.04%	11.505498506500002	0.0012571660882468394	0.0002596338414409078	2.2088380840726233e-06
ENSG00000116133_54876020_54852233_378	DHCR24	1	-	1134	49	0	4	5.82%	25.354709671731488	1.492853633392576e-05	0.00014421184258919996	1.7393667732461633e-08
ENSG00000138029_26254255_26289953_474	HADHB	2	+	1422	66	3	1	5.27%	27.234534439436715	2.5755317874190847e-05	6.675207065473808e-06	1.4097157339252867e-09
ENSG00000269893_118279412_118279714_36	SNHG8	4	+	108	22	5	0	16.67%	119.52934559530559	0.013428347753762199	0.013217226988443315	0.0008596769774033651
ENSG00000101220_3760100_3754058_199	C20orf27	20	-	597	8	0	5	2.51%	7.863054255698494	0.016947426762344633	0.21450858727089922	0.01973597174099189
ENSG00000115364_75652215_75655285_194	MRPL19	2	+	582	54	0	0	8.25%	54.443544479211354	0.00020377058636723502	0.00020377058636723502	2.8745633454040183e-07
ENSG00000156642_73633215_73561910_282	NPTN	15	-	846	106	0	2	8.87%	73.52095144933689	5.796878194903567e-06	5.755313834952151e-06	2.777566296885456e-10
ENSG00000119777_27034117_27040837_622	TMEM214	2	+	1866	11	0	0	0.96%	3.4590485863593257	0.012798402692974279	0.012798402692974279	0.0007972436888943567
ENSG00000183696_48094803_48108357_173	UPP1	7	+	519	12	0	0	2.89%	13.56717742963006	0.020613416668581838	0.020613416668581838	0.001946208561389314
ENSG00000186010_19526190_19528126_110	NDUFA13	19	+	330	235	25	8	30.00%	417.8587866678865	2.359591772346921e-05	3.468231131136563e-06	6.969200779526344e-10
ENSG00000197747_151986218_151983163_93	S100A10	1	-	279	406	25	15	48.39%	853.8811902350863	1.0520970707073368e-08	1.002770181025531e-08	1.0956173937957959e-15
ENSG00000132305_86195382_86144268_758	IMMT	2	-	2274	55	1	2	3.30%	14.192138659073223	6.688252526459028e-06	1.717267842574393e-05	9.398810624366696e-10
ENSG00000068120_42562412_42565968_593	COASY	17	+	1779	25	2	4	1.69%	8.245930632820407	0.008474269282996836	0.017689614858767277	0.0007461117900426938
ENSG00000167085_49413264_49404992_272	PHB	17	-	816	102	4	2	6.25%	73.34755297893751	0.00014002510398755815	0.0001854842840713172	1.8263145419340512e-07
ENSG00000088325_31757477_31801080_747	TPX2	20	+	2241	82	8	0	3.88%	21.470769635958504	8.493133221262696e-06	1.1014642104900248e-06	8.361698351165099e-11
ENSG00000110367_118786251_118754712_483	DDX6	11	-	1449	61	4	4	3.73%	24.702281472547625	0.00046913508627863303	0.00032923200806922443	1.0270503321449986e-06
ENSG00000070087_149968535_149966167_91	PFN2	3	-	273	61	3	8	14.29%	131.11210935429125	0.0015566408075271248	0.004937172922083453	4.521822044850897e-05
ENSG00000108846_50663766_50664129_90	ABCC3	17	+	270	10	0	1	5.56%	21.73260829005556	0.019679754444124863	0.04223451612662591	0.003715148010809822
ENSG00000160410_40583624_40591207_257	SHKBP1	19	+	771	9	2	0	1.95%	6.849576931885216	0.06032271266817251	0.020613416668581838	0.005529087794823047
ENSG00000105426_5286095_5159099_1519	PTPRS	19	-	4557	7	1	1	0.33%	0.901352417559908	0.0477903522728147	0.0477903522728147	0.009211062727049486

ENSG00000141349_44070966_44071628_72	G6PC3	17	+	216	20	1	0	12.50%	54.3315207251389	0.0033030736763512156	0.0034782380676658805	6.433817793110031e-05
ENSG00000141349_44074700_44076043_231	G6PC3	17	+	693	34	0	0	5.19%	28.788649942671004	0.000991050613121974	0.000991050613121974	6.098881496726224e-06
ENSG00000156875_100058702_100082209_418	MFSD14A	1	+	1254	27	0	0	4.31%	12.634028264314596	4.027253539844791e-05	4.027253539844791e-05	1.23110933795418e-08
ENSG00000182973_32685461_32773611_717	CNOT10	3	+	2151	11	0	0	0.84%	3.0007367095055795	0.012798402692974279	0.012798402692974279	0.0007972436888943567
ENSG00000065357_55938942_55953768_593	DGKA	12	+	1779	5	1	0	0.84%	1.6491861265640813	0.051235217429874705	0.012673659338734126	0.0031110695322510533
ENSG00000048828_93452345_93564540_975	FAM120A	9	+	2925	37	8	2	2.05%	7.4225216006035915	0.002610806916008967	0.00016235116706230171	3.138373422883761e-06
ENSG00000100650_69768157_69771461_272	SRSF5	14	+	816	131	12	9	12.50%	94.20126902196877	2.755377662040912e-06	4.418590755201586e-06	1.0381031063600711e-10
ENSG00000124593_41787698_41789328_74	AL365205.1	6	+	222	79	0	2	21.62%	208.8092499220203	0.0002001100642622418	0.00033129571046353263	4.5413923219901546e-07
ENSG00000005889_24179200_24211376_730	ZFX	X	+	2190	11	1	4	0.68%	2.947298932486987	0.03577298629450316	0.13379195373514313	0.019788666377424467
ENSG00000135390_53672614_53665314_141	ATP5MC2	12	-	423	104	13	2	17.73%	144.26752737228372	4.4036283112694194e-05	8.843606187363953e-06	3.163887414050723e-09
ENSG00000162910_228108260_228106760_164	MRPL55	1	-	492	35	2	1	6.10%	41.742509825411595	0.004407715900925203	0.0030814981005195803	7.570158080683164e-05
ENSG00000162910_228108260_228106760_128	MRPL55	1	-	384	35	2	1	7.81%	53.482590713808605	0.004407715900925203	0.0030814981005195803	7.570158080683164e-05
ENSG00000162910_228106918_228106760_52	MRPL55	1	-	156	34	1	1	17.31%	127.8880410914808	0.004355956481189797	0.004355956481189797	0.0001037507970309169
ENSG00000162910_228106918_228106760_52	MRPL55	1	-	156	34	1	1	17.31%	127.8880410914808	0.004355956481189797	0.004355956481189797	0.0001037507970309169
ENSG00000162910_228108260_228106760_128	MRPL55	1	-	384	35	2	1	7.81%	53.482590713808605	0.004407715900925203	0.0030814981005195803	7.570158080683164e-05
ENSG00000162910_228108260_228106760_128	MRPL55	1	-	384	35	2	1	7.81%	53.482590713808605	0.004407715900925203	0.0030814981005195803	7.570158080683164e-05
ENSG00000162910_228108260_228106760_128	MRPL55	1	-	384	35	2	1	7.81%	53.482590713808605	0.004407715900925203	0.0030814981005195803	7.570158080683164e-05
ENSG00000162910_228108260_228106760_128	MRPL55	1	-	384	35	2	1	7.81%	53.482590713808605	0.004407715900925203	0.0030814981005195803	7.570158080683164e-05
ENSG00000196352_207321766_207359610_550	CD55	1	+	1650	48	1	0	4.18%	17.069975966007277	2.161281327836658e-05	1.0254342719271262e-05	1.7793829783297854e-09
ENSG00000196352_207321766_207359610_381	CD55	1	+	1143	48	1	0	6.04%	24.641697588724416	2.161281327836658e-05	1.0254342719271262e-05	1.7793829783297854e-09
ENSG00000196352_207321766_207340644_444	CD55	1	+	1332	46	1	0	4.95%	20.26418881099775	3.3444954428221596e-05	1.5342867843440252e-05	4.040973303292243e-09
ENSG00000070669_97869093_97852259_540	ASNS	7	-	1620	50	1	0	4.07%	18.110506908379634	2.241375551321108e-05	1.434164336419077e-05	2.5471399262259043e-09
ENSG00000185658_39313491_39187026_2320	BRWD1	21	-	6960	16	0	2	0.39%	1.3489205145551728	0.0034529206320349946	0.014991380638102441	0.0002857513737328192
ENSG00000105755_43527177_43506850_254	ETHE1	19	-	762	11	0	0	2.76%	8.470583546124018	0.0078820576498062	0.0078820576498062	0.00031963642907451164
ENSG00000168216_69719072_69676158_325	LMBRD1	6	-	975	10	3	0	1.85%	6.018260757246155	0.08791701959130671	0.012798402692974279	0.0056117368578515354
ENSG00000143612_154213923_154207453_150	C1orf43	1	-	450	27	1	0	6.00%	35.20682542989001	0.0056974540925233165	0.0035804840969031955	0.00011176434211829552
ENSG00000112983_138168063_138157208_659	BRD8	5	-	1977	9	2	0	0.91%	2.6712310644833086	0.07134538375819317	0.011772051206276392	0.0041729868335021346
ENSG00000177302_18308367_18274802_902	TOP3A	17	-	2706	6	0	1	0.55%	1.3010652413115302	0.016947426762344633	0.0477903522728147	0.0036975118224100542
ENSG00000105281_46787965_46775510_541	SLC1A5	19	-	1623	176	5	2	9.06%	63.63114885665066	2.0845950069768537e-09	5.052851611147517e-10	1.1781942991860953e-17
ENSG00000241370_30345172_30346810_154	RPP21	6	+	462	26	2	1	3.25%	33.022274934240265	0.05742480463164306	0.02108409854857798	0.005360236623058286
ENSG00000119969_94554132_94601622_785	HELLS	10	+	2355	26	2	1	1.40%	6.4782552100293005	0.004817859085151192	0.0018842494102203086	5.2471250921023827e-05
ENSG00000150459_21140940_21147170_60	SAP18	13	+	180	15	6	0	8.33%	48.89836865262501	0.169362073879829	0.020613416668581838	0.017009049174009975
ENSG00000150459_21147208_21147342_44	SAP18	13	+	132	19	0	1	15.91%	84.46081858180683	0.00847367416878415	0.011825808327677989	0.0005036791746826746
ENSG00000108587_30477434_30490221_145	GOSR1	17	+	435	7	2	4	3.45%	9.44244360188621	0.0477903522728147	0.169362073879829	0.03179863257475834
ENSG00000106268_2244575_2251001_156	NUDT1	7	+	468	34	3	0	7.05%	42.6293470304936	0.003453048488940946	0.0016186486669924592	3.275367415488609e-05
ENSG00000180398_46907920_46905463_80	MCFD2	2	-	240	18	2	0	6.25%	44.00853178736251	0.06539853090342929	0.02108409854857798	0.006119906528703008
ENSG00000115540_197540381_197550646_126	MOB4	2	+	378	13	1	0	4.76%	20.180279126480162	0.023672501042627626	0.012798402692974279	0.0014375227604752335
ENSG00000099246_27504370_27538051_161	RAB18	10	+	483	21	0	1	5.59%	25.512192340500008	0.0035291660242640502	0.005642787686764809	0.00010924478547767898
ENSG00000240682_129159155_129130184_244	ISY1	3	-	732	10	2	1	2.46%	8.016126008627051	0.06583400801140711	0.028889785561798602	0.008027906358301883
ENSG00000240682_129159155_129130081_277	ISY1	3	-	831	11	2	1	2.53%	7.767249894279785	0.0416322583317752	0.017404239405933563	0.003292339026149851
ENSG00000089280_31190755_31191438_91	FUS	16	+	273	14	0	2	5.49%	30.091303786230775	0.020613416668581838	0.030784922059949493	0.0028441549054876347
ENSG00000185624_81860471_81843513_106	P4HB	17	-	318	20	3	0	8.49%	36.90442917179246	0.021866443808835736	0.00327844299461777	0.00040656532034450245
ENSG00000185359_81684067_81690288_173	HGS	17	+	519	11	1	0	2.89%	12.436579310494222	0.03577298629450316	0.02108409854857798	0.003354604907114977
ENSG00000103544_19555679_19700476_980	VPS35L	16	+	2940	46	1	6	2.35%	9.180918196003063	1.4960920976649777e-05	0.00010088481234045592	1.2048478962554937e-08
ENSG00000129103_56064312_56078159_150	SUMF2	7	+	450	63	4	3	6.00%	82.14925933641001	0.017782189529096822	0.013141913862703638	0.0011162015856618051
ENSG00000129103_56078432_56079612_53	SUMF2	7	+	159	12	0	0	9.43%	44.28531500615095	0.019679754444124863	0.019679754444124863	0.0017849636115922674
ENSG00000104763_18071333_18057534_324	ASAH1	8	-	972	53	3	2	7.41%	31.995228871470687	6.8176351904891705e-06	2.8957473970768623e-05	1.6190192581539036e-09
ENSG00000071082_101004219_101023641_64	RPL31	2	+	192	159	4	0	9.38%	485.92753848546107	0.013995907742832873	0.013853924679039932	0.0009341050772126533
ENSG00000111640_6534833_6536794_79	GAPDH	12	+	237	198	4	2	12.66%	490.22161990986086	0.0023628488670607213	0.0022409900782254734	3.070240847348849e-05
ENSG00000111640_6536885_6538170_268	GAPDH	12	+	804	655	55	25	15.67%	478.0362905592445	2.768145651595611e-07	3.9696829724310974e-07	1.0240198889931081e-12
ENSG00000144381_197498818_197495282_163	HSPD1	2	-	489	54	1	0	4.91%	64.79783821452149	0.009288069496478249	0.005306578019052857	0.0002590037054391842
ENSG00000257923_101816052_102283090_625	CUX1	7	+	1875	10	8	0	1.12%	3.129495593768001	0.23232849479515721	0.007593099278532274	0.012747240945536078
ENSG00000158623_130652909_130506676_777	COPG2	7	-	2331	12	0	0	0.77%	3.0207486426332055	0.012798402692974279	0.012798402692974279	0.0007972436888943567
ENSG00000122966_119876168_119688232_2069	CIT	12	-	6207	22	1	1	0.58%	2.07977595042581	0.0015356410015604012	0.0015356410015604012	1.4145984830283815e-05
ENSG00000179222_51894305_51901930_778	MAGED1	X	+	2334	18	0	2	1.03%	4.525298898443446	0.005554612275049112	0.018318557839637383	0.0005316522383050482
ENSG00000131462_42609738_42615041_451	TUBG1	17	+	1353	39	0	0	2.22%	16.913848137049893	0.0023628488670607213	0.0023628488670607213	3.2295294885504634e-05
ENSG00000174744_66341573_66337668_241	BRMS1	11	-	723	13	3	3	2.49%	10.550685352433613	0.07429615675292256	0.07429615675292256	0.020533049897397012
ENSG00000198431_104287428_104348415_547	TXNRD1	12	+	1641	259	19	13	10.97%	92.6119011409863	4.344541045840949e-09	1.0118539604345782e-09	4.834021682643186e-17
ENSG00000113240_178623416_178603617_481	CLK4	5	-	1443	10	2	2	1.04%	4.066392403544699	0.06032271266817251	0.06945717695039447	0.016020910955657944
ENSG00000139116_39358192_39294424_1262	KIF21A	12	-	3786	8	0	0	0.48%	1.239895243172742	0.011570465654371866	0.011570465654371866	0.0006594843062896773
ENSG00000104763_18063207_18057534_235	ASAH1	8	-	705	46	3	2	8.09%	38.28638226418299	5.488892743562522e-05	0.0001965696589835854	7.965536585833072e-08
ENSG00000213190_151067273_151067497_74	MLLT11	1	+	222	19	0	1	6.76%	50.21994618377704	0.019679754444124863	0.022004978195124387	0.0019818329449194675
ENSG00000026103_88990877_89014450_314	FAS	10	+	942	13	0	1	1.91%	8.097819012536625	0.012798402692974279	0.019795881618354192	0.0012079910998532793
ENSG00000169592_29996311_29999283_72	INO80E	16	+	216	22	5	0	9.72%	59.76467279765279	0.03031622373531018	0.00847367416878415	0.0012844157635113584
ENSG00000198356_12738656_12748104_246	ASNA1	19	+	738	18	0	0	2.85%	14.311717654426833	0.00847367416878415	0.00847367416878415	0.0003665024264111182
ENSG00000131368_15065188_15053376_108	MRPS25	3	-	324	10	3	3	6.48%	18.110506908379634	0.06556525143482543	0.06556525143482543	0.01638132253942991
ENSG00000015479_139307593_139329395_788	MATR3	5	+	2364	99	12	7	4.82%	24.573291860963838	2.978342675931118e-06	5.535559437143131e-07	1.5012471142621105e-11

ENSG00000089009_112408455_112405224_221	AC004086.1	12	-	663	37	0	0	2.26%	32.74641882619232	0.02108409854857798	0.02108409854857798	0.002029891263157455
ENSG00000132470_75724704_75757555_1752	ITGB4	17	+	5256	89	8	7	2.40%	9.935969886035675	2.3433006485323964e-07	2.450374282504324e-07	5.405332579885013e-13
ENSG00000105856_107182423_107201431_441	HBP1	7	+	1323	14	0	0	1.81%	6.209316654301588	0.004687384229717438	0.004687384229717438	0.00011928172701435494
ENSG00000188529_23972001_23971142_167	SRSF10	1	-	501	21	0	0	4.79%	24.59558662766767	0.005454749182134642	0.005454749182134642	0.0001591085901575364
ENSG00000115310_55050300_54973156_373	RTN4	2	-	1119	281	18	4	17.69%	147.35058006850002	1.2253672720491169e-11	1.235280245290815e-12	1.9424360090446078e-22
ENSG00000128789_12706586_12725531_233	PSMG2	18	+	699	27	2	4	3.86%	22.66533825958584	0.010026873424650893	0.010318779121986092	0.0005173850726394671
ENSG00000188677_44100073_44168678_267	PARVB	22	+	801	19	0	0	2.62%	13.918636770035583	0.00797940233269714	0.00797940233269714	0.000327142761693177
ENSG00000178149_49018564_49015588_543	DALRD3	3	-	1629	6	1	1	0.92%	2.1612538630994482	0.0477903522728147	0.0477903522728147	0.00921106272704948 6
ENSG00000184117_29581082_29555935_284	NIPSNAP1	22	-	852	53	1	1	7.39%	36.50159913505811	2.3263459991447717e-05	3.504059173599917e-05	6.311164625103103e-09
ENSG00000100297_35400439_35424255_734	MCM5	22	+	2202	36	2	0	1.77%	9.593140444111718	0.001640090834694659	0.0006909589238230294	7.103582645883212e-06
ENSG00000101608_3253248_3255918_171	MYL12A	18	+	513	80	1	0	8.19%	91.50571911602341	0.0004725673117534 86	0.00047395113928563794	1.4673862973554392e-06
ENSG00000109929_121306436_121307512_168	SC5D	11	+	504	13	0	0	3.57%	15.135209344860122	0.013007239811447113	0.013007239811447113	0.0008218654148058079
ENSG00000171617_74636266_74634716_516	ENC1	5	-	1548	29	1	0	2.52%	10.992656518807173	0.0009958682880916293	0.0006424109906750971	4.052020686628797e-06
ENSG00000112695_75243734_75241134_49	COX7A2	6	-	147	80	17	15	24.49%	319.33628507836744	0.005265875520493234	0.006610814250748408	0.00018492569923527974
ENSG00000249884_86542279_86505804_196	RNF103-CHMP3	2	-	588	17	0	0	4.08%	16.96474014478827	0.005405141725979036	0.005405141725979036	0.00015637213408961292
ENSG00000196776_108057558_108047288_47	CD47	3	-	141	12	0	0	12.77%	49.938759475021286	0.012178957859018494	0.012178957859018494	0.0007262454987071801
ENSG00000280987_139316089_139329395_504	MATR3	5	+	1512	78	11	2	4.96%	30.270418689720245	0.00019955724280967596	1.235424167238163e-05	2.0578307785244712e-08
ENSG00000105355_4861394_4839192_434	PLIN3	19	-	1302	28	2	0	3.23%	12.618933845838713	0.00036930943616753716	0.0004030602366301852	9.890178257390305e-07
ENSG00000197122_37397851_37403379_251	SRC	20	+	753	28	5	2	6.37%	21.81919238682869	0.0022688321121859907	0.0002817783606398257	4.386818488314242e-06
ENSG00000095319_128987665_129003616_751	NUP188	9	+	2253	11	2	0	0.93%	2.8648844483561926	0.03522780890193623	0.007029302661868638	0.0012822660609675817
ENSG00000108262_29581348_29574702_511	GIT1	17	-	1533	10	0	2	0.98%	3.827660951281801	0.020613416668581838	0.051235217429874684	0.004687384229717438
ENSG00000064490_19193947_19201719_260	RFXANK	19	+	780	17	3	0	1.92%	12.78880410914808	0.02784981298332479	0.020613416668581838	0.0025842615800075876
ENSG00000064607_19031002_18994366_1059	SUGP2	19	-	3177	20	0	1	0.94%	3.693927754683665	0.0020082573300163734	0.00392533363377418	4.538242985436214e-05
ENSG00000127955_80199276_80217493_236	GNAI1	7	+	708	20	2	0	2.54%	16.575718187330512	0.02034245439037237	0.013428347753762199	0.0012955184805712684
ENSG00000183283_51238908_51242652_207	DAZAP2	12	+	621	42	7	0	2.90%	39.68563252966668	0.060942872008231785	0.013853924679039932	0.00401920997888228
ENSG00000112851_66021326_66078530_1123	ERBIN	5	+	3369	28	7	0	0.98%	4.876774077554765	0.01465917395857711	0.001484234770590415	0.00013541356774210257
ENSG00000168884_2747732_2742257_266	TNIP2	4	-	798	10	0	2	1.88%	7.353138143251881	0.019679754444124863	0.06556525143482543	0.00579448895258773
ENSG00000151491_15669717_15621317_718	EPS8	12	-	2154	15	0	0	1.11%	4.086214650637188	0.005405141725979036	0.005405141725979036	0.00015637213408961292
ENSG00000135506_57694162_57720909_612	OS9	12	+	1836	75	3	6	5.88%	23.96978855520834	4.710589032573995e-07	2.298807928202195e-06	9.905140196363282e-12
ENSG00000138382_169822052_169812313_206	METTL5	2	-	618	12	0	0	2.91%	11.393794637504858	0.013007239811447113	0.013007239811447113	0.0008218654148058079
ENSG00000160796_46988048_47009320_2720	NBEAL2	3	+	8160	14	2	5	0.22%	1.0067311193187503	0.044040755831095145	0.11684743169084172	0.020273376447258438
ENSG00000233276_49358278_49358132_48	GPX1	3	-	144	16	0	0	12.50%	65.19782487016668	0.013428347753762199	0.013428347753762199	0.0008725593497644525
ENSG00000166888_57107239_57097083_610	STAT6	12	-	1830	22	3	0	1.48%	7.054190887591805	0.009836708040914554	0.0035291660242640502	0.00018946323603825 1
ENSG00000040341_73688813_73552006_473	STAU2	8	-	1419	16	0	0	1.48%	6.616269754266386	0.00797940233269714	0.00797940233269714	0.000327142761693177
ENSG00000092199_21231004_21211337_185	HNRNPC	14	-	555	15	1	8	3.24%	15.858930373824329	0.012619539009162606	0.11411551976453232	0.007459848152910935 5
ENSG00000075790_107595976_107628866_93	BCAP29	7	+	279	11	0	0	5.38%	23.134712050704305	0.019679754444124863	0.019679754444124863	0.0017849636115922674
ENSG00000142676_23692636_23695932_165	RPL11	1	+	495	276	8	3	26.67%	327.1745393484728	3.498557337707287e-09	4.950634444904532e-09	1.8457137986707628e-16
ENSG00000040341_73688813_73552006_473	STAU2	8	-	1419	16	0	0	1.48%	6.616269754266386	0.00797940233269714	0.00797940233269714	0.000327142761693177
ENSG00000124226_49945442_49952141_111	RNF114	20	+	333	16	0	0	5.41%	28.193653997909916	0.013007239811447113	0.013007239811447113	0.0008218654148058079
ENSG00000120253_149746495_149726969_380	NUP43	6	-	1140	19	0	0	1.84%	9.779673730525003	0.00847367416878415	0.00847367416878415	0.0003665024264111182
ENSG00000039523_67540585_67546463_992	RIPOR1	16	+	2976	10	3	0	0.50%	1.9717084134122989	0.12312584984626351	0.020613416668581838	0.011809609471937859
ENSG00000102763_41960952_41568197_1884	VWA8	13	-	5652	10	1	0	0.37%	1.0381819246841828	0.016854695534768073	0.007029302661868638	0.0006017499134389055
ENSG00000144118_120278719_120293260_188	RALB	2	+	564	25	0	0	6.38%	26.009770559906922	0.0008664586529590942	0.0008664586529590942	4.7094196839841995e-06
ENSG00000080822_98521358_98516659_231	CLDND1	3	-	693	34	2	0	4.33%	28.788649942671004	0.004691997894403566	0.0023628488670607213	6.289248615457574e-05
ENSG00000141522_81869930_81868876_204	ARHGDIA	17	-	612	196	7	0	17.16%	187.9231422728334	3.0930198244530585e-07	1.0062128811291726e-07	2.99248989215105e-13
ENSG00000168256_42022407_42023893_157	NKIRAS2	17	+	471	14	1	1	3.82%	17.441456334694273	0.012798402692974279	0.01634402150441562	0.0010049622802917066
ENSG00000130702_62314425_62310240_670	LAMA5	20	-	2010	47	16	9	2.54%	13.720736278647019	0.021706178877272435	0.005944126746496252	0.00067099447106914
ENSG00000112208_57182123_57184190_143	BAG2	6	+	429	40	1	3	11.89%	54.7114614295105	0.00017662137050100 88	0.0008327467823122039	1.0191167977103284e-06
ENSG00000139926_51702536_51728029_516	FRMD6	14	+	1548	10	3	0	1.16%	3.7905712133817837	0.08424673392120513	0.011772051206276392	0.005012526315124349
ENSG00000138757_75662019_75645430_480	G3BP2	4	-	1440	39	2	0	2.71%	15.89196981210313	0.0013761736105699968	0.0006629342244537616	5.740839419390654e-06
ENSG00000141510_7676594_7673219_346	TP53	17	-	1038	30	1	4	3.47%	16.958971787037576	0.0013600423278798564	0.006911511910188328	5.6581348914953786e-05
ENSG00000145730_102866196_103029065_905	PAM	5	+	2715	9	0	2	0.77%	1.9451284767895032	0.006937202941512724	0.0350911293701988	0.001262969770953026
ENSG00000071994_170584581_170577559_311	PDCD2	6	-	933	13	3	0	1.61%	8.17593302230386	0.11777945996203976	0.016947426762344633	0.009674391494749069
ENSG00000089057_4932559_4856972_649	SLC23A2	20	-	1947	9	0	0	0.77%	2.712390248835902	0.020613416668581838	0.020613416668581838	0.001946208561389314
ENSG00000108774_42128702_42126630_131	RAB5C	17	-	393	35	0	0	8.40%	52.25779856005726	0.0015806110104534784	0.0015806110104534784	1.495132122473684e-05
ENSG00000115484_61888507_61868692_539	CCT4	2	-	1617	222	25	10	8.91%	80.55983555386088	2.252519015726876e-07	1.7712254022109487e-08	4.1329611229331426e-14
ENSG00000143761_228097115_228098013_181	ARF1	1	+	543	246	3	2	24.31%	265.8342251612321	3.89149000448416e-09	3.5896094622681653e-09	1.4917525667184776e-16
ENSG00000100030_21867329_21769204_323	MAPK1	22	-	969	33	7	3	4.02%	19.983234248131584	0.012287140942460776	0.0026519075377804207	0.00018472582751774482
ENSG00000100138_41675154_41674933_73	SNU13	22	-	219	46	0	1	17.81%	123.250682631274	0.0006492051171574 64	0.0010037877862843834	4.1244594600580285e-06
ENSG00000134283_42335903_42446618_458	PPHLN1	12	+	1374	14	1	1	1.31%	5.97883983525546	0.02034245439037237	0.02034245439037237	0.0018987606724399325
ENSG00000163918_186804713_186789969_363	RFC4	3	-	1089	17	1	0	1.93%	9.160024981758955	0.016854695534768073	0.007029302661868638	0.0006017499134389055
ENSG00000103811_78935727_78922130_251	CTSH	15	-	753	17	0	0	2.39%	13.247366806288847	0.013428347753762199	0.013428347753762199	0.0008725593497644525
ENSG00000114354_100713686_100747448_284	TFG	3	+	852	43	1	0	6.34%	29.61450495863205	0.00012299303607804317	7.193802539044595e-05	6.440184934737959e-08
ENSG00000125630_112550943_112575729_718	POLR1B	2	+	2154	22	0	0	1.11%	5.993114820934542	0.005306578019052857	0.005306578019052857	0.00015100114740534233
ENSG00000138069_65129906_65088493_138	RAB1A	2	-	414	45	0	1	10.14%	63.78048085125001	0.00043622442149098933	0.0006399719954500673	1.8198932790453535e-06
ENSG00000142546_49560691_49555751_301	NOSIP	19	-	903	38	0	2	5.32%	24.69286390431562	0.00016524195568436628	0.0005033193936638034	5.750141298884926e-07
ENSG00000068400_49002193_48978251_613	GRIPAP1	X	-	1839	5	0	0	0.82%	1.5953790751264278	0.012673659338734126	0.012673659338734126	0.000782701129001274

ENSG00000085365_78416628_78475668_197	SCAMP1	5	+	591	13	0	0	2.54%	12.90718360373858	0.019679754444124863	0.019679754444124863	0.0017849636115922674
ENSG00000134759_36129934_36138369_120	ELP2	18	+	360	11	0	0	5.00%	17.92940183929584	0.012798402692974279	0.012798402692974279	0.0007972436888943567
ENSG00000120708_136049469_136049630_53	TGFBI	5	+	159	53	0	7	9.43%	195.59347461050004	0.021557223391537678	0.08647745899421033	0.008337010244447522
ENSG00000178057_49022473_49023172_116	NDUFAF3	3	+	348	9	0	1	4.31%	15.175355788745692	0.020613416668581838	0.03537493353818881	0.003252526348827943
ENSG00000101294_31514552_31569219_377	HM13	20	+	1131	238	16	4	11.41%	123.47810864005042	3.699605453423625e-07	7.65382553879486e-09	3.155645051514749e-14
ENSG00000135916_230873504_230878099_198	ITM2C	2	+	594	25	4	2	4.55%	24.696145784154044	0.02585604397786897	0.00798797567349413	0.0010369470907610767
ENSG00000108518_4946844_4945900_104	PFN1	17	-	312	289	7	4	14.42%	543.5241746387934	0.0004053966121081213	0.0003850566678273776	1.0354199802305458e-06
ENSG00000141456_4682537_4671439_928	PELP1	17	-	2784	36	1	0	0.97%	7.587677894372846	0.003684287313187465	0.003684287313187465	7.541963121699725e-05
ENSG00000166979_32412854_32515190_393	EVA1C	21	+	1179	14	1	0	1.78%	6.967706474674302	0.013892891352654344	0.00847367416878415	0.0005886798845679606
ENSG00000184076_29767399_29767587_62	UQCR10	22	+	186	77	0	0	16.13%	242.9144765323952	0.0024742386374296595	0.0024742386374296595	3.526932738104487e-05
ENSG00000111640_6534833_6536794_79	GAPDH	12	+	237	198	4	2	12.66%	490.22161990986086	0.0023628488670607213	0.0022409900782254734	3.070240847348849e-05
ENSG00000111640_6536885_6538170_268	GAPDH	12	+	804	655	55	25	15.67%	478.0362905592445	2.768145651595611e-07	3.9696829724310974e-07	1.0240198889931081e-12
ENSG00000112697_75284632_75256102_323	TMEM30A	6	-	969	65	1	1	9.29%	39.360915943289484	5.810721989179383e-07	8.828402763029427e-07	4.651807115106005e-12
ENSG00000081320_196163311_196137447_348	STK17B	2	-	1044	9	0	0	1.72%	5.0584519295818975	0.012178957859018494	0.012178957859018494	0.0007262454987071801
ENSG00000119041_196799611_196764563_886	GTF3C3	2	-	2658	11	0	0	0.56%	2.428361422929459	0.019679754444124863	0.019679754444124863	0.0017849636115922674
ENSG00000122545_35873720_35904293_285	SEPT7	7	+	855	20	7	0	1.75%	13.725857867403512	0.13072302616481507	0.021084098548577798	0.01284698802284646
ENSG00000104763_18084801_18057534_386	ASAH1	8	-	1158	54	3	2	6.22%	27.362817691624358	7.011690335458207e-06	2.7920572240514455e-05	1.602252394706242e-09
ENSG00000106976_128219081_128234066_274	DNM1	9	+	822	30	2	6	2.92%	21.41534393545621	0.00619104693109874	0.038744331104783855	0.001276960296931301
ENSG00000146648_55155892_55205617_893	EGFR	7	+	2679	37	1	4	1.90%	8.104096932349945	0.00017255179924853436	0.0011881644300165512	1.4393548671329007e-06
ENSG00000079616_29797027_29805310_597	KIF22	16	+	1791	37	8	2	3.18%	12.122208644201846	0.0032600418858822295	3.554863000883066e-05	1.1079724976205542e-06
ENSG00000110713_3695538_3676159_441	NUP98	11	-	1323	13	1	0	1.59%	5.76579403613719	0.013794764262559246	0.00837382380159347	0.0005783443517979597
ENSG00000042445_85350872_85343719_313	RETSAT	2	-	939	11	0	0	1.60%	6.8738920789632605	0.019679754444124863	0.019679754444124863	0.0017849636115922674
ENSG00000197713_210016633_210019791_72	RPE	2	+	216	11	0	0	6.94%	29.882336398826396	0.020613416668581838	0.020613416668581838	0.001946208561389314
ENSG00000138035_55693823_55684994_71	PNPT1	2	-	213	11	0	1	7.04%	30.303214376274656	0.021084098548577798	0.03577298629450316	0.003354604907114977
ENSG00000136521_179618481_179624040_53	NDUFB5	3	+	159	37	2	0	22.64%	136.5463879356321	0.0017658144955514316	0.0010405263639310788	1.1198112383099405e-05
ENSG00000137106_37430612_37432558_63	GRHPR	9	+	189	14	3	1	7.94%	43.46521658011112	0.08410206952409	0.019679754444124863	0.007521960853378093
ENSG00000100129_37874420_37888464_297	EIF3L	22	+	891	13	0	1	2.36%	8.561330538506736	0.008175610787902942	0.013599199788808391	0.0005580304998096723
ENSG00000165516_49768598_49782859_314	KLHDC2	14	+	942	16	0	0	1.59%	9.966546476968155	0.021084098548577798	0.021084098548577798	0.002029891263157455
ENSG00000142208_104776259_104770341_418	AKT1	14	-	1254	19	0	0	1.91%	8.890612482295456	0.005355751137763424 5	0.0053557511377634245	0.00015366972994913032
ENSG00000138796_108023489_108034357_127	HADH	4	+	381	30	0	0	7.87%	46.20318297885828	0.002046028879997488	0.002046028879997488	2.4518070358754455e-05
ENSG00000054654_63978007_64225564_6353	SYNE2	14	+	19059	15	6	1	0.11%	0.4618136501113648	0.15952699612641635	0.015516259750072458	0.012888680152838944
ENSG00000149792_65122359_65125681_136	MRPL49	11	+	408	16	0	0	4.41%	23.010997013000004	0.012798402692974279	0.012798402692974279	0.0007972436888943567
ENSG00000196498_124398137_124325402_1886	NCOR2	12	-	5658	34	2	22	1.06%	3.5260753641341473	8.12826489821597e-05	0.029873450661875123	3.197089663720459e-05
ENSG00000204628_181243800_181243681_39	RACK1	5	-	117	67	1	1	15.38%	336.01955894624365	0.013995907742832873	0.013853924679039932	0.0009341050772126533
ENSG00000204628_181242331_181236977_276	RACK1	5	-	828	1471	30	30	30.07%	1042.4565259132087	4.713574592626915e-15	6.7953089207812174e-15	4.4937323581208455e-28
ENSG00000169567_131165205_131162382_103	HINT1	5	-	309	47	2	2	11.65%	89.25139132712138	0.0013282749526633097	0.0023453309689915928	1.8603609104278156e-05
ENSG00000169567_131159596_131159447_49	HINT1	5	-	147	123	1	0	24.49%	490.9795383079899	0.0011466916564029902	0.0010786302489720287	7.612984240091 6915e-06
ENSG00000145916_178138120_178148032_393	RMND5B	5	+	1179	11	1	1	1.27%	5.4746265158155225	0.028542519635534256	0.028542519635534256	0.003564405341035 0844
ENSG00000165792_20990003_20996890_447	METTL17	14	+	1341	32	2	0	2.68%	14.002217421780763	0.0026957942880510015	0.0010405263639310788	1.7035015215455722e-05
ENSG00000184903_111521447_110663602_175	IMMP2L	7	-	525	16	0	0	5.14%	17.882831964388576	0.0033278027414746945	0.0033278027414746945	6.211806171561056e-05
ENSG00000140365_75336090_75338334_91	COMMD4	15	+	273	28	0	3	5.49%	60.18260757246155	0.019679754444124863	0.08410206952409	0.007521960853378093
ENSG00000125841_349647_353635_203	NRSN2	20	+	609	28	4	0	5.91%	26.978410291103454	0.007740067497657663	0.0009549346450480869	4.6790769355716326e-05
ENSG00000151835_23411239_23330136_4579	SACS	13	-	13737	10	2	1	0.13%	0.42715325313496405	0.04223451612662591	0.023672501042627626	0.004366626912467234
ENSG00000166888_57107239_57096572_737	STAT6	12	-	2211	23	3	0	1.36%	6.10400259978494	0.007867321313276006	0.0023218226288164415	0.00010390540483540199
ENSG00000146802_112784567_112767197_611	TMEM168	7	-	1833	8	0	0	0.82%	2.560962024360066	0.019679754444124863	0.019679754444124863	0.0017849636115922674
ENSG00000134014_28110379_28189725_413	ELP3	8	+	1239	21	0	1	2.66%	9.945430912398308	0.0013984928336429232	0.002484757332982506	2.0661357227484502e-05
ENSG00000074527_95787412_95659086_568	NTN4	12	-	1704	18	0	0	1.06%	6.198384758783452	0.013007239811447113	0.013007239811447113	0.0008218654148058079
ENSG00000197226_179866024_179863448_324	TBC1D9B	5	-	972	10	6	0	1.54%	6.036835636126544	0.17004230409153215	0.019679754444124863	0.016523685284367155
ENSG00000112118_52284649_52264588_762	MCM3	6	-	2286	47	0	11	2.49%	12.064164444479662	5.2494528509358865e-05	0.004993752824121904	2.5047256486173585e-06
ENSG00000196072_100286127_100275462_95	BLOC1S2	10	-	285	13	0	0	5.26%	26.76542284143685	0.020613416668581838	0.020613416668581838	0.001946208561389314
ENSG00000180008_55043042_55044364_440	SOCS4	14	+	1320	12	4	1	1.36%	5.334367489377274	0.08266357711566169	0.026602764905793736	0.009436337707064906
ENSG00000092148_31114288_31100930_720	HECTD1	14	-	2160	15	2	3	1.39%	4.074864054385418	0.012430495409378758	0.017497919476433	0.001044417297476116
ENSG00000197756_216499339_216529273_88	RPL37A	2	+	264	255	25	11	20.45%	566.7765457463354	9.152174643962531e-05	0.0003369099689742 2923	2.2125306702080885e-07
ENSG00000112335_108260921_108212149_130	SNX3	6	-	390	29	0	1	10.00%	43.632390490034624	0.0006768518708446028	0.0012297437010111859	5.237411928464366e-06
ENSG00000136193_29969067_29923957_414	SCRN1	7	-	1242	20	0	1	2.90%	9.448960126111114	0.0007764301431553706	0.0016353363669355965	7.894496093549459e-06
ENSG00000013375_83191012_83169234_542	PGM3	6	-	1626	10	0	0	0.92%	3.608735693920665	0.019679754444124863	0.019679754444124863	0.0017849636115922674
ENSG00000006534_68015298_68028140_230	ALDH3B1	11	+	690	35	5	3	3.91%	29.764224397250008	0.021979756220936595	0.005134531980829637	0.000599043544242399
ENSG00000123130_23706692_23703894_189	ACOT9	X	-	567	17	4	0	3.17%	17.593063853854503	0.055917760918677194	0.013428347753762199	0.0035774737192599405
ENSG00000123130_23730970_23703894_379	ACOT9	X	-	1137	30	5	0	3.69%	15.482333082625333	0.004525936675743194	0.00042454308224345244	1.3070548654943797e-05
ENSG00000089063_5109427_5100791_119	TMEM230	20	-	357	33	0	0	5.88%	54.240207244928584	0.00877616296920843	0.00877616296920843	0.0003916113566756457 7
ENSG00000145391_139556119_139511663_360	SETD7	4	-	1080	28	0	2	4.17%	15.212825803038893	0.00023196689917540181	0.0006659184076607086	1.0449151161860308e-06
ENSG00000163710_142888896_142818335_415	PCOLCE2	3	-	1245	17	0	0	1.93%	8.012262815369882	0.005454749182134642	0.005454749182134642	0.00015910859015753364
ENSG00000115306_54558779_54660086_2155	SPTBN1	2	+	6465	87	21	9	1.90%	7.896349091003946	3.118490343979677e-05	1.0760284598111411e-07	4.073560136259495e-11
ENSG00000124783_7313120_7303559_88	SSR1	6	-	264	37	0	12	11.36%	82.23816546123297	0.0023766146074493525	0.02081081638491048	0.0002941514941744 6994
ENSG00000111639_6492895_6492271_76	MRPL51	12	-	228	151	10	0	19.74%	388.61335087086195	0.0010230374893540142	0.0003201165486586971	2.1702003887314694e-06
ENSG00000111276_12717885_12721207_190	CDKN1B	12	+	570	27	4	0	7.37%	27.79486218149211	0.0034487610570488048	0.00042454308224345244	9.794225096245417e-06
ENSG00000140990_1959625_1961906_172	NDUFB10	16	+	516	74	5	4	13.95%	84.1506809370756	2.5199898278265692e-05	2.532139675009792e-05	4.959899932301653e-09

ENSG00000074582_218660988_218663386_419	BCS1L	2	+	1257	20	0	0	2.86%	9.336204038687352	0.00091966234969362	0.00091966234969362	5.2817946186748165e-06
ENSG00000074582_218660988_218663386_419	BCS1L	2	+	1257	20	0	0	2.86%	9.336204038687352	0.00091966234969362	0.00091966234969362	5.2817946186748165e-06
ENSG00000107372_72360778_72355953_213	ZFAND5	9	-	639	22	0	1	4.23%	20.202142917516436	0.0035291660242640502	0.004333802856335622	8.458999530518808e-05
ENSG00000107372_72360778_72355953_213	ZFAND5	9	-	639	23	0	1	4.23%	21.12042214103991	0.0035547763465261306	0.004355956481189797	8.558737951575692e-05
ENSG00000120875_29350251_29337026_385	DUSP4	8	-	1155	54	0	9	5.97%	27.433889945368836	1.0897962237138453e-05	0.000558331884749841	5.5771006690745924e-08
ENSG00000120875_29345430_29337026_270	DUSP4	8	-	810	43	0	1	6.67%	31.15007188241297	8.24188126256247e-05	0.0001789774272272485	1.0631190042137735e-07
ENSG00000168000_62705595_62702488_118	BSCL2	11	-	354	14	0	6	5.08%	23.206005462262716	0.013428347753762199	0.16970534604944526	0.012520549700299675
ENSG00000168000_62707195_62690367_464	BSCL2	11	-	1392	17	17	2	2.37%	7.166140233574355	0.45573689993954414	0.00550296840388912	0.030297291298871185
ENSG00000065150_98003017_98021822_605	IPO5	13	+	1815	64	0	1	4.13%	20.6908799587967	4.883230566509808e-06	6.88023006635177e-06	2.8003506628379944e-10
ENSG00000164051_48435035_48432408_380	CCDC51	3	-	1140	14	1	0	1.84%	7.206075380386844	0.013892891352654344	0.00847367416878415	0.0005886798845679606
ENSG00000284194_50524411_50523611_266	SCO2	22	-	798	17	8	4	3.76%	12.500334843528199	0.08854587809459352	0.02077003350494258	0.0082970343288543
ENSG00000198363_61714371_61661907_113	ASPH	8	-	339	104	6	10	15.93%	180.01523326984073	0.0005748788488204014	0.0013609137672942508	4.9713558406531076e-06
ENSG00000173085_83284533_83267173_251	COQ2	4	-	753	36	0	0	4.78%	28.05324735449403	0.0010447178140462346	0.0010447178140462346	6.749952978822125e-06
ENSG00000173085_83284533_83264199_294	COQ2	4	-	882	37	0	0	4.42%	24.615505308124156	0.0006768518708446028	0.0006768518708446028	2.9265495608176655e-06
ENSG00000122783_135168814_135167772_33	CYREN	7	-	99	10	0	0	18.18%	59.270749881969714	0.009815328628645334	0.009815328628645334	0.0004837142251238724
ENSG00000188554_43186415_43209914_734	NBR1	17	+	2202	16	1	1	1.50%	4.263617975160764	0.0025320062747194163	0.0025320062747194163	3.6860401525304375e-05
ENSG00000176697_27658504_27657821_227	BDNF	11	-	681	10	1	0	2.64%	8.616452626013217	0.023672501042627626	0.012798402692974279	0.0014375227604752335
ENSG00000103512_14864669_14896111_774	NOMO1	16	+	2322	13	0	0	0.65%	3.2851617182642125	0.020613416668581838	0.020613416668581838	0.001946208561389314
ENSG00000273749_22947285_22870028_1253	CYFIP1	15	-	3759	51	5	3	1.92%	7.961107107051478	8.115520353640984e-05	3.764722865303054e-05	2.299234600726723e-08
ENSG00000105655_18437003_18434913_430	ISYNA1	19	-	1290	12	2	0	1.86%	5.458422547269769	0.01943569092740073	0.004826937407846361	0.0004999687388256805
ENSG00000013275_39971203_39981305_387	PSMC4	19	+	1161	127	5	0	9.30%	64.18700587993153	8.285923099718348e-08	7.633730909347596e-08	6.176337725669424e-14
ENSG00000164941_94828985_94859647_520	INTS8	8	+	1560	16	0	0	1.35%	6.018260757246155	0.00847367416878415	0.00847367416878415	0.0003665024264111182
ENSG00000166788_18106041_18080399_474	SAAL1	11	-	1422	24	0	1	1.69%	9.903467068886078	0.005604866691947602	0.007225456388766877	0.00021353222845814395
ENSG00000143228_163327512_163355469_368	NUF2	1	+	1104	19	0	1	2.72%	10.098576134781252	0.0022276761777358703	0.003768080243855145	4.799485657507121e-05
ENSG00000133318_63681637_63758201_1032	RTN3	11	+	3096	92	8	7	1.94%	17.436627581556206	0.0003207014050824132	4.2495854754003497e-05	1.0372854354069738e-07
ENSG00000204899_72727602_72710322_82	MZT1	13	-	246	29	0	3	13.41%	69.17330199639636	0.0015061527253727427	0.007887772776385514	7.101070983366639e-05
ENSG00000125844_17660363_17614182_1362	RRBP1	20	-	4086	46	2	6	1.54%	6.605947013276801	9.456181311419808e-05	0.0006522131501343225	4.4831860739923035e-07
ENSG00000147123_47144679_47142317_153	NDUFB11	X	-	459	87	2	5	16.34%	111.21981889616669	5.5104407320523845e-06	9.600746911240074e-06	4.375270756993103e-10
ENSG00000072518_63895204_63909237_699	MARK2	11	+	2097	9	0	0	0.86%	2.5183709177317604	0.011772051206276392	0.011772051206276392	0.000681272511389508
ENSG00000100364_45203952_45197176_221	KIAA0930	22	-	663	11	3	0	2.26%	9.73542181319231	0.08350359792828288	0.019216965118390855	0.0073235312725521175
ENSG00000169359_155853997_155828210_447	SLC33A1	3	-	1341	19	10	0	1.79%	8.31381659418233	0.12527348858865706	0.005554612275049112	0.004553675172822361
ENSG00000099956_23787179_23791950_92	SMARCB1	22	+	276	8	0	2	5.43%	17.008128227000004	0.019679754444124863	0.10105102209468636	0.009164497669452725
ENSG00000140694_14630125_14540838_529	PARN	16	-	1587	18	0	2	1.51%	6.655354523608697	0.0053557511377634245	0.016228925652561413	0.00045523357042063324
ENSG00000125753_45517710_45526177_213	VASP	19	+	639	8	0	2	3.29%	7.346233788187795	0.005706018193000826	0.06180520118411761	0.0020032236666657145
ENSG00000013306_44321554_44320001_227	SLC25A39	17	-	681	31	3	1	7.05%	26.711003140640976	0.001100063306212375	0.0002860451348991949	2.105817534158903e-06
ENSG00000198561_57795613_57816308_840	CTNND1	11	+	2520	62	7	2	2.74%	14.436661221251194	0.0003827488158090153	2.3314382156310368e-05	7.254834957190183e-08
ENSG00000141376_60709305_61392125_813	BCAS3	17	+	2439	17	0	5	0.74%	4.089900453110087	0.011772051206276392	0.1004125613475727	0.006109270445167818
ENSG00000152904_235342032_235342772_246	GGPS1	1	+	738	13	1	0	2.44%	10.336240528197157	0.02034245439037237	0.013428347753762199	0.0012955184805712684
ENSG00000155097_103040837_103068747_382	ATP6V1C1	8	+	1146	28	1	3	2.88%	14.336694474068066	0.0022960440039733582	0.011821942446875977	0.0001565172319901872
ENSG00000167118_128371381_128389734_101	URM1	9	+	303	69	6	4	16.83%	133.62326483291588	0.0008381652722208987	0.0007312163512578176	3.8750623686153544e-06
ENSG00000164733_11853454_11845125_339	CTSB	8	-	1017	303	14	0	20.94%	174.82248615628765	1.089651732784085e-12	9.990376595038133e-14	1.4630170378218959e-24
ENSG00000168036_41224069_41233459_399	CTNNB1	3	+	1197	45	4	3	4.26%	22.059414429755645	0.0004533241809757994	0.0006143436866977971	1.8138630817816383e-06
ENSG00000168036_41233535_41239342_384	CTNNB1	3	+	1152	72	4	0	6.60%	36.67377648946876	1.688159752929848e-05	1.5078143545877652e-06	2.2731512732504024e-10
ENSG00000102974_67611577_67637872_477	CTCF	16	+	1431	8	0	0	1.26%	3.28039370415933	0.011570465654371866	0.011570465654371866	0.0006594843062896773
ENSG00000114346_172757007_172820237_805	ECT2	3	+	2415	106	1	1	4.72%	25.75516560088572	3.760462660003336e-08	3.760462660003336e-08	1.4134601572500778e-14
ENSG00000115919_142885368_143042172_465	KYNU	2	+	1395	87	4	0	6.45%	36.59490815293227	5.3070172887045495e-06	6.097417034113448e-07	2.965095924277716e-11
ENSG00000196924_154359074_154351161_1011	FLNA	X	-	3033	47	17	4	2.67%	9.092871717797728	0.006550845888627988	3.9691142553857814e-05	2.749993691140354e-06
ENSG00000196924_154350226_154348849_268	FLNA	X	-	804	21	1	0	4.10%	15.32635435380784	0.0024673358700277245	0.0013845019718837994	2.032715794165102e-05
ENSG00000100280_29358923_29328821_840	AP1B1	22	-	2520	24	0	3	1.07%	5.58838498887143	0.003684287313187465	0.017782189529096822	0.00036099358464052045
ENSG00000145247_48904549_48885531_99	OCIAD2	4	-	297	12	0	7	6.06%	23.708299952787883	0.011772051206276392	0.3212384956040438	0.026834343646100805
ENSG00000141552_81894478_81900065_84	ANAPC11	17	+	252	127	10	2	20.24%	295.7187056611132	0.00039118706886775756	0.00018946255112486357	5.081891006409044e-07
ENSG00000108298_39201214_39202471_86	RPL19	17	+	258	12	2	0	5.81%	27.292112736348844	0.05202961726446396	0.02108409854857798	0.004853002110546164
ENSG00000108298_39203018_39204648_108	RPL19	17	+	324	113	17	0	15.74%	204.64872806468986	0.0015950709364670578	0.00013535602770218042	1.5789511443195689e-06
ENSG00000145833_134758939_134828706_1032	DDX46	5	+	3096	40	0	0	1.65%	7.581142426763567	0.00012869633121932495	0.00012869633121932495	1.1789331714510463e-07
ENSG00000092621_119727007_119744040_395	PHGDH	1	+	1185	26	0	0	3.80%	12.874507189551903	0.0002582331582969843	0.0002582331582969843	4.5481323920964005e-07
ENSG00000092621_119712023_119724460_165	PHGDH	1	+	495	15	0	0	3.64%	17.781224964590912	0.012798402692974279	0.012798402692974279	0.0007972436888943567
ENSG00000103978_42273398_42258949_181	TMEM87A	15	-	543	12	0	0	2.76%	12.967523178596688	0.019679754444124863	0.019679754444124863	0.0017849636115922674
ENSG00000267855_8321358_8316610_76	NDUFA7	19	-	228	24	4	1	7.89%	61.7663604033158	0.037440157020026926	0.02034245439037237	0.003397262391305248
ENSG00000147010_19645418_19536417_404	SH3KBP1	X	-	1212	26	0	0	2.23%	12.587698861071786	0.003684287313187465	0.003684287313187465	7.541963121699725e-05
ENSG00000101247_13785069_13817210_317	NDUFAF5	20	+	951	26	1	0	3.47%	16.042367002753945	0.00236147777460975	0.0015061527253727427	2.1068444318500428e-05
ENSG00000069424_6082210_6098574_295	KCNAB2	1	+	885	8	0	0	1.69%	5.304229819945764	0.019679754444124863	0.019679754444124863	0.0017849636115922674
ENSG00000069424_6082210_6098574_295	KCNAB2	1	+	885	8	0	0	1.69%	5.304229819945764	0.019679754444124863	0.019679754444124863	0.0017849636115922674
ENSG00000072756_3129041_3146449_213	TRNT1	3	+	639	9	0	2	2.82%	8.26451301171127	0.012178957859018494	0.07022909110143051	0.004215016872891795
ENSG00000115525_85863399_85847466_196	ST3GAL5	2	-	588	12	1	2	3.06%	11.975110690438779	0.026602764905793736	0.06032271266817251	0.006860364960240205
ENSG00000110955_56644929_56643891_52	ATP5F1B	12	-	156	71	1	5	21.15%	267.0603211027981	0.0016340149330375125	0.006329842630159708	6.082017180448717e-05
ENSG00000186298_110730738_110720134_192	PPP1CC	12	-	576	36	0	0	6.77%	36.67377648946876	0.0006026251277497241	0.0006026251277497241	2.339296094881936e-06
ENSG00000174437_110332597_110334155_111	ATP2A2	12	+	333	16	4	8	7.21%	28.193653997909916	0.04099061537131024	0.2097150537537466	0.035867261227588564

ENSG00000140545_88912172_88899395_379	MFGE8	15	-	1137	18	2	0	2.64%	9.2893998495752	0.010678932720565162	0.0022011839993586477	0.0001357017294392655
ENSG00000130164_11089549_11131316_858	LDLR	19	+	2574	144	16	3	6.76%	32.8268768577063	3.288165162112553e-08	3.6357101243047854e-11	1.7758864373357156e-17
ENSG00000157823_89893949_89835515_205	AP3S2	15	-	615	16	2	0	2.93%	15.265832164721955	0.03031622373531018	0.013007239811447113	0.001861849989128658
ENSG00000160752_155318926_155320609_138	FDPS	1	+	414	19	3	0	7.25%	26.929536359416673	0.007456024827607967	0.00228120298834518	9.684751958738432e-05
ENSG00000128654_176270410_176337664_253	MTX2	2	+	759	15	1	0	3.95%	11.59645106386364	0.0065000503081102355	0.0014903523101034196	5.758212848423214e-05
ENSG00000101773_22936852_23026247_867	RBBP8	18	+	2601	18	3	0	1.15%	4.060764178764707	0.018949244379329343	0.0021227925778456486	0.00024172798388748533
ENSG00000214944_73780715_73941013_1438	ARHGEF28	5	+	4314	11	5	1	0.49%	1.496194868369611	0.07786981947941429	0.015516259750072458	0.00572336355703091
ENSG00000136603_170360425_170392417_653	SKIL	3	+	1959	31	1	2	1.68%	9.285448258691426	0.002353241622354279	0.005609982661338475	7.481743476331415e-05
ENSG00000057294_32896731_32792424_837	PKP2	12	-	2511	10	0	0	0.60%	2.336839601081243	0.019679754444124863	0.019679754444124863	0.0017849636115922674
ENSG00000085760_55262331_55236648_622	MTIF2	2	-	1866	24	1	0	2.25%	7.547015097511256	0.0006399774234965108	0.00039077502422413024	1.639360727156464e-06
ENSG00000115234_27374720_27376719_256	SNX17	2	+	768	23	1	0	3.52%	17.572851234537115	0.0050845836060656145	0.0036581908865286695	0.00010208315094899304
ENSG00000131495_140647583_140645703_76	NDUFA2	5	-	228	42	4	0	11.84%	108.09113070580266	0.010741328273627723	0.0037631575832289435	0.00021926637865774917
ENSG00000151881_43453969_43446222_215	TMEM267	5	-	645	47	0	1	2.79%	42.75764328694652	0.011772051206276392	0.026832695913080365	0.0015103817605807263
ENSG00000142507_4796737_4797732_68	PSMB6	17	+	204	113	0	0	10.29%	325.0303328086251	0.00877616296920843	0.00877616296920843	0.00039161135667564577
ENSG00000166881_57078733_57059879_440	NEMP1	12	-	1320	20	0	0	2.95%	8.890612482295456	0.0004762745294806779	0.0004762745294806779	1.4854959883925712e-06
ENSG00000150990_124989022_124947802_1157	DHX37	12	-	3471	17	2	0	0.86%	2.873888563853501	0.010187216365941177	0.0021227925778456486	0.000125180854499238
ENSG00000134313_8796836_8730720_1408	KIDINS220	2	-	4224	13	0	0	0.78%	1.8059056604662647	0.0008831683242050062	0.0008831683242050062	4.885807922518899e-06
ENSG00000114867_184329016_184334908_252	EIF4G1	3	+	756	38	1	0	4.37%	29.494254107932548	0.0016186486669924592	0.0016033688297201391	1.550737781191408e-05
ENSG00000145781_116087498_116292489_188	COMMD10	5	+	564	15	0	1	3.72%	15.605862335944153	0.00837382380159347	0.011738695074494007	0.000494661429975749
ENSG00000114784_40309942_40311083_73	EIF1B	3	+	219	16	5	0	8.22%	42.86980265435617	0.1029516053660342	0.013640585738808999	0.00703747379914186
ENSG00000099968_17702273_17727534_323	BCL2L13	22	+	969	14	1	0	1.55%	8.47773574163158	0.028889785561798623	0.02108409854857798	0.002731532589007073
ENSG00000015475_17750116_17735580_195	BID	22	-	585	19	3	0	3.59%	19.057825731279493	0.036371020034052744	0.00847367416878415	0.0015515874441493025
ENSG00000135624_73234382_73252861_338	CCT7	2	+	1014	251	10	8	14.50%	145.2484086604601	8.249767380760358e-09	9.333228035994056e-10	8.555762118286288e-17
ENSG00000214517_74203783_74253510_348	PPME1	11	+	1044	45	2	0	5.46%	25.29225964790949	0.00024002934710101625	4.950915307313248e-05	8.863406467124693e-08
ENSG00000151176_113358613_113388626_553	PLBD2	12	+	1659	33	1	0	2.89%	11.671943331187164	0.0002833883315400153	0.00017744757238953088	3.472812223617562e-07
ENSG00000114021_100334792_100348841_149	NIT2	3	+	447	43	1	12	5.37%	56.446438981553705	0.007356796211402075	0.06504720462003527	0.002592967701186 9152
ENSG00000011478_45692758_45703049_364	QPCTL	19	+	1092	14	1	0	2.20%	7.522825946557694	0.010020958156399907	0.004826937407846361	0.00025599508163266543
ENSG00000108883_44886750_44851274_937	EFTUD2	17	-	2811	71	5	3	3.42%	14.820850263975991	1.2612321871750657e-06	2.378326916760193e-06	2.6359690021912304e-11
ENSG00000155304_14383119_14373617_471	HSPA13	21	-	1413	84	4	3	5.94%	34.882912669388546	7.103770766121354e-06	5.105132029430858e-06	3.0174138574875293e-10
ENSG00000117054_75734848_75762763_273	ACADM	1	+	819	21	0	0	3.66%	15.045651893115387	0.0022409900782254734	0.0022409900782254734	2.9184102569313086e-05
ENSG00000082153_200813218_200822178_419	BZW1	2	+	1257	45	9	0	2.39%	21.006459087046544	0.029237630782826433	0.002460156992221257	0.000439785329035633
ENSG00000065911_74198642_74214242_267	MTHFD2	2	+	801	40	0	0	5.62%	29.302393200074913	0.0002994905732097556	0.0002994905732097556	6.05933306828899e-07
ENSG00000110075_68567023_68613117_516	PPP6R3	11	+	1548	16	3	0	1.74%	6.064913941410854	0.025616855544554156	0.0033030736763512156	0.00048502874613574003
ENSG00000140859_57771650_57759149_684	KIFC3	16	-	2052	31	5	3	2.19%	8.864616539364768	0.006894428981681446	0.0020295981827775276	8.055352678869601e-05
ENSG00000077684_128831759_128872262_842	JADE1	4	+	2526	10	0	2	0.83%	2.322962881359858	0.007029302661868638	0.04912109528954385	0.0018325026027891263
ENSG00000099995_30356792_30334594_793	SF3A1	22	-	2379	36	1	1	2.27%	8.879401117248426	0.0001185681716428482	0.0001185681716428482	1.0054948938218535e-07
ENSG00000143398_151231761_151247865_425	PIP5K1A	1	+	1275	12	0	1	1.18%	5.522639283120001	0.019679754444124863	0.02784981298332479	0.002477909089056847
ENSG00000162976_11155547_11177828_188	PQLC3	2	+	564	20	2	1	5.32%	20.807816447925536	0.011698770487832223	0.00397428314897603	0.0002509293845041528
ENSG00000101367_32825985_32848728_249	MAPRE1	20	+	747	46	3	0	7.23%	36.133734265393585	0.00028398238388319374	8.287246183407378e-05	1.697071607859955e-07
ENSG00000114805_155586073_155480968_1488	PLCH1	3	-	4464	9	0	0	0.34%	1.1830250480473794	0.020613416668581838	0.020613416668581838	0.001946208561389314
ENSG00000137575_58565034_58581740_297	SDCBP	8	+	891	120	4	1	10.10%	79.02766650929294	2.844226847274897e-06	1.106318704355805e-06	2.7762318096690534e-11
ENSG00000147416_20197422_20212935_313	ATP6V1B2	8	+	939	22	0	1	2.88%	13.747784157926521	0.0036321914711260127	0.005059698366439319	0.00010092379077801036
ENSG00000123349_53297850_53299345_85	PFDN5	12	+	255	53	6	0	10.59%	121.95828416890002	0.02256365863631257	0.0037896409716948643	0.0004751831865071967
ENSG00000141503_4884369_4897287_1228	MINK1	17	+	3684	20	1	5	0.65%	3.185561475741043	0.005160369442893307	0.07941064379354501	0.0024770715527741216
ENSG00000196715_65873231_65954300_223	VKORC1L1	7	+	669	21	0	0	4.48%	18.41911644314126	0.0022011839993586477	0.0022011839993586477	2.8200182015552967e-05
ENSG00000175224_46657603_46672332_292	ATG13	11	+	876	11	6	1	1.71%	7.368247331217467	0.172059248067656	0.03577298629450316	0.02599789514137937
ENSG00000145191_184135386_184141919_402	EIF2B5	3	+	1206	14	1	0	1.24%	6.811713046136817	0.0391240932165479	0.02108409854857798	0.00366011816787233
ENSG00000197912_89508547_89530693_85	SPG7	16	+	255	12	0	0	5.88%	27.613196415600008	0.019679754444124863	0.019679754444124863	0.0017849636115922674
ENSG00000138685_122827175_122892396_155	FGF2	4	+	465	20	1	0	4.52%	25.237867691677426	0.00847367416878415	0.00847367416878415	0.0003665024264111182
ENSG00000168092_117152520_117175962_131	PAFAH1B2	11	+	393	19	3	0	3.82%	28.368519218316802	0.0705580690856681	0.021557223391537678	0.006740452327400666
ENSG00000177239_137088108_137107361_466	MAN1B1	9	+	1398	30	5	2	2.79%	12.591854588658801	0.008457479729681213	0.00095416369115764	5.1393004291688025e-05
ENSG00000177239_137107370_137108591_137	MAN1B1	9	+	411	13	0	0	3.65%	18.559964744062047	0.020613416668581838	0.020613416668581838	0.001946208561389314
ENSG00000118181_119018284_119015845_125	RPS25	11	-	375	125	26	6	8.00%	195.59347461050004	0.09747853171310311	0.00908882258619153	0.004846121434778369
ENSG00000164904_126595114_126544965_444	ALDH7A1	5	-	1332	25	0	0	2.25%	11.01314609293356	0.0022409900782254734	0.0022409900782254734	2.9184102569313086e-05
ENSG00000243989_51986441_51989075_254	ACY1	3	+	762	7	1	0	1.97%	5.390371347533466	0.0477903522728147	0.016947426762344633	0.0036975118224100542
ENSG00000165782_20461325_20458559_277	PIP4P1	14	-	831	11	2	1	2.89%	7.767249894279785	0.03318103322835444	0.010499663139468709	0.001693572322031727
ENSG00000156113_77637360_77108332_328	KCNMA1	10	-	984	9	3	0	1.52%	5.366894120410062	0.12180769416495907	0.019679754444124863	0.011261990492355435
ENSG00000184047_122226014_122208557_209	DIABLO	12	-	627	9	0	1	2.87%	8.422685509543063	0.011772051206276392	0.026832695913080365	0.0015103817605807263
ENSG00000139687_48345141_48480071_781	RB1	13	+	2343	19	1	0	1.41%	4.758355976439822	0.002550198508962866	0.0012558331315113088	1.9177754206419204e-05
ENSG00000051180_40698771_40731178_336	RAD51	15	+	1008	14	3	0	1.79%	8.149728108770836	0.06032271266817251	0.011570465654371866	0.0034318131775285545
ENSG00000171421_1798911_1798624_95	MRPL36	5	-	285	9	0	1	5.26%	18.52990812099474	0.019679754444124863	0.06539853090342929	0.005779266745814885
ENSG00000164904_126554349_126544965_160	ALDH7A1	5	-	480	8	0	0	3.12%	9.779673730525003	0.016947426762344633	0.016947426762344633	0.0013498980316300959
ENSG00000083896_68337877_68314099_676	YTHDC1	4	-	2028	46	0	6	2.66%	13.30961513621746	8.348020546734336e-05	0.0015883879442000993	1.027939760352332e-06
ENSG00000142453_10871703_10921757_585	CARM1	19	+	1755	23	0	1	2.39%	7.689999856481198	0.00038956071474286695	0.00036814110674581835	9.539865471344388e-07
ENSG00000196177_123009030_123053765_432	ACADSB	10	+	1296	12	0	0	1.62%	5.43315207251389	0.008175610787902942	0.008175610787902942	0.00034251843268961324
ENSG00000135317_85593697_85505967_939	SNX14	6	-	2817	19	0	2	0.85%	3.957695439403089	0.005604866691947602	0.012088529595134127	0.00035289342940057376
ENSG00000072518_63895204_63909237_745	MARK2	11	+	2235	9	0	0	0.81%	2.362874189925504	0.011772051206276392	0.011772051206276392	0.0006812725113895 08

ENSG00000143621_153662792_153662396_82	ILF2	1	-	246	23	1	0	7.32%	54.861584341969525	0.013853924679039932	0.013640585738808999	0.000911832052528562
ENSG00000156709_130156550_130129557_560	AIFM1	X	-	1680	41	0	1	3.21%	14.320236533983039	8.594427494319189e-05	0.00010858901496822852	6.759693023984608e-08
ENSG00000132153_47843178_47850120_907	DHX30	3	+	2721	17	2	4	1.10%	3.6660298438572223	0.0073980154002197245	0.03063645427690382	0.0011563803149223198
ENSG00000094631_48806387_48824612_1063	HDAC6	X	+	3189	21	0	0	0.66%	3.8640291315338673	0.00797940233269714	0.00797940233269714	0.000327142761693177
ENSG00000198211_89919886_89935804_588	AC092143.1	16	+	1764	67	8	13	3.40%	22.28701156276106	0.0005642316196974205	0.0012745682963653385	4.576785704604193e-06
ENSG00000165525_49795940_49785216_187	NEMF	14	-	561	9	0	0	2.67%	9.413589687136366	0.020613416668581838	0.020613416668581838	0.001946208561389314
ENSG00000120053_99403566_99397547_126	GOT1	10	-	378	34	0	1	6.35%	52.7791915615635	0.00575691403842271	0.007356796211402075	0.00022276510181128367
ENSG00000167965_2206613_2208877_227	MLST8	16	+	681	13	0	4	3.52%	11.201388413817183	0.004826937407846361	0.05158844004651438	0.0014303885399334592
ENSG00000138629_74458867_74446090_380	UBL7	15	-	1140	13	0	0	1.84%	6.691355710359212	0.008175610787902942	0.008175610787902942	0.00034251843268961324
ENSG00000165806_113721653_113729540_220	CASP7	10	+	660	14	0	0	3.64%	12.446857475213639	0.005160369442893307	0.005160369442893307	0.00014319601891785326
ENSG00000182196_122981140_122982676_170	ARL6IP4	12	+	510	19	3	1	6.47%	21.860447162350006	0.011862047307812774	0.0025593219000189936	0.00017265706510873147
ENSG00000196083_190609068_190648705_429	IL1RAP	3	+	1287	15	0	0	1.40%	6.8389326786888125	0.013428347753762199	0.013428347753762199	0.0008725593497644525
ENSG00000135390_53672614_53665314_141	ATP5MC2	12	-	423	104	13	2	17.73%	144.26752737228372	4.4036283112694194e-05	8.843606187363953e-06	3.163887414050723e-09
ENSG00000138796_108004852_108034357_292	HADH	4	+	876	38	0	0	4.79%	25.453945326023977	0.00037874750290730355	0.00037874750290730355	9.541676172656993e-07
ENSG00000104763_18063207_18061715_74	ASAH1	8	-	222	9	3	0	8.11%	23.788395560736493	0.0477903522728147	0.011772051206276392	0.002720069141611748
ENSG00000183283_51240919_51242458_108	DAZAP2	12	+	324	38	0	0	7.41%	68.8199262518426	0.005655335537214311	0.005655335537214311	0.00017039952270558162
ENSG00000148450_23095609_23120862_182	MSRB2	10	+	546	28	5	0	3.30%	30.091303786230775	0.06341282918854552	0.013640585738808999	0.0041393610649128765
ENSG00000062485_56283836_56273084_392	CS	12	-	1176	39	0	0	3.83%	19.459554871963014	0.0002902893921209972	0.0002902893921209972	5.704281701590603e-07
ENSG00000117450_45519043_45511329_97	PRDX1	1	-	291	243	28	7	23.71%	489.99190031290215	0.00028255376325861295	3.73657815459283e-05	8.089096612304592e-08
ENSG00000186010_19516266_19527356_73	NDUFA13	19	+	219	98	12	14	31.51%	262.57754125793156	0.0010132485277050188	0.0018313330281384383	1.1321307960910053e-05
ENSG00000186010_19527711_19528126_59	NDUFA13	19	+	177	124	18	0	22.03%	411.0778110457967	0.006496567068686283	0.0007246167279913337	3.057624238402419e-05
ENSG00000087245_55483044_55505442_564	MMP2	16	+	1692	11	2	1	1.42%	3.8147663487863483	0.03318103322835444	0.010499663139468709	0.001693572322031727
ENSG00000143537_155051387_155062502_838	ADAM15	1	+	2514	46	13	0	2.27%	10.736634644490456	0.009314527945786596	5.328371459388349e-05	5.3170083957120194e-06
ENSG00000117984_1763859_1753503_405	CTSD	11	-	1215	429	16	2	18.52%	207.18419903186302	1.6909440416263497e-12	3.330352454300139e-14	7.950578142406847e-25
ENSG00000100116_37807968_37816718_445	GCAT	22	+	1335	8	1	0	1.12%	3.5162871840089895	0.04223451612662591	0.019679754444124863	0.003715148010809822
ENSG00000131469_43000020_43002920_80	RPL27	17	+	240	50	3	1	13.75%	122.24592163156252	0.008500829569974952	0.002493493840886608	0.00011981378883962093
ENSG00000138759_78058010_78541124_4012	FRAS1	4	+	12036	16	4	2	0.20%	0.7800337970508476	0.05423379212399942	0.02071580916834944	0.004984368886667794
ENSG00000129116_168711760_168926385_559	PALLD	4	+	1677	28	3	0	2.50%	9.797168674586764	0.0027707726756871684	0.00037518914374882155	6.9758339868607375e-06
ENSG00000157916_2402238_2403124_64	RER1	1	+	192	16	0	1	7.81%	48.89836865262501	0.019679754444124863	0.04223451612662591	0.003715148010809822
ENSG00000150753_10258133_10261871_250	CCT5	5	+	750	281	10	6	10.40%	219.84706546220204	5.441214665151731e-06	4.248744136687675e-06	1.9413012146226887e-10
ENSG00000177084_132676560_132624697_1988	POLE	12	-	5964	14	2	0	0.40%	1.3774188352852115	0.01943569092740073	0.004826937407846361	0.0004999687388256805
ENSG00000143870_10819289_10784258_439	PDIA6	2	-	1317	602	19	19	27.56%	268.2170198531231	3.82016279060415e-21	4.275049111014097e-20	2.764494454277351e-39
ENSG00000073350_75563053_75574906_826	LLGL2	17	+	2478	11	0	0	0.73%	2.6047557151519376	0.012798402692974279	0.012798402692974279	0.0007972436888943567
ENSG00000103496_31033806_31039803_297	STX4	16	+	891	16	2	0	2.69%	10.53702220123906	0.02379615584997438	0.005257622967929454	0.0006640442507048208
ENSG00000063241_55456486_55453308_135	ISOC2	19	-	405	20	0	0	6.67%	28.976811053407413	0.0035547763465261306	0.0035547763465261306	7.044676146010672e-05
ENSG00000100029_30584668_30577046_449	PES1	22	-	1347	39	2	3	3.56%	16.989188217838535	0.00046327533465069797	0.0007062068652445297	2.1221654999490226e-06
ENSG00000100029_30591833_30577046_571	PES1	22	-	1713	53	4	3	3.85%	18.154910953338884	9.98614348231814e-05	5.299266498840988e-05	3.913677847245043e-08
ENSG00000144036_72718111_72179335_595	EXOC6B	2	-	1785	16	0	1	1.01%	5.259656460114287	0.013640585738808999	0.020525443280229434	0.0013255139945858052
ENSG00000131171_81202201_81297227_114	SH3BGRL	X	+	342	20	0	0	5.26%	34.31464466850878	0.013428347753762199	0.013428347753762199	0.0008725593497644525
ENSG00000168036_41224069_41239342_781	CTNNB1	3	+	2343	117	8	3	5.76%	29.301455223339957	6.403151454483205e-08	1.1032790393731706e-08	7.305397733044446e-15
ENSG00000168036_41224069_41239342_781	CTNNB1	3	+	2343	117	8	3	5.76%	29.301455223339957	6.403151454483205e-08	1.1032790393731706e-08	7.305397733044446e-15
ENSG00000177426_3456398_3457940_252	TGIF1	18	+	756	34	0	2	2.78%	26.38959578078175	0.008574016250729465	0.013648824643852793	0.0005848802985757839
ENSG00000113269_180040465_179955654_276	RNF130	5	-	828	13	3	1	3.62%	9.212736122958335	0.02207925122323651	0.004004657782222479	0.0004866555319559463
ENSG00000121022_67059390_67045808_241	COPS5	8	-	723	28	0	0	6.22%	22.72455306678009	0.00025893283687359583	0.00025893283687359583	4.5720213171193383e-07
ENSG00000044115_138924641_138931013_232	CTNNA1	5	+	696	48	0	4	8.19%	40.46761543665518	5.839722503546666e-05	0.0003100011296740675	1.3409343662254076e-07
ENSG00000168487_22176562_22199310_563	BMP1	8	+	1689	18	0	0	1.24%	6.253432580797515	0.0078820576498062	0.0078820576498062	0.00031963642907451164
ENSG00000111843_10724614_10730666_112	TMEM14C	6	+	336	83	0	2	11.61%	144.9487356488527	0.0007052562366071395	0.0008884494972709903	3.961101715993978e-06
ENSG00000108219_80489234_80517976_253	TSPAN14	10	+	759	93	4	3	7.91%	71.89799659595457	0.00018707853009467164	6.793373103529052e-05	9.256993693435907e-08
ENSG00000092036_22951647_22946525_239	HAUS4	14	-	717	12	1	0	2.93%	9.820592867472806	0.013599199788808391	0.008175610787902942	0.0005580304998096723
ENSG00000114054_136255955_136330026_388	PCCB	3	+	1164	17	0	0	1.55%	8.569817186542528	0.013428347753762199	0.013428347753762199	0.0008725593497644525
ENSG00000185722_4263941_4167779_1170	ANKFY1	17	-	3510	35	2	1	1.37%	5.851086847322651	0.0005018492373769886	0.0002871188111466556	9.63461383505656e-07
ENSG00000164587_150446935_150445460_119	RPS14	5	-	357	67	14	0	7.56%	110.12405713364288	0.00934169773149884	0.0035547763465261306	0.00018105413413905744
ENSG00000105341_41438243_41433484_98	DMAC2	19	-	294	11	4	1	5.10%	21.95436959913776	0.24555942524033636	0.04223451612662591	0.04392468383442931
ENSG00000137815_41417236_41480687_670	RTF1	15	+	2010	11	0	0	0.90%	3.2112361503216427	0.011772051206276392	0.011772051206276392	0.000681272511389508
ENSG00000110651_2386595_2396866_229	CD81	11	+	687	191	5	2	18.78%	163.13691550482756	2.456062352528191e-08	7.548823057206886e-09	1.9294274512603784e-15
ENSG00000122257_24540726_24572445_919	RBBP6	16	+	2757	12	1	0	0.65%	2.5539953159151256	0.02015986069794795	0.013217226988443315	0.0012659333861537747
ENSG00000173915_103392376_103392194_31	ATP5MD	10	-	93	101	2	0	22.58%	637.256159214855	0.008980238763039383	0.008980238763039383	0.0004089866599971986
ENSG00000168488_28826345_28836777_878	ATXN2L	16	+	2634	50	7	8	1.94%	11.138580558684513	0.001435957343153687	0.0010673266102214833	9.370824648744565e-06
ENSG00000108439_47944638_47946782_166	PNPO	17	+	498	21	0	0	4.82%	24.743752812171692	0.0053557511377634245	0.0053557511377634245	0.00015366972994913032
ENSG00000108439_47944638_47946782_148	PNPO	17	+	444	21	0	0	5.41%	27.753128154192574	0.0053557511377634245	0.0053557511377634245	0.00015366972994913032
ENSG00000214078_35632923_35626241_481	CPNE1	20	-	1443	97	10	3	7.90%	39.444006314383586	7.863683324805115e-07	1.0570278052828432e-07	8.056852000662528e-13
ENSG00000100462_22928599_22920904_595	PRMT5	14	-	1785	45	7	0	4.20%	14.792783794071433	0.00019100914974351442	4.014129735738276e-06	7.197795323371274e-09
ENSG00000159335_6766706_6770442_102	PTMS	12	+	306	106	12	9	28.43%	203.26380694816672	1.8176808080565085e-05	2.1624599447604932e-05	3.093061109606383e-09
ENSG00000127955_80135161_80217493_354	GNAI1	7	+	1062	26	4	0	2.54%	14.365622429019778	0.015528142895806445	0.0036062891729786097	0.0003077713301577895
ENSG00000175376_66000389_65998599_165	EIF1AD	11	-	495	11	0	0	3.64%	13.039564974033336	0.012798402692974279	0.012798402692974279	0.0007972436888943567
ENSG00000131871_101277417_101275249_107	SELENOS	15	-	321	8	4	0	4.67%	14.623811185831778	0.19427189192379535	0.019679754444124863	0.019384065375599255
ENSG00000196961_49767161_49806758_946	AP2A1	19	+	2838	34	1	0	1.90%	7.029786613908035	0.00011592007149080553	7.870973464443577e-05	6.622085795723275e-08

ENSG00000171004_132957277_132628223_486	HS6ST2	X	-	1458	14	0	0	1.44%	5.634379927051442	0.00797940233269714	0.00797940233269714	0.000327142761693177
ENSG00000117054_75724788_75762763_454	ACADM	1	+	1362	24	0	0	2.86%	10.339743151215862	0.0005460508606305346	0.0005460508606305346	1.9341343681665648e-06
ENSG00000171004_132957277_132628223_446	HS6ST2	X	-	1338	14	0	0	1.57%	6.139705481047087	0.00797940233269714	0.00797940233269714	0.000327142761693177
ENSG00000196419_41622005_41663815_609	XRCC6	22	+	1827	46	10	6	3.45%	14.773891349889986	0.0017348806905588586	0.00027598514109279714	3.254294054705697e-06
ENSG00000067064_1049003_1041187_284	IDI1	10	-	852	104	5	4	13.03%	71.625779434831	9.09479102888368e-08	9.053460208369271e-08	8.005664989042483e-14
ENSG00000113161_75342627_75360342_828	HMGCR	5	+	2484	107	2	12	5.68%	25.275968337347226	1.4129268184731312e-09	2.7247019981283795e-07	4.8299639239796125e-15
ENSG00000204628_181243800_181236977_269	RACK1	5	-	807	970	26	26	26.39%	705.299889859424	2.9664427105341975e-13	6.597406458863746e-13	2.5567722579603654e-24
ENSG00000113593_65569985_65587396_477	PPWD1	5	+	1431	13	2	0	1.26%	5.330639769258911	0.044040755831095145	0.012798402692974279	0.0026804992160797854
ENSG00000075785_128795368_128813422_160	RAB7A	3	+	480	45	0	3	9.38%	55.01066473420314	0.0003026020628493325	0.0008949864876454083	1.800462191750564e-06
ENSG00000170248_33798729_33866525_873	PDCD6IP	3	+	2619	39	0	0	2.06%	8.737852817651206	8.317592195240937e-05	8.317592195240937e-05	5.049519016010041e-08
ENSG00000123737_121801809_121816856_423	EXOSC9	4	+	1269	23	0	0	3.78%	10.635106184495275	0.0001415909997498741	0.0001415909997498741	1.4189685379808827e-07
ENSG00000156469_96257085_96239491_296	MTERF3	8	-	888	12	0	2	2.03%	7.929465186912164	0.013007239811447113	0.060667625179241055	0.0037986472884049276
ENSG00000108654_64504108_64503956_50	DDX5	17	-	150	15	4	3	10.00%	58.678042383150014	0.08588638879785576	0.020613416668581838	0.007983738891631635
ENSG00000266412_46016680_46006592_614	NCOA4	10	-	1842	50	0	0	2.93%	15.927807378705216	7.625943357267001e-05	7.625943357267001e-05	4.2652882223459296e-08
ENSG00000172725_67442628_67440744_282	CORO1B	11	-	846	26	0	0	2.48%	18.033440921535465	0.008574016250729465	0.008574016250729465	0.0003747459614100891
ENSG00000163798_27663915_27694683_742	SLC4A1AP	2	+	2226	27	0	1	1.75%	7.117282768845689	0.0006606329705481882	0.0009282178722589528	3.88426112483076e-06
ENSG00000108298_39201214_39204648_194	RPL19	17	+	582	128	19	0	11.86%	129.05136469146396	0.00023490963595251618	1.236195501581318e-05	2.4469965317013604e-08
ENSG00000149554_125627722_125635541_181	CHEK1	11	+	543	13	0	0	3.31%	14.048150110146413	0.013428347753762199	0.013428347753762199	0.0008725593497644525
ENSG00000109971_123061324_123061049_91	HSPA8	11	-	273	65	0	0	13.19%	139.70962472178576	0.0010197273511756433	0.0010197273511756433	6.442859155158972e-06
ENSG00000100504_50944403_50923948_226	PYGL	14	-	678	21	0	0	4.42%	18.174614897435845	0.002046028879997488	0.002046028879997488	2.4518070358754455e-05
ENSG00000172428_240136284_240131051_57	COPS9	2	-	171	65	0	3	24.56%	223.04519034530708	0.00046432135692914766	0.0019709495986684534	5.937495296652284e-06
ENSG00000105254_36116080_36125672_60	TBCB	19	+	180	36	1	11	11.67%	117.35608476630003	0.012176559331126819	0.17798271378568276	0.012391266068887321
ENSG00000105290_35871651_35879441_458	APLP1	19	+	1374	8	0	0	1.09%	3.4164799058602626	0.019679754444124863	0.019679754444124863	0.0017849636115922674
ENSG00000147955_34637697_34635632_223	SIGMAR1	9	-	669	117	6	1	12.11%	102.62079161178701	1.9832361740714642e-05	2.8723559595784297e-06	4.890715748208014e-10
ENSG00000157593_44255605_44254706_299	SLC35B2	6	-	897	12	0	0	3.01%	7.849905335538463	0.002874854311667729	0.002874854311667729	4.6982854837323694e-05
ENSG00000273703_27815044_27815424_126	HIST1H2BM	6	+	378	193	1	1	12.70%	299.59952857005163	0.0002224705907394276	0.0002131294310221524	3.269046946257035e-07
ENSG00000100823_20455646_20457508_318	APEX1	14	+	954	109	11	4	9.43%	67.0430463287563	1.257751652788525e-05	2.805509405993715e-06	3.0087131975870506e-10
ENSG00000040633_7236536_7235626_273	PHF23	17	-	819	14	3	1	2.56%	10.03043459541026	0.04363948305378087	0.015516259750072458	0.00312766395889963
ENSG00000213523_140557452_140550700_224	SRA1	5	-	672	14	0	0	3.12%	12.224592163156252	0.008175610787902942	0.008175610787902942	0.00034251843268961324
ENSG00000151503_134223926_134152944_1498	NCAPD3	11	-	4494	19	1	1	0.60%	2.4808251118821767	0.006432154271512831	0.006432154271512831	0.00021751960674370818
ENSG00000135108_117190429_117146087_619	FBXO21	12	-	1857	35	0	0	2.58%	11.059404864890956	0.00019160400119445356	0.00019160400119445356	2.55124648444939e-07
ENSG00000148019_78240031_78270851_602	CEP78	9	+	1806	21	0	0	1.83%	6.823028184087211	0.0012558331315113088	0.0012558331315113088	9.614604369954505e-06
ENSG00000115415_191009922_190970703_723	STAT1	2	-	2169	45	8	4	2.77%	12.173867714346477	0.0013950305237650524	0.0003026526610799105	2.8253310383760704e-06
ENSG00000188677_44100073_44168678_290	PARVB	22	+	870	22	0	0	2.76%	14.8381256601069	0.005306578019052857	0.005306578019052857	0.00015100114740534233
ENSG00000128609_123550481_123542119_59	NDUFA5	7	-	177	13	1	2	8.47%	43.09686728705933	0.02108409854857798	0.0700082515985845	0.006564739044797468
ENSG00000108262_29581348_29574950_444	GIT1	17	-	1332	10	0	2	1.13%	4.4052584371734245	0.020613416668581838	0.051235217429874684	0.004687384229717438
ENSG00000169371_75609991_75601108_160	SNUPN	15	-	480	20	0	0	5.62%	24.449184326312505	0.0034529206320349946	0.0034529206320349946	6.664817351595063e-05
ENSG00000130227_21966293_22005088_1088	XPO7	8	+	3264	26	2	0	1.29%	4.674108768265626	0.00195670001083769	0.0003353510003294976	4.392229340842254e-06
ENSG00000127774_3669147_3669479_110	EMC6	17	+	330	21	1	0	5.45%	37.340572425640914	0.013640585738808999	0.013428347753762199	0.0008855098933240206
ENSG00000010256_48600720_48599128_118	UQCRC1	3	-	354	32	0	1	7.63%	53.04229819945764	0.0036321914711260127	0.005059698366439319	0.00010092379077801036
ENSG00000120694_31161582_31137318_814	HSPH1	13	-	2442	89	6	1	4.55%	21.38552732227826	1.3718911248964808e-06	3.650219388518709e-08	5.299269638539756e-13
ENSG00000142230_47130931_47209251_346	SAE1	19	+	1038	54	9	2	6.07%	30.526149216667637	0.00022669783984938897	6.303668889100666e-05	1.0470243399522495e-07
ENSG00000163463_155172824_155169440_112	KRTCAP2	1	-	336	60	1	1	12.50%	104.7822185413393	0.0006106561369186716	0.0006106561369186716	2.3997955259749722e-06
ENSG00000196975_69788129_69810691_114	ANXA4	2	+	342	56	3	0	11.40%	96.08100507182458	0.002293196040126747	0.0007052562366071395	1.013519372398099e-05
ENSG00000090061_99492678_99510782_580	CCNK	14	+	1740	14	0	0	1.38%	4.721221800943105	0.004873903550586949	0.004873903550586949	0.00012846764509679302
ENSG00000174840_57556380_57560004_609	PDE12	3	+	1827	22	1	0	1.48%	7.065774123860429	0.005059698366439319	0.0036321914711260127	0.00010092379077801036
ENSG00000130177_114239476_114272443_447	CDC16	13	+	1341	34	0	5	3.13%	14.877356010642062	0.0004323067881491664	0.0037401700898596867	1.0841548675644574e-05
ENSG00000110066_68190049_68165914_393	KMT5B	11	-	1179	5	0	1	1.27%	2.4884665980979648	0.012673659338734126	0.051235217429874705	0.0031110695322510533
ENSG00000168291_58431954_58431404_121	PDHB	3	-	363	13	0	0	4.13%	21.014174958152896	0.020613416668581838	0.020613416668581838	0.001946208561389314
ENSG00000137203_10414985_10398417_437	TFAP2A	6	-	1311	9	2	2	1.37%	4.028240895868422	0.04178282491678763	0.0416322583317752	0.007176695144763618
ENSG00000023330_52199350_52214180_604	ALAS1	3	+	1812	32	2	4	2.15%	10.36256819128477	0.0026030014435915734	0.0015639623262203096	2.4008739039704547e-05
ENSG00000105669_18919348_18899679_308	COPE	19	-	924	60	4	0	6.17%	38.10262492412338	0.0002841149068428368	5.934787645966978e-05	1.245027709245959e-07
ENSG00000115275_74465247_74462717_294	MOGS	2	-	882	9	0	1	2.04%	5.9875553452193895	0.011772051206276392	0.026832695913080365	0.0015103817605807263
ENSG00000115275_74462225_74461275_316	MOGS	2	-	948	19	0	3	2.85%	11.760367144302219	0.0033030736763512156	0.024710983489837978	0.0004665285142020836
ENSG00000128944_40383253_40393597_238	KNSTRN	15	+	714	15	7	0	2.94%	12.32731982839286	0.08502160687168953	0.00847367416878415	0.003922604800908926
ENSG00000100749_96833565_96881208_365	VRK1	14	+	1095	10	0	0	2.19%	5.358725331794521	0.00414960799764038	0.00414960799764038	9.45964903272165e-05
ENSG00000162191_62678923_62676590_297	UBXN1	11	-	891	86	0	0	9.76%	56.63649433165995	1.0282381626427154e-06	1.0282381626427154e-06	9.442453934460063e-12
ENSG00000119760_27662192_27653485_279	SUPT7L	2	-	837	14	0	2	2.15%	9.81472632454122	0.013428347753762199	0.037178613843887286	0.0023492041693324566
ENSG00000172534_153957004_153949347_1277	HCFC1	X	-	3831	17	0	1	0.63%	2.603828557853172	0.005454749182134642	0.008219046535586472	0.00023581017309990104
ENSG00000169242_155127978_155133693_192	EFNA1	1	+	576	10	1	1	3.12%	10.187160135963545	0.023672501042627626	0.023672501042627626	0.0025184098398201636
ENSG00000196586_75832877_75915012_1153	MYO6	6	+	3459	13	0	0	0.43%	2.205303703327407	0.02108409854857798	0.02108409854857798	0.002029891263157455
ENSG00000006282_50548842_50555662_671	SPATA20	17	+	2013	8	3	0	0.89%	2.3319639297824146	0.09373101630823749	0.009815328628645334	0.004911899492075861
ENSG00000177556_151758551_151746325_68	ATOX1	5	-	204	43	3	7	11.76%	123.68410894487502	0.007121295578054772	0.028462570115697007	0.0010369866735897414
ENSG00000115875_38751256_38745133_238	SRSF7	2	-	714	180	6	3	16.39%	147.9278379407143	4.6818303992575346e-08	4.22597978718481e-08	1.9676117970096296e-14
ENSG00000037474_6620212_6599926_531	NSUN2	5	-	1593	61	2	3	4.71%	22.469306876159138	1.801218945375667e-05	2.3525749339974676e-05	3.3301312306983467e-09
ENSG00000135535_109381552_109368851_155	CD164	6	-	465	65	3	1	12.90%	82.02306999795164	0.00011806040044846176	3.839907090312695e-05	3.4079023028341796e-08
ENSG00000071655_1585156_1578486_185	MBD3	19	-	555	11	0	1	2.70%	11.62988227413784	0.019216965118390855	0.04178282491678763	0.003600525600033436

ENSG00000166226_69585522_69589731_230	CCT2	12	+	690	127	10	5	13.91%	108.00161424145003	4.064739902926829e-07	2.2907520772485206e-06	8.579834329228287e-12
ENSG00000116044_177234268_177230785_589	NFE2L2	2	−	1767	17	1	0	1.19%	5.645312509980477	0.011825808327677989	0.00847367416878415	0.0005036791746826746
ENSG00000164941_94832070_94880234_762	INTS8	8	+	2286	18	2	0	1.05%	4.620318297885828	0.020990660982539537	0.0053557511377634245	0.0005922972719933773
ENSG00000197081_160089060_160105084_400	IGF2R	6	+	1200	11	0	1	1.25%	5.378820551788751	0.016947426762344633	0.020239728056605077	0.001595379919513119
ENSG00000197081_160061815_160088238_980	IGF2R	6	+	2940	24	2	0	0.92%	4.790044276175511	0.01052924788556975	0.003684287313187465	0.00021087137401632993
ENSG00000169045_179617909_179615546_159	HNRNPH1	5	−	477	68	1	0	5.03%	83.65003945606291	0.006307833610902286	0.00575691403842271	0.0001922616017969111
ENSG00000060982_24948932_24818008_325	BCAT1	12	−	975	29	0	0	2.77%	17.45295619601385	0.0034782380676658805	0.0034782380676658805	6.758309314600117e-05
ENSG00000105928_24749774_24699026_496	GSDME	7	−	1488	11	0	1	1.21%	4.337758509507058	0.012798402692974279	0.019795881618345192	0.0012079910998532793
ENSG00000104408_108240001_108201885_352	EIF3E	8	−	1056	62	5	0	6.82%	34.4511233688949	4.753018224745257e-05	7.527794548535961e-06	2.942175977332029e-09
ENSG00000072041_84886036_84861632_623	SLC6A15	12	−	1869	20	0	3	1.77%	6.27908425715891	0.0014481341328822738	0.011821942446875977	0.00010518227444157728
ENSG00000133083_35855528_35774535_433	DCLK1	13	−	1299	9	0	0	1.15%	4.06545328289723	0.019679754444124863	0.019679754444124863	0.0017849636115922674
ENSG00000111371_46239755_46196127_488	SLC38A1	12	−	1464	35	1	0	4.10%	14.02822051509734	7.977567501134817e-05	3.106876052321276e-05	1.8831791554240613e-08
ENSG00000204209_33321925_33318747_688	DAXX	6	−	2064	12	5	0	0.73%	3.4115140920436056	0.14592027257189422	0.02108409854857798	0.014551245568658585
ENSG00000178694_94063127_94126490_340	NSUN3	3	+	1020	10	4	2	1.47%	5.752749253250001	0.17004230409153215	0.06556525143482543	0.04074822827482303
ENSG00000055044_202265942_202303436_529	NOP58	2	+	1587	33	0	1	3.21%	12.201483293282612	0.00011226201234273827	0.00018608344908209348	1.4825504535560468e-07
ENSG00000089157_120200783_120196773_317	RPLP0	12	−	951	61	1	4	3.15%	37.63786104492272	0.0025025372014526115	0.004791578323725913	6.770248138606176e-05
ENSG00000089159_120221730_120212314_350	PXN	12	−	1050	21	0	0	3.14%	11.735608476630002	0.001092092816906976	0.001092092816906976	7.350803965414657e-06
ENSG00000100596_77597311_77535896_403	SPTLC2	14	−	1209	25	0	0	2.73%	12.133590236383377	0.0015505692382983198	0.0015505692382983198	1.4410974035783556e-05
ENSG00000254996_140401968_140549237_2617	ANKHD1-EIF4EBP3	5	+	7851	27	0	5	0.57%	2.0179685955229276	0.00020884357926031504	0.004391170382365921	6.867882329755922e-06
ENSG00000255292_112086908_112170527_140	AP002884.3	11	+	420	16	0	1	4.29%	22.35353995548572	0.013007239811447113	0.016503128830616266	0.001029548967667127
ENSG00000107020_5431914_5358239_126	PLGRKT	9	−	378	30	1	0	9.52%	46.56987490726191	0.0010073743004618205	0.000978917897162665	6.122602232868213e-06
ENSG00000134283_42335903_42446618_434	PPHLN1	12	+	1302	12	1	1	1.15%	5.408114505359448	0.03577298629450316	0.03577298629450316	0.005410795288624905
ENSG00000143570_153960697_153960098_199	SLC39A1	1	−	597	33	0	7	8.54%	32.43509880475629	0.00011682594516430408	0.001443728563113205	1.2469064792834003e-06
ENSG00000112304_24687682_24701615_117	ACOT13	6	+	351	34	0	0	10.26%	56.83912937399147	0.0010238660768343774	0.0010238660768343774	6.493246270986479e-06
ENSG00000111581_68687066_68709245_202	NUP107	12	+	606	17	2	0	3.47%	16.460836972170796	0.023672501042627626	0.008175610787902942	0.0009673284272160682
ENSG00000111665_6850485_6849521_118	CDCA3	12	−	354	17	2	0	7.63%	28.17872091846187	0.014936572009614516	0.0033030736763512156	0.0002738986320205498
ENSG00000123240_13110300_13136866_513	OPTN	10	+	1539	26	4	3	1.95%	9.913119570902536	0.01790347956991311	0.01184944301685081	0.0010220276812614802
ENSG00000050426_51048357_51058072_155	LETMD1	12	+	465	17	0	0	3.87%	21.452187537925813	0.013007239811447113	0.013007239811447113	0.0008218654148058079
ENSG00000131165_89657588_89646066_187	CHMP1A	16	−	561	15	1	1	3.74%	15.689316145227277	0.011738695074494007	0.011738695074494007	0.0006776446936291177
ENSG00000058272_79934925_79775929_972	PPP1R12A	12	−	2916	22	0	2	1.23%	4.427012799826133	0.0007660506977098094	0.003206113631917875	1.536359415844302e-05
ENSG00000130713_130698228_130703774_181	EXOSC2	9	+	543	22	5	0	6.08%	23.77379249409393	0.0025593219000189936	0.0012624143894574274	1.9339285108591855e-05
ENSG00000130713_130693798_130695597_73	EXOSC2	9	+	219	18	0	0	6.85%	48.228527986150695	0.02108409854857798	0.02108409854857798	0.002029891263157455
ENSG00000139644_49752494_49762896_237	TMBIM6	12	+	711	661	23	17	32.49%	545.5159777111414	4.4607759695857914e-14	1.475365366222195e-13	8.888324263736637e-26
ENSG00000160124_122371785_122359869_122	CCDC58	3	−	366	9	0	0	4.92%	14.429026815528692	0.011772051206276392	0.011772051206276392	0.000681272511389508
ENSG00000183291_86914111_86880690_95	SELENOF	1	−	285	222	2	3	18.95%	457.07106698453697	9.617268880422018e-05	8.931821586475278e-05	6.23200399238289e-08
ENSG00000108523_4941098_4945016_315	RNF167	17	+	945	27	4	0	2.86%	16.76515496661429	0.0033030736763512156	0.0033278027414746945	6.167781147582356e-05
ENSG00000156787_123072770_123151939_970	TBC1D31	8	+	2910	13	0	0	0.72%	2.6213558452953616	0.0078820576498062	0.0078820576498062	0.00031963642907451164
ENSG00000124067_67957957_67944840_942	SLC12A4	16	−	2826	9	0	1	0.53%	1.868727464431529	0.019216965118390855	0.04178282491678763	0.003600525600033436
ENSG00000125630_112550943_112575729_901	POLR1B	2	+	2703	23	0	0	1.00%	4.9929521820660385	0.0034782380676658805	0.0034782380676658805	6.758309314600117e-05
ENSG00000108848_50741124_50750743_356	LUC7L3	17	+	1068	55	1	1	6.46%	30.218092987577254	1.0353844738560667e-05	1.0353844738560667e-05	8.695172811905806e-10
ENSG00000138614_65598879_65579408_319	INTS14	15	−	957	11	0	0	1.88%	6.744602572775864	0.012798402692974279	0.012798402692974279	0.0007972436888943567
ENSG00000160124_122383224_122368167_110	CCDC58	3	−	330	12	0	0	6.36%	21.337469957509096	0.0078820576498062	0.0078820576498062	0.00031963642907451164
ENSG00000131389_14443635_14485007_620	SLC6A6	3	+	1860	80	10	3	4.68%	25.237867691677426	1.3105914637044646e-05	6.2887884200256284e-06	6.767351074608523e-10
ENSG00000197170_67366519_67340843_436	PSMD12	17	−	1308	57	1	7	6.19%	25.570706543115833	2.9879715676052896e-06	3.406636886839298e-05	8.859562321776163e-10
ENSG00000064042_41360841_41697185_1056	LIMCH1	4	+	3168	22	3	6	1.23%	4.074864054385418	0.005301672691061329	0.009535600913472842	0.0002655394963642886
ENSG00000160789_156136930_156139106_171	LMNA	1	+	513	31	0	0	6.43%	35.45846615745907	0.00151350169064872	0.00151350169064872	1.3757299738441384e-05
ENSG00000080822_98521358_98516856_130	CLDND1	3	−	390	18	0	0	4.62%	27.082173407607698	0.013007239811447113	0.013007239811447113	0.0008218654148058079
ENSG00000138674_82881936_82819074_1205	SEC31A	4	−	3615	28	0	0	1.00%	4.544910613356017	0.0008664586529590942	0.0008664586529590942	4.7094196839841995e-06
ENSG00000082641_48051119_48059641_761	NFE2L1	17	+	2283	48	4	0	3.02%	12.337039134433644	0.00014645970963619303	1.0359460454137243e-05	1.26693106383586e-08
ENSG00000160752_155310161_155318757_160	FDPS	1	+	480	94	3	3	13.12%	114.91116633366877	6.925833928252037e-05	6.200870144336857e-05	3.176815581592885e-08
ENSG00000132507_7309636_7311640_154	EIF5A	17	+	462	179	71	8	11.69%	227.34566204726954	0.007938263751100936	9.685105774909149e-05	7.0979032624909975e-06
ENSG00000091039_76410578_76355889_798	OSBPL8	12	−	2394	18	0	1	1.38%	4.411882885951129	0.0008884494972709903	0.0024579339080672683	1.3429802402146636e-05
ENSG00000111799_75194894_75087557_3020	COL12A1	6	−	9060	27	4	0	0.43%	1.748683382279305	0.010340900302280928	0.0005460508606305346	3.9861314150013366e-05
ENSG00000134910_125614099_125620810_183	STT3A	11	+	549	34	2	0	6.01%	36.3397712391093	0.00447458916152286	0.001558047347358915	4.0977392991800375e-05
ENSG00000163170_74145285_74135593_83	BOLA3	2	−	249	39	0	8	6.02%	91.90536758806628	0.02108409854857798	0.24871115394169385	0.027647718863847367
ENSG00000155957_66160192_66137960_215	TMBIM4	12	−	645	49	0	4	6.98%	44.577117469369774	0.0003026020628493325	0.001576668176329556	3.203251411610351e-06
ENSG00000104635_22404711_22419698_492	SLC39A14	8	+	1476	33	1	4	3.25%	13.11907451655793	0.00027048932411835087	0.0015146672631477993	2.776937546067962e-06
ENSG00000174840_57556380_57559420_472	PDE12	3	+	1416	13	1	0	1.27%	5.3871084108824165	0.02034245439037237	0.013428347753762199	0.0012955184805712684
ENSG00000176153_64942726_64939488_189	GPX2	14	−	567	82	14	1	3.17%	84.86066094212171	0.16200327800095415	0.016503128830616266	0.013728928102175093
ENSG00000125746_45638587_45609663_617	EML2	19	−	1851	19	0	0	1.62%	6.023137791895463	0.0022543491824521134	0.0022543491824521134	2.9517890247939808e-05
ENSG00000167461_16111902_16127661_182	RAB8A	19	+	546	32	0	1	7.69%	34.39006146997803	0.0003386460863230731	0.0006764943839929669	1.5125996257610516e-06
ENSG00000160285_46228745_46191104_721	LSS	21	−	2163	34	6	2	2.08%	9.223548039884884	0.007223235120512989	0.0004157276149849715	2.1311208584235123e-05
ENSG00000136068_58008565_58170762_2591	FLNB	3	+	7773	126	16	17	2.08%	9.511685758750678	7.410892035135557e-08	8.76675846503856e-08	6.342385039057957e-14
ENSG00000160209_43741744_43756063_239	PDXK	21	+	717	65	7	1	9.62%	53.194878032144366	0.0001351468025584792	1.1534864121266443e-05	1.284318564725418e-08
ENSG00000166206_26621357_26547793_334	GABRB3	15	−	1002	27	0	1	2.99%	15.811448546357788	0.0024321290225875155	0.0031727927861210656	4.407497946231007e-05
ENSG00000083896_68337877_68314099_684	YTHDC1	4	−	2052	47	0	6	2.78%	13.439902495165938	5.372600417609808e-05	0.0011847243965794093	5.093733261254563e-07
ENSG00000204256_32975417_32980718_679	BRD2	6	+	2037	21	2	4	1.77%	6.049282719912372	0.0029618375832235755	0.010411316862582036	0.00017166830783797216

ENSG00000134330_9481342_9488329_135	IAH1	2	+	405	18	0	0	5.19%	26.079129948066672	0.008674846900444944	0.008674846900444944	0.00038311532916634116
ENSG00000109452_142431259_142028782_924	INPP4B	4	−	2772	8	2	0	0.76%	1.6934499966277061	0.06180520118411761	0.005706018193000826	0.0020032236666657145
ENSG00000145920_175878740_175879737_70	CPLX2	5	+	210	22	0	2	7.14%	61.47223487758573	0.019679754444124863	0.04223451612662591	0.003715148010809822
ENSG00000135535_109382378_109368279_157	CD164	6	−	471	49	6	1	10.83%	61.04509717142995	0.002157477545980476	0.00013266285020134932	2.1429150631263794e−06
ENSG00000064042_41360841_41697185_1069	LIMCH1	4	+	3207	22	3	6	1.22%	4.0253100481113195	0.005301672691061329	0.009535600913472842	0.0002655394963642886
ENSG00000138777_105449391_105405758_188	PPA2	4	−	564	16	6	0	3.72%	16.64625315834043	0.0677965006331511	0.007029302661868638	0.002620981619745068
ENSG00000006534_68015298_68027939_468	ALDH3B1	11	+	1404	58	5	9	3.85%	24.240216938908127	0.0005962423705106502	0.0013386730596341172	5.059401949948585e−06
ENSG00000128585_131414689_131487728_460	MKLN1	7	+	1380	11	0	0	1.52%	4.677235262425001	0.0078820576498062	0.0078820576498062	0.00031963642907451164
ENSG00000074201_77625050_77619628_109	CLNS1A	11	−	327	17	0	0	4.59%	30.505404297050465	0.02108409854857798	0.02108409854857798	0.002029891263157455
ENSG00000166206_26621357_26547793_334	GABRB3	15	−	1002	27	0	1	2.99%	15.811448546357788	0.0024321290225875155	0.0031727927861210656	4.407497946231007e−05
ENSG00000113580_143400839_143281889_778	NR3C1	5	−	2334	12	5	2	1.16%	3.0168659322956306	0.07471635411946746	0.014696946993710543	0.005240911805500765
ENSG00000092036_22955118_22946525_306	HAUS4	14	−	918	18	1	2	2.94%	11.505498506500002	0.005588370640926919	0.015046251877931842	0.00043792386055833824
ENSG00000105974_116525063_116526839_114	CAV1	7	+	342	45	11	2	8.77%	77.20795050414475	0.03154446286322828	0.003768080243855145	0.0006783320643524425
ENSG00000143771_224356925_224375822_92	CNIH4	1	+	276	21	0	3	7.61%	44.64633659587501	0.00847367416878415	0.04544439262286352	0.001963364310668532
ENSG00000143771_224356925_224379090_137	CNIH4	1	+	411	24	1	3	8.03%	34.26455029672994	0.001783135576100216	0.01157547599262317	0.00012298119508918844
ENSG00000143771_224356925_224379090_90	CNIH4	1	+	270	14	0	3	6.67%	30.425651606077786	0.012798402692974279	0.08620755130569058	0.005490655705532311
ENSG00000087460_58891727_58910829_379	GNAS	20	+	1137	322	38	16	17.15%	166.1770417535119	7.255826837916426e−09	1.814040287566488e−11	1.855038147230084e−18
ENSG00000124225_57659647_57652053_234	PMEPA1	20	−	702	56	5	5	5.13%	46.808694778581206	0.00638574031296502	0.00396067672340455	0.0001371656018651211
ENSG00000105852_95396293_95359973_335	PON3	7	−	1005	38	0	1	3.88%	22.18672249313135	0.0006768518708446028	0.0009455931686164678	4.047434072351853e−06
ENSG00000143815_225424075_225403303_615	LBR	1	−	1845	78	13	1	5.85%	24.806977267673176	2.3838632615582214e−05	5.188227973846246e−08	1.5960586005856595e−11
ENSG00000166747_71789479_71733058_822	AP1G1	16	−	2466	43	0	2	2.43%	10.231775435829078	3.592951532853599e−05	7.887251914198805e−05	2.1376767587154857e−08
ENSG00000109180_48832625_48860762_245	OCIAD1	4	+	735	64	0	2	5.31%	51.09380561253879	0.0006791898698869142	0.0006791898698869142	2.9460669171009034e−06
ENSG00000164062_49674402_49683342_732	APEH	3	+	2196	33	6	0	2.32%	8.817738609489757	0.002215835380605914	0.00012002575157059987	2.021565418185597e−06
ENSG00000115966_175114798_175074609_329	ATF2	2	−	987	18	1	0	1.52%	10.70116274464742	0.029237630782826433	0.021557223391537678	0.002819005644529471
ENSG00000125450_75231967_75235679_195	NUP85	17	+	585	22	0	0	5.64%	22.06695610990257	0.0013430411379358334	0.0013430411379358334	1.0937773364376948e−05
ENSG00000105974_116526588_116559287_147	CAV1	7	+	441	215	20	3	27.21%	286.07208871603746	7.477091847878022e−07	1.5384592190056337e−08	1.2608926631122026e−13
ENSG00000126773_60115422_60125844_640	PCNX4	14	+	1920	7	0	3	0.78%	2.1393036285523443	0.019216965118390855	0.169362073879829	0.016163991620339734
ENSG00000112514_33417668_33416650_156	CUTA	6	−	468	163	2	6	16.03%	204.37010488148402	6.966821039712242e−06	3.1649250629192755e−05	1.808117256872564e−09
ENSG00000183291_86914111_86863547_123	SELENOF	1	−	369	223	6	3	15.45%	354.61255965968707	0.00020362850050586174	6.330845917447371e−05	9.433724748256515e−08
ENSG00000112081_36596763_36601989_164	SRSF3	6	+	492	114	23	9	18.90%	135.9613177170549	4.910866830745771e−05	3.821106563651327e−06	1.6251474483041573e−09
ENSG00000010704_26090867_26094411_303	HFE	6	+	909	23	0	5	3.63%	14.84702942587954	0.0014988252558711152	0.01983887091810603	0.00019024488125369727
ENSG00000085978_233264025_233294350_491	ATG16L1	2	+	1473	34	0	0	3.05%	13.544150991358455	0.00028426559431428834	0.00028426559431428834	5.47742908020375e−07
ENSG00000085978_233251828_233294350_588	ATG16L1	2	+	1764	35	0	0	2.72%	11.642468726815478	0.0001842144602393462	0.0001842144602393462	2.3639666231890906e−07
ENSG00000116260_180154908_180178849_155	QSOX1	1	+	465	12	0	0	3.23%	15.142720615006455	0.02108409854857798	0.02108409854857798	0.002029891263157455
ENSG00000167565_40441765_40441490_91	SERTAD3	19	−	273	52	0	3	10.99%	111.7676997774286	0.002446120450917513	0.005134531980829637	7.09837241422139e−05
ENSG00000204628_181243800_181236977_273	RACK1	5	−	819	1389	25	25	25.64%	995.1624037874892	3.952333287511675e−13	3.8016776140858613e−13	1.9619281127447064e−24
ENSG00000120942_11273532_11286131_338	UBIAD1	1	+	1014	19	0	0	2.07%	10.994899460353553	0.00797940233269714	0.00797940233269714	0.000327142761693177
ENSG00000133028_10697501_10695832_89	SCO1	17	−	267	7	0	1	5.62%	15.383756430039329	0.019216965118390855	0.04178282491678763	0.003600525600033436
ENSG00000162734_160211545_160213486_130	PEA15	1	+	390	31	3	0	8.46%	46.6415208686577	0.005080496801168777	0.0013845019718837994	4.188667743299567e−05
ENSG00000160014_46608271_46609153_113	CALM3	19	+	339	45	2	1	15.04%	77.89120670329648	0.0001889056949786359	0.00018618114937238455	2.447366381606584e−07
ENSG00000118680_3272899_3277937_172	MYL12B	18	+	516	55	6	0	5.23%	62.54442502079943	0.006172075136861155	0.0037896409716948643	0.00012737069552876224
ENSG00000142327_240575050_240577892_289	RNPEPL1	2	+	867	16	0	0	2.08%	10.828704476705886	0.013007239811447113	0.013007239811447113	0.0008218654148058079
ENSG00000107175_35733181_35736726_290	CREB3	9	+	870	19	0	2	3.45%	12.814744888274141	0.0022011839993586477	0.010678932720565162	0.0001357017294392655
ENSG00000074800_8874908_8870218_123	ENO1	1	−	369	152	10	8	14.63%	241.70900927476427	0.0002826983987097233	0.00047281371754217643	8.952391724974047e−07
ENSG00000213079_154803550_154833395_1008	SCAF8	6	+	3024	7	0	0	0.50%	1.3582880181284724	0.019216965118390855	0.019216965118390855	0.0017073955890589052
ENSG00000181029_7682254_7682820_188	TRAPPC5	19	+	564	20	8	0	5.85%	20.807816447925536	0.08243509129588328	0.0012624143894574274	0.0009110501663464096
ENSG00000070061_108912405_108869115_983	ELP1	9	−	2949	28	0	0	1.42%	5.571329897348933	0.00040182005258836655	0.00040182005258836655	1.0696965984890887e−06
ENSG00000188243_75537805_75526589_85	COMMD6	13	−	255	41	0	3	11.76%	94.34508775330002	0.0022409900782254734	0.013819019308084528	0.00018056055434049928
ENSG00000105722_42254999_42248465_548	ERF	19	−	1644	19	12	6	1.46%	6.781525579561133	0.2262256881413594	0.05255849808827775	0.046754979633612206
ENSG00000213593_57712619_57738725_103	TMX2	11	+	309	47	12	0	11.65%	89.25139132712138	0.024331435649611596	0.0010658336519988904	0.00018138477791255583
ENSG00000137074_33001604_32973498_302	APTX	9	−	906	19	7	3	3.64%	12.305549727150664	0.02387123336711234	0.01157547599262317	0.0013252018702315468
ENSG00000159352_151262180_151266156_253	PSMD4	1	+	759	19	11	0	2.77%	14.688838014227276	0.1175222253765591	0.00847367416878415	0.005733631208538938
ENSG00000257341_105487260_105488511_77	AL928654.3	14	+	231	250	20	12	28.57%	635.0437487353897	4.829947205692244e−05	3.108342685307635e−05	1.1448317702416725e−08
ENSG00000147955_34637697_34635632_192	SIGMAR1	9	−	576	82	5	1	11.46%	83.53471311490107	0.00014330972901034227	2.2632976634642962e−05	2.5340272271843576e−08
ENSG00000149273_75399548_75404865_243	RPS3	11	+	729	404	22	14	18.11%	325.1842129326832	1.1222818510483995e−08	4.443184394310116e−09	5.264848949559217e−16
ENSG00000092621_119727007_119744014_219	PHGDH	1	+	657	17	0	2	4.57%	15.183055106751144	0.002046028879997488	0.011698770487832223	0.00014006520345963755
ENSG00000183258_177513451_177511791_245	DDX41	5	−	735	17	1	4	3.27%	13.571792115830615	0.01007916461776185	0.04753395219010738	0.0023771889737864704
ENSG00000109320_102597543_102616594_463	NFKB1	4	+	1389	21	0	0	2.16%	8.87141029550864	0.0023218226288164415	0.0023218226288164415	3.1231198880462635e−05
ENSG00000124570_2968811_2948298_332	SERPINB6	6	−	996	54	1	2	5.72%	31.813396472792174	6.293293459921025e−05	0.0001311217014807635	6.040197107585146e−08
ENSG00000114439_107728780_107805457_751	BBX	3	+	2253	16	4	0	1.07%	4.167104652154461	0.04727912037993979	0.005454749182134642	0.0014311075230184615
ENSG00000242265_94664609_94665682_357	PEG10	7	+	1071	31	3	0	2.80%	16.98430731911905	0.008605408492104586	0.0022543491824521134	0.00011091378991179106
ENSG00000242265_94663557_94664534_325	PEG10	7	+	975	49	0	2	6.15%	29.48947771050616	3.77282131074845e−05	4.8247556065654664e−05	1.3786811572392767e−08
ENSG00000131844_71592941_71643794_281	MCCC2	5	+	843	18	0	0	3.20%	12.529119370067619	0.0034782380676658805	0.0034782380676658805	6.758309314600117e−05
ENSG00000124571_43567285_43523868_965	XPO5	6	−	2895	12	2	1	0.93%	2.4322504614777207	0.02207925122323651	0.0065000503081102355	0.0007379030744458475
ENSG00000164258_53646350_53658659_54	NDUFS4	5	+	162	18	0	0	9.26%	65.19782487016668	0.019679754444124863	0.019679754444124863	0.0017849636115922674
ENSG00000065000_2121856_2102173_827	AP3D1	19	−	2481	28	2	0	1.57%	6.622269998904476	0.0017865357182168743	0.0005983074123552221	6.775349462899382e−06
ENSG00000257923_101816052_102249042_1453	CUX1	7	+	4359	6	2	2	0.34%	0.8076812440901585	0.1029516053660342	0.1029516053660342	0.036819135060151394

ENSG00000109180_48849958_48851992_103	OCIAD1	4	+	309	19	0	2	5.83%	36.08034968543205	0.011772051206276392	0.011772051206276392	0.000681272511389508
ENSG00000140043_73860574_73884177_327	PTGR2	14	+	981	10	4	0	1.83%	5.981451822951072	0.11368416502207135	0.011570465654371866	0.006956434060967934
ENSG00000131966_58215281_58234551_219	ACTR10	14	+	657	20	1	2	3.20%	17.862417772648406	0.012088529595134127	0.020779353648479937	0.0012024586295609598
ENSG00000122406_92832115_92833798_63	RPL5	1	+	189	123	2	2	15.87%	381.8729742395477	0.0025025372014526115	0.0024742386374296595	3.5654968698922646e-05
ENSG00000217555_66552782_66566011_130	CKLF	16	+	390	20	0	2	4.62%	30.091303786230775	0.013428347753762199	0.024135905425855194	0.0015294273112737603
ENSG00000111859_11213721_11200981_168	NEDD9	6	−	504	15	0	0	3.57%	17.46370309022322	0.013428347753762199	0.013428347753762199	0.0008725593497644525
ENSG00000100811_100276523_100277600_102	YY1	14	+	306	14	0	0	4.90%	26.84616318183334	0.02108409854857798	0.02108409854857798	0.002029891263157455
ENSG00000144713_12840237_12839345_93	RPL32	3	−	279	41	3	0	8.60%	86.22938127989786	0.012176559331126819	0.00575691403842271	0.000364134589963107
ENSG00000169032_66481874_66490615_164	MAP2K1	15	+	492	8	0	0	3.05%	9.541145102951221	0.019216965118390855	0.019216965118390855	0.0017073955890589052
ENSG00000143294_156787094_156800460_125	PRCC	1	+	375	9	1	1	4.00%	14.082730171956003	0.04178282491678763	0.04178282491678763	0.007200520635230478
ENSG00000145495_10353899_10433684_862	6-Mar	5	+	2586	38	4	4	2.55%	8.622450156843389	0.00017665241896447223	0.00019367824162986244	2.383105656538059e-07
ENSG00000143537_155051387_155062502_862	ADAM15	1	+	2586	46	13	0	2.20%	10.437702821441997	0.009314527945786596	5.328371459388349e-05	5.3170083957120194e-06
ENSG00000137807_69444775_69447807_172	AC027237.1	15	+	516	17	0	0	4.07%	19.331913188247096	0.008175610787902942	0.008175610787902942	0.0003425184326896132
ENSG00000132623_10038590_10055660_680	ANKEF1	20	+	2040	10	0	0	0.74%	2.8763746266250005	0.019679754444124863	0.019679754444124863	0.0017849636115922674
ENSG00000157916_2402238_2403124_64	RER1	1	+	192	16	0	1	7.81%	48.89836865262501	0.019679754444124863	0.04223451612662591	0.003715148010809822
ENSG00000174444_66504790_66501367_227	RPL4	15	−	681	66	8	2	3.96%	56.86858733168724	0.003710480643933249	0.005134531980829637	0.00010443102032728217
ENSG00000177192_131932280_131943586_291	PUS1	12	+	873	8	0	0	2.06%	5.377140195477664	0.011570465654371866	0.011570465654371866	0.0006594843062896773
ENSG00000172936_38138779_38141286_270	MYD88	3	+	810	26	1	3	4.81%	18.834927184714818	0.0009723463632442102	0.0039558943403557294	2.3612861239842443e-05
ENSG00000077232_182756443_182777132_199	DNAJC10	2	+	597	13	0	0	3.02%	12.777463165510053	0.013428347753762199	0.013428347753762199	0.0008725593497644525
ENSG00000072518_63895204_63909237_699	MARK2	11	+	2097	9	0	0	0.86%	2.5183709177317604	0.011772051206276392	0.011772051206276392	0.000681272511389508
ENSG00000118816_77066263_77048219_344	CCNI	4	−	1032	38	0	1	4.94%	21.60625591627617	0.00010397439549526847	0.00018746639396660767	1.3880925736845516e-07
ENSG00000103197_2060673_2088610_1412	TSC2	16	+	4236	10	5	3	0.57%	1.3852229080063743	0.07635947366271362	0.04922925118879209	0.01464496403362635
ENSG00000157593_44255605_44254706_299	SLC35B2	6	−	897	12	0	0	3.01%	7.849905335538463	0.002874854311667729	0.002874854311667729	4.6982854837323694e-05
ENSG00000135452_57745215_57746424_108	TSPAN31	12	+	324	10	0	2	5.56%	18.110506908379634	0.012798402692974279	0.061356297364084836	0.0037940651817215715
ENSG00000139620_48681632_48654044_454	KANSL2	12	−	1362	24	2	0	2.42%	10.339743151215862	0.005006301263112021	0.001484234770590415	4.3930092952350055e-05
ENSG00000121073_49707944_49701458_359	SLC35B1	17	−	1077	24	0	2	3.62%	13.075886882039	0.0006069633386880125	0.0029991865458758355	1.1635651180309215e-05
ENSG00000072778_7223870_7224528_107	ACADVL	17	+	321	78	3	5	18.69%	142.58215906185984	0.00012197867544768428	0.00011166216326712384	9.751804367969783e-08
ENSG00000072778_7222818_7224645_184	ACADVL	17	+	552	75	12	5	11.41%	79.72560106406252	0.0010429865450067596	9.541395249618329e-05	7.382661609919258e-07
ENSG00000144028_96304727_96275012_2074	SNRNP200	2	−	6222	67	16	1	1.40%	6.318593442094263	0.00043281472326906514	1.3598755271372222e-06	7.044985209176152e-09
ENSG00000104419_133256848_133238878_239	NDRG1	8	−	717	21	1	1	2.51%	17.18603751807741	0.013301727469301692	0.013301727469301692	0.0008571698582519322
ENSG00000241878_31623797_31619612_340	PISD	22	−	1020	8	1	0	1.47%	4.602199402600001	0.03418265644467716	0.019216965118390855	0.0029560527921161815
ENSG00000106263_2366556_2371914_143	EIF3B	7	+	429	22	1	0	5.59%	30.091303786230775	0.008364861494636245	0.005604866691947602	0.00024596970711672695
ENSG00000165688_136410669_136416532_257	PMPCA	9	+	771	28	0	0	4.28%	21.309794899198447	0.00151350169064872	0.00151350169064872	1.3757299738441384e-05
ENSG00000108588_63756251_63746881_171	CCDC47	17	−	513	15	1	3	5.26%	17.15732233425439	0.006083074359664965	0.02555710920085966	0.0008103757961915687
ENSG00000108588_63764112_63754490_174	CCDC47	17	−	522	32	1	1	10.92%	35.97121372147127	7.717848815635669e-05	7.717848815635669e-05	4.365804279096419e-08
ENSG00000102317_48575181_48575814_118	RBM3	X	+	354	53	2	2	8.47%	87.85130639285171	0.0061547572324231374	0.0062201494882096935	0.00020209328853378674
ENSG00000217128_131796921_131644685_1121	FNIP1	5	−	3363	16	0	3	0.71%	2.7916999052346125	0.005306578019052857	0.03149539866061481	0.0008997191470395496
ENSG00000170633_121416210_121423576_353	RNF34	12	+	1059	8	0	0	1.70%	4.432713305620398	0.011570465654371866	0.011570465654371866	0.0006594843062896773
ENSG00000152558_102452623_102398867_189	TMEM123	11	−	567	65	0	3	10.58%	67.26759708826721	4.117406115269451e-05	0.0001201996442578728	3.7057706681821837e-08
ENSG00000112640_43007042_43011286_451	PPP2R5D	6	+	1353	22	2	0	2.00%	9.541145102951221	0.011768186811045352	0.0036062891729786097	0.00023156194506613595
ENSG00000137497_72022389_72003527_872	NUMA1	11	−	2616	24	1	3	1.15%	5.383306640655965	0.003133490598659634	0.00796980489516242	0.00013781154241186144
ENSG00000187024_127715639_127714150_182	PTRH1	9	−	546	15	0	3	4.40%	16.1203413140522	0.004193160480899131	0.056043582888960386	0.0014062628675739213
ENSG00000139263_58890111_58872572_938	LRIG3	12	−	2814	9	0	0	0.85%	1.8766964514866742	0.0033278027414746884	0.0033278027414746884	6.211806171561044e-05
ENSG00000118855_158804334_158828982_368	MFSD1	3	+	1104	17	0	0	2.17%	9.035568120593751	0.005405141725979036	0.005405141725979036	0.00015637213408961292
ENSG00000116754_70237501_70250805_262	SRSF11	1	+	786	19	7	2	3.05%	14.184259609158401	0.12780399893705963	0.02671499548383061	0.015018989223513957
ENSG00000138646_88486220_88505878_410	HERC5	4	+	1230	10	7	2	1.22%	4.7705725514756105	0.17139085557395572	0.06945717695039447	0.04296862163966939
ENSG00000089127_112907043_112919553_399	AC004551.1	12	+	1197	125	1	4	7.27%	61.27615119376568	1.3352908125979871e-06	4.880270062754391e-06	5.7141230561497103e-11
ENSG00000182199_57229779_57234361_504	SHMT2	12	+	1512	77	10	2	5.56%	29.882336398826396	0.00012110712879965657	2.233331381752029e-06	2.6128937042648212e-09
ENSG00000267228_47176277_47156177_82	AC012254.2	18	−	246	38	9	1	9.76%	90.64087847803661	0.010275150823238127	0.006346985027636522	0.00033687584863086295
ENSG00000140564_90875741_90881878_794	FURIN	15	+	2382	7	4	1	0.63%	1.7243757207474815	0.169362073879829	0.04178282491678763	0.02871445979022244
ENSG00000152683_32165901_32220713_438	SLC30A6	2	+	1314	28	5	0	3.20%	12.503692440853884	0.008686213303541933	0.00042454308224345244	2.659831953216553e-05
ENSG00000058272_79796884_79781843_197	PPP1R12A	12	−	591	10	0	0	2.54%	9.9286027721066	0.019679754444124863	0.019679754444124863	0.0017849636115922674
ENSG00000258315_7014858_7017321_157	C17orf49	17	+	471	38	1	0	7.64%	47.34109576559874	0.002197839395551526	0.0010155991509472172	1.3589100397475408e-05
ENSG00000075415_98593979_98594347_122	SLC25A3	12	+	366	36	6	2	4.92%	57.71610726211477	0.1526275292331874	0.032320015382316665	0.021102369076557558
ENSG00000075415_98595699_98601528_318	SLC25A3	12	+	954	336	20	9	19.18%	206.6648033620378	9.017090358169785e-10	1.1582578584797781e-10	1.2169451379338966e-18
ENSG00000077782_38424619_38413628_547	FGFR1	8	−	1641	10	0	0	1.65%	3.57574907880256	0.001946208561389314	0.001946208561389314	2.2278545302028032e-05
ENSG00000114209_167720136_167684308_205	PDCD10	3	−	615	18	0	0	3.41%	17.174061185312198	0.00847367416878415	0.00847367416878415	0.0003665024264111182
ENSG00000197448_143265006_143268837_127	GSTK1	7	+	381	43	1	1	7.87%	66.22456226969686	0.0024042815392806777	0.002446120450917513	3.394260134797701e-05
ENSG00000113048_72226107_72220909_152	MRPS27	5	−	456	12	0	0	3.95%	15.44159010082895	0.012178957859018494	0.012178957859018494	0.0007262454987071801
ENSG00000166788_18106041_18083713_353	SAAL1	11	−	1059	24	0	1	2.27%	13.298139916861192	0.005604866691947602	0.007225456388766877	0.00021353222845814395
ENSG00000110330_102350683_102378183_342	BIRC2	11	+	1026	12	0	0	1.75%	6.862928933701756	0.011772051206276392	0.011772051206276392	0.000681272511389508
ENSG00000152422_83104920_83353242_334	XRCC4	5	+	1002	11	5	0	2.10%	6.441701259627247	0.06583400801140711	0.007029302661868638	0.0025354904771632734
ENSG00000162521_32652003_32680546_375	RBBP4	1	+	1125	27	2	0	2.67%	14.082730171956003	0.00946955908897248	0.0022543491824521134	0.0001223697458287481
ENSG00000143398_151231761_151247865_404	PIP5K1A	1	+	1212	12	0	1	1.24%	5.809707166648516	0.019679754444124863	0.02784981298332479	0.002477909089056847
ENSG00000131828_19350021_19359653_330	PDHA1	X	+	990	38	1	0	5.15%	22.52288495514849	0.0001766213705010088	0.0001253290466518004	1.564941815559266e-07
ENSG00000175550_65919502_65921435_212	DRAP1	11	+	636	23	3	0	6.13%	21.220046773780666	0.0035574736217214766	0.0006069633386880125	1.3884903021488936e-05
ENSG00000156482_98042750_98041801_51	RPL30	8	−	153	163	11	5	17.65%	625.1320855198335	0.022643693555890774	0.030362948313965823	0.0030553704311476805

ENSG00000100567_58244921_58257751_36	PSMA3	14	+	108	10	6	1	16.67%	54.3315207251389	0.169362073879829	0.011772051206276392	0.011371739581240074
ENSG00000036257_224557733_224474245_705	CUL3	2	-	2115	25	2	3	1.99%	6.935938815975179	0.0019573865167617018	0.0033854124258957835	3.8307544722804655e-05
ENSG00000035403_73998208_74118169_1066	VCL	10	+	3198	81	6	2	3.47%	14.862168333443249	1.140442210918127e-06	5.3827441018819736e-08	6.263881739969658e-13
ENSG00000084073_40258278_40292669_473	ZMPSTE24	1	+	1419	52	3	0	5.07%	21.502876701365757	4.878313441828309e-05	7.6128113346902785e-06	3.0533923507959577e-09
ENSG00000159228_36070116_36071197_178	CBR1	21	+	534	91	9	1	12.36%	99.99441679525565	0.0001278538856941533	1.8130571119295507e-05	1.8360490984814848e-08
ENSG00000166888_57107239_57096572_737	STAT6	12	-	2211	23	3	0	1.36%	6.10400259978494	0.007867321313276006	0.0023218226288164415	0.00010390540483540199
ENSG00000198931_88809835_88809698_45	APRT	16	-	135	38	9	0	20.00%	165.16782300442225	0.029420544192244574	0.0037896409716948643	0.0006317346207603694
ENSG00000166340_6619400_6614546_500	TPP1	11	-	1500	30	0	3	3.00%	11.735608476630002	0.0002812865659102998	0.002191140628632457	4.222804636768014e-06
ENSG00000147044_41665429_41520420_735	CASK	X	-	2205	19	0	0	0.95%	5.056157847074151	0.00847367416878415	0.00847367416878415	0.0003665024264111182
ENSG00000255439_31091310_31091134_58	AC135050.2	16	-	174	56	0	12	15.52%	188.8488720377242	0.0035547763465261306	0.06638642199807827	0.001507226391593381
ENSG00000159720_67481086_67445920_115	ATP6V0D1	16	-	345	18	1	0	5.22%	30.61463080860001	0.02034245439037237	0.013428347753762199	0.0012955184805712684
ENSG00000092621_119712023_119744040_533	PHGDH	1	+	1599	41	0	0	3.94%	15.045651893115387	2.2606885438602453e-05	2.2606885438602453e-05	3.994412047840312e-09
ENSG00000091140_107891251_107904873_115	DLD	7	+	345	26	6	1	8.70%	44.22113339020001	0.041503828325407145	0.0037834851205969236	0.000921877301934226
ENSG00000100836_23321470_23324299_296	PABPN1	14	+	888	18	0	3	3.72%	11.894197780368247	0.0012427339549726212	0.010874198155266036	8.43832315623922e-05
ENSG00000060339_68722505_68791266_1150	CCAR1	10	+	3450	31	3	2	1.39%	5.272519750370001	0.0010357778271376215	0.00045252173331933966	3.030348957682848e-06
ENSG00000149925_30069571_30070212_132	ALDOA	16	+	396	153	35	7	21.21%	226.71061829853414	0.00038299838656496126	1.8379770207793107e-05	5.87088609513672e-08
ENSG00000111642_6587838_6578113_517	CHD4	12	-	1551	9	0	0	0.97%	3.4049154187514517	0.016947426762344633	0.016947426762344633	0.0013498980316300959
ENSG00000284946_91009831_90984800_475	AC068831.7	15	-	1425	11	0	0	1.47%	4.529533096243159	0.0078820576498062	0.0078820576498062	0.00031963642907451164
ENSG00000284946_90984125_90969101_510	AC068831.7	15	-	1530	73	3	2	4.51%	27.99671303248334	4.1950664451468485e-05	3.711982512760291e-05	1.1835034324178249e-08
ENSG00000125505_54183527_54180703_145	MBOAT7	19	-	435	14	1	0	3.45%	18.88488720377242	0.022600676488261057	0.020613416668581838	0.0021211989024304354
ENSG00000117054_75724788_75762763_425	ACADM	1	+	1275	28	0	0	3.29%	12.886158327280002	0.00037874750290730355	0.00037874750290730355	9.541676172656993e-07
ENSG00000184162_19203060_19201474_167	NR2C2AP	19	-	501	8	1	0	3.59%	9.369747286730542	0.026602764905793736	0.011570465654371866	0.001474889218184677
ENSG00000115486_85559067_85558643_56	GGCX	2	-	168	18	0	0	8.93%	62.86933112480359	0.02108409854857798	0.02108409854857798	0.002029891263157455
ENSG00000181744_143972414_143989841_403	DIPK2A	3	+	1209	8	0	0	1.49%	3.882748875642681	0.011570465654371866	0.011570465654371866	0.0006594843062896773
ENSG00000131143_85804978_85805115_45	COX4I1	16	+	135	48	1	1	17.78%	208.63303958453338	0.006386247275100883	0.006386247275100883	0.00021458612494678405
ENSG00000103353_23557967_23559787_176	UBFD1	16	+	528	19	0	0	3.41%	21.11520464545171	0.011772051206276392	0.011772051206276392	0.000681272511389508
ENSG00000172270_572635_582577_269	BSG	19	+	807	1323	23	21	28.62%	961.970880705173	1.2369273809587741e-14	2.345673311819235e-13	4.069219428363745e-26
ENSG00000148019_78240031_78266762_619	CEP78	9	+	1857	21	0	0	1.78%	6.635642918934574	0.0012558331315113088	0.0012558331315113088	9.614604369954505e-06
ENSG00000117020_243843170_243505249_479	AKT3	1	-	1437	14	3	0	1.46%	5.716719508448853	0.05989746521295916	0.008175610787902942	0.002573001276540562
ENSG00000035687_244451817_244409586_456	ADSS	1	-	1368	27	0	1	3.51%	11.581192575621714	0.00012890076524792922	0.0002741275686995999	2.480696456344315e-07
ENSG00000157557_38810059_38822889_461	ETS2	21	+	1383	52	3	1	5.42%	22.062604511379615	3.568529010364622e-05	6.668585730439088e-06	1.960520580519893e-09
ENSG00000176946_241637017_241584606_577	THAP4	2	-	1731	10	1	5	0.87%	3.389834915259966	0.02784981298332479	0.17004230409153215	0.021303539010859313
ENSG00000137100_34620464_34613782_186	DCTN3	9	-	558	40	4	0	6.99%	42.063112819462376	0.005593414947727874	0.0006424109906750971	2.348465047092721e-05
ENSG00000177106_720080_726981_654	EPS8L2	11	+	1962	14	0	1	1.07%	4.1870162760657506	0.00847367416878415	0.013892891352654344	0.0005886798845679606
ENSG00000122406_92832115_92841865_297	RPL5	1	+	891	137	4	2	5.39%	90.22325259810945	0.0004109516762323294	0.00021408175522948358	5.989525344593417e-07
ENSG00000151327_35053278_35054670_61	FAM177A1	14	+	183	6	0	0	8.20%	19.23870242070492	0.016947426762344633	0.016947426762344633	0.0013498980316300959
ENSG00000198015_93469286_93501221_142	MRPL42	12	+	426	53	0	4	10.56%	73.00319827011621	0.0003026020628493325	0.0015043197483827605	3.052159989377238e-06
ENSG00000125954_64914496_65061312_471	CHURC1-FNTB	14	+	1413	50	22	0	4.67%	20.763638493683658	0.0003911305796778262	1.3895906163457093e-05	4.684808713827431e-08
ENSG00000088986_120496422_120498210_89	DYNLL1	12	+	267	251	5	9	15.73%	551.6175519914102	0.00048083181382083286	0.000745376529342948	2.3179087696108e-06
ENSG00000176101_137188727_137189914_119	SSNA1	9	+	357	15	0	1	7.56%	24.65463965678572	0.002874854311667729	0.00308494966027208	5.026164262036141e-05
ENSG00000278588_26272976_26273356_126	HIST1H2BI	6	+	378	236	4	3	17.46%	366.3496826037937	1.861291078010239e-05	4.988868139390561e-05	7.240818080430953e-09
ENSG00000132436_50446933_50445263_556	FIGNL1	7	-	1668	11	0	0	0.90%	3.8696550732293176	0.020613416668581838	0.020613416668581838	0.001946208561389314
ENSG00000218739_37199705_37201724_80	CEBPZOS	2	+	240	11	0	0	7.50%	26.894102758943756	0.009815328628645334	0.009815328628645334	0.0004837142251238724
ENSG00000153048_8859328_8855164_147	CARHSP1	16	-	441	23	0	4	4.76%	30.603060653343544	0.00877616296920843	0.04584514077471458	0.0020400732494421717
ENSG00000044115_138827710_138887696_98	CTNNA1	5	+	294	9	0	0	7.14%	17.962666035658167	0.005706018193000826	0.005706018193000826	0.0001733096755673334
ENSG00000120896_22566421_22574728_329	SORBS3	8	+	987	12	0	0	1.82%	7.134108496431613	0.011570465654371866	0.011570465654371866	0.0006594843062896773
ENSG00000204463_31651763_31639131_1077	BAG6	6	-	3231	27	4	3	1.30%	4.903457580764625	0.004323854527232739	0.002608298974881378	6.359766818065422e-05
ENSG00000082641_48051119_48059641_772	NFE2L1	17	+	2316	51	4	0	3.24%	12.921330576600392	6.932916241866984e-05	4.511155229672303e-06	2.7126418074631862e-09
ENSG00000140416_63042857_63071172_275	TPM1	15	+	825	171	9	9	13.09%	121.62357875780185	7.091969157861833e-07	1.9984652195701008e-06	1.2720112296347732e-11
ENSG00000105438_48391358_48389535_122	KDELR1	19	-	366	511	1	16	18.85%	819.2480780816846	1.3467675527572699e-05	0.00023639552072572247	2.660489382242959e-08
ENSG00000171793_40983291_41010245_591	CTPS1	1	+	1773	25	3	2	2.20%	8.273835643422167	0.004587372372998262	0.0025860288909179195	6.686082635979315e-05
ENSG00000065427_75644428_75627895_625	KARS	16	-	1875	76	4	1	4.80%	23.784166512636805	3.797894522359459e-06	8.890006527197744e-07	3.0117234862137525e-11
ENSG00000067182_6333514_6329312_347	TNFRSF1A	12	-	1041	71	13	3	7.20%	40.020566851139776	0.0003271090037829848	1.4415444336371848e-05	3.994938537398264e-08
ENSG00000116062_47783234_47806860_1230	MSH6	2	+	3690	47	0	5	1.22%	7.47389699731179	0.00020704336734043597	0.006773176113680622	1.104481430250076e-05
ENSG00000149084_43680828_43855248_312	HSD17B12	11	+	936	86	0	1	8.01%	53.91358595033014	5.128714732243465e-06	1.011413470694664e-05	4.299244915835132e-10
ENSG00000087274_2881994_2928171_535	ADD1	4	+	1605	12	0	1	1.12%	4.387143355749534	0.013007239811447113	0.023878473930440184	0.0014709138084317821
ENSG00000141425_36067404_35993151_312	RPRD1A	18	-	936	16	0	0	2.24%	10.03043459541026	0.008175610787902942	0.008175610787902942	0.00034251843268961324
ENSG00000196535_29166826_29074770_2000	MYO18A	17	-	6000	10	0	1	0.25%	0.9779673730525003	0.019679754444124863	0.02784981298332479	0.002477909089056847
ENSG00000188243_75530239_75526589_58	COMMD6	13	-	174	38	0	3	13.79%	128.1474488827414	0.0053557511377634245	0.027805252533285658	0.0007943520777641052
ENSG00000136527_185922105_185917715_107	TRA2B	3	-	321	30	0	0	10.28%	54.839291946869174	0.001484234770590415	0.001484234770590415	1.3251387882263029e-05
ENSG00000138430_174246815_174075426_396	OLA1	2	-	1188	32	1	1	2.27%	15.80553330185859	0.004333802856335622	0.004333802856335622	0.0001027489724492267
ENSG00000159423_18889430_18872845_503	ALDH4A1	1	-	1509	13	0	3	0.99%	5.0550997414244545	0.020613416668581838	0.06945717695039447	0.006391023138248565
ENSG00000072501_53421937_53380103_1211	SMC1A	X	-	3633	30	4	1	1.16%	4.845420510582165	0.0023847795666952836	0.0006310955412434601	9.530319595764203e-06
ENSG00000148110_94440998_94459412_370	MFSD14B	9	+	1110	42	0	0	4.32%	22.20250252335406	0.00019829743686022551	0.00019829743686022551	2.7268184690767617e-07
ENSG00000074054_121605760_121340861_1432	CLASP1	2	-	4296	13	0	0	0.70%	1.7756390851511876	0.0014903523101034196	0.0014903523101034196	1.3356391343355004e-05
ENSG00000174437_110343359_110345091_93	ATP2A2	12	+	279	15	0	0	6.45%	31.54733461459678	0.011772051206276392	0.011772051206276392	0.000681272511389508
ENSG00000113161_75359985_75360342_69	HMGCR	5	+	207	11	0	0	7.25%	31.181568416166673	0.020613416668581838	0.020613416668581838	0.001946208561389314

ENSG00000129422_17715785_17645926_415	MTUS1	8	-	1245	11	2	0	1.20%	5.184405351121688	0.08350359792828288	0.019216965118390855	0.0073235312725521175
ENSG00000134759_36129934_36136324_51	ELP2	18	+	153	11	0	0	9.80%	42.186827857166676	0.020613416668581838	0.020613416668581838	0.001946208561389314
ENSG00000146416_143061010_143335091_217	AIG1	6	+	651	20	0	0	3.69%	18.02704835119816	0.005604866691947602	0.005604866691947602	0.00016752456572620382
ENSG00000257671_52245587_52245342_81	KRT7-AS	12	-	243	122	6	39	20.99%	294.5975790429754	0.000475084930342192	0.009885779789334408	3.375069491780341e-05
ENSG00000165661_136245803_136208728_698	QSOX2	9	-	2094	39	2	1	3.30%	10.928575228953441	2.5444216682513155e-05	1.4829016742866657e-05	2.9816949751804476e-09
ENSG00000197837_14771084_14770773_103	HIST4H4	12	-	309	140	10	4	20.39%	265.85520820844664	0.00026071680269953887	9.597779911861294e-05	1.7868177656905507e-07
ENSG00000137868_74195675_74180080_476	STRA6	15	-	1428	47	2	8	3.15%	19.312801064482148	0.0010266911004198547	0.00727779966686158	4.668091370280168e-05
ENSG00000110090_68807631_68757644_677	CPT1A	11	-	2031	44	1	4	3.10%	12.712131289308719	2.116023157680266e-05	0.000186161329078746	3.1202902669590814e-08
ENSG00000127311_66314022_66338102_515	HELB	12	+	1545	14	0	0	0.97%	5.317104164168933	0.020613416668581838	0.020613416668581838	0.001946208561389314
ENSG00000162378_52756611_52805476_505	ZYG11B	1	+	1515	7	0	0	0.99%	2.7111966777693075	0.016947426762344633	0.016947426762344633	0.0013498980316300959
ENSG00000153310_129879461_129842142_324	FAM49B	8	-	972	28	0	1	3.70%	16.903139781154326	0.000978917897162665	0.0013147568071904994	7.922904919322548e-06
ENSG00000124767_38686953_38677295_149	GLO1	6	-	447	53	2	0	8.05%	69.57351781447316	0.0017556302545386716	0.0010615893185374377	1.1346218659563242e-05
ENSG00000183696_48094803_48108357_173	UPP1	7	+	519	12	0	0	2.89%	13.56717742963006	0.020613416668581838	0.020613416668581838	0.001946208561389314
ENSG00000183696_48101854_48108357_246	UPP1	7	+	738	9	1	0	2.44%	7.155858827213416	0.02305674932606751	0.012178957859018494	0.0013400639730601711
ENSG00000134283_42335903_42446618_458	PPHLN1	12	+	1374	14	1	1	1.31%	5.97883983525546	0.02034245439037237	0.02034245439037237	0.0018987606724399325
ENSG00000146109_26596983_26598645_272	ABT1	6	+	816	16	0	0	2.57%	11.505498506500002	0.008674846900444944	0.008674846900444944	0.00038311532916634116
ENSG00000136238_6391949_6402446_148	RAC1	7	+	444	30	0	2	4.05%	39.64732593456082	0.013428347753762199	0.03101958831192484	0.001958810244408279
ENSG00000163479_156020896_156009540_186	SSR2	1	-	558	483	4	5	19.35%	507.9120872950082	2.7589975114299486e-07	2.1162504174658848e-07	5.498127879444063e-13
ENSG00000126088_45012992_45013706_100	UROD	1	+	300	11	1	0	6.00%	21.515282207155003	0.023672501042627626	0.012798402692974279	0.0014375227604752335
ENSG00000166452_8911450_8919480_210	AKIP1	11	+	630	12	0	0	2.86%	11.17676997774286	0.011772051206276392	0.011772051206276392	0.000681272511389508
ENSG00000103197_2060673_2088610_1457	TSC2	16	+	4371	12	5	3	0.62%	1.6109277250006868	0.04946560907745733	0.033570409067276055	0.006921950394855025
ENSG00000124588_3006553_3019655_231	NQO2	6	+	693	12	4	2	3.03%	10.160699979766235	0.08195766739919752	0.03781105849985612	0.012525790834751529
ENSG00000124588_3006553_3019655_193	NQO2	6	+	579	12	3	2	3.63%	12.161252307388605	0.058506512523593034	0.03781105849985612	0.0090237716975301
ENSG00000080503_2104121_2192739_509	SMARCA2	9	+	1527	14	4	4	0.98%	5.37978122700786	0.10163622319758386	0.1029516053660342	0.03640120526961381
ENSG00000130402_38647839_38729432_661	ACTN4	19	+	1983	179	22	24	7.87%	52.96706801101287	2.504658802832966e-07	1.5989552903120557e-06	3.761126029553992e-12
ENSG00000120948_11016999_11023234_138	TARDBP	1	+	414	12	0	0	4.35%	17.008128227000004	0.013007239811447113	0.013007239811447113	0.0008218654148058079
ENSG00000149925_30067255_30070116_361	ALDOA	16	+	1083	606	42	29	26.87%	328.3369684597314	1.6902544981203353e-15	3.235312307028756e-16	7.990973461762927e-30
ENSG00000196562_47757363_47658362_870	SULF2	20	-	2610	19	0	0	1.38%	4.2715816294247135	0.0007764301431553706	0.0007764301431553706	3.8125731154589255e-06
ENSG00000178035_49025035_49024491_123	IMPDH2	3	-	369	6	0	0	4.07%	9.541145102951221	0.016947426762344633	0.016947426762344633	0.0013498980316300959
ENSG00000115216_27433274_27441812_543	NRBP1	2	+	1629	30	9	1	2.95%	10.80626931549724	0.007983496269485775	0.00028024904858585246	1.7106587723629186e-05
ENSG00000261832_28491759_28479730_363	AC138894.1	16	-	1089	21	2	0	2.20%	11.315324977466945	0.018151375140008384	0.005604866691947602	0.0005308776856007569
ENSG00000107201_32489380_32457122_671	DDX58	9	-	2013	28	6	0	1.79%	8.161873754238451	0.022003523599005054	0.0009549346450480869	0.00014791777707120728
ENSG00000134013_23368351_23298043_774	LOXL2	8	-	2322	65	3	4	4.13%	16.42580859132106	1.592743770682931e-06	2.904891919508144e-06	4.029415395541892e-11
ENSG00000103042_58676021_58667385_195	SLC38A7	16	-	585	7	0	0	2.56%	7.021304216787181	0.019216965118390855	0.019216965118390855	0.0017073955890589052
ENSG00000074582_218660988_218663386_419	BCS1L	2	+	1257	20	0	0	2.86%	9.336204038687352	0.00091966234969362	0.00091966234969362	5.2817946186748165e-06
ENSG00000124783_7313120_7289864_286	SSR1	6	-	858	113	2	5	12.59%	77.27993926918359	7.991166815367668e-08	2.712069423649562e-07	2.100377621768647e-13
ENSG00000197226_179876034_179863448_638	TBC1D9B	5	-	1914	12	7	0	1.10%	3.6788741306050166	0.0935056295832879	0.008175610787902942	0.004259743896695977
ENSG00000135316_85641439_85612882_562	SYNCRIP	6	-	1686	58	21	4	4.09%	20.185803429553385	0.011939252279154475	0.00011183021767937667	1.2932835787678265e-05
ENSG00000120756_142678036_142712007_462	PLS1	3	+	1386	27	0	2	2.81%	11.430787477237015	0.000609140140830996	0.0030056054444520318	1.1698852710189299e-05
ENSG00000137845_58749486_58655345_202	ADAM10	15	-	606	19	0	0	2.48%	18.3974060277203	0.02108409854857798	0.02108409854857798	0.002029891263157455
ENSG00000113389_32712236_32786345_387	NPR3	5	+	1161	5	0	0	1.29%	2.527047475587856	0.012673659338734126	0.012673659338734126	0.000782701129001274
ENSG00000137845_58749486_58597547_732	ADAM10	15	-	2196	73	6	3	4.64%	19.50590662099249	1.8542051138428257e-06	8.201719824803529e-07	1.357605031509973e-11
ENSG00000153827_229880079_229807609_822	TRIP12	2	-	2466	11	0	0	0.85%	2.6174309254446477	0.007593099278532274	0.007593099278532274	0.00029783286705462064
ENSG00000125691_38853710_38850132_140	RPL23	17	-	420	224	17	13	20.00%	312.9495593768001	1.3996993674188494e-05	9.105639046034765e-06	1.0319021944824536e-09
ENSG00000106992_127871924_127868008_120	AK1	9	-	360	17	3	0	6.67%	27.70907556982084	0.03585926825421706	0.005405141725979036	0.0010501039328310528
ENSG00000153214_112060007_112116143_490	TMEM87B	2	+	1470	15	0	0	1.84%	5.9875553452193895	0.002967235347054297	0.002967235347054297	4.9907879201100895e-05
ENSG00000183735_64455871_64501381_729	TBK1	12	+	2187	20	0	0	1.23%	5.366076121001373	0.0036062891729786097	0.0036062891729786097	7.240558534568731e-05
ENSG00000011376_45419682_45547530_747	LARS2	3	+	2241	13	0	0	0.80%	3.4039025032617145	0.012798402692974279	0.012798402692974279	0.0007972436888943567
ENSG00000271723_54716624_54741513_307	MROH7-TTC4	1	+	921	18	0	0	1.63%	11.468021312667755	0.020613416668581838	0.020613416668581838	0.001946208561389314
ENSG00000146112_30686018_30677269_613	PPP1R18	6	-	1839	19	4	2	1.79%	6.062440485480425	0.002549573717490699	0.007962492813783481	0.0001143653150129499
ENSG00000169139_48043161_48060828_97	UBE2V2	8	+	291	10	3	0	5.15%	20.16427573304124	0.12180769416495907	0.019679754444124863	0.011261990492355435
ENSG00000143106_109426330_109413029_109	PSMA5	1	-	327	18	4	0	6.42%	32.299839843935786	0.05258119297243839	0.00847367416878415	0.0022969807838458776
ENSG00000178425_116100931_116244024_455	NT5DC1	6	+	1365	18	0	0	2.20%	7.737763830745057	0.0017901637611834264	0.0017901637611834264	1.8982104156792192e-05
ENSG00000185630_164559844_164821583_340	PBX1	1	+	1020	31	3	0	2.35%	17.833522685075003	0.013648824643852793	0.005604866691947602	0.00039820635657586146
ENSG00000148737_112950757_113165972_579	TCF7L2	10	+	1737	11	0	0	1.04%	3.715938205035407	0.012798402692974279	0.012798402692974279	0.0007972436888943567
ENSG00000103512_14864669_14896111_774	NOMO1	16	+	2322	13	0	0	0.65%	3.2851617182642125	0.020613416668581838	0.020613416668581838	0.001946208561389314
ENSG00000132463_70836185_70824319_318	GRSF1	4	-	954	41	4	3	3.46%	25.218026600724848	0.0027029703238042042	0.004224170501915509	6.426303143589004e-05
ENSG00000164211_111507433_111500919_159	STARD4	5	-	477	14	0	0	3.77%	17.22206694683648	0.012798402692974279	0.012798402692974279	0.0007972436888943567
ENSG00000134352_55976278_55941082_918	IL6ST	5	-	2754	42	1	3	1.96%	8.948721060611113	0.00011552752953057298	0.0005414231194405095	4.445530033470899e-07
ENSG00000111371_46197800_46197657_47	SLC38A1	12	-	141	15	0	0	14.89%	62.42344934377661	0.00837382380159347	0.00837382380159347	0.0003583838894600799
ENSG00000123353_55819102_55820395_119	ORMDL2	12	+	357	23	0	0	8.40%	37.80378080707144	0.0023354528714849923	0.0023354528714849923	3.158285405866755e-05
ENSG00000106853_111586005_111564239_177	PTGR1	9	-	531	46	5	1	10.73%	50.832202441146904	0.0006049228908724183	7.673237711562165e-05	3.4303863279307e-07
ENSG00000106853_111597422_111589370_73	PTGR1	9	-	219	12	0	0	6.85%	32.15235199076713	0.020613416668581838	0.020613416668581838	0.001946208561389314
ENSG00000060982_24948932_24818008_349	BCAT1	12	-	1047	29	0	0	2.58%	16.252752904597425	0.0034782380676658805	0.0034782380676658805	6.758309314600117e-05
ENSG00000087088_48956199_48961097_140	BAX	19	+	420	75	1	1	11.43%	104.7822185413393	0.0002028510352551382	0.0003665275989911912	5.082700333559607e-07
ENSG00000122786_134891621_134968345_532	CALD1	7	+	1596	35	0	2	2.63%	12.867991750690793	0.0004156054716904614	0.00115701054860631	3.127308461959799e-06
ENSG00000131174_77902663_77905261_60	COX7B	X	+	180	110	1	2	20.00%	358.58803678591676	0.0010700885808033968	0.0017920611887025132	1.1664294749921331e-05

ENSG00000023839_99782845_99796587_236	ABCC2	10	+	708	14	7	1	2.54%	11.603002731131358	0.33632999628228655	0.026832695913080365	0.04814272760090725
ENSG00000118257_205683297_205776526_904	NRP2	2	+	2712	43	6	1	2.43%	9.303671911782635	0.00018343869857815974	2.078120617164616e-05	3.0225434820810336e-08
ENSG00000167900_78187120_78174759_309	TK1	17	-	927	96	6	2	7.44%	60.76690473335924	0.0002569984551636973	1.7466659193405162e-05	3.672715047841074e-08
ENSG00000153310_129879461_129842142_324	FAM49B	8	-	972	28	0	1	3.70%	16.903139781154326	0.000978917897162665	0.001314756807190499 4	7.922904919322548e-06
ENSG00000167863_75042650_75040015_81	ATP5PD	17	-	243	18	5	0	11.11%	43.46521658011112	0.038744331104783855	0.0033030736763512156	0.0007650515271477136
ENSG00000148337_128179348_128166197_638	CIZ1	9	-	1914	21	2	0	1.57%	6.438029728558779	0.01044836432889887	0.002046028879997488	0.0001244475177269439 7
ENSG00000271723_54716624_54741513_342	MROH7-TTC4	1	+	1026	18	0	0	1.46%	10.294393400552634	0.020613416668581838	0.020613416668581838	0.001946208561389314
ENSG00000178982_38624180_38635129_124	EIF3K	19	+	372	260	23	4	22.58%	410.11534998975816	4.330474937912603e-05	6.136920381315798e-06	2.2111142104043397e-09
ENSG00000196072_100286127_100275462_95	BLOC1S2	10	-	285	13	0	0	5.26%	26.76542284143685	0.020613416668581838	0.020613416668581838	0.001946208561389314
ENSG00000101191_62911348_62905786_474	DIDO1	20	-	1422	11	0	0	1.05%	4.539089073239452	0.020613416668581838	0.020613416668581838	0.001946208561389314
ENSG00000102181_150898588_150769034_262	CD99L2	X	-	786	24	0	0	3.44%	17.916959506305346	0.003684287313187465	0.003684287313187465	7.541963121699725e-05
ENSG00000070087_149970856_149968350_110	PFN2	3	-	330	113	7	8	19.09%	200.92784209987732	0.0002007775159515276	0.0002074350473618961	2.8830533048683205e-07
ENSG00000110092_69648023_69651282_94	CCND1	11	+	282	18	0	0	6.38%	37.45406960626597	0.013428347753762199	0.013428347753762199	0.0008725593497644525
ENSG00000204463_31651763_31639131_903	BAG6	6	-	2709	27	4	3	1.55%	5.848309872074752	0.004323854527232739	0.002608298974881378	6.359766818065422e-05
ENSG00000164733_11853454_11850698_164	CTSB	8	-	492	68	9	0	10.37%	81.09973337508538	0.0014545096389273744	0.0001329304023902354	1.4091186835065798e-06
ENSG00000174173_101564899_101565993_364	TRMT10C	3	+	1092	25	0	0	3.02%	13.433617761710169	0.0015356776430442238	0.0015356776430442238	1.41466323862755e-05
ENSG00000137161_42929580_42937777_140	CNPY3	6	+	420	13	1	0	4.29%	18.162251213832146	0.023672501042627626	0.012798402692974279	0.0014375227604752335
ENSG00000114021_100334792_100355268_276	NIT2	3	+	828	74	2	2	6.16%	52.44172869991668	0.00018195398980347797	0.0003652193633644823	4.569186895037056e-07
ENSG00000134248_110406378_110401523_79	LAMTOR5	1	-	237	18	0	0	10.13%	44.56560180998735	0.0053557511377634245	0.0053557511377634245	0.00015366972994913032
ENSG00000103145_3023941_3022863_119	HCFC1R1	16	-	357	19	1	1	5.88%	31.22921023192858	0.013892891352654344	0.013892891352654344	0.0009300961024180418
ENSG00000134375_201957321_201969554_149	TIMM17A	1	+	447	61	2	2	8.72%	80.07518088080874	0.001251551271000974	0.001251551271000974	9.551731304272604e-06
ENSG00000176393_201982667_202005716_611	RNPEP	1	+	1833	57	4	7	3.93%	18.24685442356547	4.854403497825543e-05	0.0001439892262622478	5.187946355454949e-08
ENSG00000163435_202011137_202015323_371	ELF3	1	+	1113	76	4	10	7.55%	40.0676659579461	5.668432318013583e-06	1.910321253549328e-05	8.934166712586255e-10
ENSG00000163435_202011137_202015323_371	ELF3	1	+	1113	76	4	10	7.55%	40.0676659579461	5.668432318013583e-06	1.910321253549328e-05	8.934166712586255e-10
ENSG00000177239_137099857_137108591_402	MAN1B1	9	+	1206	37	3	0	3.48%	18.002384479075875	0.0023378132945477614	0.0004575241474660434	6.98094951309893e-06
ENSG00000137133_35813753_35813054_157	HINT2	9	-	471	53	3	5	8.92%	66.02837040991403	0.002094424825050526	0.0036455838927912905	4.3872759239703255e-05
ENSG00000165813_114163268_114125143_898	CCDC186	10	-	2694	7	0	0	0.56%	1.52467073749833	0.016947426762344633	0.016947426762344633	0.0013498980316300959
ENSG00000134255_111147787_111182316_256	CEPT1	1	+	768	26	0	0	4.69%	19.86496226512891	0.0009430837296224684	0.0009430837296224684	5.543685104868835e-06
ENSG00000079459_11802833_11838609_417	FDFT1	8	+	1251	250	17	10	15.59%	117.26227494634296	1.2802270129991288e-10	6.055999232395061e-12	9.787871393417142e-21
ENSG00000095261_120842909_120817906_504	PSMD5	9	-	1512	48	1	1	4.76%	18.627949962904765	9.796137473130914e-06	9.796137473130914e-06	7.803401830030882e-10
ENSG00000211455_27297757_27322455_452	STK38L	12	+	1356	10	2	0	1.11%	4.327289261294249	0.08350359792828288	0.020613416668581838	0.007749259071793911
ENSG00000115414_215435802_215361555_2296	FN1	2	-	6888	105	6	10	2.48%	8.94482353401677	1.8206326957627449e-10	1.9309074127734127e-09	4.074000358432147e-18
ENSG00000133318_63681637_63758201_255	RTN3	11	+	765	88	8	7	7.06%	67.49892457146669	0.0006743190549973731	9.482646538282103e-05	4.6525414814092407e-07
ENSG00000137700_119027649_119024910_228	SLC37A4	11	-	684	10	0	0	2.19%	8.578661167127194	0.020613416668581838	0.020613416668581838	0.001946208561389314
ENSG00000163636_64013479_64010668_71	PSMD6	3	-	213	14	2	0	8.45%	38.567727387985926	0.05258119297243839	0.013007239811447113	0.0032664690612771 1
ENSG00000184277_101650135_101633677_144	TM2D3	15	-	432	10	5	0	4.17%	13.582880181284725	0.11411551976453232	0.011772051206276392	0.007078637642502531
ENSG00000177410_49279128_49279205_25	ZFAS1	20	+	75	60	3	2	28.00%	469.4243390652001	0.027657845067597595	0.023857440118675605	0.002933789275920174
ENSG00000198563_31540532_31530434_428	DDX39B	6	-	1284	133	23	13	6.78%	60.78021524111333	8.674315149873927e-05	5.255637551304787e-06	3.944361950351243e-09
ENSG00000141543_80137480_80135490_115	EIF4A3	17	-	345	18	1	0	7.83%	30.61463080860001	0.0064006895615519015	0.002943990679779778	0.00010465593861806192
ENSG00000196072_100286127_100275462_95	BLOC1S2	10	-	285	13	0	0	5.26%	26.76542284143685	0.020613416668581838	0.020613416668581838	0.001946208561389314
ENSG00000159131_33539315_33531411_224	GART	21	-	672	13	0	0	2.23%	11.351407008645092	0.02108409854857798	0.02108409854857798	0.002029891263157455
ENSG00000283782_132579932_132642364_1105	AC116366.3	5	+	3315	34	3	1	1.45%	6.018260757246155	0.0009282671483213431	0.00025342656963789123	1.5856976771058425e-06
ENSG00000132507_7309636_7309884_82	EIF5A	17	+	246	169	70	8	18.29%	403.1133805996891	0.0158684317527454	0.0003250121193590314	4.2512425905242323e-05
ENSG00000187118_28263338_28319629_84	CMC1	3	+	252	18	1	0	5.95%	41.912887416535725	0.04223451612662591	0.019679754444124863	0.003715148010809822
ENSG00000143933_47176443_47160776_83	CALM2	2	-	249	177	14	2	21.69%	417.1089759766085	0.0001556611380424777	9.685105774909149e-05	1.0797212608379623e-07
ENSG00000120533_109336137_109340498_48	ENY2	8	+	144	55	1	2	22.92%	224.11752299119797	0.0019534759119410605	0.0032429057496866946	3.665724911465891e-05
ENSG00000168827_158644635_158691467_751	GFM1	3	+	2253	42	1	0	2.93%	10.938649711905462	1.2623898414380606e-05	1.3773078124060501e-05	1.3946793848296891e-09
ENSG00000232388_18567479_18568739_73	SMIM26	20	+	219	20	7	3	9.59%	53.58725331794522	0.1051474467696889	0.03350388737713736	0.014592307848098307
ENSG00000131467_42833632_42841578_198	PSME3	17	+	594	48	3	1	8.08%	47.416599905575765	0.0001842144602393462	0.00026058368327187955	3.314644779286142e-07
ENSG00000104763_18067256_18057534_280	ASAH1	8	-	840	49	3	2	7.50%	34.2288580568375 1	2.3967087618982578e-05	9.128754267453394e-05	1.6881896835191243e-08
ENSG00000179091_144095100_144097336_325	CYC1	8	+	975	122	6	10	9.85%	73.42278123840309	2.839485428508834e-06	1.4473173694206806e-05	3.508810819689091e-10
ENSG00000107554_99930763_99887151_1209	DNMBP	10	-	3627	31	6	8	0.99%	5.0152172977051 3	0.014987968494 23233	0.021466363864824912	0.0015077582489813122
ENSG00000102805_76995908_77000969_243	CLN5	13	+	729	12	0	2	2.47%	9.65893701780247	0.011772051206276392	0.060667625179241055	0.0035017256847258756
ENSG00000241468_99466141_99460057_49	ATP5MF	7	-	147	26	0	0	10.20%	103.78429265046941	0.021557223391537678	0.021557223391537678	0.002115616449879074
ENSG00000128585_131327900_131478787_731	MKLN1	7	+	2193	19	0	0	1.50%	5.0838249214767455	0.0013845019718837994	0.0013845019718837994	1.1595255170738555e-05
ENSG00000109814_39521512_39514175_55	UGDH	4	-	165	39	8	1	14.55%	138.69355472380911	0.03356284476030215	0.007356796211402075	0.0012660602295357357
ENSG00000132199_712506_674305_416	ENOSF1	18	-	1248	10	1	0	1.68%	4.701766216598559	0.016854695534768073	0.007029302661868638	0.0006017499134389055
ENSG00000006015_18588802_18592180_179	REX1BD	19	+	537	20	0	1	5.03%	21.854019509553076	0.0035291660242640502	0.004333802856335622	8.458999530518808e-05
ENSG00000103197_2083787_2088610_482	TSC2	16	+	1446	6	0	0	1.04%	2.434773542869295	0.016947426762344633	0.016947426762344633	0.0013498980316300959
ENSG00000215301_41343231_41347719_475	DDX3X	X	+	1425	82	1	0	7.58%	33.76561035381264	6.964665585111948e-08	4.0857694212614696e-08	2.8200919065482846e-14
ENSG00000138744_75940949_75917771_323	NAAA	4	-	969	49	0	4	4.64%	29.672075095710532	0.0002820292751200387	0.0018744168113352109	3.5959393606580097e-06
ENSG00000183258_177513451_177511791_245	DDX41	5	-	735	17	1	4	3.27%	13.571792115830615	0.01007916461776185	0.04753395219010738	0.0023771889737864704
ENSG00000151693_9207105_9403327_961	ASAP2	2	+	2883	18	6	0	0.73%	3.663561439114465	0.09390162377375488	0.0078820576498062	0.004163385456649045
ENSG00000185359_81684067_81701618_777	HGS	17	+	2331	23	4	5	1.42%	5.789768231713643	0.011956911202292093	0.02406622995975122	0.0013749674921400253
ENSG00000181610_57850010_57839783_190	MRPS23	17	-	570	54	1	3	11.05%	55.58972436298422	3.503641263936661e-05	0.00012933019366439098	3.427724672988216e-08
ENSG00000114353_50252497_50257690_268	GNAI2	3	+	804	20	0	4	2.99%	14.596527956007465	0.005306578019052857	0.019778610614370123	0.0005526454465689531
ENSG00000161939_7013669_7014286_71	RNASEK-C17orf49	17	+	213	86	4	4	23.94%	236.9160396690564	0.000449965669174599	0.00030104622191380663	9.05267180070782e-07

ENSG00000177425_79689956_79604658_152	PAWR	12	-	456	12	0	1	3.95%	15.44159010082895	0.011772051206276392	0.015555382717448078	0.0008874794703752884
ENSG00000120159_26892715_26869929_237	CAAP1	9	-	711	8	1	1	2.53%	6.602311379257386	0.026602764905793736	0.026602764905793736	0.003128245973917467 5
ENSG00000072803_171957620_171868635_501	FBXW11	5	-	1503	21	1	0	1.80%	8.198528875889224	0.005034872532813168	0.0036062891729786097	9.977408550613472e-05
ENSG00000113732_172983861_173020331_65	ATP6V0E1	5	+	195	27	0	0	16.92%	81.2465202228231	0.0014055202957965589	0.0014055202957965589	1.1935530040246081e-05
ENSG00000140391_77070954_77046835_228	TSPAN3	15	-	684	95	3	2	11.84%	81.49728108770836	2.6598479278066645e-06	2.665499089949705e-06	6.098658898401317e-11
ENSG00000165304_36589641_36677337_568	MELK	9	+	1704	37	1	0	2.46%	12.741124226388207	0.0004271204035778915	0.00043491588733509306	1.2248704772630042e-06
ENSG00000135404_55726940_55725561_145	CD63	12	-	435	128	17	3	25.52%	172.66182586306212	9.787700055082263e-07	2.0792420187236854e-07	1.9151658325959747e-12
ENSG00000141499_7688649_7701666_287	WRAP53	17	+	861	19	2	1	2.09%	12.9486969254338	0.02553836591027338	0.016983116543564363	0.001995648896998549
ENSG00000108312_44213282_44207242_606	UBTF	17	-	1818	33	4	3	2.31%	10.65112980552228	0.0038766098248878157	0.002532570608603844	5.56157809650073e-05
ENSG00000131626_70324901_70343732_443	PPFIA1	11	+	1329	8	0	3	1.13%	3.5321620697155764	0.016947426762344633	0.11777945996203976	0.009674391494749069
ENSG00000131626_70339211_70382146_675	PPFIA1	11	+	2025	11	0	0	0.89%	3.1874492158748153	0.011772051206276392	0.011772051206276392	0.000681272511389508
ENSG00000109854_20364238_20367380_133	HTATIP2	11	+	399	42	3	0	9.02%	61.7663604033158	0.0021367610743209236	0.0010197273511756433	1.326422536870925e-05
ENSG00000148335_129634156_129635464_135	NTMT1	9	+	405	28	4	0	5.19%	40.56753547477038	0.08387828324766944	0.00847367416878415	0.003862246457080268
ENSG00000005889_24179200_24211376_780	ZFX	X	+	2340	13	1	4	0.77%	3.259891243508334	0.02034245439037237	0.09009720761581791	0.008312714083466195
ENSG00000050393_13814429_13790809_359	MCUR1	6	-	1077	12	1	0	1.67%	6.5379434410195	0.02015986069794795	0.013217226988443315	0.0012659333861537747
ENSG00000129103_56073037_56079612_213	SUMF2	7	+	639	85	1	1	9.86%	78.05373399949532	3.5722957519171646e-05	3.5722957519171646e-05	9.746930957628094e-09
ENSG00000146731_56051849_56063085_531	CCT6A	7	+	1593	257	12	5	9.98%	94.66576831430982	4.544629416503036e-10	1.9381253883786937e-10	1.0070114407947812e-18
ENSG00000177410_49279128_49279205_25	ZFAS1	20	+	75	60	3	2	28.00%	469.4243390652001	0.027657845067597595	0.023857440118675605	0.002933789275920174
ENSG00000157593_44255605_44254706_299	SLC35B2	6	-	897	12	0	0	3.01%	7.849905335538463	0.002874854311667729	0.002874854311667729	4.6982854837323694e-05
ENSG00000155380_112929308_112913891_500	SLC16A1	1	-	1500	81	2	0	6.00%	31.686142886901006	1.2426114377822172e-06	6.947723824150777e-07	7.76521840936032e-12
ENSG00000094914_53309004_53308048_127	AAAS	12	-	381	23	0	0	4.72%	35.422440283791346	0.013640585738808999	0.013640585738808999	0.00089863692579514
ENSG00000204220_33289857_33290845_129	PFDN6	6	+	387	31	0	1	9.30%	47.00308304593412	0.0010155991509472172	0.0014942378687529357	9.293758677126684e-06
ENSG00000204220_33289857_33290454_87	PFDN6	6	+	261	28	0	0	10.34%	62.94962401257472	0.0037631575832289435	0.0037631575832289435	7.852614211537582e-05
ENSG00000068001_50320429_50318129_453	HYAL2	3	-	1359	6	0	0	1.10%	2.5906420478211927	0.016947426762344633	0.016947426762344633	0.0013498980316300959
ENSG00000050820_75236980_75229511_599	BCAR1	16	-	1797	18	0	0	1.34%	5.877600238712856	0.005554612275049112	0.005554612275049112	0.00016468457691007306
ENSG00000022556_54970034_55000898_1032	NLRP2	19	+	3096	17	2	0	0.87%	3.221985531374516	0.013292571329153055	0.0034529206320349946	0.0002524038378411522
ENSG00000145817_144165549_144160397_202	YIPF5	5	-	606	16	0	0	4.46%	15.492552444396043	0.0033030736763512156	0.0033030736763512156	6.124050385493983e-05
ENSG00000103415_4507809_4509756_216	HMOX2	16	+	648	12	0	0	2.78%	10.86630414502778	0.012798402692974279	0.012798402692974279	0.0007972436888943567
ENSG00000006327_3020747_3021826_67	TNFRSF12A	16	+	201	98	1	0	22.39%	286.09194793774634	0.00036804788788593267	0.0003144697103273026	7.756998943382397e-07
ENSG00000070814_150357747_150383755_958	TCOF1	5	+	2874	21	0	4	0.73%	4.28753963133664	0.00877616296920843	0.04209057871058814	0.0018631442046486153
ENSG00000125991_35542098_35543012_145	ERGIC3	20	+	435	40	0	1	9.66%	53.95682058220691	0.0004093002068662083	0.0006629387121614735	1.773333733167258e-06
ENSG00000166598_103930516_103942805_550	HSP90B1	12	+	1650	451	11	6	17.09%	160.38664918061005	3.020477635570719e-17	3.258859756486516e-17	1.48897341885146e-32
ENSG00000166598_103943293_103947662_182	HSP90B1	12	+	546	218	11	12	25.27%	234.28229376422533	1.387917615776834e-08	4.2181500304566776e-08	5.9857376097058856e-15
ENSG00000124570_2956782_2948298_350	SERPINB6	6	-	1050	54	1	2	5.43%	30.177278939905722	6.293293459921025e-05	0.0001311217014807635	6.040197107585146e-08
ENSG00000144827_111979062_111990741_205	ABHD10	3	+	615	12	0	1	3.90%	11.449374123541466	0.004826937407846361	0.010020958156399907	0.0002559950816326654 3
ENSG00000204371_31884953_31880084_408	EHMT2	6	-	1224	16	1	0	1.23%	7.670332337666668	0.04223451612662591	0.019679754444124863	0.003715148010809822
ENSG00000089820_153934904_153921704_163	ARHGAP4	X	-	489	8	5	0	3.68%	9.599679735484665	0.11368416502207135	0.011570465654371866	0.006956434060967934
ENSG00000164611_160422313_160422923_101	PTTG1	5	+	303	22	80	0	4.95%	42.60451922208912	0.24610349632790784	0.019679754444124863	0.026033963007913108
ENSG00000078140_39774844_39778434_97	UBE2K	4	+	291	18	0	0	7.22%	36.29569631947424	0.008175610787902942	0.008175610787902942	0.0003425184326896132 4
ENSG00000147684_124547045_124549892_66	NDUFB9	8	+	198	14	2	2	12.12%	41.489524917378795	0.016854695534768073	0.022868511308519305	0.0017828375111446518
ENSG00000114805_155586073_155480968_1488	PLCH1	3	-	4464	9	0	0	0.34%	1.1830250480473794	0.020613416668581838	0.020613416668581838	0.001946208561389314
ENSG00000120158_4841257_4860275_170	RCL1	9	+	510	11	0	0	4.12%	12.656048357150002	0.0078820576498062	0.0078820576498062	0.00031963642907451164
ENSG00000090266_140691330_140704934_93	NDUFB2	7	+	279	101	2	0	9.68%	212.418719738285	0.005387810551254694 5	0.0038428970276066315	0.00011306125532763197
ENSG00000110917_120687297_120696545_292	MLEC	12	+	876	27	3	0	4.45%	18.085697994806512	0.004658089743314183	0.0006424109906750971	1.9353752788868475e-05
ENSG00000103266_681311_682489_197	STUB1	16	+	591	22	0	0	5.08%	21.84292609863452	0.0023218226288164415	0.0023218226288164415	3.1231198880462635e-05
ENSG00000138760_76213543_76163210_470	SCARB2	4	-	1410	89	2	1	8.09%	37.03791327730745	2.9017619533945115e-08	3.6365873129834784e-08	1.05989119828751e-14
ENSG00000172845_173955656_173909941_496	SP3	2	-	1488	22	0	0	1.61%	8.675517019014116	0.005604866691947602	0.005604866691947602	0.00016752456572620382
ENSG00000134910_125595916_125609627_384	STT3A	11	+	1152	74	7	1	5.73%	37.69249250306511	0.0001619996851053046	2.077791655640556e-05	2.657186059342941e-08
ENSG00000137547_54147438_54147719_93	MRPL15	8	+	279	32	1	3	9.68%	67.3009805111398	0.005034872532813168	0.015528142895806445	0.000412219691349819
ENSG00000162734_160211545_160213486_130	PEA15	1	+	390	31	3	0	8.46%	46.6415208686577	0.005080496801168777	0.0013845019718837994	4.188667743299567e-05
ENSG00000171148_9791466_9784024_369	TADA3	3	-	1107	20	2	2	2.71%	10.60127233661247	0.007398015400219724 5	0.012926124733057015	0.0004848234653350558
ENSG00000140416_63048576_63069943_245	TPM1	15	+	735	109	8	7	9.39%	87.01913768385512	0.0001262282821358246	0.000150482303530231	1.3472379671804562e-07
ENSG00000143418_150967454_150967053_70	CERS2	1	-	210	38	3	0	15.71%	106.17931478855716	0.0036776636726157915	0.0014915192737735051	3.234610845106501e-05
ENSG00000143418_150966853_150964460_160	CERS2	1	-	480	33	6	0	8.12%	40.34115413841563	0.008096559011484128	0.0006629342244537616	3.589083703821225e-05
ENSG00000169814_15635440_15642138_159	BTD	3	+	477	10	0	0	3.77%	12.301476390597488	0.012798402692974279	0.012798402692974279	0.0007972436888943567
ENSG00000167460_16086484_16095346_139	TPM4	19	+	417	27	0	3	5.04%	37.99297708261512	0.00847367416878415	0.03299602752967379	0.001401919804466075 7
ENSG00000198860_184051756_184095070_130	TSEN15	1	+	390	13	0	0	4.62%	19.559347461050006	0.013007239811447113	0.013007239811447113	0.0008218654148058079
ENSG00000115875_38751256_38749516_132	SRSF7	2	-	396	124	3	3	17.42%	183.7393246341061	2.3334555990505223e-05	2.681120551891437e-05	4.866556242016747e-09
ENSG00000150527_39294000_39350264_639	MIA2	14	+	1917	17	0	0	1.41%	5.203582266633021	0.002967235347054297	0.002967235347054297	4.9907879201100895e-05
ENSG00000125746_45638587_45616824_395	EML2	19	-	1185	9	0	0	1.27%	4.456560180998736	0.020613416668581838	0.020613416668581838	0.001946208561389314
ENSG00000159176_201496303_201485366_40	CSRP1	1	-	120	66	2	5	12.50%	322.72923310732506	0.029237630782826433	0.05202961726446396	0.006435460209766285 6
ENSG00000149792_65122359_65125872_162	MRPL49	11	+	486	25	0	0	5.56%	30.184178180632724	0.0035547763465261306	0.0035547763465261306	7.044676146010672e-05
ENSG00000116641_62688264_62455414_2109	DOCK7	1	-	6327	16	0	0	0.28%	1.4838765262057851	0.013007239811447113	0.013007239811447113	0.0008218654148058079
ENSG00000084754_26244596_26191250_792	HADHA	2	-	2376	89	2	1	3.79%	21.979569747897102	8.060720038481549e-07	8.154771907763202e-07	5.9234632713205364e-12
ENSG00000133835_119479012_119541994_532	HSD17B4	5	+	1596	38	0	0	2.82%	13.970962472178575	0.00029563251185049877	0.00029563251185049877	5.909204346842462e-07
ENSG00000185627_244210_247465_108	PSMD13	11	+	324	24	6	1	6.48%	43.46521658011112	0.060777753348899316	0.012088529595134127	0.003585135084738235
ENSG00000092020_35087995_35085590_77	PPP2R3C	14	-	231	8	0	0	6.49%	20.32139995953247	0.019216965118390855	0.019216965118390855	0.0017073955890589052

ENSG00000181090_137744064_137846574_803	EHMT1	9	+	2409	21	0	2	0.87%	5.1151469076220435	0.0078820576498062	0.028902805874428627	0.0011492025149225664
ENSG00000162385_53238448_53233497_100	MAGOH	1	-	300	40	1	0	6.00%	78.23738984420002	0.013640585738808999	0.013640585738808999	0.00089863692579514
ENSG00000139624_50167297_50135469_351	CERS5	12	-	1053	11	0	0	1.99%	6.129710030528491	0.005706018193000826	0.005706018193000826	0.0001733096755673334
ENSG00000134285_48925428_48924219_106	FKBP11	12	-	318	15	2	0	5.66%	27.678321878844347	0.03537493353818881	0.012798402692974279	0.0021433541193402405
ENSG00000071462_73683626_73697886_281	BUD23	7	+	843	76	4	1	10.68%	52.90072622917439	8.116167051946057e-06	8.723194954653699e-07	6.415097923814551e-11
ENSG00000006704_74508081_74602433_959	GTF2IRD1	7	+	2877	13	6	0	0.73%	2.651423534866007	0.3189063506976081	0.007029302661868638	0.019256003434193907
ENSG00000106682_74174384_74195308_248	EIF4H	7	+	744	29	1	4	4.44%	22.871817595582666	0.00187740676320226	0.0033869280463461717	3.686552297980832e-05
ENSG00000105974_116526588_116559287_147	CAV1	7	+	441	215	20	3	27.21%	286.07208871603746	7.477091847878022e-07	1.5384592190056337e-08	1.2608926631122026e-13
ENSG00000204852_110628829_110647892_395	TCTN1	12	+	1185	18	0	0	2.03%	8.913120361997471	0.005160369442893307	0.005160369442893307	0.00014319601891785326
ENSG00000010278_6200500_6237828_228	CD9	12	+	684	225	12	26	22.37%	193.01987626036188	3.0947290438040336e-09	5.712446715057884e-09	1.886678929190764e-16
ENSG00000196730_87645916_87707364_1086	DAPK1	9	+	3258	15	0	4	0.64%	2.70156732887431	0.0078820576498062	0.059287764867537715	0.0024707045821742487
ENSG00000104823_38815982_38815613_76	ECH1	19	-	228	23	3	0	11.84%	59.192762053177645	0.011179354118128544	0.003684287313187465	0.00022402973088262996
ENSG00000106609_66945081_66951216_198	TMEM248	7	+	594	17	1	8	3.54%	16.79337913322475	0.015625008558487224	0.08272817174309494	0.006144370996230344
ENSG00000152700_134612730_134606950_130	SAR1B	5	-	390	54	0	0	13.08%	81.2465202228231	0.00013106574077567133	0.00013106574077567133	1.221428801656259e-07
ENSG00000189067_11556730_11549637_161	LITAF	16	-	483	34	1	0	7.45%	41.30545426557144	0.0010073743004618205	0.0010155991509472172	6.342998511872531e-06
ENSG00000164120_174522451_174492056_178	HPGD	4	-	534	22	5	3	6.18%	24.174474390061803	0.016114278516965504	0.009331102573597311	0.0007425518204544101
ENSG00000164120_174522451_174491956_266	HPGD	4	-	798	53	6	3	7.52%	38.97163215923497	0.0003343022993874415	0.0002070447000007143	4.733014918990921e-07
ENSG00000138061_38075388_38070722_543	CYP1B1	2	-	1629	6	0	0	0.92%	2.1612538630994482	0.016947426762344633	0.016947426762344633	0.0013498980316300959
ENSG00000143537_155051387_155062502_839	ADAM15	1	+	2517	46	13	0	2.26%	10.723837702125152	0.009314527945786596	5.328371459388349e-05	5.3170083957120194e-06
ENSG00000092931_76741970_76778352_310	MFSD11	17	+	930	10	0	0	1.94%	6.3094669229193565	0.012798402692974279	0.012798402692974279	0.0007972436888943567
ENSG00000243678_51169976_51171604_63	NME2	17	+	189	75	1	2	15.87%	232.84937453630957	0.0024742386374296595	0.004580613746693	6.408837893308616e-05
ENSG00000114956_73926962_73958736_260	DGUOK	2	+	780	10	2	9	2.69%	7.522825946557694	0.04912109528954385	0.1990553367779263	0.03864249705345155
ENSG00000105974_116526588_116559287_147	CAV1	7	+	441	215	20	3	27.21%	286.07208871603746	7.477091847878022e-07	1.5384592190056337e-08	1.2608926631122026e-13
ENSG00000105974_116526588_116559287_147	CAV1	7	+	441	215	20	3	27.21%	286.07208871603746	7.477091847878022e-07	1.5384592190056337e-08	1.2608926631122026e-13
ENSG00000140829_72096158_72112497_1227	DHX38	16	+	3681	26	4	0	0.81%	4.144605003971476	0.015911563693245086	0.0024042815392806777	0.00022267397200383376
ENSG00000157020_10312690_10301261_254	SEC13	3	-	762	51	0	4	6.69%	39.27270553202953	0.0001294826452477558	0.000711119951210592	6.525943473714536e-07
ENSG00000158417_99361298_99399414_1088	EIF5B	2	+	3264	48	4	3	1.47%	8.629123879875001	0.0009779607528883226	0.0005854100116629538	3.6457916621055834e-06
ENSG00000141526_82236009_82238976_465	SLC16A3	17	+	1395	72	7	1	5.59%	30.28544123001291	1.1849891463284232e-05	4.637421080669856e-06	4.5704910601567277e-10
ENSG00000105676_19042826_19057628_453	ARMC6	19	+	1359	13	0	4	1.99%	5.613057770279251	0.00308494966027208	0.046217795284775376	0.0008850096456078009
ENSG00000105676_19042826_19057628_453	ARMC6	19	+	1359	13	0	4	1.99%	5.613057770279251	0.00308494966027208	0.046217795284775376	0.0008850096456078009
ENSG00000162736_160343397_160358271_709	NCSTN	1	+	2127	125	1	5	4.65%	34.4840399524859	2.8111520282027415e-07	1.7125981462789182e-06	4.4997932614728465e-12
ENSG00000175166_184299267_184301662_160	PSMD2	3	+	480	9	1	0	3.12%	11.002132946840627	0.03537493353818881	0.020613416668581838	0.003252526348827943
ENSG00000012963_93207292_93227035_425	UBR7	14	+	1275	15	3	0	1.18%	6.903299103900002	0.08620755130569058	0.020613416668581838	0.008015435028723247
ENSG00000115421_60760249_60781888_234	PAPOLG	2	+	702	6	1	0	2.14%	5.01521729770513	0.0477903522728147	0.016947426762344633	0.0036975118224100542
ENSG00000241468_99466141_99458327_49	ATP5MF	7	-	147	34	0	0	14.29%	135.71792115830615	0.00877616296920843	0.00877616296920843	0.00039161135667564577
ENSG00000188690_125816499_125788868_265	UROS	10	-	795	12	1	0	2.64%	8.857063001230191	0.015516259750072458	0.0078820576498062	0.0006142571802709984
ENSG00000091129_108232428_108149910_1103	NRCAM	7	-	3309	20	2	5	1.09%	3.5465725224025393	0.004302923267342423	0.014861317392387344	0.0003432852811583412
ENSG00000101558_9936202_9954211_141	VAPA	18	+	423	25	1	0	6.38%	34.679694079875894	0.004355956481189797	0.0035547763465261306	8.558737951575692e-05
ENSG00000128641_191276953_191423960_1059	MYO1B	2	+	3177	22	2	1	1.23%	4.063320530152031	0.0032701130750291308	0.001015856855223422	2.0240423793271705e-05
ENSG00000105701_18541970_18532169_413	FKBP8	19	-	1239	28	5	3	2.91%	13.26057454986441	0.00874233720526068	0.0050592589238414535	0.0002336436379047641
ENSG00000149187_47488963_47472230_465	CELF1	11	-	1395	19	1	0	1.51%	7.991991435697852	0.015516259750072458	0.0078820576498062	0.0006142571802709984
ENSG00000146834_100430028_100433554_686	MEPCE	7	+	2058	10	0	0	0.87%	2.8512168310568518	0.012798402692974279	0.012798402692974279	0.0007972436888943567
ENSG00000115204_27322517_27309912_191	MPV17	2	-	573	22	1	3	6.81%	22.529091316392677	0.0010442431046425516	0.005573589289617817	3.58324891837677e-05
ENSG00000106541_16801190_16792951_117	AGR2	7	-	351	92	0	0	16.24%	153.79999712962396	6.246827902376977e-05	6.246827902376977e-05	2.8936600271513838e-08
ENSG00000147475_37737923_37744893_206	ERLIN2	8	+	618	13	0	0	3.40%	12.343277523963595	0.0078820576498062	0.0078820576498062	0.00031963642907451164
ENSG00000171720_141634920_141621468_371	HDAC3	5	-	1113	6	0	3	1.35%	3.1632367861536395	0.016947426762344633	0.165899918248885	0.014398895220848395
ENSG00000051180_40706201_40731178_256	RAD51	15	+	768	14	3	0	2.34%	10.696518142761722	0.06032271266817251	0.011570465654371866	0.0034318131775285545
ENSG00000168958_227340328_227357117_89	MFF	2	+	267	14	2	1	6.74%	30.767512860078657	0.013007239811447113	0.013007239811447113	0.0008218654148058079
ENSG00000119537_63359759_63331782_255	KDSR	18	-	765	10	0	0	2.35%	7.670332337666668	0.012798402692974279	0.012798402692974279	0.0007972436888943567
ENSG00000143933_47162588_47160776_113	CALM2	2	-	339	138	7	1	12.39%	238.86636722344252	0.0003909674708303042	0.00047118620257580814	1.215777600715846e-06
ENSG00000143612_154212583_154207453_126	C1orf43	1	-	378	26	1	0	6.35%	40.360558252960324	0.008413704741378425	0.005655335537214311	0.00024942285766697933
ENSG00000105185_32584960_32587300_87	PDCD5	19	+	261	18	1	2	5.75%	40.46761543665518	0.022600676488261057	0.02108409854857798	0.0021660048121727006
ENSG00000062822_50398852_50417184_1009	POLD1	19	+	3027	34	2	1	1.49%	6.590860393218039	0.0009502830449746255	0.000405860922741421	2.512435789199588e-06
ENSG00000104824_38846077_38836722_493	HNRNPL	19	-	1479	118	9	8	5.68%	46.8154766816207	1.4231592959075099e-05	3.946485846766709e-05	4.437515975741084e-09
ENSG00000059804_7933033_7921413_422	SLC2A3	12	-	1266	10	0	0	1.18%	4.634916459964456	0.019679754444124863	0.019679754444124863	0.0017849636115922674
ENSG00000164081_51674305_51704016_313	TEX264	3	+	939	23	1	0	3.19%	14.372683437832272	0.003133490598659634	0.002390425500591921	4.28420906379313e-05
ENSG00000070770_58197736_58164071_350	CSNK2A2	16	-	1050	61	1	1	5.71%	34.08914843211572	5.3316394277849575e-05	5.3316394277849575e-05	2.1260111080270267e-08
ENSG00000197713_210016633_210019791_72	RPE	2	+	216	11	0	0	6.94%	29.882336398826396	0.020613416668581838	0.020613416668581838	0.001946208561389314
ENSG00000135525_136550408_136344228_712	MAP7	6	-	2136	7	0	0	0.70%	1.922969553754916	0.019216965118390855	0.019216965118390855	0.0017073955890589052
ENSG00000169359_155853997_155845054_265	SLC33A1	3	-	795	18	0	0	2.64%	13.285594501845287	0.008674846900444944	0.008674846900444944	0.00038311532916634116
ENSG00000108439_47941676_47946782_218	PNPO	17	+	654	17	0	0	3.67%	15.252702148525232	0.005306578019052857	0.005306578019052857	0.00015100114740534233
ENSG00000138600_50765533_50707800_520	SPPL2A	15	-	1560	70	0	3	4.42%	26.32989081295193	1.0500947211644856e-05	3.736705740802518e-05	3.1491877910251115e-09
ENSG00000007341_112619113_112540834_555	ST7L	1	-	1665	8	0	0	1.08%	2.8193653997909918	0.009815328628645334	0.009815328628645334	0.0004837142251238724
ENSG00000168487_22176562_22201203_576	BMP1	8	+	1728	18	0	0	1.22%	6.112296081578126	0.0078820576498062	0.0078820576498062	0.00031963642907451164
ENSG00000129103_56064312_56079612_232	SUMF2	7	+	696	125	5	3	8.19%	105.38441519962286	0.00038804113162733305	0.0001683940165550828	4.5109392841344923e-07
ENSG00000182985_115231410_115169589_219	CADM1	11	-	657	8	0	0	2.28%	7.144967109059363	0.019679754444124863	0.019679754444124863	0.0017849636115922674
ENSG00000134294_46370789_46361111_406	SLC38A2	12	-	1218	268	7	4	15.02%	129.1109635359951	3.590735224218046e-11	1.9033694411192753e-11	8.234030662225177e-21

ENSG00000134419_18789104_18783009_127	RPS15A	16	-	381	43	14	4	4.72%	66.22456226969686	0.13042902411515872	0.05352897298295963	0.026520273205808225
ENSG00000023228_206153678_206124185_727	NDUFS1	2	-	2181	55	1	0	3.44%	14.797305506984184	4.827952539080958e-06	4.925024046154609e-06	1.9941444209025355e-10
ENSG00000100911_24145273_24143409_162	PSME2	14	-	486	76	1	6	5.56%	91.75990166912348	0.0037896409716948643	0.014105683279870995	0.00029108618517596367
ENSG00000077782_38424619_38413628_547	FGFR1	8	-	1641	10	0	0	1.65%	3.57574907880256	0.001946208561389314	0.001946208561389314	2.2278545302028032e-05
ENSG00000074842_4670334_4659803_189	MYDGF	19	-	567	23	0	0	3.70%	23.80238050815609	0.008574016250729465	0.008574016250729465	0.0003747459614100891
ENSG00000135940_97646087_97648108_129	COX5B	2	+	387	126	4	1	15.50%	191.0447891544419	4.286188796909697e-05	4.304580202464658e-05	1.3957858971002244e-08
ENSG00000244038_20661350_20652379_439	DDOST	1	-	1317	111	4	8	8.43%	49.455297680559234	1.9374023593402796e-07	1.447225209722416e-06	2.664241197115936e-12
ENSG00000274290_26183796_26184176_126	HIST1H2BE	6	+	378	24	0	0	5.56%	37.25589992580953	0.00877616296920843	0.00877616296920843	0.00039161135667564577
ENSG00000170854_97967530_97945184_443	RIOX2	3	-	1329	10	3	0	1.58%	4.415202587144471	0.07708712836172119	0.007593099278532274	0.0032211598000607736
ENSG00000196290_200892025_200903678_350	NIF3L1	2	+	1050	29	0	0	1.71%	16.206316467727145	0.013428347753762199	0.013428347753762199	0.0008725593497644525
ENSG00000096384_44248702_44253598_700	HSP90AB1	6	+	2100	209	17	13	8.57%	58.398623133706444	5.600961997648614e-11	8.916411341006866e-11	5.88230326242746e-20
ENSG00000163932_53184882_53192266_411	PRKCD	3	+	1233	5	0	0	1.22%	2.3794826594951344	0.012673659338734126	0.012673659338734126	0.000782701129001274
ENSG00000054523_10261953_10378402_1672	KIF1B	1	+	5016	8	1	1	0.30%	0.935853945504785	0.0477903522728147	0.0477903522728147	0.009211062727049486
ENSG00000136271_44573612_44566002_483	DDX56	7	-	1449	70	5	0	4.55%	28.34688037833334	0.000274154548097287	1.5266870230847838e-05	3.484125911686077e-08
ENSG00000127445_9835345_9849199_163	PIN1	19	+	489	15	0	0	4.29%	17.999399504033747	0.00797940233269714	0.00797940233269714	0.000327142761693177
ENSG00000131446_180792971_180791634_445	MGAT1	5	-	1335	9	0	0	1.57%	3.9558230820101135	0.006937202941512724	0.006937202941512724	0.00025101522741487095
ENSG00000198742_99143780_99030584_757	SMURF1	7	-	2271	8	6	1	0.79%	2.067038040797887	0.2828598995720949	0.028889785561798602	0.04025112963270498
ENSG00000133114_44989655_45028003_338	GPALPP1	13	+	1014	10	0	0	2.07%	5.786789189659765	0.007593099278532274	0.007593099278532274	0.00029783286705462064
ENSG00000072518_63895204_63909237_686	MARK2	11	+	2058	9	0	0	0.87%	2.5660951479511667	0.011772051206276392	0.011772051206276392	0.000681272511389508
ENSG00000259112_78079744_78038664_134	NDUFC2-KCTD14	11	-	402	173	4	15	20.15%	252.51993363892916	2.8741749484516152e-06	1.4831766004695903e-05	3.638568363463898e-10
ENSG00000114346_172757007_172820237_805	ECT2	3	+	2415	106	1	1	4.72%	25.75516560088572	3.760462660003336e-08	3.760462660003336e-08	1.4134601572500778e-14
ENSG00000203879_154437255_154442672_447	GDI1	X	+	1341	39	1	4	2.91%	17.065202482795307	0.0009331614137809266	0.006759882772062428	3.9732797216215845e-05
ENSG00000057608_5773913_5766071_126	GDI2	10	-	378	13	3	8	5.56%	20.180279126480162	0.03970531299947108	0.08387828324766944	0.013353813514968269
ENSG00000185624_81860471_81844012_464	P4HB	17	-	1392	376	17	20	18.97%	158.49816046023278	1.6725061297971517e-15	9.239666103066457e-15	2.204409293756452e-28
ENSG00000111667_6852180_6866077_858	USP5	12	+	2574	37	1	1	2.68%	8.434683637049536	1.0400008237688723e-05	1.0400008237688723e-05	8.771093304690495e-10
ENSG00000111665_6850952_6849043_243	CDCA3	12	-	729	19	2	0	4.94%	15.293316944853913	0.005380042077471066	0.0007764301431553706	2.6597637939514903e-05
ENSG00000111666_101697862_101728945_406	CHPT1	12	+	1218	37	3	0	3.20%	17.825021085193352	0.0034429763734983116	0.0006514802459581769	1.4295939283146878e-05
ENSG00000167515_88857151_88859368_75	TRAPPC2L	16	+	225	28	0	2	12.00%	73.02156385458669	0.0037631575832289435	0.004490071680569804	9.29822359294349e-05
ENSG00000114062_25375498_25354349_676	UBE3A	15	-	2028	8	13	0	0.74%	2.314715675863906	0.3331867010986109	0.019216965118390855	0.038471517829580006
ENSG00000163565_159014750_159054901_650	IFI16	1	+	1950	9	0	0	0.77%	2.7082173407607697	0.020613416668581838	0.020613416668581838	0.001946208561389314
ENSG00000103978_42273276_42211708_494	TMEM87A	15	-	1482	11	0	0	1.62%	4.355320284849191	0.004687384229717438	0.004687384229717438	0.00011928172701435494
ENSG00000134243_109397892_109314043_831	SORT1	1	-	2493	38	0	0	1.81%	8.944105938867631	0.0002994905732097556	0.0002994905732097556	6.05933306828899e-07
ENSG00000168394_32852497_32845579_547	TAP1	6	-	1641	28	5	0	2.01%	10.01209742064717	0.0037834851205969236	0.00151350169064872	3.3725420018176794e-05
ENSG00000110696_16744670_16755104_139	C11orf58	11	+	417	16	4	0	4.32%	22.514356789697846	0.07916783876068934	0.013007239811447113	0.005062572325237441
ENSG00000108883_44853375_44851274_145	EFTUD2	17	-	435	12	4	0	5.52%	16.187046174662072	0.028551546089151347	0.004826937407846361	0.0007484902020293999
ENSG00000183780_233905533_234323243_471	SLC35F3	1	+	1413	14	0	0	1.27%	5.813818778231424	0.013007239811447113	0.013007239811447113	0.0008218654148058079
ENSG00000183780_234214499_234323243_421	SLC35F3	1	+	1263	13	0	0	1.19%	6.039703491535631	0.02108409854857798	0.02108409854857798	0.002029891263157455
ENSG00000118894_5097739_5085632_330	EEF2KMT	16	-	990	13	0	0	2.12%	7.705197484656062	0.0078820576498062	0.0078820576498062	0.00031963642907451164
ENSG00000168275_234374246_234383818_79	COA6	1	+	237	24	0	5	10.13%	59.42080241331647	0.0053557511377634245	0.03196954465377191	0.0009212659198961023
ENSG00000168275_234374246_234383818_79	COA6	1	+	237	24	0	5	10.13%	59.42080241331647	0.0053557511377634245	0.03196954465377191	0.0009212659198961023
ENSG00000168264_234609494_234607137_571	IRF2BP2	1	-	1713	21	3	1	2.28%	7.193455283398426	0.005573589289617817	0.0010442431046425516	3.58324891837677e-05
ENSG00000089597_62646599_62625815_852	GANAB	11	-	2556	294	12	6	10.80%	67.49352292897537	2.7116006778423486e-16	2.3811029554376624e-16	9.486341777361198e-31
ENSG00000110536_47565942_47571629_131	PTPMT1	11	+	393	14	0	2	5.34%	20.903119424022904	0.00847367416878415	0.03299602752967379	0.0014019198044660757
ENSG00000111678_6944529_6945922_62	C12orf57	12	+	186	49	3	0	14.52%	154.58193961152423	0.0035547763465261306	0.0036321914711260127	7.190915230658967e-05
ENSG00000138029_26254255_26289953_351	HADHB	2	+	1053	38	0	0	5.13%	21.17536192364388	6.99138909308556e-05	6.99138909308556e-05	3.6022735069195e-08
ENSG00000184047_122218361_122208381_166	DIABLO	12	-	498	17	0	0	3.61%	20.030657038424703	0.013007239811447113	0.013007239811447113	0.0008218654148058079
ENSG00000204356_31954314_31952301_90	NELFE	6	-	270	12	0	0	8.89%	26.079129948066672	0.004873903550586949	0.004873903550586949	0.00012846764509679302
ENSG00000101444_34290436_34281034_143	AHCY	20	-	429	34	2	0	9.09%	46.50474221508393	0.0015782393174957076	0.0006047916691041674	6.042175486152561e-06
ENSG00000101444_34303270_34290547_285	AHCY	20	-	855	106	2	0	9.82%	72.74704669723862	3.988342894102345e-06	1.6996910885276309e-06	5.880951085974811e-11
ENSG00000100644_61736971_61740577_57	HIF1A	14	+	171	19	4	0	14.04%	65.19782487016668	0.04167809076821618	0.005554612275049112	0.0012643001137217715
ENSG00000168092_117152520_117163979_141	PAFAH1B2	11	+	423	19	3	0	3.55%	26.35656750070568	0.0705580690856681	0.021557223391537678	0.006740452327400666
ENSG00000144713_12840237_12836094_135	RPL32	3	-	405	30	3	0	5.19%	43.46521658011112	0.020525443280229434	0.00877616296920843	0.0008921776838278579
ENSG00000141696_41811816_41802957_404	P3H4	17	-	1212	24	0	0	2.97%	11.619414333297032	0.000978917897162665	0.000978917897162665	5.956062365741233e-06
ENSG00000166888_57107239_57096572_737	STAT6	12	-	2211	23	3	0	1.36%	6.10400259978494	0.007867321313276006	0.0023218226288164415	0.00010390540483540199
ENSG00000100714_64415361_64458303_854	MTHFD1	14	+	2562	49	4	1	3.16%	11.22257641207787	2.3938002210861648e-05	2.9027527199669267e-06	5.99076290729707e-10
ENSG00000131844_71592941_71643794_281	MCCC2	5	+	843	18	0	0	3.20%	12.529119370067619	0.0034782380676658805	0.0034782380676658805	6.758309314600117e-05
ENSG00000103429_14655148_14667827_210	BFAR	16	+	630	19	0	0	4.29%	17.696552464759527	0.0034529206320349946	0.0034529206320349946	6.664817351595063e-05
ENSG00000149923_30082782_30085062_228	PPP4C	16	+	684	31	6	1	6.58%	26.593849618094303	0.003979706689969856	0.00040502216050484493	1.0936921062085188e-05
ENSG00000137404_30690139_30688661_183	NRM	6	-	549	12	0	5	4.92%	12.825801613803282	0.002874854311667729	0.10974091431798999	0.0023930003940198863
ENSG00000184371_109914343_109925189_215	CSF1	1	+	645	7	2	0	2.33%	6.368159638481397	0.1191346346731873	0.019216965118390855	0.010787784428637617
ENSG00000172845_173955656_173909941_496	SP3	2	-	1488	22	0	0	1.61%	8.675517019014116	0.005604866691947602	0.005604866691947602	0.00016752456572620382
ENSG00000099624_1244171_1244437_45	ATP5F1D	19	+	135	115	7	5	22.22%	499.8499906712779	0.0028151052444788517	0.013900915060036254	0.00022123606969677142
ENSG00000129351_10670980_10689312_891	ILF3	19	+	2673	63	4	8	2.69%	13.829841639126267	4.715073818687368e-05	3.588355153095289e-05	1.284152373971558e-08
ENSG00000158019_27894557_28327433_415	BABAM2	2	+	1245	12	2	0	1.93%	5.655714928496387	0.030313543781140352	0.004826937407846361	0.0007979355218377862
ENSG00000171858_62387339_62388001_90	RPS21	20	+	270	240	7	9	13.33%	521.5825989613335	0.0033021427446191217	0.0110656055465517	0.00020116086972894994
ENSG00000184220_100162372_100178308_75	CMSS1	3	+	225	12	0	0	6.67%	31.294955937680008	0.02108409854857798	0.02108409854857798	0.002029891263157455
ENSG00000158850_161175991_161171816_370	B4GALT3	1	-	1110	10	0	0	1.35%	5.286310124608109	0.019679754444124863	0.019679754444124863	0.0017849636115922674

ENSG00000159461_56369309_56362909_177	AMFR	16	-	531	12	0	0	3.39%	13.26057454986441	0.009815328628645334	0.009815328628645334	0.0004837142251238724
ENSG00000164961_125083898_125024617_1159	WASHC5	8	-	3477	11	3	3	0.69%	1.856366023050475	0.05174126704647503	0.05111742236774389	0.010532862688759005
ENSG00000155368_119367052_119372318_129	DBI	2	+	387	103	6	4	16.28%	156.17153399132948	0.00014145917115575762	8.387869876068085e-05	8.562985301249247e-08
ENSG00000133104_36335467_36304365_545	SPART	13	-	1635	12	0	0	1.10%	4.306645312524772	0.011772051206276392	0.011772051206276392	0.000681272511389508
ENSG00000111615_75511597_75506728_148	KRR1	12	-	444	21	0	0	5.41%	27.753128154192574	0.005454749182134642	0.005454749182134642	0.00015910859015753364
ENSG00000087191_63829923_63831969_327	PSMC5	17	+	981	139	12	3	13.15%	83.1421803390199	1.9000534763159954e-07	6.538927111584896e-09	1.3586299916706076e-14
ENSG00000136450_58007137_58005747_201	SRSF1	17	-	603	120	0	22	13.93%	116.77222364805972	1.5853614593892364e-06	7.80495470659366e-05	1.197920011383821e-09
ENSG00000108465_47975891_47981502_281	CDK5RAP3	17	+	843	12	0	0	2.14%	8.352746246711746	0.009815328628645334	0.009815328628645334	0.0004837142251238724
ENSG00000183495_132037776_132050752_481	EP400	12	+	1443	11	4	0	1.04%	4.473031643899169	0.12312584984626351	0.020613416668581838	0.011809609471937859
ENSG00000198561_57795613_57816308_861	CTNND1	11	+	2583	62	7	2	2.67%	14.084547532927994	0.0003827488158090153	2.3314382156310368e-05	7.254834957190183e-08
ENSG00000135900_223957473_223967034_332	MRPL44	2	+	996	33	0	1	3.92%	19.441520066706328	0.0006629342244537616	0.0006940860426134196	2.93900851029603e-06
ENSG00000135899_230216783_230176665_501	SP110	2	-	1503	7	1	0	1.20%	2.732842958629741	0.028889785561798602	0.009815328628645334	0.0013877953586383081
ENSG00000135899_230216783_230169124_665	SP110	2	-	1995	8	1	1	1.05%	2.353004205840602	0.017404239405933563	0.017404239405933563	0.0014187724443961607
ENSG00000124214_49166105_49114878_545	STAU1	20	-	1635	36	5	0	2.75%	12.919935937574316	0.003777991055085985	0.00026886811829191485	7.243741759240639e-06
ENSG00000055130_148730123_148800582_776	CUL1	7	+	2328	27	0	1	1.16%	6.805443059901419	0.0035547763465261306	0.005670089768331009	0.00011049933550063637
ENSG00000132591_28855035_28860553_437	ERAL1	17	+	1311	35	0	4	2.52%	15.66538126171053	0.001565547007472977	0.009965665945239083	9.382696552887332e-05
ENSG00000221926_15651591_15628615_558	TRIM16	17	-	1674	13	0	0	1.08%	4.556837222108424	0.013007239811447113	0.013007239811447113	0.0008218654148058079
ENSG00000196141_200389245_200478031_558	SPATS2L	2	+	1674	21	2	0	1.79%	7.361044743405916	0.005670089768331009	0.0022409900782254734	7.232548112602907e-05
ENSG00000006625_30504709_30497092_94	GGCT	7	-	282	21	5	1	5.32%	43.69641454064363	0.10041256134757276	0.020613416668581838	0.009439548653788753
ENSG00000180398_46907920_46905463_80	MCFD2	2	-	240	18	2	0	6.25%	44.00853178736251	0.06539853090342929	0.02108409854857798	0.006119906528703008
ENSG00000157349_70324649_70333582_328	DDX19B	16	+	984	14	0	0	1.52%	8.348501965082319	0.019679754444124863	0.019679754444124863	0.0017849636115922674
ENSG00000136861_120550836_120389236_1727	CDK5RAP2	9	-	5181	12	4	4	0.35%	1.3590745195865666	0.08266357711566169	0.14908097407465987	0.043018381094693095
ENSG00000197798_126235002_126262133_350	FAM118B	11	+	1050	20	0	0	1.71%	11.17676997774286	0.013428347753762199	0.013428347753762199	0.0008725593497644525
ENSG00000166333_6608898_6610611_298	ILK	11	+	894	35	0	0	4.70%	22.972387957609065	0.0004284130286064981	0.0004284130286064981	1.2106972447307754e-06
ENSG00000074201_77637714_77619628_167	CLNS1A	11	-	501	41	0	1	5.99%	48.01995484449402	0.0024742386374296595	0.003941085002384085	5.530224864202724e-05
ENSG00000091129_108232428_108149910_1081	NRCAM	7	-	3243	20	2	5	1.11%	3.6187506865957455	0.004302923267342423	0.014861317392387344	0.0003432852811583412
ENSG00000122687_2241303_2235122_246	MRM2	7	-	738	15	0	0	2.85%	11.926431378689028	0.00847367416878415	0.00847367416878415	0.0003665024264111182
ENSG00000145920_175878740_175880045_134	CPLX2	5	+	402	35	0	5	8.96%	51.08784784602613	0.0009470236307512361	0.006392297441380809	3.7944780494471626e-05
ENSG00000115207_27343497_27326675_892	GTF3C2	2	-	2676	21	0	0	1.01%	4.604779110785315	0.0033278027414746945	0.0033278027414746945	6.211806171561056e-05
ENSG00000089053_121327194_121308480_308	ANAPC5	12	-	924	13	0	0	2.27%	8.255568733560066	0.0078820576498062	0.0078820576498062	0.00031963642907451164
ENSG00000189067_11556730_11549637_161	LITAF	16	-	483	34	1	0	7.45%	41.30545426557144	0.0010073743004618205	0.0010155991509472172	6.342998511872531e-06
ENSG00000156802_123396357_123321134_1390	ATAD2	8	-	4170	44	2	6	1.44%	6.191448117166908	0.00016254048686914515	0.0009348900789086237	1.063948097469627e-06
ENSG00000133961_73355751_73276578_603	NUMB	14	-	1809	19	0	0	1.66%	6.162978470314264	0.0022011839993586477	0.0022011839993586477	2.8200182015552967e-05
ENSG00000146678_45888836_45893091_198	IGFBP1	7	+	594	14	0	0	4.04%	13.829841639126267	0.004873903550586949	0.004873903550586949	0.00012846764509679302
ENSG00000146674_45921140_45914820_291	IGFBP3	7	-	873	149	7	8	10.65%	100.1492361407715	3.0967396523317614e-06	6.373656333363763e-06	1.671352176618229e-10
ENSG00000169359_155853388_155828210_234	SLC33A1	3	-	702	11	1	0	2.14%	9.194565045792737	0.04223451612662591	0.019679754444124863	0.003715148010809822
ENSG00000198668_90399082_90404717_150	CALM1	14	+	450	122	5	2	23.33%	159.08269268320672	5.838853150623411e-07	1.4025768871339777e-07	7.801033500390575e-13
ENSG00000136044_105203713_105177184_339	APPL2	12	-	1017	5	0	4	1.47%	2.8848595075294994	0.012673659338734126	0.15865525393145707	0.011061610944025602
ENSG00000182899_197950968_197951315_55	RPL35A	3	+	165	89	1	6	16.36%	316.50580436971825	0.003710480643933249	0.018085401378232165	0.0003696764468647276
ENSG00000196305_92289419_92210866_1242	IARS	9	-	3726	129	5	13	4.75%	20.315264271138894	1.1788901678220095e-11	3.4513312225339325e-09	5.655956012220754e-19
ENSG00000198791_17243059_17232421_163	CNOT7	8	-	489	9	1	0	4.91%	10.799639702420247	0.010460667668897016	0.0033278027414746884	0.00019130306338196331
ENSG00000159202_48908504_48927134_354	UBE2Z	17	+	1062	43	4	6	5.08%	23.758529401840402	0.0007238505195158527	0.0006518860872334505	3.011712459882249e-06
ENSG00000104133_44663647_44563121_2330	SPG11	15	-	6990	20	5	2	0.34%	1.6789139451545068	0.040339912027649666	0.015646180920032563	0.002909766083943559
ENSG00000113163_75511207_75379346_596	COL4A3BP	5	-	1788	14	4	0	1.17%	4.594477591521813	0.0750151653743942	0.008175610787902942	0.0033080285523474006
ENSG00000127990_94618900_94585465_278	SGCE	7	-	834	11	0	2	2.52%	7.739310146458635	0.007029302661868638	0.03522780890193623	0.0012822660609675817
ENSG00000008394_16354274_16376164_80	MGST1	12	+	240	540	13	7	21.25%	1320.2559536208753	0.00016188098650472748	0.00013305494748860932	1.5221430338451802e-07
ENSG00000128739_24976387_24978444_161	SNRPN	15	+	483	25	0	0	4.35%	30.371657548214294	0.00877616296920843	0.00877616296920843	0.00039161135667564577
ENSG00000067141_73176525_73302696_1075	NEO1	15	+	3225	16	0	1	0.74%	2.91115869187721	0.004873903550586949	0.015625008558487224	0.000403121801006346
ENSG00000270757_197500437_197550646_261	HSPE1-MOB4	2	+	783	150	9	1	6.51%	112.41004287959773	0.00021169651038003775	0.00016485590990204216	2.4314618215323077e-07
ENSG00000105852_95372278_95359973_267	PON3	7	-	801	23	0	0	4.12%	16.848876090043074	0.001484234770590415	0.001484234770590415	1.3251387882263029e-05
ENSG00000185627_237050_247278_113	PSMD13	11	+	339	19	1	1	5.31%	32.88739838583629	0.020525443280229434	0.020525443280229434	0.0019307453316667101
ENSG00000239830_9683718_9683533_61	RPS4XP22	19	-	183	49	5	2	8.20%	157.1160697690902	0.03736774915294124	0.021557223391537678	0.003569200999645275
ENSG00000159842_1091688_1006080_690	ABR	17	-	2070	24	1	11	1.30%	6.803251290800001	0.004445139713096192	0.13389133575829926	0.004224641413841655
ENSG00000125971_34534648_34540624_63	DYNLRB1	20	+	189	60	1	3	9.52%	186.27949962904765	0.017143983986795872	0.025747275800495253	0.0020285680161856415
ENSG00000163159_151190121_151176644_364	VPS72	1	-	1092	19	1	0	2.75%	10.209549498899728	0.003415127931522041	0.00228120298834518	4.457419682640551e-05
ENSG00000186501_27330745_27334285_107	TMEM222	1	+	321	8	1	0	4.67%	14.623811185831778	0.04223451612662591	0.019679754444124863	0.003715148010809822
ENSG00000124570_2959176_2948298_324	SERPINB6	6	-	972	54	1	2	5.86%	32.59891243508334	6.293293459921025e-05	0.0001311217014807635	6.040197107585146e-08
ENSG00000110619_3057367_3001114_831	CARS	11	-	2493	9	0	0	0.60%	2.118340880258123	0.019679754444124863	0.019679754444124863	0.0017849636115922674
ENSG00000124570_2959176_2948298_324	SERPINB6	6	-	972	54	1	2	5.86%	32.59891243508334	6.293293459921025e-05	0.0001311217014807635	6.040197107585146e-08
ENSG00000155876_19049726_19050601_291	RRAGA	9	+	873	60	0	1	7.56%	40.32855146608248	1.5863663608784762e-05	2.309696283698584e-05	2.8921304831278516e-09
ENSG00000283496_133309432_133352440_310	ZNF511-PRAP1	10	+	930	19	1	1	3.23%	11.987987153546777	0.00397428314897603	0.00397428314897603	8.713148241777404e-05
ENSG00000111906_125301932_125297555_107	HDDC2	6	-	321	11	4	0	5.61%	20.107740380518695	0.1493069711690196	0.011772051206276392	0.009740173639615848
ENSG00000109099_15239585_15230917_92	PMP22	17	-	276	16	0	0	5.43%	34.01625645400001	0.02108409854857798	0.02108409854857798	0.002029891263157455
ENSG00000068366_109683363_109644029_670	ACSL4	X	-	2010	36	1	3	2.39%	10.509500128325376	0.00027655323682364736	0.0009450514886518204	1.7500984682627828e-06
ENSG00000177700_842508_840372_67	POLR2L	11	-	201	190	20	6	43.28%	554.6680623282837	9.351844648399558e-06	5.375819584381181e-06	4.1623196695931515e-10
ENSG00000088035_63396530_63415821_268	ALG6	1	+	804	9	0	0	2.61%	6.568437580203359	0.006937202941512724	0.006937202941512724	0.00025101522741487095
ENSG00000088035_63428929_63437020_131	ALG6	1	+	393	8	0	0	5.34%	11.944639670870231	0.005706018193000826	0.005706018193000826	0.0001733096755673334

ENSG00000132153_47843178_47850120_907	DHX30	3	+	2721	17	2	4	1.10%	3.6660298438572223	0.0073980154002197245	0.03063645427690382	0.0011563803149223198
ENSG00000168291_58431954_58428034_317	PDHB	3	-	951	29	0	0	3.79%	17.893409349225557	0.0010405263639310788	0.0010405263639310788	6.697970672456085e-06
ENSG00000120533_109336137_109343481_96	ENY2	8	+	288	145	1	4	29.17%	295.4276439429428	1.9038365037503747e-06	6.4844824125302795e-06	1.0667943006982838e-10
ENSG00000113407_33457290_33459889_135	TARS	5	+	405	14	1	0	4.44%	20.28376773738519	0.023878473930440184	0.013007239811447113	0.0014709138084317821
ENSG00000104946_49877724_49888624_648	TBC1D17	19	+	1944	8	4	0	0.77%	2.4147342544506176	0.21453803860113568	0.019679754444124863	0.02189527136926916
ENSG00000119541_63422259_63396986_379	VPS4B	18	-	1137	27	0	6	2.11%	13.9340997743628	0.005706018193000826	0.042503047233143215	0.0013203279708978932
ENSG00000106628_44122053_44114785_469	POLD2	7	-	1407	57	1	0	5.12%	23.77148838549787	1.0054561647833067e-05	6.503806951040267e-06	5.37545342681054e-10
ENSG00000140575_90388342_90500108_1657	IQGAP1	15	+	4971	51	4	0	1.63%	6.02007676833766	2.3575939526038945e-05	2.027367833152339e-06	4.22644478251645e-10
ENSG00000187498_110307027_110191626_519	COL4A1	13	-	1557	17	0	0	1.35%	6.406722675103084	0.008674846900444944	0.008674846900444944	0.00038311532916634116
ENSG00000166226_69586816_69601325_488	CCT2	12	+	1464	228	16	10	10.86%	91.38383649834839	3.1689235517123985e-10	3.0803222400284716e-09	1.1150908129709376e-17
ENSG00000104442_65627257_65604394_235	ARMC1	8	-	705	16	0	0	2.55%	13.317002526672344	0.013007239811447113	0.013007239811447113	0.0008218654148058079
ENSG00000038427_83490172_83512419_306	VCAN	5	+	918	21	1	1	4.58%	13.42308159091667	0.0006700061001850382	0.0006700061001850382	2.869758972620885e-06
ENSG00000129103_56064312_56079612_301	SUMF2	7	+	903	141	5	3	8.64%	91.62352132917111	2.8393160413368106e-05	1.0124970165731674e-05	2.3083584118077667e-09
ENSG00000114354_100713686_100748531_400	TFG	3	+	1200	50	4	4	4.75%	24.449184326312505	0.00043927481975472	0.00037023034910173925	1.077756252494911e-06
ENSG00000198231_63807823_63818398_623	DDX42	17	+	1869	22	0	0	1.93%	6.906992682874801	0.0007799100084460569	0.0007799100084460569	3.845555587596494e-06
ENSG00000166226_69585522_69586860_61	CCT2	12	+	183	16	4	0	8.20%	51.30320645521313	0.03577298629450316	0.021557223391537678	0.0034214162395858295
ENSG00000155508_154863393_154875439_254	CNOT8	5	+	762	18	0	0	3.15%	13.860954893657484	0.005554612275049112	0.005554612275049112	0.00016468457691007306
ENSG00000189171_153626472_153618895_98	S100A13	1	-	294	51	39	2	15.31%	101.78844086872961	0.00774017645579024	0.00029563251185049877	1.727517427423317e-05
ENSG00000106477_130428012_130398891_272	CEP41	7	-	816	8	0	2	1.84%	5.752749253250001	0.016947426762344633	0.11777945996203976	0.009674391494749069
ENSG00000136450_58005932_58005406_108	SRSF1	17	-	324	46	0	0	11.11%	83.30833177854632	0.0010197273511756433	0.0010197273511756433	6.442859155158972e-06
ENSG00000136450_58007137_58006940_65	SRSF1	17	-	195	74	0	22	18.46%	222.67564801810775	0.0010786302489720287	0.02312540627886428	0.0001727785056696475
ENSG00000119314_112268156_112223851_443	PTBP3	9	-	1329	30	1	4	2.93%	13.245607761433412	0.0006606329705481882	0.005221500909681797	2.2372145234106156e-05
ENSG00000119314_112268156_112223851_443	PTBP3	9	-	1329	30	1	4	2.93%	13.245607761433412	0.0006606329705481882	0.005221500909681797	2.2372145234106156e-05
ENSG00000100644_61697851_61747085_850	HIF1A	14	+	2550	170	14	6	7.65%	39.11869492210001	1.37050230541643e-09	6.077601540226734e-12	1.151829239966011e-19
ENSG00000113851_3174246_3150865_335	CRBN	3	-	1005	9	0	3	1.49%	5.254750064162688	0.019216965118390855	0.1191346346731873	0.010787784428637617
ENSG00000107581_119080676_119036039_1382	EIF3A	10	-	4146	107	7	11	3.47%	15.14363370718054	2.092500174220995e-08	4.68537536528311e-08	9.903593743667191e-15
ENSG00000096384_44248702_44253598_700	HSP90AB1	6	+	2100	209	17	13	8.57%	58.398623133706444	5.600961997648614e-11	8.916411341006866e-11	5.88230326242746e-20
ENSG00000077721_119574739_119583255_143	UBE2A	X	+	429	58	0	1	9.09%	79.33161907279023	0.0006862354746923267	0.0006885945587055891	3.015214816289631e-06
ENSG00000094916_54257650_54241755_191	CBX5	12	-	573	29	7	3	8.38%	29.697438553426707	0.0016400929380019878	0.00043872953614622595	4.675554956550797e-06
ENSG00000177156_747482_760257_154	TALDO1	11	+	462	125	0	4	14.94%	158.76093718384743	1.236195501581318e-05	5.673552722653153e-05	5.602876717971638e-09
ENSG00000117519_94926894_94897742_329	CNN3	1	-	987	36	2	0	4.56%	21.40232548929484	0.0007434794588380547	0.0002596338414409078	1.2961847533454468e-06
ENSG00000144029_95101694_95087357_166	MRPS5	2	-	498	26	0	0	3.61%	30.635122529355428	0.013853924679039932	0.013853924679039932	0.0009252050120140923
ENSG00000129292_132794453_132845923_975	PHF20L1	8	+	2925	14	0	2	0.51%	2.8085216867148723	0.02108409854857798	0.055917760918677194	0.00521801263590997
ENSG00000136942_124861912_124858469_96	RPL35	9	-	288	35	3	2	8.33%	71.3101209517448	0.012070269314219385	0.008808686117259405	0.0005324252474387162
ENSG00000136942_124858481_124857918_57	RPL35	9	-	171	373	14	1	14.04%	1279.9362461353776	0.040643964021588805	0.0053963910816940885	0.0012013795769869825
ENSG00000112473_33201246_33203813_469	SLC39A7	6	+	1407	84	3	3	5.54%	35.03166709441792	3.800011204784303e-06	1.4202491659066792e-05	4.5344776802063415e-10
ENSG00000112473_33201246_33203813_469	SLC39A7	6	+	1407	84	3	3	5.54%	35.03166709441792	3.800011204784303e-06	1.4202491659066792e-05	4.5344776802063415e-10
ENSG00000181827_56103591_56093345_1300	RFX7	15	-	3900	8	1	0	0.38%	1.203652151449231	0.0477903522728147	0.016947426762344633	0.0036975118224100542
ENSG00000149503_62128795_62151976_863	INCENP	11	+	2589	15	5	3	0.93%	3.3996548310052153	0.05423379212399942	0.0385659205926511	0.008542915557582598
ENSG00000119969_94554132_94601622_831	HELLS	10	+	2493	26	2	1	1.32%	6.1196514318568	0.004817859085151192	0.0018842494102203086	5.2471250921023827e-05
ENSG00000131370_15269736_15256086_298	SH3BP5	3	-	894	23	1	0	4.03%	15.096140657857386	0.0016120252826907794	0.0008664586529590942	8.62301436870289e-06
ENSG00000091164_56626417_56603027_243	TXNL1	18	-	729	30	0	0	4.94%	24.147342544506177	0.000978917897162665	0.000978917897162665	5.956062365741233e-06
ENSG00000005100_5462477_5444205_534	DHX33	17	-	1602	19	3	0	2.06%	6.9593183850177915	0.010251872179285306	0.0013775385517804596	8.65610277291482e-05
ENSG00000165060_69035783_69072762_210	FXN	9	+	630	18	0	0	3.81%	16.76515496661429	0.005306578019052857	0.005306578019052857	0.00015100114740534233
ENSG00000132300_86106248_86137559_689	PTCD3	2	+	2067	34	1	0	2.47%	9.651927629545721	0.0001814611398669728	9.157988150005313e-05	1.1911596081040839e-07
ENSG00000136827_129824085_129813972_332	TOR1A	9	-	996	39	2	0	3.61%	22.97634189701657	0.0033248780160496675	0.0010032776369550557	2.0351779040362854e-05
ENSG00000104341_97776016_97851474_224	LAPTM4B	8	+	672	98	10	3	12.95%	85.57214514209377	9.018439932227367e-05	4.82058214579656e-06	3.799154944459226e-09
ENSG00000181090_137716682_137834953_1251	EHMT1	9	+	3753	25	0	2	0.64%	3.908742498211432	0.005306578019052857	0.016170007243128042	0.0004498844289484224
ENSG00000167632_140451355_139731061_1142	TRAPPC9	8	-	3426	9	0	4	0.61%	1.541454703585377	0.006937202941512724	0.18065521426308934	0.008531843872917413
ENSG00000107625_68906819_68946626_666	DDX50	10	+	1998	7	0	0	0.90%	2.0557872706809315	0.009815328628645334	0.009815328628645334	0.0004837142251238724
ENSG00000148290_133353936_133351913_191	SURF1	9	-	573	16	1	0	2.62%	16.384793684649217	0.02784981298332479	0.019679754444124863	0.002477909089056847
ENSG00000165219_125298922_125362746_1478	GAPVD1	9	+	4434	10	1	3	0.34%	1.323365863399865	0.03537493353818881	0.12114404826813535	0.017662684075641494
ENSG00000163931_53255942_53242053_98	TKT	3	-	294	30	4	0	11.22%	59.87555345219389	0.009821676566158627	0.001558047347358915	9.20153756607732e-05
ENSG00000166197_102159553_102162269_418	NOLC1	10	+	1254	106	0	9	5.02%	49.6002591117536	2.3200298509694487e-05	0.0012787427712268279	2.67882882501554e-07
ENSG00000177674_11747543_11754428_103	AGTRAP	1	+	309	18	0	3	6.80%	34.18138391251457	0.00847367416878415	0.00837382380159347	0.000362422040249726
ENSG00000090006_40593166_40629550_1587	LTBP4	19	+	4761	16	2	7	0.57%	1.9719568958840583	0.01292879023172146	0.13801304074353182	0.009490053674322586
ENSG00000120708_136049469_136054879_208	TGFBI	5	+	624	202	3	10	15.87%	189.95135515058178	4.1996105009398927e-07	3.752773448337038e-06	1.4643481873777723e-11
ENSG00000123444_47577999_47572930_518	KBTBD4	11	-	1554	14	3	0	0.97%	5.286310124608109	0.1029516053660342	0.02108409854857798	0.009872287793463467
ENSG00000159322_72752775_72752341_144	ADPGK	15	-	432	17	2	0	5.56%	23.090896308184032	0.013018137044106636	0.005160369442893307	0.0003527402747546314
ENSG00000070761_58129115_58115212_173	CFAP20	16	-	519	14	0	0	3.47%	15.828373667901738	0.013007239811447113	0.013007239811447113	0.0008218654148058079
ENSG00000054116_36140070_36137203_134	TRAPPC3	1	-	402	25	0	0	6.72%	36.491319890018666	0.003684287313187465	0.003684287313187465	7.541963121699725e-05
ENSG00000100811_100276523_100277600_102	YY1	14	+	306	14	0	0	4.90%	26.84616318183334	0.02108409854857798	0.02108409854857798	0.002029891263157455
ENSG00000172053_49103350_49096029_600	QARS	3	-	1800	27	0	3	2.67%	8.801706357472503	0.00016643457103121306	0.0013368055239091048	1.5755689716000913e-06
ENSG00000261832_28489291_28477516_338	AC138894.1	16	-	1014	16	0	5	2.07%	9.258862703455623	0.00847367416878415	0.06059069429271301	0.002681660320446393
ENSG00000088367_36207493_36229340_456	EPB41L1	20	+	1368	22	0	6	1.97%	9.436527283839915	0.0035547763465261306	0.03525671152904061	0.0007308815383483617
ENSG00000160691_154970196_154963803_474	SHC1	1	-	1422	17	0	4	1.90%	7.014955840460972	0.002943990679779778	0.01815489508770333	0.0003044408248345521
ENSG00000160691_154970196_154963803_473	SHC1	1	-	1419	17	0	4	1.90%	7.029786613908035	0.002943990679779778	0.01815489508770333	0.0003044408248345521

ENSG00000184640_77320327_77498658_568	SEPT9	17	+	1704	55	3	6	3.17%	18.93950898517166	0.000442321637794036	0.0014426254751151526	4.136427255035133e-06
ENSG00000128534_118184224_118192002_96	LSM8	7	+	288	42	3	3	13.54%	85.57214514209377	0.0006311913977015671	0.004081271281976878	1.6588772874149273e-05
ENSG00000133597_140673331_140695804_590	ADCK2	7	+	1770	16	0	7	1.02%	5.304229819945764	0.013640585738808999	0.14081442098989871	0.010110099915137491
ENSG00000084234_130069978_130143448_751	APLP2	11	+	2253	145	2	6	7.72%	37.76438591014981	1.7541027497337622e-11	1.3388236953785959e-10	2.8501863811994904e-20
ENSG00000131051_35740847_35707190_414	RBM39	20	-	1242	30	1	0	3.14%	14.17344018916667	0.0009086099620780121	0.0006424109906750971	3.7043534070101014e-06
ENSG00000090316_1289914_1339169_328	MAEA	4	+	984	26	4	0	3.05%	15.504360792295735	0.012181225872892837	0.0024042815392806777	0.00016808527397305602
ENSG00000146674_45921140_45914820_263	IGFBP3	7	-	789	146	6	0	11.41%	108.58040795868064	4.231027273024064e-06	8.176786336581406e-07	3.10321575578913e-11
ENSG00000100316_39316750_39312940_251	RPL3	22	-	753	135	6	5	7.17%	105.19967757935261	0.00014568836401649625	0.00019133729237301805	1.955813407519041e-07
ENSG00000100316_39318439_39316844_128	RPL3	22	-	384	259	21	0	17.19%	395.7711712821837	5.017800315001901e-05	1.8768467850450943e-05	7.340640507038093e-09
ENSG00000116171_52927457_53050704_503	SCP2	1	+	1509	33	1	0	2.39%	12.832176266692846	0.0014539914977685167	0.000978917897162665	8.739992886708085e-06
ENSG00000124226_49936413_49952242_206	RNF114	20	+	618	20	0	1	2.91%	18.989657729174763	0.012798402692974279	0.023672501042627626	0.0014375227604752335
ENSG00000113328_163441222_163443703_105	CCNG1	5	+	315	15	1	1	5.71%	27.94192494435715	0.02034245439037237	0.02034245439037237	0.0018987606724399325
ENSG00000284194_50524411_50523611_266	SCO2	22	-	798	17	8	4	3.76%	12.500334843528199	0.08854587809459352	0.02077003350494258	0.0082970343288543
ENSG00000134333_18396843_18407281_332	LDHA	11	+	996	576	24	9	15.96%	339.3428957097832	1.0678239339972586e-10	4.5319795175477673e-10	5.61667038708755e-19
ENSG00000111328_123267253_123261736_87	CDK2AP1	12	-	261	16	0	0	8.05%	35.97121372147127	0.00847367416878415	0.00847367416878415	0.0003665024264111182
ENSG00000131370_15269736_15256086_298	SH3BP5	3	-	894	23	1	0	4.03%	15.096140657857386	0.0016120252826907794	0.0008664586529590942	8.62301436870289e-06
ENSG00000135317_85593697_85505967_658	SNX14	6	-	1974	16	0	2	1.06%	4.756072330954408	0.00847367416878415	0.02034245439037237	0.0008571698582519303
ENSG00000140612_84716075_84670019_179	SEC11A	15	-	537	166	19	0	17.32%	181.38836192929054	4.155305712748523e-05	4.64563659604018e-07	2.0290627192344187e-10
ENSG00000148337_128179348_128166197_612	CIZ1	9	-	1836	21	2	0	1.63%	6.711540795458335	0.01044836432889887	0.002046028879997488	0.00012444751772694397
ENSG00000132170_12379727_12434145_470	PPARG	3	+	1410	13	0	0	1.49%	5.4100322764606394	0.00797940233269714	0.00797940233269714	0.000327142761693177
ENSG00000180921_143730321_143725921_1092	FAM83H	8	-	3276	6	7	1	0.46%	1.0746894209368134	0.165899918248885	0.0477903522728147	0.03110788599674642
ENSG00000183735_64464361_64501381_610	TBK1	12	+	1830	14	0	0	0.98%	4.489030564831149	0.013428347753762199	0.013428347753762199	0.0008725593497644525
ENSG00000166949_67165004_67190536_320	SMAD3	15	+	960	17	2	5	2.19%	10.390903338682815	0.023857440118675605	0.07942646894547645	0.008281420578649067
ENSG00000122140_135501096_135504133_249	MRPS2	9	+	747	46	3	5	5.62%	36.133734265393585	0.002161000987616309	0.004657727720839925	5.7512551960463115e-05
ENSG00000178449_50120044_50120217_57	COX14	12	+	171	21	1	0	12.28%	72.06075380386844	0.00797940233269714	0.00797940233269714	0.000327142761693177
ENSG00000123349_53297850_53297954_34	PFDN5	12	+	102	31	2	0	14.71%	178.33522685075005	0.028889785561798623	0.02108409854857798	0.002731532589007073
ENSG00000163320_88055976_88055473_167	CGGBP1	3	-	501	14	1	0	4.19%	16.397057751778448	0.016854695534768073	0.007029302661868638	0.0006017499134389055
ENSG00000112339_135039741_134965279_597	HBS1L	6	-	1791	30	0	0	2.18%	9.828817819623119	0.000596156229600823	0.000596156229600823	2.2911026310734046e-06
ENSG00000189067_11556730_11551723_152	LITAF	16	-	456	19	0	0	3.95%	24.449184326312505	0.013640585738808999	0.013640585738808999	0.00089863692579514
ENSG00000130779_122380446_122272875_1436	CLIP1	12	-	4308	17	6	2	0.56%	2.315521635361073	0.06699482590911521	0.016170007243128042	0.00504143104966337
ENSG00000070669_97864400_97852259_446	ASNS	7	-	1338	37	1	0	4.04%	16.226364485624444	0.00011765802228039974	6.806337496646363e-05	5.8463901086995966e-08
ENSG00000136560_161231165_161232848_146	TANK	2	+	438	15	0	0	3.42%	20.095219994229456	0.02108409854857798	0.02108409854857798	0.002029891263157455
ENSG00000149115_57320305_57300540_1562	TNKS1BP1	11	-	4686	24	4	3	0.70%	3.005277458804098	0.013696866554086288	0.010332180556073796	0.0006965721008113797
ENSG00000183255_44861832_44851581_159	PTTG1IP	21	-	477	44	0	0	9.43%	54.12649611862894	0.00028877520139654507	0.00028877520139654507	5.646841491566603e-07
ENSG00000183255_44873616_44851581_107	PTTG1IP	21	-	321	26	0	0	8.41%	47.52738635395328	0.0035291660242640502	0.0035291660242640502	6.948232509684344e-05
ENSG00000080608_2838507_2804331_648	PUM3	9	-	1944	19	2	4	1.54%	5.7349938543202175	0.008105529771034738	0.023814920846593857	0.0009659344609939381
ENSG00000103145_3023324_3022863_75	HCFC1R1	16	-	225	16	0	0	6.67%	41.72660791690667	0.02108409854857798	0.02108409854857798	0.002029891263157455
ENSG00000143476_212044668_212068647_191	DTL	1	+	573	7	0	0	2.62%	7.168347237034033	0.019216965118390855	0.019216965118390855	0.0017073955890589052
ENSG00000143476_212068679_212102940_431	DTL	1	+	1293	17	0	0	1.39%	7.714823824544085	0.012798402692974279	0.012798402692974279	0.0007972436888943567
ENSG00000109919_47642402_47617471_124	MTCH2	11	-	372	18	9	0	8.06%	28.392601153137104	0.08284191945650154	0.0022011839993586477	0.001377230079539875
ENSG00000076043_114439529_114449975_222	REXO2	11	+	666	44	8	2	6.76%	38.766274247126134	0.00715018638591889	0.0009639335707776476	4.336927673047974e-05
ENSG00000132768_43970185_43972539_258	DPH2	1	+	774	10	2	0	1.94%	7.581142426763567	0.08350359792828288	0.020613416668581838	0.007749259071793911
ENSG00000166333_6608898_6610611_298	ILK	11	+	894	35	0	0	4.70%	22.972387957609065	0.0004284130286064981	0.0004284130286064981	1.2106972447307754e-06
ENSG00000257103_34172643_34226431_463	LSM14A	19	+	1389	31	0	0	2.81%	13.095891388607994	0.0006629342244537616	0.0006629342244537616	2.811652200271938e-06
ENSG00000135679_68816839_68839849_430	MDM2	12	+	1290	9	0	0	1.16%	4.093816910452326	0.019679754444124863	0.019679754444124863	0.0017849636115922674
ENSG00000135677_64759276_64716741_552	GNS	12	-	1656	94	0	0	6.88%	33.307584444541675	2.6236431290532118e-08	2.6236431290532118e-08	6.95566304205585e-15
ENSG00000136718_130342933_130346468_291	IMP4	2	+	873	28	1	0	3.44%	18.819990684171824	0.0026278853289981806	0.0022276761777358703	3.381389658291476e-05
ENSG00000136715_128000315_127942006_720	SAP130	2	-	2160	8	0	0	0.69%	2.173260829005556	0.019679754444124863	0.019679754444124863	0.0017849636115922674
ENSG00000136715_128000315_127942006_686	SAP130	2	-	2058	8	0	0	0.73%	2.2809734648454816	0.019679754444124863	0.019679754444124863	0.0017849636115922674
ENSG00000166260_54968646_54961265_231	COX11	17	-	693	34	0	3	4.33%	28.788649942671004	0.002446120450917513	0.0072608768093428535	0.00010037784624098265
ENSG00000172922_65720758_65719783_164	RNASEH2C	11	-	492	13	0	0	3.05%	15.504360792295735	0.020613416668581838	0.020613416668581838	0.001946208561389314
ENSG00000115286_1383927_1393407_236	NDUFS7	19	+	708	34	3	0	5.51%	28.17872091846187	0.0017439832781934702	0.0006026251277497241	6.6568752911666554e-06
ENSG00000134574_47216251_47238849_224	DDB2	11	+	672	7	0	0	2.23%	6.112296081578126	0.019216965118390855	0.019216965118390855	0.0017073955890589052
ENSG00000204673_49873295_49869917_256	AKT1S1	19	-	768	21	3	0	3.12%	16.04477721414258	0.03185093770301228	0.005306578019052857	0.0009105731017613844
ENSG00000204673_49873295_49869917_256	AKT1S1	19	-	768	21	3	0	3.12%	16.04477721414258	0.03185093770301228	0.005306578019052857	0.0009105731017613844
ENSG00000204673_49873295_49869917_256	AKT1S1	19	-	768	21	3	0	3.12%	16.04477721414258	0.03185093770301228	0.005306578019052857	0.0009105731017613844
ENSG00000204673_49873295_49869917_256	AKT1S1	19	-	768	21	3	0	3.12%	16.04477721414258	0.03185093770301228	0.005306578019052857	0.0009105731017613844
ENSG00000204673_49873295_49869917_256	AKT1S1	19	-	768	21	3	0	3.12%	16.04477721414258	0.03185093770301228	0.005306578019052857	0.0009105731017613844
ENSG00000132155_12612023_12584514_566	RAF1	3	-	1698	16	5	0	1.06%	5.529144158600708	0.08873763529811646	0.011772051206276392	0.005312171954116946
ENSG00000122033_27440124_27435675_170	MTIF3	13	-	510	13	1	0	3.53%	14.957148058450004	0.023878473930440184	0.013007239811447113	0.0014709138084317821
ENSG00000241258_66114829_66152357_141	CRCP	7	+	423	12	0	3	4.96%	16.64625315834043	0.008175610787902942	0.023672501042627626	0.0009673284272160682
ENSG00000151292_123573443_123614396_311	CSNK1G3	5	+	933	8	0	0	1.93%	5.031343398340837	0.009815328628645334	0.009815328628645334	0.0004837142251238724
ENSG00000099204_114491803_114436260_485	ABLIM1	10	-	1455	8	1	2	1.24%	3.226284117286599	0.026602764905793736	0.08266357711566169	0.009436337707064906
ENSG00000099204_114491803_114436260_483	ABLIM1	10	-	1449	8	1	2	1.24%	3.2396434718095244	0.026602764905793736	0.08266357711566169	0.009436337707064906
ENSG00000099204_114445395_114436260_197	ABLIM1	10	-	591	7	0	0	2.54%	6.950021940474621	0.019216965118390855	0.019216965118390855	0.0017073955890589052
ENSG00000146263_97282477_97146806_1203	MMS22L	6	-	3609	17	0	2	0.58%	2.763997563074398	0.008175610787902942	0.027965089620958453	0.0011463419454493871
ENSG00000131778_147242704_147295509_897	CHD1L	1	+	2691	20	0	1	1.11%	4.361058519743591	0.001801896632038876	0.006432154271512831	6.747259634841345e-05

ENSG00000131778_147242704_147295509_693	CHD1L	1	+	2079	19	0	1	1.30%	5.362591655987735	0.002967235347054297	0.01007916461776185	0.000166354917991791
ENSG00000080503_2086868_2192739_717	SMARCA2	9	+	2151	16	4	4	0.98%	4.364707941099025	0.05995084452706934	0.05237010832359605	0.012325812363490476
ENSG00000104450_100184720_100241022_697	SPAG1	8	+	2091	13	0	4	1.15%	3.6480848923048788	0.005160369442893307	0.05268573235456097	0.0015446541073930182
ENSG00000187720_71547410_71777374_658	THSD4	15	+	1974	16	0	0	1.37%	4.756072330954408	0.0033030736763512156	0.0033030736763512156	6.124050385493983e-05
ENSG00000115446_98609916_98618304_207	UNC50	2	+	621	49	1	1	7.73%	46.299904617944536	0.00025057736413350534	0.00025057736413350534	4.290682187398661e-07
ENSG00000132716_160240344_160217592_572	DCAF8	1	-	1716	12	0	0	1.40%	4.103359607213288	0.004826937407846361	0.004826937407846361	0.00012612451166366307
ENSG00000174780_56467636_56501861_610	SRP72	4	+	1830	28	4	1	1.48%	8.978061129662297	0.013577461948370874	0.004535229864886203	0.00032802727111032304
ENSG00000113328_163441222_163442565_159	CCNG1	5	+	477	35	1	1	5.66%	43.05516736709121	0.004535229864886203	0.004535229864886203	0.00011202547754054781
ENSG00000147526_38745468_38848023_731	TACC1	8	+	2193	19	3	0	1.64%	5.0838249214767455	0.006073451221574228	0.0006577637618416096	2.6167522447195022e-05
ENSG00000127990_94656098_94585499_429	SGCE	7	-	1287	26	3	2	2.80%	11.854149976393941	0.005454749182134642	0.0026311212314026142	8.05834708867572e-05
ENSG00000172053_49099226_49096029_228	QARS	3	-	684	10	0	0	3.07%	8.578661167127194	0.007593099278532274	0.007593099278532274	0.00029783286705462064
ENSG00000134779_36818914_36798422_179	TPGS2	18	-	537	8	0	0	2.79%	8.741607803821232	0.019216965118390855	0.019216965118390855	0.0017073955890589052
ENSG00000105171_29610472_29615380_179	POP4	19	+	537	12	0	0	3.91%	13.112411705731846	0.0078820576498062	0.0078820576498062	0.00031963642907451164
ENSG00000111361_123633557_123630157_126	EIF2B1	12	-	378	15	1	0	4.76%	23.284937453630956	0.022004978195124387	0.013007239811447113	0.001358223331354383
ENSG00000155545_56928856_56923128_272	MIER3	5	-	816	12	1	4	2.21%	8.629123879875001	0.023878473930440184	0.10041256134757276	0.010603761142319451
ENSG00000106609_66941953_66955522_285	TMEM248	7	+	855	32	1	11	4.21%	21.96137258784562	0.0014807488005297608	0.029252016701462123	0.000291836043000113
ENSG00000104671_30156384_30183173_190	DCTN6	8	+	570	32	0	0	5.79%	32.94205888176843	0.0016033688297201391	0.0016033688297201391	1.536694271268808e-05
ENSG00000108604_63837221_63832938_392	SMARCD2	17	-	1176	19	0	1	3.32%	9.480295963264032	0.0005280076130035933	0.00047074547413106	1.6229707001080104e-06
ENSG00000108602_19745129_19738221_453	ALDH3A1	17	-	1359	1460	90	42	29.14%	630.3895649698236	3.0830000355996146e-20	4.26820705775165e-22	2.336312221601433e-40
ENSG00000106608_43879012_43876667_781	URGCP	7	-	2343	15	0	2	0.90%	3.7565968235051224	0.00847367416878415	0.027657845067597595	0.0011690047180828366
ENSG00000110321_10804047_10803521_72	EIF4G2	11	-	216	20	0	3	9.72%	54.3315207251389	0.008674846900444944	0.020779353648479937	0.000893938215340485
ENSG00000110321_10803521_10797816_650	EIF4G2	11	-	1950	98	2	1	6.00%	29.48947771050616	6.683999657642746e-08	2.5043965068970413e-08	1.681434228220179e-14
ENSG00000133318_63681637_63758201_1013	RTN3	11	+	3039	92	8	7	1.97%	17.763671929087863	0.0003207014050824132	4.2495854754003497e-05	1.0372854354069738e-07
ENSG00000094631_48802943_48824612_1160	HDAC6	X	+	3480	25	0	0	0.69%	4.215376607984915	0.005306578019052857	0.005306578019052857	0.00015100114740534233
ENSG00000113328_163439257_163442565_295	CCNG1	5	+	885	59	4	2	6.44%	39.11869492210001	0.0003001544741977667	0.00010633743604202221	2.265662457194439e-07
ENSG00000144824_111884390_111974563_1149	PHLDB2	3	+	3447	16	7	1	0.61%	2.72366892408007	0.10514110692308698	0.007258496433688866	0.004472182841009268
ENSG00000134333_18396843_18407281_332	LDHA	11	+	996	576	24	9	15.96%	339.3428957097832	1.0678239339972586e-10	4.5319795175477673e-10	5.61667038708755e-19
ENSG00000172354_100676266_100678801_340	GNB2	7	+	1020	96	10	3	9.12%	55.226392831200016	1.3190375598500069e-05	1.991941274990916e-06	2.2871148676631544e-10
ENSG00000144824_111884390_111974563_1106	PHLDB2	3	+	3318	16	7	1	0.63%	2.829562019681737	0.10514110692308698	0.007258496433688866	0.004472182841009268
ENSG00000106355_32490365_32487261_91	LSM5	7	-	273	66	6	1	21.98%	141.85900356365937	7.328263060222736e-05	5.0814294837104226e-05	2.7699931713753095e-08
ENSG00000124380_69893985_69904308_155	SNRNP27	2	+	465	14	0	0	4.52%	17.6665073841742	0.00837382380159347	0.00837382380159347	0.0003583838894600799
ENSG00000130255_5690508_5691621_105	RPL36	19	+	315	146	19	7	23.81%	271.96806945840956	7.68794642724014e-05	5.608071145489347e-06	3.6873382543846206e-09
ENSG00000254505_24213439_24209877_222	CHMP4A	14	-	666	27	3	6	5.86%	23.788395560736493	0.003897705227460077	0.012383447100975772	0.0002612565341663802
ENSG00000100906_35404608_35402653_173	NFKBIA	14	-	519	21	0	4	5.78%	23.742560501852605	0.0022011839993586477	0.013770268269996456	0.00017713700148663133
ENSG00000255508_62574838_62559679_487	AP002990.1	11	-	1461	177	0	2	1.44%	71.08838810278954	0.00877616296920843	0.00877616296920843	0.00039161135667564577
ENSG00000104518_143559815_143562904_399	GSDMD	8	+	1197	13	3	0	2.26%	6.37271972415163	0.030443420019796913	0.00308494966027208	0.0005534707948519848
ENSG00000178449_50120044_50120217_57	COX14	12	+	171	21	1	0	12.28%	72.06075380386844	0.00797940233269714	0.00797940233269714	0.000327142761693177
ENSG00000186468_82278323_82276311_123	RPS23	5	-	369	823	27	15	11.38%	1308.7270699548092	0.015447474250996157	0.009170306816233516	0.0007014192833257137
ENSG00000173540_49723726_49721752_387	GMPPB	3	-	1161	11	0	2	1.29%	5.559504446293283	0.019216965118390855	0.055414058431395546	0.004780438930026009
ENSG00000143337_179882866_179918239_462	TOR1AIP1	1	+	1386	31	0	0	3.03%	13.12423747386472	0.00043491588733509306	0.00043491588733509306	1.2464499370452549e-06
ENSG00000197912_89508547_89536981_464	SPG7	16	+	1392	13	0	0	1.29%	5.479989590380389	0.011772051206276392	0.011772051206276392	0.000681272511389508
ENSG00000092199_21231004_21211223_190	HNRNPC	14	-	570	21	2	9	4.21%	21.61822614116053	0.005642787686764809	0.04407391494785414	0.0013614158727831508
ENSG00000072274_196074120_196051942_679	TFRC	3	-	2037	120	5	2	7.36%	34.5673298280707	2.6996470015073354e-09	4.213919486488874e-10	1.2830291524692826e-17
ENSG00000101608_3253248_3255918_171	MYL12A	18	+	513	80	1	0	8.19%	91.50571911602341	0.000472567311753486	0.00047395113928563794	1.4673862973554392e-06
ENSG00000125733_6739762_6751326_593	TRIP10	19	+	1779	16	2	0	1.35%	5.2773956050050606	0.02319724882453354	0.004826937407846361	0.0006010349372949749
ENSG00000163806_28783930_28816040_184	SPDYA	2	+	552	35	4	1	8.15%	37.20528049656251	0.002405063290143513	0.0006660403445293448	1.0095678347110753e-05
ENSG00000141699_42592110_42581813_369	RETREG3	17	-	1107	13	0	0	1.63%	6.890827018798104	0.011772051206276392	0.011772051206276392	0.000681272511389508
ENSG00000117877_45406697_45409501_510	CD3EAP	19	+	1530	11	6	0	1.37%	4.218682785716668	0.25452096168535077	0.007593099278532274	0.01449162980351053
ENSG00000167460_16075707_16101346_247	TPM4	19	+	741	32	0	3	3.64%	25.340045293668023	0.0036321914711260127	0.015376780629637295	0.0003065683871243863
ENSG00000078674_17935644_18027662_1849	PCM1	8	+	5547	64	8	8	1.62%	6.770136492737698	6.092332320218848e-05	3.429381114351296e-05	1.5824631676329043e-08
ENSG00000055130_148730123_148800582_759	CUL1	7	+	2277	22	0	1	0.92%	5.669376075666668	0.0078820576498062	0.015516259750072458	0.0006142571802709984
ENSG00000168439_64186262_64204126_543	STIP1	11	+	1629	138	14	7	8.10%	49.708838851287304	2.5908658299220287e-07	3.267443732202941e-08	8.521641180983517e-14
ENSG00000228830_234607288_234607560_90	AL160408.2	1	+	270	9	0	1	6.67%	19.559347461050006	0.012178957859018494	0.02305674932606751	0.0013400639730601711
ENSG00000148843_103405181_103445549_1685	PDCD11	10	+	5055	15	2	0	0.53%	1.7411882012804158	0.01102972334328111	0.00327844299461777	0.00019922031439862552
ENSG00000124570_2959176_2948298_324	SERPINB6	6	-	972	54	1	2	5.86%	32.59891243508334	6.293293459921025e-05	0.0001311217014807635	6.040197107585146e-08
ENSG00000244005_35697701_35669622_355	NFS1	20	-	1065	24	0	0	1.97%	13.223220818738032	0.00877616296920843	0.00877616296920843	0.00039161135667564577
ENSG00000117335_207752282_207793591_376	CD46	1	+	1128	39	2	0	5.85%	20.287621036727398	7.639996631620973e-05	1.3529991062581404e-05	8.254540628586197e-09
ENSG00000100722_88563134_88611751_711	ZC3H14	14	+	2133	28	3	1	2.53%	7.702696609133616	0.0006720187443280254	0.00011503032606539441	5.526668492135103e-07
ENSG00000166747_71789479_71733058_825	AP1G1	16	-	2475	43	0	2	2.42%	10.19456897969879	3.592951532853599e-05	7.887251914198805e-05	2.1376767587154857e-08
ENSG00000214078_35632923_35632254_146	CPNE1	20	-	438	23	7	3	6.85%	30.8126706578185	0.031335693934481226	0.012925525021785452	0.0019137651600981288
ENSG00000214078_35632195_35626241_396	CPNE1	20	-	1188	69	3	0	6.82%	34.08068118213259	4.185792871158531e-06	2.392356609050559e-06	8.581251688521367e-11
ENSG00000185686_22556832_22550763_115	PRAME	22	-	345	13	0	0	4.35%	22.105666695100005	0.02108409854857798	0.02108409854857798	0.002029891263157455
ENSG00000104763_18061423_18057534_149	ASAH1	8	-	447	29	3	0	6.04%	38.06852861546645	0.0035547763465261306	0.0037104860643933249	7.338809221316118e-05
ENSG00000104763_18084058_18062204_240	ASAH1	8	-	720	68	0	0	5.83%	55.41815113964168	0.00036930943616753716	0.00036930943616753716	9.087391537321962e-07
ENSG00000089280_31189689_31191438_206	FUS	16	+	618	92	1	2	8.25%	87.3524255542039	0.0001435126345526971	0.0001619409311799793	1.6380520438280447e-07
ENSG00000089280_31180215_31185183_255	FUS	16	+	765	81	7	6	8.63%	62.12969193510001	0.00014740658983888644	0.00012777112820887222	1.3359559342551206e-07
ENSG00000078403_21534705_21741983_1048	MLLT10	10	+	3144	10	0	0	0.57%	1.8663499485734738	0.009815328628645334	0.009815328628645334	0.0004837142251238724

ENSG00000177674_11747543_11750192_97	AGTRAP	1	+	291	18	0	3	7.22%	36.29569631947424	0.00847367416878415	0.00837382380159347	0.000362422040249726
ENSG00000204592_30489532_30492581_358	HLA-E	6	+	1074	27	4	2	3.63%	14.751463168948327	0.0028093570647764778	0.0026030014435915734	4.180545240011107e-05
ENSG00000160932_143021325_143021789_110	LY6E	8	+	330	67	2	1	11.82%	119.13420726275912	0.0007373308535318079	0.0007313336786544864	3.424294031634491e-06
ENSG00000054983_87986531_87933973_512	GALC	14	-	1536	12	0	1	1.76%	4.584222061183595	0.002874854311667729	0.006306718652620871	0.00010091605209104467
ENSG00000256591_61430147_61481813_133	AP003108.2	11	+	399	12	0	0	3.76%	17.647531543804515	0.02108409854857798	0.02108409854857798	0.002029891263157455
ENSG00000144320_175995584_175929967_459	LNPK	2	-	1377	14	0	0	1.31%	5.965814040407409	0.013428347753762199	0.013428347753762199	0.0008725593497644525
ENSG00000126247_36141012_36149839_94	CAPNS1	19	+	282	134	3	3	24.47%	278.82474040220217	1.2085150412269163e-05	1.2085150412269163e-05	1.1761982275609733e-09
ENSG00000130024_169721057_169704003_451	PHF10	6	-	1353	16	0	1	1.33%	6.9390146203281615	0.013007239811447113	0.023878473930440184	0.0014709138084317821
ENSG00000162729_160094094_160091823_440	IGSF8	1	-	1320	15	3	0	1.36%	6.667959361721592	0.05321326914949129	0.013428347753762199	0.0033962782826269635
ENSG00000127554_1984714_1986028_130	GFER	16	+	390	9	2	2	3.85%	13.541086703803849	0.08350359792828288	0.04223451612662591	0.014005638704950658
ENSG00000112983_138168063_138157208_605	BRD8	5	-	1815	10	2	0	1.16%	3.2329499935619843	0.04912109528954385	0.007029302661868638	0.0018325026027891263
ENSG00000113621_134893498_134899685_161	TXNDC15	5	+	483	17	0	0	3.73%	20.65272713278572	0.013428347753762199	0.013428347753762199	0.0008725593497644525
ENSG00000182899_197950968_197955773_110	RPL35A	3	+	330	326	22	11	16.36%	579.6679338456638	9.152174643962531e-05	0.0010625902542210125	7.255013513371004e-07
ENSG00000143862_202144512_202134467_166	ARL8A	1	-	498	15	0	2	3.01%	17.67410915155121	0.02108409854857798	0.0700082515985845	0.006564739044797468
ENSG00000115525_85848138_85840144_290	ST3GAL5	2	-	870	11	0	3	1.72%	7.41906283005345	0.019216965118390855	0.13115678761539135	0.012020477827111095
ENSG00000073712_52919198_52858377_593	FERMT2	14	-	1779	19	0	0	2.02%	6.266907280943509	0.0008627354604389072	0.0008627354604389072	4.670539650158736e-06
ENSG00000136319_20305934_20295308_352	TTC5	14	-	1056	10	0	1	1.42%	5.55663280143466	0.019679754444124863	0.04223451612662591	0.003715148010809822
ENSG00000100591_77458190_77462257_122	AHSA1	14	+	366	77	0	0	19.67%	123.44834053285659	7.777249916520277e-06	7.777249916520277e-06	4.969942913148055e-10
ENSG00000136811_128471335_128500255_680	ODF2	9	+	2040	15	0	0	1.32%	4.314561939937501	0.002967235347054297	0.002967235347054297	4.9907879201100895e-05
ENSG00000127990_94656026_94587779_431	SGCE	7	-	1293	17	3	2	2.09%	7.714823824544085	0.020955738116168132	0.01292879023172146	0.0012882305750462103
ENSG00000168036_41224552_41239342_768	CTNNB1	3	+	2304	116	8	0	5.73%	29.542764394294277	8.699215501017508e-08	2.9105570714308586e-09	2.8276345500981447e-15
ENSG00000119760_27661249_27653485_363	SUPT7L	2	-	1089	15	0	2	1.93%	8.08237498390496	0.00847367416878415	0.028426896806120847	0.0012022752110812016
ENSG00000119487_125672439_125438887_441	MAPKAP1	9	-	1323	10	0	0	1.13%	4.4352261816439915	0.016947426762344633	0.016947426762344633	0.0013498980316300959
ENSG00000041982_115086281_115021157_1718	TNC	9	-	5154	12	0	0	0.41%	1.3661942347648433	0.008175610787902942	0.008175610787902942	0.00034251843268961324
ENSG00000011304_797498_810826_531	PTBP1	19	+	1593	105	2	5	6.97%	38.67667577043786	1.41009540067656e-07	3.597649406746905e-07	4.822312312539587e-13
ENSG00000117632_25904676_25885723_174	STMN1	1	-	522	111	3	13	12.07%	124.77514759635348	6.14933927678558e-05	0.00029963100012494375	1.3583429456951833e-07
ENSG00000182134_151783022_151774452_561	TDRKH	1	-	1683	9	0	0	0.89%	3.1378632290454553	0.020613416668581838	0.020613416668581838	0.001946208561389314
ENSG00000108854_64661880_64545848_748	SMURF2	17	-	2244	13	2	0	0.67%	3.3993518314659097	0.04584514077471458	0.02108409854857798	0.004277928753731027
ENSG00000162775_110339538_110342279_913	RBM15	1	+	2739	22	0	3	1.10%	4.713095773746989	0.0021880057261679567	0.007664059176943723	9.5950101060544e-05
ENSG00000131778_147266075_147295509_603	CHD1L	1	+	1809	18	0	1	1.33%	5.838611182402986	0.004873903550586949	0.015625008558487224	0.000403121801006346
ENSG00000104964_3054064_3053819_81	TLE5	19	-	243	12	2	7	6.17%	28.976811053407413	0.06556525143482543	0.17004230409153215	0.04074822827482303
ENSG00000048162_176388539_176383981_232	NOP16	5	-	696	32	5	2	7.33%	26.978410291103454	0.0019220844833477847	0.0005818042179531959	7.10946639641572e-06
ENSG00000167863_75042650_75038932_137	ATP5PD	17	-	411	75	8	3	11.68%	107.07671967728105	0.0039771035546031785	0.0009058228042885615	2.2278917471267893e-05
ENSG00000151923_119588163_119575665_336	TIAL1	10	-	1008	13	4	3	2.98%	7.567604672430061	0.021861499269432903	0.02530858902615888	0.002490672398251629
ENSG00000134851_55417158_55425452_151	TMEM165	4	+	453	20	0	0	7.28%	25.906420478211928	0.0013775385517804596	0.0013775385517804596	1.1483554366703679e-05
ENSG00000185686_22556832_22548067_509	PRAME	22	-	1527	45	1	1	3.54%	17.292153943953835	0.00011105827337505001	0.00011105827337505001	8.855184710094631e-08
ENSG00000185686_22556832_22548067_509	PRAME	22	-	1527	45	1	1	3.54%	17.292153943953835	0.00011105827337505001	0.00011105827337505001	8.855184710094631e-08
ENSG00000075790_107594044_107621953_243	BCAP29	7	+	729	22	0	0	3.29%	17.70805119930453	0.005306578019052857	0.005306578019052857	0.00015100114740534233
ENSG00000167774_8321358_8311505_113	AC010323.1	19	-	339	54	3	1	13.27%	93.46944804395578	0.00033620229360644755	0.0003354983092902608	7.562899237154315e-07
ENSG00000111780_120438384_120459959_155	AL021546.1	12	+	465	73	0	0	9.03%	92.1182170746226	0.000472567311753486	0.000472567311753486	1.46324919219222e-06
ENSG00000152556_48131382_48145708_674	PFKM	12	+	2022	36	0	0	2.97%	10.447129207682496	1.8298518738994393e-05	1.8298518738994393e-05	2.6441018379261105e-09
ENSG00000204386_31862776_31859719_415	NEU1	6	-	1245	18	1	1	2.17%	8.48357239274458	0.004911637253759624	0.004911637253759624	0.00013036481642765842
ENSG00000116337_109625404_109631152_761	AMPD2	1	+	2283	7	0	1	0.66%	1.7991515404382397	0.019216965118390855	0.04178282491678763	0.003600525600033436
ENSG00000112514_33416969_33416650_58	CUTA	6	-	174	38	1	0	15.52%	128.1474488827414	0.003710480643933249	0.003710480643933249	7.644477796348072e-05
ENSG00000089685_78214317_78223657_137	BIRC5	17	+	411	36	7	0	8.03%	51.396825445094905	0.00979775812676883	0.001565547007472977	9.216270660291179e-05
ENSG00000105193_39433667_39433273_66	RPS16	19	-	198	63	1	47	9.09%	186.7028621282046	0.017143983986795872	0.359674527872447	0.03999063535325043
ENSG00000105193_39435636_39433409_62	RPS16	19	-	186	377	11	5	27.42%	1189.3345149702986	0.00020649660536145654	0.00022454643003309613	3.1993638380828357e-07
ENSG00000105364_10252254_10258738_263	MRPL4	19	+	789	36	4	0	4.56%	26.773251277482895	0.005421488241340597	0.0009549346450480869	3.2170208058226516e-05
ENSG00000152234_46098231_46084282_531	ATP5F1A	18	-	1593	286	7	18	13.18%	105.3478978128117	8.522510213422693e-13	3.661005615873691e-12	4.0014171931586904e-23
ENSG00000006530_141555467_141652924_422	AGK	7	+	1266	17	0	0	1.66%	7.879357981939576	0.00797940233269714	0.00797940233269714	0.000327142761693177
ENSG00000115540_197540381_197550646_126	MOB4	2	+	378	13	1	0	4.76%	20.180279126480162	0.023672501042627626	0.012798402692974279	0.0014375227604752335
ENSG00000196290_200892025_200903678_350	NIF3L1	2	+	1050	29	0	0	1.71%	16.206316467727145	0.013428347753762199	0.013428347753762199	0.0008725593497644525
ENSG00000007392_229321_189198_365	LUC7L	16	-	1095	12	1	0	1.64%	6.430470398153426	0.023878473930440184	0.013007239811447113	0.0014709138084317821
ENSG00000012660_53295699_53269127_326	ELOVL5	6	-	978	56	6	1	6.44%	33.598879074196326	0.0004571839711376916	2.736941844189311e-05	1.0112062350323723e-07
ENSG00000115525_85848138_85847466_124	ST3GAL5	2	-	372	11	0	2	4.03%	17.35103403802823	0.019216965118390855	0.08350359792828288	0.0073235312725521175
ENSG00000231925_33314041_33301760_361	TAPBP	6	-	1083	70	5	3	5.54%	37.92671252835181	0.00014099745920215513	0.00011779988008061188	1.1827396126148578e-07
ENSG00000275700_36989298_37021018_116	AATF	17	+	348	8	2	1	5.17%	13.489205145551727	0.0477903522728147	0.028889785561798602	0.005865206870874011
ENSG00000244462_35655274_35652524_916	RBM12	20	-	2748	19	2	0	0.87%	4.057069888209062	0.013091824048534366	0.005554612275049112	0.00037891795282519336
ENSG00000100075_19178673_19176130_311	SLC25A1	22	-	933	52	15	3	8.04%	32.70373208921544	0.0006872564024528944	2.986453694873718e-05	1.6897879028792847e-07
ENSG00000064012_201258232_201286594_538	CASP8	2	+	1614	21	2	0	2.23%	7.634689529406136	0.003545596016926051	0.0006608884069239612	1.4927156541185968e-05
ENSG00000134313_8796836_8730720_1427	KIDINS220	2	-	4281	13	0	0	0.77%	1.781860665687807	0.0008831683242050062	0.0008831683242050062	4.885807922518899e-06
ENSG00000177054_19117250_19175960_622	ZDHHC13	11	+	1866	15	0	0	0.96%	4.716884435944535	0.013007239811447113	0.013007239811447113	0.0008218654148058079
ENSG00000111711_21501924_21515707_138	GOLT1B	12	+	414	84	2	0	8.70%	119.05689758900003	0.0032325254326216507	0.0010073743004618205	1.9853098421906118e-05
ENSG00000265241_145927378_145925882_158	RBM8A	1	-	474	13	0	0	5.06%	16.093133986939876	0.005160369442893307	0.005160369442893307	0.00014319601891785326
ENSG00000164609_160408403_160406390_206	SLU7	5	-	618	11	1	0	2.43%	10.444311751046119	0.03537493353818881	0.020613416668581838	0.003252526348827943
ENSG00000173334_125436046_125436471_141	TRIB1	8	+	423	15	0	0	4.26%	20.807816447925536	0.013428347753762199	0.013428347753762199	0.0008725593497644525
ENSG00000152223_45955164_45852467_2500	EPG5	18	-	7500	16	0	0	0.24%	1.2517982375072003	0.013007239811447113	0.013007239811447113	0.0008218654148058079

ENSG00000100567_58252240_58257855_37	PSMA3	14	+	111	20	2	0	16.22%	105.72620249216219	0.044040755831095145	0.013428347753762199	0.0027917083341200354
ENSG00000129422_17715785_17645926_415	MTUS1	8	−	1245	11	2	0	1.20%	5.184405351121688	0.08350359792828288	0.019216965118390855	0.007323531272552175
ENSG00000108055_110567817_110604302_1217	SMC3	10	+	3651	45	6	0	1.56%	7.232297746485211	0.0007151176852164741	5.6254241454829154e-05	3.092154484382156e-07
ENSG00000129422_17755807_17645926_1216	MTUS1	8	−	3648	36	5	0	1.23%	5.790596287810857	0.0037831568864232245	0.00029257101221602755	7.805446381757098e-06
ENSG00000083845_58388138_58393646_201	RPS5	19	+	603	306	10	9	12.94%	297.7691703025523	4.856335713157403e-06	3.9857150284698616e-06	1.6311501519880673e-10
ENSG00000108883_44886750_44851274_937	EFTUD2	17	−	2811	71	5	4	3.42%	14.820850263975991	1.2612321871750657e-06	3.3365429726544585e-06	3.693814629760399e-11
ENSG00000188603_28491759_28479730_253	CLN3	16	−	759	18	2	0	2.77%	13.915741276636366	0.03248889098803485	0.00847367416878415	0.0013795926531524484
ENSG00000175203_57547063_57530689_401	DCTN2	12	−	1203	34	4	0	4.24%	16.58398537844639	0.0012938644918315683	0.00011226201234273827	1.0715358491567707e-06
ENSG00000077235_27549890_27461350_2084	GTF3C1	16	−	6252	24	3	5	0.62%	2.252516022385797	0.0036543159405919446	0.0104827207134568	0.00020840451555593497
ENSG00000089006_17954069_17942357_299	SNX5	20	−	897	21	1	0	4.35%	13.73733433719231	0.001038231149674533	0.0004762745294806779	3.1862098168137684e-06
ENSG00000188186_100153490_100153964_41	LAMTOR4	7	+	123	21	0	0	14.63%	100.18202358098783	0.013640585738808999	0.013640585738808999	0.00089863692579514
ENSG00000147684_124543115_124579586_95	NDUFB9	8	+	285	18	2	2	9.47%	37.05981624198948	0.015046251877931842	0.009197853169380737	0.0006855503356692256
ENSG00000164104_173333649_173332080_209	HMGB2	4	−	627	85	4	1	8.13%	79.54758536790672	0.00024144924403469368	0.0001584151477028702	2.6622050706451913e-07
ENSG00000091073_76480516_76505601_620	DTX2	7	+	1860	8	4	0	0.81%	2.5237867691677427	0.169362073879829	0.019216965118390855	0.016163991620339734
ENSG00000174915_488205_490582_278	PTDSS2	11	+	834	14	0	0	2.16%	9.850031095492808	0.013007239811447113	0.013007239811447113	0.0008218654148058079
ENSG00000183207_48993912_49015842_463	RUVBL2	19	+	1389	67	3	1	5.18%	28.304023323765666	1.9075211966221044e-05	7.856546471177677e-06	1.2175265516164304e-09
ENSG00000134283_42335903_42441509_367	PPHLN1	12	+	1101	15	1	1	1.91%	7.994283703426432	0.013892891352654344	0.013892891352654344	0.0009300961024180418
ENSG00000126432_64318216_64321691_125	PRDX5	11	+	375	107	0	5	21.60%	167.42801426658804	2.481688024092517e-06	2.3807459474142074e-05	5.149329246776446e-10
ENSG00000086758_53654107_53584278_1672	HUWE1	X	−	5016	21	2	2	0.60%	2.4566166069500603	0.008577310921410378	0.009257958062480545	0.0004031328540372165
ENSG00000174442_66518919_66546679_471	ZWILCH	15	+	1413	25	0	0	1.70%	10.381819246841829	0.005706018193000826	0.005706018193000826	0.0001733096755673334
ENSG00000275410_37744884_37731661_293	HNF1B	17	−	879	27	3	3	2.05%	18.023972063083622	0.05989746521295916	0.07429615675292256	0.01694277415908056
ENSG00000175756_1374756_1373801_199	AURKAIP1	1	−	597	71	5	5	9.55%	69.78460651932414	0.00036318786265457023	0.0004249631561444351	1.0245332642003257e-06
ENSG00000106367_101156603_101160566_154	AP1S1	7	+	462	108	0	2	16.23%	137.1694497268442	5.812840407340944e-06	1.1130272536517737e-05	5.333791321401286e-10
ENSG00000113163_75425466_75379346_403	COL4A3BP	5	−	1209	7	3	0	1.24%	3.3974052661873455	0.169362073879829	0.019216965118390855	0.016163991620339734
ENSG00000285382_32743337_32640582_193	FO393400.1	20	−	579	8	0	0	3.11%	8.107501538259068	0.011570465654371866	0.011570465654371866	0.0006594843062896773
ENSG00000077782_38424619_38413628_545	FGFR1	8	−	1635	10	0	0	1.65%	3.588871093770643	0.001946208561389314	0.001946208561389314	2.2278545302028032e-05
ENSG00000075415_98593979_98601528_361	SLC25A3	12	+	1083	357	26	10	18.28%	193.42623389459422	9.890655008128518e-10	2.258805719105101e-11	2.8081063550720807e-19
ENSG00000134352_55960410_55941082_612	IL6ST	5	−	1836	29	1	3	1.80%	9.268318241347224	0.00196749583514115	0.00799036934523467	9.110246415414719e-05
ENSG00000134352_55976278_55959643_333	IL6ST	5	−	999	13	0	0	2.10%	7.635781291100602	0.0078820576498062	0.0078820576498062	0.00031963642907451164
ENSG00000138772_78604313_78610115_48	ANXA3	4	+	144	25	3	0	16.67%	101.87160135963543	0.032657852871826265	0.004873903550586949	0.0008714009653636729
ENSG00000078304_101761894_101925272_555	PPP2R5C	14	+	1665	12	1	0	1.26%	4.229048099686487	0.015516259750072458	0.0078820576498062	0.0006142571802709984
ENSG00000105221_40255258_40233872_419	AKT2	19	−	1257	12	4	4	1.43%	5.601722423212411	0.12894951764616974	0.10041256134757276	0.04414068170626992
ENSG00000197170_67366519_67345741_303	PSMD12	17	−	909	39	1	7	6.27%	25.175397722143572	8.076903470455387e-05	0.0008189089309249383	4.930554530553869e-07
ENSG00000127952_76030523_75996468_313	STYXL1	7	−	939	14	4	0	1.92%	8.748589918680514	0.10069662721979156	0.013007239811447113	0.0066169769657669466
ENSG00000117362_150267233_150266522_97	APH1A	1	−	291	13	0	0	6.19%	26.213558452953613	0.012798402692974279	0.012798402692974279	0.0007972436888943567
ENSG00000066044_7991725_7963483_296	ELAVL1	19	−	888	37	0	0	6.42%	24.449184326312505	5.2494528509358865e-05	5.2494528509358865e-05	2.0626862340810485e-08
ENSG00000100243_42636837_42619773_291	CYB5R3	22	−	873	47	0	1	4.81%	31.590698648431278	0.00045482414116276956	0.0006064187916096173	1.7966830332614766e-06
ENSG00000197622_151055730_151054247_79	CDC42SE1	1	−	237	9	2	0	7.59%	22.282800904993675	0.07022909110143051	0.012178957859018494	0.004215016872891795
ENSG00000071553_154428759_154429291_112	ATP6AP1	X	+	336	13	0	3	4.46%	22.702814017290184	0.020613416668581838	0.08350359792828288	0.007749259071793911
ENSG00000136010_105068721_105024424_726	ALDH1L2	12	−	2178	7	2	2	0.83%	1.8858874962444907	0.1029516053660342	0.09373101630823749	0.03388885712342489
ENSG00000100796_91509647_91458759_833	PPP4R3A	14	−	2499	17	8	1	1.68%	3.9917035634795925	0.06191469475189823	0.0005593438005584256	0.00034627387638462906
ENSG00000134644_31059467_30933211_1058	PUM1	1	−	3174	27	2	0	1.23%	4.991515892706523	0.003031355522137688	0.0005983074123552221	1.1613785618560066e-05
ENSG00000134644_31059467_30933211_1155	PUM1	1	−	3465	28	2	0	1.21%	4.741659990557577	0.0020767912373559883	0.0003763727141378971	5.1823113164462156e-06
ENSG00000149636_36771458_36752788_284	DSN1	20	−	852	22	0	0	3.17%	15.151607188137328	0.0035547763465261306	0.0035547763465261306	7.044676146010672e-05
ENSG00000134644_31059467_30933211_1156	PUM1	1	−	3468	28	2	0	1.21%	4.737558208558824	0.0020767912373559883	0.0003763727141378971	5.1823113164462156e-06
ENSG00000005187_20775896_20796972_494	ACSM3	16	+	1482	12	0	0	1.21%	4.751258492562754	0.011570465654371866	0.011570465654371866	0.0006594843062896773
ENSG00000182774_82540428_82539977_52	RPS17	15	−	156	69	9	8	15.38%	259.5374951562404	0.01200801488064321	0.0218201783429237	0.001253653100544156
ENSG00000137807_69414466_69447807_810	AC027237.1	15	+	2430	47	0	2	3.21%	11.349250995917904	2.4361580478405375e-06	1.4592695357258795e-05	3.063200117190264e-10
ENSG00000143436_151763479_151762482_122	MRPL9	1	−	366	12	5	0	5.74%	19.23870242070492	0.13318996167124125	0.007029302661868638	0.0058279945612632715
ENSG00000145050_51385352_51389089_179	MANF	3	+	537	130	10	0	13.97%	142.051126812095	2.6707307614615587e-05	5.717595954774521e-06	1.2637299361878367e-09
ENSG00000213593_57738362_57740245_172	TMX2	11	+	516	26	0	0	6.98%	29.566455464377913	0.000991050613121974	0.000991050613121974	6.098881496726224e-06
ENSG00000213593_57712619_57737933_115	TMX2	11	+	345	47	0	0	10.43%	79.93820266690001	0.0010658336519988904	0.0010658336519988904	7.014744445236256e-06
ENSG00000122218_160307171_160290157_793	COPA	1	−	2379	34	5	0	2.40%	8.386101055179068	0.00035554045139049687	5.085749902382852e-05	1.3599205713714354e-07
ENSG00000106976_128219081_128248034_528	DNM1	9	+	1584	46	2	7	2.65%	17.040340591066293	0.0005037932350194342	0.003995344569083216	1.3297243525760514e-05
ENSG00000156515_69343848_69401135_889	HK1	10	+	2667	60	7	3	2.70%	13.200909422530936	0.00020078835655311822	4.3403317049897516e-05	6.539831550755316e-08
ENSG00000154723_25729794_25724640_108	ATP5PF	21	−	324	43	6	1	12.96%	77.87517970603243	0.004732992222340745	0.000675450826132064	2.0568376277725097e-05
ENSG00000140043_73860574_73884177_327	PTGR2	14	+	981	10	4	0	1.83%	5.981451822951072	0.11368416502207135	0.011570465654371866	0.006956434060967934
ENSG00000102081_147912180_147948844_586	FMR1	X	+	1758	7	0	3	0.85%	2.3364408229923215	0.019216965118390855	0.1700871372700561	0.016246064094152867
ENSG00000184640_77482176_77498658_335	SEPT9	17	+	1005	45	3	3	3.88%	26.273750320813438	0.0029815187184188178	0.0029815187184188178	5.0367565560955816e-05
ENSG00000093167_37148983_37053851_424	LRRFIP2	3	−	1272	18	2	0	1.89%	8.303496563653304	0.018318557839637383	0.005554612275049112	0.0005316522383050482
ENSG00000062822_50398852_50417947_1107	POLD1	19	+	3321	34	2	1	1.36%	6.007387657413732	0.0009502830449746255	0.000405860922741421	2.512435789199588e-06
ENSG00000127955_80135161_80217493_354	GNAI1	7	+	1062	26	4	0	2.54%	14.365622429019778	0.015528142895806445	0.0036062891729786097	0.0003077713301577895
ENSG00000188229_137241361_137243556_445	TUBB4B	9	+	1335	169	12	9	9.89%	74.28156676218991	1.431523619142434e-08	2.3806729162700347e-08	3.486456814443742e-15
ENSG00000009307_114739890_114718169_798	CSDE1	1	−	2394	90	11	5	4.39%	22.059414429755645	9.267998481696954e-06	8.096231702821984e-07	6.8610803470884e-11
ENSG00000151353_675603_669580_112	TMEM18	2	−	336	19	0	0	8.93%	33.181035871424115	0.0021880057261679567	0.0021880057261679567	2.7877966332435854e-05
ENSG00000137693_102110849_102229940_504	YAP1	11	+	1512	17	2	4	1.98%	6.597398945195438	0.010350136863208615	0.018344283626974538	0.0009255963303532005
ENSG00000168488_28826345_28835621_778	ATXN2L	16	+	2334	50	6	2	2.19%	12.57027471789846	0.0014101810454236296	0.00012584544091628916	1.2990200430959167e-06

ENSG00000074054_121605760_121340861_1493	CLASP1	2	−	4479	13	3	0	0.67%	1.7030912055837244	0.0182999617455631	0.0014903523101034196	0.0001731805207519894
ENSG00000118482_63646552_63713708_2039	PHF3	6	+	6117	25	2	2	0.54%	2.398154421413684	0.0037346640008052404	0.0037346640008052404	7.739704037213834e−05
ENSG00000140365_75336090_75338334_91	COMMD4	15	+	273	28	0	3	5.49%	60.18260757246155	0.019679754444124863	0.08410206952409	0.007521960853378093
ENSG00000168036_41235736_41236507_88	CTNNB1	3	+	264	29	0	0	11.36%	64.45694049664206	0.0023354528714849923	0.0023354528714849923	3.158285405866755e−05
ENSG00000163507_108581486_108551149_746	CIP2A	3	−	2238	10	0	1	0.67%	2.6218964425000006	0.019679754444124863	0.04223451612662591	0.003715148010809822
ENSG00000101856_119236364_119243254_143	PGRMC1	X	+	429	31	17	3	7.69%	42.40138260787064	0.15538107353546626	0.007146612126194863	0.007162341740679877
ENSG00000156958_49201181_49328159_434	GALK2	15	+	1302	9	0	3	1.15%	4.056085879019586	0.016947426762344633	0.165899918248885	0.014398895220848395
ENSG00000175455_123948968_123914779_733	CCDC14	3	−	2199	9	0	5	0.68%	2.4015569870320608	0.019216965118390855	0.2463583386135439	0.025652408423363686
ENSG00000135452_57749009_57749422_137	TSPAN31	12	+	411	32	0	3	5.11%	45.68606706230658	0.008877961307018026	0.028462570115697007	0.0012532059812813028
ENSG00000135452_57745215_57747290_106	TSPAN31	12	+	318	12	0	2	4.72%	22.142657503075476	0.02108409854857798	0.0700082515985845	0.006564739044797468
ENSG00000272325_34392362_34288753_172	NUDT3	6	−	516	11	0	0	4.07%	12.508885004159886	0.0078820576498062	0.0078820576498062	0.00031963642907451164
ENSG00000214046_16659990_16647246_63	SMIM7	19	−	189	20	3	0	11.11%	62.09316654301589	0.05989746521295916	0.00847367416878415	0.0026479354932912483
ENSG00000108465_47971147_47981502_506	CDK5RAP3	17	+	1518	21	0	0	2.17%	8.117515744704548	0.0010860768436549261	0.0010860768436549261	7.273150737364793e−06
ENSG00000153113_96742661_96771679_422	CAST	5	+	1266	94	0	1	7.82%	43.56821472366589	1.8986597045405824e−07	4.028205529135115e−07	7.199962048671089e−13
ENSG00000138772_78554474_78582296_105	ANXA3	4	+	315	21	3	8	6.67%	39.11869492210001	0.0424352593247778	0.23213831478624525	0.04129143269519758
ENSG00000112576_41940540_41935851_195	CCND3	6	−	585	17	2	2	3.59%	17.05173881219744	0.019778610614370123	0.019778610614370123	0.0018017358906253808
ENSG00000082641_48054726_48059641_584	NFE2L1	17	+	1752	37	2	0	3.42%	12.392052329774831	0.00016625032885512364	3.297819501010363e−05	4.1806499809954696e−08
ENSG00000061273_47797459_47783841_824	HDAC7	12	−	2472	18	0	3	0.73%	4.272672989064321	0.013007239811447113	0.06272892732212154	0.003936255353260731
ENSG00000033867_27385993_27376764_129	SLC4A7	3	−	387	9	0	0	3.88%	13.646056368174422	0.016947426762344633	0.016947426762344633	0.0013498980316300959
ENSG00000204435_31666832_31668128_110	CSNK2B	6	+	330	42	5	0	11.82%	74.68114485128183	0.0033089026865655274	0.0006933285739671933	1.4508073422599898e−05
ENSG00000221823_68217104_68180963_160	PPP3R1	2	−	480	50	4	0	10.62%	61.12296081578126	0.0009026671614782853	0.00013186253945817638	8.487718741337922e−07
ENSG00000129460_23469716_23478026_315	NGDN	14	+	945	25	0	0	2.86%	15.523291635753973	0.0035547763465261306	0.0035547763465261306	7.044676146010672e−05
ENSG00000164171_53042155_53090599_1105	ITGA2	5	+	3315	33	2	0	1.81%	5.841253087915386	0.0001831509473774287	2.514927655891717e−05	3.594738051902089e−08
ENSG00000118482_63680152_63713708_1907	PHF3	6	+	5721	23	2	2	0.52%	2.359019358175931	0.006387515641543697	0.006387515641543697	0.0002146669244491845
ENSG00000143013_87332016_87344796_165	LMO4	1	+	495	25	0	0	3.03%	29.635374940984857	0.019679754444124863	0.019679754444124863	0.0017849636115922674
ENSG00000143013_87332016_87344796_165	LMO4	1	+	495	25	0	0	3.03%	29.635374940984857	0.019679754444124863	0.019679754444124863	0.0017849636115922674
ENSG00000141295_47840843_47837844_425	SCRN2	17	−	1275	23	0	0	2.59%	10.585058625980002	0.0012624143894574274	0.0012624143894574274	9.711623744831373e−06
ENSG00000068400_49002193_48974193_784	GRIPAP1	X	−	2352	9	0	0	0.77%	2.245333254457271	0.009815328628645334	0.009815328628645334	0.0004837142251238724
ENSG00000141522_81869930_81868876_249	ARHGDIA	17	−	747	178	7	3	13.65%	139.82184128782734	4.692992227922891e−07	7.839575748172191e−07	3.35938279372123e−12
ENSG00000177302_18308889_18274802_899	TOP3A	17	−	2697	6	0	1	0.56%	1.305406949569522	0.016947426762344633	0.0477903522728147	0.0036975118224100542
ENSG00000140950_84498086_84479913_456	MEAK7	16	−	1368	13	0	0	1.97%	5.576129758632677	0.002943990679779778	0.002943990679779778	4.916404313974209e−05
ENSG00000047315_57011050_57030988_591	POLR2B	4	+	1773	20	0	0	2.03%	6.619068514737734	0.0008852284070317903	0.0008852284070317903	4.90776869367864e−06
ENSG00000042753_46846085_46838447_122	AP2S1	19	−	366	43	7	1	10.66%	68.93868367419263	0.00598105518663113	0.0008767407814908394	3.307224816267442e−05
ENSG00000177156_747482_756006_74	TALDO1	11	+	222	13	0	2	9.46%	34.36101580995271	0.008175610787902942	0.03744183116399598	0.001552076439258323
ENSG00000184990_104753202_104766829_169	SIVA1	14	+	507	26	4	3	7.69%	30.091303786230775	0.003975543265698643	0.004220201682030066	9.229357116153692e−05
ENSG00000072042_67693048_67684963_205	RDH11	14	−	615	136	0	1	10.24%	129.75957340013662	2.5322361694693923e−05	2.5356142810338793e−05	4.9900691294388435e−09
ENSG00000164104_173333649_173332080_209	HMGB2	4	−	627	85	4	1	8.13%	79.54758536790672	0.00024144924403469368	0.0001584151477028702	2.6622050706451913e−07
ENSG00000197045_54483671_54478088_109	GMFB	14	−	327	10	1	1	4.59%	17.944355468853214	0.020613416668581838	0.02784981298332479	0.0025842615800075876
ENSG00000174437_110327898_110332043_63	ATP2A2	12	+	189	16	0	0	11.11%	49.674533234412706	0.00847367416878415	0.00847367416878415	0.0003665024264111182
ENSG00000183527_39180416_39175590_201	PSMG1	21	−	603	19	0	0	3.48%	18.48893541094279	0.00797940233269714	0.00797940233269714	0.000327142761693177
ENSG00000113161_75342627_75360342_881	HMGCR	5	+	2643	112	2	14	5.68%	24.86545874730534	4.238720230587428e−10	2.82457611766688e−07	1.6880877088974284e−15
ENSG00000143442_151442183_151404802_1403	POGZ	1	−	4209	12	0	7	0.50%	1.672930645278689	0.0078820576498062	0.10248820237021045	0.00461455943846696
ENSG00000013306_44323562_44320001_359	SLC25A39	17	−	1077	39	4	1	5.57%	21.248316183313374	0.00032124723708661584	5.271582575184717e−05	1.265865098096616e−07
ENSG00000131508_139614609_139626801_110	UBE2D2	5	+	330	12	6	1	4.55%	21.337469957509096	0.1530680197004018	0.03537493353818881	0.022673278827518507
ENSG00000165672_119178790_119168480_256	PRDX3	10	−	768	526	28	11	18.36%	401.88346736376184	1.6710757145558307e−07	1.089510009705908e−08	1.921675573136514e−14
ENSG00000141564_80791457_80857936_235	RPTOR	17	+	705	8	2	0	2.55%	6.658501263336172	0.08266357711566169	0.011570465654371866	0.004841719827405853
ENSG00000189067_11556730_11549637_161	LITAF	16	−	483	34	1	0	7.45%	41.30545426557144	0.0010073743004618205	0.0010155991509472172	6.342998511872531e−06
ENSG00000095059_12681562_12675838_303	DHPS	19	−	909	23	0	0	1.98%	14.84702942587954	0.013640585738808999	0.013640585738808999	0.00089863692579514
ENSG00000165533_88824720_88877410_527	TTC8	14	+	1581	17	1	1	2.09%	6.3094669229193565	0.00241548406293102	0.00241548406293102	3.368517373808125e−05
ENSG00000182220_40581066_40600832_262	ATP6AP2	X	+	786	33	0	4	7.25%	24.63581932116985	4.059835149508202e−05	0.0003895927467372698	1.2181482000366677e−07
ENSG00000182220_40599707_40605755_109	ATP6AP2	X	+	327	9	1	1	4.59%	16.149919921967893	0.03537493353818881	0.03537493353818881	0.005300298232677315
ENSG00000113643_168486499_168492922_147	RARS	5	+	441	12	3	0	3.40%	15.96681425391837	0.11497452839710681	0.020613416668581838	0.010945600217888082
ENSG00000095906_1783021_1788207_251	NUBP2	16	+	753	16	4	4	3.59%	12.46810993533068	0.05211780486661547	0.031335693934481226	0.006848817262428956
ENSG00000117395_43172118_43171388_137	EBNA1BP2	1	−	411	29	9	0	8.03%	41.40299827521534	0.01790347956991311	0.0015061527253727427	0.0001705426601949098
ENSG00000111142_95474180_95513904_455	METAP2	12	+	1365	103	1	0	8.13%	44.277204142596716	5.660376161185916e−08	4.4928943704717294e−08	2.5201450924676847e−14
ENSG00000120948_11016999_11022654_283	TARDBP	1	+	849	29	0	0	3.89%	20.043147574927566	0.001565547007472977	0.001565547007472977	1.467919054732344e−05
ENSG00000138594_51889106_51908810_200	TMOD3	15	+	600	21	0	0	4.50%	20.537314834102503	0.0032294771334464936	0.0032294771334464936	5.8663648728941325e−05
ENSG00000257923_101913334_102283090_692	CUX1	7	+	2076	10	8	0	1.01%	2.826495297839596	0.23232849479515721	0.007593099278532274	0.012747240945536078
ENSG00000156113_77403927_76877869_1024	KCNMA1	10	−	3072	9	1	2	0.49%	1.719083272943848	0.03537493353818881	0.08350359792828288	0.012068607709980346
ENSG00000103275_1314031_1324793_158	UBE2I	16	+	474	91	7	4	14.56%	112.65193790857914	4.1653514996278555e−05	4.2145511149793747e−05	1.32979772114665e−08
ENSG00000198121_110941739_110875421_206	LPAR1	9	−	618	14	0	0	3.88%	13.292760410422334	0.005160369442893307	0.005160369442893307	0.00014319601891785326
ENSG00000135148_112134853_112152791_528	TRAFD1	12	+	1584	13	0	0	1.33%	4.815748427910039	0.00797940233269714	0.00797940233269714	0.000327142761693177
ENSG00000110917_120687297_120696555_217	MLEC	12	+	651	11	0	0	2.76%	9.914876593158988	0.012798402692974279	0.012798402692974279	0.0007972436888943567
ENSG00000070367_57235746_57208609_500	EXOC5	14	−	1500	15	1	0	1.00%	5.867804238315001	0.028889785561798623	0.02108409854857798	0.002731532589007073
ENSG00000104763_18084058_18067074_175	ASAH1	8	−	525	60	0	0	4.57%	67.06061986645716	0.005160369442893307	0.005160369442893307	0.00014319601891785326
ENSG00000101444_34303270_34281034_432	AHCY	20	−	1296	144	4	0	9.95%	65.19782487016668	2.1658554247084668e−08	4.415010928776022e−09	1.0126030708792521e−15
ENSG00000175198_100102913_100530166_456	PCCA	13	+	1368	8	0	0	1.32%	3.431464466850878	0.011570465654371866	0.011570465654371866	0.0006594843062896773

ENSG00000101901_111681219_111713281_296	ALG13	X	+	888	10	3	0	1.69%	6.607887655760137	0.11497452839710681	0.020613416668581838	0.010945600217888082
ENSG00000197324_22875363_22877527_575	LRP10	14	+	1725	114	3	7	5.22%	38.77853235756001	2.5263517702349227e-06	6.418624060084663e-06	1.38399646260563e-10
ENSG00000043143_134578682_134579317_211	JADE2	5	+	633	11	0	0	2.37%	10.196816211921803	0.02108409854857798	0.02108409854857798	0.002029891263157455
ENSG00000122952_56361236_56358418_230	ZWINT	10	-	690	33	1	3	4.78%	28.063411574550006	0.00151350169064872	0.00678005960038948	6.09680973531223e-05
ENSG00000072274_196077096_196072145_80	TFRC	3	-	240	19	2	0	7.50%	46.45345021999376	0.037440157020026926	0.013640585738808999	0.0023980863030810032
ENSG00000175592_65896949_65892886_219	FOSL1	11	-	657	13	1	9	2.28%	11.610571552221463	0.028889785561798623	0.172059248067656	0.02218357037204162
ENSG00000018408_149657306_149520805_400	WWTR1	3	-	1200	52	4	3	6.25%	25.427151699365005	5.8577042392145836e-05	9.480696975793493e-06	4.517357339069053e-09
ENSG00000163125_150364901_150473334_1399	RPRD2	1	+	4197	18	0	0	0.57%	2.516570795560401	0.005554612275049112	0.005554612275049112	0.00016468457691007306
ENSG00000143374_150487451_150507064_718	TARS2	1	+	2154	7	0	0	0.84%	1.9069001702973543	0.009815328628645334	0.009815328628645334	0.0004837142251238724
ENSG00000112305_70668024_70860334_440	SMAP1	6	+	1320	16	1	0	1.14%	7.1124899858363655	0.028889785561798623	0.02108409854857798	0.002731532589007073
ENSG00000092964_26514434_26655706_641	DPYSL2	8	+	1923	164	15	4	8.58%	50.042636249800324	4.5261623346453655e-09	1.8292959401083973e-10	9.789584878907087e-18
ENSG00000104763_18061782_18057534_193	ASAH1	8	-	579	34	3	2	5.70%	34.45688153760104	0.0015061527253727427	0.00357218960850817	3.170309406106661e-05
ENSG00000049618_157174031_157207891_1286	ARID1B	6	+	3858	16	0	0	0.54%	2.433511348186626	0.00847367416878415	0.00847367416878415	0.0003665024264111182
ENSG00000104763_18071320_18064259_93	ASAH1	8	-	279	7	0	0	5.38%	14.722089486811832	0.019216965118390855	0.019216965118390855	0.0017073955890589052
ENSG00000104763_18063207_18057534_235	ASAH1	8	-	705	46	3	2	8.09%	38.28638226418299	5.488892743562522e-05	0.0001965696589835854	7.965536585833072e-08
ENSG00000143801_226883858_226895579_350	PSEN2	1	+	1050	12	0	2	2.00%	6.706061986645715	0.008175610787902942	0.03744183116399598	0.001552076439258323
ENSG00000143801_226883858_226895579_350	PSEN2	1	+	1050	12	0	2	2.00%	6.706061986645715	0.008175610787902942	0.03744183116399598	0.001552076439258323
ENSG00000080845_36500221_36527031_285	DLGAP4	20	+	855	14	0	1	2.11%	9.608100507182458	0.013428347753762199	0.02034245439037237	0.0012955184805712684
ENSG00000144029_95121791_95104524_292	MRPS5	2	-	876	22	2	0	3.77%	14.736494662434934	0.005578142294983723	0.001484234770590415	4.904388375137372e-05
ENSG00000132463_70827882_70824319_112	GRSF1	4	-	336	14	0	0	4.46%	24.449184326312505	0.02108409854857798	0.02108409854857798	0.002029891263157455
ENSG00000144048_73778345_73762661_227	DUSP11	2	-	681	11	0	0	2.20%	9.47809788861454	0.019679754444124863	0.019679754444124863	0.0017849636115922674
ENSG00000138107_102488209_102484136_141	ACTR1A	10	-	423	19	0	1	4.96%	26.35656750070568	0.008674846900444944	0.012000726946169103	0.0005220387231428462
ENSG00000103707_65029613_65003062_389	MTFMT	15	-	1167	14	0	0	1.80%	7.039353842023138	0.00847367416878415	0.00847367416878415	0.0003665024264111182
ENSG00000089685_78214317_78222851_120	BIRC5	17	+	360	36	7	0	8.33%	58.678042383150014	0.010115653169763636	0.002446120450917513	0.00014075096960426497
ENSG00000116221_54200244_54225382_483	MRPL37	1	+	1449	17	0	3	1.86%	6.88424237759524	0.002943990679779778	0.009656372839907584	0.00015814857567522978
ENSG00000148344_129752934_129739611_126	PTGES	9	-	378	57	5	1	12.70%	88.48276232379764	0.00046359495955322985	0.0002519671529549621	7.872796763391223e-07
ENSG00000105372_41860775_41871377_145	RPS19	19	+	435	553	34	48	25.52%	745.9530445490105	4.854444670314789e-07	6.585681452917502e-07	2.922004871846743e-12
ENSG00000216490_18175178_18177373_148	IFI30	19	+	444	11	0	0	3.38%	14.5373528426723	0.02108409854857798	0.02108409854857798	0.002029891263157455
ENSG00000171530_77776257_77693410_59	TBCA	5	-	177	78	4	0	16.95%	258.581203722356	0.003418871784254595	0.0025167541003031247	4.8932821474765326e-05
ENSG00000105185_32581262_32586889_65	PDCD5	19	+	195	104	0	3	9.23%	312.9495593768001	0.013640585738808999	0.024275373211574098	0.0015598167171334096
ENSG00000104904_2269599_2271445_68	OAZ1	19	+	204	25	6	0	11.76%	71.90936566562502	0.039104403811496304	0.0053557511377634245	0.0011447568947324828
ENSG00000136448_45061387_45105639_477	NMT1	17	+	1431	19	4	0	2.31%	7.790935047378409	0.019243135633285505	0.0014409783181973444	0.00017803274274212172
ENSG00000105971_116499782_116500451_113	CAV2	7	+	339	38	2	2	14.16%	65.77479677167258	0.0004405878379892636	0.0008082499550292343	2.3110431664839363e-06
ENSG00000165060_69046445_69072762_135	FXN	9	+	405	17	0	0	5.19%	24.630289395396304	0.00847367416878415	0.00847367416878415	0.0003665024264111182
ENSG00000166508_100101294_100092932_719	MCM7	7	-	2157	187	4	12	8.48%	50.87062552456677	6.995436904014321e-12	6.725559657822672e-10	6.227432617023672e-20
ENSG00000092201_21354485_21352673_142	SUPT16H	14	-	426	25	0	0	6.34%	34.43547088213029	0.003580484096903955	0.003580484096903955	7.142116083921267e-05
ENSG00000255339_100529851_100524089_172	AL133352.1	10	-	516	102	1	1	15.70%	115.99147912948258	3.0069264253400697e-06	3.0069264253400697e-06	7.738804226946091e-11
ENSG00000126756_47658927_47651842_157	UXT	X	-	471	46	1	0	9.55%	57.30764224256689	0.00036124044826812014	0.00030731120605623235	7.450628969252732e-07
ENSG00000137936_93584078_93562241_501	BCAR3	1	-	1503	18	1	0	2.00%	7.027310465047906	0.0033728746429155087	0.0022409900782254734	4.330926822416902e-05
ENSG00000114796_183650441_183679286_572	KLHL24	3	+	1716	8	0	0	0.87%	2.735573071475525	0.019679754444124863	0.019679754444124863	0.0017849636115922674
ENSG00000172340_67654586_67408957_379	SUCLG2	3	-	1137	23	1	0	2.90%	11.869788696679421	0.0024673358700277245	0.0013845019718837994	2.032715794165102e-05
ENSG00000197622_151055730_151054247_79	CDC42SE1	1	-	237	9	2	0	7.59%	22.282800904993675	0.07022909110143051	0.012178957859018494	0.004215016872891795
ENSG00000166130_98644701_98625930_377	IKBIP	12	-	1131	35	0	2	2.65%	18.15854538824271	0.0024321290225875155	0.005479012991939057	7.531219954511901e-05
ENSG00000059588_234457720_234391577_1065	TARBP1	1	-	3195	9	0	0	0.47%	1.652902602342254	0.016947426762344633	0.016947426762344633	0.0013498980316300959
ENSG00000136875_113275744_113291660_522	PRPF4	9	+	1566	34	5	0	3.07%	12.739804859687743	0.0020671763086911954	0.0001837862173985288	2.7241879608450814e-06
ENSG00000078808_1228772_1218621_247	SDF4	1	-	741	26	8	5	4.86%	20.588786801105268	0.03038145671462883	0.009915671890931099	0.001473559784796807
ENSG00000164120_174508711_174491956_131	HPGD	4	-	393	37	1	0	8.40%	55.243958477774825	0.001588175367824666	0.0015806110104534784	1.5019944328400419e-05
ENSG00000188554_43186415_43210074_842	NBR1	17	+	2526	17	3	1	1.43%	3.9490368983117587	0.009745921382577108	0.001620025777606367	9.441403448178248e-05
ENSG00000119616_74723298_74734930_92	FCF1	14	+	276	16	0	3	7.61%	34.01625645400001	0.0078820576498062	0.059202726829632964	0.002466792732126766
ENSG00000137106_37422751_37434257_358	GRHPR	9	+	1074	77	10	5	7.82%	42.06898755588968	6.788822833318642e-05	9.555346279138338e-06	5.293673025681837e-09
ENSG00000168488_28829425_28831031_163	ATXN2L	16	+	489	10	2	4	3.07%	11.999599669355831	0.051235217429874684	0.17004230409153215	0.03367890709093125
ENSG00000111481_54325164_54350523_154	COPZ1	12	+	462	95	4	1	16.88%	120.65831225972406	6.916003708982932e-06	4.643833617878754e-06	2.680756807485722e-10
ENSG00000076043_114439529_114440757_82	REXO2	11	+	246	15	8	2	9.76%	35.77929413606708	0.1539609423056087	0.02389626957043539	0.016983116543564356
ENSG00000196968_73772334_73775685_492	FUT11	10	+	1476	11	0	0	1.22%	4.373024838852643	0.012798402692974279	0.012798402692974279	0.0007972436888943567
ENSG00000131051_35740847_35704481_521	RBM39	20	-	1563	31	1	0	2.69%	11.637999448993284	0.0006179331360801681	0.0004168743852437731	1.684212386012298e-06
ENSG00000152700_134612730_134606950_130	SAR1B	5	-	390	54	0	0	13.08%	81.2465202228231	0.00013106574077567133	0.00013106574077567133	1.221428801656259e-07
ENSG00000067334_93879148_93872194_601	DNTTIP2	1	-	1803	35	1	1	2.50%	11.390634960678039	0.000405860922741421	0.000405860922741421	1.0905817698103266e-06
ENSG00000183853_157993677_158095120_757	KIRREL1	1	+	2271	37	2	4	2.11%	9.560050938690226	0.0008752860098322594	0.0018879002145992226	1.0178160117136353e-05
ENSG00000148690_93700106_93669826_315	FRA10AC1	10	-	945	12	6	0	1.59%	7.4511799851619065	0.1432110113889294	0.016947426762344633	0.01210505737557178
ENSG00000144674_37296123_37366103_1989	GOLGA4	3	+	5967	17	0	4	0.35%	1.6717390992350432	0.008674846900444944	0.052998774212472885	0.00236139011758887
ENSG00000132470_75731932_75739898_276	ITGB4	17	+	828	31	0	0	4.71%	21.968832293208337	0.0006652407155885319	0.0006652407155885319	2.8305411988850537e-06
ENSG00000104613_19820468_19851805_580	INTS10	8	+	1740	17	0	1	1.21%	5.732912186859484	0.00797940233269714	0.015625008558487224	0.0006253789790324684
ENSG00000078747_34393812_34507794_830	ITCH	20	+	2490	16	6	0	0.72%	3.7704766189975913	0.10041256134757276	0.013007239811447113	0.006595870280092993
ENSG00000055130_148756998_148800582_666	CUL1	7	+	1998	26	0	0	1.20%	7.635781291100602	0.005604866691947602	0.005604866691947602	0.00016752456572620382
ENSG00000146909_156949801_156969703_839	NOM1	7	+	2517	16	0	0	0.72%	3.7300305050870093	0.013428347753762199	0.013428347753762199	0.0008725593497644525
ENSG00000231925_33314041_33301760_466	TAPBP	6	-	1398	75	5	3	4.94%	31.479636471647	4.402326033587937e-05	3.727999369941126e-05	1.24584200824792e-08
ENSG00000168216_69790322_69705581_244	LMBRD1	6	-	732	13	2	0	2.46%	10.420963811215167	0.060667625179241055	0.011772051206276392	0.0035017256847258756

ENSG00000256861_122263600_122218375_449	AC048338.1	12	-	1347	18	1	1	1.78%	7.841163792848554	0.005454749182134642	0.0069659089849165145	0.00020098479551741233
ENSG00000256861_122218361_122208381_166	AC048338.1	12	-	498	17	0	0	3.61%	20.030657038424703	0.013007239811447113	0.013007239811447113	0.0008218654148058079
ENSG00000129562_22589157_22575158_65	DAD1	14	-	195	60	73	2	21.54%	180.54782271738466	0.305456048541467	0.0010927575603436111	0.005765461814671 7575
ENSG00000178209_143935297_143916177_4341	PLEC	8	-	13023	130	33	39	1.38%	5.857441073339094	2.796321955810114e-06	1.2083355211512743e-05	2.8801264455689585e-10
ENSG00000124067_67957957_67945046_925	SLC12A4	16	-	2775	9	0	1	0.54%	1.9030716448589193	0.019216965118390855	0.04178282491678763	0.003600525600033436
ENSG00000160783_156213136_156239601_167	PMF1	1	+	501	26	0	0	5.39%	30.45167868187426	0.0035291660242640502	0.0035291660242640502	6.948232509684344e-05
ENSG00000160783_156232372_156239601_134	PMF1	1	+	402	29	0	0	8.21%	42.32993107242165	0.001484234770590415	0.001484234770590415	1.3251387882263029e-05
ENSG00000260238_156232372_156243083_149	PMF1-BGLAP	1	+	447	8	0	2	3.36%	10.501663066335572	0.019216965118390855	0.09718295540155453	0.008620840042435133
ENSG00000160783_156232372_156239587_109	PMF1	1	+	327	8	0	5	4.59%	14.355484375082572	0.019216965118390855	0.28185143082538655	0.030596495671179075
ENSG00000113448_58993456_58974664_499	PDE4D	5	-	1497	37	1	0	3.61%	14.502922967111227	0.0001185681716428482	8.091994981866961e-05	6.953223258948248e-08
ENSG00000182220_40581066_40598678_186	ATP6AP2	X	+	558	16	0	0	6.45%	16.82524512778495	0.0006577637618416096	0.0006577637618416096	2.769528725862714e-06
ENSG00000182220_40600774_40605755_100	ATP6AP2	X	+	300	9	1	0	5.00%	17.603412714945005	0.03537493353818881	0.020613416668581838	0.003252526348827943
ENSG00000079246_216109437_216205202_732	XRCC5	2	+	2196	126	12	1	5.46%	33.66772923623361	1.9552876373210196e-06	1.7040353875419224e-08	3.843329887844177e-13
ENSG00000196655_119019284_119023399_79	TRAPPC4	11	+	237	26	0	1	11.39%	64.3725359477595	0.0034782380676658805	0.005588370640926919	0.00010676685949938237
ENSG00000113448_58993456_58974664_499	PDE4D	5	-	1497	37	1	0	3.61%	14.502922967111227	0.0001185681716428482	8.091994981866961e-05	6.953223258948248e-08
ENSG00000138078_44339308_44321356_458	PREPL	2	-	1374	18	0	0	3.06%	7.687079788185591	0.00024035368145103084	0.00024035368145103084	3.9581571392385245e-07
ENSG00000171603_9823733_9730508_971	CLSTN1	1	-	2913	89	7	3	3.09%	17.927723213526782	1.317816552236354e-05	2.199315159844212e-06	2.5077037963359434e-10
ENSG00000183010_81936814_81933214_319	PYCR1	17	-	957	30	8	1	4.08%	18.39437065302508	0.02387747459605924	0.0008605146384484945	0.0001494637719288805
ENSG00000137449_15004186_15066380_492	CPEB2	4	+	1476	11	1	0	1.42%	4.373024838852643	0.016854695534768073	0.007029302661868638	0.0006017499134389055
ENSG00000083444_11972929_11974808_74	PLOD1	1	+	222	16	0	0	6.76%	42.29048099686487	0.02108409854857798	0.02108409854857798	0.002029891263157455
ENSG00000123213_65780203_65822915_510	NLN	5	+	1530	23	2	1	2.16%	8.820882188316668	0.004981126742780332	0.002568463469732168	7.205037994814023e-05
ENSG00000182473_76101342_76083648_547	EXOC7	17	-	1641	9	0	0	0.91%	3.218174170922304	0.019679754444124863	0.019679754444124863	0.0017849636115922674
ENSG00000172262_43122054_43175133_589	ZNF131	5	+	1767	7	1	0	1.02%	2.3245404452860785	0.028889785561798602	0.009815328628645334	0.0013877953586383081
ENSG00000145740_69094256_69129617_765	SLC30A5	5	+	2295	35	2	7	1.70%	8.948721060611113	0.002390058766287232	0.017208615801730855	0.00024085063808575258
ENSG00000168000_62705595_62702488_118	BSCL2	11	-	354	14	0	6	5.08%	23.206005462262716	0.013428347753762199	0.16970534604944526	0.012520549700299675
ENSG00000168002_62761649_62766507_172	POLR2G	11	+	516	101	0	5	12.79%	114.85430776546805	1.881309885574092e-05	0.00012539290996078527	1.8621867514955183e-08
ENSG00000073712_52950547_52858377_680	FERMT2	14	-	2040	35	0	1	2.50%	10.067311193187502	0.00011297380978831068	0.00018702083254192264	1.498932361695684e-07
ENSG00000110713_3695538_3676159_441	NUP98	11	-	1323	13	1	0	1.59%	5.76579403613719	0.013794764262559246	0.00837382380159347	0.0005783443517979597
ENSG00000110713_3782117_3696763_1348	NUP98	11	-	4044	26	2	0	0.89%	3.7725744361075675	0.003291134468783262	0.0010155991509472172	2.036736926868007e-05
ENSG00000176014_12308293_12311100_107	TUBB6	18	+	321	16	1	0	4.67%	29.247622371663557	0.03577298629450316	0.02108409854857798	0.003354604907114977
ENSG00000167460_16076566_16088101_152	TPM4	19	+	456	26	10	5	6.58%	33.45677855179606	0.07171658053824743	0.019439702257615755	0.0062963105272569805
ENSG00000143575_154273565_154275701_185	HAX1	1	+	555	10	1	0	3.24%	10.572620249216218	0.026832695913080365	0.011772051206276392	0.0015103817605807263
ENSG00000172809_74203956_74209829_67	RPL38	17	+	201	112	23	11	20.90%	326.96222621456724	0.004783851601676504	0.0059448713639815316	0.0001526140224882 5746
ENSG00000134910_125595916_125620810_705	STT3A	11	+	2115	151	9	1	6.24%	41.89307044849008	5.8747299195070665e-08	3.515628446861874e-09	2.25155047665 1329e-15
ENSG00000122705_36191057_36211774_248	CLTA	9	+	744	177	11	4	11.69%	139.59695566959076	2.048454080571646e-05	4.711542246598215e-06	8.047163936813439e-10
ENSG00000100600_92732786_92716132_135	LGMN	14	-	405	41	0	5	7.41%	59.402462659485195	0.0021227925778456486	0.0231829132189225	0.00030131201280442655
ENSG00000168056_65548055_65539080_733	LTBP3	11	-	2199	25	14	2	1.64%	6.670991630644613	0.10177692382604403	0.0018731751028398648	0.0015949641217173221
ENSG00000092020_35108148_35085590_289	PPP2R3C	14	-	867	9	0	0	2.08%	6.09114626814706	0.012178957859018494	0.012178957859018494	0.0007262454987071801
ENSG00000074416_127821818_127692198_303	MGLL	3	-	909	19	2	0	3.30%	12.264937351813535	0.01044836432889887	0.002046028879997488	0.00012444751772694397
ENSG00000012061_45423374_45409675_273	ERCC1	19	-	819	13	9	0	1.83%	9.313974981452382	0.1973134331215476	0.02108409854857798	0.02074155215028262
ENSG00000100644_61695805_61746939_735	HIF1A	14	+	2205	154	14	6	7.76%	40.98148991839049	2.6102285755461856e-08	1.36035889603921e-10	4.6327448982099324e-17
ENSG00000221983_18573301_18575150_128	UBA52	19	+	384	33	4	0	9.38%	50.426442673019544	0.00236147777460975	0.0009589057725548893	1.3840982015979587e-05
ENSG00000129515_34604190_34563122_294	SNX6	14	-	882	58	4	2	8.84%	38.58646778030273	3.0908511880131716e-05	4.528101658053882e-06	1.1787236985709098e-09
ENSG00000166228_70888533_70883950_104	PCBD1	10	-	312	49	4	0	10.58%	92.15461784533176	0.005689958798670841	0.0015806110104534784	5.2950781800948645e-05
ENSG00000115355_55362358_55291711_1650	CCDC88A	2	-	4950	20	1	0	0.61%	2.3708299952787883	0.003386928046346 1717	0.0022543491824521134	4.372784971036962e-05
ENSG00000130703_62263635_62293887_413	OSBPL2	20	+	1239	11	0	2	1.21%	5.209511430303875	0.019679754444124863	0.10105102209468636	0.009164497669452725
ENSG00000278540_37263858_37087316_1998	ACACA	17	-	5994	42	1	5	0.90%	4.111574541361863	0.00012278702112867795	0.0003211449410709515	2.774801896023291e-07
ENSG00000136930_124415425_124353598_277	PSMB7	9	-	831	112	12	2	13.72%	79.08472619630327	2.7409551884129724e-06	3.5629092949117514e-08	1.0781380199155845e-12
ENSG00000141424_36127007_36109593_755	SLC39A6	18	-	2265	35	1	1	2.25%	9.067247167374175	0.00018129710408843058	0.00018129710408843058	2.2919265383054332e-07
ENSG00000127947_77537643_77639280_748	PTPN12	7	+	2244	12	0	1	1.20%	3.1378632290454553	0.002874854311667729	0.006306718652620871	0.00010091605209104467
ENSG00000124226_49945442_49952141_111	RNF114	20	+	333	16	0	0	5.41%	28.193653997909916	0.013007239811447113	0.013007239811447113	0.0008218654148058079
ENSG00000005108_11831946_11375794_1657	THSD7A	7	-	4971	20	0	2	0.72%	2.360814418955945	0.0007764301431553706	0.003276402312815604	1.5900255387266077e-05
ENSG00000130731_636290_634526_205	METTL26	16	-	615	13	4	0	3.41%	12.403488633836588	0.1117471228039768	0.007029302661868638	0.004698816577020771
ENSG00000064607_19010212_18994993_432	SUGP2	19	-	1296	14	0	1	1.85%	6.338677417932872	0.004193160480899131	0.010499663139468709	0.00023613604789861 67
ENSG00000171421_1798911_1798624_95	MRPL36	5	-	285	9	0	1	5.26%	18.52990812099474	0.019679754444124863	0.06539853090342929	0.005779266745814885
ENSG00000130731_636290_634526_185	METTL26	16	-	555	13	4	0	3.78%	13.744406323981083	0.1117471228039768	0.007029302661868638	0.004698816577020771
ENSG00000138796_107990044_108034357_280	HADH	4	+	840	36	0	0	4.64%	25.147732449921435	0.0005460508606305346	0.0005460508606305346	1.9341343681665648e-06
ENSG00000100348_36480837_36467804_166	TXN2	22	-	498	11	1	0	4.82%	12.96101337780422	0.009847127393243164	0.004687384229717438	0.0002449884871127 1347
ENSG00000172922_65720758_65719783_164	RNASEH2C	11	-	492	13	0	0	3.05%	15.504360792295735	0.020613416668581838	0.020613416668581838	0.001946208561389314
ENSG00000122545_35832669_35904293_417	SEPT7	7	+	1251	26	10	3	1.68%	12.195276594419667	0.10581804224798252	0.024135905425855194	0.011311393888965189
ENSG00000122545_35873720_35904293_285	SEPT7	7	+	855	20	7	0	1.75%	13.725857867403512	0.13072302616481507	0.02108409854857798	0.01284698802284646
ENSG00000138029_26261021_26289953_452	HADHB	2	+	1356	53	3	1	4.87%	22.93463308485952	9.170783746108725e-05	2.2867465441111084e-05	1.622961324585564e-08
ENSG00000147533_41490855_41507106_137	GOLGA7	8	+	411	12	2	0	4.38%	17.13227514836497	0.03537493353818881	0.012798402692974279	0.002143354119340 2405
ENSG00000108179_79347549_79351609_145	PPIF	10	+	435	24	4	1	6.21%	32.374092349324144	0.01007916461776185	0.0033030736763512156	0.00018301566053 7603
ENSG00000138413_208239983_208237079_124	IDH1	2	-	372	77	1	2	14.52%	121.4572382661976	0.00012089867747187387	0.0002751182007047 6035	2.34330923074983e-07
ENSG00000169710_82096352_82079143_2478	FASN	17	-	7434	233	37	35	3.75%	18.391153988800045	1.06586491676882e-12	1.0527222552373566e-11	1.4437785381077121e-22
ENSG00000178252_49011688_49015288_1070	WDR6	3	+	3210	21	0	2	1.03%	3.838750436280842	0.0013984928336429232	0.0067530256680052286	5.6613811115239e-05

ENSG00000269893_118279412_118279714_36	SNHG8	4	+	108	22	5	0	16.67%	119.52934559530559	0.013428347753762199	0.013217226988443315	0.0008596769774033651
ENSG00000154305_222621164_222645775_1186	MIA3	1	+	3558	16	0	3	0.76%	2.6386978025025303	0.0025000845279814113	0.025100479631792585	0.0003764481700398674
ENSG00000077684_128831759_128872262_830	JADE1	4	+	2490	10	0	2	0.84%	2.3565478868734946	0.007029302661868638	0.04912109528954385	0.0018325026027891263
ENSG00000130255_5690526_5691621_99	RPL36	19	+	297	144	19	7	24.24%	284.4995994334546	0.00010977385607262625	8.295996882913001e-06	7.651396897599448e-09
ENSG00000182473_76101342_76083648_620	EXOC7	17	-	1860	9	0	0	0.81%	2.8392601153137105	0.019679754444124863	0.019679754444124863	0.0017849636115922674
ENSG00000139163_22625164_22644304_258	ETNK1	12	+	774	11	2	0	2.33%	8.339256669439925	0.053594824633710306	0.011772051206276392	0.003068672569093115
ENSG00000171298_80104587_80119331_952	GAA	17	+	2856	25	0	2	1.47%	5.136383261830359	0.0003981150787954058	0.0019055409341788827	4.989136850098823e-06
ENSG00000049860_74713551_74721175_284	HEXB	5	+	852	78	6	2	10.21%	53.71933457612325	3.978514080622567e-06	3.7939054598731192e-06	1.2782087366311134e-10
ENSG00000127990_94656026_94585499_441	SGCE	7	-	1323	16	3	2	1.81%	7.096361890630387	0.030785654306124375	0.019504170898671437	0.002706393878223981
ENSG00000101347_36919456_36904145_251	SAMHD1	20	-	753	8	0	0	1.99%	6.23405496766534	0.019216965118390855	0.019216965118390855	0.0017073955890589052
ENSG00000085662_134447435_134442728_87	AKR1B1	7	-	261	147	4	3	20.69%	330.4855260660173	9.460772736956512e-05	0.0002592321843555918	1.752782133501671e-07
ENSG00000085662_134459062_134447929_263	AKR1B1	7	-	789	1364	64	22	19.39%	1014.408742846852	2.5082608676805146e-09	9.085155830797446e-10	2.5087689513879232e-17
ENSG00000038382_14387570_14508422_1863	TRIO	5	+	5589	16	2	0	0.32%	1.6798151335308646	0.023857440118675605	0.013007239811447113	0.00146964612208996
ENSG00000167642_38264893_38292006_195	SPINT2	19	+	585	132	3	10	14.36%	132.4017366594154	1.8390032344988886e-06	1.7572843939176264e-05	2.84935429741873e-10
ENSG00000127990_94639350_94587779_413	SGCE	7	-	1239	16	3	2	1.94%	7.577471171351092	0.030785654306124375	0.019504170898671437	0.002706393878223981
ENSG00000133835_119479012_119541994_532	HSD17B4	5	+	1596	38	0	0	2.82%	13.970962472178575	0.00029563251185049877	0.00029563251185049877	5.909204346842462e-07
ENSG00000284946_90984125_90981472_179	AC068831.7	15	-	537	29	0	2	5.59%	31.688328288851963	0.0024042815392806777	0.006881839805563927	9.362814707606752e-05
ENSG00000284946_91009831_90984800_475	AC068831.7	15	-	1425	11	0	0	1.47%	4.529533096243159	0.0078820576498062	0.0078820576498062	0.00031963642907451164
ENSG00000124762_36684102_36685800_164	CDKN1A	6	+	492	29	2	8	6.71%	34.58665099819818	0.007205323565060537	0.012371379225907928	0.00045344217813502347
ENSG00000136279_44044738_44060916_430	DBNL	7	+	1290	13	2	1	1.63%	5.913291092875583	0.022898969405409888	0.013599199788808391	0.001469595755550733
ENSG00000131828_19350021_19351439_82	PDHA1	X	+	246	10	1	0	6.10%	23.852862757378055	0.03537493353818881	0.020613416668581838	0.003252526348827943
ENSG00000077942_45518729_45563266_641	FBLN1	22	+	1923	32	2	2	1.56%	9.764416829229331	0.004781414996256699	0.004781414996256699	0.00012387271853120556
ENSG00000124155_45420349_45425826_343	PIGT	20	+	1029	52	2	0	3.79%	29.65265504299126	0.0022083206986541785	0.0006768518708446028	9.39609878359303e-06
ENSG00000136942_124861912_124858469_96	RPL35	9	-	288	35	3	2	8.33%	71.3101209517448	0.012070269314219385	0.008808686117259405	0.0005324252474387162
ENSG00000159352_151266385_151267343_97	PSMD4	1	+	291	11	0	0	5.15%	22.180703306345364	0.020613416668581838	0.020613416668581838	0.001946208561389314
ENSG00000163755_149129724_149172222_1004	HPS3	3	+	3012	12	5	0	0.70%	2.3377706128745026	0.13995080206115273	0.0078820576498062	0.006732128388594889
ENSG00000163762_149333382_149321478_201	TM4SF18	3	-	603	141	1	3	11.94%	137.20736278647018	8.163791924687944e-06	2.678104035760154e-05	1.7742024011977063e-09
ENSG00000071127_10088363_10077116_377	WDR1	4	-	1131	74	1	0	5.57%	38.3923531065703	2.857956701709019e-05	2.425964890545029e-05	5.379934573821803e-09
ENSG00000134330_9481342_9487473_80	IAH1	2	+	240	14	0	0	6.25%	34.22885805683751	0.02108409854857798	0.02108409854857798	0.002029891263157455
ENSG00000115966_175121492_175074609_357	ATF2	2	-	1071	16	1	0	1.40%	8.766094100190479	0.03577298629450316	0.02108409854857798	0.003354604907114977
ENSG00000006625_30504709_30497819_166	GGCT	7	-	498	20	5	1	3.01%	23.565478868734946	0.13159953908062888	0.019679754444124863	0.012285429850268035
ENSG00000138032_44201305_44230001_345	PPM1B	2	+	1035	23	1	1	2.32%	13.039564974033336	0.0073129010928764775	0.0073129010928764775	0.0002773765668225625
ENSG00000204843_74370732_74361499_961	DCTN1	2	-	2883	37	9	0	1.87%	7.530654069290844	0.004706327301402772	8.409124798936147e-05	3.451299602800358e-06
ENSG00000155438_121736850_121727724_293	NIFK	2	-	879	38	0	6	3.41%	25.367071792488062	0.0023354528714849923	0.012600507772939716	0.0001698008312845689
ENSG00000072501_53415155_53380103_1192	SMC1A	X	-	3576	29	4	1	1.09%	4.758566076933306	0.0032995531421861958	0.0009306875619951529	1.8864209682888326e-05
ENSG00000100938_24233254_24238778_348	GMPR2	14	+	1044	11	2	1	1.72%	6.182552358377875	0.0319825310473083	0.019795881618345192	0.0028454869840150642
ENSG00000134900_102634053_102678316_813	TPP2	13	+	2439	18	3	0	0.74%	4.330482832704798	0.06272892732212154	0.013007239811447113	0.003936255353260731
ENSG00000171552_31721972_31665949_88	BCL2L1	20	-	264	12	0	1	6.82%	26.671837446886368	0.012798402692974279	0.03537493353818881	0.0021433541193402405
ENSG00000136108_52455557_52475141_682	CKAP2	13	+	2046	25	2	2	1.47%	7.169848776044723	0.011698770487832223	0.011698770487832223	0.0006733142386616558
ENSG00000105953_44624344_44707937_1001	OGDH	7	+	3003	46	7	2	2.70%	8.988311520562439	0.0002159190192892263	9.081675197610674e-06	1.696968813507591e-08
ENSG00000160214_43797450_43803774_311	RRP1	21	+	933	6	0	0	1.61%	3.773507548755628	0.016947426762344633	0.016947426762344633	0.0013498980316300959
ENSG00000162783_181088903_181089886_327	IER5	1	+	981	14	2	6	2.75%	8.374032552131501	0.01288881088065249	0.10237214469055689	0.006694625147298221
ENSG00000135521_143843478_143863527_475	LTV1	6	+	1425	29	0	3	3.16%	11.941496344641056	0.0002582331582969843	0.0008550837723306223	1.4847714728639393e-06
ENSG00000044115_138810106_138934089_783	CTNNA1	5	+	2349	99	8	5	6.26%	24.7302094335115	3.586508589667153e-08	3.178181444471347e-09	1.2335735376655032e-15
ENSG00000023697_15911384_16036746_275	DERA	12	+	825	44	0	2	5.82%	31.294955937680008	0.00019829743686022551	0.0004540917148719461	6.152641120077487e-07
ENSG00000116473_111697468_111709235_133	RAP1A	1	+	399	28	1	0	9.02%	41.177573602210536	0.0019464877837825903	0.0010197273511756433	1.2094964810796119e-05
ENSG00000104660_30101945_30104922_134	LEPROTL1	8	+	402	18	0	1	5.97%	26.273750320813438	0.004873903550586949	0.01007916461776185	0.0002597390186955864
ENSG00000153774_75433352_75293952_299	CFDP1	16	-	897	41	0	0	3.01%	26.820509896423083	0.003684287313187465	0.003684287313187465	7.541963121699725e-05
ENSG00000123066_115997159_115961266_1330	MED13L	12	-	3990	15	0	0	0.45%	2.2059414429755644	0.012798402692974279	0.012798402692974279	0.0007972436888943567
ENSG00000145191_184135386_184144943_721	EIF2B5	3	+	2163	21	1	0	1.39%	5.696897318752429	0.005670089768331009	0.0022409900782254734	7.232548112602907e-05
ENSG00000137700_119029267_119024910_395	SLC37A4	11	-	1185	17	0	0	2.28%	8.417947008553167	0.0034782380676658805	0.0034782380676658805	6.758309314600117e-05
ENSG00000164144_152829634_152910219_373	ARFIP1	4	+	1119	13	2	0	1.88%	6.816930750500002	0.022898969405409888	0.008175610787902942	0.0009353765476651278
ENSG00000171530_77776257_77691418_131	TBCA	5	-	393	104	4	0	12.21%	155.280315721313	0.0003397374742370762	0.00021123378834695988	4.901340339131649e-07
ENSG00000122783_135168814_135167772_33	CYREN	7	-	99	10	0	0	18.18%	59.270749881969714	0.009815328628645334	0.009815328628645334	0.0004837142251238724
ENSG00000140262_56919935_57282587_699	TCF12	15	+	2097	16	0	0	1.29%	4.477103853745351	0.002967235347054297	0.002967235347054297	4.9907879201100895e-05
ENSG00000168679_110389323_110363766_439	SLC16A4	1	-	1317	43	4	1	2.96%	19.158358560937362	0.002591458187623629	0.0008128177682286688	1.3056590898592814e-05
ENSG00000286070_24557675_24572026_62	GGT1	22	+	186	21	2	5	8.06%	66.24940269065324	0.02108409854857798	0.10267143333503442	0.009843169644325755
ENSG00000215021_6970543_6970331_70	PHB2	12	-	210	18	2	3	7.14%	50.29546489984287	0.0391240932165479	0.055917760918677194	0.008906759045815992
ENSG00000073060_124863630_124782683_451	SCARB1	12	-	1353	49	1	2	4.21%	21.250732274754995	8.068268196270137e-05	0.00018238551396083748	1.0616351244808664e-07
ENSG00000145604_36152868_36182031_389	SKP2	5	+	1167	10	0	0	1.54%	5.028109887159384	0.012798402692974279	0.012798402692974279	0.0007972436888943567
ENSG00000152795_82429324_82426059_298	HNRNPDL	4	-	894	114	3	1	11.41%	74.82434934764096	2.8068292164810636e-07	1.8169410532370092e-07	4.81845735322702e-13
ENSG00000131446_180792971_180791634_445	MGAT1	5	-	1335	9	0	0	1.57%	3.9558230820101135	0.006937202941512724	0.006937202941512724	0.00025101522741487095
ENSG00000182774_82540428_82538222_136	RPS17	15	-	408	146	9	9	11.03%	209.97534774362504	0.0006000802399128133	0.0014566623805867178	5.53662286405284e-06
ENSG00000132716_160240344_160217592_572	DCAF8	1	-	1716	12	0	0	1.40%	4.103359607213288	0.004826937407846361	0.004826937407846361	0.00012612451166366307
ENSG00000135926_218281949_218275475_247	TMBIM1	2	-	741	15	0	0	2.83%	11.878146231406886	0.00847367416878415	0.00847367416878415	0.0003665024264111182
ENSG00000145901_151056834_151030713_450	TNIP1	5	-	1350	10	0	0	1.11%	4.346521658011112	0.019679754444124863	0.019679754444124863	0.0017849636115922674
ENSG00000113273_78985248_78885529_398	ARSB	5	-	1194	11	0	0	1.76%	5.405849800792715	0.007029302661868638	0.007029302661868638	0.0002573639808771639

ENSG00000115525_85863399_85846391_175	ST3GAL5	2	-	525	12	1	2	3.43%	13.41212397329143	0.026602764905793736	0.06032271266817251	0.006860364960240205
ENSG00000104341_97775737_97851474_317	LAPTM4B	8	+	951	100	10	4	9.46%	61.70141154905364	6.151209471659141e-05	4.618783793986918e-06	2.445751372201625e-09
ENSG00000115415_190999658_190975808_512	STAT1	2	-	1536	25	3	4	2.34%	9.550462627465823	0.004220201682030066	0.00799021973021164	0.00018113708436900733
ENSG00000102978_57462725_57469775_150	POLR2C	16	+	450	15	0	0	4.67%	19.559347461050006	0.0078820576498062	0.0078820576498062	0.00031963642907451164
ENSG00000179958_30429980_30424233_71	DCTPP1	16	-	213	17	0	0	11.27%	46.83224039969719	0.004873903550586949	0.004873903550586949	0.00012846764509679302
ENSG00000165272_33443892_33441990_201	AQP3	9	-	603	9	0	1	3.48%	8.75791677360448	0.006937202941512724	0.016735684067288745	0.000590529598856171
ENSG00000125247_100668740_100604994_741	TMTC4	13	-	2223	16	4	0	1.21%	4.223340882278004	0.027614347364305233	0.0032294771334464936	0.0005164706090267083
ENSG00000126858_32192201_32224733_438	RHOT1	17	+	1314	7	0	1	1.14%	3.125923110213471	0.019216965118390855	0.04178282491678763	0.003600525600033436
ENSG00000132780_45614150_45618141_268	NASP	1	+	804	27	2	0	3.36%	19.70531274061008	0.013494345032394814	0.0035547763465261306	0.00026296295714225076
ENSG00000134905_110663486_110641537_247	CARS2	13	-	741	16	0	3	2.83%	12.670022646834012	0.008674846900444944	0.04524396496563379	0.001992364361831615
ENSG00000067225_72219097_72199650_531	PKM	15	-	1593	1683	112	80	24.86%	619.9318602061611	3.476784005898616e-21	8.281107185501635e-21	4.850751206084504e-40
ENSG00000034713_75568127_75577369_57	GABARAPL2	16	+	171	38	0	1	14.04%	130.39564974033337	0.00575691403842271	0.007356796211402075	0.00022276510181128367
ENSG00000103197_2060673_2088610_1414	TSC2	16	+	4242	10	5	3	0.57%	1.3832636111067895	0.07635947366271362	0.04922925118879209	0.01464496403362635
ENSG00000065000_2121856_2102173_877	AP3D1	19	-	2631	35	3	4	1.71%	7.805896934284494	0.0010795612484085625	0.002020802647119262	1.3236839687112236e-05
ENSG00000084754_26244596_26199259_499	HADHA	2	-	1497	50	4	0	3.21%	19.598544550150304	0.00044045998227980814	0.00020701231900031988	6.215584287042554e-07
ENSG00000084754_26195196_26191250_258	HADHA	2	-	774	39	0	1	5.43%	29.566455464377913	0.0004441315507112266	0.0005444500874584297	1.581205649667966e-06
ENSG00000078618_51840459_51789236_1019	NRDC	1	-	3057	33	0	0	1.37%	6.334234212116292	0.00042454308224345244	0.00042454308224345244	1.1896574514138388e-06
ENSG00000126088_45012992_45015498_286	UROD	1	+	858	34	0	1	5.24%	23.252371107541965	0.0002732064791285261 5	0.0004209495667581447	7.730741880533374e-07
ENSG00000163507_108581486_108551149_746	CIP2A	3	-	2238	10	0	1	0.67%	2.6218964425000006	0.019679754444124863	0.04223451612662591	0.003715148010809822
ENSG00000009844_142169631_142218643_184	VTA1	6	+	552	30	5	0	5.43%	31.890240425625006	0.014094678151315428	0.0023766146074493525	0.00019392272823799724
ENSG00000172354_100676729_100678801_296	GNB2	7	+	888	85	10	3	8.45%	56.16704507396116	0.00012182267339257282	2.1245207444956663e-05	2.0232348006038713e-08
ENSG00000104824_38840176_38836722_205	HNRNPL	19	-	615	80	0	2	9.76%	76.32916082360977	3.9825936162184115e-05	0.00011194503879329248	3.343818295096618e-08
ENSG00000072682_132218599_132193010_526	P4HA2	5	-	1578	34	2	0	3.61%	12.642924214366923	4.430797606548033e-05	4.8597989611994695e-05	1.6225649837067363e-08
ENSG00000196531_56712866_56712538_31	NACA	12	-	93	47	1	2	25.81%	296.54494537720973	0.00575691403842271	0.010216256954394064	0.00030633528987432585
ENSG00000170892_54191365_54193671_312	TSEN34	19	+	936	33	5	0	3.85%	20.687771353033657	0.008356254428487338	0.0010155991509472172	5.354085463802975e-05
ENSG00000026508_35139304_35229333_361	CD44	11	+	1083	362	22	24	18.84%	196.1352847894765	1.5517509657302538e-11	8.813673743103588e-11	1.6590819491570265e-20
ENSG00000100744_96382284_96385684_127	GSKIP	14	+	381	24	1	1	9.45%	36.962546383086625	0.0016353363669355965	0.0016353363669355965	1.5959896251026578e-05
ENSG00000148019_78240031_78270851_586	CEP78	9	+	1758	20	0	0	1.71%	6.675545208549489	0.002033393162560146	0.002033393162560146	2.4228963598721857e-05
ENSG00000125257_95247698_95021575_1282	ABCC4	13	-	3846	26	6	0	1.17%	3.966794336874416	0.0034555441908205656	0.0002554476795345014	6.294044387237091e-06
ENSG00000142002_4704003_4685545_486	DPP9	19	-	1458	11	0	8	1.44%	4.427012799826133	0.006937202941512724	0.15710216847693848	0.007113201434144746
ENSG00000138696_95104425_95154673_502	BMPR1B	4	+	1506	16	0	1	1.59%	6.23405496766534	0.004873903550586949	0.01007916461776185	0.0002597390186955864
ENSG00000124882_74365327_74381141_87	EREG	4	+	261	16	0	0	10.34%	35.97121372147127	0.0032294771334464936	0.0032294771334464936	5.8663648728941325e-05
ENSG00000086504_370218_367675_256	MRPL28	16	-	768	46	2	0	7.81%	35.14570246907423	7.237715554886367e-05	3.592951532853599e-05	1.96263786912504e-08
ENSG00000149485_61813305_61802411_360	FADS1	11	-	1080	133	6	14	7.78%	72.26092256443474	1.5160076052832811e-05	2.2859346735468922e-05	2.7400335148722413e-09
ENSG00000138496_122559608_122536115_706	PARP9	3	-	2118	6	1	0	0.71%	1.662267489607649	0.0477903522728147	0.016947426762344633	0.0036975118224100542
ENSG00000169714_129171757_129170453_172	CNBP	3	-	516	259	23	7	27.33%	294.5273832797646	1.3906307342092838e-08	1.898624118077459e-08	2.7078936197306147e-15
ENSG00000186185_44936344_44926080_852	KIF18B	17	-	2556	6	0	1	0.59%	1.3774188352852115	0.016947426762344633	0.0477903522728147	0.0036975118224100542
ENSG00000137955_75786272_75794650_331	RABGGTB	1	+	993	29	1	0	4.83%	17.13658840998339	0.00028425713480484475	0.0001480809710132869	2.93269791698029e-07
ENSG00000122884_73074883_73008222_534	P4HA1	10	-	1602	61	6	3	4.12%	22.343074815057122	0.00018501039400454754	4.486373432579194e-05	6.205972399254342e-08
ENSG00000116954_38857067_38839553_315	RRAGC	1	-	945	15	0	2	2.22%	9.313974981452382	0.00847367416878415	0.027657845067597595	0.0011690047180828366
ENSG00000185624_81860471_81859146_128	P4HB	17	-	384	39	3	0	8.59%	59.594886795386735	0.008479013361307032	0.0013984928336429232	7.176883980625285e-05
ENSG00000134330_9481342_9488329_135	IAH1	2	+	405	18	0	0	5.19%	26.079129948066672	0.008674846900444944	0.008674846900444944	0.00038311532916634116
ENSG00000148840_102139106_102150029_1465	PPRC1	10	+	4395	28	1	0	0.82%	3.7383053167877143	0.0011547957782248217	0.0009470236307512361	6.768235413376606e-06
ENSG00000115234_27374720_27376719_256	SNX17	2	+	768	23	1	0	3.52%	17.572851234537115	0.0050845836060656145	0.0036581908865286695	0.00010208315094899304
ENSG00000242372_35279705_35279197_49	EIF6	20	-	147	48	2	10	20.41%	191.60177104702046	0.0022409900782254734	0.006112543793728684	7.798427274601149e-05
ENSG00000130713_130693798_130703774_261	EXOSC2	9	+	783	35	5	0	5.36%	26.22901000523947	0.0006754508261320 64	0.0003763727141378971	1.6684700400220385e-06
ENSG00000130713_130693798_130703774_291	EXOSC2	9	+	873	47	5	0	6.53%	31.590698648431278	8.076903470455387e-05	4.950915307313248e-05	2.9731955296261546e-08
ENSG00000126088_45012992_45013372_62	UROD	1	+	186	11	0	0	9.68%	34.70206807605646	0.012798402692974279	0.012798402692974279	0.0007972436888943567
ENSG00000138303_72203481_72097334_272	ASCC1	10	-	816	13	0	0	2.57%	9.348217536531251	0.00837382380159347	0.00837382380159347	0.00035838388946007 99
ENSG00000198668_90397231_90404717_83	CALM1	14	+	249	88	7	1	26.51%	207.3762140448675	7.103949014678404e-05	2.208062338766634e-05	1.2135692440702944e-08
ENSG00000125991_35542098_35557504_388	ERGIC3	20	+	1164	107	0	4	10.31%	53.93943758588532	1.137806245743416e-08	7.76637492594302e-08	9.150466587051923e-15
ENSG00000054654_63978007_64225564_6359	SYNE2	14	+	19077	15	6	1	0.11%	0.4613779083436862	0.15952699612641635	0.015516259750072458	0.012888680152838944
ENSG00000106105_30595084_30622466_484	GARS	7	+	1452	96	5	1	4.75%	38.79539992274381	0.0001429257763440441	1.515307708016231e-05	1.7469788148123357e-08
ENSG00000198561_57791641_57816308_879	CTNND1	11	+	2637	64	7	2	2.73%	14.241163111572245	0.0002556913665467353	1.5363660848022635e-05	3.2544366602591786e-08
ENSG00000168958_227340328_227357117_89	MFF	2	+	267	14	2	1	6.74%	30.767512860078657	0.013007239811447113	0.013007239811447113	0.0008218654148058079
ENSG00000114867_184320681_184334908_1404	EIF4G1	3	+	4212	86	7	5	2.71%	11.980796877851143	7.385814168744835e-07	2.993515018880905e-07	2.0477869100525147e-12
ENSG00000092621_119712023_119744040_445	PHGDH	1	+	1335	34	0	0	3.82%	14.944220532038205	0.00012102304356057305	0.00012102304356057305	1.0463087141192928e-07
ENSG00000115267_162318307_162267200_986	IFIH1	2	-	2958	23	0	0	1.12%	4.562525269818966	0.0014055202957965589	0.0014055202957965589	1.1935530040246081e-05
ENSG00000149136_57328335_57327422_100	SSRP1	11	-	300	9	5	0	6.00%	17.603412714945005	0.23864467013471535	0.011772051206276392	0.017699960186670707
ENSG00000149136_57335121_57332749_343	SSRP1	11	-	1029	15	11	1	2.62%	8.553650493170556	0.28940078266192815	0.006083074359664965	0.015176201000587591
ENSG00000149136_57332927_57330723_320	SSRP1	11	-	960	43	1	1	4.38%	26.282873150785942	0.0005208242783633627	0.0005208242783633627	1.765397052434068e-06
ENSG00000111843_10728672_10730666_35	TMEM14C	6	+	105	24	0	0	14.29%	134.12123973291432	0.021557223391537678	0.021557223391537678	0.002115616449879074
ENSG00000015171_180013_252470_548	ZMYND11	10	+	1644	16	0	0	1.28%	5.710758382788322	0.008674846900444944	0.008674846900444944	0.00038311532916634116
ENSG00000155368_119367052_119372318_87	DBI	2	+	261	103	6	4	24.14%	231.56468833197133	0.00014145917115575762	8.387869876068085e-05	8.562985301249247e-08
ENSG00000015171_180013_252470_522	ZMYND11	10	+	1566	14	0	0	1.15%	5.245802001047894	0.013428347753762199	0.013428347753762199	0.0008725593497644525
ENSG00000115310_55050300_54973156_1192	RTN4	2	-	3576	270	18	4	5.29%	44.3038910611032	4.2434411103570285e-11	4.146690427066568e-12	2.2073315562955326e-21
ENSG00000110075_68537665_68614677_791	PPP6R3	11	+	2373	31	7	5	2.28%	7.66548383429267	0.004823571330713604	0.0009114593787008354	2.7339250645509414e-05

ENSG00000011485_46387113_46390346_191	PPP5C	19	+	573	16	0	0	3.14%	16.384793684649217	0.013428347753762199	0.013428347753762199	0.0008725593497644525
ENSG00000147457_23246786_23260599_423	CHMP7	8	+	1269	17	1	5	2.36%	7.8607306581052026	0.005561255870711926	0.023814920846593857	0.0006973237161995367
ENSG00000138760_76213543_76161738_449	SCARB2	4	-	1347	85	2	1	8.24%	37.02771791067373	4.216736558117416e-08	5.371868189415899e-08	2.248815043798071e-14
ENSG00000233822_27838662_27865747_126	HIST1H2BN	6	+	378	16	4	1	3.97%	24.837266617206353	0.08588638879785576	0.02108409854857798	0.008130108632843268
ENSG00000132388_4366316_4282835_170	UBE2G1	17	-	510	24	1	0	4.71%	27.613196415600008	0.00934169773149884	0.0053557511377634245	0.00026267894186613697
ENSG00000180596_26123904_26123524_126	HIST1H2BC	6	-	378	237	10	2	18.25%	367.9020117673691	1.3051182819688529e-05	1.0917961931988644e-05	1.1486309934405817e-09
ENSG00000115525_85863399_85840144_362	ST3GAL5	2	-	1086	12	1	3	1.66%	6.483761589298344	0.026602764905793736	0.09783387127182075	0.011244525270099857
ENSG00000072803_172006460_171868635_494	FBXW11	5	-	1482	21	1	0	1.82%	8.31470236198482	0.005034872532813168	0.0036062891729786097	9.977408550613472e-05
ENSG00000110075_68537665_68613117_873	PPP6R3	11	+	2619	35	7	5	2.29%	7.841662785071594	0.002080913587518237	0.00040947059703959126	5.602597070136581e-06
ENSG00000110075_68537665_68613117_879	PPP6R3	11	+	2637	35	7	5	2.28%	7.788136076641071	0.002080913587518237	0.00040947059703959126	5.602597070136581e-06
ENSG00000115525_85863399_85840144_362	ST3GAL5	2	-	1086	12	1	3	1.66%	6.483761589298344	0.026602764905793736	0.09783387127182075	0.011244525270099857
ENSG00000140157_22860765_22866847_219	NIPA2	15	+	657	30	2	0	4.11%	26.79362665897261	0.009335735749052325	0.003736770770438521	0.00018925956129292707
ENSG00000142192_26112071_25881670_651	APP	21	-	1953	257	3	2	12.29%	77.21585710429879	4.170611303523564e-15	5.021185775406545e-15	2.94665750627112e-28
ENSG00000134574_47215137_47238849_427	DDB2	11	+	1281	12	0	0	1.64%	5.496772120201406	0.0078820576498062	0.0078820576498062	0.00031963642907451164
ENSG00000134575_47248789_47240116_423	ACP2	11	-	1269	16	1	0	2.60%	7.398334737040191	0.0024579339080672683	0.0008884494972709903	1.3429802402146636e-05
ENSG00000070010_19471785_19454650_251	UFD1	22	-	753	43	0	1	7.17%	33.508045451201205	8.83028806972547e-05	0.00010345227670696391	6.61782596792799e-08
ENSG00000145391_139556119_139496353_356	SETD7	4	-	1068	25	0	2	3.65%	13.735496812535116	0.0005399915898224664	0.0016120252826907794	5.558958727896411e-06
ENSG00000119705_77708112_77717561_107	SLIRP	14	+	321	133	3	2	20.56%	243.12086096445333	2.310482322909139e-05	1.8746183408550337e-05	3.400835151591253e-09
ENSG00000121073_49705189_49701458_168	SLC35B1	17	-	504	18	0	0	5.36%	20.956443708267862	0.0034529206320349946	0.0034529206320349946	6.664817351595063e-05
ENSG00000109332_102809680_102797415_110	UBE2D3	4	-	330	19	0	1	5.45%	33.78432743272273	0.013428347753762199	0.013428347753762199	0.0008725593497644525
ENSG00000140365_75339779_75340005_46	COMMD4	15	+	138	10	0	0	10.87%	42.52032056750001	0.020613416668581838	0.020613416668581838	0.001946208561389314
ENSG00000115677_241262839_241229601_1161	HDLBP	2	-	3483	54	5	0	1.81%	9.097370912116281	0.0002484843715678419	2.4620977378132598e-05	4.8380732440387274e-08
ENSG00000039560_34686920_34830765_951	RAI14	5	+	2853	30	2	1	1.37%	6.170141154905364	0.003031355522137688	0.0010322727522116193	1.9025352038280622e-05
ENSG00000075292_71349159_71395273_850	ZNF638	2	+	2550	19	0	1	0.82%	4.372089432470001	0.00847367416878415	0.013892891352654344	0.0005886798845679606
ENSG00000132199_706638_691130_181	ENOSF1	18	-	543	8	0	0	2.76%	8.645015452397793	0.019679754444124863	0.019679754444124863	0.0017849636115922674
ENSG00000167553_49265182_49273227_449	TUBA1C	12	+	1347	70	0	5	3.56%	30.4934147499666	0.00019965580804963987	0.001745752242454467	2.452088265146553e-06
ENSG00000103018_69424696_69462534_131	CYB5B	16	+	393	109	37	6	12.21%	162.74571551560692	0.0508475943575662	0.00020701231900031988	0.00012904584195035662
ENSG00000283765_183884846_183817356_408	AC131160.1	3	-	1224	8	0	0	1.47%	3.835166168833334	0.009815328628645334	0.009815328628645334	0.0004837142251238724
ENSG00000156113_77637360_76887266_1114	KCNMA1	10	-	3342	15	4	2	0.72%	2.63366437985413	0.048994651353958696	0.02389626957043539	0.005091569865673637
ENSG00000163902_128650800_128641112_154	RPN1	3	-	462	19	1	6	4.55%	24.13166245194481	0.010042643336235583	0.052370108323589605	0.0026274130286747
ENSG00000054148_136849431_136850789_124	PHPT1	9	+	372	164	18	7	24.19%	258.6881438396936	7.347621497956601e-06	1.230465089864599e-06	8.003180922141827e-11
ENSG00000147164_71068306_71061016_162	SNX12	X	-	486	17	0	3	4.94%	20.525241162830252	0.005160369442893307	0.0385659205926511	0.0010937528243987265
ENSG00000099817_1091860_1089486_117	POLR2E	19	-	351	18	0	0	4.27%	30.091303786230775	0.019216965118390855	0.019216965118390855	0.0017073955890589052
ENSG00000117151_84574415_84554999_385	CTBS	1	-	1155	8	0	0	1.56%	4.064279991906495	0.009815328628645334	0.009815328628645334	0.0004837142251238724
ENSG00000155252_97640743_97673742_479	PI4K2A	10	+	1437	11	2	1	1.04%	4.491708185209813	0.08350359792828288	0.03537493353818881	0.012068607709980346
ENSG00000265241_145927378_145925882_158	RBM8A	1	-	474	13	0	0	5.06%	16.093133986939876	0.005160369442893307	0.005160369442893307	0.00014319601891785326
ENSG00000161513_74872944_74862817_497	FDXR	17	-	1491	13	7	0	1.41%	5.116127102487929	0.23624880700034423	0.0078820576498062	0.013373491090098295
ENSG00000164032_99950170_99949447_98	H2AFZ	4	-	294	110	94	14	8.16%	219.5436959913776	0.3168971863731953	0.010275150823238127	0.024155458178858683
ENSG00000182544_53252836_53254188_450	MFSD5	12	+	1350	18	0	2	2.67%	7.823738984420002	0.0007729603088974596	0.001620025777606367	7.78860056060297e-06
ENSG00000100722_88568098_88611751_559	ZC3H14	14	+	1677	21	3	3	2.15%	7.347876505940073	0.007443125733849675	0.007963342261428788	0.00030576808039074245
ENSG00000196712_31201100_31374155_2461	NF1	17	+	7383	19	0	2	0.41%	1.5100674594065424	0.0022011839993586477	0.006387515641543697	8.020473513973342e-05
ENSG00000198258_9828388_9830008_27	UBL5	19	+	81	191	34	0	51.85%	1383.642727800204	0.01220012423109503	0.0004823113094284696	4.3285161738674235e-05
ENSG00000168615_38997064_39090052_628	ADAM9	8	+	1884	167	8	8	9.39%	52.01291442667756	7.478228524144883e-11	5.441651357530984e-10	4.784211728138782e-19
ENSG00000168615_39090049_39103700_129	ADAM9	8	+	387	43	0	0	7.75%	65.19782487016668	0.0022543491824521134	0.0022543491824521134	2.9517890247939808e-05
ENSG00000144746_69085048_69104636_188	ARL6IP5	3	+	564	74	4	2	10.64%	76.98892085732449	0.00021665776725833996	0.00011819081825936175	1.809455336697872e-07
ENSG00000056097_32444299_32406747_330	ZFR	5	-	990	18	1	1	1.52%	10.668734978754548	0.03577298629450316	0.03577298629450316	0.005410795288624905
ENSG00000123472_46658162_46635776_177	ATPAF1	1	-	531	7	0	0	3.39%	7.735335154087572	0.009815328628645334	0.009815328628645334	0.0004837142251238724
ENSG00000164120_174522451_174492084_143	HPGD	4	-	429	16	5	3	6.29%	21.8845845718042	0.04292732827649999	0.025100479631792585	0.004671049007300601
ENSG00000103274_10743864_10769105_309	NUBP1	16	+	927	10	0	4	2.59%	6.32988590972492	0.00414960799764038	0.04922925118879209	0.0012030081715228372
ENSG00000106009_2538944_2538069_291	BRAT1	7	-	873	12	0	0	1.72%	8.065710293216497	0.020613416668581838	0.020613416668581838	0.001946208561389314
ENSG00000138382_169822052_169810339_160	METTL5	2	-	480	12	0	0	3.75%	14.669510595787504	0.013007239811447113	0.013007239811447113	0.0008218654148058079
ENSG00000275052_55598964_55549911_693	PPP4R3B	2	-	2079	14	2	3	1.01%	3.9513833254646475	0.02750441681463286	0.03282689904363471	0.003925293300512299
ENSG00000149554_125627722_125655320_416	CHEK1	11	+	1248	13	0	0	1.44%	6.112296081578126	0.013428347753762199	0.013428347753762199	0.0008725593497644525
ENSG00000164062_49674402_49683342_737	APEH	3	+	2211	33	6	0	2.31%	8.75791677360448	0.002215835380605914	0.00012002575157059987	2.021565418185597e-06
ENSG00000156928_23299395_23309543_220	MALSU1	7	+	660	26	4	2	5.91%	23.115592453968187	0.010500707027057524	0.0005854768899833019	4.298010167241181e-05
ENSG00000099783_8445050_8488854_713	HNRNPM	19	+	2139	87	7	2	5.47%	23.866244447564522	6.848398248756243e-07	3.475770271304628e-08	2.4527058924076137e-13
ENSG00000006744_13015830_12992818_703	ELAC2	17	-	2109	29	0	4	1.71%	8.068578611243955	0.0008739357725210369	0.010411316862582036	5.9659373416971355e-05
ENSG00000106853_111597422_111563121_329	PTGR1	9	-	987	85	6	2	12.46%	50.53326851639059	1.330931660265237e-07	9.622770584463436e-09	1.3524272853957849e-14
ENSG00000006715_38869163_38726246_779	VPS41	7	-	2337	21	0	2	1.28%	5.272738083209886	0.0022276761777358703	0.008577310921410378	0.00010938174272448573
ENSG00000084623_32222442_32231196_325	EIF3I	1	+	975	149	12	6	13.85%	89.67208528296771	4.343965919697891e-08	6.9380420164204665e-09	3.1607221131522955e-15
ENSG00000108107_55386379_55386752_80	RPL28	19	+	240	418	54	21	21.25%	1021.9759048398628	0.0009190739260373504	0.00048443517260985205	2.8689647365565095e-06
ENSG00000162144_61356632_61350399_215	CYB561A3	11	-	645	7	0	0	2.79%	6.368159638481397	0.009815328628645334	0.009815328628645334	0.0004837142251238724
ENSG00000069424_6082210_6098574_295	KCNAB2	1	+	885	8	0	0	1.69%	5.304229819945764	0.019679754444124863	0.019679754444124863	0.0017849636115922674
ENSG00000116560_35192911_35184456_661	SFPQ	1	-	1983	95	2	6	5.30%	28.11101374886158	3.3254576335231036e-07	9.196424628683734e-07	2.8219264320921297e-12
ENSG00000132199_712506_683012_342	ENOSF1	18	-	1026	9	1	0	1.75%	5.147196700276317	0.026832695913080365	0.011772051206276392	0.0015103817605807263
ENSG00000117335_207752282_207793591_347	CD46	1	+	1041	39	2	0	6.34%	21.983128270344384	7.639996631620973e-05	1.3529991062581404e-05	8.254540628586197e-09
ENSG00000101224_3787132_3804961_489	CDC25B	20	+	1467	65	12	17	6.13%	25.999132616937633	0.0002293816785940497	0.0007363193815128565	1.1441541893463115e-06

ENSG00000108651_31887295_31863359_410	UTP6	17	-	1230	21	1	1	2.68%	10.018202358098783	0.002559321900018936	0.002559321900018936	3.76242980579141e-05
ENSG00000166441_8682814_8685806_113	RPL27A	11	+	339	59	2	0	4.42%	102.12402656654427	0.029237630782826433	0.021557223391537678	0.002819005644529471
ENSG00000140400_75368583_75355906_1057	MAN2C1	15	-	3171	8	0	1	0.47%	1.480366884469253	0.019216965118390855	0.03418265644467716	0.002956052792116815
ENSG00000134077_9365069_9384688_507	THUMPD3	3	+	1521	23	4	2	1.97%	8.873076757478305	0.019264875824957298	0.011064573844522778	0.0010312247960341252
ENSG00000113558_134174494_134157733_174	SKP1	5	-	522	65	8	1	8.62%	73.06652787173853	0.00123924787469707	0.0003672872050780424	2.9898626710592256e-06
ENSG00000114446_108222322_108162477_429	IFT57	3	-	1287	28	0	0	2.33%	12.766007666885784	0.002404281539280677	0.002404281539280677	3.3387040459710164e-05
ENSG00000028116_58048901_58154762_373	VRK2	2	+	1119	14	2	1	1.88%	7.3413100390000015	0.03744183116399598	0.013599199788808391	0.002391925567846126
ENSG00000113141_140652089_140662329_498	IK	5	+	1494	16	2	5	2.01%	6.284127698329319	0.011663199792667247	0.03031039435646347	0.001692298879084381
ENSG00000131143_85806797_85806874_25	COX4I1	16	+	75	128	0	0	44.00%	1001.4385900057603	0.0016572264983485026	0.0016572264983485026	1.6372075009059705e-05
ENSG00000131143_85804978_85805115_45	COX4I1	16	+	135	48	1	1	17.78%	208.63303958453338	0.006386247275100883	0.006386247275100883	0.0002145861249467840 5
ENSG00000131143_85805594_85805872_92	COX4I1	16	+	276	97	2	0	18.48%	206.22355475237504	0.00025616893534566494	0.0001358993853317198 8	2.4386791746338747e-07
ENSG00000196792_30955638_30895411_566	STRN3	14	-	1698	24	0	0	2.30%	8.293716237901062	0.0006334249406157621	0.0006334249406157621	2.5753349202917255e-06
ENSG00000139436_109996224_109932978_681	GIT2	12	-	2043	15	0	0	1.32%	4.3082263130066085	0.0033278027414746945	0.0033278027414746945	6.211806171561056e-05
ENSG00000067064_1044083_1043970_37	IDI1	10	-	111	14	0	0	13.51%	74.00834174451353	0.019679754444124863	0.019679754444124863	0.0017849636115922674
ENSG00000083312_72848394_72872694_128	TNPO1	5	+	384	7	0	0	3.91%	10.696518142761722	0.019216965118390855	0.019216965118390855	0.0017073955890589052
ENSG00000115806_170929341_170966130_452	GORASP2	2	+	1356	14	1	2	1.77%	6.058204965811949	0.007930666369886513	0.022898969405409888	0.0009107991204150371
ENSG00000156113_77637360_76877869_1090	KCNMA1	10	-	3270	15	4	2	0.73%	2.6916533203279824	0.048994651353958696	0.02389626957043539	0.005091569865673637
ENSG00000003989_17543340_17565146_657	SLC7A2	8	+	1971	17	0	0	1.37%	5.061018368917049	0.0033030736763512156	0.0033030736763512156	6.124050385493983e-05
ENSG00000126226_113195112_113178198_292	PCID2	13	-	876	16	0	0	2.74%	10.717450663589043	0.005306578019052857	0.005306578019052857	0.0001510011474053423 3
ENSG00000033627_42478512_42521120_646	ATP6V0A1	17	+	1938	8	2	0	0.77%	2.4222102118947375	0.10105102209468636	0.019679754444124863	0.009164497669452725
ENSG00000035403_74072741_74075008_125	VCL	10	+	375	19	0	1	6.40%	29.730208140796005	0.0053555751137763424 5	0.00934169773149884	0.0002626789418661369 7
ENSG00000035403_74082466_74118169_801	VCL	10	+	2403	61	5	0	3.50%	14.89538321003808	3.0141221654393955e-05	1.4564117308665593e-06	4.0297645114507894e-10
ENSG00000107249_4125774_3828272_745	GLIS3	9	-	2235	13	0	2	0.81%	3.413040496559061	0.012798402692974279	0.05067994629101479	0.003100990187173963
ENSG00000137073_33996330_33922504_1059	UBAP2	9	-	3177	19	0	0	0.85%	3.5092313669494812	0.0035291660242640502	0.0035291660242640502	6.948232509684344e-05
ENSG00000184047_122218361_122208381_166	DIABLO	12	-	498	17	0	0	3.61%	20.030657038424703	0.013007239811447113	0.013007239811447113	0.0008218654148058079
ENSG00000132676_155709783_155738242_397	DAP3	1	+	1191	41	1	0	4.53%	20.199829871613353	0.00012004008880381689	7.569124107156332e-05	6.60130439958492e-08
ENSG00000204713_28908823_28904070_212	TRIM27	6	-	636	11	2	0	3.30%	10.148718022242926	0.02305674932606751	0.007593099278532274	0.0008829677069027922
ENSG00000171603_9744584_9730508_633	CLSTN1	1	-	1899	66	7	3	3.48%	20.393632423843606	0.0003146078987317475	5.563698968961584e-05	1.3019794958728496e-07
ENSG00000082701_120093359_119826788_445	GSK3B	3	-	1335	24	0	0	3.60%	10.548861552026969	0.00014480970487777632	0.00014480970487777632	1.4822276924058578e-07
ENSG00000167600_41193265_41203462_165	CYP2S1	19	+	495	12	0	0	3.64%	14.224979971672731	0.012178957859018494	0.012178957859018494	0.0007262454987071801
ENSG00000104067_29761691_29701595_1431	TJP1	15	-	4293	20	1	1	0.77%	2.733661420132775	0.0012624143894574274	0.0024673358700277245	1.8651743405009573e-05
ENSG00000136156_48233361_48261224_160	ITM2B	13	+	480	94	8	7	13.12%	114.91116633366877	0.00015112654033270804	0.00046884857877254444	4.926469794307586e-07
ENSG00000160113_17240683_17232352_284	NR2F6	19	-	852	11	0	0	1.76%	7.575803594068664	0.019679754444124863	0.019679754444124863	0.0017849636115922674
ENSG00000074054_121605760_121340861_1433	CLASP1	2	-	4299	13	3	0	0.70%	1.7743999790205867	0.0182999617455631	0.0014903523101034196	0.0001731805207519894
ENSG00000171310_104457442_104757803_337	CHST11	12	+	1011	9	0	0	1.48%	5.223564603841248	0.019679754444124863	0.019679754444124863	0.0017849636115922674
ENSG00000181929_49005362_49002899_247	PRKAG1	12	-	741	29	0	0	4.05%	22.964416047386646	0.0023628488670607213	0.0023628488670607213	3.2295294885504634e-05
ENSG00000176261_32650529_32621862_167	ZBTB8OS	1	-	501	7	0	0	2.99%	8.198528875889224	0.019216965118390855	0.019216965118390855	0.0017073955890589052
ENSG00000204427_31691845_31687212_325	ABHD16A	6	-	975	6	0	0	1.54%	3.610956454347693	0.016947426762344633	0.016947426762344633	0.0013498980316300959
ENSG00000123130_23730970_23707915_136	ACOT9	X	-	408	9	1	0	3.68%	12.943685819812503	0.05123521742987468 4	0.020613416668581838	0.004687384229717438
ENSG00000173486_64242388_64244029_142	FKBP2	11	+	426	62	4	3	14.08%	85.39996778768312	0.00016569421277282895	0.00014894787451579541	1.736266846401335e-07
ENSG00000117410_43976115_43977411_214	ATP6V0B	1	+	642	48	3	0	8.41%	43.87143355749534	0.0001789774272272485	8.751142344759464e-05	1.1253437756231516e-07
ENSG00000076242_36993650_37050653_653	MLH1	3	+	1959	21	0	3	1.68%	6.290142368790966	0.001484234770590415	0.010728371185261619	9.686994068872613e-05
ENSG00000165060_69046445_69072762_135	FXN	9	+	405	17	0	0	5.19%	24.630289395396304	0.00847367416878415	0.00847367416878415	0.0003665024264111182
ENSG00000076043_114439529_114446035_95	REXO2	11	+	285	15	8	2	8.42%	30.8831802016579	0.1539609423056087	0.02389626957043539	0.016983116543564356
ENSG00000175567_73981586_73981512_24	UCP2	11	-	72	19	0	0	20.83%	154.84483406664586	0.02108409854857798	0.02108409854857798	0.002029891263157455
ENSG00000197747_151986218_151984118_61	S100A10	1	-	183	108	0	3	19.67%	346.2966435726886	0.0010573555742419644	0.001779122956135253	1.145217792472409e-05
ENSG00000168827_158644635_158682169_591	GFM1	3	+	1773	37	1	0	3.55%	12.245276752264807	1.788379863796444e-05	1.9703256797614847e-05	2.779451340220311 4e-09
ENSG00000163430_120450746_120396952_308	FSTL1	3	-	924	48	0	0	7.14%	30.48209993929871	1.4724699406439145e-05	1.4724699406439145e-05	1.730005367946513e-09
ENSG00000133858_71663610_71611782_1910	ZFC3H1	12	-	5730	17	0	2	0.31%	1.7408843289939795	0.012798402692974279	0.023672501042627626	0.0014375227604752335
ENSG00000101132_54215423_54219150_49	PFDN4	20	+	147	16	2	0	10.20%	63.86725701567348	0.07864960352514258	0.016947426762344633	0.006209665325776152
ENSG00000144827_111979062_111991721_306	ABHD10	3	+	918	16	0	1	3.59%	10.227109783555559	0.0012427339549726212	0.0025320062747194163	1.8857845948475982e-05
ENSG00000198668_90397231_90401545_106	CALM1	14	+	318	65	7	1	12.26%	119.9393948083255	0.0018664915068243 37	0.0008019778103825313	9.278430172001293e-06
ENSG00000198805_20475108_20476601_120	PNP	14	+	360	37	0	2	10.00%	60.30798800490418	0.0010155991509472172	0.0023453309689915928	1.4495747037441908e-05
ENSG00000151718_183245429_183315729_987	WWC2	4	+	2961	8	0	0	0.51%	1.5853574436514695	0.019216965118390855	0.019216965118390855	0.0017073955890589052
ENSG00000114439_107728780_107805457_801	BBX	3	+	2403	16	4	0	1.00%	3.9069857600099884	0.04727912037993979	0.005454749182134642	0.001431107523018461 5
ENSG00000214046_16660110_16647246_75	SMIM7	19	-	225	25	3	0	10.67%	65.19782487016668	0.04140309663709693	0.005355751137763424 5	0.001218378129224801
ENSG00000185722_4263941_4167779_1169	ANKFY1	17	-	3507	35	2	1	1.37%	5.856092054206588	0.0005018492373769886	0.0002871188111466556	9.63461383505656e-07
ENSG00000177885_75393628_75320368_217	GRB2	17	-	651	22	2	0	5.07%	19.829753186317976	0.005530543042558543	0.00151350169064872	4.947549134344851e-05
ENSG00000232119_120604237_120612264_181	MCTS1	X	+	543	17	2	1	3.87%	18.37065783634531	0.03299602752967379	0.013892891352654344	0.002146120390806649 7
ENSG00000140455_63537128_63590826_435	USP3	15	+	1305	9	3	0	1.15%	4.046761543665518	0.1191346346731873	0.019216965118390855	0.010787784428637617
ENSG00000187109_76067450_76048429_322	NAP1L1	12	-	966	77	10	4	5.28%	46.77235262425001	0.0008981259909197917	0.000457673737066691	2.658404556194875e-06
ENSG00000166226_69585522_69587648_95	CCT2	12	+	285	34	10	0	9.47%	70.00187512375791	0.005670089768331009	0.0037896409716948643	0.0001172684818723252 6
ENSG00000134575_47248502_47240116_391	ACP2	11	-	1173	15	1	0	2.56%	7.5035859825000015	0.004004657782222479	0.0014903523101034196	3.520021001122424e-05
ENSG00000114745_39107541_39098236_285	GORASP1	3	-	855	10	2	0	1.75%	6.862928933701756	0.06539853090342929	0.020613416668581838	0.006006025205075414
ENSG00000101266_489860_483961_177	CSNK2A1	20	-	531	9	0	0	2.82%	9.945430912398308	0.019216965118390855	0.019216965118390855	0.0017073955890589052
ENSG00000141127_18877732_18930698_278	PRPSAP2	17	+	834	14	0	0	3.24%	9.850031095492808	0.002943990679779778	0.002943990679779778	4.916404313974209e-05
ENSG00000170291_7252551_7258788_263	ELP5	17	+	789	34	0	2	4.94%	25.285848428733846	0.0006698694257094773	0.0013845845069509028	5.832045150346985e-06

ENSG00000080503_2029239_2182174_1356	SMARCA2	9	+	4068	10	4	2	0.52%	1.4424297537647497	0.10186826994475356	0.049338281967443096	0.019397008212484922
ENSG00000196230_30720507_30724397_444	TUBB	6	+	1332	335	24	11	8.33%	147.5761576453097	2.75083302959803e-06	3.8120552282922505e-07	9.728087087195689e-12
ENSG00000275835_23031961_22999820_866	TUBGCP5	15	-	2598	12	0	0	0.69%	2.710302188598153	0.011772051206276392	0.011772051206276392	0.000681272511389508
ENSG00000171530_77776257_77693122_129	TBCA	5	-	387	90	4	0	8.53%	136.46056368174422	0.0022000046091025264	0.0016650069558729898	2.1612248589266253e-05
ENSG00000065665_12129788_12164454_476	SEC61A2	10	+	1428	14	0	0	1.26%	5.752749253250001	0.013428347753762199	0.013428347753762199	0.0008725593497644525
ENSG00000071282_8532737_8537512_138	LMCD1	3	+	414	7	0	0	3.62%	9.92140813241667	0.016947426762344633	0.016947426762344633	0.0013498980316300959
ENSG00000184009_81512354_81511434_198	ACTG1	17	-	594	144	82	125	15.66%	142.2497997167273	0.004750321861637258	0.03006533168808229	0.0007804394475385115
ENSG00000127955_80135161_80217493_354	GNAI1	7	+	1062	26	4	0	2.54%	14.365622429019778	0.015528142895806445	0.0036062891729786097	0.0003077713301577895
ENSG00000255639_4654363_4772597_527	AC005833.1	12	+	1581	50	4	3	2.85%	18.557255655645164	0.0020698205712825087	0.0014529944780787229	1.790917693707681e-05
ENSG00000075239_108121607_108147390_427	ACAT1	11	+	1281	56	0	4	5.62%	25.651603227606564	7.375167250191021e-06	5.0348237186136366e-05	3.0620893268234025e-09
ENSG00000153922_98896228_98856380_1141	CHD1	5	-	3423	7	0	2	0.44%	1.1999599669355832	0.016947426762344633	0.11777945996203976	0.009674391494749069
ENSG00000169604_69013500_69145376_333	ANTXR1	2	+	999	134	4	0	5.11%	78.70728407749851	0.0012819264959411905	0.0001263582208913867	1.1787927496929023e-06
ENSG00000087087_100875591_100888549_872	SRRT	7	+	2616	42	6	7	2.06%	9.420786621147938	0.001743706597532283	0.0026175142796489408	2.6731077080476952e-05
ENSG00000145191_184135386_184144943_729	EIF2B5	3	+	2187	21	1	0	1.37%	5.634379927051442	0.005670089768331009	0.0022409900782254734	7.232548112602907e-05
ENSG00000196139_5077963_5107503_300	AKR1C3	10	+	900	778	30	33	21.33%	507.2390774898968	1.115871738903263e-11	2.948851201637969e-11	4.006459921014171e-21
ENSG00000125733_6739762_6751211_545	TRIP10	19	+	1635	16	2	0	1.47%	5.742193750033029	0.02319724882453354	0.004826937407846361	0.0006010349372949749
ENSG00000013810_1723785_1745013_765	TACC3	4	+	2295	33	0	2	2.35%	8.437365571433336	6.99138909308556e-05	0.00020753571005996044	1.0554092732354401e-07
ENSG00000116586_156054890_156058371_125	LAMTOR2	1	+	375	50	8	1	10.40%	78.23738984420002	0.006396122678036902	0.0008128177682286688	3.322857385525337e-05
ENSG00000116586_156054890_156058371_95	LAMTOR2	1	+	285	27	2	1	8.42%	55.58972436298422	0.012088529595134127	0.007225456388766877	0.0004444545540374498
ENSG00000116586_156054890_156058409_150	LAMTOR2	1	+	450	50	8	1	8.67%	65.19782487016668	0.006396122678036902	0.0008128177682286688	3.322857385525337e-05
ENSG00000160789_156114919_156137764_572	LMNA	1	+	1716	232	20	11	12.41%	79.33161907279023	1.61714722671505e-11	4.2240645767133704e-12	8.48920342212921e-22
ENSG00000160789_156114919_156139106_664	LMNA	1	+	1992	237	20	11	10.99%	69.81273114862728	7.490004894240365e-12	1.908361821843222e-12	1.8044740563619138e-22
ENSG00000164032_99950170_99949356_34	H2AFZ	4	-	102	110	94	14	23.53%	632.8024178575001	0.3168971863731953	0.010275150823238127	0.024155458178858683
ENSG00000259316_64381371_64366005_65	AC087632.1	15	-	195	101	9	16	10.77%	303.92216824093083	0.008877961307018026	0.08357182984467554	0.003987317265369605
ENSG00000103550_19714855_19706910_398	KNOP1	16	-	1194	13	2	1	1.76%	6.388731582755026	0.03744183116399598	0.013599199788808391	0.002391925567846126
ENSG00000147536_41530203_41542087_223	GINS4	8	+	669	10	0	0	2.69%	8.771007830067267	0.011570465654371866	0.011570465654371866	0.0006594843062896773
ENSG00000054118_36282564_36304017_955	THRAP3	1	+	2865	26	0	2	1.15%	5.325057947510996	0.001484234770590415	0.004803510583362958	4.2126168520311525e-05
ENSG00000143321_156751429_156744511_120	HDGF	1	-	360	42	0	4	8.33%	68.45771611367502	0.0023766146074493525	0.014134202735441947	0.0001949612211687396
ENSG00000108219_80489234_80509501_159	TSPAN14	10	+	477	59	4	3	10.06%	72.57871070452518	0.0005983449737856036	0.0003864879047887105	1.5185664274167091e-06
ENSG00000111077_53050186_53063882_1344	TNS2	12	+	4032	12	0	5	0.37%	1.746370309022322	0.019679754444124863	0.1741008397065631	0.016992325807804815
ENSG00000116288_7962786_7985054_189	PARK7	1	+	567	159	14	0	19.58%	164.5468913389921	1.2818357415835378e-06	4.611197172071847e-08	6.127179820803827e-13
ENSG00000152422_83104920_83353242_336	XRCC4	5	+	1008	11	5	0	2.08%	6.403357799748513	0.06583400801140711	0.007029302661868638	0.0025354904771632734
ENSG00000100813_23071542_23059148_614	ACIN1	14	-	1842	36	11	5	1.95%	11.468021312667755	0.03788578172153152	0.005441582120040429	0.0011206124069290926
ENSG00000156976_186785967_186787487_161	EIF4A2	3	+	483	8	2	0	3.11%	9.718930415428574	0.06448902149598212	0.019216965118390855	0.005584917655007069
ENSG00000161204_184186208_184193698_709	ABCF3	3	+	2127	21	4	3	1.69%	5.793318712017632	0.006868294398631195	0.006358125177535369	0.0002289843735996333
ENSG00000071553_154428759_154434218_122	ATP6AP1	X	+	366	14	3	3	4.92%	22.44515282415574	0.061356297364084836	0.061356297364084836	0.014524511080970309
ENSG00000089157_120199407_120196773_273	RPLP0	12	-	819	49	1	1	2.93%	35.1065210839359	0.005808022449631236	0.006346985027636522	0.00019501864856894435
ENSG00000115677_241262839_241229601_1092	HDLBP	2	-	3276	51	5	0	1.74%	9.134860077962914	0.0005365087398156134	5.6254241454829154e-05	2.2857915155022076e-07
ENSG00000146112_30686018_30677269_613	PPP1R18	6	-	1839	19	4	2	1.25%	6.062440485480425	0.002549573717490699	0.007962492813783481	0.0001143653150129499
ENSG00000140284_50182428_50236096_567	SLC27A2	15	+	1701	26	3	5	2.29%	8.969012945102294	0.003975389419798935	0.007795957541432644	0.00016738859108894694
ENSG00000150527_39294000_39350264_639	MIA2	14	+	1917	17	0	0	1.41%	5.203582266633021	0.002967235347054297	0.002967235347054297	4.9907879201100895e-05
ENSG00000124225_57659647_57652053_234	PMEPA1	20	-	702	56	5	5	5.13%	46.808694778581206	0.00638574031296502	0.00396067672340455	0.0001371656018651211
ENSG00000123143_14440291_14471784_948	PKN1	19	+	2844	29	7	1	1.69%	5.983344687452005	0.006675442645532525	0.00027661276299603757	1.3849078365108538e-05
ENSG00000149782_64255551_64267556_1057	PLCB3	11	+	3171	9	1	0	0.57%	1.6654127450279095	0.02305674932606751	0.012178957859018494	0.0013400639730601711
ENSG00000115234_27370744_27376719_445	SNX17	2	+	1335	30	1	0	3.15%	13.186076940033711	0.0006613914452745182	0.00040804699845214287	1.764130391499901e-06
ENSG00000122966_119758688_119688232_1243	CIT	12	-	3729	19	0	0	0.80%	2.989763489621481	0.0022011839993586477	0.0022011839993586477	2.8200182015552967e-05
ENSG00000108826_50373170_50368092_148	MRPL27	17	-	444	33	0	0	8.78%	43.6120585280169	0.0006838816678496783	0.0006838816678496783	2.9854207730648617e-06
ENSG00000149273_75404048_75404865_117	RPS3	11	+	351	241	7	6	19.66%	402.8891229156454	1.1369612993097455e-05	1.5723465776201874e-05	1.4347240633892047e-09
ENSG00000150779_112085450_112085295_51	TIMM8B	11	-	153	22	0	0	15.69%	84.37365571433335	0.0053557511377634245	0.0053557511377634245	0.00015366972994913032
ENSG00000131844_71592941_71641095_315	MCCC2	5	+	945	18	0	0	2.86%	11.17676997774286	0.0034782380676658805	0.0034782380676658805	6.758309314600117e-05
ENSG00000161513_74872838_74862817_489	FDXR	17	-	1467	13	8	0	1.43%	5.199826523387527	0.23559043810812774	0.0078820576498062	0.013321720827107576
ENSG00000102317_48575181_48575814_118	RBM3	X	+	354	53	2	2	8.47%	87.85130639285171	0.0061547572324231374	0.0062201494882096935	0.00020209328853378674
ENSG00000156697_129906211_129929608_719	UTP14A	X	+	2157	13	1	0	0.97%	3.5364605979645347	0.015191410988288745	0.007593099278532274	0.0005816659162195647
ENSG00000143614_153828215_153810177_533	GATAD2B	1	-	1599	13	0	0	1.13%	4.7705725514756105	0.012798402692974279	0.012798402692974279	0.0007972436888943567
ENSG00000037637_16315343_16250670_692	FBXO42	1	-	2076	15	0	0	1.30%	4.239742946759394	0.0033278027414746945	0.0033278027414746945	6.211806171561056e-05
ENSG00000100888_21431499_21385613_2533	CHD8	14	-	7599	25	0	5	0.36%	1.9304527695469802	0.0036062891729786097	0.040339912027649666	0.0008591420312047067
ENSG00000172845_173955656_173909941_496	SP3	2	-	1488	22	0	0	1.61%	8.675517019014116	0.005604866691947602	0.005604866691947602	0.00016752456572620382
ENSG00000023839_99782845_99796384_217	ABCC2	10	+	651	14	7	1	2.76%	12.618933845838713	0.33632999628228655	0.026832695913080365	0.04814272760090725
ENSG00000139734_60112072_59666584_1084	DIAPH3	13	-	3252	10	0	0	0.46%	1.8043678469603326	0.019679754444124863	0.019679754444124863	0.0017849636115922674
ENSG00000139734_60112072_59666584_1049	DIAPH3	13	-	3147	10	0	0	0.48%	1.8645707779837946	0.019679754444124863	0.019679754444124863	0.0017849636115922674
ENSG00000175029_125003077_124989518_365	CTBP2	10	-	1095	10	0	0	1.64%	5.358725331794521	0.012798402692974279	0.012798402692974279	0.0007972436888943567
ENSG00000100522_52784650_52778311_184	GNPNAT1	14	-	552	21	2	0	5.98%	22.323168297937507	0.006423650881882899	0.0013984928336429232	5.3761815209204905e-05
ENSG00000174485_65756450_65661851_1863	DENND4A	15	-	5589	26	0	0	0.48%	2.729699591987655	0.003684287313187465	0.003684287313187465	7.541963121699725e-05
ENSG00000196531_56724521_56712538_215	NACA	12	-	645	209	14	5	14.88%	190.13505206323026	2.4168959547478946e-06	1.161173264099816e-06	2.4727756758011836e-11
ENSG00000165688_136417068_136419202_202	PMPCA	9	+	606	13	6	2	3.47%	12.587698861071786	0.091912668396609	0.04363948305378087	0.015821324234917434
ENSG00000139436_109996224_109948823_421	GIT2	12	-	1263	12	0	0	1.90%	5.575110915263659	0.004873903550586949	0.004873903550586949	0.00012846764509679302
ENSG00000131652_3026086_3027657_260	THOC6	16	+	780	28	0	1	4.23%	21.063912650361544	0.0015505692382983198	0.002167177068466985	1.9913191269291018e-05

ENSG00000184500_93898546_93874245_426	PROS1	3	-	1278	13	0	0	1.17%	5.968814952902584	0.020613416668581838	0.020613416668581838	0.001946208561389314
ENSG00000138802_109494617_109539675_852	SEC24B	4	+	2556	7	0	0	0.70%	1.60698864116608	0.009815328628645334	0.009815328628645334	0.0004837142251238724
ENSG00000130703_62263635_62293887_413	OSBPL2	20	+	1239	11	0	2	1.21%	5.209511430303875	0.019679754444124863	0.10105102209468636	0.009164497669452725
ENSG00000104763_18084801_18067074_191	ASAH1	8	-	573	8	0	0	2.62%	8.192396842324609	0.019216965118390855	0.019216965118390855	0.0017073955890589052
ENSG00000156261_29073590_29066883_189	CCT8	21	-	567	35	6	0	5.82%	36.22101381675927	0.011380896472064226	0.0016033688297201391	0.00011022446416510084
ENSG00000124155_45420633_45425826_254	PIGT	20	+	762	45	0	0	4.33%	34.65238723414371	0.00151350169064872	0.00151350169064872	1.3757299738441384e-05
ENSG00000138760_76179654_76161713_320	SCARB2	4	-	960	72	5	0	9.38%	44.00853178736251	4.09601459398266e-06	6.893507041361946e-07	2.554533397957012e-11
ENSG00000138760_76213543_76180849_175	SCARB2	4	-	525	13	0	1	4.57%	14.529800971065718	0.005160369442893307	0.00912793029372963	0.0002481924023507605 6
ENSG00000169045_179623133_179620012_140	HNRNPH1	5	-	420	101	6	7	15.00%	141.1067209690036	5.218963683194363e-05	0.000352797469487041	1.38137441163045e-07
ENSG00000148824_133395724_133420165_293	MTG1	10	+	879	18	4	2	2.73%	12.015981375389082	0.03588324695817727	0.018318557839637383	0.0029714513955091536
ENSG00000166797_64093672_64075518_70	CIAO2A	15	-	210	5	0	0	7.14%	13.970962472178575	0.012673659338734126	0.012673659338734126	0.000782701129001274
ENSG00000168958_227347269_227357117_130	MFF	2	+	390	12	2	1	3.85%	18.054782271738464	0.019679754444124863	0.019679754444124863	0.0017849636115922674
ENSG00000127054_1324608_1319816_75	INTS11	1	-	225	7	0	2	6.67%	18.255390963646672	0.019216965118390855	0.1191346346731873	0.010787784428637617
ENSG00000179818_70088801_70088577_74	PCBP1-AS1	2	-	222	25	2	5	10.81%	66.07887655760136	0.005554612275049112	0.021174362825021963	0.0006167941659507972
ENSG00000104388_60584796_60618687_79	RAB2A	8	+	237	15	0	1	8.86%	37.138001508322795	0.0078820576498062	0.015516259750072458	0.0006142571802709984
ENSG00000018408_149527879_149520805_113	WWTR1	3	-	339	33	4	3	12.39%	57.12021824908408	0.004211963786197448	0.0010322727522116193	2.6568708173615947e-05
ENSG00000074201_77637714_77619628_237	CLNS1A	11	-	711	56	0	1	7.17%	46.2161796548017	0.00013535602770218042	0.0002552309950560562	2.4206154131009565e-07
ENSG00000108578_30291370_30249017_368	BLMH	17	-	1104	14	0	0	2.17%	7.441056099312502	0.005160369442893307	0.005160369442893307	0.00014319601891785326
ENSG00000111700_20855085_20916247_655	SLCO1B3	12	+	1965	10	0	0	1.07%	2.9861599177175577	0.007029302661868638	0.007029302661868638	0.0002573639808771639
ENSG00000060982_24948932_24818008_386	BCAT1	12	-	1158	33	0	0	2.85%	16.72172192265933	0.001484234770590415	0.001484234770590415	1.3251387882263029e-05
ENSG00000169764_63856320_63891227_497	UGP2	2	+	1491	40	8	0	4.23%	15.741929546116705	0.0010006558440764162	2.032182481103021e-05	1.8207368289573541e-07
ENSG00000108602_19745129_19738221_453	ALDH3A1	17	-	1359	1460	90	42	29.14%	630.3895649698236	3.0830000355996146e-20	4.26820705775165e-22	2.336312221601433e-40
ENSG00000105819_103300226_103329052_383	PMPCB	7	+	1149	20	2	1	3.66%	10.213758465300263	0.0010296184977138137	0.0006619531157664456	4.306972454960475e-06
ENSG00000119977_95693899_95664067_607	TCTN3	10	-	1821	47	0	0	3.13%	15.144799516793249	5.745807750721616e-05	5.745807750721616e-05	2.45922963507537e-08
ENSG00000095564_91951489_92028933_1687	BTAF1	10	+	5061	11	0	1	0.53%	1.2753575700743927	0.001946208561389314	0.006277459298483267	7.053443163459546e-05
ENSG00000196591_113970908_113941058_488	HDAC2	6	-	1464	47	2	0	4.51%	18.83789612027357	6.490634218901057e-05	1.3738142124962996e-05	7.091210246476639e-09
ENSG00000005339_3850743_3727718_2287	CREBBP	16	-	6861	27	1	1	0.57%	2.3091490225113693	0.0008263938593182232	0.0008263938593182232	4.2991338318617794e-06
ENSG00000157193_53271061_53247018_498	LRP8	1	-	1494	15	9	0	1.20%	5.8913697171837365	0.2405700776118731	0.012798402692974279	0.018919609472776648
ENSG00000135480_52248596_52248760_54	KRT7	12	+	162	125	7	10	37.04%	452.76267270949086	3.757398726066812e-05	0.0003365783525345705	9.748978693557187e-08
ENSG00000126456_49664838_49659589_452	IRF3	19	-	1356	19	5	0	1.99%	8.221849596459073	0.048657759782558534	0.003529166024264050 2	0.0010441032749023465
ENSG00000115966_175121492_175092614_345	ATF2	2	-	1035	12	0	0	1.45%	6.803251290800001	0.02108409854857798	0.02108409854857798	0.002029891263157455
ENSG00000092036_22955118_22947993_188	HAUS4	14	-	564	18	0	2	3.72%	18.727034803132984	0.00847367416878415	0.020402682033483024	0.0008597039727527121
ENSG00000099956_23787179_23834180_336	SMARCB1	22	+	1008	23	7	4	4.17%	13.388839035837801	0.022731438634345433	0.008784088447361777	0.000988881924296293
ENSG00000139579_56224857_56229213_211	NABP2	12	+	633	19	1	0	3.79%	17.61268254786493	0.009288069496478249	0.005306578019052857	0.0002590037054391842
ENSG00000077782_38421911_38413628_500	FGFR1	8	-	1500	9	0	0	1.60%	3.5206825429890007	0.0033278027414746884	0.0033278027414746884	6.211806171561044e-05
ENSG00000184922_45230610_45246596_1055	FMNL1	17	+	3165	13	1	1	0.66%	2.4101565591815173	0.013794764262559246	0.013794764262559246	0.00091780185964856
ENSG00000154277_41256977_41268073_207	UCHL1	4	+	621	395	4	3	27.54%	373.23392498138895	3.6683649611617057e-11	2.38145085271208e-11	1.0485246521976584e-20
ENSG00000126226_113195112_113178198_292	PCID2	13	-	876	16	0	0	2.74%	10.717450663589043	0.005306578019052857	0.005306578019052857	0.00015100114740534233
ENSG00000063245_55678802_55695356_492	EPN1	19	+	1476	23	4	2	2.44%	9.143597390328255	0.008780371388832966	0.003975543265698643	0.00018829814118630052
ENSG00000167972_2288167_2276674_750	ABCA3	16	-	2250	5	0	0	0.67%	1.3039564974033335	0.012673659338734126	0.012673659338734126	0.000782701129001274
ENSG00000167775_8308275_8305702_76	CD320	19	-	228	11	0	0	7.89%	28.309581851519745	0.011772051206276392	0.011772051206276392	0.000681272511389508
ENSG00000196235_39459039_39476399_946	SUPT5H	19	+	2838	32	2	3	2.01%	6.616269754266386	0.0001389057379015588	0.0004512865513599377	4.3825258532272856e-07
ENSG00000115275_74462963_74461275_562	MOGS	2	-	1686	22	0	3	1.96%	7.656684059485767	0.0013845019718837994	0.01157547599262317	9.8960288269354e-05
ENSG00000111540_55986961_55992212_215	AC034102.1	12	+	645	12	0	0	3.26%	10.916845094539537	0.008175610787902942	0.008175610787902942	0.0003425184326896132 4
ENSG00000197756_216499339_216520753_60	RPL37A	2	+	180	255	25	11	30.00%	831.2722670946251	9.152174643962531e-05	0.0003369099689742292 3	2.2125306702080885e-07
ENSG00000175054_142566169_142449429_2499	ATR	3	-	7497	13	0	0	0.36%	1.0174930652006806	0.002943990679779778	0.002943990679779778	4.916404313974209e-05
ENSG00000126858_32192201_32224733_479	RHOT1	17	+	1437	10	0	1	1.25%	4.083371077463466	0.012798402692974279	0.023672501042627626	0.0014375227604752335
ENSG00000104529_143586845_143581107_136	EEF1D	8	-	408	43	0	0	6.62%	61.84205447243751	0.003710480643933249	0.003710480643933249	7.644477796348072e-05
ENSG00000100266_42912080_42871357_486	PACSIN2	22	-	1458	8	3	2	1.23%	3.219645672600824	0.09373101630823749	0.0802647675719614 9	0.027156458056844114
ENSG00000154743_12496532_12532721_370	TSEN2	3	+	1110	9	0	0	1.35%	4.757679112147298	0.020613416668581838	0.020613416668581838	0.001946208561389314
ENSG00000134318_11217124_11183437_929	ROCK2	2	-	2787	31	0	2	1.40%	6.526800552126481	0.000649205117157464	0.002390058766287232	9.800683060424628e-06
ENSG00000115652_106166060_106094026_381	UXS1	2	-	1143	23	4	1	2.89%	11.807480094597116	0.011987288894911035	0.0023450217334965687	0.00016174504289948947
ENSG00000197448_143263514_143268837_214	GSTK1	7	+	642	46	9	9	7.94%	42.04345715926637	0.004109312069827537	0.006962996228477849	0.00015443548036893825
ENSG00000143442_151442183_151404802_1394	POGZ	1	-	4182	12	0	7	0.50%	1.683731488756098	0.0078820576498062	0.10248820237021045	0.00461455943846696
ENSG00000221914_26359008_26370413_340	PPP2R2A	8	+	1020	21	0	0	3.24%	12.080773431825003	0.0013430411379358334	0.0013430411379358334	1.0937773364376948e-05
ENSG00000169679_110641719_110637964_236	BUB1	2	-	708	9	0	0	2.12%	7.45907318429873	0.019216965118390855	0.019216965118390855	0.0017073955890589052
ENSG00000101901_111681219_111759999_1137	ALG13	X	+	3411	10	4	0	0.44%	1.720259231402815	0.1592709555222523	0.020613416668581838	0.015829670090686688
ENSG00000196419_41622042_41663815_559	XRCC6	22	+	1677	40	15	1	3.40%	13.995955249409663	0.013826087482469898	7.805734269599954e-05	1.164064112598279e-05
ENSG00000083857_186709110_186588592_4349	FAT1	4	-	13047	109	7	11	0.92%	4.902204813185676	3.4976471422289744e-07	1.2196311103537715e-06	3.932090433934067e-12
ENSG00000138629_74458867_74446090_380	UBL7	15	-	1140	13	0	0	1.84%	6.691355710359212	0.008175610787902942	0.008175610787902942	0.0003425184326896132 4
ENSG00000107951_30349275_30313609_582	MTPAP	10	-	1746	18	6	1	1.89%	6.049282719912372	0.03056701560917638	0.002458650919525015	0.0004619283894048296 3
ENSG00000108433_46929545_46963884_192	GOSR2	17	+	576	11	0	0	2.60%	11.205876149559899	0.016947426762344633	0.016947426762344633	0.0013498980316300959
ENSG00000118961_20801463_20684896_370	LDAH	2	-	1110	8	0	0	1.35%	4.229048099686487	0.019679754444124863	0.019679754444124863	0.0017849636115922674
ENSG00000141198_54912675_54960626_399	TOM1L1	17	+	1197	17	3	1	2.51%	8.333556562352133	0.01664033820237559	0.00397428314897603	0.0003597475883221794
ENSG00000198561_57795613_57816308_832	CTNND1	11	+	2496	62	7	2	2.76%	14.575475271455533	0.0003827488158090153	2.3314382156310368e-05	7.254834957190183e-08
ENSG00000115685_241157830_241167068_212	PPP1R7	2	+	636	20	0	0	3.77%	18.45221458589623	0.005306578019052857	0.005306578019052857	0.00015100114740534233
ENSG00000023839_99782845_99794011_195	ABCC2	10	+	585	14	7	1	3.08%	14.042608433574362	0.33632999628228655	0.026832695913080365	0.04814272760090725

ENSG00000145191_184135386_184141928_389	EIF2B5	3	+	1167	14	1	0	1.29%	7.039353842023138	0.0391240932165479	0.02108409854857798	0.00366011816787233
ENSG00000285839_52028617_52020933_115	AL445685.3	1	-	345	74	0	1	6.96%	125.86014887980002	0.005604866691947602	0.012088529595134127	0.00035289342940057376
ENSG00000175895_95154045_95154794_249	PLEKHF2	8	+	747	21	0	0	2.01%	16.49583520811446	0.02108409854857798	0.02108409854857798	0.002029891263157455
ENSG00000147439_22630576_22621422_199	BIN3	8	-	597	8	0	0	3.02%	7.863054255698494	0.011570465654371866	0.011570465654371866	0.0006594843062896773
ENSG00000134333_18396843_18407132_241	LDHA	11	+	723	288	14	7	14.52%	233.73826011545233	1.1279738371723337e-07	5.912422903882768e-07	6.414489826677433e-13
ENSG00000115414_215435802_215361555_2240	FN1	2	-	6720	109	7	10	2.54%	9.517718184171654	3.0534199084709574e-10	1.8364920686751289e-09	6.3688848188218295e-18
ENSG00000115241_27387116_27381599_492	PPM1G	2	-	1476	88	4	7	6.91%	34.98419871082115	6.602091307463113e-07	2.769097892935369e-06	1.648833382773843e-11
ENSG00000136045_107688670_107712220_439	PWP1	12	+	1317	32	0	1	3.42%	14.257383115116177	0.0002610398334131 02	0.0004393245722899426	7.720662262985669e-07
ENSG00000100722_88563134_88611751_736	ZC3H14	14	+	2208	28	3	3	2.45%	7.441056099312502	0.0006720187443280254	0.0007645086132399178	3.269339617940824e-06
ENSG00000132780_45584153_45618141_786	NASP	1	+	2358	43	2	2	2.29%	10.700406371821249	0.00035097825675520125	0.00035097825675520125	8.235522520959779e-07
ENSG00000169814_15635440_15645488_523	BTD	3	+	1569	13	0	0	1.15%	4.861788087832697	0.013428347753762199	0.013428347753762199	0.0008725593497644525
ENSG00000079459_11802847_11838609_250	FDFT1	8	+	750	120	8	5	12.00%	93.88486781304002	1.531972360261985e-05	4.050363744449223e-06	5.19972966323571e-10
ENSG00000164168_147617957_147634434_438	TMEM184C	4	+	1314	8	5	0	1.37%	3.5724835545296814	0.21886412946940276	0.011570465654371866	0.015594623583470883
ENSG00000138442_202897354_202880860_290	WDR12	2	-	870	28	2	0	4.14%	18.88488720377242	0.002674329485664681	0.0010280153344583009	1.6712315643600486e-05
ENSG00000100888_21415594_21385613_1932	CHD8	14	-	5796	24	0	3	0.41%	2.4297326038571434	0.005554612275049112	0.024700093995601816	0.0007236013026813561
ENSG00000167642_38264893_38292006_252	SPINT2	19	+	756	158	4	10	13.10%	122.63400392245637	2.7819786610178883e-07	2.259849513408874e-06	5.911619387999046e-12
ENSG00000241468_99466141_99458327_98	ATP5MF	7	-	294	81	0	1	14.29%	161.6639943209235	0.000472567311753486	0.0005719734963971691	1.7605100106746918e-06
ENSG00000165280_35061609_35060777_114	VCP	9	-	342	33	0	0	7.02%	56.61916370303949	0.005554612275049112	0.005554612275049112	0.0001646845769100 7306
ENSG00000103275_1314031_1324793_158	UBE2I	16	+	474	91	7	4	14.56%	112.65193790857914	4.1653514996278555e-05	4.2145511149793747e-05	1.32979772114665e-08
ENSG00000129103_56064312_56079647_352	SUMF2	7	+	1056	135	11	3	6.82%	75.01454281936792	0.0003756302393955671	2.2773204007700768e-05	6.963798606940528e-08
ENSG00000198836_193637210_193692127_642	OPA1	3	+	1926	25	0	1	2.02%	7.616568325954051	0.0006223089785121674	0.000806344341637709	3.1989545673191004e-06
ENSG00000258881_70994748_70942315_194	AC007040.2	2	-	582	20	0	1	5.15%	20.16427573304124	0.002122792577845 6486	0.0021227925778456486	2.6309315010939275e-05
ENSG00000125457_75269377_75266740_205	MIF4GD	17	-	615	9	0	0	2.44%	8.587030592656099	0.016947426762344633	0.016947426762344633	0.0013498980316300959
ENSG00000125458_75131677_75130488_191	NT5C	17	-	573	14	1	2	3.66%	14.336694474068066	0.007029302661868638	0.04912109528954385	0.0018325026027891263
ENSG00000100567_58244921_58267524_197	PSMA3	14	+	591	239	24	3	17.77%	237.29360625334778	1.8506454718217028e-05	1.0313769518071171e-07	2.206471196130081e-11
ENSG00000162298_65132346_65131444_83	SYVN1	11	-	249	11	1	0	6.02%	25.92202675560844	0.04223451612662591	0.019679754444124863	0.003715148010809822
ENSG00000206527_123585027_123494888_254	HACD2	3	-	762	30	4	0	5.12%	23.10159148942914	0.006559179018921906	0.000609140140830996	2.65903587934989e-05
ENSG00000197885_23901047_23893095_160	NKIRAS1	3	-	480	16	0	1	5.00%	19.559347461050006	0.005306578019052857	0.009288069496478249	0.0002590037054391842
ENSG00000243678_51166831_51171604_152	NME2	17	+	456	301	13	6	25.00%	387.32655169579283	1.2638481888959088e-07	1.1359927279737081e-07	1.3834799338967196e-13
ENSG00000132432_54757588_54755775_66	SEC61G	7	-	198	101	0	2	25.76%	299.31728690394704	0.0001375389481522447	0.0003468173162106 4607	3.3336730618183494e-07
ENSG00000124357_71068684_71078508_344	NAGK	2	+	1032	17	2	0	2.03%	9.665956594123548	0.04912109528954385	0.007029302661868638	0.0018325026027891263
ENSG00000177200_53222720_53324895_2278	CHD9	16	+	6834	12	0	3	0.22%	1.030343149835821	0.020613416668581838	0.09783387127182075	0.009177765633874927
ENSG00000137106_37422751_37436782_328	GRHPR	9	+	984	88	11	6	9.15%	52.47629806623172	3.076092715032158e-05	4.771686749322835e-06	1.231773762836079e-09
ENSG00000184056_91009831_90998975_493	VPS33B	15	-	1479	11	0	0	1.42%	4.364154605913794	0.0078820576498062	0.0078820576498062	0.0003196364290745 1164
ENSG00000011304_797498_810826_197	PTBP1	19	+	591	65	0	2	10.66%	64.53591801869291	2.6142789501994117e-05	4.735339972598898e-05	9.506718342742637e-09
ENSG00000143418_150967837_150966148_230	CERS2	1	-	690	80	9	0	12.61%	68.03251290800002	3.376746903000881e-05	1.0713725137466392e-06	3.4362075881160587e-10
ENSG00000118193_200618492_200553388_1571	KIF14	1	-	4713	26	6	0	0.76%	3.2370657796772764	0.013219332612197623	0.0009991914723789358	8.671783710435585e-05
ENSG00000100949_24271115_24265615_567	RABGGTA	14	-	1701	9	0	0	0.88%	3.104658327150794	0.019679754444124863	0.019679754444124863	0.0017849636115922674
ENSG00000150782_112155053_112143596_193	IL18	11	-	579	60	0	6	8.81%	60.80626153694302	9.260308916294388e-05	0.0001827208711679 4644	1.2120896812938756e-07
ENSG00000049618_157174031_157207891_1286	ARID1B	6	+	3858	16	0	0	0.54%	2.433511348186626	0.00847367416878415	0.00847367416878415	0.0003665024264111182
ENSG00000170142_23810476_23889378_167	UBE2E1	3	+	501	25	0	1	3.59%	29.28046027103294	0.013428347753762199	0.01371683287978855	0.0008901624618536816
ENSG00000113448_58993456_58974664_499	PDE4D	5	-	1497	37	1	0	3.61%	14.502922967111227	0.0001185681716428482	8.091994981866961e-05	6.953223258948248e-08
ENSG00000112996_44809113_44815202_389	MRPS30	5	+	1167	31	5	0	3.60%	15.58714065019409	0.003777282907900914	0.0004168743852437731	1.0611583329332023e-05
ENSG00000130479_17720400_17734428_1030	MAP1S	19	+	3090	14	4	0	0.68%	2.6585520820844666	0.08736059003872232	0.00797940233269714	0.003864285406208 3823
ENSG00000113460_34915739_34922291_129	BRIX1	5	+	387	17	1	0	6.20%	25.77588425099613	0.008170681041463092	0.005405141725979036	0.0002324897789243 5937
ENSG00000039560_34688204_34830765_983	RAI14	5	+	2949	30	2	1	1.32%	5.969282032873857	0.003031355522137688	0.0010322727522116193	1.9025352038280622e-05
ENSG00000147536_41530203_41542087_223	GINS4	8	+	669	10	0	0	2.69%	8.771007830067267	0.011570465654371866	0.011570465654371866	0.0006594843062896773
ENSG00000169727_82053289_82057127_475	GPS1	17	+	1425	37	6	9	3.58%	15.235702232817898	0.0036153727960830124	0.005325788655027384	0.0001056095470972 8684
ENSG00000141522_81869930_81868379_235	ARHGDIA	17	-	705	113	7	0	10.21%	94.05133034462342	2.5038603510923497e-05	7.620578087781505e-06	1.5532777709261638e-09
ENSG00000102393_101398612_101397809_177	GLA	X	-	531	21	2	0	6.21%	23.206005462262716	0.008474269282996836	0.001092092816906976	5.786447861430024e-05
ENSG00000070061_108926583_108869115_1197	ELP1	9	-	3591	32	1	0	1.42%	5.228898235201338	0.0001529756704662 8207	0.0001148880751151 2702	1.2503580343413566e-07
ENSG00000187109_76074219_76049463_383	NAP1L1	12	-	1149	91	12	3	6.01%	46.4726010171162	0.0002376322401470 0557	1.5548142148013118e-05	3.044601116463459e-08
ENSG00000111684_6981056_6977146_203	LPCAT3	12	-	609	20	2	6	5.91%	19.270293065073897	0.002467335870027 7245	0.034048574204743325	0.0005229378202293401
ENSG00000177239_137107370_137108591_137	MAN1B1	9	+	411	13	0	0	3.65%	18.559964744062047	0.020613416668581838	0.020613416668581838	0.001946208561389314
ENSG00000003402_201130085_201145385_170	CFLAR	2	+	510	11	0	0	2.94%	12.656048357150002	0.019679754444124863	0.019679754444124863	0.0017849636115922674
ENSG00000100650_69770482_69771461_145	SRSF5	14	+	435	30	2	4	8.28%	40.46761543665518	0.003182737791905174	0.006765523591417148	0.0001187342466505311
ENSG00000108559_5419650_5386206_741	NUP88	17	-	2223	38	1	0	1.89%	10.03043459541026	0.0005444500874584297	0.0004441315507112266	1.581205649667966e-06
ENSG00000149792_65122359_65125872_162	MRPL49	11	+	486	25	0	0	5.56%	30.184178180632724	0.0035547763465261306	0.0035547763465261306	7.044676146010672e-05
ENSG00000014216_65182702_65211286_714	CAPN1	11	+	2142	30	5	5	2.38%	8.218213218928573	0.0033811758088108748	0.0017177785361068316	3.389112207118646e-05
ENSG00000103187_84590215_84566845_73	COTL1	16	-	219	65	0	0	21.92%	174.15857328332197	0.00020608240359398683	0.00020608240359398683	2.9380978096451203e-07
ENSG00000105135_15122742_15115252_410	ILVBL	19	-	1230	12	5	0	1.95%	5.724687061770733	0.07962816006291146	0.004826937407846361	0.0023618906866966134
ENSG00000131143_85801206_85805115_83	COX4I1	16	+	249	116	18	4	19.28%	273.35955487732537	0.0012803358715245219	0.0005756616192752859	4.684157119402114e-06
ENSG00000175606_73976282_73978869_107	TMEM70	8	+	321	16	0	0	6.54%	29.247622371663557	0.0078820576498062	0.0078820576498062	0.0003196364290745 1164
ENSG00000110660_107815808_107792659_268	SLC35F2	11	-	804	25	1	2	4.85%	18.245659945009333	0.0009061776457653113	0.002546666671256 2532	1.4171498675249201e-05
ENSG00000084754_26244596_26191250_763	HADHA	2	-	2289	89	4	1	3.93%	22.814966238970516	1.7122260248073914e-06	8.154771907763202e-07	1.2470936655583645e-11
ENSG00000172409_57659597_57661436_385	CLP1	11	+	1155	15	2	0	1.82%	7.620524984824677	0.017818254085047065	0.00837382380159347	0.0007434988581310937
ENSG00000122033_27440124_27435675_170	MTIF3	13	-	510	13	1	0	3.53%	14.957148058450004	0.023878473930440184	0.013007239811447113	0.0014709138084317821

ENSG00000121940_108939653_108934670_210	CLCC1	1	-	630	9	0	0	2.86%	8.382577483307145	0.012178957859018494	0.012178957859018494	0.0007262454987071801
ENSG00000121940_108941429_108934670_294	CLCC1	1	-	882	11	0	0	2.38%	7.318123199712587	0.007593099278532274	0.007593099278532274	0.00029783286705462064
ENSG00000077150_102395960_102402376_900	NFKB2	10	+	2700	23	0	0	1.00%	4.998499906712779	0.0035547763465261306	0.0035547763465261306	7.044676146010672e-05
ENSG00000122378_80420507_80432099_216	PRXL2A	10	+	648	49	0	0	6.02%	44.370741925530105	0.0006862354746923267	0.0006862354746923267	3.005258142461633e-06
ENSG00000123992_219386772_219374292_410	DNPEP	2	-	1230	17	2	2	1.95%	8.109973337508539	0.02671499548383061	0.02671499548383061	0.003152782508864385
ENSG00000163507_108589375_108551149_906	CIP2A	3	-	2718	12	0	2	0.66%	2.5906420478211927	0.013007239811447113	0.0424352593247778	0.002615673534585255
ENSG00000132507_7309636_7309884_82	EIF5A	17	+	246	169	70	8	18.29%	403.1133805996891	0.0158684317527454	0.0003250121193590314	4.2512425905242323e-05
ENSG00000116133_54887119_54879791_133	DHCR24	1	-	399	10	0	0	4.51%	14.706276286503762	0.011570465654371866	0.011570465654371866	0.0006594843062896773
ENSG00000116133_54879763_54852233_392	DHCR24	1	-	1176	50	0	4	5.87%	24.948147271747455	9.575030103382214e-06	9.53194825725167e-05	7.530174693379461e-09
ENSG00000186575_29604095_29683055_475	NF2	22	+	1425	13	0	0	1.26%	5.35308456828737	0.011772051206276392	0.011772051206276392	0.000681272511389508
ENSG00000144591_219501485_219506798_371	GMPPA	2	+	1113	15	0	2	1.35%	7.908091965384099	0.02108409854857798	0.0700082515985845	0.006564739044797468
ENSG00000102471_79533424_79551120_140	NDFIP2	13	+	420	13	0	0	3.57%	18.162251213832146	0.02108409854857798	0.02108409854857798	0.002029891263157455
ENSG00000053371_19311974_19304225_309	AKR7A2	1	-	927	7	0	0	1.94%	4.430920136807444	0.009815328628645334	0.009815328628645334	0.0004837142251238724
ENSG00000132780_45584153_45618141_447	NASP	1	+	1341	41	2	2	3.58%	17.940341071656604	0.0007300672788143355	0.0007300672788143355	3.3861259192495386e-06
ENSG00000198642_21334589_21333006_527	KLHL9	9	-	1581	12	1	0	1.52%	4.45374135735484	0.010437296944230427	0.00414960799764038	0.00023253924594002017
ENSG00000104064_50309723_50286020_323	GABPB1	15	-	969	14	0	7	1.86%	8.47773574163158	0.012798402692974279	0.2405700776118731	0.018919609472776648
ENSG00000196961_49767161_49806758_968	AP2A1	19	+	2904	34	1	0	1.86%	6.870018736319216	0.00011592007149080553	7.870973464443577e-05	6.622085795723275e-08
ENSG00000198546_133309432_133311950_197	ZNF511	10	+	591	19	1	1	5.08%	18.86434526700254	0.00397428314897603	0.00397428314897603	8.713148241777404e-05
ENSG00000119048_134371623_134390353_134	UBE2B	5	+	402	15	1	1	6.72%	21.8947919340112	0.006024333578509648	0.006024333578509648	0.00019211261281100973
ENSG00000132470_75724704_75757555_1752	ITGB4	17	+	5256	89	8	7	2.40%	9.935969886035675	2.3433006485323964e-07	2.450374282504324e-07	5.405332579885013e-13
ENSG00000175390_7987353_7996022_357	EIF3F	11	+	1071	31	0	10	2.52%	16.98430731911905	0.0033278027414746945	0.13400371787331594	0.0034457488676848075
ENSG00000113522_132579932_132594589_399	RAD50	5	+	1197	17	1	0	2.26%	8.333556562352133	0.005588370640926919	0.0034782380676658805	0.00010676685949938237
ENSG00000198189_87372791_87337276_142	HSD17B11	4	-	426	29	0	0	7.04%	39.94514622327114	0.0022276761777358703	0.0022276761777358703	2.8853233252577778e-05
ENSG00000155229_97498384_97458692_987	MMS19	10	-	2961	27	4	0	1.01%	5.35058137232371	0.023573842698425523	0.0023218226288164415	0.00033052785286200547
ENSG00000197451_178204865_178210623_276	HNRNPAB	5	+	828	102	20	7	10.14%	72.28454496475001	0.0005272710709344839	1.3691309628042567e-05	6.391815525073465e-08
ENSG00000197747_151986218_151983163_93	S100A10	1	-	279	406	25	15	48.39%	853.8811902350863	1.0520970707073368e-08	1.002770181025531e-08	1.0956173937957959e-15
ENSG00000167088_21612430_21629138_119	SNRPD1	18	+	357	49	2	2	5.88%	80.53848954550001	0.00877616296920843	0.0173950697634955	0.0007572669587723205
ENSG00000148356_127454591_127502899_702	LRSAM1	9	+	2106	8	2	0	0.71%	2.228985465646724	0.11777945996203976	0.016947426762344633	0.009674391494749069
ENSG00000144036_72718111_72465094_461	EXOC6B	2	-	1383	16	0	1	1.30%	6.788493695809112	0.013640585738808999	0.020525443280229434	0.0013255139945858052
ENSG00000088682_57447506_57460624_207	COQ9	16	+	621	23	0	0	5.31%	21.73260829005556	0.0014055202957965589	0.0014055202957965589	1.1935530040246081e-05
ENSG00000172936_38138779_38140608_201	MYD88	3	+	603	22	0	3	4.98%	21.408241002144283	0.00228120298834518	0.011739578714767718	0.00015450514653894077
ENSG00000145685_78510213_78488897_228	LHFPL2	5	-	684	10	0	0	3.07%	8.578661167127194	0.007029302661868638	0.007029302661868638	0.0002573639808771639
ENSG00000123136_14413220_14410592_208	DDX39A	19	-	624	22	7	6	5.77%	20.687771353033657	0.02628191594397717	0.027312817400366944	0.003169936918525003
ENSG00000164291_95555279_95600879_395	ARSK	5	+	1185	7	0	0	1.27%	3.4662134741101274	0.016947426762344633	0.016947426762344633	0.0013498980316300959
ENSG00000134480_87399503_87394446_69	CCNH	5	-	207	16	0	0	8.70%	45.355008605333346	0.013007239811447113	0.013007239811447113	0.0008218654148058079
ENSG00000136143_47988672_47943371_329	SUCLA2	13	-	987	13	0	0	2.74%	7.728617537800914	0.002478942540635376	0.002478942540635376	3.5397643206479886e-05
ENSG00000090266_140702960_140704934_41	NDUFB2	7	+	123	97	0	0	17.07%	462.74553749313424	0.008980238763039383	0.008980238763039383	0.0004089866599971986
ENSG00000090266_140696745_140700880_34	NDUFB2	7	+	102	94	1	1	32.35%	540.7584298055001	0.0016109979480538772	0.0016109979480538772	1.550748317303123e-05
ENSG00000090266_140702960_140704934_41	NDUFB2	7	+	123	97	0	0	17.07%	462.74553749313424	0.008980238763039383	0.008980238763039383	0.0004089866599971986
ENSG00000123159_14478708_14478416_58	GIPC1	19	-	174	20	0	0	15.52%	67.44602572775864	0.0033030736763512156	0.0033030736763512156	6.124050385493983e-05
ENSG00000123200_46042238_45963822_1476	ZC3H13	13	-	4428	17	2	0	0.41%	2.2527703715301497	0.02491347480025195	0.013007239811447113	0.0015333653594885373
ENSG00000166136_100529851_100529376_71	NDUFB8	10	-	213	41	1	0	12.68%	112.94834449338735	0.004557893626254153	0.0037896409716948643	9.495750423643499e-05
ENSG00000166136_100526536_100524089_62	NDUFB8	10	-	186	26	0	0	14.52%	82.02306999795164	0.0034529206320349946	0.0034529206320349946	6.664817351595063e-05
ENSG00000172845_173955656_173909941_496	SP3	2	-	1488	22	0	0	1.61%	8.675517019014116	0.005604866691947602	0.005604866691947602	0.00016752456572620382
ENSG00000133895_64810109_64804334_615	MEN1	11	-	1845	22	4	1	1.95%	6.996839742164229	0.01878413720018969	0.0016120252826907794	0.0001902478323907079
ENSG00000133895_64810109_64804334_615	MEN1	11	-	1845	22	4	1	1.95%	6.996839742164229	0.01878413720018969	0.0016120252826907794	0.0001902478323907079
ENSG00000145868_148414216_148442147_722	FBXO38	5	+	2166	17	0	0	0.97%	4.605386521299862	0.0078820576498062	0.0078820576498062	0.00031963642907451164
ENSG00000167005_56451202_56447785_106	NUDT21	16	-	318	13	0	0	6.60%	23.9878789616651	0.00797940233269714	0.00797940233269714	0.000327142761693177
ENSG00000089685_78214680_78216062_68	BIRC5	17	+	204	24	5	0	8.82%	69.03299103900001	0.029237630782826433	0.013640585738808999	0.001872687038404406
ENSG00000169045_179623133_179614841_472	HNRNPH1	5	-	1416	257	17	9	11.65%	106.49898935359855	1.1617800006254185e-09	9.257241552365015e-10	1.1877883915833602e-17
ENSG00000063177_48619143_48616582_146	RPL18	19	-	438	66	2	7	10.96%	88.41896797460961	0.000268372558905113	0.0016434039859274943	3.0000819239898347e-06
ENSG00000143774_228145513_228148755_242	GUK1	1	+	726	7	0	0	2.07%	5.6576624887334725	0.019216965118390855	0.019216965118390855	0.0017073955890589052
ENSG00000215301_41334253_41364342_733	DDX3X	X	+	2199	94	12	3	6.14%	25.082928531223743	5.81627248979138e-07	8.039453835617377e-09	5.3199907532461864e-14
ENSG00000033867_27403173_27376764_461	SLC4A7	3	-	1383	15	0	0	1.95%	6.364212839821042	0.002874854311667729	0.002874854311667729	4.6982854837323694e-05
ENSG00000033867_27434087_27404954_265	SLC4A7	3	-	795	9	0	2	1.89%	6.642797250922643	0.020613416668581838	0.08350359792828288	0.007749259071793911
ENSG00000173039_65662832_65654952_246	RELA	11	-	738	18	1	5	3.25%	14.311717654426833	0.01007916461776185	0.04753395219010738	0.0023771889737864704
ENSG00000141526_82237209_82261095_293	SLC16A3	17	+	879	28	3	1	4.10%	18.691526583938572	0.0054114546266510665	0.0015734716984481248	5.011014200213529e-05
ENSG00000075188_102077500_102074354_145	NUP37	12	-	435	21	0	0	5.52%	28.327330805658626	0.005554612275049112	0.005554612275049112	0.00016468457691007306
ENSG00000139116_39358192_39294424_1237	KIF21A	12	-	3711	8	0	0	0.49%	1.2649537565755864	0.011570465654371866	0.011570465654371866	0.0006594843062896773
ENSG00000285815_39380385_39405984_173	WRB-SH3BGR	21	+	519	19	0	0	4.62%	21.48136426358093	0.005405141725979036	0.005405141725979036	0.00015637213408961292
ENSG00000145191_184140487_184144943_417	EIF2B5	3	+	1251	7	0	0	1.20%	3.2833436984976028	0.016947426762344633	0.016947426762344633	0.0013498980316300959
ENSG00000156671_75150529_75176692_415	SAMD8	10	+	1245	20	2	0	2.17%	9.426191547493978	0.012991839772286901	0.0034782380676658805	0.00024813008800201436
ENSG00000100526_54397069_54420078_172	CDKN3	14	+	516	17	1	3	6.40%	19.331913188247096	0.002558966410643687	0.010372145937058125	0.00015028040810348176
ENSG00000136942_124861912_124857918_123	RPL35	9	-	369	408	17	3	13.01%	648.797867000683	0.002362045486731161	0.0002613566507245454	4.281409266201683e-06
ENSG00000239900_40349871_40366522_420	ADSL	22	+	1260	42	0	0	4.05%	19.559347461050006	0.00013239568919489033	0.00013239568919489033	1.245599941315653e-07
ENSG00000117385_42762325_42746833_492	P3H1	1	-	1476	22	0	3	1.83%	8.746049677705287	0.003684287313187465	0.017782189529096822	0.00036099358464052045
ENSG00000106355_32489303_32487261_62	LSM5	7	-	186	49	4	0	27.42%	154.58193961152423	0.0002473554500538834	0.00013319834607924987	2.311148598512169e-07

ENSG00000014641_63589044_63606987_169	MDH1	2	+	507	143	3	3	16.57%	165.50217082426926	1.6449104547372236e-05	1.1532688333734049e-05	1.5207330752586155e-09
ENSG00000103363_2777239_2771990_118	ELOB	16	-	354	749	59	5	23.73%	1241.5212922310554	6.664554313962327e-06	3.096326659089507e-06	1.747028849359234e-10
ENSG00000142534_49496457_49499635_158	RPS11	19	+	474	822	45	13	29.75%	1017.5812413280445	1.1264917441989338e-09	6.696636476348421e-09	8.296702505254324e-17
ENSG00000087191_63828138_63831969_398	PSMC5	17	+	1194	144	14	3	11.56%	70.76748830128645	1.634647098621326e-07	2.0668185553905446e-09	3.978615250315918e-15
ENSG00000111665_6850952_6846788_219	CDCA3	12	-	657	18	2	0	4.57%	16.076175995383565	0.01044836432889887	0.002046028879997488	0.00012444751772694397
ENSG00000067560_49375589_49360366_187	RHOA	3	-	561	177	6	0	16.58%	185.13393051368186	4.745267202507306e-06	4.979143408534445e-07	2.1859709652028875e-11
ENSG00000067560_49360352_49360209_47	RHOA	3	-	141	128	0	3	44.68%	532.6801010668937	2.7555717747565283e-05	9.717543140995877e-05	2.0499922745792747e-08
ENSG00000168288_149587097_149569974_330	MMADHC	2	-	990	53	3	4	6.06%	31.413497437443947	0.00014769757371358336	0.0003416426969924909	3.511782852147847e-07
ENSG00000118785_87977833_87982896_289	SPP1	4	+	867	305	8	14	22.49%	206.42217908720593	1.711762903811837e-11	2.7703102692940294e-11	5.730050061220136e-21
ENSG00000142949_43569670_43622003_1745	PTPRF	1	+	5235	26	2	0	0.69%	2.9142867277209175	0.002619468406317666	0.0009470236307512361	1.5180240942830124e-05
ENSG00000160888_13153187_13153858_223	IER2	19	+	669	49	10	0	5.38%	42.97793836732961	0.013967204762937563	0.0010573555742419644	9.659782915611994e-05
ENSG00000179409_748009_747226_173	GEMIN4	17	-	519	10	3	2	3.47%	11.305981191358384	0.11368416502207135	0.05685341913966222	0.024301342007733352
ENSG00000161203_184176994_184179157_124	AP2M1	3	+	372	11	0	0	4.03%	17.35103403802823	0.016947426762344633	0.016947426762344633	0.0013498980316300959
ENSG00000149136_57335121_57326407_709	SSRP1	11	-	2127	94	11	7	5.64%	25.9319980442694	1.7444822969800511e-06	2.424135591635435e-07	3.984901440903666e-12
ENSG00000104388_60584796_60620769_98	RAB2A	8	+	294	17	0	1	9.18%	33.92948028957654	0.002943990679779778	0.0064006895615519015	0.00010465593861806192
ENSG00000154001_63453790_63376009_378	PPP2R5E	14	-	1134	19	0	0	1.85%	9.831418035977515	0.00847367416878415	0.00847367416878415	0.0003665024264111182
ENSG00000114867_184320681_184334908_1403	EIF4G1	3	+	4209	90	7	5	2.78%	12.546979839590167	4.60942195086692e-07	1.9011841143914044e-07	8.246422000810537e-13
ENSG00000213145_105487260_105488511_77	CRIP1	14	+	231	250	20	12	28.57%	635.0437487353897	4.829947205692244e-05	3.108342685307635e-05	1.1448317702416725e-08
ENSG00000103932_41536954_41520238_1258	RPAP1	15	-	3774	17	0	0	0.56%	2.6431550623040545	0.00847367416878415	0.00847367416878415	0.0003665024264111182
ENSG00000164828_838871_873331_735	SUN1	7	+	2205	22	0	0	0.95%	5.854498559770069	0.0078820576498062	0.0078820576498062	0.00031963642907451164
ENSG00000112029_152972406_152971163_128	FBXO5	6	-	384	11	2	0	5.47%	16.808814224339848	0.0416322583317752	0.007593099278532274	0.0016329495508977223
ENSG00000160014_46608271_46608618_68	CALM3	19	+	204	24	0	1	13.24%	69.03299103900001	0.002967235347054297	0.006432154271512831	0.00010592475669358982
ENSG00000160014_46608888_46609153_40	CALM3	19	+	120	10	2	0	12.50%	48.89836865262501	0.04223451612662591	0.020613416668581838	0.0038681811577380596
ENSG00000100473_30878908_30894920_382	COCH	14	+	1146	17	0	0	2.88%	8.704421644969898	0.0012558331315113088	0.0012558331315113088	9.614604369954505e-06
ENSG00000145919_173616436_173609239_185	BOD1	5	-	555	8	0	1	2.70%	8.458096199372974	0.019679754444124863	0.04223451612662591	0.003715148010809822
ENSG00000143418_150969090_150966148_380	CERS2	1	-	1140	122	13	6	11.84%	62.79579974337107	9.274309105767741e-08	2.3215391495753192e-08	2.1706759421677818e-14
ENSG00000143321_156751429_156743449_240	HDGF	1	-	720	101	2	4	11.67%	82.31225389858544	3.6294620226253088e-06	5.436123694587638e-06	1.6642546176290767e-10
ENSG00000105726_19663666_19645422_1204	ATP13A1	19	-	3612	15	0	2	0.66%	2.436795780031147	0.005160369442893307	0.022898969405409888	0.0006278347384112434
ENSG00000131652_3026086_3027657_260	THOC6	16	+	780	28	0	1	4.23%	21.063912650361544	0.0015505692382983198	0.002167177068466985	1.9913191269291018e-05
ENSG00000105197_39486289_39489820_155	TIMM50	19	+	465	21	5	0	5.16%	26.499761076261297	0.040065279221313775	0.005554612275049112	0.0012110652168332507
ENSG00000100292_35386691_35393598_238	HMOX1	22	+	714	24	1	0	4.20%	19.723711725428576	0.0030814981005195803	0.0023354528714849923	4.1239111951852285e-05
ENSG00000134480_87411304_87394446_270	CCNH	5	-	810	24	0	0	2.96%	17.38608663204445	0.005655335537214311	0.005655335537214311	0.00017039952270558162
ENSG00000134779_36818914_36796805_252	TPGS2	18	-	756	8	0	0	1.98%	6.209316654301588	0.019216965118390855	0.019216965118390855	0.0017073955890589052
ENSG00000179950_143821895_143816520_499	PUF60	8	-	1497	66	0	0	5.41%	25.870078806198403	1.916420072849888e-06	1.916420072849888e-06	3.2014646159581766e-11
ENSG00000171940_53582172_53571744_830	ZNF217	20	-	2490	21	0	1	1.45%	4.948750562434339	0.0008852284070317903	0.0014891271583564725	8.137399560608084e-06
ENSG00000127415_987085_1004393_653	IDUA	4	+	1959	13	0	0	0.92%	3.8938976568705983	0.013428347753762199	0.013428347753762199	0.0008725593497644525
ENSG00000221914_26363861_26370413_133	PPP2R2A	8	+	399	17	0	0	5.26%	25.000669687056398	0.0078820576498062	0.0078820576498062	0.00031963642907451164
ENSG00000109332_102809680_102797415_110	UBE2D3	4	-	330	19	0	1	5.45%	33.78432743272273	0.013428347753762199	0.013428347753762199	0.0008725593497644525
ENSG00000164889_151061988_151076367_1241	SLC4A2	7	+	3723	59	26	5	2.18%	9.298964546349318	0.01641305528749142	5.228841052443753e-05	1.057051460316207e-05
ENSG00000041357_78544194_78548944_190	PSMA4	15	+	570	221	0	8	9.47%	227.50609415221322	8.735376412917216e-05	0.0014627342080213868	9.78739406124214e-07
ENSG00000105341_41439899_41432231_257	DMAC2	19	-	771	24	8	2	3.89%	18.265538485027243	0.0637201447313876	0.009257958062480545	0.0030425768460727643
ENSG00000114125_141738342_141742795_108	RNF7	3	+	324	22	0	0	8.33%	39.84311519843519	0.0036062891729786097	0.0036062891729786097	7.240558534568731e-05
ENSG00000100029_30591833_30577046_583	PES1	22	-	1749	54	4	3	3.95%	18.11671977524357	7.270193601083459e-05	3.695702943025601e-05	2.0251747674380822e-08
ENSG00000169241_155137696_155138281_82	SLC50A1	1	+	246	7	0	0	6.10%	16.697003930164637	0.019216965118390855	0.019216965118390855	0.0017073955890589052
ENSG00000119689_74889332_74902330_349	DLST	14	+	1047	32	2	0	3.72%	17.934072170590262	0.0013600423278798564	0.0006652407155885319	5.692578211673844e-06
ENSG00000101266_489860_483961_177	CSNK2A1	20	-	531	9	0	0	2.82%	9.945430912398308	0.019216965118390855	0.019216965118390855	0.0017073955890589052
ENSG00000168036_41233346_41233960_176	CTNNB1	3	+	528	29	8	0	6.25%	32.22847024832103	0.020090055490004644	0.001558047347358915	0.00019919496610202849
ENSG00000110719_68043589_68050819_614	TCIRG1	11	+	1842	12	4	5	1.63%	3.8226737708892515	0.07131960316528611	0.08361556339140147	0.022043295418559098
ENSG00000003402_201130085_201163080_389	CFLAR	2	+	1167	11	1	0	1.29%	5.5309208758753226	0.04223451612662591	0.019679754444124863	0.003715148010809822
ENSG00000117868_158829418_158734207_866	ESYT2	7	-	2598	42	5	3	2.31%	9.486057660093536	4.983000130598301e-05	0.00023625467940204776	8.773628016212268e-08
ENSG00000129197_5421311_5428083_113	RPAIN	17	+	339	21	2	0	5.31%	36.34922979487169	0.037178613843887286	0.013428347753762199	0.0023492041693324566
ENSG00000089157_120200783_120196773_317	RPLP0	12	-	951	61	1	4	3.15%	37.63786104492272	0.0025025372014526115	0.004791578323725913	6.770248138606176e-05
ENSG00000065357_55938942_55953768_593	DGKA	12	+	1779	5	1	0	0.84%	1.6491861265640813	0.051235217429874705	0.012673659338734126	0.0031110695322510533
ENSG00000101972_124047365_124100597_1005	STAG2	X	+	3015	15	0	1	1.00%	2.919305591201493	0.0020082573300163734	0.00392533363377418	4.538242985436214e-05
ENSG00000145979_13326898_13305101_293	TBC1D7	6	-	879	17	0	0	1.71%	11.348426854534132	0.02108409854857798	0.02108409854857798	0.002029891263157455
ENSG00000123130_23743144_23703894_448	ACOT9	X	-	1344	32	5	0	3.57%	13.970962472178575	0.0024774008637122387	0.00017495645137376598	3.1656393133230364e-06
ENSG00000008283_63437460_63434402_222	CYB561	17	-	666	14	0	3	2.70%	12.334723624085589	0.013217226988443315	0.06307108214216126	0.0040111611771127525
ENSG00000125166_58734228_58708171_387	GOT2	16	-	1161	61	1	0	4.65%	30.82997920217184	0.0001127271246253546	8.99016647834184e-05	7.322784784629293e-08
ENSG00000004799_95596185_95585641_375	PDK4	7	-	1125	72	4	3	6.40%	37.553947125216006	1.6626066702354336e-05	1.892280898636615e-05	2.4886435040449815e-09
ENSG00000013441_200861712_200853306_434	CLK1	2	-	1302	36	1	0	4.15%	16.224343516078346	0.00011644620007245363	7.914814395228409e-05	6.68720263805529e-08
ENSG00000104635_22404711_22434004_481	SLC39A14	8	+	1443	31	1	4	2.91%	12.605816450988568	0.0005855227822530266	0.002703861664531204	1.0125509206082202e-05
ENSG00000213246_58351454_58346246_76	SUPT4H1	17	-	228	18	0	0	6.58%	46.324770302486854	0.021557223391537678	0.021557223391537678	0.002115616449879074
ENSG00000240682_129159155_129130081_299	ISY1	3	-	897	11	2	1	2.34%	7.195746557576925	0.0416322583317752	0.017404239405933563	0.003292339026149851
ENSG00000141198_54912675_54915925_183	TOM1L1	17	+	549	13	0	0	3.28%	13.894618414953555	0.013428347753762199	0.013428347753762199	0.0008725593497644525
ENSG00000142875_84182305_84235305_280	PRKACB	1	+	840	14	0	0	2.14%	9.779673730525003	0.013428347753762199	0.013428347753762199	0.0008725593497644525
ENSG00000142875_84182305_84235305_280	PRKACB	1	+	840	14	0	0	2.14%	9.779673730525003	0.013428347753762199	0.013428347753762199	0.0008725593497644525
ENSG00000142875_84182305_84235305_280	PRKACB	1	+	840	14	0	0	2.14%	9.779673730525003	0.013428347753762199	0.013428347753762199	0.0008725593497644525

ENSG00000090621_39571256_39561698_488	PABPC4	1	-	1464	10	0	1	1.02%	4.0080630043135255	0.020613416668581838	0.03537493353818881	0.003252526348827943
ENSG00000025770_50508338_50523259_589	NCAPH2	22	+	1767	11	1	3	1.02%	3.652849271163838	0.023672501042627626	0.08503348072695238	0.008834625609373653
ENSG00000149084_43680828_43754886_98	HSD17B12	11	+	294	15	0	1	6.12%	29.937776726096946	0.012798402692974279	0.02784981298332479	0.0016870839615509433
ENSG00000135924_219279834_219285997_277	DNAJB2	2	+	831	15	0	0	1.81%	10.591704401290617	0.02108409854857798	0.02108409854857798	0.002029891263157455
ENSG00000068903_38883633_38879155_181	SIRT2	19	-	543	16	0	0	2.76%	17.290030904795586	0.020613416668581838	0.020613416668581838	0.001946208561389314
ENSG00000127824_219252188_219250352_433	TUBA4A	2	-	1299	35	0	0	3.23%	15.810096100155892	0.00037874750290730355	0.00037874750290730355	9.541676172656993e-07
ENSG00000115641_105386516_105361283_279	FHL2	2	-	837	24	2	0	3.23%	16.82524512778495	0.01052924788556975	0.003684287313187465	0.00021087137401632993
ENSG00000247315_297587_298798_403	ZCCHC3	20	+	1209	12	0	0	1.99%	5.824123313464021	0.004193160480899131	0.004193160480899131	9.649553577281792e-05
ENSG00000164120_174508711_174491956_131	HPGD	4	-	393	37	1	0	8.40%	55.243958477774825	0.0015881753678246666	0.0015806110104534784	1.5019944328400419e-05
ENSG00000163347_190322206_190308277_211	CLDN1	3	-	633	26	2	1	6.64%	24.10156559181517	0.0019774936324837536	0.0010499093600357017	1.2624279789573373e-05
ENSG00000143793_228103038_228101131_228	C1orf35	1	-	684	12	0	4	2.19%	10.294393400552634	0.019679754444124863	0.15040137116388086	0.014313628319454642
ENSG00000168000_62705512_62690367_398	BSCL2	11	-	1194	16	15	2	2.51%	7.863054255698494	0.3857046718699725	0.008474269282996836	0.029120313164340397
ENSG00000168000_62705595_62702488_118	BSCL2	11	-	354	14	0	6	5.08%	23.206005462262716	0.013428347753762199	0.16970534604944526	0.012520549700299675
ENSG00000177628_155240684_155234995_467	GBA	1	-	1401	43	2	0	1.93%	18.009677533729125	0.009297184653683213	0.003710480643933249	0.00018728191086118568
ENSG00000106070_50626921_50592952_407	GRB10	7	-	1221	12	0	0	1.47%	5.766883772299756	0.013217226988443315	0.013217226988443315	0.0008469697265466234
ENSG00000106070_50626921_50592952_407	GRB10	7	-	1221	12	0	0	1.47%	5.766883772299756	0.013217226988443315	0.013217226988443315	0.0008469697265466234
ENSG00000092036_22955118_22946525_351	HAUS4	14	-	1053	24	1	2	3.13%	13.373912793880345	0.0021519382791564774	0.005035606171387428	6.19116467518456e-05
ENSG00000160710_154586283_154584806_193	ADAR	1	-	579	53	0	3	8.29%	53.712197690966335	0.00019829743686022551	0.0007824961798974837	1.0636756757335573e-06
ENSG00000151914_56642496_56615517_2591	DST	6	-	7773	8	1	1	0.23%	0.6039165561111541	0.028889785561798602	0.028889785561798602	0.0036451790457678215
ENSG00000162616_78005111_78016247_337	DNAJB4	1	+	1011	15	0	0	2.37%	8.70594100640208	0.005306578019052857	0.005306578019052857	0.00015100114740534233
ENSG00000134851_55403333_55425452_257	TMEM165	4	+	771	25	0	0	5.06%	19.026602588570043	0.0006289630342484525	0.0006289630342484525	2.5404679232419015e-06
ENSG00000166888_57107239_57096572_737	STAT6	12	-	2211	23	3	0	1.36%	6.10400259978494	0.007867321313276006	0.0023218226288164415	0.00010390540483540199
ENSG00000166801_59152092_59153504_470	FAM111A	11	+	1410	8	0	0	1.06%	3.329250631668086	0.019679754444124863	0.019679754444124863	0.0017849636115922674
ENSG00000214063_862679_866630_174	TSPAN4	11	+	522	50	6	2	12.64%	56.20502143979886	0.00037908809303956073	3.476132325118705e-05	1.0286581531374531e-07
ENSG00000214063_850341_866630_226	TSPAN4	11	+	678	58	8	2	11.06%	50.19655543101329	0.00019441792049279118	9.897872049539573e-06	1.639486903430763e-08
ENSG00000126773_60114713_60168748_873	PCNX4	14	+	2619	13	0	3	0.69%	2.9126176058837348	0.012798402692974279	0.08620755130569058	0.005490655705532311
ENSG00000149485_61803755_61802411_146	FADS1	11	-	438	86	0	7	8.22%	115.21259463358221	0.0010872143717490798	0.0010872143717490798	7.287803622448181e-06
ENSG00000121211_153363020_153414857_115	MND1	4	+	345	11	0	0	5.22%	18.708941049700005	0.012798402692974279	0.012798402692974279	0.0007972436888943567
ENSG00000140395_78288374_78278251_102	WDR61	15	-	306	15	0	0	5.88%	28.763746266250006	0.013428347753762199	0.013428347753762199	0.0008725593497644525
ENSG00000101294_31568169_31569219_51	HM13	20	+	153	31	1	1	13.73%	118.89015123383336	0.010275150823238127	0.010275150823238127	0.0005273288931938515
ENSG00000101294_31514552_31564139_365	HM13	20	+	1095	200	15	3	9.32%	107.17450663589044	1.2160970649635597e-05	2.5720573657730357e-07	3.180869277886326e-11
ENSG00000182093_39390698_39390907_69	WRB	21	+	207	17	0	0	8.70%	48.18969664316668	0.012798402692974279	0.012798402692974279	0.0007972436888943567
ENSG00000170445_140691304_140674257_489	HARS	5	-	1467	66	1	1	4.09%	26.39911927258283	4.812783160904289e-05	4.812783160904289e-05	1.7418305196102364e-08
ENSG00000143575_154272724_154273657_124	HAX1	1	+	372	10	0	0	4.03%	15.77366730729839	0.019679754444124863	0.019679754444124863	0.0017849636115922674
ENSG00000115944_42361161_42351219_114	COX7A2L	2	-	342	25	14	0	7.89%	42.89330583563598	0.13201477310855114	0.0036581908865286695	0.0036123670377061616
ENSG00000089685_78214317_78214793_74	BIRC5	17	+	222	33	7	0	10.81%	87.2241170560338	0.01882253325647002	0.005655335537214311	0.0005552396394223762
ENSG00000152234_46091840_46089784_123	ATP5F1A	18	-	369	123	3	1	13.82%	195.59347461050004	0.00026095534406300096	0.00015718001048987173	2.8523211692691823e-07
ENSG00000108559_5419650_5388771_557	NUP88	17	-	1671	23	1	0	1.97%	8.076570764885998	0.0021519382791564774	0.0015356776430442238	1.959765111893205e-05
ENSG00000106541_16801796_16794847_188	AGR2	7	-	564	141	0	0	17.02%	146.69510595787503	3.578829702385202e-07	3.578829702385202e-07	1.188970156833874e-12
ENSG00000128309_37024216_37029514_297	MPST	22	+	891	13	1	2	2.69%	8.561330538506736	0.010020958156399907	0.030313543781140352	0.0014836836722180672
ENSG00000127483_20780440_20744797_553	HP1BP3	1	-	1659	29	4	2	1.99%	10.257162321346295	0.010778133380008164	0.0038108971419065062	0.0002225164781485849
ENSG00000175376_66000389_65998599_165	EIF1AD	11	-	495	11	0	0	3.64%	13.039564974033336	0.012798402692974279	0.012798402692974279	0.0007972436888943567
ENSG00000101811_100820417_100837409_438	CSTF2	X	+	1314	17	0	1	1.14%	7.591527553375572	0.02108409854857798	0.028889785561798623	0.002731532589007073
ENSG00000153187_244863998_244854450_697	HNRNPU	1	-	2091	189	25	12	7.60%	53.037541895817085	1.0091259207953188e-06	2.1346169843538475e-08	2.338712094124885e-13
ENSG00000119004_203239063_203296908_470	CYP20A1	2	+	1410	15	2	1	1.70%	6.242344934377661	0.02389626957043539	0.009288069496478249	0.0010920267704436078
ENSG00000177830_903742_869858_369	CHID1	11	-	1107	14	0	3	1.90%	7.420890635628728	0.007029302661868638	0.04912109528954385	0.0018325026027891263
ENSG00000204843_74378227_74361499_1256	DCTN1	2	-	3768	38	9	2	1.51%	5.917636970699842	0.0033918133431191906	0.00020713935581772233	5.14464272896285e-06
ENSG00000169371_75621051_75598358_360	SNUPN	15	-	1080	29	0	0	3.33%	15.756141010290282	0.0010032776369550557	0.0010032776369550557	6.244443566103777e-06
ENSG00000104763_18069848_18057534_313	ASAH1	8	-	939	51	3	2	7.35%	31.8698632751933	1.0081031668924613e-05	4.339047084990489e-05	3.524512317448131e-09
ENSG00000130635_134642188_134842303_1838	COL5A1	9	+	5514	13	8	2	0.33%	1.3834141294540265	0.2728812359313196	0.03639452886275914	0.04495854477582206
ENSG00000063177_48619143_48615873_164	RPL18	19	-	492	236	3	7	20.73%	281.46378053706104	2.3459350807284704e-07	1.2570802182955269e-06	2.7682136326496367e-12
ENSG00000167792_67608700_67611931_230	NDUFV1	11	+	690	29	4	0	4.78%	24.661785929150007	0.004400313145429775	0.0014055202957965589	3.6695114437754866e-05
ENSG00000130429_99385715_99394489_372	ARPC1B	7	+	1116	114	5	7	10.48%	59.939935767733886	1.1109155435768817e-07	3.273677265832452e-07	3.485077842819282e-13
ENSG00000132294_131946499_132010895_744	EFR3A	8	+	2232	59	2	9	3.23%	15.510772852176752	1.412135627885162e-05	0.00038488881812705244	4.670392296146739e-08
ENSG00000169398_140846306_140659466_824	PTK2	8	-	2472	15	6	4	1.21%	3.5605608242202678	0.0820328997370387	0.023029368248455667	0.00832076987426179
ENSG00000102805_76995908_77000969_243	CLN5	13	+	729	12	0	2	2.47%	9.65893701780247	0.011772051206276392	0.060667625179241055	0.0035017256847258756
ENSG00000114062_25375498_25340081_724	UBE3A	15	-	2172	8	13	0	0.69%	2.1612538630994482	0.3331867010986109	0.019216965118390855	0.038471517829580006
ENSG00000162910_228106918_228106760_52	MRPL55	1	-	156	34	1	1	17.31%	127.8880410914808	0.004355956481189797	0.004355956481189797	0.0001037507970309169
ENSG00000117523_171522225_171591847_2553	PRRC2C	1	+	7659	17	10	3	0.35%	1.3024242335990994	0.15039870036150454	0.019224948125267174	0.014067798201048379
ENSG00000139637_53305908_53307149_213	C12orf10	12	+	639	17	3	2	4.69%	15.610746799899065	0.01664033820237559	0.011698770487832223	0.0009413727934111041
ENSG00000197912_89508547_89529209_187	SPG7	16	+	561	12	0	0	2.67%	12.551452916181821	0.019679754444124863	0.019679754444124863	0.0017849636115922674
ENSG00000143376_151612202_151693601_407	SNX27	1	+	1221	15	0	2	1.47%	7.208604715374695	0.011772051206276392	0.060667625179241055	0.0035017256847258756
ENSG00000186298_110722672_110721076_141	PPP1CC	12	-	423	36	0	0	9.22%	49.938759475021286	0.0006026251277497241	0.0006026251277497241	2.339296094881936e-06
ENSG00000139644_49753061_49758539_115	TMBIM6	12	+	345	259	5	3	25.22%	440.5105210793001	3.126854677694789e-06	1.2074416764977363e-06	3.319711396102164e-11
ENSG00000104763_18084058_18057534_370	ASAH1	8	-	1110	106	3	2	7.30%	56.03488732084596	2.1209243094296095e-06	8.259411896664993e-06	1.5086207056338041e-10
ENSG00000143179_165896284_165907823_111	UCK2	1	+	333	23	0	0	8.11%	40.5283776219955	0.0036062891729786097	0.0036062891729786097	7.240558534568731e-05
ENSG00000257923_101816052_102283090_671	CUX1	7	+	2013	11	8	0	1.19%	3.2064504034508206	0.18176997233392067	0.004687384229717438	0.006576065488566696

ENSG00000125520_63741404_63742990_282	SLC2A4RG	20	+	846	12	0	2	2.13%	8.323126579170214	0.013007239811447113	0.05258119297243839	0.00326646906127711
ENSG00000157168_32722003_32763338_308	NRG1	8	+	924	16	0	0	1.62%	10.160699979766235	0.021557223391537678	0.021557223391537678	0.002115616449879074
ENSG00000103351_3504767_3536271_409	CLUAP1	16	+	1227	10	0	0	1.71%	4.782236543044011	0.007029302661868638	0.007029302661868638	0.0002573639808771639
ENSG00000126214_103675572_103685018_149	KLC1	14	+	447	11	0	0	3.36%	14.439786716211414	0.021084098548577898	0.021084098548577898	0.002029891263157455
ENSG00000100938_24233254_24238778_320	GMPR2	14	+	960	9	2	0	1.56%	5.501066473420313	0.06032271266817251	0.020613416668581838	0.005529087794823047
ENSG00000048649_77820684_77666917_1431	RSF1	11	−	4293	11	1	0	0.42%	1.5035137810730261	0.026832695913080365	0.011772051206276392	0.0015103817605807263
ENSG00000100902_35292477_35317272_203	PSMA6	14	+	609	79	3	2	7.88%	76.11765760704189	0.0002953471520703521	0.0002074783909516633	4.199236596901837e-07
ENSG00000100612_60165309_60152864_235	DHRS7	14	−	705	37	1	2	5.96%	30.795568342929794	0.000496609694897612	0.0006940860426134196	2.2290338882851133e-06
ENSG00000143321_156751429_156743449_240	HDGF	1	−	720	101	2	4	11.67%	82.31225389858544	3.6294620226253088e-06	5.436123694587638e-06	1.6642546176290767e-10
ENSG00000070159_109463434_109379556_913	PTPN3	9	−	2739	9	0	0	0.55%	1.9280846347146774	0.020613416668581838	0.020613416668581838	0.001946208561389314
ENSG00000168944_123418486_123346519_960	CEP120	5	−	2880	12	0	0	0.83%	2.4449184326312507	0.004826937407846361	0.004826937407846361	0.00012612451166366307
ENSG00000204209_33321925_33318743_740	DAXX	6	−	2220	17	0	0	0.81%	4.493363605916893	0.013428347753762199	0.013428347753762199	0.0008725593497644525
ENSG00000182944_29282517_29288790_145	EWSR1	22	+	435	22	0	0	4.14%	29.6762513202138	0.013428347753762199	0.013428347753762199	0.0008725593497644525
ENSG00000128609_123557795_123542119_101	NDUFA5	7	−	303	17	3	2	5.94%	32.92167394434159	0.013428347753762199	0.037440157020026926	0.002365921094328065
ENSG00000127922_96709763_96645933_57	SEM1	7	−	171	34	0	11	17.54%	116.66979187292985	0.0023766146074493525	0.10274152173808476	0.0019162447551253363
ENSG00000149428_119056160_119045593_999	HYOU1	11	−	2997	89	3	1	3.40%	17.425244484819324	5.816796237368974e-07	1.6284037999071413e-07	8.967828762845731e-13
ENSG00000166452_8911450_8919480_183	AKIP1	11	+	549	12	0	0	3.28%	12.825801613803282	0.011772051206276392	0.011772051206276392	0.000681272511389508
ENSG00000204316_75904875_75898850_380	MRPL38	17	−	1140	46	0	6	4.74%	23.677104821271058	8.782737371870633e-05	0.0018981863084467764	1.3031585365405299e-06
ENSG00000072864_15664779_15724251_335	NDE1	16	+	1005	9	4	0	1.49%	5.254750064162688	0.1700871372700561	0.019216965118390855	0.016246064094152867
ENSG00000072864_15664779_15724251_335	NDE1	16	+	1005	9	4	0	1.49%	5.254750064162688	0.1700871372700561	0.019216965118390855	0.016246064094152867
ENSG00000141012_88842766_88814439_461	GALNS	16	−	1383	22	1	1	1.52%	9.33417883173753	0.009583874861041503	0.009583874861041503	0.00046242146588181525
ENSG00000135250_105388806_105117838_699	SRPK2	7	−	2097	13	0	2	0.86%	3.637646881168098	0.012798402692974279	0.044040755831095145	0.0026804992160797854
ENSG00000157349_70324649_70333582_328	DDX19B	16	+	984	14	0	0	1.52%	8.348501965082319	0.019679754444124863	0.019679754444124863	0.0017849636115922674
ENSG00000099956_23787179_23834180_391	SMARCB1	22	+	1173	24	4	4	3.58%	12.005737572000003	0.004909317959302256	0.008784088447361777	0.0002283466035886222
ENSG00000172270_580434_582577_176	BSG	19	+	528	833	13	21	30.68%	925.7350247190144	6.190942331300489e-11	1.504974989271497e-09	1.12678231073978e-18
ENSG00000141556_82768540_82942463_1045	TBCD	17	+	3135	23	3	8	1.15%	4.30492814932201	0.002234548513461168	0.045262276060680215	0.000670214776361679
ENSG00000143514_223802146_223780853_736	TP53BP2	1	−	2208	7	0	0	0.68%	1.8602640248281255	0.019216965118390855	0.019216965118390855	0.0017073955890589052
ENSG00000005448_74423343_74425657_193	WDR54	2	+	579	17	1	0	3.63%	17.22844076880052	0.013892891352654344	0.00847367416878415	0.0005886798845679606
ENSG00000181555_47123969_47017093_2342	SETD2	3	−	7026	15	0	0	0.30%	1.2527336119374468	0.00847367416878415	0.00847367416878415	0.0003665024264111182
ENSG00000147642_109579914_109574906_457	SYBU	8	−	1371	9	0	0	1.09%	3.8519502658522984	0.020613416668581838	0.020613416668581838	0.001946208561389314
ENSG00000166557_79311250_79411504_173	TMED3	15	+	519	9	0	0	2.89%	10.175383072222546	0.020613416668581838	0.020613416668581838	0.001946208561389314
ENSG00000116685_11926237_11920315_634	KIAA2013	1	−	1902	12	2	2	0.79%	3.7020846929432185	0.06945717695039447	0.06032271266817251	0.016020910955657944
ENSG00000170889_54201185_54206526_156	RPS9	19	+	468	135	11	0	7.69%	169.2635837975481	0.004183873134198697	0.001100170263453246	2.7943281629777465e-05
ENSG00000006744_13015830_12996418_472	ELAC2	17	−	1416	13	0	4	1.69%	5.3871084108824165	0.004826937407846361	0.05830098158568198	0.0016433245845561082
ENSG00000109452_142431259_142028782_924	INPP4B	4	−	2772	8	2	0	0.76%	1.6934499966277061	0.06180520118411761	0.005706018193000826	0.0020032236666657145
ENSG00000101266_489860_483961_177	CSNK2A1	20	−	531	9	0	0	2.82%	9.945430912398308	0.019216965118390855	0.019216965118390855	0.0017073955890589052
ENSG00000168944_123418486_123346519_960	CEP120	5	−	2880	12	0	0	0.83%	2.4449184326312507	0.004826937407846361	0.004826937407846361	0.00012612451166366307
ENSG00000173801_41771836_41755744_739	JUP	17	−	2217	62	0	3	2.71%	16.409736706158327	4.058371258354367e-05	0.00011112278459049561	3.378833332041556e-08
ENSG00000145901_151065095_151030713_572	TNIP1	5	−	1716	11	2	0	0.87%	3.761412973278847	0.0477903522728147	0.016947426762344633	0.0036975118224100542
ENSG00000109390_139295798_139292550_76	NDUFC1	4	−	228	19	2	0	7.89%	48.89836865262501	0.016822681477512567	0.013428347753762199	0.0010798218650012886
ENSG00000120533_109334469_109340498_53	ENY2	8	+	159	56	1	3	22.64%	206.6648033620378	0.0013600423278798564	0.003261738042631143	2.636254622534456e-05
ENSG00000196591_113946098_113941058_191	HDAC2	6	−	573	25	0	0	5.76%	25.601240132264405	0.0014409783181973444	0.0014409783181973444	1.2520176346377507e-05
ENSG00000145901_151065095_151030642_635	TNIP1	5	−	1905	10	2	0	0.79%	3.080212198590552	0.06556525143482543	0.019679754444124863	0.00579448895258773
ENSG00000168385_241326104_241350174_321	SEPT2	2	+	963	74	6	1	10.90%	45.090084489647985	2.5859254594989955e-07	7.981522422512355e-08	1.999971846101505e-13
ENSG00000010278_6235323_6237828_81	CD9	12	+	243	77	7	1	20.99%	185.93453759269758	0.002710882253903909	0.00023349597112665984	4.486946918727257e-06
ENSG00000010278_6200500_6236202_182	CD9	12	+	546	145	51	27	17.58%	155.82996603583794	0.00036932596748593527	1.036890029555778e-05	3.400249608179956e-08
ENSG00000097033_86704900_86743235_365	SH3GLB1	1	+	1095	23	1	0	2.47%	12.3250682631274	0.005670089768331009	0.0035547763465261306	0.00011049933550063637
ENSG00000182180_73250959_73250852_35	MRPS16	10	−	105	21	0	1	28.57%	117.35608476630003	0.0022409900782254734	0.005670089768331009	7.232548112602907e-05
ENSG00000168002_62765397_62766507_42	POLR2G	11	+	126	70	0	5	33.33%	325.98912435083344	0.00046843213071395124	0.0031185037294365974	9.613998767368705e-06
ENSG00000137710_110272594_110231869_447	RDX	11	−	1341	20	4	1	2.01%	8.751385888612978	0.03582012146929187	0.00426768422311763	0.0008635007970486224
ENSG00000132155_12618588_12584514_566	RAF1	3	−	1698	16	5	0	1.06%	5.529144158600708	0.08873763529811646	0.011772051206276392	0.005312171954116946
ENSG00000074582_218662227_218663386_189	BCS1L	2	+	567	12	0	0	4.23%	12.418633308603177	0.004826937407846361	0.004826937407846361	0.00012612451166366307
ENSG00000004897_47189172_47120935_823	CDC27	17	−	2469	13	0	2	0.97%	3.089568857760025	0.004826937407846361	0.026606355080852302	0.0006944327269402791
ENSG00000149806_65121813_65120681_133	FAU	11	−	399	197	10	5	16.54%	289.71364284412414	5.147669781924341e-05	2.584577234195794e-05	1.0214756268404264e-08
ENSG00000095002_47410181_47607456_787	MSH2	2	+	2361	30	1	2	1.27%	7.455913898748413	0.0031859520777271916	0.006198316675603911	0.00010898907104230859
ENSG00000111530_67292692_67312830_1136	CAND1	12	+	3408	83	0	5	3.17%	14.29072041608407	4.838292635566865e-08	1.4150720083717559e-06	7.10402114954021e-13
ENSG00000123159_14482976_14478416_333	GIPC1	19	−	999	26	3	3	3.90%	15.271562582201204	0.00346840949707983	0.00436789375498805	8.384841920484692e-05
ENSG00000113643_168486499_168494570_129	RARS	5	+	387	12	1	0	3.88%	18.194741824232562	0.03537493353818881	0.020613416668581838	0.003252526348827943
ENSG00000113643_168495354_168519190_454	RARS	5	+	1362	49	1	0	5.29%	21.110308933732384	1.0005003478380114e-05	6.860381480352942e-06	5.633048328684357e-10
ENSG00000103353_23557967_23570590_295	UBFD1	16	+	885	29	0	0	3.73%	19.227833097303396	0.0013845019718837994	0.0013845019718837994	1.1595255170738555e-05
ENSG00000215301_41334253_41345417_420	DDX3X	X	+	1260	37	12	3	5.95%	17.23085371568691	0.0017977780560661303	3.0360303229622683e-05	4.932263651350944e-07
ENSG00000215301_41345404_41347719_272	DDX3X	X	+	816	59	0	0	8.09%	42.42652574271876	1.4055095028526564e-05	1.4055095028526564e-05	1.579705563707443e-09
ENSG00000095002_47410181_47632186_767	MSH2	2	+	2301	30	1	2	1.30%	7.650331471075621	0.0031859520777271916	0.006198316675603911	0.00010898907104230859
ENSG00000177426_3456398_3457940_252	TGIF1	18	+	756	34	0	2	2.78%	26.38959578078175	0.008574016250729465	0.013648824643852793	0.0005848802985757839
ENSG00000178209_143935297_143916177_4341	PLEC	8	−	13023	130	33	39	1.38%	5.857441073339094	2.796321955810114e-06	1.2083355211512743e-05	2.8801264455689585e-10
ENSG00000166340_6619400_6617622_127	TPP1	11	−	381	14	0	0	4.72%	21.561485390133864	0.012798402692974279	0.012798402692974279	0.0007972436888943567
ENSG00000101266_489860_483961_177	CSNK2A1	20	−	531	9	0	0	2.82%	9.945430912398308	0.019216965118390855	0.019216965118390855	0.0017073955890589052

ENSG00000120948_11022340_11022654_104	TARDBP	1	+	312	17	0	0	4.81%	31.9720102728702	0.021084098548577798	0.021084098548577798	0.002029891263157455
ENSG00000128708_171922501_171983352_419	HAT1	2	+	1257	60	0	0	5.25%	28.008612116062057	1.5096957790992848e-05	1.5096957790992848e-05	1.8164356658901269e-09
ENSG00000091409_172427789_172501879_1073	ITGA6	2	+	3219	85	0	6	2.61%	15.494357261782389	1.4498153181488628e-06	4.097369601144186e-05	5.549685729188893e-10
ENSG00000149311_108227625_108365508_3056	ATM	11	+	9168	26	0	0	0.39%	1.6640806085971862	0.0009829516685359422	0.0009829516685359422	6.0033663169184795e-06
ENSG00000170315_16381908_16382597_229	UBB	17	+	687	204	22	3	8.73%	174.2404751988734	0.0003677098512070003	4.177093299346451e-05	1.1769370889349975e-07
ENSG00000166803_64381371_64373707_115	PCLAF	15	-	345	151	12	4	12.17%	256.82273622770003	0.0009939604220181157	0.0021373669800862794	1.2956270448965783e-05
ENSG00000101247_13785069_13814443_267	NDUFAF5	20	+	801	19	1	0	2.62%	13.918636770035583	0.013892891352654344	0.00847367416878415	0.0005886798845679606
ENSG00000069424_6082210_6098574_310	KCNAB2	1	+	930	8	0	0	1.61%	5.047573538335485	0.019679754444124863	0.019679754444124863	0.0017849636115922674
ENSG00000103429_14644574_14667827_306	BFAR	16	+	918	21	0	0	3.27%	13.42308159091667	0.00228120298834518	0.00228120298834518	3.0194288573932866e-05
ENSG00000168036_41233535_41233960_141	CTNNB1	3	+	423	15	4	0	4.96%	20.807816447925536	0.06565845108178825	0.00847367416878415	0.002930684821567823
ENSG00000263001_74689207_74758838_972	GTF2I	7	+	2916	19	0	0	1.13%	3.8233292362134783	0.0012624143894574274	0.0012624143894574274	9.711623744831373e-06
ENSG00000173638_45531578_45514976_236	SLC19A1	21	-	708	7	0	0	2.12%	5.801501365565679	0.019216965118390855	0.019216965118390855	0.0017073955890589052
ENSG00000182718_60386075_60347630_339	ANXA2	15	-	1017	453	19	26	8.55%	261.3682713821726	3.759316366201967e-06	1.2105372558515973e-05	3.821431147987569e-10
ENSG00000135480_52245529_52251778_46	KRT7	12	+	138	75	3	4	32.61%	318.90240425625007	0.0003120708621194	0.0003128690666030151	6.577674053314938e-07
ENSG00000114391_101686676_101681179_121	RPL24	3	-	363	51	14	3	7.44%	82.44022483583059	0.0204156028385188	0.0037896409716948643	0.0004274779298826572
ENSG00000197771_119853104_119843174_186	MCMBP	10	-	558	13	0	2	3.23%	13.670511666325272	0.011772051206276392	0.060667625179241055	0.0035017256847258756
ENSG00000258643_23307903_23325286_288	BCL2L2-PABPN1	14	+	864	15	0	0	3.12%	10.187160135963545	0.00327844299461777	0.00327844299461777	6.0372290137947024e-05
ENSG00000113580_143400839_143281889_777	NR3C1	5	-	2331	12	5	2	1.16%	3.0207486426332055	0.07471635411946746	0.014696946993710543	0.005240911805500765
ENSG00000104419_133256848_133238878_239	NDRG1	8	-	717	21	1	1	2.51%	17.18603751807741	0.013301727469301692	0.013301727469301692	0.0008571698582519322
ENSG00000120438_159779908_159779045_76	TCP1	6	-	228	46	0	2	9.21%	118.38552410635529	0.008877961307018026	0.0173950697634955	0.0007651590344002274
ENSG00000120438_159788141_159779250_411	TCP1	6	-	1233	50	0	1	3.65%	23.794826594951342	0.0002948650613319232	0.00041261355168930957	8.152932968510409e-07
ENSG00000100883_35000958_35029152_440	SRP54	14	+	1320	22	2	0	2.73%	9.779673730525003	0.004359748848448076	0.0009430837296224684	2.5386113327095592e-05
ENSG00000168303_40134567_40133059_179	MPLKIP	7	-	537	11	2	0	3.35%	12.019710730254193	0.0319825310473083	0.012798402692974279	0.0019365420928339585
ENSG00000113328_163439257_163442565_295	CCNG1	5	+	885	59	4	2	6.44%	39.11869492210001	0.0003001544741977667	0.00010633743604202221	2.265662457194439e-07
ENSG00000065883_39950642_40094980_1452	CDK13	7	+	4356	18	1	8	0.55%	2.4247124951714882	0.0069659089849165145	0.1386446290442363	0.006087887711934443
ENSG00000115355_55362358_55291711_1651	CCDC88A	2	-	4953	20	1	0	0.40%	2.3693939989158093	0.0033869280463461717	0.0022543491824521134	4.372784971036962e-05
ENSG00000136068_58008565_58170762_2602	FLNB	3	+	7806	126	16	17	2.08%	9.471474942706767	7.410892035135557e-08	8.76675846503856e-08	6.342385039057957e-14
ENSG00000105223_40366649_40378173_468	PLD3	19	+	1404	34	1	0	3.63%	14.209782343497867	0.00017709925282996202	0.0001151290146190202	1.4464965079969512e-07
ENSG00000144136_112646898_112663025_656	SLC20A1	2	+	1968	56	14	3	4.88%	16.697003930164637	0.00013311541598833555	1.647350026583807e-06	2.21435931101325e-09
ENSG00000165916_47426231_47418835_423	PSMC3	11	-	1269	56	2	3	6.86%	25.894171579640666	3.0151512582610386e-06	4.360324577882111e-06	1.1181617717196137e-10
ENSG00000179933_23097773_23098081_102	C14orf119	14	+	306	29	0	0	6.86%	55.60990944808334	0.00877616296920843	0.00877616296920843	0.00039161135667564577
ENSG00000108592_63826282_63819802_782	FTSJ3	17	-	2346	19	0	0	1.41%	4.752271122250001	0.0013845019718837994	0.0013845019718837994	1.1595255170738555e-05
ENSG00000126214_103654586_103685643_544	KLC1	14	+	1632	52	6	7	3.86%	18.696435073062503	0.0005764071508637824	0.0006376289743136012	2.366923025010413e-06
ENSG00000134333_18396843_18400670_94	LDHA	11	+	282	33	0	1	8.51%	68.66579427815427	0.005604866691947602	0.0053557511377634245	0.0001604572327171668
ENSG00000136819_129833550_129828987_277	C9orf78	9	-	831	13	0	0	2.89%	9.179477147785201	0.005160369442893307	0.005160369442893307	0.00014319601891785326
ENSG00000129636_47461045_47155719_612	ITFG1	16	-	1836	59	3	0	4.25%	18.85623366343056	2.0616604192103226e-05	2.9089442248430728e-06	5.149449773004906e-10
ENSG00000197894_99076847_99072417_234	ADH5	4	-	702	26	0	6	4.70%	21.73260829005556	0.0013430411379358334	0.011651814340914361	9.710472547923025e-05
ENSG00000213983_23567140_23559749_727	AP1G2	14	-	2181	11	2	2	1.10%	2.959461101396837	0.033944577430914495	0.019230617045588212	0.0029378519685176155
ENSG00000062485_56283836_56274820_223	CS	12	-	669	14	2	0	2.69%	12.279410962094174	0.04375184787808565	0.013217226988443315	0.0027359276556426933
ENSG00000062485_56275043_56273503_145	CS	12	-	435	7	0	0	3.45%	9.44244360188621	0.019216965118390855	0.019216965118390855	0.0017073955890589052
ENSG00000155463_22766717_22770407_212	OXA1L	14	+	636	44	11	0	6.13%	40.594872088971705	0.0032876702414337345	0.0007100644329059803	1.4724138630909662e-05
ENSG00000164346_74770733_74776651_41	NSA2	5	+	123	17	0	0	12.20%	81.09973337508538	0.020613416668581838	0.020613416668581838	0.001946208561389314
ENSG00000243678_51166831_51171604_152	NME2	17	+	456	303	13	6	25.66%	389.900150045931	8.797986037150864e-08	8.006540688929941e-08	6.866574535207844e-14
ENSG00000011052_51155655_51171604_267	NME1-NME2	17	+	801	580	34	9	26.59%	424.88470140108626	2.0910081065672603e-11	4.1088143236107227e-13	1.165434872525707e-22
ENSG00000117305_23820578_23804346_177	HMGCL	1	-	531	7	0	0	2.82%	7.735335154087572	0.019216965118390855	0.019216965118390855	0.0017073955890589052
ENSG00000104723_15540431_15733399_289	TUSC3	8	+	867	30	0	0	4.15%	20.303820893823534	0.0010405263639310788	0.0010405263639310788	6.697970672456085e-06
ENSG00000125870_16731742_16741005_212	SNRPB2	20	+	636	51	2	2	8.49%	47.05314719403539	0.0001817182897104588	0.0001817182897104588	2.3022607162508642e-07
ENSG00000173039_65661862_65654378_498	RELA	11	-	1494	22	5	8	2.21%	8.640675585202814	0.015652231761283	0.022234889799829897	0.0016223270445432194
ENSG00000141279_47603963_47619182_262	NPEPPS	17	+	786	18	0	1	3.44%	13.437719629729012	0.0034529206320349946	0.005561255870711926	0.00010554338560930125
ENSG00000015475_17739423_17735580_99	BID	22	-	297	12	0	0	5.05%	23.708299952787883	0.020613416668581838	0.020613416668581838	0.001946208561389314
ENSG00000015475_17739423_17735580_99	BID	22	-	297	12	0	0	5.05%	23.708299952787883	0.020613416668581838	0.020613416668581838	0.001946208561389314
ENSG00000141646_51054795_51078467_396	SMAD4	18	+	1188	13	0	0	1.52%	6.4209979038800515	0.012798402692974279	0.012798402692974279	0.0007972436888943567
ENSG00000132463_70839827_70824319_424	GRSF1	4	-	1272	52	4	3	4.01%	23.9878789616651	0.00023611244043024747	0.000442321637794036	7.067246555246459e-07
ENSG00000198925_219228024_219220447_839	ATG9A	2	-	2517	9	0	0	0.95%	2.098142159111443	0.0033278027414746884	0.0033278027414746884	6.211806171561044e-05
ENSG00000140264_43793010_43793773_45	SERF2	15	+	135	75	5	0	17.78%	325.98912435083344	0.026239749779623293	0.005808022449631236	0.0008006752930530814
ENSG00000140264_43792377_43800501_40	SERF2	15	+	120	50	0	0	17.50%	244.49184326312505	0.00847367416878415	0.00847367416878415	0.0003665024264111182
ENSG00000140264_43800623_43801806_121	SERF2	15	+	363	44	1	0	12.40%	71.12489985836365	0.0006786479905456451	0.00023719413513965303	1.0872976294357274e-06
ENSG00000115762_131120942_131146773_222	PLEKHB2	2	+	666	27	4	0	7.21%	23.788395560736493	0.004020532480401752	0.0001469846801210205	4.620862033087646e-06
ENSG00000170525_6213664_6231305_487	PFKFB3	10	+	1461	50	13	3	4.31%	20.081465565759757	0.0012488256715441548	0.00012803126463754768	1.160281498178595e-06
ENSG00000040341_73673120_73421372_476	STAU2	8	-	1428	16	0	0	1.47%	6.574570575142858	0.00797940233269714	0.00797940233269714	0.000327142761693177
ENSG00000113558_134174022_134157733_163	SKP1	5	-	489	58	8	1	7.98%	69.59767808226383	0.00326556549244236455	0.0008884494972709903	1.7898087465695128e-05
ENSG00000149591_117203041_117204359_192	TAGLN	11	+	576	14	1	0	4.69%	14.262024190348962	0.0065000503081102355	0.0025000845279814113	9.158895334168734e-05
ENSG00000150782_112155053_112148613_189	IL18	11	-	567	52	0	6	7.94%	53.81407767061377	0.00021004986723785753	0.00043215126715083924	6.183552199578226e-07
ENSG00000148943_27506752_27498649_173	LIN7C	11	-	519	9	0	0	2.89%	10.175383072222546	0.019679754444124863	0.019679754444124863	0.0017849636115922674
ENSG00000101290_5127093_5190234_445	CDS2	20	+	1335	26	3	6	3.15%	11.427933348029216	0.003121831368066678	0.0034181738847840126	5.997028203546671e-05
ENSG00000074266_86245272_86277168_370	EED	11	+	1110	7	3	0	1.62%	3.7004170872256763	0.15232096101830483	0.009815328628645334	0.008755902507845
ENSG00000110321_10804047_10803521_72	EIF4G2	11	-	216	20	0	3	9.72%	54.3315207251389	0.008674846900444944	0.020779353648479937	0.000893938215340485

ENSG00000163710_142888896_142818432_401	PCOLCE2	3	-	1203	16	0	0	1.75%	7.804228413386536	0.00847367416878415	0.00847367416878415	0.0003665024264111182
ENSG00000100938_24233254_24238778_348	GMPR2	14	+	1044	12	2	0	2.01%	6.744602572775864	0.022898969405409888	0.008175610787902942	0.0009353765476651278
ENSG00000154518_175181393_175178288_142	ATP5MC3	2	-	426	138	31	7	21.83%	190.0837992693592	6.092060945556509e-05	2.2032051986119076e-06	1.2346752126045435e-09
ENSG00000151136_107318941_107657718_1104	BTBD11	12	+	3312	27	6	1	0.91%	4.783536063843751	0.02361430724871239	0.0034718995429267267	0.0004632788085350782
ENSG00000170540_18801466_18793252_88	ARL6IP1	16	-	264	23	0	2	10.23%	51.121021773198876	0.003684287313187465	0.009067227940747451	0.00018148670210371202
ENSG00000117593_173825230_173857705_624	DARS2	1	+	1872	30	0	1	2.88%	9.403532433197118	4.880853176346471e-05	0.00010937685577000501	3.9612105019506515e-08
ENSG00000077152_202333278_202331835_83	UBE2T	1	-	249	27	1	2	14.46%	63.62679294558435	0.0009235399941089087	0.004265403350967943	2.43602958278845e-05
ENSG00000117479_169485766_169465849_464	SLC19A2	1	-	1392	7	2	0	1.08%	2.9507636255894405	0.11777945996203976	0.016947426762344633	0.009674391494749069
ENSG00000131941_32996197_32979996_270	RHPN2	19	-	810	13	1	0	2.22%	9.417463592357409	0.026832695913080365	0.011772051206276392	0.0015103817605807263
ENSG00000140391_77052426_77046835_44	TSPAN3	15	-	132	18	1	1	11.36%	80.01551234065911	0.02108409854857798	0.019679754444124863	0.0019038513263291816
ENSG00000159176_201490405_201484713_176	CSRP1	1	-	528	83	2	1	6.82%	92.24010450381536	0.0013780655586762929	0.0011466916564029902	9.639415015841312e-06
ENSG00000129559_24232267_24217127_81	NEDD8	14	-	243	27	10	3	6.17%	65.19782487016668	0.15481445136531652	0.05352897298295963	0.03158695817877253
ENSG00000129562_22589157_22575103_113	DAD1	14	-	339	165	8	4	29.20%	285.60109124542043	1.365675839954964e-06	6.838647034103645e-07	8.399235877613966e-12
ENSG00000100225_32483984_32498530_354	FBXO7	22	+	1062	41	3	0	3.11%	22.653481522685034	0.00799036934523467	0.0015806110104534784	7.508909326457793e-05
ENSG00000013588_12909072_12912181_65	GPRC5A	12	+	195	21	0	0	10.77%	63.19173795108463	0.00847367416878415	0.00847367416878415	0.0003665024264111182
ENSG00000103035_74296915_74305733_324	PSMD7	16	+	972	126	16	11	7.10%	76.06412901519447	1.4400794555770834e-05	0.0002129427003416898	2.5307582983064897e-08
ENSG00000143322_179229397_179107718_1161	ABL2	1	-	3483	14	2	1	0.69%	2.3585776438819988	0.01599363345933208	0.00912793029372963	0.0007225562286945759
ENSG00000167642_38283671_38292006_202	SPINT2	19	+	606	153	4	0	14.36%	148.14753274953716	1.164934443723582e-06	1.1494936665597707e-06	1.1905447438997993e-11
ENSG00000182326_7062524_7067014_312	C1S	12	+	936	8	0	1	1.60%	5.01521729770513	0.016947426762344633	0.0477903522728147	0.0036975118224100542
ENSG00000182326_7067021_7070651_365	C1S	12	+	1095	20	0	0	2.47%	10.717450663589043	0.002943990679779778	0.002943990679779778	4.916404313974209e-05
ENSG00000178921_8253938_8269264_1338	PFAS	17	+	4014	23	0	0	0.90%	3.362219668192452	0.0009235399941089087	0.0009235399941089087	5.324735186779001e-06
ENSG00000138801_107701282_107614249_603	PAPSS1	4	-	1809	20	0	3	1.99%	6.4873457582255405	0.0008852284070317903	0.0033971548649953435	1.8571559899647074e-05
ENSG00000188352_20740279_20995629_1691	FOCAD	9	+	5073	21	2	0	0.47%	2.429014173164105	0.018151375140008384	0.005306578019052857	0.0005060696292973746
ENSG00000089280_31180215_31191438_525	FUS	16	+	1575	193	8	10	8.95%	71.90388685681239	1.0539123172849828e-08	7.456051191311929e-09	8.201367580456936e-16
ENSG00000136068_58008565_58170762_2633	FLNB	3	+	7899	126	16	17	2.05%	9.359961185310674	7.410892035135557e-08	8.76675846503856e-08	6.342385039057957e-14
ENSG00000143549_154183119_154157639_248	TPM3	1	-	744	83	1	5	5.65%	65.46071932528832	0.0005257599192501259	0.0018512044310315418	6.238201644005689e-06
ENSG00000277791_38762447_38764167_35	PSMB3	17	+	105	131	0	0	22.86%	732.0784335421573	0.00575691403842271	0.00575691403842271	0.0001762552514680027
ENSG00000134824_61840382_61840564_60	FADS2	11	+	180	29	2	0	11.67%	94.53684606174168	0.00847367416878415	0.008574016250729465	0.0003706029064635438
ENSG00000105281_46782522_46775510_313	SLC1A5	19	-	939	121	1	2	10.54%	75.61281286859587	3.853689253368675e-07	2.5874694891720296e-07	9.310312160974592e-13
ENSG00000183010_81936814_81933214_319	PYCR1	17	-	957	30	8	1	4.08%	18.39437065302508	0.02387747459605924	0.0008605146384484945	0.0001494637719288805
ENSG00000164548_23516395_23507416_113	TRA2A	7	-	339	22	0	0	7.08%	38.08014549938939	0.0053557511377634245	0.0053557511377634245	0.00015366972994913032
ENSG00000156515_69343848_69384547_566	HK1	10	+	1698	34	6	1	2.47%	11.749431337026504	0.007710563253712815	0.0006399719954500673	3.3030545126423115e-05
ENSG00000115128_24076223_24067762_123	SF3B6	2	-	369	41	1	1	12.20%	65.19782487016668	0.0003801290169235231	0.0003801290169235231	9.609065696424175e-07
ENSG00000138119_93482194_93306963_2061	MYOF	10	-	6183	113	11	5	2.38%	10.723950815616936	5.31551996302833e-08	5.632750296231521e-09	3.1795105709711013e-15
ENSG00000160305_46532166_46567622_1160	DIP2A	21	+	3480	11	3	0	0.52%	1.8547657075133626	0.10000264635955686	0.012798402692974279	0.006484128903061078
ENSG00000198755_35468435_35469043_58	RPL10A	6	+	174	28	3	1	10.34%	94.4244360188621	0.03577298629450316	0.013007239811447113	0.002198223167318211
ENSG00000169814_15635440_15642138_159	BTD	3	+	477	10	0	0	3.77%	12.301476390597488	0.012798402692974279	0.012798402692974279	0.0007972436888943567
ENSG00000133318_63681637_63758201_236	RTN3	11	+	708	132	8	8	9.32%	109.39974003638137	0.00012112784143779584	1.937801655336985e-05	1.8470392448066008e-08
ENSG00000127481_19076779_19074832_34	UBR4	1	-	102	25	3	1	20.59%	143.81873133125004	0.028462570115697007	0.008877961307018026	0.0012532059812813028
ENSG00000112578_41921563_41932706_437	BYSL	6	+	1311	27	0	0	2.52%	12.084722687605266	0.0015356776430442238	0.0015356776430442238	1.41466323862755e-05
ENSG00000178982_38619287_38626156_129	EIF3K	19	+	387	201	7	4	12.40%	304.7619255558954	0.001216496151769452	0.0007532718901981798	5.730981237418765e-06
ENSG00000160633_5623206_5651046_251	SAFB	19	+	753	12	1	7	2.39%	9.35108245149801	0.02015986069794795	0.15439520988099065	0.015020081633012095
ENSG00000070010_19465339_19451990_149	UFD1	22	-	447	30	0	1	8.05%	39.3812364987584	0.0010155991509472172	0.001093842117104189	6.866441555161833e-06
ENSG00000104853_44955056_44992897_655	CLPTM1	19	+	1965	165	14	11	7.63%	49.2716386423397	8.29193108161171e-09	8.652030919492055e-09	7.490952833641285e-16
ENSG00000065518_120596360_120596569_69	NDUFB4	3	+	207	18	2	3	10.14%	51.024384681000015	0.02034245439037237	0.03350388737713736	0.0030495386397099617
ENSG00000101040_47298917_47210761_1100	ZMYND8	20	-	3300	6	0	0	0.45%	1.0668734978754548	0.016947426762344633	0.016947426762344633	0.0013498980316300959
ENSG00000157734_64156036_64156287_83	SNX22	15	+	249	84	5	35	10.84%	197.95002249737354	0.0220856724542213	0.0706906638683072	0.006890087056981816
ENSG00000157734_64156726_64156857_43	SNX22	15	+	129	21	2	4	16.28%	95.52239457722095	0.00847367416878415	0.013991246267585163	0.0005927351821891016
ENSG00000163931_53255942_53225756_623	TKT	3	-	1869	525	67	26	16.69%	164.82596175042138	8.012371654125739e-14	1.0749619350488686e-17	1.6754596357069326e-29
ENSG00000165304_36607614_36677337_449	MELK	9	+	1347	25	1	0	2.45%	10.890505267845214	0.0014409783181973444	0.001484234770590415	1.2880848012053486e-05
ENSG00000177119_45216355_45429311_899	ANO6	12	+	2697	22	4	4	1.22%	4.786492148421581	0.012914280349163228	0.012821549509893823	0.0008053923563397052
ENSG00000124783_7313120_7289864_291	SSR1	6	-	873	113	2	5	12.37%	75.95210526112201	7.991166815367668e-08	2.712069423649562e-07	2.100377621768647e-13
ENSG00000162910_228108260_228106760_128	MRPL55	1	-	384	35	2	1	7.81%	53.482590713808605	0.004407715900925203	0.0030814981005195803	7.570158080683164e-05
ENSG00000101150_63865366_63889945_186	TPD52L2	20	+	558	43	3	2	12.90%	45.21784628092205	5.242639664992008e-05	3.122167277164887e-05	1.2466281101107478e-08
ENSG00000148737_112950757_113160661_476	TCF7L2	10	+	1428	11	0	0	1.26%	4.520017270410715	0.012798402692974279	0.012798402692974279	0.0007972436888943567
ENSG00000166741_114312268_114312477_69	NNMT	11	+	207	34	2	4	20.29%	96.37939328633335	0.001373376896918719	0.0004156054716904614	3.716678856638805e-06
ENSG00000166908_57594058_57601606_352	PIP4K2C	12	+	1056	14	0	5	2.27%	7.779285922008524	0.005160369442893307	0.05268573235456097	0.0015446541073930182
ENSG00000136877_127804381_127813604_509	FPGS	9	+	1527	20	0	0	1.38%	7.685401752868371	0.008574016250729465	0.008574016250729465	0.0003747459614100891
ENSG00000136877_127804381_127810095_297	FPGS	9	+	891	16	0	1	1.68%	10.53702220123906	0.02108409854857798	0.03577298629450316	0.003354604907114977
ENSG00000124207_49058464_49096438_915	CSE1L	20	+	2745	83	4	5	3.83%	17.74235889909454	8.381510446449585e-07	1.5217957196178004e-06	1.1390086892240475e-11
ENSG00000106153_56106413_56101851_151	CHCHD2	7	-	453	36	1	0	5.30%	46.631556860781465	0.007356796211402075	0.00575691403842271	0.00022276510181128367
ENSG00000071564_1622056_1611707_436	TCF3	19	-	1308	7	0	0	1.15%	3.1402622070493127	0.016947426762344633	0.016947426762344633	0.0013498980316300959
ENSG00000126787_55189179_55148454_842	DLGAP5	14	-	2526	29	2	0	1.90%	6.736592355943588	0.0004209495667581447	0.00017495645137376598	5.071402924144588e-07
ENSG00000141012_88837632_88814439_337	GALNS	16	-	1011	21	1	0	1.78%	12.188317408962911	0.012619539009162606	0.011772051206276392	0.0007274184967276076
ENSG00000185088_63157405_63155553_97	RPS27L	15	-	291	81	11	0	14.43%	163.33063343763405	0.0009562326943231171	0.00044016598898382844	2.7273134233375386e-06
ENSG00000124688_43635876_43640533_274	MAD2L1BP	6	+	822	19	4	0	2.19%	13.563051159122265	0.013428347753762199	0.013640585738808999	0.0008855098933240206
ENSG00000162909_223712641_223774857_700	CAPN2	1	+	2100	110	7	10	7.00%	30.736117438792864	1.2571117228721673e-08	7.638263691124957e-08	9.898433946613383e-15

ENSG00000185875_25023512_25025455_647	THNSL1	10	+	1941	13	0	0	1.55%	3.9300079906282854	0.0017552353687121413	0.0017552353687121413	1.827860114218897e-05
ENSG00000149499_62609433_62602475_670	EML3	11	-	2010	5	2	0	0.75%	1.4596527956007466	0.15865525393145707	0.012673659338734126	0.011061610944025602
ENSG00000095739_28677952_28681583_115	BAMBI	10	+	345	14	0	0	4.35%	23.811379517800006	0.019679754444124863	0.019679754444124863	0.0017849636115922674
ENSG00000114354_100713686_100748531_400	TFG	3	+	1200	50	4	4	4.75%	24.449184326312505	0.00043927481975472	0.00037023034910173925	1.07775625249491e-06
ENSG00000173915_103392376_103392194_31	ATP5MD	10	-	93	101	2	0	22.58%	637.256159214855	0.008980238763039383	0.008980238763039383	0.0004089866599971986
ENSG00000176153_64942726_64942607_39	GPX2	14	-	117	85	3	1	15.38%	426.293470304936	0.0682897090290637	0.020525443280229434	0.006257767283596938
ENSG00000173915_103392376_103392194_31	ATP5MD	10	-	93	101	2	0	22.58%	637.256159214855	0.008980238763039383	0.008980238763039383	0.0004089866599971986
ENSG00000138796_108004852_108034357_292	HADH	4	+	876	38	0	0	4.79%	25.453945326023977	0.00037874750290730355	0.00037874750290730355	9.541676172656993e-07
ENSG00000197879_1483656_1465726_963	MYO1C	17	-	2889	71	10	0	3.63%	14.42070269713967	1.85668876691052e-05	7.500225446215369e-08	1.670861245916329e-11
ENSG00000127980_92517434_92487457_923	PEX1	7	-	2769	8	2	0	0.54%	1.6952847203510297	0.10105102209468636	0.019679754444124863	0.009164497669452725
ENSG00000140395_78295686_78283432_212	WDR61	15	-	636	27	0	0	5.19%	24.91048969095991	0.001558047347358915	0.001558047347358915	1.4544595481456893e-05
ENSG00000139641_56128359_56143862_1091	ESYT1	12	+	3273	71	1	3	3.48%	12.728814571352432	3.395093314933653e-08	1.5937986764350073e-07	5.399978661493248e-14
ENSG00000137942_93448282_93551125_551	FNBP1L	1	+	1653	7	3	0	1.09%	2.4848535794437394	0.1097457788509939	0.009815328628645334	0.005898503241319083
ENSG00000137936_93674723_93562241_756	BCAR3	1	-	2268	22	2	0	1.72%	5.691873599776456	0.0015384793293873654	0.0006069633386880125	5.90945903854469e-06
ENSG00000137944_88969464_88936183_420	KYAT3	1	-	1260	8	0	0	1.43%	3.7255899925809532	0.011570465654371866	0.011570465654371866	0.0006594843062896773
ENSG00000109861_88337672_88326377_141	CTSC	11	-	423	22	3	0	6.38%	30.518130790290787	0.015321538789749097	0.0034782380676658805	0.000294139505510845
ENSG00000164828_852875_873331_460	SUN1	7	+	1380	17	0	0	1.09%	7.228454496475002	0.019216965118390855	0.019216965118390855	0.0017073955890589052
ENSG00000145833_134758939_134828706_1031	DDX46	5	+	3093	40	0	0	1.65%	7.588495620193988	0.00012869633121932495	0.00012869633121932495	1.1789331714510463e-07
ENSG00000134330_9481342_9484586_86	IAH1	2	+	258	14	0	0	5.81%	31.840798192406982	0.02108409854857798	0.02108409854857798	0.002029891263157455
ENSG00000143437_150846291_150812021_708	ARNT	1	-	2124	12	0	0	0.71%	3.3151436374661025	0.02108409854857798	0.02108409854857798	0.002029891263157455
ENSG00000175334_66003293_66003772_75	BANF1	11	+	225	197	14	8	18.67%	513.7588599769134	0.00048652826472334255	0.0008793782949311993	2.757750418467631e-06
ENSG00000107874_102424544_102424165_91	CUEDC2	10	-	273	18	0	0	6.59%	38.688819153725284	0.012798402692974279	0.012798402692974279	0.0007972436888943567
ENSG00000137955_75786272_75790101_152	RABGGTB	1	+	456	9	0	0	3.95%	11.581192575621714	0.009815328628645334	0.009815328628645334	0.0004837142251238724
ENSG00000182446_81636930_81559259_608	NPLOC4	17	-	1824	13	3	0	0.82%	4.182097318974508	0.13159953908062888	0.019679754444124863	0.012285429850268035
ENSG00000197265_30653598_30578921_291	GTF2E2	8	-	873	21	0	2	3.78%	14.114993013128869	0.0012690167775026367	0.004981126742780332	3.796380708341253e-05
ENSG00000143933_47162588_47160776_113	CALM2	2	-	339	138	7	1	12.39%	238.86636722344252	0.0003909674708303042	0.00047112862025758081 4	1.215777600715846e-06
ENSG00000143401_150235780_150220691_134	ANP32E	1	-	402	26	2	1	4.48%	37.95097268561941	0.03101958831192484	0.02034245439037237	0.002831109456962054
ENSG00000198363_61689688_61626273_256	ASPH	8	-	768	240	11	25	16.80%	183.3688824473438	1.3067781838055203e-07	1.1652356729413298e-06	1.4715152360428695e-12
ENSG00000113643_168486499_168493922_113	RARS	5	+	339	12	3	0	4.42%	20.770988454212393	0.11497452839710681	0.020613416668581838	0.010945600217888082
ENSG00000198542_101452834_101715654_445	ITGBL1	13	+	1335	35	0	3	4.27%	15.383756430039329	4.4847209281215234e-05	0.0003851896958162796	1.3176156271407322e-07
ENSG00000100504_50944403_50905392_847	PYGL	14	-	2541	83	9	4	4.37%	19.166774961831763	2.389169534235617e-07	3.286083162813544e-07	7.35738423455426e-13
ENSG00000277775_26250605_26250195_136	HIST1H3F	6	-	408	114	4	0	11.76%	163.95335371762505	0.0005052650208883981	0.0001978461102466251	6.829869187169484e-07
ENSG00000114021_100334792_100345707_152	NIT2	3	+	456	46	1	2	7.24%	59.192762053177645	0.0023697301888609516	0.004528451082822598	6.089168788763513e-05
ENSG00000114021_100346216_100355268_121	NIT2	3	+	363	28	1	0	4.96%	45.26129990986778	0.013640585738808999	0.013640585738808999	0.00089863692579514
ENSG00000168061_65041332_65044727_345	SAC3D1	11	+	1035	19	1	0	1.74%	10.77181454376667	0.02034245439037237	0.013428347753762199	0.0012955184805712684
ENSG00000130402_38647839_38730020_71	ACTN4	19	+	213	52	5	2	18.31%	143.251558869662	0.007193226003081166	0.002535482166444355	0.000102700102517247
ENSG00000182093_39380385_39390907_103	WRB	21	+	309	19	0	0	7.77%	36.08034968543205	0.005405141725979036	0.005405141725979036	0.00015637213408961292
ENSG00000148840_102141743_102150029_586	PPRC1	10	+	1758	20	1	0	1.37%	6.675545208549489	0.008364861494636245	0.005604866691947602	0.00024596970711672695
ENSG00000102030_153934904_153930269_169	NAA10	X	-	507	11	5	0	4.73%	12.730936217251482	0.05111742236774389	0.004687384229717438	0.0013830119492932043
ENSG00000148634_68073108_67922931_1057	HERC4	10	-	3171	27	2	1	1.14%	4.996238235083728	0.0028873670906769807	0.0012031805969473861	2.0853802272196706e-05
ENSG00000102882_30123047_30116837_303	MAPK3	16	-	909	13	0	0	2.31%	8.391799240714523	0.00797940233269714	0.00797940233269714	0.000327142761693177
ENSG00000132507_7309636_7311640_154	EIF5A	17	+	462	179	71	8	11.69%	227.34566204726954	0.007938263751100936	9.685105774909149e-05	7.0979032624909975e-06
ENSG00000111678_6944529_6945922_91	C12orf57	12	+	273	51	3	0	12.09%	109.61832093555498	0.0014055202957965589	0.0014915192773735051	1.2636475669781728e-05
ENSG00000119812_33599206_33584776_513	FAM98A	2	-	1539	20	0	1	1.75%	7.6254765930019515	0.0033030736763512156	0.006112543793728684	0.00011113261345270447
ENSG00000120708_136029056_136063226_683	TGFBI	5	+	2049	477	23	29	15.23%	136.60042077482947	1.1671637794684118e-16	6.111801729935041e-16	1.0577117887347606e-30
ENSG00000100650_69768157_69769126_99	SRSF5	14	+	297	80	10	5	18.18%	158.05533301858588	0.0005759641158097748	0.0005025909556147243	1.8803032497408369e-06
ENSG00000067248_55289387_55256482_1054	DHX29	5	-	3162	16	0	0	1.04%	2.9691609049032266	0.0012427339549726212	0.0012427339549726212	9.422880418492092e-06
ENSG00000140262_57231251_57282587_480	TCF12	15	+	1440	13	0	0	1.46%	5.2973232707010425	0.007029302661868638	0.007029302661868638	0.0002573639808771639
ENSG00000131023_149695083_149661729_968	LATS1	6	-	2904	17	0	0	0.52%	3.435009368159608	0.02108409854857798	0.02108409854857798	0.002029891263157455
ENSG00000105819_103297460_103309125_340	PMPCB	7	+	1020	31	2	1	5.59%	17.833522685075003	0.0001159434964184692	7.706915127420958e-05	6.490808866473943e-08
ENSG00000120265_149749728_149810628_286	PCMT1	6	+	858	45	1	4	6.99%	30.775197054099657	5.185709832851827e-05	0.00035548993793247866	1.3841409122193912e-07
ENSG00000120265_149749728_149802379_250	PCMT1	6	+	750	44	1	4	7.60%	34.42445153144801	7.797505503656233e-05	0.0005141533505933988	2.941070708282081e-07
ENSG00000113249_157055447_157029588_357	HAVCR1	5	-	1071	127	5	8	8.40%	69.58087192026193	1.965862297170231e-06	6.051295078329439e-06	1.0261299644962253e-10
ENSG00000082898_61526431_61478820_999	XPO1	2	-	2997	94	7	9	3.40%	18.404190804191195	2.8597618265040248e-06	5.055987184425535e-06	1.2297773581093105e-10
ENSG00000175602_65890919_65891392_157	CCDC85B	11	+	471	12	0	0	3.18%	14.949819715452232	0.02108409854857798	0.02108409854857798	0.002029891263157455
ENSG00000213983_23567140_23559749_727	AP1G2	14	-	2181	11	2	2	1.19%	2.959461101396837	0.033944577430914495	0.019230617045588212	0.0029378519685176155
ENSG00000132356_40798162_40762778_550	PRKAA1	5	-	1650	19	3	0	1.64%	6.756865486544547	0.02736362956991709	0.002967235347054297	0.000476754742153865
ENSG00000104067_29761691_29701595_1511	TJP1	15	-	4533	22	1	3	0.79%	2.8478202789086704	0.0008664586529590942	0.006880387941862762	3.795289366585291e-05
ENSG00000256646_42924697_42912534_65	AC010132.3	7	-	195	32	7	0	10.77%	96.29217211593848	0.046045457964455916	0.008776162969209843	0.0020495800739836604
ENSG00000256646_42932158_42924769_48	AC010132.3	7	-	144	48	4	1	10.42%	195.59347461050004	0.021557223391537678	0.029237630782826433	0.002819005644529471
ENSG00000130202_44846526_44888379_538	NECTIN2	19	+	1614	24	0	7	2.42%	8.72535946217844	0.000596156229600823	0.01907291812977645	8.578797820436054e-05
ENSG00000115641_105386516_105361283_279	FHL2	2	-	837	24	2	0	3.23%	16.82524512778495	0.01052924788556975	0.003684287313187465	0.00021087137401632993
ENSG00000116539_155481270_155337660_2431	ASH1L	1	-	7293	22	0	8	0.37%	1.7700766933076928	0.0035291660242640502	0.10513476682431783	0.002628225900902591
ENSG00000068400_49002193_48974193_798	GRIPAP1	X	-	2394	9	0	0	0.75%	2.2059414429755644	0.009815328628645334	0.009815328628645334	0.0004837142251238724
ENSG00000162298_65133580_65128382_609	SYVN1	11	-	1827	20	1	5	1.64%	6.423431021691298	0.00397428314897603	0.043341461536463954	0.0010067842203199241
ENSG00000111142_95512882_95513904_95	METAP2	12	+	285	34	1	0	12.63%	70.00187512375791	0.0013893479493583228	0.001053132412899783	8.960332326514618e-06
ENSG00000111142_95474180_95512823_328	METAP2	12	+	984	48	0	0	5.49%	28.623435308853665	7.24851034273329e-05	7.24851034273329e-05	3.864393654082707e-08

ENSG00000158042_6683296_6682118_175	MRPL17	11	-	525	25	0	5	8.57%	27.94192494435715	0.00024925744678760264	0.004948691747922397	9.106726448597117e-06
ENSG00000173786_41971846_41973924_211	CNP	17	+	633	19	1	1	4.74%	17.61268254786493	0.0036460193534901884	0.0036460193534901884	7.39336293426026e-05
ENSG00000005007_18832210_18866163_1118	UPF1	19	+	3354	21	6	5	0.98%	3.6739382529700366	0.03337069516002332	0.030355651698054653	0.004362293029214473
ENSG00000105879_107744164_107758148_123	CBLL1	7	+	369	18	1	0	4.88%	28.623435308853665	0.016983116543564363	0.013640585738808999	0.0011055102295404414
ENSG00000184428_143329415_143309441_503	TOP1MT	8	-	1509	11	0	0	0.99%	4.277392088897615	0.02108409854857798	0.02108409854857798	0.002029891263157455
ENSG00000109320_102597543_102616594_463	NFKB1	4	+	1389	21	0	0	2.16%	8.87141029550864	0.0023218226288164415	0.0023218226288164415	3.1231198880462635e-05
ENSG00000168078_27828112_27810305_274	PBK	8	-	822	72	8	4	7.66%	51.396825445094905	2.377357299925333e-05	0.00014578310744264934	2.6988623278082068e-08
ENSG00000188603_28489349_28477516_360	CLN3	16	-	1080	17	1	0	2.22%	9.236358523273614	0.013892891352654344	0.005306578019052857	0.0003860137809582807
ENSG00000111642_6595423_6570676_1251	CHD4	12	-	3753	14	5	1	0.64%	2.1888957989984017	0.11158492712740181	0.005930764660659649	0.00413790451668508
ENSG00000112855_140696966_140698552_223	HARS2	5	+	669	12	1	0	2.69%	10.52520939608072	0.019795881618345192	0.012798402692974279	0.0012079910998532793
ENSG00000187720_71215155_71777374_945	THSD4	15	+	2835	19	0	1	1.16%	3.932567214391006	0.0013845019718837994	0.0024673358700277245	2.032715794165102e-05
ENSG00000089157_120200783_120196773_255	RPLP0	12	-	765	58	1	4	3.53%	44.48792755846668	0.0037896409716948643	0.005348311750988237	0.0001108044151879065
ENSG00000100201_38506000_38485935_650	DDX17	22	-	1950	106	5	5	5.54%	31.89678201340462	4.642416269712811e-07	4.5479550956233605e-07	1.9426156433990175e-12
ENSG00000170445_140679874_140674257_406	HARS	5	-	1218	48	1	1	4.19%	23.124351678088676	0.00015267201677270214	0.00015267201677270214	1.6423430888992484e-07
ENSG00000099250_33263694_33249421_166	NRP1	10	-	498	21	0	2	6.02%	24.743752812171692	0.0023218226288164415	0.00228120298834518	3.07086036194621e-05
ENSG00000273345_134302673_134205868_115	AC104109.4	5	-	345	16	0	0	5.22%	27.213005163200005	0.013217226988443315	0.013217226988443315	0.0008469697265466234
ENSG00000164919_99892021_99887505_75	COX6C	8	-	225	398	3	3	32.00%	1037.9493719330535	1.963911548577778e-05	1.0960740445380867e-05	1.7249443418630812e-09
ENSG00000187601_55452477_55453034_185	MAGEH1	X	+	555	9	0	0	2.70%	9.515358224294596	0.020613416668581838	0.020613416668581838	0.001946208561389314
ENSG00000015475_17739423_17735580_99	BID	22	-	297	12	0	0	5.05%	23.708299952787883	0.020613416668581838	0.020613416668581838	0.001946208561389314
ENSG00000145555_16764369_16666692_1656	MYO10	5	-	4968	33	3	3	1.09%	3.897696052020834	0.000702394804095236	0.0005191986277685761	2.352718666705488e-06
ENSG00000182923_134507167_134561535_669	CEP63	3	+	2007	10	0	0	0.90%	2.923669276689089	0.011772051206276392	0.011772051206276392	0.000681272511389508
ENSG00000162194_62667660_62663050_156	LBHD1	11	-	468	36	3	3	7.05%	45.136955679346165	0.001988634903193983	0.0020028004154862638	2.337634404283484e-05
ENSG00000078618_51840459_51789236_1087	NRDC	1	-	3261	33	0	0	1.29%	5.937980369959983	0.00042454308224345244	0.00042454308224345244	1.1896574514138388e-06
ENSG00000065613_103990753_104025720_1128	SLK	10	+	3384	37	3	2	1.42%	6.41574340477704	0.001252275242318572	0.0008252803903793908	6.4270413783261505e-06
ENSG00000188010_38881442_38882515_79	MORN2	2	+	237	8	0	0	7.59%	19.806934137772156	0.009815328628645334	0.009815328628645334	0.0004837142251238724
ENSG00000170802_48346302_48375443_402	FOXN2	2	+	1206	8	1	2	1.49%	3.8924074549353245	0.026602764905793736	0.08266357711566169	0.009436337707064906
ENSG00000123240_13110300_13136866_507	OPTN	10	+	1521	26	4	5	1.97%	10.03043459541026	0.01790347956991311	0.030396003057272823	0.0024761070165161886
ENSG00000159055_32279014_32269037_233	MIS18A	21	-	699	12	3	1	2.58%	10.073483670927041	0.08620755130569058	0.019795881618345192	0.007759185451282548
ENSG00000163964_196716869_196733902_217	PIGX	3	+	651	17	1	0	3.69%	15.322991098518436	0.005585354798807341	0.004826937407846361	0.00014496567577559376
ENSG00000072274_196077096_196051942_759	TFRC	3	-	2277	142	7	2	7.51%	36.59324557930304	4.739677167691646e-10	2.6828389625454316e-11	1.5520703034019612e-19
ENSG00000129255_7583863_7586857_155	MPDU1	17	+	465	17	0	3	3.23%	21.452187537925813	0.020613416668581838	0.044040755831095145	0.004031490413620829
ENSG00000197045_54483671_54478088_109	GMFB	14	-	327	10	1	1	4.59%	17.944355468853214	0.020613416668581838	0.02784981298332479	0.0025842615800075876
ENSG00000126453_49666693_49673748_250	BCL2L12	19	+	750	11	0	0	2.00%	8.606112882862002	0.02108409854857798	0.02108409854857798	0.002029891263157455
ENSG00000188641_97699487_97078976_844	DPYD	1	-	2532	9	0	3	0.59%	2.085712406984005	0.020613416668581838	0.10105102209468636	0.009504589763467653
ENSG00000164053_48466710_48467600_296	ATRIP	3	+	888	11	0	1	1.69%	7.26867642133615	0.020613416668581838	0.03537493353818881	0.003252526348827943
ENSG00000164053_48460123_48465554_435	ATRIP	3	+	1305	15	0	0	1.15%	6.744602572775864	0.02108409854857798	0.02108409854857798	0.002029891263157455
ENSG00000009307_114739890_114718169_798	CSDE1	1	-	2394	90	11	5	4.39%	22.059414429755645	9.267998481696954e-06	8.096231702821984e-07	6.8610803470884e-11
ENSG00000106009_2543798_2538069_623	BRAT1	7	-	1869	17	0	0	1.12%	5.337221618585073	0.00837382380159347	0.00837382380159347	0.0003583838894600799
ENSG00000127022_179705682_179728644_592	CANX	5	+	1776	809	23	24	25.68%	267.28905567549754	2.5128693307178184e-25	3.9393706079733635e-25	1.83499292970765e-48
ENSG00000164713_98281796_98291243_125	BRI3	7	+	375	34	3	2	6.40%	53.20142509405601	0.008219046535586472	0.01732176123716967	0.0007110158483636684
ENSG00000100714_64388428_64440136_535	MTHFD1	14	+	1605	33	1	0	3.55%	12.064644228311218	7.86073439274704e-05	4.438470604278364e-05	2.6068329528989587e-08
ENSG00000133884_65340413_65341586_142	DPF2	11	+	426	8	0	0	3.52%	11.019350682281692	0.019679754444124863	0.019679754444124863	0.0017849636115922674
ENSG00000160688_154983986_154993037_490	FLAD1	1	+	1470	32	1	0	2.65%	12.773451403134697	0.0009381216109661054	0.0006698694257094773	3.976835882658254e-06
ENSG00000017260_130934676_131001350_823	ATP2C1	3	+	2469	17	3	1	1.22%	4.040205429378494	0.011168917508652896	0.00324779312858951	0.00020011016427610653
ENSG00000141971_17420136_17424993_273	MVB12A	19	+	819	20	0	0	2.56%	14.329192279157512	0.00877616296920843	0.00877616296920843	0.0003916113566756457
ENSG00000185515_155071528_155120150_316	BRCC3	X	+	948	8	0	0	2.22%	4.951733534443039	0.005706018193000826	0.005706018193000826	0.0001733096755673334
ENSG00000116830_117075104_117101524_989	TTF2	1	+	2967	12	0	0	0.51%	2.373227194465117	0.020613416668581838	0.020613416668581838	0.001946208561389314
ENSG00000100804_23034881_23026089_263	PSMB5	14	-	789	153	11	1	14.45%	113.78631792930231	4.6050698544438123e-07	3.170594202686764e-08	1.493136997850396e-13
ENSG00000151881_43453969_43446222_215	TMEM267	5	-	645	47	0	1	2.79%	42.75764328694652	0.011772051206276392	0.026832695913080365	0.0015103817605807263
ENSG00000091039_76410578_76355889_798	OSBPL8	12	-	2394	18	0	1	1.38%	4.411882885951129	0.0008884494972709903	0.0024579339080672683	1.3429802402146636e-05
ENSG00000138078_44339308_44321356_458	PREPL	2	-	1374	18	0	0	3.06%	7.687079788185591	0.00024035368145103084	0.00024035368145103084	3.9581571392385245e-07
ENSG00000176697_27658504_27657821_227	BDNF	11	-	681	10	1	0	2.64%	8.616452626013217	0.023672501042627626	0.012798402692974279	0.0014375227604752335
ENSG00000079459_11808548_11838609_466	FDFT1	8	+	1398	218	12	8	12.23%	91.50081001092062	1.5645980642916244e-09	1.2631884318185338e-10	2.31609260057506e-18
ENSG00000100347_43976046_43996383_256	SAMM50	22	+	768	15	1	0	2.73%	11.460555152958987	0.013892891352654344	0.00847367416878415	0.0005886798845679606
ENSG00000159210_48944734_48937068_53	SNF8	17	-	159	12	0	0	11.32%	44.28531500615095	0.011772051206276392	0.011772051206276392	0.000681272511389508
ENSG00000175203_57547063_57532083_315	DCTN2	12	-	945	33	4	0	5.08%	20.490744959195244	0.0018719653023208534	0.00017619886174203892	2.362474342372684e-06
ENSG00000104763_18061423_18057534_149	ASAH1	8	-	447	29	3	0	6.04%	38.06852861546645	0.0035547763465261306	0.003710480643933249	7.338809221316118e-05
ENSG00000115355_55362358_55291711_1678	CCDC88A	2	-	5034	20	1	0	0.60%	2.331269065679381	0.0033869280463461717	0.0022543491824521134	4.372784971036962e-05
ENSG00000136522_179604624_179588772_250	MRPL47	3	-	750	33	2	0	5.20%	25.818338648586007	0.0021935318410404503	0.0006698694257094773	9.244393167965786e-06
ENSG00000134779_36818914_36783042_200	TPGS2	18	-	600	8	0	0	2.50%	7.823738984420002	0.019216965118390855	0.019216965118390855	0.0017073955890589052
ENSG00000175931_76398948_76390943_736	UBE2O	17	-	2208	17	0	2	0.95%	4.517784060296876	0.00797940233269714	0.022200630790666427	0.000887484071868763
ENSG00000055163_157319804_157393000_787	CYFIP2	5	+	2361	8	4	0	0.76%	1.9882437063329101	0.23639154306007898	0.011570465654371866	0.017275440155518785
ENSG00000283782_132587682_132595107_306	AC116366.3	5	+	918	14	1	0	2.61%	8.948721060611113	0.00912793029372963	0.005160369442893307	0.0002481924235076056
ENSG00000283782_132603920_132642364_513	AC116366.3	5	+	1539	17	2	1	1.36%	6.481655104051659	0.027657845067597595	0.011825808327677989	0.001562883405286683
ENSG00000143514_223806902_223780853_995	TP53BP2	1	-	2985	13	0	0	0.70%	2.5554926331020105	0.0078820576498062	0.0078820576498062	0.0003196364290745 1164
ENSG00000155366_112703799_112703587_70	RHOC	1	-	210	15	4	5	8.57%	41.912887416535725	0.0865972937396412	0.14351487215180903	0.043088778062005 92
ENSG00000104805_48900797_48922424_461	NUCB1	19	+	1383	67	0	0	6.29%	28.426817351200658	8.926251709647316e-07	8.926251709647316e-07	7.154188182400702e-12

ENSG00000032444_7540198_7561562_1165	PNPLA6	19	+	3495	25	0	0	0.94%	4.197284862886267	0.001484234770590415	0.001484234770590415	1.3251387882263029e-05
ENSG00000064545_19134839_19119930_359	TMEM161A	19	-	1077	9	1	0	1.39%	4.903457580764625	0.04223451612662591	0.019679754444124863	0.003715148010809822
ENSG00000005884_50076638_50090318_216	ITGA3	17	+	648	34	0	3	5.09%	30.787861744245376	0.001565547007472977	0.008052197636152732	7.505875649486829e-05
ENSG00000082153_200813218_200822178_419	BZW1	2	+	1257	45	9	0	2.39%	21.006459087046544	0.029237630782826433	0.002460156992221257	0.000439785329035633
ENSG00000157181_186379786_186419076_422	ODR4	1	+	1266	17	0	0	1.90%	7.879357981939576	0.005306578019052857	0.005306578019052857	0.00015100114740534233
ENSG00000177189_20187899_20155398_506	RPS6KA3	X	-	1518	20	2	0	2.37%	7.730967375909093	0.004498799248433914	0.0008852284070317903	2.4782952419168947e-05
ENSG00000111845_10694986_10709452_392	PAK1IP1	6	+	1176	29	0	0	3.06%	14.469925417613524	0.001032175130546352	0.001032175130546352	6.594970400795017e-06
ENSG00000105323_41276293_41306565_535	HNRNPUL1	19	+	1605	68	4	3	4.30%	24.860479015914024	3.909687559760479e-05	3.261376853454941e-05	9.743458217329787e-09
ENSG00000027697_137207006_137198031_437	IFNGR1	6	-	1311	23	0	1	2.29%	10.294393400552634	0.001801896632038876	0.0040705821972625445	4.2571970118849174e-05
ENSG00000180488_77783253_77875064_601	MIGA1	1	+	1803	15	0	0	0.83%	4.881700697433446	0.019679754444124863	0.019679754444124863	0.0017849636115922674
ENSG00000027697_137207006_137198031_437	IFNGR1	6	-	1311	23	0	1	2.29%	10.294393400552634	0.001801896632038876	0.0040705821972625445	4.2571970118849174e-05
ENSG00000065000_2121856_2102173_889	AP3D1	19	-	2667	35	3	4	1.69%	7.70053049647638	0.0010795612484085625	0.002020802647119262	1.3236839687112236e-05
ENSG00000015171_180013_252470_517	ZMYND11	10	+	1551	11	0	0	0.97%	4.161563289585107	0.02108409854857798	0.02108409854857798	0.002029891263157455
ENSG00000197858_144083224_144084570_214	GPAA1	8	+	642	28	4	0	5.14%	25.591669575205614	0.009726140309244114	0.0014915192737735051	8.769016224859227e-05
ENSG00000182220_40591300_40599762_96	ATP6AP2	X	+	288	17	0	4	7.29%	34.63634446227605	0.008574016250729465	0.044329616041373636	0.0019307453316667101
ENSG00000182220_40600774_40605755_100	ATP6AP2	X	+	300	9	1	0	5.00%	17.603412714945005	0.03537493353818881	0.020613416668581838	0.003252526348827943
ENSG00000147804_144413369_144409915_143	SLC39A4	8	-	429	26	2	0	4.90%	35.562449929181824	0.03299602752967379	0.00847367416878415	0.0014019198044660757
ENSG00000198589_150867772_150265722_1964	LRBA	4	-	5892	18	5	0	0.36%	1.7926082194445014	0.07942646894547645	0.00847367416878415	0.0036293722074778505
ENSG00000183735_64455871_64490226_515	TBK1	12	+	1545	14	0	0	1.17%	5.317104164168933	0.013428347753762199	0.013428347753762199	0.0008725593497645525
ENSG00000100991_35092781_35003146_797	TRPC4AP	20	-	2391	33	0	1	1.63%	8.098600579857592	0.0006698694257094773	0.0007742304923760376	3.299459557941386e-06
ENSG00000278637_26021679_26021990_103	HIST1H4A	6	+	309	498	16	1	27.18%	945.6849549129031	3.1876381486978807e-06	1.83402991602191e-06	5.064701316934504e-11
ENSG00000135250_105268843_105117838_688	SRPK2	7	-	2064	13	0	2	0.87%	3.695806933047239	0.012798402692974279	0.044040755831095145	0.0026804992160797854
ENSG00000139436_109996224_109932978_631	GIT2	12	-	1893	15	0	0	1.43%	4.649607161897783	0.0033278027414746945	0.0033278027414746945	6.211806171561056e-05
ENSG00000111540_55986961_55992212_215	AC034102.1	12	+	645	12	0	0	3.26%	10.916845094539537	0.008175610787902942	0.008175610787902942	0.00034251843268961324
ENSG00000164904_126595114_126544965_447	ALDH7A1	5	-	1341	24	0	0	2.01%	10.501663066335572	0.0035547763465261306	0.0035547763465261306	7.044676146010672e-05
ENSG00000135390_53672614_53665314_141	ATP5MC2	12	-	423	104	13	2	17.73%	144.26752737228372	4.4036283112694194e-05	8.843606187363953e-06	3.163887414050723e-09
ENSG00000143771_224365930_224375822_76	CNIH4	1	+	228	29	1	0	11.84%	74.63435215400659	0.003760782331962294	0.0035291660242640502	7.382944917623143e-05
ENSG00000143756_224130484_224157746_275	FBXO28	1	+	825	33	4	0	4.00%	23.471216953260004	0.011063329961164447	0.001558047347358915	0.0001043552053149375
ENSG00000067829_153789745_153786149_276	IDH3G	X	-	828	12	0	0	2.17%	8.504064113500002	0.011772051206276392	0.011772051206276392	0.000681272511389508
ENSG00000142396_58312274_58314783_74	ERVK3-1	19	+	222	30	0	1	12.16%	79.29465186912164	0.0035547763465261306	0.00378059991914173	7.47127591689191e-05
ENSG00000004864_96234853_96121191_583	SLC25A13	7	-	1749	27	1	1	1.89%	9.058359887621785	0.0016965121740976229	0.0016965121740976229	1.712430827849048e-05
ENSG00000110075_68537665_68613117_827	PPP6R3	11	+	2481	32	7	5	2.30%	7.568308570176543	0.003622420583891007	0.0005933294589348272	1.3865891050279807e-05
ENSG00000110075_68537665_68613117_793	PPP6R3	11	+	2379	31	7	5	2.27%	7.646150962075033	0.004823571330713604	0.0009114593787008354	2.7339250645509414e-05
ENSG00000134996_75088693_75146741_214	OSTF1	9	+	642	8	0	0	2.80%	7.311905592915889	0.011570465654371866	0.011570465654371866	0.0006594843062896773
ENSG00000131389_14443635_14472313_359	SLC6A6	3	+	1077	34	14	5	5.01%	18.524173082888584	0.0063730122972834894	0.0009107006179919566	3.655267001923536e-05
ENSG00000166260_54968646_54963261_230	COX11	17	-	690	34	0	3	4.35%	28.913817985900007	0.002446120450917513	0.0072608768093428535	0.00010037784624098265
ENSG00000185787_78873018_78897067_323	MORF4L1	15	+	969	33	3	2	2.79%	19.983234248131584	0.01695752242623858	0.012991839772286901	0.0010554380324329336
ENSG00000148175_121370187_121339621_168	STOM	9	-	504	49	0	0	7.14%	57.04809676139585	0.0009589057725548893	0.0009589057725548893	5.724023106877083e-06
ENSG00000137700_119028355_119024910_356	SLC37A4	11	-	1068	15	0	0	2.25%	8.24129808752107	0.005306578019052857	0.005306578019052857	0.00015100114740534233
ENSG00000167460_16086484_16101346_139	TPM4	19	+	417	27	0	3	5.04%	37.99297708261512	0.00847367416878415	0.03299602752967379	0.0014019198044660757
ENSG00000104723_15623143_15650850_86	TUSC3	8	+	258	9	0	0	5.81%	20.469084552261634	0.019679754444124863	0.019679754444124863	0.0017849636115922674
ENSG00000131467_42833632_42841578_170	PSME3	17	+	510	45	3	1	8.24%	51.77474327925001	0.00043491588733509306	0.0005483269449780406	1.5605106156804662e-06
ENSG00000186501_27322258_27335466_188	TMEM222	1	+	564	9	1	0	3.19%	9.363517401566492	0.026832695913080365	0.011772051206276392	0.0015103817605807263
ENSG00000146701_76048161_76066410_296	MDH2	7	+	888	208	15	8	18.24%	137.44406323981084	4.046072923093785e-09	1.3273078216221343e-09	5.856091750192829e-17
ENSG00000047579_15662869_15522975_316	DTNBP1	6	-	948	12	6	0	1.58%	7.427600301664559	0.23029830935238566	0.02108409854857798	0.025086352391888767
ENSG00000114745_39107541_39098236_345	GORASP1	3	-	1035	11	2	1	1.74%	6.236313683233335	0.04223451612662591	0.023672501042627626	0.004366626912467234
ENSG00000110660_107815808_107792615_285	SLC35F2	11	-	855	27	1	0	4.91%	18.52990812099474	0.000565108330078236	0.0004156054716904614	1.5387906409916872e-06
ENSG00000168958_227330816_227357117_221	MFF	2	+	663	17	2	1	3.62%	15.045651893115387	0.005306578019052857	0.005306578019052857	0.00015100114740534233
ENSG00000206503_29942563_29945455_368	HLA-A	6	+	1104	67	1	3	4.35%	35.610768475281255	0.00024827301248527657	0.00019965580804963987	3.4153240183593913e-07
ENSG00000206503_29942563_29945455_296	HLA-A	6	+	888	48	1	3	4.73%	31.717860747648658	0.0006007992072967827	0.0004481211664877703	1.7553784301879126e-06
ENSG00000206503_29942563_29945455_362	HLA-A	6	+	1086	69	1	3	4.97%	37.281629138465476	0.00011621995324350891	8.56333565309349e-05	7.199417312878499e-08
ENSG00000010244_32350286_32360971_184	ZNF207	17	+	552	43	0	2	5.98%	45.70934461006251	0.0016263209931528667	0.003274169447268781	3.120876447037022e-05
ENSG00000142192_25897626_25881670_100	APP	21	-	300	27	1	0	10.00%	52.81023814483501	0.0031465620335393613	0.0024042815392806777	4.325011185788818e-05
ENSG00000111237_110502051_110492992_144	VPS29	12	-	432	63	0	5	15.28%	85.57214514209377	1.772192605277682e-05	7.4465522816381e-05	1.0348654104157737e-08
ENSG00000167118_128371381_128387901_63	URM1	9	+	189	33	6	4	15.87%	102.45372479597621	0.015528142895806445	0.014991380638102441	0.0011088416938546152
ENSG00000196950_195680169_195735041_789	SLC39A10	2	+	2367	51	4	3	2.92%	12.642924214366923	0.00010271693332470929	6.743242744434434e-05	5.068255003386595e-08
ENSG00000091409_172427789_172504252_1091	ITGA6	2	+	3273	91	0	6	2.84%	16.314396140747483	4.3876988440771667e-07	1.2934267084192172e-05	5.5227396237404276e-11
ENSG00000130669_39172987_39178579_500	PAK4	19	+	1500	29	0	0	3.00%	11.344421527409002	0.000268868811829191485	0.000268868811829191485	4.91773397748167e-07
ENSG00000128739_24976387_24978444_161	SNRPN	15	+	483	25	0	0	4.35%	30.371657548214294	0.00877616296920843	0.00877616296920843	0.0003916135667564577
ENSG00000069275_205749973_205718280_243	NUCKS1	1	-	729	100	15	6	8.64%	80.4911418150206	0.0016922048903729044	0.0001551638541859011	1.8970169759622167e-06
ENSG00000145901_151065095_151030713_572	TNIP1	5	-	1716	11	0	0	0.87%	3.761412973278847	0.016947426762344633	0.016947426762344633	0.0013498980316300959
ENSG00000105699_35249023_35267859_601	LSR	19	+	1803	13	0	1	1.00%	4.230807271108986	0.011772051206276392	0.026832695913080365	0.0015103817605807263
ENSG00000198561_57791641_57816308_885	CTNND1	11	+	2655	64	7	2	2.71%	14.144612853188704	0.0002556913665467353	1.5363660848022635e-05	3.2544366602591786e-08
ENSG00000133119_33818179_33966125_305	RFC3	13	+	915	18	0	1	3.28%	11.543221452422953	0.001801896632038876	0.0040705821972625445	4.2571970118849174e-05
ENSG00000111596_70278227_70353915_540	AC092881.1	12	+	1620	24	0	0	2.22%	8.693043316022225	0.000991050613121974	0.000991050613121974	6.098881496726224e-06
ENSG00000144554_10028658_10098950_1471	FANCD2	3	+	4413	9	0	5	0.34%	1.1966969894592117	0.020613416668581838	0.19688431732149636	0.02035859654822594
ENSG00000119408_124301986_124350947_306	NEK6	9	+	918	18	0	3	1.96%	11.505498506500002	0.013007239811447113	0.044329616041373636	0.00273578248190731

ENSG00000100280_29358923_29328821_810	AP1B1	22	-	2430	24	0	0	1.11%	5.795362210681483	0.003684287313187465	0.003684287313187465	7.541963121699725e-05
ENSG00000015475_17773665_17735580_233	BID	22	-	699	21	3	0	3.43%	17.62859642412232	0.022572793433991795	0.005604866691947602	0.0006640639638462718
ENSG00000101266_489860_483961_177	CSNK2A1	20	-	531	9	0	0	2.82%	9.945430912398308	0.019216965118390855	0.019216965118390855	0.0017073955890589052
ENSG00000043143_134578682_134579317_211	JADE2	5	+	633	11	0	0	2.37%	10.196816211921803	0.02108409854857798	0.02108409854857798	0.002029891263157455
ENSG00000178057_49022473_49023172_116	NDUFAF3	3	+	348	9	0	1	4.31%	15.175355788745692	0.020613416668581838	0.03537493353818881	0.003252526348827943
ENSG00000127990_94618900_94585499_255	SGCE	7	-	765	11	0	2	2.75%	8.437365571433336	0.007029302661868638	0.03522780890193623	0.0012822660609675817
ENSG00000113163_75511207_75411005_311	COL4A3BP	5	-	933	12	4	0	1.93%	7.547015097511256	0.11379025261811354	0.012798402692974279	0.007514858560785578
ENSG00000197912_89507690_89557093_751	SPG7	16	+	2253	13	0	0	0.80%	3.3857725298755	0.011772051206276392	0.011772051206276392	0.000681272511389508
ENSG00000163762_149333382_149321478_201	TM4SF18	3	-	603	141	1	3	11.94%	137.20736278647018	8.163791924687944e-06	2.678104035760154e-05	1.7742024011977063e-09
ENSG00000105723_42242465_42230794_483	GSK3A	19	-	1449	65	4	1	4.55%	26.322103208452386	9.295530582708931e-05	2.0330004469380697e-05	1.4738570004187963e-08
ENSG00000137100_34620464_34613782_176	DCTN3	9	-	528	36	4	0	6.25%	40.00775617032956	0.012042586532450569	0.0015061527253727427	0.00011094877274825198
ENSG00000204316_75904684_75900978_203	MRPL38	17	-	609	28	0	3	5.42%	26.978410291103454	0.0014988252558711152	0.00872026670279408	7.851711812863793e-05
ENSG00000068400_49002193_48974193_829	GRIPAP1	X	-	2487	9	0	0	0.72%	2.1234514734553684	0.009815328628645334	0.009815328628645334	0.0004837142251238724
ENSG00000168002_62765397_62766507_42	POLR2G	11	+	126	70	0	5	33.33%	325.98912435083344	0.00046843213071395124	0.0031185037294365974	9.613998767368705e-06
ENSG00000119707_73077513_73119805_743	RBM25	14	+	2229	30	0	3	1.75%	7.897448503788696	0.000596156229600823	0.004225046164915315	1.635559735018257e-05
ENSG00000139637_53299861_53307149_284	C12orf10	12	+	852	21	9	0	3.87%	14.462897770494722	0.06279311226126572	0.0013845019718837994	0.0006896031064594562
ENSG00000175387_47896756_47845234_431	SMAD2	18	-	1293	18	0	3	1.86%	8.168636990693738	0.0053557511377634245	0.03142348392573343	0.0009044752776837991
ENSG00000053371_19311974_19304225_274	AKR7A2	1	-	822	7	0	0	2.19%	4.996913584939782	0.009815328628645334	0.009815328628645334	0.0004837142251238724
ENSG00000109099_15260727_15230917_160	PMP22	17	-	480	43	1	0	8.12%	52.565746301571885	0.0010459108907576228	0.0006446704936422262	4.265786271834212e-06
ENSG00000124733_43013378_43012470_172	MEA1	6	-	516	46	2	2	9.88%	52.30988274466862	0.0005393137985452151	0.0005393137985452151	1.8883487944536844e-06
ENSG00000113163_75511207_75374043_605	COL4A3BP	5	-	1815	14	4	0	1.16%	4.526129990986778	0.0750151653743942	0.008175610787902942	0.0033080285523474006
ENSG00000179818_70088801_70051267_106	PCBP1-AS1	2	-	318	14	2	4	5.66%	25.833100420254723	0.012798402692974279	0.07356204933788085	0.004612581755044152
ENSG00000072364_132934902_132881059_1109	AFF4	5	-	3327	19	0	3	0.90%	3.3510153449950413	0.0022276761777358703	0.015358647673375043	0.00020093250298549382
ENSG00000130811_10119859_10118417_146	EIF3G	19	-	438	18	1	0	4.79%	24.114263993075348	0.012000726946169103	0.008674846900444944	0.0005220387231428462
ENSG00000179335_74619197_74629883_490	CLK3	15	+	1470	7	0	0	1.22%	2.794192494435715	0.009815328628645334	0.009815328628645334	0.0004837142251238724
ENSG00000163754_148991641_149026933_350	GYG1	3	+	1050	9	0	0	1.43%	5.029546489984287	0.020613416668581838	0.020613416668581838	0.001946208561389314
ENSG00000075624_5529657_5529045_125	ACTB	7	-	375	421	15	5	12.00%	658.7588224881641	0.00139366094242993	0.0010286746874834437	8.789184600531728e-06
ENSG00000111642_6601370_6570676_1673	CHD4	12	-	5019	21	5	1	0.66%	2.4551482168682015	0.02946433998583899	0.001323351237915142	0.00026896173468470523
ENSG00000112992_43644650_43704404_707	NNT	5	+	2121	7	0	0	0.71%	1.9365690555495054	0.019216965118390855	0.019216965118390855	0.0017073955890589052
ENSG00000133961_73355751_73276578_651	NUMB	14	-	1953	19	0	1	1.54%	5.708565311212751	0.0022011839993586477	0.0037373685375237473	4.708421183416152e-05
ENSG00000058272_79934925_79775929_993	PPP1R12A	12	-	2979	25	0	2	1.31%	4.924307014363042	0.0005379818162649481	0.0019648567937019914	6.767654423560422e-06
ENSG00000176209_42546494_42552608_100	SMIM19	8	+	300	7	0	0	5.00%	13.691543222735003	0.016947426762344633	0.016947426762344633	0.0013498980316300959
ENSG00000149380_74311611_74267248_544	P4HA3	11	-	1632	17	0	1	1.29%	6.112296081578126	0.008674846900444944	0.012000726946169103	0.0005220387231428462
ENSG00000167526_89560981_89561796_147	RPL13	16	+	441	82	1	1	7.48%	109.1065640684422	0.00201702417617476	0.0016417304930345215	1.9605440729082122e-05
ENSG00000133961_73355751_73276578_592	NUMB	14	-	1776	19	0	0	1.69%	6.27749327297213	0.0022011839993586477	0.0022011839993586477	2.8200182015552967e-05
ENSG00000100902_35308980_35317306_167	PSMA6	14	+	501	49	1	2	5.99%	57.38970213122457	0.0025061435727443374	0.0024742386374296595	3.570411499116179e-05
ENSG00000276043_4910907_4960803_786	UHRF1	19	+	2358	26	0	2	1.27%	6.470013155054708	0.0020082573300163734	0.009197853169380737	0.00010725224857862305
ENSG00000145495_10353899_10433684_910	6-Mar	5	+	2730	40	4	4	2.53%	8.597515367494507	0.00011794655806817046	0.00012758244000304582	1.0742282639254168e-07
ENSG00000122884_73074883_73008222_534	P4HA1	10	-	1602	52	6	3	3.56%	19.046555580048693	0.0007714575261437182	0.00017486467114188932	9.339465873984023e-07
ENSG00000104133_44663647_44573512_2079	SPG11	15	-	6237	22	5	2	0.43%	2.0697722181005296	0.02633284015351082	0.010026873424650893	0.0012874779080855213
ENSG00000067225_72219097_72210423_69	PKM	15	-	207	69	31	0	14.49%	195.59347461050004	0.053663778880805944	0.0025167541003031247	0.0008980724580173977
ENSG00000067225_72221201_72199650_516	PKM	15	-	1548	1602	95	147	22.09%	607.2495083837617	4.588542629396154e-19	8.013065333385794e-14	9.179681874425083e-31
ENSG00000140395_78295686_78283432_305	WDR61	15	-	915	78	0	2	6.56%	50.0206262938328	4.0701262642950556e-05	9.794668315674687e-05	2.98644402202463e-08
ENSG00000132623_10038590_10055660_680	ANKEF1	20	+	2040	10	0	0	0.74%	2.8763746266250005	0.019679754444124863	0.019679754444124863	0.0017849636115922674
ENSG00000133226_24648928_24672286_803	SRRM1	1	+	2409	15	2	2	0.75%	3.653676362587174	0.024135905425855194	0.03031622373531018	0.003233007463082675
ENSG00000205903_6642410_6654611_1004	ZNF316	7	+	3012	12	0	0	0.50%	2.3377706128745026	0.019216965118390855	0.019216965118390855	0.0017073955890589052
ENSG00000196235_39459039_39476399_946	SUPT5H	19	+	2838	32	2	3	2.01%	6.616269754266386	0.0001389057379015588	0.0004512865513599377	4.3825258532272856e-07
ENSG00000092841_56158402_56160174_124	MYL6	12	+	372	847	13	12	28.23%	1336.0296209281737	1.4287512713975842e-07	2.8858785212374194e-07	3.9201101556400547e-13
ENSG00000092841_56160251_56160331_26	MYL6	12	+	78	262	0	7	42.31%	1970.980397998116	0.0016340149330375125	0.005303083112260659	5.078993352132664e-05
ENSG00000119681_74611944_74500884_1777	LTBP2	14	-	5331	16	1	0	0.39%	1.7611117578885767	0.009583874861041503	0.00797940233269714	0.0003893623151447896
ENSG00000136536_159729023_159767342_704	7-Mar	2	+	2112	17	2	0	1.42%	4.723137881219461	0.010645877633714064	0.0021880057261679567	0.0001345568947401665
ENSG00000005486_75879083_75888349_364	RHBDD2	7	+	1092	12	2	0	1.65%	6.448136525620881	0.04375184787808565	0.013217226988443315	0.0027359272655426933
ENSG00000127554_1984297_1986028_179	GFER	16	+	537	11	3	1	3.35%	12.019710730254193	0.07760924484234204	0.012798402692974279	0.004890251693990683
ENSG00000100225_32475003_32498530_522	FBXO7	22	+	1566	42	3	0	2.30%	15.73740600314368	0.005630074349578456	0.001032175130546352	3.583971133418495e-05
ENSG00000111321_6375556_6390937_416	AC005840.1	12	+	1248	49	8	1	4.57%	23.03865446133294	0.001540933378346842	7.560248252737187e-05	9.12325510568034e-07
ENSG00000167658_3985380_3984132_73	EEF2	19	-	219	18	6	0	6.85%	48.228527986150695	0.03577298629450316	0.02108409854857798	0.003354604907114977
ENSG00000155463_22766717_22771558_430	OXA1L	14	+	1290	88	13	3	6.74%	40.02843201331164	1.3415032380162829e-05	3.729670428313194e-06	4.2058270446536635e-10
ENSG00000117632_25901583_25901449_44	STMN1	1	-	132	22	0	3	15.91%	97.79673730525002	0.00847367416878415	0.008175610787902942	0.00035432190916051797
ENSG00000171320_27775515_27803438_601	ESCO2	8	+	1803	13	0	0	1.00%	4.230807271108986	0.011772051206276392	0.011772051206276392	0.000681272511389508
ENSG00000103363_2777239_2772040_66	ELOB	16	-	198	71	2	4	30.30%	210.41116208099248	8.154797557606704e-05	0.0002229551772274552	1.3106165688639122e-07
ENSG00000129103_56064312_56079612_301	SUMF2	7	+	903	141	5	3	8.64%	91.62352132917111	2.8393160413368106e-05	1.0124970165731674e-05	2.3083584118077667e-09
ENSG00000217128_131796921_131644685_1138	FNIP1	5	-	3414	16	0	3	0.70%	2.749996128091389	0.005306578019052857	0.03149539866061481	0.0008997191470395496
ENSG00000168090_100089385_100089787_67	COPS6	7	+	201	19	0	2	7.46%	55.46680623282837	0.02108409854857798	0.0700082515985845	0.006564739044797468
ENSG00000239672_51155655_51160183_109	NME1	17	+	327	169	14	0	16.51%	303.25960742361934	0.001399549156265313	9.070903305645646e-05	9.64909255603878e-07
ENSG00000155256_97744827_97759300_294	ZFYVE27	10	+	882	5	0	2	1.70%	3.326419636232994	0.012673659338734126	0.15865525393145707	0.011061610944025602
ENSG00000089327_35155551_35169615_178	FXYD5	19	+	534	101	4	3	12.92%	110.98281424528373	9.80386385557854e-05	3.0510438660264174e-05	2.276384256667706e-08
ENSG00000089006_17968425_17942357_404	SNX5	20	-	1212	24	2	0	3.71%	11.619414333297032	0.0008562249375319555	0.00020764221034884014	1.2161319549105282e-06

ENSG00000138674_82881936_82819074_1067	SEC31A	4	-	3201	25	0	0	1.03%	4.582789939327555	0.0012427339549726212	0.0012427339549726212	9.422880418492092e-06
ENSG00000080815_73170959_73219289_384	PSEN1	14	+	1152	27	1	0	3.65%	13.752666183550785	0.0006786479905456451	0.00037874750290730355	1.686250983246043e-06
ENSG00000149016_62581416_62575094_688	TUT1	11	-	2064	16	1	0	0.87%	4.5486854560581405	0.020525443280229434	0.013640585738808999	0.0013255139945858052
ENSG00000133835_119493868_119625993_453	HSD17B4	5	+	1359	23	0	0	2.43%	9.930794516647905	0.0014915192737735051	0.0014915192737735051	1.337646624223462e-05
ENSG00000215301_41334253_41347719_662	DDX3X	X	+	1986	97	12	3	7.25%	28.659466823592908	1.8411069105942975e-07	2.168363292173117e-09	4.7152014957107245e-15
ENSG00000116337_109625635_109631152_760	AMPD2	1	+	2280	7	0	1	0.66%	1.801518845096711	0.019216965118390855	0.04178282491678763	0.003600525600033436
ENSG00000258947_89923402_89934499_148	TUBB3	16	+	444	50	6	2	9.46%	66.07887655760136	0.002881754508607864	0.00095416369115764	1.682544927174563e-05
ENSG00000084090_96208434_96186730_370	STARD7	2	-	1110	14	0	0	1.62%	7.400834174451353	0.013007239811447113	0.013007239811447113	0.0008218654148058079
ENSG00000177156_764251_764845_71	TALDO1	11	+	213	69	5	6	15.49%	190.0837992693592	0.012563987137771532	0.002409760966390446	0.00017394784195504904
ENSG00000079459_11808887_11838609_353	FDFT1	8	+	1059	211	12	7	15.01%	116.91281343573799	5.823304590514566e-09	4.985113558253148e-10	3.292241988068417e-17
ENSG00000162521_32652003_32679705_390	RBBP4	1	+	1170	33	5	0	3.33%	16.550217082426926	0.0032701130750291308	0.0006311913977015671	1.3176275336358013e-05
ENSG00000119487_125672439_125438887_477	MAPKAP1	9	-	1431	10	0	0	1.05%	4.100492130199163	0.016947426762344633	0.016947426762344633	0.0013498980316300959
ENSG00000141756_41813035_41822408_582	FKBP10	17	+	1746	69	1	0	6.19%	23.18891709299743	1.0476388400758902e-07	3.9807038664992497e-08	4.1298647718362087e-14
ENSG00000060138_10723111_10701288_372	YBX3	12	-	1116	76	0	5	3.76%	39.959957178489255	0.0004561727980981236	0.002742298879542603	8.212716772877843e-06
ENSG00000164252_77030767_77063252_714	AGGF1	5	+	2142	13	0	2	1.12%	3.561225728202382	0.004826937407846361	0.01943569092740073	0.0004999687388256805
ENSG00000115548_86442048_86492119_1321	KDM3A	2	+	3963	11	2	3	0.45%	1.6287117492168814	0.05067994629101479	0.10000264635955686	0.0194788414689791
ENSG00000204574_30571488_30590701_845	ABCF1	6	+	2535	16	3	1	1.18%	3.7035450813822495	0.018462046284954254	0.003957931943710776	0.0003993137606956889
ENSG00000147140_71290638_71300076_471	NONO	X	+	1413	42	1	3	3.82%	17.441456334694273	0.00012070538323268708	0.0005818042179531959	4.981421812408915e-07
ENSG00000121022_67059390_67043233_268	COPS5	8	-	804	39	0	0	6.34%	28.463229514214557	0.00012152394927894852	0.00012152394927894852	1.0547329526488542e-07
ENSG00000013441_200858106_200853306_307	CLK1	2	-	921	22	0	0	4.23%	14.01647049326059	0.0005854768899833019	0.0005854768899833019	2.212593397499504e-06
ENSG00000172115_25124083_25123701_93	CYCS	7	-	279	29	0	2	8.60%	60.99151358822044	0.005706018193000826	0.010402771787637745	0.00030929694644272603
ENSG00000163939_52679711_52548063_1597	PBRM1	3	-	4791	14	1	0	0.50%	1.7146578863788358	0.01007916461776185	0.004873903550586949	0.0002597390186955864
ENSG00000168883_85616196_85647969_488	USP39	2	+	1464	20	9	1	1.64%	8.016126008627051	0.10867564933999213	0.005960500748457015	0.004021135078528911
ENSG00000148337_128179348_128166197_612	CIZ1	9	-	1836	21	2	0	1.63%	6.711540795458335	0.01044836432889887	0.002046028879997488	0.00012444751772694397
ENSG00000126088_45013615_45014115_127	UROD	1	+	381	8	0	0	3.94%	12.320848794362208	0.019679754444124863	0.019679754444124863	0.0017849636115922674
ENSG00000130177_114239476_114272443_498	CDC16	13	+	1494	36	0	5	3.01%	14.139287321240968	0.00028877520139654507	0.0025792363906108322	5.131036494031878e-06
ENSG00000151491_15669717_15621317_718	EPS8	12	-	2154	15	0	0	1.11%	4.086214650637188	0.005405141725979036	0.005405141725979036	0.00015637213408961292
ENSG00000140416_63059609_63061730_78	TPM1	15	+	234	95	0	9	14.10%	238.22282164099366	0.0016494676794180302	0.01253092217194181	0.00012512190574386016
ENSG00000156642_73633215_73561910_278	NPTN	15	-	834	90	0	2	7.91%	63.32162847102519	1.8790772995134137e-05	2.0436069670547633e-05	3.0226366623665172e-09
ENSG00000103479_53454256_53490300_839	RBL2	16	+	2517	11	0	0	0.72%	2.564395972247319	0.012798402692974279	0.012798402692974279	0.0007972436888943567
ENSG00000131143_85805768_85806874_77	COX4I1	16	+	231	227	1	0	36.36%	576.6197238517339	1.7670986448811327e-06	1.7683913714861395e-06	2.732753662508527e-11
ENSG00000137497_72022389_72003527_872	NUMA1	11	-	2616	24	1	3	1.15%	5.383306640655965	0.003133490598659634	0.00796980489516242	0.00013781154241186144
ENSG00000139324_88153390_88172987_310	TMTC3	12	+	930	19	0	0	2.26%	11.987987153546777	0.008574016250729465	0.008574016250729465	0.0003747459614100891
ENSG00000283761_100058702_100082209_418	AC118553.2	1	+	1254	27	0	0	4.31%	12.634028264314596	4.027253539844791e-05	4.027253539844791e-05	1.23110933795418e-08
ENSG00000152700_134624019_134606950_198	SAR1B	5	-	594	70	0	0	11.62%	69.14920819563133	1.1975596777994732e-05	1.1975596777994732e-05	1.1554586455257723e-09
ENSG00000100227_42614857_42585791_392	POLDIP3	22	-	1176	18	0	0	1.79%	8.981333017829083	0.00847367416878415	0.00847367416878415	0.0003665024264111182
ENSG00000115919_142918683_142967328_166	KYNU	2	+	498	29	0	0	6.63%	34.16994435966567	0.0014915192737735051	0.0014915192737735051	1.337646624223462e-05
ENSG00000104687_30708100_30687595_245	GSR	8	-	735	13	2	1	3.27%	10.378429265046941	0.02682370818175567	0.00912793029372963	0.0012085513616569776
ENSG00000104687_30680936_30679520_60	GSR	8	-	180	13	0	2	8.33%	42.37858616560834	0.020613416668581838	0.056923149003329024	0.005212417464661139
ENSG00000028310_878410_864468_192	BRD9	5	-	576	12	0	0	2.60%	12.224592163156252	0.019679754444124863	0.019679754444124863	0.0017849636115922674
ENSG00000096433_33658679_33695780_2545	ITPR3	6	+	7635	20	15	4	0.43%	1.5370803505736743	0.28221976165292784	0.021183503835977 27	0.03267682752849782
ENSG00000169814_15635440_15645488_523	BTD	3	+	1569	13	0	0	1.15%	4.861788087832697	0.013428347753762199	0.013428347753762199	0.0008725593497644525
ENSG00000117859_51617123_51787789_732	OSBPL9	1	+	2196	17	0	1	1.09%	4.542471404888662	0.005306578019052857	0.009288069496478249	0.0002590037054391842
ENSG00000139324_88153390_88195649_818	TMTC3	12	+	2454	42	0	0	1.71%	10.042696740392422	0.0004588781914451711	0.0004588781914451711	1.382498018603181e-06
ENSG00000184047_122226014_122208381_195	DIABLO	12	-	585	17	0	1	3.08%	17.05173881219744	0.013007239811447113	0.023878473930440184	0.0014709138084317821
ENSG00000249915_271721_314515_189	PDCD6	5	+	567	56	0	4	11.11%	57.95362210681483	2.348557756166878e-05	0.00016296318333800002	2.992452301248373e-08
ENSG00000136450_58007137_58005747_201	SRSF1	17	-	603	120	0	22	13.93%	116.77222364805972	1.5853614593892364e-06	7.80495470659366e-05	1.197920011383821e-09
ENSG00000143622_155910498_155900388_181	RIT1	1	-	543	13	1	0	3.31%	14.048150110146413	0.013007239811447113	0.012798402692974279	0.000809468393769003
ENSG00000172590_22833438_22834321_64	MRPL52	14	+	192	27	1	5	17.19%	82.5159971013047	0.0021748195570013406	0.012088529595134127	0.00015284085456361443
ENSG00000181019_69718478_69710976_253	NQO1	16	-	759	1147	57	40	39.53%	886.741958016773	3.289096005677169e-15	1.511028645207384e-16	7.452620310577443e-30
ENSG00000163072_168802647_168864970_563	NOSTRIN	2	+	1689	9	0	0	0.89%	3.1267162903987575	0.016947426762344633	0.016947426762344633	0.0013498980316300959
ENSG00000158406_26285499_26285188_103	HIST1H4H	6	-	309	786	40	9	34.95%	1492.5870975131363	5.651924870219209e-07	1.1367975501194819e-07	6.174281713338003e-13
ENSG00000101040_47298917_47210761_1019	ZMYND8	20	-	3057	6	0	0	0.49%	1.1516789476575076	0.016947426762344633	0.016947426762344633	0.0013498980316300959
ENSG00000272886_53347517_53287580_582	DCP1A	3	-	1746	7	0	0	0.86%	2.352498835521478	0.019216965118390855	0.019216965118390855	0.0017073955890589052
ENSG00000156467_96231935_96231553_76	UQCRB	8	-	228	318	1	7	23.68%	818.4042753439344	9.600364516278243e-05	0.0002641474933126208	1.810835102932693e-07
ENSG00000118680_3272899_3277937_172	MYL12B	18	+	516	55	6	0	5.23%	62.54442502079943	0.006172075136861155	0.0037896409716948643	0.00012737069552876224
ENSG00000120549_24513334_24546324_1251	KIAA1217	10	+	3753	8	0	4	0.40%	1.2507975994276581	0.019679754444124863	0.17004230409153215	0.016523685284367155
ENSG00000120549_24513334_24545118_1090	KIAA1217	10	+	3270	8	0	0	0.46%	1.4355484375082572	0.019679754444124863	0.019679754444124863	0.0017849636115922674
ENSG00000152683_32165901_32220713_461	SLC30A6	2	+	1383	30	5	0	3.25%	12.728425679642084	0.005975744103077381	0.0002812865659102998	1.2422237301200548e-05
ENSG00000128609_123550481_123542119_59	NDUFA5	7	-	177	13	1	2	8.47%	43.09686728705933	0.02108409854857798	0.0700082515985845	0.006564739044797468
ENSG00000160214_43795198_43803774_338	RRP1	21	+	1014	7	0	0	1.78%	4.050752432761835	0.009815328628645334	0.009815328628645334	0.0004837142251238724
ENSG00000155957_66169858_66137960_207	TMBIM4	12	-	621	49	0	4	7.25%	46.299904617944456	0.0003026020628493325	0.001576668176329556	3.203251411610351e-06
ENSG00000136802_128907165_128916371_810	LRRC8A	9	+	2430	20	3	1	1.48%	4.829468508901235	0.008727216264005591	0.0015309509177879102	8.005137351093444e-05
ENSG00000077150_102395960_102402376_899	NFKB2	10	+	2697	23	0	0	1.00%	5.004059973349834	0.0035547763465261306	0.0035547763465261306	7.044676146010672e-05
ENSG00000170270_93207037_93203688_100	GON7	14	-	300	13	1	0	6.00%	25.427151699365005	0.023672501042627626	0.012798402692974279	0.0014375227604752335
ENSG00000162521_32669128_32679705_173	RBBP4	1	+	519	11	5	0	3.47%	12.436579310494222	0.08266357711566169	0.012798402692974279	0.005241450380307 1884
ENSG00000151726_184803424_184757125_668	ACSL1	4	-	2004	18	0	0	1.20%	5.270482848785929	0.005454749182134642	0.005454749182134642	0.00015910859015753364

ENSG00000154518_175181393_175178288_142	ATP5MC3	2	-	426	138	31	7	21.83%	190.0837992693592	6.092060945556509e-05	2.2032051986119076e-06	1.2346752126045435e-09
ENSG00000152795_82429324_82426059_298	HNRNPDL	4	-	894	114	3	1	11.41%	74.82434934764096	2.8068292164810636e-07	1.8169410532370092e-07	4.81845735322702e-13
ENSG00000078140_39698328_39778434_149	UBE2K	4	+	447	34	0	0	8.05%	44.63206803192618	0.0010073743004618205	0.0010073743004618205	6.293580389004731e-06
ENSG00000129128_176320131_176328330_162	SPCS3	4	+	486	37	0	1	9.88%	44.67258370733643	0.0001924870242054139	0.00024054947992775683	3.197181953766169e-07
ENSG00000089693_6752334_6750515_155	MLF2	12	-	465	13	1	0	5.16%	16.404613999590325	0.004826937407846361	0.004826937407846361	0.00012612451166366307
ENSG00000015479_139322767_139326168_177	MATR3	5	+	531	26	0	21	3.95%	28.731244858039556	0.008574016250729465	0.5140188448859375	0.04840202231039189
ENSG00000104852_49104447_49108443_341	SNRNP70	19	+	1023	83	7	1	8.21%	47.60779587293696	1.9782258819560762e-05	1.961880122406878e-06	3.4192240211750377e-10
ENSG00000135111_114683008_114671841_679	TBX3	12	-	2037	10	0	0	0.88%	2.880610819005892	0.011570465654371866	0.011570465654371866	0.0006594843062896773
ENSG00000146409_132790236_132770270_383	SLC18B1	6	-	1149	8	3	0	1.31%	4.085503386120106	0.14389369507707506	0.019679754444124863	0.013602114125134476
ENSG00000137210_10749246_10755347_135	TMEM14B	6	+	405	21	0	0	5.19%	30.425651606077786	0.00877616296920843	0.00877616296920843	0.00039161135667564577
ENSG00000175061_16439055_16440252_53	SNHG29	17	+	159	22	3	0	16.98%	81.18974417794341	0.01772171524267468	0.0036581908865286695	0.0003575151330641259
ENSG00000167491_19465346_19505474_633	GATAD2A	19	+	1899	10	2	0	1.11%	3.0899443066429706	0.049338281967443096	0.007593099278532274	0.0019605633087212975
ENSG00000198604_34874604_34753508_1524	BAZ1A	14	-	4572	9	3	1	0.52%	1.155079574471457	0.07229263996867695	0.010460667668897016	0.003853881152483681
ENSG00000072571_163469713_163491164_610	HMMR	5	+	1830	26	1	0	1.15%	8.336771048972134	0.010197420091687953	0.00877616296920843	0.0004515216795882782
ENSG00000131732_81304758_81312662_271	ZCCHC9	5	+	813	11	0	0	2.95%	7.939218526625463	0.004687384229717438	0.004687384229717438	0.00011928172701435494
ENSG00000151366_78079744_78069873_88	NDUFC2	11	-	264	71	3	5	17.05%	157.80837156074435	0.00039453730762884214	0.0019794610128320875	5.1460700234593595e-06
ENSG00000145907_151786627_151791089_123	G3BP1	5	+	369	18	1	0	7.32%	28.623435308853665	0.002967235347054297	0.0033030736763512156	5.530095551685825e-05
ENSG00000161013_179801456_179798007_336	MGAT4B	5	-	1008	20	1	9	2.38%	11.642468726815478	0.008364861494636245	0.12211003001358411	0.005947433949770055
ENSG00000109016_21172043_21191153_310	DHRS7B	17	+	930	9	2	0	1.61%	5.678520230627421	0.10105102209468636	0.019679754444124863	0.009164497669452725
ENSG00000086062_33167130_33104752_212	B4GALT1	9	-	636	51	5	1	8.49%	47.05314719403539	0.0012257744443895519	0.0001226024805824952	1.0946115650439794e-06
ENSG00000174780_56467636_56501861_671	SRP72	4	+	2013	32	4	1	1.64%	9.327855719129658	0.006263309918074338	0.001988634903193983	7.175199281327647e-05
ENSG00000127990_94656098_94597920_436	SGCE	7	-	1308	25	3	2	2.52%	11.215222168033259	0.007887772776385514	0.0038108971419065062	0.00016292425881891603
ENSG00000206053_1678313_1698998_218	JPT2	16	+	654	22	0	5	5.05%	19.738791015738535	0.0012690167775026367	0.03615784735065794	0.0003303939651969694
ENSG00000133997_70600637_70584892_181	MED6	14	-	543	10	3	0	3.31%	10.80626931549724	0.09783387127182075	0.011570465654371866	0.005852729696011089
ENSG00000153066_11742730_11679195_958	TXNDC11	16	-	2874	15	3	4	0.63%	3.0625283080976	0.044241358585871825	0.07916783876068934	0.01383142491069238
ENSG00000140612_84716075_84670757_164	SEC11A	15	-	492	114	13	1	14.63%	135.9613177170549	0.00010743891859535151	1.000644152070349e-05	8.879339940177268e-09
ENSG00000067141_73052676_73302696_1408	NEO1	15	+	4224	22	0	1	0.85%	3.056148040789063	0.0007764301431553706	0.0025593219000189936	1.2365068093582474e-05
ENSG00000028528_64130012_64134858_170	SNX1	15	+	510	9	0	3	2.94%	10.354948655850002	0.016947426762344633	0.165899918248885	0.014398895220848395
ENSG00000141504_7627855_7626447_249	SAT2	17	-	747	12	5	5	3.21%	9.426191547493978	0.04938683320192602	0.06904305126011098	0.013348489610371488
ENSG00000215305_2840775_2866574_695	VPS16	20	+	2085	11	0	1	1.01%	3.095724058583454	0.007593099278532274	0.013018137044106636	0.0004999111921735253
ENSG00000166851_23678933_23679454_173	PLK1	16	+	519	9	0	0	2.89%	10.175383072222546	0.019679754444124863	0.019679754444124863	0.0017849636115922674
ENSG00000085721_15094233_15060168_638	RRN3	16	-	1914	12	2	3	0.94%	3.6788741306050166	0.06032271266817251	0.06032271266817251	0.014083278824088103
ENSG00000108439_47942095_47946782_228	PNPO	17	+	684	22	0	0	3.95%	18.87305456767983	0.0033278027414746945	0.0033278027414746945	6.211806171561056e-05
ENSG00000013364_29830550_29847989_893	MVP	16	+	2679	69	5	5	2.91%	15.113045630598547	3.5064313186673395e-05	2.413580229981598e-05	6.543708222377824e-09
ENSG00000113368_126777539_126819146_377	LMNB1	5	+	1131	24	0	0	2.92%	12.451573980509286	0.0015356776430442238	0.0015356776430442238	1.41466323862755e-05
ENSG00000188186_100153490_100153964_41	LAMTOR4	7	+	123	21	0	0	14.63%	100.18202358098783	0.013640585738808999	0.013640585738808999	0.00089863692579514
ENSG00000104763_18063207_18057534_235	ASAH1	8	-	705	46	3	2	8.09%	38.28638226418299	5.488892743562522e-05	0.0001965696589835854	7.965536585833072e-08
ENSG00000111300_112078669_112029531_789	NAA25	12	-	2367	28	3	2	1.39%	6.941213294162233	0.007928743640974104	0.00456905887799821	0.00019335970712184186
ENSG00000086666_80120378_80137628_197	ZFAND6	15	+	591	20	0	3	5.58%	19.8572055442132	0.0014481341328822738	0.011821942446875977	0.00010518227444157728
ENSG00000152558_102402045_102398867_102	TMEM123	11	-	306	19	0	0	4.90%	36.434078603916674	0.019679754444124863	0.019679754444124863	0.0017849636115922674
ENSG00000065833_83430954_83211924_572	ME1	6	-	1716	91	2	4	4.90%	31.117143688034098	2.8441264327705253e-06	5.8597706939390365e-06	1.4162974661283165e-10
ENSG00000116473_111697468_111709235_133	RAP1A	1	+	399	28	1	0	9.02%	41.177573602210536	0.0019464877837825903	0.0010197273511756433	1.2094964810796119e-05
ENSG00000124795_18263987_18225719_341	DEK	6	-	1023	32	2	5	3.91%	18.35481286667449	0.0021690009861618943	0.005689030396290439	7.044557031939336e-05
ENSG00000156970_40165129_40220759_1027	BUB1B	15	+	3081	21	6	0	1.07%	3.9994770855116855	0.03471218668272105	0.0012427339549726212	0.0003097508753469615
ENSG00000169641_23093913_23088895_960	LUZP1	1	-	2880	10	0	0	0.83%	2.037432027192709	0.00414960799764038	0.00414960799764038	9.45964903272165e-05
ENSG00000168488_28826345_28836800_854	ATXN2L	16	+	2562	50	7	2	1.99%	11.45160858375293	0.001435957343153687	0.00012584544091628916	1.3241823717153865e-06
ENSG00000175832_43545310_43528519_445	ETV4	17	-	1335	18	0	1	2.02%	7.911646164020227	0.002967235347054297	0.002943990679779778	4.953464307974475e-05
ENSG00000152240_46118204_46128125_202	HAUS1	18	+	606	23	0	0	2.97%	22.27054413881931	0.013428347753762199	0.013428347753762199	0.0008725593497644525
ENSG00000185896_113306517_113322421_386	LAMP1	13	+	1158	194	0	9	10.10%	98.30345615139122	2.200938134315088e-08	7.398822467446333e-07	1.736957369707412e-13
ENSG00000183735_64464361_64501381_644	TBK1	12	+	1932	18	0	0	1.24%	5.466898358678573	0.005554612275049112	0.005554612275049112	0.00016468457691007306
ENSG00000237188_147252712_147295509_768	AC242426.2	1	+	2304	13	0	1	1.04%	3.310827044188152	0.004193160480899131	0.016854695534768073	0.0003819250574820159
ENSG00000213246_58352135_58346246_117	SUPT4H1	17	-	351	36	0	3	8.55%	60.18260757246155	0.0023766146074493525	0.00789471430737301	0.00010646932543934645
ENSG00000108946_68515440_68530449_381	PRKAR1A	17	+	1143	159	2	3	9.97%	81.62562326264963	4.363518371891827e-08	4.3268258388075745e-08	1.8788011721226132e-14
ENSG00000105220_34394016_34400036_221	GPI	19	+	663	132	9	1	12.22%	116.82506175830773	1.4902330648812101e-05	2.737935397021113e-06	3.4927181258953293e-10
ENSG00000037474_6604760_6599926_213	NSUN2	5	-	639	26	0	0	4.69%	23.875259811610334	0.0024042815392806777	0.0024042815392806777	3.3387040459710164e-05
ENSG00000143198_165632240_165655504_166	MGST3	1	+	498	268	11	5	24.10%	315.77741684104825	9.214140078261415e-08	2.224106865033278e-08	2.0692716130967128e-14
ENSG00000143198_165649848_165655504_152	MGST3	1	+	456	259	11	5	25.00%	333.28098634289154	1.986995211673844e-07	4.996581120954683e-08	9.776089761827312e-14
ENSG00000143183_165768742_165728023_185	TMCO1	1	-	555	125	4	13	18.38%	132.15775311520272	1.8486385112133776e-07	2.6109012305360913e-06	4.6733436335802924e-12
ENSG00000143198_165632240_165655504_166	MGST3	1	+	498	268	11	5	24.10%	315.77741684104825	9.214140078261415e-08	2.224106865033278e-08	2.0692716130967128e-14
ENSG00000143198_165649848_165655504_152	MGST3	1	+	456	283	11	5	25.66%	364.1641665445494	1.270287480232934e-07	3.3439986500329884e-08	4.234014700356746e-14
ENSG00000143198_165649848_165661496_129	MGST3	1	+	387	167	9	3	20.16%	253.21015705390315	2.091839762480174e-05	3.775357135995119e-06	6.672635601362294e-10
ENSG00000171793_40983291_41008797_451	CTPS1	1	+	1353	20	5	1	2.00%	8.673768275410202	0.04011189309666471	0.005034872532813168	0.0011189735043589642
ENSG00000077514_74612937_74640766_360	POLD3	11	+	1080	12	0	0	1.39%	6.519782487016668	0.019679754444124863	0.019679754444124863	0.0017849636115922674
ENSG00000137942_93448282_93551125_609	FNBP1L	1	+	1827	7	3	0	0.99%	2.2482008575919545	0.1097457788509939	0.009815328628645334	0.005898503241319083
ENSG00000166471_9314441_9283424_291	TMEM41B	11	-	873	10	1	1	2.41%	6.72142524434708	0.016854695534768073	0.016854695534768073	0.0013361081351157898
ENSG00000136156_48233361_48261224_266	ITM2B	13	+	798	183	18	8	16.17%	134.56242802150942	1.193206591984365e-06	2.3445999505718923e-07	2.624310085223165e-12
ENSG00000076928_41894238_41907413_741	ARHGEF1	19	+	2223	16	1	3	1.35%	4.223340882278004	0.0036460193534901884	0.021084098548578	0.0004279540139786225

ENSG00000147044_41665429_41520420_724	CASK	X	-	2172	19	0	0	0.97%	5.132977924861189	0.00847367416878415	0.00847367416878415	0.0003665024264111182
ENSG00000147044_41665429_41520420_723	CASK	X	-	2169	19	0	0	0.97%	5.140077479390734	0.00847367416878415	0.00847367416878415	0.0003665024264111182
ENSG00000102554_73061873_73075886_366	KLF5	13	+	1098	21	6	1	3.01%	11.22257641207787	0.014185330784655574	0.0022157162847038963	0.00018382649744872658
ENSG00000174744_66341573_66338493_243	BRMS1	11	-	729	12	4	3	2.06%	9.65893701780247	0.11710064162938172	0.1029516053660342	0.04131679168203548
ENSG00000167770_63986457_63997546_271	OTUB1	11	+	813	41	1	0	7.38%	29.591632690149453	3.145332252154177e-05	3.404299617394451e-05	8.215888711579203e-09
ENSG00000135297_73466259_73500735_628	MTO1	6	+	1884	8	0	8	0.80%	2.4916366192420387	0.016947426762344633	0.165899918248885	0.014398895220848395
ENSG00000135297_73461855_73500735_717	MTO1	6	+	2151	10	2	8	0.84%	2.7279424631868903	0.07134538375819317	0.11411551976453232	0.02947800993301872
ENSG00000123444_47577999_47572930_518	KBTBD4	11	-	1554	14	3	0	0.97%	5.286310124608109	0.1029516053660342	0.02108409854857798	0.009872287793463467
ENSG00000204256_32974691_32980718_750	BRD2	6	+	2250	22	2	4	1.73%	5.737408588574668	0.0019774936324837536	0.007183997053108228	8.20703915407641e-05
ENSG00000099246_27504370_27538051_142	RAB18	10	+	426	13	0	1	4.93%	17.90644485870775	0.00837382380159347	0.013794764262559246	0.0005783443517979597
ENSG00000132780_45584153_45618141_420	NASP	1	+	1260	39	2	2	3.33%	18.162251213832146	0.0014501041461665893	0.0014501041461665893	1.2672797129722473e-05
ENSG00000080815_73148065_73173782_169	PSEN1	14	+	507	10	0	0	4.14%	11.57357837931953	0.007029302661868638	0.007029302661868638	0.0002573639808771639
ENSG00000140937_65004824_64948091_678	CDH11	16	-	2034	18	0	0	1.62%	5.192747113553098	0.0012427339549726212	0.0012427339549726212	9.422880418492092e-06
ENSG00000186501_27330745_27335466_160	TMEM222	1	+	480	8	1	0	3.12%	9.779673730525003	0.04223451612662591	0.019679754444124863	0.003715148010809822
ENSG00000124942_62536098_62433884_149	AHNAK	11	-	447	10	2	0	4.03%	13.127078832919466	0.061356297364084836	0.012798402692974279	0.0037940651817215715
ENSG00000134809_57530189_57528717_90	TIMM10	11	-	270	118	8	13	24.44%	256.4447778226556	2.785638855085842e-05	0.0004849677896341693	1.0937705086186082e-07
ENSG00000102974_67616855_67637872_371	CTCF	16	+	1113	7	0	0	1.35%	3.6904429171792463	0.019216965118390855	0.019216965118390855	0.0017073955890589052
ENSG00000140284_50182428_50236096_620	SLC27A2	15	+	1860	28	3	5	2.42%	8.8332536920871	0.0020269077718480222	0.0037160416954276137	4.3365891491343226e-05
ENSG00000148908_119500247_119500113_44	RGS10	10	-	132	11	0	0	13.64%	48.89836865262501	0.012798402692974279	0.012798402692974279	0.0007972436888943567
ENSG00000198363_61646807_61626273_112	ASPH	8	-	336	172	10	18	16.96%	300.37569315183936	0.00042704584530036006	0.001329543869373327	3.6880131635937774e-06
ENSG00000120694_31140297_31137318_236	HSPH1	13	-	708	41	3	1	5.51%	33.98022228402755	0.003918955930507044	0.0006629342244537616	1.6607943669001863e-05
ENSG00000120694_31151026_31141065_360	HSPH1	13	-	1080	21	1	0	2.78%	11.409619352279169	0.003457660202585786	0.0023218226288164415	4.58668892290874e-05
ENSG00000165322_31908573_31807658_747	ARHGAP12	10	-	2241	24	1	1	1.34%	6.284127698329319	0.0034718995429267267	0.0034718995429267267	6.734844907553302e-05
ENSG00000130520_18323020_18307464_125	LSM4	19	-	375	47	5	0	10.40%	73.54314645354802	0.004314308702745973	0.0007052562366071395	1.939278677175654e-05
ENSG00000198860_184051756_184072863_129	TSEN15	1	+	387	13	0	0	4.65%	19.710970309585274	0.013007239811447113	0.013007239811447113	0.0008218654148058079
ENSG00000113163_75425466_75379346_430	COL4A3BP	5	-	1290	7	3	0	1.16%	3.1840798192406985	0.169362073879829	0.019216965118390855	0.016163991620339734
ENSG00000149428_119056160_119055746_62	HYOU1	11	-	186	8	0	0	8.06%	25.237867691677426	0.019679754444124863	0.019679754444124863	0.0017849636115922674
ENSG00000184009_81512354_81510690_375	ACTG1	17	-	1125	686	106	22	21.33%	357.80566288747474	9.755374408544912e-11	1.0362837595840047e-13	1.601133678667311e-22
ENSG00000141543_80146961_80135490_390	EIF4A3	17	-	1170	76	5	3	7.44%	38.115651462558986	2.811666357532789e-06	6.299618447560476e-06	1.5053993793236088e-10
ENSG00000169714_129171757_129170453_179	CNBP	3	-	537	258	23	7	25.70%	281.9168516732347	2.002440947481587e-08	2.7403139435589005e-08	5.567744773780436e-15
ENSG00000177674_11747543_11750192_104	AGTRAP	1	+	312	18	0	3	6.73%	33.852716759509626	0.00847367416878415	0.00837382380159347	0.000362422040249726
ENSG00000140553_90934999_90953716_961	UNC45A	15	+	2883	26	2	3	1.46%	5.291810967609782	0.0016931352282804095	0.0036482233473974857	3.6055762566959285e-05
ENSG00000153187_244860372_244854450_491	HNRNPU	1	-	1473	122	22	12	7.94%	48.59960061605093	7.980721359266331e-05	5.741849097790464e-06	3.917809384021959e-09
ENSG00000110906_109469554_109451595_254	KCTD10	12	-	762	13	0	0	3.15%	10.010689645419294	0.004826937407846361	0.004826937407846361	0.00012612451166366307
ENSG00000132361_2703447_2690594_1233	CLUH	17	-	3699	28	9	2	1.38%	4.441700964390917	0.009970898787836703	0.0005456143505417452	3.825479896560591e-05
ENSG00000122882_73163910_73134583_592	ECD	10	-	1776	10	0	5	1.18%	3.3039438278800684	0.007029302661868638	0.11069588287817317	0.004645432679727841
ENSG00000146648_55019278_55205617_1165	EGFR	7	+	3495	49	1	4	1.80%	8.226678331257084	3.145600943717007e-05	0.00025972902470557466	6.33369466831687e-08
ENSG00000138674_82881936_82819074_1220	SEC31A	4	-	3660	28	0	0	0.98%	4.489030564831149	0.0008664586529590942	0.0008664586529590942	4.7094196839841995e-06
ENSG00000119787_38377260_38296483_579	ATL2	2	-	1737	36	2	0	3.11%	12.161252307388605	0.00035725766419111426	7.085312003219173e-05	1.85295863416304e-07
ENSG00000168040_70203460_70206473_208	FADD	11	+	624	29	3	2	4.33%	27.27024405627164	0.020822301124399242	0.013141913862703638	0.0012993709375762057
ENSG00000137710_110272598_110200362_579	RDX	11	-	1737	24	4	0	2.25%	8.107501538259068	0.00721048140476193	0.0006223089785121674	2.9992429400450293e-05
ENSG00000075624_5529657_5527748_375	ACTB	7	-	1125	955	44	33	16.53%	498.11138200807346	1.8241793141731438e-10	4.564440690072044e-10	9.53223743448052e-19
ENSG00000182809_105474863_105479653_208	CRIP2	14	+	624	12	2	1	2.88%	11.284238919836541	0.04375184787808565	0.02015986069794795	0.003928694794497386
ENSG00000256053_103563002_103587334_177	APOPT1	14	+	531	11	0	3	2.82%	12.155526670709042	0.019679754444124863	0.04178282491678763	0.00367583974676279
ENSG00000069849_141902161_141921998_193	ATP1B3	3	+	579	108	9	1	9.84%	109.45127076649743	0.00013335529858829194	6.43783413045692e-05	6.276455949284798e-08
ENSG00000069849_141922019_141925701_71	ATP1B3	3	+	213	26	0	0	8.45%	71.625779434831	0.013640585738808999	0.013640585738808999	0.00089863692579514
ENSG00000134248_110406378_110401523_79	LAMTOR5	1	-	237	18	0	0	10.13%	44.56560180998735	0.0053557511377634245	0.0053557511377634245	0.00015366972994913032
ENSG00000112851_66021326_66078530_431	ERBIN	5	+	1293	15	0	0	1.16%	6.807197492244781	0.02108409854857798	0.02108409854857798	0.002029891263157455
ENSG00000090266_140702960_140704934_41	NDUFB2	7	+	123	97	0	0	17.07%	462.74553749313424	0.008980238763039383	0.008980238763039383	0.0004089866599971986
ENSG00000258289_64914496_64932230_113	CHURC1	14	+	339	50	23	0	14.16%	86.54578522588497	0.004018004424882606	0.00020239236636571483	6.0654610624108315e-06
ENSG00000107372_72360631_72355953_164	ZFAND5	9	-	492	19	0	1	4.88%	22.660219619509153	0.005306578019052857	0.0069659089849165145	0.00019587836168716277
ENSG00000126226_113185526_113178198_232	PCID2	13	-	696	15	0	0	3.02%	12.646129823954745	0.00847367416878415	0.00847367416878415	0.0003665024264111182
ENSG00000187109_76067450_76048429_349	NAP1L1	12	-	1047	92	11	6	6.30%	51.560457490447	0.00015693298428385555	7.045884100291688e-05	8.039562620315599e-08
ENSG00000131143_85801206_85806874_169	COX4I1	16	+	507	357	21	4	28.40%	413.1767481417072	7.671319482569737e-09	2.2242278867119043e-09	1.8363164231604172e-16
ENSG00000150316_94966377_94963385_70	CWC15	11	-	210	13	0	1	8.57%	36.32450242766429	0.011772051206276392	0.015555382717448078	0.0008874794703752884
ENSG00000138758_77005630_77036794_375	SEPT11	4	+	1125	16	0	0	2.13%	8.345321583381335	0.005405141725979036	0.005405141725979036	0.00015637213408961292
ENSG00000109743_15707574_15723456_164	BST1	4	+	492	8	1	0	3.05%	9.541145102951221	0.03418265644467716	0.019216965118390855	0.0029560527921161815
ENSG00000056097_32444299_32355760_1052	ZFR	5	-	3156	43	1	4	2.00%	7.994790312026143	3.44550204506708e-05	0.00026104129223185335	6.919739521733992e-08
ENSG00000075785_128795368_128813422_207	RAB7A	3	+	621	69	3	3	11.11%	65.19782487016668	1.168892403477553e-05	3.596597760143498e-05	3.353977140426602e-09
ENSG00000095002_47410181_47562111_773	MSH2	2	+	2319	30	1	2	1.29%	7.590949855517466	0.0031859520777271916	0.006198316675603911	0.00010898907104230859
ENSG00000186468_82278323_82275254_134	RPS23	5	-	402	828	27	20	10.45%	1208.5925147574183	0.012180114527427986	0.014519426903687729	0.0008576173033568209
ENSG00000070087_149968535_149966489_91	PFN2	3	-	273	64	3	8	17.58%	137.56024587991212	0.0006674249601086224	0.001736918071338517	7.284967500522852e-06
ENSG00000143321_156745364_156743449_208	HDGF	1	-	624	81	2	4	12.02%	76.16861270889665	1.0702020624377367e-05	1.5021122042592045e-05	1.293509195057473e-09
ENSG00000164405_132866882_132867109_75	UQCRQ	5	+	225	10	1	2	6.67%	26.079129948066672	0.03537493353818881	0.06032271266817251	0.008767877784015958
ENSG00000140416_63059609_63069943_141	TPM1	15	+	423	98	4	7	9.93%	135.94440079311352	0.000469807809610967	0.0034160674538945943	1.0603831148400669e-05
ENSG00000134480_87411304_87392860_272	CCNH	5	-	816	15	0	0	1.84%	10.786404849843752	0.021557223391537678	0.021557223391537678	0.002115616449879074
ENSG00000149428_119056160_119045114_678	HYOU1	11	-	2034	41	1	0	2.95%	11.827923980870947	4.441415812564126e-05	3.4197238319135e-05	1.1559158704370502e-08

ENSG00000077097_25643754_25598307_1536	TOP2B	3	-	4608	36	0	4	1.37%	4.584222061183595	1.962005849075374e-05	0.0002350044648393195	3.713615366833325e-08
ENSG00000108953_1400110_1345447_255	YWHAE	17	-	765	221	13	13	15.69%	169.51434466243336	5.1772946147711686e-08	2.435112578870297e-07	1.241807386573992e-13
ENSG00000259316_64381371_64300704_48	AC087632.1	15	-	144	101	9	1	14.58%	411.5612694929272	0.008877961307018026	0.008877961307018026	0.00040023486315785033
ENSG00000120889_23068894_23022671_411	TNFRSF10B	8	-	1233	81	1	8	4.38%	38.547619083821175	0.0001140753585416825	0.0014068810995928915	1.187815784493955e-06
ENSG00000132646_5119798_5115283_261	PCNA	20	-	783	350	18	13	23.37%	262.2901000523947	6.253645033670741e-11	1.7783641736001606e-10	1.3047738787450618e-19
ENSG00000185104_50959787_50441440_490	FAF1	1	-	1470	7	0	0	1.43%	2.794192494435715	0.00407548579675135	0.00407548579675135	9.14053164909177e-05
ENSG00000173486_64242388_64243556_129	FKBP2	11	+	387	51	4	3	11.63%	77.32765275298839	0.0010574379041354276	0.0009071831790816377	5.964859088103189e-06
ENSG00000241837_33907388_33903612_59	ATP5PO	21	-	177	33	1	12	16.95%	109.39974003638137	0.0022409900782254734	0.05430206290119184	0.0008326842043706653
ENSG00000241837_33903615_33903526_29	ATP5PO	21	-	87	14	1	0	20.69%	94.4244360188621	0.012798402692974279	0.013428347753762199	0.0008341197643438865
ENSG00000038358_67877237_67884148_1244	EDC4	16	+	3732	14	8	0	0.48%	2.2012127367741163	0.24084172041503737	0.013428347753762199	0.019567424388346417
ENSG00000155366_112703799_112701540_193	RHOC	1	-	579	87	4	5	15.03%	88.16907922856738	6.357679160154451e-06	1.1512870382308943e-05	6.012907082345096e-10
ENSG00000064012_201266643_201277805_226	CASP8	2	+	678	17	1	0	3.54%	14.712783488400445	0.01007916461776185	0.004873903550586949	0.0002597390186955864
ENSG00000064012_201266643_201286594_412	CASP8	2	+	1236	20	1	0	2.67%	9.494828864587381	0.002558966410643687	0.001092092816906976	1.6908373131935995e-05
ENSG00000111615_75511597_75506469_177	KRR1	12	-	531	30	0	0	6.21%	33.151436374661024	0.001558047347358915	0.001558047347358915	1.4544595481456893e-05
ENSG00000115525_85863399_85840144_362	ST3GAL5	2	-	1086	12	1	3	1.66%	6.483761589298344	0.026602764905793736	0.09783387127182075	0.011244525270099857
ENSG00000138760_76179654_76161713_320	SCARB2	4	-	960	72	5	0	9.38%	44.00853178736251	4.09601459398266e-06	6.893507041361946e-07	2.554533397957012e-11
ENSG00000179632_144105684_144107109_286	MAF1	8	+	858	20	8	0	3.50%	13.677865357377625	0.03995989181594016	0.0022011839993586477	0.0005730923856194446
ENSG00000084234_130069978_130120810_116	APLP2	11	+	348	22	0	1	6.03%	37.09531415026725	0.00847367416878415	0.011825808327677989	0.0005036791746826746
ENSG00000070423_649726_648128_135	AC004156.1	19	-	405	12	0	0	3.70%	17.38608663204445	0.019679754444124863	0.019679754444124863	0.0017849636115922674
ENSG00000197879_1483656_1465726_963	MYO1C	17	-	2889	71	10	0	3.63%	14.42070269713967	1.85668876691052e-05	7.500225446215369e-08	1.670861245916329e-11
ENSG00000005100_5461088_5444205_474	DHX33	17	-	1422	16	0	0	1.69%	6.602311379257386	0.005405141725979036	0.005405141725979036	0.00015637213408961292
ENSG00000143570_153962715_153960098_324	SLC39A1	1	-	972	45	1	7	6.79%	27.16576036256945	1.946122850477892e-05	0.0001930553194167021	3.001873045404824e-08
ENSG00000158769_161021073_160998871_299	F11R	1	-	897	14	2	0	1.67%	9.158222891461541	0.020613416668581838	0.020613416668581838	0.001946208561389314
ENSG00000158773_161042214_161039920_251	USF1	1	-	753	10	1	0	2.39%	7.792568709581675	0.026832695913080365	0.011772051206276392	0.0015103817605807263
ENSG00000158773_161042214_161039920_251	USF1	1	-	753	10	1	0	2.39%	7.792568709581675	0.026832695913080365	0.011772051206276392	0.0015103817605807263
ENSG00000113282_157817534_157787646_625	CLINT1	5	-	1875	49	1	1	3.52%	15.334528409463204	2.2857236768867948e-05	2.2857236768867948e-05	4.081157079754527e-09
ENSG00000104760_17874426_17868532_151	FGL1	8	-	453	19	1	1	7.28%	24.61109945430133	0.0025593219000189936	0.0012624143894574274	1.9339285108591855e-05
ENSG00000198959_38132918_38130219_288	TGM2	20	-	864	80	4	10	10.07%	54.3315207251389	9.132602985950441e-07	2.008187777254202e-06	1.6306671427377046e-11
ENSG00000198805_20469525_20476601_289	PNP	14	+	867	114	5	2	9.34%	77.15451939652942	2.4328052481074442e-06	6.404512183489293e-06	1.3318895047630472e-10
ENSG00000122068_197750065_197773408_98	FYTTD1	3	+	294	5	0	0	5.10%	9.979258908698982	0.012673659338734126	0.012673659338734126	0.000782701129001274
ENSG00000250151_9803888_9810665_56	ARPC4-TTLL3	3	+	168	10	0	0	8.93%	34.92740618044644	0.020613416668581838	0.020613416668581838	0.001946208561389314
ENSG00000180900_143815372_143791163_1630	SCRIB	8	-	4890	12	3	9	0.55%	1.4399519603226998	0.016254722322859756	0.2087027175654277	0.01852228469542362
ENSG00000138796_108033208_108034357_67	HADH	4	+	201	21	0	0	10.45%	61.30541741523136	0.007029302661868638	0.007029302661868638	0.0002573639808771639
ENSG00000164171_53042155_53080514_866	ITGA2	5	+	2598	23	2	0	1.50%	5.1947458614797934	0.00346840949707983	0.0005399915898224664	1.2188419424074013e-05
ENSG00000164171_53080592_53090599_178	ITGA2	5	+	534	10	0	0	3.93%	10.988397450028092	0.007029302661868638	0.007029302661868638	0.0002573639808771639
ENSG00000078674_17935644_17955657_519	PCM1	8	+	1557	15	7	0	1.73%	5.652990595679192	0.18990531686869888	0.002943990679779778	0.005109142742463914
ENSG00000109814_39521512_39500143_494	UGDH	4	-	1482	452	15	10	14.37%	178.9640698865304	5.908996713676991e-12	2.6853055225160395e-12	1.988725379820943e-22
ENSG00000105849_19709016_19698316_338	TWISTNB	7	-	1014	18	0	0	1.78%	10.416220541387576	0.013007239811447113	0.013007239811447113	0.0008218654148058079
ENSG00000156521_70146535_70139924_549	TYSND1	10	-	1647	19	0	0	1.46%	6.769173073951732	0.004873903550586949	0.004873903550586949	0.00012846764509679302
ENSG00000100401_41281044_41246603_587	RANGAP1	22	-	1761	32	0	2	2.21%	10.662676639754688	0.0006446704936422262	0.0015919262655107664	6.465887001051626e-06
ENSG00000124228_49236348_49243707_252	DDX27	20	+	756	11	2	0	3.17%	8.537810399664684	0.022868511308519305	0.004193160480899131	0.0005251843016725186
ENSG00000110921_109574823_109596577_396	MVK	12	+	1188	56	1	4	5.30%	27.659683278252533	3.564215514287635e-05	0.00016114339175358615	4.353089130015296e-08
ENSG00000137106_37422751_37424897_35	GRHPR	9	+	105	11	0	2	17.14%	61.47223487758573	0.012798402692974279	0.05067994629101479	0.003100990187173963
ENSG00000113194_176479209_176506950_417	FAF2	5	+	1251	53	1	5	6.00%	24.859602288624707	6.612540685552464e-06	3.98312382139373e-05	2.1759385649180677e-09
ENSG00000110435_34916656_34995172_501	PDHX	11	+	1503	20	1	0	1.60%	7.808122738942117	0.009288069496478249	0.005306578019052857	0.0002590037054391842
ENSG00000143294_156787094_156800460_189	PRCC	1	+	567	10	1	1	3.17%	10.348861090502648	0.026602764905793736	0.026602764905793736	0.0031282459739174675
ENSG00000114796_183650441_183679286_572	KLHL24	3	+	1716	8	0	0	0.87%	2.735573071475525	0.019679754444124863	0.019679754444124863	0.0017849636115922674
ENSG00000103254_721275_722557_235	FAM173A	16	+	705	25	1	0	4.68%	20.807816447925536	0.002336818109389262	0.001484234770590415	2.0568290278350056e-05
ENSG00000127990_94656098_94587779_482	SGCE	7	-	1446	26	3	2	2.49%	10.550685352433613	0.005454749182134642	0.0026311212314026142	8.05834708867572e-05
ENSG00000131149_85655817_85672539_921	GSE1	16	+	2763	10	1	0	0.65%	2.123707650494029	0.023672501042627626	0.012798402692974279	0.0014375227604752335
ENSG00000114062_25375498_25339137_763	UBE3A	15	-	2289	8	13	0	0.66%	2.050783482154653	0.3331867010986109	0.019216965118390855	0.038471517829580006
ENSG00000258643_23307903_23325286_288	BCL2L2-PABPN1	14	+	864	15	0	0	3.12%	10.187160135963545	0.00327844299461777	0.00327844299461777	6.0372290137947024e-05
ENSG00000116337_109625404_109631152_761	AMPD2	1	+	2283	7	0	1	0.66%	1.7991515404382397	0.019216965118390855	0.04178282491678763	0.003600525600033436
ENSG00000110075_68587954_68613117_326	PPP6R3	11	+	978	11	0	0	1.84%	6.5997798181457075	0.012798402692974279	0.012798402692974279	0.0007972436888943567
ENSG00000137807_69423187_69447807_673	AC027237.1	15	+	2019	44	0	2	3.57%	12.787686304401191	5.891986681449955e-06	3.390638122567748e-05	1.6571995538272996e-09
ENSG00000213626_30234430_30257621_88	LBH	2	+	264	19	0	0	11.36%	42.23040929090342	0.0020082573300163734	0.0020082573300163734	2.3658688350241813e-05
ENSG00000186480_155298286_155308270_277	INSIG1	7	+	831	48	0	2	5.78%	33.89345408412997	0.00020377058636723502	0.0005044284266681147	7.009698902327871e-07
ENSG00000138459_112563452_112582736_408	SLC35A5	3	+	1224	21	1	0	2.70%	10.067311193187502	0.0012427339549726212	0.0010860768436549261	8.281785567176126e-06
ENSG00000150093_32935558_32933573_25	ITGB1	10	-	75	148	41	0	24.00%	1157.9133696941603	0.029237630782826433	0.013640585738808999	0.001872687038404406
ENSG00000127990_94618900_94585499_264	SGCE	7	-	792	12	0	2	3.03%	8.890612482295456	0.004193160480899131	0.022868511308519305	0.0005251843016725186
ENSG00000147155_48523772_48528457_230	EBP	X	+	690	82	1	0	7.83%	69.73332573070002	0.00010035536823964925	9.283325738969487e-05	6.743430955794319e-08
ENSG00000144824_111911674_111974563_730	PHLDB2	3	+	2190	14	7	1	0.68%	3.7511077322561652	0.17838283139301825	0.022004978195124387	0.018958940685546533
ENSG00000206503_29943288_29945513_262	HLA-A	6	+	786	57	1	3	3.82%	42.5527788274752	0.002430995894238001	0.0023766146074493525	3.3370697785285276e-05
ENSG00000206503_29942563_29943034_113	HLA-A	6	+	339	10	0	0	5.31%	17.309157045176995	0.011772051206276392	0.011772051206276392	0.000681272511389508
ENSG00000186591_129952525_129834937_142	UBE2H	7	-	426	10	0	0	5.63%	13.774188352852116	0.00414960799764038	0.00414960799764038	9.45964903272165e-05
ENSG00000135506_57694162_57715841_298	OS9	12	+	894	29	2	5	3.69%	19.034264307733224	0.005952040056363293	0.01799077827457437	0.0005546014674181435
ENSG00000100316_39318439_39316844_128	RPL3	22	-	384	259	21	0	17.19%	395.7711712821837	5.017800315001901e-05	1.8768467850450943e-05	7.340640507038093e-09

ENSG00000169241_155137696_155138281_82	SLC50A1	1	+	246	7	0	0	6.10%	16.697003930164637	0.019216965118390855	0.019216965118390855	0.0017073955890589052
ENSG00000168291_58431954_58428034_317	PDHB	3	-	951	29	0	0	3.79%	17.893409349225557	0.0010405263639310788	0.0010405263639310788	6.697970672456085e-06
ENSG00000197956_153535339_153535142_65	S100A6	1	-	195	315	50	17	20.00%	947.8760692662695	0.0014221215104371048	0.0010408454571312028	9.06438972251585e-06
ENSG00000105819_103297460_103308968_202	PMPCB	7	+	606	25	2	2	7.43%	24.207113194368816	0.0006716243423686093	0.0007978635973091095	3.40557628155321e-06
ENSG00000089327_35155551_35169615_178	FXYD5	19	+	534	101	4	3	12.92%	110.98281424528373	9.80386385557854e-05	3.0510438660264174e-05	2.276384256667706e-08
ENSG00000143476_212044668_212102940_668	DTL	1	+	2004	24	0	0	1.65%	7.027310465047906	0.001484234770590415	0.001484234770590415	1.3251387882263029e-05
ENSG00000143493_212035437_211941824_942	INTS7	1	-	2826	16	1	0	0.96%	3.322182158989385	0.005561255870711926	0.0034529206320349946	0.00010554338560930125
ENSG00000143493_212035437_211941824_948	INTS7	1	-	2844	16	1	0	0.95%	3.301155689628693	0.005561255870711926	0.0034529206320349946	0.00010554338560930125
ENSG00000143493_212035437_211941824_962	INTS7	1	-	2886	16	1	0	0.94%	3.2531139228357597	0.005561255870711926	0.0034529206320349946	0.00010554338560930125
ENSG00000123684_211829224_211749899_346	LPGAT1	1	-	1038	15	0	0	1.73%	8.479485893518788	0.011772051206276392	0.011772051206276392	0.000681272511389508
ENSG00000123684_211829224_211749899_346	LPGAT1	1	-	1038	15	0	0	1.73%	8.479485893518788	0.011772051206276392	0.011772051206276392	0.000681272511389508
ENSG00000117650_211675479_211667062_384	NEK2	1	-	1152	23	2	2	2.34%	11.715234156358076	0.010026873424650893	0.010026873424650893	0.0005035630638330185
ENSG00000167985_61430147_61438169_141	SDHAF2	11	+	423	12	0	0	3.55%	16.64625315834043	0.02108409854857798	0.02108409854857798	0.002029891263157455
ENSG00000111652_6724657_6731039_275	COPS7A	12	+	825	16	0	0	3.27%	11.379983977338185	0.0032294771334464936	0.0032294771334464936	5.8663648728941325e-05
ENSG00000178999_8207605_8204871_287	AURKB	17	-	861	17	2	3	2.79%	11.585676196440769	0.016170007243128042	0.015625008558487224	0.0011972788582631131
ENSG00000141480_4710722_4721039_406	ARRB2	17	+	1218	15	0	6	1.72%	7.226359899402711	0.0078820576498062	0.12508587557927525	0.0058624916154086425
ENSG00000113558_134174494_134173744_50	SKP1	5	-	150	18	8	0	10.00%	70.41365085978002	0.10041256134757276	0.02108409854857798	0.009609042473219148
ENSG00000083857_186709110_186588592_4351	FAT1	4	-	13053	109	7	11	0.92%	4.899951443931166	3.4976471422289744e-07	1.2196311103537715e-06	3.932090433934067e-12
ENSG00000122786_134891621_134968345_557	CALD1	7	+	1671	35	0	2	2.51%	12.290433772652605	0.0004156054716904614	0.00115701054860631	3.127308461959799e-06
ENSG00000168411_74661374_74623928_749	RFWD3	16	-	2247	33	5	2	1.60%	8.617603020222298	0.009700139251047473	0.0027029703238042042	0.00014726552354281044
ENSG00000197766_860679_863238_214	CFD	19	+	642	30	0	0	4.67%	27.419645973434587	0.002033393162560146	0.002033393162560146	2.4228963598721857e-05
ENSG00000129055_134482904_134478590_74	ANAPC13	3	-	222	14	0	0	10.81%	37.004170872256765	0.005160369442893307	0.005160369442893307	0.00014319601891785326
ENSG00000148180_121317107_121332603_473	GSN	9	+	1419	17	0	0	1.06%	7.029786613908035	0.02108409854857798	0.02108409854857798	0.002029891263157455
ENSG00000164111_121696589_121668468_320	ANXA5	4	-	960	421	8	7	23.44%	257.3276650344391	2.4167096206658833e-13	6.294767585948028e-14	2.043297450833816e-25
ENSG00000118961_20801463_20686903_283	LDAH	2	-	849	8	0	0	1.77%	5.529144158600708	0.019679754444124863	0.019679754444124863	0.0017849636115922674
ENSG00000174547_66438754_66436837_181	MRPL11	11	-	543	22	4	0	5.52%	23.77379249409393	0.02437920970750698	0.0023354528714849923	0.00034461905735642576
ENSG00000185686_22556832_22548067_509	PRAME	22	-	1527	45	1	1	3.54%	17.292153943953835	0.00011105827337505001	0.00011105827337505001	8.855184710094631e-08
ENSG00000175387_47896756_47841827_467	SMAD2	18	-	1401	37	4	3	3.21%	15.496699273208783	0.002020802647119262	0.0015846582505280478	1.899274279269736e-05
ENSG00000167114_128343133_128360491_643	SLC27A4	9	+	1929	12	0	4	0.93%	3.6502670222799387	0.013007239811447113	0.10069662721979156	0.0066169769657669466
ENSG00000206560_15724423_15670270_836	ANKRD28	3	-	2508	13	2	0	0.84%	3.0415253228905508	0.04363948305378087	0.0078820576498062	0.0017710903275458955
ENSG00000155636_178112547_178126176_476	RBM45	2	+	1428	6	0	0	1.05%	2.465463965678572	0.016947426762344633	0.016947426762344633	0.0013498980316300959
ENSG00000135404_55728323_55725561_232	CD63	12	-	696	246	22	6	26.29%	207.3965291128578	2.6552528959412224e-10	2.5295184939073248e-11	8.125620299060758e-20
ENSG00000107164_130622759_130631184_159	FUBP3	9	+	477	11	0	0	3.14%	13.531624029657236	0.020613416668581838	0.020613416668581838	0.001946208561389314
ENSG00000134684_32781140_32775981_179	YARS	1	-	537	9	2	0	3.91%	9.834308779298885	0.053594824633710306	0.005706018193000826	0.0017059082182193615
ENSG00000164091_52261463_52257490_199	WDR82	3	-	597	16	0	0	5.03%	15.726108511396989	0.0021227925778456486	0.0021227925778456486	2.6309315010939275e-05
ENSG00000261582_35279705_35272097_62	AL121753.1	20	-	186	48	2	10	16.13%	151.42720615006456	0.0022409900782254734	0.006112543793728684	7.798427274601149e-05
ENSG00000156113_77637360_76877869_1034	KCNMA1	10	-	3102	14	4	2	0.68%	2.6482675479177957	0.067234255889369	0.03299602752967379	0.009178574298006526
ENSG00000188690_125816499_125788868_265	UROS	10	-	795	12	1	0	2.64%	8.857063001230191	0.015516259750072458	0.0078820576498062	0.0006142571802709984
ENSG00000138685_122827175_122892396_155	FGF2	4	+	465	20	1	0	4.52%	25.237867691677426	0.00847367416878415	0.00847367416878415	0.0003665024264111182
ENSG00000110911_51010704_50981726_560	SLC11A2	12	-	1680	22	0	0	1.61%	7.684029359698216	0.0033030736763512156	0.0033030736763512156	6.124050385493983e-05
ENSG00000197912_89508547_89541011_413	SPG7	16	+	1239	12	0	0	1.21%	5.6831033785133185	0.019679754444124863	0.019679754444124863	0.0017849636115922674
ENSG00000063244_55655105_55674068_471	U2AF2	19	+	1413	131	11	9	8.92%	54.40073285345118	2.301339199512164e-07	2.5611941259085713e-07	5.546585704647803e-13
ENSG00000169139_48008455_48060828_145	UBE2V2	8	+	435	21	14	0	6.90%	28.327330805658626	0.10421915560328554	0.0022409900782254734	0.001871144303758135
ENSG00000157193_53271061_53247018_557	LRP8	1	-	1671	15	9	0	1.08%	5.26732875970826	0.2405700776118731	0.012798402692974279	0.018919609472776648
ENSG00000086061_33036599_33037136_70	DNAJA1	9	+	210	46	2	0	12.86%	128.53285474404288	0.004490071680569804	0.003710480643933249	9.175448395681885e-05
ENSG00000078747_34393812_34507794_903	ITCH	20	+	2709	16	6	0	0.66%	3.465665109377631	0.10041256134757276	0.013007239811447113	0.006595870280092993
ENSG00000116918_231529344_231542637_95	TSNAX	1	+	285	15	0	0	5.26%	30.8831802016579	0.02108409854857798	0.02108409854857798	0.002029891263157455
ENSG00000140497_74851356_74845083_173	SCAMP2	15	-	519	42	2	2	3.47%	47.48512100370521	0.03031622373531018	0.03031622373531018	0.003985451574990196
ENSG00000124733_43013378_43012470_172	MEA1	6	-	516	46	2	2	9.88%	52.30988274466862	0.0005393137985452151	0.0005393137985452151	1.8883487944536844e-06
ENSG00000149925_30067255_30070212_364	ALDOA	16	+	1092	714	70	30	29.67%	383.6641232744424	2.1584910851290338e-16	3.7216843132467856e-18	1.2870459985292045e-32
ENSG00000100528_54441327_54427814_121	CNIH1	14	-	363	122	5	2	21.49%	197.20994960728103	3.755331300162224e-06	3.852644714214543e-06	1.22625768870526e-10
ENSG00000080603_30704079_30739733_3207	SRCAP	16	+	9621	7	0	5	0.19%	0.4269268232845339	0.009815328628645334	0.3133740834924025	0.023087605743781926
ENSG00000087245_55479492_55505442_656	MMP2	16	+	1968	15	5	1	1.52%	4.472411767008385	0.05856790736200192	0.0040534433873287605	0.0014407806237584245
ENSG00000109332_102809680_102797415_110	UBE2D3	4	-	330	19	0	1	5.45%	33.78432743272273	0.013428347753762199	0.013428347753762199	0.0008725593497644525
ENSG00000101246_63707058_63700443_190	ARFRP1	20	-	570	16	1	0	2.63%	16.471029440884216	0.02784981298332479	0.019679754444124863	0.002477909089056847
ENSG00000215301_41343231_41347719_476	DDX3X	X	+	1428	83	1	0	7.56%	34.10558485855358	6.958679763556736e-08	4.081835640353621e-08	2.8150375102779016e-14
ENSG00000168036_41224069_41239342_781	CTNNB1	3	+	2343	117	8	3	5.76%	29.301455223339957	6.403151454483205e-08	1.1032790393731706e-08	7.305397733044446e-15
ENSG00000115525_85848138_85840144_290	ST3GAL5	2	-	870	11	0	3	1.72%	7.41906283005345	0.019216965118390855	0.13115678761539135	0.012020477827111095
ENSG00000108433_46923193_46940613_213	GOSR2	17	+	639	12	0	0	2.82%	11.019350682281692	0.011772051206276392	0.011772051206276392	0.000681272511389508
ENSG00000197122_37384154_37403379_536	SRC	20	+	1608	46	6	4	3.92%	16.786007149408587	0.0003884290151169211	0.00016054896953742018	4.3172723051176966e-07
ENSG00000241370_30345172_30346773_144	RPP21	6	+	432	26	1	1	3.47%	35.31548847134029	0.03980790073005672	0.02108409854857798	0.0037226566061864964
ENSG00000170889_54201185_54206565_139	RPS9	19	+	417	135	11	0	8.63%	189.9648854130756	0.004183873134198697	0.001100170263453246	2.7943281629777465e-05
ENSG00000150093_32935558_32933573_25	ITGB1	10	-	75	148	41	0	24.00%	1157.9133696941603	0.029237630782826433	0.013640585738808999	0.001872687038404406
ENSG00000105404_41958772_41956846_75	RABAC1	19	-	225	36	1	1	10.67%	93.88486781304002	0.008413704741378425	0.008413704741378425	0.000361616358215097
ENSG00000144455_4467245_4362144_242	SUMF1	3	-	726	12	0	1	2.48%	9.698849980685953	0.011772051206276392	0.026832695913080365	0.0015103817605807263
ENSG00000135404_55728323_55725561_232	CD63	12	-	696	246	22	6	26.29%	207.3965291128578	2.6552528959412224e-10	2.5295184939073248e-11	8.125620299060758e-20
ENSG00000005020_26864429_26670100_359	SKAP2	7	-	1077	20	0	0	2.79%	10.896572401699167	0.0022276761777358703	0.0022276761777358703	2.8853233252577778e-05

ENSG00000104825_38900033_38907707_338	NFKBIB	19	+	1014	7	0	0	1.48%	4.050752432761835	0.016947426762344633	0.016947426762344633	0.0013498980316300959
ENSG00000092199_21233974_21211223_232	HNRNPC	14	-	696	32	2	9	4.31%	26.978410291103454	0.0019307453316667101	0.01515278463046571	0.000175341018721809
ENSG00000085978_233251828_233294350_444	ATG16L1	2	+	1332	29	0	0	2.70%	12.775249467802931	0.0010073743004618205	0.0010073743004618205	6.293580389004731e-06
ENSG00000128944_40383253_40393597_238	KNSTRN	15	+	714	15	7	0	2.94%	12.32731982839286	0.08502160687168953	0.00847367416878415	0.003922604800908926
ENSG00000124067_67957957_67944840_942	SLC12A4	16	-	2826	9	0	1	0.53%	1.868727464431529	0.019216965118390855	0.04178282491678763	0.003600525600033436
ENSG00000113851_3179687_3150865_442	CRBN	3	-	1326	10	0	3	1.36%	4.425191733269232	0.011570465654371866	0.08266357711566169	0.004841719827405853
ENSG00000170860_14178861_14198116_102	LSM3	3	+	306	45	9	4	4.90%	86.29123879875002	0.11411551976453232	0.08620755130569058	0.03462010578048691
ENSG00000166347_74291875_74256845_98	CYB5A	18	-	294	45	4	0	9.18%	89.81333017829084	0.008830848114324176	0.003816220824102754	0.00018248060063567366
ENSG00000166598_103938307_103941559_139	HSP90B1	12	+	417	116	1	1	17.99%	163.2290867253094	6.446554945146614e-06	9.341610901697043e-06	4.956473257357282e-10
ENSG00000135956_96265381_96253808_238	TMEM127	2	-	714	11	0	0	2.10%	9.04003454082143	0.020613416668581838	0.020613416668581838	0.001946208561389314
ENSG00000160710_154601756_154601037_239	ADAR	1	-	717	17	2	0	3.77%	13.91250656225314	0.016881135945258162	0.0033030736763512156	0.0003111891849301449
ENSG00000131467_42833632_42841578_254	PSME3	17	+	762	59	3	1	7.09%	45.433129929210644	8.641221921650504e-05	0.00010392058179106278	6.509470880944243e-08
ENSG00000196365_5719790_5692032_829	LONP1	19	-	2487	17	2	1	1.33%	4.010963894304584	0.007962492813783481	0.002550198508962866	0.00011439042603247118
ENSG00000151491_15669717_15621317_718	EPS8	12	-	2154	15	0	0	1.11%	4.086214650637188	0.005405141725979036	0.005405141725979036	0.00015637213408961292
ENSG00000257923_101816052_102249042_1509	CUX1	7	+	4527	6	2	2	0.33%	0.7777076525268392	0.1029516053660342	0.1029516053660342	0.036819135060151394
ENSG00000104529_143589937_143580019_583	EEF1D	8	-	1749	173	0	5	4.63%	58.04060224290996	2.5213715056597438e-06	1.2909652315362702e-05	2.792956634712577e-10
ENSG00000077549_19485438_19342762_277	CAPZB	1	-	831	348	8	10	12.27%	245.7275421099423	1.0556109215175274e-06	1.582266963390918e-07	1.5944352832856359e-12
ENSG00000077549_19419717_19339530_260	CAPZB	1	-	780	346	8	11	13.85%	260.28977775089623	5.528600246353339e-07	7.948828161521429e-08	4.293404419067047e-13
ENSG00000125877_3209552_3223462_194	ITPA	20	+	582	32	1	5	5.67%	32.26284117286599	0.001988634903193983	0.010115653169763636	0.0001173683986419945
ENSG00000160752_155310161_155318336_144	FDPS	1	+	432	73	0	4	11.81%	99.1550253233785	0.00013891616214042798	0.00039475350870333604	3.8307694579386786e-07
ENSG00000154743_12496532_12532721_370	TSEN2	3	+	1110	9	0	0	1.35%	4.757679112147298	0.020613416668581838	0.020613416668581838	0.001946208561389314
ENSG00000204130_68341286_68341203_27	RUFY2	10	-	81	15	0	3	25.93%	108.6630414502778	0.00797940233269714	0.04444193652437137	0.0018243500037866422
ENSG00000150093_32935558_32926080_128	ITGB1	10	-	384	210	46	48	17.19%	320.89554428285163	6.030376247319467e-05	0.016258235096238915	1.1656391756474655e-05
ENSG00000129197_5421311_5426246_75	RPAIN	17	+	225	22	2	0	9.33%	57.37408588574668	0.028426896806120847	0.00847367416878415	0.0012022752110812016
ENSG00000254505_24210743_24209877_94	CHMP4A	14	-	282	17	0	1	9.57%	35.37328796147341	0.0033030736763512156	0.006112543793728684	0.00011113261345270447
ENSG00000115525_85848138_85847466_124	ST3GAL5	2	-	372	11	0	2	4.03%	17.35103403802823	0.019216965118390855	0.08350359792828288	0.0073235312725521175
ENSG00000100162_41947076_41939056_180	CENPM	22	-	540	30	1	1	6.67%	32.59891243508334	0.001080010432913501	0.001080010432913501	7.195244741682991e-06
ENSG00000049245_7777172_7779645_72	VAMP3	1	+	216	64	0	1	23.61%	173.86086632044447	0.0001297455242311012	0.00018165475342050424	1.6630239591960388e-07
ENSG00000111669_6867609_6870383_235	TPI1	12	+	705	289	10	3	11.91%	240.5383581380192	5.455330855611919e-06	1.6950419017174195e-06	8.025709236916846e-11
ENSG00000111674_6917077_6922800_341	ENO2	12	+	1023	22	0	1	2.93%	12.618933845838713	0.0023218226288164415	0.0030685733490663438	4.084557731917468e-05
ENSG00000107959_3166244_3138031_837	PITRM1	10	-	2511	24	1	1	1.31%	5.6084150425949835	0.00187740676320226	0.00151350169064872	1.6931802814287563e-05
ENSG00000143924_42169612_42330207_923	EML4	2	+	2769	33	0	1	1.52%	6.993049471447997	0.0004441315507112266	0.0005952140785536965	1.7246665473088204e-06
ENSG00000147586_80003120_79918980_96	MRPS28	8	-	288	31	0	3	11.46%	63.16039284297397	0.0014481341328822738	0.0067530256680052286	5.8388157991235136e-05
ENSG00000104549_125009289_125020875_160	SQLE	8	+	480	42	3	0	8.12%	51.343287085256264	0.0027761216894033104	0.0007052562366071395	1.2306420929548679e-05
ENSG00000151914_56642496_56624416_1085	DST	6	-	3255	7	1	0	0.46%	1.2618933845838713	0.0477903522728147	0.016947426762344633	0.0036975118224100542
ENSG00000076554_80171443_80038116_207	TPD52	8	-	621	45	5	0	6.76%	42.52032056750001	0.002462368798132946	0.00046705916373637345	7.5029210566117364e-06
ENSG00000149187_47488963_47472230_466	CELF1	11	-	1398	19	1	0	1.50%	7.974841239483907	0.015516259750072458	0.0078820576498062	0.0006142571802709984
ENSG00000122786_134867785_134968345_521	CALD1	7	+	1563	30	0	2	2.30%	11.262580111928985	0.0009430837296224684	0.0028873670906769807	1.667964675043473e-05
ENSG00000198836_193593378_193692127_997	OPA1	3	+	2991	32	1	1	1.81%	6.2778246615205635	0.0001048543557987398	0.0001048543557987398	7.919728780723367e-08
ENSG00000140931_66604806_66612637_182	CMTM3	16	+	546	8	0	0	2.75%	8.597515367494507	0.019679754444124863	0.019679754444124863	0.0017849636115922674
ENSG00000069329_46677332_46672458_79	VPS35	16	-	237	12	0	3	6.33%	29.710401206658236	0.020613416668581838	0.08529346643572072	0.00792527975686362
ENSG00000110697_67504111_67492033_1221	PITPNM1	11	-	3663	16	0	2	0.57%	2.563059454355447	0.0078820576498062	0.019654369976907376	0.0007767617569840121
ENSG00000041357_78544194_78548944_190	PSMA4	15	+	570	221	0	8	9.47%	227.50609415221322	8.735376412917216e-05	0.0014627342080213868	9.78739406124214e-07
ENSG00000270106_231528811_231616693_138	TSNAX-DISC1	1	+	414	16	0	0	4.35%	22.677504302666673	0.013428347753762199	0.013428347753762199	0.0008725593497644525
ENSG00000215301_41334253_41347719_662	DDX3X	X	+	1986	96	12	3	7.25%	28.364008402731123	1.935160314504737e-07	2.169376474014155e-09	4.972312673151123e-15
ENSG00000115355_55332660_55291711_850	CCDC88A	2	-	2550	6	1	0	0.59%	1.3806598207800003	0.0477903522728147	0.016947426762344633	0.0036975118224100542
ENSG00000136631_150068645_150144796_534	VPS45	1	+	1602	15	0	3	1.50%	5.494198725014046	0.005306578019052857	0.03497523187724695	0.0010067653727672797
ENSG00000151136_107318941_107649307_1007	BTBD11	12	+	3021	26	5	1	0.89%	5.050079781403179	0.024096744146595385	0.005059698366439319	0.0006511241884944809
ENSG00000104325_90006269_90051897_315	DECR1	8	+	945	41	5	0	5.71%	25.458198282636513	0.0007989627392442813	8.226800648516007e-05	4.884499446636161e-07
ENSG00000107959_3172772_3138031_1037	PITRM1	10	-	3111	28	4	1	1.45%	5.281212429213116	0.0028612383254294944	0.0002554476795345014	5.142102490072575e-06
ENSG00000204852_110634747_110647892_324	TCTN1	12	+	972	16	0	0	2.16%	9.65893701780247	0.0078820576498062	0.0078820576498062	0.00031963642907451164
ENSG00000138363_215334930_215347638_195	ATIC	2	+	585	21	0	0	5.64%	21.063912650361544	0.001484234770590415	0.001484234770590415	1.3251387882263029e-05
ENSG00000177105_3828138_3827563_191	RHOG	11	-	573	27	0	1	5.24%	27.649339342845558	0.0023628488670607213	0.0035004709947243163	4.7187726630981656e-05
ENSG00000120451_130915495_130905829_655	SNX19	11	-	1965	6	0	5	0.76%	1.7916959506305348	0.016947426762344633	0.165899918248885	0.014398895220848395
ENSG00000099204_114491803_114436260_455	ABLIM1	10	-	1365	8	1	2	1.32%	3.439006146997803	0.026602764905793736	0.08266357711566169	0.009436337707064906
ENSG00000181019_69718478_69718003_119	NQO1	16	-	357	348	30	7	27.73%	571.9876400374287	9.52250996812838e-05	1.0569650075914238e-06	1.0381543778303481e-09
ENSG00000188603_28491759_28477516_390	CLN3	16	-	1170	23	2	0	2.56%	11.534999784721798	0.00740512872812498	0.0022543491824521134	9.516194975080656e-05
ENSG00000100603_77735012_77718088_300	SNW1	14	-	900	20	0	0	3.00%	13.039564974033336	0.0033278027414746945	0.0033278027414746945	6.211806171561056e-05
ENSG00000197798_126235002_126262133_275	FAM118B	11	+	825	17	0	0	1.82%	12.091232975921821	0.02108409854857798	0.02108409854857798	0.002029891263157455
ENSG00000140365_75336090_75338334_91	COMMD4	15	+	273	28	0	3	5.49%	60.18260757246155	0.019679754444124863	0.08410206952409	0.007521960853378093
ENSG00000111145_96247009_96267180_315	ELK3	12	+	945	9	1	1	1.59%	5.58838498887143	0.03537493353818881	0.03537493353818881	0.005300298232677315
ENSG00000215301_41344365_41347719_331	DDX3X	X	+	993	65	0	0	8.46%	38.40959471203173	1.0077168445509615e-06	1.0077168445509615e-06	9.076267751723256e-12
ENSG00000106615_151471431_151467119_70	RHEB	7	-	210	16	0	0	8.57%	44.70707991097144	0.013007239811447113	0.013007239811447113	0.0008218654148058079
ENSG00000003056_8946404_8941818_191	M6PR	12	-	573	48	7	15	8.90%	49.15438105394765	0.001055465719730038	0.01175145716701725	7.985253841210302e-05
ENSG00000113712_149525134_149496853_276	CSNK1A1	5	-	828	30	0	0	4.71%	21.260160283750004	0.0006289630342484525	0.0006289630342484525	2.5404679232419015e-06
ENSG00000139926_51702536_51728029_516	FRMD6	14	+	1548	10	3	0	1.16%	3.7905712133817837	0.08424673392120513	0.011772051206276392	0.005012526315124349
ENSG00000141349_44075330_44076043_161	G6PC3	17	+	483	18	0	0	4.97%	21.86759343471429	0.004873903550586949	0.004873903550586949	0.00012846764509679302

ENSG00000125107_58599337_58528091_2150	CNOT1	16	-	6450	38	3	5	0.88%	3.457000946604187	0.00041901277480991617	0.0006897760808808691	1.885314417365881e-06
ENSG00000165688_136416305_136423264_343	PMPCA	9	+	1029	27	6	3	3.21%	15.396570887707	0.012914280349163228	0.008798119198845685	0.0005676380989905498
ENSG00000165688_136410669_136416294_146	PMPCA	9	+	438	13	0	0	4.11%	17.415857328332194	0.013007239811447113	0.013007239811447113	0.0008218654148058079
ENSG00000152700_134612730_134606950_130	SAR1B	5	-	390	54	0	0	13.08%	81.2465202228231	0.00013106574077567133	0.00013106574077567133	1.221428801656259e-07
ENSG00000080822_98521358_98516834_230	CLDND1	3	-	690	28	0	0	3.04%	23.811379517800006	0.008574016250729465	0.008574016250729465	0.0003747459614100891
ENSG00000048162_176388539_176388429_36	NOP16	5	-	108	13	5	0	19.44%	70.63097694268058	0.12533303590662392	0.007029302661868638	0.00540528361133355
ENSG00000161960_7577836_7578142_40	EIF4A1	17	+	120	137	4	0	12.50%	669.9076505409627	0.046295797875114966	0.021557223391537678	0.004403396695964728
ENSG00000105852_95363975_95359973_160	PON3	7	-	480	8	0	0	3.12%	9.779673730525003	0.019679754444124863	0.019679754444124863	0.0017849636115922674
ENSG00000106049_27662788_27526214_336	HIBADH	7	-	1008	26	0	0	3.27%	15.135209344860122	0.0015208721599504675	0.0015208721599504675	1.3886126472521594e-05
ENSG00000106052_27748525_27828829_747	TAX1BP1	7	+	2241	40	8	2	2.14%	10.473546163882197	0.002040495191437342	0.0004122651324805293	5.524411911762451e-06
ENSG00000186298_110742707_110722406_270	PPP1CC	12	-	810	71	4	0	8.52%	51.43383961979816	8.691612568466429e-05	1.0465432500631416e-05	7.427570755296397e-09
ENSG00000175061_16439055_16439685_39	SNHG29	17	+	117	12	3	0	12.82%	60.18260757246155	0.1029516053660342	0.02108409854857798	0.009872287793463467
ENSG00000112984_138179747_138181770_116	KIF20A	5	+	348	6	1	0	4.31%	10.116903859163795	0.0477903522728147	0.016947426762344633	0.0036975118224100542
ENSG00000124228_49219449_49243834_765	DDX27	20	+	2295	24	3	4	2.09%	6.136265870133335	0.0009747456371656623	0.0019340424811117715	1.1523237442356582e-05
ENSG00000131778_147266075_147295509_603	CHD1L	1	+	1809	18	0	1	1.33%	5.838611182402986	0.004873903550586949	0.015625008558487224	0.000403121801006346
ENSG00000048392_102232190_102208133_297	RRM2B	8	-	891	18	0	0	2.02%	11.854149976393941	0.013217226988443315	0.013217226988443315	0.0008469697265466234
ENSG00000117448_45561834_45569956_312	AKR1A1	1	+	936	32	0	0	5.13%	20.06086919082052	0.00016405548854949773	0.00016405548854949773	1.8881802569129578e-07
ENSG00000117318_23559426_23558960_119	ID3	1	-	357	186	18	6	19.33%	305.71753174414295	0.0006437726054395274	4.922474283456086e-05	2.455756602018691e-07
ENSG00000130414_240025301_240018494_201	NDUFA10	2	-	603	19	0	0	3.48%	18.48893541094279	0.00877616296920843	0.00877616296920843	0.00039161135667564577
ENSG00000104823_38831772_38817349_191	ECH1	19	-	573	102	4	0	13.61%	104.45305973963876	5.347809470446742e-06	3.7484984614400756e-06	1.689663585104152e-10
ENSG00000136270_45109171_45100325_609	TBRG4	7	-	1827	48	8	0	3.45%	15.416234452059117	0.0011513765331522398	2.1814045225814675e-05	2.260979456382319e-07
ENSG00000126602_3717508_3658129_704	TRAP1	16	-	2112	74	5	3	4.83%	20.55954136530824	1.9670963539757345e-06	4.1126793201009607e-07	7.434511418011761e-12
ENSG00000150753_10254154_10264783_503	CCT5	5	+	1509	401	22	14	13.72%	155.93038433163125	1.8293902923444076e-11	3.569095696106475e-12	8.15420653478722e-22
ENSG00000265681_49492316_49491779_37	RPL17	18	-	111	8	3	0	13.51%	42.29048099686487	0.07864960352514258	0.019679754444124863	0.007006522949969347
ENSG00000170653_53601000_53543240_117	ATF7	12	-	351	14	5	1	4.27%	23.404347389290603	0.2614777938727097	0.04223451612662591	0.04729316614615979
ENSG00000132471_75855297_75846734_261	WBP2	17	-	783	34	0	1	4.98%	25.479609719375485	0.0006629342244537616	0.0009306875619951529	3.907239258273379e-06
ENSG00000183161_22625324_22624686_212	FANCF	11	-	636	15	0	0	3.30%	13.839160939422174	0.00847367416878415	0.00847367416878415	0.0003665024264111182
ENSG00000163507_108589375_108563122_545	CIP2A	3	-	1635	12	0	2	1.10%	4.306645312524772	0.013007239811447113	0.042435593247778	0.002615673534585255
ENSG00000011454_122984551_123103213_997	RABGAP1	9	+	2991	16	2	0	0.90%	3.1389123307602818	0.016881135945258162	0.0033030736763512156	0.0003111891849301449
ENSG00000161960_7576539_7576700_53	EIF4A1	17	+	159	29	1	1	13.21%	107.02284459819813	0.00847367416878415	0.00797940233269714	0.0003463032305947362
ENSG00000029534_41715660_41661466_1349	ANK1	8	-	4047	9	0	0	0.37%	1.3049231071123057	0.020613416668581838	0.020613416668581838	0.001946208561389314
ENSG00000196715_65954076_65954300_74	VKORC1L1	7	+	222	20	0	0	12.16%	52.863101246081094	0.0034782380676658805	0.0034782380676658805	6.758309314600117e-05
ENSG00000126858_32192201_32221064_445	RHOT1	17	+	1335	9	0	1	1.12%	3.9558230820101135	0.020613416668581838	0.03537493353818881	0.003252526348827943
ENSG00000215301_41343817_41347719_409	DDX3X	X	+	1227	72	0	0	7.82%	34.43210310991688	1.8060166406889672e-07	1.8060166406889672e-07	3.100108887739879e-13
ENSG00000154845_9559424_9547789_276	PPP4R1	18	-	828	6	4	0	1.81%	4.252032056750001	0.165899918248885	0.016947426762344633	0.014398895220848395
ENSG00000101384_10672964_10639498_1177	JAG1	20	-	3531	145	4	9	4.84%	24.096052522109183	1.277267868922201e-10	8.856399430158281e-10	1.3132138609033661e-18
ENSG00000132612_69311512_69324309_437	VPS4A	16	+	1311	23	2	2	2.29%	10.294393400552634	0.006441753363760071	0.008605408492104586	0.00028764299811096465
ENSG00000101310_18510836_18560740_767	SEC23B	20	+	2301	42	2	3	2.74%	10.710464059505869	9.993194084537427e-05	0.0001424141019315285	1.0194420312747164e-07
ENSG00000136536_159729023_159767342_704	7-Mar	2	+	2112	17	2	0	1.42%	4.723137881219461	0.010645877633714064	0.0021880057261679567	0.0001345568947401665
ENSG00000115641_105386516_105361283_279	FHL2	2	-	837	24	2	0	3.23%	16.82524512778495	0.01052924788556975	0.003684287313187465	0.00021087137401632993
ENSG00000173744_227491587_227554495_491	AGFG1	2	+	1473	18	0	0	2.04%	7.170432877778006	0.0021227925778456486	0.0021227925778456486	2.6309315010939275e-05
ENSG00000131149_85655817_85672539_921	GSE1	16	+	2763	10	1	0	0.65%	2.123707650494029	0.023672501042627626	0.012798402692974279	0.0014375227604752335
ENSG00000131143_85801206_85806874_169	COX4I1	16	+	507	371	22	4	29.59%	429.3797578727545	3.2747393497168896e-09	1.0211939817064629e-09	3.6715764850989334e-17
ENSG00000131148_85799295_85779708_210	EMC8	16	-	630	11	3	5	3.33%	10.245372479597622	0.061356297364084836	0.07786981947941429	0.01807742885044581
ENSG00000108651_31901627_31880547_330	UTP6	17	-	990	8	1	0	1.52%	4.741659990557577	0.04223451612662591	0.019679754444124863	0.003715148010809822
ENSG00000000419_50958523_50940883_188	DPM1	20	-	564	35	6	4	9.04%	36.41367878386969	0.004063707016760359	0.0010756817844974774	2.658033686989162e-05
ENSG00000136261_16689838_16706088_225	BZW2	7	+	675	10	0	0	3.56%	8.693043316022225	0.00414960799764038	0.00414960799764038	9.45964903272165e-05
ENSG00000123374_55967009_55971625_238	CDK2	12	+	714	19	2	0	2.94%	15.61460511596429	0.03744183116399598	0.008175610787902942	0.001552076439258323
ENSG00000147642_109579914_109574906_457	SYBU	8	-	1371	9	0	0	1.09%	3.8519502658522984	0.020613416668581838	0.020613416668581838	0.001946208561389314
ENSG00000198074_134527948_134541089_304	AKR1B10	7	+	912	511	35	7	16.12%	328.77718923014976	1.9442608346364464e-09	4.240331626008552e-10	9.267605970856452e-18
ENSG00000169504_24745566_24840937_249	CLIC4	1	+	747	10	2	1	2.41%	7.855159622911648	0.08026476757196149	0.026832695913080365	0.009220061968845724
ENSG00000196363_134139878_134157993_334	WDR5	9	+	1002	23	0	0	3.89%	13.469011724675152	0.0006069633386880125	0.0006069633386880125	2.3718849540264015e-06
ENSG00000165533_88824720_88841526_182	TTC8	14	+	546	11	0	1	3.85%	11.821583630304948	0.0078820576498062	0.015516259750072458	0.0006142571802709984
ENSG00000172572_20572127_20680271_819	PDE3A	12	+	2457	32	0	0	1.47%	7.6422358822173395	0.0008814539243352768	0.0008814539243352768	4.867568044090692e-06
ENSG00000133216_22781429_22891149_459	EPHB2	1	+	1377	13	0	0	1.74%	5.539684466092594	0.004826937407846361	0.004826937407846361	0.00012612451166366307
ENSG00000026025_17229462_17237271_453	VIM	10	+	1359	847	43	41	26.49%	365.712302417425	7.46657357325229e-20	8.046924215316331e-20	9.800747085166055e-38
ENSG00000168610_42345636_42315745_672	STAT3	17	-	2016	31	1	0	2.08%	9.022913263281996	0.0005938231711225875	0.00044280702707823286	1.7158342196872716e-06
ENSG00000156795_123416850_123441415_205	WDYHV1	8	+	615	7	2	0	2.44%	6.678801572065855	0.1191346346731873	0.019216965118390855	0.010787784428637617
ENSG00000158941_22605774_22619682_923	CCAR2	8	+	2769	21	2	0	0.98%	4.450122390921453	0.00600341887269475	0.0036062891729786097	0.00011840703590302771
ENSG00000156787_123072770_123151939_1066	TBC1D31	8	+	3198	13	0	0	0.66%	2.3852862757378053	0.0078820576498062	0.0078820576498062	0.00031963642907451164
ENSG00000125826_419618_430430_296	RBCK1	20	+	888	13	7	4	1.69%	8.590253952488178	0.24754287813003473	0.020613416668581838	0.0270650614500049
ENSG00000129250_5007486_5024151_625	KIF1C	17	+	1875	8	1	0	0.96%	2.5035964750144006	0.0477903522728147	0.009815328628645334	0.0023388674905236288
ENSG00000168653_39028725_39034496_106	NDUFS5	1	+	318	175	10	13	20.75%	322.91375525318404	3.095223887384876e-05	0.00017897042368448322	4.250899264974502e-08
ENSG00000196655_119018796_119023399_176	TRAPPC4	11	+	528	30	0	1	7.39%	33.339796808607964	0.0005399915898224664	0.0010442431046425516	3.604565821948577e-06
ENSG00000114416_180947925_180976292_535	FXR1	3	+	1605	13	0	0	1.50%	4.752738635395328	0.004687384229717438	0.004687384229717438	0.00011928172701435494
ENSG00000089280_31180215_31191438_526	FUS	16	+	1578	196	8	16	9.13%	72.88273958870344	6.825921249335542e-09	3.064580693084873e-08	2.187013092129838e-15
ENSG00000174485_65756450_65661851_1906	DENND4A	15	-	5718	26	0	0	0.47%	2.66811665260913	0.003684287313187465	0.003684287313187465	7.541963121699725e-05

ENSG00000156510_69227261_69266757_878	HKDC1	10	+	2634	48	2	1	2.62%	10.693037336337133	4.383737281623799e-05	1.4941806215212725e-05	5.163737956160449e-09
ENSG00000169045_179621263_179620884_57	HNRNPH1	5	-	171	30	4	1	15.79%	102.94393400552634	0.0034782380676658805	0.005059698366439319	9.69217750210964e-05
ENSG00000253719_74538113_74538406_97	ATXN7L3B	12	+	291	15	0	1	7.22%	30.246413599561862	0.00847367416878415	0.016503128830616266	0.0006966866551102258
ENSG00000100804_23034716_23016631_114	PSMB5	14	-	342	84	3	1	18.42%	144.12150760773687	2.3326754951162877e-05	2.3613239148262888e-05	4.297146570502968e-09
ENSG00000115947_147958839_147935510_352	ORC4	2	-	1056	14	0	0	1.99%	7.779285922008524	0.007029302661868638	0.007029302661868638	0.0002573639808771639
ENSG00000111642_6601370_6570676_1688	CHD4	12	-	5064	21	5	1	0.65%	2.4333311414813394	0.02946433998583899	0.001323351237915142	0.00026896173468470523
ENSG00000142192_26170620_25881670_770	APP	21	-	2310	414	7	8	13.51%	105.16324479058055	1.3133076695754875e-18	1.1331726377189472e-18	2.3483072771145317e-35
ENSG00000080822_98521358_98516659_231	CLDND1	3	-	693	34	2	0	4.33%	28.788649942671004	0.004691997894403566	0.0023628488670607213	6.289248615457574e-05
ENSG00000122952_56359749_56358418_110	ZWINT	10	-	330	21	0	2	5.45%	37.340572425640914	0.013640585738808999	0.037440157020026926	0.0023980863030810032
ENSG00000168906_85541275_85542409_204	MAT2A	2	+	612	72	4	2	8.82%	69.03299103900001	0.0003684667290494535	0.00010360820579762472	2.718514870742077e-07
ENSG00000048707_12279615_12509024_2865	VPS13D	1	+	8595	9	0	0	0.21%	0.6144297631743457	0.009815328628645334	0.009815328628645334	0.0004837142251238724
ENSG00000111358_123645512_123659859_191	GTF2H3	12	+	573	19	4	3	4.19%	19.456942500520945	0.04471043695002634	0.028023386503852714	0.0053546812962637375
ENSG00000173692_231057026_231170712_953	PSMD1	2	+	2859	52	1	5	2.31%	10.67246661043652	2.318900554996275e-05	0.00032427121235073774	6.058520602478171e-08
ENSG00000137776_58933565_58879999_1034	SLTM	15	-	3102	42	8	0	1.84%	7.944802643753387	0.0006766942253105029	5.5159969420047564e-05	2.866100587605571e-07
ENSG00000079616_29797027_29805310_597	KIF22	16	+	1791	37	8	2	3.18%	12.122208644201846	0.0032600418858822295	3.554863000883066e-05	1.1079724976205542e-06
ENSG00000143549_154172070_154157639_85	TPM3	1	-	255	58	0	0	9.41%	133.46378267540004	0.00575691403842271	0.00575691403842271	0.0001762552514680027
ENSG00000127884_133373333_133362868_290	ECHS1	10	-	870	60	4	0	8.28%	40.46761543665518	1.0193861914695746e-05	7.026239171075649e-06	5.87226389238453e-10
ENSG00000152904_235342032_235342772_246	GGPS1	1	+	738	13	1	0	2.44%	10.336240528197157	0.02034245439037237	0.013428347753762199	0.0012955184805712684
ENSG00000168701_67228552_67229113_85	TMEM208	16	+	255	12	0	0	8.24%	27.613196415600008	0.0078820576498062	0.0078820576498062	0.00031963642907451164
ENSG00000125124_56519811_56484761_704	BBS2	16	-	2112	10	1	0	0.85%	2.77831640071733	0.026832695913080365	0.011772051206276392	0.0015103817605807263
ENSG00000163832_47504433_47496069_193	ELP6	3	-	579	10	0	0	3.11%	10.134376922823837	0.011772051206276392	0.011772051206276392	0.000681272511389508
ENSG00000114988_96740040_96711973_169	LMAN2L	2	-	507	11	0	0	2.96%	12.730936217251482	0.019216965118390855	0.019216965118390855	0.0017073955890589052
ENSG00000115761_10682031_10572071_588	NOL10	2	-	1764	18	2	0	1.53%	5.9875553452193895	0.007456024827607967	0.0035291660242640502	0.00014366719176556365
ENSG00000133026_8623072_8475804_1927	MYH10	17	-	5781	23	0	0	0.47%	2.334535503913597	0.0035547763465261306	0.0035547763465261306	7.044676146010672e-05
ENSG00000155366_112703799_112701540_193	RHOC	1	-	579	87	4	5	15.03%	88.16907922856738	6.357679160154451e-06	1.1512870382308943e-05	6.012907082345096e-10
ENSG00000155366_112701683_112701540_47	RHOC	1	-	141	20	0	0	12.77%	83.23126579170214	0.013007239811447113	0.013007239811447113	0.0008218654148058079
ENSG00000155366_112703799_112701461_199	RHOC	1	-	597	62	4	25	9.55%	60.93867048166333	0.00027439032260447667	0.03271397772121476	8.973191109061109e-05
ENSG00000155366_112703799_112701540_193	RHOC	1	-	579	87	4	5	15.03%	88.16907922856738	6.357679160154451e-06	1.1512870382308943e-05	6.012907082345096e-10
ENSG00000155366_112703799_112701540_193	RHOC	1	-	579	87	4	5	15.03%	88.16907922856738	6.357679160154451e-06	1.1512870382308943e-05	6.012907082345096e-10
ENSG00000155366_112703799_112701540_193	RHOC	1	-	579	87	4	5	15.03%	88.16907922856738	6.357679160154451e-06	1.1512870382308943e-05	6.012907082345096e-10
ENSG00000175110_139348202_139357014_233	MRPS22	3	+	699	14	0	6	2.58%	11.752397616081547	0.013217226988443315	0.11710064162938172	0.007958958069389534
ENSG00000119335_128689583_128694664_277	SET	9	+	831	13	3	1	2.17%	9.179477147785201	0.06032271266817251	0.023672501042627626	0.006212924252457367
ENSG00000085491_108200138_108136653_477	SLC25A24	1	-	1431	23	0	4	3.14%	9.431131899458073	0.00020704336734043597	0.0028143556762708437	4.204636032568585e-06
ENSG00000095383_98213212_98199381_468	TBC1D2	9	-	1404	14	2	0	1.71%	5.851086847322651	0.019504170898671437	0.004873903550586949	0.0005060140340053911
ENSG00000197965_167722152_167773372_202	MPZL1	1	+	606	38	0	1	7.43%	36.7948120554406	0.0003033834228180314	0.00038889063296026935	7.9072884068841e-07
ENSG00000183011_7857423_7856731_173	NAA38	17	-	519	111	24	8	11.56%	125.49639122407807	0.003996564699959848	0.0015537412816747345	3.6491424901556987e-05
ENSG00000143179_165827834_165907823_261	UCK2	1	+	783	33	0	1	4.98%	24.7302094335115	0.0006791898698869142	0.0008628208183719401	3.714078072945139e-06
ENSG00000172409_57659597_57661436_321	CLP1	11	+	963	15	2	0	2.18%	9.139881991144861	0.017818254085047065	0.00837382380159347	0.0007434988581310937
ENSG00000173482_7888114_8406162_1397	PTPRM	18	+	4191	33	2	3	0.93%	4.620318297885828	0.0019848805137266086	0.0033021427446191217	3.7872484143859e-05
ENSG00000134352_55960471_55956011_125	IL6ST	5	-	375	18	1	0	4.00%	28.165460343912006	0.029237630782826433	0.021557223391537678	0.002819005644529471
ENSG00000065809_14530459_14521190_131	FAM107B	10	-	393	21	0	0	6.11%	31.35467913603436	0.005604866691947602	0.005604866691947602	0.00016752456572620382
ENSG00000103051_70523531_70481010_785	COG4	16	-	2355	33	2	0	1.66%	8.222400843498727	0.0023847795666952836	0.0006629342244537616	9.966692237144255e-06
ENSG00000204852_110634747_110647892_324	TCTN1	12	+	972	16	0	0	2.16%	9.65893701780247	0.0078820576498062	0.0078820576498062	0.00031963642907451164
ENSG00000138796_108023489_108034357_127	HADH	4	+	381	30	0	0	7.87%	46.20318297885828	0.002046028879997488	0.002046028879997488	2.4518070358754455e-05
ENSG00000181029_7682254_7682820_188	TRAPPC5	19	+	564	20	8	0	5.85%	20.807816447925536	0.08243509129588328	0.0012624143894574274	0.0009110501663464096
ENSG00000071282_8532737_8549894_233	LMCD1	3	+	699	7	0	0	2.15%	5.876198808040773	0.016947426762344633	0.016947426762344633	0.0013498980316300959
ENSG00000146457_159753494_159755611_234	WTAP	6	+	702	17	4	0	3.85%	14.209782343497867	0.028023386503852714	0.0033278027414746945	0.0005380123252020438
ENSG00000124795_18263987_18226147_380	DEK	6	-	1140	27	2	5	3.16%	13.897431090746055	0.003604785382371263	0.009994241782726202	0.00019621036725596744
ENSG00000060237_827064_908792_2515	WNK1	12	+	7545	27	1	4	0.60%	2.099810661822466	0.00034995587926105035	0.0025810035995757507	6.087871601150127e-06
ENSG00000120211_5231524_5233877_139	INSL4	9	+	417	43	0	0	7.91%	60.50733387231296	0.001595761305259402	0.001595761305259402	1.5227405548207672e-05
ENSG00000103544_19601693_19700476_712	VPS35L	16	+	2136	33	0	0	2.39%	9.065427896273176	0.00011014631109049817	0.00011014631109049817	8.714511530515982e-08
ENSG00000111652_6724657_6731039_275	COPS7A	12	+	825	16	0	0	3.27%	11.379983977338185	0.0032294771334464936	0.0032294771334464936	5.8663648728941325e-05
ENSG00000023839_99782845_99794011_195	ABCC2	10	+	585	14	7	1	3.08%	14.042608433574362	0.33632999628228655	0.026832695913080365	0.04814272760090725
ENSG00000274290_26183796_26184176_126	HIST1H2BE	6	+	378	24	0	0	5.56%	37.25589992580953	0.00877616296920843	0.00877616296920843	0.00039161135667564577
ENSG00000168216_69790322_69676158_467	LMBRD1	6	-	1401	21	3	0	2.14%	8.795423911821201	0.01424822060979566	0.0022409900782254734	0.00018649561876446586
ENSG00000104904_2269599_2273108_226	OAZ1	19	+	678	39	6	2	4.42%	33.75285623809514	0.013646340364237132	0.007635351959044894	0.0005261303871423992
ENSG00000178971_8248036_8228180_1217	CTC1	17	-	3651	6	0	1	0.41%	0.9643063661980281	0.016947426762344633	0.0477903522728147	0.0036975118224100542
ENSG00000171903_15929580_15913877_381	CYP4F11	19	-	1143	11	0	3	1.31%	5.647055697416012	0.019679754444124863	0.10105102209468636	0.009164497669452725
ENSG00000177119_45216355_45439938_918	ANO6	12	+	2754	22	4	4	1.20%	4.687425317462964	0.012914280349163228	0.012821549509893823	0.0008053923563397052
ENSG00000104522_143616712_143612910_321	TSTA3	8	-	963	29	0	5	3.43%	17.670438516213398	0.001558047347358915	0.013577461948370874	0.00012987996908748637
ENSG00000108465_47975891_47981502_281	CDK5RAP3	17	+	843	12	0	0	2.14%	8.352746246711746	0.009815328628645334	0.009815328628645334	0.0004837142251238724
ENSG00000108439_47941784_47946782_212	PNPO	17	+	636	18	0	0	4.25%	16.60699312730661	0.002967235347054297	0.002967235347054297	4.9907879201100895e-05
ENSG00000152556_48131382_48145708_619	PFKM	12	+	1857	36	0	0	3.23%	11.375387861030697	1.8298518738994393e-05	1.8298518738994393e-05	2.6441018379261105e-09
ENSG00000078668_42394212_42405462_284	VDAC3	8	+	852	183	37	2	13.03%	126.03382342859686	2.4306934703417023e-05	1.1600059286357198e-07	3.3031672802923816e-11
ENSG00000109971_123061324_123060047_210	HSPA8	11	-	630	92	0	0	8.10%	85.68856982936192	0.00013400452932326877	0.00013400452932326877	1.275145451879141e-07
ENSG00000099622_1270934_1272443_172	CIRBP	19	+	516	27	1	0	6.40%	30.703626828392448	0.002493493840886608	0.0014055202957965589	2.0830049893778164e-05
ENSG00000196535_29166826_29074770_2001	MYO18A	17	-	6003	10	0	1	0.25%	0.9774786337356324	0.019679754444124863	0.02784981298332479	0.002477909089056847

ENSG00000096060_35642824_35575835_457	FKBP5	6	-	1371	16	0	0	1.31%	6.847911583737419	0.013007239811447113	0.013007239811447113	0.0008218654148058079
ENSG00000136536_159729023_159767352_635	7-Mar	2	+	1905	17	2	0	1.57%	5.236360737603938	0.010645877633714064	0.0021880057261679567	0.0001345568947401665
ENSG00000115286_1390886_1395488_132	NDUFS7	19	+	396	26	3	0	9.09%	38.52598742328031	0.00387261209348426	0.0010073743004618205	2.38528981796687e-05
ENSG00000112531_163415257_163564710_296	QKI	6	+	888	20	0	0	3.72%	13.215775311520273	0.0014481341328822738	0.0014481341328822738	1.2639776101338432e-05
ENSG00000066855_65670010_65708125_329	MTFR1	8	+	987	23	0	0	2.74%	13.673707951493924	0.0035291660242640502	0.0035291660242640502	6.948232509684344e-05
ENSG00000123472_46668322_46635776_328	ATPAF1	1	-	984	23	0	0	2.74%	13.715396085492381	0.0033278027414746945	0.0033278027414746945	6.211806171561056e-05
ENSG00000149925_30067255_30070212_368	ALDOA	16	+	1104	713	70	30	29.08%	378.96235705784386	2.8434265653329284e-16	5.0305764798834974e-18	2.2821492036410582e-32
ENSG00000134265_10539849_10550220_197	NAPG	18	+	591	16	0	1	3.05%	15.885764435370563	0.013428347753762199	0.016822681477512567	0.0010798218650012886
ENSG00000122642_32957574_33005351_623	FKBP9	7	+	1869	35	1	1	2.09%	10.988397450028092	0.0009985037337807711	0.0009985037337807711	6.187415572756679e-06
ENSG00000206503_29942563_29943034_113	HLA-A	6	+	339	10	0	0	5.31%	17.309157045176995	0.011772051206276392	0.011772051206276392	0.000681272511389508
ENSG00000197122_37384154_37403379_536	SRC	20	+	1608	46	6	4	3.92%	16.786007149408587	0.0003884290151169211	0.00016054896953742018	4.3172723051176966e-07
ENSG00000108515_4951836_4957047_432	ENO3	17	+	1296	16	0	0	1.85%	7.244202763351853	0.005257622967929454	0.005257622967929454	0.0001483661556465691
ENSG00000136381_78438338_78473390_343	IREB2	15	+	1029	20	2	1	1.75%	11.404867324227407	0.029237630782826433	0.029237630782826433	0.0037269006366701394
ENSG00000156958_49201181_49328159_434	GALK2	15	+	1302	9	0	3	1.15%	4.056085879019586	0.016947426762344633	0.165899918248885	0.014398895220848395
ENSG00000167792_67608469_67609836_212	NDUFV1	11	+	636	38	1	0	5.19%	35.05920771320284	0.00220554346506264	0.001588175367824666	2.071934180454389e-05
ENSG00000075239_108134253_108139065_110	ACAT1	11	+	330	21	0	1	9.09%	37.340572425640914	0.0022543491824521134	0.0022011839993586477	2.885191208804781e-05
ENSG00000139613_56189461_56163689_1152	SMARCC2	12	-	3456	15	1	0	0.52%	2.546790033990886	0.015555382717448078	0.011772051206276392	0.0008874794703752884
ENSG00000119723_73955892_73962999_320	COQ6	14	+	960	25	0	0	2.19%	15.280740203945316	0.008877961307018026	0.008877961307018026	0.00040023486315785033
ENSG00000117899_80989791_80979219_234	MESD	15	-	702	38	7	3	7.26%	31.76304288546582	0.000764267771632909	0.0010102842767105284	4.845736183686482e-06
ENSG00000032444_7540198_7561562_1138	PNPLA6	19	+	3414	23	0	0	0.88%	3.953119434131372	0.0023218226288164415	0.0023218226288164415	3.1231198880462635e-05
ENSG00000070081_17295324_17317598_338	NUCB2	11	+	1014	18	0	1	2.96%	10.416220541387576	0.0022011839993586477	0.0037373685375237473	4.708421183416152e-05
ENSG00000141696_41806873_41802957_81	P3H4	17	-	243	11	0	0	7.41%	26.562076798956795	0.012798402692974279	0.012798402692974279	0.0007972436888943567
ENSG00000213625_65420725_65430201_143	LEPROT	1	+	429	29	0	0	9.09%	39.665809536395116	0.0006026251277497241	0.0006026251277497241	2.339296094881936e-06
ENSG00000130429_99390975_99394489_178	ARPC1B	7	+	534	46	5	2	8.99%	50.546628270129226	0.0014719922646461731	0.0003706101014865687	3.590017235794361e-06
ENSG00000136854_127653778_127690857_544	STXBP1	9	+	1632	9	2	0	0.92%	3.2359214549531257	0.08350359792828288	0.020613416668581838	0.007749259071793911
ENSG00000147140_71291892_71300076_382	NONO	X	+	1146	41	1	3	4.45%	20.993016908456813	0.00018651559025433358	0.0008591708244044518	1.1060335313988607e-06
ENSG00000181031_319509_213852_199	RPH3AL	17	-	597	12	0	0	2.51%	11.794581383547742	0.019679754444124863	0.019679754444124863	0.0017849636115922674
ENSG00000162384_53218923_53214659_130	CZIB	1	-	390	11	0	0	3.85%	16.550217082426926	0.020613416668581838	0.020613416668581838	0.001946208561389314
ENSG00000144840_120742507_120689823_212	RABL3	3	-	636	12	0	1	3.77%	11.071328751537738	0.004826937407846361	0.010020958156399907	0.0002559950816326654
ENSG00000180340_44557689_44559386_565	FZD2	17	+	1695	13	1	0	1.06%	4.5003808317460186	0.026832695913080365	0.011772051206276392	0.0015103817605807263
ENSG00000128581_101321709_101315134_185	IFT22	7	-	555	9	0	0	3.24%	9.515358224294596	0.012178957859018494	0.012178957859018494	0.0007262454987071801
ENSG00000130429_99385715_99394102_352	ARPC1B	7	+	1056	108	3	6	10.51%	60.01163425549433	7.906098564594512e-08	5.812653707897395e-07	4.495042350107638e-13
ENSG00000214655_73786080_73801519_1770	ZSWIM8	10	+	5310	8	0	0	0.34%	0.884038303324294	0.011570465654371866	0.011570465654371866	0.0006594843062896773
ENSG00000151292_123545664_123614396_423	CSNK1G3	5	+	1269	16	0	0	2.13%	7.398334737040191	0.002943990679779778	0.002943990679779778	4.916404313974209e-05
ENSG00000164253_77492220_77433135_404	WDR41	5	-	1212	27	0	0	2.97%	13.071841124959162	0.0006608884069239612	0.0006608884069239612	2.7949486515898395e-06
ENSG00000134056_69228289_69229225_38	MRPS36	5	+	114	24	0	0	13.16%	123.5327208066316	0.02108409854857798	0.02108409854857798	0.002029891263157455
ENSG00000113621_134887796_134899685_292	TXNDC15	5	+	876	27	3	0	4.45%	18.085697994806512	0.004676575434377382	0.0005460508606305346	1.685429606172708e-05
ENSG00000101132_54215423_54219150_49	PFDN4	20	+	147	16	2	0	10.20%	63.86725701567348	0.07864960352514258	0.016947426762344633	0.006209665325776152
ENSG00000180398_46909165_46908893_90	MCFD2	2	-	270	17	0	0	6.67%	36.945434093094455	0.013007239811447113	0.013007239811447113	0.0008218654148058079
ENSG00000120509_70289341_70287082_140	PDZD11	X	-	420	10	0	0	4.29%	13.970962472178575	0.012798402692974279	0.012798402692974279	0.0007972436888943567
ENSG00000099795_14571966_14566133_125	NDUFB7	19	-	375	49	0	0	8.80%	76.67264204731602	0.00151350169064872	0.00151350169064872	1.3757299738441384e-05
ENSG00000176986_73759740_73771095_975	SEC24C	10	+	2925	50	5	4	2.56%	10.03043459541026	5.410543665017846e-05	2.9306621768175134e-05	1.2103598921629691e-08
ENSG00000110075_68544920_68551204_108	PPP6R3	11	+	324	7	1	5	4.63%	12.677354835865744	0.04178282491678763	0.169362073879829	0.02871445979022244
ENSG00000205336_57650288_57663582_687	ADGRG1	16	+	2061	71	4	0	4.37%	20.21417277633989	3.4901523427252256e-06	6.614649994567748e-07	2.087834633512807e-11
ENSG00000140264_43792377_43793972_133	SERF2	15	+	399	50	0	29	5.26%	73.53138143251881	0.00847367416878415	0.1057548992775299	0.005056518355234598
ENSG00000198954_68988833_69016416_646	KIF1BP	10	+	1938	28	3	0	2.17%	8.47773574163158	0.0027474448481791879	0.00037874750290730355	6.9725200976450386e-06
ENSG00000165322_31908573_31807658_752	ARHGAP12	10	-	2256	24	1	1	1.33%	6.242344934377661	0.0034718995429267267	0.0034718995429267267	6.734844907553302e-05
ENSG00000131778_147266075_147295509_603	CHD1L	1	+	1809	18	0	1	1.33%	5.838611182402986	0.004873903550586949	0.015625008558487224	0.000403121801006346
ENSG00000131778_147275365_147295509_470	CHD1L	1	+	1410	7	0	1	1.28%	2.9130943027095753	0.009815328628645334	0.0477903522728147	0.0023388674905236288
ENSG00000136827_129824181_129818194_315	TOR1A	9	-	945	15	2	0	2.54%	9.313974981452382	0.02671499548383061	0.004873903550586949	0.0007031623440447889
ENSG00000136827_129814118_129813972_48	TOR1A	9	-	144	25	0	0	10.42%	101.87160135963543	0.02108409854857798	0.02108409854857798	0.002029891263157455
ENSG00000162402_55214948_55069045_2565	USP24	1	-	7695	16	0	0	0.39%	1.2200762548803121	0.002033393162560146	0.002033393162560146	2.4228963598721857e-05
ENSG00000123159_14478708_14478416_58	GIPC1	19	-	174	20	0	0	15.52%	67.44602572775864	0.0033030736763512156	0.0033030736763512156	6.124050385493983e-05
ENSG00000105171_29610472_29615380_179	POP4	19	+	537	12	0	0	3.91%	13.112411705731846	0.00788205764980062	0.00788205764980062	0.0003196364290745164
ENSG00000108946_68515400_68523761_131	PRKAR1A	17	+	393	21	0	12	5.34%	31.35467913603436	0.00797940233269714	0.15482795152292833	0.007709038975975287
ENSG00000182944_29292142_29300161_317	EWSR1	22	+	951	104	6	8	5.68%	64.16946801101578	0.0015143143267861746	0.001564483162889613	1.4209166874967526e-05
ENSG00000188153_108573595_108696378_1529	COL4A5	X	+	4587	5	0	0	0.33%	0.6396124087982343	0.012673659338734126	0.012673659338734126	0.000782701129001274
ENSG00000114439_107728780_107805457_771	BBX	3	+	2313	16	4	0	1.04%	4.059008552228276	0.04727912037993979	0.005454749182134642	0.001431075230184615
ENSG00000144381_197498818_197487046_563	HSPD1	2	-	1689	207	7	6	5.68%	71.91447467917142	3.8279354707081292e-07	9.681079734561403e-07	3.4022552296226068e-12
ENSG00000070018_12203396_12121126_1462	LRP6	12	-	4386	17	2	1	0.68%	2.2743427280290702	0.006369482933362988	0.0037220617515305076	0.00012918474591820854
ENSG00000151726_184776947_184757125_527	ACSL1	4	-	1581	15	0	0	1.33%	5.56717669669355	0.00847367416878415	0.00847367416878415	0.0003665024264111182
ENSG00000169045_179618370_179617960_108	HNRNPH1	5	-	324	56	10	2	15.74%	101.41883868692595	0.001867416853427377	0.00028515737378700517	3.61756383588949e-06
ENSG00000111481_54325164_54347848_132	COPZ1	12	+	396	116	9	1	23.48%	171.88517465771216	7.527352934826526e-06	6.224764817239588e-07	4.3325727667756984e-11
ENSG00000133657_194460735_194405919_1180	ATP13A3	3	-	3540	37	0	1	1.02%	6.133015729312289	0.0010447178140462346	0.0017534708537449623	1.1163276398224378e-05
ENSG00000010278_6232667_6237828_158	CD9	12	+	474	139	7	1	20.25%	172.07274032189562	6.613736944929179e-06	5.631038869139985e-07	3.452409511008813e-11
ENSG00000124733_43013378_43012470_172	MEA1	6	-	516	46	2	2	9.88%	52.30988274466862	0.0005393137985452151	0.0005393137985452151	1.8883487944536844e-06
ENSG00000148337_128179348_128166197_556	CIZ1	9	-	1668	17	2	0	1.62%	5.98037602226349	0.018399683928041276	0.002967235347054297	0.00031087262125642094

ENSG00000166557_79311250_79314564_146	TMED3	15	+	438	9	0	0	3.42%	12.057131996537674	0.020613416668581838	0.020613416668581838	0.001946208561389314
ENSG00000182180_73252482_73250852_137	MRPS16	10	−	411	25	0	6	9.49%	35.69223989242702	0.0005983074123552221	0.025745290591051732	0.00012232263610794313
ENSG00000110801_121888857_121903048_179	PSMD9	12	+	537	23	2	7	3.35%	25.13212243598604	0.02034245439037237	0.07538138751332264	0.0068840887708533265
ENSG00000119537_63367067_63331782_315	KDSR	18	−	945	14	0	0	2.54%	8.693043316022225	0.005257622967929454	0.005257622967929454	0.00014836615564656991
ENSG00000168036_41224069_41239342_781	CTNNB1	3	+	2343	117	8	3	5.76%	29.301455223339957	6.403151454483205e−08	1.1032790393731706e−08	7.305397733044446e−15
ENSG00000153310_129871436_129849289_217	FAM49B	8	−	651	12	0	6	2.30%	10.816229010718896	0.02108409854857798	0.24029758967682546	0.026464846241199956
ENSG00000104783_43780861_43774274_129	KCNN4	19	−	387	5	3	0	3.88%	7.581142426763567	0.36944134018176367	0.012673659338734126	0.03462072451183559
ENSG00000114120_140942055_140976453_311	SLC25A36	3	+	933	10	0	1	2.57%	6.289179247926047	0.00414960799764038	0.010437296944230427	0.00023253924594002017
ENSG00000118515_134173142_134170268_288	SGK1	6	−	864	11	3	1	1.74%	7.470584099706599	0.06539853090342929	0.03537493353818881	0.009482640652036243
ENSG00000197965_167772389_167787844_85	MPZL1	1	+	255	18	0	1	10.59%	41.41979462340001	0.0034782380676658805	0.005588370640926919	0.00010676685949938237
ENSG00000118515_134173142_134170268_288	SGK1	6	−	864	11	3	1	1.74%	7.470584099706599	0.06539853090342929	0.03537493353818881	0.009482640652036243
ENSG00000170876_14129505_14141795_365	TMEM43	3	+	1095	30	4	6	4.11%	16.076175995383565	0.004414380476409315	0.0030217567445692886	7.443736238861179e−05
ENSG00000091129_108232428_108149910_1196	NRCAM	7	−	3588	20	2	5	1.00%	3.270793889807693	0.004302923267342423	0.014861317392387344	0.0003432852811583412
ENSG00000127564_2975767_2972953_358	PKMYT1	16	−	1074	13	2	0	2.23%	7.1025563406047505	0.01943569092740073	0.004826937407846361	0.0004999687388256805
ENSG00000077684_128831759_128872262_842	JADE1	4	+	2526	10	0	2	0.83%	2.322962881359858	0.007029302661868638	0.04912109528954385	0.0018325026027891263
ENSG00000196712_31163201_31374155_2738	NF1	17	+	8214	23	2	6	0.44%	1.6430423360268447	0.004359748848448076	0.01964131194755714	0.00046292460174369505
ENSG00000006576_77908873_77954878_610	PHTF2	7	+	1830	20	4	0	0.98%	6.412900806901641	0.08424673392120513	0.011772051206276392	0.005012526315124349
ENSG00000130414_240025301_239983517_429	NDUFA10	2	−	1287	29	1	0	2.80%	13.221936512131705	0.0013683766156140878	0.001032175130546352	8.661907654447743e−06
ENSG00000154719_25607460_25585751_348	MRPL39	21	−	1044	18	0	0	3.16%	10.116903859163795	0.0013775385517804596	0.0013775385517804596	1.1483554366703679e−05
ENSG00000106245_99411093_99419441_144	BUD31	7	+	432	91	2	0	13.89%	123.604209649691	7.79038924815374e−05	4.011609959528814e−05	2.3456094754023457e−08
ENSG00000168090_100089385_100091789_270	COPS6	7	+	810	150	6	4	11.85%	108.6630414502778	9.70638981631239e−07	8.296765206286212e−07	7.231093242504799e−12
ENSG00000143569_154257405_154270919_264	UBAP2L	1	+	792	12	1	5	2.27%	8.890612482295456	0.019795881618345192	0.17808035639161363	0.01752998634990791
ENSG00000113448_58993456_58974664_499	PDE4D	5	−	1497	37	1	0	3.61%	14.502922967111227	0.0001185681716428482	8.091994981866961e−05	6.953223258948248e−08
ENSG00000179409_748009_744866_1047	GEMIN4	17	−	3141	39	10	3	1.43%	7.285716819302294	0.02169658182459074	0.0010518076417120354	0.00015741372066537928
ENSG00000106305_6009433_6023691_228	AIMP2	7	+	684	58	5	5	9.21%	49.75623476933773	0.0002216977995945237	0.00017300823528471786	2.6644568605676426e−07
ENSG00000082212_50908093_50940313_434	ME2	18	+	1302	25	0	0	2.53%	11.26690521949885	0.0014915192737735051	0.0014915192737735051	1.337646624223462e−05
ENSG00000124155_45420349_45425826_316	PIGT	20	+	948	52	2	0	4.11%	32.18626797387975	0.0022083206986541785	0.0006768518708446028	9.39609878359303e−06
ENSG00000153187_244859284_244857574_176	HNRNPU	1	−	528	30	8	7	8.52%	33.339796808607964	0.020791797637945246	0.013553239405677453	0.001334299951365701
ENSG00000137700_119027649_119024910_228	SLC37A4	11	−	684	10	0	0	2.19%	8.578661167127194	0.020613416668581838	0.020613416668581838	0.001946208561389314
ENSG00000136938_97983562_98015431_249	ANP32B	9	+	747	99	6	14	14.46%	77.76608026682531	5.336505086930901e−07	1.0546527261338006e−05	5.3343728129859905e−11
ENSG00000138760_76166264_76161713_70	SCARB2	4	−	210	16	3	0	7.14%	44.70707991097144	0.056923149003329024	0.020613416668581838	0.005212417464661139
ENSG00000156467_96235530_96231105_140	UQCRB	8	−	420	340	1	8	12.86%	475.01272405407155	9.634195171794265e−05	0.00015670653198069615	1.0814009756487191e−07
ENSG00000198959_38165198_38130219_687	TGM2	20	−	2061	380	18	25	14.85%	108.18853035224166	3.3183552244724876e−18	5.3410030144867615e−17	2.7826013502413522e−33
ENSG00000188603_28491759_28477516_438	CLN3	16	−	1314	26	2	0	2.51%	11.610571552221463	0.004407715900925203	0.0015208721599504675	3.948464526818382e−05
ENSG00000152952_146124173_146070717_703	PLOD2	3	−	2109	157	5	3	7.25%	43.6816152401828	6.773524720109577e−10	5.533521704199033e−10	4.1937780127485715e−18
ENSG00000127054_1324608_1311859_499	INTS11	1	−	1497	10	0	2	1.40%	3.919708910030061	0.007593099278532274	0.049338281967443096	0.0019605633087212975
ENSG00000147044_41665429_41520420_712	CASK	X	−	2136	19	0	0	0.98%	5.219488788763344	0.00847367416878415	0.00847367416878415	0.0003665024264111182
ENSG00000198836_193637210_193692127_694	OPA1	3	+	2082	25	0	1	1.87%	7.0458744456232	0.0006223089785121674	0.000806344341637709	3.1989545673191004e−06
ENSG00000140025_89954660_89797243_163	EFCAB11	14	−	489	12	0	1	3.07%	14.399519603226997	0.020613416668581838	0.03537493353818881	0.003252526348827943
ENSG00000096746_68338645_68342054_215	HNRNPH3	10	+	645	91	3	1	12.09%	82.78607530025816	1.5502390583192025e−05	4.496782845038374e−06	5.811110446196424e−10
ENSG00000144559_11846636_11790505_316	TAMM41	3	−	948	9	0	0	1.90%	5.570700226248419	0.011772051206276392	0.011772051206276392	0.000681272511389508
ENSG00000132471_75855297_75846747_231	WBP2	17	−	693	33	0	1	5.19%	27.94192494435715	0.0010197273511756433	0.0013558867987602367	8.486686545361988e−06
ENSG00000104763_18071320_18057534_305	ASAH1	8	−	915	52	3	2	7.54%	33.347084195888534	1.0477260120960575e−05	4.2062176516136585e−05	3.541478753783154e−09
ENSG00000162910_228108260_228106760_128	MRPL55	1	−	384	35	2	1	7.81%	53.482590713808605	0.004407715900925203	0.0030814981005195803	7.570158080683164e−05
ENSG00000116337_109625635_109631152_760	AMPD2	1	+	2280	7	0	1	0.66%	1.801518845096711	0.019216965118390855	0.04178282491678763	0.003600525600033436
ENSG00000102119_154379485_154381197_254	EMD	X	+	762	36	2	1	4.72%	27.721909787314967	0.0036685121388185782	0.0014807488005297608	3.20519878233733e−05
ENSG00000021574_32063832_32154496_616	SPAST	2	+	1848	12	3	0	1.14%	3.8102624924123387	0.051745348319195604	0.008175610787902942	0.002192614907007965
ENSG00000133706_146135664_146114106_460	LARS	5	−	1380	26	2	0	3.26%	11.055283347550002	0.001405504862529688	0.00023522430645143535	2.265670116061457e−06
ENSG00000145882_149364095_149368654_376	PCYOX1L	5	+	1128	8	0	3	1.33%	4.161563289585107	0.019679754444124863	0.15040137116388086	0.014313628319454642
ENSG00000108599_19958449_19906227_515	AKAP10	17	−	1545	9	0	0	0.97%	3.418138391251457	0.019216965118390855	0.019216965118390855	0.0017073955890589052
ENSG00000106682_74174384_74189935_141	EIF4H	7	+	423	18	0	4	4.26%	24.969379737510643	0.013428347753762199	0.03577298629450316	0.002259558529646323
ENSG00000115364_75655076_75655285_69	MRPL19	2	+	207	17	0	0	10.14%	48.18969664316668	0.00847367416878415	0.00847367416878415	0.0003665024264111182
ENSG00000105426_5286095_5206774_1933	PTPRS	19	−	5799	10	2	1	0.31%	1.0118648453724783	0.0533594081646912	0.026602764905793736	0.006076671267284093
ENSG00000186185_44936344_44926400_833	KIF18B	17	−	2499	6	0	1	0.60%	1.4088365518163268	0.016947426762344633	0.0477903522728147	0.0036975118224100542
ENSG00000177697_836070_838192_253	CD151	11	+	759	213	23	10	17.00%	164.66960510686368	5.628074008217504e−07	7.179261916658316e−08	3.96916788384252e−13
ENSG00000131238_40097238_40074061_203	PPT1	1	−	609	48	0	0	8.87%	46.24870335617735	7.414496580508643e−05	7.414496580508643e−05	4.038337914182223e−08
ENSG00000197563_62157141_62045856_788	PIGN	18	−	2364	12	0	0	0.76%	2.97858083163198	0.011772051206276392	0.011772051206276392	0.000681272511389508
ENSG00000182199_57230833_57234361_483	SHMT2	12	+	1449	69	8	2	5.18%	27.94192494435715	0.00021707216054561796	7.388005576868282e−06	1.4195700185393147e−08
ENSG00000141552_81894478_81900065_84	ANAPC11	17	+	252	127	10	2	20.24%	295.7187056611132	0.00039118706886775756	0.00018946255112486357	5.081891006409044e−07
ENSG00000167965_2206086_2208877_326	MLST8	16	+	978	19	0	4	3.37%	11.39961968588804	0.0013775385517804596	0.01572186727294618	0.00013725983080102293
ENSG00000213614_72375972_72346662_373	HEXA	15	−	1119	75	0	5	8.04%	39.32844663750001	6.808389018186618e−07	5.340842444172843e−06	3.310105539403331e−11
ENSG00000029534_41715660_41655727_1350	ANK1	8	−	4050	9	0	0	0.37%	1.3039564974033335	0.020613416668581838	0.020613416668581838	0.001946208561389314
ENSG00000128944_40383253_40391989_163	KNSTRN	15	+	489	12	7	0	3.07%	14.399519603226997	0.1754079647031212	0.02108409854857798	0.01802001171292494
ENSG00000100225_32493041_32498530_221	FBXO7	22	+	663	31	3	0	2.71%	27.436188746269238	0.044040755831095145	0.013428347753762199	0.0027917083341200354
ENSG00000140105_100369185_100334875_471	WARS	14	−	1413	60	6	3	4.88%	24.916366192420387	0.00017457506911736429	5.349366132029235e−05	6.898497935895578e−08
ENSG00000184752_94996976_94971440_113	NDUFA12	12	−	339	69	2	0	15.93%	119.43318361172126	0.0002820961601313486	8.688204745348054e−05	1.7617538944783414e−07
ENSG00000111596_70330426_70353915_365	AC092881.1	12	+	1095	22	0	0	3.01%	11.789195729947949	0.001484234770590415	0.001484234770590415	1.3251387882263029e−05

ENSG00000063177_48619143_48615372_133	RPL18	19	-	399	72	2	9	13.53%	105.8851892628271	0.00011866732593941614	0.0018302529826180365	1.6287361770789166e-06
ENSG00000112378_138107340_138092042_193	PERP	6	-	579	146	6	4	20.21%	147.961903073228	5.3387450470309126e-08	9.461450207212906e-08	4.9628381142445075e-14
ENSG00000248643_66639712_66644132_364	RBM14-RBM4	11	+	1092	15	6	0	2.75%	8.0601706570261	0.06856615097740389	0.0017901637611834264	0.0009325288179663202
ENSG00000120733_138417503_138435700_652	KDM3B	5	+	1956	11	1	5	0.92%	3.2998899090728537	0.014014727539149415	0.1097457788509939	0.007724738414448233
ENSG00000240065_32856177_32859532_186	PSMB9	6	+	558	15	0	0	3.76%	15.77366730729839	0.0078820576498062	0.0078820576498062	0.00031963642907451164
ENSG00000174953_154324227_154276171_916	DHX36	3	-	2748	30	2	8	1.42%	6.405899823487993	0.0009728868468695743	0.01270978408730525	8.121592525257742e-05
ENSG00000094631_48802943_48824612_1160	HDAC6	X	+	3480	25	0	0	0.69%	4.215376607984915	0.005306578019052857	0.005306578019052857	0.00015100114740534233
ENSG00000115355_55332660_55291711_849	CCDC88A	2	-	2547	6	1	0	0.59%	1.382286039650177	0.0477903522728147	0.016947426762344633	0.0036975118224100542
ENSG00000013306_44323562_44320001_351	SLC25A39	17	-	1053	39	4	1	5.70%	21.73260829005556	0.00032124723708661584	5.271582575184717e-05	1.265865098096616e-07
ENSG00000137074_32989888_32973498_341	APTX	9	-	1023	20	7	3	3.23%	11.471758041671556	0.024096744146595385	0.011821942446875977	0.0013628509401148498
ENSG00000145414_163166727_163128897_494	NAF1	4	-	1482	11	0	0	1.21%	4.355320284849191	0.012798402692974279	0.012798402692974279	0.0007972436888943567
ENSG00000133142_103586676_103587323_215	TCEAL4	X	+	645	18	0	0	3.26%	16.375267641809305	0.00847367416878415	0.00847367416878415	0.0003665024264111182
ENSG00000142541_49487630_49490915_130	RPL13A	19	+	390	42	14	0	4.62%	63.19173795108463	0.08647745899421033	0.013853924679039932	0.005863614208897366
ENSG00000205336_57650288_57663582_687	ADGRG1	16	+	2061	71	4	0	4.37%	20.21417277633989	3.4901523427252256e-06	6.614649994567748e-07	2.087834633512807e-11
ENSG00000167526_89562377_89563042_57	RPL13	16	+	171	102	1	6	12.28%	350.00937561878953	0.008877961307018026	0.010353108236244411	0.0004630494296106523
ENSG00000167526_89560981_89561506_120	RPL13	16	+	360	143	111	53	10.00%	233.0822239108459	0.03637137457764638	0.046521888739526465	0.007015145074721836
ENSG00000112306_132814769_132815191_77	RPS12	6	+	231	32	8	15	9.09%	81.28559983812988	0.05346792862759031	0.06845323997737227	0.014200631288570085
ENSG00000100316_39318439_39317457_70	RPL3	22	-	210	259	21	0	31.43%	723.6958560588502	5.017800315001901e-05	1.8768467850450943e-05	7.340640507038093e-09
ENSG00000244005_35697701_35669622_355	NFS1	20	-	1065	24	0	0	1.97%	13.223220818738032	0.00877616296920843	0.00877616296920843	0.00039161135667564577
ENSG00000115275_74465079_74461275_687	MOGS	2	-	2061	26	0	3	2.04%	7.402373129363903	0.00036930943616753716	0.003539467718397726	8.906402084963395e-06
ENSG00000163950_1699682_1693597_150	SLBP	4	-	450	41	0	0	11.33%	53.46221639353668	0.00012077315611212433	0.00012077315611212433	1.0421183092217773e-07
ENSG00000007923_6701800_6635675_559	DNAJC11	1	-	1677	21	2	0	2.15%	7.347876505940073	0.0029488083751021267	0.0008664586529590942	1.5772708037927043e-05
ENSG00000129084_14517954_14505192_238	PSMA1	11	-	714	94	6	3	9.24%	77.25120425792859	0.0002752372154973131	4.470682380518355e-05	9.280871926425765e-08
ENSG00000180900_143815372_143791163_1655	SCRIB	8	-	4965	12	3	9	0.54%	1.4182004201365561	0.016254722322859756	0.2087027175654277	0.01852228469542362
ENSG00000177674_11736209_11750192_152	AGTRAP	1	+	456	23	0	4	6.58%	29.596381026588823	0.0023218226288164415	0.0033728746429155087	4.477174645253263e-05
ENSG00000177674_11747543_11750192_104	AGTRAP	1	+	312	18	0	3	6.73%	33.852716759509626	0.00847367416878415	0.00837382380159347	0.000362422040249726
ENSG00000182944_29282517_29300161_475	EWSR1	22	+	1425	126	6	8	5.05%	51.88374273878527	0.00012487302501014574	0.0001440551817339119	1.2777049173942254e-07
ENSG00000175063_45812696_45816767_161	UBE2C	20	+	483	44	0	0	6.21%	53.454117284857155	0.002460156992221257	0.002460156992221257	3.4886517907067876e-05
ENSG00000120533_109334469_109343481_101	ENY2	8	+	303	146	1	5	28.71%	282.7390821102278	1.3060495209591968e-06	5.885669548807485e-06	6.759868121948275e-11
ENSG00000175581_73844903_73864370_113	MRPL48	11	+	339	26	0	0	5.31%	45.003808317460184	0.013640585738808999	0.013640585738808999	0.00089863692579514
ENSG00000187109_76056041_76048429_208	NAP1L1	12	-	624	53	10	3	5.77%	49.83872189594472	0.009337308690898131	0.0033503060952949962	0.00017158932169682655
ENSG00000161011_179820937_179821567_73	SQSTM1	5	+	219	26	0	4	8.22%	69.66342931332878	0.012798402692974279	0.04223451612662591	0.0025674639266022752
ENSG00000258881_70994748_70948370_48	AC007040.2	2	-	144	15	0	1	12.50%	61.12296081578126	0.011772051206276392	0.012798402692974279	0.0007371592163134675
ENSG00000068912_53794387_53817969_415	ERLEC1	2	+	1245	27	0	1	3.61%	12.72535858911687	0.0002582331582969843	0.0003716399344246037	6.483412863293416e-07
ENSG00000078674_17935644_18027662_2005	PCM1	8	+	6015	69	8	14	1.60%	6.7311470065458865	2.554274401115327e-05	0.0002237792097068832	4.486414886301421e-08
ENSG00000158710_159920509_159918800_199	TAGLN2	1	-	597	269	22	22	21.61%	264.3951993478619	7.442845536908087e-07	3.390316985711192e-07	2.3274339170761862e-12
ENSG00000158710_159920509_159918800_199	TAGLN2	1	-	597	269	22	22	21.61%	264.3951993478619	7.442845536908087e-07	3.390316985711192e-07	2.3274339170761862e-12
ENSG00000154582_73959768_73946630_112	ELOC	8	-	336	8	4	1	4.46%	13.970962472178575	0.2618042821861254	0.04223451612662591	0.04736319147700863
ENSG00000080822_98518942_98516659_138	CLDND1	3	-	414	25	2	0	4.35%	35.43360047291667	0.02553836591027338	0.013640585738808999	0.0016391236133375881
ENSG00000197912_89508547_89557093_737	SPG7	16	+	2211	13	0	0	0.81%	3.450088425965401	0.011772051206276392	0.011772051206276392	0.000681272511389508
ENSG00000243147_27774440_27779482_46	MRPL33	2	+	138	74	2	0	28.26%	314.65037219950005	0.0013809361561184436	0.0006768518708446028	5.87344888251601e-06
ENSG00000101150_63865366_63889945_209	TPD52L2	20	+	627	39	3	2	10.05%	36.49830387468661	0.0001703216465405105	0.00010187626107045325	1.2383489804634146e-07
ENSG00000078808_1218796_1217512_126	SDF4	1	-	378	23	1	0	6.35%	35.703570762234136	0.007181907427766128	0.005554612275049112	0.0002105152421252746
ENSG00000143870_10812696_10784258_440	PDIA6	2	-	1320	614	21	19	28.18%	272.9418032064705	1.2129328379981673e-21	1.2907787647533918e-20	2.6819224978807765e-40
ENSG00000143401_150235780_150220691_266	ANP32E	1	-	798	93	5	4	10.90%	68.3841847322425	1.1636037477503736e-05	6.818461444530895e-06	6.500909348331256e-10
ENSG00000133835_119479012_119541994_532	HSD17B4	5	+	1596	38	0	0	2.82%	13.970962472178575	0.00029563251185049877	0.00029563251185049877	5.909204346842462e-07
ENSG00000147044_41665429_41520420_713	CASK	X	-	2139	19	0	0	0.98%	5.212168327629033	0.00847367416878415	0.00847367416878415	0.0003665024264111182
ENSG00000215301_41334253_41347468_641	DDX3X	X	+	1923	94	12	3	7.02%	28.682974435861162	5.81627248979138e-07	8.039453835617377e-09	5.3199907532461864e-14
ENSG00000151491_15669717_15621317_726	EPS8	12	-	2178	15	0	0	1.10%	4.04118749195248	0.005405141725979036	0.005405141725979036	0.00015637213408961292
ENSG00000141867_15265488_15238377_1124	BRD4	19	-	3372	14	5	1	0.62%	2.436217655290926	0.091912668396609	0.015516259750072458	0.006854886692768062
ENSG00000105968_44847971_44834501_102	H2AFV	7	-	306	67	1	1	8.82%	128.4780666559167	0.004557893626254153	0.0037896409716948643	9.495750423643499e-05
ENSG00000103197_2060673_2088610_1458	TSC2	16	+	4374	12	5	3	0.62%	1.609822836300412	0.04946560907745733	0.033570409067276055	0.006921950394855025
ENSG00000168701_67227219_67229113_173	TMEM208	16	+	519	25	0	0	6.36%	28.26495297839596	0.0015505692382983198	0.0015505692382983198	1.4410974035783556e-05
ENSG00000117868_158830038_158734207_921	ESYT2	7	-	2763	42	5	3	2.17%	8.91957213207492	4.983000130598301e-05	0.00023625467940204776	8.773628016212268e-08
ENSG00000065883_39950642_40069346_938	CDK13	7	+	2814	8	0	4	0.53%	1.6681746235437105	0.019216965118390855	0.169362073879829	0.016163991620339734
ENSG00000168385_241324233_241350174_361	SEPT2	2	+	1083	75	6	1	9.97%	40.63576342323408	1.7455265122155713e-07	5.171310977525534e-08	8.870577633093306e-14
ENSG00000103187_84617914_84566845_142	COTL1	16	-	426	84	1	3	17.61%	115.70318216395778	6.684769440272103e-06	2.012844624432275e-05	1.1013894935334609e-09
ENSG00000096063_35870416_35835304_370	SRPK1	6	-	1110	14	0	1	2.43%	7.400834174451353	0.0032294771334464936	0.006024333578509648	0.00010730535955833177
ENSG00000182220_40581066_40605755_350	ATP6AP2	X	+	1050	42	1	4	6.86%	23.471216953260004	9.706969571762432e-06	4.881320999582746e-05	3.8337854993427554e-09
ENSG00000099622_1270934_1274311_168	CIRBP	19	+	504	27	1	0	6.55%	31.434665562401793	0.002493493840886608	0.0014055202957965589	2.0830049893778164e-05
ENSG00000047579_15662869_15522975_351	DTNBP1	6	-	1053	18	6	0	2.28%	10.03043459541026	0.0750151653743942	0.005554612275049112	0.0024514621214718943
ENSG00000002549_17577559_17607589_488	LAP3	4	+	1464	38	4	3	3.69%	15.230639416391396	0.0008701861546363455	0.0005853750266995722	3.251076934972306e-06
ENSG00000099282_69495669_69506978_150	TSPAN15	10	+	450	12	0	0	4.00%	15.647477968840004	0.013007239811447113	0.013007239811447113	0.0008218654148058079
ENSG00000213923_38314157_38293287_416	CSNK1E	22	-	1248	27	1	0	1.20%	12.694768784816109	0.029237630782826433	0.021557223391537678	0.002819005644529471
ENSG00000108654_64504108_64499923_509	DDX5	17	-	1527	254	15	19	11.98%	97.60460226142831	1.581853372908017e-10	2.699384476567586e-10	4.910456554443996e-19
ENSG00000086189_62403341_62398492_126	DIMT1	5	-	378	7	0	0	3.97%	10.86630414502778	0.019216965118390855	0.019216965118390855	0.0017073955890589052
ENSG00000055332_37147776_37107273_500	EIF2AK2	2	-	1500	41	1	2	4.20%	16.038664918061002	3.483426394569797e-05	5.7030968798874024e-05	1.5049175604359762e-08

ENSG00000267261_42130502_42120326_161	AC099811.2	17	-	483	32	0	21	8.07%	38.875721661714294	0.0006311913977015671	0.17578995542174075	0.0016480472016401546
ENSG00000167645_38309608_38305352_248	YIF1B	19	-	744	66	0	0	3.63%	52.05310211408469	0.003684287313187465	0.003684287313187465	7.541963121699725e-05
ENSG00000182093_39390698_39396939_140	WRB	21	+	420	20	0	0	5.71%	27.94192494435715	0.005405141725979036	0.005405141725979036	0.00015637213408961292
ENSG00000152234_46098231_46084282_553	ATP5F1A	18	-	1659	312	7	19	13.38%	110.35291876758772	1.748164185774474e-13	8.752711903801398e-13	2.0214085795663137e-24
ENSG00000164211_111507433_111499886_205	STARD4	5	-	615	18	0	2	3.90%	17.174061185312198	0.005454749182134642	0.02071580916834944	0.0005936342691250666
ENSG00000138772_78604313_78610115_48	ANXA3	4	+	144	25	3	0	16.67%	101.87160135963543	0.032657852871826265	0.004873903550586949	0.0008714009653636729
ENSG00000075624_5529657_5527748_375	ACTB	7	-	1125	955	44	33	16.53%	498.11138200807346	1.8241793141731438e-10	4.564440690072044e-10	9.53223743448052e-19
ENSG00000175029_125003077_124989518_365	CTBP2	10	-	1095	10	0	0	1.64%	5.358725331794521	0.012798402692974279	0.012798402692974279	0.0007972436888943567
ENSG00000125166_58716156_58709243_155	GOT2	16	-	465	21	0	0	3.23%	26.499761076261297	0.02108409854857798	0.02108409854857798	0.002029891263157455
ENSG00000085449_223912255_223878671_318	WDFY1	2	-	954	13	0	0	2.20%	7.995959653888367	0.00797940233269714	0.00797940233269714	0.000327142761693177
ENSG00000076108_56615278_56598618_1748	BAZ2A	12	-	5244	27	2	8	0.74%	3.0211806719013166	0.0017936202733988364	0.025438491335356538	0.0002908813190580962
ENSG00000221914_26362710_26370413_226	PPP2R2A	8	+	678	19	0	0	3.98%	16.443699192918146	0.0033278027414746945	0.0033278027414746945	6.211806171561056e-05
ENSG00000106028_141739167_141750354_148	SSBP1	7	+	444	109	3	4	17.57%	144.05195089557097	1.3567009445878335e-05	3.4303114258667915e-06	3.929288911145101e-10
ENSG00000137210_10749202_10756518_103	TMEM14B	6	+	309	25	0	0	8.74%	47.4741443229369	0.0036321914711260127	0.0036321914711260127	7.340009712338726e-05
ENSG00000142534_49497931_49499635_79	RPS11	19	+	237	493	8	1	39.24%	1220.6023162402091	5.703516416371565e-07	5.112362712138789e-07	2.667508397423284e-12
ENSG00000140564_90875741_90881878_794	FURIN	15	+	2382	7	4	1	0.63%	1.7243757207474815	0.169362073879829	0.04178282491678763	0.02871445979022244
ENSG00000132031_20012631_19993111_444	MATN3	2	-	1332	9	0	0	1.35%	3.964732593456082	0.011772051206276392	0.011772051206276392	0.000681272511389508
ENSG00000108219_80489234_80517976_253	TSPAN14	10	+	759	93	4	3	7.91%	71.89799659595457	0.00018707853009467164	6.793373103529052e-05	9.256993693435907e-08
ENSG00000188603_28491759_28477516_341	CLN3	16	-	1023	24	2	0	2.93%	13.766109650005868	0.007558271381162128	0.0023354528714849923	0.00010028418779467875
ENSG00000104960_49851497_49860279_360	PTOV1	19	+	1080	32	5	0	4.17%	17.38608663204445	0.0035763217077886345	0.00028351878813536103	7.149954426385882e-06
ENSG00000141646_51054795_51078467_396	SMAD4	18	+	1188	13	0	0	1.52%	6.4209979038800515	0.012798402692974279	0.012798402692974279	0.0007972436888943567
ENSG00000141429_35683456_35709770_377	GALNT1	18	+	1131	24	0	0	2.39%	12.451573980509286	0.0035547763465261306	0.0035547763465261306	7.044676146010672e-05
ENSG00000129083_14498883_14457824_938	COPB1	11	-	2814	39	2	0	1.42%	8.132351289775588	0.00021852315413304935	5.3992704767724636e-05	8.731581146046835e-08
ENSG00000141526_82236009_82238976_465	SLC16A3	17	+	1395	72	7	1	5.59%	30.28544123001291	1.1849891463284232e-05	4.637421080669856e-06	4.5704910601567277e-10
ENSG00000169604_69013500_69172384_368	ANTXR1	2	+	1104	142	4	0	5.71%	75.47356900731252	0.0002819600622069734	2.4620977378132598e-05	5.521359011229967e-08
ENSG00000115084_113750514_113742677_145	SLC35F5	2	-	435	14	0	0	3.45%	18.88488720377242	0.02108409854857798	0.02108409854857798	0.002029891263157455
ENSG00000115446_98609916_98618304_207	UNC50	2	+	621	49	1	1	7.73%	46.299904617944456	0.00025057736413350534	0.00025057736413350534	4.290682187398661e-07
ENSG00000157483_59372500_59137380_1108	MYO1E	15	-	3324	37	0	3	1.53%	6.531551047462546	0.00012177496818858337	0.0005853750266995722	5.053467791992213e-07
ENSG00000161980_53586_47430_108	POLR3K	16	-	324	11	0	0	6.48%	19.921557599217596	0.0078820576498062	0.0078820576498062	0.00031963642907451164
ENSG00000168291_58431954_58429886_209	PDHB	3	-	627	23	0	0	3.83%	21.524640746610054	0.005706018193000826	0.005706018193000826	0.0001733096755673334
ENSG00000177239_137106191_137108591_259	MAN1B1	9	+	777	22	0	0	2.70%	16.61411753448263	0.00877616296920843	0.00877616296920843	0.00039161135667564577
ENSG00000159403_7086394_7080532_338	C1R	12	-	1014	14	0	0	2.07%	8.10150486552367	0.00837382380159347	0.00837382380159347	0.0003583838894600799
ENSG00000167491_19465346_19505474_633	GATAD2A	19	+	1899	10	2	0	1.11%	3.0899443066429706	0.049338281967443096	0.007593099278532274	0.0019605633087212975
ENSG00000198604_34874604_34753508_1556	BAZ1A	14	-	4668	9	3	1	0.51%	1.1313247246108615	0.07229263996867695	0.010460667668897016	0.003853881152483681
ENSG00000065150_97985562_98021822_993	IPO5	13	+	2979	90	2	10	3.32%	17.72750525170695	6.863358279914768e-07	3.084588439101575e-06	1.9079962804815285e-11
ENSG00000196924_154371164_154348849_2612	FLNA	X	-	7836	166	37	7	3.10%	12.43051944308691	1.2525976993412299e-08	2.5928160559868572e-14	9.924675692259457e-21
ENSG00000107537_13302007_13278301_321	PHYH	10	-	963	21	6	2	2.18%	12.795834787602807	0.07558292134845049	0.020779353648479937	0.007024251439427237
ENSG00000166197_102159553_102162269_418	NOLC1	10	+	1254	106	0	9	5.02%	49.6002591117536	2.3200298509694487e-05	0.0012787427712268279	2.67882882501554e-07
ENSG00000145860_159203617_159158670_663	RNF145	5	-	1989	34	2	1	2.71%	10.03043459541026	0.00041617781144845243	0.00011977551446593921	3.5184401231069343e-07
ENSG00000164754_116852016_116847500_164	RAD21	8	-	492	33	0	0	6.10%	39.35722354967379	0.0022543491824521134	0.0022543491824521134	2.9517890247939808e-05
ENSG00000120885_27610571_27608842_113	CLU	8	-	339	33	1	0	9.73%	57.12021824908408	0.001779142780631133	0.0016109979480538772	1.705970569913741e-05
ENSG00000120756_142678036_142712007_462	PLS1	3	+	1386	27	0	2	2.81%	11.430787477237015	0.000609140140830996	0.0030056054444520318	1.1698852710189299e-05
ENSG00000256500_103563002_103568018_719	AL139300.1	14	+	2157	63	6	10	3.62%	17.138232128597362	8.046650302611864e-05	0.00012982740717191115	7.561717902840828e-08
ENSG00000127990_94618900_94585465_278	SGCE	7	-	834	11	0	2	2.52%	7.739310146458635	0.007029302661868638	0.03522780890193623	0.0012822660609675817
ENSG00000182134_151783022_151774452_561	TDRKH	1	-	1683	9	0	0	0.89%	3.1378632290454553	0.020613416668581838	0.020613416668581838	0.001946208561389314
ENSG00000179010_6640863_6641246_127	MRFAP1	4	+	381	76	1	3	14.96%	117.04806354644097	5.6254241454829154e-05	0.0001733650811203729	7.181689729777536e-08
ENSG00000148229_113410293_113408811_147	POLE3	9	-	441	57	5	0	11.56%	75.84236770611226	0.0008512616127049016	0.0001297455242311012	7.871132705232902e-07
ENSG00000167632_140439100_139731061_912	TRAPPC9	8	-	2736	7	0	4	0.66%	1.501265704247259	0.009815328628645334	0.28185143082538655	0.019787191117097723
ENSG00000083312_72848394_72872694_128	TNPO1	5	+	384	7	0	0	3.91%	10.696518142761722	0.019216965118390855	0.019216965118390855	0.0017073955890589052
ENSG00000163159_151190121_151176644_375	VPS72	1	-	1125	13	1	0	1.87%	6.780573786497335	0.013794764262559246	0.00837382380159347	0.0005783443517979597
ENSG00000087088_48955571_48961636_124	BAX	19	+	372	79	1	4	13.71%	124.61197172765729	0.00013535602770218042	0.000705360111007344	6.739380643944618e-07
ENSG00000174547_66438754_66436007_192	MRPL11	11	-	576	38	4	0	7.29%	38.711208516661465	0.003908675642856669	0.0004467886182089563	1.1692354439766053e-05
ENSG00000182196_122981140_122982676_226	ARL6IP4	12	+	678	39	3	1	7.52%	33.75285623809514	0.0007987558521470434	0.00018702083254192264	1.0292491114519113e-06
ENSG00000164244_127523483_127548050_417	PRRC1	5	+	1251	17	1	1	2.40%	7.973834696351321	0.0036460193534901884	0.0036460193534901884	7.39336293426026e-05
ENSG00000155508_154870770_154875439_152	CNOT8	5	+	456	12	0	0	3.29%	15.44159010082895	0.02108409854857798	0.02108409854857798	0.002029891263157455
ENSG00000147526_38820520_38848023_391	TACC1	8	+	1173	7	3	0	1.28%	3.501673458500001	0.13974268412556323	0.016947426762344633	0.011764960919844317
ENSG00000066855_65670010_65709044_281	MTFR1	8	+	843	23	0	0	3.20%	16.009430306197512	0.0035291660242640502	0.0035291660242640502	6.948232509684344e-05
ENSG00000145860_159203617_159158670_663	RNF145	5	-	1989	34	2	1	2.71%	10.03043459541026	0.00041617781144845243	0.00011977551446593921	3.5184401231069343e-07
ENSG00000110651_2393896_2396866_242	CD81	11	+	726	171	3	0	14.88%	138.2086122247748	1.6371366340023164e-07	7.479694926966041e-08	1.1889718748468135e-13
ENSG00000035928_39327547_39288761_968	RFC1	4	-	2904	18	0	0	0.72%	3.6370687427572324	0.008574016250729465	0.008574016250729465	0.0003747459614100891
ENSG00000108010_130145263_130179392_287	GLRX3	10	+	861	69	0	3	6.97%	47.02421515025959	3.942240149922151e-05	0.00017763310340949173	5.2792693157387635e-08
ENSG00000167196_75914127_75933102_275	FBXO22	15	+	825	19	0	1	4.00%	13.513730973089094	0.0008884494972709903	0.0024579339080672683	1.3429802402146636e-05
ENSG00000149923_30076378_30085062_307	PPP4C	16	+	921	40	8	6	6.84%	25.484491805928343	0.000674573746487692	0.00042744270133535626	1.8796403195632223e-06
ENSG00000144224_135770661_135782887_259	UBXN4	2	+	777	17	1	1	3.47%	12.838181731191122	0.0064006895615519015	0.0064006895615519015	0.00021550699013712693
ENSG00000136877_127804381_127807766_195	FPGS	9	+	585	16	0	0	2.56%	16.048695352656413	0.02108409854857798	0.02108409854857798	0.002029891263157455
ENSG00000122705_36191057_36265473_260	CLTA	9	+	780	162	8	4	9.62%	121.86978033423465	3.6242609689812406e-05	2.064795566791557e-05	5.817250829843229e-09
ENSG00000065809_14530459_14521190_131	FAM107B	10	-	393	21	0	0	6.11%	31.35467913603436	0.005604866691947602	0.005604866691947602	0.00016752456572620382

ENSG00000119523_99221047_99217934_323	ALG2	9	-	969	12	0	0	1.55%	7.266630635684212	0.019679754444124863	0.019679754444124863	0.0017849636115922674
ENSG00000143811_225922380_225920455_199	PYCR2	1	-	597	6	0	0	3.02%	5.897290691773871	0.007152939217714824	0.007152939217714824	0.0002660027525696252
ENSG00000011422_43667671_43648890_261	PLAUR	19	-	783	34	4	0	7.28%	25.479609719375485	0.00011433018679532622	4.942578964878572e-05	4.189596943205669e-08
ENSG00000156976_186786559_186787886_132	EIF4A2	3	+	396	19	2	0	6.06%	28.153606193935612	0.015516259750072458	0.005160369442893307	0.00042094872630173483
ENSG00000140299_59682457_59678806_107	BNIP2	15	-	321	9	1	0	4.67%	16.451787584060753	0.03537493353818881	0.020613416668581838	0.003252526348827943
ENSG00000100528_54432178_54427814_80	CNIH1	14	-	240	111	4	2	26.25%	271.3859460220688	2.8765194065111864e-05	2.5341096079599938e-05	5.648408106896459e-09
ENSG00000165533_88824795_88877410_418	TTC8	14	+	1254	16	1	1	2.39%	7.48683156403828	0.0036460193534901884	0.0036460193534901884	7.39336293426026e-05
ENSG00000100796_91509647_91458759_820	PPP4R3A	14	-	2460	17	8	1	1.71%	4.054986668754269	0.06191469475189823	0.0005593438005584256	0.00034627387638462906
ENSG00000184432_139389550_139357863_906	COPB2	3	-	2718	51	1	2	2.98%	11.010228703240069	2.8839408258457105e-06	7.981914834483447e-06	1.953916494329706e-10
ENSG00000137177_17987463_17764110_1757	KIF13A	6	-	5271	8	1	0	0.34%	0.8905792810950486	0.028889785561798602	0.009815328628645334	0.0013877953586383081
ENSG00000183049_12349819_12825725_357	CAMK1D	10	+	1071	13	0	0	1.40%	7.122451456404764	0.02108409854857798	0.02108409854857798	0.002029891263157455
ENSG00000167397_31091310_31091134_58	VKORC1	16	-	174	56	0	12	15.52%	188.8488720377242	0.0035547763465261306	0.06638642199807827	0.001507226391593381
ENSG00000130175_11436148_11449412_515	PRKCSH	19	+	1545	153	8	10	10.49%	58.10835265127477	1.708509627995029e-09	1.2071460570932025e-09	2.26005863529839e-17
ENSG00000117410_43975041_43976383_93	ATP6V0B	1	+	279	50	0	8	11.83%	105.15778204865593	0.0016494676794180302	0.00557880781788349	5.392524653205357e-05
ENSG00000215301_41343231_41347719_476	DDX3X	X	+	1428	83	1	0	7.56%	34.10558485855358	6.958679763556736e-08	4.081835640353621e-08	2.8150375102779016e-14
ENSG00000118564_15655236_15612252_653	FBXL5	4	-	1959	12	0	0	0.92%	3.594367067880552	0.012798402692974279	0.012798402692974279	0.0007972436888943567
ENSG00000123374_55967009_55971625_298	CDK2	12	+	894	29	2	0	3.69%	19.034264307733224	0.0062082853022021895	0.0014915192737735051	5.496436396844801e-05
ENSG00000102081_147912180_147948844_592	FMR1	X	+	1776	7	0	3	0.84%	2.312760679516048	0.019216965118390855	0.1700871372700561	0.016246064094152867
ENSG00000104904_2269599_2271445_68	OAZ1	19	+	204	25	6	0	11.76%	71.90936566562502	0.039104403811496304	0.0053557511377634245	0.0011447568947324828
ENSG00000104889_12810092_12813466_155	RNASEH2A	19	+	465	19	2	1	7.10%	23.975974307093555	0.0037251911965256856	0.0022879379939880367	4.865470762826102e-05
ENSG00000112305_70668024_70860234_436	SMAP1	6	+	1308	16	1	0	1.15%	7.177742187541286	0.028889785561798623	0.02108409854857798	0.002731532589007073
ENSG00000112305_70668024_70860334_467	SMAP1	6	+	1401	16	1	0	1.07%	6.701275361387582	0.028889785561798623	0.02108409854857798	0.002731532589007073
ENSG00000272822_48941032_48903660_189	AC073610.2	12	-	567	44	0	4	6.88%	45.53498879821165	0.0006838816678496783	0.0032656549244236455	1.4137749239483728e-05
ENSG00000100902_35308980_35317306_167	PSMA6	14	+	501	49	1	2	5.99%	57.38970213122457	0.0025061435727443374	0.0024742386374296595	3.570411499116179e-05
ENSG00000163636_64022521_64010668_340	PSMD6	3	-	1020	72	5	0	5.88%	41.41979462340001	0.000602543381100086	3.45592214554821e-05	1.670045363763243e-07
ENSG00000184903_111521447_111483647_106	IMMP2L	7	-	318	13	0	0	6.60%	23.9878789616651	0.008175610787902942	0.008175610787902942	0.00034251843268961324
ENSG00000099624_1244171_1244437_45	ATP5F1D	19	+	135	115	7	5	22.22%	499.8499906712779	0.0028151052444788517	0.013900915060036254	0.00022123606969677142
ENSG00000143418_150969090_150966148_380	CERS2	1	-	1140	122	13	1	11.84%	62.79579974337107	9.274309105767741e-08	2.3768576749846654e-09	2.5004010241501566e-15
ENSG00000168487_22176562_22211728_832	BMP1	8	+	2496	26	0	0	1.08%	6.112296081578126	0.0034782380676658805	0.0034782380676658805	6.758309314600117e-05
ENSG00000136261_16689838_16706088_225	BZW2	7	+	675	10	0	0	3.56%	8.69304331602225	0.00414960799764038	0.00414960799764038	9.45964903272165e-05
ENSG00000144959_172635990_172633475_230	NCEH1	3	-	690	15	0	1	3.48%	12.756096170250004	0.005160369442893307	0.007930666369886513	0.00021637657341139557
ENSG00000185236_8390417_8403558_218	RAB11B	19	+	654	50	2	3	7.80%	44.860888672133036	0.00015002426089994736	0.0005886262585681982	6.158627151589229e-07
ENSG00000259040_8026413_7883144_360	BLOC1S5-TXNDC5	6	-	1080	157	3	8	8.61%	85.30048753846808	5.907994537635511e-07	4.0272195077250355e-06	2.1703803411040562e-11
ENSG00000164305_184635393_184629272_251	CASP3	4	-	753	9	0	1	1.99%	7.013311838623507	0.020613416668581838	0.03537493353818881	0.003252526348827943
ENSG00000136986_123023718_123019191_89	DERL1	8	-	267	45	3	0	14.61%	98.89557705025283	0.001581381895184553	0.0007052562366071395	6.981921433965871e-06
ENSG00000163166_127526208_127481044_819	IWS1	2	-	2457	20	2	4	1.10%	4.776397426385837	0.013141913862703638	0.032506134594470416	0.002014254730681511
ENSG00000163170_74145285_74135522_77	BOLA3	2	-	231	40	0	7	7.79%	101.60699979766235	0.013007239811447113	0.1754079647031212	0.012749674501614912
ENSG00000102226_47233044_47247930_920	USP11	X	+	2760	25	1	0	0.98%	5.315040070937501	0.006112543793728684	0.0033030736763512156	0.00011113261345270447
ENSG00000175198_100089196_100530166_656	PCCA	13	+	1968	8	0	0	0.91%	2.3852862757378053	0.011570465654371866	0.011570465654371866	0.0006594843062896773
ENSG00000145247_48904549_48885531_99	OCIAD2	4	-	297	12	0	7	6.06%	23.708299952787883	0.011772051206276392	0.3212384956040438	0.026834343646100805
ENSG00000106052_27769694_27828829_590	TAX1BP1	7	+	1770	32	8	2	2.54%	10.608459639891528	0.003223480975443246	0.0006589470154116208	1.3492589463426083e-05
ENSG00000114209_167720136_167687230_179	PDCD10	3	-	537	18	0	0	3.91%	19.66861755859777	0.00847367416878415	0.00847367416878415	0.0003665024264111182
ENSG00000149636_36771458_36752788_249	DSN1	20	-	747	20	0	0	3.21%	15.710319245823296	0.005604866691947602	0.005604866691947602	0.00016752456572620382
ENSG00000185630_164559844_164846676_332	PBX1	1	+	996	24	3	0	1.81%	14.139287321240968	0.03031622373531018	0.013428347753762199	0.0019146142599917133
ENSG00000163382_156592168_156592688_94	NAXE	1	+	282	10	0	0	5.32%	20.807816447925536	0.020613416668581838	0.020613416668581838	0.001946208561389314
ENSG00000164346_74769221_74776651_123	NSA2	5	+	369	20	0	0	4.88%	31.803817009837406	0.013217226988443315	0.013217226988443315	0.0008469697265466234
ENSG00000164347_74751461_74721655_667	GFM2	5	-	2001	31	0	5	1.65%	9.090551293741381	0.0015208721599504675	0.013147104497242925	0.00012290835639369062
ENSG00000160948_144423903_144423805_32	VPS28	8	-	96	16	0	0	18.75%	97.79673730525002	0.013007239811447113	0.013007239811447113	0.0008218654148058079
ENSG00000160948_144425732_144423887_185	VPS28	8	-	555	52	4	0	9.19%	54.977625295924334	0.0008031626276856301	0.00013400452932326877	7.632532354872831e-07
ENSG00000172409_57659597_57661436_385	CLP1	11	+	1155	15	2	0	1.82%	7.620524984824677	0.017818254085047065	0.00837382380159347	0.0007434988581310937
ENSG00000166136_100529851_100526951_111	NDUFB8	10	-	333	64	1	5	15.32%	112.77461599163966	0.0001654009931304681	0.0006983206845962714	8.017136639663946e-07
ENSG00000125991_35548618_35557485_145	ERGIC3	20	+	435	21	0	1	6.21%	28.327330805658626	0.002967235347054297	0.01007916461776185	0.000166354917991791
ENSG00000172269_119100804_119096998_301	DPAGT1	11	-	903	45	0	0	4.98%	29.24154936037376	0.00030026635541046905	0.00030026635541046905	6.089739818410118e-07
ENSG00000104738_47961668_47976778_789	MCM4	8	+	2367	57	4	3	4.06%	14.130327063115972	2.9324177990319606e-06	8.856783463782354e-07	2.312536887112216e-11
ENSG00000163956_3532412_3516630_279	LRPAP1	4	-	837	129	1	3	6.81%	90.4356925618441	6.108831601487675e-05	0.0001508558279104481	6.752219995609528e-08
ENSG00000060339_68757372_68791266_512	CCAR1	10	+	1536	18	1	1	1.76%	6.876333091775392	0.005642787686764809	0.005642787686764809	0.0001696825972573206
ENSG00000060339_68722505_68757375_568	CCAR1	10	+	1704	13	2	1	1.23%	4.476611214676938	0.0319825310473083	0.015191410988288745	0.002250081498979975
ENSG00000188186_100153791_100153964_57	LAMTOR4	7	+	171	14	0	0	8.77%	48.04050253591229	0.02108409854857798	0.02108409854857798	0.002029891263157455
ENSG00000188186_100148973_100153729_137	LAMTOR4	7	+	411	29	1	1	5.11%	41.40299827521534	0.00877616296920843	0.012088529595134127	0.0005313814658942758
ENSG00000064607_19031002_18994993_1017	SUGP2	19	-	3051	18	0	0	0.79%	3.461831409035399	0.005160369442893307	0.005160369442893307	0.00014319601891785326
ENSG00000065057_2044728_2039924_163	NTHL1	16	-	489	10	0	0	4.29%	11.999599669355831	0.005706018193000826	0.005706018193000826	0.0001733096755673334
ENSG00000127463_19251509_19219303_992	EMC1	1	-	2976	54	3	1	2.52%	10.647225432426414	3.3948671561060086e-05	6.679592927180221e-06	1.8663229281686025e-09
ENSG00000085662_134459062_134442728_316	AKR1B1	7	-	948	1511	68	25	21.84%	935.258671317929	2.6489841314029303e-12	1.919163314244164e-12	6.426287011277166e-23
ENSG00000177105_3828138_3827563_191	RHOG	11	-	573	27	0	1	5.24%	27.649339342845558	0.0023628488670607213	0.0035004709947243163	4.7187726630981656e-05
ENSG00000170917_122922536_122892828_304	NUDT6	4	-	912	8	0	1	1.64%	5.147196700276317	0.019679754444124863	0.04223451612662591	0.003715148010809822
ENSG00000012660_53295699_53269127_299	ELOVL5	6	-	897	56	6	1	7.02%	36.632891565846165	0.0004571839711376916	2.736941844189311e-05	1.0112062350323723e-07
ENSG00000136068_58008565_58170762_2578	FLNB	3	+	7734	126	16	17	2.09%	9.55965003914779	7.410892035135557e-08	8.76675846503856e-08	6.342385039057957e-14

ENSG00000005483_105073671_105113333_1675	KMT2E	7	+	5025	11	0	0	0.36%	1.284494460128657	0.012798402692974279	0.012798402692974279	0.0007972436888943567
ENSG00000083896_68337877_68314099_658	YTHDC1	4	−	1974	45	0	6	2.58%	13.376453430809274	0.00012922015363027575	0.0021027232080245203	2.0371126385249337e-06
ENSG00000079805_10775852_10831047_692	DNM2	19	+	2076	19	1	0	1.01%	5.370341065895232	0.011825808327677989	0.00847367416878415	0.0005036791746826746
ENSG00000132603_69340204_69341652_129	NIP7	16	+	387	26	2	0	9.30%	39.42194061917055	0.0035416441995153296	0.0009829516685359422	2.130032403102833e-05
ENSG00000114867_184328740_184334908_245	EIF4G1	3	+	735	41	1	0	4.90%	32.73196922053266	0.0010658336519988904	0.0010573555742419644	6.9610995220593235e-06
ENSG00000119912_92514944_92480826_356	IDE	10	−	1068	11	0	0	1.97%	6.043618597515451	0.006937202941512724	0.006937202941512724	0.00025101522741487095
ENSG00000124789_17675687_17616097_1336	NUP153	6	−	4008	19	2	1	0.75%	2.7816437257481295	0.01044836432889887	0.00397428314897603	0.00022393280292430794
ENSG00000049860_74720698_74721175_35	HEXB	5	+	105	23	2	0	14.29%	128.53285474404288	0.055917760918677194	0.02108409854857798	0.00521801263590997
ENSG00000127184_86618056_86619469_63	COX7C	5	+	189	92	1	1	12.70%	285.6285660978731	0.00575691403842271	0.007400806090490468	0.0002240461484226741
ENSG00000076928_41894238_41906786_687	ARHGEF1	19	+	2061	16	1	3	1.46%	4.555306541147017	0.0036460193534901884	0.021084098548578	0.0004279540139786225
ENSG00000109736_2932440_2930838_254	MFSD10	4	−	762	21	3	1	2.36%	16.171114042600397	0.051745348319195604	0.020525443280229434	0.004716982285816912
ENSG00000067082_3784999_3779539_278	KLF6	10	−	834	16	0	4	3.24%	11.257178394848923	0.002967235347054297	0.04248171122805103	0.0007792293048803088
ENSG00000088833_1466824_1443749_339	NSFL1C	20	−	1017	33	0	2	3.83%	19.040072749694694	0.0005983074123552221	0.003031355522137688	1.1613785618560066e-05
ENSG00000196139_5094445_5097598_138	AKR1C3	10	+	414	174	16	4	15.22%	246.61785929150005	9.576594468149801e-05	4.472969316820264e-05	3.19419420553714e-08
ENSG00000008282_106112208_106091805_241	SYPL1	7	−	723	245	12	2	13.69%	198.83983933432577	2.317110709525402e-06	3.3841173188800266e-07	7.297137615885193e-12
ENSG00000062716_59735462_59839911_172	VMP1	17	+	516	8	1	3	4.07%	9.097370912116281	0.017404239405933563	0.10587257754383972	0.008764339474420521
ENSG00000100650_69768157_69769561_138	SRSF5	14	+	414	98	10	5	14.49%	138.89971385383336	0.00020622244759692734	0.0002165592947110879	3.0848500546799e-07
ENSG00000003056_8943899_8943401_77	M6PR	12	−	231	22	1	0	11.69%	55.8838498887143	0.006112543793728684	0.0033030736763512156	0.00011113261345270447
ENSG00000136718_130345474_130346468_193	IMP4	2	+	579	22	1	0	3.63%	22.29562923021244	0.012000726946169103	0.008674846900444944	0.0005220387231428462
ENSG00000138758_77005630_77034512_372	SEPT11	4	+	1116	16	0	0	2.15%	8.412622563892475	0.005405141725979036	0.005405141725979036	0.00015637213408961292
ENSG00000078747_34507813_34540624_127	ITCH	20	+	381	80	2	4	8.66%	123.20848794362207	0.0018115419044284426	0.003777991055085985	3.972844003097169e-05
ENSG00000168056_65557917_65539080_1242	LTBP3	11	−	3726	40	15	2	1.69%	6.299306750740742	0.007241026036168108	5.292302315479159e-05	3.906733611023767e-06
ENSG00000105552_48811007_48795426_392	BCAT2	19	−	1176	69	0	2	4.59%	34.428443235011486	8.83028806972547e-05	0.00024781325086388353	1.570284568037866e-07
ENSG00000163110_94575691_94664067_474	PDLIM5	4	+	1422	91	2	12	7.38%	37.55064596952638	8.976557121876814e-08	1.3999896692044456e-06	1.2488650112397084e-12
ENSG00000064419_129018073_128957255_889	TNPO3	7	−	2667	28	0	0	1.57%	6.160424397181104	0.0003981150787954058	0.0003981150787954058	1.0507179780062227e-06
ENSG00000107745_72475261_72368195_219	MICU1	10	−	657	13	1	0	3.65%	11.610571552221463	0.00912793029372963	0.005160369442893307	0.00024819240235076056
ENSG00000182473_76101342_76083648_582	EXOC7	17	−	1746	9	0	0	0.86%	3.024641359956186	0.019679754444124863	0.019679754444124863	0.0017849636115922674
ENSG00000124193_43458034_43463350_276	SRSF6	20	+	828	44	2	9	5.43%	31.181568416166673	0.00025054484926030667	0.002614349599501924	4.592569200215246e-06
ENSG00000135506_57694162_57694771_75	OS9	12	+	225	22	0	0	8.00%	57.37408588574668	0.013007239811447113	0.013007239811447113	0.0008218654148058079
ENSG00000145912_178153817_178149713_153	NHP2	5	−	459	34	3	0	7.19%	43.46521658011112	0.0021824774412010223	0.001565547007472977	2.0232669183499854e-05
ENSG00000119977_95693899_95683393_443	TCTN3	10	−	1329	46	0	0	4.29%	20.309931900864566	5.5796325045909546e-05	5.5796325045909546e-05	2.322710366798558e-08
ENSG00000072274_196065548_196062562_131	TFRC	3	−	393	19	1	1	4.58%	28.368519218316802	0.016503128830616266	0.016503128830616266	0.0012844157635113584
ENSG00000279605_143580648_143580761_37	AC067930.5	8	+	111	23	0	2	16.22%	121.58513286598651	0.013007239811447113	0.05258119297243839	0.00326646906127711
ENSG00000002586_2691361_2733364_160	CD99	X	+	480	52	6	1	8.75%	63.56787924841252	0.001368321372009316	0.00044945639405704485	3.979749443618499e-06
ENSG00000132466_73177491_73076231_2458	ANKRD17	4	−	7374	9	1	3	0.33%	0.7161681332361678	0.010460667668897016	0.07229263996867695	0.003853881152483681
ENSG00000183291_86914111_86880690_95	SELENOF	1	−	285	222	2	3	18.95%	457.07106698453697	9.617268880422018e-05	8.931821586475278e-05	6.23200399238289e-08
ENSG00000153936_87084230_87104696_223	HS2ST1	1	+	669	10	0	0	2.24%	8.771007830067267	0.019679754444124863	0.019679754444124863	0.0017849636115922674
ENSG00000011422_43667671_43648890_310	PLAUR	19	−	930	38	4	0	6.77%	23.975974307093555	5.050506164817064e-05	2.2146792118947398e-05	8.648658456644847e-09
ENSG00000173821_80294947_80393498_4641	AC124319.1	17	+	13923	52	18	13	0.67%	2.1915235250476197	0.0036793750888163366	0.00024128078536283745	6.4104439430515e-06
ENSG00000185515_155071528_155120150_293	BRCC3	X	+	879	7	0	0	2.05%	4.672881645984643	0.009815328628645334	0.009815328628645334	0.0004837142251238724
ENSG00000172500_65888418_65885797_178	FIBP	11	−	534	12	1	0	3.93%	13.186076940033711	0.015516259750072458	0.0078820576498062	0.0006142571802709984
ENSG00000157450_59031054_59094900_917	RNF111	15	+	2751	11	0	0	0.98%	2.346268506778081	0.002478942540635376	0.002478942540635376	3.5397643206479886e-05
ENSG00000110330_102350683_102378183_342	BIRC2	11	+	1026	12	0	0	1.75%	6.862928933701756	0.011772051206276392	0.011772051206276392	0.000681272511389508
ENSG00000254986_66487301_66509251_560	DPP3	11	+	1680	10	2	0	0.89%	3.492740618044644	0.08026476757196149	0.019679754444124863	0.007158563601160521
ENSG00000007202_28642954_28615064_2092	KIAA0100	17	−	6276	11	0	2	0.38%	1.0284551724261475	0.004687384229717438	0.033944577430914495	0.0008796663632127183
ENSG00000090520_186570898_186585408_358	DNAJB11	3	+	1074	87	8	3	6.42%	47.532492433277945	0.00021126911468117307	4.658806620064333e-05	7.355680469454738e-08
ENSG00000010244_32350286_32373409_493	ZNF207	17	+	1479	58	4	3	3.04%	23.010997013000004	0.0016687198628102912	0.0007650509232230277	7.944487524641043e-06
ENSG00000167632_140451355_139731061_1142	TRAPPC9	8	−	3426	9	0	4	0.61%	1.541454703585377	0.006937202941512724	0.18065521426308934	0.008531843872917413
ENSG00000121064_56981156_57006235_402	SCPEP1	17	+	1206	34	0	2	3.23%	16.54273168347513	0.0006791898698869142	0.0016207143154899155	6.908867689334616e-06
ENSG00000189067_11553630_11549637_68	LITAF	16	−	204	23	1	0	13.24%	66.15661641237502	0.0035547763465261306	0.0035547763465261306	7.044676146010672e-05
ENSG00000155463_22767037_22771558_419	OXA1L	14	+	1257	84	12	3	6.68%	39.21205696248688	1.5987408189021976e-05	5.82419431868612e-06	7.667219592922941e-10
ENSG00000104687_30727706_30679520_426	GSR	8	−	1278	47	1	5	5.40%	21.57956175280165	2.315767526637638e-05	0.00016008322696248542	2.9001876369534095e-08
ENSG00000175390_7992114_7996022_202	EIF3F	11	+	606	30	0	10	3.96%	29.048535833242582	0.0053557511377634245	0.162589740049164	0.006205844656775128
ENSG00000145191_184135386_184142809_530	EIF2B5	3	+	1590	15	1	0	1.13%	5.535664375768869	0.028889785561798623	0.013428347753762199	0.0018252172022209389
ENSG00000028116_58048901_58154770_374	VRK2	2	+	1122	14	2	1	1.87%	7.321680867772729	0.03744183116399598	0.013599199788808391	0.002391925567846126
ENSG00000112245_63576890_63580174_170	PTP4A1	6	+	510	33	0	1	7.65%	37.96814507145001	0.0006424109906750971	0.0006698694257094773	2.7552909673861137e-06
ENSG00000112245_63532525_63576531_33	PTP4A1	6	+	99	82	1	2	30.30%	486.0201490321516	0.0022543491824521134	0.006459879053210272	8.288720459767728e-05
ENSG00000136156_48253859_48261224_210	ITM2B	13	+	630	169	12	8	17.62%	157.40617718654528	4.000132270571673e-06	2.4373769502837046e-06	8.35359386367274e-11
ENSG00000121152_96364490_96373351_178	NCAPH	2	+	534	8	0	2	2.81%	8.790717960022475	0.016947426762344633	0.11777945996203976	0.009674391494749069
ENSG00000002834_38870190_38918778_261	LASP1	17	+	783	114	0	8	11.11%	85.43163258849427	9.803275463287148e-07	1.5720720116184197e-05	1.417551573230477e-10
ENSG00000118600_63779961_63809092_443	RXYLT1	12	+	1329	22	0	2	2.48%	9.713445691717835	0.00151350169064872	0.005530543042558543	4.947549134344851e-05
ENSG00000163607_112991000_113000919_307	GTPBP8	3	+	921	7	0	0	1.95%	4.45978606603746	0.009815328628645334	0.009815328628645334	0.0004837142251238724
ENSG00000100462_22922554_22920904_109	PRMT5	14	−	327	8	5	0	5.50%	14.355484375082572	0.1097457788509939	0.011570465654371866	0.006677619190092414
ENSG00000091140_107891251_107919259_509	DLD	7	+	1527	69	11	1	5.50%	26.51463604739588	0.00017041041997235105	2.0264452402002208e-06	3.4828789752522884e-09
ENSG00000100473_30878908_30889791_438	COCH	14	+	1314	18	0	0	2.74%	8.038087997691783	0.0007729603088974596	0.0007729603088974596	3.7798209788587765e-06
ENSG00000149925_30067255_30067440_61	ALDOA	16	+	183	47	4	8	18.03%	150.70316896218856	0.004488024406907798	0.008897470729103343	0.00021303388065318946
ENSG00000184588_66355569_66372678_473	PDE4B	1	+	1419	32	1	0	2.54%	13.232539508532772	0.0013767418916647864	0.0010405263639310788	8.780312937977671e-06

ENSG00000177239_137107370_137108591_137	MAN1B1	9	+	411	13	0	0	3.65%	18.559964744062047	0.020613416668581838	0.020613416668581838	0.001946208561389314
ENSG00000144824_111884390_111974563_1106	PHLDB2	3	+	3318	16	7	1	0.63%	2.829562019681737	0.10514110692308698	0.007258496433688866	0.004472182841009268
ENSG00000213923_38314157_38296561_336	CSNK1E	22	−	1008	27	1	0	1.49%	15.717332781200897	0.029237630782826433	0.021557223391537678	0.002819005644529471
ENSG00000143374_150487451_150507064_588	TARS2	1	+	1764	6	0	0	0.85%	1.9958517817397963	0.016947426762344633	0.016947426762344633	0.0013498980316300959
ENSG00000243989_51986441_51989075_254	ACY1	3	+	762	7	1	0	1.97%	5.390371347533466	0.0477903522728147	0.016947426762344633	0.0036975118224100542
ENSG00000153048_8859328_8855164_147	CARHSP1	16	−	441	23	0	4	4.76%	30.603060653343544	0.00877616296920843	0.04584514077471458	0.0020400732494421717
ENSG00000172172_120419914_120396104_68	MRPL13	8	−	204	12	0	0	8.82%	34.516495519500005	0.013007239811447113	0.013007239811447113	0.0008218654148058079
ENSG00000144354_173358772_173367664_423	CDCA7	2	+	1269	22	0	0	1.42%	10.172710263430263	0.013640585738808999	0.013640585738808999	0.00089863692579514
ENSG00000139350_96917657_96952053_571	NEDD1	12	+	1713	12	0	1	1.05%	4.110545876227672	0.013007239811447113	0.023878473930440184	0.0014709138084317821
ENSG00000135018_83707571_83661787_525	UBQLN1	9	−	1575	52	6	8	4.57%	19.373067961420958	0.0002054838780814908	0.00026048084003786414	3.679497327913369e-07
ENSG00000122566_26196593_26195800_75	HNRNPA2B1	7	−	225	154	15	0	36.00%	401.61860120022675	2.2219043411585865e-05	2.702209111073101e-06	5.190938440840651e-10
ENSG00000078269_157981962_158096364_1496	SYNJ2	6	+	4488	16	3	4	0.53%	2.091908819363637	0.028023386503852714	0.04727912037993979	0.005651162519258192
ENSG00000137207_43516807_43512167_356	YIPF3	6	−	1068	62	5	2	5.62%	34.064032095087086	0.00034507815500485066	6.800413984331272e-05	1.7219780116013005e-07
ENSG00000106355_32490365_32488621_57	LSM5	7	−	171	42	3	1	21.05%	144.12150760773687	0.0017534708537449623	0.0012450833151434304	1.3169840305271018e-05
ENSG00000112514_33417668_33417247_83	CUTA	6	−	249	116	1	6	15.66%	273.35955487732537	0.001006436396730239	0.005421488241340597	3.3699460502274616e-05
ENSG00000101343_20047861_20036195_512	CRNKL1	20	−	1536	22	3	1	2.54%	8.404407112169924	0.004909317959302256	0.001015856855223422	3.0659672620044405e-05
ENSG00000196139_5094445_5099647_190	AKR1C3	10	+	570	92	3	2	6.84%	94.70841928508423	0.0017309905491085032	0.001401683495487929	1.4551348458882258e-05
ENSG00000115963_150487372_150469987_229	RND3	2	−	687	55	5	1	7.42%	46.97659870557861	0.0003802473661612438	0.00014852201280131984	3.928342352568908e-07
ENSG00000118965_19990015_19913558_1170	WDR35	2	−	3510	27	1	0	0.94%	4.513695567934616	0.0016902520194187448	0.0015061527253727427	1.5227828847849133e-05
ENSG00000167779_53100855_53102167_81	IGFBP6	12	+	243	42	0	0	11.11%	101.41883868692595	0.003710480643933249	0.003710480643933249	7.644477796348072e-05
ENSG00000120948_11016999_11023183_153	TARDBP	1	+	459	12	0	0	3.92%	15.340664675333336	0.013007239811447113	0.013007239811447113	0.0008218654148058079
ENSG00000102804_44434862_44434626_78	TSC22D1	13	−	234	19	3	0	8.97%	47.644564328198726	0.00797940233269714	0.00797940233269714	0.000327142761693177
ENSG00000110108_60919751_60922165_224	TMEM109	11	+	672	60	0	0	8.04%	52.39110927066965	8.51685422527664e-05	8.51685422527664e-05	5.287317816903535e-08
ENSG00000160199_43007491_43030101_419	PKNOX1	21	+	1257	8	0	0	1.19%	3.7344816154749414	0.019216965118390855	0.019216965118390855	0.0017073955890589052
ENSG00000125844_17636630_17614182_649	RRBP1	20	−	1947	22	2	0	1.85%	6.630287274932205	0.005380042077471066	0.0007764301431553706	2.6597637939514903e-05
ENSG00000086189_62403341_62373015_289	DIMT1	5	−	867	40	2	1	5.19%	27.07176119176471	0.0006660403445293448	0.00042978108473948845	1.8585197475478207e-06
ENSG00000126261_34460491_34469221_166	UBA2	19	+	498	13	0	1	4.22%	15.317561264677714	0.007029302661868638	0.016854695534768073	0.0006017499134389055
ENSG00000136603_170360425_170392417_653	SKIL	3	+	1959	31	1	2	1.68%	9.285448258691426	0.002353241622354279	0.005609982661338475	7.481743476331415e-05
ENSG00000131165_89657588_89647038_181	CHMP1A	16	−	543	12	1	0	3.31%	12.967523178596688	0.02015986069794795	0.013217226988443315	0.0012659333861537747
ENSG00000095752_55370310_55366007_199	IL11	19	−	597	14	2	0	4.02%	13.760344947472365	0.01611115222852496	0.005257622967929454	0.000444576531620782
ENSG00000168610_42345636_42316827_624	STAT3	17	−	1872	31	1	0	2.24%	9.716983514303688	0.0005938231711225875	0.00044280702707823286	1.7158342196872716e-06
ENSG00000050130_59495136_59504072_188	JKAMP	14	+	564	61	1	2	11.17%	63.463840166172886	3.16311290393987e-05	6.695136793639445e-05	1.6084855235608698e-08
ENSG00000136436_48848100_48863005_374	CALCOCO2	17	+	1122	18	0	0	2.67%	9.413589687136366	0.002046028879997488	0.002046028879997488	2.4518070358754455e-05
ENSG00000100906_35403755_35402013_227	NFKBIA	14	−	681	18	0	2	4.41%	15.509614726823791	0.002046028879997488	0.007459848152910933	8.7920393864264e-05
ENSG00000177485_120253416_120255434_672	ZBTB33	X	+	2016	11	0	0	0.89%	3.2016788998742567	0.012798402692974279	0.012798402692974279	0.0007972436888943567
ENSG00000204842_111599514_111452812_1271	ATXN2	12	−	3813	23	0	0	0.63%	3.5394570543206143	0.005604866691497602	0.005604866691497602	0.00016752456572620382
ENSG00000127946_75582124_75538172_873	HIP1	7	−	2619	10	2	0	0.69%	2.240475081449027	0.061356297364084836	0.012798402692974279	0.0037940651817215715
ENSG00000135624_73234382_73244004_82	CCT7	2	+	246	19	2	0	8.54%	45.320439239018306	0.029020025081745005	0.00797940233269714	0.0011662184072477957
ENSG00000197930_52695481_52657949_257	ERO1A	14	−	771	48	1	0	6.61%	36.531076970054485	0.0012644225059219485	0.0010658336519988904	8.271599490222998e-06
ENSG00000166136_100529851_100523837_186	NDUFB8	10	−	558	114	1	1	16.13%	119.87987153546777	9.119998148167766e-07	9.119998148167766e-07	7.462085398811575e-12
ENSG00000102893_47461351_47699366_1093	PHKB	16	+	3279	22	0	2	1.19%	3.936922636258921	0.0006069633386880125	0.0015384793293873654	5.90945903854469e-06
ENSG00000240972_23894481_23895005_111	MIF	22	+	333	457	5	6	27.03%	805.2812423153019	7.950867661350179e-07	7.971187629051549e-07	5.715090834524945e-12
ENSG00000110906_109469554_109451595_231	KCTD10	12	−	693	8	0	0	2.60%	6.7737999865108245	0.011570465654371866	0.011570465654371866	0.0006594843062896773
ENSG00000154582_73959768_73946630_112	ELOC	8	−	336	8	4	1	4.46%	13.970962472178575	0.2618042821861254	0.04223451612662591	0.04736319147700863
ENSG00000276043_4910907_4960803_786	UHRF1	19	+	2358	26	0	2	1.27%	6.470013155054708	0.0020082573300163734	0.009197853169380737	0.00010725224857862305
ENSG00000106976_128203486_128253122_846	DNM1	9	+	2538	54	2	7	2.36%	12.484689868755321	5.0604056647534704e-05	0.0009696113333780397	3.894133597030637e-07
ENSG00000179115_12933696_12922748_477	FARSA	19	−	1431	46	0	0	3.35%	18.862263798916146	0.0002028510352551382	0.0002028510352551382	2.849477956609537e-07
ENSG00000123562_103677027_103676161_288	MORF4L2	X	−	864	67	3	4	10.76%	45.50264860730383	2.6778889786849916e-06	6.382017302541668e-06	1.4552401796346898e-10
ENSG00000134686_33334242_33324865_323	PHC2	1	−	969	18	3	6	2.17%	10.899945953526318	0.013171727738572194	0.05346792862759031	0.0033590577316883704
ENSG00000084234_130140481_130143448_111	APLP2	11	+	333	16	0	0	8.11%	28.193653997909916	0.0033278027414746945	0.0033278027414746945	6.211806171561056e-05
ENSG00000185475_62787513_62789901_117	TMEM179B	11	+	351	30	0	1	10.26%	50.15217297705129	0.001032175130546352	0.0012335676370081575	7.832627816756785e-06
ENSG00000142186_65525154_65538540_787	SCYL1	11	+	2361	6	1	1	0.76%	1.4911827797496826	0.02939086067767943	0.02939086067767943	0.003763157583228937
ENSG00000185686_22556832_22548067_509	PRAME	22	−	1527	45	1	1	3.54%	17.292153943953835	0.00011105827337505001	0.00011105827337505001	8.855184710094631e-08
ENSG00000068024_239176542_239053097_969	HDAC4	2	−	2907	7	0	0	0.52%	1.4129559569385968	0.019216965118390855	0.019216965118390855	0.0017073955890589052
ENSG00000121578_119230099_119212549_344	B4GALT4	3	−	1032	23	0	0	2.33%	13.077470686167155	0.005454749182134642	0.005454749182134642	0.00015910859015753364
ENSG00000140105_100369185_100334285_471	WARS	14	−	1413	60	6	3	4.78%	24.916366192420387	0.00017457506911736429	5.349366132029235e-05	6.898497935895578e-08
ENSG00000109390_139295798_139292550_76	NDUFC1	4	−	228	19	2	0	7.89%	48.89836865262501	0.016822681477512567	0.013428347753762199	0.0010798218650012886
ENSG00000141959_44306713_44326862_741	PFKL	21	+	2223	26	2	0	1.75%	6.862928933701756	0.0032750890440651926	0.0004781271981472485	1.0304806018649936e-05
ENSG00000015171_180013_252470_602	ZMYND11	10	+	1806	18	0	0	1.50%	5.848309872074752	0.0035291660242640502	0.0035291660242640502	6.948232509684344e-05
ENSG00000103351_3523279_3536271_135	CLUAP1	16	+	405	9	0	0	4.44%	13.039564974033336	0.011772051206276392	0.011772051206276392	0.000681272511389508
ENSG00000147475_37737923_37745603_152	ERLIN2	8	+	456	13	0	0	4.61%	16.72838927589803	0.0078820576498062	0.0078820576498062	0.00031963642907451164
ENSG00000114416_180947925_180976292_564	FXR1	3	+	1692	16	0	0	1.60%	5.548751052780143	0.0032294771334464936	0.0032294771334464936	5.8663648728941325e-05
ENSG00000002586_2691361_2733364_160	CD99	X	+	480	52	6	1	8.75%	63.56787924841252	0.001368321372009316	0.0004494563945704485	3.979749443618499e-06
ENSG00000113758_177467675_177457433_555	DBN1	5	−	1665	24	0	0	1.62%	8.458096199372974	0.0034782380676658805	0.0034782380676658805	6.758309314600117e-05
ENSG00000078269_158071633_158096364_839	SYNJ2	6	+	2517	12	3	0	0.72%	2.797522878815257	0.07786981947941429	0.013007239811447113	0.0049717523581729916
ENSG00000187118_28263338_28319629_84	CMC1	3	+	252	18	1	0	5.95%	41.912887416535725	0.04223451612662591	0.019679754444124863	0.003715148010809822
ENSG00000138796_108023489_108034357_127	HADH	4	+	381	30	0	0	7.87%	46.20318297885828	0.002046028879997488	0.002046028879997488	2.4518070358754455e-05

ENSG00000164904_126554349_126544965_160	ALDH7A1	5	-	480	8	0	0	3.12%	9.779673730525003	0.016947426762344633	0.016947426762344633	0.0013498980316300959
ENSG00000100591_77458190_77468612_288	AHSA1	14	+	864	153	4	2	15.97%	103.90903338682814	1.6464557633249268e-09	3.949339527896889e-09	7.050542310400052e-17
ENSG00000213983_23566001_23559749_632	AP1G2	14	-	1896	9	0	2	1.11%	2.7853501131242093	0.006937202941512724	0.0350911293701988	0.001262969770953026
ENSG00000080824_102086291_102081712_703	HSP90AA1	14	-	2109	446	24	22	13.37%	124.08917450395877	2.0782847351969592e-16	2.5828625729642765e-15	7.951553247159537e-30
ENSG00000243989_51986441_51989075_254	ACY1	3	+	762	7	1	0	1.97%	5.390371347533466	0.0477903522728147	0.016947426762344633	0.0036975118224100542
ENSG00000102763_41960952_41719587_1018	VWA8	13	-	3054	6	1	0	0.49%	1.1528102629302557	0.0477903522728147	0.016947426762344633	0.0036975118224100542
ENSG00000164631_6697718_6690848_623	ZNF12	7	-	1869	13	1	0	0.80%	4.081404767153291	0.04223451612662591	0.019679754444124863	0.003715148010809822
ENSG00000178209_143935297_143916177_4341	PLEC	8	-	13023	130	33	39	1.38%	5.857441073339094	2.796321955810114e-06	1.2083355211512743e-05	2.8801264455689585e-10
ENSG00000182196_122981140_122982676_229	ARL6IP4	12	+	687	42	3	1	7.86%	35.87303901153276	0.00048070449453883614	0.00012186633930364116	4.1373486007659405e-07
ENSG00000172725_67442628_67441011_289	CORO1B	11	-	867	26	0	0	2.42%	17.596644774647064	0.008574016250729465	0.008574016250729465	0.0003747459614100891
ENSG00000124155_45420349_45425826_316	PIGT	20	+	948	52	2	0	4.11%	32.18626797387975	0.0022083206986541785	0.0006768518708446028	9.39609878359303e-06
ENSG00000138796_108004852_108023617_207	HADH	4	+	621	9	0	0	2.42%	8.504064113500002	0.019679754444124863	0.019679754444124863	0.0017849636115922674
ENSG00000091136_107951301_107942444_83	LAMB1	7	-	249	9	4	0	6.02%	21.20893098186145	0.14389369507707506	0.019679754444124863	0.013602114125134476
ENSG00000143321_156745364_156743449_208	HDGF	1	-	624	84	2	4	12.50%	78.98967243885579	6.901149552498872e-06	9.576909903257872e-06	5.426479337941795e-10
ENSG00000152894_128520358_127970227_1472	PTPRK	6	-	4416	26	1	1	0.95%	3.4547760461093757	0.0006179331360801681	0.0006179331360801681	2.4552533592415123e-06
ENSG00000109113_28717266_28714643_237	RAB34	17	-	711	75	7	2	8.44%	61.89666918053799	0.0008160159367655577	0.00012914063444101055	7.502356321592999e-07
ENSG00000109113_28718165_28717320_49	RAB34	17	-	147	17	3	0	10.20%	67.85896057915308	0.0682897090290637	0.021557223391537678	0.006517186294745621
ENSG00000177410_49279128_49279205_25	ZFAS1	20	+	75	60	3	1	28.00%	469.4243390652001	0.027657845067597595	0.016503128830616266	0.0021005858213874354
ENSG00000125743_45688538_45687553_108	SNRPD2	19	-	324	228	16	5	32.41%	412.91955751105564	2.1762257060309274e-07	1.81362136574898e-07	3.7403146226291825e-13
ENSG00000074603_65507278_65446884_670	DPP8	15	-	2010	5	0	0	0.75%	1.4596527956007466	0.012673659338734126	0.012673659338734126	0.000782701129001274
ENSG00000198060_92291519_92351207_270	5-Mar	10	+	810	15	1	5	2.59%	10.86630414502778	0.015625008558487224	0.06804322570028769	0.004983399768417791
ENSG00000011422_43667671_43646457_256	PLAUR	19	-	768	24	4	0	5.47%	18.33688824473438	0.0010414171786762797	0.00037518914374882155	2.5591984477994843e-06
ENSG00000123136_14413220_14409910_267	DDX39A	19	-	801	33	7	6	5.99%	24.174474390061803	0.0031050027908403072	0.003966301615612612	6.883974414016248e-05
ENSG00000112237_99550995_99544222_137	CCNC	6	-	411	9	0	1	3.65%	12.849206361273726	0.019679754444124863	0.04223451612662591	0.003715148010809822
ENSG00000101346_32228372_32231091_118	POFUT1	20	+	354	9	0	0	4.24%	14.91814636859746	0.019679754444124863	0.019679754444124863	0.0017849636115922674
ENSG00000204525_31271857_31269169_344	HLA-C	6	-	1032	18	2	4	1.74%	10.234542276130817	0.03535057243299149	0.0865972937396412	0.01250960075939979
ENSG00000175470_131943995_131955963_285	PPP2R2D	10	+	855	6	2	0	1.75%	4.117757360221054	0.13974268412556323	0.016947426762344633	0.011764960919844317
ENSG00000111300_112078669_112040439_676	NAA25	12	-	2028	21	3	0	1.18%	6.076128649142753	0.027695922668752454	0.005554612275049112	0.000815836010617027
ENSG00000183527_39178620_39175590_127	PSMG1	21	-	381	6	0	0	3.94%	9.240636595771656	0.016947426762344633	0.016947426762344633	0.0013498980316300959
ENSG00000197256_11194511_11169853_841	KANK2	19	-	2523	9	0	2	0.59%	2.093152522585613	0.019679754444124863	0.10105102209468636	0.009164497669452725
ENSG00000205155_35745931_35746651_97	PSENEN	19	+	291	14	14	2	7.22%	28.229986026257738	0.07551307739531453	0.04912109528954385	0.014462694973637112
ENSG00000205155_35746701_35746847_48	PSENEN	19	+	144	13	0	0	12.50%	52.97323270701043	0.013007239811447113	0.013007239811447113	0.0008218654148058079
ENSG00000119048_134371623_134390238_130	UBE2B	5	+	390	12	1	1	5.38%	18.054782271738464	0.015516259750072458	0.015516259750072458	0.0011443157696259844
ENSG00000185658_39313491_39194723_2199	BRWD1	21	-	6597	16	0	2	0.41%	1.423144881204184	0.0034529206320349946	0.014991380638102441	0.0002857513737328192
ENSG00000117868_158829418_158734207_845	ESYT2	7	-	2535	42	5	3	2.37%	9.721805838628404	4.983000130598301e-05	0.00023625467940204776	8.773628016212268e-08
ENSG00000108946_68530294_68530449_51	PRKAR1A	17	+	153	36	1	0	13.73%	138.06598207800002	0.012176559331126819	0.008877961307018026	0.0005408334713909414
ENSG00000139372_103982846_103987090_235	TDG	12	+	705	12	0	0	2.13%	9.987751895004257	0.019679754444124863	0.019679754444124863	0.0017849636115922674
ENSG00000099622_1270934_1272443_297	CIRBP	19	+	891	27	1	0	3.70%	17.781224964590912	0.002493493840886608	0.0014055202957965589	2.0830049893778164e-05
ENSG00000148824_133394221_133420165_283	MTG1	10	+	849	21	4	2	2.83%	14.514003416326858	0.032678162153413765	0.016025708091071147	0.0024091337154673693
ENSG00000060749_32893126_32976474_1864	QSER1	11	+	5592	18	1	3	0.27%	1.8887781882988202	0.022899794555593333	0.0700082515985845	0.007029404108641898
ENSG00000164347_74751461_74721655_667	GFM2	5	-	2001	31	0	5	1.65%	9.090551293741381	0.0015208721599504675	0.013147104497242925	0.00012290835639369062
ENSG00000131507_142108975_142131903_52	NDFIP1	5	+	156	9	4	0	9.62%	33.852716759509626	0.1975540342952461	0.020613416668581838	0.020442591980743177
ENSG00000177565_177050593_177025498_366	TBL1XR1	3	-	1098	11	4	0	2.19%	5.8784924063265045	0.07206351740800761	0.004687384229717438	0.0020529246237639028
ENSG00000141295_47838673_47837844_138	SCRN2	17	-	414	12	0	0	5.07%	17.008128227000004	0.007029302661868638	0.007029302661868638	0.0002573639808771639
ENSG00000125691_38853710_38850333_122	RPL23	17	-	366	260	17	13	19.67%	416.83855244860666	5.801068441724047e-05	4.523764157258184e-05	1.9685515372232413e-08
ENSG00000133316_62839570_62835241_210	WDR74	11	-	630	13	9	3	3.81%	12.108167475888099	0.27504865861519845	0.03961598513640774	0.048089941565447575
ENSG00000133997_70600637_70584813_253	MED6	14	-	759	17	2	0	4.35%	13.142644539045458	0.007962492813783481	0.0012558331315113088	6.116682160194365e-05
ENSG00000160888_13153187_13153858_223	IER2	19	+	669	49	10	0	5.38%	42.97793836732961	0.013967204762937563	0.0010573555742419644	9.659782915611994e-05
ENSG00000214078_35626794_35626516_92	CPNE1	20	-	276	17	2	0	7.61%	36.142272482375006	0.02034245439037237	0.008674846900444944	0.0008751884318457689
ENSG00000100664_103336078_103341052_393	EIF5	14	+	1179	23	1	0	2.54%	11.446946351250638	0.0030814981005195803	0.0023354528714849923	4.1239111951852285e-05
ENSG00000108946_68515400_68530449_381	PRKAR1A	17	+	1143	159	2	3	9.97%	81.62562326264963	4.363518371891827e-08	4.3268258388075745e-08	1.8788011721226132e-14
ENSG00000108946_68524032_68530449_229	PRKAR1A	17	+	687	126	2	3	12.66%	107.61911703459828	1.4017650229823044e-06	1.3878483501044505e-06	1.717197270747381e-11
ENSG00000143367_151562606_151581707_308	TUFT1	1	+	924	22	0	0	3.57%	13.970962472178575	0.0012427339549726212	0.0012427339549726212	9.422880418492092e-06
ENSG00000091490_25862749_25748425_1103	SEL1L3	4	-	3309	9	0	2	0.45%	1.5959576350811426	0.019216965118390855	0.08350359792828288	0.0073235312725521175
ENSG00000144867_133805849_133819766_271	SRPRB	3	+	813	54	1	0	4.80%	38.97434549434318	0.000883754130767968	0.0007004692736203066	3.915224658583823e-06
ENSG00000110651_2394127_2396866_165	CD81	11	+	495	153	3	0	20.00%	181.36849463882731	5.737735194735298e-07	2.4033686813656327e-07	1.2871857065132192e-12
ENSG00000172590_22830249_22834321_51	MRPL52	14	+	153	13	0	5	11.76%	49.85716019483335	0.013007239811447113	0.1140827265444288	0.007629990289950498
ENSG00000165410_34713513_34712865_149	CFL2	14	-	447	53	0	0	8.72%	69.57351781447316	0.0007004692736203066	0.0007004692736203066	3.1265561041742324e-06
ENSG00000198805_20469525_20474632_113	PNP	14	+	339	71	5	0	9.73%	122.89501502075666	0.0016109979480538772	0.0016340149330375125	1.5719946693098273e-05
ENSG00000136295_2632156_2661739_523	TTYH3	7	+	1569	11	0	0	0.96%	4.11382068970459	0.020613416668581838	0.020613416668581838	0.001946208561389314
ENSG00000126749_6974567_6975809_99	EMG1	12	+	297	6	0	1	5.05%	11.854149976393941	0.016947426762344633	0.0477903522728147	0.0036975118224100542
ENSG00000075151_20981092_20807319_1566	EIF4G3	1	-	4698	11	0	0	0.45%	1.3739005240839721	0.007593099278532274	0.007593099278532274	0.00029783286705462064
ENSG00000142227_48326845_48327634_63	EMP3	19	+	189	193	7	0	26.98%	599.1990571401033	0.0003872421465994906	0.00014226219545339003	3.842347183833656e-07
ENSG00000076928_41896424_41928954_650	ARHGEF1	19	+	1950	13	1	3	1.23%	3.911869492210001	0.00912793029372963	0.046396937147164995	0.002134426878753118
ENSG00000119638_75097223_75086997_242	NEK9	14	-	726	12	1	0	2.07%	9.698849980685953	0.016947426762344633	0.016947426762344633	0.0013498980316300959
ENSG00000077232_182718162_182777132_722	DNAJC10	2	+	2166	21	0	1	1.39%	5.689006879252771	0.0023218226288164415	0.0023218226288164415	3.1231198880462635e-05
ENSG00000278053_37643420_37614296_599	DDX52	17	-	1797	40	4	1	2.84%	13.061333863806347	0.0009922631355087487	0.00018859061491912913	1.2950090630800739e-06

ENSG00000127922_96709763_96688924_70	SEM1	7	-	210	44	0	11	17.14%	122.94446975517145	0.0010573555742419644	0.043430789637218754	0.0003570775041158087
ENSG00000127920_93922186_93926216_57	GNG11	7	+	171	321	17	9	35.09%	1101.5000938591318	5.8341570798572216e-05	5.754249221428216e-05	2.499589389991457e-08
ENSG00000219200_7013669_7013746_25	RNASEK	17	+	75	48	2	4	24.00%	375.53947125216007	0.021261238999025815	0.0291989533746507	0.0027803964083144245
ENSG00000157538_37267294_37232571_168	VPS26C	21	-	504	10	1	0	2.98%	11.642468726815478	0.03537493353818881	0.020613416668581838	0.003252526348827943
ENSG00000055950_100987443_100986972_44	MRPL43	10	-	132	12	3	0	11.36%	53.343674893772736	0.08678408327796083	0.020613416668581838	0.008072389047184183
ENSG00000108433_46923193_46955562_214	GOSR2	17	+	642	12	0	0	2.80%	10.967858389373834	0.011772051206276392	0.011772051206276392	0.000681272511389508
ENSG00000052723_114780607_114774271_207	SIKE1	1	-	621	14	0	0	2.90%	13.228544176555559	0.013007239811447113	0.013007239811447113	0.0008218654148058079
ENSG00000143761_228097115_228098013_181	ARF1	1	+	543	246	3	2	24.31%	265.8342251612321	3.89149000448416e-09	3.5896094622681653e-09	1.4917525667184776e-16
ENSG00000141378_59697957_59697439_172	PTRH2	17	-	516	44	2	1	10.47%	50.035540016639544	0.00012769218138170972	0.00010113397357209863	9.26681018554367e-08
ENSG00000023287_52674111_52623782_1346	RB1CC1	8	-	4038	21	0	2	0.67%	3.05160695900483	0.0035291660242640502	0.01344380615658633	0.000260294942673285
ENSG00000120656_28613361_28589750_112	TAF12	1	-	336	15	0	0	5.36%	26.195554635334826	0.012178957859018494	0.012178957859018494	0.0007262454987071801
ENSG00000198431_104215803_104348415_647	TXNRD1	12	+	1941	259	19	13	9.27%	78.2978515055943	4.344541045840949e-09	1.0118539604345782e-09	4.834021682643186e-17
ENSG00000203705_212796574_212815156_221	TATDN3	1	+	663	6	0	0	2.26%	5.310230079923078	0.016947426762344633	0.016947426762344633	0.0013498980316300959
ENSG00000174165_66546003_66539529_284	ZDHHC24	11	-	852	12	0	1	1.76%	8.26451301171127	0.019679754444124863	0.02784981298332479	0.002477909089056847
ENSG00000143537_155057085_155062502_486	ADAM15	1	+	1458	25	5	0	2.26%	10.061392726877575	0.017867215592848443	0.001484234770590415	0.00016809892502198752
ENSG00000065427_75648640_75634110_362	KARS	16	-	1086	19	0	0	1.66%	10.265955849722378	0.013428347753762199	0.013428347753762199	0.0008725593497644525
ENSG00000168036_41224552_41239342_768	CTNNB1	3	+	2304	116	8	0	5.73%	29.542764394294277	8.699215501017508e-08	2.9105570714308586e-09	2.8276345500981447e-15
ENSG00000082515_154941111_154966897_232	MRPL22	5	+	696	40	0	0	5.60%	33.72301286387932	0.0006311913977015671	0.0006311913977015671	2.5578527397951546e-06
ENSG00000215301_41340781_41347719_622	DDX3X	X	+	1866	91	3	3	7.07%	28.61576557806351	1.3918695140175526e-08	1.1261251676471362e-08	1.6190827680347769e-15
ENSG00000127990_94618900_94585465_287	SGCE	7	-	861	12	0	2	2.79%	8.17812437395819	0.004193160480899131	0.022868511308519305	0.0005251843016725186
ENSG00000100605_93115163_92941561_414	ITPK1	14	-	1242	15	1	0	1.93%	7.086720094583335	0.007930666369886513	0.005160369442893307	0.00021637657341139557
ENSG00000115641_105386516_105361283_279	FHL2	2	-	837	24	2	0	3.23%	16.82524512778495	0.01052924788556975	0.003684287313187465	0.00021087137401632993
ENSG00000143376_151638856_151693601_435	SNX27	1	+	1305	16	0	2	1.38%	7.194242744294255	0.013007239811447113	0.05258119297243839	0.00326646906127711
ENSG00000143569_154227312_154270295_1047	UBAP2L	1	+	3141	42	13	5	2.01%	7.846156574633239	0.005538146750059835	0.0005279745362177505	1.9599354329655325e-05
ENSG00000171858_62387339_62388368_81	RPS21	20	+	243	285	9	11	24.69%	688.199262518426	9.43865819293397e-05	0.0003501593979130858	2.3675169737559605e-07
ENSG00000176087_140564844_140566762_103	SLC35A4	5	+	309	25	0	1	4.85%	47.4741443229369	0.02108409854857798	0.028889785561798623	0.002731532589007073
ENSG00000197930_52653100_52643570_127	ERO1A	14	-	381	9	0	0	3.94%	13.860954893657484	0.019679754444124863	0.019679754444124863	0.0017849636115922674
ENSG00000197930_52695481_52657949_257	ERO1A	14	-	771	48	1	0	4.67%	36.531076970054485	0.0012644225054167608	0.0010658336519988904	8.271599490222998e-06
ENSG00000126804_64521934_64523646_570	ZBTB1	14	+	1710	14	0	0	0.88%	4.804050253591229	0.02108409854857798	0.02108409854857798	0.002029891263157455
ENSG00000132912_150756451_150711149_403	DCTN4	5	-	1209	29	0	0	3.72%	14.074964674204718	0.0002582331582969843	0.0002582331582969843	4.5481323920964005e-07
ENSG00000130402_38647839_38729432_490	ACTN4	19	+	1470	133	7	15	7.76%	53.08965739427858	2.6216194404325027e-07	1.7650585863784056e-05	4.8207595161184145e-11
ENSG00000048828_93550581_93564540_351	FAM120A	9	+	1053	8	2	2	1.71%	4.457970931293448	0.0533594081646912	0.08266357711566169	0.016932737880899308
ENSG00000125944_23337834_23310454_494	HNRNPR	1	-	1482	55	0	1	2.43%	21.776601424245957	0.0010197273511756433	0.001097317208428726	6.914311341606604e-06
ENSG00000156113_77637360_76887266_1113	KCNMA1	10	-	3339	15	4	2	0.72%	2.636030655128033	0.048994651353958696	0.02389626957043539	0.005091569865673637
ENSG00000075275_46536321_46363178_2731	CELSR1	22	-	8193	16	9	3	0.40%	1.1459156330164777	0.14379799863380072	0.014654763774774537	0.010925700667127102
ENSG00000146674_45921140_45914820_270	IGFBP3	7	-	810	147	6	0	11.48%	106.48978062127225	3.21993475663908e-06	5.464751138522367e-07	1.6053333144352504e-11
ENSG00000131469_42998763_43002920_132	RPL27	17	+	396	441	17	11	14.39%	653.460017448716	0.00034175999527323304	0.001372342566410722	3.104046494904808e-06
ENSG00000197912_89508547_89520042_103	SPG7	16	+	309	12	0	0	4.85%	22.787589275009715	0.019679754444124863	0.019679754444124863	0.0017849636115922674
ENSG00000117523_171522225_171591847_2553	PRRC2C	1	+	7659	17	10	3	0.35%	1.3024242335990994	0.15039870036150454	0.019224948125267174	0.014067798201048379
ENSG00000131368_15065188_15053376_108	MRPS25	3	-	324	10	3	3	6.48%	18.110506908379634	0.06556525143482543	0.06556525143482543	0.01638132253942991
ENSG00000124795_18263987_18244508_285	DEK	6	-	855	25	2	5	3.86%	17.15732233425439	0.00543592712689352	0.014914789744379387	0.00042354077910386267
ENSG00000163931_53255942_53225756_631	TKT	3	-	1893	525	67	26	16.48%	162.7362506664224	8.012371654125739e-14	1.0749619350488686e-17	1.6754596357069326e-29
ENSG00000173821_80294947_80393498_4641	AC124319.1	17	+	13923	52	18	13	0.67%	2.1915235250476197	0.0036793750888163366	0.00024128078536283745	6.4104439430515e-06
ENSG00000196141_200309073_200478031_588	SPATS2L	2	+	1764	23	2	0	1.87%	7.650765163335886	0.0034718995429267267	0.0014915192737735051	3.054066176404144e-05
ENSG00000144034_73737304_73729171_145	TPRKB	2	-	435	15	0	0	3.45%	20.23380771832759	0.019679754444124863	0.019679754444124863	0.0017849636115922674
ENSG00000170606_133101732_133104436_170	HSPA4	5	+	510	24	1	6	5.29%	27.613196415600008	0.004355956481189797	0.03004189642534358	0.0007254253758805535
ENSG00000154582_73959768_73946630_112	ELOC	8	-	336	8	4	1	4.46%	13.970962472178575	0.2618042821861254	0.04223451612662591	0.04736319147700863
ENSG00000172757_65858099_65855931_104	CFL1	11	-	312	795	51	12	31.73%	1495.1616568783418	9.216808915531523e-07	8.602591938778798e-07	7.120194426874872e-12
ENSG00000015475_17750116_17735580_195	BID	22	-	585	19	3	0	3.59%	19.057825731279493	0.036371020034052744	0.00847367416878415	0.0015515874441493025
ENSG00000166206_26621357_26547793_334	GABRB3	15	-	1002	27	0	1	2.99%	15.811448546357788	0.0024321290225875155	0.0031727927861210656	4.407497946231007e-05
ENSG00000172115_25124083_25123701_93	CYCS	7	-	279	29	0	2	8.60%	60.99151358822044	0.005706018193000826	0.010402771787637745	0.00030929694644272603
ENSG00000013441_200861712_200854685_305	CLK1	2	-	915	26	1	0	3.61%	16.673542097944267	0.0021748195570013406	0.001558047347358915	2.0072435939476812e-05
ENSG00000013441_200854632_200853306_83	CLK1	2	-	249	9	0	0	7.23%	21.20893098186145	0.012178957859018494	0.012178957859018494	0.0007262454987071801
ENSG00000173933_66639712_66644132_364	RBM4	11	+	1092	15	6	0	2.75%	8.0601706570261	0.06856615097740389	0.0017901637611834264	0.0009325288179663202
ENSG00000141456_4673654_4671439_596	PELP1	17	-	1788	25	1	0	1.01%	8.204424270574666	0.013428347753762199	0.013428347753762199	0.0008725593497644525
ENSG00000137133_35814979_35813258_135	HINT2	9	-	405	30	7	0	6.67%	43.46521658011112	0.03342660139382465	0.003710480643933249	0.0007137052189749978
ENSG00000151657_7787933_7756080_393	KIN	10	-	1179	16	0	2	1.78%	7.963093113913488	0.00797940233269714	0.022200630790666427	0.000887484071868763
ENSG00000197448_143263514_143268837_183	GSTK1	7	+	549	53	3	10	9.29%	56.64729046096449	0.0004218576446894213	0.004485785010211988	1.2875388661386852e-05
ENSG00000263001_74689207_74758838_931	GTF2I	7	+	2793	19	0	0	1.18%	3.9917035634795925	0.0012624143894574274	0.0012624143894574274	9.711623744831373e-06
ENSG00000099797_14529697_14565043_248	TECR	19	+	744	90	8	1	6.45%	70.98150288284276	0.0018081082813629175	0.00023962802153351347	2.9935607927464093e-06
ENSG00000168066_64778392_64765389_571	SF1	11	-	1713	71	9	5	4.20%	24.32072976768039	0.00013764886944903955	5.8157147011588306e-05	5.8819977773674186e-08
ENSG00000168066_64778392_64765818_639	SF1	11	-	1917	70	10	7	3.91%	21.426515215547735	0.0001855399422845024	0.00015308070185822943	1.990497469280764e-07
ENSG00000175390_7987353_7996022_357	EIF3F	11	+	1071	31	0	10	2.52%	16.98430731911905	0.0033278027414746945	0.13400371787331594	0.0034457488676848075
ENSG00000152818_144421902_144850997_3378	UTRN	6	+	10134	7	0	0	0.15%	0.4053150746813204	0.019216965118390855	0.019216965118390855	0.0017073955890589052
ENSG00000124155_45420349_45425826_316	PIGT	20	+	948	52	2	0	4.11%	32.18626797387975	0.0022083206986541785	0.0006768518708446028	9.39609878359303e-06
ENSG00000170043_7931829_7930606_145	TRAPPC1	17	-	435	52	6	0	11.03%	70.14386675686899	0.0012682043809137623	0.00019920228515162423	1.7530996256218918e-06
ENSG00000140538_88256153_87979345_612	NTRK3	15	-	1836	14	6	4	1.14%	4.474360530305557	0.1398844737482668	0.06927320754946001	0.03501991084660459

ENSG00000182220_40589096_40605755_301	ATP6AP2	X	+	903	37	1	4	6.64%	24.043051696307316	5.27823020285109e-05	0.00024353236528075492	9.545093995671736e-08
ENSG00000090006_40605416_40629550_1406	LTBP4	19	+	4218	13	2	6	0.57%	1.8084745163133005	0.022868511308519305	0.17645041170550768	0.01924712335493658
ENSG00000110911_51009138_50988325_557	SLC11A2	12	-	1671	22	0	0	1.62%	7.725415514238781	0.0033030736763512156	0.0033030736763512156	6.124050385493983e-05
ENSG00000125826_408739_430430_468	RBCK1	20	+	1404	14	7	4	1.28%	5.851086847322651	0.19807195457603705	0.012798402692974279	0.014686027802829961
ENSG00000165458_72228228_72238130_1137	INPPL1	11	+	3411	5	1	2	0.44%	0.8601296157014074	0.051235217429874705	0.12841962897892828	0.025204067182957087
ENSG00000166741_114312268_114312477_69	NNMT	11	+	207	34	2	4	20.29%	96.37939328633335	0.001373376896918719	0.0004156054716904614	3.716678856638805e-06
ENSG00000076924_7628265_7619586_827	XAB2	19	-	2481	22	10	0	0.85%	5.2032121419963735	0.19809812002768817	0.00877616296920843	0.011325749763237948
ENSG00000133895_64810109_64804334_575	MEN1	11	-	1725	22	4	1	2.09%	7.483576419880002	0.01878413720018969	0.0016120252826907794	0.0001902478323907079
ENSG00000065665_12129788_12169327_437	SEC61A2	10	+	1311	12	0	0	1.14%	5.370987861157896	0.02108409854857798	0.02108409854857798	0.002029891263157455
ENSG00000126858_32192201_32224733_511	RHOT1	17	+	1533	10	0	1	1.17%	3.827660951281801	0.012798402692974279	0.023672501042627626	0.0014375227604752335
ENSG00000131051_35725100_35704481_367	RBM39	20	-	1101	22	1	0	2.18%	11.724949431692101	0.008364861494636245	0.005604866691947602	0.00024596970711672695
ENSG00000087191_63829467_63831969_383	PSMC5	17	+	1149	144	12	3	12.01%	73.5390609501619	6.421566081584028e-08	2.0668185553905446e-09	1.4966142751574784e-15
ENSG00000129255_7583863_7587444_259	MPDU1	17	+	777	21	0	5	3.09%	15.858930373824329	0.0053557511377634245	0.03172800529576679	0.0009138333828318776
ENSG00000089902_102707360_102726506_316	RCOR1	14	+	948	12	0	3	2.22%	7.427600301664559	0.008175610787902942	0.06853594973600552	0.002988282660209286
ENSG00000116001_70236084_70212719_246	TIA1	2	-	738	9	0	0	2.44%	7.155858827213416	0.011772051206276392	0.011772051206276392	0.000681272511389508
ENSG00000140688_31508373_31490835_423	C16orf58	16	-	1269	24	4	0	2.84%	11.097502105560286	0.008403274891555457	0.0009509739334972347	5.0894006428203135e-05
ENSG00000182253_99126783_99133058_921	SYNM	15	+	2763	11	0	0	0.54%	2.3360784155434318	0.020613416668581838	0.020613416668581838	0.001946208561389314
ENSG00000185215_103130020_103135360_323	TNFAIP2	14	+	969	55	6	3	4.33%	33.30539041355264	0.003702347862178866	0.0016673058435266576	3.6072409171206726e-05
ENSG00000115355_55332660_55291711_951	CCDC88A	2	-	2853	6	1	0	0.53%	1.2340282309810728	0.0477903522728147	0.016947426762344633	0.0036975118224100542
ENSG00000148019_78243471_78270851_502	CEP78	9	+	1506	17	0	0	1.59%	6.623683403144423	0.004826937407846361	0.004826937407846361	0.00012612451166366307
ENSG00000113163_75511207_75379346_567	COL4A3BP	5	-	1701	14	4	0	1.23%	4.829468508901235	0.0750151653743942	0.008175610787902942	0.0033080285523474006
ENSG00000221926_15651591_15628615_558	TRIM16	17	-	1674	13	0	0	1.08%	4.556837222108424	0.013007239811447113	0.013007239811447113	0.0008218654148058079
ENSG00000171793_40983291_41008718_451	CTPS1	1	+	1353	20	5	1	2.00%	8.673768275410202	0.04011189309666471	0.005034872532813168	0.0011189735043589642
ENSG00000003402_201130085_201141433_148	CFLAR	2	+	444	11	0	0	3.38%	14.5373528426723	0.019679754444124863	0.019679754444124863	0.0017849636115922674
ENSG00000241837_33906487_33903526_79	ATP5PO	21	-	237	57	1	0	17.72%	141.1244057316266	0.00044945639405704485	0.0004575241474660434	1.3512229108765406e-06
ENSG00000174442_66518919_66546679_471	ZWILCH	15	+	1413	25	0	0	1.70%	10.381819246841829	0.005706018193000826	0.005706018193000826	0.0001733096755673334
ENSG00000121749_71861494_71923204_665	TBC1D15	12	+	1995	14	3	0	1.20%	4.117757360221054	0.046396937147164995	0.005160369442893307	0.0013402346425884413
ENSG00000221983_18573301_18575150_128	UBA52	19	+	384	29	4	0	8.59%	44.314146591441414	0.003768080243855145	0.0014055202957965589	3.138862417155242e-05
ENSG00000175061_16439055_16439470_49	SNHG29	17	+	147	21	3	0	12.24%	83.82577483307145	0.05352897298295963	0.013640585738808999	0.0034621625354375684
ENSG00000112118_52273354_52264664_316	MCM3	6	-	948	15	2	3	3.16%	9.284500377080699	0.012544274337212767	0.01782474427444472	0.0010720591924173765
ENSG00000105135_15122742_15115252_410	ILVBL	19	-	1230	12	5	0	1.95%	5.724687061770733	0.07962816006291146	0.004826937407846361	0.0023618906866966134
ENSG00000105011_14136456_14120459_202	ASF1B	19	-	606	31	2	0	5.45%	30.016820361017334	0.005094465778387268	0.001565547007472977	4.691850280781464e-05
ENSG00000105058_16185443_16190955_112	FAM32A	19	+	336	17	0	0	8.93%	29.688295253379472	0.001801896632038876	0.001801896632038876	1.9221247299282243e-05
ENSG00000140740_21962771_21976247_242	UQCRC2	16	+	726	31	3	10	7.02%	25.055362450105378	0.0002780814637306785	0.01759426291355606	4.241537858161786e-05
ENSG00000067064_1044083_1041187_208	IDI1	10	-	624	95	5	2	16.35%	89.33355811537261	2.8056621783320065e-07	1.2891654733083578e-07	3.449659433067601e-13
ENSG00000114480_81646453_81490407_462	GBE1	3	-	1386	9	0	0	1.30%	3.8102624924123387	0.012178957859018494	0.012178957859018494	0.0007262454987071801
ENSG00000183624_129279733_129304825_312	HMCES	3	+	936	14	3	0	2.56%	8.776630270983976	0.034881440664207244	0.005257622967929454	0.0009961798610865866
ENSG00000065883_39988230_40094980_854	CDK13	7	+	2562	17	1	8	0.82%	3.8935469184759963	0.009583874861041503	0.1608142242740624	0.009210430778519922
ENSG00000167004_43746540_43771218_505	PDIA3	15	+	1515	147	2	2	4.16%	56.935130233155455	2.8063932813508764e-05	2.7881603561729963e-05	6.051164590134268e-09
ENSG00000089006_17950309_17947481_128	SNX5	20	-	384	11	1	0	3.91%	16.808814224339848	0.04223451612662591	0.019679754444124863	0.003715148010809822
ENSG00000101166_59037211_59035007_104	PRELID3B	20	-	312	23	0	9	7.69%	43.25624919270674	0.005706018193000826	0.05635854237047183	0.0018048610472522419
ENSG00000117479_169485766_169465849_497	SLC19A2	1	-	1491	7	2	0	1.01%	2.754837670570423	0.11777945996203976	0.016947426762344633	0.009674391494749069
ENSG00000022840_120534854_120576666_802	RNF10	12	+	2406	15	2	2	0.87%	3.6582320687749386	0.03799572790022047	0.03799572790022047	0.0060463421217767785
ENSG00000129128_176322126_176328330_147	SPCS3	4	+	441	29	0	1	8.84%	38.58646778030273	0.0006401566880482122	0.0009061776457653113	3.6823488174641324e-06
ENSG00000108424_47677101_47678418_93	KPNB1	17	+	279	19	0	0	7.53%	39.959957178489255	0.00847367416878415	0.00847367416878415	0.0003665024264111182
ENSG00000113070_140346328_140334676_208	HBEGF	5	-	624	14	0	0	2.88%	13.164945406475965	0.013007239811447113	0.013007239811447113	0.0008218654148058079
ENSG00000183431_37989965_37958186_501	SF3A3	1	-	1503	65	3	1	4.79%	25.37639890156188	3.463551942608206e-05	7.180468754705515e-06	2.0384993443402033e-09
ENSG00000183520_38017750_38023628_184	UTP11	1	+	552	35	11	0	6.52%	37.20528049656251	0.043039091886448146	0.001053132412899783	0.0003520873362000192
ENSG00000264772_7572842_7577934_377	AC016876.2	17	+	1131	210	7	5	6.63%	108.95127232945626	3.2657606588264e-05	1.0782458024799038e-05	2.819796540653389e-09
ENSG00000168159_228487816_228493885_126	RNF187	1	+	378	34	1	3	10.32%	52.7791915615635	0.0006698694257094773	0.0035156470961704048	1.4976062642096571e-05
ENSG00000147439_22630576_22621422_199	BIN3	8	-	597	8	0	0	3.02%	7.863054255698494	0.011570465654371866	0.011570465654371866	0.0006594843062896773
ENSG00000108094_35071242_35010311_720	CUL2	10	-	2160	24	0	0	1.67%	6.519782487016668	0.0008739357725210369	0.0008739357725210369	4.787965623198429e-06
ENSG00000108094_35071242_35010311_681	CUL2	10	-	2043	23	0	0	1.62%	6.605947013276801	0.0013984928336429232	0.0013984928336429232	1.1821238358622835e-05
ENSG00000161016_144792129_144789804_257	RPL8	8	-	771	860	54	28	26.07%	654.5151290468095	1.5061677631743077e-12	1.7940116575495218e-12	3.4351652844413365e-23
ENSG00000162441_9942735_9942631_34	LZIC	1	-	102	15	1	0	14.71%	86.29123879875002	0.019679754444124863	0.019679754444124863	0.0017849636115922674
ENSG00000133773_82358376_82353151_241	CCDC59	12	-	723	23	3	5	3.73%	18.66659716199793	0.016881135945258162	0.0064006895615519015	0.0005545513529377137
ENSG00000141552_81894478_81900100_196	ANAPC11	17	+	588	92	28	4	4.59%	91.80918196003063	0.09058867107711571	0.0053878105512546945	0.0029930116433432605
ENSG00000141552_81899524_81900065_54	ANAPC11	17	+	162	20	0	0	9.26%	72.44202763351853	0.021557223391537678	0.021557223391537678	0.002115616449879074
ENSG00000141425_36067404_35993151_312	RPRD1A	18	-	936	16	0	0	2.24%	10.03043459541026	0.008175610787902942	0.008175610787902942	0.00034251843268961324
ENSG00000113810_160402022_160433809_1206	SMC4	3	+	3618	102	8	3	3.57%	16.54273168347513	1.9782058533149353e-07	2.8601838525598117e-08	5.70455157126797e-14
ENSG00000104687_30727706_30679520_450	GSR	8	-	1350	49	3	6	5.33%	21.297956124254448	6.471395361352189e-05	0.000150447248739949	7.111650832307864e-08
ENSG00000171634_67825725_67982288_3046	BPTF	17	+	9138	20	1	2	0.16%	1.2842644426165466	0.028889785561798623	0.044040755831095145	0.005421998832161893
ENSG00000123352_49494861_49526255_417	SPATS2	12	+	1251	13	0	1	2.16%	6.0976382972098335	0.001946208561389314	0.006277459298483267	7.053443163459546e-05
ENSG00000103356_23557343_23525138_506	EARS2	16	-	1518	10	0	0	0.99%	3.8654836879545464	0.020613416668581838	0.020613416668581838	0.001946208561389314
ENSG00000120656_28621991_28603539_131	TAF12	1	-	393	19	0	0	6.11%	28.368519218316802	0.005160369442893307	0.005160369442893307	0.00014319601891785326
ENSG00000101294_31514552_31567643_345	HM13	20	+	1035	206	15	3	10.14%	116.78914715873336	7.5750608841212355e-06	1.6537999197845017e-07	1.2869933270663358e-11
ENSG00000188690_125816499_125802999_139	UROS	10	-	417	10	1	0	3.60%	14.071472993561155	0.03537493353818881	0.020613416668581838	0.003252526348827943

ENSG00000159140_33543093_33557551_2108	SON	21	+	6324	63	3	0	1.33%	5.845535531528228	8.169877164644909e-06	1.3820338985610477e-06	9.948228571329459e-11
ENSG00000153187_244864307_244848809_804	HNRNPU	1	-	2412	203	27	13	7.34%	49.384919584491925	2.778004265593307e-07	2.2053718895192142e-09	7.407954170762384e-15
ENSG00000138160_92607193_92653796_942	KIF11	10	+	2826	43	0	1	2.23%	8.928364552283972	2.2699789131724214e-05	3.432902348927335e-05	6.0402534230695134e-09
ENSG00000172531_67401782_67401033_73	PPP1CA	11	-	219	39	3	14	15.07%	104.49514396999318	0.005404561673025823	0.08727241690401005	0.002869338308477355
ENSG00000115520_197453561_197473924_188	COQ10B	2	+	564	17	0	0	3.72%	17.686643980736704	0.008674846900444944	0.008674846900444944	0.00038311532916634116
ENSG00000108825_42961207_42950593_307	PTGES3L-AARSD1	17	-	921	26	2	1	2.93%	16.564919673853424	0.010402771787637745	0.005642787686764809	0.0003061675497078111
ENSG00000213145_105487260_105488511_77	CRIP1	14	+	231	250	20	12	28.57%	635.0437487353897	4.829947205692244e-05	3.108342685307635e-05	1.1448317702416725e-08
ENSG00000068120_42562623_42565968_564	COASY	17	+	1692	24	2	4	1.60%	8.323126579170214	0.01292879023172146	0.026799084696674903	0.001638717780202175
ENSG00000157593_44255605_44254706_299	SLC35B2	6	-	897	12	0	0	3.01%	7.849905335538463	0.002874854311667729	0.002874854311667729	4.6982854837323694e-05
ENSG00000110090_68807631_68754816_660	CPT1A	11	-	1980	40	1	4	3.03%	11.854149976393941	3.0310500299891545e-05	0.00028327162729008254	6.700811886114469e-08
ENSG00000170889_54201185_54231754_77	RPS9	19	+	231	89	11	0	7.79%	226.07557454979874	0.044329616041373636	0.013640585738808999	0.002848092740928625 3
ENSG00000005889_24179200_24211376_780	ZFX	X	+	2340	13	1	4	0.77%	3.259891243508334	0.02034245439037237	0.09009720761581791	0.008312714083466195
ENSG00000109917_116787640_116778925_372	ZPR1	11	-	1116	9	1	2	1.34%	4.732100192189518	0.03537493353818881	0.08350359792828288	0.012068607709980346
ENSG00000148737_112950757_113165634_489	TCF7L2	10	+	1467	11	0	0	1.23%	4.399853212097138	0.012798402692974279	0.012798402692974279	0.0007972436888943567
ENSG00000163939_52679711_52548063_1609	PBRM1	3	-	4827	14	1	0	0.50%	1.7018698847402116	0.01007916461776185	0.004873903550586949	0.0002597390186955864
ENSG00000092621_119712023_119723445_119	PHGDH	1	+	357	15	0	0	5.04%	24.65463965678572	0.012798402692974279	0.012798402692974279	0.0007972436888943567
ENSG00000167325_4102013_4138383_779	RRM1	11	+	2337	73	0	3	3.34%	18.329041908301033	3.6565794610453736e-06	7.783485396183328e-06	2.3926886163361557e-10
ENSG00000231500_33272120_33276271_128	RPS18	6	+	384	78	8	0	7.03%	119.18977359077347	0.017322097050858944	0.003736770770438521	0.00035556838822001897
ENSG00000115525_85863399_85840144_362	ST3GAL5	2	-	1086	12	1	3	1.66%	6.483761589298344	0.026602764905793736	0.09783387127182075	0.011244525270099857
ENSG00000054983_87993116_87963321_391	GALC	14	-	1173	11	0	1	2.30%	5.502629720500002	0.002478942540635376	0.006467046632624 22	9.042414316359671e-05
ENSG00000129292_132794453_132845923_975	PHF20L1	8	+	2925	14	0	2	0.51%	2.8085216867148723	0.02108409854857798	0.055917760918677194	0.00521801263590997
ENSG00000119986_97679905_97677869_147	AVPI1	10	-	441	9	8	0	4.76%	11.975110690438779	0.4270380304967267	0.006937202941512724	0.030749786102305814
ENSG00000165650_117375212_117283268_1149	PDZD8	10	-	3447	26	8	7	1.48%	4.425962001630114	0.018809832122149275	0.003204987245677556	0.00033982826468722854
ENSG00000065809_14530459_14521190_131	FAM107B	10	-	393	21	0	0	6.11%	31.35467913603436	0.005604866691947602	0.005604866691947602	0.00016752456572620382
ENSG00000130749_47085148_47066356_881	AC008755.1	19	-	2643	11	1	0	0.57%	2.4421432698246317	0.03577298629450316	0.02108409854857798	0.003354604907114977
ENSG00000161960_7576539_7577934_257	EIF4A1	17	+	771	177	5	1	6.61%	134.7083463270759	0.0007517711932480938	0.00013027244556866954	6.948584554767835e-07
ENSG00000213246_58351550_58346246_108	SUPT4H1	17	-	324	34	0	0	7.41%	61.57572348849075	0.00575691403842271	0.00575691403842271	0.0001762552514680027
ENSG00000099622_1270934_1272443_297	CIRBP	19	+	891	27	1	0	3.70%	17.781224964590912	0.002493493840886608	0.0014055202957965589	2.0830049893778164e-05
ENSG00000094804_40289421_40302001_560	CDC6	17	+	1680	31	2	4	1.79%	10.827495915938396	0.009621867080838033	0.028352861400377415	0.0013383263540335357
ENSG00000183648_92117583_92116193_40	NDUFB1	14	-	120	169	7	3	30.00%	826.3824302293627	0.00108291700051257	0.0030976334765319066	2.0314095821141805e-05
ENSG00000151465_12237258_12250337_134	CDC123	10	+	402	14	0	0	5.22%	20.435139138410452	0.008175610787902942	0.008175610787902942	0.0003425184326896132 4
ENSG00000116096_72888544_72891537_83	SPR	2	+	249	16	0	0	8.43%	37.70476618997591	0.00847367416878415	0.00847367416878415	0.0003665024264111182
ENSG00000113368_126819018_126836264_241	LMNB1	5	+	723	21	2	0	4.15%	17.043414800085067	0.010187216365941177	0.0021227925778456486	0.000125180854499238
ENSG00000113368_126777539_126815220_319	LMNB1	5	+	957	20	0	0	2.51%	12.262913768683388	0.005454749182134642	0.005454749182134642	0.0001591085901575336 4
ENSG00000163516_219231975_219234365_361	ANKZF1	2	+	1083	15	0	0	1.39%	8.127152684646816	0.02108409854857798	0.02108409854857798	0.002029891263157455
ENSG00000148248_133366008_133363493_96	SURF4	9	-	288	46	18	1	12.50%	93.7218732508646	0.033590309857166684	0.00220554346506264	0.0004705272927290935
ENSG00000160633_5623206_5668291_916	SAFB	19	+	2748	38	1	6	2.18%	8.114139776418124	5.012229235663673e-05	0.0007133092711558117	2.782141960148045e-07
ENSG00000115306_54526419_54668569_2366	SPTBN1	2	+	7098	89	23	9	1.86%	7.357489112567415	2.601944907884968e-05	4.71986561986566e-08	1.621265330093391e-11
ENSG00000005893_120469169_120439154_410	LAMP2	X	-	1230	21	0	0	3.17%	10.018202358098783	0.0005399915898224664	0.0005399915898224664	1.8929313262929258e-06
ENSG00000119969_94562707_94601622_700	HELLS	10	+	2100	23	2	1	1.29%	6.426642737202144	0.010026873424650893	0.004355956481189797	0.0002334014788582348 3
ENSG00000017260_130934676_131016247_853	ATP2C1	3	+	2559	17	3	1	1.17%	3.898111451791912	0.011168917508652896	0.00324779312858951	0.0002001101642761065 3
ENSG00000115641_105386516_105361283_279	FHL2	2	-	837	24	2	0	3.23%	16.82524512778495	0.01052924788556975	0.003684287313187465	0.0002108713740163299 3
ENSG00000180398_46907962_46905463_94	MCFD2	2	-	282	19	2	0	6.38%	39.53485125105852	0.04223451612662591	0.013007239811447113	0.0026029828050034526
ENSG00000111859_11213721_11185162_828	NEDD9	6	-	2484	35	5	0	2.05%	8.267840110347224	0.0009367090916572945	0.0001248167544030276	8.393567413677706e-07
ENSG00000227500_1915020_1924284_229	SCAMP4	19	+	687	13	4	7	3.93%	11.103559694045854	0.0338510027225088	0.0338510027225088	0.004886670814259379
ENSG00000148737_112950757_113165972_607	TCF7L2	10	+	1821	11	0	0	0.99%	3.5445275464835264	0.012798402692974279	0.012798402692974279	0.0007972436888943567
ENSG00000164898_139341385_139345704_113	FMC1	7	+	339	18	5	0	7.96%	31.15648268131859	0.018219707656727348	0.0034782380676658805	0.0003524253498654976 4
ENSG00000167699_783265_760173_502	GLOD4	17	-	1506	33	8	5	2.39%	12.857738370809765	0.011782222721347909	0.008400953123688435	0.0004979131569927121
ENSG00000115947_147958839_147935510_352	ORC4	2	-	1056	14	0	0	1.99%	7.779285922008524	0.007029302661868638	0.007029302661868638	0.0002573639808771639
ENSG00000165533_88824720_88877410_471	TTC8	14	+	1413	17	1	1	2.34%	7.059637087852443	0.00241548406293102	0.00241548406293102	3.368517373808125e-05
ENSG00000165124_110549936_110366539_3338	SVEP1	9	-	10014	16	0	0	0.24%	0.9375361275518277	0.005306578019052857	0.005306578019052857	0.0001510011474053423 3
ENSG00000169826_43155630_43183542_382	CSGALNACT2	10	+	1146	10	0	0	1.31%	5.120248026452881	0.016947426762344633	0.016947426762344633	0.0013498980316300959
ENSG00000099250_33334382_33180076_906	NRP1	10	-	2718	134	11	15	5.30%	28.928836200669984	9.807932236891332e-08	1.3169797617710608e-07	1.247623533268653e-13
ENSG00000197756_216499339_216520753_60	RPL37A	2	+	180	255	25	11	30.00%	831.2722670946251	9.152174643962531e-05	0.00033690996897422923	2.2125306702080885e-07
ENSG00000077312_40751409_40763086_203	SNRPA	19	+	609	24	2	0	2.96%	23.124351678088676	0.0424352593247778	0.013007239811447113	0.002615673534585255
ENSG00000127603_39357657_39485794_3596	MACF1	1	+	10788	18	1	9	0.28%	0.9790552121771415	0.0033728746429155087	0.08512160584679956	0.001954614867589968 5
ENSG00000051523_88651013_88647097_68	CYBA	16	-	204	30	1	2	7.35%	86.29123879875002	0.03537493353818881	0.08350359792828288	0.012068607709980346
ENSG00000149657_62129823_62133461_230	LSM14B	20	+	690	12	0	0	3.04%	10.204876936200002	0.0078820576498062	0.0078820576498062	0.0003196364290745116 4
ENSG00000112992_43644650_43704404_707	NNT	5	+	2121	7	0	0	0.71%	1.9365690555495054	0.019216965118390855	0.019216965118390855	0.0017073955890589052
ENSG00000156411_103915153_103912666_46	ATP5MPL	14	-	138	66	5	2	19.57%	280.6341157455001	0.0081271044488283	0.003816220824102754	0.0001680317681842806
ENSG00000108559_5416586_5386206_626	NUP88	17	-	1878	35	1	0	2.08%	10.935737398350641	0.0008628208183719401	0.0006791898698869142	3.714078072945139e-06
ENSG00000104763_18084058_18067074_175	ASAH1	8	-	525	60	0	0	4.57%	67.06061986645716	0.005160369442893307	0.005160369442893307	0.0001431960189178532 6
ENSG00000057663_106316102_106046821_153	ATG5	6	-	459	11	0	0	3.27%	14.062275952388893	0.02108409854857798	0.02108409854857798	0.002029891263157455
ENSG00000096872_27029083_27062736_256	IFT74	9	+	768	8	0	0	1.95%	6.112296081578126	0.019679754444124863	0.019679754444124863	0.0017849636115922674
ENSG00000096060_35642824_35575835_457	FKBP5	6	-	1371	16	0	0	1.31%	6.847911583737419	0.013007239811447113	0.013007239811447113	0.0008218654148058079
ENSG00000104964_3054064_3053819_81	TLE5	19	-	243	12	2	7	6.17%	28.976811053407413	0.06556525143482543	0.17004230409153215	0.04074822827482303
ENSG00000169684_78565720_78593253_468	CHRNA5	15	+	1404	99	0	8	4.49%	41.375542706067314	2.6983639552617383e-05	0.0005790438422856566	1.2851001735262682e-07

ENSG00000147862_14313508_14093461_444	NFIB	9	−	1332	8	1	0	1.13%	3.5242067497387395	0.03418265644467716	0.019216965118390855	0.0029560527921161815
ENSG00000164924_100924973_100920693_125	YWHAZ	8	−	375	11	0	0	4.00%	17.212225765724003	0.019679754444124863	0.019679754444124863	0.0017849636115922674
ENSG00000072803_171914393_171868635_510	FBXW11	5	−	1530	21	1	0	1.76%	8.053848954550002	0.005034872532813168	0.0036062891729786097	9.977408550613472e−05
ENSG00000160932_143021325_143023697_45	LY6E	8	+	135	55	2	1	22.22%	239.05869119061117	0.002851000228491492	0.002827042522519993	4.587005910581118e−05
ENSG00000119541_63410299_63396986_261	VPS4B	18	−	783	20	0	0	2.30%	14.988005717279696	0.013428347753762199	0.013428347753762199	0.0008725593497644525
ENSG00000139289_76030976_76030536_146	PHLDA1	12	−	438	23	9	3	4.11%	30.8126706578185	0.1543801567637662	0.03736774915294124	0.023860979254184938
ENSG00000170315_16382364_16382597_77	UBB	17	+	231	70	6	1	7.79%	177.81224964590913	0.013640585738808999	0.013640585738808999	0.00089863692579514
ENSG00000170142_23807270_23887768_134	UBE2E1	3	+	402	22	4	0	5.22%	32.11236150321643	0.046045457964455916	0.00877616296920843	0.0020495800739836604
ENSG00000164081_51674305_51704016_313	TEX264	3	+	939	23	1	0	3.19%	14.372683437832272	0.003133490598659634	0.002390425500591921	4.28420906379313e−05
ENSG00000168872_70346992_70371986_478	DDX19A	16	+	1434	8	0	0	1.05%	3.2735309558242687	0.019679754444124863	0.019679754444124863	0.0017849636115922674
ENSG00000185630_164559823_164849402_420	PBX1	1	+	1260	27	5	0	1.90%	12.573866224960717	0.03265524478991762	0.005706018193000826	0.000993589204822162
ENSG00000111142_95474180_95513904_442	METAP2	12	+	1326	103	1	0	8.37%	45.57947485267309	5.660376161185916e−08	4.4928943704717294e−08	2.5201450924676847e−14
ENSG00000127990_94656098_94585465_485	SGCE	7	−	1455	26	3	2	2.47%	10.485423381181446	0.005454749182134642	0.0026311212314026142	8.05834708867572e−05
ENSG00000215301_41343231_41347719_476	DDX3X	X	+	1428	83	1	0	7.56%	34.10558485855358	6.958679763556736e−08	4.081835640353621e−08	2.8150375102779016e−14
ENSG00000215301_41334253_41342657_148	DDX3X	X	+	444	12	11	3	6.76%	15.858930373824329	0.2253218638402108	0.020489116813065835	0.023933338958737164
ENSG00000198042_33485207_33489143_131	MAK16	8	+	393	24	2	1	5.34%	35.83391901261069	0.016983116543564363	0.010042643336235583	0.0008354226420605304
ENSG00000169139_48043161_48060828_97	UBE2V2	8	+	291	10	3	0	5.15%	20.16427573304124	0.12180769416495907	0.019679754444124863	0.011261990492355435
ENSG00000176095_49748022_49727122_435	IP6K1	3	−	1305	7	1	3	1.15%	3.1474812006287363	0.06448902149598212	0.169362073879829	0.040061615358839356
ENSG00000101266_489860_483961_177	CSNK2A1	20	−	531	9	0	0	2.82%	9.945430912398308	0.019216965118390855	0.019216965118390855	0.0017073955890589052
ENSG00000166340_6616817_6614546_320	TPP1	11	−	960	19	0	0	3.12%	11.61336255499844	0.0017901637611834264	0.0017901637611834264	1.8982104156792192e−05
ENSG00000133657_194441331_194419892_576	ATP13A3	3	−	1728	29	0	1	1.39%	9.847588131431426	0.005706018193000826	0.010197420091687953	0.0003033095104916256
ENSG00000127990_94618900_94524260_254	SGCE	7	−	762	11	0	2	2.76%	8.470583546124018	0.007029302661868638	0.03522780890193623	0.0012822660609675817
ENSG00000179262_12946003_12953049_344	RAD23A	19	+	1032	33	0	0	4.36%	18.76332750623983	0.00028877520139654507	0.00028877520139654507	5.646841491566603e−07
ENSG00000105011_14136456_14120459_143	ASF1B	19	−	429	29	2	0	6.99%	39.665809536395116	0.007816515653272151	0.0023766146074493525	0.00010539817287955899
ENSG00000166946_43190120_43192884_201	CCNDBP1	15	+	603	22	0	0	4.98%	21.408241002144283	0.002046028879997488	0.002046028879997488	2.4518070358754455e−05
ENSG00000261067_28982291_28984299_228	AC109460.3	16	+	684	19	0	1	3.51%	16.29945621754167	0.005306578019052857	0.0069659089849165145	0.00019587836168716277
ENSG00000261067_28977925_28981619_162	AC109460.3	16	+	486	7	0	1	3.70%	8.451569890577163	0.009815328628645334	0.0477903522728147	0.0023388674905236288
ENSG00000181924_73873378_73873115_87	COA4	11	−	261	43	15	7	5.75%	96.67263687645405	0.08588638879785576	0.08588638879785576	0.026644917313437298
ENSG00000183605_119155112_119141242_110	SFXN4	10	−	330	12	0	0	4.55%	21.337469957509096	0.020613416668581838	0.020613416668581838	0.001946208561389314
ENSG00000285130_69035922_69099934_92	AL358113.1	9	+	276	7	0	0	5.43%	14.882112198625004	0.019216965118390855	0.019216965118390855	0.0017073955890589052
ENSG00000131508_139561792_139626801_147	UBE2D2	5	+	441	38	6	1	5.44%	50.56157847074151	0.03274340904245237	0.007356796211402075	0.0012336742649452252
ENSG00000174891_158122105_158544275_334	RSRC1	3	+	1002	24	3	1	1.50%	14.054620930095812	0.13601972394786777	0.04223451612662591	0.022931112790783643
ENSG00000198663_36899445_36923441_347	C6orf89	6	+	1041	7	0	0	1.44%	3.9456896895489924	0.019216965118390855	0.019216965118390855	0.0017073955890589052
ENSG00000037474_6632654_6599926_701	NSUN2	5	−	2103	69	4	3	4.28%	19.252424747681175	8.708963997464472e−06	3.4139231024345645e−06	2.50566853000206e−10
ENSG00000132780_45607467_45618141_603	NASP	1	+	1809	32	2	0	2.16%	10.379753213160864	0.003031355522137688	0.0005983074123552221	1.1613785618560066e−05
ENSG00000205208_158671801_158669613_113	C4orf46	4	−	339	14	0	0	4.42%	24.232819863247794	0.02108409854857798	0.02108409854857798	0.002029891263157455
ENSG00000120539_27159688_27186536_709	MASTL	10	+	2127	23	0	2	1.83%	6.345063351257406	0.000583356306045086	0.0032632366911302837	1.2253440031507521e−05
ENSG00000184640_77281536_77498658_567	SEPT9	17	+	1701	55	3	6	3.17%	18.972911999254855	0.000442321637794036	0.0014426254751151526	4.136427255035133e−06
ENSG00000149474_18142865_18187459_714	KAT14	20	+	2142	10	0	0	0.70%	2.7394044063095246	0.019679754444124863	0.019679754444124863	0.0017849636115922674
ENSG00000100372_40794548_40776282_190	SLC25A17	22	−	570	23	0	0	3.68%	23.677104821271058	0.007029302661868638	0.007029302661868638	0.0002573639808771639
ENSG00000114030_122451605_122426985_311	KPNA1	3	−	933	24	2	1	3.54%	15.094030195022512	0.005625935045480705	0.00236147777460975	7.526908837398504e−05
ENSG00000128567_131556359_131504311_558	PODXL	7	−	1674	10	0	0	1.08%	3.505259401621865	0.009815328628645334	0.009815328628645334	0.0004837142251238724
ENSG00000138032_44201305_44232345_352	PPM1B	2	+	1056	23	1	1	2.27%	12.780255443299719	0.0073129010928764775	0.0073129010928764775	0.0002773765668225625
ENSG00000125457_75270195_75266740_170	MIF4GD	17	−	510	13	0	0	3.53%	14.957148058450004	0.011772051206276392	0.011772051206276392	0.000681272511389508
ENSG00000258947_89923402_89933236_87	TUBB3	16	+	261	23	0	0	8.05%	51.708619724614955	0.0078820576498062	0.0078820576498062	0.00031963642907451164
ENSG00000198862_28992805_28930448_1766	LTN1	21	−	5298	15	0	0	0.57%	1.6613262282885055	0.00199575720716401	0.00199575720716401	2.3377487150540766e−05
ENSG00000163428_120349243_120331200_371	LRRC58	3	−	1113	20	0	0	2.70%	10.544122620512132	0.0020587098026626535	0.0020587098026626535	2.4809845078521198e−05
ENSG00000151092_25783390_25737305_343	NGLY1	3	−	1029	13	0	0	1.46%	7.413163760747815	0.020613416668581838	0.020613416668581838	0.001946208561389314
ENSG00000134001_67364768_67374556_109	EIF2S1	14	+	327	19	0	3	7.34%	34.09427539082111	0.005306578019052857	0.03497523187724695	0.0010067653727672797
ENSG00000198668_90404421_90404717_40	CALM1	14	+	120	33	0	0	30.00%	161.36461655366253	0.0010447178140462346	0.0010447178140462346	6.749952978822125e−06
ENSG00000097033_86704900_86743235_394	SH3GLB1	1	+	1182	23	1	0	2.28%	11.41789318792259	0.005670089768331009	0.0035547763465261306	0.00011049933550063637
ENSG00000117410_43976115_43978007_158	ATP6V0B	1	+	474	48	3	0	11.39%	59.42080241331647	0.0001789774272272485	8.751142344759464e−05	1.1253437756231516e−07
ENSG00000055163_157319804_157393000_812	CYFIP2	5	+	2436	8	4	0	0.74%	1.9270293065073896	0.23639154306007898	0.011570465654371866	0.017275440155518785
ENSG00000141510_7676594_7669609_393	TP53	17	−	1179	109	1	6	4.83%	54.24857183853564	6.857358883697343e−05	0.0007313507128457925	3.780271356515748e−07
ENSG00000149084_43830976_43855248_145	HSD17B12	11	+	435	21	0	0	6.90%	28.327330805658626	0.0023218226288164415	0.0023218226288164415	3.1231198880462635e−05
ENSG00000134371_193141905_193250712_342	CDC73	1	+	1026	15	0	0	2.05%	8.578661167127194	0.00847367416878415	0.00847367416878415	0.0003665024264111182
ENSG00000109854_20383104_20383205_33	HTATIP2	11	+	99	11	0	0	18.18%	65.19782487016668	0.012798402692974279	0.012798402692974279	0.0007972436888943567
ENSG00000112118_52284674_52264588_808	MCM3	6	−	2424	55	3	11	2.60%	13.31391225690285	0.00016523502770687375	0.0018426548085934298	2.1950330175683155e−06
ENSG00000101255_388011_396690_358	TRIB3	20	+	1074	98	8	0	7.26%	53.54234779840504	4.752487226358163e−05	3.650308449239545e−06	1.5059769789961688e−09
ENSG00000090273_26921849_26946181_331	NUDC	1	+	993	46	7	0	6.34%	27.182174719283996	0.0005179315122922545	2.2730822885583496e−05	9.795191628669849e−08
ENSG00000084072_39741919_39753355_235	PPIE	1	+	705	19	0	0	4.26%	15.813940500423408	0.002046028879997488	0.002046028879997488	2.4518070358754455e−05
ENSG00000188986_137255510_137272928_580	NELFB	9	+	1740	21	0	4	1.55%	7.0818327014146565	0.0033030736763512156	0.0360990988508288	0.0007068562907806174
ENSG00000197885_23901047_23893095_160	NKIRAS1	3	−	480	16	0	1	5.00%	19.559347461050006	0.005306578019052857	0.009288069496478249	0.0002590037054391842
ENSG00000110048_59610396_59576577_622	OSBP	11	−	1866	20	6	5	1.29%	6.289179247926047	0.05158844004651438	0.05158844004651438	0.010592703807868874
ENSG00000124422_21043760_21003031_513	USP22	17	−	1539	42	2	2	3.70%	16.013500845304097	0.00020419716377313488	0.00020419716377313488	2.8862363757069656e−07
ENSG00000077549_19484279_19339530_301	CAPZB	1	−	903	349	8	11	12.62%	226.78446059489872	2.6404430716085847e−07	3.60870533568439e−08	9.548977441942281e−14
ENSG00000251357_23894805_23895106_68	AP000350.4	22	+	204	470	17	4	38.24%	1351.8960745137504	8.06630639247374e−06	3.988223592346426e−06	2.6952009996165263e−10

ENSG00000124155_45419234_45425826_434	PIGT	20	+	1302	69	4	0	4.84%	31.09665840581683	0.00016569421277282895	2.583275382380064e-05	3.3235265447170386e-08
ENSG00000124155_45420349_45425826_316	PIGT	20	+	948	52	2	0	4.11%	32.18626797387975	0.0022083206986541785	0.0006768518708446028	9.39609878359303e-06
ENSG00000283761_100058702_100082209_418	AC118553.2	1	+	1254	27	0	0	4.31%	12.634028264314596	4.027253539844791e-05	4.027253539844791e-05	1.23110933795418e-08
ENSG00000168159_228487816_228493885_126	RNF187	1	+	378	34	1	3	10.32%	52.7791915615635	0.0006698694257094773	0.0035156470961704048	1.4976062642096571e-05
ENSG00000131389_14472288_14485007_227	SLC6A6	3	+	681	31	8	0	3.52%	26.711003140640976	0.015765166103784645	0.00575691403842271	0.00047124852588542913
ENSG00000131389_14443635_14472313_359	SLC6A6	3	+	1077	34	14	5	5.01%	18.524173082888584	0.0063730122972834894	0.0009107006179919566	3.655267001923536e-05
ENSG00000147140_71294272_71297465_212	NONO	X	+	636	22	1	4	4.72%	20.297436044485853	0.0040705821972625445	0.030308563384185347	0.0006922921711919004
ENSG00000106392_7234320_7243727_363	C1GALT1	7	+	1089	10	0	0	1.65%	5.388249989269974	0.011772051206276392	0.011772051206276392	0.000681272511389508
ENSG00000135480_52233297_52248760_469	KRT7	12	+	1407	1001	69	46	27.51%	417.46069954181354	6.743195792563088e-22	2.371588151770765e-21	2.742708795776788e-41
ENSG00000026508_35139304_35176744_78	CD44	11	+	234	173	6	12	25.64%	433.81629625149367	4.953489653357821e-05	0.000311353650550448	1.1576901870726388e-07
ENSG00000049618_157174031_157207891_1233	ARID1B	6	+	3699	16	0	0	0.57%	2.53811483679481	0.00847367416878415	0.00847367416878415	0.0003665024264111182
ENSG00000146409_132790236_132770270_383	SLC18B1	6	-	1149	8	3	0	1.31%	4.085503386120106	0.14389369507707506	0.019679754444124863	0.013602114125134476
ENSG00000168216_69790322_69676158_467	LMBRD1	6	-	1401	21	3	0	2.14%	8.795423911821201	0.01424822060979566	0.0022409900782254734	0.00018649561876446586
ENSG00000103423_4425992_4455568_300	DNAJA3	16	+	900	36	6	4	4.67%	23.471216953260004	0.0038333091638150267	0.0038089915011381885	8.084724590990909e-05
ENSG00000108433_46923193_46938760_210	GOSR2	17	+	630	12	0	0	2.86%	11.17676997774286	0.011772051206276392	0.011772051206276392	0.000681272511389508
ENSG00000184009_81511106_81510690_107	ACTG1	17	-	321	272	18	4	24.30%	497.2095803182805	1.0089767718949073e-05	7.283122306280141e-06	6.019099417021277e-10
ENSG00000184009_81512354_81511129_286	ACTG1	17	-	858	414	88	18	18.88%	283.1318128977168	7.699429894939255e-07	1.1981720906171204e-09	1.2819844530288115e-14
ENSG00000108433_46923193_46966724_257	GOSR2	17	+	771	12	0	0	2.33%	9.132769242513621	0.011772051206276392	0.011772051206276392	0.000681272511389508
ENSG00000140297_59618296_59619555_419	GCNT3	15	+	1257	43	0	3	3.58%	20.07283868317781	0.00029563251185049877	0.0017490684155374503	3.4913052889215228e-06
ENSG00000108107_55386379_55388216_156	RPL28	19	+	468	437	56	21	14.74%	547.9124897742854	0.0001798645505738252	5.292524753533204e-05	7.038375442223417e-08
ENSG00000196535_29166826_29074770_2016	MYO18A	17	-	6048	10	0	1	0.25%	0.9702057272346233	0.019679754444124863	0.02784981298332479	0.002477909089056847
ENSG00000010278_6200500_6237828_228	CD9	12	+	684	225	12	26	22.37%	193.01987626036188	3.0947290438040336e-09	5.712446715057884e-09	1.886678929190764e-16
ENSG00000120549_24513334_24545118_557	KIAA1217	10	+	1671	8	0	0	0.90%	2.8092420051777385	0.019679754444124863	0.019679754444124863	0.0017849636115922674
ENSG00000120727_139363893_139368798_31	PAIP2	5	+	93	22	0	0	16.13%	138.80827230422585	0.02108409854857798	0.02108409854857798	0.002029891263157455
ENSG00000125505_54183527_54174044_310	MBOAT7	19	-	930	31	1	0	4.19%	19.559347461050006	0.0005654071426656465	0.0005854768899833019	2.1394375775186603e-06
ENSG00000090263_141010603_141006430_96	MRPS33	7	-	288	46	11	2	11.46%	93.7218732508646	0.0644728091714328	0.0031465620335393613	0.0013258518394647205
ENSG00000150527_39236945_39350264_1293	MIA2	14	+	3879	18	0	0	0.77%	2.7228789968979124	0.001801896632038876	0.001801896632038876	1.9221247299282243e-05
ENSG00000205133_92916989_92886622_86	TRIQK	8	-	258	7	0	0	5.81%	15.920399096203491	0.019216965118390855	0.019216965118390855	0.0017073955890589052
ENSG00000130770_28236184_28246637_75	ATP5IF1	1	+	225	32	0	0	17.33%	83.45321583381335	0.0006311913977015671	0.0006311913977015671	2.5578527397951546e-06
ENSG00000105618_54118279_54131322_491	PRPF31	19	+	1473	33	1	5	2.85%	13.145793609259677	0.0006384869324604317	0.004575099335829988	1.8889284785867128e-05
ENSG00000147274_136879427_136872338_290	RBMX	X	-	870	6	1	0	1.72%	4.046761543665518	0.0477903522728147	0.016947426762344633	0.0036975118224100542
ENSG00000183283_51240919_51242458_108	DAZAP2	12	+	324	38	0	0	7.41%	68.8199262518426	0.005655335537214311	0.005655335537214311	0.00017039952270558162
ENSG00000126432_64318216_64321691_214	PRDX5	11	+	642	229	5	9	24.30%	209.30329759721732	5.004014051711365e-10	3.344716246859963e-09	1.880774996126428e-17
ENSG00000124920_61766142_61786143_1014	MYRF	11	+	3042	12	1	0	0.59%	2.314715675863906	0.019795881618345192	0.012798402692974279	0.0012079910998532793
ENSG00000100605_93115163_92941561_414	ITPK1	14	-	1242	15	1	0	1.93%	7.086720094583335	0.007930666369886513	0.005160369442893307	0.00021637657341139557
ENSG00000175387_47896756_47841827_467	SMAD2	18	-	1401	37	4	3	3.21%	15.496699273208783	0.002020802647119262	0.0015846582505280478	1.899274279269736e-05
ENSG00000130726_58544758_58550553_753	TRIM28	19	+	2259	257	18	7	7.97%	66.75633861208301	4.980912625603854e-10	4.690535231852462e-11	2.7901515464457435e-19
ENSG00000168575_42472108_42417803_558	SLC20A2	8	-	1674	14	0	0	1.08%	4.90736316227061	0.012798402692974279	0.012798402692974279	0.0007972436888943567
ENSG00000119655_74493274_74480618_174	NPC2	14	-	522	72	10	0	12.07%	80.93523087331036	0.0001380777919487111	2.7375946223247435e-05	2.9098331983747132e-08
ENSG00000182871_45495370_45512398_525	COL18A1	21	+	1575	15	2	0	1.71%	5.58838498887143	0.01652445925752544	0.0033278027414746945	0.0003062742250611212
ENSG00000173230_121698635_121664480_2635	GOLGB1	3	-	7905	23	7	0	0.46%	1.7072675203193552	0.01039256944151647	0.0008776897154483972	5.9759235036696684e-05
ENSG00000164237_10290570_10280453_181	CMBL	5	-	543	13	2	0	3.31%	14.048150110146413	0.05258119297243839	0.013007239811447113	0.00326646906127711
ENSG00000134294_46365266_46361111_344	SLC38A2	12	-	1032	241	7	3	15.41%	137.0291493637515	8.535582850552234e-10	3.407453597279063e-10	3.2805611798105094e-18
ENSG00000168000_62705512_62690367_398	BSCL2	11	-	1194	16	15	2	2.51%	7.863054255698494	0.3857046718699725	0.008474269282996836	0.029120313164340397
ENSG00000168000_62705595_62702488_118	BSCL2	11	-	354	14	0	6	5.08%	23.206005462262716	0.013428347753762199	0.16970534604944526	0.012520549700299675
ENSG00000108773_42120311_42113649_663	KAT2A	17	-	1989	18	1	1	1.51%	5.310230079923078	0.0037373685375237473	0.0037373685375237473	7.750388117310624e-05
ENSG00000169714_129171757_129170453_177	CNBP	3	-	531	271	23	7	26.55%	299.4679752511046	1.2937999542621098e-08	1.7109348863420996e-08	2.275784413274624e-15
ENSG00000135473_56333046_56317609_1186	PAN2	12	-	3558	8	0	4	0.42%	1.3193489012512651	0.019679754444124863	0.2463583386135439	0.026068547507937054
ENSG00000108788_42567125_42571603_214	MLX	17	+	642	12	1	2	2.34%	10.967858389373834	0.051235217429874684	0.08350359792828288	0.01650998562589935
ENSG00000136754_26823167_26748570_394	ABI1	10	-	1182	20	1	0	2.28%	9.9286027721066	0.005588370640926919	0.0034782380676658805	0.00010676685949938237
ENSG00000108094_35071242_35010311_720	CUL2	10	-	2160	24	0	0	1.67%	6.519782487016668	0.0008739357725210369	0.0008739357725210369	4.787965623198429e-06
ENSG00000072041_84873319_84861859_316	SLC6A15	12	-	948	10	0	3	1.90%	6.189666918053799	0.012798402692974279	0.10000264635955686	0.006484128903061078
ENSG00000138085_27213252_27215396_40	ATRAID	2	+	120	78	0	0	12.50%	381.4072754904751	0.021557223391537678	0.021557223391537678	0.002115616449879074
ENSG00000170471_38492942_38574967_1425	RALGAPB	20	+	4275	5	1	0	0.35%	0.6862928933701756	0.051235217429874705	0.012673659338734126	0.0031110695322510533
ENSG00000170471_38492942_38574967_1428	RALGAPB	20	+	4284	5	1	0	0.35%	0.6848511015773812	0.051235217429874705	0.012673659338734126	0.0031110695322510533
ENSG00000197563_62157141_62045856_788	PIGN	18	-	2364	12	0	0	0.76%	2.97858083163198	0.011772051206276392	0.011772051206276392	0.000681272511389508
ENSG00000128524_128862905_128865578_147	ATP6V1F	7	+	441	24	2	0	6.80%	31.93362850783674	0.00931822015308154	0.0020587098026626535	0.00011110314649289232
ENSG00000106268_2244575_2251001_156	NUDT1	7	+	468	34	3	0	7.05%	42.6293470304936	0.003453048488940946	0.0016186486669924592	3.275367415488609e-05
ENSG00000141552_81894478_81900065_84	ANAPC11	17	+	252	127	10	2	20.24%	295.7187056611132	0.00039118706886775756	0.00018946255112486357	5.081891006409044e-07
ENSG00000164713_98281796_98292690_98	BRI3	7	+	294	9	1	0	5.10%	17.962666035658167	0.019216965118390855	0.016947426762344633	0.0015190811725471732
ENSG00000076248_109098327_109105098_258	UNG	12	+	774	25	2	4	3.49%	18.952856066908918	0.008413704741378425	0.021542863694648147	0.0009021109423852869
ENSG00000060982_24902988_24818008_398	BCAT1	12	-	1194	33	0	0	2.76%	16.217549402378143	0.001484234770590415	0.001484234770590415	1.3251387882263029e-05
ENSG00000148834_104255213_104267405_213	GSTO1	10	+	639	103	1	2	13.15%	94.58276002291787	1.998320446936423e-06	2.478752623735077e-06	4.294055395081671e-11
ENSG00000149600_32743337_32703382_182	COMMD7	20	-	546	8	0	0	3.30%	8.597515367494507	0.011570465654371866	0.011570465654371866	0.0006594843062896773
ENSG00000083845_58388138_58394750_204	RPS5	19	+	612	525	12	15	21.08%	503.3655596593751	6.36195112857163e-09	4.974003188196502e-09	3.343268422650572e-16
ENSG00000068796_62306473_62385569_744	KIF2A	5	+	2232	25	0	1	1.88%	6.572361378040996	0.0003353510003294976	0.0006716243423686093	1.4880509536321035e-06
ENSG00000005022_119470375_119471058_98	SLC25A5	X	+	294	208	5	3	18.37%	415.13717060187764	0.00012830498881185334	0.00016284809821805806	1.4783283720471343e-07

ENSG00000013441_200859774_200853306_333	CLK1	2	-	999	26	0	0	4.50%	15.271562582201204	0.0002582331582969843	0.0002582331582969843	4.5481323920964005e-07
ENSG00000105854_95434951_95416188_84	PON2	7	-	252	214	7	7	17.86%	498.2976615077025	0.0020951569491565744	0.0016955003789966925	2.0971188500252076e-05
ENSG00000156261_29073590_29056475_548	CCT8	21	-	1644	157	8	2	8.76%	56.03681663111041	5.388589530921917e-09	6.167169475354342e-10	3.729750770685513e-17
ENSG00000091542_18184292_18208396_378	ALKBH5	17	+	1134	22	4	0	2.91%	11.383747199552912	0.013696866554086288	0.0014055202957965589	0.00012014733367509039
ENSG00000130164_11089549_11131316_682	LDLR	19	+	2046	121	12	2	6.16%	34.70206807605646	2.293709178316566e-06	1.0282233410664766e-08	2.8932017711882993e-13
ENSG00000164327_38991075_38942304_1556	RICTOR	5	-	4668	7	0	1	0.39%	0.8799192302528922	0.009815328628645334	0.028889785561798602	0.0013877953586383081
ENSG00000175221_890233_868373_800	MED16	19	-	2400	8	1	0	0.62%	1.9559347461050005	0.04223451612662591	0.019679754444124863	0.003715148010809822
ENSG00000089177_16528406_16273253_1239	KIF16B	20	-	3717	19	0	0	0.65%	2.99941567199314	0.005454749182134642	0.005454749182134642	0.00015910859015753364
ENSG00000188603_28491759_28482459_307	CLN3	16	-	921	21	2	0	2.61%	13.379358198112381	0.018151375140008384	0.005604866691947602	0.0005308776856007569
ENSG00000105676_19051622_19057628_408	ARMC6	19	+	1224	12	0	4	1.96%	5.752749253250001	0.004873903550586949	0.06582357386012391	0.0019037538527061307
ENSG00000068024_239176542_239053097_964	HDAC4	2	-	2892	7	0	0	0.52%	1.4202845666737556	0.019216965118390855	0.019216965118390855	0.0017073955890589052
ENSG00000196704_68251115_68256969_186	AMZ2	17	+	558	27	0	0	5.38%	28.392601153137104	0.0023218226288164415	0.0023218226288164415	3.1231198880462635e-05
ENSG00000115091_113916937_113957455_367	ACTR3	2	+	1101	62	0	0	7.08%	33.04303930749592	3.4812544889409737e-06	3.4812544889409737e-06	1.0309951424027688e-10
ENSG00000134330_9481342_9488329_135	IAH1	2	+	405	18	0	0	5.19%	26.079129948066672	0.008674846900444944	0.008674846900444944	0.00038311532916634116
ENSG00000117298_21260288_21219955_571	ECE1	1	-	1713	50	3	3	4.20%	17.127274484281966	2.597019403851631e-05	5.2349357447643746e-05	1.0434486324538327e-08
ENSG00000077549_19448914_19339567_260	CAPZB	1	-	780	297	13	10	10.00%	223.4279306127635	0.00011048624775174703	3.6785970682872193e-06	3.6932207013979282e-09
ENSG00000077549_19485438_19339530_272	CAPZB	1	-	816	359	8	11	14.34%	258.15462273959383	1.6818361878037985e-07	2.4416874222578906e-08	4.159592379626624e-14
ENSG00000175193_183884846_183829598_379	PARL	3	-	1137	14	0	0	1.85%	7.225088771891822	0.007029302661868638	0.007029302661868638	0.0002573639808771639
ENSG00000091039_76410578_76355889_798	OSBPL8	12	-	2394	18	0	1	1.38%	4.411882885951129	0.0008884494972709903	0.0024579339080672683	1.3429802402146636e-05
ENSG00000187109_76074219_76048429_391	NAP1L1	12	-	1173	94	12	6	6.14%	47.02247215700001	0.00015072380328551603	3.6411670473104e-05	4.149127284218979e-08
ENSG00000140396_70216745_70113632_1464	NCOA2	8	-	4392	10	0	0	0.34%	1.3360210014378417	0.019679754444124863	0.019679754444124863	0.0017849636115922674
ENSG00000025800_32154662_32160639_160	KPNA6	1	+	480	7	0	0	3.75%	8.557214514209377	0.009815328628645334	0.009815328628645334	0.0004837142251238724
ENSG00000025800_32161975_32170894_311	KPNA6	1	+	933	10	0	0	1.61%	6.289179247926047	0.019679754444124863	0.019679754444124863	0.0017849636115922674
ENSG00000114054_136255955_136333476_456	PCCB	3	+	1368	18	0	1	1.54%	7.720795050414475	0.00847367416878415	0.013892891352654344	0.0005886798845679606
ENSG00000159176_201496303_201490172_94	CSRP1	1	-	282	122	4	5	11.70%	253.85536066469155	0.0030191375341050296	0.002851000228491492	4.8845430887746786e-05
ENSG00000197535_52528806_52313696_1828	MYO5A	15	-	5484	14	0	1	0.38%	1.4979806589425606	0.0078820576498062	0.015516259750072458	0.0006142571802709984
ENSG00000119705_77708112_77717494_92	SLIRP	14	+	276	112	3	2	18.48%	238.11379517800006	0.00018664599259042051	0.00013836408333450526	1.8165617297358609e-07
ENSG00000275714_26020490_26020900_136	HIST1H3A	6	+	408	53	3	4	13.24%	76.22392760556252	0.00047386423480427233	0.0006114786663785357	1.8836624710765269e-06
ENSG00000124357_71068684_71078508_344	NAGK	2	+	1032	17	2	0	2.03%	9.665956594123548	0.04912109528954385	0.007029302661868638	0.0018325026027891263
ENSG00000137714_110430121_110462468_184	FDX1	11	+	552	15	0	0	2.72%	15.945120212812503	0.020613416668581838	0.020613416668581838	0.001946208561389314
ENSG00000072958_16198027_16234435_435	AP1M1	19	+	1305	14	2	0	1.61%	6.2949624012574725	0.03299602752967379	0.00847367416878415	0.0014019198044660757
ENSG00000163072_168802647_168864970_563	NOSTRIN	2	+	1689	9	0	0	0.89%	3.1267162903987575	0.016947426762344633	0.016947426762344633	0.0013498980316300959
ENSG00000119912_92514944_92454444_766	IDE	10	-	2298	17	0	0	1.31%	4.340847347752612	0.0020082573300163734	0.0020082573300163734	2.3658688350241813e-05
ENSG00000168476_22141470_22139155_221	REEP4	8	-	663	19	3	1	5.43%	16.81572858642308	0.011794527126255325	0.0025320062747194163	0.00017003622858608236
ENSG00000099341_38374791_38383390_287	PSMD8	19	+	861	67	4	3	5.92%	45.66119442126656	0.00021754394475824391	0.000557069202053668	8.229714661227028e-07
ENSG00000112759_44229610_44233528_412	SLC29A1	6	+	1236	49	6	0	4.61%	23.262330718239085	0.0003953272125394652	5.6254241454829154e-05	1.6639839158165156e-07
ENSG00000164347_74751461_74736764_401	GFM2	5	-	1203	12	0	0	1.50%	5.853171310039902	0.012798402692974279	0.012798402692974279	0.0007972436888943567
ENSG00000067369_43492337_43407383_1931	TP53BP1	15	-	5793	17	1	0	0.52%	1.7219518738366135	0.0037220617515305076	0.0021880057261679567	4.6633814146144326e-05
ENSG00000100503_50821942_50725872_1339	NIN	14	-	4017	11	6	5	0.52%	1.6068171924686336	0.07786981947941429	0.16840675538759814	0.046193992236766436
ENSG00000135535_109382378_109368279_189	CD164	6	-	567	59	6	1	10.58%	61.05828043396562	0.0006472167783162078	3.936500948162073e-05	2.023492121826453e-07
ENSG00000167779_53100855_53102167_81	IGFBP6	12	+	243	42	0	0	11.11%	101.41883868692595	0.003710480643933249	0.003710480643933249	7.644477796348072e-05
ENSG00000099998_24244725_24219970_554	GGT5	22	-	1662	12	2	4	1.08%	4.236681760516246	0.05258119297243839	0.10069662721979156	0.02022434976052801
ENSG00000099991_24042910_24178196_2103	CABIN1	22	+	6309	6	0	1	0.24%	0.5580412970342369	0.016947426762344633	0.0477903522728147	0.0036975118224100542
ENSG00000185347_105491376_105498946_426	TEDC1	14	+	1278	10	3	0	1.88%	4.591396117617372	0.06188259712708487	0.00414960799764038	0.001564036282935533
ENSG00000185347_105491376_105498946_495	TEDC1	14	+	1485	10	3	0	1.62%	3.9513833254646475	0.06188259712708487	0.00414960799764038	0.001564036282935533
ENSG00000185347_105491103_105498946_352	TEDC1	14	+	1056	10	3	0	2.27%	5.55663280143466	0.06188259712708487	0.00414960799764038	0.001564036282935533
ENSG00000100796_91509647_91466415_540	PPP4R3A	14	-	1620	13	6	0	2.04%	4.7087317961787045	0.08128002369891074	0.0006703205586147392	0.0005664216636048183
ENSG00000064042_41605996_41697185_1467	LIMCH1	4	+	4401	18	5	8	0.75%	2.3999199338711663	0.03804190072700041	0.05524769284829258	0.008589813277144354
ENSG00000149806_65121813_65121492_75	FAU	11	-	225	85	0	3	12.00%	221.67260455856672	0.0037631575832289435	0.006346985027636522	0.00013005064677948632
ENSG00000104388_60517208_60620769_212	RAB2A	8	+	636	22	0	1	5.66%	20.297436044485853	0.0007660506977098094	0.001620025777606367	7.723690688708469e-06
ENSG00000102125_154411916_154421004_224	TAZ	X	+	672	13	1	0	2.68%	11.351407008645092	0.015555382717448078	0.011772051206276392	0.0008874794703752884
ENSG00000148180_121317107_121332603_473	GSN	9	+	1419	17	0	0	1.06%	7.029786613908035	0.02108409854857798	0.02108409854857798	0.002029891263157455
ENSG00000129055_134482904_134478590_74	ANAPC13	3	-	222	14	0	0	10.81%	37.004170872256765	0.005160369442893307	0.005160369442893307	0.00014319601891785326
ENSG00000127948_75982288_75986481_415	POR	7	+	1245	65	10	2	4.82%	30.635122529355428	0.001047612343505962	6.632932927897572e-05	5.371273304583372e-07
ENSG00000109099_15260727_15239412_125	PMP22	17	-	375	29	1	0	7.20%	45.37768610963601	0.00562235203092679	0.0033030736763512156	0.00010238030643782358
ENSG00000096063_35921056_35835304_655	SRPK1	6	-	1965	16	1	1	1.68%	4.777855868348093	0.0025320062747194163	0.0025320062747194163	3.6860401525304375e-05
ENSG00000187109_76074219_76048429_391	NAP1L1	12	-	1173	94	12	6	6.14%	47.02247215700001	0.00015072380328551603	3.6411670473104e-05	4.149127284218979e-08
ENSG00000167978_2756593_2770867_2676	SRRM2	16	+	8028	88	20	14	1.72%	6.432072408715995	2.654949857609676e-05	3.1432776588688094e-06	7.176668031457734e-10
ENSG00000120708_136055749_136056455_24	TGFBI	5	+	72	40	0	2	37.50%	325.98912435083344	0.003710480643933249	0.006229864304675134	0.0001260498053663043
ENSG00000140688_31508373_31490835_466	C16orf58	16	-	1398	33	4	0	3.43%	13.85104004752468	0.0016335188667720405	0.00017828355461003195	2.0673971369923424e-06
ENSG00000067225_72206790_72203018_77	PKM	15	-	231	192	2	5	6.49%	487.71359902877936	0.0700082515985845	0.11245794200798093	0.028610771066532883
ENSG00000131389_14443635_14485007_620	SLC6A6	3	+	1860	80	10	3	4.68%	25.237867691677426	1.3105914637044646e-05	6.2887884200256284e-06	6.767351074608523e-10
ENSG00000147677_116755797_116645006_352	EIF3H	8	-	1056	106	12	2	9.94%	58.9003076952074	2.7373909625867942e-06	1.5290348771350275e-07	4.112348019776196e-12
ENSG00000147684_124543115_124549892_136	NDUFB9	8	+	408	29	4	2	11.03%	41.70743208606251	0.0032364592914596087	0.0008056452657918871	1.6233811652763675e-05
ENSG00000214063_850341_866630_226	TSPAN4	11	+	678	58	8	2	11.06%	50.19655543101329	0.00019441792049279118	9.897872049539573e-06	1.639486903430763e-08
ENSG00000091409_172467516_172501879_954	ITGA6	2	+	2862	63	0	6	2.20%	12.916550210127362	2.1514936418488064e-05	0.000816242864672838	1.528571388549824e-07
ENSG00000116337_109625404_109631152_761	AMPD2	1	+	2283	7	0	1	0.66%	1.7991515404382397	0.019216965118390855	0.04178282491678763	0.003600525600033436

ENSG00000173039_65662832_65654378_551	RELA	11	-	1653	23	5	8	2.18%	8.164518903886572	0.010777829388223404	0.0150551319960408	0.0007930107050930395
ENSG00000143801_226883858_226895579_350	PSEN2	1	+	1050	12	0	2	2.00%	6.706061986645715	0.008175610787902942	0.03744183116399598	0.001552076439258323
ENSG00000124155_45420633_45425826_187	PIGT	20	+	561	8	0	0	2.67%	8.367635277454548	0.019679754444124863	0.019679754444124863	0.0017849636115922674
ENSG00000124155_45416169_45420543_296	PIGT	20	+	888	32	2	0	5.07%	21.145240498432436	0.0005367511400985738	0.00028426559431428834	1.019917887006844e-06
ENSG00000116750_193059230_193020343_316	UCHL5	1	-	948	13	1	0	1.58%	8.046566993469938	0.03577298629450316	0.02108409854857798	0.003354604907114977
ENSG00000149480_62601438_62593875_664	MTA2	11	-	1992	31	11	4	1.81%	9.13162306163479	0.04282066216758641	0.006549171093867908	0.0014899520499011747
ENSG00000221968_61891381_61873814_445	FADS3	11	-	1335	14	0	0	1.35%	6.153502572015732	0.013428347753762199	0.013428347753762199	0.0008725593497644525
ENSG00000204356_31958446_31952301_380	NELFE	6	-	1140	51	0	2	5.53%	26.250703171409217	2.1874253386572725e-05	0.00010279671943510007	1.7481791484830737e-08
ENSG00000204356_31958446_31952301_380	NELFE	6	-	1140	51	0	2	5.53%	26.250703171409217	2.1874253386572725e-05	0.00010279671943510007	1.7481791484830737e-08
ENSG00000163938_52686792_52694275_537	GNL3	3	+	1611	32	0	1	2.98%	11.655477071761641	0.00017619886174203892	0.00028181564603640876	3.430664841479143e-07
ENSG00000196968_73772334_73775515_476	FUT11	10	+	1428	9	0	0	1.05%	3.698195948517858	0.019216965118390855	0.019216965118390855	0.0017073955890589052
ENSG00000132470_75724704_75757555_1822	ITGB4	17	+	5466	84	8	7	2.25%	9.017481815193197	3.782798880545537e-07	3.9425282639437177e-07	1.3807467766845968e-12
ENSG00000120727_139363893_139368798_91	PAIP2	5	+	273	72	1	0	17.58%	154.75527661490113	0.00021123378834695988	0.00021123378834695988	3.082076744927865e-07
ENSG00000143061_116600343_116577312_652	IGSF3	1	-	1956	11	0	1	1.07%	3.2998899090728537	0.007029302661868638	0.016854695534768073	0.0006017499134389055
ENSG00000139684_46781549_46777435_113	ESD	13	-	339	51	4	1	9.73%	88.27670093040267	0.0016263209931528667	0.0015806110104534784	1.5366000304906956e-05
ENSG00000146674_45921140_45914820_297	IGFBP3	7	-	891	149	7	8	10.44%	98.12601924903875	3.0967396523317614e-06	6.373656333363763e-06	1.671352176618229e-10
ENSG00000162337_68312715_68403486_471	LRP5	11	+	1413	13	0	5	1.27%	5.398546008357751	0.012798402692974279	0.11625384694841989	0.007703066966718039
ENSG00000162337_68406631_68449070_979	LRP5	11	+	2937	28	4	0	1.12%	5.594093247287029	0.00663533126580401	0.0015806110104534784	6.197025505198053e-05
ENSG00000163104_94233997_94289534_889	SMARCAD1	4	+	2667	7	1	0	0.67%	1.540106099295276	0.028889785561798602	0.009815328628645334	0.0013877953586383081
ENSG00000126858_32192201_32224733_438	RHOT1	17	+	1314	7	0	1	1.14%	3.125923110213471	0.019216965118390855	0.04178282491678763	0.003600525600033436
ENSG00000103202_397223_400342_187	NME4	16	+	561	11	2	0	3.21%	11.505498506500002	0.011772051206276392	0.011772051206276392	0.000681272511389508
ENSG00000103197_2060673_2088610_1481	TSC2	16	+	4443	12	5	3	0.61%	1.5848222115638086	0.04946560907745733	0.033570409067276055	0.006921950394855025
ENSG00000133265_55279608_55262608_359	HSPBP1	19	-	1077	46	2	1	6.13%	25.062116523908085	3.3624968141452445e-05	2.158727805327666e-05	5.638215995335599e-09
ENSG00000099204_114491803_114436260_448	ABLIM1	10	-	1344	8	1	2	1.34%	3.492740618044644	0.026602764905793736	0.08266357711566169	0.009436337707064906
ENSG00000149591_117203041_117204359_192	TAGLN	11	+	576	14	1	0	4.69%	14.262024190348962	0.0065000503081102355	0.0025000845279814113	9.158895334168734e-05
ENSG00000099204_114491803_114436260_455	ABLIM1	10	-	1365	8	1	2	1.32%	3.439006146997803	0.026602764905793736	0.08266357711566169	0.009436337707064906
ENSG00000100347_43955578_43996383_469	SAMM50	22	+	1407	37	4	0	3.84%	15.430615267779322	0.0003901122591641076	8.287246183407378e-05	2.3424148132128374e-07
ENSG00000176454_34367100_34359127_524	LPCAT4	15	-	1572	10	0	0	1.72%	3.7326998971469476	0.001946208561389314	0.001946208561389314	2.2278545302028032e-05
ENSG00000165264_32573060_32553876_113	NDUFB6	9	-	339	236	14	9	21.24%	408.4961062661771	3.5625929038692126e-05	3.058265349216846e-05	8.358065131281246e-09
ENSG00000049618_157174031_157207891_1233	ARID1B	6	+	3699	16	0	0	0.57%	2.53811483679481	0.00847367416878415	0.00847367416878415	0.0003665024264111182
ENSG00000197345_68896655_68891331_120	MRPL21	11	-	360	42	7	0	10.00%	68.45771611367502	0.01548920352033037	0.0009589057725548893	9.984900912010116e-05
ENSG00000072134_19283294_19334254_583	EPN2	17	+	1749	9	0	0	1.03%	3.019453295873929	0.012178957859018494	0.012178957859018494	0.0007262454987071801
ENSG00000150753_10263194_10264783_82	CCT5	5	+	246	44	4	6	18.29%	104.95259613246344	0.001653042079250274	0.0017286642245792514	1.7007534741748086e-05
ENSG00000132432_54757588_54752411_68	SEC61G	7	-	204	108	1	2	27.94%	310.6484596755001	7.348101949952925e-05	0.00016691480729352244	8.895761390022922e-08
ENSG00000176974_18347600_18328750_345	SHMT1	17	-	1035	19	0	0	2.03%	10.77181454376667	0.008574016250729465	0.008574016250729465	0.0003747459614100891
ENSG00000122861_73911892_73916565_414	PLAU	10	+	1242	65	3	2	6.04%	30.709120409861118	2.2741950236119856e-05	1.6358871612942217e-05	2.9343040825764965e-09
ENSG00000157593_44257410_44254706_432	SLC35B2	6	-	1296	25	2	0	2.78%	11.319066817737271	0.004359748848448076	0.0009430837296224684	2.5386113327095592e-05
ENSG00000112695_75243734_75241134_49	COX7A2	6	-	147	80	17	15	24.49%	319.33628507836744	0.005265875520493234	0.006610814250748408	0.00018492569923527974
ENSG00000134825_61792558_61789795_79	TMEM258	11	-	237	85	10	2	18.99%	210.44867521382915	0.0003152721518633181	0.0006000802399128133	1.255801040244497e-06
ENSG00000142192_26170620_25881670_751	APP	21	-	2253	401	7	8	13.58%	104.43806034462119	2.9070212329313007e-18	2.4856162279993556e-18	1.12890947007472e-34
ENSG00000108107_55386379_55388332_130	RPL28	19	+	390	485	65	27	22.31%	729.7141168160963	0.0001822311627020114	2.968844876012001e-05	4.166765513448663e-08
ENSG00000178741_74926861_74923657_69	COX5A	15	-	207	34	0	0	14.49%	96.37939328633335	0.002418182715931848	0.002418182715931848	3.375718052237727e-05
ENSG00000065057_2044728_2039924_162	NTHL1	16	-	486	10	0	0	4.32%	12.073671272253089	0.005706018193000826	0.005706018193000826	0.0001733096755673334
ENSG00000121390_19782754_19703175_522	PSPC1	13	-	1566	19	1	2	1.53%	7.119302715707856	0.007181907427766128	0.015570605297898355	0.0005669161536572745
ENSG00000101447_38926443_38952520_585	FAM83D	20	+	1755	71	4	1	4.62%	23.738695209137614	1.1421742562142165e-05	3.054605489091399e-06	2.9585900655168326e-10
ENSG00000168275_234374246_234383818_79	COA6	1	+	237	24	0	5	10.13%	59.42080241331647	0.0053557511377634245	0.03196954465377191	0.0009212659198961023
ENSG00000101266_489860_483961_177	CSNK2A1	20	-	531	9	0	0	2.82%	9.945430912398308	0.019216965118390855	0.019216965118390855	0.0017073955890589052
ENSG00000111786_120469609_120464812_119	SRSF9	12	-	357	67	17	3	12.61%	110.12405713364288	0.0020844322791246667	0.00043786382916130237	5.962593471792616e-06
ENSG00000111786_120464742_120462019_108	SRSF9	12	-	324	23	0	0	12.04%	41.65416588927316	0.000583356306045086	0.000583356306045086	2.197160003967981e-06
ENSG00000023228_206153678_206124185_727	NDUFS1	2	-	2181	55	1	0	3.44%	14.797305506984184	4.827952539080958e-06	4.925024046154609e-06	1.9941444209025355e-10
ENSG00000149179_46987231_47161638_308	C11orf49	11	+	924	17	0	0	3.57%	10.795743728501625	0.0012558331315113088	0.0012558331315113088	9.614604369954505e-06
ENSG00000115457_216661906_216664104_85	IGFBP2	2	+	255	15	0	0	9.41%	34.516495519500005	0.004193160480899131	0.004193160480899131	9.649553577281792e-05
ENSG00000104419_133256848_133238878_239	NDRG1	8	-	717	21	1	1	2.51%	17.18603751807741	0.013301727469301692	0.013301727469301692	0.0008571698582519322
ENSG00000125450_75205762_75235679_656	NUP85	17	+	1968	39	3	0	2.74%	11.628270594221801	0.0005179428059491108	8.091994981866961e-05	3.0653929032473984e-07
ENSG00000182199_57231904_57234361_346	SHMT2	12	+	1038	47	4	1	5.20%	26.5690557996922	0.0005021442139254169	0.00010439044196235416	3.7497909488849795e-07
ENSG00000166938_66293597_66333312_1054	DIS3L	15	+	3162	22	6	0	0.95%	4.082596244241937	0.02846543027925588	0.0023628488670607213	0.00041303410457268683
ENSG00000108828_43018763_43016061_252	VAT1	17	-	756	54	0	1	8.33%	41.912887416535725	2.6246772046413107e-05	2.6455697038791432e-05	5.386007614031533e-09
ENSG00000140365_75339779_75340005_46	COMMD4	15	+	138	10	0	0	10.87%	42.52032056750001	0.020613416668581838	0.020613416668581838	0.001946208561389314
ENSG00000159720_67439291_67438528_144	ATP6V0D1	16	-	432	26	3	0	6.25%	35.31548847134029	0.01002233431131372	0.003684287313187465	0.00020065090816454291
ENSG00000120256_149864020_149842612_325	LRP11	6	-	975	23	0	3	3.08%	13.841999741666157	0.0021227925778456486	0.018949244379329343	0.00024172798388748533
ENSG00000198563_31540532_31535229_290	DDX39B	6	-	870	81	19	4	5.17%	54.631280839484496	0.012574248081650233	0.0008231177872211322	6.977244585748771e-05
ENSG00000198563_31532908_31530434_182	DDX39B	6	-	546	45	0	2	7.14%	48.361023942156606	0.0007100644329059803	0.0007124765031541974	3.220125068842593e-06
ENSG00000126602_3677574_3658129_495	TRAP1	16	-	1485	56	5	3	5.05%	22.127746622602025	8.41513921138342e-05	1.7399829895753393e-05	1.1517722422893719e-08
ENSG00000150787_112230644_112233555_77	PTS	11	+	231	14	1	0	6.49%	35.562449929181824	0.021708440108399722	0.019216965118390855	0.0019141264286724178
ENSG00000078618_51794503_51789236_237	NRDC	1	-	711	11	0	0	2.11%	9.078178146478905	0.020613416668581838	0.020613416668581838	0.001946208561389314
ENSG00000078618_51840459_51792073_817	NRDC	1	-	2451	22	3	0	1.10%	5.266898949119952	0.022360378399288262	0.0035291660242640502	0.00044311298448702793
ENSG00000146281_89149400_89162263_236	PM20D2	6	+	708	9	0	0	2.54%	7.45907318429873	0.011772051206276392	0.011772051206276392	0.000681272511389508

ENSG00000105618_54128315_54131322_130	PRPF31	19	+	390	13	0	0	4.62%	19.559347461050006	0.013007239811447113	0.013007239811447113	0.0008218654148058079
ENSG00000105618_54118453_54128204_300	PRPF31	19	+	900	19	0	5	2.33%	12.38758672533167	0.00847367416878415	0.07942646894547645	0.0036293722074778505
ENSG00000072518_63895204_63909237_691	MARK2	11	+	2073	9	0	0	0.87%	2.5475271656939222	0.011772051206276392	0.011772051206276392	0.000681272511389508
ENSG00000142634_15409972_15428724_240	EFHD2	1	+	720	32	0	0	5.00%	26.079129948066672	0.0008776897154483972	0.0008776897154483972	4.827634122642857e-06
ENSG00000133216_22781429_22913570_964	EPHB2	1	+	2892	23	0	0	1.45%	4.666649290499483	0.00036930943616753716	0.00036930943616753716	9.087391537321962e-07
ENSG00000133216_22781429_22913570_963	EPHB2	1	+	2889	23	0	0	1.45%	4.671495239918485	0.00036930943616753716	0.00036930943616753716	9.087391537321962e-07
ENSG00000116133_54887119_54852233_528	DHCR24	1	−	1584	59	0	4	5.30%	21.85608901897633	1.3168995500762979e-06	1.3926528413036871e-05	1.642992871843085e-10
ENSG00000141219_73235867_73247341_481	C17orf80	17	+	1443	9	2	0	1.25%	3.6597531631902296	0.07134538375819317	0.011772051206276392	0.0041729868335021346
ENSG00000138071_65246584_65268734_321	ACTR2	2	+	963	62	7	1	5.61%	37.77817889673209	0.00011849127689191459	8.79856650028905e-05	7.531222124892845e-08
ENSG00000166801_59152092_59153504_470	FAM111A	11	+	1410	8	0	0	1.06%	3.329250631668086	0.019679754444124863	0.019679754444124863	0.0017849636115922674
ENSG00000070814_150357747_150399048_1451	TCOF1	5	+	4353	29	0	4	0.83%	3.9091735104786363	0.001011481469578233	0.00882464045628309	5.655081206426826e-05
ENSG00000101843_108091520_108084974_185	PSMD10	X	−	555	10	3	0	2.70%	10.572620249216218	0.12312584984626351	0.020613416668581838	0.011809609471937859
ENSG00000149782_64255551_64267556_1057	PLCB3	11	+	3171	9	1	0	0.57%	1.6654127450279095	0.02305674932606751	0.012178957859018494	0.0013400639730601711
ENSG00000149554_125627722_125655320_416	CHEK1	11	+	1248	13	0	0	1.44%	6.112296081578126	0.013428347753762199	0.013428347753762199	0.0008725593497644525
ENSG00000171204_85634105_85635782_96	TMEM126B	11	+	288	11	1	0	5.21%	22.411752299119797	0.019679754444124863	0.019679754444124863	0.0017849636115922674
ENSG00000164904_126595114_126544965_526	ALDH7A1	5	−	1578	25	0	0	1.90%	9.29626780468156	0.0022409900782254734	0.0022409900782254734	2.9184102569313086e-05
ENSG00000183605_119155112_119141242_110	SFXN4	10	−	330	12	0	0	4.55%	21.337469957509096	0.020613416668581838	0.020613416668581838	0.001946208561389314
ENSG00000117360_150325066_150352979_642	PRPF3	1	+	1926	16	1	0	0.93%	4.874603728610593	0.02034245439037237	0.013428347753762199	0.0012955184805712684
ENSG00000176454_34362580_34360009_155	LPCAT4	15	−	465	7	0	0	3.87%	8.8332536920871	0.009815328628645334	0.009815328628645334	0.0004837142251238724
ENSG00000034713_75577286_75577369_27	GABARAPL2	16	+	81	16	0	1	18.52%	115.90724421362965	0.021557223391537678	0.029237630782826433	0.002819005644529471
ENSG00000167978_2757518_2771357_311	SRRM2	16	+	933	11	4	2	1.93%	6.918097172718651	0.11290941015783806	0.052264829583131125	0.022520058696434957
ENSG00000143537_155051387_155058474_633	ADAM15	1	+	1899	39	7	0	2.53%	12.050782795907585	0.006111399706205241	0.00018810075197750367	9.079593432521678e-06
ENSG00000105518_11345583_11342815_177	TMEM205	19	−	531	78	6	9	11.86%	86.19373457411866	0.0002837053614922544	0.0003036328747503824	5.827449871051712e-07
ENSG00000074201_77637714_77619628_179	CLNS1A	11	−	537	43	0	1	7.82%	46.98614194553912	0.00045888781914451711	0.0008884494972709903	2.63656854774153e-06
ENSG00000148926_10305701_10306641_185	ADM	11	+	555	16	0	0	3.24%	16.91619239874595	0.013428347753762199	0.013428347753762199	0.0008725593497644525
ENSG00000052749_97401231_97357094_1197	RRP12	10	−	3591	15	0	1	0.58%	2.4510460477506273	0.0078820576498062	0.011306643944114857	0.0004511737406738057
ENSG00000026025_17229462_17230788_220	VIM	10	+	660	315	9	14	18.64%	280.0542931923069	5.8850522801184346e-08	2.2813348955411096e-08	1.3523964226300428e-14
ENSG00000136518_179570091_179588010_387	ACTL6A	3	+	1161	46	4	1	4.91%	23.248836775408275	0.00041632727507850433	7.746725325434995e-05	2.3527920765168633e-07
ENSG00000075239_108121607_108136123_162	ACAT1	11	+	486	16	0	1	4.32%	19.31787403560494	0.00847367416878415	0.00847367416878415	0.0003665024264111182
ENSG00000165533_88824720_88877410_511	TTC8	14	+	1533	17	1	1	2.15%	6.507023617179062	0.00241548406293102	0.00241548406293102	3.368517373808125e-05
ENSG00000121940_108950323_108934670_463	CLCC1	1	−	1389	21	2	0	1.94%	8.87141029550864	0.012991839772286901	0.0034782380676658805	0.00024813008800201436
ENSG00000196586_75832877_75915012_1143	MYO6	6	+	3429	13	0	0	0.44%	2.2245976989820653	0.02108409854857798	0.02108409854857798	0.002029891263157455
ENSG00000196586_75832877_75915012_1111	MYO6	6	+	3333	13	0	0	0.45%	2.28867252019487	0.02108409854857798	0.02108409854857798	0.002029891263157455
ENSG00000134686_33334242_33324865_323	PHC2	1	−	969	18	3	6	2.17%	10.899945953526318	0.013171727738572194	0.05346792862759031	0.0033590577316883704
ENSG00000122406_92832115_92834928_112	RPL5	1	+	336	123	2	2	8.93%	214.80354800974558	0.0025025372014526115	0.0024742386374296595	3.5654968698922646e-05
ENSG00000123066_115997159_115961266_1330	MED13L	12	−	3990	15	0	0	0.45%	2.2059414429755644	0.012798402692974279	0.012798402692974279	0.0007972436888943567
ENSG00000266412_46016680_46009147_634	NCOA4	10	−	1902	50	0	0	2.84%	15.42535288726341	7.625943357267001e-05	7.625943357267001e-05	4.2652882223459296e-08
ENSG00000138036_43774139_43809767_351	DYNC2LI1	2	+	1053	9	0	0	1.71%	5.01521729770513	0.011772051206276392	0.011772051206276392	0.000681272511389508
ENSG00000138035_55693823_55680832_179	PNPT1	2	−	537	32	0	0	7.82%	34.96643121528493	0.0004093002068662083	0.0004093002068662083	1.108510359686022e-06
ENSG00000138035_55672039_55643198_380	PNPT1	2	−	1140	12	0	3	1.84%	6.17663604033158	0.0078820576498062	0.058506512523593034	0.002434811297588452
ENSG00000138031_24918987_24819932_1144	ADCY3	2	−	3432	15	4	0	0.61%	2.5645997545083046	0.06927320754946001	0.00847367416878415	0.0031109847893480494
ENSG00000137575_58565034_58581740_298	SDCBP	8	+	894	120	4	1	10.07%	78.7624729975168	2.844226847274897e-06	1.106318704355805e-06	2.7762318096690534e-11
ENSG00000139289_76030976_76030536_146	PHLDA1	12	−	438	23	9	3	4.11%	30.8126706578185	0.1543801567637062	0.03736774915294124	0.023860979254184938
ENSG00000163719_9662322_9701973_417	MTMR14	3	+	1251	19	1	0	2.16%	8.911932895922064	0.004911637253759624	0.0034782380676658805	9.416876808028951e-05
ENSG00000196655_119018796_119023399_181	TRAPPC4	11	+	543	28	0	1	6.08%	30.25755408339227	0.0013845019718837994	0.0024673358700277245	2.032715794165102e-05
ENSG00000109390_139295798_139292550_76	NDUFC1	4	−	228	19	2	0	7.89%	48.89836865262501	0.016822681477512567	0.013428347753762199	0.0010798218650012886
ENSG00000006695_14069606_14209528_315	COX10	17	+	945	9	0	0	1.59%	5.58838498887143	0.019679754444124863	0.019679754444124863	0.0017849636115922674
ENSG00000143549_154191290_154171486_168	TPM3	1	−	504	25	1	5	3.57%	29.106171817038696	0.023878473930440184	0.1166778501254116	0.012466590856758575
ENSG00000143549_154191290_154167937_239	TPM3	1	−	717	53	1	5	3.77%	43.374285164671555	0.00510952817114065	0.02299889500569315	0.0006253630702124706
ENSG00000152422_83104920_83353242_336	XRCC4	5	+	1008	11	5	0	2.08%	6.403357799748513	0.06583400801140711	0.007029302661868638	0.0025354904771632734
ENSG00000165092_72930953_72923989_179	ALDH1A1	9	−	537	1125	65	23	35.75%	1229.2885974123606	4.468314941361676e-11	9.909445722993449e-12	5.41265523275926e-21
ENSG00000154723_25729794_25724640_108	ATP5PF	21	−	324	43	6	1	12.96%	77.87517970603243	0.004732992222340745	0.000675450826132064	2.0568376277725097e-05
ENSG00000177189_20187899_20155398_506	RPS6KA3	X	−	1518	20	2	0	2.37%	7.730967375909093	0.004498799248433914	0.0008852284070317903	2.4782952419168947e-05
ENSG00000062716_59731489_59839911_392	VMP1	17	+	1176	42	2	5	4.59%	20.956443708267862	0.00012107880093671411	0.0019510281878484426	1.7755135285717446e-06
ENSG00000068489_59169753_59201631_360	PRR11	17	+	1080	19	0	0	3.06%	10.32298893777639	0.0013775385517804596	0.0013775385517804596	1.1483554366703679e-05
ENSG00000163349_113940384_113972038_1075	HIPK1	1	+	3225	6	0	0	0.47%	1.0916845094539538	0.016947426762344633	0.016947426762344633	0.0013498980316300959
ENSG00000136631_150068645_150098868_398	VPS45	1	+	1194	12	0	3	1.51%	5.897290691773871	0.013007239811447113	0.07786981947941429	0.0049717523581729916
ENSG00000196642_136808326_136840522_687	RABL6	9	+	2061	25	6	4	1.46%	7.117666470542214	0.022335497433757793	0.014881989021557816	0.001556353048701133
ENSG00000054148_136849431_136850789_124	PHPT1	9	+	372	166	18	7	25.00%	261.8428773011533	5.197493477332335e-06	8.221683690380237e-07	3.845136283385412e-11
ENSG00000131724_118727639_118791854_427	IL13RA1	X	+	1281	8	0	0	1.41%	3.6645147468009376	0.011570465654371866	0.011570465654371866	0.0006594843062896773
ENSG00000105254_36115561_36116316_129	TBCB	19	+	387	36	1	0	5.43%	54.58422547269769	0.012176559331126819	0.008877961307018026	0.0005408334719909414
ENSG00000113448_58993456_58974664_499	PDE4D	5	−	1497	37	1	0	3.61%	14.502922967111227	0.0001185681716428482	8.091994981866961e-05	6.953223258948248e-08
ENSG00000164111_121696589_121668468_220	ANXA5	4	−	660	196	6	6	20.00%	174.25600465299095	2.04421682927846e-08	9.951970842784638e-09	2.1008550304910427e-15
ENSG00000122359_80172861_80155853_505	ANXA11	10	−	1515	35	2	4	2.97%	13.555983388846538	0.001101133767808773	0.0035763217077886345	2.3843511628047896e-05
ENSG00000074054_121605760_121340861_1493	CLASP1	2	−	4479	13	3	0	0.67%	1.7030912055837244	0.0182999617455631	0.0014903523101034196	0.0001731805207519894
ENSG00000128585_131388945_131487728_611	MKLN1	7	+	1833	15	0	0	1.64%	4.801803795675124	0.0020082573300163734	0.0020082573300163734	2.3658688350241813e-05
ENSG00000109790_39081185_39121066_493	KLHL5	4	+	1479	9	0	0	1.01%	3.570671950293104	0.019216965118390855	0.019216965118390855	0.0017073955890589052

ENSG00000121067_49611406_49600378_197	SPOP	17	-	591	11	0	0	2.54%	10.92146304931726	0.020613416668581838	0.020613416668581838	0.001946208561389314
ENSG00000070010_19475590_19450670_302	UFD1	22	-	906	51	0	1	6.95%	33.03068610972021	2.5935781044435533e-05	2.8428260534270372e-05	5.7110557495888096e-09
ENSG00000134815_47372753_47382113_546	DHX34	19	+	1638	14	3	0	1.10%	5.01521729770513	0.06307108214216126	0.013217226988443315	0.0040111611771127525
ENSG00000175470_131901149_131955963_453	PPP2R2D	10	+	1359	7	3	0	1.32%	3.022415722458058	0.15232096101830483	0.009815328628645334	0.008755902507845
ENSG00000105401_10393423_10391551_123	CDC37	19	-	369	49	0	0	8.13%	77.91935167410165	0.0023628488670607213	0.0023628488670607213	3.2295294885504634e-05
ENSG00000105254_36116080_36118560_45	TBCB	19	+	135	35	1	0	13.33%	152.12825803038893	0.017143983986795872	0.013853924679039932	0.0011315679034796663
ENSG00000152253_168889519_168871431_224	SPC25	2	-	672	33	0	3	4.91%	28.81511009886831	0.00151350169064872	0.00808812032268059	7.320214738294993e-05
ENSG00000137700_119027649_119024910_228	SLC37A4	11	-	684	10	0	0	2.19%	8.578661167127194	0.020613416668581838	0.020613416668581838	0.001946208561389314
ENSG00000149273_75399548_75404780_91	RPS3	11	+	273	97	13	8	16.48%	208.4897476617418	0.0015120179381525396	0.0003432981470903991	3.440890842732202e-06
ENSG00000070047_606536_611777_1133	PHRF1	11	+	3399	6	0	0	0.44%	1.0357995125004416	0.016947426762344633	0.016947426762344633	0.0013498980316300959
ENSG00000135390_53672614_53665314_141	ATP5MC2	12	-	423	104	13	2	17.73%	144.26752737228372	4.4036283112694194e-05	8.843606187363953e-06	3.163887414050723e-09
ENSG00000167645_38309608_38305352_283	YIF1B	19	-	849	66	0	0	3.18%	45.61543930845584	0.003684287313187465	0.003684287313187465	7.541963121699725e-05
ENSG00000136811_128460559_128494731_576	ODF2	9	+	1728	15	0	0	1.39%	5.093580067981772	0.004826937407846361	0.004826937407846361	0.00012612451166366307
ENSG00000137700_119029267_119024910_395	SLC37A4	11	-	1185	17	0	0	2.28%	8.417947008553167	0.0034782380676658805	0.0034782380676658805	6.758309314600117e-05
ENSG00000159840_143381740_143390682_516	ZYX	7	+	1548	38	0	1	4.07%	14.404170610850779	1.765320032351358e-05	5.105009430698233e-05	7.046723956326861e-09
ENSG00000121067_49611406_49600378_197	SPOP	17	-	591	11	0	0	2.54%	10.92146304931726	0.020613416668581838	0.020613416668581838	0.001946208561389314
ENSG00000152795_82429324_82426059_298	HNRNPDL	4	-	894	114	3	1	11.41%	74.82434934764096	2.8068292164810636e-07	1.8169410532370092e-07	4.81845735322702e-13
ENSG00000143740_227747737_227780673_419	SNAP47	1	+	1257	13	0	0	1.19%	6.068532625146779	0.02108409854857798	0.02108409854857798	0.002029891263157455
ENSG00000160633_5623206_5668291_915	SAFB	19	+	2745	36	1	6	2.08%	7.695480968281969	7.765474117981868e-05	0.0010821105868375448	6.392267689608755e-07
ENSG00000123836_207063386_207072371_367	PFKFB2	1	+	1101	13	2	0	1.63%	6.928379209636241	0.052264829583131125	0.012798402692974279	0.003202548019818642
ENSG00000184014_9193637_9139671_956	DENND5A	11	-	2868	10	0	0	0.52%	2.0459568473901677	0.019679754444124863	0.019679754444124863	0.0017849636115922674
ENSG00000119596_74763490_74835411_2146	YLPM1	14	+	6438	14	1	0	0.33%	1.276005892146785	0.013892891352654344	0.00847367416878415	0.0005886798845679606
ENSG00000050426_51049080_51059431_109	LETMD1	12	+	327	16	0	0	4.59%	28.710968750165144	0.02108409854857798	0.02108409854857798	0.002029891263157455
ENSG00000110955_56643918_56640165_145	ATP5F1B	12	-	435	291	1	80	19.31%	392.53586973555525	2.979221540301505e-06	0.020403429938821657	1.6753790952868428e-06
ENSG00000100030_21867329_21769204_279	MAPK1	22	-	837	26	7	3	3.58%	18.227348888433696	0.045043503820603986	0.010728371185261619	0.00236593462751607
ENSG00000169221_30370183_30357944_808	TBC1D10B	16	-	2424	12	0	0	0.87%	2.904853583324258	0.006937202941512724	0.006937202941512724	0.00025101522741487095
ENSG00000163975_197029702_197003372_738	MELTF	3	-	2214	28	0	0	1.36%	7.420890635628728	0.0021227925778456486	0.0021227925778456486	2.6309315010939275e-05
ENSG00000150527_39294000_39350264_639	MIA2	14	+	1917	17	0	0	1.41%	5.203582266633021	0.002967235347054297	0.002967235347054297	4.9907879201100895e-05
ENSG00000213965_32691961_32711957_375	NUDT19	19	+	1125	21	3	0	2.93%	10.953234578188003	0.01157547599262317	0.0013845019718837994	9.8960288269354e-05
ENSG00000116044_177234268_177230785_589	NFE2L2	2	-	1767	17	1	0	1.19%	5.645312509980477	0.011825808327677989	0.00847367416878415	0.0005036791746826746
ENSG00000116044_177234268_177230785_589	NFE2L2	2	-	1767	17	1	0	1.19%	5.645312509980477	0.011825808327677989	0.00847367416878415	0.0005036791746826746
ENSG00000218739_37199705_37201724_80	CEBPZOS	2	+	240	16	0	0	10.00%	39.11869492210001	0.004826937407846361	0.004826937407846361	0.00012612451166366307
ENSG00000162063_2429482_2457020_786	CCNF	16	+	2358	11	0	0	0.89%	2.7373132579077613	0.0078820576498062	0.0078820576498062	0.00031963642907451164
ENSG00000187498_110307027_110150363_1669	COL4A1	13	-	5007	37	1	1	1.14%	4.336104589927203	7.466131303189086e-05	7.466131303189086e-05	4.0932040839079915e-08
ENSG00000164111_121671628_121668468_107	ANXA5	4	-	321	120	3	2	24.30%	219.3571677874767	6.829623287082529e-06	6.797917988279537e-06	3.8372924089845753e-10
ENSG00000164548_23516395_23505559_180	TRA2A	7	-	540	34	0	0	8.33%	36.945434093094455	0.00027539387036832887	0.00027539387036832887	5.151399376569847e-07
ENSG00000088986_120496422_120498210_89	DYNLL1	12	+	267	251	5	9	15.73%	551.6175519914102	0.00048083181382083286	0.000745376529342948	2.3179087696108e-06
ENSG00000088986_120496422_120498210_89	DYNLL1	12	+	267	251	5	9	15.73%	551.6175519914102	0.00048083181382083286	0.000745376529342948	2.3179087696108e-06
ENSG00000138448_186668894_186675957_130	ITGAV	2	+	390	23	0	0	4.62%	34.60499935416539	0.013640585738808999	0.013640585738808999	0.00089863692579514
ENSG00000213593_57712619_57740245_296	TMX2	11	+	888	73	0	0	8.11%	48.237579887048994	8.0901699496228e-06	8.0901699496228e-06	5.368433166356741e-10
ENSG00000172053_49104733_49103886_100	QARS	3	-	300	13	1	0	5.00%	25.427151699365005	0.03577298629450316	0.02108409854857798	0.003354604907114977
ENSG00000154380_225567380_225497775_789	ENAH	1	-	2367	22	2	3	1.01%	5.453810445413183	0.016025708091071147	0.024498661976836228	0.0018193556908024401
ENSG00000270647_35809570_35846945_589	TAF15	17	+	1767	42	1	0	3.57%	13.947242671716472	3.4277784798780443e-05	2.116023157680266e-05	5.6367016014772224e-09
ENSG00000075188_102118518_102074354_326	NUP37	12	-	978	58	0	1	6.13%	34.79883904113191	3.8737898727308975e-05	4.628699659366991e-05	1.3580661597432277e-08
ENSG00000077254_77734378_77697317_747	USP33	1	-	2241	16	0	0	0.94%	4.189418465552879	0.00837382380159347	0.00837382380159347	0.0003583838894600799
ENSG00000114302_48783065_48751585_250	PRKAR2A	3	-	750	7	0	0	2.00%	5.476617289094001	0.016947426762344633	0.016947426762344633	0.0013498980316300959
ENSG00000127481_19076779_19074832_34	UBR4	1	-	102	25	3	1	20.59%	143.81873133125004	0.028462570115697007	0.008877961307018026	0.0012532059812813028
ENSG00000196517_44008606_43997545_521	SLC6A9	1	-	1563	11	0	0	0.96%	4.129612707707294	0.019679754444124863	0.019679754444124863	0.0017849636115922674
ENSG00000261832_28491759_28479730_363	AC138894.1	16	-	1089	21	2	0	2.20%	11.315324977466945	0.018151375140008384	0.005604866691947602	0.0005308776856007569
ENSG00000139579_56224857_56229213_211	NABP2	12	+	633	19	1	0	3.79%	17.61268254786493	0.009288069496478249	0.005306578019052857	0.0002590037054391842
ENSG00000131732_81304758_81312662_271	ZCCHC9	5	+	813	11	0	0	2.95%	7.939218526625463	0.004687384229717438	0.004687384229717438	0.00011928172701435494
ENSG00000180488_77783253_77875064_600	MIGA1	1	+	1800	15	0	0	0.83%	4.8898368652625015	0.019679754444124863	0.019679754444124863	0.0017849636115922674
ENSG00000129255_7583925_7587551_160	MPDU1	17	+	480	12	5	0	4.38%	14.669510595787504	0.07864960352514258	0.008175610787902942	0.003490498801610728
ENSG00000164930_103300114_103329666_465	FZD6	8	+	1395	19	0	0	1.94%	7.991991435697852	0.0034529206320349946	0.0034529206320349946	6.664817351595063e-05
ENSG00000114867_184320681_184334908_1404	EIF4G1	3	+	4212	86	7	5	2.71%	11.980796877851143	7.385814168744835e-07	2.993515018880905e-07	2.0477869100525147e-12
ENSG00000185033_90201594_90228643_832	SEMA4B	15	+	2496	46	0	0	2.28%	10.814062298176685	4.06701403799178e-05	4.06701403799178e-05	1.2548961177492392e-08
ENSG00000186635_72710549_72685664_1033	ARAP1	11	-	3099	7	1	1	0.48%	1.3254156072347534	0.04178282491678763	0.04178282491678763	0.007200520635230478
ENSG00000125691_38852646_38850132_79	RPL23	17	-	237	105	9	3	21.52%	259.96601055825954	0.0006059827437326382	0.00013946983484782184	5.930742218858907e-07
ENSG00000214078_35632923_35626241_537	CPNE1	20	-	1611	101	10	3	7.45%	36.78759950774768	3.647459440559311e-07	4.844179399097695e-08	1.7557256615158434e-13
ENSG00000079246_216109437_216117760_112	XRCC5	2	+	336	18	2	0	5.36%	31.434665562401793	0.03031622373531018	0.013428347753762199	0.0019146142599917133
ENSG00000159692_1241433_1241335_32	CTBP1	4	-	96	17	0	0	15.62%	103.90903338682814	0.019679754444124863	0.019679754444124863	0.0017849636115922674
ENSG00000138768_75724841_75813295_955	USO1	4	+	2865	18	0	0	1.36%	3.686578579046074	0.00047074547413106	0.00047074547413106	1.4523754767299686e-06
ENSG00000135637_74472660_74472119_112	CCDC142	2	-	336	59	4	1	16.96%	103.035848232317	0.00045208122059449304	8.03541330359969e-05	2.6478191410469155e-07
ENSG00000087269_2963319_2938831_857	NOP14	4	-	2571	6	0	2	0.58%	1.36938255269895	0.016947426762344633	0.1029516053660342	0.008323174032180154
ENSG00000167972_2288167_2276674_750	ABCA3	16	-	2250	5	0	0	0.67%	1.3039564974033335	0.012673659338734126	0.012673659338734126	0.000782701129001274
ENSG00000204264_32843032_32840959_208	PSMB8	6	-	624	25	4	0	4.81%	23.508831082992796	0.004400434476856543	0.002390425500591921	5.967362184433355e-05
ENSG00000117139_202755365_202729036_1063	KDM5B	1	-	3189	23	5	0	1.03%	4.232031905965664	0.015673363221486523	0.001484234770590415	0.00014563006287121952

ENSG00000010704_26090867_26094226_208	HFE	6	+	624	14	0	5	3.37%	13.164945406475965	0.00847367416878415	0.10069662721979156	0.004772963284717595
ENSG00000119669_77027792_77025402_796	IRF2BPL	14	-	2388	11	3	0	0.88%	2.7029249003963574	0.07962816006291146	0.007029302661868638	0.0031495291952635916
ENSG00000090316_1289914_1339169_434	MAEA	4	+	1302	35	0	0	3.23%	15.77366730729839	0.0004561727980981236	0.0004561727980981236	1.3667994923812405e-06
ENSG00000113621_134893498_134899685_161	TXNDC15	5	+	483	17	0	0	3.73%	20.65272713278572	0.013428347753762199	0.013428347753762199	0.0008725593497644525
ENSG00000008294_51047454_50989652_775	SPAG9	17	-	2325	20	0	0	1.55%	5.047573538335485	0.0008442728493916082	0.0008442728493916082	4.480017286136276e-06
ENSG00000087053_95923954_95835290_643	MTMR2	11	-	1929	11	2	0	0.93%	3.3460781037566103	0.05067994629101479	0.012798402692974279	0.003100990187173963
ENSG00000070814_150357747_150383755_994	TCOF1	5	+	2982	21	0	4	0.70%	4.132256505855635	0.00877616296920843	0.04209057871058814	0.0018631442046486153
ENSG00000113569_37370950_37291900_1318	NUP155	5	-	3954	42	0	2	0.99%	6.232872483794387	0.000681533134233071	0.0020214219157901094	8.648439546629165e-06
ENSG00000167460_16086484_16101346_139	TPM4	19	+	417	27	0	3	5.04%	37.99297708261512	0.00847367416878415	0.03299602752967379	0.0014019198044660757
ENSG00000006534_68018608_68027939_387	ALDH3B1	11	+	1161	52	1	8	3.88%	26.2812937461137	0.00041346295719931213	0.0032442879998225726	8.97193284683029e-06
ENSG00000105655_18437979_18434913_504	ISYNA1	19	-	1512	17	3	0	1.79%	6.597398945195438	0.019264875824957298	0.0033030736763512156	0.0003576062290707871
ENSG00000167792_67608700_67612458_363	NDUFV1	11	+	1089	36	5	2	4.68%	19.397699961371906	0.0009992727214952726	0.0003526372300916182	2.318045036860568e-06
ENSG00000104522_143616712_143614602_161	TSTA3	8	-	483	21	0	5	4.97%	25.512192340500008	0.005454749182134642	0.04181558783514158	0.0012502076894015317
ENSG00000165916_47426231_47424414_139	PSMC3	11	-	417	20	0	0	8.63%	28.14294598712231	0.0006577637618416096	0.0006577637618416096	2.769528725862714e-06
ENSG00000162607_62444940_62450981_532	USP1	1	+	1596	20	2	0	1.50%	7.353138143251881	0.015797663760347944	0.005454749182134642	0.00045021431474288393
ENSG00000101972_124047365_124100597_1042	STAG2	X	+	3126	14	0	1	0.86%	2.6279353594500967	0.0032294771334464936	0.006024333578509648	0.00010730535955833177
ENSG00000101972_124047365_124100597_1005	STAG2	X	+	3015	15	0	1	1.00%	2.919305591201493	0.0020082573300163734	0.00392533363377418	4.538242985436214e-05
ENSG00000149532_61429235_61410943_471	CPSF7	11	-	1413	16	1	0	1.70%	6.64436431797877	0.006542767527223707	0.004826937407846361	0.0001687841081301107
ENSG00000077147_96586835_96522263_589	TM9SF3	10	-	1767	129	4	5	8.49%	42.837959634557734	9.904882660401043e-10	2.4468299405545486e-09	2.6623629168725244e-17
ENSG00000174938_29899019_29871699_879	SEZ6L2	16	-	2637	6	0	0	0.57%	1.3351090417098979	0.016947426762344633	0.016947426762344633	0.0013498980316300959
ENSG00000123131_23667571_23675191_186	PRDX4	X	+	558	92	0	2	13.98%	96.74515948476346	3.5267143672048792e-06	1.0780765863822602e-05	3.2007058448786594e-10
ENSG00000089057_4932559_4856972_649	SLC23A2	20	-	1947	9	0	0	0.77%	2.712390248835902	0.020613416668581838	0.020613416668581838	0.001946208561389314
ENSG00000164104_173333649_173333065_99	HMGB2	4	-	297	60	4	1	6.06%	118.54149976393943	0.029237630782826433	0.017143983986795872	0.002293865321994621
ENSG00000113441_96979362_97028533_944	LNPEP	5	+	2832	31	0	0	1.80%	6.423090797590573	0.00011488807511512702	0.00011488807511512702	9.457822244500565e-08
ENSG00000119383_129136511_129137827_106	PTPA	9	+	318	13	3	0	6.60%	23.9878789616651	0.023672501042627626	0.00837382380159347	0.0009877981595025045
ENSG00000103145_3023941_3022863_138	HCFC1R1	16	-	414	20	2	3	5.07%	28.34688037833334	0.023296851685565965	0.015625008558487224	0.0016940249279937029
ENSG00000092208_39118597_39135988_152	GEMIN2	14	+	456	17	0	0	3.95%	21.875585976174346	0.011772051206276392	0.011772051206276392	0.000681272511389508
ENSG00000104133_44663647_44563258_2265	SPG11	15	-	6795	22	5	2	0.40%	1.899804168402208	0.02633284015351082	0.010026873424650893	0.0012874779080855213
ENSG00000112992_43644650_43704404_707	NNT	5	+	2121	7	0	0	0.71%	1.9365690555495054	0.019216965118390855	0.019216965118390855	0.0017073955890589052
ENSG00000152683_32165901_32220713_432	SLC30A6	2	+	1296	30	5	0	3.47%	13.582880181284725	0.005975744103077381	0.0002812865659102998	1.2422237301200548e-05
ENSG00000163902_128650800_128620411_607	RPN1	3	-	1821	124	3	11	7.91%	39.95649234217793	1.388052985578206e-09	1.5510962245864763e-08	2.3793080642812273e-16
ENSG00000125818_1118774_1166254_263	PSMF1	20	+	789	45	1	2	6.46%	33.46656409685362	0.0001798667136015901	0.0001798667136015901	2.256998005176214e-07
ENSG00000052723_114780607_114774271_211	SIKE1	1	-	633	14	0	0	2.84%	12.977766087900477	0.013007239811447113	0.013007239811447113	0.0008218654148058079
ENSG00000010165_171781968_171796756_699	EEF1AKNMT	1	+	2097	16	1	0	1.29%	4.477103853745351	0.006112543793728684	0.0033030736763512156	0.00011113261345270447
ENSG00000176396_39539767_39539369_132	EID2	19	-	396	9	0	1	4.55%	13.335918723443184	0.011772051206276392	0.04223451612662591	0.0023920425764481982
ENSG00000141376_60709305_61392125_828	BCAS3	17	+	2484	17	0	5	0.72%	4.015808053597223	0.011772051206276392	0.1004125613475727	0.006109270445167818
ENSG00000099968_17638694_17727534_461	BCL2L13	22	+	1383	12	1	0	1.08%	5.091370271856834	0.03537493353818881	0.020613416668581838	0.003252526348827943
ENSG00000096063_35891039_35835304_639	SRPK1	6	-	1917	15	1	1	1.56%	4.591396117617372	0.00392533363377418	0.00392533363377418	8.509888062156286e-05
ENSG00000101972_124047365_124100597_1005	STAG2	X	+	3015	15	0	1	1.00%	2.919305591201493	0.0020082573300163734	0.00392533363377418	4.538242985436214e-05
ENSG00000116005_70258165_70277392_505	PCYOX1	2	+	1515	57	2	0	4.36%	22.07688723326436	5.8313036325659564e-05	1.6844195871163673e-05	7.705593086755319e-09
ENSG00000128245_31956262_31956792_176	YWHAH	22	+	528	10	0	11	3.98%	11.11326560286932	0.007593099278532274	0.47947610046037925	0.039785224825561294
ENSG00000143321_156745364_156744511_88	HDGF	1	-	264	25	0	4	9.09%	55.5663280143466	0.00575691403842271	0.021542863694648147	0.0006476010016061797
ENSG00000180398_46909171_46907669_149	MCFD2	2	-	447	24	0	0	6.04%	31.50498919900672	0.0036062891729786097	0.0036062891729786097	7.240558534568731e-05
ENSG00000170248_33798729_33825194_113	PDCD6IP	3	+	339	10	0	0	5.31%	17.309157045176995	0.012798402692974279	0.012798402692974279	0.0007972436888943567
ENSG00000101255_388011_396690_358	TRIB3	20	+	1074	98	8	0	7.26%	53.54234779840504	4.752487226358163e-05	3.650308449239545e-06	1.5059769789961688e-09
ENSG00000177917_152718625_152759840_226	ARL6IP6	2	+	678	43	2	0	3.98%	37.21468764713054	0.0036321914711260127	0.003684287313187465	7.440341320821008e-05
ENSG00000143622_155910498_155900388_182	RIT1	1	-	546	13	1	0	3.30%	13.970962472178575	0.013007239811447113	0.012798402692974279	0.000809468393769003
ENSG00000114982_96637135_96598143_903	KANSL3	2	-	2709	8	0	0	0.55%	1.7328325546888155	0.019679754444124863	0.019679754444124863	0.0017849636115922674
ENSG00000112992_43644650_43704404_707	NNT	5	+	2121	7	0	0	0.71%	1.9365690555495054	0.019216965118390855	0.019216965118390855	0.0017073955890589052
ENSG00000127445_9835345_9848196_145	PIN1	19	+	435	14	0	0	4.14%	18.88488720377242	0.013007239811447113	0.013007239811447113	0.0008218654148058079
ENSG00000116138_15529106_15566190_595	DNAJC16	1	+	1785	10	0	0	0.84%	3.287285287571429	0.020613416668581838	0.020613416668581838	0.001946208561389314
ENSG00000138668_82358757_82355334_181	HNRNPD	4	-	543	20	0	0	5.52%	21.61253863099448	0.0022409900782254734	0.0022409900782254734	2.9184102569313086e-05
ENSG00000138668_82373678_82358793_86	HNRNPD	4	-	258	27	0	0	11.63%	61.407253656784896	0.0023628488670607213	0.0023628488670607213	3.2295294885504634e-05
ENSG00000249915_306604_314515_119	PDCD6	5	+	357	49	0	4	15.97%	80.53848954550001	4.9927568186110207e-05	0.0004145266145085789	1.5678268270530424e-07
ENSG00000115084_113750514_113717775_414	SLC35F5	2	-	1242	18	1	0	2.17%	8.504064113500002	0.006432154271512831	0.002967235347054297	0.00010592475669358982
ENSG00000167397_31091310_31091134_58	VKORC1	16	-	174	56	0	12	15.52%	188.8488720377242	0.0035547763465261306	0.06638642199807827	0.001507226391593381
ENSG00000167397_31094729_31091237_149	VKORC1	16	-	447	26	53	3	6.04%	34.130404965590614	0.3523439820043579	0.01204568828101519	0.0312316868439282
ENSG00000012061_45423374_45409675_297	ERCC1	19	-	891	18	12	2	2.69%	11.854149976393941	0.16470045294401847	0.020990660982539537	0.016680854478167306
ENSG00000104529_143586845_143579792_281	EEF1D	8	-	843	172	0	4	9.25%	119.72269620286835	3.816226438010809e-06	1.4269275421894181e-05	4.574424447029569e-10
ENSG00000104626_9008096_9030034_271	ERI1	8	+	813	20	1	0	2.95%	14.43494277568266	0.009234565224265509	0.005257622967929454	0.00025536431448355644
ENSG00000101654_13734581_13759979_298	RNMT	18	+	894	15	0	0	2.35%	9.8453091246896	0.0078820576498062	0.0078820576498062	0.00031963642907451164
ENSG00000102805_76992135_77000969_346	CLN5	13	+	1038	14	0	2	2.02%	7.914186833950869	0.00797940233269714	0.03799572790022047	0.0015444991742588488
ENSG00000140694_14630125_14436717_664	PARN	16	-	1992	19	0	2	1.36%	5.59680123132455	0.0033278027414746945	0.011099465161221279	0.00020317731502769463
ENSG00000143322_179229397_179107718_1079	ABL2	1	-	3237	14	2	1	0.74%	2.5378208012483787	0.01599363345933208	0.00912793029372963	0.0007225562286945759
ENSG00000070756_100713158_100709644_111	PABPC1	8	-	333	18	0	1	6.31%	31.717860747648658	0.00847367416878415	0.011825808327677989	0.0005036791746826746
ENSG00000120694_31152870_31137318_688	HSPH1	13	-	2064	81	4	1	4.36%	23.027720121294337	5.338423016832602e-06	6.746235644553921e-07	3.280178726113765e-11
ENSG00000172071_88588899_88557736_727	EIF2AK3	2	-	2181	7	1	0	0.69%	1.8832934281616236	0.0477903522728147	0.016947426762344633	0.0036975118224100542

ENSG00000187522_14838403_14871606_509	HSPA14	10	+	1527	25	1	0	2.55%	9.606752191085464	0.0009879882513538032	0.0006356636671888767	3.980616343459717e-06
ENSG00000067560_49362504_49360209_60	RHOA	3	-	180	138	0	3	36.67%	449.8649916041501	1.867945416818258e-05	6.233241129939043e-05	9.085447588305868e-09
ENSG00000139644_49759248_49762896_57	TMBIM6	12	+	171	112	2	7	31.58%	384.3240202872983	0.00010171885600757616	0.0005423870787726692	3.9623573268503386e-07
ENSG00000102030_153930774_153929987_82	NAA10	X	-	246	24	0	1	8.54%	57.24687061770733	0.0078820576498062	0.023672501042627626	0.0009369425085439399
ENSG00000102030_153934904_153932300_118	NAA10	X	-	354	8	5	0	5.08%	13.26057454986441	0.11368416502207135	0.011570465654371866	0.006956434060967934
ENSG00000101193_62943019_62944912_178	GID8	20	+	534	14	0	0	3.93%	15.383756430039329	0.007029302661868638	0.007029302661868638	0.0002573639808771639
ENSG00000116171_53015021_53047967_121	SCP2	1	+	363	16	0	0	4.13%	25.863599948495875	0.020613416668581838	0.020613416668581838	0.001946208561389314
ENSG00000072756_3129041_3148154_434	TRNT1	3	+	1302	12	0	2	1.84%	5.408114505359448	0.004873903550586949	0.029607078753113344	0.0007843881175907335
ENSG00000149428_119056160_119045114_677	HYOU1	11	-	2031	41	1	0	2.95%	11.84539506503767	4.441415812564126e-05	3.4197238319135e-05	1.1559158704370502e-08
ENSG00000157851_26898500_26946995_564	DPYSL5	2	+	1692	20	0	0	0.89%	6.935938815975179	0.02108409854857798	0.02108409854857798	0.002029891263157455
ENSG00000143545_153983523_153982099_122	RAB13	1	-	366	28	1	0	7.38%	44.89030564831148	0.004911637253759624	0.0034782380676658805	9.416876808028951e-05
ENSG00000163565_159014750_159054901_706	IFI16	1	+	2118	9	0	0	0.71%	2.4934012344114738	0.020613416668581838	0.020613416668581838	0.001946208561389314
ENSG00000197102_102033284_102043027_859	DYNC1H1	14	+	2577	34	3	3	2.21%	7.7417673303341115	0.00024061312443602098	0.00045119717515961275	7.337393641233198e-07
ENSG00000106608_43879012_43876667_781	URGCP	7	-	2343	15	0	2	0.90%	3.7565968235051224	0.00847367416878415	0.027657845067597595	0.0011690047180828366
ENSG00000138326_78033902_78037480_143	RPS24	10	+	429	108	13	2	10.49%	147.72094585967835	0.0013010442815015533	0.000393727242440075	3.348109036281904e-06
ENSG00000006327_3020398_3021826_94	TNFRSF12A	16	+	282	237	5	0	24.47%	493.1452498158352	1.27380764436131e-05	1.2952905594172416e-05	1.3249975098466169e-09
ENSG00000113712_149511835_149496853_114	CSNK1A1	5	-	342	9	0	0	5.26%	15.44159010082895	0.012178957859018494	0.012178957859018494	0.0007262454987071801
ENSG00000169047_226798666_226795010_1218	IRS1	2	-	3654	10	2	0	0.57%	1.6058577554228246	0.03522780890193623	0.007029302661868638	0.0012822660609675817
ENSG00000168778_123671530_123707713_661	TCTN2	12	+	1983	10	0	0	0.76%	2.959054078827535	0.020613416668581838	0.020613416668581838	0.001946208561389314
ENSG00000092199_21233974_21211287_158	HNRNPC	14	-	474	18	0	8	3.16%	22.282800904993675	0.016947426762344633	0.11777945996203976	0.009674391494749069
ENSG00000149177_47980913_48167362_1342	PTPRJ	11	+	4026	20	5	4	0.75%	2.9149549122280187	0.02387123336711234	0.021399249315173112	0.002311502123040317
ENSG00000033627_42478512_42521120_646	ATP6V0A1	17	+	1938	8	2	0	0.77%	2.4222102118947375	0.10105102209468636	0.019679754444124863	0.009164497669452725
ENSG00000107789_87504916_87552185_312	MINPP1	10	+	936	16	2	1	3.21%	10.03043459541026	0.011663199792667247	0.0036460193534901884	0.00023170328030554097
ENSG00000107789_87552209_87552478_89	MINPP1	10	+	267	10	0	0	6.74%	21.976794900056184	0.011772051206276392	0.011772051206276392	0.000681272511389508
ENSG00000115457_216633740_216664104_253	IGFBP2	2	+	759	29	2	0	6.32%	22.419805390136368	0.0005584916486254665	0.0001282392546064565	5.051391868219266e-07
ENSG00000110717_68032173_68033427_164	NDUFS8	11	+	492	34	7	2	9.15%	40.54986668754269	0.0026396893348161607	0.0012658469904766267	1.999096483119431e-05
ENSG00000167601_41219414_41259904_887	AXL	19	+	2661	93	2	0	4.51%	20.50754581598253	4.0203044021105216e-08	1.0783759994197337e-08	4.468616743028718e-15
ENSG00000179715_47235064_47236362_432	PCED1B	12	+	1296	20	3	1	2.55%	9.055253454189817	0.0040705821972625445	0.0025593219000189936	5.890442307735102e-05
ENSG00000137074_33001604_32987654_99	APTX	9	-	297	10	3	2	5.05%	19.756916627323236	0.10105102209468636	0.08410206952409	0.030302490514899416
ENSG00000165060_69035783_69099934_171	FXN	9	+	513	18	0	0	4.68%	20.588786801105268	0.005306578019052857	0.005306578019052857	0.00015100114740534233
ENSG00000165060_69035783_69072712_196	FXN	9	+	588	18	0	0	4.08%	17.962666035658167	0.005306578019052857	0.005306578019052857	0.00015100114740534233
ENSG00000166783_15635952_15596693_1564	MARF1	16	-	4692	26	0	12	0.58%	3.2515539257500006	0.003710480643933249	0.09637930362891867	0.0024453678769249306
ENSG00000124783_7313120_7287913_288	SSR1	6	-	864	113	2	5	12.50%	76.7432730242587	7.991166815367668e-08	2.712069423649562e-07	2.100377621768647e-13
ENSG00000079999_10499409_10486652_416	KEAP1	19	-	1248	19	3	0	2.64%	8.933355811537261	0.011088249102336101	0.0012624143894574274	8.733152613708329e-05
ENSG00000174851_66288985_66284637_293	YIF1A	11	-	879	30	2	2	5.46%	20.026635625648467	0.0007409968934050745	0.0007409968934050745	3.4844774759867696e-06
ENSG00000275379_27872315_27871905_136	HIST1H3I	6	-	408	132	0	0	7.35%	189.84072535725005	0.002460156992221257	0.002460156992221257	3.4886517907067876e-05
ENSG00000141526_82236009_82237182_127	SLC16A3	17	+	381	23	5	1	6.30%	35.422440283791346	0.004873903550586949	0.009288069496478249	0.0002396171204823838
ENSG00000085721_15083566_15061744_447	RRN3	16	-	1341	11	2	3	1.12%	4.8132622387371375	0.08350359792828288	0.08350359792828288	0.02533407572296648
ENSG00000106355_32489303_32487261_62	LSM5	7	-	186	49	4	0	27.42%	154.58193961152423	0.0002473554500538834	0.00013319834607924987	2.311148598512169e-07
ENSG00000176407_84971452_85053409_381	KCMF1	2	+	1143	10	11	0	1.84%	5.13368699765092	0.2535612276799126	0.007029302661868638	0.013708537499209457
ENSG00000138069_65129906_65088493_202	RAB1A	2	-	606	66	1	1	10.40%	63.90677883313368	2.6281503917673907e-05	3.443854328470227e-05	6.980466965962083e-09
ENSG00000101150_63865366_63890042_222	TPD52L2	20	+	666	27	3	2	6.76%	23.788395560736493	0.002324716909904271	0.0006589470154116208	9.658669237050791e-06
ENSG00000171262_38454195_38481555_319	FAM98B	15	+	957	11	0	2	1.88%	6.744602572775864	0.012798402692974279	0.011772051206276392	0.0007371592163134675
ENSG00000099860_2476359_2477601_160	GADD45B	19	+	480	23	1	0	8.12%	28.11656197525938	0.0009723463632442102	0.0006223089785121674	3.840867094721006e-06
ENSG00000283900_38314157_38293287_416	TPTEP2-CSNK1E	22	-	1248	27	1	0	1.20%	12.694768784816109	0.029237630782826433	0.021557223391537678	0.002819005644529471
ENSG00000185236_8390417_8402594_179	RAB11B	19	+	537	46	1	0	7.82%	50.26424487197208	0.0004534781744779963	0.00045482414116276956	1.3551220708216137e-06
ENSG00000072506_53434345_53431571_169	HSD17B10	X	-	507	32	2	1	8.88%	37.03545081382249	0.0006296240393168069	0.00040502216050484493	1.669058847898387e-06
ENSG00000079819_130956485_130858136_935	EPB41L2	6	-	2805	19	1	6	0.96%	3.97462675679091	0.006142079104304129	0.03535057243299149	0.0011498351620616329
ENSG00000077721_119574956_119583255_119	UBE2A	X	+	357	54	0	1	10.08%	88.7567027644286	0.0010197273511756433	0.0010238660768343774	6.468005899943356e-06
ENSG00000138674_82881936_82819074_1106	SEC31A	4	-	3318	28	0	0	1.08%	4.951733534443039	0.0008664586529590942	0.0008664586529590942	4.7094196839841995e-06
ENSG00000113312_160043145_160065065_120	TTC1	5	+	360	32	3	4	5.83%	52.158259896133345	0.039104403811496304	0.05315521400506113	0.008485924368449567
ENSG00000156471_96299703_96333850_301	PTDSS1	8	+	903	29	0	3	4.98%	18.84455403224087	0.00023587963149241382	0.0032364592914596087	5.4733617697197015e-06
ENSG00000104299_28859500_28771151_501	INTS9	8	-	1503	13	0	0	1.20%	5.075279780312377	0.013428347753762199	0.013428347753762199	0.0008725593497644525
ENSG00000061987_62501630_62592749_1477	MON2	12	+	4431	5	3	0	0.34%	0.6621309228520651	0.15865525393145707	0.012673659338734126	0.011061610944025602
ENSG00000160014_46601435_46609153_83	CALM3	19	+	249	17	4	2	13.25%	40.061314076849406	0.017818381924954024	0.0054893863033663335	0.0005115393994008742
ENSG00000157349_70324649_70333582_328	DDX19B	16	+	984	14	0	0	1.52%	8.348501965082319	0.019679754444124863	0.019679754444124863	0.0017849636115922674
ENSG00000162616_78013254_78016247_199	DNAJB4	1	+	597	11	0	0	3.02%	10.81169960158543	0.011772051206276392	0.011772051206276392	0.000681272511389508
ENSG00000176102_33161325_33085087_749	CSTF3	11	-	2247	42	0	1	1.47%	10.967858389373834	0.00151350169064872	0.0023697301888609516	2.1237244050853162e-05
ENSG00000065911_74198642_74206004_102	MTHFD2	2	+	306	14	0	0	4.90%	26.84616318183334	0.02108409854857798	0.02108409854857798	0.002029891263157455
ENSG00000174437_110346029_110346470_119	ATP2A2	12	+	357	27	0	1	6.72%	44.3783513822143	0.00575691403842271	0.00575691403842271	0.0001762552514680027
ENSG00000101311_6119554_6077173_677	FERMT1	20	-	2031	11	2	0	0.74%	3.1780328223271797	0.0700082515985845	0.02108409854857798	0.006564739044797468
ENSG00000010278_6232667_6237828_158	CD9	12	+	474	139	7	1	20.25%	172.07274032189562	6.613736944929179e-06	5.631038869139985e-07	3.452409511008813e-11
ENSG00000100983_34951852_34941806_124	GSS	20	-	372	15	0	0	5.65%	23.660500960947587	0.0078820576498062	0.0078820576498062	0.00031963642907451164
ENSG00000116209_54053119_54045644_122	TMEM59	1	-	366	12	2	0	4.92%	19.23870242070492	0.05258119297243839	0.013007239811447113	0.00326646906127711
ENSG00000134759_36129934_36136324_51	ELP2	18	+	153	11	0	0	9.80%	42.186827857166676	0.020613416668581838	0.020613416668581838	0.001946208561389314
ENSG00000101146_57356500_57378099_285	RAE1	20	+	855	16	0	3	3.51%	10.98068629392281	0.0020082573300163734	0.02002743130790103	0.0002450882969218505
ENSG00000101146_57356500_57378099_285	RAE1	20	+	855	16	0	3	3.51%	10.98068629392281	0.0020082573300163734	0.02002743130790103	0.0002450882969218505

ENSG00000105185_32584960_32587300_87	PDCD5	19	+	261	18	1	2	5.75%	40.46761543665518	0.022600676488261057	0.02108409854857798	0.0021660048121727006
ENSG00000085063_33722445_33710328_91	CD59	11	−	273	33	5	2	8.79%	70.92950178182969	0.04366060473514472	0.015646180920032563	0.003151583157314635
ENSG00000085063_33713388_33710126_75	CD59	11	−	225	29	0	1	13.33%	75.62947684939336	0.0022543491824521134	0.0033869280463461717	4.372784971036962e−05
ENSG00000131051_35740847_35704481_515	RBM39	20	−	1545	31	1	0	2.72%	11.773587792088351	0.0006179331360801681	0.0004168743852437731	1.684212386012298e−06
ENSG00000041802_194672114_194642068_642	LSG1	3	−	1926	11	2	2	1.09%	3.351290063419783	0.04363948305378087	0.04363948305378087	0.007798642042119184
ENSG00000196352_207321766_207359669_439	CD55	1	+	1317	46	3	0	5.01%	20.494988227979505	0.00013526789672648355	1.5342867843440252e−05	1.668931369873416e−08
ENSG00000168036_41224069_41239342_781	CTNNB1	3	+	2343	117	8	3	5.76%	29.301455223339957	6.403151454483205e−08	1.1032790393731706e−08	7.305397733044446e−15
ENSG00000101343_20047861_20036195_512	CRNKL1	20	−	1536	22	3	1	2.54%	8.404407112169924	0.004909317959302256	0.001015856855223422	3.0659672620044405e−05
ENSG00000122566_26197458_26192516_301	HNRNPA2B1	7	−	903	493	16	17	35.22%	320.35741854809476	2.0686521528754775e−18	1.3831759838043311e−17	4.48310802352614e−34
ENSG00000143740_227747737_227780673_419	SNAP47	1	+	1257	13	0	0	1.19%	6.068532625146779	0.02108409854857798	0.02108409854857798	0.002029891263157455
ENSG00000115966_175121492_175074609_455	ATF2	2	−	1365	19	1	0	1.32%	8.167639599119783	0.020525443280229434	0.013640585738808999	0.0013255139945858052
ENSG00000162775_110339538_110346354_925	RBM15	1	+	2775	22	0	3	1.08%	4.651952909655137	0.0021880057261679567	0.007664059176943723	9.5950101060544e−05
ENSG00000103769_65869586_65887752_155	RAB11A	15	+	465	41	4	2	8.39%	51.73762876793872	0.002243910108980661	0.0010174708267004099	1.389574881847499e−05
ENSG00000074696_65556886_65576379_245	HACD3	15	+	735	67	0	1	8.57%	53.48882775062654	2.5935781044435533e−05	3.416865027958123e−05	6.838556857470237e−09
ENSG00000173848_5446766_5456994_542	NET1	10	+	1626	42	1	0	2.95%	15.156689914466794	0.0002771340729883236	0.00017249046415619826	3.3068081356265e−07
ENSG00000134014_28110379_28189725_413	ELP3	8	+	1239	21	0	1	2.66%	9.945430912398308	0.0013984928336429232	0.002484757332982506	2.0661357227484502e−05
ENSG00000131238_40097238_40075138_301	PPT1	1	−	903	55	0	0	7.64%	35.73967144045682	9.564019873338271e−06	9.564019873338271e−06	7.446112729828008e−10
ENSG00000131238_40074162_40074061_33	PPT1	1	−	99	26	0	0	15.15%	154.10394969312125	0.019679754444124863	0.019679754444124863	0.0017849636115922674
ENSG00000108312_44213282_44207242_569	UBTF	17	−	1707	33	4	3	2.46%	11.34373402837698	0.0038766098248878157	0.002532570608603844	5.56157809650073e−05
ENSG00000125691_38853710_38847939_114	RPL23	17	−	342	199	8	10	10.53%	341.43071445166237	0.0018172566841077903	0.004295162393546244	4.5261622740756707e−05
ENSG00000184640_77402556_77498658_395	SEPT9	17	+	1185	48	3	5	3.54%	23.768320965326588	0.001956288004330115	0.0046517634603797245	5.243032915395539e−05
ENSG00000172270_572635_582540_55	BSG	19	+	165	425	9	0	20.00%	1511.4041219902276	0.002305925096779131	0.0016650069558729898	2.2622071915815264e−05
ENSG00000172270_579563_582577_24	BSG	19	+	72	47	1	1	29.17%	383.03722111222925	0.010275150823238127	0.008877961307018026	0.00045972819701380264
ENSG00000131778_147275365_147295509_470	CHD1L	1	+	1410	7	0	1	1.28%	2.9130943027095753	0.009815328628645334	0.0477903522728147	0.0023388674905236288
ENSG00000172046_49119145_49108412_1372	USP19	3	−	4116	20	0	0	0.66%	2.8512168310568518	0.0034782380676658805	0.0034782380676658805	6.758309314600117e−05
ENSG00000162813_220074026_220067290_106	BPNT1	1	−	318	10	1	0	4.72%	18.45221458589623	0.019679754444124863	0.020613416668581838	0.001864001753513706
ENSG00000115648_237510536_237553592_496	MLPH	2	+	1488	43	0	9	2.62%	16.95669235534577	0.0007052562366071395	0.011084753933732204	5.333329704317463e−05
ENSG00000184277_101645142_101642479_73	TM2D3	15	−	219	11	0	0	6.85%	29.472989324869868	0.019679754444124863	0.019679754444124863	0.0017849636115922674
ENSG00000139697_123350441_123295908_1393	SBNO1	12	−	4179	47	6	3	1.36%	6.5993491074612365	0.0007820454136382059	0.00032416673522850386	1.6769857499877671e−06
ENSG00000100650_69770482_69771461_145	SRSF5	14	+	435	30	2	4	8.28%	40.46761543665518	0.003182737791905174	0.006765523591417148	0.0001187342466505311
ENSG00000100650_69768157_69769561_138	SRSF5	14	+	414	98	10	5	14.49%	138.89971385383336	0.00020622244759692734	0.0002165592947110879	3.0848500546799e−07
ENSG00000105372_41860775_41871377_145	RPS19	19	+	435	696	42	48	28.28%	938.8486781304002	1.2051174925481255e−07	1.451025061362703e−07	1.6798907765211764e−13
ENSG00000092931_76741970_76778352_362	MFSD11	17	+	1086	13	0	0	2.21%	7.024075055073206	0.005160369442893307	0.005160369442893307	0.00014319601891785326
ENSG00000114988_96740040_96707256_348	LMAN2L	2	−	1044	17	0	0	2.30%	9.554853644765807	0.005160369442893307	0.005160369442893307	0.00014319601891785326
ENSG00000079462_42302240_42297078_184	PAFAH1B3	19	−	552	37	0	1	5.43%	39.33129652493751	0.002460156992221257	0.00319914062860709	4.491170301715699e−05
ENSG00000138078_44339308_44321356_458	PREPL	2	−	1374	18	0	0	3.06%	7.687079788185591	0.00024035368145103084	0.00024035368145103084	3.9581571392385245e−07
ENSG00000133997_70600637_70592872_157	MED6	14	−	471	10	2	0	3.82%	12.458183096210194	0.06032271266817251	0.011570465654371866	0.0034318131775285545
ENSG00000100596_77597311_77578951_94	SPTLC2	14	−	282	13	0	0	5.32%	27.050161382303198	0.020613416668581838	0.020613416668581838	0.001946208561389314
ENSG00000258790_35278700_35317306_227	AL121594.1	14	+	681	94	3	2	7.49%	80.99465468452425	0.00018794081008863267	0.00013974726970550526	1.8464591887511134e−07
ENSG00000258790_35123687_35273566_436	AL121594.1	14	+	1308	20	2	2	1.83%	8.972177734426607	0.013717442413887062	0.013717442413887062	0.0009081672528942985
ENSG00000167315_49813820_49783847_393	ACAA2	18	−	1179	34	2	0	3.31%	16.92157286706616	0.0014807488005297608	0.0006629342244537616	6.173936140520713e−06
ENSG00000170312_60780166_60792147_224	CDK1	10	+	672	25	2	0	4.46%	21.82962886277902	0.0034715542798333917	0.0022276761777358703	4.429711321425767e−05
ENSG00000170312_60792014_60793975_100	CDK1	10	+	300	42	0	5	9.00%	82.14925933641001	0.003580484096903955	0.03624304254342322	0.0007580126380034337
ENSG00000166855_65180181_65150823_599	CLPX	15	−	1797	26	0	0	2.17%	8.489867011474125	0.0006311913977015671	0.0006311913977015671	2.5578527397951546e−06
ENSG00000106211_76302713_76303273_186	HSPB1	7	+	558	180	13	1	13.44%	189.2840076875807	6.551153940751174e−05	5.633993761331117e−06	3.1337206528779318e−09
ENSG00000106211_76304060_76304173_37	HSPB1	7	+	111	16	1	0	13.51%	84.58096199372974	0.021557223391537678	0.02108409854857798	0.0020723516977995955
ENSG00000143569_154227312_154263152_936	UBAP2L	1	+	2808	46	13	5	2.24%	9.612499820601498	0.005124207874005909	0.000528336445990485	1.804818163388961e−05
ENSG00000136270_45109171_45100325_499	TBRG4	7	−	1497	32	6	0	2.81%	12.543068512096195	0.008195816100623025	0.00040182005258836655	2.3810526426963284e−05
ENSG00000164258_53560678_53683221_170	NDUFS4	5	+	510	58	0	20	11.76%	66.73189133770002	4.011609959528814e−05	0.0020371889960313625	7.18959434837228e−07
ENSG00000040633_7236536_7235626_273	PHF23	17	−	819	14	3	1	2.56%	10.03043459541026	0.04363948305378087	0.015516259750072458	0.00312766395889963
ENSG00000110911_51010704_50981726_560	SLC11A2	12	−	1680	22	0	0	1.61%	7.684029359698216	0.0033030736763512156	0.0033030736763512156	6.124050385493983e−05
ENSG00000092841_56158402_56159789_170	MYL6	12	+	510	174	6	3	7.06%	200.19567401310005	0.0010615893185374377	0.0010101077082154788	6.636565409653654e−06
ENSG00000188529_23972001_23971569_69	SRSF10	1	−	207	16	0	0	7.25%	45.355008605333346	0.02108409854857798	0.02108409854857798	0.002029891263157455
ENSG00000100813_23061439_23059148_189	ACIN1	14	−	567	22	0	0	3.70%	22.767494399105825	0.00837382380159347	0.00837382380159347	0.0003583838894600799
ENSG00000116898_36456327_36455788_92	MRPS15	1	−	276	7	0	0	5.43%	14.882112198625004	0.019216965118390855	0.019216965118390855	0.0017073955890589052
ENSG00000243678_51166831_51171507_82	NME2	17	+	246	200	6	0	28.05%	477.0572551475611	4.125039049561602e−05	1.292493402683512e−05	4.234058907598031e−09
ENSG00000136997_127738590_127740958_330	MYC	8	+	990	74	7	1	8.18%	43.860354912657584	2.370806319628599e−05	3.009127783884032e−06	6.133872979550775e−10
ENSG00000134910_125602808_125620810_613	STT3A	11	+	1839	140	9	1	6.53%	44.670614103539975	2.5686997484041683e−07	1.6398638084513643e−08	4.3990711441167415e−14
ENSG00000100280_29358923_29328821_830	AP1B1	22	−	2490	24	0	3	1.08%	5.655714928496387	0.003684287313187465	0.017782189529096822	0.00036099358464052045
ENSG00000198000_92321744_92297836_1099	NOL8	9	−	3297	10	1	1	0.64%	1.779740442315742	0.016854695534768073	0.016854695534768073	0.0013361081351157898
ENSG00000149273_75399548_75401660_44	RPS3	11	+	132	74	9	8	25.00%	328.95266184493187	0.004747109690211661	0.0018115419044284426	4.987801668802467e−05
ENSG00000149273_75400758_75404865_196	RPS3	11	+	588	307	11	7	14.80%	306.36324849705875	1.1291498956243786e−06	1.5107067421749245e−06	1.510793691821115e−11
ENSG00000177697_836070_838192_253	CD151	11	+	759	213	23	10	17.00%	164.66960510686368	5.628074008217504e−07	7.179261916658316e−08	3.96916788384252e−13
ENSG00000159596_45691069_45693905_247	TMEM69	1	+	741	16	0	0	2.43%	12.670022646834012	0.013640585738808999	0.013640585738808999	0.00089863692579514
ENSG00000101040_47347880_47210761_1105	ZMYND8	20	−	3315	6	0	0	0.45%	1.0620460159846157	0.016947426762344633	0.016947426762344633	0.0013498980316300959
ENSG00000115419_190927345_190932862_169	GLS	2	+	507	15	0	0	5.33%	17.360367568979292	0.0032294771334464936	0.0032294771334464936	5.8663648728941325e−05
ENSG00000168036_41224069_41239342_781	CTNNB1	3	+	2343	117	8	3	5.76%	29.301455223339957	6.403151454483205e−08	1.1032790393731706e−08	7.305397733044446e−15

ENSG00000099795_14572000_14566133_137	NDUFB7	19	-	411	50	3	0	7.30%	71.38447978485404	0.004528451082822598	0.002418182715931848	6.204123035105927e-05
ENSG00000114491_124730472_124731550_66	UMPS	3	+	198	10	0	0	7.58%	29.635374940984857	0.019679754444124863	0.019679754444124863	0.0017849636115922674
ENSG00000117385_42762325_42755161_203	P3H1	1	-	609	15	0	0	2.46%	14.452719798805422	0.021557223391537678	0.021557223391537678	0.002115616449879074
ENSG00000161016_144791514_144791248_88	RPL8	8	-	264	442	34	1	22.73%	982.4126792936479	7.385360151578949e-05	4.3353659318283884e-05	2.396376485469562e-08
ENSG00000161016_144792129_144791768_94	RPL8	8	-	282	227	10	9	27.66%	472.3374333679097	1.864757259356777e-05	1.3987967486836834e-05	2.0742562751397988e-09
ENSG00000160746_43600486_43366906_582	ANO10	3	-	1746	7	0	0	0.86%	2.352498835521478	0.019216965118390855	0.019216965118390855	0.0017073955890589052
ENSG00000075945_169992184_169921676_374	KIFAP3	1	-	1122	8	0	0	1.34%	4.183817638727274	0.019679754444124863	0.019679754444124863	0.0017849636115922674
ENSG00000075945_169992184_169921676_374	KIFAP3	1	-	1122	8	0	0	1.34%	4.183817638727274	0.019679754444124863	0.019679754444124863	0.0017849636115922674
ENSG00000085063_33722445_33710275_119	CD59	11	-	357	33	6	4	6.72%	54.240207244928584	0.05621651956210631	0.03525671152904061	0.008168485530807177
ENSG00000215301_41334253_41345417_420	DDX3X	X	+	1260	37	12	3	5.95%	17.23085371568691	0.0017977780560661303	3.0360303229622683e-05	4.932263651350944e-07
ENSG00000215301_41345404_41347719_271	DDX3X	X	+	813	58	0	0	8.12%	41.861334049479716	1.4055095028526564e-05	1.4055095028526564e-05	1.579705563707443e-09
ENSG00000168216_69719072_69676158_325	LMBRD1	6	-	975	10	3	0	1.85%	6.018260757246155	0.08791701959130671	0.012798402692974279	0.0056117368578515354
ENSG00000168216_69790322_69676158_467	LMBRD1	6	-	1401	21	3	0	2.14%	8.795423911821201	0.01424822060979566	0.0022409900782254734	0.00018649561876446586
ENSG00000167965_2206086_2208877_326	MLST8	16	+	978	19	0	4	3.37%	11.39961968588804	0.0013775385517804596	0.01572186727294618	0.00013725983080102293
ENSG00000168216_69790322_69676158_467	LMBRD1	6	-	1401	21	3	0	2.14%	8.795423911821201	0.01424822060979566	0.0022409900782254734	0.00018649561876446586
ENSG00000100138_41675154_41674933_73	SNU13	22	-	219	46	0	1	17.81%	123.250682631274	0.000649205117157464	0.0010037877862843834	4.1244594600580285e-06
ENSG00000137700_119027649_119024910_228	SLC37A4	11	-	684	10	0	0	2.19%	8.578661167127194	0.020613416668581838	0.020613416668581838	0.001946208561389314
ENSG00000167508_88663080_88652525_400	MVD	16	-	1200	32	2	10	3.75%	15.647477968840004	0.0005964540801110259	0.022490287069040917	0.00010401404519069548
ENSG00000108433_46923193_46963884_210	GOSR2	17	+	630	12	0	0	2.86%	11.17676997774286	0.011772051206276392	0.011772051206276392	0.000681272511389508
ENSG00000283761_100058702_100082209_418	AC118553.2	1	+	1254	27	0	0	4.31%	12.634028264314596	4.027253539844791e-05	4.027253539844791e-05	1.23110933795418e-08
ENSG00000073008_44647366_44661811_343	PVR	19	+	1029	17	5	1	2.92%	9.694137225593296	0.036335183767386564	0.00397428314897603	0.0008275473095198716
ENSG00000177963_209296_214350_524	RIC8A	11	+	1572	18	3	1	1.91%	6.718859814864506	0.010960145167579947	0.0037220617515305076	0.00022159278179O8156
ENSG00000147162_71536192_71573794_1029	OGT	X	+	3087	13	1	0	0.78%	2.471054586915938	0.010020958156399907	0.004826937407846361	0.00025599508163266543
ENSG00000197756_216498875_216499958_58	RPL37A	2	+	174	40	0	4	15.52%	134.89205145551728	0.003710480643933249	0.018024647789873605	0.0003683785285852921
ENSG00000197756_216499955_216501404_46	RPL37A	2	+	138	413	32	19	47.83%	1756.0892394377504	1.9792895150559914e-05	0.00010059742109512844	1.557879026316028e-08
ENSG00000197563_62157141_62045856_727	PIGN	18	-	2181	12	0	0	0.83%	3.22850301970564	0.011772051206276392	0.011772051206276392	0.000681272511389508
ENSG00000180185_1827239_1839306_223	FAHD1	16	+	669	26	0	0	4.93%	22.804620358174894	0.0013845019718837994	0.0013845019718837994	1.1595255170738555e-05
ENSG00000180185_1827239_1838126_245	FAHD1	16	+	735	26	0	0	4.49%	20.756858530093883	0.0013845019718837994	0.0013845019718837994	1.1595255170738555e-05
ENSG00000172500_65888418_65887822_131	FIBP	11	-	393	10	1	0	4.58%	14.93079958858779	0.026832695913080365	0.011772051206276392	0.0015103817605807263
ENSG00000105983_156892993_156684078_490	LMBR1	7	-	1470	11	0	1	1.22%	4.390873919827552	0.012798402692974279	0.023672501042627626	0.0014375227604752335
ENSG00000169567_131165205_131159447_126	HINT1	5	-	378	170	3	2	19.05%	263.8959578078175	9.70482587088322e-06	1.56577310295924e-05	1.2259539183057494e-09
ENSG00000109111_28675120_28701625_1561	SUPT6H	17	+	4683	19	2	3	0.70%	2.3807021253039724	0.006279669515525777	0.014028501358900838	0.00045314590533802423
ENSG00000166803_64381371_64366005_65	PCLAF	15	-	195	101	9	16	10.77%	303.92216824093083	0.008877961307018026	0.08357182984467554	0.003987317265369605
ENSG00000100722_88563134_88611751_605	ZC3H14	14	+	1815	23	3	3	2.31%	7.435784985192564	0.003468409497O7983	0.0038547319688416013	7.435844836576693e-05
ENSG00000133997_70600637_70584813_246	MED6	14	-	738	18	4	0	4.88%	14.311717654426833	0.021412135282300855	0.0007729603088974596	0.000120880508277367
ENSG00000095794_35148378_35179329_135	CREM	10	+	405	7	3	0	3.70%	10.141883868692595	0.16699912791099897	0.019216965118390855	0.01589743055190296
ENSG00000160752_155319665_155320609_154	FDPS	1	+	462	16	2	0	5.19%	20.32139995953247	0.011738695074494007	0.005405141725979036	0.0003317203066785242
ENSG00000149925_30069625_30070212_114	ALDOA	16	+	342	152	35	7	23.68%	260.79129948066674	0.00047836134882154355	2.422326146460831e-05	9.51795205750582e-08
ENSG00000107362_71875080_71866787_288	ABHD17B	9	-	864	31	2	0	3.47%	21.053464280991324	0.007790523468944856	0.0023628488670607213	0.00010449788798782673
ENSG00000174437_110296650_110346470_917	ATP2A2	12	+	2751	208	9	16	8.29%	44.365804491803715	2.2444016765299717e-13	5.717258554521762e-12	1.7265737879385225e-23
ENSG00000171566_154547798_154536685_448	PLRG1	4	-	1344	38	0	1	3.35%	16.590517935712057	0.00026886811829191485	0.0004157276149849715	7.521021178385541e-07
ENSG00000272540_30723832_30723725_35	AL662797.1	6	-	105	73	0	22	14.29%	407.9521041876144	0.021557223391537678	0.11245794200798093	0.011059731054231003
ENSG00000076554_80044171_80038116_57	TPD52	8	-	171	9	0	0	8.77%	30.8831802016579	0.016947426762344633	0.016947426762344633	0.0013498980316300959
ENSG00000106829_79627422_79725144_684	TLE4	9	+	2052	8	0	0	0.73%	2.2876429779005854	0.019679754444124863	0.019679754444124863	0.0017849636115922674
ENSG00000099956_23787179_23801397_259	SMARCB1	22	+	777	14	1	2	2.70%	10.572620249216218	0.015625008558487224	0.03799572790022047	0.002736976542475398
ENSG00000127955_80189190_80217493_267	GNAI1	7	+	801	23	4	0	3.00%	16.848876090043074	0.028023386503852714	0.005454749182134642	0.0008134260429016822
ENSG00000161011_179823917_179833291_217	SQSTM1	5	+	651	227	23	10	16.59%	204.60699878609913	2.931500424722683e-06	1.0086439366514393e-07	3.0042571112103264e-12
ENSG00000178105_108665322_108940423_819	DDX10	11	+	2457	19	0	0	0.98%	4.537577555066545	0.005306578019052857	0.005306578019052857	0.0001510011474O534233
ENSG00000087365_66055178_66068745_775	SF3B2	11	+	2325	109	4	5	5.42%	27.509275783928395	3.949421197182057e-08	9.527427635099006e-08	3.727423664406201e-14
ENSG00000091483_241519593_241497828_467	FH	1	-	1401	60	0	3	4.50%	25.12978260520343	2.3326754951162877e-05	0.00013526789672648355	2.455997215826865e-08
ENSG00000185721_31400620_31433971_353	DRG1	22	+	1059	24	0	0	2.27%	13.298139916861192	0.005604866691947602	0.005604866691947602	0.00016752456572620382
ENSG00000147642_109579914_109574906_457	SYBU	8	-	1371	9	0	0	1.09%	3.8519502658522984	0.020613416668581838	0.020613416668581838	0.001946208561389314
ENSG00000069345_46971461_46957029_329	DNAJA2	16	-	987	69	0	6	8.81%	41.02112385448177	9.900363017176685e-07	1.6488567333441712e-05	1.503437599074599e-10
ENSG00000168291_58431954_58428034_317	PDHB	3	-	951	29	0	0	3.79%	17.893409349225557	0.0010405263639310788	0.0010405263639310788	6.697970672456085e-06
ENSG00000160410_40586917_40591207_270	SHKBP1	19	+	810	9	2	0	2.22%	6.519782487016668	0.053594824633710306	0.011772051206276392	0.003068672569093115
ENSG00000124795_18255838_18237103_236	DEK	6	-	708	16	0	4	2.97%	13.26057454986441	0.00797940233239714	0.05128931843854215	0.002129167107140446
ENSG00000112983_138168063_138157208_659	BRD8	5	-	1977	9	2	0	0.91%	2.6712310644833086	0.07134538375819317	0.011772051206276392	0.0041729868335021346
ENSG00000106723_88448967_88475277_236	SPIN1	9	+	708	23	0	2	3.39%	19.06207591543009	0.005454749182134642	0.015797663760347944	0.00045021431474288393
ENSG00000005448_74423343_74425701_231	WDR54	2	+	693	18	1	0	3.46%	15.241049969649355	0.009288069496478249	0.005306578019052857	0.0002590037054391842
ENSG00000153048_8859328_8855164_147	CARHSP1	16	-	441	23	0	4	4.76%	30.603060653343544	0.00877616296920843	0.04584514077471458	0.0020400732494421717
ENSG00000074319_18526816_18484063_152	TSG101	11	-	456	7	2	0	3.29%	9.007594225483555	0.1191346346731873	0.019216965118390855	0.010787784428637617
ENSG00000132382_4555324_4539415_1328	MYBBP1A	17	-	3984	14	4	0	0.53%	2.0619794010143075	0.04910688221045026	0.00837382380159347	0.002112891589592773
ENSG00000132383_1844583_1897175_560	RPA1	17	+	1680	38	6	1	3.75%	13.272414348569646	0.0003660481250520678	3.33487708824674e-05	9.551146850246299e-08
ENSG00000064042_41360841_41697185_1083	LIMCH1	4	+	3249	22	3	6	1.20%	3.9732746458273325	0.005301672691061329	0.009535600913472842	0.0002655394963642886
ENSG00000009307_114739890_114718169_798	CSDE1	1	-	2394	90	11	5	4.39%	22.059414429755645	9.267998481696954e-06	8.096231702821984e-07	6.8610803470884e-11
ENSG00000123485_233854479_233837577_656	HJURP	2	-	1968	30	1	0	1.83%	8.94482353401677	0.0013767418916647864	0.0010405263639310788	8.780312937977671e-06
ENSG00000257103_34172643_34226431_422	LSM14A	19	+	1266	28	0	0	2.61%	12.977766087900477	0.001558047347358915	0.001558047347358915	1.4544595481456893e-05

ENSG00000180817_70233327_70203155_289	PPA1	10	-	867	25	0	0	4.15%	16.919850744852944	0.0008664586529590942	0.0008664586529590942	4.7094196839841995e-06
ENSG00000126067_35641432_35603267_201	PSMB2	1	-	603	92	6	5	8.96%	89.52537146351246	0.0004688424238804269	0.0004722470567701311	1.451162779427316e-06
ENSG00000079332_70161667_70157746_78	SAR1A	10	-	234	34	0	1	16.67%	85.2586940609872	0.0006560461581947184	0.0007616358238233979	3.1832011613819197e-06
ENSG00000079332_70161667_70152476_155	SAR1A	10	-	465	57	0	1	12.90%	71.92792292128067	4.0232615489265806e-05	4.479439896941e-05	1.3644348512002314e-08
ENSG00000129103_56064312_56079612_217	SUMF2	7	+	651	114	4	3	8.29%	102.75417560182952	0.00047905572924371163	0.00026612130312053076	8.563930459662114e-07
ENSG00000129103_56064312_56079612_286	SUMF2	7	+	858	130	4	3	8.74%	88.90612482295457	3.317859714981013e-05	1.5575645104956635e-05	4.0670996866079406e-09
ENSG00000136631_150068645_150144921_538	VPS45	1	+	1614	14	0	3	1.30%	5.08979301960409	0.00847367416878415	0.046544029217479216	0.002014207749665457
ENSG00000206560_15724423_15670270_836	ANKRD28	3	-	2508	13	2	0	0.84%	3.0415253228905508	0.04363948305378087	0.0078820576498062	0.0017710903275458955
ENSG00000167283_118401666_118431041_76	ATP5MG	11	+	228	87	10	1	6.58%	223.90305646201978	0.1973134331215476	0.02108409854857798	0.02074155215028262
ENSG00000162972_199956126_199963811_182	MAIP1	2	+	546	15	0	0	3.30%	16.1203413140522	0.013428347753762199	0.013428347753762199	0.0008725593497644525
ENSG00000156261_29071954_29056475_529	CCT8	21	-	1587	151	7	2	8.70%	55.8310296147174	9.471792432582565e-09	1.3108420079153866e-09	1.3636724125296766e-16
ENSG00000105700_18557907_18562105_25	KXD1	19	+	75	23	1	1	20.00%	179.94599664166003	0.02108409854857798	0.02108409854857798	0.002029891263157455
ENSG00000144746_69085048_69101846_93	ARL6IP5	3	+	279	20	1	0	6.45%	42.063112819462376	0.020525443280229434	0.013640585738808999	0.0013255139945858052
ENSG00000105341_41439899_41433268_172	DMAC2	19	-	516	15	4	2	3.49%	17.057570460218027	0.12835325026041705	0.060667625179241055	0.028884111462696958
ENSG00000006695_14069606_14207213_443	COX10	17	+	1329	18	0	0	1.81%	7.947364656860047	0.005160369442893307	0.005160369442893307	0.00014319601891785326
ENSG00000072310_17836817_17812622_1147	SREBF1	17	-	3441	84	7	18	3.23%	14.324195176357458	1.2985094596972424e-06	3.177457264205154e-05	3.836727907419007e-10
ENSG00000184371_109914343_109925189_513	CSF1	1	+	1539	8	2	0	1.17%	3.0501906372007803	0.08266357711566169	0.011570465654371866	0.004841719827405853
ENSG00000184371_109914343_109925189_397	CSF1	1	+	1191	8	2	0	1.51%	3.9414302188513863	0.08266357711566169	0.011570465654371866	0.004841719827405853
ENSG00000070018_12266735_12121126_1613	LRP6	12	-	4839	18	2	1	0.68%	2.1826922151202734	0.004356893100389425	0.002458650919525015	6.065372924591701e-05
ENSG00000270617_43881673_43866699_159	URGCP-MRPS24	7	-	477	27	5	1	6.29%	33.21398625461322	0.015528142895806445	0.0023218226288164415	0.00021060506759671452
ENSG00000214114_38866933_38864670_32	MYCBP	1	-	96	11	0	0	15.62%	67.23525689735939	0.02108409854857798	0.02108409854857798	0.002029891263157455
ENSG00000197563_62157141_62045856_768	PIGN	18	-	2304	12	0	0	0.78%	3.056148040789063	0.011772051206276392	0.011772051206276392	0.000681272511389508
ENSG00000124155_45416169_45424321_425	PIGT	20	+	1275	75	4	0	5.41%	34.516495519500005	6.051873075356466e-05	1.181854822373525e-05	5.736528091018088e-09
ENSG00000179010_6640863_6641246_127	MRFAP1	4	+	381	76	1	3	14.96%	117.04806354644097	5.6254241454829154e-05	0.0001733650811203729	7.181689729777536e-08
ENSG00000131373_15589566_15560865_460	HACL1	3	-	1380	9	0	0	1.09%	3.826828851075001	0.019679754444124863	0.019679754444124863	0.0017849636115922674
ENSG00000090487_64983569_64963620_281	SPG21	15	-	843	33	2	2	4.27%	22.9700521784573	0.0010197273511756433	0.0026814738672924004	1.66328217535521e-05
ENSG00000171843_20621775_20346443_565	MLLT3	9	-	1695	7	1	0	0.88%	2.4232819863247794	0.04178282491678763	0.019216965118390855	0.003600525600033436
ENSG00000130119_54532567_54560602_582	GNL3L	X	+	1746	27	1	0	2.06%	9.07392407986856	0.0015592205693390887	0.0009430837296224684	9.035662816596504e-06
ENSG00000181789_129249710_129277424_874	COPG1	3	+	2622	51	4	0	2.29%	11.413349204960529	0.0002748363328174478	3.82866330551027e-05	8.037930612789419e-08
ENSG00000164070_127783673_127832874_806	HSPA4L	4	+	2418	19	3	2	1.24%	4.610764289825683	0.015358647673375043	0.009621867080838033	0.0007286998036562927
ENSG00000111684_7018424_6981166_158	LPCAT3	12	-	474	13	0	0	3.16%	16.093133986939876	0.02108409854857798	0.02108409854857798	0.002029891263157455
ENSG00000101182_62143303_62139679_149	PSMA7	20	-	447	109	8	4	11.41%	143.08515927882218	0.00038939156998806537	0.00017606549270215196	4.720553114173496e-07
ENSG00000130731_636290_634597_127	METTL26	16	-	381	14	1	1	5.51%	21.561485390133864	0.015625008558487224	0.015625008558487224	0.0011593907271672923
ENSG00000110066_68190049_68157688_876	KMT5B	11	-	2628	7	0	1	0.80%	1.5629615551067355	0.00407548579675135	0.016947426762344623	0.0003747122194962002
ENSG00000137207_43516029_43513468_137	YIPF3	6	-	411	38	0	1	8.03%	54.25220463648906	0.0016263209931528667	0.0020099050143758913	1.936438837227613e-05
ENSG00000115738_8682166_8682899_134	ID2	2	+	402	74	7	9	14.93%	108.01430687445524	0.00044068318690335015	0.0001588118650064621	4.848563687370304e-07
ENSG00000184270_149887916_149887524_130	HIST2H2AB	1	-	390	360	7	2	16.15%	541.643468152154	0.00011193010679996882	2.8765194065111864e-05	2.462026491677325e-08
ENSG00000159199_48893418_48895769_136	ATP5MC1	17	+	408	86	1	1	11.76%	123.68410894487502	0.00041346295719931213	0.00041346295719931213	1.1303979870669889e-06
ENSG00000101166_59042730_59035007_194	PRELID3B	20	-	582	44	2	13	7.22%	44.36140661269073	0.00046432135692914766	0.009092742361888844	3.0167093417490815e-05
ENSG00000097033_86704909_86743235_383	SH3GLB1	1	+	1149	21	1	0	2.09%	10.724446388565276	0.009288069496478249	0.005306578019052857	0.0002590037054391842
ENSG00000176697_27658504_27657821_227	BDNF	11	-	681	10	1	0	2.64%	8.616452626013217	0.023672501042627626	0.012798402692974279	0.0014375227604752335
ENSG00000162129_72329736_72293367_396	CLPB	11	-	1188	16	0	0	1.26%	7.902766650929295	0.019679754444124863	0.019679754444124863	0.0017849636115922674
ENSG00000162819_222715700_222732714_379	BROX	1	+	1137	39	0	0	5.01%	20.127033007412933	5.597915316361888e-05	5.597915316361888e-05	2.337545340249931e-08
ENSG00000005889_24172743_24211376_844	ZFX	X	+	2532	14	1	4	0.83%	3.2444415219751193	0.013892891352654344	0.067234255889369	0.004472097786560951
ENSG00000265354_45972625_46003318_209	TIMM23	10	+	627	34	1	0	3.83%	31.819034147162686	0.011825808327677989	0.005454749182134642	0.00033692926567688086
ENSG00000103051_70508440_70481010_447	COG4	16	-	1341	19	0	0	1.34%	8.31381659418233	0.013428347753762199	0.013428347753762199	0.0008725593497644525
ENSG00000103051_70517694_70509207_241	COG4	16	-	723	11	2	0	2.07%	8.927502990520749	0.0700082515985845	0.02108409854857798	0.006564739044797468
ENSG00000102030_153934904_153929987_220	NAA10	X	-	660	35	5	1	6.82%	31.117143688034098	0.002557734800548535	0.0006738559393031653	1.0862454565344298e-05
ENSG00000116266_108753114_108808877_542	STXBP3	1	+	1626	20	6	0	1.48%	7.21747138784133	0.04534666772420983	0.005554612275049112	0.0013868444848912668
ENSG00000159140_33557200_33576424_358	SON	21	+	1074	9	0	1	1.40%	4.9171543896494425	0.016947426762344633	0.0477903522728147	0.0036975118224100542
ENSG00000166197_102159553_102162269_418	NOLC1	10	+	1254	106	0	9	5.02%	49.6002591117536	2.3200298509694487e-05	0.0012787427712268279	2.67882882501554e-07
ENSG00000172301_31856811_31852139_133	COPRS	17	-	399	40	1	1	9.77%	58.82510514601505	0.0009985037337807711	0.0009985037337807711	6.187415572756679e-06
ENSG00000137449_15004186_15066380_503	CPEB2	4	+	1509	12	1	0	1.59%	4.666245915161035	0.010499663139468709	0.004193160480899131	0.0002361360478986167
ENSG00000137054_37493559_37503202_285	POLR1E	9	+	855	25	2	5	3.86%	17.15732233425439	0.004933404332508489	0.01572186727294618	0.0004100204633648438
ENSG00000141510_7676594_7673307_341	TP53	17	-	1023	30	0	4	3.52%	17.207637062507334	0.0010238660768343774	0.006911511910188328	4.410159297683948e-05
ENSG00000142186_65536618_65538566_246	SCYL1	11	+	738	6	0	0	2.03%	4.7705725514756105	0.016947426762344633	0.016947426762344633	0.0013498980316300959
ENSG00000111237_110501503_110492005_214	VPS29	12	-	642	64	0	11	10.75%	58.49524474332711	1.1598859020456752e-05	0.0003995223902392225	4.0868810868669265e-08
ENSG00000132305_86195382_86144268_757	IMMT	2	-	2271	55	1	2	3.30%	14.210886530485473	6.688252526459028e-06	1.717267842574393e-05	9.398810624366696e-10
ENSG00000072042_67685124_67678321_70	RDH11	14	-	210	26	0	6	14.29%	72.64900485532858	0.0022409900782254734	0.015400968107160708	0.00020254936092241094
ENSG00000072042_67691200_67690988_70	RDH11	14	-	210	17	0	0	7.14%	47.50127240540716	0.019679754444124863	0.019679754444124863	0.0017849636115922674
ENSG00000171135_9892988_9893377_129	JAGN1	3	+	387	21	0	0	3.88%	31.840798192406982	0.02108409854857798	0.02108409854857798	0.002029891263157455
ENSG00000137074_32989891_32973498_342	APTX	9	-	1026	21	7	3	3.51%	12.010125633978072	0.016059575308391173	0.007971974128487071	0.0006419765354178032
ENSG00000256269_119092389_119093283_149	HMBS	11	+	447	23	0	0	5.37%	30.192281315714773	0.005604866691947602	0.005604866691947602	0.00016752456572620382
ENSG00000077721_119574739_119583255_113	UBE2A	X	+	339	58	0	1	11.50%	100.39311086202657	0.0006862354746923267	0.0006885945587055891	3.015214816289631e-06
ENSG00000178209_143935297_143916177_4341	PLEC	8	-	13023	130	33	39	1.38%	5.857441073339094	2.796321955810114e-06	1.2083355211512743e-05	2.8801264455689585e-10
ENSG00000172500_65888418_65885634_146	FIBP	11	-	438	10	2	0	4.11%	13.396813329486305	0.053594824633710306	0.011772051206276392	0.003068672569093115
ENSG00000113318_80744512_80875862_584	MSH3	5	+	1752	10	1	2	0.86%	3.349203332371576	0.02784981298332479	0.06556525143482543	0.007750594856566061

ENSG00000128309_37024216_37029514_297	MPST	22	+	891	13	1	2	2.69%	8.561330538506736	0.010020958156399907	0.030313543781140352	0.0014836836722180672
ENSG00000157020_10315424_10301106_278	SEC13	3	−	834	45	0	4	5.76%	31.660814235512596	0.00017954280306204412	0.0013997216573861052	1.7693578653504246e−06
ENSG00000077782_38424619_38413628_547	FGFR1	8	−	1641	10	0	0	1.65%	3.57574907880256	0.001946208561389314	0.001946208561389314	2.2278545302028032e−05
ENSG00000134905_110706093_110641537_564	CARS2	13	−	1692	22	0	3	1.77%	7.629532697572697	0.0023628488670607213	0.013976320728530797	0.00019124783671400775
ENSG00000075785_128795368_128813422_134	RAB7A	3	+	402	42	0	3	9.70%	61.30541741523136	0.0006909589238230294	0.001779122956135253	7.705053750716641e−06
ENSG00000135535_109382378_109368851_184	CD164	6	−	552	73	9	1	15.76%	77.59958503568751	6.318407701361028e−05	9.631878146120184e−07	6.115742478072153e−10
ENSG00000175455_123948968_123914779_733	CCDC14	3	−	2199	9	0	5	0.68%	2.4015569870320608	0.019216965118390855	0.2463583386135439	0.025652408423363686
ENSG00000066926_57586599_57550712_416	FECH	18	−	1248	15	0	0	2.16%	7.052649324897838	0.0033278027414746945	0.0033278027414746945	6.211806171561056e−05
ENSG00000167085_49413264_49408934_205	PHB	17	−	615	43	4	0	4.88%	41.02692394269025	0.002446120450917513	0.002446120450917513	3.450690187599895e−05
ENSG00000073921_86014950_85959046_497	PICALM	11	−	1491	38	0	5	2.82%	14.954833068810869	0.0004481211664877703	0.003929042651386831	1.1787616094891344e−05
ENSG00000131446_180792971_180791634_445	MGAT1	5	−	1335	9	0	0	1.57%	3.9558230820101135	0.006937202941512724	0.006937202941512724	0.00025101522741487095
ENSG00000182196_122981140_122982586_215	ARL6IP4	12	+	645	31	3	0	6.05%	28.201849827560473	0.00458450705388845	0.0005983074123552221	1.7879347088619252e−05
ENSG00000198836_193593378_193692127_978	OPA1	3	+	2934	32	1	1	1.84%	6.39978649032311	0.0001048543557987398	0.0001048543557987398	7.919728780723367e−08
ENSG00000181649_2929364_2928906_152	PHLDA2	11	−	456	15	0	7	3.95%	19.301987626036187	0.012798402692974279	0.17808035639161363	0.012843613314113225
ENSG00000173273_9580197_9776736_1090	TNKS	8	+	3270	13	0	2	0.46%	2.332766210950918	0.020613416668581838	0.056923149003329024	0.005212417464661139
ENSG00000137312_30730565_30728116_110	FLOT1	6	−	330	14	1	0	5.45%	24.893714950427277	0.02034245439037237	0.013428347753762199	0.0012955184805712684
ENSG00000137312_30740729_30730915_161	FLOT1	6	−	483	17	0	0	3.73%	20.65272713278572	0.013640585738808999	0.013640585738808999	0.00089863692579514
ENSG00000183011_7857442_7856731_118	NAA38	17	−	354	123	25	1	21.19%	203.8813337041653	0.0009199037630120651	9.780777069682743e−06	8.85827670819452e−08
ENSG00000250644_1763859_1735280_557	AC068580.4	11	−	1671	329	16	2	10.95%	115.5300774629345	3.403507226483371e−10	7.449244420375941e−12	3.2585062895910803e−20
ENSG00000104671_30179425_30183173_90	DCTN6	8	+	270	10	0	0	5.56%	21.73260829005556	0.019679754444124863	0.019679754444124863	0.0017849636115922674
ENSG00000141002_89873668_89911238_676	TCF25	16	+	2028	29	7	2	2.66%	8.39084432500666	0.004450405279219605	0.00021989273129921432	7.283186957841822e−06
ENSG00000130669_39177765_39178579_66	PAK4	19	+	198	12	0	0	7.58%	35.562449929181824	0.02108409854857798	0.02108409854857798	0.002029891263157455
ENSG00000049618_157174031_157207891_1286	ARID1B	6	+	3858	16	0	0	0.54%	2.433511348186626	0.00847367416878415	0.00847367416878415	0.0003665024264111182
ENSG00000152620_36241798_36195144_410	NADK2	5	−	1230	12	0	0	1.46%	5.724687061770733	0.012798402692974279	0.012798402692974279	0.0007972436888943567
ENSG00000145741_73502922_73505238_99	BTF3	5	+	297	401	5	5	15.15%	792.2523567556618	0.0002880086553510197	0.00033376502694317683	6.481610733390738e−07
ENSG00000124570_2954670_2948298_259	SERPINB6	6	−	777	52	1	2	6.95%	39.26973235423167	9.707904216161918e−05	0.0001955312910985679	1.3557960306751437e−07
ENSG00000168036_41224069_41239342_781	CTNNB1	3	+	2343	117	8	3	5.76%	29.301455223339957	6.403151454483205e−08	1.1032790393731706e−08	7.305397733044446e−15
ENSG00000132570_134905221_134960681_103	PCBD2	5	+	309	8	0	2	4.85%	15.19172618333981	0.019679754444124863	0.10105102209468636	0.009164497669452725
ENSG00000134779_36818914_36798422_179	TPGS2	18	−	537	8	0	0	2.79%	8.741607803821232	0.019216965118390855	0.019216965118390855	0.0017073955890589052
ENSG00000134333_18396843_18407281_274	LDHA	11	+	822	554	23	10	16.42%	395.47001800809136	2.376544549839141e−09	8.864456996146075e−09	2.264020224922316e−16
ENSG00000132541_98106314_98102842_76	RIDA	8	−	228	14	4	0	11.84%	36.03037690193422	0.04077810018967558	0.002943990679779778	0.0007391052320154406
ENSG00000067533_218285317_218331091_282	RRP15	1	+	846	27	1	0	2.84%	18.727034803132984	0.0073129010928764775	0.005706018193000826	0.0002196570096149794
ENSG00000092969_218346798_218441362_382	TGFB2	1	+	1146	17	0	0	1.83%	8.704421644969898	0.008674846900444944	0.008674846900444944	0.00038311532916634116
ENSG00000140105_100361879_100354443_134	WARS	14	−	402	15	0	0	3.73%	21.8947919340112	0.02108409854857798	0.02108409854857798	0.002029891263157455
ENSG00000183258_177513451_177511791_245	DDX41	5	−	735	17	1	4	3.27%	13.571792115830615	0.01007916461776185	0.04753395219010738	0.0023771889737864704
ENSG00000169045_179623133_179621168_108	HNRNPH1	5	−	324	72	2	6	12.04%	130.39564974033337	0.0015782393174957076	0.006715248226692483	6.265410609736626e−05
ENSG00000112514_33416969_33416650_58	CUTA	6	−	174	38	1	0	15.52%	128.1474488827414	0.003710480643933249	0.003710480643933249	7.644477796348072e−05
ENSG00000112514_33417668_33417247_83	CUTA	6	−	249	116	1	6	15.66%	273.35955487732537	0.001006436396730239	0.005421488241340597	3.3699460502274616e−05
ENSG00000196531_56724521_56712538_925	NACA	12	−	2775	209	11	5	3.46%	44.19355264172379	9.91904999642488e−07	1.161173264099816e−06	1.0272139787411823e−11
ENSG00000102974_67616855_67637872_403	CTCF	16	+	1209	7	0	0	1.24%	3.3974052661873455	0.019216965118390855	0.019216965118390855	0.0017073955890589052
ENSG00000113194_176494230_176496721_93	FAF2	5	+	279	14	1	0	6.45%	29.444178973623664	0.026832695913080365	0.011772051206276392	0.0015103817605807263
ENSG00000133835_119479012_119541994_532	HSD17B4	5	+	1596	38	0	0	2.82%	13.970962472178575	0.00029563251185049877	0.00029563251185049877	5.909204346842462e−07
ENSG00000125863_10420737_10420546_63	MKKS	20	−	189	38	2	7	17.46%	117.97701643173019	0.001565547007472977	0.0028475905029315958	2.6248108435057554e−05
ENSG00000145247_48904549_48885531_99	OCIAD2	4	−	297	12	0	7	6.06%	23.708299952787883	0.011772051206276392	0.3212384956040438	0.026834343646100805
ENSG00000036257_224557733_224474245_705	CUL3	2	−	2115	25	2	3	1.99%	6.935938815975179	0.0019573865167617018	0.0033854124258957835	3.8307544722804655e−05
ENSG00000065665_12129788_12164454_410	SEC61A2	10	+	1230	11	0	0	1.22%	5.247629806623172	0.020613416668581838	0.020613416668581838	0.001946208561389314
ENSG00000091129_108232428_108149910_1196	NRCAM	7	−	3588	20	2	5	1.00%	3.270793889807693	0.004302923267342423	0.014861317392387344	0.0003432852811583412
ENSG00000091129_108232428_108149910_1084	NRCAM	7	−	3252	20	2	5	1.11%	3.608735693920665	0.004302923267342423	0.014861317392387344	0.0003432852811583412
ENSG00000091129_108232428_108149910_1196	NRCAM	7	−	3588	20	2	5	1.00%	3.270793889807693	0.004302923267342423	0.014861317392387344	0.0003432852811583412
ENSG00000182704_76795617_76796678_353	TSKU	11	+	1059	18	9	1	2.83%	9.973604937645895	0.1368354269974691	0.0033728746429155087	0.0035759678615782473
ENSG00000164692_94395032_94430393_1366	COL1A2	7	+	4098	28	1	1	1.24%	4.009236668443632	0.00018150266028862277	0.00018150266028862277	2.2969672481630137e−07
ENSG00000257103_34215168_34227388_269	LSM14A	19	+	807	14	0	0	2.60%	10.17958603920818	0.00847367416878415	0.00847367416878415	0.0003665024264111182
ENSG00000108679_78976139_78973324_108	LGALS3BP	17	−	324	48	0	0	13.89%	86.93043316022224	0.00029333431268700034	0.00029333431268700034	5.820635966874735e−07
ENSG00000156261_29060636_29056475_57	CCT8	21	−	171	27	0	0	14.04%	92.64954060497371	0.005706018193000826	0.005706018193000826	0.0001733096755673334
ENSG00000101558_9936202_9954211_186	VAPA	18	+	558	21	0	0	4.84%	22.08313423021775	0.0034782380676658805	0.0034782380676658805	6.758309314600117e−05
ENSG00000182149_71921346_71923392_139	IST1	16	+	417	17	0	0	4.32%	23.92150408905396	0.013007239811447113	0.013007239811447113	0.0008218654148058079
ENSG00000082641_48055019_48059641_616	NFE2L1	17	+	1848	37	2	0	3.25%	11.74830935160471	0.00016625032885512364	3.297819501010363e−05	4.1806499809954696e−08
ENSG00000152990_22515784_22387705_1321	ADGRA3	4	−	3963	9	0	0	0.38%	1.3325823402683576	0.020613416668581838	0.020613416668581838	0.001946208561389314
ENSG00000120805_101405934_101395640_164	ARL1	12	−	492	22	0	2	6.71%	26.23814903311586	0.0014409783181973444	0.003092538603222271	2.637025864246733e−05
ENSG00000141552_81894478_81900065_84	ANAPC11	17	+	252	127	10	2	20.24%	295.7187056611132	0.00039118706886775756	0.00018946255112486357	5.081891006409044e−07
ENSG00000079459_11808887_11838609_353	FDFT1	8	+	1059	211	12	7	15.01%	116.91281343573799	5.823304590514566e−09	4.985113558253148e−10	3.292241988068417e−17
ENSG00000125691_38853710_38852596_77	RPL23	17	−	231	199	8	10	15.58%	505.49482399337023	0.0018172566841077903	0.004295162393546244	4.5261622740756707e−05
ENSG00000168003_62885528_62886520_54	SLC3A2	11	+	162	13	0	0	9.26%	47.08731796178705	0.019679754444124863	0.019679754444124863	0.0017849636115922674
ENSG00000139613_56189461_56163689_1245	SMARCC2	12	−	3735	15	1	0	0.48%	2.3565478868734946	0.015555382717448078	0.011772051206276392	0.0008874794703752884
ENSG00000221983_18573301_18575150_128	UBA52	19	+	384	136	4	0	9.38%	207.81806677365628	0.00236147777460975	0.0009589057725548893	1.3840982015979587e−05
ENSG00000184990_104753202_104756764_157	SIVA1	14	+	471	26	4	3	8.28%	32.3912760501465	0.003975543265698643	0.004220201682030066	9.229357116153692e−05
ENSG00000137868_74196092_74180080_560	STRA6	15	−	1680	53	2	9	3.39%	18.51152527563661	0.00021186981035795232	0.0026742133664407067	4.061495374133696e−06

ENSG00000154723_25729794_25724640_108	ATP5PF	21	-	324	43	6	1	12.96%	77.87517970603243	0.004732992222340745	0.000675450826132064	2.0568376277725097e-05
ENSG00000128739_24976387_24978444_161	SNRPN	15	+	483	25	0	0	4.35%	30.371657548214294	0.00877616296920843	0.00877616296920843	0.00039161135667564577
ENSG00000154723_25729794_25724640_108	ATP5PF	21	-	324	43	6	1	12.96%	77.87517970603243	0.004732992222340745	0.000675450826132064	2.0568376277725097e-05
ENSG00000154723_25729794_25724640_108	ATP5PF	21	-	324	43	6	1	12.96%	77.87517970603243	0.004732992222340745	0.000675450826132064	2.0568376277725097e-05
ENSG00000154723_25729794_25717689_119	ATP5PF	21	-	357	36	6	1	10.08%	59.17113517628573	0.012730752221812172	0.0016353363669355965	0.00012632044941473906
ENSG00000198931_88809835_88809698_45	APRT	16	-	135	37	9	0	17.78%	160.82130134641116	0.04264052945664037	0.005808022449631236	0.0013442947795583938
ENSG00000089876_125841920_125836687_288	DHX32	10	-	864	13	0	0	2.08%	8.828872117835072	0.013428347753762199	0.013428347753762199	0.0008725593497644525
ENSG00000197956_153535339_153534696_90	S100A6	1	-	270	2776	88	58	50.00%	6032.972061319424	3.2080755063846185e-09	2.6004311897614428e-09	8.97263545200942e-17
ENSG00000188153_108573595_108696378_1523	COL4A5	X	+	4569	5	0	0	0.33%	0.6421322213082733	0.012673659338734126	0.012673659338734126	0.000782701129001274
ENSG00000112855_140694028_140698552_414	HARS2	5	+	1242	15	1	0	2.17%	7.086720094583335	0.006024333578509648	0.0032294771334464936	0.00010730535955833177
ENSG00000108061_110964875_111011818_410	SHOC2	10	+	1230	13	0	0	1.46%	6.201744316918294	0.012798402692974279	0.012798402692974279	0.0007972436888943567
ENSG00000185515_155071528_155120150_247	BRCC3	X	+	741	5	0	0	2.02%	3.9593820771356283	0.012673659338734126	0.012673659338734126	0.000782701129001274
ENSG00000146540_1000487_997625_65	C7orf50	7	-	195	11	5	0	7.69%	33.10043416485385	0.19774078372971998	0.02108409854857798	0.020795917079366868
ENSG00000077782_38424619_38413628_547	FGFR1	8	-	1641	10	0	0	1.65%	3.57574907880256	0.001946208561389314	0.001946208561389314	2.2278545302028032e-05
ENSG00000114902_52706674_52707812_80	SPCS1	3	+	240	76	7	2	23.75%	185.81380087997505	0.00046324249190235744	0.00011969550530628595	3.919124007656001e-07
ENSG00000187446_41262784_41272182_54	CHP1	15	+	162	17	0	0	14.81%	61.57572348849075	0.005160369442893307	0.005160369442893307	0.00014319601891785326
ENSG00000145741_73499134_73505238_162	BTF3	5	+	486	630	12	7	12.96%	760.6412901519446	2.6023379003580606e-05	2.7426286549419726e-05	5.5324725093672956e-09
ENSG00000124635_27132750_27132370_126	HIST1H2BJ	6	-	378	101	25	0	9.52%	156.78524552111512	0.15816042627465982	0.0009549346450480869	0.0018460039225789491
ENSG00000073111_127604781_127619121_534	MCM2	3	+	1602	31	0	0	2.25%	11.354677365029028	0.0009549346450480869	0.0009549346450480869	5.678501630038347e-06
ENSG00000187134_4963445_4967115_146	AKR1C1	10	+	438	141	0	1	6.85%	188.8950679457569	0.0024883653771069682	0.003961387960289652	5.587640157578619e-05
ENSG00000187134_4968897_4977742_149	AKR1C1	10	+	447	19	5	0	3.36%	24.941449782546986	0.028889785561798623	0.02108409854857798	0.002731532589007073
ENSG00000101365_2664188_2658509_387	IDH3B	20	-	1161	81	4	0	7.75%	40.93816910452327	1.5478984385807574e-06	7.337335776570539e-07	1.018440612884783e-11
ENSG00000103197_2071615_2088610_1158	TSC2	16	+	3474	10	5	3	0.69%	1.6890628204706395	0.07635947366271362	0.04922925118879209	0.01464496403362635
ENSG00000002549_17577559_17607589_488	LAP3	4	+	1464	38	4	3	3.69%	15.230639416391396	0.0008701861546363455	0.0005853750266995722	3.251076934972306e-06
ENSG00000132294_131946499_132010895_744	EFR3A	8	+	2232	59	2	9	3.23%	15.510772852176752	1.412135627885162e-05	0.00038488881812705244	4.670392296146739e-08
ENSG00000204628_181243800_181236977_317	RACK1	5	-	951	1557	31	32	28.08%	960.6909778187652	4.1509230429145434e-16	5.821569099152168e-16	3.517896881483283e-30
ENSG00000164828_838871_873331_632	SUN1	7	+	1896	20	0	0	0.95%	6.189666918053799	0.011570465654371866	0.011570465654371866	0.0006594843062896773
ENSG00000106628_44122053_44114785_455	POLD2	7	-	1365	56	1	0	5.05%	24.07304302898462	1.5160323594503475e-05	1.0081031668924613e-05	1.231944966409922e-09
ENSG00000163466_218217471_218253915_300	ARPC2	2	+	900	62	3	3	6.67%	40.422651419503346	0.00012307905159007396	9.224956238958888e-05	8.181208507545852e-08
ENSG00000121741_20031328_20086014_757	ZMYM2	13	+	2271	11	0	0	0.79%	2.8421773060970943	0.012798402692974279	0.012798402692974279	0.0007972436888943567
ENSG00000121741_20031328_20086014_757	ZMYM2	13	+	2271	11	0	0	0.79%	2.8421773060970943	0.012798402692974279	0.012798402692974279	0.0007972436888943567
ENSG00000160948_144424253_144423805_82	VPS28	8	-	246	33	0	0	14.63%	78.71444709934758	0.0010197273511756433	0.0010197273511756433	6.442859155158972e-06
ENSG00000152464_15103315_15104166_283	RPP38	10	+	849	18	0	1	2.12%	12.440574356851593	0.011772051206276392	0.015555382717448078	0.0008874794703752884
ENSG00000152464_15103315_15104166_283	RPP38	10	+	849	18	0	1	2.12%	12.440574356851593	0.011772051206276392	0.015555382717448078	0.0008874794703752884
ENSG00000233276_49358278_49358132_48	GPX1	3	-	144	16	0	0	12.50%	65.19782487016668	0.013428347753762199	0.013428347753762199	0.0008725593497644525
ENSG00000133104_36335467_36304365_545	SPART	13	-	1635	12	0	0	1.10%	4.306645312524772	0.011772051206276392	0.011772051206276392	0.000681272511389508
ENSG00000197912_89508547_89545958_468	SPG7	16	+	1404	13	0	0	1.28%	5.43315207251389	0.011772051206276392	0.011772051206276392	0.000681272511389508
ENSG00000074410_63340783_63340207_100	CA12	15	-	300	38	2	1	11.00%	74.32552035199002	0.0029234385313636584	0.001981573988058776	3.357102258741097e-05
ENSG00000258311_55719107_55721706_44	AC009779.3	12	+	132	11	0	0	11.36%	48.89836865262501	0.02108409854857798	0.02108409854857798	0.002029891263157455
ENSG00000148337_128179348_128162939_539	CIZ1	9	-	1617	17	2	0	1.67%	6.168996416286643	0.018399683928041276	0.002967235347054297	0.00031087262125642094
ENSG00000025708_50529306_50525770_400	TYMP	22	-	1200	11	0	0	1.25%	5.378820551788751	0.02108409854857798	0.02108409854857798	0.002029891263157455
ENSG00000215021_6970543_6965692_267	PHB2	12	-	801	113	8	4	10.86%	82.77926079021162	1.3775732931235347e-05	2.8366854541987133e-06	3.334094263708255e-10
ENSG00000101166_59042730_59035007_164	PRELID3B	20	-	492	37	2	11	7.32%	44.1277961011494	0.0010573555742419644	0.012981845878104827	8.914709364559112e-05
ENSG00000180957_27919191_27854892_271	PITPNB	22	-	813	26	0	2	4.06%	18.765425608387456	0.0014055202957965589	0.006088081514183966	5.109689293194805e-05
ENSG00000162129_72434474_72293367_648	CLPB	11	-	1944	17	0	0	0.93%	5.131310290707563	0.011772051206276392	0.011772051206276392	0.000681272511389508
ENSG00000135441_55719107_55719609_75	BLOC1S1	12	+	225	14	1	0	9.33%	36.510781927293344	0.013892891352654344	0.00847367416878415	0.0005886798845679606
ENSG00000136010_105058159_105031882_350	ALDH1L2	12	-	1050	6	1	0	1.43%	3.3530309933228577	0.07864960352514258	0.016947426762344633	0.006209665325776152
ENSG00000141349_44070966_44071187_73	G6PC3	17	+	219	20	1	0	12.33%	53.58725331794522	0.0033030736763512156	0.0034782380676658805	6.433817793110031e-05
ENSG00000144320_175995584_175929967_428	LNPK	2	-	1284	14	0	0	1.40%	6.397917393801404	0.013428347753762199	0.013428347753762199	0.0008725593497644525
ENSG00000152082_130182283_130190626_158	MZT2B	2	+	474	25	4	0	4.43%	30.948334590268995	0.016724852853975118	0.00797940233269714	0.0006687959809311652
ENSG00000110090_68807631_68757644_677	CPT1A	11	-	2031	44	1	4	3.10%	12.712131289308719	2.116023157680266e-05	0.000186161329078746	3.1202902669590814e-08
ENSG00000180185_1827239_1827904_221	FAHD1	16	+	663	26	0	0	4.98%	23.010997013000004	0.0013845019718837994	0.0013845019718837994	1.1595255170738555e-05
ENSG00000131459_180338586_180301564_675	GFPT2	5	-	2025	53	5	0	3.41%	15.35770985830593	0.00011301917416022748	1.0776417898158317e-05	1.0017161950070467e-08
ENSG00000101150_63865366_63889945_206	TPD52L2	20	+	618	44	3	2	11.65%	41.777247004184474	5.49444442415296e-05	3.082883802222531e-05	1.2900473921190551e-08
ENSG00000106853_111597422_111549773_301	PTGR1	9	-	903	79	6	2	11.96%	51.33516443265616	9.024271742974549e-07	7.35065564252991e-08	6.582762010934858e-13
ENSG00000119326_109013442_108942769_734	CTNNAL1	9	-	2202	75	1	2	3.54%	19.985709258566082	4.336067237760262e-06	2.8113679727103686e-06	1.0389975569668392e-10
ENSG00000159069_136944091_136944026_21	FBXW5	9	-	63	12	0	0	28.57%	111.7676997774286	0.011772051206276392	0.011772051206276392	0.000681272511389508
ENSG00000115365_210472019_210434487_353	LANCL1	2	-	1059	32	0	0	4.25%	17.730853222481592	0.00023522430645143535	0.00023522430645143535	3.796194994189714e-07
ENSG00000247077_132710877_132720828_289	PGAM5	12	+	867	10	0	2	1.73%	6.767940297941178	0.020613416668581838	0.06945717695039447	0.006391023138248565
ENSG00000115738_8682166_8682899_134	ID2	2	+	402	73	7	9	14.18%	106.5546540788545	0.0006081022933862213	0.00022647487885317342	9.328599714590898e-07
ENSG00000117054_75749428_75762763_182	ACADM	1	+	546	11	0	0	3.85%	11.821583630304948	0.007029302661868638	0.007029302661868638	0.0002573639808771639
ENSG00000036257_224557733_224474245_705	CUL3	2	-	2115	25	2	3	1.99%	6.935938815975179	0.0019573865167617018	0.0033854124258957835	3.8307544722804655e-05
ENSG00000155368_119367585_119372318_104	DBI	2	+	312	103	6	4	20.19%	193.71276812386063	0.00014145917115575762	8.387869876068085e-05	8.562985301249247e-08
ENSG00000138674_82881936_82819074_1251	SEC31A	4	-	3753	31	0	0	1.04%	4.846840697782175	0.000596156229600823	0.000596156229600823	2.2911026310734046e-06
ENSG00000135972_105038093_105099761_396	MRPS9	2	+	1188	6	0	1	1.26%	2.9635374940984853	0.016947426762344633	0.0477903522728147	0.0036975118224100542
ENSG00000156958_49201181_49328159_434	GALK2	15	+	1302	9	0	3	1.15%	4.056085879019586	0.016947426762344633	0.165899918248885	0.014398895220848395
ENSG00000156802_123396357_123344782_939	ATAD2	8	-	2817	29	2	4	1.28%	6.040693039088926	0.004676575434377382	0.009603828063251378	0.00023849919929173522

ENSG00000123562_103677027_103676161_288	MORF4L2	X	−	864	67	3	4	10.76%	45.50264860730383	2.6778889786849916e−06	6.382017302541668e−06	1.4552401796346898e−10
ENSG00000268790_16659990_16647246_63	AC008764.4	19	−	189	18	3	0	9.52%	55.8838498887143	0.10041256134757276	0.013007239811447113	0.006595870280092993
ENSG00000130311_17315804_17319656_100	DDA1	19	+	300	16	0	2	9.00%	31.294955937680008	0.0032294771334464936	0.014772524220114101	0.00026552696214677065
ENSG00000064012_201266643_201276997_224	CASP8	2	+	672	17	1	0	3.57%	14.844147626689736	0.01007916461776185	0.004873903550586949	0.0002597390186955864
ENSG00000154429_229342432_229325235_259	CCSAP	1	−	777	11	0	0	1.93%	8.307058767241315	0.020613416668581838	0.020613416668581838	0.001946208561389314
ENSG00000145996_20548666_21231039_497	CDKAL1	6	+	1491	15	2	0	1.21%	5.903223579793764	0.044040755831095145	0.013428347753762199	0.0027917083341200354
ENSG00000163545_205308700_205303450_500	NUAK2	1	−	1500	12	0	1	1.60%	4.694243390652001	0.004193160480899131	0.010499663139468709	0.0002361360478986167
ENSG00000143621_153670922_153667496_150	ILF2	1	−	450	52	5	0	11.33%	67.80573786497335	0.002444361331516568	0.00011392800213026252	2.149780829018921e−06
ENSG00000160679_153638350_153645269_208	CHTOP	1	+	624	15	0	3	2.88%	14.105298649795676	0.013007239811447113	0.023878473930440184	0.0014709138084317821
ENSG00000134824_61857477_61865689_168	FADS2	11	+	504	70	8	4	16.07%	81.49728108770836	7.093902035563334e−05	3.0707973409091526e−06	1.959198640511705e−09
ENSG00000155229_97498348_97458692_1018	MMS19	10	−	3054	26	4	0	0.88%	4.995511139364441	0.0293371185312488	0.0036062891729786097	0.000604440848518855
ENSG00000169641_23093913_23088895_960	LUZP1	1	−	2880	10	0	0	0.83%	2.037432027192709	0.00414960799764038	0.00414960799764038	9.45964903272165e−05
ENSG00000213920_24215955_24214024_176	MDP1	14	−	528	15	0	1	3.41%	16.669898404303982	0.013428347753762199	0.02034245439037237	0.0012955184805712684
ENSG00000198561_57801746_57816308_610	CTNND1	11	+	1830	50	4	2	2.62%	16.032252017254102	0.0013582198574350129	0.00041261355168930957	3.650882652566793e−06
ENSG00000167792_67611168_67612458_173	NDUFV1	11	+	519	8	2	2	4.05%	9.044784953086706	0.06180520118411761	0.0416322583317752	0.010346370125474175
ENSG00000167792_67608700_67610611_145	NDUFV1	11	+	435	18	3	0	4.83%	24.280569261993108	0.015625008558487224	0.00797940233269714	0.0006253789790324684
ENSG00000052841_43396736_43445965_726	TTC17	11	+	2178	11	0	0	0.96%	2.9635374940984853	0.0078820576498062	0.0078820576498062	0.00031963642907451164
ENSG00000127990_94618900_94587779_273	SGCE	7	−	819	11	0	2	2.56%	7.881055753536632	0.007029302661868638	0.03522780890193623	0.0012822660609675817
ENSG00000150593_110876028_110898088_469	PDCD4	10	+	1407	20	2	0	3.41%	8.340873117718552	0.0010559073030188334	8.753035227130121e−05	6.926370138218015e−07
ENSG00000103197_2083787_2088610_482	TSC2	16	+	1446	6	0	0	1.04%	2.434773542869295	0.016947426762344633	0.016947426762344633	0.0013498980316300959
ENSG00000115525_85863399_85847466_196	ST3GAL5	2	−	588	12	1	2	3.06%	11.975110690438779	0.026602764905793736	0.06032271266817251	0.006860364960240205
ENSG00000278615_62665169_62663050_123	C11orf98	11	−	369	41	3	2	9.76%	65.19782487016668	0.001268312326064897	0.0010743541110924547	8.359492535112299e−06
ENSG00000162191_62678697_62677769_146	UBXN1	11	−	438	32	0	0	9.59%	42.86980265435617	0.00041307551203580774	0.00041307551203580774	1.1283521396441709e−06
ENSG00000075239_108138919_108142582_171	ACAT1	11	+	513	22	0	0	5.85%	25.16407275690644	0.0023218226288164415	0.0023218226288164415	3.1231198880462635e−05
ENSG00000075239_108121607_108138989_141	ACAT1	11	+	423	16	3	1	4.96%	22.195004211120573	0.03598716594496433	0.00847367416878415	0.0015344700145845673
ENSG00000083845_58388138_58394750_225	RPS5	19	+	675	525	12	15	19.11%	456.3847740911668	6.36195112857163e−09	4.974003188196502e−09	3.343268422650572e−16
ENSG00000140694_14628215_14555696_390	PARN	16	−	1170	14	0	2	1.54%	7.021304216787181	0.013007239811447113	0.0424352593247778	0.002615673534585255
ENSG00000103197_2060673_2088610_1434	TSC2	16	+	4302	10	5	3	0.56%	1.3639712315934451	0.07635947366271362	0.04922925118879209	0.01464496403362635
ENSG00000127990_94639350_94597920_412	SGCE	7	−	1236	17	3	2	2.18%	8.070604534899275	0.020955738116168132	0.01292879023172146	0.0012882305750462103
ENSG00000077684_128831759_128872262_842	JADE1	4	+	2526	10	0	2	0.83%	2.322962881359858	0.007029302661868638	0.04912109528954385	0.0018325026027891263
ENSG00000100242_38752628_38736267_717	SUN2	22	−	2151	24	1	3	1.81%	6.547061911648537	0.0010295252912250277	0.004890190720947476	3.090191282706422e−05
ENSG00000119048_134371596_134390353_152	UBE2B	5	+	456	16	1	1	6.58%	20.588786801105268	0.00392533363377418	0.00392533363377418	8.509888062156286e−05
ENSG00000213585_133993012_133972771_283	VDAC1	5	−	849	60	0	0	3.89%	41.46858118950531	0.0014915192737735051	0.0014915192737735051	1.337646624223462e−05
ENSG00000197102_102018536_102042059_1211	DYNC1H1	14	+	3633	42	9	6	1.82%	6.78358871481503	0.0022105401852253407	0.0005317257867869461	7.549974234932373e−06
ENSG00000197102_102042037_102050563_604	DYNC1H1	14	+	1812	20	2	4	1.66%	6.476605119552982	0.011698770487832223	0.022448209392418098	0.0012592222951511114
ENSG00000285920_41358244_41380186_225	AC087721.2	15	+	675	12	3	1	2.67%	10.431651979226668	0.02784981298332479	0.015555382717448078	0.002005853335013532
ENSG00000153113_96742661_96771679_399	CAST	5	+	1197	93	0	1	8.02%	45.589456488161666	2.9105967594210546e−07	6.016454218304325e−07	1.6243458137832645e−12
ENSG00000196961_49767161_49796199_287	AP2A1	19	+	861	17	0	0	2.79%	11.585676196440769	0.005454749182134642	0.005454749182134642	0.00015910859015753364
ENSG00000135862_183023867_183142790_1559	LAMC1	1	+	4677	140	3	2	3.66%	17.564519849563826	7.745700647827728e−11	4.0956771916954586e−11	3.7593588585468036e−20
ENSG00000062822_50398852_50417947_1133	POLD1	19	+	3399	34	2	1	1.32%	5.869530570835836	0.0009502830449746255	0.000405860922741421	2.512435789199588e−06
ENSG00000143545_153983523_153982099_122	RAB13	1	−	366	28	1	0	7.38%	44.89030564831148	0.004911637253759624	0.0034782380676658805	9.416876808028951e−05
ENSG00000204520_31403633_31412431_332	MICA	6	+	996	15	4	0	1.81%	8.837054575775605	0.0319825310473083	0.013428347753762199	0.00201944237183233
ENSG00000171241_46621359_46603930_378	SHCBP1	16	−	1134	22	0	0	2.91%	11.383747199552912	0.0012624143894574274	0.0012624143894574274	9.711623744831373e−06
ENSG00000149925_30067255_30068229_123	ALDOA	16	+	369	134	16	11	24.39%	213.0855739659106	1.1970365748366949e−05	3.367522327008567e−06	3.402952953596963e−10
ENSG00000089159_120216326_120212314_332	PXN	12	−	996	21	0	0	3.31%	12.371876406085846	0.001092092816906976	0.001092092816906976	7.350803965414657e−06
ENSG00000087274_2881994_2928171_566	ADD1	4	+	1698	10	0	1	0.88%	3.4557150991254426	0.019679754444124863	0.04223451612662591	0.003715148010809822
ENSG00000213523_140552041_140550700_126	SRA1	5	−	378	8	0	0	3.97%	12.418633308603177	0.019679754444124863	0.019679754444124863	0.0017849636115922674
ENSG00000136527_185922105_185917715_87	TRA2B	3	−	261	26	0	0	10.34%	58.453222297390816	0.0033030736763512156	0.0033030736763512156	6.124050385493983e−05
ENSG00000041357_78542509_78548944_237	PSMA4	15	+	711	256	5	8	10.97%	211.27396413623634	6.6208765513498455e−06	3.011138781180906e−05	1.6385863812713446e−09
ENSG00000176087_140564844_140566762_103	SLC35A4	5	+	309	25	0	1	4.85%	47.4741443229369	0.02108409854857798	0.028889785561798623	0.002731532589007073
ENSG00000099875_2050851_2037739_414	MKNK2	19	−	1242	23	5	2	2.66%	10.86630414502778	0.010728371185261619	0.003107435676029586	0.0001846931615845698
ENSG00000165959_95319792_95191564_1002	CLMN	14	−	3006	8	0	1	0.60%	1.5616245477884234	0.011570465654371866	0.026602764905793736	0.001474889218184677
ENSG00000128534_118184224_118188409_67	LSM8	7	+	201	36	3	3	14.93%	105.09500128325375	0.0023354528714849923	0.012563987137771532	0.00016928552099578897
ENSG00000163430_120450746_120396952_273	FSTL1	3	−	819	48	0	0	8.06%	34.39006146997803	1.4724699406439145e−05	1.4724699406439145e−05	1.730005367946513e−09
ENSG00000167772_8364322_8373886_406	ANGPTL4	19	+	1218	8	1	0	1.72%	3.854058613014779	0.00407548579675135	0.005706018193000826	0.0001262636872816894
ENSG00000163634_63863790_63834132_204	THOC7	3	−	612	38	3	0	7.35%	36.434078603916674	0.0006644883448370029	0.0002798052361462181	1.2421450771693592e−06
ENSG00000108010_130145263_130179392_287	GLRX3	10	+	861	69	0	3	6.97%	47.02421515025959	3.942240149922151e−05	0.00017763310340949173	5.2792693157387635e−08
ENSG00000108439_47941676_47946782_243	PNPO	17	+	729	25	0	0	4.53%	20.12278545375515	0.0014055202957965589	0.0014055202957965589	1.1935530040246081e−05
ENSG00000198917_128828822_128822765_336	SPOUT1	9	−	1008	10	1	0	1.79%	5.821234363407739	0.026832695913080365	0.011772051206276392	0.0015103817605807263
ENSG00000139620_48681632_48671925_155	KANSL2	12	−	465	17	0	1	4.52%	21.452187537925813	0.00847367416878415	0.011825808327677989	0.0005036791746826746
ENSG00000110880_108652315_108647403_155	CORO1C	12	−	465	29	0	0	5.81%	36.59490815293227	0.003736770770438521	0.003736770770438521	7.748026083818498e−05
ENSG00000204389_31815757_31817682_641	HSPA1A	6	+	1923	39	0	1	1.09%	11.900383010623248	0.00847367416878415	0.013892891352654344	0.0005886798845679606
ENSG00000169223_177351647_177332202_325	LMAN2	5	−	975	120	7	7	10.46%	72.21912908695386	1.216799385666966e−06	1.1140506322521483e−06	1.2050132190172676e−11
ENSG00000100889_24098330_24103964_506	PCK2	14	+	1518	26	0	0	2.37%	10.05025758868182	0.0009235399941089087	0.0009235399941089087	5.324735186779001e−06
ENSG00000137497_72022389_72003527_2008	NUMA1	11	−	6024	37	5	3	0.90%	3.6040630281815247	0.0010297636725339206	0.0004366288061172337	2.913957752380149e−06
ENSG00000119917_89328074_89340128_490	IFIT3	10	+	1470	46	0	2	3.47%	18.361836392006126	0.0001313309506374033	0.0003296392542250699	3.0340503072784886e−07
ENSG00000132522_7314514_7311579_244	GPS2	17	−	732	9	5	2	2.87%	7.214513407764346	0.07524961812757737	0.0350911293701988	0.01080828922997042

ENSG00000104529_143586845_143580630_130	EEF1D	8	-	390	97	0	0	8.46%	145.94282336321928	0.0016340149330375125	0.0016340149330375125	1.593517422383077e-05
ENSG00000149269_77379253_77323137_380	PAK1	11	-	1140	8	0	3	1.32%	4.117757360221054	0.019679754444124863	0.13601972394786777	0.012754723264859217
ENSG00000254692_24213439_24189415_815	AL136295.1	14	-	2445	88	7	6	4.42%	21.11929541806626	1.9690595315902233e-06	6.490136616291408e-07	1.1509506008821172e-11
ENSG00000132792_37802994_37872013_177	CTNNBL1	20	+	531	9	0	0	2.82%	9.945430912398308	0.020613416668581838	0.020613416668581838	0.001946208561389314
ENSG00000115310_55050300_54973156_345	RTN4	2	-	1035	262	18	3	16.81%	148.5376531824667	2.671011707474315e-10	2.384168190740431e-11	7.722005189300842e-20
ENSG00000100243_42636837_42619773_291	CYB5R3	22	-	873	47	0	1	4.81%	31.590698648431278	0.00045482414116276956	0.0006064187916096173	1.7966830332614766e-06
ENSG00000100983_34951852_34942859_140	GSS	20	-	420	15	0	0	5.00%	20.956443708267862	0.0078820576498062	0.0078820576498062	0.00031963642907451164
ENSG00000075151_20981092_20807319_1530	EIF4G3	1	-	4590	11	0	0	0.46%	1.4062275952388892	0.007593099278532274	0.007593099278532274	0.00029783286705462064
ENSG00000104131_44537195_44561149_204	EIF3J	15	+	612	13	1	6	3.92%	12.464290048708337	0.010499663139468709	0.09936484761062991	0.005530608059979668
ENSG00000171004_132957277_132628223_446	HS6ST2	X	-	1338	14	0	0	1.57%	6.139705481047087	0.00797940233269714	0.00797940233269714	0.000327142761693177
ENSG00000131653_2172538_2176574_426	TRAF7	16	+	1278	16	0	1	2.11%	7.346233788187795	0.0033030736763512156	0.006112543793728684	0.0001113261345270447
ENSG00000013583_13000105_12975308_186	HEBP1	12	-	558	24	0	0	5.38%	25.237867691677426	0.0022409900782254734	0.0022409900782254734	2.9184102569313086e-05
ENSG00000166479_68714981_68691257_197	TMX3	18	-	591	10	6	0	3.55%	9.9286027721066	0.07708712836172119	0.007593099278532274	0.003221598000607736
ENSG00000197043_151147901_151101448_460	ANXA6	5	-	1380	20	0	0	1.52%	8.504064113500002	0.00797940233269714	0.00797940233269714	0.000327142761693177
ENSG00000106399_7639126_7637000_82	RPA3	7	-	246	94	0	0	9.76%	224.2169099193537	0.005655335537214311	0.005655335537214311	0.00017039952270558162
ENSG00000197694_128566741_128633334_2477	SPTAN1	9	+	7431	76	4	6	1.33%	6.0012531572054915	1.556229315321248e-06	4.240796765082592e-06	5.74504648209071e-11
ENSG00000124145_45348384_45327264_198	SDC4	20	-	594	40	0	4	9.09%	39.51383325464647	7.783833532730189e-05	0.000995836474107808	5.864507963895375e-07
ENSG00000138069_65129906_65088493_170	RAB1A	2	-	510	50	0	1	9.41%	57.52749253250001	0.0001894090731644577	0.0002770628028076844846	3.61497692104533e-07
ENSG00000221823_68217104_68180963_160	PPP3R1	2	-	480	50	4	0	10.62%	61.12296081578126	0.0009026671614782853	0.00013186253945817638	8.487718741337922e-07
ENSG00000108298_39201214_39204648_194	RPL19	17	+	582	128	19	0	11.86%	129.05136469146396	0.00023490963595251618	1.236195501581318e-05	2.4469965317013604e-08
ENSG00000130640_133298090_133279766_743	TUBGCP2	10	-	2229	15	3	1	1.35%	3.948724251894348	0.020613416668581838	0.00392533363377418	0.0004450089172968374
ENSG00000130638_45672106_45843671_461	ATXN10	22	+	1383	129	7	4	6.94%	54.732230422460965	6.270272926949079e-07	4.0684457062231364e-07	2.3431865387748366e-12
ENSG00000073350_75543427_75574878_1019	LLGL2	17	+	3057	14	0	0	0.69%	2.687250877867518	0.00837382380159347	0.00837382380159347	0.0003583838894600799
ENSG00000100865_102362851_102350033_105	CINP	14	-	315	9	13	2	5.71%	16.76515496661429	0.17004230409153215	0.08026476757196149	0.04777148144492673
ENSG00000122729_32405507_32450111_889	ACO1	9	+	2667	13	0	0	1.01%	2.8601970415483695	0.002943990679779778	0.002943990679779778	4.916404313974209e-05
ENSG00000184164_49918770_49927307_402	CRELD2	22	+	1206	25	0	0	2.99%	12.163773296672888	0.0009235399941089087	0.0009235399941089087	5.324735186779001e-06
ENSG00000140416_63048576_63071172_248	TPM1	15	+	744	161	9	9	12.90%	126.97802182375204	3.3497473325584192e-06	8.913595671298703e-06	2.5183598610147725e-10
ENSG00000006007_19507752_19503470_141	GDE1	16	-	423	16	0	0	4.26%	22.195004211120573	0.013640585738808999	0.013640585738808999	0.00089863692579514
ENSG00000111358_123645512_123648140_75	GTF2H3	12	+	225	17	3	3	8.00%	44.33452091171334	0.07356204933788085	0.044241358585871825	0.01288487733638965
ENSG00000050426_51049080_51058072_99	LETMD1	12	+	297	16	0	0	5.05%	31.61106660371718	0.02108409854857798	0.02108409854857798	0.002029891263157455
ENSG00000167770_63986457_63997546_280	OTUB1	11	+	840	41	1	0	7.14%	28.640473067966077	3.145332252154177e-05	3.404299617394451e-05	8.215888711579203e-09
ENSG00000092621_119712023_119727234_213	PHGDH	1	+	639	19	0	0	3.76%	17.447305246946012	0.005554612275049112	0.005554612275049112	0.00016468457691007306
ENSG00000092621_119712023_119724800_123	PHGDH	1	+	369	15	0	0	4.88%	23.852862757378055	0.012798402692974279	0.012798402692974279	0.0007972436888943567
ENSG00000108825_42961207_42950593_307	PTGES3L-AARSD1	17	-	921	26	2	1	2.93%	16.564919673853424	0.010402771787637745	0.005642787686764809	0.0003061675497078111
ENSG00000003989_17543340_17565146_658	SLC7A2	8	+	1974	17	0	0	1.37%	5.053326851639059	0.0033030736763512156	0.0033030736763512156	6.124050385493983e-05
ENSG00000115525_85848138_85840144_290	ST3GAL5	2	-	870	11	0	3	1.72%	7.41906283005345	0.019216965118390855	0.13115678761539135	0.012020477827111095
ENSG00000171960_42658856_42659348_57	PPIH	1	+	171	29	1	0	12.28%	99.51246953867546	0.00847367416878415	0.00847367416878415	0.0003665024264111182
ENSG00000116977_236539066_236548161_210	LGALS8	1	+	630	11	1	0	2.86%	10.245372479597622	0.023672501042627626	0.012798402692974279	0.0014375227604752335
ENSG00000004700_21483475_21470194_449	RECQL	12	-	1347	12	1	3	1.56%	5.227442528565703	0.013599199788808391	0.06853594973600552	0.004486614508126413
ENSG00000133597_140673331_140694803_626	ADCK2	7	+	1878	16	0	7	0.96%	4.999194239246007	0.013640585738808999	0.14081442098989871	0.010110099915137491
ENSG00000206053_1685521_1698998_148	JPT2	16	+	444	21	0	4	6.76%	27.753128154192574	0.0020587098026626535	0.03347891029545733	0.0004435301633944256
ENSG00000206053_1685521_1698998_148	JPT2	16	+	444	21	0	4	6.76%	27.753128154192574	0.0020587098026626535	0.03347891029545733	0.0004435301633944256
ENSG00000087263_56453280_56476205_485	OGFOD1	16	+	1455	22	0	1	2.06%	8.872281322538146	0.0020587098026626535	0.003990669019770486	4.7198559455707632e-05
ENSG00000070669_97869093_97864200_160	ASNS	7	-	480	14	0	0	3.12%	17.114429028418755	0.02108409854857798	0.02108409854857798	0.002029891263157455
ENSG00000127837_218269517_218264533_388	AAMP	2	-	1164	75	1	0	5.41%	37.80801699945233	3.539339565224249e-05	2.522122750237898e-05	6.890710905347536e-09
ENSG00000101266_489860_483961_177	CSNK2A1	20	-	531	9	0	0	2.82%	9.945430912398308	0.019216965118390855	0.019216965118390855	0.0017073955890589052
ENSG00000215301_41343231_41347719_476	DDX3X	X	+	1428	83	1	0	7.56%	34.10558485855358	6.958679763556736e-08	4.081835640353621e-08	2.8150375102779016e-14
ENSG00000136754_26823167_26777005_88	ABI1	10	-	264	12	0	0	5.68%	26.671837446886368	0.02108409854857798	0.02108409854857798	0.002029891263157455
ENSG00000165416_52652921_52687835_365	SUGT1	13	+	1095	10	0	0	1.64%	5.358725331794521	0.012798402692974279	0.012798402692974279	0.0007972436888943567
ENSG00000139644_49752494_49762896_237	TMBIM6	12	+	711	661	23	17	32.49%	545.5159777111414	4.4607759695857914e-14	1.475365366222195e-13	8.888324263736637e-26
ENSG00000145439_169010089_168990150_237	CBR4	4	-	711	10	0	0	2.11%	8.252889224071732	0.019679754444124863	0.019679754444124863	0.0017849636115922674
ENSG00000150093_32912060_32908533_106	ITGB1	10	-	318	137	4	9	20.75%	252.79533982677836	3.2241459690708464e-05	8.153484436366413e-05	1.9935223720652605e-08
ENSG00000126088_45013615_45014115_127	UROD	1	+	381	8	0	0	3.94%	12.320848794362208	0.019679754444124863	0.019679754444124863	0.0017849636115922674
ENSG00000108465_47975891_47981734_316	CDK5RAP3	17	+	948	12	0	0	1.90%	7.427600301664559	0.009815328628645334	0.009815328628645334	0.0004837142251238724
ENSG00000172270_572635_582577_385	BSG	19	+	1155	1313	23	21	19.48%	667.0499536716534	2.758967742426988e-14	4.991218394259621e-13	1.9052967911501122e-25
ENSG00000125871_17969866_17990109_342	MGME1	20	+	1026	32	1	0	4.39%	18.301143823204683	0.0006355235948337754	0.000277593445288976	1.179379397482956e-06
ENSG00000117500_93156458_93154670_125	TMED5	1	-	375	43	0	0	6.40%	67.28415526601202	0.005604866691947602	0.005604866691947602	0.00016752456572620382
ENSG00000133895_64810109_64804334_610	MEN1	11	-	1830	22	4	1	1.97%	7.054190887591805	0.01878413720018969	0.0016120253826907794	0.0001902478323907079
ENSG00000104904_2269599_2271445_68	OAZ1	19	+	204	25	6	0	11.76%	71.90936566562502	0.039104403811496304	0.0053557511377634245	0.0011447568947324828
ENSG00000165105_83062867_82982677_740	RASEF	9	-	2220	15	9	1	1.35%	3.964732593456082	0.1916431178827469	0.003496400579249009	0.005806426023475721
ENSG00000065000_2110177_2108692_144	AP3D1	19	-	432	14	0	0	3.47%	19.016032253798617	0.02108409854857798	0.02108409854857798	0.002029891263157455
ENSG00000204560_30665502_30653242_742	DHX16	6	-	2226	12	0	0	0.94%	3.1632367861536395	0.006937202941512724	0.006937202941512724	0.00025101522741487095
ENSG00000100316_39313888_39313826_20	RPL3	22	-	60	41	18	3	25.00%	400.9666229515251	0.13552063980517154	0.046295797875114966	0.024559327072987594
ENSG00000166681_103377711_103377827_38	BEX3	X	+	114	6	0	0	15.79%	30.8831802016579	0.007152939217714824	0.007152939217714824	0.0002660027525696252
ENSG00000067048_12904937_12918122_660	DDX3Y	Y	+	1980	22	1	3	1.82%	6.519782487016668	0.0015356410015604012	0.006880387941862762	6.270070317857036e-05
ENSG00000141552_81899865_81900065_66	ANAPC11	17	+	198	20	0	0	7.58%	59.270749881969714	0.021557223391537678	0.021557223391537678	0.002115616449879074
ENSG00000119408_124301986_124350947_306	NEK6	9	+	918	18	0	3	1.96%	11.505498506500002	0.013007239811447113	0.044329616041373636	0.00273578248190731

ENSG00000127527_16471945_16361967_756	EPS15L1	19	-	2268	8	0	0	0.66%	2.0697722181005296	0.019679754444124863	0.019679754444124863	0.0017849636115922674
ENSG00000054654_63978007_64225526_6442	SYNE2	14	+	19326	15	6	4	0.11%	0.45543342427157724	0.15952699612641635	0.0581241478854986	0.03474437533153787
ENSG00000071462_73683626_73692799_220	BUD23	7	+	660	57	2	1	9.55%	50.6764911490841	7.141512933486306e-05	3.0092684367208587e-05	1.6357379797321908e-08
ENSG00000071462_73693377_73697886_95	BUD23	7	+	285	17	2	0	7.37%	35.000937561878956	0.04363948305378087	0.0078820576498062	0.0017710903275458955
ENSG00000140740_21953424_21959405_132	UQCRC2	16	+	396	31	2	0	7.58%	45.93483115852653	0.0023628488670607213	0.0023766146074493525	3.247523061546013e-05
ENSG00000135114_121038962_121020561_511	OASL	12	-	1533	17	8	2	1.96%	6.507023617179062	0.10241930311425046	0.006369482933362988	0.003931193047667653
ENSG00000168385_241324233_241337520_159	SEPT2	2	+	477	29	1	0	8.81%	35.674281532732714	0.000565108330078236	0.0004156054716904614	1.5387906409916872e-06
ENSG00000126821_63727944_63686105_441	SGPP1	14	-	1323	22	2	1	2.95%	9.757497599616782	0.0032701130750291308	0.001015856855223422	2.0240423793271705e-05
ENSG00000090266_140696745_140721744_96	NDUFB2	7	+	288	94	1	1	11.46%	191.51861055611462	0.0016109979480538772	0.0016109979480538772	1.550748317303123e-05
ENSG00000161011_179823005_179836593_356	SQSTM1	5	+	1068	734	39	20	24.16%	403.274186416031	1.4597292307828136e-14	2.157512376301075e-15	4.481196465231996e-28
ENSG00000115355_55362358_55291711_1604	CCDC88A	2	-	4812	20	1	0	0.62%	2.4388213791832922	0.0033869280463461717	0.0022543491824521134	4.372784971036962e-05
ENSG00000136631_150068645_150144796_534	VPS45	1	+	1602	15	0	3	1.50%	5.494198725014046	0.005306578019052857	0.03497523187724695	0.0010067653727672797
ENSG00000111276_12717885_12718946_96	CDKN1B	12	+	288	16	0	1	9.38%	32.59891243508334	0.0034529206320349946	0.00327844299461777	6.343712121069989e-05
ENSG00000172269_119100804_119097520_169	DPAGT1	11	-	507	19	0	2	2.96%	21.989798920707106	0.02108409854857798	0.03696871792607606	0.0034634377619234 1
ENSG00000143614_153828215_153810177_549	GATAD2B	1	-	1647	13	0	0	1.09%	4.631539471651185	0.012798402692974279	0.012798402692974279	0.0007972436888943567
ENSG00000134371_193122201_193156975_370	CDC73	1	+	1110	31	0	0	2.43%	16.38756138628514	0.0035547763465261306	0.0035547763465261306	7.044676146010672e-05
ENSG00000123066_115997159_115961266_1330	MED13L	12	-	3990	15	0	0	0.45%	2.2059414429755644	0.012798402692974279	0.012798402692974279	0.0007972436888943567
ENSG00000115275_74462963_74461275_562	MOGS	2	-	1686	22	0	3	1.96%	7.656684059485767	0.0013845019718837994	0.011575475992623 17	9.8960288269354e-05
ENSG00000170322_129892774_129864725_1274	NFRKB	11	-	3822	21	0	0	0.39%	3.2240682628104405	0.02108409854857798	0.02108409854857798	0.002029891263157455
ENSG00000128463_34225115_34229847_149	EMC4	15	+	447	21	20	3	5.37%	27.566865549130878	0.5	0.005306578019052857	0.035397654034058104
ENSG00000134453_6097066_6115556_401	RBM17	10	+	1203	25	2	1	2.00%	12.194106895916462	0.010318779121986092	0.007225456388766877	0.0003811748795003534
ENSG00000107201_32526166_32457122_925	DDX58	9	-	2775	33	6	0	1.73%	6.977929364482704	0.00620101965479469	0.00016405548854949773	8.234437500958699e-06
ENSG00000148834_104254929_104267405_241	GSTO1	10	+	723	105	1	2	12.03%	85.21707400042533	1.3254840125053664e-06	1.6168456468084585e-06	1.8888471804650402e-11
ENSG00000148834_104254929_104267405_208	GSTO1	10	+	624	80	1	1	11.06%	75.22825946557694	1.4988956867794067e-05	1.4988956867794067e-05	1.7911490274622001e-09
ENSG00000058729_97179181_97163061_526	RIOK2	5	-	1578	7	0	0	0.95%	2.602954985310837	0.019216965118390855	0.019216965118390855	0.0017073955890589052
ENSG00000101972_124047365_124100597_1042	STAG2	X	+	3126	14	0	1	0.86%	2.6279353594500967	0.0032294771334464936	0.006024333578509648	0.0001073053595583317 7
ENSG00000168488_28826345_28836777_872	ATXN2L	16	+	2616	50	7	8	1.95%	11.215222168033259	0.001435957343153687	0.0010673266102214833	9.370824648744565e-06
ENSG00000185104_50584793_50452086_372	FAF1	1	-	1116	5	0	0	1.34%	2.6289445512163985	0.012673659338734126	0.012673659338734126	0.000782701129001274
ENSG00000166979_32412854_32515190_438	EVA1C	21	+	1314	14	1	0	1.60%	6.251846220426942	0.013892891352654344	0.00847367416878415	0.0005886798845679606
ENSG00000143569_154257405_154270295_300	UBAP2L	1	+	900	12	1	5	2.00%	7.823738984420002	0.019795881618345192	0.17808035639161363	0.01752998634990791
ENSG00000104325_90001493_90051897_335	DECR1	8	+	1005	42	5	0	5.67%	24.52216696609254	0.0005546900658196059	5.3019663895075235e-05	2.2438898536188557e-07
ENSG00000131469_42998763_42998939_58	RPL27	17	+	174	355	13	7	8.62%	1197.1669566677158	0.021557223391537678	0.05742480463164306	0.00546177326240234
ENSG00000131778_147266075_147295509_603	CHD1L	1	+	1809	18	0	1	1.33%	5.838611182402986	0.004873903550586949	0.015625008558487224	0.000403121801006346
ENSG00000141510_7676594_7669609_393	TP53	17	-	1179	109	1	6	4.83%	54.24857183853564	6.857358883697343e-05	0.0007313507128457925	3.780271356515748e-07
ENSG00000078124_76860977_77020327_267	ACER3	11	+	801	9	0	0	2.25%	6.593038470016856	0.011772051206276392	0.011772051206276392	0.000681272511389508
ENSG00000126457_49680535_49688245_325	PRMT1	19	+	975	170	9	1	10.46%	102.31043287318464	2.077678379817867e-06	1.9042978068302973e-07	3.796528804798302e-12
ENSG00000105221_40255258_40233872_376	AKT2	19	-	1128	9	2	4	1.33%	4.681758700783246	0.10105102209468636	0.14389369507707506	0.04910224496431627
ENSG00000182154_137552133_137552495_120	MRPL41	9	+	360	46	2	4	11.67%	74.97749860069169	0.0009455931686164678	0.0027105289142414562	1.568886610852488e-05
ENSG00000147010_19645418_19588411_248	SH3KBP1	X	-	744	17	0	0	2.02%	13.407617211203632	0.02108409854857798	0.02108409854857798	0.002029891263157455
ENSG00000018699_32758514_32820938_285	TTC27	2	+	855	6	0	0	2.11%	4.117757360221054	0.007152939217714824	0.007152939217714824	0.0002660027525696252
ENSG00000088035_63411255_63437020_306	ALG6	1	+	918	13	0	0	3.59%	8.30952669913889	0.0008831683242050062	0.0008831683242050062	4.885807922518899e-06
ENSG00000147010_19645418_19536417_404	SH3KBP1	X	-	1212	26	0	0	2.23%	12.587698861071786	0.003684287313187465	0.003684287313187465	7.541963121699725e-05
ENSG00000170190_75100099_75106033_360	SLC16A5	17	+	1080	9	0	1	1.39%	4.8898368652625015	0.019216965118390855	0.04178282491678763	0.003600525600033436
ENSG00000137124_38395749_38397302_517	ALDH1B1	9	+	1551	35	3	0	2.51%	13.241337739588978	0.0039818987331168895	0.0006311913977015671	1.61670531972368e-05
ENSG00000111652_6724657_6731152_262	COPS7A	12	+	786	13	0	0	2.67%	9.705019732582063	0.0078820576498062	0.0078820576498062	0.0003196364290745116 4
ENSG00000010278_6200500_6225725_121	CD9	12	+	363	70	5	25	10.74%	113.15324977466945	0.0007433598070309323	0.011439320522227195	5.771026777003216e-05
ENSG00000149187_47488963_47472230_468	CELF1	11	-	1404	19	1	0	1.50%	7.940760721366455	0.015516259750072458	0.0078820576498062	0.0006142571802709984
ENSG00000119977_95693899_95664067_459	TCTN3	10	-	1377	38	0	0	3.05%	16.192923823962968	0.0004521348958584617 7	0.0004521348958584617 7	1.3435289073568847e-06
ENSG00000175581_73787972_73864370_121	MRPL48	11	+	363	40	0	0	6.61%	64.65899987123969	0.005808022449631236	0.005808022449631236	0.0001792364769885134 3
ENSG00000091317_32502655_32483960_153	CMTM6	3	-	459	15	1	5	5.23%	19.175830844166672	0.009288069496478249	0.05315521400506113	0.002504473938649158
ENSG00000100938_24234191_24238778_357	GMPR2	14	+	1071	10	1	0	1.40%	5.478808812619049	0.02784981298332479	0.019679754444124863	0.002477909089056847
ENSG00000134255_111147787_111184310_392	CEPT1	1	+	1176	39	0	0	4.08%	19.459554871963014	0.0001782835546100319 5	0.0001782835546100319 5	2.2186407606589736e-07
ENSG00000089157_120200783_120198735_142	RPLP0	12	-	426	54	1	4	5.63%	74.38061710540143	0.005808022449631236	0.009175245078463236	0.0002780762357321736
ENSG00000140391_77070954_77056028_96	TSPAN3	15	-	288	50	2	0	13.54%	101.87160135963543	0.0008084705596873852	0.0007004692736203066	3.591164211716913e-06
ENSG00000131504_141583543_141516851_1114	DIAPH1	5	-	3342	26	2	5	1.35%	4.565018258413825	0.0006738559393031653	0.003803083599965396	1.633323860690344e-05
ENSG00000177628_155238621_155234995_375	GBA	1	-	1125	37	0	0	1.60%	19.298556161569337	0.013428347753762199	0.013428347753762199	0.0008725593497644525
ENSG00000150527_39279472_39350264_729	MIA2	14	+	2187	18	0	0	1.37%	4.829468508901235	0.001801896632038876	0.001801896632038876	1.9221247299282243e-05
ENSG00000185104_50959787_50441440_642	FAF1	1	-	1926	8	0	0	1.25%	2.4373018643052964	0.0023388674905236288	0.0023388674905236288	3.16712418331198e-05
ENSG00000113273_78985248_78816086_413	ARSB	5	-	1239	11	0	0	1.69%	5.209511430303875	0.007029302661868638	0.007029302661868638	0.0002573639808771639
ENSG00000168090_100089385_100090756_138	COPS6	7	+	414	33	0	2	6.52%	46.77235262425001	0.003710480643933249	0.009297184653683213	0.0001872819108611856 8
ENSG00000072849_5480531_5477957_92	DERL2	17	-	276	19	0	0	10.87%	40.39430453912501	0.0021880057261679567	0.0021880057261679567	2.7877966332435854e-05
ENSG00000133318_63681637_63758201_920	RTN3	11	+	2760	92	8	7	2.17%	19.559347461050006	0.0003207014050824132	4.2495854754003497e-05	1.0372854354069738e-07
ENSG00000134759_36129934_36160949_101	ELP2	18	+	303	11	0	0	5.94%	21.30225961104456	0.012798402692974279	0.012798402692974279	0.0007972436888943567
ENSG00000088367_36175560_36229340_779	EPB41L1	20	+	2337	47	3	6	2.57%	11.800889995755458	0.0001701960794612542 4	0.0003817880236155185 4	4.483271452642457e-07
ENSG00000168894_85595764_85597196_139	RNF181	2	+	417	25	1	0	6.47%	35.17868248390288	0.005134531980829637	0.003710480643933249	0.0001044310203272821 7
ENSG00000117859_51617123_51787789_715	OSBPL9	1	+	2145	15	0	1	0.98%	4.103359607213288	0.0079794023326971 4	0.015625008558487224	0.000625378979032468 4
ENSG00000176340_63974681_63976320_69	COX8A	11	+	207	257	13	3	36.23%	728.5148257231668	2.3432067318831253e-05	9.566215697465356e-06	1.8046100744775884e-09

ENSG00000166441_8682814_8685806_148	RPL27A	11	+	444	59	2	0	3.38%	77.9730743379696	0.029237630782826433	0.021557223391537678	0.002819005644529471
ENSG00000114744_149752444_149750377_135	COMMD2	3	-	405	18	1	0	5.19%	26.079129948066672	0.013892891352654344	0.00847367416878415	0.0005886798845679606
ENSG00000174886_5901347_5894742_113	NDUFA11	19	-	339	22	1	1	7.08%	38.08014549938939	0.008364861494636245	0.008364861494636245	0.00035765932356324415
ENSG00000107745_72566793_72368195_480	MICU1	10	-	1440	46	1	0	3.12%	18.744374650172922	0.0004008460213753435	0.00028426559431428834	7.652897942382661e-07
ENSG00000050820_75236980_75229511_599	BCAR1	16	-	1797	18	0	0	1.34%	5.877600238712856	0.005554612275049112	0.005554612275049112	0.00016468457691007306
ENSG00000205336_57650288_57663582_687	ADGRG1	16	+	2061	71	4	0	4.37%	20.21417277633989	3.4901523427252256e-06	6.614649994567748e-07	2.087834633512807e-11
ENSG00000171208_47128397_47083221_344	NETO2	16	-	1032	10	0	0	1.45%	5.685856820072676	0.019679754444124863	0.019679754444124863	0.0017849636115922674
ENSG00000151491_15669717_15621317_718	EPS8	12	-	2154	15	0	0	1.11%	4.086214650637188	0.005405141725979036	0.005405141725979036	0.00015637213408961292
ENSG00000100600_92732786_92704319_433	LGMN	14	-	1299	85	4	10	4.85%	38.39594767180717	5.520116496558956e-05	0.001800655504581463	8.235236159168947e-07
ENSG00000167397_31091310_31091134_58	VKORC1	16	-	174	56	0	12	15.52%	188.8488720377242	0.0035547763465261306	0.06638642199807827	0.001507226391593381
ENSG00000167397_31094729_31091237_92	VKORC1	16	-	276	26	48	3	9.78%	55.27641673775001	0.1519415333234279	0.01204568828101519	0.010116336193794073
ENSG00000161526_75667373_75702950_163	SAP30BP	17	+	489	18	2	0	4.29%	21.599279404840495	0.019778610614370123	0.00847367416878415	0.0008334713975769906
ENSG00000145907_151786627_151804091_464	G3BP1	5	+	1392	84	4	13	7.11%	35.40916350707328	1.2430920413536388e-06	3.721489883809389e-05	4.3590835456923123e-10
ENSG00000214253_101245004_101239806_152	FIS1	7	-	456	30	4	1	6.58%	38.603975252072374	0.012673659338734126	0.0035004709947243163	0.0002432657949324486
ENSG00000120948_11016999_11018930_68	TARDBP	1	+	204	10	0	0	7.35%	28.763746266250006	0.019679754444124863	0.019679754444124863	0.0017849636115922674
ENSG00000168028_39407654_39412368_295	RPSA	3	+	885	132	0	3	3.05%	87.5197920291051	0.0037896409716948643	0.007400806090490468	0.00015220579843420285
ENSG00000177189_20187899_20155398_506	RPS6KA3	X	-	1518	20	2	0	2.37%	7.730967375909093	0.004498799248433914	0.0008852284070317903	2.4782952419168947e-05
ENSG00000119977_95693899_95692912_168	TCTN3	10	-	504	30	0	0	7.14%	34.92740618044644	0.0010032776369550557	0.0010032776369550557	6.244443566103777e-06
ENSG00000114805_155586073_155480968_1508	PLCH1	3	-	4524	9	0	0	0.33%	1.1673350606727457	0.020613416668581838	0.020613416668581838	0.001946208561389314
ENSG00000113575_134199117_134197772_34	PPP2CA	5	-	102	21	1	0	14.71%	120.80773431825003	0.03980790073005672	0.021557223391537678	0.0037960059236543955
ENSG00000108654_64500303_64499923_126	DDX5	17	-	378	58	3	5	11.11%	90.03509148737304	0.00047533768731052587	0.0014977049368909707	4.5899949001426435e-06
ENSG00000108654_64506119_64502005_406	DDX5	17	-	1218	180	12	7	10.10%	86.71631879283254	4.976808168050046e-07	2.6954218182362552e-08	1.3889922125032738e-13
ENSG00000120694_31138481_31137089_86	HSPH1	13	-	258	11	0	0	6.98%	25.017770008319772	0.012798402692974279	0.012798402692974279	0.0007972436888943567
ENSG00000156515_69343848_69401135_889	HK1	10	+	2667	60	7	3	2.70%	13.200909422530936	0.00020078835655311822	4.3403317049897516e-05	6.539831550755316e-08
ENSG00000104529_143580715_143579792_147	EEF1D	8	-	441	75	0	4	10.20%	99.79258908698982	0.00030731120605623235	0.0014004785486803105	2.8763404750661345e-06
ENSG00000121057_57105813_57120324_787	AKAP1	17	+	2361	22	5	0	1.14%	5.467670192415503	0.05150015748280293	0.0033030736763512156	0.0010579038602935387
ENSG00000149503_62128795_62151976_859	INCENP	11	+	2577	15	5	3	0.93%	3.4154855869121077	0.05423379212399942	0.0385659205926511	0.008542915557582598
ENSG00000130638_45738548_45843671_180	ATXN10	22	+	540	58	0	1	6.67%	63.02456404116113	0.0010197273511756433	0.0014987505965367745	9.356970090829691e-06
ENSG00000106144_143291559_143303787_312	CASP2	7	+	936	9	0	7	1.92%	5.642119459918271	0.009815328628645334	0.1097457788509939	0.005898503241319083
ENSG00000104529_143586845_143579792_281	EEF1D	8	-	843	172	0	4	9.25%	119.72269620286835	3.816226438010809e-06	1.4269275421894181e-05	4.574424447029569e-10
ENSG00000163956_3532412_3523464_128	LRPAP1	4	-	384	92	0	1	5.47%	140.58280987629692	0.00877616296920843	0.012088529595134127	0.0005313814658942758
ENSG00000171793_40983291_40996120_307	CTPS1	1	+	921	10	3	0	1.63%	6.371122951482086	0.12114404826813535	0.020613416668581838	0.011598140826794287
ENSG00000113389_32712236_32786345_388	NPR3	5	+	1164	5	0	0	1.29%	2.520534466630155	0.012673659338734126	0.012673659338734126	0.000782701129001274
ENSG00000135525_136383755_136344228_565	MAP7	6	-	1695	7	0	0	0.88%	2.4232819863247794	0.019216965118390855	0.019216965118390855	0.0017073955890589052
ENSG00000103510_31117682_31131259_458	KAT8	16	+	1374	14	2	2	1.53%	5.97883983525546	0.03299602752967379	0.03299602752967379	0.004661188023718761
ENSG00000215301_41344365_41347016_260	DDX3X	X	+	780	58	0	0	9.23%	43.632390490034624	5.63132504724852e-06	5.63132504724852e-06	2.64302915466999e-10
ENSG00000115355_55362358_55301816_1383	CCDC88A	2	-	4149	20	1	0	0.72%	2.8285390399204635	0.0033869280463461717	0.0022543491824521134	4.372784971036962e-05
ENSG00000109919_47627109_47618833_86	MTCH2	11	-	258	16	0	0	12.79%	36.389483648465124	0.0012427339549726212	0.0012427339549726212	9.422880418492092e-06
ENSG00000235173_144137836_144139552_390	HGH1	8	+	1170	12	2	0	2.05%	6.018260757246155	0.030313543781140352	0.004826937407846361	0.0007979355218377862
ENSG00000144354_173363298_173367664_298	CDCA7	2	+	894	16	0	0	1.68%	10.501663066335572	0.02108409854857798	0.02108409854857798	0.002029891263157455
ENSG00000141452_23503619_23531684_561	RMC1	18	+	1683	9	0	0	0.89%	3.1378632290454553	0.020613416668581838	0.020613416668581838	0.001946208561389314
ENSG00000133114_44989655_45028003_340	GPALPP1	13	+	1020	10	0	0	2.06%	5.752749253250001	0.007593099278532274	0.007593099278532274	0.00029783286705462064
ENSG00000125746_45638587_45609663_617	EML2	19	-	1851	19	0	0	1.62%	6.023137791895463	0.0022543491824521134	0.0022543491824521134	2.9517890247939808e-05
ENSG00000164654_7572476_7607092_875	MIOS	7	+	2625	8	0	0	0.69%	1.7882831964388575	0.011570465654371866	0.011570465654371866	0.0006594843062896773
ENSG00000169429_73740659_73742464_99	CXCL8	4	+	297	78	3	9	14.14%	154.10394969312125	0.00045482414116276956	0.010402808958502903	3.445768182961708e-05
ENSG00000170312_60780166_60785799_109	CDK1	10	+	327	15	1	0	4.59%	26.916533203279823	0.022004978195124387	0.019679754444124863	0.0019818329449194675
ENSG00000124228_49219449_49243834_765	DDX27	20	+	2295	24	3	4	2.09%	6.136265870133335	0.0009747456371656623	0.0019340424811117715	1.1523237442356582e-05
ENSG00000124160_46079424_46062300_579	NCOA5	20	-	1737	27	0	0	1.90%	9.120939230541453	0.0012690167775026367	0.0012690167775026367	9.80942059614775e-06
ENSG00000137055_26947045_26905511_795	PLAA	9	-	2385	42	1	0	2.64%	10.33324016810189	3.366721444312314e-05	2.2177236488341457e-05	5.793859795751208e-09
ENSG00000064012_201266643_201276939_183	CASP8	2	+	549	14	2	0	3.28%	14.963435216103829	0.060667625179241055	0.011772051206276392	0.0035017256847258756
ENSG00000111859_11213721_11185162_828	NEDD9	6	-	2484	35	5	0	2.05%	8.267840110347224	0.0009367090916572945	0.0001248167544030276	8.393567413677706e-07
ENSG00000101945_48696785_48707570_412	SUV39H1	X	+	1236	8	0	0	1.21%	3.7979315458349525	0.016947426762344633	0.016947426762344633	0.0013498980316300959
ENSG00000147416_20197422_20220402_506	ATP6V1B2	8	+	1518	31	0	1	2.77%	11.982999432659094	0.0004284130286064981	0.0006310955412434601	1.7645799714593236e-06
ENSG00000162704_183635659_183627532_151	ARPC5	1	-	453	145	57	6	17.88%	187.82154846703648	0.001744172782902307	4.826694637345914e-06	1.0274079773240887e-07
ENSG00000284431_40350019_40400704_324	AL022238.4	22	+	972	30	0	0	3.70%	18.110506908379634	0.0010405263639310788	0.0010405263639310788	6.697970672456085e-06
ENSG00000270800_34425077_34288753_243	RPS10-NUDT3	6	-	729	9	0	0	2.06%	7.244202763351853	0.020613416668581838	0.020613416668581838	0.001946208561389314
ENSG00000120948_11016999_11023248_167	TARDBP	1	+	501	12	0	0	3.59%	14.054620930095812	0.013007239811447113	0.013007239811447113	0.0008218654148058079
ENSG00000242265_94663557_94664534_325	PEG10	7	+	975	49	0	2	6.15%	29.48947771050616	3.77282131074845e-05	4.8247556065654664e-05	1.3786811572392767e-08
ENSG00000242265_94664609_94665682_357	PEG10	7	+	1071	31	3	0	2.80%	16.98430731911905	0.008605408492104586	0.0022543491824521134	0.00011091378991179106
ENSG00000091640_4960547_4959716_154	SPAG7	17	-	462	18	1	1	5.19%	22.86157495447403	0.008316137131635013	0.008316137131635013	0.00035373208051571366
ENSG00000108953_1400110_1361898_124	YWHAE	17	-	372	80	6	10	10.48%	126.18933845838713	0.003042349403049352	0.005731544663142747	9.670614408573852e-05
ENSG00000175567_73977955_73975007_220	UCP2	11	-	660	19	1	0	2.73%	16.892163716361367	0.023878473930440184	0.013007239811447113	0.0014709138084317821
ENSG00000175567_73981586_73981512_24	UCP2	11	-	72	19	0	0	20.83%	154.84483406664586	0.02108409854857798	0.02108409854857798	0.002029891263157455
ENSG00000100360_36767356_36760917_113	IFT27	22	-	339	16	0	0	7.08%	27.694651272283192	0.005306578019052857	0.005306578019052857	0.00015100114740534233
ENSG00000089597_62639071_62625815_847	GANAB	11	-	2541	315	13	7	11.22%	72.74137485514464	8.378776174174828e-17	8.067490256025824e-17	1.009053197098447e-31
ENSG00000121022_67047741_67043233_96	COPS5	8	-	288	18	0	0	5.21%	36.67377648946876	0.02108409854857798	0.02108409854857798	0.002029891263157455
ENSG00000198546_133309432_133312866_187	ZNF511	10	+	561	22	1	1	6.42%	23.010997013000004	0.001621945894645527	0.001621945894645527	1.571022352093035e-05

ENSG00000170266_33072698_33058265_84	GLB1	3	-	252	12	3	0	5.95%	27.94192494435715	0.07356204933788085	0.02108409854857798	0.006910583573522876
ENSG00000198171_3195245_3190814_38	DDRGK1	20	-	114	7	0	0	13.16%	36.03037690193422	0.019216965118390855	0.019216965118390855	0.0017073955890589052
ENSG00000105856_107182423_107201431_441	HBP1	7	+	1323	14	0	0	1.81%	6.209316654301588	0.004687384229717438	0.004687384229717438	0.00011928172701435494
ENSG00000105854_95435014_95405330_375	PON2	7	-	1125	441	12	9	17.33%	230.01792614194804	2.199773929273865e-11	4.7628156924550425e-12	1.3001091419103812e-21
ENSG00000188313_146533542_146516045_311	PLSCR1	3	-	933	36	1	0	5.14%	22.641045292533768	0.00028338833315400153	0.00017744757238953088	3.472812223617562e-07
ENSG00000134014_28097245_28189725_532	ELP3	8	+	1596	27	0	1	2.26%	9.92673649339004	0.0009549346450480869	0.0015734716984481248	9.224426685502696e-06
ENSG00000108518_4948394_4945900_140	PFN1	17	-	420	1266	113	23	30.00%	1768.7238489778076	2.2396379874617514e-08	1.8325040274563 51e-08	4.180397463846852e-15
ENSG00000196083_190609068_190648705_429	IL1RAP	3	+	1287	15	0	0	1.40%	6.8389326786888125	0.013428347753762199	0.013428347753762199	0.0008725593497644525
ENSG00000119681_74611944_74500884_1821	LTBP2	14	-	5463	16	1	0	0.38%	1.7185588104162552	0.009583874861041503	0.00797940233269714	0.0003893623151447896
ENSG00000205413_93105929_93101328_1533	SAMD9	7	-	4599	18	3	0	0.78%	2.296596570769081	0.0025320062747194163	0.0008442728493916082	1.320482778495691e-05
ENSG00000156113_77637360_76877869_1146	KCNMA1	10	-	3438	15	4	2	0.70%	2.5601240132264405	0.048994651353958696	0.02389626957043539	0.005091569865673637
ENSG00000119203_9423774_9473017_684	CPSF3	2	+	2052	55	1	2	2.78%	15.727545473066524	8.123247908069087e-05	0.00020664691546861568	1.2102541393845173e-07
ENSG00000077721_119574739_119576988_55	UBE2A	X	+	165	33	0	0	9.09%	117.35608476630003	0.021557223391537678	0.021557223391537678	0.002115616449879074
ENSG00000131724_118740153_118791854_425	IL13RA1	X	+	1275	8	0	0	1.41%	3.681759522080001	0.011570465654371866	0.011570465654371866	0.0006594843062896773
ENSG00000065268_984366_994344_405	WDR18	19	+	1215	38	8	0	4.94%	18.351980333824695	0.0017289832101357312	2.8656551254862736e-05	4.4948899367602487e-07
ENSG00000198682_87659982_87745970_619	PAPSS2	10	+	1857	19	0	0	0.97%	6.00367692665509	0.013640585738808999	0.013640585738808999	0.00089863692579514
ENSG00000132406_4248202_4237836_165	TMEM128	4	-	495	14	0	0	3.64%	16.59580996695152	0.013428347753762199	0.013428347753762199	0.0008725593497644525
ENSG00000169490_38995428_38991332_146	TM2D2	8	-	438	31	0	0	7.53%	41.53012132140754	0.0014055202957965589	0.0014055202957965589	1.193553004024608 1e-05
ENSG00000117118_17033135_17027665_137	SDHB	1	-	411	24	0	0	6.57%	34.26455029672994	0.0034782380676658805	0.0034782380676658805	6.758309314600117e-05
ENSG00000135318_85450140_85471469_264	NT5E	6	+	792	38	0	0	7.58%	28.153606193935612	3.327977637974103e-05	3.327977637974103e-05	8.490069064475856e-09
ENSG00000073578_225446_235435_338	SDHA	5	+	1014	20	1	1	2.37%	11.57357837931953	0.008316137131635013	0.008316137131635013	0.00035373208051571366
ENSG00000155366_112703799_112701540_193	RHOC	1	-	579	87	4	5	15.03%	88.16907922856738	6.357679160154451e-06	1.1512870382308943e-05	6.012907082345096e-10
ENSG00000159322_72752775_72752341_144	ADPGK	15	-	432	17	2	0	5.56%	23.090896308184032	0.013018137044106636	0.005160369442893307	0.0003527402747546314
ENSG00000168036_41224069_41236742_702	CTNNB1	3	+	2106	98	8	3	5.41%	27.30507195417237	1.0471752281441074e-06	1.697052242985141e-07	1.6902179079025326e-12
ENSG00000111642_6601370_6580630_1413	CHD4	12	-	4239	19	5	0	0.71%	2.6300608758665964	0.04412263738957799	0.0017901637611834264	0.0005469523947526553
ENSG00000100519_52707220_52727617_389	PSMC6	14	+	1167	36	0	0	3.08%	18.101195593773785	0.0010197273511756433	0.0010197273511756433	6.442859155158972e-06
ENSG00000107929_885721_812926_738	LARP4B	10	-	2214	20	0	0	1.22%	5.300636168306235	0.0036062891729786097	0.0036062891729786097	7.240558534568731e-05
ENSG00000125821_18628147_18744252_79	DTD1	20	+	237	31	0	2	11.39%	76.75186978386711	0.003736770770438521	0.007400806090490468	0.00015026100263372736
ENSG00000103202_399666_400342_65	NME4	16	+	195	10	2	0	7.69%	30.091303786230775	0.019679754444124863	0.019679754444124863	0.0017849636115922674
ENSG00000091140_107891251_107904670_114	DLD	7	+	342	26	6	1	8.77%	44.60903806906141	0.041503828325407145	0.0037834851205969236	0.000921877301934226
ENSG00000152620_36225612_36195144_301	NADK2	5	-	903	8	0	0	1.66%	5.198497664066446	0.019216965118390855	0.019216965118390855	0.0017073955890589052
ENSG00000135506_57694162_57720909_667	OS9	12	+	2001	76	3	6	5.55%	22.286512849172418	3.1323528810920676e-07	1.6230674168790844e-06	4.722340860379946e-12
ENSG00000131143_85804978_85805115_45	COX4I1	16	+	135	48	1	1	17.78%	208.63303958453338	0.006386247275100883	0.006386247275100883	0.0002145861249467 8405
ENSG00000100632_69394831_69380538_76	ERH	14	-	228	495	11	6	23.68%	1273.9311833183885	0.0005870797137989368	0.00028827692576097245	1.1294036042514793e-06
ENSG00000168883_85612326_85648808_487	USP39	2	+	1461	17	9	1	1.44%	6.827698292358318	0.17089730187428281	0.009583874861041503	0.009945008634743948
ENSG00000168488_28826345_28836777_854	ATXN2L	16	+	2562	50	7	2	1.99%	11.45160858375293	0.001435957343153687	0.00012584544091628916	1.3241823711538 65e-06
ENSG00000168679_110381667_110363766_371	SLC16A4	1	-	1113	55	1	1	4.31%	28.996337206408363	0.00025243161447129205	0.00025243161447129205	4.352374274938186e-07
ENSG00000136527_185925496_185917715_188	TRA2B	3	-	564	41	1	3	8.51%	42.65602371824735	0.00022282222326679875	0.001013197191032157	1.5404611290049819e-06
ENSG00000071127_10116666_10075378_606	WDR1	4	-	1818	87	3	0	5.45%	28.08025130546783	7.458395907570739e-07	1.9985778497703959e-07	1.4017973607944687e-12
ENSG00000071127_10116666_10075378_466	WDR1	4	-	1398	70	1	0	5.15%	29.38099404020387	9.28369932835357e-06	7.543195326261127e-06	5.738048499589578e-10
ENSG00000167965_2206086_2208877_325	MLST8	16	+	975	17	0	4	3.08%	10.231043287318464	0.0020082573300163734	0.023029368248455667	0.00028571595868184737
ENSG00000161203_184176994_184183616_433	AP2M1	3	+	1299	65	2	7	5.54%	29.36160704314666	1.9476261111745904e-05	0.0001547348909454494	2.387872630864365e-08
ENSG00000132170_12379727_12434145_470	PPARG	3	+	1410	13	0	0	1.49%	5.4100322764606394	0.00797940233269714	0.00797940233269714	0.000327142761693177
ENSG00000105197_39480854_39489820_157	TIMM50	19	+	471	18	0	5	4.46%	22.42472957317835	0.067234255889369	0.0084736741687 8415	0.003009010068621 3806
ENSG00000121749_71839782_71923204_691	TBC1D15	12	+	2073	14	3	0	1.16%	3.962820035523879	0.046396937147164995	0.005160369442893307	0.0013402346425884413
ENSG00000161813_50401011_50475864_653	LARP4	12	+	1959	20	3	0	1.53%	5.99061177980092	0.014216764456991368	0.0022276761777358703	0.0001851108649729137
ENSG00000162704_183633147_183627532_101	ARPC5	1	-	303	94	2	3	11.88%	182.0374912216535	0.0024967744609976712	0.0023000413718151508	3.318285179317672e-05
ENSG00000101365_2664188_2660613_204	IDH3B	20	-	612	44	3	0	8.33%	42.186827857166676	0.00024735545005388 34	0.00013400452932326877	2.3244397848138355e-07
ENSG00000173915_103392376_103392194_31	ATP5MD	10	-	93	101	2	0	22.58%	637.256159214855	0.00898023876303 9383	0.00898023876303 9383	0.0004089866599971986
ENSG00000132170_12379727_12434145_470	PPARG	3	+	1410	13	0	0	1.49%	5.4100322764606394	0.00797940233269714	0.00797940233269714	0.000327142761693177
ENSG00000117632_25904676_25901016_149	STMN1	1	-	447	233	7	18	23.49%	305.86093680702356	2.043479731736036e-07	1.4662072863005735e-06	2.8395761137540576e-12
ENSG00000215301_41343231_41347719_476	DDX3X	X	+	1428	83	1	0	7.56%	34.10558485855358	6.958679763556736e-08	4.081835640353621e-08	2.8150375102779016e-14
ENSG00000116717_67685495_67687774_131	GADD45A	1	+	393	38	6	0	9.16%	56.737038436633604	0.004981126742780332	0.0008814539243352768	2.744125459780358e-05
ENSG00000277791_38760525_38764167_75	PSMB3	17	+	225	168	1	1	20.00%	438.1293831275201	0.0003418708136139773	0.0003418708136139773	7.827289718747123e-07
ENSG00000162772_212615025_212619555_180	ATF3	1	+	540	16	2	0	3.89%	17.38608663204445	0.03299602752967379	0.0084736741687 8415	0.0014019198044660757
ENSG00000146678_45888836_45893091_155	IGFBP1	7	+	465	9	0	0	3.23%	11.357040461254842	0.019679754444124863	0.019679754444124863	0.0017849636115922674
ENSG00000117691_212432944_212446006_172	NENF	1	+	516	23	0	0	4.65%	26.15494137233431	0.005355751137763 4245	0.005355751137763 4245	0.00015366972994913032
ENSG00000170892_54191365_54193362_310	TSEN34	19	+	930	33	5	0	3.87%	20.821240845633877	0.008356254428487338	0.0010155991509472172	5.354085463802975e-05
ENSG00000079805_10775852_10831047_691	DNM2	19	+	2073	19	1	0	1.01%	5.378112905353836	0.011825808327677989	0.0084736741687 8415	0.000503679174626 6746
ENSG00000141699_42592110_42581813_369	RETREG3	17	-	1107	13	0	0	1.63%	6.890827018798104	0.011772051206276392	0.011772051206276392	0.000681272511389508
ENSG00000168291_58431954_58428034_299	PDHB	3	-	897	29	0	0	4.01%	18.97060456088462	0.0010405263639310788	0.0010405263639310788	6.697970672456085e-06
ENSG00000131368_15065188_15052441_171	MRPS25	3	-	513	13	3	3	4.68%	14.869679356353805	0.03961598513640774	0.03961598513640774	0.006529232268371349
ENSG00000159063_78139588_78102894_467	ALG8	11	-	1401	53	5	0	3.85%	22.197974634596363	0.0007799372344069463	8.332796070245443e-05	4.817754366714235e-07
ENSG00000100911_24146581_24143409_254	PSME2	14	-	762	106	1	7	7.09%	81.62562326264963	9.365996500916305e-05	0.0004314251208999809	2.9060245554957137e-07
ENSG00000154518_175181393_175180068_49	ATP5MC3	2	-	147	57	4	1	16.33%	227.52710311833678	0.018067938367053574	0.005706018193000826	0.0005367449530804556
ENSG00000071894_144401009_144393318_1292	CPSF1	8	-	3876	9	5	2	0.62%	1.3624932441907898	0.1278877087947798	0.026101817670657335	0.014762966464186279
ENSG00000204580_30888730_30899296_913	DDR1	6	+	2739	26	4	1	0.99%	5.570022278064624	0.02387123336711234	0.005670089768331009	0.0007107549611358789

ENSG00000119431_113374354_113373599_251	HDHD3	9	-	753	6	2	1	1.99%	4.675541225749005	0.1029516053660342	0.0477903522728147	0.019089002044397063
ENSG00000164104_173333649_173332080_209	HMGB2	4	-	627	85	4	1	8.13%	79.54758536790672	0.00024144924403469368	0.0001584151477028702	2.6622050706451913e-07
ENSG00000009307_114739890_114718169_798	CSDE1	1	-	2394	90	11	5	4.39%	22.059414429755645	9.267998481696954e-06	8.096231702821984e-07	6.8610803470884e-11
ENSG00000108468_48077004_48071435_185	CBX1	17	-	555	36	4	1	4.32%	38.061432897178385	0.013952015397585121	0.006346985027636522	0.0004550309184833047
ENSG00000115286_1383927_1395488_213	NDUFS7	19	+	639	47	3	0	8.92%	43.1591235056033	0.00015518018174202626	5.488892743562522e-05	6.278587505600271e-08
ENSG00000182326_7064377_7070651_521	C1S	12	+	1563	27	0	1	2.50%	10.136322100736086	0.00047627452948067799	0.001038231149674533	3.1862098168137684e-06
ENSG00000099995_30356792_30346255_149	SF3A1	22	-	447	9	0	0	3.36%	11.81437094962752	0.020613416668581838	0.020613416668581838	0.001946208561389314
ENSG00000198546_133310236_133312866_85	ZNF511	10	+	255	14	0	0	8.24%	32.21539581820001	0.00797940233269714	0.00797940233269714	0.000327142761693177
ENSG00000158373_26158170_26158550_126	HIST1H2BD	6	+	378	123	13	5	15.08%	190.93648711977386	0.00019158148951032086	9.00886142045281e-05	1.2376604711048995e-07
ENSG00000274641_27893463_27893843_126	HIST1H2BO	6	+	378	130	11	8	9.52%	201.80279126480164	0.0010230721480519933	0.000911854467393528	5.805714284204345e-06
ENSG00000043143_134559882_134579317_670	JADE2	5	+	2010	14	1	2	1.04%	4.087027827682091	0.013892891352654344	0.03299602752967379	0.002146120390806497
ENSG00000143621_153670922_153662396_390	ILF2	1	-	1170	155	6	1	10.77%	77.73586811442951	8.945748451907353e-08	7.755690543875306e-09	7.33583163158286e-15
ENSG00000180398_46907920_46905463_80	MCFD2	2	-	240	18	2	0	6.25%	44.00853178736251	0.06539853090342929	0.02108409854857798	0.006119906528703008
ENSG00000156113_77637360_76887266_1084	KCNMA1	10	-	3252	15	4	2	0.74%	2.7065517704404987	0.048994651353958696	0.02389626957043539	0.005091569865673637
ENSG00000115525_85848138_85840356_104	ST3GAL5	2	-	312	11	0	2	4.81%	20.687771353033657	0.019216965118390855	0.08350359792828288	0.007323531272552175
ENSG00000159322_72783691_72752341_496	ADPGK	15	-	1488	25	2	3	2.42%	9.858542067061494	0.0020543872447033253	0.006506282245906878	7.678558181767462e-05
ENSG00000182473_76101342_76083648_592	EXOC7	17	-	1776	9	0	0	0.84%	2.9735494450920616	0.019679754444124863	0.019679754444124863	0.0017849636115922674
ENSG00000184220_99817980_100178388_279	CMSS1	3	+	837	31	0	7	5.38%	21.73260829005556	0.00028802016930314084	0.0033478801539048786	6.75370596065142e-06
ENSG00000153310_129854286_129842142_159	FAM49B	8	-	477	16	0	0	3.77%	19.68236222495598	0.012798402692974279	0.012798402692974279	0.0007972436888943567
ENSG00000073060_124863630_124778467_476	SCARB1	12	-	1428	46	1	2	3.78%	18.90189040353572	0.00012374249556490374	0.00031567046080049535	2.747431735905352e-07
ENSG00000154124_14664826_14693048_352	OTULIN	5	+	1056	7	0	0	1.42%	3.889642961004262	0.016947426762344633	0.016947426762344633	0.0013498980316300959
ENSG00000154127_122777208_122809886_516	UBASH3B	11	+	1548	9	2	0	1.36%	3.4115140920436056	0.0350911293701988	0.006937202941512724	0.001262969770953026
ENSG00000186566_44400189_44397568_873	GPATCH8	17	-	2619	16	0	0	0.69%	3.584760130318443	0.013640585738808999	0.013640585738808999	0.00089863692579514
ENSG00000134419_18789104_18784734_97	RPS15A	16	-	291	43	14	4	6.19%	86.70638565207734	0.13042902411515872	0.05352897298295963	0.026520273205808225
ENSG00000285238_6601370_6578908_1439	AC006064.6	12	-	4317	19	5	0	0.69%	2.5825406654617797	0.04412263738957799	0.0017901637611834264	0.0005469523947526553
ENSG00000285238_6567918_6556993_808	AC006064.6	12	-	2424	26	4	2	2.10%	6.293849430535893	0.0010891986948716168	0.0002707893389517208	1.9824595550127703e-06
ENSG00000116906_231265310_231277542_485	GNPAT	1	+	1455	21	0	1	2.47%	8.468995807877322	0.0008664586529590942	0.0016120252826907794	8.62301436870289e-06
ENSG00000133872_30069825_30063888_143	SARAF	8	-	429	36	2	3	12.59%	49.24031528655945	0.0003839034557620171	0.0003114890598217295	8.008419892350254e-07
ENSG00000166562_59139949_59158650_161	SEC11C	18	+	483	30	3	0	5.59%	36.445989057857155	0.00699534207690609	0.003684287313187465	0.00014031246620721993
ENSG00000141376_60889721_61392125_684	BCAS3	17	+	2052	14	0	5	0.73%	4.003375211326024	0.019679754444124863	0.1526275292331874	0.014559368415092097
ENSG00000149016_62581416_62575094_688	TUT1	11	-	2064	16	1	0	0.87%	4.5486854560581405	0.020525443280229434	0.013640585738808999	0.0013255139945858052
ENSG00000256053_103563002_103574175_129	APOPT1	14	+	387	12	0	3	4.65%	18.194741824232562	0.011772051206276392	0.026602764905793736	0.001497510566720742
ENSG00000100902_35292477_35310287_107	PSMA6	14	+	321	45	2	0	6.54%	82.25893792030375	0.013892891352654344	0.00847367416878415	0.0005886798845679606
ENSG00000215301_41340781_41347719_622	DDX3X	X	+	1866	91	3	3	7.07%	28.61576557806351	1.3918695140175526e-08	1.1261251676471362e-08	1.6190827680347769e-15
ENSG00000170445_140679874_140674257_406	HARS	5	-	1218	48	1	1	4.19%	23.124351678088676	0.00015267201677270214	0.00015267201677270214	1.6423430888992484e-07
ENSG00000104824_38846077_38836722_456	HNRNPL	19	-	1368	118	9	8	6.14%	50.61410088605045	1.4231592959075099e-05	3.946485846766709e-05	4.437515975741084e-09
ENSG00000104872_49448006_49446382_126	PIH1D1	19	-	378	6	0	0	3.97%	9.313974981452382	0.016947426762344633	0.016947426762344633	0.0013498980316300959
ENSG00000285130_69046445_69099934_96	AL358113.1	9	+	288	17	0	0	7.29%	34.63634446227605	0.00847367416878415	0.00847367416878415	0.0003665024264111182
ENSG00000197102_102008259_102016143_543	DYNC1H1	14	+	1629	12	3	0	0.92%	4.3225077261988964	0.08385215180161965	0.019679754444124863	0.007498199469051051
ENSG00000092621_119712023_119744040_526	PHGDH	1	+	1578	41	0	0	3.99%	15.245879199677761	2.2606885438602453e-05	2.2606885438602453e-05	3.994412047840312e-09
ENSG00000171132_45984578_46184881_497	PRKCE	2	+	1491	11	0	3	1.01%	4.329030625182093	0.019679754444124863	0.10105102209468636	0.009164497669452725
ENSG00000172725_67442628_67438376_489	CORO1B	11	-	1467	36	2	2	2.66%	14.399519603226997	0.0027852197205094193	0.0017971687341926767	2.9217951810824196e-05
ENSG00000065057_2047802_2039924_297	NTHL1	16	-	891	24	6	5	4.71%	15.80553330185859	0.013243555427667874	0.00436789375498805	0.00030937578781533053
ENSG00000119383_129136511_129142900_213	PTPA	9	+	639	15	3	0	4.23%	13.774188352852116	0.010020958156399907	0.00327844299461777	0.0001807348713395933
ENSG00000140983_668200_671768_322	RHOT2	16	+	966	9	1	3	2.17%	5.466898358678573	0.017404239405933563	0.09510738879508468	0.007788568812190347
ENSG00000127054_1324608_1311859_389	INTS11	1	-	1167	11	0	2	2.06%	5.5309208758753226	0.004687384229717438	0.033944577430914495	0.0008796663632127183
ENSG00000109606_24584393_24527924_795	DHX15	4	-	2385	70	0	3	3.27%	17.22206694683648	3.3477301940231204e-06	2.3032069702246652e-05	6.577688127704486e-10
ENSG00000054267_235326919_235168525_1312	ARID4B	1	-	3936	14	0	0	0.53%	2.0871254912705797	0.00847367416878415	0.00847367416878415	0.0003665024264111182
ENSG00000101407_38013816_37983456_1089	TTI1	20	-	3267	21	2	0	0.92%	3.7717749924889814	0.010645877633714064	0.0021880057261679567	0.0001345568947401665
ENSG00000177830_904816_869858_393	CHID1	11	-	1179	14	0	3	1.78%	6.967706474674302	0.007029302661868638	0.04912109529854385	0.0018325026027891263
ENSG00000155957_66169858_66138145_130	TMBIM4	12	-	390	39	0	1	8.46%	58.678042383150014	0.001588175367824666	0.001759868229841518	1.6653375375657385e-05
ENSG00000100823_20455646_20457508_318	APEX1	14	+	954	109	11	4	9.43%	67.0430463287563	1.257751652788525e-05	2.805509405993715e-06	3.0087131975870506e-10
ENSG00000143761_228097115_228098013_181	ARF1	1	+	543	246	3	2	24.31%	265.8342251612321	3.89149000448416e-09	3.5896094622681653e-09	1.4917525667184776e-16
ENSG00000149428_119056160_119045593_937	HYOU1	11	-	2811	86	3	1	3.42%	17.952015812703312	1.2574717104166116e-06	3.6511892842525544e-07	4.2252682724029906e-12
ENSG00000198712_7894_7995_33	MT-CO2	MT	+	99	51	25	20	21.21%	302.2808243980455	0.027657845067597595	0.11765687773853717	0.014107912102157146
ENSG00000121060_56908536_56891700_422	TRIM25	17	-	1266	15	2	0	1.66%	6.952374689946684	0.017901377192768454	0.00847367416878415	0.0007548976371881338
ENSG00000107438_95290915_95237925_329	PDLIM1	10	-	987	43	0	5	5.78%	25.563888778879946	5.5341223974656866e-05	0.0008501967676584522	3.6617653068811154e-07
ENSG00000137145_19341102_19372173_961	DENND4C	9	+	2883	5	2	1	0.52%	1.0176559553095736	0.15865525393145707	0.051235217429874705	0.0313151272044354
ENSG00000135919_224001900_223975867_398	SERPINE2	2	-	1194	67	4	0	7.04%	32.926539695737446	1.4297196710699094e-05	1.2083345548513282e-06	1.5585590041173699e-10
ENSG00000204852_110634747_110647892_324	TCTN1	12	+	972	16	0	0	2.16%	9.65893701780247	0.0078820576498062	0.0078820576498062	0.00031963642907451164
ENSG00000166908_57594058_57601606_334	PIP4K2C	12	+	1002	14	0	5	2.40%	8.198528875889224	0.005160369442893307	0.05268573235456097	0.0015446541073930182
ENSG00000106397_101217969_101212894_333	PLOD3	7	-	999	33	7	0	3.30%	19.383137123563067	0.040842264374640405	0.001558047347358915	0.00044797973699658506
ENSG00000115875_38751256_38749319_137	SRSF7	2	-	411	124	3	3	16.79%	177.033509866438	2.3334559990505223e-05	2.68112055189137e-05	4.866556242016747e-09
ENSG00000115875_38748932_38745133_118	SRSF7	2	-	354	41	1	0	8.47%	67.9604445680551	0.0024203653230594926	0.0023766146074493525	3.323100869682161e-05
ENSG00000105323_41276293_41306565_532	HNRNPUL1	19	+	1596	83	5	3	5.64%	30.515523294495306	2.5349936190846526e-06	1.9066683571442567e-06	4.193633978817017e-11
ENSG00000173120_67215866_67255055_961	KDM2A	11	+	2883	25	6	0	1.25%	5.088279776547868	0.02120642283783385	0.0009829516685359422	0.00014517098847388396
ENSG00000123472_46668322_46635776_328	ATPAF1	1	-	984	23	0	0	2.74%	13.715396085492381	0.0033278027414746945	0.0033278027414746945	6.211806171561056e-05

ENSG00000115464_61380198_61188102_3218	USP34	2	-	9654	19	2	7	0.40%	1.1548402789308578	0.0021377078453811284	0.04120406791801686	0.0005802191941536071
ENSG00000081177_69209471_69241100_621	EXD2	14	+	1863	9	0	0	0.97%	2.834688037833334	0.012178957859018494	0.012178957859018494	0.0007262454987071801
ENSG00000204843_74378227_74361499_1236	DCTN1	2	-	3708	38	9	2	1.54%	6.0133916142386745	0.0033918133431191906	0.00020713935581772233	5.14464272896285e-06
ENSG00000068724_46956974_47073923_697	TTC7A	2	+	2091	16	3	0	0.86%	4.489950636682928	0.06341282918854552	0.013428347753762199	0.004086769345423957
ENSG00000243449_2042771_2043926_39	C4orf48	4	+	117	23	0	0	20.51%	115.34999784721798	0.0053557511377634245	0.0053557511377634245	0.00015366972994913032
ENSG00000147065_65667842_65739893_577	MSN	X	+	1731	50	2	3	4.16%	16.949174576299832	3.0151795258011407e-05	4.1955319042663064e-05	9.678202471931484e-09
ENSG00000257218_120446481_120459959_136	GATC	12	+	408	20	0	0	5.88%	28.763746266250006	0.005554612275049112	0.005554612275049112	0.00016468457691007306
ENSG00000140612_84716075_84670019_129	SEC11A	15	-	387	132	15	0	19.38%	200.14216006655818	0.000523734753610826	4.336027588575596e-06	2.3455249128712497e-08
ENSG00000099998_24244725_24219970_586	GGT5	22	-	1758	12	2	4	1.02%	4.005327125129694	0.05258119297243839	0.10069662721979156	0.02022434976052801
ENSG00000133606_140479344_140454517_482	MKRN1	7	-	1446	9	1	1	1.45%	3.6521603143039427	0.016735684067288745	0.026602764905793736	0.0020485104298708923
ENSG00000130414_240011687_239961118_129	NDUFA10	2	-	387	15	1	1	4.65%	22.743427280290703	0.02034245439037237	0.02034245439037237	0.0018987606724399325
ENSG00000130414_240025301_240014697_236	NDUFA10	2	-	708	21	0	0	3.39%	17.40450409669704	0.005604866691947602	0.005604866691947602	0.00016752456572620382
ENSG00000136937_97666803_97669814_141	NCBP1	9	+	423	15	0	0	4.26%	20.807816447925536	0.013428347753762199	0.013428347753762199	0.0008725593497644525
ENSG00000132507_7309636_7311640_186	EIF5A	17	+	558	187	71	8	10.22%	196.6450524309866	0.0049868505638379	6.47859756483761e-05	2.9727350416935365e-06
ENSG00000157734_64156726_64156857_43	SNX22	15	+	129	21	2	4	16.28%	95.52239457722095	0.00847367416878415	0.013991246267585163	0.0005927351821891016
ENSG00000157734_64156036_64156287_83	SNX22	15	+	249	84	5	35	10.84%	197.95002249737354	0.0220856724542213	0.0706906638683072	0.006890087056981816
ENSG00000005187_20775896_20786251_346	ACSM3	16	+	1038	8	0	0	1.45%	4.522392476543353	0.016947426762344633	0.016947426762344633	0.0013498980316300959
ENSG00000196531_56712866_56712759_35	NACA	12	-	105	22	1	0	17.14%	122.94446975517145	0.013428347753762199	0.013640585738808999	0.0008855098933240206
ENSG00000086589_150696802_150691751_300	RBM22	5	-	900	25	0	2	3.33%	16.29945621754167	0.0022276761777358703	0.004515676995858069	5.735615488377851e-05
ENSG00000143437_150846291_150812021_723	ARNT	1	-	2169	13	0	0	0.83%	3.516895117477871	0.013428347753762199	0.013428347753762199	0.0008725593497644525
ENSG00000141030_17276159_17247098_345	COPS3	17	-	1035	40	2	5	4.64%	22.677504302666673	0.000716369705445885	0.0017661069627062194	7.908598144253648e-06
ENSG00000149187_47488963_47472230_469	CELF1	11	-	1407	19	1	0	1.49%	7.923829461832624	0.015516259750072458	0.0078820576498062	0.0006142571802709984
ENSG00000197111_53454801_53479442_365	PCBP2	12	+	1095	162	7	5	10.96%	86.81135037507126	1.5222071908818342e-07	1.6804004177476402e-08	2.6326057733437794e-14
ENSG00000108946_68515400_68530449_381	PRKAR1A	17	+	1143	161	2	3	10.24%	82.6523606621798	2.8854180147143214e-08	2.854877492738632e-08	8.301483419201996e-15
ENSG00000111602_56433648_56417716_1122	TIMELESS	12	-	3366	23	1	3	0.98%	4.009491903780304	0.0021291955662021897	0.007306944804361946	8.920186517297622e-05
ENSG00000148358_130086423_130135121_411	GPR107	9	+	1233	17	1	0	2.19%	8.090241042283457	0.004669667620227399	0.0032294771334464936	8.365569901249867e-05
ENSG00000089053_121327194_121308480_308	ANAPC5	12	-	924	13	0	0	2.27%	8.255568733560066	0.0078820576498062	0.0078820576498062	0.00031963642907451164
ENSG00000134759_36129934_36138479_165	ELP2	18	+	495	13	0	0	4.24%	15.410394969312124	0.0078820576498062	0.0078820576498062	0.00031963642907451164
ENSG00000089916_76154364_76201851_477	GPATCH2L	14	+	1431	9	0	0	1.05%	3.6904429171792463	0.020613416668581838	0.020613416668581838	0.001946208561389314
ENSG00000100938_24233254_24238778_348	GMPR2	14	+	1044	12	2	0	2.01%	6.744602572775864	0.022898969405409888	0.008175610787902942	0.0009353765476651278
ENSG00000086666_80120378_80137628_197	ZFAND6	15	+	591	20	0	3	5.58%	19.8572055442132	0.0014481341328822738	0.011821942446875977	0.00010518227444157728
ENSG00000140416_63059609_63061730_78	TPM1	15	+	234	95	0	9	14.10%	238.22282164099366	0.0016494676794180302	0.01253092217194181	0.00012512190574386016
ENSG00000055163_157319804_157393000_787	CYFIP2	5	+	2361	8	4	0	0.76%	1.9882437063329101	0.23639154306007898	0.011570465654371866	0.017275440155518785
ENSG00000067955_67029408_67098778_187	CBFB	16	+	561	35	1	0	4.81%	36.60840433886364	0.0033278027414746945	0.003580484096903955	6.661675796445952e-05
ENSG00000051108_56940238_56943290_92	HERPUD1	16	+	276	15	0	0	7.61%	31.890240425625006	0.00847367416878415	0.00847367416878415	0.0003665024264111182
ENSG00000102858_4650391_4686299_492	MGRN1	16	+	1476	15	0	2	1.83%	5.963215689344514	0.0033278027414746945	0.01652445925752544	0.0003062742250611212
ENSG00000055044_202265942_202284485_145	NOP58	2	+	435	11	0	1	3.45%	14.8381256601069	0.02108409854857798	0.03577298629450316	0.003354604907114977
ENSG00000156411_103915153_103913964_87	ATP5MPL	14	-	261	51	1	2	5.75%	114.65824373718968	0.023199730935452297	0.021557223391537678	0.002265826071202869
ENSG00000117308_23799007_23796166_360	GALE	1	-	1080	34	0	5	3.06%	18.472717046547228	0.001558047347358915	0.009946374827215387	9.324655672019411e-05
ENSG00000164305_184635393_184629436_156	CASP3	4	-	468	9	0	1	3.21%	11.284238919836541	0.020613416668581838	0.03537493353818881	0.003252526348827943
ENSG00000076864_21613636_21597720_526	RAP1GAP	1	-	1578	15	0	5	1.14%	5.577760682808937	0.012178957859018494	0.11497452839710681	0.007325749448687799
ENSG00000166741_114312268_114312477_69	NNMT	11	+	207	34	2	4	20.29%	96.37939328633335	0.001373376896918719	0.0004156054716904614	3.716678856638805e-06
ENSG00000104824_38840176_38838890_68	HNRNPL	19	-	204	25	0	2	13.24%	71.90936566562502	0.003710480643933249	0.010571535755182448	0.00021307036092134356
ENSG00000124299_33521760_33387344_493	PEPD	19	-	1479	56	3	1	2.84%	22.217514357379315	0.0004467886182089563	0.000464967292513729	1.36460816978523e-06
ENSG00000111331_112938531_112969973_1087	OAS3	12	+	3261	85	6	8	3.50%	15.294797922624198	8.01185275224442e-07	1.569816795456069e-06	1.1244896535745209e-11
ENSG00000141499_7688649_7703486_548	WRAP53	17	+	1644	20	2	0	1.64%	7.138447978485403	0.013494345032394814	0.0035547763465261306	0.00026296295714225076
ENSG00000181929_49005362_49002899_247	PRKAG1	12	-	741	28	0	0	3.64%	22.17253963195952	0.003684287313187465	0.003684287313187465	7.541963121699725e-05
ENSG00000151552_17501836_17487131_138	QDPR	4	-	414	9	0	0	3.62%	12.756096170250004	0.020613416668581838	0.020613416668581838	0.001946208561389314
ENSG00000096092_52677103_52686049_99	TMEM14A	6	+	297	45	6	2	10.10%	88.90612482295457	0.0037834851205969236	0.009257958062480545	0.00018979851107685274
ENSG00000198836_193593378_193648799_569	OPA1	3	+	1707	14	1	0	1.58%	4.8124932241599305	0.005162103456864908	0.00308494966027208	8.839506087016058e-05
ENSG00000198836_193654944_193692127_317	OPA1	3	+	951	15	0	1	2.21%	9.255211732358045	0.00837382380159347	0.011738695074494007	0.000494661429975749
ENSG00000111642_6601370_6570676_1674	CHD4	12	-	5022	21	5	1	0.66%	2.453681581135305	0.02946433998583899	0.001323351237915142	0.00026896173468470523
ENSG00000215301_41343231_41347719_475	DDX3X	X	+	1425	82	1	0	7.58%	33.76561035381264	6.964665585111948e-08	4.0857694212614696e-08	2.8200919065482846e-14
ENSG00000215301_41334253_41342657_148	DDX3X	X	+	444	12	11	3	6.76%	15.858930373824329	0.2253218638402108	0.020489116813065835	0.023933338958737164
ENSG00000137337_30713697_30700465_1910	MDC1	6	-	5730	15	1	1	0.47%	1.5360744079358641	0.00542662340375733	0.00542662340375733	0.00015755438270506302
ENSG00000083312_72848394_72905410_890	TNPO1	5	+	2670	74	4	3	3.26%	16.262828226041577	8.601389492772949e-06	5.945460507496746e-06	4.224228075481936e-10
ENSG00000198925_219228024_219220447_839	ATG9A	2	-	2517	9	0	0	0.95%	2.098142159111443	0.0033278027414746884	0.0033278027414746884	6.211806171561044e-05
ENSG00000131732_81301590_81312662_170	ZCCHC9	5	+	510	8	0	0	2.94%	9.204398805200002	0.019216965118390855	0.019216965118390855	0.0017073955890589052
ENSG00000168701_67227219_67228635_100	TMEM208	16	+	300	21	0	0	9.00%	41.074629668205006	0.0036581908865286695	0.0036581908865286695	7.440475834640714e-05
ENSG00000140365_75336090_75340005_113	COMMD4	15	+	339	40	0	3	8.85%	69.23662818070798	0.002418182715931848	0.010146833869036963	0.00013977670892684782
ENSG00000136868_113258732_113260473_110	SLC31A1	9	+	330	10	0	0	6.36%	17.781224964590912	0.007593099278532274	0.007593099278532274	0.00029783286705462064
ENSG00000063177_48617357_48615372_136	RPL18	19	-	408	229	2	9	24.26%	329.3448947485626	2.74637929582453e-07	5.1994055681244e-06	1.3804057535571269e-11
ENSG00000138674_82881936_82863297_509	SEC31A	4	-	1527	12	0	0	1.38%	4.611241051721023	0.006937202941512724	0.006937202941512724	0.00025101522741487095
ENSG00000115414_215379132_215361555_573	FN1	2	-	1719	30	3	6	2.44%	10.240496052905762	0.0006629387121614735	0.005613246330330595	2.422757178037215e-05
ENSG00000064419_129018073_128957255_855	TNPO3	7	-	2565	29	0	0	1.75%	6.634164635911698	0.0002582331582969843	0.0002582331582969843	4.5481323920964005e-07
ENSG00000127054_1324608_1313787_278	INTS11	1	-	834	8	0	2	2.16%	5.6285891974244615	0.011570465654371866	0.08266357711566169	0.004841719827405853
ENSG00000136518_179570091_179588010_387	ACTL6A	3	+	1161	46	4	1	4.91%	23.248836775408275	0.00041632727507850433	7.746725325434995e-05	2.3527920765168633e-07

ENSG00000005700_82220607_82171425_943	IBTK	6	-	2829	25	0	9	1.38%	5.185404947256099	0.0005440261073422468	0.03224050198770741	0.00014888093047244966
ENSG00000072110_68877156_68874859_77	ACTN1	14	-	231	14	1	1	9.09%	35.562449929181824	0.015516259750072458	0.015516259750072458	0.0011443157696259844
ENSG00000163808_44761866_44852735_1388	KIF15	3	+	4164	6	0	0	0.36%	0.8455049334747841	0.016947426762344633	0.016947426762344633	0.0013498980316300959
ENSG00000101294_31514552_31569219_426	HM13	20	+	1278	238	16	4	10.09%	109.27522759929346	3.699605453423625e-07	7.65382553879486e-09	3.155645051514749e-14
ENSG00000021574_32063832_32154496_615	SPAST	2	+	1845	12	3	0	1.14%	3.8164580411804887	0.051745348319195604	0.008175610787902942	0.002192614907007965
ENSG00000204217_202377475_202559702_530	BMPR2	2	+	1590	9	0	1	0.94%	3.3213986254613217	0.019679754444124863	0.04223451612662591	0.003715148010809822
ENSG00000187109_76067450_76049738_326	NAP1L1	12	-	978	89	11	3	6.44%	53.39821852863345	0.00024281149894461168	3.377290424524866e-05	6.304346886768158e-08
ENSG00000051128_18938982_18929443_361	HOMER3	19	-	1083	13	0	2	1.39%	7.043532326693907	0.020613416668581838	0.056923149003329024	0.005212417464661139
ENSG00000106070_50626921_50592952_407	GRB10	7	-	1221	12	0	0	1.47%	5.766883772299756	0.013217226988443315	0.013217226988443315	0.0008469697265466234
ENSG00000145293_82430830_82460120_261	ENOPH1	4	+	783	21	1	0	2.68%	15.73740600314368	0.012088529595134127	0.00877616296920843	0.0005313814658942758
ENSG00000104388_60584796_60620769_98	RAB2A	8	+	294	17	0	1	9.18%	33.92948028957654	0.002943990679779778	0.0064006895615519015	0.00010465593861806192
ENSG00000198818_166328344_166324533_55	SFT2D1	6	-	165	14	2	0	9.09%	49.787429900854555	0.028915638670995947	0.016947426762344633	0.002246052661730742
ENSG00000140157_22860765_22866847_219	NIPA2	15	+	657	32	2	0	4.57%	28.57986843623745	0.0062201494882096935	0.002460156992221257	8.636895811385565e-05
ENSG00000162368_47333946_47376745_228	CMPK1	1	+	684	21	0	0	3.07%	18.01518845096711	0.00797940233269714	0.00797940233269714	0.000327142761693177
ENSG00000131778_147275365_147295509_470	CHD1L	1	+	1410	7	0	1	1.28%	2.9130943027095753	0.009815328628645334	0.0477903522728147	0.0023388674905236288
ENSG00000204370_112086908_112094893_85	SDHD	11	+	255	15	0	1	5.88%	34.516495519500005	0.019679754444124863	0.022004978195124387	0.0019818329449194675
ENSG00000132603_69339863_69341652_122	NIP7	16	+	366	23	2	0	7.38%	36.874179639684435	0.009067227940747451	0.003684287313187465	0.00018148670210371202
ENSG00000132603_69339863_69341652_169	NIP7	16	+	507	31	2	0	8.28%	35.87809297589054	0.0013761736105699968	0.0004284130286064981	3.82979492686478e-06
ENSG00000132604_69385430_69356898_397	TERF2	16	-	1191	14	0	0	1.26%	6.897502882989926	0.02108409854857798	0.02108409854857798	0.002029891263157455
ENSG00000134759_36129934_36139675_152	ELP2	18	+	456	13	0	0	4.61%	16.72838927589803	0.0078820576498062	0.0078820576498062	0.00031963642907451164
ENSG00000182809_105474863_105479653_280	CRIP2	14	+	840	15	2	1	2.50%	10.478221854133931	0.0237184464124327	0.011738695074494007	0.0013333408968070467
ENSG00000092841_56158402_56161415_151	MYL6	12	+	453	1273	19	25	36.42%	1648.943663438189	7.453965936112657e-11	2.4974067551776686e-09	2.2645985689122854e-18
ENSG00000100162_41947076_41946413_46	CENPM	22	-	138	17	3	1	15.22%	72.28454496475001	0.016503128830616266	0.009965599646782678	0.0008067722732487133
ENSG00000160299_46363711_46445327_2541	PCNT	21	+	7623	19	0	0	0.39%	1.4625249970875642	0.00228120298834518	0.00228120298834518	3.0194288573932866e-05
ENSG00000106105_30595084_30633860_685	GARS	7	+	2055	119	5	1	4.82%	33.97901237759052	2.4437528916854315e-06	2.420944153824251e-07	5.6197751167676765e-12
ENSG00000168003_62882768_62884024_55	SLC3A2	11	+	165	58	10	0	14.55%	206.26220958925458	0.024275373211574098	0.00575691403842271	0.0007327513147156588
ENSG00000008838_40035741_40019529_887	MED24	17	-	2661	14	2	1	1.01%	3.0871574346640367	0.01288881088065249	0.0064006895615519015	0.0004239112468696499
ENSG00000169241_155136321_155138281_187	SLC50A1	1	+	561	11	0	1	4.81%	11.505498506500002	0.002478942540635376	0.00646704663262422	9.042414316359671e-05
ENSG00000163875_37514740_37496592_179	MEAF6	1	-	537	13	0	0	4.47%	14.205112681209501	0.004826937407846361	0.004826937407846361	0.00012612451166366307
ENSG00000069849_141903710_141925701_69	ATP1B3	3	+	207	25	0	0	7.25%	70.86720094583335	0.02108409854857798	0.02108409854857798	0.002029891263157455
ENSG00000129691_38107048_38139071_534	ASH2L	8	+	1602	24	0	0	2.06%	8.790717960022475	0.0014055202957965589	0.0014055202957965589	1.1935530040246081e-05
ENSG00000131844_71604445_71641095_125	MCCC2	5	+	375	14	0	0	4.00%	21.906469156376005	0.019679754444124863	0.019679754444124863	0.0017849636115922674
ENSG00000091947_44014922_44011928_247	TMEM101	17	-	741	11	0	0	2.43%	8.710640569698382	0.011772051206276392	0.011772051206276392	0.000681272511389508
ENSG00000213614_72356657_72355449_102	HEXA	15	-	306	18	0	0	6.86%	34.516495519500005	0.00847367416878415	0.00847367416878415	0.0003665024264111182
ENSG00000213614_72351228_72346299_198	HEXA	15	-	594	44	0	3	8.08%	43.46521658011112	0.00020239236636571483	0.00046103896994722474	6.367612280593842e-07
ENSG00000135686_84650871_84662133_615	KLHL36	16	+	1845	9	0	0	0.81%	2.8623435308853664	0.020613416668581838	0.020613416668581838	0.001946208561389314
ENSG00000067225_72206868_72206398_156	PKM	15	-	468	269	2	5	8.33%	337.27336327066996	0.001043499966109752	0.002077244288437711	1.3177795541922734e-05
ENSG00000110801_121888857_121903022_85	PSMD9	12	+	255	23	4	3	7.06%	52.92529312990001	0.03248889098803485	0.037178613843887286	0.005134009427672865
ENSG00000111674_6917077_6922800_341	ENO2	12	+	1023	22	0	1	2.93%	12.618933845838713	0.0023218226288164415	0.0030685733490663438	4.084557731917468e-05
ENSG00000167965_2206086_2208877_326	MLST8	16	+	978	19	0	4	3.37%	11.39961968588804	0.0013775385517804596	0.01572186727294618	0.00013725983080102293
ENSG00000179818_70088003_70051267_110	PCBP1-AS1	2	-	330	24	0	7	9.09%	42.67493991501819	0.0023218226288164415	0.023814920846593857	0.0003342402705462041
ENSG00000132763_45511087_45511437_116	MMACHC	1	+	348	34	1	20	5.17%	57.32912186859484	0.013856139512579892	0.18077353572602145	0.013841187707810836
ENSG00000197448_143263514_143264196_60	GSTK1	7	+	180	15	2	9	13.33%	48.89836865262501	0.02682370818175567	0.20526787258486068	0.025803417298024496
ENSG00000051180_40706201_40731123_197	RAD51	15	+	591	13	3	0	2.54%	12.90718360373858	0.08350359792828288	0.019216965118390855	0.0073235312725521175
ENSG00000164961_125073174_125024617_783	WASHC5	8	-	2349	10	1	3	0.89%	2.4980009528799494	0.015191410988288745	0.07708712836172119	0.005566177142988587
ENSG00000106976_128203486_128254714_859	DNM1	9	+	2577	53	2	7	2.21%	12.068049073756114	7.448194265828549e-05	0.001199678750155866	6.877941490576349e-07
ENSG00000102054_16858763_16853678_122	RBBP7	X	-	366	17	0	0	4.92%	27.254828429331972	0.013428347753762199	0.013428347753762199	0.0008725593497644525
ENSG00000089685_78214317_78222843_78	BIRC5	17	+	234	33	7	0	10.26%	82.75108541213463	0.01882253325647002	0.005655335537214311	0.0005552396394223762
ENSG00000169592_29996311_29996621_51	INO80E	16	+	153	19	2	0	9.80%	72.86815720783335	0.02108409854857798	0.019679754444124863	0.0019038513263291816
ENSG00000147874_19096765_19056343_911	HAUS6	9	-	2733	10	0	0	0.55%	2.147019479807904	0.019679754444124863	0.019679754444124863	0.0017849636115922674
ENSG00000143545_153986236_153982099_203	RAB13	1	-	609	33	1	0	5.42%	31.79598355737193	0.0018842494102203086	0.0014915192737735051	1.6757239882151976e-05
ENSG00000115204_27312694_27309912_88	MPV17	2	-	264	12	0	1	7.95%	26.671837446886368	0.007029302661868638	0.016854695534768073	0.0006017499134389055
ENSG00000103502_29862857_29859275_157	CDIPT	16	-	471	20	0	8	6.37%	24.916366192420387	0.002046028879997488	0.07219470529229986	0.0010985556915534824
ENSG00000141994_5791141_5785203_650	DUS3L	19	-	1950	14	3	0	1.38%	4.2127825300723085	0.031072488949796268	0.0032294771334464936	0.0005877479115185361
ENSG00000151292_123545664_123614396_423	CSNK1G3	5	+	1269	16	0	0	2.13%	7.398334737040191	0.002943990679779778	0.002943990679779778	4.916404313974209e-05
ENSG00000163466_218249839_218253977_85	ARPC2	2	+	255	15	1	1	5.88%	34.516495519500005	0.04223451612662591	0.04223451612662591	0.007344095972923886
ENSG00000213145_105487260_105488511_77	CRIP1	14	+	231	250	20	12	28.57%	635.0437487353897	4.829947205692244e-05	3.108342685307635e-05	1.1448317702416725e-08
ENSG00000135317_85593697_85505967_930	SNX14	6	-	2790	19	0	2	0.86%	3.995995717848926	0.005604866691947602	0.012088529595134127	0.00035289342940057376
ENSG00000155380_112929308_112913891_500	SLC16A1	1	-	1500	81	2	0	6.00%	31.686142886901006	1.2426114377822172e-06	6.947723824150777e-07	7.76521840936032e-12
ENSG00000135316_85641439_85614756_623	SYNCRIP	6	-	1869	58	21	4	3.69%	18.20934434576084	0.011939252279154475	0.00011183021767937667	1.2932835787678265e-05
ENSG00000160211_154546155_154532000_561	G6PD	X	-	1683	374	17	12	15.15%	130.39564974033337	7.895570369686585e-15	4.128796370961724e-15	4.57812807587751e-28
ENSG00000184897_129315902_129315261_213	H1FX	3	-	639	19	2	0	5.16%	17.447305246946012	0.006279669515525777	0.0012039422000035016	4.599507779105085e-05
ENSG00000163545_205308700_205303450_500	NUAK2	1	-	1500	12	0	1	1.60%	4.694243390652001	0.004193160480899131	0.010499663139468709	0.0002361360478986167
ENSG00000197451_178204865_178210623_276	HNRNPAB	5	+	828	102	20	7	10.14%	72.28454496475001	0.0005272710709344839	1.3691309628042567e-05	6.391815525073465e-08
ENSG00000105197_39480854_39489820_353	TIMM50	19	+	1059	37	6	0	4.25%	20.501299038494338	0.003261459880928516	0.000261039833413102	6.027546718483403e-06
ENSG00000104964_3062927_3053819_264	TLE5	19	-	792	40	2	14	6.06%	29.635374940984857	0.0006152005395151786	0.017339793977529612	7.884060079193574e-05
ENSG00000104969_2769068_2757343_313	SGTA	19	-	939	28	1	4	2.88%	17.497179837361028	0.005134531980829637	0.019761506805970185	0.0005365874254733387

ENSG00000142227_48329378_48330470_94	EMP3	19	+	282	70	3	0	19.15%	145.65471513547877	0.00012253155467421766	8.424452531401259e-05	7.464675359916606e-08
ENSG00000142227_48326845_48327634_63	EMP3	19	+	189	193	7	0	26.98%	599.1990571401033	0.0003872421465994906	0.00014226219545339003	3.842347183833656e-07
ENSG00000092841_56158402_56160331_145	MYL6	12	+	435	1111	13	19	32.41%	1498.650691670797	1.4817857354790871e-09	1.273329913166416e-08	2.071430596708457e-16
ENSG00000185033_90201594_90228643_832	SEMA4B	15	+	2496	46	0	0	2.28%	10.814062298176685	4.06701403799178e-05	4.06701403799178e-05	1.2548961177492392e-08
ENSG00000186834_45149191_45150270_359	HEXIM1	17	+	1077	92	7	6	9.47%	50.12423304781617	1.8547860674402013e-06	1.3998734553642478e-06	2.2807453618573876e-11
ENSG00000113318_80744512_80875862_584	MSH3	5	+	1752	10	1	2	0.86%	3.349203332371576	0.02784981298332479	0.06556525143482543	0.007750594856566061
ENSG00000119912_92514944_92454444_766	IDE	10	-	2298	17	0	0	1.31%	4.340847347752612	0.0020082573300163734	0.0020082573300163734	2.3658688350241813e-05
ENSG00000131503_140401968_140472312_581	ANKHD1	5	+	1743	17	0	2	1.20%	5.7230448681213435	0.0078820576498062	0.028902805874428627	0.0011492025149225664
ENSG00000133318_63681637_63750202_117	RTN3	11	+	351	60	7	1	7.69%	100.30434595410259	0.02307007353645424	0.003710480643933249	0.0004779201318577655
ENSG00000189159_75148589_75147538_58	JPT1	17	-	174	78	6	7	27.59%	263.0395003382587	0.0012891350587872076	0.0002065469900829085	1.842325861084642e-06
ENSG00000107949_125823558_125836274_314	BCCIP	10	+	942	12	0	0	2.87%	7.474909857726116	0.0025000845279814113	0.0025000845279814113	3.597708735589818e-05
ENSG00000172936_38138779_38141286_270	MYD88	3	+	810	26	1	3	4.81%	18.834927184714818	0.0009723463632442102	0.0039558943403557294	2.3612861239842443e-05
ENSG00000157168_32647829_32763338_485	NRG1	8	+	1455	16	0	0	1.03%	6.452568234573198	0.021557223391537678	0.021557223391537678	0.002115616449879074
ENSG00000157168_32595851_32854547_355	NRG1	8	+	1065	22	4	1	1.97%	12.121285750509863	0.03928927557292159	0.012000726946169103	0.00225701047848129
ENSG00000077782_38424619_38413628_545	FGFR1	8	-	1635	10	0	0	1.65%	3.588871093770643	0.001946208561389314	0.001946208561389314	2.2278545302028032e-05
ENSG00000075239_108139039_108144062_147	ACAT1	11	+	441	14	0	0	3.40%	18.627949962904765	0.02108409854857798	0.02108409854857798	0.002029891263157455
ENSG00000182979_105464517_105470215_284	MTA1	14	+	852	15	2	0	2.11%	10.330641264639087	0.05258119297243839	0.013007239811447113	0.00326646906127711
ENSG00000152404_107429448_107345464_481	CWF19L2	11	-	1443	10	0	0	1.04%	4.066392403544699	0.020613416668581838	0.020613416668581838	0.001946208561389314
ENSG00000005007_18832210_18866163_1129	UPF1	19	+	3387	21	6	5	0.97%	3.638142574686006	0.03337069516002332	0.030355651698054653	0.004362293029214473
ENSG00000115204_27313101_27312392_132	MPV17	2	-	396	14	0	3	5.30%	20.744762458689397	0.00797940233269714	0.058290625655499875	0.0024489077996833817
ENSG00000111684_7018424_6977146_487	LPCAT3	12	-	1461	50	2	6	5.13%	20.081465565759757	9.532324018753114e-06	0.00013574083367285996	1.0846377690522244e-08
ENSG00000138744_75940949_75914904_359	NAAA	4	-	1077	49	0	4	4.18%	26.696602384162958	0.0002820292751200387	0.0018744168113352109	3.5959393606580097e-06
ENSG00000110057_68004043_67999515_185	UNC93B1	11	-	555	9	0	0	3.24%	9.515358224294596	0.012178957859018494	0.012178957859018494	0.0007262454987071801
ENSG00000137522_71982779_71996315_240	RNF121	11	+	720	11	0	1	2.92%	8.96470091964792	0.007029302661868638	0.016854695534768073	0.0006017499134389055
ENSG00000107789_87552209_87552478_89	MINPP1	10	+	267	10	0	0	6.74%	21.976794900056184	0.011772051206276392	0.011772051206276392	0.000681272511389508
ENSG00000163479_156020896_156011768_163	SSR2	1	-	489	443	4	5	18.40%	531.5822653524633	3.113077934529443e-06	2.380619492390147e-06	6.373988624992119e-11
ENSG00000213639_28752125_28799303_327	PPP1CB	2	+	981	155	9	6	11.93%	92.71250325574161	1.530997901648864e-07	4.474563023063923e-07	6.494503469312218e-13
ENSG00000156976_186783611_186789269_407	EIF4A2	3	+	1221	37	2	3	3.44%	17.781224964590912	0.0009306875619951529	0.0019988780011558314	1.1404326938421266e-05
ENSG00000106399_7640418_7637000_121	RPA3	7	-	363	120	0	0	9.09%	193.97699961371904	0.001588175367824666	0.001588175367824666	1.5088866706348812e-05
ENSG00000140299_59682457_59677897_161	BNIP2	15	-	483	16	1	0	4.97%	19.437860830857147	0.008219046535586472	0.005454749182134642	0.00023581017309990104
ENSG00000124067_67957957_67945365_868	SLC12A4	16	-	2604	9	0	0	0.58%	2.028042939509793	0.019216965118390855	0.019216965118390855	0.0017073955890589052
ENSG00000243678_51168317_51171604_85	NME2	17	+	255	182	7	6	22.35%	418.8001456366001	6.802234984171572e-05	0.0001664110458161859	8.244263305021595e-08
ENSG00000108107_55386379_55388332_130	RPL28	19	+	390	485	65	27	22.31%	729.7141168160963	0.0001822311627020114	2.968844876012001e-05	4.166765513448663e-08
ENSG00000100387_40951399_40972488_108	RBX1	22	+	324	47	17	3	14.81%	85.11938246938428	0.011911127421001252	0.0010528062381012057	8.086039590045971e-05
ENSG00000074201_77637714_77619628_237	CLNS1A	11	-	711	56	0	1	7.17%	46.2161796548017	0.00013535602770218042	0.0002552309950560562	2.4206154131009565e-07
ENSG00000205531_2964682_2954521_145	NAP1L4	11	-	435	14	0	0	3.45%	18.88488720377242	0.019679754444124863	0.019679754444124863	0.0017849636115922674
ENSG00000165916_47426231_47424586_120	PSMC3	11	-	360	20	0	0	10.00%	32.59891243508334	0.0006577637618416096	0.0006577637618416096	2.769528725862714e-06
ENSG00000100097_37675703_37677525_30	LGALS1	22	+	90	111	1	7	33.33%	723.6958560588502	0.002390425500591921	0.01991630962237072	0.0002818506926820679
ENSG00000198886_11557_11856_99	MT-ND4	MT	+	297	491	168	110	26.26%	970.0646064015709	0.1225641801982098	0.11413700846922298	0.047087048640504656
ENSG00000132792_37694123_37872013_563	CTNNBL1	20	+	1689	15	0	0	1.60%	5.211193817331262	0.0033278027414746945	0.0033278027414746945	6.211806171561056e-05
ENSG00000185803_144359354_144361015_425	SLC52A2	8	+	1275	20	1	0	2.59%	9.204398805200002	0.0012558331315113088	0.0013775385517804596	1.0509596336390694e-05
ENSG00000129197_5421311_5428078_74	RPAIN	17	+	222	21	2	0	8.11%	55.50625630838515	0.037178613843887286	0.013428347753762199	0.0023492041693324566
ENSG00000121774_32014284_32042624_347	KHDRBS1	1	+	1041	131	1	4	10.09%	73.84076419013114	1.5646548331313916e-07	2.3070473232588066e-07	3.429475132441642e-13
ENSG00000017260_130934676_131001350_823	ATP2C1	3	+	2469	17	3	1	1.22%	4.040205429378494	0.011168917508652896	0.00324779312858951	0.00020011016427610653
ENSG00000141682_59900178_59902753_54	PMAIP1	18	+	162	30	3	0	18.52%	108.6630414502778	0.009067227940747451	0.0024042815392806777	0.00012388997584410576
ENSG00000100316_39315411_39314080_110	RPL3	22	-	330	84	5	3	10.00%	149.36228970256366	0.002484541834108666	0.003305593496988668	4.6790409438600785e-05
ENSG00000188603_28489291_28477516_338	CLN3	16	-	1014	16	0	5	2.07%	9.258862703455623	0.00847367416878415	0.06059069429271301	0.002681660320446393
ENSG00000198954_68989719_69016416_487	KIF1BP	10	+	1461	21	3	0	2.26%	8.434215537619098	0.009920634900645505	0.0012624143894574274	7.753726787359189e-05
ENSG00000188603_28491759_28474155_370	CLN3	16	-	1110	20	2	0	1.89%	10.572620249216218	0.024275373211574098	0.00877616296920843	0.0010556658063702027
ENSG00000198954_68989845_69016416_484	KIF1BP	10	+	1452	21	3	0	2.27%	8.48649373310021	0.009920634900645505	0.0012624143894574274	7.753726787359189e-05
ENSG00000116337_109621108_109631152_804	AMPD2	1	+	2412	7	0	1	0.62%	1.7029282615342043	0.019216965118390855	0.04178282491678763	0.003600525600033436
ENSG00000197535_52528806_52313696_1828	MYO5A	15	-	5484	14	0	1	0.38%	1.4979806589425606	0.0078820576498062	0.015516259750072458	0.0006142571802709984
ENSG00000133316_62839142_62835608_112	WDR74	11	-	336	11	2	3	5.36%	19.21007339924554	0.052264829583131125	0.08503348072695238	0.017088614627780895
ENSG00000017260_130934676_131001350_823	ATP2C1	3	+	2469	17	3	1	1.22%	4.040205429378494	0.011168917508652896	0.00324779312858951	0.00020011016427610653
ENSG00000084754_26232162_26212501_157	HADHA	2	-	471	42	2	0	7.01%	52.32436900408281	0.0013887899417379728	0.0016033688297201391	1.3394466165596107e-05
ENSG00000173418_20026786_20033187_121	NAA20	20	+	363	91	0	0	19.01%	147.09922470707028	1.1471149284826326e-05	1.1471149284826326e-05	1.0622823044333803e-09
ENSG00000129103_56064312_56079871_339	SUMF2	7	+	1017	120	5	9	5.31%	69.23662818070798	0.0006413312588009767	0.0017341039835456417	7.010063428374115e-06
ENSG00000114850_156555089_156543250_174	SSR3	3	-	522	81	2	0	13.79%	91.05213473247416	1.1931380382067736e-05	8.539974513442116e-06	8.285028529936203e-10
ENSG00000130414_240011687_239961118_129	NDUFA10	2	-	387	15	1	1	4.65%	22.743427280290703	0.02034245439037237	0.02034245439037237	0.0018987606724399325
ENSG00000106443_10974324_11169490_948	PHF14	7	+	2844	23	1	3	0.95%	4.745411303841246	0.005670089768331009	0.017322097050858944	0.0005113995283599821
ENSG00000130175_11448275_11449412_142	PRKCSH	19	+	426	22	0	5	5.63%	30.303214376274656	0.005306578019052857	0.027805252533285658	0.0007881853308038498
ENSG00000147526_38757333_38840338_209	TACC1	8	+	627	7	3	0	2.39%	6.550977618533494	0.13974268412556323	0.016947426762344633	0.011764960919844317
ENSG00000134108_5122466_5178713_177	ARL8B	3	+	531	10	6	0	3.39%	11.050478791553674	0.25881705929167276	0.009815328628645334	0.017540961353816963
ENSG00000157601_41432071_41458758_662	MX1	21	+	1986	11	1	0	0.76%	3.250042629479608	0.020613416668581838	0.020613416668581838	0.001946208561389314
ENSG00000154305_222621164_222665619_1861	MIA3	1	+	5583	30	1	10	0.91%	3.1530382795889316	0.0001814611398669728	0.006331237320996696	9.169197580384731e-06
ENSG00000131778_147252712_147294454_816	CHD1L	1	+	2448	19	0	1	1.10%	4.554259825489584	0.002967235347054297	0.01007916461776185	0.000166354917991791
ENSG00000188157_1034625_1054981_1940	AGRN	1	+	5820	104	12	22	2.37%	10.485423381181446	1.0932700426938786e-07	1.3891301681676028e-06	1.4901361836981474e-12

ENSG00000001036_143511634_143495707_467	FUCA2	6	-	1401	67	2	2	6.21%	28.0615905758105	2.0001995930932634e-06	2.0201755697964947e-06	3.515617972362541e-11
ENSG00000104723_15540431_15743570_276	TUSC3	8	+	828	30	0	0	4.35%	21.260160283750004	0.0010405263639310788	0.0010405263639310788	6.697970672456085e-06
ENSG00000163931_53231526_53225899_289	TKT	3	-	867	138	3	46	11.76%	93.39757611158825	4.023956149609838e-07	0.005951965402222071	6.919727886074125e-08
ENSG00000163931_53255942_53230566_334	TKT	3	-	1002	268	48	23	15.27%	156.94326705273656	1.0560155056146695e-06	6.982448313864758e-09	8.832315004565527e-14
ENSG00000139613_56189461_56163689_1130	SMARCC2	12	-	3390	15	1	0	0.53%	2.596373556776549	0.015555382717448078	0.011772051206276392	0.0008874794703752884
ENSG00000163170_74145285_74135522_77	BOLA3	2	-	231	40	0	7	7.79%	101.60699979766235	0.013007239811447113	0.1754079647031212	0.012749674501614912
ENSG00000184924_24793335_24790411_126	PTRHD1	2	-	378	11	0	0	5.56%	17.075620799329368	0.006937202941512724	0.006937202941512724	0.00025101522741487095
ENSG00000008988_56074383_56069738_142	RPS20	8	-	426	83	2	1	7.04%	114.32576332867256	0.002460156992221257	0.00321235842540502	4.509123567224043e-05
ENSG00000138796_108033208_108034357_67	HADH	4	+	201	21	0	0	10.45%	61.30541741523136	0.007029302661868638	0.007029302661868638	0.0002573639808771639
ENSG00000138796_107990044_108027967_208	HADH	4	+	624	9	0	0	2.40%	8.463179189877406	0.019679754444124863	0.019679754444124863	0.0017849636115922674
ENSG00000160131_151397309_151405058_101	VMA21	X	+	303	9	0	0	4.95%	17.42912149994555	0.019216965118390855	0.019216965118390855	0.0017073955890589052
ENSG00000109332_102809680_102797415_110	UBE2D3	4	-	330	19	0	1	5.45%	33.78432743272273	0.013428347753762199	0.013428347753762199	0.0008725593497644525
ENSG00000142676_23692636_23696373_167	RPL11	1	+	501	276	8	3	26.35%	323.2562813922037	3.498557337707287e-09	4.950634444904532e-09	1.8457137986707628e-16
ENSG00000185414_99186204_99195705_161	MRPL30	2	+	483	9	0	0	3.11%	10.933796717357145	0.019679754444124863	0.019679754444124863	0.0017849636115922674
ENSG00000170677_70324669_70326276_535	SOCS6	18	+	1605	16	1	0	1.87%	5.8495244743327115	0.0040705821972625445	0.001801896632038876	4.2571970118849174e-05
ENSG00000248919_99466141_99441444_54	ATP5MF-PTCD1	7	-	162	26	0	0	9.26%	94.1746359235741	0.021557223391537678	0.021557223391537678	0.002115616449879074
ENSG00000115211_27369534_27364400_492	EIF2B4	2	-	1476	51	0	0	3.46%	20.274933343771345	0.0001294826452477558	0.0001294826452477558	1.1929552110054982e-07
ENSG00000049656_1341724_1318369_369	CLPTM1L	5	-	1107	40	4	0	4.34%	21.20254467322494	0.0006730592418038804	0.00019160400119445356	8.84504601064243e-07
ENSG00000117523_171522225_171591847_2553	PRRC2C	1	+	7659	17	10	3	0.35%	1.3024242335990994	0.15039870036150454	0.019224948125267174	0.014067798201048379
ENSG00000198467_35685547_35683159_158	TPM2	9	-	474	66	6	2	10.13%	81.70360331831014	0.001353265381634888	0.0002369862977522709	2.193436607334992e-06
ENSG00000198824_114323843_114326281_812	CHAMP1	13	+	2436	13	1	2	0.86%	3.131422623074508	0.015516259750072458	0.04363948305378087	0.00312766395889963
ENSG00000134324_11771429_11824791_811	LPIN1	2	+	2433	25	11	1	2.34%	6.029391942370531	0.016766006532464923	6.790538910674993e-05	1.3277829615613356e-05
ENSG00000122705_36191057_36211774_230	CLTA	9	+	690	177	11	4	12.61%	150.52193480895002	2.048454080571646e-05	4.711542246598215e-06	8.047163936813439e-10
ENSG00000157193_53271061_53247018_557	LRP8	1	-	1671	15	9	0	1.08%	5.26732875970826	0.2405700776118731	0.012798402692974279	0.018919609472776648
ENSG00000127022_179705682_179708253_61	CANX	5	+	183	97	2	10	31.15%	311.0256891347296	0.00011441425137092887	0.0011873494012083255	9.952192288190206e-07
ENSG00000017260_130964044_130992947_204	ATP2C1	3	+	612	7	0	0	2.45%	6.711540795458335	0.019216965118390855	0.019216965118390855	0.0017073955890589052
ENSG00000113580_143400839_143281889_778	NR3C1	5	-	2334	12	5	2	1.16%	3.0168659322956306	0.07471635411946746	0.014696946993710543	0.005240911805500765
ENSG00000120254_150877652_151092556_869	MTHFD1L	6	+	2607	21	1	1	1.15%	4.726654737422901	0.0037373685375237473	0.0037373685375237473	7.750388117310624e-05
ENSG00000023228_206153678_206124185_727	NDUFS1	2	-	2181	55	1	0	3.44%	14.797305506984184	4.827952539080958e-06	4.925024046154609e-06	1.9941444209025355e-10
ENSG00000112855_140694028_140698552_381	HARS2	5	+	1143	15	1	0	2.36%	7.70053049647638	0.006024333578509648	0.0032294771334464936	0.00010730535955833177
ENSG00000127990_94618900_94585512_245	SGCE	7	-	735	12	0	2	3.27%	9.580088552351022	0.004193160480899131	0.022868511308519305	0.0005251843016725186
ENSG00000127990_94656098_94597920_436	SGCE	7	-	1308	25	3	2	2.52%	11.215222168033259	0.007887772776385514	0.0038108971419065062	0.00016292425881891603
ENSG00000141756_41821026_41822408_137	FKBP10	17	+	411	12	1	0	5.84%	17.13227514836497	0.016854695534768073	0.004193160480899131	0.0003819250574820159
ENSG00000148337_128179348_128166197_556	CIZ1	9	-	1668	17	2	0	1.62%	5.98037602226349	0.018399683928041276	0.002967235347054297	0.00031087262125642094
ENSG00000109390_139295798_139292550_76	NDUFC1	4	-	228	19	2	0	7.89%	48.89836865262501	0.016822681477512567	0.013428347753762199	0.0010798218650012886
ENSG00000124570_2959176_2948298_324	SERPINB6	6	-	972	54	1	2	5.86%	32.59891243508334	6.293293459921025e-05	0.0001311217014807635	6.040197107585146e-08
ENSG00000140990_1959625_1961734_168	NDUFB10	16	+	504	63	5	4	12.50%	73.34755297893751	8.992178401293835e-05	9.019371947824221e-05	5.893183588315603e-08
ENSG00000142892_77219402_77154436_332	PIGK	1	-	996	35	0	0	3.92%	20.619794010143078	0.0006933285739671933	0.0006933285739671933	3.065417257202339e-06
ENSG00000118257_205683297_205743579_553	NRP2	2	+	1659	35	6	1	3.25%	12.379333836107598	0.0010988015585354652	0.00011194167591548285	8.995106762614531e-07
ENSG00000162909_223771817_223772269_65	CAPN2	1	+	195	14	0	0	7.69%	42.12782530072309	0.020613416668581838	0.020613416668581838	0.001946208561389314
ENSG00000143164_167993255_168075435_712	DCAF6	1	+	2136	20	0	2	1.54%	5.494198725014046	0.0014409783181973444	0.003092538603222271	2.637025864246733e-05
ENSG00000143164_167993255_168075435_641	DCAF6	1	+	1923	20	0	2	1.72%	6.102760518268332	0.0014409783181973444	0.003092538603222271	2.637025864246733e-05
ENSG00000143158_167935775_167918323_105	MPC2	1	-	315	27	0	0	8.57%	50.29546489984287	0.003684287313187465	0.003684287313187465	7.541963121699725e-05
ENSG00000077150_102395960_102402376_900	NFKB2	10	+	2700	23	0	0	1.00%	4.998499906712779	0.0035547763465261306	0.0035547763465261306	7.044676146010672e-05
ENSG00000257923_101816052_102283090_669	CUX1	7	+	2007	11	8	0	1.20%	3.2160362043579975	0.18176997233392067	0.004687384229717438	0.006576065488566696
ENSG00000091640_4967804_4959534_227	SPAG7	17	-	681	25	1	1	4.41%	21.541131565033044	0.0035004709947243163	0.0035004709947243163	6.84091658136962e-05
ENSG00000161813_50401011_50475864_653	LARP4	12	+	1959	22	3	1	1.84%	6.589672957781012	0.007352086581507323	0.001621945894645527	7.047187728606626e-05
ENSG00000072778_7220628_7225097_579	ACADVL	17	+	1737	127	5	10	6.91%	42.90219563995424	8.178541174624566e-08	4.029888968772649e-07	3.201061380776908e-13
ENSG00000170291_7254674_7258930_170	ELP5	17	+	510	10	0	0	2.94%	11.505498506500002	0.019679754444124863	0.019679754444124863	0.0017849636115922674
ENSG00000117335_207752282_207793595_326	CD46	1	+	978	36	2	1	5.83%	21.599279404840495	0.00024258085037912188	7.797505503656233e-05	1.3677180114845038e-07
ENSG00000089195_5950405_5938535_497	TRMT6	20	-	1491	39	0	0	2.21%	15.348381307463786	0.0014055202957965589	0.0014055202957965589	1.1935530040246081e-05
ENSG00000168385_241324233_241336177_139	SEPT2	2	+	417	20	0	0	7.19%	28.14294598712231	0.002033393162560146	0.002033393162560146	2.4228963598721857e-05
ENSG00000197713_210002737_210019791_203	RPE	2	+	609	16	0	1	3.94%	15.416234452059117	0.005257622967929454	0.006923060740356555	0.00019303953989409622
ENSG00000135698_82170175_82148731_160	MPHOSPH6	16	-	480	6	0	3	3.12%	7.334755297893752	0.016947426762344633	0.21450858727089922	0.01973597174099189
ENSG00000221968_61891381_61873814_417	FADS3	11	-	1251	13	0	0	1.20%	6.0976382972098335	0.02108409854857798	0.02108409854857798	0.002029891263157455
ENSG00000134809_57530189_57528717_90	TIMM10	11	-	270	118	8	13	24.44%	256.4447778226556	2.785638855085842e-05	0.0004849677896341693	1.0937705086186082e-07
ENSG00000188342_45120656_45283561_249	GTF2F2	13	+	747	17	1	5	3.21%	13.353771358949801	0.009288069496478249	0.054588706672672294	0.0025774222633569393
ENSG00000047315_56994753_57030988_1020	POLR2B	4	+	3060	29	5	0	1.67%	5.560990944808335	0.0021301889646226013	0.00010991304945921688	1.7944698229304764e-06
ENSG00000196497_24187138_24186203_135	IPO4	14	-	405	10	0	0	3.70%	14.488405526703707	0.020613416668581838	0.020613416668581838	0.001946208561389314
ENSG00000196497_24186173_24180442_743	IPO4	14	-	2229	27	5	5	1.75%	7.107703653409827	0.0026136527696882916	0.007309596406889314	0.00010726947524524964
ENSG00000243989_51986441_51989075_254	ACY1	3	+	762	7	1	0	1.97%	5.390371347533466	0.0477903522728147	0.016947426762344633	0.0036975118224100542
ENSG00000137693_102110849_102229940_454	YAP1	11	+	1362	17	2	4	2.20%	7.323984732111235	0.010350136863208615	0.018344283626974538	0.0009255963303532005
ENSG00000065518_120596360_120601293_120	NDUFB4	3	+	360	77	2	3	10.83%	125.50581287507086	0.0011431200524508536	0.0017109824466264843	1.1861774089250169e-05
ENSG00000160633_5623206_5668291_848	SAFB	19	+	2544	31	0	6	1.89%	7.150233152034789	0.00017744757238953088	0.003366733226746175	4.465936000960435e-06
ENSG00000158716_159781101_159782338_150	DUSP23	1	+	450	22	0	1	7.33%	28.68704294287334	0.0014055202957965589	0.002493493840886608	2.0830049893778164e-05
ENSG00000158716_159781101_159782338_150	DUSP23	1	+	450	22	0	1	7.33%	28.68704294287334	0.0014055202957965589	0.002493493840886608	2.0830049893778164e-05
ENSG00000014123_96523264_96553503_729	UFL1	6	+	2187	11	0	0	1.10%	2.951341866550755	0.004193160480899131	0.004193160480899131	9.649553577281792e-05

ENSG00000126088_45012266_45015358_298	UROD	1	+	894	21	0	1	4.03%	13.783432774565439	0.0009313263635146231	0.0015450469930285433	8.849983904714244e-06
ENSG00000198408_101813583_101786451_842	OGA	10	-	2526	32	2	0	1.31%	7.4334812203515455	0.005641908870879185	0.00151350169064872	5.049205084370289e-05
ENSG00000104763_18084058_18057534_376	ASAH1	8	-	1128	106	3	2	7.18%	55.14071358700267	2.1209243094296095e-06	8.259411896664993e-06	1.5086207056338041e-10
ENSG00000188603_28489291_28477516_338	CLN3	16	-	1014	16	0	5	2.07%	9.258862703455623	0.00847367416878415	0.06059069429271301	0.002681660320446393
ENSG00000103363_2777115_2771990_136	ELOB	16	-	408	68	3	2	15.44%	97.79673730525002	0.00013792218304021245	5.3584965685641065e-05	5.45427244474405e-08
ENSG00000132507_7309636_7311640_154	EIF5A	17	+	462	179	71	8	11.69%	227.34566204726954	0.007938263751100936	9.685105774909149e-05	7.0979032624909975e-06
ENSG00000102974_67611577_67637872_479	CTCF	16	+	1437	8	0	0	1.25%	3.266696861970773	0.011570465654371866	0.011570465654371866	0.0006594843062896773
ENSG00000168036_41224069_41238381_715	CTNNB1	3	+	2145	102	8	3	5.73%	27.902845329050358	3.848110861563986e-07	5.3975231483360274e-08	2.0549670857037851e-13
ENSG00000177830_904816_869858_362	CHID1	11	-	1086	14	0	3	1.93%	7.564388520848068	0.007029302661868638	0.04912109528954385	0.0018325026027891263
ENSG00000088833_1466824_1463429_71	NSFL1C	20	-	213	13	0	2	8.45%	35.8128897174155	0.012798402692974279	0.061356297364084836	0.0037940651817215715
ENSG00000104687_30727706_30679520_479	GSR	8	-	1437	50	3	6	5.22%	20.416855387317334	4.511463656377517e-05	0.00010930560731824569	3.673773685804692e-08
ENSG00000026103_88990877_89014450_335	FAS	10	+	1005	18	0	1	2.09%	10.509500128325376	0.00847367416878415	0.011825808327677989	0.0005036791746826746
ENSG00000103253_727510_729092_227	HAGHL	16	+	681	8	3	0	2.64%	6.893162100810574	0.11368416502207135	0.011570465654371866	0.006956434060967934
ENSG00000030582_44350711_44352652_368	GRN	17	+	1104	186	5	4	9.78%	98.85974531943752	7.33487071200475e-08	4.06092426189075e-07	2.907242231473992e-13
ENSG00000185591_53381682_53411240_775	SP1	12	+	2325	11	0	0	1.16%	2.7761654460845167	0.002478942540635376	0.002478942540635376	3.5397643206479886e-05
ENSG00000113851_3179687_3150865_441	CRBN	3	-	1323	10	0	3	1.36%	4.4352261816439915	0.011570465654371866	0.08266357711566169	0.0048417927405853
ENSG00000171617_74636266_74634716_516	ENC1	5	-	1548	29	1	0	2.52%	10.992656518807173	0.0009958682880916293	0.0006424109906750971	4.052020686628797e-06
ENSG00000108587_30477434_30522378_250	GOSR1	17	+	750	17	2	4	3.60%	13.300356273514003	0.0065000503081102355	0.026717773946956125	0.0008981863076292358
ENSG00000108588_63766175_63746881_483	CCDC47	17	-	1449	82	1	7	8.07%	33.206345586047625	2.116266785070952e-08	1.1067054278810206e-06	2.5573258824620425e-13
ENSG00000108591_18093920_18107240_307	DRG2	17	+	921	30	1	1	2.93%	19.113368854446257	0.0033030736763512156	0.003584867146401604	6.622403703300922e-05
ENSG00000108679_78976126_78971576_459	LGALS3BP	17	-	1377	97	3	12	9.59%	41.33456870853705	1.3816146586039891e-08	2.733282372227404e-07	3.992408195087321e-14
ENSG00000101266_489860_483961_177	CSNK2A1	20	-	531	9	0	0	2.82%	9.945430912398308	0.019216965118390855	0.019216965118390855	0.0017073955890589052
ENSG00000134313_8796836_8730720_1370	KIDINS220	2	-	4110	13	0	0	0.80%	1.8559964744062047	0.0008831683242050062	0.0008831683242050062	4.885807922518899e-06
ENSG00000059378_140056973_140024560_487	PARP12	7	-	1461	21	2	1	1.64%	8.434215537619098	0.018151375140008384	0.009288069496478249	0.0008309403832967919
ENSG00000116213_3649999_3630975_460	WRAP73	1	-	1380	15	0	2	1.96%	6.378048085125002	0.002967235347054297	0.018399683928041276	0.00031087262125642094
ENSG00000058272_79934925_79775929_1028	PPP1R12A	12	-	3084	25	0	2	1.26%	4.756650647142511	0.0005379818162649481	0.0019648567937019914	6.767654423560422e-06
ENSG00000077232_182756443_182777132_199	DNAJC10	2	+	597	13	0	0	3.02%	12.777463165510053	0.013428347753762199	0.013428347753762199	0.0008725593497644525
ENSG00000137776_58894166_58879999_566	SLTM	15	-	1698	30	0	0	2.12%	10.367145297376327	0.0009430837296224684	0.0009430837296224684	5.543685104868835e-06
ENSG00000005893_120469169_120431323_410	LAMP2	X	-	1230	21	0	0	3.17%	10.018202358098783	0.0005399915898224664	0.0005399915898224664	1.8929313262929258e-06
ENSG00000166598_103942563_103943415_191	HSP90B1	12	+	573	120	8	2	16.75%	122.88595263486914	6.075679933676445e-07	8.217338412454136e-07	4.525957597950004e-12
ENSG00000131165_89657588_89647038_181	CHMP1A	16	-	543	12	1	0	3.31%	12.967523178596688	0.02015986069794795	0.013217226988443315	0.0012659333861537747
ENSG00000166226_69585522_69588272_151	CCT2	12	+	453	80	10	4	12.58%	103.62568191284771	7.348101949952925e-05	0.0002850788918386375	1.52309554871177e-07
ENSG00000164654_7572476_7607092_875	MIOS	7	+	2625	8	0	0	0.69%	1.7882831964388575	0.011570465654371866	0.011570465654371866	0.0006594843062896773
ENSG00000129484_20352324_20357797_378	PARP2	14	+	1134	15	0	2	1.85%	7.761645817876986	0.00847367416878415	0.027657845067597595	0.0011690047180828366
ENSG00000125971_34516775_34540624_129	DYNLRB1	20	+	387	80	2	4	8.53%	121.29827882821708	0.0018115419044284426	0.003777991055085985	3.972844003097169e-05
ENSG00000113845_119498734_119523756_151	TIMMDC1	3	+	453	23	1	0	5.30%	29.792383549943715	0.005454749182134642	0.005454749182134642	0.00015910859015753364
ENSG00000198805_20469525_20472569_90	PNP	14	+	270	70	5	0	11.11%	152.12825803038893	0.002460156992221257	0.0025025372014526115	3.546108526767075e-05
ENSG00000141349_44070966_44076043_346	G6PC3	17	+	1038	76	1	0	7.23%	42.96272852716186	4.532898578170701e-06	4.759567921528711e-06	1.8131909399685636e-10
ENSG00000087191_63828138_63831969_398	PSMC5	17	+	1194	144	14	3	11.56%	70.76748830128645	1.634647098621326e-07	2.0668185553905446e-09	3.978615250319518e-15
ENSG00000139163_22625164_22684954_452	ETNK1	12	+	1356	19	2	0	2.43%	8.221849596459073	0.0054893863033663335	0.0010860768436549261	3.651973182960827e-05
ENSG00000130726_58550255_58550553_68	TRIM28	19	+	204	22	2	0	7.35%	63.280241785750015	0.06945717695039447	0.020613416668581838	0.006391023138248565
ENSG00000129667_76477283_76473001_365	RHBDF2	17	-	1095	10	0	1	1.92%	5.358725331794521	0.007029302661868638	0.016854695534768073	0.0006017499134389055
ENSG00000196704_68250296_68256969_324	AMZ2	17	+	972	29	0	0	3.09%	17.50682334476698	0.0009430837296224684	0.0009430837296224684	5.543685104868835e-06
ENSG00000105220_34399246_34399512_88	GPI	19	+	264	42	0	1	5.68%	93.3514310641023	0.021557223391537678	0.023199730935452297	0.002265826071202869
ENSG00000133112_45339619_45337386_80	TPT1	13	-	240	41	6	5	7.50%	100.24165573788127	0.013640585738808999	0.05742480463164306	0.003726900636670136
ENSG00000130429_99385715_99390016_167	ARPC1B	7	+	501	65	0	5	11.98%	76.12919670468564	4.0937141508087716e-05	0.0002163581746594846	6.685263786949392e-08
ENSG00000171793_40983291_41009593_564	CTPS1	1	+	1692	23	3	2	2.13%	7.976329638371456	0.006549171093867908	0.0039559866451434845	0.00014043786861695157
ENSG00000140443_98649582_98957442_1366	IGF1R	15	+	4098	43	3	4	1.17%	6.157042026538435	0.0006394447605033927	0.0007933619615132744	3.23158037643184e-06
ENSG00000168411_74661449_74623928_774	RFWD3	16	-	2322	38	5	2	1.68%	9.602780407233853	0.005522028406875141	0.0016517825634750807	5.343542046647286e-05
ENSG00000173193_122700559_122720490_1012	PARP14	3	+	3036	14	5	0	0.59%	2.7058385815681825	0.14502352150466868	0.013428347753762199	0.010350889218535215
ENSG00000115286_1383927_1393407_206	NDUFS7	19	+	618	34	3	0	6.31%	32.2824181395971	0.0017439832781934702	0.0006026251277497241	6.6568752911666554e-06
ENSG00000273088_155169547_155169440_35	AL713999.1	1	-	105	12	0	0	14.29%	67.06061986645716	0.020613416668581838	0.020613416668581838	0.001946208561389314
ENSG00000215301_41334253_41347719_662	DDX3X	X	+	1986	97	12	3	7.25%	28.659466823592908	1.8411069105942975e-07	2.168363292173117e-09	4.7152014957107245e-15
ENSG00000138073_27134421_27131414_359	PREB	2	-	1077	7	0	1	1.39%	3.8138003405947085	0.016947426762344633	0.0477903522728147	0.0036975118224100542
ENSG00000173852_35037394_34931573_748	DPY19L1	7	-	2244	34	4	0	2.14%	8.890612482295456	0.002091891133506428	0.00017249046415619826	2.6090196998376473e-06
ENSG00000175334_66003293_66003772_75	BANF1	11	+	225	197	14	8	18.67%	513.7588599769134	0.00048652826472334255	0.0008793782949311993	2.757750418467631e-06
ENSG00000142634_15425905_15428720_162	EFHD2	1	+	486	27	0	0	4.94%	32.59891243508334	0.0053557511377634245	0.0053557511377634245	0.00015366972994913032
ENSG00000157168_32595851_32763338_421	NRG1	8	+	1263	29	0	1	2.14%	13.473184711887177	0.003736770770438521	0.0045126225279669635	9.281320660291148e-05
ENSG00000124762_36684102_36685800_164	CDKN1A	6	+	492	68	2	8	7.32%	81.09973337508538	0.004245579444686589	0.007076478868397069	0.00016165526835226757
ENSG00000198522_27629137_27650200_348	GPN1	2	+	1044	26	0	0	2.87%	14.613305574347704	0.0024042815392806777	0.0024042815392806777	3.3387040459710164e-05
ENSG00000147853_4722566_4712976_157	AK3	9	-	471	16	0	0	4.46%	19.93309295393631	0.00847367416878415	0.00847367416878415	0.0003665024264111182
ENSG00000188690_125816499_125796854_160	UROS	10	-	480	11	1	0	3.75%	13.447051379471878	0.023672501042627626	0.012798402692974279	0.0014375227604752335
ENSG00000023734_15883552_15902978_309	STRAP	12	+	927	94	3	0	8.09%	59.50092755141425	1.1542166166670309e-05	5.759770332045563e-06	5.481214356828984e-10
ENSG00000100983_34951852_34928828_474	GSS	20	-	1422	38	0	0	3.38%	15.68048952573629	0.00018810075197750367	0.00018810075197750367	2.4616022383928534e-07
ENSG00000197102_102039237_102048175_640	DYNC1H1	14	+	1920	24	1	5	1.88%	7.334755297893752	0.0015356410015604012	0.013890748332252284	0.00013148498491260117
ENSG00000157193_53327912_53247018_790	LRP8	1	-	2370	11	9	0	0.76%	2.7234534439436717	0.3362635552170612	0.012798402692974279	0.03023589517231469
ENSG00000198363_61684117_61680939_58	ASPH	8	-	174	91	6	2	24.14%	306.8794170613018	0.002573466062604956	0.0007519736033842511	1.2076766394570286e-05

ENSG00000107951_30365333_30313609_712	MTPAP	10	−	2136	16	6	1	1.40%	4.395358980011237	0.04689162522292638	0.003957931943710776	0.0010965012363251278
ENSG00000101365_2664188_2660613_204	IDH3B	20	−	612	43	3	0	7.84%	41.228036314958345	0.00035600579356901785	0.00020239236636571483	4.92801331147905e-07
ENSG00000101365_2660659_2658751_231	IDH3B	20	−	693	38	1	0	6.06%	32.17554993592641	0.0005993996978725321	0.0004481211664877703	1.7513886233350606e-06
ENSG00000140043_73860574_73884177_327	PTGR2	14	+	981	10	4	0	1.83%	5.981451822951072	0.11368416502207135	0.011570465654371866	0.006956434060967934
ENSG00000149554_125629246_125655320_375	CHEK1	11	+	1125	12	0	0	1.33%	6.258991187536002	0.02108409854857798	0.02108409854857798	0.002029891263157455
ENSG00000127990_94618900_94585465_278	SGCE	7	−	834	11	0	2	2.52%	7.739310146458635	0.007029302661868638	0.03522780890193623	0.0012822660609675817
ENSG00000001617_50182319_50188115_559	SEMA3F	3	+	1677	20	3	0	1.61%	6.9979776247048315	0.009167859863837249	0.003229477133446936	0.00016299320232631272
ENSG00000114503_196941958_196937011_138	NCBP2	3	−	414	20	2	0	8.70%	28.34688037833334	0.002549573717490699	0.0006577637618416096	1.0592243560232986e-05
ENSG00000111057_52949174_52950414_167	KRT18	12	+	501	104	2	5	7.19%	121.80671472749704	0.001153855561434436	0.0010615893185374377	7.542842681441396e-06
ENSG00000111057_52950769_52952346_218	KRT18	12	+	654	909	29	96	11.93%	815.5709560593787	9.403148010757479e-06	9.650364460230358e-06	7.388356734108364e-10
ENSG00000065357_55938942_55953768_593	DGKA	12	+	1779	5	1	0	0.84%	1.6491861265640813	0.051235217429874705	0.012673659338734126	0.0031110695322510533
ENSG00000168036_41224552_41239342_746	CTNNB1	3	+	2238	110	8	0	5.76%	28.840860867500005	1.3783924859146478e-07	4.229699917184914e-09	6.4743380738092134e-15
ENSG00000177189_20187899_20155398_506	RPS6KA3	X	−	1518	20	2	0	2.37%	7.730967375909093	0.004498799248433914	0.0008852284070317903	2.4782952419168947e-05
ENSG00000143878_20447466_20448056_196	RHOB	2	+	588	29	4	4	6.12%	28.93985083522705	0.01031091966776115	0.007509295436624617	0.0003947190774922549
ENSG00000042753_46846085_46838447_122	AP2S1	19	−	366	43	7	1	10.66%	68.93868367419263	0.00598105518663113	0.0008767407814908394	3.307224816267442e-05
ENSG00000198431_104309938_104348415_563	TXNRD1	12	+	1689	254	16	13	10.30%	88.24288197347605	2.687490931222515e-09	2.244971880808802e-09	6.516440818400921e-17
ENSG00000127990_94656098_94587779_446	SGCE	7	−	1338	25	3	2	2.47%	10.963759787584083	0.007887772776385514	0.0038108971419065062	0.00016292425881891603
ENSG00000090013_40465688_40447889_206	BLVRB	19	−	618	161	6	4	14.08%	152.86674471985683	4.747084556845331e-06	4.255654209563462e-06	1.7003898879446298e-10
ENSG00000074266_86245272_86278525_427	EED	11	+	1281	11	3	0	1.64%	5.03870777685129	0.07962816006291146	0.007029302661868638	0.0031495291952635916
ENSG00000105323_41276293_41306565_596	HNRNPUL1	19	+	1788	83	5	3	5.03%	27.238688578307894	2.5349936190846526e-06	1.9066683571442567e-06	4.193633978817017e-11
ENSG00000204316_75904684_75901577_207	MRPL38	17	−	621	21	0	3	3.86%	19.84281626483334	0.005554612275049112	0.02824321405900014	0.0008328330622014512
ENSG00000115355_55332660_55295064_1107	CCDC88A	2	−	3321	6	1	0	0.45%	1.0601272336612468	0.0477903522728147	0.016947426762344633	0.0036975118224100542
ENSG00000188157_1034625_1054981_1917	AGRN	1	+	5751	104	12	22	2.40%	10.611226582937926	1.0932700426938786e-07	1.3891301681676028e-06	1.4901361836981474e-12
ENSG00000067955_67066700_67098778_87	CBFB	16	+	261	33	0	0	9.20%	74.1906283005345	0.0053557511377634245	0.0053557511377634245	0.00015366972994913032
ENSG00000183527_39180416_39175590_201	PSMG1	21	−	603	19	0	0	3.48%	18.48893541094279	0.00797940233269714	0.00797940233269714	0.000327142761693177
ENSG00000145592_40835185_40832504_97	RPL37	5	−	291	487	32	14	21.65%	982.0002281991085	6.23998701282055e-05	0.00014587207147177605	6.662244061191489e-08
ENSG00000092931_76741970_76778352_310	MFSD11	17	+	930	10	0	0	1.94%	6.3094669229193565	0.012798402692974279	0.012798402692974279	0.0007972436888943567
ENSG00000163946_56633619_56623037_613	TASOR	3	−	1839	8	0	1	0.82%	2.5526065202022843	0.019216965118390855	0.03418265644467716	0.0029560527921161815
ENSG00000164466_175492104_175511550_177	SFXN1	5	+	531	40	6	0	7.91%	44.2019151662147	0.0017825173154265793	0.0004507943031154868	5.216577928785211e-06
ENSG00000161956_7563077_7571483_574	SENP3	17	+	1722	14	2	1	1.92%	4.7705725514756105	0.009446187005438042	0.0024579339080672683	0.00013174815516271736
ENSG00000129116_168898667_168926385_368	PALLD	4	+	1104	20	2	0	2.45%	10.630080141875002	0.011860429764611099	0.0035291660242640502	0.000228982799894572
ENSG00000123144_12734441_12730961_78	TRIR	19	−	234	15	0	0	8.97%	37.61412973278847	0.00797940233269714	0.00797940233269714	0.000327142761693177
ENSG00000055130_148730123_148800582_678	CUL1	7	+	2034	27	0	1	1.33%	7.789120670329647	0.0035547763465261306	0.005670089768331009	0.00011049933550063637
ENSG00000138796_107990044_108034357_294	HADH	4	+	882	36	0	0	4.42%	23.950221380877558	0.0005460508606305346	0.0005460508606305346	1.9341343681665648e-06
ENSG00000087460_58903762_58909635_123	GNAS	20	+	369	155	22	11	21.95%	246.4795818262399	9.310476775917055e-05	1.01225519091334e-05	7.734177080485668e-09
ENSG00000125875_441682_438586_226	TBC1D20	20	−	678	15	0	0	2.21%	12.981867783882747	0.02108409854857798	0.02108409854857798	0.002029891263157455
ENSG00000125954_64924066_64932230_75	CHURC1-FNTB	14	+	225	16	2	0	10.67%	41.72660791690667	0.017901377192768454	0.005454749182134642	0.0005111246077595201
ENSG00000015479_139307527_139329395_246	MATR3	5	+	738	45	3	2	4.88%	35.77929413606708	0.0032751767503367245	0.0024633983720734258	4.600163005874518e-05
ENSG00000141503_4884369_4889985_415	MINK1	17	+	1245	15	1	4	1.20%	7.069643660620484	0.019679754444124863	0.21453803860113568	0.02189527136926916
ENSG00000175354_12884141_12785823_410	PTPN2	18	−	1230	8	1	0	1.22%	3.8164580411804887	0.04223451612662591	0.019679754444124863	0.003715148010809822
ENSG00000136048_101877811_101921260_231	DRAM1	12	+	693	16	0	0	3.03%	13.547599973021649	0.00847367416878415	0.00847367416878415	0.0003665024264111182
ENSG00000178982_38619287_38636920_212	EIF3K	19	+	636	303	31	4	17.45%	279.5510509763279	2.4420177733335807e-06	1.6670500602408155e-07	3.959697802658379e-12
ENSG00000158604_44582206_44579479_178	TMED4	7	−	534	42	0	0	7.30%	46.15126929011799	0.0006768518708446028	0.0006768518708446028	2.9265495608176655e-06
ENSG00000136754_26823167_26748570_423	ABI1	10	−	1269	20	1	0	2.13%	9.247918421300238	0.005588370640926919	0.0034782380676658805	0.00010676685949938237
ENSG00000137133_35814979_35813054_163	HINT2	9	−	489	63	10	0	9.20%	75.59747791694174	0.007110331649323444	0.0003065228477605667	1.619614559798326e-05
ENSG00000146918_158701899_158641464_1156	NCAPG2	7	−	3468	50	0	3	1.90%	8.459925372426472	1.4965834940431001e-05	0.0001066924919191799	1.2766188626507607e-08
ENSG00000104320_89981448_89935582_672	NBN	8	−	2016	17	2	1	1.64%	4.948049208896578	0.007962492813783481	0.002550198508962866	0.00011439042603247118
ENSG00000105568_52212720_52225981_410	PPP2R1A	19	+	1230	78	1	7	8.29%	37.210465901509764	2.3985456129107117e-07	1.0615599814775205e-06	2.382808611425758e-12
ENSG00000168003_62881024_62888693_529	SLC3A2	11	+	1587	657	46	11	21.93%	242.92044011171745	9.431690964375726e-19	3.8644495570591555e-20	6.065325871090569e-37
ENSG00000044115_138886260_138934089_536	CTNNA1	5	+	1608	79	3	5	6.72%	28.828142713114744	4.179830179336049e-07	5.365410861317036e-07	2.0623548334814216e-12
ENSG00000196531_56724521_56712538_215	NACA	12	−	645	209	14	5	14.88%	190.13505206323026	2.4168959547478946e-06	1.161173264099816e-06	2.4727756758011836e-11
ENSG00000104611_19333014_19394642_374	SH2D4A	8	+	1122	11	0	3	1.60%	5.752749253250001	0.012798402692974279	0.05111742236774389	0.003128977431720179
ENSG00000131504_141583543_141516851_1114	DIAPH1	5	−	3342	26	2	5	1.35%	4.565018258413825	0.0006738559393031653	0.003803083599965396	1.633323860690344e-05
ENSG00000106268_2244575_2251001_156	NUDT1	7	+	468	34	3	0	7.05%	42.6293470304936	0.003453048488940946	0.0016186486669924592	3.275367415488609e-05
ENSG00000030582_44349165_44352798_593	GRN	17	+	1779	260	13	6	8.94%	85.75767858133223	1.835510848519117e-09	1.3133816402015605e-09	2.6363242867329608e-17
ENSG00000138399_169538100_169529825_147	FASTKD1	2	−	441	10	0	0	3.40%	13.305678544931975	0.019679754444124863	0.019679754444124863	0.0017849636115922674
ENSG00000136854_127653778_127690857_544	STXBP1	9	+	1632	9	2	0	0.92%	3.2359214549531257	0.08350359792828288	0.020613416668581838	0.007749259071793911
ENSG00000065911_74198642_74205939_111	MTHFD2	2	+	333	14	0	0	4.50%	24.669447248171178	0.02108409854857798	0.02108409854857798	0.002029891263157455
ENSG00000148229_113410293_113408811_147	POLE3	9	−	441	57	5	0	11.56%	75.84236770611226	0.0008512616127049016	0.0001297455242311012	7.871132705232902e-07
ENSG00000198715_156294893_156293044_325	GLMP	1	−	975	14	0	1	2.46%	8.425565060144617	0.005160369442893307	0.00912793029372963	0.00024819240235076056
ENSG00000089157_120200783_120198800_172	RPLP0	12	−	516	54	1	4	4.65%	61.407253656784896	0.005808022449631236	0.009175245078463236	0.0002780762357321736
ENSG00000134324_11771429_11824791_775	LPIN1	2	+	2325	24	11	1	2.32%	6.057088246002582	0.02300587965136625	0.00010796340825013603	2.825820597833666e-05
ENSG00000138413_208251500_208237079_397	IDH1	2	−	1191	134	3	2	9.32%	66.01895616576073	7.800330351856624e-08	1.5614034113292325e-07	1.1813902497174013e-13
ENSG00000126254_35629154_35637554_458	RBM42	19	+	1374	40	6	5	3.49%	17.082399529301313	0.002548497010389099	0.002096928865005929	3.099819838896916e-05
ENSG00000087470_32679364_32743410_699	DNM1L	12	+	2097	33	12	0	2.86%	9.234026698349787	0.0015372245518336012	3.0310500299891545e-05	4.1338318752620734e-07
ENSG00000163110_94523808_94664067_565	PDLIM5	4	+	1695	91	2	12	6.19%	31.50266582222213	8.976557121876814e-08	1.3999896692044456e-06	1.2488650112397084e-12
ENSG00000158941_22614456_22619400_592	CCAR2	8	+	1776	16	1	0	1.01%	5.286310124608109	0.016503128830616266	0.013007239811447113	0.001029548967667127

ENSG00000116729_68155212_68126226_357	WLS	1	-	1071	20	0	0	2.52%	10.957617625238099	0.0035291660242640502	0.0035291660242640502	6.948232509684344e-05
ENSG00000136942_124861912_124858469_96	RPL35	9	-	288	35	3	2	8.33%	71.3101209517448	0.012070269314219385	0.008808686117259405	0.0005324252474387162
ENSG00000156113_77637360_76877739_1167	KCNMA1	10	-	3501	15	4	2	0.69%	2.514054943579692	0.048994651353958696	0.02389626957043539	0.005091569865673637
ENSG00000124155_45416169_45424583_491	PIGT	20	+	1473	75	4	0	4.68%	29.876803657408356	6.051873075356466e-05	1.181854822373525e-05	5.736528091018088e-09
ENSG00000128564_101164141_101162996_381	VGF	7	-	1143	16	4	4	2.10%	8.213899196241472	0.05423379212399942	0.04727912037993979	0.010247738400701924
ENSG00000072778_7224441_7225097_133	ACADVL	17	+	399	10	1	0	3.76%	14.706276286503762	0.03537493353818881	0.020613416668581838	0.003252526348827943
ENSG00000161513_74872944_74862817_534	FDXR	17	-	1602	14	8	0	1.50%	5.12791881001311	0.18446498823826807	0.005160369442893307	0.0071631748089246074
ENSG00000150593_110887761_110890694_120	PDCD4	10	+	360	8	2	0	5.83%	13.039564974033336	0.06180520118411761	0.005706018193000826	0.0020032236666657145
ENSG00000084207_67584484_67584783_61	GSTP1	11	+	183	154	0	16	34.43%	493.79336213142636	2.5695877403898546e-05	0.0003543769227346193	7.297186085012358e-08
ENSG00000065911_74208652_74214242_186	MTHFD2	2	+	558	37	0	0	6.99%	38.9083793580027	0.0006933285739671933	0.0006933285739671933	3.065417257202339e-06
ENSG00000187555_8921168_8893998_932	USP7	16	-	2796	44	0	1	1.61%	9.234026698349787	0.0003033834228180314	0.0003574960659478307	7.284839681605404e-07
ENSG00000197622_151055730_151054247_79	CDC42SE1	1	-	237	9	2	0	7.59%	22.282800904993675	0.07022909110143051	0.012178957859018494	0.004215016872891795
ENSG00000100503_50821942_50725872_2052	NIN	14	-	6156	14	6	5	0.39%	1.3344584037753415	0.052998774212472885	0.09865097884783247	0.0199560565611326
ENSG00000167460_16081341_16095346_205	TPM4	19	+	615	32	0	3	4.39%	30.53166432944391	0.0036321914711260127	0.015376780629637295	0.0003065683871243863
ENSG00000044115_138886260_138904456_97	CTNNA1	5	+	291	10	0	0	6.19%	20.16427573304124	0.011570465654371866	0.011570465654371866	0.0006594843062896773
ENSG00000198363_61689688_61626273_298	ASPH	8	-	894	366	19	26	19.46%	240.22554264242623	3.927503861104185e-10	2.557836638701447e-09	1.1351471479747843e-17
ENSG00000070367_57235746_57208609_497	EXOC5	14	-	1491	15	1	0	1.01%	5.903223579793764	0.028889785561798623	0.02108409854857798	0.002731532589007073
ENSG00000137944_88962097_88936183_287	KYAT3	1	-	861	7	0	0	1.74%	4.7705725514756105	0.019216965118390855	0.019216965118390855	0.0017073955890589052
ENSG00000179409_748009_744866_1047	GEMIN4	17	-	3141	39	10	3	1.43%	7.285716819302294	0.02169658182459074	0.0010518076417120354	0.00015741372066537928
ENSG00000177156_747482_764845_337	TALDO1	11	+	1011	291	11	11	17.21%	168.89525552420034	5.787632381240798e-10	1.4685564578168595e-10	9.788859067862173e-19
ENSG00000108654_64506119_64499923_535	DDX5	17	-	1605	247	11	18	10.84%	90.30203407251123	1.324525106517775e-10	1.1176538365759745e-09	1.7216205439646401e-18
ENSG00000137804_41332958_41380186_378	NUSAP1	15	+	1134	46	3	2	4.50%	23.80238050815609	0.00024099906677261195	0.00019869373203914534	3.3023133334942463e-07
ENSG00000103544_19569589_19700476_869	VPS35L	16	+	2607	45	0	0	2.53%	10.128545865906217	1.3790572374667199e-05	1.3790572374667199e-05	1.5221640363264113e-09
ENSG00000169045_179618370_179617500_193	HNRNPH1	5	-	579	118	11	2	12.44%	119.58564768932128	8.904143521645122e-05	1.6162535005161726e-05	1.138969783715916e-08
ENSG00000095906_1786889_1788714_182	NUBP2	16	+	546	11	2	0	3.85%	11.821583630304948	0.04363948305378087	0.0078820576498062	0.0017710903275458955
ENSG00000127527_16471945_16361967_754	EPS15L1	19	-	2262	8	0	0	0.66%	2.075262330084881	0.019679754444124863	0.019679754444124863	0.0017849636115922674
ENSG00000154710_66771900_66809284_491	RABGEF1	7	+	1473	10	0	0	1.22%	3.983573820987781	0.012798402692974279	0.012798402692974279	0.0007972436888943567
ENSG00000168385_241337698_241350174_166	SEPT2	2	+	498	18	1	0	6.02%	21.20893098186145	0.006432154271512831	0.001801896632038876	6.747259634841345e-05
ENSG00000145725_103148006_103201634_1039	PPIP5K2	5	+	3117	13	0	3	0.87%	2.447271578379693	0.00308494966027208	0.03441276307238556	0.0006340181607701747
ENSG00000145901_151065095_151030713_636	TNIP1	5	-	1908	12	2	0	0.94%	3.6904429171792463	0.03970531299947108	0.011772051206276392	0.002244512946580312
ENSG00000070961_89655775_89590984_1183	ATP2B1	12	-	3549	26	1	0	1.01%	4.298757683747254	0.001343466641814883	0.0010073743004618205	8.31430019743034e-06
ENSG00000004059_127590135_127591299_71	ARF5	7	+	213	16	0	1	7.04%	44.07740272912677	0.019679754444124863	0.04223451612662591	0.003715148010809822
ENSG00000004059_127588499_127589621_94	ARF5	7	+	282	41	0	1	19.15%	85.3120474364947	7.914814395228409e-05	0.00011644620007245363	6.68720263805529e-08
ENSG00000109572_169689081_169719997_666	CLCN3	4	+	1998	8	2	0	0.90%	2.349471166492493	0.08266357711566169	0.011570465654371866	0.004841719827405853
ENSG00000197756_216498875_216529273_72	RPL37A	2	+	216	270	25	15	31.94%	733.4755297893752	1.2443108043233659e-05	0.00019025636053522364	1.969515653838464e-08
ENSG00000196683_22822779_22817987_54	TOMM7	7	-	162	20	0	2	14.81%	72.44202763351853	0.005604866691947602	0.01840230248560155	0.0005383581833854688
ENSG00000156261_29062187_29056475_164	CCT8	21	-	492	65	0	0	10.98%	77.52180396147868	8.751142344759464e-05	8.751142344759464e-05	5.5736829768171844e-08
ENSG00000127980_92528435_92487457_1283	PEX1	7	-	3849	9	2	0	0.47%	1.3720508741188624	0.07134538375819317	0.011772051206276392	0.0041729868335021346
ENSG00000113407_33454984_33467708_559	TARS	5	+	1677	88	9	13	6.98%	30.79110154870126	3.920155159499126e-07	2.55527640947914e-06	9.265034338875877e-12
ENSG00000116584_155965093_155947942_780	ARHGEF2	1	-	2340	7	0	5	0.64%	1.7553260541967952	0.019216965118390855	0.169362073879829	0.016163991620339734
ENSG00000178982_38619287_38630851_161	EIF3K	19	+	483	201	7	4	9.94%	244.18812668764292	0.001216496151769452	0.0007532718901981798	5.730981237418765e-06
ENSG00000122482_90940963_90916798_1197	ZNF644	1	-	3591	12	0	0	0.42%	1.9608368382005017	0.02108409854857798	0.02108409854857798	0.002029891263157455
ENSG00000182087_1020996_1011092_406	TMEM259	19	-	1218	11	0	2	1.23%	5.299330592895322	0.02108409854857798	0.0700082515985845	0.006564739044797468
ENSG00000177054_19149296_19175960_461	ZDHHC13	11	+	1383	12	0	0	1.08%	5.091370271856834	0.019679754444124863	0.019679754444124863	0.0017849636115922674
ENSG00000160209_43719295_43743697_91	PDXK	21	+	273	22	1	0	7.69%	47.28633452121979	0.010197420091687953	0.00877616296920843	0.0004515216795882782
ENSG00000171155_120627097_120626210_295	C1GALT1C1	X	-	885	36	1	3	4.75%	23.869034189755936	0.0006325693003332578	0.002713573254137601	1.0896677684161084e-05
ENSG00000117118_17054019_17032947_132	SDHB	1	-	396	26	0	0	5.30%	38.52598742328031	0.00797940233269714	0.00797940233269714	0.000327142761693177
ENSG00000198000_92321744_92297836_1099	NOL8	9	-	3297	10	1	1	0.64%	1.779740442315742	0.016854695534768073	0.016854695534768073	0.0013361081351157898
ENSG00000087191_63830397_63830949_81	PSMC5	17	+	243	26	0	0	13.58%	62.78309061571606	0.0013984928336429232	0.0013984928336429232	1.1821238358622835e-05
ENSG00000123131_23667571_23671431_92	PRDX4	X	+	276	40	0	2	9.78%	85.04064113500002	0.0037896409716948643	0.0081271044488283	0.00016696601944312815
ENSG00000162191_62678923_62678218_136	UBXN1	11	-	408	25	0	0	5.15%	35.95468283281251	0.008877961307018026	0.008877961307018026	0.00040023486315785033
ENSG00000137710_110247751_110199612_257	RDX	11	-	771	11	4	0	1.95%	8.37170513897082	0.17139085557395572	0.020613416668581838	0.01724926236372712
ENSG00000278311_36554826_36589411_664	GGNBP2	17	+	1992	12	4	0	0.75%	3.534821830310242	0.10105102209468636	0.019679754444124863	0.009164497669452725
ENSG00000115053_231458474_231455191_350	NCL	2	-	1050	104	3	0	8.86%	58.11920388426287	1.4547908854964419e-06	4.4175630167817633e-07	5.872480430011314e-12
ENSG00000115053_231463238_231458993_358	NCL	2	-	1074	82	5	6	6.70%	44.80073999458381	4.099625344154047e-05	1.528455217378632e-05	4.933508172984664e-09
ENSG00000214046_16660110_16652862_103	SMIM7	19	-	309	23	3	0	6.80%	43.67621277710195	0.0682897090290637	0.00797940233269714	0.0029197462565442433
ENSG00000126456_49664838_49659648_427	IRF3	19	-	1281	21	0	3	2.11%	9.61935121035246	0.0036581908865286695	0.014055189556040475	0.0002811788838551419
ENSG00000130731_636290_634597_184	METTL26	16	-	552	16	1	1	4.89%	17.008128227000004	0.006432154271512831	0.006432154271512831	0.00021751960674370818
ENSG00000090520_186570898_186585408_358	DNAJB11	3	+	1074	87	8	3	6.42%	47.532492433277945	0.00021126911468117307	4.658806620064333e-05	7.355680469454738e-08
ENSG00000113013_138556845_138556037_96	HSPA9	5	-	288	37	5	1	13.54%	75.38498500613022	0.004837227893505887	0.0008720849937359773	2.6366524991668415e-05
ENSG00000134014_28110379_28189725_413	ELP3	8	+	1239	21	0	1	2.66%	9.945430912398308	0.0013984928336429232	0.002484757332982506	2.0661357227484502e-05
ENSG00000160752_155310161_155318336_144	FDPS	1	+	432	73	0	4	11.81%	99.1550253233785	0.00013891616214042798	0.00039475350870333604	3.8307694579386786e-07
ENSG00000132823_44206957_44197052_292	OSER1	20	-	876	16	0	0	2.40%	10.717450663589043	0.00847367416878415	0.00847367416878415	0.0003665024264111182
ENSG00000165119_83978252_83969407_464	HNRNPK	9	-	1392	206	5	5	5.82%	86.83675812448925	1.015882989989372e-05	8.29273540037101e-06	6.873991583164199e-10
ENSG00000125166_58734228_58716168_237	GOT2	16	-	711	46	1	0	5.91%	37.96329043072997	0.0004549569228770849	0.0004481211664877703	1.3400425077769636e-06
ENSG00000167515_88857151_88859368_75	TRAPPC2L	16	+	225	28	0	2	12.00%	73.02156385458669	0.0037631575832289435	0.004490071680569804	9.29822359294349e-05
ENSG00000148187_122270919_122322594_192	MRRF	9	+	576	9	0	6	2.60%	9.16844412236719	0.020613416668581838	0.2618042821861254	0.029088402804692926

ENSG00000148187_122280532_122322617_171	MRRF	9	+	513	17	2	1	4.68%	19.444965312154974	0.02071580916834944	0.008219046535586472	0.0008496570697932704
ENSG00000161057_103347712_103368054_433	PSMC2	7	+	1299	83	14	1	5.77%	37.49251360894112	0.00042670678534371807	6.332363126702123e-06	2.5872920010490292e-08
ENSG00000100889_24094069_24103867_509	PCK2	14	+	1527	33	1	0	2.55%	12.680912892232813	0.0009086099620780121	0.0006424109906750971	3.7043534070101014e-06
ENSG00000140455_63537128_63590826_435	USP3	15	+	1305	9	3	0	1.15%	4.046761543665518	0.1191346346731873	0.019216965118390855	0.010787784428637617
ENSG00000185215_103133368_103136003_199	TNFAIP2	14	+	597	11	6	1	3.02%	10.81169960158543	0.11625384694841989	0.023672501042627626	0.012333872096178604
ENSG00000134255_111182208_111184310_171	CEPT1	1	+	513	16	0	0	3.51%	18.301143823204683	0.013007239811447113	0.013007239811447113	0.0008218654148058079
ENSG00000139496_25301774_25340134_587	NUP58	13	+	1761	34	2	0	2.04%	11.329093929739356	0.002476718586969124	0.0010405263639310788	1.5651736172102553e-05
ENSG00000133112_45340184_45337386_138	TPT1	13	−	414	46	8	5	6.52%	65.19782487016668	0.009288069496478249	0.018151375140008384	0.0008309403832967919
ENSG00000138796_108027733_108034357_87	HADH	4	+	261	29	0	0	10.34%	65.19782487016668	0.0033030736763512156	0.0033030736763512156	6.124050385493983e-05
ENSG00000138796_107990044_108023617_192	HADH	4	+	576	9	0	0	2.60%	9.16844412236719	0.019679754444124863	0.019679754444124863	0.0017849636115922674
ENSG00000142867_85276352_85267627_233	BCL10	1	−	699	17	1	0	3.00%	14.270768533813309	0.016854695534768073	0.007029302661868638	0.0006017499134389055
ENSG00000106399_7640418_7637000_121	RPA3	7	−	363	120	0	0	9.09%	193.97699961371904	0.001588175367824666	0.001588175367824666	1.5088866706348812e-05
ENSG00000204569_30606818_30601549_800	PPP1R10	6	−	2400	24	0	0	1.12%	5.867804238315001	0.0036321914711260127	0.0036321914711260127	7.340009712338726e-05
ENSG00000257046_20855085_21090106_701	AC011604.2	12	+	2103	8	0	0	0.71%	2.2321651881369475	0.019679754444124863	0.019679754444124863	0.0017849636115922674
ENSG00000175063_45812696_45816767_150	UBE2C	20	+	450	121	3	0	13.33%	157.77873618580338	4.0525034736360485e-05	4.0525034736360485e-05	1.2461895536420793e-08
ENSG00000196531_56713680_56712538_136	NACA	12	−	408	155	7	3	14.71%	222.91903356343755	4.323031604313141e-05	7.547702702290602e-05	2.4417558722358517e-08
ENSG00000154710_66771900_66809284_491	RABGEF1	7	+	1473	10	0	0	1.22%	3.983573820987781	0.012798402692974279	0.012798402692974279	0.0007972436888943567
ENSG00000123159_14478708_14478416_58	GIPC1	19	−	174	20	0	0	15.52%	67.44602572775864	0.0033030736763512156	0.0033030736763512156	6.124050385493983e-05
ENSG00000136603_170360425_170392417_607	SKIL	3	+	1821	31	0	2	1.81%	9.989123085544483	0.0014988252558711152	0.005609982661338475	4.976399469025566e-05
ENSG00000047315_56994753_57030988_1020	POLR2B	4	+	3060	29	5	0	1.67%	5.560990944808335	0.0021301889646226013	0.00010991304945921688	1.7944698229304764e-06
ENSG00000181019_69718478_69710976_219	NQO1	16	−	657	895	50	39	38.36%	799.3431953260161	1.849948437045586e-12	7.728178966437419e-14	1.955615948123593e-24
ENSG00000104331_56993576_56963793_356	IMPAD1	8	−	1068	43	0	0	5.62%	23.6250545175604	3.1598473442192024e-05	3.1598473442192024e-05	7.674144019852283e-09
ENSG00000102931_57246138_57252267_91	ARL2BP	16	+	273	12	0	0	5.49%	25.792546102483524	0.019679754444124863	0.019679754444124863	0.0017849636115922674
ENSG00000110911_51010704_50988325_553	SLC11A2	12	−	1659	22	0	0	1.63%	7.7812955541247755	0.0033030736763512156	0.0033030736763512156	6.124050385493983e-05
ENSG00000146963_139398602_139422340_339	LUC7L2	7	+	1017	28	0	7	5.31%	16.155213242165196	5.634389248581345e-05	0.001963690465134474	9.222651616384024e-07
ENSG00000186416_119592452_119589352_675	NKRF	X	−	2025	11	0	0	0.74%	3.1874492158748153	0.02108409854857798	0.02108409854857798	0.002029891263157455
ENSG00000120526_109334010_109243009_583	NUDCD1	8	−	1749	10	2	0	1.37%	3.3549481065265874	0.03755618289012509	0.00414960799764038	0.0008890075989244122
ENSG00000080371_71773989_71785673_106	RAB21	12	+	318	14	1	1	5.66%	25.833100420254723	0.013007239811447113	0.02034245439037237	0.0012585798064549353
ENSG00000101150_63865366_63889945_200	TPD52L2	20	+	600	39	3	2	10.50%	38.14072754904751	0.0001703216465405105	0.00010187626107045325	1.2383489804634146e-07
ENSG00000107815_100988358_100993510_635	TWNK	10	+	1905	18	0	0	0.94%	5.544381957462994	0.013640585738808999	0.013640585738808999	0.00089863692579514
ENSG00000092203_21488653_21495396_475	TOX4	14	+	1425	8	0	0	1.05%	3.294205888176843	0.019679754444124863	0.019679754444124863	0.0017849636115922674
ENSG00000116016_46297912_46384660_870	EPAS1	2	+	2610	57	8	4	2.87%	12.814744888274141	7.006199212422604e-05	3.283580308332821e-05	1.743846153648635e-08
ENSG00000106537_16753968_16783491_204	TSPAN13	7	+	612	12	0	0	2.94%	11.505498506500002	0.011772051206276392	0.011772051206276392	0.000681272511389508
ENSG00000111481_54325164_54350523_177	COPZ1	12	+	531	123	9	1	19.21%	135.9208891361102	2.373413040653174e-06	1.848215521193233e-07	4.234034659341311e-12
ENSG00000140937_64998706_64947603_670	CDH11	16	−	2010	17	0	0	1.34%	4.962819505042538	0.0033278027414746945	0.0033278027414746945	6.211806171561056e-05
ENSG00000188603_28489349_28482512_262	CLN3	16	−	786	18	1	0	2.29%	13.437719629729012	0.029237630782826433	0.013640585738808999	0.001872687038404406
ENSG00000138326_78035415_78040626_111	RPS24	10	+	333	93	15	2	9.91%	163.8756138628514	0.005706018193000826	0.002377998863656553	7.682465500360151e-05
ENSG00000136830_127568874_127506845_746	FAM129B	9	−	2238	77	1	2	3.75%	20.188602607250004	1.920106734637258e-06	5.144196331814339e-06	8.525782730962909e-11
ENSG00000204628_181243800_181241574_116	RACK1	5	−	348	237	27	17	21.55%	399.6177024369699	0.000701829233747013	7.045887262302434e-05	3.709427955529044e-07
ENSG00000204628_181242204_181241483_63	RACK1	5	−	189	535	12	12	19.05%	1660.992205025675	0.009752974559067457	0.005337436885683857	0.0002731502778314808
ENSG00000138796_107990044_108023617_192	HADH	4	+	576	9	0	0	2.60%	9.16844412236719	0.019679754444124863	0.019679754444124863	0.0017849636115922674
ENSG00000141552_81894478_81894612_44	ANAPC11	17	+	132	98	8	1	22.73%	435.64001163247735	0.004326528353113636	0.0025167541003031247	6.155846001043173e-05
ENSG00000125691_38850368_38850132_29	RPL23	17	−	87	44	0	0	17.24%	296.762513202138	0.021557223391537678	0.021557223391537678	0.002115616449879074
ENSG00000089280_31180215_31191438_527	FUS	16	+	1581	189	8	10	8.73%	70.14642637833873	1.4566474402011096e-08	1.124861263595642e-08	1.6917267863924949e-15
ENSG00000108107_55386379_55403131_163	RPL28	19	+	489	437	56	21	14.11%	524.3825055508498	0.0001798645505738252	5.292524753533204e-05	7.038375442223417e-08
ENSG00000151353_669768_669580_35	TMEM18	2	−	105	10	0	0	14.29%	55.8838498887143	0.020613416668581838	0.020613416668581838	0.001946208561389314
ENSG00000116977_236539066_236548161_210	LGALS8	1	+	630	11	1	0	2.86%	10.245372479597622	0.023672501042627626	0.012798402692974279	0.0014375227604752335
ENSG00000173540_49723726_49721752_360	GMPPB	3	−	1080	11	0	2	1.39%	5.976467279765279	0.019216965118390855	0.055414058431395546	0.004780438930026009
ENSG00000169241_155135953_155138281_214	SLC50A1	1	+	642	10	1	1	3.74%	9.139881991144861	0.010437296944230427	0.010437296944230427	0.0005431233129110158
ENSG00000105404_41959292_41956846_151	RABAC1	19	−	453	23	1	0	6.62%	29.792383549943715	0.0035004709947243163	0.0023628488670607213	4.7187726630981656e-05
ENSG00000119013_201078883_201085615_98	NDUFB3	2	+	294	38	6	9	9.18%	75.84236770611226	0.0076106684850624365	0.02876187041677918	0.0011095992622971113
ENSG00000092203_21488653_21496606_494	TOX4	14	+	1482	8	0	0	1.01%	3.167505661708503	0.019679754444124863	0.019679754444124863	0.0017849636115922674
ENSG00000100526_54397069_54418273_203	CDKN3	14	+	609	18	1	3	6.40%	17.343263758566508	0.0009931305439351676	0.004801362767940752	2.936605092714173e-05
ENSG00000052841_43379245_43493904_1084	TTC17	11	+	3252	14	0	0	0.83%	2.5261149857444654	0.0032294771334464936	0.0032294771334464936	5.8663648728941325e-05
ENSG00000105552_48806618_48799671_166	BCAT2	19	−	498	17	0	2	3.61%	20.030657038424703	0.013428347753762199	0.044040755831095145	0.0027917083341200354
ENSG00000154277_41261759_41264227_93	UCHL1	4	+	279	99	1	1	16.13%	208.21240845633875	0.0002861705430093419	0.0002861705430093419	5.548689392255158e-07
ENSG00000159692_1241433_1241335_32	CTBP1	4	−	96	17	0	0	15.62%	103.90903338682814	0.019679754444124863	0.019679754444124863	0.0017849636115922674
ENSG00000241837_33903615_33903526_29	ATP5PO	21	−	87	14	1	0	20.69%	94.4244360188621	0.012798402692974279	0.013428347753762199	0.0008341197643438865
ENSG00000112062_36052814_36108447_283	MAPK14	6	+	849	19	1	1	3.18%	13.131717376676681	0.006112543793728684	0.009288069496478249	0.0002949766129424608
ENSG00000247596_52239016_52229197_254	TWF2	3	−	762	35	0	0	6.30%	26.951856737667327	0.0001842144602393462	0.0001842144602393462	2.3639666231890906e-07
ENSG00000100902_35292477_35310287_107	PSMA6	14	+	321	45	2	0	6.54%	82.25893792030375	0.013892891352654344	0.00847367416878415	0.0005886798845679606
ENSG00000100138_41675154_41674933_73	SNU13	22	−	219	46	0	1	17.81%	123.250682631274	0.000649205117157464	0.0010037877862843834	4.1244594600580285e-06
ENSG00000018236_40908439_41070035_1016	CNTN1	12	+	3048	87	4	1	3.54%	16.748653829836126	4.7065801126129457e-07	8.058044997009374e-08	3.693287659042921e-13
ENSG00000184640_77320327_77498658_568	SEPT9	17	+	1704	55	3	6	3.17%	18.93950898517166	0.000442321637794036	0.0014426254751151526	4.136427255035133e-06
ENSG00000119977_95693899_95664067_607	TCTN3	10	−	1821	47	0	0	3.13%	15.144799516793249	5.745807750721616e-05	5.745807750721616e-05	2.45922963507537e-08
ENSG00000115204_27313101_27309912_118	MPV17	2	−	354	13	0	2	7.63%	21.548433643529666	0.002943990679779778	0.020677326986596276	0.00034970851831863124
ENSG00000177707_111072018_111133692_366	NECTIN3	3	+	1098	19	4	0	2.46%	10.153759610927597	0.03497523187724695	0.0034782380676658805	0.0007116935409828717

ENSG00000070404_676502_681708_237	FSTL3	19	+	711	15	0	0	2.95%	12.379333836107598	0.00837382380159347	0.00837382380159347	0.0003583838894600799
ENSG00000149311_108227625_108365508_3056	ATM	11	+	9168	26	0	0	0.39%	1.6640806085971862	0.0009829516685359422	0.0009829516685359422	6.0033663169184795e-06
ENSG00000242265_94663557_94665682_707	PEG10	7	+	2121	73	7	2	3.96%	20.195648722159127	3.660035217086396e-05	2.013535543408069e-06	6.643981862910677e-10
ENSG00000118418_79234560_79201688_130	HMGN3	6	-	390	7	6	0	4.62%	10.531956325180772	0.1705668419458189	0.009815328628645334	0.010088762169828331
ENSG00000143457_150694694_150648321_237	GOLPH3L	1	-	711	12	0	2	2.11%	9.903467068886078	0.02108409854857798	0.060667625179241055	0.005667715763110205
ENSG00000099194_100347505_100360933_359	SCD	10	+	1077	418	41	18	21.45%	227.7383631955126	3.779905837392004e-12	2.6150138760901744e-13	1.311978948614191e-23
ENSG00000072756_3129041_3148154_434	TRNT1	3	+	1302	12	0	2	1.84%	5.408114505359448	0.004873903550586949	0.029607078753113344	0.0007843881175907335
ENSG00000007341_112619113_112526013_575	ST7L	1	-	1725	8	0	0	1.04%	2.721300516320001	0.009815328628645334	0.009815328628645334	0.0004837142251238724
ENSG00000033867_27434087_27376764_1044	SLC4A7	3	-	3132	25	0	2	1.44%	4.683751786649905	0.00023917554320093795	0.0012356032184771147	2.013922539906876e-06
ENSG00000145191_184135734_184144943_661	EIF2B5	3	+	1983	21	1	0	1.51%	6.214013565537823	0.005670089768331009	0.0022409900782254734	7.232548112602907e-05
ENSG00000147044_41665429_41520420_707	CASK	X	-	2121	19	0	0	0.99%	5.2564017222058	0.00847367416878415	0.00847367416878415	0.0003665024264111182
ENSG00000100526_54397069_54420078_207	CDKN3	14	+	621	23	1	3	6.76%	21.73260829005556	0.0006748684829181083	0.0030780475554645728	1.3145404537177733e-05
ENSG00000119013_201078922_201085615_85	NDUFB3	2	+	255	31	2	9	9.41%	71.33409074030001	0.012834473343944776	0.04154059348323694	0.002530235953906631
ENSG00000134108_5122466_5178713_186	ARL8B	3	+	558	17	8	0	3.76%	17.87682294827151	0.2617390908898303	0.007029302661868638	0.01434881016164812
ENSG00000026508_35139304_35229333_493	CD44	11	+	1479	372	22	25	14.40%	147.5877739454483	4.9167581300353395e-12	2.984105520995165e-11	1.823160404296044e-21
ENSG00000117118_17054019_17018881_280	SDHB	1	-	840	86	0	3	9.29%	60.075138630367874	3.142794709437514e-06	2.932455149675853e-06	7.885442585475852e-11
ENSG00000078618_51840459_51789236_1087	NRDC	1	-	3261	33	0	0	1.29%	5.937980369959983	0.00042454308224345244	0.00042454308224345244	1.1896574514138388e-06
ENSG00000147526_38787583_38848023_805	TACC1	8	+	2415	20	3	0	1.61%	4.859465207714287	0.003960944875547292	0.0003978237664344016	1.0712921469115462e-05
ENSG00000126814_60980973_60975109_509	TRMT5	14	-	1527	20	0	0	2.16%	7.685401752868371	0.0013775385517804596	0.0013775385517804596	1.1483554366703679e-05
ENSG00000020426_60734926_60968349_288	MNAT1	14	+	864	18	0	0	2.78%	12.224592163156252	0.005257622967929454	0.005257622967929454	0.00014836615564656991
ENSG00000137574_55785832_55824593_646	TGS1	8	+	1938	15	0	2	0.77%	4.541644147302633	0.020613416668581838	0.030784922059949493	0.0028441549054876347
ENSG00000104765_26391263_26505319_167	BNIP3L	8	+	501	17	1	0	5.39%	19.9107129843024	0.006083074359664965	0.00327844299461777	0.00010984599638310821
ENSG00000198663_36899445_36923441_347	C6orf89	6	+	1041	7	0	0	1.44%	3.9456896895489924	0.019216965118390855	0.019216965118390855	0.0017073955890589052
ENSG00000204842_111554184_111452812_1004	ATXN2	12	-	3012	20	0	0	0.70%	3.8962843547908377	0.00877616296920843	0.00877616296920843	0.00039161135667564577
ENSG00000173692_231060356_231170712_953	PSMD1	2	+	2859	50	1	5	2.20%	10.26198712541973	3.4776101863616385e-05	0.0004632182687560305	1.2705258177527566e-07
ENSG00000137804_41332958_41380186_441	NUSAP1	15	+	1323	48	3	2	4.08%	21.28908567189116	0.00015267201677270214	0.00012290668625881772	1.3316840793902767e-07
ENSG00000087053_95845039_95835290_210	MTMR2	11	-	630	10	2	0	2.38%	9.313974981452382	0.06556525143482543	0.019679754444124863	0.00579448895258773
ENSG00000134882_99200909_99385335_344	UBAC2	13	+	1032	24	1	0	2.91%	13.646056368174422	0.003107435676029586	0.0023628488670607213	4.203480668093069e-05
ENSG00000197712_38878136_38943557_544	FAM114A1	4	+	1632	17	6	0	1.10%	6.112296081578126	0.08620755130569058	0.012798402692974279	0.005490655705532311
ENSG00000171204_85634105_85636229_156	TMEM126B	11	+	468	12	1	0	3.85%	15.045651893115387	0.012798402692974279	0.012798402692974279	0.0007972436888943567
ENSG00000167767_52191902_52171523_422	KRT80	12	-	1266	61	2	7	4.74%	28.272990405783183	0.00016287896179824948	0.0024796990634472845	2.9771713786052052e-06
ENSG00000179085_155140240_155139962_92	DPM3	1	-	276	56	0	0	14.13%	119.05689758900003	0.0007028601028765856	0.0007028601028765856	3.1471554975732053e-06
ENSG00000168487_22177929_22201203_461	BMP1	8	+	1383	17	0	0	1.30%	7.212774551797182	0.012798402692974279	0.012798402692974279	0.0007972436888943567
ENSG00000166979_32412854_32515190_441	EVA1C	21	+	1323	14	1	0	1.59%	6.209316654301588	0.013892891352654344	0.00847367416878415	0.0005886798845679606
ENSG00000172500_65888418_65883974_357	FIBP	11	-	1071	45	3	5	4.76%	24.65463965678572	0.0008014823932862815	0.001238271318614919	6.1832960422298e-06
ENSG00000141510_7676594_7669609_393	TP53	17	-	1179	109	1	6	4.83%	54.24857183853564	6.857358883697343e-05	0.0007313507128457925	3.780271356515748e-07
ENSG00000119820_32278156_32305626_244	YIPF4	2	+	732	30	0	0	4.92%	24.048378025881153	0.0008664586529590942	0.0008664586529590942	4.7094196839841995e-06
ENSG00000130724_58554212_58551649_222	CHMP2A	19	-	666	59	6	4	10.81%	51.98204955864641	4.0252863518770574e-05	2.5996912534775306e-05	8.053411106274734e-09
ENSG00000136003_108562623_108568916_167	ISCU	12	+	501	52	1	2	5.99%	60.90335736374852	0.002516983716564381	0.0025025372014526115	3.624350259415304e-05
ENSG00000172590_22834199_22834321_40	MRPL52	14	+	120	12	0	5	12.50%	58.678042383150014	0.02108409854857798	0.15350694901691259	0.01542296770981773
ENSG00000109736_2933923_2931907_242	MFSD10	4	-	726	16	4	4	3.72%	12.931799974247937	0.03582012146929187	0.030259101754271298	0.00464736148215842
ENSG00000132294_131985022_132000798_118	EFR3A	8	+	354	14	0	1	5.08%	23.206005462262716	0.012798402692974279	0.023672501042627626	0.0014375227604752335
ENSG00000170266_33097085_32997045_546	GLB1	3	-	1638	43	7	0	3.66%	15.403881700094326	0.0010752341924744806	3.523926415704706e-05	3.173349370636792e-07
ENSG00000159377_151399588_151400899_209	PSMB4	1	+	627	85	5	3	12.44%	79.54758536790672	8.868774796247663e-06	1.646722769462151e-05	1.1815030108521125e-09
ENSG00000113013_138575318_138570956_137	HSPA9	5	-	411	37	0	2	10.95%	52.82451504079198	0.00020944610772571289	0.0013206781946010849	1.912961843109268e-06
ENSG00000107959_3155702_3138031_534	PITRM1	10	-	1602	17	0	1	1.87%	6.226758555015919	0.0021227925778456486	0.0020082573300163734	2.4950658261319527e-05
ENSG00000070087_149970856_149965191_188	PFN2	3	-	564	101	7	8	10.64%	105.07947306202396	0.0003343471810376137	0.0003402136324842599	7.624793996016567e-07
ENSG00000168000_62705595_62702488_118	BSCL2	11	-	354	14	0	6	5.08%	23.206005462262716	0.013428347753762199	0.16970534604944526	0.012520549700299675
ENSG00000160948_144424801_144423805_115	VPS28	8	-	345	49	0	0	13.91%	83.33982831230001	0.0001956004280718289	0.0001956004280718289	2.6553971138428216e-07
ENSG00000075420_172226891_172397475_1135	FNDC3B	3	+	3405	27	0	4	1.06%	4.652884418047138	0.0008739357725210369	0.007045996527915517	3.921453735198883e-05
ENSG00000139624_50143075_50130545_248	CERS5	12	-	744	13	1	0	3.63%	10.252883749743955	0.006277459298483267	0.001946208561389314	7.053443163459546e-05
ENSG00000006744_13013234_12992818_624	ELAC2	17	-	1872	28	0	4	1.76%	8.776630270983976	0.0013984928336429232	0.014914789744379387	0.00013123649998910444
ENSG00000211460_121755780_121765207_148	TSN	2	+	444	21	5	0	5.41%	27.753128154192574	0.06816078244095183	0.005554612275049112	0.0021939178336270048
ENSG00000135047_87727604_87731107_333	CTSL	9	+	999	587	16	17	22.22%	344.7848936827734	4.706364451384451e-13	1.238948330436374e-12	7.552603161219588e-24
ENSG00000116747_193069142_193084731_509	RO60	1	+	1527	26	0	0	2.95%	9.991022278728883	0.0002582331582969843	0.0002582331582969843	4.5481323920964005e-07
ENSG00000171617_74636266_74634716_516	ENC1	5	-	1548	29	1	0	2.52%	10.992656518807173	0.0009958682880916293	0.0006424109906750971	4.052020686628797e-06
ENSG00000132780_45615198_45618141_128	NASP	1	+	384	12	2	0	5.47%	18.33688824473438	0.04363948305378087	0.0078820576498062	0.0017710903275458955
ENSG00000075415_98593979_98601528_361	SLC25A3	12	+	1083	357	26	10	18.28%	193.42623389459422	9.890655008128518e-10	2.258805719105101e-11	2.8081063550720807e-19
ENSG00000113649_146458971_146510070_857	TCERG1	5	+	2571	15	0	4	0.82%	3.4234563817473753	0.00797940233269714	0.10392556251212837	0.004734720909678899
ENSG00000033627_42478512_42521120_652	ATP6V0A1	17	+	1956	8	2	0	0.77%	2.3999199338711663	0.10105102209468636	0.019679754444124863	0.009164497669452725
ENSG00000011114_93294026_93242273_706	BTBD7	14	-	2118	15	0	0	0.85%	4.155668724019122	0.013428347753762199	0.013428347753762199	0.0008725593497644525
ENSG00000160691_154970526_154963803_584	SHC1	1	-	1752	23	14	4	2.05%	7.703167664454625	0.05274044890240762	0.006417498004051308	0.0018409653586200223
ENSG00000149636_36771179_36752788_340	DSN1	20	-	1020	21	1	0	2.65%	12.080773431825003	0.005059698366439319	0.0036321914711260127	0.00010092379077801036
ENSG00000011454_123073591_123103213_395	RABGAP1	9	+	1185	8	2	0	1.27%	3.9613868275544313	0.10105102209468636	0.019679754444124863	0.009164497669452725
ENSG00000197756_216499339_216501404_68	RPL37A	2	+	204	442	32	19	41.18%	1271.3575849682502	1.828705992343819e-06	1.2567447325831347e-05	2.0089761647904422e-10
ENSG00000141510_7676239_7669609_350	TP53	17	-	1050	108	1	6	5.14%	60.354557879811445	9.811283348709951e-05	0.000972102382868709	7.028233303176368e-07
ENSG00000120802_98515868_98547858_454	TMPO	12	+	1362	51	0	2	5.73%	21.971954196333705	2.653088520569039e-06	1.4673849820553943e-05	3.337850866049076e-10

ENSG00000119718_75003090_75005920_173	EIF2B2	14	+	519	20	0	4	4.62%	22.61196238271677	0.005306578019052857	0.040065279221313775	0.0011665972595063037
ENSG00000055950_100987443_100981463_202	MRPL43	10	−	606	80	9	8	8.42%	77.46276222198021	0.0006859646665343167	0.0011696436724044363	5.0483553287219605e−06
ENSG00000165282_35092536_35090241_515	PIGO	9	−	1545	13	0	0	1.17%	4.9373110095854384	0.012798402692974279	0.012798402692974279	0.0007972436888943567
ENSG00000165282_35095565_35094157_237	PIGO	9	−	711	23	1	0	3.38%	18.981645215364985	0.007181907427766128	0.005554612275049112	0.0002105152421252746
ENSG00000116062_47798890_47806860_1058	MSH6	2	+	3174	46	0	3	1.32%	8.504064113500002	0.0003342575629110139	0.0024353838450188407	5.499714282630621e−06
ENSG00000144591_219501485_219506798_371	GMPPA	2	+	1113	15	0	2	1.35%	7.908091965384099	0.02108409854857798	0.0700082515985845	0.006564739044797468
ENSG00000144559_11846636_11790505_337	TAMM41	3	−	1011	9	0	0	1.78%	5.223564603841248	0.011772051206276392	0.011772051206276392	0.000681272511389508
ENSG00000055163_157319804_157393000_787	CYFIP2	5	+	2361	8	4	0	0.76%	1.9882437063329101	0.23639154306007898	0.011570465654371866	0.017275440155518785
ENSG00000159228_36070116_36072445_222	CBR1	21	+	666	91	9	1	9.91%	80.17570355655633	0.0001278538856941533	1.8130571119295507e−05	1.8360490984814848e−08
ENSG00000131467_42834817_42841578_193	PSME3	17	+	579	53	3	0	8.81%	53.712197690966335	0.00012558576601528435	0.0001308009201890298	1.169564989540249e−07
ENSG00000140545_88913319_88899395_335	MFGE8	15	−	1005	12	2	0	2.39%	7.0063334188835835	0.030313543781140352	0.004826937407846361	0.0007979355218377862
ENSG00000140545_88913319_88899395_387	MFGE8	15	−	1161	18	2	0	2.58%	9.097370912116281	0.010678932720565162	0.0022011839993586477	0.0001357017294392655
ENSG00000196683_22822779_22817340_55	TOMM7	7	−	165	17	0	2	12.73%	60.456164879609105	0.00847367416878415	0.03299602752967379	0.0014019198044660757
ENSG00000140632_4847265_4805236_472	GLYR1	16	−	1416	14	0	0	1.48%	5.801501365565679	0.008175610787902942	0.008175610787902942	0.00034251843268961324
ENSG00000119777_27034117_27040837_622	TMEM214	2	+	1866	11	0	0	0.96%	3.4590485863593257	0.012798402692974279	0.012798402692974279	0.0007972436888943567
ENSG00000111231_110465196_110453034_262	GPN3	12	−	786	35	0	0	4.58%	26.12889928002863	0.0010073743004618205	0.0010073743004618205	6.293580389004731e−06
ENSG00000111321_6384359_6386420_181	AC005840.1	12	+	543	13	7	2	2.76%	14.048150110146413	0.22096984023559663	0.056923149003329024	0.048282070174702645
ENSG00000177410_49279128_49279205_25	ZFAS1	20	+	75	60	3	2	28.00%	469.4243390652001	0.027657845067597595	0.023857440118675605	0.002933789275920174
ENSG00000159335_6766706_6770442_102	PTMS	12	+	306	106	12	9	28.43%	203.26380694816672	1.8176808080565085e−05	2.1624599447604932e−05	3.093061109606383e−09
ENSG00000285723_10420737_10420546_63	AL034430.2	20	−	189	36	2	6	15.87%	111.7676997774286	0.002418182715931848	0.0038403772236827336	5.27810418431744e−05
ENSG00000091073_76480516_76505601_620	DTX2	7	+	1860	8	4	0	0.81%	2.5237867691677427	0.169362073879829	0.019216965118390855	0.016163991620339734
ENSG00000105723_42240179_42230794_401	GSK3A	19	−	1203	56	4	3	4.74%	27.314799446852874	0.0003406692596502022	0.00018443883641972277	4.320131925689194e−07
ENSG00000006530_141594800_141652924_418	AGK	7	+	1254	16	0	0	1.44%	7.48683156403828	0.013007239811447113	0.013007239811447113	0.0008218654148058079
ENSG00000159176_201496303_201484713_193	CSRP1	1	−	579	158	4	6	9.84%	160.12315538061662	9.373706360862351e−05	0.00011939345589185465	8.064598906831972e−08
ENSG00000231925_33314041_33304268_412	TAPBP	6	−	1236	56	5	1	4.61%	26.585520820844668	0.00023895547138354508	5.6162432427137455e−05	9.917914698168181e−08
ENSG00000015171_180013_252470_517	ZMYND11	10	+	1551	11	0	0	0.97%	4.161563289585107	0.02108409854857798	0.02108409854857798	0.002029891263157455
ENSG00000121067_49611406_49600378_197	SPOP	17	−	591	11	0	0	2.54%	10.92146304931726	0.020613416668581838	0.020613416668581838	0.001946208561389314
ENSG00000175061_16439055_16439603_38	SNHG29	17	+	114	12	3	0	13.16%	61.7663604033158	0.1029516053660342	0.02108409854857798	0.009872287793463467
ENSG00000115275_74462963_74461275_562	MOGS	2	−	1686	22	0	3	1.96%	7.656684059485767	0.0013845019718837994	0.01157547599262317	9.8960288269354e−05
ENSG00000172053_49104733_49104202_128	QARS	3	−	384	13	1	0	3.91%	19.86496226512891	0.03577298629450316	0.02108409854857798	0.003354604907114977
ENSG00000164919_99892021_99887505_75	COX6C	8	−	225	398	3	3	32.00%	1037.9493719330535	1.963911548567778e−05	1.0960740445380867e−05	1.7249443418630812e−09
ENSG00000100865_102362851_102348557_212	CINP	14	−	636	25	13	2	4.72%	23.06526823237029	0.03619603686939757	0.009288069496478249	0.0016682816117574197
ENSG00000144674_37251425_37366103_2209	GOLGA4	3	+	6627	18	0	4	0.36%	1.5937901960113177	0.005554612275049112	0.04471043695002634	0.0013654474274588616
ENSG00000023608_61762461_61794983_368	SNAPC1	14	+	1104	27	6	0	4.89%	14.350608191531252	0.0019103435864441566	6.114625879970644e−05	9.597682521295058e−07
ENSG00000104408_108248702_108240076_75	EIF3E	8	−	225	29	2	0	10.67%	75.62947684939336	0.007225456388766877	0.005604866691947602	0.00021353222845814395
ENSG00000142910_31577149_31583245_156	TINAGL1	1	+	468	6	1	0	3.21%	7.522825946557694	0.0477903522728147	0.016947426762344633	0.0036975118224100542
ENSG00000163902_128650800_128644915_109	RPN1	3	−	327	19	1	6	6.42%	34.09427539082111	0.010042643336235583	0.052370108323589605	0.0026274130286747
ENSG00000143870_10819289_10784258_439	PDIA6	2	−	1317	602	19	19	27.56%	268.2170198531231	3.82016279060415e−21	4.275049111014097e−20	2.764494454277351e−39
ENSG00000003436_187503756_187478676_247	TFPI	2	−	741	31	4	0	6.07%	24.548168878240897	0.001081087078822254	0.0002681497654631663	1.949844281316839e−06
ENSG00000142546_49560691_49555751_304	NOSIP	19	−	912	38	0	2	5.26%	24.449184326312505	0.00016524195568436628	0.0005033193936638034	5.750141298884926e−07
ENSG00000112531_163415257_163565971_298	QKI	6	+	894	21	0	0	4.03%	13.783432774565439	0.0009235399941089087	0.0009235399941089087	5.324735186779001e−06
ENSG00000115525_85863399_85840356_176	ST3GAL5	2	−	528	12	1	2	3.41%	13.335918723443184	0.026602764905793736	0.06032271266817251	0.006860364960240205
ENSG00000143319_156727097_156723349_168	ISG20L2	1	−	504	10	0	1	2.98%	11.642468726815478	0.020613416668581838	0.03537493353818881	0.003252526348827943
ENSG00000143314_156738704_156737398_216	MRPL24	1	−	648	87	3	3	10.65%	78.7807050514514	3.747101137361674e−05	1.575548754843906e−05	4.641782502622811e−09
ENSG00000049860_74685336_74705410_261	HEXB	5	+	783	20	0	2	3.07%	14.988005717279696	0.005306578019052857	0.016170007243128042	0.0004498844289484224
ENSG00000006468_13989019_13895866_448	ETV1	7	−	1344	11	0	1	1.12%	4.802518349811385	0.020613416668581838	0.028542519635534256	0.0026455223939317236
ENSG00000280148_143857434_143884331_140	AL049844.3	6	+	420	7	0	0	3.57%	9.779673730525003	0.019216965118390855	0.019216965118390855	0.0017073955890589052
ENSG00000139131_32755874_32747204_477	YARS2	12	−	1431	20	0	0	1.89%	8.200984260398325	0.0034782380676658805	0.0034782380676658805	6.758309314600117e−05
ENSG00000144824_111884390_111974563_1106	PHLDB2	3	+	3318	16	7	1	0.63%	2.829562019681737	0.10514110692308698	0.007258496433688866	0.004472182841009268
ENSG00000051128_18938982_18929443_358	HOMER3	19	−	1074	13	0	2	1.40%	7.1025563406047505	0.020613416668581838	0.056923149003329024	0.005212417464661139
ENSG00000172466_35340650_35339060_193	ZNF24	18	−	579	15	0	1	2.59%	15.201565384235755	0.020613416668581838	0.02108409854857798	0.001987650065908466
ENSG00000127540_1605409_1599440_56	UQCR11	19	−	168	142	2	2	32.14%	495.9691677623394	8.862094166460118e−05	0.00023470857500707915	1.492130862233656e−07
ENSG00000014919_99732049_99714587_410	COX15	10	−	1230	45	3	0	3.90%	21.467576481640247	0.0005870166795382963	0.00020331044171977792	8.142077103917332e−07
ENSG00000140280_51737622_51723607_215	LYSMD2	15	−	645	9	0	0	2.33%	8.187633820904653	0.016947426762344633	0.016947426762344633	0.0013498980316300959
ENSG00000176623_86508620_86474308_314	RMDN1	8	−	942	13	0	1	1.91%	8.097819012536625	0.012798402692974279	0.023672501042627626	0.0014375227604752335
ENSG00000085231_69369675_69352061_169	AK6	5	−	507	16	0	4	4.14%	18.517725406911246	0.008674846900444944	0.052370108323589605	0.002331093272636393
ENSG00000100241_50474840_50447142_1893	SBF1	22	−	5679	11	0	2	0.48%	1.1365706395750135	0.002478942540635376	0.024779191546489752	0.0003685640769040464
ENSG00000136997_127738590_127740958_330	MYC	8	+	990	70	7	1	7.88%	41.489524917378795	3.883483280678225e−05	4.517740773020254e−06	1.4872135334928209e−09
ENSG00000179262_12946003_12953049_345	RAD23A	19	+	1035	33	0	0	4.35%	18.708941049700005	0.00028877520139654507	0.00028877520139654507	5.646841491566603e−07
ENSG00000163820_45967980_45921765_1027	FYCO1	3	−	3081	9	0	0	0.49%	1.7140616080764366	0.019679754444124863	0.019679754444124863	0.0017849636115922674
ENSG00000163818_45838003_45826314_282	LZTFL1	3	−	846	14	0	0	2.13%	9.710314342365251	0.012798402692974279	0.012798402692974279	0.0007972436888943567
ENSG00000198931_88811899_88809702_134	APRT	16	−	402	93	8	5	14.18%	135.74770999086942	0.0003634707508242938	0.0003291212387702471	8.006659831292054e−07
ENSG00000196233_96949067_96985134_1554	LCOR	10	+	4662	9	0	0	0.32%	1.132780740987452	0.020613416668581838	0.020613416668581838	0.001946208561389314
ENSG00000132376_1516499_1495823_448	INPP5K	17	−	1344	10	0	0	1.12%	4.365925772555805	0.020613416668581838	0.020613416668581838	0.001946208561389314
ENSG00000130779_122380446_122272875_1390	CLIP1	12	−	4170	17	6	2	0.58%	2.392150408905396	0.06699482590911521	0.016170007243128042	0.00504143104966337
ENSG00000111684_6978659_6978337_75	LPCAT3	12	−	225	13	0	0	9.33%	33.902868932486676	0.0078820576498062	0.0078820576498062	0.00031963642907451164
ENSG00000108094_35071242_35010311_720	CUL2	10	−	2160	24	0	0	1.67%	6.519782487016668	0.0008739357725210369	0.0008739357725210369	4.787965623198429e−06

ENSG00000108691_34255350_34256827_99	CCL2	17	+	297	80	1	1	16.16%	158.05533301858588	0.00021265439553880942	0.00022205705531260086	3.2561162005337056e-07
ENSG00000139725_121793525_121780830_134	RHOF	12	-	402	34	0	0	4.48%	49.62819505042538	0.013853924679039932	0.013853924679039932	0.0009252050120140923
ENSG00000134371_193122201_193237739_482	CDC73	1	+	1446	42	0	0	2.90%	17.043414800085067	0.0004481211664877703	0.0004481211664877703	1.3205877300278812e-06
ENSG00000254772_62571557_62559679_317	EEF1G	11	-	951	174	0	2	1.89%	107.36045609535334	0.013640585738808999	0.013640585738808999	0.00089863692579514
ENSG00000196704_68250296_68256969_324	AMZ2	17	+	972	29	0	0	3.70%	17.50682334476698	0.0009430837296224684	0.0009430837296224684	5.543685104868835e-06
ENSG00000143442_151424271_151404802_1010	POGZ	1	-	3030	11	0	1	0.59%	2.130225961104456	0.012798402692974279	0.023672501042627626	0.0014375227604752335
ENSG00000110921_109574823_109596577_344	MVK	12	+	1032	46	0	4	4.94%	26.15494137233431	0.00012278282495802782	0.00082197727627415	7.220656270611895e-07
ENSG00000101856_119236364_119243254_195	PGRMC1	X	+	585	40	17	3	8.21%	40.12173838164104	0.053568588128584	0.0010466504734268915	0.0004585139137806009
ENSG00000175567_73983001_73981512_133	UCP2	11	-	399	20	2	0	4.51%	29.412552573007524	0.03101958831192484	0.013428347753762199	0.001958810244408279
ENSG00000175567_73977955_73975007_220	UCP2	11	-	660	19	1	0	2.73%	16.892163716361367	0.023878473930440184	0.013007239811447113	0.0014709138084317821
ENSG00000089693_6752334_6748795_248	MLF2	12	-	744	71	7	0	11.29%	55.99651894090929	6.266083036366892e-06	1.432345712974874e-06	7.861429959638495e-11
ENSG00000178202_108486309_108474827_330	POGLUT3	11	-	990	13	0	0	1.52%	7.705197484656062	0.02108409854857798	0.02108409854857798	0.002029891263157455
ENSG00000116750_193059230_193016351_346	UCHL5	1	-	1038	14	1	0	1.73%	7.914186833950869	0.023878473930440184	0.013007239811447113	0.0014709138084317821
ENSG00000116750_193059230_193016351_345	UCHL5	1	-	1035	14	1	0	1.74%	7.937126505933335	0.023878473930440184	0.013007239811447113	0.0014709138084317821
ENSG00000116750_193059230_193016351_318	UCHL5	1	-	954	14	1	0	1.89%	8.61103347341824	0.023878473930440184	0.013007239811447113	0.0014709138084317821
ENSG00000116750_193059230_193016351_319	UCHL5	1	-	957	14	1	0	1.88%	8.584039638078371	0.023878473930440184	0.013007239811447113	0.0014709138084317821
ENSG00000168385_241324233_241350174_361	SEPT2	2	+	1083	75	6	1	9.97%	40.63576342323408	1.7455265122155713e-07	5.171310977525534e-08	8.870577633093306e-14
ENSG00000146674_45921140_45914820_194	IGFBP3	7	-	582	100	6	0	12.89%	100.82137866520621	3.657830636984195e-05	5.7650597058383796e-06	1.7546597247556737e-09
ENSG00000141030_17267929_17247098_291	COPS3	17	-	873	32	2	5	4.81%	21.508560781910656	0.0019165825227435488	0.004482050696313214	4.958387400677629e-05
ENSG00000173638_45531578_45515658_338	SLC19A1	21	-	1014	8	0	0	1.48%	4.629431351727812	0.019216965118390855	0.019216965118390855	0.0017073955890589052
ENSG00000126756_47657801_47651842_81	UXT	X	-	243	31	1	0	12.35%	74.85676188796916	0.002744064162451129	0.0023628488670607213	3.7279262481759005e-05
ENSG00000185896_113306517_113320614_308	LAMP1	13	+	924	173	0	9	9.42%	109.86256853122242	1.1558158866017217e-06	2.7499921717321935e-05	2.962787193211633e-10
ENSG00000161203_184176994_184181049_209	AP2M1	3	+	627	34	1	7	5.26%	31.819034147162686	0.0017534708537449623	0.018875778537041504	0.00020514193455088982
ENSG00000064490_19193947_19201719_260	RFXANK	19	+	780	17	3	0	1.92%	12.78880410914808	0.02784981298332479	0.020613416668581838	0.0025842615800075876
ENSG00000070756_100721448_100704298_502	PABPC1	8	-	1506	50	7	6	4.58%	19.48142177395419	0.00014540928233658506	0.00038466426193033745	3.895809032796238e-07
ENSG00000132432_54757588_54752411_68	SEC61G	7	-	204	108	1	2	27.94%	310.6484596755001	7.348101949952925e-05	0.00016691480729352244	8.895761390022922e-08
ENSG00000026508_35139304_35229333_340	CD44	11	+	1020	338	19	23	18.24%	194.44292475985006	9.84854942457517e-11	8.638610102015618e-10	9.96626059782687e-19
ENSG00000182871_45510103_45512398_161	COL18A1	21	+	483	10	2	0	3.11%	12.148663019285717	0.06556525143482543	0.019679754444124863	0.00579448895258773
ENSG00000078674_17935644_18027662_1849	PCM1	8	+	5547	64	8	8	1.62%	6.770136492737698	6.092332320218848e-05	3.429381114351296e-05	1.5824631676329043e-08
ENSG00000071282_8532737_8567598_351	LMCD1	3	+	1053	8	0	0	1.71%	4.457970931293448	0.009815328628645334	0.009815328628645334	0.0004837142251238724
ENSG00000138363_215312143_215325272_84	ATIC	2	+	252	26	1	4	8.33%	60.54083737944049	0.010042643336235583	0.04564478511300255	0.0022706292917759373
ENSG00000198242_28722772_28723718_91	RPL23A	17	+	273	152	140	45	13.19%	326.7055839647913	0.27506838540693507	0.038732610581141004	0.04736146888622022
ENSG00000166946_43185499_43194089_309	CCNDBP1	15	+	927	39	0	2	5.50%	24.68655504792719	0.00011368890353703816	0.0005302436077569723	4.288042893434021e-07
ENSG00000153815_81652287_81709799_586	CMIP	16	+	1758	51	6	4	3.58%	17.0226402818012	0.00019472172638378385	0.00017687841580861182	2.3985497459705644e-07
ENSG00000039068_68810245_68815528_221	CDH1	16	+	663	7	0	1	2.26%	6.1952684265769244	0.019216965118390855	0.04178282491678763	0.003600525600033436
ENSG00000074696_65556886_65576379_245	HACD3	15	+	735	67	0	1	8.57%	53.48882775062654	2.5935781044435533e-05	3.416865027958123e-05	6.838556857470237e-09
ENSG00000143727_264965_272332_85	ACP1	2	+	255	22	3	3	5.88%	50.62419342860001	0.05202961726446396	0.021557223391537678	0.004945949454720384
ENSG00000109917_116787640_116778925_401	ZPR1	11	-	1203	9	2	2	1.25%	4.389878482529927	0.06032271266817251	0.08350359792828288	0.019010920333203073
ENSG00000145632_58457294_58456895_103	PLK2	5	-	309	15	0	1	5.83%	28.484486593762142	0.013007239811447113	0.023878473930440184	0.0014709138084317821
ENSG00000137331_30744518_30743936_156	IER3	6	-	468	42	11	6	9.62%	52.65978162590386	0.004051592760295134	0.00038568882576422547	1.0681835869022328e-05
ENSG00000023287_52674111_52623782_1349	RB1CC1	8	-	4047	21	0	2	0.67%	3.0448205832620467	0.0035291660242640502	0.01344380615658633	0.000260294942673285
ENSG00000169641_23093913_23090890_910	LUZP1	1	-	2730	5	0	0	0.55%	1.0746894209368134	0.012673659338734126	0.012673659338734126	0.000782701129001274
ENSG00000153140_90405689_90394064_78	CETN3	5	-	234	8	3	2	7.69%	20.06086919082052	0.11368416502207135	0.08266357711566169	0.03328115424858219
ENSG00000204568_30617866_30625797_258	MRPS18B	6	+	774	37	0	6	5.81%	28.0502269790252	0.00023587963149241382	0.002356051840367436	3.900032969906179e-06
ENSG00000172775_57173854_57154290_254	FAM192A	16	-	762	24	0	0	4.72%	18.481273191543313	0.0009313263635146231	0.0009313263635146231	5.411461678703094e-06
ENSG00000243927_34073737_34142600_113	MRPS6	21	+	339	17	0	1	7.96%	29.42556697680089	0.0033278027414746945	0.007930666369886513	0.00014480970487777632
ENSG00000123349_53295568_53299345_109	PFDN5	12	+	327	56	6	0	10.09%	100.488390625578	0.013640585738808999	0.0016263209931528667	0.00013537566486704274
ENSG00000153140_90409661_90394064_191	CETN3	5	-	573	13	3	2	4.71%	13.31264486877749	0.030022290370960176	0.020677326986596276	0.0027843047542440305
ENSG00000163938_52686792_52688201_126	GNL3	3	+	378	14	0	1	6.35%	21.73260829005556	0.005160369442893307	0.00912793029372963	0.00024819240235076056
ENSG00000162521_32669128_32679705_173	RBBP4	1	+	519	11	5	0	3.47%	12.436579310494222	0.08266357711566169	0.012798402692974279	0.0052414503803071884
ENSG00000074800_8874908_8871880_63	ENO1	1	-	189	152	10	8	28.57%	471.90806572692077	0.0002826983987097233	0.00047281371754217643	8.952391724974047e-07
ENSG00000020426_60808372_60968586_157	MNAT1	14	+	471	11	0	0	3.18%	13.704001405831214	0.020613416668581838	0.020613416668581838	0.001946208561389314
ENSG00000131389_14443635_14485007_620	SLC6A6	3	+	1860	80	10	3	4.68%	25.237867691677426	1.3105914637044646e-05	6.2887884200256284e-06	6.767351074608523e-10
ENSG00000117450_45519043_45511329_199	PRDX1	1	-	597	977	49	23	32.16%	960.2755009771786	1.136835137365289e-09	1.6823377230520907e-11	2.4565153567559364e-19
ENSG00000162772_212615193_212619555_124	ATF3	1	+	372	15	0	0	4.84%	23.660500960947587	0.013428347753762199	0.013428347753762199	0.0008725593497644525
ENSG00000185803_144359354_144361015_425	SLC52A2	8	+	1275	20	1	0	2.59%	9.204398805200002	0.0012558331315113088	0.0013775385517804596	1.0509596336390694e-05
ENSG00000103197_2060673_2088610_1456	TSC2	16	+	4368	12	5	3	0.62%	1.6120341314052202	0.04946560907745733	0.033570409067276055	0.006921950394855025
ENSG00000138078_44339308_44321356_458	PREPL	2	-	1374	18	0	0	3.06%	7.687079788185591	0.00024035368145103084	0.00024035368145103084	3.9581571392385245e-07
ENSG00000172757_65858099_65855336_166	CFL1	11	-	498	811	51	16	25.30%	955.580168127202	4.060318142563395e-08	1.255068054445319e-07	5.044460439427906e-14
ENSG00000104960_49851497_49860279_360	PTOV1	19	+	1080	32	5	0	4.17%	17.38608663204445	0.0035763217077886345	0.00028351878813536103	7.149954426385882e-06
ENSG00000138744_75940949_75936103_167	NAAA	4	-	501	32	0	4	5.39%	37.47898914692217	0.0033278027414746945	0.02200732127736772	0.0004145611180116456
ENSG00000090316_1338561_1339169_50	MAEA	4	+	150	14	0	0	10.00%	54.76617289094001	0.02108409854857798	0.02108409854857798	0.002029891263157455
ENSG00000182858_49913412_49903838_399	ALG12	22	-	1197	14	3	0	1.25%	6.862928933701756	0.08588638879785576	0.02108409854857798	0.008130108632843268
ENSG00000120306_140194466_140243411_97	CYSTM1	5	+	291	47	4	1	12.37%	94.77209594529384	0.0018120733740713878	0.0010786302489720287	1.1880510518164743e-05
ENSG00000048162_176388539_176383964_237	NOP16	5	-	711	31	6	2	6.75%	25.583956594622368	0.004071547892375548	0.000859170824044518	2.1768183886872667e-05
ENSG00000198055_177430916_177441827_557	GRK6	5	+	1671	6	4	1	0.90%	2.106931503883304	0.165899918248885	0.0477903522728147	0.03110788599674642
ENSG00000160710_154586283_154585744_74	ADAR	1	-	222	14	0	0	6.76%	37.004170872256765	0.02108409854857798	0.02108409854857798	0.002029891263157455

ENSG00000164053_48451738_48465554_634	ATRIP	3	+	1902	19	0	0	0.95%	5.8616340971600955	0.013428347753762199	0.013428347753762199	0.0008725593497644525
ENSG00000138760_76179654_76173872_263	SCARB2	4	−	789	38	2	0	6.08%	28.260654126231945	0.000193372941328359	0.00019604810642485452	2.6319190328233624e−07
ENSG00000105223_40366649_40378173_468	PLD3	19	+	1404	34	1	0	3.63%	14.209782343497867	0.00017709925282996202	0.0001151290146190202	1.4464965079969512e−07
ENSG00000138078_44339308_44321356_458	PREPL	2	−	1374	18	0	0	3.06%	7.687079788185591	0.00024035368145103084	0.00024035368145103084	3.9581571392385245e−07
ENSG00000126067_35609342_35603267_84	PSMB2	1	−	252	35	2	0	5.95%	81.49728108770836	0.044040755831095145	0.02108409854857798	0.004111363880405617
ENSG00000179222_51894305_51901930_778	MAGED1	X	+	2334	18	0	2	1.03%	4.525298898443446	0.005554612275049112	0.018318557839637383	0.0005316522383050482
ENSG00000105953_44624344_44707999_1034	OGDH	7	+	3102	47	7	2	2.71%	8.890612482295456	0.00015218123858451484	5.9691612864838124e−06	8.023613401683988e−09
ENSG00000149311_108292632_108365508_1573	ATM	11	+	4719	18	0	0	0.38%	2.238196149389066	0.013640585738808999	0.013640585738808999	0.00089863692579514
ENSG00000196655_119021660_119023399_101	TRAPPC4	11	+	303	26	0	1	8.91%	50.350795444287144	0.0034782380676658805	0.005588370640926919	0.00010676685949938237
ENSG00000170322_129892774_129864725_1286	NFRKB	11	−	3858	21	0	0	0.39%	3.193983644494946	0.02108409854857798	0.02108409854857798	0.002029891263157455
ENSG00000168066_64778227_64765454_618	SF1	11	−	1854	68	10	8	3.72%	21.52161209306473	0.00030820690004427836	0.00034533786888916567	7.151700298269759e−07
ENSG00000152291_85327962_85322736_437	TGOLN2	2	−	1311	56	2	3	5.03%	25.064610018736847	3.503787992526855e−05	8.434738688455117e−05	2.231575800975992e−08
ENSG00000011478_45692758_45695701_121	QPCTL	19	+	363	13	0	0	5.79%	21.014174958152896	0.0078820576498062	0.0078820576498062	0.00031963642907451164
ENSG00000099875_2050851_2039613_465	MKNK2	19	−	1395	20	5	2	2.15%	8.412622563892475	0.01718512520643334	0.005034872532813168	0.0004570485918098071
ENSG00000196655_119018796_119023399_219	TRAPPC4	11	+	657	31	0	1	6.39%	27.68674754760503	0.0003353510003294976	0.0006716243423686093	1.4880509536321035e−06
ENSG00000117298_21260288_21219955_571	ECE1	1	−	1713	50	3	3	4.20%	17.127274484281966	2.597019403851631e−05	5.2349357447643746e−05	1.0434486324538327e−08
ENSG00000139726_122753702_122769078_198	DENR	12	+	594	26	0	0	3.03%	25.683991615520206	0.011772051206276392	0.011772051206276392	0.000681272511389508
ENSG00000127955_80135161_80217493_354	GNAI1	7	+	1062	26	4	0	2.54%	14.365622429019778	0.015528142895806445	0.0036062891729786097	0.0003077713301577895
ENSG00000026103_89002809_89014450_374	FAS	10	+	1122	18	0	0	1.87%	9.413589687136366	0.00847367416878415	0.00847367416878415	0.0003665024264111182
ENSG00000197563_62157141_62045856_788	PIGN	18	−	2364	12	0	0	0.76%	2.97858083163198	0.011772051206276392	0.011772051206276392	0.000681272511389508
ENSG00000013588_12908250_12912181_339	GPRC5A	12	+	1017	150	4	15	13.57%	86.54578522588497	2.4523450863774506e−09	8.890633562558292e−09	2.3408683452808043e−16
ENSG00000169718_82065059_82058115_473	DUS1L	17	−	1419	14	0	2	1.48%	5.789236034983088	0.007029302661868638	0.03522780890193623	0.0012822660609675817
ENSG00000155508_154863393_154875439_200	CNOT8	5	+	600	14	0	0	3.50%	13.691543222735003	0.00847367416878415	0.00847367416878415	0.0003665024264111182
ENSG00000112667_43229444_43225962_144	DNPH1	6	−	432	71	4	3	11.81%	96.43844928712154	0.00039776729316394607	0.000560139871198347	1.4630384750469772e−06
ENSG00000126746_6679520_6667714_577	ZNF384	12	−	1731	12	4	0	1.21%	4.067801898311959	0.10186826994475356	0.008175610787902942	0.004709405731244299
ENSG00000196943_24300428_24304958_446	NOP9	14	+	1338	6	0	0	1.12%	2.63130234902018	0.016947426762344633	0.016947426762344633	0.0013498980316300959
ENSG00000135926_218281949_218275475_247	TMBIM1	2	−	741	15	0	0	2.83%	11.878146231406886	0.00847367416878415	0.00847367416878415	0.0003665024264111182
ENSG00000113811_53891788_53885558_93	SELENOK	3	−	279	20	0	0	6.45%	42.063112819462376	0.013007239811447113	0.013007239811447113	0.0008218654148058079
ENSG00000109534_109816165_109824431_217	GAR1	4	+	651	16	0	1	4.15%	14.421638680958528	0.0034529206320349946	0.005561255870711926	0.00010554338560930125
ENSG00000110435_34931445_34995172_434	PDHX	11	+	1302	16	1	0	1.61%	7.210819340479264	0.015625008558487224	0.00797940233269714	0.0006253789790324684
ENSG00000131238_40097238_40074061_282	PPT1	1	−	846	81	0	0	9.93%	56.18110440939895	1.4345198332063826e−06	1.4345198332063826e−06	1.81443087270272e−11
ENSG00000146457_159736266_159755611_396	WTAP	6	+	1188	27	4	0	2.78%	13.335918723443184	0.009821676566158627	0.0015208721599504675	9.010826720143868e−05
ENSG00000162368_47333946_47376745_179	CMPK1	1	+	537	9	0	0	2.79%	9.834308779298885	0.019679754444124863	0.019679754444124863	0.0017849636115922674
ENSG00000122952_56359749_56358418_110	ZWINT	10	−	330	21	0	2	5.45%	37.340572425640914	0.013640585738808999	0.037440157020026926	0.002398086303081003
ENSG00000159352_151262180_151267343_365	PSMD4	1	+	1095	31	11	0	3.56%	16.61204852856302	0.011127572954820556	0.0006629342244537616	5.085518031899158e−05
ENSG00000123505_110875106_110893616_214	AMD1	6	+	642	29	1	0	2.80%	26.5056577743201	0.012798402692974279	0.012798402692974279	0.0007972436888943567
ENSG00000143398_151231761_151247865_453	PIP5K1A	1	+	1359	12	0	1	1.10%	5.181284095642385	0.019679754444124863	0.02784981298332479	0.002477909089056847
ENSG00000172977_65712268_65719181_461	KAT5	11	+	1383	9	0	2	1.08%	3.8185277038926255	0.020613416668581838	0.06032271266817251	0.005529087794823047
ENSG00000134049_47176277_47156177_82	IER3IP1	18	−	246	42	9	1	10.98%	100.18202358098783	0.006229864304675134	0.003920835779450732	0.0001326510610907540 8
ENSG00000148444_22316454_22320062_165	COMMD3	10	+	495	17	0	2	5.45%	20.1520549598697	0.0033030736763512156	0.014936572009614516	0.0002738986320205498
ENSG00000118965_19990015_19913558_1181	WDR35	2	−	3543	27	1	0	0.93%	4.471654372975022	0.0016902520194187448	0.0015061527253727427	1.5227828847849133e−05
ENSG00000100811_100239290_100277600_399	YY1	14	+	1197	38	6	5	2.76%	18.627949962904765	0.009851978645844589	0.010110470438853885	0.0004991797616392003
ENSG00000006125_35605733_35723699_880	AP2B1	17	+	2640	53	1	7	2.84%	11.78006153904148	6.573705050248402e−06	0.00011376689561157966	6.429578434254303e−09
ENSG00000188603_28491759_28484033_181	CLN3	16	−	543	17	2	0	3.31%	18.37065783634531	0.044040755831095145	0.013428347753762199	0.0027917083341200354
ENSG00000067225_72200690_72199650_107	PKM	15	−	321	283	22	8	21.50%	517.3173206987992	6.241612241465782e−05	4.570762081638441e−05	2.1363188435827172e−08
ENSG00000167508_88656314_88652525_269	MVD	16	−	807	17	1	10	3.35%	12.360925904752792	0.006112543793728684	0.18398076989739892	0.008013077888104255
ENSG00000176248_137188532_137187477_247	ANAPC2	9	−	741	15	0	0	2.43%	11.878146231406886	0.011772051206276392	0.011772051206276392	0.000681272511389508
ENSG00000185630_164792559_164846676_320	PBX1	1	+	960	21	0	0	1.88%	12.835821771314066	0.013640585738808999	0.013640585738808999	0.00089863692579514
ENSG00000130731_636290_634597_107	METTL26	16	−	321	16	1	1	7.48%	29.247622371663557	0.009288069496478249	0.009288069496478249	0.00043585636963420864
ENSG00000136270_45105650_45103282_233	TBRG4	7	−	699	29	5	0	5.15%	24.34425220474035	0.010798573069263205	0.0009589057725548893	6.715624073655132e−05
ENSG00000105290_35871651_35879441_459	APLP1	19	+	1377	8	0	0	1.09%	3.4090365945185193	0.019679754444124863	0.019679754444124863	0.0017849636115922674
ENSG00000166780_15434654_15586753_151	BMERB1	16	+	453	10	1	0	3.31%	12.953210239105964	0.03537493353818881	0.020613416668581838	0.003252526348827943
ENSG00000140545_88913319_88899395_387	MFGE8	15	−	1161	18	2	0	2.58%	9.097370912116281	0.010678932720565162	0.0022011839993586477	0.0001357017294392655
ENSG00000055950_100987443_100981491_235	MRPL43	10	−	705	88	9	0	7.66%	73.24351389669789	0.00042046454754264 49	9.054713245598514e−05	2.743826929636447e−07
ENSG00000169991_18921081_18908561_235	IFFO2	1	−	705	15	0	0	2.98%	12.484689868755321	0.007029302661868638	0.007029302661868638	0.0002573639808771639
ENSG00000172053_49104733_49103358_174	QARS	3	−	522	13	4	0	2.87%	14.613305574347704	0.14351487215180903	0.02108409854857798	0.01427779647895453
ENSG00000170142_23810476_23890606_160	UBE2E1	3	+	480	52	0	1	7.50%	63.56787924841252	0.0010489198282625518	0.00100366442382108	6.520071851930225e−06
ENSG00000122068_197756524_197781909_251	FYTTD1	3	+	753	11	0	0	3.19%	8.571825580539842	0.004687384229717438	0.004687384229717438	0.00011928172701435494
ENSG00000105373_47754755_47757005_60	NOP53	19	+	180	6	0	0	8.33%	19.559347461050006	0.016947426762344633	0.016947426762344633	0.0013498980316300959
ENSG00000051128_18938982_18929443_325	HOMER3	19	−	975	13	0	0	1.54%	7.823738984420002	0.020613416668581838	0.020613416668581838	0.001946208561389314
ENSG00000064042_41605996_41697185_910	LIMCH1	4	+	2730	17	3	6	1.10%	3.6539440311851656	0.01904707903054789	0.03056701560917638	0.002631323313079151 7
ENSG00000033178_67682143_67663822_272	UBA6	4	−	816	16	0	0	2.21%	11.505498506500002	0.013217226988443315	0.013217226988443315	0.0008469697265466234
ENSG00000104824_38846077_38836722_456	HNRNPL	19	−	1368	118	9	8	6.14%	50.61410088605045	1.4231592959075099e−05	3.946485846766709e−05	4.437515975741084e−09
ENSG00000139350_96917657_96952053_571	NEDD1	12	+	1713	12	0	1	1.05%	4.110545876227672	0.013007239811447113	0.023878473930440184	0.0014709138084317821
ENSG00000167005_56451202_56432712_227	NUDT21	16	−	681	35	0	0	6.61%	30.15758419104626	0.0002610398334131 02	0.0002610398334131 02	4.644323699828953e−07
ENSG00000100116_37807968_37816718_419	GCAT	22	+	1257	8	1	0	1.19%	3.7344816154749414	0.04223451612662591	0.019679754444124863	0.003715148010809822
ENSG00000124783_7301363_7295384_103	SSR1	6	−	309	16	0	0	5.83%	30.38345236667962	0.013007239811447113	0.013007239811447113	0.0008218654148058079

ENSG00000120805_101405934_101395640_164	ARL1	12	-	492	22	0	2	6.71%	26.23814903311586	0.0014409783181973444	0.003092538603222271	2.637025864246733e-05
ENSG00000172270_572635_582577_189	BSG	19	+	567	1212	20	18	31.22%	1254.281964168921	1.2386865019838455e-11	1.0966905668946333e-10	1.663766628928982e-20
ENSG00000173482_7888114_8406162_1384	PTPRM	18	+	4152	33	2	3	0.94%	4.663717241435333	0.0019848805137266086	0.0033021427446191217	3.7872484143859e-05
ENSG00000164300_80177968_80143663_364	SERINC5	5	-	1092	10	3	0	1.65%	5.373447104684067	0.08791701959130671	0.012798402692974279	0.0056117368578515354
ENSG00000105819_103300226_103312216_262	PMPCB	7	+	786	18	2	1	4.58%	13.437719629729012	0.0025320062747194163	0.001582480966117931	2.3626091829657855e-05
ENSG00000105819_103297460_103300215_122	PMPCB	7	+	366	17	0	0	8.20%	27.254828429331972	0.0018018966320388876	0.0018018966320388876	1.9221247299282243e-05
ENSG00000196950_195680553_195717037_528	SLC39A10	2	+	1584	33	4	0	2.27%	12.224592163156252	0.006252299888704362	0.0010405263639310788	4.026455175153929e-05
ENSG00000149100_32583888_32589604_123	EIF3M	11	+	369	47	8	3	12.20%	74.7389699731179	0.003934305922594096	0.0016317067119313	3.757027883404765e-05
ENSG00000148339_128068320_128107444_515	SLC25A25	9	+	1545	10	3	1	1.36%	3.7979315458349525	0.06556525143482543	0.016854695534768073	0.00509884188515607
ENSG00000148339_128091991_128107444_461	SLC25A25	9	+	1383	8	0	1	1.08%	3.394246847904556	0.019679754444124863	0.04223451612662591	0.003715148010809822
ENSG00000054523_10261953_10378402_1660	KIF1B	1	+	4980	8	1	1	0.30%	0.9426191547493978	0.0477903522728147	0.0477903522728147	0.009211062727049486
ENSG00000107897_27240394_27197430_501	ACBD5	10	-	1503	32	0	1	2.59%	12.492996382307387	0.0006424109906750971	0.0009958682880916293	4.052020686628797e-06
ENSG00000088826_4175056_4187407_555	SMOX	20	+	1665	50	10	1	2.88%	17.621033748693698	0.0054035682678658945	0.00023176445685600347	9.43317090891406e-06
ENSG00000204435_31666832_31669926_215	CSNK2B	6	+	645	71	12	5	10.23%	64.5913334760256	0.0006666452877691515	0.00010937320379062025	5.23484437691494e-07
ENSG00000204435_31666832_31669926_215	CSNK2B	6	+	645	71	12	5	10.23%	64.5913334760256	0.0006666452877691515	0.00010937320379062025	5.23484437691494e-07
ENSG00000263020_31666832_31671864_228	AL662899.2	6	+	684	65	12	5	8.33%	55.76129758632677	0.0019475751524943912	0.0003445528786214961	4.478462135732285e-06
ENSG00000177731_18256989_18245138_1183	FLII	17	-	3549	47	4	3	2.03%	7.770831197543113	7.603552193335797e-05	5.398906517019921e-05	3.0448324935766147e-08
ENSG00000104529_143586845_143579792_257	EEF1D	8	-	771	158	0	4	9.34%	120.24812835976267	8.279378352254905e-06	3.52747712715908e-05	2.3742621826643e-09
ENSG00000133112_45338743_45337476_53	TPT1	13	-	159	22	6	4	9.43%	81.18974417794341	0.02108409854857798	0.05202961726446396	0.004853002110546164
ENSG00000164818_756902_785653_396	DNAAF5	7	+	1188	29	2	4	3.79%	14.323764554809348	0.0008082499550292343	0.003008558643275781	1.5112770250654428e-05
ENSG00000108094_35060890_35010311_645	CUL2	10	-	1935	23	0	0	1.71%	6.974651032622482	0.0013984928336429232	0.0013984928336429232	1.1821238358622835e-05
ENSG00000108094_35071242_35010311_720	CUL2	10	-	2160	24	0	0	1.67%	6.519782487016668	0.0008739357725210369	0.0008739357725210369	4.787965623198429e-06
ENSG00000269711_7682254_7682820_188	AC008763.3	19	+	564	20	8	0	5.85%	20.807816447925536	0.08243509129588328	0.0012624147894574274	0.0009110560463464096
ENSG00000197746_71851221_71817441_524	PSAP	10	-	1572	859	54	11	25.95%	320.6389211649228	4.399441121491606e-23	5.806362791980091e-24	4.607441712136435e-45
ENSG00000198242_28722772_28723881_70	RPL23A	17	+	210	113	19	2	8.57%	315.74375187123576	0.013439622348536238	0.013856139512579892	0.0008993698691259064
ENSG00000197746_71851221_71817441_527	PSAP	10	-	1581	848	54	11	25.43%	314.731055919742	9.852410973573922e-23	1.2590406449157446e-23	2.2211412166471297e-44
ENSG00000138696_95104425_95154673_502	BMPR1B	4	+	1506	16	0	1	1.59%	6.23405496766534	0.004873903550586949	0.01007916461776185	0.0002597390186955864
ENSG00000087206_176982615_176905282_719	UIMC1	5	-	2157	8	0	0	0.70%	2.176283444901252	0.019679754444124863	0.019679754444124863	0.0017849636115922674
ENSG00000198242_28722772_28723881_70	RPL23A	17	+	210	113	19	2	8.57%	315.74375187123576	0.013439622348536238	0.013856139512579892	0.0008993698691259064
ENSG00000117697_212791763_212739544_213	NSL1	1	-	639	15	0	0	3.29%	13.774188352852116	0.007593099278532274	0.007593099278532274	0.00029783286705462064
ENSG00000184903_111521447_110963063_110	IMMP2L	7	-	330	13	0	0	6.36%	23.115592453968187	0.008175610787902942	0.008175610787902942	0.00034251843268961324
ENSG00000147535_38267333_38264444_132	PLPP5	8	-	396	10	0	0	3.79%	14.817687470492428	0.019679754444124863	0.019679754444124863	0.0017849636115922674
ENSG00000166347_74263384_74253584_60	CYB5A	18	-	180	11	0	0	10.00%	35.85880367859168	0.012798402692974279	0.012798402692974279	0.0007972436888943567
ENSG00000050426_51048357_51058022_147	LETMD1	12	+	441	17	0	0	4.08%	22.619653526384358	0.013007239811447113	0.013007239811447113	0.0008218654148058079
ENSG00000164176_84137262_83943419_331	EDIL3	5	-	993	11	0	0	1.51%	6.500085258959216	0.020613416668581838	0.020613416668581838	0.001946208561389314
ENSG00000143882_10784971_10785045_24	ATP6V1C2	2	+	72	32	4	3	20.83%	260.79129948066674	0.046295797875114966	0.023199730935452297	0.004693283096906252
ENSG00000175198_100102913_100530166_610	PCCA	13	+	1830	8	0	0	0.98%	2.5651603227606565	0.011570465654371866	0.011570465654371866	0.0006594843062896773
ENSG00000177565_177050593_177037750_254	TBL1XR1	3	-	762	6	4	0	1.97%	4.620318297885828	0.29434406697594495	0.016947426762344633	0.029893606618547678
ENSG00000100902_35292477_35317306_246	PSMA6	14	+	738	94	3	2	6.91%	74.7389699731179	0.00018794081008863267	0.00013974726970550526	1.8464591887511134e-07
ENSG00000065427_75636467_75627895_441	KARS	16	-	1323	60	4	1	5.90%	26.61135708986395	2.1748079095900168e-05	4.409171032095565e-06	8.032538805839145e-10
ENSG00000188603_28489349_28482323_267	CLN3	16	-	801	19	1	0	2.62%	13.918636770035583	0.020525443280229434	0.008574016250729465	0.0008739357725210369
ENSG00000204138_28459117_28496549_686	PHACTR4	1	+	2058	11	4	0	1.02%	3.1363385141625373	0.091912668396609	0.0078820576498062	0.0040606651277917995
ENSG00000204138_28459117_28496549_686	PHACTR4	1	+	2058	11	4	0	1.02%	3.1363385141625373	0.091912668396609	0.0078820576498062	0.0040606651277917995
ENSG00000099204_114491803_114436260_511	ABLIM1	10	-	1533	8	1	2	1.17%	3.062128761025441	0.026602764905793736	0.08266357711566169	0.009436337707064906
ENSG00000077147_96576799_96522263_545	TM9SF3	10	-	1635	127	4	5	8.81%	45.578662890887166	1.9756070697989512e-09	5.097569046778809e-09	1.0867027667238493e-16
ENSG00000006530_141594800_141651645_384	AGK	7	+	1152	15	0	0	1.30%	7.640370101972658	0.02108409854857798	0.02108409854857798	0.002029891263157455
ENSG00000149179_47137656_47161638_234	C11orf49	11	+	702	15	0	0	3.85%	12.538043244262823	0.00327844299461777	0.00327844299461777	6.0372290137947024e-05
ENSG00000085733_70429152_70436408_232	CTTN	11	+	696	27	0	6	2.59%	22.76303368311854	0.013640585738808999	0.1029516053660342	0.00703747379914186
ENSG00000197798_126235002_126262133_275	FAM118B	11	+	825	17	0	0	1.82%	12.091232975921821	0.02108409854857798	0.02108409854857798	0.002029891263157455
ENSG00000204852_110634747_110647892_324	TCTN1	12	+	972	16	0	0	2.16%	9.65893701780247	0.0078820576498062	0.0078820576498062	0.00031963642907451164
ENSG00000127948_75954002_75983528_208	POR	7	+	624	59	9	0	4.81%	55.48084135586299	0.02014906884320511	0.0022543491824521134	0.0002717316256421768
ENSG00000130559_135850224_135811309_1283	CAMSAP1	9	-	3849	13	1	0	0.39%	1.9818512626161344	0.03577298629450316	0.02108409854857798	0.003354604907114977
ENSG00000196141_200416411_200478031_498	SPATS2L	2	+	1494	20	2	0	1.81%	7.855159622911648	0.008364861494636245	0.0035547763465261306	0.00016212294748301082
ENSG00000148688_90871987_90903262_322	RPP30	10	+	966	14	0	0	2.80%	8.504064113500002	0.002943990679779778	0.002943990679779778	4.916404313974209e-05
ENSG00000257923_101816052_102283090_632	CUX1	7	+	1896	10	7	0	1.11%	3.0948334590268995	0.17965032695128647	0.007593099278532274	0.009015184583118783
ENSG00000033627_42478512_42521120_646	ATP6V0A1	17	+	1938	8	2	0	0.77%	2.4222102118947375	0.10105102209468636	0.019679754444124863	0.009164497669452725
ENSG00000130731_636290_635847_147	METTL26	16	-	441	11	2	0	3.40%	14.636246399425174	0.11777945996203976	0.019679754444124863	0.010847351889472574
ENSG00000138757_75662019_75645430_480	G3BP2	4	-	1440	39	2	0	2.71%	15.89196981210313	0.0013761736105699968	0.0006629342244537616	5.740839419390654e-06
ENSG00000163565_159014750_159054971_706	IFI16	1	+	2118	9	0	0	0.71%	2.4934012344114738	0.020613416668581838	0.020613416668581838	0.001946208561389314
ENSG00000100888_21431499_21385613_2533	CHD8	14	-	7599	25	0	5	0.36%	1.9304527695469802	0.0036062891729786097	0.040339912027649666	0.0008591420312047067
ENSG00000105723_42242465_42230794_483	GSK3A	19	-	1449	65	4	1	4.55%	26.322103208452386	9.295530582708931e-05	2.0330004469380697e-05	1.4738570004187963e-08
ENSG00000161013_179801456_179798045_403	MGAT4B	5	-	1209	30	2	12	3.47%	14.560308283660053	0.000971426378142793	0.052496307575546707	0.0004226670570746395
ENSG00000065268_984366_994344_426	WDR18	19	+	1278	35	8	0	4.46%	16.0698864116608	0.0027126661505484343	4.059835149508202e-05	1.0031035462619275e-06
ENSG00000015479_139316089_139329395_552	MATR3	5	+	1656	74	10	2	4.17%	26.22086434995834	0.00036346350304509875	2.6948883025916046e-05	7.822668948588309e-08
ENSG00000164258_53560678_53604851_68	NDUFS4	5	+	204	30	1	20	14.71%	86.29123879875002	0.0028151052444788517	0.08416498417658174	0.0016851219795831656
ENSG00000108846_50679831_50691200_281	ABCC3	17	+	843	21	3	2	3.91%	14.617305931745555	0.007922415784188109	0.0037251911965256856	0.00016027471044301945
ENSG00000108846_50659238_50678082_1013	ABCC3	17	+	3039	112	6	3	5.43%	21.625339739759138	3.9060103270668656e-10	5.6872200140719284e-11	2.625702403318433e-19

ENSG00000047579_15662869_15522975_334	DTNBP1	6	-	1002	13	6	0	1.80%	7.612919670468565	0.1741008397065631	0.013428347753762199	0.012921179834843385
ENSG00000204070_45363532_45367115_156	SYS1	20	+	468	8	0	0	3.85%	10.03043459541026	0.011570465654371866	0.011570465654371866	0.0006594843062896773
ENSG00000260916_55385699_55359499_732	CCPG1	15	-	2196	39	0	1	2.73%	10.420963811215167	3.368517373808133e-05	3.145332252154177e-05	8.131556884151293e-09
ENSG00000175573_65919031_65917459_292	C11orf68	11	-	876	6	0	0	1.71%	4.019043998845891	0.016947426762344633	0.016947426762344633	0.0013498980316300959
ENSG00000143322_179143044_179107718_1043	ABL2	1	-	3129	14	2	1	0.77%	2.625415766583893	0.01599363345933208	0.00912793029372963	0.0007225562286945759
ENSG00000117399_43359342_43363129_457	CDC20	1	+	1371	55	7	0	4.81%	23.539696069097378	0.00012095671017772633	1.63781969658517e-05	1.5788685795417643e-08
ENSG00000108433_46929545_46938760_194	GOSR2	17	+	582	11	0	0	2.58%	11.090351653172682	0.016947426762344633	0.016947426762344633	0.0013498980316300959
ENSG00000188010_38881442_38882515_79	MORN2	2	+	237	8	0	0	7.59%	19.806934137772156	0.009815328628645334	0.009815328628645334	0.0004837142251238724
ENSG00000163479_156020896_156009623_89	SSR2	1	-	267	44	3	169	13.48%	96.69789756024721	0.003623755840117038	0.11625866286625514	0.0030442722933034026
ENSG00000174851_66284965_66284637_79	YIF1A	11	-	237	14	0	0	10.13%	34.662134741101276	0.005160369442893307	0.005160369442893307	0.00014319601891785326
ENSG00000160789_156114919_156139106_634	LMNA	1	+	1902	232	20	11	11.04%	71.57363739690223	2.266811862557301e-11	5.830245142153368e-12	1.631944519888118e-21
ENSG00000157557_38810059_38822889_461	ETS2	21	+	1383	52	3	1	5.42%	22.062604511379615	3.568529010364622e-05	6.668585730439088e-06	1.960520580519893e-09
ENSG00000080845_36462569_36527031_453	DLGAP4	20	+	1359	17	3	1	1.99%	7.340152468826712	0.024710983489837978	0.006112543793728684	0.0007857918783425606
ENSG00000132199_712506_693170_115	ENOSF1	18	-	345	8	0	0	4.35%	13.606502581600003	0.019679754444124863	0.019679754444124863	0.0017849636115922674
ENSG00000125630_112550943_112575729_901	POLR1B	2	+	2703	23	0	0	1.00%	4.9929521820660385	0.0034782380676658805	0.0034782380676658805	6.758309314600117e-05
ENSG00000219200_7012684_7014286_98	RNASEK	17	+	294	99	5	3	22.45%	197.58932639223985	7.209434834108462e-05	2.316730259140134e-05	1.2888437364854621e-08
ENSG00000181929_49005362_49002899_192	PRKAG1	12	-	576	27	0	0	4.69%	27.50533236710157	0.0035547763465261306	0.0035547763465261306	7.044676146010672e-05
ENSG00000147164_71068306_71056332_172	SNX12	X	-	516	17	0	3	4.65%	19.331913188247096	0.005160369442893307	0.0385659205926511	0.0010937528243987265
ENSG00000155256_97744827_97759300_287	ZFYVE27	10	+	861	5	0	2	1.74%	3.407551822482579	0.012673659338734126	0.15865525393145707	0.011061610944025602
ENSG00000197111_53454801_53479442_318	PCBP2	12	+	954	136	7	5	10.38%	83.65003945606291	2.684328031612175e-06	2.694295709433714e-07	6.842903447663729e-12
ENSG00000183207_49007020_49015650_310	RUVBL2	19	+	930	21	3	2	3.55%	13.249880538130649	0.00756007632796937	0.007443125733849675	0.0002910636718876283
ENSG00000108654_64503241_64502922_76	DDX5	17	-	228	125	8	1	25.00%	321.6997937672698	0.00065093332880904930	5.887110733694651e-05	2.916549924988003e-07
ENSG00000108883_44853605_44851274_148	EFTUD2	17	-	444	11	4	0	4.73%	14.5373528426723	0.0416322583317752	0.0078820576498062	0.001684286202785131
ENSG00000175224_46657603_46672332_255	ATG13	11	+	765	11	6	1	1.96%	8.437365571433336	0.172059248067656	0.03577298629450316	0.02599789514137937
ENSG00000130935_67724076_67743859_537	NOL11	17	+	1611	17	1	6	1.49%	6.191972194373372	0.008316137131635013	0.067772724847226	0.0029911215645674312
ENSG00000156515_69343848_69401135_889	HK1	10	+	2667	60	7	3	2.70%	13.200909422530936	0.00020078835655311822	4.3403317049897516e-05	6.539831550755316e-08
ENSG00000142494_19542408_19577336_393	SLC47A1	17	+	1179	14	0	0	1.53%	6.967706474674302	0.013217226988443315	0.013217226988443315	0.0008469697265466234
ENSG00000100300_43159263_43162991_161	TSPO	22	+	483	31	2	3	9.32%	37.66085535978572	0.0006164818938994256	0.0021016973734692764	8.205574567920397e-06
ENSG00000148444_22316418_22319998_162	COMMD3	10	+	486	19	5	2	6.79%	22.93997541728087	0.017597438308343550	0.0024586509195250150	0.00025266743613694200
ENSG00000085662_134449116_134447929_119	AKR1B1	7	-	357	149	6	5	10.92%	244.90275392407148	0.001527289136916534	0.0007124765031541974	6.808835563851866e-06
ENSG00000129103_56064312_56074260_141	SUMF2	7	+	423	74	5	3	7.09%	102.65189447643264	0.013577461948370874	0.007635351959044894	0.0005235313929847659
ENSG00000138326_78033902_78037310_131	RPS24	10	+	393	108	13	2	11.45%	161.25263555674812	0.0013010442815015533	0.000393727242440075	3.348109036281904e-06
ENSG00000160194_42896930_42909021_456	NDUFV3	21	+	1368	18	1	0	1.97%	7.720795050414475	0.009288069496478249	0.0033030736763512156	0.0001685180267781662
ENSG00000106588_42924697_42917574_117	PSMA2	7	-	351	64	7	2	11.11%	106.99130235104276	0.004462641388715142	0.0018493817471908023	4.7782444967540276e-05
ENSG00000163516_219231975_219234365_361	ANKZF1	2	+	1083	15	0	0	1.39%	8.127152684646816	0.021084098548577980	0.021084098548577980	0.00202989126315745500
ENSG00000168036_41234271_41236507_101	CTNNB1	3	+	303	30	0	0	10.89%	58.097071666485164	0.0014915192737735051	0.0014915192737735051	1.337646624223462e-05
ENSG00000129084_14517954_14514353_105	PSMA1	11	-	315	14	2	2	8.57%	26.079129948066672	0.01782474427444472	0.01782474427444472	0.0014835529493238105
ENSG00000141510_7676239_7669609_339	TP53	17	-	1017	101	1	6	4.72%	58.27416205209588	0.00023306224115712893	0.0023656552595847120	3.875584957361342e-06
ENSG00000093144_127331028_127314886_125	ECHDC1	6	-	375	10	0	1	4.00%	15.647477968840004	0.019679754444124863	0.02784981298332479	0.002477909089056847
ENSG00000086504_370218_367675_256	MRPL28	16	-	768	46	2	0	7.81%	35.14570246907423	7.237715554886367e-05	3.592951532853599e-05	1.96263786912504e-08
ENSG00000165629_7788208_7806980_298	ATP5F1C	10	+	894	254	8	5	15.10%	166.7139011780772	9.474040692339726e-09	2.5018986997389888e-09	2.5440584461148106e-16
ENSG00000072518_63895204_63909237_745	MARK2	11	+	2235	9	0	0	0.81%	2.362874189925504	0.011772051206276392	0.011772051206276392	0.000681272511389508
ENSG00000158290_120560638_120526761_895	CUL4B	X	-	2685	31	0	2	1.56%	6.774746047961454	0.0004441315507112266	0.0008462728807417305	2.4367334153721876e-06
ENSG00000074582_218662227_218663386_189	BCS1L	2	+	567	12	0	0	4.23%	12.418633308603177	0.004826937407846361	0.004826937407846361	0.00012612451166366307
ENSG00000136436_48848100_48863005_374	CALCOCO2	17	+	1122	18	0	0	2.67%	9.413589687136366	0.002046028879997488	0.002046028879997488	2.4518070358754455e-05
ENSG00000197150_151034375_151045349_548	ABCB8	7	+	1644	29	0	1	2.37%	10.350749568803835	0.0006629342244537616	0.0009306875619951529	3.907239258273379e-06
ENSG00000110911_51000365_50988325_400	SLC11A2	12	-	1200	15	0	0	2.00%	7.334755297893752	0.004826937407846361	0.004826937407846361	0.00012612451166366307
ENSG00000110921_109574823_109579811_117	MVK	12	+	351	15	0	0	4.27%	25.076086488525647	0.021084098548577980	0.021084098548577980	0.00202989126315745500
ENSG00000011304_804625_808386_179	PTBP1	19	+	537	29	2	0	5.59%	31.688328288851963	0.008661717511597958	0.0023354528714849923	0.00011521534222637746
ENSG00000115107_119245509_119257645_453	STEAP3	2	+	1359	9	0	2	1.10%	3.885963071731789	0.016947426762344633	0.11777945996203976	0.009674391494749069
ENSG00000111678_6944529_6944735_68	C12orf57	12	+	204	40	0	0	10.29%	115.05498506500003	0.00847367416878415	0.00847367416878415	0.0003665024264111182
ENSG00000134684_32817244_32780251_388	YARS	1	-	1164	21	2	0	2.84%	10.586244759846652	0.008090339536935701	0.0013430411379358334	6.595165381462159e-05
ENSG00000085871_139666020_139704148_147	MGST2	4	+	441	35	3	1	7.48%	46.56987490726191	0.00808812032268059	0.0023697301888609516	0.00010883980745233609
ENSG00000089327_35155551_35166423_194	FXYD5	19	+	582	99	5	3	11.34%	99.81316487855415	0.0002559344838384169	4.7579444987819155e-05	9.122436101222974e-08
ENSG00000101654_13734581_13759979_298	RNMT	18	+	894	15	0	0	2.35%	9.8453091246896	0.0078820576498062	0.0078820576498062	0.00031963642907451164
ENSG00000119333_128640693_128636138_137	WDR34	9	-	411	14	1	2	4.38%	19.98765433975913	0.026832695913080365	0.060667625179241055	0.006950196351539762
ENSG00000283103_58312344_58312081_87	AC010642.2	19	-	261	15	0	12	6.90%	33.72301286387932	0.013007239811447113	0.172059248067656	0.012449010896424265
ENSG00000272398_106974646_106971661_80	CD24	6	-	240	41	6	0	8.75%	100.24165573788127	0.032657852871826265	0.00877616296920843	0.0014291000018004636
ENSG00000086289_37948877_37950396_122	EPDR1	7	+	366	19	0	0	6.56%	30.461278832782792	0.005554612275049112	0.005554612275049112	0.00016468457691007306
ENSG00000176697_27658504_27657821_227	BDNF	11	-	681	10	1	0	2.64%	8.616452626013217	0.023672501042627626	0.012798402692974279	0.0014375227604752335
ENSG00000176697_27658504_27657821_227	BDNF	11	-	681	10	1	0	2.64%	8.616452626013217	0.023672501042627626	0.012798402692974279	0.0014375227604752335
ENSG00000176697_27658504_27657821_227	BDNF	11	-	681	10	1	0	2.64%	8.616452626013217	0.023672501042627626	0.012798402692974279	0.0014375227604752335
ENSG00000143379_150927823_150964364_1255	SETDB1	1	+	3765	11	0	0	0.48%	1.7143651161079685	0.012798402692974279	0.012798402692974279	0.0007972436888943567
ENSG00000175166_184301576_184308890_776	PSMD2	3	+	2328	89	1	1	4.90%	22.43275675300838	3.4129856697517863e-08	3.4129856697517863e-08	1.1677590882796056e-14
ENSG00000162129_72434474_72293367_707	CLPB	11	-	2121	17	0	0	0.85%	4.703096277763085	0.011772051206276392	0.011772051206276392	0.000681272511389508
ENSG00000188976_954081_944694_521	NOC2L	1	-	1563	32	0	0	2.30%	12.013418786057585	0.0010073743004618205	0.0010073743004618205	6.293580389004731e-06
ENSG00000204256_32974691_32980718_715	BRD2	6	+	2145	22	2	4	1.82%	6.018260757246155	0.0019774936324837536	0.007183997053108228	8.20703915407641e-05

ENSG00000182220_40581066_40605755_318	ATP6AP2	X	+	954	39	1	4	7.23%	23.9878789616651	1.4382195839923245e-05	7.658455977631608e-05	8.759557247706125e-09
ENSG00000133119_33818179_33836295_356	RFC3	13	+	1068	21	0	1	3.37%	11.537817322529497	0.0007764301431553706	0.0016353363669355965	7.894496093549459e-06
ENSG00000055163_157319804_157393000_787	CYFIP2	5	+	2361	8	4	0	0.76%	1.9882437063329101	0.23639154306007898	0.011570465654371866	0.017275440155518785
ENSG00000006530_141555467_141652924_422	AGK	7	+	1266	17	0	0	1.66%	7.879357981939576	0.00797940233269714	0.00797940233269714	0.000327142761693177
ENSG00000145191_184137579_184142809_437	EIF2B5	3	+	1311	14	0	0	1.14%	6.266152504684212	0.02108409854857798	0.02108409854857798	0.002029891263157455
ENSG00000166452_8911450_8919480_183	AKIP1	11	+	549	12	0	0	3.28%	12.825801613803282	0.011772051206276392	0.011772051206276392	0.000681272511389508
ENSG00000170017_105520147_105545190_279	ALCAM	3	+	837	70	0	0	8.60%	49.0736316227061	6.565625114555139e-06	6.565625114555139e-06	3.5687416513061913e-10
ENSG00000112305_70668629_70860334_457	SMAP1	6	+	1371	22	1	0	1.31%	9.415878427638951	0.016983116543564363	0.013640585738808999	0.0011055102295404414
ENSG00000140416_63048576_63065899_248	TPM1	15	+	744	109	8	7	9.27%	85.96648682477623	0.0001262282821358246	0.000150482303530231	1.3472379671804562e-07
ENSG00000170962_104163927_103909694_364	PDGFD	11	-	1092	9	0	3	1.37%	4.8361023942156605	0.020613416668581838	0.14524829880653062	0.014232744618850459
ENSG00000197912_89508547_89530841_296	SPG7	16	+	888	12	0	0	1.69%	7.929465186912164	0.019679754444124863	0.019679754444124863	0.0017849636115922674
ENSG00000005100_5468859_5444205_707	DHX33	17	-	2121	24	5	0	1.84%	6.63966533331259	0.0104827207134568	0.0006223089785121674	4.514743094980773e-05
ENSG00000104660_30095804_30105862_154	LEPROTL1	8	+	462	31	3	1	8.44%	39.372712421594166	0.0005460508606305346	0.0010527496933334325	3.671867446159757e-06
ENSG00000011260_51260609_51296989_548	UTP18	17	+	1644	16	0	1	1.82%	5.710758382788322	0.0020082573300163734	0.00392533363377418	4.538242985436214e-05
ENSG00000139644_49752494_49762896_237	TMBIM6	12	+	711	661	23	17	32.49%	545.5159777111414	4.4607759695857914e-14	1.475365366222195e-13	8.888324263736637e-26
ENSG00000118564_15644531_15605723_572	FBXL5	4	-	1716	8	0	0	0.87%	2.735573071475525	0.019216965118390855	0.019216965118390855	0.0017073955890589052
ENSG00000135404_55728323_55725561_232	CD63	12	-	696	246	22	6	26.29%	207.3965291128578	2.6552528959412224e-10	2.5295184939073248e-11	8.125620299060758e-20
ENSG00000131899_18234867_18242821_753	LLGL1	17	+	2259	18	0	0	1.20%	4.675541225749005	0.0033030736763512156	0.0033030736763512156	6.124050385493983e-05
ENSG00000111481_54325164_54350523_160	COPZ1	12	+	480	123	9	1	21.25%	150.3624836068219	2.373413040653174e-06	1.848215521193233e-07	4.234034659341311e-12
ENSG00000285043_30067255_30070212_364	AC093512.2	16	+	1092	714	70	30	29.67%	383.6641232744424	2.1584910851290338e-16	3.7216843132467856e-18	1.2870459985292045e-32
ENSG00000140525_89285159_89316459_741	FANCI	15	+	2223	9	0	0	0.81%	2.375629246281377	0.011772051206276392	0.011772051206276392	0.000681272511389508
ENSG00000005194_57440928_57429170_246	CIAPIN1	16	-	738	6	0	0	2.03%	4.7705725514756105	0.016947426762344633	0.016947426762344633	0.0013498980316300959
ENSG00000170540_18801466_18795446_141	ARL6IP1	16	-	423	137	6	4	17.02%	190.0447235577199	8.396268532167667e-06	1.7965759934406335e-05	1.2222761595395175e-09
ENSG00000170779_105011929_105011204_241	CDCA4	14	-	723	17	1	1	3.73%	13.797050076259339	0.004669667620227399	0.004669667620227399	0.0001184258353255006
ENSG00000275740_146203766_146339240_1027	AC091959.3	5	+	3081	27	0	6	1.17%	5.14218482422931	0.0010280153344583009	0.011168955746117197	7.389561266477443e-05
ENSG00000159069_136944091_136944026_21	FBXW5	9	-	63	12	0	0	28.57%	111.7676997774286	0.011772051206276392	0.011772051206276392	0.000681272511389508
ENSG00000121578_119230099_119216339_246	B4GALT4	3	-	738	13	0	0	2.44%	10.336240528197157	0.012798402692974279	0.012798402692974279	0.0007972436888943567
ENSG00000175061_16439055_16439470_49	SNHG29	17	+	147	21	3	0	12.24%	83.82577483307145	0.05352897298295963	0.013640585738808999	0.0034621625354375684
ENSG00000071655_1584884_1578340_270	MBD3	19	-	810	16	0	1	1.85%	11.590724421362966	0.020613416668581838	0.03537493353818881	0.003252526348827943
ENSG00000105011_14136456_14121657_81	ASF1B	19	-	243	24	2	2	7.41%	57.95362210681483	0.025747275800495253	0.025747275800495253	0.0029440564502117394
ENSG00000112992_43644650_43704404_707	NNT	5	+	2121	7	0	0	0.71%	1.9365690555495054	0.019216965118390855	0.019216965118390855	0.0017073955890589052
ENSG00000013588_12908250_12909281_343	GPRC5A	12	+	1029	146	4	15	12.83%	83.25553146686008	5.394176856496585e-09	1.898956246588768e-08	1.0763635069857895e-15
ENSG00000167985_61430147_61438326_133	SDHAF2	11	+	399	12	0	0	3.76%	17.647531543804515	0.02108409854857798	0.02108409854857798	0.002029891263157455
ENSG00000205213_27472302_27367867_951	LGR4	11	-	2853	8	1	0	0.63%	1.6453709746414305	0.028889785561798602	0.009815328628645334	0.0013877953586383081
ENSG00000103174_5033388_5025478_339	NAGPA	16	-	1017	10	0	0	1.47%	5.769719015058999	0.019679754444124863	0.019679754444124863	0.0017849636115922674
ENSG00000132824_44521951_44500296_473	SERINC3	20	-	1419	103	0	0	6.98%	42.59223654308986	2.1086219761622706e-07	2.1086219761622706e-07	4.2038889425931844e-13
ENSG00000064115_27014168_26973965_570	TM7SF3	12	-	1710	35	2	0	2.81%	12.010125633978072	0.0007129716010360364	0.0001863663641484606	9.139709771274696e-07
ENSG00000140848_57121096_57147658_320	CPNE2	16	+	960	8	0	1	1.88%	4.8898368652625015	0.011570465654371866	0.026602764905793736	0.001474889218184677
ENSG00000166508_100101294_100092932_389	MCM7	7	-	1167	107	1	9	9.25%	53.800775792605414	8.488675083732291e-08	3.92544999872585e-06	3.487309372664515e-12
ENSG00000120686_38349920_38360778_85	UFM1	13	+	255	11	1	0	5.88%	25.312096714300004	0.04223451612662591	0.019679754444124863	0.003715148010809822
ENSG00000130520_18323020_18312268_159	LSM4	19	-	477	40	5	0	8.18%	49.20590556238995	0.00500277944419397	0.0006838816678496783	2.2042196630895807e-05
ENSG00000171497_158723288_158709736_370	PPID	4	-	1110	12	1	0	2.16%	6.343572149529731	0.010020958156399907	0.004826937407846361	0.0002559950816326654З
ENSG00000078403_21534705_21741983_1048	MLLT10	10	+	3144	10	0	0	0.57%	1.8663499485734738	0.009815328628645334	0.009815328628645334	0.0004837142251238724
ENSG00000143476_212044668_212102940_668	DTL	1	+	2004	24	0	0	1.65%	7.027310465047906	0.001484234770590415	0.001484234770590415	1.3251387882263029e-05
ENSG00000123297_57782802_57807895_200	TSFM	12	+	600	16	4	4	5.00%	15.647477968840004	0.033371777158467175	0.017641636971747657	0.0026769434285169994
ENSG00000120948_11016999_11022654_283	TARDBP	1	+	849	29	0	0	3.89%	20.043147574927566	0.001565547007472977	0.001565547007472977	1.467919054732344e-05
ENSG00000168216_69790322_69676158_467	LMBRD1	6	-	1401	21	3	0	2.14%	8.795423911821201	0.01424822060979566	0.0022409900782254734	0.00018649561876446586
ENSG00000114742_39068815_39094743_502	WDR48	3	+	1506	17	0	0	1.59%	6.623683403144423	0.005454749182134642	0.005454749182134642	0.00015910859015753364
ENSG00000253729_47960126_47774173_4097	PRKDC	8	-	12291	163	10	6	1.81%	7.78172720564108	2.3868536550551e-12	4.817830187220715e-13	1.4915905606011676e-23
ENSG00000172500_65888418_65883974_364	FIBP	11	-	1092	45	3	5	4.67%	24.180511971078303	0.0008014823932862815	0.001238271318614919	6.1832960422298e-06
ENSG00000112855_140696966_140698552_223	HARS2	5	+	669	12	1	0	2.69%	10.52520939608072	0.019795881618345192	0.012798402692974279	0.0012079910998532793
ENSG00000143727_264965_277304_158	ACP1	2	+	474	37	3	3	7.59%	45.80353519359811	0.0021901507208165597	0.0010405263639310788	1.385022639065595e-05
ENSG00000143727_264965_277304_158	ACP1	2	+	474	53	8	3	10.76%	65.61046933137027	0.0020281690811773884	0.00012922015363027575	1.9594695430042934e-06
ENSG00000100650_69768157_69768924_107	SRSF5	14	+	321	80	10	5	16.82%	146.23811185831778	0.0005759641158097748	0.0005025909556147243	1.8803032497408369e-06
ENSG00000100505_51094930_50977291_707	TRIM9	14	-	2121	8	3	0	0.71%	2.213221777770863	0.14389369507707506	0.019679754444124863	0.013602114125134476
ENSG00000187498_110307027_110178915_821	COL4A1	13	-	2463	21	0	1	1.22%	5.002999959586481	0.0023218226288164415	0.003457660202585786	4.58668892290874e-05
ENSG00000113758_177468173_177457433_586	DBN1	5	-	1758	28	0	0	1.88%	9.345763291969286	0.001484234770590415	0.001484234770590415	1.3251387882263029e-05
ENSG00000186205_220791522_220813418_252	MARC1	1	+	756	5	0	0	1.98%	3.880822908938493	0.012673659338734126	0.012673659338734126	0.000782701129001274
ENSG00000115414_215435802_215361555_2265	FN1	2	-	6795	110	7	10	2.56%	9.49902084201104	2.047867936532335e-10	1.2483331839700447e-09	2.9313629260743297e-18
ENSG00000141560_82716756_82727171_309	FN3KRP	17	+	927	21	3	0	3.24%	13.292760410422334	0.009197853169380737	0.0023218226288164415	0.00012189422965487374
ENSG00000006530_141555467_141652924_422	AGK	7	+	1266	17	0	0	1.66%	7.879357981939576	0.00797940233269714	0.00797940233269714	0.000327142761693177
ENSG00000160752_155317974_155320609_248	FDPS	1	+	744	66	6	2	7.66%	52.05310211408469	0.0005355536484359015	0.00011289501303220994	4.3040803786619567e-07
ENSG00000121057_57105813_57120324_787	AKAP1	17	+	2361	22	5	0	1.14%	5.467670192415503	0.05150015748280293	0.0033030736763512156	0.0010579038602935387
ENSG00000148498_34814935_34111169_1320	PARD3	10	-	3960	19	0	1	0.61%	2.8153606193935614	0.005405141725979036	0.005554612275049112	0.00016047798536882382
ENSG00000120948_11016999_11023327_170	TARDBP	1	+	510	12	0	0	3.53%	13.806598207800004	0.013007239811447113	0.013007239811447113	0.0008218654148058079
ENSG00000086848_111871482_111786397_618	ALG9	11	-	1854	8	4	0	0.81%	2.531954363889968	0.19427189192379535	0.019679754444124863	0.019384065375599255
ENSG00000147642_109579914_109574906_457	SYBU	8	-	1371	9	0	0	1.09%	3.8519502658522984	0.020613416668581838	0.020613416668581838	0.001946208561389314

ENSG00000121064_56998441_57006235_140	SCPEP1	17	+	420	22	0	0	5.00%	30.736117438792864	0.00877616296920843	0.00877616296920843	0.00039161135667564577
ENSG00000121064_56981156_56998384_246	SCPEP1	17	+	738	12	0	2	2.44%	9.541145102951221	0.013217226988443315	0.04375184787808565	0.0027359272655426933
ENSG00000100462_22928599_22920904_595	PRMT5	14	-	1785	45	7	0	4.20%	14.792783794071433	0.00019100914974351442	4.014129735738276e-06	7.197795323371274e-09
ENSG00000170291_7252551_7254886_163	ELP5	17	+	489	28	0	2	6.13%	33.598879074196326	0.0024042815392806777	0.00447458916152286	6.09885447150312e-05
ENSG00000000003_100635246_100630798_123	TSPAN6	X	-	369	30	0	1	8.94%	47.70572551475611	0.0015061527253727427	0.00236147777460975	2.106844318500428e-05
ENSG00000166037_95799268_95831256_473	CEP57	11	+	1419	22	0	0	1.48%	9.097370912116281	0.008574016250729465	0.008574016250729465	0.0003747459614100891
ENSG00000163479_156020896_156018248_94	SSR2	1	-	282	287	1	3	23.40%	597.1843320554628	2.2985161501133994e-05	6.140828680895134e-05	1.08911622536981e-08
ENSG00000068654_86105722_86027423_1702	POLR1A	2	-	5106	21	3	6	0.71%	2.4133154916689197	0.005516113884530951	0.026064745174772808	0.0007609302532879782
ENSG00000115561_86542279_86505804_196	CHMP3	2	-	588	17	0	0	4.08%	16.96474014478827	0.005405141725979036	0.005405141725979036	0.00015637213408961292
ENSG00000001497_65534670_65512775_719	LAS1L	X	-	2157	39	1	0	2.36%	10.609381793893604	0.00017868169825138985	0.00012713412764493257	1.6047129032860347e-07
ENSG00000136521_179618481_179624040_53	NDUFB5	3	+	159	37	2	0	22.64%	136.5463879356321	0.0017658144955514316	0.0010405263639310788	1.1198112383099405e-05
ENSG00000157601_41432071_41458758_662	MX1	21	+	1986	11	1	0	0.76%	3.250042629479608	0.020613416668581838	0.020613416668581838	0.001946208561389314
ENSG00000177674_11747543_11750192_104	AGTRAP	1	+	312	18	0	3	6.73%	33.852716759509626	0.00847367416878415	0.00837382380159347	0.000362422040249726
ENSG00000000971_196673109_196701331_386	CFH	1	+	1158	7	0	0	1.81%	3.5470319229883427	0.00407548579675135	0.00407548579675135	9.14053164909177e-05
ENSG00000184983_42087119_42086183_63	NDUFA6	22	-	189	45	3	0	20.63%	139.70962472178576	0.0006026251277497241	0.0006424109906750971	2.4882418785897563e-06
ENSG00000170017_105520147_105524516_82	ALCAM	3	+	246	34	0	0	14.63%	81.09973337508538	0.0010155991509472172	0.0010155991509472172	6.392786756915814e-06
ENSG00000184500_93898546_93874245_426	PROS1	3	-	1278	13	0	0	1.17%	5.968814952902584	0.020613416668581838	0.020613416668581838	0.001946208561389314
ENSG00000144741_66243286_66377807_183	SLC25A26	3	+	549	10	0	0	2.73%	10.688168011502734	0.019679754444124863	0.019679754444124863	0.0017849636115922674
ENSG00000182196_122981140_122982676_218	ARL6IP4	12	+	654	39	3	1	7.80%	34.99149316426377	0.0007987558521470434	0.00018702083254192264	1.0292491114519113e-06
ENSG00000064601_45891569_45898450_463	CTSA	20	+	1389	104	2	5	5.40%	43.93460336823327	8.220090956320345e-06	3.885258826455451e-05	2.6010433724839864e-09
ENSG00000139974_60982577_61083712_463	SLC38A6	14	+	1389	11	1	0	1.51%	4.6469292024092885	0.016854695534768073	0.007029302661868638	0.0006017499134389055
ENSG00000277157_26189076_26188765_103	HIST1H4D	6	-	309	243	9	14	20.39%	461.4486828189467	4.945644963556789e-05	0.00014896273127643764	5.462867158708055e-08
ENSG00000102158_77895410_77829220_335	MAGT1	X	-	1005	25	5	1	3.28%	14.596527956007465	0.01475453632861615	0.00236147777460975	0.00020240892325058517
ENSG00000175701_110212525_110212355_56	MTLN	2	-	168	28	0	2	17.86%	97.79673730525002	0.0024042815392806777	0.002708918507878203	3.743268655641858e-05
ENSG00000073921_86014950_85959046_490	PICALM	11	-	1470	38	0	5	2.86%	15.168473541222452	0.0004481211664877703	0.003929042651386831	1.1787616094891344e-05
ENSG00000055332_37147806_37107273_551	EIF2AK2	2	-	1653	52	1	2	4.36%	18.458912304439206	1.0227094929764169e-05	1.6066295977898533e-05	1.3228477549572443e-09
ENSG00000153187_244864307_244854450_806	HNRNPU	1	-	2418	203	27	13	7.32%	49.26237635971651	2.778004265593307e-07	2.2053718895192142e-09	7.407954170762384e-15
ENSG00000152234_46091840_46084282_503	ATP5F1A	18	-	1509	274	7	15	13.32%	106.54594839617697	2.7452805277496704e-12	2.51392818949791e-12	8.693982046132606e-23
ENSG00000113638_40747018_40716145_218	TTC33	5	-	654	7	0	0	2.75%	6.280524414098625	0.009815328628645334	0.009815328628645334	0.0004837142251238724
ENSG00000131778_147266075_147295509_603	CHD1L	1	+	1809	18	0	1	1.33%	5.838611182402986	0.004873903550586949	0.015625008558487224	0.000403121801006346
ENSG00000110651_2394127_2396866_165	CD81	11	+	495	153	3	0	20.00%	181.36849463882731	5.737735194735298e-07	2.4033686813656327e-07	1.2871857065132192e-12
ENSG00000109320_102597543_102616594_463	NFKB1	4	+	1389	21	0	0	2.16%	8.87141029550864	0.0023218226288164415	0.0023218226288164415	3.1231198880462635e-05
ENSG00000183258_177514993_177511791_382	DDX41	5	-	1146	20	1	5	2.36%	10.240496052905762	0.006142079104304129	0.04227848696273928	0.0013942017966237796
ENSG00000175334_66003293_66003772_75	BANF1	11	+	225	197	14	8	18.67%	513.7588599769134	0.00048652826472334255	0.0008793782949311993	2.757750418467631e-06
ENSG00000079387_48074748_48045322_444	SENP1	12	-	1332	10	0	0	1.13%	4.4052584371734245	0.020613416668581838	0.020613416668581838	0.001946208561389314
ENSG00000187109_76074219_76049738_368	NAP1L1	12	-	1104	91	12	3	6.25%	48.36686464553126	0.00023763224014700557	1.5548142148013118e-05	3.044601116463459e-08
ENSG00000147684_124543115_124549892_136	NDUFB9	8	+	408	29	4	2	11.03%	41.70743208606251	0.0032364592914596087	0.0008056452657918871	1.6233811652763675e-05
ENSG00000104760_17885509_17864592_297	FGL1	8	-	891	33	1	1	5.39%	21.73260829005556	0.00028658162324229414	0.00016484578434305903	3.2734509361577746e-07
ENSG00000184117_29581082_29570158_91	NIPSNAP1	22	-	273	19	0	1	7.69%	40.83819799559891	0.008574016250729465	0.013991246267585163	0.000591351246454473
ENSG00000172590_22833438_22834321_64	MRPL52	14	+	192	27	1	5	17.19%	82.5159971013047	0.0021748195570013406	0.012088529595134127	0.00015284085456361443
ENSG00000106028_141743665_141750354_85	SSBP1	7	+	255	31	2	0	15.29%	71.33409074030001	0.0014891271583564725	0.0005770250423907659	5.457984496189206e-06
ENSG00000161513_74872944_74862817_491	FDXR	17	-	1473	13	8	0	1.43%	5.178645967284115	0.23559043810812774	0.0078820576498062	0.013321720827107576
ENSG00000123992_219386772_219374292_410	DNPEP	2	-	1230	17	2	2	1.95%	8.109973337508539	0.02671499548383061	0.02671499548383061	0.003152782508864385
ENSG00000198668_90403900_90404717_77	CALM1	14	+	231	66	4	0	27.27%	167.6515496661429	0.00017125867357046663	2.6912780514484047e-05	3.570715200798569e-08
ENSG00000205336_57650288_57663582_693	ADGRG1	16	+	2079	71	4	0	4.33%	20.039158293427853	3.4901523427252256e-06	6.614649994567748e-07	2.087834633512807e-11
ENSG00000205336_57650288_57663582_687	ADGRG1	16	+	2061	71	4	0	4.37%	20.21417277633989	3.4901523427252256e-06	6.614649994567748e-07	2.087834633512807e-11
ENSG00000118007_136630898_136338254_1258	STAG1	3	-	3774	7	0	0	0.40%	1.0883579668310814	0.019216965118390855	0.019216965118390855	0.0017073955890589052
ENSG00000119718_75003090_75009188_318	EIF2B2	14	+	954	41	0	0	5.97%	25.218026600724848	5.4081826622593156e-05	5.4081826622593156e-05	2.1858222664711426e-08
ENSG00000285565_31829595_31835419_55	AL671762.1	6	+	165	8	1	0	9.09%	28.449959943345462	0.019216965118390855	0.019216965118390855	0.0017073955890589052
ENSG00000136261_16689838_16706088_225	BZW2	7	+	675	10	0	0	3.56%	8.693043316022225	0.00414960799764038	0.00414960799764038	9.45964903272165e-05
ENSG00000133773_82358376_82356929_164	CCDC59	12	-	492	23	3	5	4.88%	27.430792170984763	0.024498661976836228	0.009288069496478249	0.0011196858224149957
ENSG00000169689_82022861_82019205_63	CENPX	17	-	189	56	1	3	20.63%	173.86086632044447	0.0006885945587055891	0.0017914270203067853	7.733845775290168e-06
ENSG00000151327_35046533_35081228_213	FAM177A1	14	+	639	29	3	0	7.04%	26.63009748218076	0.0005610828647514753	0.00027685889791379426	1.039467627697229e-06
ENSG00000166377_79096588_79377383_1070	ATP9B	18	+	3210	9	1	1	0.56%	1.645178758406075	0.02305674932606751	0.02305674932606751	0.002397884865356477
ENSG00000184009_81512354_81510690_375	ACTG1	17	-	1125	686	106	22	21.33%	357.80566288747474	9.755374408544912e-11	1.0362837595840047e-13	1.601133678667311e-22
ENSG00000163110_94523808_94664067_565	PDLIM5	4	+	1695	91	2	12	6.19%	31.50266582222213	8.976557121876814e-08	1.3999896692044456e-06	1.2488650112397084e-12
ENSG00000101654_13734581_13759979_298	RNMT	18	+	894	15	0	0	2.35%	9.8453091246896	0.0078820576498062	0.0078820576498062	0.00031963642907451164
ENSG00000131467_42833632_42841578_267	PSME3	17	+	801	55	3	1	6.74%	40.290790650103006	8.332796070245443e-05	0.00010924350985083219	6.599888983271902e-08
ENSG00000145715_87268455_87390883_1046	RASA1	5	+	3138	12	5	3	0.57%	2.243902194384322	0.11379025261811354	0.08620755130569058	0.0345288686728861
ENSG00000145247_48897834_48885484_92	OCIAD2	4	-	276	20	1	2	8.70%	42.52032056750001	0.009341697731498884	0.016228925652561413	0.0007484770126118359
ENSG00000140950_84497490_84479913_429	MEAK7	16	-	1287	6	0	0	1.40%	2.735573071475525	0.007152939217714824	0.007152939217714824	0.0002660027525696252
ENSG00000111859_11191130_11185162_588	NEDD9	6	-	1764	20	5	0	1.87%	6.652839272465988	0.018103472727687364	0.0013845019718837994	0.00016097545794732337
ENSG00000134333_18396843_18407281_332	LDHA	11	+	996	576	24	9	15.96%	339.3428957097832	1.0678239339972586e-10	4.5319795175477673e-10	5.61667038708755e-19
ENSG00000140443_98649582_98957442_1367	IGF1R	15	+	4101	43	3	4	1.17%	6.152537972385883	0.0006394447605033927	0.0007933619615132744	3.23158037643184e-06
ENSG00000116717_67685495_67687697_60	GADD45A	1	+	180	17	9	1	8.33%	55.41815113964168	0.1995375982741186	0.028889785561798623	0.026316151282870313
ENSG00000136045_107685900_107712220_501	PWP1	12	+	1503	33	0	1	3.19%	12.883402519254494	0.00016484578434305903	0.00028765669904259547	3.285569460041191e-07
ENSG00000136522_179598701_179588872_125	MRPL47	3	-	375	21	2	0	7.20%	32.85970373456401	0.01344380615658633	0.0035291660242640502	0.000260294942673285

ENSG00000118855_158802153_158828982_465	MFSD1	3	+	1395	19	0	0	1.94%	7.991991435697852	0.0034529206320349946	0.0034529206320349946	6.664817351595063e-05
ENSG00000128739_24976387_24978444_161	SNRPN	15	+	483	25	0	0	4.35%	30.371657548214294	0.00877616296920843	0.00877616296920843	0.00039161135667564577
ENSG00000127922_96709763_96673760_89	SEM1	7	-	267	34	0	11	11.24%	74.72110266019102	0.0023766146074493525	0.10274152173808476	0.0019162447551253363
ENSG00000115233_161318826_161411400_310	PSMD14	2	+	930	67	4	4	8.39%	42.27342838355969	2.5288549577691826e-05	1.533060453589004e-05	3.06012960688112e-09
ENSG00000187514_231708707_231712851_107	PTMA	2	+	321	178	8	9	9.35%	325.37979888475707	0.0025025372014526115	0.004926212446124825	6.957895768210636e-05
ENSG00000068654_86105722_86027423_1641	POLR1A	2	-	4923	21	3	6	0.73%	2.5030243551617923	0.005516113884530951	0.026064745174772808	0.0007609302532879782
ENSG00000122550_23125124_23174298_455	KLHL7	7	+	1365	14	0	0	1.32%	6.018260757246155	0.011772051206276392	0.011772051206276392	0.000681272511389508
ENSG00000141985_4400368_4361600_304	SH3GL1	19	-	912	7	0	0	1.64%	4.503797112741777	0.019216965118390855	0.019216965118390855	0.0017073955890589052
ENSG00000148926_10305701_10306641_185	ADM	11	+	555	16	0	0	3.24%	16.91619239874595	0.013428347753762199	0.013428347753762199	0.0008725593497644525
ENSG00000090686_21756576_21679417_856	USP48	1	-	2568	8	4	1	0.70%	1.8279763982289723	0.11368416502207135	0.026602764905793736	0.013188743089541226
ENSG00000121390_19782754_19677800_392	PSPC1	13	-	1176	19	1	2	2.04%	9.480295963264032	0.007181907427766128	0.015570605297898355	0.0005669161536572745
ENSG00000182199_57230105_57234361_513	SHMT2	12	+	1539	52	6	16	4.09%	19.826239141805072	0.0006457820660227714	0.0021961434262326132	8.950935731874315e-06
ENSG00000173744_227491587_227554495_493	AGFG1	2	+	1479	18	0	0	2.03%	7.141343900586208	0.0021227925778456486	0.0021227925778456486	2.6309315010939275e-05
ENSG00000135454_57632132_57626744_478	B4GALNT1	12	-	1434	12	0	0	1.05%	4.910296433736403	0.020613416668581838	0.020613416668581838	0.001946208561389314
ENSG00000077232_182756443_182777132_199	DNAJC10	2	+	597	13	0	0	3.02%	12.777463165510053	0.013428347753762199	0.013428347753762199	0.0008725593497644525
ENSG00000130414_240025301_239961118_390	NDUFA10	2	-	1170	36	1	1	3.59%	18.054782271738464	0.0005574441649685308	0.0005574441649685308	2.0127594615230986e-06
ENSG00000148019_78240031_78266762_618	CEP78	9	+	1854	21	0	0	1.78%	6.646380205211167	0.0012558331315113088	0.0012558331315113088	9.614604369954505e-06
ENSG00000101361_2656887_2658294_170	NOP56	20	+	510	7	0	0	2.94%	8.053848954550002	0.016947426762344633	0.016947426762344633	0.0013498980316300959
ENSG00000120438_159788141_159779045_534	TCP1	6	-	1602	108	0	3	4.68%	39.55823082010113	5.696602288596049e-06	1.4876608012511523e-05	6.984851170914464e-10
ENSG00000184752_95003680_94971440_145	NDUFA12	12	-	435	101	3	2	21.38%	136.24097197007245	1.9411955000405834e-06	1.5203893429944198e-06	2.58545831773849e-11
ENSG00000155508_154863393_154875439_254	CNOT8	5	+	762	18	0	0	3.15%	13.860954893657484	0.005554612275049112	0.005554612275049112	0.00016468457691007306
ENSG00000166165_103522406_103519864_352	CKB	14	-	1056	11	4	0	1.70%	6.112296081578126	0.10000264635955686	0.012798402692974279	0.006484128903061078
ENSG00000068366_109683363_109644029_670	ACSL4	X	-	2010	36	1	3	2.39%	10.509500128325376	0.0002765532368236473 6	0.0009450514886518204	1.7500984682627828e-06
ENSG00000164904_126582881_126544965_377	ALDH7A1	5	-	1131	23	0	0	2.39%	11.932758397988067	0.0033278027414746945	0.0033278027414746945	6.211806171561056e-05
ENSG00000142168_31659770_31668578_135	SOD1	21	+	405	350	11	1	27.41%	507.09419343462974	9.031103472522433e-08	7.38163987753338e-08	6.505814287475193e-14
ENSG00000204264_32843996_32840959_276	PSMB8	6	-	828	27	5	3	3.99%	19.134144255375006	0.003549695279765918	0.007908246895360361	0.0001531162308011883
ENSG00000003056_8942469_8941818_58	M6PR	12	-	174	9	3	0	8.62%	30.350711577491385	0.14524829880653062	0.020613416668581838	0.014232744618850459
ENSG00000003056_8946404_8942484_133	M6PR	12	-	399	30	6	15	6.77%	44.11882885951129	0.021136549012736098	0.1519415333234279	0.015270057611866584
ENSG00000084092_56977585_56963450_698	NOA1	4	-	2094	20	0	0	1.43%	5.6043975533094565	0.002046028879997488	0.002046028879997488	2.4518070358754455e-05
ENSG00000082701_120093359_119826788_395	GSK3B	3	-	1185	24	0	0	4.05%	11.884160482663294	0.00014480970487777632	0.00014480970487777632	1.4822276924058578e-07
ENSG00000145592_40835185_40832488_74	RPL37	5	-	222	164	12	11	10.81%	433.477430217865	0.012070269314219385	0.045982552676424815	0.0026680981546566393
ENSG00000168906_85541611_85542062_122	MAT2A	2	+	366	45	4	1	9.84%	72.14513407764346	0.005079719694443635	0.0009829516685359422	3.084542292842951e-05
ENSG00000143106_109413127_109402013_164	PSMA5	1	-	492	58	5	2	9.15%	69.17330199639636	0.0007806331660549522	0.0005330961663265602	2.6748466647885183e-06
ENSG00000186625_149632871_149595036_422	KATNA1	6	-	1266	9	0	0	1.42%	4.17142481396801	0.011772051206276392	0.011772051206276392	0.000681272511389508
ENSG00000089280_31180215_31188352_263	FUS	16	+	789	82	7	6	8.75%	60.983516798711044	0.00011137210608356469	8.873706848134339e-05	7.146220371541651e-08
ENSG00000089280_31185038_31191438_307	FUS	16	+	921	114	1	39	8.14%	72.63080164689578	6.080941165609422e-06	0.014035767869427632	1.6891849710757947e-06
ENSG00000139684_46781549_46775612_133	ESD	13	-	399	51	4	1	8.27%	75.00200906116919	0.0016263209931528667	0.0015806110104534784	1.5366000304906956e-05
ENSG00000234745_31356946_31354483_334	HLA-B	6	-	1002	21	4	0	2.69%	12.297793313833836	0.028844092512703905	0.0036581908865286695	0.0006004738415469464
ENSG00000163939_52679711_52589249_856	PBRM1	3	-	2568	9	1	0	0.70%	2.056473448007594	0.026832695913080365	0.011772051206276392	0.0015103817605807263
ENSG00000197563_62157141_62045856_827	PIGN	18	-	2481	12	0	0	0.73%	2.8381157138162036	0.011772051206276392	0.011772051206276392	0.000681272511389508
ENSG00000137145_19276175_19372173_1909	DENND4C	9	+	5727	14	2	3	0.52%	1.4344204528795184	0.013900737735337839	0.016262090632663734	0.001079581874877004
ENSG00000139620_48681632_48654044_492	KANSL2	12	-	1476	24	2	0	2.24%	9.541145102951221	0.005006301263112021	0.001484234770590415	4.3930092952350055e-05
ENSG00000152556_48131382_48145708_705	PFKM	12	+	2115	47	0	0	3.12%	13.039564974033336	9.836921317677237e-06	9.836921317677237e-06	7.867021274290272e-10
ENSG00000092010_24138540_24138816_33	PSME1	14	+	99	19	0	1	24.24%	112.61442477574245	0.005554612275049112	0.005554612275049112	0.00016468457691007306
ENSG00000092010_24138214_24138478_37	PSME1	14	+	111	24	6	1	13.51%	126.87144299059463	0.12378039365238508	0.02108409854857798	0.01208667083877082
ENSG00000135940_97647117_97648108_78	COX5B	2	+	234	101	0	1	17.95%	253.26847353410903	0.0004851198761851069	0.0004851198761851069	1.539226456240155e-06
ENSG00000086200_62437280_62579410_863	IPO11	5	+	2589	18	0	0	0.70%	4.079585797206258	0.013428347753762199	0.013428347753762199	0.0008725593497644525
ENSG00000175931_76453141_76390943_1292	UBE2O	17	-	3876	22	3	2	0.85%	3.330539041355264	0.011821942446875977	0.005952040056363293	0.00036448562555597974
ENSG00000167085_49413264_49404992_272	PHB	17	-	816	102	4	2	6.25%	73.34755297893751	0.00014002510398755815	0.0001854842840713172	1.8263145419340512e-07
ENSG00000108091_59906358_59792917_452	CCDC6	10	-	1356	6	0	0	1.11%	2.596373556776549	0.016947426762344633	0.016947426762344633	0.0013498980316300959
ENSG00000133961_73355751_73286994_245	NUMB	14	-	735	10	0	0	2.45%	7.983407126959185	0.012798402692974279	0.012798402692974279	0.0007972436888943567
ENSG00000127184_86618056_86619469_63	COX7C	5	+	189	92	1	1	12.70%	285.6285660978731	0.00575691403842271	0.007400806090490468	0.0002240461484226741
ENSG00000049656_1323866_1318369_138	CLPTM1L	5	-	414	21	0	0	5.07%	29.764224397250008	0.008574016250729465	0.008574016250729465	0.0003747459614100891
ENSG00000153187_244864307_244854450_806	HNRNPU	1	-	2418	203	27	13	7.32%	49.26237635971651	2.778004265593307e-07	2.2053718895192142e-09	7.407954170762384e-15
ENSG00000134371_193122201_193237074_508	CDC73	1	+	1524	42	0	0	2.76%	16.171114042600397	0.0004481211664877703	0.0004481211664877703	1.3205877300278812e-06
ENSG00000102181_150898588_150769034_272	CD99L2	X	-	816	24	0	0	3.31%	17.258247759750002	0.003684287313187465	0.003684287313187465	7.541963121699725e-05
ENSG00000082781_124859278_124763623_691	ITGB5	3	-	2073	138	5	2	6.51%	39.06208320730681	1.0728105969214392e-08	3.2633152925485685e-09	3.7287311541972963e-16
ENSG00000104870_49513401_49526119_365	FCGRT	19	+	1095	13	0	0	1.64%	6.966342931332878	0.012798402692974279	0.012798402692974279	0.0007972436888943567
ENSG00000108107_55386379_55387289_62	RPL28	19	+	186	418	54	21	27.42%	1318.6785868901454	0.0009190739260373504	0.00048443517260985205	2.8689647365565095e-06
ENSG00000166780_15434654_15586829_204	BMERB1	16	+	612	10	1	0	2.45%	9.587915422083336	0.03537493353818881	0.020613416668581838	0.003252526348827943
ENSG00000196923_177496512_177490028_191	PDLIM7	5	-	573	9	0	4	3.14%	9.216446447615185	0.011772051206276392	0.14044376265978525	0.009046049034002456
ENSG00000018510_177437018_177558786_429	AGPS	2	+	1287	26	3	0	2.80%	11.854149976393941	0.00556715574294182	0.000991050613121974	3.4173181281555684e-05
ENSG00000041982_115086281_115021157_1445	TNC	9	-	4335	10	0	0	0.42%	1.3535880595882357	0.012798402692974279	0.012798402692974279	0.0007972436888943567
ENSG00000105887_135977082_135929926_112	MTPN	7	-	336	66	0	4	18.75%	115.26044039547324	2.3581273365087796e-05	0.00017114371649090893	3.159198651738125e-08
ENSG00000261460_72351140_72351259_39	AC009690.2	15	+	117	17	0	1	12.82%	85.2586940609872	0.02108409854857798	0.028889785561798623	0.002731532589007073
ENSG00000079616_29790760_29797092_89	KIF22	16	+	267	10	0	0	6.74%	21.976794900056184	0.011570465654371866	0.011570465654371866	0.0006594843062896773
ENSG00000099282_69451595_69506978_294	TSPAN15	10	+	882	28	0	0	3.74%	18.627949962904765	0.0014915192737735051	0.0014915192737735051	1.337646624223462e-05

ENSG00000144591_219501485_219506736_350	GMPPA	2	+	1050	15	0	2	1.43%	8.382577483307145	0.02108409854857798	0.0700082515985845	0.006564739044797468
ENSG00000115677_241262752_241229601_959	HDLBP	2	-	2877	28	5	0	1.36%	5.710758382788322	0.007942117638539853	0.0006424109906750971	3.4218256453608685e-05
ENSG00000136854_127653778_127690857_544	STXBP1	9	+	1632	9	2	0	0.92%	3.2359214549531257	0.08350359792828288	0.020613416668581838	0.007749259071793911
ENSG00000135387_34071753_34097781_613	CAPRIN1	11	+	1839	50	2	0	4.40%	15.953790751264277	6.524192347581214e-06	1.940453335491528e-06	1.0930444540327216e-10
ENSG00000186132_119339959_119302472_126	C2orf76	2	-	378	10	0	0	3.97%	15.523291635753973	0.020613416668581838	0.020613416668581838	0.001946208561389314
ENSG00000198522_27628708_27650200_362	GPN1	2	+	1086	24	0	0	2.49%	12.967523178596688	0.003684287313187465	0.003684287313187465	7.541963121699725e-05
ENSG00000163349_113940384_113973512_1165	HIPK1	1	+	3495	6	0	0	0.43%	1.0073483670927041	0.016947426762344633	0.016947426762344633	0.0013498980316300959
ENSG00000164924_100924973_100920693_125	YWHAZ	8	-	375	11	0	0	4.00%	17.212225765724003	0.019679754444124863	0.019679754444124863	0.0017849636115922674
ENSG00000003989_17543340_17565146_658	SLC7A2	8	+	1974	17	0	0	1.37%	5.053326851639059	0.0033030736763512156	0.0033030736763512156	6.124050385493983e-05
ENSG00000124920_61766142_61786143_1045	MYRF	11	+	3135	12	1	0	0.57%	2.2460494692114836	0.019795881618345192	0.012798402692974279	0.0012079910998532793
ENSG00000179051_17438514_17409090_522	RCC2	1	-	1566	67	6	12	4.79%	25.104909576443493	6.174797939587089e-05	0.0003662984066582837	1.6751271798113142e-07
ENSG00000179051_17438514_17409090_522	RCC2	1	-	1566	67	6	12	4.79%	25.104909576443493	6.174797939587089e-05	0.0003662984066582837	1.6751271798113142e-07
ENSG00000152894_128520358_128000272_876	PTPRK	6	-	2628	16	1	1	0.80%	3.5724835545296814	0.011306643944114857	0.011306643944114857	0.0006314637693596251
ENSG00000166801_59152092_59153504_470	FAM111A	11	+	1410	8	0	0	1.06%	3.329250631668086	0.019679754444124863	0.019679754444124863	0.0017849636115922674
ENSG00000135018_83666430_83661787_172	UBQLN1	9	-	516	24	0	0	6.40%	27.292112736348844	0.0014915192737735051	0.0014915192737735051	1.337646624223462e-05
ENSG00000135018_83679918_83669173_230	UBQLN1	9	-	690	20	5	2	4.78%	17.008128227000004	0.031014378990889855	0.007480508837587838	0.0011823292938527442
ENSG00000175063_45814437_45816767_50	UBE2C	20	+	150	28	5	0	12.00%	109.53234578188003	0.14532313704036504	0.013007239811447113	0.010137492882366636
ENSG00000175063_45812696_45815877_79	UBE2C	20	+	237	18	0	1	6.33%	44.56560180998735	0.02108409854857798	0.022899794555593333	0.0021928557012350514
ENSG00000117410_43975041_43978007_252	ATP6V0B	1	+	756	51	3	0	7.54%	39.58439367117263	0.00011708974100859733	5.934787645966978e-05	5.105199252329913e-08
ENSG00000171202_85654228_85656501_104	TMEM126A	11	+	312	26	0	0	5.77%	48.89836865262501	0.013428347753762199	0.013428347753762199	0.0008725593497644525
ENSG00000125611_112584648_112586533_158	CHCHD5	2	+	474	18	0	0	5.06%	22.282800904993675	0.004873903550586949	0.004873903550586949	0.00012846764509679302
ENSG00000005175_47702768_47663505_607	RPAP3	12	-	1821	27	0	0	2.64%	8.700203977732292	0.00017086036933386465	0.00017086036933386465	2.0430238215851987e-07
ENSG00000156113_77637360_76877869_1087	KCNMA1	10	-	3261	15	4	2	0.74%	2.6990819863454467	0.048994651353958696	0.02389626957043539	0.005091569865673637
ENSG00000198276_63945680_63939976_340	UCKL1	20	-	1020	10	0	1	1.76%	5.752749253250001	0.011570465654371866	0.01871402756203153	0.0010439183847388118
ENSG00000171310_104457442_104757803_342	CHST11	12	+	1026	9	0	0	1.46%	5.147196700276317	0.019679754444124863	0.019679754444124863	0.0017849636115922674
ENSG00000033867_27403173_27376764_461	SLC4A7	3	-	1383	15	0	0	1.95%	6.364212839821042	0.002874854311667729	0.002874854311667729	4.6982854837323694e-05
ENSG00000087460_58907985_58910829_237	GNAS	20	+	711	201	17	6	17.72%	165.88307340384182	3.597550509915525e-06	4.816267963584106e-08	1.895682211843631e-12
ENSG00000006468_13989019_13895866_466	ETV1	7	-	1398	11	0	1	1.07%	4.6170133491748935	0.020613416668581838	0.028542519635534256	0.0026455223939317236
ENSG00000129197_5421311_5421547_78	RPAIN	17	+	234	20	2	0	6.41%	50.15217297705129	0.04584514077471458	0.02108409854857798	0.004277928753731027
ENSG00000148737_112950757_113165972_619	TCF7L2	10	+	1857	11	0	0	0.97%	3.4758129575371575	0.012798402692974279	0.012798402692974279	0.0007972436888943567
ENSG00000157349_70324649_70333582_328	DDX19B	16	+	984	14	0	0	1.52%	8.348501965082319	0.019679754444124863	0.019679754444124863	0.0017849636115922674
ENSG00000135297_73461855_73500735_732	MTO1	6	+	2196	10	2	8	0.82%	2.6720420028756835	0.07134538375819317	0.11411551976453232	0.02947800993301872
ENSG00000038274_163505687_163518363_334	MAT2B	5	+	1002	24	6	6	3.89%	14.054620930095812	0.011510770535392872	0.0177349877353449	0.0009863240002409624
ENSG00000153395_1501594_1463651_486	LPCAT1	5	-	1458	25	0	1	3.50%	10.061392726877575	7.902559150230045e-05	0.00017287710693640943	9.869440830278292e-08
ENSG00000134153_34101839_34084334_242	EMC7	15	-	726	168	4	2	11.16%	135.78389972960335	1.6983159681743925e-05	6.722130385638495e-06	9.340125143732927e-10
ENSG00000055130_148730123_148800582_732	CUL1	7	+	2196	22	0	1	1.09%	5.8784924063265045	0.0053557511377634245	0.00934169773149884	0.00026267894186613697
ENSG00000167193_1456117_1423513_304	CRK	17	-	912	11	0	0	2.30%	7.077395462879936	0.0078820576498062	0.0078820576498062	0.00031963642907451164
ENSG00000082898_61494079_61478820_718	XPO1	2	-	2154	56	4	9	2.92%	15.255201362378834	0.00014419167706224479	0.0008000147913871459	8.126146420286765e-07
ENSG00000166946_43190380_43194841_199	CCNDBP1	15	+	597	21	0	0	4.52%	20.640517421208546	0.0033030736763512156	0.0033030736763512156	6.124050385493983e-05
ENSG00000166946_43185499_43190989_142	CCNDBP1	15	+	426	17	0	3	4.93%	23.416120199848596	0.008574016250729465	0.04140309663709693	0.001795778436935961
ENSG00000168036_41224552_41239342_768	CTNNB1	3	+	2304	116	8	0	5.73%	29.542764394294277	8.699215501017508e-08	2.9105570714308586e-09	2.8276345500981447e-15
ENSG00000175073_66666958_66634501_1222	VCPIP1	8	-	3666	13	0	0	0.65%	2.0807816447925536	0.005160369442893307	0.005160369442893307	0.00014319601891785326
ENSG00000143537_155051387_155062502_863	ADAM15	1	+	2589	46	13	0	2.20%	10.425608148415993	0.009314527945786596	5.328371459388349e-05	5.3170083957120194e-06
ENSG00000164924_100948658_100923943_152	YWHAZ	8	-	456	23	0	0	3.95%	29.596381026588823	0.013007239811447113	0.013007239811447113	0.0008218654148058079
ENSG00000075624_5529657_5529394_87	ACTB	7	-	261	28	9	5	6.90%	62.94962401257472	0.05352897298295963	0.08776167540602747	0.01797735625151174
ENSG00000132432_54757588_54755775_66	SEC61G	7	-	198	107	0	2	27.27%	317.09851186853797	9.135877515977258e-05	0.00023189001247815545	1.5170594668409738e-07
ENSG00000166477_51971736_51938156_603	LEO1	15	-	1809	19	0	2	1.66%	6.162978470314264	0.0022276761777358703	0.009621867080838033	0.00012310157985546497
ENSG00000177426_3456398_3457940_252	TGIF1	18	+	756	34	0	2	2.78%	26.38959578078175	0.008574016250729465	0.013648824643852793	0.0005848802985757839
ENSG00000075415_98599983_98601528_138	SLC25A3	12	+	414	119	13	2	16.67%	168.66393825108338	0.00027361052923396495	2.5572940677403147e-05	5.5376198426901035e-08
ENSG00000114867_184320681_184334908_1403	EIF4G1	3	+	4209	90	7	5	2.78%	12.546979839590167	4.60942195086692e-07	1.9011841143914044e-07	8.246422000810537e-13
ENSG00000125445_75261901_75265923_242	MRPS7	17	+	726	27	1	1	6.61%	21.822412456543393	0.000279948838573249	0.0004331864327387125	8.138110542891507e-07
ENSG00000165410_34713513_34712865_149	CFL2	14	-	447	53	0	0	8.72%	69.57351781447316	0.0007004692736203066	0.0007004692736203066	3.1265561041742324e-06
ENSG00000100902_35308980_35317306_167	PSMA6	14	+	501	49	1	2	5.99%	57.38970213122457	0.0025061435727443374	0.0024742386374296595	3.570411499116179e-05
ENSG00000089737_94079613_94057798_627	DDX24	14	-	1881	24	1	0	1.44%	7.48683156403828	0.006432154271512831	0.002967235347054297	0.00010592475669358982
ENSG00000100603_77761127_77718063_571	SNW1	14	-	1713	28	0	1	1.93%	9.5912737111979	0.0015208721599504675	0.0021367610743209236	1.92860157369266e-05
ENSG00000136986_123023718_123015447_134	DERL1	8	-	402	48	4	0	11.19%	70.06333418883584	0.0009065557339205618	0.000309684704389587	1.8634234647643323e-06
ENSG00000116212_53952168_53967939_372	LRRC42	1	+	1116	8	0	1	1.61%	4.206311281946237	0.009815328628645334	0.028889785561798602	0.0013877953586383081
ENSG00000082515_154941111_154966897_206	MRPL22	5	+	618	40	0	0	6.31%	37.979315458349525	0.0006311913977015671	0.0006311913977015671	2.5578527397951546e-06
ENSG00000142867_85276352_85267627_222	BCL10	1	-	666	7	0	0	2.25%	6.167361812042794	0.016947426762344633	0.016947426762344633	0.0013498980316300959
ENSG00000114209_167697030_167684308_130	PDCD10	3	-	390	14	0	0	3.85%	21.063912650361544	0.02108409854857798	0.02108409854857798	0.002029891263157455
ENSG00000133997_70600637_70584094_257	MED6	14	-	771	18	4	0	4.67%	13.699153863770432	0.021412135282300855	0.0007729603088974596	0.000120880508277367
ENSG00000120265_149749728_149802379_285	PCMT1	6	+	855	45	1	4	7.02%	30.8831802016579	5.185709832851827e-05	0.00035548993793247866	1.3841409122193912e-07
ENSG00000019186_54173579_54157179_514	CYP24A1	20	-	1542	422	35	19	13.81%	160.58452584753115	6.91523622745467e-11	1.5646792094491102e-11	1.3100063554315418e-20
ENSG00000135655_62302891_62404375_875	USP15	12	+	2625	17	1	2	1.14%	3.800101792432572	0.0037220617515305076	0.006369482933362988	0.00012918474591820854
ENSG00000115310_55025605_54973156_361	RTN4	2	-	1083	210	11	2	10.53%	113.78013758505543	2.294705829532471e-07	5.231659427479232e-08	1.1813286012580124e-13
ENSG00000163159_151190121_151176644_364	VPS72	1	-	1092	19	1	0	2.75%	10.209549498899728	0.003415127931522041	0.00228120298834518	4.457419682640551e-05
ENSG00000108561_5438193_5433015_178	C1QBP	17	-	534	190	8	8	16.29%	208.77955155053377	9.501567493411681e-06	1.836613851270535e-05	1.4068361432126016e-09

ENSG00000126214_103654586_103700726_566	KLC1	14	+	1698	52	6	7	3.71%	17.9697185154523	0.0005764071508637824	0.0006376289743136012	2.366923025010413e-06
ENSG00000102858_4650391_4686299_514	MGRN1	16	+	1542	15	0	2	1.75%	5.707980776571013	0.0033278027414746945	0.01652445925752544	0.0003062742250611212
ENSG00000160948_144424253_144423805_82	VPS28	8	-	246	33	0	0	14.63%	78.71444709934758	0.0010197273511756433	0.0010197273511756433	6.442859155158972e-06
ENSG00000087365_66061927_66068745_259	SF3B2	11	+	777	28	0	2	4.63%	21.145240498432436	0.0010280153344583009	0.002674329485664681	1.6712315643600486e-05
ENSG00000177311_141442398_141445976_1192	ZBTB38	3	+	3576	27	0	2	0.92%	4.4303891061103196	0.00151350169064872	0.005530543042558543	4.947549134344851e-05
ENSG00000116679_185300283_185299684_132	IVNS1ABP	1	-	396	19	2	0	4.55%	28.153606193935612	0.037440157020026926	0.013640585738808999	0.002398086303081032
ENSG00000149273_75399548_75404865_259	RPS3	11	+	777	350	18	14	16.22%	264.31550623040545	2.6256668953332315e-08	9.824024933221953e-09	2.6634335943439783e-15
ENSG00000196730_87499078_87707364_1430	DAPK1	9	+	4290	17	0	4	0.56%	2.3252371107541965	0.004826937407846361	0.03906554526712903	0.001050233689227362
ENSG00000204463_31651763_31639131_1126	BAG6	6	-	3378	30	4	3	1.42%	5.211193817331262	0.001994982063403954	0.001165552400368026	1.4041745202165171e-05
ENSG00000163811_28894699_28946779_677	WDR43	2	+	2031	31	5	0	2.22%	8.956274317467505	0.0018343943883925903	0.0002596338414409078	3.2648966373270473e-06
ENSG00000181704_68513360_68531999_163	YIPF6	X	+	489	18	1	0	3.68%	21.599279404840495	0.02034245439037237	0.013428347753762199	0.0012955184805712684
ENSG00000099956_23787179_23834180_373	SMARCB1	22	+	1119	24	4	4	3.75%	12.585102924000003	0.004909317959302256	0.008784088447361777	0.0002283466035886222
ENSG00000198492_28737121_28768952_579	YTHDF2	1	+	1737	24	2	1	1.38%	8.107501538259068	0.004873903550586949	0.005998643116553665	0.00015665812899927624
ENSG00000120733_138417503_138435700_652	KDM3B	5	+	1956	11	1	5	0.92%	3.2998899090728537	0.014014727539149415	0.1097457788509939	0.007724738414448233
ENSG00000073060_124817617_124782683_437	SCARB1	12	-	1311	48	1	1	4.12%	21.483951444631582	0.00012089867747187387	0.00012089867747187387	1.0442221824633747e-07
ENSG00000221914_26293274_26370413_448	PPP2R2A	8	+	1344	28	0	0	2.90%	12.224592163156252	0.0005854768899833019	0.0005854768899833019	2.212593397499504e-06
ENSG00000149485_61813305_61802411_360	FADS1	11	-	1080	133	6	14	7.78%	72.26092256443474	1.5160076052832811e-05	2.2859346735468922e-05	2.7400335148722413e-09
ENSG00000163110_94523808_94666026_423	PDLIM5	4	+	1269	84	2	12	7.57%	38.841257369461005	2.9787941996864795e-07	4.448589776860371e-06	1.2657962288066218e-11
ENSG00000110628_2903346_2925197_424	SLC22A18	11	+	1272	13	0	0	1.42%	5.996969740416275	0.011772051206276392	0.011772051206276392	0.000681272511389508
ENSG00000269955_139341385_139422340_458	FMC1-LUC7L2	7	+	1374	38	5	7	5.02%	16.22827955283625	9.086501925144255e-05	0.0002508235171719083	1.6326952509234065e-07
ENSG00000164611_160422313_160428681_202	PTTG1	5	+	606	87	81	1	9.90%	84.24075391640349	0.0006872427350830977	4.064245520376573e-05	2.219234233011689e-07
ENSG00000079462_42302240_42297078_208	PAFAH1B3	19	-	624	47	0	1	6.25%	44.19660243602645	0.0007124765031541974	0.0008128177682286688	3.669126715936747e-06
ENSG00000108433_46923193_46935280_194	GOSR2	17	+	582	12	0	0	3.09%	12.098565439824744	0.011772051206276392	0.011772051206276392	0.000681272511389508
ENSG00000068079_43012218_43014299_266	IFI35	17	+	798	13	0	0	3.01%	9.559079586227446	0.004826937407846361	0.004826937407846361	0.00012612451166366307
ENSG00000114503_196939285_196937011_81	NCBP2	3	-	243	15	2	0	11.11%	36.22101381675927	0.010499663139468709	0.0025000845279814113	0.00014909639159692154
ENSG00000074800_8874908_8873957_33	ENO1	1	-	99	48	2	0	18.18%	284.4995994334546	0.013853924679039932	0.013640585738808999	0.000911832052528562
ENSG00000160326_133477121_133473855_261	SLC2A6	9	-	783	22	1	0	3.45%	16.486806289007667	0.004911637253759624	0.0034782380676658805	9.416876808028951e-05
ENSG00000164885_151057848_151054009_292	CDK5	7	-	876	17	0	0	3.08%	11.38729133006336	0.0033030736763512156	0.0033030736763512156	6.124050385493983e-05
ENSG00000104765_26383131_26410412_219	BNIP3L	8	+	657	25	1	0	5.48%	22.328022215810506	0.0014407231495320988	0.0009668793038582057	8.56135849638478e-06
ENSG00000159377_151401314_151401379_21	PSMB4	1	+	63	19	0	1	23.81%	176.9655246475953	0.02108409854857798	0.02108409854857798	0.002029891263157455
ENSG00000159377_151399588_151400191_116	PSMB4	1	+	348	25	1	3	10.34%	42.15376607984915	0.002484757332982506	0.005728072827781676	8.02731214801202e-05
ENSG00000198276_63945680_63939976_340	UCKL1	20	-	1020	10	0	1	1.76%	5.752749253250001	0.011570465654371866	0.01871402756203153	0.0010439183847388118
ENSG00000120699_37000806_37009299_276	EXOSC8	13	+	828	67	6	1	3.62%	47.481024633708344	0.010053403314622913	0.0027558349459111645	0.00015539364357887065
ENSG00000144591_219501485_219506798_371	GMPPA	2	+	1113	15	0	2	1.35%	7.908091965384099	0.02108409854857798	0.0700082515985845	0.006564739044797468
ENSG00000080845_36439782_36527031_569	DLGAP4	20	+	1707	17	3	1	1.58%	5.8437417721942015	0.024710983489837978	0.006112543793728684	0.0007857918783425606
ENSG00000095380_98056932_98083055_318	NANS	9	+	954	31	1	0	3.46%	19.067288405426105	0.002167177068466985	0.0015505692382983198	1.9913191269291018e-05
ENSG00000196497_24187138_24183586_450	IPO4	14	-	1350	25	0	5	2.89%	10.86630414502778	0.0006289630342484525	0.0072475799089764485	3.0435330234948232e-05
ENSG00000173821_80294947_80336504_984	AC124319.1	17	+	2952	18	8	3	1.02%	3.577929413606708	0.09485622571796531	0.010874198155266036	0.0053870282726036405
ENSG00000140374_76311388_76231315_299	ETFA	15	-	897	188	2	2	12.04%	122.98185025676926	7.978050037597041e-08	1.3255448869134695e-07	1.0262315899400684e-13
ENSG00000181610_57850010_57840887_152	MRPS23	17	-	456	17	0	0	4.61%	21.875585976174346	0.008574016250729465	0.008574016250729465	0.0003747459614100891
ENSG00000153113_96662423_96771679_791	CAST	5	+	2373	121	0	4	5.44%	29.920114320948805	4.0077545563218955e-09	4.42202571771872e-08	1.9053817941938333e-15
ENSG00000114544_126050972_126007016_370	SLC41A3	3	-	1110	9	0	0	1.35%	4.757679112147298	0.020613416668581838	0.020613416668581838	0.001946208561389314
ENSG00000175054_142566169_142449429_2563	ATR	3	-	7689	13	0	0	0.35%	0.9920855130458449	0.002943990679779778	0.002943990679779778	4.916404313974209e-05
ENSG00000065427_75647639_75627895_597	KARS	16	-	1791	76	6	1	5.03%	24.899671809711897	1.056791066219392e-05	8.890006527197744e-07	8.57247410047012e-11
ENSG00000143437_150846291_150812021_721	ARNT	1	-	2163	13	0	0	0.83%	3.5266507211324556	0.013428347753762199	0.013428347753762199	0.0008725593497644525
ENSG00000169604_69013500_69245485_564	ANTXR1	2	+	1692	200	5	0	6.38%	69.35938815975179	9.147422220805801e-07	6.187115286023347e-08	5.677941940996279e-13
ENSG00000163513_30644854_30691599_500	TGFBR2	3	+	1500	47	6	0	3.00%	18.385786613387005	0.002288823876695954	0.00029563251185049877	4.620324153630056e-06
ENSG00000196586_75832877_75915012_1120	MYO6	6	+	3360	13	0	0	0.45%	2.2702814017290183	0.02108409854857798	0.02108409854857798	0.002029891263157455
ENSG00000107862_102351360_102382336_1726	GBF1	10	+	5178	13	5	0	0.52%	1.47318376010226	0.046217795284775376	0.00308494966027208	0.0008850096456078009
ENSG00000104408_108229111_108228263_56	EIF3E	8	-	168	13	0	0	8.93%	45.40562803458037	0.020613416668581838	0.020613416668581838	0.001946208561389314
ENSG00000125630_112550943_112575729_901	POLR1B	2	+	2703	23	0	0	1.00%	4.9929521820660385	0.0034782380676658805	0.0034782380676658805	6.758309314600117e-05
ENSG00000169398_140846306_140659466_870	PTK2	8	-	2610	15	6	4	1.15%	3.372301286387932	0.0820328997370387	0.023029368248455667	0.00832076987426179
ENSG00000148400_136545786_136496071_2555	NOTCH1	9	-	7665	12	3	1	0.20%	0.9186386283076323	0.07356204933788085	0.028889785561798623	0.008971813318439464
ENSG00000113407_33441123_33467708_711	TARS	5	+	2133	95	9	15	6.05%	26.134149209560483	8.967003924683803e-08	1.809091735111514e-06	1.6275565000097462e-12
ENSG00000072310_17836817_17812622_1177	SREBF1	17	-	3531	84	7	18	3.14%	13.959092495566699	1.2985094596972424e-06	3.177457264205154e-05	3.836727907419007e-10
ENSG00000121064_56981156_57006235_402	SCPEP1	17	+	1206	34	0	2	3.23%	16.54273168347513	0.0006791898698869142	0.0016207143154899155	6.908867689334616e-06
ENSG00000125730_6719354_6677882_1622	C3	19	-	4866	73	6	2	1.91%	8.802912235860976	1.4529861547585328e-05	1.7487103254777452e-06	2.2355239593505751e-10
ENSG00000165792_20990003_20992637_180	METTL17	14	+	540	23	2	0	4.44%	24.992499533563894	0.013786173822478529	0.005706018193000826	0.0004086985969104885
ENSG00000122566_26200577_26192516_341	HNRNPA2B1	7	-	1023	613	27	22	34.31%	351.6093839772332	7.432075641399788e-20	2.9932281443250103e-19	3.6217577802253484e-37
ENSG00000072682_132218599_132193010_524	P4HA2	5	-	1572	34	2	0	3.63%	12.69117965029962	4.430797606548033e-05	4.8597989611994695e-05	1.6225649837067363e-08
ENSG00000075790_107580773_107618363_241	BCAP29	7	+	723	32	0	0	5.81%	25.970917790605814	0.0004156054716904614	0.0004156054716904614	1.1417433411599823e-06
ENSG00000268173_18173842_18177911_250	AC007192.1	19	+	750	16	0	1	2.80%	12.517982375072004	0.00847367416878415	0.013892891352654344	0.0005886798845679606
ENSG00000006451_39686668_39706245_206	RALA	7	+	618	13	0	1	4.37%	12.343277523963595	0.00308494966027208	0.0058497041193523325	9.994046760347681e-05
ENSG00000105518_11345583_11342815_177	TMEM205	19	-	531	78	6	9	11.86%	86.19373457411866	0.0002837053614922544	0.0003036328747503824	5.827449871051712e-07
ENSG00000115286_1383927_1391089_148	NDUFS7	19	+	444	29	1	0	6.76%	38.325748403408795	0.0023155019563311486	0.0022409900782254734	3.0112473260437535e-05
ENSG00000135108_117189349_117146087_477	FBXO21	12	-	1431	31	0	0	2.94%	12.711525603617403	0.0004441315507112266	0.0004441315507112266	1.2979723423445215e-06
ENSG00000092841_56159661_56160654_116	MYL6	12	+	348	745	10	17	36.21%	1256.1822291795047	9.650415573208686e-09	8.18577860008708e-08	8.248545996355498e-15

ENSG00000184752_94994225_94971440_78	NDUFA12	12	-	234	68	2	0	21.79%	170.5173881219744	0.00039718811007476005	0.00013319834607924987	3.705977798409796e-07
ENSG00000117543_101025443_100990408_285	DPH5	1	-	855	10	2	1	3.16%	6.862928933701756	0.026101817670657335	0.006277459298483267	0.0008509360463365077
ENSG00000079819_130956485_130858136_1005	EPB41L2	6	-	3015	21	1	6	1.09%	4.087027827682091	0.002568463469732168	0.015186081405644649	0.0002242723246544596
ENSG00000125247_100668740_100604994_630	TMTC4	13	-	1890	15	1	0	1.27%	4.656987490726191	0.005257622967929454	0.005160369442893307	0.0001457596151645891
ENSG00000143337_179882866_179918239_463	TOR1AIP1	1	+	1389	31	0	0	3.02%	13.095891388607994	0.00043491588733509306	0.00043491588733509306	1.2464499370452549e-06
ENSG00000143570_153962715_153960098_324	SLC39A1	1	-	972	45	1	7	6.79%	27.16576036256945	1.946122850477892e-05	0.0001930553194167021	3.001873045404824e-08
ENSG00000125841_349647_353635_203	NRSN2	20	+	609	28	4	0	5.91%	26.978410291103454	0.007740067497657663	0.0009549346450480869	4.6790769355716326e-05
ENSG00000039068_68737416_68834281_647	CDH1	16	+	1941	15	0	1	1.70%	4.534624604571098	0.0012039422000035016	0.0024778661586389522	1.7923014500004964e-05
ENSG00000162910_228108260_228106760_128	MRPL55	1	-	384	35	2	1	7.81%	53.482590713808605	0.004407715900925203	0.0030814981005195803	7.570158080683164e-05
ENSG00000162910_228108260_228106760_128	MRPL55	1	-	384	35	2	1	7.81%	53.482590713808605	0.004407715900925203	0.0030814981005195803	7.570158080683164e-05
ENSG00000162910_228108260_228106760_128	MRPL55	1	-	384	35	2	1	7.81%	53.482590713808605	0.004407715900925203	0.0030814981005195803	7.570158080683164e-05
ENSG00000162910_228108260_228106760_128	MRPL55	1	-	384	35	2	1	7.81%	53.482590713808605	0.004407715900925203	0.0030814981005195803	7.570158080683164e-05
ENSG00000162910_228108260_228106760_128	MRPL55	1	-	384	35	2	1	7.81%	53.482590713808605	0.004407715900925203	0.0030814981005195803	7.570158080683164e-05
ENSG00000162910_228108260_228106760_128	MRPL55	1	-	384	35	2	1	7.81%	53.482590713808605	0.004407715900925203	0.0030814981005195803	7.570158080683164e-05
ENSG00000148180_121317107_121332603_473	GSN	9	+	1419	17	0	0	1.06%	7.029786613908035	0.02108409854857798	0.02108409854857798	0.002029891263157455
ENSG00000254536_133395724_133420165_293	AL360181.3	10	+	879	18	4	2	2.73%	12.015981375389082	0.03588324695817727	0.018318557839637383	0.0029714513955091536
ENSG00000143256_161118026_161100683_83	PFDN2	1	-	249	40	0	0	12.05%	94.26191547493978	0.0023354528714849923	0.0023354528714849923	3.158285405866755e-05
ENSG00000165752_132331736_132330478_102	STK32C	10	-	306	9	0	9	5.88%	17.258247759750002	0.011772051206276392	0.11411551976453232	0.007078637642502531
ENSG00000170515_56106707_56113878_215	PA2G4	12	+	645	24	0	1	3.26%	21.833690189079075	0.008877961307018026	0.012176559331126819	0.0005408334713909414
ENSG00000110958_56687263_56666156_165	PTGES3	12	-	495	43	50	5	5.45%	50.97284489849395	0.26908356298492286	0.019390513203712073	0.028945400868498037
ENSG00000126088_45012266_45013401_132	UROD	1	+	396	10	0	0	3.79%	14.817687470492428	0.020613416668581838	0.020613416668581838	0.001946208561389314
ENSG00000105879_107744164_107759178_490	CBLL1	7	+	1470	25	6	0	1.84%	9.979258908698982	0.02614799453092737	0.0035547763465261306	0.0005272417427595446
ENSG00000042493_85402145_85394893_333	CAPG	2	-	999	28	0	0	4.20%	16.446298165447452	0.00042454308224345244	0.00042454308224345244	1.1896574514138388e-06
ENSG00000196975_69788129_69825515_271	ANXA4	2	+	813	247	4	0	18.08%	178.27154327968086	1.6098684970355589e-09	4.816482458383826e-10	8.675370257040188e-18
ENSG00000160710_154601756_154584806_931	ADAR	1	-	2793	107	6	3	4.19%	22.47959375222718	7.29368051916334e-07	9.227426656624114e-08	6.559954505852833e-13
ENSG00000117528_94418479_94478550_236	ABCD3	1	+	708	10	0	0	2.54%	8.287859093665256	0.011772051206276392	0.011772051206276392	0.000681272511389508
ENSG00000122566_26200577_26192516_341	HNRNPA2B1	7	-	1023	613	27	22	34.31%	351.6093839772332	7.432075641399788e-20	2.9932281443250103e-19	3.6217577802253484e-37
ENSG00000118762_88056176_88075694_366	PKD2	4	+	1098	15	0	2	1.91%	8.016126008627051	0.00847367416878415	0.028426896806120847	0.0012022752110812016
ENSG00000109736_2933923_2933037_146	MFSD10	4	-	438	11	4	1	3.42%	14.736494662434934	0.17139085557395572	0.028542519635534256	0.021889347154574825
ENSG00000164815_104207904_104126848_435	ORC5	7	-	1305	10	4	0	1.15%	4.496401715183909	0.19427189192379535	0.020613416668581838	0.020032097720265936
ENSG00000188243_75537805_75526589_98	COMMD6	13	-	294	41	0	3	10.20%	81.82992305133165	0.0022409900782254734	0.013819019308084528	0.00018056055434049928
ENSG00000103275_1314031_1324793_158	UBE2I	16	+	474	91	7	4	14.56%	112.65193790857914	4.1653514996278555e-05	4.2145511149793747e-05	1.32979772114665e-08
ENSG00000185344_123712566_123758032_856	ATP6V0A2	12	+	2568	12	0	0	0.58%	2.7419645973434585	0.019679754444124863	0.019679754444124863	0.0017849636115922674
ENSG00000169490_38995428_38991332_146	TM2D2	8	-	438	31	0	0	7.53%	41.53012132140754	0.0014055202957965589	0.0014055202957965589	1.1935530040246081e-05
ENSG00000116138_15529106_15566190_595	DNAJC16	1	+	1785	10	0	0	0.84%	3.287285287571429	0.020613416668581838	0.020613416668581838	0.001946208561389314
ENSG00000111142_95474180_95513904_478	METAP2	12	+	1434	103	1	0	7.74%	42.14671105623746	5.660376161185916e-08	4.4928943704717294e-08	2.5201450924676847e-14
ENSG00000154001_63453790_63376009_383	PPP2R5E	14	-	1149	19	0	0	1.83%	9.70307054203525	0.00847367416878415	0.00847367416878415	0.0003665024264111182
ENSG00000128534_118185686_118192002_75	LSM8	7	+	225	36	3	0	12.00%	93.88486781304002	0.0035547763465261306	0.0033030736763512156	6.569182429188685e-05
ENSG00000169567_131165205_131162551_78	HINT1	5	-	234	47	2	2	15.38%	117.85760649607055	0.0013282749526633097	0.0023453309689915928	1.8603609104278156e-05
ENSG00000158864_161209857_161214193_275	NDUFS2	1	+	825	40	0	3	5.82%	28.449959943345462	0.00019694564903112173	0.00027655323682364736	3.7457401835257923e-07
ENSG00000089157_120200783_120196773_317	RPLP0	12	-	951	61	1	4	3.15%	37.63786104492272	0.0025025372014526115	0.004791578323725913	6.770248138606176e-05
ENSG00000100320_35809971_35744205_361	RBFOX2	22	-	1083	12	0	0	1.66%	6.501722147717453	0.013217226988443315	0.013217226988443315	0.0008469697265466234
ENSG00000057252_179339478_179353641_407	SOAT1	1	+	1221	31	0	0	3.19%	14.897783078441035	0.0006311913977015671	0.0006311913977015671	2.5578527397951546e-06
ENSG00000157399_2960400_2934832_535	ARSE	X	-	1605	21	4	0	2.43%	7.677500872561684	0.007786176826479684	0.0005399915898224664	2.8884530273725324e-05
ENSG00000169592_29996311_30001229_137	INO80E	16	+	411	41	5	5	8.76%	58.53527342358031	0.004105719329788636	0.007597837651723933	0.00016809397763060268
ENSG00000169592_30005221_30005442_73	INO80E	16	+	219	12	0	0	8.22%	32.15235199076713	0.011772051206276392	0.011772051206276392	0.000681272511389508
ENSG00000163814_45146287_45110465_343	CDCP1	3	-	1029	14	1	0	2.33%	7.983407126959185	0.007930666369886513	0.005160369442893307	0.00021637657341139557
ENSG00000187778_49563530_49558643_271	MCRS1	12	-	813	14	1	0	2.21%	10.104459942977861	0.023878473930440184	0.013007239811447113	0.0014709138084317821
ENSG00000057608_5800654_5766006_368	GDI2	10	-	1104	74	4	5	7.34%	39.33129652493751	1.0685331876483796e-05	1.4581466828895188e-05	1.2543299075774233e-09
ENSG00000104763_18084801_18067074_191	ASAH1	8	-	573	8	0	0	2.62%	8.192396842324609	0.019216965118390855	0.019216965118390855	0.0017073955890589052
ENSG00000139624_50137760_50133808_228	CERS5	12	-	684	10	0	0	2.63%	8.578661167127194	0.009815328628645334	0.009815328628645334	0.0004837142251238724
ENSG00000088812_3471108_3646847_1429	ATRN	20	+	4287	33	2	1	1.47%	4.516854207240379	0.00010975056285908883	2.9610398476553646e-05	2.4799639625789015e-08
ENSG00000068323_49043226_49030158_575	TFE3	X	-	1725	11	2	0	1.39%	3.741788209940001	0.03318103322835444	0.004193160480899131	0.0007828122868454912
ENSG00000167088_21612430_21623918_44	SNRPD1	18	+	132	45	2	2	11.36%	200.03878085164777	0.02108409854857798	0.03736774915294124	0.0034998002586384648
ENSG00000076554_80080438_80038116_242	TPD52	8	-	726	45	5	0	5.79%	36.37068742757232	0.002462368798132946	0.0004670591637363745	7.5029210566117364e-06
ENSG00000163072_168802647_168864970_506	NOSTRIN	2	+	1518	9	0	0	0.99%	3.4789353191590915	0.016947426762344633	0.016947426762344633	0.0013498980316300959
ENSG00000215301_41334253_41342854_186	DDX3X	X	+	558	14	11	3	6.45%	14.722089486811832	0.1318072353628263	0.008289087595049986	0.006484008759670153
ENSG00000215301_41344365_41347719_332	DDX3X	X	+	996	66	0	0	8.43%	38.883040133412656	1.0077168445509615e-06	1.0077168445509615e-06	9.076267751723256e-12
ENSG00000151491_15669717_15621317_718	EPS8	12	-	2154	15	0	0	1.11%	4.086214650637188	0.005405141725979036	0.005405141725979036	0.00015637213408961292
ENSG00000126214_103654586_103701203_611	KLC1	14	+	1833	52	6	7	3.44%	16.64625315834043	0.0005764071508637824	0.0006376289743136012	2.366923025010413e-06
ENSG00000188313_146533538_146516045_237	PLSCR1	3	-	711	33	1	0	5.91%	27.234534439436715	0.0006629387121614735	0.0004093002068662083	1.773333733167258e-06
ENSG00000186010_19516266_19526264_49	NDUFA13	19	+	147	85	10	15	36.73%	339.2948028957654	0.0024298635553492215	0.009561174510657014	0.00013204530193759224
ENSG00000132170_12351593_12406147_294	PPARG	3	+	882	15	0	0	2.38%	9.979258908698982	0.00847367416878415	0.00847367416878415	0.0003665024264111182
ENSG00000120539_27169974_27186536_469	MASTL	10	+	1407	13	0	2	1.49%	5.421567526517059	0.008175610787902942	0.03744183116399598	0.001552076439258323
ENSG00000136628_220046388_220025202_194	EPRS	1	-	582	11	2	0	2.58%	11.090351653172682	0.0700082515985845	0.02108409854857798	0.006564739044797468
ENSG00000109323_102760894_102632057_925	MANBA	4	-	2775	8	0	7	0.54%	1.691619239874595	0.019216965118390855	0.19558626140506974	0.01921731642938781
ENSG00000126768_48897749_48893729_172	TIMM17B	X	-	516	6	0	1	2.91%	6.823028184087211	0.016947426762344633	0.0477903522728147	0.0036975118224100542

ENSG00000104765_26391263_26410412_179	BNIP3L	8	+	537	19	1	0	5.59%	20.761318534075425	0.0037220617515305076	0.0021880057261679567	4.6633814146144326e-05
ENSG00000157168_32595851_32742768_166	NRG1	8	+	498	19	0	1	3.01%	22.3872049252982	0.02108409854857798	0.028889785561798623	0.002731532589007073
ENSG00000139687_48345141_48480037_776	RB1	13	+	2328	19	1	0	1.42%	4.789015486597295	0.002550198508962866	0.0012558331315113088	1.9177754206419204e-05
ENSG00000115919_142954836_142971203_120	KYNU	2	+	360	17	0	0	5.83%	27.70907556982084	0.00847367416878415	0.00847367416878415	0.0003665024264111182
ENSG00000164347_74751461_74721655_620	GFM2	5	−	1860	31	0	5	1.77%	9.779673730525003	0.0015208721599504675	0.013147104497242925	0.00012290835639369062
ENSG00000164111_121696589_121683464_35	ANXA5	4	−	105	18	1	3	14.29%	100.59092979968574	0.03577298629450316	0.03577298629450316	0.005410795288624905
ENSG00000164111_121683414_121681657_51	ANXA5	4	−	153	77	2	0	35.29%	295.3077950001667	0.0002166508755893562	9.03854454941855e-05	1.4032475585630475e-07
ENSG00000258674_19516266_19535810_264	AC011448.1	19	+	792	171	12	15	12.88%	126.69122787271026	7.988967423775599e-06	1.5005780708483578e-05	9.74403654138291e-10
ENSG00000100567_58252240_58260977_65	PSMA3	14	+	195	23	8	0	10.77%	69.20999870833079	0.1057548992775299	0.00877616296920843	0.005191516656837364
ENSG00000100888_21405431_21385613_1561	CHD8	14	−	4683	16	0	3	0.45%	2.004801789729661	0.00837382380159347	0.04524396496563379	0.001935093610214004
ENSG00000184260_149886975_149887364_129	HIST2H2AC	1	+	387	59	5	1	7.75%	89.4574806358101	0.01181427592813292	0.0030814981005195803	0.00020233721848821377
ENSG00000259332_79889300_79845210_146	ST20-MTHFS	15	−	438	5	0	0	3.42%	6.698406664743152	0.012673659338734126	0.012673659338734126	0.000782701129001274
ENSG00000073921_86014950_85959046_455	PICALM	11	−	1365	38	0	5	3.08%	16.335279198239565	0.0004481211664877703	0.003929042651386831	1.1787616094891344e-05
ENSG00000159176_201496192_201488783_123	CSRP1	1	−	369	58	2	0	5.69%	92.23106932852848	0.013855018329567799	0.008877961307018026	0.0006126639634572191
ENSG00000167460_16086484_16101346_139	TPM4	19	+	417	27	0	3	5.04%	37.99297708261512	0.00847367416878415	0.03299602752967379	0.0014019198044660757
ENSG00000135387_34096637_34099367_88	CAPRIN1	11	+	264	10	1	0	5.68%	22.22653120573864	0.051235217429874684	0.020613416668581838	0.004687384229717438
ENSG00000179335_74622114_74629883_369	CLK3	15	+	1107	6	0	0	1.36%	3.1803817009837405	0.016947426762344633	0.016947426762344633	0.0013498980316300959
ENSG00000135679_68816839_68839849_430	MDM2	12	+	1290	9	0	0	1.16%	4.093816910452326	0.019679754444124863	0.019679754444124863	0.0017849636115922674
ENSG00000135480_52241589_52243320_118	KRT7	12	+	354	237	33	8	15.25%	392.84452103973314	0.0003112612311656194	8.203283381336422e-05	1.844419497099444e-07
ENSG00000026508_35176703_35222378_229	CD44	11	+	687	167	8	9	14.85%	142.63803606966596	1.1220852508471112e-06	2.201753889018482e-06	2.1804288486662594e-11
ENSG00000184743_63659244_63629319_523	ATL3	11	−	1569	63	7	4	3.82%	23.56097304103538	0.00011219365649543519	0.00023695714496868413	1.8820742010091813e-07
ENSG00000148444_22316418_22318310_117	COMMD3	10	+	351	20	1	1	8.55%	33.43478198470086	0.002276761777358703	0.002276761777358703	2.8853233252577778e-05
ENSG00000102900_56818721_56844609_637	NUP93	16	+	1911	27	0	0	1.88%	8.290461247226846	0.0008776897154483972	0.0008776897154483972	4.827634122642857e-06
ENSG00000163739_73869471_73870536_107	CXCL1	4	+	321	56	4	0	9.35%	102.36667830082246	0.012325483512267905	0.002460156992221257	0.0001735960218436989
ENSG00000107371_37783921_37780679_69	EXOSC3	9	−	207	31	0	0	7.25%	87.87532917283335	0.02108409854857798	0.02108409854857798	0.002029891263157455
ENSG00000099817_1095315_1089526_204	POLR2E	19	−	612	38	3	0	3.92%	36.434078603916674	0.008219046535586472	0.005454749182134642	0.00023581017309990104
ENSG00000099812_756947_763590_679	MISP	19	+	2037	22	6	4	1.77%	6.3373438018129615	0.02576233377891242	0.012749624636220407	0.0015567752711832428
ENSG00000077312_40751409_40765167_282	SNRPA	19	+	846	31	4	0	3.19%	21.501410329523054	0.02149159670096621	0.003632191471126012	0.0004353151193509418
ENSG00000090006_40611945_40629550_844	LTBP4	19	+	2532	9	0	2	0.59%	2.085712406984005	0.016947426762344633	0.1029516053660342	0.008323174032180154
ENSG00000114126_142000353_141952513_418	TFDP2	3	−	1254	9	0	0	1.20%	4.211342754771532	0.019679754444124863	0.019679754444124863	0.0017849636115922674
ENSG00000092010_24136263_24138816_249	PSME1	14	+	747	114	8	3	15.66%	89.5488197011928	2.77030389452321e-07	2.160231528913744e-08	6.165831675525458e-14
ENSG00000188042_234496535_234496008_175	ARL4C	2	−	525	7	0	0	2.86%	7.823738984420002	0.016947426762344633	0.016947426762344633	0.0013498980316300959
ENSG00000138069_65129906_65088493_202	RAB1A	2	−	606	66	1	1	10.40%	63.90677883313368	2.6281503917673907e-05	3.443854328470227e-05	6.980466965962083e-09
ENSG00000169813_43387881_43386637_414	HNRNPF	10	−	1242	101	4	4	8.45%	47.71724863686112	4.932031907248019e-07	8.552842395818309e-07	3.843530684890113e-12
ENSG00000129187_182915511_182891399_159	DCTD	4	−	477	18	0	0	4.40%	22.142657503075476	0.00797940233269714	0.00797940233269714	0.000327142761693177
ENSG00000064042_41619253_41697185_833	LIMCH1	4	+	2499	15	3	6	0.96%	3.522091379540817	0.041265600891389115	0.06128749994915507	0.010184520773394703
ENSG00000156976_186785967_186789269_263	EIF4A2	3	+	789	24	2	0	3.80%	17.848834184988597	0.005588370640926919	0.0022543491824521134	7.166979570209328e-05
ENSG00000009307_114739890_114718169_767	CSDE1	1	−	2301	89	11	5	4.43%	22.695983364191008	1.2651144433362197e-05	1.2057861126918465e-06	1.3707486370165965e-10
ENSG00000146282_87545625_87514413_403	RARS2	6	−	1209	13	0	0	1.24%	6.3094669229193565	0.016947426762344633	0.016947426762344633	0.0013498980316300959
ENSG00000168476_22141470_22138350_247	REEP4	8	−	741	30	13	4	6.07%	23.75629246281377	0.023908016651798578	0.004428466751180383	0.0005765471011256886
ENSG00000158019_27894557_28310124_376	BABAM2	2	+	1128	12	2	0	2.13%	6.242344934377661	0.030313543781140352	0.004826937407846361	0.0007979355218377862
ENSG00000221983_18573301_18575150_128	UBA52	19	+	384	29	4	0	8.59%	44.314146591441414	0.003768080243855145	0.0014055202957965589	3.138862417155242e-05
ENSG00000242028_43800623_43801806_121	HYPK	15	+	363	44	1	0	12.40%	71.12489985836365	0.0006786479905456451	0.00023719413513965303	1.0872976294357274e-06
ENSG00000104529_143589937_143579792_599	EEF1D	8	−	1797	173	0	5	4.51%	56.49026896096245	2.5213715056597438e-06	1.2909652315362702e-05	2.792956634712577e-10
ENSG00000028528_64127762_64137618_268	SNX1	15	+	804	19	0	3	4.10%	13.866701558207092	0.0013430411379358334	0.014028501358900838	0.00011866004684692356
ENSG00000104064_50309723_50286020_322	GABPB1	15	−	966	14	0	7	1.86%	8.504064113500002	0.012798402692974279	0.2405700776118731	0.018919609472776648
ENSG00000100354_40262062_40323241_1608	TNRC6B	22	+	4824	9	0	0	0.31%	1.09473959670056	0.016947426762344633	0.016947426762344633	0.0013498980316300959
ENSG00000092621_119712023_119724460_165	PHGDH	1	+	495	15	0	0	3.64%	17.781224964590912	0.012798402692974279	0.012798402692974279	0.0007972436888943567
ENSG00000092621_119727007_119744040_395	PHGDH	1	+	1185	26	0	0	3.80%	12.874507189551903	0.0002582331582969843	0.0002582331582969843	4.5481323920964005e-07
ENSG00000140694_14630125_14436717_614	PARN	16	−	1842	19	0	2	1.47%	6.052566803907982	0.0033278027414746945	0.011099465161221279	0.00020317731502769463
ENSG00000145907_151786627_151795496_120	G3BP1	5	+	360	18	1	0	7.50%	29.339021191575007	0.002967235347054297	0.0033030736763512156	5.530095551685825e-05
ENSG00000145907_151797234_151804091_284	G3BP1	5	+	852	57	0	10	6.69%	39.25643680562853	6.031011859453544e-05	0.0014838816918955997	7.19293351139654e-07
ENSG00000168575_42472108_42417803_558	SLC20A2	8	−	1674	14	0	0	1.08%	4.90736316227061	0.012798402692974279	0.012798402692974279	0.0007972436888943567
ENSG00000157851_26898500_26946995_564	DPYSL5	2	+	1692	20	0	0	0.89%	6.935938815975179	0.02108409854857798	0.02108409854857798	0.002029891263157455
ENSG00000178467_48990449_49006907_438	P4HTM	3	+	1314	21	7	4	2.51%	9.377769330640414	0.0523161259852883	0.01313178205197555	0.0032750644748262827
ENSG00000100938_24233254_24238778_348	GMPR2	14	+	1044	12	2	0	2.01%	6.744602572775864	0.022898969405409888	0.008175610787902942	0.0009353765476651278
ENSG00000134077_9365069_9384688_507	THUMPD3	3	+	1521	23	4	2	1.97%	8.873076757478305	0.019264875824957298	0.011064573844522778	0.0010312247960341252
ENSG00000104299_28859500_28768146_613	INTS9	8	−	1839	18	0	0	1.31%	5.74336467045514	0.005554612275049112	0.005554612275049112	0.00016468457691007306
ENSG00000105341_41438243_41432231_194	DMAC2	19	−	582	22	7	1	4.12%	22.180703306345364	0.054588706672672294	0.008316137131635013	0.0023562926152911018
ENSG00000082212_50908093_50942723_490	ME2	18	+	1470	27	0	0	2.45%	10.7775996213949	0.0009829516685359422	0.0009829516685359422	6.0033663169184795e-06
ENSG00000124155_45420349_45427437_237	PIGT	20	+	711	45	2	0	3.80%	37.138001508322795	0.01052924788556975	0.003684287313187465	0.00021087137401632993
ENSG00000136261_16685956_16706088_267	BZW2	7	+	801	11	0	0	3.37%	8.058158130020601	0.002478942540635376	0.002478942540635376	3.5397643206479886e-05
ENSG00000196498_124483709_124325402_2415	NCOR2	12	−	7245	36	9	23	0.91%	2.9156791246285723	0.0012956169751729947	0.021274507517138642	0.00018245967258346169
ENSG00000147475_37737923_37744893_206	ERLIN2	8	+	618	13	0	0	3.40%	12.343277523963595	0.0078820576498062	0.0078820576498062	0.00031963642907451164
ENSG00000112096_159693167_159692464_140	SOD2	6	−	420	13	2	0	4.29%	18.162251213832146	0.061356297364084836	0.012798402692974279	0.0037940651817215715
ENSG00000177963_208909_214350_513	RIC8A	11	+	1539	18	3	1	1.95%	6.862929933701756	0.010960145167579947	0.0037220617515305076	0.00022215927817908156
ENSG00000110651_2396712_2396866_21	CD81	11	+	63	16	0	0	23.81%	149.02359970323812	0.019216965118390855	0.019216965118390855	0.0017073955890589052

ENSG00000167491_19465346_19505474_634	GATAD2A	19	+	1902	10	2	0	1.10%	3.085070577452682	0.049338281967443096	0.007593099278532274	0.0019605633087212975
ENSG00000110429_33774488_33747121_407	FBXO3	11	−	1221	14	0	0	1.23%	6.728031067683048	0.019679754444124863	0.019679754444124863	0.0017849636115922674
ENSG00000071564_1622056_1611707_378	TCF3	19	−	1134	7	0	0	1.32%	3.6221013816759267	0.016947426762344633	0.016947426762344633	0.0013498980316300959
ENSG00000030582_44349165_44352798_436	GRN	17	+	1308	167	10	4	8.72%	74.91768408246217	5.101580467959961e−07	1.7587347038544392e−07	8.465855955152095e−13
ENSG00000140694_14630125_14436717_581	PARN	16	−	1743	18	0	2	1.38%	6.059694566246129	0.0053557511377634245	0.016228925652561413	0.00045523357042063324
ENSG00000286235_5952016_6037158_1009	AL035461.3	20	+	3027	13	0	1	0.79%	2.520034856230427	0.005160369442893307	0.00912793029372963	0.00024819240235076056
ENSG00000094916_54257650_54241755_191	CBX5	12	−	573	29	7	3	8.38%	29.697438553426707	0.0016400929380019878	0.00043872953614622595	4.675554956550797e−06
ENSG00000094914_53321465_53307489_546	AAAS	12	−	1638	57	2	3	3.66%	20.419098997799455	0.00011858270277495242	0.0001926526083748436	1.616511173608218e−07
ENSG00000103415_4507809_4509756_216	HMOX2	16	+	648	12	0	0	2.78%	10.86630414502778	0.012798402692974279	0.012798402692974279	0.0007972436888943567
ENSG00000127990_94656026_94597920_421	SGCE	7	−	1263	17	3	2	2.14%	7.8980737966235175	0.020955738116168132	0.01292879023172146	0.0012882305750462103
ENSG00000111481_54325164_54342373_84	COPZ1	12	+	252	37	5	0	13.10%	86.15426857843454	0.015856692345517474	0.0014125691255928705	0.0001414935010418394
ENSG00000144233_127874234_127865094_310	AMMECR1L	2	−	930	8	0	0	1.61%	5.047573538335485	0.019216965118390855	0.019216965118390855	0.0017073955890589052
ENSG00000099817_1095315_1090121_149	POLR2E	19	−	447	26	0	0	3.36%	34.130404965590614	0.019679754444124863	0.019679754444124863	0.0017849636115922674
ENSG00000134001_67364900_67382656_251	EIF2S1	14	+	753	37	0	5	5.58%	28.832504225452197	0.0004388494700883803	0.004258728227950203	1.26075993853867e−05
ENSG00000170348_75139079_75134885_84	TMED10	14	−	252	52	3	2	8.33%	121.08167475888098	0.04444193652437137	0.03799572790022047	0.006991003306294263
ENSG00000170348_75176579_75139121_153	TMED10	14	−	459	60	5	1	13.07%	76.70332337666669	0.00010574058228990304	7.694288680697315e−05	5.919944823072599e−08
ENSG00000136997_127738590_127740958_330	MYC	8	+	990	70	7	1	7.88%	41.489524917378795	3.883483280678225e−05	4.517740773020254e−06	1.4872135334928209e−09
ENSG00000168938_123036625_123023875_212	PPIC	5	−	636	5	3	0	2.36%	4.613053646474057	0.15865525393145707	0.012673659338734126	0.011061610944025602
ENSG00000134644_31059467_30933211_1155	PUM1	1	−	3465	28	2	0	1.21%	4.741659990557577	0.0020767912373559883	0.0003763727141378971	5.1823113164462156e−06
ENSG00000061987_62501630_62592749_1477	MON2	12	+	4431	5	3	0	0.34%	0.6621309228520651	0.15865525393145707	0.012673659338734126	0.011061610944025602
ENSG00000039068_68737416_68833499_903	CDH1	16	+	2709	20	0	1	1.55%	4.332081386722039	0.00034085514668541917	0.0006271482673124025	1.412042964497456e−06
ENSG00000123240_13110300_13136866_507	OPTN	10	+	1521	26	4	5	1.97%	10.03043459541026	0.01790347956991311	0.030396003057272823	0.0024761070165161886
ENSG00000133639_92145505_92144080_161	BTG1	12	−	483	25	2	0	6.21%	30.371657548214294	0.0030685733490663438	0.0023218226288164415	4.084557731917468e−05
ENSG00000197111_53464814_53479442_138	PCBP2	12	+	414	93	0	5	9.42%	131.81299375925002	0.0007028601028765856	0.0008860998327423869	3.938109021436656e−06
ENSG00000086300_26346443_26372572_201	SNX10	7	+	603	5	0	0	2.49%	4.865509318669155	0.012673659338734126	0.012673659338734126	0.000782701129001274
ENSG00000135919_223984879_223975867_145	SERPINE2	2	−	435	36	3	0	8.28%	48.561138523986216	0.005430946793605584	0.0007799100084460569	2.6967900916859873e−05
ENSG00000155636_178112547_178126176_474	RBM45	2	+	1422	6	0	0	1.05%	2.4758667672215195	0.016947426762344633	0.016947426762344633	0.0013498980316300959
ENSG00000213741_49586346_49583667_56	RPS29	14	−	168	120	15	3	10.71%	419.1288741653572	0.013640585738808999	0.017143983986795872	0.0011154796180721693
ENSG00000135046_73158536_73170107_346	ANXA1	9	+	1038	1154	68	17	31.21%	652.3551147413788	6.508356967796867e−17	7.642107337009581e−19	8.18814254356542e−34
ENSG00000054116_36149378_36137203_188	TRAPPC3	1	−	564	30	0	5	5.85%	31.211724671888305	0.001558047347358915	0.013577461948370874	0.00012987996908748637
ENSG00000106070_50626921_50592952_407	GRB10	7	−	1221	12	0	0	1.47%	5.766883772299756	0.013217226988443315	0.013217226988443315	0.0008469697265466234
ENSG00000174938_29899019_29871699_809	SEZ6L2	16	−	2427	5	0	0	0.62%	1.208859546418418	0.012673659338734126	0.012673659338734126	0.000782701129001274
ENSG00000136888_114587839_114597743_118	ATP6V1G1	9	+	354	53	0	0	14.41%	87.85130639285171	0.00011368890353703816	0.00011368890353703816	9.267065049664284e−08
ENSG00000106355_32490365_32487720_64	LSM5	7	−	192	37	3	2	15.62%	113.07747750919533	0.0038004716283322327	0.003594758625082528	7.589020838675016e−05
ENSG00000196141_200389245_200478031_558	SPATS2L	2	+	1674	21	2	0	1.79%	7.361044743405916	0.005670089768331009	0.0022409900782254734	7.232548112602907e−05
ENSG00000112695_75243734_75237866_76	COX7A2	6	−	228	80	17	53	15.79%	205.8878680110527	0.005265875520493234	0.03998272178132884	0.0011566646576120765
ENSG00000153879_33379411_33379692_93	CEBPG	19	+	279	11	0	0	5.38%	23.134712050704305	0.02108409854857798	0.02108409854857798	0.002029891263157455
ENSG00000009844_142147288_142218643_307	VTA1	6	+	921	35	9	0	4.56%	22.2989303301873	0.014990099012597163	0.00041055602707030847	4.802655147598213e−05
ENSG00000075239_108121607_108134390_135	ACAT1	11	+	405	16	0	1	5.19%	23.18144884272593	0.00847367416878415	0.00847367416878415	0.0003665024264111182
ENSG00000100926_24194758_24189415_519	TM9SF1	14	−	1557	60	4	0	4.43%	22.61196238271677	0.0001015940903691073	1.1458439848907443e−05	9.488143575773584e−09
ENSG00000112081_36596763_36599854_124	SRSF3	6	+	372	103	17	9	23.39%	162.4687732651734	1.942439202719079e−05	8.307973949626923e−06	1.307812701818994e−09
ENSG00000146425_158644708_158636827_113	DYNLT1	6	−	339	31	0	1	7.96%	53.658386840048685	0.0037896409716948643	0.0037631575832289435	7.905265261082401e−05
ENSG00000101266_489860_483961_177	CSNK2A1	20	−	531	9	0	0	2.82%	9.945430912398308	0.019216965118390855	0.019216965118390855	0.0017073955890589052
ENSG00000049618_157174031_157207891_1286	ARID1B	6	+	3858	16	0	0	0.54%	2.433511348186626	0.00847367416878415	0.00847367416878415	0.0003665024264111182
ENSG00000163932_53184882_53192266_411	PRKCD	3	+	1233	5	0	0	1.22%	2.3794826594951344	0.012673659338734126	0.012673659338734126	0.000782701129001274
ENSG00000141027_16101554_16032296_1526	NCOR1	17	−	4578	9	0	6	0.39%	1.1535657087119924	0.011772051206276392	0.15741437844908046	0.010389467034322046
ENSG00000085063_33722445_33710126_128	CD59	11	−	384	62	5	3	14.06%	94.74058926446097	0.00102540667708157	0.0003583833865847517	2.4148597638421077e−06
ENSG00000175387_47896756_47841827_437	SMAD2	18	−	1311	34	0	3	2.75%	15.217798939947372	0.0010447178140462346	0.003942234527428402	2.5100692658404202e−05
ENSG00000175467_65961781_65979030_800	SART1	11	+	2400	21	0	1	1.50%	5.134328708525626	0.00093915422288674	0.0014100492328341582	8.155839521565571e−06
ENSG00000143384_150579530_150577375_197	MCL1	1	−	591	38	1	2	8.63%	37.72869053400508	0.00018796157908136063	0.0005302436077569723	6.832715439216683e−07
ENSG00000103202_399666_400342_65	NME4	16	+	195	10	2	0	7.69%	30.091303786230775	0.019679754444124863	0.019679754444124863	0.0017849636115922674
ENSG00000183624_129279733_129304825_354	HMCES	3	+	1062	21	3	0	2.82%	11.603002731131358	0.010728371185261619	0.0023218226288164415	0.00014287115526610158
ENSG00000043143_134559882_134579137_669	JADE2	5	+	2007	14	1	2	1.05%	4.0931369873647245	0.013892891352654344	0.03299602752967379	0.0021461203908066497
ENSG00000197912_89508547_89528710_235	SPG7	16	+	705	12	0	0	2.13%	9.987751895004257	0.019679754444124863	0.019679754444124863	0.0017849636115922674
ENSG00000136108_52460972_52475141_606	CKAP2	13	+	1818	24	0	2	1.49%	7.746276222198022	0.0033030736763512156	0.016881135945258162	0.0003111891849301449
ENSG00000163875_37513472_37494099_139	MEAF6	1	−	417	11	0	0	5.04%	15.47862029291727	0.006937202941512724	0.006937202941512724	0.00025101522741487095
ENSG00000085491_108161274_108136653_338	SLC25A24	1	−	1014	18	0	4	3.25%	10.416220541387576	0.0012427339549726212	0.015053030435227769	0.00012006203002810468
ENSG00000145685_78510213_78488897_228	LHFPL2	5	−	684	10	0	0	3.07%	8.578661167127194	0.007029302661868638	0.007029302661868638	0.0002573639808771639
ENSG00000138613_63279210_63305781_203	APH1B	15	+	609	6	1	0	2.46%	5.781087919522169	0.0477903522728147	0.016947426762344633	0.0036975118224100542
ENSG00000138613_63277624_63305781_216	APH1B	15	+	648	8	1	0	2.31%	7.244202763351853	0.04223451612662591	0.019679754444124863	0.003715148010809822
ENSG00000170525_6213664_6232942_481	PFKFB3	10	+	1443	54	13	3	4.57%	21.958518979141378	0.0007624027321826563	7.934543476937361e−05	4.501702025837454e−07
ENSG00000171490_11841975_11837787_270	RSL1D1	16	−	810	59	0	0	5.93%	42.74079630377594	0.0001978461102466251	0.0001978461102466251	2.7148029154684727e−07
ENSG00000145349_113759386_113457331_447	CAMK2D	4	−	1341	8	0	0	1.34%	3.500554355445191	0.011570465654371866	0.011570465654371866	0.0006594843062896773
ENSG00000163069_52033610_52023957_297	SGCB	4	−	891	27	2	1	5.05%	17.781224964590912	0.00040286545213605605	0.0003716399344246037	9.945643565034057e−07
ENSG00000172340_67654586_67375744_432	SUCLG2	3	−	1296	36	1	0	3.24%	16.29945621754167	0.0006644883448370029	0.00041055602707030847	1.7825765893568471e−06
ENSG00000122642_32957574_32988747_377	FKBP9	7	+	1131	29	0	1	2.65%	15.045651893115387	0.0022409900782254734	0.0037834851205969236	4.845513352116639e−05
ENSG00000105323_41302406_41306565_390	HNRNPUL1	19	+	1170	29	0	3	2.05%	14.544130163344875	0.00575691403842271	0.012185301878589709	0.00036439343170220357

ENSG00000099991_24042910_24178196_2024	CABIN1	22	+	6072	6	0	1	0.25%	0.579822553193182	0.016947426762344633	0.0477903522728147	0.0036975118224100542
ENSG00000105700_18557907_18562105_25	KXD1	19	+	75	23	1	1	20.00%	179.94599664166003	0.02108409854857798	0.02108409854857798	0.002029891263157455
ENSG00000087470_32679364_32711269_181	DNM1L	12	+	543	6	2	0	2.76%	6.483761589298344	0.13974268412556323	0.016947426762344633	0.011764960919844317
ENSG00000131002_19587267_19588482_117	TXLNGY	Y	+	351	10	0	0	4.27%	16.71739099235043	0.020613416668581838	0.020613416668581838	0.001946208561389314
ENSG00000142494_19555513_19577553_324	SLC47A1	17	+	972	12	0	0	1.54%	7.244202763351853	0.020613416668581838	0.020613416668581838	0.001946208561389314
ENSG00000155463_22770608_22771558_163	OXA1L	14	+	489	43	2	3	9.20%	51.59827857823007	0.0010376102755361187	0.0013919855294422787	8.851235358247057e-06
ENSG00000155463_22766717_22770407_258	OXA1L	14	+	774	45	11	0	5.43%	34.115140920436055	0.002513773846554814	0.00046705916373637345	7.664790090554796e-06
ENSG00000109332_102809680_102797415_110	UBE2D3	4	−	330	19	0	1	5.45%	33.78432743272273	0.013428347753762199	0.013428347753762199	0.0008725593497644525
ENSG00000172354_100676266_100678801_340	GNB2	7	+	1020	96	10	3	9.12%	55.226392831200016	1.3190375598500069e-05	1.991941274990916e-06	2.2871148676631544e-10
ENSG00000166260_54968646_54961265_231	COX11	17	−	693	34	0	3	4.33%	28.788649942671004	0.002446120450917513	0.0072608768093428535	0.00010037784624098265
ENSG00000143537_155051387_155062502_814	ADAM15	1	+	2442	46	13	0	2.33%	11.053193896907866	0.009314527945786596	5.328371459388349e-05	5.3170083957120194e-06
ENSG00000001630_92127610_92113665_346	CYP51A1	7	−	1038	86	3	0	3.76%	48.61571912284105	0.0017995462278608356	0.0007148938932365558	8.04285368988614e-06
ENSG00000172590_22833438_22834278_52	MRPL52	14	+	156	17	13	0	9.62%	63.9440205457404	0.3770620196976409	0.02108409854857798	0.048637581181073294
ENSG00000172590_22834199_22834321_40	MRPL52	14	+	120	12	0	5	12.50%	58.678042383150014	0.02108409854857798	0.15350694901691259	0.01542296770981773
ENSG00000165792_20992569_20996883_320	METTL17	14	+	960	11	0	0	1.56%	6.723525689735939	0.019679754444124863	0.019679754444124863	0.0017849636115922674
ENSG00000256206_14513696_14505192_124	AC018523.2	11	−	372	79	4	1	10.48%	124.61197172765729	0.0030557506494497556	0.0006838816678496783	1.32043342222491e-05
ENSG00000256206_14517954_14510943_139	AC018523.2	11	−	417	14	3	25	6.47%	19.700062190985616	0.024663957215093197	0.1878932019458327	0.02188599883226073
ENSG00000119685_75690241_75954446_1141	TTLL5	14	+	3423	13	0	0	0.44%	2.2284970814517973	0.02108409854857798	0.02108409854857798	0.002029891263157455
ENSG00000186522_109593083_109545425_466	SEPT10	2	−	1398	8	1	0	1.29%	3.3578278903090135	0.009815328628645334	0.011570465654371866	0.0005653151658108352
ENSG00000136718_130342933_130346364_248	IMP4	2	+	744	16	1	0	2.42%	12.618933845838713	0.011293444082089835	0.009815328628645334	0.0005524292941436505
ENSG00000138078_44339308_44321356_458	PREPL	2	−	1374	18	0	0	3.06%	7.687079788185591	0.00024035368145103084	0.00024035368145103084	3.9581571392385245e-07
ENSG00000105290_35871651_35879441_459	APLP1	19	+	1377	8	0	0	1.09%	3.4090365945185193	0.019679754444124863	0.019679754444124863	0.0017849636115922674
ENSG00000105281_46784597_46775510_365	SLC1A5	19	−	1095	124	1	2	9.32%	66.44819411425208	2.5916830432815443e-07	1.7451872588878565e-07	4.280939693249412e-13
ENSG00000163694_40438758_40425904_548	RBM47	4	−	1644	14	0	0	1.28%	4.996913584939782	0.00797940233269714	0.00797940233269714	0.000327142761693177
ENSG00000241878_31623797_31619612_340	PISD	22	−	1020	8	1	0	1.47%	4.602199402600001	0.03418265644467716	0.019216965118390855	0.0029560527921161815
ENSG00000239672_51155655_51161845_152	NME1	17	+	456	508	29	5	36.18%	653.6939809350922	3.4475848714650074e-09	4.903157486537709e-11	2.132159649588751e-18
ENSG00000184640_77281536_77498658_586	SEPT9	17	+	1758	57	3	6	3.24%	19.025303844366046	0.00031034636673037916	0.000987392842142568	2.0348119158311122e-06
ENSG00000133961_73355751_73276578_640	NUMB	14	−	1920	19	0	1	1.56%	5.80668127749922	0.0022011839993586477	0.0037373685375237473	4.708421183416152e-05
ENSG00000064012_201266643_201286594_427	CASP8	2	+	1281	20	1	0	2.58%	9.161286867002344	0.002558966410643687	0.001092092816906976	1.6908373131935995e-05
ENSG00000127125_42456566_42457195_209	PPCS	1	+	627	10	3	0	2.39%	9.358539455047849	0.08350359792828288	0.020613416668581838	0.007749259071793911
ENSG00000139318_89352000_89349254_368	DUSP6	12	−	1104	20	0	1	2.99%	10.630080141875002	0.0012624143894574274	0.0025593219000189936	1.9339285108591855e-05
ENSG00000136240_6484057_6474094_93	KDELR2	7	−	279	79	0	0	11.83%	166.1492956368764	0.0016572264983485026	0.0016572264983485026	1.6372075009059705e-05
ENSG00000175548_38320341_38321213_290	ALG10B	12	+	870	12	0	0	2.41%	8.093523087331036	0.0078820576498062	0.0078820576498062	0.00031963642907451164
ENSG00000061987_62501630_62588893_1435	MON2	12	+	4305	5	3	0	0.35%	0.6815103644965158	0.15865525393145707	0.012673659338734126	0.011061610944025602
ENSG00000205323_55817701_55757512_210	SARNP	12	−	630	41	5	8	6.67%	38.18729742395477	0.0036128142112999487	0.01635557282465551	0.000325315688932636
ENSG00000143222_161153998_161157697_111	UFC1	1	+	333	28	10	4	9.91%	49.338894496342355	0.0566406603933506	0.007459848152910933	0.0022478334037315728
ENSG00000118007_136630898_136338254_1224	STAG1	3	−	3672	7	0	0	0.41%	1.1185901325763892	0.019216965118390855	0.019216965118390855	0.0017073955890589052
ENSG00000177200_53156537_53324895_2732	CHD9	16	+	8196	14	0	5	0.22%	1.002309167110908	0.013217226988443315	0.11669065552020402	0.007926800932849956
ENSG00000187608_1014047_1014478_143	ISG15	1	+	429	28	4	1	8.39%	38.29802300065735	0.007509295436624617	0.0014807488005297608	6.646977536659495e-05
ENSG00000256269_119088273_119093283_338	HMBS	11	+	1014	27	0	0	2.96%	15.624330812081364	0.0023354528714849923	0.0023354528714849923	3.158285405866755e-05
ENSG00000104522_143616712_143612910_321	TSTA3	8	−	963	29	0	5	3.43%	17.670438516213398	0.001558047347358915	0.013577461948370874	0.00012987996908748637
ENSG00000180992_44116611_44113843_145	MRPL14	6	−	435	24	1	0	6.21%	32.374092349324144	0.005670089768331009	0.0035547763465261306	0.00011049933550063637
ENSG00000143889_38602867_38564182_537	HNRNPLL	2	−	1611	35	3	6	1.86%	12.748178047239294	0.009821676566158627	0.03375807224221634	0.0016279040644707498
ENSG00000115966_175118118_175074609_399	ATF2	2	−	1197	18	1	0	1.25%	8.823765771902258	0.029237630782826433	0.021557223391537678	0.002819005644529471
ENSG00000204370_112086908_112094970_159	SDHD	11	+	477	16	0	1	3.77%	19.68236222495598	0.013007239811447113	0.016503128830616266	0.001029548967667127
ENSG00000137693_102114357_102229940_326	YAP1	11	+	978	13	0	1	2.45%	7.79973978508129	0.004826937407846361	0.004826937407846361	0.00012612451166366307
ENSG00000179889_15017348_15036275_492	PDXDC1	16	+	1476	12	0	0	1.42%	4.7705725514756105	0.008175610787902942	0.008175610787902942	0.00034251843268961324
ENSG00000004897_47138759_47120935_263	CDC27	17	−	789	10	0	0	2.28%	7.437014243745248	0.011570465654371866	0.011570465654371866	0.0006594843062896773
ENSG00000023191_502162_494691_461	RNH1	11	−	1383	58	1	4	6.72%	24.60828964730803	4.0021555061397233e-07	6.5177990801153216e-06	2.4756288940905907e-11
ENSG00000175216_46763083_46744023_771	CKAP5	11	−	2313	22	0	4	1.17%	5.581136759313879	0.0036321914711260127	0.02570283571280724	0.0005269848238663837
ENSG00000130204_44891416_44903169_361	TOMM40	19	+	1083	23	1	0	2.22%	12.461634116458452	0.007181907427766128	0.005554612275049112	0.0002105152421252746
ENSG00000221926_15651591_15628615_558	TRIM16	17	−	1674	13	0	0	1.08%	4.556837222108424	0.013007239811447113	0.013007239811447113	0.0008218654148058079
ENSG00000128641_191276953_191423960_1088	MYO1B	2	+	3264	22	2	1	1.19%	3.955015111609376	0.0032701130750291308	0.001015856855223422	2.0240423793271705e-05
ENSG00000167526_89562377_89563042_57	RPL13	16	+	171	102	1	6	12.28%	350.00937561878953	0.008877961307018026	0.010353108236244411	0.0004630494296106523
ENSG00000140740_21953424_21961346_116	UQCRC2	16	+	348	30	2	0	7.76%	50.58451929581898	0.003684287313187465	0.003710480643933249	7.59305759620077e-05
ENSG00000092621_119712023_119724460_165	PHGDH	1	+	495	15	0	0	3.64%	17.781224964590912	0.012798402692974279	0.012798402692974279	0.0007972436888943567
ENSG00000171848_10122799_10142261_374	RRM2	2	+	1122	40	0	1	3.48%	20.91908819363637	0.0006026251277497241	0.00103778146287135	3.972184375698158e-06
ENSG00000124155_45416169_45420776_367	PIGT	20	+	1101	38	4	0	4.63%	20.25218538201363	0.0007168585048009968	0.00012869633121932495	6.543617886399178e-07
ENSG00000135506_57694162_57720909_406	OS9	12	+	1218	67	0	4	7.39%	32.277740883998774	5.932739832345998e-07	5.59240431002644e-06	3.049655432681014e-11
ENSG00000114742_39068815_39094743_502	WDR48	3	+	1506	17	0	0	1.59%	6.623683403144423	0.005454749182134642	0.005454749182134642	0.00015910859015753364
ENSG00000095485_100267593_100233227_538	CWF19L1	10	−	1614	6	0	0	0.93%	2.18133986554461	0.016947426762344633	0.016947426762344633	0.0013498980316300959
ENSG00000146674_45916649_45914820_75	IGFBP3	7	−	225	58	2	0	14.67%	151.2589536987867	0.002052838453648385	0.0016340149330375125	1.9853260068582068e-05
ENSG00000185627_239063_252600_311	PSMD13	11	+	933	56	17	2	6.11%	35.21940378838586	0.00913402444084107	0.00010621786803924688	9.021861802914776e-06
ENSG00000179115_12933696_12922748_508	FARSA	19	−	1524	53	0	0	3.54%	20.406405815662406	9.087114746133616e-05	9.087114746133616e-05	5.997075583396284e-08
ENSG00000091732_130028904_130018664_296	ZC3HC1	7	−	888	21	0	0	3.38%	13.876564077096287	0.0017901637611834264	0.0017901637611834264	1.8982104156792192e-05
ENSG00000104823_38831772_38817349_191	ECH1	19	−	573	102	4	0	13.61%	104.45305973963876	5.347809470446742e-06	3.7484984614400756e-06	1.689663585104152e-10
ENSG00000104852_49090342_49102139_100	SNRNP70	19	+	300	10	0	2	5.00%	19.559347461050006	0.020613416668581838	0.08350359792828288	0.007749259071793911

ENSG00000104852_49108075_49108443_122	SNRNP70	19	+	366	22	1	0	5.74%	35.27095443795903	0.010197420091687953	0.00877616296920843	0.0004515216795882782
ENSG00000120725_139127780_138947117_440	SIL1	5	−	1320	9	0	0	1.82%	4.000775617032955	0.0033278027414746884	0.0033278027414746884	6.211806171561044e-05
ENSG00000070756_100721583_100704298_636	PABPC1	8	−	1908	54	8	6	3.93%	16.60699312730661	9.149015046940165e-05	0.0001705511630397554	1.1191445947132568e-07
ENSG00000124228_49219449_49243834_765	DDX27	20	+	2295	24	3	4	2.09%	6.136265870133335	0.0009747456371656623	0.001934042481117715	1.1523237442356582e-05
ENSG00000185624_81845290_81844012_75	P4HB	17	−	225	107	7	6	33.33%	279.0466904443134	8.263990294912092e-06	8.798715467854478e-06	5.950232858901878e-10
ENSG00000167930_254414_268853_540	FAM234A	16	+	1620	31	0	1	2.59%	11.228514283195373	0.00040182005258836655	0.0006536905545227689	1.7187018856253374e-06
ENSG00000175061_16439055_16440252_53	SNHG29	17	+	159	22	3	0	16.98%	81.18974417794341	0.01772171524267468	0.0036581908865286695	0.0003575151330641259
ENSG00000065268_984366_994344_391	WDR18	19	+	1173	36	8	0	4.86%	18.008606358000005	0.0025813700353149146	4.074202602784786e-05	9.510286350937302e-07
ENSG00000103018_69424696_69448195_123	CYB5B	16	+	369	114	16	6	14.63%	181.28175695607322	0.00039255943793907	9.283325738969487e-05	2.6180588023363114e-07
ENSG00000121067_49611406_49600378_197	SPOP	17	−	591	11	0	0	2.54%	10.92146304931726	0.020613416668581838	0.020613416668581838	0.001946208561389314
ENSG00000103415_4507809_4509756_216	HMOX2	16	+	648	12	0	0	2.78%	10.86630414502778	0.012798402692974279	0.012798402692974279	0.0007972436888943567
ENSG00000124831_237692456_237764140_752	LRRFIP1	2	+	2256	28	1	7	1.46%	7.2827357567739375	0.0021519382791564774	0.04554459336807182	0.0006554929825905563
ENSG00000102572_98521852_98453173_443	STK24	13	−	1329	41	0	0	2.93%	18.10233060729233	0.0006698694257094773	0.0006698694257094773	2.868630583058064e-06
ENSG00000106829_79627422_79725144_583	TLE4	9	+	1749	8	0	0	0.86%	2.68395848522127	0.019679754444124863	0.019679754444124863	0.0017849636115922674
ENSG00000204574_30571488_30590701_807	ABCF1	6	+	2421	16	3	1	1.24%	3.877937538745974	0.018462046284954254	0.003957931943710776	0.0003993137606956889
ENSG00000100997_25390703_25294973_404	ABHD12	20	−	1212	25	4	0	3.47%	12.103556597184408	0.004559701316823627	0.0003386460863230731	1.0832931975553587e-05
ENSG00000149257_75566350_75569192_324	SERPINH1	11	+	972	101	5	7	8.02%	60.9720399248781	2.576858229190474e-05	2.4496209342856002e-05	4.908024255576602e-09
ENSG00000141905_3381826_3456600_367	NFIC	19	+	1101	43	3	3	3.27%	22.916946616489106	0.0027101569075239007	0.0010032776369550557	1.6561952297087845e-05
ENSG00000113712_149525134_149496853_279	CSNK1A1	5	−	837	29	0	0	4.30%	20.330504529406813	0.000978917897162665	0.000978917897162665	5.956062365741233e-06
ENSG00000168528_31424824_31434199_341	SERINC2	1	+	1023	16	0	0	1.47%	9.177406433337245	0.02108409854857798	0.02108409854857798	0.002029891263157455
ENSG00000169359_155853997_155833538_335	SLC33A1	3	−	1005	18	9	0	2.09%	10.509500128325376	0.12953833035117723	0.008674846900444944	0.00656210637445099
ENSG00000169359_155853312_155828210_258	SLC33A1	3	−	774	11	1	0	1.94%	8.339256669439925	0.04223451612662591	0.019679754444124863	0.003715148010809822
ENSG00000188186_100153490_100153964_41	LAMTOR4	7	+	123	21	0	0	14.63%	100.18202358098783	0.013640585738808999	0.013640585738808999	0.00089863692579514
ENSG00000124155_45416169_45425826_518	PIGT	20	+	1554	76	4	0	4.63%	28.69711210501545	4.402326033587937e-05	7.832689905173982e-06	2.82322008883783e-09
ENSG00000166557_79311250_79314564_146	TMED3	15	+	438	9	0	0	3.42%	12.057131996537674	0.020613416668581838	0.020613416668581838	0.001946208561389314
ENSG00000166557_79321978_79322214_78	TMED3	15	+	234	24	2	0	14.10%	60.18260757246155	0.011064573844522778	0.0012624143894574274	8.713148241777389e-05
ENSG00000164543_43608335_43624842_248	STK17A	7	+	744	16	0	0	4.03%	12.618933845838713	0.0020082573300163734	0.0020082573300163734	2.3658688350241813e-05
ENSG00000120885_27610571_27599699_414	CLU	8	−	1242	347	8	3	13.53%	163.93945818802783	5.606170291860397e-11	1.0224195827327958e-10	6.746525246741049e-20
ENSG00000038382_14387570_14531774_1579	TRIO	5	+	4737	16	2	0	0.38%	1.9819478111260296	0.023857440118675605	0.013007239811447113	0.00146964612208996
ENSG00000054277_241640254_241594428_402	OPN3	1	−	1206	17	7	4	2.49%	8.271365841737564	0.0860522389173572	0.024631852554464408	0.009238428685478722
ENSG00000110172_90222954_90202405_332	CHORDC1	11	−	996	12	0	0	1.81%	7.069643660620484	0.012798402692974279	0.012798402692974279	0.0007972436888943567
ENSG00000175115_66193529_66243280_830	PACS1	11	+	2490	20	0	0	0.84%	4.713095773746989	0.00847367416878415	0.00847367416878415	0.0003665024264111182
ENSG00000101079_36721735_36653520_388	NDRG3	20	−	1164	26	1	0	2.06%	13.106779226476807	0.008364861494636245	0.005604866691947602	0.00024596970711672695
ENSG00000169727_82051932_82057127_490	GPS1	17	+	1470	39	6	9	3.67%	15.567643897570411	0.002468906503015184	0.003585008937736941	5.032478504846074e-05
ENSG00000148180_121317107_121332603_473	GSN	9	+	1419	17	0	0	1.06%	7.029786613908035	0.02108409854857798	0.02108409854857798	0.002029891263157455
ENSG00000170312_60780166_60793975_240	CDK1	10	+	720	79	1	1	8.75%	64.3828520592896	3.443854328470227e-05	5.068977653237062e-05	1.3251921946741904e-08
ENSG00000131149_85655817_85672539_921	GSE1	16	+	2763	10	1	0	0.65%	2.123707650494029	0.023672501042627626	0.012798402692974279	0.0014375227604752335
ENSG00000197976_1593463_1601594_695	AKAP17A	X	+	2085	11	0	0	0.72%	3.095724058583454	0.02108409854857798	0.02108409854857798	0.002029891263157455
ENSG00000129484_20352324_20357524_326	PARP2	14	+	978	13	0	0	1.84%	7.79973978508129	0.013428347753762199	0.013428347753762199	0.0008725593497644525
ENSG00000161016_144792129_144789804_148	RPL8	8	−	444	322	18	26	23.65%	425.5479650309528	2.5191807253293443e-07	5.188589492702603e-07	1.2186449489332845e-12
ENSG00000141905_3381826_3462769_460	NFIC	19	+	1380	43	3	3	2.61%	18.283737844025005	0.0027101569075239007	0.0010032776369550557	1.6561952297087845e-05
ENSG00000168439_64186262_64193371_100	STIP1	11	+	300	38	9	4	9.00%	74.32552035199002	0.052910752944514895	0.019439702257615755	0.004606074003574909
ENSG00000178252_49011688_49015288_1070	WDR6	3	+	3210	21	0	2	1.03%	3.838750436280842	0.0013984928336429232	0.0067530256680052286	5.6613811115239e-05
ENSG00000255730_41378310_41424608_479	AC011462.1	19	+	1437	8	0	0	1.25%	3.266696861970773	0.011570465654371866	0.011570465654371866	0.0006594843062896773
ENSG00000136877_127804381_127813604_509	FPGS	9	+	1527	20	0	0	1.38%	7.685401752868371	0.008574016250729465	0.008574016250729465	0.0003747459614100891
ENSG00000042286_70123973_70114178_336	AIFM2	10	−	1008	36	0	2	5.06%	20.956443708267862	0.00012077315611212433	0.0005080074149423065	4.3399894519320936e-07
ENSG00000122952_56361236_56358418_277	ZWINT	10	−	831	39	1	3	5.05%	27.538431443355602	0.00043622442149098933	0.0018832899751346501	5.355328816048756e-06
ENSG00000196704_68250296_68256969_266	AMZ2	17	+	798	28	0	0	4.14%	20.588786801105268	0.001484234770590415	0.001484234770590415	1.3251387882263029e-05
ENSG00000184009_81512354_81510690_375	ACTG1	17	−	1125	676	106	22	21.07%	352.5898368978614	1.5005167573807522e-10	1.5983683066564432e-13	3.7807222952344665e-22
ENSG00000160948_144426945_144423887_233	VPS28	8	−	699	55	4	0	8.15%	46.170133491748935	0.00038381556184962756	5.8776100278201664e-05	1.6799871986435287e-07
ENSG00000160948_144423903_144423805_32	VPS28	8	−	96	16	0	0	18.75%	97.79673730525002	0.013007239811447113	0.013007239811447113	0.0008218654148058079
ENSG00000151348_44108115_44116066_168	EXT2	11	+	504	11	0	0	3.57%	12.806715599497027	0.012798402692974279	0.012798402692974279	0.0007972436888943567
ENSG00000100353_36526040_36510987_521	EIF3D	22	−	1563	64	2	5	4.03%	24.02683757211517	8.204348628700177e-05	0.00022694159317023126	1.3417225830610453e-07
ENSG00000135720_66751591_66732335_271	DYNC1LI2	16	−	813	17	0	0	2.58%	12.269701359330261	0.008674846900444944	0.008674846900444944	0.00038311532916634116
ENSG00000089597_62639071_62634817_90	GANAB	11	−	270	78	3	1	14.44%	169.51434466243336	0.0008531384257998691	0.0006592978435953202	3.5708293565321865e-06
ENSG00000119408_124301986_124350947_306	NEK6	9	+	918	18	0	3	1.96%	11.505498506500002	0.013007239811447113	0.044329616041373636	0.00273578248190731
ENSG00000092621_119727007_119744040_395	PHGDH	1	+	1185	26	0	0	3.80%	12.874507189551903	0.0002582331582969843	0.0002582331582969843	4.5481323920964005e-07
ENSG00000285932_99727012_99679798_216	AL133353.2	10	−	648	21	3	0	3.70%	19.016032253798617	0.025320519897108592	0.005604866691947602	0.0007483196699512048
ENSG00000182093_39380385_39390907_103	WRB	21	+	309	19	0	0	7.77%	36.08034968543205	0.005405141725979036	0.005405141725979036	0.00015637213408961292
ENSG00000273003_65014208_65027320_113	ARL2-SNX15	11	+	339	41	0	3	9.73%	70.96754388522568	0.0015208721599504675	0.005542082247366204	4.979728931750281e-05
ENSG00000086504_370218_367675_256	MRPL28	16	−	768	46	2	0	7.81%	35.14570246907423	7.237715554886367e-05	3.592951532853599e-05	1.96263786912504e-08
ENSG00000140443_98649582_98916955_759	IGF1R	15	+	2277	13	0	1	0.66%	3.3500858628939403	0.02108409854857798	0.028889785561798623	0.002731532589007073
ENSG00000184277_101650135_101633677_144	TM2D3	15	−	432	10	5	0	4.17%	13.582880181284725	0.11411551976453232	0.011772051206276392	0.007078637642502531
ENSG00000175110_139344027_139357014_360	MRPS22	3	+	1080	15	1	6	1.94%	8.149728108770836	0.013794764262559246	0.07916783876068934	0.005304542044665636
ENSG00000283761_100058702_100082209_418	AC118553.2	1	+	1254	27	0	0	4.31%	12.634028264314596	4.027253539844791e-05	4.027253539844791e-05	1.23110933795418e-08
ENSG00000158042_6682827_6682118_121	MRPL17	11	−	363	16	1	4	7.44%	25.863599948495875	0.006112543793728684	0.03624304254342325	0.001176177993073994
ENSG00000158042_6683245_6683030_71	MRPL17	11	−	213	8	0	1	7.04%	22.038701364563384	0.019216965118390855	0.03418265644467716	0.0029560527921161815

ENSG00000116586_156054890_156055599_134	LAMTOR2	1	+	402	29	2	1	6.72%	42.32993107242165	0.007225456388766877	0.004355956481189797	0.00016897055837894996
ENSG00000113161_75350110_75351368_205	HMGCR	5	+	615	42	1	3	6.83%	40.07280943239513	0.0006399719955500673	0.0022916949559961732	9.269314349844266e-06
ENSG00000153113_96722678_96771679_636	CAST	5	+	1908	115	0	4	6.29%	35.36674462296777	1.3070320824253267e-08	1.4148588299346265e-07	1.92439610425533e-14
ENSG00000137074_33001604_32988108_63	APTX	9	-	189	10	0	0	7.94%	31.046583271507945	0.019679754444124863	0.019679754444124863	0.0017849636115922674
ENSG00000173486_64242388_64244029_142	FKBP2	11	+	426	62	4	3	14.08%	85.39996778768312	0.00016569421277282895	0.00014894787451579541	1.736266846401335e-07
ENSG00000167699_778692_760173_275	GLOD4	17	-	825	30	8	2	4.00%	21.337469957509096	0.017469647593621265	0.00447458916152286	0.0004193428366505549
ENSG00000088986_120496422_120496625_67	DYNLL1	12	+	201	47	2	2	7.46%	137.20736278647018	0.029237630782826433	0.021557223391537678	0.002819005644529471
ENSG00000135506_57694162_57694771_75	OS9	12	+	225	22	0	0	8.00%	57.37408588574668	0.013007239811447113	0.013007239811447113	0.0008218654148058079
ENSG00000117054_75724788_75762763_421	ACADM	1	+	1263	28	0	0	3.33%	13.008592135615205	0.00037874750290730355	0.00037874750290730355	9.541676172656993e-07
ENSG00000173230_121698635_121664480_2635	GOLGB1	3	-	7905	23	7	0	0.46%	1.7072675203193552	0.01039256944151647	0.0008776897154483972	5.9759235036696684e-05
ENSG00000163636_64022521_64010668_340	PSMD6	3	-	1020	72	5	0	5.88%	41.41979462340001	0.000602543381100086	3.45592214554821e-05	1.670045363763243e-07
ENSG00000112576_41940540_41936544_160	CCND3	6	-	480	20	0	2	5.00%	24.449184326312505	0.005454749182134642	0.01133090110388836	0.0003229626448716648
ENSG00000106976_128203486_128253122_846	DNM1	9	+	2538	54	2	7	2.36%	12.484689868755321	5.0604056647534704e-05	0.0009696113333780397	3.89413359703063 7e-07
ENSG00000069329_46682109_46660472_740	VPS35	16	-	2220	75	0	5	4.32%	19.82366296728041	2.539100626534125e-07	3.7461440237701814e-06	9.129518999647536e-12
ENSG00000008394_16354274_16364041_148	MGST1	12	+	444	871	34	10	29.73%	1151.0940296334159	2.1707182870174733e-08	9.355012467178048e-09	2.1000262057422205e-15
ENSG00000178741_74938014_74920320_153	COX5A	15	-	459	44	1	1	8.50%	56.24910380955557	0.0007233867859870795	0.0007233867859870795	3.3266741301337226e-06
ENSG00000060491_62804860_62813649_677	OGFR	20	+	2031	17	7	1	1.77%	4.911505270869277	0.02477571070412006	0.0015948498531084352	0.00025659862208291705
ENSG00000241837_33915763_33903526_213	ATP5PO	21	-	639	149	2	0	14.55%	136.82360430499767	4.64563659604018e-07	4.6741172831175084e-07	1.9968758907777217e-12
ENSG00000109854_20364238_20383205_196	HTATIP2	11	+	588	62	5	0	11.73%	61.871405233933686	4.9917421597482345e-05	1.0728102490110545e-05	4.307389351223169e-09
ENSG00000153406_4474132_4461780_299	NMRAL1	16	-	897	13	2	2	2.01%	8.504064113500002	0.044040755831095145	0.044040755831095145	0.00793066636988651
ENSG00000162191_62678923_62678484_76	UBXN1	11	-	228	14	0	0	6.58%	36.03037690193422	0.02108409854857798	0.02108409854857798	0.002029891263157455
ENSG00000140950_84498086_84487003_193	MEAK7	16	-	579	9	0	1	2.59%	9.120939230541453	0.020613416668581838	0.03537493353818881	0.003252526348827943
ENSG00000084731_25981815_25928978_759	KIF3C	2	-	2277	16	0	0	0.79%	4.123182600484849	0.013007239811447113	0.013007239811447113	0.0008218654148058079
ENSG00000115839_135133996_135168781_501	RAB3GAP1	2	+	1503	17	0	0	1.20%	6.6369043281008	0.013428347753762199	0.013428347753762199	0.0008725593497644525
ENSG00000079785_15599686_15630906_648	DDX1	2	+	1944	53	3	1	4.01%	15.997614435735343	1.0400968689976693e-05	4.5072028892119814e-06	3.904511913824903e-10
ENSG00000070495_76726475_76720448_361	JMJD6	17	-	1083	11	0	3	1.39%	5.95991196874099 85	0.020613416668581838	0.12312584984626351	0.011809609471937859
ENSG00000132128_46303151_46278865_755	LRRC41	1	-	2265	23	0	1	1.19%	5.9584767099887435	0.0035291660242640502	0.004333802856335622	8.458999530518808e-05
ENSG00000275410_37744884_37687372_531	HNF1B	17	-	1593	34	3	3	1.88%	12.523875963760831	0.01424822060979566	0.013819019308084528	0.0009476796271540015
ENSG00000100603_77735012_77730962_116	SNW1	14	-	348	14	0	0	4.31%	23.606109004715524	0.019679754444124863	0.019679754444124863	0.0017849636115922674
ENSG00000257727_56311354_56310552_94	CNPY2	12	-	282	40	4	0	13.83%	83.23126579170214	0.0030765917645763823	0.0007076576786684489	1.3712349168963352e-05
ENSG00000198561_57791641_57816308_879	CTNND1	11	+	2637	64	7	2	2.73%	14.241163111572245	0.0002556913665467353	1.5363660848022635e-05	3.2544366602591786e-08
ENSG00000095002_47410181_47482949_783	MSH2	2	+	2349	30	1	2	1.28%	7.494002858639848	0.0031859520777271916	0.006198316675603911	0.00010898907104230859
ENSG00000060971_38129378_38123047_272	ACAA1	3	-	816	21	0	0	2.94%	15.100966789781253	0.005604866691947602	0.005604866691947602	0.00016752456572620382
ENSG00000146963_139398602_139422340_339	LUC7L2	7	+	1017	28	0	7	5.31%	16.155213242165196	5.634389248581345e-05	0.001963690465134474	9.222651616384024e-07
ENSG00000142676_23691824_23694797_133	RPL11	1	+	399	230	6	3	27.07%	338.2443545895865	7.646473524697801e-08	1.0247523700656556e-07	7.629945048239531e-14
ENSG00000162695_100896263_100974857_376	SLC30A7	1	+	1128	13	0	0	1.60%	6.7625403455757995	0.012798402692974279	0.012798402692974279	0.0007972436888943567
ENSG00000159840_143381740_143390682_485	ZYX	7	+	1455	34	0	0	3.71%	13.711707498468044	6.422159002560852e-05	6.422159002560852e-05	3.053760610838711e-08
ENSG00000107984_52314438_52316807_265	DKK1	10	+	795	402	25	3	22.64%	296.71161054121137	3.0704257608183525e-10	1.5848554796986854e-11	6.01614672103646e-20
ENSG00000139218_45964137_45922048_1453	SCAF11	12	-	4359	24	4	5	0.83%	3.230724976360634	0.011715480431495331	0.023343299718182477	0.0013102485441989073
ENSG00000157020_10315424_10311830_174	SEC13	3	-	522	24	0	1	5.17%	26.978410291103454	0.0035291660242640502	0.005642787686764809	0.00010924478547767898
ENSG00000077782_38419583_38413628_411	FGFR1	8	-	1233	8	0	0	1.70%	3.807172255192215	0.005706018193000826	0.005706018193000826	0.0001733096755673334
ENSG00000146540_1000487_997625_65	C7orf50	7	-	195	11	5	0	7.69%	33.10043416485385	0.19774078372971998	0.02108409854857798	0.020795917079366868
ENSG00000157020_10315424_10301261_302	SEC13	3	-	906	52	0	4	5.96%	33.6783466216755	8.363265641277529e-05	0.0005277913847560486	3.221045008074421e-07
ENSG00000077782_38424619_38413628_547	FGFR1	8	-	1641	10	0	0	1.65%	3.57574907880256	0.001946208561389314	0.001946208561389314	2.2278545302028032e-05
ENSG00000119383_129120587_129147464_288	PTPA	9	+	864	25	5	0	4.86%	16.978600226605906	0.004748305419133081	0.00037518914374882155	1.236400975172076e-05
ENSG00000171204_85634105_85636229_156	TMEM126B	11	+	468	12	1	0	3.85%	15.045651893115387	0.012798402692974279	0.012798402692974279	0.0007972436888943567
ENSG00000108439_47944638_47946782_166	PNPO	17	+	498	21	0	0	4.82%	24.743752812171692	0.005355751137763 4245	0.0053557511377634245	0.00015366972994193032
ENSG00000167699_782171_771301_160	GLOD4	17	-	480	24	8	2	4.38%	29.339021191575007	0.06059069429271301	0.017507490509831244	0.004846260759894 5065
ENSG00000067955_67066700_67098732_82	CBFB	16	+	246	33	0	0	9.76%	78.71444709934758	0.0053557511377634245	0.0053557511377634245	0.00015366972994193032
ENSG00000111077_53050186_53063882_1409	TNS2	12	+	4227	12	0	5	0.35%	1.6658067390532296	0.019679754444124863	0.1741008397065631	0.016992325807804815
ENSG00000066322_43365239_43363916_218	ELOVL1	1	-	654	56	1	0	10.55%	50.244195312789	1.4069570834807505e-05	1.1909960387592219e-05	1.3456146463793288e-09
ENSG00000092964_26578255_26655706_572	DPYSL2	8	+	1716	166	15	4	9.79%	56.763141233117146	3.054918643714188e-09	1.2294907634067458e-10	4.479758946449172e-18
ENSG00000086300_26346443_26372572_201	SNX10	7	+	603	5	0	0	2.49%	4.865509318669155	0.012673659338734126	0.012673659338734126	0.000782701129001274
ENSG00000170242_11903301_11956175_1096	USP47	11	+	3288	12	0	0	0.73%	2.141534393545621	0.004193160480899131	0.004193160480899131	9.649553577281792e-05
ENSG00000170542_2900611_2890163_376	SERPINB9	6	-	1128	24	0	0	2.66%	12.484689868755321	0.0021880057261679567	0.0021880057261679567	2.7877966332435854e-05
ENSG00000172936_38138779_38141152_219	MYD88	3	+	657	19	0	4	4.57%	16.969296884015986	0.0021880057261679567	0.022853480259233 11	0.0003041272231193027
ENSG00000100320_35809971_35744205_338	RBFOX2	22	-	1014	11	0	0	1.48%	6.365468108625741	0.020613416668581838	0.020613416668581838	0.001946208561389314
ENSG00000167508_88663080_88656054_217	MVD	16	-	651	20	2	9	4.15%	18.02704835119816	0.007532653098952397	0.1648596486563395	0.008019181618715867
ENSG00000143569_154227312_154270295_1047	UBAP2L	1	+	3141	42	13	5	2.01%	7.846156574633239	0.005538146750059835	0.0005279745362177505	1.9599354329655325e-05
ENSG00000137807_69444775_69446945_168	AC027237.1	15	+	504	17	0	0	4.17%	19.792196835586314	0.008175610787902942	0.008175610787902942	0.00034251843268961324
ENSG00000112992_43644650_43704404_707	NNT	5	+	2121	7	0	0	0.71%	1.9365690555495054	0.019216965118390855	0.019216965118390855	0.0017073955890589052
ENSG00000198431_104291009_104348415_541	TXNRD1	12	+	1623	254	16	13	10.72%	91.83131709993903	2.687490931222515e-09	2.244971880808802e-09	6.516440818400921e-17
ENSG00000089597_62646599_62634817_73	GANAB	11	-	219	51	2	0	9.59%	136.6474959607603	0.010327712670190173	0.008877961307018026	0.00046196764897730986
ENSG00000185187_406427_405896_80	SIGIRR	11	-	240	21	2	1	8.75%	51.343287085256264	0.023878473930440184	0.015625008558487224	0.0017347488047933673
ENSG00000261832_28491759_28477516_438	AC138894.1	16	-	1314	26	2	0	2.51%	11.610571552221463	0.004407715900925203	0.0015208721599504675	3.948464526818382e-05
ENSG00000188603_28489291_28477516_338	CLN3	16	-	1014	16	0	5	2.07%	9.258862703455623	0.00847367416878415	0.06059069429271301	0.002681660320446393
ENSG00000114019_134371433_134357705_779	AMOTL2	3	-	2337	42	4	13	2.57%	10.545476166419771	0.00036942355516503625	0.014762025815636752	4.331749224354655e-05

ENSG00000152795_82427543_82426059_155	HNRNPDL	4	-	465	41	1	1	9.03%	51.73762876793872	0.0005444500874584297	0.0005444500874584297	1.9232077828672115e-06
ENSG00000164615_134738630_134750763_77	CAMLG	5	+	231	8	0	0	6.49%	20.32139995953247	0.019216965118390855	0.019216965118390855	0.0017073955890589052
ENSG00000006534_68015298_68021514_169	ALDH3B1	11	+	507	24	5	3	4.14%	27.77658811036687	0.05362420239623477	0.012088529595134127	0.0031383996376146807
ENSG00000006534_68021511_68027939_272	ALDH3B1	11	+	816	23	0	6	3.31%	16.539154103093754	0.0035547763465261306	0.048153582793014404	0.0010376666387331335
ENSG00000101474_24983982_24963813_380	APMAP	20	-	1140	102	5	7	8.16%	52.501406342818434	3.890891921242002e-06	1.0943503384116791e-05	3.568664999127242e-10
ENSG00000144840_120742507_120694200_135	RABL3	3	-	405	9	0	1	4.44%	13.039564974033336	0.011772051206276392	0.026832695913080365	0.0015103817605807263
ENSG00000114446_108222322_108213919_198	IFT57	3	-	594	13	0	0	2.53%	12.841995807760103	0.02108409854857798	0.02108409854857798	0.002029891263157455
ENSG00000168090_100089596_100089787_63	COPS6	7	+	189	19	0	2	7.94%	58.988508215865096	0.02108409854857798	0.0700082515985845	0.006564739044797468
ENSG00000100983_34951852_34928828_363	GSS	20	-	1089	31	0	0	3.58%	16.70357496673692	0.0006424109906750971	0.0006424109906750971	2.6462455329680906e-06
ENSG00000188529_23980255_23971142_262	SRSF10	1	-	786	67	0	1	5.73%	50.018178621769096	0.00030026635541046905	0.0003250600599851574	6.576238098407666e-07
ENSG00000119787_38376179_38295994_565	ATL2	2	-	1695	37	2	0	3.36%	12.808776213430976	0.0002418058900770233	4.4847209281215234e-05	8.149827001673648e-08
ENSG00000109445_141222372_141233989_320	ZNF330	4	+	960	17	1	0	3.75%	10.390903338682815	0.001620025777606367	0.0007660506977098094	7.723690688708469e-06
ENSG00000158545_88577124_88631299_953	ZC3H18	16	+	2859	10	0	0	0.52%	2.052397425083946	0.019679754444124863	0.019679754444124863	0.0017849636115922674
ENSG00000131469_42998763_43002920_132	RPL27	17	+	396	441	17	11	14.39%	653.460017448716	0.00034175999527323304	0.001372342566410722	3.104046494904808e-06
ENSG00000103335_88784964_88715605_2521	PIEZO1	16	-	7563	32	1	3	0.60%	2.48274144686077	0.0004262158040685525	0.0015781317627790912	4.378741665580246e-06
ENSG00000122642_32976433_32992762_147	FKBP9	7	+	441	13	0	0	3.40%	17.29738210841157	0.02108409854857798	0.02108409854857798	0.002029891263157455
ENSG00000136628_220046388_219968806_1512	EPRS	1	-	4536	90	5	0	2.65%	11.642468726815478	9.034705243244963e-08	1.257894109314286e-08	1.174406626556778e-14
ENSG00000067704_220094217_220147635_1012	IARS2	1	+	3036	150	4	5	5.14%	28.991127659659096	8.238161439277789e-10	2.074140104224431e-09	1.8858197588476165e-17
ENSG00000105058_16185443_16189495_107	FAM32A	19	+	321	7	0	0	5.61%	12.795834787602807	0.009815328628645334	0.009815328628645334	0.0004837142251238724
ENSG00000129351_10677289_10681056_119	ILF3	19	+	357	22	1	4	5.88%	36.16013816328572	0.012000726946169103	0.008674846900444944	0.0005220387231428462
ENSG00000099817_1091860_1089486_117	POLR2E	19	-	351	18	0	0	4.27%	30.091303786230775	0.019216965118390855	0.019216965118390855	0.0017073955890589052
ENSG00000123143_14469835_14471784_229	PKN1	19	+	687	6	0	0	2.18%	5.124719858790394	0.016947426762344633	0.016947426762344633	0.0013498980316300959
ENSG00000123143_14433522_14467645_603	PKN1	19	+	1809	19	7	1	1.49%	6.162978470314264	0.07589534913612099	0.005642787686764809	0.0025153013409982576
ENSG00000124151_47622248_47653417_1420	NCOA3	20	+	4260	23	2	1	0.92%	3.1680633211559868	0.0030056054444520318	0.0009568617188207251	1.7600708154182596e-05
ENSG00000120306_140194466_140243411_97	CYSTM1	5	+	291	47	4	1	12.37%	94.77209594529384	0.0018120733740713878	0.0010786302489720287	1.1880510518164743e-05
ENSG00000124733_43013378_43012470_72	MEA1	6	-	516	46	2	2	9.88%	52.30988274466862	0.0005393137985452151	0.0005393137985452151	1.8883487944536844e-06
ENSG00000187109_76067450_76053104_194	NAP1L1	12	-	582	37	0	3	4.64%	37.303910106126295	0.0037631575832289435	0.004535229864886203	9.38808655657263e-05
ENSG00000197150_151034375_151045349_548	ABCB8	7	+	1644	29	0	1	2.37%	10.350749568803835	0.0006629342244537616	0.0009306875619951529	3.907239258273379e-06
ENSG00000123066_115997159_115961266_1330	MED13L	12	-	3990	15	0	0	0.45%	2.2059414429755644	0.012798402692974279	0.012798402692974279	0.0007972436888943567
ENSG00000079459_11808887_11826102_113	FDFT1	8	+	339	77	4	2	16.81%	133.28050924786285	0.00031555689732807603	6.0343527006082885e-05	1.4074473491426774e-07
ENSG00000138674_82870399_82819074_928	SEC31A	4	-	2784	20	0	0	0.75%	4.215376607984915	0.00797940233269714	0.00797940233269714	0.000327142761693177
ENSG00000106052_27800010_27828829_210	TAX1BP1	7	+	630	12	0	2	2.38%	11.17676997774286	0.019679754444124863	0.04317391049183127	0.0037970045606445157
ENSG00000138663_83035261_83075353_340	COPS4	4	+	1020	14	3	0	2.94%	8.053848954550002	0.019679754444124863	0.0017901637611834264	0.00021847779822210372
ENSG00000163479_156020896_156011887_93	SSR2	1	-	279	287	1	3	23.66%	603.6056689592851	2.2985161501133994e-05	6.140828680895134e-05	1.089111622536981e-08
ENSG00000023228_206147824_206124185_611	NDUFS1	2	-	1833	45	0	0	3.44%	14.40541138702537	2.4325037092551905e-05	2.4325037092551905e-05	4.607943600628182e-09
ENSG00000122359_80169133_80155853_373	ANXA11	10	-	1119	26	2	1	3.75%	13.633861501000004	0.0018061760845665517	0.0009061776457653113	1.0061129210387768e-05
ENSG00000151743_31709409_31672304_240	AMN1	12	-	720	9	0	0	2.08%	7.334755297893752	0.016947426762344633	0.016947426762344633	0.0013498980316300959
ENSG00000118600_63779961_63785088_147	RXYLT1	12	+	441	10	0	2	3.40%	13.305678544931975	0.020613416668581838	0.06945717695039447	0.006391023138248565
ENSG00000129515_34605639_34563122_290	SNX6	14	-	870	61	4	2	9.66%	41.14207569393277	1.4477111217474092e-05	1.9934896674075532e-06	2.5174388176657424e-10
ENSG00000107371_37783921_37780679_69	EXOSC3	9	-	207	31	0	0	7.25%	87.87532917283335	0.02108409854857798	0.02108409854857798	0.002029891263157455
ENSG00000159079_32612243_32601863_290	CFAP298	21	-	870	31	0	0	5.17%	20.908267975605177	0.0002561420663320581	0.0002561420663320581	4.4770960258782436e-07
ENSG00000108424_47650245_47682404_876	KPNB1	17	+	2628	224	12	12	7.88%	50.014769763415536	4.121801950857531e-12	1.8495321163323976e-11	9.485681657080199e-22
ENSG00000177410_49279128_49279205_25	ZFAS1	20	+	75	68	3	1	32.00%	532.0142509405601	0.015646180920032563	0.010042643336235583	0.0007714669080894274
ENSG00000135316_85640301_85612882_464	SYNCRIP	6	-	1392	28	4	4	3.02%	11.803054502357762	0.003575527586573016	0.00446415347681005	8.811533233150764e-05
ENSG00000161960_7572842_7576700_173	EIF4A1	17	+	519	62	3	5	8.67%	70.09708338642199	0.0003574960659478307	0.0007150129185897719	1.681474765078715e-06
ENSG00000088986_120496422_120496625_67	DYNLL1	12	+	201	47	2	2	7.46%	137.20736278647018	0.029237630782826433	0.021557223391537678	0.002819005644529471
ENSG00000111481_54340590_54350523_126	COPZ1	12	+	378	74	4	18	13.49%	114.87235810457939	0.00025382860003781874	0.006480796454346327	1.2465613026177647e-05
ENSG00000159720_67481086_67452423_101	ATP6V0D1	16	-	303	18	1	0	5.94%	34.8582429998911	0.02034245439037237	0.013428347753762199	0.0012955184805712684
ENSG00000154723_25729794_25724640_108	ATP5PF	21	-	324	43	6	1	12.96%	77.87517970603243	0.004732992222340745	0.000675450826132064	2.0568376277725097e-05
ENSG00000094914_53321465_53307489_513	AAAS	12	-	1539	57	2	3	3.90%	21.73260829005556	0.00011858270277495242	0.0001926526083748436	1.616511173608218e-07
ENSG00000197956_153535339_153534696_90	S100A6	1	-	270	2776	88	58	50.00%	6032.972061319424	3.2080755063846185e-09	2.6004311897614428e-09	8.97263545200942e-17
ENSG00000188015_153548485_153547682_101	S100A3	1	-	303	55	1	0	14.85%	106.5112980552228	0.0004411551276150803	0.0002624511439355449	7.792140170284656e-07
ENSG00000196154_153544794_153543759_101	S100A4	1	-	303	1149	89	31	45.54%	2225.1178448263818	2.935379469644316e-09	1.4818842270205308e-08	4.658591980648176e-16
ENSG00000196154_153544794_153543759_101	S100A4	1	-	303	1149	89	31	45.54%	2225.1178448263818	2.935379469644316e-09	1.4818842270205308e-08	4.658591980648176e-16
ENSG00000196154_153544794_153543759_101	S100A4	1	-	303	1149	89	31	45.54%	2225.1178448263818	2.935379469644316e-09	1.4818842270205308e-08	4.658591980648176e-16
ENSG00000120705_138542918_138508305_437	ETF1	5	-	1311	44	0	0	3.66%	19.69362215757895	0.0001978461102466251	0.0001978461102466251	2.7148029154684727e-07
ENSG00000155508_154870770_154875439_152	CNOT8	5	+	456	12	0	0	3.29%	15.44159010082895	0.02108409854857798	0.02108409854857798	0.002029891263157455
ENSG00000109332_102809680_102797415_110	UBE2D3	4	-	330	19	0	1	5.45%	33.78432743272273	0.013428347753762199	0.013428347753762199	0.0008725593497644525
ENSG00000127990_94656098_94585499_491	SGCE	7	-	1473	25	3	2	2.24%	9.958934552469453	0.007887772776385514	0.0038108971419065062	0.00016292425881891603
ENSG00000127586_792984_797975_445	CHTF18	16	+	1335	10	0	0	1.12%	4.395358980011237	0.019679754444124863	0.019679754444124863	0.0017849636115922674
ENSG00000169241_155136321_155138281_181	SLC50A1	1	+	543	10	0	0	4.42%	10.80626931549724	0.00414960799764038	0.00414960799764038	9.45964903272165e-05
ENSG00000140416_63048576_63062809_275	TPM1	15	+	825	153	8	9	10.55%	108.82109678329638	9.375120023785914e-06	2.9726612038368743e-05	2.2467759299320734e-09
ENSG00000115355_55336743_55291711_1265	CCDC88A	2	-	3795	8	1	0	0.47%	1.2369547801454548	0.026602764905793736	0.011570465654371866	0.001474889218184677
ENSG00000148019_78240031_78266762_596	CEP78	9	+	1788	20	0	0	1.68%	6.563539416459733	0.002033393162560146	0.002033393162560146	2.4228963598721857e-05
ENSG00000072518_63895204_63909237_755	MARK2	11	+	2265	9	0	0	0.79%	2.3315778430390734	0.011772051206276392	0.011772051206276392	0.000681272511389508
ENSG00000175470_131943995_131955963_285	PPP2R2D	10	+	855	6	2	0	1.75%	4.117757360221054	0.13974268412556323	0.016947426762344633	0.011764960919844317
ENSG00000077150_102395960_102402376_899	NFKB2	10	+	2697	23	0	0	1.00%	5.004059973349834	0.0035547763465261306	0.0035547763465261306	7.044676146010672e-05

ENSG00000101158_58988935_58994676_442	NELFCD	20	+	1326	21	1	3	1.36%	9.292902639865387	0.02034245439037237	0.05989746521295916	0.005428711624617369
ENSG00000131778_147271162_147295509_522	CHD1L	1	+	1566	12	0	1	1.34%	4.496401715183909	0.007029302661868638	0.026832695913080365	0.0009647702379235399
ENSG00000149273_75404048_75404865_117	RPS3	11	+	351	241	7	6	19.66%	402.8891229156454	1.1369612993097455e-05	1.5723465776201874e-05	1.4347240633892047e-09
ENSG00000258674_19516266_19532721_213	AC011448.1	19	+	639	259	30	15	19.25%	237.83431889257986	5.247706530348127e-06	1.0063582714445163e-06	4.7004215891379304e-11
ENSG00000172009_2796066_2813276_568	AC006538.1	19	+	1704	33	4	0	1.94%	11.363705391102995	0.010985690497494479	0.001558047347358915	0.00010357838117651425
ENSG00000166260_54968646_54962733_276	COX11	17	-	828	40	0	3	4.35%	28.34688037833334	0.0010447178140462346	0.0032953901782040457	2.0925237526607307e-05
ENSG00000049860_74720698_74721175_35	HEXB	5	+	105	23	2	0	14.29%	128.53285474404288	0.055917760918677194	0.02108409854857798	0.00521801263590997
ENSG00000153048_8859328_8855164_147	CARHSP1	16	-	441	23	0	4	4.76%	30.603060653343544	0.00877616296920843	0.04584514077471458	0.0020400732494421717
ENSG00000133961_73355751_73276578_603	NUMB	14	-	1809	19	0	0	1.66%	6.162978470314264	0.0022011839993586477	0.0022011839993586477	2.8200182015552967e-05
ENSG00000072958_16198027_16234435_370	AP1M1	19	+	1110	14	2	0	1.89%	7.400834174451353	0.03299602752967379	0.00847367416878415	0.0014019198044660757
ENSG00000070214_105356336_105389056_449	SLC44A1	9	+	1347	22	0	0	2.90%	9.583644635703788	0.0004762745294806779	0.0004762745294806779	1.4854959883925712e-06
ENSG00000094880_138206669_138201056_151	CDC23	5	-	453	13	0	0	3.97%	16.83917331083775	0.012798402692974279	0.012798402692974279	0.0007972436888943567
ENSG00000137845_58610395_58597547_106	ADAM10	15	-	318	10	5	0	5.66%	18.45221458589623	0.17188837764917897	0.011772051206276392	0.011583370046088282
ENSG00000134375_201963648_201969554_97	TIMM17A	1	+	291	47	0	2	8.25%	94.77209594529384	0.005655335537214311	0.00865969128055974	0.00025649559259445493
ENSG00000124762_36684102_36685800_164	CDKN1A	6	+	492	29	2	8	6.71%	34.58665099819818	0.007205323565060537	0.012371379225907928	0.00045344217813502347
ENSG00000205302_122816966_122829648_209	SNX2	5	+	627	10	0	0	2.39%	9.358539455047849	0.020613416668581838	0.020613416668581838	0.001946208561389314
ENSG00000047579_15660399_15524425_290	DTNBP1	6	-	870	18	2	0	2.76%	12.140284630996554	0.013892891352654344	0.005554612275049112	0.0004020752376677488
ENSG00000109534_109816165_109822514_198	GAR1	4	+	594	16	0	1	4.55%	15.80553330185859	0.0034529206320349946	0.005561255870711926	0.00010554338560930125
ENSG00000130770_28236184_28237978_106	ATP5IF1	1	+	318	141	0	0	21.70%	260.17622566113687	1.1547640668375227e-05	1.1547640668375227e-05	1.0761655520040726e-09
ENSG00000124593_41787698_41789328_74	AL365205.1	6	+	222	79	0	2	21.62%	208.8092499220203	0.0002001100642622418	0.00033129571046353263	4.5413923219901546e-07
ENSG00000089597_62639071_62634817_90	GANAB	11	-	270	78	3	1	14.44%	169.51434466243336	0.0008531384257998691	0.0006592978435953202	3.5708293565321865e-06
ENSG00000137210_10749246_10756518_114	TMEM14B	6	+	342	25	0	0	7.89%	42.89330583563598	0.0036321914711260127	0.0036321914711260127	7.340009712338726e-05
ENSG00000141503_4884369_4897287_1199	MINK1	17	+	3597	20	1	5	0.67%	3.262610085246039	0.005160369442893307	0.07941064379354501	0.0024770715527741216
ENSG00000204356_31953771_31952301_46	NELFE	6	-	138	7	0	0	10.87%	29.764224397250008	0.019216965118390855	0.019216965118390855	0.0017073955890589052
ENSG00000137440_15936632_15935928_234	FGFBP1	4	-	702	19	0	0	3.42%	15.88152144273291	0.005554612275049112	0.005554612275049112	0.00016468457691007306
ENSG00000114867_184320681_184334908_1403	EIF4G1	3	+	4209	90	7	5	2.78%	12.546979839590167	4.60942195086692e-07	1.9011841143914044e-07	8.246422000810537e-13
ENSG00000048162_176388539_176386872_95	NOP16	5	-	285	14	6	0	8.42%	28.824301521547376	0.13318996167124125	0.004193160480899131	0.004025606069812367
ENSG00000124217_50958843_50960225_460	MOCS3	20	+	1380	23	3	5	1.96%	9.779673730525003	0.016228925652561413	0.03179577763392218	0.0023713157095832894
ENSG00000139842_113239480_113263582_438	CUL4A	13	+	1314	11	2	0	1.37%	4.912164887478312	0.04223451612662591	0.012798402692974279	0.0025674639266022752
ENSG00000118894_5097739_5090428_159	EEF2KMT	16	-	477	11	0	0	3.14%	13.531624029657236	0.020613416668581838	0.020613416668581838	0.001946208561389314
ENSG00000131469_42998763_43002920_132	RPL27	17	+	396	441	17	11	14.39%	653.460017448716	0.00034175999527323304	0.001372342566410722	3.104046494904808e-06
ENSG00000110429_33774488_33741908_468	FBXO3	11	-	1404	14	0	0	1.07%	5.851086847322651	0.019679754444124863	0.019679754444124863	0.0017849636115922674
ENSG00000100600_92712832_92706375_238	LGMN	14	-	714	11	2	4	2.94%	9.04003454082143	0.006937202941512724	0.07786981947941429	0.0030384159844079146
ENSG00000138685_122827175_122892396_155	FGF2	4	+	465	20	1	0	4.52%	25.237867691677426	0.00847367416878415	0.00847367416878415	0.0003665024264111182
ENSG00000099860_2476359_2476634_49	GADD45B	19	+	147	11	0	0	12.24%	43.90873919827552	0.012798402692974279	0.012798402692974279	0.0007972436888943567
ENSG00000175061_16439055_16439553_39	SNHG29	17	+	117	12	3	0	12.82%	60.18260757246155	0.1029516053660342	0.02108409854857798	0.009872287793463467
ENSG00000060688_31271463_31260072_127	SNRNP40	1	-	381	19	0	2	4.72%	29.262015886610243	0.013428347753762199	0.044040755831095145	0.0027917083341200354
ENSG00000136877_127804381_127813604_483	FPGS	9	+	1449	19	0	0	1.24%	7.6941532455476205	0.013640585738808999	0.013640585738808999	0.00089863692579514
ENSG00000130638_45729444_45740592_159	ATXN10	22	+	477	82	7	4	12.58%	100.87210640289939	8.145317493450215e-05	5.0132298531531764e-05	3.034227497123452e-08
ENSG00000182149_71915641_71927799_360	IST1	16	+	1080	27	2	5	2.50%	14.669510595787504	0.009181916080321966	0.029977737148348938	0.0013603405452221253
ENSG00000110955_56639268_56638323_87	ATP5F1B	12	-	261	86	0	0	22.99%	193.3452737529081	3.8455384355631195e-05	3.8455384355631195e-05	1.1252125337168915e-08
ENSG00000101040_47347880_47210761_1161	ZMYND8	20	-	3483	6	0	0	0.43%	1.0108189902351423	0.016947426762344633	0.016947426762344633	0.0013498980316300959
ENSG00000085978_233290310_233294350_145	ATG16L1	2	+	435	9	0	0	3.45%	12.140284630996554	0.020613416668581838	0.020613416668581838	0.001946208561389314
ENSG00000085978_233251828_233290063_470	ATG16L1	2	+	1410	26	0	0	2.34%	10.820064552921279	0.0014915192737735051	0.0014915192737735051	1.337646624223462e-05
ENSG00000115211_27370314_27364400_520	EIF2B4	2	-	1560	60	0	0	3.85%	22.568477839673083	3.8568200545620204e-05	3.8568200545620204e-05	1.1316524295322383e-08
ENSG00000115548_86442048_86492119_1321	KDM3A	2	+	3963	11	2	3	0.45%	1.6287117492168814	0.05067994629101479	0.10000264635955686	0.0194788414689791
ENSG00000141279_47592506_47621920_457	NPEPPS	17	+	1371	27	0	1	2.84%	11.555850797556895	0.0006356636671888767	0.0009879882513538032	3.980616343459717e-06
ENSG00000114126_142000353_141952513_418	TFDP2	3	-	1254	9	0	0	1.20%	4.211342754771532	0.019679754444124863	0.019679754444124863	0.0017849636115922674
ENSG00000104964_3062197_3053819_196	TLE5	19	-	588	40	2	14	8.16%	39.91703563479593	0.0006152005395151786	0.017339793977529612	7.884060079193574e-05
ENSG00000171004_132957277_132628223_486	HS6ST2	X	-	1458	14	0	0	1.44%	5.634379927051442	0.00797940233269714	0.00797940233269714	0.000327142761693177
ENSG00000078269_158071633_158096364_839	SYNJ2	6	+	2517	12	3	0	0.72%	2.797522878815257	0.07786981947941429	0.013007239811447113	0.0049717523581729916
ENSG00000175582_73760635_73677898_208	RAB6A	11	-	624	18	0	0	4.33%	16.926358379754813	0.0033030736763512156	0.0033030736763512156	6.124050385493983e-05
ENSG00000160679_153636589_153645269_248	CHTOP	1	+	744	20	2	3	2.82%	15.77366730729839	0.00847367416878415	0.013892891352654344	0.0005886798845679606
ENSG00000160679_153636589_153645269_249	CHTOP	1	+	747	20	2	3	2.81%	15.710319245823296	0.00847367416878415	0.013892891352654344	0.0005886798845679606
ENSG00000131626_70372302_70382146_218	PPFIA1	11	+	654	8	0	0	2.29%	7.177742187541286	0.016947426762344633	0.016947426762344633	0.0013498980316300959
ENSG00000103512_14864669_14895645_729	NOMO1	16	+	2187	13	0	0	0.69%	3.487949478650892	0.020613416668581838	0.020613416668581838	0.001946208561389314
ENSG00000137693_102110849_102229940_470	YAP1	11	+	1410	17	2	4	2.13%	7.074657592294683	0.010350136863208615	0.018344283626974538	0.0009255963303532005
ENSG00000099956_23801047_23834180_230	SMARCB1	22	+	690	15	3	2	3.48%	12.756096170250004	0.03744183116399598	0.02682370818175567	0.004345618068718719
ENSG00000225921_13615410_13620827_240	NOL7	6	+	720	28	0	0	4.58%	22.819238704558337	0.0012624143894574274	0.0012624143894574274	9.711623744831373e-06
ENSG00000167460_16086484_16101346_139	TPM4	19	+	417	27	0	3	5.04%	37.99297708261512	0.00847367416878415	0.03299602752967379	0.0014019198044660757
ENSG00000142186_65525154_65538350_775	SCYL1	11	+	2325	9	1	0	1.03%	2.2714080922509683	0.010460667668897016	0.0033278027414746884	0.00019130306338196331
ENSG00000079819_130956485_130858136_935	EPB41L2	6	-	2805	19	1	6	0.96%	3.97462675679091	0.006142079104304129	0.03535057243299149	0.0011498351620616329
ENSG00000143537_155057085_155062502_485	ADAM15	1	+	1455	25	5	0	2.27%	10.08213786652062	0.017867215592848443	0.001484234770590415	0.00016809892502198752
ENSG00000139291_71686789_71700995_300	TMEM19	12	+	900	13	0	0	3.00%	8.475717233121669	0.00308494966027208	0.00308494966027208	5.3755588364750274e-05
ENSG00000139289_76030976_76030536_146	PHLDA1	12	-	438	23	9	3	4.11%	30.8126706578185	0.1543801567637662	0.03736774915294124	0.023860979254184938
ENSG00000111605_69239647_69262559_588	CPSF6	12	+	1764	23	0	6	2.55%	7.650765163335886	0.00020764221034884014	0.002818594382603812	4.222052649395771e-06
ENSG00000051523_88651013_88645166_210	CYBA	16	-	630	90	8	4	8.10%	83.82577483307145	0.000767764139684436	0.0006086408084211703	2.9859622635684733e-06

ENSG00000164924_100948658_100920693_168	YWHAZ	8	−	504	23	0	0	3.57%	26.777678071675602	0.013007239811447113	0.013007239811447113	0.0008218654148058079
ENSG00000134371_193122201_193250712_531	CDC73	1	+	1593	42	0	0	2.64%	15.470670308175144	0.0004481211664877703	0.0004481211664877703	1.3205877300278812e−06
ENSG00000134333_18402941_18407281_159	LDHA	11	+	477	494	20	8	21.38%	607.6929336955159	1.6871854087431992e−07	5.793694942921583e−07	9.241568657874308e−13
ENSG00000188313_146533542_146521797_196	PLSCR1	3	−	588	14	0	0	4.08%	13.970962472178575	0.005257622967929454	0.005257622967929454	0.00014836615564656991
ENSG00000092020_35087995_35085590_77	PPP2R3C	14	−	231	8	0	0	6.49%	20.32139995953247	0.019216965118390855	0.019216965118390855	0.0017073955890589052
ENSG00000156136_70993836_71029452_317	DCK	4	+	951	21	0	2	2.84%	12.957296425301264	0.0036581908865286695	0.007583927325470684	0.00015097343227454333
ENSG00000117385_42762325_42746912_599	P3H1	1	−	1797	22	0	3	1.50%	7.183733625093491	0.003684287313187465	0.017782189529096822	0.00036099358464052045
ENSG00000108021_5730687_5763032_2201	TASOR2	10	+	6603	10	0	3	0.23%	0.8886573130872333	0.020613416668581838	0.12312584984626351	0.011809609471937859
ENSG00000104738_47961145_47976778_749	MCM4	8	+	2247	54	2	2	3.87%	14.101532214909216	1.94771820250471e−06	1.9200891721536845e−06	3.258808442229013e−11
ENSG00000162775_110339538_110346354_896	RBM15	1	+	2688	22	0	3	1.12%	4.802518349811385	0.0021880057261679567	0.007664059176943723	9.5950101060544e−05
ENSG00000172046_49116596_49108412_1005	USP19	3	−	3015	18	0	0	0.80%	3.5031667094417918	0.005454749182134642	0.005454749182134642	0.00015910859015753364
ENSG00000204256_32974691_32977265_315	BRD2	6	+	945	9	0	0	1.59%	5.58838498887143	0.020613416668581838	0.020613416668581838	0.001946208561389314
ENSG00000100320_35809971_35744205_360	RBFOX2	22	−	1080	12	0	0	1.67%	6.519782487016668	0.013217226988443315	0.013217226988443315	0.0008469697265466234
ENSG00000135829_182842669_182887434_1236	DHX9	1	+	3708	51	2	0	1.94%	8.070604534899275	1.412135627885162e−05	7.210151149583218e−06	8.31187961104987e−10
ENSG00000137331_30744109_30743936_57	IER3	6	−	171	27	8	6	14.04%	92.64954060497371	0.013648824643852793	0.007225456388766877	0.0005005250855746109
ENSG00000055130_148730123_148800582_776	CUL1	7	+	2328	27	0	1	1.16%	6.805443059901419	0.0035547763465261306	0.005670089768331009	0.00011049933550063637
ENSG00000110717_68032934_68033029_31	NDUFS8	11	+	93	22	0	2	19.35%	138.80827230422585	0.013428347753762199	0.044040755831095145	0.0027917083341200354
ENSG00000168488_28826345_28836265_885	ATXN2L	16	+	2655	50	7	8	1.92%	11.050478791553674	0.001435957343153687	0.0010673266102214833	9.370824648744565e−06
ENSG00000067182_6333514_6331888_110	TNFRSF1A	12	−	330	15	9	1	5.45%	26.671837446886368	0.26746260656921983	0.02034245439037237	0.029646270713916262
ENSG00000072274_196077096_196051942_759	TFRC	3	−	2277	142	7	2	7.51%	36.59324557930304	4.739677167691646e−10	2.6828389625454316e−11	1.5520703034019612e−19
ENSG00000141030_17276159_17247098_403	COPS3	17	−	1209	43	2	5	4.47%	20.86977520657941	0.00032999515838639013	0.0008280970490423662	1.8053823275441823e−06
ENSG00000014641_63589328_63606987_352	MDH1	2	+	1056	338	11	4	15.06%	187.81418868849153	2.0377094687814834e−09	4.100846544759926e−10	9.40695538242557e−18
ENSG00000163625_84837066_84672868_3380	WDFY3	4	−	10140	10	3	1	0.15%	0.5786789189659765	0.11497452839710681	0.03537493353818881	0.016723633890736285
ENSG00000006118_60927137_60933748_509	TMEM132A	11	+	1527	8	1	2	1.38%	3.0741607011473486	0.017404239405933563	0.0416322583317752	0.003292339026149851
ENSG00000018408_149542361_149525966_106	WWTR1	3	−	318	9	4	0	5.66%	16.60699312730661	0.169362073879829	0.012178957859018494	0.011649760594361461
ENSG00000102316_54809755_54815682_580	MAGED2	X	+	1740	26	0	0	2.07%	8.767983344608623	0.0008739357725210369	0.0008739357725210369	4.787965623198429e−06
ENSG00000129255_7586750_7587551_127	MPDU1	17	+	381	10	0	0	4.72%	15.40106099295276	0.012798402692974279	0.012798402692974279	0.0007972436888943567
ENSG00000138594_51862885_51908810_352	TMOD3	15	+	1056	33	2	0	4.55%	18.33688824473438	0.0008081111075299929	0.00014734945683592696	8.374520733964951e−07
ENSG00000140859_57803012_57759149_848	KIFC3	16	−	2544	33	6	3	1.89%	7.611538516682195	0.005988781451706615	0.0012748355907334584	4.606531483331719e−05
ENSG00000122884_73074883_73008222_516	P4HA1	10	−	1548	57	6	2	3.88%	21.60625591627617	0.0003929310530894001	9.023683734855572e−05	2.5527144820081737e−07
ENSG00000170776_85579527_85744677_2331	AKAP13	15	+	6993	15	7	6	0.43%	1.2586452677638356	0.14305881271338705	0.0820328997370387	0.04108668187794306
ENSG00000198925_219228024_219220447_839	ATG9A	2	−	2517	9	0	0	0.95%	2.098142159111443	0.0033278027414746884	0.0033278027414746884	6.211806171561044e−05
ENSG00000116584_155965093_155947942_780	ARHGEF2	1	−	2340	7	0	5	0.64%	1.7553260541967952	0.019216965118390855	0.169362073879829	0.016163991620339734
ENSG00000198752_102999704_102933712_1559	CDC42BPB	14	−	4677	27	4	7	1.03%	3.3874431138444523	0.0022688321121859907	0.00936001342702443	0.00012159754436964941
ENSG00000115419_190881109_190962986_661	GLS	2	+	1983	53	2	8	3.93%	15.682986617785934	2.969336201391047e−06	7.847250784985821e−05	2.1153791602712277e−09
ENSG00000168439_64186262_64204126_519	STIP1	11	+	1557	131	14	7	7.90%	49.36945120226494	7.880792738167525e−07	9.880271854956636e−08	7.57429430101104e−13
ENSG00000066084_50706580_50744839_760	DIP2B	12	+	2280	11	1	0	0.92%	2.8309581851519745	0.015516259750072458	0.0078820576498062	0.0006142571802709984
ENSG00000111674_6919628_6922800_191	ENO2	12	+	573	13	0	1	3.14%	13.31264486877749	0.013428347753762199	0.02034245439037237	0.0012955184805712684
ENSG00000131876_101295178_101281724_255	SNRPA1	15	−	765	39	1	0	1.96%	29.914296116900008	0.02108409854857798	0.02108409854857798	0.002029891263157455
ENSG00000182087_1020996_1010886_408	TMEM259	19	−	1224	11	0	2	1.23%	5.2733534821458345	0.02108409854857798	0.0700082515985845	0.006564739044797468
ENSG00000115652_106166060_106094026_386	UXS1	2	−	1158	23	4	1	2.85%	11.654533461247413	0.011987288894911035	0.0023450217334965687	0.00016174504289948947
ENSG00000149499_62609433_62602475_670	EML3	11	−	2010	5	2	0	0.75%	1.4596527956007466	0.15865525393145707	0.012673659338734126	0.011061610944025602
ENSG00000147140_71290638_71300076_471	NONO	X	+	1413	42	1	3	3.82%	17.441456334694273	0.00012070538323268708	0.0005818042179531959	4.981421812408915e−07
ENSG00000147133_71366375_71464046_1873	TAF1	X	+	5619	18	3	0	0.37%	1.8797023721243997	0.04544439262286352	0.00847367416878415	0.001963364310668532
ENSG00000134684_32817244_32775981_528	YARS	1	−	1584	30	4	0	3.41%	11.11326560286932	0.0022327055155805073	5.634389248581345e−05	1.063372296286992e−06
ENSG00000132792_37802994_37872013_177	CTNNBL1	20	+	531	9	0	0	2.82%	9.945430912398308	0.020613416668581838	0.020613416668581838	0.001946208561389314
ENSG00000095002_47410181_47512434_770	MSH2	2	+	2310	30	1	2	1.30%	7.620524984824677	0.0031859520777271916	0.006198316675603911	0.00010898907104230859
ENSG00000115286_1390886_1393407_125	NDUFS7	19	+	375	13	3	0	4.80%	20.341721359492006	0.05989746521295916	0.013428347753762199	0.003846583043074148
ENSG00000198000_92324161_92297836_1129	NOL8	9	−	3387	11	1	1	0.71%	1.9056937295974319	0.010499663139468709	0.010499663139468709	0.000549255679315805
ENSG00000122565_26206374_26211710_91	CBX3	7	+	273	21	6	2	5.49%	45.136955679346165	0.1035802244557304	0.04584514077471458	0.018556329999926704
ENSG00000115762_131120942_131146750_170	PLEKHB2	2	+	510	17	4	3	5.88%	19.559347461050006	0.03950234393976166	0.013383828821373808	0.002491043730068833
ENSG00000067182_6341814_6331888_218	TNFRSF1A	12	−	654	20	12	1	4.59%	17.944355468853214	0.19167299705412816	0.003768080243855145	0.006106472968629397
ENSG00000167508_88663080_88657895_91	MVD	16	−	273	15	1	0	6.59%	32.2406826281044	0.016822681477512567	0.013428347753762199	0.0010798218650012886
ENSG00000177700_842508_840372_67	POLR2L	11	−	201	190	20	6	43.28%	554.6680623282837	9.351844648399558e−06	5.375819584381181e−06	4.1623196695931515e−10
ENSG00000127990_94618900_94585465_278	SGCE	7	−	834	11	0	2	2.52%	7.739310146458635	0.007029302661868638	0.03522780890193623	0.0012822660609675817
ENSG00000140632_4847265_4805236_553	GLYR1	16	−	1659	15	0	0	1.45%	5.305428786903256	0.005160369442893307	0.005160369442893307	0.00014319601891785326
ENSG00000146729_55964610_55999072_247	NIPSNAP2	7	+	741	36	1	7	5.67%	28.507550955376523	0.0004441315507112266	0.01024944125544436	3.322488986442508e−05
ENSG00000261236_144291370_144262164_746	BOP1	8	−	2238	64	2	6	3.89%	16.780137232000005	3.104714245968104e−06	1.3335750775849784e−05	3.513574145987675e−10
ENSG00000133112_45338743_45337386_28	TPT1	13	−	84	22	6	5	17.86%	153.68058719396433	0.02108409854857798	0.11140049559056725	0.010758629910005835
ENSG00000260729_72375972_72320627_227	AC009690.1	15	−	681	19	0	0	4.85%	16.371259989425113	0.0013775385517804596	0.0013775385517804596	1.1483554366703679e−05
ENSG00000168615_38997064_39090052_655	ADAM9	8	+	1965	212	8	9	10.53%	63.30659025561223	1.313678689778135e−12	9.368753776906347e−12	1.5714293541369874e−22
ENSG00000168615_39090049_39103700_129	ADAM9	8	+	387	43	0	0	7.75%	65.19782487016668	0.0022543491824521134	0.0022543491824521134	2.9517890247939808e−05
ENSG00000141543_80146961_80135490_411	EIF4A3	17	−	1233	87	5	3	8.03%	41.40299827521534	5.247688003250481e−07	1.2687121683844718e−06	6.042802401697213e−12
ENSG00000187498_110213967_110198387_390	COL4A1	13	−	1170	11	0	0	1.28%	5.5167390274756425	0.020613416668581838	0.020613416668581838	0.001946208561389314
ENSG00000122218_160332529_160306468_276	COPA	1	−	828	9	0	0	2.17%	6.378048085125002	0.011772051206276392	0.011772051206276392	0.000681272511389508
ENSG00000122218_160309103_160290157_791	COPA	1	−	2373	34	5	0	2.40%	8.40730485051454	0.00035554045139049687	5.085749902382852e−05	1.3599205713714354e−07
ENSG00000218739_37199705_37201724_80	CEBPZOS	2	+	240	11	0	0	7.50%	26.894102758943756	0.009815328628645334	0.009815328628645334	0.0004837142251238724

ENSG00000100350_36506290_36490008_640	FOXRED2	22	-	1920	32	4	1	1.88%	9.779673730525003	0.005391414071672784	0.000871358954992987	2.9501686946772053e-05
ENSG00000128309_37024216_37029514_297	MPST	22	+	891	13	1	2	2.69%	8.561330538506736	0.010020958156399907	0.030313543781140352	0.0014836836722180672
ENSG00000153187_244864307_244854450_825	HNRNPU	1	-	2475	203	27	13	7.15%	48.12784890415941	2.778004265593307e-07	2.2053718895192142e-09	7.407954170762384e-15
ENSG00000068903_38883633_38879155_181	SIRT2	19	-	543	16	0	0	2.76%	17.290030904795586	0.020613416668581838	0.020613416668581838	0.001946208561389314
ENSG00000255339_100529851_100516949_107	AL133352.1	10	-	321	64	1	1	15.89%	116.99048948665423	0.0001654009931304681	0.0001654009931304681	1.918329799768428e-07
ENSG00000102967_72008765_72024199_395	DHODH	16	+	1185	12	2	5	2.03%	5.942080241331647	0.015516259750072458	0.05158844004651438	0.0037107121880202653
ENSG00000092010_24136263_24138644_250	PSME1	14	+	750	95	8	2	12.40%	74.32552035199002	6.238555408105777e-06	5.094696486450436e-07	2.960765148293362e-11
ENSG00000149925_30068931_30069446_62	ALDOA	16	+	186	68	3	4	20.97%	214.52187537925812	0.0008149969909848477	0.0006980837449708158	3.6074462084714233e-06
ENSG00000168036_41224069_41239342_781	CTNNB1	3	+	2343	117	8	3	5.76%	29.301455223339957	6.403151454483205e-08	1.1032790393731706e-08	7.305397733044446e-15
ENSG00000163832_47504433_47496069_193	ELP6	3	-	579	10	0	0	3.11%	10.134376922823837	0.011772051206276392	0.011772051206276392	0.000681272511389508
ENSG00000139343_95859074_95866071_86	SNRPF	12	+	258	100	4	0	12.79%	227.43427280290703	0.002305925096779131	0.0016494676794180302	2.242285138605241e-05
ENSG00000120802_98515868_98534342_694	TMPO	12	+	2082	56	0	4	4.03%	15.78275875819597	8.883355118806814e-07	8.563644934347708e-06	6.895504727466577e-11
ENSG00000108861_43777530_43769609_144	DUSP3	17	-	432	14	0	0	4.17%	19.016032253798617	0.012798402692974279	0.012798402692974279	0.0007972436888943567
ENSG00000099622_1270934_1271629_135	CIRBP	19	+	405	12	1	2	3.70%	17.38608663204445	0.04223451612662591	0.10105102209468636	0.016920389188621314
ENSG00000126457_49677311_49684987_234	PRMT1	19	+	702	59	7	52	8.12%	49.31630342743377	0.0008549111909355715	0.0008423366803494044	4.5243563545708995e-06
ENSG00000113580_143400839_143281889_777	NR3C1	5	-	2331	12	5	2	1.16%	3.0207486426332055	0.07471635411946746	0.014696946993710543	0.005240911805500765
ENSG00000106829_79576166_79725144_668	TLE4	9	+	2004	8	0	0	0.75%	2.3424368216826355	0.019679754444124863	0.019679754444124863	0.0017849636115922674
ENSG00000113643_168486499_168519190_660	RARS	5	+	1980	66	4	0	4.55%	19.559347461050006	4.827290513372417e-06	6.320149411913668e-07	2.7830696095932178e-11
ENSG00000167996_61965419_61964727_113	FTH1	11	-	339	53	5	5	5.31%	91.73853233943808	0.08385215180161965	0.08424673392120513	0.025631820909149765
ENSG00000172172_120445094_120396104_178	MRPL13	8	-	534	32	1	1	9.55%	35.1628718400899	0.00017168856529097966	0.00017168856529097966	2.062270878068706e-07
ENSG00000265681_49491571_49488519_184	RPL17	18	-	552	36	2	0	2.72%	38.26828851075001	0.03980790073005672	0.021557223391537678	0.0037960059236543955
ENSG00000072210_19648972_19652647_161	ALDH3A2	17	+	483	13	6	0	3.73%	15.793261925071432	0.17808035639161363	0.012798402692974279	0.012843613314113225
ENSG00000119471_112380164_112470544_345	HSDL2	9	+	1035	26	0	0	2.61%	14.740377796733336	0.0035547763465261306	0.0035547763465261306	7.044676146010672e-05
ENSG00000163694_40438758_40425904_548	RBM47	4	-	1644	14	0	0	1.28%	4.996913584939782	0.00797940233269714	0.00797940233269714	0.000327142761693177
ENSG00000004779_23596218_23582284_132	NDUFAB1	16	-	396	225	5	11	31.82%	333.39796808607963	2.0469302697238314e-08	7.356461747733742e-08	1.5229218894484512e-14
ENSG00000125871_17969866_17990109_262	MGME1	20	+	786	30	1	0	5.34%	22.396199382881683	0.0009958682880916293	0.0004093002068662083	2.653442324795289e-06
ENSG00000184207_2214777_2213728_321	PGP	16	-	963	28	5	1	4.05%	17.061113050137074	0.009048123305391056	0.0009801476264495266	5.6536020429725805e-05
ENSG00000107338_38068321_37919821_401	SHB	9	-	1203	13	0	1	1.75%	6.34093558587656	0.00837382380159347	0.013794764262559246	0.0005783443517979597
ENSG00000125871_17969866_17990186_250	MGME1	20	+	750	13	5	0	2.40%	10.170860679746003	0.19807195457603705	0.012798402692974279	0.014686027802829961
ENSG00000120948_11016999_11023248_164	TARDBP	1	+	492	12	0	0	3.66%	14.311717654426833	0.013007239811447113	0.013007239811447113	0.0008218654148058079
ENSG00000175701_110212525_110212355_56	MTLN	2	-	168	28	0	2	17.86%	97.79673730525002	0.0024042815392806777	0.002708918507878203	3.743268655641858e-05
ENSG00000117500_93180089_93154670_178	TMED5	1	-	534	45	1	0	5.62%	49.44778852512641	0.0034718995429267267	0.0023354528714849923	4.63040222851508e-05
ENSG00000272657_34073737_34262842_70	AP000317.2	21	+	210	15	0	1	11.43%	41.912887416535725	0.005160369442893307	0.013599199788808391	0.000368527319865593
ENSG00000143612_154220359_154207453_185	C1orf43	1	-	555	36	2	0	5.95%	38.061432897178385	0.0027029703238042042	0.0016033688297201391	2.5499085628679756e-05
ENSG00000213246_58352135_58347238_59	SUPT4H1	17	-	177	35	0	3	15.25%	116.03002731131359	0.003710480643933249	0.010698784348459516	0.00021565736966781135
ENSG00000006534_68015298_68027939_468	ALDH3B1	11	+	1404	58	5	9	3.85%	24.240216938908127	0.0005962423705106502	0.0013386730596341172	5.059401949948585e-06
ENSG00000198258_9828338_9828897_66	UBL5	19	+	198	88	13	1	10.61%	260.79129948066674	0.008877961307018026	0.010275150823238127	0.00045972819701380264
ENSG00000198804_6902_7444_180	MT-CO1	MT	+	540	317	143	137	8.33%	344.4618413973806	0.08417711942797734	0.0433339075734075	0.014426344671708334
ENSG00000131236_40069713_40071533_198	CAP1	1	+	594	26	0	0	4.04%	25.683991615520206	0.00575691403842271	0.00575691403842271	0.0001762552514680027
ENSG00000172269_119098084_119097851_77	DPAGT1	11	-	231	14	0	0	6.49%	35.562449929181824	0.02108409854857798	0.02108409854857798	0.002029891263157455
ENSG00000138448_186666746_186667791_79	ITGAV	2	+	237	13	0	0	7.59%	32.18626797387975	0.013428347753762199	0.013428347753762199	0.0008725593497644525
ENSG00000133142_103586676_103587323_215	TCEAL4	X	+	645	18	0	0	3.26%	16.375267641809305	0.00847367416878415	0.00847367416878415	0.0003665024264111182
ENSG00000115204_27322517_27311623_171	MPV17	2	-	513	20	1	3	5.85%	22.876429779005854	0.003768080243855145	0.014216764456991368	0.00029197494105209104
ENSG00000101457_45793943_45811195_263	DNTTIP1	20	+	789	16	0	7	3.04%	11.899222789992399	0.004873903550586949	0.07206351740800761	0.00211541037343695
ENSG00000127564_2975767_2972953_358	PKMYT1	16	-	1074	13	2	0	2.23%	7.1025563406047505	0.01943569092740073	0.004826937407846361	0.0004999687388256805
ENSG00000148290_133353936_133351913_179	SURF1	9	-	537	18	1	0	3.35%	19.66861755859777	0.015555382717448078	0.011772051206276392	0.0008874794703752884
ENSG00000156535_73696216_73823633_1428	CD109	6	+	4284	96	13	3	3.15%	13.149141150285717	1.3298147890182806e-06	9.875691891942078e-09	1.5494322564315558e-13
ENSG00000197321_29550907_29458247_2010	SVIL	10	-	6030	11	0	0	0.25%	1.070412050107214	0.02108409854857798	0.02108409854857798	0.002029891263157455
ENSG00000156515_69318948_69401135_917	HK1	10	+	2751	62	7	3	2.73%	13.224422492749184	0.00013761595298308717	2.834430847032052e-05	2.995187343596802e-08
ENSG00000126883_131129336_131233487_1940	NUP214	9	+	5820	23	1	0	0.52%	2.3188917092997428	0.0035004709947243163	0.0023628488670607213	4.7187726630981656e-05
ENSG00000257103_34172643_34227388_463	LSM14A	19	+	1389	31	0	0	2.81%	13.095891388607994	0.0006629342244537616	0.0006629342244537616	2.811652200271938e-06
ENSG00000160285_46228745_46191104_732	LSS	21	-	2196	36	6	2	2.19%	9.61935121035246	0.004872789343862693	0.00026353281753581636	9.38917134882687e-06
ENSG00000143537_155051387_155062502_824	ADAM15	1	+	2472	47	8	0	2.31%	11.156423915890173	0.002270051558325673	5.561394738214947e-05	1.0712191690138388e-06
ENSG00000101247_13801528_13817210_158	NDUFAF5	20	+	474	12	1	0	3.80%	14.855200603329118	0.023878473930440184	0.013007239811447113	0.0014709138084317821
ENSG00000132912_150756451_150711149_403	DCTN4	5	-	1209	29	0	0	3.72%	14.074964674204718	0.0002582331582969843	0.0002582331582969843	4.5481323920964005e-07
ENSG00000028310_892657_864468_597	BRD9	5	-	1791	14	1	1	1.01%	4.586781649157455	0.01634402150441562	0.01634402150441562	0.0012613283443022277
ENSG00000081087_108074651_108073754_148	OSTM1	6	-	444	13	3	0	3.38%	17.180507904976356	0.08350359792828288	0.019216965118390855	0.0073235312725521175
ENSG00000114978_74178674_74156568_216	MOB1A	2	-	648	86	5	2	11.57%	77.87517970603243	4.335789394068854e-05	4.990610562694879e-06	1.8272031306601476e-09
ENSG00000125166_58734228_58718338_261	GOT2	16	-	783	43	1	0	4.98%	32.22421229215135	0.0007057118220565012	0.0006768518708446028	3.0468192682322283e-06
ENSG00000125630_112550943_112575729_901	POLR1B	2	+	2703	23	0	0	1.00%	4.9929521820660385	0.0034782380676658805	0.0034782380676658805	6.758309314600117e-05
ENSG00000119650_75988894_76083577_187	IFT43	14	+	561	18	0	0	3.74%	18.82717937427273	0.0078820576498062	0.0078820576498062	0.00031963642907451164
ENSG00000132432_54757588_54752411_68	SEC61G	7	-	204	102	1	2	26.47%	293.39021191575006	0.00011323133856440072	0.00024980220152187344	2.000849288569143e-07
ENSG00000108599_19958449_19906227_457	AKAP10	17	-	1371	9	0	0	1.09%	3.8519502658522984	0.019216965118390855	0.019216965118390855	0.0017073955890589052
ENSG00000128487_20156197_20314065_987	SPECC1	17	+	2961	17	0	0	1.01%	3.3688845677593724	0.002046028879997488	0.002046028879997488	2.4518070358754455e-05
ENSG00000085063_33722445_33710126_128	CD59	11	-	384	62	5	3	14.06%	94.74058926446097	0.00102540667708157	0.0003583833865847517	2.4148597638421077e-06
ENSG00000091140_107891251_107919259_486	DLD	7	+	1458	55	11	1	5.14%	22.135063999130665	0.000669431635242198	6.6992207385925154e-06	4.484993352984415e-08
ENSG00000135473_56333046_56317609_1182	PAN2	12	-	3546	8	0	4	0.42%	1.3238137029475467	0.019679754444124863	0.2463583386135439	0.026068547507937054

ENSG00000130731_636290_634902_103	METTL26	16	−	309	11	1	1	4.85%	20.888623502092237	0.04223451612662591	0.04223451612662591	0.007344095972923886
ENSG00000188603_28491759_28482512_316	CLN3	16	−	948	20	2	0	2.22%	12.379333836107598	0.024275373211574098	0.00877616296920843	0.0010556658063702027
ENSG00000071655_1585156_1578486_185	MBD3	19	−	555	11	0	1	2.70%	11.62988227413784	0.019216965118390855	0.04178282491678763	0.003600525600033436
ENSG00000125037_9986661_9964069_261	EMC3	3	−	783	46	1	0	6.13%	34.4724131497433	0.0002519671529549621	0.00020377058636723502	3.533538062473344e−07
ENSG00000110047_64855450_64854333_217	EHD1	11	−	651	7	0	2	2.30%	6.3094669229193565	0.019216965118390855	0.1191346346731873	0.010787784428637617
ENSG00000075624_5529657_5529045_125	ACTB	7	−	375	421	15	5	12.00%	658.7588224881641	0.00139366094242993	0.0010286746874834437	8.789184600531728e−06
ENSG00000105202_39840438_39834664_228	FBL	19	−	684	177	2	5	17.11%	151.84230265815134	2.288692743367176e−08	1.8076094653894796e−07	4.20850350938848e−14
ENSG00000182220_40591300_40605755_272	ATP6AP2	X	+	816	35	1	4	6.62%	25.168277982968757	0.00012186633930364116	0.0005279920463075931	4.5515488091230315e−07
ENSG00000198954_68999728_69016416_508	KIF1BP	10	+	1524	21	3	0	2.17%	8.085557021300199	0.009920634900645505	0.0012624143894574274	7.753726787359189e−05
ENSG00000064726_83067151_83018067_482	BTBD1	15	−	1446	32	0	0	2.28%	12.985458895302907	0.001558047347358915	0.001558047347358915	1.4544595481456893e−05
ENSG00000071553_154428759_154435891_448	ATP6AP1	X	+	1344	89	1	5	6.03%	38.85673937574666	3.054010994025094e−06	2.2511476813243364e−05	5.897199179203898e−10
ENSG00000100129_37850054_37888464_540	EIF3L	22	+	1620	41	6	1	2.59%	14.850615664871299	0.009301464950567872	0.0006644883448370029	4.183029195470548e−05
ENSG00000082898_61526431_61478820_999	XPO1	2	−	2997	94	7	9	3.40%	18.404190804191195	2.8597618265040248e−06	5.055987184425535e−06	1.2297773581093105e−10
ENSG00000105583_12669388_12668359_99	WDR83OS	19	−	297	63	5	0	20.20%	124.46857475213639	0.00028663786130319756	3.8737898727308975e−05	8.485463167558208e−08
ENSG00000185009_74138379_74123810_418	AP3M1	10	−	1254	22	2	0	2.63%	10.294393400552634	0.007443125733849675	0.0013845019718837994	6.20801023785377e−05
ENSG00000110713_3782117_3676159_1726	NUP98	11	−	5178	35	3	0	0.98%	3.9662639695060844	0.0005606273783293826	0.00012610035281603184	4.993125573738249e−07
ENSG00000113240_178623416_178603617_373	CLK4	5	−	1119	10	2	2	1.34%	5.243792885000001	0.06032271266817251	0.06945717695039447	0.016020910955657944
ENSG00000266472_150294403_150308228_75	MRPS21	1	+	225	56	5	2	22.67%	146.04312770917338	0.0008517967108605519	0.00023306224115712893	1.3453852255506281e−06
ENSG00000175471_95017427_94931935_150	MCTP1	5	−	450	6	1	0	3.33%	7.823738984420002	0.0477903522728147	0.016947426762344633	0.0036975118224100542
ENSG00000182180_73250959_73250852_35	MRPS16	10	−	105	21	0	1	28.57%	117.35608476630003	0.0022409900782254734	0.005670089768331009	7.232548112602907e−05
ENSG00000117868_158829418_158734207_845	ESYT2	7	−	2535	42	5	3	2.37%	9.721805838628404	4.983000130598301e−05	0.00023625467940204776	8.773628016212268e−08
ENSG00000104964_3055981_3053819_204	TLE5	19	−	612	21	2	9	3.92%	20.134622386375003	0.01732176123716967	0.08147709756895899	0.006563186845139556
ENSG00000198258_9828338_9830008_73	UBL5	19	+	219	212	34	1	24.66%	568.0248851702194	0.00349644985133044	0.00010324976142615792	2.9322108478769698e−06
ENSG00000115648_237518520_237553592_430	MLPH	2	+	1290	26	0	3	2.33%	11.826582185751166	0.0023628488670607213	0.009929351914350733	0.0001339318614411771
ENSG00000100097_37675703_37677069_30	LGALS1	22	+	90	112	6	7	33.33%	730.2156385458668	0.011952930790615185	0.017541748908947063	0.0010100848806274727
ENSG00000121892_39928140_39825485_1283	PDS5A	4	−	3849	21	8	0	1.09%	3.201452039610679	0.021961533137259858	0.0003342575629110139	6.396813528347796e−05
ENSG00000115204_27313020_27312392_105	MPV17	2	−	315	10	0	3	4.76%	18.627949962904765	0.019679754444124863	0.15040137116388086	0.014313628319454642
ENSG00000172262_43122054_43175133_589	ZNF131	5	+	1767	7	1	0	1.02%	2.3245404452860785	0.028889785561798602	0.009815328628645334	0.0013877953586383081
ENSG00000104332_41309159_41265167_314	SFRP1	8	−	942	18	0	0	1.91%	11.212364786589175	0.013428347753762199	0.013428347753762199	0.0008725593497644525
ENSG00000119969_94562865_94601622_697	HELLS	10	+	2091	23	2	1	1.29%	6.454304040231709	0.010026873424650893	0.004355956481189797	0.00023340147885823483
ENSG00000107554_99909066_99877151_797	DNMBP	10	−	2391	29	5	2	1.25%	7.116952024723339	0.016699570039232777	0.006881839805563927	0.0005850670080306801
ENSG00000131778_147266075_147295509_603	CHD1L	1	+	1809	18	0	1	1.33%	5.838611182402986	0.004873903550586949	0.015625008558487224	0.000403121801006346
ENSG00000171456_32358776_32438438_625	ASXL1	20	+	1875	11	0	0	0.80%	3.4424451531448006	0.019679754444124863	0.019679754444124863	0.0017849636115922674
ENSG00000062822_50402016_50417947_947	POLD1	19	+	2841	32	2	1	1.48%	6.609283196975714	0.0014271065596448318	0.0006310955412434601	5.684867372284401e−06
ENSG00000169714_129171757_129171375_95	CNBP	3	−	285	67	8	0	18.95%	137.9448715674053	0.0011110308651439797	7.870973464443577e−05	6.653339876128778e−07
ENSG00000173436_19597046_19626401_62	MICOS10	1	+	186	24	0	0	9.68%	75.71360307503228	0.013640585738808999	0.013640585738808999	0.00089863692579514
ENSG00000167085_49413264_49404992_272	PHB	17	−	816	102	4	2	6.25%	73.34755297893751	0.00014002510398755815	0.0001854842840713172	1.8263145419340512e−07
ENSG00000183726_25340487_25360705_157	TMEM50A	1	+	471	39	2	9	10.83%	48.58691407521976	0.0005011152378431419	0.00100592494141216	3.2384211799565086e−06
ENSG00000152240_46118204_46128125_202	HAUS1	18	+	606	23	0	0	2.97%	22.27054413881931	0.013428347753762199	0.013428347753762199	0.0008725593497644525
ENSG00000157193_53327912_53247018_904	LRP8	1	−	2712	17	9	0	0.77%	3.6781958721001113	0.18607691510208607	0.00847367416878415	0.010183475765332767
ENSG00000164904_126582881_126544965_377	ALDH7A1	5	−	1131	23	0	0	2.39%	11.932758397988067	0.0033278027414746945	0.0033278027414746945	6.211806171561056e−05
ENSG00000123737_121801425_121816856_456	EXOSC9	4	+	1368	23	0	0	3.51%	9.865460342196274	0.0001415909997498741	0.0001415909997498741	1.4189685379808827e−07
ENSG00000108424_47680028_47682404_89	KPNB1	17	+	267	28	1	4	12.36%	61.535025720157314	0.00236147777460975	0.00874233720526068	0.0001174624148574889
ENSG00000166136_100529851_100524089_172	NDUFB8	10	−	516	102	1	1	15.70%	115.99147912948258	3.0069264253400697e−06	3.0069264253400697e−06	7.738804226946091e−11
ENSG00000166136_100529423_100523837_130	NDUFB8	10	−	390	98	0	1	19.23%	147.4473885525308	5.5104407320523845e−06	6.572665321543611e−06	3.010931943233488e−10
ENSG00000206190_25725972_25679341_1180	ATP10A	15	−	3540	7	0	3	0.42%	1.1603002731131358	0.016947426762344633	0.13974268412556323	0.011764960919844317
ENSG00000153956_82349627_81950392_1052	CACNA2D1	7	−	3156	24	0	1	1.24%	4.462208546247149	0.0004762745294806779	0.001038231149674533	3.1862098168137684e−06
ENSG00000171867_4699242_4699982_246	PRNP	20	+	738	93	0	0	8.54%	73.94387454787197	2.6246772046413107e−05	2.6246772046413107e−05	5.344526997695175e−09
ENSG00000083312_72848394_72905410_890	TNPO1	5	+	2670	74	4	3	3.26%	16.262828226041577	8.601389492772949e−06	5.945460507496746e−06	4.224228075481936e−10
ENSG00000173113_64317311_64316861_81	TRMT112	11	−	243	85	0	0	19.75%	205.25241162830253	0.00021408175522948358	0.00021408175522948358	3.163100203905902e−07
ENSG00000127527_16471945_16361967_754	EPS15L1	19	−	2262	8	0	0	0.66%	2.075262330084881	0.019679754444124863	0.019679754444124863	0.0017849636115922674
ENSG00000102910_48244473_48351802_780	LONP2	16	+	2340	41	3	0	1.67%	10.281195460295516	0.00215310643496043	0.0007052562366071395	9.510215769526577e−06
ENSG00000155368_119367585_119372318_104	DBI	2	+	312	103	6	4	20.19%	193.71276812386063	0.00014145917115575762	8.387869876068085e−05	8.562985301249247e−08
ENSG00000116747_193069142_193085002_489	RO60	1	+	1467	25	0	0	2.86%	9.999666391129859	0.0003981150787954058	0.0003981150787954058	1.0507179780062227e−06
ENSG00000189067_11556730_11549637_161	LITAF	16	−	483	34	1	0	7.45%	41.30545426557144	0.0010073743004618205	0.0010155991509472172	6.342998511872531e−06
ENSG00000134759_36129934_36174641_891	ELP2	18	+	2673	25	0	0	1.57%	5.488032396478677	0.0003981150787954058	0.0003981150787954058	1.0507179780062227e−06
ENSG00000162194_62667660_62663050_156	LBHD1	11	−	468	36	3	3	7.05%	45.136955679346165	0.001988634903193983	0.0020028004154862638	2.337634404283484e−05
ENSG00000028528_64127762_64137618_268	SNX1	15	+	804	19	0	3	4.10%	13.866701558207092	0.0013430411379358334	0.014028501358900838	0.00011866004684692356
ENSG00000114850_156549056_156543203_116	SSR3	3	−	348	60	1	0	14.66%	101.16903859163796	0.00021999752059115895	0.00013974726970550526	2.1551097651367336e−07
ENSG00000135052_86079290_86027817_391	GOLM1	9	−	1173	43	7	0	4.35%	21.510279816500006	0.0015944032073887035	0.00011368890353703816	1.3531327877350338e−06
ENSG00000135052_86079290_86027817_391	GOLM1	9	−	1173	43	7	0	4.35%	21.510279816500006	0.0015944032073887035	0.00011368890353703816	1.3531327877350338e−06
ENSG00000112983_138168063_138157208_574	BRD8	5	−	1722	9	2	0	1.05%	3.066796640234321	0.07134538375819317	0.011772051206276392	0.0041729868335021346
ENSG00000175216_46821231_46744023_2032	CKAP5	11	−	6096	70	1	9	1.38%	6.737964184416832	1.9773946824889005e−06	8.871077060996727e−05	1.6748457889077919e−09
ENSG00000135829_182878055_182887434_526	DHX9	1	+	1578	22	1	0	1.90%	8.180715668119774	0.0033869280463461717	0.0022543491824521134	4.372784971036962e−05
ENSG00000198732_69879679_70030258_435	SMOC1	14	+	1305	23	0	6	2.30%	10.341723944922991	0.0022011839993586477	0.019243135633285505	0.0002533889729906116
ENSG00000054267_235161211_235131713_425	ARID4B	1	−	1275	21	0	3	2.12%	9.664618745460002	0.0035291660242640502	0.012200824781023672	0.00023568507339691614
ENSG00000057019_98812554_98808065_181	DCBLD2	3	−	543	17	0	3	4.97%	18.37065783634531	0.0033030736763512156	0.024710983489837978	0.0004665285142020836

ENSG00000116209_54036675_54032150_73	TMEM59	1	−	219	43	1	0	23.29%	115.21259463358221	0.00017709925282996202	0.0001151290146190202	1.4464965079969512e−07
ENSG00000164904_126595114_126544965_451	ALDH7A1	5	−	1353	14	0	0	1.77%	6.071637792787141	0.004873903550586949	0.004873903550586949	0.00012846764509679302
ENSG00000153827_229880079_229767554_1722	TRIP12	2	−	5166	30	0	4	0.93%	3.407551822482579	0.00018250575362727147	0.0020325392531191206	2.6592817112142196e−06
ENSG00000153827_229880079_229767554_2040	TRIP12	2	−	6120	34	0	4	0.88%	3.259891243508334	7.973550974633635e−05	0.0009177192316615867	5.495447938168758e−07
ENSG00000050130_59485071_59504072_319	JKAMP	14	+	957	77	5	5	9.40%	47.21221800943105	6.0874644722481565e−06	1.1115049296679773e−05	5.568793004618189e−10
ENSG00000169813_43387881_43386637_414	HNRNPF	10	−	1242	101	4	4	8.45%	47.71724863686112	4.932031907248019e−07	8.552842395818309e−07	3.843530684890113e−12
ENSG00000197111_53454801_53479442_362	PCBP2	12	+	1086	151	7	5	10.22%	81.58733333200416	5.054304745247685e−07	5.406005520657781e−08	2.7180737229998643e−13
ENSG00000141127_18865870_18930698_317	PRPSAP2	17	+	951	18	0	0	3.47%	11.106254078829656	0.0013430411379358334	0.0013430411379358334	1.0937773364376948e−05
ENSG00000117650_211675479_211658657_388	NEK2	1	−	1164	23	2	2	2.32%	11.594458546498714	0.010026873424650893	0.010026873424650893	0.0005035630638330185
ENSG00000088367_36175560_36229340_779	EPB41L1	20	+	2337	47	3	6	2.57%	11.800889995755458	0.00017019607946125424	0.00038178802361551854	4.483271452642457e−07
ENSG00000135913_218558611_218454930_965	USP37	2	−	2895	13	0	0	0.62%	2.6349379999341975	0.012798402692974279	0.012798402692974279	0.0007972436888943567
ENSG00000105404_41958370_41956886_63	RABAC1	19	−	189	28	2	0	9.52%	86.93043316022224	0.032657852871826265	0.013640585738808999	0.0020904521300403643
ENSG00000158201_21704638_21664098_204	ABHD3	18	−	612	7	0	0	2.94%	6.711540795458335	0.009815328628645334	0.009815328628645334	0.0004837142251238724
ENSG00000108946_68515400_68515600_66	PRKAR1A	17	+	198	18	0	0	7.58%	53.343674893772736	0.019679754444124863	0.019679754444124863	0.0017849636115922674
ENSG00000180530_14967961_14964716_1081	NRIP1	21	−	3243	20	0	3	0.93%	3.6187506865957455	0.0022276761777358703	0.015358647673375043	0.00020093250298549382
ENSG00000138085_27216525_27216948_66	ATRAID	2	+	198	45	0	1	21.21%	133.35918723443186	0.00043491588733509306	0.0005855227822530266	1.6636196221710345e−06
ENSG00000265681_49491571_49488519_184	RPL17	18	−	552	36	2	0	2.72%	38.26828851075001	0.03980790073005672	0.021557223391537678	0.0037960059236543955
ENSG00000106392_7234320_7243727_363	C1GALT1	7	+	1089	10	0	0	1.65%	5.388249989269974	0.011772051206276392	0.011772051206276392	0.000681272511389508
ENSG00000106397_101217274_101206281_738	PLOD3	7	−	2214	66	8	8	3.25%	17.492099355410573	0.00039382578929151663	8.634013571541536e−05	2.4563727617726646e−07
ENSG00000265681_49492316_49491779_37	RPL17	18	−	111	8	3	0	13.51%	42.29048099686487	0.07864960352514258	0.019679754444124863	0.007006522949969347
ENSG00000265681_49491571_49488519_184	RPL17	18	−	552	36	2	0	2.72%	38.26828851075001	0.03980790073005672	0.021557223391537678	0.0037960059236543955
ENSG00000134108_5122466_5178783_163	ARL8B	3	+	489	17	8	0	4.29%	20.399319437904914	0.2617390908898303	0.007029302661868638	0.01434881016164812
ENSG00000164733_11845760_11845125_65	CTSB	8	−	195	64	5	0	24.62%	192.58434423187697	0.0003082577812411811	0.00020935012119566927	4.4168983060279205e−07
ENSG00000111642_6599910_6570676_1463	CHD4	12	−	4389	18	5	1	0.68%	2.406481574155161	0.049730516107242	0.0019601722425773736	0.0006762773414982664
ENSG00000127990_94618900_94585465_278	SGCE	7	−	834	11	0	2	2.52%	7.739310146458635	0.007029302661868638	0.03522780890193623	0.0012822660609675817
ENSG00000168827_158644635_158662664_453	GFM1	3	+	1359	29	1	1	3.75%	12.521436564469099	0.00010197470416588956	0.00018298304573899598	1.3304275972114245e−07
ENSG00000183258_177514993_177511791_382	DDX41	5	−	1146	20	1	5	2.36%	10.240496052905762	0.006142079104304129	0.04227848696273928	0.0013942017966237796
ENSG00000176697_27658504_27657821_227	BDNF	11	−	681	10	1	0	2.64%	8.616452626013217	0.023672501042627626	0.012798402692974279	0.0014375227604752335
ENSG00000059377_139953368_140020099_383	TBXAS1	7	+	1149	11	0	0	1.31%	5.617567155915145	0.016947426762344633	0.016947426762344633	0.0013498980316300959
ENSG00000113638_40747018_40716145_262	TTC33	5	−	786	7	0	0	2.29%	5.225779856005726	0.009815328628645334	0.009815328628645334	0.0004837142251238724
ENSG00000116288_7962786_7985054_189	PARK7	1	+	567	159	14	0	19.58%	164.5468913389921	1.2818357415835378e−06	4.611197172071847e−08	6.127179820803827e−13
ENSG00000159377_151401314_151401634_44	PSMB4	1	+	132	19	0	1	11.36%	84.46081858180683	0.02108409854857798	0.02108409854857798	0.002029891263157455
ENSG00000159377_151399681_151400447_85	PSMB4	1	+	255	22	1	3	12.94%	50.62419342860001	0.00397428314897603	0.00895222003271217	0.00019190433382141147
ENSG00000111711_21508458_21515707_74	GOLT1B	12	+	222	73	2	0	9.46%	192.95031954819598	0.024275373211574098	0.00877616296920843	0.0010556658063702027
ENSG00000257727_56315217_56311351_82	CNPY2	12	−	246	11	1	24	8.54%	26.23814903311586	0.015516259750072458	0.3253064819663768	0.032501170894773765
ENSG00000050820_75236980_75229511_599	BCAR1	16	−	1797	18	0	0	1.34%	5.877600238712856	0.005554612275049112	0.005554612275049112	0.00016468457691007306
ENSG00000072042_67693048_67678321_279	RDH11	14	−	837	167	0	7	11.47%	117.07566401417027	3.0553715917383984e−07	1.2504558549554672e−06	3.5426764443431557e−12
ENSG00000101843_108091520_108087682_127	PSMD10	X	−	381	10	3	0	3.94%	15.40106099295276	0.12312584984626351	0.020613416668581838	0.011809609471937859
ENSG00000219200_7013669_7014286_71	RNASEK	17	+	213	86	4	2	23.94%	236.9160396690564	0.000449965669174599	0.00015666089602381063	4.888330309678694e−07
ENSG00000013375_83191012_83169234_501	PGM3	6	−	1503	10	0	0	1.00%	3.9040613694710586	0.019679754444124863	0.019679754444124863	0.0017849636115922674
ENSG00000162496_12617318_12568340_292	DHRS3	1	−	876	136	3	7	7.19%	91.09833064050687	4.753417061228364e−05	0.0003089157875591553	1.1057241635063116e−07
ENSG00000114850_156555089_156543203_185	SSR3	3	−	555	83	2	0	12.97%	87.75274806849461	1.3457868176494376e−05	8.591160490260707e−06	9.383759773964053e−10
ENSG00000100526_54397069_54417878_148	CDKN3	14	+	444	16	1	3	7.43%	21.145240498432436	0.002549573717490699	0.01145762988930082	0.00016600381219738253
ENSG00000185480_102123922_102196023_172	PARPBP	12	+	516	19	0	0	4.07%	21.60625591627617	0.00877616296920843	0.00877616296920843	0.00039161135667564577
ENSG00000184047_122218361_122208381_166	DIABLO	12	−	498	17	0	0	3.61%	20.030657038424703	0.013007239811447113	0.013007239811447113	0.0008218654148058079
ENSG00000215301_41344365_41347719_331	DDX3X	X	+	993	65	0	0	8.46%	38.40959471203173	1.0077168445509615e−06	1.0077168445509615e−06	9.076267751723256e−12
ENSG00000010278_6232667_6236484_158	CD9	12	+	474	116	5	0	17.72%	143.60027249884814	1.6864292402005362e−05	1.6810037984717075e−06	2.5120325157468856e−10
ENSG00000108021_5730687_5763032_2201	TASOR2	10	+	6603	10	0	3	0.23%	0.8886573130872333	0.020613416668581838	0.12312584984626351	0.011809609471937859
ENSG00000147044_41665429_41520420_736	CASK	X	−	2208	19	0	0	0.95%	5.049288067390626	0.00847367416878415	0.00847367416878415	0.0003665024264111182
ENSG00000165476_63566351_63620869_240	REEP3	10	+	720	27	2	2	6.25%	22.004265893681254	0.0011705283430067086	0.0009505490124674304	6.881964800301074e−06
ENSG00000109180_48832625_48860762_245	OCIAD1	4	+	735	64	0	2	5.31%	51.09380561253879	0.0006791898698869142	0.0006791898698869142	2.9460669171009034e−06
ENSG00000171848_10122676_10129386_430	RRM2	2	+	1290	57	1	1	4.19%	25.9275070995314	8.317592195240937e−05	0.00012126015959033826	7.298537411493792e−08
ENSG00000132646_5119798_5115283_261	PCNA	20	−	783	350	18	13	23.37%	262.2901000523947	6.253645033670741e−11	1.7783641736001606e−10	1.3047738787450618e−19
ENSG00000139263_58890111_58872572_938	LRIG3	12	−	2814	9	0	0	0.85%	1.8766964514866742	0.0033278027414746884	0.0033278027414746884	6.211806171561044e−05
ENSG00000087074_48873309_48875973_649	PPP1R15A	19	+	1947	13	1	1	1.08%	3.917897026096303	0.013599199788808391	0.013599199788808391	0.0008935241583747976
ENSG00000087077_100867498_100873303_476	TRIP6	7	+	1428	28	1	1	2.73%	11.505498506500002	0.0008152162100056033	0.0008152162100056033	4.187859324889015e−06
ENSG00000161920_4731490_4733187_117	MED11	17	+	351	24	0	0	5.98%	40.12173838164104	0.008574016250729465	0.008574016250729465	0.0003747459614100891
ENSG00000106976_128203486_128253122_846	DNM1	9	+	2538	53	2	7	2.25%	12.253491908222816	7.448194265828549e−05	0.001199678750155866	6.877941490576349e−07
ENSG00000164733_11853454_11845125_339	CTSB	8	−	1017	303	14	0	20.94%	174.82248615628765	1.089651732784085e−12	9.990376595038133e−14	1.4630170378218959e−24
ENSG00000228144_66169858_66138087_137	AC078927.1	12	−	411	39	1	1	8.03%	55.67989423218614	0.001759868229841518	0.001759868229841518	1.8371187347528584e−05
ENSG00000070087_149968535_149966489_91	PFN2	3	−	273	64	3	8	17.58%	137.56024587991212	0.0006674249601086224	0.001736918071338517	7.284967500522852e−06
ENSG00000179889_15017348_15036275_492	PDXDC1	16	+	1476	12	0	0	1.42%	4.7705725514756105	0.008175610787902942	0.008175610787902942	0.00034251843268961324
ENSG00000135677_64752715_64716741_474	GNS	12	−	1422	83	0	0	7.17%	34.249490279897685	1.1661654974984182e−07	1.1661654974984182e−07	1.3114986241366903e−13
ENSG00000096872_27029083_27062736_256	IFT74	9	+	768	8	0	0	1.95%	6.112296081578126	0.019679754444124863	0.019679754444124863	0.0017849636115922674
ENSG00000134575_47247721_47240116_351	ACP2	11	−	1053	13	1	0	2.56%	7.244202763351853	0.006277459298483267	0.001946208561389314	7.053443163459546e−05
ENSG00000007202_28642954_28615064_2092	KIAA0100	17	−	6276	11	0	2	0.38%	1.0284551724261475	0.004687384229717438	0.033944577430914495	0.0008796663632127183
ENSG00000215021_6970543_6968532_112	PHB2	12	−	336	40	3	4	11.61%	69.85481236089288	0.0013315596295692745	0.0019985383115843874	1.5940596572636573e−05

ENSG00000215021_6968587_6967857_113	PHB2	12	−	339	46	6	0	9.73%	79.62212240781417	0.011219216521103996	0.0016033688297201391	0.00010856363384302963
ENSG00000003402_201130085_201136427_177	CFLAR	2	+	531	11	5	0	2.82%	12.155526670709042	0.12180769416495907	0.019679754444124863	0.011261990492355435
ENSG00000138760_76213543_76161713_174	SCARB2	4	−	522	25	3	1	5.75%	28.10251071989943	0.011246719313271002	0.0035004709947243163	0.00021537205478367794
ENSG00000080824_102086291_102081712_703	HSP90AA1	14	−	2109	446	24	22	13.37%	124.08917450395877	2.0782847351969592e−16	2.5828625729642765e−15	7.951553247159537e−30
ENSG00000074319_18526816_18480546_390	TSG101	11	−	1170	38	2	0	5.13%	19.057825731279493	0.00013502576859051514	3.404299617394451e−05	3.485380474868625e−08
ENSG00000145555_16711009_16666692_1369	MYO10	5	−	4107	27	3	3	1.02%	3.8575776584978096	0.0033854124258957835	0.002608298974881378	5.012719993238035e−05
ENSG00000115307_74527775_74527146_125	AUP1	2	−	375	30	0	1	5.60%	46.94243390652001	0.008574016250729465	0.010042643336235583	0.00043540252252504097
ENSG00000158796_161124462_161122147_348	DEDD	1	−	1044	12	0	0	1.72%	6.744602572775864	0.013007239811447113	0.013007239811447113	0.0008218654148058079
ENSG00000158796_161124462_161122147_318	DEDD	1	−	954	12	0	0	1.89%	7.380885834358493	0.013007239811447113	0.013007239811447113	0.0008218654148058079
ENSG00000143222_161153998_161158492_167	UFC1	1	+	501	30	10	4	7.19%	35.13655232523953	0.04198543924994409	0.004367727785308368	0.001048458846888566
ENSG00000157168_32595851_32760410_379	NRG1	8	+	1137	29	0	1	2.37%	14.966255313204488	0.003736770770438521	0.0045126225279669635	9.281320660291148e−05
ENSG00000097021_6339454_6264597_238	ACOT7	1	−	714	16	1	2	3.36%	13.149141150285717	0.008219046535586472	0.02071580916834944	0.0008496570697932704
ENSG00000126581_42818656_42810760_325	BECN1	17	−	975	10	6	1	1.85%	6.018260757246155	0.14351487215180903	0.023672501042627626	0.015574533941414816
ENSG00000113448_58993456_58974664_499	PDE4D	5	−	1497	37	1	0	3.61%	14.502922967111227	0.0001185681716428482	8.091994981866961e−05	6.953223258948248e−08
ENSG00000215301_41343231_41347719_476	DDX3X	X	+	1428	83	1	0	7.56%	34.10558485855358	6.958679763556736e−08	4.081835640353621e−08	2.8150375102779016e−14
ENSG00000146416_143061010_143337994_203	AIG1	6	+	609	20	0	0	3.94%	19.270293065073897	0.005604866691947602	0.005604866691947602	0.00016752456572620382
ENSG00000179218_12938680_12943913_417	CALR	19	+	1251	712	30	26	27.34%	333.9629590471847	5.473279215681177e−19	3.615449190194388e−18	3.1518957733742697e−35
ENSG00000105825_93890678_93886820_235	TFPI2	7	−	705	356	16	9	25.53%	296.3033062184596	3.9248015905340364e−11	3.464871216398847e−11	1.6229319798910794e−20
ENSG00000182979_105460864_105470215_435	MTA1	14	+	1305	17	2	0	1.84%	7.643882915812646	0.02671499548383061	0.004873903550586949	0.0007031623440447889
ENSG00000064419_129018073_128957255_889	TNPO3	7	−	2667	28	0	0	1.57%	6.160424397181104	0.0003981150787954058	0.0003981150787954058	1.0507179780062227e−06
ENSG00000178252_49011688_49014120_810	WDR6	3	+	2430	20	0	2	1.23%	4.829468508901235	0.0022276761777358703	0.009621867080838033	0.00012310157985546497
ENSG00000160932_143021325_143021789_110	LY6E	8	+	330	67	2	1	11.82%	119.13420726275912	0.0007373308535318079	0.0007313336786544864	3.424294031634491e−06
ENSG00000108528_4939211_4937741_282	SLC25A11	17	−	846	32	0	0	4.26%	22.195004211120573	0.0010447178140462346	0.0010447178140462346	6.749952978822125e−06
ENSG00000182923_134507167_134561535_669	CEP63	3	+	2007	10	0	0	0.90%	2.923669276689089	0.011772051206276392	0.011772051206276392	0.000681272511389508
ENSG00000153187_244860415_244848809_511	HNRNPU	1	−	1533	127	22	12	7.83%	48.61129408127888	4.908286529999873e−05	3.4479342709277845e−06	1.4781091094746038e−09
ENSG00000105852_95372278_95359973_267	PON3	7	−	801	23	0	0	4.12%	16.848876090043074	0.001484234770590415	0.001484234770590415	1.3251387882263029e−05
ENSG00000168036_41224552_41239342_768	CTNNB1	3	+	2304	116	8	0	5.73%	29.542764394294277	8.699215501017508e−08	2.9105570714308586e−09	2.8276345500981447e−15
ENSG00000215301_41334253_41347719_716	DDX3X	X	+	2148	97	12	3	6.70%	26.497998655333106	1.8411069105942975e−07	2.168363292173117e−09	4.7152014957107245e−15
ENSG00000231007_87011916_87013151_411	CDC20P1	9	+	1233	10	0	0	1.22%	4.758965318990269	0.020613416668581838	0.020613416668581838	0.001946208561389314
ENSG00000205531_2976103_2945608_355	NAP1L4	11	−	1065	19	2	0	2.25%	10.468383148167607	0.02682370818175567	0.005160369442893307	0.0007411199362065436
ENSG00000106355_32489303_32487261_62	LSM5	7	−	186	49	4	0	27.42%	154.58193961152423	0.0002473554500538834	0.00013319834607924987	2.311148598512169e−07
ENSG00000181381_168472468_168390522_1288	DDX60L	4	−	3864	11	1	5	0.47%	1.670441165151786	0.019795881618345192	0.126368739272204	0.011788600186521595
ENSG00000037474_6620212_6599926_531	NSUN2	5	−	1593	61	2	3	4.71%	22.469306876159138	1.801218945375667e−05	2.3525749339974676e−05	3.3301312306983467e−09
ENSG00000064042_41613075_41697185_890	LIMCH1	4	+	2670	19	3	6	1.24%	4.175591031010675	0.013013784588570694	0.019903044927659083	0.0012328590309843733
ENSG00000135446_57752292_57752176_38	CDK4	12	−	114	19	0	2	18.42%	97.79673730525002	0.008674846900444944	0.024135905425855194	0.0010389365785287079
ENSG00000168002_62765397_62766507_42	POLR2G	11	+	126	70	0	5	33.33%	325.98912435083344	0.00046843213071395124	0.0031185037294365974	9.613998767368705e−06
ENSG00000166557_79321978_79322214_78	TMED3	15	+	234	24	2	0	14.10%	60.18260757246155	0.011064573844522778	0.0012624143894574274	8.713148241777389e−05
ENSG00000104689_23225061_23191694_310	TNFRSF10A	8	−	930	19	0	0	2.90%	11.987987153546777	0.0034782380676658805	0.0034782380676658805	6.758309314600117e−05
ENSG00000117592_173477398_173487863_224	PRDX6	1	+	672	131	0	2	12.05%	114.38725524096208	2.5676489710131477e−06	3.55131294271747e−06	7.811921954748982e−11
ENSG00000198604_34874604_34753508_1556	BAZ1A	14	−	4668	9	3	1	0.51%	1.1313247246108615	0.07229263996867695	0.010460667668897016	0.003853881152483681
ENSG00000111641_6567918_6556993_845	NOP2	12	−	2535	26	4	2	2.01%	6.018260757246155	0.0010891986948716168	0.0002707893389517208	1.9824595550127703e−06
ENSG00000013810_1739999_1745013_152	TACC3	4	+	456	15	0	2	5.92%	19.301987626036187	0.0032294771334464936	0.010960145167579947	0.00019531721591777918
ENSG00000172009_2806827_2807881_221	AC006538.1	19	+	663	20	4	0	2.71%	17.700766933076928	0.07429615675292256	0.013428347753762199	0.004845296888780459
ENSG00000163714_143012252_143056450_989	U2SURP	3	+	2967	9	1	0	0.61%	1.7799203958488377	0.026832695913080365	0.011772051206276392	0.0015103817605807263
ENSG00000144118_120278719_120293260_188	RALB	2	+	564	25	0	0	6.38%	26.009770559906922	0.0008664586529590942	0.0008664586529590942	4.7094196839841995e−06
ENSG00000095794_35148378_35211398_230	CREM	10	+	690	9	3	0	2.61%	7.653657702150002	0.10105102209468636	0.011772051206276392	0.00615362087940124
ENSG00000115286_1390886_1395488_132	NDUFS7	19	+	396	26	3	0	9.09%	38.52598742328031	0.00387261209348426	0.0010073743004618205	2.38528981796687e−05
ENSG00000142864_67430300_67413207_408	SERBP1	1	−	1224	48	0	7	3.19%	23.010997013000004	0.0006652407155885319	0.010132552959945132	4.5992887540844404e−05
ENSG00000142864_67430300_67413207_387	SERBP1	1	−	1161	48	0	7	3.36%	24.259655765643416	0.0006652407155885319	0.010132552959945132	4.5992887540844404e−05
ENSG00000052749_97401231_97357094_1297	RRP12	10	−	3891	16	0	1	0.62%	2.4128724701372404	0.005160369442893307	0.007930666369886513	0.00021637657341139557
ENSG00000142864_67430300_67413207_402	SERBP1	1	−	1206	48	0	7	3.23%	23.354444729611945	0.0006652407155885319	0.010132552959945132	4.5992887540844404e−05
ENSG00000140688_31508373_31490835_468	C16orf58	16	−	1404	33	4	0	3.42%	13.791847568689105	0.0016335188667720405	0.00017828355461003195	2.0673971369923424e−06
ENSG00000155229_97498348_97458692_920	MMS19	10	−	2760	24	4	0	0.76%	5.102438468100001	0.04002830022536342	0.00847367416878415	0.0017159085326906823
ENSG00000183735_64455871_64499124_731	TBK1	12	+	2193	19	0	0	1.09%	5.0838249214767455	0.005554612275049112	0.005554612275049112	0.00016468457691007306
ENSG00000166797_64093672_64075518_70	CIAO2A	15	−	210	5	0	0	7.14%	13.970962472178575	0.012673659338734126	0.012673659338734126	0.000782701129001274
ENSG00000280987_139307593_139329395_788	MATR3	5	+	2364	99	12	7	4.82%	24.573291860963838	2.978342675931118e−06	5.535559437143131e−07	1.5012471142621105e−11
ENSG00000144744_69071581_69055437_363	UBA3	3	−	1089	23	0	0	3.03%	12.39297497532094	0.0014481341328822738	0.0014481341328822738	1.2639776101338432e−05
ENSG00000204422_31691845_31687212_325	AL662899.1	6	−	975	6	0	0	1.54%	3.610956454347693	0.016947426762344633	0.016947426762344633	0.0013498980316300959
ENSG00000131051_35725100_35704481_367	RBM39	20	−	1101	22	1	0	2.18%	11.724949431692101	0.008364861494636245	0.005604866691947602	0.00024596970711672695
ENSG00000143624_153740684_153773270_981	INTS3	1	+	2943	25	2	2	1.12%	4.9845431857925595	0.0041798898569932064	0.0024699148363837185	5.848323136957794e−05
ENSG00000135506_57694162_57720909_560	OS9	12	+	1680	75	3	6	6.43%	26.195554635334826	4.710589032573995e−07	2.298807928202195e−06	9.905140196363282e−12
ENSG00000111237_110501503_110492005_214	VPS29	12	−	642	64	0	11	10.75%	58.49524474332711	1.1598859020456752e−05	0.0003995223902392225	4.0868810868669265e−08
ENSG00000170421_52900665_52897428_279	KRT8	12	−	837	523	6	12	11.47%	366.650133409647	5.378491755130599e−07	1.1095576056170031e−06	5.414293976247711e−12
ENSG00000170421_52904981_52901860_178	KRT8	12	−	534	205	36	10	7.30%	225.2621477255759	0.0031207049338327648	0.0013949106480501364	2.5822337670666416e−05
ENSG00000130770_28236184_28237797_71	ATP5IF1	1	+	213	32	0	0	18.31%	88.15480545825353	0.0006311913977015671	0.0006311913977015671	2.5578527397951546e−06
ENSG00000130770_28236368_28237978_100	ATP5IF1	1	+	300	28	0	16	8.00%	54.76617289094001	0.005604866691947602	0.29311644564288997	0.014696759452302546
ENSG0000099783_8445050_8488854_713	HNRNPM	19	+	2139	94	7	2	5.89%	25.786516989322585	2.0804894262303012e−07	1.0383379122784742e−08	2.3024171826196435e−14

ENSG00000168036_41224069_41239342_781	CTNNB1	3	+	2343	117	8	3	5.76%	29.301455223339957	6.403151454483205e-08	1.1032790393731706e-08	7.305397733044446e-15
ENSG00000135679_68815643_68839849_465	MDM2	12	+	1395	11	0	0	1.29%	4.626942410140861	0.012798402692974279	0.012798402692974279	0.0007972436888943567
ENSG00000085063_33722445_33710126_128	CD59	11	-	384	62	5	3	14.06%	94.74058926446097	0.00102540667708157	0.0003583833865847517	2.4148597638421077e-06
ENSG00000152700_134624019_134606950_198	SAR1B	5	-	594	70	0	0	11.62%	69.14920819563133	1.1975596777994732e-05	1.1975596777994732e-05	1.1554586455257723e-09
ENSG00000131016_151240563_151353740_1782	AKAP12	6	+	5346	56	10	0	1.46%	6.146596284056118	0.0001828101181634415	2.7312900469374966e-06	4.887709699872272e-09
ENSG00000115524_197418565_197392303_1158	SF3B1	2	-	3474	53	5	0	2.07%	8.95203294849439	0.00011907465617200065	7.2548590762310375e-06	7.3767003929979675e-09
ENSG00000064042_41613075_41697185_902	LIMCH1	4	+	2706	19	3	6	1.22%	4.120039930819845	0.013013784588570694	0.019903044927659083	0.0012328590309843733
ENSG00000159921_36249271_36217365_694	GNE	9	-	2082	17	0	3	1.44%	4.7911946230237765	0.0021880057261679567	0.01904707903054789	0.00024933432573122474
ENSG00000153048_8859328_8855164_147	CARHSP1	16	-	441	23	0	4	4.76%	30.603060653343544	0.00877616296920843	0.04584514077471458	0.0020400732494421717
ENSG00000124356_73847513_73862259_257	STAMBP	2	+	771	15	0	0	3.11%	11.415961553142026	0.005405141725979036	0.005405141725979036	0.00015637213408961292
ENSG00000173660_46303791_46316584_83	UQCRH	1	+	249	37	1	3	9.64%	87.1922718143193	0.005604866691947602	0.012088529595134127	0.00035289342940057376
ENSG00000115380_55874991_55867073_175	EFEMP1	2	-	525	20	0	0	4.57%	22.35353995548572	0.005554612275049112	0.005554612275049112	0.00016468457691007306
ENSG00000104419_133256848_133238878_239	NDRG1	8	-	717	21	1	1	2.51%	17.18603751807741	0.013301727469301692	0.013301727469301692	0.0008571698582519322
ENSG00000131069_34876646_34927214_701	ACSS2	20	+	2103	24	8	0	1.43%	6.696495564410843	0.07890888349271175	0.0023628488670607213	0.0013657496381190898
ENSG00000113648_135389093_135345974_223	H2AFY	5	-	669	37	1	3	5.38%	32.45272897124889	0.001032175130546352	0.0029690360456949214	1.8630052217835324e-05
ENSG00000140262_56919935_57282587_675	TCF12	15	+	2025	16	0	0	1.33%	4.636289768545186	0.002967235347054297	0.002967235347054297	4.9907879201100895e-05
ENSG00000140259_43817380_43805094_390	MFAP1	15	-	1170	11	0	0	1.79%	5.5167390274756425	0.0078820576498062	0.0078820576498062	0.00031963642907451164
ENSG00000186480_155298286_155308358_178	INSIG1	7	+	534	25	0	2	5.62%	27.470993625070232	0.0023354528714849923	0.007558271381162128	0.00010028418779467875
ENSG00000105429_42334007_42375648_2308	MEGF8	19	+	6924	11	1	8	0.35%	0.9322046016964907	0.010499663139468709	0.3089002125664514	0.023581961233983657
ENSG00000258289_64914496_64932230_112	CHURC1	14	+	336	48	23	0	13.39%	83.82577483307145	0.006197250200377523	0.00030416618013389714	1.3816785432125349e-05
ENSG00000135404_55728323_55725561_232	CD63	12	-	696	246	22	6	26.29%	207.3965291128578	2.6552528959412224e-10	2.5295184939073248e-11	8.125620299060758e-20
ENSG00000111481_54325164_54350523_119	COPZ1	12	+	357	97	9	1	19.33%	159.43333644721432	0.00014781183975611854	1.5704846637853805e-05	1.86927181384664e-08
ENSG00000177410_49279128_49279205_25	ZFAS1	20	+	75	60	3	2	28.00%	469.4243390652001	0.027657845067597595	0.023857440118675605	0.002933789275920174
ENSG00000055917_20311519_20251585_821	PUM2	2	-	2463	22	1	0	1.22%	5.241238052900123	0.0037834851205969236	0.0022409900782254734	4.845513352116639e-05
ENSG00000125779_3889497_3910584_149	PANK2	20	+	447	11	0	4	3.36%	14.439786716211414	0.020613416668581838	0.17139085557395572	0.01724926236372712
ENSG00000125779_3912531_3923294_134	PANK2	20	+	402	13	0	0	4.48%	18.975486342809706	0.012798402692974279	0.012798402692974279	0.0007972436888943567
ENSG00000119689_74889332_74902330_349	DLST	14	+	1047	32	2	0	3.72%	17.934072170590262	0.0013600423278798564	0.0006652407155885319	5.692578211673844e-06
ENSG00000111276_12717885_12718946_183	CDKN1B	12	+	549	34	4	1	9.84%	36.3397712391093	0.0006845597953738475	7.625943357267001e-05	3.8807085961378077e-07
ENSG00000158406_26285499_26285188_103	HIST1H4H	6	-	309	806	40	9	35.92%	1530.5664129714858	3.5788032684303076e-07	7.586554145585192e-08	2.642131348744708e-13
ENSG00000103423_4443044_4447086_128	DNAJA3	16	+	384	9	0	1	3.91%	13.752666183550785	0.019216965118390855	0.04178282491678763	0.003600525600033436
ENSG00000138326_78034374_78035786_129	RPS24	10	+	387	75	12	1	9.30%	113.71713640145352	0.005089012094854324	0.0010073743004618205	3.1584573945156126e-05
ENSG00000107341_33817797_33917237_225	UBE2R2	9	+	675	39	4	3	5.78%	33.902868932486676	0.0010499093600357017	0.0030574666656489327	1.9489734499849677e-05
ENSG00000142676_23691824_23696373_178	RPL11	1	+	534	277	8	3	25.28%	304.3786093657782	2.4062822328757686e-09	3.500724908796212e-09	9.064835906628387e-17
ENSG00000110104_60842125_60850325_360	CCDC86	11	+	1080	12	0	2	1.94%	6.519782487016668	0.008175610787902942	0.03744183116399598	0.001552076439258323
ENSG00000110108_60919751_60922165_224	TMEM109	11	+	672	60	0	0	8.04%	52.39110927066965	8.51685422527664e-05	8.51685422527664e-05	5.287317170903535e-08
ENSG00000110107_60902872_60891166_419	PRPF19	11	-	1257	90	6	0	7.16%	42.01291817409309	1.404489321272174e-05	7.294736112174368e-07	9.597953069558437e-11
ENSG00000198242_28722772_28723881_70	RPL23A	17	+	210	113	19	2	8.57%	315.74375187123576	0.013439622348536238	0.013856139512579892	0.0008993698691259064
ENSG00000100473_30878908_30889791_457	COCH	14	+	1371	18	0	0	2.63%	7.703900531704597	0.0007729603088974596	0.0007729603088974596	3.7798209788587765e-06
ENSG00000139719_122263600_122232246_548	VPS33A	12	-	1644	20	1	3	1.82%	7.138447978485403	0.0022011839993586477	0.012837471289382485	0.0001645072545682577
ENSG00000143889_38602867_38581872_275	HNRNPLL	2	-	825	13	3	4	2.55%	9.246236981587275	0.058290625655499875	0.10392556251212837	0.022736565507324002
ENSG00000106554_133081937_132785637_227	CHCHD3	7	-	681	22	2	0	3.96%	18.95619577722908	0.00817113761938053	0.0033030736763512156	0.00014818970257535655
ENSG00000187514_231708707_231712851_111	PTMA	2	+	333	181	9	9	11.71%	318.9407108513559	0.0012800257995343638	0.0027996699912639944	2.1412447721174e-05
ENSG00000127990_94656098_94585465_444	SGCE	7	-	1332	25	3	2	2.48%	11.01314609293356	0.007887772776385514	0.0038108971419065062	0.00016292425881891603
ENSG00000103591_67254631_67202821_315	AAGAB	15	-	945	38	5	1	4.76%	23.595403286346038	0.0028900246157174264	0.00037933925179087024	7.362507485253584e-06
ENSG00000075131_66352947_66336958_301	TIPIN	15	-	903	12	0	0	2.33%	7.79774649609967	0.006937202941512724	0.006937202941512724	0.00025101522741487095
ENSG00000103671_64387864_64455064_581	TRIP4	15	+	1743	10	0	0	0.86%	3.3664969812478494	0.020613416668581838	0.020613416668581838	0.001946208561389314
ENSG00000028528_64096014_64138197_557	SNX1	15	+	1671	19	0	3	1.97%	6.671949762297129	0.0013430411379358334	0.014028501358900838	0.00011866004684692356
ENSG00000205250_67194953_67198123_153	E2F4	16	+	459	15	0	0	3.27%	19.175830844166672	0.020613416668581838	0.020613416668581838	0.001946208561389314
ENSG00000091651_46695593_46697585_92	ORC6	16	+	276	12	0	4	6.52%	25.512192340500008	0.013007239811447113	0.04223451612662591	0.0026029828050034526
ENSG00000146729_55983761_55999072_127	NIPSNAP2	7	+	381	27	1	0	8.66%	41.58286468097245	0.0015356776430442238	0.0015356776430442238	1.41466323862755e-05
ENSG00000121774_32014056_32042624_384	KHDRBS1	1	+	1152	119	1	3	7.29%	60.61360280898309	1.974166248087294e-06	2.1862290759419604e-06	3.750516055098153e-11
ENSG00000110697_67504111_67492033_1220	PITPNM1	11	-	3660	16	0	2	0.57%	2.5651603227606565	0.0078820576498062	0.019654369976907376	0.0007767617569840121
ENSG00000156642_73633215_73561910_398	NPTN	15	-	1194	108	0	2	6.53%	53.075616225964836	3.861928555414659e-06	3.7462418761579247e-06	1.226241433378412e-10
ENSG00000165782_20461325_20458559_284	PIP4P1	14	-	852	11	2	1	2.82%	7.575803594068664	0.03318103322835444	0.010499663139468709	0.001693572322031727
ENSG00000101337_32133049_32165444_625	TM9SF4	20	+	1875	35	9	5	2.40%	10.953234578188003	0.014867810601794082	0.0028038351964853017	0.00023652946316941574
ENSG00000066322_43365239_43363916_218	ELOVL1	1	-	654	56	1	0	10.55%	50.244195312789	1.4069570834807505e-05	1.1909960387592219e-05	1.3456146463793288e-09
ENSG00000162298_65133580_65128382_610	SYVN1	11	-	1830	20	1	5	1.64%	6.412900806901641	0.00397428314897603	0.043341461536463954	0.0010067842203199241
ENSG00000088930_21303399_21389338_950	XRN2	20	+	2850	62	7	2	3.16%	12.765047816685266	1.2563429202614738e-05	1.2875150957369303e-06	1.4466528978645605e-10
ENSG00000034510_84906018_84906423_44	TMSB10	2	+	132	181	12	10	38.64%	804.6004296477388	0.000626423983046506	0.00014142027782850438	6.213676168559304e-07
ENSG00000095002_47410181_47482949_783	MSH2	2	+	2349	30	1	2	1.28%	7.494002858639848	0.0031859520777271916	0.006198316675603911	0.00010898907104230859
ENSG00000135926_218281949_218275475_247	TMBIM1	2	-	741	15	0	0	2.83%	11.878146231406886	0.00847367416878415	0.00847367416878415	0.0003665024264111182
ENSG00000132466_73177491_73076231_2457	ANKRD17	4	-	7371	9	1	3	0.33%	0.7164596139578756	0.010460667668897016	0.07229263996867695	0.003853881152483681
ENSG00000140319_40039116_40038760_70	SRP14	15	-	210	90	0	3	7.14%	251.47732449921435	0.021557223391537678	0.046295797875114966	0.004403396695964728
ENSG00000206503_29942563_29943034_113	HLA-A	6	+	339	10	0	0	5.31%	17.309157045176995	0.011772051206276392	0.011772051206276392	0.000681272511389508
ENSG00000100129_37850054_37888464_540	EIF3L	22	+	1620	41	6	1	2.59%	14.850615664871299	0.009301464950567872	0.0006644883448370029	4.183029195470548e-05
ENSG00000141101_69754910_69742332_412	NOB1	16	-	1236	22	3	2	2.67%	10.444311751046119	0.011088249102336101	0.004968901071354418	0.0002905361484413382
ENSG00000152082_130182283_130190626_218	MZT2B	2	+	654	25	4	0	3.21%	22.430444336066518	0.016724852853975118	0.00797940233269714	0.0006687959809311652

ENSG00000132305_86195382_86171359_147	IMMT	2	-	441	12	0	0	4.08%	15.96681425391837	0.012798402692974279	0.012798402692974279	0.0007972436888943567
ENSG00000125755_45823860_45815560_439	SYMPK	19	-	1317	7	0	0	1.37%	3.1188025564316635	0.009815328628645334	0.009815328628645334	0.0004837142251238724
ENSG00000139350_96917657_96952053_571	NEDD1	12	+	1713	12	0	1	1.05%	4.110545876227672	0.013007239811447113	0.023878473930440184	0.0014709138084317821
ENSG00000049656_1323866_1318369_138	CLPTM1L	5	-	414	21	0	0	5.07%	29.764224397250008	0.008574016250729465	0.008574016250729465	0.0003747459614100891
ENSG00000141552_81894478_81900065_84	ANAPC11	17	+	252	127	10	2	20.24%	295.7187056611132	0.00039118706886775756	0.00018946255112486357	5.081891006409044e-07
ENSG00000157349_70324649_70333582_328	DDX19B	16	+	984	14	0	0	1.52%	8.348501965082319	0.019679754444124863	0.019679754444124863	0.0017849636115922674
ENSG00000134419_18789104_18783009_127	RPS15A	16	-	381	43	14	4	4.72%	66.22456226969686	0.13042902411515872	0.05352897298295963	0.026520273205808225
ENSG00000114450_179419493_179401213_304	GNB4	3	-	912	20	0	5	3.29%	12.867991750690793	0.0014903523101034196	0.016360242415786763	0.00015313761876917885
ENSG00000143575_154272724_154275237_191	HAX1	1	+	573	9	0	2	2.62%	9.216446447615185	0.016947426762344633	0.1029516053660342	0.008323174032180154
ENSG00000072062_14107984_14093112_427	PRKACA	19	-	1281	15	0	1	1.87%	6.870965150251758	0.005160369442893307	0.007930666369886513	0.00021637657341139557
ENSG00000111615_75511597_75499809_381	KRR1	12	-	1143	38	0	0	4.46%	19.508010591073496	0.00012584286796367173	0.00012584286796367173	1.1287214964574215e-07
ENSG00000131966_58200218_58234551_417	ACTR10	14	+	1251	44	1	2	2.64%	20.638160390556358	0.001988634903193983	0.0032951010080974528	3.786005601889957e-05
ENSG00000163739_73869471_73869993_103	CXCL1	4	+	309	33	4	0	6.80%	62.66587050627671	0.05352897298295963	0.00877616296920843	0.0024096315144322635
ENSG00000177189_20187899_20155398_506	RPS6KA3	X	-	1518	20	2	0	2.37%	7.730967375909093	0.004498799248433914	0.0008852284070317903	2.4782952419168947e-05
ENSG00000159210_48944734_48934495_167	SNF8	17	-	501	32	0	0	6.59%	37.47898914692217	0.0015061527253727427	0.0015061527253727427	1.3629417347018274e-05
ENSG00000015171_180013_252470_571	ZMYND11	10	+	1713	13	0	0	1.23%	4.453091365913311	0.00797940233269714	0.00797940233269714	0.000327142761693177
ENSG00000155508_154870770_154875439_152	CNOT8	5	+	456	12	0	0	3.29%	15.44159010082895	0.02108409854857798	0.02108409854857798	0.002029891263157455
ENSG00000168487_22177929_22201203_461	BMP1	8	+	1383	17	0	0	1.30%	7.212774551797182	0.012798402692974279	0.012798402692974279	0.0007972436888943567
ENSG00000079819_130956485_130858136_935	EPB41L2	6	-	2805	19	1	6	0.96%	3.97462675679091	0.006142079104304129	0.03535057243299149	0.0011498351620616329
ENSG00000144674_37251425_37361272_2196	GOLGA4	3	+	6588	18	0	4	0.36%	1.6032252017254103	0.005554612275049112	0.04471043695002634	0.0013654474274588616
ENSG00000157349_70324649_70333582_328	DDX19B	16	+	984	14	0	0	1.52%	8.348501965082319	0.019679754444124863	0.019679754444124863	0.0017849636115922674
ENSG00000100567_58244921_58271895_248	PSMA3	14	+	744	222	21	3	14.92%	175.08770711101215	1.4047062898829936e-05	4.750271130166021e-08	8.208955549314333e-12
ENSG00000159720_67439291_67438528_144	ATP6V0D1	16	-	432	26	3	0	6.25%	35.31548847134029	0.01002233431131372	0.003684287313187465	0.00020065090816454291
ENSG00000164631_6705173_6690848_697	ZNF12	7	-	2091	14	1	0	0.86%	3.9287068070975617	0.026832695913080365	0.011772051206276392	0.0015103817605807263
ENSG00000136560_161231165_161235390_170	TANK	2	+	510	15	0	0	2.94%	17.258247759750002	0.02108409854857798	0.02108409854857798	0.002029891263157455
ENSG00000141503_4884369_4897287_1191	MINK1	17	+	3573	20	1	5	0.67%	3.2845251823761554	0.005160369442893307	0.07941064379354501	0.0024770715527741216
ENSG00000127947_77585552_77639280_650	PTPN12	7	+	1950	11	0	1	1.23%	3.3100434164853856	0.004687384229717438	0.009847127393243164	0.00024498848711271347
ENSG00000112514_33417668_33416672_132	CUTA	6	-	396	119	14	7	11.36%	176.3304808988599	0.012087716311245814	0.003741986492465975	0.00024587341543500787
ENSG00000090061_99492678_99534683_378	CCNK	14	+	1134	11	0	0	1.59%	5.691873599776456	0.011772051206276392	0.011772051206276392	0.000681272511389508
ENSG00000119725_73892176_73931541_478	ZNF410	14	+	1434	8	0	0	1.05%	3.2735309558242687	0.019216965118390855	0.019216965118390855	0.0017073955890589052
ENSG00000120137_168568981_168557571_355	PANK3	5	-	1065	29	3	0	3.38%	15.978058489308454	0.0021519382791564774	0.0010073743004618205	1.3207298689024939e-05
ENSG00000158941_22605774_22619682_923	CCAR2	8	+	2769	21	2	0	0.98%	4.450122390921453	0.00600341887269475	0.0036062891729786097	0.00011840703590302771
ENSG00000151491_15669717_15621317_718	EPS8	12	-	2154	15	0	0	1.11%	4.086214650637188	0.005405141725979036	0.005405141725979036	0.00015637213408961292
ENSG00000128050_56436313_56441904_85	PAICS	4	+	255	12	0	0	7.06%	27.613196415600008	0.012798402692974279	0.012798402692974279	0.0007972436888943567
ENSG00000109775_185425868_185400392_469	UFSP2	4	-	1407	12	0	0	1.28%	5.0045238706311315	0.011772051206276392	0.011772051206276392	0.000681272511389508
ENSG00000102081_147912180_147948844_611	FMR1	X	+	1833	7	0	3	0.82%	2.2408417713150577	0.019216965118390855	0.1700871372700561	0.016246064094152867
ENSG00000104960_49851497_49860279_360	PTOV1	19	+	1080	32	5	0	4.17%	17.38608663204445	0.0035763217077886345	0.00028351878813536103	7.149954426385882e-06
ENSG00000015676_44490600_44386011_361	NUDCD3	7	-	1083	9	0	4	1.66%	4.87629161078809	0.011772051206276392	0.11411551976453232	0.007078637642502531
ENSG00000160014_46608271_46609153_113	CALM3	19	+	339	45	2	1	15.04%	77.89120670329648	0.0001889056949786359	0.00018618114937238455	2.447366381606584e-07
ENSG00000117713_26766258_26780756_1145	ARID1A	1	+	3435	12	4	1	0.61%	2.0498879435161577	0.057109797348681025	0.016854695534768073	0.00441659119964 7673
ENSG00000213619_47580667_47584481_243	NDUFS3	11	+	729	73	0	2	7.82%	58.75853352496503	5.944357765789079e-05	0.00016691480729352244	7.281488498831857e-08
ENSG00000148303_133349594_133351366_151	RPL7A	9	+	453	75	2	6	3.31%	97.14907679329473	0.021557223391537678	0.04584514077471458	0.00436099002980892
ENSG00000177697_837286_838192_124	CD151	11	+	372	75	11	2	14.52%	118.30250480473794	0.001198541136783148	0.00021401787103109384	1.764510186009213e-06
ENSG00000177697_836070_836574_135	CD151	11	+	405	107	6	8	13.33%	155.02593913572966	0.0001902876011544047	0.00043315234247867423	5.646756660764465e-07
ENSG00000161939_7014275_7017321_186	RNASEK-C17orf49	17	+	558	44	1	0	9.14%	46.26942410140861	0.00028496711628707346	0.00011368890353703816	2.2903155808076546e-07
ENSG00000183684_81891541_81888131_251	ALYREF	17	-	753	289	8	3	15.94%	225.2052357069104	1.2036435732403148e-07	1.69131332429671e-08	2.0855149418528037e-14
ENSG00000197535_52528806_52313696_1855	MYO5A	15	-	5565	13	0	1	0.32%	1.3707359406665771	0.012798402692974279	0.023672501042627626	0.0014375227604752335
ENSG00000197535_52528806_52313696_1855	MYO5A	15	-	5565	14	0	1	0.38%	1.4761771668716985	0.0078820576498062	0.015516259750072458	0.0006142571802709984
ENSG00000107362_71875080_71862581_293	ABHD17B	9	-	879	31	2	0	3.41%	20.694190146503416	0.007790523468944856	0.0023628488670607213	0.00010449788798782673
ENSG00000129103_56078432_56079612_53	SUMF2	7	+	159	12	0	0	9.43%	44.28531500615095	0.019679754444124863	0.019679754444124863	0.0017849636115922674
ENSG00000100462_22928599_22920904_551	PRMT5	14	-	1653	42	7	0	4.17%	14.909121476662435	0.0004116794586798226	9.249081916035159e-06	3.46108189490799e-08
ENSG00000154277_41256977_41268073_223	UCHL1	4	+	669	399	4	3	26.01%	349.96321241968394	2.4563964943034244e-11	1.6060151220740705e-11	4.774824478333106e-21
ENSG00000189050_59962581_59952977_252	RNFT1	17	-	756	10	0	0	1.98%	7.761645817876986	0.020613416668581838	0.020613416668581838	0.001946208561389314
ENSG00000165533_88824720_88877410_501	TTC8	14	+	1503	17	1	1	2.20%	6.6369043281008	0.00241548406293102	0.00241548406293102	3.368517373808125e-05
ENSG00000196730_87499078_87707364_1430	DAPK1	9	+	4290	17	0	4	0.56%	2.3252371107541965	0.004826937407846361	0.03906554526712903	0.001050233689227362
ENSG00000110711_67483159_67490993_330	AIP	11	+	990	13	0	0	2.42%	7.705197484656062	0.005160369442893307	0.005160369442893307	0.00014319601891785326
ENSG00000273841_69365737_69364943_264	TAF9	5	-	792	35	0	0	3.03%	25.93095307336175	0.005604866691947602	0.005604866691947602	0.00016752456572620382
ENSG00000273841_69369490_69365750_41	TAF9	5	-	123	8	1	4	12.20%	38.164580411804884	0.03418265644467716	0.22648320137553352	0.03442252230376013
ENSG00000165209_123179131_123125597_639	STRBP	9	-	1917	12	0	0	1.10%	3.6731168940938974	0.0078820576498062	0.0078820576498062	0.00031963642907451164
ENSG00000102265_47585205_47586691_140	TIMP1	X	+	420	51	8	3	12.86%	71.25190860811072	0.0024075449030187	0.00029080051227485244	4.801490498045177e-06
ENSG00000164535_6446076_6409837_631	DAGLB	7	-	1893	7	0	0	0.79%	2.1698166755522985	0.016947426762344633	0.016947426762344633	0.0013498980316300959
ENSG00000185787_78873018_78897067_362	MORF4L1	15	+	1086	32	2	2	2.21%	17.290030904795586	0.01732176123716967	0.01732176123716967	0.0014062213434849191
ENSG00000080839_37095928_36998759_1068	RBL1	20	-	3204	19	1	0	0.56%	3.4796591925088958	0.023672501042627626	0.012798402692974279	0.0014375227604752335
ENSG00000136143_47954253_47943371_132	SUCLA2	13	-	396	9	0	0	5.30%	13.335918723443184	0.005706018193000826	0.005706018193000826	0.0001733096755673334
ENSG00000162302_64361508_64371480_562	RPS6KA4	11	+	1686	12	1	5	0.89%	4.176373123355873	0.03537493353818881	0.24754287813003473	0.03918556126692674
ENSG00000211460_121756641_121765367_223	TSN	2	+	669	52	0	0	6.73%	45.60924071634979	0.00030182209906671564	0.00030182209906671564	6.150937717279372e-07
ENSG00000108465_47975891_47981502_281	CDK5RAP3	17	+	843	12	0	0	2.14%	8.352746246711746	0.009815328628645334	0.009815328628645334	0.0004837142251238724

ENSG00000173486_64242388_64244029_142	FKBP2	11	+	426	64	4	3	14.79%	88.15480545825353	0.00010806191022412408	9.696609516997229e-05	7.559710286990966e-08
ENSG00000143815_225404548_225403303_101	LBR	1	-	303	12	3	0	5.94%	23.238828666594063	0.08581700149962773	0.013217226988443315	0.005602990945683561
ENSG00000115365_210472019_210434487_353	LANCL1	2	-	1059	32	0	0	4.25%	17.730853222481592	0.00023522430645143535	0.00023522430645143535	3.796194994189714e-07
ENSG00000125107_58599337_58543385_1551	CNOT1	16	-	4653	26	3	4	0.84%	3.2788074402791754	0.004862286191257199	0.005808621511948061	0.00015155177025343995
ENSG00000116977_236539066_236548161_252	LGALS8	1	+	756	12	1	0	2.78%	9.313974981452382	0.015516259750072458	0.0078820576498062	0.0006142571802709984
ENSG00000168924_1855950_1834836_294	LETM1	4	-	882	11	0	0	1.70%	7.318123199712587	0.020613416668581838	0.020613416668581838	0.001946208561389314
ENSG00000089157_120200783_120196773_281	RPLP0	12	-	843	61	1	4	3.56%	42.459793420784706	0.0025025372014526115	0.004791578323725913	6.770248138606176e-05
ENSG00000168066_64778392_64771471_100	SF1	11	-	300	25	0	1	8.00%	48.89836865262501	0.005306578019052857	0.009288069496478249	0.0002590037054391842
ENSG00000023191_498818_494691_218	RNH1	11	-	654	33	0	0	8.26%	29.608186523607806	7.870973464443577e-05	7.870973464443577e-05	4.535791134868777e-08
ENSG00000134330_9481342_9488329_135	IAH1	2	+	405	18	0	0	5.19%	26.079129948066672	0.008674846900444944	0.008674846900444944	0.0003831153291663416
ENSG00000175224_46657603_46672332_292	ATG13	11	+	876	11	6	1	1.71%	7.368247331217467	0.172059248067656	0.03577298629450316	0.02599789514137937
ENSG00000072518_63895204_63909237_676	MARK2	11	+	2028	9	0	0	0.89%	2.604055135346894	0.011772051206276392	0.011772051206276392	0.000681272511389508
ENSG00000134759_36129934_36174641_821	ELP2	18	+	2463	23	0	0	1.46%	5.479476146213765	0.0009235399941089087	0.0009235399941089087	5.324735186779001e-06
ENSG00000079805_10775852_10831047_692	DNM2	19	+	2076	23	1	0	1.16%	6.500939185031071	0.007225456388766877	0.005604866691947602	0.00021353222845814395
ENSG00000180891_57885306_57866477_300	CUEDC1	17	-	900	10	1	3	1.67%	6.519782487016668	0.02784981298332479	0.14389369507707506	0.017662684075641494
ENSG00000112245_63576890_63580174_145	PTP4A1	6	+	435	21	0	0	5.52%	28.327330805658626	0.004873903550586949	0.004873903550586949	0.00012846764509679302
ENSG00000168724_34937437_34954714_361	DNAJC21	5	+	1083	12	0	0	1.66%	6.501722147717453	0.013217226988443315	0.013217226988443315	0.0008469697265466234
ENSG00000061987_62501630_62592749_1471	MON2	12	+	4413	5	3	0	0.34%	0.6648316608106731	0.15865525393145707	0.012673659338734126	0.011061610944025602
ENSG00000136068_58132891_58168726_979	FLNB	3	+	2937	41	0	1	1.63%	8.191350826384578	0.00019028488239266955	0.0002780838975359565	3.644087174015043e-07
ENSG00000148344_129739844_129739611_77	PTGES	9	-	231	49	4	0	16.88%	124.46857475213639	0.0014043310921045605	0.0006838816678496783	6.029980119200531e-06
ENSG00000143811_225921941_225920455_168	PYCR2	1	-	504	12	2	0	3.57%	13.970962472178575	0.07134538375819317	0.011772051206276392	0.0041729868335021346
ENSG00000143933_47176443_47160776_149	CALM2	2	-	447	251	15	2	17.45%	329.4896787062786	6.194146472294571e-06	3.981493930367033e-06	2.0710992593478474e-10
ENSG00000167881_76067278_76039706_526	SRP68	17	-	1578	64	1	0	4.18%	23.798445579984797	1.9265609889438935e-05	1.8000736937174306e-05	2.7363779275191477e-09
ENSG00000130816_10180431_10133667_1511	DNMT1	19	-	4533	20	10	0	0.79%	2.5889275262806093	0.0150551319960408	0.0007764301431553706	8.123820541617567e-05
ENSG00000130640_133298090_133279766_743	TUBGCP2	10	-	2229	15	3	1	1.35%	3.948724251894348	0.020613416668581838	0.00392533363377418	0.0004450089172968374
ENSG00000130640_133289962_133279766_495	TUBGCP2	10	-	1485	10	3	1	1.41%	3.9513833254646475	0.07708712836172119	0.015191410988288745	0.005566177142988587
ENSG00000240344_200882366_200871395_112	PPIL3	2	-	336	12	0	0	4.46%	20.956443708267862	0.019679754444124863	0.019679754444124863	0.0017849636115922674
ENSG00000133142_103586676_103587323_215	TCEAL4	X	+	645	18	0	0	3.26%	16.375267641809305	0.00847367416878415	0.00847367416878415	0.0003665024264111182
ENSG00000004897_47189172_47120935_824	CDC27	17	-	2472	13	0	2	0.97%	3.0858193809908987	0.004826937407846361	0.026606355080852302	0.0006944327269402791
ENSG00000175063_45814384_45816767_136	UBE2C	20	+	408	125	3	0	18.38%	179.77341416406253	4.9717999447271865e-06	4.9717999447271865e-06	2.071325054074169e-10
ENSG00000072778_7219985_7220863_124	ACADVL	17	+	372	23	1	3	4.84%	36.2794348067863	0.023878473930440184	0.0981223633022556	0.010346292632117097
ENSG00000161960_7577674_7578164_55	EIF4A1	17	+	165	140	4	0	12.73%	497.87429900854556	0.0291989533746507	0.008980238763039383	0.0012991156119657764
ENSG00000138668_82359635_82355334_208	HNRNPD	4	-	624	112	4	1	10.58%	105.31956325180772	1.8544589859207252e-05	1.8837272099629194e-05	2.7557411418497156e-09
ENSG00000134058_69234976_69277135_309	CDK7	5	+	927	12	1	0	2.91%	7.595863091669905	0.006306718652620871	0.002874854311667729	0.00010091605209104467
ENSG00000127125_42456566_42457380_213	PPCS	1	+	639	13	3	0	3.29%	11.937629905805167	0.03744183116399598	0.008175610787902942	0.001552076439258323
ENSG00000127125_42456566_42459926_311	PPCS	1	+	933	15	3	0	2.89%	9.43376887188907	0.018785663926082854	0.0032294771334464936	0.00034157783724479517
ENSG00000108963_2035995_2041857_326	DPH1	17	+	978	11	0	0	1.84%	6.5997798181457075	0.009815328628645334	0.009815328628645334	0.0004837142251238724
ENSG00000204628_181241686_181240313_69	RACK1	5	-	207	537	6	4	18.84%	1522.2274763165003	0.0015309397813934378	0.0007221780205579514	6.911579843828089e-06
ENSG00000113387_32588513_32601084_127	SUB1	5	+	381	63	6	7	7.87%	97.02668425560239	0.004585801871278292	0.008525693657702423	0.000208327805194355
ENSG00000145623_38869045_38933444_979	OSMR	5	+	2937	73	1	4	2.55%	14.584600251855468	6.587316430636338e-06	3.375720799499365e-05	1.8315528003360363e-09
ENSG00000147457_23249241_23260599_343	CHMP7	8	+	1029	13	1	5	2.33%	7.413163760747815	0.015516259750072458	0.05158844004651438	0.0037107121880202653
ENSG00000160392_40341842_40321882_380	C19orf47	19	-	1140	9	0	0	1.58%	4.632477030248685	0.011772051206276392	0.011772051206276392	0.000681272511389508
ENSG00000114491_124730472_124738286_342	UMPS	3	+	1026	16	1	2	2.92%	9.150571911602341	0.00542662340375733	0.011663199792667247	0.00033078808038876356
ENSG00000072274_196077096_196074327_113	TFRC	3	-	339	19	2	0	5.31%	32.88739838583629	0.037440157020026926	0.013640585738808999	0.0023980863030810032
ENSG00000171793_40983291_41010245_591	CTPS1	1	+	1773	25	3	2	2.20%	8.273835643422167	0.004587372372998262	0.0025860288909179195	6.686082635979315e-05
ENSG00000105948_139134655_139168565_389	TTC26	7	+	1167	11	0	0	1.54%	5.5309208758753226	0.012798402692974279	0.012798402692974279	0.0007972436888943567
ENSG00000188313_146533542_146516045_311	PLSCR1	3	-	933	36	1	0	5.14%	22.641045292533768	0.0002833883315400153	0.00017744757238953088	3.472812223617562e-07
ENSG00000099968_17702273_17727534_323	BCL2L13	22	+	969	14	1	0	1.55%	8.47773574163158	0.028889785561798623	0.02108409854857798	0.002731532589007073
ENSG00000107651_119892783_119915807_486	SEC23IP	10	+	1458	13	0	0	1.23%	5.2319242179763386	0.012798402692974279	0.012798402692974279	0.0007972436888943567
ENSG00000162910_228108260_228106760_164	MRPL55	1	-	492	35	2	1	6.10%	41.742509825411595	0.004407715900925203	0.0030814981005195803	7.570158080683164e-05
ENSG00000176697_27658504_27657821_227	BDNF	11	-	681	10	1	0	2.64%	8.616452626013217	0.023672501042627626	0.012798402692974279	0.0014375227604752335
ENSG00000140694_14627330_14436717_549	PARN	16	-	1647	15	0	2	1.28%	5.344084005751367	0.00797940233269714	0.029020025081745005	0.0011662184072477957
ENSG00000101361_2652661_2657390_478	NOP56	20	+	1434	32	7	6	3.77%	13.094123823297075	0.002751215209031313	0.0021352618666157126	3.3955596852472125e-05
ENSG00000105677_35547152_35547447_70	TMEM147	19	+	210	16	0	0	11.43%	44.70707991097144	0.005454749182134642	0.005454749182134642	0.00015910859015753364
ENSG00000105677_35545740_35546911_148	TMEM147	19	+	444	50	8	2	10.81%	66.07887655760136	0.003319597487424367	0.0002464402706279408	5.842970998732901e-06
ENSG00000125730_6682180_6677882_256	C3	19	-	768	16	2	0	3.12%	12.224592163156252	0.02389626957043539	0.005306578019052857	0.0006722688034155889
ENSG00000115875_38748932_38745133_121	SRSF7	2	-	363	47	1	0	9.92%	75.97432484870663	0.0010101077082154788	0.0010573555742419644	6.6110628068585484e-06
ENSG00000115875_38751256_38749319_137	SRSF7	2	-	411	124	3	3	16.79%	177.033509866438	2.3334555990505223e-05	2.681120551891437e-05	4.866556242016747e-09
ENSG00000118855_158802153_158828982_426	MFSD1	3	+	1278	19	0	0	2.11%	8.723652623473006	0.0034529206320349946	0.0034529206320349946	6.664817351595063e-05
ENSG00000138326_78033902_78035786_114	RPS24	10	+	342	107	13	2	12.28%	183.58334897652196	0.001837345062632954	0.0005706423989581065	6.673180174535586e-06
ENSG00000125743_45687717_45687553_54	SNRPD2	19	-	162	138	11	3	33.33%	499.8499906712779	0.000239322815548449	9.135877515977258e-05	1.5656884248905503e-07
ENSG00000100300_43161182_43162991_65	TSPO	22	+	195	10	0	0	9.23%	30.091303786230775	0.012798402692974279	0.012798402692974279	0.0007972436888943567
ENSG00000132912_150753626_150711149_388	DCTN4	5	-	1164	24	0	0	3.09%	12.098565439824744	0.0009430837296224684	0.0009430837296224684	5.543685104868835e-06
ENSG00000115685_241157830_241182823_292	PPP1R7	2	+	876	36	0	0	4.11%	24.114263993075348	0.001053132412899783	0.001053132412899783	6.854889198107451e-06
ENSG00000099875_2042016_2040130_129	MKNK2	19	-	387	9	0	0	3.88%	13.646056368174422	0.020613416668581838	0.020613416668581838	0.001946208561389314
ENSG00000105426_5286095_5206774_1895	PTPRS	19	-	5685	10	2	1	0.32%	1.032155538841689	0.0533594081646912	0.026602764905793736	0.006076671267284093
ENSG00000186416_119592452_119589352_675	NKRF	X	-	2025	11	0	0	0.74%	3.1874492158748153	0.02108409854857798	0.02108409854857798	0.002029891263157455

ENSG00000185187_409874_405896_410	SIGIRR	11	-	1230	31	3	6	3.17%	14.788774909574393	0.002619468406317666	0.011525312719715134	0.0001710210613826226
ENSG00000163946_56646789_56623037_980	TASOR	3	-	2940	10	1	2	0.61%	1.9958517817397963	0.01871402756203153	0.030972360806982763	0.00262334409652499
ENSG00000149100_32583888_32602399_242	EIF3M	11	+	726	116	0	1	10.74%	93.75554981329755	3.3215330747881025e-06	4.5599076484664974e-06	1.2838549877137622e-10
ENSG00000109854_20364238_20383205_242	HTATIP2	11	+	726	88	5	0	10.74%	71.12489985836365	1.332055539276809e-05	3.403588463047415e-06	3.828731031802331e-10
ENSG00000213928_24162145_24166196_393	IRF9	14	+	1179	35	0	5	3.05%	17.419266186685753	0.0010032776369550557	0.011414899784380185	7.411878122820631e-05
ENSG00000115415_190999658_190970703_550	STAT1	2	-	1650	36	7	4	2.55%	12.802481974505458	0.008046283469837703	0.0026716800954716354	0.00012053388376697416
ENSG00000061794_27755220_27755450_76	MRPS35	12	+	228	12	0	0	7.89%	30.8831802016579	0.013217226988443315	0.013217226988443315	0.0008469697265466234
ENSG00000061794_27710844_27737601_194	MRPS35	12	+	582	50	1	0	8.76%	50.410689332603106	0.00018433876575066528	0.00012102304356057305	1.5782782520252772e-07
ENSG00000107625_68901415_68946630_727	DDX50	10	+	2181	9	0	1	1.10%	2.42137726477923	0.0033278027414746884	0.010460667668897016	0.00019130306338196331
ENSG00000185630_164792559_164846676_320	PBX1	1	+	960	21	0	0	1.88%	12.835821771314066	0.013640585738808999	0.013640585738808999	0.00089863692579514
ENSG00000180530_14967961_14964716_1081	NRIP1	21	-	3243	20	0	3	0.93%	3.6187506865957455	0.0022276761777358703	0.015358647673375043	0.00020093250298549382
ENSG00000133884_65340413_65351759_385	DPF2	11	+	1155	28	0	0	3.90%	14.224979971672731	0.0002681497654631663	0.0002681497654631663	4.892331229526857e-07
ENSG00000196531_56724521_56713554_86	NACA	12	-	258	29	7	2	8.14%	65.95593911284304	0.08245741127665074	0.01371683287978855	0.005521157195977387
ENSG00000204843_74378227_74361499_1254	DCTN1	2	-	3762	37	8	2	1.44%	5.771099330612841	0.004198794703165439	0.0002950509054422764	8.798140545161517e-06
ENSG00000106400_101218196_101223958_154	ZNHIT1	7	+	462	41	0	3	11.04%	52.07358739630196	0.00012102304356057305	0.0006003689505754606	5.156549015086668e-07
ENSG00000180035_30395795_30399107_618	ZNF48	16	+	1854	7	0	0	0.81%	2.215460068403722	0.019216965118390855	0.019216965118390855	0.0017073955890589052
ENSG00000099219_5811162_5787144_479	ERMP1	9	-	1437	39	1	1	4.38%	15.925147202107519	3.346692701179964e-05	3.346692701179964e-05	8.583366146370407e-09
ENSG00000198561_57791641_57816308_887	CTNND1	11	+	2661	64	7	2	2.71%	14.11271970132131	0.0002556913665467353	1.5363660848022635e-05	3.2544366602591786e-08
ENSG00000196792_30955638_30895411_650	STRN3	14	-	1950	24	0	0	2.00%	7.221912908695386	0.0006334249406157621	0.0006334249406157621	2.5753349202917255e-06
ENSG00000107581_119080676_119036039_1382	EIF3A	10	-	4146	107	7	11	3.47%	15.14363370718054	2.092500174220995e-08	4.68537536528311e-08	9.903593743667191e-15
ENSG00000166295_72223915_72233116_110	ANAPC16	10	+	330	20	3	0	6.36%	35.562449929181824	0.027941506428179053	0.00877616296920843	0.0012176006631866223
ENSG00000172053_49100019_49098238_282	QARS	3	-	846	8	0	0	1.77%	5.548751052780143	0.019216965118390855	0.019216965118390855	0.0017073955890589052
ENSG00000204574_30583162_30590701_549	ABCF1	6	+	1647	8	3	1	1.09%	2.850178136400729	0.09373101630823749	0.028889785561798602	0.011492152405141842
ENSG00000114520_125497947_125447779_305	SNX4	3	-	915	9	0	0	1.97%	5.771610726211477	0.011772051206276392	0.011772051206276392	0.000681272511389508
ENSG00000168487_22176562_22196284_468	BMP1	8	+	1404	17	0	0	1.28%	7.104891171748934	0.012798402692974279	0.012798402692974279	0.0007972436888943567
ENSG00000137955_75791515_75794650_157	RABGGTB	1	+	471	17	1	0	5.73%	21.17891126355733	0.006112543793728684	0.0033030736763512156	0.00011113261345270447
ENSG00000169905_179852665_179846071_470	TOR1AIP2	1	-	1410	29	0	1	2.34%	12.068533539796812	0.001573068226067653	0.0017470872109163853	1.6386720308046277e-05
ENSG00000116586_156055423_156058371_49	LAMTOR2	1	+	147	23	6	0	10.20%	91.80918196003063	0.08647745899421033	0.021557223391537678	0.008337010244447522
ENSG00000242265_94664609_94665682_357	PEG10	7	+	1071	31	3	0	2.80%	16.98430731911905	0.008605408492104586	0.0022543491824521134	0.00011091378991179106
ENSG00000242265_94663557_94664534_325	PEG10	7	+	975	49	0	2	6.15%	29.48947771050616	3.77282131074845e-05	4.8247556065654664e-05	1.3786811572392767e-08
ENSG00000189043_10940057_10933636_81	NDUFA4	7	-	243	554	23	26	24.69%	1337.7627769656424	6.104755652305633e-05	4.8888426677839263e-05	2.2307239681950503e-08
ENSG00000105063_55247103_55230528_881	PPP6R1	19	-	2643	20	5	6	1.25%	4.440260490590239	0.025291128564067122	0.02118350383597727	0.002418053026064387
ENSG00000080845_36439782_36527031_566	DLGAP4	20	+	1698	17	3	1	1.59%	5.874715668513252	0.024710983489837978	0.006112543793728684	0.0007857918783425606
ENSG00000125868_17600786_17605129_118	DSTN	20	+	354	123	8	1	16.95%	203.8813337041653	0.00032866746870422836	4.91724319750471e-05	1.2158626209121111e-07
ENSG00000125868_17605175_17607146_48	DSTN	20	+	144	411	14	0	27.08%	1674.7691263524066	0.0006057074883987301	0.0007270607742110769	2.818979820093719e-06
ENSG00000127586_792984_797975_445	CHTF18	16	+	1335	10	0	0	1.12%	4.395358980011237	0.019679754444124863	0.019679754444124863	0.0017849636115922674
ENSG00000131778_147275365_147295509_470	CHD1L	1	+	1410	7	0	1	1.28%	2.9130943027095753	0.009815328628645334	0.0477903522728147	0.0023388674905236288
ENSG00000196730_87499078_87707364_1364	DAPK1	9	+	4092	14	0	4	0.51%	2.0075576572925224	0.007029302661868638	0.06548277849422955	0.002520255333567344
ENSG00000151718_183245429_183315729_1011	WWC2	4	+	3033	8	0	0	0.49%	1.5477228455825918	0.019216965118390855	0.019216965118390855	0.0017073955890589052
ENSG00000100567_58270326_58271895_89	PSMA3	14	+	267	21	1	0	7.87%	46.15126929011799	0.010042643336235583	0.008574016250729465	0.00043540252252504097
ENSG00000182899_197950968_197952624_82	RPL35A	3	+	246	89	1	6	10.98%	212.29047854066468	0.003710480643933249	0.018085401378232165	0.0003696764468647276
ENSG00000182158_138001705_137880476_520	CREB3L2	7	-	1560	24	0	0	2.12%	9.027391135869232	0.0015208721599504675	0.0015208721599504675	1.3886126472521594e-05
ENSG00000121741_20031328_20086014_757	ZMYM2	13	+	2271	11	0	0	0.79%	2.8421773060970943	0.012798402692974279	0.012798402692974279	0.0007972436888943567
ENSG00000177576_49483748_49482345_76	C18orf32	18	-	228	18	3	0	6.58%	46.324770302486854	0.09783387127182075	0.020613416668581838	0.009177765633874927
ENSG00000177674_11747543_11750192_97	AGTRAP	1	+	291	18	0	3	7.22%	36.29569631947424	0.00847367416878415	0.00837382380159347	0.000362422040249726
ENSG00000169045_179623133_179618166_184	HNRNPH1	5	-	552	108	8	58	13.04%	114.80486553225002	5.0050612623388566e-05	0.2654460648142067	0.0007015925737530442
ENSG00000169045_179618250_179616986_190	HNRNPH1	5	-	570	80	4	0	8.42%	82.35514720442107	0.0007960416953513527	0.00018810075197750367	1.0311233395192524e-06
ENSG00000132199_712362_674305_402	ENOSF1	18	-	1206	10	1	0	1.74%	4.865509318669155	0.016854695534768073	0.007029302661868638	0.0006017499134389055
ENSG00000110628_2903346_2925197_326	SLC22A18	11	+	978	11	0	0	1.53%	6.5997798181457075	0.019679754444124863	0.019679754444124863	0.0017849636115922674
ENSG00000167460_16076566_16093060_183	TPM4	19	+	549	33	10	5	7.10%	35.27095443795903	0.024107743687997098	0.006197383421659556	0.0007750763162008691
ENSG00000127990_94618900_94588678_253	SGCE	7	-	759	11	0	2	2.77%	8.504064113500002	0.007029302661868638	0.03522780890193623	0.0012822660609675817
ENSG00000148019_78240031_78266762_640	CEP78	9	+	1920	21	0	0	1.72%	6.417910885657033	0.0012558331315113088	0.0012558331315113088	9.614604369954505e-06
ENSG00000166471_9314441_9283424_291	TMEM41B	11	-	873	10	1	1	2.41%	6.72142524434708	0.016854695534768073	0.016854695534768073	0.0013361081351157898
ENSG00000091039_76410578_76355889_798	OSBPL8	12	-	2394	18	0	1	1.38%	4.411882885951129	0.0008884494972709903	0.0024579339080672683	1.3429802402146636e-05
ENSG00000145741_73499134_73504432_156	BTF3	5	+	468	630	12	7	13.46%	789.8967243885579	2.6023379003580606e-05	2.7426286549419726e-05	5.5324725093672956e-09
ENSG00000132780_45584153_45606611_140	NASP	1	+	420	11	0	2	3.57%	15.368058719396432	0.02108409854857798	0.0700082515985845	0.006564739044797468
ENSG00000138674_82881936_82819074_1067	SEC31A	4	-	3201	25	0	0	1.03%	4.582789939327555	0.0012427339549726212	0.0012427339549726212	9.422880418492092e-06
ENSG00000198189_87366003_87340486_109	HSD17B11	4	-	327	28	0	0	8.26%	50.244195312789	0.0035291660242640502	0.0035291660242640502	6.948232509684344e-05
ENSG00000151835_23365181_23330136_4432	SACS	13	-	13296	9	1	1	0.11%	0.3971889150483981	0.03537493353818881	0.03537493353818881	0.005300298232677315
ENSG00000086666_80120378_80137628_197	ZFAND6	15	+	591	20	0	3	5.58%	19.8572055442132	0.0014481341328822738	0.011821942446875977	0.00010518227444157728
ENSG00000168653_39028725_39034496_106	NDUFS5	1	+	318	174	10	13	20.75%	321.06853379459443	3.2495386787536644e-05	0.00018700811918797254	4.6512049100001946e-08
ENSG00000197885_23901047_23893095_160	NKIRAS1	3	-	480	16	0	1	5.00%	19.559347461050006	0.005306578019052857	0.009288069496478249	0.0002590037054391842
ENSG00000101294_31514552_31538328_143	HM13	20	+	429	29	12	0	5.59%	39.665809536395116	0.14132450907367944	0.005306578019052857	0.005136606310018997
ENSG00000152382_166862280_166857567_181	TADA1	1	-	543	8	0	0	2.76%	8.645015452397793	0.019679754444124863	0.019679754444124863	0.0017849636115922674
ENSG00000143157_166847517_166850409_515	POGK	1	+	1545	16	0	0	1.36%	6.0766904733359235	0.007029302661868638	0.007029302661868638	0.0002573639808771639
ENSG00000143157_166847517_166850409_515	POGK	1	+	1545	16	0	0	1.36%	6.0766904733359235	0.007029302661868638	0.007029302661868638	0.0002573639808771639
ENSG00000157193_53327912_53247018_963	LRP8	1	-	2889	17	9	0	0.73%	3.4528443077658366	0.18607691510208607	0.00847367416878415	0.010183475765332767

ENSG00000171456_32359747_32437338_1536	ASXL1	20	+	4608	24	4	0	0.72%	3.056148040789063	0.011823316657575232	0.0015061527253727427	0.00010879167933478646
ENSG00000172531_67399140_67398535_148	PPP1CA	11	−	444	82	2	1	15.54%	108.36935755446625	2.204249949405992e−05	1.3344210250438053e−05	2.3371943840383437e−09
ENSG00000198189_87391070_87337276_300	HSD17B11	4	−	900	107	1	1	9.67%	69.76167261107835	8.549824919679852e−07	1.3620900843830119e−06	1.0403411484970525e−11
ENSG00000169429_73740659_73742036_95	CXCL8	4	+	285	71	3	9	11.58%	146.1803862878474	0.001595761305259402	0.03160943490200885	0.00033868880623128824
ENSG00000151923_119588163_119577026_265	TIAL1	10	−	795	12	4	2	3.40%	8.857063001230191	0.033570409067276055	0.022974702734688237	0.003406938546080682
ENSG00000129255_7586750_7587551_127	MPDU1	17	+	381	10	0	0	4.72%	15.40106099295276	0.012798402692974279	0.012798402692974279	0.0007972436888943567
ENSG00000131174_77899554_77902785_60	COX7B	X	+	180	48	3	0	11.67%	156.47477968840005	0.017620111912137252	0.00877616296920843	0.0007669208289534906
ENSG00000111669_6867609_6870383_235	TPI1	12	+	705	289	10	3	11.91%	240.5383581380192	5.455330855611919e−06	1.6950419017174195e−06	8.025709236916846e−11
ENSG00000104823_38831772_38816280_244	ECH1	19	−	732	148	5	0	14.75%	118.63866492768035	1.204311245703574e−07	7.276325044800611e−08	8.52921042898175e−14
ENSG00000161800_50016715_49990268_632	RACGAP1	12	−	1896	91	8	2	5.38%	28.162984477144786	5.25541479825437e−06	2.520538012295458e−07	1.2893675243698857e−11
ENSG00000163882_184365001_184368294_86	POLR2H	3	+	258	13	1	7	6.98%	29.566455464377913	0.03577298629450316	0.2507237357435369	0.04009964211340507
ENSG00000082212_50921158_50947184_242	ME2	18	+	726	15	0	0	2.48%	12.12356247585744	0.013428347753762199	0.013428347753762199	0.0008725593497644525
ENSG00000163565_159014750_159054901_706	IFI16	1	+	2118	9	0	0	0.71%	2.4934012344114738	0.020613416668581838	0.020613416668581838	0.001946208561389314
ENSG00000163565_159014750_159054901_706	IFI16	1	+	2118	9	0	0	0.71%	2.4934012344114738	0.020613416668581838	0.020613416668581838	0.001946208561389314
ENSG00000187051_39532738_39529492_136	RPS19BP1	22	−	408	41	0	0	8.09%	58.96567984581252	0.0013845019718837994	0.0013845019718837994	1.1595255170738555e−05
ENSG00000143198_165649848_165653789_113	MGST3	1	+	339	79	4	2	14.16%	136.74234065689825	0.0010053033289375173	0.00023262907903552502	1.58961883112022e−06
ENSG00000154743_12496532_12532721_311	TSEN2	3	+	933	9	0	0	1.61%	5.660261323133442	0.020613416668581838	0.020613416668581838	0.001946208561389314
ENSG00000243678_51166831_51167166_111	NME2	17	+	333	46	0	0	7.21%	81.056755243991	0.005306578019052857	0.005306578019052857	0.00015100114740534233
ENSG00000113732_172983861_173020331_81	ATP6V0E1	5	+	243	55	0	0	17.28%	132.810383994784	0.0004388494700883803	0.0004388494700883803	1.268319071721425e−06
ENSG00000099204_114445395_114436260_197	ABLIM1	10	−	591	7	0	0	2.54%	6.950021940474621	0.019216965118390855	0.019216965118390855	0.0017073955890589052
ENSG00000077254_77734378_77697317_747	USP33	1	−	2241	16	0	0	0.94%	4.189418465552879	0.00837382380159347	0.00837382380159347	0.0003583838894600799
ENSG00000186575_29636751_29683055_511	NF2	22	+	1533	14	0	0	1.37%	5.358725331794521	0.007029302661868638	0.007029302661868638	0.0002573639808771639
ENSG00000104763_18071320_18057534_330	ASAH1	8	−	990	53	3	2	7.27%	31.413497437443947	6.8176351904891705e−06	2.8957473970768623e−05	1.6190192581539036e−09
ENSG00000149792_65122359_65127072_95	MRPL49	11	+	285	15	0	0	5.26%	30.8831802016579	0.020613416668581838	0.020613416668581838	0.001946208561389314
ENSG00000003989_17543340_17565146_657	SLC7A2	8	+	1971	17	0	0	1.37%	5.061018368917049	0.0033030736763512156	0.0033030736763512156	6.124050385493983e−05
ENSG00000148187_122291788_122322617_96	MRRF	9	+	288	13	2	1	6.25%	26.486616353505216	0.05067994629101479	0.019795881618345192	0.004478851500628176
ENSG00000100243_42631082_42619773_257	CYB5R3	22	−	771	36	0	1	3.50%	27.398307727540864	0.003684287313187465	0.00510952817114065	0.00010325221210932265
ENSG00000059377_139953368_140020040_309	TBXAS1	7	+	927	11	0	0	1.62%	6.962874500697413	0.016947426762344633	0.016947426762344633	0.0013498980316300959
ENSG00000140694_14630125_14558411_446	PARN	16	−	1338	18	0	2	1.79%	7.89390704706054	0.0053557511377634245	0.016228925652561413	0.00045523357042063324
ENSG00000197912_89508547_89545958_468	SPG7	16	+	1404	13	0	0	1.28%	5.43315207251389	0.011772051206276392	0.011772051206276392	0.000681272511389508
ENSG00000198898_116862612_116910231_174	CAPZA2	7	+	522	43	7	1	8.62%	48.336318438227025	0.006540261968451337	0.00041261355168930957	1.899011618243904e−05
ENSG00000085662_134459062_134450883_102	AKR1B1	7	−	306	72	0	6	11.76%	138.06598207800002	0.0010447178140462346	0.006557658047460363	4.247547070520692e−05
ENSG00000134352_55960410_55941082_569	IL6ST	5	−	1707	21	0	3	1.58%	7.218739836239896	0.0036062891729786097	0.01975957366273817	0.00039589275246000024
ENSG00000134352_55976278_55956219_329	IL6ST	5	−	987	13	0	0	2.13%	7.728617537800914	0.0078820576498062	0.0078820576498062	0.00031963642907451164
ENSG00000081177_69209471_69241100_621	EXD2	14	+	1863	9	0	0	0.97%	2.834688037833334	0.012178957859018494	0.012178957859018494	0.0007262454987071801
ENSG00000135446_57752292_57752176_38	CDK4	12	−	114	19	0	2	18.42%	97.79673730525002	0.008674846900444944	0.024135905425855194	0.0010389365785287079
ENSG00000135446_57750669_57748525_97	CDK4	12	−	291	50	2	0	16.49%	100.82137866520621	0.000266410292493954	0.00019829743686022551	3.634959756323822e−07
ENSG00000198732_69879679_70030258_434	SMOC1	14	+	1302	22	0	6	2.07%	9.914876593158988	0.0034782380676658805	0.028515402634699986	0.0005687569814023099
ENSG00000108582_30379392_30464814_1243	CPD	17	+	3729	60	0	2	2.09%	9.441358388278362	3.1818196403583947e−06	6.257721656729047e−06	1.6845581808243292e−10
ENSG00000099956_23787179_23834180_382	SMARCB1	22	+	1146	28	7	4	3.93%	14.336694474068066	0.012730851315274157	0.004999209642455344	0.0003352925029903522
ENSG00000159079_32612243_32601863_258	CFAP298	21	−	774	29	0	0	5.04%	21.985313037614347	0.0006311913977015671	0.0006311913977015671	2.5578527397951546e−06
ENSG00000134982_112767311_112844126_2729	APC	5	+	8187	14	0	1	0.26%	1.0034110093613047	0.007029302661868638	0.008838362722921076	0.00032011746987943644
ENSG00000163399_116373512_116384977_105	ATP1A1	1	+	315	9	0	0	6.67%	16.76515496661429	0.006937202941512724	0.006937202941512724	0.00025101522741487095
ENSG00000151743_31709409_31672304_240	AMN1	12	−	720	9	0	0	2.08%	7.334755297893752	0.016947426762344633	0.016947426762344633	0.0013498980316300959
ENSG00000150991_124913771_124911714_685	UBC	12	−	2055	42	16	6	1.90%	11.992592603855478	0.024066422912456	0.004243599845194191	0.0005599777061919586
ENSG00000234857_62727156_62707247_746	HNRNPUL2-BSCL2	11	−	2238	59	8	6	4.02%	15.469189010750004	1.917483988636437e−05	2.7614637140838583e−05	4.1418260895281564e−09
ENSG00000234857_62705595_62702488_118	HNRNPUL2-BSCL2	11	−	354	14	0	6	5.08%	23.206005462262716	0.013428347753762199	0.16970534604944526	0.012520549700299675
ENSG00000234857_62707195_62690367_462	HNRNPUL2-BSCL2	11	−	1386	17	17	2	2.38%	7.197162485667751	0.45573689993954414	0.00550296840388912	0.030297291298871185
ENSG00000142910_31577149_31586979_467	TINAGL1	1	+	1401	25	2	2	2.36%	10.470742752168096	0.004400313145429775	0.006771542009076099	0.00016010923667865434
ENSG00000102898_67865131_67870913_127	NUTF2	16	+	381	42	0	0	5.51%	64.68445617040159	0.008877961307018026	0.008877961307018026	0.00040023486315785033
ENSG00000188603_28491759_28477516_438	CLN3	16	−	1314	26	2	0	2.51%	11.610571552221463	0.004407715900925203	0.0015208721599504675	3.948464526818382e−05
ENSG00000105854_95434951_95416188_84	PON2	7	−	252	214	6	7	17.86%	498.2976615077025	0.001378973783002602	0.0016955003789966925	1.404270945860997e−05
ENSG00000114416_180947925_180976138_453	FXR1	3	+	1359	13	0	0	1.77%	5.613057770279251	0.004687384229717438	0.004687384229717438	0.00011928172701435494
ENSG00000101365_2664188_2660905_133	IDH3B	20	−	399	32	3	0	10.53%	47.06008411681204	0.0009086099620780121	0.00043491588733509306	2.563447471756744e−06
ENSG00000138668_82373678_82355334_306	HNRNPD	4	−	918	154	4	1	12.09%	98.43593166672224	5.3242387479675336e−08	5.399052639268365e−08	2.8419590530610048e−14
ENSG00000172046_49117460_49108958_1225	USP19	3	−	3675	19	0	0	0.65%	3.0336947082444903	0.005306578019052857	0.005306578019052857	0.00015100114740534233
ENSG00000136003_108562623_108567704_154	ISCU	12	+	462	42	1	2	5.19%	53.343674893772736	0.006346985027636522	0.005808022449631236	0.00019501864856894435
ENSG00000140859_57771650_57759149_687	KIFC3	16	−	2061	31	5	3	2.18%	8.825906423472345	0.006894428981681446	0.0020295981827775276	8.055352678869601e−05
ENSG00000099250_33263760_33180076_735	NRP1	10	−	2205	117	6	6	5.99%	31.135287795140822	3.775062577620317e−08	1.2258227511860406e−08	4.748626217357416e−15
ENSG00000103549_30762546_30769455_838	RNF40	16	+	2514	16	2	4	1.31%	3.7344816154749414	0.009446187005438042	0.019533210169997005	0.0009068893338487036
ENSG00000116171_53015021_53050704_143	SCP2	1	+	429	16	0	0	3.50%	21.8845845718042	0.020613416668581838	0.020613416668581838	0.001946208561389314
ENSG00000133027_17577012_17505834_196	PEMT	17	−	588	17	4	0	3.06%	16.96474014478827	0.0873743558752767	0.013428347753762199	0.005787156622962468
ENSG00000285130_69035783_69099934_171	AL358113.1	9	+	513	18	0	0	4.68%	20.588786801105268	0.005306578019052857	0.005306578019052857	0.00015100114740534233
ENSG00000132128_46286112_46278705_617	LRRC41	1	−	1851	17	0	1	1.13%	5.389123287485415	0.00847367416878415	0.011825808327677989	0.0005036791746826746
ENSG00000112514_33417668_33416650_156	CUTA	6	−	468	163	2	6	16.03%	204.37010488148402	6.966821039712242e−06	3.1649250629192755e−05	1.808117256872564e−09
ENSG00000277957_7572842_7578486_406	SENP3-EIF4A1	17	+	1218	212	7	5	6.40%	102.13255324489165	2.216511804978622e−05	7.1775503821723165e−06	1.2981956091804373e−09
ENSG00000277957_7563464_7572395_481	SENP3-EIF4A1	17	+	1443	11	2	1	1.66%	4.473031643899169	0.03318103322835444	0.010499663139468709	0.001693572322031727

ENSG00000259112_78079744_78038664_86	NDUFC2-KCTD14	11	-	258	63	3	5	13.95%	143.28359186583143	0.0014987505965367745	0.005655335537214311	5.0172599989173626e-05
ENSG00000171988_63207708_63177711_950	JMJD1C	10	-	2850	8	4	0	0.53%	1.6471029440884215	0.17004230409153215	0.019679754444124863	0.016523685284367155
ENSG00000100714_64388428_64459935_964	MTHFD1	14	+	2892	52	5	1	3.11%	10.550685352433613	1.1147445449505758e-05	8.158381672037628e-07	8.365813824253027e-11
ENSG00000171720_141636790_141621468_428	HDAC3	5	-	1284	9	0	3	1.64%	4.112946896015188	0.006937202941512724	0.07524961812757737	0.0029212478031785537
ENSG00000067334_93879148_93869851_756	DNTTIP2	1	-	2268	41	2	2	2.25%	10.607582617765214	0.0002285314373396388	0.0002285314373396388	3.5897701454782663e-07
ENSG00000168028_39407654_39408799_108	RPSA	3	+	324	36	0	1	4.63%	65.19782487016668	0.021557223391527678	0.03980790073005672	0.0037960056923654355
ENSG00000162736_160344590_160358271_689	NCSTN	1	+	2067	124	1	5	4.64%	35.20114782540204	4.1982093850167133e-07	2.3676354330365974e-06	9.148504942478631e-12
ENSG00000102119_154379971_154381197_182	EMD	X	+	546	16	2	1	3.85%	17.195030734989015	0.03299602752967379	0.013892891352654344	0.0021461203908066497
ENSG00000111057_52949174_52952842_430	KRT18	12	+	1290	1059	31	101	10.47%	481.7057897965571	8.375690291877957e-09	1.1586575176866824e-08	1.0097463044095238e-15
ENSG00000111057_52949174_52952842_430	KRT18	12	+	1290	1059	31	101	10.47%	481.7057897965571	8.375690291877957e-09	1.1586575176866824e-08	1.0097463044095238e-15
ENSG00000132669_19975025_20000936_562	RIN2	20	+	1686	28	2	1	2.31%	9.744870621163704	0.003181324321652496	0.0010499093600357017	2.0285467582265932e-05
ENSG00000258289_64914496_64932230_112	CHURC1	14	+	336	48	23	0	13.39%	83.82577483307145	0.006197250200377523	0.00030416618013389714	1.3816785432125349e-05
ENSG00000144895_150552379_150583911_507	EIF2A	3	+	1521	25	7	1	2.56%	9.644648649432941	0.03477146179107616	0.0010322727522116193	0.0002683312982183043
ENSG00000134107_4979719_4983692_412	BHLHE40	3	+	1236	76	5	8	6.55%	36.08034968543205	1.527771579977133e-05	6.93484284044965e-05	8.377605784900168e-09
ENSG00000134108_5122466_5178713_186	ARL8B	3	+	558	17	8	0	3.76%	17.87682294827151	0.2617390908898303	0.007029302661868638	0.01434881016164812
ENSG00000166206_26621357_26547793_334	GABRB3	15	-	1002	27	0	1	2.99%	15.811448546357788	0.0024321290225855155	0.0031727927861210656	4.407497946231007e-05
ENSG00000198668_90397231_90404717_149	CALM1	14	+	447	141	12	2	24.83%	185.09181154416447	8.159490752671504e-07	6.532200639335521e-08	5.311029901629442e-13
ENSG00000106588_42932158_42917574_164	PSMA2	7	-	492	66	7	2	9.15%	78.71444709934758	0.0020970238279720637	0.0009656485456496491	1.2376916447494947e-05
ENSG00000121940_108950323_108934670_513	CLCC1	1	-	1539	22	2	0	1.95%	8.388024252302147	0.00946955908897248	0.0022543491824521134	0.0001223697458287481
ENSG00000002586_2691361_2740804_185	CD99	X	+	555	61	6	1	9.73%	64.49298352021893	0.00023580046066088578	8.893984923091428e-05	1.50415514072071e-07
ENSG00000069329_46682109_46660472_740	VPS35	16	-	2220	75	0	5	4.32%	19.82366296728041	2.539100626534125e-07	3.7461440237701814e-06	9.129518999647536e-12
ENSG00000120533_109336137_109343481_96	ENY2	8	+	288	145	1	4	29.17%	295.4276439429428	1.9038365037503747e-06	6.4844824125302795e-06	1.0667943006982838e-10
ENSG00000196923_177496512_177483644_423	PDLIM7	5	-	1269	16	0	4	2.36%	7.398334737040191	0.0020333931625601446	0.02689622310494778	0.00034298044785809853
ENSG00000103254_721275_721971_145	FAM173A	16	+	435	15	0	0	4.14%	20.23380771832759	0.013428347753762199	0.013428347753762199	0.0008725593497644525
ENSG00000109861_88337672_88294006_463	CTSC	11	-	1389	147	12	6	10.58%	62.09987206856049	3.6115280951439485e-08	2.964845814087886e-09	1.1628914456561082e-15
ENSG00000138399_169560769_169529825_651	FASTKD1	2	-	1953	19	0	0	1.38%	5.708565311212751	0.0036062891729786097	0.0036062891729786097	7.240558534568731e-05
ENSG00000177556_151751803_151746325_74	ATOX1	5	-	222	31	3	7	6.76%	81.9378069314257	0.03770178143563168	0.08588638879785576	0.013090394844661306
ENSG00000138614_65607272_65579408_482	INTS14	15	-	1446	14	0	0	1.45%	5.681138266695022	0.00837382380159347	0.00837382380159347	0.0003583838894600799
ENSG00000066322_43365239_43363916_191	ELOVL1	1	-	573	40	1	0	8.90%	40.96198421162305	0.00017035554405485864	0.00013106574077567133	1.5769200789774585e-07
ENSG00000066322_43368009_43365268_87	ELOVL1	1	-	261	6	0	0	5.75%	13.489205145551727	0.016947426762344633	0.016947426762344633	0.0013498980316300959
ENSG00000126214_103654586_103693618_621	KLC1	14	+	1863	52	4	7	3.38%	16.37819755192593	0.00018571932730564617	0.0006376289743136012	8.13594806396098e-07
ENSG00000156515_69343848_69401135_889	HK1	10	+	2667	60	7	3	2.70%	13.200909422530936	0.00020078835655311822	4.3403317049897516e-05	6.539831550755316e-08
ENSG00000101365_2660289_2659949_84	IDH3B	20	-	252	7	1	0	5.95%	16.29945621754167	0.04178282491678763	0.019216965118390855	0.003600525600033436
ENSG00000160752_155318926_155320609_117	FDPS	1	+	351	19	3	0	8.55%	31.76304288546582	0.007456024827607967	0.00228120298834518	9.684751958738432e-05
ENSG00000150459_21147208_21147342_44	SAP18	13	+	132	19	0	1	15.91%	84.46081858180683	0.00847367416878415	0.011825808327677989	0.0005036791746826746
ENSG00000152291_85327962_85324879_447	TGOLN2	2	-	1341	56	2	3	4.92%	24.503880488116337	3.503787992526855e-05	8.434738688455117e-05	2.231575800975992e-08
ENSG00000100612_60156135_60149110_354	DHRS7	14	-	1062	42	1	0	3.11%	23.206005462262716	0.0019534759119410605	0.001565547007472977	1.8169309371855918e-05
ENSG00000177576_49483748_49482345_76	C18orf32	18	-	228	18	3	0	6.58%	46.324770302486854	0.09783387127182075	0.020613416668581838	0.009177765633874927
ENSG00000179085_155140240_155139962_92	DPM3	1	-	276	56	0	0	14.13%	119.05689758900003	0.0007028601028765856	0.0007028601028765856	3.1471554975732053e-06
ENSG00000189077_75994570_75987172_343	TMEM120A	7	-	1029	15	4	4	2.04%	8.553650493170556	0.059434471961489266	0.059434471961489266	0.013708788036758825
ENSG00000165092_72930953_72927095_95	ALDH1A1	9	-	285	719	51	21	38.95%	1480.3337709994687	9.066679005540261e-07	3.1694098946150004e-07	2.6562249088075358e-12
ENSG00000172340_67654586_67360629_440	SUCLG2	3	-	1320	30	1	0	2.73%	13.335918723443184	0.0015639623262203096	0.0009470236307512361	9.098239545706659e-06
ENSG00000132436_50446933_50445263_556	FIGNL1	7	-	1668	11	0	0	0.90%	3.8696550732293176	0.020613416668581838	0.020613416668581838	0.001946208561389314
ENSG00000148634_67955130_67922931_374	HERC4	10	-	1122	12	0	1	1.87%	6.275726458090911	0.008175610787902942	0.013599199788808391	0.0005580304998096723
ENSG00000168259_41997186_41976733_421	DNAJC7	17	-	1263	46	5	3	4.04%	21.371258508510692	0.0019323170733234999	0.0006999552747219783	8.470534155928047e-06
ENSG00000174915_450456_490582_487	PTDSS2	11	+	1461	17	0	0	1.16%	6.827698292358318	0.005306578019052857	0.005306578019052857	0.00015100114740534233
ENSG00000174243_48845782_48830469_820	DDX23	12	-	2460	33	2	0	1.34%	7.8714447099347575	0.0026957942880510015	0.001595761305259402	2.531988771243731e-05
ENSG00000105677_35545740_35546116_101	TMEM147	19	+	303	30	5	1	8.91%	58.097071666485164	0.020021357285181146	0.0035547763465261306	0.00039653063614353707
ENSG00000151694_9555605_9523325_258	ADAM17	2	-	774	27	3	0	3.10%	20.469084552261634	0.02163521358803487	0.005706018193000826	0.0006454818958744852
ENSG00000159720_67481086_67438528_351	ATP6V0D1	16	-	1053	44	4	0	4.27%	24.518840122113964	0.0009915726665809088	0.0003010435303081121	1.986855280261905e-06
ENSG00000183255_44873616_44851581_180	PTTG1IP	21	-	540	60	0	0	10.56%	65.19782487016668	5.8491771070895074e-05	5.8491771070895074e-05	2.5460565826646392e-08
ENSG00000090615_132808453_132775111_1185	GOLGA3	12	-	3555	11	5	0	0.42%	1.815635629295781	0.17808035639161363	0.02108409854857798	0.01834527900567475
ENSG00000171848_10122799_10123909_163	RRM2	2	+	489	14	0	1	4.29%	16.799439537098163	0.00847367416878415	0.013892891352654344	0.0005886798845679606
ENSG00000162736_160351737_160353331_137	NCSTN	1	+	411	28	0	1	6.57%	39.97530867951826	0.0033030736763512156	0.006112543793728684	0.0001113261345270447
ENSG00000015171_180013_252470_547	ZMYND11	10	+	1641	16	0	0	1.28%	5.7211985260840965	0.008674846900444944	0.008674846900444944	0.00038311532916634116
ENSG00000167526_89562377_89563042_57	RPL13	16	+	171	102	1	6	12.28%	350.00937561878953	0.008877961307018026	0.010353108236244411	0.0004630492946106523
ENSG00000111641_6567918_6556993_808	NOP2	12	-	2424	26	4	2	2.10%	6.293849430535893	0.0010891986948716168	0.0002707893389517208	1.9824595550127703e-06
ENSG00000215041_7329312_7316123_1562	NEURL4	17	-	4686	11	2	0	0.45%	1.3774188352852115	0.04363948305378087	0.0078820576498062	0.0017710903275458955
ENSG00000108561_5439073_5433015_282	C1QBP	17	-	846	261	16	17	14.89%	181.02800309695218	7.303800097410925e-07	2.754645405434158e-06	1.806048825447839e-11
ENSG00000168066_64778392_64765389_638	SF1	11	-	1914	69	10	7	3.76%	21.153526250978846	0.00024054445896067397	0.00021049759311091902	3.4846980072473654e-07
ENSG00000179262_12946003_12953049_290	RAD23A	19	+	870	32	0	0	4.83%	21.582728232882765	0.0004441315507112266	0.0004441315507112266	1.2979723423445215e-06
ENSG00000089737_94062219_94051191_485	DDX24	14	-	1455	10	0	0	1.24%	4.032855146608249	0.011570465654371866	0.011570465654371866	0.0006594843062896773
ENSG00000101246_63707058_63700443_190	ARFRP1	20	-	570	16	1	0	2.63%	16.471029440884216	0.02784981298332479	0.019679754444124863	0.002477909089056847
ENSG00000163762_149333382_149324876_137	TM4SF18	3	-	411	41	1	3	8.03%	58.53527342358031	0.001595761305259402	0.005089012094854324	4.7683448559002785e-05
ENSG00000142208_104776759_104770341_418	AKT1	14	-	1254	19	0	0	1.91%	8.890612482295456	0.0053557511377634245	0.0053557511377634245	0.00015366972994913032
ENSG00000163946_56682928_56623788_1486	TASOR	3	-	4458	12	3	5	0.54%	1.5794897007577393	0.042056994326668116	0.06582357386012391	0.011084902001483645
ENSG00000128708_171925548_171983352_413	HAT1	2	+	1239	59	0	0	5.08%	27.94192494435715	2.348557756166878e-05	2.348557756166878e-05	4.302860942433267e-09

ENSG00000123268_50814087_50819779_136	ATF1	12	+	408	27	1	2	4.41%	38.83105745943751	0.013428347753762199	0.044040755831095145	0.0027917083341200354
ENSG00000151881_43453969_43446222_215	TMEM267	5	-	645	47	0	1	2.79%	42.75764328694652	0.011772051206276392	0.026832695913080365	0.0015103817605807263
ENSG00000177697_836070_838192_253	CD151	11	+	759	213	23	10	17.00%	164.66960510686368	5.628074008217504e-07	7.179261916658316e-08	3.96916788384252e-13
ENSG00000095787_28590832_28619606_444	WAC	10	+	1332	12	0	0	1.58%	5.286310124608109	0.008175610787902942	0.008175610787902942	0.00034251843268961324
ENSG00000204394_31785726_31777594_895	VARS	6	-	2685	42	1	2	1.45%	9.178688194012292	0.0007096150488534449	0.0007096150488534449	3.2057057589297512e-06
ENSG00000204392_31806757_31797757_95	LSM2	6	-	285	15	6	4	7.37%	30.8831802016579	0.1990553367779263	0.067234255889369	0.04887712954565338
ENSG00000102471_79533424_79551120_140	NDFIP2	13	+	420	13	0	0	3.57%	18.162251213832146	0.02108409854857798	0.02108409854857798	0.002029891263157455
ENSG00000254772_62573842_62559679_437	EEF1G	11	-	1311	177	0	2	1.60%	79.22207095207897	0.00877616296920843	0.00877616296920843	0.00039161135667564577
ENSG00000169100_1392009_1386602_298	SLC25A6	X	-	894	73	15	11	8.39%	47.913837740156055	0.0015724362773696912	0.00023994000431142186	2.591135257069784e-06
ENSG00000159063_78139588_78102894_467	ALG8	11	-	1401	53	5	0	3.85%	22.197974634596363	0.0007799372344069463	8.332796070245443e-05	4.817754366714235e-07
ENSG00000170525_6213664_6225187_416	PFKFB3	10	+	1248	46	13	3	4.57%	21.62812459635337	0.002674329485664681	0.0002944342621990559	5.423138123191577e-06
ENSG00000187514_231707766_231712851_131	PTMA	2	+	393	178	8	9	7.63%	265.7682326768627	0.0025025372014526115	0.004926212446124825	6.957895768210636e-05
ENSG00000071082_101002702_101006091_121	RPL31	2	+	363	182	8	8	9.92%	294.19844941414055	0.0011610472579390045	0.004747109690211661	3.33708560158967e-05
ENSG00000143889_38602626_38564182_508	HNRNPLL	2	-	1524	32	3	6	1.57%	12.320848794362208	0.020086935144256027	0.054053662918446446	0.0048393408464938855
ENSG00000169045_179623133_179620884_134	HNRNPH1	5	-	402	101	6	7	15.67%	147.4249323556754	5.218963683194363e-05	0.000352797469487041	1.38137441163045e-07
ENSG00000168256_42022407_42023893_157	NKIRAS2	17	+	471	14	1	1	3.82%	17.441456334694273	0.012798402692974279	0.01634402150441562	0.0010049622802917066
ENSG00000168256_42022407_42023893_157	NKIRAS2	17	+	471	14	1	1	3.82%	17.441456334694273	0.012798402692974279	0.01634402150441562	0.0010049622802917066
ENSG00000168256_42022407_42023893_157	NKIRAS2	17	+	471	14	1	1	3.82%	17.441456334694273	0.012798402692974279	0.01634402150441562	0.0010049622802917066
ENSG00000173786_41968125_41973924_401	CNP	17	+	1203	35	3	1	4.74%	17.071749654283046	0.00019105397311604708	7.701705110099018e-05	1.0641296742635947e-07
ENSG00000175354_12884141_12785823_387	PTPN2	18	-	1161	8	1	0	1.29%	4.043275960940569	0.04223451612662591	0.019679754444124863	0.003715148010809822
ENSG00000087470_32679364_32743410_710	DNM1L	12	+	2130	36	12	0	3.10%	9.917415614053523	0.0007125289164387594	1.3171720342437143e-05	8.600839255139515e-08
ENSG00000175567_73981586_73981512_24	UCP2	11	-	72	19	0	0	20.83%	154.84483406664586	0.02108409854857798	0.02108409854857798	0.002029891263157455
ENSG00000133142_103586676_103587323_215	TCEAL4	X	+	645	18	0	0	3.26%	16.375267641809305	0.00847367416878415	0.00847367416878415	0.0003665024264111182
ENSG00000099622_1270934_1272443_172	CIRBP	19	+	516	28	1	0	6.98%	31.840798192406982	0.0016269198037485093	0.0008776897154483972	8.807164582131405e-06
ENSG00000072210_19648972_19655358_182	ALDH3A2	17	+	546	13	6	0	3.30%	13.970962472178575	0.17808035639161363	0.012798402692974279	0.012843613314113225
ENSG00000240065_32857958_32858570_127	PSMB9	6	+	381	12	0	0	3.94%	18.481273191543313	0.019679754444124863	0.019679754444124863	0.0017849636115922674
ENSG00000105429_42334007_42376775_2728	MEGF8	19	+	8184	13	1	10	0.37%	0.932080340885814	0.004004657782222479	0.33811242438423683	0.014991278359044154
ENSG00000158089_31138086_30910901_552	GALNT14	2	-	1656	10	0	0	0.91%	3.5433600472916673	0.020613416668581838	0.020613416668581838	0.001946208561389314
ENSG00000266086_58007137_58005747_201	AC015813.2	17	-	603	120	0	22	13.93%	116.77222364805972	1.5853614593892364e-06	7.80495470659366e-05	1.197920011383821e-09
ENSG00000105854_95434951_95416238_50	PON2	7	-	150	49	3	4	22.00%	191.68160511829004	0.006279669515525777	0.012926124733057015	0.0004178513437537143
ENSG00000128564_101164141_101162996_381	VGF	7	-	1143	16	4	4	2.10%	8.213899196241472	0.05423379212399942	0.04727912037993979	0.010247738400701924
ENSG00000156113_77637360_77090430_309	KCNMA1	10	-	927	11	3	0	1.94%	6.962874500697413	0.08620755130569058	0.012798402692974279	0.005490655705532311
ENSG00000156113_77637360_76877869_1083	KCNMA1	10	-	3249	15	4	2	0.74%	2.7090508948822722	0.048994651353958696	0.02389626957043539	0.005091569865673637
ENSG00000156113_77637360_76877869_1087	KCNMA1	10	-	3261	15	4	2	0.74%	2.6990819863454467	0.048994651353958696	0.02389626957043539	0.005091569865673637
ENSG00000073350_75543427_75564542_356	LLGL2	17	+	1068	9	0	0	1.40%	4.944778852512641	0.020613416668581838	0.020613416668581838	0.001946208561389314
ENSG00000147669_100151356_100153316_58	POLR2K	8	+	174	12	1	0	12.07%	40.46761543665518	0.026832695913080365	0.007029302661868638	0.0009647702379235399
ENSG00000044115_138781925_138933823_841	CTNNA1	5	+	2523	98	8	5	5.71%	22.79210524593223	5.097274940946299e-08	4.742927035014322e-09	2.5861596824591767e-15
ENSG00000179958_30424382_30424233_49	DCTPP1	16	-	147	16	0	0	14.29%	63.86725701567348	0.00797940233269714	0.00797940233269714	0.000327142761693177
ENSG00000126214_103654586_103701203_602	KLC1	14	+	1806	52	4	7	3.49%	16.89511740821595	0.00018571932730564617	0.0006376289743136012	8.13594806396098e-07
ENSG00000197535_52528806_52313696_1853	MYO5A	15	-	5559	14	0	1	0.38%	1.477770450376147	0.0078820576498062	0.015516259750072458	0.0006142571802709984
ENSG00000103035_74301126_74305733_244	PSMD7	16	+	732	57	1	6	8.20%	45.69191824917419	4.948520556132759e-05	0.0004777140390012938	1.8031238186048593e-07
ENSG00000101266_489860_483961_177	CSNK2A1	20	-	531	9	0	0	2.82%	9.945430912398308	0.019216965118390855	0.019216965118390855	0.0017073955890589052
ENSG00000113163_75511207_75379346_598	COL4A3BP	5	-	1794	14	4	0	1.17%	4.579111445730771	0.0750151653743942	0.008175610787902942	0.0033080285523474006
ENSG00000145979_13326898_13305101_293	TBC1D7	6	-	879	17	0	0	1.71%	11.348426854534132	0.02108409854857798	0.02108409854857798	0.002029891263157455
ENSG00000197885_23901047_23893095_160	NKIRAS1	3	-	480	16	0	1	5.00%	19.559347461050006	0.005306578019052857	0.009288069496478249	0.0002590037054391842
ENSG00000088812_3471108_3615845_1272	ATRN	20	+	3816	24	2	1	1.26%	3.6904429171792463	0.0010105108356577906	0.00025750057329094263	1.7539774912624636e-06
ENSG00000184277_101650135_101642479_182	TM2D3	15	-	546	20	5	0	5.49%	21.49378841873627	0.020822301124399242	0.0020587098026626535	0.0002610826352726224
ENSG00000126945_101412265_101413338_357	HNRNPH2	X	+	1071	11	0	0	1.96%	6.026689693880954	0.0078820576498062	0.0078820576498062	0.00031963642907451164
ENSG00000133657_194428845_194405960_184	ATP13A3	3	-	552	23	0	3	3.26%	24.449184326312505	0.013640585738808999	0.06341282918854552	0.0041393610649128765
ENSG00000157168_32647829_32760410_446	NRG1	8	+	1338	16	0	0	1.12%	7.016806264053813	0.021557223391537678	0.021557223391537678	0.002115616449879074
ENSG00000088766_6009811_6037158_187	CRLS1	20	+	561	7	0	0	2.67%	7.321680867772729	0.019216965118390855	0.019216965118390855	0.0017073955890589052
ENSG00000140416_63048576_63060943_152	TPM1	15	+	456	69	6	7	10.53%	88.78914307976646	0.002408131853863617	0.0027952235058118125	3.863785555762664e-05
ENSG00000088766_6009811_6037158_187	CRLS1	20	+	561	7	0	0	2.67%	7.321680867772729	0.019216965118390855	0.019216965118390855	0.0017073955890589052
ENSG00000205133_92916989_92886622_86	TRIQK	8	-	258	7	0	0	5.81%	15.920399096203491	0.019216965118390855	0.019216965118390855	0.0017073955890589052
ENSG00000082212_50908093_50947039_433	ME2	18	+	1299	25	0	0	2.54%	11.292925785825638	0.0014915192737735051	0.0014915192737735051	1.337646624223462e-05
ENSG00000114786_51986441_51989075_254	ABHD14A-ACY1	3	+	762	7	1	0	1.97%	5.390371347533466	0.0477903522728147	0.016947426762344633	0.0036975118224100542
ENSG00000166508_100098685_100098120_92	MCM7	7	-	276	22	0	0	11.96%	46.77235262425001	0.0012690167775026367	0.0012690167775026367	9.80942059614775e-06
ENSG00000166508_100101294_100099577_95	MCM7	7	-	285	30	1	2	7.37%	61.7663604033158	0.010353108236244411	0.014886438171519604	0.0007560214316058348
ENSG00000146066_176388758_176388841_27	HIGD2A	5	+	81	19	0	2	18.52%	137.6398525036852	0.02108409854857798	0.04584514077471458	0.004277928753731027
ENSG00000146066_176388820_176389497_106	HIGD2A	5	+	318	37	11	2	9.43%	68.27319396781606	0.04474275303150902	0.005459156962569303	0.00134745681282221
ENSG00000113712_149525134_149496853_264	CSNK1A1	5	-	792	30	0	0	4.92%	22.22653120573864	0.0006289630342484525	0.0006289630342484525	2.5404679232419015e-06
ENSG00000130985_47198803_47214929_1058	UBA1	X	+	3174	80	3	3	2.74%	14.789676719130439	4.81084946039956e-06	1.2929724131792992e-06	5.4619632483004856e-11
ENSG00000117410_43976115_43978007_158	ATP6V0B	1	+	474	48	3	0	11.39%	59.42080241331647	0.0001789774272272485	8.751142344759464e-05	1.1253437756231516e-07
ENSG00000152795_82429324_82426059_298	HNRNPDL	4	-	894	114	3	1	11.41%	74.82434934764096	2.8068292164810636e-07	1.8169410532370092e-07	4.81845735322702e-13
ENSG00000184840_177592278_177593793_121	TMED9	5	+	363	118	3	0	20.66%	190.74404962015706	5.502524763219458e-06	5.654801326551865e-06	2.594435231415209e-10
ENSG00000184840_177594091_177595416_114	TMED9	5	+	342	36	5	3	8.77%	61.7663604033158	0.03154446286322828	0.006112543793728684	0.0010146316358673928
ENSG00000102081_147912180_147948844_632	FMR1	X	+	1896	7	0	3	0.79%	2.1663834213188298	0.019216965118390855	0.1700871372700561	0.016246064094152867

ENSG00000102081_147912180_147948844_582	FMR1	X	+	1746	7	0	3	0.86%	2.352498835521478	0.019216965118390855	0.1700871372700561	0.016246064094152867
ENSG00000197879_1483656_1465726_963	MYO1C	17	−	2889	71	10	0	3.63%	14.42070269713967	1.85668876691052e-05	7.500225446215369e-08	1.670861245916329e-11
ENSG00000107796_88948930_88941209_211	ACTA2	10	−	633	21	0	0	2.37%	19.466649131850716	0.019679754444124863	0.019679754444124863	0.0017849636115922674
ENSG00000085365_78360672_78475668_338	SCAMP1	5	+	1014	22	0	0	2.66%	12.730936217251482	0.0034782380676658805	0.0034782380676658805	6.758309314600117e-05
ENSG00000119004_203278578_203296908_234	CYP20A1	2	+	702	11	0	0	2.56%	9.194565045792737	0.012798402692974279	0.012798402692974279	0.0007972436888943567
ENSG00000131732_81304758_81312662_271	ZCCHC9	5	+	813	11	0	0	2.95%	7.939218526625463	0.004687384229717438	0.004687384229717438	0.0001192817270143 5494
ENSG00000197451_178204865_178210623_274	HNRNPAB	5	+	822	102	20	7	10.22%	72.81216938055111	0.0005272710709344839	1.3691309628042567e-05	6.391815525073465e-08
ENSG00000146729_55964610_55999072_286	NIPSNAP2	7	+	858	42	1	1	5.59%	28.723517250493014	0.00019160400119445356	0.00022282222326679875	2.954165305707878e-07
ENSG00000141295_47838673_47837844_160	SCRN2	17	−	480	12	0	0	4.38%	14.669510595787504	0.007029302661868638	0.007029302661868638	0.0002573639808771639
ENSG00000111237_110493141_110492005_87	VPS29	12	−	261	31	0	11	12.64%	69.69422658535059	0.001573068226067653	0.041293795919607114	0.00045724949912209686
ENSG00000011485_46347103_46391174_484	PPP5C	19	+	1452	20	0	7	1.86%	8.08237498390496	0.0034529206320349946	0.09499524564278244	0.002277815931089166
ENSG00000134255_111147787_111184310_392	CEPT1	1	+	1176	39	0	0	4.08%	19.459554871963014	0.00017828355461003195	0.00017828355461003195	2.2186407606589736e-07
ENSG00000162772_212615193_212619555_124	ATF3	1	+	372	15	0	0	4.84%	23.660500960947587	0.013428347753762199	0.013428347753762199	0.0008725593497644525
ENSG00000130559_135850224_135811309_1283	CAMSAP1	9	−	3849	13	1	0	0.39%	1.9818512626161344	0.03577298629450316	0.02108409854857798	0.003354604907114977
ENSG00000103051_70514452_70481010_647	COG4	16	−	1941	28	2	0	1.39%	8.464632595199383	0.009067227940747451	0.003684287313187465	0.00018148670210371202
ENSG00000112081_36596763_36601989_164	SRSF3	6	+	492	114	23	9	18.90%	135.9613177170549	4.910866830745771e-05	3.821106563651327e-06	1.6251474483041573e-09
ENSG00000168528_31424824_31434199_341	SERINC2	1	+	1023	16	0	0	1.47%	9.177406433337245	0.02108409854857798	0.02108409854857798	0.002029891263157455
ENSG00000124762_36684102_36685800_164	CDKN1A	6	+	492	29	2	8	6.71%	34.58665099819818	0.007205323565060537	0.012371379225907928	0.0004534421781350234 7
ENSG00000151694_9555541_9490177_783	ADAM17	2	−	2349	29	11	4	1.53%	7.244202763351853	0.06552994569900335	0.008796285366707597	0.0030125141411416986
ENSG00000182093_39390698_39391468_135	WRB	21	+	405	13	0	0	3.70%	18.834927184714818	0.020613416668581838	0.020613416668581838	0.001946208561389314
ENSG00000127955_80135161_80217493_354	GNAI1	7	+	1062	26	4	0	2.54%	14.365622429019778	0.015528142895806445	0.0036062891729786097	0.0003077713301577895
ENSG00000120158_4841257_4860275_170	RCL1	9	+	510	11	0	0	4.12%	12.656048357150002	0.0078820576498062	0.0078820576498062	0.00031963642907451164
ENSG00000115525_85848138_85847466_124	ST3GAL5	2	−	372	11	0	2	4.03%	17.35103403802823	0.019216965118390855	0.08350359792828288	0.007323531272552117 5
ENSG00000185630_164792559_164846676_320	PBX1	1	+	960	21	0	0	1.88%	12.835821771314066	0.013640585738808999	0.013640585738808999	0.00089863692579514
ENSG00000147862_14313508_14088309_419	NFIB	9	−	1257	8	1	0	1.19%	3.7344816154749414	0.03418265644467716	0.019216965118390855	0.0029560527921161815
ENSG00000134480_87411304_87394446_270	CCNH	5	−	810	24	0	0	2.96%	17.38608663204445	0.005655335537214311	0.005655335537214311	0.00017039952270558162
ENSG00000100138_41682358_41674933_128	SNU13	22	−	384	51	2	2	11.72%	77.93177504012111	0.0002798052361462181	0.000679565994910149	1.2702632058293115e-06
ENSG00000171824_11099762_11066718_837	EXOSC10	1	−	2511	21	3	0	1.08%	4.90736316227061	0.025244179843020544	0.0034782380676658805	0.0004984738509773108
ENSG00000169764_63856320_63891227_497	UGP2	2	+	1491	40	8	0	4.23%	15.741929546116705	0.0010006558440764162	2.032182481103021e-05	1.8207368289573541e-07
ENSG00000124193_43460126_43461063_186	SRSF6	20	+	558	16	2	0	4.30%	16.82524512778495	0.008170681041463092	0.005405141725979036	0.0002324897789 2435937
ENSG00000124357_71070780_71078508_293	NAGK	2	+	879	16	2	0	2.05%	10.680872333679183	0.07134538375819317	0.011772051206276392	0.004172988335021346
ENSG00000172053_49104733_49103886_100	QARS	3	−	300	13	1	0	5.00%	25.427151699365005	0.03577298629450316	0.02108409854857798	0.003354604907114977
ENSG00000101224_3796532_3804961_539	CDC25B	20	+	1617	66	12	16	5.94%	23.950221380877558	0.00011918856764145169	0.000380920683222518	3.2031198499322335e-07
ENSG00000104763_18084058_18057534_395	ASAH1	8	−	1185	107	3	2	7.09%	52.98354881854052	1.3915529916405591e-06	5.690060522923631e-06	6.938368705859939e-11
ENSG00000041353_54877586_54889413_218	RAB27B	18	+	654	21	2	1	4.59%	18.841573242295876	0.009621867080838033	0.003768080243855145	0.00019653720836328292
ENSG00000168036_41224069_41239342_781	CTNNB1	3	+	2343	117	8	3	5.76%	29.301455223339957	6.403151454483205e-08	1.1032790393731706e-08	7.305397733044446e-15
ENSG00000166946_43185499_43189324_124	CCNDBP1	15	+	372	17	0	2	5.65%	26.815234422407265	0.008574016250729465	0.028581075458438136	0.001221280685253724
ENSG00000141425_36040849_35993151_276	RPRD1A	18	−	828	12	0	0	2.54%	8.504064113500002	0.008175610787902942	0.008175610787902942	0.0003425184326896132 4
ENSG00000167461_16111902_16121948_127	RAB8A	19	+	381	23	0	0	6.30%	35.422440283791346	0.005306578019052857	0.005306578019052857	0.00015100114740534233
ENSG00000090263_141010603_141010415_62	MRPS33	7	−	186	12	7	2	11.29%	37.85680153751614	0.14351487215180903	0.023672501042627626	0.015574533941414816
ENSG00000163320_88055976_88055473_167	CGGBP1	3	−	501	14	1	0	4.19%	16.397057751778448	0.016854695534768073	0.007029302661868638	0.0006017499134389055
ENSG00000122958_69132979_69171184_223	VPS26A	10	+	669	26	6	0	5.83%	22.804620358174894	0.008478974447796736	0.0005460508606305346	3.2011587677781556e-05
ENSG00000055917_20327318_20251585_1050	PUM2	2	−	3150	32	1	0	1.43%	5.960943988129525	0.0004235771399443867	0.0002753938703632887	7.837420488361 42e-07
ENSG00000164053_48451738_48465554_661	ATRIP	3	+	1983	19	0	0	0.91%	5.622202749772316	0.013428347753762199	0.013428347753762199	0.0008725593497644525
ENSG00000112245_63576466_63576531_21	PTP4A1	6	+	63	77	1	1	38.10%	717.1760735718335	0.0053557511377634245	0.00934169773149884	0.0002626789418661369 7
ENSG00000182054_90087202_90084266_160	IDH2	15	−	480	12	0	0	3.75%	14.669510595787504	0.013217226988443315	0.013217226988443315	0.0008469697265466234
ENSG00000108219_80489234_80517976_213	TSPAN14	10	+	639	89	2	3	9.86%	81.72685089358922	3.362201902229704e-05	3.9627284348366385e-05	1.0168661766816096e-08
ENSG00000074603_65507278_65446884_770	DPP8	15	−	2310	5	0	0	0.65%	1.2700874974707794	0.012673659338734126	0.012673659338734126	0.000782701129001274
ENSG00000169682_28975152_28984299_476	SPNS1	16	+	1428	28	0	2	3.15%	11.505498506500002	0.0002582331582969843	0.000924854567236639	1.607372876378084e-06
ENSG00000168476_22141470_22138689_169	REEP4	8	−	507	19	3	5	7.10%	21.989798920707106	0.011794527126255325	0.0257879318104385	0.0014544118605536
ENSG00000131778_147275365_147295509_470	CHD1L	1	+	1410	7	0	1	1.28%	2.9130943027095753	0.009815328628645334	0.0477903522728147	0.002338867490523628 8
ENSG00000105355_4861394_4839192_433	PLIN3	19	−	1299	28	2	0	3.23%	12.648076880124714	0.00036930943616753716	0.0004030602366301852	9.890178257390305e-07
ENSG00000062822_50413769_50415728_137	POLD1	19	+	411	13	0	0	3.65%	18.559964744062047	0.02108409854857798	0.02108409854857798	0.002029891263157455
ENSG00000062822_50402016_50413484_602	POLD1	19	+	1806	16	5	1	1.33%	5.198497664066446	0.07477566955963268	0.009288069496478249	0.003642757640968572
ENSG00000123143_14433522_14471784_942	PKN1	19	+	2826	30	7	1	1.80%	6.229091548105097	0.004706093564504747	0.00018298304573899598	6.625668378962383e-06
ENSG00000123144_12734441_12730961_104	TRIR	19	−	312	27	3	3	13.46%	50.779075139264435	0.0010499093600357017	0.002747448181791879	1.750572093836995e-05
ENSG00000123146_14381524_14408121_835	ADGRE5	19	+	2505	88	4	0	4.79%	20.61344403080719	1.0925247204044555e-07	1.1653223005960751e-08	1.3249646480684923e-14
ENSG00000166986_57514727_57516581_205	MARS	12	+	615	18	1	4	2.93%	17.174061185312198	0.016503128830616266	0.0865972937396412	0.006743508880899142
ENSG00000126088_45012992_45013706_100	UROD	1	+	300	11	1	0	6.00%	21.515282207155003	0.023672501042627626	0.012798402692974279	0.0014375227604752335
ENSG00000185630_164792559_164846676_320	PBX1	1	+	960	21	0	0	1.88%	12.835821771314066	0.013640585738808999	0.013640585738808999	0.00089863692579514
ENSG00000110344_118396518_118396440_943	UBE4A	11	+	2829	29	0	0	1.27%	6.015069738817075	0.0010032776369550557	0.0010032776369550557	6.244443566103777e-06
ENSG00000141582_79839251_79833959_560	CBX4	17	−	1680	32	0	0	1.79%	11.17676997774286	0.002446120450917513	0.002446120450917513	3.450690187599895e-05
ENSG00000164111_121671628_121668468_107	ANXA5	4	−	321	120	3	2	24.30%	219.3571677874767	6.829623287082529e-06	6.797917988279537e-06	3.8372924089845753e-10
ENSG00000164111_121696589_121678397_163	ANXA5	4	−	489	250	3	4	23.93%	299.98999173389575	6.377198686805677e-08	3.336633307727165e-08	2.1205145073060317e-14
ENSG00000086189_62403341_62391971_247	DIMT1	5	−	741	32	2	1	4.86%	25.340045293668023	0.002484757332982506	0.0015592205693390887	2.2874853984014616e-05
ENSG00000148444_22316454_22318310_105	COMMD3	10	+	315	16	0	0	7.62%	29.804719940647626	0.005306578019052857	0.005306578019052857	0.00015100114740534233
ENSG00000156136_70993836_71026715_199	DCK	4	+	597	14	0	1	3.02%	13.760344947472365	0.013428347753762199	0.02034245439037237	0.0012955184805712684

ENSG00000141905_3381826_3456600_391	NFIC	19	+	1173	43	3	3	3.07%	21.510279816500006	0.0027101569075239007	0.0010032776369550557	1.6561952297087845e-05
ENSG00000116133_54887119_54852233_516	DHCR24	1	-	1548	60	0	4	5.62%	22.743427280290703	8.562155290090387e-07	9.231964907111184e-06	7.199084724192089e-11
ENSG00000151332_36320588_36305297_298	MBIP	14	-	894	17	1	0	3.36%	11.158017007981545	0.001801896632038876	0.002046028879997488	2.1716970494348987e-05
ENSG00000166130_98644701_98613585_350	IKBIP	12	-	1050	38	1	2	6.00%	21.235862957711433	3.1741589596316336e-05	8.437897964260508e-05	2.032733796604195e-08
ENSG00000162434_64883427_64834562_1136	JAK1	1	-	3408	68	0	9	2.29%	11.708060099924298	3.6284287822867264e-06	1.8825641902534283e-05	5.774344777922703e-10
ENSG00000017260_130934676_131016247_843	ATP2C1	3	+	2529	17	3	1	1.19%	3.9443523942805467	0.011168917508652896	0.00324779312858951	0.00020011016427610653
ENSG00000167397_31091310_31091134_58	VKORC1	16	-	174	56	0	12	15.52%	188.8488720377242	0.0035547763465261306	0.06638642199807827	0.001507226391593381
ENSG00000167397_31094729_31091237_156	VKORC1	16	-	468	29	48	3	6.41%	36.360325408362186	0.10910614416461617	0.007868611133155495	0.004964567759925797
ENSG00000186635_72726879_72685664_1356	ARAP1	11	-	4068	13	2	1	0.44%	1.8751586798941744	0.023672501042627626	0.023672501042627626	0.0025184098398201636
ENSG00000114491_124730472_124731550_66	UMPS	3	+	198	10	0	0	7.58%	29.635374940984857	0.019679754444124863	0.019679754444124863	0.0017849636115922674
ENSG00000114491_124735213_124744084_388	UMPS	3	+	1164	10	1	2	1.80%	5.04106893326031	0.026832695913080365	0.04912109528954385	0.0056458782638956085
ENSG00000131370_15256922_15256086_95	SH3BP5	3	-	285	7	0	0	5.26%	14.412150760773688	0.016947426762344633	0.016947426762344633	0.0013498980316300959
ENSG00000104213_17577253_17642801_375	PDGFRL	8	+	1125	14	1	0	2.40%	7.302156385458669	0.0064006895615519015	0.002943990679779778	0.00010465593861806192
ENSG00000067248_55289387_55256488_1053	DHX29	5	-	3159	18	0	0	1.14%	3.3434781984700863	0.0008442728493916082	0.0008442728493916082	4.480017286136276e-06
ENSG00000171148_9791466_9780357_432	TADA3	3	-	1296	22	2	2	2.55%	9.960778799608798	0.004302923267342423	0.008090339536935701	0.00018665864242267414
ENSG00000150768_112026225_112062535_418	DLAT	11	+	1254	16	0	3	2.15%	7.48683156403828	0.002967235347054297	0.02365112532335208	0.00040680116263351043
ENSG00000213463_70416963_70372991_145	SYNJ2BP	14	-	435	9	0	1	3.45%	12.140284630996554	0.016947426762344633	0.0477903522728147	0.0036975118224100542
ENSG00000184056_91009831_90998975_493	VPS33B	15	-	1479	11	0	0	1.42%	4.364154605913794	0.0078820576498062	0.0078820576498062	0.00031963642907451164
ENSG00000111328_123267253_123261736_87	CDK2AP1	12	-	261	16	0	0	8.05%	35.97121372147127	0.00847367416878415	0.00847367416878415	0.0003665024264111182
ENSG00000047621_4536194_4489807_552	C12orf4	12	-	1656	10	0	2	0.91%	3.5433600472916673	0.020613416668581838	0.06945717695039447	0.006391023138248565
ENSG00000137168_36871871_36855813_147	PPIL1	6	-	441	64	0	3	6.80%	85.15634268756465	0.0023628488670607213	0.010805326709914683	0.000146158664627034
ENSG00000272398_106974646_106971661_80	CD24	6	-	240	41	6	0	8.75%	100.24165573788127	0.032657852871826265	0.00877616296920843	0.0014291000018004636
ENSG00000196497_24187138_24183013_591	IPO4	14	-	1773	28	1	5	2.71%	9.266695920632827	0.0004320536919546781 3	0.002053904701162150 5	5.8024133979673115e-06
ENSG00000115762_131120942_131146773_236	PLEKHB2	2	+	708	27	4	0	6.78%	22.377219552896193	0.004020532480401752	0.000146984680121020 5	4.620862033087646e-06
ENSG00000093010_19962677_19968736_221	COMT	22	+	663	44	2	1	6.79%	38.94168725276924	0.0002617448232484793	0.00044023920707255256	7.756332432131463e-07
ENSG00000096746_68337893_68342054_297	HNRNPH3	10	+	891	138	4	1	13.47%	90.88181648568688	6.351109318260742e-08	1.7126556029015377e-08	1.1061018665298157e-14
ENSG00000198000_92321744_92297836_1099	NOL8	9	-	3297	10	1	1	0.64%	1.779740442315742	0.016854695534768073	0.016854695534768073	0.0013361081351157898
ENSG00000108384_58696694_58734222_242	RAD51C	17	+	726	15	0	1	4.13%	12.12356247585744	0.0017901637611834264	0.004053443387328760 5	4.213999707786468e-05
ENSG00000111907_125153952_125262843_131	TPD52L1	6	+	393	5	0	0	3.82%	7.465399794293895	0.012673659338734126	0.012673659338734126	0.000782701129001274
ENSG00000115525_85848138_85840144_290	ST3GAL5	2	-	870	11	0	3	1.72%	7.41906283005345	0.019216965118390855	0.13115678761539135	0.012020477827111095
ENSG00000104738_47964595_47976778_581	MCM4	8	+	1743	51	3	3	4.82%	17.169134604364032	9.386699493371726e-06	4.3620794767387785e-06	3.416033078293889e-10
ENSG00000129696_33512382_33499173_400	TTI2	8	-	1200	16	2	0	1.75%	7.823738984420002	0.04912109528954385	0.007029302661868638	0.0018325026027891263
ENSG00000057663_106316102_106078293_159	ATG5	6	-	477	11	0	0	3.14%	13.531624029657236	0.02108409854857798	0.02108409854857798	0.002029891263157455
ENSG00000145860_159203617_159158670_663	RNF145	5	-	1989	34	2	1	2.71%	10.03043459541026	0.00041617781144845243	0.00011977551446593921	3.5184401231069343e-07
ENSG00000167272_120581362_120579318_163	POP5	12	-	489	29	0	1	6.75%	34.79883904113191	0.0014915192737735051	0.0018842494102203086	1.6757239882151976e-05
ENSG00000140688_31508373_31490835_468	C16orf58	16	-	1404	33	4	0	3.42%	13.791847568689105	0.0016335188667720405	0.00017828355461003195	2.0673971369923424e-06
ENSG00000079819_130956485_130858136_747	EPB41L2	6	-	2241	19	1	0	1.20%	4.974934427844044	0.006142079104304129	0.0033278027414746945	0.00011243016581512157
ENSG00000061273_47797459_47783841_824	HDAC7	12	-	2472	18	0	3	0.73%	4.272672989064321	0.013007239811447113	0.06272892732212154	0.003936255353260731
ENSG00000134440_57602474_57601652_83	NARS	18	-	249	18	0	0	9.64%	42.4178619637229	0.005454749182134642	0.005454749182134642	0.00015910859015753364
ENSG00000005884_50070855_50075549_294	ITGA3	17	+	882	51	7	0	5.44%	33.92948028957654	0.0018254975572948215	0.0001876660804935657	2.432608097223915e-06
ENSG00000005884_50056503_50070858_121	ITGA3	17	+	363	27	4	2	6.61%	43.64482491308679	0.032563251835678174	0.012088529595134127	0.0018764760318108222
ENSG00000107959_3172772_3138031_1038	PITRM1	10	-	3114	28	4	1	1.45%	5.276124555967246	0.0028612383254294944	0.0002554476795345014	5.142102490072575e-06
ENSG00000198563_31540519_31531292_248	DDX39B	6	-	744	84	4	32	8.47%	66.24940269065324	3.932261952824847e-05	0.008265048871407773	3.612716775288172e-06
ENSG00000186184_27622933_27623250_105	POLR1D	13	+	315	15	2	0	5.71%	27.94192494435715	0.044040755831095145	0.013428347753762199	0.0027917083341200354
ENSG00000123213_65758595_65822915_681	NLN	5	+	2043	40	2	1	2.50%	11.488603501350957	0.00028963977910633935	0.0001852583231847433	3.696982800547629e-07
ENSG00000087460_58895650_58910829_320	GNAS	20	+	960	302	38	15	19.69%	184.59134166365942	1.6503038806255647e-08	3.509861194884339e-11	8.183374169022172e-18
ENSG00000136527_185925496_185917715_188	TRA2B	3	-	564	41	1	3	8.51%	42.65602371824735	0.00022282222326679875	0.001013197191032157	1.5404611290049819e-06
ENSG00000007255_45178218_45163192_173	TRAPPC6A	19	-	519	8	0	0	2.89%	9.044784953086706	0.019216965118390855	0.019216965118390855	0.0017073955890589052
ENSG00000115207_27343497_27326675_892	GTF3C2	2	-	2676	21	0	0	1.01%	4.604779110785315	0.0033278027414746945	0.0033278027414746945	6.211806171561056e-05
ENSG00000182093_39380385_39406629_172	WRB	21	+	516	19	0	0	4.65%	21.60625591627617	0.005405141725979036	0.005405141725979036	0.00015637213408961292
ENSG00000108825_42961207_42950593_307	PTGES3L-AARSD1	17	-	921	26	2	1	2.93%	16.564919673853424	0.010402771787637745	0.005642787686764809	0.0003061675497078111
ENSG00000145725_103148006_103201634_983	PPIP5K2	5	+	2949	13	0	3	0.92%	2.5866888809120048	0.00308494966027208	0.03441276307238556	0.0006340181607701747
ENSG00000164941_94828985_94866163_582	INTS8	8	+	1746	16	0	0	1.20%	5.377140195477664	0.00847367416878415	0.00847367416878415	0.0003665024264111182
ENSG00000106268_2244575_2251001_156	NUDT1	7	+	468	34	3	0	7.05%	42.6293470304936	0.003453048488940946	0.0016186486669924592	3.275367415488609e-05
ENSG00000136450_58005932_58005406_108	SRSF1	17	-	324	46	0	0	11.11%	83.30833177854632	0.0010197273511756433	0.0010197273511756433	6.442859155158972e-06
ENSG00000161526_75667373_75706521_308	SAP30BP	17	+	924	24	3	0	3.57%	15.241049969649355	0.00592586945981591	0.001484234770590415	5.217299829332742e-05
ENSG00000137713_111766361_111741596_556	PPP2R1B	11	-	1668	16	6	0	1.26%	5.6285891974244615	0.059434471961489266	0.00797940233269714	0.0025019228726421154
ENSG00000161671_50476593_50482728_355	EMC10	19	+	1065	60	2	3	5.07%	33.05805204684508	0.00027730861539238227	0.00018648948553842 59	3.564784065792814e-07
ENSG00000160209_43719295_43756063_312	PDXK	21	+	936	86	8	1	9.29%	53.91358595033014	1.3237746793483629e-05	1.1244914719207622e-06	1.3462516683410196e-10
ENSG00000160213_43776266_43774202_97	CSTB	21	-	291	194	2	1	17.53%	391.1869492210001	0.0001431077334901677 8	0.000156920298718341 4	1.5842697113989606e-07
ENSG00000179262_12946003_12951527_264	RAD23A	19	+	792	30	0	0	4.92%	22.22653120573864	0.0006698694257094773	0.0006698694257094773	2.868630583058064e-06
ENSG00000164828_886593_895422_241	SUN1	7	+	723	13	0	0	2.07%	10.550685352433613	0.02108409854857798	0.02108409854857798	0.002029891263157455
ENSG00000215301_41334253_41347719_661	DDX3X	X	+	1983	97	12	3	7.26%	28.702824564627086	1.8411069105942975e-07	2.168363292173117e-09	4.7152014957107245e-15
ENSG00000135506_57716187_57719469_333	OS9	12	+	999	36	0	2	5.11%	21.145240498432436	0.00012177496818858337	0.0005029797262019704	4.3292016791638394e-07
ENSG00000135506_57720182_57720909_106	OS9	12	+	318	6	0	0	4.72%	11.071328751537738	0.016947426762344633	0.016947426762344633	0.0013498980316300959
ENSG00000258947_89923402_89933796_164	TUBB3	16	+	492	50	8	2	8.54%	59.632156893445135	0.005294700847588866 6	0.00095416369115764	3.136506323248823e-05
ENSG00000198668_90401333_90404717_113	CALM1	14	+	339	91	5	2	25.66%	157.51332911111066	6.726673991946336e-06	1.6026805246815267e-06	9.396668403386252e-11

ENSG00000137942_93448282_93552416_605	FNBP1L	1	+	1815	7	3	0	0.99%	2.263064995493389	0.1097457788509939	0.009815328628645334	0.005898503241319083
ENSG00000170190_75100099_75106033_360	SLC16A5	17	+	1080	9	0	1	1.39%	4.8898368652625015	0.019216965118390855	0.04178282491678763	0.003600525600033436
ENSG00000101439_23637745_23633916_107	CST3	20	−	321	235	6	5	26.17%	429.5744535838085	2.855582722436658e−06	1.900663467930204e−06	4.702211580522806e−11
ENSG00000140694_14630125_14436717_464	PARN	16	−	1392	15	0	0	1.72%	6.323064911977372	0.004873903550586949	0.004873903550586949	0.00012846764509679302
ENSG00000121057_57105813_57120324_787	AKAP1	17	+	2361	22	5	0	1.14%	5.467670192415503	0.05150015748280293	0.0033030736763512156	0.0010579038602935387
ENSG00000174437_110281790_110346470_1042	ATP2A2	12	+	3126	278	13	16	8.06%	52.183287851937635	3.644137479150839e−14	2.422899263333288e−13	1.2006899187622587e−25
ENSG00000076043_114439529_114449854_79	REXO2	11	+	237	15	8	2	10.13%	37.138001508322795	0.1539609423056087	0.02389626957043539	0.016983116543564356
ENSG00000148358_130086423_130128765_332	GPR107	9	+	996	15	0	0	2.11%	8.837054575775605	0.007029302661868638	0.007029302661868638	0.0002573639808771639
ENSG00000155957_66169858_66144749_131	TMBIM4	12	−	393	39	0	1	8.40%	58.23011839549238	0.001588175367824666	0.001759868229841518	1.6653375375657385e−05
ENSG00000214114_38872573_38864670_74	MYCBP	1	−	222	14	0	0	8.11%	37.004170872256765	0.013428347753762199	0.013428347753762199	0.0008725593497644525
ENSG00000160789_156129898_156139106_549	LMNA	1	+	1647	187	12	10	10.75%	66.62291393836705	1.3886043932350163e−10	3.2067672992944225e−10	5.130839405609402e−19
ENSG00000135454_57632132_57626744_533	B4GALNT1	12	−	1599	13	0	0	1.13%	4.7705725514756105	0.012798402692974279	0.012798402692974279	0.0007972436888943567
ENSG00000188529_23972001_23967663_88	SRSF10	1	−	264	21	0	0	7.95%	46.67571553205115	0.00847367416878415	0.00847367416878415	0.0003665024264111182
ENSG00000167112_128322408_128309550_331	TRUB2	9	−	993	33	4	0	3.02%	19.500255776877648	0.007459848152910933	0.002046028879997488	8.7920393864264e−05
ENSG00000175854_128284597_128288716_64	SWI5	9	+	192	15	0	0	10.94%	45.84222061183595	0.00847367416878415	0.00847367416878415	0.0003665024264111182
ENSG00000167969_2246975_2239979_243	ECI1	16	−	729	58	2	0	6.58%	46.684862252711945	0.0003656024900126997	0.0002051554554784822	5.12466363037456e−07
ENSG00000137868_74196092_74180080_560	STRA6	15	−	1680	53	2	9	3.39%	18.51152527563661	0.00021186981035795232	0.0026742133664407067	4.061495374133696e−06
ENSG00000130164_11106660_11131316_597	LDLR	19	+	1791	98	12	1	5.86%	32.10747154410218	4.1217997060369484e−05	1.2145633119755274e−07	6.178659406039773e−11
ENSG00000177426_3456398_3457940_252	TGIF1	18	+	756	34	0	2	2.78%	26.38959578078175	0.008574016250729465	0.013648824643852793	0.0005848802985757839
ENSG00000089248_112022170_112022652_160	ERP29	12	+	480	32	3	2	10.62%	39.11869492210001	0.0007977588895183646	0.0005818042179531959	2.969077245756971e−06
ENSG00000169991_18921081_18908561_282	IFFO2	1	−	846	15	0	0	2.48%	10.403908223962768	0.007029302661868638	0.007029302661868638	0.0002573639808771639
ENSG00000149084_43834084_43855248_147	HSD17B12	11	+	441	20	0	0	6.12%	26.61135708986395	0.0036062891729786097	0.0036062891729786097	7.240558534568731e−05
ENSG00000172893_71438902_71435136_202	DHCR7	11	−	606	60	4	3	10.89%	58.097071666485164	5.691956773028159e−05	7.589008455545774e−05	3.19797153423464e−08
ENSG00000017260_130934676_131016189_792	ATP2C1	3	+	2376	16	2	1	1.14%	3.9513833254646475	0.007152939217714809	0.004669667620227399	0.00017843109580844045
ENSG00000150316_94966377_94963385_70	CWC15	11	−	210	13	0	1	8.57%	36.32450242766429	0.011772051206276392	0.015555382717448078	0.0008874794703752884
ENSG00000161956_7563077_7571483_574	SENP3	17	+	1722	14	2	1	1.92%	4.7705725514756105	0.009446187005438042	0.0024579339080672683	0.00013174815516271736
ENSG00000180357_64674256_64681382_944	ZNF609	15	+	2832	10	0	0	0.53%	2.071964773416314	0.020613416668581838	0.020613416668581838	0.001946208561389314
ENSG00000139644_49753028_49762896_200	TMBIM6	12	+	600	628	23	17	35.00%	614.1635102769701	5.86889946281294e−13	1.8413772565497805e−12	1.394250288303154e−23
ENSG00000167699_771387_760173_109	GLOD4	17	−	327	9	0	2	4.59%	16.149919921967893	0.019216965118390855	0.08350359792828288	0.0073235312725521175
ENSG00000196504_152694610_152656306_854	PRPF40A	2	−	2562	43	1	1	1.99%	9.848383382027519	0.00016188098650472748	0.00016188098650472748	1.8399422460768125e−07
ENSG00000101182_62143303_62139130_166	PSMA7	20	−	498	109	15	4	10.24%	128.43185983460546	0.0029136331567189634	0.00017606549270215196	3.791311287112626e−06
ENSG00000101182_62139707_62136857_136	PSMA7	20	−	408	75	5	0	17.65%	107.86404849843753	2.599647901600642e−05	6.746866635113938e−06	1.4374834868097512e−09
ENSG00000162909_223759210_223769969_208	CAPN2	1	+	624	16	1	0	2.88%	15.045651893115387	0.02015986069794795	0.013217226988443315	0.0012659333861537747
ENSG00000162909_223771817_223774857_63	CAPN2	1	+	189	15	0	0	9.52%	46.56987490726191	0.012798402692974279	0.012798402692974279	0.0007972436888943567
ENSG00000125868_17600786_17607146_148	DSTN	20	+	444	534	22	1	22.30%	705.7224016351826	1.2950821190747694e−06	2.493208979762966e−07	3.023290449207889e−12
ENSG00000109390_139295798_139292550_76	NDUFC1	4	−	228	19	2	0	7.89%	48.89836865262501	0.016822681477512567	0.013428347753762199	0.0010798218650012886
ENSG00000014216_65182702_65211286_714	CAPN1	11	+	2142	30	5	5	2.38%	8.218213218928573	0.0033811758088108748	0.0017177785361068316	3.389112207118646e−05
ENSG00000166415_53699831_53517699_541	WDR72	15	−	1623	20	0	2	1.48%	7.230812370073939	0.005306578019052857	0.02389626957043539	0.0006722688034155889
ENSG00000131023_149695083_149661729_968	LATS1	6	−	2904	17	0	0	0.52%	3.435009368159608	0.02108409854857798	0.02108409854857798	0.002029891263157455
ENSG00000156113_77637360_76877739_1140	KCNMA1	10	−	3420	15	4	2	0.70%	2.5735983501381585	0.048994651353958696	0.02389626957043539	0.005091569865673637
ENSG00000138760_76213543_76161713_438	SCARB2	4	−	1314	77	5	1	8.22%	34.38515421234818	4.1973368744213245e−07	8.065083516506614e−08	3.2914084380969047e−13
ENSG00000131016_151240563_151353740_1782	AKAP12	6	+	5346	56	10	0	1.46%	6.146596284056118	0.0001828101181634415	2.7312900469374966e−06	4.887709699872272e−09
ENSG00000118855_158805389_158828982_384	MFSD1	3	+	1152	17	0	0	2.08%	8.659086115569012	0.005405141725979036	0.005405141725979036	0.00015637213408961292
ENSG00000197586_25207334_25225597_380	ENTPD6	20	+	1140	17	2	2	2.37%	8.750234390469739	0.01292879023172146	0.018399683928041276	0.0011362162494091641
ENSG00000089177_16528406_16273253_1290	KIF16B	20	−	3870	19	0	0	0.62%	2.880834122170156	0.005454749182134642	0.005454749182134642	0.00015910859015753364
ENSG00000113575_134225861_134197772_309	PPP2CA	5	−	927	156	3	5	12.62%	98.74622019170876	7.734539680885756e−08	1.215634237814575e−07	9.137471817279289e−14
ENSG00000093009_19479969_19518908_566	CDC45	22	+	1698	20	0	2	1.94%	6.911430198250885	0.0014481341328822738	0.0067530256680052286	5.8388157991235136e−05
ENSG00000109332_102809680_102797415_110	UBE2D3	4	−	330	19	0	1	5.45%	33.78432743272273	0.013428347753762199	0.013428347753762199	0.0008725593497644525
ENSG00000255302_48878231_48878740_147	EID1	15	+	441	91	0	7	14.29%	121.08167475888098	2.7304291930629852e−05	4.139480324534568e−05	8.679326368284707e−09
ENSG00000104529_143586845_143580630_106	EEF1D	8	−	318	83	0	0	8.49%	153.1533810629387	0.003736770770438521	0.003736770770438521	7.748026083818498e−05
ENSG00000152894_128520358_127970227_1440	PTPRK	6	−	4320	26	1	1	0.97%	3.5315488471340286	0.0006179331360801681	0.0006179331360801681	2.4552533592415123e−06
ENSG00000197586_25207334_25225597_380	ENTPD6	20	+	1140	17	2	2	2.37%	8.750234390469739	0.01292879023172146	0.018399683928041276	0.0011362162494091641
ENSG00000175029_125003077_124989518_365	CTBP2	10	−	1095	10	0	0	1.64%	5.358725331794521	0.012798402692974279	0.012798402692974279	0.0007972436888943567
ENSG00000119013_201078922_201085615_85	NDUFB3	2	+	255	31	2	9	9.41%	71.33409074030001	0.012834473343944776	0.04154059348323694	0.002530235953906631
ENSG00000113387_32588513_32594630_84	SUB1	5	+	252	42	3	7	9.52%	97.79673730525002	0.015646180920032563	0.0407959993643421	0.0029428665299278934
ENSG00000128567_131509070_131504311_231	PODXL	7	−	693	9	0	0	2.16%	7.620524984824677	0.016947426762344633	0.016947426762344633	0.0013498980316300959
ENSG00000155097_103040837_103068747_382	ATP6V1C1	8	+	1146	28	1	3	2.88%	14.336694474068066	0.0022960440039733582	0.011821942446875977	0.0001565172319901872
ENSG00000148498_34814935_34111169_1290	PARD3	10	−	3870	19	0	1	0.62%	2.880834122170156	0.005405141725979036	0.005554612275049112	0.00016047798536882382
ENSG00000260916_55371795_55359499_482	CCPG1	15	−	1446	28	0	1	3.11%	11.362276533390045	0.0002681497654631663	0.0002554476795345014	4.6680192937444826e−07
ENSG00000134291_47964237_47968229_179	TMEM106C	12	+	537	14	0	4	2.79%	15.297813656687154	0.02108409854857798	0.10163622319758386	0.009735731963679783
ENSG00000077348_41397328_41386633_235	EXOSC5	19	−	705	22	5	0	4.26%	18.310878474174473	0.01934135566922576	0.0022276761777358703	0.00025736151836636346
ENSG00000088038_54142979_54155407_753	CNOT3	19	+	2259	8	3	0	0.80%	2.0780183225551134	0.11368416502207135	0.011570465654371866	0.006956434060967934
ENSG00000188554_43186415_43209643_813	NBR1	17	+	2439	17	3	1	1.48%	4.089900453110087	0.009745921382577108	0.001620025777606367	9.441403448178248e−05
ENSG00000048707_12234267_12509024_4363	VPS13D	1	+	13089	15	0	0	0.21%	0.6724506346911531	0.002478942540635376	0.002478942540635376	3.5397643206479886e−05
ENSG00000125746_45638587_45607009_741	EML2	19	−	2223	19	0	0	1.35%	5.01521729770513	0.0022543491824521134	0.0022543491824521134	2.9517890247939808e−05
ENSG00000108883_44886750_44851274_937	EFTUD2	17	−	2811	71	5	4	3.42%	14.820850263975991	1.2612321871750657e−06	3.3365429726544585e−06	3.693814629760399e−11
ENSG00000182979_105460864_105470215_435	MTA1	14	+	1305	17	2	0	1.84%	7.643882915812646	0.02671499548383061	0.004873903550586949	0.0007031623440447889

ENSG00000164975_15422880_15459866_411	SNAPC3	9	+	1233	14	0	0	1.95%	6.662551446586376	0.005257622967929454	0.005257622967929454	0.00014836615564656991
ENSG00000085231_69369490_69352061_172	AK6	5	-	516	16	1	4	4.07%	18.194741824232562	0.012000726946169103	0.052370108323589605	0.003042637799777812
ENSG00000152234_46084649_46084323_108	ATP5F1A	18	-	324	20	0	9	6.48%	36.22101381675927	0.00797940233269714	0.3585092873457507	0.02497955363525581
ENSG00000152234_46086245_46085949_98	ATP5F1A	18	-	294	21	0	0	6.12%	41.912887416535725	0.013640585738808999	0.013640585738808999	0.00089863692579514
ENSG00000213639_28752125_28799303_327	PPP1CB	2	+	981	155	9	6	11.93%	92.71250325574161	1.530997901648864e-07	4.474563023063923e-07	6.494503469312218e-13
ENSG00000163814_45146287_45085638_836	CDCP1	3	-	2508	29	1	2	1.79%	6.784941104909691	0.0003523988826580711	0.0010368094939864315	2.4040487983194343e-06
ENSG00000121691_34439047_34471433_516	CAT	11	+	1548	37	0	0	2.71%	14.0251134895126	0.0004561727980981236	0.0004561727980981236	1.3667994923812405e-06
ENSG00000100280_29358923_29328821_830	AP1B1	22	-	2490	24	0	3	1.08%	5.655714928496387	0.003684287313187465	0.017782189529096822	0.00036099358464052045
ENSG00000165819_21511223_21503220_153	METTL3	14	-	459	20	5	0	3.92%	25.567774458888895	0.06341282918854552	0.013428347753762199	0.004086769345423957
ENSG00000110906_109469554_109451595_255	KCTD10	12	-	765	13	0	0	3.14%	9.971432038966668	0.004826937407846361	0.004826937407846361	0.00012612451166366307
ENSG00000108666_32342115_32331763_388	C17orf75	17	-	1164	17	0	1	1.29%	8.569817186542528	0.02108409854857798	0.03577298629450316	0.003354604907114977
ENSG00000163956_3532412_3512974_357	LRPAP1	4	-	1071	139	1	3	6.16%	76.15544249540478	1.8282969234632734e-05	4.460000345921091e-05	6.364328663209483e-09
ENSG00000127952_76030523_75999096_282	STYXL1	7	-	846	14	4	0	2.13%	9.710314342365251	0.10069662721979156	0.013007239811447113	0.0066169769657669466
ENSG00000113407_33461056_33466004_173	TARS	5	+	519	25	0	2	8.67%	28.26495297839596	0.0002533724336195327	0.0006690860449594656	1.1399285139925363e-06
ENSG00000133313_74510849_74516527_236	CNDP2	18	+	708	6	0	2	2.54%	4.972715456199154	0.007152939217714824	0.1029516053660342	0.004313678482395006
ENSG00000133028_10697501_10681119_268	SCO1	17	-	804	15	0	1	3.36%	10.9473959670056	0.0032294771334464936	0.006024333578509648	0.00010730535955833177
ENSG00000166471_9314441_9283570_196	TMEM41B	11	-	588	8	0	0	2.55%	7.983407126959185	0.019679754444124863	0.019679754444124863	0.0017849636115922674
ENSG00000134283_42335903_42441509_348	PPHLN1	12	+	1044	15	1	1	2.01%	8.43075321596983	0.013892891352654344	0.013892891352654344	0.0009300961024180418
ENSG00000126249_34404431_34426120_358	PDCD2L	19	+	1074	19	3	0	1.96%	10.380659267037712	0.051745348319195604	0.00797940233269714	0.002149744678485897
ENSG00000071054_101823961_101891249_1201	MAP4K4	2	+	3603	23	5	3	1.08%	3.7457534688105754	0.007949119477744631	0.004175186551296234	0.00017845908989950217
ENSG00000099341_38374791_38383390_287	PSMD8	19	+	861	67	4	3	5.92%	45.66119442126656	0.00021754394475824391	0.000557069202053668	8.229714661227028e-07
ENSG00000137575_58565034_58581740_297	SDCBP	8	+	891	120	4	1	10.10%	79.02766650929294	2.844226847274897e-06	1.106318704355805e-06	2.7762318096690534e-11
ENSG00000155561_135557945_135648556_2012	NUP205	7	+	6036	52	3	1	1.09%	5.0550997414244545	6.654374809707275e-05	1.981397842966006e-05	1.0258908872129734e-08
ENSG00000196655_119018796_119023399_165	TRAPPC4	11	+	495	31	0	1	8.48%	36.74786492682122	0.0003353510003294976	0.0006716243423686093	1.4880509536321035e-06
ENSG00000213465_65014208_65018979_194	ARL2	11	+	582	41	0	3	5.67%	41.336765252734544	0.0015208721599504675	0.005542082247366204	4.979728931750281e-05
ENSG00000136271_44573895_44566002_547	DDX56	7	-	1641	78	5	0	4.57%	27.89084281465997	7.516307092440293e-05	4.656235523973952e-06	3.039019381178132e-09
ENSG00000136270_45109171_45100325_609	TBRG4	7	-	1827	48	8	0	3.45%	15.416234452059117	0.0011513765331522398	2.1814045225814675e-05	2.260979456382319e-07
ENSG00000175166_184299267_184308890_908	PSMD2	3	+	2724	96	2	1	4.63%	20.679486302431723	8.900489055389234e-09	7.045293184468855e-09	6.56232629824295e-16
ENSG00000183207_48993912_49010171_255	RUVBL2	19	+	765	36	1	0	4.31%	27.613196415600008	0.0017791427806311133	0.0016109979480538772	1.705970569913741e-05
ENSG00000111711_21501924_21507955_42	GOLT1B	12	+	126	11	0	0	11.90%	51.22686239798811	0.019679754444124863	0.019679754444124863	0.0017849636115922674
ENSG00000177565_177050593_177026462_326	TBL1XR1	3	-	978	8	4	0	1.84%	4.799839867742333	0.1700871372700561	0.011570465654371866	0.011293071913932045
ENSG00000116747_193069142_193091671_505	RO60	1	+	1515	25	0	0	2.77%	9.682845277747527	0.0003981150787954058	0.0003981150787954058	1.0507179780062227e-06
ENSG00000116747_193069142_193091685_496	RO60	1	+	1488	25	0	0	2.82%	9.858542067061494	0.0003981150787954058	0.0003981150787954058	1.0507179780062227e-06
ENSG00000116747_193069142_193084731_509	RO60	1	+	1527	26	0	0	2.95%	9.991022278728883	0.0002582331582969843	0.0002582331582969843	4.5481323920964005e-07
ENSG00000023572_193101203_193096625_124	GLRX2	1	-	372	31	2	2	8.06%	48.89836865262501	0.007790523468944856	0.00510952817114065	0.00021072141659941136
ENSG00000089351_35009228_35026141_681	GRAMD1A	19	+	2043	25	0	2	1.32%	7.180377188344348	0.0035291660242640502	0.0046742147278768555	9.098972624008064e-05
ENSG00000116750_193059230_193018812_306	UCHL5	1	-	918	13	1	0	1.63%	8.30952669913889	0.03577298629450316	0.02108409854857798	0.003354604907114977
ENSG00000120885_27610571_27598241_449	CLU	8	-	1347	460	12	4	17.15%	200.38529692835195	1.5830288136621456e-14	2.9827041344232324e-14	6.49000258331343e-27
ENSG00000120889_23028589_23022671_248	TNFRSF10B	8	-	744	65	1	3	5.65%	51.26441874871977	0.0006444407754932549	0.0018744168113352109	7.613687579508107e-06
ENSG00000153310_129879461_129842142_324	FAM49B	8	-	972	28	0	1	3.70%	16.903139781154326	0.000978917897162665	0.0013147568071904994	7.922904919322548e-06
ENSG00000170525_6213664_6225187_416	PFKFB3	10	+	1248	46	13	3	4.57%	21.62812459635337	0.002674329485664681	0.0002944342621990559	5.423138123191577e-06
ENSG00000054116_36140070_36137733_115	TRAPPC3	1	-	345	16	0	0	4.35%	27.213005163200005	0.02108409854857798	0.02108409854857798	0.002029891263157455
ENSG00000147642_109579914_109574906_457	SYBU	8	-	1371	9	0	0	1.09%	3.8519502658522984	0.020613416668581838	0.020613416668581838	0.001946208561389314
ENSG00000172466_35340650_35337232_368	ZNF24	18	-	1104	19	0	1	2.17%	10.098576134781252	0.005306578019052857	0.005306578019052857	0.00015100114740534233
ENSG00000151353_675603_669580_112	TMEM18	2	-	336	19	0	0	8.93%	33.181035871424115	0.0021880057261679567	0.0021880057261679567	2.7877966332435854e-05
ENSG00000182220_40597536_40599762_117	ATP6AP2	X	+	351	21	0	4	8.55%	35.1065210839359	0.0022409900782254734	0.014991380638102441	0.00019682638437717605
ENSG00000119523_99221894_99221069_138	ALG2	9	-	414	7	0	1	3.62%	9.92140813241667	0.019216965118390855	0.04178282491678763	0.003600525600033436
ENSG00000119523_99221047_99217934_323	ALG2	9	-	969	12	0	0	1.55%	7.266630635684212	0.019679754444124863	0.019679754444124863	0.0017849636115922674
ENSG00000161533_75973689_75946748_592	ACOX1	17	-	1776	15	0	0	1.35%	4.955915741820102	0.004873903550586949	0.004873903550586949	0.00012846764509679302
ENSG00000278259_36527583_36496251_681	MYO19	17	-	2043	13	3	0	0.88%	3.733796137939061	0.07134538375819317	0.012798402692974279	0.004461830587596429
ENSG00000100106_37751802_37759527_360	TRIOBP	22	+	1080	12	2	3	1.39%	6.519782487016668	0.06945717695039447	0.08529346643572072	0.02193284716703279
ENSG00000183853_158076244_158095120_593	KIRREL1	1	+	1779	24	2	2	2.02%	7.91609340750759	0.00511207214703033	0.00511207214703033	0.00014066041004820107
ENSG00000007341_112619113_112607266_260	ST7L	1	-	780	7	0	0	1.92%	5.265978162590386	0.016947426762344633	0.016947426762344633	0.0013498980316300959
ENSG00000161203_184176994_184183616_433	AP2M1	3	+	1299	65	2	7	5.54%	29.36160704314666	1.9476261111745904e-05	0.0001547348909454494	2.387872630864365e-08
ENSG00000034713_75566496_75568213_85	GABARAPL2	16	+	255	81	5	1	18.82%	186.38907580530005	0.0002001100642622418	0.00019965580804963987	2.769228308974791e-07
ENSG00000100902_35292477_35310924_145	PSMA6	14	+	435	66	3	2	8.28%	89.0287539606414	0.0013480573084962576	0.0010573555742419644	8.734696542495579e-06
ENSG00000150782_112150219_112143596_167	IL18	11	-	501	50	0	6	7.78%	58.56092054206588	0.0005480806164703292	0.0010527496933334325	3.684681404463176e-06
ENSG00000092010_24138214_24138478_37	PSME1	14	+	111	24	6	1	13.51%	126.87144299059463	0.12378039365238508	0.02108409854857798	0.01208667083877082
ENSG00000092010_24138540_24138816_33	PSME1	14	+	99	19	0	1	24.24%	112.61442477574245	0.005554612275049112	0.005554612275049112	0.00016468457691007306
ENSG00000141736_39707049_39717484_589	ERBB2	17	+	1767	9	0	0	0.85%	2.9886948582249584	0.020613416668581838	0.020613416668581838	0.001946208561389314
ENSG00000137767_45658924_45691030_450	SQOR	15	+	1350	69	3	1	6.67%	29.990999440276674	1.8536762582917239e-06	8.861895121520649e-07	1.4625694787674506e-11
ENSG00000140497_74851356_74845083_173	SCAMP2	15	-	519	42	2	2	3.47%	47.48512100370521	0.03031622373531018	0.03031622373531018	0.003985451574990196
ENSG00000166033_122458556_122514359_374	HTRA1	10	+	1122	28	0	0	2.41%	14.643361735545458	0.0033278027414746945	0.0033278027414746945	6.211806171561056e-05
ENSG00000147586_80003120_79918980_96	MRPS28	8	-	288	32	0	3	12.50%	65.19782487016668	0.0009235399941089087	0.0047328325455606455	2.7121800617259456e-05
ENSG00000103356_23557343_23524371_523	EARS2	16	-	1569	12	0	0	1.15%	4.487804388768644	0.013217226988443315	0.013217226988443315	0.0008469697265466234
ENSG00000010704_26091349_26094226_209	HFE	6	+	627	17	0	2	3.35%	15.909517073581343	0.00837382380159347	0.02413590542585519	0.0010073561300188419
ENSG00000114745_39102761_39098236_352	GORASP1	3	-	1056	13	2	1	2.27%	7.223622641865059	0.016854695534768073	0.010020958156399907	0.0008276455671405646

ENSG00000179958_30429980_30424233_170	DCTPP1	16	-	510	43	6	0	7.65%	49.473643577950014	0.004378270345045809	0.000681533134233071	1.9106150240739215e-05
ENSG00000197102_102033284_102034501_196	DYNC1H1	14	+	588	7	0	2	2.55%	6.985481236089288	0.019216965118390855	0.1191346346731873	0.010787784428637617
ENSG00000204842_111554184_111470070_861	ATXN2	12	-	2583	20	0	0	0.81%	4.543402429976772	0.00877616296920843	0.00877616296920843	0.00039161135667564577
ENSG00000123505_110890281_110893616_217	AMD1	6	+	651	9	0	0	3.23%	8.112171758039173	0.006937202941512724	0.006937202941512724	0.0002510152274148709‌5
ENSG00000072042_67691200_67678321_174	RDH11	14	-	522	48	0	6	9.77%	53.95682058220691	0.00011440730225837537	0.0007274417225341049	5.967917620171261e-07
ENSG00000198522_27631059_27650200_295	GPN1	2	+	885	23	0	0	2.71%	15.249660732344072	0.005706018193000826	0.005706018193000826	0.0001733096755673334
ENSG00000136631_150068645_150144796_534	VPS45	1	+	1602	15	0	3	1.50%	5.494198725014046	0.005306578019052857	0.03497523187724695	0.0010067653727672797
ENSG00000168036_41224069_41239342_781	CTNNB1	3	+	2343	117	8	3	5.76%	29.301455223339957	6.403151454483205e-08	1.1032790393731706e-08	7.305397733044446e-15
ENSG00000085662_134447363_134442728_63	AKR1B1	7	-	189	144	4	3	26.98%	447.0707991097144	0.00014496049037061087	0.0003883664866035581	3.921970234426077e-07
ENSG00000085662_134459062_134447929_263	AKR1B1	7	-	789	1364	64	22	19.39%	1014.408742846852	2.5082608676805146e-09	9.085155830797446e-10	2.5087689513879232e-17
ENSG00000129636_47311289_47155719_272	ITFG1	16	-	816	21	0	0	4.41%	15.100966789781253	0.0008442728493916082	0.0008442728493916082	4.480017286136276e-06
ENSG00000142186_65525154_65538566_791	SCYL1	11	+	2373	9	1	0	1.01%	2.2254630486656137	0.010460667668897016	0.003327802741474688‌4	0.0001913030633819633‌1
ENSG00000141510_7676594_7673307_341	TP53	17	-	1023	30	0	4	3.52%	17.207637062507334	0.0010238660768343774	0.006911511910188328	4.410159297683948e-05
ENSG00000166801_59152092_59153504_470	FAM111A	11	+	1410	8	0	0	1.06%	3.329250631668086	0.019679754444124863	0.019679754444124863	0.0017849636115922674
ENSG00000003402_201130085_201163973_372	CFLAR	2	+	1116	11	1	0	1.34%	5.783678012676076	0.04223451612662591	0.019679754444124863	0.00371514801080982‌2
ENSG00000132471_75855297_75846747_231	WBP2	17	-	693	33	0	1	5.19%	27.94192494435715	0.0010197273511756433	0.0013558867987602367	8.486686545361988e-06
ENSG00000076242_37001037_37050653_658	MLH1	3	+	1974	21	0	3	1.67%	6.242344934377661	0.001484234770590415	0.010728371185261619	9.686994068872613e-05
ENSG00000105968_44847971_44834501_128	H2AFV	7	-	384	76	1	1	8.59%	116.1336255499844	0.0017920611887025132	0.0016263209931528667	1.7334867057987664e-05
ENSG00000041357_78541928_78548944_230	PSMA4	15	+	690	245	11	8	10.43%	208.34957078075004	0.00021025475014341177	9.060247417322352e-05	1.3650249863795117e-07
ENSG00000102900_56818721_56844609_637	NUP93	16	+	1911	27	0	0	1.88%	8.290461247226846	0.0008776897154483972	0.0008776897154483972	4.827634122642857e-06
ENSG00000051108_56932245_56943290_390	HERPUD1	16	+	1170	42	5	0	4.36%	21.063912650361544	0.0011345297860857772	0.00012791349488564853	1.0481486550354235e-06
ENSG00000164163_145104413_145121606_421	ABCE1	4	+	1263	20	0	2	1.66%	9.291851525439432	0.008175610787902942	0.013599199788808391	0.00055803049980967‌23
ENSG00000049656_1321793_1318369_91	CLPTM1L	5	-	273	20	0	0	6.59%	42.98757683747254	0.013640585738808999	0.013640585738808999	0.00089863692579514
ENSG00000164111_121671628_121669518_115	ANXA5	4	-	345	54	1	2	14.78%	91.84389242580002	0.00025389937360947086	0.00026353281753581636	4.563187129293613‌6e-07
ENSG00000111665_6850485_6849194_105	CDCA3	12	-	315	17	2	0	8.57%	31.6675149369381	0.014936572009614516	0.0033030736763512156	0.0002738986320205498
ENSG00000109390_139295798_139292550_76	NDUFC1	4	-	228	19	2	0	7.89%	48.89836865262501	0.016822681477512567	0.013428347753762199	0.0010798218650012886
ENSG00000103275_1314031_1320659_184	UBE2I	16	+	552	87	7	4	11.96%	92.48169723431252	6.541542848391905e-05	6.762754633260803e-05	3.269269447906433e-08
ENSG00000106608_43879012_43876667_781	URGCP	7	-	2343	15	0	2	0.90%	3.7565968235051224	0.00847367416878415	0.027657845067597595	0.0011690047180828366
ENSG00000084207_67583844_67586577_210	GSTP1	11	+	630	411	16	21	26.67%	382.8043717376929	2.2053363750583546e-10	1.3741503960353618e-09	3.469736882256924e-18
ENSG00000084207_67583844_67586577_174	GSTP1	11	+	522	318	9	19	26.44%	357.46393635712076	1.0621063827902208e-08	2.931362786209297e-08	3.20761556715274e-15
ENSG00000173418_20018025_20033187_166	NAA20	20	+	498	100	0	0	15.66%	117.82739434367473	3.48427022202856e-06	3.48427022202856e-06	1.032744793565972e-10
ENSG00000115541_197500437_197503176_101	HSPE1	2	+	303	139	7	0	10.89%	269.18309872138127	0.0016572264983485026	0.0016572264983485026	1.6372075009059705e-05
ENSG00000055130_148730123_148800582_776	CUL1	7	+	2328	27	0	1	1.16%	6.805443059901419	0.0035547763465261306	0.005670089768331009	0.0001104993355006363‌7
ENSG00000115415_191009922_190970703_723	STAT1	2	-	2169	45	8	4	2.77%	12.173867714346477	0.0013950305237650524	0.0003026526610799105	2.8253310383760704e-06
ENSG00000112667_43229444_43225733_170	DNPH1	6	-	510	76	4	4	12.35%	87.44178864940002	9.202218186154535e-05	0.00016599101035605533	1.0955737162561409e-07
ENSG00000115129_24084326_24077634_248	TP53I3	2	-	744	9	5	0	2.82%	7.098150288284276	0.20870646171529367	0.005706018193000826	0.009081953977665104
ENSG00000196923_177491100_177483644_309	PDLIM7	5	-	927	10	0	1	2.27%	6.32988590972492	0.007029302661868638	0.016854695534768073	0.0006017499134389055
ENSG00000121067_49611406_49600378_197	SPOP	17	-	591	11	0	0	2.54%	10.92146304931726	0.020613416668581838	0.020613416668581838	0.001946208561389314
ENSG00000196083_190609068_190648705_429	IL1RAP	3	+	1287	15	0	0	1.40%	6.8389326786888125	0.013428347753762199	0.013428347753762199	0.0008725593497644525
ENSG00000023191_502162_499825_148	RNH1	11	-	444	15	1	2	5.41%	19.82366296728041	0.005257622967929454	0.02389626957043539	0.0006669614278904383
ENSG00000149182_47176853_47165482_521	ARFGAP2	11	-	1563	11	0	2	1.15%	4.129612707707294	0.012798402692974279	0.05067994629101479	0.003100990187173963
ENSG00000168385_241324233_241336863_139	SEPT2	2	+	417	20	0	0	7.19%	28.14294598712231	0.002033393162560146	0.002033393162560146	2.4228963598721857e-05
ENSG00000126934_4117430_4090598_303	MAP2K2	19	-	909	16	0	0	3.63%	10.32836829626403	0.0010860768436549261	0.0010860768436549261	7.273150737364793e-06
ENSG00000115685_241150541_241167068_259	PPP1R7	2	+	777	24	0	0	3.86%	18.12449185579923	0.0023628488670607213	0.0023628488670607213	3.2295294885504634e-05
ENSG00000108262_29578972_29574702_511	GIT1	17	-	1533	10	0	2	0.98%	3.827660951281801	0.020613416668581838	0.05123521742987468‌4	0.004687384229717438
ENSG00000188223_35745931_35752177_55	AD000671.1	19	+	165	14	14	2	12.73%	49.787429900854555	0.07551307739531453	0.04912109528954385	0.014462694973637112
ENSG00000151348_44107836_44244287_677	EXT2	11	+	2031	49	1	1	2.66%	14.156691663093799	0.00011910304516311777	0.00011910304516311777	1.014320640544434e-07
ENSG00000213593_57712619_57737790_84	TMX2	11	+	252	47	0	0	14.29%	109.4392060320655	0.0010658336519988904	0.0010658336519988904	7.014744445236256e-06
ENSG00000149257_75566350_75572083_418	SERPINH1	11	+	1254	133	13	7	7.66%	62.234287376068195	1.5092259326469038e-05	2.0097836860962044e-06	2.647185579577165e-10
ENSG00000204264_32843032_32840959_208	PSMB8	6	-	624	25	4	0	4.81%	23.508831082992796	0.004400434476856543	0.002390425500591921	5.967362184433355e-05
ENSG00000127054_1324608_1319816_89	INTS11	1	-	267	7	0	2	5.62%	15.383756430039329	0.019216965118390855	0.1191346346731873	0.010787784428637617
ENSG00000061273_47797459_47783841_787	HDAC7	12	-	2361	18	0	3	0.76%	4.473548339249048	0.013007239811447113	0.06272892732212154	0.003936255353260731
ENSG00000099942_20918037_20949845_269	CRKL	22	+	807	12	1	3	2.23%	8.72535946217844	0.023878473930440184	0.07356204933788085	0.007656850418168591
ENSG00000196154_153544794_153543759_101	S100A4	1	-	303	1149	89	31	45.54%	2225.1178448263818	2.935379469644316e-09	1.4818842270205308e-08	4.658591980648176e-16
ENSG00000135624_73234382_73252861_455	CCT7	2	+	1365	343	10	12	15.16%	147.4473885525308	3.5866107110789213e-12	2.3550132621183942e-12	1.0628313656404882e-22
ENSG00000147526_38819845_38848023_604	TACC1	8	+	1812	13	3	0	1.49%	4.209793327709438	0.024779191546489752	0.002478942540635376	0.0003685640769040464
ENSG00000169398_140846306_140659466_870	PTK2	8	-	2610	15	6	4	1.15%	3.372301286387932	0.0820328997370387	0.023029368248455667	0.0083207698742617‌9
ENSG00000113597_65636006_65664611_359	TRAPPC13	5	+	1077	9	1	0	1.39%	4.903457580764625	0.03537493353818881	0.020613416668581838	0.003252526348827943
ENSG00000213923_38314157_38293287_416	CSNK1E	22	-	1248	27	1	0	1.20%	12.694768784816109	0.029237630782826433	0.021557223391537678	0.002819005644529471
ENSG00000108312_44213282_44207242_569	UBTF	17	-	1707	33	4	3	2.46%	11.34373402837698	0.0038766098248878157	0.002532570608603844	5.56157809650073e-05
ENSG00000032444_7540198_7561562_1165	PNPLA6	19	+	3495	25	0	0	0.94%	4.197284862886267	0.001484234770590415	0.001484234770590415	1.3251387882263029e-05
ENSG00000115525_85848138_85840144_290	ST3GAL5	2	-	870	11	0	3	1.72%	7.41906283005345	0.019216965118390855	0.13115678761539135	0.012020477827111095
ENSG00000138078_44339308_44321356_458	PREPL	2	-	1374	18	0	0	3.06%	7.687079788185591	0.00024035368145103084	0.00024035368145103084	3.9581571392385245e-07
ENSG00000023734_15889962_15902978_256	STRAP	12	+	768	86	2	0	8.59%	65.70718287696486	3.859972537601811e-05	1.896996103042005e-05	5.708913605564418e-09
ENSG00000103202_399666_400342_65	NME4	16	+	195	10	2	0	7.69%	30.091303786230775	0.019679754444124863	0.019679754444124863	0.0017849636115922674
ENSG00000163931_53255942_53235040_153	TKT	3	-	459	164	39	22	16.34%	209.65575056288893	0.003986569303607465	0.00014071149938461175	4.411220060075061e-06
ENSG00000163931_53235017_53225756_425	TKT	3	-	1275	316	28	3	16.00%	145.42950112216002	1.0828882245757083e-10	3.316178275338737e-13	5.276822060004583e-22

ENSG00000117899_80989791_80979219_234	MESD	15	-	702	38	7	3	7.26%	31.76304288546582	0.000764267771632909	0.0010102842767105284	4.845736183686482e-06
ENSG00000084234_130069978_130143448_763	APLP2	11	+	2289	141	2	6	7.34%	36.14505887297576	3.7916035566903175e-11	2.8643103666563556e-10	1.296524660361252e-19
ENSG00000110063_126304081_126345613_337	DCPS	11	+	1011	11	1	2	2.37%	6.384356738028191	0.015191410988288745	0.033944577430914495	0.002386812976484452
ENSG00000086598_123584637_123596729_201	TMED2	12	+	603	183	4	3	17.41%	178.07764106329108	5.640011992435725e-07	3.717141552349798e-07	1.9322274947743437e-12
ENSG00000134057_69167263_69177631_433	CCNB1	5	+	1299	104	2	6	9.70%	46.97857126903465	1.0258434512233877e-08	1.5098800514282163e-07	1.6355863422446172e-14
ENSG00000134058_69234976_69277135_346	CDK7	5	+	1038	12	1	0	2.60%	6.78358871481503	0.006306718652620871	0.002874854311667729	0.00010091605209104467
ENSG00000134056_69228289_69229225_38	MRPS36	5	+	114	24	0	0	13.16%	123.5327208066316	0.02108409854857798	0.02108409854857798	0.002029891263157455
ENSG00000137309_34240781_34244884_107	HMGA1	6	+	321	34	3	0	5.61%	62.15119753978506	0.044040755831095145	0.013428347753762199	0.0027917083341200354
ENSG00000067225_72230987_72199650_605	PKM	15	-	1815	1686	113	82	21.98%	545.0753689145505	2.4592107458427927e-21	9.110112853897879e-21	3.789739423564993e-40
ENSG00000166747_71789479_71765520_236	AP1G1	16	-	708	13	0	1	2.54%	10.774216821764833	0.013428347753762199	0.02034245439037237	0.0012955184805712684
ENSG00000185825_153723244_153715538_114	BCAP31	X	-	342	33	1	3	13.16%	56.61916370303949	0.0003801290169235231	0.00028426559431428834	7.26588358637063e-07
ENSG00000167721_2336427_2324196_804	TSR1	17	-	2412	27	0	1	2.11%	6.568437580203359	9.036308349764196e-05	0.00017958319452132361	1.1640441351870632e-07
ENSG00000166197_102152411_102162269_700	NOLC1	10	+	2100	148	6	9	4.71%	41.35404891764858	4.092992156126415e-06	9.067146650735788e-06	3.104355683107411e-10
ENSG00000145632_58459959_58458964_132	PLK2	5	-	396	14	0	0	5.30%	20.744762458689397	0.00797940233269714	0.00797940233269714	0.000327142761693177
ENSG00000126777_55612133_55684103_1278	KTN1	14	+	3834	34	0	0	1.49%	5.203582266633021	4.942578964878572e-05	4.942578964878572e-05	1.834455077736299e-08
ENSG00000184640_77482176_77496266_290	SEPT9	17	+	870	41	3	3	3.79%	27.652870548381042	0.005641908870879185	0.005641908870879185	0.00016963243881650024
ENSG00000196531_56712866_56712759_35	NACA	12	-	105	22	1	0	17.14%	122.94446975517145	0.013428347753762199	0.013640585738808999	0.0008855098933240206
ENSG00000091073_76480516_76505601_573	DTX2	7	+	1719	8	4	0	0.87%	2.7307989474415364	0.169362073879829	0.019216965118390855	0.016163991620339734
ENSG00000178502_41865370_41853740_708	KLHL11	17	-	2124	11	0	1	0.85%	3.0388816676772605	0.012798402692974279	0.019795881618345192	0.0012079910998532793
ENSG00000137804_41332958_41380186_441	NUSAP1	15	+	1323	48	3	2	4.08%	21.28908567189116	0.00015267201677270214	0.00012290668625881772	1.3316840793902767e-07
ENSG00000095059_12681035_12675838_301	DHPS	19	-	903	23	0	0	1.99%	14.945680784191033	0.013640585738808999	0.013640585738808999	0.00089863692579514
ENSG00000111144_96029086_96000989_525	LTA4H	12	-	1575	79	1	3	4.95%	29.43216094138953	4.5810888689605946e-06	1.715814696264805e-05	6.55130209481541e-10
ENSG00000172340_67520580_67375744_141	SUCLG2	3	-	423	21	0	0	4.96%	29.13094302709575	0.00877616296920843	0.00877616296920843	0.00039161135667564577
ENSG00000087269_2963319_2938271_788	NOP14	4	-	2364	6	0	1	0.63%	1.48929041581599	0.016947426762344633	0.0477903522728147	0.0036975118224100542
ENSG00000143322_179229397_179107718_1182	ABL2	1	-	3546	14	2	1	0.68%	2.316673980158207	0.01599363345933208	0.00912793029372963	0.0007225562286945759
ENSG00000162910_228108260_228106760_128	MRPL55	1	-	384	35	2	1	7.81%	53.482590713808605	0.004407715900925203	0.0030814981005195803	7.570158080683164e-05
ENSG00000033867_27434087_27376764_897	SLC4A7	3	-	2691	27	0	2	1.78%	5.887429001653848	0.00015252522453692948	0.0007701916578329232	8.226488299996088e-07
ENSG00000033867_27434087_27404954_265	SLC4A7	3	-	795	9	0	2	1.89%	6.642797250922643	0.020613416668581838	0.08350359792828288	0.007749259071793911
ENSG00000033867_27403173_27376764_497	SLC4A7	3	-	1491	15	0	0	1.81%	5.903223579793764	0.002874854311667729	0.002874854311667729	4.6982854837323694e-05
ENSG00000157107_72956103_73088090_775	FCHO2	5	+	2325	13	0	0	0.65%	3.2809227999180655	0.02108409854857798	0.02108409854857798	0.002029891263157455
ENSG00000176986_73768885_73769523_114	SEC24C	10	+	342	17	0	2	7.02%	29.167447968232462	0.005306578019052857	0.02389626957043539	0.0006722688034155889
ENSG00000091732_130028904_130018664_296	ZC3HC1	7	-	888	21	0	0	3.38%	13.876564077096287	0.0017901637611834264	0.0017901637611834264	1.8982104156792192e-05
ENSG00000169764_63856320_63891227_497	UGP2	2	+	1491	40	8	0	4.23%	15.741929546116705	0.0010006558440764162	2.032182481103021e-05	1.8207368289573541e-07
ENSG00000106052_27748525_27828829_813	TAX1BP1	7	+	2439	41	8	2	2.09%	9.86387756338315	0.0013527752314266856	0.0002846471448417044	2.5890903697371525e-06
ENSG00000159692_1241433_1241335_32	CTBP1	4	-	96	17	0	0	15.62%	103.90903338682814	0.019679754444124863	0.019679754444124863	0.0017849636115922674
ENSG00000004487_23030484_23083364_736	KDM1A	1	+	2208	15	0	1	1.49%	3.9862800532031257	0.0010860768436549261	0.002549573717490699	1.6760814380175745e-05
ENSG00000126088_45012266_45015498_321	UROD	1	+	963	36	0	1	4.98%	21.93571677874767	0.0001842144602393462	0.00027099107140879646	3.4444091938493246e-07
ENSG00000125835_2470690_2461929_231	SNRPB	20	-	693	262	16	20	20.35%	221.8419495582295	5.355382931887235e-09	9.082610365444878e-08	5.247574683757744e-15
ENSG00000104763_18083928_18057534_379	ASAH1	8	-	1137	55	3	2	6.60%	28.384277318146445	4.58542909915364e-06	1.926540703241544e-05	7.37125132342833e-10
ENSG00000117713_26766258_26780756_1361	ARID1A	1	+	4083	12	4	1	0.51%	1.7245567195635565	0.057109797348681025	0.016854695534768073	0.004416591199647673
ENSG00000138760_76213543_76166190_432	SCARB2	4	-	1296	71	2	1	7.87%	32.14614976237385	1.2783768833362086e-07	1.722451992888563e-07	2.1082580208750053e-13
ENSG00000163875_37514740_37493769_190	MEAF6	1	-	570	13	0	0	4.21%	13.382711420718424	0.004826937407846361	0.004826937407846361	0.00012612451166366307
ENSG00000163875_37514740_37494099_199	MEAF6	1	-	597	13	0	0	4.02%	12.777463165510053	0.004826937407846361	0.004826937407846361	0.00012612451166366307
ENSG00000075218_46316118_46326658_196	GTSE1	22	+	588	12	0	0	3.06%	11.975110690438779	0.013217226988443315	0.013217226988443315	0.0008469697265466234
ENSG00000197150_151034568_151045349_509	ABCB8	7	+	1527	26	0	1	1.96%	9.991022278728883	0.0023628488670607213	0.002744064162451129	3.7279262481759005e-05
ENSG00000115286_1388690_1393407_213	NDUFS7	19	+	639	26	3	0	4.23%	23.875259811610334	0.011179354118128544	0.003684287313187465	0.00022402973088262996
ENSG00000107897_27240394_27197430_490	ACBD5	10	-	1470	32	0	1	2.65%	12.773451403134697	0.0006424109906750971	0.0009958682880916293	4.052020686628797e-06
ENSG00000076864_21613636_21597720_508	RAP1GAP	1	-	1524	15	0	5	1.18%	5.775397872357285	0.012178957859018494	0.11497452839710681	0.007325749448687799
ENSG00000172893_71444952_71435375_475	DHCR7	11	-	1425	227	10	3	11.37%	93.47309207701791	1.3573680883774385e-09	2.6354727696978287e-10	4.071163788948371e-18
ENSG00000076864_21613636_21597720_593	RAP1GAP	1	-	1779	15	0	5	1.01%	4.947558379692244	0.012178957859018494	0.11497452839710681	0.007325749448687799
ENSG00000076864_21613636_21597720_508	RAP1GAP	1	-	1524	15	0	5	1.18%	5.775397872357285	0.012178957859018494	0.11497452839710681	0.007325749448687799
ENSG00000079785_15595156_15630906_731	DDX1	2	+	2193	62	3	1	3.97%	16.589323427976748	2.996239415418512e-06	1.3400865838641015e-06	3.516972762400955e-11
ENSG00000116337_109625404_109631152_761	AMPD2	1	+	2283	7	0	1	0.66%	1.7991515404382397	0.019216965118390855	0.04178282491678763	0.003600525600033436
ENSG00000100926_24195019_24189415_589	TM9SF1	14	-	1767	65	4	1	4.07%	21.585018420513588	6.300236447879929e-05	9.089826356408796e-06	4.680690391555862e-09
ENSG00000072042_67693048_67678321_292	RDH11	14	-	876	168	0	7	11.30%	112.53323196768496	1.9877396341292258e-07	8.487597540983129e-07	1.5885659062266477e-12
ENSG00000155561_135617647_135648556_767	NUP205	7	+	2301	8	0	1	0.78%	2.0400883922868323	0.011570465654371866	0.026602764905793736	0.001474889218184677
ENSG00000135506_57694162_57720909_538	OS9	12	+	1614	67	3	4	5.58%	24.358295165248148	5.093601341966135e-06	1.0362735745734785e-05	4.375233972404395e-10
ENSG00000132341_130874563_130875926_128	RAN	12	+	384	179	6	0	11.72%	273.52524965062116	0.00048312743288401577	0.0003201165486586971	1.0291391939946253e-06
ENSG00000277791_38752827_38760535_107	PSMB3	17	+	321	105	9	5	14.95%	191.9375218140421	0.002069489755581042	0.001083310337977956	1.3595452778194462e-05
ENSG00000277791_38760525_38764167_75	PSMB3	17	+	225	168	1	1	20.00%	438.1293831275201	0.0003418708136139773	0.0003418708136139773	7.827289718747123e-07
ENSG00000085872_16541966_16519159_882	CHERP	19	-	2646	13	1	0	0.68%	2.882897018068595	0.012798402692974279	0.013007239811447113	0.000809468393769003
ENSG00000058272_79832450_79775929_854	PPP1R12A	12	-	2562	22	0	2	1.29%	5.03870777685129	0.0012558331315113088	0.004166527304844592	3.135602378839528e-05
ENSG00000185088_63156431_63154032_52	RPS27L	15	-	156	62	11	0	21.15%	233.20760434328852	0.0038480782014917924	0.00151350169064872	3.430123487290639e-05
ENSG00000166337_6612189_6610922_218	TAF10	11	-	654	12	0	0	2.29%	10.76661328131193	0.020613416668581838	0.020613416668581838	0.001946208561389314
ENSG00000166340_6619400_6614546_563	TPP1	11	-	1689	36	0	3	3.20%	12.50686516159503	7.870973464443577e-05	0.0006845597953738475	3.9930073224240944e-07
ENSG00000166333_6608898_6610611_298	ILK	11	+	894	35	0	0	4.70%	22.972387957609065	0.0004284130286064981	0.0004284130286064981	1.2106972447307754e-06
ENSG00000197694_128566741_128599034_1176	SPTAN1	9	+	3528	40	1	0	1.70%	6.652839272465988	5.158252009557849e-05	3.523926415704706e-05	1.3783541859280755e-08

ENSG00000198721_4127816_4115874_222	ECI2	6	-	666	29	0	0	3.15%	25.550498935605862	0.00847367416878415	0.00847367416878415	0.0003665024264111182
ENSG00000147862_14307922_14088197_478	NFIB	9	-	1434	8	1	0	1.05%	3.2735309558242687	0.03418265644467716	0.019216965118390855	0.0029560527921161815
ENSG00000009307_114739890_114718169_767	CSDE1	1	-	2301	89	11	5	4.43%	22.695983364191008	1.2651144433362197e-05	1.2057861126918465e-06	1.3707486370165965e-10
ENSG00000179222_51894305_51901930_778	MAGED1	X	+	2334	18	0	2	1.03%	4.525298898443446	0.005554612275049112	0.018318557839637383	0.0005316522383050482
ENSG00000089289_70133948_70165981_339	IGBP1	X	+	1017	15	4	0	2.06%	8.654578522588498	0.058290625655499875	0.007029302661868638	0.00221264198215177
ENSG00000162910_228108260_228106760_128	MRPL55	1	-	384	35	2	1	7.81%	53.482590713808605	0.004407715900925203	0.0030814981005195803	7.570158080683164e-05
ENSG00000156671_75150529_75176129_295	SAMD8	10	+	885	19	2	0	2.71%	12.59754582237119	0.01732176123716967	0.005306578019052857	0.000482474482019965
ENSG00000133961_73355751_73276578_456	NUMB	14	-	1368	16	0	0	1.75%	6.862928933701756	0.005306578019052857	0.005306578019052857	0.00015100114740534233
ENSG00000068489_59169753_59201631_360	PRR11	17	+	1080	19	0	0	3.06%	10.32298893777639	0.0013775385517804596	0.0013775385517804596	1.1483554366703679e-05
ENSG00000141552_81894478_81894612_44	ANAPC11	17	+	132	98	8	1	22.73%	435.64001163247735	0.004326528353113636	0.0025167541003031247	6.155846001043173e-05
ENSG00000178999_8207605_8204871_255	AURKB	17	-	765	13	2	3	2.75%	9.971432038966668	0.028902805874428627	0.026832695913080365	0.003406149252095427
ENSG00000123505_110888867_110893616_265	AMD1	6	+	795	10	0	0	3.02%	7.380885834358493	0.00414960799764038	0.00414960799764038	9.45964903272165e-05
ENSG00000112531_163415257_163563745_298	QKI	6	+	894	21	0	0	4.03%	13.783432774565439	0.0009235399941089087	0.0009235399941089087	5.324735186779001e-06
ENSG00000118200_200807409_200858059_1318	CAMSAP2	1	+	3954	12	2	1	0.38%	1.780820709655539	0.06556525143482543	0.02784981298332479	0.007750594856566061
ENSG00000197150_151034375_151037319_261	ABCB8	7	+	783	17	0	1	2.68%	12.739804859687743	0.008574016250729465	0.013991246267585163	0.0005991351246454473
ENSG00000110429_33774488_33747121_412	FBXO3	11	-	1236	14	0	0	1.21%	6.646380205211167	0.019679754444124863	0.019679754444124863	0.0017849636115922674
ENSG00000143933_47162588_47160776_113	CALM2	2	-	339	138	7	1	12.39%	238.86636722344252	0.0003909674708303042	0.00047118620257580814	1.215777600715846e-06
ENSG00000125818_1118774_1165080_271	PSMF1	20	+	813	44	3	2	5.90%	31.75687410650185	0.0008370797728924139	0.00026543292893292496	1.4906856844334798e-06
ENSG00000134291_47964237_47968229_250	TMEM106C	12	+	750	16	0	4	2.40%	12.517982375072004	0.013007239811447113	0.0695184692773032	0.004395185670641208
ENSG00000022556_54970034_55000898_1053	NLRP2	19	+	3159	24	2	0	1.04%	4.457970931293448	0.005035606171387428	0.0015356776430442238	4.557004617261039e-05
ENSG00000091140_107891251_107919259_509	DLD	7	+	1527	69	11	1	5.50%	26.51463604739588	0.00017041041997235105	2.0264452402002208e-06	3.4828789752522884e-09
ENSG00000127946_75582124_75538172_873	HIP1	7	-	2619	10	2	0	0.69%	2.240475081449027	0.061356297364084836	0.012798402692974279	0.0037940651817215715
ENSG00000125450_75209834_75235679_610	NUP85	17	+	1830	31	3	0	2.62%	9.939996250697543	0.0012077785613956338	0.00017208190038811133	1.4604670393809444e-06
ENSG00000169359_155853997_155853194_267	SLC33A1	3	-	801	18	0	0	2.62%	13.186076940033711	0.008674846900444944	0.008674846900444944	0.00038311532916634116
ENSG00000136861_120550836_120389236_1806	CDK5RAP2	9	-	5418	12	4	4	0.33%	1.2996244160166115	0.08266357711566169	0.14908097407465987	0.043018381094693095
ENSG00000169599_69437422_69396246_254	NFU1	2	-	762	33	0	0	7.48%	25.41175063837205	4.0169645523667093e-05	4.0169645523667093e-05	1.2249899171546548e-08
ENSG00000105058_16185670_16190955_72	FAM32A	19	+	216	16	0	0	12.50%	43.46521658011112	0.002967235347054297	0.002967235347054297	4.9907879201100895e-05
ENSG00000160789_156135194_156137091_257	LMNA	1	+	771	51	7	6	7.39%	38.814269280682886	0.0002686155635799695	0.0005050675515745223	9.103223220948592e-07
ENSG00000196712_31163201_31299606_1521	NF1	17	+	4563	19	2	5	0.66%	2.443310991189678	0.010678932720565162	0.032024696723185885	0.0016574421096634785
ENSG00000118680_3272899_3277937_172	MYL12B	18	+	516	55	6	0	5.23%	62.54442502079943	0.006172075136861155	0.0037896409716948643	0.00012737069552876224
ENSG00000184220_100162372_100178388_181	CMSS1	3	+	543	26	0	0	7.18%	28.096300220292825	0.0006560461581947184	0.0006560461581947184	2.755602860048755e-06
ENSG00000133835_119479012_119541994_532	HSD17B4	5	+	1596	38	0	0	2.82%	13.970962472178575	0.00029563251185049877	0.00029563251185049877	5.909204346842462e-07
ENSG00000178982_38624180_38626156_48	EIF3K	19	+	144	169	3	4	16.67%	688.6520251911355	0.013991246267585163	0.024297543496444415	0.0015971477399863874
ENSG00000198901_90974623_90966579_184	PRC1	15	-	552	32	1	0	3.80%	34.01625645400001	0.020525443280229434	0.008574016250729465	0.0008739357725210369
ENSG00000100462_22924125_22921047_159	PRMT5	14	-	477	20	5	0	5.03%	24.602952781194976	0.03662704988910695	0.005306578019052857	0.0010582113206215205
ENSG00000065809_14530459_14521190_131	FAM107B	10	-	393	21	0	0	6.11%	31.35467913603436	0.005604866691947602	0.005604866691947602	0.00016752456572620382
ENSG00000173409_230995813_230997263_104	ARV1	1	+	312	13	3	0	4.81%	24.449184326312505	0.09718295540155453	0.019216965118390855	0.008620840042435133
ENSG00000104960_49850881_49860319_374	PTOV1	19	+	1122	20	5	0	2.67%	10.459544096818185	0.0293371185312488	0.0023218226288164415	0.0004211151703108785
ENSG00000111665_6849703_6849043_102	CDCA3	12	-	306	12	2	0	7.84%	23.010997013000004	0.030313543781140352	0.004826937407846361	0.0007979355218377862
ENSG00000105355_4861394_4839192_422	PLIN3	19	-	1266	24	2	0	2.61%	11.123799503914695	0.0013845019718837994	0.0014915192737735051	1.2455606331158933e-05
ENSG00000072778_7219985_7221978_167	ACADVL	17	+	501	29	1	9	5.39%	33.96533391439821	0.006142079104304129	0.15108907193380217	0.006208248298524754
ENSG00000071626_1407774_1434912_406	DAZAP1	19	+	1218	23	5	5	2.46%	11.08041851241749	0.022448209392418098	0.051388429793604085	0.005057665769100759
ENSG00000152291_85327962_85322665_479	TGOLN2	2	-	1437	56	2	3	4.59%	22.866878033795413	3.503787992526855e-05	8.434738688455117e-05	2.231575800975992e-08
ENSG00000092203_21477279_21496606_621	TOX4	14	+	1863	10	0	0	1.13%	3.149653375370371	0.007029302661868638	0.007029302661868638	0.0002573639808771639
ENSG00000163636_64022521_64010668_340	PSMD6	3	-	1020	72	5	0	5.88%	41.41979462340001	0.000602543381100086	3.45592214554821e-05	1.670045363763243e-07
ENSG00000103202_399666_400342_65	NME4	16	+	195	10	2	0	7.69%	30.091303786230775	0.019679754444124863	0.019679754444124863	0.0017849636115922674
ENSG00000133706_146182493_146114106_1176	LARS	5	-	3528	54	3	3	2.21%	8.981333017829083	1.967365218906857e-05	1.967365218906857e-05	3.045738923194256e-09
ENSG00000115084_113750514_113742677_145	SLC35F5	2	-	435	14	0	0	3.45%	18.88488720377242	0.02108409854857798	0.02108409854857798	0.002029891263157455
ENSG00000084207_67584484_67585081_72	GSTP1	11	+	216	154	0	16	29.17%	418.35270958356955	2.5695877403898546e-05	0.0003543769227346193	7.297186085012358e-08
ENSG00000084207_67585179_67586577_119	GSTP1	11	+	357	204	14	4	23.53%	335.3030993322858	1.167709203215424e-05	6.362023127114081e-06	6.101955064853252e-10
ENSG00000169738_82037682_82035960_244	DCXR	17	-	732	44	4	1	6.56%	35.27095443795903	0.000496726323837006	0.00016170093096774888	5.560978152561381e-07
ENSG00000163931_53229340_53228863_111	TKT	3	-	333	58	0	0	10.81%	102.20199574242345	0.00108291700051257	0.00108291700051257	7.232521514942848e-06
ENSG00000101365_2664188_2658751_385	IDH3B	20	-	1155	82	4	0	8.05%	41.65886991704157	1.0850441418293873e-06	4.886518220880421e-07	4.825951805722707e-12
ENSG00000130826_154769182_154774789_158	DKC1	X	+	474	11	0	0	3.80%	13.617267219718357	0.012798402692974279	0.012798402692974279	0.0007972436888943567
ENSG00000130204_44891416_44903169_361	TOMM40	19	+	1083	23	1	0	2.22%	12.461634116458452	0.007181907427766128	0.005554612275049112	0.0002105152421252746
ENSG00000116580_155827017_155750584_2069	GON4L	1	-	6207	16	2	0	0.29%	1.5125643275824072	0.044040755831095145	0.013428347753762199	0.0027917083341200354
ENSG00000175203_57547063_57535020_132	DCTN2	12	-	396	16	1	0	6.06%	23.708299952787883	0.009288069496478249	0.005306578019052857	0.0002590037054391842
ENSG00000100138_41682358_41674933_128	SNU13	22	-	384	51	2	2	11.72%	77.93177504012111	0.0002798052361462181	0.000679565994910149	1.2702632058293115e-06
ENSG00000106268_2244575_2251001_156	NUDT1	7	+	468	34	3	0	7.05%	42.6293470304936	0.003453048488940946	0.0016186486669924592	3.275367415488609e-05
ENSG00000117543_101025443_100990408_284	DPH5	1	-	852	9	2	1	2.82%	6.198384758783452	0.0416322583317752	0.010460667668897016	0.0021333624110880687
ENSG00000169567_131165205_131162382_103	HINT1	5	-	309	47	2	2	11.65%	89.25139132712138	0.0013282749526633097	0.0023453309689915928	1.8603609104278156e-05
ENSG00000139718_121810458_121830239_1419	SETD1B	12	+	4257	8	0	0	0.35%	1.1027116257110643	0.019679754444124863	0.019679754444124863	0.0017849636115922674
ENSG00000182985_115504394_115176474_414	CADM1	11	-	1242	18	6	0	2.42%	8.504064113500002	0.055579408403928485	0.002046028879997488	0.0007983402781655223
ENSG00000164885_151057848_151054009_260	CDK5	7	-	780	15	0	0	3.08%	11.284238919836541	0.004873903550586949	0.004873903550586949	0.00012846764509679302
ENSG00000248167_30329576_30346810_417	TRIM39-RPP21	6	+	1251	28	2	1	1.68%	13.133374793990411	0.032657852871826265	0.00847367416878415	0.0013870266618523912
ENSG00000087111_28570954_28554220_494	PIGS	17	-	1482	27	5	2	3.24%	10.690331608266197	0.003374005176131343	0.0008737614202411996	1.822654100925673e-05
ENSG00000134318_11344109_11183437_1379	ROCK2	2	-	4137	33	2	2	1.09%	4.680627021135969	0.001164427877724285	0.001164427877724285	8.315212452002076e-06

ENSG00000182985_115231410_115176474_285	CADM1	11	-	855	9	0	0	2.11%	6.17663604033158	0.011772051206276392	0.011772051206276392	0.000681272511389508
ENSG00000011028_62664700_62676548_526	AC080038.1	17	+	1578	20	4	0	1.33%	7.437014243745248	0.01975957366273817	0.008175610787902942	0.0008066304929418462
ENSG00000136371_79896919_79845210_180	MTHFS	15	-	540	8	0	0	3.33%	8.693043316022225	0.009815328628645334	0.009815328628645334	0.0004837142251238724
ENSG00000084234_130070613_130143448_761	APLP2	11	+	2283	145	2	6	7.62%	37.26813905193496	1.7541027497337622e-11	1.3388236953785959e-10	2.8501863811994904e-20
ENSG00000141198_54912675_54960626_399	TOM1L1	17	+	1197	17	3	1	2.51%	8.333556562352133	0.01664033820237559	0.00397428314897603	0.0003597475883221794
ENSG00000173473_47738079_47588209_961	SMARCC1	3	-	2883	37	5	2	1.56%	7.530654069290844	0.0026483996600820516	0.0010357853790658362	1.6665132175167482e-05
ENSG00000114120_140968715_140976453_188	SLC25A36	3	+	564	6	0	0	2.66%	6.242344934377661	0.016947426762344633	0.016947426762344633	0.0013498980316300959
ENSG00000102054_16869134_16845035_391	RBBP7	X	-	1173	65	2	2	4.35%	32.51553925750001	0.00013535602770218042	0.000239322815548449	2.2713971572179392e-07
ENSG00000102054_16869134_16845035_391	RBBP7	X	-	1173	65	2	2	4.35%	32.51553925750001	0.00013535602770218042	0.000239322815548449	2.2713971572179392e-07
ENSG00000080503_2029239_2192739_1500	SMARCA2	9	+	4500	22	4	6	0.73%	2.868704294287334	0.014015870411971724	0.03286917352493041	0.0021544021968327126
ENSG00000141522_81869930_81868876_160	ARHGDIA	17	-	480	95	3	0	15.62%	116.1336255499844	5.085528497282444e-06	5.085528497282444e-06	2.165060536621522e-10
ENSG00000078808_1228772_1217512_355	SDF4	1	-	1065	47	8	4	5.35%	25.895474103361977	0.0017282002293757697	0.00044982325727408205	5.044838693846214e-06
ENSG00000132305_86173719_86144268_598	IMMT	2	-	1794	43	1	2	3.18%	14.06441372617308	7.529564399720783e-05	0.00017380946227089694	9.478246527617716e-08
ENSG00000120254_150877652_151092556_869	MTHFD1L	6	+	2607	21	1	1	1.15%	4.726654737422901	0.0037373685375237473	0.0037373685375237473	7.750388117310624e-05
ENSG00000120265_149749728_149802379_285	PCMT1	6	+	855	45	1	4	7.02%	30.8831802016579	5.185709832851827e-05	0.00035548993793247866	1.3841409122193912e-07
ENSG00000250151_9793122_9834821_625	ARPC4-TTLL3	3	+	1875	80	1	7	2.88%	25.035964750144007	0.00010660377176095898	0.00015306160015459227	1.1642472826723993e-07
ENSG00000103249_1474974_1446631_781	CLCN7	16	-	2343	19	0	2	1.15%	4.758355976439822	0.0034782380676658805	0.011768186811045352	0.00022426489914806197
ENSG00000148187_122291788_122322617_96	MRRF	9	+	288	13	2	1	6.25%	26.486616353505216	0.05067994629101479	0.019795881618345192	0.004478851500628176
ENSG00000156469_96258690_96239829_361	MTERF3	8	-	1083	13	0	2	1.94%	7.043532326693907	0.00797940233269714	0.0424352593247778	0.0017364963264733548
ENSG00000197563_62157141_62045856_704	PIGN	18	-	2112	9	0	0	0.71%	2.5004847606455973	0.016947426762344633	0.016947426762344633	0.0013498980316300959
ENSG00000156113_77637360_76877739_1140	KCNMA1	10	-	3420	15	4	2	0.70%	2.5735983501381585	0.048994651353958696	0.02389626957043539	0.005091569865673637
ENSG00000182985_115504394_115169589_387	CADM1	11	-	1161	17	6	0	2.33%	8.59196141699871	0.07496934455568298	0.0033030736763512156	0.0016484444894582333
ENSG00000071553_154432941_154435891_281	ATP6AP1	X	+	843	63	1	0	6.41%	43.851917795236666	0.00011373720035898656	9.087114746133616e-05	7.463786495767001e-08
ENSG00000100196_38468279_38482536_199	KDELR3	22	+	597	20	0	0	3.02%	19.657635639246237	0.011772051206276392	0.011772051206276392	0.000681272511389508
ENSG00000166206_26621357_26553491_230	GABRB3	15	-	690	18	0	1	2.61%	15.307315404300004	0.013428347753762199	0.02034245439037237	0.0012955184805712684
ENSG00000153310_129879461_129842142_324	FAM49B	8	-	972	28	0	1	3.70%	16.903139781154326	0.000978917897162665	0.0013147568071904994	7.922904919322548e-06
ENSG00000156482_98045367_98025633_90	RPL30	8	-	270	98	11	5	16.67%	212.9795612425445	0.0008568559278198205	0.000309684704389587	1.7608478415181632e-06
ENSG00000165102_43146980_43168049_172	HGSNAT	8	+	516	18	0	0	2.91%	20.469084552261634	0.02108409854857798	0.02108409854857798	0.002029891263157455
ENSG00000213339_10712596_10713270_127	QTRT1	19	+	381	16	0	0	3.94%	24.641697588724416	0.02108409854857798	0.02108409854857798	0.002029891263157455
ENSG00000074800_8874908_8863202_402	ENO1	1	-	1206	831	50	48	27.61%	404.3238243814068	9.055240199112954e-18	9.845858981642282e-19	1.4131037291240412e-34
ENSG00000284934_122226014_122208381_239	DIABLO	12	-	717	18	0	1	2.93%	14.730889301209208	0.00797940233269714	0.015625008558487224	0.0006253789790324684
ENSG00000139644_49752494_49762896_237	TMBIM6	12	+	711	661	23	17	32.49%	545.5159777111414	4.4607759695857914e-14	1.475365366222195e-13	8.888324263736637e-26
ENSG00000135899_230216783_230176665_499	SP110	2	-	1497	6	1	0	1.00%	2.3518253460180367	0.0477903522728147	0.016947426762344633	0.0036975118224100542
ENSG00000114209_167720136_167684308_205	PDCD10	3	-	615	18	0	0	3.41%	17.174061185312198	0.00847367416878415	0.00847367416878415	0.0003665024264111182
ENSG00000124225_57659647_57652053_234	PMEPA1	20	-	702	56	5	5	5.13%	46.808694778581206	0.00638574031296502	0.00396067672340455	0.0001371656018651211
ENSG00000021574_32064090_32154496_498	SPAST	2	+	1494	10	3	0	1.20%	3.927579811455824	0.08791701959130671	0.012798402692974279	0.0056117368578515354
ENSG00000198836_193615695_193655039_550	OPA1	3	+	1650	19	0	0	2.18%	6.756865486544547	0.0008852284070317903	0.0008852284070317903	4.90776869367864e-06
ENSG00000198836_193657119_193692127_276	OPA1	3	+	828	11	0	1	1.81%	7.795392104041668	0.020613416668581838	0.028542519635534256	0.0026455223939317236
ENSG00000215301_41343231_41347719_476	DDX3X	X	+	1428	83	1	0	7.56%	34.10558485855358	6.958679763556736e-08	4.081835640353621e-08	2.8150375102779016e-14
ENSG00000105821_103344622_103312569_621	DNAJC2	7	-	1863	12	0	4	1.13%	3.7795840504444453	0.008175610787902942	0.07864960352514258	0.003490498801610728
ENSG00000168438_110180445_110230131_579	CDC40	6	+	1737	12	2	0	1.21%	4.053750769129534	0.03781105849985612	0.0078820576498062	0.001520872574988137
ENSG00000171503_158685160_158708527_435	ETFDH	4	+	1305	8	0	0	1.15%	3.5971213721471273	0.019679754444124863	0.019679754444124863	0.0017849636115922674
ENSG00000161960_7572842_7575402_162	EIF4A1	17	+	486	33	2	4	4.94%	39.84311519843519	0.007225456388766877	0.013786173822478529	0.0005054747501467518
ENSG00000110880_108701318_108647403_474	CORO1C	12	-	1422	60	4	0	4.43%	24.758667672215196	0.00010410568222945417	2.6701066368151088e-05	2.1337412292728915e-08
ENSG00000072134_19283294_19334254_526	EPN2	17	+	1578	9	0	0	1.14%	3.346656409685362	0.012178957859018494	0.012178957859018494	0.0007262454987071801
ENSG00000072134_19283294_19335448_426	EPN2	17	+	1278	9	0	0	1.41%	4.132256505855635	0.012178957859018494	0.012178957859018494	0.0007262454987071801
ENSG00000120438_159781025_159779045_262	TCP1	6	-	786	98	0	2	7.63%	73.16091798408017	4.243508135080653e-05	8.358446385742584e-05	2.6538515701902975e-08
ENSG00000133657_194460735_194405919_1177	ATP13A3	3	-	3531	37	0	1	1.02%	6.148647884951998	0.0010447178140462346	0.0017534708537449623	1.1163276398224378e-05
ENSG00000137824_40754783_40736541_470	RMDN3	15	-	1410	6	0	0	1.06%	2.496937973751064	0.016947426762344633	0.016947426762344633	0.0013498980316300959
ENSG00000175756_1374756_1373801_199	AURKAIP1	1	-	597	71	5	5	9.55%	69.78460651932414	0.00036318786265457023	0.0004249631561444351	1.0245332642003257e-06
ENSG00000176393_201982667_202005716_650	RNPEP	1	+	1950	74	5	7	4.62%	22.267564801810774	5.045347666206196e-06	1.3301471780101773e-05	5.561063255761415e-10
ENSG00000100591_77468895_77469249_87	AHSA1	14	+	261	38	0	0	8.05%	85.43163258849427	0.008877961307018026	0.008877961307018026	0.00040023486315785033
ENSG00000090060_96502593_96531696_238	PAPOLA	14	+	714	18	0	1	2.94%	14.792783794071433	0.00797940233269714	0.015625008558487224	0.0006253789790324684
ENSG00000115762_131120942_131146750_171	PLEKHB2	2	+	513	17	4	3	5.85%	19.444965312154974	0.03950234393976166	0.013383828821373808	0.002491043730068833
ENSG00000123136_14413220_14408936_427	DDX39A	19	-	1281	69	7	6	7.03%	31.606439691158087	9.889430814937904e-06	1.459776839959068e-05	1.1650174034388367e-09
ENSG00000160710_154627995_154584806_1235	ADAR	1	-	3705	110	8	7	3.32%	17.421281139396765	1.0440789895332017e-06	2.7557510489182665e-07	2.6760137837556876e-12
ENSG00000261796_129159155_129095025_323	ISY1-RAB43	3	-	969	11	2	1	2.17%	6.661078082710528	0.0416322583317752	0.017404239405933563	0.003292339026149851
ENSG00000089159_120221730_120212314_350	PXN	12	-	1050	21	0	0	3.14%	11.735608476630002	0.001092092816906976	0.001092092816906976	7.350803965414657e-06
ENSG00000117592_173477398_173481509_92	PRDX6	1	+	276	57	0	1	7.61%	121.18291361737502	0.008877961307018026	0.012176559331126819	0.0005408334713909414
ENSG00000092841_56158402_56160654_151	MYL6	12	+	453	777	13	19	31.13%	1006.4644355785333	1.4364899689827779e-09	1.2743310073030253e-08	2.0123650479473058e-16
ENSG00000097021_6339454_6281241_183	ACOT7	1	-	549	8	1	0	2.73%	8.550534409202188	0.04223451612662591	0.019679754444124863	0.003715148010809822
ENSG00000184226_67228440_67225342_1032	PCDH9	13	-	3096	7	0	1	0.48%	1.3266999246836244	0.016947426762344633	0.0477903522728147	0.0036975118224100542
ENSG00000184226_67228440_66304655_1237	PCDH9	13	-	3711	11	0	1	0.57%	1.7393114152914313	0.006937202941512724	0.016735684067288745	0.000590529598856171
ENSG00000141425_36040849_35993151_276	RPRD1A	18	-	828	12	0	0	2.54%	8.504064113500002	0.008175610787902942	0.008175610787902942	0.00034251843268961324
ENSG00000119650_75988894_76083577_187	IFT43	14	+	561	18	0	0	3.74%	18.82717937427273	0.0078820576498062	0.0078820576498062	0.00031963642907451164
ENSG00000111716_21637217_21635542_104	LDHB	12	-	312	248	8	0	25.00%	466.41520868657705	7.02437887523143e-06	4.0118501991435526e-06	2.362921543768097e-10
ENSG00000112742_80005892_80042202_840	TTK	6	+	2520	11	0	0	0.95%	2.5613431198994054	0.004193160480899131	0.004193160480899131	9.649553577281792e-05

ENSG00000082516_154938133_154888210_1508	GEMIN5	5	−	4524	20	4	0	0.86%	2.5940779126061013	0.006880387941862762	0.000564499309058709	2.6225564658237746e−05
ENSG00000183010_81936814_81933214_288	PYCR1	17	−	864	30	5	1	4.51%	20.37432027192709	0.008109984001678278	0.0008605146384484945	4.495667212437756e−05
ENSG00000065548_186486383_186508733_426	ZC3H15	2	+	1278	12	2	2	2.58%	5.509675341140846	0.010450088497317475	0.010450088497317475	0.00054437848984498
ENSG00000073060_124863630_124786096_523	SCARB1	12	−	1569	46	1	2	3.44%	17.203250156946467	0.00012374249556490374	0.00031567046080049535	2.747431735905352e−07
ENSG00000155463_22770608_22771558_163	OXA1L	14	+	489	43	2	3	9.20%	51.59827857823007	0.0010376102755361187	0.0013919855294422787	8.851235358247057e−06
ENSG00000110921_109586077_109596577_202	MVK	12	+	606	11	0	0	2.97%	10.65112980552228	0.012798402692974279	0.012798402692974279	0.0007972436888943567
ENSG00000104763_18084058_18057534_366	ASAH1	8	−	1098	103	3	2	6.83%	55.04406525923908	4.832539619711528e−06	1.7945646473521957e−05	7.210754651504709e−10
ENSG00000151176_113358613_113388626_585	PLBD2	12	+	1755	35	1	0	2.91%	11.702173694645301	0.0001834224710074371	0.00012027451043711783	1.561264616469967e−07
ENSG00000008282_106096295_106091805_108	SYPL1	7	−	324	162	4	0	14.81%	293.39021191575006	0.0006212154707631996	0.00020701231900031988	8.764945859625247e−07
ENSG00000186132_119339959_119302472_126	C2orf76	2	−	378	10	0	0	3.97%	15.523291635753973	0.020613416668581838	0.020613416668581838	0.001946208561389314
ENSG00000042493_85402145_85394893_348	CAPG	2	−	1044	30	0	0	4.31%	16.86150643193966	0.00028426559431428834	0.00028426559431428834	5.47742908020375e−07
ENSG00000159210_48944734_48933341_36	SNF8	17	−	108	12	0	0	16.67%	65.19782487016668	0.011772051206276392	0.011772051206276392	0.000681272511389508
ENSG00000172046_49117460_49108412_1190	USP19	3	−	3570	20	0	0	0.76%	3.287285287571429	0.0034782380676658805	0.0034782380676658805	6.758309314600117e−05
ENSG00000161011_179820937_179836593_440	SQSTM1	5	+	1320	867	41	24	22.95%	385.408051107508	3.636194378348849e−17	1.6962409662399367e−17	9.37775314841159e−33
ENSG00000143252_161328430_161362433_132	SDHC	1	+	396	15	1	0	4.55%	22.22653120573864	0.012798402692974279	0.012798402692974279	0.0007972436888943567
ENSG00000122705_36191057_36211774_230	CLTA	9	+	690	177	11	4	12.61%	150.52193480895002	2.048454080571646e−05	4.711542246598215e−06	8.047163936813439e−10
ENSG00000106976_128203486_128254078_859	DNM1	9	+	2577	54	2	7	2.33%	12.295748112883588	5.0604056647534704e−05	0.0009696113333780397	3.894133597030637e−07
ENSG00000269893_118279412_118279714_36	SNHG8	4	+	108	22	5	0	16.67%	119.52934559530559	0.013428347753762199	0.013217226988443315	0.0008596769774033651
ENSG00000168159_228493062_228493885_71	RNF187	1	+	213	19	0	3	12.68%	52.34191574083804	0.0035291660242640502	0.022360378399288262	0.00044311298448702793
ENSG00000164754_116850749_116847500_135	RAD21	8	−	405	32	0	0	6.67%	46.36289768545186	0.0034782380676658805	0.0034782380676658805	6.758309314600117e−05
ENSG00000112237_99550995_99543555_138	CCNC	6	−	414	9	0	1	3.62%	12.756096170250004	0.019679754444124863	0.04223451612662591	0.003715148010809822
ENSG00000153187_244856835_244856443_96	HNRNPU	1	−	288	49	0	5	8.33%	99.83416933244273	0.005604866691947602	0.036675066252427384	0.0011086612201768738
ENSG00000153187_244856177_244855391_163	HNRNPU	1	−	489	23	13	0	7.36%	27.59907923951841	0.07938308143334022	0.0009430837296224684	0.0007018733438413496
ENSG00000158545_88577124_88631299_977	ZC3H18	16	+	2931	10	0	0	0.51%	2.001980292840328	0.019679754444124863	0.019679754444124863	0.0017849636115922674
ENSG00000153113_96722678_96771679_686	CAST	5	+	2058	118	0	4	6.12%	33.64435860647085	5.853191170022466e−09	6.665252736283171e−08	4.1552551561798035e−15
ENSG00000105928_24749774_24708046_356	GSDME	7	−	1068	9	0	1	1.40%	4.944778852512641	0.020613416668581838	0.03537493353818881	0.003252526348827943
ENSG00000162923_224431702_224389835_494	WDR26	1	−	1482	18	0	0	1.62%	7.126887738844131	0.005306578019052857	0.005306578019052857	0.00015100114740534233
ENSG00000116288_7962786_7969355_88	PARK7	1	+	264	61	4	0	15.91%	135.5818403550057	0.001052136180361582	0.00041307551203580774	2.8272893896968226e−06
ENSG00000116288_7965412_7971013_85	PARK7	1	+	255	31	1	0	9.41%	71.33409074030001	0.005706018193000826	0.005706018193000826	0.0001733096755673334
ENSG00000123444_47577999_47572930_518	KBTBD4	11	−	1554	14	3	0	0.97%	5.286310124608109	0.1029516053660342	0.02108409854857798	0.009872287793463467
ENSG00000131069_34882901_34927214_606	ACSS2	20	+	1818	21	8	0	1.32%	6.777991694423269	0.12092248052568716	0.005554612275049112	0.004356608937343701
ENSG00000159403_7092388_7080532_705	C1R	12	−	2115	34	0	6	2.13%	9.432876789726244	0.00028877520139654507	0.0030936958978407523	6.222146906924512e−06
ENSG00000270647_35809570_35824202_202	TAF15	17	+	606	15	0	1	3.96%	14.524267916621291	0.005405141725979036	0.008170681041463092	0.00023248977892435937
ENSG00000100997_25390703_25300845_398	ABHD12	20	−	1194	25	4	2	3.52%	12.286022274528897	0.004559701316823627	0.00214973468486707	5.6042172567140295e−05
ENSG00000078304_101882173_101925272_422	PPP2R5C	14	+	1266	8	1	0	1.18%	3.707933167971565	0.04223451612662591	0.019679754444124863	0.003715148010809822
ENSG00000115525_85848138_85840144_237	ST3GAL5	2	−	711	13	0	3	2.53%	10.728755991293252	0.012798402692974279	0.07760924484234204	0.004890251693990683
ENSG00000131508_139614609_139626801_110	UBE2D2	5	+	330	12	6	1	4.55%	21.337469957509096	0.1530680197004018	0.03537493353818881	0.022673278827518507
ENSG00000153310_129879461_129842142_324	FAM49B	8	−	972	28	0	1	3.70%	16.903139781154326	0.000978917897162665	0.0013147568071904994	7.922904919322548e−06
ENSG00000087111_28571496_28554220_555	PIGS	17	−	1665	29	5	2	3.06%	10.220199574242345	0.0020816078341534825	0.0005793435147328683	7.677924913035978e−06
ENSG00000171848_10122799_10129386_389	RRM2	2	+	1167	52	1	1	4.37%	26.1461714132288	0.00012152394927894852	0.00018495144114449941	1.589708426019472e−07
ENSG00000165983_16484475_16511256_319	PTER	10	+	957	14	0	0	1.88%	8.584039638078371	0.012798402692974279	0.012798402692974279	0.0007972436888943567
ENSG00000152683_32184277_32197756_132	SLC30A6	2	+	396	10	0	6	4.55%	14.817687470492428	0.012798402692974279	0.2855630337600017	0.023910390215371852
ENSG00000152683_32197810_32220713_245	SLC30A6	2	+	735	14	0	0	1.90%	11.17676997774286	0.008175610787902942	0.008175610787902942	0.00034251843268961324
ENSG00000092148_31205828_31100930_2574	HECTD1	14	−	7722	49	5	9	0.85%	3.723418902841687	0.00018963874744821557	0.0014876542978302075	1.980385353934105e−06
ENSG00000133083_35855528_35774642_422	DCLK1	13	−	1266	9	0	0	1.18%	4.17142481396801	0.019679754444124863	0.019679754444124863	0.0017849636115922674
ENSG00000122359_80169133_80155853_373	ANXA11	10	−	1119	26	2	1	3.75%	13.633861501000004	0.0018061760845665517	0.0009061776457653113	1.0061129210387768e−05
ENSG00000066855_65670010_65709044_314	MTFR1	8	+	942	23	0	0	2.87%	14.326910560641723	0.0035291660242640502	0.0035291660242640502	6.948232509684344e−05
ENSG00000177576_49483748_49482345_76	C18orf32	18	−	228	18	3	0	6.58%	46.324770302486854	0.09783387127182075	0.020613416668581838	0.009177765633874927
ENSG00000164211_111507433_111499886_205	STARD4	5	−	615	18	0	2	3.90%	17.174061185312198	0.005454749182134642	0.02071580916834944	0.0005936342691250666
ENSG00000092199_21234154_21211223_213	HNRNPC	14	−	639	21	2	14	3.76%	19.283863693992963	0.005642787686764809	0.16149615651168414	0.006378704820560942
ENSG00000140416_63059609_63061730_78	TPM1	15	+	234	95	0	9	14.10%	238.22282164099366	0.0016494676794180302	0.01253092217194181	0.00012512190574386016
ENSG00000122378_80420507_80432099_216	PRXL2A	10	+	648	49	0	0	6.02%	44.370741925530105	0.0006862354746923267	0.0006862354746923267	3.005258142461633e−06
ENSG00000147862_14313508_14088309_493	NFIB	9	−	1479	8	1	0	1.01%	3.1739306224827595	0.03418265644467716	0.019216965118390855	0.0029560527921161815
ENSG00000077152_202335718_202333168_116	UBE2T	1	−	348	48	0	4	10.34%	80.93523087331036	0.00108291700051257	0.0010573555742419644	7.068406058778556e−06
ENSG00000144895_150552379_150583911_568	EIF2A	3	+	1704	27	7	1	2.46%	9.297577138175178	0.022993437733051677	0.0006552260109690562	0.0001150461046045796
ENSG00000161638_54419198_54396293_1049	ITGA5	12	−	3147	31	1	0	1.14%	5.780169411749763	0.0013683766156140878	0.001032175130546352	8.661907654447743e−06
ENSG00000182196_122981140_122981774_92	ARL6IP4	12	+	276	8	0	5	5.43%	17.008128227000004	0.019679754444124863	0.2192890130404999	0.02250016639352502
ENSG00000120708_136060885_136063226_65	TGFBI	5	+	195	32	3	0	13.85%	96.29217211593848	0.003710480643933249	0.003710480643933249	7.644477796348072e−05
ENSG00000111912_125885201_125928771_695	NCOA7	6	+	2085	90	4	3	5.32%	25.328651388410076	3.000729572110403e−07	1.150747799011701e−07	3.3048058005417396e−13
ENSG00000198015_93469306_93501221_142	MRPL42	12	+	426	49	0	3	10.56%	67.49352292897537	0.00029180909274801665	0.0013919855294422787	2.7269977818995094e−06
ENSG00000035862_78871006_78855667_143	TIMP2	17	−	429	45	3	0	11.89%	61.55039410819931	0.00018181311632258342	0.00010000342828152886	1.2976591849339404e−07
ENSG00000130749_47112584_47066356_1303	AC008755.1	19	−	3909	18	1	0	0.84%	2.7019819976891792	0.0025593219000189936	0.0012624143894574274	1.9339285108591855e−05
ENSG00000188186_100148973_100153896_82	LAMTOR4	7	+	246	29	1	1	8.54%	69.17330199639636	0.00877616296920843	0.012088529595134127	0.0005313814658942758
ENSG00000126456_49664838_49659648_300	IRF3	19	−	900	15	0	3	2.33%	9.779673730525003	0.00847367416878415	0.041265600891389115	0.0017720059071908959
ENSG00000122786_134891621_134968345_558	CALD1	7	+	1674	39	0	2	2.69%	13.670511666325272	0.00028351878813536103	0.0007217664708893913	1.365787103429489e−06
ENSG00000146409_132790236_132770951_347	SLC18B1	6	−	1041	8	3	0	1.44%	4.509359645198848	0.14389369507707506	0.019679754444124863	0.013602114125134476
ENSG00000117593_173825230_173853651_548	DARS2	1	+	1644	26	0	1	2.55%	9.279982372031023	0.00033754525954144115	0.0006748684829181083	1.504379838610738e−06

ENSG00000138382_169822052_169811820_171	METTL5	2	-	513	13	0	0	4.09%	14.869679356353805	0.00797940233269714	0.00797940233269714	0.000327142761693177
ENSG00000065357_55938942_55953768_593	DGKA	12	+	1779	5	1	0	0.84%	1.6491861265640813	0.051235217429874705	0.012673659338734126	0.0031110695322510533
ENSG00000101911_12799286_12822796_254	PRPS2	X	+	762	8	6	0	1.97%	6.160424397181104	0.3752427096712272	0.016947426762344633	0.04227639730288046
ENSG00000131018_152284010_152122436_2739	SYNE1	6	-	8217	7	2	0	0.18%	0.4998737941852867	0.11777945996203976	0.016947426762344633	0.009674391494749069
ENSG00000175455_123948968_123914779_733	CCDC14	3	-	2199	9	0	5	0.68%	2.4015569870320608	0.019216965118390855	0.2463583386135439	0.025652408423363686
ENSG00000136518_179563093_179570267_100	ACTL6A	3	+	300	7	4	0	5.00%	13.691543222735003	0.29434406697594495	0.019216965118390855	0.03243088401287838
ENSG00000128309_37024216_37029514_297	MPST	22	+	891	13	1	2	2.69%	8.561330538506736	0.010020958156399907	0.030313543781140352	0.0014836836722180672
ENSG00000038427_83490172_83580434_3348	VCAN	5	+	10044	223	16	16	2.69%	13.02788077602793	6.642101753069708e-14	2.32686055362522e-14	2.1098282865994446e-26
ENSG00000163938_52686792_52694275_537	GNL3	3	+	1611	32	0	1	2.98%	11.655477071761641	0.00017619886174203892	0.0002818156460364 0876	3.430664841479143e-07
ENSG00000113552_142012035_142002029_289	GNPDA1	5	-	867	14	0	2	3.11%	9.475116417117649	0.00308494966027208	0.00881104548116 2216	0.00015029880372022516
ENSG00000073050_43575458_43543392_602	XRCC1	19	-	1806	8	0	2	0.83%	2.599248832033223	0.019216965118390855	0.09718295540155453	0.008620840042435133
ENSG00000167770_63988369_63997546_241	OTUB1	11	+	723	40	1	0	7.88%	32.463647238257266	4.942578964878572e-05	5.3019663895075235e-05	1.964301260235025e-08
ENSG00000198160_66925029_66984900_565	MIER1	1	+	1695	13	1	2	1.59%	4.5003808317460186	0.005849704119323 3325	0.020607861465604144	0.0006273817384862227
ENSG00000198160_66930392_66986473_433	MIER1	1	+	1299	15	1	2	1.85%	6.7757554714953825	0.007930666369886513	0.022898969405409888	0.0009107991204150371
ENSG00000113384_32173848_32126212_236	GOLPH3	5	-	708	15	1	0	3.81%	12.431788640497885	0.0025000845279814113	0.002967235347054297	4.240505170776202e-05
ENSG00000090266_140702960_140704934_41	NDUFB2	7	+	123	97	0	0	17.07%	462.74553749313424	0.008980238763039383	0.008980238763039383	0.0004089866599971986
ENSG00000151413_31561530_31859180_289	NUBPL	14	+	867	16	0	0	1.73%	10.828704476705886	0.02108409854857798	0.02108409854857798	0.002029891263157455
ENSG00000172053_49104733_49103886_100	QARS	3	-	300	13	1	0	5.00%	25.427151699365005	0.03577298629450316	0.02108409854857798	0.00335460490711 4977
ENSG00000101040_47298917_47210761_1118	ZMYND8	20	-	3354	6	0	0	0.45%	1.0496966437057247	0.016947426762344633	0.016947426762344633	0.0013498980316300959
ENSG00000067955_67029408_67098732_182	CBFB	16	+	546	35	1	0	4.95%	37.61412973278847	0.0033278027414746945	0.003580484096903955	6.661675796445952e-05
ENSG00000099783_8445050_8488854_674	HNRNPM	19	+	2022	95	7	2	6.08%	27.568813186939916	2.816928504629793e-07	1.5099924714536297e-08	4.477141256828353e-14
ENSG00000175455_123948968_123914779_733	CCDC14	3	-	2199	9	0	5	0.68%	2.4015569870320608	0.019216965118390855	0.2463583386135439	0.025652408423363686
ENSG00000104763_18069848_18057534_313	ASAH1	8	-	939	51	3	2	7.35%	31.8698632751933	1.0081031668924613e-05	4.339047084990489e-05	3.524512317448131e-09
ENSG00000100983_34951852_34928828_363	GSS	20	-	1089	30	0	0	3.31%	16.16474996780992	0.0010032776369550557	0.0010032776369550557	6.244443566103777e-06
ENSG00000114353_50252497_50257690_268	GNAI2	3	+	804	20	0	4	2.99%	14.596527956007465	0.005306578019052857	0.019778610614370123	0.0005526454465689531
ENSG00000040341_73688813_73421372_532	STAU2	8	-	1596	17	0	0	1.50%	6.2501674217640995	0.004873903550586949	0.004873903550586949	0.00012846764509679302
ENSG00000128050_56436313_56459538_425	PAICS	4	+	1275	49	1	0	5.41%	22.550777072740004	1.202122360114406e-05	9.932719860816285e-06	9.665014861240016e-10
ENSG00000128059_56435477_56395352_517	PPAT	4	-	1551	8	0	0	0.97%	3.0265914833346237	0.019679754444124863	0.019679754444124863	0.0017849636115922674
ENSG00000019186_54173063_54172905_52	CYP24A1	20	-	156	28	1	3	19.23%	105.31956325180772	0.002376614607 4493525	0.01128045782890445	0.0001536097771 6467975
ENSG00000135778_232950711_232978231_120	NTPCR	1	+	360	35	2	0	7.50%	57.04809676139585	0.011952930790615185	0.003580484096903955	0.00023376975231837705
ENSG00000066117_50085370_50099000_474	SMARCD1	12	+	1422	10	0	0	1.48%	4.126444612035866	0.007593099278532274	0.007593099278532274	0.0002978328670546 2064
ENSG00000068001_50320429_50318129_453	HYAL2	3	-	1359	6	0	0	1.10%	2.5906420478211927	0.016947426762344633	0.016947426762344633	0.0013498980316300959
ENSG00000159352_151266385_151267343_97	PSMD4	1	+	291	11	0	0	5.15%	22.180703306345364	0.020613416668581838	0.020613416668581838	0.001946208561389314
ENSG00000075790_107580773_107618605_348	BCAP29	7	+	1044	31	1	0	3.74%	17.423556646337648	0.0009061776457653113	0.0006401566880482122	3.6823488174641324e-06
ENSG00000139842_113239480_113263582_438	CUL4A	13	+	1314	11	2	0	1.37%	4.912164887478312	0.04223451612662591	0.012798402692974279	0.0025674639266022752
ENSG00000173334_125436046_125436471_141	TRIB1	8	+	423	15	0	0	4.26%	20.807816447925536	0.013428347753762199	0.013428347753762199	0.0008725593497644525
ENSG00000010270_38207604_38228854_201	STARD3NL	7	+	603	13	0	2	3.98%	12.650324228539803	0.004193160480899131	0.03522780890193623	0.0008357165082995001
ENSG00000131143_85801206_85805115_83	COX4I1	16	+	249	116	18	4	19.28%	273.35955487732537	0.0012803358715245219	0.0005756616192752859	4.684157119402114e-06
ENSG00000039068_68737416_68822049_537	CDH1	16	+	1611	14	0	1	1.86%	5.099271218895718	0.0019339343333298487	0.0038283733559 73562	4.275798019266722e-05
ENSG00000204370_112086908_112088294_72	SDHD	11	+	216	15	0	1	6.94%	40.74864054385418	0.019679754444124863	0.022004978195124387	0.0019818329449194675
ENSG00000118508_146543872_146554605_225	RAB32	6	+	675	14	0	1	3.56%	12.170260642431113	0.004687384229717438	0.009847127393243164	0.0002449884871 1271347
ENSG00000050426_51049080_51058072_99	LETMD1	12	+	297	16	0	0	5.05%	31.61106660371718	0.02108409854857798	0.02108409854857798	0.002029891263157455
ENSG00000111331_112938531_112944687_223	OAS3	12	+	669	27	0	0	5.83%	23.68172114118162	0.0005460508606305346	0.0005460508606305346	1.9341343681665648e-06
ENSG00000129691_38114228_38139071_421	ASH2L	8	+	1263	15	0	0	2.14%	6.968888644079574	0.002967235347054297	0.002967235347054297	4.9907879201100895e-05
ENSG00000149485_61816758_61811820_223	FADS1	11	-	669	31	3	9	4.93%	27.190124273208525	0.005079719694443635	0.06086138968360902	0.0017966388823062675
ENSG00000163932_53184882_53192266_411	PRKCD	3	+	1233	5	0	0	1.22%	2.3794826594951344	0.012673659338734126	0.012673659338734126	0.000782701129001274
ENSG00000142002_4704003_4676564_648	DPP9	19	-	1944	17	1	8	1.39%	5.131310290707563	0.006112543793728684	0.06699482590911521	0.002317603761511 6186
ENSG00000183258_177514993_177511791_382	DDX41	5	-	1146	20	1	5	2.36%	10.240496052905762	0.006142079104304129	0.04227848696273928	0.0013942017966237796
ENSG00000139197_7191313_7210223_549	PEX5	12	+	1647	13	0	0	1.28%	4.631539471651185	0.007029302661868638	0.007029302661868638	0.0002573639808771639
ENSG00000176890_657743_672997_313	TYMS	18	+	939	77	3	2	7.35%	48.117244552742825	2.4539384506914935e-05	1.5630813368268775e-05	3.026634757605723e-09
ENSG00000126247_36141012_36149839_268	CAPNS1	19	+	804	542	21	21	24.63%	395.56590760780233	7.582643319929246e-13	7.696921295911204e-12	7.55989125983609e-23
ENSG00000184983_42090744_42086183_128	NDUFA6	22	-	384	66	3	2	14.84%	100.85288534603909	5.0264223003119184e-05	0.00019330044329667593	7.213440131483771e-08
ENSG00000166788_18106041_18080399_473	SAAL1	11	-	1419	24	0	1	1.69%	9.924404631399579	0.005604866691947602	0.007225456388766877	0.00021353222845814395
ENSG00000167986_61332968_61300136_1140	DDB1	11	-	3420	91	14	4	3.16%	15.613163324171495	8.644851396572901e-06	4.6546303745396545e-07	3.82054899496771e-11
ENSG00000167985_61430147_61446071_166	SDHAF2	11	+	498	17	0	0	5.42%	20.030657038424703	0.0034782380676658805	0.0034782380676658805	6.758309314600117e-05
ENSG00000113048_72297685_72228198_197	MRPS27	5	-	591	23	0	1	5.08%	22.83578637584518	0.002390425500591921	0.003133490598659634	4.28420906379313e-05
ENSG00000167526_89560981_89563042_204	RPL13	16	+	612	184	2	7	8.82%	176.41764376633338	9.633259387622616e-05	0.00014401435018527767	9.950423633065643e-08
ENSG00000177200_53156537_53324895_2748	CHD9	16	+	8244	14	0	5	0.22%	0.99647330587591	0.013217226988443315	0.11669065552020402	0.007926800932849956
ENSG00000113657_147413634_147394035_403	DPYSL3	5	-	1209	15	2	0	2.23%	7.280154141830026	0.016821537760974333	0.003278442994 61777	0.0003080469631192784
ENSG00000103145_3023324_3022863_75	HCFC1R1	16	-	225	16	0	0	6.67%	41.72660791690667	0.02108409854857798	0.02108409854857798	0.002029891263157455
ENSG00000088833_1466824_1446110_274	NSFL1C	20	-	822	37	0	2	4.74%	26.41225752039599	0.0006446704936422262	0.00260867269305024	1.0646997672603425e-05
ENSG00000089220_118139479_118144803_96	PEBP1	12	+	288	55	4	3	14.58%	112.05876149559899	0.0020810102006077586	0.0015021311196991417	1.8577555160358326e-05
ENSG00000070495_76726475_76720448_361	JMJD6	17	-	1083	11	0	3	1.39%	5.9599119687409985	0.020613416668581838	0.12312584984626351	0.011809609471937859
ENSG00000135048_71730049_71685197_724	CEMIP2	9	-	2172	40	6	0	2.62%	10.80626931549724	0.00045618483746905 41	5.3019663895075235e-05	1.829208803361143e-07
ENSG00000135048_71750373_71730250_580	CEMIP2	9	-	1740	26	2	1	2.07%	8.767983344608623	0.003316424919377475	0.0014762688094080156	2.890450985722491e-05
ENSG00000050820_75236980_75229511_599	BCAR1	16	-	1797	18	0	0	1.34%	5.877600238712856	0.005554612275049112	0.005554612275049112	0.0001646845769100 7306
ENSG00000105221_40255258_40233872_376	AKT2	19	-	1128	9	2	4	1.33%	4.681758700783246	0.10105102209468636	0.14389369507707506	0.04910224496431627

ENSG00000081870_53940082_53921651_144	HSPB11	1	-	432	8	0	5	3.47%	10.86630414502778	0.019679754444124863	0.17004230409153215	0.016523685284367155
ENSG00000149499_62609433_62602475_670	EML3	11	-	2010	5	2	0	0.75%	1.4596527956007466	0.15865525393145707	0.012673659338734126	0.011061610944025602
ENSG00000134824_61828391_61865689_444	FADS2	11	+	1332	197	20	15	13.96%	86.78359121231647	6.920756109805933e-10	4.715342049834562e-11	3.920671787858485e-19
ENSG00000139842_113209628_113263582_759	CUL4A	13	+	2277	13	2	0	1.05%	3.3500858628939403	0.01943569092740073	0.004826937407846361	0.0004999687388256805
ENSG00000162976_11155547_11177828_202	PQLC3	2	+	606	20	2	1	4.95%	19.365690555495053	0.011698770487832223	0.00397428314897603	0.0002509293845041528
ENSG00000182325_144356510_144355531_201	FBXL6	8	-	603	8	0	0	3.48%	7.784814909870649	0.005706018193000826	0.005706018193000826	0.0001733096755673334
ENSG00000116679_185309157_185298035_600	IVNS1ABP	1	-	1800	28	3	7	1.83%	9.127695481823336	0.00678005960038948	0.031366318898922906	0.0011006958601886057
ENSG00000257923_101816052_102283090_671	CUX1	7	+	2013	11	8	0	1.19%	3.2064504034508206	0.18176997233392067	0.004687384229717438	0.006576065488566696
ENSG00000172590_22829913_22834321_123	MRPL52	14	+	369	46	3	7	13.82%	73.14877912262604	0.000574079775045688	0.002659913808842404	9.780954137223355e-06
ENSG00000101361_2655284_2656044_163	NOP56	20	+	489	10	2	1	3.68%	11.999599669355831	0.061356297364084836	0.023672501042627626	0.006320164737030106
ENSG00000115525_85863399_85840144_362	ST3GAL5	2	-	1086	12	1	3	1.66%	6.483761589298344	0.026602764905793736	0.09783387127182075	0.011244525270099857
ENSG00000125875_441682_438586_226	TBC1D20	20	-	678	15	0	0	2.21%	12.981867783882747	0.02108409854857798	0.02108409854857798	0.002029891263157455
ENSG00000172269_119101631_119096998_400	DPAGT1	11	-	1200	50	0	0	4.50%	24.449184326312505	8.83028806972547e-05	8.83028806972547e-05	5.672071890219966e-08
ENSG00000132356_40798162_40762778_565	PRKAA1	5	-	1695	19	3	0	1.59%	6.577479677167259	0.02736362956991709	0.002967235347054297	0.000476754742153865
ENSG00000132424_99412620_99400540_736	PNISR	6	-	2208	16	0	0	1.22%	4.252032056750001	0.0032294771334464936	0.0032294771334464936	5.8663648728941325e-05
ENSG00000138018_26364341_26389070_354	SELENOI	2	+	1062	25	0	0	2.82%	13.813098489442094	0.0023628488670607213	0.0023628488670607213	3.2295294885504634e-05
ENSG00000176209_42546494_42552608_100	SMIM19	8	+	300	8	0	0	6.00%	15.647477968840004	0.009815328628645334	0.009815328628645334	0.0004837142251238724
ENSG00000125485_132669934_132594866_746	DDX31	9	-	2238	9	0	0	0.80%	2.3597067982500004	0.012178957859018494	0.012178957859018494	0.0007262454987071801
ENSG00000154781_14661595_14671194_267	CCDC174	3	+	801	8	0	0	1.87%	5.860478640014983	0.016947426762344633	0.016947426762344633	0.0013498980316300959
ENSG00000010278_6232667_6237828_158	CD9	12	+	474	139	7	1	20.25%	172.07274032189562	6.613736944929179e-06	5.631038869139985e-07	3.452409511008813e-11
ENSG00000010278_6200500_6237828_228	CD9	12	+	684	225	12	26	22.37%	193.01987626036188	3.0947290438040336e-09	5.712446715057884e-09	1.886678929190764e-16
ENSG00000010278_6200500_6237828_251	CD9	12	+	753	205	12	26	19.52%	159.74765854642433	7.50656186143687e-09	1.2442019806107201e-08	9.733530744265433e-16
ENSG00000138796_108033208_108034157_67	HADH	4	+	201	21	0	0	10.45%	61.30541741523136	0.007029302661868638	0.007029302661868638	0.0002573639808771639
ENSG00000133872_30069825_30065935_176	SARAF	8	-	528	48	2	0	11.36%	53.343674893772736	0.0001363236617681562	3.592951532853599e-05	3.70143209306826e-08
ENSG00000168036_41224069_41239342_781	CTNNB1	3	+	2343	117	8	3	5.76%	29.301455223339957	6.403151454483205e-08	1.1032790393731706e-08	7.305397733044446e-15
ENSG00000165118_83955434_83939511_160	C9orf64	9	-	480	10	3	0	3.75%	12.224592163156252	0.10000264635955686	0.012798402692974279	0.006484128903061078
ENSG00000140105_100369185_100354443_181	WARS	14	-	543	16	0	0	3.31%	17.290030904795586	0.013428347753762199	0.013428347753762199	0.0008725593497644525
ENSG00000100902_35292477_35317306_252	PSMA6	14	+	756	94	3	2	6.75%	72.95947068804367	0.00018794081008863267	0.00013974726970550526	1.8464591887511134e-07
ENSG00000214078_35626794_35626149_154	CPNE1	20	-	462	17	2	0	4.55%	21.59148745700325	0.02034245439037237	0.008674846900444944	0.0008751884318457689
ENSG00000141480_4710722_4721039_430	ARRB2	17	+	1290	20	1	6	2.33%	9.097370912116281	0.00392533363377418	0.041715644099625256	0.0009550238449207022
ENSG00000013375_83191012_83166387_566	PGM3	6	-	1698	10	0	0	0.88%	3.4557150991254426	0.019679754444124863	0.019679754444124863	0.0017849636115922674
ENSG00000130429_99385715_99390994_196	ARPC1B	7	+	588	65	1	5	10.20%	64.86518290654338	4.25491196367864e-05	0.0002163581746594846	6.928535181552735e-08
ENSG00000067829_153794326_153786149_380	IDH3G	X	-	1140	13	0	0	1.84%	6.691355710359212	0.0078820576498062	0.0078820576498062	0.00031963642907451164
ENSG00000067829_153794326_153786351_340	IDH3G	X	-	1020	13	0	0	2.06%	7.478574029225002	0.0078820576498062	0.0078820576498062	0.00031963642907451164
ENSG00000058262_128051886_128069662_482	SEC61A1	3	+	1446	61	3	3	5.60%	24.753531019171167	9.137791467800357e-06	1.2865920656238535e-05	9.52871282015569e-10
ENSG00000089157_120199407_120199240_55	RPLP0	12	-	165	34	1	1	9.09%	120.91232975921821	0.021557223391537678	0.023199730935452297	0.002265826071202869
ENSG00000013441_200861712_200853306_404	CLK1	2	-	1212	32	1	0	3.96%	15.492552444396043	0.00026353281753581636	0.0001778652122394772	3.2408119596924833e-07
ENSG00000115204_27322517_27312392_158	MPV17	2	-	474	19	1	3	5.70%	23.520734288604437	0.005642787686764809	0.019194073369826602	0.000565339831017376
ENSG00000125991_35542346_35557504_361	ERGIC3	20	+	1083	96	0	4	10.25%	52.01377718173963	3.663050562822937e-08	2.7008514134119287e-07	9.911388056692168e-14
ENSG00000092820_158789350_158766914_575	EZR	6	-	1725	170	13	13	9.22%	57.827635971800014	3.487391094699976e-09	1.0934349168888382e-08	4.053550315311633e-16
ENSG00000107798_89247642_89214828_397	LIPA	10	-	1191	137	13	3	12.85%	67.49699249782999	2.0986298849682558e-08	1.0109834816569461e-09	2.400326742329277e-16
ENSG00000134057_69177320_69177631_45	CCNB1	5	+	135	11	0	0	11.11%	47.81173823812223	0.020613416668581838	0.020613416668581838	0.001946208561389314
ENSG00000117984_1754065_1753503_112	CTSD	11	-	336	126	0	0	16.96%	220.04265893681256	6.118611840318758e-05	6.118611840318758e-05	2.7792276704520703e-08
ENSG00000111799_75194894_75086547_3021	COL12A1	6	-	9063	27	4	0	0.43%	1.748104539716485	0.010340900302280928	0.0005460508606305346	3.9861314150013366e-05
ENSG00000183853_158086692_158095120_571	KIRREL1	1	+	1713	24	0	0	1.93%	8.221091752455344	0.0014055202957965589	0.0014055202957965589	1.1935530040246081e-05
ENSG00000257727_56315217_56314800_84	CNPY2	12	-	252	10	1	23	7.14%	23.284937453630956	0.023672501042627626	0.3597187722116957	0.0488269977673272
ENSG00000033178_67682143_67618997_984	UBA6	4	-	2952	19	0	0	0.91%	3.7767032699181917	0.00327844299461777	0.00327844299461777	6.0372290137947024e-05
ENSG00000071054_101823961_101891249_973	MAP4K4	2	+	2919	21	5	3	1.13%	4.221441898068346	0.01708852610271173	0.009176002286762741	0.0007746066087055626
ENSG00000174307_201468786_201468403_127	PHLDA3	1	-	381	10	1	0	3.94%	15.40106099295276	0.0477903522728147	0.016947426762344633	0.0036975118224100542
ENSG00000159176_201496303_201484713_193	CSRP1	1	-	579	158	4	6	9.84%	160.12315538061662	9.373706360862351e-05	0.00011939345589185465	8.064598906831972e-08
ENSG00000134369_201648669_201819932_1817	NAV1	1	+	5451	14	0	0	0.50%	1.5070493365696207	0.002943990679779778	0.002943990679779778	4.916404313974209e-05
ENSG00000120008_120880879_120908713_652	WDR11	10	+	1956	7	1	1	0.92%	2.0999299421372704	0.028889785561798602	0.028889785561798602	0.0036451790457678215
ENSG00000059377_139953368_140016416_312	TBXAS1	7	+	936	11	0	0	1.60%	6.895923784344553	0.016947426762344633	0.016947426762344633	0.0013498980316300959
ENSG00000132670_2964278_3038133_793	PTPRA	20	+	2379	15	3	3	1.13%	3.6997504655201774	0.022973464169004504	0.03142348392573343	0.003198441971580262
ENSG00000088833_1466824_1443749_370	NSFL1C	20	-	1110	38	0	2	3.78%	20.087978473510816	0.00041055602707030847	0.0018236796286870123	4.904354613402144e-06
ENSG00000114850_156555089_156544289_169	SSR3	3	-	507	81	2	0	14.20%	93.7459848724882	1.1931380382067736e-05	8.539974513442116e-06	8.285028529936203e-10
ENSG00000089737_94062589_94051191_609	DDX24	14	-	1827	16	1	0	1.48%	5.138744817353039	0.00542662340375733	0.0033278027414746945	9.957894592038278e-05
ENSG00000169813_43387881_43386637_414	HNRNPF	10	-	1242	101	4	4	8.45%	47.71724863686112	4.932031907248019e-07	8.552842395818309e-07	3.843530684890113e-12
ENSG00000129636_47454100_47155768_464	ITFG1	16	-	1392	52	4	0	4.53%	21.919958361521555	0.0001991658979379247	2.442337559670031e-05	3.81613929299931e-08
ENSG00000100503_50821942_50723463_2095	NIN	14	-	6285	14	6	5	0.38%	1.3070685654162295	0.052998774212472885	0.09865097884783247	0.0199560565611326
ENSG00000254505_24211416_24209877_103	CHMP4A	14	-	309	20	0	1	9.71%	37.979315458349525	0.0022011839993586477	0.0037373685375237473	4.708421183416152e-05
ENSG00000132199_712506_690554_152	ENOSF1	18	-	456	8	0	0	3.29%	10.294393400552634	0.019679754444124863	0.019679754444124863	0.0017849636115922674
ENSG00000122786_134941149_134968345_312	CALD1	7	+	936	17	0	0	2.56%	10.6573367576234	0.005160369442893307	0.005160369442893307	0.00014319601891785326
ENSG00000143761_228097115_228098013_181	ARF1	1	+	543	246	3	2	24.31%	265.8342251612321	3.89149000448416e-09	3.5896094622681653e-09	1.4917525667184776e-16
ENSG00000122882_73163910_73134583_668	ECD	10	-	2004	10	0	5	1.05%	2.928046027103294	0.007029302661868638	0.11069588287817317	0.004645432679727841
ENSG00000141510_7676594_7673307_341	TP53	17	-	1023	30	0	4	3.52%	17.207637062507334	0.0010238660768343774	0.006911511910188328	4.410159297683948e-05
ENSG00000149269_77379252_77323299_411	PAK1	11	-	1233	8	0	3	1.22%	3.807172255192215	0.019679754444124863	0.13601972394786777	0.012754723264859217

ENSG00000204463_31651763_31639131_1132	BAG6	6	-	3396	30	4	3	1.41%	5.1835726486881635	0.001994982063403954	0.001165552400368026	1.4041745202165171e-05
ENSG00000125944_23337834_23310454_535	HNRNPR	1	-	1605	62	0	1	2.99%	22.66690733803926	0.0001791223493998751	0.00019256488680101648	2.4018138913807657e-07
ENSG00000227057_33289170_33279276_610	WDR46	6	-	1830	15	1	2	1.64%	4.80967560517623	0.004004657782222479	0.009367992579282357	0.00020216237368725587
ENSG00000184500_93898546_93874245_426	PROS1	3	-	1278	13	0	0	1.17%	5.968814952902584	0.020613416668581838	0.020613416668581838	0.001946208561389314
ENSG00000100109_26506405_26492013_698	TFIP11	22	-	2094	10	0	1	0.72%	2.8021987766547283	0.019679754444124863	0.02784981298332479	0.002477909089056847
ENSG00000140545_88913319_88899395_343	MFGE8	15	-	1029	18	2	0	2.92%	10.264380591804667	0.010678932720565162	0.0022011839993586477	0.0001357017294392655
ENSG00000041982_115086281_115021157_1355	TNC	9	-	4065	10	0	0	0.44%	1.443494277568266	0.012798402692974279	0.012798402692974279	0.0007972436888943567
ENSG00000128595_128748605_128769094_217	CALU	7	+	651	169	8	0	13.82%	152.32855856762447	6.734041737950367e-06	8.058032552433406e-07	4.91689014965101e-11
ENSG00000163918_186804713_186789969_363	RFC4	3	-	1089	17	1	0	1.93%	9.160024981758955	0.016854695534768073	0.007029302661868638	0.0006017499134389055
ENSG00000161547_76737124_76736161_209	SRSF2	17	-	627	99	6	4	16.75%	92.64954060497371	4.015689770399338e-07	5.525591407366016e-07	2.0416473965633404e-12
ENSG00000151632_5003835_4989996_297	AKR1C2	10	-	891	496	26	17	13.80%	326.64768823841086	7.124509936089185e-08	5.719166085606851e-08	4.0079245323228954e-14
ENSG00000181019_69718478_69710976_181	NQO1	16	-	543	756	46	38	39.78%	816.9539602515913	1.0709409553328282e-10	7.48656806692064e-12	9.997316876533186e-21
ENSG00000114316_49310637_49278447_552	USP4	3	-	1656	13	0	1	1.27%	4.606368061479167	0.007029302661868638	0.016854695534768073	0.0006017499134389055
ENSG00000087088_48955571_48961636_160	BAX	19	+	480	98	1	1	15.00%	119.80100319893128	8.049498772756595e-06	1.2938129277419723e-05	8.475122510343651e-10
ENSG00000102805_76995908_77000969_243	CLN5	13	+	729	12	0	2	2.47%	9.65893701780247	0.011772051206276392	0.060667625179241055	0.0035017256847258756
ENSG00000075142_88226914_88206478_155	SRI	7	-	465	38	0	6	10.32%	47.95194861418711	0.00018897224769700208	0.005066266124291195	7.389845401112817e-06
ENSG00000075420_172226891_172397475_1135	FNDC3B	3	+	3405	27	0	4	1.06%	4.652884418047138	0.0008739357725210369	0.007045996527915517	3.921453735198883e-05
ENSG00000159842_1091688_1006080_690	ABR	17	-	2070	24	1	11	1.30%	6.803251290800001	0.004445139713096192	0.13389133575829926	0.004224641413841655
ENSG00000106246_99435119_99419967_659	PTCD1	7	-	1977	7	1	0	0.76%	2.0776241612647954	0.04178282491678763	0.019216965118390855	0.003600525600033436
ENSG00000241468_99466141_99458327_94	ATP5MF	7	-	282	82	0	1	15.96%	170.6240948729894	0.0003088921332160845	0.00039453730762884214	8.158770260640019e-07
ENSG00000166260_54968646_54962733_276	COX11	17	-	828	40	0	3	4.35%	28.34688037833334	0.0010447178140462346	0.0032953901782040457	2.0925237526607307e-05
ENSG00000196083_190609068_190656607_546	IL1RAP	3	+	1638	13	0	0	0.92%	4.656987490726191	0.02108409854857798	0.02108409854857798	0.002029891263157455
ENSG00000114867_184321467_184334908_1305	EIF4G1	3	+	3915	87	7	5	2.76%	13.039564974033336	1.2883430284708553e-06	5.547471395647561e-07	6.474080287680731e-12
ENSG00000213339_10701620_10713270_350	QTRT1	19	+	1050	38	0	0	3.14%	21.235862957711433	0.0016109979480538772	0.0016109979480538772	1.550748317303123e-05
ENSG00000021574_32063832_32154496_616	SPAST	2	+	1848	12	3	0	1.14%	3.8102624924123387	0.051745348319195604	0.008175610787902942	0.002192614907007965
ENSG00000164649_21916816_21902322_374	CDCA7L	7	-	1122	7	0	0	1.60%	3.6608404338863645	0.009815328628645334	0.009815328628645334	0.0004837142251238724
ENSG00000131043_36239884_36255745_379	AAR2	20	+	1137	19	5	1	2.64%	9.805477618996045	0.04227848696273928	0.0040705821972625445	0.0009982242249968432
ENSG00000156113_77637360_76877739_1140	KCNMA1	10	-	3420	15	4	2	0.70%	2.5735983501381585	0.048994651353958696	0.02389626957043539	0.005091569865673637
ENSG00000133961_73355751_73276578_651	NUMB	14	-	1953	19	0	1	1.54%	5.708565311212751	0.0022011839993586477	0.0037373685375237473	4.708421183416152e-05
ENSG00000138614_65607272_65579408_482	INTS14	15	-	1446	14	0	0	1.45%	5.681138266695022	0.00837382380159347	0.00837382380159347	0.0003583838894600799
ENSG00000270106_231528811_231616709_186	TSNAX-DISC1	1	+	558	17	1	0	3.76%	17.87682294827151	0.013892891352654344	0.00847367416878415	0.0005886798845679606
ENSG00000141510_7676239_7674820_193	TP53	17	-	579	18	0	4	2.59%	18.241878461082905	0.02108409854857798	0.08588638879785576	0.008130108632843268
ENSG00000182220_40581066_40591542_158	ATP6AP2	X	+	474	9	0	0	5.06%	11.141400452496837	0.0033278027414746884	0.0033278027414746884	6.211806171561044e-05
ENSG00000174132_100535531_100586184_190	FAM174A	5	+	570	16	5	0	3.16%	16.471029440884216	0.12378039365238508	0.013428347753762199	0.008589103006023845
ENSG00000138107_102502647_102472973_221	ACTR1A	10	-	663	20	0	4	3.62%	17.700766933076928	0.005554612275049112	0.03600804981277603	0.0010788777123559284
ENSG00000175768_37592532_37588878_98	TOMM5	9	-	294	109	53	0	11.22%	217.54784420963782	0.22373273041614722	0.0016263209931528667	0.004422058886495139
ENSG00000204628_181242331_181239385_167	RACK1	5	-	501	772	12	20	24.55%	904.1806131694972	2.2233243086281224e-08	8.710385899005934e-08	1.954722080325139e-14
ENSG00000204628_181243800_181243681_39	RACK1	5	-	117	67	1	1	15.38%	336.01955894624365	0.013995907742832873	0.013853924679039932	0.0009341050772126533
ENSG00000079616_29798939_29799575_185	KIF22	16	+	555	11	0	0	3.24%	11.62988227413784	0.012798402692974279	0.012798402692974279	0.0007972436888943567
ENSG00000168259_42017386_41976733_484	DNAJC7	17	-	1452	50	5	3	4.13%	20.205937459762403	0.000657074651330092	0.00022384167652469728	9.973232233474765e-07
ENSG00000107223_136866258_136862871_139	EDF1	9	-	417	68	4	2	10.79%	95.68601635621584	0.001169888954089309	0.0005741546194468325	4.272433733062865e-06
ENSG00000107223_136866258_136862456_141	EDF1	9	-	423	70	4	2	11.35%	97.1031434236525	0.0007662353277193262	0.00039105794466626177	1.959360123532922e-06
ENSG00000096384_44248702_44253598_700	HSP90AB1	6	+	2100	209	17	13	8.57%	58.398623133706444	5.600961997648614e-11	8.916411341006866e-11	5.88230326242746e-20
ENSG00000141456_4682537_4671439_928	PELP1	17	-	2784	36	1	0	0.97%	7.587677894372846	0.003684287313187465	0.003684287313187465	7.541963121699725e-05
ENSG00000076554_80080438_80038116_219	TPD52	8	-	657	45	5	0	6.39%	40.19043998845891	0.002462368798132946	0.00046705916373637345	7.5029210566117364e-06
ENSG00000076554_80171443_80038116_184	TPD52	8	-	552	45	5	0	7.61%	47.835360638437514	0.002462368798132946	0.00046705916373637345	7.5029210566117364e-06
ENSG00000197712_38914999_38943557_273	FAM114A1	4	+	819	8	0	0	1.83%	5.731676911663005	0.019216965118390855	0.019216965118390855	0.0017073955890589052
ENSG00000151726_184803424_184757125_668	ACSL1	4	-	2004	18	0	0	1.20%	5.270482848785929	0.005454749182134642	0.005454749182134642	0.00015910859015753364
ENSG00000079459_11808548_11809895_190	FDFT1	8	+	570	56	1	2	8.95%	57.64860304309475	0.0001533166966314766	0.0001643121177648815	1.7710103147982963e-07
ENSG00000152240_46118204_46128125_202	HAUS1	18	+	606	23	0	0	2.97%	22.27054413881931	0.013428347753762199	0.013428347753762199	0.0008725593497644525
ENSG00000143106_109413127_109402013_164	PSMA5	1	-	492	55	5	2	7.93%	65.59537258278965	0.0014447818515996	0.00100672181717514	8.919035997171275e-06
ENSG00000204628_181238227_181237185_124	RACK1	5	-	372	182	3	1	16.13%	287.0807449928307	4.1280781501046566e-05	4.2495854754003497e-05	1.3288906153334029e-08
ENSG00000176101_137188727_137189914_81	SSNA1	9	+	243	10	0	0	7.41%	24.147342544506177	0.011570465654371866	0.011570465654371866	0.0006594843062896773
ENSG00000167515_88857151_88860959_146	TRAPPC2L	16	+	438	39	0	2	9.59%	52.24757198499659	0.0004388494700883803	0.0006399719954500673	1.8303114714294788e-06
ENSG00000183479_153470557_153447878_358	TREX2	X	-	1074	15	0	0	2.23%	8.195257316082404	0.004826937407846361	0.004826937407846361	0.00012612451166366307
ENSG00000116337_109625404_109631152_761	AMPD2	1	+	2283	7	0	1	0.66%	1.7991515404382397	0.019216965118390855	0.04178282491678763	0.003600525600033436
ENSG00000197102_102033284_102037591_264	DYNC1H1	14	+	792	10	0	2	2.65%	7.408843735246214	0.007593099278532274	0.049338281967443096	0.0019605633087212975
ENSG00000123416_49129620_49127958_416	TUBA1B	12	-	1248	58	4	5	2.40%	27.27024405627164	0.009060088632836347	0.012930838315446122	0.0005838894657401317
ENSG00000070087_149968427_149966167_55	PFN2	3	-	165	34	0	1	12.73%	120.91232975921821	0.008877961307018026	0.008576800850100302	0.00038741434111749746
ENSG00000070087_149970856_149968490_55	PFN2	3	-	165	52	4	0	14.55%	184.9247396317455	0.0204156028385188	0.005808022449631236	0.0006175306982988044
ENSG00000140374_76274447_76217588_90	ETFA	15	-	270	32	0	0	7.78%	69.5443465281778	0.008574016250729465	0.008574016250729465	0.0003747459614100891
ENSG00000088035_63428929_63437020_131	ALG6	1	+	393	8	0	0	5.34%	11.944639670870231	0.005706018193000826	0.005706018193000826	0.0001733096755673334
ENSG00000138613_63279210_63305781_203	APH1B	15	+	609	6	1	0	2.46%	5.781087919522169	0.0477903522728147	0.016947426762344633	0.0036975118224100542
ENSG00000113013_138566691_138556037_377	HSPA9	5	-	1131	173	5	4	8.75%	89.7550957761711	1.3599133298441578e-06	8.9250407297059e-07	1.0829926842577023e-11
ENSG00000113013_138575318_138567554_180	HSPA9	5	-	540	40	0	2	8.89%	43.46521658011112	0.00014734945683592696	0.0008081111075299929	8.374520733964951e-07
ENSG00000196236_40881826_40926435_428	XPNPEP3	22	+	1284	10	0	0	1.64%	4.569940995572431	0.007593099278532274	0.007593099278532274	0.00029783286705462064
ENSG00000197702_12473768_12513346_206	PARVA	11	+	618	12	0	0	2.43%	11.393794637504858	0.019679754444124863	0.019679754444124863	0.0017849636115922674

ENSG00000167325_4107491_4138383_678	RRM1	11	+	2034	65	0	3	3.54%	18.751586798941744	7.92044824908934e-06	1.8867290912509913e-05	1.2136468465155665e-09
ENSG00000106355_32489303_32487261_62	LSM5	7	−	186	49	4	0	27.42%	154.58193961152423	0.0002473554500538834	0.00013319834607924987	2.311148598512169e-07
ENSG00000176697_27658504_27657821_227	BDNF	11	−	681	10	1	0	2.64%	8.616452626013217	0.023672501042627626	0.012798402692974279	0.0014375227604752335
ENSG00000182087_1020996_1010350_620	TMEM259	19	−	1860	11	0	2	0.81%	3.470206807605646	0.02108409854857798	0.0700082515985845	0.006564739044797468
ENSG00000099622_1270934_1272443_172	CIRBP	19	+	516	27	1	0	6.40%	30.703626828392448	0.002493493840886608	0.0014055202957965589	2.0830049893778164e-05
ENSG00000167881_76050487_76039706_288	SRP68	17	−	864	17	0	0	2.43%	11.545448154092016	0.008674846900444944	0.008674846900444944	0.00038311532916634116
ENSG00000148737_112950757_113161585_459	TCF7L2	10	+	1377	11	0	0	1.31%	4.687425317462964	0.012798402692974279	0.012798402692974279	0.0007972436888943567
ENSG00000151491_15658081_15621317_456	EPS8	12	−	1368	11	0	0	1.32%	4.7182636419199575	0.012798402692974279	0.012798402692974279	0.0007972436888943567
ENSG00000132170_12379727_12433915_243	PPARG	3	+	729	11	0	0	2.06%	8.854025599652266	0.02108409854857798	0.02108409854857798	0.002029891263157455
ENSG00000182180_73250959_73250852_35	MRPS16	10	−	105	21	0	1	28.57%	117.35608476630003	0.0022409900782254734	0.005670089768331009	7.232548112602907e-05
ENSG00000054267_235224715_235181550_803	ARID4B	1	−	2409	10	0	0	0.75%	2.435784241724783	0.012798402692974279	0.012798402692974279	0.0007972436888943567
ENSG00000185608_19433344_19435962_162	MRPL40	22	+	486	23	0	0	4.94%	27.769443926182106	0.005604866691947602	0.005604866691947602	0.00016752456572620382
ENSG00000101347_36941044_36892932_512	SAMHD1	20	−	1536	9	0	0	1.17%	3.438166545887696	0.012178957859018494	0.012178957859018494	0.0007262454987071801
ENSG00000120742_150546135_150545626_78	SERP1	3	−	234	23	3	0	7.69%	57.67499892360899	0.05258119297243839	0.013007239811447113	0.00326646906127711
ENSG00000177885_75393628_75320368_217	GRB2	17	−	651	22	2	0	5.07%	19.829753186317976	0.005530543042558543	0.00151350169064872	4.947549134344851e-05
ENSG00000111816_116060311_115942414_505	FRK	6	−	1515	8	0	7	0.99%	3.0985104888792088	0.019679754444124863	0.17004230409153215	0.016523685284367155
ENSG00000177885_75393628_75320368_217	GRB2	17	−	651	22	2	0	5.07%	19.829753186317976	0.005530543042558543	0.00151350169064872	4.947549134344851e-05
ENSG00000177885_75393628_75320368_176	GRB2	17	−	528	20	2	0	5.68%	22.22653120573864	0.008633544044986457	0.0023218226288164415	0.0001142366932985535
ENSG00000138430_174123669_174075426_211	OLA1	2	−	633	21	1	0	2.84%	19.466649131850716	0.016503128830616266	0.013007239811447113	0.001029548967667127
ENSG00000186815_113267897_113296076_660	TPCN1	12	+	1980	11	5	1	1.06%	3.259891243508334	0.1670934997604609	0.016854695534768073	0.014465463506112032
ENSG00000134440_57621717_57607649_174	NARS	18	−	522	16	1	2	5.75%	17.985606860735636	0.003044943557155862	0.010752400113165628	0.0001818138336614375
ENSG00000196411_100822526_100803461_803	EPHB4	7	−	2409	13	5	2	1.12%	3.1665195142422173	0.09417654855689778	0.020677326986596276	0.008830673516152602
ENSG00000162909_223759210_223774857_281	CAPN2	1	+	843	44	2	4	5.69%	30.626736237943067	0.0004177761426647793	0.0012026240249285258	3.2668543822608645e-06
ENSG00000077152_202335718_202333168_138	UBE2T	1	−	414	49	0	5	9.42%	69.44985692691668	0.0007076576786684489	0.0009531022847370905	4.255106128999559e-06
ENSG00000077152_202331945_202331835_36	UBE2T	1	−	108	17	1	2	13.89%	92.36358523273613	0.02108409854857798	0.055917760918677194	0.00521801263590997
ENSG00000006744_12998452_12992818_333	ELAC2	17	−	999	19	0	0	1.80%	11.159988040839341	0.012798402692974279	0.012798402692974279	0.0007972436888943567
ENSG00000241553_9793122_9803547_113	ARPC4	3	+	339	84	1	7	16.81%	145.39691917948676	7.007977131478223e-05	0.00010050619916455365	5.147960069493998e-08
ENSG00000241553_9803888_9806215_43	ARPC4	3	+	129	23	0	0	18.60%	104.61976548933724	0.005306578019052857	0.005306578019052857	0.00015100114740534233
ENSG00000165832_114938344_114975379_319	TRUB1	10	+	957	27	2	1	3.45%	16.554933587722573	0.004803510583362958	0.00187740676320226	5.214157315808195e-05
ENSG00000138031_24918987_24819932_1145	ADCY3	2	−	3435	15	4	0	0.61%	2.562359929395197	0.06927320754946001	0.00847367416878415	0.0031109847893480494
ENSG00000153187_244860523_244858720_136	HNRNPU	1	−	408	41	0	4	7.35%	58.96567984581252	0.0024321290225875155	0.007999495149173846	0.00011015539794682304
ENSG00000124155_45420633_45421675_117	PIGT	20	+	351	38	0	0	5.98%	63.52608577093164	0.008574016250729465	0.008574016250729465	0.0003747459614100891
ENSG00000134318_11344109_11183437_1379	ROCK2	2	−	4137	33	2	2	1.09%	4.680627021135969	0.001164427877724285	0.001164427877724285	8.315212452002076e-06
ENSG00000188603_28491759_28484033_181	CLN3	16	−	543	17	2	0	3.31%	18.37065783634531	0.044040755831095145	0.013428347753762199	0.0027917083341200354
ENSG00000164930_103300279_103331509_649	FZD6	8	+	1947	34	0	0	2.16%	10.246807606713407	0.00037518914374882155	0.00037518914374882155	9.369150750814024e-07
ENSG00000164941_94828985_94880234_819	INTS8	8	+	2457	20	2	0	1.10%	4.776397426385837	0.013494345032394814	0.0035547763465261306	0.00026296295714225076
ENSG00000096717_67906255_67916593_444	SIRT1	10	+	1332	13	0	0	1.58%	5.726835968325452	0.0078820576498062	0.0078820576498062	0.00031963642907451164
ENSG00000129625_112922178_112887096_95	REEP5	5	−	285	74	2	7	14.74%	152.35702232817897	0.0005574441649685308	0.004690929750820717	1.7217224340400157e-05
ENSG00000100097_37675703_37678493_38	LGALS1	22	+	114	111	1	7	26.32%	571.3388337306711	0.002390425500591921	0.01991630962237072	0.0002818506926820679
ENSG00000101347_36919456_36892932_373	SAMHD1	20	−	1119	8	0	0	1.34%	4.195034308000001	0.019216965118390855	0.019216965118390855	0.0017073955890589052
ENSG00000168036_41224069_41239342_781	CTNNB1	3	+	2343	117	8	3	5.76%	29.301455223339957	6.403151454483205e-08	1.1032790393731706e-08	7.305397733044446e-15
ENSG00000139343_95859074_95879762_67	SNRPF	12	+	201	68	1	0	10.45%	198.51278020170153	0.008576800850100302	0.008877961307018026	0.00038741434111749746
ENSG00000257727_56311354_56310552_94	CNPY2	12	−	282	40	4	0	13.83%	83.23126579170214	0.0030765917645763823	0.0007076576786684489	1.3712349168963352e-05
ENSG00000115204_27322517_27309912_176	MPV17	2	−	528	26	1	3	8.52%	28.894490567460235	0.0004378023748130596	0.002254446938826655	6.462878484388623e-06
ENSG00000113810_160402022_160412457_245	SMC4	3	+	735	26	2	0	4.49%	20.756858530093883	0.0037917638115023676	0.0014915192737735051	3.33493668815224e-05
ENSG00000168216_69719072_69676158_325	LMBRD1	6	−	975	10	3	0	1.85%	6.018260757246155	0.08791701959130671	0.012798402692974279	0.0056117368578515354
ENSG00000119383_129127964_129147464_286	PTPA	9	+	858	25	5	0	4.90%	17.097331696722033	0.004748305419133081	0.00037518914374882155	1.236400975172076e-05
ENSG00000109133_41939383_41953811_112	TMEM33	4	+	336	58	0	0	6.25%	101.28947792329467	0.00877616296920843	0.00877616296920843	0.00039161135667564577
ENSG00000204568_30617866_30620013_125	MRPS18B	6	+	375	17	0	0	6.40%	26.600712547028007	0.004873903550586949	0.004873903550586949	0.00012846764509679302
ENSG00000105193_39435895_39435538_72	RPS16	19	−	216	203	16	4	15.28%	551.4649353601599	0.029722275852917902	0.0016263209931528667	0.0003204481972181041
ENSG00000114125_141738342_141745277_113	RNF7	3	+	339	24	0	0	9.73%	41.541976908424786	0.001484234770590415	0.001484234770590415	1.3251387882263029e-05
ENSG00000085063_33722445_33710126_128	CD59	11	−	384	62	5	3	14.06%	94.74058926446097	0.00102540667708157	0.0003583833865847517	2.4148597638421077e-06
ENSG00000066926_57573334_57550712_348	FECH	18	−	1044	12	0	0	2.30%	6.744602572775864	0.004873903550586949	0.004873903550586949	0.00012846764509679302
ENSG00000155463_22767037_22768218_145	OXA1L	14	+	435	40	10	0	8.28%	53.95682058220691	0.0038649158102149425	0.0010658336519988904	2.504749064142115e-05
ENSG00000108479_75763051_75758056_171	GALK1	17	−	513	8	0	0	2.92%	9.150571911602341	0.019679754444124863	0.019679754444124863	0.0017849636115922674
ENSG00000146678_45888836_45893091_196	IGFBP1	7	+	588	11	0	0	2.55%	10.97718479956888	0.02108409854857798	0.02108409854857798	0.002029891263157455
ENSG00000134058_69234976_69255696_154	CDK7	5	+	462	12	1	0	3.90%	15.241049969649355	0.019795881618345192	0.012798402692974279	0.0012079910998532793
ENSG00000115414_215391764_215361555_1014	FN1	2	−	3042	37	3	6	1.68%	7.1370400005803765	0.0001834224710074371	0.0016951283021485475	2.204582954034169e-06
ENSG00000198408_101813583_101797930_603	OGA	10	−	1809	12	2	0	1.16%	3.8924074549353245	0.04363948305378087	0.0078820576498062	0.0017710903275458955
ENSG00000145494_1801418_1815916_124	NDUFS6	5	+	372	78	3	3	12.90%	123.03460499692744	0.00032273965163335844	0.00032273965163335844	7.002297410584018e-07
ENSG00000203705_212796574_212815156_229	TATDN3	1	+	687	6	0	0	2.18%	5.124719858790394	0.016947426762344633	0.016947426762344633	0.0013498980316300959
ENSG00000131236_40059356_40071533_471	CAP1	1	+	1413	86	1	0	5.94%	35.71345820913589	1.6797640363981946e-06	1.6797640363981946e-06	2.4724907928301373e-11
ENSG00000137575_58565034_58581740_292	SDCBP	8	+	876	116	4	1	9.93%	77.70151731102057	4.405629274117141e-06	1.661763445746148e-06	6.356253652813504e-11
ENSG00000148019_78240031_78248917_283	CEP78	9	+	849	16	0	0	3.53%	11.058288317201416	0.0017901637611834264	0.0017901637611834264	1.8982104156792192e-05
ENSG00000065883_39988230_40094980_863	CDK13	7	+	2589	12	1	8	0.70%	2.719723864804172	0.015555382717448078	0.2910650221082247	0.027849658145045288
ENSG00000167468_1104044_1106572_196	GPX4	19	+	588	35	7	4	7.14%	34.92740618044644	0.007200144708739569	0.004077829543963717	0.00015845737483450431
ENSG00000167778_53077242_53065832_428	SPRYD3	12	−	1284	9	0	0	1.17%	4.112946896015188	0.020613416668581838	0.020613416668581838	0.001946208561389314

ENSG00000072310_17817418_17812622_666	SREBF1	17	-	1998	62	2	18	3.75%	18.20840154031682	6.792301223675473e-06	0.00201928888006137	1.6170068401372103e-07
ENSG00000072310_17836817_17811755_1071	SREBF1	17	-	3213	81	7	16	3.27%	14.792783794071433	2.7894683202327617e-05	2.3461249035565923e-05	5.654561639937663e-10
ENSG00000072310_17817418_17812622_666	SREBF1	17	-	1998	62	2	18	3.75%	18.20840154031682	6.792301223675473e-06	0.00201928888006137	1.6170068401372103e-07
ENSG00000072310_17819403_17817583_216	SREBF1	17	-	648	15	2	0	3.70%	13.582880181284725	0.022898969405409888	0.005160369442893307	0.0006278347384112434
ENSG00000160075_1574557_1542066_194	SSU72	1	-	582	22	0	0	6.19%	22.180703306345364	0.0008664586529590942	0.0008664586529590942	4.7094196839841995e-06
ENSG00000106976_128203486_128254686_851	DNM1	9	+	2553	54	2	7	2.35%	12.4113368142973	5.0604056647534704e-05	0.0009696113333780397	3.89413359703063 7e-07
ENSG00000116641_62688264_62455414_2100	DOCK7	1	-	6300	16	0	0	0.29%	1.4902359970323813	0.013007239811447113	0.013007239811447113	0.0008218654148058079
ENSG00000134287_48941032_48938947_160	ARF3	12	-	480	54	0	3	9.38%	66.01279768104376	0.0003081009674896864 6	0.001089527314876726	2.2326759774577627e-06
ENSG00000187193_56682541_56684049_61	MT1X	16	+	183	60	0	1	9.84%	192.38702420704922	0.013853924679039932	0.017143983986795872	0.0011315679034796663
ENSG00000165092_72953000_72901208_501	ALDH1A1	9	-	1503	3481	149	92	42.12%	1359.0037627128756	3.243914860345707e-33	2.0267660774878423e-34	1.4386585495871958e-65
ENSG00000163743_75514286_75482538_261	RCHY1	4	-	783	8	0	0	1.92%	5.995202286911879	0.019216965118390855	0.019216965118390855	0.0017073955890589052
ENSG00000091073_76480516_76505601_620	DTX2	7	+	1860	8	4	0	0.81%	2.5237867691677427	0.169362073879829	0.019216965118390855	0.016163991620339734
ENSG00000184743_63659244_63629319_523	ATL3	11	-	1569	63	7	4	3.82%	23.56097304103538	0.00011219365649543519	0.00023695714496868413	1.8820742010091813e-07
ENSG00000166206_26621357_26547793_334	GABRB3	15	-	1002	27	0	1	2.99%	15.811448546357788	0.0024321290225875155	0.003172792786121065 6	4.407497946231007e-05
ENSG00000004487_23050431_23083364_669	KDM1A	1	+	2007	14	0	1	1.49%	4.0931369873647245	0.0017901637611834264	0.0040534433873287605	4.213999707786468e-05
ENSG00000180398_46909171_46905570_109	MCFD2	2	-	327	24	0	0	8.26%	43.06645312524772	0.0036062891729786097	0.0036062891729786097	7.240558534568731e-05
ENSG00000117298_21260288_21219955_571	ECE1	1	-	1713	50	3	3	4.20%	17.127274484281966	2.597019403851631e-05	5.2349357447643746e-05	1.0434486324538327e-08
ENSG00000138674_82881936_82844378_877	SEC31A	4	-	2631	26	0	0	1.25%	5.7986662940399105	0.0013845019718837994	0.0013845019718837994	1.1595255170738555e-05
ENSG00000109332_102809680_102797415_110	UBE2D3	4	-	330	19	0	1	5.45%	33.78432743272273	0.013428347753762199	0.013428347753762199	0.0008725593497644525
ENSG00000125870_16731742_16741005_212	SNRPB2	20	+	636	51	2	2	8.49%	47.05314719403539	0.0001817182897104588	0.0001817182897104588	2.3022607162508642e-07
ENSG00000169813_43387881_43386637_414	HNRNPF	10	-	1242	101	4	4	8.45%	47.71724863686112	4.932031907248019e-07	8.552842395818309e-07	3.843530684890113e-12
ENSG00000138363_215318188_215349655_533	ATIC	2	+	1599	86	5	0	6.75%	31.559172263607888	1.0487445945631443e-06	5.870280736659544e-08	6.226034182633726e-13
ENSG00000116752_114581591_114568130_225	BCAS2	1	-	675	36	0	2	5.78%	31.294955937680008	0.0006289630342484525	0.0013147568071904994	5.223611450468446e-06
ENSG00000123992_219386772_219374292_410	DNPEP	2	-	1230	17	2	2	1.95%	8.109973337508539	0.02671499548383061	0.02671499548383061	0.003152782508864385
ENSG00000153310_129879461_129852147_215	FAM49B	8	-	645	16	0	1	3.72%	14.55579345938605	0.005405141725979036	0.008170681041463092	0.00023248977892435937
ENSG00000198836_193593378_193692127_979	OPA1	3	+	2937	32	1	1	1.84%	6.3932494254708905	0.0001048543557987398	0.0001048543557987398	7.919728780723367e-08
ENSG00000112335_108260921_108212149_140	SNX3	6	-	420	33	0	1	10.71%	46.1041761581893	0.00028802016930314084	0.0004847901396444878	9.339504306716138e-07
ENSG00000142227_48329378_48330470_94	EMP3	19	+	282	70	3	0	19.15%	145.65471513547877	0.00012253155467421766	8.424452531401259e-05	7.464675359916606e-08
ENSG00000184500_93898546_93874245_426	PROS1	3	-	1278	13	0	0	1.17%	5.968814952902584	0.020613416668581838	0.020613416668581838	0.001946208561389314
ENSG00000275052_55598964_55549911_640	PPP4R3B	2	-	1920	12	2	2	0.94%	3.667377648946876	0.04375184787808565	0.04375184787808565	0.007835513351106278
ENSG00000188243_75537805_75526589_85	COMMD6	13	-	255	40	0	3	10.59%	92.04398805200002	0.0035547763465261306	0.01995583193976384	0.0003951603994987857 6
ENSG00000108654_64505789_64499923_614	DDX5	17	-	1842	250	15	20	9.93%	79.63903689352608	1.7165048591755142e-10	3.4560808278790264e-10	6.804261973709769e-19
ENSG00000117906_76943866_76948612_101	RCN2	15	+	303	21	0	0	4.95%	40.66795016653961	0.019679754444124863	0.019679754444124863	0.0017849636115922674
ENSG00000261771_55385699_55356220_782	DNAAF4-CCPG1	15	-	2346	38	0	1	2.43%	9.504542244500001	5.2407389423840785e-05	4.942578964878572e-05	1.9421790558505986e-08
ENSG00000103415_4507809_4509756_216	HMOX2	16	+	648	12	0	0	2.78%	10.86630414502778	0.012798402692974279	0.012798402692974279	0.0007972436888943567
ENSG00000089327_35155551_35169615_213	FXYD5	19	+	534	101	4	3	12.92%	110.98281424528373	9.80386385517854e-05	3.0510434866026417 4e-05	2.276384256667706e-08
ENSG00000141560_82719041_82727171_217	FN3KRP	17	+	651	15	3	0	3.69%	13.52028626339862	0.029020025081745005	0.005306578019052857	0.0008246758582037483
ENSG00000081087_108074651_108044785_334	OSTM1	6	-	1002	19	3	0	2.69%	11.126574902992518	0.02186644380835736	0.00327844299461777	0.00040656532034450245
ENSG00000129235_6641083_6642966_80	TXNDC17	17	+	240	46	4	2	12.50%	112.46624790103752	0.008052197636152732	0.0023766146074493525	0.0001086291086812655
ENSG00000129235_6641796_6643019_35	TXNDC17	17	+	105	26	0	2	17.14%	145.29800971065717	0.013640585738808999	0.03125445809822415	0.0020008781889257073
ENSG00000137575_58565034_58581740_239	SDCBP	8	+	717	85	4	1	9.21%	69.56253281126571	6.483422006267064e-05	2.698569302079388e-05	1.3391357933363732e-08
ENSG00000140264_43793010_43793773_45	SERF2	15	+	135	75	5	0	17.78%	325.98912435083344	0.026239749779623293	0.005808022449631236	0.0008006752930530814
ENSG00000177426_3456398_3457940_252	TGIF1	18	+	756	34	0	2	2.78%	26.38959578078175	0.008574016250729465	0.013648824643852793	0.0005848802985757839
ENSG00000106399_7639126_7637000_82	RPA3	7	-	246	94	0	0	9.76%	224.2169099193537	0.005655335537214311	0.005655335537214311	0.00017039952270558162
ENSG00000167670_4409592_4443025_692	CHAF1A	19	+	2076	10	0	0	0.87%	2.826495297839596	0.012798402692974279	0.012798402692974279	0.0007972436888943567
ENSG00000144029_95101694_95087357_166	MRPS5	2	-	498	26	0	0	3.61%	30.635122529355428	0.013853924679039932	0.013853924679039932	0.0009252050120140923
ENSG00000144029_95121791_95100858_251	MRPS5	2	-	753	15	0	2	3.19%	11.688853064372513	0.005306578019052857	0.02389626957043539	0.0006722688034155889
ENSG00000115504_62707273_63045500_1204	EHBP1	2	+	3612	27	5	0	1.41%	4.386232404056064	0.0009758619863385294	0.00010000342828152886	7.17841410011521e-07
ENSG00000182220_40581066_40589155_68	ATP6AP2	X	+	204	13	0	0	7.35%	37.392870146125006	0.019679754444124863	0.019679754444124863	0.0017849636115922674
ENSG00000151092_25789865_25719460_612	NGLY1	3	-	1836	10	0	3	0.82%	3.195971807361112	0.019679754444124863	0.13159953908062888	0.012285429850268035
ENSG00000197563_62157141_62088729_655	PIGN	18	-	1965	12	0	0	0.92%	3.5833919012610695	0.011772051206276392	0.011772051206276392	0.00068127251138950 8
ENSG00000156113_77637360_76877739_1194	KCNMA1	10	-	3582	15	4	2	0.67%	2.4572044549057797	0.048994651353958696	0.02389626957043539	0.005091569865673637
ENSG00000114316_49310637_49278293_651	USP4	3	-	1953	14	0	1	1.23%	4.206311281946237	0.004193160480899131	0.010499663139468709	0.0002361360478986167
ENSG00000006468_13989019_13895866_466	ETV1	7	-	1398	11	0	1	1.07%	4.6170133491748935	0.020613416668581838	0.028542519635534256	0.0026455223939317236
ENSG00000179010_6640863_6641246_127	MRFAP1	4	+	381	76	1	3	14.96%	117.04806354644097	5.6254241454829154e-05	0.0001733650811203729	7.181689729777536e-08
ENSG00000113387_32588513_32601084_127	SUB1	5	+	381	63	6	7	7.87%	97.02668425560239	0.004585801871278292	0.008525693657702423	0.0002083278051943 55
ENSG00000124357_71068684_71072839_159	NAGK	2	+	477	15	0	0	3.14%	18.45221458589623	0.019679754444124863	0.019679754444124863	0.0017849636115922674
ENSG00000113407_33445388_33467708_619	TARS	5	+	1857	89	9	13	6.46%	28.122486656437	2.8101658559250745e-07	1.7972622951594e-06	4.724594987784353e-12
ENSG00000049618_157206653_157207891_412	ARID1B	6	+	1236	12	0	0	1.21%	5.696897318752429	0.021084098541 57798	0.021084098541 57798	0.002029891263157455
ENSG00000138814_101346796_101025865_289	PPP3CA	4	-	867	12	0	0	2.08%	8.121528357529414	0.011570465654371866	0.011570465654371866	0.0006594843062896773
ENSG00000198836_193615695_193692127_854	OPA1	3	+	2562	30	0	1	1.99%	6.870965150251758	0.00011585404928284773	0.00016573236184519563	1.3634101730193225e-07
ENSG00000070081_17295324_17331419_390	NUCB2	11	+	1170	21	0	1	2.56%	10.531956325180772	0.002240990078225473 4	0.0037834851205969236	4.845513352116639e-05
ENSG00000076108_56615278_56598618_1718	BAZ2A	12	-	5154	27	2	8	0.76%	3.073937028220897	0.0017936202733988364	0.025438491335356538	0.0002908813190580962
ENSG00000213593_57712619_57740245_258	TMX2	11	+	774	73	0	0	9.30%	55.342339715374045	8.0901699496228e-06	8.0901699496228e-06	5.368433166356741e-10
ENSG00000188603_28491651_28482512_331	CLN3	16	-	993	20	1	0	2.11%	11.818336834471301	0.016983116543564363	0.00877616296920843	0.0007396122037230692
ENSG00000165280_35062054_35057117_463	VCP	9	-	1389	85	9	6	7.13%	35.9080892913445	1.1487262332496753e-05	3.3229281476135027e-06	3.2235669238575655e-10
ENSG00000165280_35068057_35062193_277	VCP	9	-	831	37	0	0	5.78%	26.126204189850185	0.0001842144602393462	0.0001842144602393462	2.3639666231890906e-07

ENSG00000132541_98106314_98102842_76	RIDA	8	-	228	14	4	0	11.84%	36.03037690193422	0.04077810018967558	0.002943990679779778	0.0007391052320154406
ENSG00000120526_109293404_109243009_390	NUDCD1	8	-	1170	8	2	0	1.79%	4.012173838164103	0.06180520118411761	0.005706018193000826	0.0020032236666657145
ENSG00000028310_878410_864468_192	BRD9	5	-	576	12	0	0	2.60%	12.224592163156252	0.019679754444124863	0.019679754444124863	0.0017849636115922674
ENSG00000110880_108701318_108654379_159	CORO1C	12	-	477	16	2	0	3.14%	19.68236222495598	0.03577298629450316	0.02108409854857798	0.003354604907114977
ENSG00000110880_108652315_108647403_155	CORO1C	12	-	465	29	0	0	5.81%	36.59490815293227	0.003736770770438521	0.003736770770438521	7.748026083818498e-05
ENSG00000104763_18083928_18057534_373	ASAH1	8	-	1119	55	3	2	6.70%	28.840860867500005	4.58542909915364e-06	1.926540703241544e-05	7.37125132342833e-10
ENSG00000204209_33321925_33318743_740	DAXX	6	-	2220	17	0	0	0.81%	4.493363605916893	0.013428347753762199	0.013428347753762199	0.0008725593497644525
ENSG00000121390_19782754_19703175_522	PSPC1	13	-	1566	19	1	2	1.53%	7.119302715707856	0.007181907427766128	0.015570605297898355	0.0005669161536572745
ENSG00000124214_49153958_49114878_471	STAU1	20	-	1413	29	4	0	2.76%	12.042910326336521	0.005851237224165338	0.0005854768899833019	2.273373872391799e-05
ENSG00000286264_64242388_64244029_142	AP001453.5	11	+	426	62	4	3	14.08%	85.39996778768312	0.00016569421277282895	0.00014894787451579541	1.736266846401335e-07
ENSG00000141905_3435108_3462769_142	NFIC	19	+	426	10	0	0	3.52%	13.774188352852116	0.020613416668581838	0.020613416668581838	0.001946208561389314
ENSG00000249915_306604_314515_121	PDCD6	5	+	363	56	0	5	17.36%	90.52259981973556	2.348557756166878e-05	0.00023430629050383736	4.359212256874465e-08
ENSG00000145868_148414216_148442147_552	FBXO38	5	+	1656	17	0	0	1.27%	6.023712080395835	0.0078820576498062	0.0078820576498062	0.00031963642907451164
ENSG00000113649_146458971_146510642_923	TCERG1	5	+	2769	19	0	6	1.08%	4.0263012108336955	0.002046028879997488	0.09422925153322642	0.0015379924355527336
ENSG00000258947_89923402_89934499_148	TUBB3	16	+	444	50	6	2	9.46%	66.07887655760136	0.002881754508607864	0.00095416369115764	1.682544927174563e-05
ENSG00000167283_118401666_118409098_103	ATP5MG	11	+	309	114	13	1	10.68%	216.48209811259227	0.022501155875582667	0.001588175367824666	0.0002295502545141123
ENSG00000267796_35748725_35754474_246	LIN37	19	+	738	15	0	1	2.44%	11.926431378689028	0.012798402692974279	0.012798402692974279	0.0007972436888943567
ENSG00000158769_161021073_160998871_250	F11R	1	-	750	14	2	0	2.00%	10.953234578188003	0.020613416668581838	0.020613416668581838	0.001946208561389314
ENSG00000141736_39707049_39728044_1211	ERBB2	17	+	3633	9	0	1	0.41%	1.4536261531746493	0.020613416668581838	0.03537493353818881	0.003252526348827943
ENSG00000101384_10658684_10639498_1059	JAG1	20	-	3177	131	4	9	4.82%	24.195226793178005	1.3802407997535192e-09	9.163575978520153e-09	1.385729365861971e-16
ENSG00000187118_28241794_28319629_76	CMC1	3	+	228	18	0	0	6.58%	46.324770302486854	0.019679754444124863	0.019679754444124863	0.0017849636115922674
ENSG00000177311_141442398_141445976_1192	ZBTB38	3	+	3576	27	0	2	0.92%	4.4303891061103196	0.00151350169064872	0.005530543042558543	4.947549134344851e-05
ENSG00000164597_107563896_107210522_792	COG5	7	-	2376	19	1	0	1.14%	4.692267698989268	0.005588370640926919	0.0034782380676658805	0.00010676685949938237
ENSG00000186635_72726879_72685664_1367	ARAP1	11	-	4101	13	2	1	0.44%	1.8600696195585227	0.023672501042627626	0.023672501042627626	0.0025184098398201636
ENSG00000145414_163166727_163136132_372	NAF1	4	-	1116	11	0	0	1.61%	5.783678012676076	0.012798402692974279	0.012798402692974279	0.0007972436888943567
ENSG00000164548_23531824_23505559_282	TRA2A	7	-	846	63	0	1	8.87%	43.69641454064363	4.995322194014732e-06	6.677298738584374e-06	2.779547209241056e-10
ENSG00000135525_136383755_136344228_565	MAP7	6	-	1695	7	0	0	0.88%	2.4232819863247794	0.019216965118390855	0.019216965118390855	0.0017073955890589052
ENSG00000130429_99385715_99388655_130	ARPC1B	7	+	390	51	0	2	11.54%	76.73282465488847	0.0003112740676895738	0.0006361888637828822	1.3147055450679545e-06
ENSG00000018236_40908439_40971513_625	CNTN1	12	+	1875	52	3	1	3.68%	16.273377087593605	7.403032745465754e-05	1.4838839790734058e-05	8.712351456153708e-09
ENSG00000170892_54191365_54193362_310	TSEN34	19	+	930	33	5	0	3.87%	20.821240845633877	0.008356254428487338	0.0010155991509472172	5.354085463802975e-05
ENSG00000196230_30722260_30724397_426	TUBB	6	+	1278	335	24	11	8.69%	153.81176994018196	2.75083302959803e-06	3.8120552282922505e-07	9.728087087195689e-12
ENSG00000122565_26206374_26212208_173	CBX3	7	+	519	54	6	0	9.25%	61.052298433335274	0.0016935394879116449	0.00019739551450717686	2.3511436678116523e-06
ENSG00000196230_30722968_30724397_372	TUBB	6	+	1116	219	17	11	7.80%	115.14777134327825	3.9580971101444664e-05	1.1716555672536487e-05	3.7013700744866613e-09
ENSG00000080845_36500221_36527031_285	DLGAP4	20	+	855	14	0	1	2.11%	9.608100507182458	0.013428347753762199	0.02034245439037237	0.0012955184805712684
ENSG00000173171_155209264_155213698_313	MTX1	1	+	939	40	3	0	2.24%	24.995971196230037	0.0424352593247778	0.007029302661868638	0.0015638435298188141
ENSG00000174996_66258604_66266956_619	KLC2	11	+	1857	10	0	5	0.97%	3.159829961397416	0.011772051206276392	0.21747454246502812	0.015647194567730636
ENSG00000101439_23637745_23633916_107	CST3	20	-	321	235	6	5	26.17%	429.5744535838085	2.855582722436658e-06	1.900663467930204e-06	4.702211580522806e-11
ENSG00000182158_137915848_137880476_359	CREB3L2	7	-	1077	20	0	0	2.51%	10.896572401699167	0.0035547763465261306	0.0035547763465261306	7.044676146010672e-05
ENSG00000143614_153828215_153810177_549	GATAD2B	1	-	1647	13	0	0	1.09%	4.631539471651185	0.012798402692974279	0.012798402692974279	0.0007972436888943567
ENSG00000102317_48575181_48575877_90	RBM3	X	+	270	53	2	2	11.11%	115.18282393729447	0.0061547572324231374	0.0062201494882096935	0.00020209328853378674
ENSG00000101266_489860_478663_171	CSNK2A1	20	-	513	9	0	0	2.92%	10.294393400552634	0.019216965118390855	0.019216965118390855	0.0017073955890589052
ENSG00000178467_48990449_49005245_359	P4HTM	3	+	1077	16	7	4	1.95%	8.717257921359334	0.17034922041731837	0.06059069429271301	0.03839526610066628
ENSG00000044115_138886260_138891191_64	CTNNA1	5	+	192	7	0	0	7.81%	21.393036285523443	0.016947426762344633	0.016947426762344633	0.0013498980316300959
ENSG00000163110_94618059_94656820_163	PDLIM5	4	+	489	34	2	11	7.36%	40.79863887580983	0.0010197273511756433	0.013733787011225951	9.19907079417941e-05
ENSG00000112851_66021326_66078530_1233	ERBIN	5	+	3699	28	7	0	0.89%	4.441700964390917	0.01465917395857711	0.001484234770590415	0.00013541356774210257
ENSG00000186283_179082129_179095218_397	TOR3A	1	+	1191	33	2	0	3.02%	16.25839965276197	0.0022805788404053587	0.0010032776369550557	1.3938975116499231e-05
ENSG00000009844_142169631_142218643_211	VTA1	6	+	633	30	5	0	4.74%	27.809498759786734	0.014094678151315428	0.0023766146074493525	0.00019392272823799724
ENSG00000117593_173826769_173857705_553	DARS2	1	+	1659	26	0	0	2.71%	9.196076563965644	0.0001780993973766097	0.0001780993973766097	2.214199536784249e-07
ENSG00000127990_94618900_94585465_303	SGCE	7	-	909	11	0	2	2.31%	7.10075320368152	0.007029302661868638	0.03522780890193623	0.0012822660609675817
ENSG00000104518_143559815_143561023_402	GSDMD	8	+	1206	8	0	0	1.24%	3.8924074549353245	0.019216965118390855	0.019216965118390855	0.0017073955890589052
ENSG00000110075_68537665_68613117_844	PPP6R3	11	+	2532	32	7	5	2.25%	7.41586633594313	0.003622420583891007	0.0005933294589348272	1.3865891050279807e-05
ENSG00000100439_22598307_22609749_285	ABHD4	14	+	855	23	0	3	2.46%	15.784736547514038	0.00877616296920843	0.027941506428179053	0.0012176006631866223
ENSG00000182149_71915641_71927813_366	IST1	16	+	1098	33	2	0	3.28%	17.635477218979513	0.0027101569075239007	0.0010197273511756433	1.6811112043185018e-05
ENSG00000176974_18355921_18328750_424	SHMT1	17	-	1272	23	0	0	2.36%	10.610023386890333	0.002046028879997488	0.002046028879997488	2.4518070358754455e-05
ENSG00000163516_219231975_219234365_361	ANKZF1	2	+	1083	15	0	0	1.39%	8.127152684646816	0.02108409854857798	0.02108409854857798	0.002029891263157455
ENSG00000213689_48466710_48467600_296	TREX1	3	+	888	11	0	1	1.69%	7.26867642133615	0.020613416668581838	0.03537493353818881	0.003252526348827943
ENSG00000112339_135002753_134965279_511	HBS1L	6	-	1533	28	0	0	2.35%	10.717450663589043	0.0008739357725210369	0.0008739357725210369	4.787965623198429e-06
ENSG00000159176_201496303_201487004_140	CSRP1	1	-	420	124	4	5	8.57%	173.23993465501434	0.001806384452599662	0.0018115419044284426	1.936593110542893e-05
ENSG00000085063_33722445_33710126_128	CD59	11	-	384	62	5	3	14.06%	94.74058926446097	0.00102540667708157	0.0003583833865847517	2.4148597638421077e-06
ENSG00000112759_44229610_44233528_412	SLC29A1	6	+	1236	49	6	0	4.61%	23.262330718239085	0.0003953272125394652	5.6254241454829154e-05	1.6639839158165156e-07
ENSG00000126883_131129336_131233487_1928	NUP214	9	+	5784	23	1	0	0.52%	2.3333246452497414	0.0035004709947243163	0.0023628488670607213	4.7187726630981656e-05
ENSG00000103353_23557967_23570590_295	UBFD1	16	+	885	29	0	0	3.73%	19.227833097303396	0.0013845019718837994	0.0013845019718837994	1.1595255170738555e-05
ENSG00000081154_101574216_101592753_178	PCNP	3	+	534	41	6	0	5.62%	45.05242954511518	0.013428347753762199	0.002446120450917513	0.00018893366792563466
ENSG00000214046_16660110_16647246_75	SMIM7	19	-	225	25	3	0	10.67%	65.19782487016668	0.04140309663709693	0.0053557511377634245	0.001218378129224801
ENSG00000115355_55343714_55332562_530	CCDC88A	2	-	1590	13	0	0	0.94%	4.79757579233302	0.02108409854857798	0.02108409854857798	0.002029891263157455
ENSG00000102738_40771043_40729372_364	MRPS31	13	-	1092	12	0	0	1.65%	6.448136525620881	0.012798402692974279	0.012798402692974279	0.0007972436888943567
ENSG00000137274_3123754_3152575_223	BPHL	6	+	669	6	0	0	2.69%	5.26260469804036	0.007152939217714824	0.007152939217714824	0.0002660027525696252

ENSG00000137274_3123754_3152575_223	BPHL	6	+	669	6	0	0	2.69%	5.26260469804036	0.007152939217714824	0.007152939217714824	0.0002660027525696252
ENSG00000250479_23767502_23766007_105	CHCHD10	22	-	315	15	5	0	8.57%	27.94192494435715	0.07455652756907821	0.00327844299461777	0.0016282502955699567
ENSG00000071553_154428759_154429291_112	ATP6AP1	X	+	336	13	0	3	4.46%	22.702814017290184	0.020613416668581838	0.08350359792828288	0.007749259071793911
ENSG00000197756_216498875_216578933_75	RPL37A	2	+	225	266	25	15	28.00%	693.7048566185734	2.795442514340845e-05	0.00035172977952708476	7.81047735534185e-08
ENSG00000067113_55534629_55425206_284	PLPP1	5	-	852	12	0	0	2.46%	8.26451301171127	0.008175610787902942	0.008175610787902942	0.00034251843268961324
ENSG00000124882_74365327_74384808_163	EREG	4	+	489	23	0	0	7.36%	27.59907923951841	0.0009235399941089087	0.0009235399941089087	5.324735186779001e-06
ENSG00000129083_14498883_14457824_938	COPB1	11	-	2814	39	2	0	2.03%	8.132351289775588	0.00021852315413304935	5.3992704767724636e-05	8.731581146046835e-08
ENSG00000141295_47840843_47837844_425	SCRN2	17	-	1275	23	0	0	2.59%	10.585058625980002	0.0012624143894574274	0.0012624143894574274	9.711623744831373e-06
ENSG00000080503_2029239_2192739_1500	SMARCA2	9	+	4500	22	4	6	0.73%	2.868704294287334	0.014015870411971724	0.03286917352493041	0.0021544021968327126
ENSG00000136527_185925496_185917715_188	TRA2B	3	-	564	41	1	3	8.51%	42.65602371824735	0.00022282222326679875	0.001013197191032157	1.5404611290049819e-06
ENSG00000187778_49563530_49558643_271	MCRS1	12	-	813	14	1	0	2.21%	10.104459942977861	0.023878473930440184	0.013007239811447113	0.0014709138084317821
ENSG00000136636_215567686_215620618_815	KCTD3	1	+	2445	42	0	5	1.84%	10.079663722258898	0.0002790666132128672	0.004093767516927554	8.158546660624852e-06
ENSG00000142208_104776759_104770341_418	AKT1	14	-	1254	19	0	0	1.91%	8.890612482295456	0.0053557511377634245	0.0053557511377634245	0.00015366972994913032
ENSG00000119718_75003090_75004987_194	EIF2B2	14	+	582	20	0	0	4.12%	20.16427573304124	0.005306578019052857	0.005306578019052857	0.00015100114740534233
ENSG00000112312_24780693_24785799_182	GMNN	6	+	546	40	1	2	6.04%	42.98757683747254	0.00151350169064872	0.0030285643754408066	2.7030838072125858e-05
ENSG00000180398_46909171_46905463_146	MCFD2	2	-	438	36	2	0	8.22%	48.228527986150695	0.0021748195570013406	0.0010405263639310788	1.3754092960537414e-05
ENSG00000163110_94618059_94664067_271	PDLIM5	4	+	813	80	2	12	11.07%	57.73977110273064	6.476670722334506e-07	1.0606894616217906e-05	6.41614398960698e-11
ENSG00000179010_6640863_6641246_103	MRFAP1	4	+	309	29	1	3	11.65%	55.070007414606806	0.0010155991509472172	0.002856573962475447	1.765694880448258e-05
ENSG00000138768_75724841_75813295_966	USO1	4	+	2898	18	0	0	1.35%	3.644598905785715	0.00047074547413106	0.00047074547413106	1.4523754767299686e-06
ENSG00000138772_78554474_78610115_323	ANXA3	4	+	969	113	7	14	11.46%	68.42743848602633	6.856536186732406e-07	1.2616254723110354e-05	8.104087845296682e-11
ENSG00000109180_48832625_48860762_245	OCIAD1	4	+	735	64	0	2	5.31%	51.09380561253879	0.0006791898698869142	0.0006791898698869142	2.9460669171009034e-06
ENSG00000102125_154411916_154421004_238	TAZ	X	+	714	13	1	0	2.52%	10.683677184607145	0.015555382717448078	0.011772051206276392	0.0008874794703752884
ENSG00000106608_43879012_43876667_781	URGCP	7	-	2343	15	0	2	0.90%	3.7565968235051224	0.00847367416878415	0.027657845067597595	0.0011690047180828366
ENSG00000119723_73955892_73959458_190	COQ6	14	+	570	16	0	0	2.63%	16.471029440884216	0.02108409854857798	0.02108409854857798	0.002029891263157455
ENSG00000170892_54191365_54193671_312	TSEN34	19	+	936	33	5	0	3.85%	20.687771353033657	0.008356254428487338	0.0010155991509472172	5.354085463802975e-05
ENSG00000164889_151063181_151076367_1227	SLC4A2	7	+	3681	59	26	5	2.20%	9.405065201319887	0.01641305528749142	5.228841052443753e-05	1.057051460316207e-05
ENSG00000204628_181243800_181240313_153	RACK1	5	-	459	780	12	19	24.84%	997.1432038966669	7.839829874602346e-08	3.003658583419622e-07	2.2850899182934405e-13
ENSG00000173113_64317526_64316861_120	TRMT112	11	-	360	79	21	2	18.33%	128.7657041185792	0.0024434495081023543	1.8458363644021042e-05	4.610271848302717e-07
ENSG00000173402_49510610_49533199_870	DAG1	3	+	2610	51	1	2	2.30%	11.465824373718968	5.300913698185325e-05	7.960803452267643e-05	3.12978990934939e-08
ENSG00000143314_156738110_156737398_115	MRPL24	1	-	345	52	0	2	11.30%	88.44226678040002	0.000649205117157464	0.000649205117157464	2.700471097049339e-06
ENSG00000114062_25375498_25339137_763	UBE3A	15	-	2289	8	13	0	0.66%	2.050783482154653	0.3331867010986109	0.019216965118390855	0.038471517829580006
ENSG00000131238_40078607_40074061_80	PPT1	1	-	240	28	0	0	8.75%	68.45771611367502	0.00847367416878415	0.00847367416878415	0.0003665024264111182
ENSG00000131238_40097238_40089569_148	PPT1	1	-	444	37	0	0	8.78%	48.89836865262501	0.0006745191331285194	0.0006745191331285194	2.90713800764924e-06
ENSG00000133112_45341069_45337386_172	TPT1	13	-	516	219	12	6	12.21%	249.0405287191832	4.394701515060627e-05	7.749520157855128e-05	2.5460826018628213e-08
ENSG00000116221_54200244_54218249_423	MRPL37	1	+	1269	21	0	3	2.60%	9.710314342365251	0.0013775385517804596	0.004262378865900335	3.4890884543666204e-05
ENSG00000130119_54532567_54560602_582	GNL3L	X	+	1746	27	1	0	2.06%	9.07392407986856	0.0015592205693390887	0.0009430837296224684	9.035662816596504e-06
ENSG00000131051_35725100_35704481_367	RBM39	20	-	1101	22	1	0	2.18%	11.724949431692101	0.008364861494636245	0.005604866691947602	0.00024596970711672695
ENSG00000133961_73355751_73276578_603	NUMB	14	-	1809	19	0	0	1.66%	6.162978470314264	0.0022011839993586477	0.0022011839993586477	2.8200182015552967e-05
ENSG00000141736_39707049_39727697_1011	ERBB2	17	+	3033	9	0	1	0.49%	1.7411882012804158	0.020613416668581838	0.03537493353818881	0.003252526348827943
ENSG00000133872_30074050_30063824_311	SARAF	8	-	933	127	7	7	13.83%	79.8725764486608	5.377026222435529e-08	3.9988544008785666e-08	2.1368738572241854e-14
ENSG00000177556_151752316_151746325_73	ATOX1	5	-	219	31	3	7	6.85%	83.06024264281508	0.03770178143563168	0.08588638879785576	0.013090394844661306
ENSG00000070081_17295324_17331419_420	NUCB2	11	+	1260	24	0	1	2.86%	11.17676997774286	0.0009470236307512361	0.0015639623262203096	9.098239545706659e-06
ENSG00000075142_88226914_88206478_183	SRI	7	-	549	44	0	8	9.84%	47.027939250612036	8.656861907854571e-05	0.003245780418868412	2.3244292897784216e-06
ENSG00000165322_31908573_31807658_700	ARHGAP12	10	-	2100	23	1	1	1.29%	6.426642737202144	0.005059698366439319	0.005059698366439319	0.000137934786740623
ENSG00000151014_139043082_139045474_365	NOCT	4	+	1095	9	4	1	1.37%	4.822852798615069	0.17139085557395572	0.03537493353818881	0.02567049216018708
ENSG00000101266_489860_483961_177	CSNK2A1	20	-	531	9	0	0	2.82%	9.945430912398308	0.019216965118390855	0.019216965118390855	0.0017073955890589052
ENSG00000132155_12612023_12584514_586	RAF1	3	-	1758	16	5	0	1.02%	5.340436166839591	0.08873763529811646	0.011772051206276392	0.005312171954116946
ENSG00000132541_98106314_98102842_76	RIDA	8	-	228	14	4	0	11.84%	36.03037690193422	0.04077810018967558	0.002943990679779778	0.0007391052320154406
ENSG00000113312_160010529_160065065_292	TTC1	5	+	876	51	4	4	4.79%	34.161873990190074	0.0035347405877527685	0.0035347405877527685	6.96917194851713e-05
ENSG00000163558_170235237_170303127_560	PRKCI	3	+	1680	20	2	0	1.96%	6.985481236089288	0.0043894378151575035	0.0013984928336429232	3.643756032304807e-05
ENSG00000204469_31626063_31637586_1863	PRRC2A	6	+	5589	27	0	2	0.59%	2.834688037833334	0.0014055202957965589	0.006088081514183966	5.109689293194805e-05
ENSG00000143324_180806519_180884066_417	XPR1	1	+	1251	12	2	4	1.68%	5.6285891974244615	0.03744183116399598	0.07864960352514258	0.011926715440658299
ENSG00000213619_47582358_47584481_92	NDUFS3	11	+	276	19	0	0	7.61%	40.39430453912501	0.00847367416878415	0.00847367416878415	0.0003665024264111182
ENSG00000149600_32743337_32703382_181	COMMD7	20	-	543	8	0	0	3.31%	8.645015452397793	0.011570465654371866	0.011570465654371866	0.0006594843062896773
ENSG00000112118_52284649_52264588_762	MCM3	6	-	2286	47	0	11	2.49%	12.064164444479662	5.2494528509358865e-05	0.004993752824121904	2.5047256486173585e-06
ENSG00000103197_2060673_2088610_1443	TSC2	16	+	4329	10	5	3	0.55%	1.3554641345148999	0.07635947366271362	0.04922925118879209	0.01464496403362635
ENSG00000151366_78079744_78069987_119	NDUFC2	11	-	357	178	4	15	24.37%	292.5683905938572	1.3138182778422497e-06	7.208461721875831e-06	8.349852085024178e-11
ENSG00000110057_68004043_68003301_197	UNC93B1	11	-	591	8	0	0	2.54%	7.942882217685281	0.019216965118390855	0.019216965118390855	0.0017073955890589052
ENSG00000168679_110389323_110363766_319	SLC16A4	1	-	957	35	16	1	3.13%	21.46009909519593	0.07593394988259367	0.0031596627670218355	0.0016190560662952258
ENSG00000162775_110339538_110345609_933	RBM15	1	+	2799	22	0	3	1.07%	4.612064781812434	0.0021880057261679567	0.007664059176943723	9.5950101060544e-05
ENSG00000089157_120200783_120198735_166	RPLP0	12	-	498	54	1	4	4.82%	63.62679294558435	0.005808022449631236	0.009175245078463236	0.0002780762357321736
ENSG00000204220_33289857_33290845_129	PFDN6	6	+	387	31	0	1	9.30%	47.00308304593412	0.0010155991509472172	0.0014942378687529357	9.293758677126684e-06
ENSG00000115525_85848138_85840144_237	ST3GAL5	2	-	711	13	0	3	2.53%	10.728755991293252	0.012798402692974279	0.07760924484234204	0.004890251693990683
ENSG00000125944_23337834_23310454_532	HNRNPR	1	-	1596	60	0	1	2.82%	22.059414429755645	0.0002805452199820376	0.0003075018333337533	5.836061363138067e-07
ENSG00000114416_180947925_180976138_453	FXR1	3	+	1359	13	0	0	1.77%	5.613057770279251	0.004687384229717438	0.004687384229717438	0.00011928172701435494
ENSG00000137522_71982779_71996315_240	RNF121	11	+	720	11	0	1	2.92%	8.96470091964792	0.007029302661868638	0.016854695534768073	0.0006017499134389055
ENSG00000185787_78873018_78894908_269	MORF4L1	15	+	807	32	3	2	2.97%	23.26762523247584	0.021725767126240147	0.01732176123716967	0.0017408846237968172

ENSG00000140391_77052426_77046835_44	TSPAN3	15	-	132	18	1	1	11.36%	80.01551234065911	0.02108409854857798	0.019679754444124863	0.0019038513263291816
ENSG00000142192_26170620_25881670_695	APP	21	-	2085	267	3	4	12.09%	75.14166578561657	8.960913548354413e-16	3.014650095336499e-15	3.882751493283315e-29
ENSG00000097033_86719593_86743235_265	SH3GLB1	1	+	795	13	1	0	2.26%	9.59515158466604	0.023672501042627626	0.012798402692974279	0.0014375227604752335
ENSG00000031698_109213993_109237888_514	SARS	1	+	1542	69	4	4	5.84%	26.25671157222666	3.7833099270326014e-06	5.480226057201275e-06	1.7465379379865084e-10
ENSG00000168286_67842555_67843499_314	THAP11	16	+	942	17	0	0	2.87%	10.589455631778664	0.0034782380676658805	0.0034782380676658805	6.758309314600117e-05
ENSG00000198961_109381590_109337231_660	PJA2	5	-	1980	16	0	0	0.91%	4.741659990557577	0.013640585738808999	0.013640585738808999	0.00089863692579514
ENSG00000147955_34637697_34637074_106	SIGMAR1	9	-	318	54	4	1	12.26%	99.64195876383964	0.0038350587622105986	0.0009531022847370905	2.2467576109501583e-05
ENSG00000147955_34637318_34635632_123	SIGMAR1	9	-	369	60	2	8	10.57%	95.41145102951222	0.0009939604220181157	0.005373211516716593	3.3021188001601206e-05
ENSG00000170468_73491018_73492943_641	RIOX1	14	+	1923	10	0	0	0.94%	3.051380259134166	0.012798402692974279	0.012798402692974279	0.0007972436888943567
ENSG00000147130_71253174_71240916_1253	ZMYM3	X	-	3759	11	0	0	0.40%	1.717101532893456	0.019679754444124863	0.019679754444124863	0.0017849636115922674
ENSG00000167930_254414_268853_540	FAM234A	16	+	1620	31	0	1	2.59%	11.228514283195373	0.00040182005258836655	0.0006536905545227689	1.7187018856253374e-06
ENSG00000129518_34536157_34516470_167	EAPP	14	-	501	10	0	5	2.99%	11.712184108413176	0.019679754444124863	0.14389369507707506	0.013602114125134476
ENSG00000113593_65569985_65587396_477	PPWD1	5	+	1431	13	2	0	1.26%	5.330639769258911	0.044040755831095145	0.012798402692974279	0.0026804992160797854
ENSG00000109016_21172043_21191153_310	DHRS7B	17	+	930	9	2	0	1.61%	5.678520230627421	0.10105102209468636	0.019679754444124863	0.009164497669452725
ENSG00000170348_75147762_75134885_115	TMED10	14	-	345	76	4	2	10.43%	129.26177452520002	0.004173829468951125	0.003042349403049352	7.094788537145463e-05
ENSG00000114019_134371433_134357705_780	AMOTL2	3	-	2340	42	4	13	2.56%	10.531956325180772	0.00036942355516503625	0.014762025815636752	4.331749224354655e-05
ENSG00000143013_87332016_87344796_165	LMO4	1	+	495	25	0	0	3.03%	29.635374940984857	0.019679754444124863	0.019679754444124863	0.0017849636115922674
ENSG00000151327_35046533_35081228_213	FAM177A1	14	+	639	29	3	0	7.04%	26.63009748218076	0.0005610828647514753	0.00027685889791379426	1.039467627697229e-06
ENSG00000174371_241853461_241889600_718	EXO1	1	+	2154	10	0	0	0.70%	2.724143100424792	0.019679754444124863	0.019679754444124863	0.0017849636115922674
ENSG00000115091_113927396_113934410_95	ACTR3	2	+	285	22	0	0	8.42%	45.29533096243159	0.005655335537214311	0.005655335537214311	0.00017039952270558162
ENSG00000152556_48131382_48145708_705	PFKM	12	+	2115	47	0	0	3.12%	13.039564974033336	9.836921317677237e-06	9.836921317677237e-06	7.867021274290272e-10
ENSG00000118785_87976896_87982896_300	SPP1	4	+	900	310	8	14	22.33%	202.1132570975167	8.461148190190012e-12	1.3318280542126079e-11	1.3819443431450789e-21
ENSG00000071462_73686664_73692633_62	BUD23	7	+	186	9	0	1	8.06%	28.392601153137104	0.020613416668581838	0.03537493353818881	0.003252526348827943
ENSG00000071462_73686853_73703529_176	BUD23	7	+	528	20	2	1	5.11%	22.22653120573864	0.018785663926082854	0.006024333578509648	0.0005856602134908656
ENSG00000173141_21176917_21177225_102	MRPL57	13	+	306	22	0	0	6.86%	42.186827857166676	0.008674846900444944	0.008674846900444944	0.00038311532916634116
ENSG00000075213_84134949_83961371_733	SEMA3A	7	-	2199	11	1	2	0.82%	2.93523631748363	0.023672501042627626	0.052264829583131125	0.005383570679128256
ENSG00000106829_79627422_79725144_652	TLE4	9	+	1956	8	0	0	0.77%	2.3999199338711663	0.019679754444124863	0.019679754444124863	0.0017849636115922674
ENSG00000102572_98466486_98453173_184	STK24	13	-	552	23	0	0	2.72%	24.449184326312505	0.021557223391537678	0.021557223391537678	0.002115616449879074
ENSG00000011405_17155554_17089738_1306	PIK3C2A	11	-	3918	12	0	0	0.38%	1.797183533940276	0.02108409854857798	0.02108409854857798	0.002029891263157455
ENSG00000151465_12196246_12198780_49	CDC123	10	+	147	30	1	0	10.20%	119.75110690438778	0.021557223391537678	0.021557223391537678	0.002115616449879074
ENSG00000119318_107302103_107329656_337	RAD23B	9	+	1011	30	3	0	4.75%	17.41188201280416	0.0012748355907334584	0.00017249046415619826	1.5482698443538261e-06
ENSG00000180628_91222872_91278316_256	PCGF5	10	+	768	8	0	0	2.34%	6.112296081578126	0.011570465654371866	0.011570465654371866	0.0006594843062896773
ENSG00000175832_43545310_43528519_445	ETV4	17	-	1335	18	0	1	2.02%	7.911646164020227	0.002967235347054297	0.002943990679779778	4.953464307974475e-05
ENSG00000157851_26898500_26946995_564	DPYSL5	2	+	1692	20	0	0	0.89%	6.935938815975179	0.02108409854857798	0.02108409854857798	0.002029891263157455
ENSG00000110619_3057367_3001188_726	CARS	11	-	2178	9	0	0	0.69%	2.4247124951714882	0.019679754444124863	0.019679754444124863	0.0017849636115922674
ENSG00000087088_48956199_48961097_140	BAX	19	+	420	75	1	1	11.43%	104.7822185413393	0.0002028510352551382	0.0003665275989911912	5.082700333559607e-07
ENSG00000113648_135389012_135359931_172	H2AFY	5	-	516	36	1	0	6.40%	40.93816910452327	0.001558047347358915	0.001558047347358915	1.4544595481456893e-05
ENSG00000174371_241853461_241889600_718	EXO1	1	+	2154	10	0	0	0.70%	2.724143100424792	0.019679754444124863	0.019679754444124863	0.0017849636115922674
ENSG00000204130_68341286_68341203_27	RUFY2	10	-	81	15	0	3	25.93%	108.6630414502778	0.00797940233269714	0.04441936522437137	0.0018243500037866422
ENSG00000054148_136850042_136850847_62	PHPT1	9	+	186	63	6	2	24.19%	198.7482080719597	0.0007464216444983301	0.00036804788788593267	1.8033213812899662e-06
ENSG00000120708_136060885_136063209_51	TGFBI	5	+	153	19	3	0	9.80%	72.86815720783335	0.02108409854857798	0.02108409854857798	0.002029891263157455
ENSG00000196305_92289419_92287743_147	IARS	9	-	441	15	0	1	4.08%	19.958517817397965	0.013428347753762199	0.02034245439037237	0.0012955184805712684
ENSG00000132109_4390037_4385285_351	TRIM21	11	-	1053	7	0	0	1.71%	3.9007245648817674	0.009815328628645334	0.009815328628645334	0.0004837142251238724
ENSG00000113328_163441222_163442565_159	CCNG1	5	+	477	36	1	1	6.29%	44.28531500615095	0.0031859520777271916	0.0031859520777271916	5.71642936696178e-05
ENSG00000215021_6970543_6968581_139	PHB2	12	-	417	34	2	9	6.47%	47.84300817810792	0.0052928676796983615	0.03625986857027159	0.0010445072256952466
ENSG00000137868_74196092_74180080_560	STRA6	15	-	1680	53	2	9	3.39%	18.51152527563661	0.00021186981035795232	0.0026742133664407067	4.061495374133696e-06
ENSG00000146477_160348420_160451056_556	SLC22A3	6	+	1668	21	0	0	1.80%	7.387523321619606	0.0022276761777358703	0.0022276761777358703	2.8853233252577778e-05
ENSG00000148498_34814935_34111169_1246	PARD3	10	-	3738	19	0	1	0.64%	2.982565022150482	0.005405141725979036	0.005554612275049112	0.00016047798536882382
ENSG00000284969_33722445_33702718_108	AL049629.2	11	-	324	28	3	3	12.96%	50.709419343462976	0.0021016973734692764	0.0021016973734692764	2.5811106592006456e-05
ENSG00000114054_136255955_136330026_426	PCCB	3	+	1278	17	0	1	1.41%	7.8053733999495325	0.013428347753762199	0.02034245439037237	0.0012955184805712684
ENSG00000128654_176329388_176337664_95	MTX2	2	+	285	14	1	0	9.47%	28.824301521547376	0.010499663139468709	0.0025000845279814113	0.00014909639159692154
ENSG00000133704_30676539_30630860_808	IPO8	12	-	2424	19	0	0	1.36%	4.599351506930075	0.0013775385517804596	0.0013775385517804596	1.1483554366703679e-05
ENSG00000147650_104588897_104490673_859	LRP12	8	-	2577	13	3	0	0.81%	2.96008750865716	0.05128931843854215	0.00797940233269714	0.002129167107140446
ENSG00000156990_9843990_9838016_343	RPUSD3	3	-	1029	10	0	2	1.75%	5.7024336621137035	0.011570465654371866	0.04178282491678763	0.002331326177745642
ENSG00000173113_64317311_64317007_62	TRMT112	11	-	186	85	0	0	25.81%	268.15234422407264	0.00021408175522948358	0.00021408175522948358	3.163100203905902e-07
ENSG00000164543_43623800_43624842_137	STK17A	7	+	411	8	0	0	3.65%	11.421516765576644	0.016947426762344633	0.016947426762344633	0.0013498980316300959
ENSG00000171204_85634105_85636229_156	TMEM126B	11	+	468	12	1	0	3.85%	15.045651893115387	0.012798402692974279	0.012798402692974279	0.0007972436888943567
ENSG00000134910_125595916_125620810_705	STT3A	11	+	2115	151	9	1	6.24%	41.89307044849008	5.8747299195070665e-08	3.515628446861874e-09	2.251550476651329e-15
ENSG00000010072_231338399_231353361_484	SPRTN	1	+	1452	8	2	0	1.03%	3.2329499935619843	0.10105102209468636	0.019679754444124863	0.009164497669452725
ENSG00000125107_58599337_58520958_2376	CNOT1	16	-	7128	45	7	5	0.93%	3.704421867623107	0.0008315867542965732	0.00020037430501765357	1.1424951378129194e-06
ENSG00000112245_63576466_63576531_21	PTP4A1	6	+	63	77	1	1	38.10%	717.1760735718335	0.0053557511377634245	0.00934169773149884	0.00026267894186613697
ENSG00000075618_5603259_5605474_215	FSCN1	7	+	645	50	6	0	10.23%	45.486854560581406	0.00044470793150527295	1.1310431733989869e-05	4.490968381381516e-08
ENSG00000182093_39390698_39396939_140	WRB	21	+	420	20	0	0	5.71%	27.94192494435715	0.005405141725979036	0.005405141725979036	0.00015637213408961292
ENSG00000182093_39380385_39396939_174	WRB	21	+	522	22	0	0	5.75%	24.7302094335115	0.00228120298834518	0.00228120298834518	3.0194288573932866e-05
ENSG00000102393_101407903_101397809_470	GLA	X	-	1410	47	4	0	5.32%	19.559347461050006	5.1297874593508184e-05	3.5161929362867004e-06	1.575929232257885e-09
ENSG00000125755_45827605_45815560_579	SYMPK	19	-	1737	9	0	0	1.21%	3.040313076847151	0.006937202941512724	0.006937202941512724	0.00025101522741487095
ENSG00000121749_71839782_71923204_674	TBC1D15	12	+	2022	14	3	0	1.19%	4.062772469654304	0.046396937147164995	0.005160369442893307	0.0013402346425884413

ENSG00000104824_38838973_38837346_157	HNRNPL	19	-	471	52	0	0	7.01%	64.782552100293	0.0015806110104534784	0.0015806110104534784	1.495132122473684e-05
ENSG00000104763_18071320_18057534_330	ASAH1	8	-	990	53	3	2	7.27%	31.413497437443947	6.8176351904891705e-06	2.8957473970768623e-05	1.6190192581539036e-09
ENSG00000113460_34915739_34919928_119	BRIX1	5	+	357	16	0	0	5.88%	26.298282300571433	0.00837382380159347	0.00837382380159347	0.0003583838894600799
ENSG00000164120_174508711_174491956_131	HPGD	4	-	393	37	1	0	8.40%	55.243958477774825	0.001588175367824666	0.0015806110104534784	1.5019944328400419e-05
ENSG00000067057_3129882_3136579_202	PFKP	10	+	606	61	4	0	11.88%	59.06535619425991	5.93592075022617e-05	5.053563307003881e-06	2.559355081885797e-09
ENSG00000102753_49746990_49701800_497	KPNA3	13	-	1491	41	1	0	2.21%	16.13547778476962	0.0019464877837825903	0.001558047347358915	1.8024065784116922e-05
ENSG00000278845_38297184_38322595_256	MRPL45	17	+	768	36	0	0	3.52%	27.50533236710157	0.002967235347054297	0.002967235347054297	4.9907879201100895e-05
ENSG00000278540_37205791_37087316_991	ACACA	17	-	2973	34	0	2	1.41%	6.710573296424825	0.0004323067881491664	0.00043491588733509306	1.2392274416256264e-06
ENSG00000134283_42335903_42446618_434	PPHLN1	12	+	1302	12	1	1	1.15%	5.408114505359448	0.03577298629450316	0.03577298629450316	0.005410795288624905
ENSG00000180776_21401672_21376648_204	ZDHHC20	13	-	612	12	0	0	2.45%	11.505498506500002	0.020613416668581838	0.020613416668581838	0.001946208561389314
ENSG00000162704_183630664_183630443_73	ARPC5	1	-	219	86	0	0	10.96%	230.42518926716443	0.005808022449631236	0.005808022449631236	0.00017923647698851343
ENSG00000137880_40765842_40767349_67	GCHFR	15	+	201	13	0	0	7.46%	37.95097268561941	0.02108409854857798	0.02108409854857798	0.002029891263157455
ENSG00000103707_65029613_65003062_389	MTFMT	15	-	1167	14	0	0	1.80%	7.039353842023138	0.00847367416878415	0.00847367416878415	0.0003665024264111182
ENSG00000259529_24162145_24166196_393	AL136295.5	14	+	1179	35	0	5	3.05%	17.419266186685753	0.0010032776369550557	0.011414899784380185	7.411878122820631e-05
ENSG00000115525_85848138_85840144_290	ST3GAL5	2	-	870	11	0	3	1.72%	7.41906283005345	0.019216965118390855	0.13115678761539135	0.012020477827111095
ENSG00000153187_244860523_244854450_549	HNRNPU	1	-	1647	134	22	12	7.65%	47.74048378471222	2.3110809700256053e-05	1.540309533619753e-06	3.2124906889294303e-10
ENSG00000153187_244864307_244862453_294	HNRNPU	1	-	882	40	5	1	5.44%	26.61135708986395	0.0025752641032110073	0.00017744757238953088	3.341171639595483e-06
ENSG00000128595_128759798_128769167_119	CALU	7	+	357	25	3	0	6.72%	41.09106609464287	0.010402771787637745	0.0053557511377634245	0.0002919438096861508
ENSG00000182718_60360946_60347630_181	ANXA2	15	-	543	314	14	26	8.84%	339.31685650661336	0.000475084930342192	0.001482642773071759	4.541208410640205e-06
ENSG00000176697_27658504_27657821_227	BDNF	11	-	681	10	1	0	2.64%	8.616452626013217	0.023672501042627626	0.012798402692974279	0.0014375227604752335
ENSG00000116584_155965093_155947942_780	ARHGEF2	1	-	2340	7	0	5	0.64%	1.7553260541967952	0.019216965118390855	0.169362073879829	0.016163991620339734
ENSG00000165219_125298922_125362746_1439	GAPVD1	9	+	4317	10	1	3	0.35%	1.3592319291904102	0.03537493353818881	0.12114404826813535	0.017662684075641494
ENSG00000087460_58891727_58910829_394	GNAS	20	+	1182	322	38	16	16.50%	159.85050463091628	7.255826837916426e-09	1.814040287566488e-11	1.855038147230084e-18
ENSG00000132507_7309636_7311640_154	EIF5A	17	+	462	179	71	8	11.69%	227.34566204726954	0.007938263751100936	9.685105774909149e-05	7.0979032624909975e-06
ENSG00000132507_7309636_7311640_154	EIF5A	17	+	462	179	71	8	11.69%	227.34566204726954	0.007938263751100936	9.685105774909149e-05	7.0979032624909975e-06
ENSG00000166140_40808316_40814226_399	ZFYVE19	15	+	1197	15	0	1	1.25%	7.353138143251881	0.02108409854857798	0.028889785561798623	0.002731532589007073
ENSG00000123728_132217268_132214168_183	RAP2C	X	-	549	8	0	0	3.83%	8.550534409202188	0.005706018193000826	0.005706018193000826	0.0001733096755673334
ENSG00000184226_67228440_66304655_1195	PCDH9	13	-	3585	11	0	1	0.59%	1.8004420257033478	0.006937202941512724	0.016735684067288745	0.000590529598856171
ENSG00000101182_62143303_62136857_248	PSMA7	20	-	744	192	13	4	17.34%	151.42720615006456	4.410321130296757e-08	5.404587772397641e-09	2.5272106368780816e-15
ENSG00000124570_2959176_2948298_324	SERPINB6	6	-	972	54	1	2	5.86%	32.59891243508334	6.293293459921025e-05	0.0001311217014807635	6.040197107585146e-08
ENSG00000072518_63895204_63909237_755	MARK2	11	+	2265	9	0	0	0.79%	2.3315778430390734	0.011772051206276392	0.011772051206276392	0.000681272511389508
ENSG00000109572_169689081_169719997_666	CLCN3	4	+	1998	8	2	0	0.90%	2.349471166492493	0.08266357711566169	0.011570465654371866	0.004841719827405853
ENSG00000145882_149364095_149368654_376	PCYOX1L	5	+	1128	8	0	3	1.33%	4.161563289585107	0.019679754444124863	0.15040137116388086	0.014313628319454642
ENSG00000260027_48610918_48607842_217	HOXB7	17	-	651	12	7	1	4.15%	10.816229010718896	0.08079292213908085	0.0065000503081102355	0.003015727495994416
ENSG00000070770_58167287_58164071_135	CSNK2A2	16	-	405	9	1	1	3.70%	13.039564974033336	0.04223451612662591	0.04223451612662591	0.007344095972923886
ENSG00000023228_206147824_206124185_611	NDUFS1	2	-	1833	45	0	0	3.44%	14.40541138702537	2.4325037092551905e-05	2.4325037092551905e-05	4.607943600628182e-09
ENSG00000116096_72887433_72891537_261	SPR	2	+	783	30	6	8	5.75%	22.482008575919544	0.0029966743598977834	0.01598706110775044	0.00027030046415628677
ENSG00000154277_41260716_41268073_142	UCHL1	4	+	426	280	3	1	26.76%	385.67727387985923	5.167348813635984e-08	3.508250166850731e-08	1.8072293358517298e-14
ENSG00000038274_163503416_163518363_316	MAT2B	5	+	948	22	5	4	3.80%	13.617267219718357	0.011862047307812774	0.008515928397032187	0.0005074909267623899
ENSG00000131626_70324901_70374700_984	PPFIA1	11	+	2952	16	0	0	0.81%	3.1803817009837405	0.004826937407846361	0.004826937407846361	0.00012612451166366307
ENSG00000116688_11989229_12011565_737	MFN2	1	+	2211	25	0	0	1.49%	6.634785434548848	0.0015356776430442238	0.0015356776430442238	1.41466323862755e-05
ENSG00000145996_20548666_21231039_497	CDKAL1	6	+	1491	15	2	0	1.21%	5.903223579793764	0.044040755831095145	0.013428347753762199	0.0027917083341200354
ENSG00000149179_46987231_47161638_314	C11orf49	11	+	942	17	0	0	3.50%	10.589455631778664	0.0012558331315113088	0.0012558331315113088	9.614604369954505e-06
ENSG00000165282_35095565_35089092_1089	PIGO	9	-	3267	81	2	3	3.12%	14.54827497102893	2.6403759053336497e-07	1.2380624369608265e-07	3.1221877534735346e-13
ENSG00000149179_46987231_47164252_303	C11orf49	11	+	909	14	0	0	3.30%	9.037322259231026	0.0017901637611834264	0.0017901637611834264	1.8982104156792192e-05
ENSG00000126254_35629154_35637554_386	RBM42	19	+	1158	19	6	5	2.59%	9.627658076682645	0.03254225401506977	0.025871143657089396	0.003682223596473654
ENSG00000170248_33798729_33813568_72	PDCD6IP	3	+	216	7	0	0	6.94%	19.016032253798617	0.019216965118390855	0.019216965118390855	0.0017073955890589052
ENSG00000105518_11345583_11342815_177	TMEM205	19	-	531	78	6	9	11.86%	86.19373457411866	0.0002837053614922544	0.0003036328747503824	5.827449871051712e-07
ENSG00000136450_58007137_58006317_134	SRSF1	17	-	402	123	0	22	14.93%	179.53729385889184	4.017432531637543e-05	0.0014291545160923483	4.856223636007619e-07
ENSG00000134243_109355396_109314043_659	SORT1	1	-	1977	33	0	0	1.82%	9.794513903105464	0.0010405263639310788	0.0010405263639310788	6.697970672456085e-06
ENSG00000134186_108696184_108700020_384	PRPF38B	1	+	1152	16	0	0	2.08%	8.149728108770836	0.005306578019052857	0.005306578019052857	0.00015100114740534233
ENSG00000134186_108692772_108700020_486	PRPF38B	1	+	1458	22	0	3	1.85%	8.854025599652266	0.0035547763465261306	0.0035291660242640502	6.996297800229744e-05
ENSG00000019186_54164539_54162696_84	CYP24A1	20	-	252	121	17	5	17.86%	281.7477431889346	0.0063727427714238664	0.0011302503791542806	4.41746220707327e-05
ENSG00000155096_102843652_102828567_448	AZIN1	8	-	1344	26	5	0	2.46%	11.351407008645092	0.015652231761283	0.0012624143894574274	0.00012697725382556204
ENSG00000132507_7309636_7311640_154	EIF5A	17	+	462	179	71	8	11.69%	227.34566204726954	0.007938263751100936	9.685105774909149e-05	7.0979032624909975e-06
ENSG00000239789_55953259_55955178_109	MRPS17	7	+	327	11	1	0	4.59%	19.738791015738535	0.04223451612662591	0.019679754444124863	0.003715148010809822
ENSG00000022556_54970034_55000898_1034	NLRP2	19	+	3102	24	2	0	1.06%	4.5398872250019355	0.005035606171387428	0.0015356776430442238	4.557004617261039e-05
ENSG00000111196_10613532_10609819_91	MAGOHB	12	-	273	15	1	0	8.79%	32.2406826281044	0.008170681041463092	0.005405141725979036	0.00023248977892435937
ENSG00000183648_92117583_92116193_40	NDUFB1	14	-	120	169	7	3	30.00%	826.3824302293627	0.00108291700051257	0.0030976334765319066	2.0314095821141805e-05
ENSG00000204435_31666832_31668696_110	CSNK2B	6	+	330	49	12	2	14.55%	87.12800232649548	0.006656029676487497	0.0004977690090443463	2.2717099521481564e-05
ENSG00000112237_99550995_99544222_137	CCNC	6	-	411	9	0	1	3.65%	12.849206361273726	0.019679754444124863	0.04223451612662591	0.003715148010809822
ENSG00000175110_139350248_139357014_169	MRPS22	3	+	507	12	0	6	2.96%	13.888294055183435	0.020613416668581838	0.17139085557395572	0.01724926236372712
ENSG00000075945_169992184_169921676_374	KIFAP3	1	-	1122	8	0	0	1.34%	4.183817638727274	0.019679754444124863	0.019679754444124863	0.0017849636115922674
ENSG00000159199_48893418_48895769_136	ATP5MC1	17	+	408	54	1	1	11.03%	77.66211491887502	0.0006399719954500673	0.0006399719954500673	2.6269077971285762e-06
ENSG00000143393_151316481_151292852_816	PI4KB	1	-	2448	7	2	0	0.61%	1.6778851988645838	0.1191346346731873	0.019216965118390855	0.010787784428637617
ENSG00000143393_151316481_151292852_801	PI4KB	1	-	2403	7	2	0	0.62%	1.70930627000437	0.1191346346731873	0.019216965118390855	0.010787784428637617
ENSG00000169045_179623133_179615546_449	HNRNPH1	5	-	1347	254	17	9	11.80%	110.64753352130738	2.343977030772386e-09	1.8681740671904346e-09	4.750060387460151e-17

ENSG00000116001_70236084_70212719_347	TIA1	2	-	1041	11	0	0	2.02%	6.200369512148416	0.0078820576498062	0.0078820576498062	0.000319636429074511 64
ENSG00000143393_151316481_151292852_816	PI4KB	1	-	2448	7	2	0	0.61%	1.6778851988645838	0.1191346346731873	0.019216965118390855	0.010787784428637617
ENSG00000114850_156555089_156543203_198	SSR3	3	-	594	75	2	0	11.62%	74.08843735246214	2.295954710616975e-05	1.2565626818475328e-05	2.2965809304057447e-09
ENSG00000146918_158655228_158631666_298	NCAPG2	7	-	894	15	0	0	2.68%	9.8453091246896	0.005306578019052857	0.005306578019052857	0.000151001147405342 33
ENSG00000146918_158693311_158655312_755	NCAPG2	7	-	2265	34	0	3	1.72%	8.808182962592054	0.0006446704936422262	0.00387261209348426	1.6001669440543304e-05
ENSG00000198721_4127816_4115874_222	ECI2	6	-	666	29	0	0	3.15%	25.550498935605862	0.00847367416878415	0.00847367416878415	0.0003665024264111182
ENSG00000108523_4941098_4945016_315	RNF167	17	+	945	27	4	0	2.86%	16.76515496661429	0.0033030736763512156	0.0033278027414746945	6.167781147582356e-05
ENSG00000111665_6850952_6849194_182	CDCA3	12	-	546	18	2	0	5.49%	19.344409576862642	0.01044836432889887	0.002046028879997488	0.00012444751772694397
ENSG00000109113_28717266_28714572_211	RAB34	17	-	633	65	7	2	6.64%	60.25391397953793	0.006123000203050806	0.0011545904039420565	4.317766457517374e-05
ENSG00000136527_185937860_185917715_288	TRA2B	3	-	864	97	4	7	8.33%	65.87696887923092	3.723709983354454e-05	0.00014931830129672562	4.1968336466055094e-08
ENSG00000112159_89789830_89644005_5203	MDN1	6	-	15609	16	5	6	0.17%	0.6014790685696715	0.068706750559974	0.04697581791875827	0.012717122376319197
ENSG00000100201_38494939_38493510_199	DDX17	22	-	597	22	2	0	3.52%	21.62339920317086	0.015625008558487224	0.00847367416878415	0.0006602594281152338
ENSG00000086758_53654107_53533309_4374	HUWE1	X	-	13122	25	8	5	0.30%	1.1179325252086194	0.028417192073345858	0.013243555427667874	0.0017738379429698841
ENSG00000182220_40600774_40605755_100	ATP6AP2	X	+	300	9	1	0	5.00%	17.603412714945005	0.03537493353818881	0.020613416668581838	0.003252526348827943
ENSG00000182220_40581066_40598977_209	ATP6AP2	X	+	627	20	0	4	7.18%	18.717078910095697	0.00017649243460590906	0.002564571218333799	3.3106427232230413e-06
ENSG00000138085_27213252_27216948_171	ATRAID	2	+	513	158	4	2	19.30%	180.72379525414624	3.923276361638616e-07	3.0047831814209654e-07	1.0966347783875862e-12
ENSG00000104375_98883744_98455842_515	STK3	8	-	1545	8	0	0	0.97%	3.0383452366679617	0.016947426762344633	0.016947426762344633	0.0013498980316300959
ENSG00000023572_193105382_193096625_164	GLRX2	1	-	492	33	2	2	6.71%	39.35722354967379	0.005079719694443635	0.0031465620335393613	8.86274125149418e-05
ENSG00000064607_19031002_18994366_1059	SUGP2	19	-	3177	20	0	1	0.94%	3.693927754683665	0.0020082573300163734	0.00392533363377418	4.538242985436214e-05
ENSG00000102804_44574913_44434626_686	TSC22D1	13	-	2058	24	3	1	1.46%	6.842920394536445	0.002046028879997488	0.0024699148363837185	2.9382506506355576e-05
ENSG00000177410_49278329_49279205_56	ZFAS1	20	+	168	67	3	4	16.07%	234.01362140899113	0.011952930790615185	0.007815503041163737	0.00047239346875713124
ENSG00000144747_69048086_69023177_890	TMF1	3	-	2670	6	0	2	0.56%	1.318607694003371	0.016947426762344633	0.13974268412556323	0.011764960919844317
ENSG00000132471_75849694_75846734_190	WBP2	17	-	570	33	0	0	6.32%	33.971498221823694	0.0010197273511756433	0.0010197273511756433	6.442859155158972e-06
ENSG00000108312_44213282_44207184_587	UBTF	17	-	1761	23	5	12	1.87%	7.663798834823681	0.023683723201275597	0.07606005236645379	0.007871317557306379
ENSG00000136044_105203713_105174314_433	APPL2	12	-	1299	5	0	6	1.15%	2.2585851571651276	0.012673659338734126	0.43037811169186835	0.04408102937307552
ENSG00000255835_225922380_225887073_312	AL117348.2	1	-	936	5	0	0	1.60%	3.134510811065706	0.012673659338734126	0.012673659338734126	0.000782701129001274
ENSG00000119760_27661249_27653485_363	SUPT7L	2	-	1089	15	0	2	1.93%	8.08237498390496	0.00847367416878415	0.028426896806120847	0.0012022752110812016
ENSG00000147274_136876515_136874142_215	RBMX	X	-	645	8	0	0	2.33%	7.277896729693025	0.019679754444124863	0.019679754444124863	0.0017849636115922674
ENSG00000110651_2394127_2396777_165	CD81	11	+	495	137	3	0	16.97%	162.401854676597	4.622384802694728e-06	1.7210770249190888e-06	6.897319869574234e-11
ENSG00000186205_220791522_220813418_235	MARC1	1	+	705	5	0	0	2.13%	4.161563289585107	0.012673659338734126	0.012673659338734126	0.000782701129001274
ENSG00000114867_184320681_184334908_1404	EIF4G1	3	+	4212	90	7	5	2.78%	12.538043244262823	4.60942195086692e-07	1.9011841143914044e-07	8.246422000810537e-13
ENSG00000123728_132217268_132214168_183	RAP2C	X	-	549	8	0	0	3.83%	8.550534409202188	0.005706018193000826	0.005706018193000826	0.0001733096755673334
ENSG00000154781_14661595_14671194_343	CCDC174	3	+	1029	8	0	0	1.46%	4.561946929690963	0.016947426762344633	0.016947426762344633	0.0013498980316300959
ENSG00000131370_15269736_15256086_298	SH3BP5	3	-	894	23	1	0	4.03%	15.096140657857386	0.0016120252826907794	0.0008664586529590942	8.62301436870289e-06
ENSG00000196704_68250296_68256969_324	AMZ2	17	+	972	29	0	0	3.70%	17.50682334476698	0.0009430837296224684	0.0009430837296224684	5.543685104868835e-06
ENSG00000180628_91222872_91278316_256	PCGF5	10	+	768	8	0	0	2.34%	6.112296081578126	0.011570465654371866	0.011570465654371866	0.0006594843062896773
ENSG00000170421_52904981_52897428_483	KRT8	12	-	1449	775	43	22	10.14%	313.8404613315477	2.7212329735660806e-09	2.641628763289803e-09	7.744562051262718e-17
ENSG00000038427_83490172_83580434_1594	VCAN	5	+	4782	45	7	10	1.69%	5.521773122630177	0.0002874417781084644	0.00019896280113672804	3.928339783005784e-07
ENSG00000105939_139109331_139047594_902	ZC3HAV1	7	-	2706	60	10	3	2.77%	13.010652413115302	0.00014277813065461436	2.985230388193324e-05	3.263337516604516e-08
ENSG00000151694_9555605_9490177_824	ADAM17	2	-	2472	44	11	4	1.82%	10.444311751046119	0.01858493415639402	0.0019746824465274337	0.0002227568790254936 8
ENSG00000162191_62678923_62678218_136	UBXN1	11	-	408	25	0	0	5.15%	35.95468283281251	0.008877961307018026	0.008877961307018026	0.00040023486315785033
ENSG00000152404_107429448_107353532_411	CWF19L2	11	-	1233	10	0	0	1.22%	4.758965318990269	0.020613416668581838	0.020613416668581838	0.001946208561389314
ENSG00000182934_126268804_126263916_610	SRPRA	11	-	1830	34	0	3	2.62%	10.90193137173279	0.0001778652122394772	0.0014719922646461731	1.8494378496163408e-06
ENSG00000110172_90222954_90202405_313	CHORDC1	11	-	939	12	0	0	1.92%	7.498791358869012	0.012798402692974279	0.012798402692974279	0.0007972436888943567
ENSG00000103275_1314031_1324793_158	UBE2I	16	+	474	91	7	4	14.56%	112.65193790857914	4.1653514996278555e-05	4.2145511149793747e-05	1.32979772114665e-08
ENSG00000113161_75358702_75360342_158	HMGCR	5	+	474	25	0	2	8.86%	30.948334590268995	0.00036930943616753716	0.0022326725999624137	5.491457524341064e-06
ENSG00000141526_82236009_82238976_465	SLC16A3	17	+	1395	72	7	1	5.59%	30.28544123001291	1.1849891463284232e-05	4.637421080669856e-06	4.5704910601567277e-10
ENSG00000060237_827064_908792_1816	WNK1	12	+	5448	27	1	4	0.83%	2.908052761279461	0.00034995587926105035	0.0025810035995757507	6.087871601150127e-06
ENSG00000005194_57440928_57429170_312	CIAPIN1	16	-	936	10	0	0	2.24%	6.269021622131412	0.007029302661868638	0.007029302661868638	0.0002573639808771639
ENSG00000188643_153608151_153607534_103	S100A16	1	-	309	49	9	1	15.53%	93.04932287295634	0.0029276013608434044	0.0002519671529549621	5.206527765482977e-06
ENSG00000188643_153608151_153607534_103	S100A16	1	-	309	49	9	1	15.53%	93.04932287295634	0.0029276013608434044	0.0002519671529549621	5.206527765482977e-06
ENSG00000111237_110502051_110492005_186	VPS29	12	-	558	64	0	11	12.37%	67.3009805111398	1.1598859020456752e-05	0.0003995223902392225	4.0868810868669265e-08
ENSG00000128944_40383253_40393231_208	KNSTRN	15	+	624	13	7	0	2.88%	12.224592163156252	0.129839126104895	0.013428347753762199	0.009081842412704622
ENSG00000115204_27324450_27309912_120	MPV17	2	-	360	13	0	3	6.67%	21.18929308280417	0.004193160480899131	0.056043582888960386	0.0014062628675739213
ENSG00000167085_49413264_49404992_155	PHB	17	-	465	43	4	0	6.45%	54.261415537106465	0.002446120450917513	0.002446120450917513	3.450690187599895e-05
ENSG00000076928_41894238_41906786_687	ARHGEF1	19	+	2061	16	1	3	1.46%	4.555306541147017	0.0036460193534901884	0.021084098548578	0.000427954013978622 5
ENSG00000114209_167720136_167684308_205	PDCD10	3	-	615	18	0	0	3.41%	17.174061185312198	0.00847367416878415	0.00847367416878415	0.0003665024264111182
ENSG00000179403_1435749_1439787_445	VWA1	1	+	1335	12	0	0	1.12%	5.274430776013484	0.02108409854857798	0.02108409854857798	0.002029891263157455
ENSG00000119787_38298473_38298117_118	ATL2	2	-	354	7	0	0	4.24%	11.603002731131358	0.016947426762344633	0.016947426762344633	0.0013498980316300959
ENSG00000138463_122826849_122879378_316	SLC49A4	3	+	948	19	0	3	2.22%	11.760367144302219	0.00877616296920843	0.039474168858000536	0.0017412572126575905
ENSG00000128463_34228437_34229788_62	EMC4	15	+	186	73	0	2	30.65%	230.2955426865565	6.226972273047339e-05	6.138207594421736e-05	2.8359142201543077e-08
ENSG00000157823_89893949_89835496_156	AP3S2	15	-	468	18	1	0	4.49%	22.568477839673083	0.013301727469301692	0.00847367416878415	0.0005643273318649336
ENSG00000242028_43800623_43801806_121	HYPK	15	+	363	44	1	0	12.40%	71.12489985836365	0.0006786479905456451	0.0002371941351396530 3	1.0872976294357274e-06
ENSG00000286070_24557675_24571977_126	GGT1	22	+	378	169	11	5	15.87%	262.34362864424213	0.00019417054298710972	0.00013154246493632366	1.7989521925952042e-07
ENSG00000079459_11802833_11838609_410	FDFT1	8	+	1230	234	15	9	14.63%	111.63139770452929	4.6391790974868225e-10	3.8468733557881155e-11	2.1451222431326902e-19
ENSG00000198561_57801746_57816308_616	CTNND1	11	+	1848	50	4	2	2.60%	15.876093718384745	0.0013582198574350129	0.00041261355168930957	3.650882652566793e-06
ENSG00000111843_10724614_10730666_112	TMEM14C	6	+	336	83	0	2	11.61%	144.9487356488527	0.0007052562366071395	0.0008884494972709903	3.961101715993978e-06

ENSG00000178896_144078729_144080601_245	EXOSC4	8	+	735	23	2	0	3.27%	18.361836392006126	0.015949584761613404	0.005554612275049112	0.0004619408601232623
ENSG00000196642_136822041_136839350_471	RABL6	9	+	1413	20	1	4	1.70%	8.305455397473462	0.008316137131635013	0.03600804981277603	0.0015112924408709608
ENSG00000130640_133298090_133283164_562	TUBGCP2	10	-	1686	13	4	0	1.42%	4.524404216968862	0.06004650948776591	0.005160369442893307	0.0017911988961098178
ENSG00000080815_73148065_73211858_359	PSEN1	14	+	1077	23	2	0	3.90%	12.531058261954042	0.0013084265593569383	0.00033754525954144115	2.9235818092876446e-06
ENSG00000274944_38866933_38864670_32	AL139260.3	1	-	96	11	0	0	15.62%	67.23525689735939	0.02108409854857798	0.02108409854857798	0.002029891263157455
ENSG00000129534_45247286_45232573_496	MIS18BP1	14	-	1488	6	0	0	1.01%	2.366050096094759	0.016947426762344633	0.016947426762344633	0.0013498980316300959
ENSG00000125743_45691888_45687553_118	SNRPD2	19	-	354	232	17	7	31.36%	384.5566619460679	1.0049039649305707e-07	1.3579233756153153e-07	1.3168933483077247e-13
ENSG00000184164_49918770_49923400_284	CRELD2	22	+	852	25	0	0	4.23%	17.217735441065145	0.0009235399941089087	0.0009235399941089087	5.324735186779001e-06
ENSG00000116161_175004728_175010079_185	CACYBP	1	+	555	30	0	0	3.78%	31.717860747648658	0.008574016250729465	0.008574016250729465	0.0003747459614100891
ENSG00000237190_134411734_134402915_76	CDKN2AIPNL	5	-	228	8	3	1	6.58%	20.588786801105268	0.0477903522728147	0.019216965118390855	0.004116507567392483
ENSG00000038002_177440367_177434482_147	AGA	4	-	441	26	0	0	6.12%	34.59476421682314	0.003327802741474945	0.003327802741474945	6.211806171561056e-05
ENSG00000068796_62347147_62385569_660	KIF2A	5	+	1980	22	0	1	1.97%	6.519782487016668	0.00047074547413106	0.0010296184977138137	3.125625275725106e-06
ENSG00000079332_70153963_70152476_80	SAR1A	10	-	240	15	0	0	7.50%	36.67377648946876	0.013428347753762199	0.013428347753762199	0.0008725593497644525
ENSG00000104979_13774478_13778198_99	C19orf53	19	+	297	27	9	0	8.08%	53.343674893772736	0.058675800694782766	0.004826937407846361	0.0016554019715233344
ENSG00000099622_1270934_1272443_297	CIRBP	19	+	891	27	1	0	3.70%	17.781224964590912	0.002493493840886608	0.0014055202957965589	2.0830049893778164e-05
ENSG00000186815_113226853_113296076_816	TPCN1	12	+	2448	15	5	1	0.98%	3.595468283281251	0.08940501323137268	0.010020958156399907	0.004728514333684338
ENSG00000115524_197434999_197392303_1304	SF3B1	2	-	3912	58	9	0	2.07%	8.699709760282978	0.00022286767513824776	2.129602572665134e-06	4.89168401904768e-09
ENSG00000146731_56051849_56063085_486	CCT6A	7	+	1458	253	12	5	10.70%	101.82129439600105	6.787964649222874e-10	2.890320227591733e-10	2.2218816719639003e-18
ENSG00000221983_18573301_18575150_128	UBA52	19	+	384	30	4	0	9.38%	45.84222061183595	0.002484757332982506	0.0008776897154483972	1.3426037390218656e-05
ENSG00000141429_35683456_35691197_205	GALNT1	18	+	615	19	0	0	3.41%	18.12817569560732	0.0078820576498062	0.0078820576498062	0.00031963642907451164
ENSG00000123352_49494861_49526255_417	SPATS2	12	+	1251	13	0	1	2.16%	6.0976382972098335	0.001946208561389314	0.006277459298483267	7.053443163459546e-05
ENSG00000134779_36818914_36796805_209	TPGS2	18	-	627	8	0	0	2.39%	7.48683156403828	0.019216965118390855	0.019216965118390855	0.0017073955890589052
ENSG00000187840_38030574_38059935_118	EIF4EBP1	8	+	354	13	1	0	6.78%	21.548433643529666	0.010020958156399907	0.004826937407846361	0.00025599508163266543
ENSG00000263020_31666832_31672282_304	AL662899.2	6	+	912	63	14	3	5.92%	40.534174014676	0.004571978973438455	0.0002341666377711511	7.915604097002622e-06
ENSG00000111641_6567918_6558028_628	NOP2	12	-	1884	25	4	2	2.55%	7.786364435131371	0.0015749519000881804	0.00041073065323788186	4.22440045232234e-06
ENSG00000127990_94618900_94599352_197	SGCE	7	-	591	9	0	2	2.54%	8.93574249489594	0.019679754444124863	0.08026476757196149	0.007158563601160521
ENSG00000165264_32573060_32553876_128	NDUFB6	9	-	384	280	16	10	22.66%	427.86072571046884	3.821633937132092e-06	3.3251758558396076e-06	1.0802089957361898e-10
ENSG00000119616_74723298_74734930_92	FCF1	14	+	276	16	0	3	7.61%	34.01625645400001	0.0078820576498062	0.059202726829632964	0.002466792732126766
ENSG00000196141_200309073_200478031_588	SPATS2L	2	+	1764	23	2	0	1.87%	7.650765163335886	0.0034718995429267267	0.0014915192737735051	3.054066176404144e-05
ENSG00000090530_189994235_189957912_481	P3H2	3	-	1443	7	0	0	1.04%	2.8464746824812894	0.019216965118390855	0.019216965118390855	0.0017073955890589052
ENSG00000061273_47797459_47783841_824	HDAC7	12	-	2472	18	0	3	0.73%	4.272672989064321	0.013007239811447113	0.06272892732212154	0.003936255353260731
ENSG00000131475_42773476_42779069_176	VPS25	17	+	528	52	4	1	6.82%	57.78898113492047	0.013696866554086288	0.002493493840886608	0.00019612387815855648
ENSG00000174442_66518919_66546679_471	ZWILCH	15	+	1413	25	0	0	1.70%	10.381819246841829	0.005706018193000826	0.005706018193000826	0.0001733096755673334
ENSG00000131966_58215281_58234551_219	ACTR10	14	+	657	20	1	2	3.20%	17.862417772648406	0.012088529595134127	0.020779353648479937	0.0012024586295609598
ENSG00000103018_69424696_69462520_146	CYB5B	16	+	438	142	16	6	17.81%	190.23474927870552	1.3970210197328605e-05	3.7581135926596835e-06	4.413025307199103e-10
ENSG00000167468_1105222_1105405_32	GPX4	19	+	96	14	0	1	21.88%	85.57214514209377	0.0078820576498062	0.011306643944114857	0.0004511737406738057
ENSG00000164024_98995754_99061317_386	METAP1	4	+	1158	28	0	0	3.37%	14.18812769195337	0.0006652407155885319	0.0006652407155885319	2.8305411988850537e-06
ENSG00000197894_99076847_99072417_234	ADH5	4	-	702	26	0	6	4.70%	21.73260829005556	0.0013430411379358334	0.011651814340914361	9.710472547923025e-05
ENSG00000164032_99950170_99948462_128	H2AFZ	4	-	384	309	191	23	18.75%	472.1748723019103	0.00043818552676915684	2.889235254972367e-05	1.01463441749233e-07
ENSG00000114315_194136396_194138233_275	HES1	3	+	825	20	0	1	3.27%	14.224979971672731	0.0035547763465261306	0.005670089768331009	0.00011049933550063637
ENSG00000028116_58048901_58159693_485	VRK2	2	+	1455	14	2	1	1.44%	5.645997205251548	0.03744183116399598	0.013599199788808391	0.002391925567846126
ENSG00000135446_57752292_57752176_38	CDK4	12	-	114	17	0	2	15.79%	87.50234390469738	0.013428347753762199	0.037178613843887286	0.0023492041693324566
ENSG00000105364_10252254_10259506_357	MRPL4	19	+	1071	33	4	0	3.08%	18.08006908164286	0.009432837192081834	0.0014055202957965589	8.06420916640711e-05
ENSG00000109180_48832625_48860762_218	OCIAD1	4	+	654	26	0	2	4.59%	23.32766210950918	0.0022011839993586477	0.0022011839993586477	2.8200182015552967e-05
ENSG00000148444_22316418_22318195_113	COMMD3	10	+	339	19	1	1	7.96%	32.88739838583629	0.0035291660242640502	0.0035291660242640502	6.948232509684344e-05
ENSG00000110700_17077632_17074408_145	RPS13	11	-	435	87	1	11	5.52%	117.35608476630003	0.005808022449631236	0.016297769181052975	0.0004911246612960676
ENSG00000124596_41071198_41067335_113	OARD1	6	-	339	24	1	0	4.42%	41.541976908424786	0.03537493353818881	0.020613416668581838	0.003252526348827943
ENSG00000123983_222908773_222941654_720	ACSL3	2	+	2160	223	3	10	8.75%	60.57964560852987	3.755165188231679e-12	1.0799559380154858e-10	5.206077401290476e-21
ENSG00000218739_37199705_37201724_80	CEBPZOS	2	+	240	16	0	0	10.00%	39.11869492210001	0.004826937407846361	0.004826937407846361	0.00012612451166366307
ENSG00000169689_82019671_82019239_57	CENPX	17	-	171	34	1	3	14.04%	116.66979187292985	0.005454749182134642	0.01728285133886564	0.0004931488542729515
ENSG00000074054_121605760_121340861_1426	CLASP1	2	-	4278	13	3	0	0.70%	1.7831102173467746	0.0182999617455631	0.0014903523101034196	0.0001731805207519894
ENSG00000159140_33543093_33560131_2303	SON	21	+	6909	64	3	1	1.26%	5.435511235376467	5.668532419523906e-06	1.330866544824565e-06	6.625054244128261e-11
ENSG00000183010_81936814_81933356_217	PYCR1	17	-	651	13	8	0	2.30%	11.717581428278805	0.24029758967682546	0.020613416668581838	0.02606066163800816
ENSG00000101773_22936852_23026240_897	RBBP8	18	+	2691	17	4	0	1.00%	3.706899741782052	0.035523672008851884	0.003327802741474945	0.0006985300827019562
ENSG00000129197_5421311_5432563_116	RPAIN	17	+	348	21	2	1	5.17%	35.40916350707328	0.037178613843887286	0.02034245439037237	0.003374091548142354
ENSG00000092841_56158402_56162322_161	MYL6	12	+	483	1111	13	19	29.19%	1349.716461442643	1.4817857354790871e-09	1.273329913166416e-08	2.071430596708457e-16
ENSG00000267855_8321358_8311505_113	NDUFA7	19	-	339	54	3	1	13.27%	93.46944804395578	0.00033620229360644755	0.0003354983092902608	7.562899237154315e-07
ENSG00000254505_24213439_24209877_222	CHMP4A	14	-	666	27	3	6	5.86%	23.788395560736493	0.003897705227460077	0.012383447100975772	0.0002612565341663802
ENSG00000088367_36175560_36229340_670	EPB41L1	20	+	2010	44	3	6	2.69%	12.84494460128657	0.0003603583757000094	0.000782701129001274	1.8531924741329877e-06
ENSG00000135775_230664484_230693393_610	COG2	1	+	1830	10	0	1	1.15%	3.2064504034508206	0.007593099278532274	0.015191410988288745	0.0005816659162195647
ENSG00000135775_230664484_230693393_611	COG2	1	+	1833	10	0	1	1.15%	3.2012025304500824	0.007593099278532274	0.015191410988288745	0.0005816659162195647
ENSG00000119280_230868467_230839852_312	C1orf198	1	-	936	9	0	0	1.92%	5.642119459918271	0.011772051206276392	0.011772051206276392	0.000681272511389508
ENSG00000143643_230978830_230906905_860	TTC13	1	-	2580	14	1	0	0.81%	3.1840798192406985	0.015625008558487224	0.00797940233269714	0.0006253789790324684
ENSG00000154174_100400949_100365564_608	TOMM70	3	-	1824	29	0	3	3.45%	9.329294019250824	1.3182862754164802e-05	0.00023866680458304977	2.6364474557643167e-08
ENSG00000198055_177431937_177441790_546	GRK6	5	+	1638	6	4	0	0.92%	2.149378841873627	0.165899918248885	0.016947426762344633	0.014398895220848395
ENSG00000150768_112026225_112062535_545	DLAT	11	+	1635	18	0	3	1.83%	6.459967968787158	0.002046028879997488	0.01664033820237559	0.00020366262896160314
ENSG00000123444_47577999_47572930_518	KBTBD4	11	-	1554	14	3	0	0.97%	5.286310124608109	0.1029516053660342	0.02108409854857798	0.009872287793463467

ENSG00000134077_9365069_9384688_507	THUMPD3	3	+	1521	23	4	2	1.97%	8.873076757478305	0.019264875824957298	0.011064573844522778	0.0010312247960341252
ENSG00000152234_46098231_46084282_553	ATP5F1A	18	-	1659	312	7	19	13.38%	110.35291876758772	1.748164185774474e-13	8.752711903801398e-13	2.0214085795663137e-24
ENSG00000126214_103654586_103685018_542	KLC1	14	+	1626	52	4	7	3.87%	18.765425608387456	0.00018571932730564617	0.0006376289743136012	8.13594806396098e-07
ENSG00000178567_36992705_36991254_483	EPM2AIP1	3	-	1449	12	3	0	1.04%	4.859465207714287	0.10105102209468636	0.019679754444124863	0.009164497669452725
ENSG00000185803_144359376_144361015_86	SLC52A2	8	+	258	9	0	0	5.81%	20.469084552261634	0.019679754444124863	0.019679754444124863	0.0017849636115922674
ENSG00000168522_43056534_43077500_214	FNTA	8	+	642	26	0	0	3.27%	23.76369317697664	0.00877616296920843	0.00877616296920843	0.0003916113566756457
ENSG00000101266_489860_483961_177	CSNK2A1	20	-	531	9	0	0	2.82%	9.945430912398308	0.019216965118390855	0.019216965118390855	0.0017073955890589052
ENSG00000168036_41224552_41239342_768	CTNNB1	3	+	2304	116	8	0	5.73%	29.542764394294277	8.699215501017508e-08	2.9105570714308586e-09	2.8276345500981447e-15
ENSG00000164144_152829634_152910219_373	ARFIP1	4	+	1119	13	2	0	1.88%	6.816930750500002	0.022898969405409888	0.008175610787902942	0.0009353765476651278
ENSG00000115414_215435802_215361555_2477	FN1	2	-	7431	111	6	10	2.42%	8.764988163813284	5.430240679044199e-11	5.795991638210809e-10	3.7472105056715723e-19
ENSG00000110075_68537665_68613117_838	PPP6R3	11	+	2514	32	7	5	2.27%	7.468963230949883	0.003622420583891007	0.0005933294589348272	1.3865891050279807e-05
ENSG00000167772_8364322_8373886_368	ANGPTL4	19	+	1104	7	1	0	1.63%	3.720528049656251	0.007152939217714824	0.009815328628645334	0.00035984443228265363
ENSG00000112514_33417668_33416650_156	CUTA	6	-	468	163	2	6	16.03%	204.37010488148402	6.966821039712242e-06	3.1649250629192755e-05	1.808117256872564e-09
ENSG00000206503_29942563_29944645_280	HLA-A	6	+	840	65	0	3	5.00%	45.40562803458037	0.0004575241474660434	0.0004561727980981236	1.370709472360254e-06
ENSG00000198467_35689790_35685265_105	TPM2	9	-	315	46	2	1	12.38%	85.68856982936192	0.0011359975802536294	0.0007124765031541974	5.084328216484943e-06
ENSG00000162729_160094094_160091823_440	IGSF8	1	-	1320	15	3	0	1.36%	6.667959361721592	0.05321326914949129	0.013428347753762199	0.0033962782826269635
ENSG00000115657_219218565_219209938_806	ABCB6	2	-	2418	17	2	3	0.99%	4.125420680370349	0.011306643944114857	0.02193685758795414	0.0011940707061197412
ENSG00000102871_67156660_67154649_312	TRADD	16	-	936	10	0	0	1.92%	6.269021622131412	0.012798402692974279	0.012798402692974279	0.0007972436888943567
ENSG00000142453_10909136_10921493_415	CARM1	19	+	1245	21	0	1	3.13%	9.897501124868677	0.000583356306045086	0.0005379818162649481	2.032314277641582e-06
ENSG00000198668_90401333_90404085_97	CALM1	14	+	291	47	5	2	13.40%	94.77209594529384	0.0029628053902178418	0.001166722555699453	2.0800836506869975e-05
ENSG00000197885_23901047_23893095_160	NKIRAS1	3	-	480	16	0	1	5.00%	19.559347461050006	0.005306578019052857	0.009288069496478249	0.0002590037054391842
ENSG00000017260_130934676_131001350_823	ATP2C1	3	+	2469	17	3	1	1.22%	4.040205429378494	0.011168917508652896	0.00324779312858951	0.00020011016427610653
ENSG00000005700_82240474_82171425_1349	IBTK	6	-	4047	40	0	9	1.48%	5.79965825383247	2.5476008567110588e-05	0.0021177151944348576	5.118685571507631e-07
ENSG00000138180_93500052_93528153_464	CEP55	10	+	1392	90	0	0	5.39%	37.938389471864234	4.897129735445945e-06	4.897129735445945e-06	2.010883163490822e-10
ENSG00000079785_15595156_15630906_731	DDX1	2	+	2193	62	3	1	3.97%	16.589323427976748	2.996239415418512e-06	1.3400865838641015e-06	3.516972762400955e-11
ENSG00000033800_68054327_68187835_651	PIAS1	15	+	1953	12	0	0	1.23%	3.605409670239632	0.004826937407846361	0.004826937407846361	0.00012612451166366307
ENSG00000137807_69414466_69427474_371	AC027237.1	15	+	1113	11	0	0	1.89%	5.799267441281672	0.006937202941512724	0.006937202941512724	0.00025101522741487095
ENSG00000140416_63061232_63071172_85	TPM1	15	+	255	90	5	2	17.65%	207.09897311700004	0.0003056514257772735	0.0004919493296827697	1.0025118378398567e-06
ENSG00000086300_26346443_26372572_201	SNX10	7	+	603	5	0	0	2.49%	4.865509318669155	0.012673659338734126	0.012673659338734126	0.000782701129001274
ENSG00000142534_49497931_49499635_79	RPS11	19	+	237	493	8	1	39.24%	1220.6023162402091	5.703516416371565e-07	5.112362712138789e-07	2.667508397423284e-12
ENSG00000142534_49496457_49497436_85	RPS11	19	+	255	314	37	6	14.12%	722.5453062082001	0.0010915223686811253	0.002299680368296036	1.5197404019650169e-05
ENSG00000278463_26033568_26033176_130	HIST1H2AB	6	-	390	117	18	4	17.69%	176.03412714945003	0.0004210669159545147	1.5174785246315587e-05	5.483072389142544e-08
ENSG00000172531_67399140_67398877_87	PPP1CA	11	-	261	53	1	0	17.24%	119.15464545237359	0.0006064187916096173	0.0002948650613319232	1.1913758122445942e-06
ENSG00000116580_155827017_155750584_2068	GON4L	1	-	6204	16	2	0	0.29%	1.5132957416673118	0.044040755831095145	0.013428347753762199	0.0027917083341200354
ENSG00000241878_31648241_31619612_349	PISD	22	-	1047	8	1	0	1.43%	4.483518042647566	0.03418265644467716	0.019216965118390855	0.0029560527921161815
ENSG00000136044_105203713_105174314_433	APPL2	12	-	1299	5	0	6	1.15%	2.2585851571651276	0.012673659338734126	0.43037811169186835	0.04408102937307552
ENSG00000198668_90401333_90404452_76	CALM1	14	+	228	44	9	2	14.47%	113.23832740607898	0.0135046255763177	0.002596438251334875	0.00020012400764995216
ENSG00000198668_90404421_90404717_40	CALM1	14	+	120	33	0	0	30.00%	161.36461655366253	0.0010447178140462346	0.0010447178140462346	6.749952978822125e-06
ENSG00000102316_54809755_54815682_580	MAGED2	X	+	1740	26	0	0	2.07%	8.767983344608623	0.0008739357725210369	0.0008739357725210369	4.787965623198429e-06
ENSG00000102316_54809755_54815682_495	MAGED2	X	+	1485	24	0	0	2.02%	9.483319981115153	0.0017901637611834264	0.0017901637611834264	1.8982104156792192e-05
ENSG00000095383_98213212_98199381_468	TBC1D2	9	-	1404	14	2	0	1.71%	5.851086847322651	0.019504170898671437	0.004873903550586949	0.0005060140340053911
ENSG00000095383_98213212_98199381_457	TBC1D2	9	-	1371	14	2	0	1.75%	5.991922635770242	0.019504170898671437	0.004873903550586949	0.0005060140340053911
ENSG00000197345_68903810_68893399_150	MRPL21	11	-	450	22	5	0	6.00%	28.68704294287334	0.02319724882453354	0.0033030736763512156	0.00043585636963421
ENSG00000151694_9526149_9490177_586	ADAM17	2	-	1758	17	8	4	1.19%	5.674213427267066	0.17806259325226725	0.06793153848426066	0.04390537220818324
ENSG00000165996_17617339_17590364_288	HACD1	10	-	864	17	0	0	2.08%	11.545448154092016	0.013428347753762199	0.013428347753762199	0.0008725593497644525
ENSG00000198791_17243059_17230720_204	CNOT7	8	-	612	11	1	0	4.41%	10.546706964291669	0.00646704663262422	0.002478942540635376	9.042414316359671e-05
ENSG00000003402_201130085_201140475_140	CFLAR	2	+	420	11	0	0	3.57%	15.368058719396432	0.019679754444124863	0.019679754444124863	0.0017849636115922674
ENSG00000147955_34637304_34635632_103	SIGMAR1	9	-	309	35	1	0	10.68%	66.46380205211166	0.0021824774412010223	0.001565547007472977	2.0232669183499854e-05
ENSG00000185627_237050_252600_376	PSMD13	11	+	1128	62	17	2	5.85%	32.25211549428458	0.0036121413216632318	3.0429755490704745e-05	1.0972752192200173e-06
ENSG00000109971_123058809_123058555_84	HSPA8	11	-	252	44	3	1	8.33%	102.45372479597621	0.013855018329567799	0.010197420091687953	0.0006958067230986645
ENSG00000109971_123058455_123057734_129	HSPA8	11	-	387	134	3	5	17.05%	203.1746170372636	2.050322088442456e-05	5.576139078602759e-05	8.872569621594097e-09
ENSG00000109971_123059884_123059014_219	HSPA8	11	-	657	109	3	7	9.13%	97.35017686093381	8.101222749739385e-05	0.0007488432921256761	4.501251140843033e-07
ENSG00000153187_244864307_244854450_744	HNRNPU	1	-	2232	172	27	13	7.53%	45.21784628092205	7.950385404367661e-07	6.821080390944838e-09	6.380391714124852e-14
ENSG00000014641_63597485_63606987_239	MDH1	2	+	717	221	3	3	15.06%	180.86258530929084	5.569978709084716e-07	4.0692571271682907e-07	2.0846421512844785e-12
ENSG00000136718_130342933_130345758_165	IMP4	2	+	495	14	0	0	3.03%	16.59580996695152	0.016947426762344633	0.016947426762344633	0.0013498980316300959
ENSG00000213782_12824008_12829554_159	DDX47	12	+	477	32	3	1	7.55%	39.36472444991196	0.0037328229080146793	0.0013683766156140878	3.0342406646763102e-05
ENSG00000213782_12813368_12823337_255	DDX47	12	+	765	24	0	2	3.92%	18.408797610400004	0.0020587098026626535	0.003990669019770486	4.719855945507632e-05
ENSG00000177106_720080_726981_654	EPS8L2	11	+	1962	14	0	1	1.07%	4.1870162760657506	0.00847367416878415	0.013892891352654344	0.0005886798845679606
ENSG00000100138_41690449_41674933_132	SNU13	22	-	396	50	2	2	11.36%	74.08843735246214	0.0002798052361462181	0.0006784704233534291	1.2682193406298224e-06
ENSG00000063177_48616815_48616582_77	RPL18	19	-	231	48	1	6	11.69%	121.92839975719484	0.0037896409716948643	0.020547428236774374	0.0004303870303576313
ENSG00000101407_38013816_37983456_1089	TTI1	20	-	3267	21	2	0	0.92%	3.7717749924889814	0.010645877633714064	0.0021880057261679567	0.0001345568947401665
ENSG00000101407_38013816_37983456_1089	TTI1	20	-	3267	21	2	0	0.92%	3.7717749924889814	0.010645877633714064	0.0021880057261679567	0.0001345568947401665
ENSG00000198542_101452834_101715654_494	ITGBL1	13	+	1482	37	0	3	4.05%	14.649713685401824	3.106876052321276e-05	0.0002487012320287892	5.986999819660672e-08
ENSG00000013588_12908250_12912539_357	GPRC5A	12	+	1071	153	4	15	13.73%	83.82577483307145	7.387941625417057e-10	2.704825369529194e-09	2.212961031883321e-17
ENSG00000135387_34071753_34099367_628	CAPRIN1	11	+	1884	50	2	0	4.30%	15.572728870262742	6.524192347581214e-06	1.940453335491528e-06	1.0930444540327216e-10
ENSG00000119333_128656726_128633744_536	WDR34	9	-	1608	45	10	3	3.92%	16.4210939505084	0.0025219163206460426	0.00012999663353939895	2.4928802244552808e-06
ENSG00000161813_50401011_50461351_445	LARP4	12	+	1335	13	0	1	1.80%	5.713966674014608	0.004193160480899131	0.010499663139468709	0.0002361360478986167

ENSG00000101811_100820417_100838361_597	CSTF2	X	+	1791	23	0	1	1.17%	7.53542699504439	0.00877616296920843	0.012088529595134127	0.0005313814658942758
ENSG00000188191_677296_550430_257	PRKAR1B	7	-	771	11	4	0	1.95%	8.37170513897082	0.14592027257189422	0.02108409854857798	0.014551245568658585
ENSG00000033867_27434087_27376764_1008	SLC4A7	3	-	3024	25	0	2	1.49%	4.851028636173116	0.00023917554320093795	0.0012356032184771147	2.013922539906876e-06
ENSG00000111077_53047007_53063882_1403	TNS2	12	+	4209	12	0	5	0.36%	1.672930645278689	0.019679754444124863	0.1741008397065631	0.016992325807804815
ENSG00000092470_43828097_43866392_562	WDR76	15	+	1686	5	0	0	0.89%	1.740155468064947	0.012673659338734126	0.012673659338734126	0.000782701129001274
ENSG00000240857_18560188_18555191_208	RDH14	2	-	624	12	0	0	3.85%	11.284238919836541	0.004826937407846361	0.004826937407846361	0.00012612451166366307
ENSG00000117593_173826769_173857705_371	DARS2	1	+	1113	15	0	0	2.96%	7.908091965384099	0.0008831683242050062	0.0008831683242050062	4.885807922518899e-06
ENSG00000070761_58115481_58114025_109	CFAP20	16	-	327	19	1	0	7.34%	34.09427539082111	0.007181907427766128	0.005554612275049112	0.0002105152421252746
ENSG00000183624_129279733_129304825_354	HMCES	3	+	1062	21	3	0	2.82%	11.603002731131358	0.010728371185261619	0.0023218226288164415	0.00014287115526610158
ENSG00000140105_100369185_100334875_471	WARS	14	-	1413	60	6	3	4.88%	24.916366192420387	0.00017457506911736429	5.349366132029235e-05	6.898497935895578e-08
ENSG00000065057_2044728_2039924_162	NTHL1	16	-	486	10	0	0	4.32%	12.073671272253089	0.005706018193000826	0.005706018193000826	0.0001733096755673334
ENSG00000258315_7015802_7017321_144	C17orf49	17	+	432	32	0	0	9.03%	43.46521658011112	0.000596156229600823	0.000596156229600823	2.2911026310734046e-06
ENSG00000039523_67540585_67546463_992	RIPOR1	16	+	2976	10	3	0	0.50%	1.9717084134122989	0.12312584984626351	0.020613416668581838	0.011809609471937859
ENSG00000100473_30878908_30889791_438	COCH	14	+	1314	18	0	0	2.74%	8.038087997691783	0.0007729603088974596	0.0007729603088974596	3.7798209788587765e-06
ENSG00000187778_49566731_49558643_462	MCRS1	12	-	1386	15	6	0	1.52%	6.350437487353897	0.08272817174309494	0.00797940233269714	0.0036292143623295452
ENSG00000167460_16076566_16101346_248	TPM4	19	+	744	42	10	8	6.45%	33.12470134532662	0.007355569109184439	0.0069257284821770465	0.00026493743887686565
ENSG00000115648_237493427_237553592_480	MLPH	2	+	1440	48	0	9	2.92%	19.559347461050006	0.00046843213071395124	0.006253208315967795	2.0153523927661433e-05
ENSG00000050820_75236980_75229511_599	BCAR1	16	-	1797	18	0	0	1.34%	5.877600238712856	0.005554612275049112	0.005554612275049112	0.00016468457691007306
ENSG00000062725_60479185_60447581_430	APPBP2	17	-	1290	14	0	0	1.63%	6.368159638481397	0.0078820576498062	0.0078820576498062	0.00031963642907451164
ENSG00000086589_150696802_150691751_300	RBM22	5	-	900	25	0	2	3.33%	16.29945621754167	0.0022276761777358703	0.004515676995858069	5.735615488377851e-05
ENSG00000166741_114296557_114298162_121	NNMT	11	+	363	67	2	1	14.05%	108.30382478432647	0.00021227517043481478	0.0001654009931304681	2.4457059089955195e-07
ENSG00000198189_87372791_87337276_142	HSD17B11	4	-	426	29	0	0	7.04%	39.94514622327114	0.0022276761777358703	0.0022276761777358703	2.8853233252577778e-05
ENSG00000185745_89402369_89403712_447	IFIT1	10	+	1341	80	3	6	8.72%	35.00554355445191	8.127534578752969e-08	6.888494304535658e-07	5.487120349278378e-13
ENSG00000267855_8321358_8311505_63	NDUFA7	19	-	189	30	2	1	14.29%	93.13974981452382	0.004490071680569804	0.004467577387460859	0.00010938927679434585
ENSG00000113645_168414520_168469017_742	WWC1	5	+	2226	18	4	0	0.94%	4.744855179230459	0.032657852871826265	0.0078820576498062	0.0013042942913966678
ENSG00000040275_169588417_169604207_605	SPDL1	5	+	1815	59	2	0	4.13%	19.074404962015706	9.207196143332367e-06	4.957729627510527e-06	3.7905341308887257e-10
ENSG00000092199_21231004_21211223_190	HNRNPC	14	-	570	21	2	9	4.21%	21.61822614116053	0.005642787686764809	0.04407391494785414	0.0013614158727831508
ENSG00000086102_33290573_33347740_833	NFX1	9	+	2499	8	1	0	0.60%	1.8784487357551025	0.04223451612662591	0.019679754444124863	0.003715148010809822
ENSG00000143924_42169612_42330207_981	EML4	2	+	2943	35	5	1	1.63%	6.978360460109584	0.002059128867186126	0.00027401520548450106	3.865533024781358e-06
ENSG00000134690_37693012_37708366_213	CDCA8	1	+	639	19	1	0	3.29%	17.447305246946012	0.013794764262559246	0.00837382380159347	0.0005783443517979597
ENSG00000039523_67540585_67546463_991	RIPOR1	16	+	2973	10	3	0	0.50%	1.9736980283602426	0.12312584984626351	0.020613416668581838	0.011809609471937859
ENSG00000185515_155071528_155120150_291	BRCC3	X	+	873	8	0	0	2.41%	5.377140195477664	0.005706018193000826	0.005706018193000826	0.0001733096755673334
ENSG00000129351_10670980_10684465_703	ILF3	19	+	2109	55	4	8	2.99%	15.30247667649716	0.00016491851939301634	0.00014059969282245537	1.6346967976074869e-07
ENSG00000091409_172427789_172504252_1086	ITGA6	2	+	3258	84	0	6	2.58%	15.128777041696136	1.3883739438226593e-06	4.184952725518285e-05	5.457363422351459e-10
ENSG00000128789_12706586_12725531_233	PSMG2	18	+	699	27	2	4	3.86%	22.66533825958584	0.010026873424650893	0.010318779121986092	0.0005173850726394671
ENSG00000158373_26158170_26158550_126	HIST1H2BD	6	+	378	123	13	5	15.08%	190.93648711977386	0.00019158148951032086	9.00886142045281e-05	1.2376604711048995e-07
ENSG00000175768_37592532_37588898_85	TOMM5	9	-	255	137	20	3	14.12%	315.25065907810006	0.006678088744345997	0.0010786302489720287	4.4522868367879575e-05
ENSG00000116161_175000181_175006771_80	CACYBP	1	+	240	13	0	0	6.25%	31.78393962420626	0.020613416668581838	0.020613416668581838	0.001946208561389314
ENSG00000101150_63865366_63889945_152	TPD52L2	20	+	456	28	3	2	10.53%	36.03037690193422	0.0012141277396384665	0.0008765049685557307	6.601757452133169e-06
ENSG00000125968_31605388_31606094_155	ID1	20	+	465	110	2	2	17.42%	138.80827230422585	3.716244462047225e-06	2.9337315965033068e-06	9.300909308077099e-11
ENSG00000079459_11802833_11838609_417	FDFT1	8	+	1251	250	17	10	15.59%	117.26227494634296	1.2802270129991288e-10	6.055999232395061e-12	9.787871393417142e-21
ENSG00000117505_93346706_93360639_156	DR1	1	+	468	18	0	0	5.13%	22.568477839673083	0.005160369442893307	0.005160369442893307	0.00014319601891785326
ENSG00000147400_152829709_152827841_154	CETN2	X	-	462	29	0	0	7.79%	36.83253742665261	0.001011481469578233	0.001011481469578233	6.343027644327304e-06
ENSG00000147383_152846349_152869116_365	NSDHL	X	+	1095	25	1	0	2.74%	13.396813329486305	0.0035004709947243163	0.0023628488670607213	4.7187726630981656e-05
ENSG00000126858_32192201_32224733_470	RHOT1	17	+	1410	7	0	1	1.06%	2.9130943027095753	0.019216965118390855	0.04178282491678763	0.003600525600033436
ENSG00000198715_156294893_156293044_325	GLMP	1	-	975	14	0	1	2.46%	8.425565060144617	0.005160369442893307	0.00912793029372963	0.00024819240235076056
ENSG00000274944_38866933_38864670_32	AL139260.3	1	-	96	11	0	0	15.62%	67.23525689735939	0.02108409854857798	0.02108409854857798	0.002029891263157455
ENSG00000118705_37179357_37241315_631	RPN2	20	+	1893	324	12	12	12.52%	100.4315146969921	8.582779249749194e-14	4.589940147860451e-14	5.319738042607397e-26
ENSG00000114648_47330000_47343941_424	KLHL18	3	+	1272	8	0	2	1.42%	3.6904429171792463	0.011570465654371866	0.08266357711566169	0.004841719827405853
ENSG00000013441_200859774_200857692_134	CLK1	2	-	402	11	0	0	4.48%	16.056180751608213	0.012798402692974279	0.012798402692974279	0.0007972436888943567
ENSG00000013441_200854632_200853306_83	CLK1	2	-	249	9	0	0	7.23%	21.20893098186145	0.012178957859018494	0.012178957859018494	0.0007262454987071801
ENSG00000164300_80177968_80113625_359	SERINC5	5	-	1077	9	3	0	1.39%	4.903457580764625	0.11497452839710681	0.020613416668581838	0.010945600217888082
ENSG00000127948_75954002_75986481_677	POR	7	+	2031	142	19	2	5.17%	41.02551461549632	2.2546480905413643e-05	1.268907762623884e-07	3.2919203835882604e-11
ENSG00000136933_125232700_125233980_112	RABEPK	9	+	336	13	0	0	4.46%	22.702814017290184	0.02108409854857798	0.02108409854857798	0.002029891263157455
ENSG00000166986_57489324_57516581_744	MARS	12	+	2232	62	5	5	3.36%	16.29945621754167	7.074140365695205e-06	5.13166956291207e-05	3.0039340838686985e-09
ENSG00000219200_7013669_7014286_71	RNASEK	17	+	213	86	4	4	23.94%	236.9160396690564	0.000449965669174599	0.00030104622191380663	9.05267180070782e-07
ENSG00000112335_108260921_108212149_162	SNX3	6	-	486	34	0	1	9.88%	41.050482325660504	0.0001911635734462366	0.0003427846808402677	4.4968742132846854e-07
ENSG00000141564_80625691_80823227_325	RPTOR	17	+	975	7	0	0	1.54%	4.2127825300723085	0.019216965118390855	0.019216965118390855	0.0017073955890589052
ENSG00000111684_6981844_6981000_84	LPCAT3	12	-	252	13	0	0	5.95%	30.270418689720245	0.020613416668581838	0.020613416668581838	0.001946208561389314
ENSG00000162144_61356632_61350399_215	CYB561A3	11	-	645	7	0	0	2.79%	6.368159638481397	0.009815328628645334	0.009815328628645334	0.0004837142251238724
ENSG00000147642_109579914_109574906_457	SYBU	8	-	1371	9	0	0	1.09%	3.8519502658522984	0.020613416668581838	0.020613416668581838	0.001946208561389314
ENSG00000079459_11809725_11838609_332	FDFT1	8	+	996	190	12	7	14.16%	111.93602462649099	6.943739138009268e-08	5.488022017727631e-09	4.076006399008511e-15
ENSG00000048828_93452345_93529733_485	FAM120A	9	+	1455	21	4	0	1.65%	8.468995807877322	0.028926347213645422	0.005706018193000826	0.0008737241085285608
ENSG00000087088_48960849_48961636_56	BAX	19	+	168	30	0	3	12.50%	104.7822185413393	0.00847367416878415	0.01728285133886564	0.0007291154164894968
ENSG00000087088_48955571_48960837_145	BAX	19	+	435	45	1	3	8.97%	60.70142315498277	0.000649205117157464	0.001585294390300587	6.480973405182851e-06
ENSG00000151131_104986353_104995996_211	C12orf45	12	+	633	9	0	0	2.84%	8.34284962793602	0.012178957859018494	0.012178957859018494	0.0007262454987071801
ENSG00000198677_95547150_95464607_1564	TTC37	5	-	4692	21	0	0	0.70%	2.6262550938750007	0.0012427339549726212	0.0012427339549726212	9.422880418492092e-06

ENSG00000083123_80106694_80343804_392	BCKDHB	6	+	1176	13	0	0	1.53%	6.486518290654338	0.012798402692974279	0.012798402692974279	0.0007972436888943567
ENSG00000118181_119017662_119015845_131	RPS25	11	−	393	101	5	5	3.82%	150.8010758447367	0.0705580690856681	0.06539853090342929	0.017471157796648762
ENSG00000125991_35542098_35548873_230	ERGIC3	20	+	690	68	0	3	10.00%	57.827635971800014	1.0910176244073849e-05	3.333827111722463e-05	2.9111606094796965e-09
ENSG00000101246_63707058_63700598_162	ARFRP1	20	−	486	16	1	0	3.09%	19.31787403560494	0.02784981298332479	0.019679754444124863	0.002477909089056847
ENSG00000123297_57796194_57796583_129	TSFM	12	+	387	14	1	0	5.43%	21.22719879493799	0.015625008558487224	0.00797940233269714	0.0006253789790324684
ENSG00000123297_57782802_57796197_167	TSFM	12	+	501	10	3	4	4.79%	11.712184108413176	0.056175598845231926	0.04922925118879209	0.010979189182267066
ENSG00000156482_98045367_98041801_96	RPL30	8	−	288	254	22	10	21.88%	517.507734906948	0.0002734680026623274	0.00015014081616729835	2.859811160642495e-07
ENSG00000111716_21654671_21635542_334	LDHB	12	−	1002	840	22	2	26.05%	491.91173255335343	8.138368970500423e-15	2.3019221157348395e-16	2.817816787640905e-29
ENSG00000162227_62778958_62787296_447	TAF6L	11	+	1341	9	0	0	1.34%	3.93812364987584	0.011772051206276392	0.011772051206276392	0.000681272511389508
ENSG00000145860_159203617_159158670_663	RNF145	5	−	1989	34	2	1	2.71%	10.03043459541026	0.00041617781144845243	0.00011977551446593921	3.5184401231069343e-07
ENSG00000147439_22630576_22622626_174	BIN3	8	−	522	7	0	0	2.87%	7.86870300157184	0.019216965118390855	0.019216965118390855	0.0017073955890589052
ENSG00000003056_8946404_8945386_124	M6PR	12	−	372	31	6	15	8.06%	48.89836865262501	0.01555382581283059	0.12180769416495907	0.00941726457538092
ENSG00000166037_95813560_95831256_342	CEP57	11	+	1026	19	0	0	1.46%	10.86630414502778	0.019679754444124863	0.019679754444124863	0.0017849636115922674
ENSG00000143727_275182_277304_67	ACP1	2	+	201	15	0	0	10.45%	43.7895838680224	0.00847367416878415	0.00847367416878415	0.0003665024264111182
ENSG00000163918_186804713_186789969_336	RFC4	3	−	1008	17	1	0	2.08%	9.896098417793157	0.016854695534768073	0.007029302661868638	0.0006017499134389055
ENSG00000128487_20096745_20236881_759	SPECC1	17	+	2277	16	0	0	1.19%	4.123182600484849	0.0033030736763512156	0.0033030736763512156	6.124050385493983e-05
ENSG00000128487_20096745_20314065_1037	SPECC1	17	+	3111	17	0	0	0.96%	3.2064504034508206	0.002046028879997488	0.002046028879997488	2.4518070358754455e-05
ENSG00000128487_20156197_20236881_709	SPECC1	17	+	2127	16	0	0	1.27%	4.413957113918196	0.0033030736763512156	0.0033030736763512156	6.124050385493983e-05
ENSG00000163584_170870167_170866380_121	RPL22L1	3	−	363	112	3	6	14.88%	181.04519963947112	0.0002561743955355881	0.000288582102202262	5.026677154334348e-07
ENSG00000128487_20156197_20232409_703	SPECC1	17	+	2109	16	0	0	1.28%	4.451629578617355	0.0033030736763512156	0.0033030736763512156	6.124050385493983e-05
ENSG00000159403_7090095_7080532_577	C1R	12	−	1731	33	0	6	2.43%	11.186455220357889	0.0004441315507112266	0.004625220748970653	1.3912158508667273e-05
ENSG00000047932_117602201_117563254_378	GOPC	6	−	1134	18	4	0	2.38%	9.313974981452382	0.032474320657059096	0.0034782380676658805	0.0006557086944889266
ENSG00000127990_94656098_94585465_451	SGCE	7	−	1353	25	3	2	2.44%	10.842210344262751	0.007887772776385514	0.0038108971419065062	0.00016292425881891603
ENSG00000103197_2060673_2088610_1437	TSC2	16	+	4311	10	5	3	0.56%	1.361123692487822	0.07635947366271362	0.04922925118879209	0.01464496403362635
ENSG00000214655_73786080_73801519_1588	ZSWIM8	10	+	4764	7	0	0	0.31%	0.8621878603737407	0.019216965118390855	0.019216965118390855	0.0017073955890589052
ENSG00000130402_38724560_38729432_238	ACTN4	19	+	714	34	2	7	6.30%	27.94192494435715	0.0008095543264922671	0.011759535387248672	6.390610565923017e-05
ENSG00000099341_38374791_38381180_264	PSMD8	19	+	792	48	2	3	5.30%	35.562449929181824	0.0008019778103825313	0.001779122956135253	8.846147611836567e-06
ENSG00000251562_65499599_65499757_52	MALAT1	11	+	156	17	3	1	9.62%	36.00758352	0.030316224	0.022899795	0.003082174
ENSG00000110713_3782117_3712543_937	NUP98	11	−	2811	26	0	0	1.28%	5.427353617794025	0.0010155991509472172	0.0010155991509472172	6.392786756915814e-06
ENSG00000164904_126582881_126549921_336	ALDH7A1	5	−	1008	22	0	0	2.38%	12.806715599497027	0.0053557511377634245	0.0053557511377634245	0.00015366972994913032
ENSG00000160688_154990430_154993037_102	FLAD1	1	+	306	10	1	0	4.90%	19.175830844166672	0.04223451612662591	0.019679754444124863	0.003715148010809822
ENSG00000103266_680526_682066_265	STUB1	16	+	795	18	3	0	1.89%	13.285594501845287	0.06307108214216126	0.02108409854857798	0.005896901601340726
ENSG00000180776_21459171_21378701_365	ZDHHC20	13	−	1095	20	0	0	1.92%	10.717450663589043	0.00847367416878415	0.00847367416878415	0.0003665024264111182
ENSG00000134440_57615969_57607381_254	NARS	18	−	762	37	1	7	7.09%	28.491962836962603	0.00011805436260872568	0.006725621822730858	6.881025892954126e-06
ENSG00000135269_116234534_116257482_412	TES	7	+	1236	8	0	3	1.46%	3.7979315458349525	0.011570465654371866	0.11368416502207135	0.006956434060967934
ENSG00000108578_30291819_30249017_455	BLMH	17	−	1365	15	0	0	1.98%	6.448136525620881	0.0032294771334464936	0.0032294771334464936	5.8663648728941325e-05
ENSG00000168679_110389323_110363766_487	SLC16A4	1	−	1461	57	16	1	3.49%	22.892870744966125	0.005383196024220725	0.00016458385254932098	7.0398597870256304e-06
ENSG00000141499_7688649_7703486_548	WRAP53	17	+	1644	20	2	0	1.64%	7.138447978485403	0.013494345032394814	0.0035547763465261306	0.00026296295714225076
ENSG00000137076_35724953_35697791_2463	TLN1	9	−	7389	79	8	5	1.62%	6.273603123925905	2.082462632322036e-06	1.269833164875973e-07	2.604577559408463e-12
ENSG00000134056_69228289_69229225_38	MRPS36	5	+	114	24	0	0	13.16%	123.5327208066316	0.02108409854857798	0.02108409854857798	0.002029891263157455
ENSG00000068120_42562623_42565968_564	COASY	17	+	1692	24	2	4	1.60%	8.323126579170214	0.01292879023172146	0.026799084696674903	0.001638717780202175
ENSG00000104835_38930724_38915606_514	SARS2	19	−	1542	11	2	2	1.17%	4.18585256948541	0.060667625179241055	0.060667625179241055	0.014229868458155282
ENSG00000146085_49459466_49431728_750	MMUT	6	−	2250	29	0	0	1.87%	7.562947684939335	0.00042454308224345244	0.00042454308224345244	1.1896574514138388e-06
ENSG00000126581_42818656_42810760_325	BECN1	17	−	975	10	6	1	1.85%	6.018260757246155	0.14351487215180903	0.023672501042627626	0.015574533941414816
ENSG00000143384_150578489_150577375_120	MCL1	1	−	360	31	1	2	10.83%	50.52831427437918	0.0010405427162388935	0.0027852197205094193	1.7601462015625278e-05
ENSG00000075420_172226891_172397475_1135	FNDC3B	3	+	3405	27	0	4	1.06%	4.652884418047138	0.0008739357725210369	0.007045996527915517	3.921453735198883e-05
ENSG00000099875_2042016_2040938_147	MKNK2	19	−	441	9	0	0	3.40%	11.975110690438779	0.020613416668581838	0.020613416668581838	0.001946208561389314
ENSG00000151491_15669717_15621317_718	EPS8	12	−	2154	15	0	0	1.11%	4.086214650637188	0.005405141725979036	0.005405141725979036	0.00015637213408961292
ENSG00000130723_131432670_131495874_1479	PRRC2B	9	+	4437	11	1	3	0.54%	1.4547182019712648	0.009847127393243164	0.05111742236774389	0.0025193211745293573
ENSG00000168528_31424824_31434199_341	SERINC2	1	+	1023	16	0	0	1.47%	9.177406433337245	0.02108409854857798	0.02108409854857798	0.002029891263157455
ENSG00000108946_68515400_68530449_381	PRKAR1A	17	+	1143	159	2	3	9.97%	81.62562326264963	4.363518371891827e-08	4.3268258388075745e-08	1.8788011721226132e-14
ENSG00000142227_48326845_48330470_163	EMP3	19	+	489	266	10	0	22.70%	319.1893512048651	2.576286696886105e-07	4.932328343902073e-08	1.2549786696660143e-13
ENSG00000131778_147275365_147295509_470	CHD1L	1	+	1410	7	0	1	1.28%	2.9130943027095753	0.009815328628645334	0.0477903522728147	0.0023388674905236288
ENSG00000118762_88056176_88075694_366	PKD2	4	+	1098	15	0	2	1.91%	8.016126008627051	0.00847367416878415	0.028426896806120847	0.0012022752110812016
ENSG00000155100_91070295_91084868_323	OTUD6B	8	+	969	12	0	0	2.17%	7.266630635684212	0.008175610787902942	0.008175610787902942	0.00034251843268961324
ENSG00000182326_7064099_7070651_556	C1S	12	+	1668	26	0	0	2.16%	9.14645744581475	0.0007764301431553706	0.0007764301431553706	3.8125731154589255e-06
ENSG00000121579_113745949_113721760_169	NAA50	3	−	507	15	0	0	4.14%	17.360367568979292	0.007029302661868638	0.007029302661868638	0.0002573639808771639
ENSG00000022556_54970034_54990196_840	NLRP2	19	+	2520	22	2	0	1.07%	5.122686239798811	0.00895312456195012	0.0036062891729786097	0.0001757758008939458
ENSG00000166347_74291875_74260867_111	CYB5A	18	−	333	45	4	0	8.11%	79.29465186912164	0.008830848114324176	0.003816220824102754	0.00018248060063567366
ENSG00000135926_218280060_218275475_200	TMBIM1	2	−	600	8	0	0	2.50%	7.823738984420002	0.019216965118390855	0.019216965118390855	0.0017073955890589052
ENSG00000105612_12878646_12875990_212	DNASE2	19	−	636	14	0	4	2.36%	12.916550210127362	0.02108409854857798	0.12378039365238508	0.01208667083877082
ENSG00000026508_35176703_35190154_186	CD44	11	+	558	133	3	9	11.83%	139.8598501247124	1.826113693614021e-05	0.000187397314381545	2.7461356886300377e-08
ENSG00000043093_182965711_182945094_244	DCUN1D1	3	−	732	13	1	0	2.87%	10.420963811215167	0.013794764262559246	0.00837382380159347	0.0005783443517979597
ENSG00000172500_65885163_65884388_112	FIBP	11	−	336	28	2	5	6.25%	48.89836865262501	0.03299602752967379	0.06059069429271301	0.00828985036369236
ENSG00000162385_53238448_53227045_109	MAGOH	1	−	327	52	2	0	6.42%	93.31064843803672	0.012000726946169103	0.00877616296920843	0.0005276681396223033
ENSG00000119314_112268156_112223851_443	PTBP3	9	−	1329	30	1	4	2.93%	13.245607761433412	0.0006606329705481882	0.005221500909681797	2.2372145234106156e-05
ENSG00000101247_13785069_13817210_345	NDUFAF5	20	+	1035	26	1	0	3.19%	14.740377796733336	0.00236147777460975	0.0015061527253727427	2.1068444318500428e-05

ENSG00000138674_82878897_82819074_1127	SEC31A	4	-	3381	27	0	0	0.98%	4.685912878867348	0.0012624143894574274	0.0012624143894574274	9.711623744831373e-06
ENSG00000133112_45338743_45337476_53	TPT1	13	-	159	22	6	4	9.43%	81.18974417794341	0.02108409854857798	0.05202961726446396	0.00485300211054616 4
ENSG00000149658_63203789_63196708_509	YTHDF1	20	-	1527	16	3	0	1.38%	6.148321402294697	0.058290625655499875	0.00797940233269714	0.0024489077996833817
ENSG00000197150_151034375_151045349_548	ABCB8	7	+	1644	29	0	1	2.37%	10.350749568803835	0.0006629342244537616	0.0009306875619951529	3.907239258273379e-06
ENSG00000062485_56291222_56273084_453	CS	12	-	1359	49	3	0	4.19%	21.156910057206407	0.0003259695436627904	5.7364792129256647e-05	1.388780755767625e-07
ENSG00000072786_172127418_172044882_860	STK10	5	-	2580	7	0	0	0.58%	1.5920399096203492	0.016947426762344633	0.016947426762344633	0.0013498980316300959
ENSG00000105854_95435014_95405330_375	PON2	7	-	1125	441	12	9	17.33%	230.01792614194804	2.199773929273865e-11	4.7628156924550425e-12	1.3001091419103812e-21
ENSG00000175063_45812696_45816767_132	UBE2C	20	+	396	37	0	0	6.06%	54.82544364082198	0.005604866691947602	0.005604866691947602	0.00016752456572620382
ENSG00000165219_125298922_125362746_1478	GAPVD1	9	+	4434	10	1	3	0.34%	1.323365863399865	0.03537493353818881	0.12114404826813535	0.017662684075641494
ENSG00000165219_125298922_125362746_1487	GAPVD1	9	+	4461	10	1	3	0.34%	1.3153562515837258	0.03537493353818881	0.12114404826813535	0.017662684075641494
ENSG00000139531_56002222_56005027_545	SUOX	12	+	1635	10	5	0	0.92%	3.588871093770643	0.17139085557395572	0.020613416668581838	0.01724926236372712
ENSG00000112339_135002753_134965279_511	HBS1L	6	-	1533	28	0	0	2.35%	10.717450663589043	0.0008739357725210369	0.0008739357725210369	4.787965623198429e-06
ENSG00000275835_23031961_22996906_868	TUBGCP5	15	-	2604	12	0	0	0.69%	2.704057252679724	0.011772051206276392	0.011772051206276392	0.000681272511389508
ENSG00000080815_73148065_73219289_448	PSEN1	14	+	1344	28	2	0	3.35%	12.224592163156252	0.0008082499550292343	0.00023719413513965303	1.2966454664305165e-06
ENSG00000117335_207752282_207793591_346	CD46	1	+	1038	39	2	0	6.36%	22.04666332314885	7.639996631620973e-05	1.3529991062581404e-05	8.254540628586197e-09
ENSG00000077147_96528119_96527236_91	TM9SF3	10	-	273	13	6	4	5.49%	27.94192494435715	0.1526275292331874	0.08678408327796083	0.04578274282100864
ENSG00000163479_156018145_156009540_103	SSR2	1	-	309	183	3	2	9.71%	347.5107364438981	0.00695314844767304	0.0023766146074493525	9.36243079795798e-05
ENSG00000163479_156020896_156018248_94	SSR2	1	-	282	287	1	3	23.40%	597.1843320554628	2.2985161501133994e-05	6.140828680895134e-05	1.089111622536981e-08
ENSG00000136270_45104655_45104006_122	TBRG4	7	-	366	16	2	0	5.74%	25.651603227606564	0.023878473930440184	0.00797940233269714	0.0009553875686822204
ENSG00000114902_52706674_52707812_80	SPCS1	3	+	240	76	7	2	23.75%	185.81380087997505	0.00046324249190235744	0.00011969550530628595	3.919124007656001e-07
ENSG00000136279_44050254_44060916_355	DBNL	7	+	1065	12	2	0	1.69%	6.611610409369016	0.0319825310473083	0.012798402692974279	0.0019365420928339585
ENSG00000162384_53218923_53214659_130	CZIB	1	-	390	11	0	0	3.85%	16.550217082426926	0.020613416668581838	0.020613416668581838	0.001946208561389314
ENSG00000103152_78310_85777_293	MPG	16	+	879	69	2	7	4.78%	46.06126191899148	0.0007823562235838132	0.002726101072049826	1.327358598232192e-05
ENSG00000283515_58312169_58314783_109	AC020915.5	19	+	327	33	12	1	11.01%	59.21637304721561	0.016570103030061338	0.0013558867987602367	0.0001434925155600661 6
ENSG00000246705_14774471_14774860_129	H2AFJ	12	+	387	109	6	7	12.40%	165.2689049034458	0.001595412498587932	0.00025382860003781874	2.767002858633509e-06
ENSG00000141905_3381826_3462769_460	NFIC	19	+	1380	43	3	3	2.61%	18.283737844025005	0.0027101569075239007	0.0010032776369550557	1.6561952297087845e-05
ENSG00000142208_104776759_104770341_418	AKT1	14	-	1254	19	0	0	1.91%	8.890612482295456	0.005355751137763424 5	0.005355751137763424 5	0.00015366972994913032
ENSG00000241370_30345172_30346810_177	RPP21	6	+	531	26	2	1	2.82%	28.731244858039556	0.05742480463164306	0.02108409854857798	0.005360236623058286
ENSG00000140950_84498086_84494773_147	MEAK7	16	-	441	9	0	0	3.40%	11.975110690438779	0.020613416668581838	0.020613416668581838	0.001946208561389314
ENSG00000086289_37920940_37950396_224	EPDR1	7	+	672	28	0	0	4.46%	24.449184326312505	0.0024321290225875155	0.0024321290225875155	3.413046187581682e-05
ENSG00000113712_149511835_149501131_154	CSNK1A1	5	-	462	9	0	0	3.90%	11.430787477237015	0.012178957859018494	0.012178957859018494	0.0007262454987071801
ENSG00000160752_155310161_155312443_77	FDPS	1	+	231	47	0	1	15.58%	119.38822476225327	0.0010573555742419644	0.0013935654826858935	9.020986797120166e-06
ENSG00000115839_135133996_135169759_492	RAB3GAP1	2	+	1476	17	0	0	1.22%	6.758311114590449	0.013428347753762199	0.013428347753762199	0.0008725593497644525
ENSG00000071462_73686643_73693338_109	BUD23	7	+	327	17	1	2	8.26%	30.505404297050465	0.006923060740356555	0.007379737683198523	0.0002656679423942265 4
ENSG00000134779_36818914_36790146_172	TPGS2	18	-	516	8	0	0	2.91%	9.097370912116281	0.019216965118390855	0.019216965118390855	0.0017073955890589052
ENSG00000105518_11345619_11342815_189	TMEM205	19	-	567	81	8	9	12.17%	83.82577483307145	0.000223516199650981	0.00014814014849330916 6	2.314515221602206e-07
ENSG00000198898_116886100_116910231_162	CAPZA2	7	+	486	36	4	0	8.02%	43.46521658011112	0.005247194031858394	0.0006446704936422262	2.201303698847903e-05
ENSG00000133313_74499974_74520068_475	CNDP2	18	+	1425	30	0	2	2.95%	12.35327208066316	0.0004323067881491664	0.0015667181870683013	4.4033979110162815e-06
ENSG00000138614_65598879_65579408_318	INTS14	15	-	954	11	0	0	1.89%	6.765812014828618	0.012798402692974279	0.012798402692974279	0.0007972436888943567
ENSG00000169359_155853997_155833538_336	SLC33A1	3	-	1008	18	0	0	2.08%	10.478221854133931	0.008674848690044494 4	0.008674848690044494 4	0.0003831153291663411 6
ENSG00000164904_126595114_126544965_471	ALDH7A1	5	-	1413	25	0	0	2.12%	10.381819246841829	0.0022409900782254734	0.0022409900782254734	2.9184102569313086e-05
ENSG00000115520_197459957_197473924_195	COQ10B	2	+	585	21	0	0	5.13%	21.063912650361544	0.0023218226288164415	0.0023218226288164415	3.1231198880462635e-05
ENSG00000139350_96917657_96952053_571	NEDD1	12	+	1713	12	0	1	1.05%	4.110545876227672	0.013007239811447713	0.023878473930440184	0.0014709138084317821
ENSG00000171617_74636266_74634716_516	ENC1	5	-	1548	29	1	0	2.52%	10.992656518807173	0.0009958682880916293	0.0006424109906750971	4.052020686628797e-06
ENSG00000149554_125627722_125655320_416	CHEK1	11	+	1248	13	0	0	1.44%	6.112296081578126	0.013428347753762199	0.013428347753762199	0.0008725593497644525
ENSG00000249915_271721_314515_191	PDCD6	5	+	573	59	0	5	11.52%	60.41892671214399	1.6040313372898932e-05	0.00014877056305080506	1.9186448467213617e-08
ENSG00000143537_155051387_155062020_822	ADAM15	1	+	2466	46	13	0	2.31%	10.945620233677618	0.009314527945786596	5.328371459388349e-05	5.3170083957120194e-06
ENSG00000085733_70407298_70435162_550	CTTN	11	+	1650	57	1	9	3.09%	20.27059645963364	0.00018195398980347797	0.004776846066244509	6.704206386268192e-06
ENSG00000167460_16086484_16101346_139	TPM4	19	+	417	27	0	3	5.04%	37.99297708261512	0.0084736741687841 5	0.03299602752967379	0.0014019198044660757
ENSG00000103197_2071615_2088610_1093	TSC2	16	+	3279	8	5	3	0.64%	1.4316082313668805	0.11797167300661582	0.07152813839304221	0.030491766962228876
ENSG00000204628_181238227_181236977_101	RACK1	5	-	303	246	6	1	25.74%	476.3959876651783	4.256876824982209e-06	3.991591885989852e-06	1.4353355679323817e-10
ENSG00000167461_16111902_16132304_207	RAB8A	19	+	621	35	0	2	7.73%	33.0713604413889	0.00014734945683592696	0.0005341760705719847	5.489275905227221e-07
ENSG00000204138_28459117_28490954_630	PHACTR4	1	+	1890	10	3	0	0.95%	3.104658327150794	0.10000264635955686	0.012798402692974279	0.006484128903061078
ENSG00000130382_6262257_6213042_477	MLLT1	19	-	1431	15	4	1	1.47%	6.150738195298744	0.07962816006291146	0.016854695534768073	0.006264736550049984
ENSG00000256269_119089087_119093283_306	HMBS	11	+	918	25	0	0	2.94%	15.979859036805559	0.0036321914711260127	0.0036321914711260127	7.340009712338726e-05
ENSG00000008283_63437460_63434402_222	CYB561	17	-	666	14	0	3	2.70%	12.334723624085589	0.013217226988443315	0.06307108214216126	0.004011161177112752 5
ENSG00000092621_119712023_119742086_465	PHGDH	1	+	1395	39	0	0	4.30%	16.404613999590325	3.445549927555771e-05	3.445549927555771e-05	9.084392181695527e-09
ENSG00000073712_52950547_52858377_680	FERMT2	14	-	2040	35	0	1	2.50%	10.067311193187502	0.00011297380978831068	0.00018702083254192264	1.498932361695684e-07
ENSG00000123374_55967009_55971625_346	CDK2	12	+	1038	28	2	0	2.89%	15.828373667901738	0.008661717511597958	0.0023354528714849923	0.00011521534222637746
ENSG00000100591_77464612_77468205_108	AHSA1	14	+	324	71	4	0	16.67%	128.5845990494954	0.00011813975087291 16	9.006271182364242e-05	7.679963160644331e-08
ENSG00000143379_150927823_150964364_1254	SETDB1	1	+	3762	11	0	0	0.48%	1.715732233425439	0.012798402692974279	0.012798402692974279	0.0007972436888943567
ENSG00000204822_74472660_74472119_147	MRPL53	2	-	441	60	4	1	12.93%	79.83407126959186	0.0004114342333053557	7.852614211537559e-05	2.3536506644062248e-07
ENSG00000115525_85863399_85840144_309	ST3GAL5	2	-	927	14	1	3	2.27%	8.861840273614888	0.015516259750072458	0.0581241478854986	0.004198806874037122
ENSG00000074416_127821818_127692198_303	MGLL	3	-	909	19	2	0	3.30%	12.264937351813535	0.01044836432889887	0.002046028879997488	0.00012444751772694397
ENSG00000105655_18436542_18434913_376	ISYNA1	19	-	1128	11	2	0	1.86%	5.722149523179523	0.028902805874428627	0.0078820576498062	0.0011492025149225664
ENSG00000073111_127599339_127621773_895	MCM2	3	+	2685	48	4	0	2.23%	10.489929364585477	0.0003073905860217978	3.6359402256064547e-05	8.610038191547308e-08
ENSG00000130811_10119859_10115114_320	EIF3G	19	-	960	56	6	0	6.56%	34.22885805683751	0.00029958714052925526	2.416182237022495e-05	5.784616008367249e-08

ENSG00000141971_17420136_17424993_273	MVB12A	19	+	819	20	0	0	2.56%	14.329192279157512	0.00877616296920843	0.00877616296920843	0.00039161135667564577
ENSG00000123349_53297850_53299345_85	PFDN5	12	+	255	53	6	0	10.59%	121.95828416890002	0.02256365863631257	0.0037896409716948643	0.0004751831865071967
ENSG00000132341_130872594_130875926_216	RAN	12	+	648	224	19	0	12.04%	202.8376773738519	0.0001788485974193862	3.921341011160596e-06	6.564894565710547e-09
ENSG00000107331_137017118_137007929_1583	ABCA2	9	-	4749	12	0	0	0.38%	1.4827047980581178	0.011772051206276392	0.011772051206276392	0.000681272511389508
ENSG00000198836_193593378_193692127_965	OPA1	3	+	2895	32	1	1	1.87%	6.486001230607255	0.0001048543557987398	0.0001048543557987398	7.919728780723367e-08
ENSG00000276966_26204645_26204956_103	HIST1H4E	6	+	309	542	140	48	33.01%	1029.239448921272	1.2071737699770179e-06	2.590304417509865e-07	2.9200701512945625e-12
ENSG00000142657_10417418_10419749_144	PGD	1	+	432	120	4	2	21.53%	162.99456217541672	3.909047960222324e-06	1.4384237862090121e-06	4.9101090162126185e-11
ENSG00000075151_20981092_20807319_1529	EIF4G3	1	-	4587	11	0	0	0.46%	1.4071472993561154	0.007593099278532274	0.007593099278532274	0.00029783286705462064
ENSG00000122783_135168814_135167772_33	CYREN	7	-	99	10	0	0	18.18%	59.270749881969714	0.009815328628645334	0.009815328628645334	0.0004837142251238724
ENSG00000166888_57107239_57096572_737	STAT6	12	-	2211	23	3	0	1.36%	6.10400259978494	0.007867321313276006	0.0023218226288164415	0.00010390540483540199
ENSG00000076242_37014478_37050653_515	MLH1	3	+	1545	17	0	3	1.75%	6.456483627919419	0.0033030736763512156	0.021725767126240147	0.00040633255960778673
ENSG00000176619_2456873_2430911_600	LMNB2	19	-	1800	45	11	0	2.83%	14.669510595787504	0.003230935807588588	0.00012102304356057305	3.0748680052283735e-06
ENSG00000086200_62437280_62627318_975	IPO11	5	+	2925	20	0	2	0.82%	4.012173838164103	0.005306578019052857	0.016170007243128042	0.0004498844289484224
ENSG00000011028_62681922_62692451_550	AC080038.1	17	+	1650	11	0	0	0.91%	3.911869492210001	0.02108409854857798	0.02108409854857798	0.002029891263157455
ENSG00000198231_63787050_63818398_938	DDX42	17	+	2814	27	0	0	1.49%	5.630089354460023	0.00037874750290730355	0.00037874750290730355	9.541676172656993e-07
ENSG00000164305_184635393_184629436_156	CASP3	4	-	468	9	0	1	3.21%	11.284238919836541	0.020613416668581838	0.03537493353818881	0.003252526348827943
ENSG00000143442_151424271_151404802_1010	POGZ	1	-	3030	11	0	1	0.59%	2.130225961104456	0.012798402692974279	0.023672501042627626	0.0014375227604752335
ENSG00000108523_4941098_4944570_125	RNF167	17	+	375	17	4	0	4.00%	26.600712547028007	0.02108409854857798	0.02108409854857798	0.002029891263157455
ENSG00000107404_1349038_1336142_686	DVL1	1	-	2058	7	0	0	0.87%	1.9958517817397963	0.009815328628645334	0.009815328628645334	0.0004837142251238724
ENSG00000182871_45468691_45512398_1154	COL18A1	21	+	3462	40	2	13	1.73%	6.779669830519932	0.0001587278030329256	0.008477751979623365	1.1460001117746155e-05
ENSG00000167186_19071969_19078158_179	COQ7	16	+	537	6	0	0	2.79%	6.556205852865923	0.016947426762344633	0.016947426762344633	0.0013498980316300959
ENSG00000241468_99466141_99458327_55	ATP5MF	7	-	165	35	0	0	14.55%	124.46857475213639	0.005706018193000826	0.005706018193000826	0.0001733096755673334
ENSG00000115525_85848138_85840144_290	ST3GAL5	2	-	870	11	0	3	1.72%	7.41906283005345	0.019216965118390855	0.13115678761539135	0.012020477827111095
ENSG00000120802_98544514_98547858_169	TMPO	12	+	507	10	0	0	4.14%	11.57357837931953	0.007593099278532274	0.007593099278532274	0.00029783286705462064
ENSG00000094631_48815339_48824612_847	HDAC6	X	+	2541	13	0	0	0.71%	3.0020249940218426	0.011772051206276392	0.011772051206276392	0.000681272511389508
ENSG00000112062_36028158_36075847_173	MAPK14	6	+	519	17	0	0	4.05%	19.220168025309253	0.00797940233269714	0.00797940233269714	0.000327142761693177
ENSG00000111445_118016828_118031278_340	RFC5	12	+	1020	16	1	1	2.65%	9.204398805200002	0.006112543793728684	0.006112543793728684	0.00019748307313365872
ENSG00000173418_20017397_20033132_111	NAA20	20	+	333	30	0	0	9.01%	52.863101246081094	0.0023354528714849923	0.0023354528714849923	3.158285405866755e-05
ENSG00000143256_161102306_161100683_106	PFDN2	1	-	318	106	4	2	21.70%	195.59347461050004	5.6215303068530866e-05	3.2627500766626656e-05	1.3933057920083333e-08
ENSG00000158773_161042214_161039920_223	USF1	1	-	669	10	0	0	2.69%	8.771007830067267	0.011772051206276392	0.011772051206276392	0.000681272511389508
ENSG00000173812_41689039_41690826_113	EIF1	17	+	339	139	25	9	20.35%	240.59728292796024	0.0007583227503585444	0.00010977385607262625	6.000790018568453e-07
ENSG00000143314_156738704_156737398_216	MRPL24	1	-	648	87	3	3	10.65%	78.7807050514514	3.747101137361674e-05	1.575548754843906e-05	4.641782502622811e-09
ENSG00000198721_4133671_4115874_364	ECI2	6	-	1092	37	0	0	2.75%	19.88175428733105	0.0023766146074493525	0.0023766146074493525	3.265611492547055e-05
ENSG00000198160_66958068_66984900_449	MIER1	1	+	1347	12	0	2	1.78%	5.227442528565703	0.004873903550586949	0.029607078753113344	0.0007843881175907335
ENSG00000156467_96235530_96231055_111	UQCRB	8	-	333	341	1	8	16.22%	600.8772508304551	9.46591699564405e-05	0.00013492753111050555	9.17766708973228e-08
ENSG00000125354_119663648_119620036_373	SEPT6	X	-	1119	22	0	0	1.34%	11.536344347000002	0.021557223391537678	0.021557223391537678	0.002115616449879074
ENSG00000003989_17543340_17565146_658	SLC7A2	8	+	1974	17	0	0	1.37%	5.053326851639059	0.0033030736763512156	0.0033030736763512156	6.124050385493983e-05
ENSG00000157193_53327912_53247018_700	LRP8	1	-	2100	17	9	0	1.00%	4.750127240540715	0.18607691510208607	0.00847367416878415	0.010183475765332767
ENSG00000040275_169588417_169595771_350	SPDL1	5	+	1050	35	1	0	3.71%	19.559347461050006	0.0010011435659054045	0.0006469352010924132	4.100197584965153e-06
ENSG00000109775_185425868_185405917_387	UFSP2	4	-	1161	12	0	0	1.55%	6.064913941410854	0.011772051206276392	0.011772051206276392	0.000681272511389508
ENSG00000153113_96662423_96771679_769	CAST	5	+	2307	121	0	4	5.59%	30.7760863821463	4.0077545563218955e-09	4.42202571771872e-08	1.9053817941938333e-15
ENSG00000113312_160043145_160065436_159	TTC1	5	+	477	32	3	4	4.40%	39.36472444991196	0.039104403811496304	0.05315521400506113	0.008485924368449567
ENSG00000161813_50429042_50461351_354	LARP4	12	+	1062	12	0	1	1.98%	6.630287274932205	0.007029302661868638	0.016854695534768073	0.0006017499134389055
ENSG00000105835_106284884_106251083_491	NAMPT	7	-	1473	22	0	0	1.63%	8.763862406173118	0.004873903550586949	0.004873903550586949	0.00012846764509679302
ENSG00000013374_151345350_151377225_615	NUB1	7	+	1845	23	0	3	1.79%	7.314877912262603	0.0013845019718837994	0.009254025112477388	7.800347852305891e-05
ENSG00000065057_2046202_2043559_137	NTHL1	16	-	411	7	0	0	3.65%	9.993827169879564	0.016947426762344633	0.016947426762344633	0.0013498980316300959
ENSG00000104419_133256848_133238878_239	NDRG1	8	-	717	21	1	1	2.51%	17.18603751807741	0.013301727469301692	0.013301727469301692	0.0008571698582519322
ENSG00000156467_96235530_96231288_161	UQCRB	8	-	483	343	1	8	11.80%	416.6991415615001	6.377049850388762e-05	0.00010337219259674415	4.834159749573331e-08
ENSG00000182149_71916578_71927813_283	IST1	16	+	849	23	0	0	3.18%	15.896289455977035	0.0035291660242640502	0.0035291660242640502	6.948232509684344e-05
ENSG00000178467_48990449_49006907_499	P4HTM	3	+	1497	21	7	4	2.20%	8.231388711063127	0.0523161259852883	0.01313178205197555	0.0032750644748262827
ENSG00000165644_75235339_75234073_177	COMTD1	10	-	531	32	0	0	6.78%	35.36153213297176	0.0010155991509472172	0.0010155991509472172	6.392786756915814e-06
ENSG00000127990_94656098_94585465_476	SGCE	7	-	1428	25	3	2	2.31%	10.272766523660717	0.007887772776385514	0.0038108971419065062	0.00016292425881891603
ENSG00000066855_65670010_65709044_281	MTFR1	8	+	843	23	0	0	3.20%	16.009430306197512	0.0035291660242640502	0.0035291660242640502	6.948232509684344e-05
ENSG00000160796_46979862_47009320_2754	NBEAL2	3	+	8262	14	2	5	0.22%	0.9943023400679014	0.044040755831095145	0.11684743169084172	0.020273376447258438
ENSG00000088682_57447506_57460624_318	COQ9	16	+	954	28	0	0	4.40%	17.22206694683648	0.00037874750290730355	0.00037874750290730355	9.541676172656993e-07
ENSG00000013561_141973589_141987790_474	RNF14	5	+	1422	32	1	0	3.16%	13.204622758514772	0.0004415189800600387	0.00023785331623704515	7.103787349602483e-07
ENSG00000163541_84432387_84423746_145	SUCLG1	2	-	435	38	4	1	7.59%	51.258979553096566	0.00151350169064872	0.0021824774412010223	1.9597693821777286e-05
ENSG00000106366_101128394_101137442_402	SERPINE1	7	+	1206	194	7	6	11.19%	94.39088078218161	2.0680319113384703e-08	1.949343627826121e-08	4.106158832789174e-15
ENSG00000124562_34757544_34773570_159	SNRPC	6	+	477	25	1	0	6.29%	30.75369097649372	0.0035004709947243163	0.0023628488670607213	4.7187726630981656e-05
ENSG00000092020_35087995_35085590_77	PPP2R3C	14	-	231	8	0	0	6.49%	20.32139995953247	0.019216965118390855	0.019216965118390855	0.0017073955890589052
ENSG00000198561_57791641_57816308_908	CTNND1	11	+	2724	64	7	2	2.64%	13.786324201621149	0.0002556913665467353	1.5363660848022635e-05	3.2544366602591786e-08
ENSG00000134285_48925428_48921984_201	FKBP11	12	-	603	22	2	0	4.48%	21.408241002144283	0.008316137131635013	0.0035291660242640502	0.0001601292768076144
ENSG00000136381_78471792_78498143_713	IREB2	15	+	2139	22	2	1	1.40%	6.035142274096776	0.005059698366439319	0.005059698366439319	0.000137934786740623
ENSG00000205413_93105929_93101328_1533	SAMD9	7	-	4599	18	3	0	0.78%	2.296596570769081	0.0025320062747194163	0.0008442728493916082	1.320482778495691e-05
ENSG00000172936_38138779_38141152_165	MYD88	3	+	495	15	0	4	5.45%	17.781224964590912	0.0032294771334464936	0.041715644099625256	0.0008166171299476855
ENSG00000084754_26236943_26191250_688	HADHA	2	-	2064	86	2	1	4.07%	24.449184326312505	1.7472267648452788e-06	1.770503586800684e-06	2.7057967764336193e-11
ENSG00000198860_184051756_184095804_155	TSEN15	1	+	465	13	0	0	3.87%	16.404613999590325	0.013007239811447113	0.013007239811447113	0.0008218654148058079

ENSG00000169814_15635440_15645488_523	BTD	3	+	1569	13	0	0	1.15%	4.861788087832697	0.013428347753762199	0.013428347753762199	0.0008725593497644525
ENSG00000091317_32502655_32487917_114	CMTM6	3	−	342	13	0	0	5.26%	22.304519034530706	0.013428347753762199	0.013428347753762199	0.0008725593497644525
ENSG00000111481_54325164_54350323_164	COPZ1	12	+	492	95	4	1	15.85%	113.30109809754576	6.916003708982932e−06	4.643833617878754e−06	2.680756807485722e−10
ENSG00000198668_90399082_90404717_175	CALM1	14	+	525	122	5	2	20.00%	136.35659372846288	5.838853150623411e−07	1.4025768871339777e−07	7.801033500390575e−13
ENSG00000108828_43018763_43016061_252	VAT1	17	−	756	54	0	1	8.33%	41.912887416535725	2.6246772046413107e−05	2.6455697038791432e−05	5.386007614031533e−09
ENSG00000102226_47233044_47247930_920	USP11	X	+	2760	25	1	0	0.98%	5.315040070937501	0.006112543793728684	0.0033030736763512156	0.00011113261345270447
ENSG00000178878_12813368_12829554_455	APOLD1	12	+	1365	57	3	3	5.05%	24.502918797359346	5.2757275294553724e−05	2.3795014530330683e−05	9.672713151591613e−09
ENSG00000106628_44122053_44114785_469	POLD2	7	−	1407	57	1	0	5.12%	23.77148838549787	1.0054561647833067e−05	6.503806951040267e−06	5.37545342681054e−10
ENSG00000167522_89316959_89268478_2643	ANKRD11	16	−	7929	11	3	0	0.30%	0.8140477566082106	0.03480468226976219	0.004687384229717438	0.00090387734074771
ENSG00000143924_42288272_42330207_592	EML4	2	+	1776	23	0	1	1.86%	7.599070804124157	0.00151350169064872	0.0021291955662021897	1.913165205264941e−05
ENSG00000163320_88055976_88055473_167	CGGBP1	3	−	501	14	1	0	4.19%	16.397057751778448	0.016854695534768073	0.007029302661868638	0.0006017499134389055
ENSG00000204262_189110408_189034070_1276	COL5A2	2	−	3828	36	0	11	1.10%	5.518311195907525	0.0004323067881491664	0.00605367091653388	1.8164182505448326e−05
ENSG00000103257_87840445_87832970_241	SLC7A5	16	−	723	70	7	4	9.13%	56.81138266695022	0.00016518296082159548	0.00010750628766608115	1.2649672835017495e−07
ENSG00000144746_69085048_69089815_59	ARL6IP5	3	+	177	14	1	0	8.47%	46.41201092452543	0.03577298629450316	0.02108409854857798	0.003354604907114977
ENSG00000117906_76935570_76938773_66	RCN2	15	+	198	25	0	5	12.12%	74.08843735246214	0.005706018193000826	0.02163521358803487	0.0006454818958744852
ENSG00000244405_186105835_186048639_499	ETV5	3	−	1497	12	1	0	1.20%	4.703650692036073	0.019795881618345192	0.012798402692974279	0.0012079910998532793
ENSG00000092199_21233974_21211223_233	HNRNPC	14	−	699	33	2	9	4.29%	27.70208009504936	0.0020841258324441106	0.015856692345517474	0.00019643434173390785
ENSG00000180957_27919191_27853613_272	PITPNB	22	−	816	23	0	2	3.68%	16.539154103093754	0.002046028879997488	0.01044836432889887	0.00012444751772694397
ENSG00000064419_129018073_128957255_855	TNPO3	7	−	2565	29	0	0	1.75%	6.634164635911698	0.0002582331582969843	0.0002582331582969843	4.5481323920964005e−07
ENSG00000170265_149195749_149224647_618	ZNF282	7	+	1854	14	0	0	1.29%	4.430920136807444	0.005257622967929454	0.005257622967929454	0.00014836615564656991
ENSG00000142875_84182305_84235305_280	PRKACB	1	+	840	14	0	0	2.14%	9.779673730525003	0.013428347753762199	0.013428347753762199	0.0008725593497644525
ENSG00000106070_50626921_50592952_407	GRB10	7	−	1221	12	0	0	1.47%	5.766883772299756	0.013217226988443315	0.013217226988443315	0.0008469697265466234
ENSG00000136279_44050254_44060916_355	DBNL	7	+	1065	12	2	0	1.69%	6.611610409369016	0.0319825310473083	0.012798402692974279	0.0019365420928339585
ENSG00000166037_95799268_95831256_447	CEP57	11	+	1341	16	0	0	1.34%	7.001108710890382	0.013007239811447113	0.013007239811447113	0.0008218654148058079
ENSG00000050820_75236980_75229511_617	BCAR1	16	−	1851	18	0	0	1.30%	5.706130539690439	0.005554612275049112	0.005554612275049112	0.00016468457691007306
ENSG00000050820_75236980_75229511_599	BCAR1	16	−	1797	18	0	0	1.34%	5.877600238712856	0.005554612275049112	0.005554612275049112	0.00016468457691007306
ENSG00000078304_101809973_101912497_439	PPP2R5C	14	+	1317	12	0	0	1.59%	5.346518668168566	0.0078820576498062	0.0078820576498062	0.00031963642907451164
ENSG00000006118_60927137_60931944_412	TMEM132A	11	+	1236	8	1	1	1.70%	3.7979315458349525	0.017404239405933563	0.017404239405933563	0.0014187724443961607
ENSG00000276043_4910907_4960803_786	UHRF1	19	+	2358	26	0	2	1.27%	6.470013155054708	0.0020082573300163734	0.009197853169380737	0.00010725224857862305
ENSG00000172531_67401782_67398535_330	PPP1CA	11	−	990	181	9	16	16.97%	107.28005728636518	7.990475364018245e−10	7.096445676701004e−09	6.325557239256397e−17
ENSG00000189067_11556730_11549437_228	LITAF	16	−	684	19	14	1	2.63%	16.29945621754167	0.32736042300928847	0.020525443280229434	0.03913121981310436
ENSG00000159131_33539315_33504124_1010	GART	21	−	3030	92	6	0	3.56%	17.81643531105545	8.145577970331606e−07	6.416136679515342e−08	5.213066704562349e−13
ENSG00000198736_1943156_1940812_94	MSRB1	16	−	282	20	0	0	6.38%	41.61563289585107	0.013007239811447113	0.013007239811447113	0.0008218654148058079
ENSG00000204843_74378227_74361499_1261	DCTN1	2	−	3783	37	8	2	1.43%	5.739063093250199	0.004198794703165439	0.0002950509054422764	8.798140545161517e−06
ENSG00000189171_153626472_153618895_98	S100A13	1	−	294	51	39	2	15.31%	101.78844086872961	0.00774017645579024	0.00029563251185049877	1.727517427423317e−05
ENSG00000080345_151416686_151475071_2337	RIF1	2	+	7011	16	1	1	0.39%	1.339108084624733	0.006112543793728684	0.006112543793728684	0.00019748307313365872
ENSG00000166797_64093672_64075484_98	CIAO2A	15	−	294	5	0	0	5.10%	9.979258908698982	0.012673659338734126	0.012673659338734126	0.000782701129001274
ENSG00000078674_17980608_18027662_723	PCM1	8	+	2169	24	1	10	1.52%	6.492729447651453	0.002336818109389262	0.0861951016664238	0.001512968574670254
ENSG00000196517_44008606_43997545_521	SLC6A9	1	−	1563	11	0	0	0.96%	4.129612707707294	0.019679754444124863	0.019679754444124863	0.0017849636115922674
ENSG00000161513_74872248_74862817_473	FDXR	17	−	1419	13	8	0	1.48%	5.375719175341439	0.23559043810812774	0.0078820576498062	0.013321720827107576
ENSG00000136986_123042122_123015447_231	DERL1	8	−	693	38	4	0	6.49%	32.17554993592641	0.0021810969700075335	0.0002790666132128672	4.1729950260130046e−06
ENSG00000151353_675603_669580_112	TMEM18	2	−	336	19	0	0	8.93%	33.181035871424115	0.0021880057261679567	0.0021880057261679567	2.7877966332435854e−05
ENSG00000111666_101697862_101726425_398	CHPT1	12	+	1194	34	3	0	4.25%	16.7089902933593	0.0042525335065667445	0.0006047916691041674	1.6677393905388735e−05
ENSG00000127990_94618900_94585465_278	SGCE	7	−	834	11	0	2	2.52%	7.739310146458635	0.007029302661868638	0.03522780890193623	0.0012822660609675817
ENSG00000109113_28717266_28714643_251	RAB34	17	−	753	70	6	3	7.57%	54.54798096707172	0.001172763320596733	0.000296469654954391	2.322409964038813e−06
ENSG00000160633_5623206_5668291_917	SAFB	19	+	2751	38	1	6	2.18%	8.105291205233371	5.012229235663673e−05	0.0007133092711558117	2.782141960148045e−07
ENSG00000104853_44973208_44987606_304	CLPTM1	19	+	912	94	10	10	8.22%	60.47956122824672	4.9891998955910695e−05	8.145867266848751e−05	3.020476034088439e−08
ENSG00000182199_57229779_57232472_213	SHMT2	12	+	639	33	7	1	5.16%	30.303214376274656	0.02164874885523164	0.001988634903193983	0.00026474119742627447
ENSG00000182199_57230833_57232472_192	SHMT2	12	+	576	25	5	1	4.17%	25.46790033990886	0.04534666772420983	0.0073129010928764775	0.0017352734887223107
ENSG00000188603_28491759_28482512_316	CLN3	16	−	948	20	2	0	2.22%	12.379333836107598	0.024275373211574098	0.00877616296920843	0.0010556658063702027
ENSG00000064601_45891569_45898450_480	CTSA	20	+	1440	106	2	5	5.42%	43.19355897648543	5.3770654556743774e−06	2.653005728463427e−05	1.1847577666367706e−09
ENSG00000088205_117814793_117830724_665	DDX18	2	+	1995	19	2	0	1.35%	5.58838498887143	0.01288881088065249	0.002943990679779778	0.00021264859682766199
ENSG00000115091_113890280_113957455_418	ACTR3	2	+	1254	65	1	0	6.46%	30.41525322890551	2.3299103948717173e−06	2.3543487063079366e−06	4.7432628183233485e−11
ENSG00000017260_130934676_131001350_823	ATP2C1	3	+	2469	17	3	1	1.22%	4.040205429378494	0.011168917508652896	0.00324779312858951	0.00020011016427610653
ENSG00000165792_20990003_20996883_478	METTL17	14	+	1434	32	2	0	2.51%	13.094123823297075	0.0026957942880510015	0.0010405263639310788	1.7035015215455722e−05
ENSG00000101310_18510836_18560740_749	SEC23B	20	+	2247	40	2	3	2.54%	10.44557941845127	0.00020862166978758353	0.0002822082734856477	4.03744396945675e−07
ENSG00000101310_18510836_18560740_767	SEC23B	20	+	2301	42	2	3	2.74%	10.710464059505869	9.993194084537427e−05	0.0001424141019315285	1.0194420312747164e−07
ENSG00000143549_154173155_154157639_144	TPM3	1	−	432	59	0	0	6.25%	80.13899306957988	0.0037896409716948643	0.0037896409716948643	7.95824840892005e−05
ENSG00000103642_63127461_63141805_306	LACTB	15	+	918	7	0	0	1.61%	4.474360530305557	0.019216965118390855	0.019216965118390855	0.0017073955890589052
ENSG00000103769_65869586_65887840_216	RAB11A	15	+	648	56	5	1	7.41%	50.709419343462976	0.0006684769570812473	0.00021835964213404212	9.916065630922874e−07
ENSG00000165516_49768598_49782410_264	KLHDC2	14	+	792	16	0	0	1.89%	11.854149976393941	0.02108409854857798	0.02108409854857798	0.002029891263157455
ENSG00000130811_10119859_10117157_149	EIF3G	19	−	447	18	2	0	4.70%	23.62874189925504	0.018067938367053574	0.008674846900444944	0.0007780168790447124
ENSG00000140157_22860765_22866847_219	NIPA2	15	+	657	32	2	0	4.57%	28.57986843623745	0.0062201494882096935	0.002460156992221257	8.636895811385565e−05
ENSG00000132471_75849694_75848541_70	WBP2	17	−	210	25	0	0	10.00%	69.85481236089288	0.008574016250729465	0.008574016250729465	0.0003747459614100891
ENSG00000104835_38930724_38915606_516	SARS2	19	−	1548	11	2	2	1.16%	4.169628334719962	0.060667625179241055	0.060667625179241055	0.014229868458155282
ENSG00000271723_54716624_54741513_342	MROH7-TTC4	1	+	1026	18	0	0	1.46%	10.294393400552634	0.020613416668581838	0.020613416668581838	0.001946208561389314
ENSG00000079785_15599686_15630906_648	DDX1	2	+	1944	53	3	1	4.01%	15.997614435735343	1.0400968689976693e−05	4.5072028892119814e−06	3.904511913824903e−10

ENSG00000161533_75978547_75946748_575	ACOX1	17	−	1725	15	0	0	1.39%	5.102438468100001	0.004873903550586949	0.004873903550586949	0.00012846764509679302
ENSG00000041357_78542509_78548807_150	PSMA4	15	+	450	238	5	3	13.33%	310.3416463819934	7.43787284094001e−05	7.14178335602628e−05	3.9058701722310284e−08
ENSG00000140455_63537128_63590826_435	USP3	15	+	1305	9	3	0	1.15%	4.046761543665518	0.1191346346731873	0.019216965118390855	0.010787784428637617
ENSG00000003436_187503756_187466936_300	TFPI	2	−	900	67	4	1	7.00%	43.682542663011674	7.887251914198805e−05	5.0604056647534704e−05	2.9659774681979362e−08
ENSG00000106682_74174384_74189767_113	EIF4H	7	+	339	18	0	4	5.31%	31.15648268131859	0.013428347753762199	0.03577298629450316	0.002259558529646323
ENSG00000114978_74178674_74165180_148	MOB1A	2	−	444	50	4	0	11.49%	66.07887655760136	0.0008567568071624047	0.00013106574077567133	7.996804468211807e−07
ENSG00000112473_33203026_33203813_117	SLC39A7	6	+	351	10	0	0	5.13%	16.71739099235043	0.012798402692974279	0.012798402692974279	0.0007972436888943567
ENSG00000013306_44321196_44320001_175	SLC25A39	17	−	525	24	3	1	7.43%	26.82424794658286	0.0038576334840820354	0.0010499093600357017	2.466191925958547e−05
ENSG00000013306_44323562_44321339_195	SLC25A39	17	−	585	11	1	0	3.08%	11.033478054951285	0.023672501042627626	0.012798402692974279	0.0014375227604752335
ENSG00000115204_27322517_27312392_158	MPV17	2	−	474	19	1	3	5.70%	23.520734288604437	0.005642787686764809	0.019194073369826602	0.000565339831017376
ENSG00000124198_48921914_49033199_1777	ARFGEF2	20	+	5331	12	2	0	0.51%	1.3208338184164325	0.012769128155103294	0.002874854311667729	0.00020627884060803262
ENSG00000121039_73295290_73323036_341	RDH10	8	+	1023	19	0	0	2.35%	10.898170139587979	0.005405141725979036	0.005405141725979036	0.00015637213408961292
ENSG00000117399_43359342_43363129_457	CDC20	1	+	1371	55	7	0	4.81%	23.539696069097378	0.00012095671017772633	1.63781969658517e−05	1.5788685795417643e−08
ENSG00000240344_200882366_200871395_112	PPIL3	2	−	336	12	0	0	4.46%	20.956443708267862	0.019679754444124863	0.019679754444124863	0.0017849636115922674
ENSG00000167658_3982268_3980752_156	EEF2	19	−	468	35	8	0	8.97%	43.88315135491988	0.01174944843020141	0.00040804699845214287	3.6142219509680676e−05
ENSG00000166598_103930516_103931720_53	HSP90B1	12	+	159	85	4	1	28.30%	313.6876479602359	0.0003642552343596574	0.0003168812800139256	7.735929406460506e−07
ENSG00000117335_207752282_207793591_332	CD46	1	+	996	39	2	0	6.63%	22.97634189701657	7.639996631620973e−05	1.3529991062581404e−05	8.254540628586197e−09
ENSG00000117632_25901583_25901016_54	STMN1	1	−	162	142	4	8	37.04%	514.3383961979816	4.2374374148069485e−05	7.457932414905961e−05	2.3672592729706925e−08
ENSG00000117632_25904676_25903488_112	STMN1	1	−	336	92	3	10	9.82%	160.6660684300536	0.0029617710283038326	0.00600714992788092	9.882409241108066e−05
ENSG00000125970_34072075_34078549_290	RALY	20	+	870	32	2	3	4.14%	21.582728232882765	0.0019464877837825903	0.005604866691947602	6.291068593467061e−05
ENSG00000198431_104311326_104348415_497	TXNRD1	12	+	1491	246	16	13	11.47%	96.81286670861773	4.301115028239964e−09	3.5953447909585565e−09	1.649442573520257e−16
ENSG00000049449_32091197_32104472_331	RCN1	11	+	993	13	0	1	1.51%	7.681918942406346	0.019679754444124863	0.022004978195124387	0.0019818329449194675
ENSG00000009830_77306345_77279680_534	POMT2	14	−	1602	16	0	0	1.50%	5.860478640014983	0.005306578019052857	0.005306578019052857	0.00015100114740534233
ENSG00000086504_370218_368972_175	MRPL28	16	−	525	25	0	0	6.86%	27.94192494435715	0.000991050613121974	0.000991050613121974	6.098881496726224e−06
ENSG00000086504_368584_367675_92	MRPL28	16	−	276	17	0	0	6.52%	36.142272482375006	0.012798402692974279	0.012798402692974279	0.0007972436888943567
ENSG00000132522_7314514_7312756_268	GPS2	17	−	804	9	5	2	2.61%	6.568437580203359	0.07524961812757737	0.0350911293701988	0.01080828922997042
ENSG00000077782_38424619_38413628_547	FGFR1	8	−	1641	10	0	0	1.65%	3.57574907880256	0.001946208561389314	0.001946208561389314	2.2278545302028032e−05
ENSG00000122545_35801267_35904293_418	SEPT7	7	+	1254	26	10	3	1.67%	12.166101291562203	0.10581804224798252	0.024135905425855194	0.011311393888965189
ENSG00000188603_28491759_28477516_339	CLN3	16	−	1017	14	9	0	2.06%	8.077606621082598	0.18936827384967286	0.008175610787902942	0.010166014512272396
ENSG00000130255_5690508_5690834_108	RPL36	19	+	324	21	8	2	5.56%	38.032064507597234	0.044040755831095145	0.013640585738808999	0.00282905808020558
ENSG00000127603_39284144_39485794_5094	MACF1	1	+	15282	26	1	9	0.27%	0.9983176953029056	0.0005610828647514753	0.013230226475135486	5.373688940890212e−05
ENSG00000184117_29581082_29564352_128	NIPSNAP1	22	−	384	21	0	1	7.03%	32.08955442828516	0.0033278027414746945	0.006142079104304129	0.00011243016581512157
ENSG00000166479_68697988_68676933_309	TMX3	18	−	927	19	6	0	2.59%	12.026783228477349	0.04099061537131024	0.005160369442893307	0.0011691093874143326
ENSG00000166946_43190120_43192884_201	CCNDBP1	15	+	603	22	0	0	4.98%	21.408241002144283	0.002046028879997488	0.002046028879997488	2.4518070358754455e−05
ENSG00000104408_108241841_108233625_118	EIF3E	8	−	354	32	4	0	8.47%	53.04229819945764	0.004799398451110185	0.0023766146074493525	6.466011935132129e−05
ENSG00000115963_150486853_150469987_218	RND3	2	−	654	50	5	0	6.88%	44.860888672133036	0.0009630468020554216	0.00030182209906671564	1.933778166896056e−06
ENSG00000166938_66308770_66333312_893	DIS3L	15	+	2679	21	6	0	1.01%	4.599622583225645	0.03769480377820191	0.0036062891729786097	0.0007964638302212087
ENSG00000167645_38309608_38305352_283	YIF1B	19	−	849	66	0	0	3.18%	45.61543930845584	0.003684287313187465	0.003684287313187465	7.541963121699725e−05
ENSG00000127554_1984297_1984947_126	GFER	16	+	378	10	1	1	3.97%	15.523291635753973	0.03537493353818881	0.020613416668581838	0.003252526348827943
ENSG00000147140_71294272_71300076_340	NONO	X	+	1020	35	1	3	4.12%	20.134622386375003	0.0006748684829181083	0.003181324321652496	1.3599137464255597e−05
ENSG00000111669_6869106_6870383_167	TPI1	12	+	501	222	8	3	9.58%	260.0104872067725	0.0006164363470607238	0.0002208197074346614	9.236265813608372e−07
ENSG00000100813_23090621_23059148_1198	ACIN1	14	−	3594	45	11	5	1.42%	7.3470002983910705	0.006607599257270876	0.0011210739524526398	4.554783231696604e−05
ENSG00000089737_94079613_94051191_816	DDX24	14	−	2448	30	1	0	1.47%	7.190936566562502	0.0015356410015604012	0.0009235399941089087	8.727894005676686e−06
ENSG00000165533_88824720_88841526_182	TTC8	14	+	546	11	0	1	3.85%	11.821583630304948	0.0078820576498062	0.015516259750072458	0.0006142571802709984
ENSG00000109323_102760894_102632057_879	MANBA	4	−	2637	8	0	7	0.57%	1.7801453889465306	0.019216965118390855	0.19558626140506974	0.01921731642938781
ENSG00000127990_94656098_94585499_500	SGCE	7	−	1500	26	3	2	2.40%	10.170860679746003	0.005454749182134642	0.0026311212314026142	8.05834708867572e−05
ENSG00000120889_23027796_23022671_205	TNFRSF10B	8	−	615	63	1	3	5.85%	60.1092141485927	0.0013935654826858935	0.0033156700022129483	2.7404117726375605e−05
ENSG00000169045_179618370_179616986_230	HNRNPH1	5	−	690	118	11	2	10.43%	100.34795653930003	8.904143521645122e−05	1.6162535005161726e−05	1.138969783715916e−08
ENSG00000042753_46846085_46838447_84	AP2S1	19	−	252	32	7	1	9.52%	74.51179985161906	0.040565318244474055	0.008413704741378425	0.0017299445475186467
ENSG00000138760_76179654_76179445_69	SCARB2	4	−	207	12	0	0	8.70%	34.01625645400001	0.013007239811447113	0.013007239811447113	0.0008218654148058079
ENSG00000138760_76213543_76180849_175	SCARB2	4	−	525	13	0	1	4.57%	14.529800971065718	0.005160369442893307	0.00912793029372963	0.00024819240235076056
ENSG00000115380_55922416_55867073_485	EFEMP1	2	−	1455	33	0	0	3.30%	13.30842198380722	0.00017703063443817632	0.00017703063443817632	2.1885094948977214e−07
ENSG00000198721_4133671_4115874_364	ECI2	6	−	1092	37	0	0	2.75%	19.88175428733105	0.0023766146074493525	0.0023766146074493525	3.265611492547055e−05
ENSG00000075415_98597967_98601528_231	SLC25A3	12	+	693	265	17	5	17.32%	224.38212455317105	1.2921920484917946e−06	9.86350542070242e−08	1.2557905723264676e−12
ENSG00000026508_35139304_35229333_742	CD44	11	+	2226	377	23	29	9.84%	99.37835569832684	2.517110752411017e−12	2.3442938558482117e−11	7.464461876359691e−22
ENSG00000132471_75855297_75846734_257	WBP2	17	−	771	34	0	1	5.06%	25.87617952045526	0.0006629342244537616	0.0009306875619951529	3.907239258273379e−06
ENSG00000243725_54715948_54741513_374	TTC4	1	+	1122	20	0	0	1.60%	10.459544096818185	0.013217226988443315	0.013217226988443315	0.0008469697265466234
ENSG00000243725_54716624_54741513_342	TTC4	1	+	1026	18	0	0	1.46%	10.294393400552634	0.020613416668581838	0.020613416668581838	0.001946208561389314
ENSG00000148444_22318624_22319998_88	COMMD3	10	+	264	9	4	1	6.82%	20.00387808516478	0.20870646171529367	0.026832695913080365	0.026324576754666044
ENSG00000148444_22316454_22318358_94	COMMD3	10	+	282	16	0	0	8.51%	33.29250631668086	0.005306578019052857	0.005306578019052857	0.00015100114740534233
ENSG00000039560_34687711_34830765_972	RAI14	5	+	2916	30	2	1	1.34%	6.036835636126544	0.003031355522137688	0.0010322727522116193	1.9025352038280622e−05
ENSG00000179010_6640863_6641246_127	MRFAP1	4	+	381	76	1	3	14.96%	117.04806354644097	5.6254241454829154e−05	0.0001733650811203729	7.181689729777536e−08
ENSG00000145982_5369021_5771429_301	FARS2	6	+	903	10	0	0	1.66%	6.498122080083058	0.019679754444124863	0.019679754444124863	0.0017849636115922674
ENSG00000102316_54809755_54815682_580	MAGED2	X	+	1740	26	0	0	2.07%	8.767983344608623	0.0008739357725210369	0.0008739357725210369	4.787965623198429e−06
ENSG00000107164_130579720_130637022_559	FUBP3	9	+	1677	41	3	0	3.22%	14.345854130644906	0.0005788381316534018	8.51685422527664e−05	3.60264644921115e−07
ENSG00000134333_18396843_18407376_274	LDHA	11	+	822	283	14	1	12.04%	202.0180777911369	2.4520818402030644e−07	2.4572580183954076e−07	5.667355864592674e−13
ENSG00000069424_6082210_6098574_295	KCNAB2	1	+	885	8	0	0	1.69%	5.304229819945764	0.019679754444124863	0.019679754444124863	0.0017849636115922674

ENSG00000114786_51986441_51989075_254	ABHD14A-ACY1	3	+	762	7	1	0	1.97%	5.390371347533466	0.0477903522728147	0.016947426762344633	0.0036975118224100542
ENSG00000076242_37014478_37050653_515	MLH1	3	+	1545	17	0	3	1.75%	6.456483627919419	0.0033030736763512156	0.021725767126240147	0.00040633255960778673
ENSG00000114023_122401532_122409831_144	FAM162A	3	+	432	26	3	1	8.33%	35.31548847134029	0.004488024406907798	0.0014539914977685167	3.860242507624909e-05
ENSG00000105341_41439899_41432231_230	DMAC2	19	-	690	22	7	2	3.48%	18.708941049700005	0.054588706672672294	0.018067938367053574	0.004469432019981973
ENSG00000183020_926049_1003733_647	AP2A2	11	+	1941	20	0	5	1.70%	6.046166139428132	0.0014409783181973444	0.018402374702323095	0.0001694437880775777
ENSG00000065809_14530459_14521190_131	FAM107B	10	-	393	21	0	0	6.11%	31.35467913603436	0.005604866691947602	0.005604866691947602	0.00016752456572620382
ENSG00000117305_23820578_23802463_300	HMGCL	1	-	900	18	0	0	2.67%	11.735608476630002	0.005257622967929454	0.005257622967929454	0.00014836615564656991
ENSG00000285043_30067255_30070212_368	AC093512.2	16	+	1104	713	70	30	29.08%	378.96235705784386	2.8434265653329284e-16	5.0305764789834974e-18	2.2821492036410582e-32
ENSG00000142676_23692636_23696373_167	RPL11	1	+	501	276	8	3	26.35%	323.2562813922037	3.498557337707287e-09	4.950634444904532e-09	1.8457137986707628e-16
ENSG00000174891_158122105_158544275_334	RSRC1	3	+	1002	24	3	1	1.50%	14.054620930095812	0.13601972394786777	0.04223451612662591	0.022931112790783643
ENSG00000258315_7014858_7017265_172	C17orf49	17	+	516	47	1	0	11.05%	53.447054108683155	0.0001224181422515567	4.950915307313248e-05	4.492436522279354e-08
ENSG00000186132_119339959_119302472_126	C2orf76	2	-	378	10	0	0	3.97%	15.523291635753973	0.020613416668581838	0.020613416668581838	0.001946208561389314
ENSG00000120948_11016999_11023248_173	TARDBP	1	+	519	12	0	0	3.47%	13.56717742963006	0.013007239811447113	0.013007239811447113	0.0008218654148058079
ENSG00000136997_127738590_127740958_330	MYC	8	+	990	74	7	1	8.18%	43.860354912657584	2.370806319628599e-05	3.009127783884032e-06	6.133872979550775e-10
ENSG00000161960_7574582_7575402_126	EIF4A1	17	+	378	28	2	4	4.76%	43.46521658011112	0.02034245439037237	0.037440157020026926	0.003397262391305248
ENSG00000178921_8263181_8269264_844	PFAS	17	+	2532	17	0	0	1.07%	3.939678990969788	0.0033278027414746945	0.0033278027414746945	6.211806171561056e-05
ENSG00000259112_78079744_78017236_114	NDUFC2-KCTD14	11	-	342	173	4	15	23.68%	296.82167638260097	2.8741749484516152e-06	1.4831766004695903e-05	3.638568363463898e-10
ENSG00000102471_79533424_79551120_120	NDFIP2	13	+	360	13	0	0	4.17%	21.18929308280417	0.02108409854857798	0.02108409854857798	0.002029891263157455
ENSG00000087460_58895650_58910829_321	GNAS	20	+	963	302	38	15	19.63%	184.01629075504988	1.6503038806255647e-08	3.509861194884339e-11	8.183374169022172e-18
ENSG00000173744_227491587_227554495_453	AGFG1	2	+	1359	17	0	0	1.99%	7.340152468826712	0.0032294771334464936	0.0032294771334464936	5.8663648728941325e-05
ENSG00000108384_58692671_58734222_367	RAD51C	17	+	1101	24	0	1	3.81%	12.790853925482292	0.00037518914374882155	0.0006738559393031653	1.6596377231462156e-06
ENSG00000182093_39390698_39391878_91	WRB	21	+	273	13	0	0	5.49%	27.94192494435715	0.020613416668581838	0.020613416668581838	0.001946208561389314
ENSG00000175376_66000092_65998599_113	EIF1AD	11	-	339	10	0	0	4.42%	17.309157045176995	0.019679754444124863	0.019679754444124863	0.0017849636115922674
ENSG00000138600_50739713_50731030_80	SPPL2A	15	-	240	15	0	0	6.25%	36.67377648946876	0.021557223391537678	0.021557223391537678	0.002115616449879074
ENSG00000131051_35740847_35704481_499	RBM39	20	-	1497	29	1	0	2.61%	11.367155839087177	0.0009827567920758283	0.0006311913977015671	3.933582706340778e-06
ENSG00000137547_54135284_54147719_296	MRPL15	8	+	888	87	1	3	8.11%	57.488622605113186	1.000743978692383e-05	3.7429563963467286e-05	3.014290871387931e-09
ENSG00000148303_133348244_133350343_172	RPL7A	9	+	516	78	3	6	4.65%	88.69936639313374	0.01253092217194181	0.02307007353645424	0.0013754485836632393
ENSG00000083857_186597212_186588592_479	FAT1	4	-	1437	27	0	1	1.25%	11.02510190915136	0.013853924679039932	0.017143983986795872	0.0011315679034796663
ENSG00000181991_88477878_88477979_33	MRPS11	15	+	99	38	0	0	15.15%	225.2288495514849	0.019679754444124863	0.019679754444124863	0.0017849636115922674
ENSG00000101474_24992688_24963813_416	APMAP	20	-	1248	103	5	7	7.69%	48.42819203096516	2.71239688701378e-06	8.050877969159443e-06	1.8586076734647386e-10
ENSG00000258289_64924066_64930898_76	CHURC1	14	+	228	9	1	0	6.58%	23.162385151243427	0.06539853090342929	0.019679754444124863	0.005779266745814885
ENSG00000185627_251546_252600_97	PSMD13	11	+	291	14	1	1	5.15%	28.229986026257738	0.03577298629450316	0.03577298629450316	0.005410795288624905
ENSG00000114867_184320681_184334908_1403	EIF4G1	3	+	4209	90	7	5	2.78%	12.546979839590167	4.60942195086692e-07	1.9011841143914044e-07	8.246422000810537e-13
ENSG00000158710_159920509_159919270_153	TAGLN2	1	-	459	227	17	22	20.92%	290.19424010838895	1.2821074267650269e-05	2.158009678398047e-05	2.202183689450467e-09
ENSG00000158710_159918868_159918800_22	TAGLN2	1	-	66	8	4	0	22.73%	71.12489985836365	0.169362073879829	0.019216965118390855	0.016163991620339734
ENSG00000122705_36191057_36211774_196	CLTA	9	+	588	133	9	2	9.69%	132.72414348569646	0.0005036416536062891	0.00011939345589185465	4.2566513729672767e-07
ENSG00000153956_82349627_81950392_1064	CACNA2D1	7	-	3192	24	0	1	1.22%	4.411882885951129	0.0004762745294806779	0.001038231149674533	3.1862098168137684e-06
ENSG00000131508_139561792_139626801_147	UBE2D2	5	+	441	38	6	1	5.44%	50.56157847074151	0.03274340904245237	0.007356796211402075	0.0012336742649452252
ENSG00000106976_128203486_128253122_846	DNM1	9	+	2538	53	2	7	2.25%	12.253491908222816	7.448194265828549e-05	0.001199678750155866	6.877941490576349e-07
ENSG00000122257_24540726_24572445_1725	RBBP6	16	+	5175	21	3	0	0.52%	2.3811379517800004	0.01157547599262317	0.0036062891729786097	0.0002277087468023593
ENSG00000151092_25783390_25719460_636	NGLY1	3	-	1908	15	0	3	1.10%	4.613053646474057	0.0078820576498062	0.05158844004651438	0.0021213996074552345
ENSG00000131016_151325861_151353740_1684	AKAP12	6	+	5052	52	10	0	1.43%	6.039703491535631	0.00037787392972408145	6.136114477444219e-06	2.2016777792351534e-08
ENSG00000103145_3023324_3022863_75	HCFC1R1	16	-	225	16	0	0	6.67%	41.72660791690667	0.02108409854857798	0.02108409854857798	0.002029891263157455
ENSG00000169398_140846306_140659466_886	PTK2	8	-	2658	15	6	4	1.13%	3.311401940358353	0.0820328997370387	0.023029368248455667	0.00832076987426179
ENSG00000140264_43792377_43793087_39	SERF2	15	+	117	50	0	0	17.95%	250.76086488525647	0.00847367416878415	0.00847367416878415	0.0003665024264111182
ENSG00000042753_46850766_46838447_92	AP2S1	19	-	276	37	6	0	10.87%	78.66259304987501	0.016168729333647557	0.002446120450917513	0.00022986057861055823
ENSG00000011422_43656578_43648890_120	PLAUR	19	-	360	10	0	0	4.17%	16.29945621754167	0.020613416668581838	0.020613416668581838	0.001946208561389314
ENSG00000114126_142005443_141952513_385	TFDP2	3	-	1155	9	0	0	1.30%	4.572314990894807	0.019679754444124863	0.019679754444124863	0.0017849636115922674
ENSG00000116337_109625404_109629824_564	AMPD2	1	+	1692	7	0	1	0.89%	2.427578585591313	0.019216965118390855	0.04178282491678763	0.003600525600033436
ENSG00000036054_100261019_100323668_699	TBC1D23	3	+	2097	9	5	0	0.72%	2.5183709177317604	0.2612156424807822	0.019216965118390855	0.027675094417377224
ENSG00000204525_31271857_31269169_338	HLA-C	6	-	1014	18	2	4	1.78%	10.416220541387576	0.03535057243299149	0.0865972937396412	0.01250960075939979
ENSG00000153113_96722678_96771679_708	CAST	5	+	2124	118	0	4	5.93%	32.59891243508334	5.853191170022466e-09	6.665252736283171e-08	4.1552551561798035e-15
ENSG00000198121_110941739_110875421_206	LPAR1	9	-	618	14	0	0	3.88%	13.292760410422334	0.005160369442893307	0.005160369442893307	0.00014319601891785326
ENSG00000198121_110941739_110875421_206	LPAR1	9	-	618	14	0	0	3.88%	13.292760410422334	0.005160369442893307	0.005160369442893307	0.00014319601891785326
ENSG00000161671_50476593_50482259_246	EMC10	19	+	738	60	2	3	7.32%	47.70572551475611	0.00027730861539238227	0.0001864894855384259	3.564784065792814e-07
ENSG00000115457_216633740_216660891_186	IGFBP2	2	+	558	13	2	0	3.76%	13.670511666325272	0.028902805874428627	0.0078820576498062	0.0011492025149225664
ENSG00000115457_216663946_216664104_52	IGFBP2	2	+	156	11	0	0	9.62%	41.375542706067314	0.016947426762344633	0.016947426762344633	0.0013498980316300959
ENSG00000143612_154220359_154212316_139	C1orf43	1	-	417	25	2	0	5.76%	35.17868248390288	0.013141913862703638	0.005604866691947602	0.00038344919015260396
ENSG00000143612_154219886_154212316_150	C1orf43	1	-	450	25	1	0	5.33%	32.59891243508334	0.008364861494636245	0.005604866691947602	0.0002459697071 1672695
ENSG00000143612_154219886_154207453_196	C1orf43	1	-	588	36	1	0	5.61%	35.92533207131633	0.001988634903193983	0.0016033688297201391	1.8909273089207902e-05
ENSG00000124596_41071198_41067335_113	OARD1	6	-	339	24	1	0	4.42%	41.541976908424786	0.03537493353818881	0.020613416668581838	0.003252526348827943
ENSG00000111859_11191130_11185162_588	NEDD9	6	-	1764	20	5	0	1.87%	6.652839272465988	0.018103472727687364	0.0013845019718837994	0.00016097545794732337
ENSG00000111859_11213721_11200323_179	NEDD9	6	-	537	15	0	0	3.35%	16.39051463216481	0.013428347753762199	0.013428347753762199	0.0008725593497644525
ENSG00000128590_108571877_108573353_173	DNAJB9	7	+	519	26	0	0	2.89%	29.395551097531797	0.02108409854857798	0.02108409854857798	0.002029891263157455
ENSG00000162813_220079777_220058844_285	BPNT1	1	-	855	27	1	0	4.56%	18.52990812099474	0.000596156229600823	0.0006289630342484525	2.4127015035650886e-06
ENSG00000059377_139953368_140020099_383	TBXAS1	7	+	1149	11	0	0	1.31%	5.617567155915145	0.016947426762344633	0.016947426762344633	0.0013498980316300959
ENSG00000159377_151399588_151400598_167	PSMB4	1	+	501	57	2	3	11.98%	66.75944941795511	0.00010455425536429166	0.00019715754743768	1.466806924630606e-07

ENSG00000256269_119088273_119093283_344	HMBS	11	+	1032	27	0	0	2.91%	15.351813414196224	0.0023354528714849923	0.0023354528714849923	3.158285405866755e-05
ENSG00000086666_80120378_80131349_166	ZFAND6	15	+	498	16	0	3	5.42%	18.852383094987957	0.0033278027414746945	0.028023386503852714	0.0005380123252020438
ENSG00000156831_125151194_125366885_187	NSMCE2	8	+	561	26	0	0	5.35%	27.194814651727278	0.0022409900782254734	0.0022409900782254734	2.9184102569313086e-05
ENSG00000157193_53327912_53247018_708	LRP8	1	-	2124	11	9	0	0.85%	3.0388816676772605	0.3362636552170612	0.012798402692974279	0.03023589517231469
ENSG00000168216_69790322_69676158_467	LMBRD1	6	-	1401	21	3	0	2.14%	8.795423911821201	0.01424822060979566	0.0022409900782254734	0.00018649561876446586
ENSG00000137161_42929580_42938791_275	CNPY3	6	+	825	29	1	0	5.09%	20.626220958925458	0.0006629387121614735	0.0004093002068662083	1.773333733167258e-06
ENSG00000172586_73782076_73782492_97	CHCHD1	10	+	291	27	0	0	12.37%	54.443544479211354	0.000978917897162665	0.000978917897162665	5.956062365741233e-06
ENSG00000111605_69257749_69262559_409	CPSF6	12	+	1227	17	0	6	2.93%	8.129802123174818	0.0007660506977098094	0.009775134546442767	4.9814609330314166e-05
ENSG00000084072_39745420_39753355_158	PPIE	1	+	474	17	0	0	5.06%	21.044867521382915	0.005306578019052857	0.005306578019052857	0.00015100114740534233
ENSG00000166913_44901540_44906438_244	YWHAB	20	+	732	197	7	8	16.80%	157.9176823699529	7.746735104569852e-08	2.40713092109698e-07	1.8084316484735223e-13
ENSG00000186480_155298286_155302829_164	INSIG1	7	+	492	30	0	3	4.88%	35.77929413606708	0.005706018193000826	0.017782189529096822	0.0005281168650189082
ENSG00000249915_306604_311525_129	PDCD6	5	+	387	41	0	1	10.85%	62.16536789946125	0.0004388494700883803	0.00064294891577167	1.8386451893262699e-06
ENSG00000184840_177592643_177593793_58	TMED9	5	+	174	30	0	0	10.34%	101.16903859163796	0.013007239811447113	0.013007239811447113	0.0008218654148058079
ENSG00000184840_177592278_177592658_126	TMED9	5	+	378	67	1	25	13.49%	104.00605395955161	0.0001369908430211749	0.020592432210695392	2.951590432224428e-05
ENSG00000103544_19569589_19616940_357	VPS35L	16	+	1071	15	0	0	2.24%	8.218213218928573	0.004193160480899131	0.004193160480899131	9.649553577281792e-05
ENSG00000134313_8796836_8757706_686	KIDINS220	2	-	2058	7	0	0	1.02%	1.9958517817397963	0.00407548579675135	0.00407548579675135	9.14053164909177e-05
ENSG00000125691_38853710_38852960_52	RPL23	17	-	156	207	7	13	23.08%	778.6124854687213	0.0015963204830409697	0.0037262067103050298	3.48797083768116e-05
ENSG00000070087_149970856_149968490_55	PFN2	3	-	165	52	4	0	14.55%	184.9247396317455	0.0204156028385188	0.005808022449631236	0.0006175306982988044
ENSG00000070087_149968427_149966489_55	PFN2	3	-	165	37	0	1	18.18%	131.58106473797275	0.0022409900782254734	0.002019278015471638	2.6421490772652897e-05
ENSG00000117632_25904676_25901016_149	STMN1	1	-	447	233	7	18	23.49%	305.86093680702356	2.043479731736036e-07	1.4662072863005735e-06	2.8395761137540576e-12
ENSG00000100804_23033563_23026089_160	PSMB5	14	-	480	119	6	1	18.75%	145.4726467415594	2.185196255107408e-06	7.370612650537491e-07	1.4435583206191696e-11
ENSG00000136003_108564132_108568916_137	ISCU	12	+	411	47	1	2	5.84%	67.10141099776278	0.006346985027636522	0.005808022449631236	0.00019501864856894435
ENSG00000162909_223771817_223772469_75	CAPN2	1	+	225	14	0	0	6.67%	36.510781927293344	0.020613416668581838	0.020613416668581838	0.001946208561389314
ENSG00000004897_47189172_47120935_830	CDC27	17	-	2490	13	0	2	0.96%	3.063512252935543	0.004826937407846361	0.026606355080852302	0.0006944327269402791
ENSG00000137673_102530622_102527429_166	MMP7	11	-	498	12	0	0	4.22%	14.139287321240968	0.008175610787902942	0.008175610787902942	0.00034251843268961324
ENSG00000120805_101407645_101395640_135	ARL1	12	-	405	16	0	2	5.93%	23.18144884272593	0.005257622967929454	0.01611115222852496	0.000444576531620782
ENSG00000116171_52927457_53050704_527	SCP2	1	+	1581	33	1	0	2.28%	12.247788732725809	0.0014539914977685167	0.000978917897162665	8.739992886708085e-06
ENSG00000020256_52160306_52152146_488	ZFP64	20	-	1464	13	0	0	1.02%	5.210481905607583	0.016947426762344633	0.016947426762344633	0.0013498980316300959
ENSG00000104388_60591850_60620769_94	RAB2A	8	+	282	15	0	1	7.45%	31.211724671888305	0.0078820576498062	0.015516259750072458	0.0006142571802709984
ENSG00000140374_76311388_76216559_284	ETFA	15	-	852	174	2	1	12.32%	119.83543866981341	1.1709106286284194e-07	1.1553659052538825e-07	1.3047542316592881e-13
ENSG00000100360_36767356_36758311_145	IFT27	22	-	435	22	0	0	6.90%	29.6762513202138	0.0022543491824521134	0.0022543491824521134	2.9517890247939808e-05
ENSG00000137868_74196092_74180080_560	STRA6	15	-	1680	53	2	9	3.39%	18.51152527563661	0.00021186981035795232	0.0026742133664407067	4.061495374133696e-06
ENSG00000092199_21231004_21211223_147	HNRNPC	14	-	441	19	2	9	4.76%	25.280789235370754	0.009775134546442773	0.06740875564089473	0.0033805363459885 05
ENSG00000101191_62911348_62879233_2152	DIDO1	20	-	6456	17	5	1	0.33%	1.545115738094099	0.06927320754946001	0.011825808327677989	0.004055849570567006
ENSG00000197563_62157141_62070368_793	PIGN	18	-	2379	12	0	0	0.76%	2.959800372416142	0.011772051206276392	0.011772051206276392	0.000681272511389508
ENSG00000101191_62911348_62890931_1101	DIDO1	20	-	3303	15	5	0	0.54%	2.6647612344754776	0.10122934795214716	0.013428347753762199	0.006821841432787573
ENSG00000283761_100080510_100082209_118	AC118553.2	1	+	354	13	0	0	7.63%	21.548433643529666	0.0025000845279814113	0.0025000845279814113	3.597708735589818e-05
ENSG00000283761_100058702_100078697_344	AC118553.2	1	+	1032	12	0	0	2.03%	6.823028184087211	0.007029302661868638	0.007029302661868638	0.0002573639808771639
ENSG00000087111_28571198_28554220_547	PIGS	17	-	1641	29	5	2	3.11%	10.369672328527425	0.0020816078341534825	0.0005793435147328683	7.677924913035978e-06
ENSG00000182054_90102390_90084266_312	IDH2	15	-	936	13	0	0	2.24%	8.149728108770836	0.00837382380159347	0.00837382380159347	0.0003583838894600799
ENSG00000067057_3067596_3136579_784	PFKP	10	+	2352	136	9	0	7.02%	33.92948028957654	5.0681472096644235e-09	1.667394506806035e-11	1.1715503230067004e-18
ENSG00000257390_55822114_55757512_236	AC023055.1	12	-	708	26	5	8	4.66%	21.548433643529666	0.013577461948370874	0.050785757073788386	0.0032647597294671645
ENSG00000197324_22875363_22879201_418	LRP10	14	+	1254	106	3	7	6.46%	49.6002591117536	8.566115769921487e-06	2.007159422424516e-05	1.3914120556780956e-09
ENSG00000093144_127331028_127314822_145	ECHDC1	6	-	435	11	0	1	4.14%	14.8381256601069	0.012798402692974279	0.019795881618345192	0.0012079910998532793
ENSG00000093144_127331028_127326987_125	ECHDC1	6	-	375	10	0	1	4.00%	15.647477968840004	0.019679754444124863	0.02784981298332479	0.002477909089056847
ENSG00000198742_99143780_99030584_731	SMURF1	7	-	2193	8	6	1	0.82%	2.140557861674419	0.2828598995720949	0.028889785561798602	0.04025112963270498
ENSG00000198730_10763817_10779105_796	CTR9	11	+	2388	13	0	0	0.88%	3.194365791377513	0.00797940233269714	0.00797940233269714	0.000327142761693177
ENSG00000198692_20582610_20592346_104	EIF1AY	Y	+	312	17	0	0	5.77%	31.9720102728702	0.013428347753762199	0.013428347753762199	0.0008725593497644525
ENSG00000049618_157206653_157207891_412	ARID1B	6	+	1236	12	0	0	1.21%	5.696897318752429	0.02108409854857798	0.02108409854857798	0.002029891263157455
ENSG00000182220_40591300_40605755_271	ATP6AP2	X	+	813	35	1	4	6.64%	25.261149857444654	0.00012186633930364116	0.0005279920463075931	4.5515488091230315e-07
ENSG00000148358_130086423_130135121_382	GPR107	9	+	1146	17	1	0	2.36%	8.704421644969898	0.004669667620227399	0.003229477133446493 6	8.365569901249867e-05
ENSG00000013364_29830550_29847989_893	MVP	16	+	2679	69	5	5	2.91%	15.113045630598547	3.5064313186673395e-05	2.413580229981598e-05	6.543708222377824e-09
ENSG00000196712_31182657_31249981_1085	NF1	17	+	3255	18	2	1	0.83%	3.244868703215669	0.015046251877931842	0.005588370640926919	0.00043792386055833824
ENSG00000166206_26621357_26547793_334	GABRB3	15	-	1002	27	0	1	2.99%	15.811448546357788	0.0024321290225875155	0.0031727927861210656	4.407497946231007e-05
ENSG00000174953_154324227_154276171_945	DHX36	3	-	2835	30	5	8	1.38%	6.209316654301588	0.0031949757085927527	0.01270978408730525	0.00022516029990253737
ENSG00000115107_119245509_119263338_484	STEAP3	2	+	1452	11	0	2	1.24%	4.445306241147728	0.011772051206276392	0.07134538375819317	0.0041729868335021346
ENSG00000103811_78935727_78922130_251	CTSH	15	-	753	17	0	0	2.39%	13.247366806288847	0.013428347753762199	0.013428347753762199	0.0008725593497644525
ENSG00000137449_15004186_15066380_522	CPEB2	4	+	1566	11	1	0	1.34%	4.121701572251917	0.016854695534768073	0.007029302661868638	0.0006017499134389055
ENSG00000072210_19648972_19673234_508	ALDH3A2	17	+	1524	83	11	1	4.92%	31.957201560376976	0.0003174237249665405	5.211096913985141e-06	1.576206940502593e-08
ENSG00000172053_49104733_49100736_264	QARS	3	-	792	20	2	1	3.41%	14.817687470492428	0.009257958062480545	0.005642787686764809	0.0002731628610857391
ENSG00000125746_45638587_45609663_617	EML2	19	-	1851	19	0	0	1.62%	6.023137791895463	0.0022543491824521134	0.0022543491824521134	2.9517890247939808e-05
ENSG00000105323_41276293_41292252_37	HNRNPUL1	19	+	111	12	0	0	13.51%	63.435721495297315	0.016947426762344633	0.016947426762344633	0.0013498980316300959
ENSG00000139197_7191313_7210223_549	PEX5	12	+	1647	13	0	0	1.28%	4.631539471651185	0.007029302661868638	0.007029302661868638	0.0002573639808771639
ENSG00000013810_1723785_1745013_765	TACC3	4	+	2295	33	0	2	2.35%	8.437365571433336	6.99138909308556e-05	0.00020753571005996044	1.0554092732354401e-07
ENSG00000197956_153535339_153534696_90	S100A6	1	-	270	2776	88	58	50.00%	6032.972061319424	3.2080755063846185e-09	2.6004311897614428e-09	8.97263545200942e-17
ENSG00000089159_120221730_120212314_316	PXN	12	-	948	21	0	0	3.48%	12.998300527912978	0.001092092816906976	0.001092092816906976	7.350803965414657e-06
ENSG00000124214_49153958_49114878_471	STAU1	20	-	1413	29	4	0	2.76%	12.042910326336521	0.005851237224165338	0.0005854768899833019	2.273373872391799e-05

ENSG00000198522_27631059_27650200_295	GPN1	2	+	885	23	0	0	2.71%	15.249660732344072	0.005706018193000826	0.005706018193000826	0.0001733096755673334
ENSG00000047579_15662869_15524661_208	DTNBP1	6	−	624	13	6	0	2.88%	12.224592163156252	0.1741008397065631	0.013428347753762199	0.012921179834843385
ENSG00000115211_27370314_27364400_523	EIF2B4	2	−	1569	60	0	0	3.82%	22.43902194384322	3.8568200545620204e-05	3.8568200545620204e-05	1.1316524295322383e-08
ENSG00000163029_17725304_17665499_865	SMC6	2	−	2595	14	0	0	0.92%	3.1656747335803477	0.005257622967929454	0.005257622967929454	0.0001483661556465699
ENSG00000185883_2514152_2519745_139	ATP6V0C	16	+	417	115	4	0	17.99%	161.82193942595327	1.2327270141267187e-05	5.8448710541996575e-06	5.934952599231161e-10
ENSG00000076043_114439655_114449975_167	REXO2	11	+	501	27	3	0	4.19%	31.622897092715576	0.033673325703933984	0.008574016250729465	0.0014461884879040322
ENSG00000164466_175492258_175522462_242	SFXN1	5	+	726	47	4	0	5.79%	37.987162424353315	0.001487949770246494	0.0004588781914451711	4.414390900776576e-06
ENSG00000166170_103561164_103559821_447	BAG5	14	−	1341	23	2	0	1.79%	10.064093771904924	0.015570605297898355	0.005554612275049112	0.00045086442503788926
ENSG00000091986_112639863_112605417_936	CCDC80	3	−	2808	11	0	0	0.85%	2.2986412614481844	0.004193160480899131	0.004193160480899131	9.649553577281792e-05
ENSG00000100401_41281044_41246603_587	RANGAP1	22	−	1761	32	0	2	2.21%	10.662676639754688	0.0006446704936422262	0.0015919262655107664	6.465887001051626e-06
ENSG00000152556_48131382_48145708_705	PFKM	12	+	2115	47	0	0	3.12%	13.039564974033336	9.836921317677237e-06	9.836921317677237e-06	7.867021274290272e-10
ENSG00000127955_80135161_80217493_354	GNAI1	7	+	1062	26	4	0	2.54%	14.365622429019778	0.015528142895806445	0.0036062891729786097	0.0003077713301577895
ENSG00000106049_27662788_27649455_89	HIBADH	7	−	267	10	0	0	5.62%	21.976794900056184	0.020613416668581838	0.020613416668581838	0.001946208561389314
ENSG00000101811_100820417_100838361_577	CSTF2	X	+	1731	23	0	1	1.21%	7.796620305097922	0.00877616296920843	0.012088529595134127	0.0005313814658942758
ENSG00000122882_73163910_73134583_635	ECD	10	−	1905	11	0	5	1.26%	3.388233418449607	0.004193160480899131	0.07650333324819984	0.0020236019016273386
ENSG00000110013_124660786_124660463_107	SIAE	11	−	321	9	0	0	5.61%	16.451787584060753	0.012178957859018494	0.012178957859018494	0.0007262454987071801
ENSG00000130733_10928410_10923291_316	YIPF2	19	−	948	12	0	0	1.58%	7.427600301664559	0.020613416668581838	0.020613416668581838	0.001946208561389314
ENSG00000130741_24054969_24076785_472	EIF2S3	X	+	1416	29	5	0	2.75%	12.017395685814622	0.005343426570972147	0.0006606329705481882	2.292517561526197e-05
ENSG00000087111_28560306_28556491_284	PIGS	17	−	852	8	0	2	1.76%	5.509675341140846	0.019679754444124863	0.10105102209468636	0.009164497669452725
ENSG00000148337_128179348_128166197_612	CIZ1	9	−	1836	21	2	0	1.63%	6.711540795458335	0.01044836432889887	0.002046028879997488	0.00012444751772694397
ENSG00000127955_80189190_80217493_267	GNAI1	7	+	801	23	4	0	3.00%	16.848876090043074	0.028023386503852714	0.005454749182134642	0.0008134260429016822
ENSG00000197965_167772389_167787921_145	MPZL1	1	+	435	30	0	1	8.28%	40.46761543665518	0.0009829516685359422	0.0014584306145854712	8.800192415597023e-06
ENSG00000059377_139953368_140020099_383	TBXAS1	7	+	1149	11	0	0	1.31%	5.617567155915145	0.016947426762344633	0.016947426762344633	0.0013498980316300959
ENSG00000276418_80080438_79958336_273	AC036214.3	8	−	819	61	5	0	6.59%	43.70403645143041	0.0006373150807818698	8.688204745348054e-05	4.053122177198919e-07
ENSG00000204822_74472660_74472119_112	MRPL53	2	−	336	59	4	1	16.96%	103.035848232317	0.00045208122059449304	8.03541330359969e-05	2.6478191410469155e-07
ENSG00000105852_95363975_95359973_160	PON3	7	−	480	8	0	0	3.12%	9.779673730525003	0.019679754444124863	0.019679754444124863	0.0017849636115922674
ENSG00000215301_41334253_41357867_647	DDX3X	X	+	1941	94	12	3	6.96%	28.416980855312218	5.81627248979138e-07	8.039453835617377e-09	5.3199907532461864e-14
ENSG00000140575_90388342_90482722_1191	IQGAP1	15	+	3573	40	4	0	1.76%	6.569050364752311	0.0002598615635756876	2.329509077470639e-05	4.821072760133524e-08
ENSG00000140575_90483523_90500108_418	IQGAP1	15	+	1254	9	0	0	1.20%	4.211342754771532	0.020613416668581838	0.020613416668581838	0.001946208561389314
ENSG00000196704_68250296_68256969_324	AMZ2	17	+	972	29	0	0	3.70%	17.50682334476698	0.0009430837296224684	0.0009430837296224684	5.543685104868835e-06
ENSG00000120333_175023408_175014669_128	MRPS14	1	−	384	40	0	0	8.59%	61.12296081578126	0.001595761305259402	0.001595761305259402	1.5227405548207672e-05
ENSG00000104763_18084058_18064392_173	ASAH1	8	−	519	61	0	0	5.20%	68.96648526728615	0.0033278027414746945	0.0033278027414746945	6.211806171561056e-05
ENSG00000079819_130956485_130858136_1005	EPB41L2	6	−	3015	21	1	6	1.09%	4.087027827682091	0.002568463469732168	0.015186081405644649	0.0002242723246544596
ENSG00000187446_41231383_41279389_195	CHP1	15	+	585	56	8	12	10.26%	56.17043373429745	0.0009347373151195707	0.0009798225270828075	5.7025696884349484e-06
ENSG00000143748_224308251_224227626_738	NVL	1	−	2214	7	0	0	0.68%	1.855222658907182	0.019216965118390855	0.019216965118390855	0.0017073955890589052
ENSG00000115216_27433274_27441812_526	NRBP1	2	+	1605	31	9	1	3.18%	11.33345366901963	0.005792608943192539	0.00018514942241825474	8.436729006999224e-06
ENSG00000116641_62688264_62455414_2129	DOCK7	1	−	6387	12	0	0	0.23%	1.1024526516326916	0.019679754444124863	0.019679754444124863	0.0017849636115922674
ENSG00000198793_11253916_11107485_2295	MTOR	1	−	6885	15	1	0	0.44%	1.2783887229444448	0.00392533363377418	0.0020082573300163734	4.538242985436214e-05
ENSG00000244509_39015611_39018387_179	APOBEC3C	22	+	537	25	0	0	6.15%	27.317524386941347	0.0014915192737735051	0.0014915192737735051	1.337646624223462e-05
ENSG00000182220_40581066_40605755_345	ATP6AP2	X	+	1035	42	1	4	6.96%	23.811379517800006	9.706969571762432e-06	4.881320999582746e-05	3.8337854993427554e-09
ENSG00000169592_29996311_30005442_244	INO80E	16	+	732	54	5	5	7.79%	43.287080446586074	0.00029776683287701603	0.0007010664838512278	1.3889116312721499e-06
ENSG00000133835_119479012_119584429_507	HSD17B4	5	+	1521	38	0	0	2.96%	14.659865947138071	0.00029563251185049877	0.00029563251185049877	5.909204346842462e-07
ENSG00000172354_100678162_100678801_153	GNB2	7	+	459	66	4	0	11.11%	84.37365571433335	0.0003761600207776246	0.00012102304356057305	3.208209902359148e-07
ENSG00000049245_7777172_7779645_72	VAMP3	1	+	216	64	0	1	23.61%	173.86086632044447	0.0001297455242311012	0.00018165475342050424	1.6630239591960388e-07
ENSG00000171960_42658856_42667419_151	PPIH	1	+	453	45	1	0	7.95%	58.289446075976834	0.0010447178140462346	0.0010447178140462346	6.749952978822125e-06
ENSG00000072062_14114155_14093112_343	PRKACA	19	−	1029	15	0	1	2.33%	8.553650493170556	0.005160369442893307	0.007930666369886513	0.00021637657341139557
ENSG00000129351_10670980_10684465_699	ILF3	19	+	2097	55	4	8	3.00%	15.390044497249646	0.00016491851939301634	0.00014059969282245537	1.6346967976074869e-07
ENSG00000143933_47162588_47160776_113	CALM2	2	−	339	138	7	1	12.39%	238.86636722344252	0.0003909674708303042	0.00047118620257580814	1.215777600715846e-06
ENSG00000086065_33265079_33280828_171	CHMP5	9	+	513	172	1	3	9.94%	196.73729609945033	0.00016376961508243266	0.00034115763570484036	3.8658379482580033e-07
ENSG00000168275_234373467_234383818_155	COA6	1	+	465	47	1	14	10.32%	59.30898907544195	0.00028549473518828597	0.021344042474557876	5.461124614643342e-05
ENSG00000104960_49858088_49860279_113	PTOV1	19	+	339	17	0	0	6.19%	29.42556697680089	0.00797940233269714	0.00797940233269714	0.000327142761693177
ENSG00000104960_49850881_49857914_237	PTOV1	19	+	711	15	2	0	3.38%	12.379333836107598	0.015625008558487224	0.005306578019052857	0.00043453029053502397
ENSG00000069424_6082210_6098574_310	KCNAB2	1	+	930	8	0	0	1.61%	5.047573538335485	0.019679754444124863	0.019679754444124863	0.0017849636115922674
ENSG00000013441_200857871_200853306_258	CLK1	2	−	774	21	0	0	4.65%	15.920399096203491	0.0009235399941089087	0.0009235399941089087	5.324735186779001e-06
ENSG00000167770_63986457_63996652_113	OTUB1	11	+	339	14	1	0	6.19%	24.232819863247794	0.00797940233269714	0.00847367416878415	0.0003463032305947362
ENSG00000167770_63996825_63997546_169	OTUB1	11	+	507	27	0	0	7.69%	31.24866162416273	0.0005983074123552221	0.0005983074123552221	2.307075671029917e-06
ENSG00000267855_8321358_8311505_113	NDUFA7	19	−	339	54	3	1	13.27%	93.46944804395578	0.00033620229360644755	0.0003354983092902608	7.562899237154315e-07
ENSG00000167772_8364322_8373886_406	ANGPTL4	19	+	1218	8	1	0	1.72%	3.854058613014779	0.00407548579675135	0.005706018193000826	0.0001262636872816894
ENSG00000162819_222715700_222732655_338	BROX	1	+	1014	36	0	0	4.44%	20.83244108277515	0.00028877520139654507	0.00028877520139654507	5.646841491566603e-07
ENSG00000167775_8308290_8302463_282	CD320	19	−	846	45	12	0	6.38%	31.211724671888305	0.013579505317004376	6.485031608499047e-05	9.849558778507936e-06
ENSG00000182481_68037133_68046596_529	KPNA2	17	+	1587	138	7	9	7.94%	51.024384681000015	7.906915555106359e-08	2.987074276209805e-08	2.359741795159071e-14
ENSG00000138382_169822052_169811820_171	METTL5	2	−	513	13	0	0	4.09%	14.869679356353805	0.00797940233269714	0.00797940233269714	0.000327142761693177
ENSG00000138385_169800988_169817756_399	SSB	2	+	1197	44	0	1	4.26%	21.569205220205518	0.0001222781397076954	0.00018587317707429726	1.6069861968432165e-07
ENSG00000134294_46371278_46361111_501	SLC38A2	12	−	1503	290	9	5	14.37%	113.21777971466071	1.500921050680341e-12	2.614290089981794e-13	5.151356973213928e-24
ENSG00000156113_77403927_76884963_1090	KCNMA1	10	−	3270	9	1	2	0.46%	1.6149919921967895	0.03537493353818881	0.08350359792828288	0.012068607709980346
ENSG00000132481_75876774_75874483_400	AC087289.1	17	−	1200	12	2	2	1.50%	5.867804238315001	0.04375184787808565	0.04375184787808565	0.007835513351106278
ENSG00000167642_38291866_38292006_46	SPINT2	19	+	138	24	2	0	17.39%	102.04876936200003	0.007181907427766128	0.005306578019052857	0.0002017304665753598

ENSG00000260916_55372066_55359499_613	CCPG1	15	-	1839	35	0	1	3.10%	11.167653525884994	4.868029355394704e-05	4.5079829736772535e-05	1.6527391850870425e-08
ENSG00000205336_57650288_57663582_692	ADGRG1	16	+	2076	71	4	0	4.34%	20.068116614661133	3.4901523427252256e-06	6.614649994567748e-07	2.087834633512807e-11
ENSG00000109099_15260727_15235247_118	PMP22	17	-	354	29	1	0	7.63%	48.069582743258486	0.00562235203092679	0.0033030736763512156	0.00010238030643782358
ENSG00000215301_41334253_41347719_661	DDX3X	X	+	1983	96	12	3	7.26%	28.406919156744333	1.935160314504737e-07	2.169376474014155e-09	4.972312673151123e-15
ENSG00000123562_103677027_103676161_288	MORF4L2	X	-	864	67	3	4	10.76%	45.50264860730383	2.6778889786849916e-06	6.382017302541668e-06	1.4552401796346898e-10
ENSG00000109016_21172043_21191153_310	DHRS7B	17	+	930	9	2	0	1.61%	5.678520230627421	0.10105102209468636	0.019679754444124863	0.009164497669452725
ENSG00000185033_90220985_90228643_675	SEMA4B	15	+	2025	31	0	0	2.07%	8.982811426556298	0.0003386460863230731	0.0003386460863230731	7.685136912859676e-07
ENSG00000017260_130934676_131016247_853	ATP2C1	3	+	2559	17	3	1	1.17%	3.898111451791912	0.011168917508652896	0.00324779312858951	0.00020011016427610653
ENSG00000170889_54201185_54207575_194	RPS9	19	+	582	162	18	0	9.28%	163.33063343763405	0.0008179544458575825	9.549782821581015e-05	5.724560240345955e-07
ENSG00000104823_38831328_38830992_64	ECH1	19	-	192	31	0	0	14.06%	94.74058926446097	0.0035547763465261306	0.0035547763465261306	7.044676146010672e-05
ENSG00000099385_30893944_30887665_217	BCL7C	16	-	651	20	5	0	2.30%	18.02704835119816	0.2463583386135439	0.019679754444124863	0.026068547507937054
ENSG00000100220_32412069_32387992_476	RTCB	22	-	1428	25	0	1	2.31%	10.272766523660717	0.001558047347358915	0.0021748195570013406	2.0072435939476812e-05
ENSG00000124172_59032251_59030306_51	ATP5F1E	20	-	153	100	1	0	19.61%	383.5166168833334	0.0024742386374296595	0.0024742386374296595	3.526932738104487e-05
ENSG00000105738_38081914_38206240_1665	SIPA1L3	19	+	4995	6	1	2	0.30%	0.7048413499477479	0.0477903522728147	0.1029516053660342	0.019089002044397063
ENSG00000189067_11553630_11549637_68	LITAF	16	-	204	23	1	0	13.24%	66.15661641237502	0.0035547763465261306	0.0035547763465261306	7.044676146010672e-05
ENSG00000111481_54325164_54347829_107	COPZ1	12	+	321	51	23	2	15.89%	93.2267963096776	0.0957187143230232	0.0005036444657169315	0.0005789973902594416
ENSG00000111481_54343312_54350523_73	COPZ1	12	+	219	25	2	0	12.33%	66.98406664743152	0.008316137131635013	0.0035291660242640502	0.0001601292768076144
ENSG00000107566_100185620_100152131_346	ERLIN1	10	-	1038	75	0	1	5.20%	42.39742946759394	8.99016647834184e-05	0.0001127271246253546	7.322784784629293e-08
ENSG00000115267_162318307_162310721_221	IFIH1	2	-	663	13	0	0	2.71%	11.505498506500002	0.013428347753762199	0.013428347753762199	0.0008725593497644525
ENSG00000054267_235326919_235176890_1153	ARID4B	1	-	3459	14	0	0	0.61%	2.374942449737208	0.00847367416878415	0.00847367416878415	0.0003665024264111182
ENSG00000099250_33334382_33180076_923	NRP1	10	-	2769	139	11	16	5.53%	29.45557201609914	3.3334522731780754e-08	4.478869538904784e-08	1.4920974883447965e-14
ENSG00000204262_189110408_189034070_1453	COL5A2	2	-	4359	38	0	11	1.10%	5.115314545904337	0.00017744757238953088	0.002853467440919741	3.7319126925757267e-06
ENSG00000136897_101398550_101390466_144	MRPL50	9	-	432	15	1	0	6.94%	20.37432027192709	0.006024333578509648	0.0020082573300163734	6.959797898348661e-05
ENSG00000078747_34393812_34507794_862	ITCH	20	+	2586	16	6	0	0.70%	3.6305053291972165	0.10041256134757276	0.013007239811447113	0.006595870280092993
ENSG00000132361_2703447_2690594_1232	CLUH	17	-	3696	28	9	2	1.38%	4.445306241147728	0.009970898787836703	0.0005456143505417452	3.825479896560591e-05
ENSG00000174106_65169597_65246325_811	LEMD3	12	+	2733	10	1	2	0.66%	2.147019479807904	0.026832695913080365	0.053594824633710306	0.006148603110732064
ENSG00000148019_78240031_78266762_624	CEP78	9	+	1872	21	0	0	1.76%	6.582472703237983	0.0012558331315113088	0.0012558331315113088	9.614604369954505e-06
ENSG00000164081_51674305_51704016_239	TEX264	3	+	717	20	1	0	3.77%	16.36765477912134	0.005034872532813168	0.0036062891729786097	9.977408550613472e-05
ENSG00000197912_89508547_89529139_83	SPG7	16	+	249	12	0	0	6.02%	28.278574642481935	0.019679754444124863	0.019679754444124863	0.0017849636115922674
ENSG00000065883_39988230_40088196_418	CDK13	7	+	1254	12	1	4	1.20%	5.61512367302871	0.02784981298332479	0.12180769416495907	0.014723589683894851
ENSG00000177469_42423097_42404687_390	CAVIN1	17	-	1170	96	4	4	9.74%	48.14608605796924	3.455252584865419e-07	3.455252584865419e-07	1.1096511480462033e-12
ENSG00000155906_151445520_151405235_352	RMND1	6	-	1056	8	0	0	1.42%	4.445306241147728	0.019679754444124863	0.019679754444124863	0.0017849636115922674
ENSG00000165282_35092536_35090977_519	PIGO	9	-	1557	12	0	0	0.96%	4.522392476543353	0.019679754444124863	0.019679754444124863	0.0017849636115922674
ENSG00000165282_35095565_35093319_346	PIGO	9	-	1038	45	1	0	5.20%	25.438457680556365	0.0001191030451631777	8.136746580010004e-05	7.021136193126235e-08
ENSG00000122966_119758688_119688232_1243	CIT	12	-	3729	19	0	0	0.80%	2.989763489621481	0.0022011839993586477	0.0022011839993586477	2.8200182015552967e-05
ENSG00000130164_11089549_11131518_948	LDLR	19	+	2844	128	16	36	5.80%	26.409245517029543	1.2486127705606373e-07	1.047790511227554e-07	1.2628043816028243e-13
ENSG00000104408_108248702_108243461_30	EIF3E	8	-	90	19	1	0	16.67%	123.8758672533167	0.020613416668581838	0.020613416668581838	0.001946208561389314
ENSG00000172270_578944_582577_432	BSG	19	+	1296	938	16	21	16.44%	424.6913870015024	8.752391343224034e-14	2.433727800930493e-12	2.917192234557471e-24
ENSG00000108953_1365026_1345447_223	YWHAE	17	-	669	192	7	13	15.70%	168.40335033729153	1.2383671676680442e-07	1.7215887102235075e-06	2.0896043278905164e-12
ENSG00000262660_81704278_81720077_406	AC139530.3	17	+	1218	81	0	2	4.93%	39.02234345677464	3.6090264246395505e-05	0.00014562154342183088	3.973714429612531e-08
ENSG00000170043_7931795_7930606_110	TRAPPC1	17	-	330	37	1	5	10.00%	65.79053236898638	0.0016186486669924592	0.00688220279286197	6.57161707674372e-05
ENSG00000112096_159693167_159688173_141	SOD2	6	-	423	13	2	0	4.26%	18.033440921535465	0.061356297364084836	0.012798402692974279	0.0037940651817215715
ENSG00000135336_87607675_87667123_568	ORC3	6	+	1704	9	0	0	0.88%	3.099192379391726	0.019216965118390855	0.019216965118390855	0.0017073955890589052
ENSG00000182944_29282517_29300161_475	EWSR1	22	+	1425	126	6	8	5.05%	51.88374273878527	0.00012487302501014574	0.0001440551817339119	1.2777049173942254e-07
ENSG00000119760_27661249_27653485_363	SUPT7L	2	-	1089	15	0	2	1.93%	8.08237498390496	0.00847367416878415	0.028426896806120847	0.0012022752110812016
ENSG00000129103_56073037_56079612_213	SUMF2	7	+	639	85	1	1	9.86%	78.05373399949532	3.5722957519171646e-05	3.5722957519171646e-05	9.746930957628094e-09
ENSG00000011304_797498_810826_557	PTBP1	19	+	1671	115	2	5	7.36%	40.38285382442999	2.8572017762581583e-08	6.773986471954645e-08	1.9365408787079888e-14
ENSG00000129691_38107048_38139071_501	ASH2L	8	+	1503	24	0	0	2.20%	9.369747286730542	0.0014055202957965589	0.0014055202957965589	1.1935530040246081e-05
ENSG00000103266_681311_682335_173	STUB1	16	+	519	13	0	0	4.05%	14.697775548765899	0.00837382380159347	0.00837382380159347	0.0003583838894600799
ENSG00000230989_83808077_83809423_76	HSBP1	16	+	228	89	3	2	15.79%	229.05025316229612	0.0016081146550637245	0.0018328587615579468	1.7525755971930474e-05
ENSG00000145349_113759386_113455755_467	CAMK2D	4	-	1401	8	0	0	1.28%	3.350637680693791	0.011570465654371866	0.011570465654371866	0.0006594843062896773
ENSG00000163527_31533068_31619819_378	STT3B	3	+	1134	19	3	1	2.65%	9.831418035977515	0.01718512520643334	0.0037373685375237473	0.00035268975452804117
ENSG00000157450_59031054_59094900_926	RNF111	15	+	2778	11	0	0	0.97%	2.3234646012046443	0.002478942540635376	0.002478942540635376	3.5397643206479886e-05
ENSG00000164611_160424237_160428681_110	PTTG1	5	+	330	65	1	1	13.64%	115.57796226984094	0.00036349992845691105	0.0003065228477605667	7.477466111124385e-07
ENSG00000135723_67238096_67229636_971	FHOD1	16	-	2913	18	5	8	0.93%	3.6258316611627195	0.060116400268406124	0.14404491314817458	0.03216577242059674
ENSG00000248919_99466141_99419967_749	ATP5MF-PTCD1	7	-	2247	33	1	0	1.34%	8.617603020222298	0.003514803071119647	0.0023766146074493525	4.76343262496996e-05
ENSG00000005486_75882128_75888349_205	RHBDD2	7	+	615	10	0	0	2.44%	9.541145102951221	0.020613416668581838	0.020613416668581838	0.001946208561389314
ENSG00000095059_12681562_12675838_301	DHPS	19	-	903	24	0	0	2.33%	15.59549299219934	0.00877616296920843	0.00877616296920843	0.00039161135667564577
ENSG00000160948_144425732_144424850_83	VPS28	8	-	249	13	0	2	7.23%	30.635122529355428	0.012798402692974279	0.044040755831095145	0.0026804992160797854
ENSG00000160948_144423903_144423805_32	VPS28	8	-	96	16	0	0	18.75%	97.79673730525002	0.013007239811447113	0.013007239811447113	0.0008218654148058079
ENSG00000197102_102036535_102050405_1072	DYNC1H1	14	+	3216	35	5	5	1.77%	6.385980980753266	0.001251551271000974	0.0009691759825094995	7.480876736042485e-06
ENSG00000135452_57745215_57746622_93	TSPAN31	12	+	279	10	0	2	6.45%	21.031556409731188	0.012798402692974279	0.061356297364084836	0.0037940651817215715
ENSG00000166340_6616817_6614546_320	TPP1	11	-	960	19	0	0	3.12%	11.61336255499844	0.0017901637611834264	0.0017901637611834264	1.8982104156792192e-05
ENSG00000172354_100677531_100678801_240	GNB2	7	+	720	70	10	1	8.33%	57.04809676139585	0.000972358176646045	5.350213422752019e-05	4.102420595090639e-07
ENSG00000189159_75148589_75136102_108	JPT1	17	-	324	185	17	9	23.15%	335.0443778050232	0.00019786512540490427	1.2910956916440569e-05	2.122231927317089e-08
ENSG00000260669_24213439_24209877_222	AL096870.3	14	-	666	27	3	6	5.86%	23.788395560736493	0.003897705227460077	0.012383447100975772	0.0002612565341663802
ENSG00000034713_75577286_75577369_27	GABARAPL2	16	+	81	16	0	1	18.52%	115.90724421362965	0.021557223391537678	0.029237630782826433	0.002819005644529471

ENSG00000095906_1786889_1788714_182	NUBP2	16	+	546	11	2	0	3.85%	11.821583630304948	0.04363948305378087	0.0078820576498062	0.0017710903275458955
ENSG00000143889_38602867_38564182_537	HNRNPLL	2	−	1611	35	3	6	1.86%	12.748178047239294	0.009821676566158627	0.03375807224221634	0.0016279040644707498
ENSG00000074201_77637714_77619628_203	CLNS1A	11	−	609	46	0	1	6.40%	44.32167404966996	0.0007028601028765856	0.0012722094802689694	5.6109541146368954e−06
ENSG00000169682_28977925_28984299_420	SPNS1	16	+	1260	28	0	2	3.81%	13.039564974033336	0.00015821478107689517	0.0007256517442685692	8.003945195112655e−07
ENSG00000167770_63986457_63997546_271	OTUB1	11	+	813	41	1	0	7.38%	29.591632690149453	3.145332252154177e−05	3.404299617394451e−05	8.215888711579203e−09
ENSG00000118564_15655236_15605723_674	FBXL5	4	−	2022	12	0	0	0.89%	3.4823764025608317	0.012798402692974279	0.012798402692974279	0.0007972436888943567
ENSG00000145725_103148006_103201634_1039	PPIP5K2	5	+	3117	13	0	3	0.87%	2.447271578379693	0.00308494966027208	0.03441276307238556	0.0006340181607701747
ENSG00000278845_38297184_38322595_306	MRPL45	17	+	918	53	0	1	3.92%	33.877301158027784	0.0009628873231322257	0.0015830155609353933	9.35200103985558e−06
ENSG00000114503_196937617_196937011_59	NCBP2	3	−	177	14	2	0	13.56%	46.41201092452543	0.016854695534768073	0.004193160480899131	0.0003819250574820159
ENSG00000249773_55953259_55955178_109	AC092647.5	7	+	327	11	1	0	4.59%	19.738791015738535	0.04223451612662591	0.019679754444124863	0.003715148010809822
ENSG00000106245_99411093_99419441_103	BUD31	7	+	309	51	0	0	9.71%	96.84725441879128	0.0022543491824521134	0.0022543491824521134	2.9517890247939808e−05
ENSG00000129625_112887162_112878786_65	REEP5	5	−	195	174	2	0	18.46%	523.5886858804155	0.0012800257995343638	0.0010872143717490798	8.5304938081734e−06
ENSG00000129625_112921293_112892773_104	REEP5	5	−	312	29	3	2	10.58%	54.54048811254328	0.005019087503143544	0.0015356776430442238	4.5418523296041846e−05
ENSG00000197702_12473768_12527925_319	PARVA	11	+	957	14	0	0	1.88%	8.584039638078371	0.012798402692974279	0.012798402692974279	0.0007972436888943567
ENSG00000187257_77696470_77779168_846	RSBN1L	7	+	2538	11	1	0	0.71%	2.5431775658575657	0.026832695913080365	0.011772051206276392	0.0015103817605807263
ENSG00000205302_122799817_122829648_402	SNX2	5	+	1206	16	2	0	1.99%	7.784814909870649	0.02071580916834944	0.005454749182134642	0.0005936342691250666
ENSG00000109534_109816165_109819165_177	GAR1	4	+	531	13	0	1	4.52%	14.365622429019778	0.005160369442893307	0.00912793029372963	0.00024819240235076056
ENSG00000214655_73786080_73801519_1775	ZSWIM8	10	+	5325	8	0	0	0.34%	0.8815480545825354	0.011570465654371866	0.011570465654371866	0.0006594843062896773
ENSG00000108561_5439073_5434864_161	C1QBP	17	−	483	78	8	9	9.94%	94.75957155042859	0.0021790720364277347	0.007202114571563827	8.974185617200301e−05
ENSG00000135046_73168981_73170107_76	ANXA1	9	+	228	100	6	2	18.42%	257.3598350138158	0.0006410319293143953	0.000873712720842268	3.5581694222000848e−06
ENSG00000147471_37762660_37778104_275	PLPBP	8	+	825	29	0	0	5.45%	20.626220958925458	0.0002596338414409078	0.0002596338414409078	4.5960159284157924e−07
ENSG00000198860_184051756_184119792_141	TSEN15	1	+	423	13	0	0	4.26%	18.033440921535465	0.013007239811447113	0.013007239811447113	0.0008218654148058079
ENSG00000127948_75985777_75986481_148	POR	7	+	444	50	6	1	7.43%	66.07887655760136	0.005213180408205425	0.002213262928959916	6.57545064955002e−05
ENSG00000115053_231460526_231457563_225	NCL	2	−	675	145	2	0	11.11%	126.04912808232226	6.467493766381814e−06	5.7544848691021985e−06	3.0915247627757433e−10
ENSG00000138029_26269966_26289953_400	HADHB	2	+	1200	52	3	1	5.25%	25.427151699365005	0.00013560821937515127	3.444684173090649e−05	3.5394269733762794e−08
ENSG00000057019_98812365_98801502_162	DCBLD2	3	−	486	22	1	4	6.17%	26.562076798956795	0.0022011839993586477	0.017397628901543883	0.0002272709811355097
ENSG00000114902_52706280_52707812_91	SPCS1	3	+	273	80	7	2	23.08%	171.95030734989015	0.0002246060989833002	5.5184627605275e−05	9.162586061576804e−08
ENSG00000169689_82022861_82019637_102	CENPX	17	−	306	34	4	0	4.90%	65.19782487016668	0.060667625179241055	0.02108409854857798	0.005667715763110205
ENSG00000136450_58007137_58005032_73	SRSF1	17	−	219	82	0	22	20.55%	219.7077386035754	0.0003144697103273026	0.008616560132786801	2.0516730802217042e−05
ENSG00000197756_216499339_216500035_48	RPL37A	2	+	144	255	25	11	37.50%	1039.0903338682815	9.152174643962531e−05	0.0003369099689742923	2.2125306702080885e−07
ENSG00000171867_4699242_4699982_246	PRNP	20	+	738	93	0	0	8.54%	73.94387454787197	2.6246772046413107e−05	2.6246772046413107e−05	5.344526997695175e−09
ENSG00000184428_143329415_143309441_503	TOP1MT	8	−	1509	11	0	0	0.99%	4.277392088897615	0.02108409854857798	0.02108409854857798	0.002029891263157455
ENSG00000171603_9823733_9730508_981	CLSTN1	1	−	2943	89	7	3	3.06%	17.744973741421514	1.317816552236354e−05	2.199315159844212e−06	2.5077037963359434e−10
ENSG00000136754_26823167_26748570_390	ABI1	10	−	1170	20	1	0	2.31%	10.03043459541026	0.005588370640926919	0.0034782380676658805	0.00010676685949938237
ENSG00000136754_26823167_26748570_361	ABI1	10	−	1083	20	1	0	2.49%	10.836203579529089	0.005588370640926919	0.0034782380676658805	0.00010676685949938237
ENSG00000170525_6213664_6225187_416	PFKFB3	10	+	1248	46	13	3	4.57%	21.62812459635337	0.002674329485664681	0.0002944342621990559	5.423138123191577e−06
ENSG00000198363_61714371_61676075_203	ASPH	8	−	609	102	6	10	8.87%	98.27849463187687	0.0005678656064409615	0.001710954571778227	6.182481515581978e−06
ENSG00000120742_150546135_150544458_66	SERP1	3	−	198	29	3	0	13.64%	85.94258732885608	0.01748038679108207	0.0035547763465261306	0.00034382947735276184
ENSG00000134809_57530189_57528717_90	TIMM10	11	−	270	118	8	13	24.44%	256.4447778226556	2.785638855085842e−05	0.0004849677896341693	1.0937705086186082e−07
ENSG00000169188_55000423_55007435_518	APEX2	X	+	1554	13	0	0	1.16%	4.908716544278959	0.012798402692974279	0.012798402692974279	0.0007972436888943567
ENSG00000173744_227491587_227554495_472	AGFG1	2	+	1416	17	0	0	1.91%	7.044680229615468	0.0032294771334464936	0.0032294771334464936	5.8663648728941325e−05
ENSG00000169679_110674154_110637964_976	BUB1	2	−	2928	27	0	1	1.43%	5.410885055823259	0.00036930943616753716	0.0006659184076607086	1.6160525083088519e−06
ENSG00000138760_76179654_76161713_320	SCARB2	4	−	960	72	5	0	9.38%	44.00853178736251	4.09601459398266e−06	6.893507041361946e−07	2.554533397957012e−11
ENSG00000140284_50202504_50236096_385	SLC27A2	15	+	1155	20	1	5	2.34%	10.160699979766235	0.005642787686764809	0.0360990988508288	0.0010960132257111213
ENSG00000157456_59105269_59124877_398	CCNB2	15	+	1194	37	0	2	4.52%	18.18331296630277	7.783833532730189e−05	0.00032828634928333024	1.8542364152958258e−07
ENSG00000204634_101050498_101007821_897	TBC1D8	2	−	2691	10	1	0	0.56%	2.1805292598717956	0.03537493353818881	0.020613416668581838	0.003252526348827943
ENSG00000161960_7572842_7578486_406	EIF4A1	17	+	1218	212	7	5	6.40%	102.13255324489165	2.216511804978622e−05	7.1775503821723165e−06	1.2981956091804373e−09
ENSG00000113645_168414520_168469017_747	WWC1	5	+	2241	18	4	0	0.94%	4.713095773746989	0.032657852871826265	0.0078820576498062	0.0013042942913966678
ENSG00000131473_41913873_41867810_1091	ACLY	17	−	3273	333	28	19	9.72%	59.6999331304276	6.536951109028016e−17	1.5133401133889646e−17	1.507319833723374e−32
ENSG00000196531_56724521_56712538_215	NACA	12	−	645	209	14	5	14.88%	190.13505206323026	2.4168959547478946e−06	1.161173264099816e−06	2.4727756758011836e−11
ENSG00000172725_67442628_67438376_489	CORO1B	11	−	1467	36	2	2	2.66%	14.399519603226997	0.0027852197205094193	0.0017971687341926767	2.9217951810824196e−05
ENSG00000176978_137111981_137110648_126	DPP7	9	−	378	30	1	1	5.56%	46.56987490726191	0.008574016250729465	0.008574016250729465	0.0003747459614100891
ENSG00000010704_26091349_26092968_174	HFE	6	+	522	14	0	2	3.45%	15.73740600314368	0.012798402692974279	0.044040755831095145	0.0026804992160797854
ENSG00000198363_61714371_61626273_270	ASPH	8	−	810	243	11	30	16.30%	176.03412714945003	8.740440559853362e−08	2.4251875115406563e−06	2.1578263420728893e−12
ENSG00000060339_68722505_68757375_568	CCAR1	10	+	1704	13	2	1	1.23%	4.476611214676938	0.0319825310473083	0.015191410988288745	0.002250081498979975
ENSG00000159131_33539315_33524765_433	GART	21	−	1299	50	6	0	3.70%	22.585851571651276	0.0017874791595539988	0.0002028510352551382	2.5495063992315097e−06
ENSG00000152556_48131382_48145708_705	PFKM	12	+	2115	47	0	0	3.12%	13.039564974033336	9.836921317677237e−06	9.836921317677237e−06	7.867021274290272e−10
ENSG00000117713_26766258_26780756_1362	ARID1A	1	+	4086	12	4	1	0.51%	1.7232905252026436	0.057109797348681025	0.016854695534768073	0.004416591199647673
ENSG00000163435_202013810_202015323_109	ELF3	1	+	327	18	0	0	8.26%	32.299839843935786	0.0034782380676658805	0.0034782380676658805	6.758309314600117e−05
ENSG00000102125_154412091_154421004_224	TAZ	X	+	672	11	1	0	2.23%	9.60503669962277	0.02784981298332479	0.019679754444124863	0.002477909089056847
ENSG00000135108_117189349_117146087_537	FBXO21	12	−	1611	33	0	0	2.79%	12.019710730254193	0.00029257101221602755	0.00029257101221602755	5.79136213531554e−07
ENSG00000116044_177234268_177230785_582	NFE2L2	2	−	1746	15	1	0	1.03%	5.04106893326031	0.02034245439037237	0.013428347753762199	0.0012955184805712684
ENSG00000182923_134507167_134574891_441	CEP63	3	+	1323	9	0	0	1.13%	3.9917035634795925	0.019679754444124863	0.019679754444124863	0.0017849636115922674
ENSG00000005339_3850743_3727718_2325	CREBBP	16	−	6975	27	1	1	0.56%	2.2714080922509683	0.0008263938593182232	0.0008263938593182232	4.2991338318617794e−06
ENSG00000087263_56453280_56476205_442	OGFOD1	16	+	1326	19	0	1	2.04%	8.40786429321154	0.002967235347054297	0.006432154271512831	0.00010592475669358982
ENSG00000140391_77070954_77055818_88	TSPAN3	15	−	264	50	2	0	14.77%	111.1326560286932	0.0008084705596873852	0.0007004692736203066	3.591164211716913e−06
ENSG00000085662_134459062_134449097_155	AKR1B1	7	−	465	942	48	12	21.29%	1188.7035682780067	8.508966704596201e−07	1.0996206499020317e−06	8.38107062679132e−12

ENSG00000155097_103048895_103068747_307	ATP6V1C1	8	+	921	23	0	3	2.61%	14.653582788408798	0.005160369442893307	0.04099061537131024	0.0011691093874143326
ENSG00000156482_98042750_98041801_51	RPL30	8	−	153	163	11	5	17.65%	625.1320855198335	0.022643693555890774	0.030362948313965823	0.0030553704311476805
ENSG00000156482_98045367_98044939_56	RPL30	8	−	168	99	11	5	28.57%	345.78132118641975	0.0006373973709768437	0.00020377058636723502	8.863778535578521e−07
ENSG00000115268_1438426_1440462_145	RPS15	19	+	435	85	1	0	4.14%	114.65824373718968	0.020525443280229434	0.013640585738808999	0.0013255139945858052
ENSG00000085415_12978777_12985252_145	SEH1L	18	+	435	12	0	0	4.14%	16.187046174662072	0.011772051206276392	0.011772051206276392	0.000681272511389508
ENSG00000198860_184051756_184070676_137	TSEN15	1	+	411	13	0	0	4.38%	18.559964744062047	0.013007239811447113	0.013007239811447113	0.0008218654148058079
ENSG00000125257_95247698_95177773_508	ABCC4	13	−	1524	13	6	0	1.18%	5.005344822709647	0.08385215180161965	0.012798402692974279	0.005324756476027482
ENSG00000125954_64914496_65012388_103	CHURC1-FNTB	14	+	309	30	27	0	9.71%	56.968973187524284	0.16207829313242916	0.0022409900782254734	0.003396454435739306
ENSG00000125954_64924121_65061312_391	CHURC1-FNTB	14	+	1173	14	0	0	2.56%	7.003346917000002	0.0017901637611834264	0.0017901637611834264	1.8982104156792192e−05
ENSG00000073111_127604982_127621773_807	MCM2	3	+	2421	36	4	0	1.98%	8.72535946217844	0.001589018185675934	0.00017828355461003195	2.008210786891129e−06
ENSG00000198363_61684117_61661907_55	ASPH	8	−	165	90	6	2	23.64%	320.06204936263646	0.0035399226636468654	0.001032612263590393	2.226152840563376e−05
ENSG00000070756_100721556_100718036_136	PABPC1	8	−	408	21	7	2	5.15%	30.201933579562507	0.06775739726339094	0.024135905425855194	0.0071029587535123275
ENSG00000130520_18323020_18310149_69	LSM4	19	−	207	40	5	0	18.84%	113.38752151333335	0.00500277944419397	0.0006838816678496783	2.2042196630895807e−05
ENSG00000100897_24115595_24123309_546	DCAF11	14	+	1638	7	0	4	0.92%	2.507608648852565	0.019216965118390855	0.21886412946940276	0.022078900761252438
ENSG00000160932_143021325_143021789_110	LY6E	8	+	330	67	2	1	11.82%	119.13420726275912	0.0007373308535318079	0.0007313336786544864	3.424294031634491e−06
ENSG00000205060_134316751_134294833_331	SLC35B4	7	−	993	8	1	0	1.51%	4.7273347337885205	0.06539853090342929	0.019679754444124863	0.005779266745814885
ENSG00000072210_19648972_19675572_485	ALDH3A2	17	+	1455	86	11	1	5.57%	34.68255426083093	0.00016788047648618558	2.296416855876984e−06	3.823263740949609e−09
ENSG00000108344_39981079_39997581_498	PSMD3	17	+	1494	55	5	5	4.62%	21.60168896300703	0.00011717619958821338	0.00011748043852590929	9.851784226088054e−08
ENSG00000108107_55386379_55391739_162	RPL28	19	+	486	442	56	21	15.43%	533.6562702335865	7.506643630697813e−05	2.235830367719763e−05	1.2980465306445974e−08
ENSG00000128951_48332321_48342078_141	DUT	15	+	423	17	0	1	6.38%	23.58219197431561	0.0034782380676658805	0.005588370640926919	0.00010676685949938237
ENSG00000175324_38176311_38163670_130	LSM1	8	−	390	12	0	0	5.38%	18.054782271738464	0.007029302661868638	0.007029302661868638	0.0002573639808771639
ENSG00000150316_94972185_94963385_229	CWC15	11	−	687	20	0	1	4.80%	17.082399529301313	0.0014481341328822738	0.0022960440039733582	1.975525661044477e−05
ENSG00000170540_18801466_18798697_57	ARL6IP1	16	−	171	18	0	2	14.04%	61.7663604033158	0.005554612275049112	0.015570605297898355	0.00045086442503788926
ENSG00000129116_168878051_168926385_619	PALLD	4	+	1857	28	3	0	2.26%	8.847523891912765	0.0027707726756871684	0.00037518914374882155	6.9758339868607375e−06
ENSG00000169689_82022861_82019205_58	CENPX	17	−	174	30	0	3	13.79%	101.16903859163796	0.005655335537214311	0.01773293231201279	0.0005225201041863723
ENSG00000122862_69088178_69104120_102	SRGN	10	+	306	59	10	0	23.53%	113.13740198058336	0.0003408585116172901	7.729980228756844e−06	2.4068564822575035e−08
ENSG00000122862_69088158_69103893_33	SRGN	10	+	99	23	0	3	15.15%	136.32272472853035	0.019679754444124863	0.08410206952409	0.007521960853378093
ENSG00000115275_74462963_74461275_562	MOGS	2	−	1686	22	0	3	1.96%	7.656684059485767	0.0013845019718837994	0.01157547599262317	9.8960288269354e−05
ENSG00000128951_48331582_48341512_204	DUT	15	+	612	17	0	1	4.41%	16.29945621754167	0.0034782380676658805	0.005588370640926919	0.00010676685949938237
ENSG00000124802_8102521_8090117_150	EEF1E1	6	−	450	21	1	2	7.33%	27.383086445470006	0.0033728746429155087	0.005483137812600607	0.00010185498177708228
ENSG00000099622_1270986_1272443_119	CIRBP	19	+	357	13	1	0	5.04%	21.36735436921429	0.026832695913080365	0.011772051206276392	0.0015103817605807263
ENSG00000060491_62808211_62813649_609	OGFR	20	+	1827	14	7	0	1.48%	4.496401715183909	0.07206351740800766	0.002943990679779778	0.0014406021265609054
ENSG00000079459_11808887_11838609_353	FDFT1	8	+	1059	211	12	7	15.01%	116.91281343573799	5.823304590514566e−09	4.985113558253148e−10	3.292241988068417e−17
ENSG00000100201_38506000_38492042_407	DDX17	22	−	1221	71	2	5	6.39%	34.12072898610689	4.528029211326047e−06	2.2082043609970337e−05	8.36468431581182e−10
ENSG00000100201_38486390_38485935_151	DDX17	22	−	453	32	3	0	5.30%	41.45027276513908	0.032206895162315674	0.005454749182134642	0.0009428869080548071
ENSG00000165819_21511223_21501771_258	METTL3	14	−	774	22	5	0	3.10%	16.67851333887985	0.032444423092575315	0.005554612275049112	0.0009648226352302006
ENSG00000104723_15540431_15757809_348	TUSC3	8	+	1044	34	0	0	4.02%	19.109707289531613	0.0004507943031154868	0.0004507943031154868	1.3358454266020458e−06
ENSG00000198522_27632663_27650200_260	GPN1	2	+	780	21	0	0	2.69%	15.797934487771158	0.00877616296920843	0.00877616296920843	0.00039161135667564577
ENSG00000078140_39774844_39778434_97	UBE2K	4	+	291	18	0	0	7.22%	36.29569631947424	0.008175610787902942	0.008175610787902942	0.00034251843268961324
ENSG00000214046_16660110_16654031_71	SMIM7	19	−	213	23	3	0	9.86%	63.36126642311973	0.0682897090290637	0.00797940233269714	0.0029197462565442433
ENSG00000087586_56388197_56370158_403	AURKA	20	−	1209	19	2	0	2.48%	9.221528579651366	0.007459848152910933	0.002046028879997488	8.7920393864264e−05
ENSG00000087586_56388197_56370158_403	AURKA	20	−	1209	19	3	0	2.48%	9.221528579651366	0.013383828821373808	0.002046028879997488	0.00016141855850843116
ENSG00000087586_56388197_56370158_403	AURKA	20	−	1209	19	2	0	2.48%	9.221528579651366	0.007459848152910933	0.002046028879997488	8.7920393864264e−05
ENSG00000087586_56388197_56370158_403	AURKA	20	−	1209	20	2	0	2.73%	9.706872189106702	0.004968901071354418	0.0012624143894574274	3.7691836973080214e−05
ENSG00000196305_92240870_92210807_189	IARS	9	−	567	22	0	0	5.29%	22.767494399105825	0.0021880057261679567	0.0021880057261679567	2.7877966332435854e−05
ENSG00000196305_92289419_92229093_1121	IARS	9	−	3363	110	5	13	4.37%	19.19293684848796	6.078035137234466e−10	1.4606383688191108e−07	1.1473368554484353e−15
ENSG00000151694_9555605_9527775_209	ADAM17	2	−	627	29	3	0	4.31%	27.139764419638762	0.015765166103784645	0.003684287313187465	0.0003185076346966345
ENSG00000118257_205743220_205743579_119	NRP2	2	+	357	14	1	0	5.88%	23.010997013000004	0.013892891352654344	0.00847367416878415	0.0005886798845679606
ENSG00000115128_24076223_24074072_48	SF3B6	2	−	144	13	0	1	12.50%	52.97323270701043	0.012798402692974279	0.019795881618345192	0.0012079910998532793
ENSG00000163162_101308588_101277238_400	RNF149	2	−	1200	31	1	3	3.00%	15.158494282313754	0.0015639623262203096	0.0016269198037485093	1.5216365882592373e−05
ENSG00000047249_53829452_53715964_384	ATP6V1H	8	−	1152	26	0	0	2.86%	13.243308176752608	0.0015356776430442238	0.0015356776430442238	1.41466323862755e−05
ENSG00000149273_75400758_75404865_90	RPS3	11	+	270	83	1	0	10.00%	180.38064880746114	0.003710480643933249	0.003580484096903955	7.38927833378911e−05
ENSG00000173039_65662832_65655587_377	RELA	11	−	1131	22	0	1	2.92%	11.413942815466847	0.0012624143894574274	0.0025593219000189936	1.9339285108591855e−05
ENSG00000214944_73780715_73911583_1358	ARHGEF28	5	+	4074	9	5	1	0.44%	1.2962748685526513	0.11411551976453232	0.026832695913080365	0.013332232705089831
ENSG00000214944_73813589_73941013_1392	ARHGEF28	5	+	4176	13	5	1	0.57%	1.826663196793463	0.05268573235456097	0.00912793029372963	0.0024456930586322554
ENSG00000167186_19071969_19078158_179	COQ7	16	+	537	6	0	0	2.79%	6.556205852865923	0.016947426762344633	0.016947426762344633	0.0013498980316300959
ENSG00000135632_73214279_73225946_414	SMYD5	2	+	1242	23	0	0	2.66%	10.86630414502778	0.0014409783181973444	0.0014409783181973444	1.2520176346377507e−05
ENSG00000118058_118473422_118522172_3215	KMT2A	11	+	9645	14	0	1	0.16%	0.851728971865319	0.020613416668581838	0.022600676488261057	0.0021211989024304354
ENSG00000147419_27765225_27736216_190	CCDC25	8	−	570	11	0	1	3.68%	11.323832740607898	0.0078820576498062	0.015516259750072458	0.0006142571802709984
ENSG00000162517_31644864_31630613_284	PEF1	1	−	852	12	0	0	2.46%	8.26451301171127	0.007029302661868638	0.007029302661868638	0.0002573639808771639
ENSG00000117697_212791763_212784354_150	NSL1	1	−	450	15	0	0	4.67%	19.559347461050006	0.007593099278532274	0.007593099278532274	0.00029783286705462064
ENSG00000147162_71533300_71573794_1036	OGT	X	+	3108	13	1	0	0.77%	2.4543582721394794	0.010020958156399907	0.004826937407846361	0.00025599508163266543
ENSG00000092621_119712023_119744040_533	PHGDH	1	+	1599	41	0	0	3.94%	15.045651893115387	2.2606885438602453e−05	2.2606885438602453e−05	3.994412047840312e−09
ENSG00000168528_31424824_31434199_341	SERINC2	1	+	1023	16	0	0	1.47%	9.177406433337245	0.02108409854857798	0.02108409854857798	0.002029891263157455
ENSG00000122218_160297421_160290157_496	COPA	1	−	1488	18	2	0	2.02%	7.098150288284276	0.003925333363377418	0.0021880057261679567	4.913282145487895e−05
ENSG00000122218_160340217_160297583_570	COPA	1	−	1710	18	1	4	2.11%	6.17663604033158	0.00241548406293102	0.013266613543334622	0.00018452543561115668
ENSG00000117139_202746239_202729036_844	KDM5B	1	−	2532	10	5	0	0.59%	2.317458229982228	0.1741008397065631	0.019679754444124863	0.016992325807804815

ENSG00000164543_43608335_43624842_248	STK17A	7	+	744	16	0	0	4.03%	12.618933845838713	0.0020082573300163734	0.0020082573300163734	2.3658688350241813e-05
ENSG00000151694_9526149_9490177_586	ADAM17	2	-	1758	17	8	4	1.19%	5.674213427267066	0.17806259325226725	0.06793153848426066	0.04390537220818324
ENSG00000074603_65507278_65446884_770	DPP8	15	-	2310	5	0	0	0.65%	1.2700874974707794	0.012673659338734126	0.012673659338734126	0.000782701129001274
ENSG00000204822_74472202_74472119_27	MRPL53	2	-	81	13	2	0	18.52%	94.1746359235741	0.056923149003329024	0.020613416668581838	0.005212417464661139
ENSG00000104763_18084801_18057534_411	ASAH1	8	-	1233	55	3	2	6.08%	26.174309254446477	4.58542909915364e-06	1.926540703241544e-05	7.37125132342833e-10
ENSG00000213593_57712619_57738725_103	TMX2	11	+	309	47	12	0	11.65%	89.25139132712138	0.024331435649611596	0.0010658336519988904	0.00018138477791255583
ENSG00000114021_100334792_100341085_92	NIT2	3	+	276	26	0	0	6.52%	55.27641673775001	0.013640585738808999	0.013640585738808999	0.00089863692579514
ENSG00000176209_42546494_42552608_100	SMIM19	8	+	300	8	0	0	6.00%	15.647477968840004	0.009815328628645334	0.009815328628645334	0.0004837142251238724
ENSG00000134575_47248717_47240116_336	ACP2	11	-	1008	13	0	0	2.38%	7.567604672430061	0.004193160480899131	0.004193160480899131	9.649553577281792e-05
ENSG00000140264_43792377_43799673_56	SERF2	15	+	168	50	0	0	12.50%	174.63703090223217	0.00847367416878415	0.00847367416878415	0.0003665024264111182
ENSG00000086200_62437280_62627318_975	IPO11	5	+	2925	19	0	2	0.72%	3.8115651462558984	0.00847367416878415	0.02319724882453354	0.000977880717745449
ENSG00000131023_149695083_149661729_968	LATS1	6	-	2904	17	0	0	0.52%	3.435009368159608	0.02108409854857798	0.02108409854857798	0.002029891263157455
ENSG00000131828_19350021_19359653_292	PDHA1	X	+	876	32	1	0	5.14%	21.434901327178085	0.0004235771399443867	0.00027539387036832887	7.83742048836142e-07
ENSG00000167985_61430147_61438264_129	SDHAF2	11	+	387	12	0	0	3.88%	18.194741824232562	0.02108409854857798	0.02108409854857798	0.002029891263157455
ENSG00000278677_27893149_27892757_130	HIST1H2AM	6	-	390	298	12	5	22.31%	448.36042641483857	1.6572407415626269e-06	1.1749561903207945e-06	1.7207237800411832e-11
ENSG00000163938_52686792_52689070_130	GNL3	3	+	390	13	0	1	5.38%	19.559347461050006	0.008175610787902942	0.013599199788808391	0.0005580304998096723
ENSG00000111906_125301932_125276146_204	HDDC2	6	-	612	43	7	0	5.88%	41.228036314958345	0.006208941657272619	0.0010155991509472172	3.9129885679133845e-05
ENSG00000100461_22911357_22904161_307	RBM23	14	-	921	22	2	2	3.58%	14.01647049326059	0.004275489618562656	0.0031006807887314905	7.392649036285615e-05
ENSG00000135318_85450140_85494004_574	NT5E	6	+	1722	70	0	0	5.57%	23.852862757378055	2.7250502744588503e-07	2.7250502744588503e-07	6.959482959521015e-13
ENSG00000223501_33271657_33250841_717	VPS52	6	-	2151	11	3	4	0.98%	3.0007367095055795	0.065131087866026	0.12775441014701422	0.030469934698935275
ENSG00000133131_106999749_106941479_903	MORC4	X	-	2709	44	5	0	2.88%	9.530579050788486	3.804852687127488e-05	2.583811225771704e-06	8.695362205548832e-10
ENSG00000129636_47454100_47426308_118	ITFG1	16	-	354	12	0	0	4.24%	19.890861824796616	0.019679754444124863	0.019679754444124863	0.0017849636115922674
ENSG00000099622_1270986_1274311_115	CIRBP	19	+	345	13	1	0	5.22%	22.110566695100005	0.026832695913080365	0.011772051206276392	0.0015103817605807263
ENSG00000156113_77637360_76877869_1118	KCNMA1	10	-	3354	15	4	2	0.72%	2.624241609264312	0.048994651353958696	0.02389626957043539	0.005091569865673637
ENSG00000115525_85863399_85840144_362	ST3GAL5	2	-	1086	12	1	3	1.66%	6.483761589298344	0.026602764905793736	0.09783387127182075	0.011244525270099857
ENSG00000070423_652285_648128_239	AC004156.1	19	-	717	15	0	0	2.51%	12.275741084341007	0.012798402692974279	0.012798402692974279	0.0007972436888943567
ENSG00000011304_797498_804307_128	PTBP1	19	+	384	14	0	2	3.91%	21.393036285523443	0.02108409854857798	0.05742480463164306	0.005360236623058286
ENSG00000122873_58277269_58287650_47	CISD1	10	+	141	10	0	0	10.64%	41.61563289585107	0.020613416668581838	0.020613416668581838	0.001946208561389314
ENSG00000121579_113723464_113721760_95	NAA50	3	-	285	13	0	0	5.26%	26.76542284143685	0.019679754444124863	0.019679754444124863	0.0017849636115922674
ENSG00000072958_16198027_16234435_423	AP1M1	19	+	1269	14	2	0	1.65%	6.4735428949101665	0.03299602752967379	0.00847367416878415	0.0014019198044660757
ENSG00000123297_57784060_57796583_285	TSFM	12	+	855	25	5	0	4.91%	17.15732233425439	0.009700627241130733	0.00029151402590056445	2.2034580553600625e-05
ENSG00000100462_22926808_22920904_485	PRMT5	14	-	1455	41	7	0	4.54%	16.534706101093818	0.0005921618210055782	1.4179899378848395e-05	7.48157556508186e-08
ENSG00000152082_130182283_130190626_156	MZT2B	2	+	468	24	4	0	3.85%	30.091303786230775	0.019433551906208595	0.011772051206276392	0.0010999473673209293
ENSG00000135404_55726940_55725561_145	CD63	12	-	435	128	17	3	25.52%	172.66182586306212	9.787700055082263e-07	2.0792420187236854e-07	1.9151658325959747e-12
ENSG00000132471_75849694_75846734_190	WBP2	17	-	570	33	0	0	6.32%	33.971498221823694	0.0010197273511756433	0.0010197273511756433	6.442859155158972e-06
ENSG00000174437_110296650_110346470_890	ATP2A2	12	+	2670	202	9	15	8.20%	44.393125698113494	6.88957659970657e-13	1.4513517265808572e-11	1.31401703612969e-22
ENSG00000101558_9936202_9954211_141	VAPA	18	+	423	25	1	0	6.38%	34.679694079875894	0.004355956481189797	0.0035547763465261306	8.558737951575692e-05
ENSG00000168439_64186262_64195852_236	STIP1	11	+	708	63	9	5	7.20%	52.21351229009112	0.0016078616808390032	0.0007308748584878709	7.337758890058988e-06
ENSG00000183258_177516945_177511791_622	DDX41	5	-	1866	25	2	5	1.93%	7.861474059907558	0.0029686363913463144	0.01340328797543303	0.0002230491042000081
ENSG00000080822_98521358_98516659_231	CLDND1	3	-	693	34	2	0	4.33%	28.788649942671004	0.004691997894403566	0.0023628488670607213	6.289248615457574e-05
ENSG00000241553_9801712_9806215_73	ARPC4	3	+	219	37	0	0	19.18%	99.13641863819865	0.0004310062186453162	0.0004310062186453162	1.2248947211367496e-06
ENSG00000054148_136849431_136850847_125	PHPT1	9	+	375	174	18	8	27.20%	272.26611665781604	1.7888246129132578e-06	2.7080479323476877e-07	4.539983500587708e-12
ENSG00000109084_28319240_28325655_92	TMEM97	17	+	276	20	5	4	7.61%	42.52032056750001	0.03585926825421706	0.019795881618345192	0.0031797771518953825
ENSG00000166333_6608898_6610611_298	ILK	11	+	894	35	0	0	4.70%	22.972387957609065	0.0004284130286064981	0.0004284130286064981	1.2106972447307754e-06
ENSG00000143751_225999312_225985302_451	SDE2	1	-	1353	23	0	3	2.66%	9.974833516721732	0.000978917897162665	0.005441582120040429	3.300997901030063e-05
ENSG00000127837_218266584_218265666_168	AAMP	2	-	504	40	1	0	4.76%	46.56987490726191	0.008413704741378425	0.005655335537214311	0.00024942285766697933
ENSG00000141569_75896937_75890779_517	TRIM65	17	-	1551	10	0	0	1.35%	3.7832393541682796	0.007593099278532274	0.007593099278532274	0.00029783286705462064
ENSG00000137074_32989891_32988108_49	APTX	9	-	147	10	0	0	10.20%	39.91703563479593	0.019679754444124863	0.019679754444124863	0.0017849636115922674
ENSG00000177917_152720457_152759840_118	ARL6IP6	2	+	354	32	0	0	4.24%	53.04229819945764	0.02108409854857798	0.02108409854857798	0.002029891263157455
ENSG00000166295_72223915_72233116_110	ANAPC16	10	+	330	20	3	0	6.36%	35.562449929181824	0.027941506428179053	0.00877616296920843	0.0012176006631866223
ENSG00000105397_10362161_10350834_624	TYK2	19	-	1872	6	0	1	0.80%	1.8807064866394234	0.016947426762344633	0.0477903522728147	0.0036975118224100542
ENSG00000169398_140846306_140659466_870	PTK2	8	-	2610	15	6	4	1.15%	3.372301286387932	0.0820328997370387	0.023029368248455667	0.00832076987426179
ENSG00000100320_35809971_35744195_370	RBFOX2	22	-	1110	9	0	0	1.35%	4.757679112147298	0.020613416668581838	0.020613416668581838	0.001946208561389314
ENSG00000268790_16659990_16647246_63	AC008764.4	19	-	189	18	3	0	9.52%	55.8838498887143	0.10041256134757276	0.013007239811447113	0.006595870280092993
ENSG00000111641_6567918_6556993_845	NOP2	12	-	2535	26	4	2	2.01%	6.018260757246155	0.0010891986948716168	0.0002707893389517208	1.9824595550127703e-06
ENSG00000106771_109119913_109020460_879	TMEM245	9	-	2637	54	3	1	2.28%	12.015981375389082	0.00014469150473349395	4.276819016713799e-05	4.628212020308305e-08
ENSG00000204217_202377475_202559946_1038	BMPR2	2	+	3114	14	0	3	0.87%	2.638062277983623	0.002943990679779778	0.033371777158467175	0.0005898405350575255
ENSG00000117410_43975041_43977411_261	ATP6V0B	1	+	783	94	3	8	10.73%	70.44362687121458	3.015385677838286e-06	9.465073544427606e-06	2.418903747385852e-10
ENSG00000198561_57791641_57816308_879	CTNND1	11	+	2637	64	7	2	2.73%	14.241163111572245	0.0002556913665467353	1.5363660848022635e-05	3.2544366602951786e-08
ENSG00000129255_7586014_7587444_180	MPDU1	17	+	540	10	0	3	2.78%	10.86630414502778	0.019679754444124863	0.08385215180161965	0.007498199469051051
ENSG00000087088_48955571_48961097_199	BAX	19	+	597	99	1	1	12.56%	97.30529641426887	5.379067290332158e-06	9.010688822659432e-06	4.01429749920101e-10
ENSG00000119318_107283630_107329656_409	RAD23B	9	+	1227	33	3	1	4.16%	15.781380592045236	0.000764267771632909	0.00018129710408843058	9.562814687146548e-07
ENSG00000186635_72710549_72685664_1033	ARAP1	11	-	3099	7	1	1	0.48%	1.3254156072347534	0.04178282491678763	0.04178282491678763	0.007200520635230478
ENSG00000108312_44213282_44207242_569	UBTF	17	-	1707	33	4	3	2.46%	11.34373402837698	0.0038766098248878157	0.002532570608603844	5.56157809650073e-05
ENSG00000134014_28097245_28189725_532	ELP3	8	+	1596	27	0	1	2.26%	9.92673649339004	0.0009549346450480869	0.0015734716984481248	9.224426685502696e-06
ENSG00000071859_154444326_154450432_309	FAM50A	X	+	927	28	2	1	3.24%	17.723680547229776	0.008661717511597958	0.0034718995429267267	0.00016432324422146306
ENSG00000283761_100007133_100082209_542	AC118553.2	1	+	1626	31	0	0	3.69%	11.187080651154062	1.8393885803062856e-05	1.8393885803062856e-05	2.671064155900456e-09

ENSG00000139974_60982577_61083712_463	SLC38A6	14	+	1389	11	1	0	1.51%	4.6469292024092885	0.016854695534768073	0.007029302661868638	0.0006017499134389055
ENSG00000148337_128179348_128166197_584	CIZ1	9	−	1752	17	2	0	1.54%	5.69364566503168	0.018399683928041276	0.002967235347054297	0.00031087262125642094
ENSG00000197601_13694766_13728774_515	FAR1	11	+	1545	13	1	0	1.17%	4.9373110095854384	0.02034245439037237	0.013428347753762199	0.0012955184805712684
ENSG00000204435_31666832_31669183_125	CSNK2B	6	+	375	65	12	3	15.20%	101.70860679746002	0.0019475751524943912	0.0001523580159507665	2.1675903008066225e−06
ENSG00000143549_154183119_154157639_227	TPM3	1	−	681	83	1	5	6.17%	71.51655679590971	0.0005257599192501259	0.0018512044310315418	6.238201644005689e−06
ENSG00000009954_73521933_73442196_1483	BAZ1B	7	−	4449	44	3	2	1.08%	5.8031779385448425	0.000642070651048262	0.0005517907460199459	2.285141187808916e−06
ENSG00000080371_71755130_71785673_225	RAB21	12	+	675	18	4	1	3.56%	15.647477968840004	0.03149539866061481	0.008316137131635013	0.0013147451223346918
ENSG00000112237_99550995_99543555_138	CCNC	6	−	414	9	0	1	3.62%	12.756096170250004	0.019679754444124863	0.04223451612662591	0.003715148010809822
ENSG00000120896_22566421_22574728_329	SORBS3	8	+	987	12	0	0	1.82%	7.134108496431613	0.011570465654371866	0.011570465654371866	0.0006594843062896773
ENSG00000113558_134167235_134157733_128	SKP1	5	−	384	26	0	1	6.25%	39.72992453025782	0.005706018193000826	0.0073129010928764775	0.0002196570096149794
ENSG00000167468_1104125_1105405_45	GPX4	19	+	135	18	0	1	20.00%	78.23738984420002	0.0034782380676658805	0.004911637253759624	9.416876808028951e−05
ENSG00000108602_19743406_19742670_77	ALDH3A1	17	−	231	281	33	14	29.87%	713.7891735785781	0.000834332798408162	0.0001270181330248376	7.562056271414726e−07
ENSG00000143183_165768742_165754273_46	TMCO1	1	−	138	58	1	10	21.74%	246.61785929150005	0.0025025372014526115	0.016365329676341956	0.00023740648418217605
ENSG00000081014_50929160_51002662_906	AP4E1	15	+	2718	10	0	0	0.55%	2.158868373184327	0.020613416668581838	0.020613416668581838	0.001946208561389314
ENSG00000100442_45134456_45116198_224	FKBP3	14	−	672	221	1	2	11.61%	192.97391914696655	4.0468086267116175e−06	6.1306945952039744e−06	2.0827231135695882e−10
ENSG00000124795_18263987_18225719_375	DEK	6	−	1125	32	2	5	3.47%	16.69064316676267	0.002169009861618943	0.005689030396290439	7.044557031939336e−05
ENSG00000112759_44229610_44233528_412	SLC29A1	6	+	1236	49	6	0	4.61%	23.262330718239085	0.0003953272125394652	5.6254241454829154e−05	1.6639839158165156e−07
ENSG00000102125_154411916_154421004_287	TAZ	X	+	861	15	1	0	2.44%	10.222655467447737	0.008838362722921076	0.007029302661868638	0.00032011746987943644
ENSG00000113580_143400839_143281889_746	NR3C1	5	−	2238	12	5	2	1.21%	3.1462757310000007	0.07471635411946746	0.014696946993710543	0.005240911805500765
ENSG00000168393_241678478_241676127_45	DTYMK	2	−	135	20	0	0	11.11%	86.93043316022224	0.02108409854857798	0.02108409854857798	0.002029891263157455
ENSG00000197150_151034375_151045349_548	ABCB8	7	+	1644	29	0	1	2.37%	10.350749568803835	0.0006629342244537616	0.0009306875619951529	3.907239258273379e−06
ENSG00000033867_27434087_27376764_1057	SLC4A7	3	−	3171	27	0	2	1.51%	4.996238235083728	0.00015252522453692948	0.0007701916578329232	8.226488299996088e−07
ENSG00000147457_23246786_23260599_423	CHMP7	8	+	1269	17	1	5	2.36%	7.8607306581052026	0.005561255870711926	0.023814920846593857	0.0006973237161995367
ENSG00000104723_15623143_15757809_281	TUSC3	8	+	843	31	0	0	4.63%	21.57792780400534	0.0006745191331285194	0.0006745191331285194	2.90713800764924e−06
ENSG00000153113_96726815_96771679_694	CAST	5	+	2082	117	0	4	5.91%	32.97469240551658	8.877373555998216e−09	9.666295517668156e−08	9.027556298029978e−15
ENSG00000140553_90935325_90953716_944	UNC45A	15	+	2832	26	2	3	1.45%	5.3871084108824165	0.0016931352282804095	0.0036482233473974857	3.6055762566959285e−05
ENSG00000173171_155209264_155213698_313	MTX1	1	+	939	40	3	0	2.24%	24.995971196230037	0.0424352593247778	0.007029302661868638	0.0015638435298188141
ENSG00000167792_67607005_67612458_457	NDUFV1	11	+	1371	73	5	2	6.13%	31.243596600801975	8.636934262206999e−06	3.2065559803742585e−06	2.3402305267661333e−10
ENSG00000143384_150579530_150577375_350	MCL1	1	−	1050	66	3	3	8.00%	36.88334092655144	8.148227735821443e−06	8.148227735821443e−06	5.444014276458433e−10
ENSG00000137563_63038768_63024068_205	GGH	8	−	615	92	1	0	9.76%	87.77853494715124	5.218963683194363e−05	3.8455384355631195e−05	1.516856243655179e−08
ENSG00000102158_77895410_77829220_335	MAGT1	X	−	1005	25	5	1	3.28%	14.596527956007465	0.01475453632861615	0.00236147777460975	0.00020240892325058517
ENSG00000110442_36289412_36274632_156	COMMD9	11	−	468	18	1	0	3.85%	22.568477839673083	0.016822681477512567	0.013428347753762199	0.0010798218650012886
ENSG00000170892_54191365_54193362_310	TSEN34	19	+	930	33	5	0	3.87%	20.821240845633877	0.008356254428487338	0.0010155991509472172	5.354085463802975e−05
ENSG00000135047_87728735_87731107_151	CTSL	9	+	453	288	9	1	21.85%	373.0524548862517	1.4308543775778015e−06	2.499002843689541e−07	3.3523907881978444e−12
ENSG00000117410_43975041_43978007_205	ATP6V0B	1	+	615	94	3	8	13.66%	89.68676396774148	3.015385677838286e−06	9.465073544427606e−06	2.418903747385852e−10
ENSG00000114125_141738342_141745277_97	RNF7	3	+	291	23	0	0	10.31%	46.37783418599486	0.0023218226288164415	0.0023218226288164415	3.1231198880462635e−05
ENSG00000146648_55019278_55156843_405	EGFR	7	+	1215	16	8	0	1.48%	7.727149614241977	0.11368416502207135	0.011570465654371866	0.006956434060967934
ENSG00000106290_100111957_100107246_423	TAF6	7	−	1269	20	2	0	1.65%	9.247918421300238	0.028426896806120847	0.00847367416878415	0.0012022752110812016
ENSG00000185515_155071528_155120150_292	BRCC3	X	+	876	7	0	0	2.05%	4.688884665320207	0.009815328628645334	0.009815328628645334	0.0004837142251238724
ENSG00000188042_234496535_234495806_184	ARL4C	2	−	552	7	0	0	2.72%	7.441056099312502	0.016947426762344633	0.016947426762344633	0.0013498980316300959
ENSG00000146409_132790236_132770270_383	SLC18B1	6	−	1149	8	3	0	1.31%	4.085503386120106	0.14389369507707506	0.019679754444124863	0.013602114125134476
ENSG00000165376_106928382_106928921_179	CLDN2	X	+	537	10	0	0	2.79%	10.927009754776538	0.020613416668581838	0.020613416668581838	0.001946208561389314
ENSG00000145191_184137938_184143258_408	EIF2B5	3	+	1224	10	0	0	1.47%	4.793957711041668	0.011772051206276392	0.011772051206276392	0.000681272511389508
ENSG00000125445_75262823_75265923_144	MRPS7	17	+	432	17	0	1	5.56%	23.090896308184032	0.005306578019052857	0.013892891352654344	0.00038601378095827807
ENSG00000092841_56158402_56160331_238	MYL6	12	+	714	764	13	19	18.91%	627.871489926143	3.3266175334312703e−09	3.22524204545709e−08	1.1560867657950006e−15
ENSG00000142494_19542408_19577553_520	SLC47A1	17	+	1560	14	0	0	1.15%	5.265978162590386	0.013217226988443315	0.013217226988443315	0.0008469697265466234
ENSG00000111077_53050186_53062664_1312	TNS2	12	+	3936	12	0	4	0.38%	1.788964706803354	0.019679754444124863	0.17004230409153215	0.016523685284367155
ENSG00000130939_10101145_10179956_1045	UBE4B	1	+	3135	22	2	0	1.15%	4.117757360221054	0.0029488083751021267	0.0008664586529590942	1.5772708037927043e−05
ENSG00000160710_154601756_154584806_931	ADAR	1	−	2793	107	6	3	4.19%	22.47959375222718	7.29368051916334e−07	9.227426656624114e−08	6.559954505852833e−13
ENSG00000159921_36246469_36217365_612	GNE	9	−	1836	14	0	3	1.31%	4.474360530305557	0.005257622967929454	0.04085312694387945	0.0011826962990484254
ENSG00000100722_88572612_88611751_580	ZC3H14	14	+	1740	21	0	3	2.24%	7.0818327014146565	0.0005280076130035933	0.005964579803964576	2.1225325314842878e−05
ENSG00000119655_74484543_74480274_73	NPC2	14	−	219	42	6	0	15.07%	112.53323196768496	0.006053937317525996	0.0016109979480538772	5.737912741585913e−05
ENSG00000062582_43869495_43866699_167	MRPS24	7	−	501	27	5	1	5.99%	31.622897092715576	0.015528142895806445	0.0023218226288164415	0.00021060506759671452
ENSG00000141452_23503619_23531704_657	RMC1	18	+	1971	12	2	3	0.91%	3.5724835545296814	0.05258119297243839	0.07356204933788085	0.014970058718206515
ENSG00000141458_23586343_23532202_1278	NPC1	18	−	3834	46	3	4	1.96%	7.04014071367997	3.118055881539828e−05	4.566877597405208e−05	1.0866403505462053e−08
ENSG00000079459_11808887_11838609_353	FDFT1	8	+	1059	211	12	7	15.01%	116.91281343573799	5.823304590514566e−09	4.985113558253148e−10	3.292241988068417e−17
ENSG00000130177_114239476_114272443_447	CDC16	13	+	1341	34	0	5	3.13%	14.877356010642062	0.0004323067881491664	0.0037401700898596867	1.0841548675644574e−05
ENSG00000130177_114239476_114272443_498	CDC16	13	+	1494	36	0	5	3.01%	14.139287321240968	0.00028877520139654507	0.0025792363906108322	5.131036494031878e−06
ENSG00000150093_32935558_32907069_801	ITGB1	10	−	2403	798	61	38	17.10%	194.86091478049818	9.518832060609776e−24	3.6515843144635014e−21	6.6344231264239206e−43
ENSG00000181704_68513360_68531999_163	YIPF6	X	+	489	18	1	0	3.68%	21.599279404840495	0.02034245439037237	0.013428347753762199	0.0012955184805712684
ENSG00000140564_90875741_90881878_794	FURIN	15	+	2382	7	4	1	0.63%	1.7243757207474815	0.169362073879829	0.04178282491678763	0.02871445979022244
ENSG00000115365_210472019_210434487_353	LANCL1	2	−	1059	32	0	0	4.25%	17.730853222481592	0.00023522430645143535	0.00023522430645143535	3.796194994189714e−07
ENSG00000131013_149546005_149505453_492	PPIL4	6	−	1476	17	0	0	1.42%	6.758311114590449	0.0078820576498062	0.0078820576498062	0.00031963642907451164
ENSG00000161960_7576539_7577547_155	EIF4A1	17	+	465	40	1	1	7.74%	50.47573538335485	0.0009470236307512361	0.0008776897154483972	5.194702683838394e−06
ENSG00000175768_37592532_37588898_51	TOMM5	9	−	153	106	20	3	19.61%	406.52761389633343	0.015037340333051557	0.0024742386374296595	0.00021494514850066922
ENSG00000152291_85327962_85322736_379	TGOLN2	2	−	1137	52	2	3	5.28%	26.83604400988391	7.960803452267643e−05	0.00019187199389134263	1.1026034304323633e−07
ENSG00000129197_5421311_5426086_110	RPAIN	17	+	330	21	2	0	5.45%	37.340572425640914	0.037178613843887286	0.013428347753762199	0.0023492041693324566

ENSG00000196642_136808326_136839730_441	RABL6	9	+	1323	18	1	3	1.59%	7.983407126959185	0.013892891352654344	0.03891955538541466	0.002533587285467915
ENSG00000182944_29282517_29300161_476	EWSR1	22	+	1428	126	6	8	5.04%	51.77474327925001	0.00012487302501014574	0.0001440551817339119	1.2777049173942254e-07
ENSG00000160014_46608271_46609153_113	CALM3	19	+	339	45	2	1	15.04%	77.89120670329648	0.0001889056949786359	0.00018618114937238455	2.447366381606584e-07
ENSG00000157168_32595851_32764402_599	NRG1	8	+	1797	29	0	1	1.50%	9.4694670512596	0.003736770770438521	0.0045126225279669635	9.281320660291148e-05
ENSG00000149177_47980913_48167362_1338	PTPRJ	11	+	4014	24	7	4	0.90%	3.5084031320269067	0.0245051549198494	0.00965975009320423	0.0011592244316170714
ENSG00000172757_65858099_65855336_166	CFL1	11	-	498	811	51	16	25.30%	955.580168127202	4.060318142563395e-08	1.255068054445319e-07	5.044460439427906e-14
ENSG00000172269_119100804_119097551_135	DPAGT1	11	-	405	19	0	0	3.70%	27.527970500737045	0.02108409854857798	0.02108409854857798	0.002029891263157455
ENSG00000168002_62765397_62766507_42	POLR2G	11	+	126	70	0	5	33.33%	325.98912435083344	0.00046843213071395124	0.0031185037294365974	9.613998767368705e-06
ENSG00000102974_67611577_67637872_479	CTCF	16	+	1437	8	0	0	1.25%	3.266696861970773	0.011570465654371866	0.011570465654371866	0.0006594843062896773
ENSG00000189067_11556730_11549637_161	LITAF	16	-	483	34	1	0	7.45%	41.30545426557144	0.0010073743004618205	0.0010155991509472172	6.342998511872531e-06
ENSG00000132541_98117096_98102842_137	RIDA	8	-	411	15	4	1	7.30%	21.41534393545621	0.028115279753387075	0.0040534433873287605	0.0006361284422476127
ENSG00000097021_6339454_6264597_238	ACOT7	1	-	714	16	1	2	3.36%	13.149141150285717	0.008219046535586472	0.02071580916834944	0.0008496570697932704
ENSG00000163382_156591805_156594084_288	NAXE	1	+	864	77	3	2	5.21%	52.294088697946194	0.0010801400526993072	0.0006337763638602917	4.332699062608505e-06
ENSG00000163382_156591805_156594056_260	NAXE	1	+	780	53	7	1	5.00%	39.87097751675578	0.017171817882917755	0.0012335676370081575	0.00013796803965043293
ENSG00000163382_156591805_156593629_245	NAXE	1	+	735	69	3	2	5.31%	55.08550917601838	0.0029628053902178418	0.0016323237767217442	2.8375409934455784e-05
ENSG00000160818_156599013_156595213_359	GPATCH4	1	-	1077	13	1	2	1.39%	7.082772061104459	0.03537493353818881	0.056923149003329024	0.008291339120283054
ENSG00000078808_1228772_1217512_355	SDF4	1	-	1065	49	8	4	5.63%	26.997409171590146	0.0011533959362000172	0.00030679325052546705	2.3562441260436685e-06
ENSG00000197771_119872584_119831474_642	MCMBP	10	-	1926	50	1	2	4.05%	15.233136651908103	4.092664829715607e-06	1.244462736205032e-05	4.2602396203460535e-10
ENSG00000182220_40597536_40605755_215	ATP6AP2	X	+	645	30	1	4	6.98%	27.292112736348844	0.0004235771399443867	0.0018201709930110535	5.035476900801534e-06
ENSG00000080503_2086868_2192739_717	SMARCA2	9	+	2151	16	4	4	0.98%	4.364707941099025	0.05995084452706934	0.052370108323589605	0.012325812363490476
ENSG00000147123_47144679_47142317_163	NDUFB11	X	-	489	85	2	5	14.11%	101.99659718952456	1.2524681740970158e-05	2.0042438260184324e-05	2.001383011567005e-09
ENSG00000104763_18061423_18057534_149	ASAH1	8	-	447	29	3	0	6.04%	38.06852861546645	0.0035547763465261306	0.003710480643933249	7.338809221316118e-05
ENSG00000205133_92916989_92886622_86	TRIQK	8	-	258	7	0	0	5.81%	15.920399096203491	0.019216965118390855	0.019216965118390855	0.0017073955890589052
ENSG00000145817_144165549_144160397_202	YIPF5	5	-	606	16	0	0	4.46%	15.492552444396043	0.0033030736763512156	0.0033030736763512156	6.124050385493983e-05
ENSG00000115540_197540381_197550646_126	MOB4	2	+	378	13	1	0	4.76%	20.180279126480162	0.023672501042627626	0.012798402692974279	0.0014375227604752335
ENSG00000106976_128203486_128222203_315	DNM1	9	+	945	29	2	4	3.17%	18.00701829747461	0.003457660202585786	0.023617360693700868	0.0004617444263088556
ENSG00000144048_73780115_73762661_377	DUSP11	2	-	1131	18	0	0	2.65%	9.338680485381966	0.0020082573300163734	0.0020082573300163734	2.3658688350241813e-05
ENSG00000149923_30076378_30082907_120	PPP4C	16	+	360	19	2	5	8.33%	30.968966813329175	0.004911637253759624	0.025871143657089396	0.0006841479597062461
ENSG00000101654_13734581_13759979_298	RNMT	18	+	894	15	0	0	2.35%	9.8453091246896	0.0078820576498062	0.0078820576498062	0.00031963642907451164
ENSG00000176974_18355921_18328750_463	SHMT1	17	-	1389	25	0	0	2.38%	10.561202732748383	0.0014055202957965589	0.0014055202957965589	1.1935530040246081e-05
ENSG00000170421_52904981_52897428_483	KRT8	12	-	1449	775	43	22	10.14%	313.8404613315477	2.7212329735660806e-09	2.641628763289803e-09	7.744562051262718e-17
ENSG00000166783_15635952_15612741_859	MARF1	16	-	2577	16	0	8	0.70%	3.6431846260395817	0.013007239811447113	0.14381801945170042	0.010011980172995838
ENSG00000101654_13734581_13759979_298	RNMT	18	+	894	15	0	0	2.35%	9.8453091246896	0.0078820576498062	0.0078820576498062	0.00031963642907451164
ENSG00000160633_5649495_5668291_535	SAFB	19	+	1605	26	0	1	2.62%	9.505477270790657	0.00039077502422413024	0.0009568617188207251	2.441942676277831e-06
ENSG00000065268_984402_989899_136	WDR18	19	+	408	14	1	0	3.68%	20.134622386375003	0.02108409854857798	0.02108409854857798	0.002029891263157455
ENSG00000130638_45729444_45823218_196	ATXN10	22	+	588	80	7	4	9.69%	79.83407126959186	0.000124270640200841	7.704672226380185e-05	6.947627997145781e-08
ENSG00000174437_110281790_110350305_997	ATP2A2	12	+	2991	274	13	16	8.22%	53.75387366426982	7.975196879814547e-14	5.009698128368371e-13	5.3591675920680345e-25
ENSG00000072110_68979020_68874859_875	ACTN1	14	-	2625	118	6	33	5.26%	26.377177147473148	1.2617677869874243e-08	0.00010438246008299231	2.657855893184278e-11
ENSG00000099797_14562396_14565871_346	TECR	19	+	1038	89	8	1	4.34%	50.31161630154481	0.002256547083596618	0.0003418708136139773	5.181847689677099e-06
ENSG00000109113_28717266_28714643_259	RAB34	17	-	777	76	7	3	8.11%	57.3942242100309	0.0006038791504987458	0.00011732921528396223	5.042084532308569e-07
ENSG00000134824_61816625_61865689_399	FADS2	11	+	1197	182	22	11	14.04%	89.21807613812283	1.8510928623203167e-08	1.7612313515040756e-10	4.0920970069878796e-17
ENSG00000143549_154173155_154157639_144	TPM3	1	-	432	59	0	0	6.25%	80.13899306957988	0.0037896409716948643	0.0037896409716948643	7.95824840892005e-05
ENSG00000128595_128748605_128769167_308	CALU	7	+	924	95	4	1	8.12%	60.32915612986203	2.4803459298984587e-05	6.608873402560172e-06	1.3443694709282272e-09
ENSG00000144021_96266351_96271351_339	CIAO1	2	+	1017	20	2	8	3.24%	11.539438030117998	0.005935135481713141	0.05179676907674221	0.0016947782921246865
ENSG00000126088_45012992_45013706_100	UROD	1	+	300	11	1	0	6.00%	21.515282207155003	0.023672501042627626	0.012798402692974279	0.0014375227604752335
ENSG00000197756_216498875_216501404_92	RPL37A	2	+	276	453	32	23	33.70%	963.0852608538752	5.659769186036882e-07	1.0866409953770624e-05	5.810105286697961e-11
ENSG00000136560_161231165_161235518_187	TANK	2	+	561	16	0	0	3.21%	16.735270554909096	0.013428347753762199	0.013428347753762199	0.0008725593497644525
ENSG00000167565_40441765_40441490_91	SERTAD3	19	-	273	52	0	3	10.99%	111.7676997774286	0.002446120450917513	0.005134531980829637	7.09837241422139e-05
ENSG00000185686_22556832_22548067_509	PRAME	22	-	1527	45	1	1	3.54%	17.292153943953835	0.00011105827337505001	0.00011105827337505001	8.855184710094631e-08
ENSG00000127955_80135161_80217493_354	GNAI1	7	+	1062	26	4	0	2.54%	14.365622429019778	0.015528142895806445	0.0036062891729786097	0.0003077713301577895
ENSG00000114353_50252497_50257690_268	GNAI2	3	+	804	20	0	4	2.99%	14.596527956007465	0.005306578019052857	0.019778610614370123	0.0005526454465689531
ENSG00000136261_16665444_16706088_419	BZW2	7	+	1257	14	0	0	2.63%	6.535342827081147	0.001068153208390582	0.001068153208390582	7.044127641813182e-06
ENSG00000106305_6015245_6023691_242	AIMP2	7	+	726	59	5	5	9.09%	47.686012405039264	0.00015080393990865712	0.00011814597527926959	1.266618772072988e-07
ENSG00000105948_139134655_139189365_486	TTC26	7	+	1458	11	0	0	1.23%	4.427012799826133	0.012798402692974279	0.012798402692974279	0.0007972436888943567
ENSG00000161203_184176994_184180275_148	AP2M1	3	+	444	24	0	2	5.41%	31.717860747648658	0.0053557511377634245	0.012834473343944776	0.0003595359162240675
ENSG00000177239_137106191_137108591_259	MAN1B1	9	+	777	22	0	0	2.70%	16.61411753448263	0.00877616296920843	0.00877616296920843	0.00039161135667564577
ENSG00000100439_22601716_22606482_157	ABHD4	14	+	471	15	2	0	3.18%	18.68727464431529	0.060667625179241055	0.02108409854857798	0.005667715763110205
ENSG00000047410_186375028_186313971_2363	TPR	1	-	7089	82	6	9	1.31%	6.7874163851294975	6.3502689983814185e-06	1.1116865741893434e-05	5.80140031593077e-10
ENSG00000157181_186379786_186419076_431	ODR4	1	+	1293	19	0	0	2.09%	8.622450156843389	0.0035291660242640502	0.0035291660242640502	6.948232590694344e-05
ENSG00000197694_128566741_128633334_2472	SPTAN1	9	+	7416	76	4	6	1.33%	6.0133916142386745	1.556229315321248e-06	4.240796765082592e-06	5.74504648209071e-11
ENSG00000156110_74200783_74708445_334	ADK	10	+	1002	32	7	8	4.19%	18.739494573461084	0.011759535387248672	0.014190628253918529	0.0008122148910489306
ENSG00000198561_57791641_57816308_914	CTNND1	11	+	2742	64	7	2	2.63%	13.695823167474838	0.0002556913665467353	1.5363660848022635e-05	3.2544366602591786e-08
ENSG00000189159_75154397_75136102_154	JPT1	17	-	462	190	17	9	17.53%	241.3166245194481	8.942764049598665e-05	5.665530273778939e-06	4.360693546071237e-09
ENSG00000176894_132695927_132704687_102	PXMP2	12	+	306	8	0	0	4.90%	15.340664675333336	0.016947426762344633	0.016947426762344633	0.0013498980316300959
ENSG00000104765_26391263_26410412_179	BNIP3L	8	+	537	19	1	0	5.59%	20.761318534075425	0.0037220617515305076	0.0021880057261679567	4.6633814146144326e-05
ENSG00000175324_38170955_38163670_99	LSM1	8	-	297	11	0	0	6.06%	21.73260829005556	0.011772051206276392	0.011772051206276392	0.000681272511389508
ENSG00000115875_38751256_38745133_235	SRSF7	2	-	705	174	6	3	15.74%	144.82240247756172	1.0931814107840405e-07	9.865761440936882e-08	1.0441239872106452e-13

ENSG00000099956_23787248_23801397_245	SMARCB1	22	+	735	16	4	0	2.45%	12.773451403134697	0.0695184692773032	0.013640585738808999	0.004566938431613105
ENSG00000099956_23816790_23834180_169	SMARCB1	22	+	507	9	3	0	3.55%	10.416220541387576	0.09718295540155453	0.012178957859018494	0.006042221727254493
ENSG00000137309_34240781_34244884_107	HMGA1	6	+	321	34	3	0	5.61%	62.15119753978506	0.044040755831095145	0.013428347753762199	0.0027917083341200354
ENSG00000102858_4650391_4681557_282	MGRN1	16	+	846	12	0	2	2.84%	8.323126579170214	0.004873903550586949	0.029607078753113344	0.0007843881175907335
ENSG00000138081_47905720_47808118_927	FBXO11	2	-	2781	7	3	1	0.54%	1.4769733789358148	0.19022756262519425	0.016947426762344633	0.01699570088362101
ENSG00000241127_39566530_39572706_189	YAE1	7	+	567	21	0	0	4.23%	21.73260829005556	0.005306578019052857	0.005306578019052857	0.00015100114740534233
ENSG00000166452_8911450_8919480_183	AKIP1	11	+	549	12	0	0	3.28%	12.825801613803282	0.011772051206276392	0.011772051206276392	0.000681272511389508
ENSG00000160014_46608271_46609153_113	CALM3	19	+	339	45	2	1	15.04%	77.89120670329648	0.0001889056949786359	0.00018618114937238455	2.447366381606584e-07
ENSG00000175061_16439055_16440145_42	SNHG29	17	+	126	12	3	0	11.90%	55.8838498887143	0.1029516053660342	0.02108409854857798	0.009872287793463467
ENSG00000103197_2060673_2088610_1415	TSC2	16	+	4245	10	5	3	0.57%	1.382286039650177	0.07635947366271362	0.04922925118879209	0.01464496403362635
ENSG00000095787_28590832_28611412_278	WAC	10	+	834	10	0	0	2.16%	7.0357364967805776	0.012798402692974279	0.012798402692974279	0.0007972436888943567
ENSG00000197746_71851221_71817441_240	PSAP	10	-	720	432	35	8	23.75%	352.06825429890006	1.227754706718647e-10	5.377450937316541e-11	7.772786316704484e-20
ENSG00000126883_131150703_131233487_1352	NUP214	9	+	4056	13	0	0	0.44%	1.8807064866394234	0.013428347753762199	0.013428347753762199	0.0008725593497644525
ENSG00000112855_140694028_140698552_414	HARS2	5	+	1242	15	1	0	2.17%	7.086720094583335	0.006024333578509648	0.0032294771334464936	0.00010730535955833177
ENSG00000166557_79311250_79383018_155	TMED3	15	+	465	9	0	0	3.23%	11.357040461254842	0.020613416668581838	0.020613416668581838	0.001946208561389314
ENSG00000274049_74455897_74459667_253	INO80B-WBP1	2	+	759	17	0	0	1.98%	13.142644539045458	0.02108409854857798	0.02108409854857798	0.002029891263157455
ENSG00000167526_89560981_89563042_157	RPL13	16	+	471	182	2	7	8.92%	226.73893235102554	0.00044168141455422886	0.0005949174369167231	1.7147483005920436e-06
ENSG00000086666_80120378_80137628_197	ZFAND6	15	+	591	20	0	3	5.58%	19.8572055442132	0.0014481341328822738	0.011821942446875977	0.00010518227444157728
ENSG00000100526_54397069_54420078_212	CDKN3	14	+	636	24	1	3	7.08%	22.142657503075476	0.0004312186824833873	0.002019188930569991	5.691763945269724e-06
ENSG00000067225_72219097_72199650_531	PKM	15	-	1593	1923	113	80	25.80%	708.3356905385906	5.610157492511679e-22	1.1873189298227106e-21	1.1445714559733268e-41
ENSG00000051523_88651013_88646751_96	CYBA	16	-	288	71	4	4	12.50%	144.65767393068234	0.0016556907807070025	0.005260035373741125	5.097491000351223e-05
ENSG00000165792_20990003_20996890_456	METTL17	14	+	1368	32	2	0	2.63%	13.725857867403512	0.0026957942880510015	0.0010405263639310788	1.7035015215455722e-05
ENSG00000147459_25308823_25374795_955	DOCK5	8	+	2865	12	8	0	0.63%	2.457719052697383	0.2827853679176493	0.013217226988443315	0.024082600958094665
ENSG00000080345_151416686_151475071_2311	RIF1	2	+	6933	16	1	1	0.39%	1.354173774888793	0.006112543793728684	0.006112543793728684	0.00019748307313365872
ENSG00000275700_36949126_37056664_560	AATF	17	+	1680	37	5	1	3.75%	12.923140286765182	0.0004090297838917604	3.3309456768330186e-05	1.0720609820332405e-07
ENSG00000125107_58599337_58520958_2371	CNOT1	16	-	7113	45	7	5	0.93%	3.712233807453607	0.0008315867542965732	0.00020037430501765357	1.1424951378129194e-06
ENSG00000140365_75339344_75340005_72	COMMD4	15	+	216	17	0	0	11.11%	46.181792616368064	0.005306578019052857	0.005306578019052857	0.00015100114740534233
ENSG00000103266_682038_682489_93	STUB1	16	+	279	13	0	0	6.45%	27.341023332650543	0.013007239811447113	0.013007239811447113	0.0008218654148058079
ENSG00000096746_68337222_68342054_346	HNRNPH3	10	+	1038	147	5	11	13.58%	83.09896175648413	6.773168428194152e-09	6.96612263320329e-08	4.994306453532507e-15
ENSG00000213585_133980895_133972771_155	VDAC1	5	-	465	56	0	0	5.16%	70.6660295366968	0.005604866691947602	0.005604866691947602	0.00016752456572620382
ENSG00000143811_225924073_225920455_307	PYCR2	1	-	921	14	3	0	2.61%	8.91957213207492	0.056043582888960386	0.004193160480899131	0.0014062628675739213
ENSG00000099817_1095315_1089486_210	POLR2E	19	-	630	41	0	0	4.29%	38.18729742395477	0.0034782380676658805	0.0034782380676658805	6.758309314600117e-05
ENSG00000100650_69768157_69769561_138	SRSF5	14	+	414	98	10	5	14.49%	138.89971385383336	0.00020622244759692734	0.0002165592947110879	3.0848500546799e-07
ENSG00000159131_33539315_33528316_306	GART	21	-	918	25	0	0	3.59%	15.979859036805559	0.001558047347358915	0.001558047347358915	1.4544595481456893e-05
ENSG00000159131_33528218_33504124_672	GART	21	-	2016	67	5	0	3.72%	19.501135117415927	4.941667822690351e-05	5.071198556446219e-06	2.122818343582949e-09
ENSG00000215301_41343817_41347719_408	DDX3X	X	+	1224	71	0	0	7.84%	34.03709974839584	1.8060166406889672e-07	1.8060166406889672e-07	3.100108887739879e-13
ENSG00000215301_41334253_41343775_193	DDX3X	X	+	579	14	11	6	6.22%	14.18812769195337	0.1318072353628263	0.04735996622374726	0.02430203731153827
ENSG00000137807_69414466_69426187_283	AC027237.1	15	+	849	11	0	0	2.47%	7.602573218075974	0.006937202941512724	0.006937202941512724	0.00025101522741487095
ENSG00000137807_69435503_69446512_345	AC027237.1	15	+	1035	20	0	2	2.61%	11.338752151333336	0.0033030736763512156	0.016881135945258162	0.0003111891849301449
ENSG00000147044_41665429_41520420_713	CASK	X	-	2139	19	0	0	0.98%	5.212168327629033	0.00847367416878415	0.00847367416878415	0.0003665024264111182
ENSG00000035862_78925088_78855667_220	TIMP2	17	-	660	58	5	0	9.55%	51.565552397313645	3.349467450681034e-05	2.057919671176267e-05	5.363785027308851e-09
ENSG00000126934_4101024_4090598_74	MAP2K2	19	-	222	7	0	0	6.76%	18.502085436128382	0.016947426762344633	0.016947426762344633	0.0013498980316300959
ENSG00000073712_52950547_52858377_673	FERMT2	14	-	2019	35	0	1	2.53%	10.172023196682765	0.00011297380978831068	0.00018702083254192264	1.498932361695684e-07
[truncated: 2,222,348 more chars]
